# Co-evolution of alpha-helical transmembrane protein residues: large-scale variant profiling and complete mutational landscape of 2277 known PDB entries representing 504 unique human protein sequences

**DOI:** 10.1007/s00239-025-10262-8

**Published:** 2025-09-24

**Authors:** Taner Karagöl, Alper Karagöl, Shuguang Zhang

**Affiliations:** 1 Istanbul University Istanbul Medical Faculty, Istanbul, Turkey; 2 Media Lab, Massachusetts Institute of Technology, 77 Massachusetts Avenue, Cambridge, MA 02139 USA

**Keywords:** Alanine transition intermediates, Hydrophobic to hydrophilic alpha-helix conversion, Molecular evolution of transmembrane proteins, QTY code

## Abstract

Membrane proteins play fundamental roles in cellular function, yet the evolutionary dynamics of their amino acid composition remain poorly understood. Our current study investigates the substitutional landscape and evolutionary patterns of hydrophilic and hydrophobic residues in membrane α-helical proteins, addressing a significant gap in our knowledge of protein evolution. We analyzed 2277 high-resolution protein structures from the RCSB Protein Data Bank corresponding to 458 unique PDB structures, 504 UniProt transmembrane entries and their AlphaMissense predicted mutational libraries including more than 5.8 million amino acid substitutions, focusing on known transmembrane α-helical proteins in *Homo sapiens*. Our analysis showed that the pathological outcome of the substitutions is diverse, as nonpolar to polar changes showed higher pathological scores in general. Notably, F <=> Y substitutions showed significantly lower pathological scores. Our further analysis revealed a significant asymmetry in the evolutionary frequencies of polar and nonpolar amino acids. We identified key residue pairs driving this asymmetry, with F <=> Y, A <=> T, V <=> T and A <=> S co-evolution diverging from the expected negative correlations (Spearman’s rho > 0.20, *p* < 0.001). The V <=> T substitution via an alanine intermediate and the G <=> N substitution via a serine intermediate lower their statistical barrier, which would otherwise require two sequential base changes. We propose two evolutionary game theory (EGT) based models to explain their diversification, with partial correlation analysis on residue frequencies in homolog sequences. These mathematical insights suggest a previously unrecognized evolutionary pressure, potentially linked to functional diversification, which could be targeted to combat drug resistance. Our results offer insights into membrane protein evolution and may inform improved methods for protein structure prediction and design.

**Supplementary Information:**

The online version contains supplementary material available at 10.1007/s00239-025-10262-8.

## Introduction

Alpha-helical membrane proteins are fundamental components of cells, involved in processes such as signal transduction, cellular transport, and cell–cell recognition (Hegde and Keenan 2022; Corradi et al. 2019; Cho and Stahelin 2005). These proteins, which comprise approximately 30% of the proteome in most organisms (Wallin and von Heijne 1998), are characterized by their distinctive structure, with α-helical domains spanning the lipid bilayer. Despite their biological significance, our understanding of the evolutionary dynamics shaping membrane protein composition remains limited, particularly regarding the variations between hydrophilic and hydrophobic residues within transmembrane regions.

The classical view of membrane α-helix composition indicates a simple dichotomy: hydrophobic residues are abundant in the transmembrane regions for maintaining interactions with membrane lipids, while hydrophilic residues are more common in extra-membranous regions (Corradi et al. 2019; Almaida 2023; White and von Heijne 2005). However, this perspective fails to capture the full complexity of residue-wise distributions observed in nature, which particularly influences the variational dynamics that can have profound effects on evolutionary fitness. For instance, polar tyrosine could be favorable when it is moved toward the ends of the TM helices (White and von Heijne 2005; Wimley and White 1996). Similar positional effects are also seen in poorly hydrophobic transmembrane domains (Ji et al. 2024). Furthermore, a fully hydrophobic α-helix is not common in transmembrane domains, as they typically contain between 81 and 91% hydrophobic amino acids (Zhang and Egli 2022). In our previous work, we investigated variant effects in artificially designed α-helices—known as QTY-code pairs—which are chemically distinct but structurally similar (Karagöl et al. 2024a; 2024b). The QTY code substitutes hydrophobic residues leucine (L), isoleucine (I), valine (V), and phenylalanine (F) with polar counterparts glutamine (Q), threonine (T), and tyrosine (Y) in membranous helices to create water-soluble variants without altering overall protein structure (Zhang et al. 2018; Zhang and Egli 2022). Evolutionary analyses revealed consistent co-occurrence of specific QTY substitutions (L > Q, I > T, V > T, F > Y) in homolog sequences (Karagöl et al. 2024a; 2024b). However, prior studies focused on a limited set of amino acids within neurotransmitter transporters, leaving broader evolutionary dynamics across diverse membrane proteins underexplored. Notably, the potential asymmetries and complex substitution networks between polar and nonpolar residues in transmembrane regions remain largely uncharacterized (Karagöl et al. 2024a). To address these knowledge gaps, we conduct a comprehensive analysis of 2277 high-resolution protein structures from the RCSB Protein Data Bank (Berman et al. 2000). Recent advances in bioinformatics have provided robust data on membrane protein structures, allowing for more accurate analyses of residue-wise distribution patterns (Corradi et al. 2019).

Knowledge of chemically altering substitutions could have significant practical applications, such as improving prediction algorithms for membrane protein structure and function, informing strategies for protein engineering. Certain substitutions may provide an evolutionary buffer, allowing for genetic variation without strong functional consequences (Karagöl et al. 2024a; Koch et al. 2024). By mimicking evolutionary mechanisms that maintain functionality despite substitutions, we can potentially design therapeutics that retain long-term efficacy when challenged by drug resistance (Flynn et al. 2023). Moreover, our application of partial correlation analysis to amino acid frequencies in homolog residues represent a methodological innovation in the field of structural bioinformatics. This approach offers a way to detect evolutionary signals after removing residue-wise conservational effects that may mislead traditional sequence analysis methods. Hence, it allows a more robust analysis of an extensive form games framework. Nodes in extensive game theory framework represent critical junctures where multiple pathways converge (Cressman 2003). When applied to evolutionary biology, these nodes can be conceptualized as variational “hubs” in sequences. For instance, the transition from threonine (T) to valine (V) usually requires at least two base changes in a codon, representing a more complex substitution path with potential intermediate states (Karagöl et al. 2024a). Studying these variational nodes could provide insights into the fitness landscape of antibiotic resistance or cancer driver variations, informing targeted therapeutic strategies that exploit these variational dynamics. Researchers can further develop more stable evolutionary therapeutics by targeting specific residues that are prone to variations altering charge profiles, thereby disrupting ligand binding.

By uncovering the relationships between polar and nonpolar residue distributions, we aim to offer a comprehensive map of residue evolution in membrane α-helical proteins, providing further understanding of the fundamental principles of membrane protein evolution. Our findings have the potential to drive advancements in methods for protein structure prediction, design, and engineering, while also providing insights into the variational dynamics of α-helical transmembrane proteins in diverse cellular environments, such as those involved in cancer progression.

## Results

### Descriptive statistics of initial data

This study presents a comprehensive analysis of 5,846,493 possible substitutions across 504 alpha-helical transmembrane proteins belonging to 458 master PDBs of 2077 RCSB entries in *Homo sapiens*, with a particular focus on hydrophilic and hydrophobic amino acids (Supplementary Tables S1, S2).

**Fig. 1:**
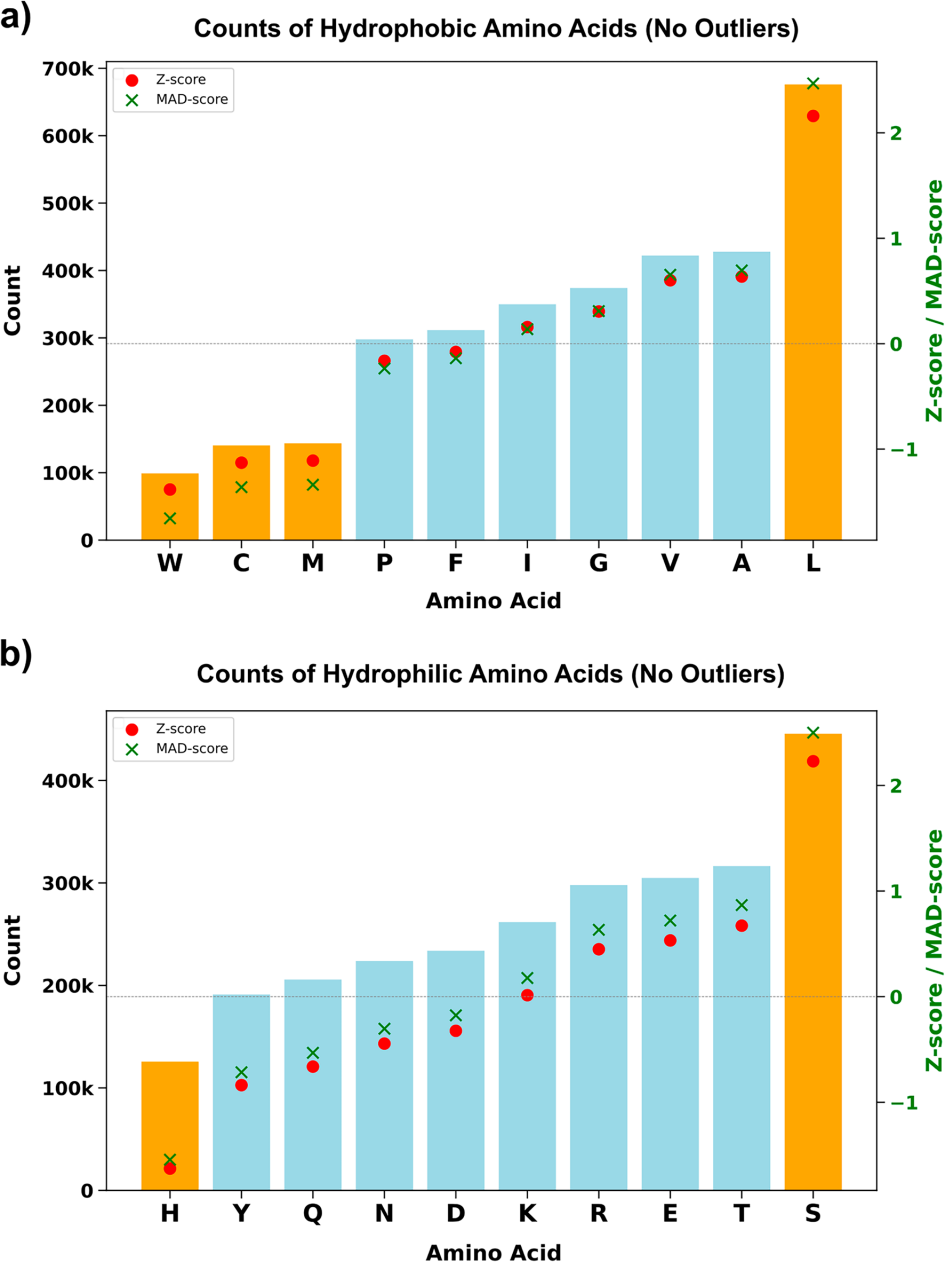
Counts of studied possible variants from 504 unique protein sequences. The distribution of 5,846,493 possible amino acid substitutions across 504 alpha-helical transmembrane proteins in *Homo sapiens* in order from low (left side) to high counts (right side). **a** Hydrophobic to other amino acid substitutions, and **b** hydrophilic to other amino acid substitutions. *Z*-scores lower than − 1 and greater than 1 are shown in orange

As expected, each amino acid has been observed in varying frequencies across protein sequences. For the changes targeting hydrophobic amino acids, the dataset shows that the counts range from 98,986 (W) to 675,713 (L), with a median of 330,641.5 and a mean of 324,112.6 (Fig. 1a, Supplementary Tables S4, S5). The result of the Shapiro–Wilk test for normality on polar and nonpolar amino acid counts shows a p-value of 0.4320 and 0.7187, respectively (Supplementary Table S6). No values fall outside the lower and upper bounds, indicating a meaningful distribution and the absence of outliers in this dataset. However, notable differences exist in the relative abundance of specific residues. A *z*-score measures how many standard deviations a particular data point is from the mean of the dataset. A *z*-score of 0 indicates that the value is exactly at the mean, positive *z*-scores represent values above the mean, and negative *z*-scores represent values below the mean. Values farther from the mean (greater than 1 or less than − 1) may indicate notable deviations. The count of L has a *z*-score greater than 1, while C, M, and W exhibit z-scores less than − 1, suggesting Leucine (L) is significantly more abundant whereas cysteine (C), methionine (M), and tryptophan (W) are less common. While the z-score is based on the mean and standard deviation, MAD is based on the median and the median absolute deviation, which is less influenced by extreme values. Given that our data follows a normal distribution, the MAD scores and *Z*-scores were in close alignment (Fig. 1), indicating that both measures of variability provided similar insights into the spread of the data. This variation can be explained by the inherent properties of these amino acids. Previous studies also suggest that leucine's high frequency in transmembrane domains reflects its optimal hydrophobic characteristics for membrane insertion and stability (Al Mughram et al. 2023), consistent with its prevalence in our dataset. On the other hand, tryptophan's low abundance is due to its large size and complex biosynthesis (Barik 2020), while cysteine and methionine play specialized roles in protein structure (Fass and Thorpe 2018).

For the hydrophilic amino acids, the dataset reveals various residue counts ranging from 125,576 (H) to 445,390 (S), with a median of 247,692 and a mean of 260,536.7 (Fig. 1b, Supplementary Table S5). The counts are all within the lower and upper bounds, indicating the absence of outliers. However, histidine (*z*-score < − 1) is less commonly found in protein structures because of its unique imidazole side chain, which can carry a charge depending on the pH, making it essential for certain enzyme functions (Liao et al. 2013). Its sensitivity to specific functional needs may limit its prevalence compared to amino acids that are more versatile and commonly used in various protein structures. In contrast, serine (*z*-score > 1) is more abundant because of its small, simple structure and polar hydroxyl group, which makes it versatile for hydrogen bonding and phosphorylation (Betts and Russell 2003). Accordingly, while no significant outliers were observed, the descriptive statistics of amino acid frequencies are largely consistent with the biochemical characteristics previously reported in the literature (Al Mughram et al. 2023; Barik 2020; Liao et al. 2013; Betts and Russell 2003).

### Patterns and trends in amino acid substitutions

AlphaMissense generates scores that estimate the probability of a specific amino acid change being harmful to protein function, using machine learning algorithms trained on extensive genomic and protein structure datasets (Cheng et al. 2023). The model leverages precomputed AlphaFold-derived structural representations as part of its input space, thereby embedding accurate 3D spatial context directly into its inference pipeline (Jumper et al. 2021). As such, the scores capture not merely the identity of the substitution, but also its topological feasibility, local residue environment, and attention-weighted evolutionary constraints (Cheng et al. 2023). In membrane proteins, this context is especially crucial, due to the requirement of hydrophobic compatibility with the lipid bilayer (Corradi et al. 2019; Almaida 2023; White and von Heijne 2005). On the other hand, conserved polar residues play key roles in facilitating protein–protein interactions, forming stabilizing hydrogen bonds, participating in substrate binding and catalysis, and mediating the transport process (Illergård 2011). The substitution pathogenicity scores range from 0 to 1, with higher scores indicating a greater probability of pathogenicity, and lower scores suggesting better tolerance (Cheng et al. 2023). The high pathogenicity scores for substitutions that significantly alter the physicochemical properties of amino acids indicate the importance of maintaining specific structural and chemical features in these proteins. Thus, the observed substitution patterns could have significant implications for understanding membrane protein function and potential disease associations.

For statistical analysis of pathogenicity scores, we analyzed single-residue substitutions individually to align with the design and scope of AlphaMissense, which is optimized for evaluating the pathogenicity of single-residue substitutions in a high-throughput, structure-aware manner (Cheng et al. 2023). This approach allows us to systematically assess the directionality, tolerance, and nonlinear effects of individual variants across diverse structural environments. This is particularly important, as linear models are often insufficient to capture the non-additive nature of epistasis (Sethi and Zhou 2024; Buda et al. 2023). It is important to note that many residues exhibit a non-linear co-evolutionary dependencies, particularly in structurally constrained or functionally critical domains (Buda et al. 2023). Our analysis serves as a complement to co-evolutionary models, which excel at capturing long-range compensatory interactions but are often limited in resolution or scalability when applied genome-wide. Even for pairwise interactions (*m* = 2) in a genome with millions of SNPs, the sheer number of tests becomes astronomically large (Tuo 2018; Niel et al. 2015). Complementary structural variant effect information is crucial for more robust and efficient analysis of epistatic networks (Buda et al. 2023). After pathogenicity profiling, we further quantified residue-level evolutionary constraints. We utilized ConSurf grades derived from a Bayesian framework applied to phylogenetically weighted MSAs (Yariv et al. 2023; Ashkenazy et al. 2016; Celniker et al. 2013; Landau et al. 2005), which are well-suited for conditioning in partial correlation models aimed at isolating context-sensitive variation (Karagöl et al. 2024a, b). As our analysis evolved, we increasingly incorporated statistical models to preserve analytical depth while maintaining statistical generalizability. To further contextualize variational networks, particularly for T-V, we employed evolutionary game theory. By integrating context-dependent pathogenicity predictions and co-evolutionary data with residue-residue couplings, molecular evolutionists can better reconstruct the global variational architecture of proteins, enabling more accurate modeling of variant effects in a multi-dimensional evolutionary context (Buda et al. 2023).

### Hydrophobicity sensitivity

The analysis of amino acid classifications based on hydrophobicity and hydrophilicity could be inconsistent when applying a binary categorization. For instance, lysine, traditionally categorized as hydrophilic, demonstrated the largest hydrophobic surface area among amino acids, as previously reported (Lins et al. 2003). Other approaches, including molecular dynamics simulations and solvent-accessible surface area (SASA) analyses, have further illustrated the context-dependent nature of hydrophobicity and residue interactions (Xi et al. 2017; Karagöl et al. 2024c). For example, residues buried within a protein’s hydrophobic core may display different behavior compared to those exposed to an aqueous solvent (Mayol et al. 2019). To incorporate these frameworks, we included Kyte-Doolittle hydropathy plots for the entire dataset. These plots allowed us to visualize the distribution of amino acid profiles across protein sequences. In our dataset, the hydropathy plots highlighted key trends, such as the clustering of residues with moderate hydrophobicity scores, which would have been obscured by a binary classification (Supplementary Fig. S6).

**Fig. 2:**
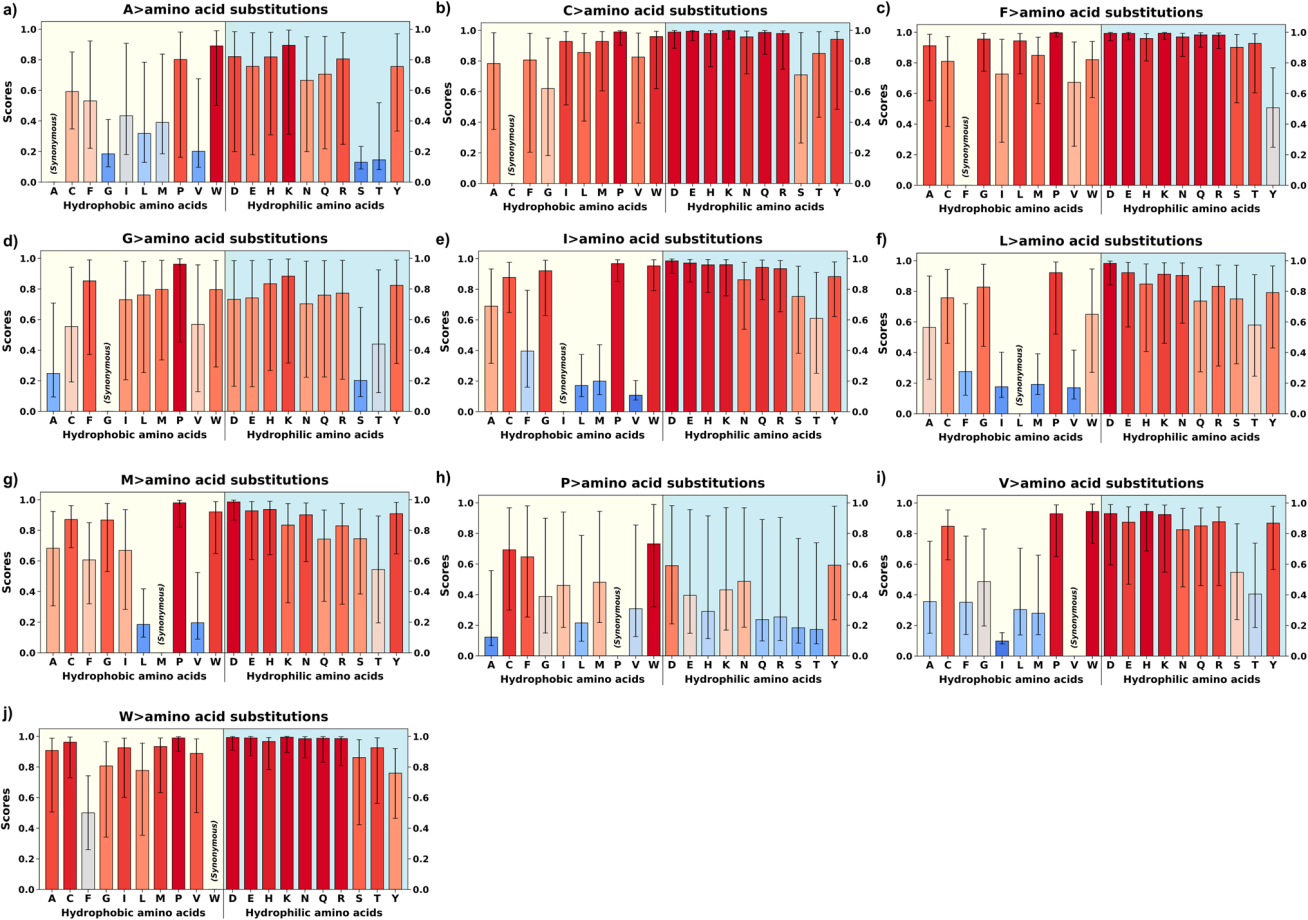
AlphaMissense pathogenicity scores of the variants from hydrophobic amino acids of structurally known alpha-helical transmembrane proteins. AlphaMissense scores, derived from machine learning algorithms trained on genomic and structural protein data, estimate the likelihood of amino acid substitutions being pathogenic to protein function. The pathogenicity scores range from 0 to 1, with darker red indicating higher pathogenicity and lighter colors to blue suggesting better tolerance. Hydrophobic to non-hydrophobic substitutions (right side of the plots) show elevated pathogenicity in general. For enlarged panels, please see Supplementary Fig. S3

**Fig. 3:**
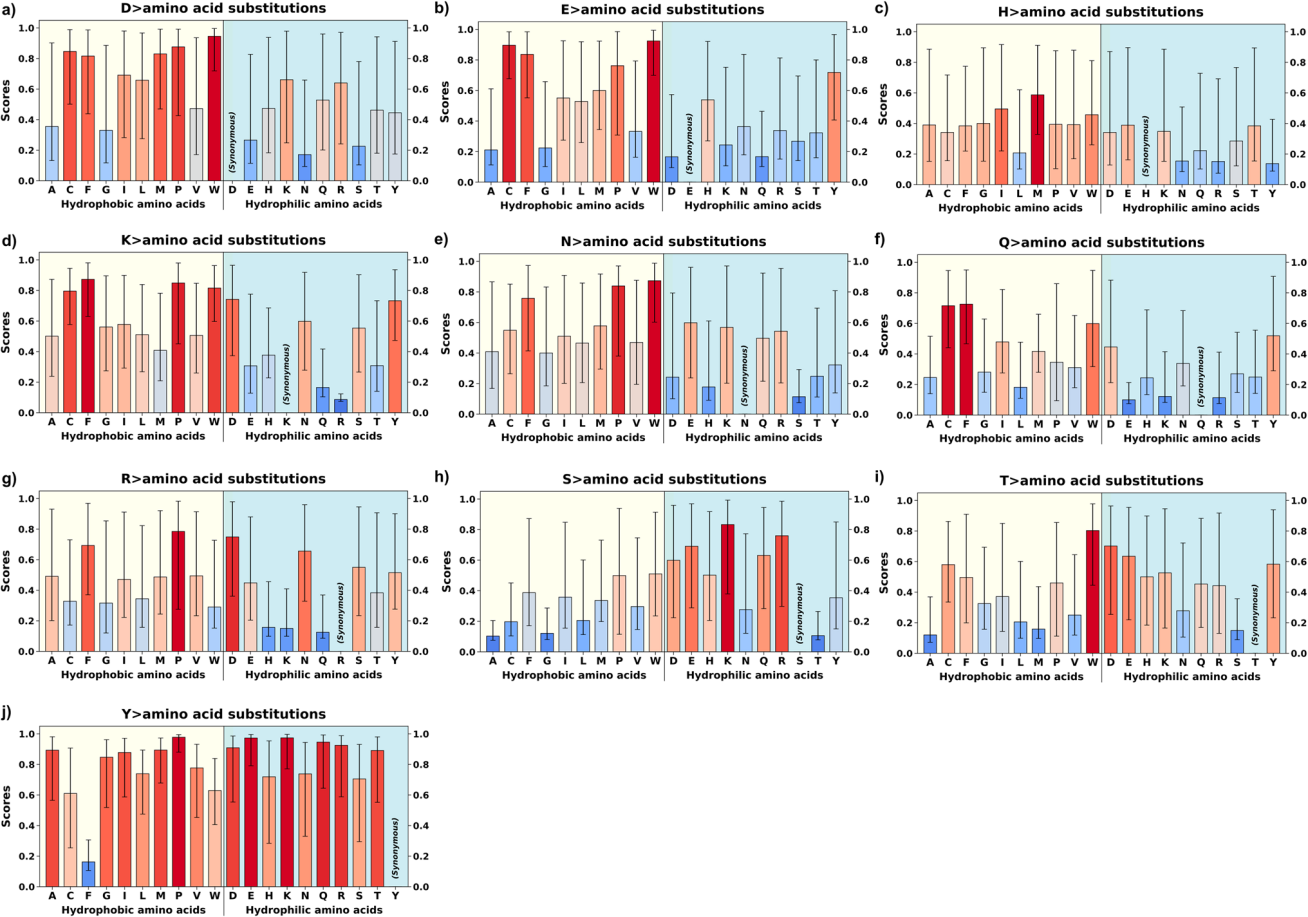
AlphaMissense pathogenicity scores of the variants from hydrophilic amino acids of structurally known alpha-helical transmembrane proteins. AlphaMissense scores, derived from machine learning algorithms trained on genomic and structural protein data, estimate the likelihood of amino acid substitutions being pathogenic to protein function. The pathogenicity scores range from 0 to 1, with darker red indicating higher pathogenicity and lighter colors to blue suggesting better tolerance. Hydrophilic to hydrophobic substitutions (left side of the plots) show elevated pathogenicity in general. For enlarged panels, please see Supplementary Fig. S4

Despite the limitations of the binary model, we used the conventional hydrophobic/hydrophilic classification terminology to examine residue changes, focusing on its simplicity and widespread application in the literature. The results indicated significant trends in the distribution and pathogenicity of hydrophobic and hydrophilic residues. Hydrophobic amino acids (A, C, F, G, I, L, M, V, and W) showed high pathogenicity scores when substituted with charged or polar residues (excluding proline) (Fig. 2, Supplementary Fig. S3). Notably, the median pathogenicity scores (M) for these substitutions predominantly fell within the “likely pathogenic” range (scores > 0.564), with the exception of eight specific substitutions: A > S (*M* = 0.130), A > T (*M* = 0.146), F > Y (*M* = 0.507), G > S (*M* = 0.202), G > T (*M* = 0.440), M > T (*M* = 0.544), V > T (*M* = 0.405), V > S (*M* = 0.547). Likewise, hydrophilic-to-hydrophobic substitutions (e.g., D, E, K, R to A, F, I, L, M, V) often resulted in higher pathogenicity scores than hydrophilic-to-hydrophilic changes, highlighting the critical role of both hydrophobic and polar residues in maintaining membrane protein structure and function (Fig. 3, Supplementary Fig. S6). Changes that cause substantial differences in amino acid polarity (e.g., I to S, F to Q) also tend to result in higher pathogenicity scores, reflecting the importance of maintaining the polarity profiles of membrane proteins (Figs. 2, 3, Supplementary Fig. S5, Supplementary Fig. S6). For certain residue pairs, AlphaMissense assigns markedly different pathogenicity scores to reciprocal substitutions, reflecting the directional nature of variational effects. For instance, in our dataset, the substitution A > D has a median score of 0.821, indicating a likely deleterious effect, whereas the reverse substitution D > A has a median score of 0.355, suggesting a benign or tolerated change. This asymmetry arises because AlphaMissense embeds each variation within the specific sequence and structural context of the protein, rather than evaluating substitutions in isolation. The model captures biochemical compatibility, evolutionary constraint, and residue-specific interactions, all of which influence the local fitness landscape (Cheng et al. 2023).

### Charge conservation

Substitutions between amino acids of similar charge (e.g., D to E, K to R) generally showed lower pathogenicity scores, indicating the importance of maintaining overall charge distribution in membrane proteins. Amino acid substitutions involving residues that are typically deprotonated at physiological pH usually resulted in benign outcomes, as reflected by their median pathogenicity scores (M). Specifically, the median pathogenicity score (M) was 0.166 for E > D and 0.266 for D > E. A similar trend was observed for amino acids that are usually protonated at physiological pH: K > R (M = 0.086) and R > K (M = 0.150). Additionally, substitutions from an uncharged amino acid to a charged one (e.g., A to D, L to K) showed higher median pathogenicity scores (Fig. 2).

### Substitutions of specific amino acids

Cysteine (C) and tyrosine (Y) substitutions often presented with high pathogenicity scores, suggesting their importance in membrane proteins, possibly due to their roles in disulfide bond formation, hydrogen bonding, and aromatic interactions. Accordingly, substitutions to proline (P) frequently resulted in high pathogenicity scores across all amino acid types, likely due to its unique structural properties that can significantly impact protein folding and flexibility. Among the 19 amino acid substitutions to proline, only four (H > P, Q > P, S > P, T > P) had median pathogenicity scores (M) that were not classified as “likely pathogenic”. The respective median scores for these substitutions were 0.394, 0.346, 0.499, and 0.460. Substitutions involving significant size changes, especially those resulting in larger amino acids (e.g., A to W, G to R), frequently yielded high pathogenicity scores.

On the other hand, some amino acids, particularly serine (S) and threonine (T), showed more variable substitution scores, indicating that their functional importance may be more context-dependent within membrane proteins. Notably, from eight specific hydrophobic to hydrophilic substitutions that were not “likely pathogenic”, seven involved S or T amino acids: A > S (*M* = 0.130), A > T (*M* = 0.146), G > S (*M* = 0.202), G > T (*M* = 0.440), M > T (*M* = 0.544), V > T (*M* = 0.405), V > S (*M* = 0.547).

### Non-pathogenic substitutions

Within the hydrophobic amino acid group, substitutions generally showed low to moderate pathogenicity scores. For instance, substitutions between isoleucine (I), leucine (L), and valine (V) consistently displayed some of the lowest pathogenicity scores across all membrane proteins analyzed: *M* = 0.108 for I > V, *M* = 0.098 for V > I, *M* = 0.170 for L > V, and *M* = 0.176 for L > I. This suggests a degree of functional plasticity within the hydrophobic amino acids of membrane proteins, possibly allowing for fine-tuning of protein dynamics without severely impacting overall function. Among polar uncharged amino acids (S, T, N, Q), there was often a higher degree of substitution tolerance. This is especially true for serine (S) and threonine (T) substitutions, which frequently exhibited lower pathogenicity scores when replaced with each other. Median scores were *M* = 0.149 for T > S, and *M* = 0.106 for S > T. This suggests that in many contexts, the hydroxyl group these amino acids provide can serve similar functional roles in membrane proteins.

While generally well-conserved, substitutions within the aromatic amino acid group (F, Y, W) often showed lower pathogenicity scores compared to exchanges with non-aromatic amino acids: *M* = 0.162 for Y > F, *M* = 0.760 for W > Y, and *M* = 0.500 for W > F. This indicates that in some contexts, the aromatic nature of these residues may be more critical than their specific identity, possibly for maintaining protein-lipid interactions or stabilizing aromatic interactions within the protein structure.

The tolerance for these substitutions may provide an evolutionary buffer, allowing for genetic variation without strong functional consequences. This could facilitate the fine-tuning of protein properties over evolutionary time. Furthermore, the variable tolerance for substitutions among different polar amino acids (e.g., the relatively lower pathogenicity scores for some serine and threonine substitutions) suggests that these residues may serve as evolutionary "nodes" for functional diversification in membrane proteins. This could provide insights into how membrane proteins have adapted to perform diverse functions across different cellular contexts and organisms. In relation to this study, pathogenicity profiles of hydrophobic to hydrophilic substitutions could reveal additional knowledge on their roles.

### Substitutions from specific hydrophobic (L, I, V, F) to polar amino acids

L > T, L > Q; V > T, V > S; I > T, I > S, and F > Y have significantly lower pathogenicity scores compared to other hydrophobic to hydrophilic substitutions. L > T and L > Q were the lowest two median scores among substitutions from L to hydrophilic acids: *M* = 0.580 and *M* = 0.737, respectively. Similarly, the median scores were *M* = 0.405 for V > T and *M* = 0.547 for V > S. Isoleucine (I) has a similar substitution profile as V, with I > T and I > S being less pathogenic: *M* = 0.610 and *M* = 0.753, respectively. Among those, L > Q, V > T, I > T, and F > Y involve relatively minor changes in the electron density maps (Zhang et al. 2018; Zhang and Egli 2022). Threonine (T) and serine (S) have polar side chains but are small and may maintain the overall hydrophobic core structure to some degree. Tyrosine (Y), though polar, has a large aromatic side chain similar to phenylalanine (F), thus preserving hydrophobic interactions while adding a hydroxyl group. Among those, T <=> V substitutions require at least two base changes in a single codon. Thus, these substitutions may serve as an evolutionary "node" for functional diversification in membrane proteins (Karagöl et al. 2024a, b).

Many of these hydrophobic residues are located in the interior of proteins, particularly in transmembrane regions (Corradi et al. 2019; White and von Heijne 2005). Substitutions with structurally similar polar residues (like I <=> T or V <=> T) may not disrupt the hydrophobic core significantly, allowing the protein to fold correctly and function normally (Zhang et al. 2018; Zhang and Egli 2022). Larger or more charged polar substitutions might disrupt the folding and structural integrity of the protein, leading to pathogenic effects. Some of these substitutions may be better tolerated evolutionarily. For example, threonine (T) and serine (S) are common substitutions for hydrophobic residues in many proteins because of their compatibility with both hydrophobic and polar environments (Betts and Russell 2003). This indicates that these substitutions are less likely to disrupt protein interactions or function in a deleterious way compared to larger, more charged residues such as lysine or glutamate.

### Substitutions from C and M amino acids

C > S, M > T, M > Q have lower pathogenicity scores compared to other substitutions with hydrophilic amino acids. For C > S, the median pathogenicity score was 0.710, which was followed by C > T (*M* = 0.850). M > T and M > Q had median pathogenicity scores of 0.544 and 0.743, respectively. Substitutions like C <=> S or M <=> T involve replacing sulfur containing amino acids (cysteine and methionine amino acids) with smaller, less reactive polar amino acids. These changes may have minimal impact on function because serine and threonine are often capable of maintaining necessary polarity or hydrogen bonding without significantly altering the protein's activity (Betts and Russell 2003).

### Evolutionary profiling and co-evolution of residues

Certain substitutions were identified as having lower pathogenicity scores, suggesting they might be tolerated or even advantageous in specific contexts. For co-evolutionary analysis, we focused on hydrophobic to hydrophilic substitutions with lower median pathogenicity scores, as these changes could result in relatively less alteration to protein functionality or structural stability. These substitutions may act as molecular "nodes” enabling the exploration of alternative structural or functional states with minimal destabilization (Karagöl et al. 2024a, c). The correlations of amino acid varieties in aligned homolog sequences could be useful for interpreting specific selection pressures acting on proteins (Karagöl et al 2024a). Yet, residue-wise conservation may create statistical biases in analyses. Since strong conservation of a residue creates a confounding effect that reduces all other amino acid frequencies, this may create false positive correlations between them (Karagöl et al. 2024a, b). In our dataset, the correlations might reflect both the conserved role of the amino acids and their natural co-occurrence due to structural or functional constraints (Supplementary Table S5). These structural descriptors could be incorporated as additional covariates in our partial correlation framework, allowing us to disentangle the relative contributions of evolutionary conservation, local structural environment, and intrinsic physicochemical constraints to the observed substitution patterns.

To quantify residue-level evolutionary constraint, we utilized ConSurf grades, which are computed through a Bayesian inference framework applied to phylogenetically weighted multiple sequence alignments (MSAs) (Yariv et al. 2023; Ashkenazy et al. 2016; Celniker et al. 2013; Landau et al. 2005). Unlike raw substitution rates or consensus-based conservation metrics, ConSurf grades are position-specific, even within the same sequence. This makes them particularly suitable for conditioning in models seeking to isolate context-sensitive variation, such as our partial correlation framework. In our dataset, evolutionary conservation grades did not follow a normal distribution, which can introduce biases in parametric statistical tests. In contrast, non-parametric tests do not rely on assumptions of specific data distributions, making them more robust for analyzing datasets with non-normal distributions (Kim et al. 2015). This characteristic makes non-parametric tests particularly useful for examining residue-wise amino acid variations in homolog sequences. To investigate how these conservation scores relate to the structural context of residues, we analyzed their relationship with relative solvent accessibility (RSA), a normalized metric indicating how exposed a residue is to the surrounding solvent.

Our analysis of the ConSurf grade distribution across RSA bins reveals a pronounced negative correlation between relative solvent accessibility and evolutionary conservation scores (Supplementary Fig. S7). Statistical analysis of the RSA-conservation relationship yields correlation coefficients that demonstrate significant departure from random expectation (rho = − 0.47, *p* < 0.001). The observed distribution pattern reflects the differential selective pressures on residues occupying distinct microenvironments within the protein fold architecture. Highly conserved residues (Grades 8–9) are predominantly found in low RSA environments ([0,10) and [10,20) bins). Conversely, the most variable residues (Grades 1–2) show enrichment in higher RSA bins ([50,60) to [90,100]), reflecting the greater evolutionary tolerance for substitutions at solvent-exposed positions. Interestingly, the steepest decline in conservation grades occurs between the [10,20) and [40,50) RSA bins, after which the median conservation levels plateau, suggesting a threshold beyond which selective pressure relaxes more uniformly across solvent-exposed residues. This trend underscores the spatial partitioning of evolutionary pressure according to the microenvironment of each residue.

The strong enrichment of conserved residues in buried regions supports the long-standing structural principle that protein cores are more conserved due to their critical role in folding and thermodynamic stability (Oberai et al 2009; Goldman et al. 1998; Eyre et al. 2004). Meanwhile, the flexibility observed in surface residues likely reflects the evolutionary drive for adaptability, such as modulating binding interfaces or facilitating interactions in membranes (Oberai et al 2009; Eyre et al. 2004). This distribution pattern aligns with the functional hypothesis that surface-exposed residues can accommodate more sequence variation without compromising essential protein functions.

**Fig. 4:**
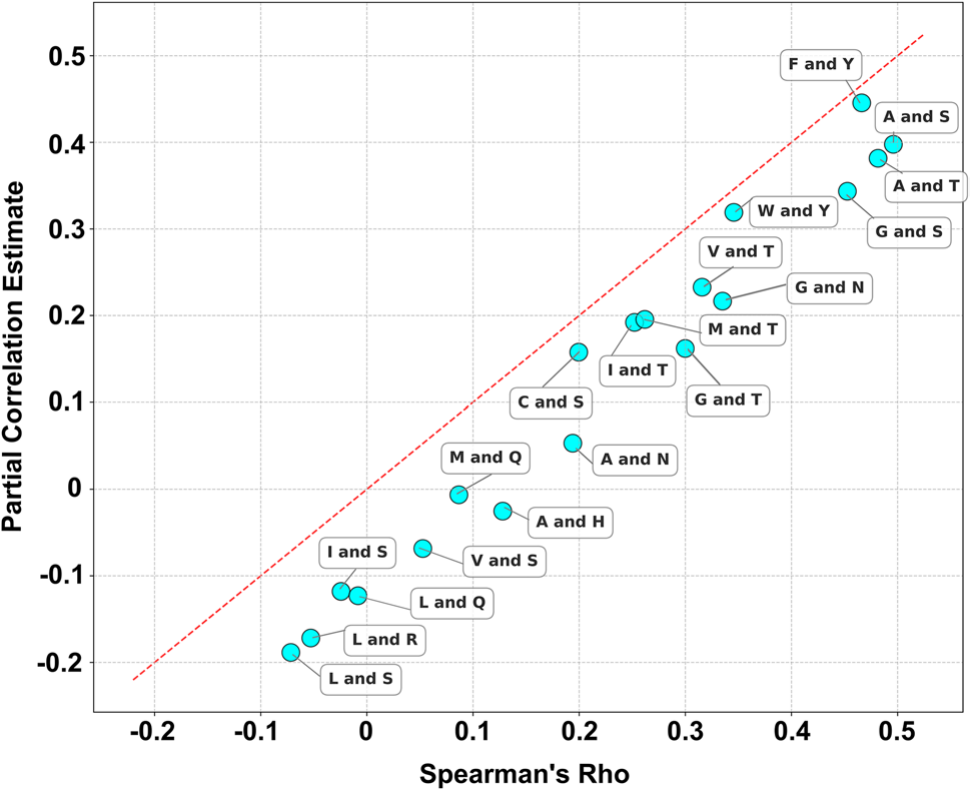
Evolutionary frequencies of sampled amino acids and the confounding effect of residue-wise conservation. The *x*-axis represents Spearman's Rho values ranging from − 0.2 to 0.5, while the *y*-axis shows Partial Correlation Estimate values from − 0.2 to 0.5. Each data point is labeled with a pair of letters representing different variables. A red dashed diagonal line indicates where values would be equal. Partial correlations generally lower than corresponding Spearman's Rho values

We next investigated how residue-level differences in evolutionary conservation may act as confounding factors in the analysis of co-occurrence patterns and substitution preferences across homologous sequences (Fig. 4). In our dataset, a small negative correlation in aligned homolog sequences (rho = − 0.0716) between L and S residues might suggest that they are less likely to substitute for each other, likely due to their polarity differences (*L* is nonpolar, S is polar). Controlling for conservation, the correlation becomes even more negative (rho = − 0.2024). This suggests a strong confounding effect of conservation on statistical evolutionary analyses (Fig. 4). This has practical implications; without accounting for conservation, algorithms may overestimate or underestimate certain substitutions, leading to misinterpretation of co-evolutionary signals. This is especially important for structural predictions that may use direct data without considering confounding effects.

Correlations involving *L* showed a stronger negative trend in general, although less profound with *Q* (rho = − 0.1230). This suggests a relative non-co-occurrence of *L*, likely due to its contrasting roles in membrane protein stability. Nevertheless, many positive correlations (12/19 of amino acid pairs) were identified in our analysis between chemically distinct amino acids with lower pathogenic scores during substitutions (Table 1). Namely, F—Y, W—Y, I—T, M—T, A—T, V—T, G—T, G—S, A—S, C—S, G—N, and A – N amino acid pairs (rho > 0, p < 0.001). This suggests the correlations were strongly affected by amino acid characteristics other than polarity, indicating a rather complex picture. When controlling for conservation, the correlations may reveal the functional flexibility of amino acids in protein structures.

**Table 1:** Partial correlations of amino acid frequencies controlled for conservation, for ranked values (Spearman’s rho)

Pair	Partial correlation estimate^a^	*t*-statistic	*p*-value
F and Y	0.4410	134.2513	< 0.001
W and Y	0.3157	90.9080	< 0.001
I and T	0.1787	49.6252	< 0.001
M and T	0.1821	50.6264	< 0.001
A and T	0.3719	109.4841	< 0.001
V and T	0.2192	61.4082	< 0.001
G and T	0.1543	42.6785	< 0.001
G and S	0.3391	98.5190	< 0.001
A and S	0.3896	115.612	< 0.001
I and S	− 0.1343	− 37.0558	< 0.001
L and S	− 0.2047	− 57.1622	< 0.001
V and S	− 0.0849	− 23.2839	< 0.001
C and S	0.1509	41.7194	< 0.001
L and R	− 0.1855	− 51.5757	< 0.001
L and Q	− 0.1377	− 38.0047	< 0.001
M and Q	− 0.0209	− 5.7181	< 0.001
G and N	0.2130	59.5837	< 0.001
A and N	0.0435	11.8895	< 0.001
A and H	− 0.0354	− 9.6851	< 0.001

^a^In the partial correlation calculations, controlling for conservation helps isolate the relationship between two amino acid frequencies by statistically removing the effect of ConSurf calculated conservation grade

### Nonpolar and polar residue frequencies with positive correlation

Phenylalanine (F) and tyrosine (Y) co-occurrence was strong, rho = 0.4410 (positive). F is nonpolar and aromatic, while Y is polar due to its hydroxyl group. Their positive correlation suggests frequent substitution, indicating some functional compatibility or less harmful effects. Previous molecular dynamics simulations on F > Y substitutions on all membrane alpha-helical residues of glutamate transporters EAA1-3 have shown that this large-scale change does not alter initial overall conformations even in different environments (Karagöl et al. 2024b, 2024c).

Tryptophan (W) and tyrosine (Y) were also positively correlated, though smaller (rho = 0.3157). W is nonpolar and aromatic; Y is polar and aromatic. The slight positive correlation suggests occasional substitution without detrimental effects in homolog proteins. Phenylalanine, tyrosine, and tryptophan are aromatic, which are crucial for stacking interactions and maintaining structural integrity.

Alanine (A) and serine (S) also co-occurred in homolog sequences (rho = 0.3719). A is nonpolar; S is polar due to its hydroxyl group. Their positive correlation implies that A can be substituted with S in certain contexts. Interestingly, G and S also showed a strong positive correlation (0.3391). Other substitutions involving S showed negative correlations (− 0.0849 to − 0.2047).

Alanine (A) and threonine (T) were also positively correlated (rho = 0.3719). It suggests that substitutions between A and T do not strongly impact protein function in some cases. Alanine, serine, and threonine are small amino acids with similar side-chain sizes, allowing them to fit into similar spatial niches within the protein structure. T and V frequencies also showed a positive correlation (rho = 0.2192). This is unexpected, as the T <=> V substitution typically requires at least two base changes within the same codon, creating a statistical barrier to its co-occurrence at aligned positions in homolog sequences. Such substitutions, while less frequent than single nucleotide changes, can arise through sequential point mutations or simultaneous double substitutions. Their substitution pathways may be of evolutionary interest. While sequential mutations traverse a fitness landscape incrementally, double point mutations bypass intermediate states, potentially affecting stability (Dieckhaus and Kuhlman 2024). Interactions between variations play another role in shaping how substitutions manifest in protein evolution (Johnson et al. 2023; Buda et al. 2023). In the case of T <=> V substitutions, epistatic interactions could reduce the variational barrier by reshaping the fitness landscape to favor intermediate or endpoint states, enabling these amino acids to appear as co-evolving residues in specific statistical contexts. Though the coefficient was smaller, a similar finding observed in the G <=> N (glycine-asparagine) relationship (rho = 0.2130). This transition represents a significant shift in amino acid properties (small/flexible to polar/H-bonding). The requirement for two base changes makes this co-occurrence statistically less likely to be seen in homolog aligned sequences when analyzed solely by codon profiles.

### T <=> V changes via alanine intermediates

After controlling for A, the correlation between V and T drops to 0.1825 (a large drop, -0.1333). Meanwhile, the correlation between I and T becomes 0.1835, with a smaller decrease (-0.0686) (Table 2). This indicates that alanine significantly moderates the V <=> T correlation, suggesting alanine's role might be critical in how valine and threonine frequencies are correlated.

**Table 2:** Partial correlations of T-V amino acid frequencies for ranked values (Spearman’s rho)

Pair	Confounding variable^a^	Correlation estimate	*p*-value	Net change^b^
A and T	None	0.4816	< 0.001	0
V and T	None	0.3158	< 0.001	0
I and T	None	0.2521	< 0.001	0
A and T	Conservation	0.3719	< 0.001	− 0.1097
V and T	Conservation	0.2192	< 0.001	− 0.0961
I and T	Conservation	0.1787	< 0.001	− 0.0734
V and T	A	0.1825	< 0.001	− 0.1333
I and T	A	0.1835	< 0.001	− 0.0686
A and T	V	0.4186	< 0.001	− 0.0630
I and T	V	0.0439	< 0.001	− 0.2082
A and T	I	0.4554	< 0.001	− 0.0262
V and T	I	0.2012	< 0.001	− 0.1146

^a^In the partial correlation calculations, controlling for confounding variables helps isolate the relationship between two variables by statistically removing the effect of a specific amino acid frequency or ConSurf calculated conservation grade

^b^Reduce of correlation coefficient compared to the standard spearman correlation with no confounding variables set

**Fig. 5:**
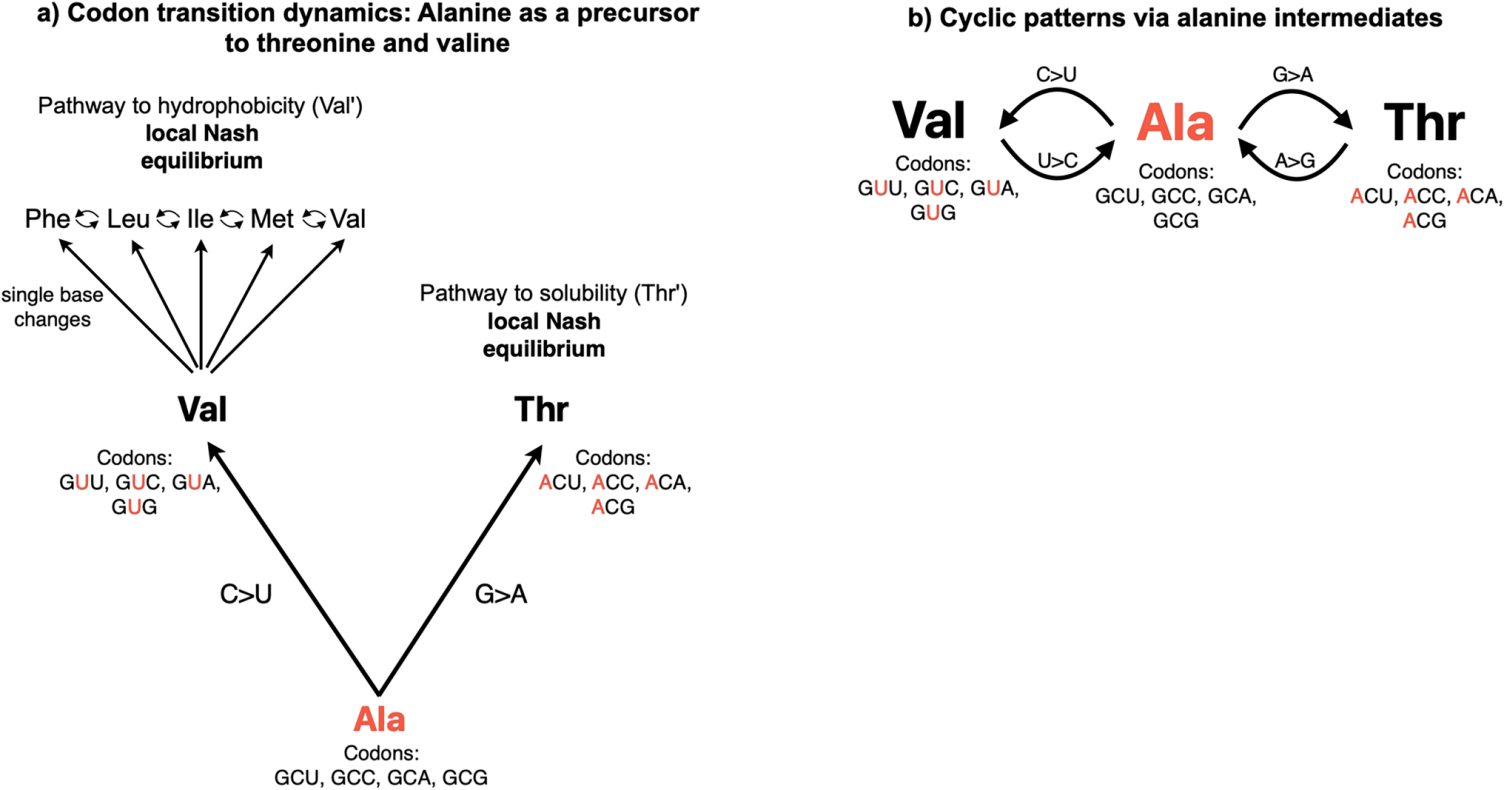
Evolution of T-V relationship in membrane alpha-helical proteins. **a** Alanine-centric model for the evolution of T-V relationship: This panel shows two evolutionary pathways centered around alanine (Ala). The "Pathway to hydrophobicity" (A > V) represents the evolution from alanine to valine and other nonpolar amino acids (F, L, I, M, V) through single base changes. The "Pathway to Solubility" shows the evolution from alanine to threonine (Thr). Both pathways are described as reaching a local Nash equilibrium. Codons for each amino acid are listed, with key base changes highlighted (C > U in 2nd position for Ala > Val, A > U in 1st position for Ala > Thr). **b** Evolution via alanine intermediates: This panel depicts a simplified linear relationship between valine (Val), alanine (Ala), and threonine (Thr). It shows the bidirectional evolutionary potential between these amino acids, with alanine serving as an intermediate. Codons for each amino acid are listed, demonstrating the base changes required for transitions between them

The significant decrease in correlation between the I and T frequencies when controlling for valine suggests that valine has a substantial effect on the I <=> T relationship, highlighting its evolutionary relevance in substitution patterns with isoleucine and threonine (Table 2). This indicates that valine plays a direct role in influencing isoleucine changes. When controlling for conservation, the drop in correlation for A and T (from 0.4816 to 0.3719) and V and T (from 0.3158 to 0.2192) may indicate that these correlations are partly driven by structural or functional constraints in conserved regions of the membrane proteins. Evolutionary constraints and codon degeneracy could help explain the limited amino acid changes at these positions (Fig. 5). This could suggest that alanine substitutions in membrane proteins allow for more flexible substitutions between valine and threonine, possibly affecting protein folding or dynamics within the lipid bilayer.

### Comparative analysis with existing substitution matrices

The observed substitution patterns within human alpha-helical transmembrane proteins were analyzed using substitution matrices, including PHAT (transmembrane-specific), BLOSUM62 (general-purpose), and JTTtm (transmembrane-specific), to assess congruence with baseline evolutionary models. We analyzed scores particularly for transitions involving threonine (T), valine (V), and isoleucine (I). Pathogenicity scores generated by AlphaMissense, a deep learning model that utilizes large-scale variant data with AlphaFold-derived structural features, indicated that while I > T (0.61) more pathogenic than T > V (0.405); T > I (0.371) were less pathogenic than T > V (0.405), indicating a non-linearity. We further quantified amino acid co-occurrence frequencies across homolog protein sequences using a non-parametric approach, subsequently through partial correlation analysis to mathematically account for site-specific evolutionary constraint. V-T correlation was stronger than I-T while controlling for site-specific conservation (rho = 0.2192 vs. 0.1787, respectively). In alanine rich context, V-T and I-T correlations became similar (rho = 0.1825 vs. 0.1835, respectively). Our data suggest a rather complex pattern that challenges the data of substitution permissiveness posited by PHAT (T-I score: − 1; T-V score: 0), BLOSUM62 (T-I: − 1; T-V: 0), and JTTtm (both substitutions scored 0) (Ng et al. 2000; Henikoff and Henikoff 1992; Jones et al. 1994, respectively).

These inconsistencies likely affected by foundational differences in data origin, structural context, and statistical modeling. Traditional substitution matrices are constructed from aggregated substitution counts across phylogenetic spectra (Henikoff and Henikoff 1992; Triverdi et al. 2020), often neglecting membrane-specific biophysical constraints, such as lipid-protein interactions and helical topology which are fundamental in alpha-helical transmembrane proteins (Triverdi et al. 2020; Corradi et al. 2019; Almaida 2023; White and von Heijne 2005). In contrast, our co-occurrence-based approach captures context-specific substitution dynamics by leveraging homolog-level residue frequencies. Moreover, the use of partial correlation analysis reveals correlated amino acid pairs. This enables the isolation of subtle evolutionary couplings that may reflect functional or structural compensatory mechanisms, often masked in standard substitution modeling (Karagöl et al. 2024a). AlphaMissense further advances this resolution by incorporating three-dimensional data and empirical pathogenicity annotations, thus aligning closer to the biophysical and functional information affecting substitution outcomes. (Cheng et al. 2023). It is also important to note that the underlying multiple sequence alignments (MSAs) informing our co-occurrence and conservation analyses were generated using HMMER and Bayesian inference pipelines, which differ from evolutionary substitution matrices. Finally, our analyses hinted at non-linear substitution trajectories, such as T > V substitutions occurring in alanine (A) rich contexts, suggesting that certain substitution events may follow multi-residue evolutionary pathways. This is especially crucial since linear models frequently fail to account for the non-additive complexity of epistatic interactions (Sethi and Zhou 2024; Buda et al. 2023). Such stepwise dynamics are inherently invisible to pairwise substitution matrices but may be biologically relevant in structurally constrained environments like membrane systems (Koch et al. 2024).

### Extensive forms and evolutionary game theory

Evolutionary game theory could be used for analyzing the underlying substitutional dynamics of T <=> V changes (Karagöl et al 2024a, b). It is important to note that these models are based on the broader, context-averaged patterns identified in our large-scale analysis. These conceptual models help frame questions for more nuanced, position-specific studies incorporating varying selective pressures and nonlinear models of epistasis. A "game" in game theory refers to any interaction where individuals (players) change states to maximize their benefits (Cressman 2003). It is important to note that the term "game" does not refer to decision-making or strategy-based choices and could be stochastic (Goeree and Holt 1999). In this case, it refers to a mathematical framework for modeling dynamic systems where outcomes depend on probabilistic events and interactions, as evolutionary models incorporate stochastic elements that mirror the randomness inherent in biological variation (Goeree and Holt 1999). Games can be cooperative or competitive, and outcomes depend on the “strategies” of all “participants” (Cressman 2003). In a stochastic game involving variational dynamics, substitutions that make a protein more polar or hydrophobic can be seen as "moves" within a game. Each substitution that changes the protein’s characteristics (such as increasing polarity or hydrophobicity) can be treated as a state transition in the game. For example, substitutions from hydrophobic amino acids to polar ones change the protein from a hydrophobic "state" to a polar "state". These transitions happen probabilistically, with different rates for different types of substitutions. The outcome of each mutation is also probabilistic, depending on factors like the environmental pressures, and the fitness consequences of being more hydrophobic or polar.

Substitutions that make a protein more hydrophobic might give it an advantage in environments with hydrophobic surfaces (like membrane-bound proteins), but a disadvantage in aqueous environments. In the context of variational dynamics, this could mean that certain polar or hydrophobic proteins become dominant in the population, depending on the environmental pressures (e.g., the presence of a membrane or aqueous environment). These dominant substitutions could be considered evolutionarily stable strategies (ESSs) if they confer a fitness advantage in the given environment (Karagöl et al 2024a). A Nash equilibrium is a fundamental concept in game theory where each player’s strategy is optimal, given the strategies of all other players (Cressman 2003). No player can improve their outcome by unilaterally changing their strategy (Cressman 2003). In a population of proteins, certain variations (e.g., those increasing hydrophobicity) might dominate in a membrane environment. Once these variations are widespread, introducing other types of variations (e.g., increasing polarity) may not confer a fitness advantage, so the hydrophobicity remains stable. This is a Nash equilibrium.

Alanine's ability to mutate into both polar (threonine) and nonpolar amino acids (valine) by single base changes makes it a unique strategy with dual potential outcomes (Karagöl et al 2024a). Alanine may serve as an evolutionary "hub" from which both polar (threonine) and nonpolar (valine and others) amino acids can evolve through single base changes (Fig. 5a). furthermore, alanine significantly complicates the correlation between threonine and valine in evolutionary analyses of amino acid varieties (Table 2). Hence, the presence of alanine in ancestral sequences can lead to divergent evolutionary outcomes, resulting in either threonine or valine (and other nonpolar amino acids) in descendant sequences.

The first model posits a dynamic game with two local Nash equilibria: a) One at the nonpolar end (for example F, L, I, M, and V), and b) another at the polar end related to T (Thr’). Purifying selection might preserve the polarity gradient (the spatial distribution of polar vs. nonpolar residues across a region) at certain sites, often by conserving residues with similar polarity profiles, even if the specific amino acid varies. In nonpolar environments, valine and nonpolar amino acids resulting from single base changes emerge as the ESS, as it offers the highest payoff, which is evidenced by our phenotypic analysis using AlphaMissense: median pathogenicity score for T > V was M = 0.250, while for V > T it increased to M = 0.405. In predominantly polar environments, threonine and other polar amino acids resulting from single base changes becomes the ESS. Moreover, this bifurcation from a common ancestral state is expected to obscure the direct relationship between threonine and valine, which could be tested using amino acid varieties in homolog sequences. After controlling for A, the correlation between V and T drops to 0.1825 (a large drop, − 0.1333). Meanwhile, the correlation between I and T becomes 0.1835, with a smaller decrease (− 0.0686) (Table 2). In line with this model, the endpoints (Val’ and Thr’) could be considered evolutionarily stable strategies (ESSs), as they may resist invasion by other strategies (amino acids) when they are optimal for their respective environments (Cressman 2003). Hence, the model suggests possible "local Nash equilibria" at the endpoints, implying stability. Furthermore, the A → V change results in a higher probability of changes to other nonpolar amino acids via single base changes, which would otherwise require two base changes, implying that the initial change of the state (A → V) influences the outcome in later stages. This type of dynamics is commonly referred to as extensive form game (Cressman 2003). Accordingly, this phenomenon could explain why the V <=> S correlation is negative (rho = − 0.0685) even if the A <=> S is strongly positive (rho = 0.3977), and both are nonpolar to polar (or vice versa) changes. Similar to this model, the G <=> N relationship could be explained by serine intermediates causing an asymmetry, as the A <=> N relationship (rho < 0) is unexpectedly small compared to similarities with the G <=> S and A <=> S coefficients (Table 1). Interestingly, serine stands out as the only amino acid encoded by two separate codon sets (TCN and AGY) that these distinct codon sets are associated with differing substitution dynamics at sites experiencing purifying and diversifying selection (Schwartz et al. 2019; Spence et al. 2021). Accordingly, the codon usage profiles could be involved in the substitutional dynamics of the observed G-N relationship.

### Cyclic patterns via alanine intermediates and prisoner’s dilemma

The second diagram shows a simpler, linear progression from V to T through A (Fig. 5b). In highly constrained regions, the game could be transformed into one with a dominant strategy, where maintaining the current amino acid is always optimal and result in extensive outcomes (as seen in the first model proposed). Hence, under weaker purifying selection, a simpler linear model might be favored. From an evolutionary game theory (EGT) perspective, substitutions can be viewed as strategic moves. The two-step process (V <=> A <=> T) represents a form of strategic commitment, where an intermediate step (A) must be adopted before reaching the opposite strategy. Alanine may serve as an evolutionary "bridge" between these amino acids. In these situations, the V <=> A <=> T relationship could be seen as a modified stochastic Prisoner's Dilemma. While “cooperation” (maintaining A) might be beneficial for overall evolutionary flexibility, there is a constant pressure to defect to either V or T for short-term fitness gains. On the other hand, maintaining A could be selectively advantageous as long as other residues also stay in A. when one part of the protein changes to become more hydrophobic the remaining part would follow for maintaining membranous interactions and stability. This could be simplified as an individualistic pragmatist has incentives to cooperate (in this case, stay in A) as long as the other parties do (Bshary et al. 2016; Axelrod 1980). Over evolutionary time, this may become a repeated game. The frequency of each amino acid in a population reflects the cumulative outcomes of many rounds of this game played across different protein positions and organisms. Thus, it is possible to test the model using organisms that have different purifying selection strengths.

Purifying selection acts as a set of rules that constrain this evolutionary game. First, it increases the payoffs for maintaining the status quo, especially in functionally critical regions. It creates high penalties (low fitness) for deviating from optimal strategies in conserved positions. Furthermore, it could generate a bias in the game, favoring certain transitions (those through A) over others. Thus, we suspect that repeating our partial correlation analysis with species with weaker purifying selection (such as eukaryotes compared to bacteria) could influence the decrease of the correlation between V and T, when controlling for A. It is important to note that the specific codons listed for each amino acid might be affected by a bias in codon usage during this evolutionary process (Iriarte et al. 2021).

### Future scopes and the potential applications

While our study primarily focuses on the direct effects of specific substitutions and their evolutionary relationships on membrane protein functionality, it is essential to expand this perspective to include compensatory variations and co-evolutionary dynamics. Our findings emphasize that while substitution matrices remain useful, they should be complemented with methods that incorporate structure, evolutionary context, and conservation-aware analysis, especially when studying specialized protein classes like transmembrane helices where the evolutionary rules are clearly different. The incorporation of structural context controls including hydropathy measurements, solvent accessibility, packing density, intrinsic disorder, and Rosetta energy calculations would complement our conservation analysis and provide a more comprehensive understanding of the factors governing substitution pathogenicity. The observed RSA-conservation correlation has important implications for our partial correlation framework. By incorporating RSA as a covariate, we can better disentangle the contributions of structural constraints from other evolutionary pressures in shaping conservation patterns. This approach allows for more accurate identification of functionally important residues that may be conserved for reasons beyond structural necessity.

Future research should also combine computational simulations of epistatic networks with in vitro and in vivo experiments, such as high-throughput mutational scanning and site-directed mutagenesis. These efforts could validate the predicted interactions and reveal new patterns of epistasis in protein evolution. Interactions between variations play a fundamental role in shaping how substitutions manifest in protein evolution (Johnson et al. 2023; Buda et al. 2023). Expanding these approaches to incorporate structural and functional data revealed in this study would provide a more comprehensive framework.

The correlations observed, and particularly their reduction when controlling for conservation or other amino acids, suggest that the evolutionary landscape of membrane proteins is shaped by selective pressures that maintain hydrophobicity, folding patterns, and function within the membrane. Our analysis indicates that the observed T <=> V and G <=> N relationships could be influenced by different substitution dynamics at sites under purifying and diversifying selection pressures. While our EGT models provide insights into the observed relationships, they may oversimplify the evolutionary landscape by not fully accounting for epistatic interactions. Hence, further analysis of differential selection pressures and codon usage biases would be beneficial. Serine could provide a basis for this analysis, as its distinct codon sets are affected by differing selection dynamics (Schwartz et al. 2019; Spence et al. 2021). Additionally, deep mutational scanning libraries could help identify networks of co-evolving residues that maintain protein stability and function (Dieckhaus and Kuhlman 2024). Statistical coupling analysis (SCA) of our sequence alignments could reveal long-range correlations between amino acid varieties, complementary structural variant effect information is crucial for efficient analysis of epistatic networks (Buda et al. 2023). Biophysical studies and molecular dynamics simulations could be incorporated into the phenotypical profiling. Previous molecular dynamic simulations assessed that the V <=> T, I <=> T and F <=> Y substitutions resulted in similar folding and conformational characteristics on glutamate transporters in membranous and solvent environments (Karagöl et al. 2024c). Membrane proteins, with their helices embedded in lipid bilayers, may have developed unique structural adaptations to interact with lipid environments (White and von Heijne 2005; Wimley and White 1996; Hegde and Keenan 2024; Ji et al. 2024). On the other hand, structural and conformational stability might have produced similar patterns in homolog sequences (Karagöl et al. 2024c). Investigating the similarities and differences between helical soluble proteins and membrane-bound proteins could reveal conserved mechanisms or distinct roles specific to the membrane environment (Karagöl et al. 2024a, 2024b). The in vivo experiments on L, I/V, F > Q, T, Y (QTY-code) variants of membranous chemokine receptors indicated they retain their ligand binding properties (Zhang et al. 2018). Site-directed mutagenesis experiments could further validate the impact of specific substitutions (e.g., T <=> V and G <=> N) on protein stability, folding efficiency, and hydrophobicity. Additionally, evolutionary simulations modeling differential codon usage biases might offer insights into how these pressures shape the adaptive landscape of membrane proteins.

Substituting hydrophobic residues with hydrophilic ones can increase protein solubility, which is essential for the expression and purification of recombinant proteins (Zhang et al. 2018; Zhang and Egli 2022). In the context of soluble protein design, leveraging these substitutions can enhance solubility, stability, and functionality without compromising the protein's structural integrity. Another key challenge involves tracing the hypothesized evolutionary pathway of membrane insertion, using a framework of increasingly sophisticated membrane protein insertion factors (Hegde and Keenan 2024). Variational dynamics could be useful to explain poorly hydrophobic transmembrane domains, which appear to minimize lipid exposure through strategic residue placement (Ji et al. 2024). These could include the evolutionary optimization of amino acid positioning to minimize unfavorable lipid-residue interactions, maximize protein–protein interface stability, facilitate membrane insertion through translocon machinery, and balance functional requirements with membrane compatibility (Ji et al. 2024; Hegde and Keenan 2024). The tolerance for conservative substitutions may provide an evolutionary buffer, allowing for genetic variation without strong functional consequences. By mimicking evolutionary strategies that maintain functionality despite variations, we can potentially design therapeutics that retain long-term efficacy, even when challenged by selective pressures like drug resistance or immune evasion. Moreover, by comparing the substitutional dynamics of membrane proteins with those of soluble helical proteins, as our Kyle Doolittle’s based framework allows, we may uncover generalizable design rules that apply to synthetic biology, such as engineering membrane mimetics or designing de novo soluble receptors. Understanding these correlation patterns allows protein engineers/designers to make informed decisions when modifying proteins for therapeutic, industrial, or research applications.

## Methods

### Sequences of structurally known alpha-helical transmembrane proteins

The transmembrane alpha-helical protein structures were sourced from the mpstruc database (https://blanco.biomol.uci.edu/mpstruc/), which curates membrane proteins with known 3D structures (White 2009). Structures belonging to *Homo sapiens* were extracted via a Python script from the XML file. To avoid redundancy, only a single representative Protein Data Bank (PDB) entry was selected for each unique or related protein, referred to as the "Master PDB ID" (Berman et al. 2000). Following this, each Master PDB ID was manually cross-referenced with the RCSB Protein Data Bank to confirm the most up-to-date identifiers. Subsequently, UniProt IDs corresponding to these proteins, including any secondary protein components, were retrieved from the RCSB database (Berman et al. 2000). Secondary structures not part of the transmembrane regions were excluded, even if they were associated with the main structure. The FASTA sequences, gene identifiers, and protein names of the selected proteins were retrieved using UniProt's batch query tool.

### Membrane protein topology and hydropathy profiles

A total of 504 UniProt entries were further analyzed to predict their transmembrane regions and hydrophobicity profiles. The Phobius web tool was used to predict transmembrane domains and topological orientation, providing insights into the membrane-spanning segments and signal peptides (Käll et al. 2004). The Phobius results were compared with existing transmembrane annotations available in the UniProt database to exclude inconsistent results (Supplementary Fig. 1). Additionally, hydropathy plots were generated using the Kyte-Doolittle method using with a web client multiple sequence queries, for the identification of hydrophobic regions typically associated with membrane-spanning domains (Kyte and Doolitle 1982; Karagöl et al. 2024d) (Supplementary Fig. S6). The combined analysis of Phobius predictions, UniProt data, and hydropathy profiles provided an overview of the structural characteristics of the selected proteins.

### AlphaMissense scores

Each UniProt ID in the list of 504 transmembrane proteins with known structures were manually submitted to the AlphaMissense database via HageLab's AlphaMissense webpage (https://alphamissense.hegelab.org) (Cheng et al. 2023). The resulting data were consolidated into a single.TSV file and subsequently split into 20 separate.CSV files based on the starting amino acid variants. Python scripts were used for processing in the Google Colab environment, using TPUv2-8 Tensor processing units, 334.6 GB RAM and 225.3 GB system storage. To improve the interpretability of the scoring system, values were categorized according to the reference paper: scores ranging from 0 to 0.34 were classified as "likely benign," those between 0.34 and 0.564 were classified as "uncertain," and scores from 0.564 to 1 were categorized as "likely pathogenic" (Cheng et al. 2023). These thresholds were determined through precision-recall curve analyses. The cutoffs were optimized to achieve a precision rate of 90% for the "likely pathogenic" and "likely benign" classes (Cheng et al. 2023).

We particularly focused on asymmetries in substitution tolerance within residue categories according to biochemical characteristics, which were later contrasted with evolutionary expectations from matrix-based substitution models. To provide evolutionary context, observed substitution patterns were compared against established amino acid replacement matrices, including BLOSUM62 (Henikoff and Henikoff 1992), PHAT (for membrane proteins) (Ng et al. 2000), and JTTtm (Jones et al. 1994). The PHAT 75/73 matrix (*H* = 0.5605), built from PHDhtm 75 target values (*H* = 0.5007) and Persson–Argos 73 background frequencies (*H* = 0.5038), was employed (Ng et al. 2000).

For each first amino acid variant, the total count, standard deviation, Z-scores, and median absolute deviation (MAD) scores were computed. To evaluate the normality of amino acid distributions, the Shapiro–Wilk test was employed separately for nonpolar and polar amino acids (Shapiro and Wilk 1965). Independent t-test was conducted to compare the means between nonpolar and polar amino acids. In case the assumption of equal variances was violated, Welch’s t-test was used to account for unequal variances between the groups (Zimmerman and Zumbo 1993). The Mann–Whitney U test was performed as a non-parametric alternative to assess differences in the ranks between the two groups. Additionally, the Kruskal–Wallis test was applied to compare the rank-based distributions of nonpolar and polar amino acids (Kruskal and Wallis 1952). The Kruskal–Wallis test acts as the non-parametric counterpart of the one-way analysis of variance (ANOVA). While the one-way ANOVA assesses whether the means of different groups are statistically different under the assumption of normality and homogeneity of variances, the Kruskal–Wallis test does not require these assumptions (McKight and Najab 2010). For a significant difference, the *p* value threshold was set to 0.05. Additionally, the pathogenicity scores for each amino acid substitution were analyzed, with the median, first quartile, third quartile, and interquartile ranges (IQR) values calculated. Statistical calculations were performed and visualized using R (The R Foundation for Statistical Computing, Vienna, Austria), version 4.4.0 (https://www.r-project.org/) (R Core Team 2016).

### Residue-wise evolutionary profiling

ConSurf Database (https://consurf.tau.ac.il/) was used to generate evolutionary conservation profiles across different residues of each protein (Yariv et al. 2023; Ashkenazy et al. 2016; Celniker et al. 2013; Landau et al. 2005). ConSurf can identify residue conservation of single proteins, a robust approach to determine which residues of individual proteins are more conserved. ConSurf scores are normalized within each protein sequence, providing intra-protein positional context rather than global residue conservation statistics. The ConSurf Database uses default parameters for homolog searches. For alignment generation, a maximum of 300 homologs are sampled to create the final list of homologs for the query protein. The database server is run using FASTA sequences from 504 UniProt entries, identifying 119 protein sequences available in the ConSurf Database. ConSurf requires sufficient homolog sequences for reliable conservation calculation, some proteins may lack adequate phylogenetic depth, and recent structural determinations may not yet be incorporated.

ConSurf utilizes a phylogenetic approach to compute conservation scores by analyzing evolutionary relationships among homolog sequences through sequence alignments using Hidden Markov Model (HMMER) (Landau et al. 2005). HMMER uses probabilistic models that represent sequence motifs, capturing evolutionary variation more effectively than simple pairwise alignments (Eddy 2011). This is particularly beneficial for detecting remote homologs that may not share strong sequence similarity but are evolutionarily related. Similar to BLAST, HMMER assigns *E*-values to alignments, the default E-value threshold was utilized (0.0001), meaning that only sequences with an *E*-value ≤ 0.0001 are considered homologs for the analysis. The *E*-value represents the expected number of matches that could occur by chance in a database search. A maximum of 300 homologs with an identity percentage of 95% and a minimum homolog threshold of 35% were selected for homolog analysis. These sequences were subsequently aligned using the MAFFT-L-INS-i method to construct a Multiple Sequence Alignment (MSA). Conservation is calculated using an empirical Bayesian method (best-fit) that considers the evolutionary distances between sequences in the phylogenetic tree. The output includes normalized conservation grades ranging from 1 (variable positions) to 9 (highly conserved positions). These conservation grades reflect position-specific constraints relative to other positions in the same protein. This within-protein normalization is critical for our downstream analyses. By removing inter-protein scale differences, effectively serves as a latent variable reflecting the relative evolutionary constraint at each site, independent of absolute substitution rates across unrelated proteins. Amino acid frequencies at aligned positions were also obtained from the ConSurf outputs for the 119 proteins available in the database.

This study also aimed to investigate the relationship between residue-level evolutionary conservation and solvent accessibility in transmembrane alpha-helical proteins. Protein structures used in Consurf analysis were retrieved using the Biopython PDB module and downloaded from the RCSB PDB using their unique PDB IDs (Cock et al. 2009). For each target structure, the specified chain was extracted from the full model and saved as an individual.pdb file using a custom subclass of Bio.PDB.Select. Evolutionary conservation scores were parsed from ConSurf. The data section of each file was located dynamically by identifying the row starting with the header "pos,SEQ". Only standard residues with defined positions were included in subsequent analyses. The Shrake-Rupley algorithm, implemented via Bio.PDB.SASA, was applied to full multichain models to ensure accurate estimation of residue exposure in a biologically relevant context (Shrake and Rupley 1973). Calculations were performed at the residue-level (level = "R"), and SASA values were extracted for the target chain only. RSA was computed using the maximum ASA values reported by Tien et al. (2013) for each standard amino acid. Per-residue ConSurf conservation grades and SASA/RSA scores were merged based on residue index. Merged results were saved as.csv files for downstream analysis. To assess the relationship between evolutionary conservation and solvent accessibility, we performed a Spearman rank correlation analysis using R (v4.4.0). This non-parametric test was chosen due to its robustness to non-linearity and non-normal distributions. Given the presence of tied ranks in the data, asymptotic p-values were used. To estimate the 95% confidence interval for Spearman’s ρ, we used the Spearman function from the RVAideMemoire package (Hervé 2020). This function employs a bootstrap approach with 10,000 resampling iterations (nrep = 10,000) to derive confidence limits for the correlation coefficient.

### Statistical analysis of evolutionary profiles

For the co-evolution data, hydrophobic to hydrophilic substitutions that have lower pathogenetic scores were selected. Namely, the data for statistical analysis consisted of the frequencies of F—Y, W—Y, I—T, M—T, A—T, V—T, G—T, G—S, A—S, I—S, L—S, V—S, C—S, L—R, L—Q, M—Q, G—N, A—N, and A—H amino acid pairs as well as the residue-wise conservation scores. Data analysis initially involved the application of the arcsine square root transformation to handle percentage data and stabilize variance. The arcsine transformation, applied to percentages converted to proportions, is typically effective for stabilizing variance in proportion data, in this case, the AA frequencies (Gabriel 1963). However, despite this transformation, our data did not achieve a normal distribution. The Anderson–Darling test (Stephens 1974) results indicated that the transformed data still deviated significantly from a normal distribution, also after BCT transformation (*p*-values < 0.001). Additionally, we applied the logit transformation, another method typically used for proportion data. However, the logit transformation also failed to normalize the data, as confirmed by subsequent normality testing.

Given the inadequacy of the transformations in normalizing the data, we chose to use Spearman’s rank correlation (Spearman’s rho) coefficient for our statistical analysis (Hervé 2020). Spearman’s rho is a non-parametric measure of rank correlation that does not assume normality of the data. This method is particularly suited for analyzing monotonic relationships when the data fail to meet the assumptions required for parametric tests (Kim et al. 2015). Evolutionary conservation is hypothesized as a possible confounding variable, since it may result in similarities of substitutions by reducing both frequencies (Karagöl et al. 2024a). For the conservation estimations, ConSurf grades were used (Yariv et al. 2023; Ashkenazy et al. 2016; Celniker et al. 2013; Landau et al. 2005). This method is particularly well-suited for studies focused on evolutionary variation and functional constraints, as it provides detailed insights into the selective pressures acting on each residue.

Partial correlations were also assessed using ranked values (Kim 2015). In this context, partial correlations were calculated by controlling for the confounding effects of specific hydrophobic amino acid frequencies, including alanine (A), isoleucine (I), and valine (V) or conservation grades. For the extensive nodes in T-V relationship, the correlations were calculated without confounding effect of certain hydrophobic amino acid (A, I, V) frequencies. By removing these confounding influences, the analysis isolated the threonine (T) and valine (V) interactions, enabling a clearer understanding of their evolutionary and functional relationships. This analysis specifically provides a more accurate depiction of how T and V co-evolve or interact in the context of selective pressures and variational constraints. The Evolutionary Game Theory (EGT) framework (Cressman 2003) was introduced as a conceptual model to explore potential mechanisms behind specific observations, such as the co-occurrence patterns of T and V (despite expectations from some substitution matrices) and the potential mediating role of alanine suggested by partial correlation analysis. We acknowledge that these models are based on the broader, context-averaged patterns identified in our large-scale analysis and that position-specific context and selective pressures are paramount. Our partial correlation analysis does not challenge exchangeability matrices but rather reveals context-dependent evolutionary dynamics that complement matrix-based models, with observed correlations reflecting compensatory evolutionary mechanisms and structural constraints operating within the fitness landscape defined by substitution matrices. Statistical calculations were performed and visualized using R (The R Foundation for Statistical Computing, Vienna, Austria), version 4.4.0 https://www.r-project.org/ (R Core Team 2016). All statistical tests were two-sided, and a p-value of < 0.05 was considered statistically significant.

## Supplementary Information

Below is the link to the electronic supplementary material.

Supplementary file1 (PDF 3122 kb)

Supplementary file2 (XLSX 252 kb)

Supplementary file3 (XLSX 182 kb)

Supplementary file4 (XLSX 40 kb)

Supplementary file5 (PDF 13642 kb)

Supplementary file6 (PDF 61643 kb)

## Data Availability

The transmembrane alpha-helical protein structures were obtained from the mpstruc database, with corresponding PDB files accessible via the RCSB Protein Data Bank. Conservation scores for these proteins are available at the ConSurf Database https://consurf.tau.ac.il/, while individual AlphaMissense scores can be found at HageLab's AlphaMissense webpage https://alphamissense.hegelab.org. A comprehensive list of proteins analyzed in this study, along with Python and R scripts used for data preparation and statistical analysis, as well as additional statistical data, can be accessed at https://github.com/karagol-taner/Transmembrane-alpha-helix-evolution.
