# Supplementary material for: Co-evolution of alpha-helical transmembrane protein residues: large-scale variant profiling and complete mutational landscape of 2277 known PDB entries representing 504 unique human protein sequences
Source: J Mol Evol. 2025 Sep 24;93(5):581–99. doi: 10.1007/s00239-025-10262-8 (PMC12579659; doi:10.1007/s00239-025-10262-8)
Supplement: Supplementary file 1 — Supplementary file1 (PDF 3122 kb) [file 239_2025_10262_MOESM1_ESM.pdf]

Supplementary Information

Co-evolution of Alpha-helical Transmembrane Protein Residues: Large-Scale Variant Profiling and Complete Mutational Landscape of 2277 known PDB Entries Representing 504 Unique Human Protein Sequences

Taner Karagöl[1,¶,*], Alper Karagöl[1,¶,*], Shuguang Zhang[2]

[1]Istanbul University Istanbul Medical Faculty, Istanbul, Turkey

[2]Laboratory of Molecular Architecture, Media Lab, Massachusetts Institute of Technology, 77 Massachusetts Avenue, Cambridge, MA, 02139, USA
[¶]These authors contribute equally.
*To whom the correspondence should be addressed.

Email:
Taner Karagöl, taner.karagol@gmail.com ORCID: 0009-0005-1011-7661
Alper Karagöl, alper.karagol@gmail.com ORCID: 0009-0001-7864-0732
Shuguang Zhang, Shuguang@MIT.EDU ORCID: 0000-0002-3856-3752

Table of Contents

1. **Supplementary Table 1.** Comprehensive Dataset of Studied 504 UniProt Entries with RCSB PDB IDs, FASTA Sequences, and Functional Annotations. (.xlsx File) Pg. 3
2. **Supplementary Table 2.** Comprehensive List of 2277 Relevant RCSB PDB Structures for Studied Proteins. (.xlsx File) Pg. 3
3. **Supplementary Table 3.** Combined AlphaMissense Pathogenicity Scores Grouped by Initial Amino Acid in Studied Proteins. (.xlsx File) Pg. 3
4. **Supplementary Figure 1.** Combined Topology Prediction Results for Studied Proteins. (.pdf File) Pg. 3
5. **Supplementary Figure 2.** Combined Hydropathy Plots for Studied Proteins. (.pdf File) Pg. 3
6. **Supplementary Table 3a.** Distribution of Potential Variants with Initial Nonpolar Amino Acids Across Studied Proteins. Pg. 4
7. **Supplementary Table 3b.** Descriptive Statistics for Data in Supplementary Table 3a. Pg. 4
8. **Supplementary Table 4a.** Distribution of Potential Variants with Initial Polar Amino Acids Across Studied Proteins. Pg. 5
9. **Supplementary Table 4b.** Descriptive Statistics for Data in Supplementary Table 4a. Pg. 5
10. **Supplementary Figure 3**. Enlarged Panels of Figure 2. Pg. 6-10
11. **Supplementary Figure 4**. Enlarged Panels of Figure 3. Pg. 11-15
12. **Supplementary Figure 5.** Re-ordered version of Figure 2, with amino acid scores arranged in ranked order instead of alphabetical order in each panel. Pg. 16
13. **Supplementary Figure 6.** Re-ordered version of Figure 3, with amino acid scores arranged in ranked order instead of alphabetical order in each panel. Pg. 17
14. **Supplementary Figure 7.** ConSurf Conservation Grade Distribution Across Relative Solvent Accessibility (RSA) Bins. Pg. 18
15. **Supplementary Table 5.** Spearman Correlation Results of 74679 Residue frequencies. Pg. 19

Supplementary Table 1. Comprehensive Dataset of Studied 504 UniProt Entries with RCSB PDB IDs, FASTA Sequences, and Functional Annotations

This material is provided as a separate Excel file named "2-Supplementary_Table_1.xlsx".

Supplementary Table 2. Comprehensive List of 2277 Relevant RCSB PDB Structures for Studied Proteins

This material is provided as a separate Excel file named "3-Supplementary_Table_2.xlsx".

Supplementary Table 3. Combined AlphaMissense Pathogenicity Scores Grouped by Initial Amino Acid in Studied Proteins

This material is provided as a separate Excel file named "4-Supplementary_Table_3.xlsx".

Supplementary Figure 1. Combined Topology Prediction Results for Studied Proteins

These results show the topology predictions for the proteins studied using the Phobius tool, with the exception of DCD and PGAM5, where predictions failed to indicate transmembrane topology and were inconsistent with UniProt data.

This material is provided as a separate .PDF file named "5-Supplementary_Figure_1.pdf".

Supplementary Figure 2. Combined Hydropathy Plots for Studied Proteins

Hydropathy plots generated using the Kyte-Doolittle scale illustrate the hydropathic properties of the protein sequences.

This material is provided as a separate .PDF file named "6-Supplementary_Figure_2.pdf".

Supplementary Table 3a. Distribution of Potential Variants with Initial Hydrophobic Amino Acids Across Studied Proteins.

Amino acid change	Count	Z-score	MAD-score
A>X	427,603	0.6357	0.6941
C>X	140,272	-1.1293	-1.3627
F>X	311,370	-0.0783	-0.1379
G>X	373,864	0.3056	0.3094
I>X	349,913	0.1585	0.1379
L>X	675,713	2.1598	2.4700
M>X	143,550	-1.1091	-1.3392
P>X	297,719	-0.1621	-0.2357
V>X	422,136	0.6021	0.6549
W>X	98,986	-1.3829	-1.6582

Supplementary Table 3b. Descriptive Statistics for Data in Supplementary Table 3a.

Statistic	Value
Total	3,241,126
Median	330,641.5
Mean	324,112.6
Standard deviation	162,795.50
Q1 (25th percentile)	182,092.25
Q3 (75th percentile)	410,068
IQR (Interquartile Range)	227,975.75
Lower Bound (5% tolerance margin)	-167,864.94
Upper Bound (5% tolerance margin)	789,633.20
MAD (Median Absolute Deviation)	139,702.43

Supplementary Table 4a. Distribution of Potential Variants with Initial Hydrophilic Amino Acids Across Studied Proteins.

Amino acid change	Count	Z-score	MAD-score
D>X	233,722	-0.3234	-0.1767
E>X	304,628	0.5318	0.7202
H>X	125,576	-1.6277	-1.5448
K>X	261,662	0.0136	0.1767
N>X	223,654	-0.4448	-0.3041
Q>X	205,534	-0.6633	-0.5333
R>X	297,756	0.4489	0.6333
S>X	445,390	2.2294	2.5009
T>X	316,328	0.6729	0.8682
Y>X	191,117	-0.8372	-0.7157

Supplementary Table 4b. Descriptive Statistics for Data in Supplementary Table 4a.

Statistic	Value
Total	2,605,367
Median	247,692
Mean	260,536.7
Standard deviation	82,916.91
Q1 (25th percentile)	210,064
Q3 (75th percentile)	302,910
IQR (Interquartile Range)	92,846
Lower Bound (5% tolerance margin)	74,334.75
Upper Bound (5% tolerance margin)	464,287.95
MAD (Median Absolute Deviation)	79,051.49

Supplementary Figure 3. Enlarged Panels of Figure 2.

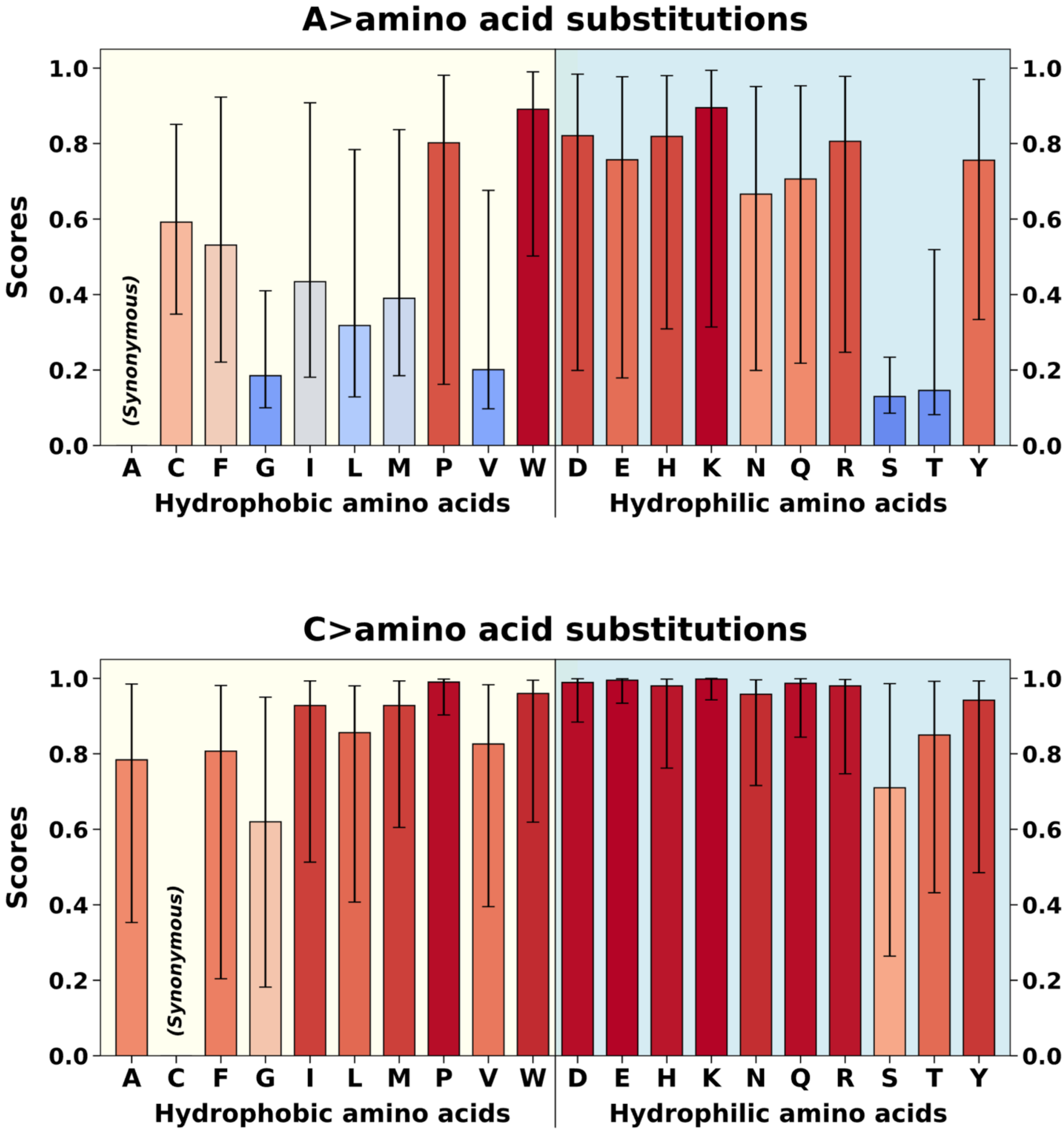

Supplementary Figure 3 (Continued). Enlarged Panels of Figure 2.

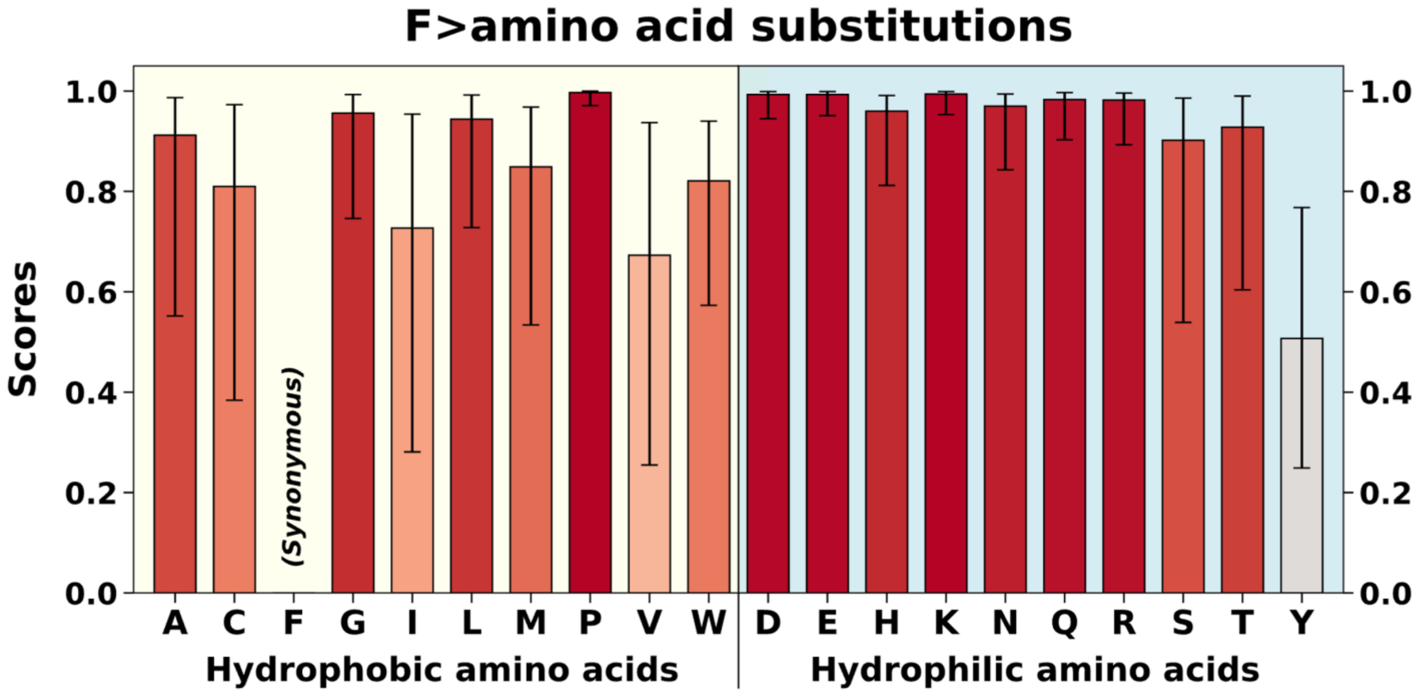

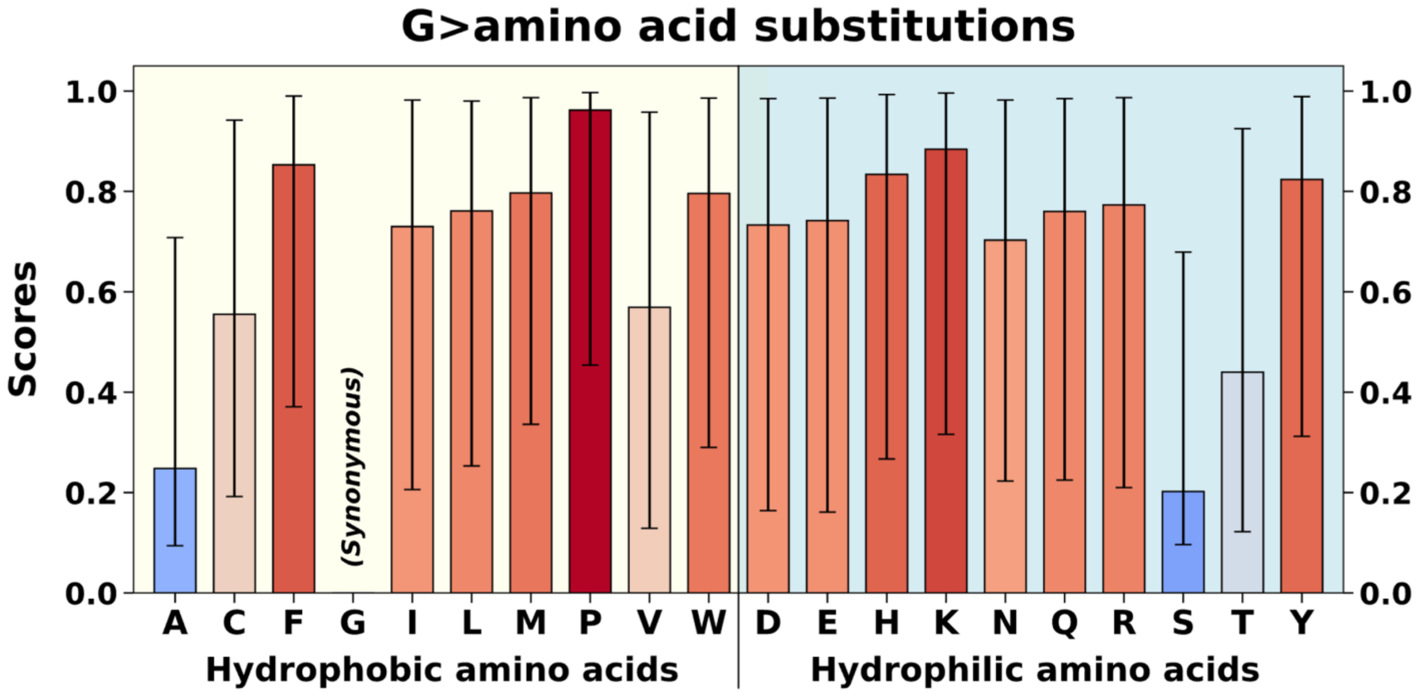

Supplementary Figure 3 (Continued). Enlarged Panels of Figure 2.

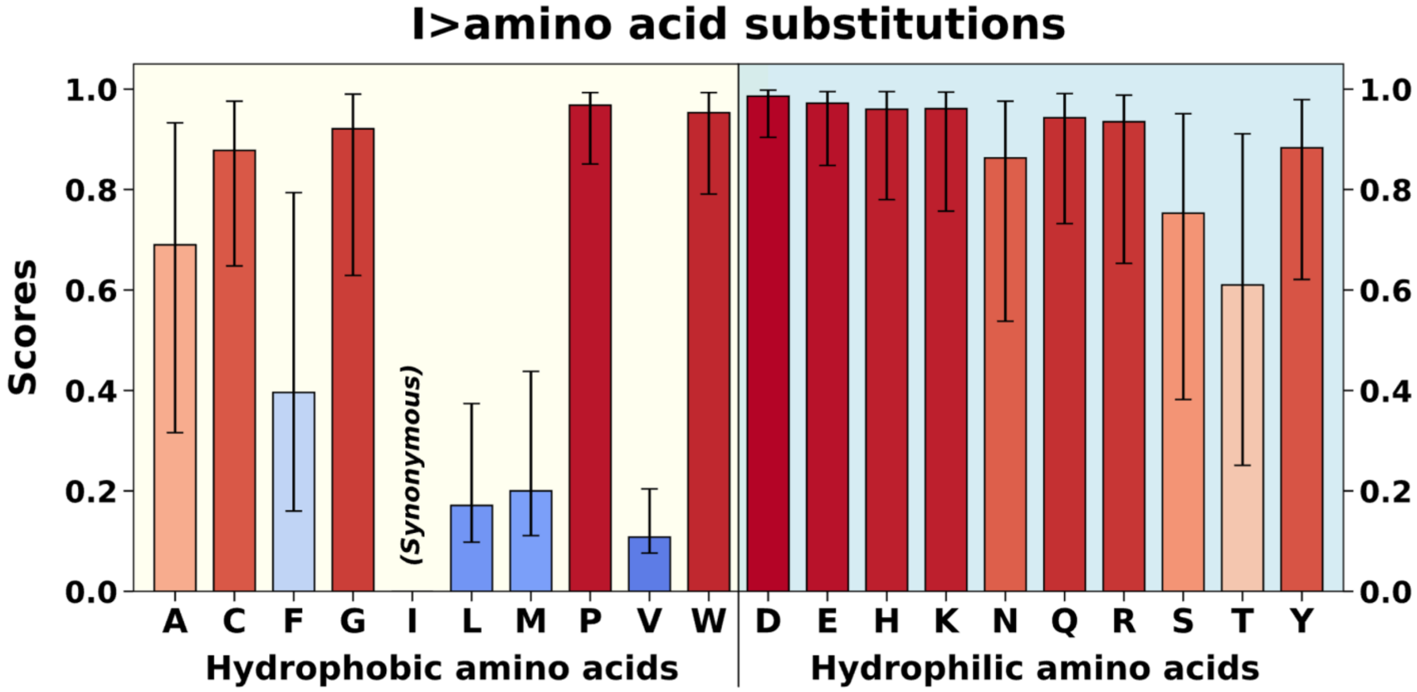

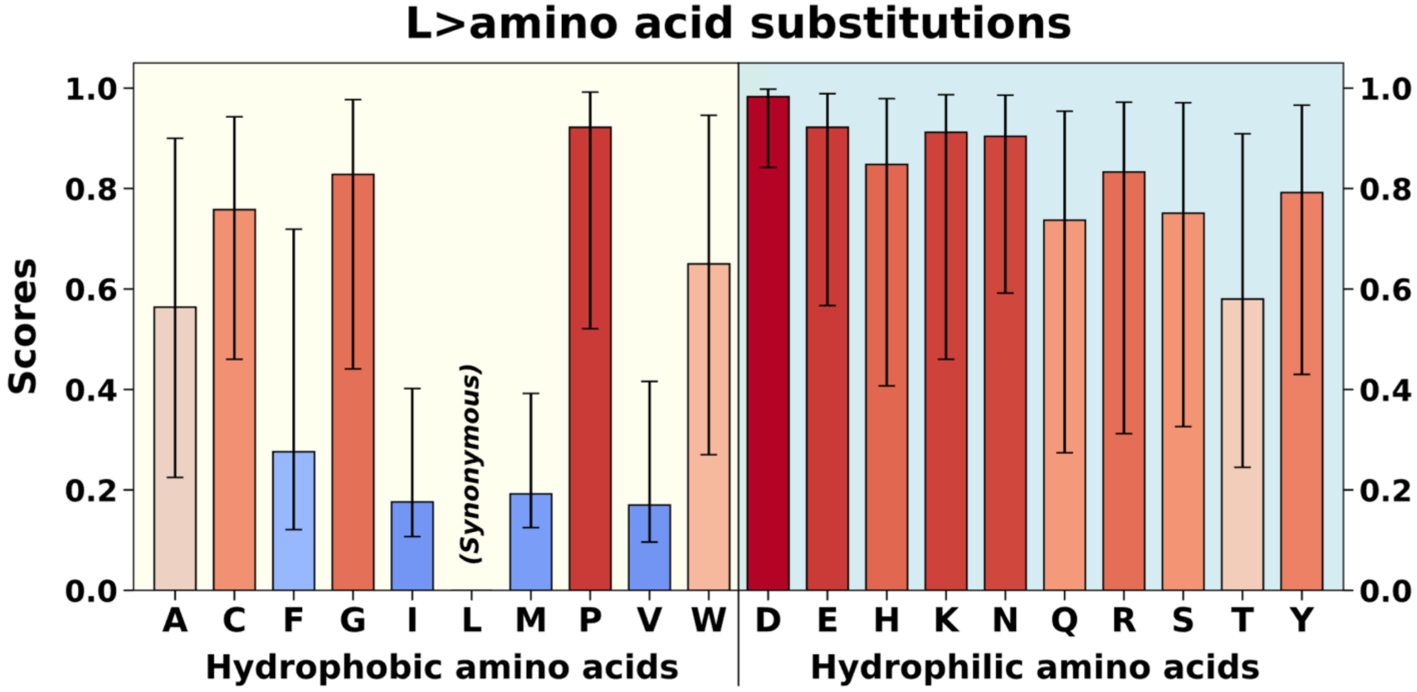

Supplementary Figure 3 (Continued). Enlarged Panels of Figure 2.

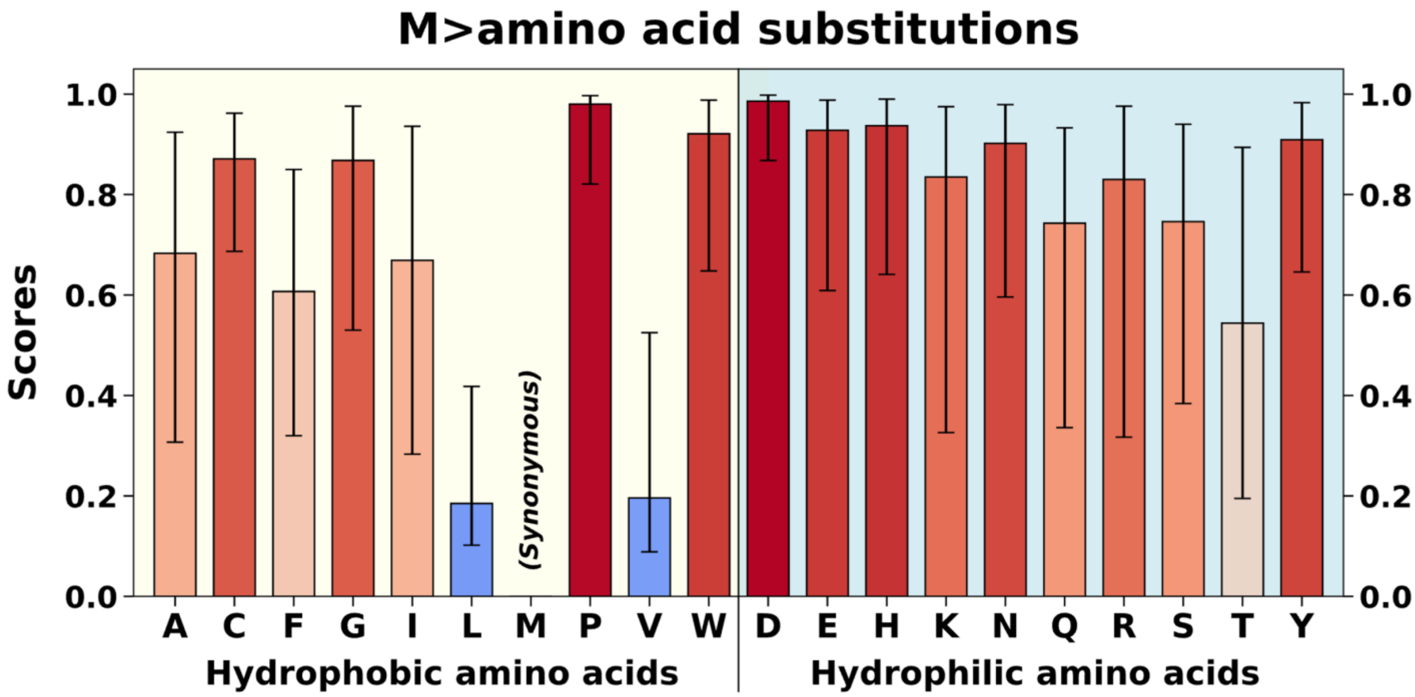

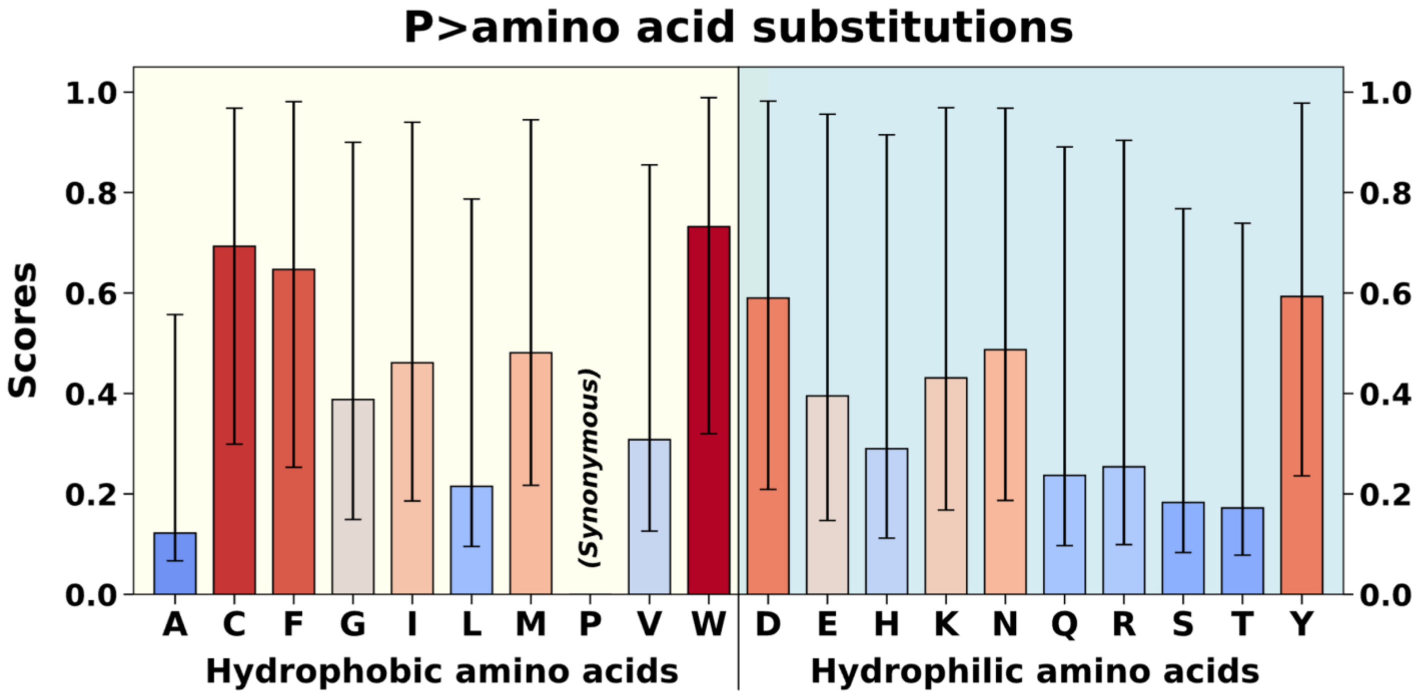

Supplementary Figure 3 (Continued). Enlarged Panels of Figure 2.

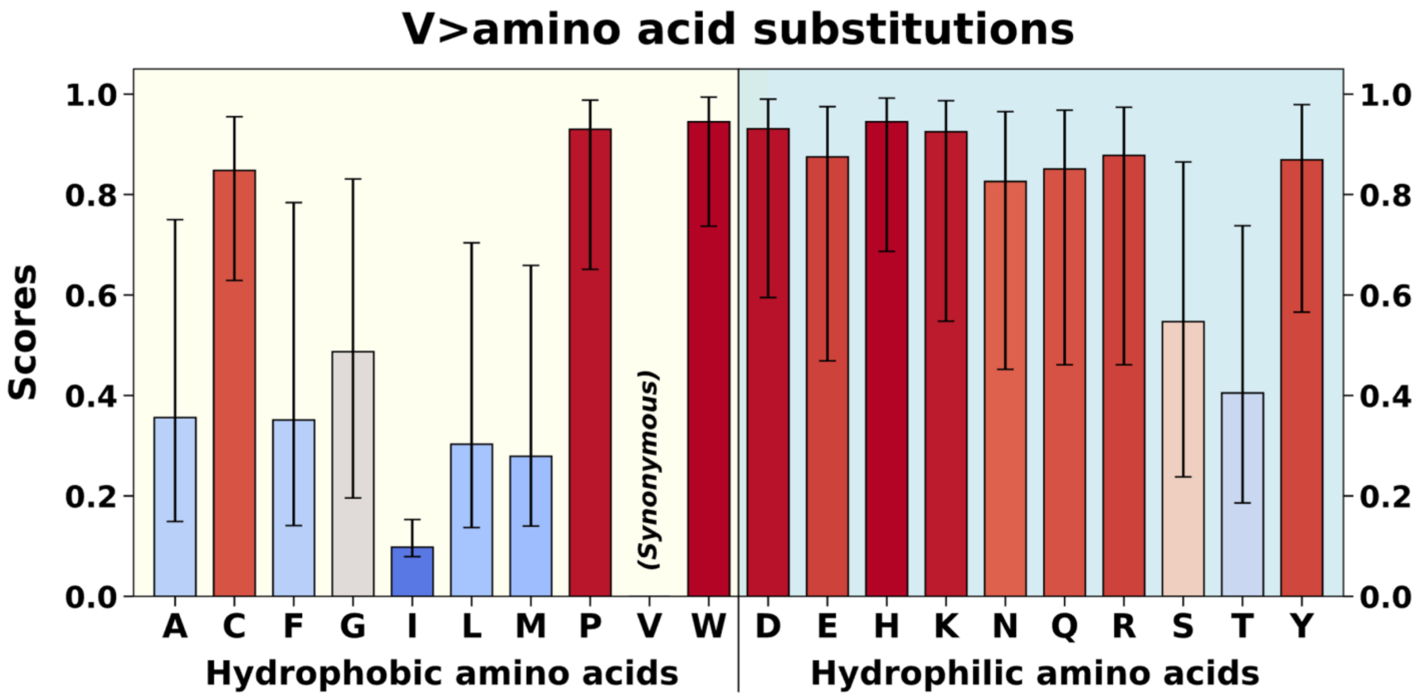

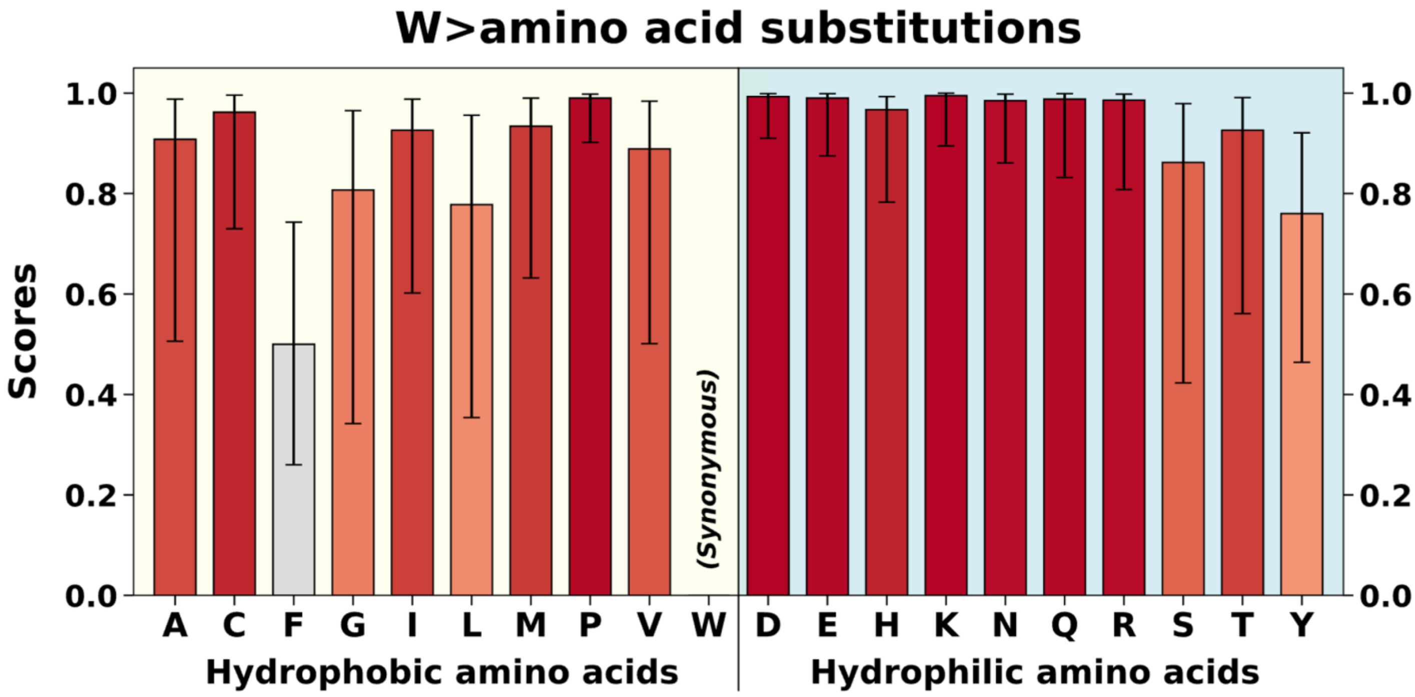

Supplementary Figure 4. Enlarged Panels of Figure 3.

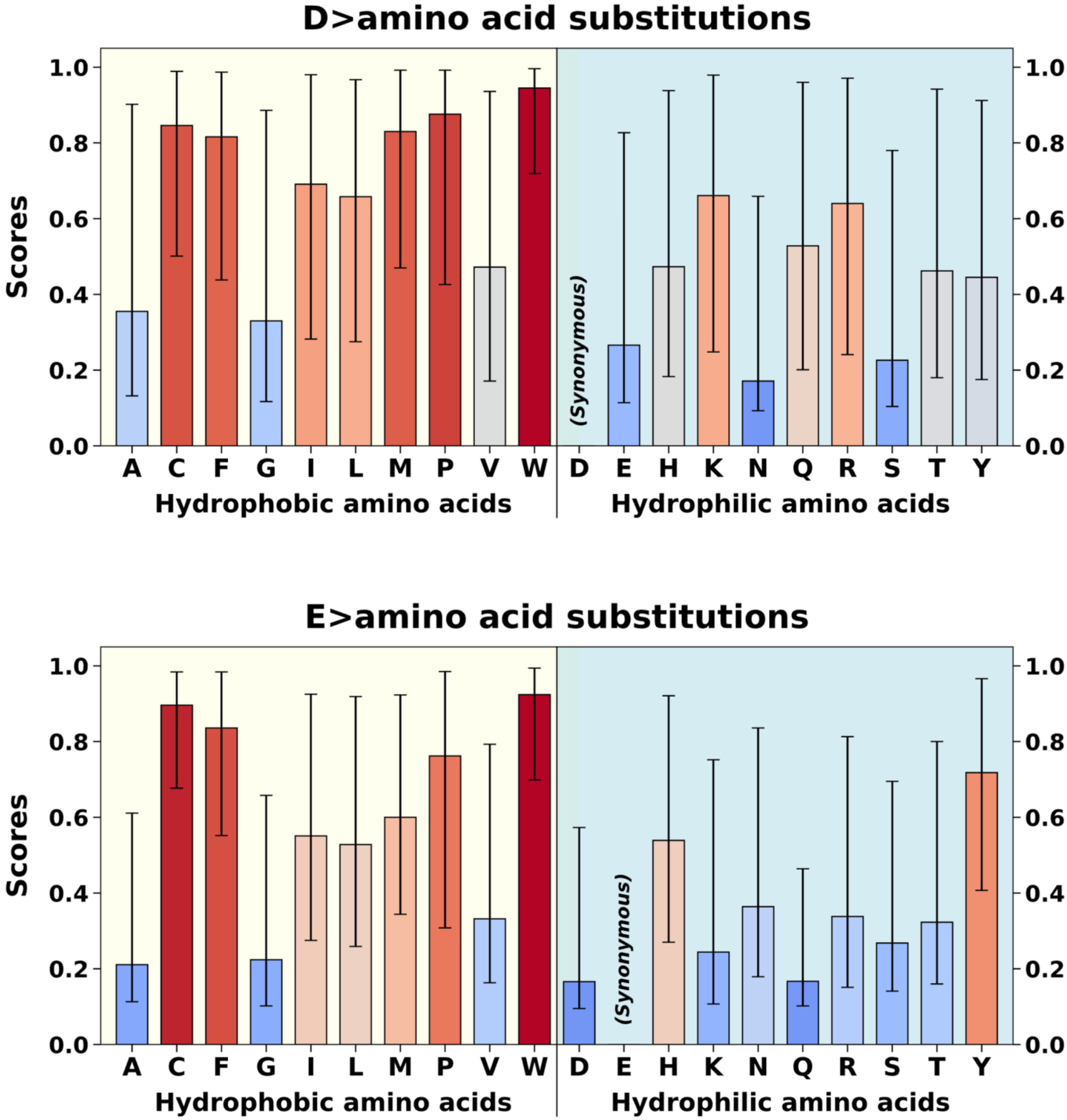

Supplementary Figure 4 (Continued). Enlarged Panels of Figure 3.

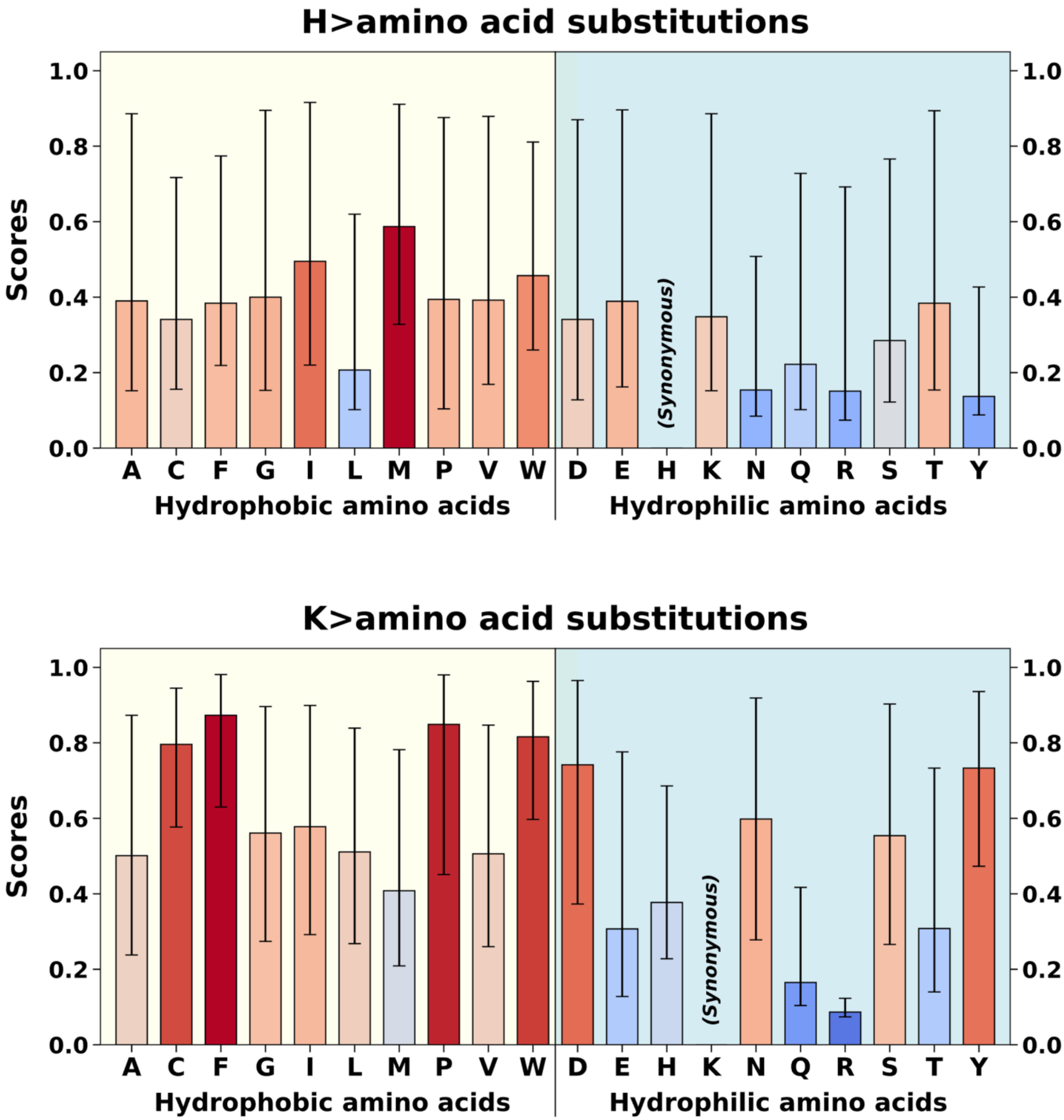

Supplementary Figure 4 (Continued). Enlarged Panels of Figure 3.

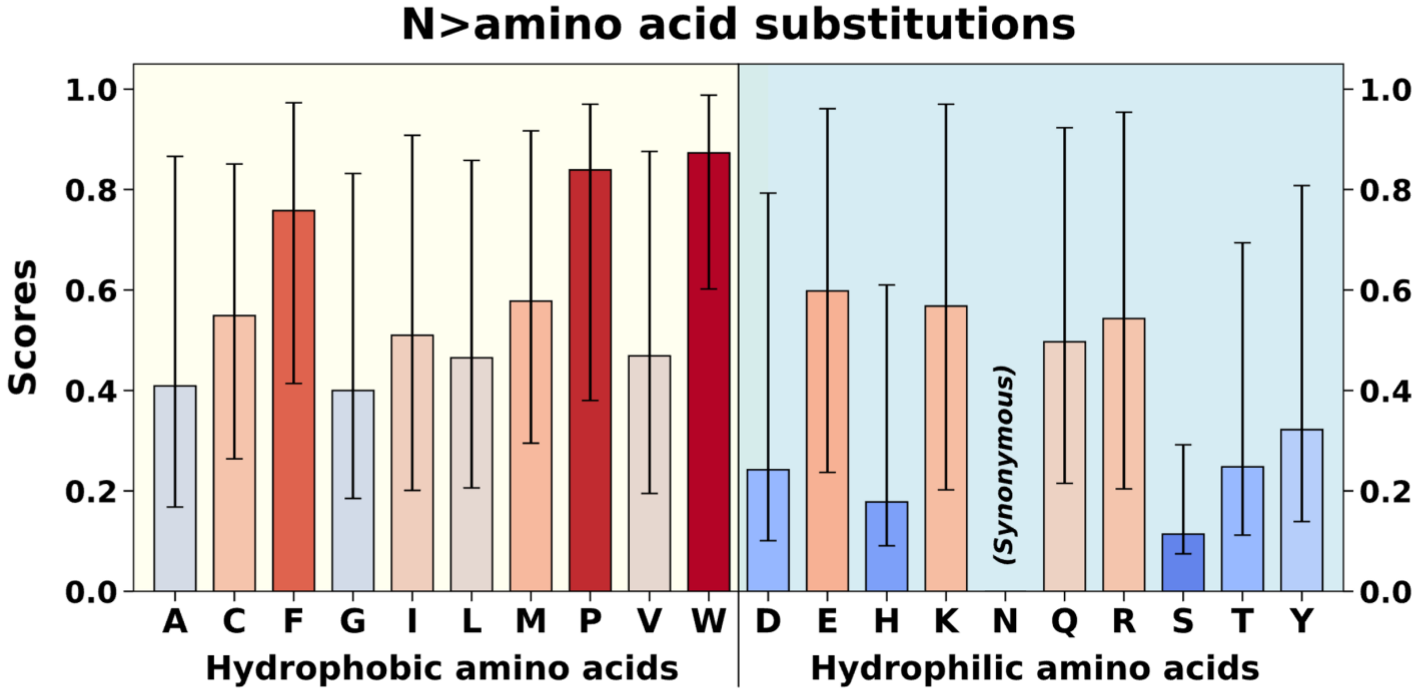

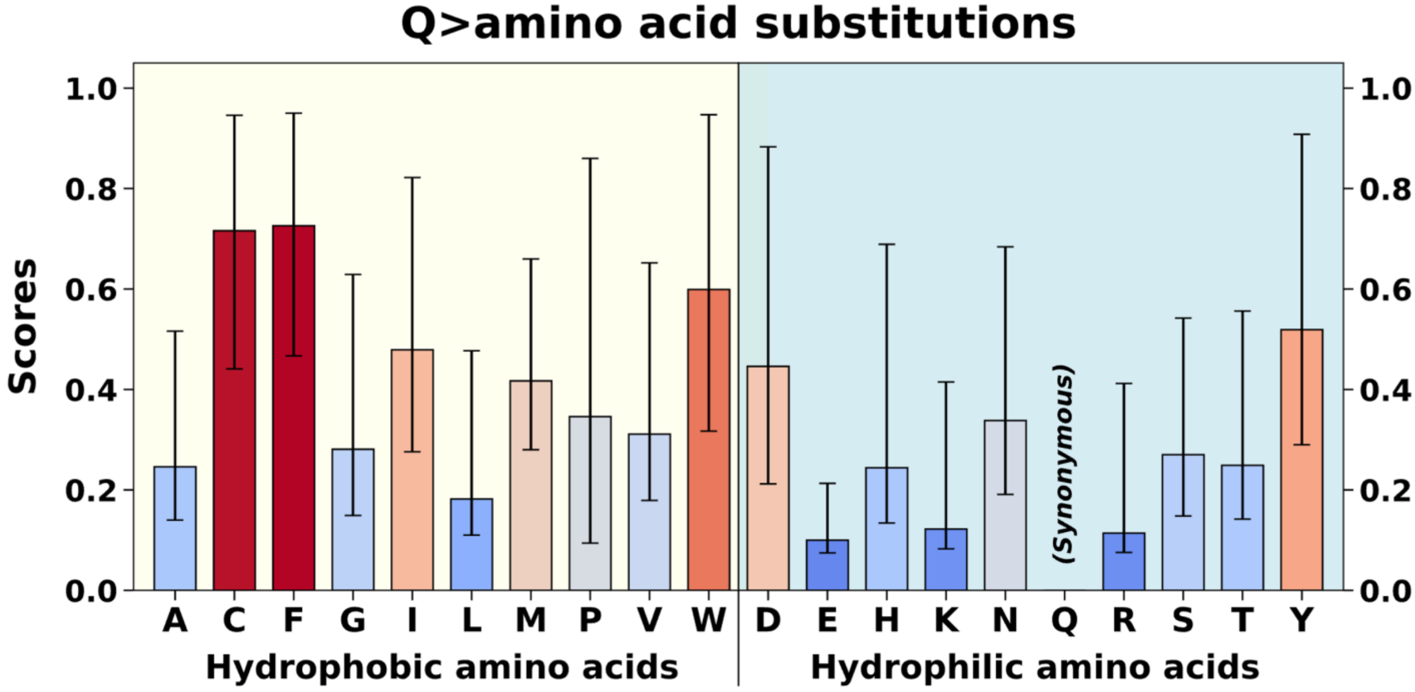

Supplementary Figure 4 (Continued). Enlarged Panels of Figure 3.

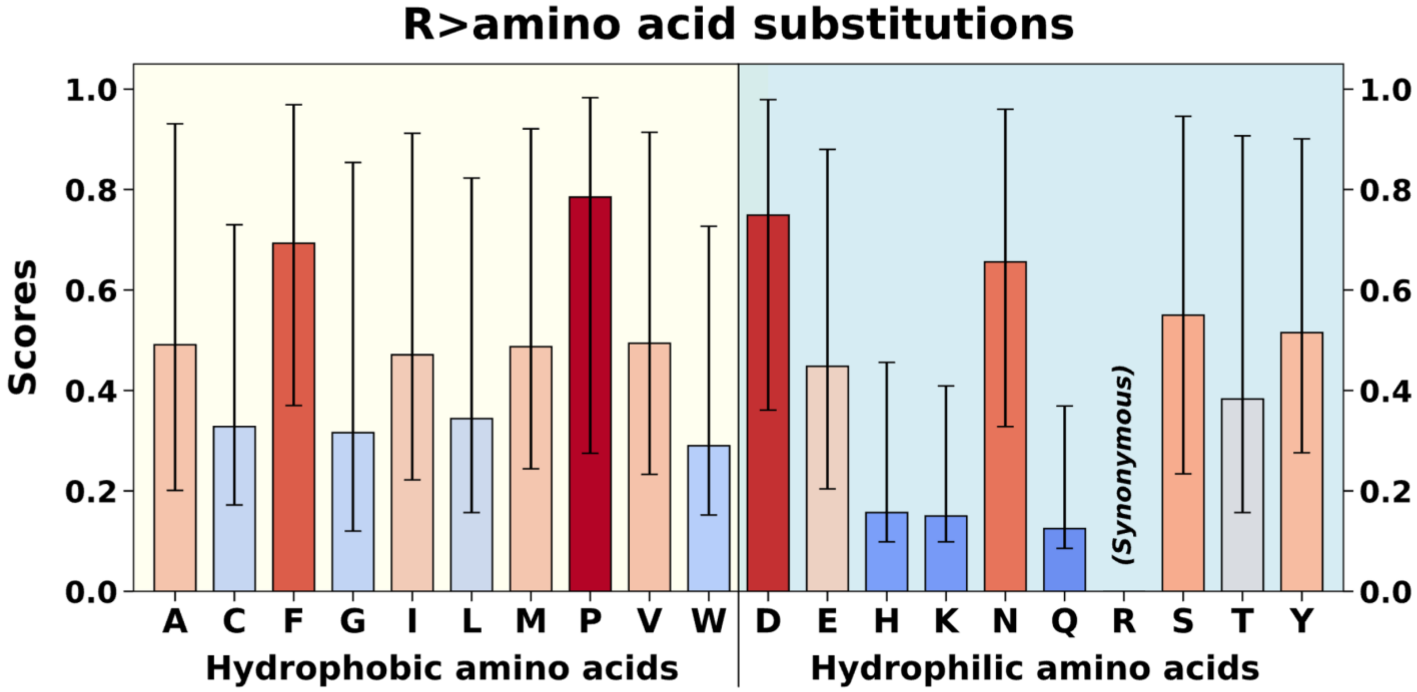

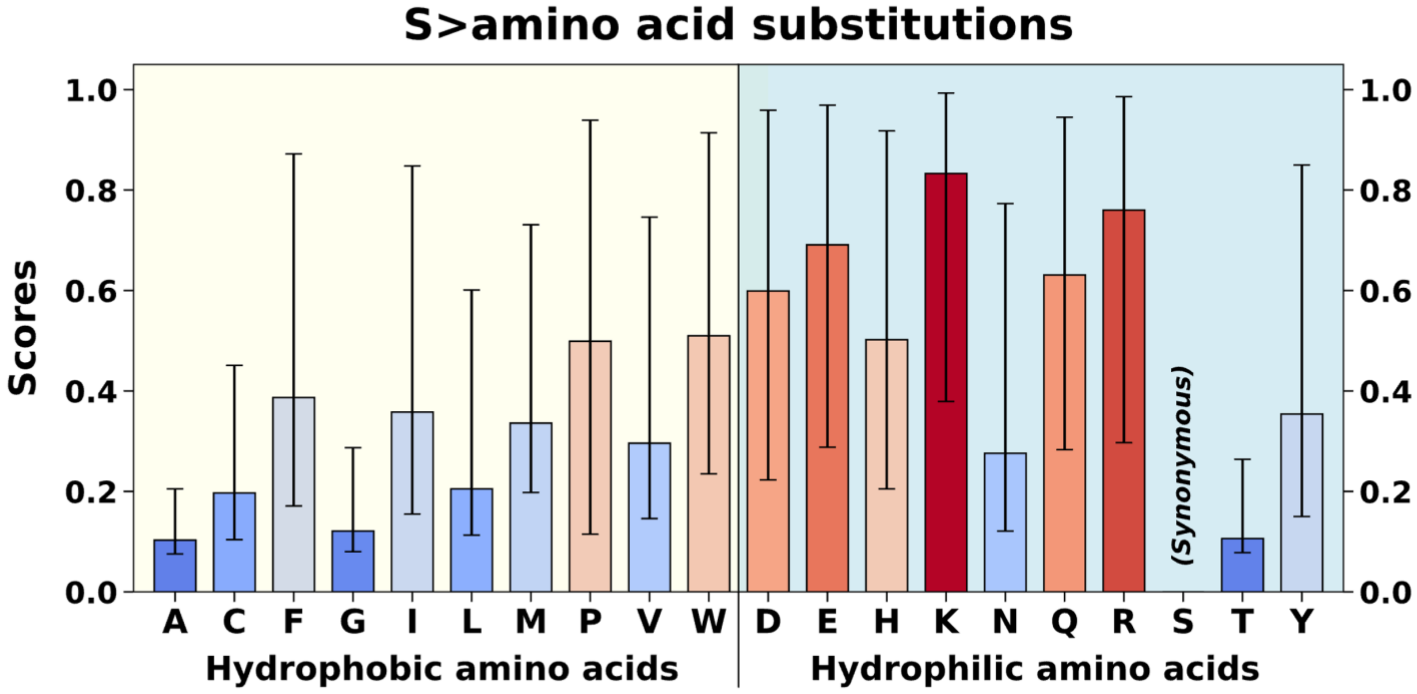

Supplementary Figure 4 (Continued). Enlarged Panels of Figure 3.

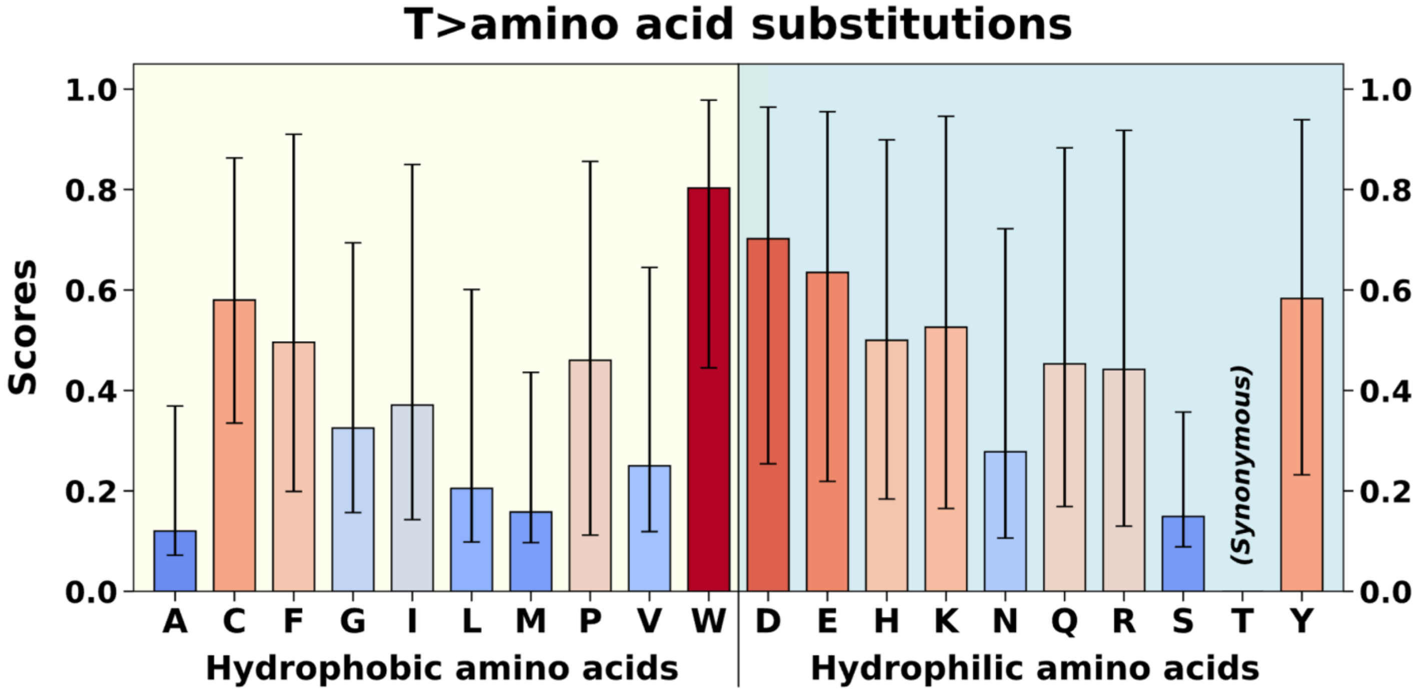

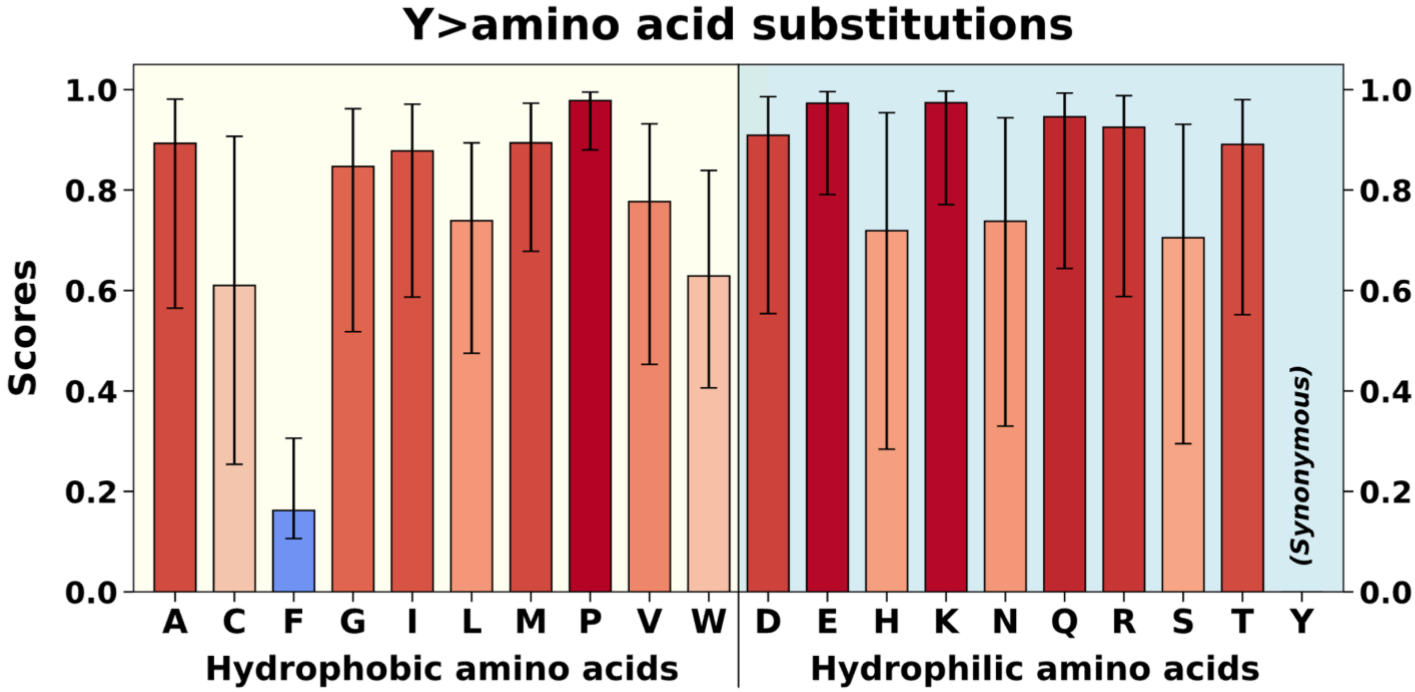

Supplementary Figure 5. Re-ordered version of Figure 2, with amino acid scores arranged in ranked order instead of alphabetical order in each panel.

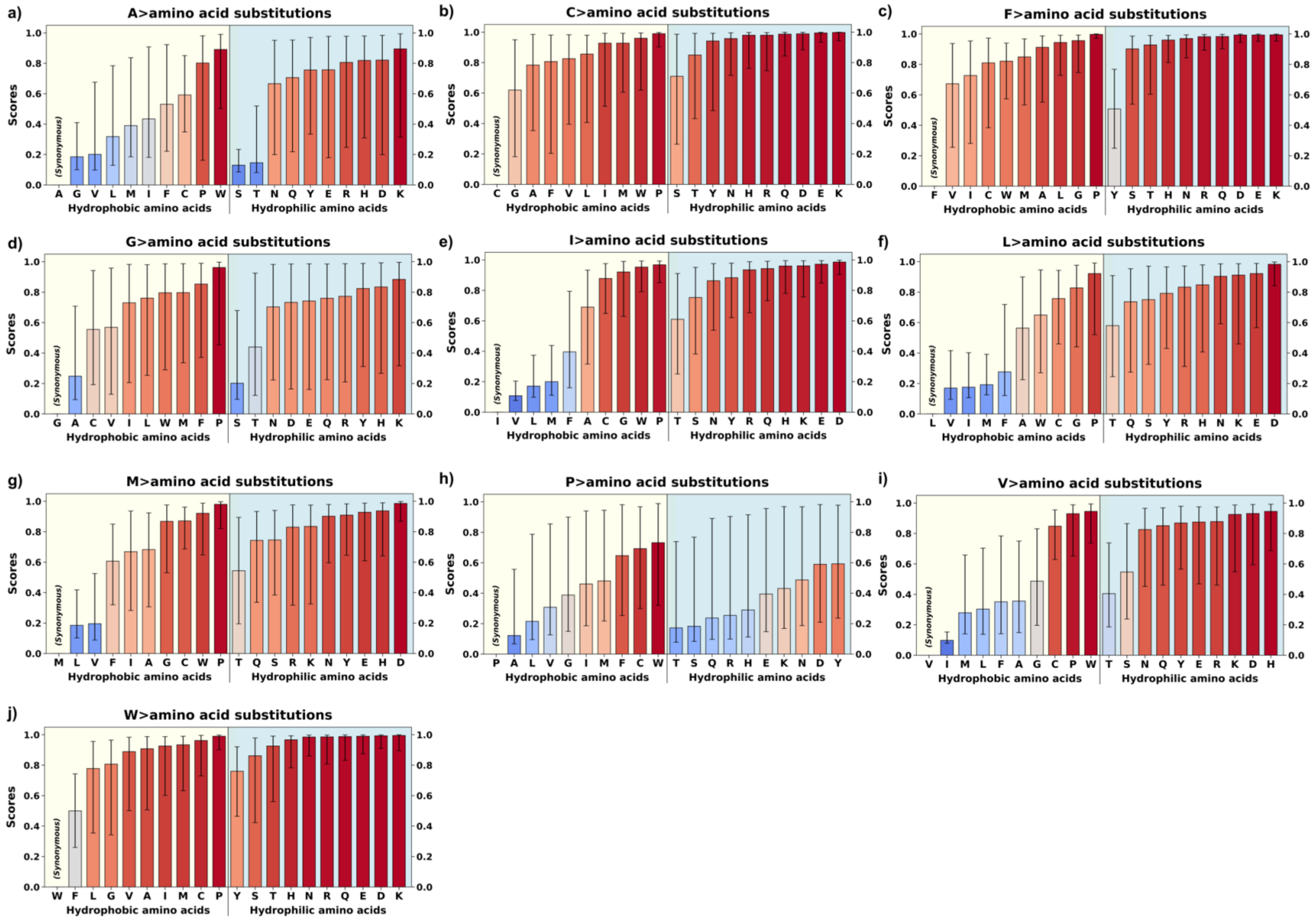

Supplementary Figure 6. Re-ordered version of Figure 3, with amino acid scores arranged in ranked order instead of alphabetical order in each panel.

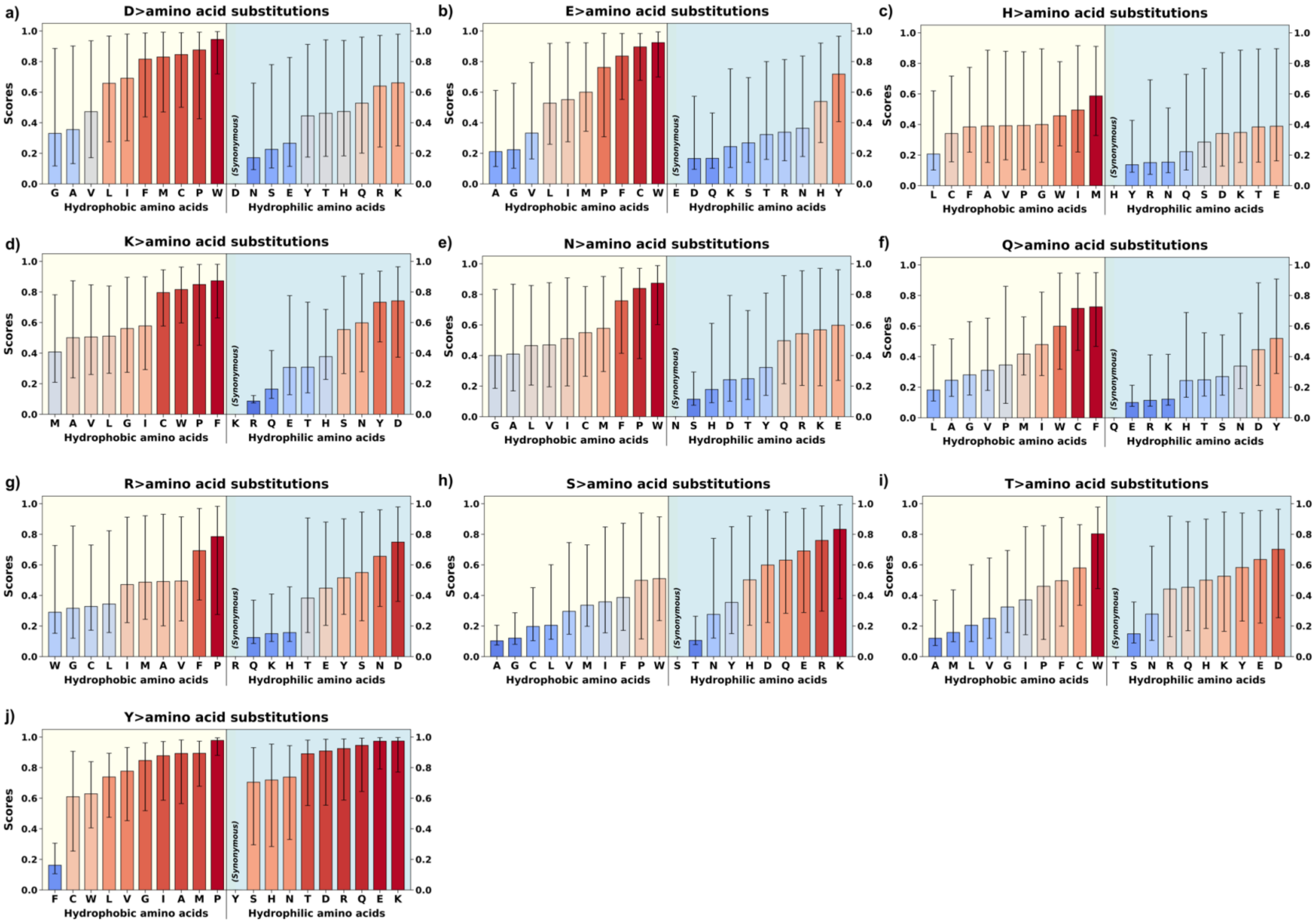

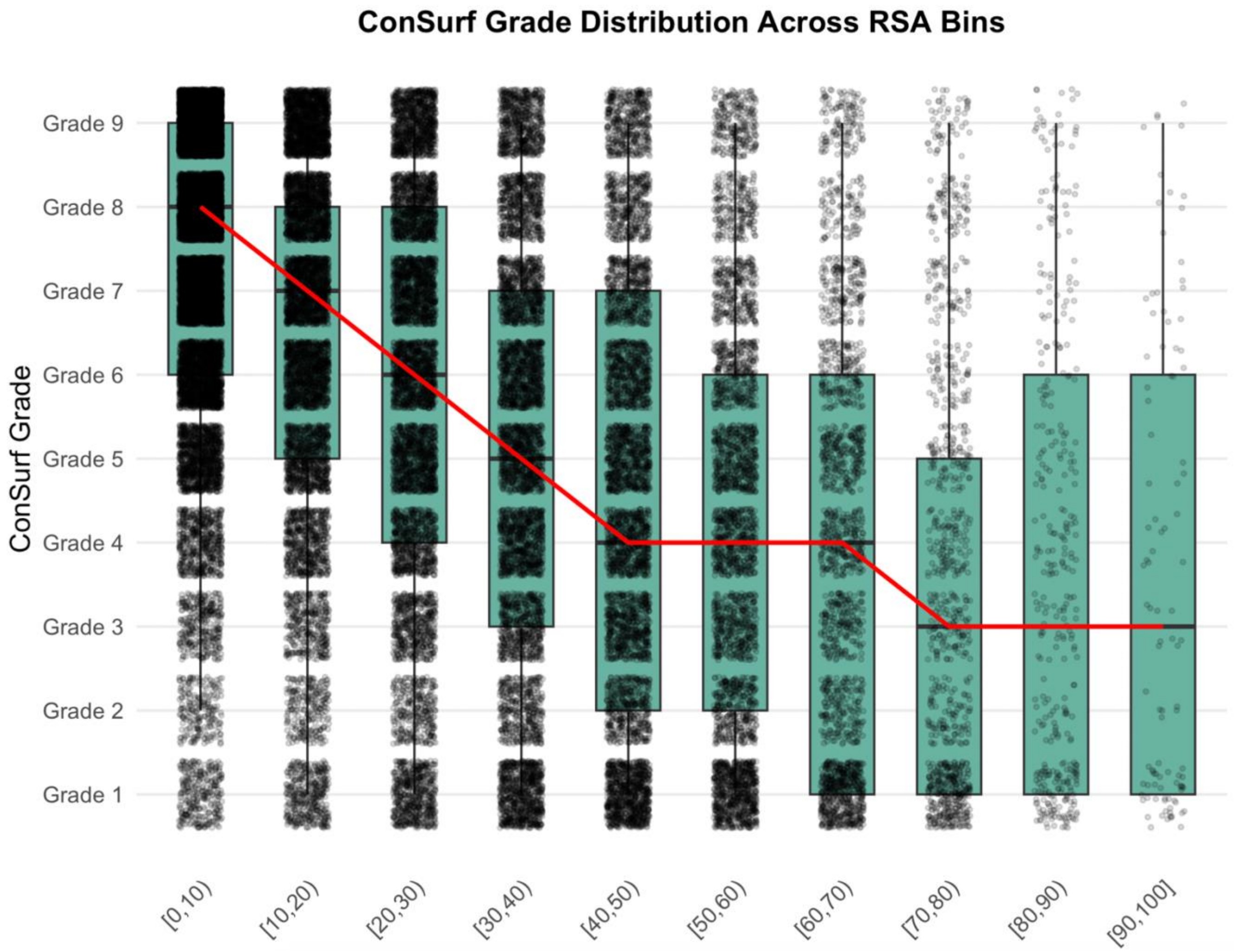

Supplementary Figure 7. ConSurf Conservation Grade Distribution Across Relative Solvent Accessibility (RSA) Bins. The distribution of ConSurf conservation grades (1 = variable, 9 = highly conserved) across bins of relative solvent accessibility (RSA, in %) for protein residues. Each RSA bin (x-axis) spans 10% intervals from 0–100%. Vertical boxplots represent the distribution of ConSurf grades (y-axis) within each RSA bin, overlaid with individual data points (black dots). The red line traces the median conservation grade across increasing RSA bins, highlighting a negative correlation between solvent accessibility and evolutionary conservation (rho= -0.47, $p < 0.001$). More buried residues (low RSA) tend to have higher conservation grades, while more exposed residues (high RSA) are generally more variable.

Supplementary Table 5. Spearman Correlation Results of 74679 Residue frequencies. (Please refer to Table 1 in the main article for partial correlation data.)

Pair	Spearman's Rho	p-value
F and Y	0.4662	< 0.001
W and Y	0.3457	< 0.001
I and T	0.2521	< 0.001
M and T	0.2618	< 0.001
A and T	0.4816	< 0.001
V and T	0.3158	< 0.001
G and T	0.2999	< 0.001
G and S	0.4527	< 0.001
A and S	0.4960	< 0.001
I and S	-0.0243	< 0.001
L and S	-0.0716	< 0.001
V and S	0.0527	< 0.001
C and S	0.1996	< 0.001
L and R	-0.0528	< 0.001
L and Q	-0.0084	0.0208
M and Q	0.0867	< 0.001
G and N	0.3351	< 0.001
A and N	0.1940	< 0.001
A and H	0.1280	< 0.001
