# Supplementary material for: Co-evolution of alpha-helical transmembrane protein residues: large-scale variant profiling and complete mutational landscape of 2277 known PDB entries representing 504 unique human protein sequences
Source: J Mol Evol. 2025 Sep 24;93(5):581–99. doi: 10.1007/s00239-025-10262-8 (PMC12579659; doi:10.1007/s00239-025-10262-8)
Supplement: Supplementary file 5 — Supplementary file5 (PDF 13642 kb) [file 239_2025_10262_MOESM5_ESM.pdf]

Supplementary Figure 1. Combined Topology Prediction Results for Studied Proteins.

These results show the topology predictions for the proteins studied using the Phobius tool, with the exception of DCD and PGAM5, where predictions failed to indicate transmembrane topology and were inconsistent with UniProt data.

Phobius prediction

Prediction of CAMP_HUMAN

```
ID    CAMP_HUMAN
FT    SIGNAL        1      34
FT    REGION        1      14       N-REGION.
FT    REGION       15      26       H-REGION.
FT    REGION       27      34       C-REGION.
FT    TOPO_DOM     35     170       NON CYTOPLASMIC.
//
```

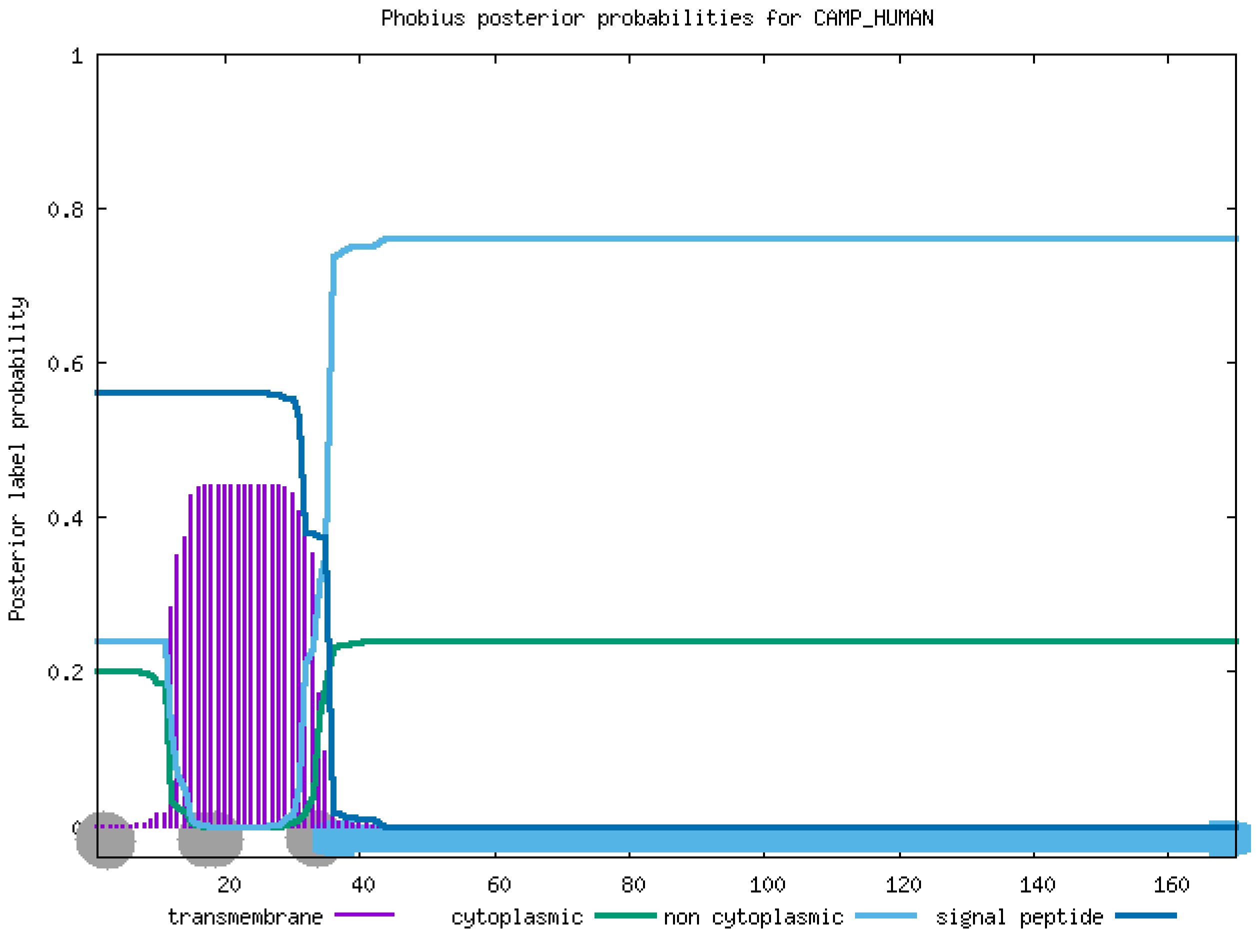

The probability data used in the plot is found here, and the gnuplot script is here.

Prediction of TTYH2_HUMAN

```
ID    TTYH2_HUMAN
FT    TOPO_DOM      1      44       NON CYTOPLASMIC.
FT    TRANSMEM     45      68
FT    TOPO_DOM     69      87       CYTOPLASMIC.
```

```
FT   TRANSMEM     88    109
FT   TOPO_DOM    110    212       NON CYTOPLASMIC.
FT   TRANSMEM    213    234
FT   TOPO_DOM    235    240       CYTOPLASMIC.
FT   TRANSMEM    241    262
FT   TOPO_DOM    263    388       NON CYTOPLASMIC.
FT   TRANSMEM    389    410
FT   TOPO_DOM    411    534       CYTOPLASMIC.
//
```

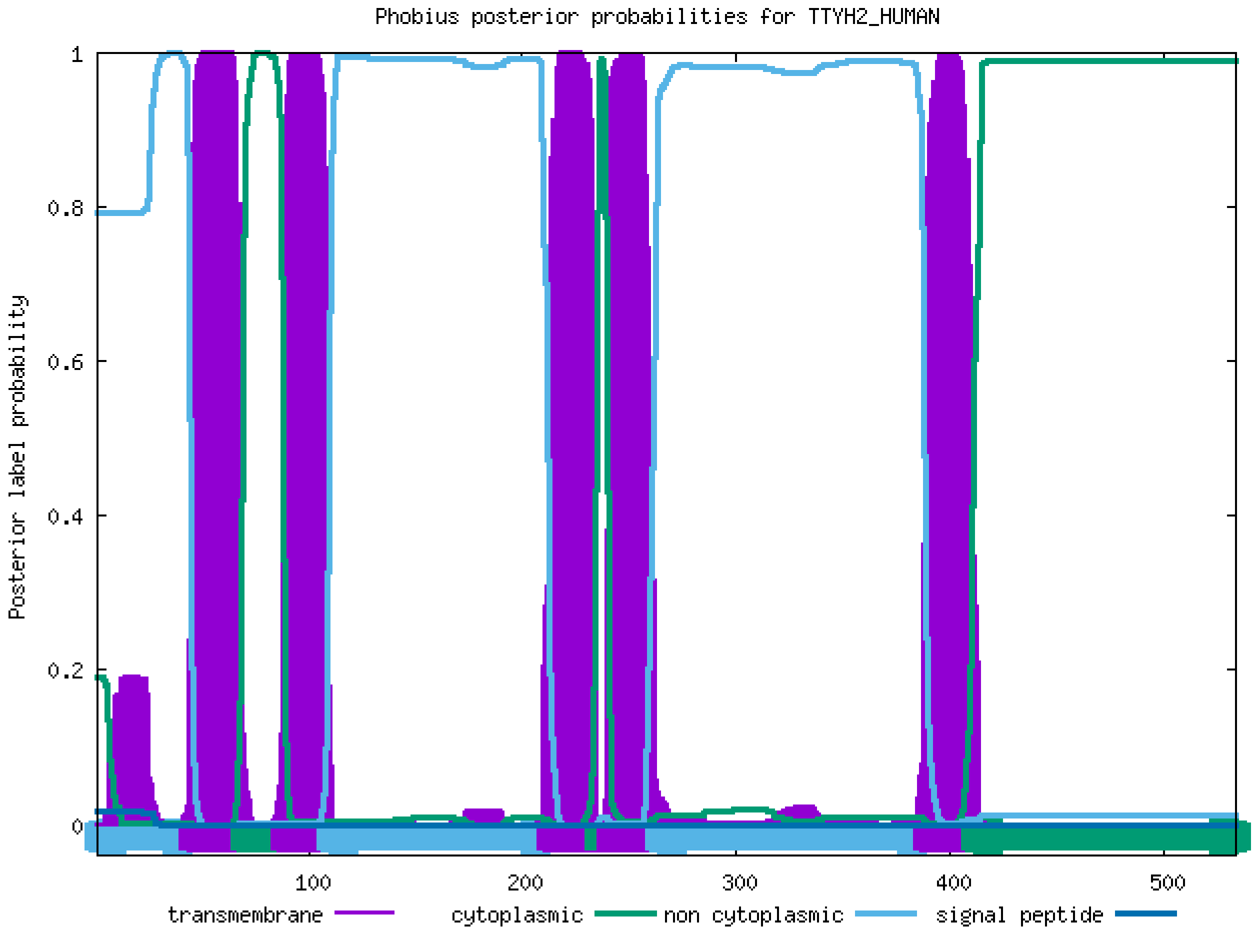

The probability data used in the plot is found here, and the gnuplot script is here.

Prediction of TACAN_HUMAN

```
ID   TACAN_HUMAN
FT   TOPO_DOM      1    135       CYTOPLASMIC.
FT   TRANSMEM    136    156
FT   TOPO_DOM    157    161       NON CYTOPLASMIC.
FT   TRANSMEM    162    181
FT   TOPO_DOM    182    192       CYTOPLASMIC.
FT   TRANSMEM    193    211
FT   TOPO_DOM    212    222       NON CYTOPLASMIC.
FT   TRANSMEM    223    240
FT   TOPO_DOM    241    266       CYTOPLASMIC.
FT   TRANSMEM    267    285
FT   TOPO_DOM    286    304       NON CYTOPLASMIC.
FT   TRANSMEM    305    329
FT   TOPO_DOM    330    343       CYTOPLASMIC.
//
```

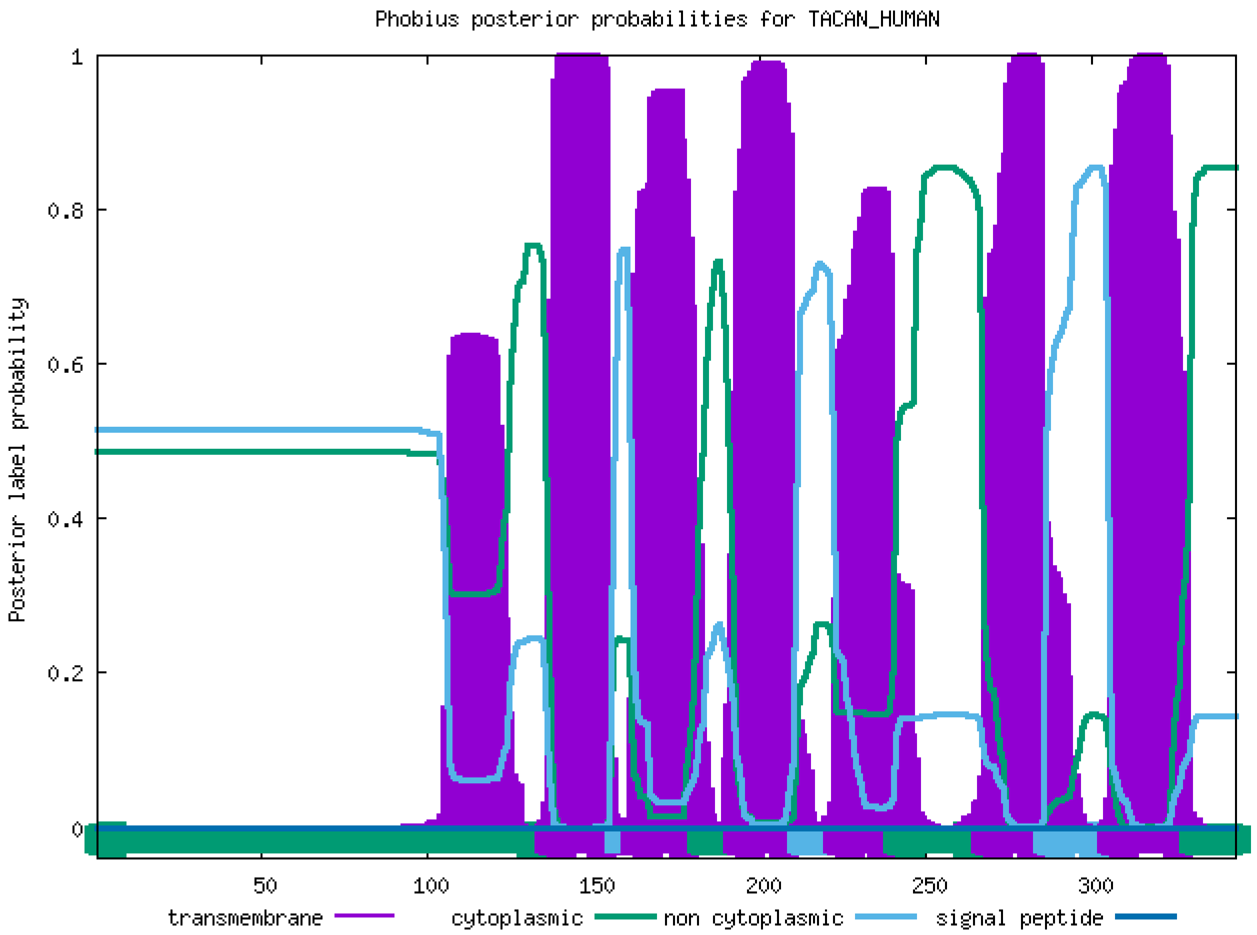

The probability data used in the plot is found here, and the gnuplot script is here.

Prediction of CD47_HUMAN

```
ID   CD47_HUMAN
FT   SIGNAL        1     20
FT   REGION        1      3       N-REGION.
FT   REGION        4     15       H-REGION.
FT   REGION       16     20       C-REGION.
FT   TOPO_DOM     21    142       NON CYTOPLASMIC.
FT   TRANSMEM    143    165
FT   TOPO_DOM    166    176       CYTOPLASMIC.
FT   TRANSMEM    177    198
FT   TOPO_DOM    199    209       NON CYTOPLASMIC.
FT   TRANSMEM    210    228
FT   TOPO_DOM    229    236       CYTOPLASMIC.
FT   TRANSMEM    237    264
FT   TOPO_DOM    265    269       NON CYTOPLASMIC.
FT   TRANSMEM    270    289
FT   TOPO_DOM    290    323       CYTOPLASMIC.
//
```

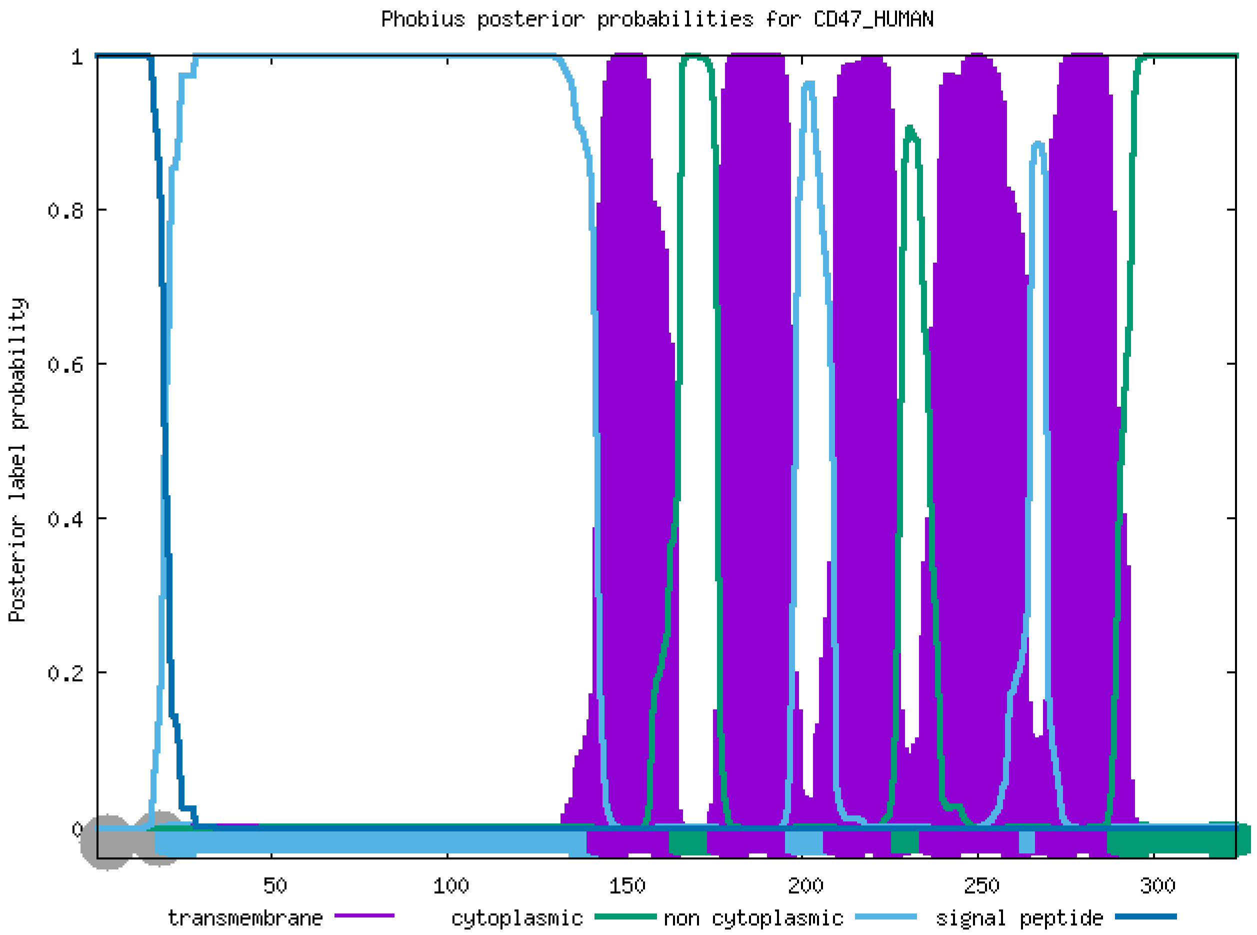

The probability data used in the plot is found here, and the gnuplot script is here.

Prediction of JAGN1_HUMAN

```
ID    JAGN1_HUMAN
FT    TOPO_DOM      1     40       NON CYTOPLASMIC.
FT    TRANSMEM     41     64
FT    TOPO_DOM     65     75       CYTOPLASMIC.
FT    TRANSMEM     76     93
FT    TOPO_DOM     94     98       NON CYTOPLASMIC.
FT    TRANSMEM     99    117
FT    TOPO_DOM    118    137       CYTOPLASMIC.
FT    TRANSMEM    138    160
FT    TOPO_DOM    161    183       NON CYTOPLASMIC.
//
```

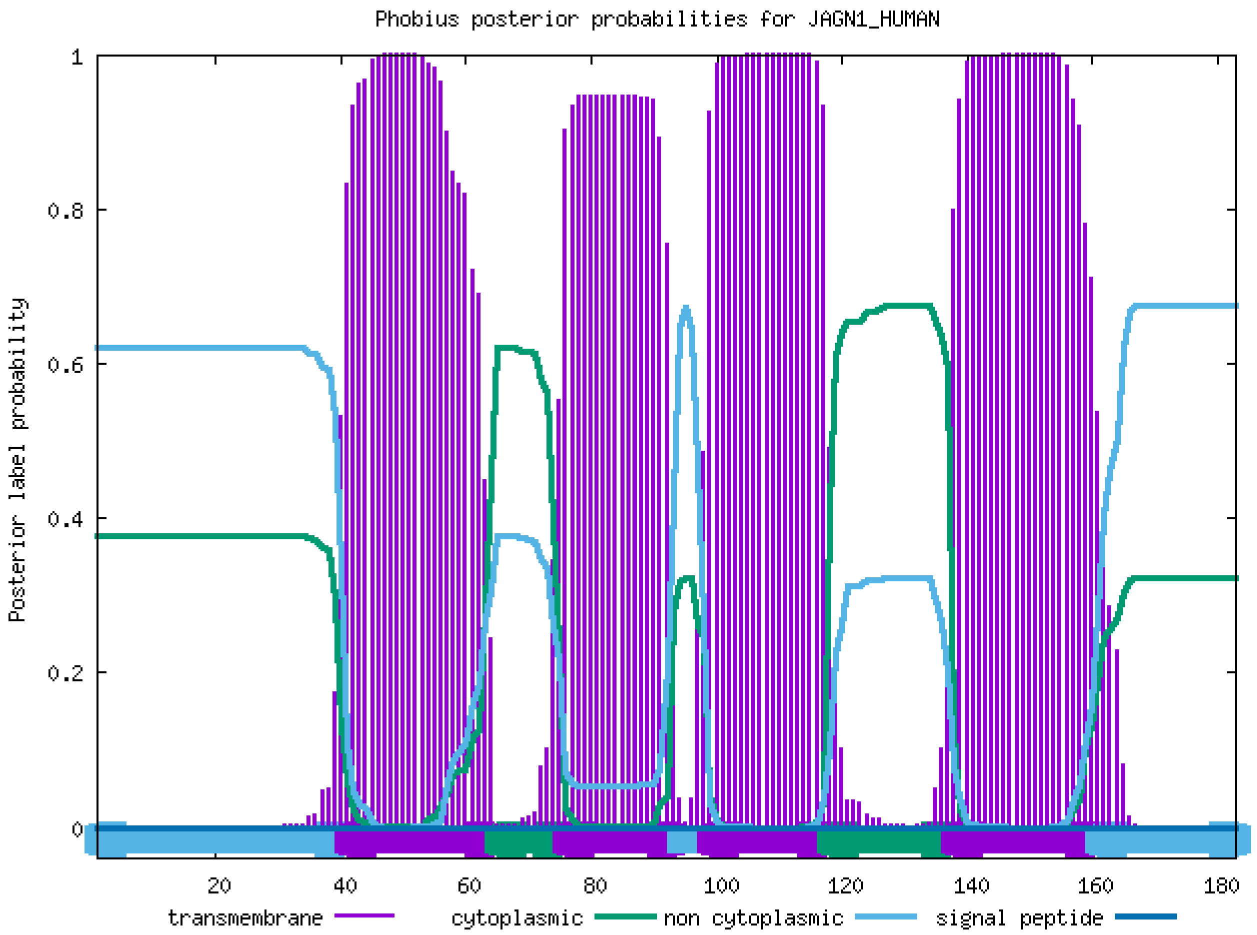

The probability data used in the plot is found here, and the gnuplot script is here.

Prediction of CD53_HUMAN

```
ID   CD53_HUMAN
FT   TOPO_DOM      1     11       CYTOPLASMIC.
FT   TRANSMEM     12     36
FT   TOPO_DOM     37     47       NON CYTOPLASMIC.
FT   TRANSMEM     48     69
FT   TOPO_DOM     70     80       CYTOPLASMIC.
FT   TRANSMEM     81    106
FT   TOPO_DOM    107    181       NON CYTOPLASMIC.
FT   TRANSMEM    182    206
FT   TOPO_DOM    207    219       CYTOPLASMIC.
//
```

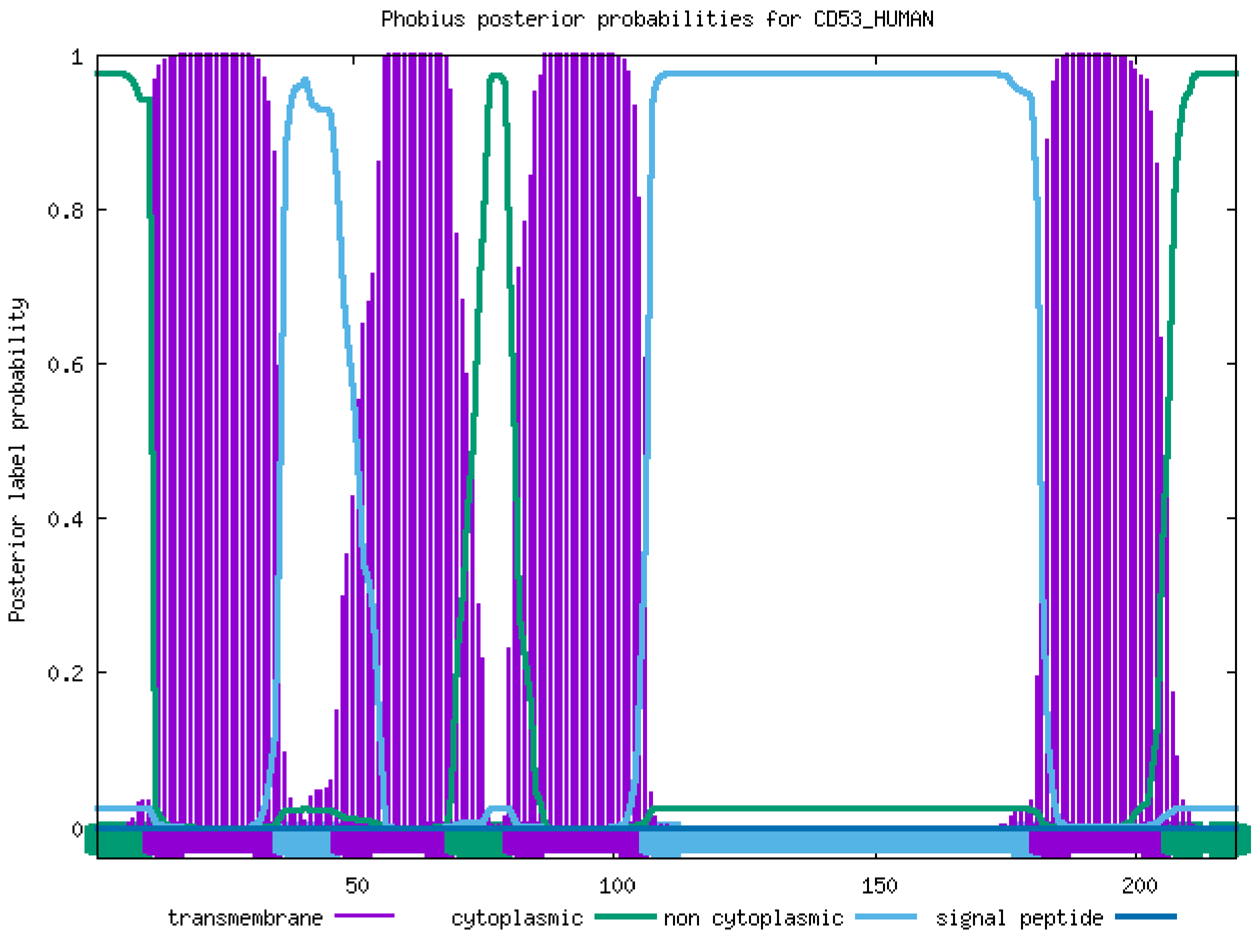

The probability data used in the plot is found here, and the gnuplot script is here.

Prediction of CD81_HUMAN

```
ID   CD81_HUMAN
FT   TOPO_DOM      1     11       CYTOPLASMIC.
FT   TRANSMEM     12     37
FT   TOPO_DOM     38     56       NON CYTOPLASMIC.
FT   TRANSMEM     57     78
FT   TOPO_DOM     79     89       CYTOPLASMIC.
FT   TRANSMEM     90    114
FT   TOPO_DOM    115    201       NON CYTOPLASMIC.
FT   TRANSMEM    202    230
FT   TOPO_DOM    231    236       CYTOPLASMIC.
//
```

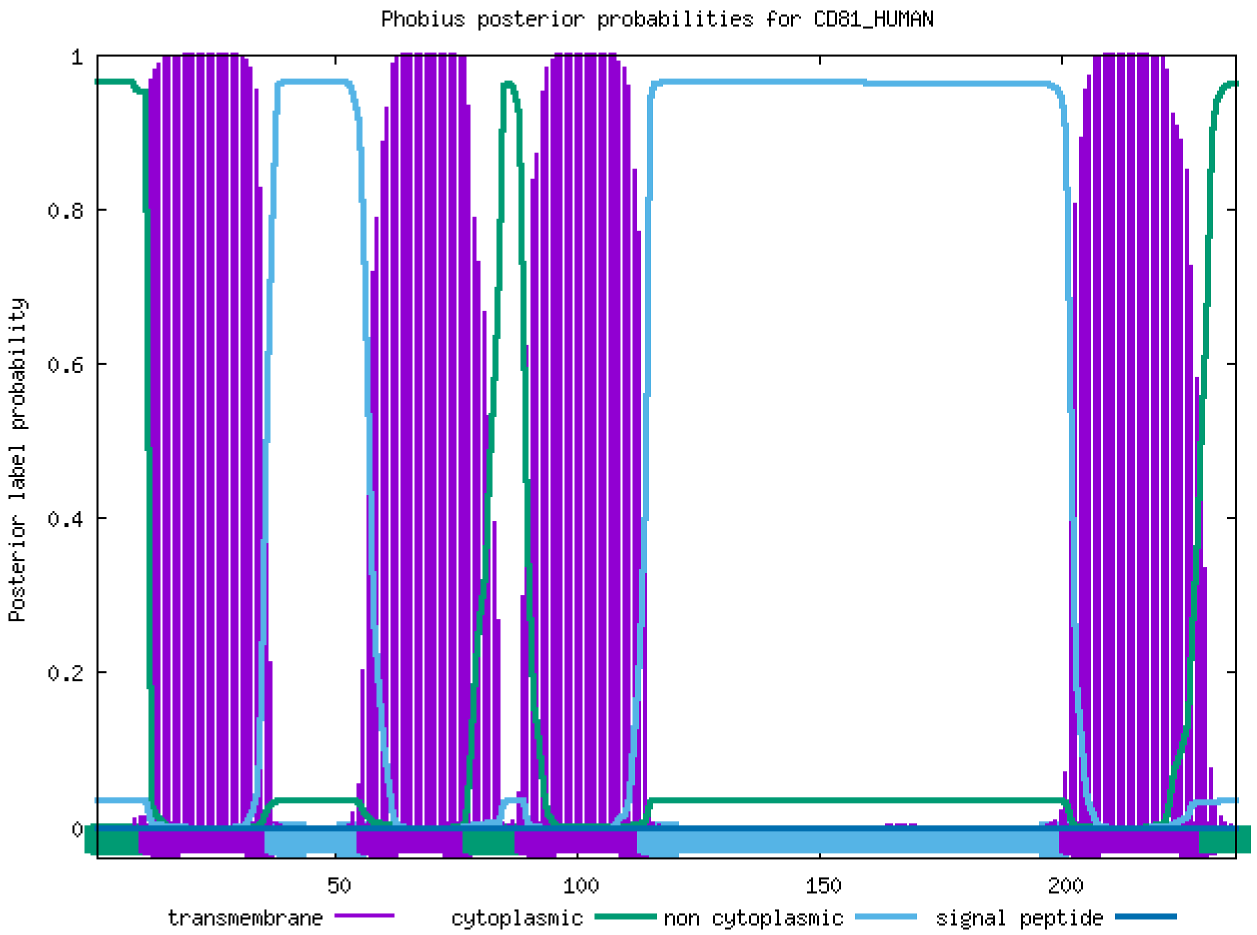

The probability data used in the plot is found here, and the gnuplot script is here.

Prediction of CD9_HUMAN

```
ID   CD9_HUMAN
FT   TOPO_DOM      1     11       CYTOPLASMIC.
FT   TRANSMEM     12     35
FT   TOPO_DOM     36     54       NON CYTOPLASMIC.
FT   TRANSMEM     55     82
FT   TOPO_DOM     83     88       CYTOPLASMIC.
FT   TRANSMEM     89    111
FT   TOPO_DOM    112    194       NON CYTOPLASMIC.
FT   TRANSMEM    195    221
FT   TOPO_DOM    222    228       CYTOPLASMIC.
//
```

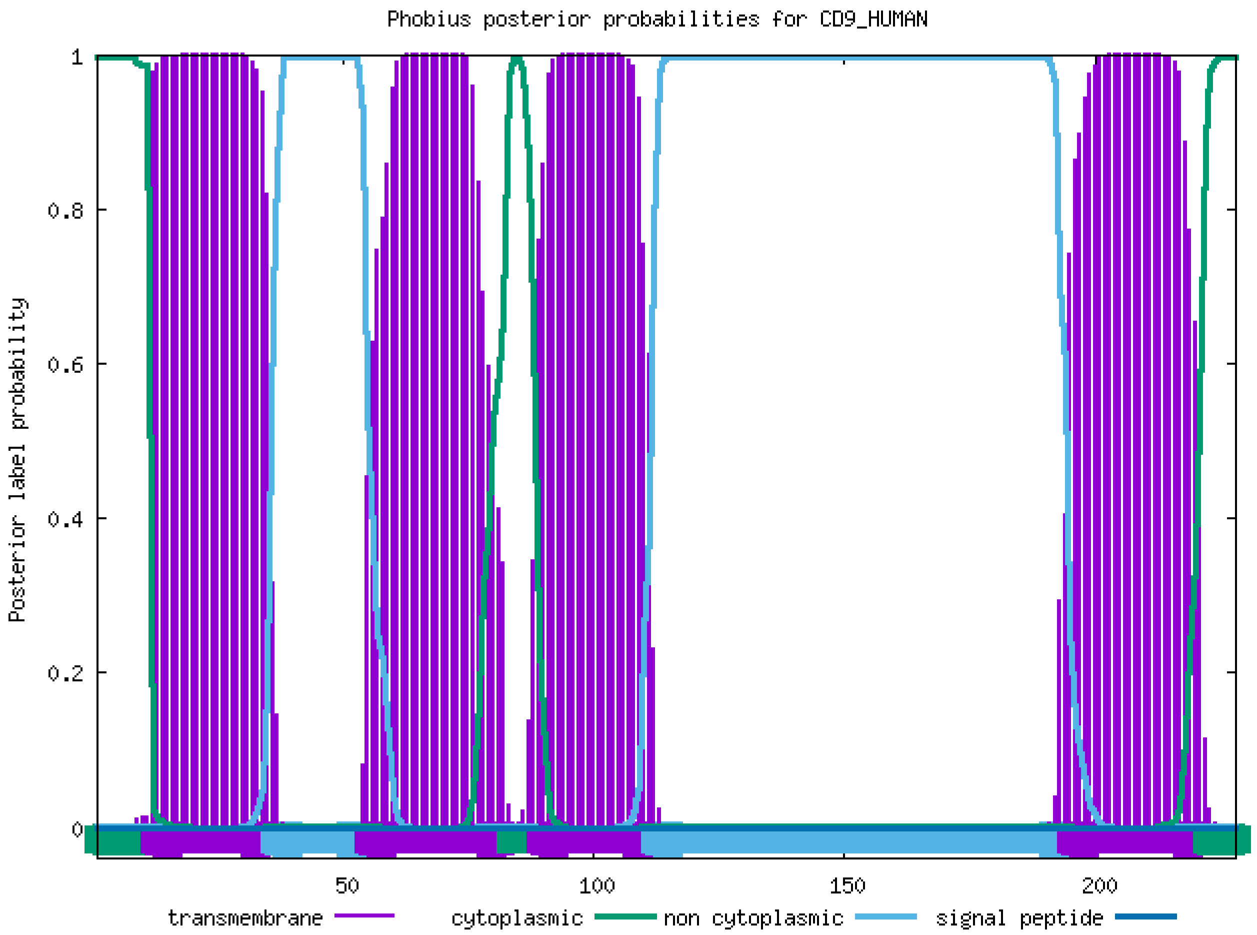

The probability data used in the plot is found here, and the gnuplot script is here.

Prediction of CD20_HUMAN

```
ID    CD20_HUMAN
FT    TOPO_DOM      1     56       CYTOPLASMIC.
FT    TRANSMEM     57     78
FT    TOPO_DOM     79     83       NON CYTOPLASMIC.
FT    TRANSMEM     84    103
FT    TOPO_DOM    104    123       CYTOPLASMIC.
FT    TRANSMEM    124    143
FT    TOPO_DOM    144    188       NON CYTOPLASMIC.
FT    TRANSMEM    189    212
FT    TOPO_DOM    213    297       CYTOPLASMIC.
//
```

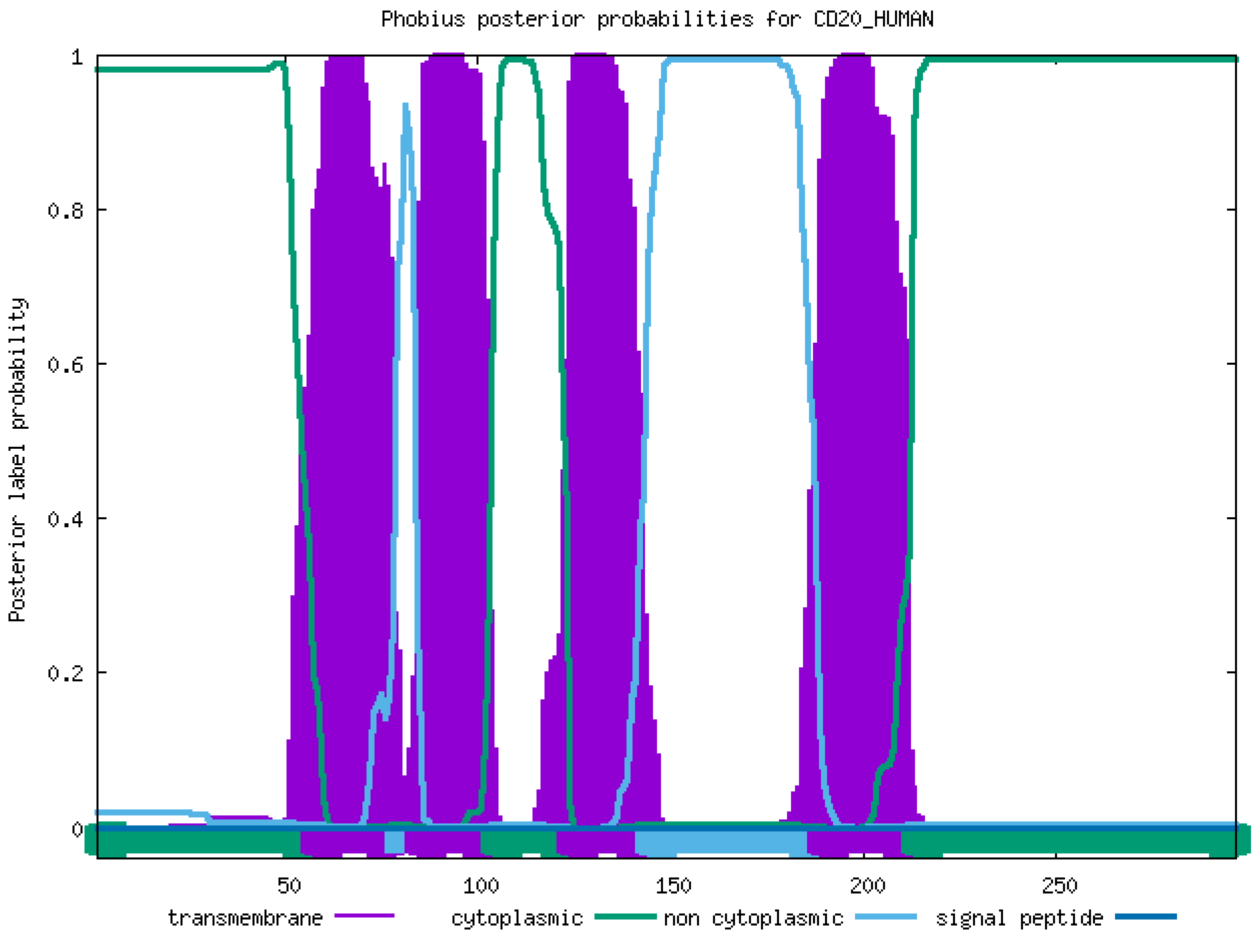

The probability data used in the plot is found here, and the gnuplot script is here.

Prediction of GLPA_HUMAN

```
ID   GLPA_HUMAN
FT   SIGNAL         1     19
FT   REGION         1      4       N-REGION.
FT   REGION         5     12       H-REGION.
FT   REGION        13     19       C-REGION.
FT   TOPO_DOM      20     91       NON CYTOPLASMIC.
FT   TRANSMEM      92    114
FT   TOPO_DOM     115    150       CYTOPLASMIC.
//
```

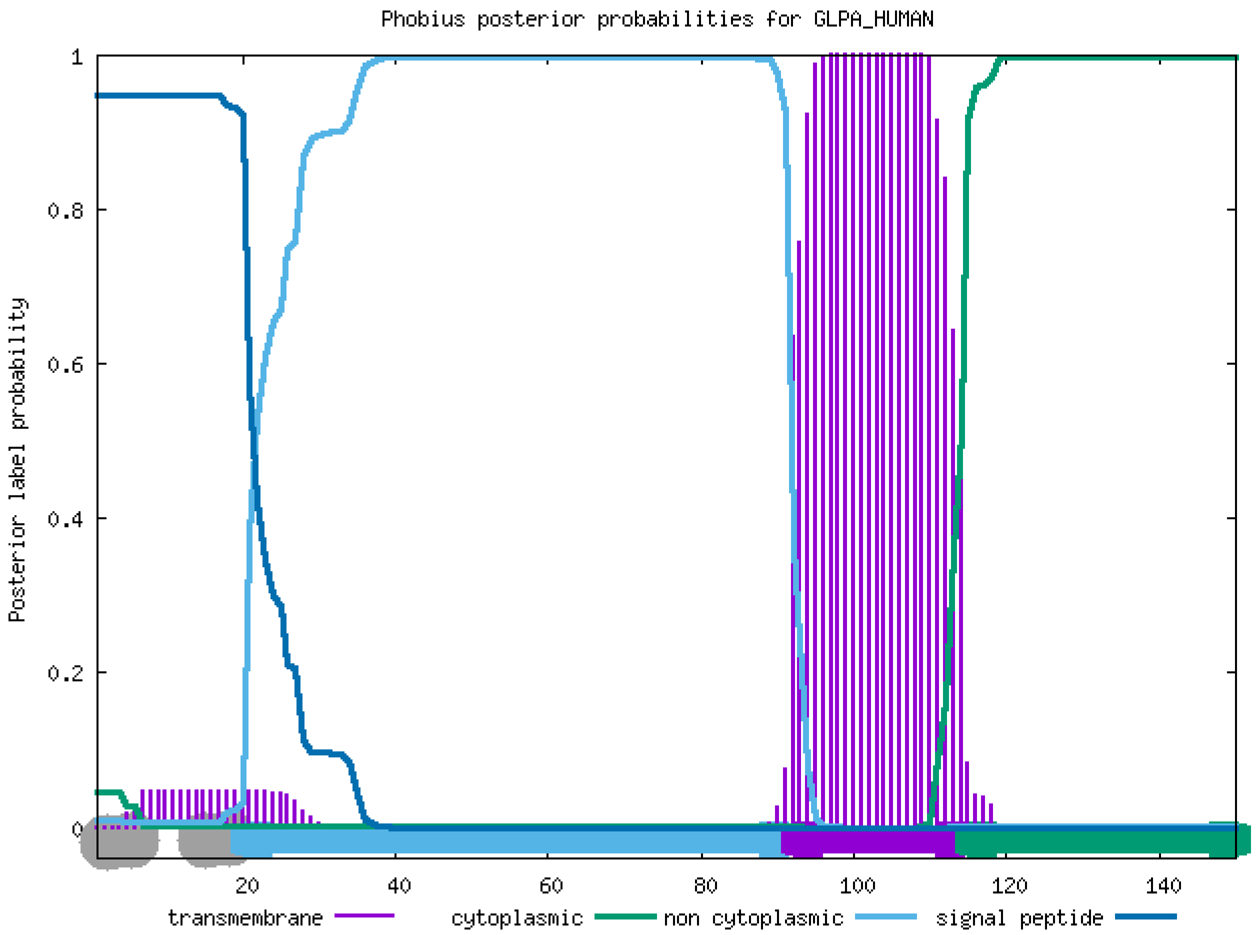

The probability data used in the plot is found here, and the gnuplot script is here.

Prediction of TNR1A_HUMAN

```
ID    TNR1A_HUMAN
FT    SIGNAL         1      29
FT    REGION         1       8       N-REGION.
FT    REGION         9      20       H-REGION.
FT    REGION        21      29       C-REGION.
FT    TOPO_DOM      30     211       NON CYTOPLASMIC.
FT    TRANSMEM     212     234
FT    TOPO_DOM     235     455       CYTOPLASMIC.
//
```

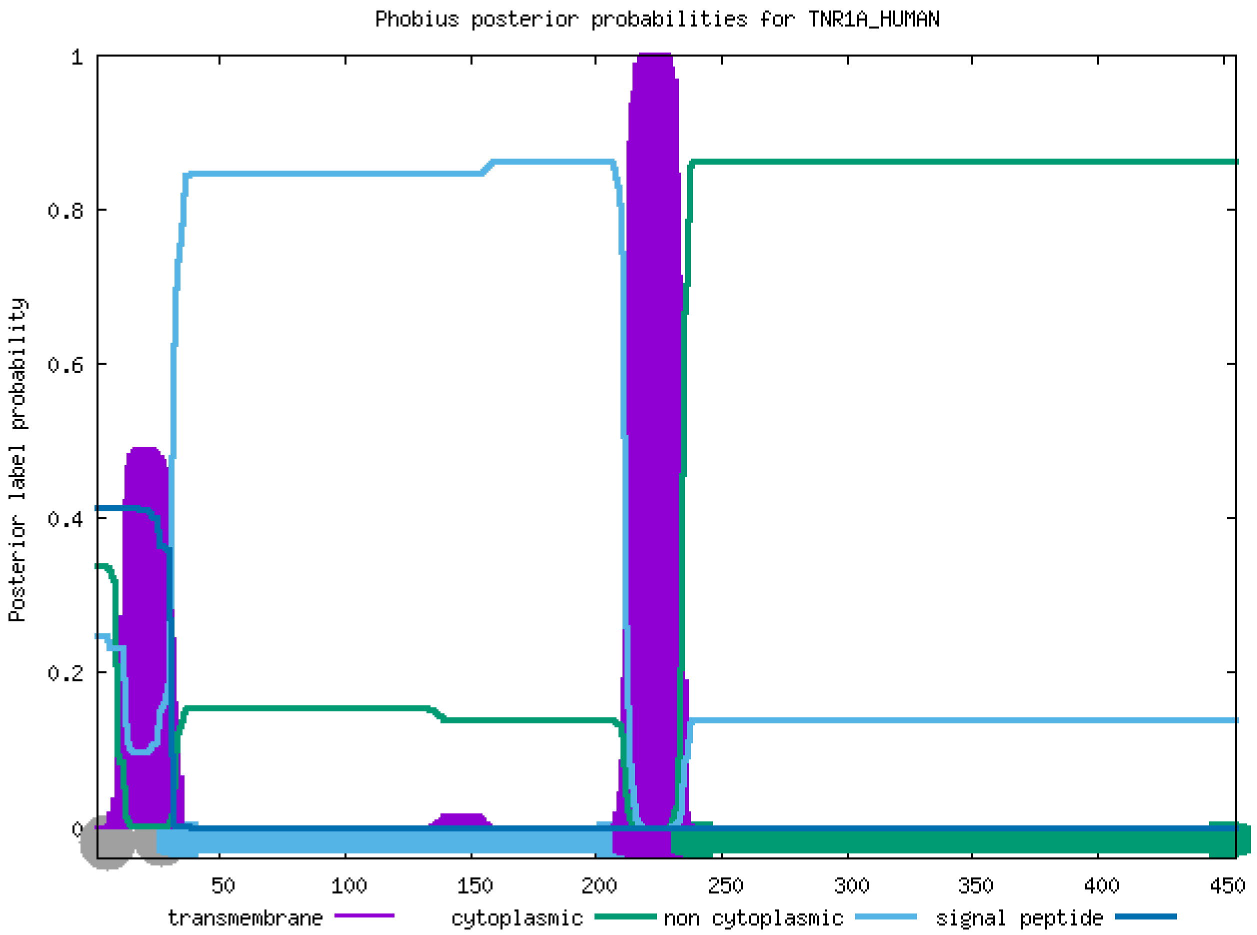

The probability data used in the plot is found here, and the gnuplot script is here.

Prediction of CD79A_HUMAN

```
ID   CD79A_HUMAN
FT   SIGNAL         1      26
FT   REGION         1       9       N-REGION.
FT   REGION        10      21       H-REGION.
FT   REGION        22      26       C-REGION.
FT   TOPO_DOM      27     143       NON CYTOPLASMIC.
FT   TRANSMEM     144     165
FT   TOPO_DOM     166     226       CYTOPLASMIC.
//
```

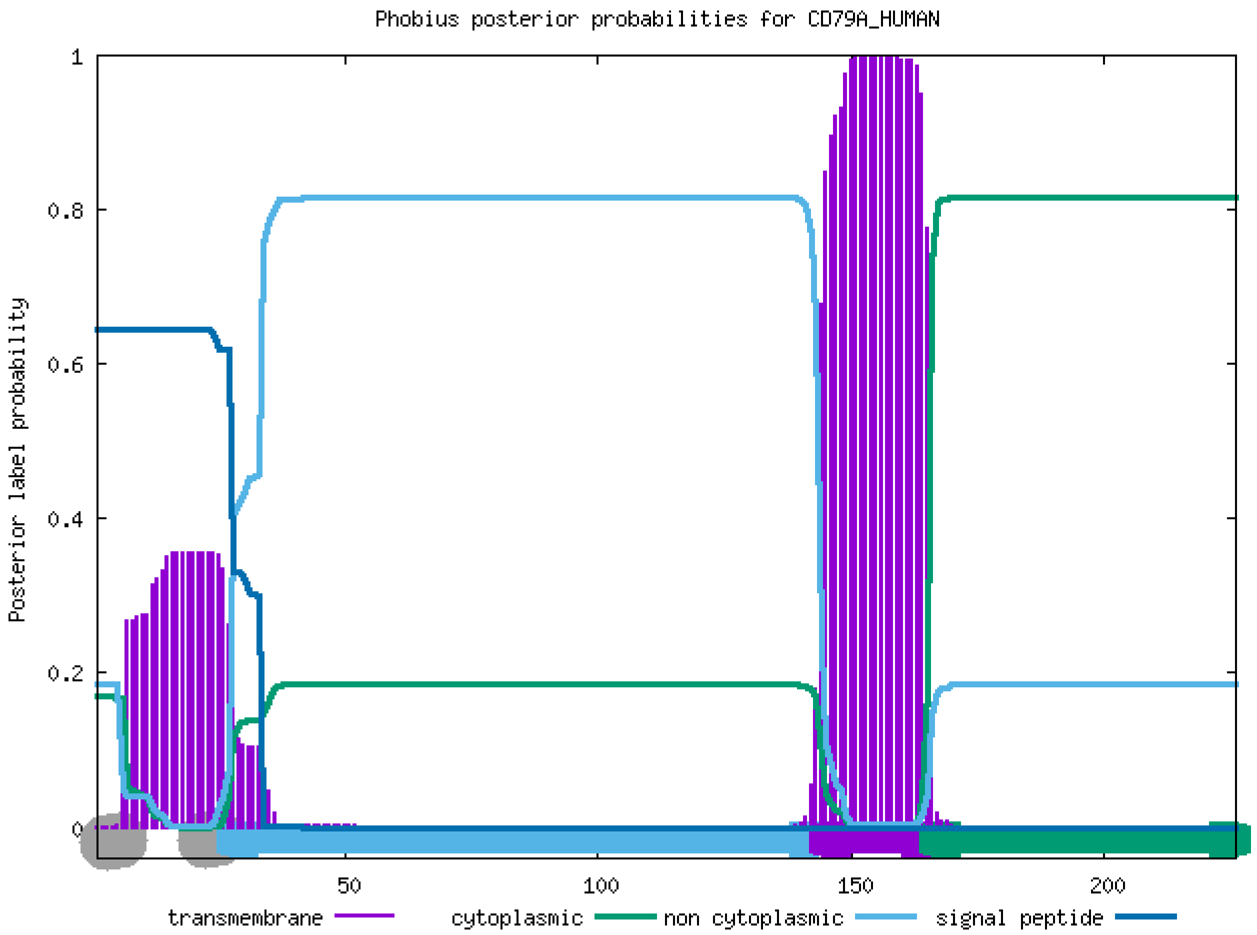

The probability data used in the plot is found here, and the gnuplot script is here.

Prediction of CD79B_HUMAN

```
ID   CD79B_HUMAN
FT   SIGNAL        1     31
FT   REGION        1     14       N-REGION.
FT   REGION       15     23       H-REGION.
FT   REGION       24     31       C-REGION.
FT   TOPO_DOM     32    159       NON CYTOPLASMIC.
FT   TRANSMEM    160    181
FT   TOPO_DOM    182    229       CYTOPLASMIC.
//
```

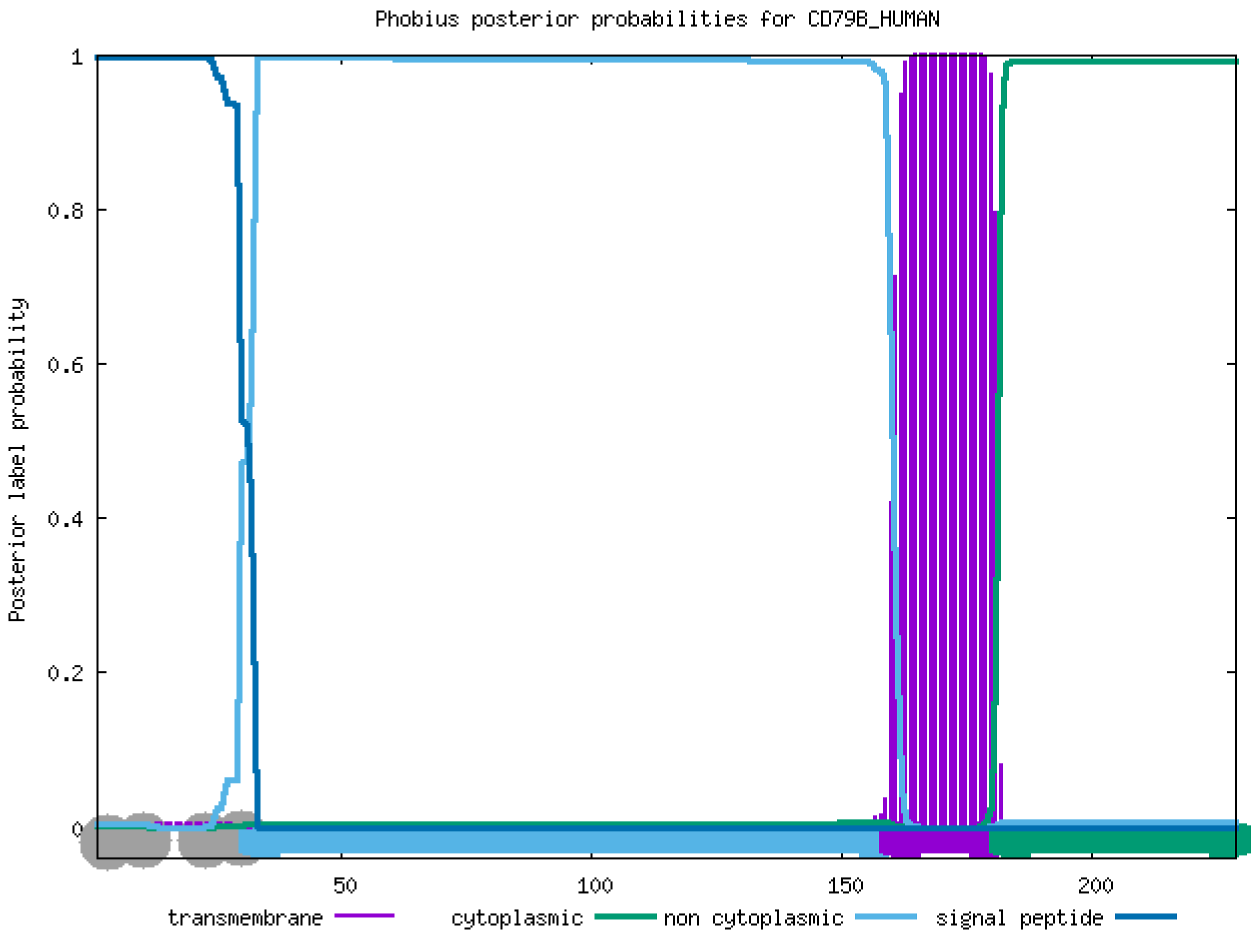

The probability data used in the plot is found here, and the gnuplot script is here.

Prediction of INSR_HUMAN

```
ID   INSR_HUMAN
FT   SIGNAL          1     27
FT   REGION          1      7       N-REGION.
FT   REGION          8     23       H-REGION.
FT   REGION         24     27       C-REGION.
FT   TOPO_DOM       28    957       NON CYTOPLASMIC.
FT   TRANSMEM      958    979
FT   TOPO_DOM      980   1382       CYTOPLASMIC.
//
```

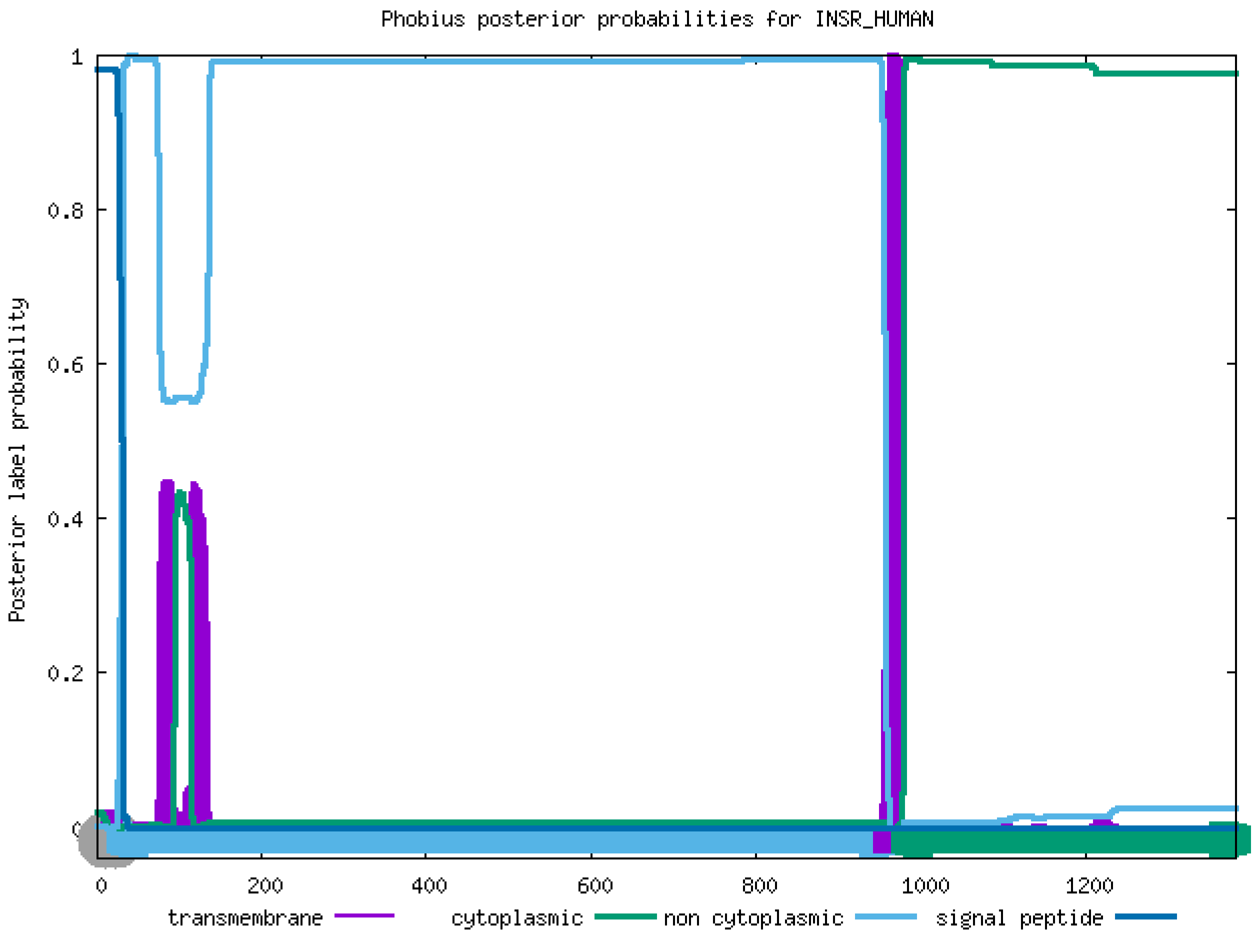

The probability data used in the plot is found here, and the gnuplot script is here.

Prediction of ERBB2_HUMAN

```
ID   ERBB2_HUMAN
FT   SIGNAL         1      21
FT   REGION         1       2       N-REGION.
FT   REGION         3      16       H-REGION.
FT   REGION        17      21       C-REGION.
FT   TOPO_DOM      22     653       NON CYTOPLASMIC.
FT   TRANSMEM     654     675
FT   TOPO_DOM     676    1255       CYTOPLASMIC.
//
```

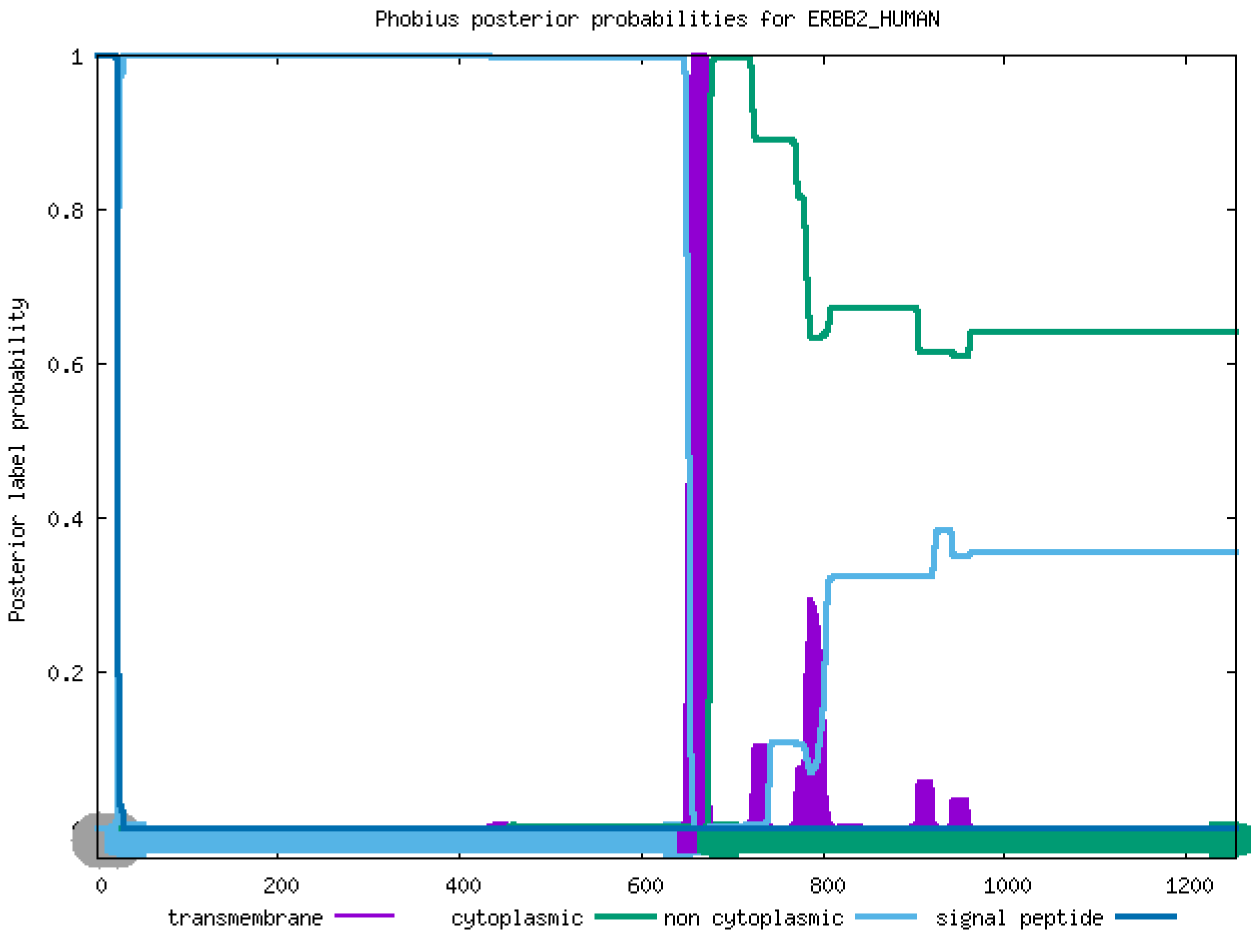

The probability data used in the plot is found here, and the gnuplot script is here.

Prediction of EGFR_HUMAN

```
ID   EGFR_HUMAN
FT   SIGNAL        1     24
FT   REGION        1      7       N-REGION.
FT   REGION        8     19       H-REGION.
FT   REGION       20     24       C-REGION.
FT   TOPO_DOM     25    645       NON CYTOPLASMIC.
FT   TRANSMEM    646    667
FT   TOPO_DOM    668   1210       CYTOPLASMIC.
//
```

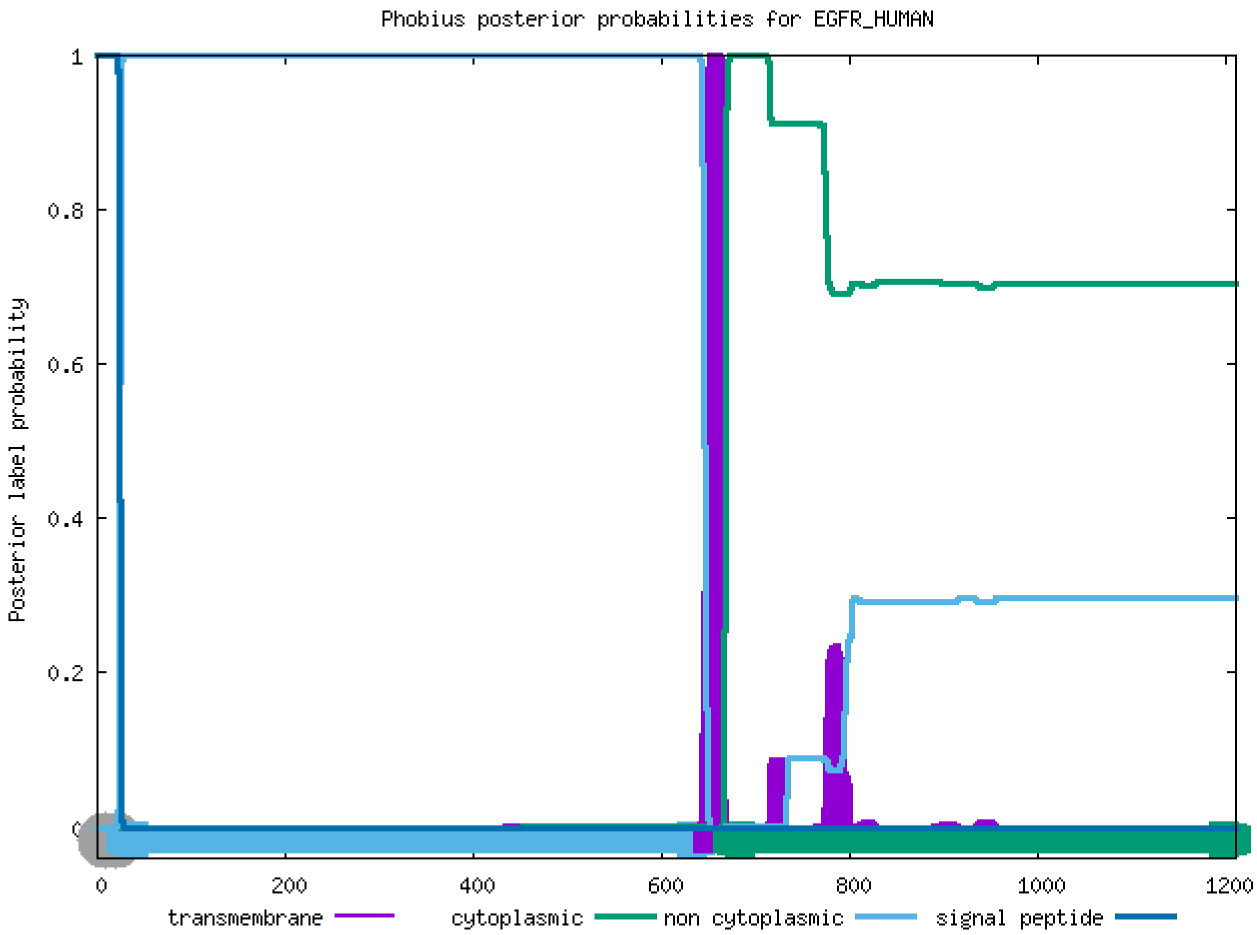

The probability data used in the plot is found here, and the gnuplot script is here.

Prediction of ERBB3_HUMAN

```
ID   ERBB3_HUMAN
FT   SIGNAL         1     19
FT   REGION         1      5       N-REGION.
FT   REGION         6     14       H-REGION.
FT   REGION        15     19       C-REGION.
FT   TOPO_DOM      20    641       NON CYTOPLASMIC.
FT   TRANSMEM     642    666
FT   TOPO_DOM     667   1342       CYTOPLASMIC.
//
```

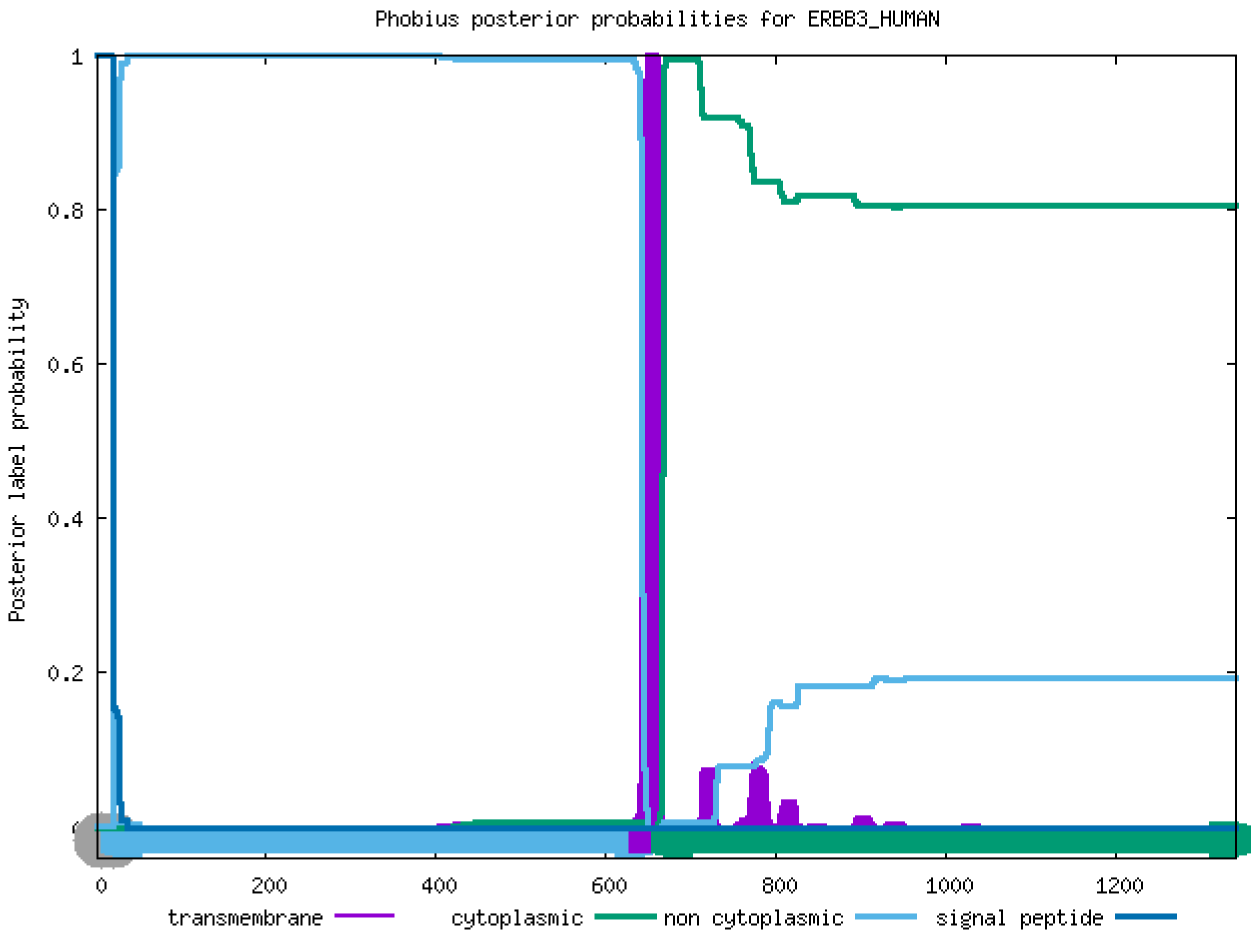

The probability data used in the plot is found here, and the gnuplot script is here.

Prediction of ERBB4_HUMAN

```
ID   ERBB4_HUMAN
FT   SIGNAL        1     22
FT   REGION        1      3       N-REGION.
FT   REGION        4     15       H-REGION.
FT   REGION       16     22       C-REGION.
FT   TOPO_DOM     23    651       NON CYTOPLASMIC.
FT   TRANSMEM    652    675
FT   TOPO_DOM    676   1308       CYTOPLASMIC.
//
```

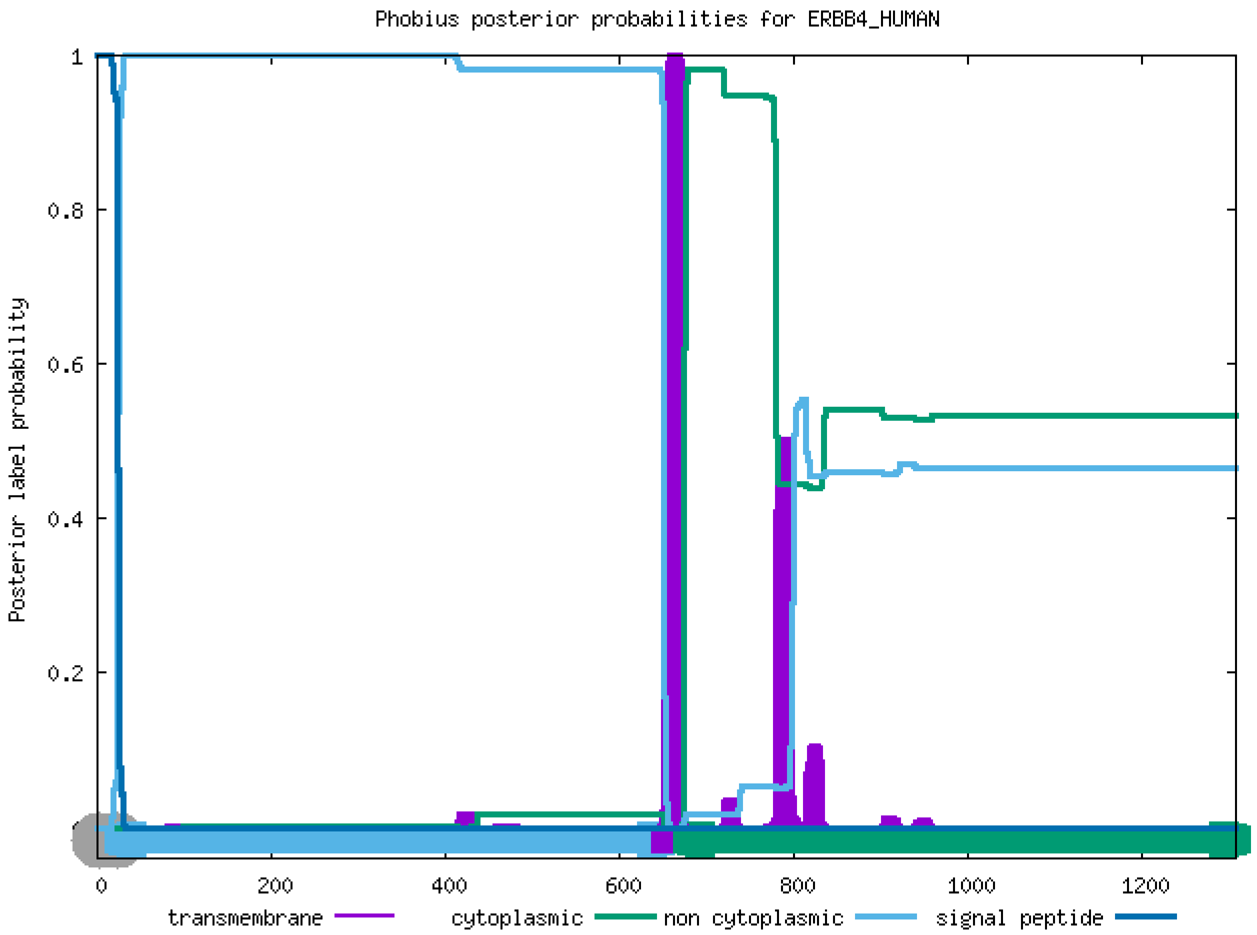

The probability data used in the plot is found here, and the gnuplot script is here.

Prediction of EPHA1_HUMAN

```
ID   EPHA1_HUMAN
FT   SIGNAL        1     25
FT   REGION        1      6       N-REGION.
FT   REGION        7     17       H-REGION.
FT   REGION       18     25       C-REGION.
FT   TOPO_DOM     26    547       NON CYTOPLASMIC.
FT   TRANSMEM    548    568
FT   TOPO_DOM    569    976       CYTOPLASMIC.
//
```

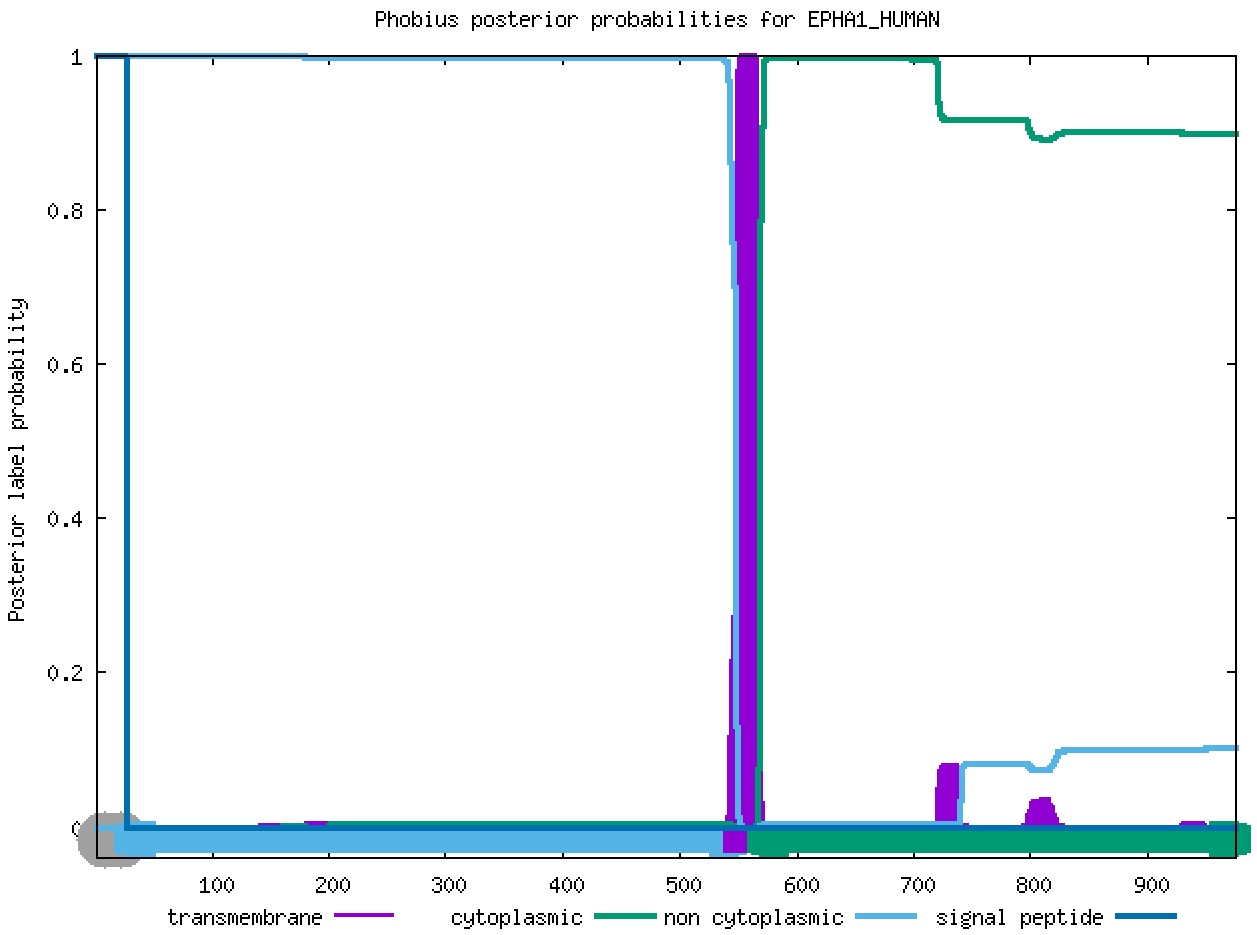

The probability data used in the plot is found here, and the gnuplot script is here.

Prediction of EPHA2_HUMAN

```
ID   EPHA2_HUMAN
FT   SIGNAL        1     26
FT   REGION        1      7       N-REGION.
FT   REGION        8     19       H-REGION.
FT   REGION       20     26       C-REGION.
FT   TOPO_DOM     27    534       NON CYTOPLASMIC.
FT   TRANSMEM    535    558
FT   TOPO_DOM    559    976       CYTOPLASMIC.
//
```

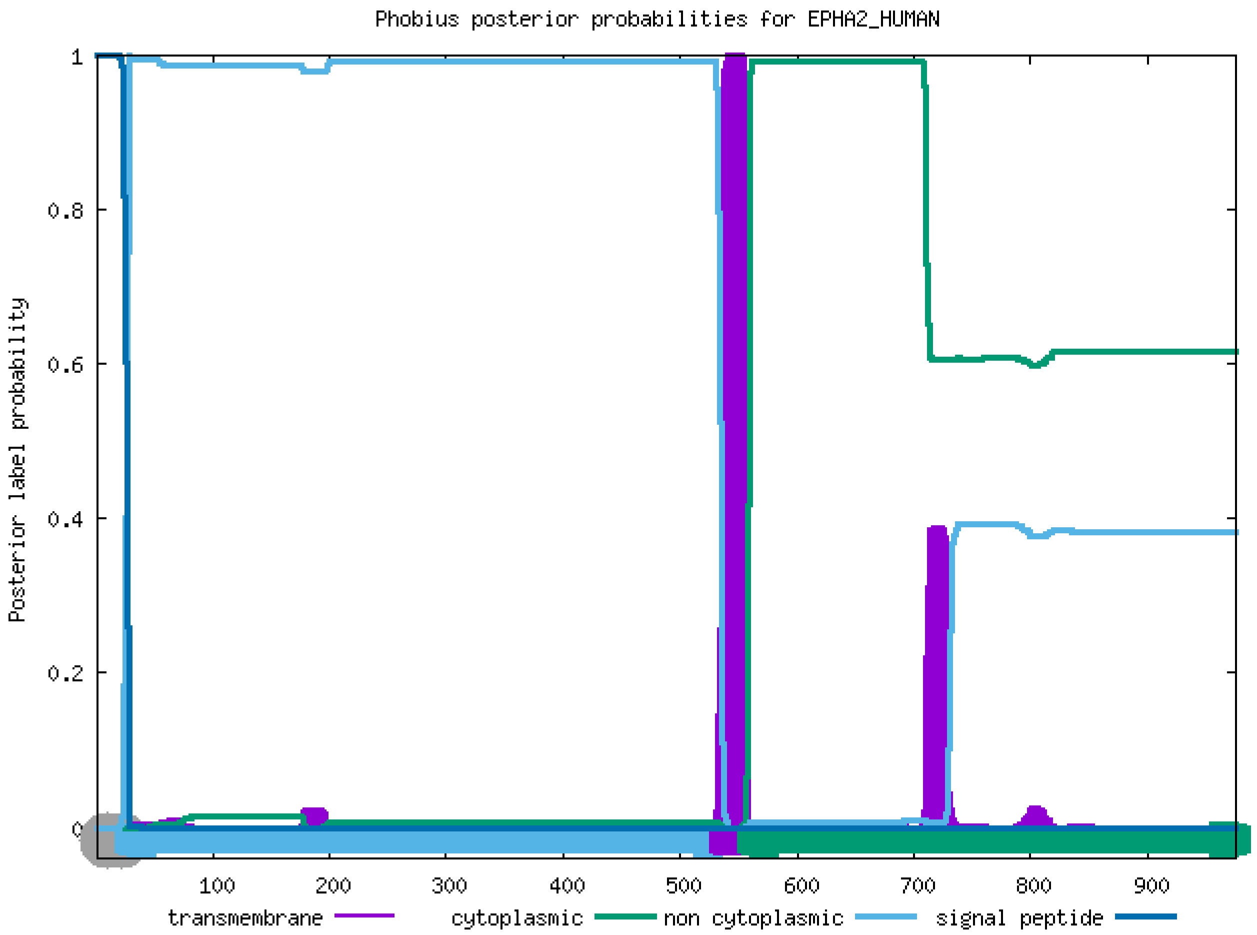

The probability data used in the plot is found here, and the gnuplot script is here.

Prediction of FGFR3_HUMAN

```
ID   FGFR3_HUMAN
FT   SIGNAL        1     22
FT   REGION        1      5       N-REGION.
FT   REGION        6     17       H-REGION.
FT   REGION       18     22       C-REGION.
FT   TOPO_DOM     23    371       NON CYTOPLASMIC.
FT   TRANSMEM    372    396
FT   TOPO_DOM    397    806       CYTOPLASMIC.
//
```

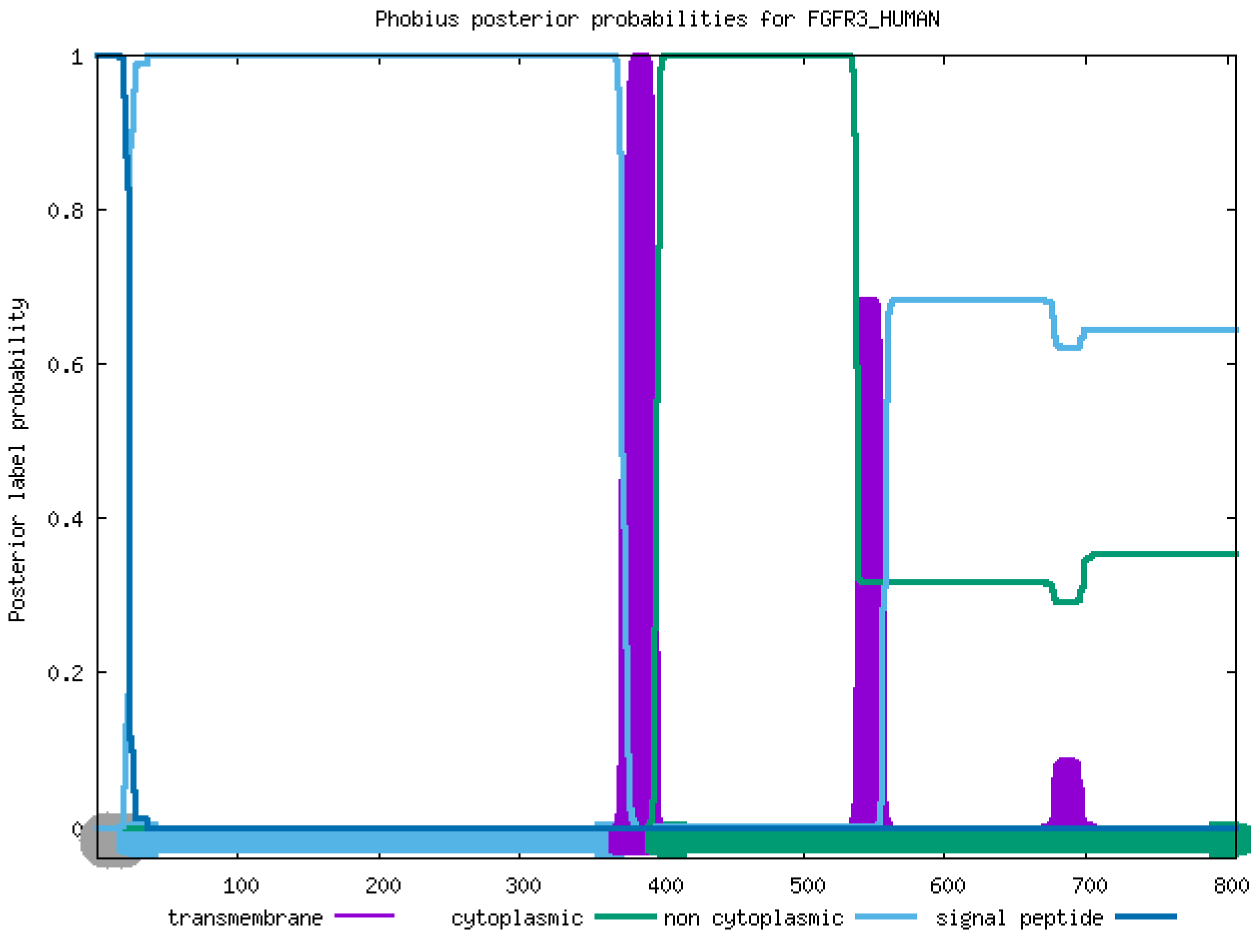

The probability data used in the plot is found here, and the gnuplot script is here.

Prediction of VGFR2_HUMAN

```
ID   VGFR2_HUMAN
FT   SIGNAL         1     19
FT   REGION         1      4       N-REGION.
FT   REGION         5     15       H-REGION.
FT   REGION        16     19       C-REGION.
FT   TOPO_DOM      20    764       NON CYTOPLASMIC.
FT   TRANSMEM     765    786
FT   TOPO_DOM     787   1356       CYTOPLASMIC.
//
```

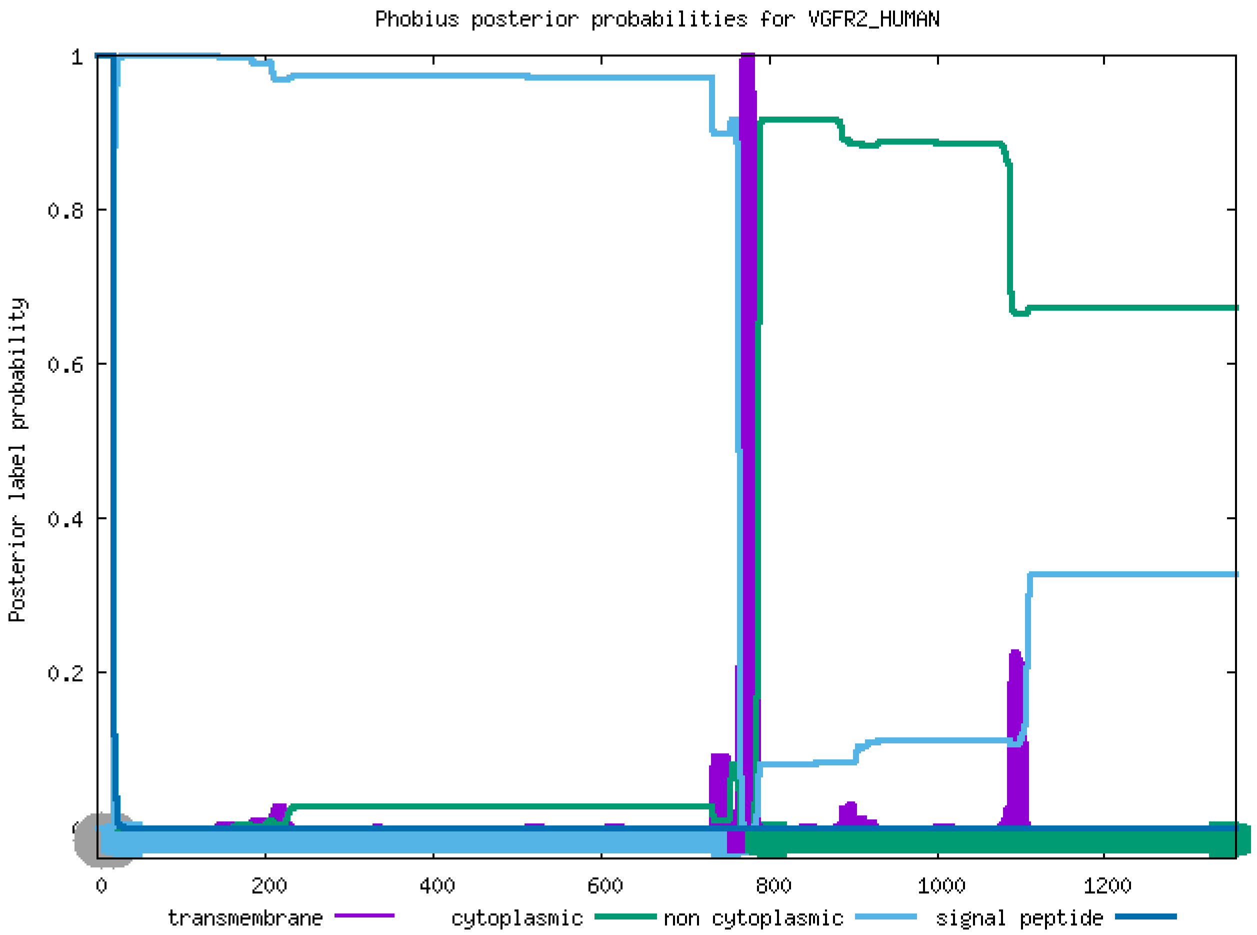

The probability data used in the plot is found here, and the gnuplot script is here.

Prediction of ITA2B_HUMAN

```
ID   ITA2B_HUMAN
FT   SIGNAL         1     31
FT   REGION         1      3       N-REGION.
FT   REGION         4     22       H-REGION.
FT   REGION        23     31       C-REGION.
FT   TOPO_DOM      32    996       NON CYTOPLASMIC.
FT   TRANSMEM     997   1019
FT   TOPO_DOM    1020   1039       CYTOPLASMIC.
//
```

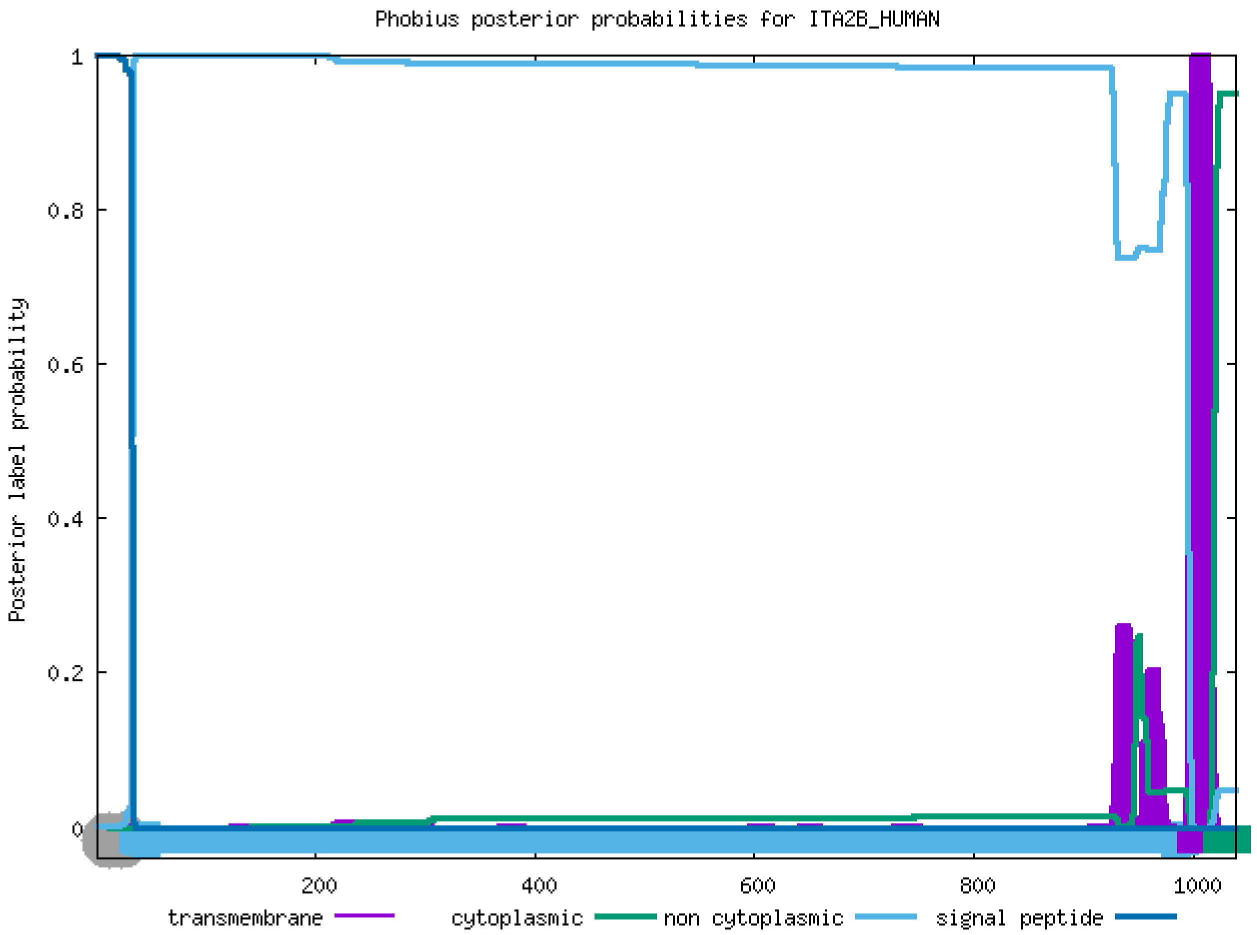

The probability data used in the plot is found here, and the gnuplot script is here.

Prediction of ITB3_HUMAN

```
ID   ITB3_HUMAN
FT   SIGNAL        1     26
FT   REGION        1      9       N-REGION.
FT   REGION       10     21       H-REGION.
FT   REGION       22     26       C-REGION.
FT   TOPO_DOM     27    718       NON CYTOPLASMIC.
FT   TRANSMEM    719    741
FT   TOPO_DOM    742    788       CYTOPLASMIC.
//
```

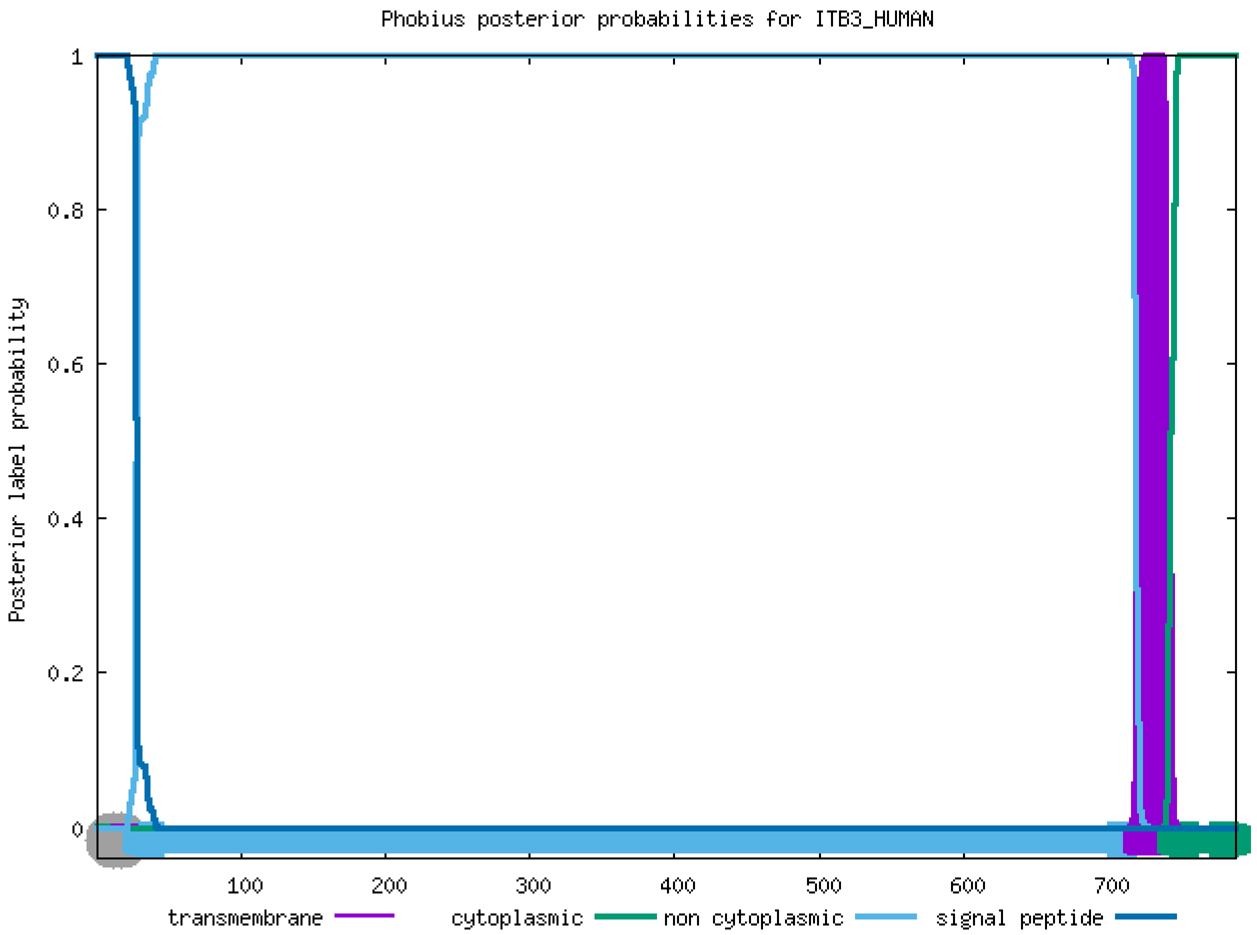

The probability data used in the plot is found here, and the gnuplot script is here.

Prediction of ITAV_HUMAN

```
ID   ITAV_HUMAN
FT   SIGNAL         1     30
FT   REGION         1     14       N-REGION.
FT   REGION        15     25       H-REGION.
FT   REGION        26     30       C-REGION.
FT   TOPO_DOM      31    993       NON CYTOPLASMIC.
FT   TRANSMEM     994   1016
FT   TOPO_DOM    1017   1048       CYTOPLASMIC.
//
```

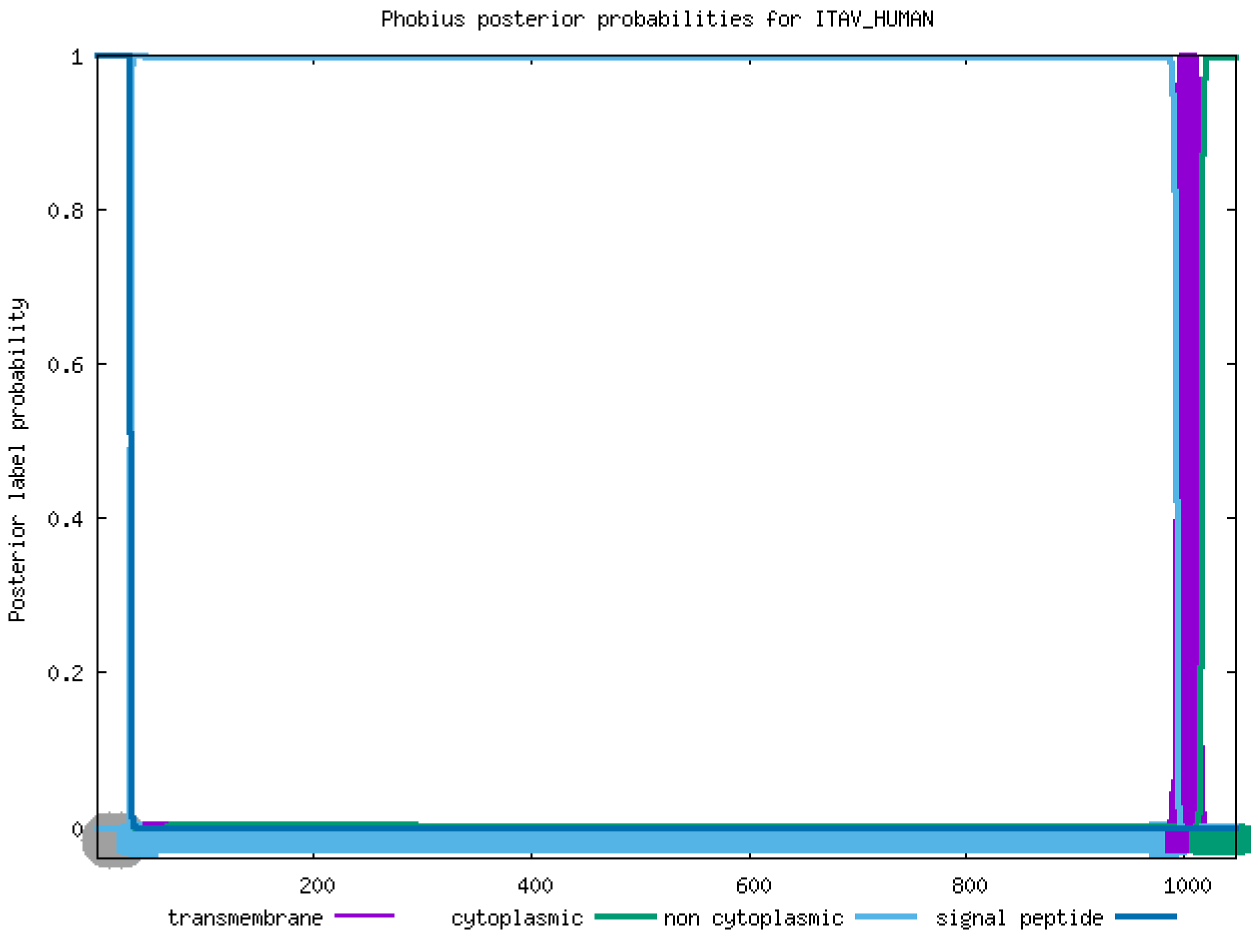

The probability data used in the plot is found here, and the gnuplot script is here.

Prediction of ITB6_HUMAN

```
ID    ITB6_HUMAN
FT    SIGNAL          1      21
FT    REGION          1       4       N-REGION.
FT    REGION          5      13       H-REGION.
FT    REGION         14      21       C-REGION.
FT    TOPO_DOM       22     709       NON CYTOPLASMIC.
FT    TRANSMEM      710     734
FT    TOPO_DOM      735     788       CYTOPLASMIC.
//
```

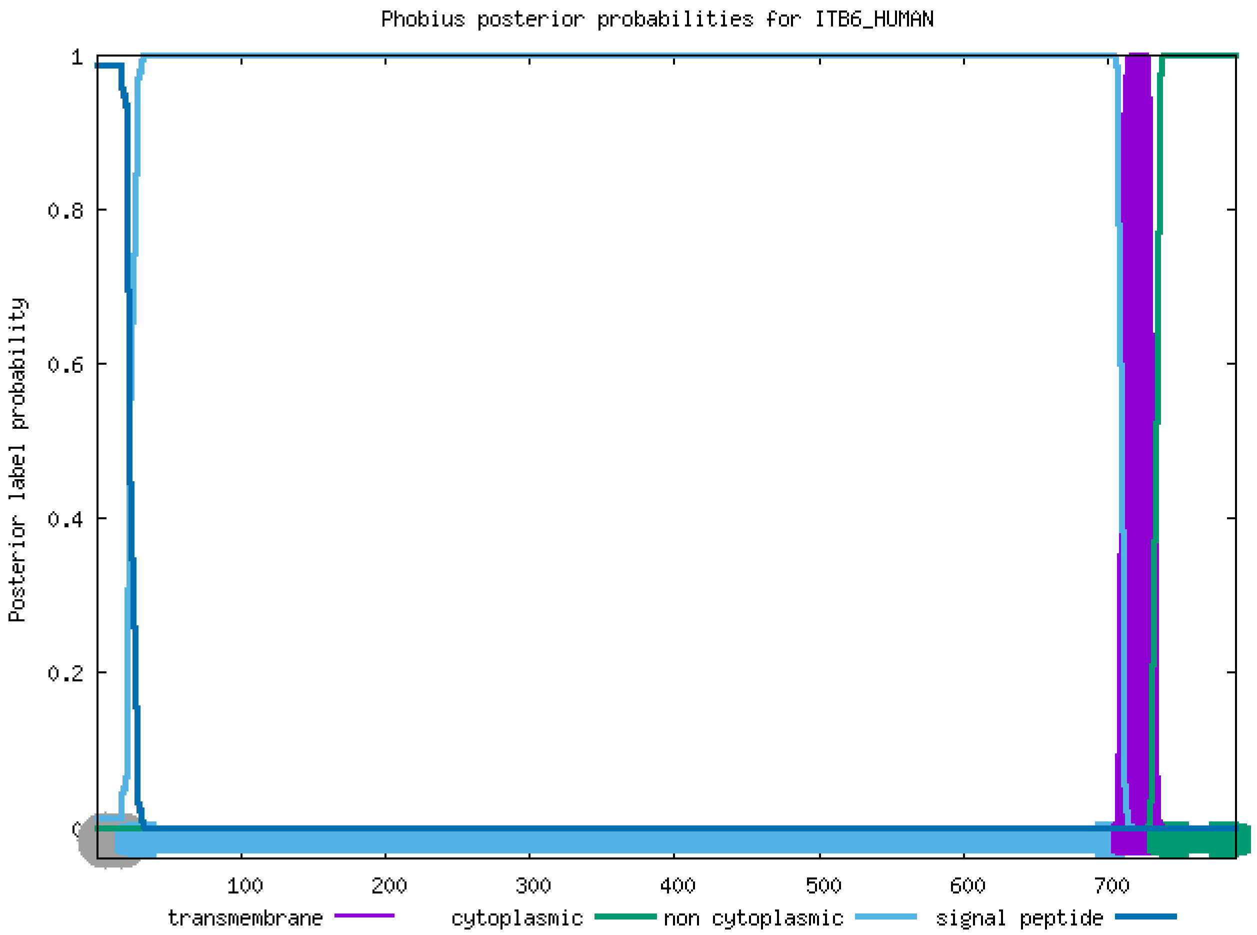

The probability data used in the plot is found here, and the gnuplot script is here.

Prediction of ITB8_HUMAN

```
ID   ITB8_HUMAN
FT   SIGNAL        1      34
FT   REGION        1       4       N-REGION.
FT   REGION        5      16       H-REGION.
FT   REGION       17      34       C-REGION.
FT   TOPO_DOM     35     681       NON CYTOPLASMIC.
FT   TRANSMEM    682     703
FT   TOPO_DOM    704     769       CYTOPLASMIC.
//
```

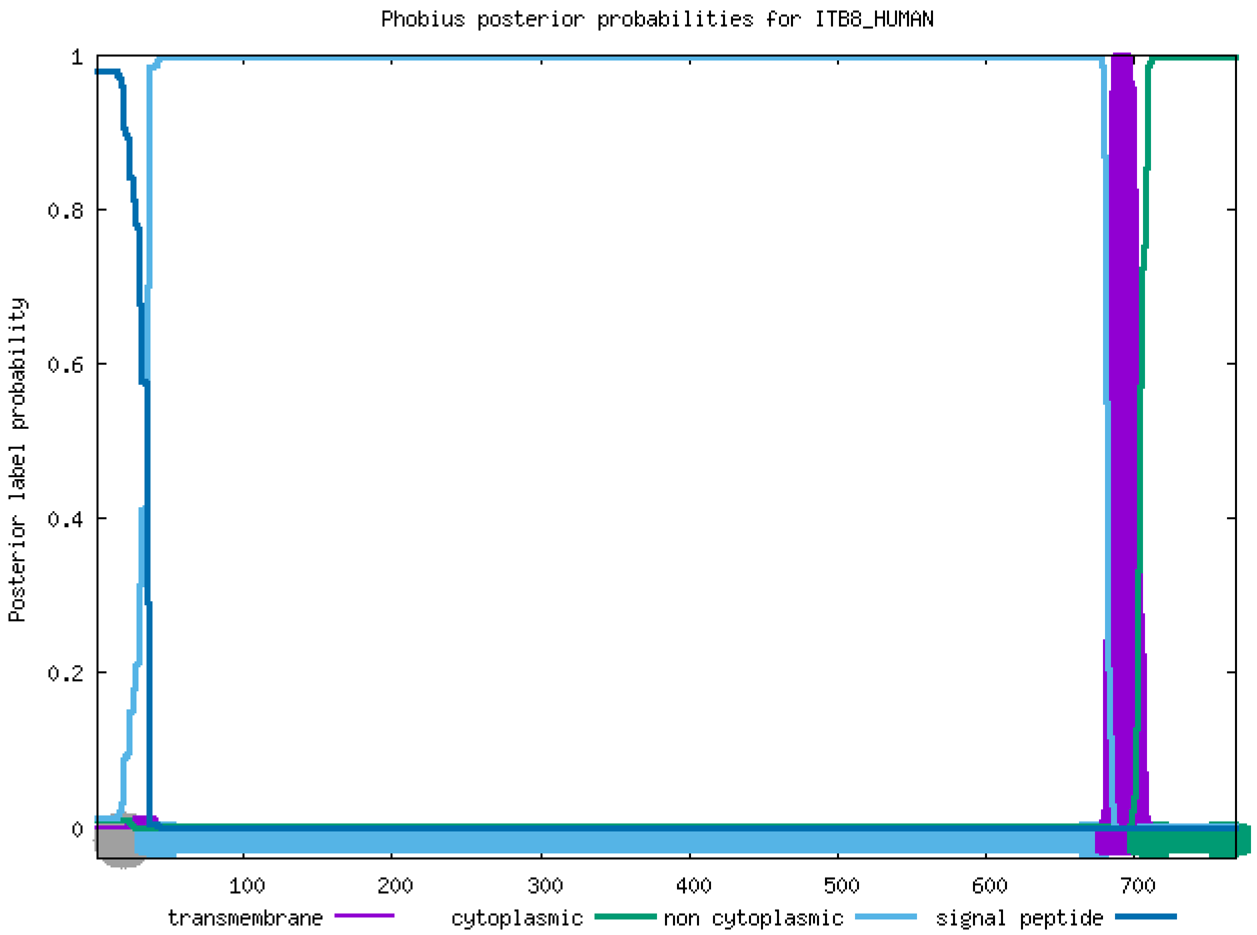

The probability data used in the plot is found here, and the gnuplot script is here.

Prediction of PAQR1_HUMAN

```
ID    PAQR1_HUMAN
FT    TOPO_DOM      1     137       CYTOPLASMIC.
FT    TRANSMEM    138     159
FT    TOPO_DOM    160     170       NON CYTOPLASMIC.
FT    TRANSMEM    171     195
FT    TOPO_DOM    196     206       CYTOPLASMIC.
FT    TRANSMEM    207     225
FT    TOPO_DOM    226     236       NON CYTOPLASMIC.
FT    TRANSMEM    237     256
FT    TOPO_DOM    257     267       CYTOPLASMIC.
FT    TRANSMEM    268     288
FT    TOPO_DOM    289     299       NON CYTOPLASMIC.
FT    TRANSMEM    300     319
FT    TOPO_DOM    320     338       CYTOPLASMIC.
FT    TRANSMEM    339     356
FT    TOPO_DOM    357     375       NON CYTOPLASMIC.
//
```

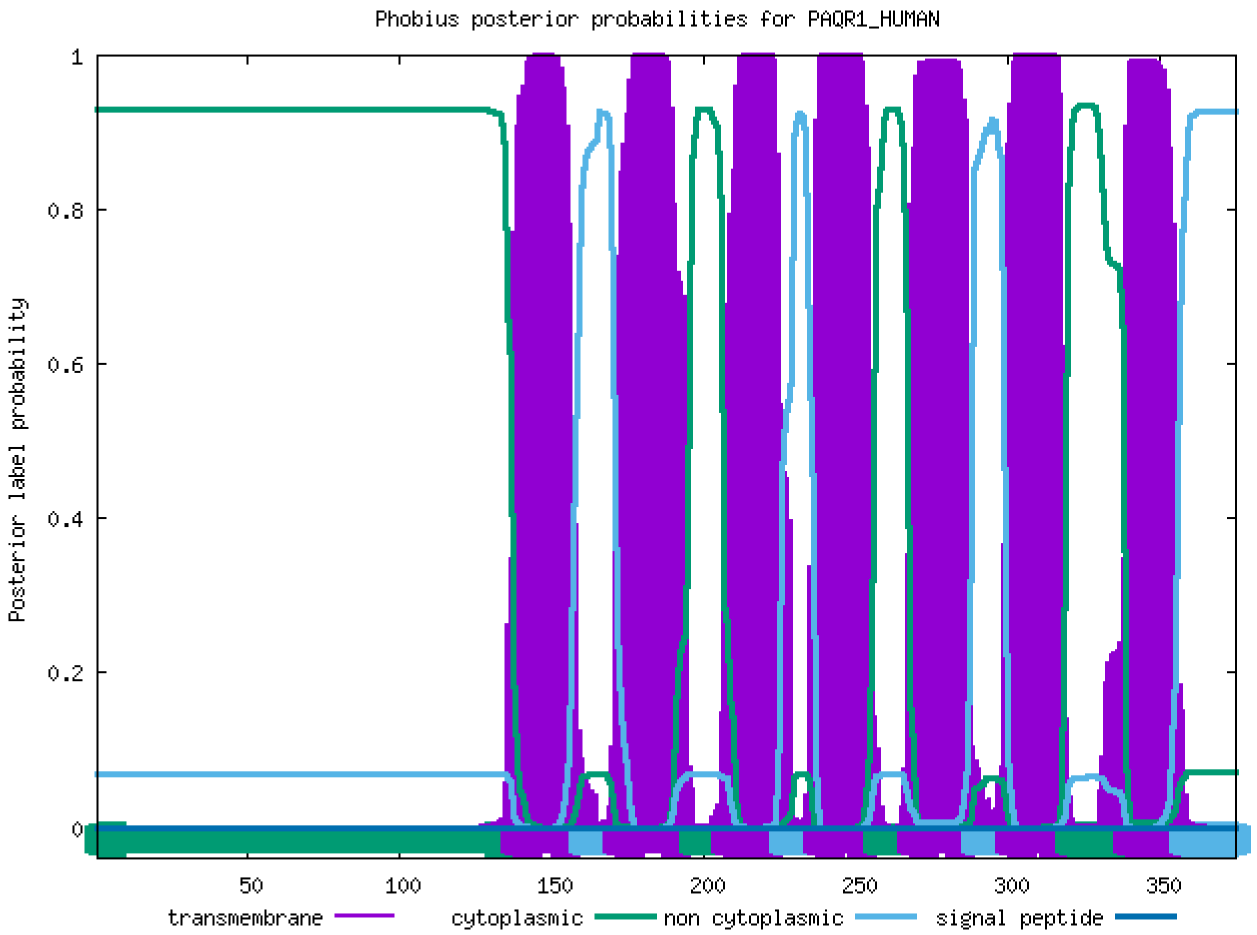

The probability data used in the plot is found here, and the gnuplot script is here.

Prediction of PAQR2_HUMAN

```
ID   PAQR2_HUMAN
FT   TOPO_DOM      1    148       NON CYTOPLASMIC.
FT   TRANSMEM    149    169
FT   TOPO_DOM    170    180       CYTOPLASMIC.
FT   TRANSMEM    181    200
FT   TOPO_DOM    201    219       NON CYTOPLASMIC.
FT   TRANSMEM    220    239
FT   TOPO_DOM    240    245       CYTOPLASMIC.
FT   TRANSMEM    246    266
FT   TOPO_DOM    267    277       NON CYTOPLASMIC.
FT   TRANSMEM    278    299
FT   TOPO_DOM    300    310       CYTOPLASMIC.
FT   TRANSMEM    311    330
FT   TOPO_DOM    331    349       NON CYTOPLASMIC.
FT   TRANSMEM    350    366
FT   TOPO_DOM    367    386       CYTOPLASMIC.
//
```

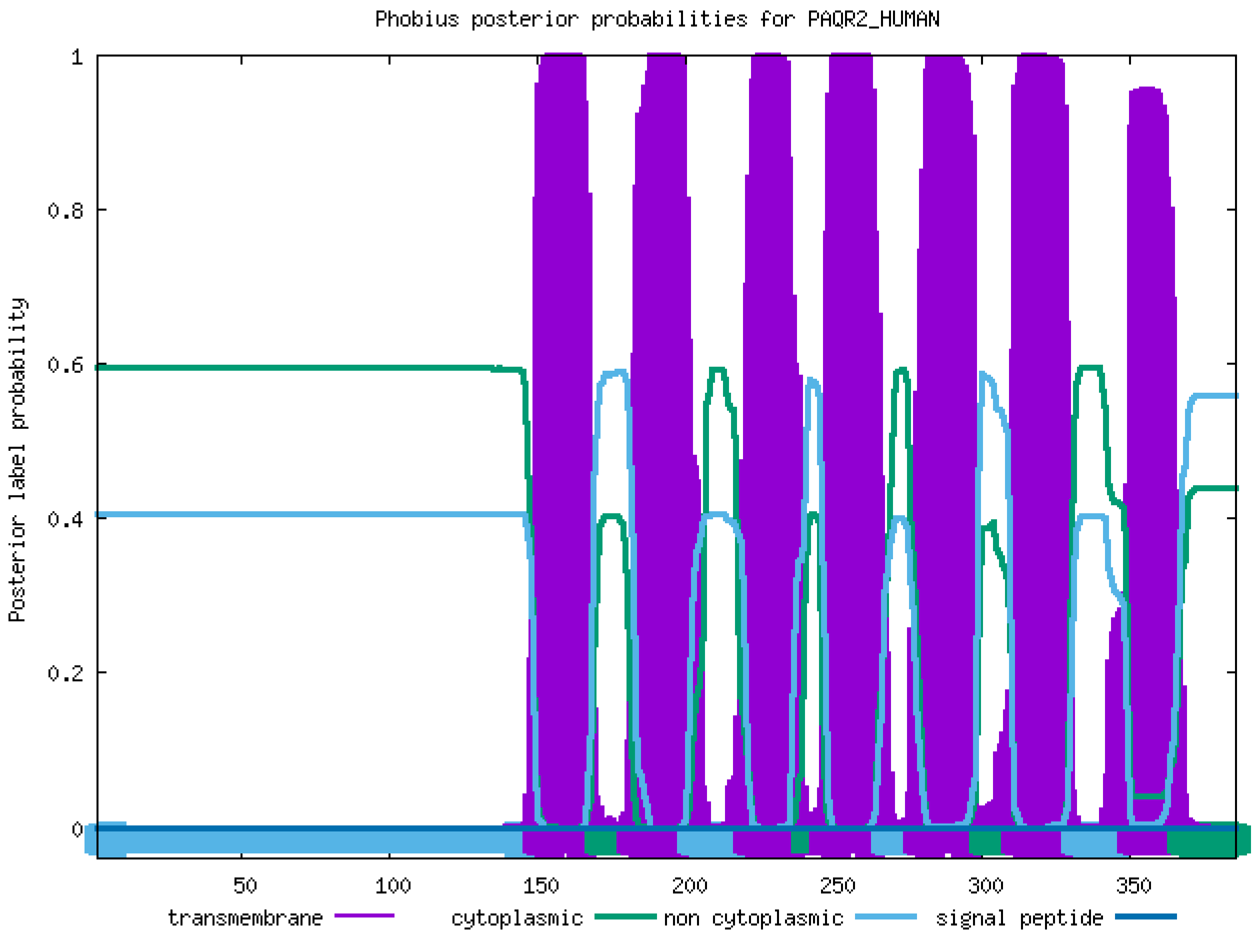

The probability data used in the plot is found here, and the gnuplot script is here.

Prediction of I17RC_HUMAN

```
ID   I17RC_HUMAN
FT   SIGNAL        1     20
FT   REGION        1      2       N-REGION.
FT   REGION        3     12       H-REGION.
FT   REGION       13     20       C-REGION.
FT   TOPO_DOM     21    538       NON CYTOPLASMIC.
FT   TRANSMEM    539    559
FT   TOPO_DOM    560    791       CYTOPLASMIC.
//
```

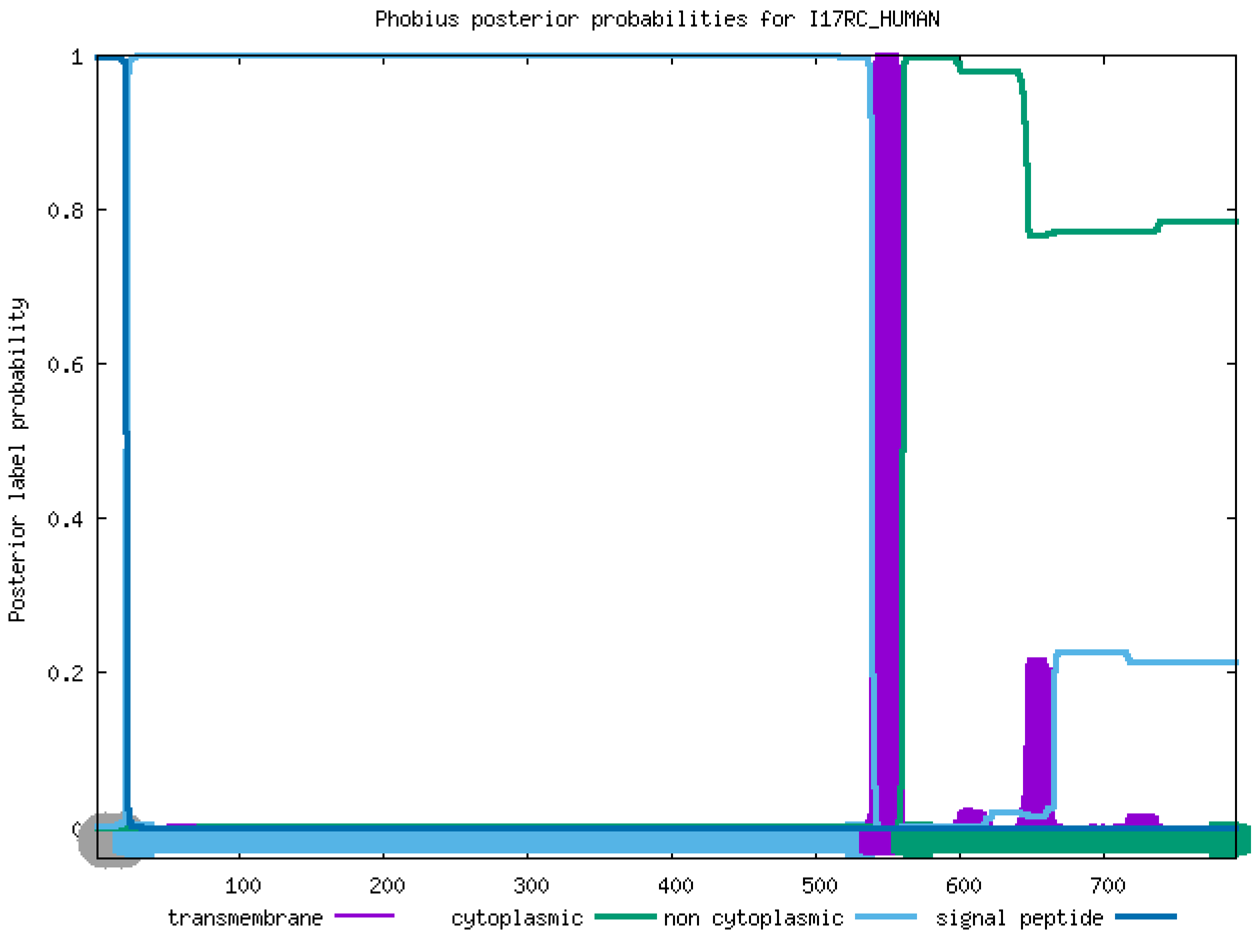

The probability data used in the plot is found here, and the gnuplot script is here.

Prediction of TLR3_HUMAN

```
ID   TLR3_HUMAN
FT   SIGNAL         1     23
FT   REGION         1      3       N-REGION.
FT   REGION         4     15       H-REGION.
FT   REGION        16     23       C-REGION.
FT   TOPO_DOM      24    703       NON CYTOPLASMIC.
FT   TRANSMEM     704    725
FT   TOPO_DOM     726    904       CYTOPLASMIC.
//
```

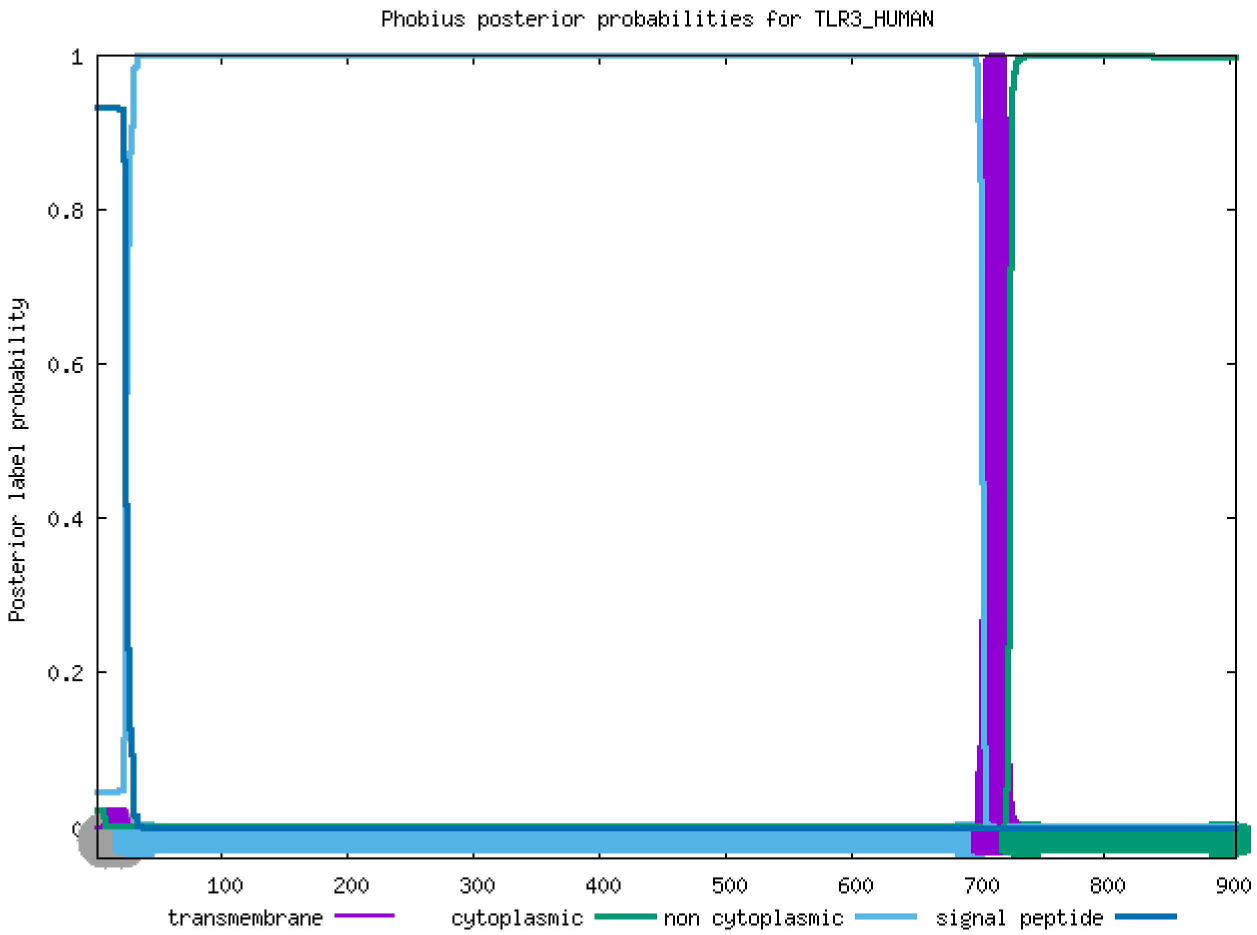

The probability data used in the plot is found here, and the gnuplot script is here.

Prediction of SGMR1_HUMAN

```
ID   SGMR1_HUMAN
FT   TOPO_DOM      1      8       CYTOPLASMIC.
FT   TRANSMEM      9     30
FT   TOPO_DOM     31     88       NON CYTOPLASMIC.
FT   TRANSMEM     89    111
FT   TOPO_DOM    112    223       CYTOPLASMIC.
//
```

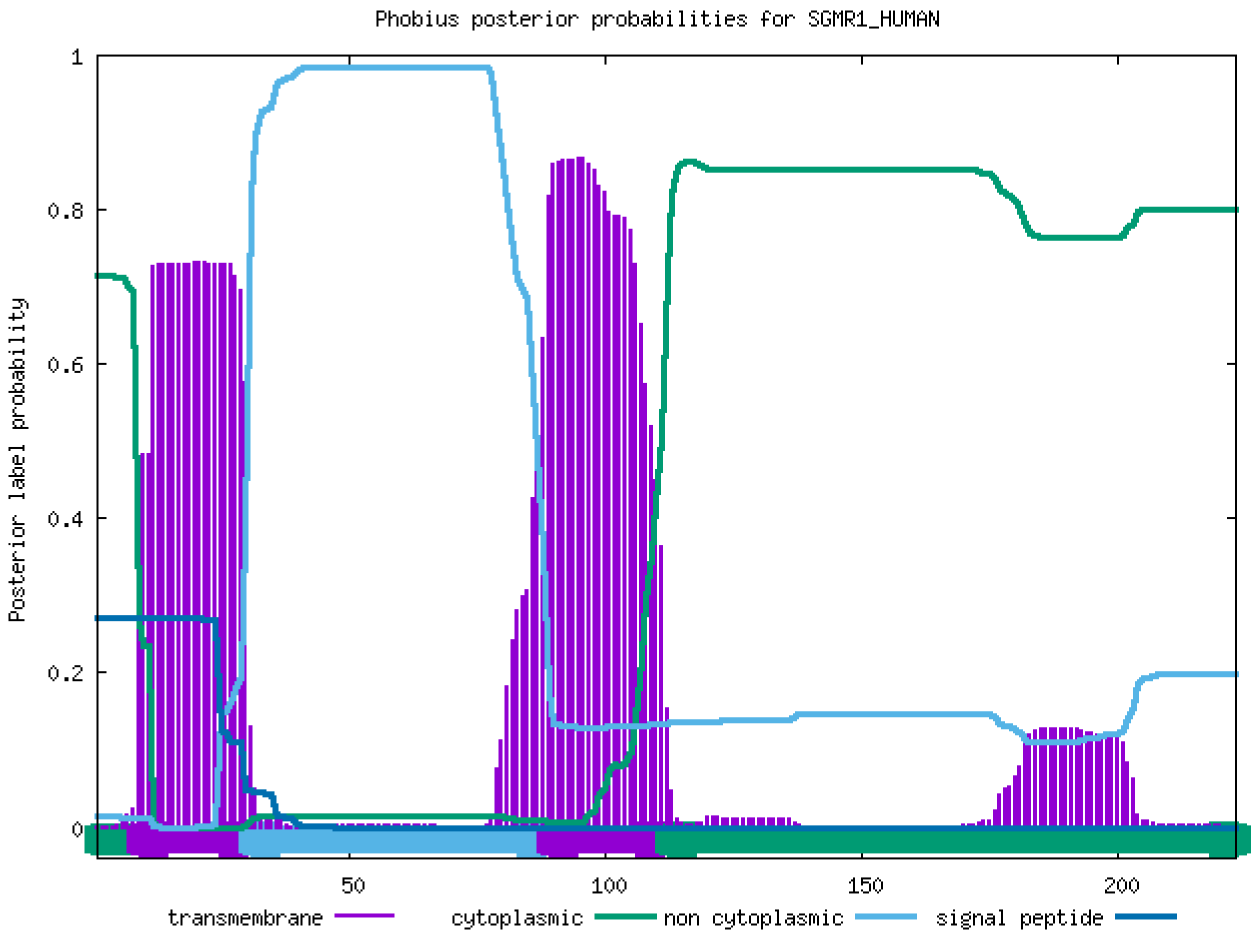

The probability data used in the plot is found here, and the gnuplot script is here.

Prediction of CD3Z_HUMAN

```
ID   CD3Z_HUMAN
FT   SIGNAL         1     21
FT   REGION         1      4       N-REGION.
FT   REGION         5     16       H-REGION.
FT   REGION        17     21       C-REGION.
FT   TOPO_DOM      22     30       NON CYTOPLASMIC.
FT   TRANSMEM      31     51
FT   TOPO_DOM      52    164       CYTOPLASMIC.
//
```

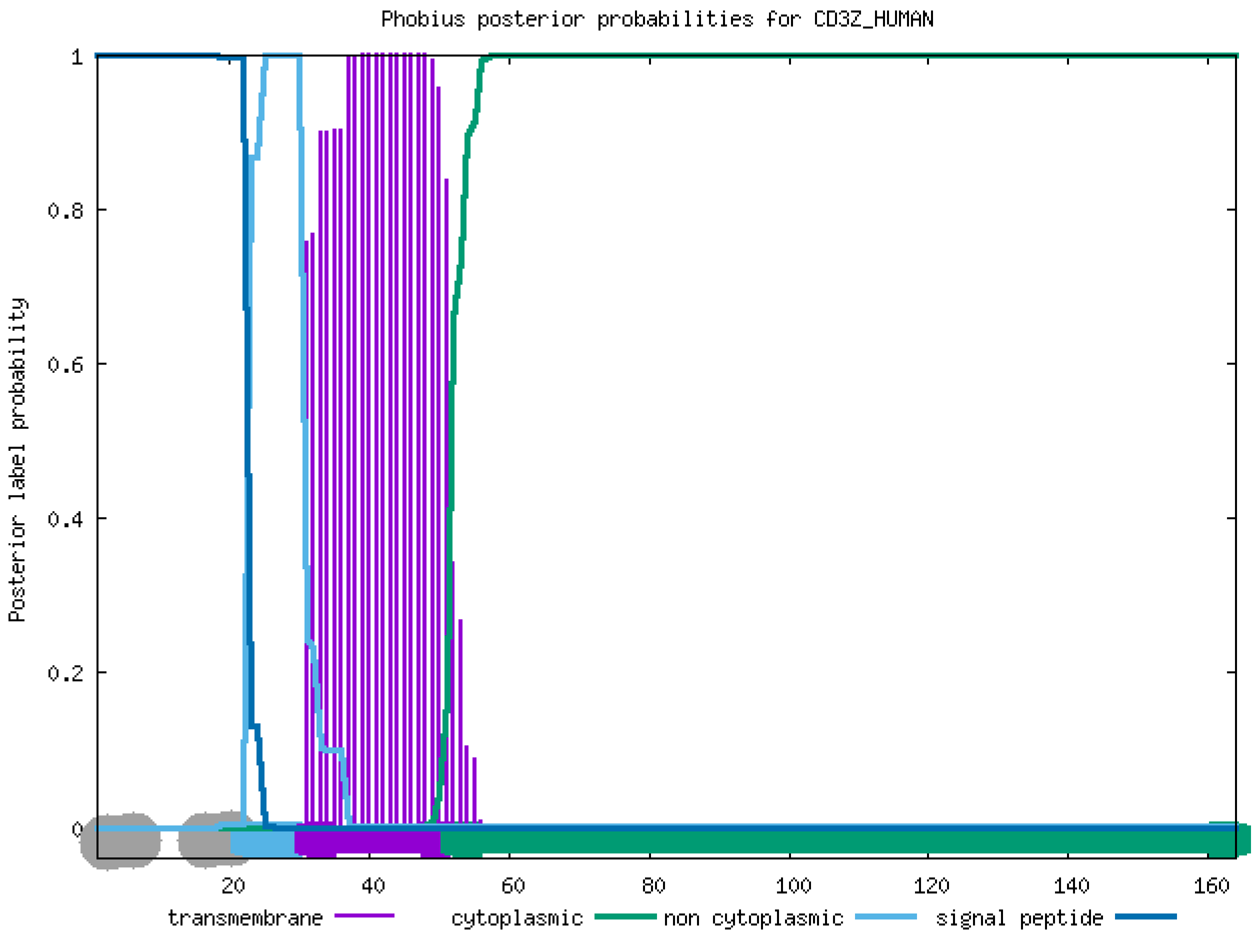

The probability data used in the plot is found here, and the gnuplot script is here.

Prediction of TYOBP_HUMAN

```
ID   TYOBP_HUMAN
FT   SIGNAL        1     27
FT   REGION        1      9       N-REGION.
FT   REGION       10     22       H-REGION.
FT   REGION       23     27       C-REGION.
FT   TOPO_DOM     28     42       NON CYTOPLASMIC.
FT   TRANSMEM     43     64
FT   TOPO_DOM     65    113       CYTOPLASMIC.
//
```

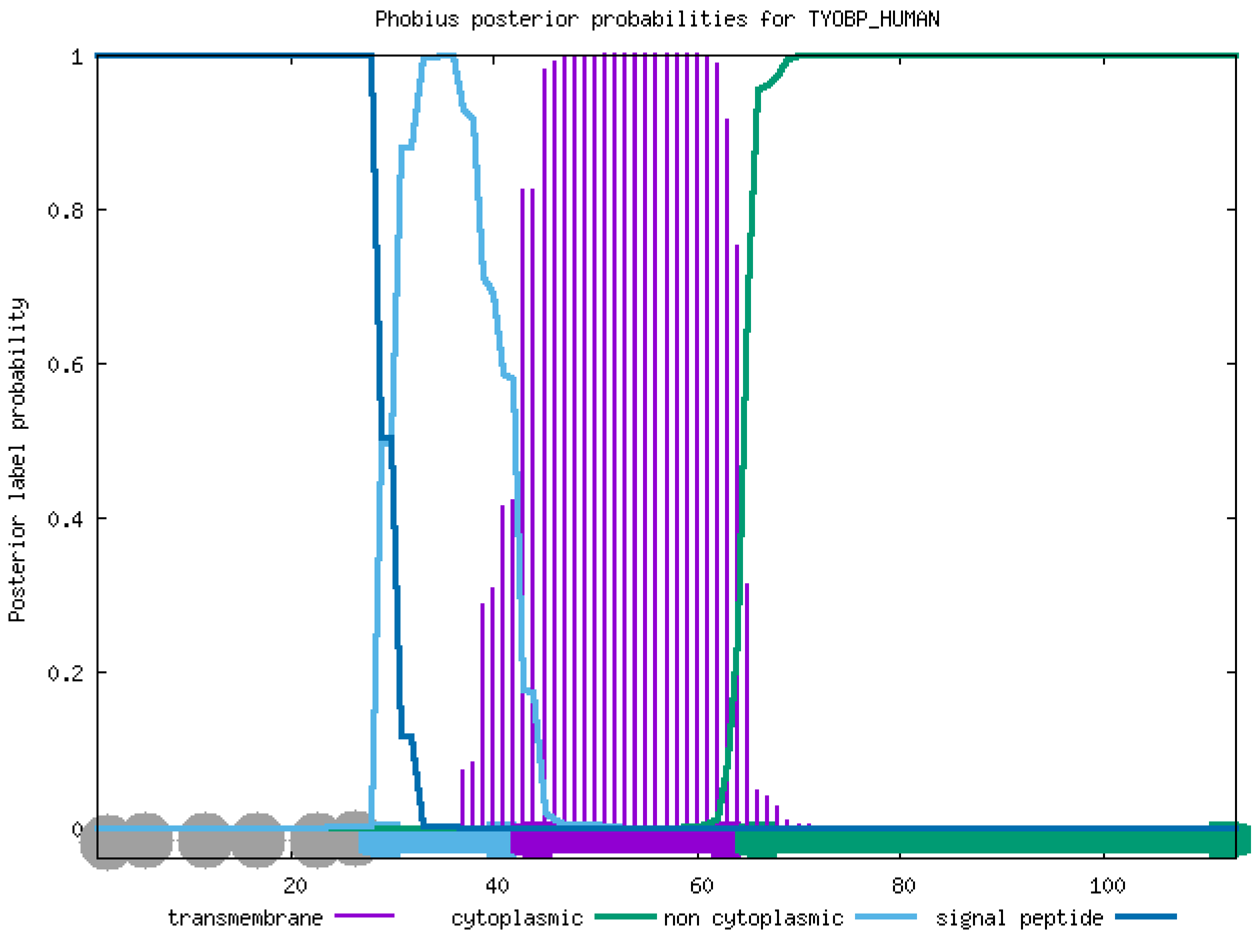

The probability data used in the plot is found here, and the gnuplot script is here.

Prediction of CD28_HUMAN

```
ID   CD28_HUMAN
FT   SIGNAL        1      18
FT   REGION        1       3       N-REGION.
FT   REGION        4      14       H-REGION.
FT   REGION       15      18       C-REGION.
FT   TOPO_DOM     19     152       NON CYTOPLASMIC.
FT   TRANSMEM    153     179
FT   TOPO_DOM    180     220       CYTOPLASMIC.
//
```

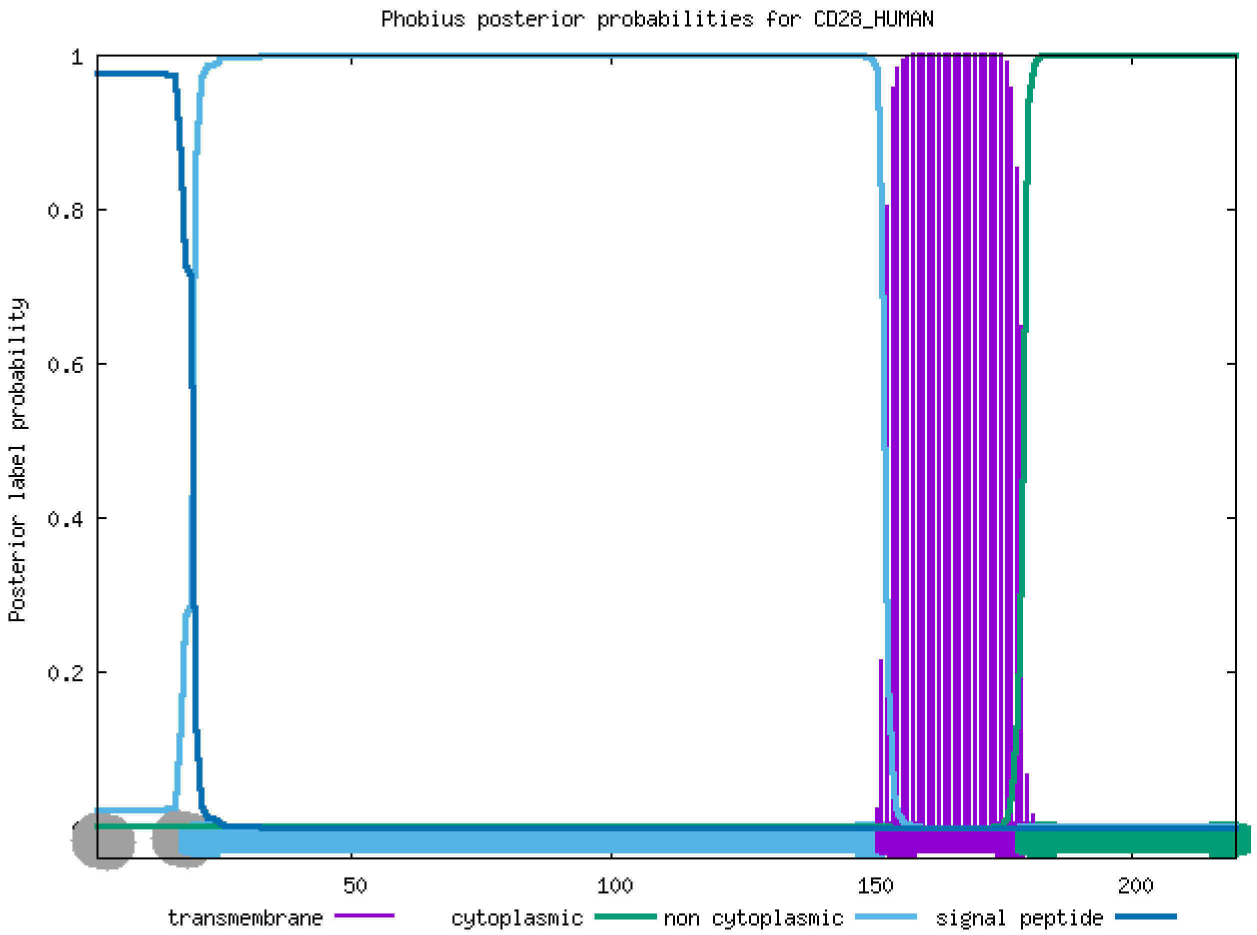

The probability data used in the plot is found here, and the gnuplot script is here.

Prediction of VAMP8_HUMAN

```
ID   VAMP8_HUMAN
FT   TOPO_DOM      1     76       CYTOPLASMIC.
FT   TRANSMEM     77     99
FT   TOPO_DOM    100    100       NON CYTOPLASMIC.
//
```

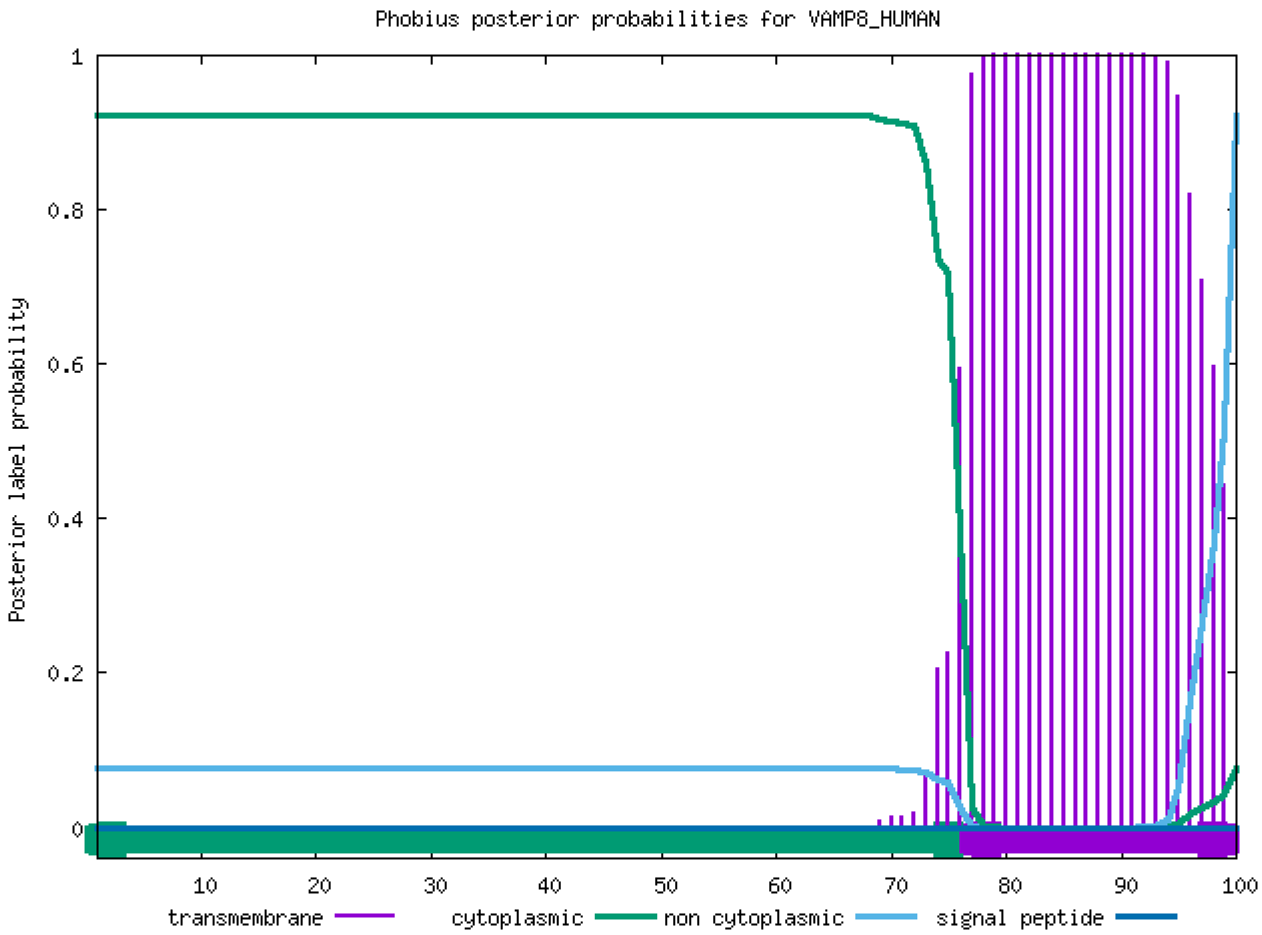

The probability data used in the plot is found here, and the gnuplot script is here.

Prediction of STX17_HUMAN

```
ID   STX17_HUMAN
FT   TOPO_DOM      1    228       CYTOPLASMIC.
FT   TRANSMEM    229    249
FT   TOPO_DOM    250    254       NON CYTOPLASMIC.
FT   TRANSMEM    255    275
FT   TOPO_DOM    276    302       CYTOPLASMIC.
//
```

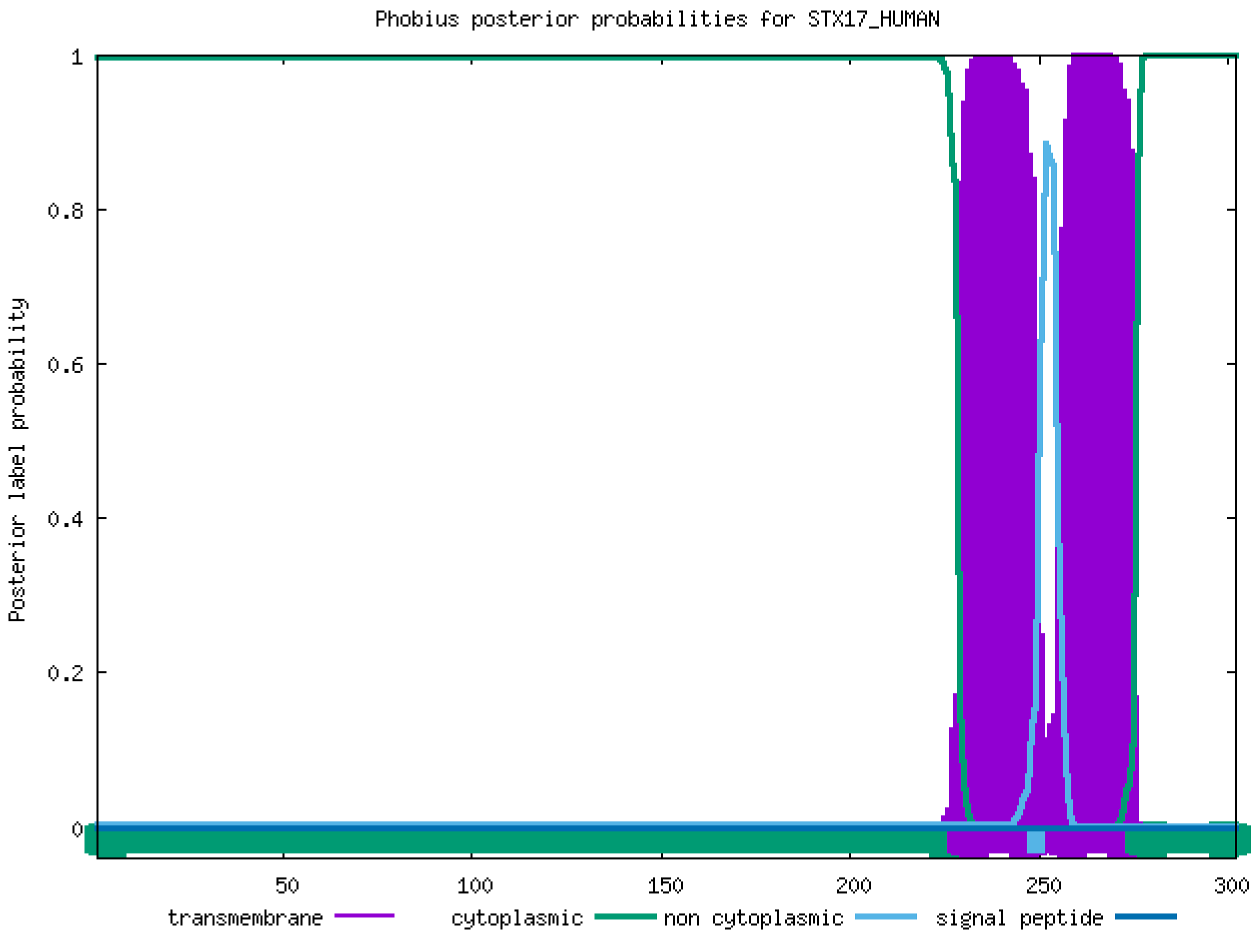

The probability data used in the plot is found here, and the gnuplot script is here.

Prediction of CLD4_HUMAN

```
ID   CLD4_HUMAN
FT   SIGNAL        1     26
FT   REGION        1      9       N-REGION.
FT   REGION       10     21       H-REGION.
FT   REGION       22     26       C-REGION.
FT   TOPO_DOM     27     78       NON CYTOPLASMIC.
FT   TRANSMEM     79     99
FT   TOPO_DOM    100    118       CYTOPLASMIC.
FT   TRANSMEM    119    140
FT   TOPO_DOM    141    159       NON CYTOPLASMIC.
FT   TRANSMEM    160    183
FT   TOPO_DOM    184    209       CYTOPLASMIC.
//
```

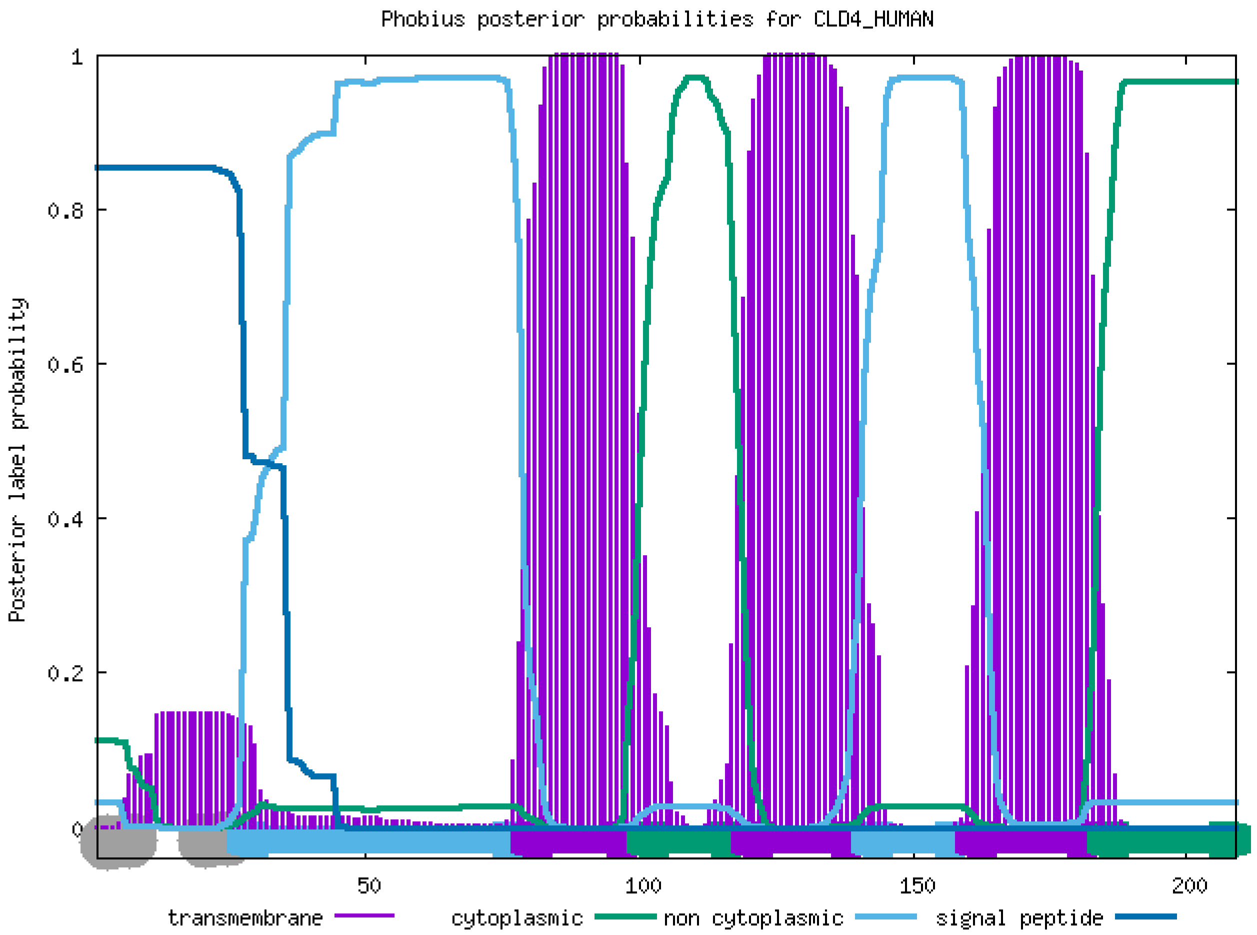

The probability data used in the plot is found here, and the gnuplot script is here.

Prediction of CLD9_HUMAN

```
ID   CLD9_HUMAN
FT   SIGNAL         1     25
FT   REGION         1      7       N-REGION.
FT   REGION         8     19       H-REGION.
FT   REGION        20     25       C-REGION.
FT   TOPO_DOM      26     78       NON CYTOPLASMIC.
FT   TRANSMEM      79    105
FT   TOPO_DOM     106    116       CYTOPLASMIC.
FT   TRANSMEM     117    144
FT   TOPO_DOM     145    163       NON CYTOPLASMIC.
FT   TRANSMEM     164    184
FT   TOPO_DOM     185    217       CYTOPLASMIC.
//
```

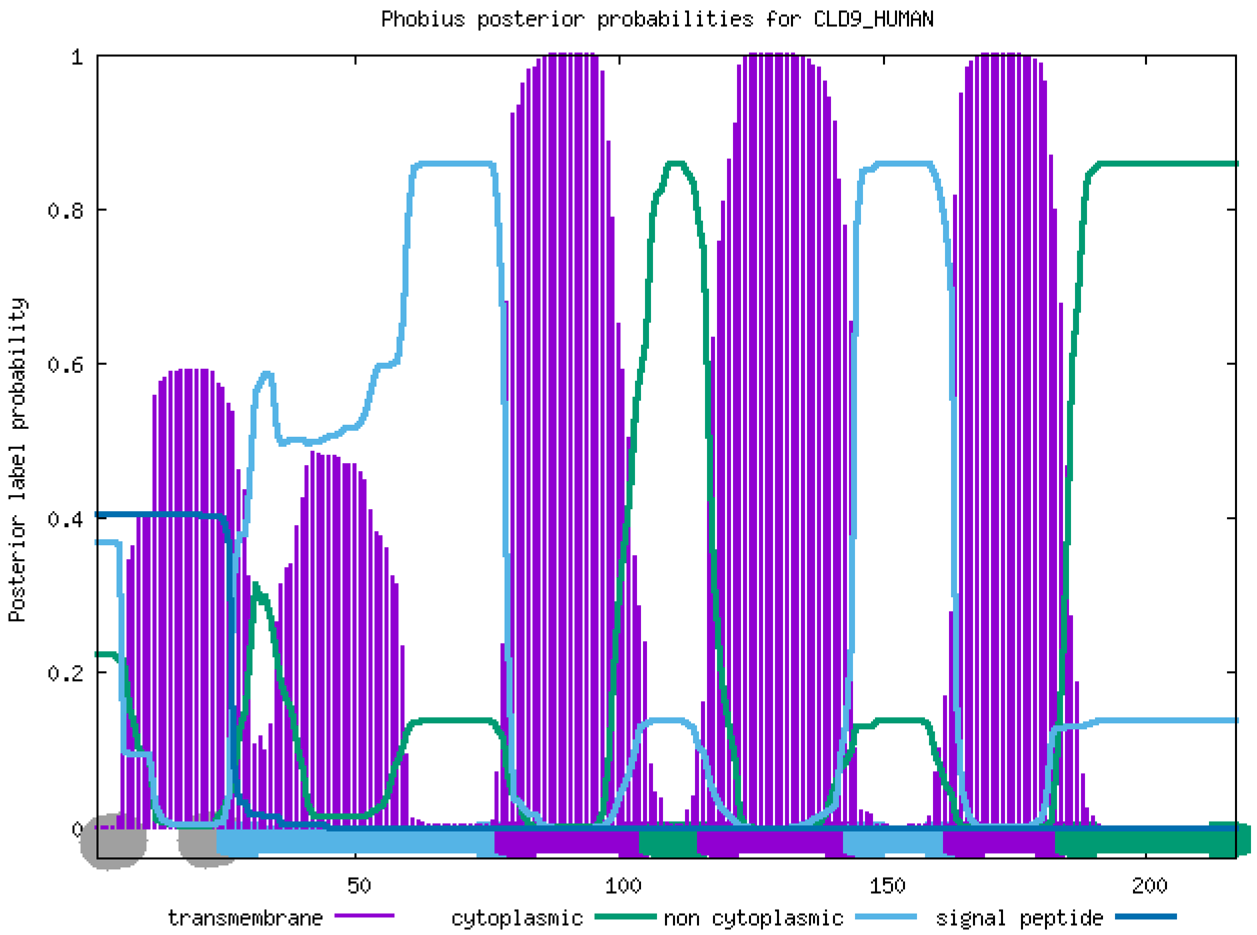

The probability data used in the plot is found here, and the gnuplot script is here.

Prediction of ANO10_HUMAN

```
ID   ANO10_HUMAN
FT   TOPO_DOM      1     207       CYTOPLASMIC.
FT   TRANSMEM    208     237
FT   TOPO_DOM    238     242       NON CYTOPLASMIC.
FT   TRANSMEM    243     261
FT   TOPO_DOM    262     313       CYTOPLASMIC.
FT   TRANSMEM    314     337
FT   TOPO_DOM    338     356       NON CYTOPLASMIC.
FT   TRANSMEM    357     377
FT   TOPO_DOM    378     399       CYTOPLASMIC.
FT   TRANSMEM    400     421
FT   TOPO_DOM    422     432       NON CYTOPLASMIC.
FT   TRANSMEM    433     455
FT   TOPO_DOM    456     500       CYTOPLASMIC.
FT   TRANSMEM    501     524
FT   TOPO_DOM    525     590       NON CYTOPLASMIC.
FT   TRANSMEM    591     613
FT   TOPO_DOM    614     660       CYTOPLASMIC.
//
```

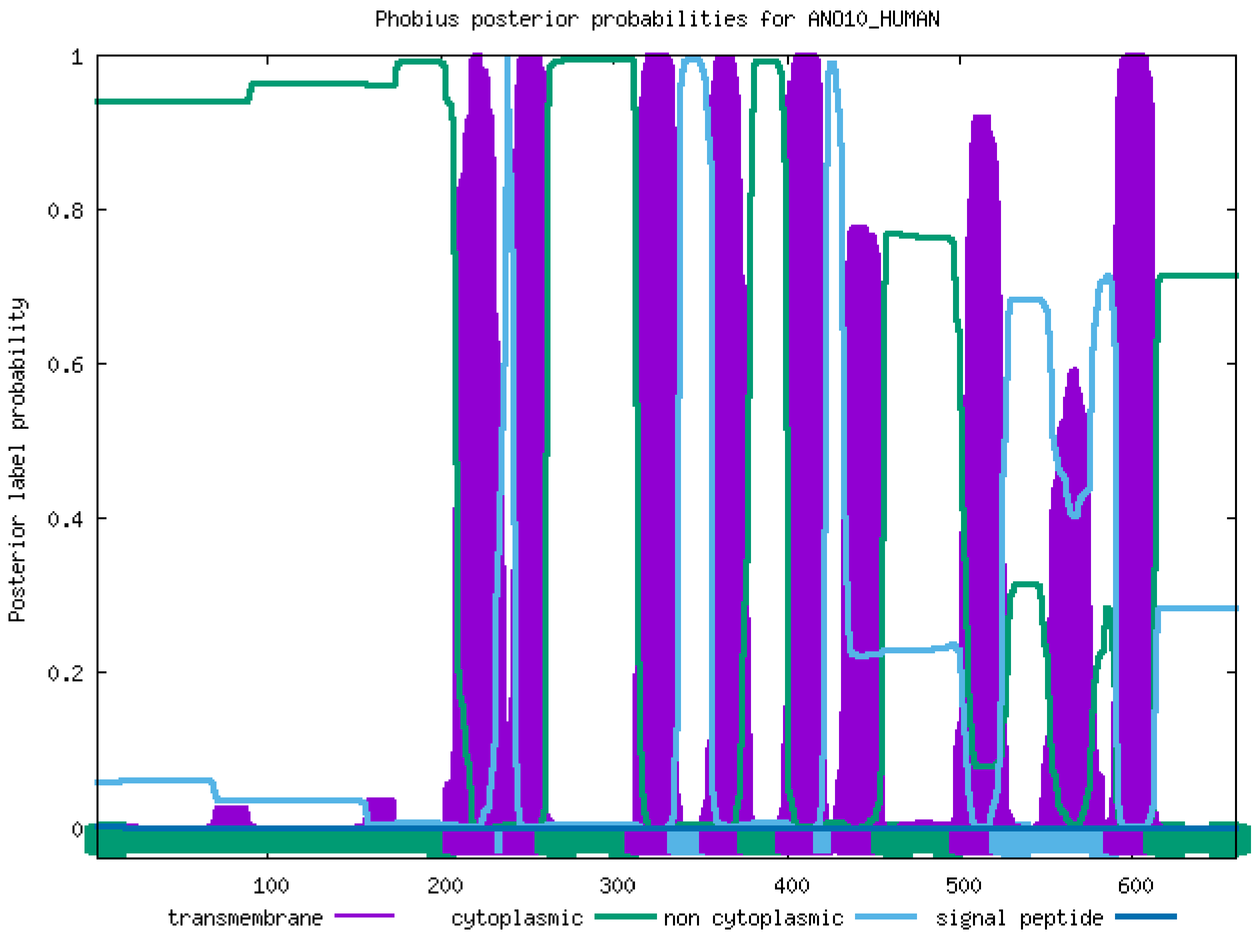

The probability data used in the plot is found here, and the gnuplot script is here.

Prediction of BASI_HUMAN

```
ID    BASI_HUMAN
FT    SIGNAL        1      21
FT    REGION        1       2       N-REGION.
FT    REGION        3      14       H-REGION.
FT    REGION       15      21       C-REGION.
FT    TOPO_DOM     22     323       NON CYTOPLASMIC.
FT    TRANSMEM    324     345
FT    TOPO_DOM    346     385       CYTOPLASMIC.
//
```

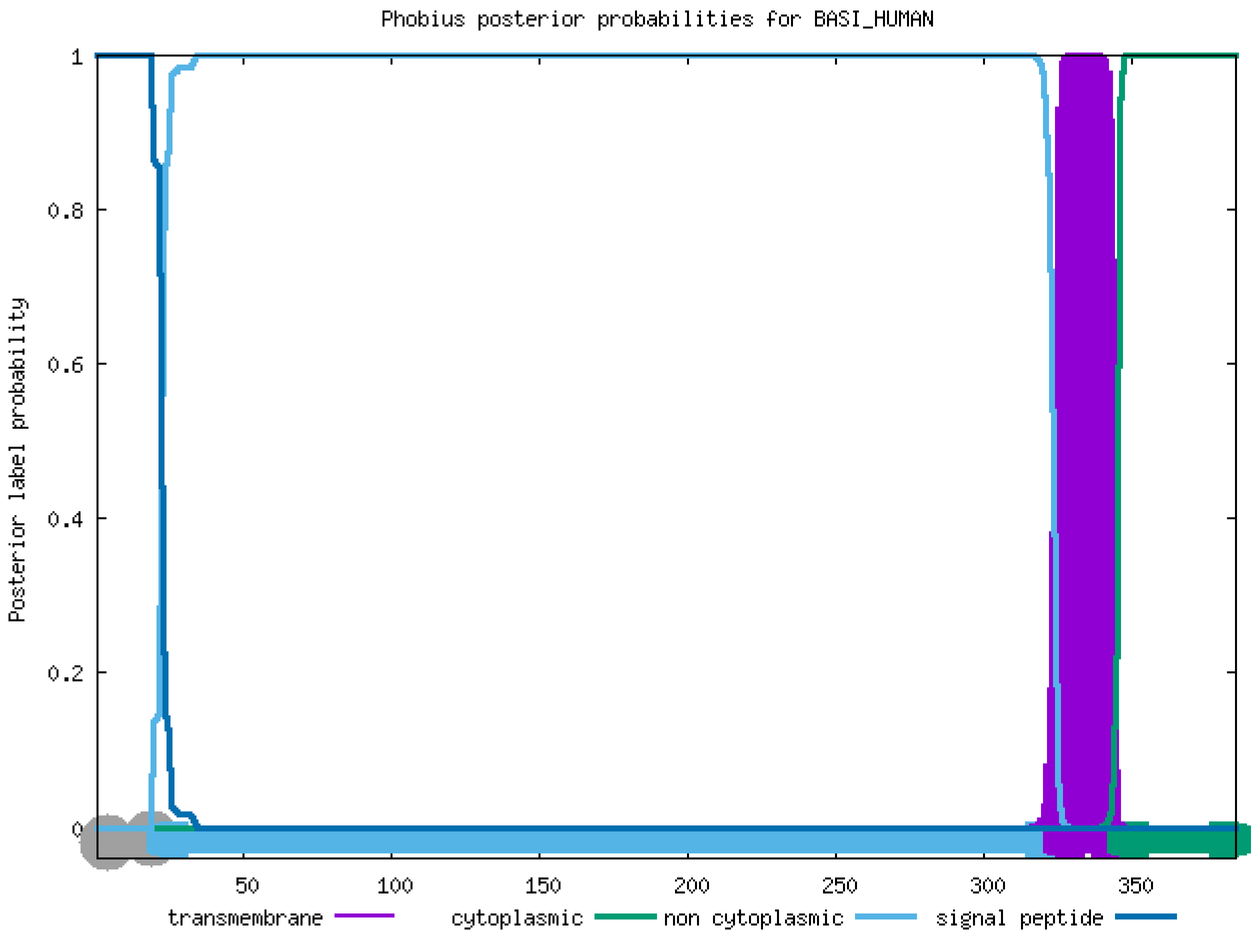

The probability data used in the plot is found here, and the gnuplot script is here.

Prediction of XKR8_HUMAN

```
ID   XKR8_HUMAN
FT   SIGNAL        1     33
FT   REGION        1     12       N-REGION.
FT   REGION       13     25       H-REGION.
FT   REGION       26     33       C-REGION.
FT   TOPO_DOM     34     42       NON CYTOPLASMIC.
FT   TRANSMEM     43     68
FT   TOPO_DOM     69    160       CYTOPLASMIC.
FT   TRANSMEM    161    181
FT   TOPO_DOM    182    200       NON CYTOPLASMIC.
FT   TRANSMEM    201    221
FT   TOPO_DOM    222    227       CYTOPLASMIC.
FT   TRANSMEM    228    247
FT   TOPO_DOM    248    258       NON CYTOPLASMIC.
FT   TRANSMEM    259    277
FT   TOPO_DOM    278    288       CYTOPLASMIC.
FT   TRANSMEM    289    306
FT   TOPO_DOM    307    317       NON CYTOPLASMIC.
FT   TRANSMEM    318    337
FT   TOPO_DOM    338    395       CYTOPLASMIC.
//
```

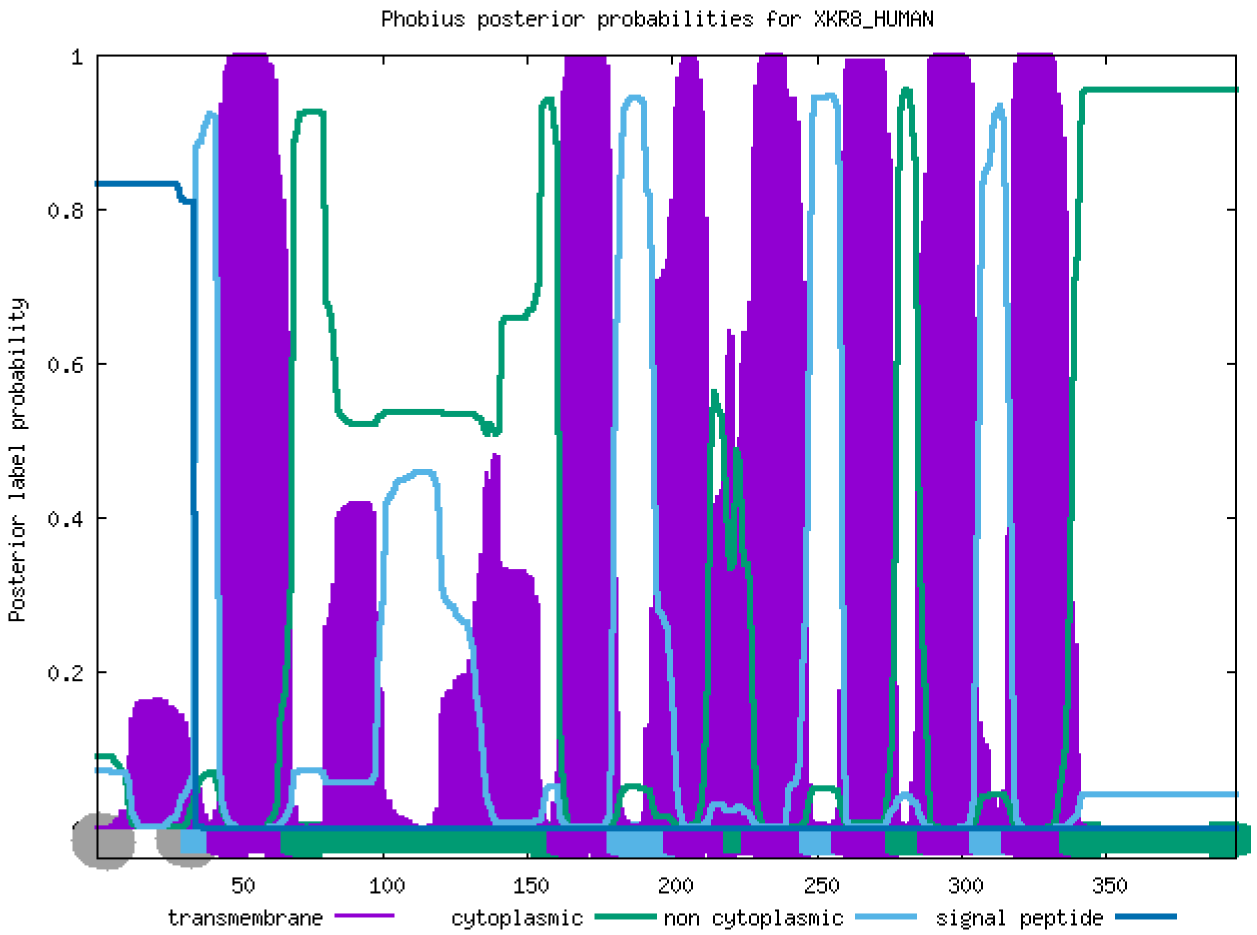

The probability data used in the plot is found here, and the gnuplot script is here.

Prediction of TM147_HUMAN

```
ID    TM147_HUMAN
FT    TOPO_DOM      1     98       CYTOPLASMIC.
FT    TRANSMEM     99    121
FT    TOPO_DOM    122    140       NON CYTOPLASMIC.
FT    TRANSMEM    141    157
FT    TOPO_DOM    158    168       CYTOPLASMIC.
FT    TRANSMEM    169    189
FT    TOPO_DOM    190    194       NON CYTOPLASMIC.
FT    TRANSMEM    195    219
FT    TOPO_DOM    220    224       CYTOPLASMIC.
//
```

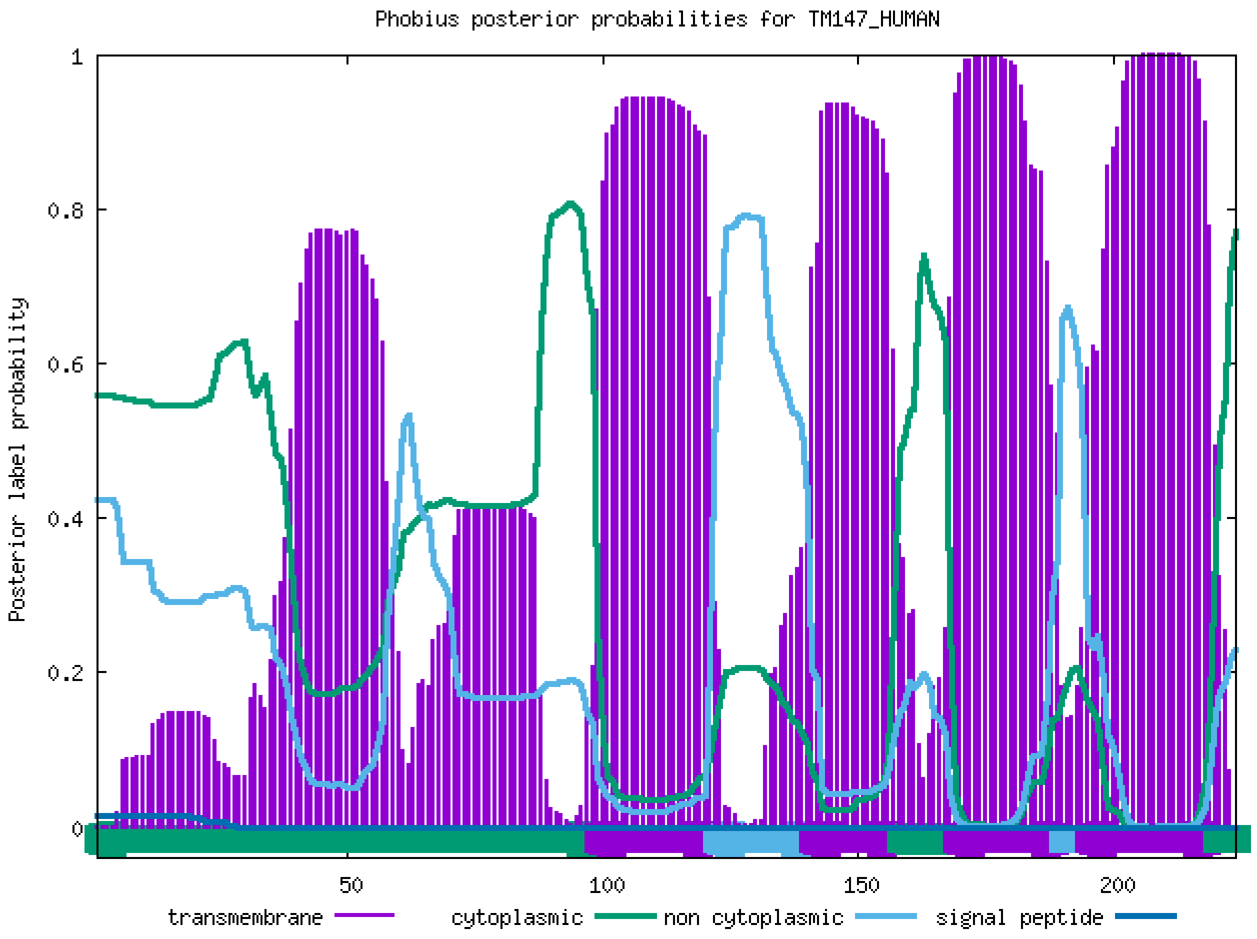

The probability data used in the plot is found here, and the gnuplot script is here.

Prediction of NCLN_HUMAN

```
ID   NCLN_HUMAN
FT   TOPO_DOM      1     11       NON CYTOPLASMIC.
FT   TRANSMEM     12     34
FT   TOPO_DOM     35    521       CYTOPLASMIC.
FT   TRANSMEM    522    543
FT   TOPO_DOM    544    563       NON CYTOPLASMIC.
//
```

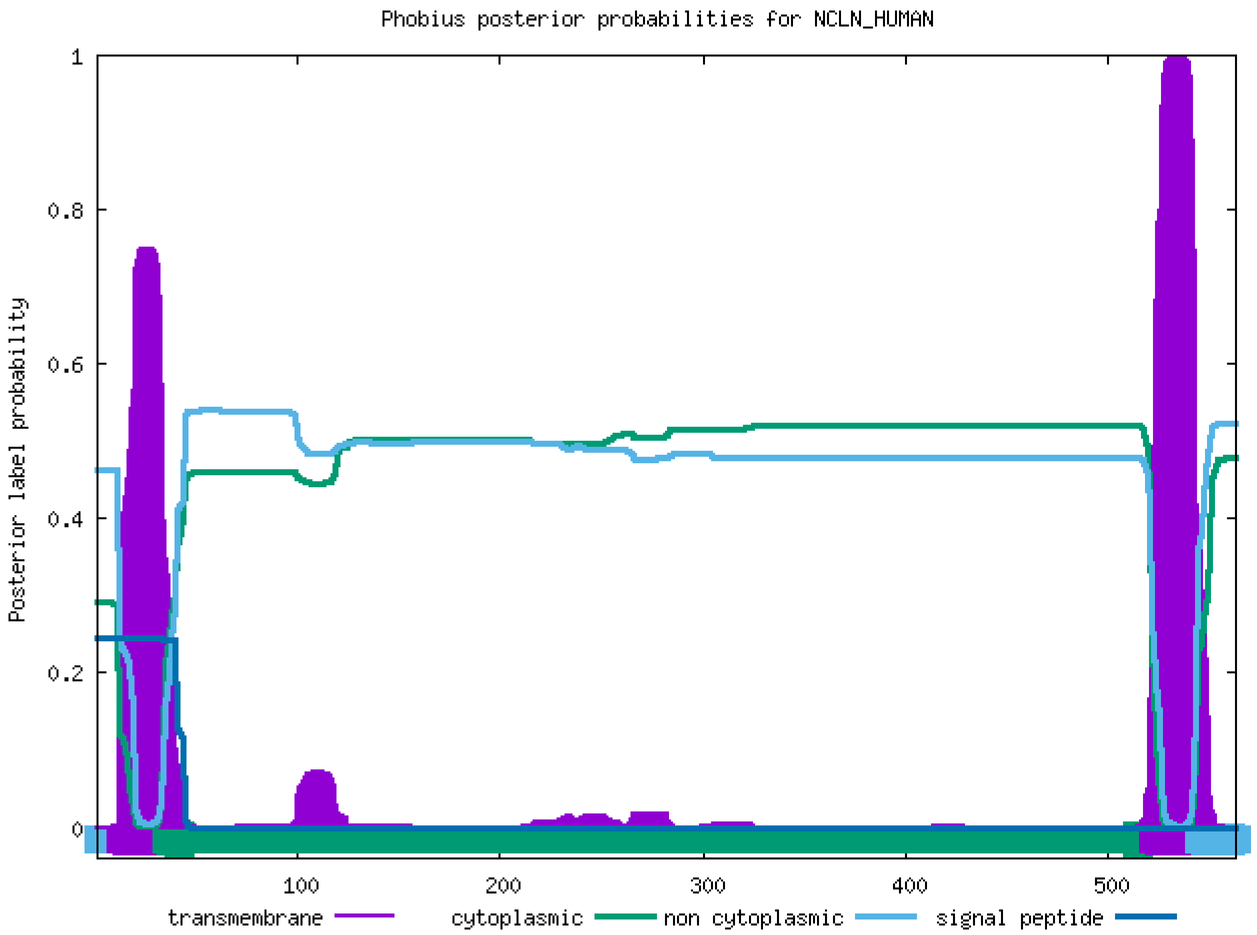

The probability data used in the plot is found here, and the gnuplot script is here.

Prediction of GET1_HUMAN

```
ID   GET1_HUMAN
FT   TOPO_DOM      1      5       NON CYTOPLASMIC.
FT   TRANSMEM      6     28
FT   TOPO_DOM     29    101       CYTOPLASMIC.
FT   TRANSMEM    102    124
FT   TOPO_DOM    125    148       NON CYTOPLASMIC.
FT   TRANSMEM    149    170
FT   TOPO_DOM    171    174       CYTOPLASMIC.
//
```

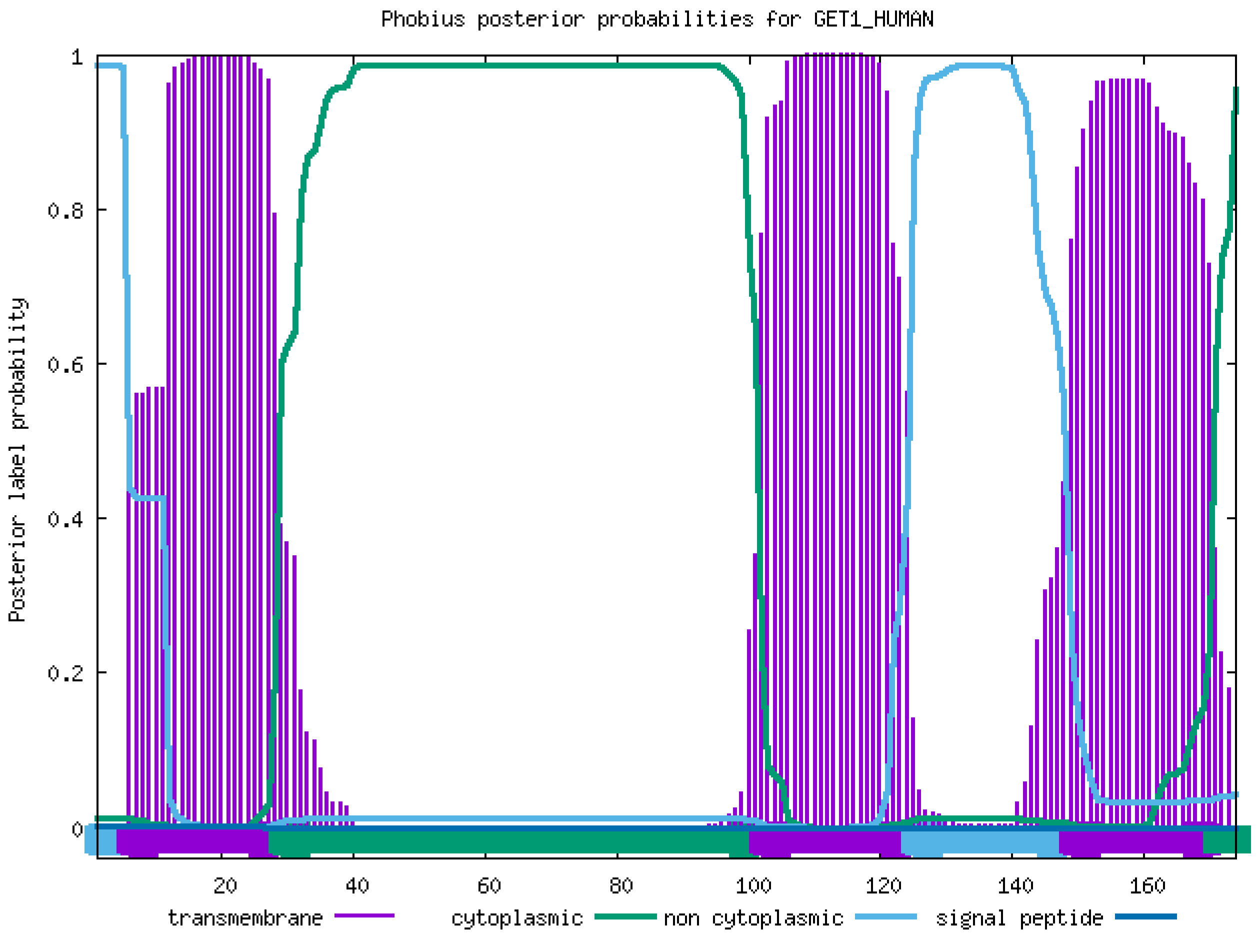

The probability data used in the plot is found here, and the gnuplot script is here.

Prediction of DERL1_HUMAN

```
ID    DERL1_HUMAN
FT    TOPO_DOM      1     16       CYTOPLASMIC.
FT    TRANSMEM     17     40
FT    TOPO_DOM     41     59       NON CYTOPLASMIC.
FT    TRANSMEM     60     80
FT    TOPO_DOM     81    100       CYTOPLASMIC.
FT    TRANSMEM    101    134
FT    TOPO_DOM    135    153       NON CYTOPLASMIC.
FT    TRANSMEM    154    186
FT    TOPO_DOM    187    251       CYTOPLASMIC.
//
```

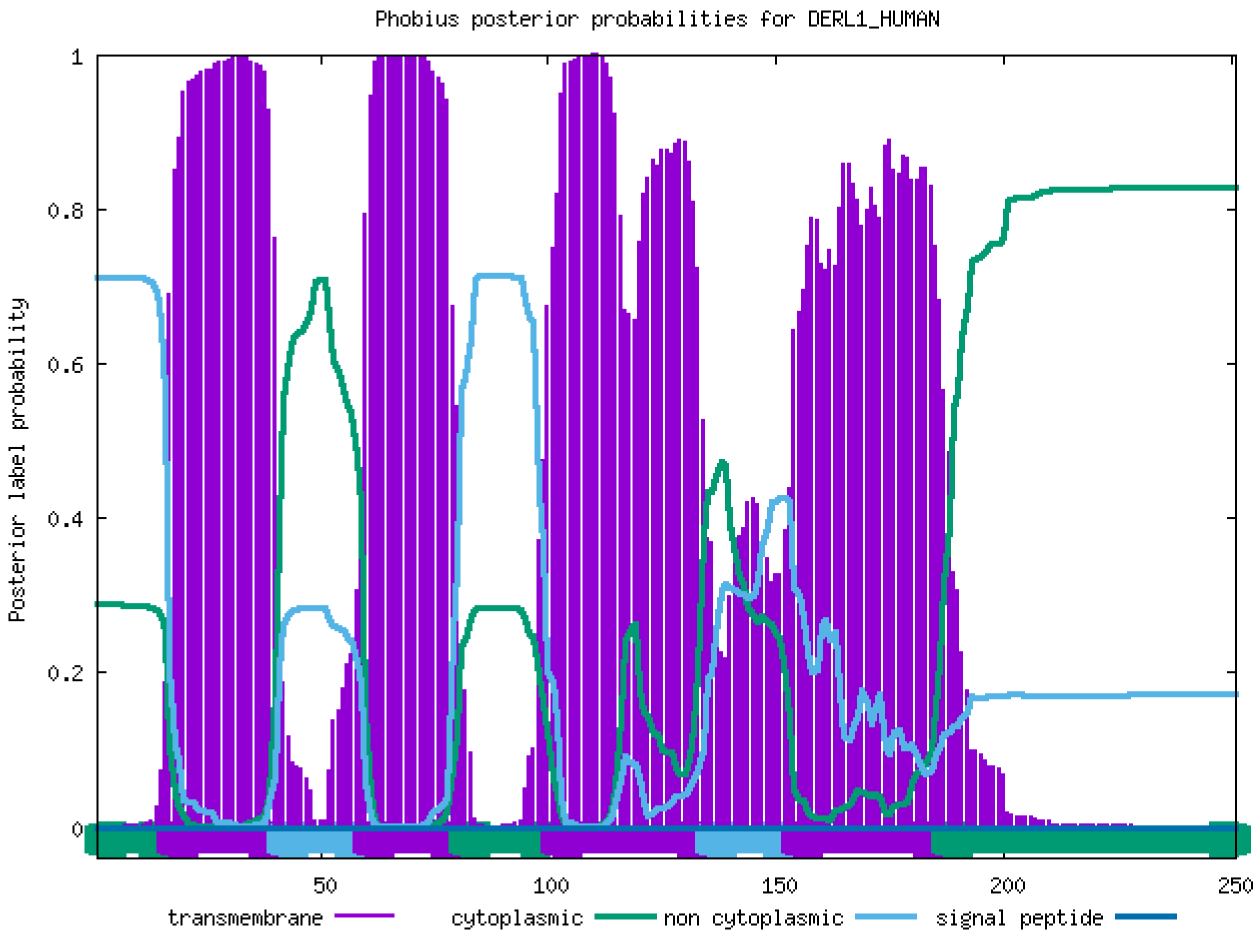

The probability data used in the plot is found here, and the gnuplot script is here.

Prediction of ATG9A_HUMAN

```
ID    ATG9A_HUMAN
FT    TOPO_DOM      1     69       NON CYTOPLASMIC.
FT    TRANSMEM     70     92
FT    TOPO_DOM     93    289       CYTOPLASMIC.
FT    TRANSMEM    290    314
FT    TOPO_DOM    315    379       NON CYTOPLASMIC.
FT    TRANSMEM    380    397
FT    TOPO_DOM    398    403       CYTOPLASMIC.
FT    TRANSMEM    404    425
FT    TOPO_DOM    426    839       NON CYTOPLASMIC.
//
```

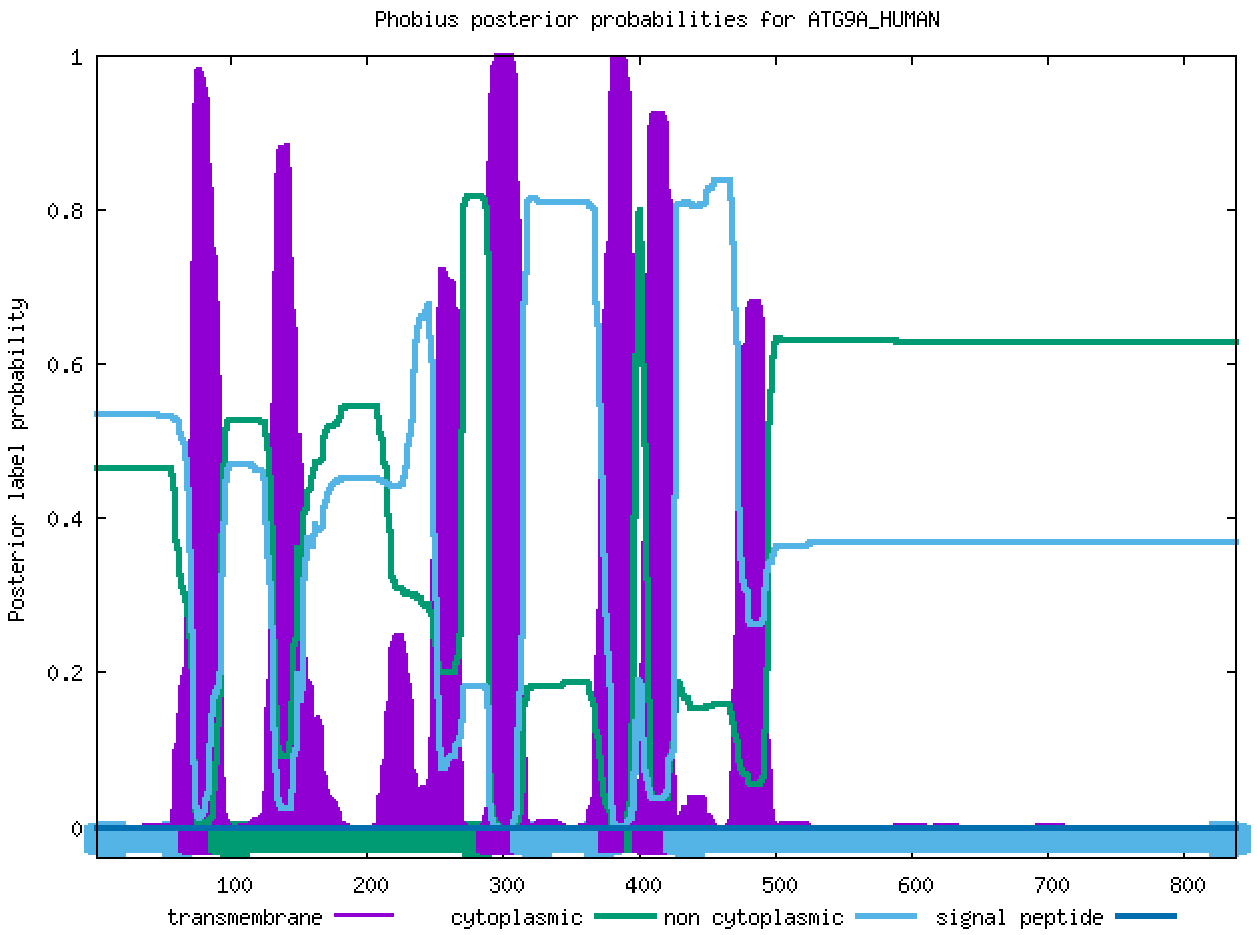

The probability data used in the plot is found here, and the gnuplot script is here.

Prediction of EMC1_HUMAN

```
ID   EMC1_HUMAN
FT   SIGNAL        1     21
FT   REGION        1      8       N-REGION.
FT   REGION        9     17       H-REGION.
FT   REGION       18     21       C-REGION.
FT   TOPO_DOM     22    961       NON CYTOPLASMIC.
FT   TRANSMEM    962    983
FT   TOPO_DOM    984    993       CYTOPLASMIC.
//
```

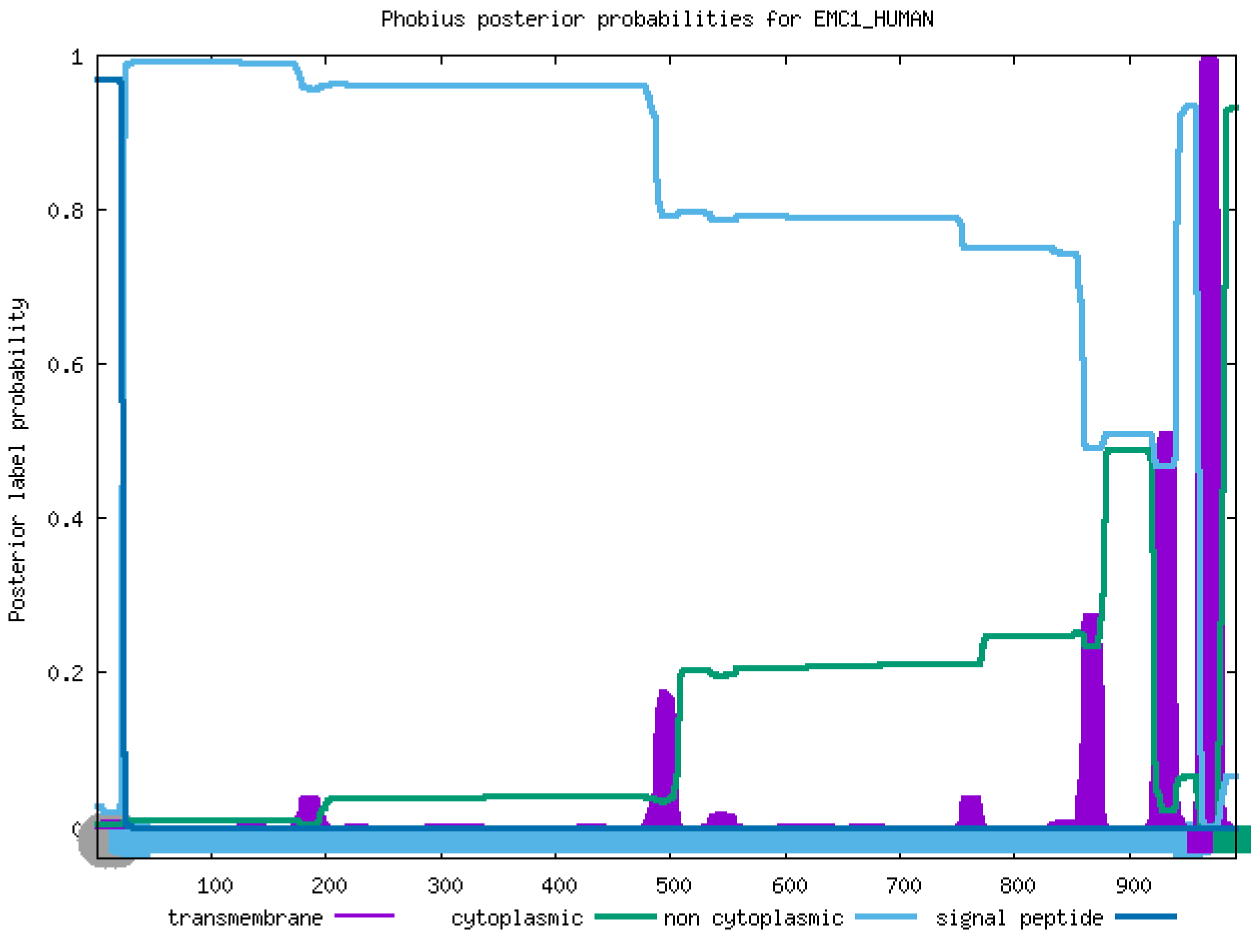

The probability data used in the plot is found here, and the gnuplot script is here.

Prediction of EMC3_HUMAN

```
ID    EMC3_HUMAN
FT    TOPO_DOM      1      11       NON CYTOPLASMIC.
FT    TRANSMEM     12      34
FT    TOPO_DOM     35     114       CYTOPLASMIC.
FT    TRANSMEM    115     136
FT    TOPO_DOM    137     168       NON CYTOPLASMIC.
FT    TRANSMEM    169     188
FT    TOPO_DOM    189     261       CYTOPLASMIC.
//
```

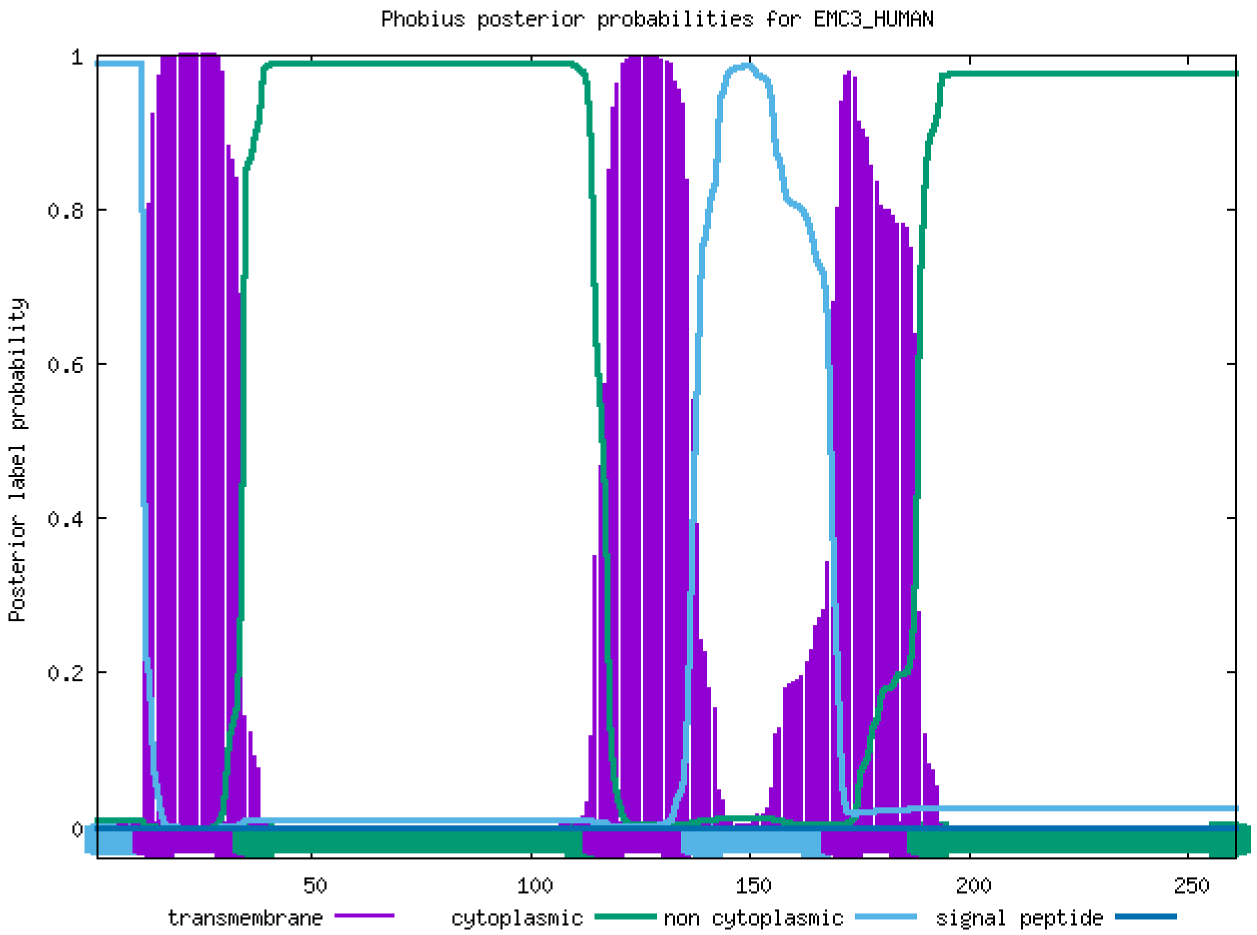

The probability data used in the plot is found here, and the gnuplot script is here.

Prediction of EMC4_HUMAN

```
ID   EMC4_HUMAN
FT   TOPO_DOM      1     84       CYTOPLASMIC.
FT   TRANSMEM     85    110
FT   TOPO_DOM    111    129       NON CYTOPLASMIC.
FT   TRANSMEM    130    149
FT   TOPO_DOM    150    183       CYTOPLASMIC.
//
```

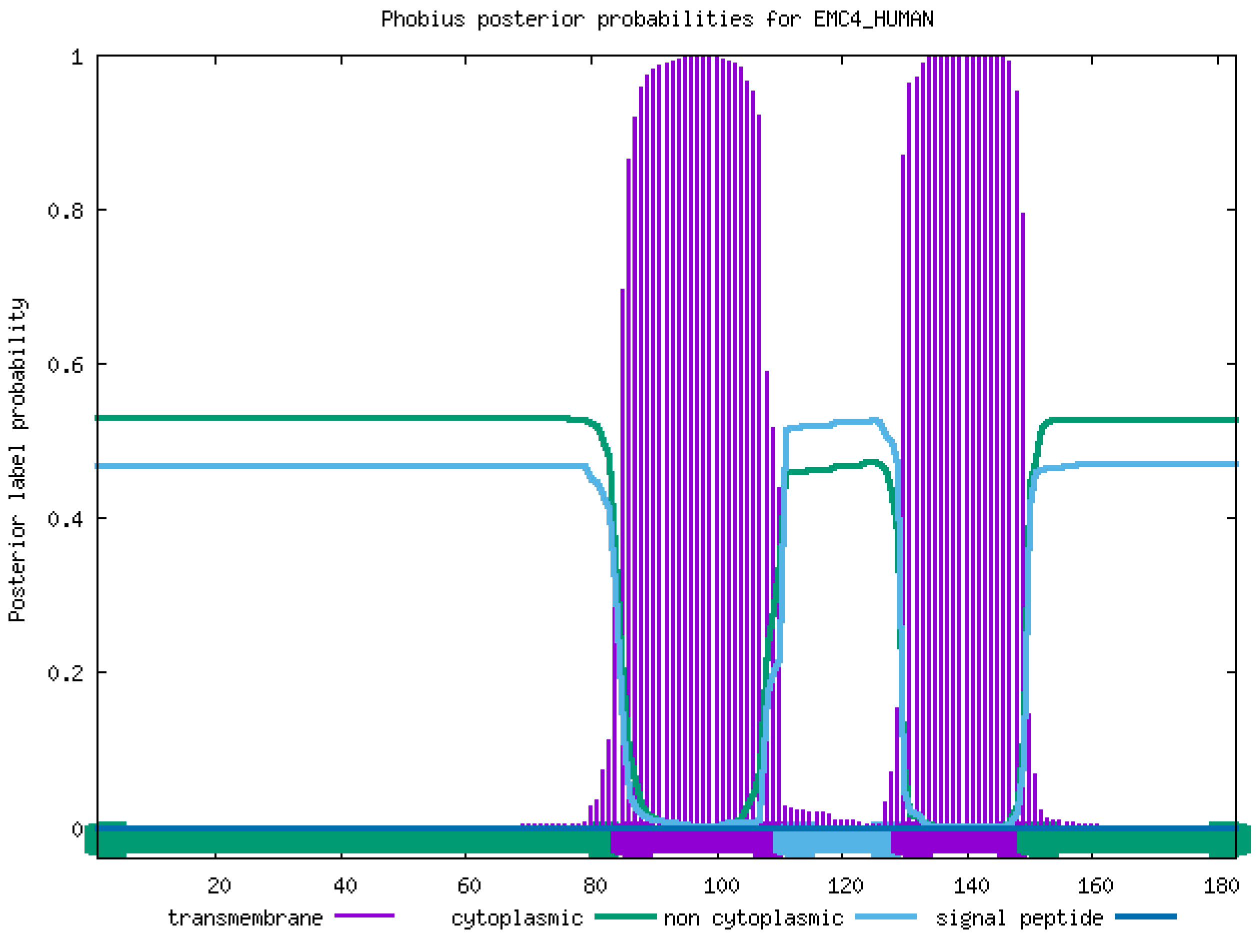

The probability data used in the plot is found here, and the gnuplot script is here.

Prediction of EMC5_HUMAN

```
ID   EMC5_HUMAN
FT   SIGNAL        1     23
FT   REGION        1      7       N-REGION.
FT   REGION        8     18       H-REGION.
FT   REGION       19     23       C-REGION.
FT   TOPO_DOM     24     46       NON CYTOPLASMIC.
FT   TRANSMEM     47     67
FT   TOPO_DOM     68    131       CYTOPLASMIC.
//
```

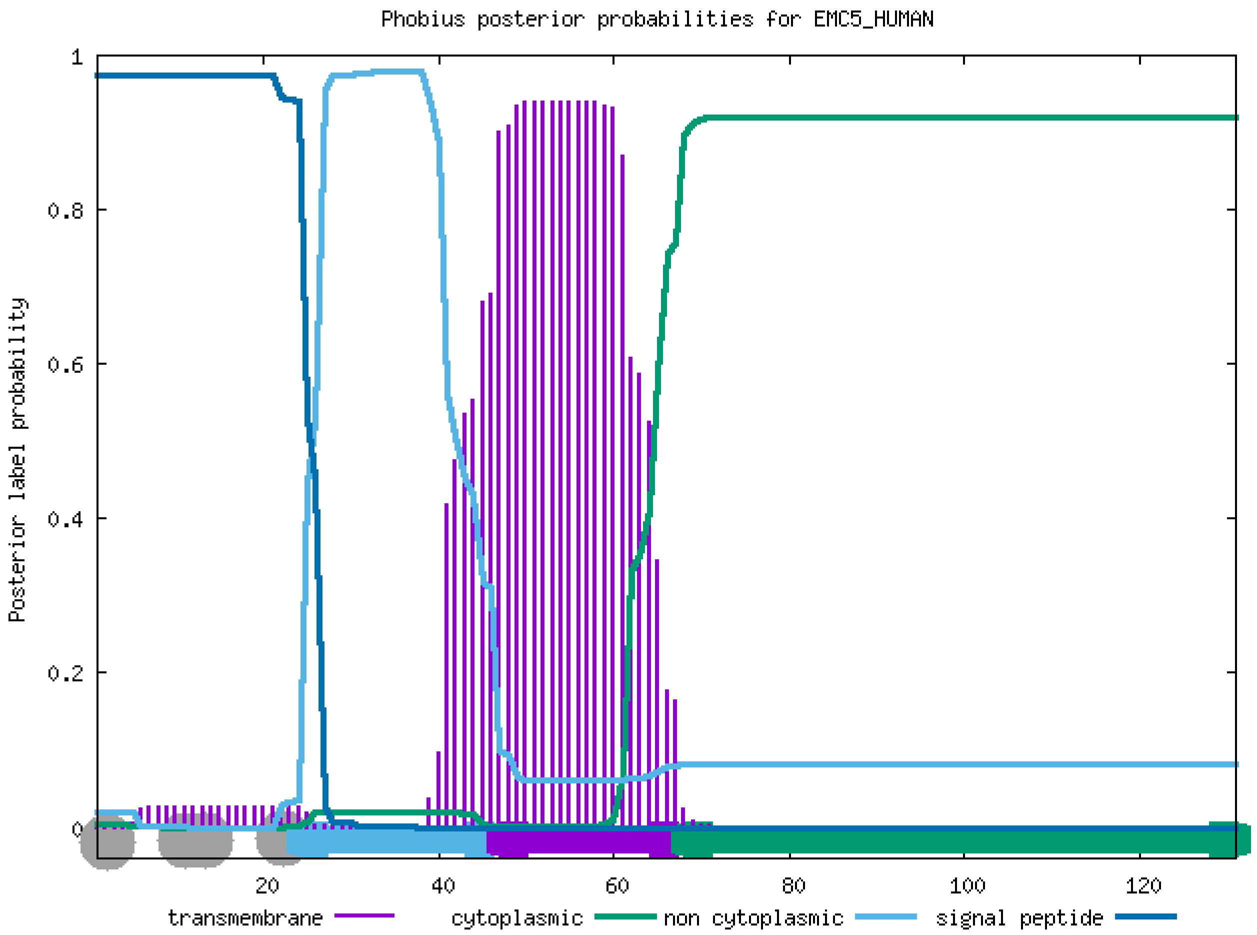

The probability data used in the plot is found here, and the gnuplot script is here.

Prediction of EMC6_HUMAN

```
ID   EMC6_HUMAN
FT   TOPO_DOM      1     51       NON CYTOPLASMIC.
FT   TRANSMEM     52     71
FT   TOPO_DOM     72     82       CYTOPLASMIC.
FT   TRANSMEM     83    107
FT   TOPO_DOM    108    110       NON CYTOPLASMIC.
//
```

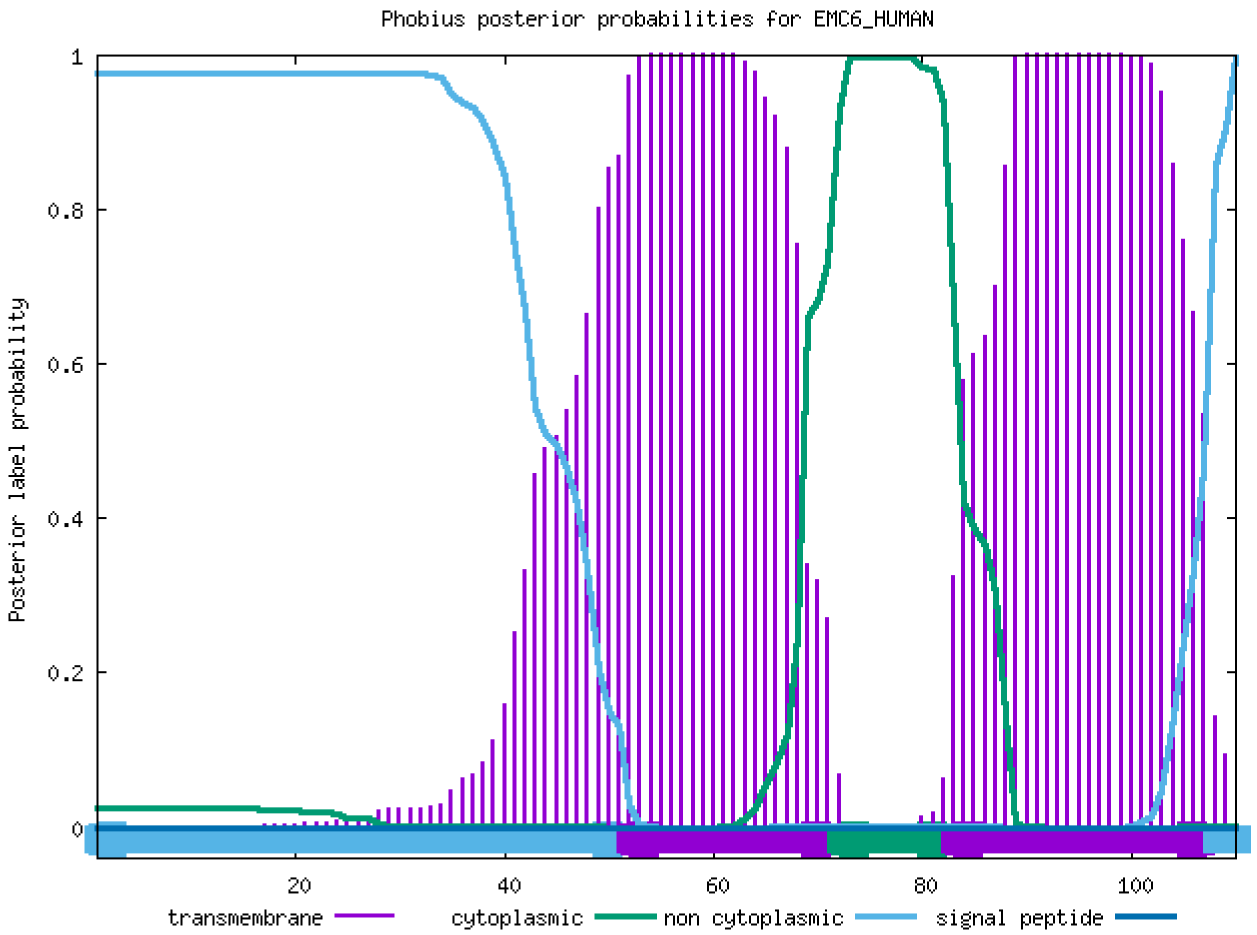

The probability data used in the plot is found here, and the gnuplot script is here.

Prediction of EMC7_HUMAN

```
ID   EMC7_HUMAN
FT   SIGNAL        1     23
FT   REGION        1      1       N-REGION.
FT   REGION        2     17       H-REGION.
FT   REGION       18     23       C-REGION.
FT   TOPO_DOM     24    164       NON CYTOPLASMIC.
FT   TRANSMEM    165    183
FT   TOPO_DOM    184    242       CYTOPLASMIC.
//
```

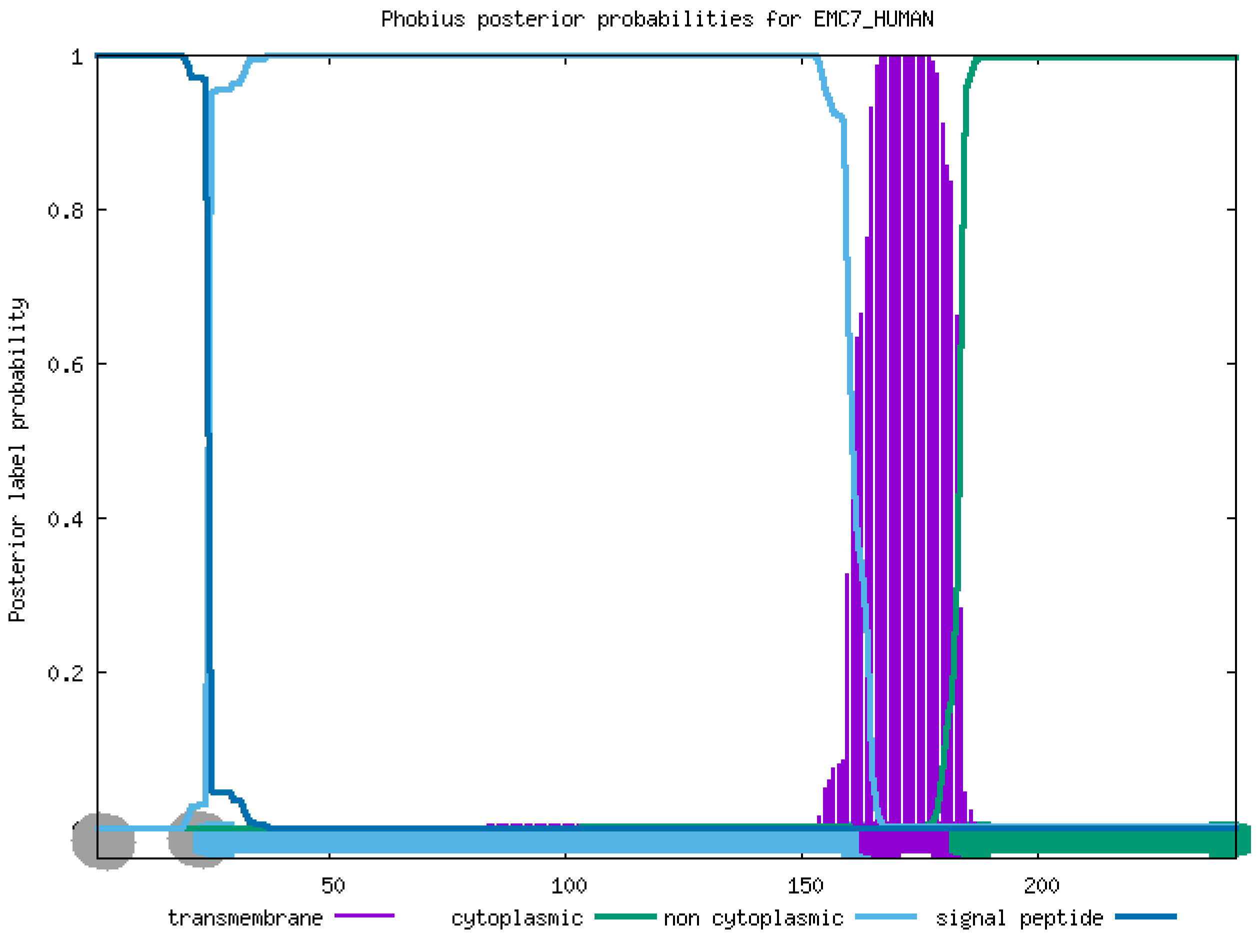

The probability data used in the plot is found here, and the gnuplot script is here.

Prediction of EMC10_HUMAN

```
ID    EMC10_HUMAN
FT    SIGNAL         1     25
FT    REGION         1     10       N-REGION.
FT    REGION        11     21       H-REGION.
FT    REGION        22     25       C-REGION.
FT    TOPO_DOM      26    262       NON CYTOPLASMIC.
//
```

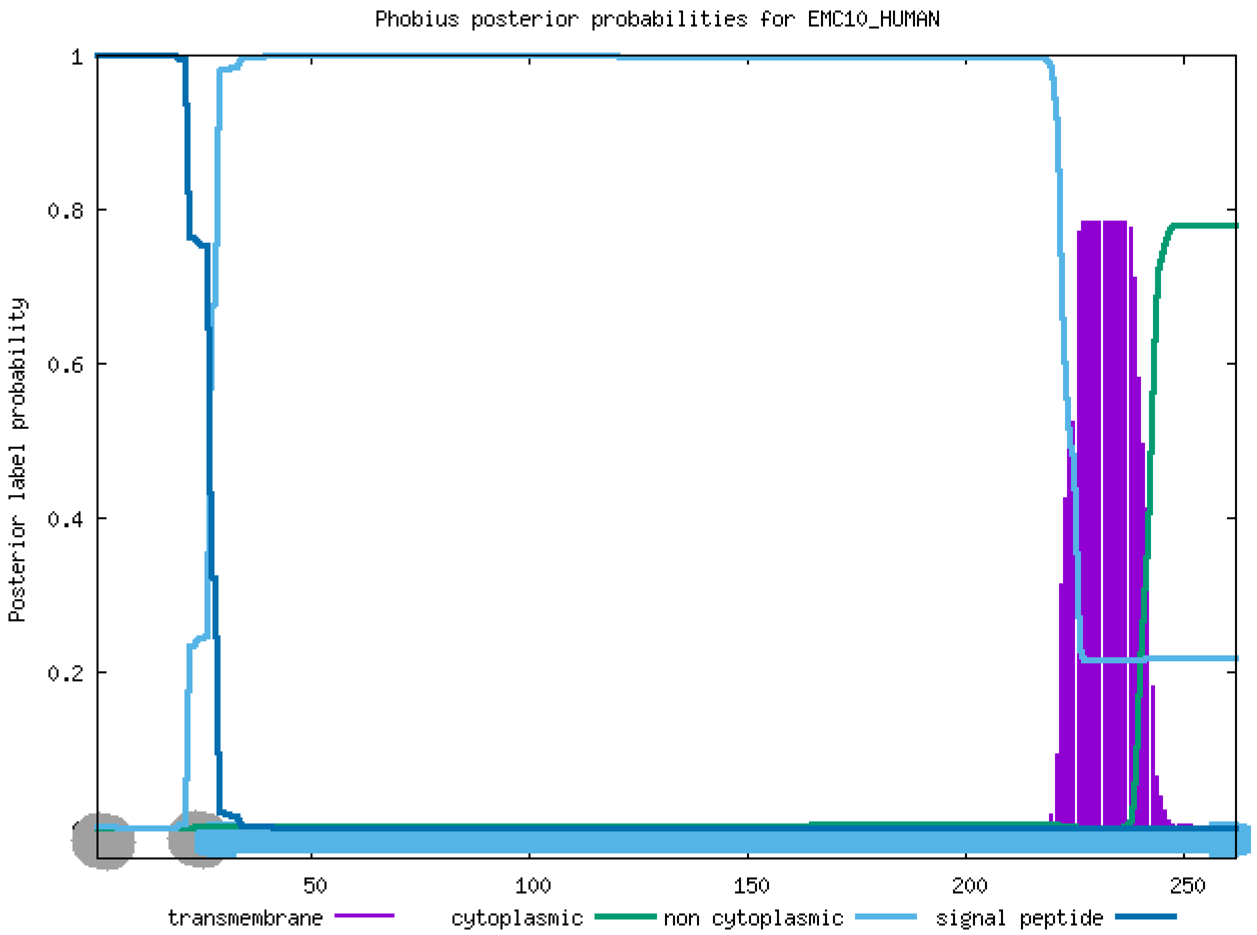

The probability data used in the plot is found here, and the gnuplot script is here.

Prediction of TIM22_HUMAN

```
ID   TIM22_HUMAN
FT   TOPO_DOM      1     68       CYTOPLASMIC.
FT   TRANSMEM     69     94
FT   TOPO_DOM     95    173       NON CYTOPLASMIC.
FT   TRANSMEM    174    193
FT   TOPO_DOM    194    194       CYTOPLASMIC.
//
```

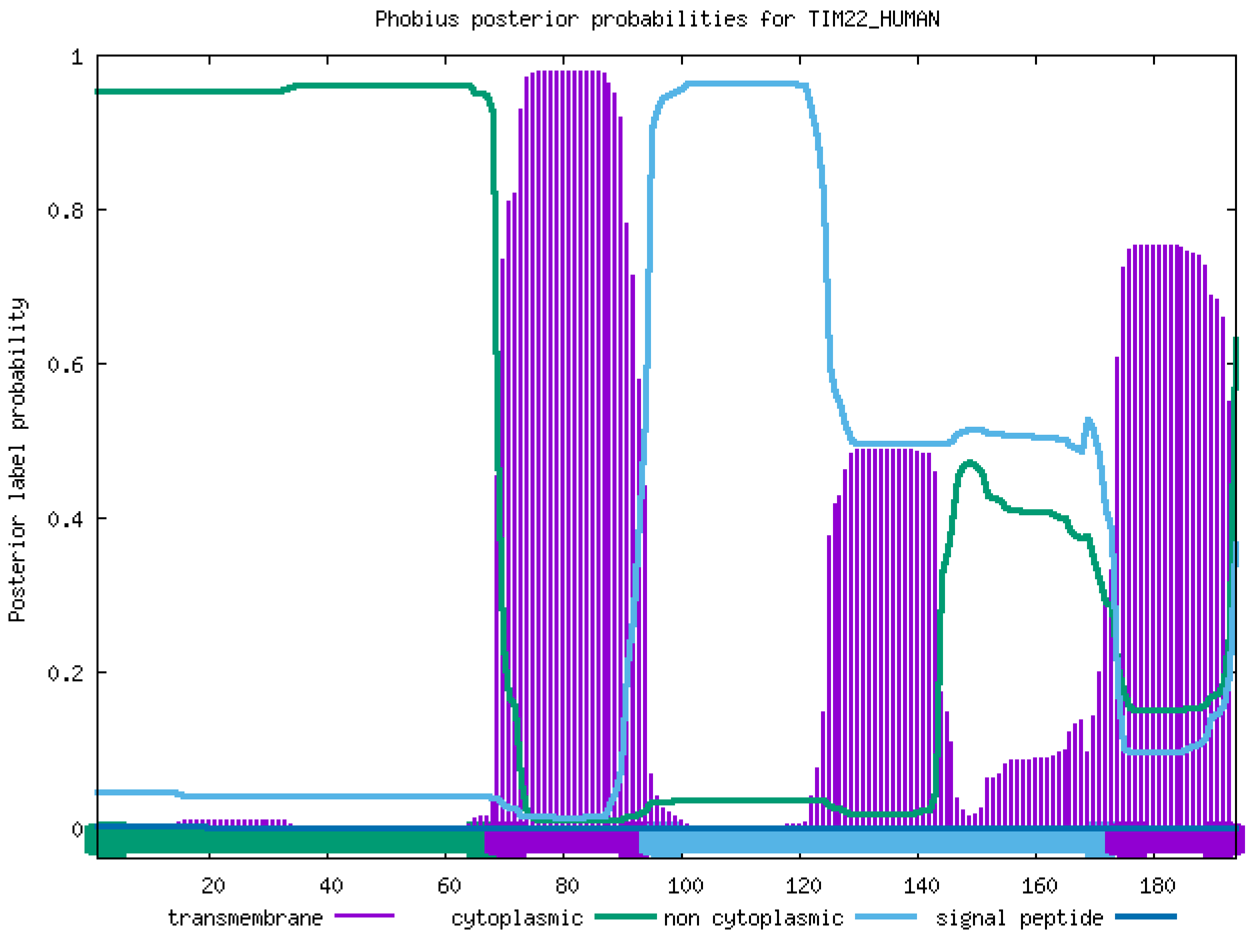

The probability data used in the plot is found here, and the gnuplot script is here.

Prediction of TIM29_HUMAN

```
ID   TIM29_HUMAN
FT   TOPO_DOM      1    260       NON CYTOPLASMIC.
//
```

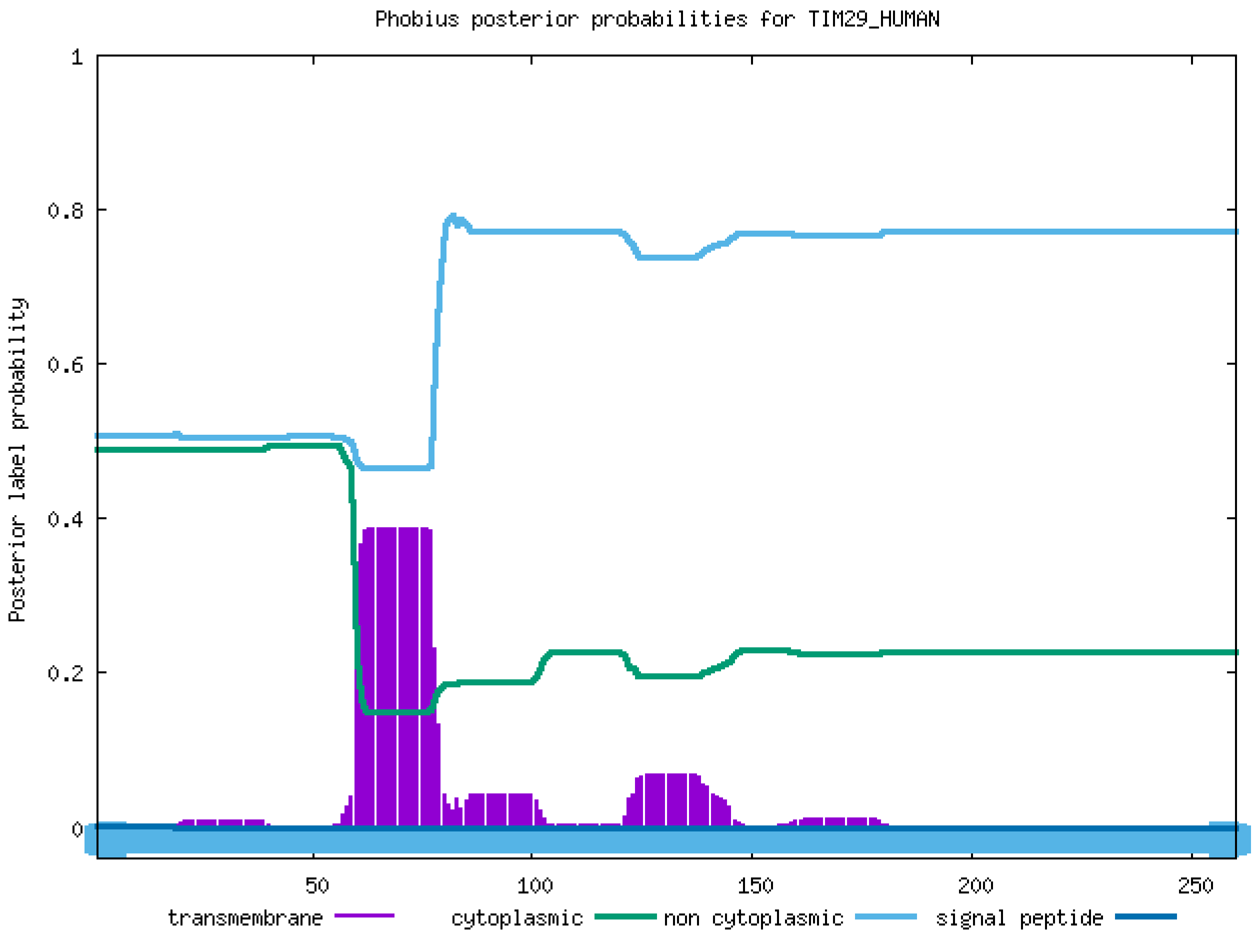

The probability data used in the plot is found here, and the gnuplot script is here.

Prediction of CSCL1_HUMAN

ID	CSCL1_HUMAN			
FT	TOPO_DOM	1	48	NON CYTOPLASMIC.
FT	TRANSMEM	49	71	
FT	TOPO_DOM	72	145	CYTOPLASMIC.
FT	TRANSMEM	146	166	
FT	TOPO_DOM	167	191	NON CYTOPLASMIC.
FT	TRANSMEM	192	211	
FT	TOPO_DOM	212	418	CYTOPLASMIC.
FT	TRANSMEM	419	442	
FT	TOPO_DOM	443	461	NON CYTOPLASMIC.
FT	TRANSMEM	462	485	
FT	TOPO_DOM	486	505	CYTOPLASMIC.
FT	TRANSMEM	506	529	
FT	TOPO_DOM	530	610	NON CYTOPLASMIC.
FT	TRANSMEM	611	641	
FT	TOPO_DOM	642	661	CYTOPLASMIC.
FT	TRANSMEM	662	684	
FT	TOPO_DOM	685	695	NON CYTOPLASMIC.
FT	TRANSMEM	696	720	
FT	TOPO_DOM	721	807	CYTOPLASMIC.
//				

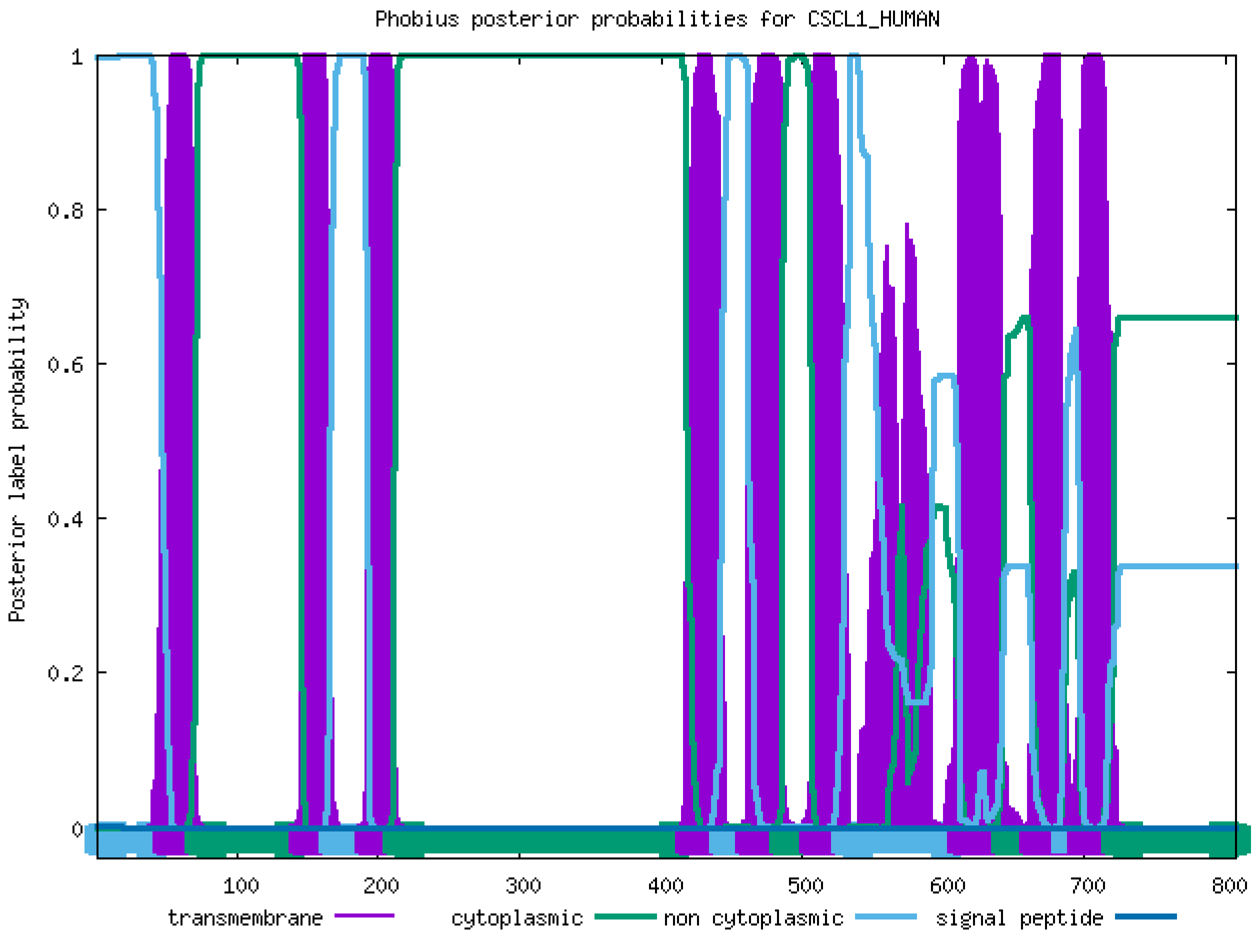

The probability data used in the plot is found here, and the gnuplot script is here.

Prediction of CSCL2_HUMAN

```
ID   CSCL2_HUMAN
FT   SIGNAL        1     19
FT   REGION        1      3       N-REGION.
FT   REGION        4     14       H-REGION.
FT   REGION       15     19       C-REGION.
FT   TOPO_DOM     20     42       NON CYTOPLASMIC.
FT   TRANSMEM     43     62
FT   TOPO_DOM     63    157       CYTOPLASMIC.
FT   TRANSMEM    158    178
FT   TOPO_DOM    179    203       NON CYTOPLASMIC.
FT   TRANSMEM    204    223
FT   TOPO_DOM    224    429       CYTOPLASMIC.
FT   TRANSMEM    430    454
FT   TOPO_DOM    455    473       NON CYTOPLASMIC.
FT   TRANSMEM    474    497
FT   TOPO_DOM    498    517       CYTOPLASMIC.
FT   TRANSMEM    518    541
FT   TOPO_DOM    542    552       NON CYTOPLASMIC.
FT   TRANSMEM    553    574
FT   TOPO_DOM    575    585       CYTOPLASMIC.
FT   TRANSMEM    586    605
FT   TOPO_DOM    606    624       NON CYTOPLASMIC.
FT   TRANSMEM    625    654
FT   TOPO_DOM    655    679       CYTOPLASMIC.
FT   TRANSMEM    680    699
FT   TOPO_DOM    700    710       NON CYTOPLASMIC.
FT   TRANSMEM    711    733
FT   TOPO_DOM    734    832       CYTOPLASMIC.
//
```

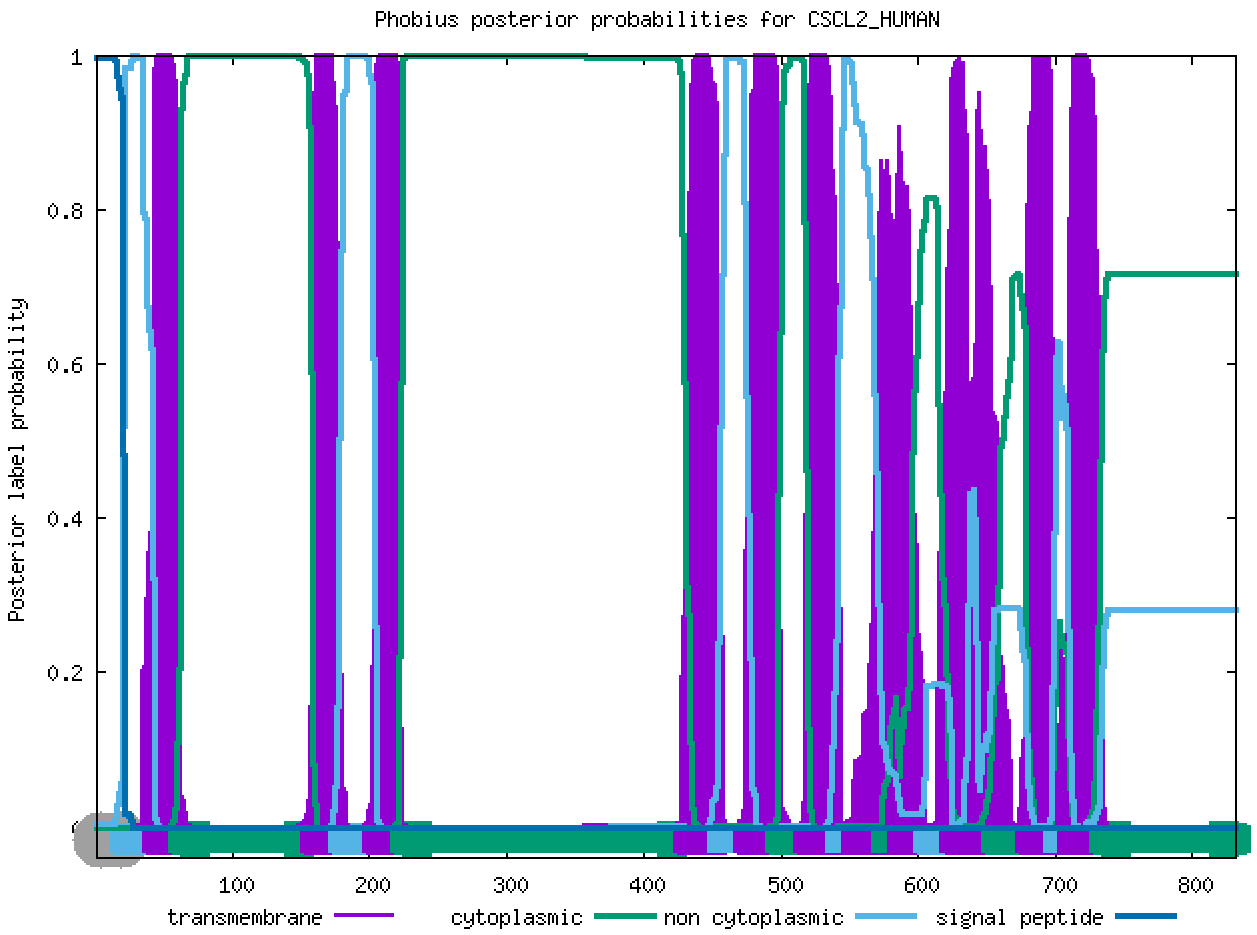

The probability data used in the plot is found here, and the gnuplot script is here.

Prediction of KCNK1_HUMAN

```
ID    KCNK1_HUMAN
FT    TOPO_DOM      1     20       CYTOPLASMIC.
FT    TRANSMEM     21     44
FT    TOPO_DOM     45    134       NON CYTOPLASMIC.
FT    TRANSMEM    135    157
FT    TOPO_DOM    158    177       CYTOPLASMIC.
FT    TRANSMEM    178    203
FT    TOPO_DOM    204    214       NON CYTOPLASMIC.
FT    TRANSMEM    215    232
FT    TOPO_DOM    233    243       CYTOPLASMIC.
FT    TRANSMEM    244    264
FT    TOPO_DOM    265    336       NON CYTOPLASMIC.
//
```

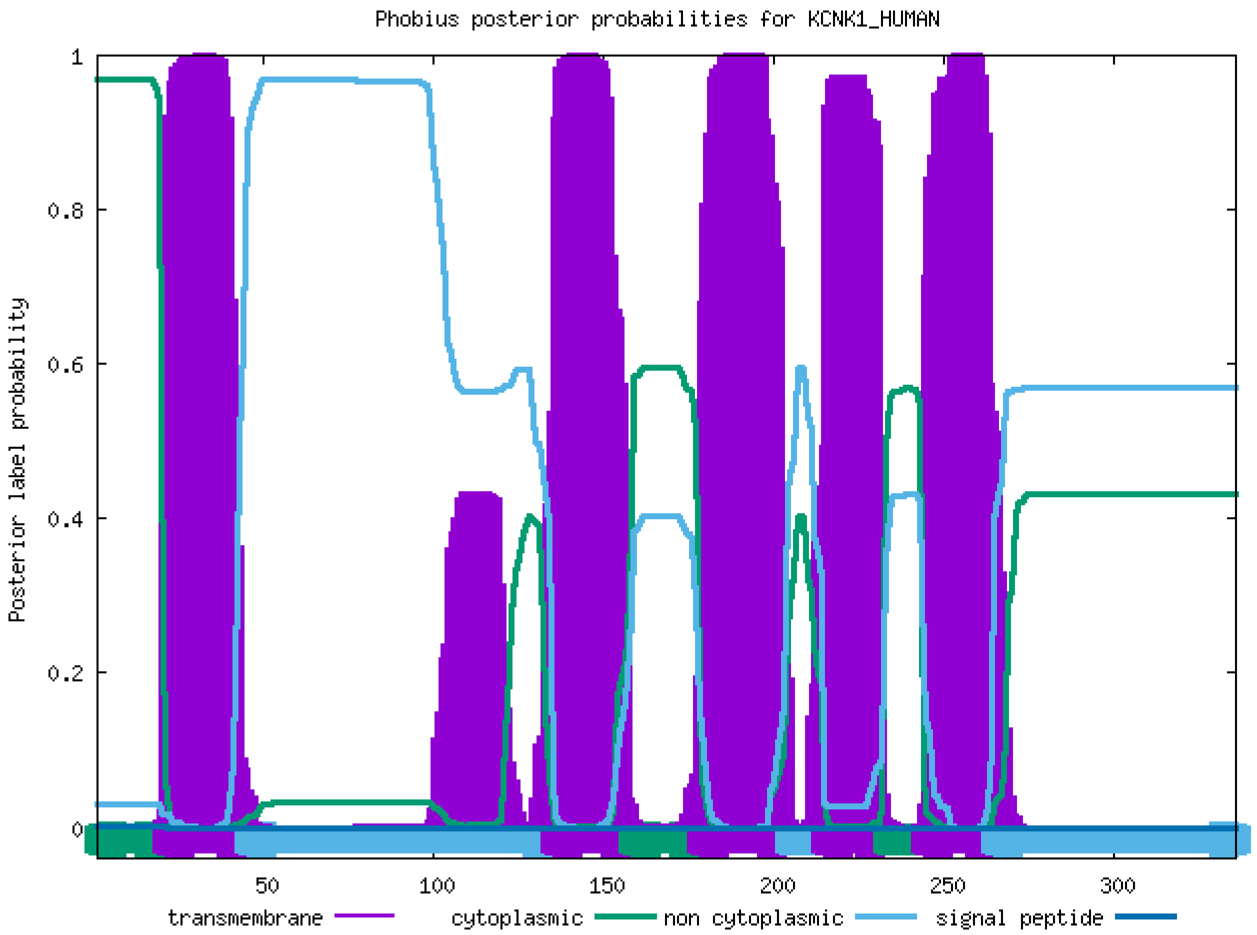

The probability data used in the plot is found here, and the gnuplot script is here.

Prediction of KCNK3_HUMAN

```
ID    KCNK3_HUMAN
FT    SIGNAL          1      23
FT    REGION          1       7       N-REGION.
FT    REGION          8      19       H-REGION.
FT    REGION         20      23       C-REGION.
FT    TOPO_DOM       24      75       NON CYTOPLASMIC.
FT    TRANSMEM       76      96
FT    TOPO_DOM       97     107       CYTOPLASMIC.
FT    TRANSMEM      108     128
FT    TOPO_DOM      129     155       NON CYTOPLASMIC.
FT    TRANSMEM      156     177
FT    TOPO_DOM      178     188       CYTOPLASMIC.
FT    TRANSMEM      189     208
FT    TOPO_DOM      209     219       NON CYTOPLASMIC.
FT    TRANSMEM      220     246
FT    TOPO_DOM      247     394       CYTOPLASMIC.
//
```

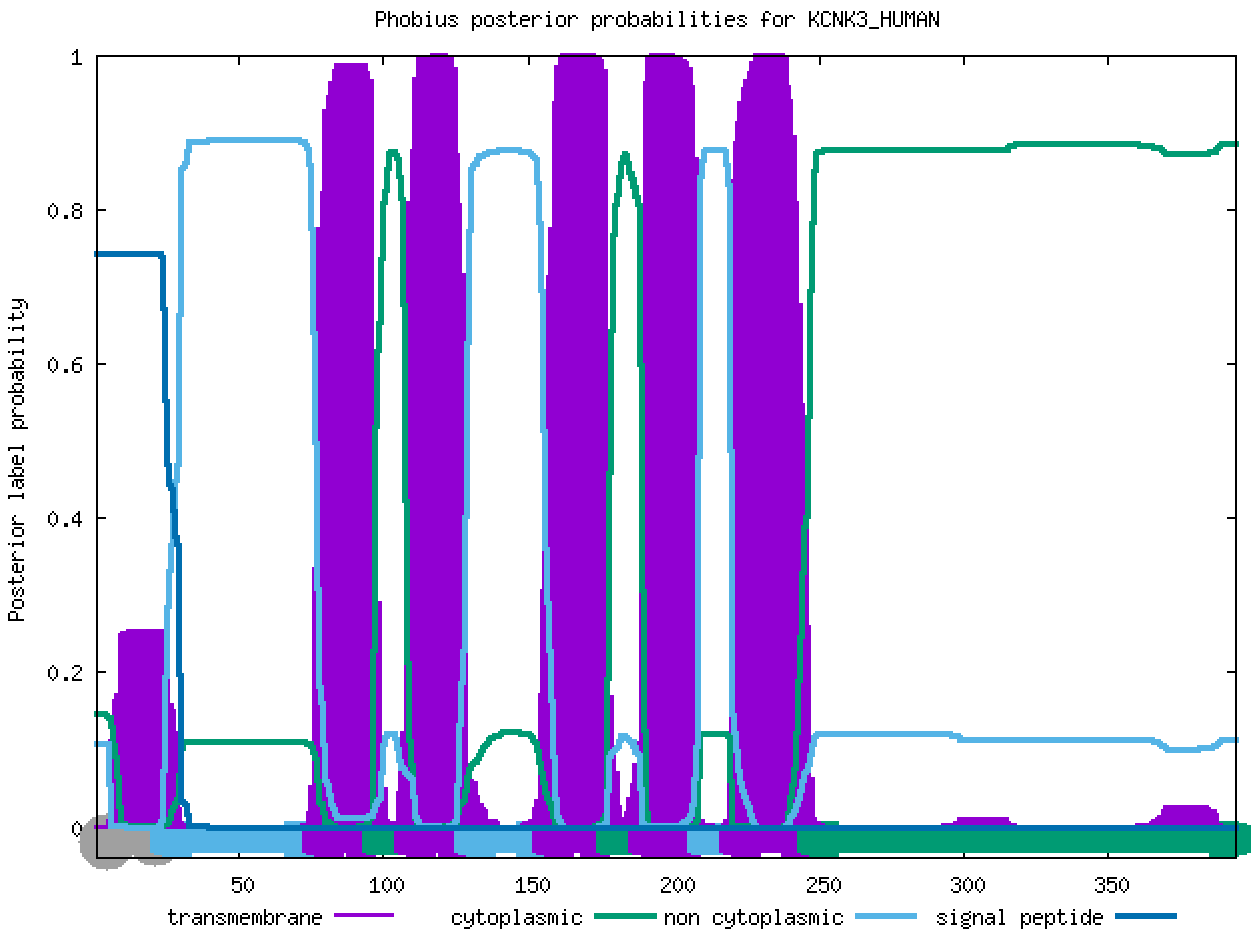

The probability data used in the plot is found here, and the gnuplot script is here.

Prediction of KCNK4_HUMAN

```
ID   KCNK4_HUMAN
FT   SIGNAL        1      20
FT   REGION        1       3       N-REGION.
FT   REGION        4      15       H-REGION.
FT   REGION       16      20       C-REGION.
FT   TOPO_DOM     21      89       NON CYTOPLASMIC.
FT   TRANSMEM     90     111
FT   TOPO_DOM    112     117       CYTOPLASMIC.
FT   TRANSMEM    118     138
FT   TOPO_DOM    139     168       NON CYTOPLASMIC.
FT   TRANSMEM    169     190
FT   TOPO_DOM    191     201       CYTOPLASMIC.
FT   TRANSMEM    202     222
FT   TOPO_DOM    223     233       NON CYTOPLASMIC.
FT   TRANSMEM    234     257
FT   TOPO_DOM    258     393       CYTOPLASMIC.
//
```

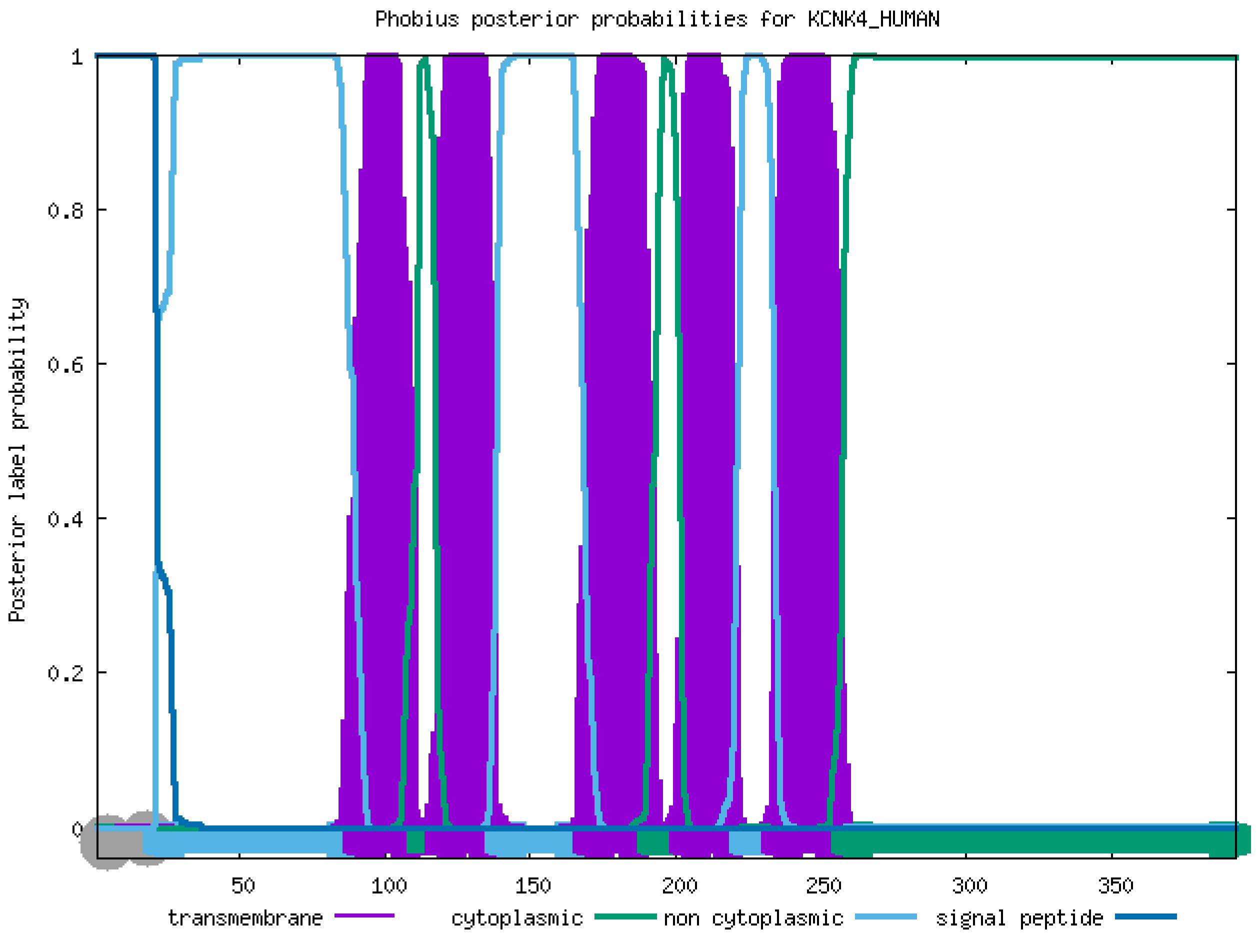

The probability data used in the plot is found here, and the gnuplot script is here.

Prediction of KCNKA_HUMAN

```
ID   KCNKA_HUMAN
FT   SIGNAL        1     24
FT   REGION        1      7       N-REGION.
FT   REGION        8     19       H-REGION.
FT   REGION       20     24       C-REGION.
FT   TOPO_DOM     25     71       NON CYTOPLASMIC.
FT   TRANSMEM     72     93
FT   TOPO_DOM     94    153       CYTOPLASMIC.
FT   TRANSMEM    154    173
FT   TOPO_DOM    174    184       NON CYTOPLASMIC.
FT   TRANSMEM    185    210
FT   TOPO_DOM    211    230       CYTOPLASMIC.
FT   TRANSMEM    231    255
FT   TOPO_DOM    256    266       NON CYTOPLASMIC.
FT   TRANSMEM    267    288
FT   TOPO_DOM    289    299       CYTOPLASMIC.
FT   TRANSMEM    300    322
FT   TOPO_DOM    323    538       NON CYTOPLASMIC.
//
```

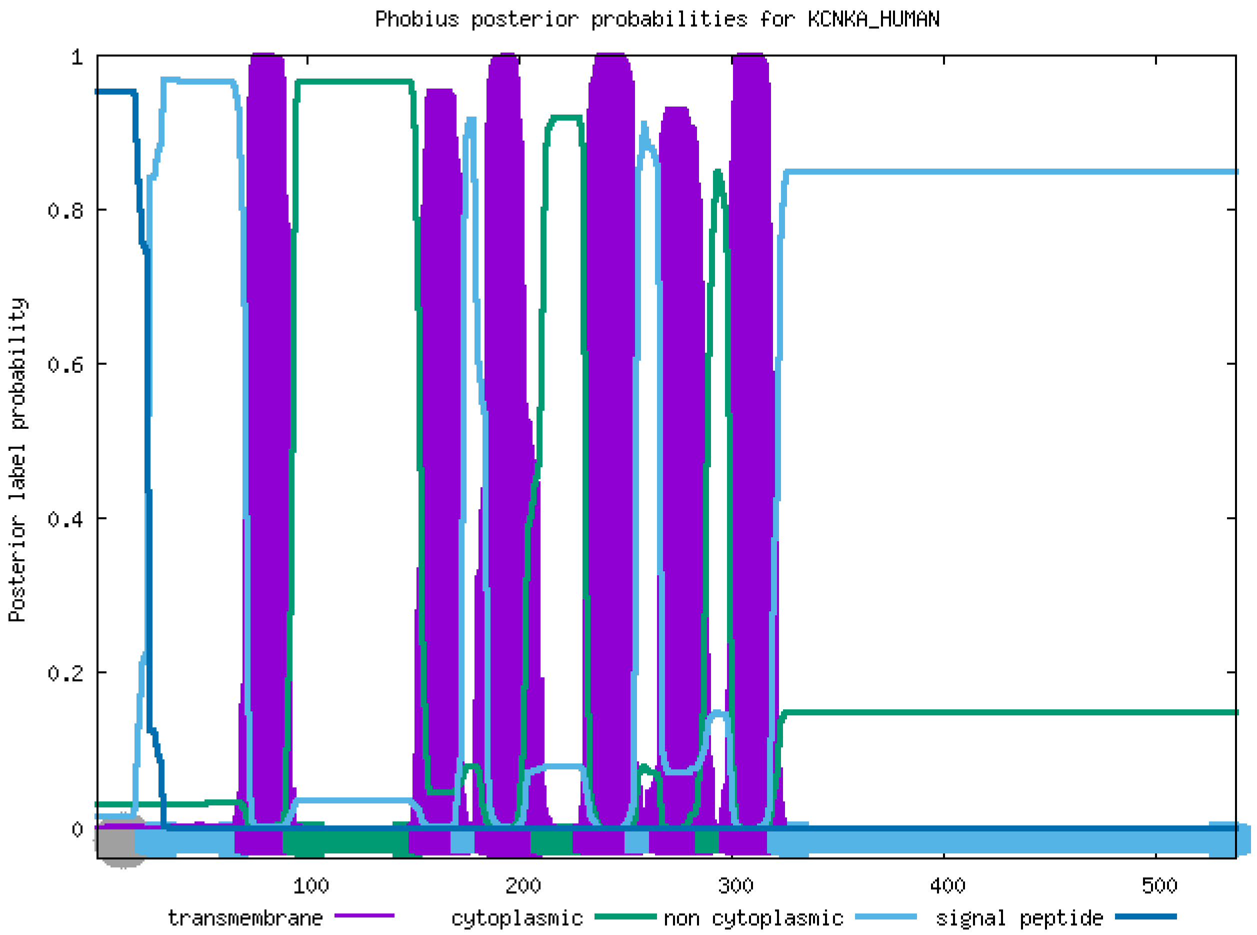

The probability data used in the plot is found here, and the gnuplot script is here.

Prediction of TPC2_HUMAN

```
ID    TPC2_HUMAN
FT    TOPO_DOM      1      80       CYTOPLASMIC.
FT    TRANSMEM     81      99
FT    TOPO_DOM    100     155       NON CYTOPLASMIC.
FT    TRANSMEM    156     179
FT    TOPO_DOM    180     218       CYTOPLASMIC.
FT    TRANSMEM    219     239
FT    TOPO_DOM    240     286       NON CYTOPLASMIC.
FT    TRANSMEM    287     312
FT    TOPO_DOM    313     435       CYTOPLASMIC.
FT    TRANSMEM    436     455
FT    TOPO_DOM    456     466       NON CYTOPLASMIC.
FT    TRANSMEM    467     486
FT    TOPO_DOM    487     506       CYTOPLASMIC.
FT    TRANSMEM    507     526
FT    TOPO_DOM    527     545       NON CYTOPLASMIC.
FT    TRANSMEM    546     569
FT    TOPO_DOM    570     580       CYTOPLASMIC.
FT    TRANSMEM    581     601
FT    TOPO_DOM    602     638       NON CYTOPLASMIC.
FT    TRANSMEM    639     662
FT    TOPO_DOM    663     673       CYTOPLASMIC.
FT    TRANSMEM    674     694
FT    TOPO_DOM    695     752       NON CYTOPLASMIC.
//
```

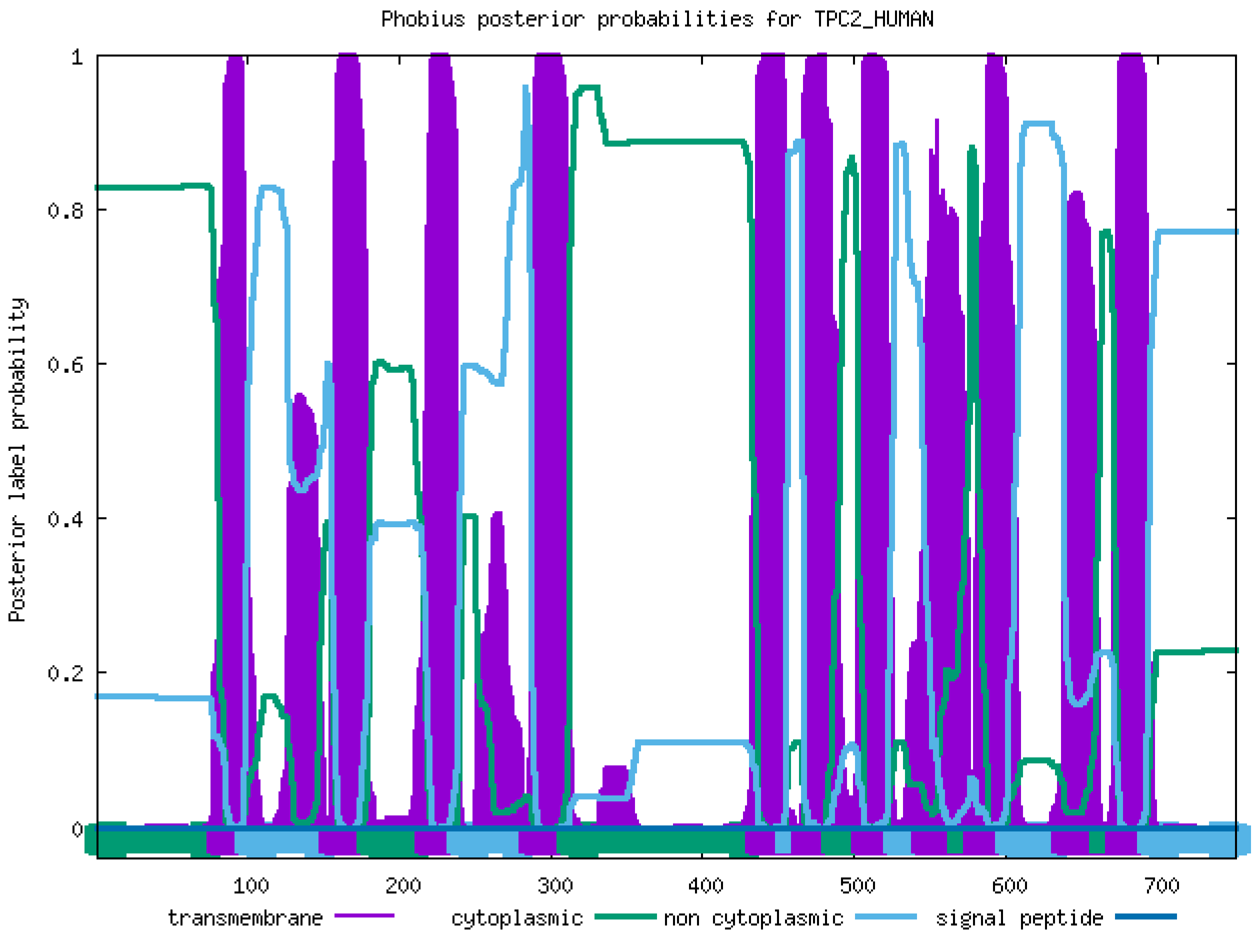

The probability data used in the plot is found here, and the gnuplot script is here.

Prediction of KCNA3_HUMAN

```
ID    KCNA3_HUMAN
FT    TOPO_DOM      1    235       CYTOPLASMIC.
FT    TRANSMEM    236    256
FT    TOPO_DOM    257    294       NON CYTOPLASMIC.
FT    TRANSMEM    295    315
FT    TOPO_DOM    316    326       CYTOPLASMIC.
FT    TRANSMEM    327    345
FT    TOPO_DOM    346    356       NON CYTOPLASMIC.
FT    TRANSMEM    357    377
FT    TOPO_DOM    378    397       CYTOPLASMIC.
FT    TRANSMEM    398    419
FT    TOPO_DOM    420    458       NON CYTOPLASMIC.
FT    TRANSMEM    459    480
FT    TOPO_DOM    481    575       CYTOPLASMIC.
//
```

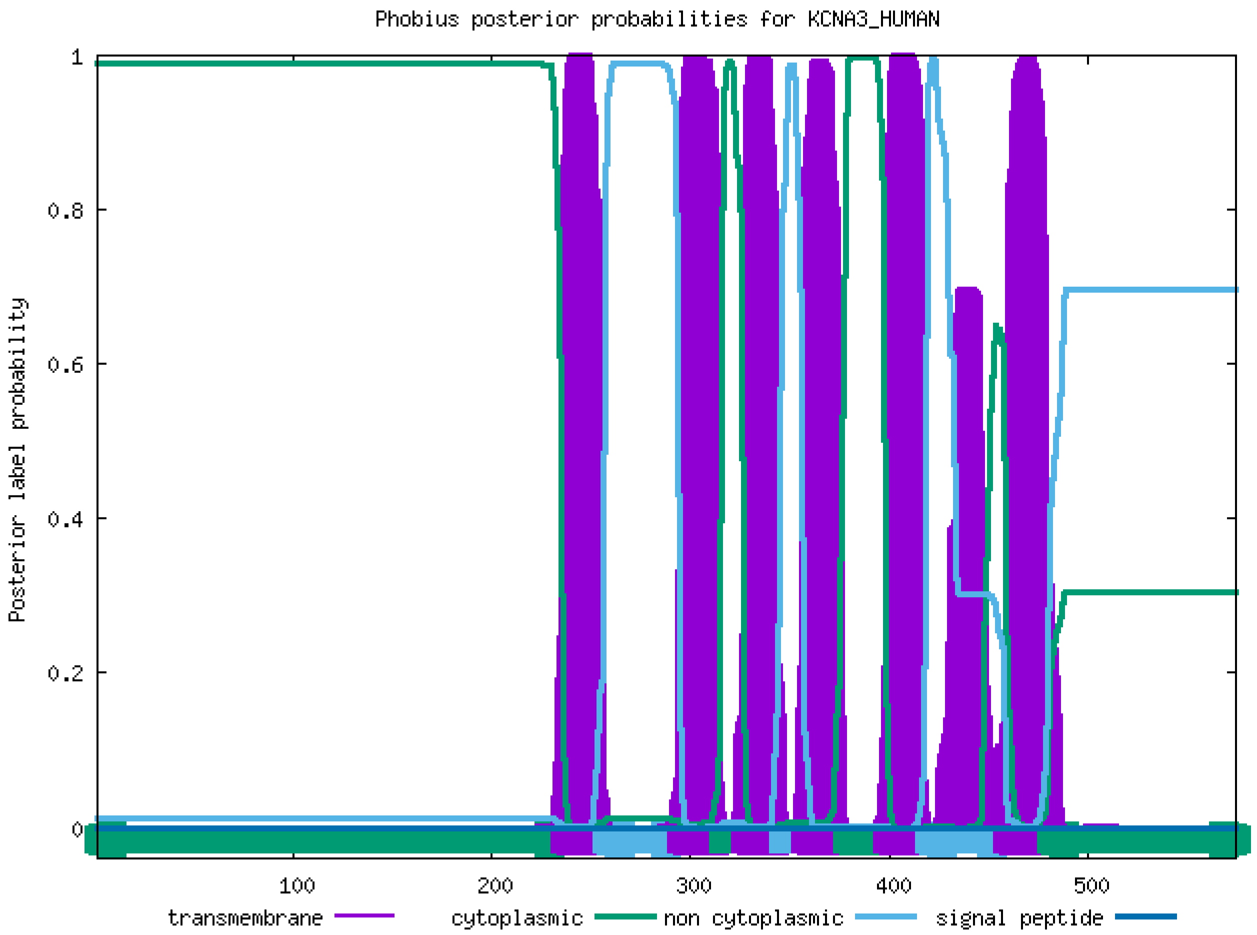

The probability data used in the plot is found here, and the gnuplot script is here.

Prediction of KCNC1_HUMAN

```
ID   KCNC1_HUMAN
FT   TOPO_DOM      1    187       CYTOPLASMIC.
FT   TRANSMEM    188    207
FT   TOPO_DOM    208    243       NON CYTOPLASMIC.
FT   TRANSMEM    244    266
FT   TOPO_DOM    267    272       CYTOPLASMIC.
FT   TRANSMEM    273    291
FT   TOPO_DOM    292    344       NON CYTOPLASMIC.
FT   TRANSMEM    345    366
FT   TOPO_DOM    367    386       CYTOPLASMIC.
FT   TRANSMEM    387    404
FT   TOPO_DOM    405    415       NON CYTOPLASMIC.
FT   TRANSMEM    416    445
FT   TOPO_DOM    446    511       CYTOPLASMIC.
//
```

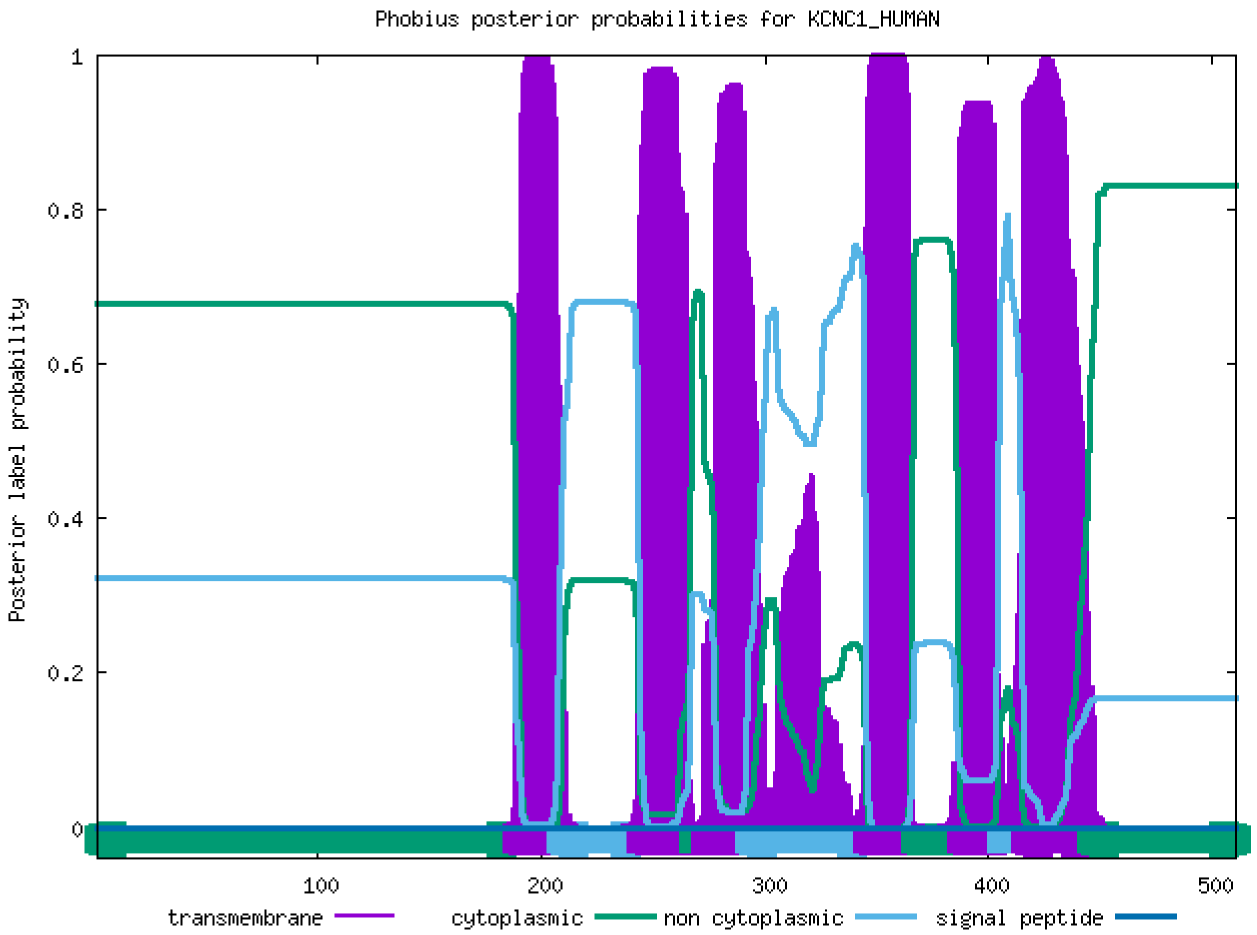

The probability data used in the plot is found here, and the gnuplot script is here.

Prediction of KCND2_HUMAN

```
ID    KCND2_HUMAN
FT    TOPO_DOM      1    182       CYTOPLASMIC.
FT    TRANSMEM    183    204
FT    TOPO_DOM    205    223       NON CYTOPLASMIC.
FT    TRANSMEM    224    245
FT    TOPO_DOM    246    256       CYTOPLASMIC.
FT    TRANSMEM    257    279
FT    TOPO_DOM    280    284       NON CYTOPLASMIC.
FT    TRANSMEM    285    303
FT    TOPO_DOM    304    323       CYTOPLASMIC.
FT    TRANSMEM    324    345
FT    TOPO_DOM    346    356       NON CYTOPLASMIC.
FT    TRANSMEM    357    373
FT    TOPO_DOM    374    384       CYTOPLASMIC.
FT    TRANSMEM    385    406
FT    TOPO_DOM    407    630       NON CYTOPLASMIC.
//
```

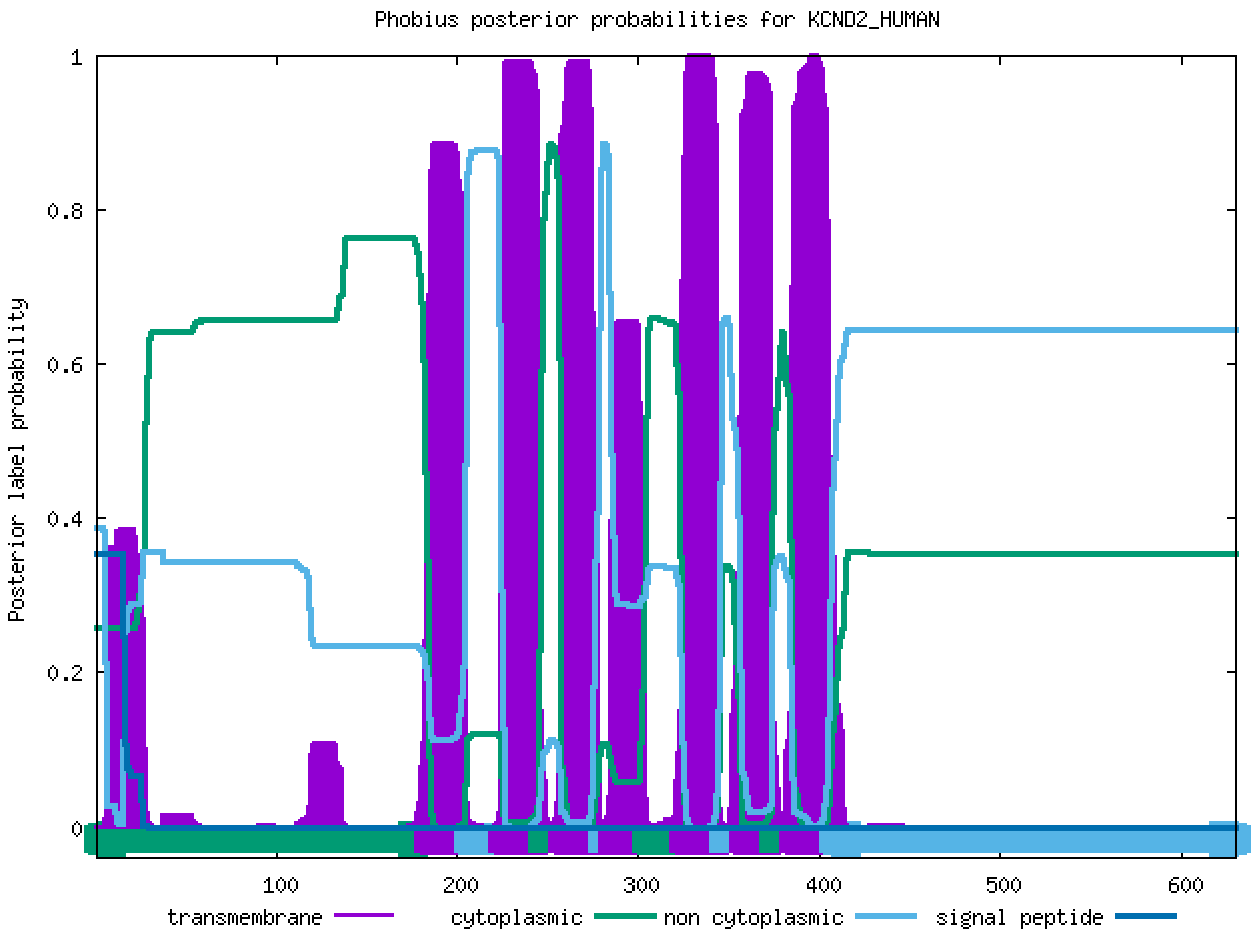

The probability data used in the plot is found here, and the gnuplot script is here.

Prediction of KCND3_HUMAN

```
ID   KCND3_HUMAN
FT   TOPO_DOM      1    180       CYTOPLASMIC.
FT   TRANSMEM    181    202
FT   TOPO_DOM    203    221       NON CYTOPLASMIC.
FT   TRANSMEM    222    243
FT   TOPO_DOM    244    254       CYTOPLASMIC.
FT   TRANSMEM    255    276
FT   TOPO_DOM    277    281       NON CYTOPLASMIC.
FT   TRANSMEM    282    300
FT   TOPO_DOM    301    320       CYTOPLASMIC.
FT   TRANSMEM    321    342
FT   TOPO_DOM    343    353       NON CYTOPLASMIC.
FT   TRANSMEM    354    370
FT   TOPO_DOM    371    381       CYTOPLASMIC.
FT   TRANSMEM    382    403
FT   TOPO_DOM    404    655       NON CYTOPLASMIC.
//
```

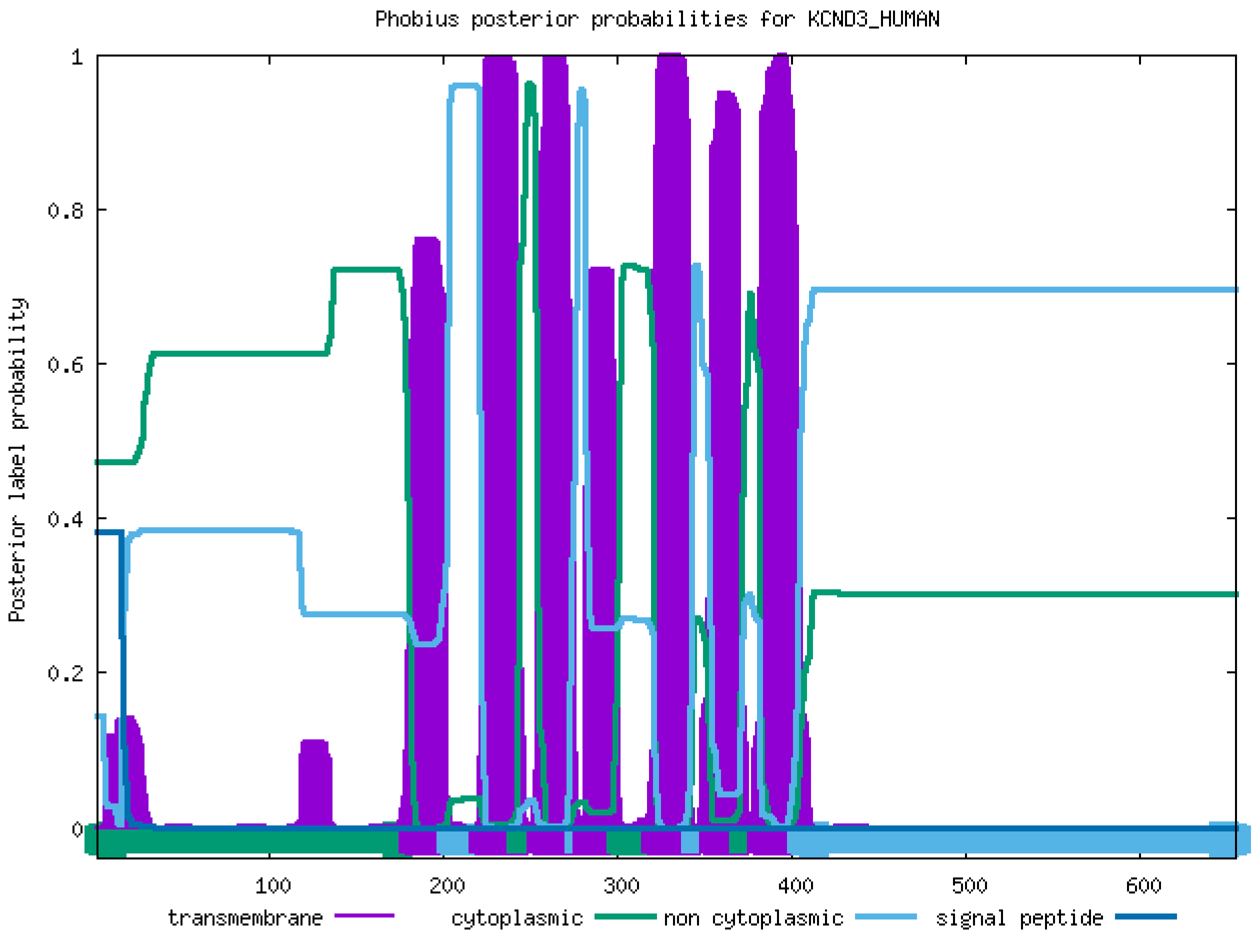

The probability data used in the plot is found here, and the gnuplot script is here.

Prediction of KCNH5_HUMAN

```
ID    KCNH5_HUMAN
FT    TOPO_DOM       1     216       NON CYTOPLASMIC.
FT    TRANSMEM     217     236
FT    TOPO_DOM     237     345       CYTOPLASMIC.
FT    TRANSMEM     346     370
FT    TOPO_DOM     371     417       NON CYTOPLASMIC.
FT    TRANSMEM     418     438
FT    TOPO_DOM     439     449       CYTOPLASMIC.
FT    TRANSMEM     450     471
FT    TOPO_DOM     472     988       NON CYTOPLASMIC.
//
```

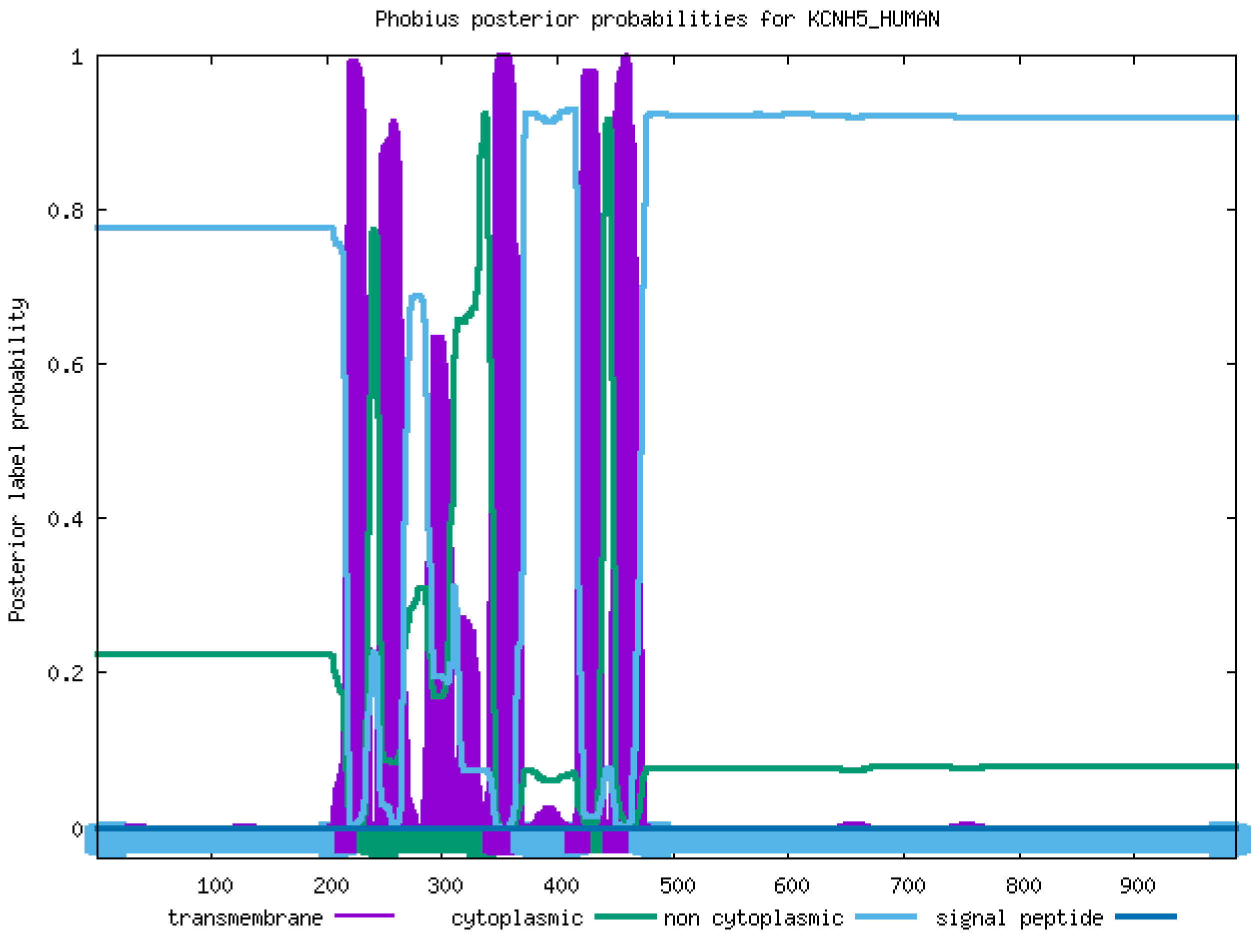

The probability data used in the plot is found here, and the gnuplot script is here.

Prediction of KCNH2_HUMAN

```
ID    KCNH2_HUMAN
FT    TOPO_DOM      1     411       NON CYTOPLASMIC.
FT    TRANSMEM    412     433
FT    TOPO_DOM    434     444       CYTOPLASMIC.
FT    TRANSMEM    445     469
FT    TOPO_DOM    470     501       NON CYTOPLASMIC.
FT    TRANSMEM    502     524
FT    TOPO_DOM    525     544       CYTOPLASMIC.
FT    TRANSMEM    545     571
FT    TOPO_DOM    572     638       NON CYTOPLASMIC.
FT    TRANSMEM    639     663
FT    TOPO_DOM    664    1159       CYTOPLASMIC.
//
```

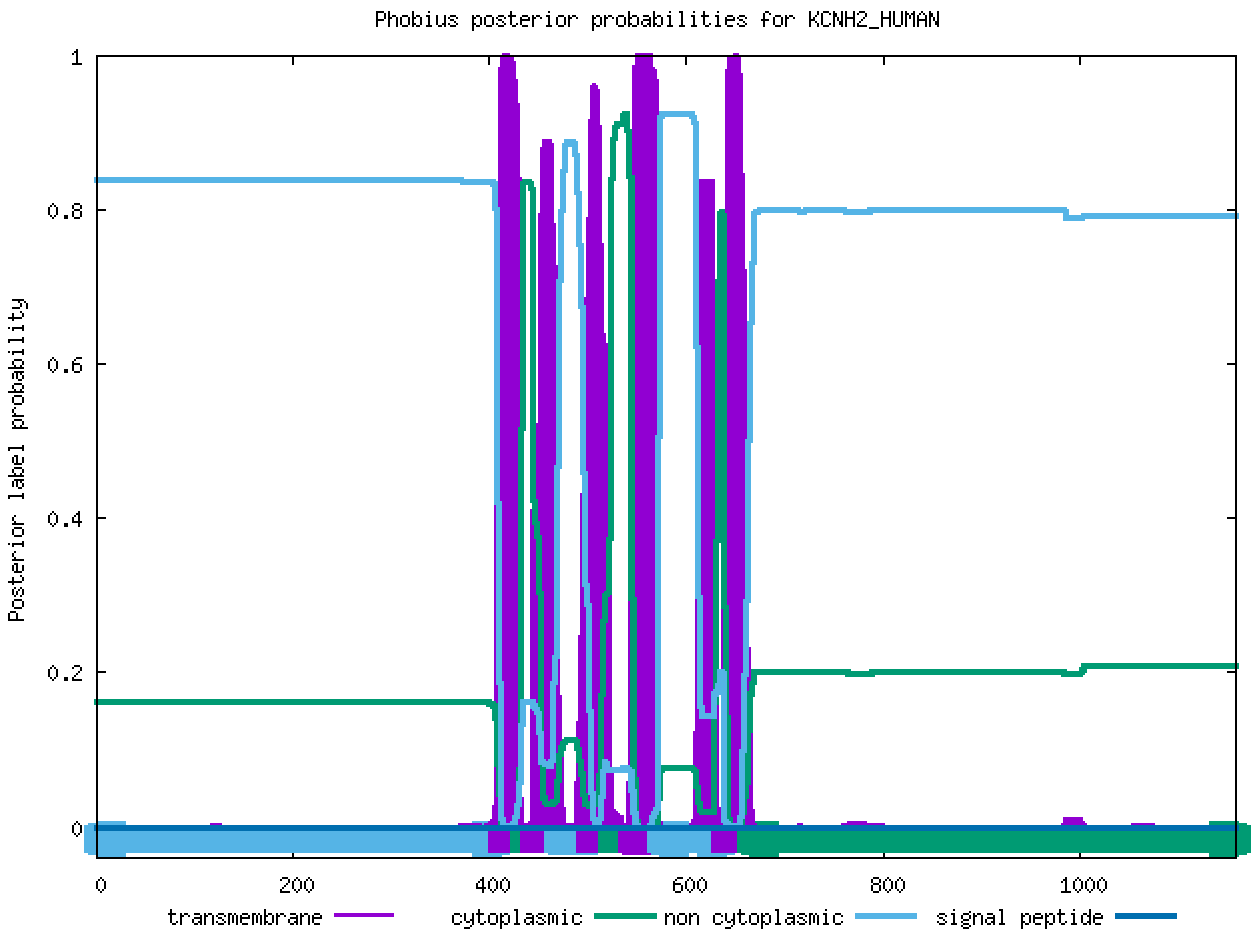

The probability data used in the plot is found here, and the gnuplot script is here.

Prediction of KCMA1_HUMAN

```
ID   KCMA1_HUMAN
FT   TOPO_DOM      1     85       NON CYTOPLASMIC.
FT   TRANSMEM     86    108
FT   TOPO_DOM    109    178       CYTOPLASMIC.
FT   TRANSMEM    179    197
FT   TOPO_DOM    198    216       NON CYTOPLASMIC.
FT   TRANSMEM    217    235
FT   TOPO_DOM    236    241       CYTOPLASMIC.
FT   TRANSMEM    242    264
FT   TOPO_DOM    265    275       NON CYTOPLASMIC.
FT   TRANSMEM    276    293
FT   TOPO_DOM    294    299       CYTOPLASMIC.
FT   TRANSMEM    300    321
FT   TOPO_DOM    322    335       NON CYTOPLASMIC.
FT   TRANSMEM    336    355
FT   TOPO_DOM    356    366       CYTOPLASMIC.
FT   TRANSMEM    367    385
FT   TOPO_DOM    386   1236       NON CYTOPLASMIC.
//
```

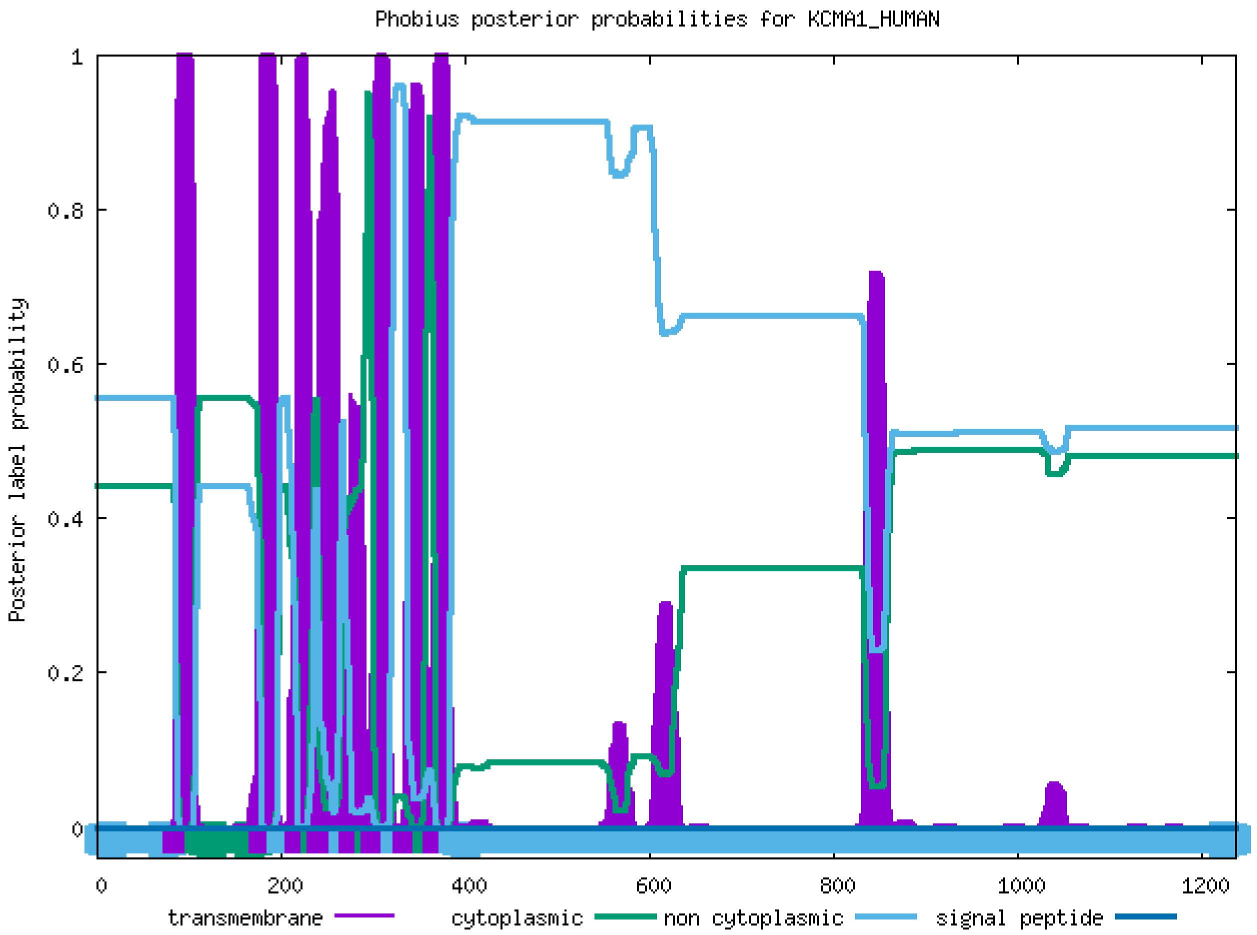

The probability data used in the plot is found here, and the gnuplot script is here.

Prediction of KCNT1_HUMAN

```
ID    KCNT1_HUMAN
FT    TOPO_DOM      1     97       CYTOPLASMIC.
FT    TRANSMEM     98    117
FT    TOPO_DOM    118    154       NON CYTOPLASMIC.
FT    TRANSMEM    155    176
FT    TOPO_DOM    177    187       CYTOPLASMIC.
FT    TRANSMEM    188    209
FT    TOPO_DOM    210    214       NON CYTOPLASMIC.
FT    TRANSMEM    215    231
FT    TOPO_DOM    232    251       CYTOPLASMIC.
FT    TRANSMEM    252    273
FT    TOPO_DOM    274    278       NON CYTOPLASMIC.
FT    TRANSMEM    279    298
FT    TOPO_DOM    299    309       CYTOPLASMIC.
FT    TRANSMEM    310    335
FT    TOPO_DOM    336   1230       NON CYTOPLASMIC.
//
```

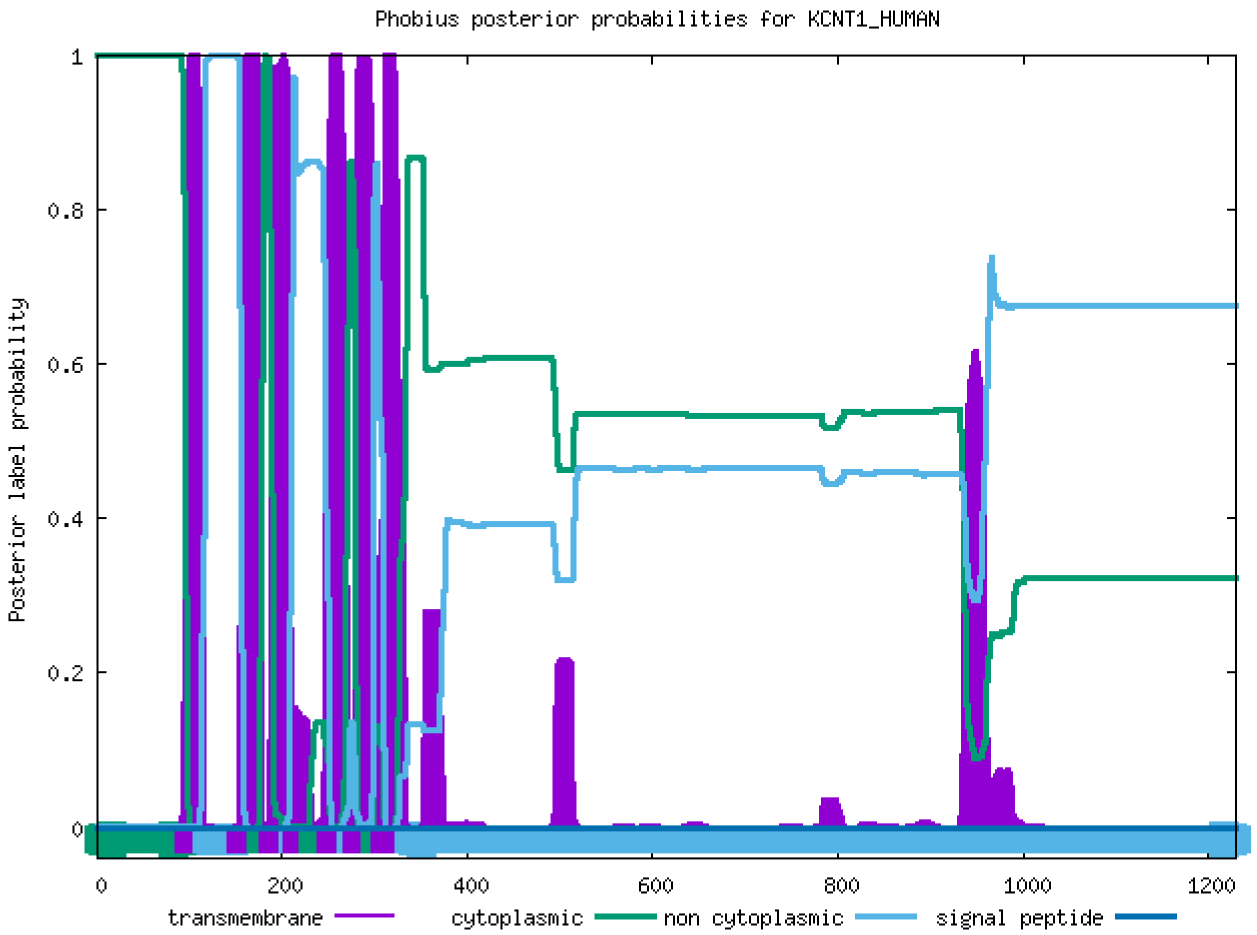

The probability data used in the plot is found here, and the gnuplot script is here.

Prediction of KCNU1_HUMAN

```
ID    KCNU1_HUMAN
FT    TOPO_DOM      1     22       NON CYTOPLASMIC.
FT    TRANSMEM     23     49
FT    TOPO_DOM     50     99       CYTOPLASMIC.
FT    TRANSMEM    100    123
FT    TOPO_DOM    124    142       NON CYTOPLASMIC.
FT    TRANSMEM    143    160
FT    TOPO_DOM    161    226       CYTOPLASMIC.
FT    TRANSMEM    227    246
FT    TOPO_DOM    247    265       NON CYTOPLASMIC.
FT    TRANSMEM    266    284
FT    TOPO_DOM    285    290       CYTOPLASMIC.
FT    TRANSMEM    291    309
FT    TOPO_DOM    310   1149       NON CYTOPLASMIC.
//
```

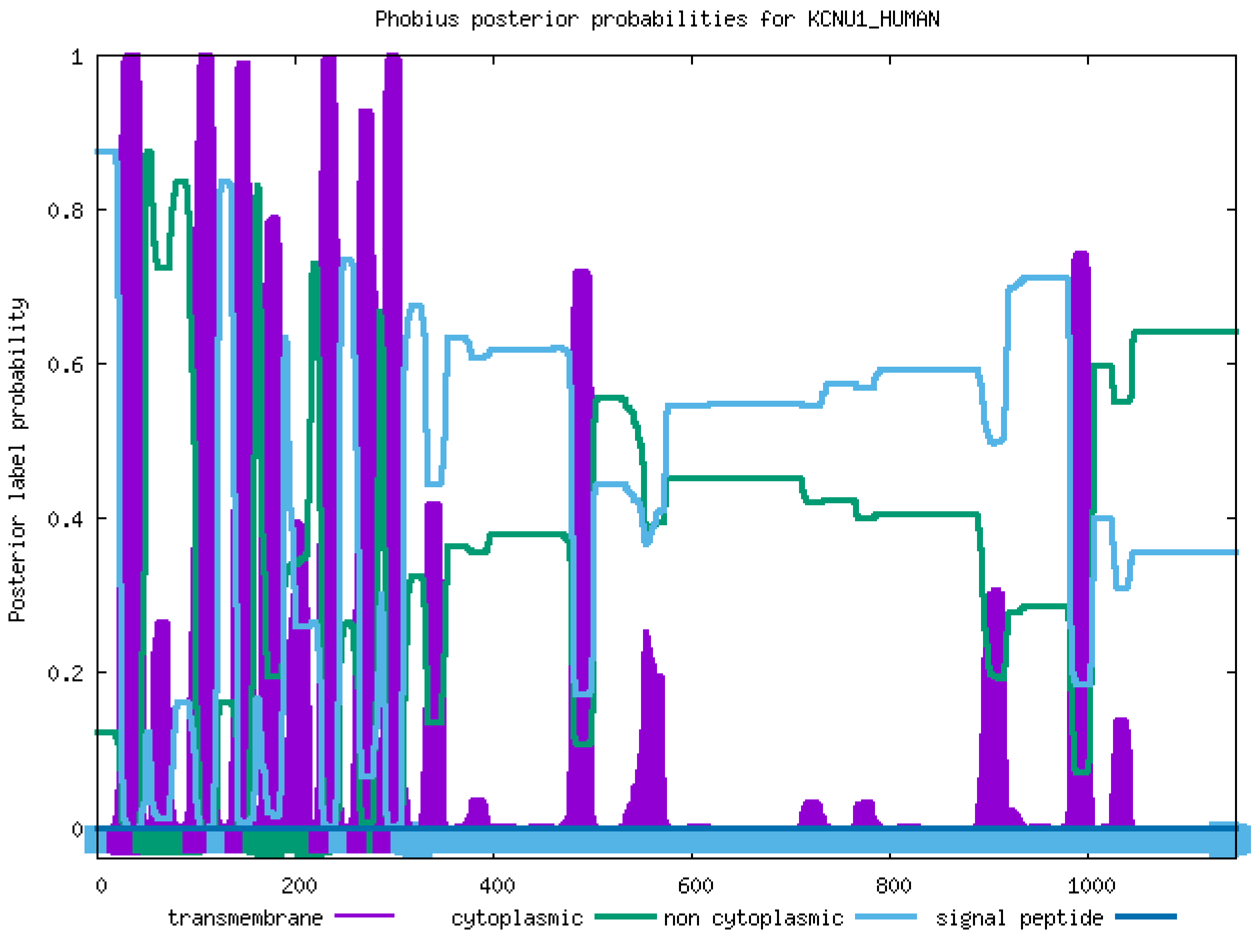

The probability data used in the plot is found here, and the gnuplot script is here.

Prediction of KCNN4_HUMAN

```
ID   KCNN4_HUMAN
FT   TOPO_DOM      1     24       CYTOPLASMIC.
FT   TRANSMEM     25     48
FT   TOPO_DOM     49     53       NON CYTOPLASMIC.
FT   TRANSMEM     54     79
FT   TOPO_DOM     80    206       CYTOPLASMIC.
FT   TRANSMEM    207    226
FT   TOPO_DOM    227    261       NON CYTOPLASMIC.
FT   TRANSMEM    262    286
FT   TOPO_DOM    287    427       CYTOPLASMIC.
//
```

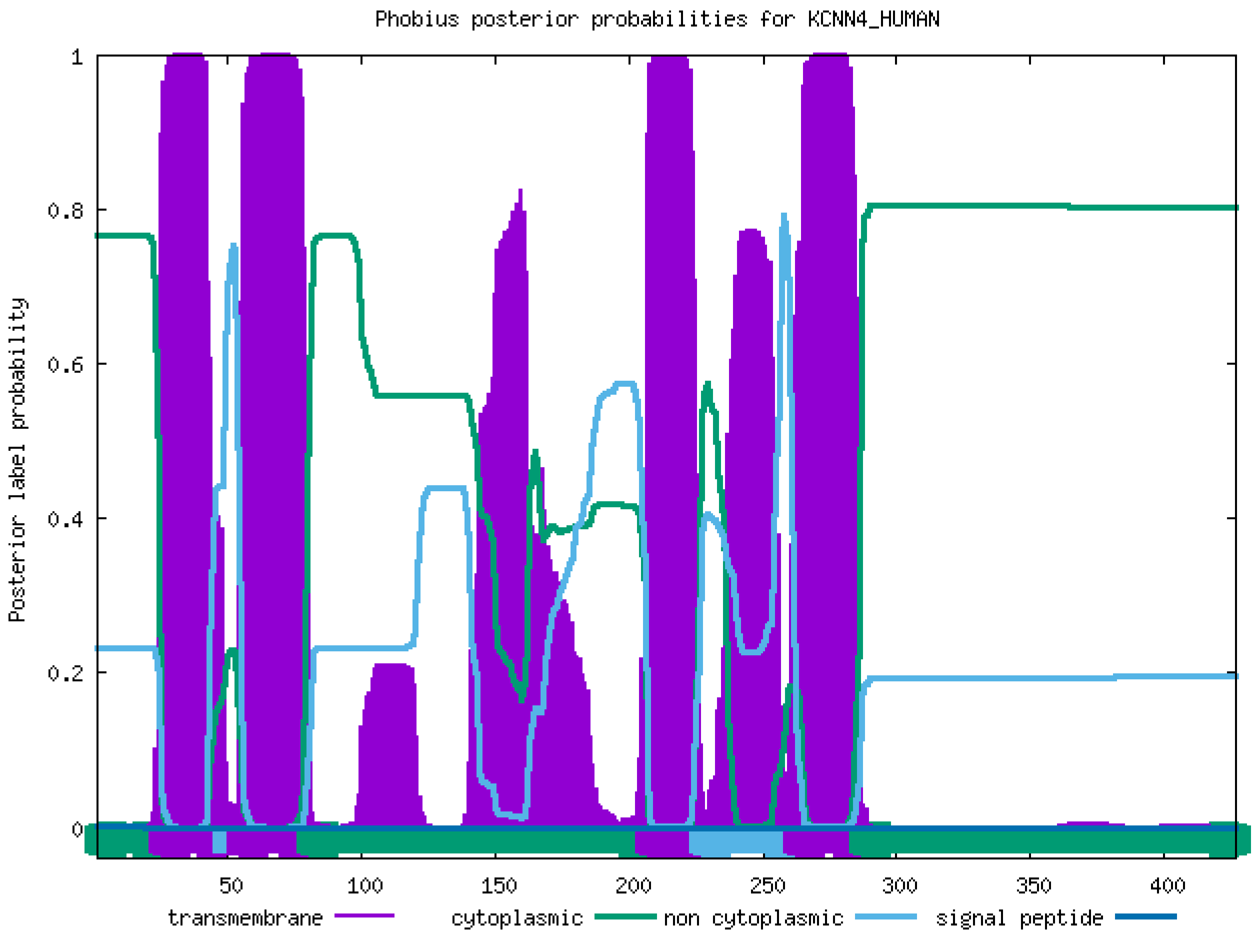

The probability data used in the plot is found here, and the gnuplot script is here.

Prediction of KCNJ2_HUMAN

```
ID   KCNJ2_HUMAN
FT   TOPO_DOM      1     82       CYTOPLASMIC.
FT   TRANSMEM     83    107
FT   TOPO_DOM    108    155       NON CYTOPLASMIC.
FT   TRANSMEM    156    181
FT   TOPO_DOM    182    427       CYTOPLASMIC.
//
```

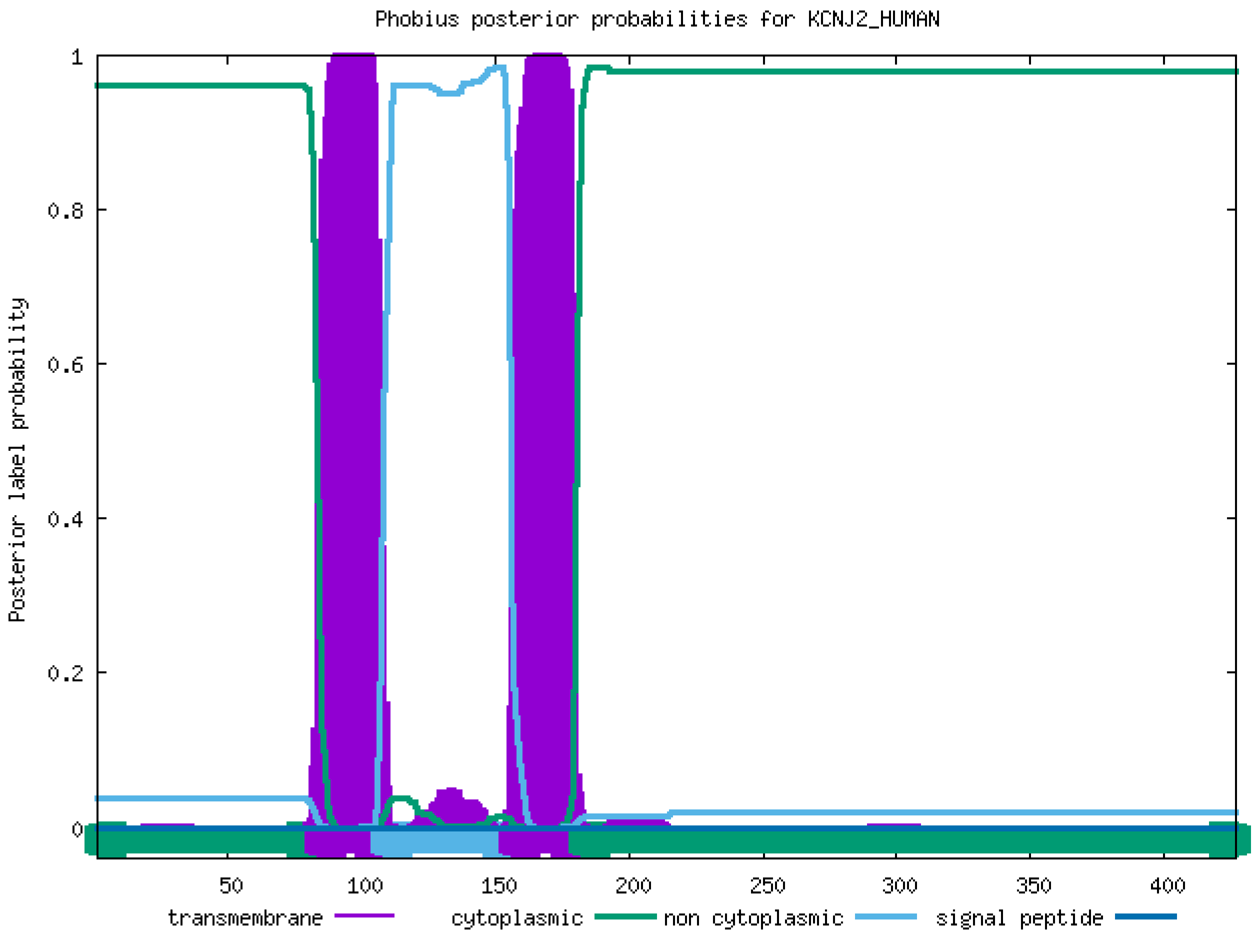

The probability data used in the plot is found here, and the gnuplot script is here.

Prediction of KCJ11_HUMAN

```
ID   KCJ11_HUMAN
FT   TOPO_DOM      1     71       CYTOPLASMIC.
FT   TRANSMEM     72     93
FT   TOPO_DOM     94    143       NON CYTOPLASMIC.
FT   TRANSMEM    144    168
FT   TOPO_DOM    169    390       CYTOPLASMIC.
//
```

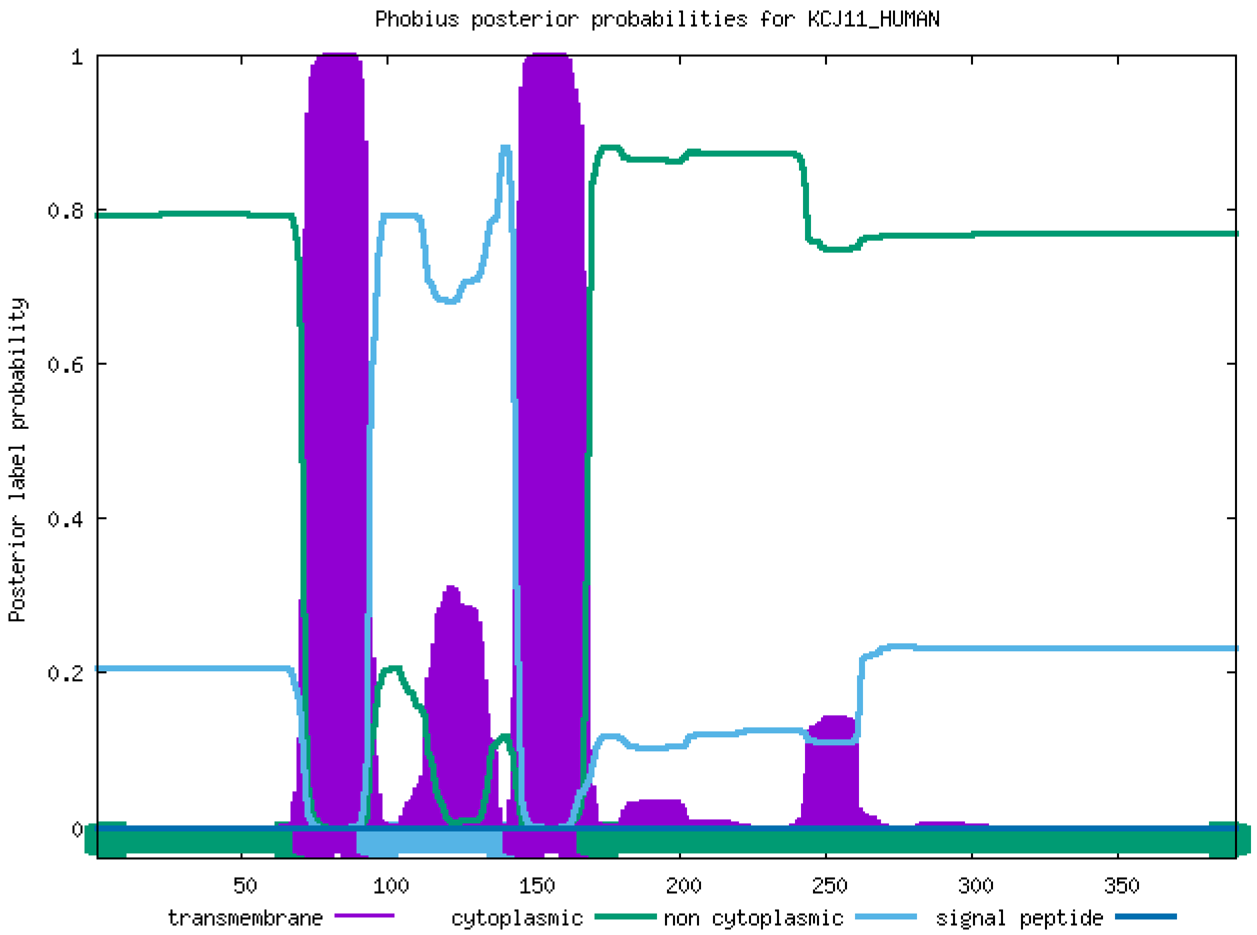

The probability data used in the plot is found here, and the gnuplot script is here.

Prediction of ABCC8_HUMAN

```
ID   ABCC8_HUMAN
FT   TOPO_DOM      1     32       NON CYTOPLASMIC.
FT   TRANSMEM     33     52
FT   TOPO_DOM     53     72       CYTOPLASMIC.
FT   TRANSMEM     73     94
FT   TOPO_DOM     95    105       NON CYTOPLASMIC.
FT   TRANSMEM    106    124
FT   TOPO_DOM    125    135       CYTOPLASMIC.
FT   TRANSMEM    136    155
FT   TOPO_DOM    156    160       NON CYTOPLASMIC.
FT   TRANSMEM    161    185
FT   TOPO_DOM    186    306       CYTOPLASMIC.
FT   TRANSMEM    307    324
FT   TOPO_DOM    325    350       NON CYTOPLASMIC.
FT   TRANSMEM    351    368
FT   TOPO_DOM    369    429       CYTOPLASMIC.
FT   TRANSMEM    430    454
FT   TOPO_DOM    455    459       NON CYTOPLASMIC.
FT   TRANSMEM    460    478
FT   TOPO_DOM    479    535       CYTOPLASMIC.
FT   TRANSMEM    536    559
FT   TOPO_DOM    560    570       NON CYTOPLASMIC.
FT   TRANSMEM    571    593
FT   TOPO_DOM    594    999       CYTOPLASMIC.
FT   TRANSMEM   1000   1021
FT   TOPO_DOM   1022   1061       NON CYTOPLASMIC.
FT   TRANSMEM   1062   1088
FT   TOPO_DOM   1089   1099       CYTOPLASMIC.
FT   TRANSMEM   1100   1121
FT   TOPO_DOM   1122   1151       NON CYTOPLASMIC.
```

```
FT   TRANSMEM   1152   1177
FT   TOPO_DOM   1178   1252       CYTOPLASMIC.
FT   TRANSMEM   1253   1271
FT   TOPO_DOM   1272   1276       NON CYTOPLASMIC.
FT   TRANSMEM   1277   1296
FT   TOPO_DOM   1297   1581       CYTOPLASMIC.
//
```

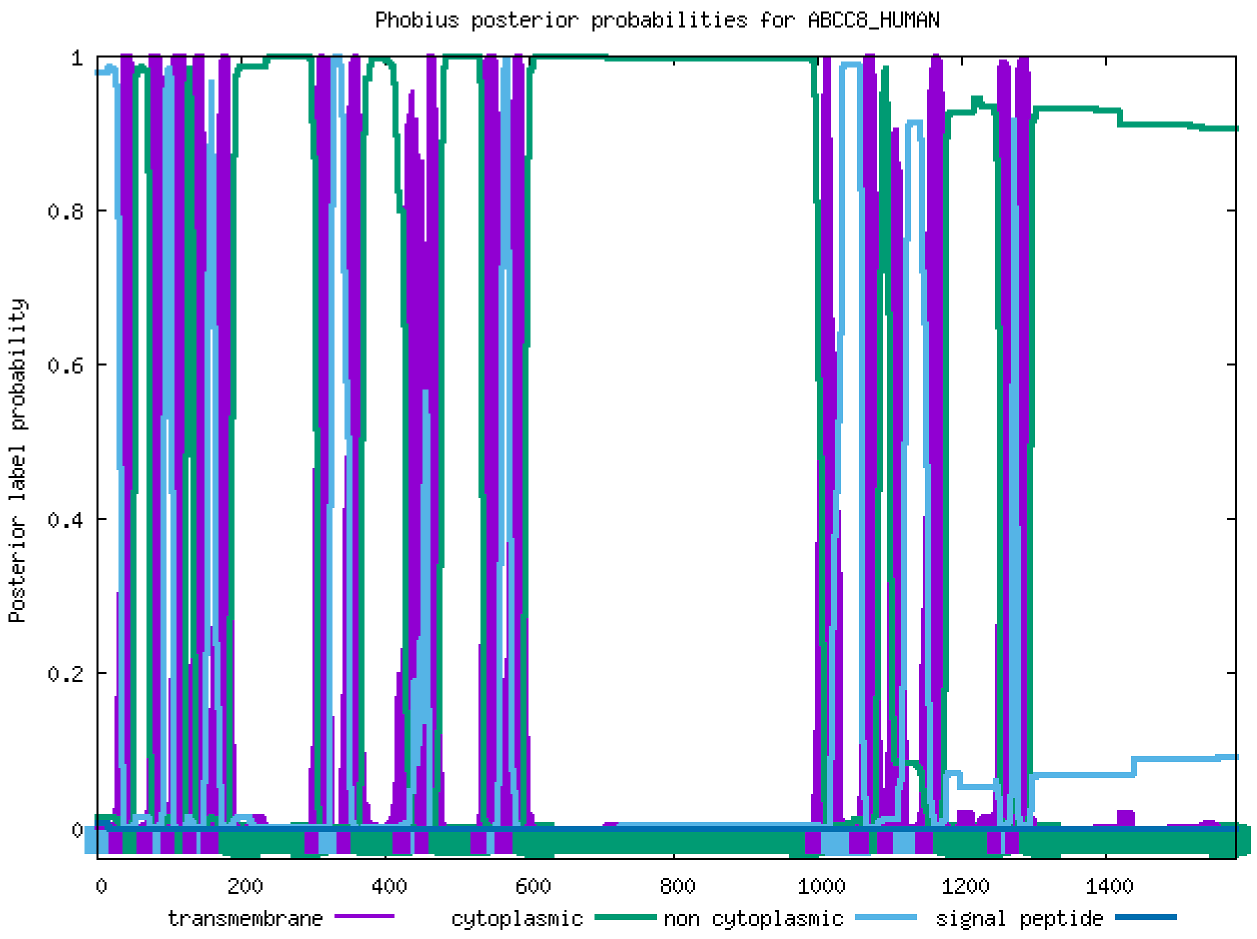

The probability data used in the plot is found here, and the gnuplot script is here.

Prediction of SCN7A_HUMAN

```
ID   SCN7A_HUMAN
FT   TOPO_DOM      1    118       NON CYTOPLASMIC.
FT   TRANSMEM    119    140
FT   TOPO_DOM    141    151       CYTOPLASMIC.
FT   TRANSMEM    152    176
FT   TOPO_DOM    177    236       NON CYTOPLASMIC.
FT   TRANSMEM    237    259
FT   TOPO_DOM    260    368       CYTOPLASMIC.
FT   TRANSMEM    369    397
FT   TOPO_DOM    398    500       NON CYTOPLASMIC.
FT   TRANSMEM    501    524
FT   TOPO_DOM    525    535       CYTOPLASMIC.
FT   TRANSMEM    536    557
FT   TOPO_DOM    558    576       NON CYTOPLASMIC.
FT   TRANSMEM    577    597
FT   TOPO_DOM    598    608       CYTOPLASMIC.
FT   TRANSMEM    609    625
FT   TOPO_DOM    626    630       NON CYTOPLASMIC.
FT   TRANSMEM    631    649
FT   TOPO_DOM    650    707       CYTOPLASMIC.
FT   TRANSMEM    708    728
FT   TOPO_DOM    729    933       NON CYTOPLASMIC.
```

```
FT   TRANSMEM    934    953
FT   TOPO_DOM    954    973       CYTOPLASMIC.
FT   TRANSMEM    974    993
FT   TOPO_DOM    994    998       NON CYTOPLASMIC.
FT   TRANSMEM    999   1017
FT   TOPO_DOM   1018   1049       CYTOPLASMIC.
FT   TRANSMEM   1050   1072
FT   TOPO_DOM   1073   1166       NON CYTOPLASMIC.
FT   TRANSMEM   1167   1193
FT   TOPO_DOM   1194   1242       CYTOPLASMIC.
FT   TRANSMEM   1243   1270
FT   TOPO_DOM   1271   1281       NON CYTOPLASMIC.
FT   TRANSMEM   1282   1300
FT   TOPO_DOM   1301   1306       CYTOPLASMIC.
FT   TRANSMEM   1307   1326
FT   TOPO_DOM   1327   1337       NON CYTOPLASMIC.
FT   TRANSMEM   1338   1357
FT   TOPO_DOM   1358   1368       CYTOPLASMIC.
FT   TRANSMEM   1369   1397
FT   TOPO_DOM   1398   1472       NON CYTOPLASMIC.
FT   TRANSMEM   1473   1499
FT   TOPO_DOM   1500   1682       CYTOPLASMIC.
//
```

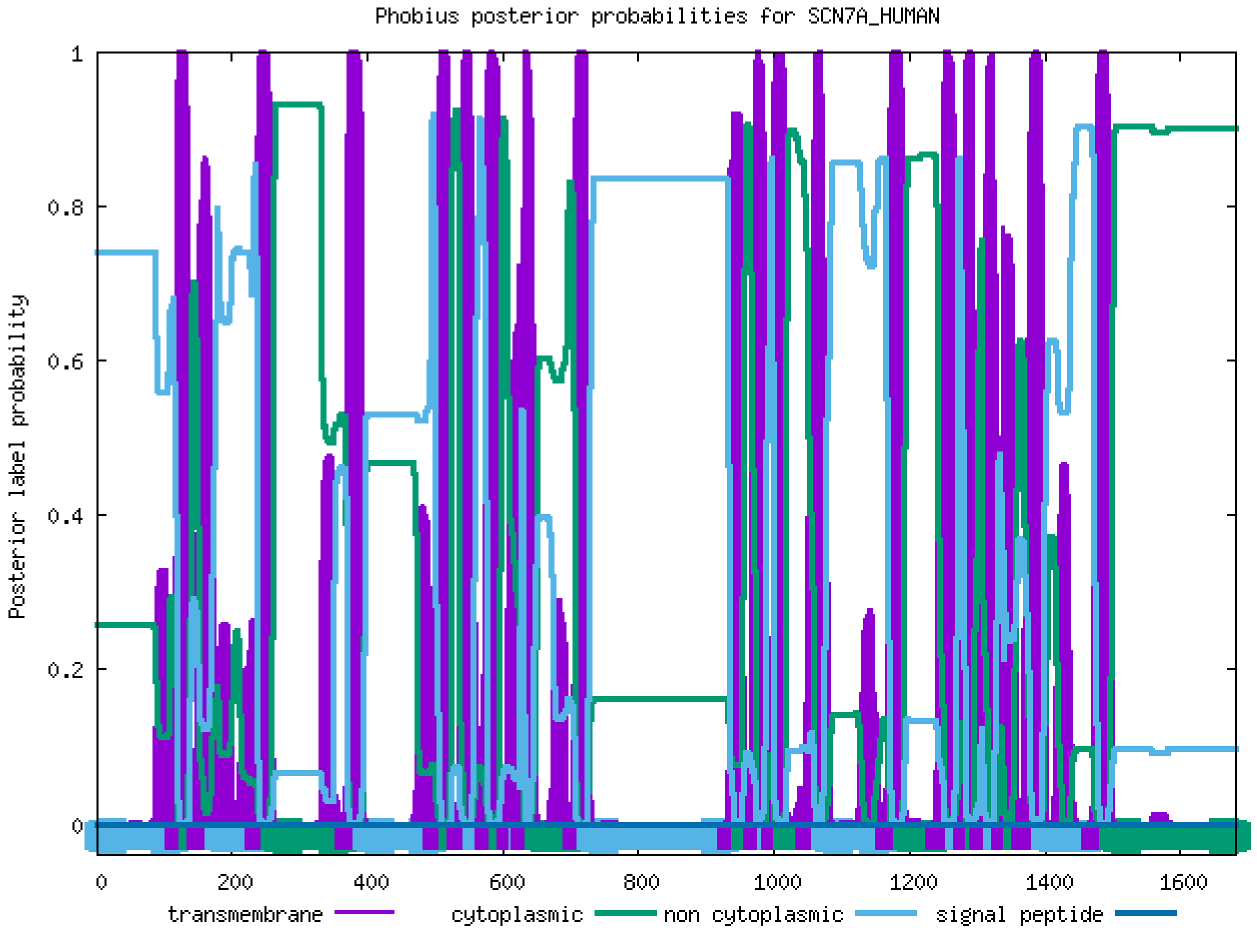

The probability data used in the plot is found here, and the gnuplot script is here.

Prediction of SCN3B_HUMAN

```
ID   SCN3B_HUMAN
FT   SIGNAL        1     22
FT   REGION        1      6       N-REGION.
FT   REGION        7     17       H-REGION.
FT   REGION       18     22       C-REGION.
FT   TOPO_DOM     23    159       NON CYTOPLASMIC.
```

```
FT   TRANSMEM    160    181
FT   TOPO_DOM    182    215       CYTOPLASMIC.
//
```

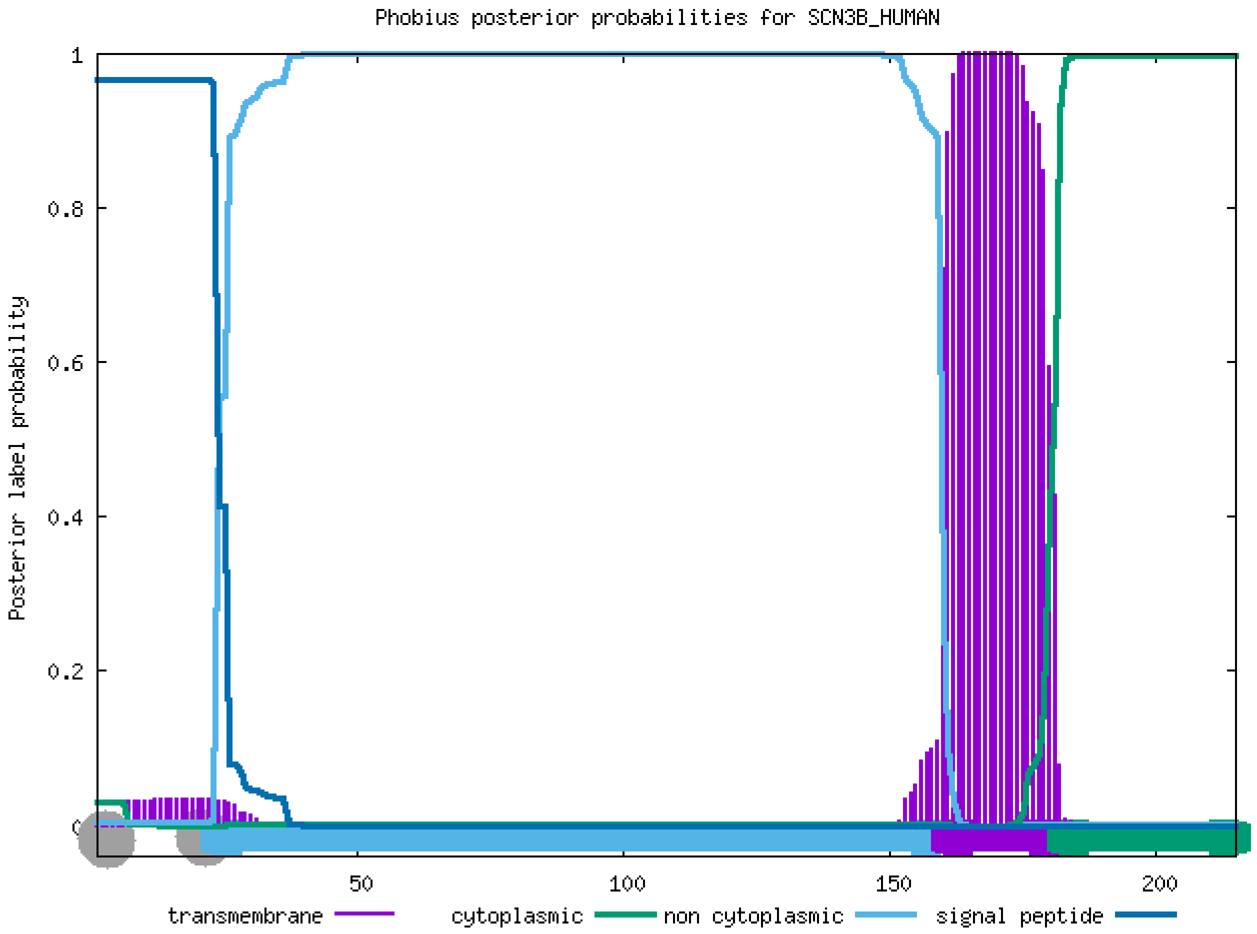

The probability data used in the plot is found here, and the gnuplot script is here.

Prediction of SCN4B_HUMAN

```
ID   SCN4B_HUMAN
FT   SIGNAL        1     30
FT   REGION        1     13       N-REGION.
FT   REGION       14     25       H-REGION.
FT   REGION       26     30       C-REGION.
FT   TOPO_DOM     31    161       NON CYTOPLASMIC.
FT   TRANSMEM    162    183
FT   TOPO_DOM    184    228       CYTOPLASMIC.
//
```

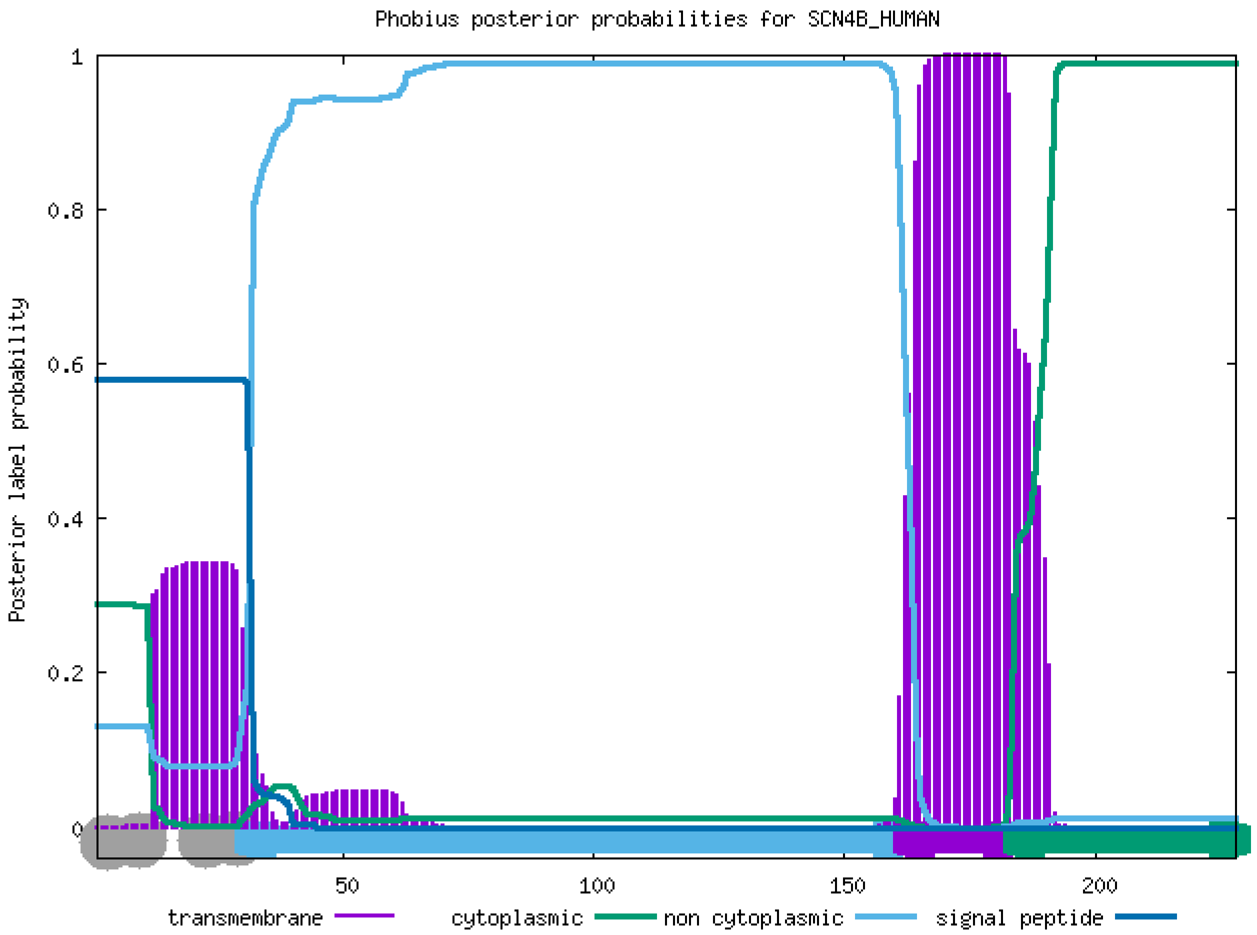

The probability data used in the plot is found here, and the gnuplot script is here.

Prediction of SCN1A_HUMAN

```
ID    SCN1A_HUMAN
FT    TOPO_DOM       1     123       CYTOPLASMIC.
FT    TRANSMEM     124     147
FT    TOPO_DOM     148     191       NON CYTOPLASMIC.
FT    TRANSMEM     192     211
FT    TOPO_DOM     212     222       CYTOPLASMIC.
FT    TRANSMEM     223     244
FT    TOPO_DOM     245     249       NON CYTOPLASMIC.
FT    TRANSMEM     250     270
FT    TOPO_DOM     271     359       CYTOPLASMIC.
FT    TRANSMEM     360     379
FT    TOPO_DOM     380     398       NON CYTOPLASMIC.
FT    TRANSMEM     399     426
FT    TOPO_DOM     427     736       CYTOPLASMIC.
FT    TRANSMEM     737     755
FT    TOPO_DOM     756     760       NON CYTOPLASMIC.
FT    TRANSMEM     761     784
FT    TOPO_DOM     785     795       CYTOPLASMIC.
FT    TRANSMEM     796     817
FT    TOPO_DOM     818     836       NON CYTOPLASMIC.
FT    TRANSMEM     837     858
FT    TOPO_DOM     859     878       CYTOPLASMIC.
FT    TRANSMEM     879     907
FT    TOPO_DOM     908     936       NON CYTOPLASMIC.
FT    TRANSMEM     937     954
FT    TOPO_DOM     955     965       CYTOPLASMIC.
FT    TRANSMEM     966     992
FT    TOPO_DOM     993    1218       NON CYTOPLASMIC.
FT    TRANSMEM    1219    1237
FT    TOPO_DOM    1238    1257       CYTOPLASMIC.
```

```
FT   TRANSMEM   1258   1277
FT   TOPO_DOM   1278   1288       NON CYTOPLASMIC.
FT   TRANSMEM   1289   1312
FT   TOPO_DOM   1313   1332       CYTOPLASMIC.
FT   TRANSMEM   1333   1361
FT   TOPO_DOM   1362   1458       NON CYTOPLASMIC.
FT   TRANSMEM   1459   1483
FT   TOPO_DOM   1484   1541       CYTOPLASMIC.
FT   TRANSMEM   1542   1560
FT   TOPO_DOM   1561   1571       NON CYTOPLASMIC.
FT   TRANSMEM   1572   1590
FT   TOPO_DOM   1591   1601       CYTOPLASMIC.
FT   TRANSMEM   1602   1622
FT   TOPO_DOM   1623   1627       NON CYTOPLASMIC.
FT   TRANSMEM   1628   1647
FT   TOPO_DOM   1648   1658       CYTOPLASMIC.
FT   TRANSMEM   1659   1687
FT   TOPO_DOM   1688   1762       NON CYTOPLASMIC.
FT   TRANSMEM   1763   1786
FT   TOPO_DOM   1787   2009       CYTOPLASMIC.
//
```

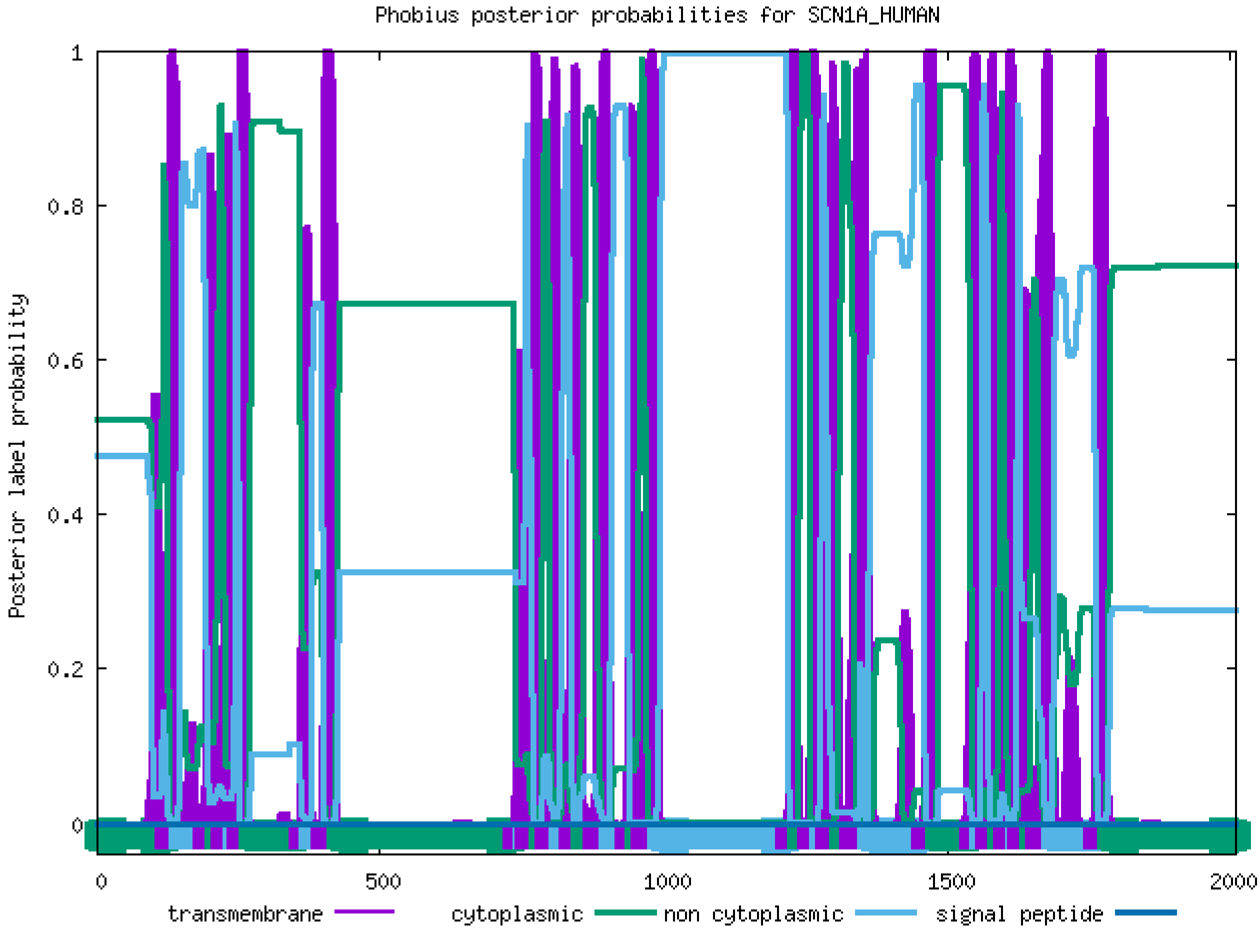

The probability data used in the plot is found here, and the gnuplot script is here.

Prediction of SCN1B_HUMAN

```
ID   SCN1B_HUMAN
FT   SIGNAL        1     19
FT   REGION        1      3       N-REGION.
FT   REGION        4     14       H-REGION.
FT   REGION       15     19       C-REGION.
FT   TOPO_DOM     20    160       NON CYTOPLASMIC.
FT   TRANSMEM    161    182
```

```
FT   TOPO_DOM    183    218       CYTOPLASMIC.
//
```

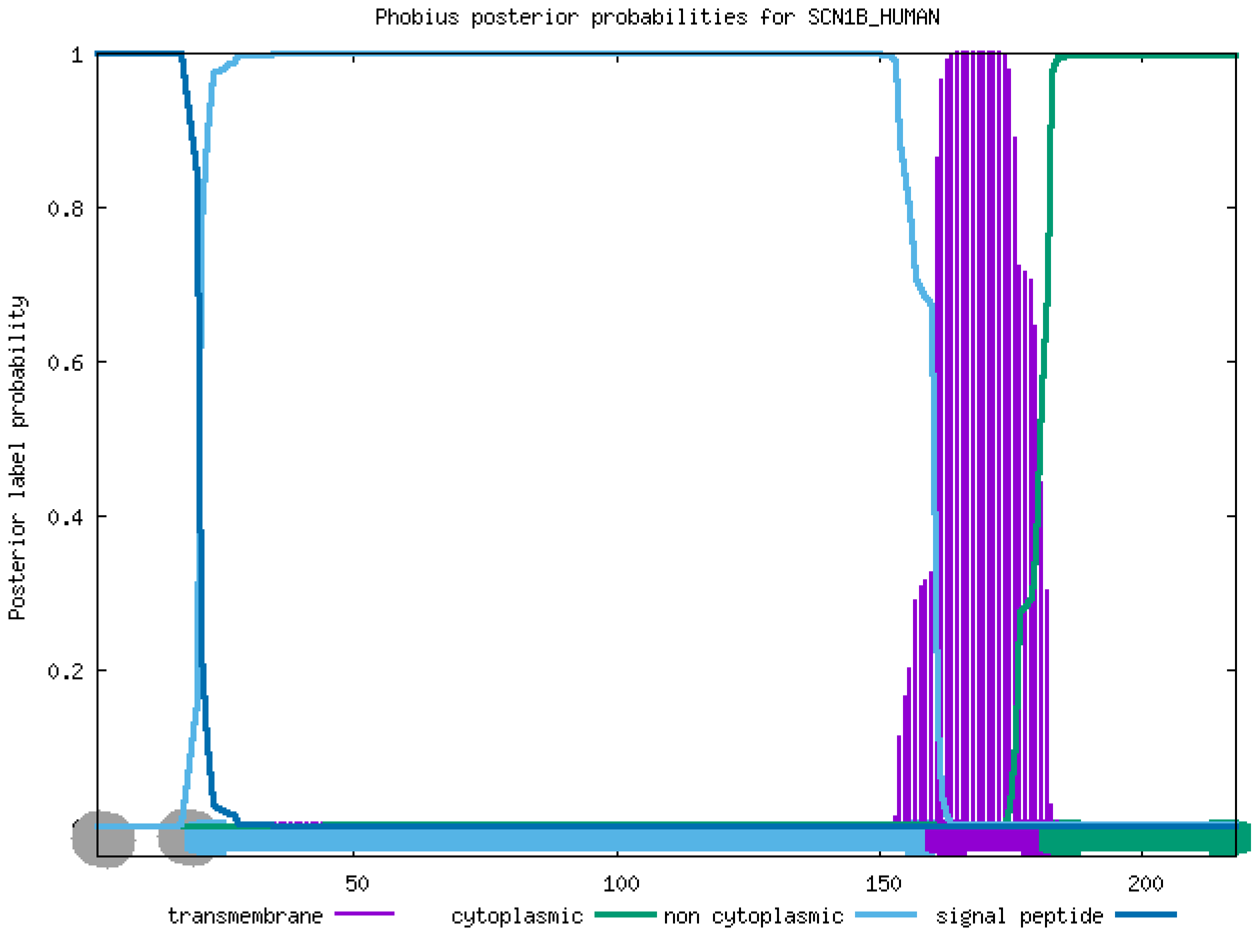

The probability data used in the plot is found here, and the gnuplot script is here.

Prediction of SCN2B_HUMAN

```
ID   SCN2B_HUMAN
FT   SIGNAL        1     29
FT   REGION        1     10       N-REGION.
FT   REGION       11     24       H-REGION.
FT   REGION       25     29       C-REGION.
FT   TOPO_DOM     30    157       NON CYTOPLASMIC.
FT   TRANSMEM    158    180
FT   TOPO_DOM    181    215       CYTOPLASMIC.
//
```

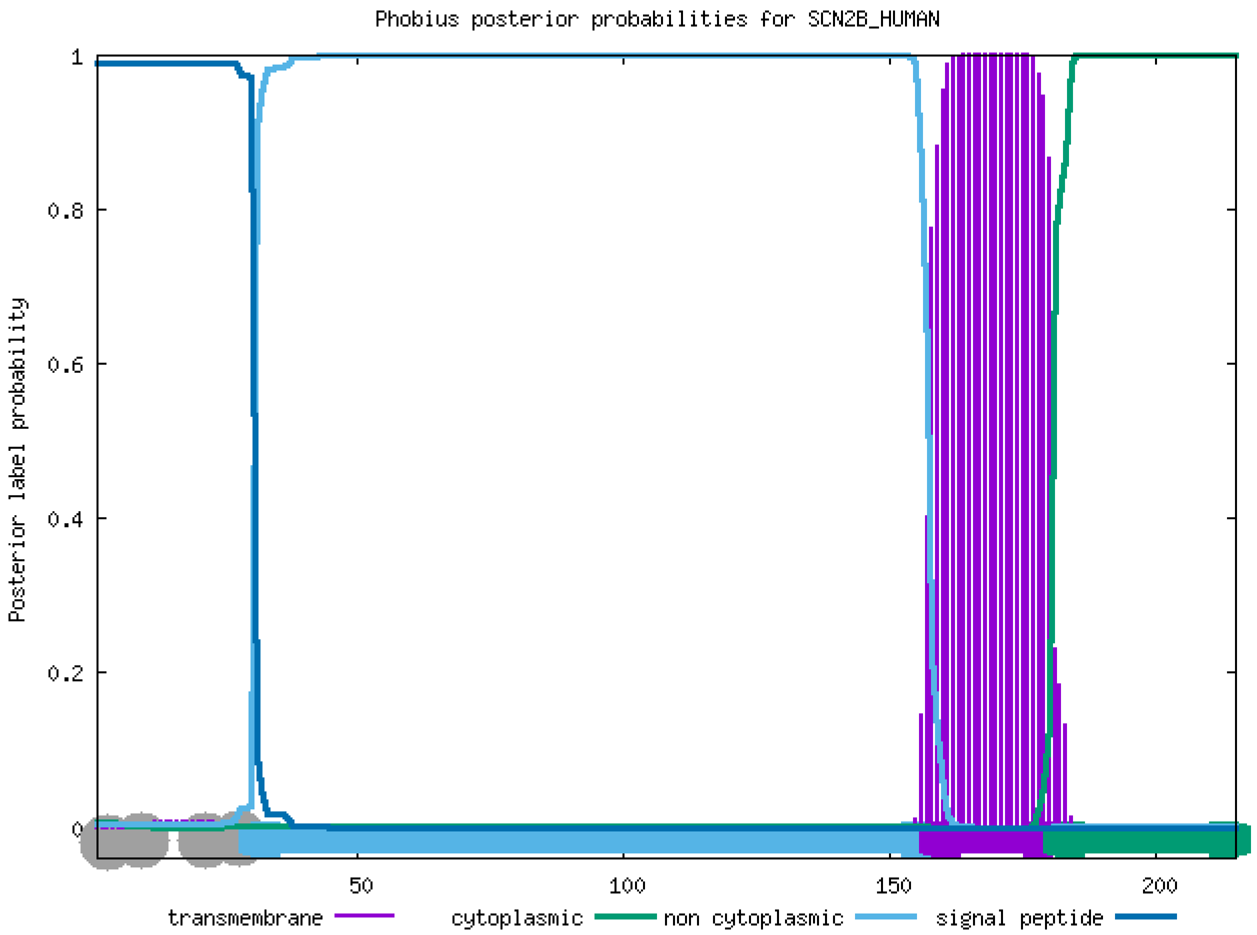

The probability data used in the plot is found here, and the gnuplot script is here.

Prediction of SCN3A_HUMAN

```
ID    SCN3A_HUMAN
FT    TOPO_DOM      1    123       CYTOPLASMIC.
FT    TRANSMEM    124    147
FT    TOPO_DOM    148    194       NON CYTOPLASMIC.
FT    TRANSMEM    195    215
FT    TOPO_DOM    216    226       CYTOPLASMIC.
FT    TRANSMEM    227    244
FT    TOPO_DOM    245    249       NON CYTOPLASMIC.
FT    TRANSMEM    250    270
FT    TOPO_DOM    271    399       CYTOPLASMIC.
FT    TRANSMEM    400    427
FT    TOPO_DOM    428    760       NON CYTOPLASMIC.
FT    TRANSMEM    761    779
FT    TOPO_DOM    780    790       CYTOPLASMIC.
FT    TRANSMEM    791    814
FT    TOPO_DOM    815    825       NON CYTOPLASMIC.
FT    TRANSMEM    826    850
FT    TOPO_DOM    851    870       CYTOPLASMIC.
FT    TRANSMEM    871    899
FT    TOPO_DOM    900    928       NON CYTOPLASMIC.
FT    TRANSMEM    929    949
FT    TOPO_DOM    950    960       CYTOPLASMIC.
FT    TRANSMEM    961    984
FT    TOPO_DOM    985   1206       NON CYTOPLASMIC.
FT    TRANSMEM   1207   1225
FT    TOPO_DOM   1226   1245       CYTOPLASMIC.
FT    TRANSMEM   1246   1268
FT    TOPO_DOM   1269   1273       NON CYTOPLASMIC.
FT    TRANSMEM   1274   1294
FT    TOPO_DOM   1295   1322       CYTOPLASMIC.
```

```
FT   TRANSMEM   1323   1349
FT   TOPO_DOM   1350   1443       NON CYTOPLASMIC.
FT   TRANSMEM   1444   1468
FT   TOPO_DOM   1469   1526       CYTOPLASMIC.
FT   TRANSMEM   1527   1545
FT   TOPO_DOM   1546   1556       NON CYTOPLASMIC.
FT   TRANSMEM   1557   1575
FT   TOPO_DOM   1576   1586       CYTOPLASMIC.
FT   TRANSMEM   1587   1607
FT   TOPO_DOM   1608   1612       NON CYTOPLASMIC.
FT   TRANSMEM   1613   1632
FT   TOPO_DOM   1633   1643       CYTOPLASMIC.
FT   TRANSMEM   1644   1672
FT   TOPO_DOM   1673   1747       NON CYTOPLASMIC.
FT   TRANSMEM   1748   1771
FT   TOPO_DOM   1772   2000       CYTOPLASMIC.
//
```

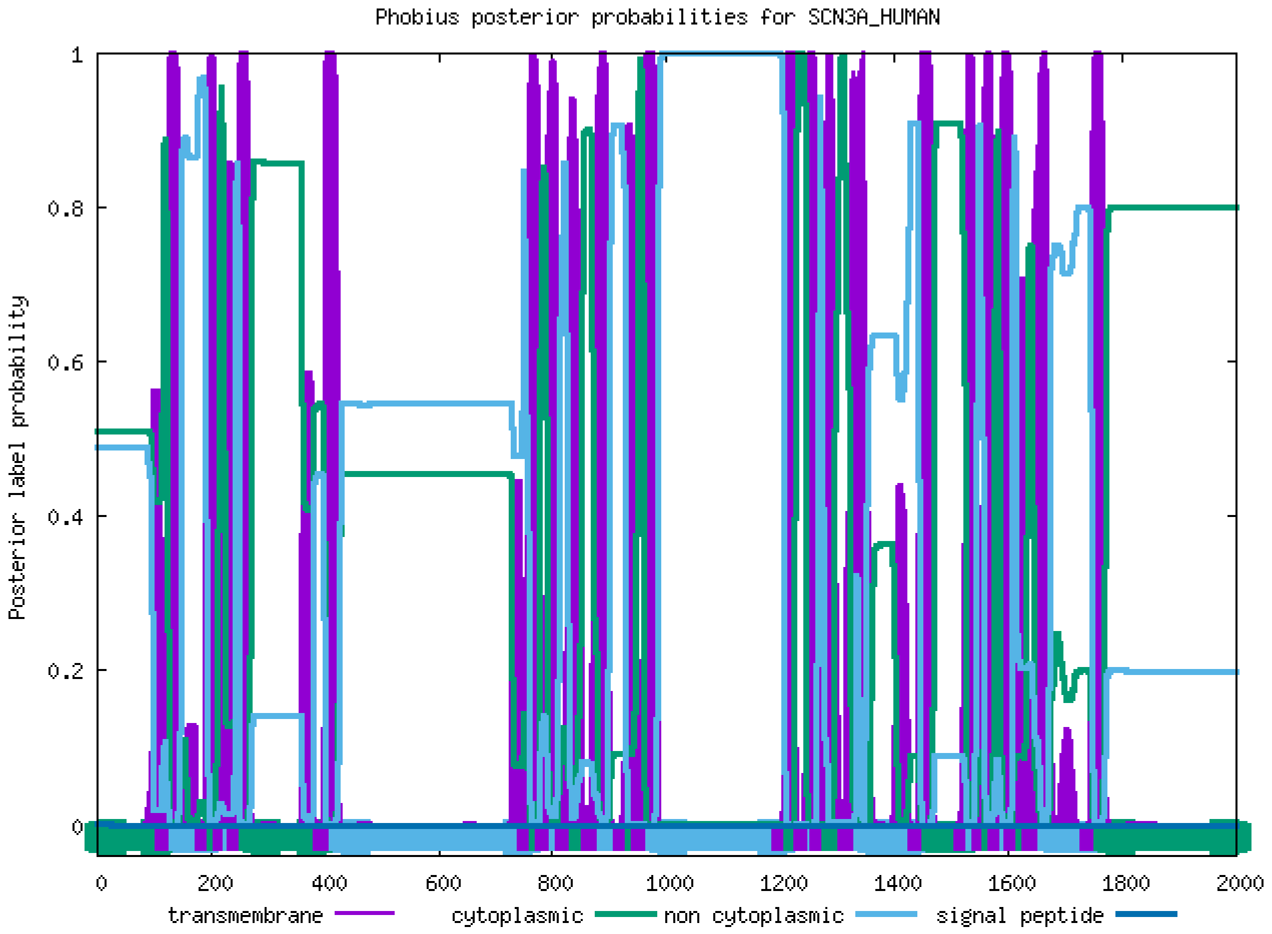

The probability data used in the plot is found here, and the gnuplot script is here.

Prediction of SCN4A_HUMAN

```
ID   SCN4A_HUMAN
FT   TOPO_DOM      1    126       CYTOPLASMIC.
FT   TRANSMEM    127    150
FT   TOPO_DOM    151    194       NON CYTOPLASMIC.
FT   TRANSMEM    195    214
FT   TOPO_DOM    215    225       CYTOPLASMIC.
FT   TRANSMEM    226    247
FT   TOPO_DOM    248    252       NON CYTOPLASMIC.
FT   TRANSMEM    253    273
FT   TOPO_DOM    274    422       CYTOPLASMIC.
FT   TRANSMEM    423    451
FT   TOPO_DOM    452    578       NON CYTOPLASMIC.
```

```
FT   TRANSMEM    579    597
FT   TOPO_DOM    598    608       CYTOPLASMIC.
FT   TRANSMEM    609    632
FT   TOPO_DOM    633    643       NON CYTOPLASMIC.
FT   TRANSMEM    644    668
FT   TOPO_DOM    669    688       CYTOPLASMIC.
FT   TRANSMEM    689    717
FT   TOPO_DOM    718    746       NON CYTOPLASMIC.
FT   TRANSMEM    747    764
FT   TOPO_DOM    765    775       CYTOPLASMIC.
FT   TRANSMEM    776    802
FT   TOPO_DOM    803   1031       NON CYTOPLASMIC.
FT   TRANSMEM   1032   1050
FT   TOPO_DOM   1051   1070       CYTOPLASMIC.
FT   TRANSMEM   1071   1093
FT   TOPO_DOM   1094   1098       NON CYTOPLASMIC.
FT   TRANSMEM   1099   1119
FT   TOPO_DOM   1120   1147       CYTOPLASMIC.
FT   TRANSMEM   1148   1174
FT   TOPO_DOM   1175   1271       NON CYTOPLASMIC.
FT   TRANSMEM   1272   1295
FT   TOPO_DOM   1296   1353       CYTOPLASMIC.
FT   TRANSMEM   1354   1372
FT   TOPO_DOM   1373   1383       NON CYTOPLASMIC.
FT   TRANSMEM   1384   1402
FT   TOPO_DOM   1403   1413       CYTOPLASMIC.
FT   TRANSMEM   1414   1434
FT   TOPO_DOM   1435   1439       NON CYTOPLASMIC.
FT   TRANSMEM   1440   1459
FT   TOPO_DOM   1460   1470       CYTOPLASMIC.
FT   TRANSMEM   1471   1499
FT   TOPO_DOM   1500   1571       NON CYTOPLASMIC.
FT   TRANSMEM   1572   1598
FT   TOPO_DOM   1599   1836       CYTOPLASMIC.
//
```

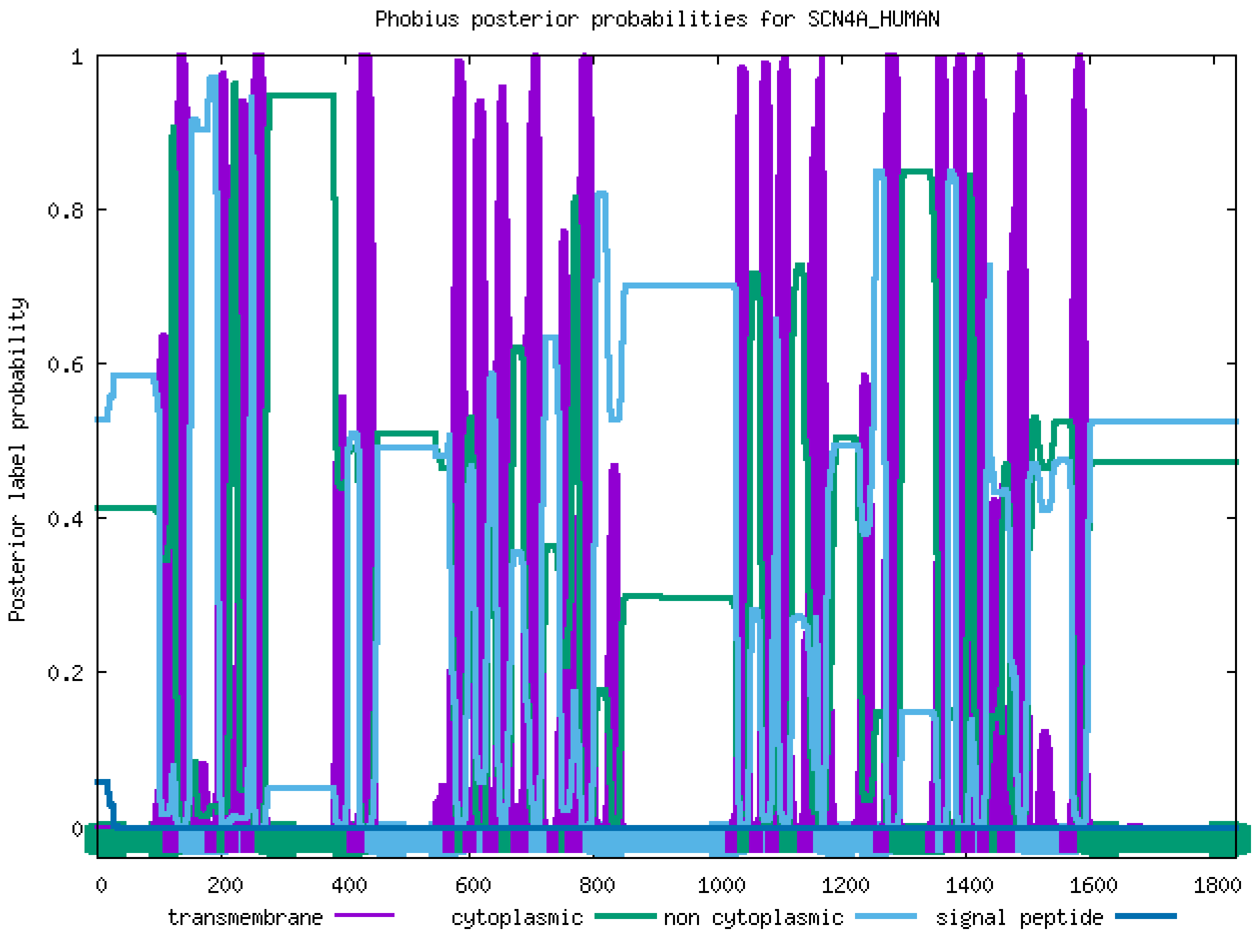

The probability data used in the plot is found here, and the gnuplot script is here.

Prediction of SCN5A_HUMAN

```
ID   SCN5A_HUMAN
FT   TOPO_DOM      1    126       CYTOPLASMIC.
FT   TRANSMEM    127    149
FT   TOPO_DOM    150    197       NON CYTOPLASMIC.
FT   TRANSMEM    198    218
FT   TOPO_DOM    219    229       CYTOPLASMIC.
FT   TRANSMEM    230    247
FT   TOPO_DOM    248    252       NON CYTOPLASMIC.
FT   TRANSMEM    253    273
FT   TOPO_DOM    274    387       CYTOPLASMIC.
FT   TRANSMEM    388    416
FT   TOPO_DOM    417    720       NON CYTOPLASMIC.
FT   TRANSMEM    721    739
FT   TOPO_DOM    740    750       CYTOPLASMIC.
FT   TRANSMEM    751    769
FT   TOPO_DOM    770    780       NON CYTOPLASMIC.
FT   TRANSMEM    781    798
FT   TOPO_DOM    799    837       CYTOPLASMIC.
FT   TRANSMEM    838    861
FT   TOPO_DOM    862    880       NON CYTOPLASMIC.
FT   TRANSMEM    881    902
FT   TOPO_DOM    903    913       CYTOPLASMIC.
FT   TRANSMEM    914    939
FT   TOPO_DOM    940   1204       NON CYTOPLASMIC.
FT   TRANSMEM   1205   1224
FT   TOPO_DOM   1225   1244       CYTOPLASMIC.
FT   TRANSMEM   1245   1261
FT   TOPO_DOM   1262   1272       NON CYTOPLASMIC.
FT   TRANSMEM   1273   1294
FT   TOPO_DOM   1295   1321       CYTOPLASMIC.
FT   TRANSMEM   1322   1348
FT   TOPO_DOM   1349   1443       NON CYTOPLASMIC.
FT   TRANSMEM   1444   1470
FT   TOPO_DOM   1471   1528       CYTOPLASMIC.
FT   TRANSMEM   1529   1547
FT   TOPO_DOM   1548   1558       NON CYTOPLASMIC.
FT   TRANSMEM   1559   1581
FT   TOPO_DOM   1582   1592       CYTOPLASMIC.
FT   TRANSMEM   1593   1609
FT   TOPO_DOM   1610   1614       NON CYTOPLASMIC.
FT   TRANSMEM   1615   1634
FT   TOPO_DOM   1635   1645       CYTOPLASMIC.
FT   TRANSMEM   1646   1674
FT   TOPO_DOM   1675   1748       NON CYTOPLASMIC.
FT   TRANSMEM   1749   1772
FT   TOPO_DOM   1773   2016       CYTOPLASMIC.
//
```

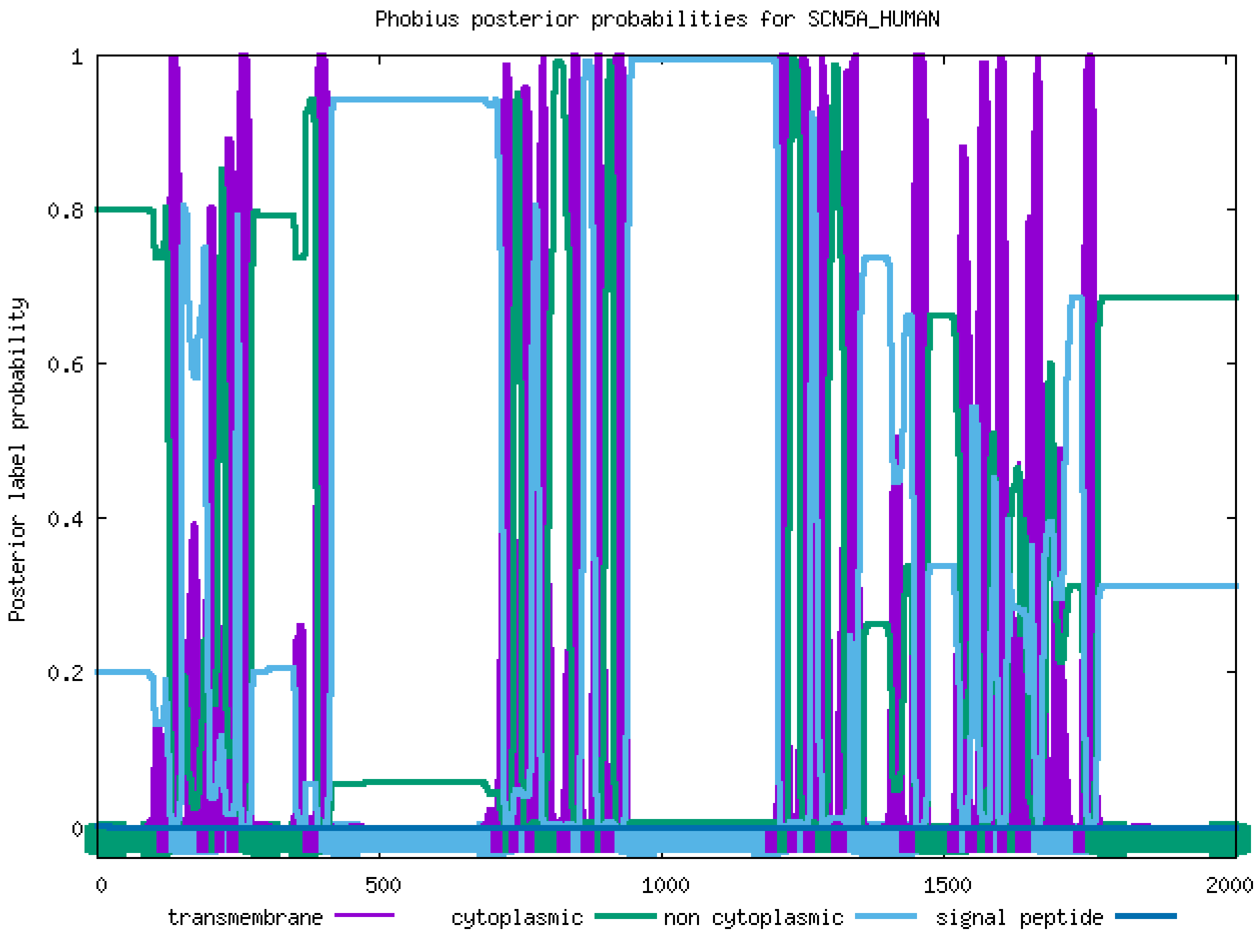

The probability data used in the plot is found here, and the gnuplot script is here.

Prediction of SCN8A_HUMAN

```
ID   SCN8A_HUMAN
FT   TOPO_DOM      1    127       CYTOPLASMIC.
FT   TRANSMEM    128    151
FT   TOPO_DOM    152    198       NON CYTOPLASMIC.
FT   TRANSMEM    199    219
FT   TOPO_DOM    220    253       CYTOPLASMIC.
FT   TRANSMEM    254    274
FT   TOPO_DOM    275    347       NON CYTOPLASMIC.
FT   TRANSMEM    348    366
FT   TOPO_DOM    367    386       CYTOPLASMIC.
FT   TRANSMEM    387    414
FT   TOPO_DOM    415    753       NON CYTOPLASMIC.
FT   TRANSMEM    754    772
FT   TOPO_DOM    773    783       CYTOPLASMIC.
FT   TRANSMEM    784    802
FT   TOPO_DOM    803    821       NON CYTOPLASMIC.
FT   TRANSMEM    822    843
FT   TOPO_DOM    844    863       CYTOPLASMIC.
FT   TRANSMEM    864    892
FT   TOPO_DOM    893    921       NON CYTOPLASMIC.
FT   TRANSMEM    922    939
FT   TOPO_DOM    940    950       CYTOPLASMIC.
FT   TRANSMEM    951    977
FT   TOPO_DOM    978   1198       NON CYTOPLASMIC.
FT   TRANSMEM   1199   1217
FT   TOPO_DOM   1218   1237       CYTOPLASMIC.
FT   TRANSMEM   1238   1257
FT   TOPO_DOM   1258   1268       NON CYTOPLASMIC.
FT   TRANSMEM   1269   1292
FT   TOPO_DOM   1293   1312       CYTOPLASMIC.
```

```
FT   TRANSMEM   1313   1341
FT   TOPO_DOM   1342   1437       NON CYTOPLASMIC.
FT   TRANSMEM   1438   1464
FT   TOPO_DOM   1465   1522       CYTOPLASMIC.
FT   TRANSMEM   1523   1541
FT   TOPO_DOM   1542   1552       NON CYTOPLASMIC.
FT   TRANSMEM   1553   1571
FT   TOPO_DOM   1572   1582       CYTOPLASMIC.
FT   TRANSMEM   1583   1603
FT   TOPO_DOM   1604   1608       NON CYTOPLASMIC.
FT   TRANSMEM   1609   1628
FT   TOPO_DOM   1629   1639       CYTOPLASMIC.
FT   TRANSMEM   1640   1668
FT   TOPO_DOM   1669   1742       NON CYTOPLASMIC.
FT   TRANSMEM   1743   1766
FT   TOPO_DOM   1767   1980       CYTOPLASMIC.
//
```

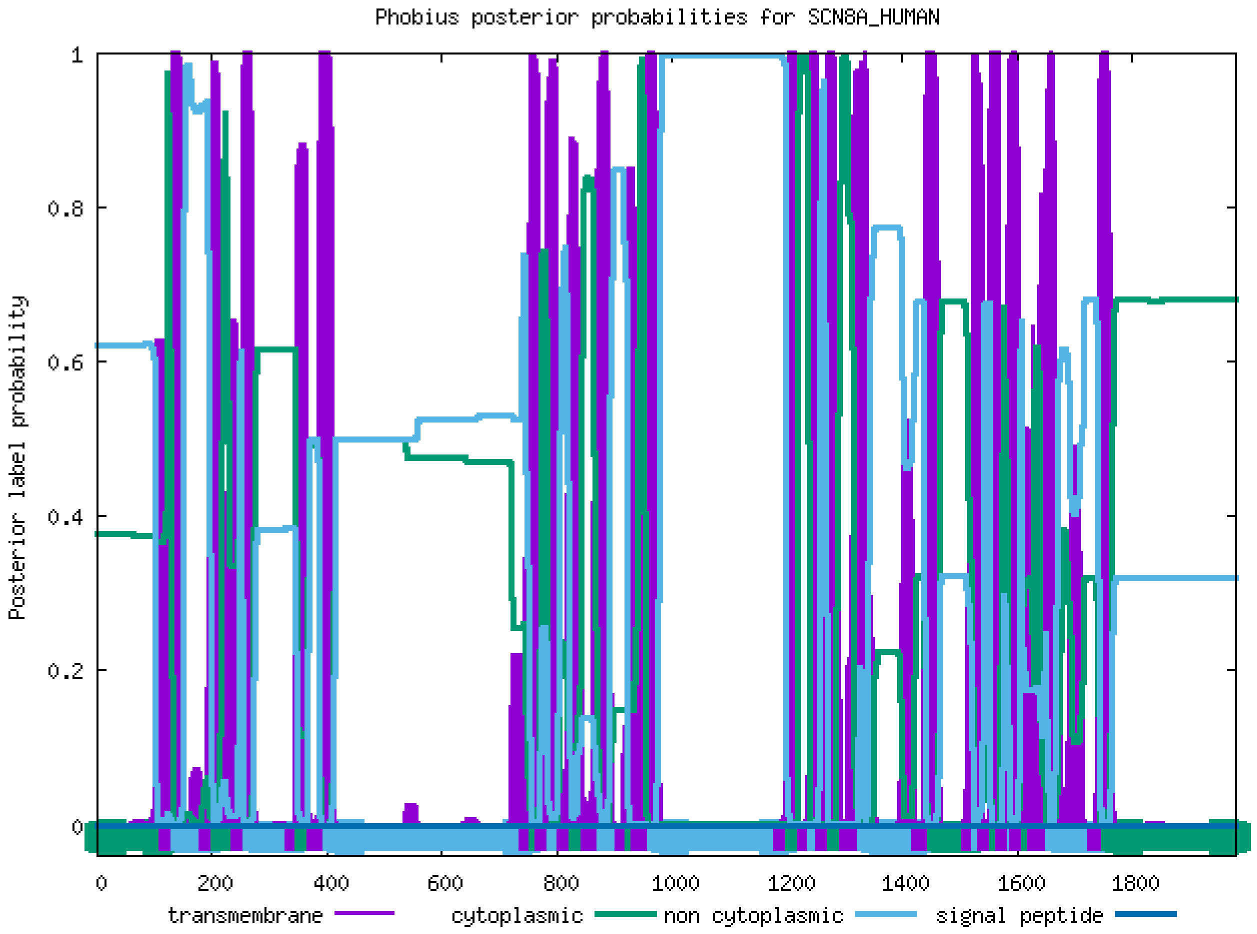

The probability data used in the plot is found here, and the gnuplot script is here.

Prediction of SCN9A_HUMAN

```
ID   SCN9A_HUMAN
FT   TOPO_DOM      1    121       CYTOPLASMIC.
FT   TRANSMEM    122    145
FT   TOPO_DOM    146    189       NON CYTOPLASMIC.
FT   TRANSMEM    190    209
FT   TOPO_DOM    210    220       CYTOPLASMIC.
FT   TRANSMEM    221    242
FT   TOPO_DOM    243    247       NON CYTOPLASMIC.
FT   TRANSMEM    248    268
FT   TOPO_DOM    269    377       CYTOPLASMIC.
FT   TRANSMEM    378    405
FT   TOPO_DOM    406    736       NON CYTOPLASMIC.
```

```
FT   TRANSMEM    737    763
FT   TOPO_DOM    764    774       CYTOPLASMIC.
FT   TRANSMEM    775    793
FT   TOPO_DOM    794    812       NON CYTOPLASMIC.
FT   TRANSMEM    813    834
FT   TOPO_DOM    835    854       CYTOPLASMIC.
FT   TRANSMEM    855    883
FT   TOPO_DOM    884    912       NON CYTOPLASMIC.
FT   TRANSMEM    913    933
FT   TOPO_DOM    934    944       CYTOPLASMIC.
FT   TRANSMEM    945    968
FT   TOPO_DOM    969   1192       NON CYTOPLASMIC.
FT   TRANSMEM   1193   1211
FT   TOPO_DOM   1212   1231       CYTOPLASMIC.
FT   TRANSMEM   1232   1248
FT   TOPO_DOM   1249   1259       NON CYTOPLASMIC.
FT   TRANSMEM   1260   1279
FT   TOPO_DOM   1280   1299       CYTOPLASMIC.
FT   TRANSMEM   1300   1318
FT   TOPO_DOM   1319   1323       NON CYTOPLASMIC.
FT   TRANSMEM   1324   1344
FT   TOPO_DOM   1345   1392       CYTOPLASMIC.
FT   TRANSMEM   1393   1414
FT   TOPO_DOM   1415   1433       NON CYTOPLASMIC.
FT   TRANSMEM   1434   1457
FT   TOPO_DOM   1458   1515       CYTOPLASMIC.
FT   TRANSMEM   1516   1534
FT   TOPO_DOM   1535   1545       NON CYTOPLASMIC.
FT   TRANSMEM   1546   1564
FT   TOPO_DOM   1565   1575       CYTOPLASMIC.
FT   TRANSMEM   1576   1596
FT   TOPO_DOM   1597   1601       NON CYTOPLASMIC.
FT   TRANSMEM   1602   1621
FT   TOPO_DOM   1622   1632       CYTOPLASMIC.
FT   TRANSMEM   1633   1661
FT   TOPO_DOM   1662   1736       NON CYTOPLASMIC.
FT   TRANSMEM   1737   1760
FT   TOPO_DOM   1761   1988       CYTOPLASMIC.
//
```

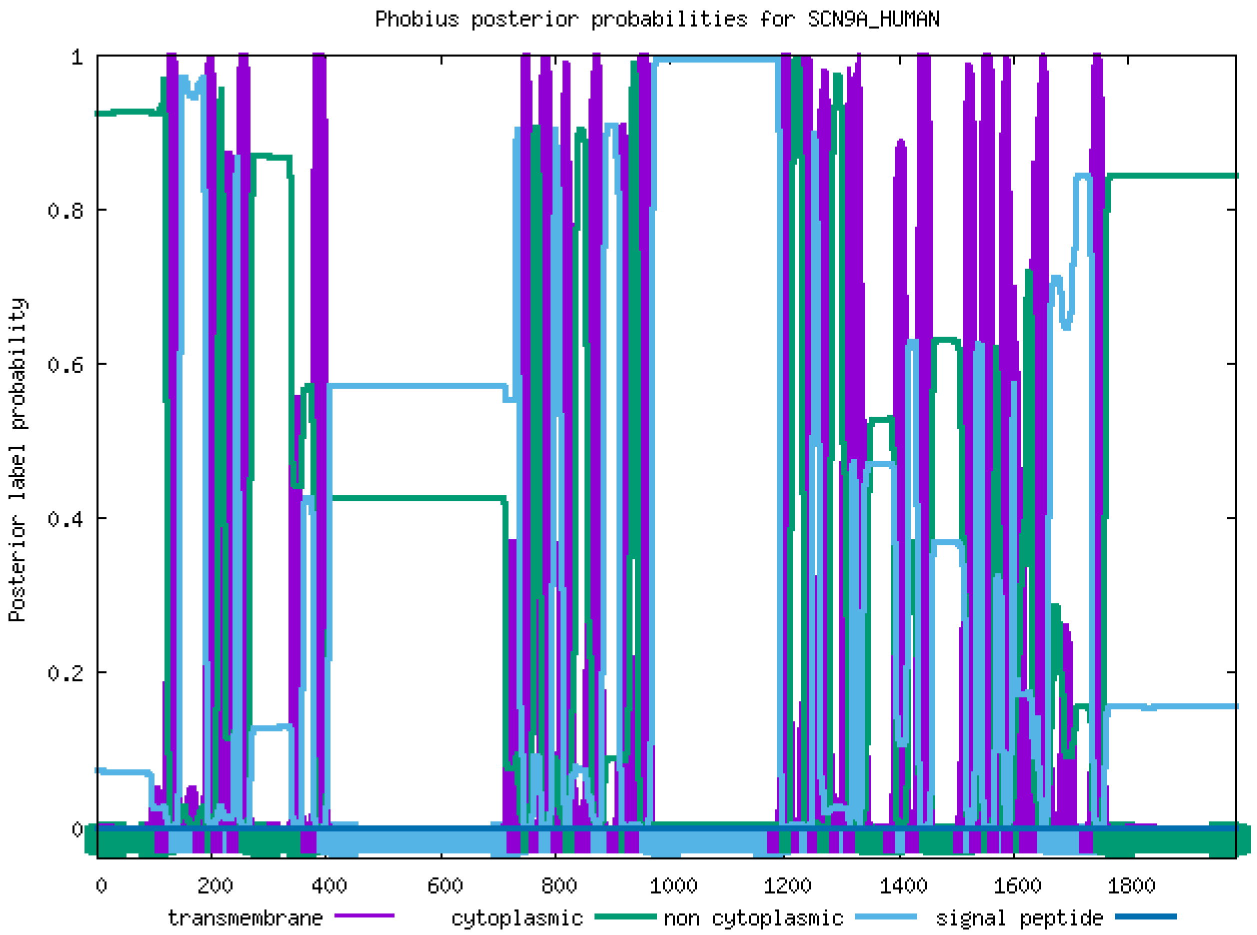

The probability data used in the plot is found here, and the gnuplot script is here.

Prediction of SCNAA_HUMAN

```
ID   SCNAA_HUMAN
FT   TOPO_DOM      1    127       NON CYTOPLASMIC.
FT   TRANSMEM    128    146
FT   TOPO_DOM    147    157       CYTOPLASMIC.
FT   TRANSMEM    158    177
FT   TOPO_DOM    178    188       NON CYTOPLASMIC.
FT   TRANSMEM    189    210
FT   TOPO_DOM    211    221       CYTOPLASMIC.
FT   TRANSMEM    222    243
FT   TOPO_DOM    244    248       NON CYTOPLASMIC.
FT   TRANSMEM    249    268
FT   TOPO_DOM    269    371       CYTOPLASMIC.
FT   TRANSMEM    372    400
FT   TOPO_DOM    401    656       NON CYTOPLASMIC.
FT   TRANSMEM    657    683
FT   TOPO_DOM    684    694       CYTOPLASMIC.
FT   TRANSMEM    695    717
FT   TOPO_DOM    718    728       NON CYTOPLASMIC.
FT   TRANSMEM    729    747
FT   TOPO_DOM    748    783       CYTOPLASMIC.
FT   TRANSMEM    784    804
FT   TOPO_DOM    805    834       NON CYTOPLASMIC.
FT   TRANSMEM    835    855
FT   TOPO_DOM    856    866       CYTOPLASMIC.
FT   TRANSMEM    867    890
FT   TOPO_DOM    891   1152       NON CYTOPLASMIC.
FT   TRANSMEM   1153   1171
FT   TOPO_DOM   1172   1191       CYTOPLASMIC.
FT   TRANSMEM   1192   1208
FT   TOPO_DOM   1209   1219       NON CYTOPLASMIC.
```

```
FT   TRANSMEM   1220   1238
FT   TOPO_DOM   1239   1268       CYTOPLASMIC.
FT   TRANSMEM   1269   1295
FT   TOPO_DOM   1296   1393       NON CYTOPLASMIC.
FT   TRANSMEM   1394   1418
FT   TOPO_DOM   1419   1467       CYTOPLASMIC.
FT   TRANSMEM   1468   1491
FT   TOPO_DOM   1492   1510       NON CYTOPLASMIC.
FT   TRANSMEM   1511   1529
FT   TOPO_DOM   1530   1540       CYTOPLASMIC.
FT   TRANSMEM   1541   1560
FT   TOPO_DOM   1561   1565       NON CYTOPLASMIC.
FT   TRANSMEM   1566   1584
FT   TOPO_DOM   1585   1595       CYTOPLASMIC.
FT   TRANSMEM   1596   1624
FT   TOPO_DOM   1625   1698       NON CYTOPLASMIC.
FT   TRANSMEM   1699   1722
FT   TOPO_DOM   1723   1956       CYTOPLASMIC.
//
```

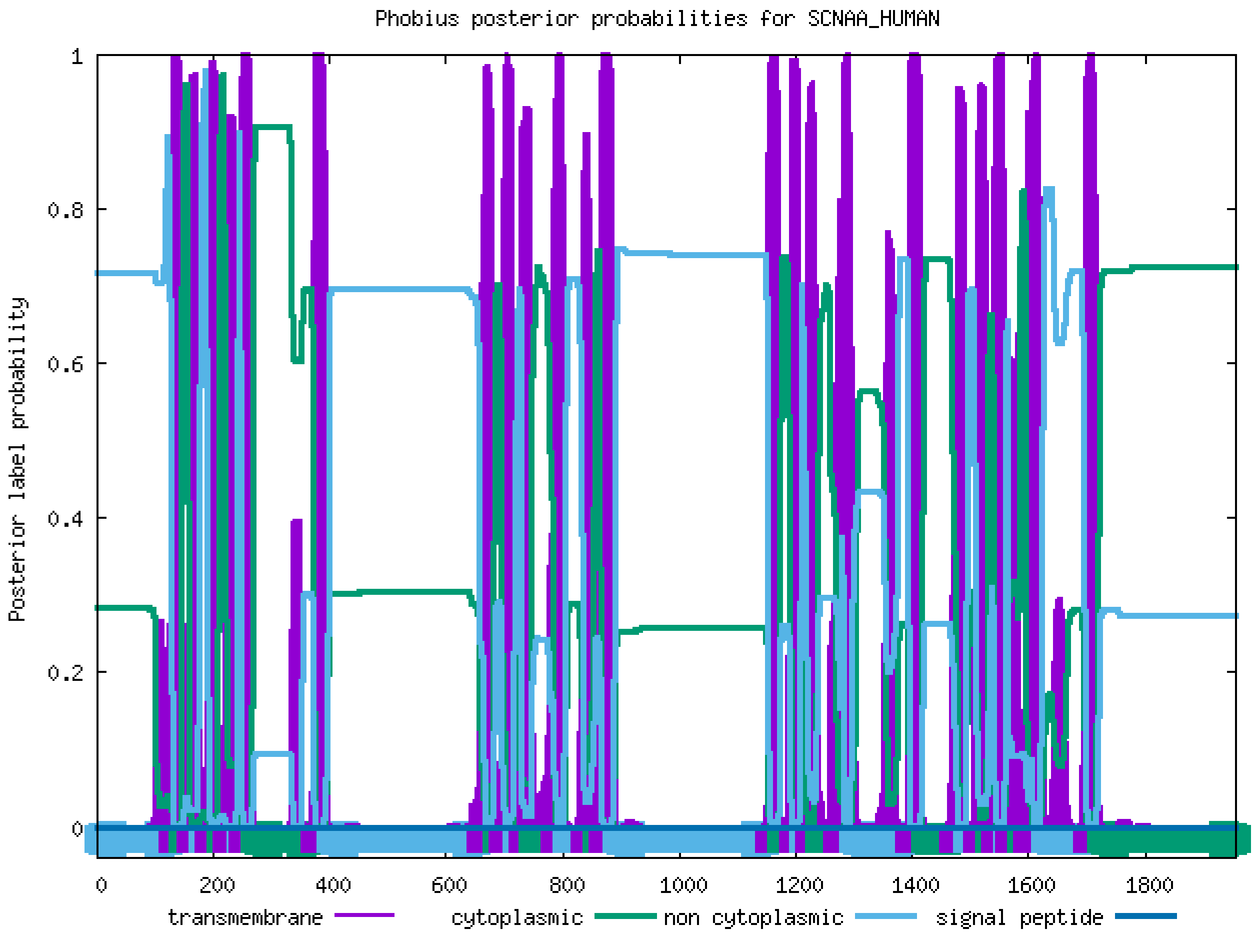

The probability data used in the plot is found here, and the gnuplot script is here.

Prediction of HVCN1_HUMAN

```
ID   HVCN1_HUMAN
FT   TOPO_DOM      1    100       CYTOPLASMIC.
FT   TRANSMEM    101    122
FT   TOPO_DOM    123    133       NON CYTOPLASMIC.
FT   TRANSMEM    134    160
FT   TOPO_DOM    161    171       CYTOPLASMIC.
FT   TRANSMEM    172    191
FT   TOPO_DOM    192    196       NON CYTOPLASMIC.
FT   TRANSMEM    197    220
```

```
FT   TOPO_DOM     221    273       CYTOPLASMIC.
//
```

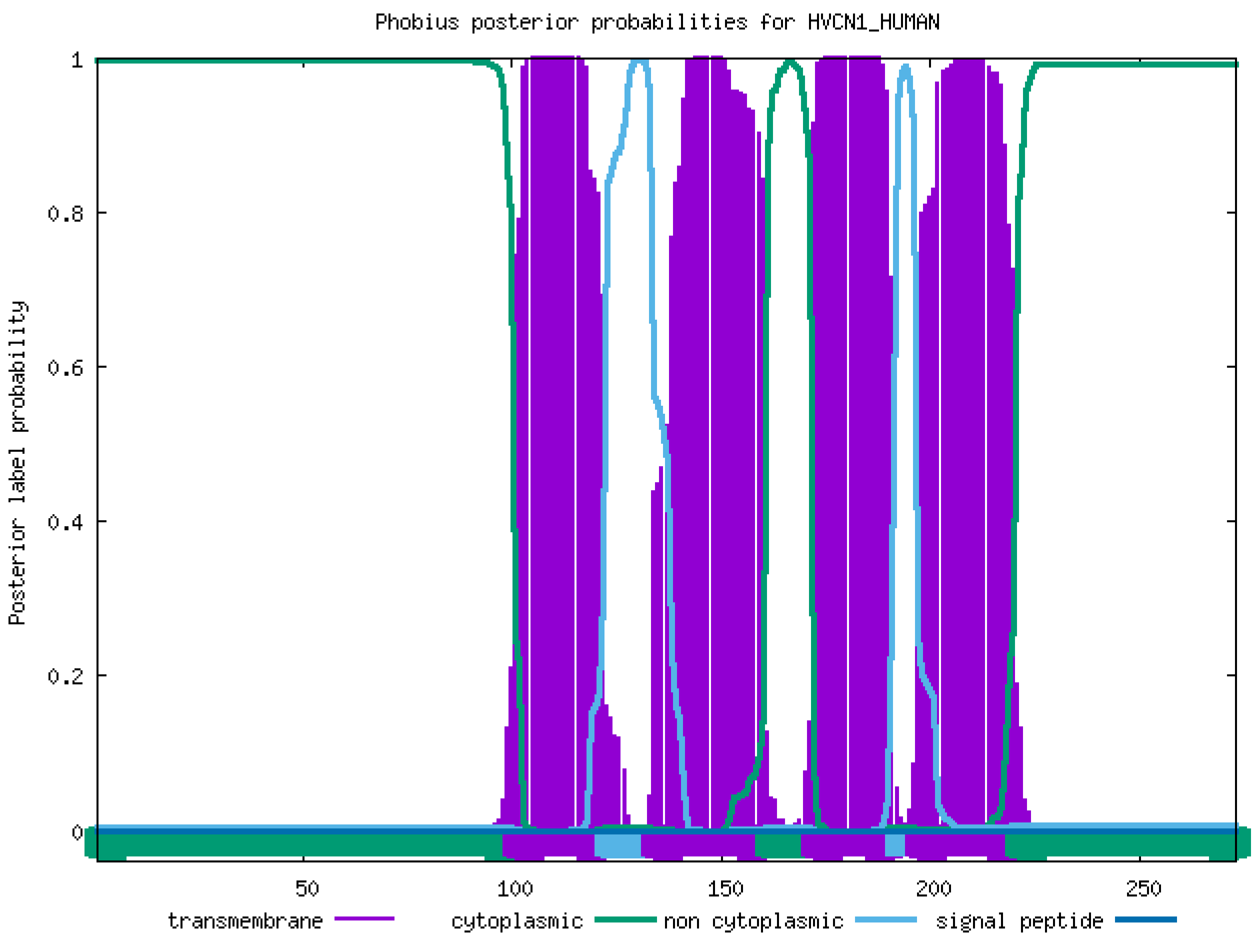

The probability data used in the plot is found here, and the gnuplot script is here.

Prediction of CNGA1_HUMAN

```
ID   CNGA1_HUMAN
FT   TOPO_DOM       1    298       CYTOPLASMIC.
FT   TRANSMEM     299    318
FT   TOPO_DOM     319    370       NON CYTOPLASMIC.
FT   TRANSMEM     371    393
FT   TOPO_DOM     394    686       CYTOPLASMIC.
//
```

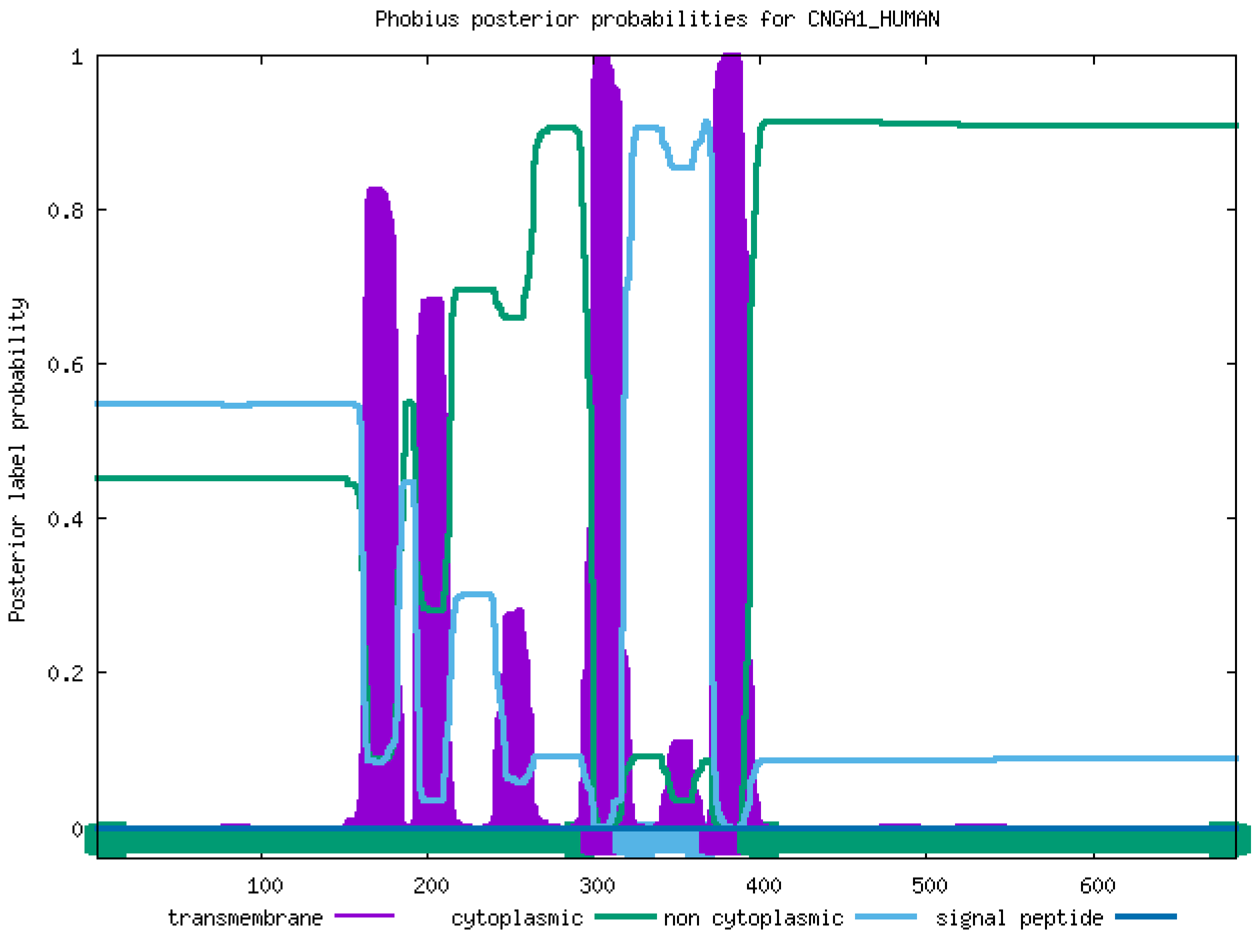

The probability data used in the plot is found here, and the gnuplot script is here.

Prediction of CNGB1_HUMAN

```
ID   CNGB1_HUMAN
FT   TOPO_DOM      1    653       NON CYTOPLASMIC.
FT   TRANSMEM    654    674
FT   TOPO_DOM    675    741       CYTOPLASMIC.
FT   TRANSMEM    742    761
FT   TOPO_DOM    762   1251       NON CYTOPLASMIC.
//
```

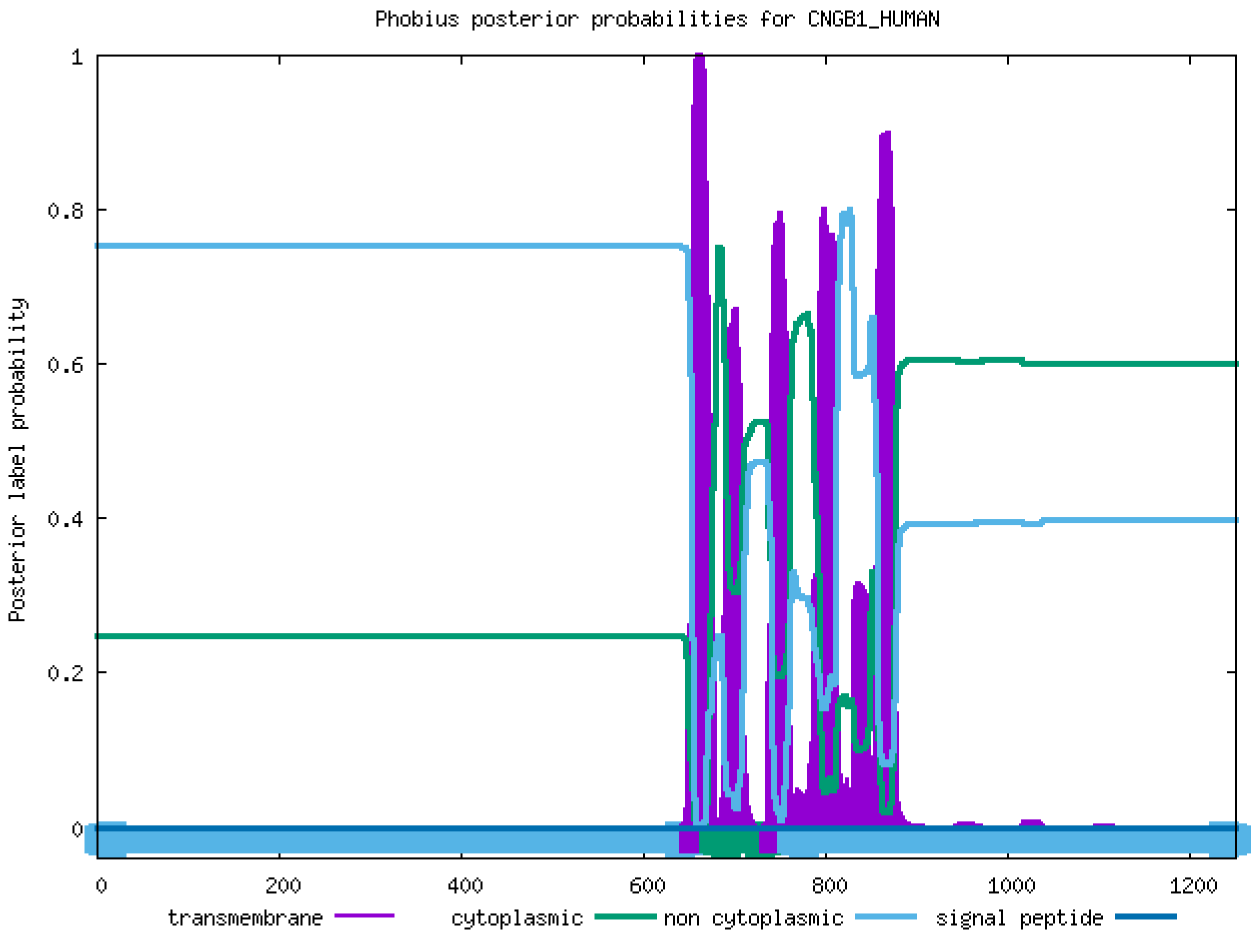

The probability data used in the plot is found here, and the gnuplot script is here.

Prediction of HCN1_HUMAN

```
ID    HCN1_HUMAN
FT    TOPO_DOM      1    142       CYTOPLASMIC.
FT    TRANSMEM    143    167
FT    TOPO_DOM    168    172       NON CYTOPLASMIC.
FT    TRANSMEM    173    191
FT    TOPO_DOM    192    220       CYTOPLASMIC.
FT    TRANSMEM    221    239
FT    TOPO_DOM    240    250       NON CYTOPLASMIC.
FT    TRANSMEM    251    269
FT    TOPO_DOM    270    288       CYTOPLASMIC.
FT    TRANSMEM    289    308
FT    TOPO_DOM    309    371       NON CYTOPLASMIC.
FT    TRANSMEM    372    397
FT    TOPO_DOM    398    890       CYTOPLASMIC.
//
```

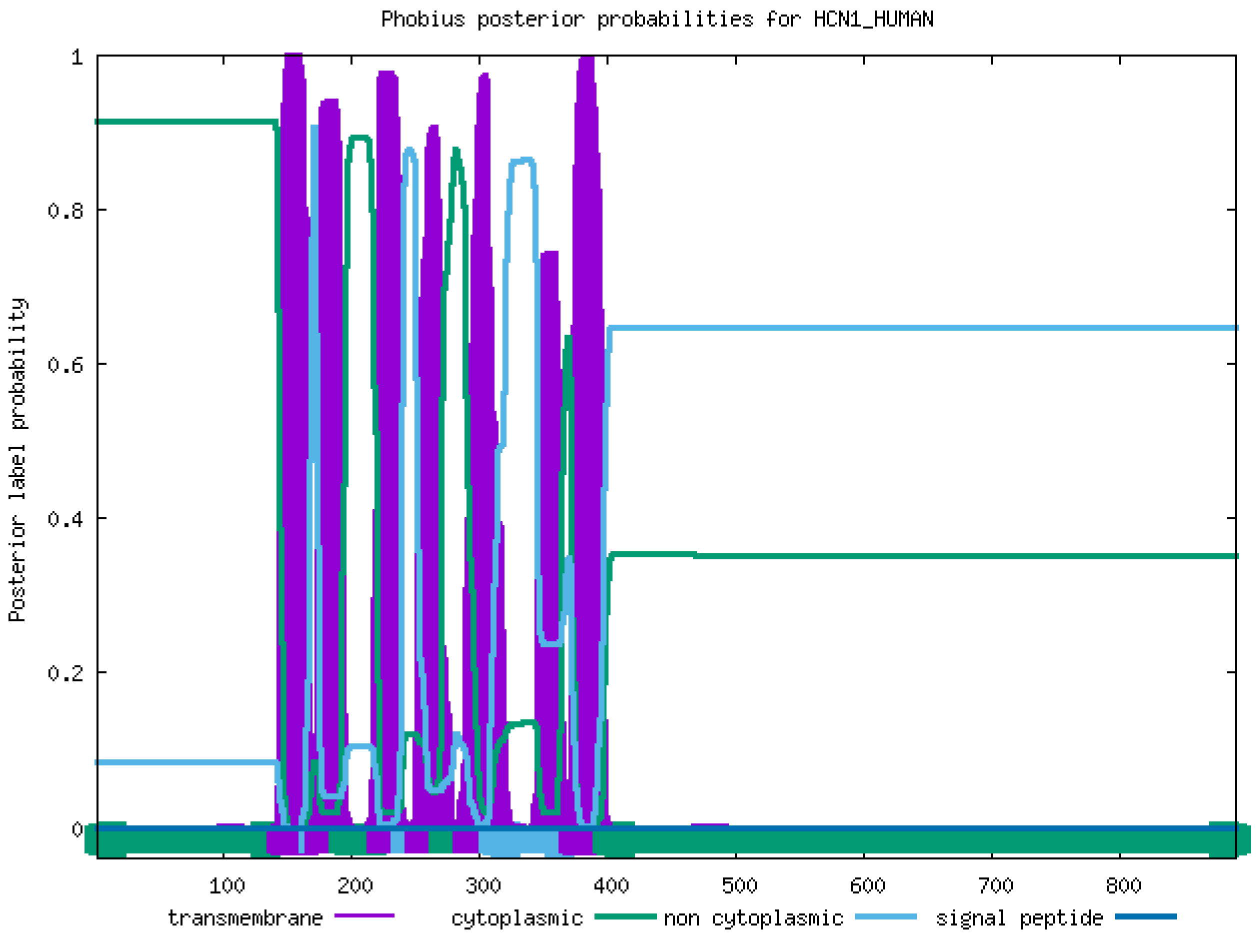

The probability data used in the plot is found here, and the gnuplot script is here.

Prediction of KCNQ1_HUMAN

```
ID   KCNQ1_HUMAN
FT   TOPO_DOM      1    122       CYTOPLASMIC.
FT   TRANSMEM    123    142
FT   TOPO_DOM    143    147       NON CYTOPLASMIC.
FT   TRANSMEM    148    173
FT   TOPO_DOM    174    199       CYTOPLASMIC.
FT   TRANSMEM    200    223
FT   TOPO_DOM    224    261       NON CYTOPLASMIC.
FT   TRANSMEM    262    284
FT   TOPO_DOM    285    295       CYTOPLASMIC.
FT   TRANSMEM    296    315
FT   TOPO_DOM    316    326       NON CYTOPLASMIC.
FT   TRANSMEM    327    353
FT   TOPO_DOM    354    676       CYTOPLASMIC.
//
```

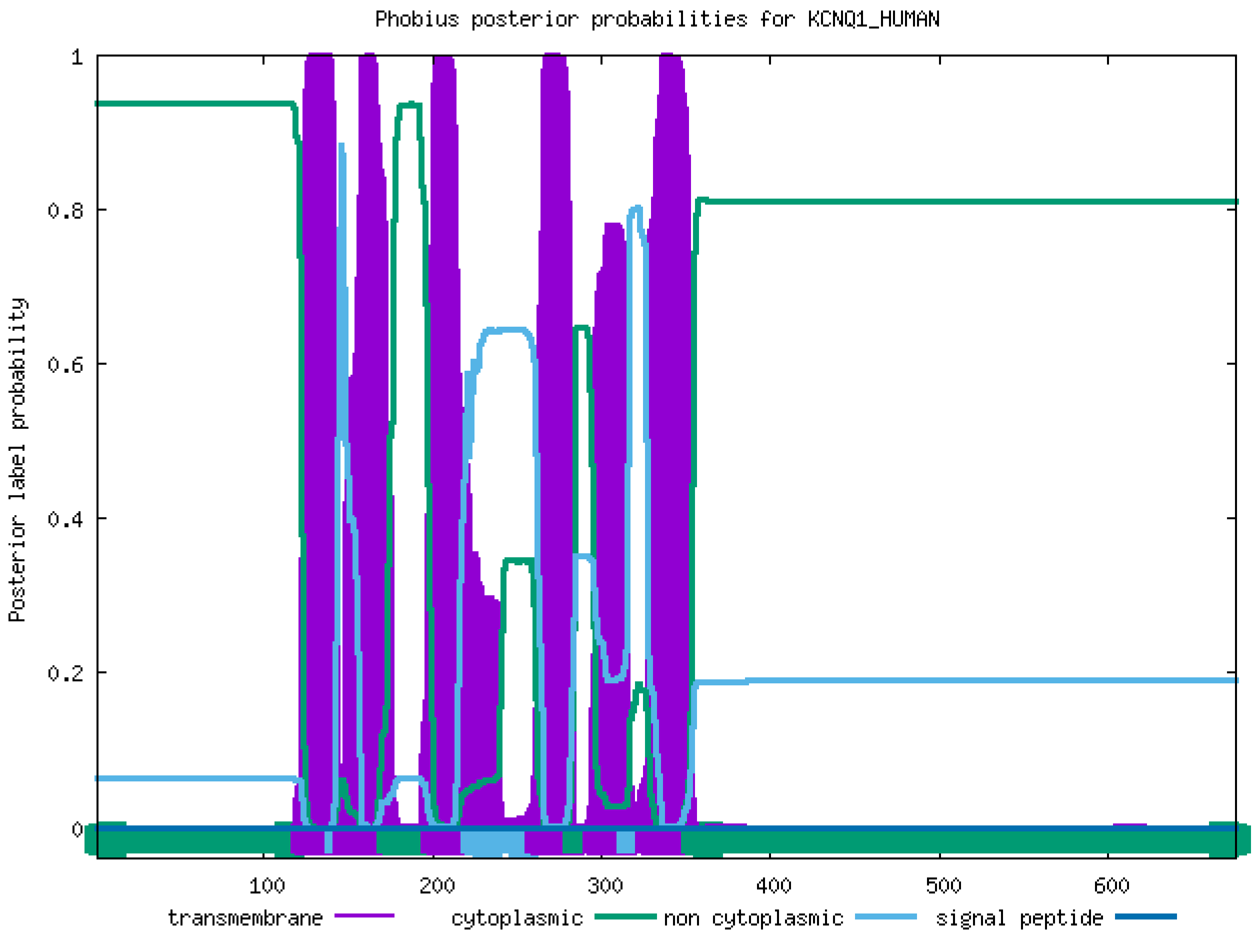

The probability data used in the plot is found here, and the gnuplot script is here.

Prediction of KCNQ2_HUMAN

```
ID   KCNQ2_HUMAN
FT   TOPO_DOM      1     92       CYTOPLASMIC.
FT   TRANSMEM     93    112
FT   TOPO_DOM    113    123       NON CYTOPLASMIC.
FT   TRANSMEM    124    147
FT   TOPO_DOM    148    167       CYTOPLASMIC.
FT   TRANSMEM    168    187
FT   TOPO_DOM    188    233       NON CYTOPLASMIC.
FT   TRANSMEM    234    252
FT   TOPO_DOM    253    263       CYTOPLASMIC.
FT   TRANSMEM    264    281
FT   TOPO_DOM    282    292       NON CYTOPLASMIC.
FT   TRANSMEM    293    318
FT   TOPO_DOM    319    872       CYTOPLASMIC.
//
```

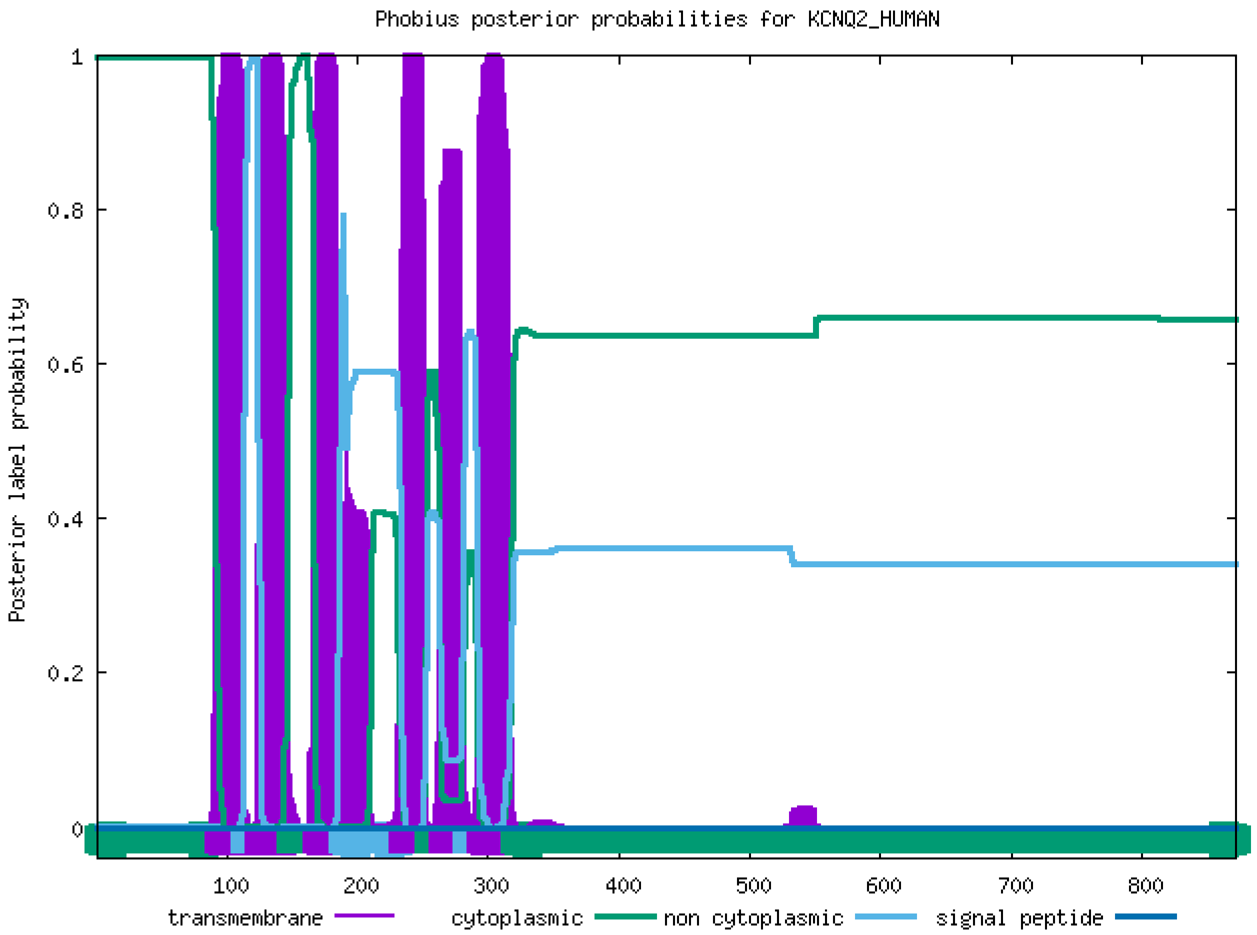

The probability data used in the plot is found here, and the gnuplot script is here.

Prediction of KCNQ4_HUMAN

```
ID   KCNQ4_HUMAN
FT   TOPO_DOM      1     98       CYTOPLASMIC.
FT   TRANSMEM     99    121
FT   TOPO_DOM    122    132       NON CYTOPLASMIC.
FT   TRANSMEM    133    153
FT   TOPO_DOM    154    173       CYTOPLASMIC.
FT   TRANSMEM    174    199
FT   TOPO_DOM    200    238       NON CYTOPLASMIC.
FT   TRANSMEM    239    258
FT   TOPO_DOM    259    269       CYTOPLASMIC.
FT   TRANSMEM    270    287
FT   TOPO_DOM    288    298       NON CYTOPLASMIC.
FT   TRANSMEM    299    324
FT   TOPO_DOM    325    695       CYTOPLASMIC.
//
```

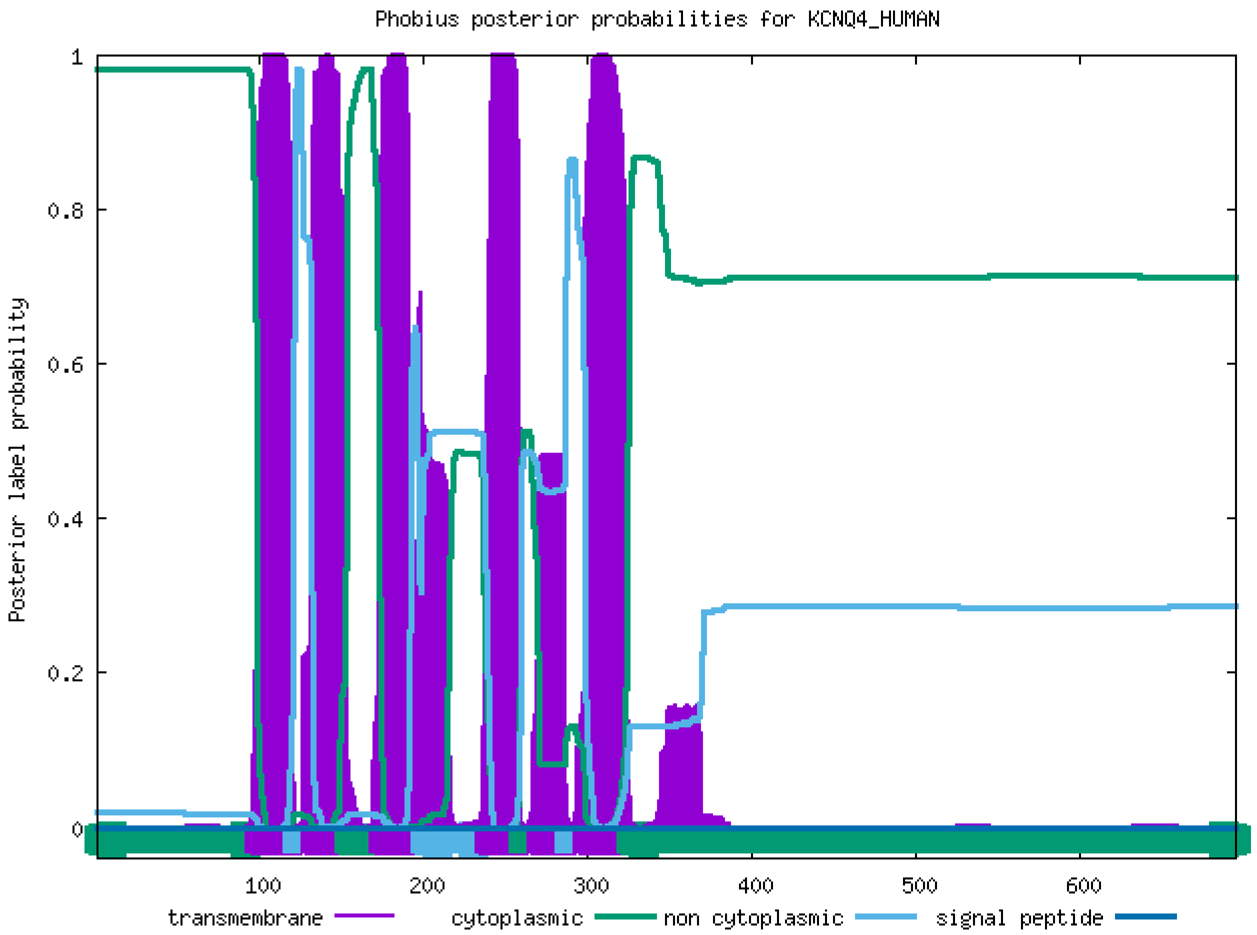

The probability data used in the plot is found here, and the gnuplot script is here.

Prediction of TM175_HUMAN

```
ID    TM175_HUMAN
FT    TOPO_DOM      1     38       CYTOPLASMIC.
FT    TRANSMEM     39     58
FT    TOPO_DOM     59     77       NON CYTOPLASMIC.
FT    TRANSMEM     78     99
FT    TOPO_DOM    100    110       CYTOPLASMIC.
FT    TRANSMEM    111    133
FT    TOPO_DOM    134    138       NON CYTOPLASMIC.
FT    TRANSMEM    139    164
FT    TOPO_DOM    165    184       CYTOPLASMIC.
FT    TRANSMEM    185    205
FT    TOPO_DOM    206    210       NON CYTOPLASMIC.
FT    TRANSMEM    211    230
FT    TOPO_DOM    231    309       CYTOPLASMIC.
FT    TRANSMEM    310    327
FT    TOPO_DOM    328    338       NON CYTOPLASMIC.
FT    TRANSMEM    339    359
FT    TOPO_DOM    360    379       CYTOPLASMIC.
FT    TRANSMEM    380    399
FT    TOPO_DOM    400    418       NON CYTOPLASMIC.
FT    TRANSMEM    419    441
FT    TOPO_DOM    442    452       CYTOPLASMIC.
FT    TRANSMEM    453    476
FT    TOPO_DOM    477    504       NON CYTOPLASMIC.
//
```

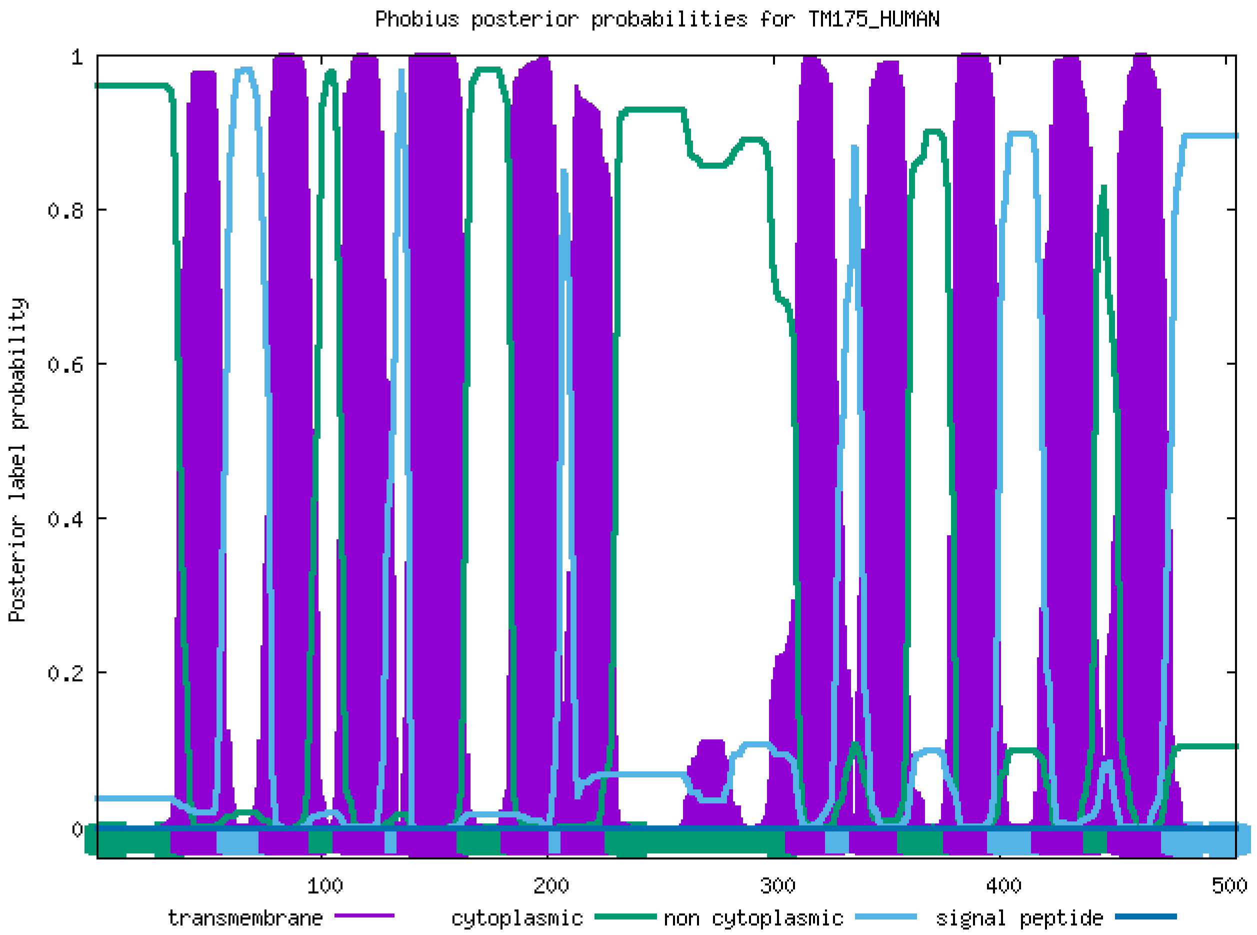

The probability data used in the plot is found here, and the gnuplot script is here.

Prediction of NALCN_HUMAN

```
ID    NALCN_HUMAN
FT    TOPO_DOM      1     40       CYTOPLASMIC.
FT    TRANSMEM     41     61
FT    TOPO_DOM     62     66       NON CYTOPLASMIC.
FT    TRANSMEM     67     88
FT    TOPO_DOM     89    107       CYTOPLASMIC.
FT    TRANSMEM    108    127
FT    TOPO_DOM    128    138       NON CYTOPLASMIC.
FT    TRANSMEM    139    158
FT    TOPO_DOM    159    177       CYTOPLASMIC.
FT    TRANSMEM    178    195
FT    TOPO_DOM    196    298       NON CYTOPLASMIC.
FT    TRANSMEM    299    322
FT    TOPO_DOM    323    384       CYTOPLASMIC.
FT    TRANSMEM    385    406
FT    TOPO_DOM    407    417       NON CYTOPLASMIC.
FT    TRANSMEM    418    446
FT    TOPO_DOM    447    505       CYTOPLASMIC.
FT    TRANSMEM    506    528
FT    TOPO_DOM    529    574       NON CYTOPLASMIC.
FT    TRANSMEM    575    599
FT    TOPO_DOM    600    880       CYTOPLASMIC.
FT    TRANSMEM    881    905
FT    TOPO_DOM    906    916       NON CYTOPLASMIC.
FT    TRANSMEM    917    935
FT    TOPO_DOM    936    955       CYTOPLASMIC.
FT    TRANSMEM    956    974
FT    TOPO_DOM    975    979       NON CYTOPLASMIC.
FT    TRANSMEM    980    997
FT    TOPO_DOM    998   1016       CYTOPLASMIC.
```

```
FT   TRANSMEM   1017   1040
FT   TOPO_DOM   1041   1133       NON CYTOPLASMIC.
FT   TRANSMEM   1134   1157
FT   TOPO_DOM   1158   1210       CYTOPLASMIC.
FT   TRANSMEM   1211   1231
FT   TOPO_DOM   1232   1236       NON CYTOPLASMIC.
FT   TRANSMEM   1237   1256
FT   TOPO_DOM   1257   1275       CYTOPLASMIC.
FT   TRANSMEM   1276   1294
FT   TOPO_DOM   1295   1299       NON CYTOPLASMIC.
FT   TRANSMEM   1300   1316
FT   TOPO_DOM   1317   1335       CYTOPLASMIC.
FT   TRANSMEM   1336   1358
FT   TOPO_DOM   1359   1422       NON CYTOPLASMIC.
FT   TRANSMEM   1423   1447
FT   TOPO_DOM   1448   1738       CYTOPLASMIC.
//
```

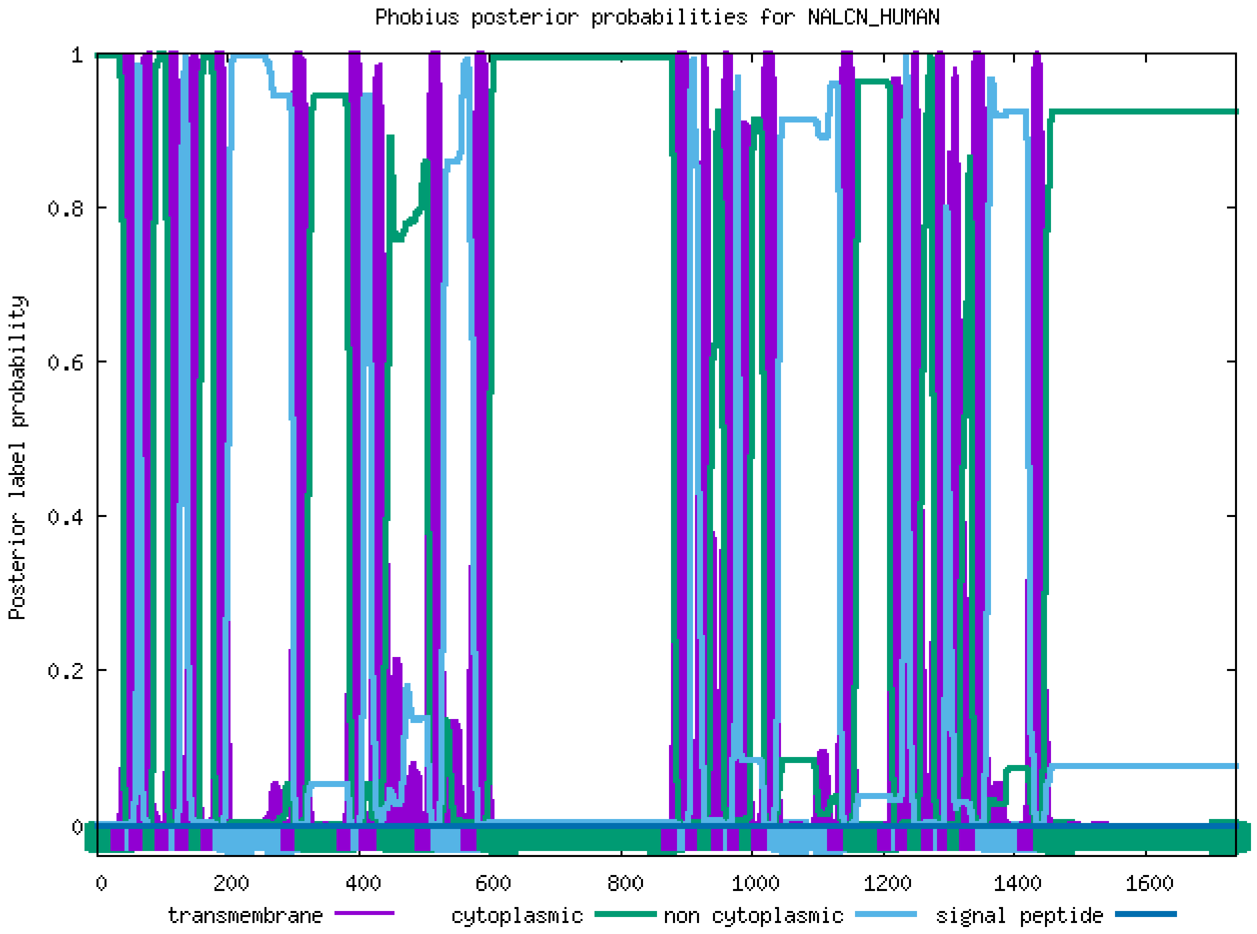

The probability data used in the plot is found here, and the gnuplot script is here.

Prediction of NALF1_HUMAN

```
ID   NALF1_HUMAN
FT   TOPO_DOM      1     39       CYTOPLASMIC.
FT   TRANSMEM     40     58
FT   TOPO_DOM     59    458       NON CYTOPLASMIC.
//
```

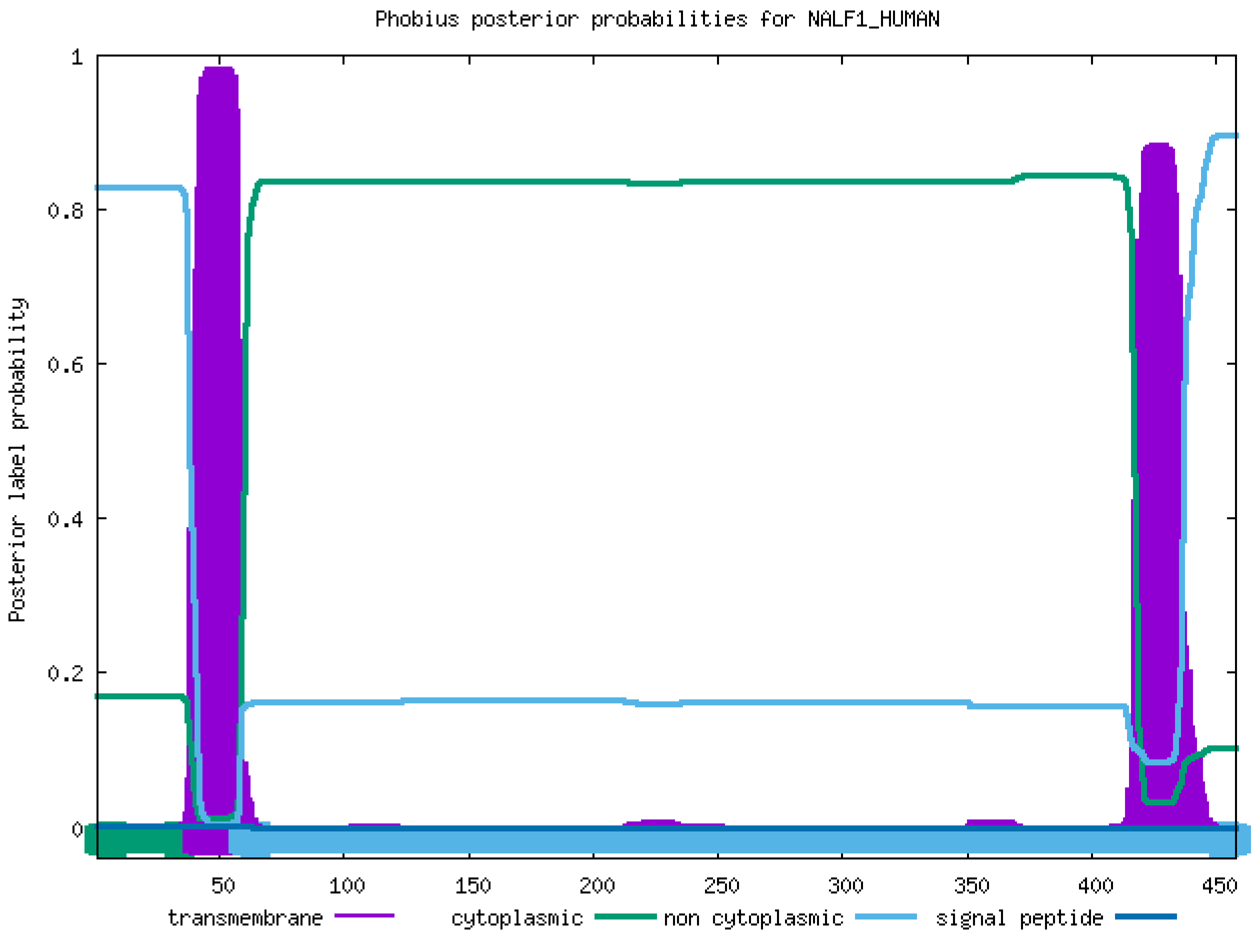

The probability data used in the plot is found here, and the gnuplot script is here.

Prediction of CAC1C_HUMAN

```
ID    CAC1C_HUMAN
FT    TOPO_DOM      1     127       CYTOPLASMIC.
FT    TRANSMEM    128     145
FT    TOPO_DOM    146     164       NON CYTOPLASMIC.
FT    TRANSMEM    165     185
FT    TOPO_DOM    186     196       CYTOPLASMIC.
FT    TRANSMEM    197     215
FT    TOPO_DOM    216     267       NON CYTOPLASMIC.
FT    TRANSMEM    268     290
FT    TOPO_DOM    291     348       CYTOPLASMIC.
FT    TRANSMEM    349     370
FT    TOPO_DOM    371     381       NON CYTOPLASMIC.
FT    TRANSMEM    382     404
FT    TOPO_DOM    405     524       CYTOPLASMIC.
FT    TRANSMEM    525     542
FT    TOPO_DOM    543     561       NON CYTOPLASMIC.
FT    TRANSMEM    562     585
FT    TOPO_DOM    586     653       CYTOPLASMIC.
FT    TRANSMEM    654     673
FT    TOPO_DOM    674     725       NON CYTOPLASMIC.
FT    TRANSMEM    726     753
FT    TOPO_DOM    754     895       CYTOPLASMIC.
FT    TRANSMEM    896     919
FT    TOPO_DOM    920     930       NON CYTOPLASMIC.
FT    TRANSMEM    931     950
FT    TOPO_DOM    951     956       CYTOPLASMIC.
FT    TRANSMEM    957     979
FT    TOPO_DOM    980     990       NON CYTOPLASMIC.
FT    TRANSMEM    991    1017
FT    TOPO_DOM   1018    1037       CYTOPLASMIC.
```

```
FT   TRANSMEM   1038   1071
FT   TOPO_DOM   1072   1120       NON CYTOPLASMIC.
FT   TRANSMEM   1121   1142
FT   TOPO_DOM   1143   1162       CYTOPLASMIC.
FT   TRANSMEM   1163   1189
FT   TOPO_DOM   1190   1239       NON CYTOPLASMIC.
FT   TRANSMEM   1240   1257
FT   TOPO_DOM   1258   1268       CYTOPLASMIC.
FT   TRANSMEM   1269   1291
FT   TOPO_DOM   1292   1302       NON CYTOPLASMIC.
FT   TRANSMEM   1303   1320
FT   TOPO_DOM   1321   1406       CYTOPLASMIC.
FT   TRANSMEM   1407   1430
FT   TOPO_DOM   1431   1499       NON CYTOPLASMIC.
FT   TRANSMEM   1500   1524
FT   TOPO_DOM   1525   2221       CYTOPLASMIC.
//
```

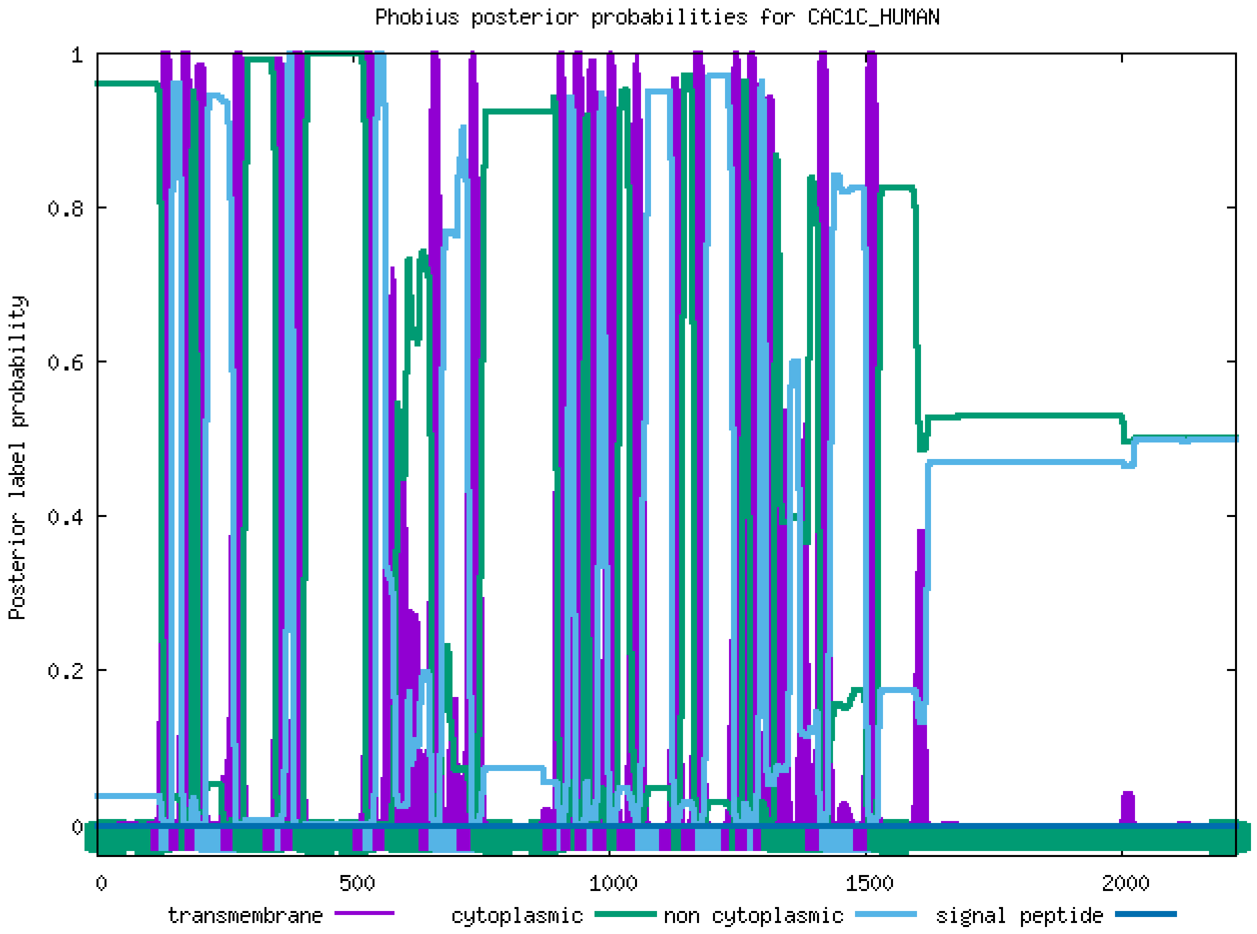

The probability data used in the plot is found here, and the gnuplot script is here.

Prediction of CA2D1_HUMAN

```
ID   CA2D1_HUMAN
FT   SIGNAL        1     18
FT   REGION        1      2       N-REGION.
FT   REGION        3     14       H-REGION.
FT   REGION       15     18       C-REGION.
FT   TOPO_DOM     19   1080       NON CYTOPLASMIC.
FT   TRANSMEM   1081   1099
FT   TOPO_DOM   1100   1103       CYTOPLASMIC.
//
```

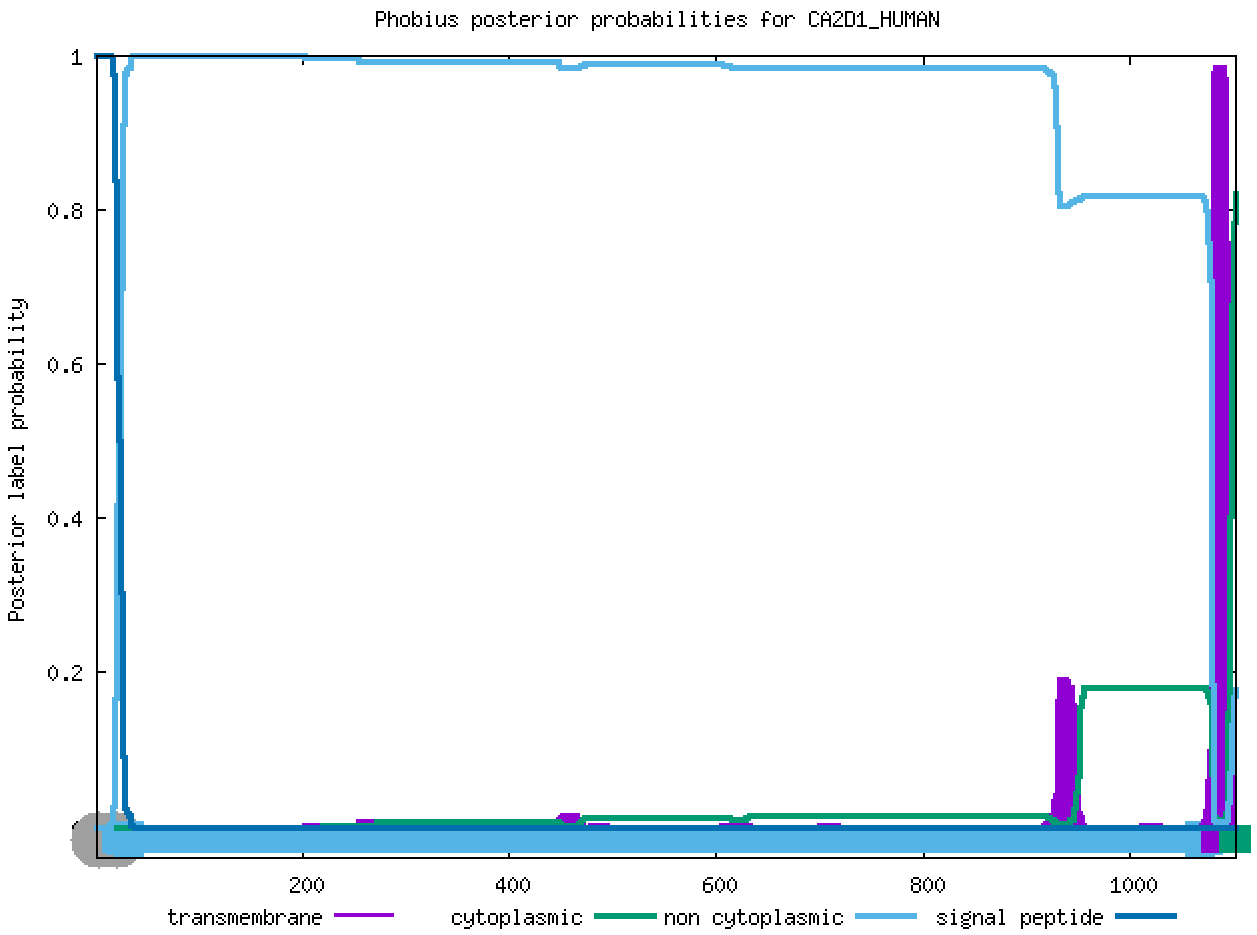

The probability data used in the plot is found here, and the gnuplot script is here.

Prediction of CAC1D_HUMAN

ID	CAC1D_HUMAN			
FT	TOPO_DOM	1	129	CYTOPLASMIC.
FT	TRANSMEM	130	147	
FT	TOPO_DOM	148	166	NON CYTOPLASMIC.
FT	TRANSMEM	167	187	
FT	TOPO_DOM	188	198	CYTOPLASMIC.
FT	TRANSMEM	199	218	
FT	TOPO_DOM	219	270	NON CYTOPLASMIC.
FT	TRANSMEM	271	294	
FT	TOPO_DOM	295	349	CYTOPLASMIC.
FT	TRANSMEM	350	371	
FT	TOPO_DOM	372	382	NON CYTOPLASMIC.
FT	TRANSMEM	383	405	
FT	TOPO_DOM	406	522	CYTOPLASMIC.
FT	TRANSMEM	523	541	
FT	TOPO_DOM	542	560	NON CYTOPLASMIC.
FT	TRANSMEM	561	584	
FT	TOPO_DOM	585	652	CYTOPLASMIC.
FT	TRANSMEM	653	672	
FT	TOPO_DOM	673	724	NON CYTOPLASMIC.
FT	TRANSMEM	725	752	
FT	TOPO_DOM	753	886	CYTOPLASMIC.
FT	TRANSMEM	887	905	
FT	TOPO_DOM	906	924	NON CYTOPLASMIC.
FT	TRANSMEM	925	945	
FT	TOPO_DOM	946	956	CYTOPLASMIC.
FT	TRANSMEM	957	983	
FT	TOPO_DOM	984	1002	NON CYTOPLASMIC.
FT	TRANSMEM	1003	1033	
FT	TOPO_DOM	1034	1129	CYTOPLASMIC.

```
FT   TRANSMEM   1130   1155
FT   TOPO_DOM   1156   1208       NON CYTOPLASMIC.
FT   TRANSMEM   1209   1227
FT   TOPO_DOM   1228   1238       CYTOPLASMIC.
FT   TRANSMEM   1239   1259
FT   TOPO_DOM   1260   1348       NON CYTOPLASMIC.
FT   TRANSMEM   1349   1367
FT   TOPO_DOM   1368   1440       CYTOPLASMIC.
FT   TRANSMEM   1441   1464
FT   TOPO_DOM   1465   2161       NON CYTOPLASMIC.
//
```

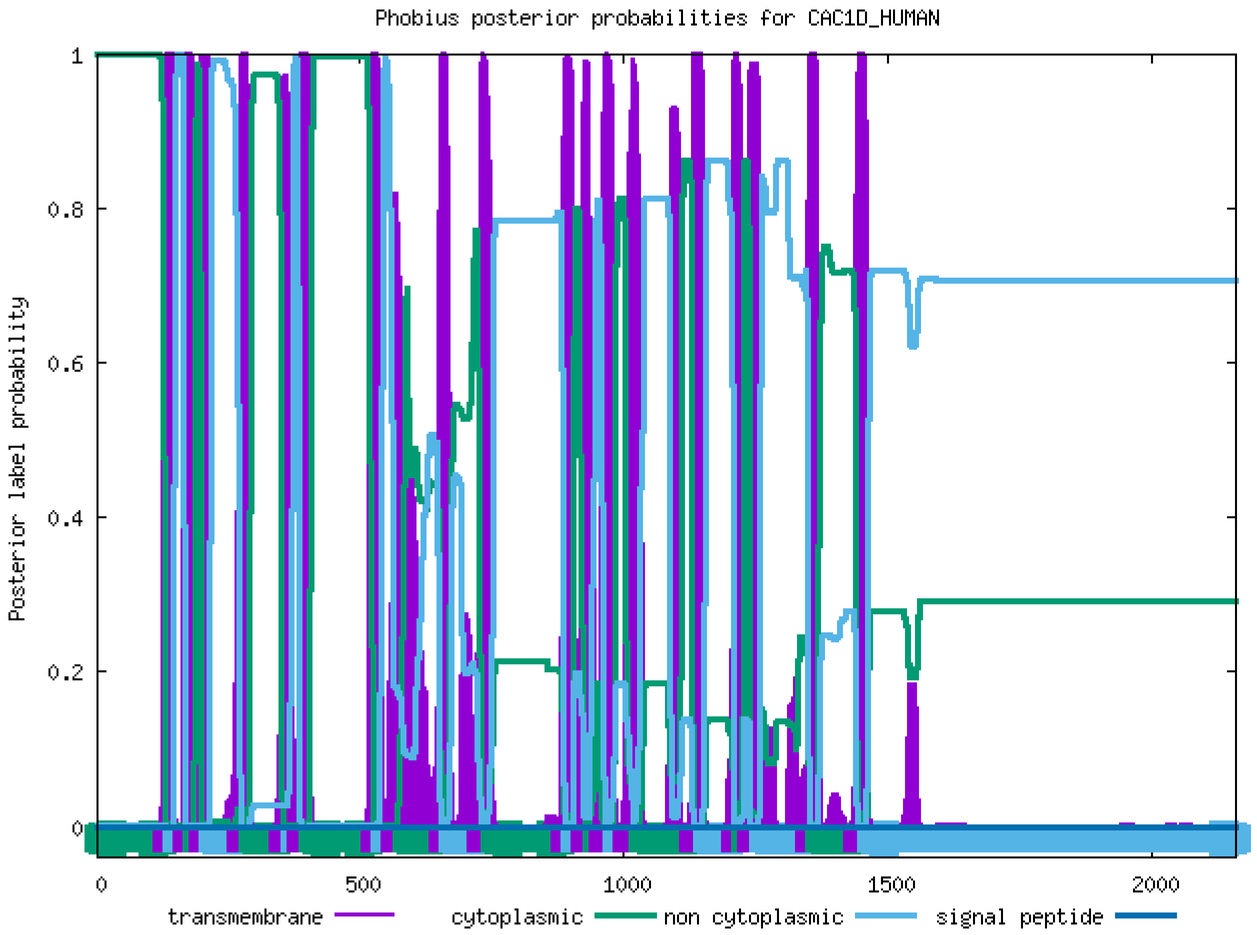

The probability data used in the plot is found here, and the gnuplot script is here.

Prediction of CAC1B_HUMAN

```
ID   CAC1B_HUMAN
FT   TOPO_DOM      1     96       NON CYTOPLASMIC.
FT   TRANSMEM     97    114
FT   TOPO_DOM    115    134       CYTOPLASMIC.
FT   TRANSMEM    135    156
FT   TOPO_DOM    157    167       NON CYTOPLASMIC.
FT   TRANSMEM    168    188
FT   TOPO_DOM    189    199       CYTOPLASMIC.
FT   TRANSMEM    200    217
FT   TOPO_DOM    218    222       NON CYTOPLASMIC.
FT   TRANSMEM    223    245
FT   TOPO_DOM    246    299       CYTOPLASMIC.
FT   TRANSMEM    300    321
FT   TOPO_DOM    322    332       NON CYTOPLASMIC.
FT   TRANSMEM    333    355
FT   TOPO_DOM    356    483       CYTOPLASMIC.
FT   TRANSMEM    484    504
FT   TOPO_DOM    505    509       NON CYTOPLASMIC.
```

```
FT   TRANSMEM    510    527
FT   TOPO_DOM    528    547       CYTOPLASMIC.
FT   TRANSMEM    548    566
FT   TOPO_DOM    567    571       NON CYTOPLASMIC.
FT   TRANSMEM    572    589
FT   TOPO_DOM    590    608       CYTOPLASMIC.
FT   TRANSMEM    609    631
FT   TOPO_DOM    632    686       NON CYTOPLASMIC.
FT   TRANSMEM    687    709
FT   TOPO_DOM    710   1150       CYTOPLASMIC.
FT   TRANSMEM   1151   1169
FT   TOPO_DOM   1170   1188       NON CYTOPLASMIC.
FT   TRANSMEM   1189   1209
FT   TOPO_DOM   1210   1220       CYTOPLASMIC.
FT   TRANSMEM   1221   1239
FT   TOPO_DOM   1240   1281       NON CYTOPLASMIC.
FT   TRANSMEM   1282   1304
FT   TOPO_DOM   1305   1393       CYTOPLASMIC.
FT   TRANSMEM   1394   1419
FT   TOPO_DOM   1420   1474       NON CYTOPLASMIC.
FT   TRANSMEM   1475   1493
FT   TOPO_DOM   1494   1504       CYTOPLASMIC.
FT   TRANSMEM   1505   1531
FT   TOPO_DOM   1532   1597       NON CYTOPLASMIC.
FT   TRANSMEM   1598   1616
FT   TOPO_DOM   1617   1684       CYTOPLASMIC.
FT   TRANSMEM   1685   1708
FT   TOPO_DOM   1709   2339       NON CYTOPLASMIC.
//
```

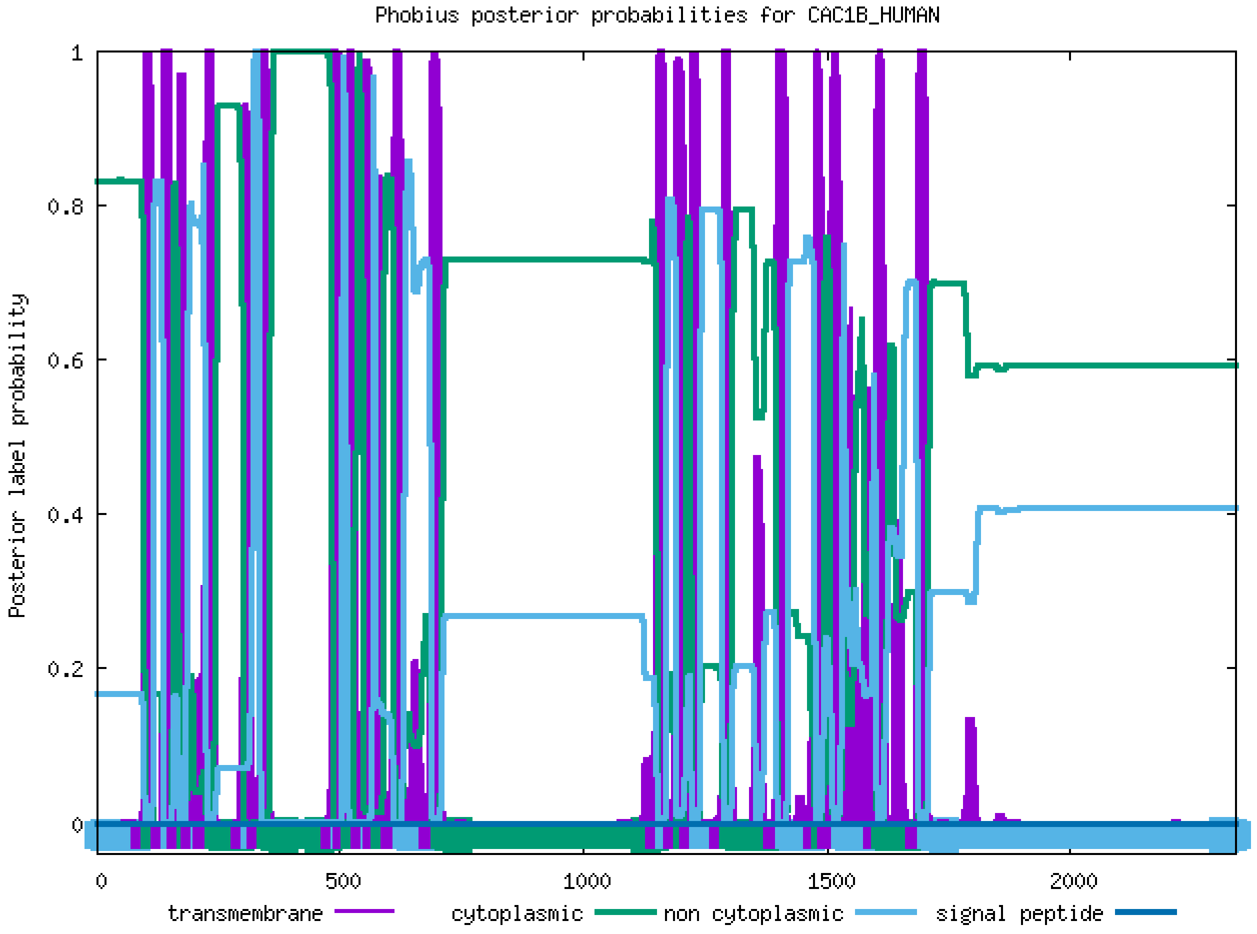

The probability data used in the plot is found here, and the gnuplot script is here.

Prediction of CAC1E_HUMAN

```
ID   CAC1E_HUMAN
FT   TOPO_DOM      1     92       CYTOPLASMIC.
FT   TRANSMEM     93    110
FT   TOPO_DOM    111    129       NON CYTOPLASMIC.
FT   TRANSMEM    130    150
FT   TOPO_DOM    151    161       CYTOPLASMIC.
FT   TRANSMEM    162    180
FT   TOPO_DOM    181    220       NON CYTOPLASMIC.
FT   TRANSMEM    221    243
FT   TOPO_DOM    244    294       CYTOPLASMIC.
FT   TRANSMEM    295    316
FT   TOPO_DOM    317    327       NON CYTOPLASMIC.
FT   TRANSMEM    328    350
FT   TOPO_DOM    351    476       CYTOPLASMIC.
FT   TRANSMEM    477    497
FT   TOPO_DOM    498    502       NON CYTOPLASMIC.
FT   TRANSMEM    503    521
FT   TOPO_DOM    522    541       CYTOPLASMIC.
FT   TRANSMEM    542    560
FT   TOPO_DOM    561    565       NON CYTOPLASMIC.
FT   TRANSMEM    566    583
FT   TOPO_DOM    584    602       CYTOPLASMIC.
FT   TRANSMEM    603    625
FT   TOPO_DOM    626    679       NON CYTOPLASMIC.
FT   TRANSMEM    680    703
FT   TOPO_DOM    704   1151       CYTOPLASMIC.
FT   TRANSMEM   1152   1171
FT   TOPO_DOM   1172   1190       NON CYTOPLASMIC.
FT   TRANSMEM   1191   1212
FT   TOPO_DOM   1213   1223       CYTOPLASMIC.
FT   TRANSMEM   1224   1243
FT   TOPO_DOM   1244   1288       NON CYTOPLASMIC.
FT   TRANSMEM   1289   1311
FT   TOPO_DOM   1312   1400       CYTOPLASMIC.
FT   TRANSMEM   1401   1426
FT   TOPO_DOM   1427   1481       NON CYTOPLASMIC.
FT   TRANSMEM   1482   1500
FT   TOPO_DOM   1501   1511       CYTOPLASMIC.
FT   TRANSMEM   1512   1535
FT   TOPO_DOM   1536   1540       NON CYTOPLASMIC.
FT   TRANSMEM   1541   1559
FT   TOPO_DOM   1560   1579       CYTOPLASMIC.
FT   TRANSMEM   1580   1600
FT   TOPO_DOM   1601   1605       NON CYTOPLASMIC.
FT   TRANSMEM   1606   1624
FT   TOPO_DOM   1625   1698       CYTOPLASMIC.
FT   TRANSMEM   1699   1723
FT   TOPO_DOM   1724   2313       NON CYTOPLASMIC.
//
```

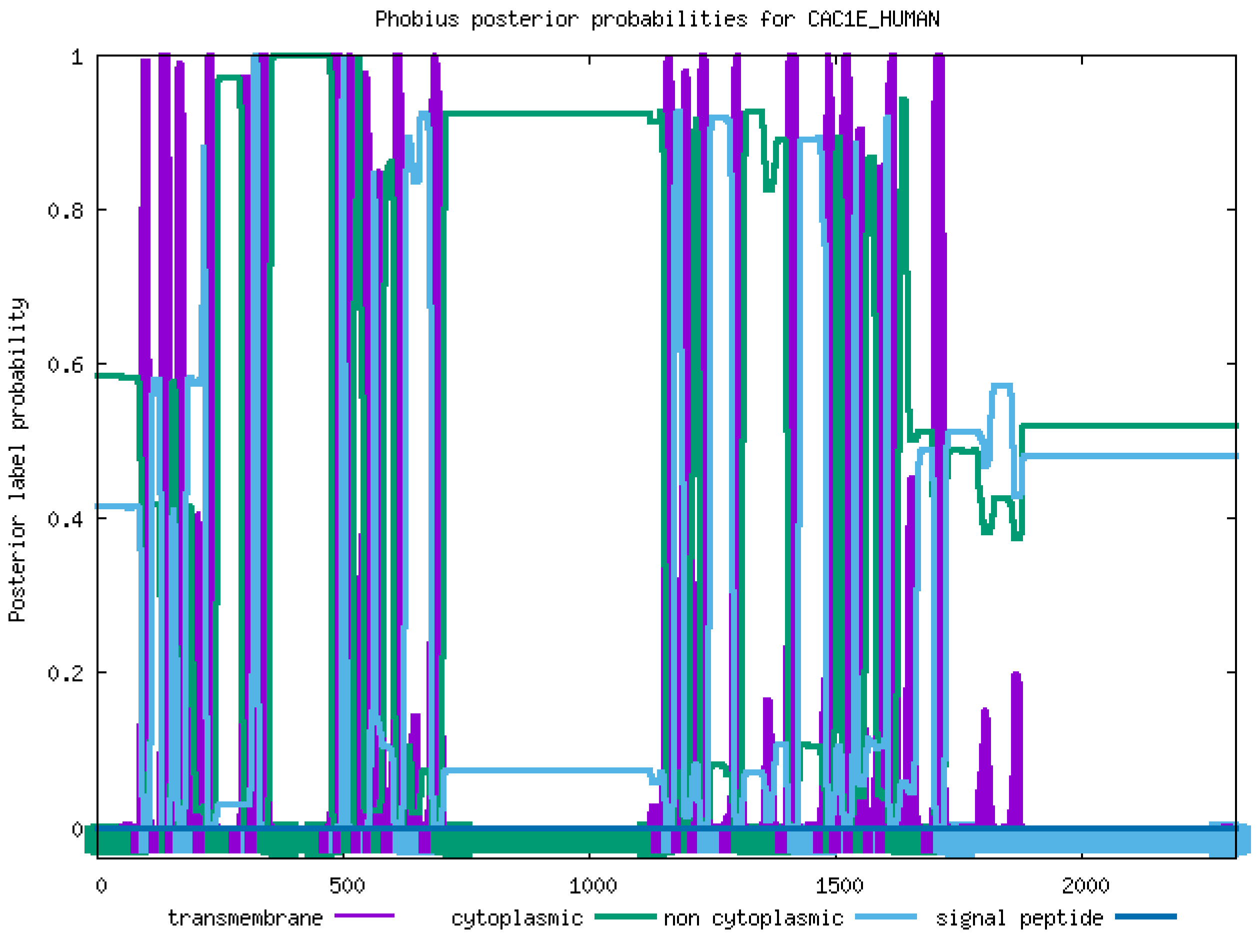

The probability data used in the plot is found here, and the gnuplot script is here.

Prediction of CAC1G_HUMAN

```
ID   CAC1G_HUMAN
FT   TOPO_DOM      1     81       NON CYTOPLASMIC.
FT   TRANSMEM     82    101
FT   TOPO_DOM    102    121       CYTOPLASMIC.
FT   TRANSMEM    122    142
FT   TOPO_DOM    143    213       NON CYTOPLASMIC.
FT   TRANSMEM    214    237
FT   TOPO_DOM    238    341       CYTOPLASMIC.
FT   TRANSMEM    342    363
FT   TOPO_DOM    364    368       NON CYTOPLASMIC.
FT   TRANSMEM    369    394
FT   TOPO_DOM    395    743       CYTOPLASMIC.
FT   TRANSMEM    744    762
FT   TOPO_DOM    763    773       NON CYTOPLASMIC.
FT   TRANSMEM    774    795
FT   TOPO_DOM    796    806       CYTOPLASMIC.
FT   TRANSMEM    807    823
FT   TOPO_DOM    824    864       NON CYTOPLASMIC.
FT   TRANSMEM    865    884
FT   TOPO_DOM    885    904       CYTOPLASMIC.
FT   TRANSMEM    905    921
FT   TOPO_DOM    922    940       NON CYTOPLASMIC.
FT   TRANSMEM    941    964
FT   TOPO_DOM    965   1276       CYTOPLASMIC.
FT   TRANSMEM   1277   1295
FT   TOPO_DOM   1296   1314       NON CYTOPLASMIC.
FT   TRANSMEM   1315   1336
FT   TOPO_DOM   1337   1347       CYTOPLASMIC.
FT   TRANSMEM   1348   1367
FT   TOPO_DOM   1368   1411       NON CYTOPLASMIC.
```

```
FT   TRANSMEM   1412   1434
FT   TOPO_DOM   1435   1513       CYTOPLASMIC.
FT   TRANSMEM   1514   1537
FT   TOPO_DOM   1538   1611       NON CYTOPLASMIC.
FT   TRANSMEM   1612   1633
FT   TOPO_DOM   1634   1644       CYTOPLASMIC.
FT   TRANSMEM   1645   1668
FT   TOPO_DOM   1669   1679       NON CYTOPLASMIC.
FT   TRANSMEM   1680   1701
FT   TOPO_DOM   1702   1721       CYTOPLASMIC.
FT   TRANSMEM   1722   1740
FT   TOPO_DOM   1741   1745       NON CYTOPLASMIC.
FT   TRANSMEM   1746   1765
FT   TOPO_DOM   1766   1828       CYTOPLASMIC.
FT   TRANSMEM   1829   1851
FT   TOPO_DOM   1852   2377       NON CYTOPLASMIC.
//
```

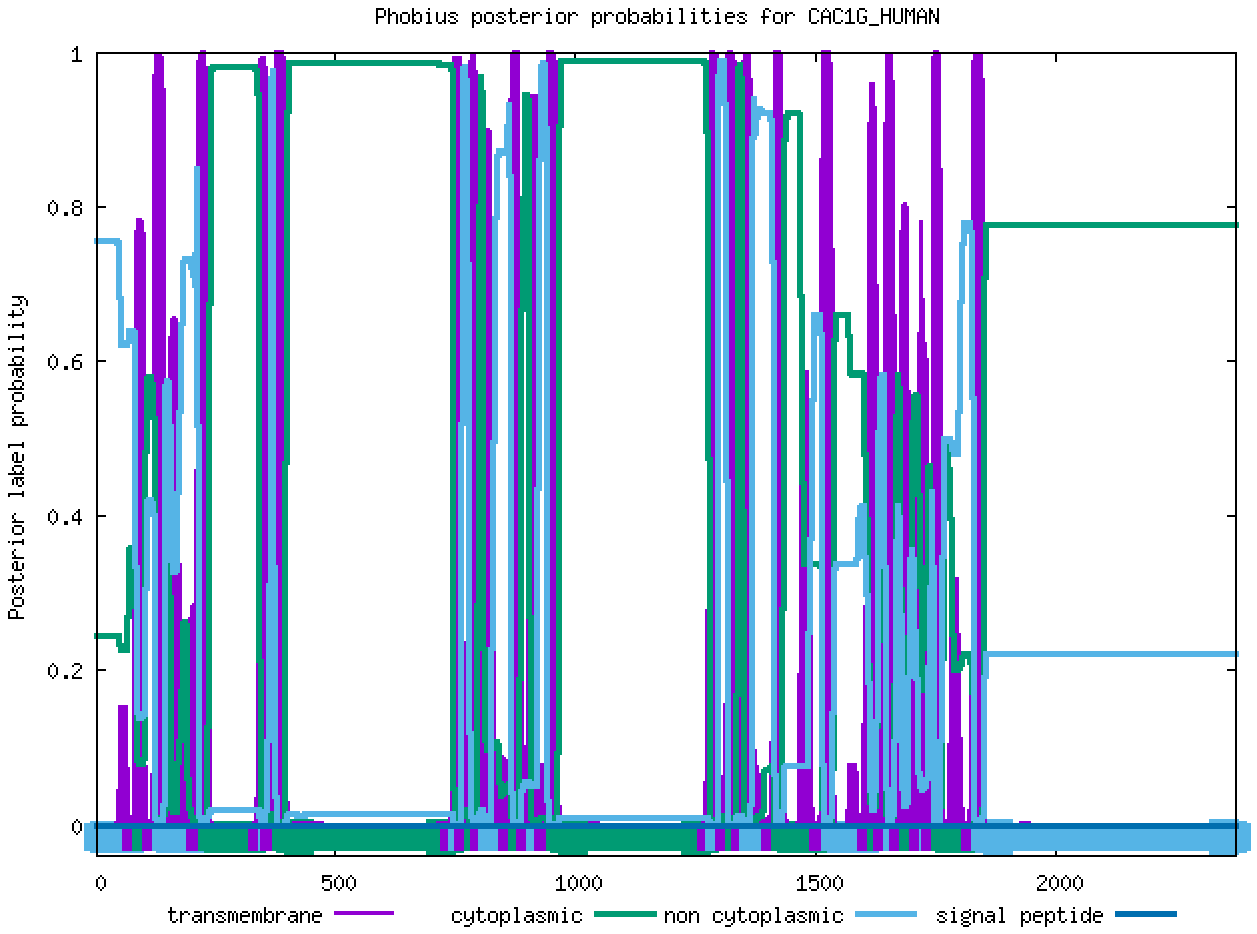

The probability data used in the plot is found here, and the gnuplot script is here.

Prediction of CAC1I_HUMAN

```
ID   CAC1I_HUMAN
FT   TOPO_DOM      1     78       CYTOPLASMIC.
FT   TRANSMEM     79    101
FT   TOPO_DOM    102    120       NON CYTOPLASMIC.
FT   TRANSMEM    121    141
FT   TOPO_DOM    142    152       CYTOPLASMIC.
FT   TRANSMEM    153    172
FT   TOPO_DOM    173    207       NON CYTOPLASMIC.
FT   TRANSMEM    208    232
FT   TOPO_DOM    233    344       CYTOPLASMIC.
FT   TRANSMEM    345    366
FT   TOPO_DOM    367    371       NON CYTOPLASMIC.
```

```
FT   TRANSMEM    372    397
FT   TOPO_DOM    398    640       CYTOPLASMIC.
FT   TRANSMEM    641    659
FT   TOPO_DOM    660    670       NON CYTOPLASMIC.
FT   TRANSMEM    671    697
FT   TOPO_DOM    698    703       CYTOPLASMIC.
FT   TRANSMEM    704    723
FT   TOPO_DOM    724    761       NON CYTOPLASMIC.
FT   TRANSMEM    762    784
FT   TOPO_DOM    785    803       CYTOPLASMIC.
FT   TRANSMEM    804    820
FT   TOPO_DOM    821    839       NON CYTOPLASMIC.
FT   TRANSMEM    840    862
FT   TOPO_DOM    863   1170       CYTOPLASMIC.
FT   TRANSMEM   1171   1189
FT   TOPO_DOM   1190   1208       NON CYTOPLASMIC.
FT   TRANSMEM   1209   1230
FT   TOPO_DOM   1231   1241       CYTOPLASMIC.
FT   TRANSMEM   1242   1268
FT   TOPO_DOM   1269   1305       NON CYTOPLASMIC.
FT   TRANSMEM   1306   1328
FT   TOPO_DOM   1329   1368       CYTOPLASMIC.
FT   TRANSMEM   1369   1387
FT   TOPO_DOM   1388   1406       NON CYTOPLASMIC.
FT   TRANSMEM   1407   1431
FT   TOPO_DOM   1432   1486       CYTOPLASMIC.
FT   TRANSMEM   1487   1504
FT   TOPO_DOM   1505   1523       NON CYTOPLASMIC.
FT   TRANSMEM   1524   1543
FT   TOPO_DOM   1544   1594       CYTOPLASMIC.
FT   TRANSMEM   1595   1616
FT   TOPO_DOM   1617   1621       NON CYTOPLASMIC.
FT   TRANSMEM   1622   1641
FT   TOPO_DOM   1642   1707       CYTOPLASMIC.
FT   TRANSMEM   1708   1730
FT   TOPO_DOM   1731   2223       NON CYTOPLASMIC.
//
```

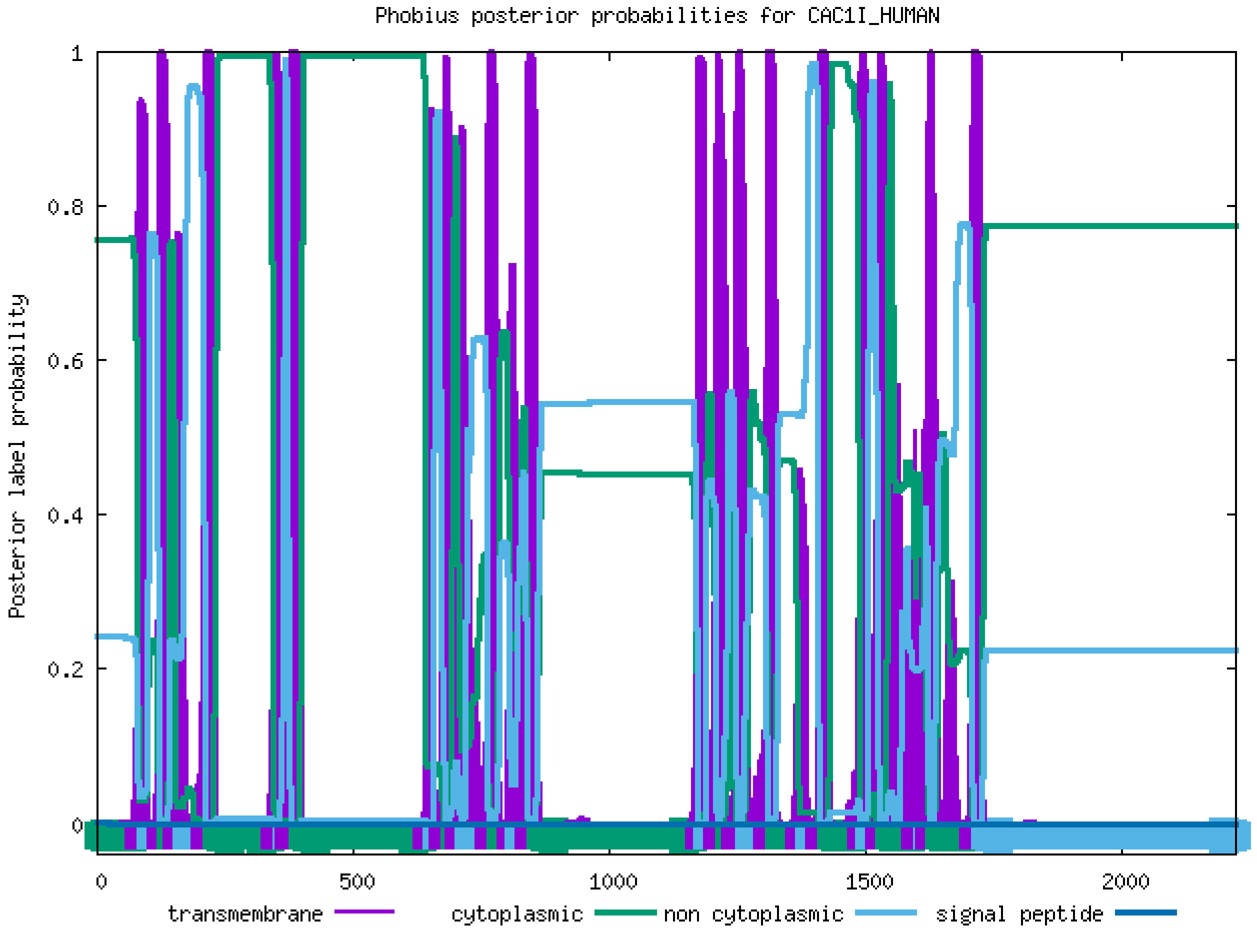

The probability data used in the plot is found here, and the gnuplot script is here.

Prediction of STIM1_HUMAN

```
ID   STIM1_HUMAN
FT   SIGNAL        1     22
FT   REGION        1      6       N-REGION.
FT   REGION        7     17       H-REGION.
FT   REGION       18     22       C-REGION.
FT   TOPO_DOM     23    213       NON CYTOPLASMIC.
FT   TRANSMEM    214    232
FT   TOPO_DOM    233    685       CYTOPLASMIC.
//
```

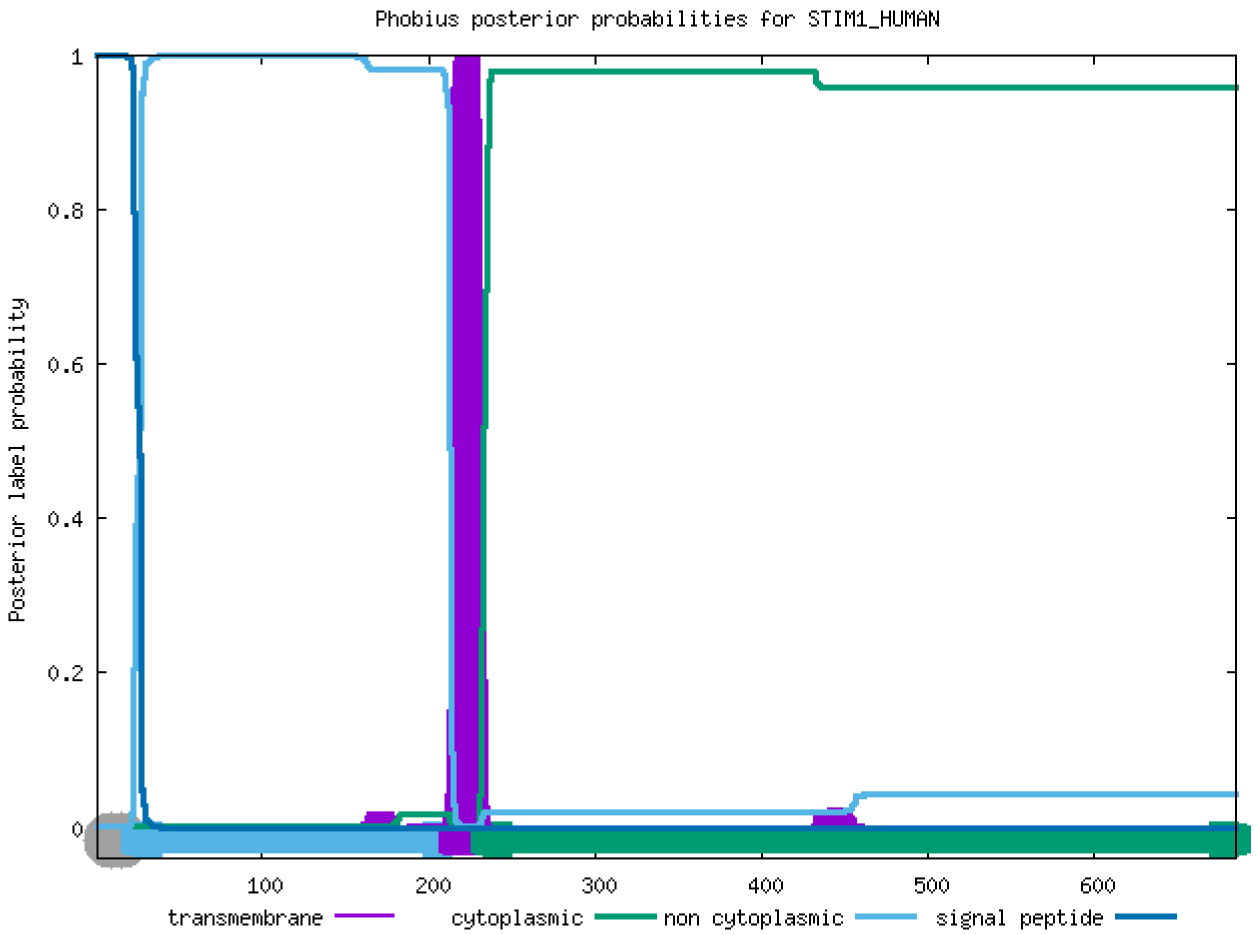

The probability data used in the plot is found here, and the gnuplot script is here.

Prediction of ITPR3_HUMAN

```
ID   ITPR3_HUMAN
FT   TOPO_DOM      1   1907       NON CYTOPLASMIC.
FT   TRANSMEM   1908   1929
FT   TOPO_DOM   1930   2200       CYTOPLASMIC.
FT   TRANSMEM   2201   2226
FT   TOPO_DOM   2227   2237       NON CYTOPLASMIC.
FT   TRANSMEM   2238   2256
FT   TOPO_DOM   2257   2267       CYTOPLASMIC.
FT   TRANSMEM   2268   2288
FT   TOPO_DOM   2289   2323       NON CYTOPLASMIC.
FT   TRANSMEM   2324   2349
FT   TOPO_DOM   2350   2368       CYTOPLASMIC.
FT   TRANSMEM   2369   2391
FT   TOPO_DOM   2392   2497       NON CYTOPLASMIC.
```

```
FT   TRANSMEM   2498   2521
FT   TOPO_DOM   2522   2671       CYTOPLASMIC.
//
```

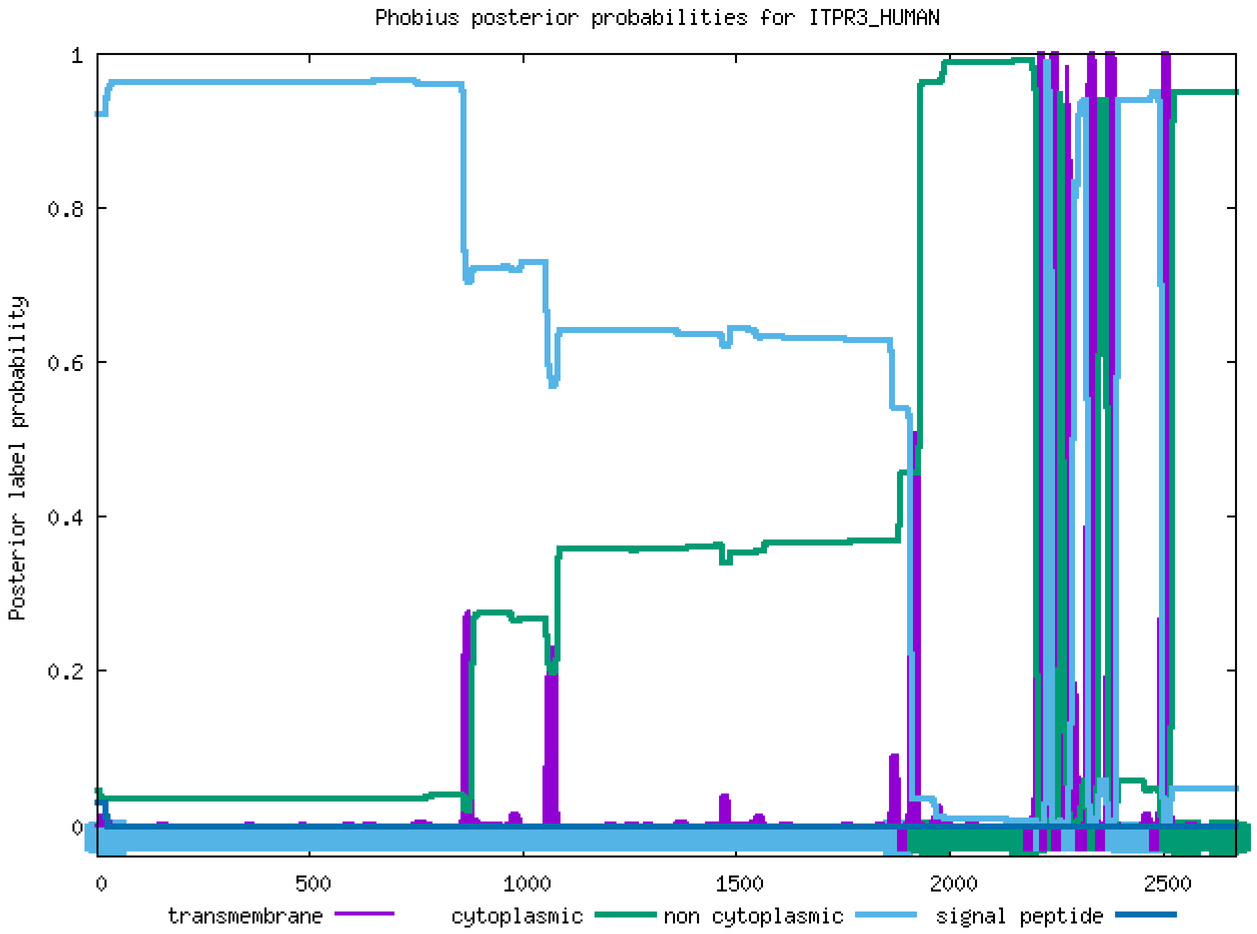

The probability data used in the plot is found here, and the gnuplot script is here.

Prediction of MCU_HUMAN

```
ID   MCU_HUMAN
FT   TOPO_DOM      1    233       CYTOPLASMIC.
FT   TRANSMEM    234    254
FT   TOPO_DOM    255    265       NON CYTOPLASMIC.
FT   TRANSMEM    266    284
FT   TOPO_DOM    285    351       CYTOPLASMIC.
//
```

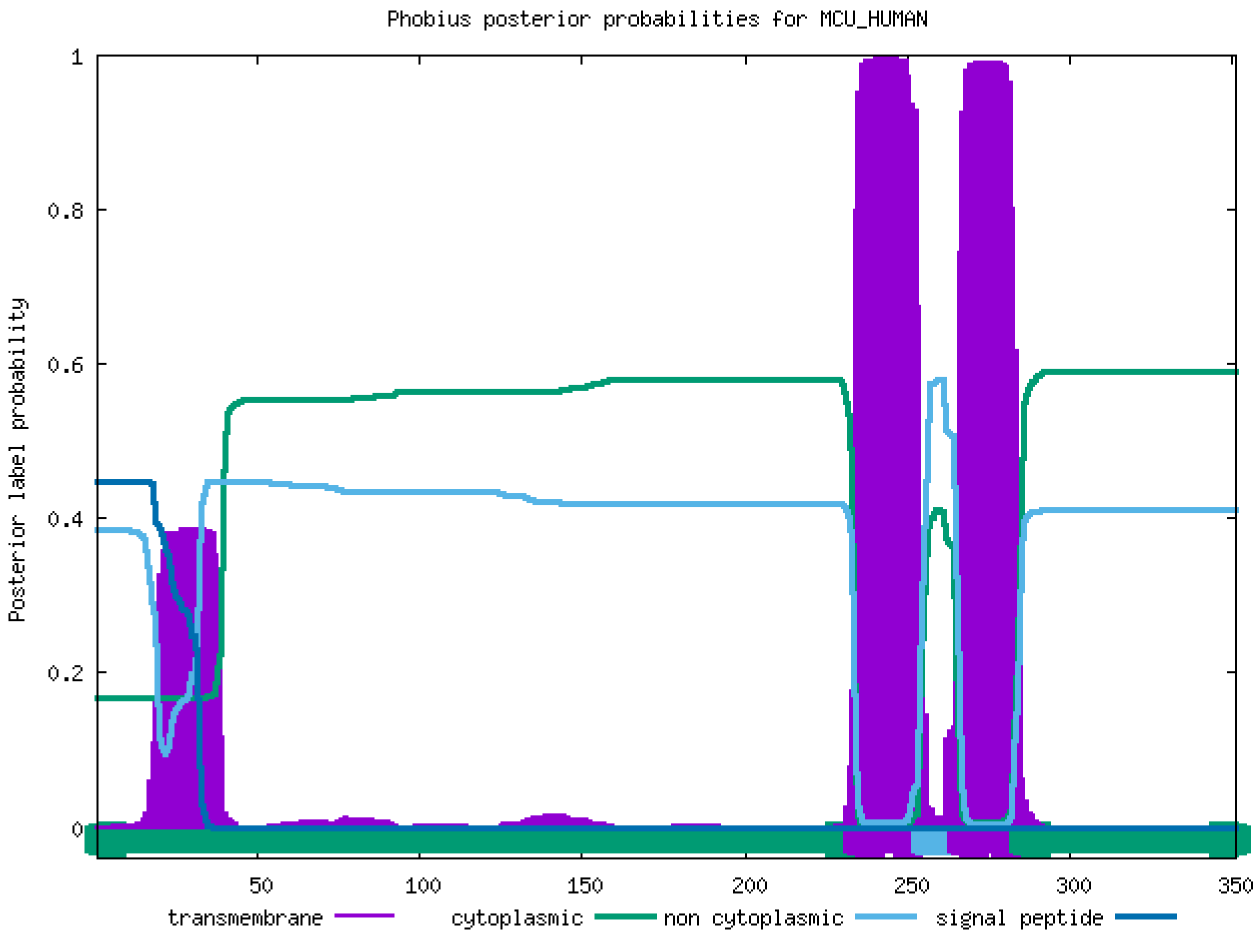

The probability data used in the plot is found here, and the gnuplot script is here.

Prediction of EMRE_HUMAN

```
ID   EMRE_HUMAN
FT   TOPO_DOM      1     65       CYTOPLASMIC.
FT   TRANSMEM     66     84
FT   TOPO_DOM     85    107       NON CYTOPLASMIC.
//
```

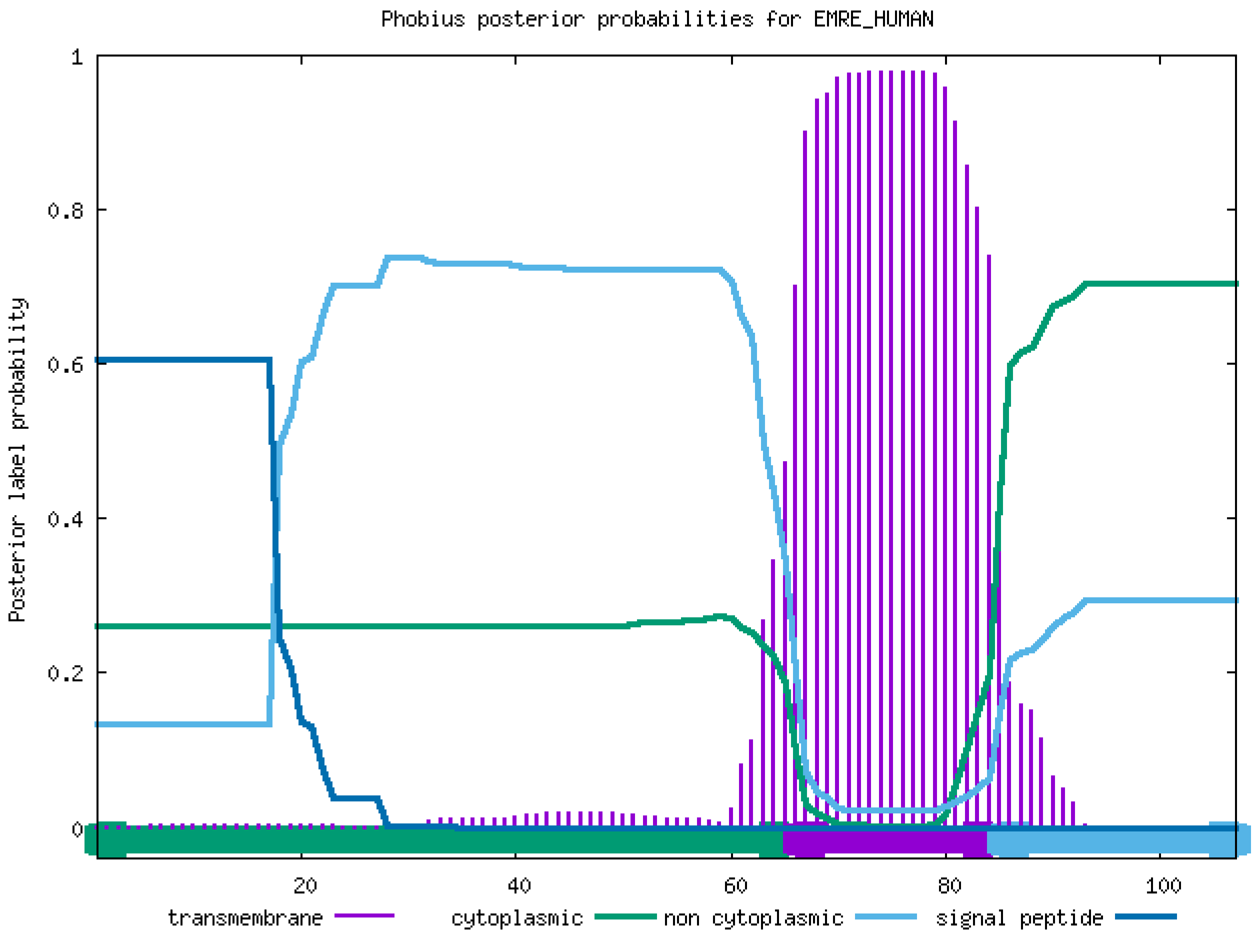

The probability data used in the plot is found here, and the gnuplot script is here.

Prediction of RYR2_HUMAN

```
ID    RYR2_HUMAN
FT    TOPO_DOM       1    4232       NON CYTOPLASMIC.
FT    TRANSMEM    4233    4259
FT    TOPO_DOM    4260    4279       CYTOPLASMIC.
FT    TRANSMEM    4280    4299
FT    TOPO_DOM    4300    4304       NON CYTOPLASMIC.
FT    TRANSMEM    4305    4322
FT    TOPO_DOM    4323    4499       CYTOPLASMIC.
FT    TRANSMEM    4500    4520
FT    TOPO_DOM    4521    4573       NON CYTOPLASMIC.
FT    TRANSMEM    4574    4593
FT    TOPO_DOM    4594    4709       CYTOPLASMIC.
FT    TRANSMEM    4710    4731
FT    TOPO_DOM    4732    4736       NON CYTOPLASMIC.
FT    TRANSMEM    4737    4756
FT    TOPO_DOM    4757    4767       CYTOPLASMIC.
FT    TRANSMEM    4768    4789
FT    TOPO_DOM    4790    4843       NON CYTOPLASMIC.
FT    TRANSMEM    4844    4867
FT    TOPO_DOM    4868    4967       CYTOPLASMIC.
//
```

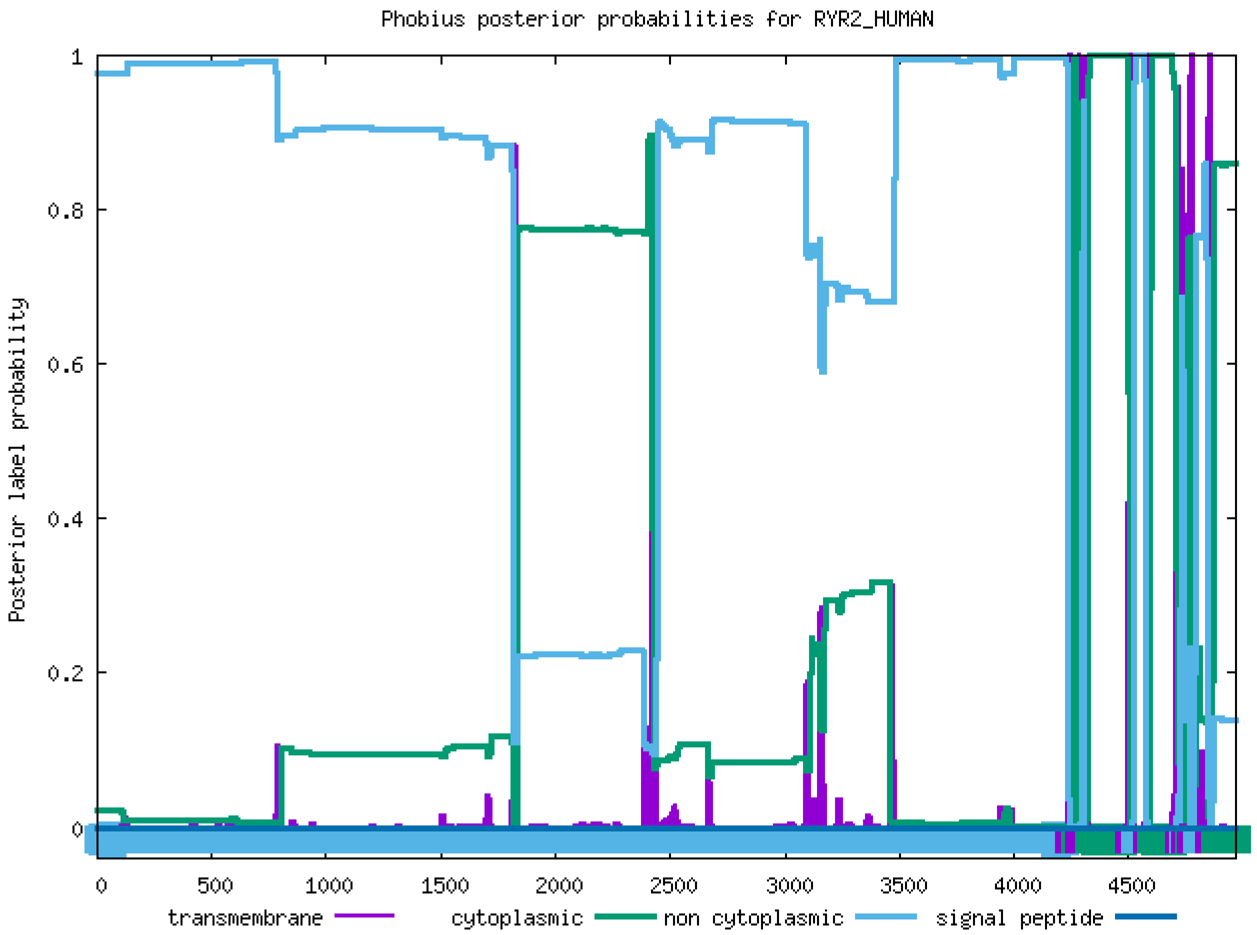

The probability data used in the plot is found here, and the gnuplot script is here.

Prediction of TRPA1_HUMAN

```
ID    TRPA1_HUMAN
FT    TOPO_DOM      1    719       CYTOPLASMIC.
FT    TRANSMEM    720    740
FT    TOPO_DOM    741    767       NON CYTOPLASMIC.
FT    TRANSMEM    768    786
FT    TOPO_DOM    787    805       CYTOPLASMIC.
FT    TRANSMEM    806    824
FT    TOPO_DOM    825    829       NON CYTOPLASMIC.
FT    TRANSMEM    830    850
FT    TOPO_DOM    851    869       CYTOPLASMIC.
FT    TRANSMEM    870    892
FT    TOPO_DOM    893    897       NON CYTOPLASMIC.
FT    TRANSMEM    898    918
FT    TOPO_DOM    919    937       CYTOPLASMIC.
FT    TRANSMEM    938    961
FT    TOPO_DOM    962   1119       NON CYTOPLASMIC.
//
```

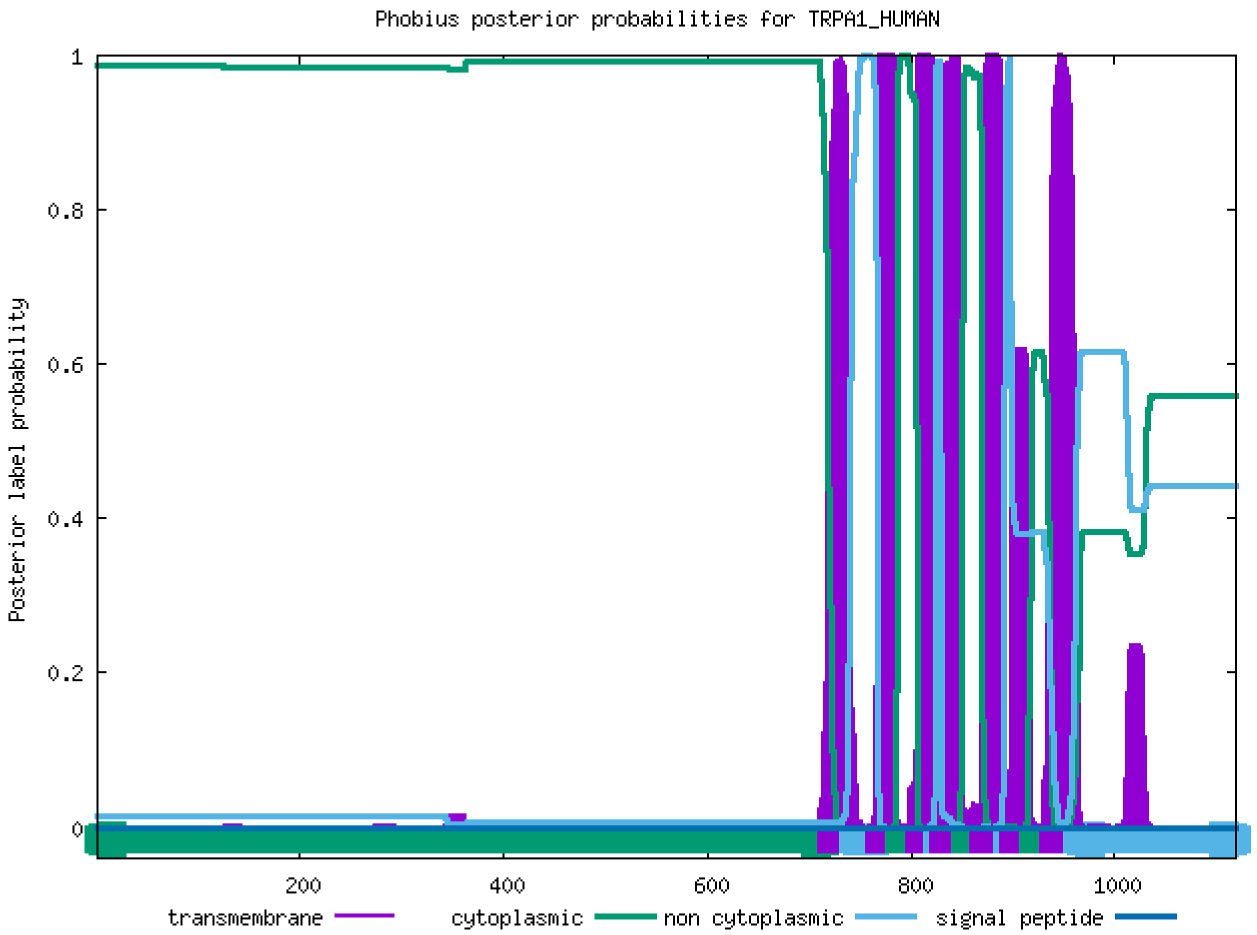

The probability data used in the plot is found here, and the gnuplot script is here.

Prediction of TRPC3_HUMAN

```
ID   TRPC3_HUMAN
FT   TOPO_DOM      1    418       CYTOPLASMIC.
FT   TRANSMEM    419    443
FT   TOPO_DOM    444    454       NON CYTOPLASMIC.
FT   TRANSMEM    455    475
FT   TOPO_DOM    476    506       CYTOPLASMIC.
FT   TRANSMEM    507    524
FT   TOPO_DOM    525    543       NON CYTOPLASMIC.
FT   TRANSMEM    544    563
FT   TOPO_DOM    564    608       CYTOPLASMIC.
FT   TRANSMEM    609    630
FT   TOPO_DOM    631    649       NON CYTOPLASMIC.
FT   TRANSMEM    650    672
FT   TOPO_DOM    673    692       CYTOPLASMIC.
FT   TRANSMEM    693    709
FT   TOPO_DOM    710    720       NON CYTOPLASMIC.
FT   TRANSMEM    721    740
FT   TOPO_DOM    741    921       CYTOPLASMIC.
//
```

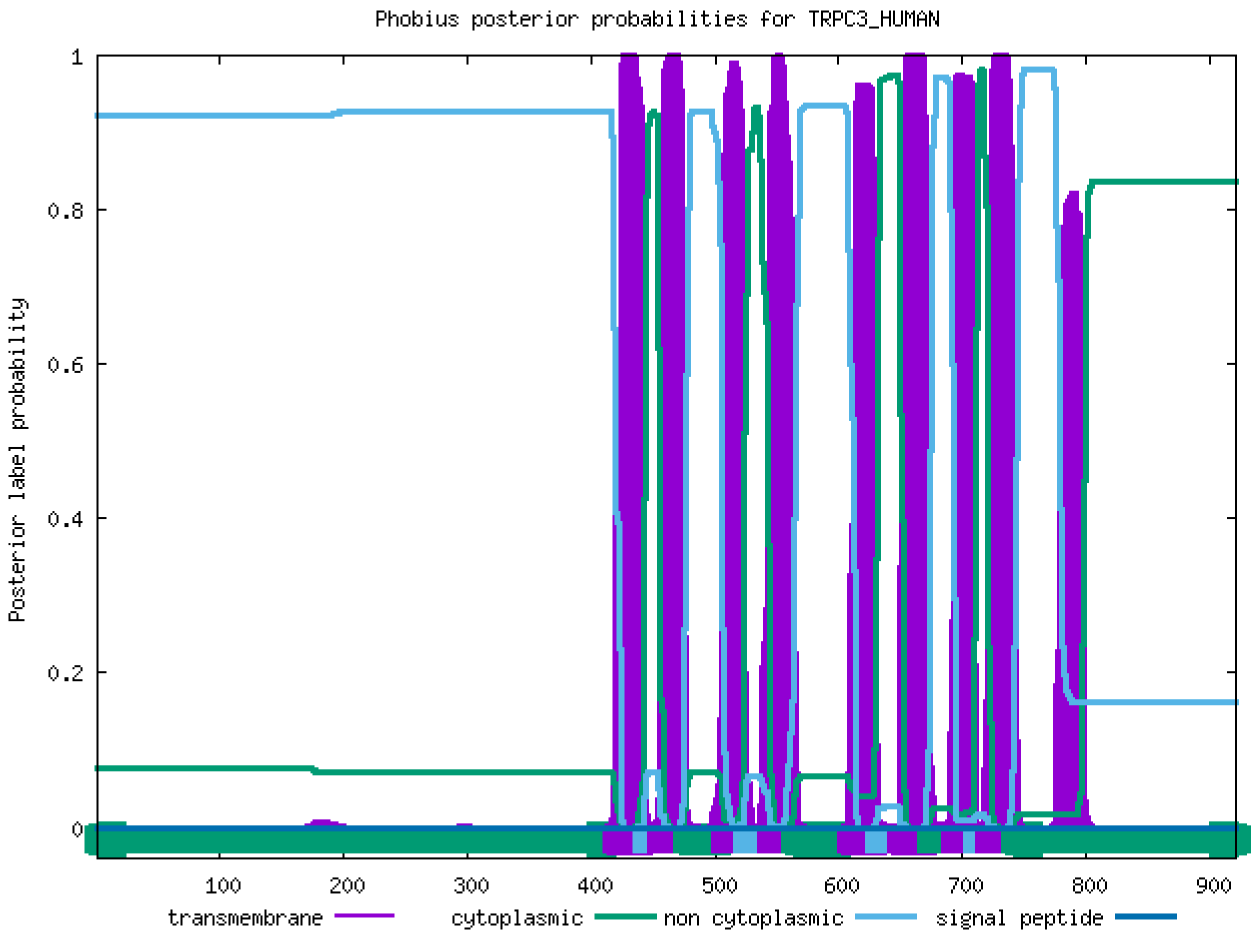

The probability data used in the plot is found here, and the gnuplot script is here.

Prediction of TRPC5_HUMAN

```
ID    TRPC5_HUMAN
FT    TOPO_DOM      1     326       NON CYTOPLASMIC.
FT    TRANSMEM    327     351
FT    TOPO_DOM    352     362       CYTOPLASMIC.
FT    TRANSMEM    363     382
FT    TOPO_DOM    383     401       NON CYTOPLASMIC.
FT    TRANSMEM    402     419
FT    TOPO_DOM    420     439       CYTOPLASMIC.
FT    TRANSMEM    440     460
FT    TOPO_DOM    461     479       NON CYTOPLASMIC.
FT    TRANSMEM    480     499
FT    TOPO_DOM    500     519       CYTOPLASMIC.
FT    TRANSMEM    520     542
FT    TOPO_DOM    543     570       NON CYTOPLASMIC.
FT    TRANSMEM    571     587
FT    TOPO_DOM    588     598       CYTOPLASMIC.
FT    TRANSMEM    599     621
FT    TOPO_DOM    622     973       NON CYTOPLASMIC.
//
```

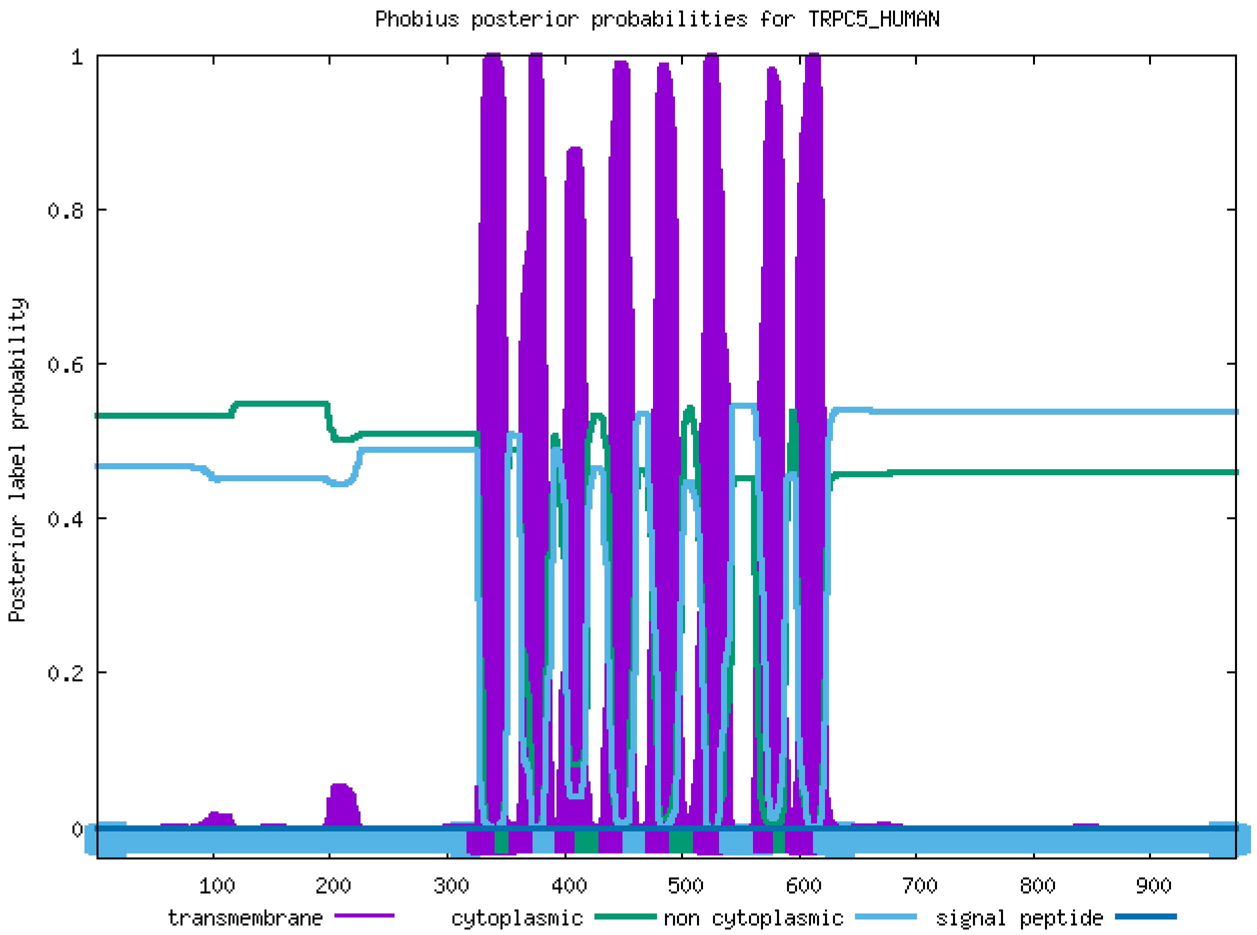

The probability data used in the plot is found here, and the gnuplot script is here.

Prediction of TRPC6_HUMAN

```
ID   TRPC6_HUMAN
FT   TOPO_DOM      1    406      CYTOPLASMIC.
FT   TRANSMEM    407    427
FT   TOPO_DOM    428    438      NON CYTOPLASMIC.
FT   TRANSMEM    439    459
FT   TOPO_DOM    460    489      CYTOPLASMIC.
FT   TRANSMEM    490    508
FT   TOPO_DOM    509    527      NON CYTOPLASMIC.
FT   TRANSMEM    528    547
FT   TOPO_DOM    548    592      CYTOPLASMIC.
FT   TRANSMEM    593    614
FT   TOPO_DOM    615    633      NON CYTOPLASMIC.
FT   TRANSMEM    634    656
FT   TOPO_DOM    657    676      CYTOPLASMIC.
FT   TRANSMEM    677    697
FT   TOPO_DOM    698    702      NON CYTOPLASMIC.
FT   TRANSMEM    703    724
FT   TOPO_DOM    725    931      CYTOPLASMIC.
//
```

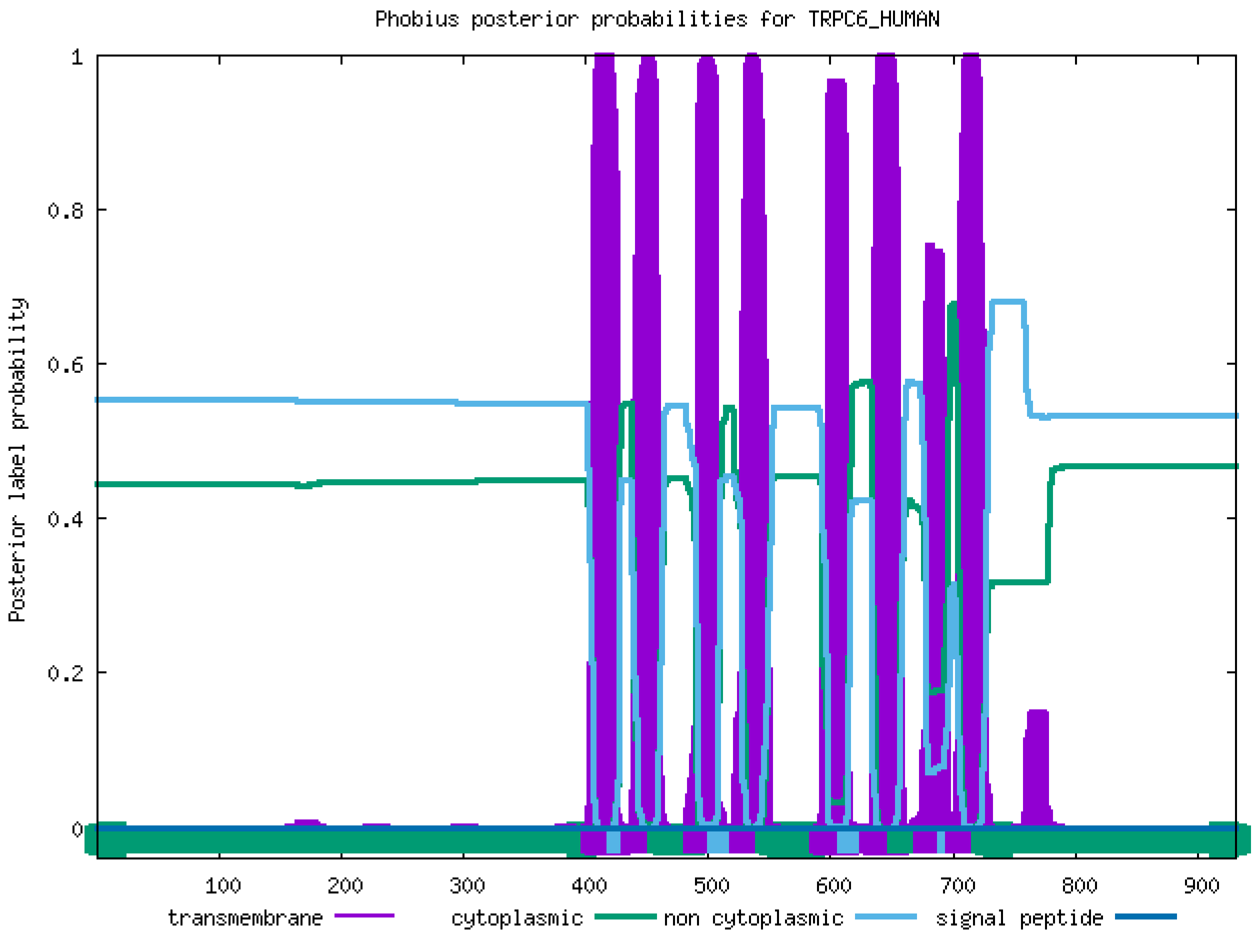

The probability data used in the plot is found here, and the gnuplot script is here.

Prediction of TRPM2_HUMAN

```
ID   TRPM2_HUMAN
FT   TOPO_DOM      1    751       NON CYTOPLASMIC.
FT   TRANSMEM    752    773
FT   TOPO_DOM    774    793       CYTOPLASMIC.
FT   TRANSMEM    794    818
FT   TOPO_DOM    819    823       NON CYTOPLASMIC.
FT   TRANSMEM    824    841
FT   TOPO_DOM    842    866       CYTOPLASMIC.
FT   TRANSMEM    867    889
FT   TOPO_DOM    890    894       NON CYTOPLASMIC.
FT   TRANSMEM    895    914
FT   TOPO_DOM    915    933       CYTOPLASMIC.
FT   TRANSMEM    934    957
FT   TOPO_DOM    958   1022       NON CYTOPLASMIC.
FT   TRANSMEM   1023   1045
FT   TOPO_DOM   1046   1503       CYTOPLASMIC.
//
```

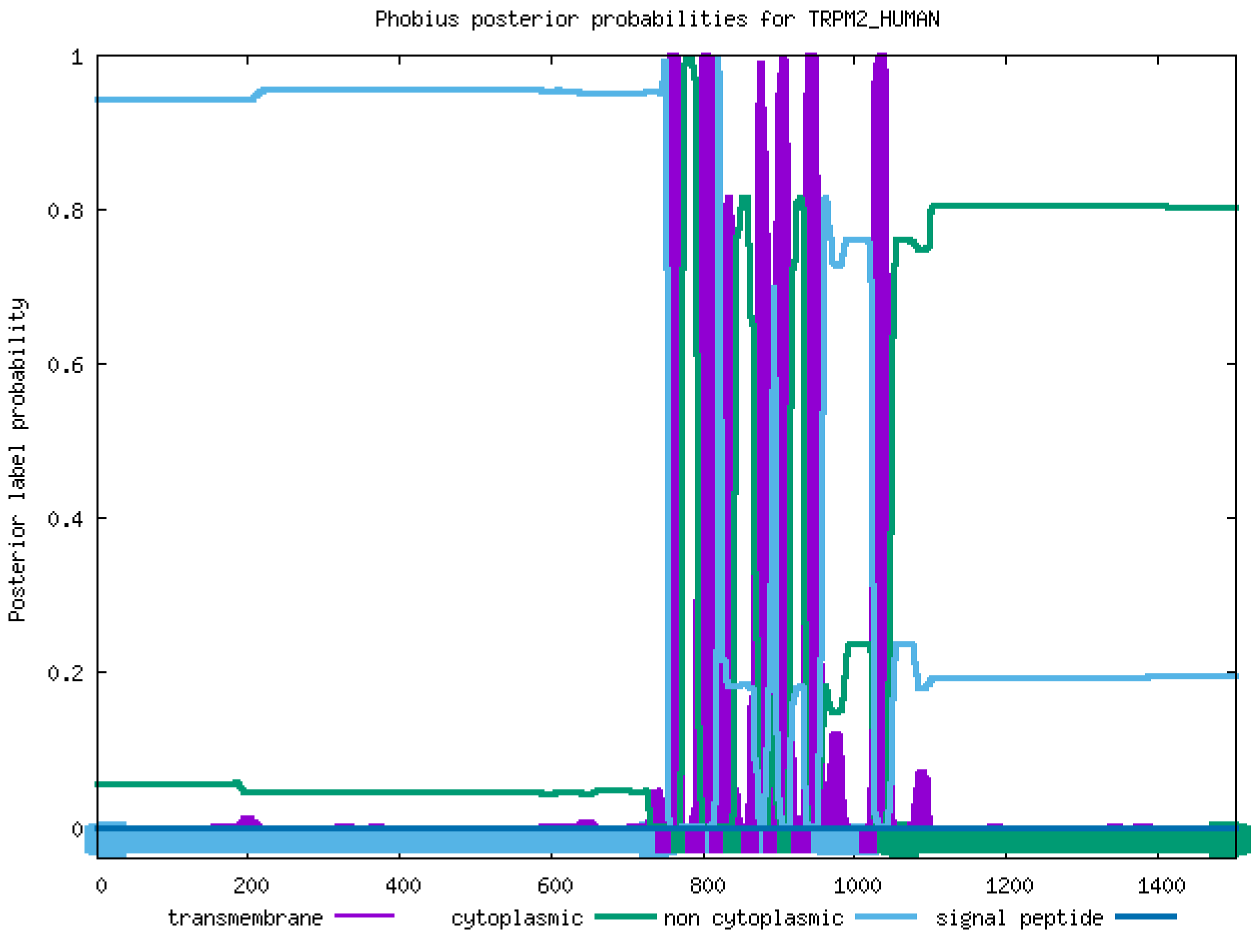

The probability data used in the plot is found here, and the gnuplot script is here.

Prediction of TRPM4_HUMAN

```
ID   TRPM4_HUMAN
FT   TOPO_DOM      1    689       NON CYTOPLASMIC.
FT   TRANSMEM    690    709
FT   TOPO_DOM    710    774       CYTOPLASMIC.
FT   TRANSMEM    775    797
FT   TOPO_DOM    798    889       NON CYTOPLASMIC.
FT   TRANSMEM    890    910
FT   TOPO_DOM    911    929       CYTOPLASMIC.
FT   TRANSMEM    930    947
FT   TOPO_DOM    948   1017       NON CYTOPLASMIC.
FT   TRANSMEM   1018   1048
FT   TOPO_DOM   1049   1068       CYTOPLASMIC.
FT   TRANSMEM   1069   1089
FT   TOPO_DOM   1090   1214       NON CYTOPLASMIC.
//
```

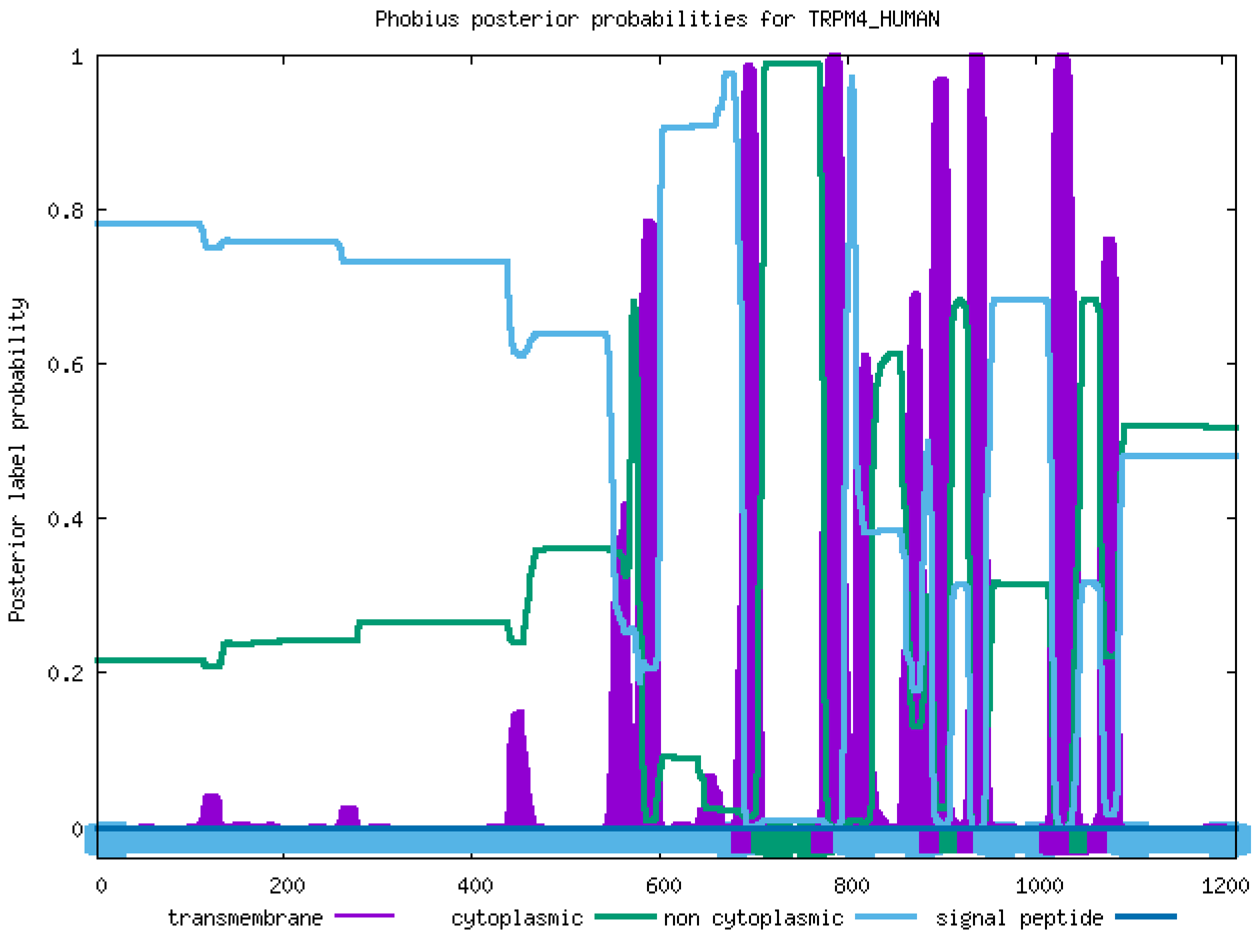

The probability data used in the plot is found here, and the gnuplot script is here.

Prediction of TRPM8_HUMAN

```
ID   TRPM8_HUMAN
FT   TOPO_DOM      1    692       NON CYTOPLASMIC.
FT   TRANSMEM    693    712
FT   TOPO_DOM    713    723       CYTOPLASMIC.
FT   TRANSMEM    724    757
FT   TOPO_DOM    758    768       NON CYTOPLASMIC.
FT   TRANSMEM    769    787
FT   TOPO_DOM    788    798       CYTOPLASMIC.
FT   TRANSMEM    799    818
FT   TOPO_DOM    819    823       NON CYTOPLASMIC.
FT   TRANSMEM    824    844
FT   TOPO_DOM    845    863       CYTOPLASMIC.
FT   TRANSMEM    864    884
FT   TOPO_DOM    885    954       NON CYTOPLASMIC.
FT   TRANSMEM    955    984
FT   TOPO_DOM    985   1004       CYTOPLASMIC.
FT   TRANSMEM   1005   1025
FT   TOPO_DOM   1026   1104       NON CYTOPLASMIC.
//
```

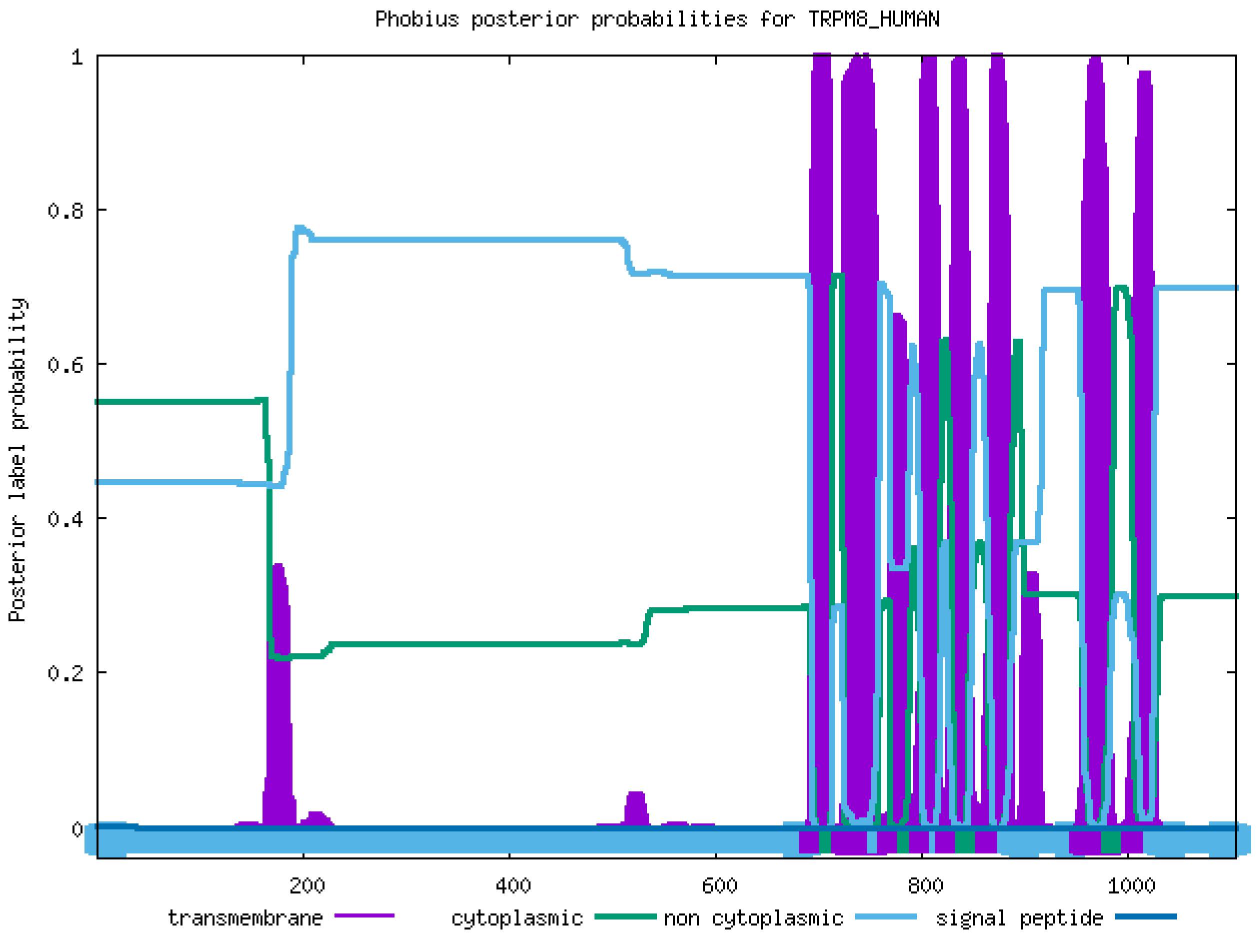

The probability data used in the plot is found here, and the gnuplot script is here.

Prediction of MCLN3_HUMAN

```
ID    MCLN3_HUMAN
FT    TOPO_DOM      1     62       NON CYTOPLASMIC.
FT    TRANSMEM     63     82
FT    TOPO_DOM     83    286       CYTOPLASMIC.
FT    TRANSMEM    287    308
FT    TOPO_DOM    309    339       NON CYTOPLASMIC.
FT    TRANSMEM    340    358
FT    TOPO_DOM    359    369       CYTOPLASMIC.
FT    TRANSMEM    370    392
FT    TOPO_DOM    393    411       NON CYTOPLASMIC.
FT    TRANSMEM    412    434
FT    TOPO_DOM    435    479       CYTOPLASMIC.
FT    TRANSMEM    480    501
FT    TOPO_DOM    502    553       NON CYTOPLASMIC.
//
```

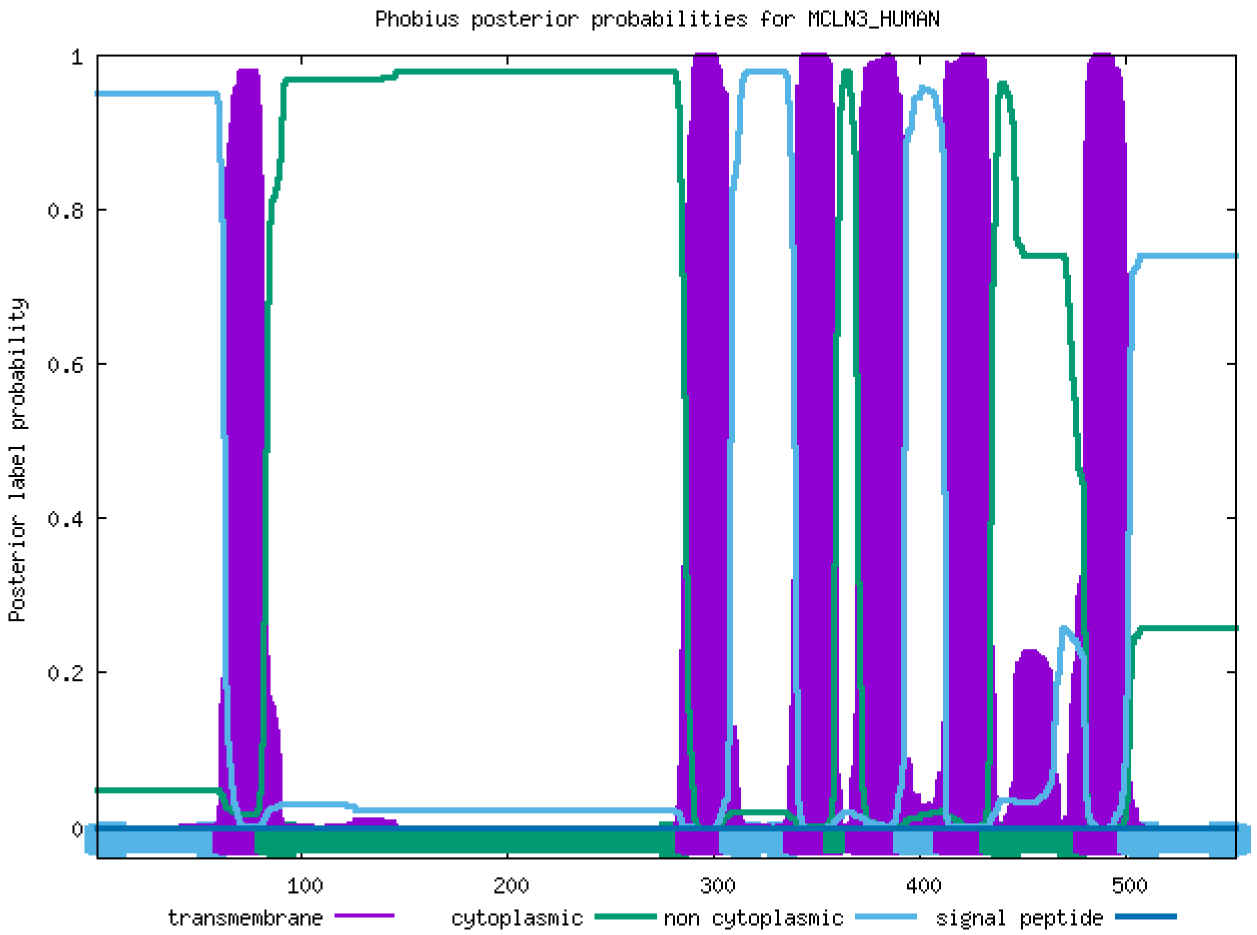

The probability data used in the plot is found here, and the gnuplot script is here.

Prediction of TRPV1_HUMAN

```
ID    TRPV1_HUMAN
FT    TOPO_DOM      1     433       CYTOPLASMIC.
FT    TRANSMEM    434     455
FT    TOPO_DOM    456     474       NON CYTOPLASMIC.
FT    TRANSMEM    475     493
FT    TOPO_DOM    494     513       CYTOPLASMIC.
FT    TRANSMEM    514     531
FT    TOPO_DOM    532     536       NON CYTOPLASMIC.
FT    TRANSMEM    537     556
FT    TOPO_DOM    557     576       CYTOPLASMIC.
FT    TRANSMEM    577     599
FT    TOPO_DOM    600     657       NON CYTOPLASMIC.
FT    TRANSMEM    658     682
FT    TOPO_DOM    683     839       CYTOPLASMIC.
//
```

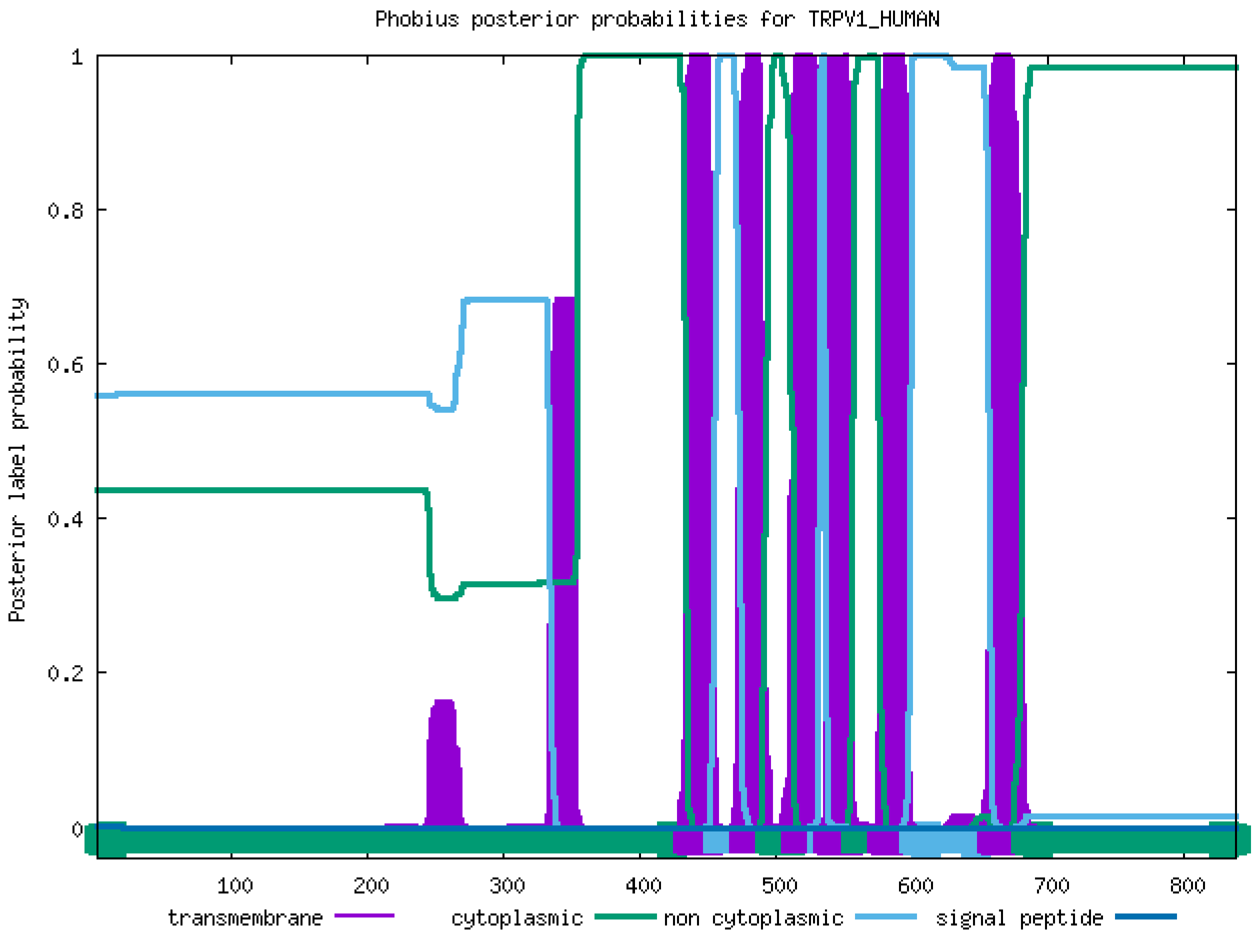

The probability data used in the plot is found here, and the gnuplot script is here.

Prediction of TRPV3_HUMAN

```
ID   TRPV3_HUMAN
FT   TOPO_DOM      1    439       CYTOPLASMIC.
FT   TRANSMEM    440    461
FT   TOPO_DOM    462    480       NON CYTOPLASMIC.
FT   TRANSMEM    481    508
FT   TOPO_DOM    509    519       CYTOPLASMIC.
FT   TRANSMEM    520    541
FT   TOPO_DOM    542    546       NON CYTOPLASMIC.
FT   TRANSMEM    547    566
FT   TOPO_DOM    567    586       CYTOPLASMIC.
FT   TRANSMEM    587    609
FT   TOPO_DOM    610    651       NON CYTOPLASMIC.
FT   TRANSMEM    652    676
FT   TOPO_DOM    677    790       CYTOPLASMIC.
//
```

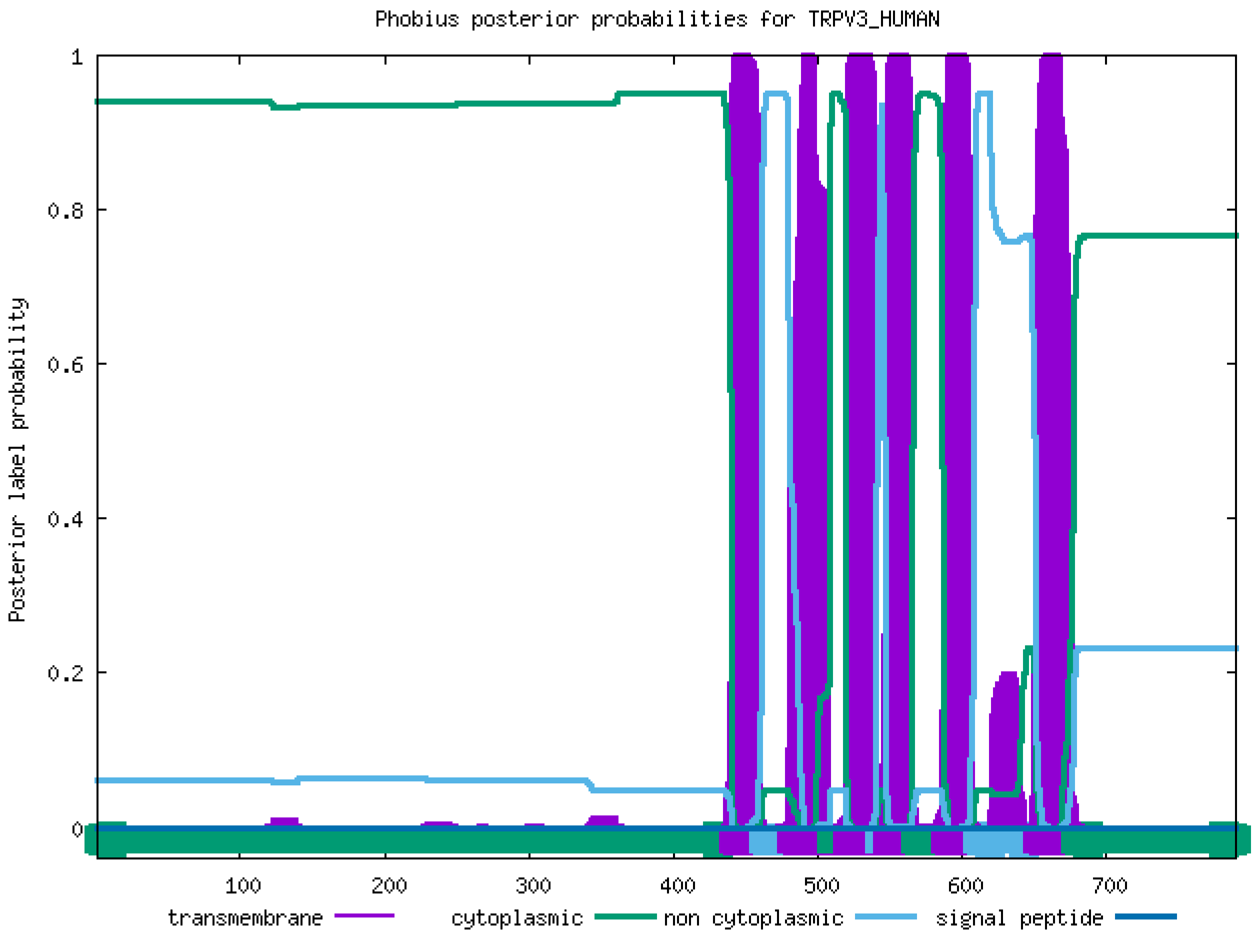

The probability data used in the plot is found here, and the gnuplot script is here.

Prediction of TRPV4_HUMAN

```
ID    TRPV4_HUMAN
FT    TOPO_DOM      1     468       CYTOPLASMIC.
FT    TRANSMEM    469     491
FT    TOPO_DOM    492     510       NON CYTOPLASMIC.
FT    TRANSMEM    511     529
FT    TOPO_DOM    530     549       CYTOPLASMIC.
FT    TRANSMEM    550     568
FT    TOPO_DOM    569     573       NON CYTOPLASMIC.
FT    TRANSMEM    574     593
FT    TOPO_DOM    594     613       CYTOPLASMIC.
FT    TRANSMEM    614     636
FT    TOPO_DOM    637     692       NON CYTOPLASMIC.
FT    TRANSMEM    693     717
FT    TOPO_DOM    718     871       CYTOPLASMIC.
//
```

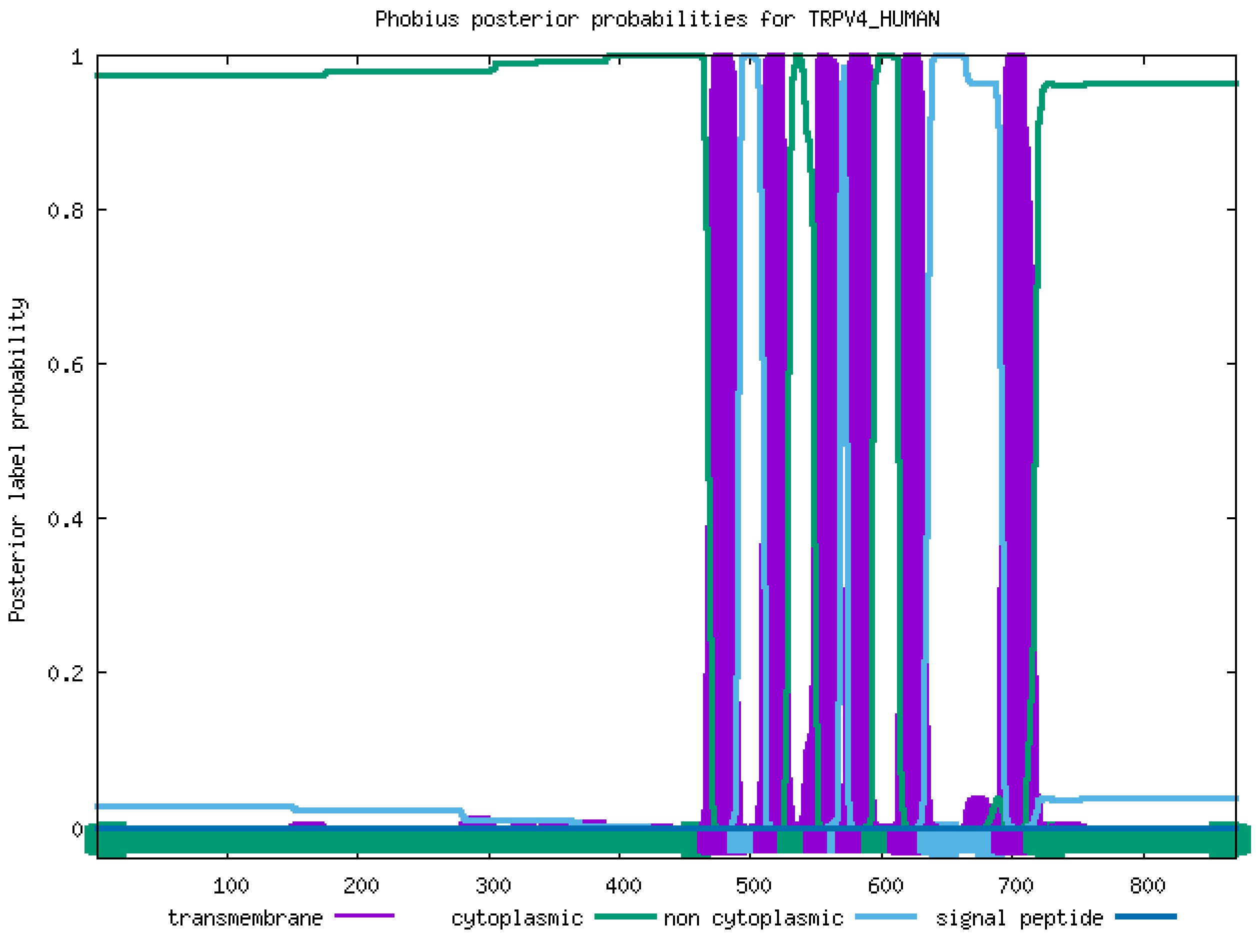

The probability data used in the plot is found here, and the gnuplot script is here.

Prediction of TRPV6_HUMAN

```
ID   TRPV6_HUMAN
FT   TOPO_DOM      1    367       CYTOPLASMIC.
FT   TRANSMEM    368    392
FT   TOPO_DOM    393    423       NON CYTOPLASMIC.
FT   TRANSMEM    424    444
FT   TOPO_DOM    445    464       CYTOPLASMIC.
FT   TRANSMEM    465    485
FT   TOPO_DOM    486    490       NON CYTOPLASMIC.
FT   TRANSMEM    491    509
FT   TOPO_DOM    510    529       CYTOPLASMIC.
FT   TRANSMEM    530    552
FT   TOPO_DOM    553    592       NON CYTOPLASMIC.
FT   TRANSMEM    593    615
FT   TOPO_DOM    616    765       CYTOPLASMIC.
//
```

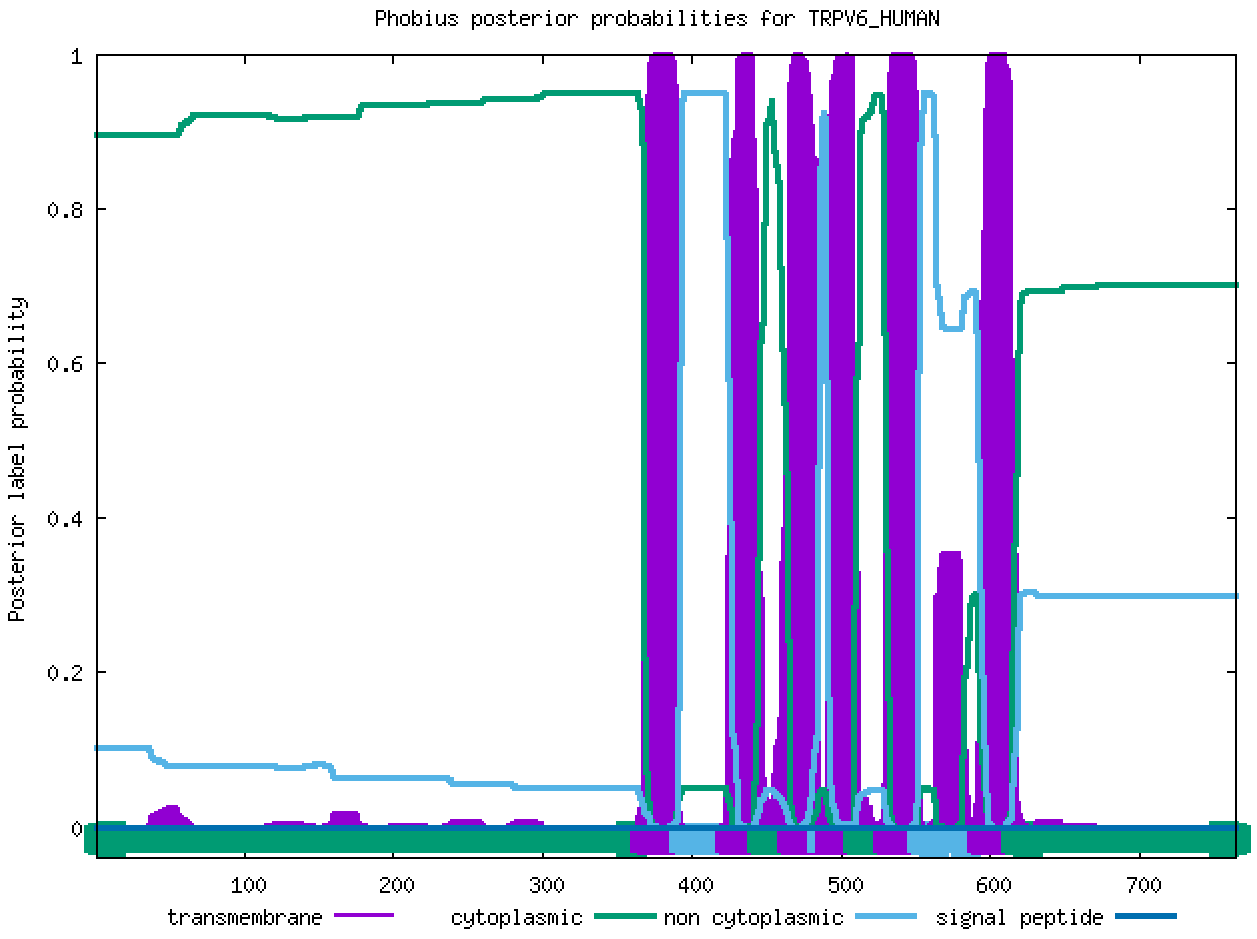

The probability data used in the plot is found here, and the gnuplot script is here.

Prediction of PKD2_HUMAN

```
ID    PKD2_HUMAN
FT    TOPO_DOM      1     223       NON CYTOPLASMIC.
FT    TRANSMEM     224    248
FT    TOPO_DOM     249    422       CYTOPLASMIC.
FT    TRANSMEM     423    443
FT    TOPO_DOM     444    462       NON CYTOPLASMIC.
FT    TRANSMEM     463    489
FT    TOPO_DOM     490    508       CYTOPLASMIC.
FT    TRANSMEM     509    527
FT    TOPO_DOM     528    555       NON CYTOPLASMIC.
FT    TRANSMEM     556    577
FT    TOPO_DOM     578    596       CYTOPLASMIC.
FT    TRANSMEM     597    619
FT    TOPO_DOM     620    658       NON CYTOPLASMIC.
FT    TRANSMEM     659    680
FT    TOPO_DOM     681    968       CYTOPLASMIC.
//
```

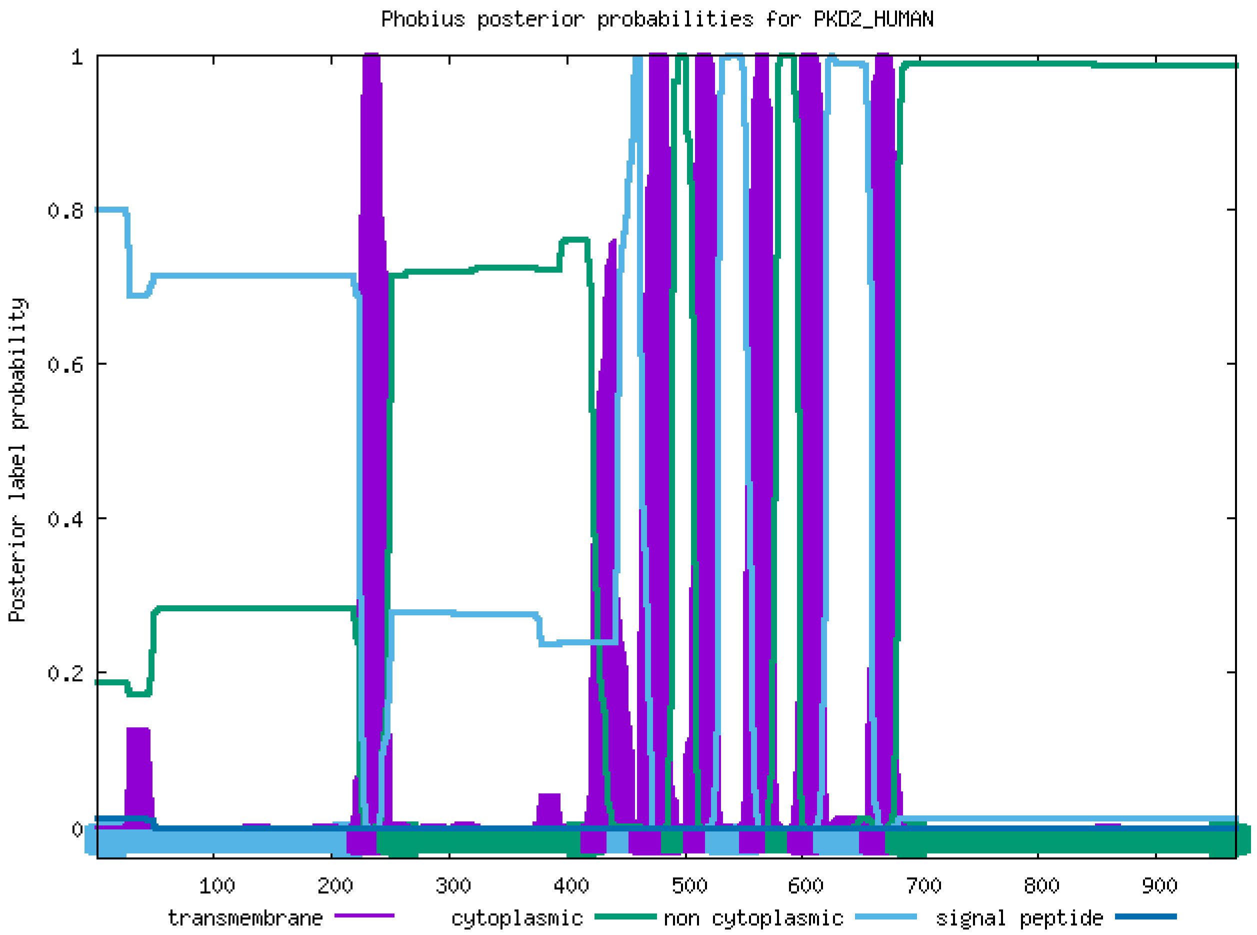

The probability data used in the plot is found here, and the gnuplot script is here.

Prediction of MCLN1_HUMAN

```
ID   MCLN1_HUMAN
FT   TOPO_DOM      1     65       NON CYTOPLASMIC.
FT   TRANSMEM     66     85
FT   TOPO_DOM     86    298       CYTOPLASMIC.
FT   TRANSMEM    299    321
FT   TOPO_DOM    322    352       NON CYTOPLASMIC.
FT   TRANSMEM    353    373
FT   TOPO_DOM    374    384       CYTOPLASMIC.
FT   TRANSMEM    385    405
FT   TOPO_DOM    406    424       NON CYTOPLASMIC.
FT   TRANSMEM    425    447
FT   TOPO_DOM    448    495       CYTOPLASMIC.
FT   TRANSMEM    496    517
FT   TOPO_DOM    518    580       NON CYTOPLASMIC.
//
```

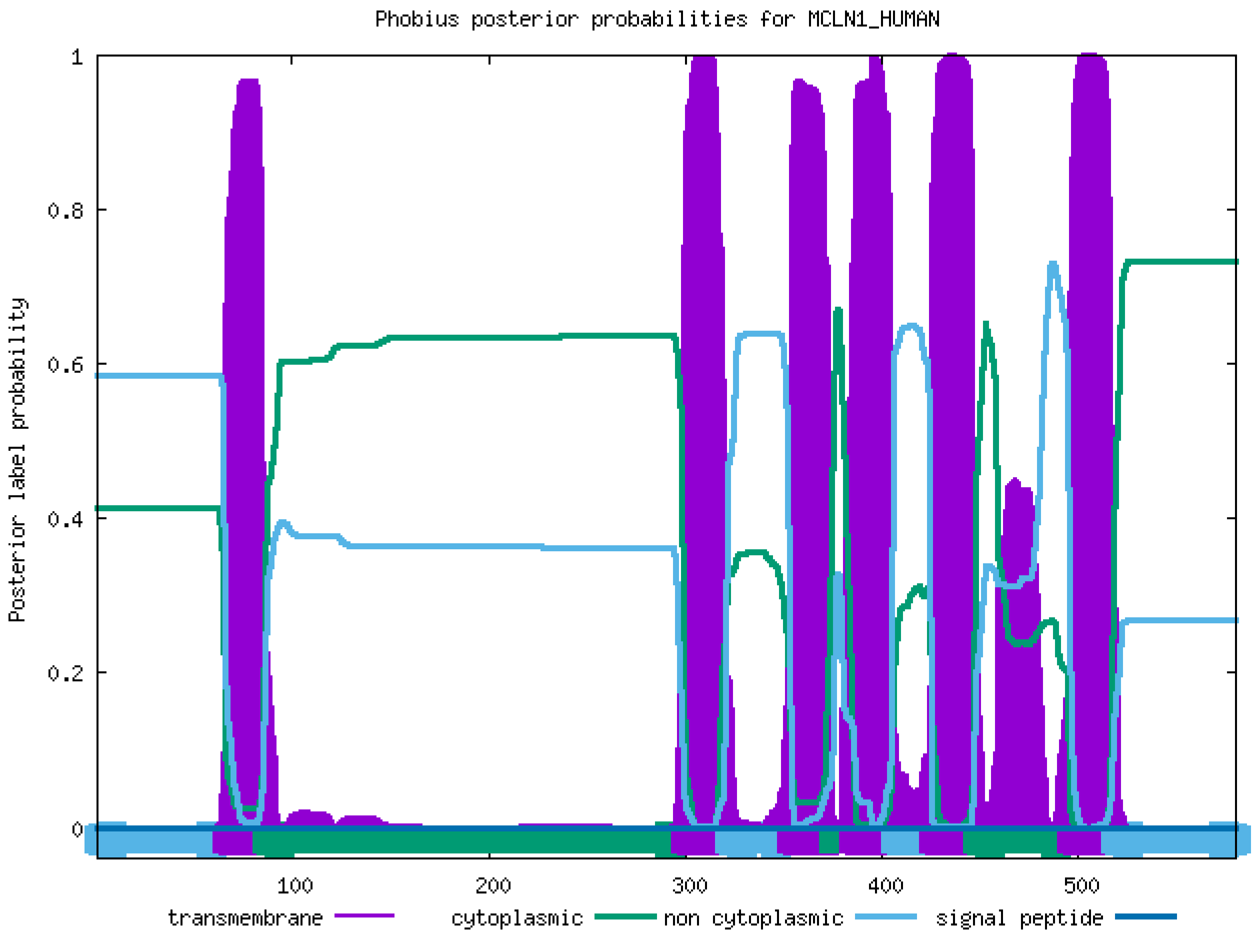

The probability data used in the plot is found here, and the gnuplot script is here.

Prediction of CXB2_HUMAN

```
ID   CXB2_HUMAN
FT   TOPO_DOM      1     22       CYTOPLASMIC.
FT   TRANSMEM     23     40
FT   TOPO_DOM     41     76       NON CYTOPLASMIC.
FT   TRANSMEM     77     97
FT   TOPO_DOM     98    131       CYTOPLASMIC.
FT   TRANSMEM    132    158
FT   TOPO_DOM    159    190       NON CYTOPLASMIC.
FT   TRANSMEM    191    215
FT   TOPO_DOM    216    226       CYTOPLASMIC.
//
```

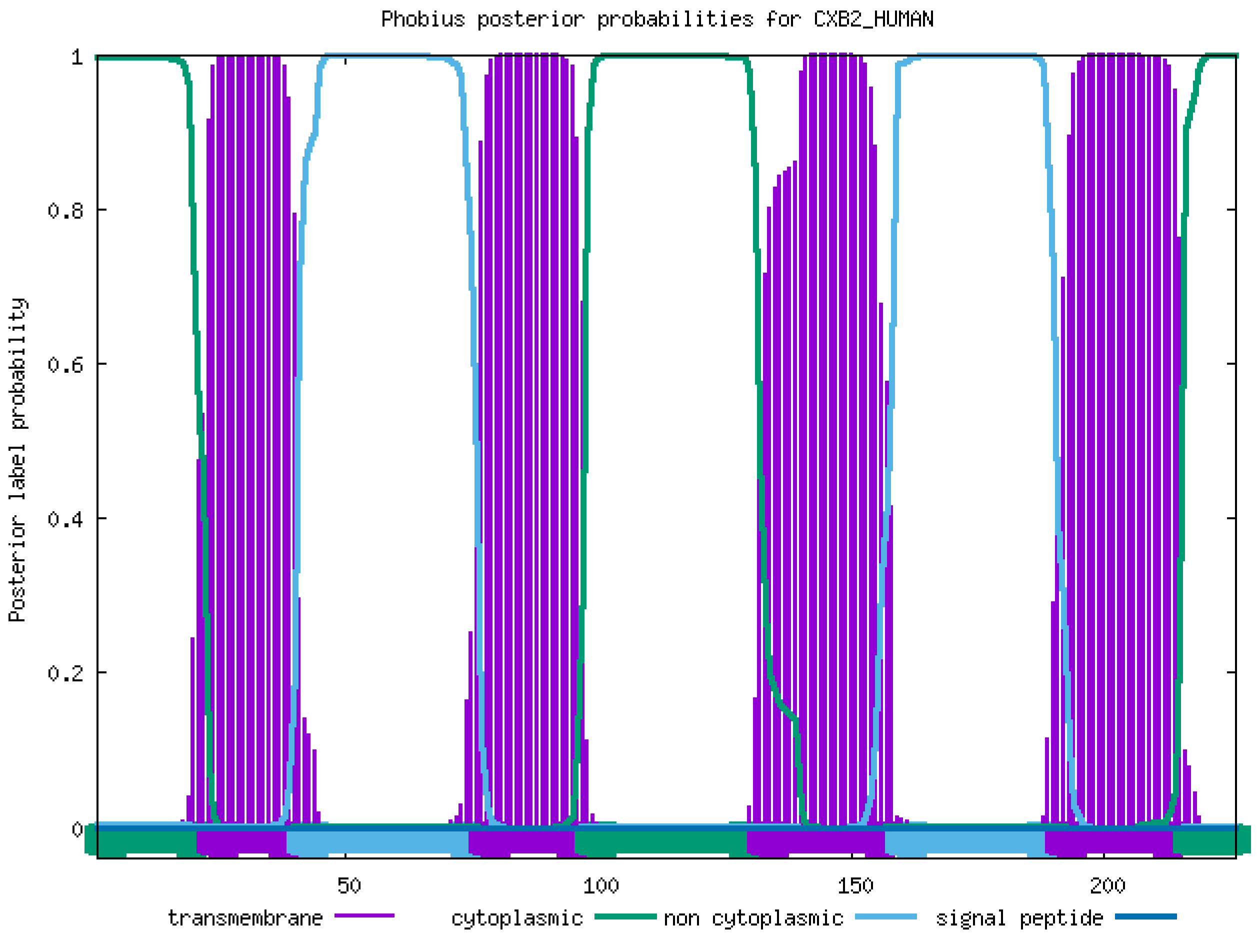

The probability data used in the plot is found here, and the gnuplot script is here.

Prediction of CXD2_HUMAN

```
ID    CXD2_HUMAN
FT    TOPO_DOM      1     20       CYTOPLASMIC.
FT    TRANSMEM     21     42
FT    TOPO_DOM     43     77       NON CYTOPLASMIC.
FT    TRANSMEM     78     98
FT    TOPO_DOM     99    197       CYTOPLASMIC.
FT    TRANSMEM    198    220
FT    TOPO_DOM    221    250       NON CYTOPLASMIC.
FT    TRANSMEM    251    270
FT    TOPO_DOM    271    321       CYTOPLASMIC.
//
```

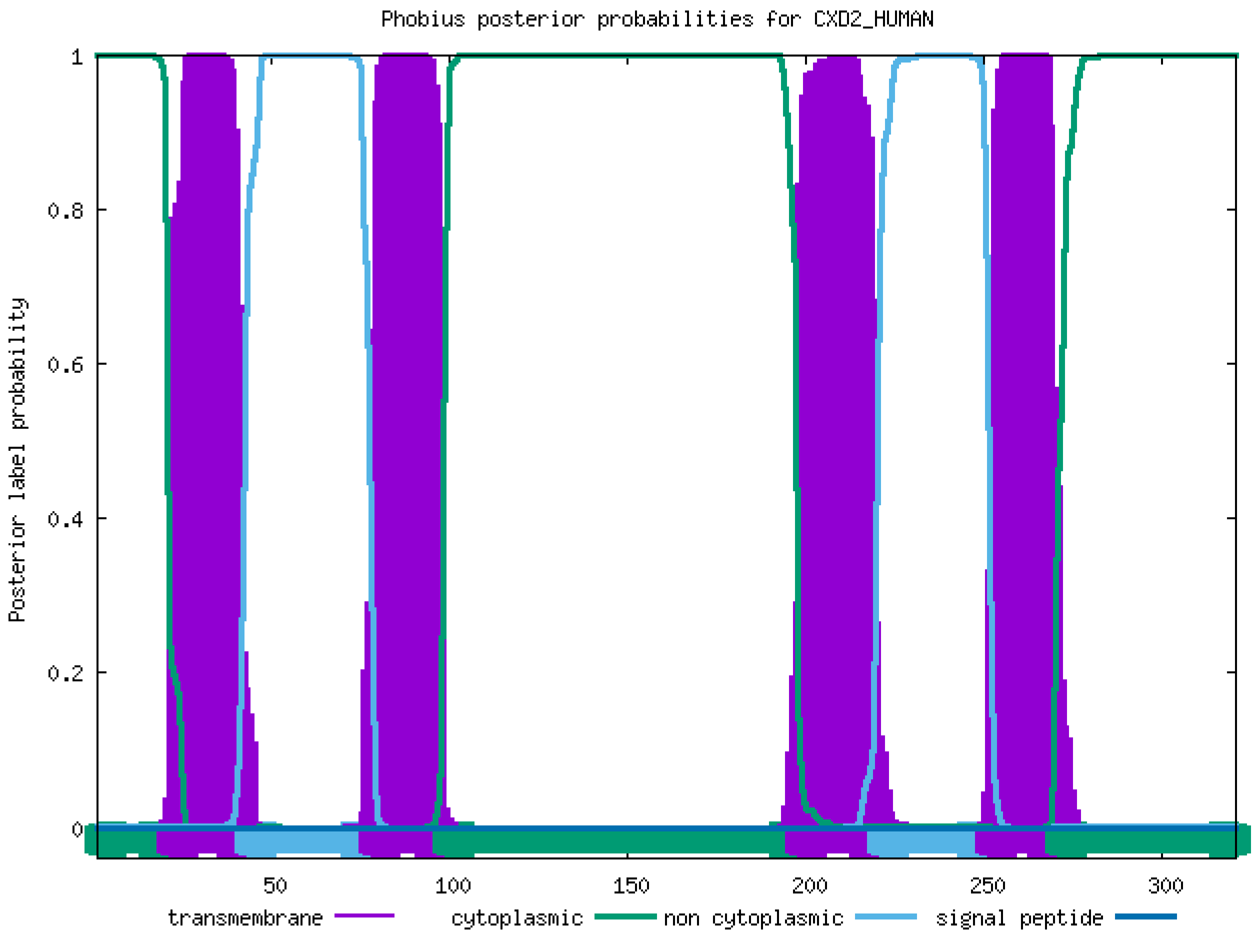

The probability data used in the plot is found here, and the gnuplot script is here.

Prediction of CXG3_HUMAN

```
ID   CXG3_HUMAN
FT   TOPO_DOM      1     19       CYTOPLASMIC.
FT   TRANSMEM     20     38
FT   TOPO_DOM     39     76       NON CYTOPLASMIC.
FT   TRANSMEM     77    100
FT   TOPO_DOM    101    193       CYTOPLASMIC.
FT   TRANSMEM    194    219
FT   TOPO_DOM    220    279       NON CYTOPLASMIC.
//
```

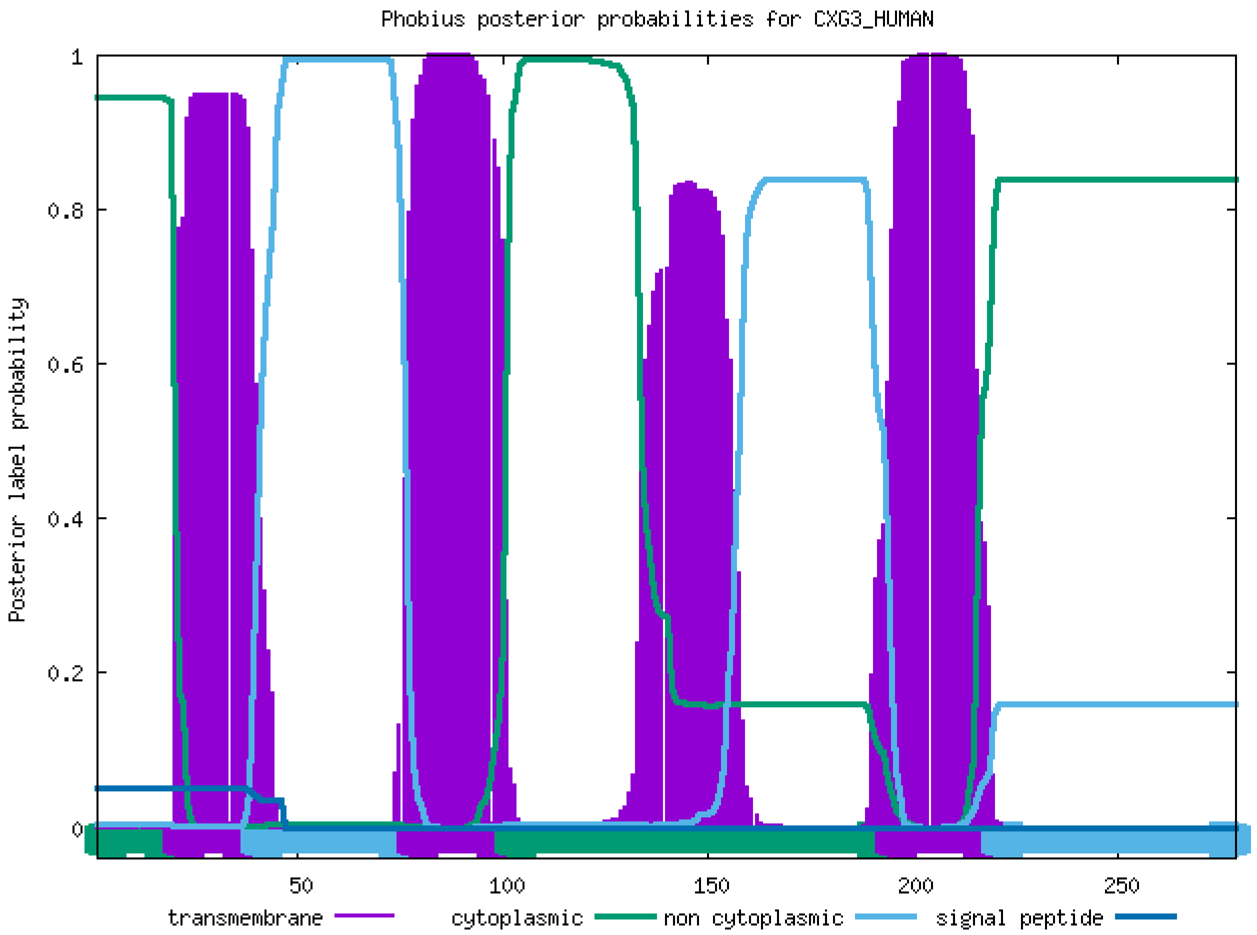

The probability data used in the plot is found here, and the gnuplot script is here.

Prediction of CAHM2_HUMAN

```
ID   CAHM2_HUMAN
FT   TOPO_DOM      1     20       CYTOPLASMIC.
FT   TRANSMEM     21     42
FT   TOPO_DOM     43     53       NON CYTOPLASMIC.
FT   TRANSMEM     54     75
FT   TOPO_DOM     76     95       CYTOPLASMIC.
FT   TRANSMEM     96    117
FT   TOPO_DOM    118    185       NON CYTOPLASMIC.
FT   TRANSMEM    186    205
FT   TOPO_DOM    206    323       CYTOPLASMIC.
//
```

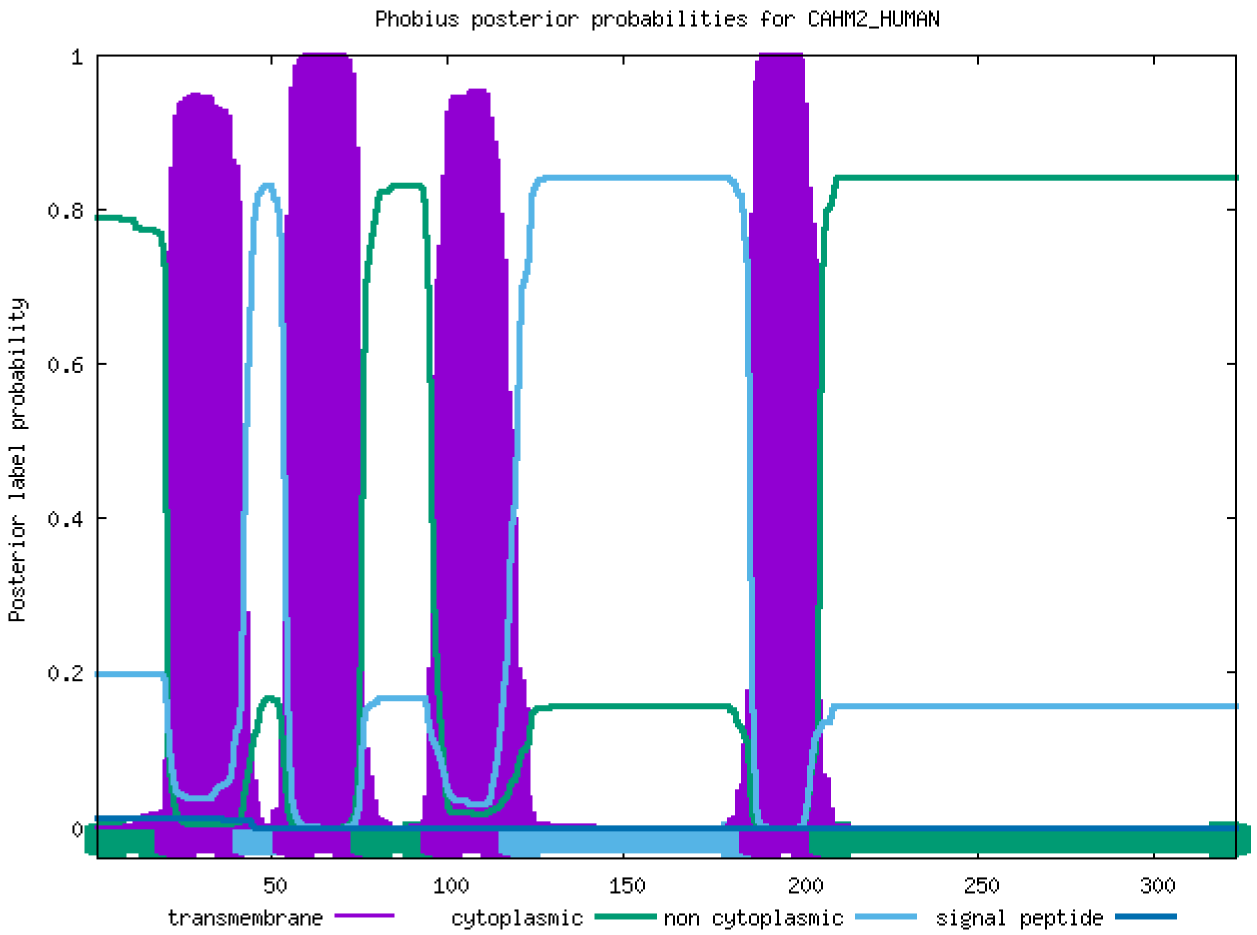

The probability data used in the plot is found here, and the gnuplot script is here.

Prediction of CAHM4_HUMAN

```
ID    CAHM4_HUMAN
FT    TOPO_DOM      1      11       CYTOPLASMIC.
FT    TRANSMEM     12      29
FT    TOPO_DOM     30      48       NON CYTOPLASMIC.
FT    TRANSMEM     49      70
FT    TOPO_DOM     71      99       CYTOPLASMIC.
FT    TRANSMEM    100     121
FT    TOPO_DOM    122     186       NON CYTOPLASMIC.
FT    TRANSMEM    187     207
FT    TOPO_DOM    208     314       CYTOPLASMIC.
//
```

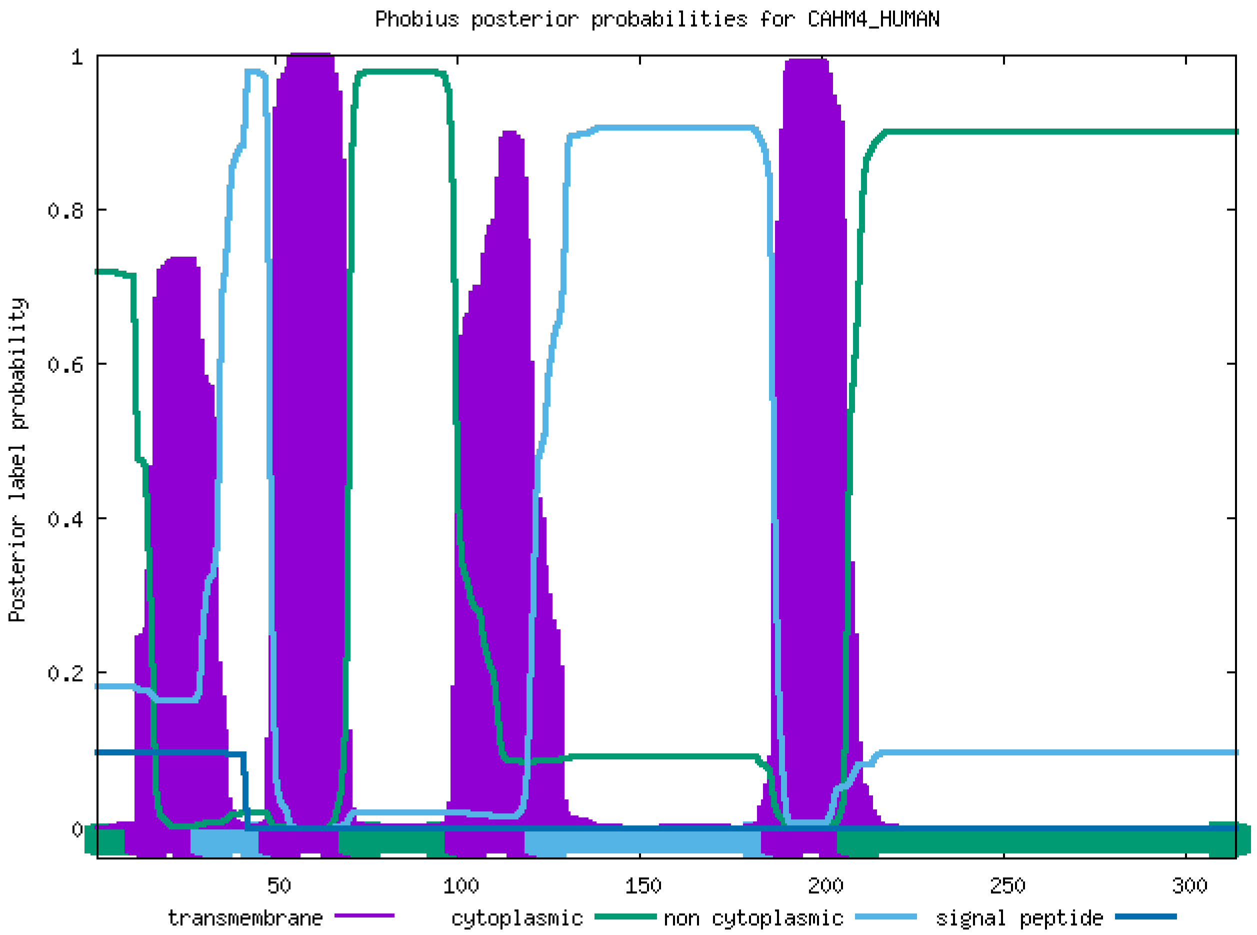

The probability data used in the plot is found here, and the gnuplot script is here.

Prediction of CAHM5_HUMAN

```
ID   CAHM5_HUMAN
FT   TOPO_DOM      1     16       CYTOPLASMIC.
FT   TRANSMEM     17     38
FT   TOPO_DOM     39     49       NON CYTOPLASMIC.
FT   TRANSMEM     50     69
FT   TOPO_DOM     70     95       CYTOPLASMIC.
FT   TRANSMEM     96    120
FT   TOPO_DOM    121    180       NON CYTOPLASMIC.
FT   TRANSMEM    181    201
FT   TOPO_DOM    202    309       CYTOPLASMIC.
//
```

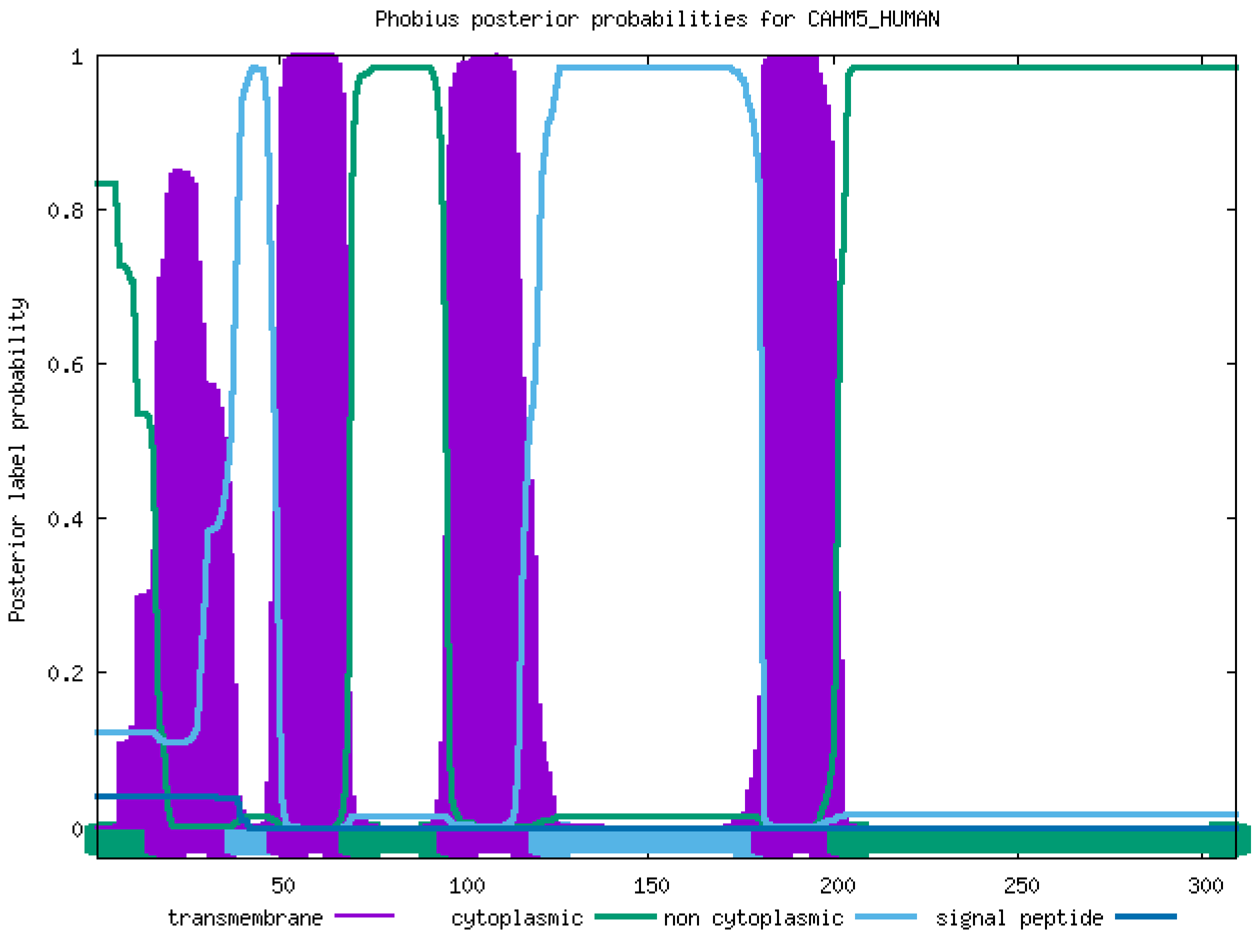

The probability data used in the plot is found here, and the gnuplot script is here.

Prediction of CAHM6_HUMAN

```
ID   CAHM6_HUMAN
FT   TOPO_DOM      1     19       CYTOPLASMIC.
FT   TRANSMEM     20     39
FT   TOPO_DOM     40     50       NON CYTOPLASMIC.
FT   TRANSMEM     51     70
FT   TOPO_DOM     71    175       CYTOPLASMIC.
FT   TRANSMEM    176    195
FT   TOPO_DOM    196    315       NON CYTOPLASMIC.
//
```

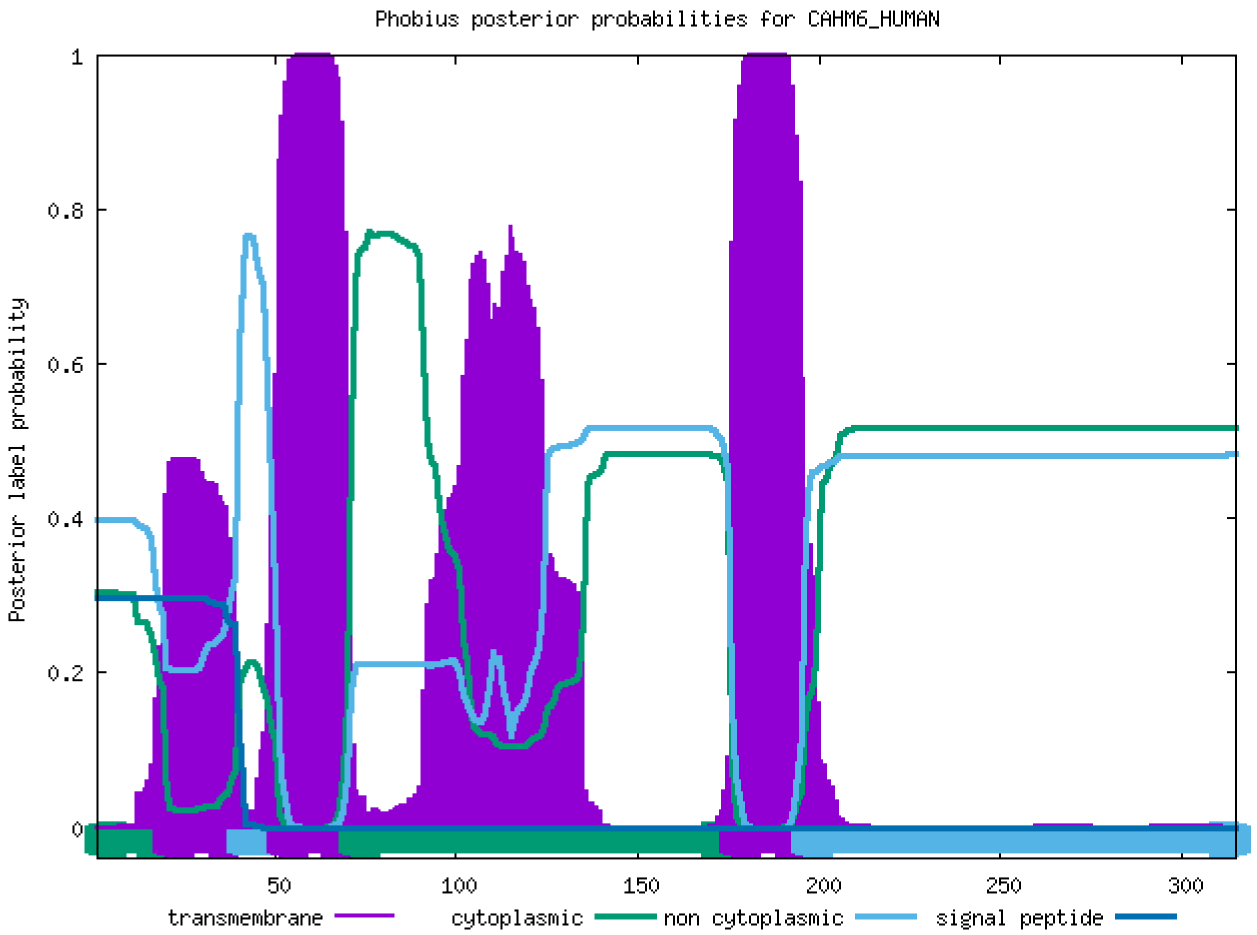

The probability data used in the plot is found here, and the gnuplot script is here.

Prediction of NMDZ1_HUMAN

```
ID   NMDZ1_HUMAN
FT   SIGNAL         1     20
FT   REGION         1      5       N-REGION.
FT   REGION         6     15       H-REGION.
FT   REGION        16     20       C-REGION.
FT   TOPO_DOM      21    561       NON CYTOPLASMIC.
FT   TRANSMEM     562    580
FT   TOPO_DOM     581    634       CYTOPLASMIC.
FT   TRANSMEM     635    657
FT   TOPO_DOM     658    812       NON CYTOPLASMIC.
FT   TRANSMEM     813    837
FT   TOPO_DOM     838    938       CYTOPLASMIC.
//
```

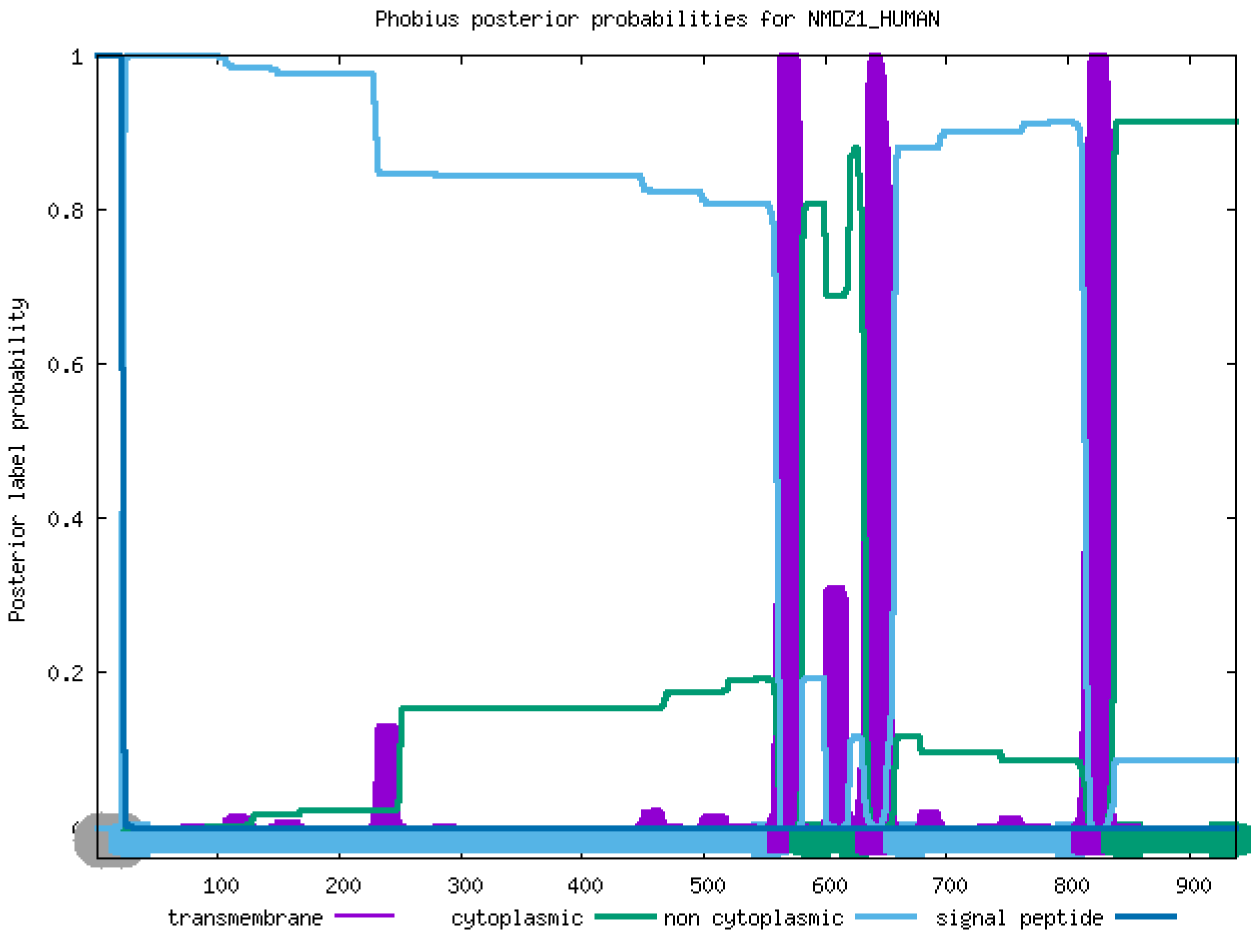

The probability data used in the plot is found here, and the gnuplot script is here.

Prediction of NMDE3_HUMAN

```
ID   NMDE3_HUMAN
FT   SIGNAL        1     19
FT   REGION        1      3       N-REGION.
FT   REGION        4     15       H-REGION.
FT   REGION       16     19       C-REGION.
FT   TOPO_DOM     20    553       NON CYTOPLASMIC.
FT   TRANSMEM    554    574
FT   TOPO_DOM    575    594       CYTOPLASMIC.
FT   TRANSMEM    595    615
FT   TOPO_DOM    616    626       NON CYTOPLASMIC.
FT   TRANSMEM    627    652
FT   TOPO_DOM    653    815       CYTOPLASMIC.
FT   TRANSMEM    816    835
FT   TOPO_DOM    836    854       NON CYTOPLASMIC.
FT   TRANSMEM    855    874
FT   TOPO_DOM    875   1233       CYTOPLASMIC.
//
```

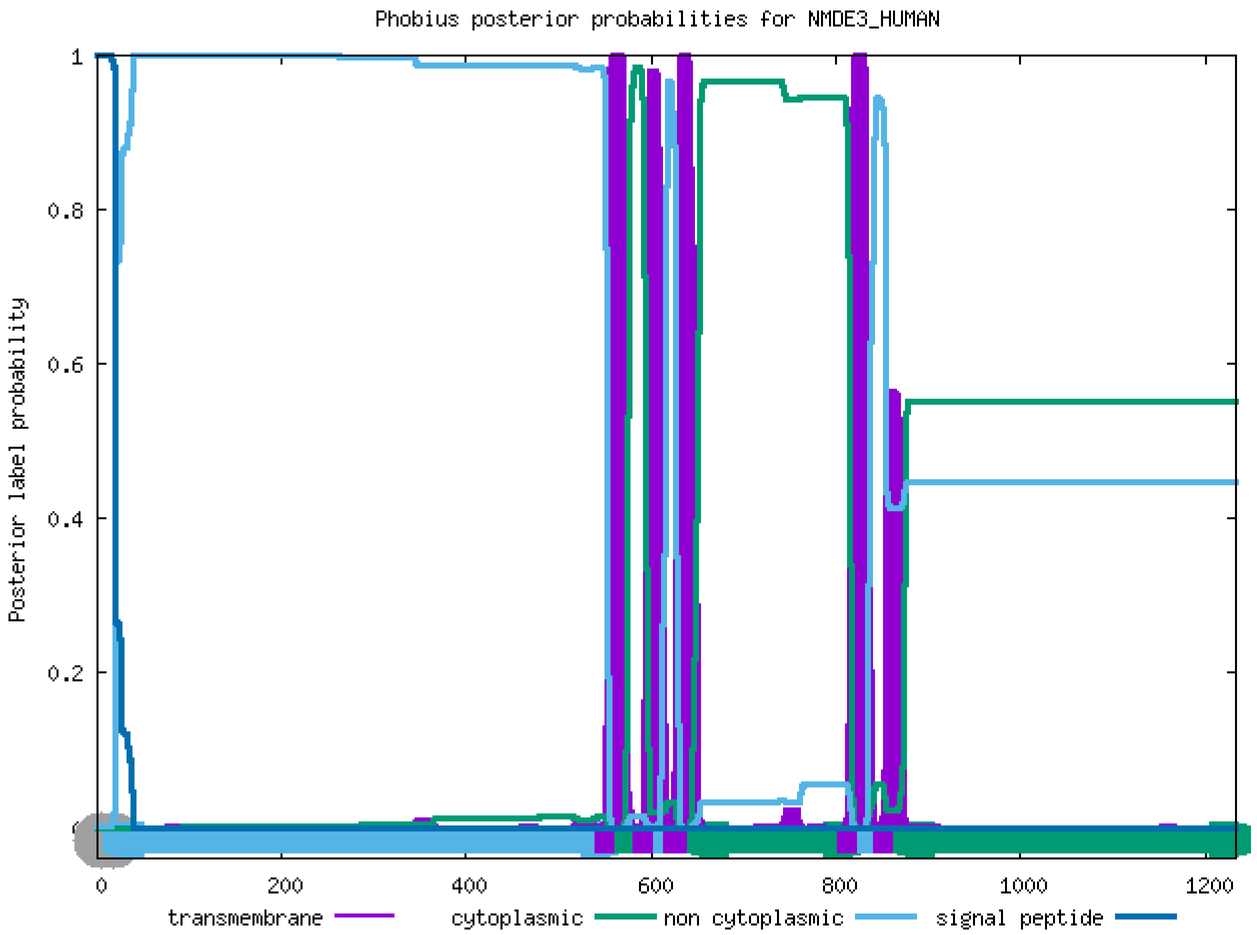

The probability data used in the plot is found here, and the gnuplot script is here.

Prediction of NMDE4_HUMAN

```
ID   NMDE4_HUMAN
FT   SIGNAL         1     46
FT   REGION         1     16       N-REGION.
FT   REGION        17     28       H-REGION.
FT   REGION        29     46       C-REGION.
FT   TOPO_DOM      47    583       NON CYTOPLASMIC.
FT   TRANSMEM     584    607
FT   TOPO_DOM     608    627       CYTOPLASMIC.
FT   TRANSMEM     628    645
FT   TOPO_DOM     646    656       NON CYTOPLASMIC.
FT   TRANSMEM     657    682
FT   TOPO_DOM     683    845       CYTOPLASMIC.
FT   TRANSMEM     846    865
FT   TOPO_DOM     866   1336       NON CYTOPLASMIC.
//
```

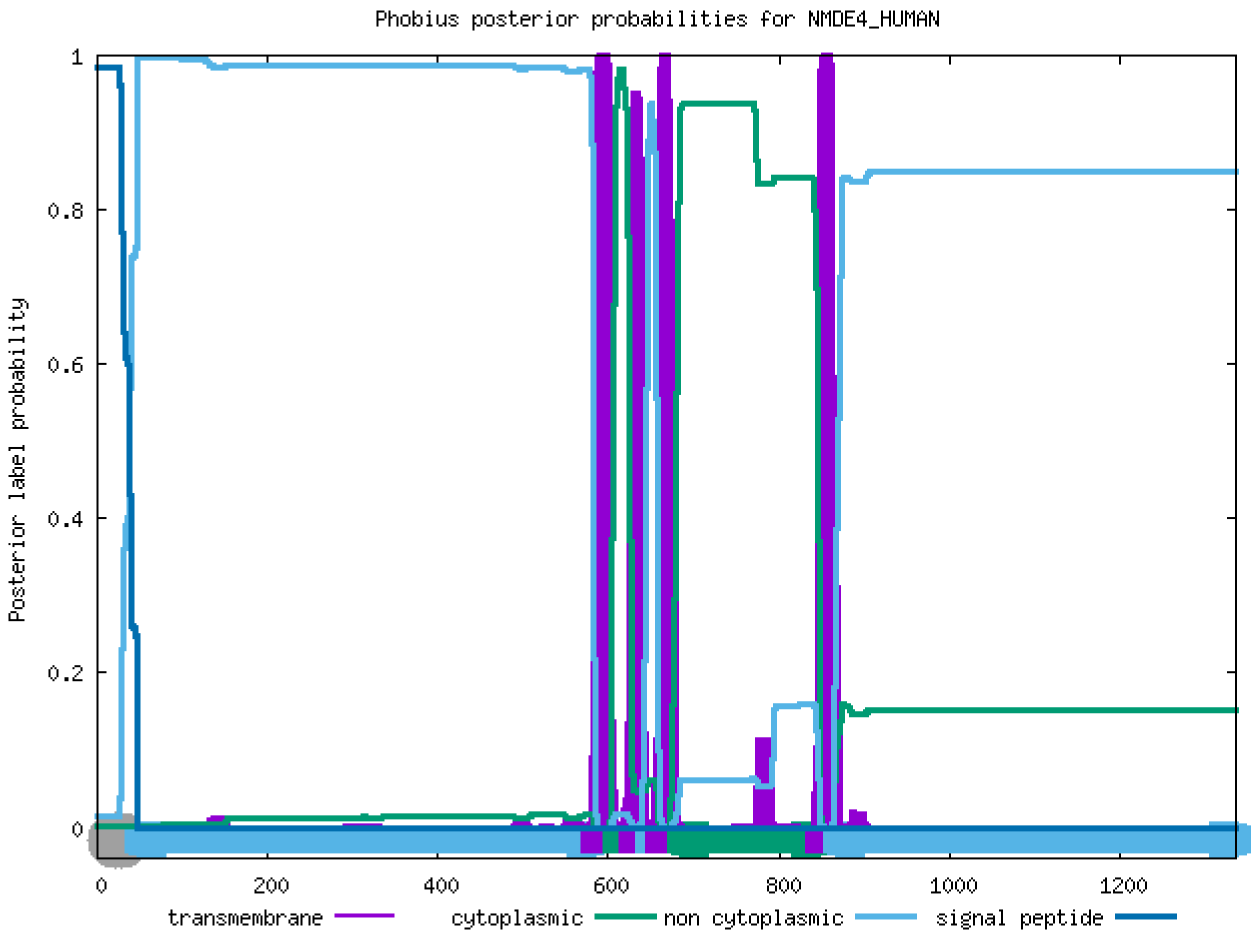

The probability data used in the plot is found here, and the gnuplot script is here.

Prediction of NMDE1_HUMAN

```
ID   NMDE1_HUMAN
FT   SIGNAL          1     26
FT   REGION          1      6       N-REGION.
FT   REGION          7     18       H-REGION.
FT   REGION         19     26       C-REGION.
FT   TOPO_DOM       27    556       NON CYTOPLASMIC.
FT   TRANSMEM      557    579
FT   TOPO_DOM      580    599       CYTOPLASMIC.
FT   TRANSMEM      600    617
FT   TOPO_DOM      618    628       NON CYTOPLASMIC.
FT   TRANSMEM      629    651
FT   TOPO_DOM      652    819       CYTOPLASMIC.
FT   TRANSMEM      820    843
FT   TOPO_DOM      844   1464       NON CYTOPLASMIC.
//
```

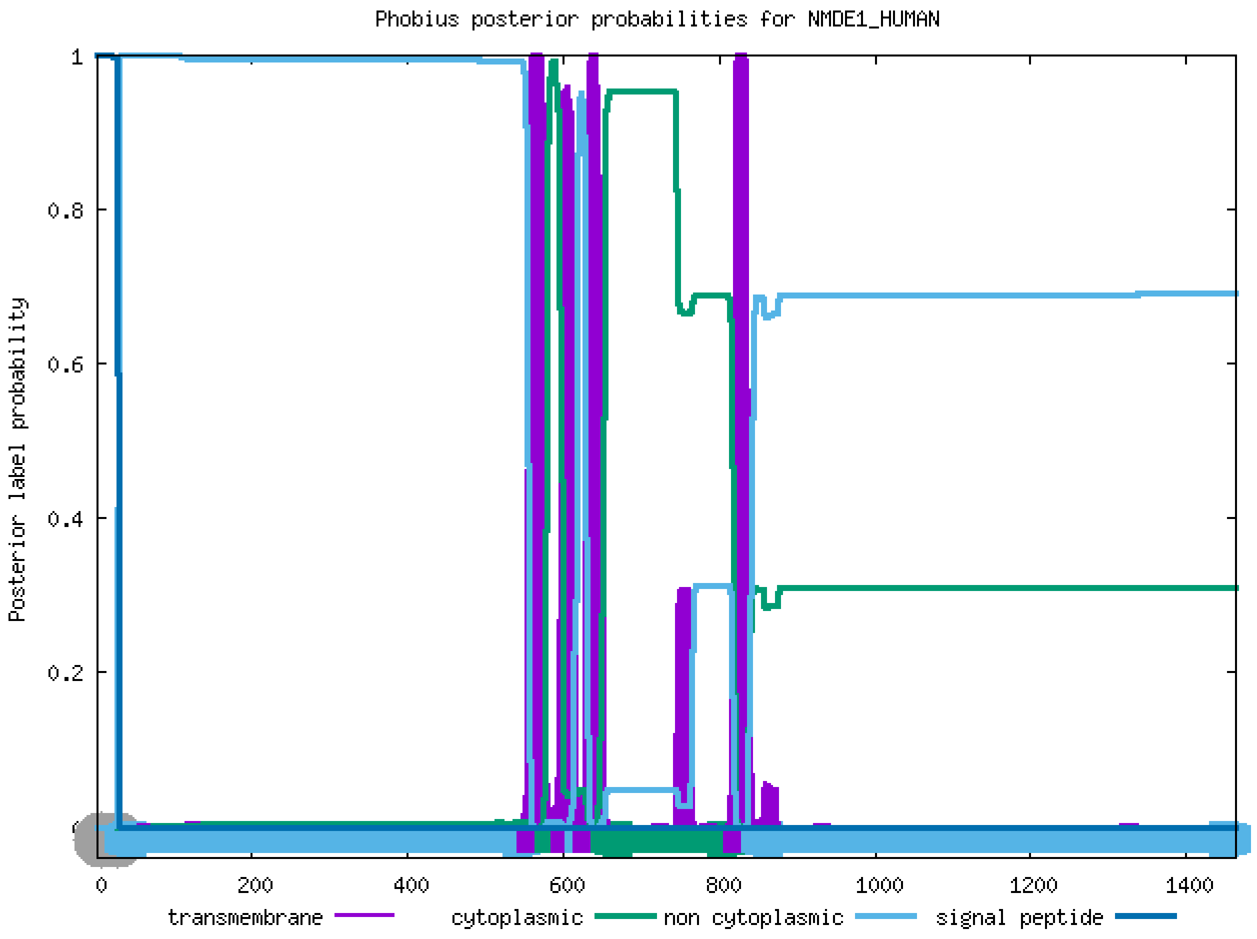

The probability data used in the plot is found here, and the gnuplot script is here.

Prediction of ASIC1_HUMAN

```
ID   ASIC1_HUMAN
FT   TOPO_DOM      1     43       NON CYTOPLASMIC.
FT   TRANSMEM     44     62
FT   TOPO_DOM     63    528       CYTOPLASMIC.
//
```

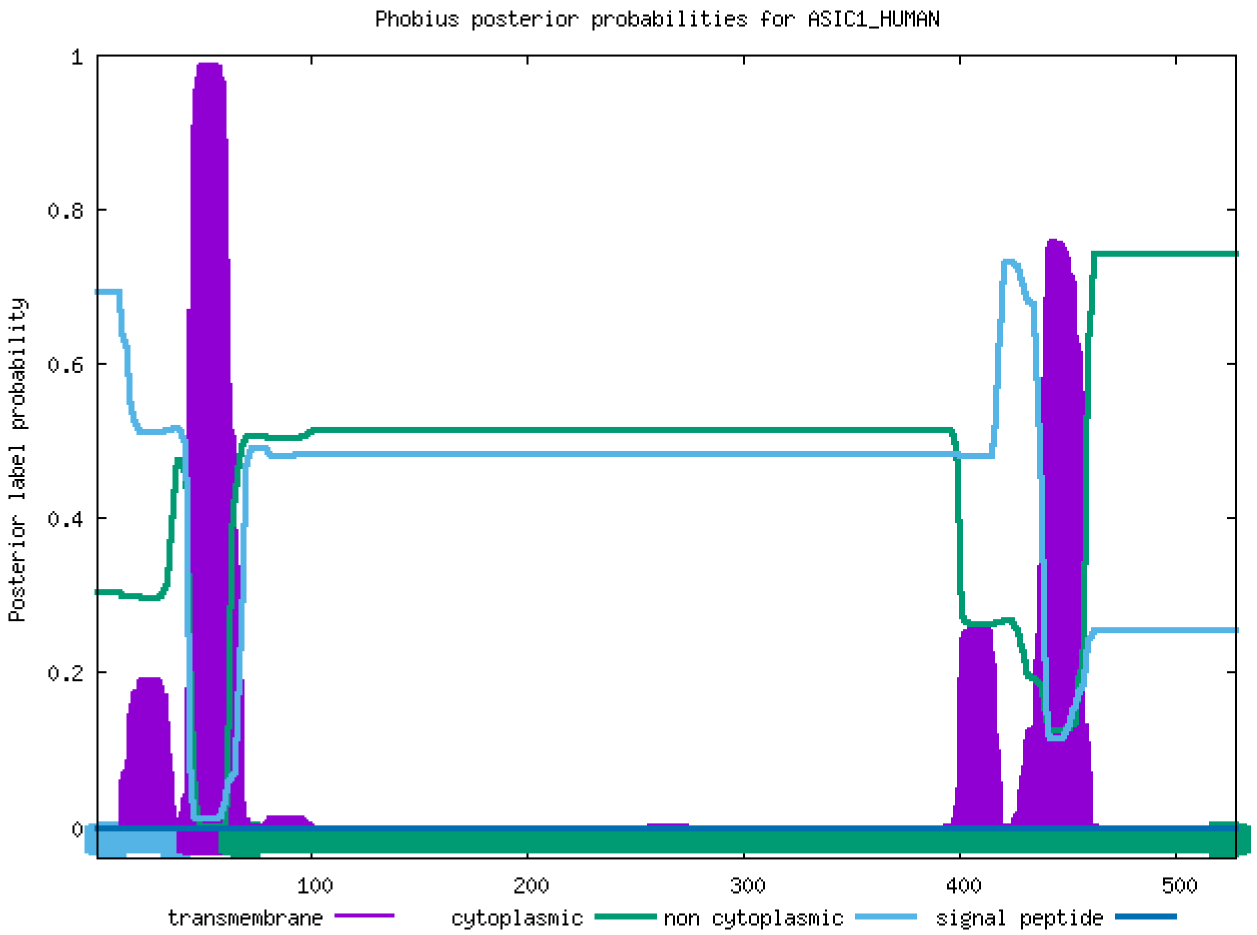

The probability data used in the plot is found here, and the gnuplot script is here.

Prediction of PACC1_HUMAN

```
ID    PACC1_HUMAN
FT    TOPO_DOM      1     64       CYTOPLASMIC.
FT    TRANSMEM     65     86
FT    TOPO_DOM     87    306       NON CYTOPLASMIC.
FT    TRANSMEM    307    332
FT    TOPO_DOM    333    350       CYTOPLASMIC.
//
```

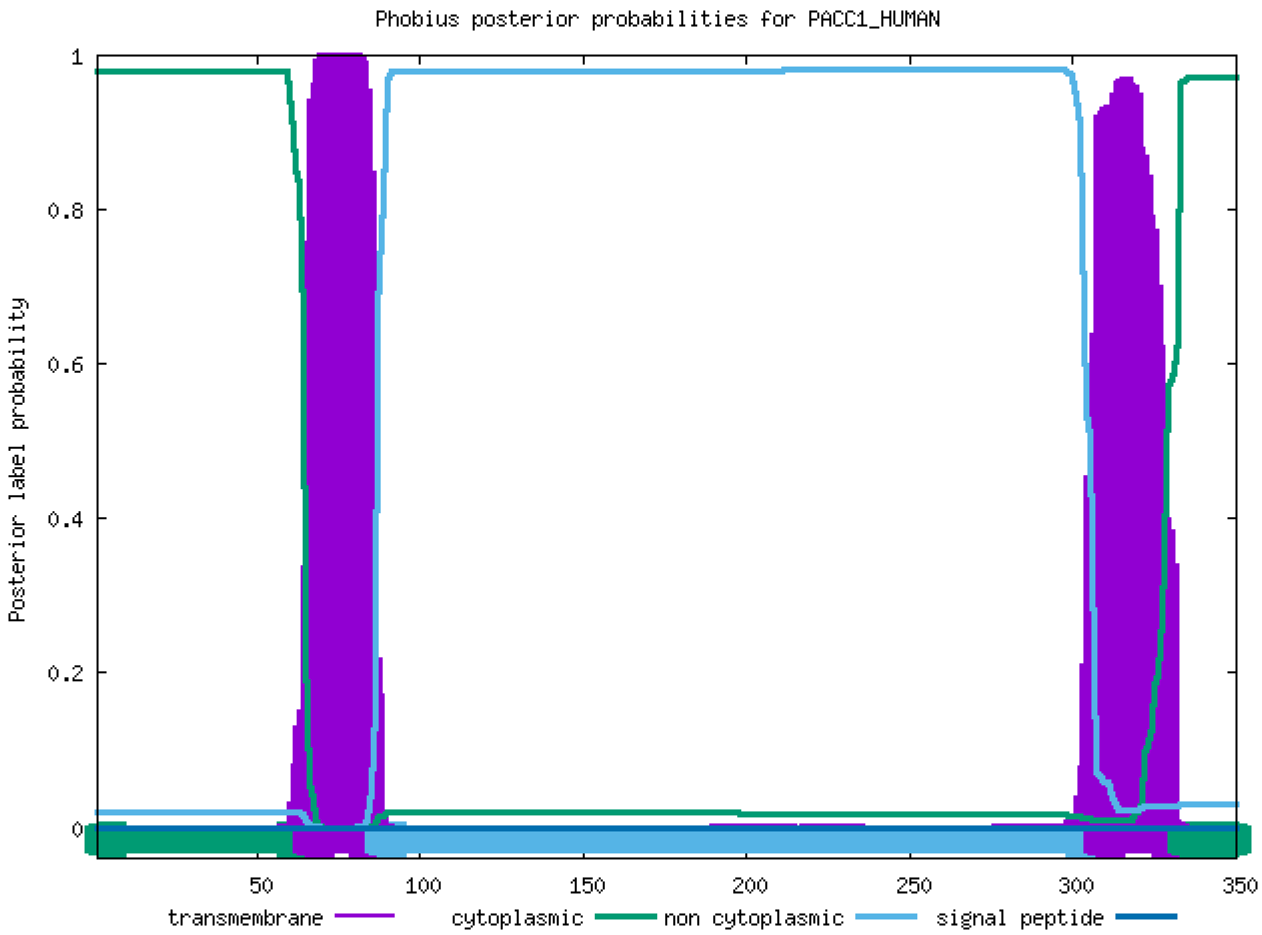

The probability data used in the plot is found here, and the gnuplot script is here.

Prediction of SCNNA_HUMAN

```
ID    SCNNA_HUMAN
FT    TOPO_DOM      1     84       CYTOPLASMIC.
FT    TRANSMEM     85    106
FT    TOPO_DOM    107    554       NON CYTOPLASMIC.
FT    TRANSMEM    555    585
FT    TOPO_DOM    586    669       CYTOPLASMIC.
//
```

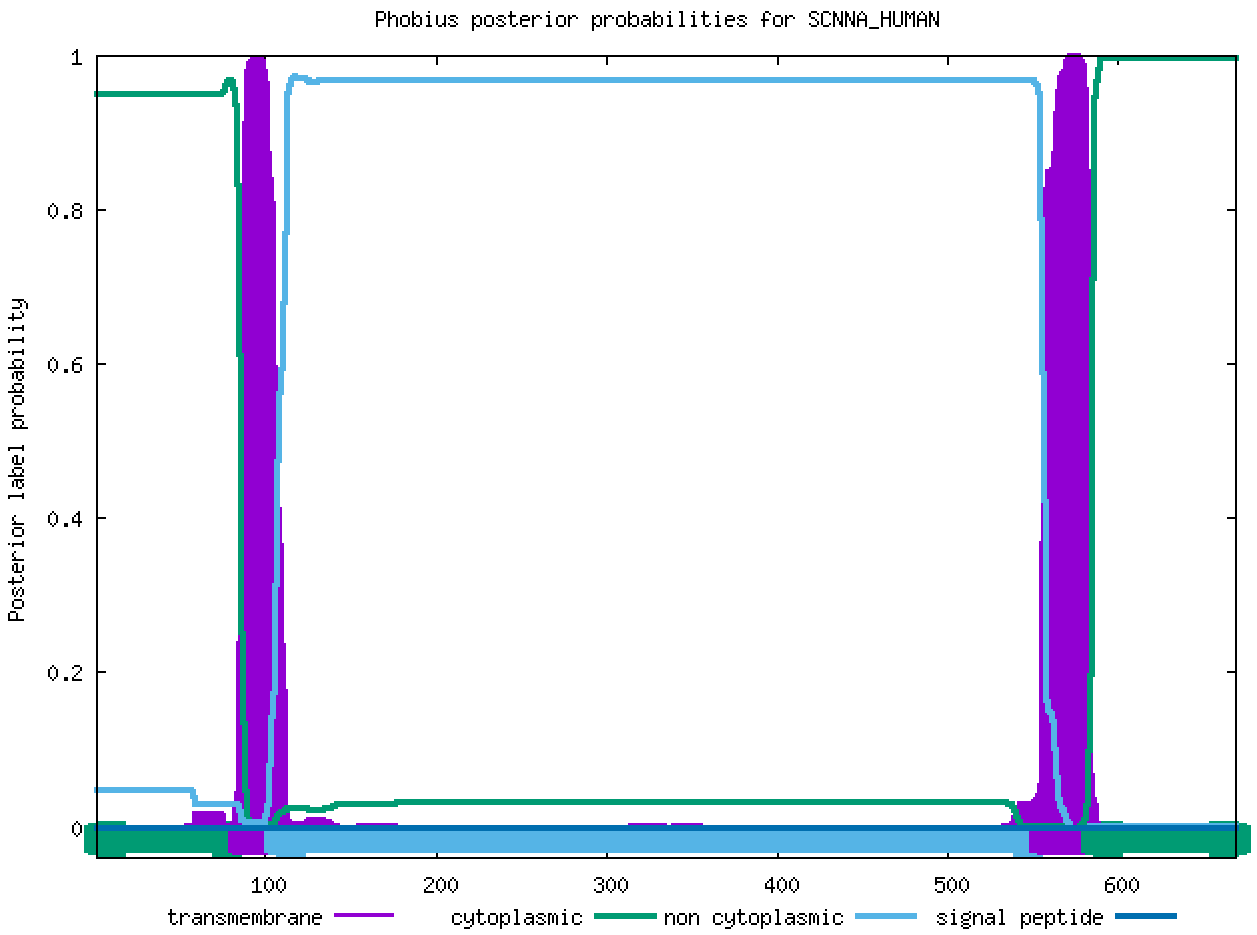

The probability data used in the plot is found here, and the gnuplot script is here.

Prediction of P2RX3_HUMAN

```
ID    P2RX3_HUMAN
FT    TOPO_DOM      1     20       CYTOPLASMIC.
FT    TRANSMEM     21     44
FT    TOPO_DOM     45    325       NON CYTOPLASMIC.
FT    TRANSMEM    326    347
FT    TOPO_DOM    348    397       CYTOPLASMIC.
//
```

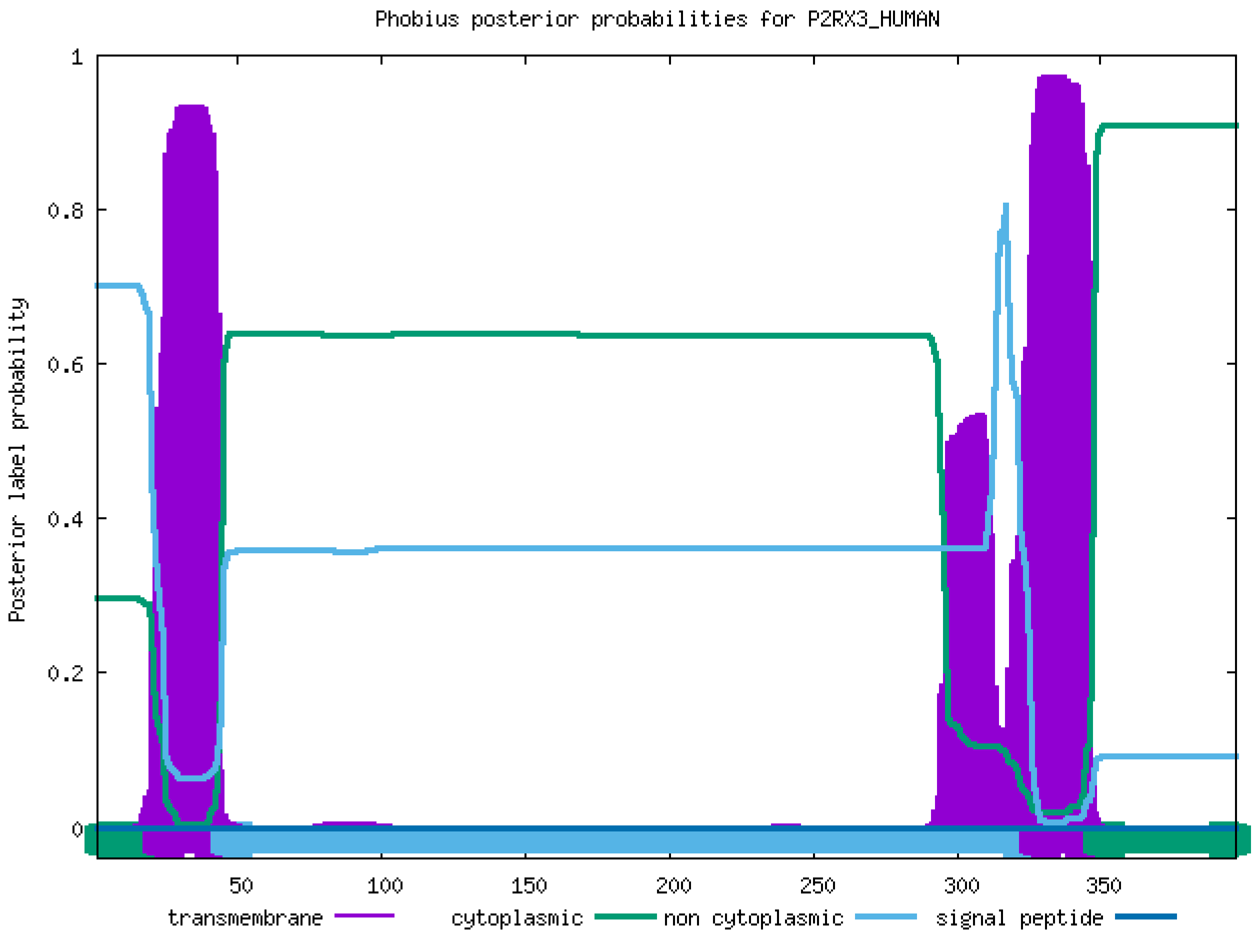

The probability data used in the plot is found here, and the gnuplot script is here.

Prediction of BEST1_HUMAN

```
ID    BEST1_HUMAN
FT    TOPO_DOM       1      30       NON CYTOPLASMIC.
FT    TRANSMEM      31      50
FT    TOPO_DOM      51      70       CYTOPLASMIC.
FT    TRANSMEM      71      94
FT    TOPO_DOM      95     234       NON CYTOPLASMIC.
FT    TRANSMEM     235     257
FT    TOPO_DOM     258     268       CYTOPLASMIC.
FT    TRANSMEM     269     287
FT    TOPO_DOM     288     585       NON CYTOPLASMIC.
//
```

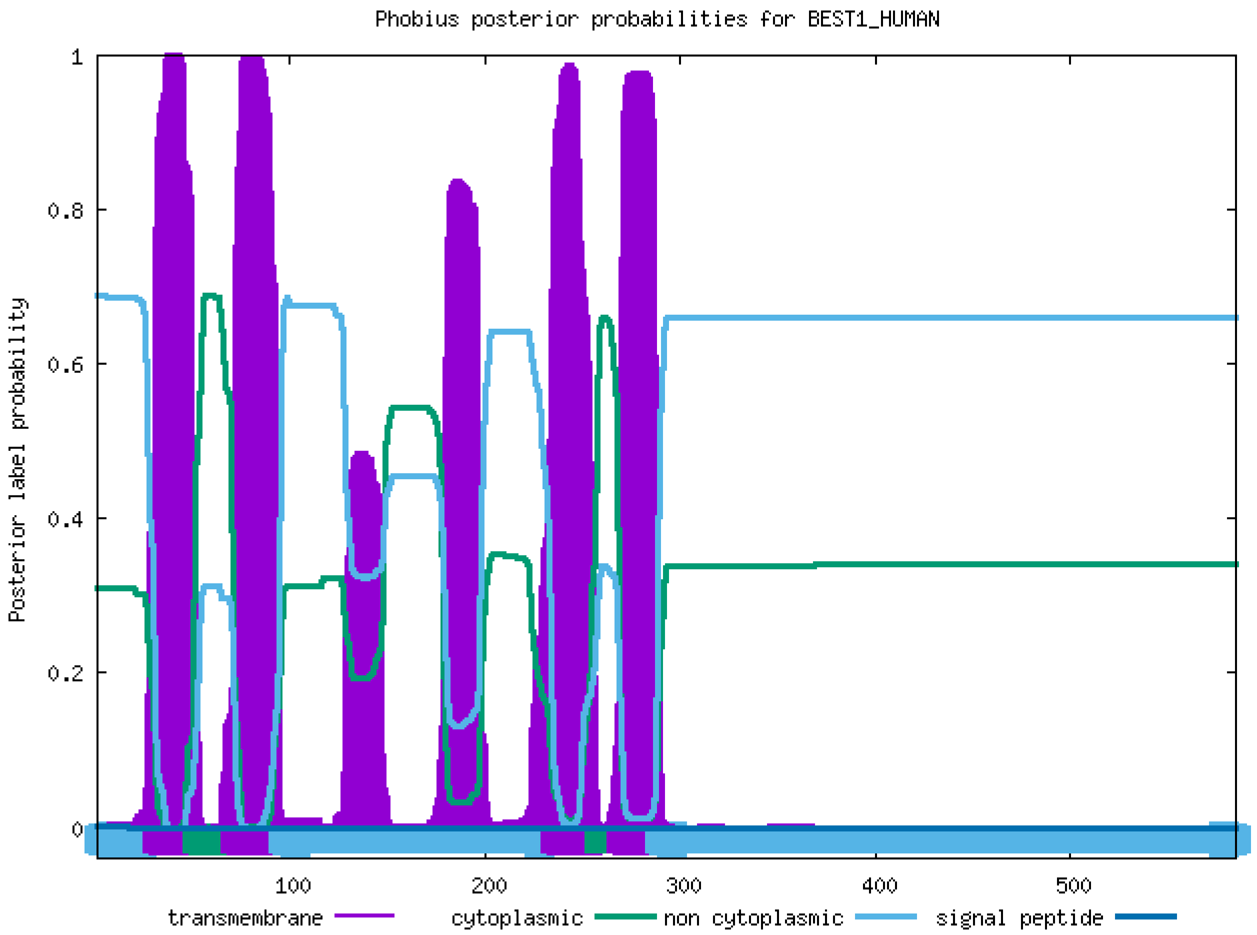

The probability data used in the plot is found here, and the gnuplot script is here.

Prediction of BEST2_HUMAN

```
ID   BEST2_HUMAN
FT   TOPO_DOM      1     30       NON CYTOPLASMIC.
FT   TRANSMEM     31     53
FT   TOPO_DOM     54     64       CYTOPLASMIC.
FT   TRANSMEM     65     88
FT   TOPO_DOM     89     93       NON CYTOPLASMIC.
FT   TRANSMEM     94    116
FT   TOPO_DOM    117    127       CYTOPLASMIC.
FT   TRANSMEM    128    148
FT   TOPO_DOM    149    234       NON CYTOPLASMIC.
FT   TRANSMEM    235    257
FT   TOPO_DOM    258    268       CYTOPLASMIC.
FT   TRANSMEM    269    287
FT   TOPO_DOM    288    509       NON CYTOPLASMIC.
//
```

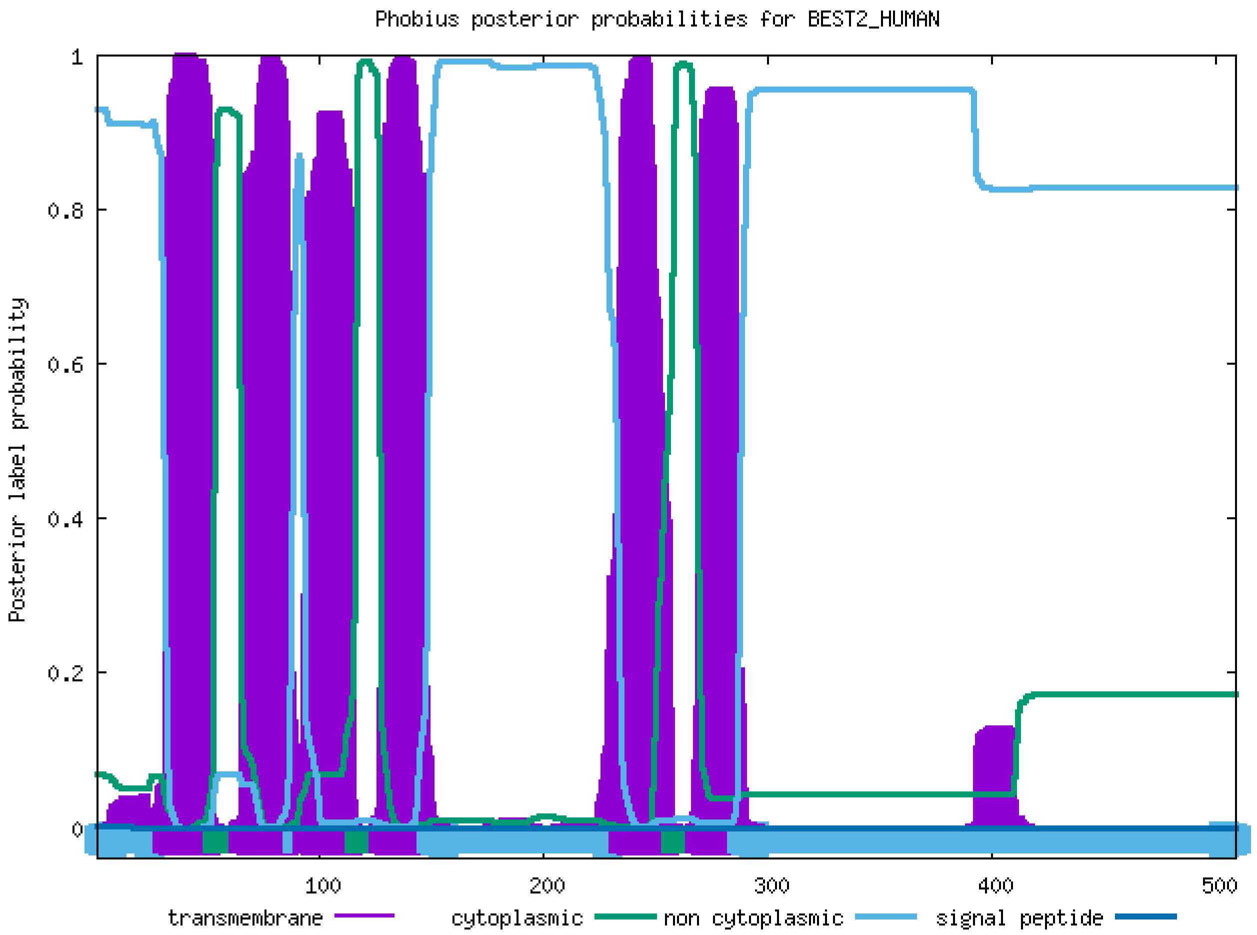

The probability data used in the plot is found here, and the gnuplot script is here.

Prediction of CLCN1_HUMAN

```
ID    CLCN1_HUMAN
FT    TOPO_DOM      1    117       CYTOPLASMIC.
FT    TRANSMEM    118    139
FT    TOPO_DOM    140    158       NON CYTOPLASMIC.
FT    TRANSMEM    159    182
FT    TOPO_DOM    183    234       CYTOPLASMIC.
FT    TRANSMEM    235    254
FT    TOPO_DOM    255    265       NON CYTOPLASMIC.
FT    TRANSMEM    266    290
FT    TOPO_DOM    291    301       CYTOPLASMIC.
FT    TRANSMEM    302    322
FT    TOPO_DOM    323    347       NON CYTOPLASMIC.
FT    TRANSMEM    348    370
FT    TOPO_DOM    371    390       CYTOPLASMIC.
FT    TRANSMEM    391    410
FT    TOPO_DOM    411    455       NON CYTOPLASMIC.
FT    TRANSMEM    456    475
FT    TOPO_DOM    476    486       CYTOPLASMIC.
FT    TRANSMEM    487    506
FT    TOPO_DOM    507    525       NON CYTOPLASMIC.
FT    TRANSMEM    526    547
FT    TOPO_DOM    548    553       CYTOPLASMIC.
FT    TRANSMEM    554    573
FT    TOPO_DOM    574    988       NON CYTOPLASMIC.
//
```

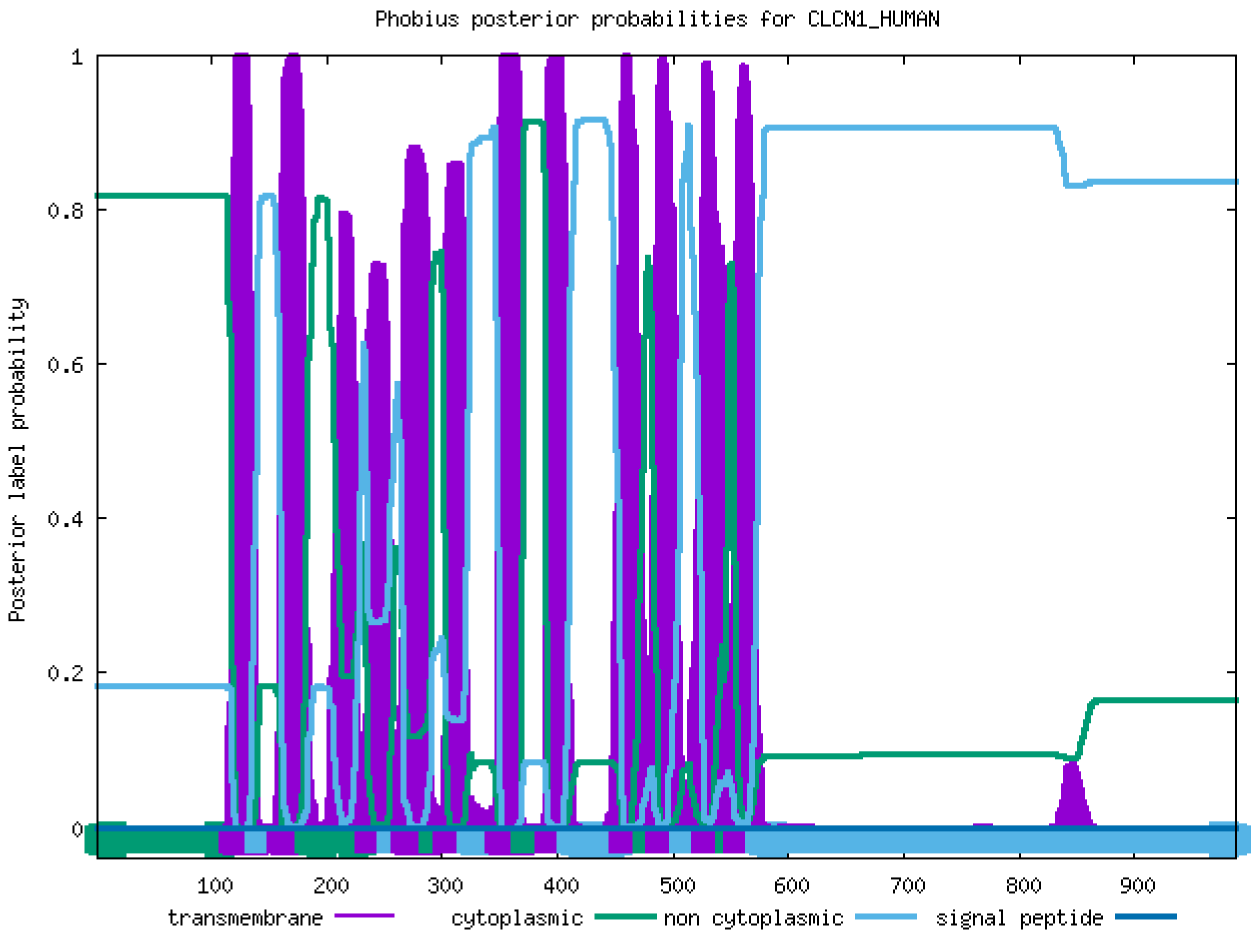

The probability data used in the plot is found here, and the gnuplot script is here.

Prediction of CLCN2_HUMAN

```
ID   CLCN2_HUMAN
FT   TOPO_DOM      1     90       NON CYTOPLASMIC.
FT   TRANSMEM     91    113
FT   TOPO_DOM    114    133       CYTOPLASMIC.
FT   TRANSMEM    134    156
FT   TOPO_DOM    157    175       NON CYTOPLASMIC.
FT   TRANSMEM    176    196
FT   TOPO_DOM    197    207       CYTOPLASMIC.
FT   TRANSMEM    208    227
FT   TOPO_DOM    228    238       NON CYTOPLASMIC.
FT   TRANSMEM    239    263
FT   TOPO_DOM    264    274       CYTOPLASMIC.
FT   TRANSMEM    275    295
FT   TOPO_DOM    296    320       NON CYTOPLASMIC.
FT   TRANSMEM    321    343
FT   TOPO_DOM    344    363       CYTOPLASMIC.
FT   TRANSMEM    364    383
FT   TOPO_DOM    384    430       NON CYTOPLASMIC.
FT   TRANSMEM    431    452
FT   TOPO_DOM    453    458       CYTOPLASMIC.
FT   TRANSMEM    459    481
FT   TOPO_DOM    482    500       NON CYTOPLASMIC.
FT   TRANSMEM    501    522
FT   TOPO_DOM    523    528       CYTOPLASMIC.
FT   TRANSMEM    529    548
FT   TOPO_DOM    549    898       NON CYTOPLASMIC.
//
```

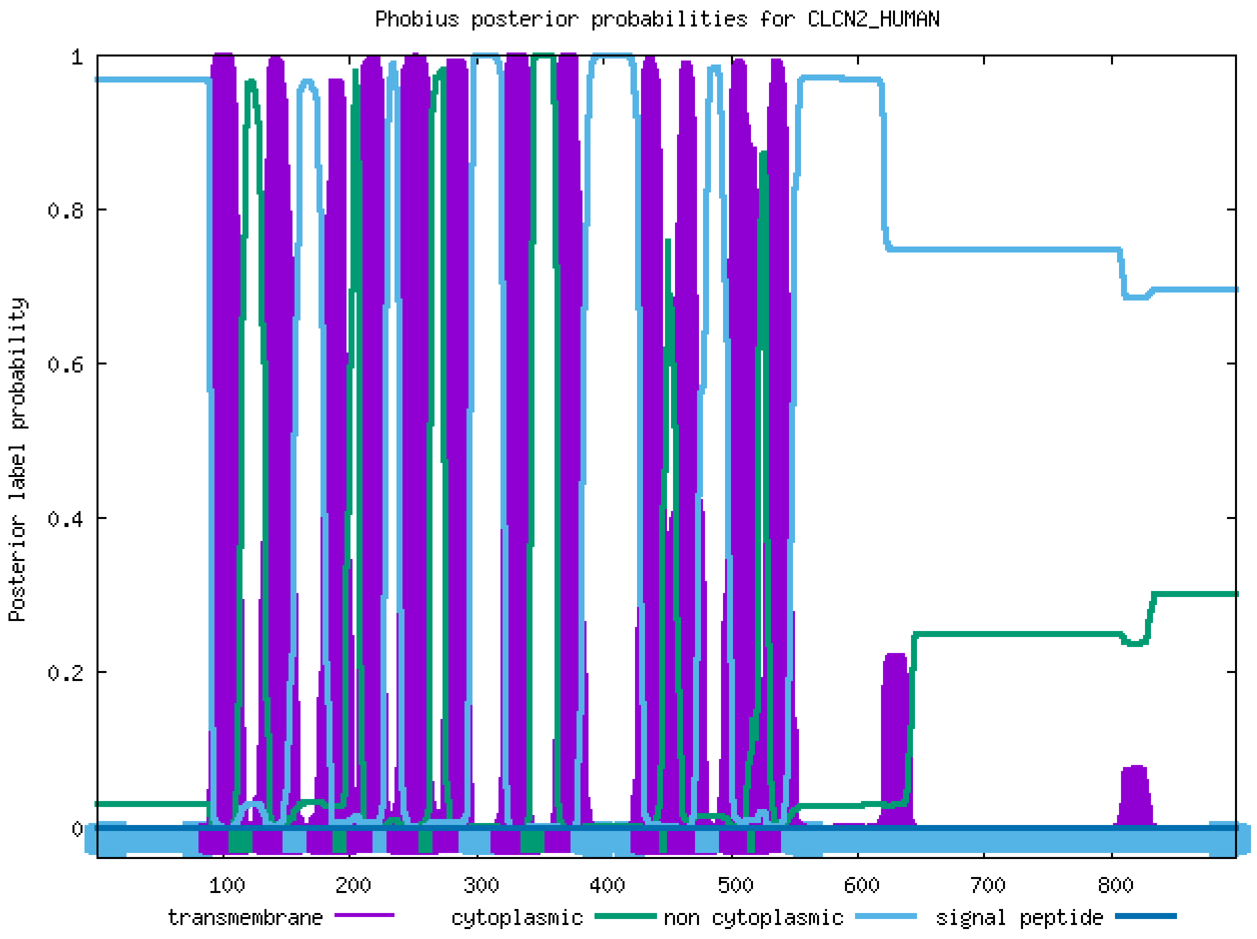

The probability data used in the plot is found here, and the gnuplot script is here.

Prediction of LRC8A_HUMAN

```
ID   LRC8A_HUMAN
FT   TOPO_DOM      1     26       CYTOPLASMIC.
FT   TRANSMEM     27     45
FT   TOPO_DOM     46    123       NON CYTOPLASMIC.
FT   TRANSMEM    124    144
FT   TOPO_DOM    145    268       CYTOPLASMIC.
FT   TRANSMEM    269    287
FT   TOPO_DOM    288    321       NON CYTOPLASMIC.
FT   TRANSMEM    322    344
FT   TOPO_DOM    345    810       CYTOPLASMIC.
//
```

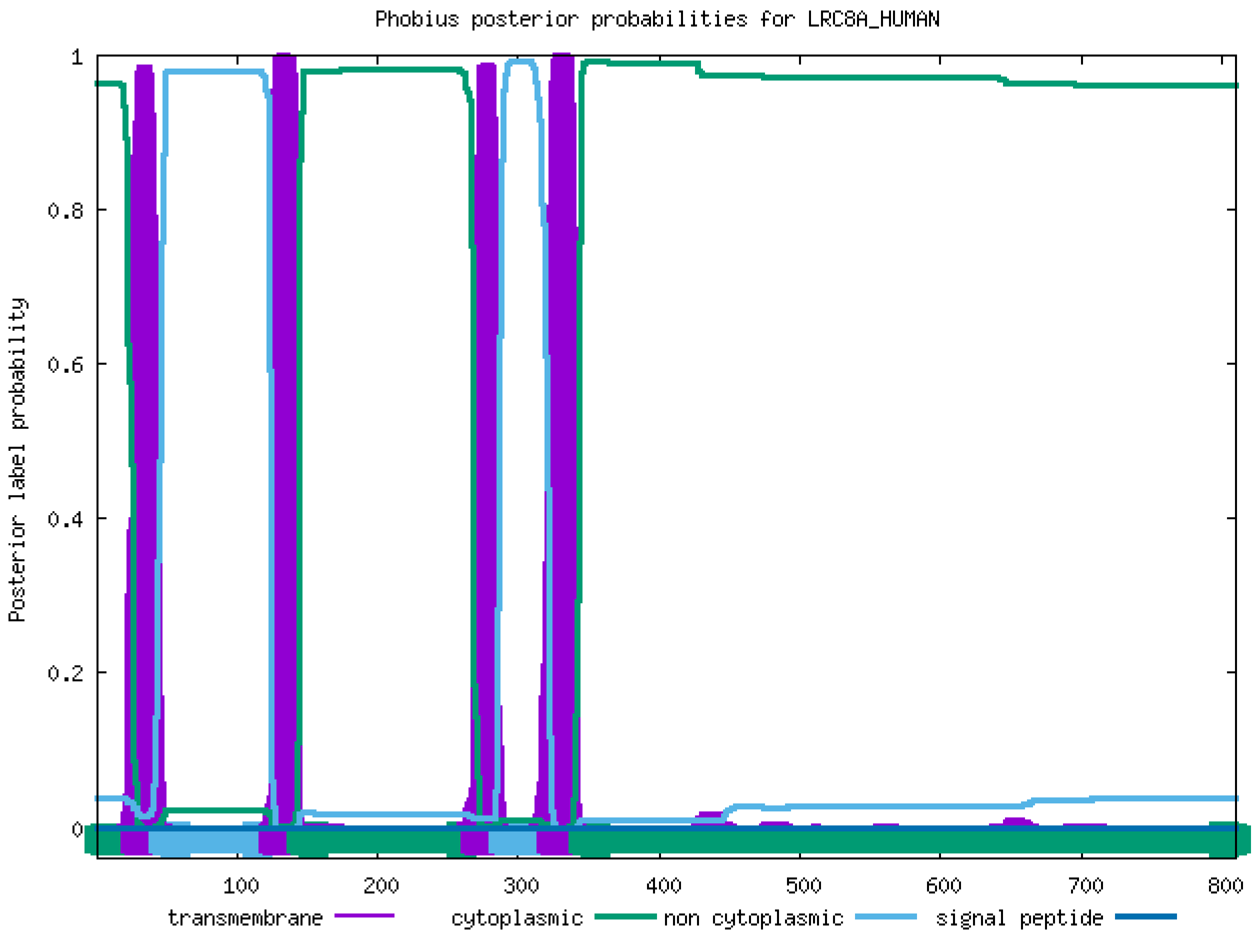

The probability data used in the plot is found here, and the gnuplot script is here.

Prediction of LRC8C_HUMAN

```
ID   LRC8C_HUMAN
FT   TOPO_DOM      1     29       NON CYTOPLASMIC.
FT   TRANSMEM     30     48
FT   TOPO_DOM     49    125       CYTOPLASMIC.
FT   TRANSMEM    126    146
FT   TOPO_DOM    147    256       NON CYTOPLASMIC.
FT   TRANSMEM    257    279
FT   TOPO_DOM    280    320       CYTOPLASMIC.
FT   TRANSMEM    321    342
FT   TOPO_DOM    343    803       NON CYTOPLASMIC.
//
```

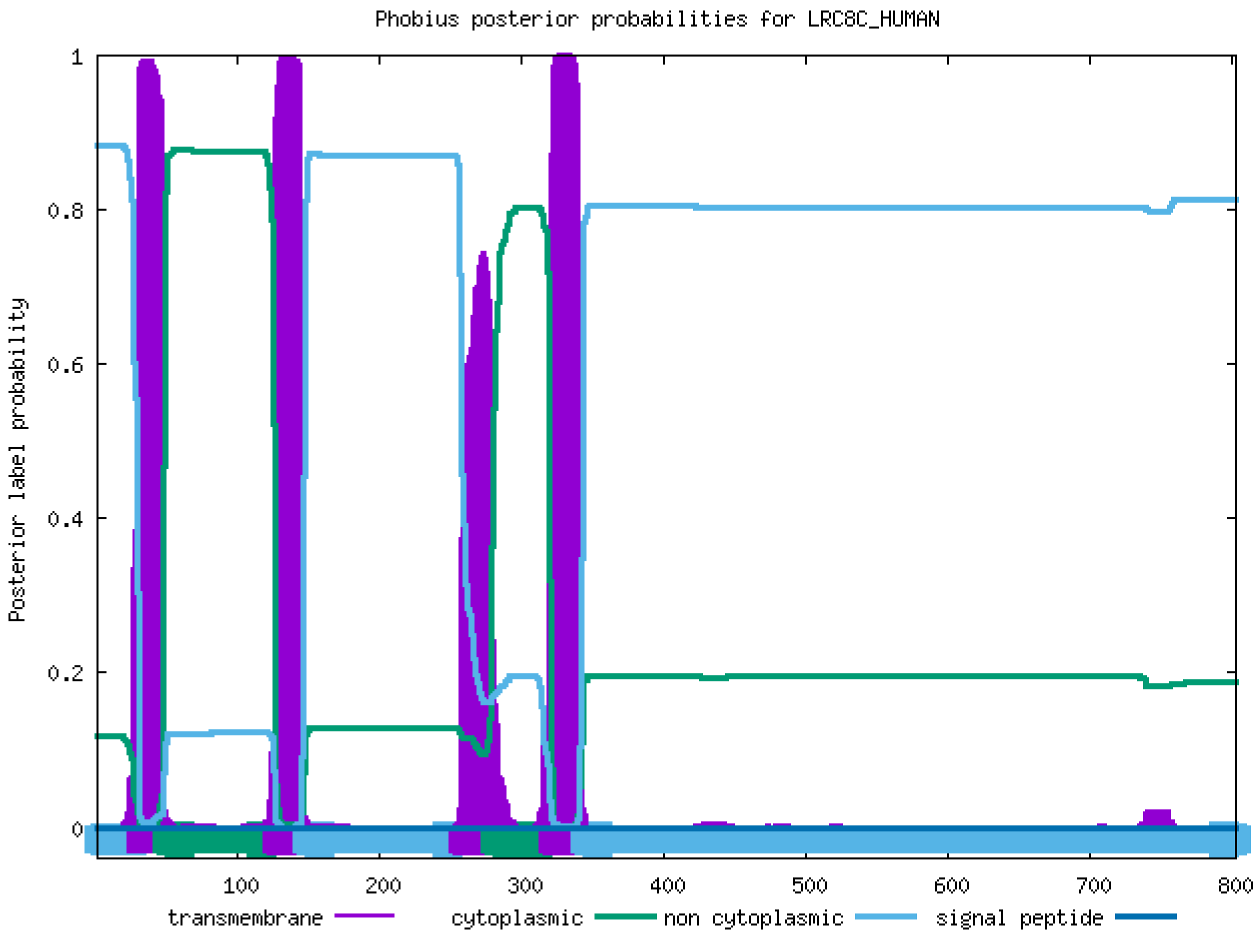

The probability data used in the plot is found here, and the gnuplot script is here.

Prediction of LRC8D_HUMAN

```
ID   LRC8D_HUMAN
FT   TOPO_DOM      1     26       CYTOPLASMIC.
FT   TRANSMEM     27     47
FT   TOPO_DOM     48    163       NON CYTOPLASMIC.
FT   TRANSMEM    164    184
FT   TOPO_DOM    185    365       CYTOPLASMIC.
FT   TRANSMEM    366    388
FT   TOPO_DOM    389    858       NON CYTOPLASMIC.
//
```

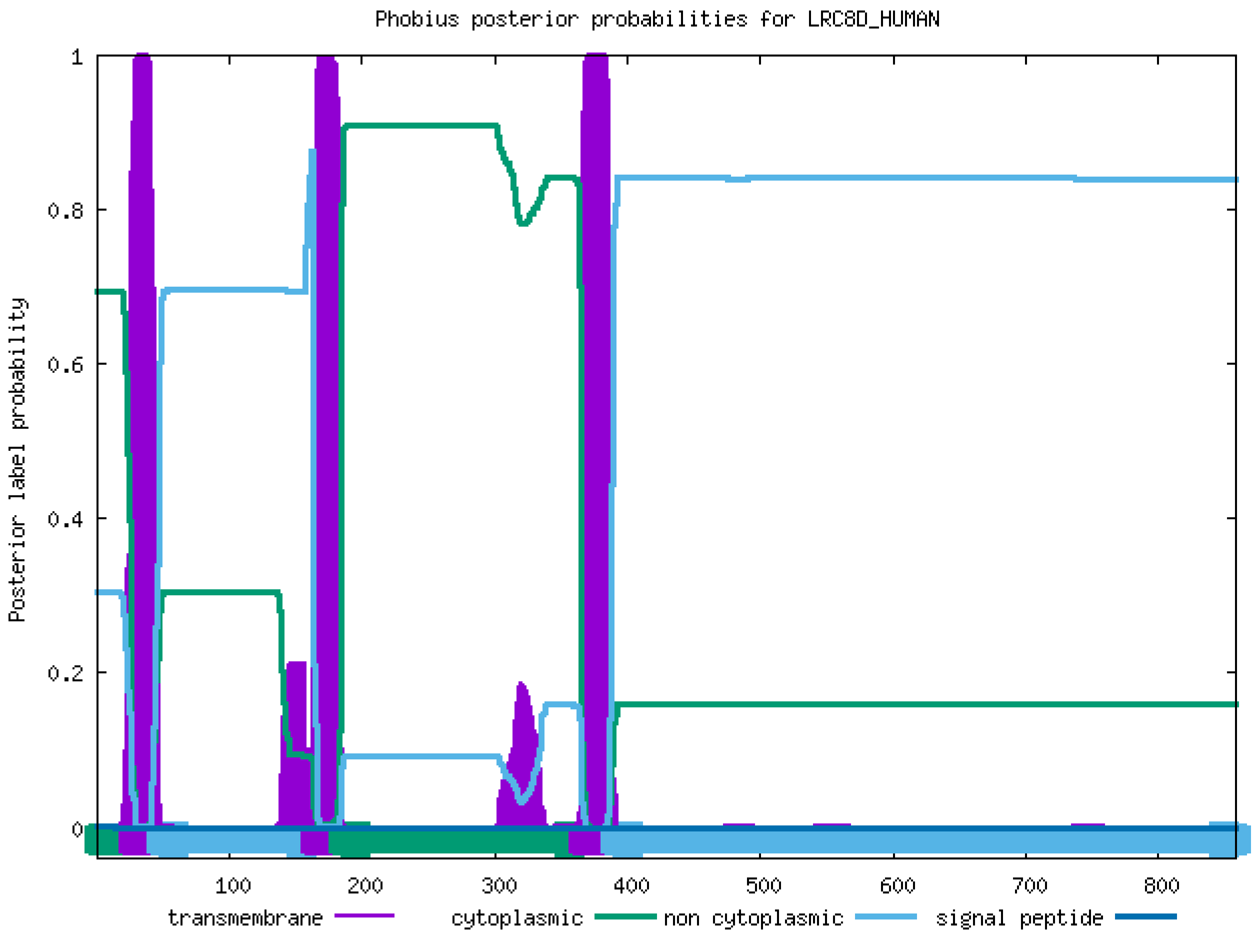

The probability data used in the plot is found here, and the gnuplot script is here.

Prediction of PANX1_HUMAN

```
ID   PANX1_HUMAN
FT   TOPO_DOM      1     32       CYTOPLASMIC.
FT   TRANSMEM     33     55
FT   TOPO_DOM     56    107       NON CYTOPLASMIC.
FT   TRANSMEM    108    127
FT   TOPO_DOM    128    209       CYTOPLASMIC.
FT   TRANSMEM    210    235
FT   TOPO_DOM    236    266       NON CYTOPLASMIC.
FT   TRANSMEM    267    297
FT   TOPO_DOM    298    426       CYTOPLASMIC.
//
```

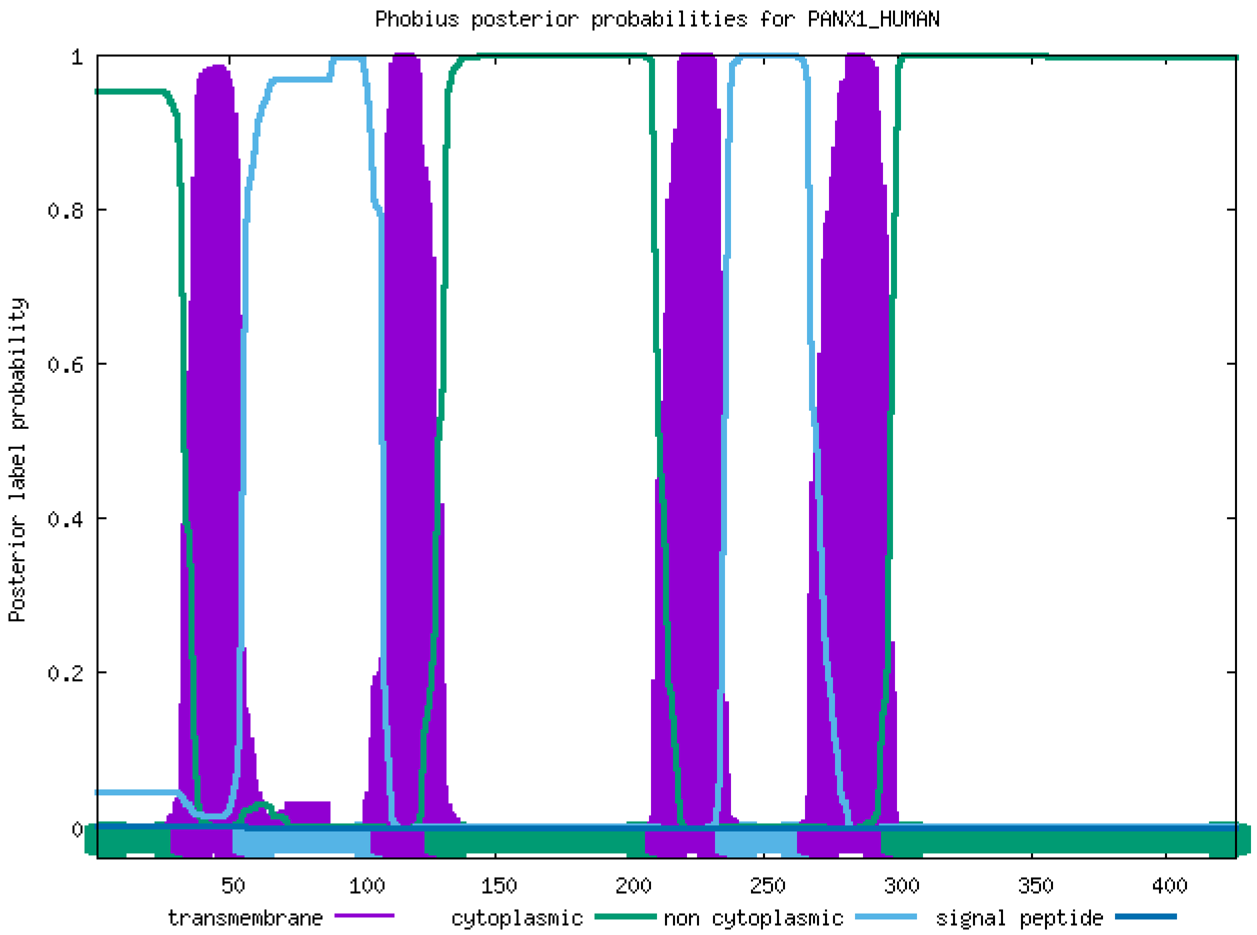

The probability data used in the plot is found here, and the gnuplot script is here.

Prediction of PANX2_HUMAN

```
ID   PANX2_HUMAN
FT   TOPO_DOM      1     52       CYTOPLASMIC.
FT   TRANSMEM     53     70
FT   TOPO_DOM     71    122       NON CYTOPLASMIC.
FT   TRANSMEM    123    145
FT   TOPO_DOM    146    226       CYTOPLASMIC.
FT   TRANSMEM    227    250
FT   TOPO_DOM    251    288       NON CYTOPLASMIC.
FT   TRANSMEM    289    315
FT   TOPO_DOM    316    677       CYTOPLASMIC.
//
```

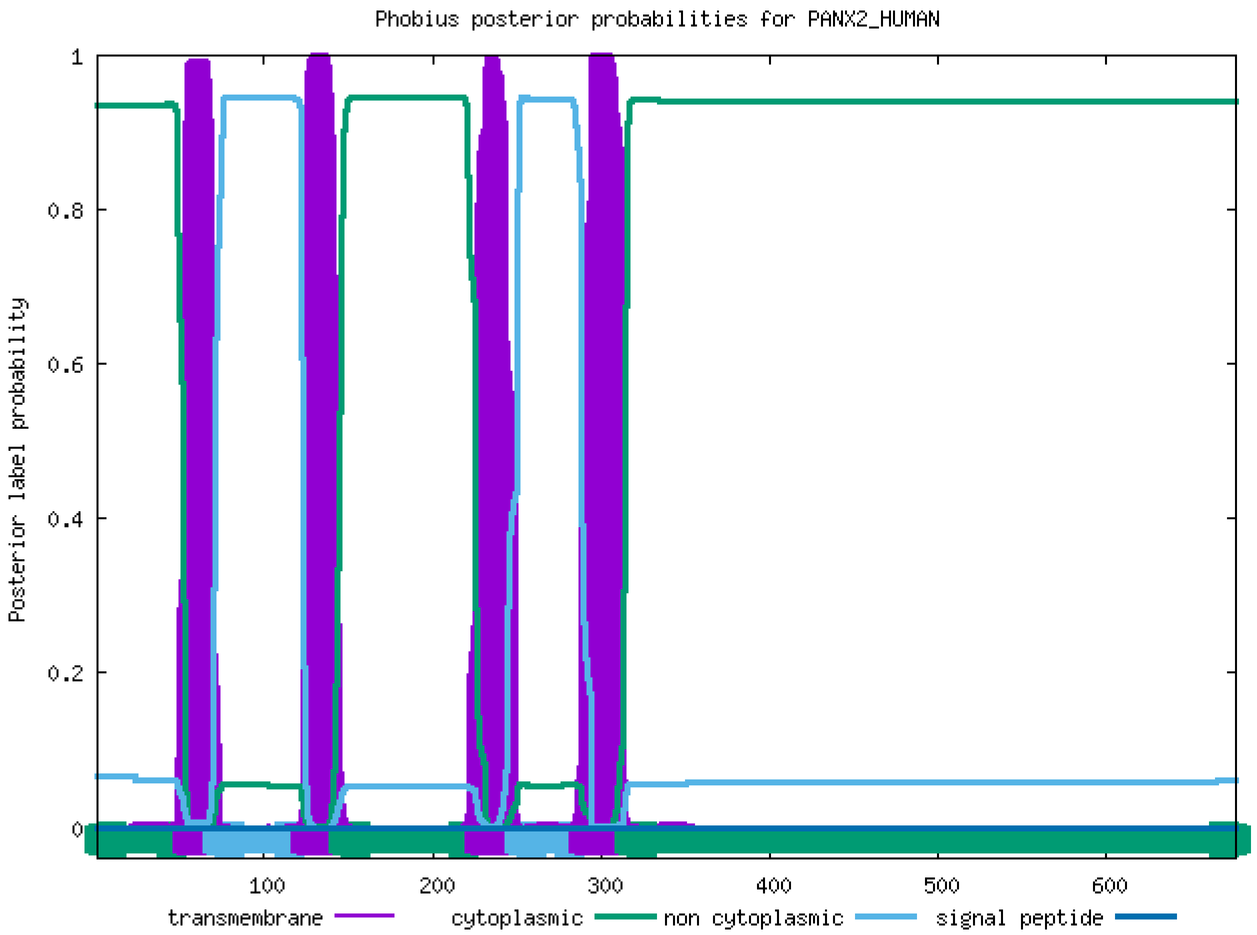

The probability data used in the plot is found here, and the gnuplot script is here.

Prediction of PANX3_HUMAN

```
ID   PANX3_HUMAN
FT   TOPO_DOM      1     36       CYTOPLASMIC.
FT   TRANSMEM     37     55
FT   TOPO_DOM     56    108       NON CYTOPLASMIC.
FT   TRANSMEM    109    128
FT   TOPO_DOM    129    208       CYTOPLASMIC.
FT   TRANSMEM    209    227
FT   TOPO_DOM    228    267       NON CYTOPLASMIC.
FT   TRANSMEM    268    291
FT   TOPO_DOM    292    392       CYTOPLASMIC.
//
```

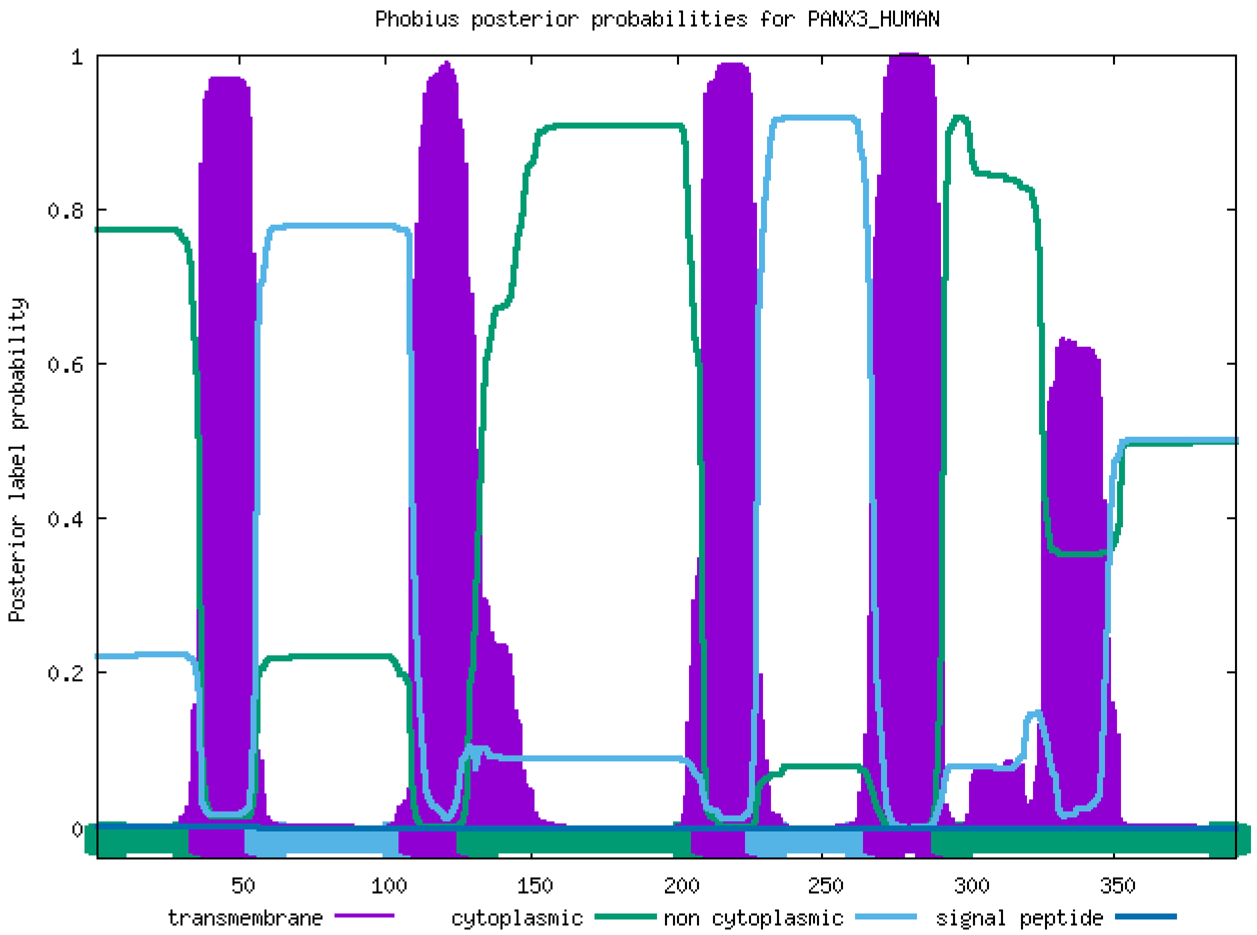

The probability data used in the plot is found here, and the gnuplot script is here.

Prediction of AQP1_HUMAN

```
ID   AQP1_HUMAN
FT   TOPO_DOM      1      8       CYTOPLASMIC.
FT   TRANSMEM      9     33
FT   TOPO_DOM     34     52       NON CYTOPLASMIC.
FT   TRANSMEM     53     73
FT   TOPO_DOM     74     93       CYTOPLASMIC.
FT   TRANSMEM     94    119
FT   TOPO_DOM    120    138       NON CYTOPLASMIC.
FT   TRANSMEM    139    157
FT   TOPO_DOM    158    163       CYTOPLASMIC.
FT   TRANSMEM    164    184
FT   TOPO_DOM    185    209       NON CYTOPLASMIC.
FT   TRANSMEM    210    231
FT   TOPO_DOM    232    269       CYTOPLASMIC.
//
```

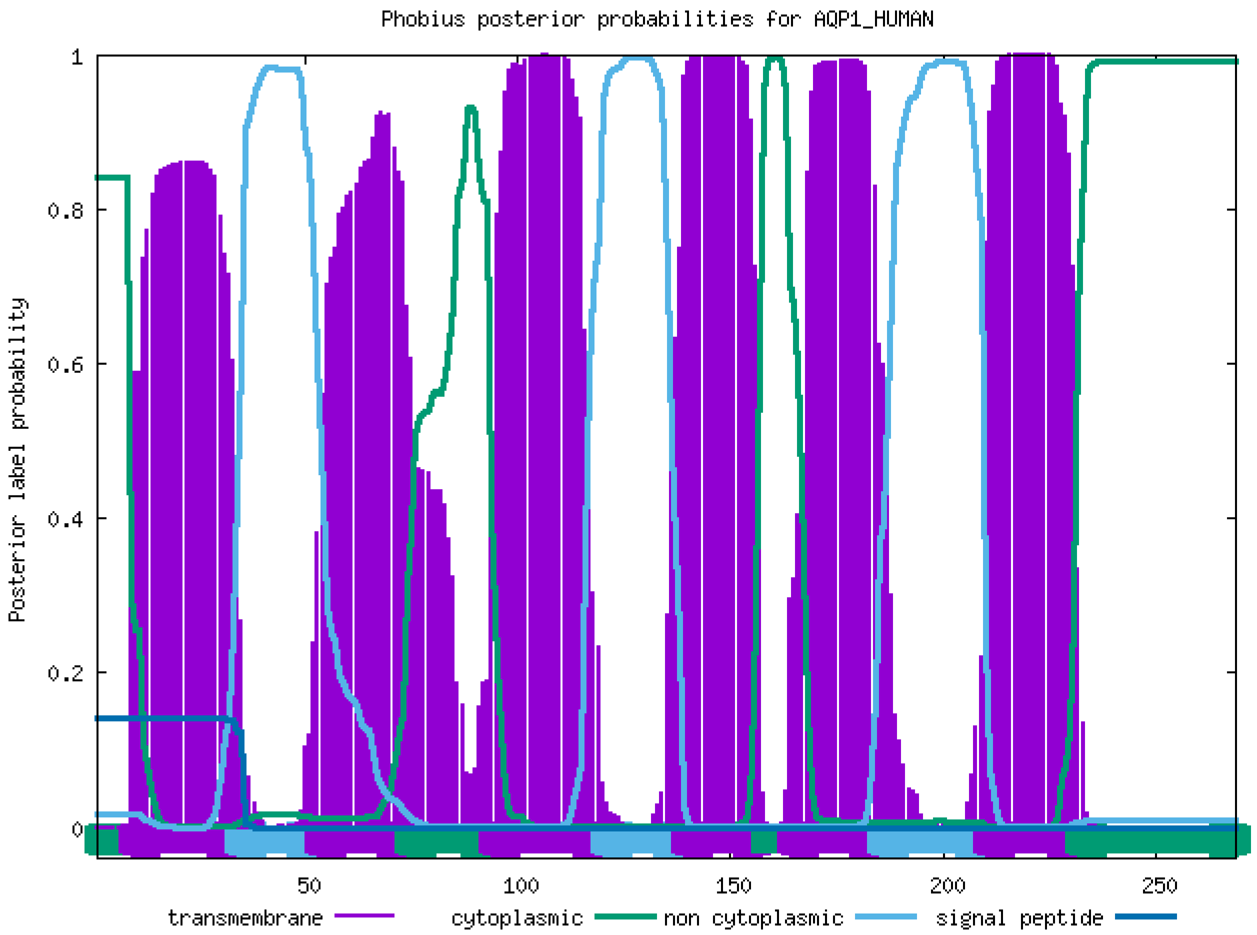

The probability data used in the plot is found here, and the gnuplot script is here.

Prediction of AQP2_HUMAN

```
ID    AQP2_HUMAN
FT    TOPO_DOM      1      11       CYTOPLASMIC.
FT    TRANSMEM     12      34
FT    TOPO_DOM     35      39       NON CYTOPLASMIC.
FT    TRANSMEM     40      59
FT    TOPO_DOM     60      70       CYTOPLASMIC.
FT    TRANSMEM     71      92
FT    TOPO_DOM     93     129       NON CYTOPLASMIC.
FT    TRANSMEM    130     149
FT    TOPO_DOM    150     160       CYTOPLASMIC.
FT    TRANSMEM    161     180
FT    TOPO_DOM    181     201       NON CYTOPLASMIC.
FT    TRANSMEM    202     224
FT    TOPO_DOM    225     271       CYTOPLASMIC.
//
```

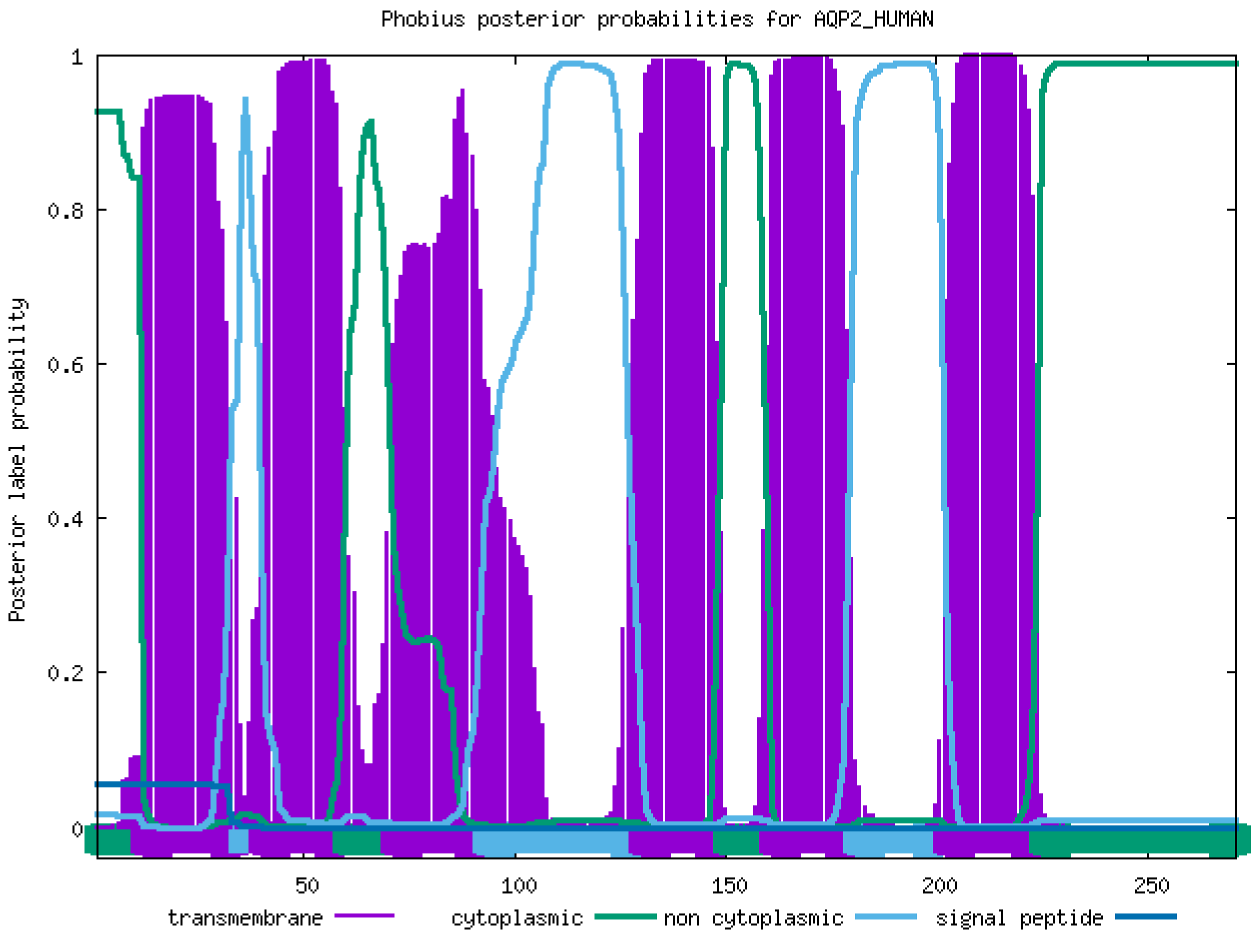

The probability data used in the plot is found here, and the gnuplot script is here.

Prediction of AQP4_HUMAN

```
ID    AQP4_HUMAN
FT    TOPO_DOM      1     32       CYTOPLASMIC.
FT    TRANSMEM     33     51
FT    TOPO_DOM     52     70       NON CYTOPLASMIC.
FT    TRANSMEM     71     94
FT    TOPO_DOM     95    114       CYTOPLASMIC.
FT    TRANSMEM    115    136
FT    TOPO_DOM    137    155       NON CYTOPLASMIC.
FT    TRANSMEM    156    177
FT    TOPO_DOM    178    188       CYTOPLASMIC.
FT    TRANSMEM    189    211
FT    TOPO_DOM    212    230       NON CYTOPLASMIC.
FT    TRANSMEM    231    253
FT    TOPO_DOM    254    323       CYTOPLASMIC.
//
```

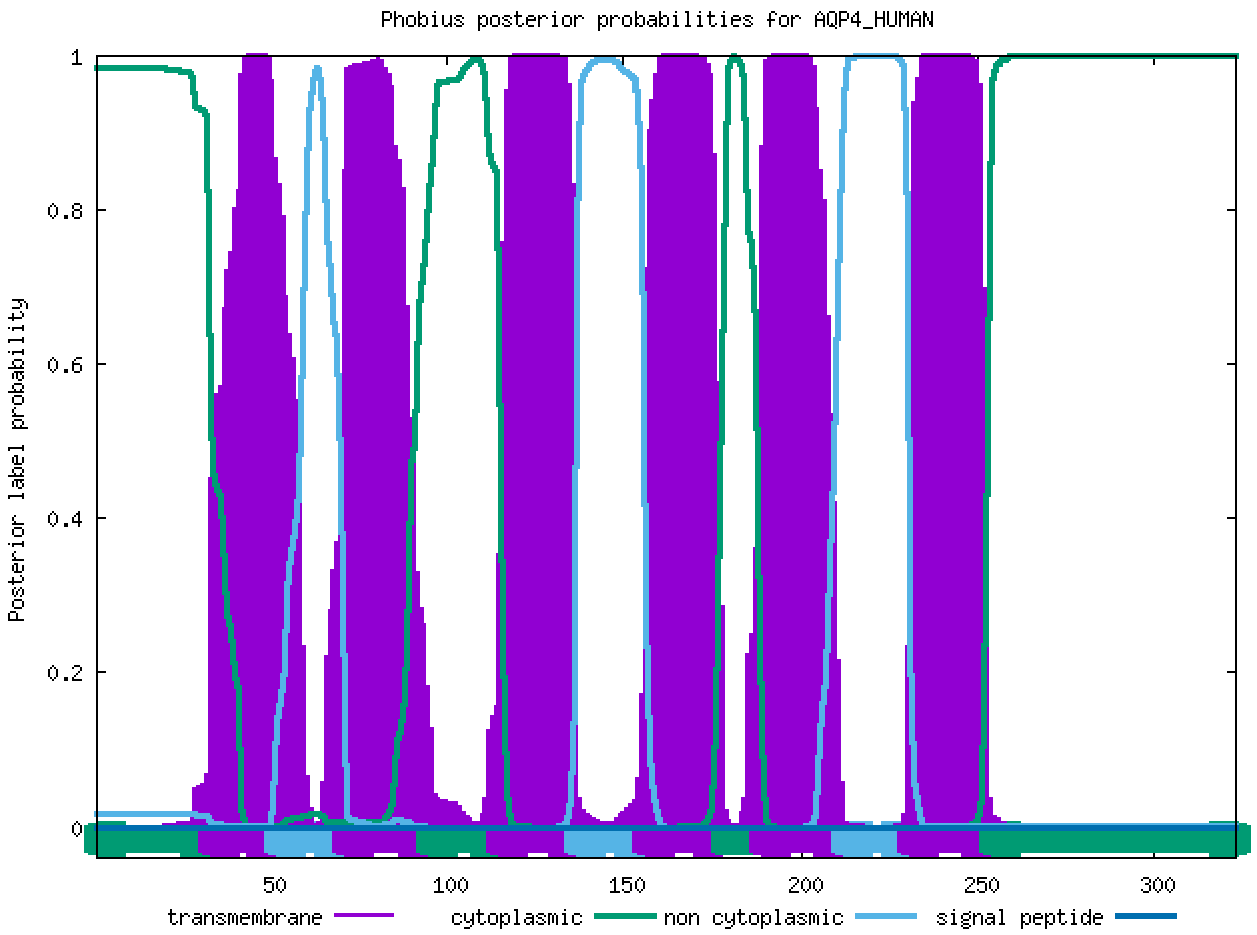

The probability data used in the plot is found here, and the gnuplot script is here.

Prediction of AQP5_HUMAN

```
ID   AQP5_HUMAN
FT   TOPO_DOM      1      8       CYTOPLASMIC.
FT   TRANSMEM      9     29
FT   TOPO_DOM     30     40       NON CYTOPLASMIC.
FT   TRANSMEM     41     63
FT   TOPO_DOM     64     83       CYTOPLASMIC.
FT   TRANSMEM     84    109
FT   TOPO_DOM    110    128       NON CYTOPLASMIC.
FT   TRANSMEM    129    150
FT   TOPO_DOM    151    161       CYTOPLASMIC.
FT   TRANSMEM    162    181
FT   TOPO_DOM    182    203       NON CYTOPLASMIC.
FT   TRANSMEM    204    226
FT   TOPO_DOM    227    265       CYTOPLASMIC.
//
```

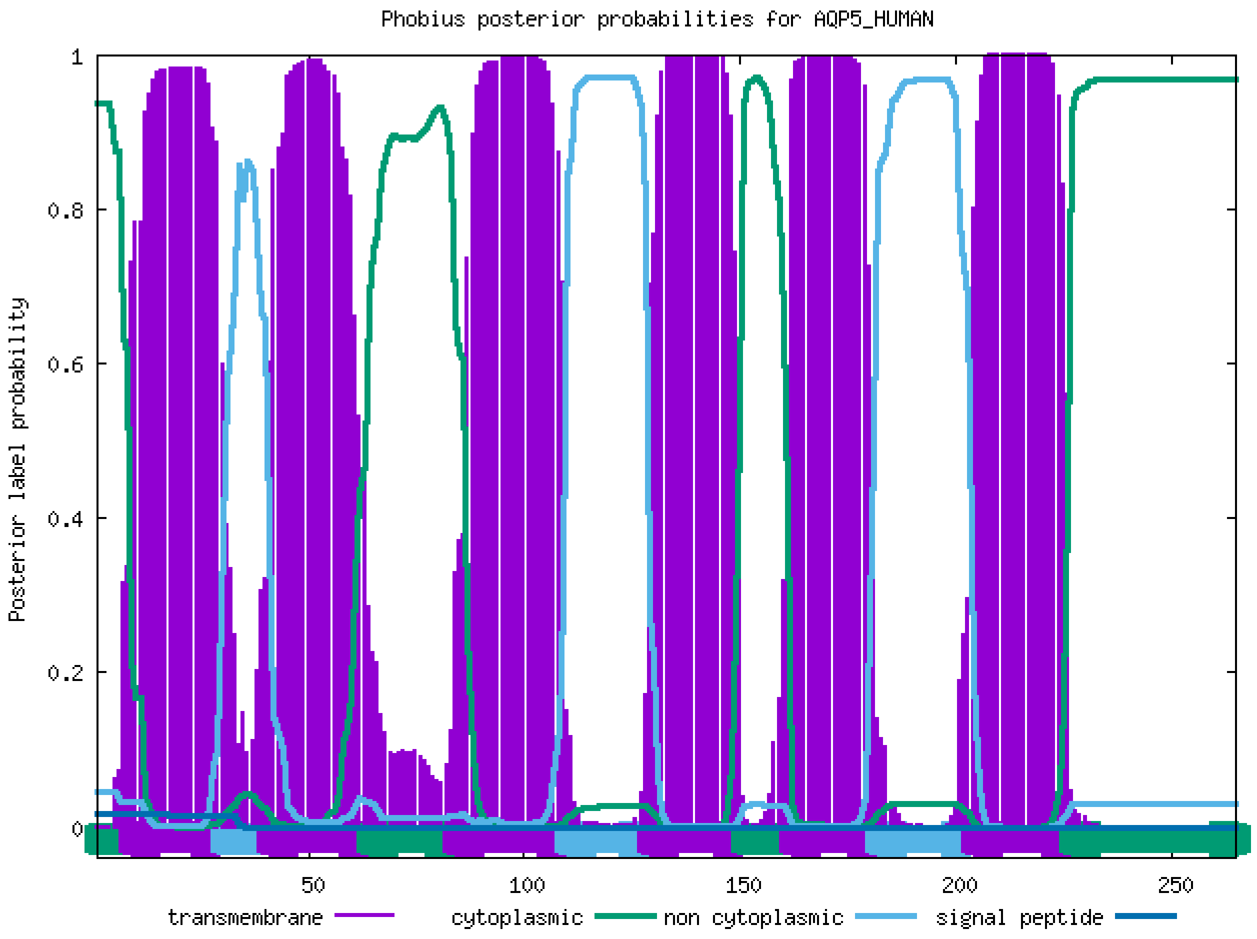

The probability data used in the plot is found here, and the gnuplot script is here.

Prediction of AQP7_HUMAN

```
ID    AQP7_HUMAN
FT    TOPO_DOM      1      36       CYTOPLASMIC.
FT    TRANSMEM     37      60
FT    TOPO_DOM     61      65       NON CYTOPLASMIC.
FT    TRANSMEM     66      85
FT    TOPO_DOM     86     113       CYTOPLASMIC.
FT    TRANSMEM    114     138
FT    TOPO_DOM    139     143       NON CYTOPLASMIC.
FT    TRANSMEM    144     162
FT    TOPO_DOM    163     202       CYTOPLASMIC.
FT    TRANSMEM    203     225
FT    TOPO_DOM    226     252       NON CYTOPLASMIC.
FT    TRANSMEM    253     276
FT    TOPO_DOM    277     342       CYTOPLASMIC.
//
```

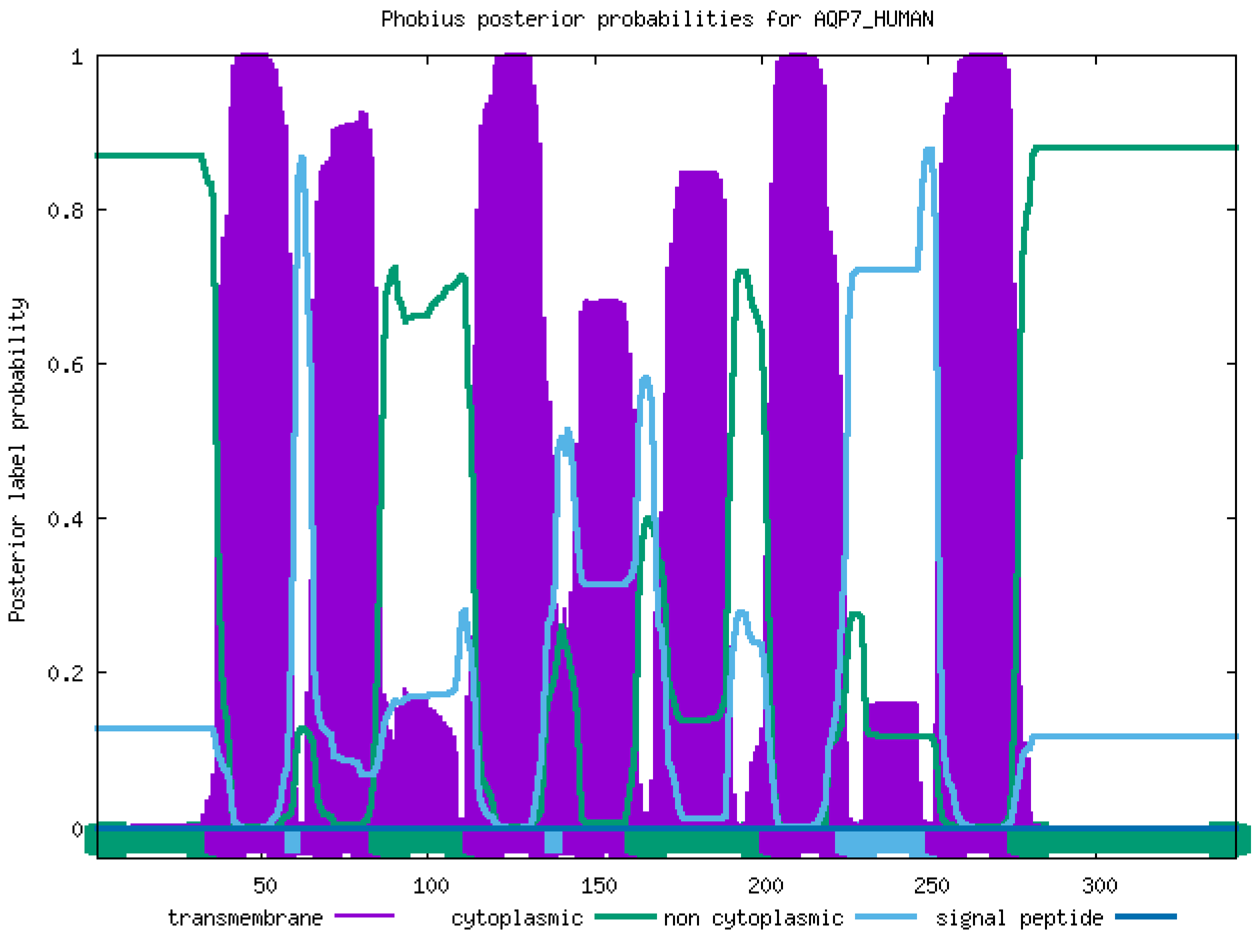

The probability data used in the plot is found here, and the gnuplot script is here.

Prediction of AQP10_HUMAN

```
ID    AQP10_HUMAN
FT    TOPO_DOM      1      19       NON CYTOPLASMIC.
FT    TRANSMEM     20      43
FT    TOPO_DOM     44      54       CYTOPLASMIC.
FT    TRANSMEM     55      73
FT    TOPO_DOM     74      78       NON CYTOPLASMIC.
FT    TRANSMEM     79      95
FT    TOPO_DOM     96     101       CYTOPLASMIC.
FT    TRANSMEM    102     122
FT    TOPO_DOM    123     159       NON CYTOPLASMIC.
FT    TRANSMEM    160     179
FT    TOPO_DOM    180     190       CYTOPLASMIC.
FT    TRANSMEM    191     213
FT    TOPO_DOM    214     239       NON CYTOPLASMIC.
FT    TRANSMEM    240     263
FT    TOPO_DOM    264     301       CYTOPLASMIC.
//
```

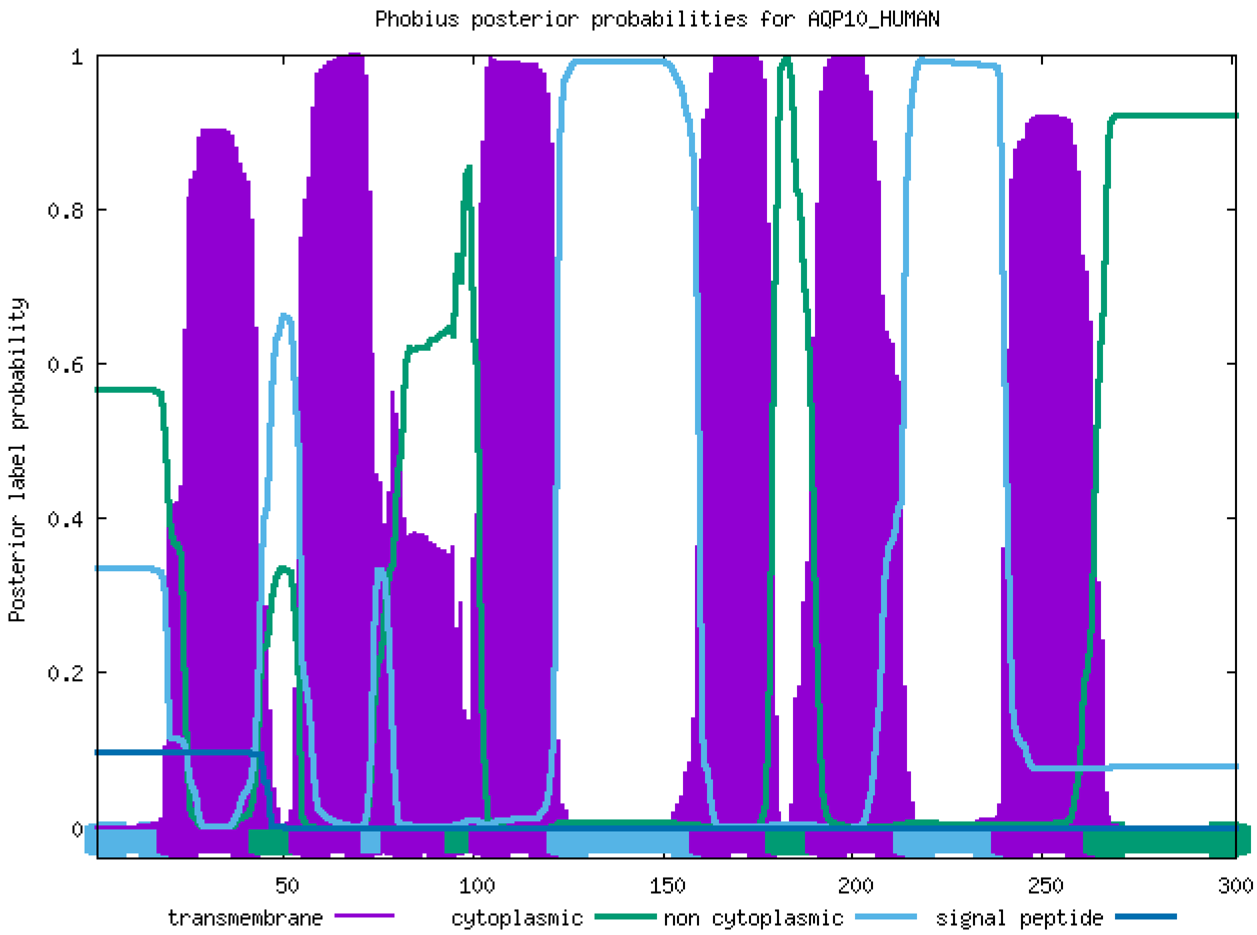

The probability data used in the plot is found here, and the gnuplot script is here.

Prediction of RHCG_HUMAN

```
ID    RHCG_HUMAN
FT    TOPO_DOM      1      11       CYTOPLASMIC.
FT    TRANSMEM     12      32
FT    TOPO_DOM     33      61       NON CYTOPLASMIC.
FT    TRANSMEM     62      81
FT    TOPO_DOM     82      92       CYTOPLASMIC.
FT    TRANSMEM     93     113
FT    TOPO_DOM    114     124       NON CYTOPLASMIC.
FT    TRANSMEM    125     144
FT    TOPO_DOM    145     150       CYTOPLASMIC.
FT    TRANSMEM    151     170
FT    TOPO_DOM    171     181       NON CYTOPLASMIC.
FT    TRANSMEM    182     200
FT    TOPO_DOM    201     211       CYTOPLASMIC.
FT    TRANSMEM    212     231
FT    TOPO_DOM    232     250       NON CYTOPLASMIC.
FT    TRANSMEM    251     271
FT    TOPO_DOM    272     303       CYTOPLASMIC.
FT    TRANSMEM    304     324
FT    TOPO_DOM    325     394       NON CYTOPLASMIC.
FT    TRANSMEM    395     416
FT    TOPO_DOM    417     479       CYTOPLASMIC.
//
```

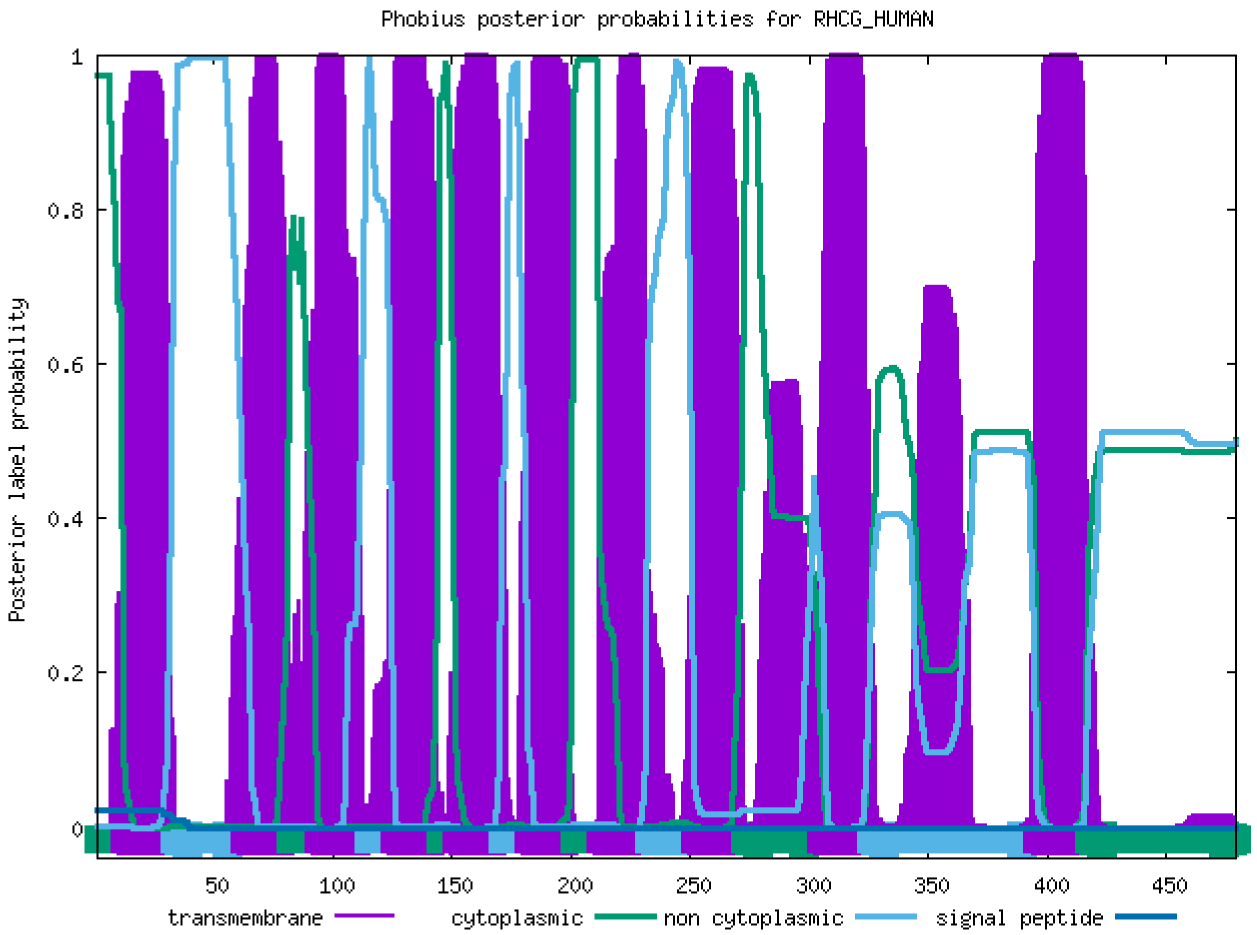

The probability data used in the plot is found here, and the gnuplot script is here.

Prediction of ACHA4_HUMAN

```
ID   ACHA4_HUMAN
FT   SIGNAL        1     36
FT   REGION        1     10       N-REGION.
FT   REGION       11     24       H-REGION.
FT   REGION       25     36       C-REGION.
FT   TOPO_DOM     37    243       NON CYTOPLASMIC.
FT   TRANSMEM    244    268
FT   TOPO_DOM    269    274       CYTOPLASMIC.
FT   TRANSMEM    275    297
FT   TOPO_DOM    298    308       NON CYTOPLASMIC.
FT   TRANSMEM    309    330
FT   TOPO_DOM    331    600       CYTOPLASMIC.
FT   TRANSMEM    601    623
FT   TOPO_DOM    624    627       NON CYTOPLASMIC.
//
```

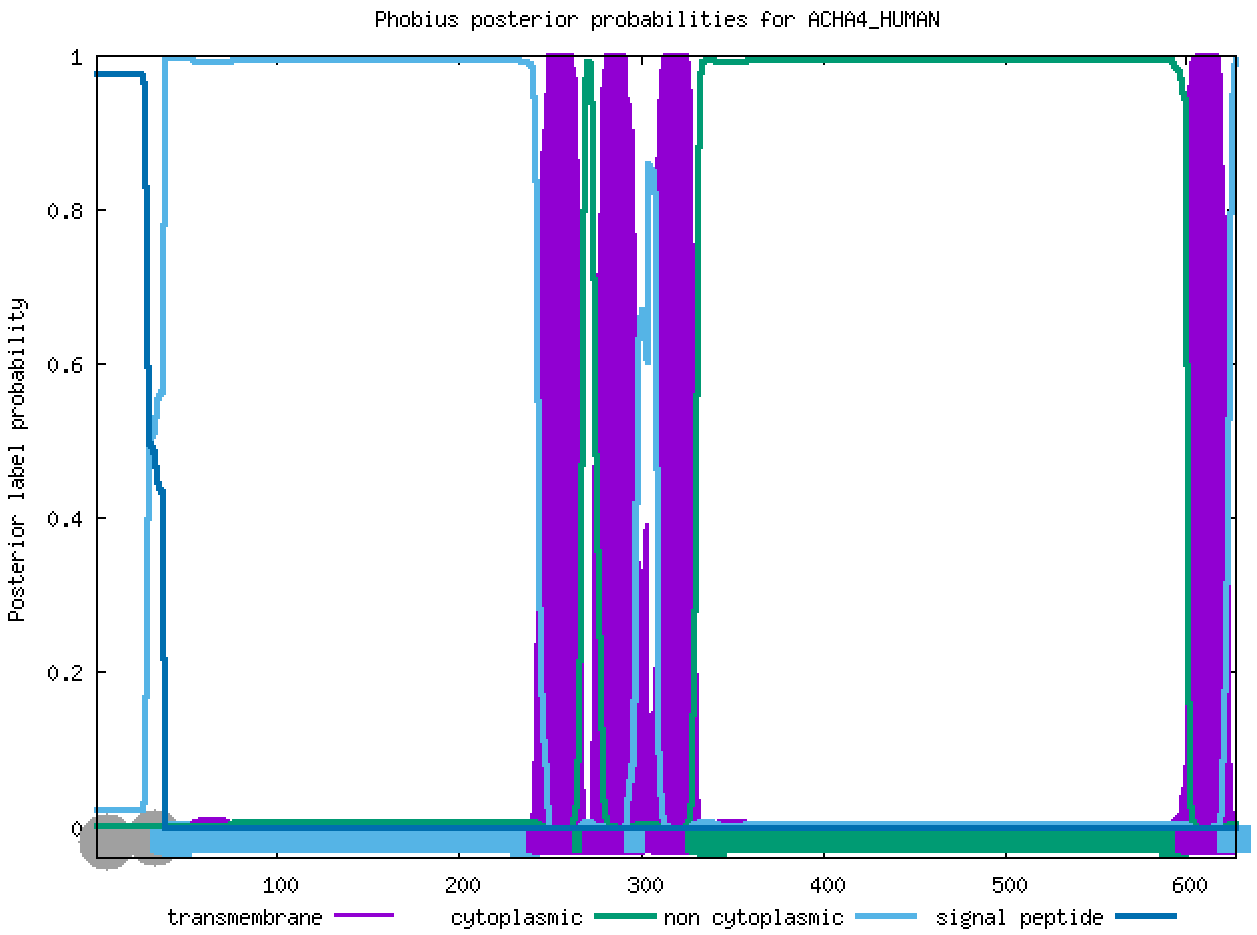

The probability data used in the plot is found here, and the gnuplot script is here.

Prediction of ACHB2_HUMAN

```
ID   ACHB2_HUMAN
FT   SIGNAL        1     25
FT   REGION        1      7       N-REGION.
FT   REGION        8     20       H-REGION.
FT   REGION       21     25       C-REGION.
FT   TOPO_DOM     26    234       NON CYTOPLASMIC.
FT   TRANSMEM    235    256
FT   TOPO_DOM    257    267       CYTOPLASMIC.
FT   TRANSMEM    268    288
FT   TOPO_DOM    289    299       NON CYTOPLASMIC.
FT   TRANSMEM    300    321
FT   TOPO_DOM    322    460       CYTOPLASMIC.
FT   TRANSMEM    461    483
FT   TOPO_DOM    484    502       NON CYTOPLASMIC.
//
```

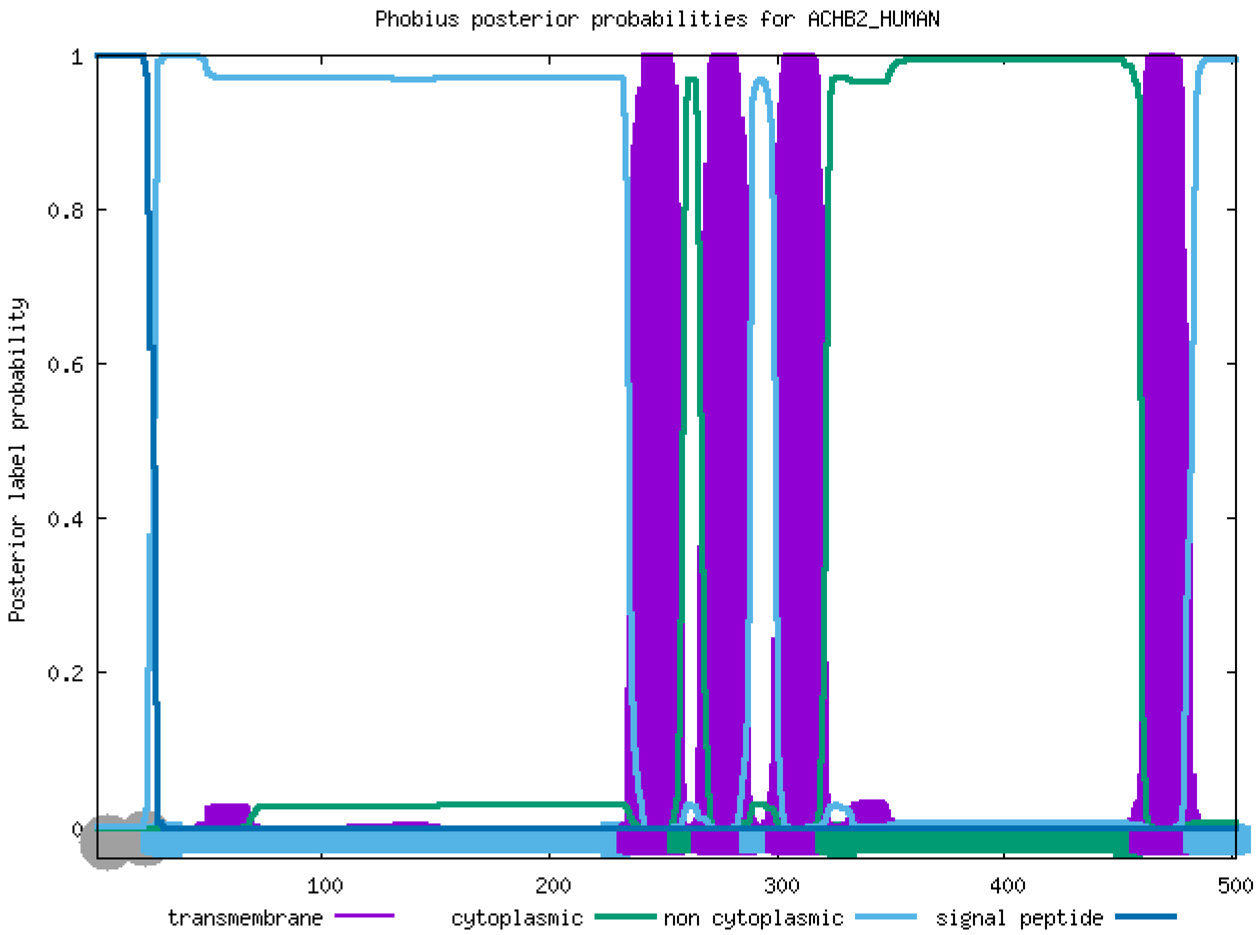

The probability data used in the plot is found here, and the gnuplot script is here.

Prediction of GLRA1_HUMAN

```
ID   GLRA1_HUMAN
FT   TOPO_DOM      1    249       CYTOPLASMIC.
FT   TRANSMEM    250    272
FT   TOPO_DOM    273    312       NON CYTOPLASMIC.
FT   TRANSMEM    313    336
FT   TOPO_DOM    337    428       CYTOPLASMIC.
FT   TRANSMEM    429    449
FT   TOPO_DOM    450    457       NON CYTOPLASMIC.
//
```

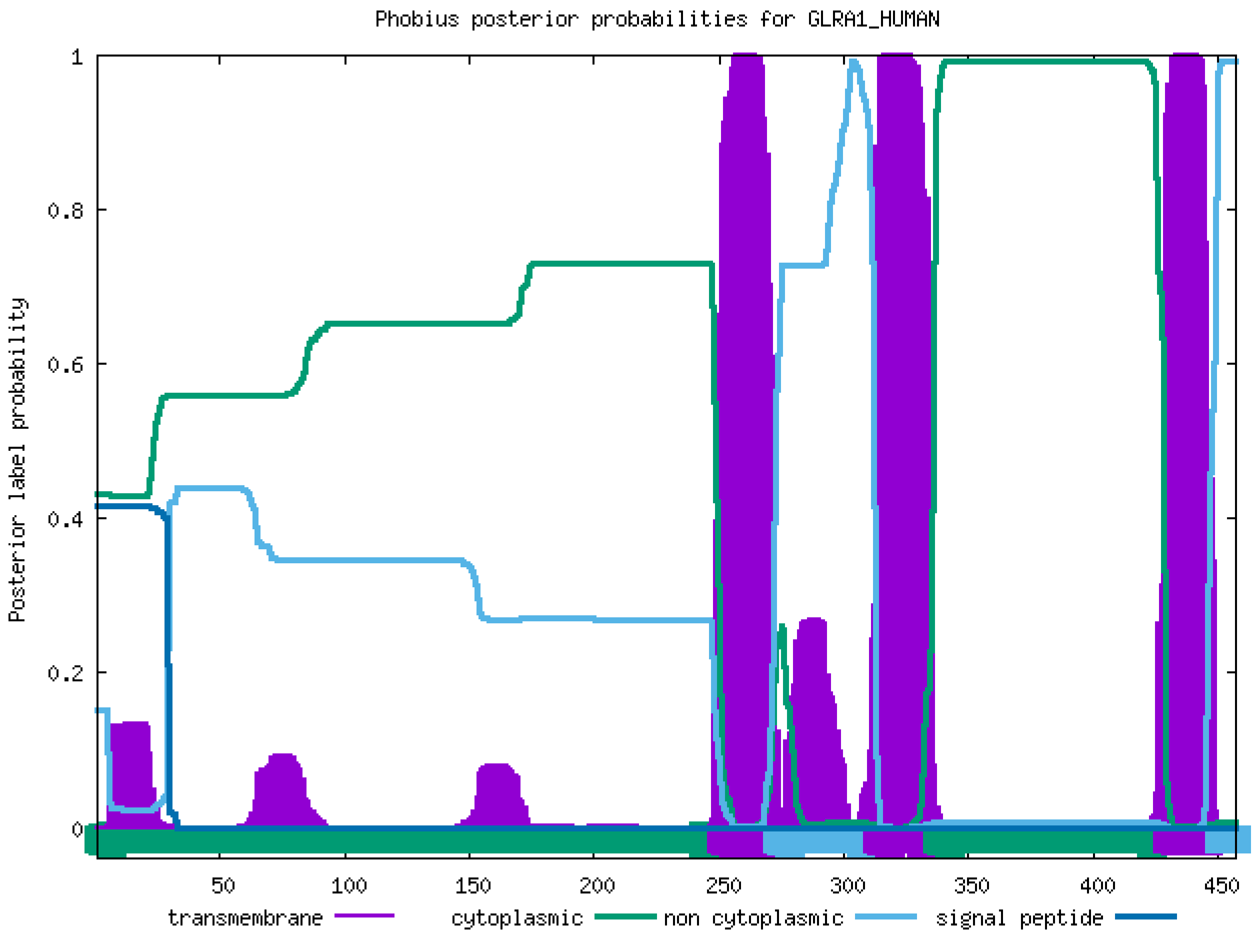

The probability data used in the plot is found here, and the gnuplot script is here.

Prediction of GLRA3_HUMAN

```
ID   GLRA3_HUMAN
FT   TOPO_DOM      1      5       NON CYTOPLASMIC.
FT   TRANSMEM      6     27
FT   TOPO_DOM     28    254       CYTOPLASMIC.
FT   TRANSMEM    255    277
FT   TOPO_DOM    278    317       NON CYTOPLASMIC.
FT   TRANSMEM    318    341
FT   TOPO_DOM    342    433       CYTOPLASMIC.
FT   TRANSMEM    434    454
FT   TOPO_DOM    455    464       NON CYTOPLASMIC.
//
```

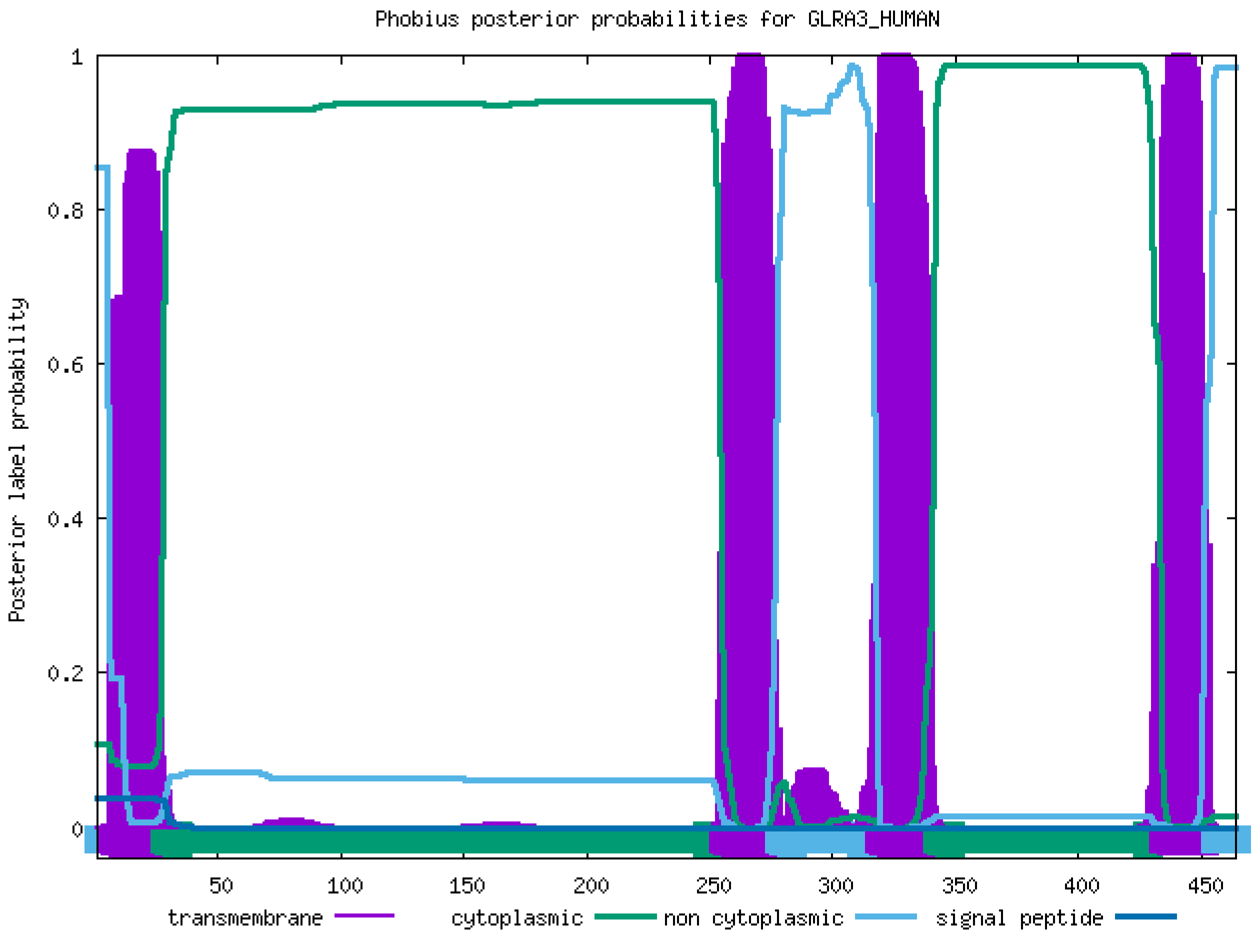

The probability data used in the plot is found here, and the gnuplot script is here.

Prediction of ACHA7_HUMAN

```
ID    ACHA7_HUMAN
FT    SIGNAL         1      22
FT    REGION         1       5       N-REGION.
FT    REGION         6      17       H-REGION.
FT    REGION        18      22       C-REGION.
FT    TOPO_DOM      23     230       NON CYTOPLASMIC.
FT    TRANSMEM     231     255
FT    TOPO_DOM     256     261       CYTOPLASMIC.
FT    TRANSMEM     262     280
FT    TOPO_DOM     281     291       NON CYTOPLASMIC.
FT    TRANSMEM     292     317
FT    TOPO_DOM     318     469       CYTOPLASMIC.
FT    TRANSMEM     470     490
FT    TOPO_DOM     491     502       NON CYTOPLASMIC.
//
```

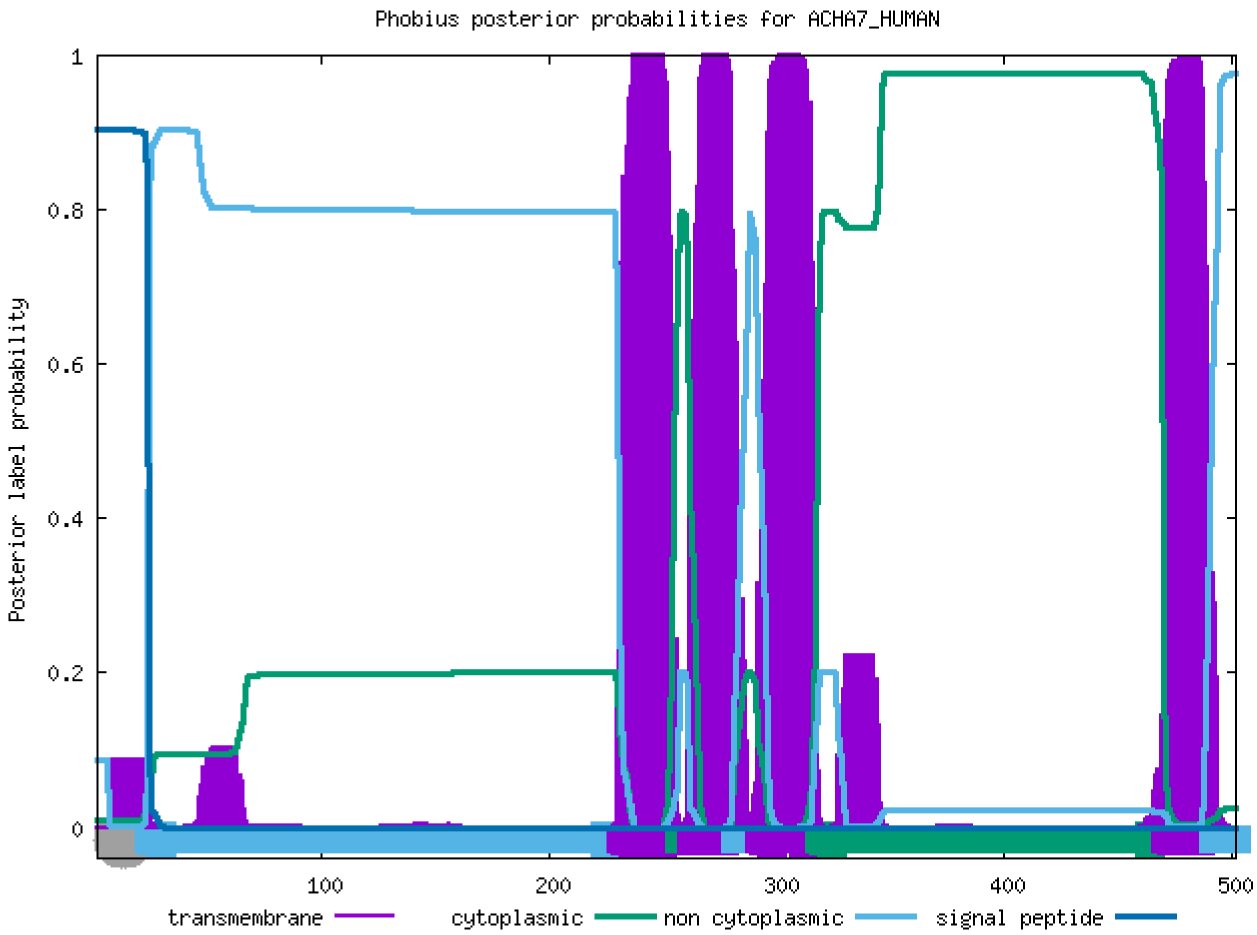

The probability data used in the plot is found here, and the gnuplot script is here.

Prediction of GBRB3_HUMAN

```
ID   GBRB3_HUMAN
FT   SIGNAL        1     25
FT   REGION        1      8       N-REGION.
FT   REGION        9     20       H-REGION.
FT   REGION       21     25       C-REGION.
FT   TOPO_DOM     26    242       NON CYTOPLASMIC.
FT   TRANSMEM    243    265
FT   TOPO_DOM    266    271       CYTOPLASMIC.
FT   TRANSMEM    272    289
FT   TOPO_DOM    290    308       NON CYTOPLASMIC.
FT   TRANSMEM    309    332
FT   TOPO_DOM    333    453       CYTOPLASMIC.
FT   TRANSMEM    454    471
FT   TOPO_DOM    472    473       NON CYTOPLASMIC.
//
```

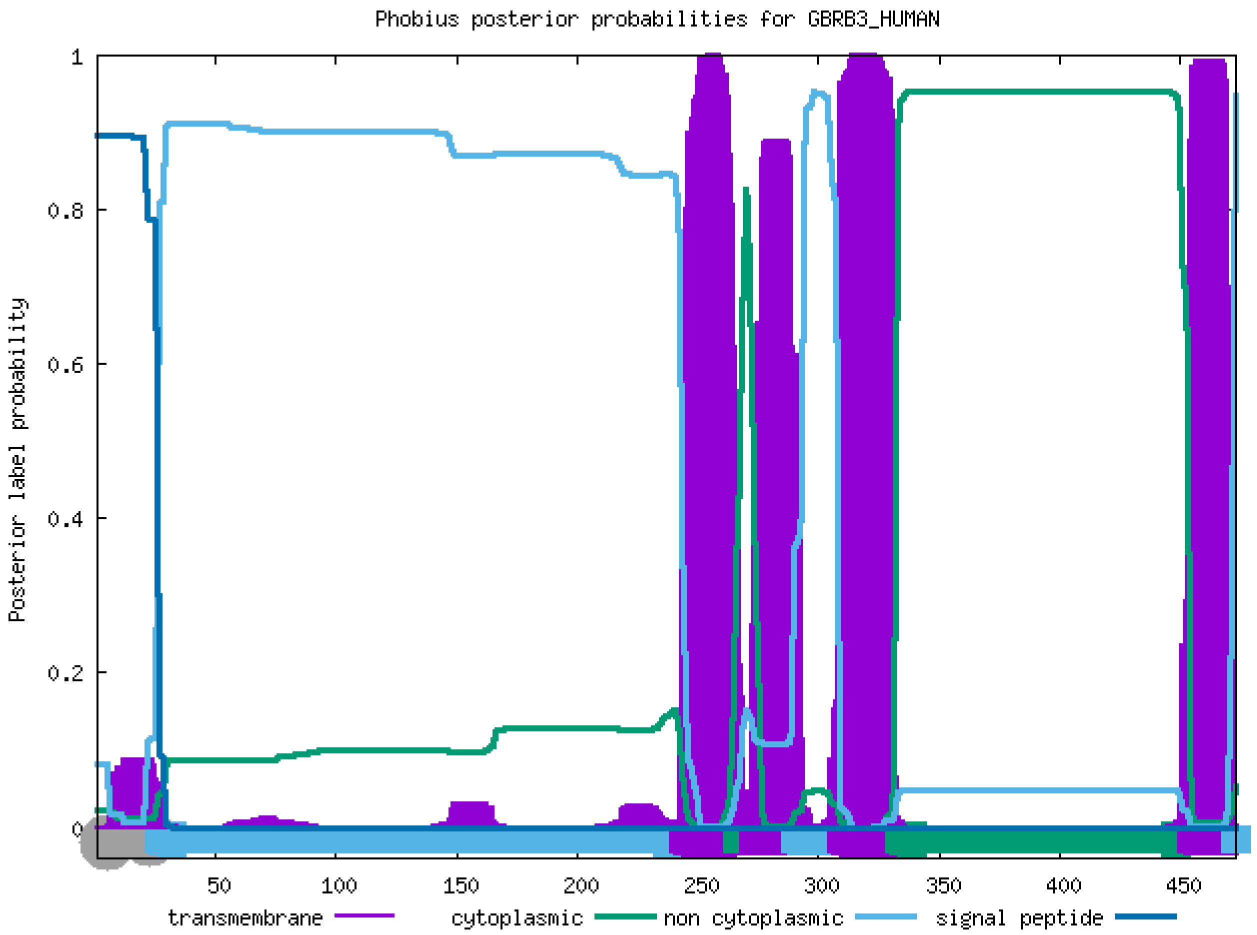

The probability data used in the plot is found here, and the gnuplot script is here.

Prediction of 5HT3A_HUMAN

```
ID   5HT3A_HUMAN
FT   SIGNAL        1     19
FT   REGION        1      2       N-REGION.
FT   REGION        3     14       H-REGION.
FT   REGION       15     19       C-REGION.
FT   TOPO_DOM     20    242       NON CYTOPLASMIC.
FT   TRANSMEM    243    266
FT   TOPO_DOM    267    277       CYTOPLASMIC.
FT   TRANSMEM    278    299
FT   TOPO_DOM    300    304       NON CYTOPLASMIC.
FT   TRANSMEM    305    327
FT   TOPO_DOM    328    451       CYTOPLASMIC.
FT   TRANSMEM    452    475
FT   TOPO_DOM    476    478       NON CYTOPLASMIC.
//
```

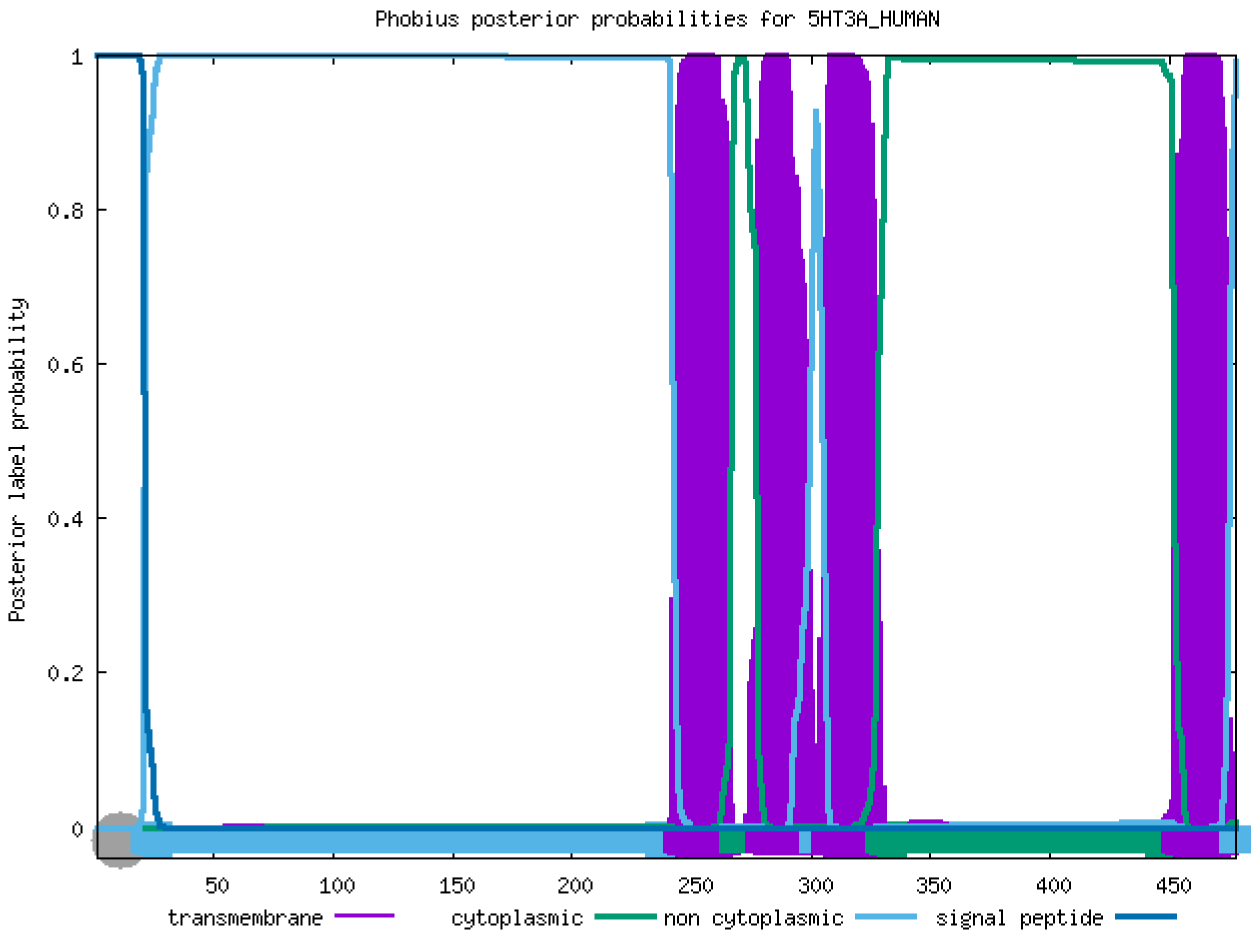

The probability data used in the plot is found here, and the gnuplot script is here.

Prediction of OPSD_HUMAN

```
ID   OPSD_HUMAN
FT   TOPO_DOM      1     39       NON CYTOPLASMIC.
FT   TRANSMEM     40     63
FT   TOPO_DOM     64     74       CYTOPLASMIC.
FT   TRANSMEM     75     95
FT   TOPO_DOM     96    114       NON CYTOPLASMIC.
FT   TRANSMEM    115    133
FT   TOPO_DOM    134    152       CYTOPLASMIC.
FT   TRANSMEM    153    173
FT   TOPO_DOM    174    202       NON CYTOPLASMIC.
FT   TRANSMEM    203    223
FT   TOPO_DOM    224    253       CYTOPLASMIC.
FT   TRANSMEM    254    277
FT   TOPO_DOM    278    282       NON CYTOPLASMIC.
FT   TRANSMEM    283    308
FT   TOPO_DOM    309    348       CYTOPLASMIC.
//
```

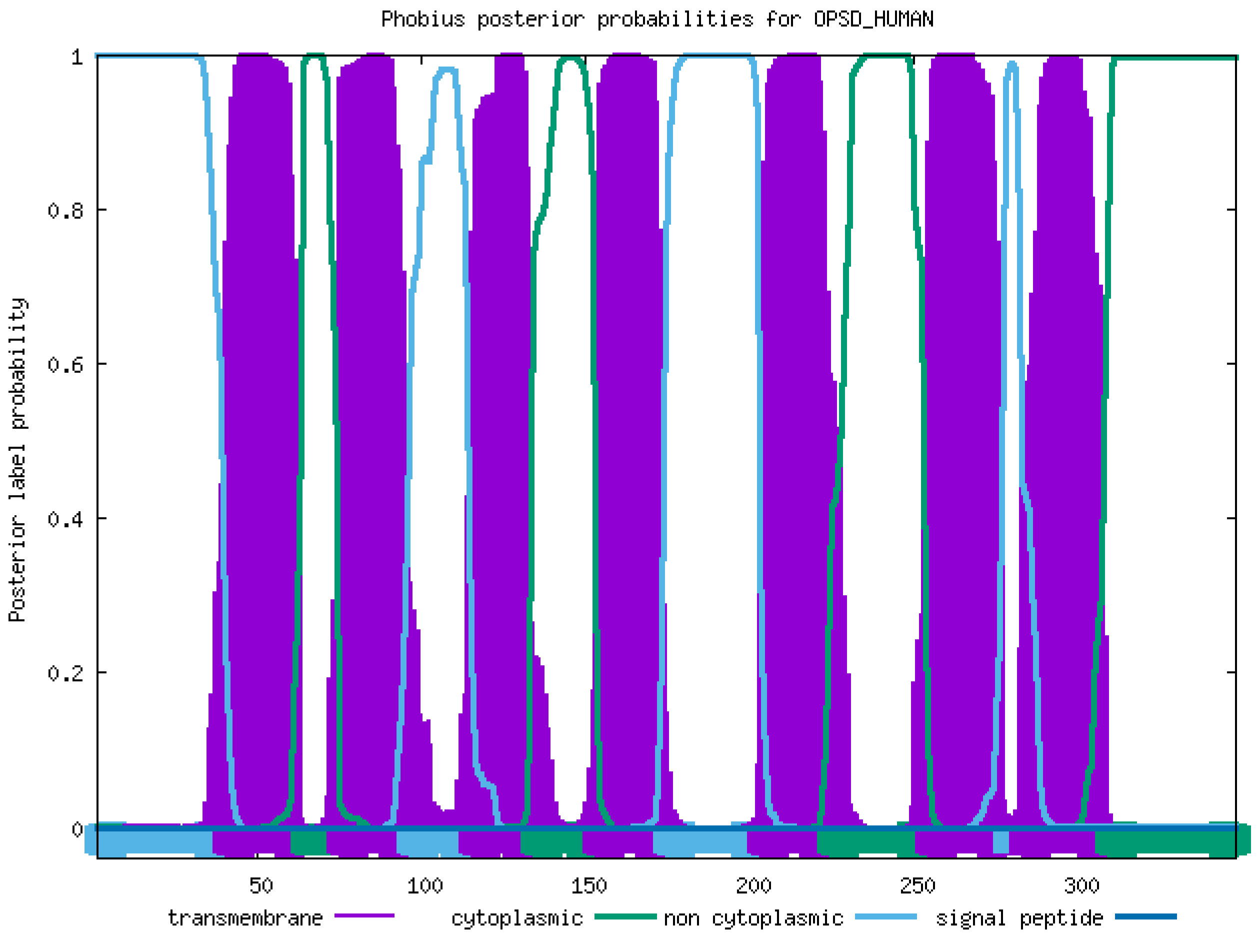

The probability data used in the plot is found here, and the gnuplot script is here.

Prediction of GPR17_HUMAN

```
ID   GPR17_HUMAN
FT   TOPO_DOM      1     64       NON CYTOPLASMIC.
FT   TRANSMEM     65     85
FT   TOPO_DOM     86     96       CYTOPLASMIC.
FT   TRANSMEM     97    118
FT   TOPO_DOM    119    137       NON CYTOPLASMIC.
FT   TRANSMEM    138    162
FT   TOPO_DOM    163    173       CYTOPLASMIC.
FT   TRANSMEM    174    196
FT   TOPO_DOM    197    220       NON CYTOPLASMIC.
FT   TRANSMEM    221    243
FT   TOPO_DOM    244    263       CYTOPLASMIC.
FT   TRANSMEM    264    281
FT   TOPO_DOM    282    367       NON CYTOPLASMIC.
//
```

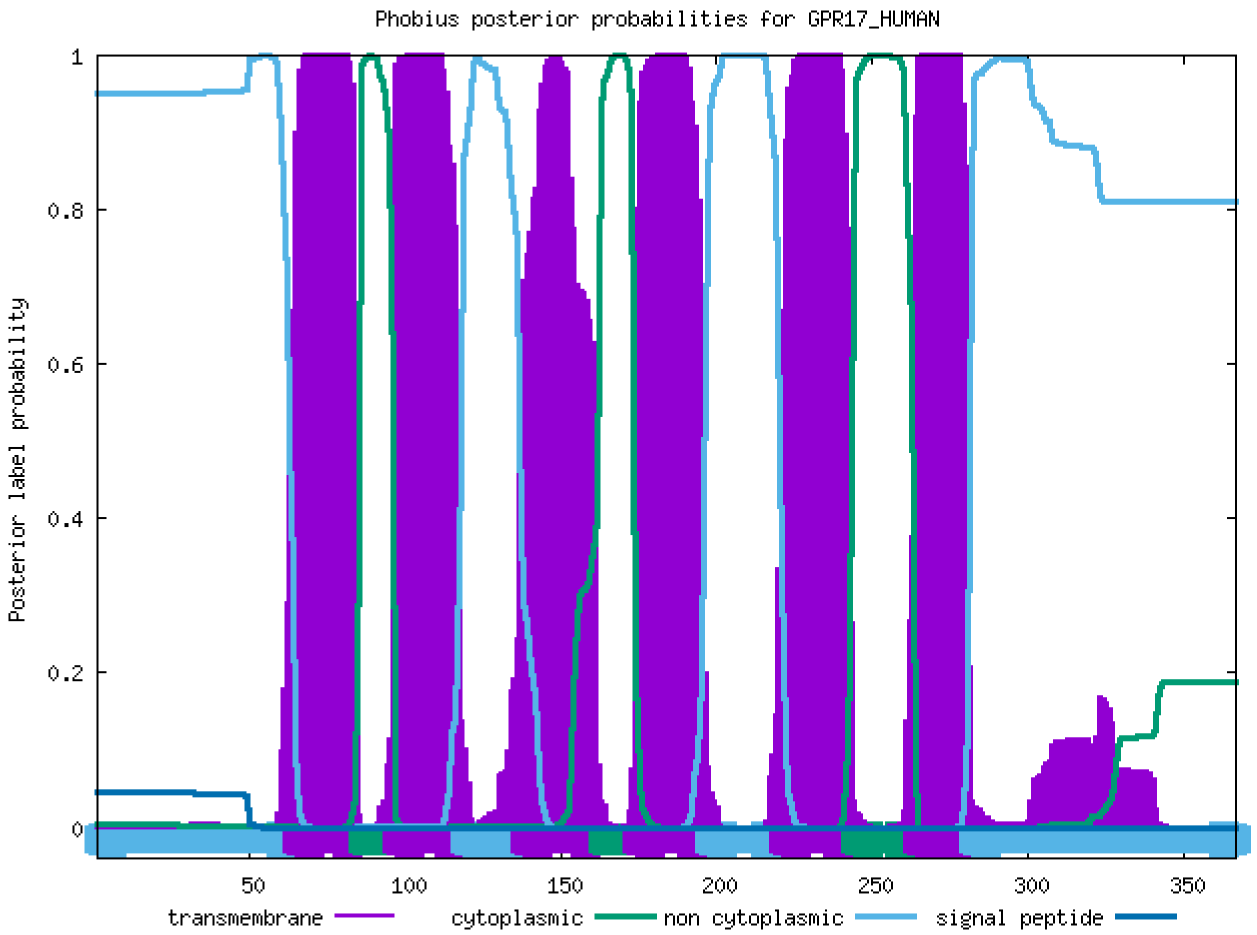

The probability data used in the plot is found here, and the gnuplot script is here.

Prediction of GPR20_HUMAN

```
ID   GPR20_HUMAN
FT   TOPO_DOM      1     51       NON CYTOPLASMIC.
FT   TRANSMEM     52     77
FT   TOPO_DOM     78     88       CYTOPLASMIC.
FT   TRANSMEM     89    107
FT   TOPO_DOM    108    126       NON CYTOPLASMIC.
FT   TRANSMEM    127    146
FT   TOPO_DOM    147    165       CYTOPLASMIC.
FT   TRANSMEM    166    186
FT   TOPO_DOM    187    197       NON CYTOPLASMIC.
FT   TRANSMEM    198    221
FT   TOPO_DOM    222    241       CYTOPLASMIC.
FT   TRANSMEM    242    259
FT   TOPO_DOM    260    278       NON CYTOPLASMIC.
FT   TRANSMEM    279    300
FT   TOPO_DOM    301    358       CYTOPLASMIC.
//
```

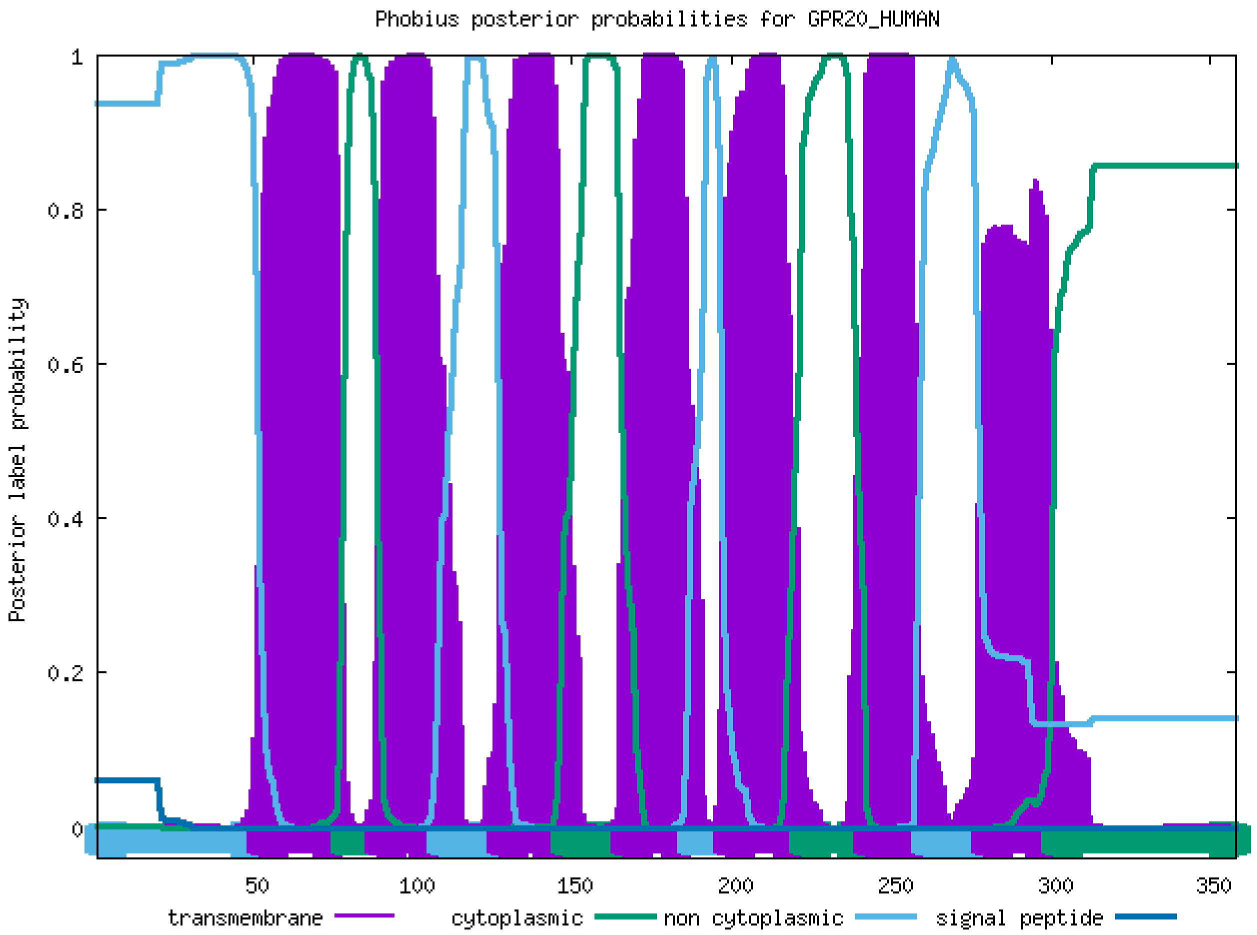

The probability data used in the plot is found here, and the gnuplot script is here.

Prediction of GPR21_HUMAN

```
ID   GPR21_HUMAN
FT   TOPO_DOM      1     31       NON CYTOPLASMIC.
FT   TRANSMEM     32     59
FT   TOPO_DOM     60     70       CYTOPLASMIC.
FT   TRANSMEM     71     92
FT   TOPO_DOM     93    103       NON CYTOPLASMIC.
FT   TRANSMEM    104    125
FT   TOPO_DOM    126    145       CYTOPLASMIC.
FT   TRANSMEM    146    169
FT   TOPO_DOM    170    188       NON CYTOPLASMIC.
FT   TRANSMEM    189    214
FT   TOPO_DOM    215    252       CYTOPLASMIC.
FT   TRANSMEM    253    274
FT   TOPO_DOM    275    285       NON CYTOPLASMIC.
FT   TRANSMEM    286    307
FT   TOPO_DOM    308    349       CYTOPLASMIC.
//
```

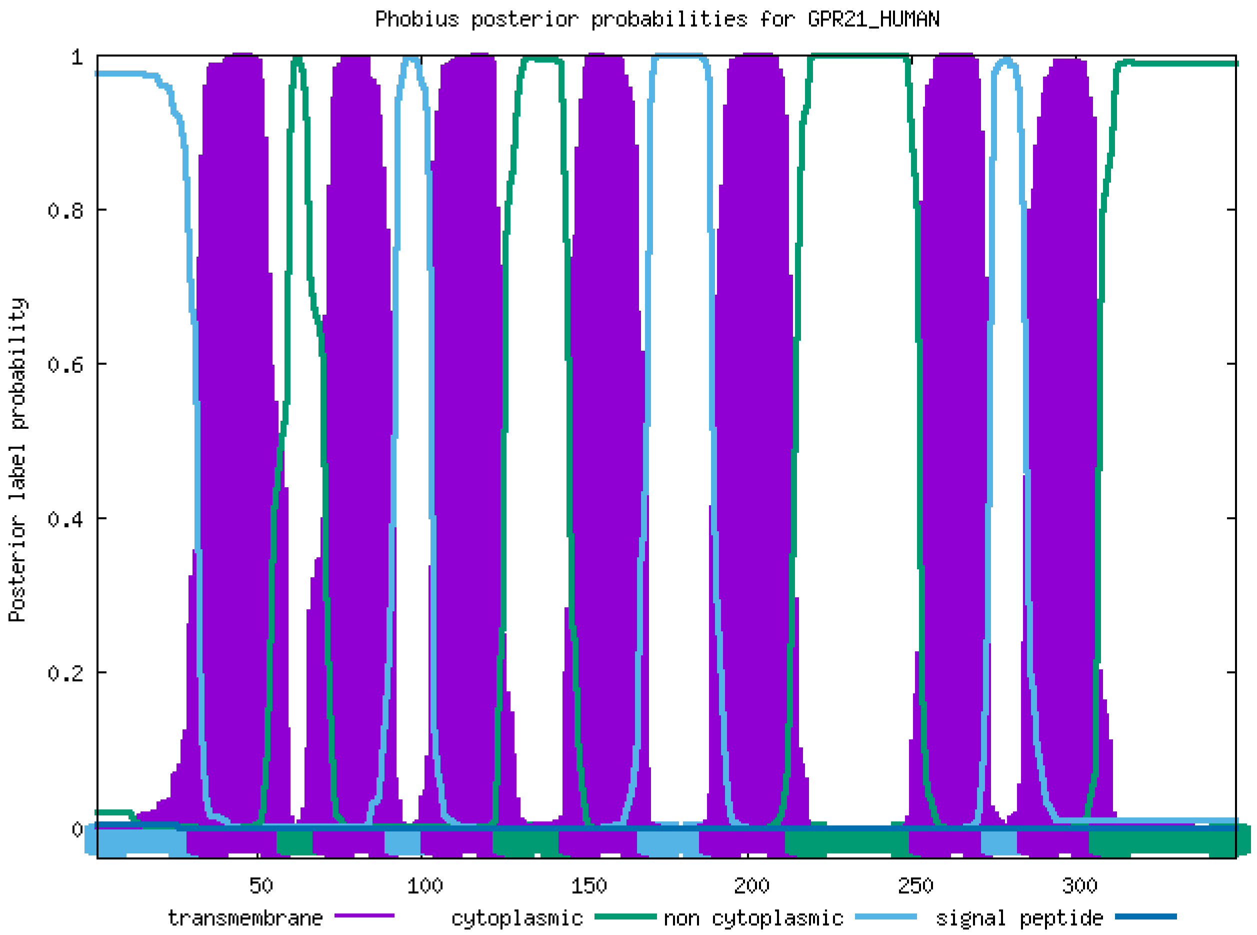

The probability data used in the plot is found here, and the gnuplot script is here.

Prediction of GPR34_HUMAN

```
ID   GPR34_HUMAN
FT   TOPO_DOM      1     60       NON CYTOPLASMIC.
FT   TRANSMEM     61     80
FT   TOPO_DOM     81     91       CYTOPLASMIC.
FT   TRANSMEM     92    113
FT   TOPO_DOM    114    132       NON CYTOPLASMIC.
FT   TRANSMEM    133    150
FT   TOPO_DOM    151    170       CYTOPLASMIC.
FT   TRANSMEM    171    192
FT   TOPO_DOM    193    216       NON CYTOPLASMIC.
FT   TRANSMEM    217    241
FT   TOPO_DOM    242    268       CYTOPLASMIC.
FT   TRANSMEM    269    291
FT   TOPO_DOM    292    310       NON CYTOPLASMIC.
FT   TRANSMEM    311    330
FT   TOPO_DOM    331    381       CYTOPLASMIC.
//
```

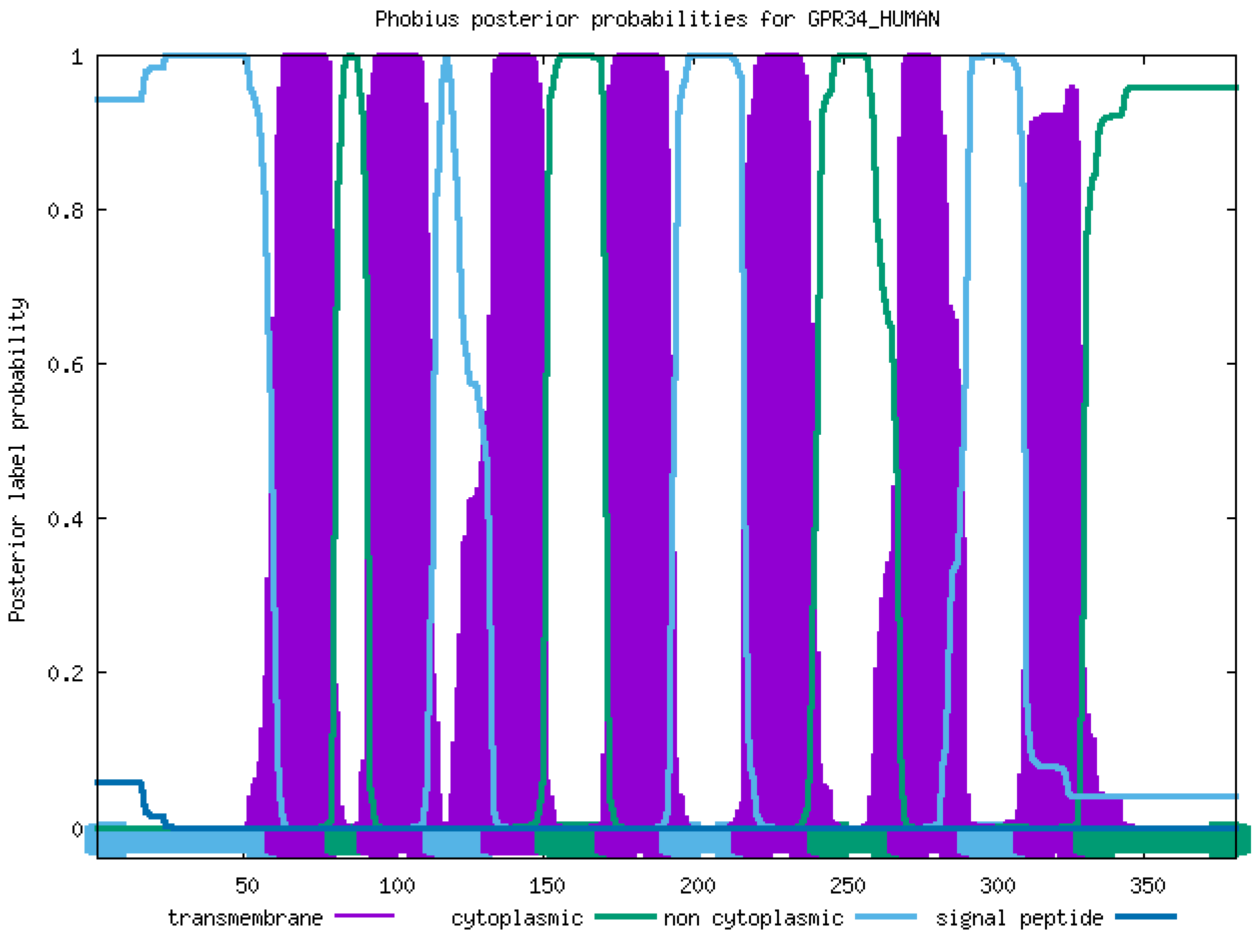

The probability data used in the plot is found here, and the gnuplot script is here.

Prediction of GPR35_HUMAN

```
ID   GPR35_HUMAN
FT   TOPO_DOM      1     22       NON CYTOPLASMIC.
FT   TRANSMEM     23     45
FT   TOPO_DOM     46     56       CYTOPLASMIC.
FT   TRANSMEM     57     75
FT   TOPO_DOM     76     94       NON CYTOPLASMIC.
FT   TRANSMEM     95    112
FT   TOPO_DOM    113    132       CYTOPLASMIC.
FT   TRANSMEM    133    154
FT   TOPO_DOM    155    173       NON CYTOPLASMIC.
FT   TRANSMEM    174    198
FT   TOPO_DOM    199    218       CYTOPLASMIC.
FT   TRANSMEM    219    243
FT   TOPO_DOM    244    309       NON CYTOPLASMIC.
//
```

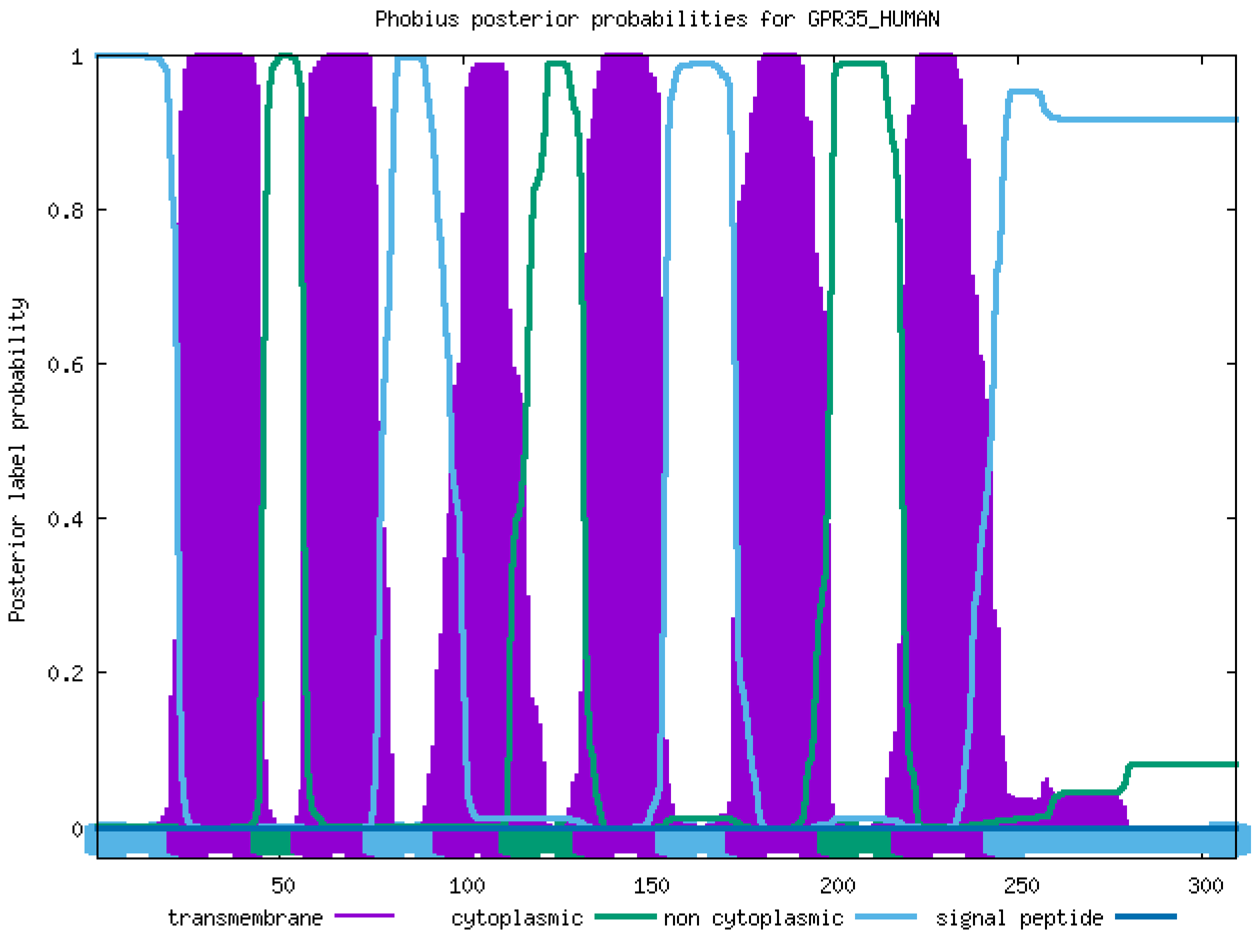

The probability data used in the plot is found here, and the gnuplot script is here.

Prediction of GPR52_HUMAN

```
ID   GPR52_HUMAN
FT   TOPO_DOM      1     44       NON CYTOPLASMIC.
FT   TRANSMEM     45     71
FT   TOPO_DOM     72     82       CYTOPLASMIC.
FT   TRANSMEM     83    104
FT   TOPO_DOM    105    115       NON CYTOPLASMIC.
FT   TRANSMEM    116    137
FT   TOPO_DOM    138    157       CYTOPLASMIC.
FT   TRANSMEM    158    181
FT   TOPO_DOM    182    200       NON CYTOPLASMIC.
FT   TRANSMEM    201    226
FT   TOPO_DOM    227    265       CYTOPLASMIC.
FT   TRANSMEM    266    287
FT   TOPO_DOM    288    298       NON CYTOPLASMIC.
FT   TRANSMEM    299    320
FT   TOPO_DOM    321    361       CYTOPLASMIC.
//
```

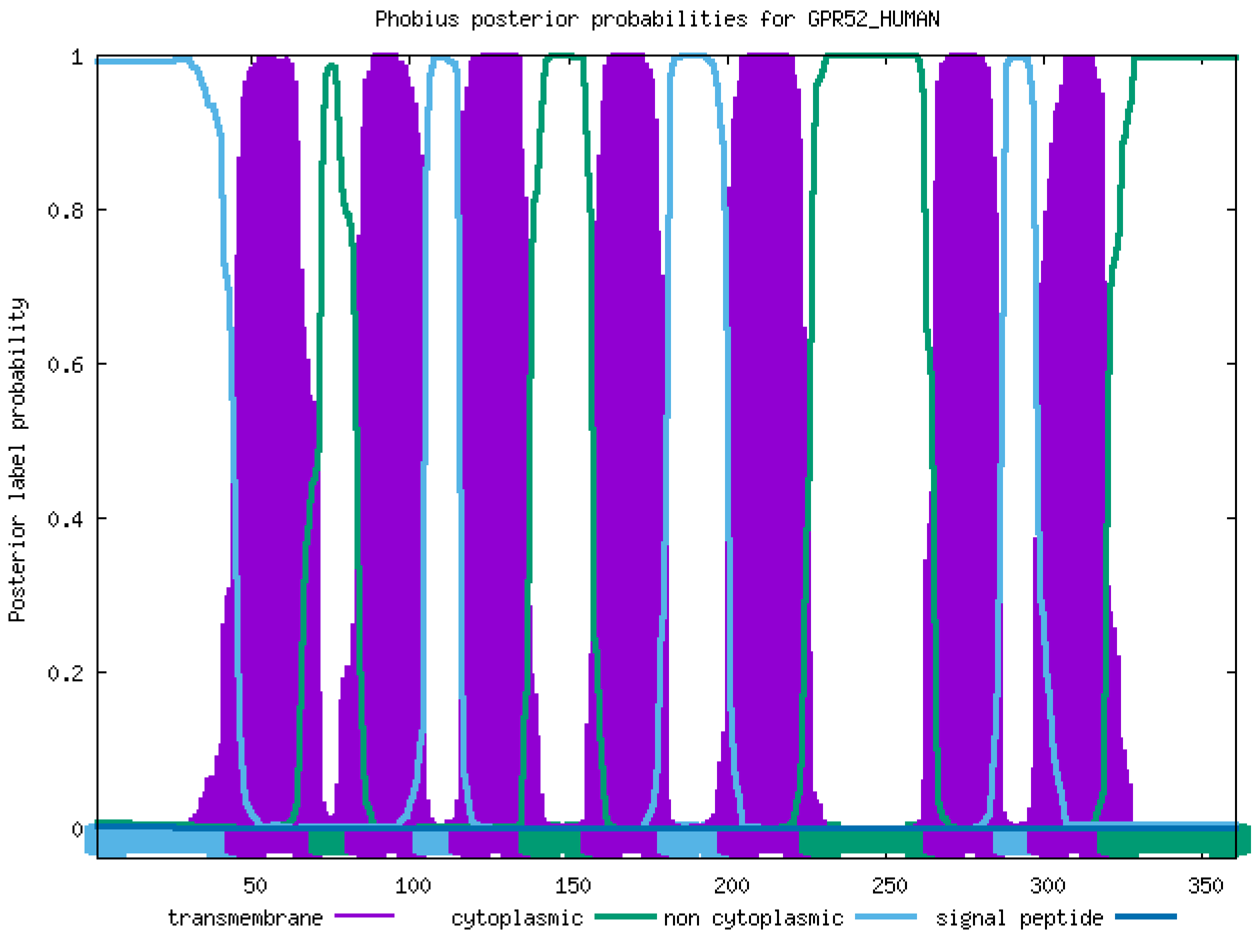

The probability data used in the plot is found here, and the gnuplot script is here.

Prediction of GPR61_HUMAN

```
ID   GPR61_HUMAN
FT   TOPO_DOM      1     43       NON CYTOPLASMIC.
FT   TRANSMEM     44     67
FT   TOPO_DOM     68     75       CYTOPLASMIC.
FT   TRANSMEM     76     97
FT   TOPO_DOM     98    116       NON CYTOPLASMIC.
FT   TRANSMEM    117    136
FT   TOPO_DOM    137    155       CYTOPLASMIC.
FT   TRANSMEM    156    179
FT   TOPO_DOM    180    206       NON CYTOPLASMIC.
FT   TRANSMEM    207    231
FT   TOPO_DOM    232    284       CYTOPLASMIC.
FT   TRANSMEM    285    305
FT   TOPO_DOM    306    324       NON CYTOPLASMIC.
FT   TRANSMEM    325    344
FT   TOPO_DOM    345    451       CYTOPLASMIC.
//
```

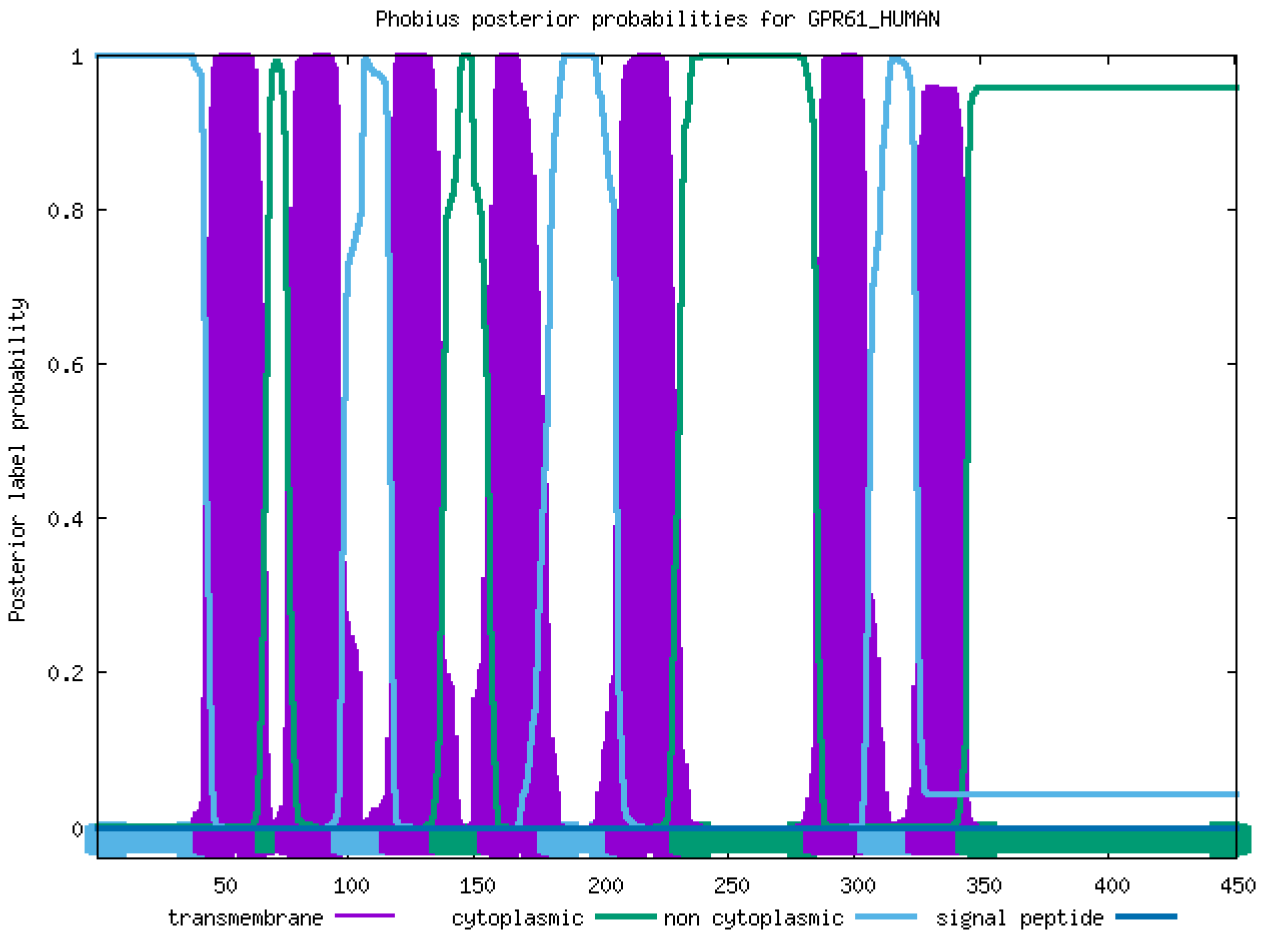

The probability data used in the plot is found here, and the gnuplot script is here.

Prediction of GPR84_HUMAN

```
ID   GPR84_HUMAN
FT   TOPO_DOM      1     22       NON CYTOPLASMIC.
FT   TRANSMEM     23     45
FT   TOPO_DOM     46     56       CYTOPLASMIC.
FT   TRANSMEM     57     76
FT   TOPO_DOM     77     95       NON CYTOPLASMIC.
FT   TRANSMEM     96    116
FT   TOPO_DOM    117    136       CYTOPLASMIC.
FT   TRANSMEM    137    161
FT   TOPO_DOM    162    180       NON CYTOPLASMIC.
FT   TRANSMEM    181    201
FT   TOPO_DOM    202    319       CYTOPLASMIC.
FT   TRANSMEM    320    341
FT   TOPO_DOM    342    352       NON CYTOPLASMIC.
FT   TRANSMEM    353    373
FT   TOPO_DOM    374    396       CYTOPLASMIC.
//
```

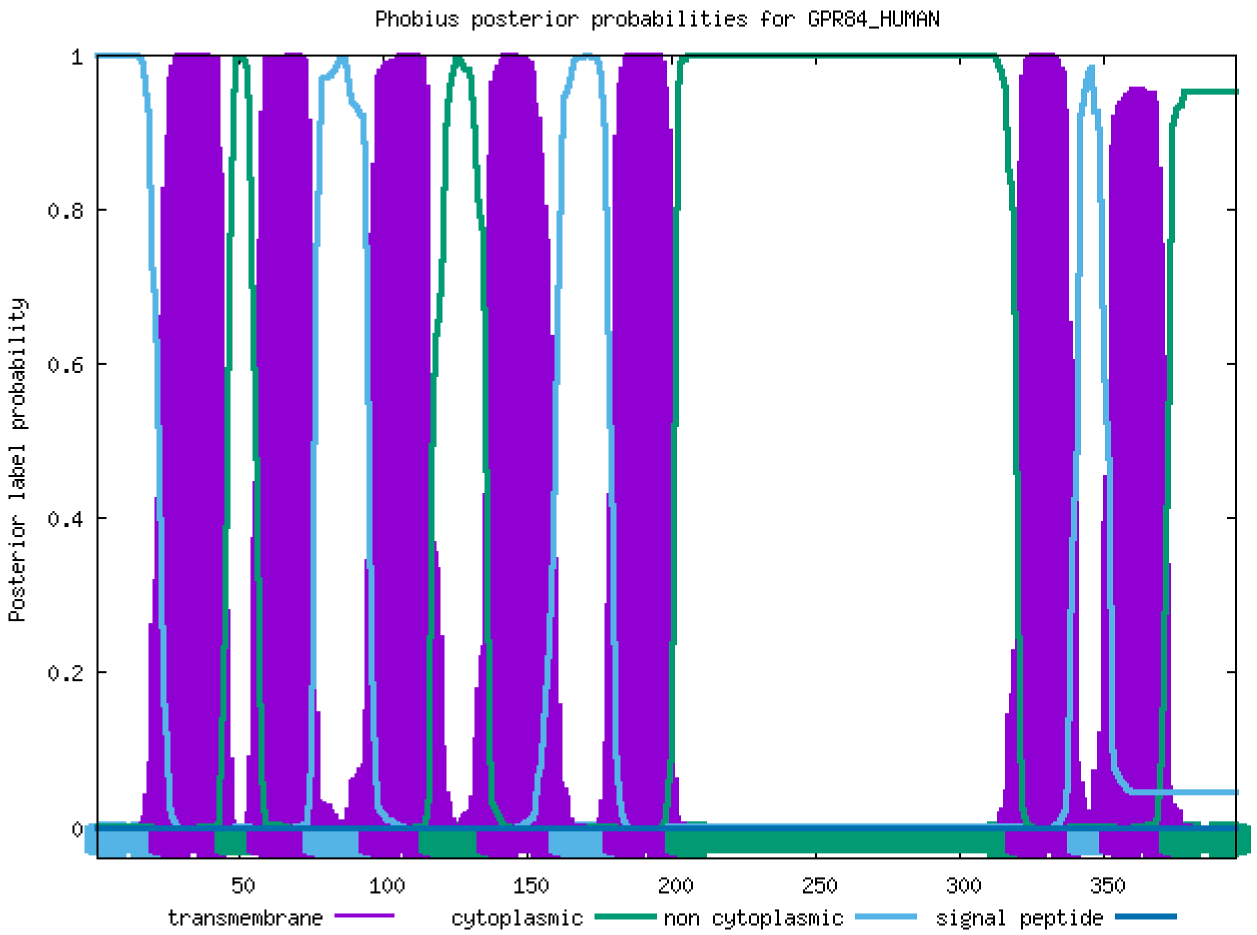

The probability data used in the plot is found here, and the gnuplot script is here.

Prediction of GPR88_HUMAN

```
ID   GPR88_HUMAN
FT   TOPO_DOM      1     35       NON CYTOPLASMIC.
FT   TRANSMEM     36     56
FT   TOPO_DOM     57     67       CYTOPLASMIC.
FT   TRANSMEM     68     85
FT   TOPO_DOM     86    118       NON CYTOPLASMIC.
FT   TRANSMEM    119    140
FT   TOPO_DOM    141    160       CYTOPLASMIC.
FT   TRANSMEM    161    180
FT   TOPO_DOM    181    191       NON CYTOPLASMIC.
FT   TRANSMEM    192    215
FT   TOPO_DOM    216    280       CYTOPLASMIC.
FT   TRANSMEM    281    303
FT   TOPO_DOM    304    314       NON CYTOPLASMIC.
FT   TRANSMEM    315    338
FT   TOPO_DOM    339    384       CYTOPLASMIC.
//
```

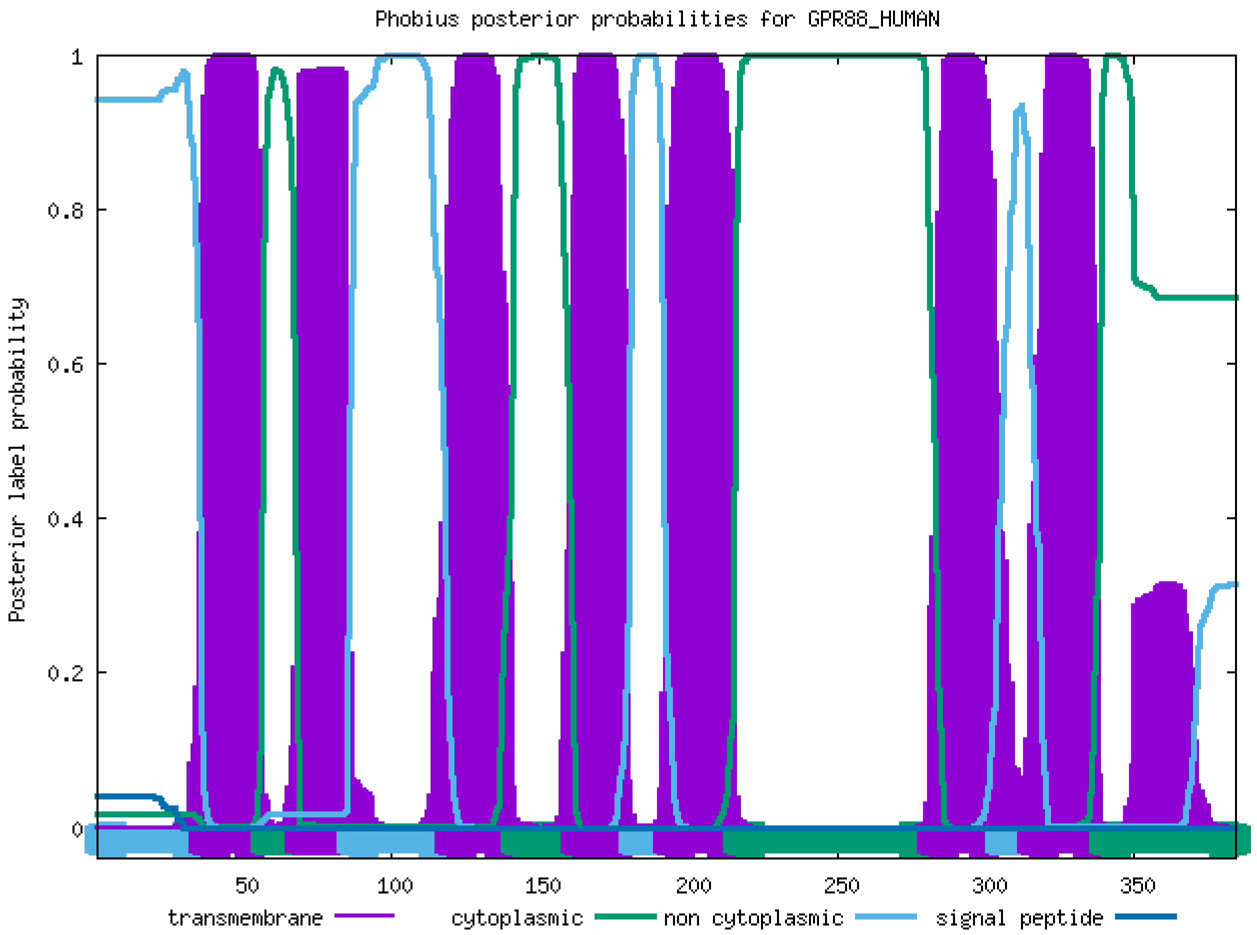

The probability data used in the plot is found here, and the gnuplot script is here.

Prediction of GP101_HUMAN

```
ID   GP101_HUMAN
FT   TOPO_DOM      1     31       NON CYTOPLASMIC.
FT   TRANSMEM     32     57
FT   TOPO_DOM     58     68       CYTOPLASMIC.
FT   TRANSMEM     69     89
FT   TOPO_DOM     90    108       NON CYTOPLASMIC.
FT   TRANSMEM    109    127
FT   TOPO_DOM    128    147       CYTOPLASMIC.
FT   TRANSMEM    148    171
FT   TOPO_DOM    172    190       NON CYTOPLASMIC.
FT   TRANSMEM    191    218
FT   TOPO_DOM    219    399       CYTOPLASMIC.
FT   TRANSMEM    400    425
FT   TOPO_DOM    426    430       NON CYTOPLASMIC.
FT   TRANSMEM    431    453
FT   TOPO_DOM    454    508       CYTOPLASMIC.
//
```

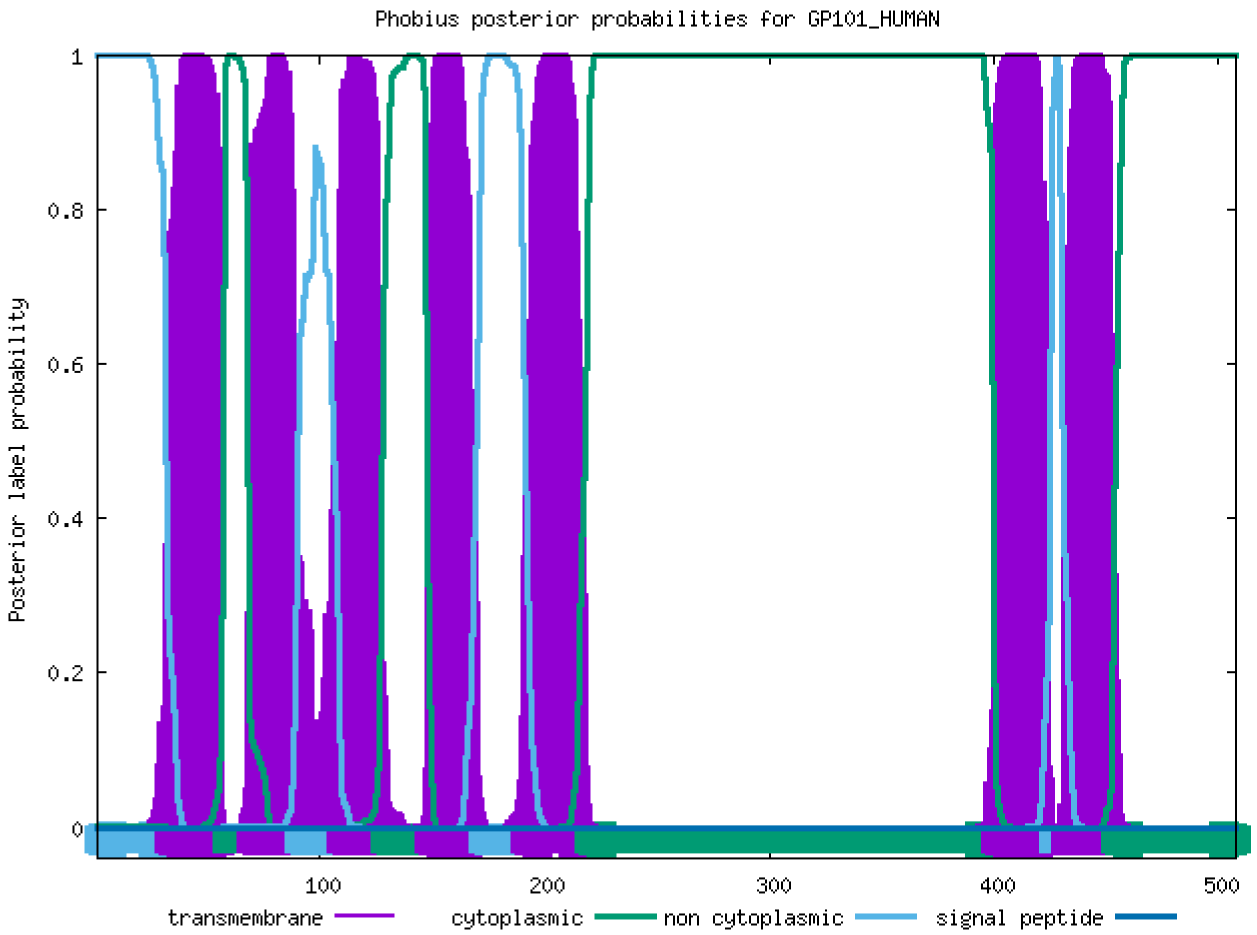

The probability data used in the plot is found here, and the gnuplot script is here.

Prediction of GP119_HUMAN

```
ID   GP119_HUMAN
FT   TOPO_DOM      1      5       NON CYTOPLASMIC.
FT   TRANSMEM      6     33
FT   TOPO_DOM     34     39       CYTOPLASMIC.
FT   TRANSMEM     40     62
FT   TOPO_DOM     63     81       NON CYTOPLASMIC.
FT   TRANSMEM     82    101
FT   TOPO_DOM    102    112       CYTOPLASMIC.
FT   TRANSMEM    113    136
FT   TOPO_DOM    137    164       NON CYTOPLASMIC.
FT   TRANSMEM    165    186
FT   TOPO_DOM    187    222       CYTOPLASMIC.
FT   TRANSMEM    223    243
FT   TOPO_DOM    244    262       NON CYTOPLASMIC.
FT   TRANSMEM    263    282
FT   TOPO_DOM    283    335       CYTOPLASMIC.
//
```

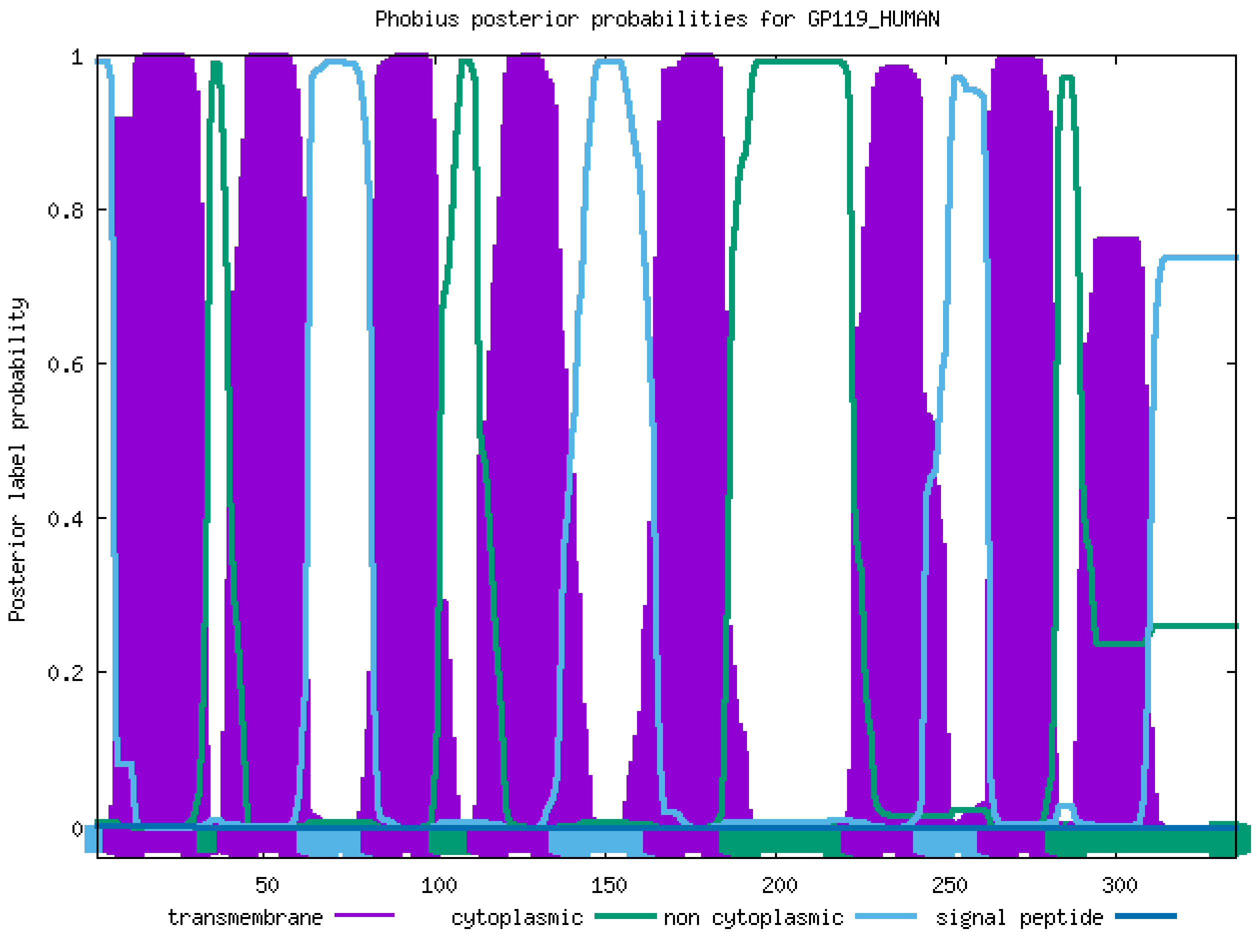

The probability data used in the plot is found here, and the gnuplot script is here.

Prediction of GP139_HUMAN

```
ID    GP139_HUMAN
FT    TOPO_DOM      1     29       NON CYTOPLASMIC.
FT    TRANSMEM     30     51
FT    TOPO_DOM     52     62       CYTOPLASMIC.
FT    TRANSMEM     63     87
FT    TOPO_DOM     88    106       NON CYTOPLASMIC.
FT    TRANSMEM    107    128
FT    TOPO_DOM    129    147       CYTOPLASMIC.
FT    TRANSMEM    148    166
FT    TOPO_DOM    167    181       NON CYTOPLASMIC.
FT    TRANSMEM    182    208
FT    TOPO_DOM    209    227       CYTOPLASMIC.
FT    TRANSMEM    228    249
FT    TOPO_DOM    250    268       NON CYTOPLASMIC.
FT    TRANSMEM    269    288
FT    TOPO_DOM    289    353       CYTOPLASMIC.
//
```

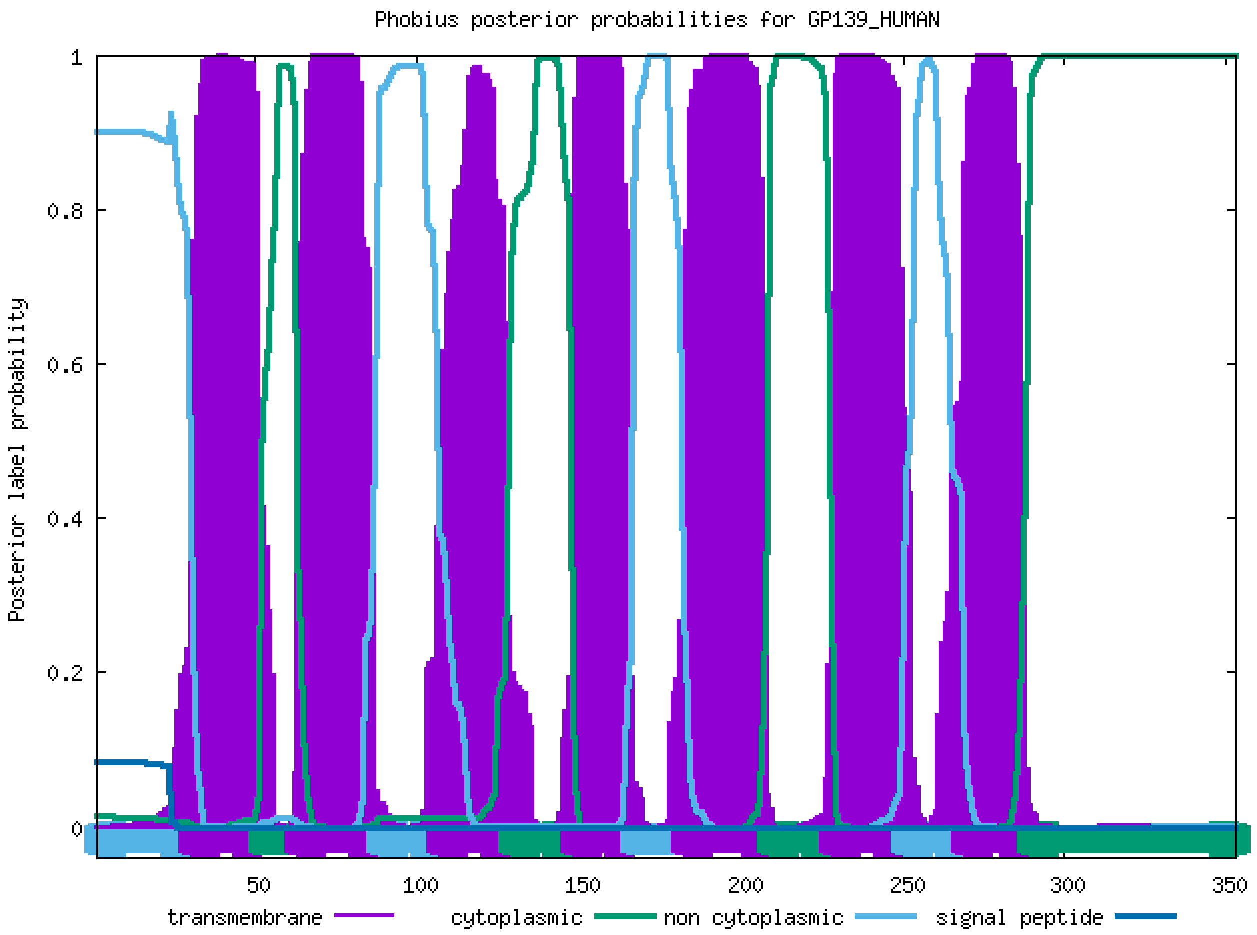

The probability data used in the plot is found here, and the gnuplot script is here.

Prediction of GP161_HUMAN

```
ID   GP161_HUMAN
FT   TOPO_DOM      1     26       NON CYTOPLASMIC.
FT   TRANSMEM     27     53
FT   TOPO_DOM     54     64       CYTOPLASMIC.
FT   TRANSMEM     65     84
FT   TOPO_DOM     85    103       NON CYTOPLASMIC.
FT   TRANSMEM    104    123
FT   TOPO_DOM    124    143       CYTOPLASMIC.
FT   TRANSMEM    144    164
FT   TOPO_DOM    165    189       NON CYTOPLASMIC.
FT   TRANSMEM    190    211
FT   TOPO_DOM    212    267       CYTOPLASMIC.
FT   TRANSMEM    268    288
FT   TOPO_DOM    289    307       NON CYTOPLASMIC.
FT   TRANSMEM    308    327
FT   TOPO_DOM    328    529       CYTOPLASMIC.
//
```

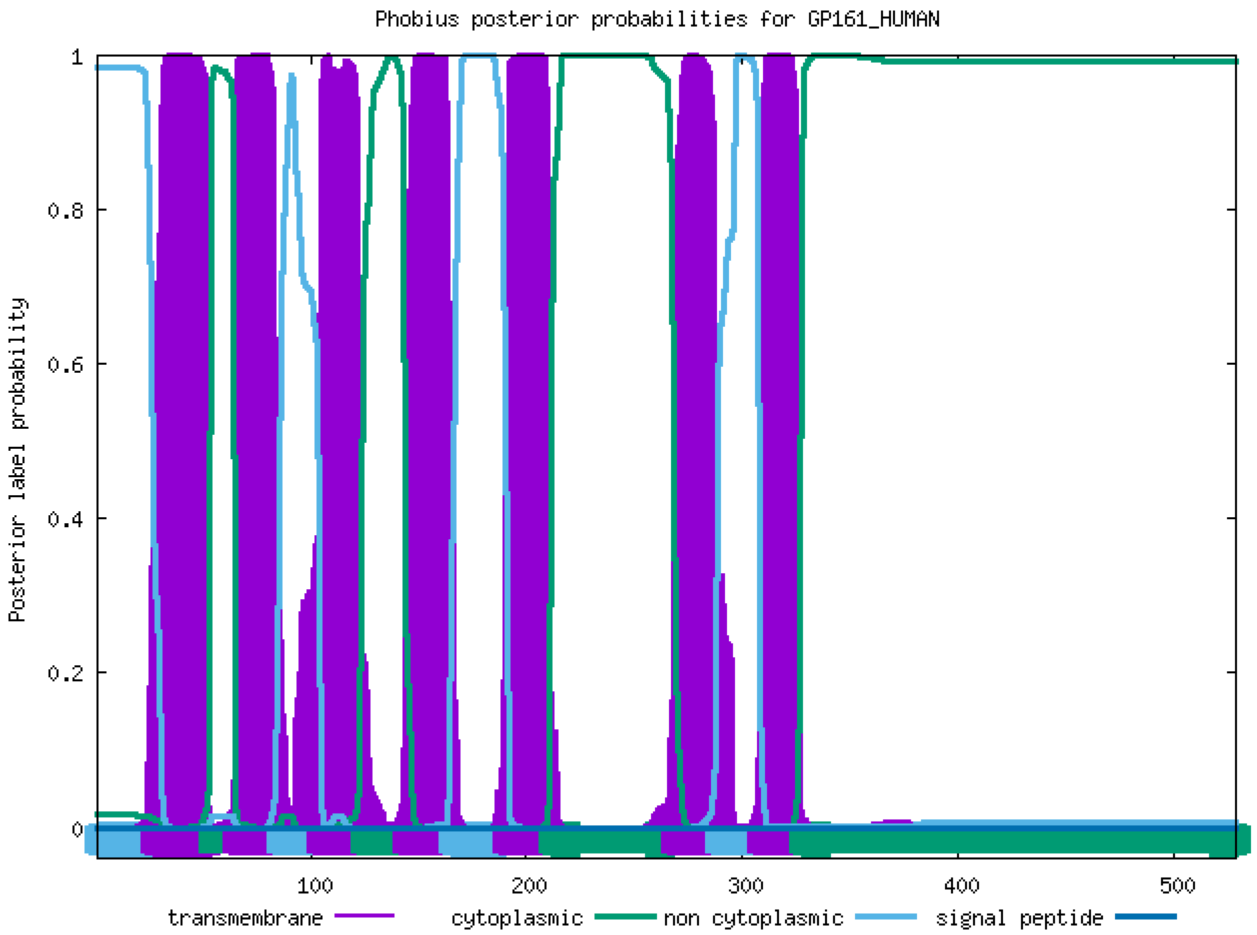

The probability data used in the plot is found here, and the gnuplot script is here.

Prediction of GP174_HUMAN

```
ID   GP174_HUMAN
FT   TOPO_DOM      1     19       NON CYTOPLASMIC.
FT   TRANSMEM     20     45
FT   TOPO_DOM     46     56       CYTOPLASMIC.
FT   TRANSMEM     57     80
FT   TOPO_DOM     81     99       NON CYTOPLASMIC.
FT   TRANSMEM    100    121
FT   TOPO_DOM    122    132       CYTOPLASMIC.
FT   TRANSMEM    133    155
FT   TOPO_DOM    156    182       NON CYTOPLASMIC.
FT   TRANSMEM    183    206
FT   TOPO_DOM    207    226       CYTOPLASMIC.
FT   TRANSMEM    227    249
FT   TOPO_DOM    250    268       NON CYTOPLASMIC.
FT   TRANSMEM    269    295
FT   TOPO_DOM    296    333       CYTOPLASMIC.
//
```

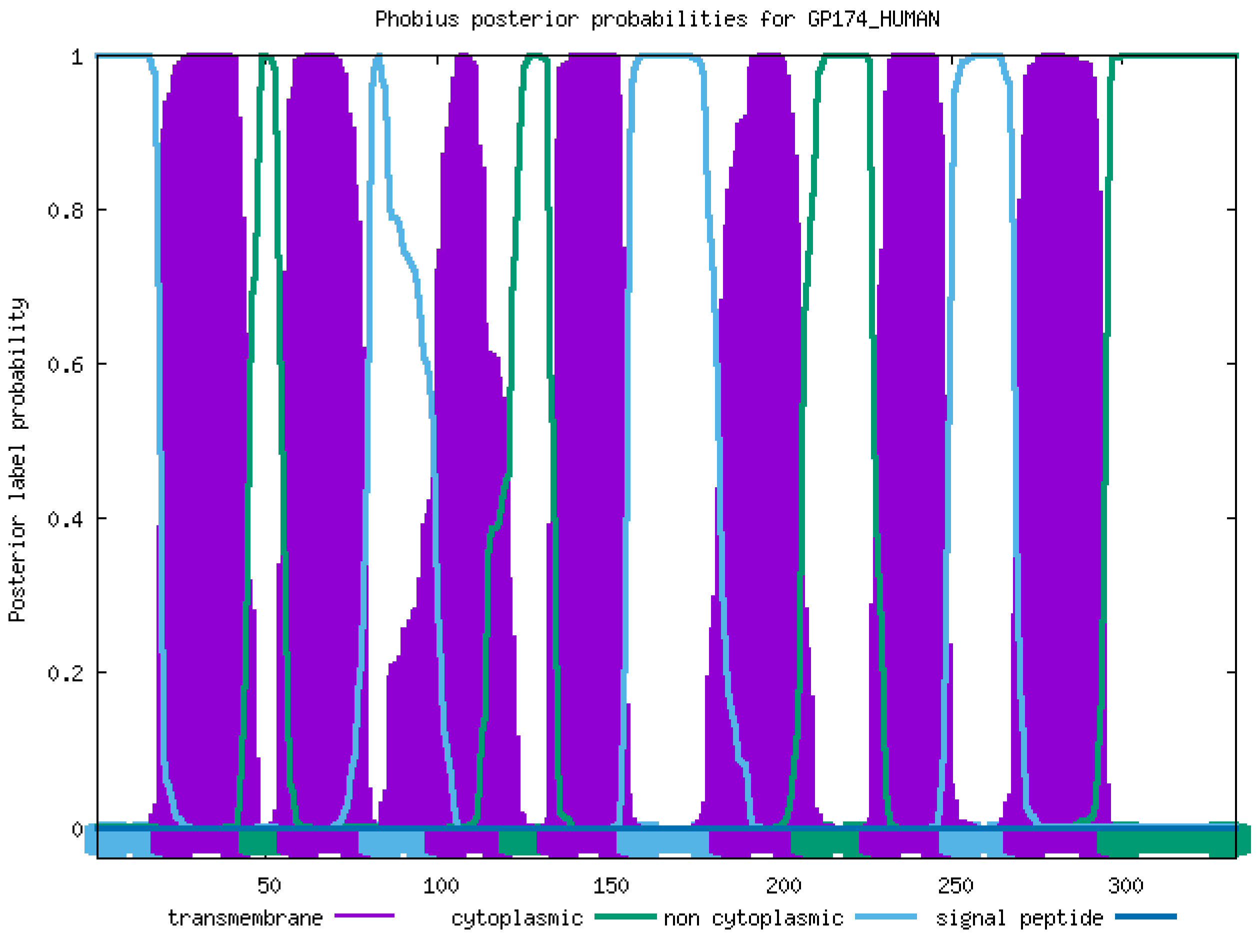

The probability data used in the plot is found here, and the gnuplot script is here.

Prediction of GP183_HUMAN

```
ID   GP183_HUMAN
FT   TOPO_DOM      1     35       NON CYTOPLASMIC.
FT   TRANSMEM     36     57
FT   TOPO_DOM     58     68       CYTOPLASMIC.
FT   TRANSMEM     69     93
FT   TOPO_DOM     94    104       NON CYTOPLASMIC.
FT   TRANSMEM    105    127
FT   TOPO_DOM    128    147       CYTOPLASMIC.
FT   TRANSMEM    148    168
FT   TOPO_DOM    169    194       NON CYTOPLASMIC.
FT   TRANSMEM    195    221
FT   TOPO_DOM    222    241       CYTOPLASMIC.
FT   TRANSMEM    242    269
FT   TOPO_DOM    270    288       NON CYTOPLASMIC.
FT   TRANSMEM    289    311
FT   TOPO_DOM    312    361       CYTOPLASMIC.
//
```

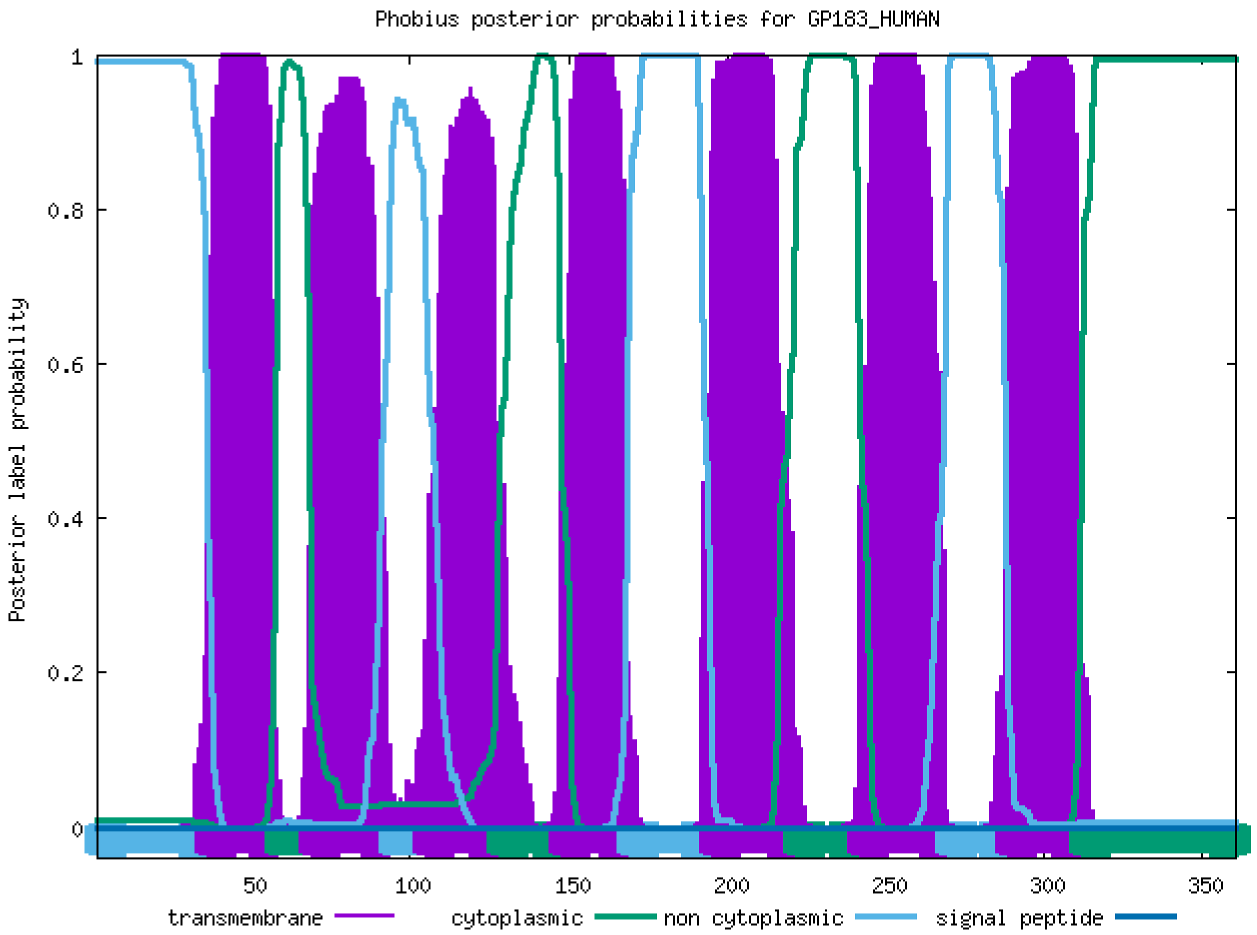

The probability data used in the plot is found here, and the gnuplot script is here.

Prediction of OX1R_HUMAN

```
ID   OX1R_HUMAN
FT   TOPO_DOM      1     46       NON CYTOPLASMIC.
FT   TRANSMEM     47     72
FT   TOPO_DOM     73     83       CYTOPLASMIC.
FT   TRANSMEM     84    104
FT   TOPO_DOM    105    123       NON CYTOPLASMIC.
FT   TRANSMEM    124    142
FT   TOPO_DOM    143    162       CYTOPLASMIC.
FT   TRANSMEM    163    182
FT   TOPO_DOM    183    213       NON CYTOPLASMIC.
FT   TRANSMEM    214    236
FT   TOPO_DOM    237    298       CYTOPLASMIC.
FT   TRANSMEM    299    320
FT   TOPO_DOM    321    334       NON CYTOPLASMIC.
FT   TRANSMEM    335    361
FT   TOPO_DOM    362    425       CYTOPLASMIC.
//
```

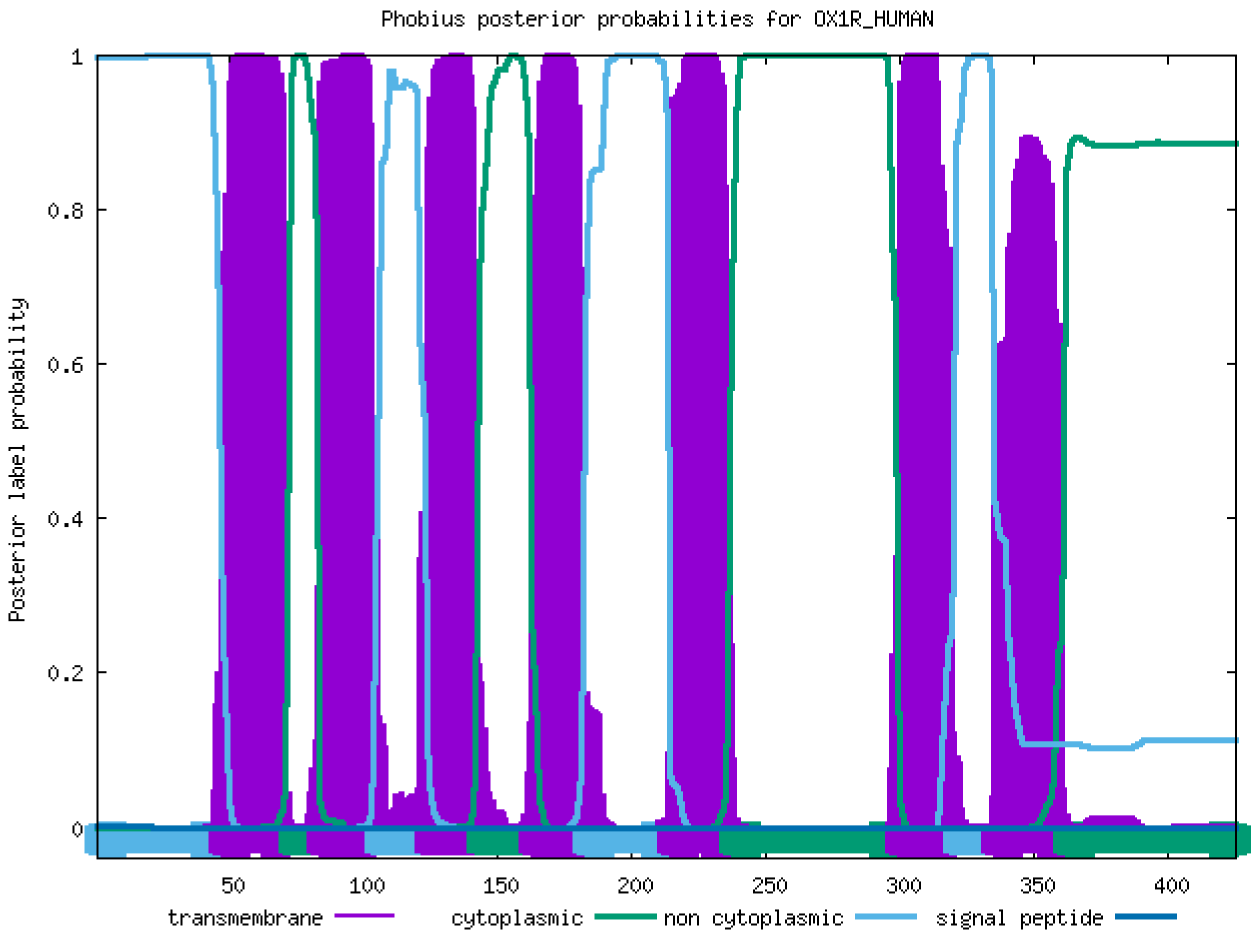

The probability data used in the plot is found here, and the gnuplot script is here.

Prediction of OX2R_HUMAN

```
ID    OX2R_HUMAN
FT    TOPO_DOM      1      54       NON CYTOPLASMIC.
FT    TRANSMEM     55      80
FT    TOPO_DOM     81      91       CYTOPLASMIC.
FT    TRANSMEM     92     112
FT    TOPO_DOM    113     131       NON CYTOPLASMIC.
FT    TRANSMEM    132     150
FT    TOPO_DOM    151     170       CYTOPLASMIC.
FT    TRANSMEM    171     190
FT    TOPO_DOM    191     221       NON CYTOPLASMIC.
FT    TRANSMEM    222     244
FT    TOPO_DOM    245     302       CYTOPLASMIC.
FT    TRANSMEM    303     322
FT    TOPO_DOM    323     341       NON CYTOPLASMIC.
FT    TRANSMEM    342     367
FT    TOPO_DOM    368     444       CYTOPLASMIC.
//
```

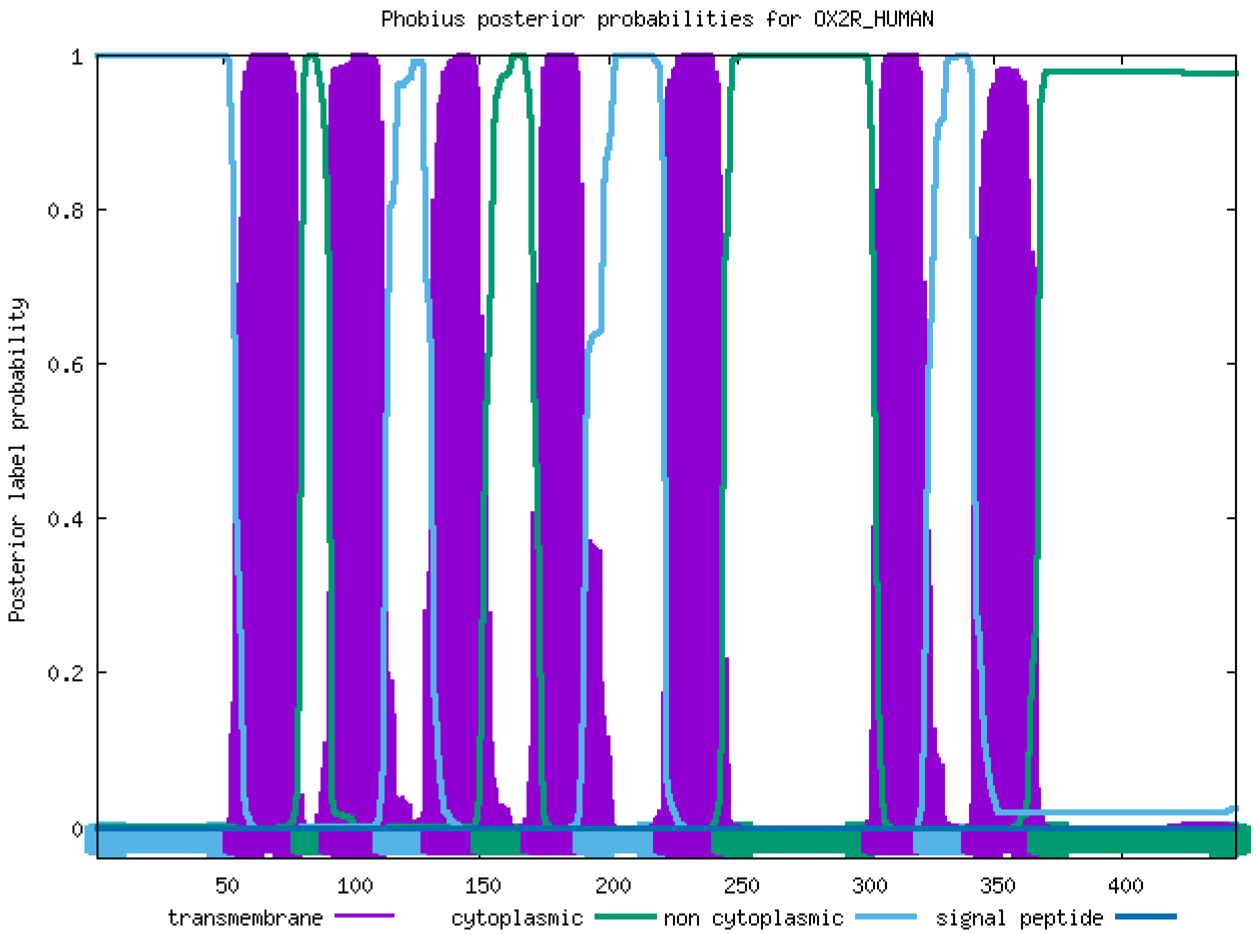

The probability data used in the plot is found here, and the gnuplot script is here.

Prediction of C3AR_HUMAN

```
ID    C3AR_HUMAN
FT    TOPO_DOM      1     22       NON CYTOPLASMIC.
FT    TRANSMEM     23     47
FT    TOPO_DOM     48     58       CYTOPLASMIC.
FT    TRANSMEM     59     77
FT    TOPO_DOM     78     96       NON CYTOPLASMIC.
FT    TRANSMEM     97    118
FT    TOPO_DOM    119    138       CYTOPLASMIC.
FT    TRANSMEM    139    160
FT    TOPO_DOM    161    340       NON CYTOPLASMIC.
FT    TRANSMEM    341    363
FT    TOPO_DOM    364    374       CYTOPLASMIC.
FT    TRANSMEM    375    396
FT    TOPO_DOM    397    415       NON CYTOPLASMIC.
FT    TRANSMEM    416    438
FT    TOPO_DOM    439    482       CYTOPLASMIC.
//
```

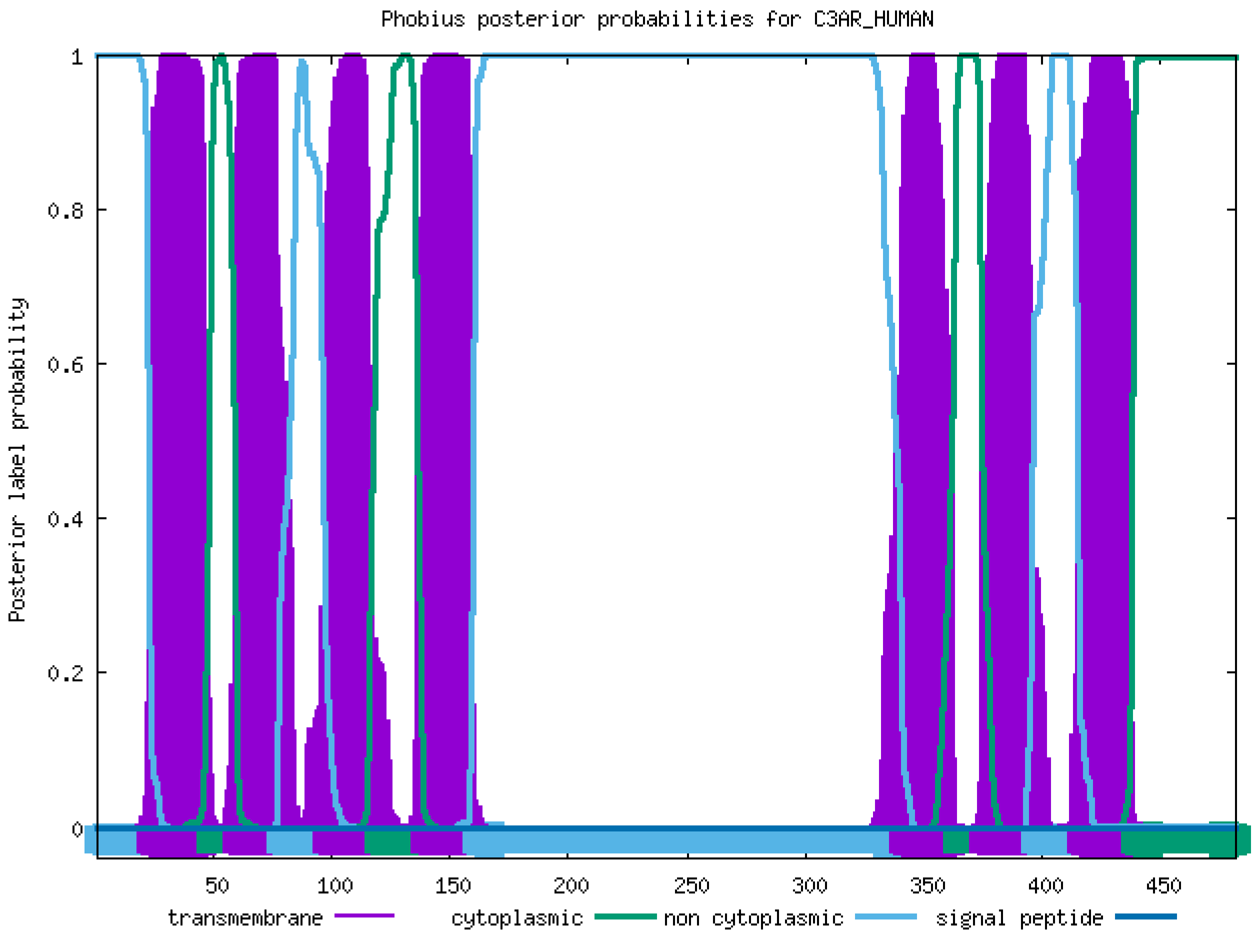

The probability data used in the plot is found here, and the gnuplot script is here.

Prediction of C5AR1_HUMAN

```
ID    C5AR1_HUMAN
FT    TOPO_DOM      1      37       NON CYTOPLASMIC.
FT    TRANSMEM     38      61
FT    TOPO_DOM     62      72       CYTOPLASMIC.
FT    TRANSMEM     73      93
FT    TOPO_DOM     94     104       NON CYTOPLASMIC.
FT    TRANSMEM    105     130
FT    TOPO_DOM    131     150       CYTOPLASMIC.
FT    TRANSMEM    151     174
FT    TOPO_DOM    175     202       NON CYTOPLASMIC.
FT    TRANSMEM    203     227
FT    TOPO_DOM    228     238       CYTOPLASMIC.
FT    TRANSMEM    239     261
FT    TOPO_DOM    262     280       NON CYTOPLASMIC.
FT    TRANSMEM    281     303
FT    TOPO_DOM    304     350       CYTOPLASMIC.
//
```

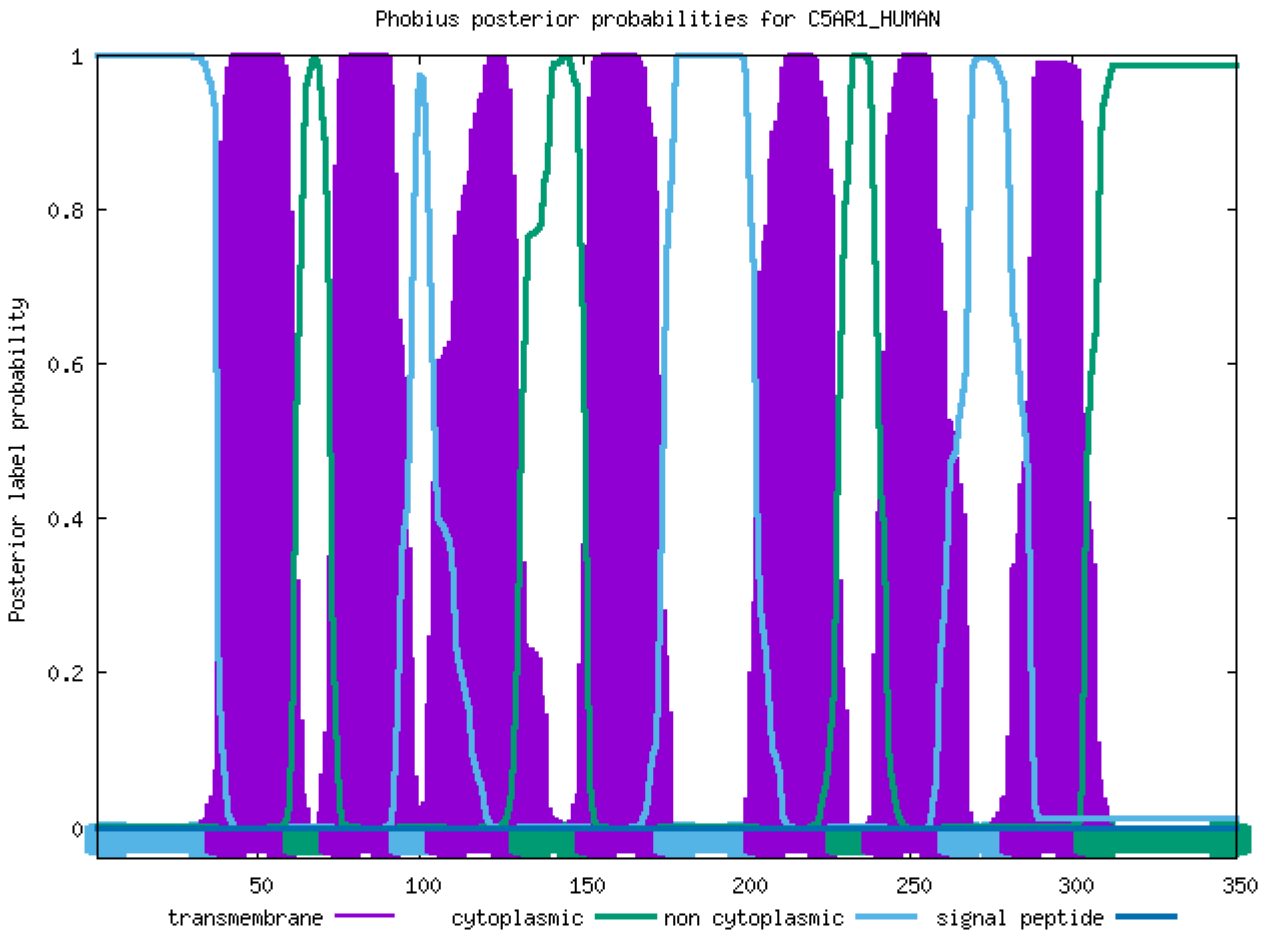

The probability data used in the plot is found here, and the gnuplot script is here.

Prediction of ADA1A_HUMAN

```
ID    ADA1A_HUMAN
FT    TOPO_DOM      1     25       NON CYTOPLASMIC.
FT    TRANSMEM     26     52
FT    TOPO_DOM     53     63       CYTOPLASMIC.
FT    TRANSMEM     64     94
FT    TOPO_DOM     95     99       NON CYTOPLASMIC.
FT    TRANSMEM    100    122
FT    TOPO_DOM    123    142       CYTOPLASMIC.
FT    TRANSMEM    143    164
FT    TOPO_DOM    165    183       NON CYTOPLASMIC.
FT    TRANSMEM    184    209
FT    TOPO_DOM    210    272       CYTOPLASMIC.
FT    TRANSMEM    273    298
FT    TOPO_DOM    299    309       NON CYTOPLASMIC.
FT    TRANSMEM    310    328
FT    TOPO_DOM    329    466       CYTOPLASMIC.
//
```

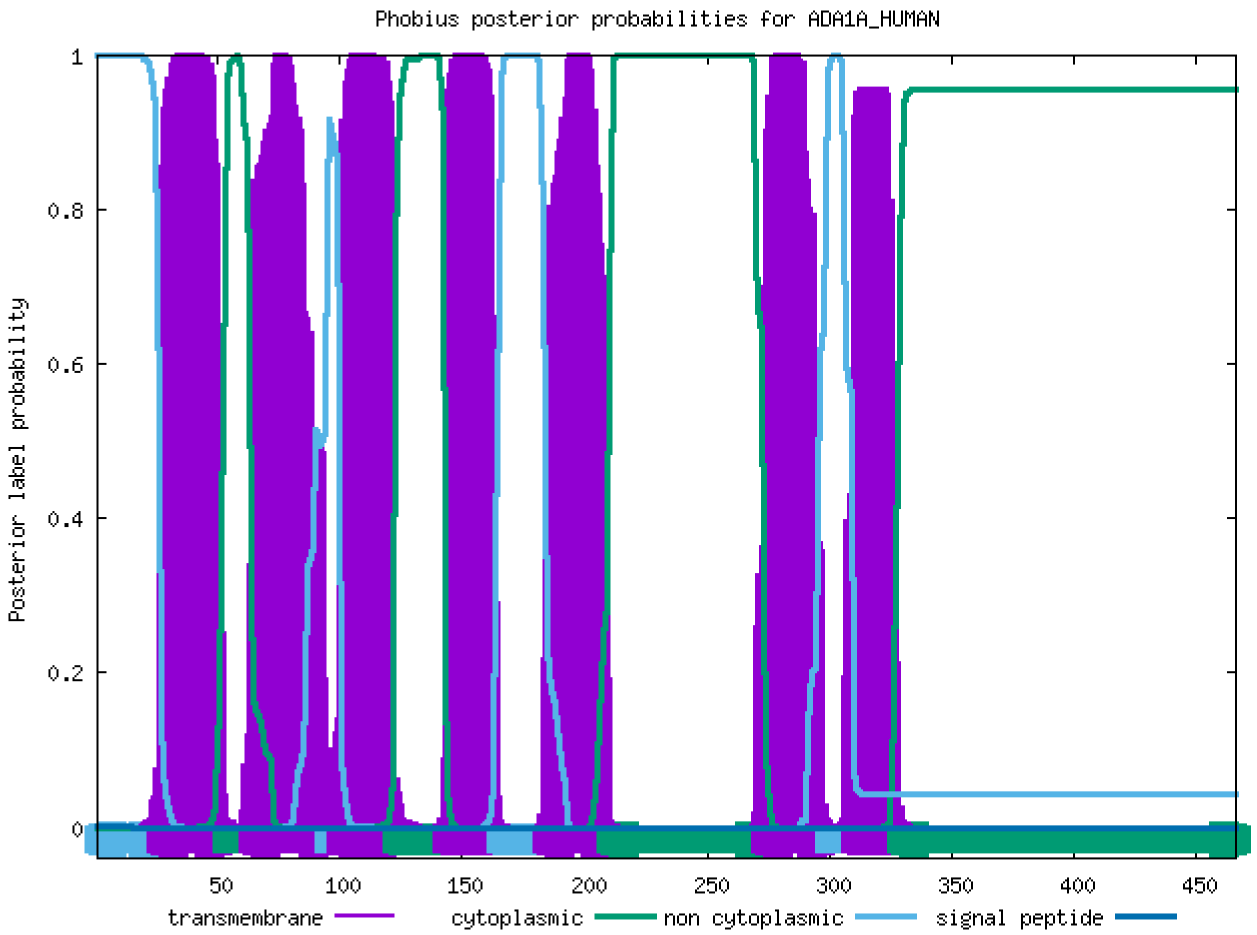

The probability data used in the plot is found here, and the gnuplot script is here.

Prediction of ADA1B_HUMAN

```
ID   ADA1B_HUMAN
FT   TOPO_DOM      1     44       NON CYTOPLASMIC.
FT   TRANSMEM     45     71
FT   TOPO_DOM     72     82       CYTOPLASMIC.
FT   TRANSMEM     83    108
FT   TOPO_DOM    109    119       NON CYTOPLASMIC.
FT   TRANSMEM    120    141
FT   TOPO_DOM    142    161       CYTOPLASMIC.
FT   TRANSMEM    162    182
FT   TOPO_DOM    183    201       NON CYTOPLASMIC.
FT   TRANSMEM    202    228
FT   TOPO_DOM    229    294       CYTOPLASMIC.
FT   TRANSMEM    295    316
FT   TOPO_DOM    317    327       NON CYTOPLASMIC.
FT   TRANSMEM    328    348
FT   TOPO_DOM    349    520       CYTOPLASMIC.
//
```

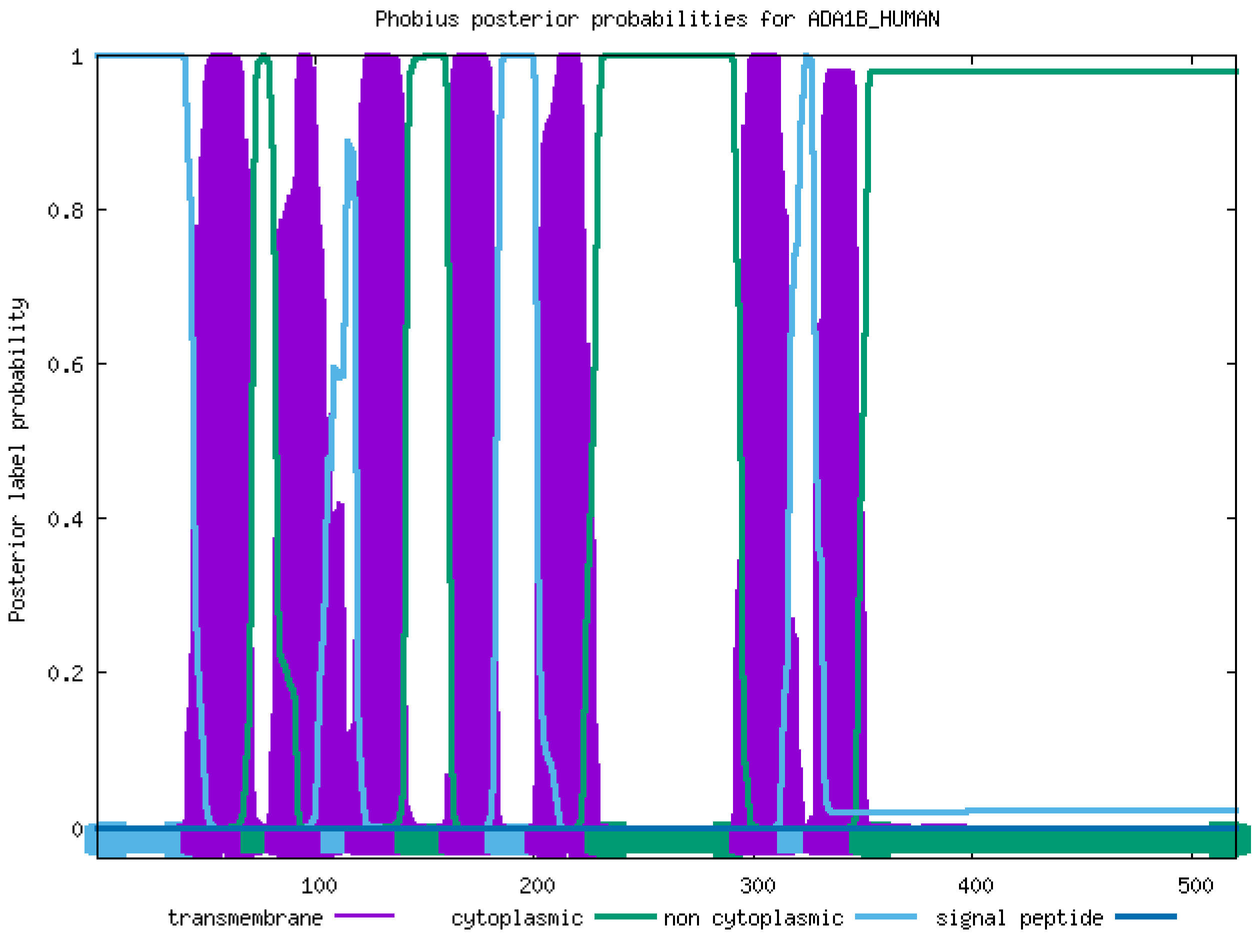

The probability data used in the plot is found here, and the gnuplot script is here.

Prediction of ADA2A_HUMAN

```
ID   ADA2A_HUMAN
FT   TOPO_DOM      1     48       NON CYTOPLASMIC.
FT   TRANSMEM     49     73
FT   TOPO_DOM     74     84       CYTOPLASMIC.
FT   TRANSMEM     85    104
FT   TOPO_DOM    105    123       NON CYTOPLASMIC.
FT   TRANSMEM    124    144
FT   TOPO_DOM    145    164       CYTOPLASMIC.
FT   TRANSMEM    165    185
FT   TOPO_DOM    186    209       NON CYTOPLASMIC.
FT   TRANSMEM    210    232
FT   TOPO_DOM    233    386       CYTOPLASMIC.
FT   TRANSMEM    387    405
FT   TOPO_DOM    406    424       NON CYTOPLASMIC.
FT   TRANSMEM    425    444
FT   TOPO_DOM    445    465       CYTOPLASMIC.
//
```

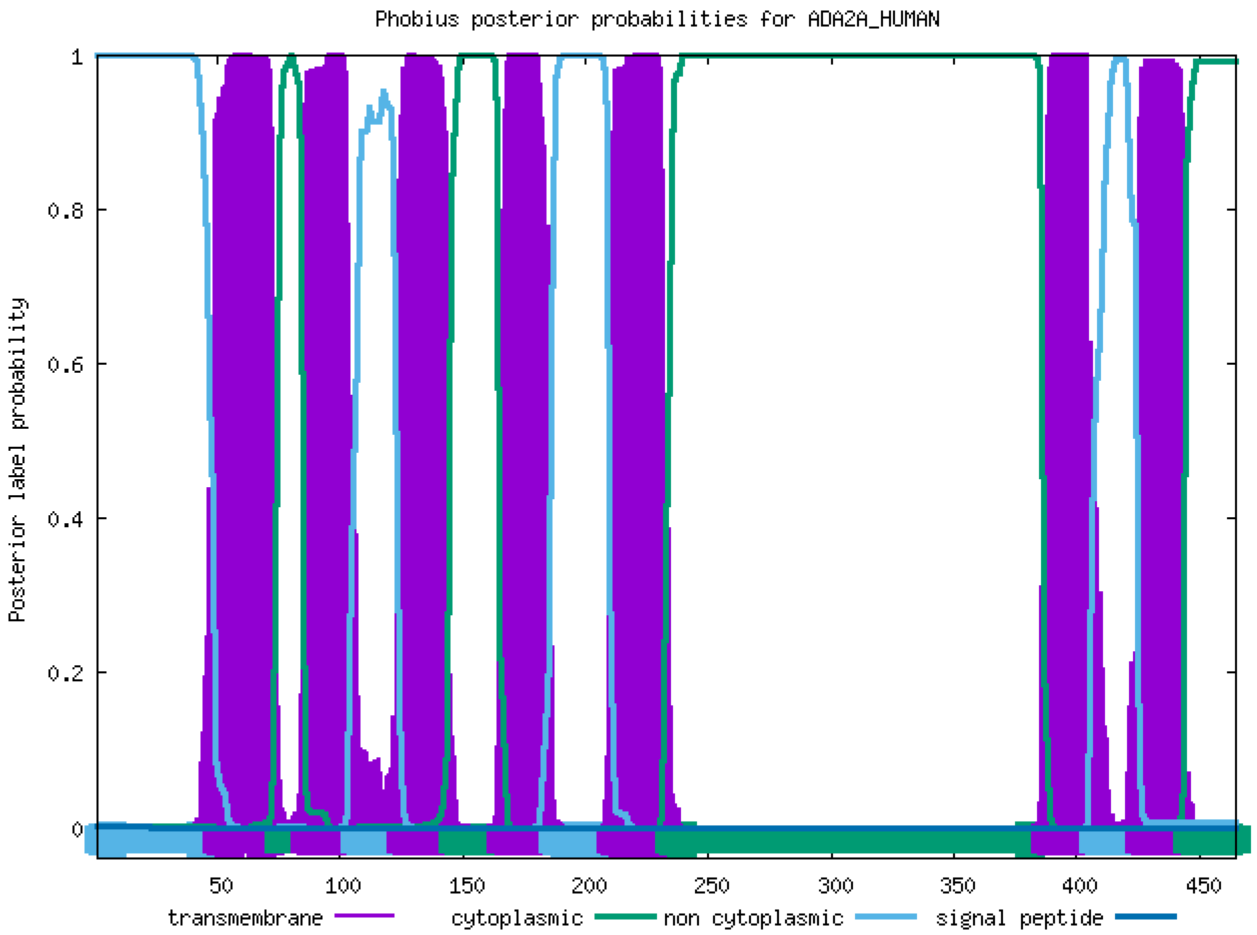

The probability data used in the plot is found here, and the gnuplot script is here.

Prediction of ADA2C_HUMAN

```
ID   ADA2C_HUMAN
FT   SIGNAL        1     34
FT   REGION        1      4       N-REGION.
FT   REGION        5     16       H-REGION.
FT   REGION       17     34       C-REGION.
FT   TOPO_DOM     35     49       NON CYTOPLASMIC.
FT   TRANSMEM     50     76
FT   TOPO_DOM     77     87       CYTOPLASMIC.
FT   TRANSMEM     88    109
FT   TOPO_DOM    110    114       NON CYTOPLASMIC.
FT   TRANSMEM    115    147
FT   TOPO_DOM    148    167       CYTOPLASMIC.
FT   TRANSMEM    168    188
FT   TOPO_DOM    189    207       NON CYTOPLASMIC.
FT   TRANSMEM    208    231
FT   TOPO_DOM    232    379       CYTOPLASMIC.
FT   TRANSMEM    380    401
FT   TOPO_DOM    402    420       NON CYTOPLASMIC.
FT   TRANSMEM    421    440
FT   TOPO_DOM    441    462       CYTOPLASMIC.
//
```

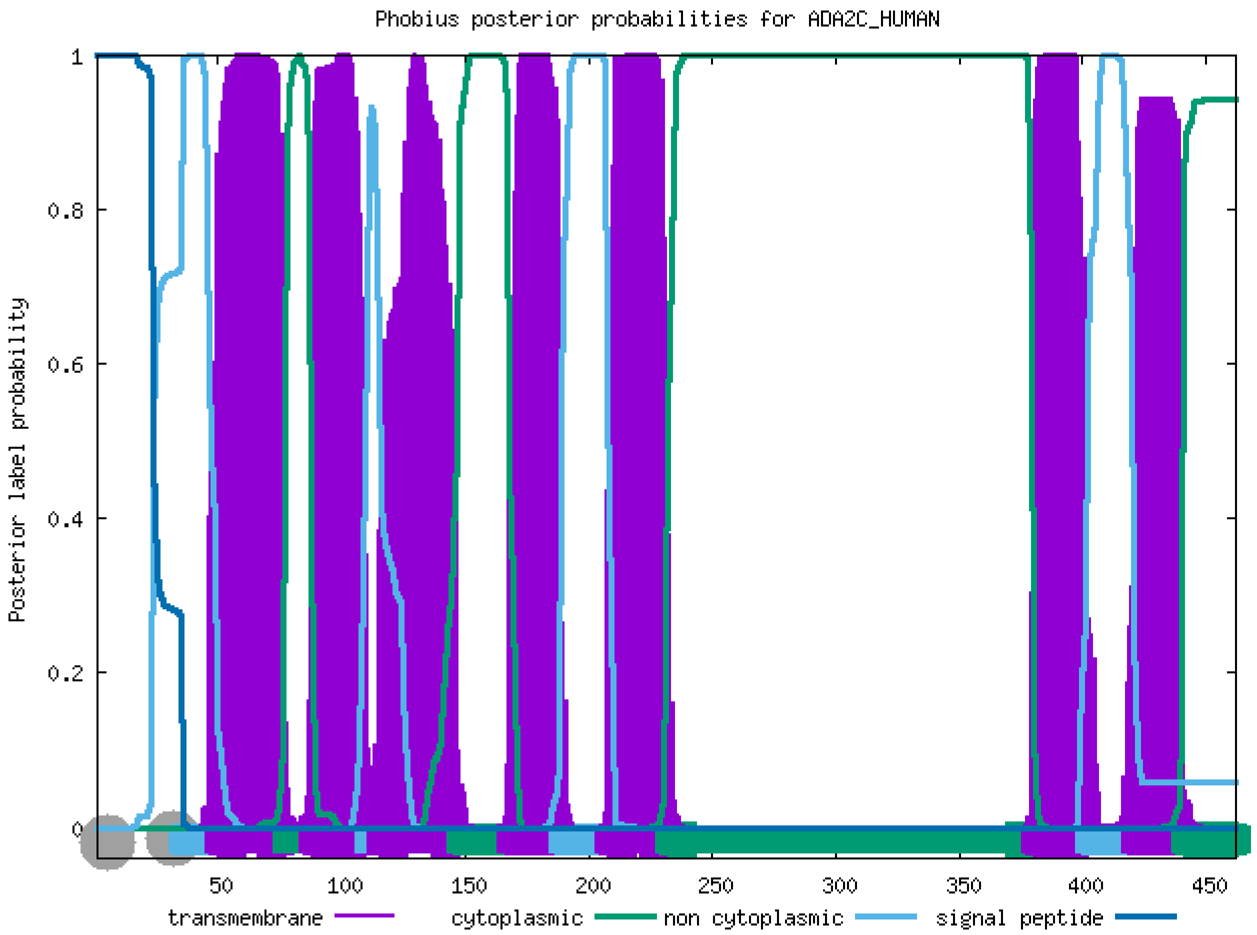

The probability data used in the plot is found here, and the gnuplot script is here.

Prediction of ADRB1_HUMAN

```
ID   ADRB1_HUMAN
FT   SIGNAL        1     19
FT   REGION        1      2       N-REGION.
FT   REGION        3     11       H-REGION.
FT   REGION       12     19       C-REGION.
FT   TOPO_DOM     20     56       NON CYTOPLASMIC.
FT   TRANSMEM     57     83
FT   TOPO_DOM     84     94       CYTOPLASMIC.
FT   TRANSMEM     95    121
FT   TOPO_DOM    122    132       NON CYTOPLASMIC.
FT   TRANSMEM    133    154
FT   TOPO_DOM    155    174       CYTOPLASMIC.
FT   TRANSMEM    175    199
FT   TOPO_DOM    200    222       NON CYTOPLASMIC.
FT   TRANSMEM    223    247
FT   TOPO_DOM    248    325       CYTOPLASMIC.
FT   TRANSMEM    326    346
FT   TOPO_DOM    347    357       NON CYTOPLASMIC.
FT   TRANSMEM    358    378
FT   TOPO_DOM    379    477       CYTOPLASMIC.
//
```

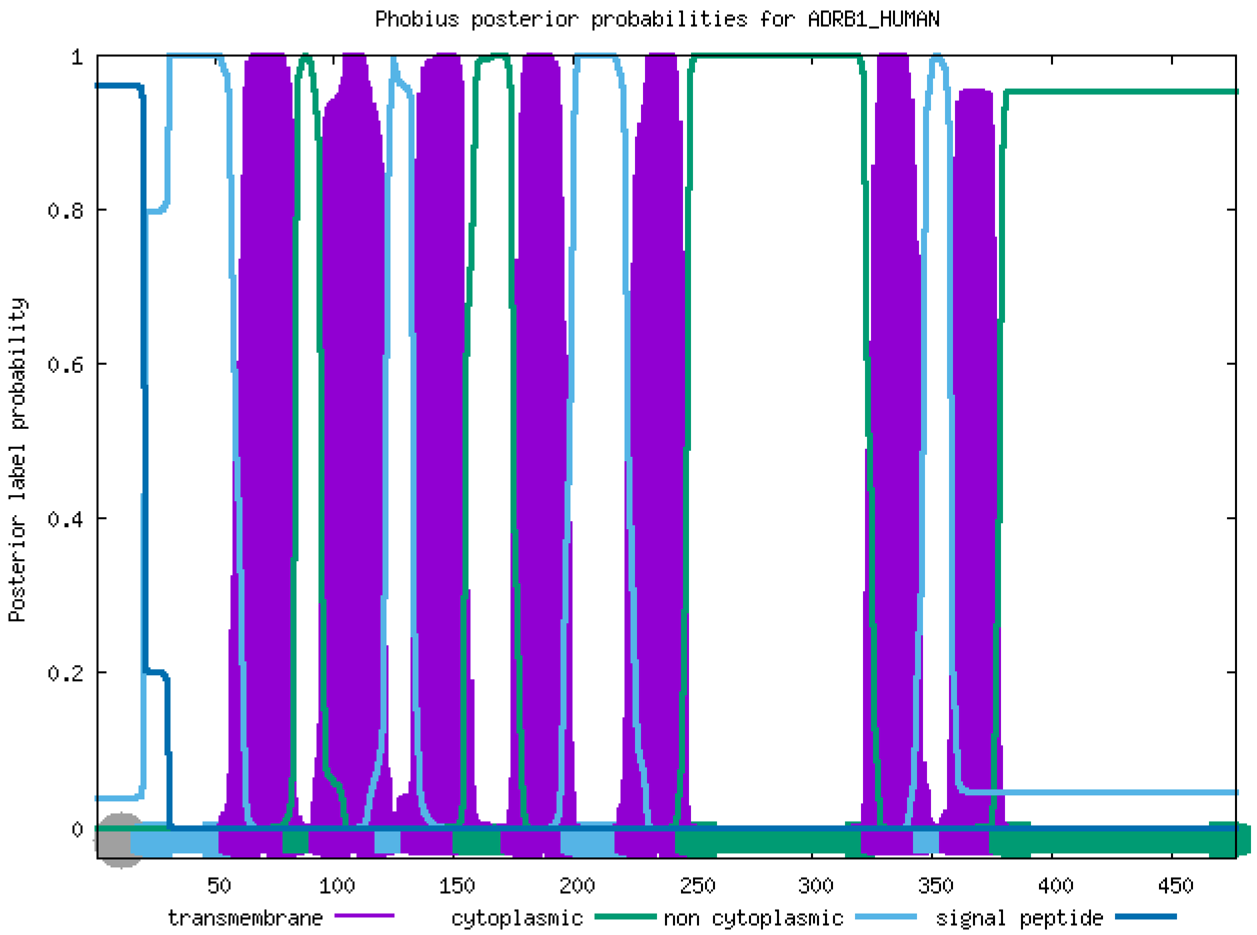

The probability data used in the plot is found here, and the gnuplot script is here.

Prediction of ADRB2_HUMAN

```
ID    ADRB2_HUMAN
FT    TOPO_DOM      1     30       NON CYTOPLASMIC.
FT    TRANSMEM     31     58
FT    TOPO_DOM     59     69       CYTOPLASMIC.
FT    TRANSMEM     70     96
FT    TOPO_DOM     97    107       NON CYTOPLASMIC.
FT    TRANSMEM    108    129
FT    TOPO_DOM    130    149       CYTOPLASMIC.
FT    TRANSMEM    150    169
FT    TOPO_DOM    170    197       NON CYTOPLASMIC.
FT    TRANSMEM    198    219
FT    TOPO_DOM    220    274       CYTOPLASMIC.
FT    TRANSMEM    275    295
FT    TOPO_DOM    296    306       NON CYTOPLASMIC.
FT    TRANSMEM    307    327
FT    TOPO_DOM    328    413       CYTOPLASMIC.
//
```

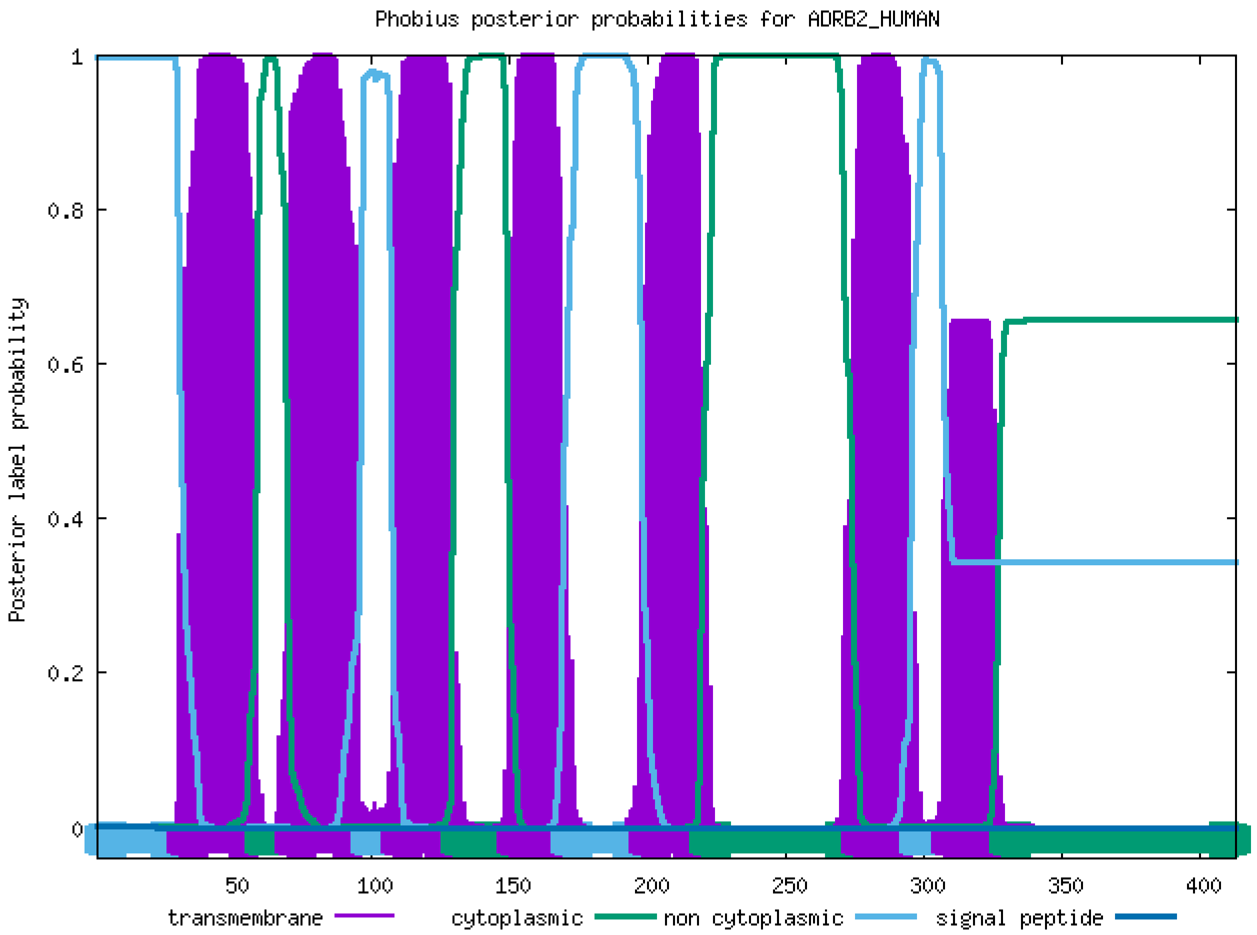

The probability data used in the plot is found here, and the gnuplot script is here.

Prediction of AA1R_HUMAN

```
ID   AA1R_HUMAN
FT   TOPO_DOM      1     11       NON CYTOPLASMIC.
FT   TRANSMEM     12     33
FT   TOPO_DOM     34     44       CYTOPLASMIC.
FT   TRANSMEM     45     69
FT   TOPO_DOM     70     74       NON CYTOPLASMIC.
FT   TRANSMEM     75    103
FT   TOPO_DOM    104    123       CYTOPLASMIC.
FT   TRANSMEM    124    146
FT   TOPO_DOM    147    184       NON CYTOPLASMIC.
FT   TRANSMEM    185    207
FT   TOPO_DOM    208    235       CYTOPLASMIC.
FT   TRANSMEM    236    259
FT   TOPO_DOM    260    270       NON CYTOPLASMIC.
FT   TRANSMEM    271    290
FT   TOPO_DOM    291    326       CYTOPLASMIC.
//
```

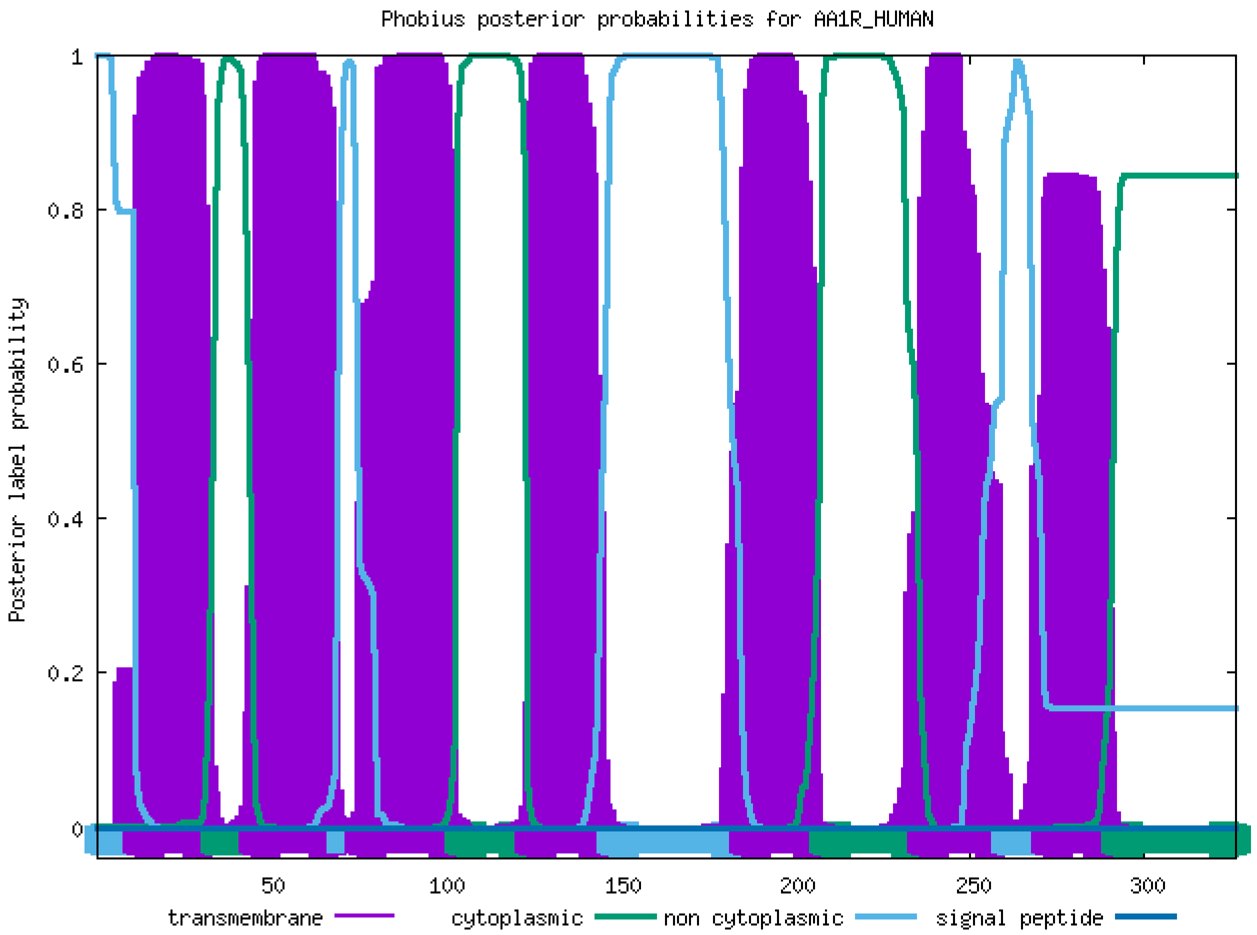

The probability data used in the plot is found here, and the gnuplot script is here.

Prediction of AA2AR_HUMAN

```
ID    AA2AR_HUMAN
FT    TOPO_DOM      1      5       NON CYTOPLASMIC.
FT    TRANSMEM      6     32
FT    TOPO_DOM     33     43       CYTOPLASMIC.
FT    TRANSMEM     44     66
FT    TOPO_DOM     67     77       NON CYTOPLASMIC.
FT    TRANSMEM     78    100
FT    TOPO_DOM    101    120       CYTOPLASMIC.
FT    TRANSMEM    121    143
FT    TOPO_DOM    144    177       NON CYTOPLASMIC.
FT    TRANSMEM    178    202
FT    TOPO_DOM    203    234       CYTOPLASMIC.
FT    TRANSMEM    235    258
FT    TOPO_DOM    259    269       NON CYTOPLASMIC.
FT    TRANSMEM    270    290
FT    TOPO_DOM    291    412       CYTOPLASMIC.
//
```

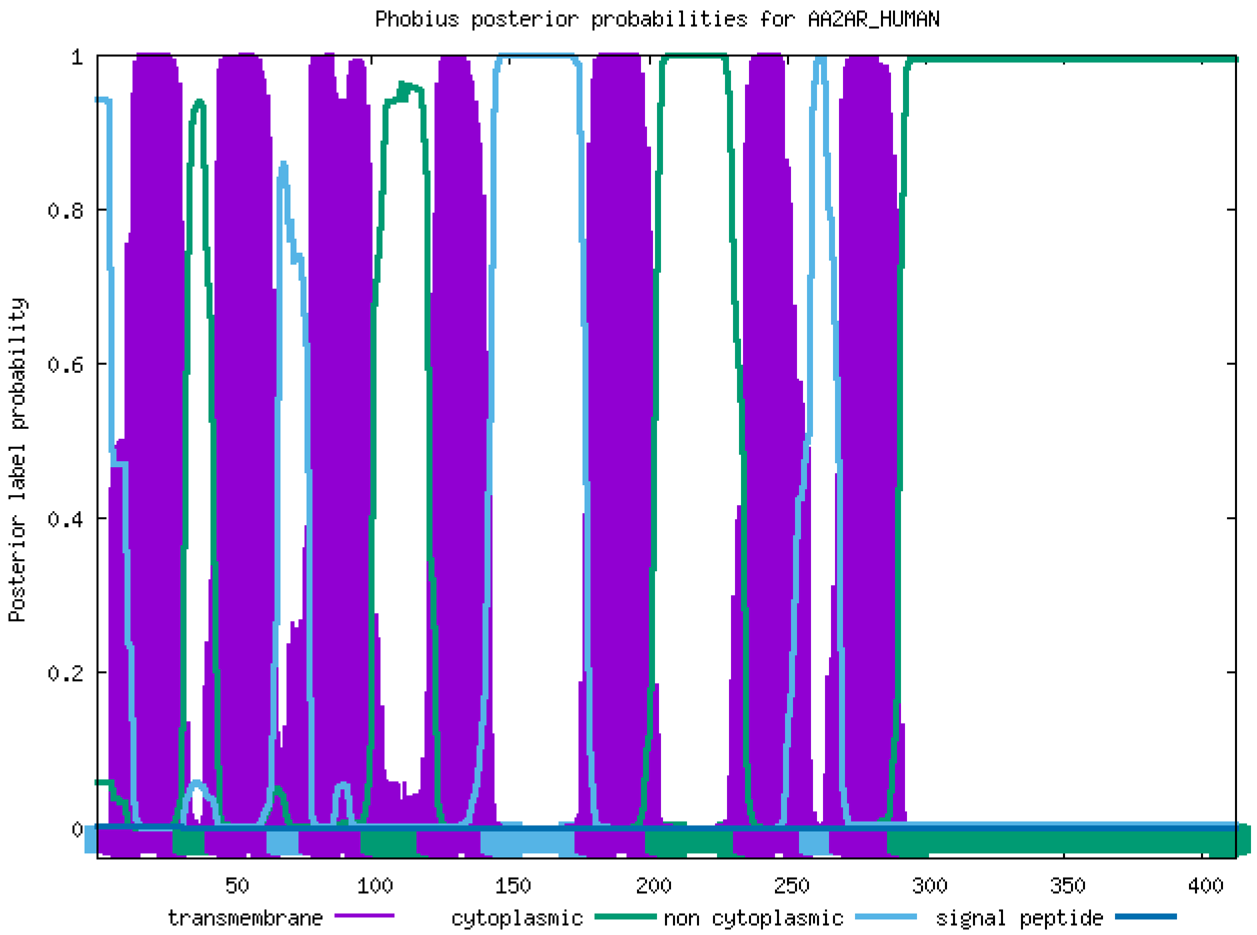

The probability data used in the plot is found here, and the gnuplot script is here.

Prediction of AA2BR_HUMAN

```
ID   AA2BR_HUMAN
FT   TOPO_DOM      1     11       NON CYTOPLASMIC.
FT   TRANSMEM     12     32
FT   TOPO_DOM     33     43       CYTOPLASMIC.
FT   TRANSMEM     44     67
FT   TOPO_DOM     68     78       NON CYTOPLASMIC.
FT   TRANSMEM     79    101
FT   TOPO_DOM    102    121       CYTOPLASMIC.
FT   TRANSMEM    122    142
FT   TOPO_DOM    143    182       NON CYTOPLASMIC.
FT   TRANSMEM    183    210
FT   TOPO_DOM    211    230       CYTOPLASMIC.
FT   TRANSMEM    231    250
FT   TOPO_DOM    251    269       NON CYTOPLASMIC.
FT   TRANSMEM    270    292
FT   TOPO_DOM    293    332       CYTOPLASMIC.
//
```

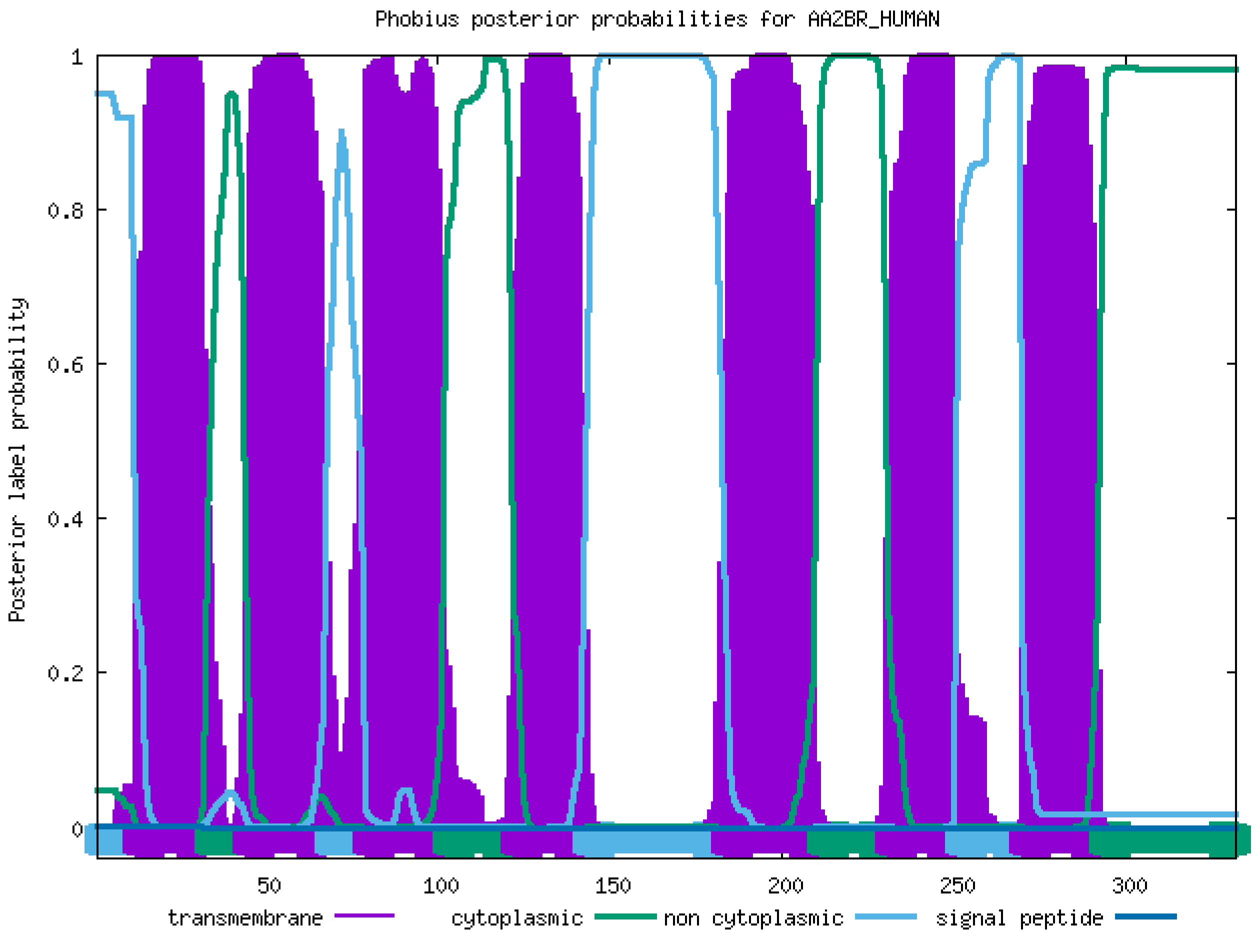

The probability data used in the plot is found here, and the gnuplot script is here.

Prediction of CXCR1_HUMAN

```
ID   CXCR1_HUMAN
FT   TOPO_DOM      1     39       NON CYTOPLASMIC.
FT   TRANSMEM     40     65
FT   TOPO_DOM     66     76       CYTOPLASMIC.
FT   TRANSMEM     77     96
FT   TOPO_DOM     97    120       NON CYTOPLASMIC.
FT   TRANSMEM    121    140
FT   TOPO_DOM    141    151       CYTOPLASMIC.
FT   TRANSMEM    152    174
FT   TOPO_DOM    175    208       NON CYTOPLASMIC.
FT   TRANSMEM    209    230
FT   TOPO_DOM    231    241       CYTOPLASMIC.
FT   TRANSMEM    242    264
FT   TOPO_DOM    265    350       NON CYTOPLASMIC.
//
```

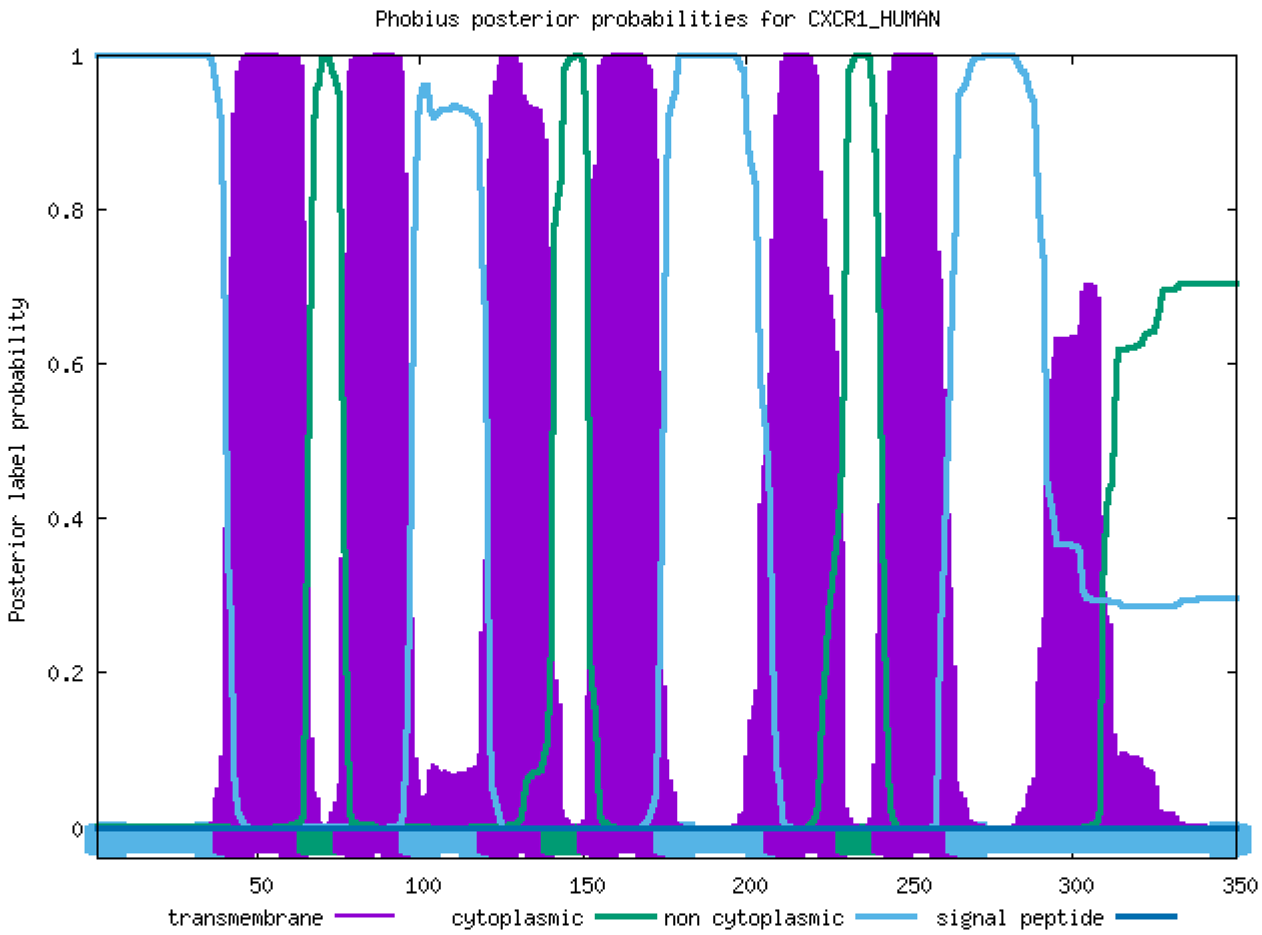

The probability data used in the plot is found here, and the gnuplot script is here.

Prediction of CXCR2_HUMAN

```
ID   CXCR2_HUMAN
FT   TOPO_DOM      1     48       NON CYTOPLASMIC.
FT   TRANSMEM     49     74
FT   TOPO_DOM     75     85       CYTOPLASMIC.
FT   TRANSMEM     86    105
FT   TOPO_DOM    106    129       NON CYTOPLASMIC.
FT   TRANSMEM    130    148
FT   TOPO_DOM    149    159       CYTOPLASMIC.
FT   TRANSMEM    160    183
FT   TOPO_DOM    184    217       NON CYTOPLASMIC.
FT   TRANSMEM    218    239
FT   TOPO_DOM    240    250       CYTOPLASMIC.
FT   TRANSMEM    251    273
FT   TOPO_DOM    274    360       NON CYTOPLASMIC.
//
```

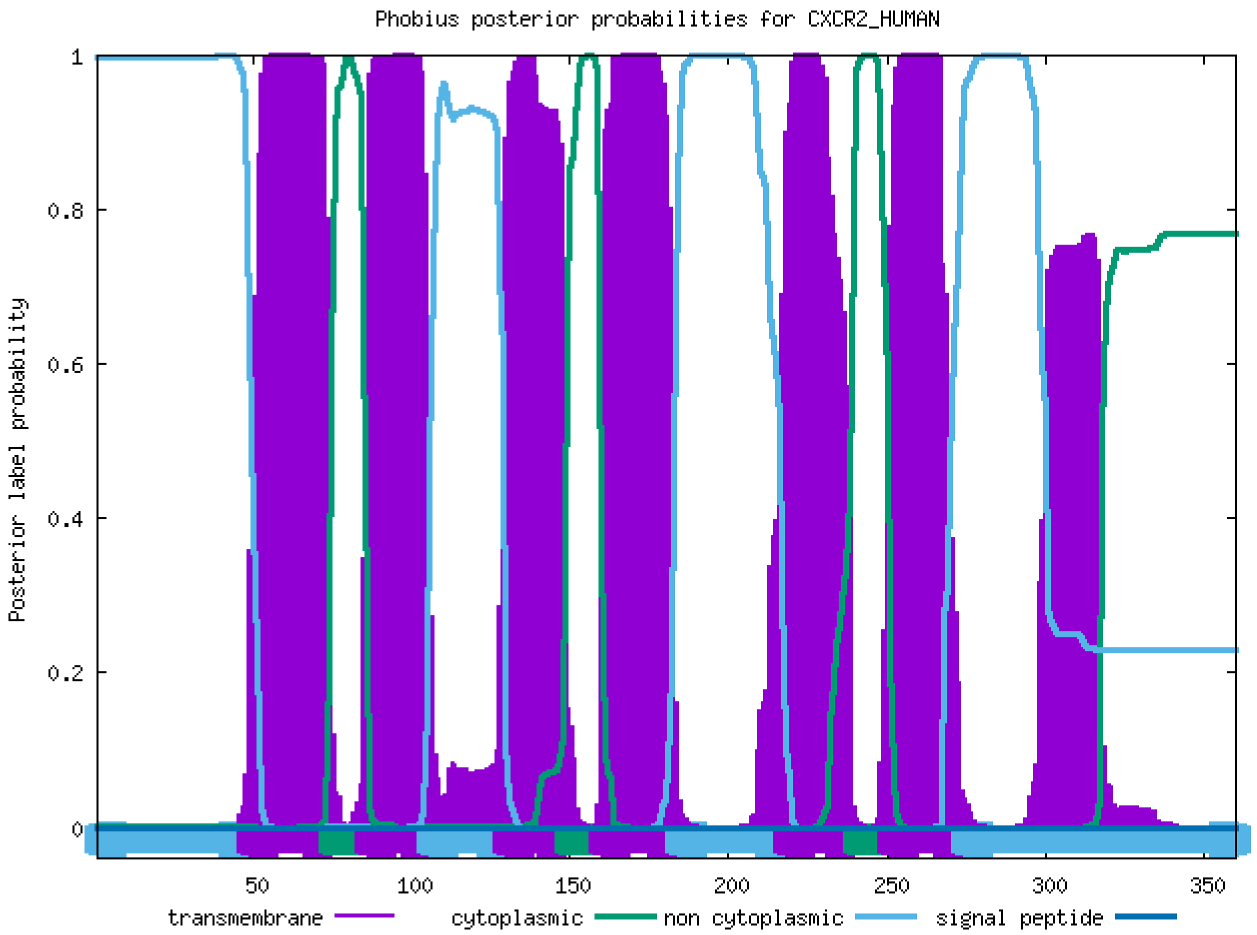

The probability data used in the plot is found here, and the gnuplot script is here.

Prediction of CCR2_HUMAN

```
ID    CCR2_HUMAN
FT    TOPO_DOM      1     47       NON CYTOPLASMIC.
FT    TRANSMEM     48     68
FT    TOPO_DOM     69     79       CYTOPLASMIC.
FT    TRANSMEM     80     99
FT    TOPO_DOM    100    118       NON CYTOPLASMIC.
FT    TRANSMEM    119    141
FT    TOPO_DOM    142    152       CYTOPLASMIC.
FT    TRANSMEM    153    178
FT    TOPO_DOM    179    207       NON CYTOPLASMIC.
FT    TRANSMEM    208    229
FT    TOPO_DOM    230    240       CYTOPLASMIC.
FT    TRANSMEM    241    259
FT    TOPO_DOM    260    374       NON CYTOPLASMIC.
//
```

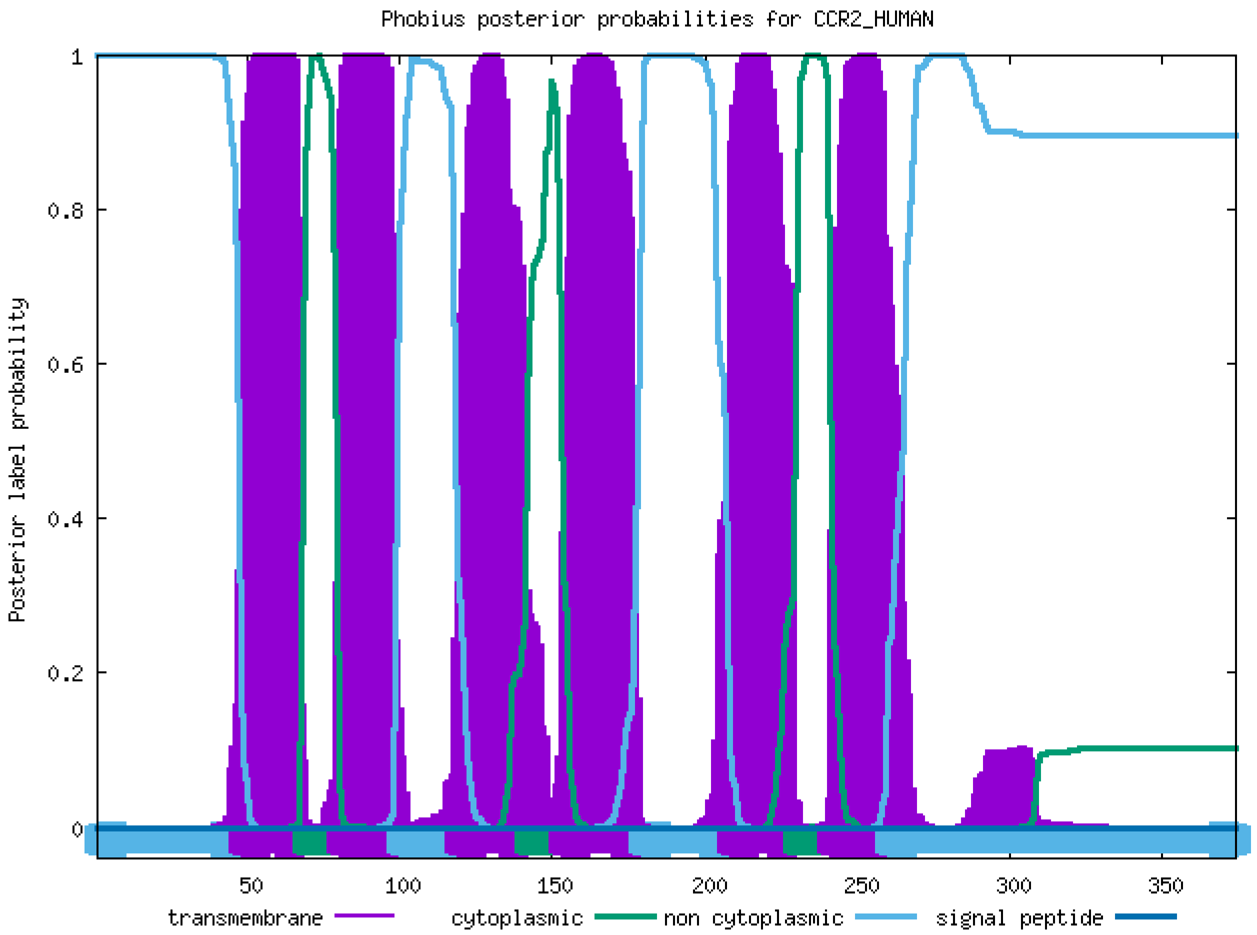

The probability data used in the plot is found here, and the gnuplot script is here.

Prediction of CCR3_HUMAN

```
ID    CCR3_HUMAN
FT    TOPO_DOM      1     39       NON CYTOPLASMIC.
FT    TRANSMEM     40     60
FT    TOPO_DOM     61     71       CYTOPLASMIC.
FT    TRANSMEM     72     91
FT    TOPO_DOM     92    110       NON CYTOPLASMIC.
FT    TRANSMEM    111    134
FT    TOPO_DOM    135    145       CYTOPLASMIC.
FT    TRANSMEM    146    166
FT    TOPO_DOM    167    202       NON CYTOPLASMIC.
FT    TRANSMEM    203    227
FT    TOPO_DOM    228    238       CYTOPLASMIC.
FT    TRANSMEM    239    261
FT    TOPO_DOM    262    280       NON CYTOPLASMIC.
FT    TRANSMEM    281    304
FT    TOPO_DOM    305    355       CYTOPLASMIC.
//
```

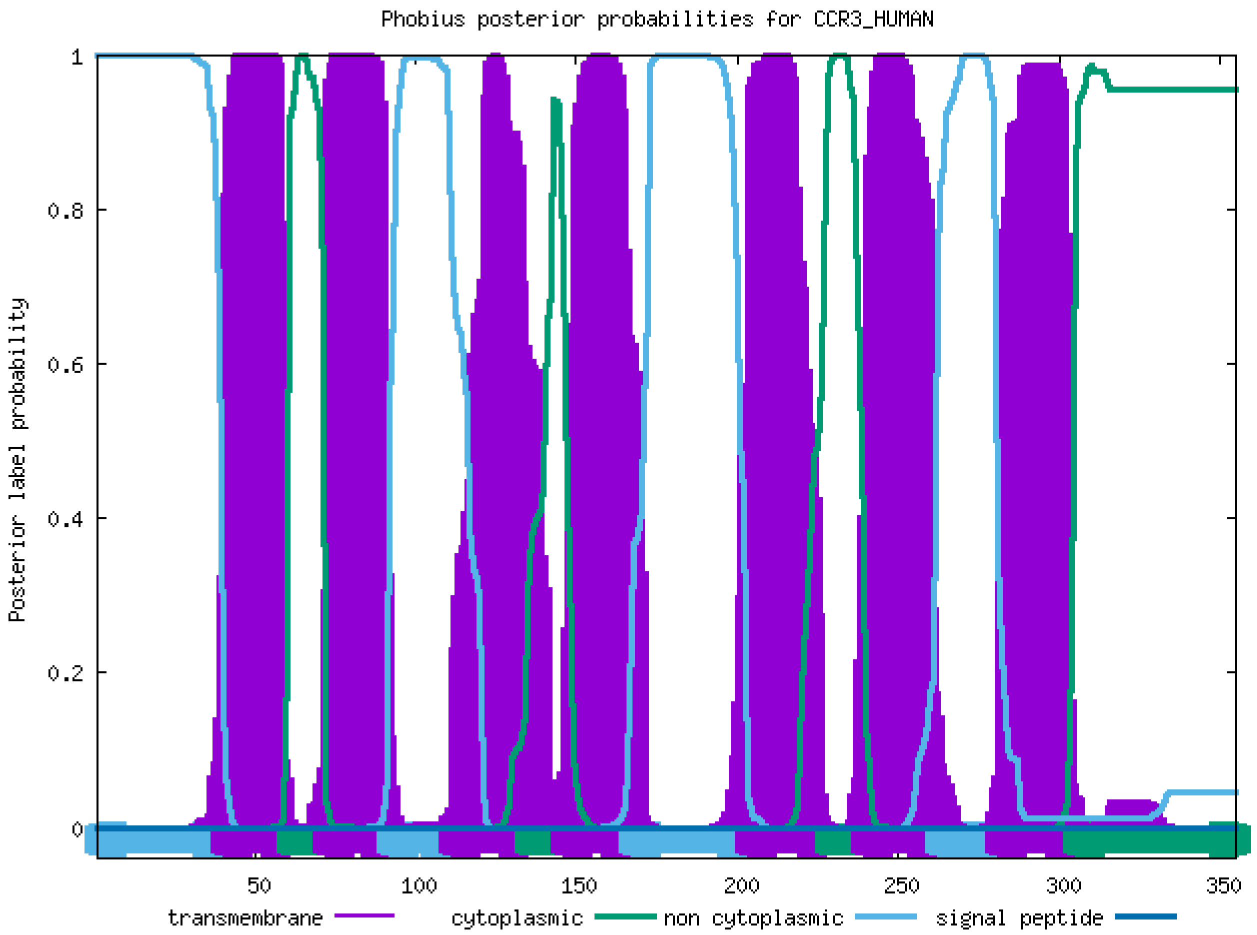

The probability data used in the plot is found here, and the gnuplot script is here.

Prediction of ACKR3_HUMAN

```
ID    ACKR3_HUMAN
FT    TOPO_DOM      1     41       NON CYTOPLASMIC.
FT    TRANSMEM     42     70
FT    TOPO_DOM     71     81       CYTOPLASMIC.
FT    TRANSMEM     82    102
FT    TOPO_DOM    103    121       NON CYTOPLASMIC.
FT    TRANSMEM    122    140
FT    TOPO_DOM    141    159       CYTOPLASMIC.
FT    TRANSMEM    160    178
FT    TOPO_DOM    179    207       NON CYTOPLASMIC.
FT    TRANSMEM    208    234
FT    TOPO_DOM    235    253       CYTOPLASMIC.
FT    TRANSMEM    254    280
FT    TOPO_DOM    281    291       NON CYTOPLASMIC.
FT    TRANSMEM    292    318
FT    TOPO_DOM    319    362       CYTOPLASMIC.
//
```

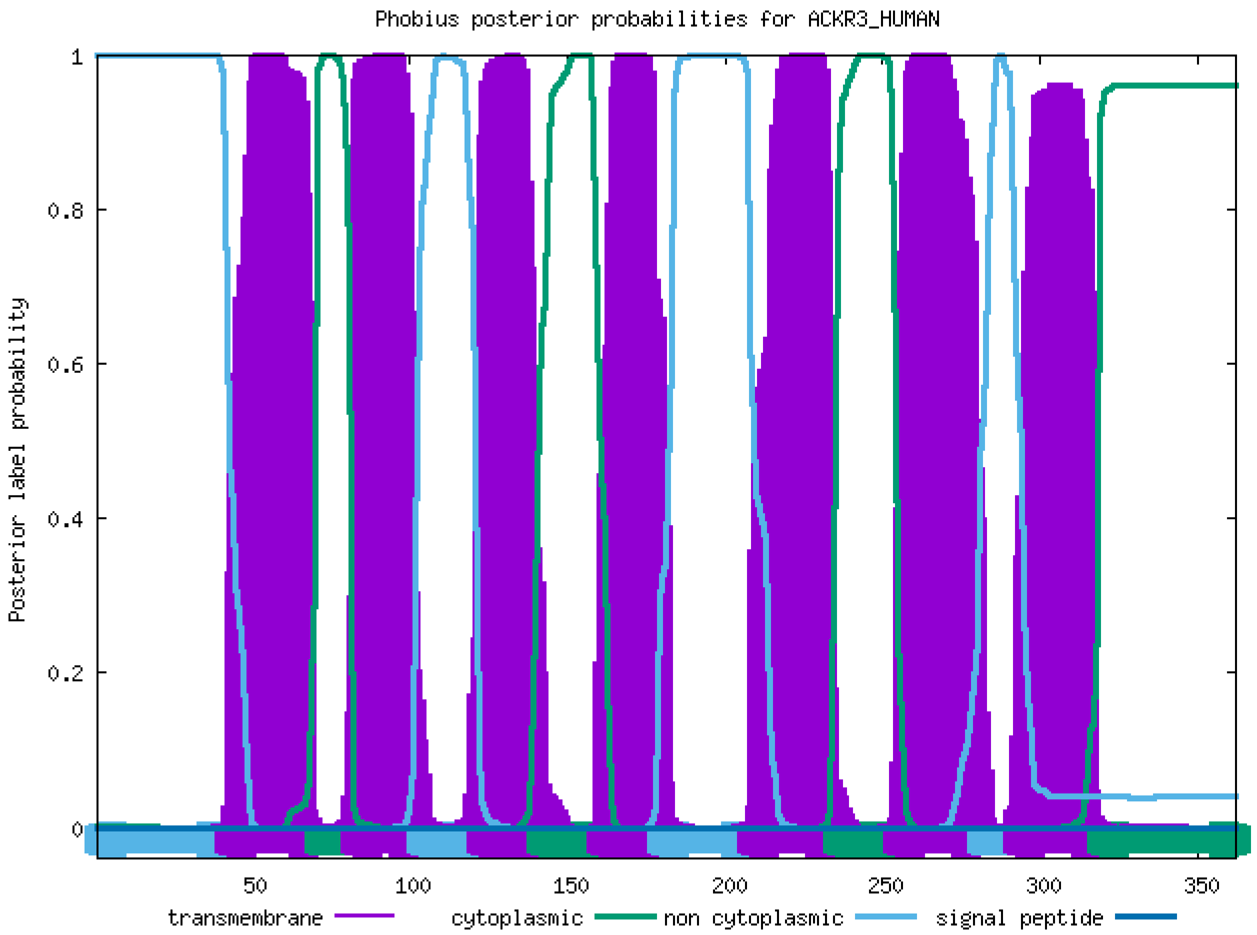

The probability data used in the plot is found here, and the gnuplot script is here.

Prediction of CXCR4_HUMAN

```
ID   CXCR4_HUMAN
FT   TOPO_DOM      1     38       NON CYTOPLASMIC.
FT   TRANSMEM     39     64
FT   TOPO_DOM     65     75       CYTOPLASMIC.
FT   TRANSMEM     76     94
FT   TOPO_DOM     95    113       NON CYTOPLASMIC.
FT   TRANSMEM    114    135
FT   TOPO_DOM    136    154       CYTOPLASMIC.
FT   TRANSMEM    155    174
FT   TOPO_DOM    175    193       NON CYTOPLASMIC.
FT   TRANSMEM    194    222
FT   TOPO_DOM    223    241       CYTOPLASMIC.
FT   TRANSMEM    242    266
FT   TOPO_DOM    267    285       NON CYTOPLASMIC.
FT   TRANSMEM    286    305
FT   TOPO_DOM    306    352       CYTOPLASMIC.
//
```

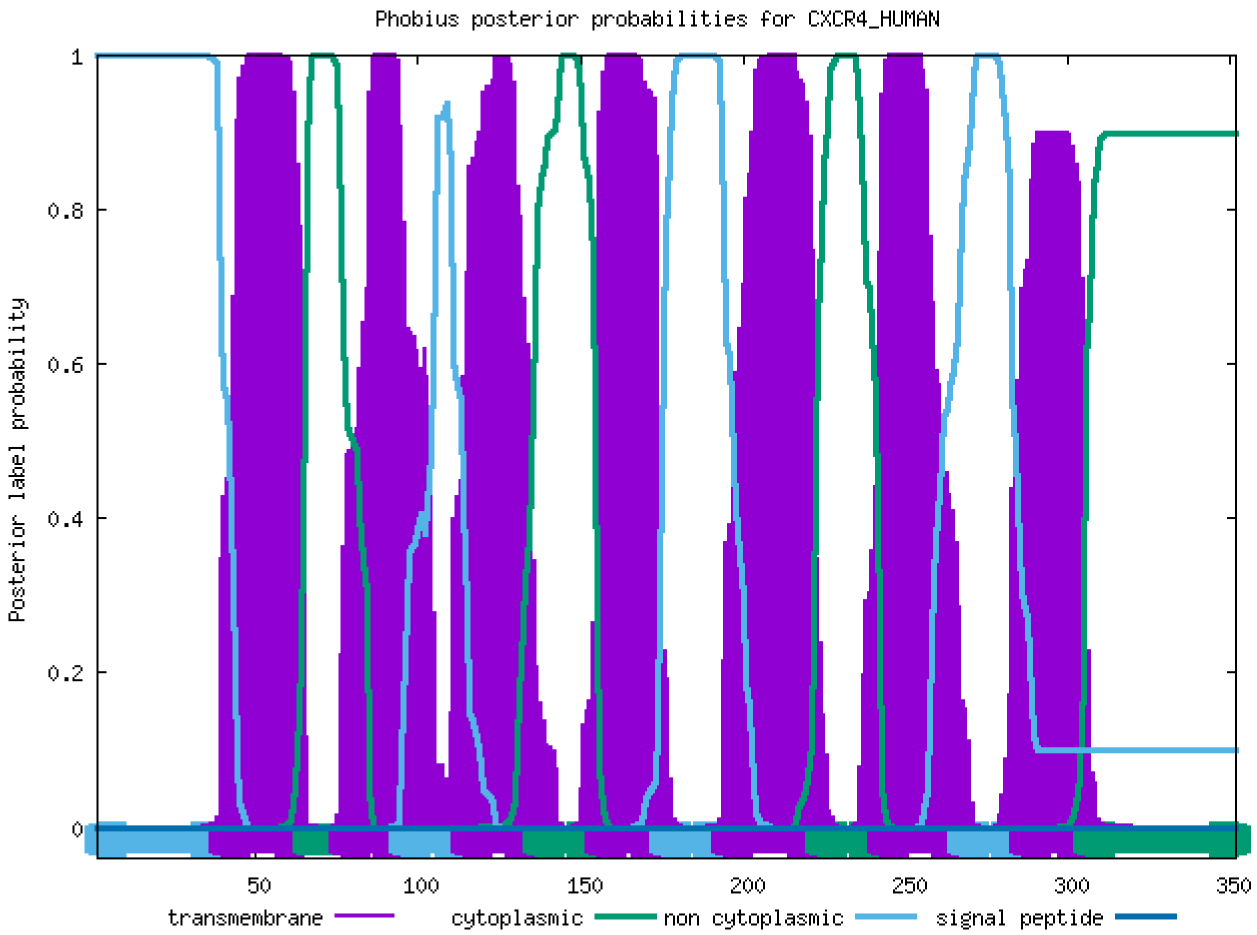

The probability data used in the plot is found here, and the gnuplot script is here.

Prediction of CX3C1_HUMAN

```
ID   CX3C1_HUMAN
FT   TOPO_DOM      1     35       NON CYTOPLASMIC.
FT   TRANSMEM     36     56
FT   TOPO_DOM     57     67       CYTOPLASMIC.
FT   TRANSMEM     68     87
FT   TOPO_DOM     88    106       NON CYTOPLASMIC.
FT   TRANSMEM    107    125
FT   TOPO_DOM    126    145       CYTOPLASMIC.
FT   TRANSMEM    146    167
FT   TOPO_DOM    168    196       NON CYTOPLASMIC.
FT   TRANSMEM    197    220
FT   TOPO_DOM    221    231       CYTOPLASMIC.
FT   TRANSMEM    232    253
FT   TOPO_DOM    254    272       NON CYTOPLASMIC.
FT   TRANSMEM    273    296
FT   TOPO_DOM    297    355       CYTOPLASMIC.
//
```

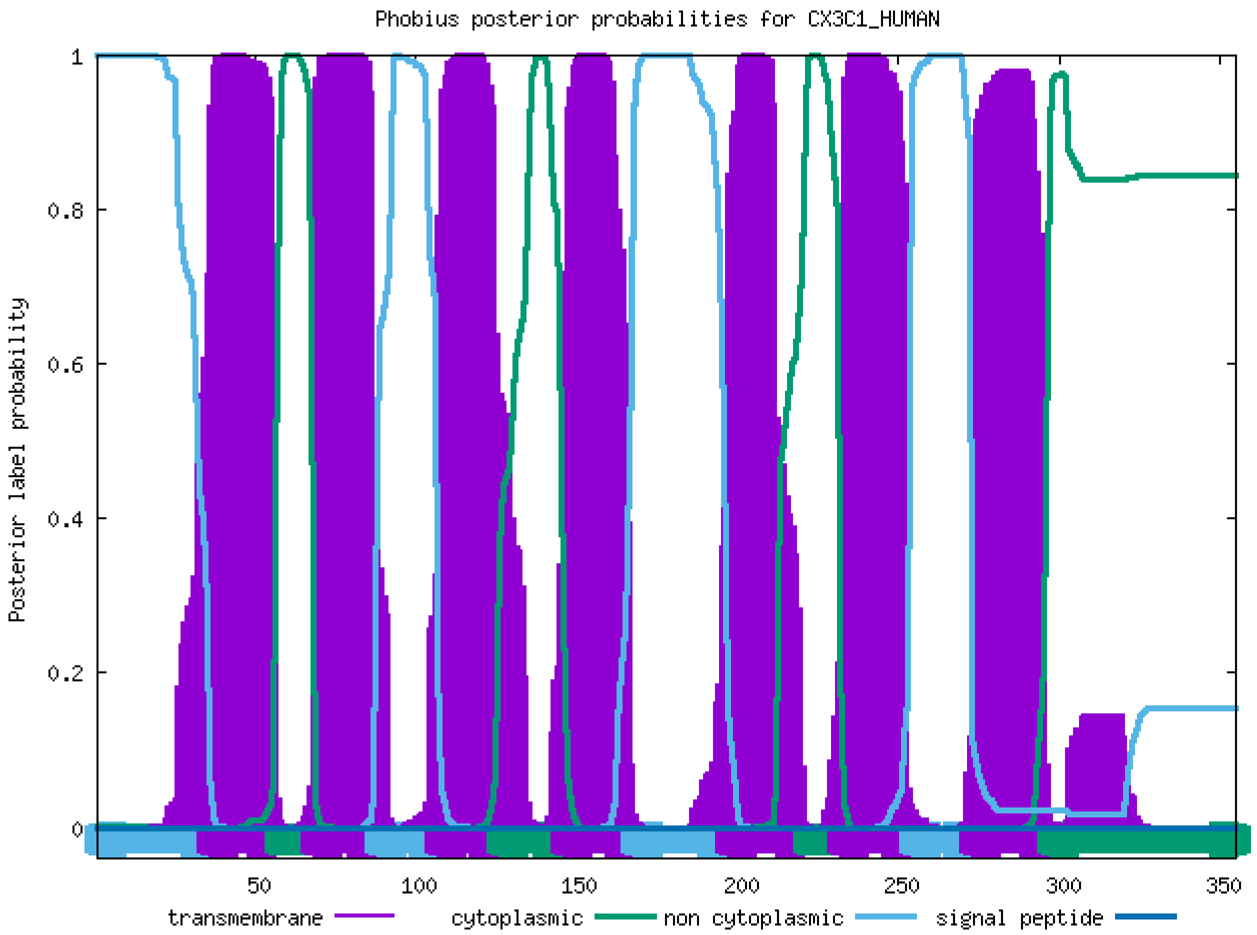

The probability data used in the plot is found here, and the gnuplot script is here.

Prediction of CCR5_HUMAN

```
ID   CCR5_HUMAN
FT   TOPO_DOM      1     35       NON CYTOPLASMIC.
FT   TRANSMEM     36     56
FT   TOPO_DOM     57     67       CYTOPLASMIC.
FT   TRANSMEM     68     87
FT   TOPO_DOM     88    106       NON CYTOPLASMIC.
FT   TRANSMEM    107    129
FT   TOPO_DOM    130    140       CYTOPLASMIC.
FT   TRANSMEM    141    166
FT   TOPO_DOM    167    197       NON CYTOPLASMIC.
FT   TRANSMEM    198    221
FT   TOPO_DOM    222    232       CYTOPLASMIC.
FT   TRANSMEM    233    251
FT   TOPO_DOM    252    352       NON CYTOPLASMIC.
//
```

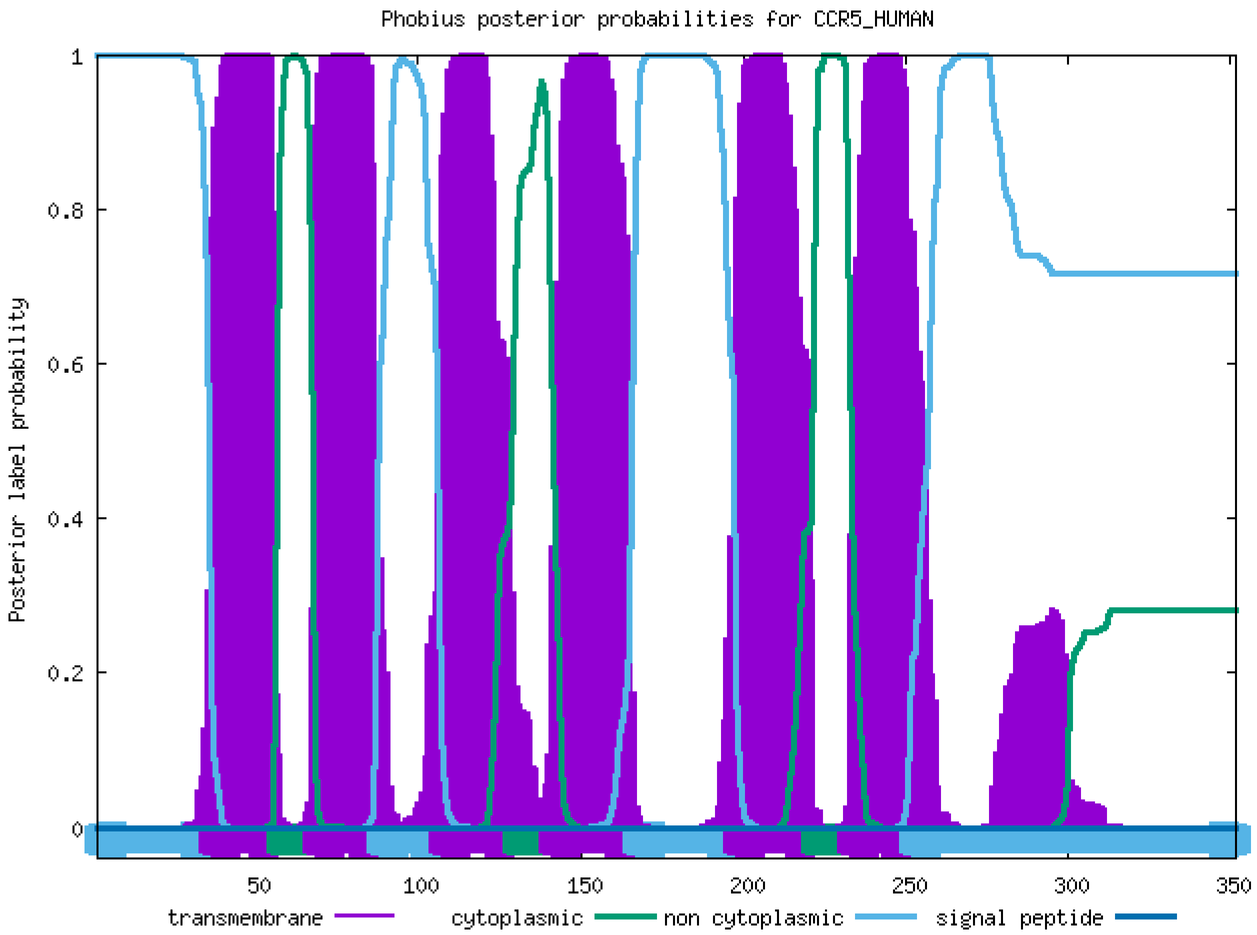

The probability data used in the plot is found here, and the gnuplot script is here.

Prediction of CCR6_HUMAN

```
ID    CCR6_HUMAN
FT    TOPO_DOM      1     50       NON CYTOPLASMIC.
FT    TRANSMEM     51     74
FT    TOPO_DOM     75     82       CYTOPLASMIC.
FT    TRANSMEM     83    104
FT    TOPO_DOM    105    123       NON CYTOPLASMIC.
FT    TRANSMEM    124    145
FT    TOPO_DOM    146    165       CYTOPLASMIC.
FT    TRANSMEM    166    185
FT    TOPO_DOM    186    218       NON CYTOPLASMIC.
FT    TRANSMEM    219    242
FT    TOPO_DOM    243    253       CYTOPLASMIC.
FT    TRANSMEM    254    279
FT    TOPO_DOM    280    298       NON CYTOPLASMIC.
FT    TRANSMEM    299    319
FT    TOPO_DOM    320    374       CYTOPLASMIC.
//
```

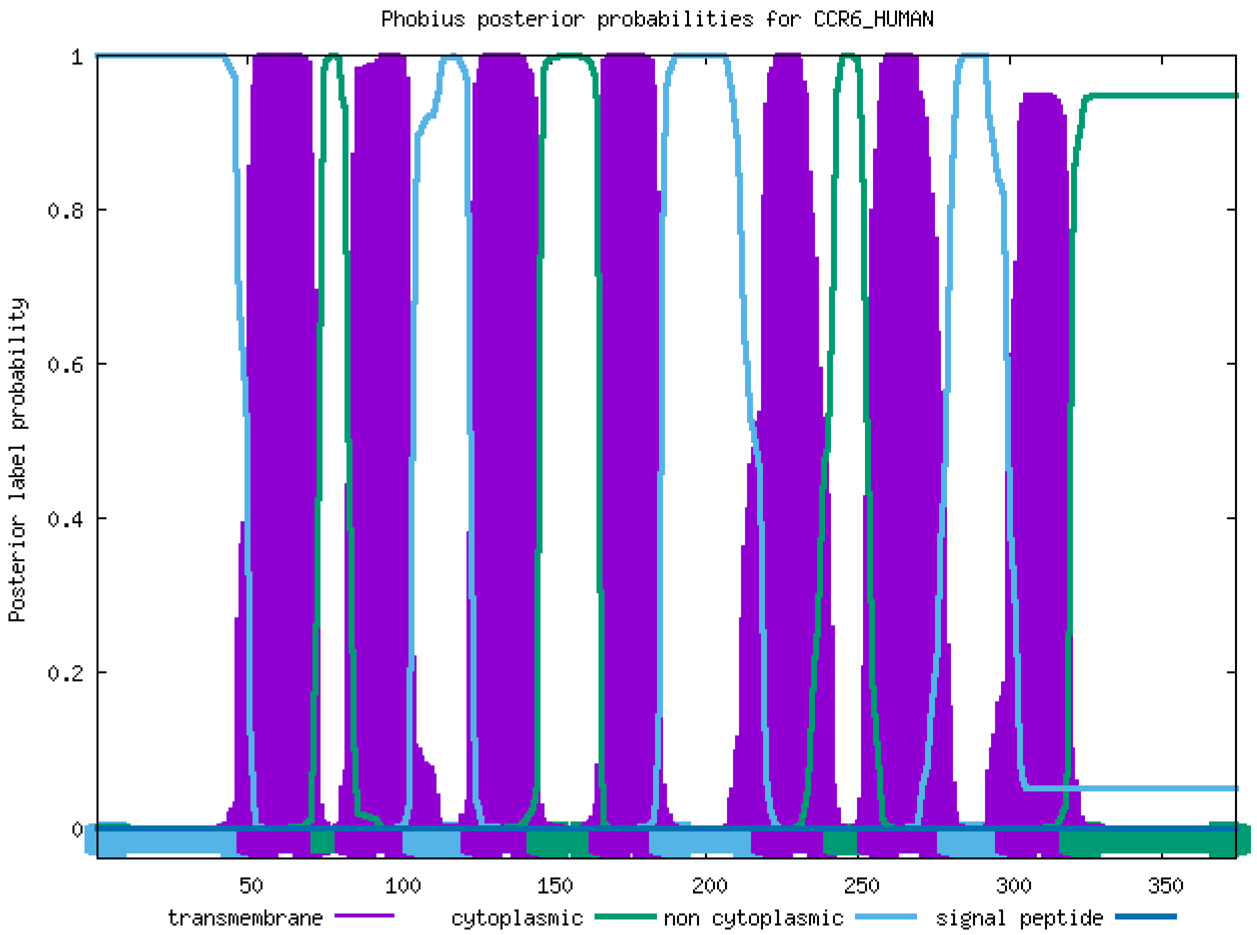

The probability data used in the plot is found here, and the gnuplot script is here.

Prediction of CCR7_HUMAN

```
ID   CCR7_HUMAN
FT   SIGNAL         1      24
FT   REGION         1       8       N-REGION.
FT   REGION         9      19       H-REGION.
FT   REGION        20      24       C-REGION.
FT   TOPO_DOM      25      58       NON CYTOPLASMIC.
FT   TRANSMEM      59      84
FT   TOPO_DOM      85      95       CYTOPLASMIC.
FT   TRANSMEM      96     116
FT   TOPO_DOM     117     121       NON CYTOPLASMIC.
FT   TRANSMEM     122     150
FT   TOPO_DOM     151     170       CYTOPLASMIC.
FT   TRANSMEM     171     191
FT   TOPO_DOM     192     224       NON CYTOPLASMIC.
FT   TRANSMEM     225     249
FT   TOPO_DOM     250     260       CYTOPLASMIC.
FT   TRANSMEM     261     279
FT   TOPO_DOM     280     309       NON CYTOPLASMIC.
FT   TRANSMEM     310     329
FT   TOPO_DOM     330     378       CYTOPLASMIC.
//
```

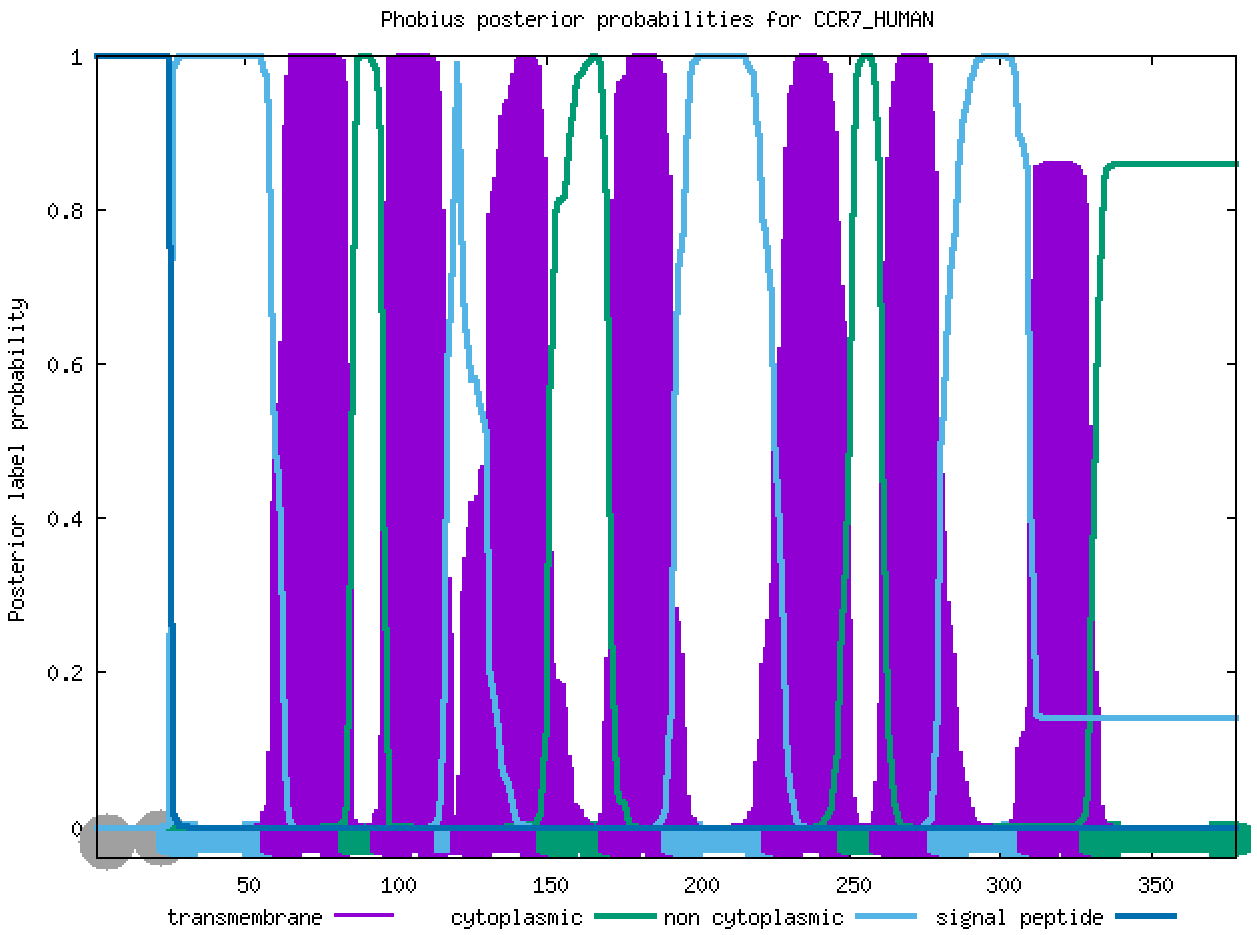

The probability data used in the plot is found here, and the gnuplot script is here.

Prediction of CCR9_HUMAN

```
ID   CCR9_HUMAN
FT   TOPO_DOM      1     53       NON CYTOPLASMIC.
FT   TRANSMEM     54     74
FT   TOPO_DOM     75     85       CYTOPLASMIC.
FT   TRANSMEM     86    108
FT   TOPO_DOM    109    127       NON CYTOPLASMIC.
FT   TRANSMEM    128    146
FT   TOPO_DOM    147    166       CYTOPLASMIC.
FT   TRANSMEM    167    186
FT   TOPO_DOM    187    218       NON CYTOPLASMIC.
FT   TRANSMEM    219    240
FT   TOPO_DOM    241    251       CYTOPLASMIC.
FT   TRANSMEM    252    270
FT   TOPO_DOM    271    369       NON CYTOPLASMIC.
//
```

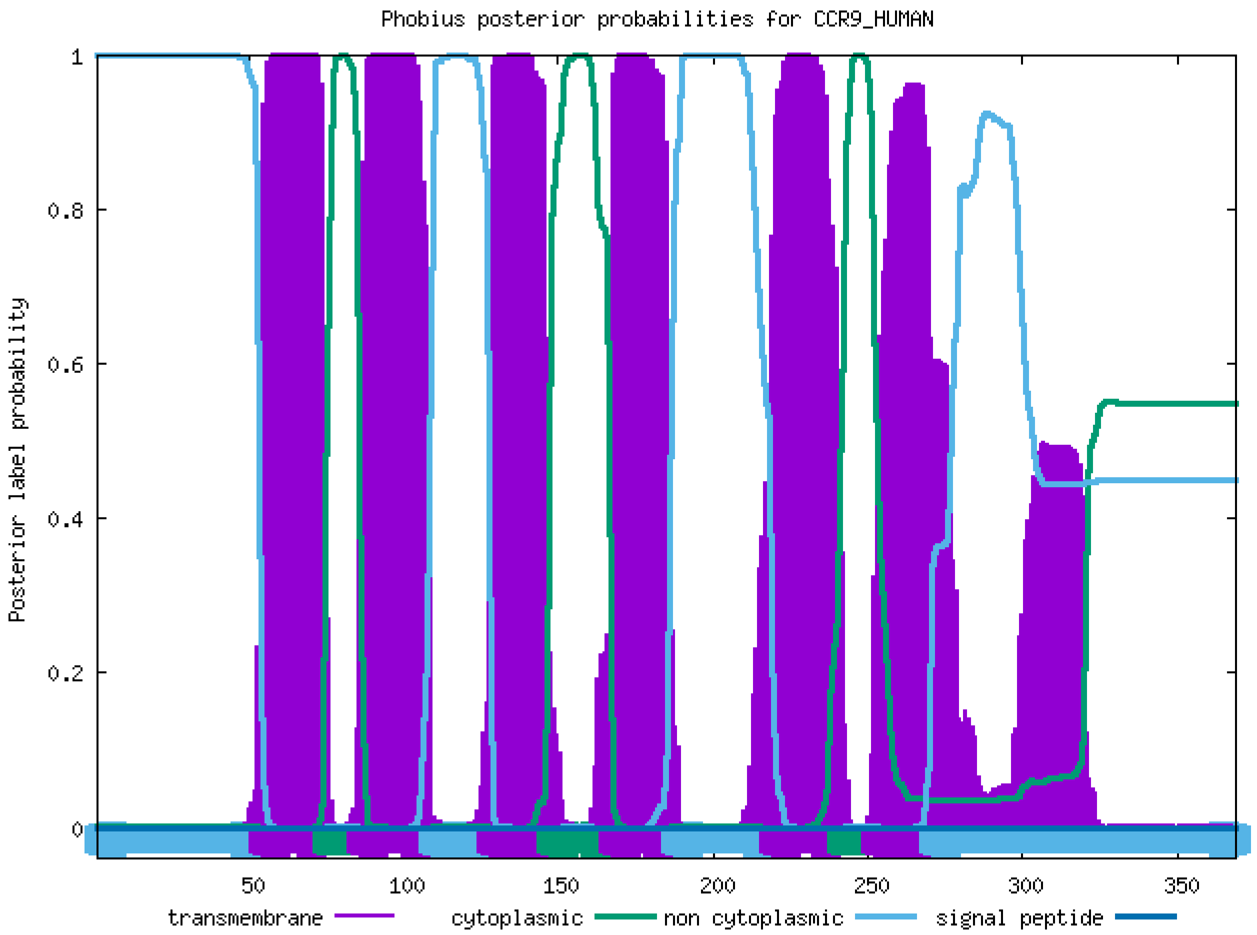

The probability data used in the plot is found here, and the gnuplot script is here.

Prediction of EDNRB_HUMAN

```
ID   EDNRB_HUMAN
FT   SIGNAL        1     26
FT   REGION        1     10       N-REGION.
FT   REGION       11     21       H-REGION.
FT   REGION       22     26       C-REGION.
FT   TOPO_DOM     27    101       NON CYTOPLASMIC.
FT   TRANSMEM    102    126
FT   TOPO_DOM    127    137       CYTOPLASMIC.
FT   TRANSMEM    138    159
FT   TOPO_DOM    160    178       NON CYTOPLASMIC.
FT   TRANSMEM    179    197
FT   TOPO_DOM    198    217       CYTOPLASMIC.
FT   TRANSMEM    218    238
FT   TOPO_DOM    239    274       NON CYTOPLASMIC.
FT   TRANSMEM    275    301
FT   TOPO_DOM    302    320       CYTOPLASMIC.
FT   TRANSMEM    321    341
FT   TOPO_DOM    342    360       NON CYTOPLASMIC.
FT   TRANSMEM    361    389
FT   TOPO_DOM    390    442       CYTOPLASMIC.
//
```

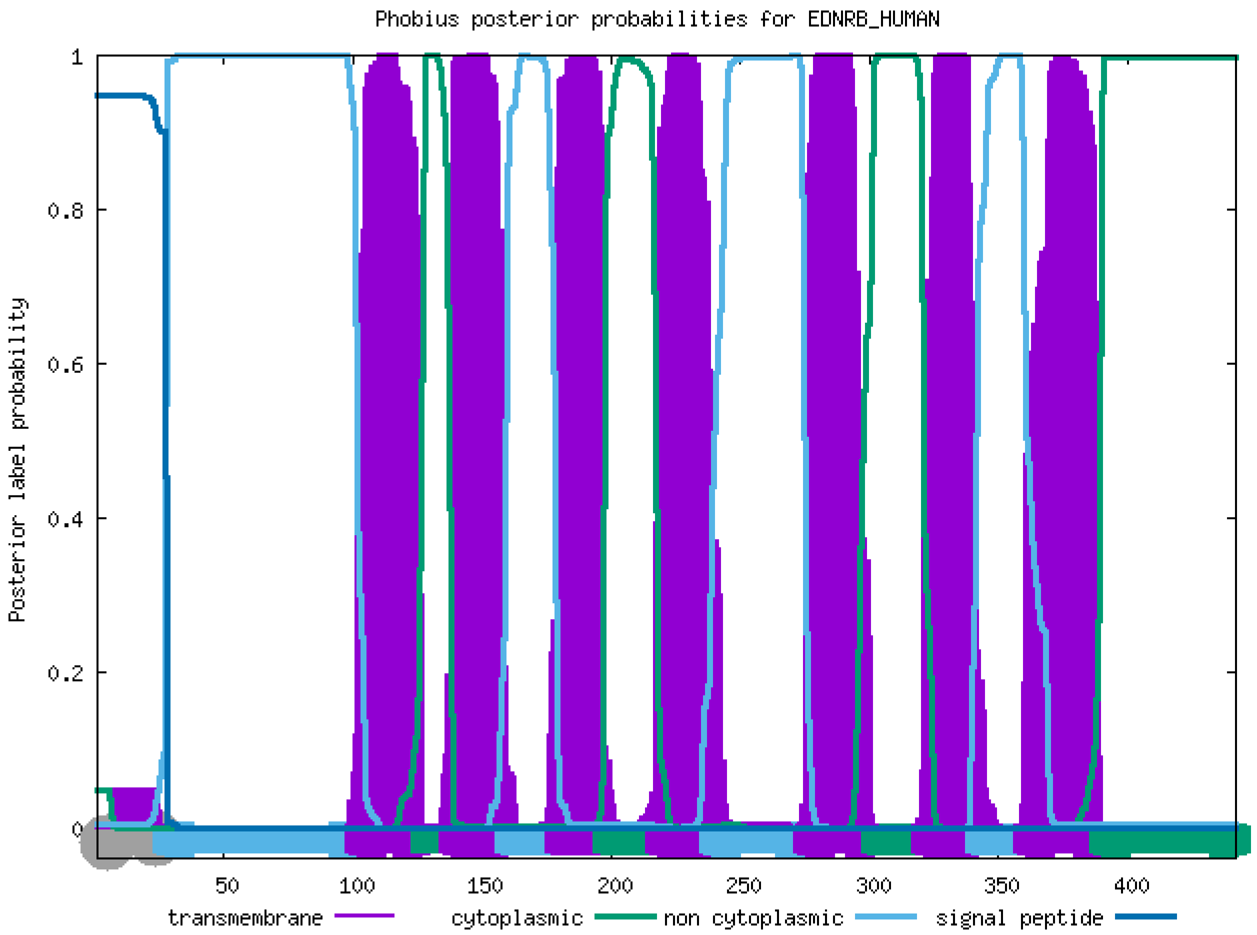

The probability data used in the plot is found here, and the gnuplot script is here.

Prediction of FPR1_HUMAN

```
ID    FPR1_HUMAN
FT    TOPO_DOM       1      26       NON CYTOPLASMIC.
FT    TRANSMEM      27      52
FT    TOPO_DOM      53      63       CYTOPLASMIC.
FT    TRANSMEM      64      82
FT    TOPO_DOM      83      93       NON CYTOPLASMIC.
FT    TRANSMEM      94     121
FT    TOPO_DOM     122     140       CYTOPLASMIC.
FT    TRANSMEM     141     162
FT    TOPO_DOM     163     202       NON CYTOPLASMIC.
FT    TRANSMEM     203     224
FT    TOPO_DOM     225     244       CYTOPLASMIC.
FT    TRANSMEM     245     267
FT    TOPO_DOM     268     278       NON CYTOPLASMIC.
FT    TRANSMEM     279     303
FT    TOPO_DOM     304     350       CYTOPLASMIC.
//
```

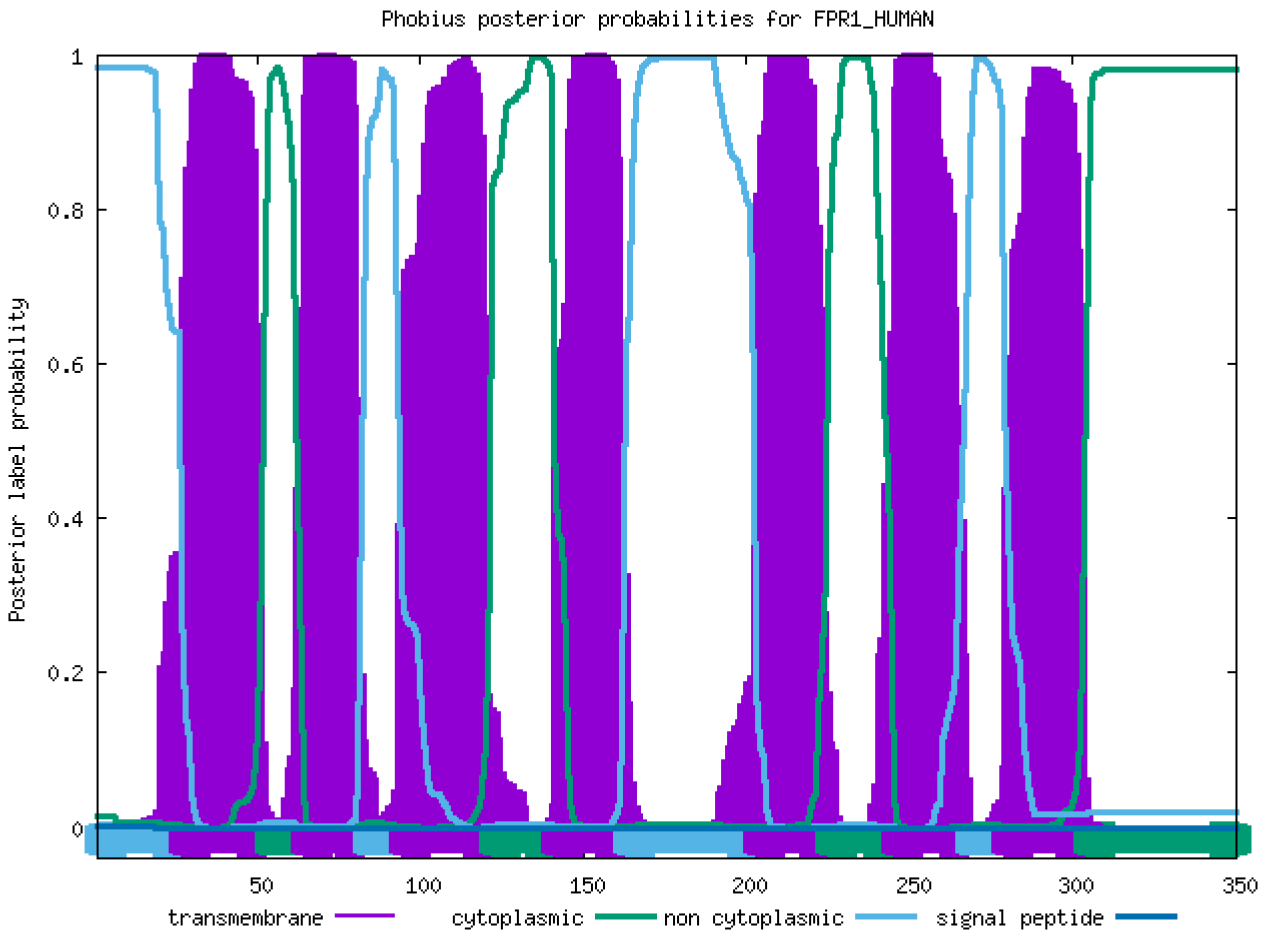

The probability data used in the plot is found here, and the gnuplot script is here.

Prediction of FPR2_HUMAN

```
ID    FPR2_HUMAN
FT    TOPO_DOM      1     26       NON CYTOPLASMIC.
FT    TRANSMEM     27     51
FT    TOPO_DOM     52     62       CYTOPLASMIC.
FT    TRANSMEM     63     87
FT    TOPO_DOM     88     92       NON CYTOPLASMIC.
FT    TRANSMEM     93    121
FT    TOPO_DOM    122    140       CYTOPLASMIC.
FT    TRANSMEM    141    164
FT    TOPO_DOM    165    202       NON CYTOPLASMIC.
FT    TRANSMEM    203    224
FT    TOPO_DOM    225    244       CYTOPLASMIC.
FT    TRANSMEM    245    268
FT    TOPO_DOM    269    287       NON CYTOPLASMIC.
FT    TRANSMEM    288    305
FT    TOPO_DOM    306    351       CYTOPLASMIC.
//
```

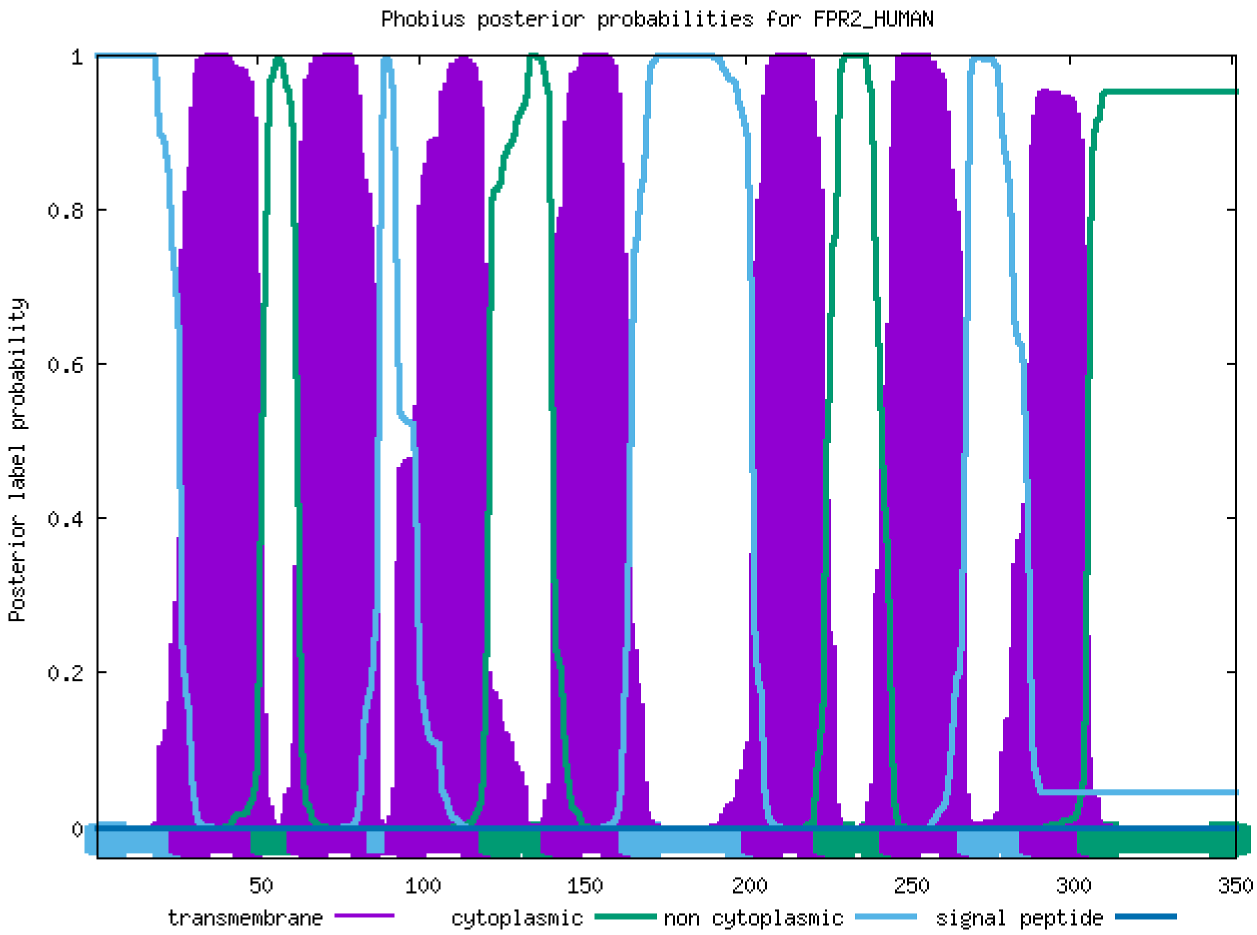

The probability data used in the plot is found here, and the gnuplot script is here.

Prediction of DRD1_HUMAN

```
ID   DRD1_HUMAN
FT   TOPO_DOM      1     19       NON CYTOPLASMIC.
FT   TRANSMEM     20     49
FT   TOPO_DOM     50     60       CYTOPLASMIC.
FT   TRANSMEM     61     78
FT   TOPO_DOM     79     97       NON CYTOPLASMIC.
FT   TRANSMEM     98    119
FT   TOPO_DOM    120    139       CYTOPLASMIC.
FT   TRANSMEM    140    161
FT   TOPO_DOM    162    193       NON CYTOPLASMIC.
FT   TRANSMEM    194    217
FT   TOPO_DOM    218    269       CYTOPLASMIC.
FT   TRANSMEM    270    290
FT   TOPO_DOM    291    446       NON CYTOPLASMIC.
//
```

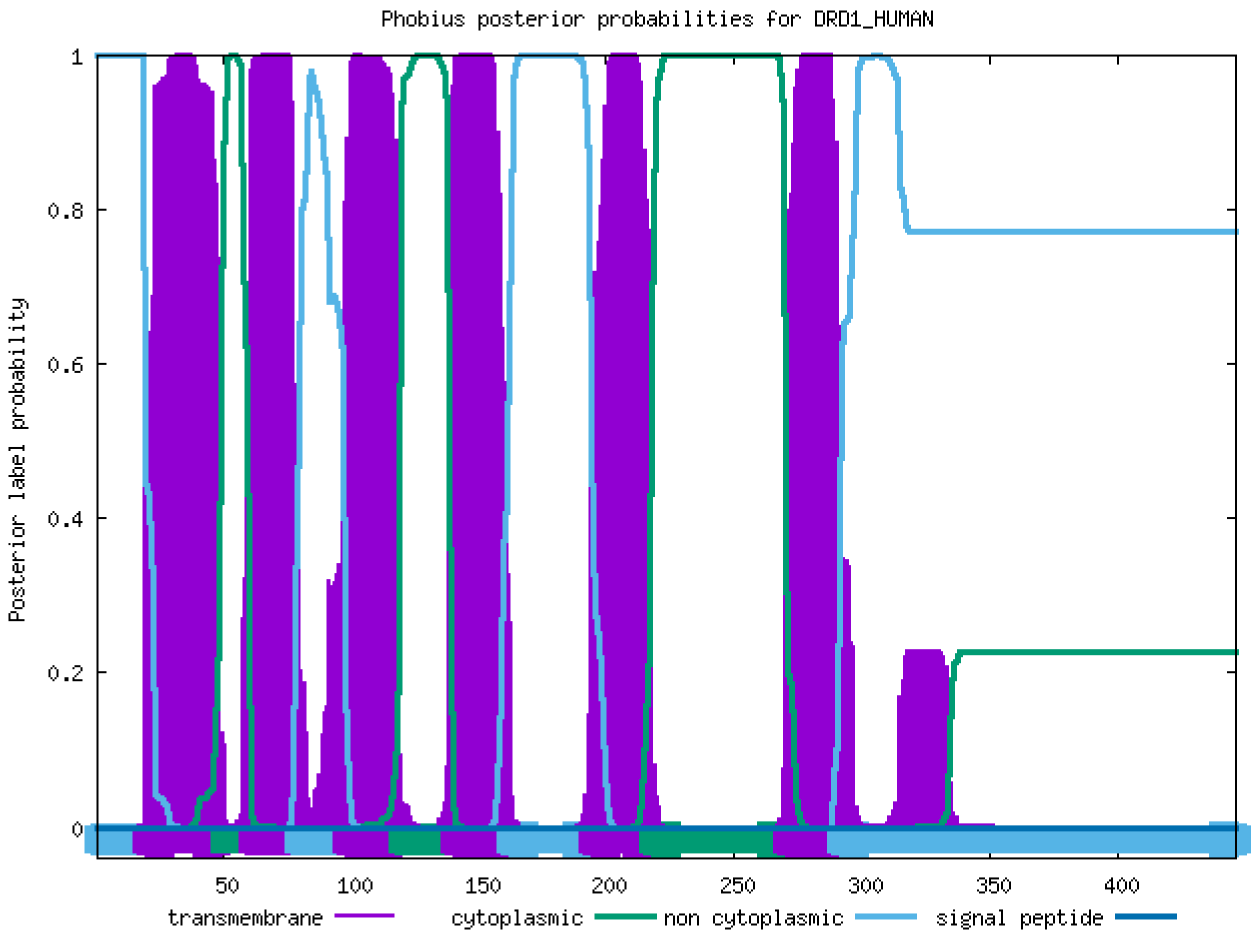

The probability data used in the plot is found here, and the gnuplot script is here.

Prediction of DRD2_HUMAN

```
ID   DRD2_HUMAN
FT   TOPO_DOM      1     35       NON CYTOPLASMIC.
FT   TRANSMEM     36     59
FT   TOPO_DOM     60     70       CYTOPLASMIC.
FT   TRANSMEM     71     93
FT   TOPO_DOM     94    108       NON CYTOPLASMIC.
FT   TRANSMEM    109    131
FT   TOPO_DOM    132    151       CYTOPLASMIC.
FT   TRANSMEM    152    172
FT   TOPO_DOM    173    191       NON CYTOPLASMIC.
FT   TRANSMEM    192    216
FT   TOPO_DOM    217    373       CYTOPLASMIC.
FT   TRANSMEM    374    395
FT   TOPO_DOM    396    406       NON CYTOPLASMIC.
FT   TRANSMEM    407    429
FT   TOPO_DOM    430    443       CYTOPLASMIC.
//
```

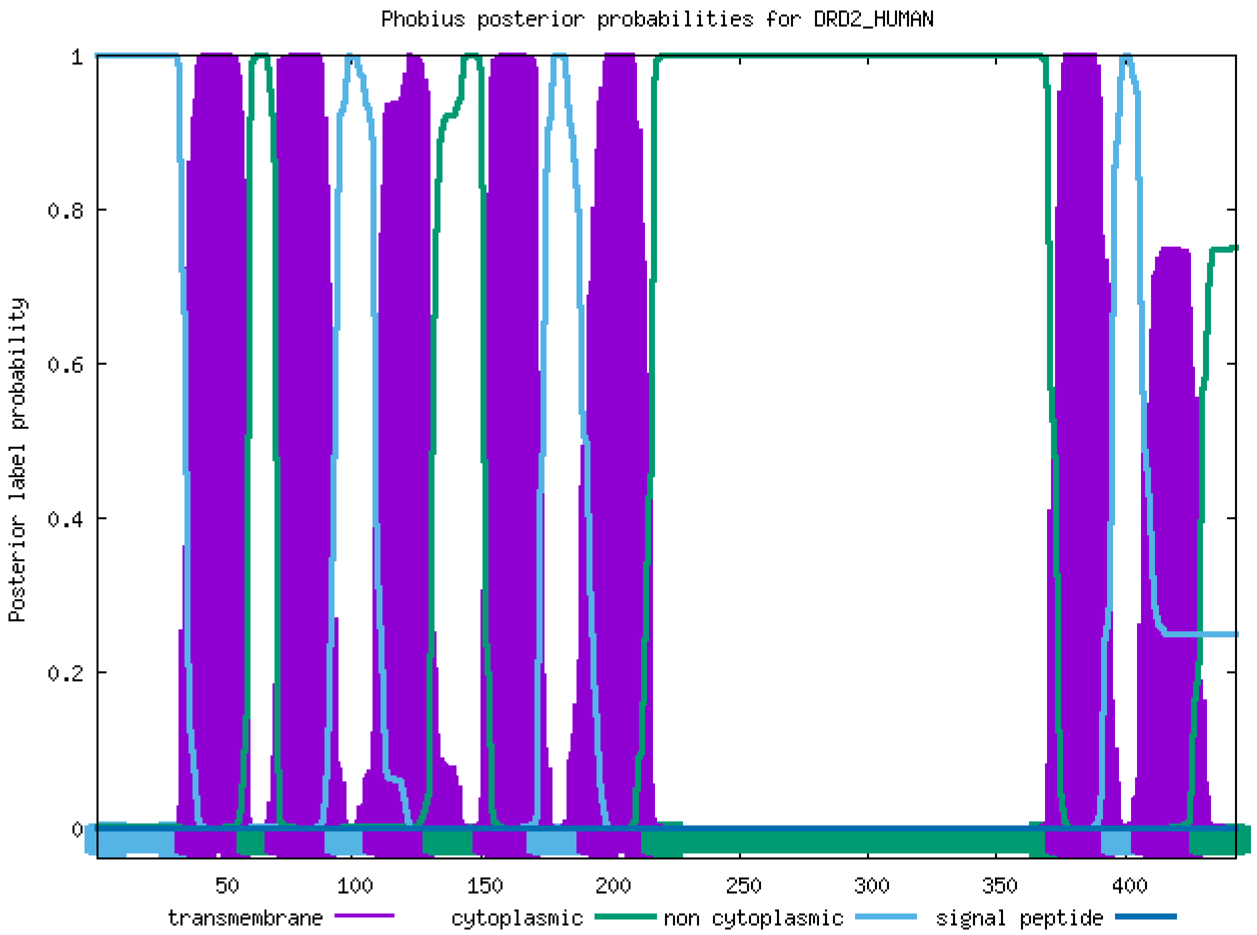

The probability data used in the plot is found here, and the gnuplot script is here.

Prediction of DRD3_HUMAN

```
ID   DRD3_HUMAN
FT   TOPO_DOM      1     29       NON CYTOPLASMIC.
FT   TRANSMEM     30     55
FT   TOPO_DOM     56     66       CYTOPLASMIC.
FT   TRANSMEM     67     87
FT   TOPO_DOM     88    106       NON CYTOPLASMIC.
FT   TRANSMEM    107    129
FT   TOPO_DOM    130    149       CYTOPLASMIC.
FT   TRANSMEM    150    170
FT   TOPO_DOM    171    189       NON CYTOPLASMIC.
FT   TRANSMEM    190    213
FT   TOPO_DOM    214    330       CYTOPLASMIC.
FT   TRANSMEM    331    351
FT   TOPO_DOM    352    400       NON CYTOPLASMIC.
//
```

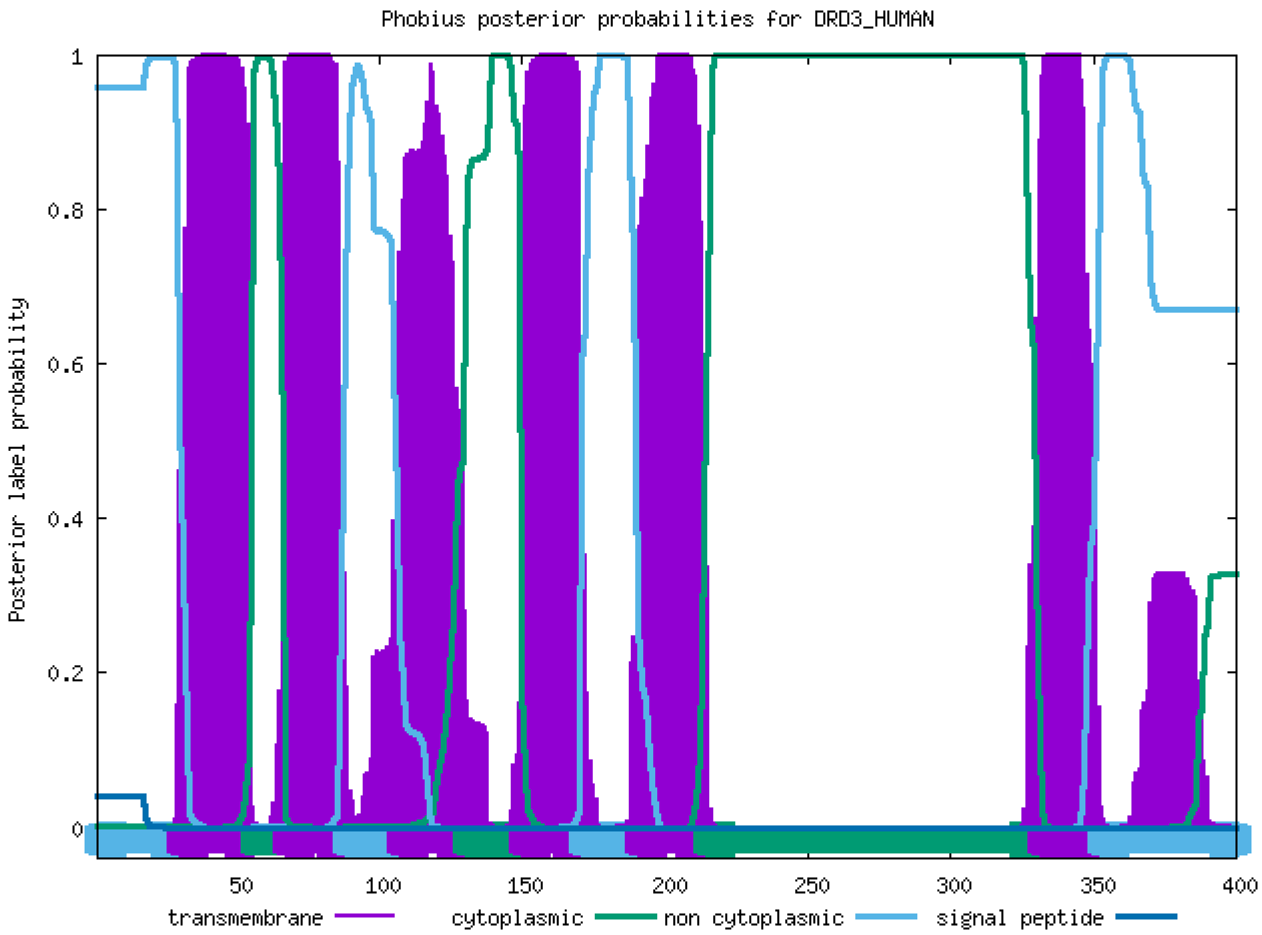

The probability data used in the plot is found here, and the gnuplot script is here.

Prediction of DRD4_HUMAN

```
ID    DRD4_HUMAN
FT    TOPO_DOM      1      34       NON CYTOPLASMIC.
FT    TRANSMEM     35      60
FT    TOPO_DOM     61      71       CYTOPLASMIC.
FT    TRANSMEM     72      92
FT    TOPO_DOM     93     111       NON CYTOPLASMIC.
FT    TRANSMEM    112     141
FT    TOPO_DOM    142     152       CYTOPLASMIC.
FT    TRANSMEM    153     172
FT    TOPO_DOM    173     191       NON CYTOPLASMIC.
FT    TRANSMEM    192     213
FT    TOPO_DOM    214     343       CYTOPLASMIC.
FT    TRANSMEM    344     364
FT    TOPO_DOM    365     383       NON CYTOPLASMIC.
FT    TRANSMEM    384     403
FT    TOPO_DOM    404     419       CYTOPLASMIC.
//
```

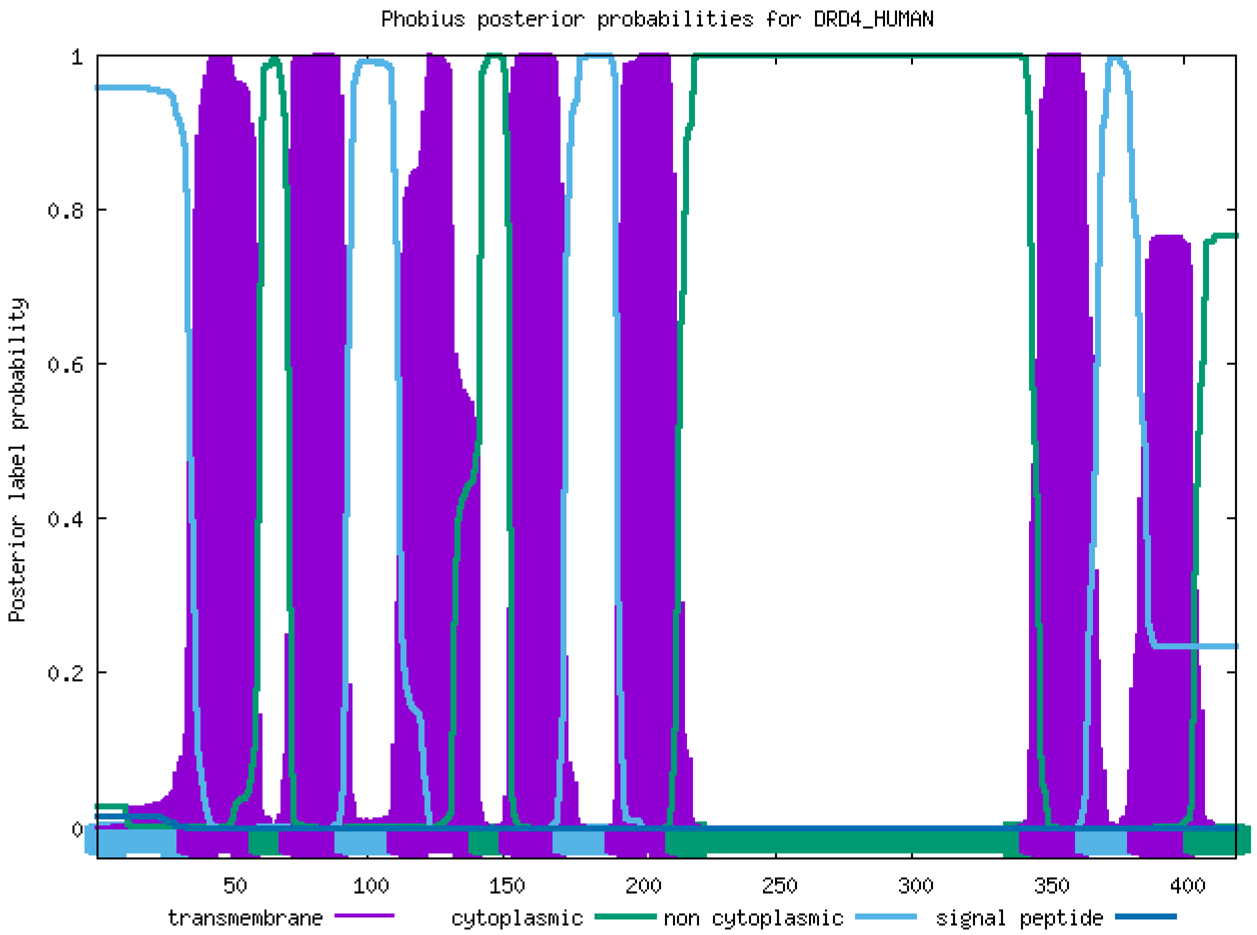

The probability data used in the plot is found here, and the gnuplot script is here.

Prediction of DRD5_HUMAN

```
ID   DRD5_HUMAN
FT   TOPO_DOM      1     40       NON CYTOPLASMIC.
FT   TRANSMEM     41     66
FT   TOPO_DOM     67     77       CYTOPLASMIC.
FT   TRANSMEM     78     95
FT   TOPO_DOM     96    114       NON CYTOPLASMIC.
FT   TRANSMEM    115    136
FT   TOPO_DOM    137    156       CYTOPLASMIC.
FT   TRANSMEM    157    178
FT   TOPO_DOM    179    224       NON CYTOPLASMIC.
FT   TRANSMEM    225    248
FT   TOPO_DOM    249    293       CYTOPLASMIC.
FT   TRANSMEM    294    315
FT   TOPO_DOM    316    477       NON CYTOPLASMIC.
//
```

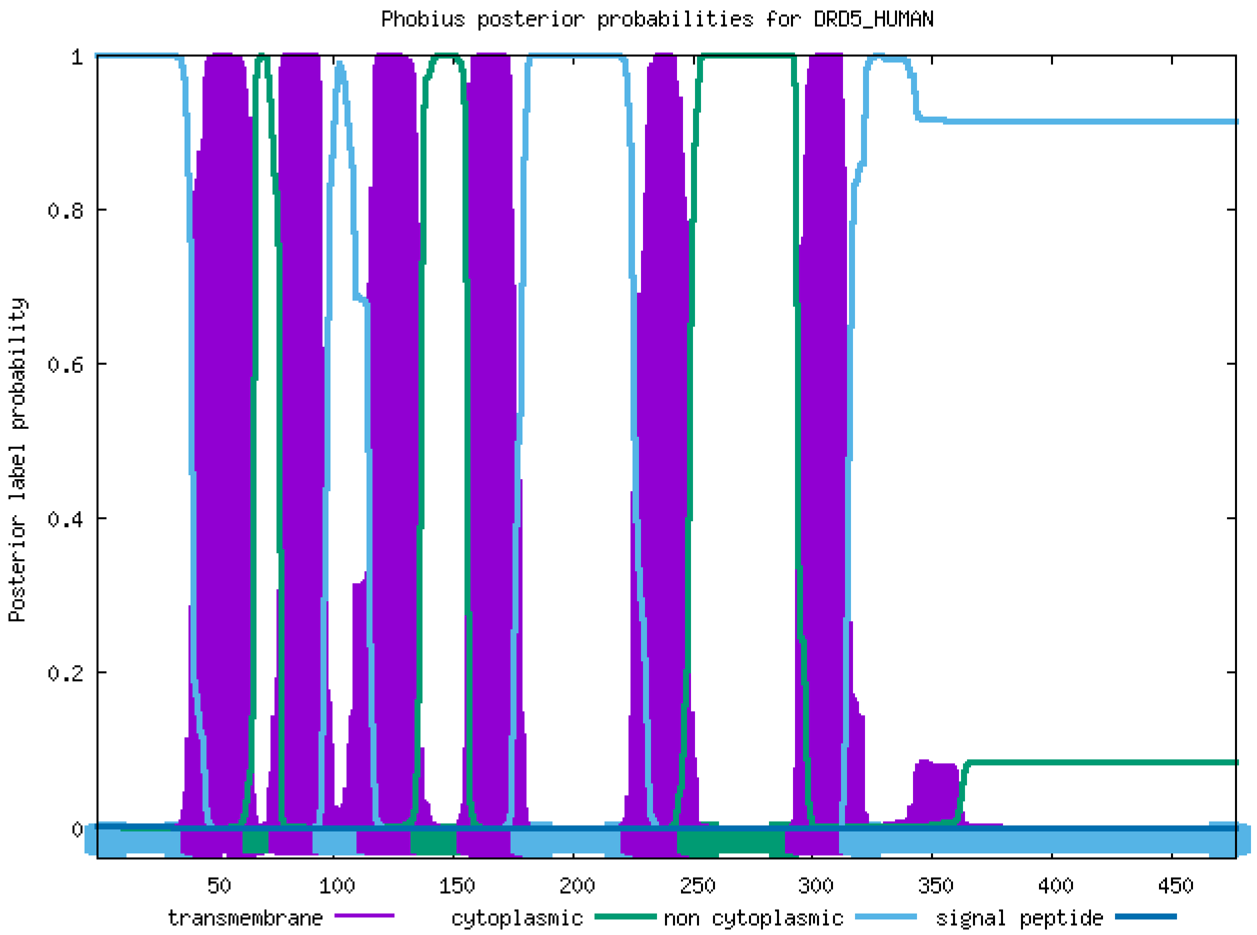

The probability data used in the plot is found here, and the gnuplot script is here.

Prediction of HRH1_HUMAN

```
ID    HRH1_HUMAN
FT    TOPO_DOM      1      26       NON CYTOPLASMIC.
FT    TRANSMEM     27      52
FT    TOPO_DOM     53      63       CYTOPLASMIC.
FT    TRANSMEM     64      89
FT    TOPO_DOM     90     100       NON CYTOPLASMIC.
FT    TRANSMEM    101     123
FT    TOPO_DOM    124     143       CYTOPLASMIC.
FT    TRANSMEM    144     168
FT    TOPO_DOM    169     187       NON CYTOPLASMIC.
FT    TRANSMEM    188     210
FT    TOPO_DOM    211     416       CYTOPLASMIC.
FT    TRANSMEM    417     440
FT    TOPO_DOM    441     451       NON CYTOPLASMIC.
FT    TRANSMEM    452     471
FT    TOPO_DOM    472     487       CYTOPLASMIC.
//
```

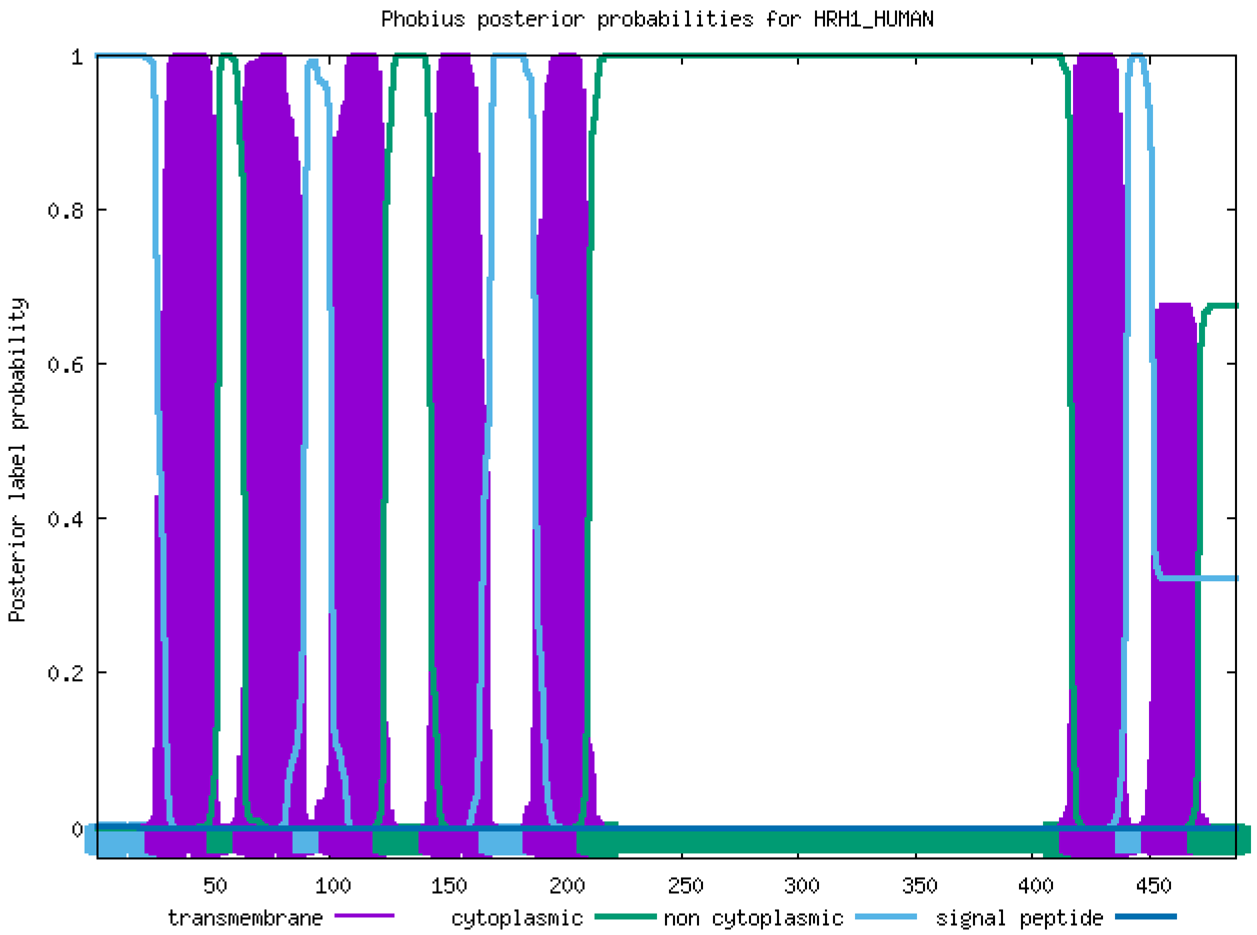

The probability data used in the plot is found here, and the gnuplot script is here.

Prediction of HRH2_HUMAN

```
ID   HRH2_HUMAN
FT   TOPO_DOM      1     19       NON CYTOPLASMIC.
FT   TRANSMEM     20     43
FT   TOPO_DOM     44     54       CYTOPLASMIC.
FT   TRANSMEM     55     81
FT   TOPO_DOM     82     92       NON CYTOPLASMIC.
FT   TRANSMEM     93    114
FT   TOPO_DOM    115    134       CYTOPLASMIC.
FT   TRANSMEM    135    158
FT   TOPO_DOM    159    188       NON CYTOPLASMIC.
FT   TRANSMEM    189    206
FT   TOPO_DOM    207    231       CYTOPLASMIC.
FT   TRANSMEM    232    252
FT   TOPO_DOM    253    271       NON CYTOPLASMIC.
FT   TRANSMEM    272    291
FT   TOPO_DOM    292    359       CYTOPLASMIC.
//
```

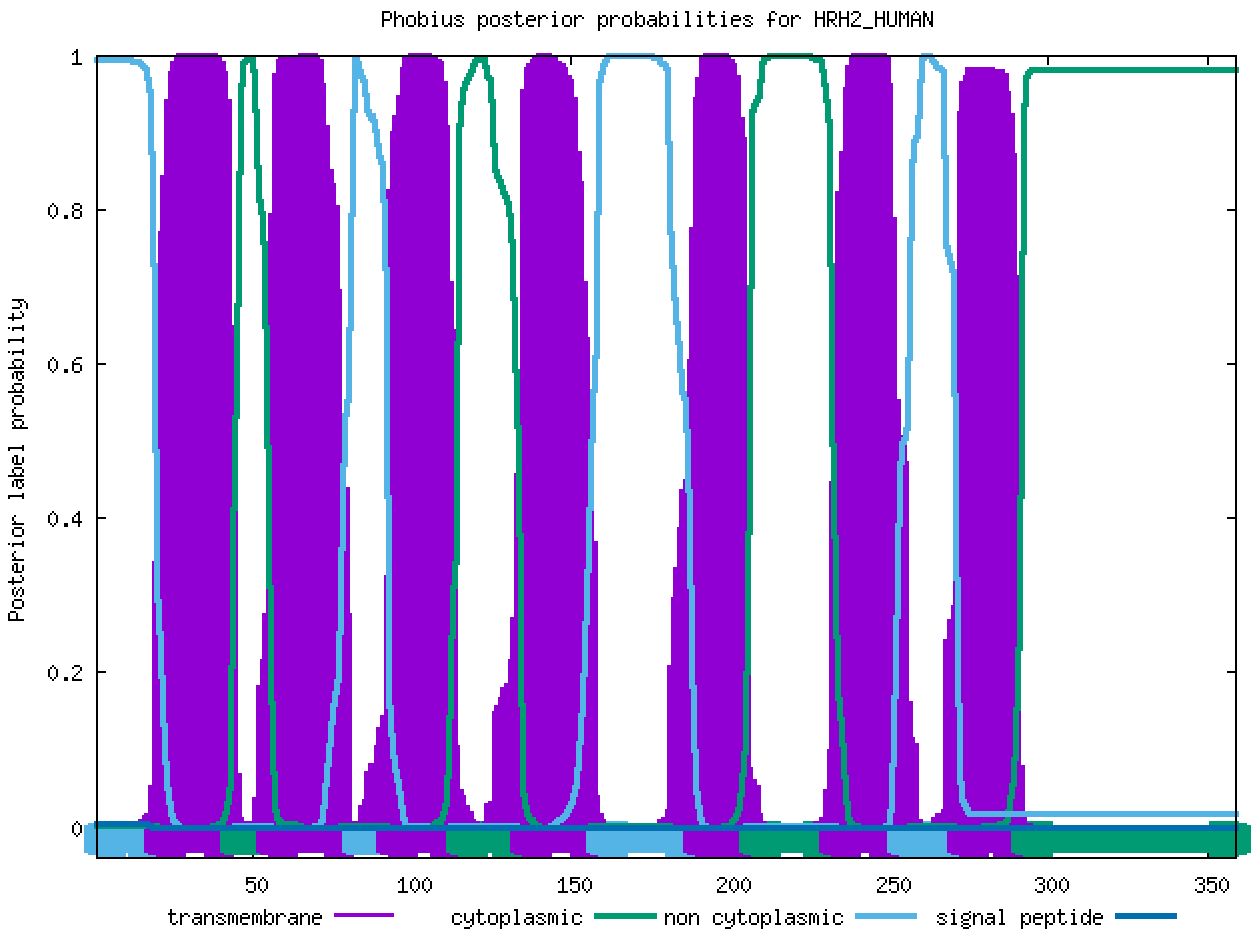

The probability data used in the plot is found here, and the gnuplot script is here.

Prediction of OPRK_HUMAN

```
ID   OPRK_HUMAN
FT   TOPO_DOM      1     56       NON CYTOPLASMIC.
FT   TRANSMEM     57     85
FT   TOPO_DOM     86     96       CYTOPLASMIC.
FT   TRANSMEM     97    119
FT   TOPO_DOM    120    138       NON CYTOPLASMIC.
FT   TRANSMEM    139    159
FT   TOPO_DOM    160    179       CYTOPLASMIC.
FT   TRANSMEM    180    199
FT   TOPO_DOM    200    223       NON CYTOPLASMIC.
FT   TRANSMEM    224    251
FT   TOPO_DOM    252    271       CYTOPLASMIC.
FT   TRANSMEM    272    296
FT   TOPO_DOM    297    310       NON CYTOPLASMIC.
FT   TRANSMEM    311    333
FT   TOPO_DOM    334    380       CYTOPLASMIC.
//
```

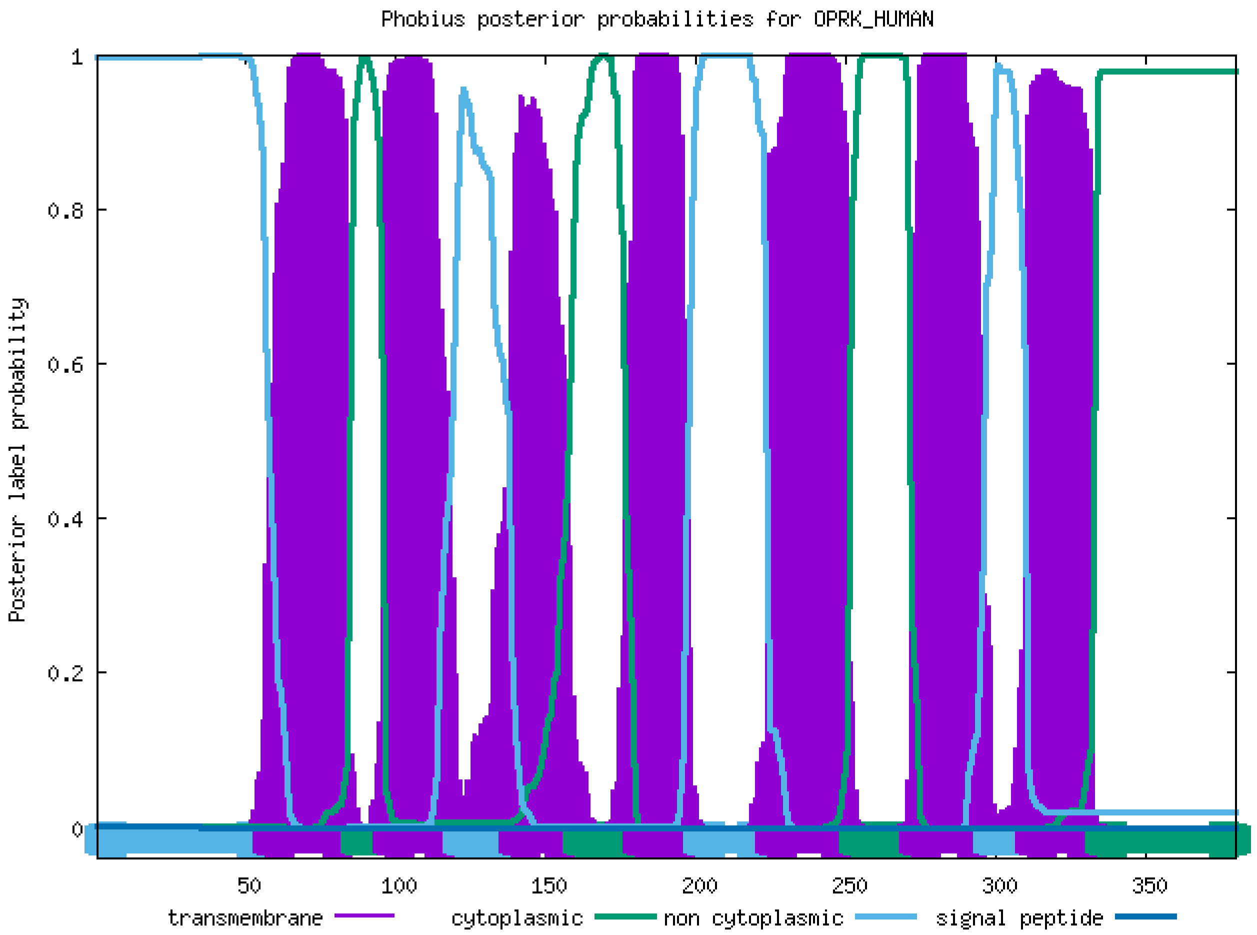

The probability data used in the plot is found here, and the gnuplot script is here.

Prediction of HRH3_HUMAN

```
ID   HRH3_HUMAN
FT   TOPO_DOM      1     34       NON CYTOPLASMIC.
FT   TRANSMEM     35     60
FT   TOPO_DOM     61     71       CYTOPLASMIC.
FT   TRANSMEM     72     92
FT   TOPO_DOM     93    111       NON CYTOPLASMIC.
FT   TRANSMEM    112    135
FT   TOPO_DOM    136    155       CYTOPLASMIC.
FT   TRANSMEM    156    176
FT   TOPO_DOM    177    195       NON CYTOPLASMIC.
FT   TRANSMEM    196    223
FT   TOPO_DOM    224    355       CYTOPLASMIC.
FT   TRANSMEM    356    380
FT   TOPO_DOM    381    391       NON CYTOPLASMIC.
FT   TRANSMEM    392    415
FT   TOPO_DOM    416    445       CYTOPLASMIC.
//
```

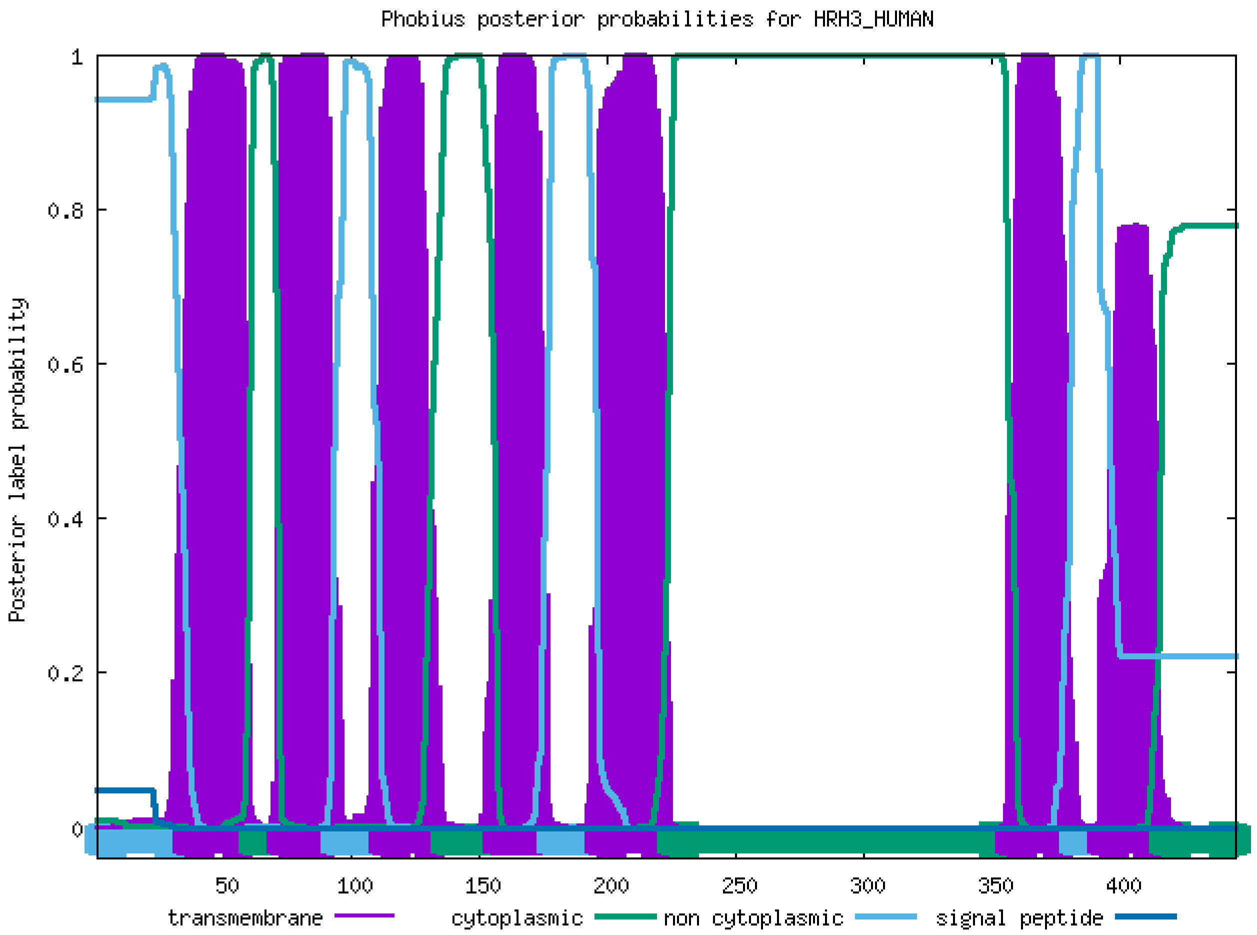

The probability data used in the plot is found here, and the gnuplot script is here.

Prediction of HRH4_HUMAN

```
ID   HRH4_HUMAN
FT   TOPO_DOM      1     19       NON CYTOPLASMIC.
FT   TRANSMEM     20     41
FT   TOPO_DOM     42     52       CYTOPLASMIC.
FT   TRANSMEM     53     73
FT   TOPO_DOM     74     87       NON CYTOPLASMIC.
FT   TRANSMEM     88    108
FT   TOPO_DOM    109    128       CYTOPLASMIC.
FT   TRANSMEM    129    152
FT   TOPO_DOM    153    171       NON CYTOPLASMIC.
FT   TRANSMEM    172    194
FT   TOPO_DOM    195    304       CYTOPLASMIC.
FT   TRANSMEM    305    330
FT   TOPO_DOM    331    341       NON CYTOPLASMIC.
FT   TRANSMEM    342    361
FT   TOPO_DOM    362    390       CYTOPLASMIC.
//
```

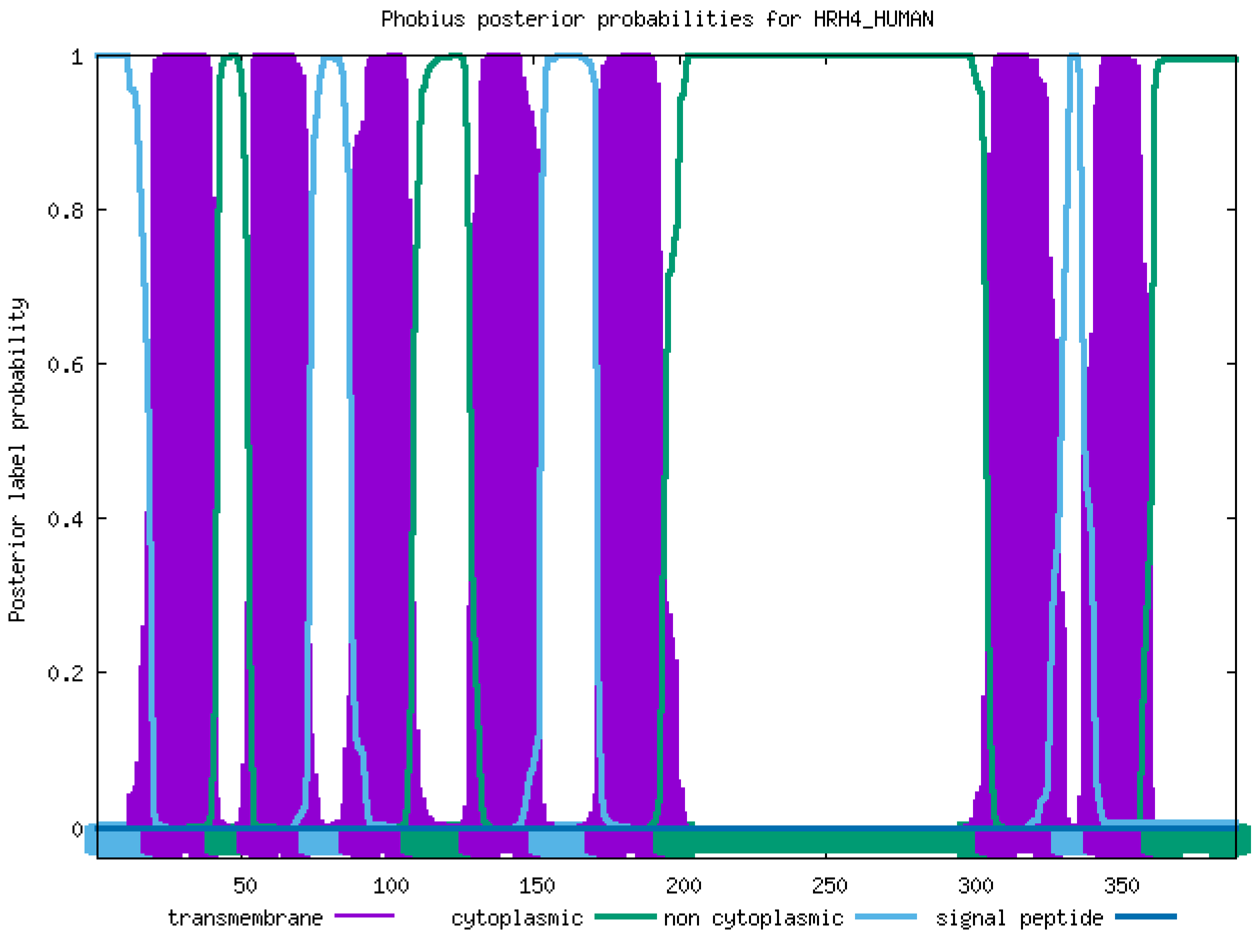

The probability data used in the plot is found here, and the gnuplot script is here.

Prediction of S1PR1_HUMAN

```
ID   S1PR1_HUMAN
FT   TOPO_DOM      1     49       NON CYTOPLASMIC.
FT   TRANSMEM     50     71
FT   TOPO_DOM     72     82       CYTOPLASMIC.
FT   TRANSMEM     83    104
FT   TOPO_DOM    105    123       NON CYTOPLASMIC.
FT   TRANSMEM    124    140
FT   TOPO_DOM    141    159       CYTOPLASMIC.
FT   TRANSMEM    160    182
FT   TOPO_DOM    183    201       NON CYTOPLASMIC.
FT   TRANSMEM    202    224
FT   TOPO_DOM    225    252       CYTOPLASMIC.
FT   TRANSMEM    253    275
FT   TOPO_DOM    276    294       NON CYTOPLASMIC.
FT   TRANSMEM    295    314
FT   TOPO_DOM    315    382       CYTOPLASMIC.
//
```

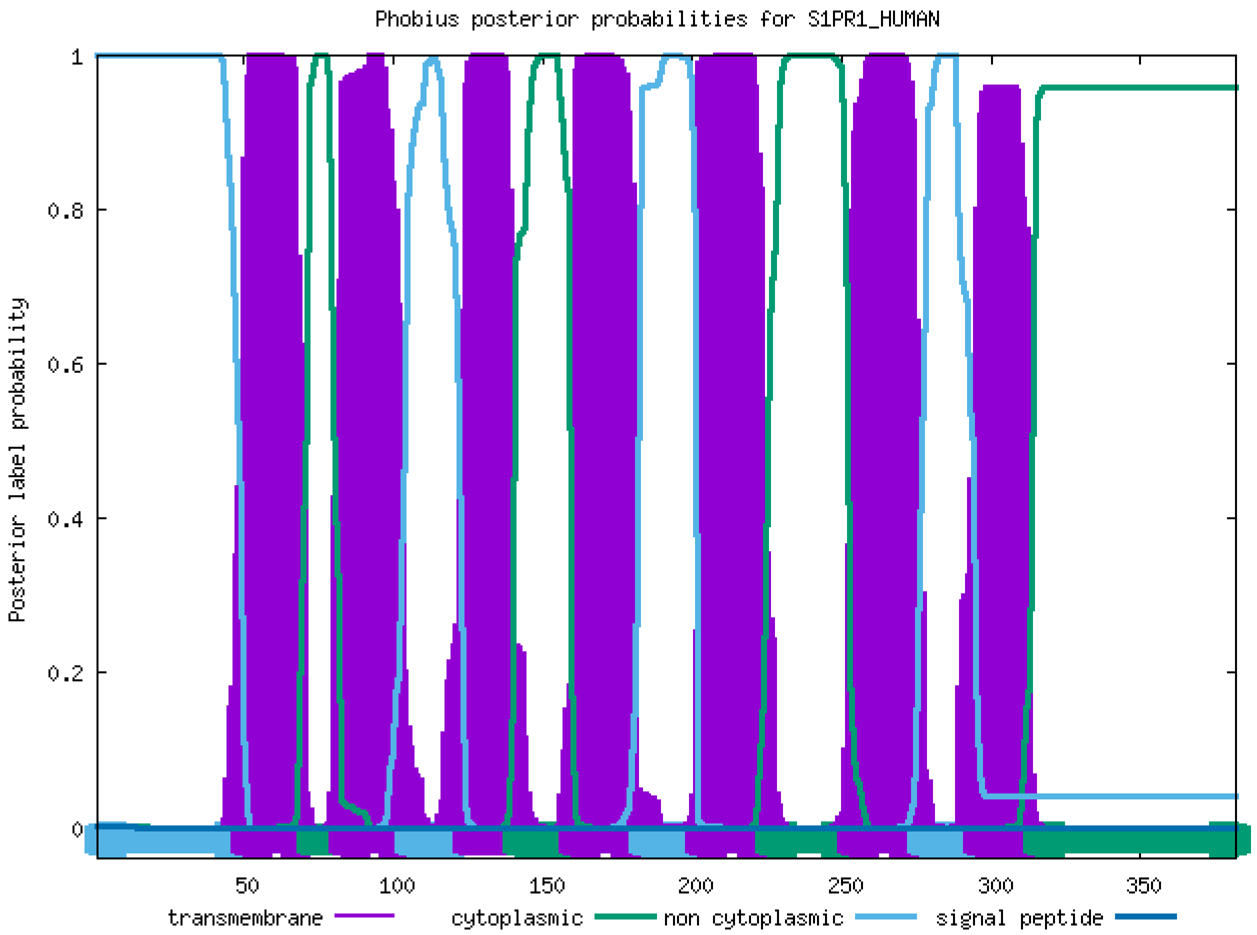

The probability data used in the plot is found here, and the gnuplot script is here.

Prediction of S1PR2_HUMAN

```
ID   S1PR2_HUMAN
FT   TOPO_DOM      1     34       NON CYTOPLASMIC.
FT   TRANSMEM     35     58
FT   TOPO_DOM     59     69       CYTOPLASMIC.
FT   TRANSMEM     70     92
FT   TOPO_DOM     93    111       NON CYTOPLASMIC.
FT   TRANSMEM    112    128
FT   TOPO_DOM    129    148       CYTOPLASMIC.
FT   TRANSMEM    149    169
FT   TOPO_DOM    170    188       NON CYTOPLASMIC.
FT   TRANSMEM    189    210
FT   TOPO_DOM    211    229       CYTOPLASMIC.
FT   TRANSMEM    230    250
FT   TOPO_DOM    251    269       NON CYTOPLASMIC.
FT   TRANSMEM    270    290
FT   TOPO_DOM    291    353       CYTOPLASMIC.
//
```

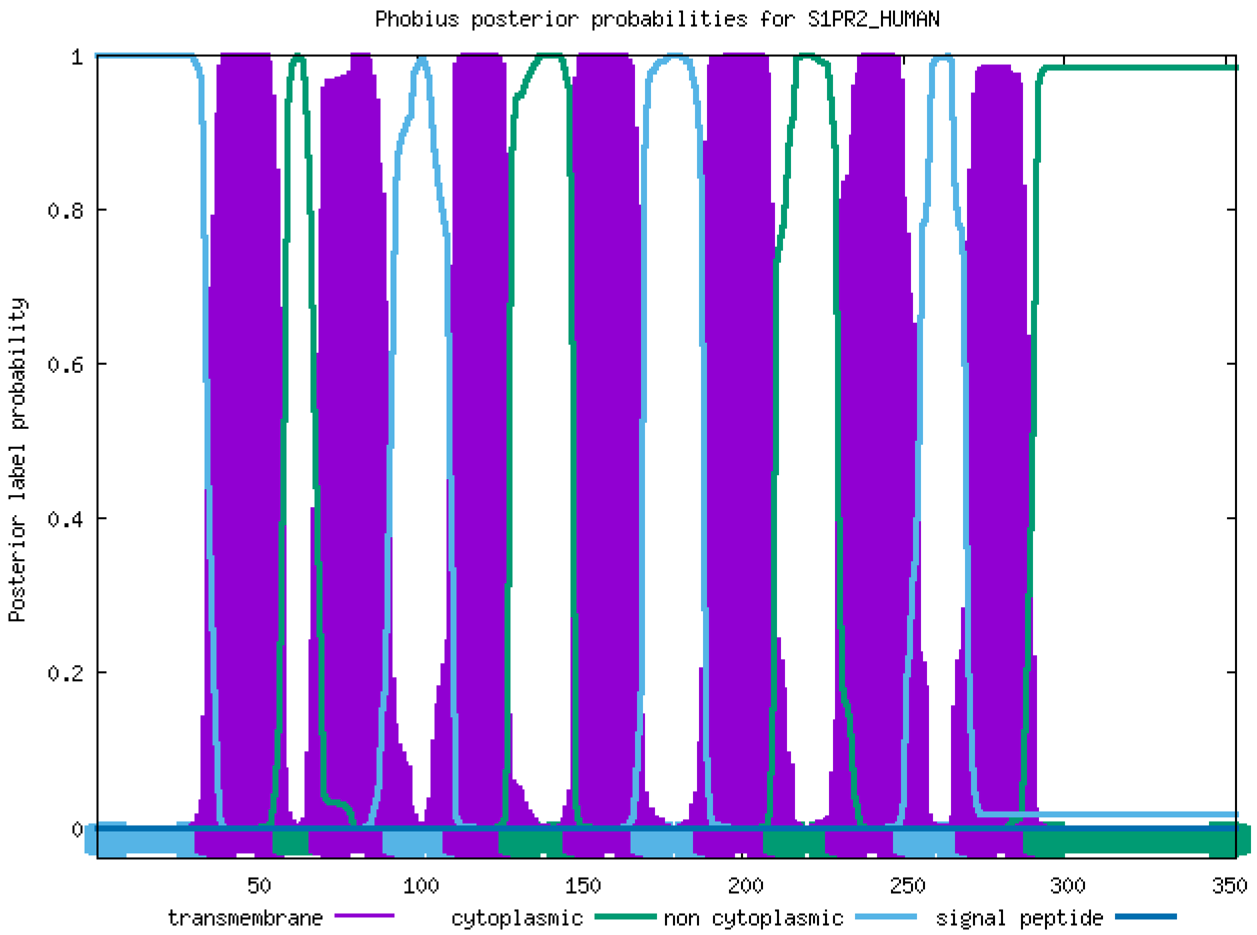

The probability data used in the plot is found here, and the gnuplot script is here.

Prediction of S1PR3_HUMAN

```
ID   S1PR3_HUMAN
FT   TOPO_DOM      1     43       NON CYTOPLASMIC.
FT   TRANSMEM     44     65
FT   TOPO_DOM     66     76       CYTOPLASMIC.
FT   TRANSMEM     77     96
FT   TOPO_DOM     97    115       NON CYTOPLASMIC.
FT   TRANSMEM    116    134
FT   TOPO_DOM    135    153       CYTOPLASMIC.
FT   TRANSMEM    154    176
FT   TOPO_DOM    177    195       NON CYTOPLASMIC.
FT   TRANSMEM    196    220
FT   TOPO_DOM    221    240       CYTOPLASMIC.
FT   TRANSMEM    241    265
FT   TOPO_DOM    266    279       NON CYTOPLASMIC.
FT   TRANSMEM    280    301
FT   TOPO_DOM    302    378       CYTOPLASMIC.
//
```

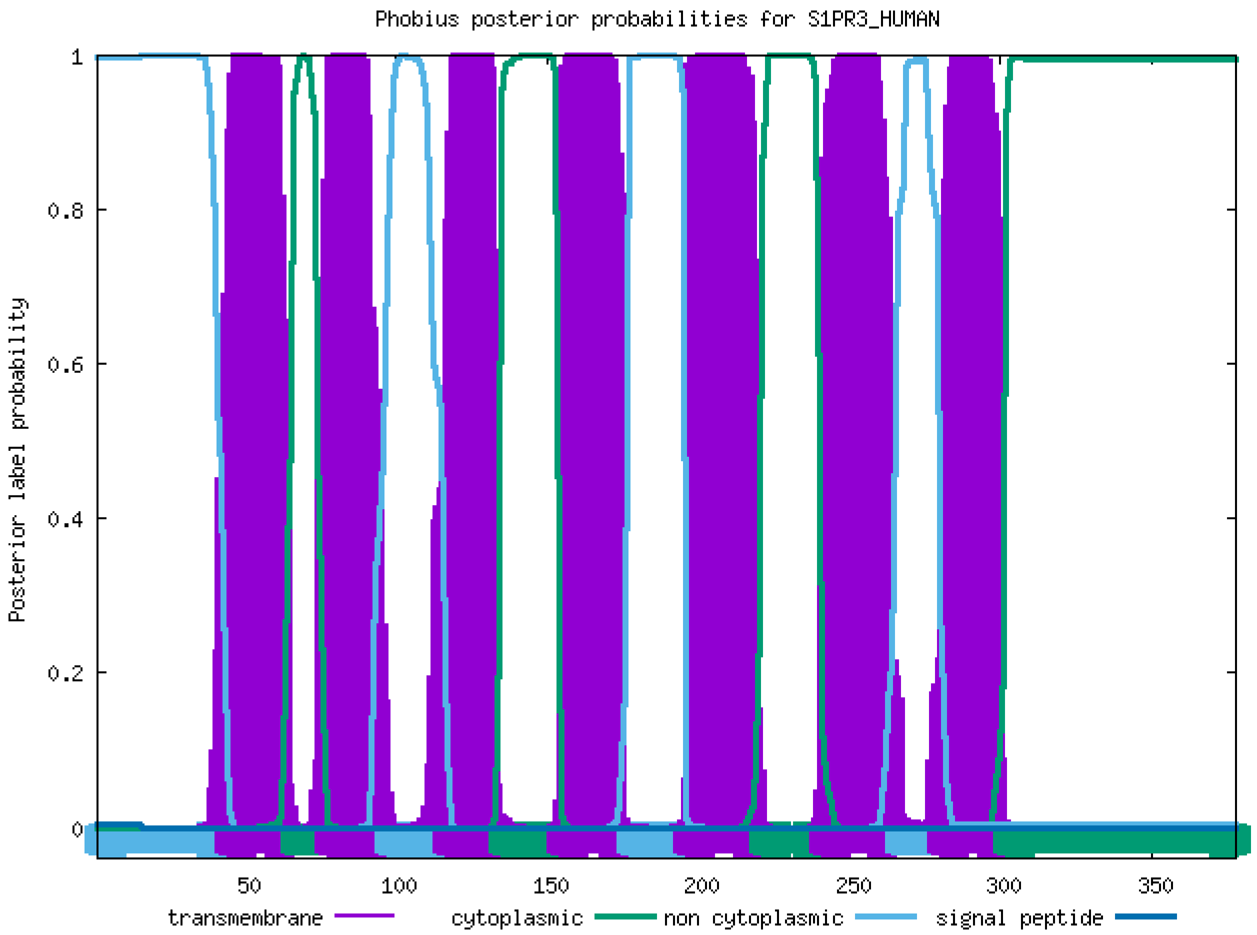

The probability data used in the plot is found here, and the gnuplot script is here.

Prediction of S1PR5_HUMAN

```
ID   S1PR5_HUMAN
FT   TOPO_DOM      1     37       NON CYTOPLASMIC.
FT   TRANSMEM     38     60
FT   TOPO_DOM     61     71       CYTOPLASMIC.
FT   TRANSMEM     72     94
FT   TOPO_DOM     95    113       NON CYTOPLASMIC.
FT   TRANSMEM    114    131
FT   TOPO_DOM    132    151       CYTOPLASMIC.
FT   TRANSMEM    152    173
FT   TOPO_DOM    174    192       NON CYTOPLASMIC.
FT   TRANSMEM    193    215
FT   TOPO_DOM    216    247       CYTOPLASMIC.
FT   TRANSMEM    248    271
FT   TOPO_DOM    272    290       NON CYTOPLASMIC.
FT   TRANSMEM    291    309
FT   TOPO_DOM    310    398       CYTOPLASMIC.
//
```

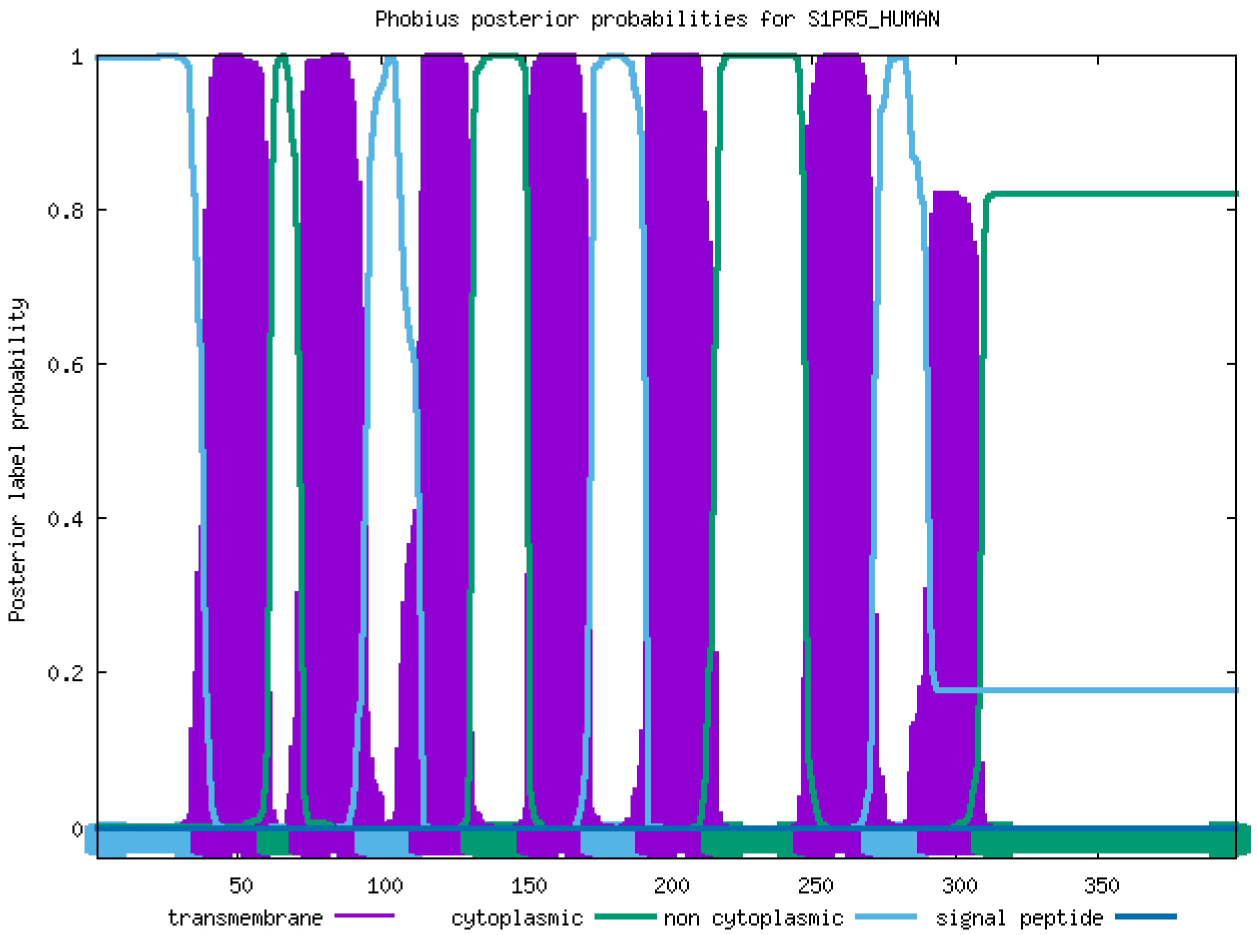

The probability data used in the plot is found here, and the gnuplot script is here.

Prediction of ACM1_HUMAN

```
ID    ACM1_HUMAN
FT    TOPO_DOM      1     24       NON CYTOPLASMIC.
FT    TRANSMEM     25     50
FT    TOPO_DOM     51     61       CYTOPLASMIC.
FT    TRANSMEM     62     88
FT    TOPO_DOM     89     99       NON CYTOPLASMIC.
FT    TRANSMEM    100    121
FT    TOPO_DOM    122    141       CYTOPLASMIC.
FT    TRANSMEM    142    167
FT    TOPO_DOM    168    186       NON CYTOPLASMIC.
FT    TRANSMEM    187    209
FT    TOPO_DOM    210    368       CYTOPLASMIC.
FT    TRANSMEM    369    390
FT    TOPO_DOM    391    401       NON CYTOPLASMIC.
FT    TRANSMEM    402    421
FT    TOPO_DOM    422    460       CYTOPLASMIC.
//
```

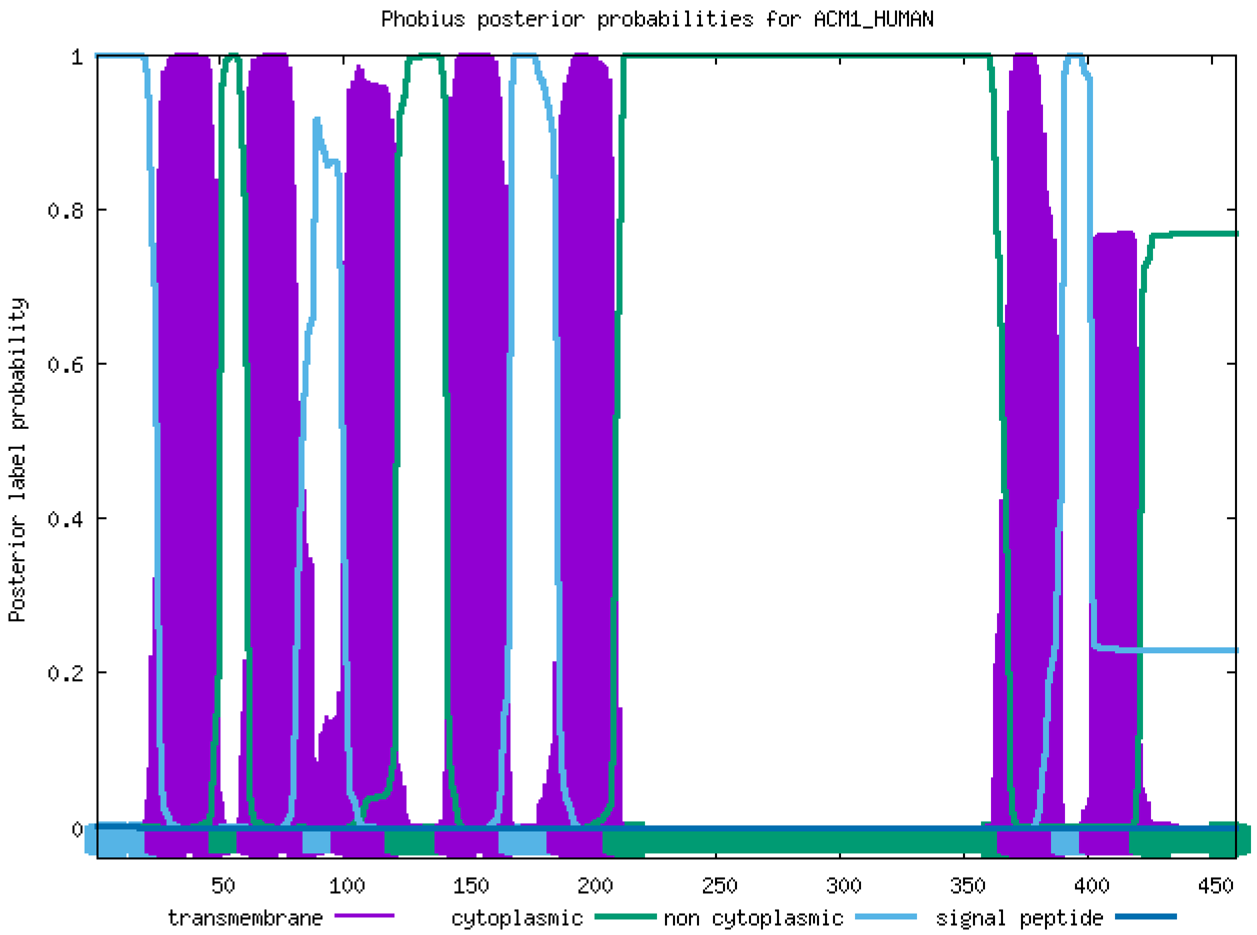

The probability data used in the plot is found here, and the gnuplot script is here.

Prediction of ACM2_HUMAN

```
ID   ACM2_HUMAN
FT   TOPO_DOM      1     22       NON CYTOPLASMIC.
FT   TRANSMEM     23     48
FT   TOPO_DOM     49     59       CYTOPLASMIC.
FT   TRANSMEM     60     86
FT   TOPO_DOM     87     97       NON CYTOPLASMIC.
FT   TRANSMEM     98    119
FT   TOPO_DOM    120    139       CYTOPLASMIC.
FT   TRANSMEM    140    165
FT   TOPO_DOM    166    184       NON CYTOPLASMIC.
FT   TRANSMEM    185    209
FT   TOPO_DOM    210    384       CYTOPLASMIC.
FT   TRANSMEM    385    403
FT   TOPO_DOM    404    466       NON CYTOPLASMIC.
//
```

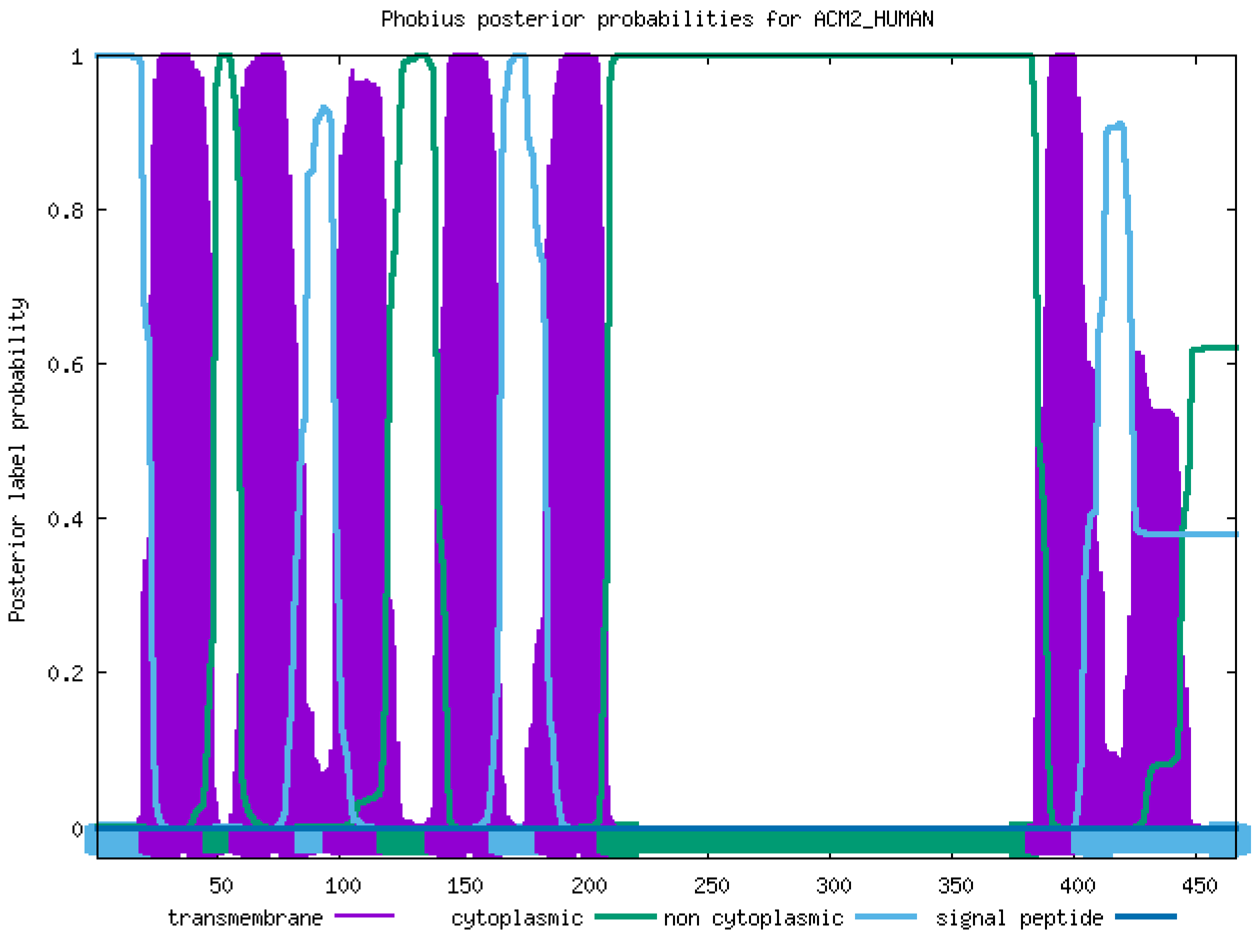

The probability data used in the plot is found here, and the gnuplot script is here.

Prediction of ACM3_HUMAN

```
ID    ACM3_HUMAN
FT    TOPO_DOM      1     64       NON CYTOPLASMIC.
FT    TRANSMEM     65     93
FT    TOPO_DOM     94    104       CYTOPLASMIC.
FT    TRANSMEM    105    131
FT    TOPO_DOM    132    142       NON CYTOPLASMIC.
FT    TRANSMEM    143    164
FT    TOPO_DOM    165    184       CYTOPLASMIC.
FT    TRANSMEM    185    210
FT    TOPO_DOM    211    229       NON CYTOPLASMIC.
FT    TRANSMEM    230    252
FT    TOPO_DOM    253    488       CYTOPLASMIC.
FT    TRANSMEM    489    509
FT    TOPO_DOM    510    528       NON CYTOPLASMIC.
FT    TRANSMEM    529    547
FT    TOPO_DOM    548    590       CYTOPLASMIC.
//
```

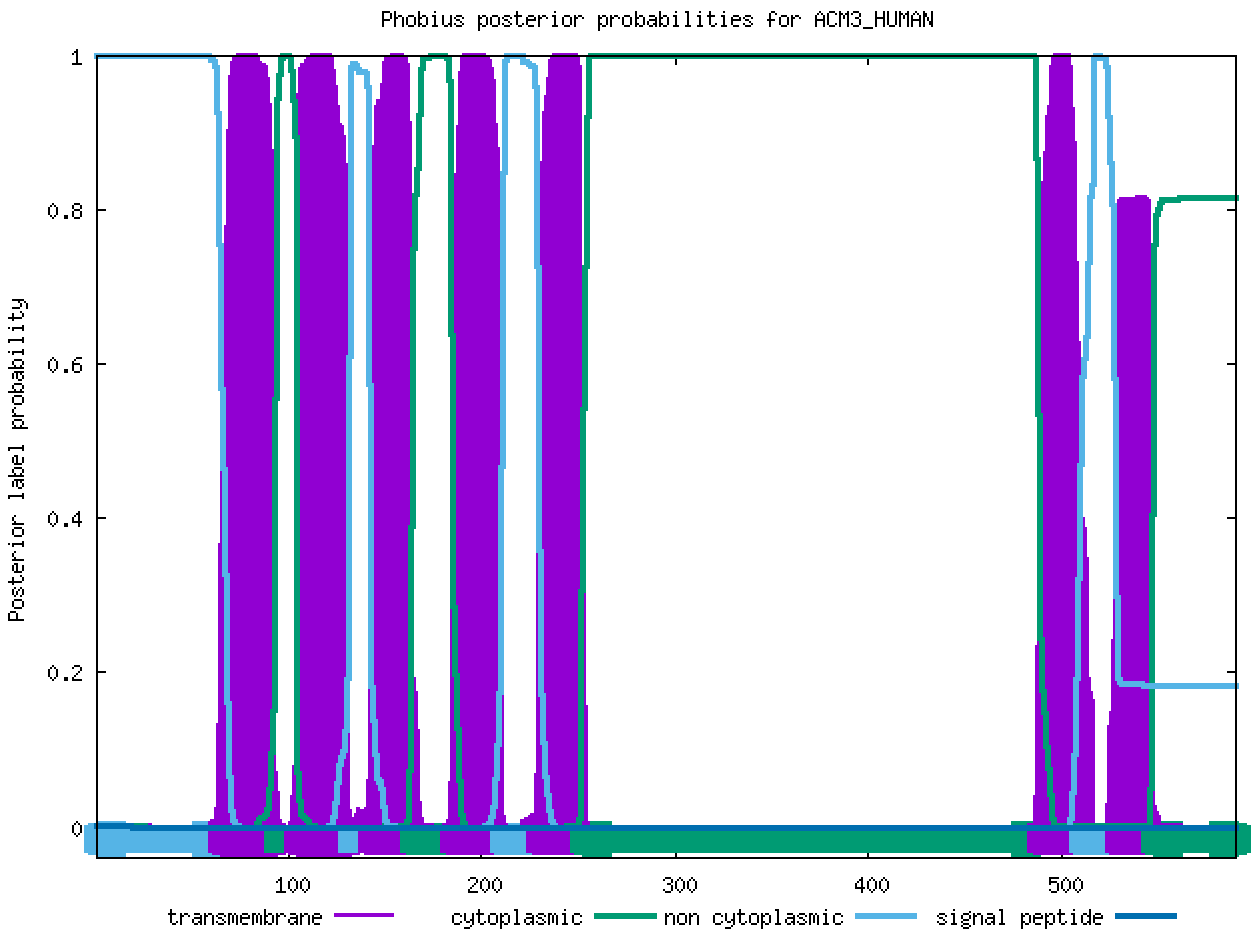

The probability data used in the plot is found here, and the gnuplot script is here.

Prediction of ACM4_HUMAN

```
ID   ACM4_HUMAN
FT   TOPO_DOM      1      31       NON CYTOPLASMIC.
FT   TRANSMEM     32      57
FT   TOPO_DOM     58      68       CYTOPLASMIC.
FT   TRANSMEM     69      95
FT   TOPO_DOM     96     106       NON CYTOPLASMIC.
FT   TRANSMEM    107     128
FT   TOPO_DOM    129     148       CYTOPLASMIC.
FT   TRANSMEM    149     174
FT   TOPO_DOM    175     193       NON CYTOPLASMIC.
FT   TRANSMEM    194     218
FT   TOPO_DOM    219     397       CYTOPLASMIC.
FT   TRANSMEM    398     416
FT   TOPO_DOM    417     479       NON CYTOPLASMIC.
//
```

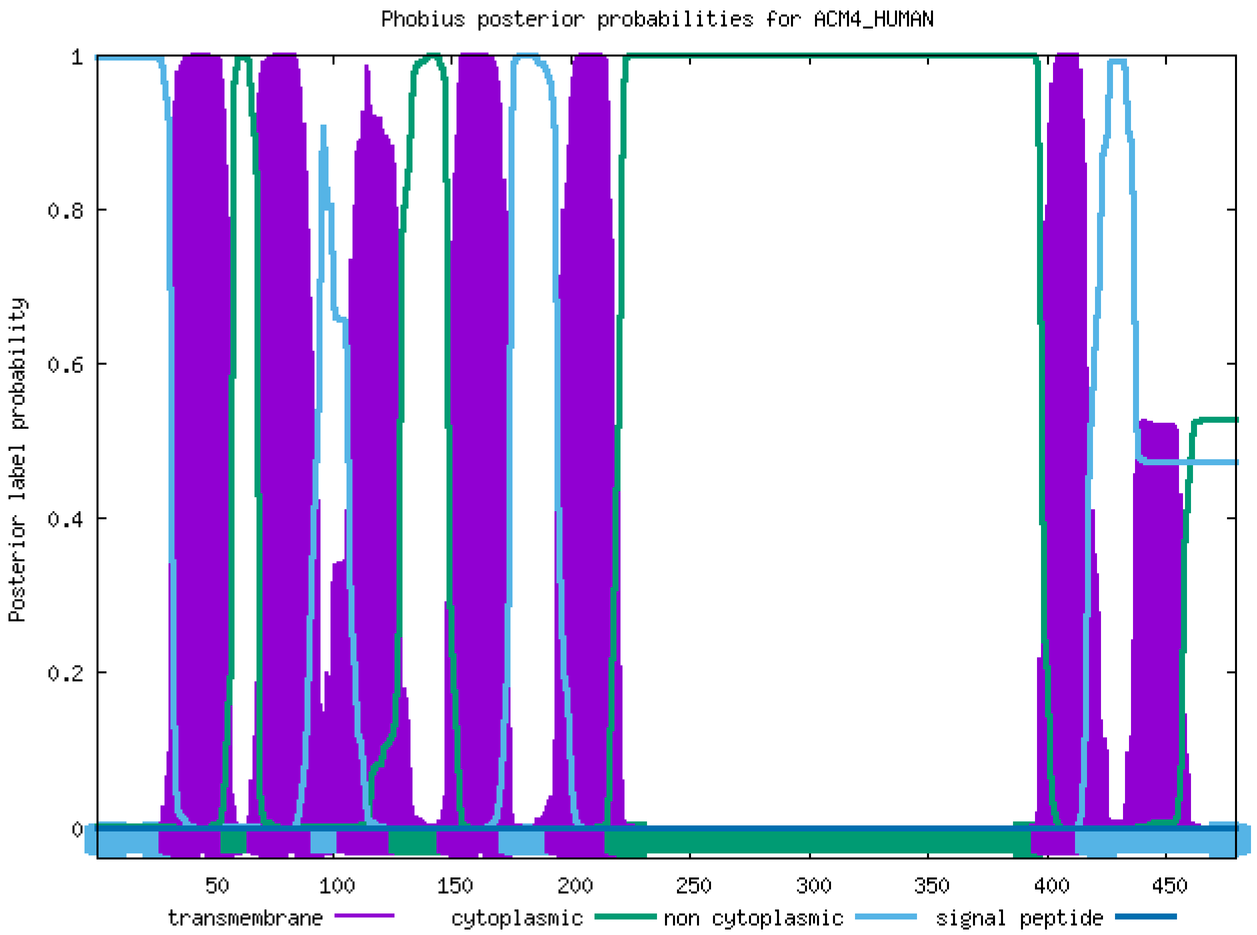

The probability data used in the plot is found here, and the gnuplot script is here.

Prediction of ACM5_HUMAN

```
ID    ACM5_HUMAN
FT    TOPO_DOM      1      29       NON CYTOPLASMIC.
FT    TRANSMEM     30      55
FT    TOPO_DOM     56      66       CYTOPLASMIC.
FT    TRANSMEM     67      93
FT    TOPO_DOM     94     104       NON CYTOPLASMIC.
FT    TRANSMEM    105     126
FT    TOPO_DOM    127     146       CYTOPLASMIC.
FT    TRANSMEM    147     172
FT    TOPO_DOM    173     191       NON CYTOPLASMIC.
FT    TRANSMEM    192     214
FT    TOPO_DOM    215     445       CYTOPLASMIC.
FT    TRANSMEM    446     467
FT    TOPO_DOM    468     478       NON CYTOPLASMIC.
FT    TRANSMEM    479     498
FT    TOPO_DOM    499     532       CYTOPLASMIC.
//
```

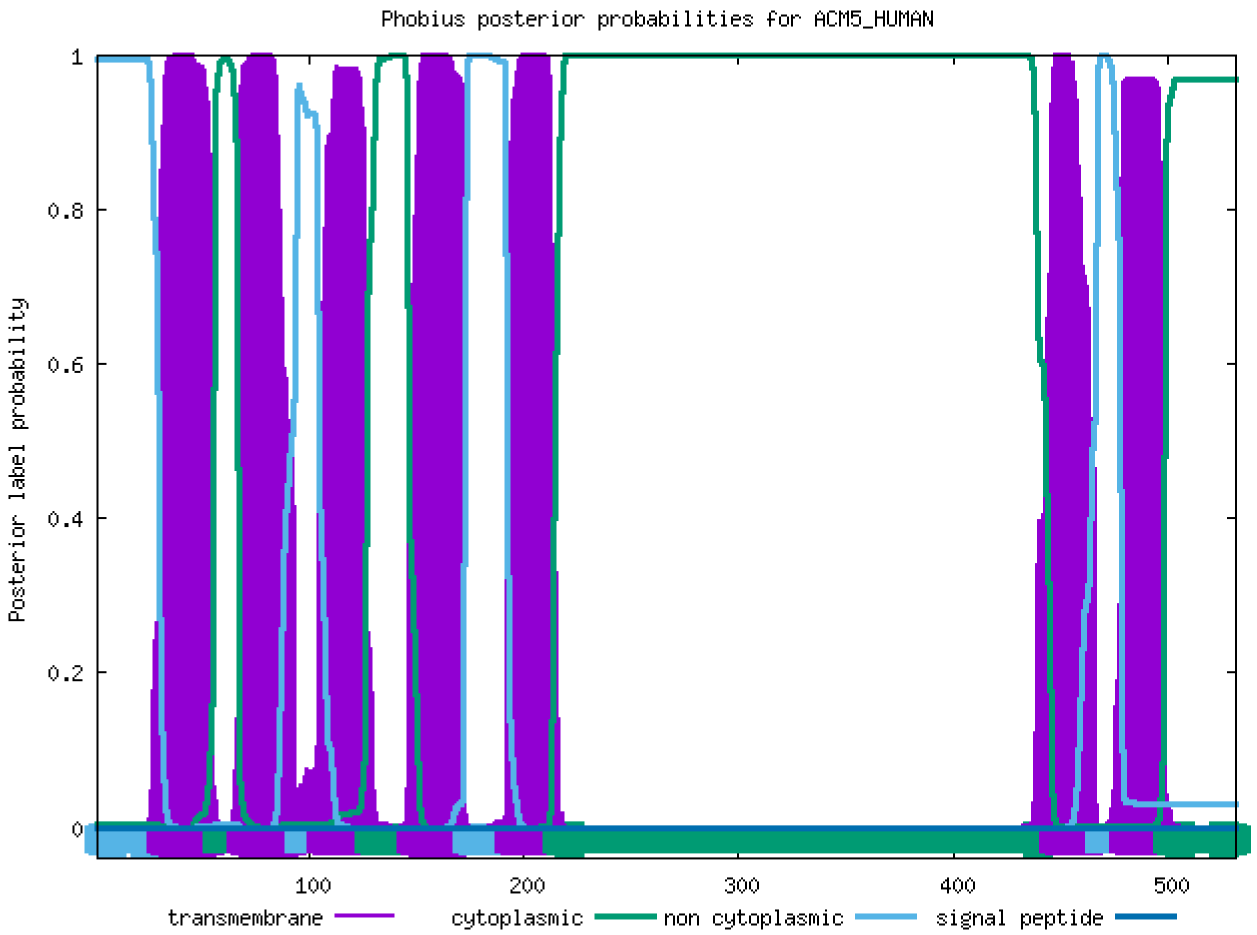

The probability data used in the plot is found here, and the gnuplot script is here.

Prediction of OPRM_HUMAN

```
ID   OPRM_HUMAN
FT   TOPO_DOM      1     66       NON CYTOPLASMIC.
FT   TRANSMEM     67     96
FT   TOPO_DOM     97    107       CYTOPLASMIC.
FT   TRANSMEM    108    130
FT   TOPO_DOM    131    149       NON CYTOPLASMIC.
FT   TRANSMEM    150    170
FT   TOPO_DOM    171    190       CYTOPLASMIC.
FT   TRANSMEM    191    208
FT   TOPO_DOM    209    232       NON CYTOPLASMIC.
FT   TRANSMEM    233    259
FT   TOPO_DOM    260    279       CYTOPLASMIC.
FT   TRANSMEM    280    304
FT   TOPO_DOM    305    318       NON CYTOPLASMIC.
FT   TRANSMEM    319    341
FT   TOPO_DOM    342    400       CYTOPLASMIC.
//
```

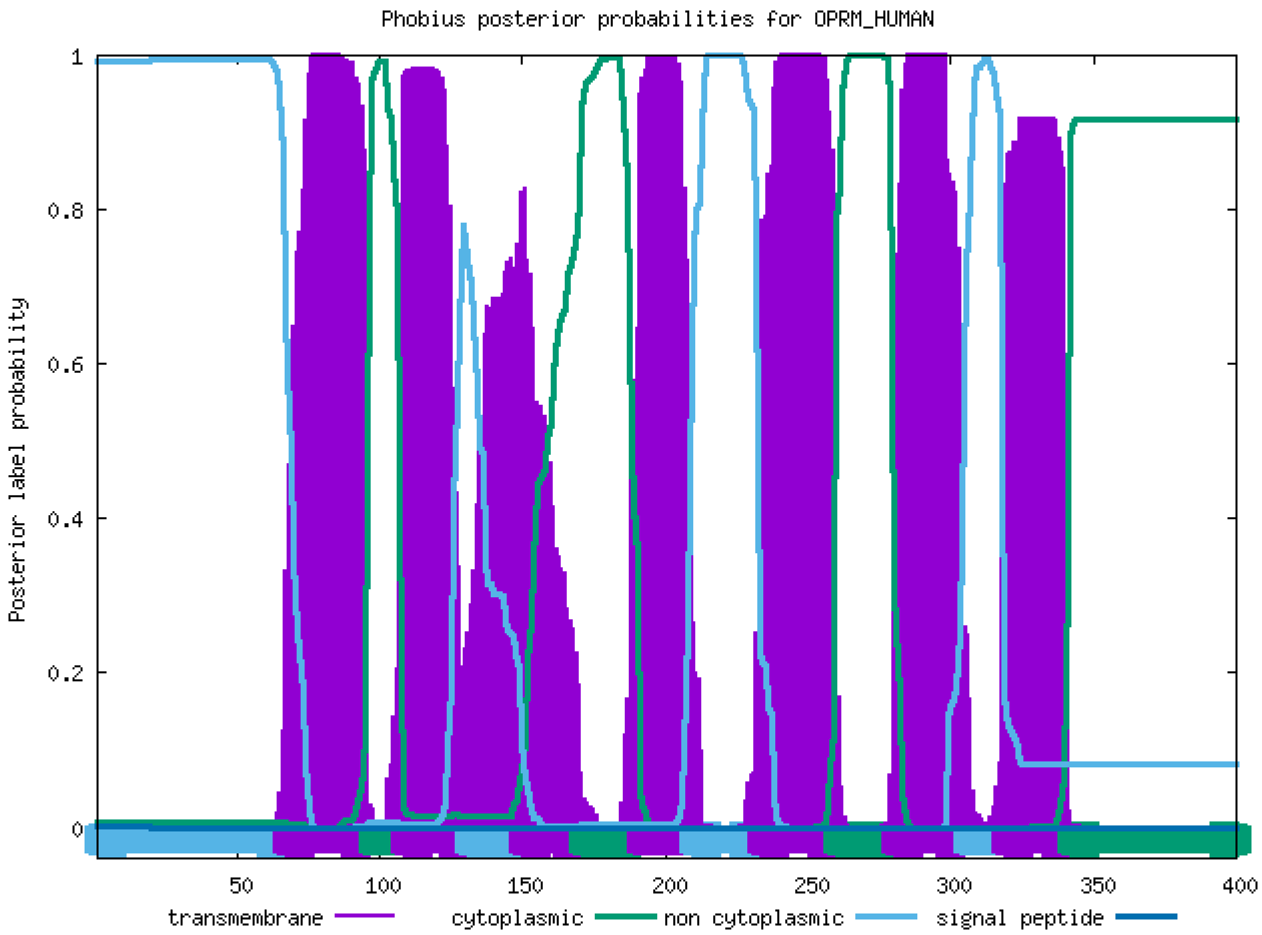

The probability data used in the plot is found here, and the gnuplot script is here.

Prediction of OPRD_HUMAN

```
ID   OPRD_HUMAN
FT   TOPO_DOM      1     45       NON CYTOPLASMIC.
FT   TRANSMEM     46     75
FT   TOPO_DOM     76     86       CYTOPLASMIC.
FT   TRANSMEM     87    107
FT   TOPO_DOM    108    126       NON CYTOPLASMIC.
FT   TRANSMEM    127    149
FT   TOPO_DOM    150    169       CYTOPLASMIC.
FT   TRANSMEM    170    191
FT   TOPO_DOM    192    210       NON CYTOPLASMIC.
FT   TRANSMEM    211    238
FT   TOPO_DOM    239    258       CYTOPLASMIC.
FT   TRANSMEM    259    287
FT   TOPO_DOM    288    298       NON CYTOPLASMIC.
FT   TRANSMEM    299    321
FT   TOPO_DOM    322    372       CYTOPLASMIC.
//
```

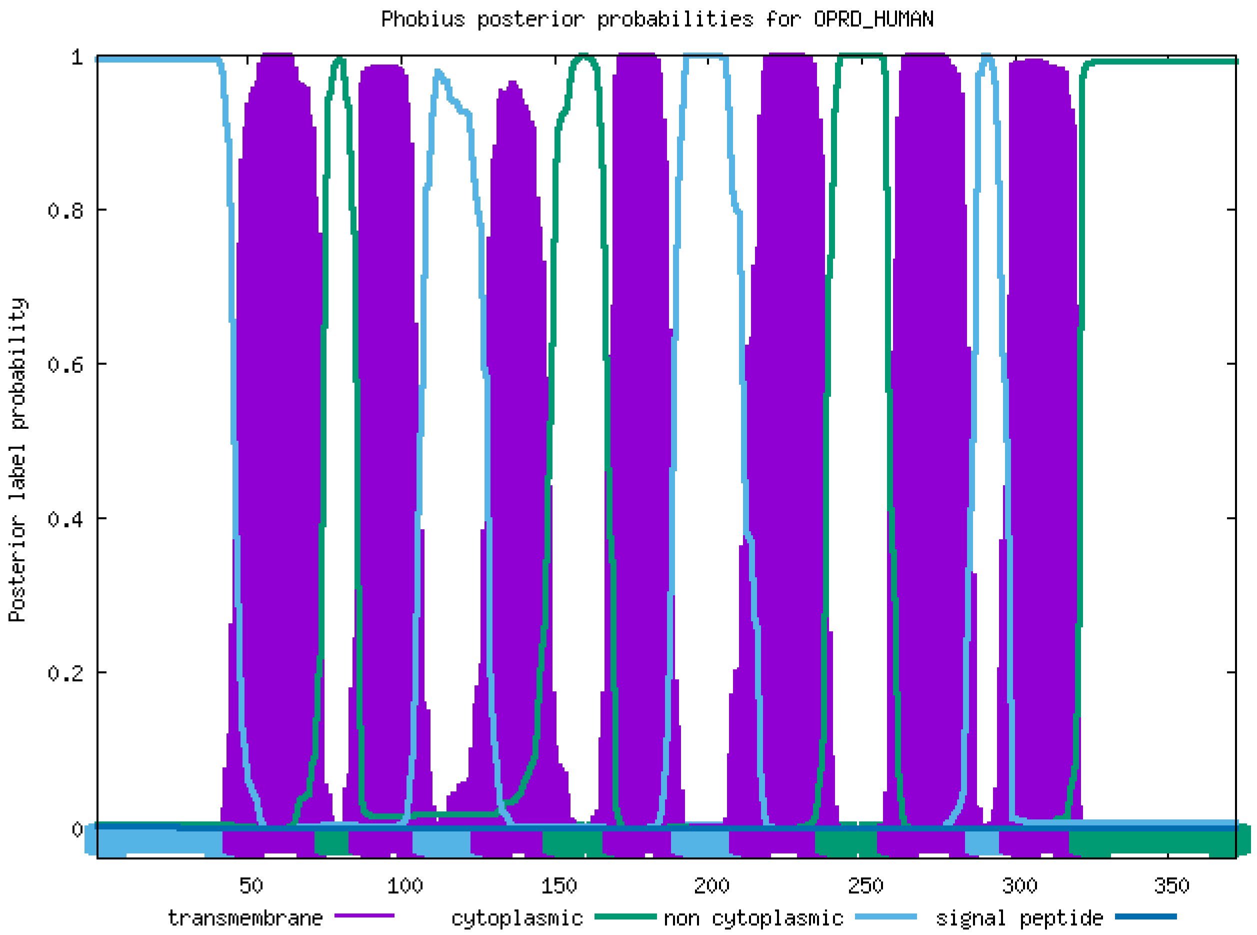

The probability data used in the plot is found here, and the gnuplot script is here.

Prediction of CNR1_HUMAN

```
ID    CNR1_HUMAN
FT    TOPO_DOM      1     116       NON CYTOPLASMIC.
FT    TRANSMEM    117     141
FT    TOPO_DOM    142     152       CYTOPLASMIC.
FT    TRANSMEM    153     175
FT    TOPO_DOM    176     186       NON CYTOPLASMIC.
FT    TRANSMEM    187     212
FT    TOPO_DOM    213     232       CYTOPLASMIC.
FT    TRANSMEM    233     255
FT    TOPO_DOM    256     274       NON CYTOPLASMIC.
FT    TRANSMEM    275     299
FT    TOPO_DOM    300     344       CYTOPLASMIC.
FT    TRANSMEM    345     365
FT    TOPO_DOM    366     376       NON CYTOPLASMIC.
FT    TRANSMEM    377     399
FT    TOPO_DOM    400     472       CYTOPLASMIC.
//
```

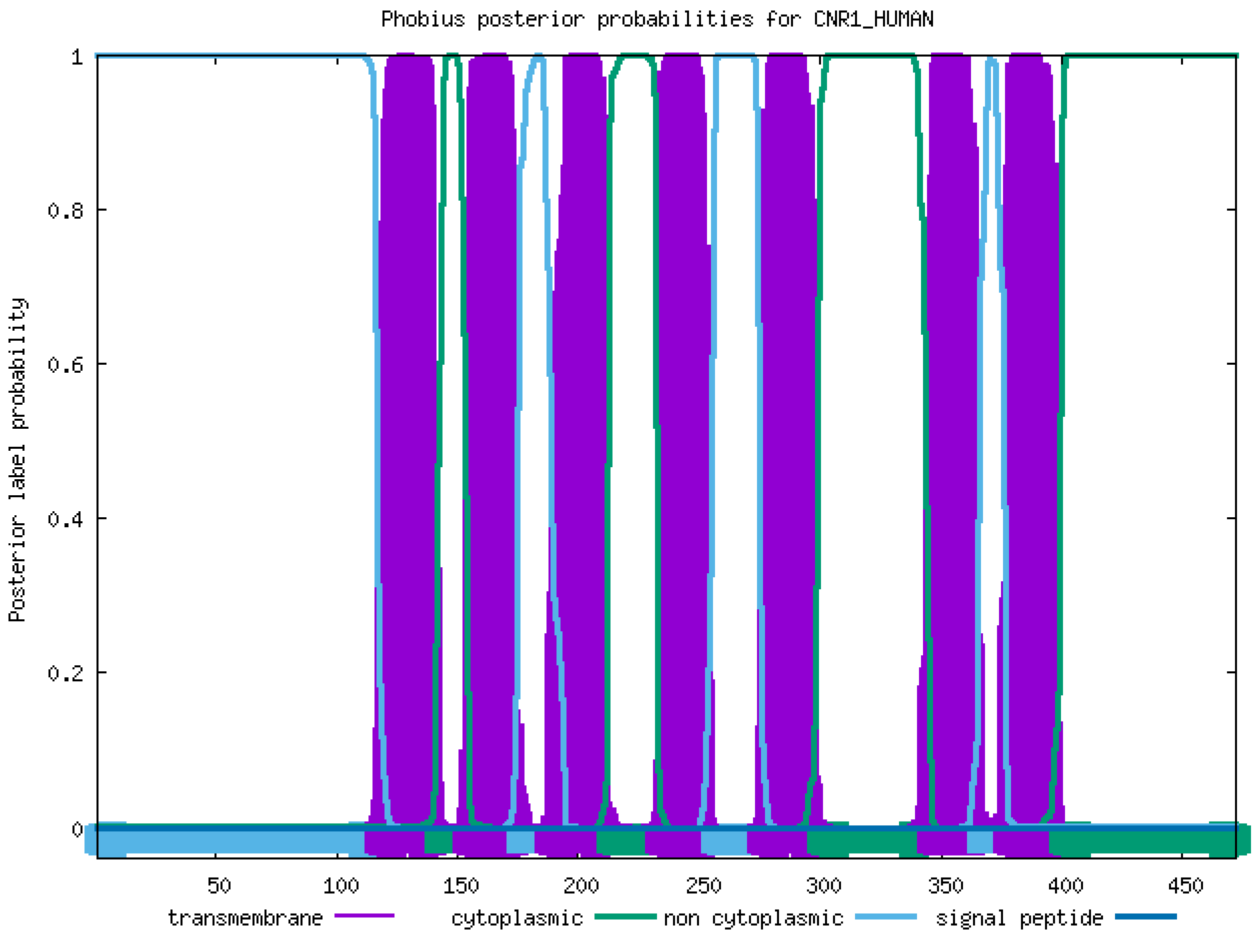

The probability data used in the plot is found here, and the gnuplot script is here.

Prediction of CNR2_HUMAN

```
ID   CNR2_HUMAN
FT   TOPO_DOM      1     34       NON CYTOPLASMIC.
FT   TRANSMEM     35     58
FT   TOPO_DOM     59     69       CYTOPLASMIC.
FT   TRANSMEM     70     94
FT   TOPO_DOM     95    105       NON CYTOPLASMIC.
FT   TRANSMEM    106    129
FT   TOPO_DOM    130    149       CYTOPLASMIC.
FT   TRANSMEM    150    170
FT   TOPO_DOM    171    189       NON CYTOPLASMIC.
FT   TRANSMEM    190    209
FT   TOPO_DOM    210    245       CYTOPLASMIC.
FT   TRANSMEM    246    268
FT   TOPO_DOM    269    279       NON CYTOPLASMIC.
FT   TRANSMEM    280    301
FT   TOPO_DOM    302    360       CYTOPLASMIC.
//
```

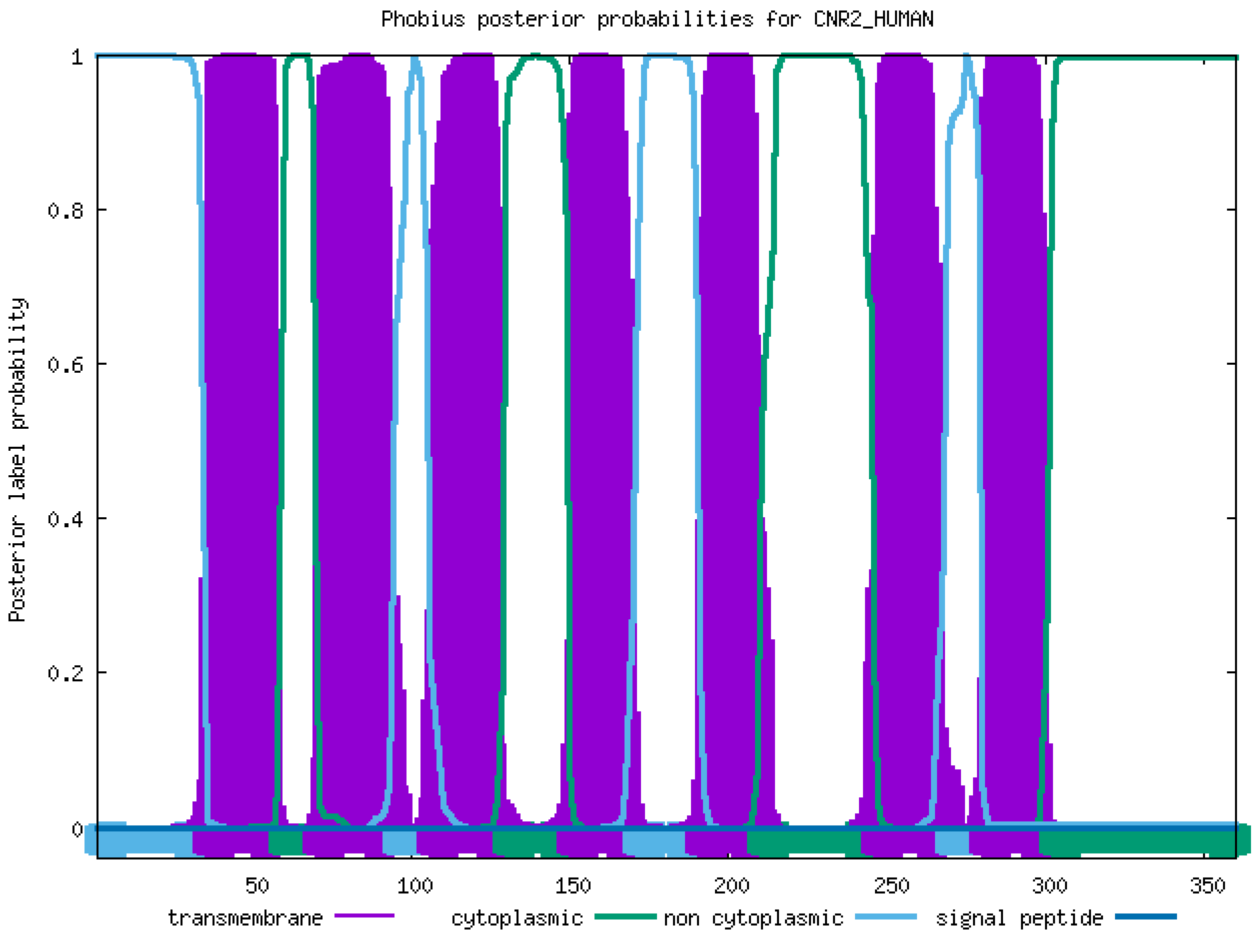

The probability data used in the plot is found here, and the gnuplot script is here.

Prediction of OPRX_HUMAN

```
ID   OPRX_HUMAN
FT   TOPO_DOM      1     54       NON CYTOPLASMIC.
FT   TRANSMEM     55     77
FT   TOPO_DOM     78     88       CYTOPLASMIC.
FT   TRANSMEM     89    107
FT   TOPO_DOM    108    112       NON CYTOPLASMIC.
FT   TRANSMEM    113    132
FT   TOPO_DOM    133    171       CYTOPLASMIC.
FT   TRANSMEM    172    191
FT   TOPO_DOM    192    210       NON CYTOPLASMIC.
FT   TRANSMEM    211    239
FT   TOPO_DOM    240    259       CYTOPLASMIC.
FT   TRANSMEM    260    279
FT   TOPO_DOM    280    298       NON CYTOPLASMIC.
FT   TRANSMEM    299    322
FT   TOPO_DOM    323    370       CYTOPLASMIC.
//
```

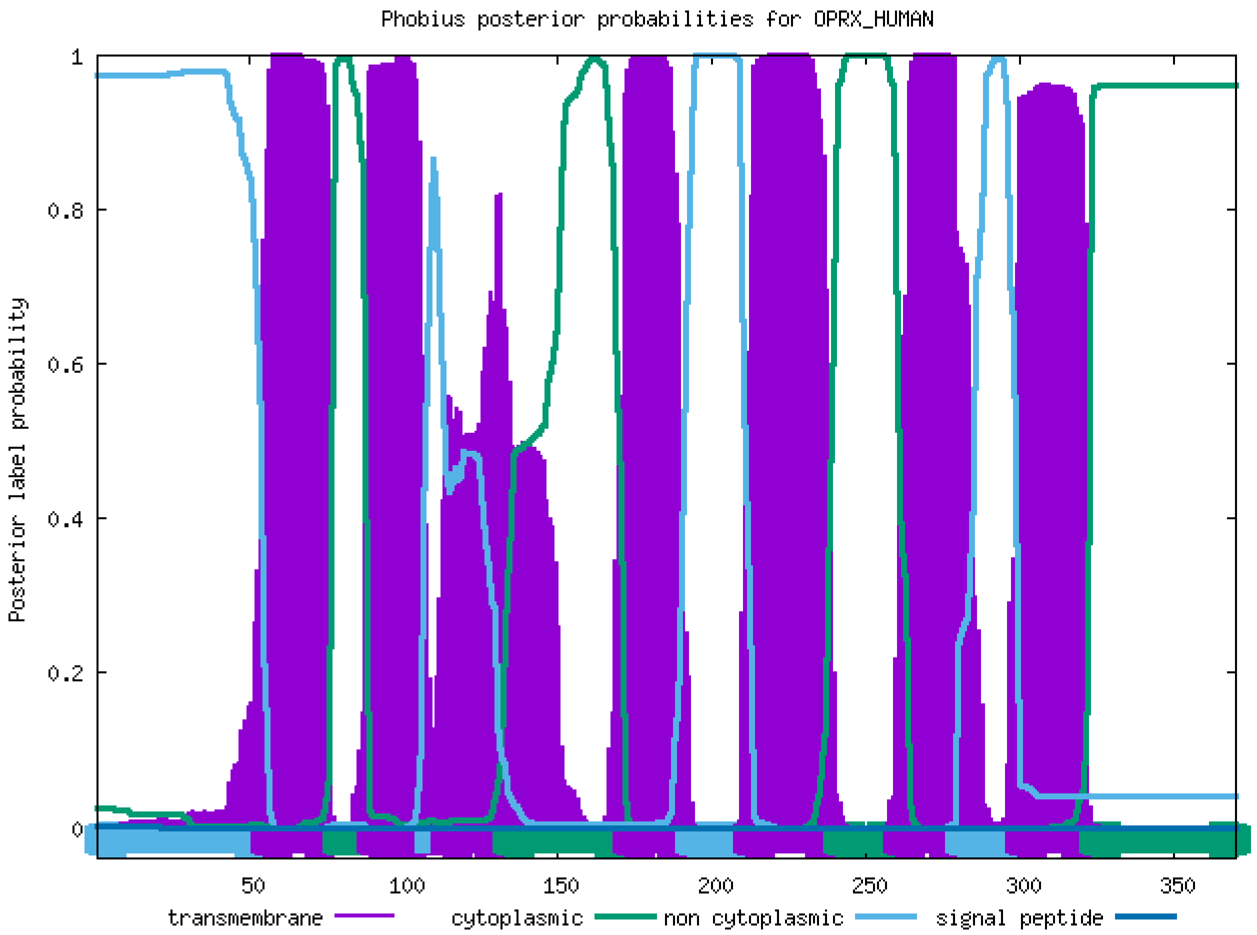

The probability data used in the plot is found here, and the gnuplot script is here.

Prediction of NTR1_HUMAN

```
ID   NTR1_HUMAN
FT   TOPO_DOM      1     63       NON CYTOPLASMIC.
FT   TRANSMEM     64     89
FT   TOPO_DOM     90    100       CYTOPLASMIC.
FT   TRANSMEM    101    125
FT   TOPO_DOM    126    144       NON CYTOPLASMIC.
FT   TRANSMEM    145    168
FT   TOPO_DOM    169    187       CYTOPLASMIC.
FT   TRANSMEM    188    205
FT   TOPO_DOM    206    241       NON CYTOPLASMIC.
FT   TRANSMEM    242    260
FT   TOPO_DOM    261    303       CYTOPLASMIC.
FT   TRANSMEM    304    321
FT   TOPO_DOM    322    340       NON CYTOPLASMIC.
FT   TRANSMEM    341    363
FT   TOPO_DOM    364    418       CYTOPLASMIC.
//
```

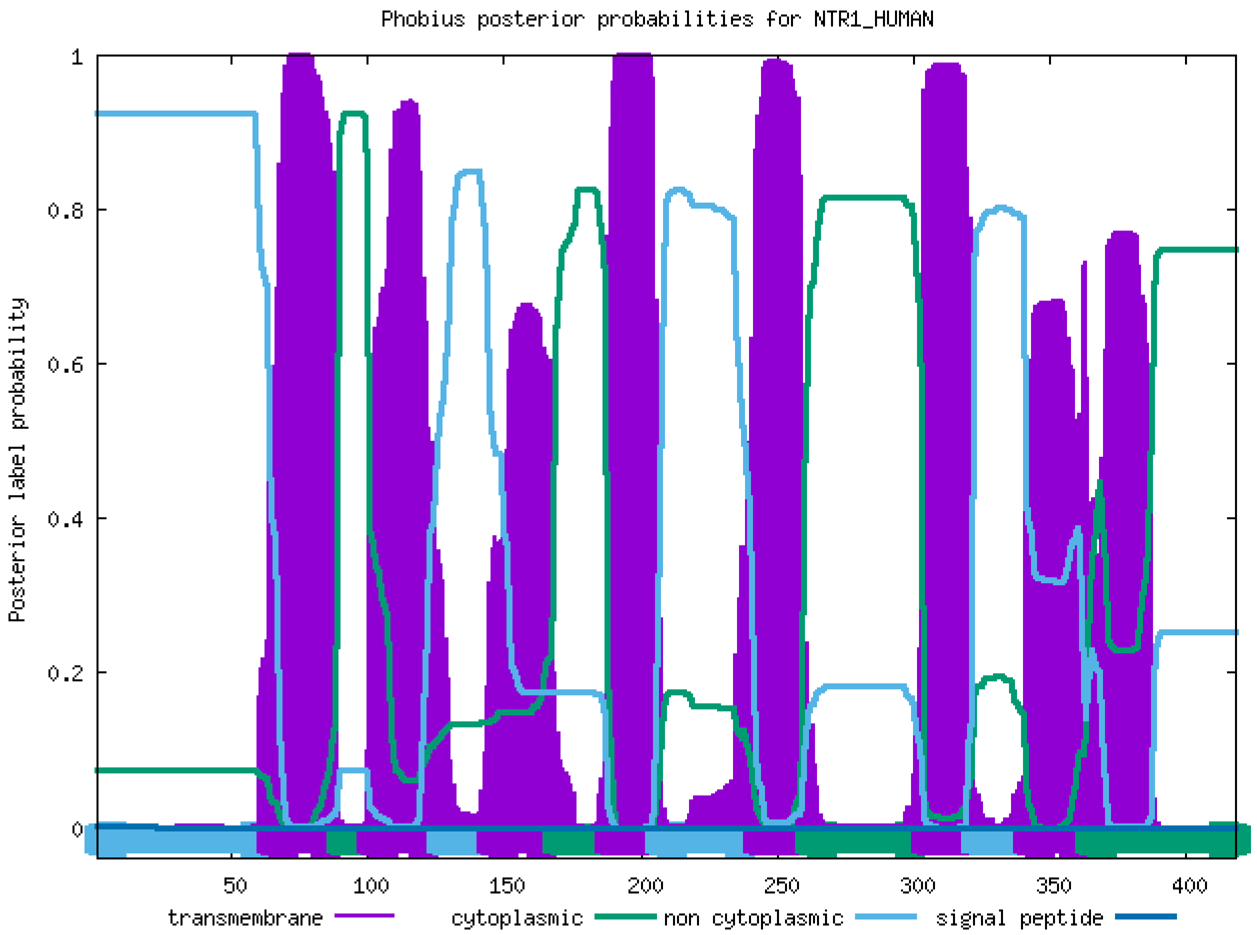

The probability data used in the plot is found here, and the gnuplot script is here.

Prediction of NPY1R_HUMAN

```
ID   NPY1R_HUMAN
FT   TOPO_DOM      1     37       NON CYTOPLASMIC.
FT   TRANSMEM     38     66
FT   TOPO_DOM     67     77       CYTOPLASMIC.
FT   TRANSMEM     78    103
FT   TOPO_DOM    104    114       NON CYTOPLASMIC.
FT   TRANSMEM    115    136
FT   TOPO_DOM    137    155       CYTOPLASMIC.
FT   TRANSMEM    156    179
FT   TOPO_DOM    180    213       NON CYTOPLASMIC.
FT   TRANSMEM    214    236
FT   TOPO_DOM    237    260       CYTOPLASMIC.
FT   TRANSMEM    261    281
FT   TOPO_DOM    282    300       NON CYTOPLASMIC.
FT   TRANSMEM    301    323
FT   TOPO_DOM    324    384       CYTOPLASMIC.
//
```

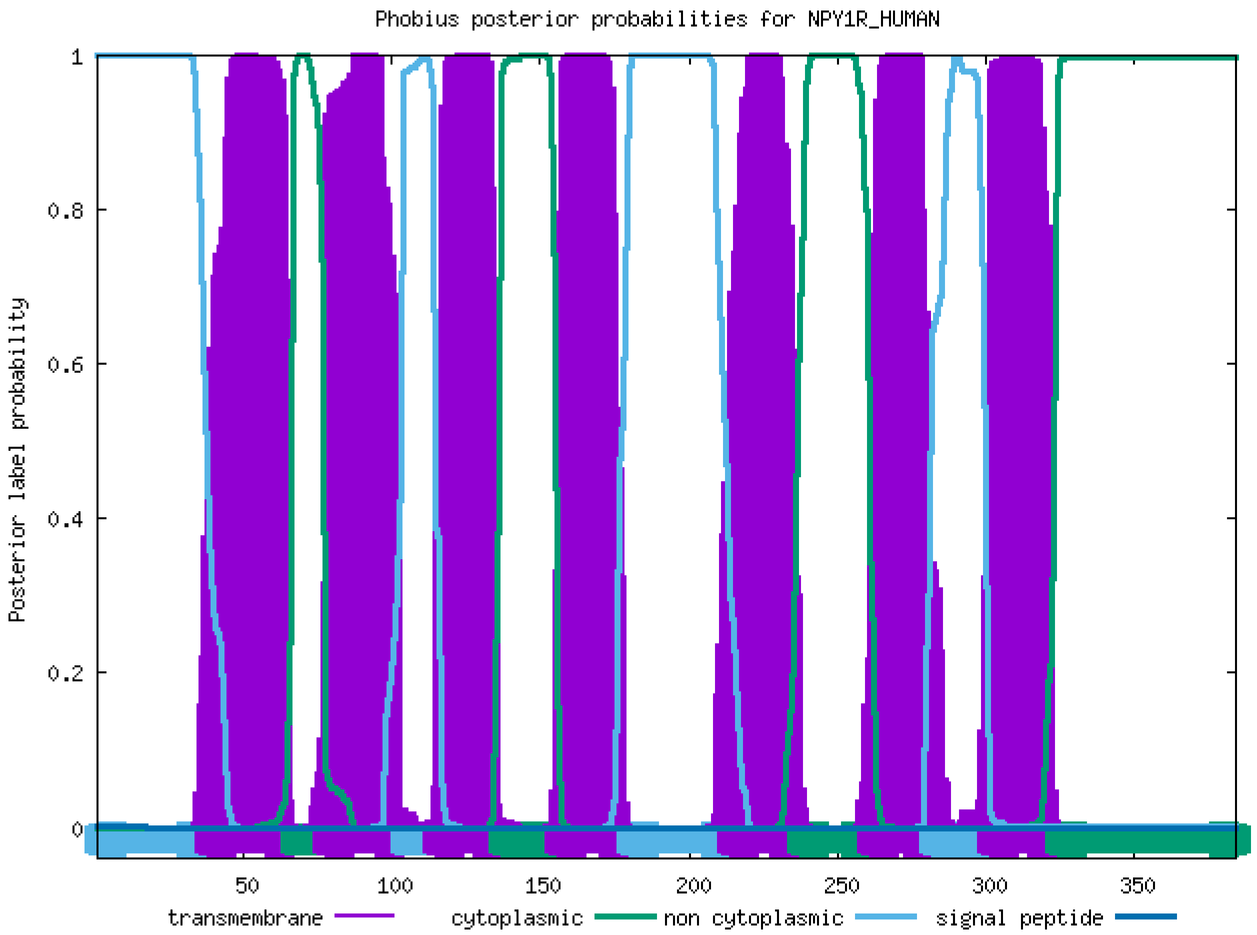

The probability data used in the plot is found here, and the gnuplot script is here.

Prediction of NPY2R_HUMAN

```
ID   NPY2R_HUMAN
FT   TOPO_DOM      1     53       NON CYTOPLASMIC.
FT   TRANSMEM     54     76
FT   TOPO_DOM     77     87       CYTOPLASMIC.
FT   TRANSMEM     88    108
FT   TOPO_DOM    109    127       NON CYTOPLASMIC.
FT   TRANSMEM    128    146
FT   TOPO_DOM    147    165       CYTOPLASMIC.
FT   TRANSMEM    166    186
FT   TOPO_DOM    187    217       NON CYTOPLASMIC.
FT   TRANSMEM    218    239
FT   TOPO_DOM    240    268       CYTOPLASMIC.
FT   TRANSMEM    269    291
FT   TOPO_DOM    292    302       NON CYTOPLASMIC.
FT   TRANSMEM    303    324
FT   TOPO_DOM    325    381       CYTOPLASMIC.
//
```

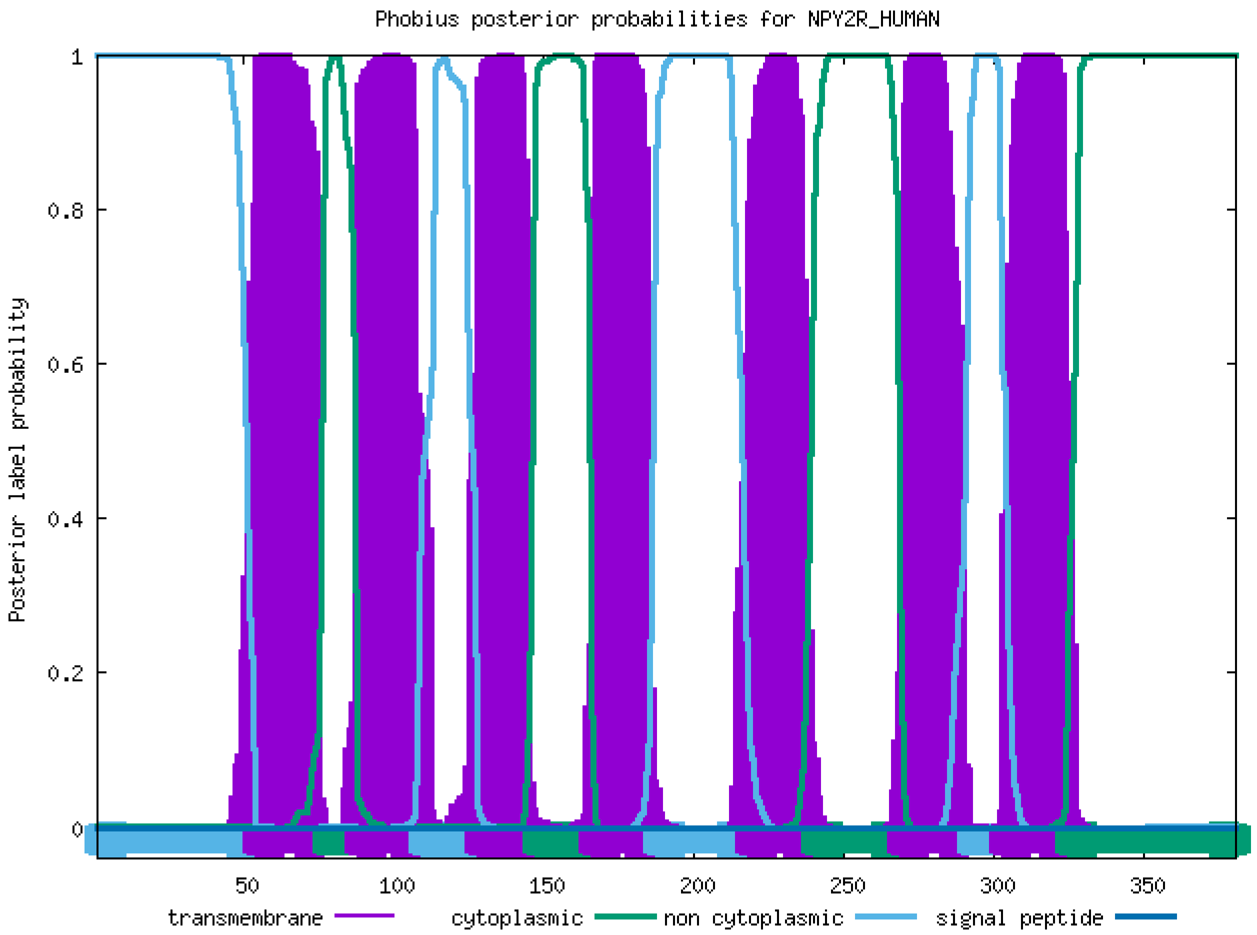

The probability data used in the plot is found here, and the gnuplot script is here.

Prediction of NPY4R_HUMAN

```
ID   NPY4R_HUMAN
FT   SIGNAL         1     16
FT   REGION         1      2       N-REGION.
FT   REGION         3     11       H-REGION.
FT   REGION        12     16       C-REGION.
FT   TOPO_DOM      17     39       NON CYTOPLASMIC.
FT   TRANSMEM      40     67
FT   TOPO_DOM      68     78       CYTOPLASMIC.
FT   TRANSMEM      79    104
FT   TOPO_DOM     105    115       NON CYTOPLASMIC.
FT   TRANSMEM     116    137
FT   TOPO_DOM     138    156       CYTOPLASMIC.
FT   TRANSMEM     157    176
FT   TOPO_DOM     177    212       NON CYTOPLASMIC.
FT   TRANSMEM     213    237
FT   TOPO_DOM     238    262       CYTOPLASMIC.
FT   TRANSMEM     263    281
FT   TOPO_DOM     282    300       NON CYTOPLASMIC.
FT   TRANSMEM     301    325
FT   TOPO_DOM     326    375       CYTOPLASMIC.
//
```

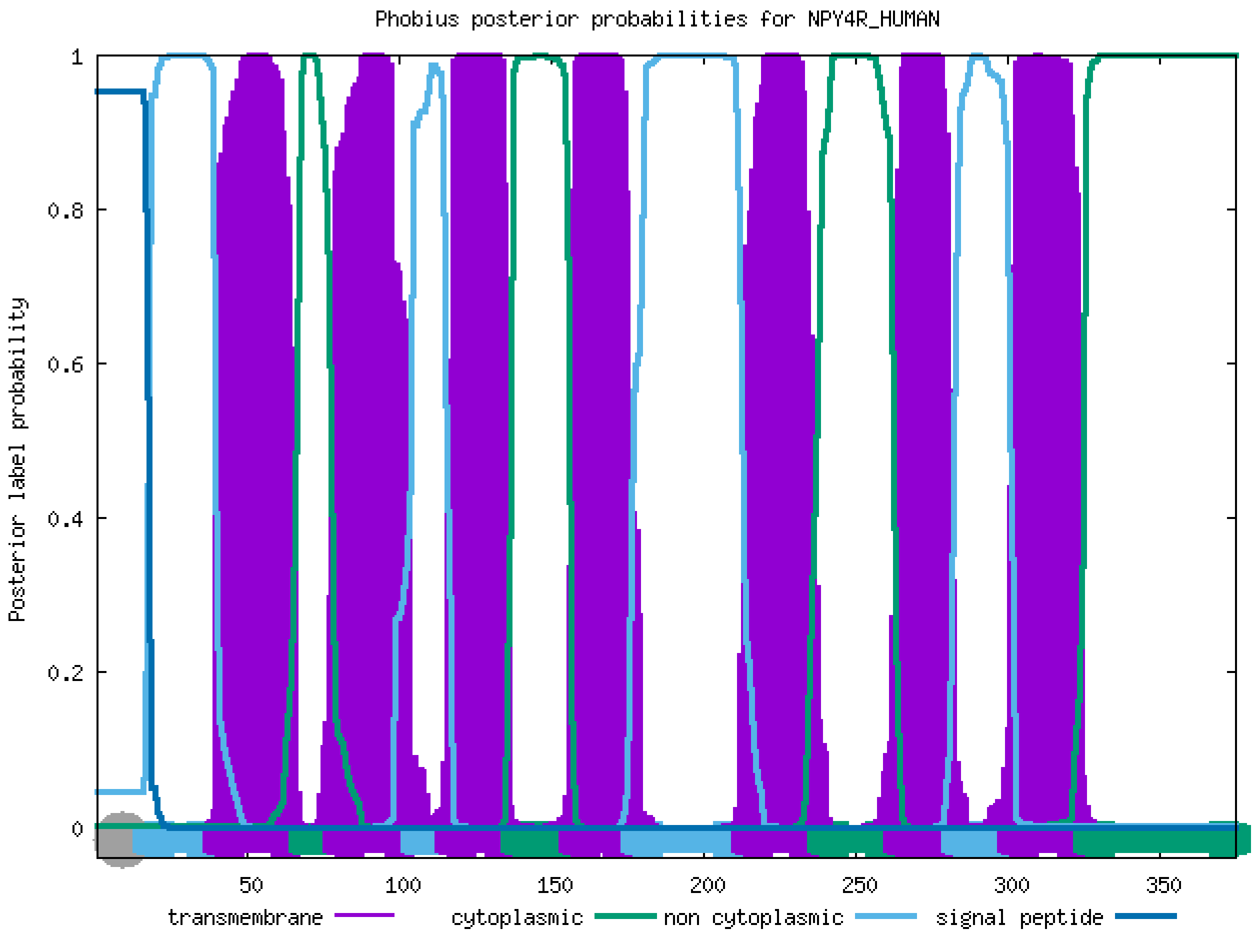

The probability data used in the plot is found here, and the gnuplot script is here.

Prediction of PAR1_HUMAN

```
ID    PAR1_HUMAN
FT    SIGNAL        1      21
FT    REGION        1       5       N-REGION.
FT    REGION        6      16       H-REGION.
FT    REGION       17      21       C-REGION.
FT    TOPO_DOM     22     107       NON CYTOPLASMIC.
FT    TRANSMEM    108     129
FT    TOPO_DOM    130     137       CYTOPLASMIC.
FT    TRANSMEM    138     157
FT    TOPO_DOM    158     176       NON CYTOPLASMIC.
FT    TRANSMEM    177     198
FT    TOPO_DOM    199     218       CYTOPLASMIC.
FT    TRANSMEM    219     239
FT    TOPO_DOM    240     266       NON CYTOPLASMIC.
FT    TRANSMEM    267     291
FT    TOPO_DOM    292     311       CYTOPLASMIC.
FT    TRANSMEM    312     338
FT    TOPO_DOM    339     349       NON CYTOPLASMIC.
FT    TRANSMEM    350     374
FT    TOPO_DOM    375     425       CYTOPLASMIC.
//
```

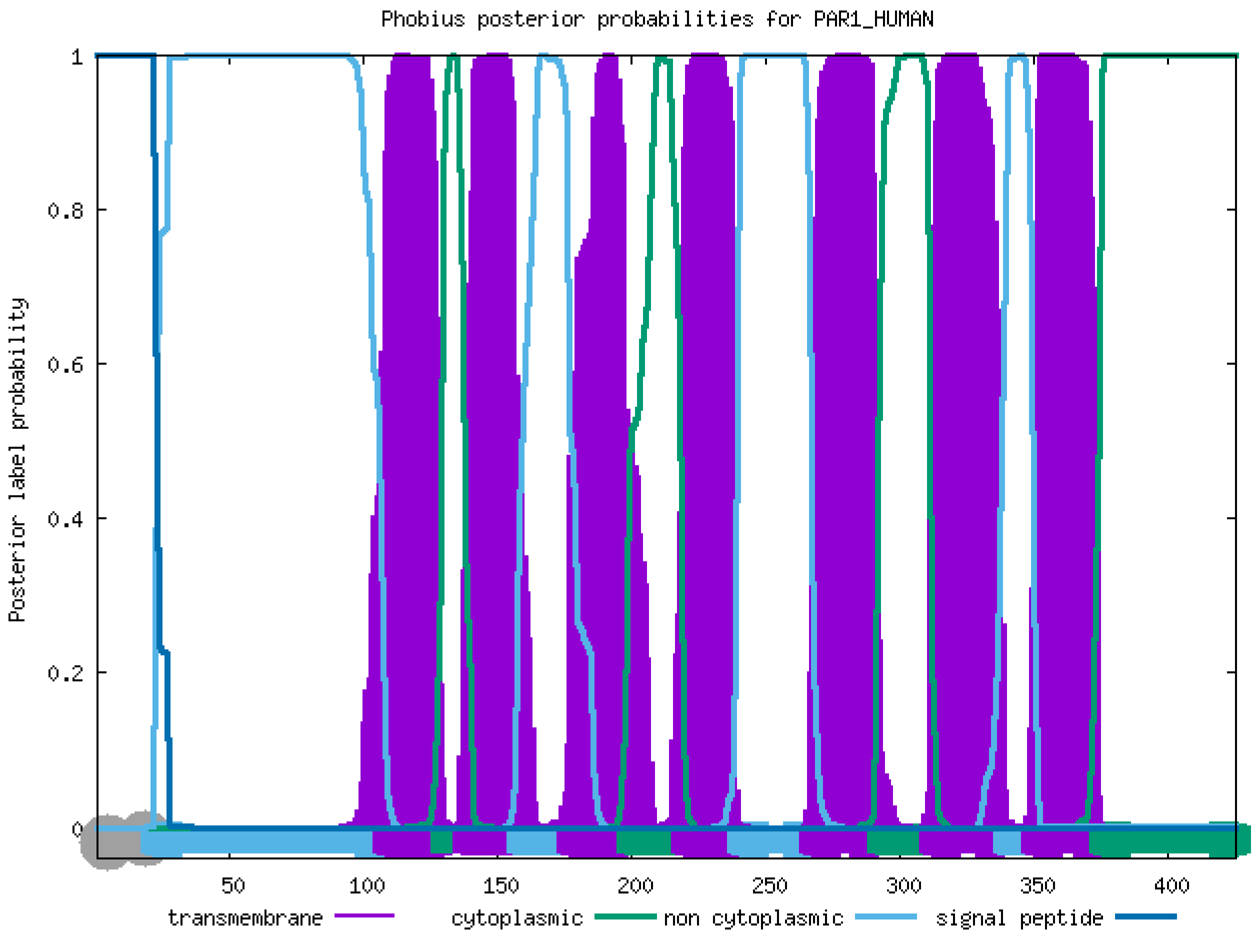

The probability data used in the plot is found here, and the gnuplot script is here.

Prediction of PAR2_HUMAN

```
ID   PAR2_HUMAN
FT   SIGNAL        1     22
FT   REGION        1      5       N-REGION.
FT   REGION        6     17       H-REGION.
FT   REGION       18     22       C-REGION.
FT   TOPO_DOM     23     75       NON CYTOPLASMIC.
FT   TRANSMEM     76    101
FT   TOPO_DOM    102    109       CYTOPLASMIC.
FT   TRANSMEM    110    130
FT   TOPO_DOM    131    149       NON CYTOPLASMIC.
FT   TRANSMEM    150    169
FT   TOPO_DOM    170    189       CYTOPLASMIC.
FT   TRANSMEM    190    211
FT   TOPO_DOM    212    241       NON CYTOPLASMIC.
FT   TRANSMEM    242    264
FT   TOPO_DOM    265    284       CYTOPLASMIC.
FT   TRANSMEM    285    313
FT   TOPO_DOM    314    324       NON CYTOPLASMIC.
FT   TRANSMEM    325    347
FT   TOPO_DOM    348    397       CYTOPLASMIC.
//
```

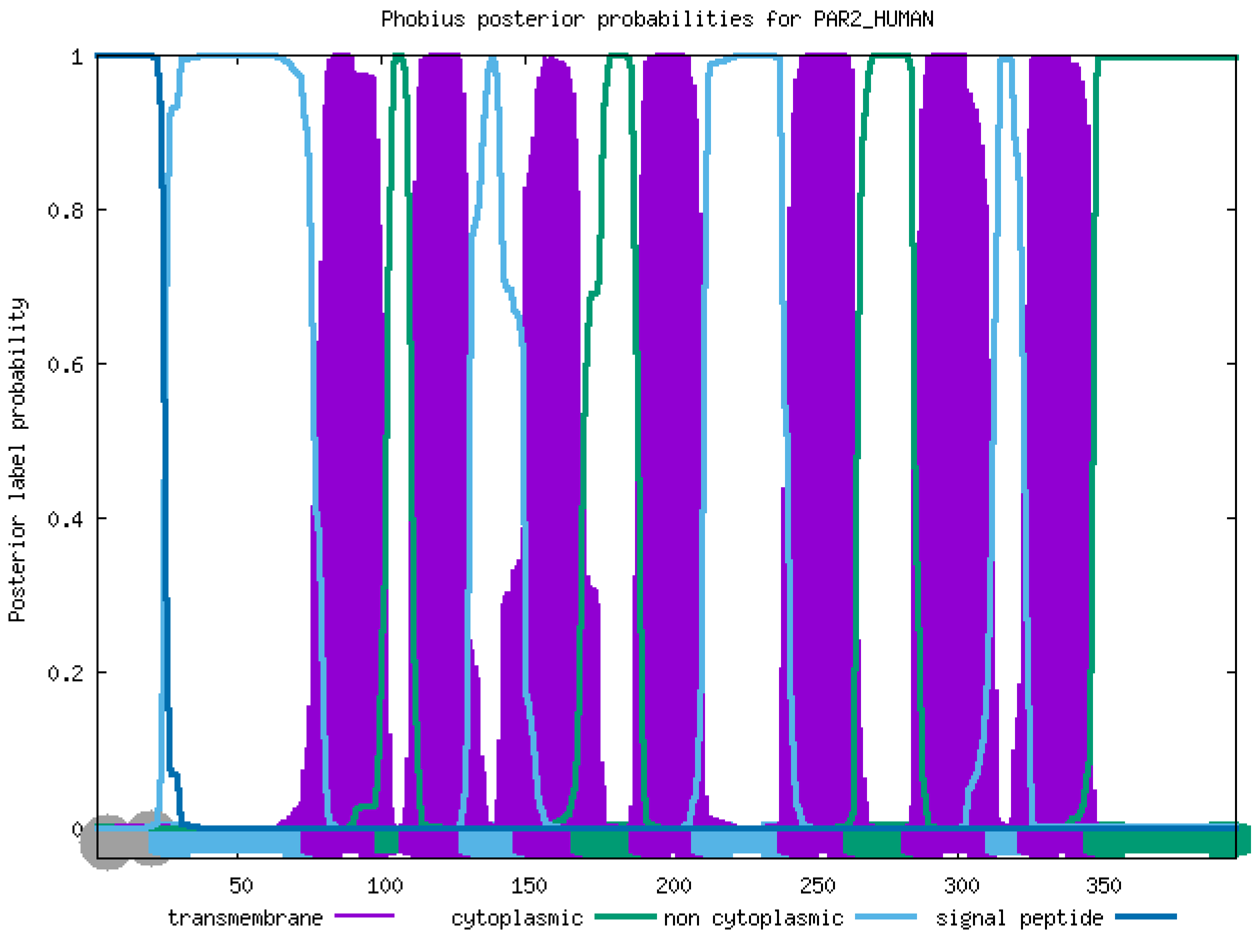

The probability data used in the plot is found here, and the gnuplot script is here.

Prediction of APJ_HUMAN

```
ID    APJ_HUMAN
FT    TOPO_DOM      1     32       NON CYTOPLASMIC.
FT    TRANSMEM     33     54
FT    TOPO_DOM     55     65       CYTOPLASMIC.
FT    TRANSMEM     66     86
FT    TOPO_DOM     87    105       NON CYTOPLASMIC.
FT    TRANSMEM    106    125
FT    TOPO_DOM    126    145       CYTOPLASMIC.
FT    TRANSMEM    146    166
FT    TOPO_DOM    167    200       NON CYTOPLASMIC.
FT    TRANSMEM    201    225
FT    TOPO_DOM    226    245       CYTOPLASMIC.
FT    TRANSMEM    246    266
FT    TOPO_DOM    267    285       NON CYTOPLASMIC.
FT    TRANSMEM    286    312
FT    TOPO_DOM    313    380       CYTOPLASMIC.
//
```

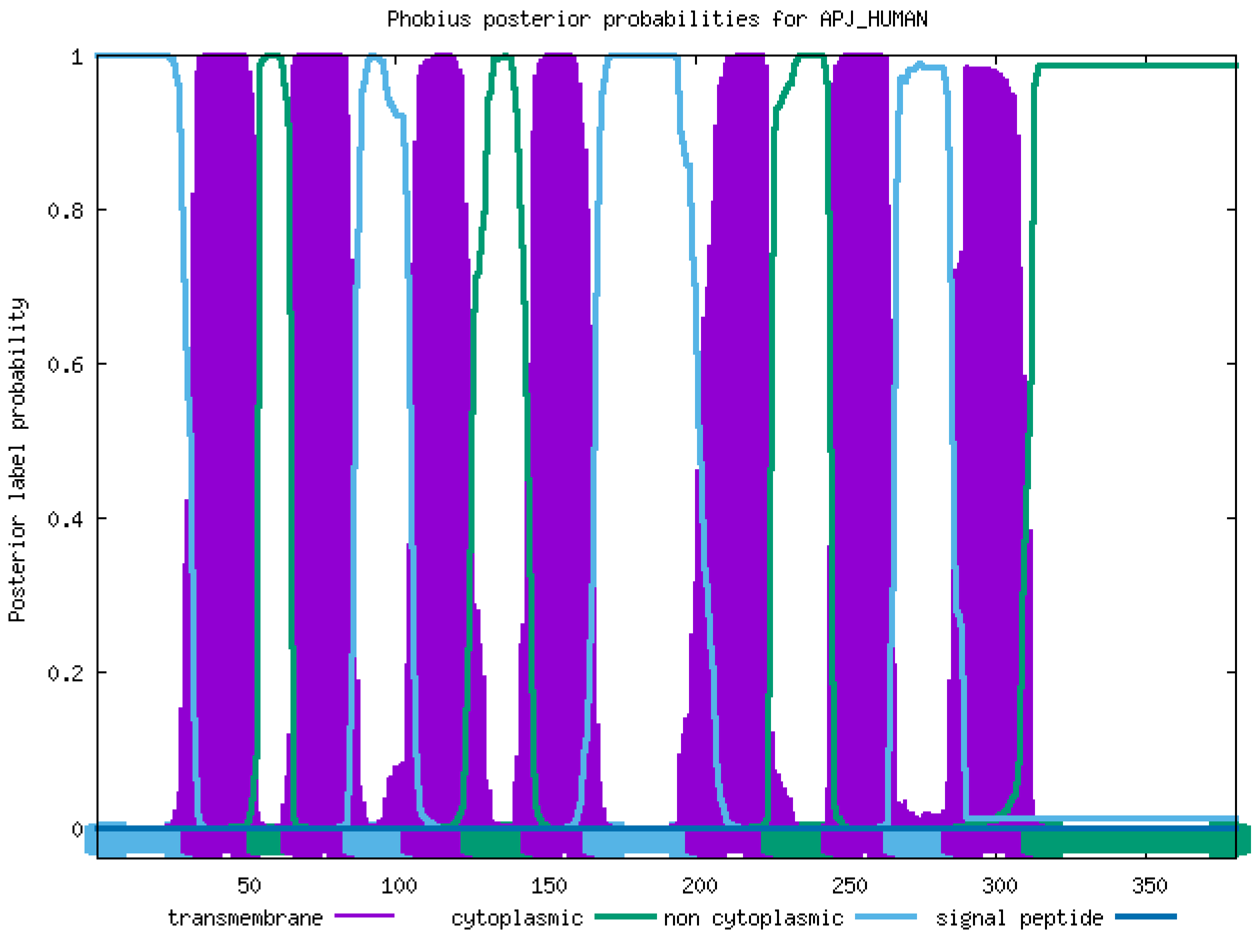

The probability data used in the plot is found here, and the gnuplot script is here.

Prediction of 5HT1A_HUMAN

```
ID   5HT1A_HUMAN
FT   TOPO_DOM      1     40       NON CYTOPLASMIC.
FT   TRANSMEM     41     63
FT   TOPO_DOM     64     69       CYTOPLASMIC.
FT   TRANSMEM     70     99
FT   TOPO_DOM    100    110       NON CYTOPLASMIC.
FT   TRANSMEM    111    132
FT   TOPO_DOM    133    152       CYTOPLASMIC.
FT   TRANSMEM    153    175
FT   TOPO_DOM    176    194       NON CYTOPLASMIC.
FT   TRANSMEM    195    218
FT   TOPO_DOM    219    346       CYTOPLASMIC.
FT   TRANSMEM    347    368
FT   TOPO_DOM    369    379       NON CYTOPLASMIC.
FT   TRANSMEM    380    403
FT   TOPO_DOM    404    422       CYTOPLASMIC.
//
```

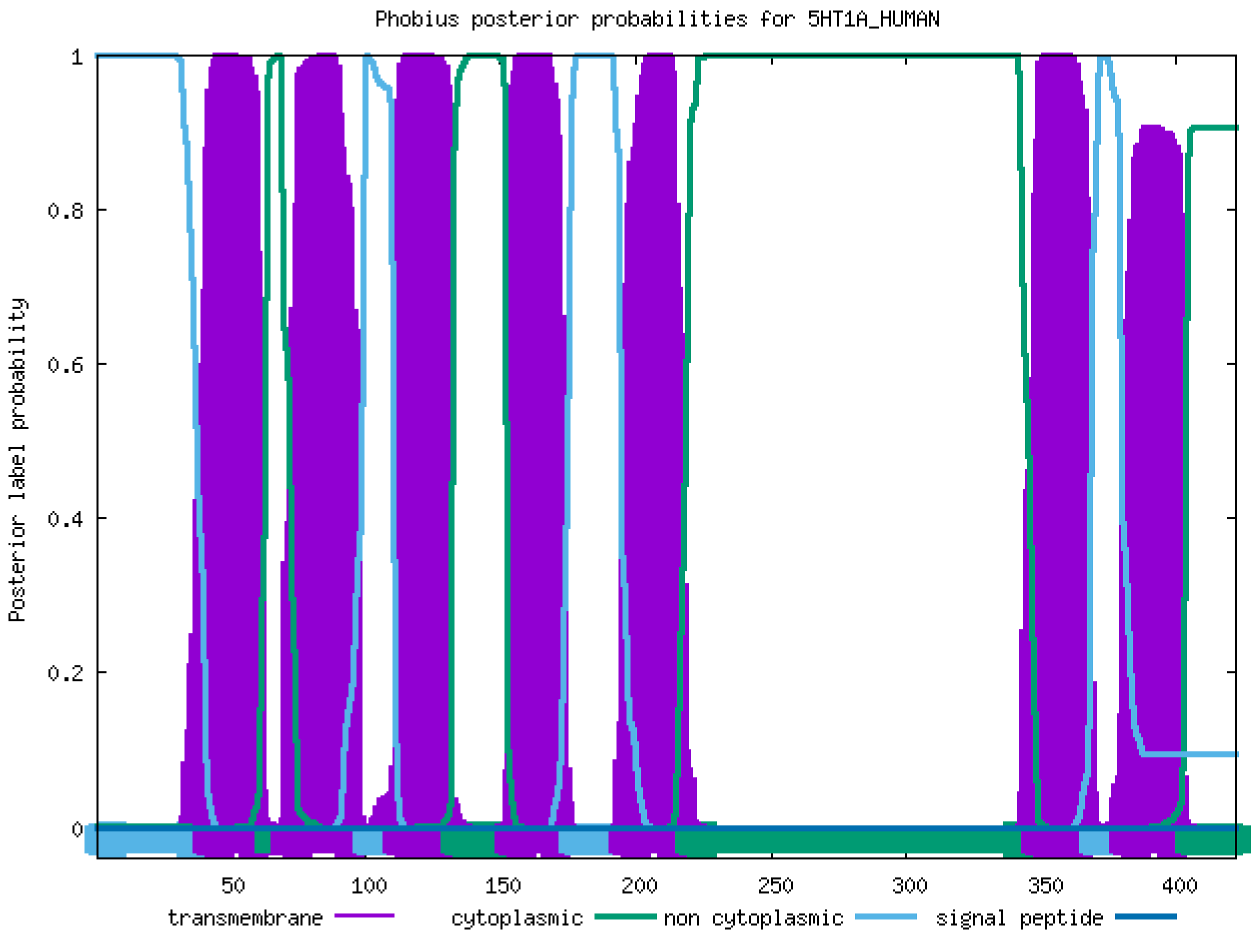

The probability data used in the plot is found here, and the gnuplot script is here.

Prediction of 5HT1B_HUMAN

```
ID   5HT1B_HUMAN
FT   TOPO_DOM      1     49       NON CYTOPLASMIC.
FT   TRANSMEM     50     74
FT   TOPO_DOM     75     85       CYTOPLASMIC.
FT   TRANSMEM     86    105
FT   TOPO_DOM    106    124       NON CYTOPLASMIC.
FT   TRANSMEM    125    145
FT   TOPO_DOM    146    165       CYTOPLASMIC.
FT   TRANSMEM    166    186
FT   TOPO_DOM    187    205       NON CYTOPLASMIC.
FT   TRANSMEM    206    228
FT   TOPO_DOM    229    314       CYTOPLASMIC.
FT   TRANSMEM    315    339
FT   TOPO_DOM    340    344       NON CYTOPLASMIC.
FT   TRANSMEM    345    364
FT   TOPO_DOM    365    390       CYTOPLASMIC.
//
```

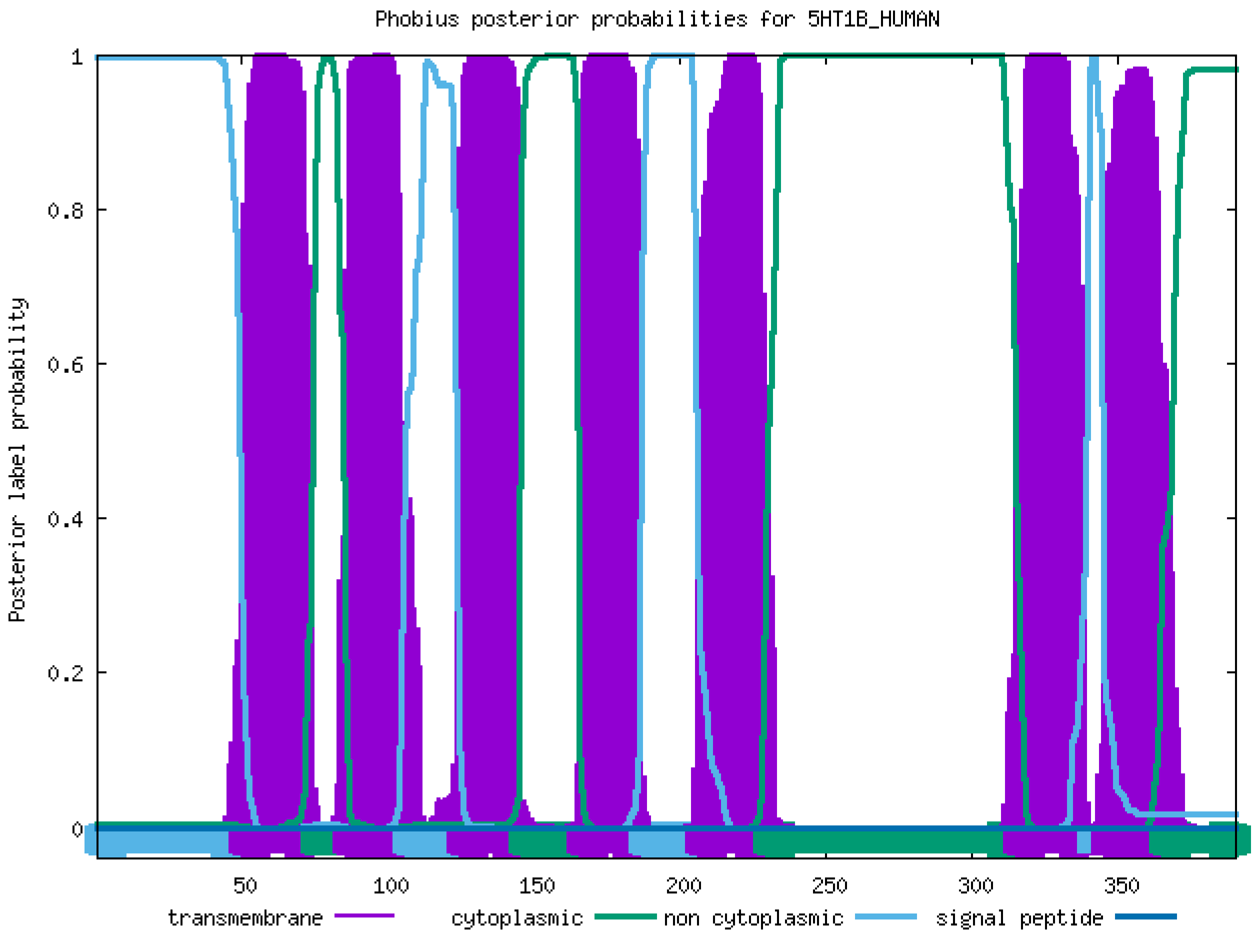

The probability data used in the plot is found here, and the gnuplot script is here.

Prediction of 5HT1D_HUMAN

```
ID   5HT1D_HUMAN
FT   TOPO_DOM      1     38       NON CYTOPLASMIC.
FT   TRANSMEM     39     65
FT   TOPO_DOM     66     76       CYTOPLASMIC.
FT   TRANSMEM     77    101
FT   TOPO_DOM    102    112       NON CYTOPLASMIC.
FT   TRANSMEM    113    134
FT   TOPO_DOM    135    154       CYTOPLASMIC.
FT   TRANSMEM    155    175
FT   TOPO_DOM    176    194       NON CYTOPLASMIC.
FT   TRANSMEM    195    217
FT   TOPO_DOM    218    301       CYTOPLASMIC.
FT   TRANSMEM    302    324
FT   TOPO_DOM    325    335       NON CYTOPLASMIC.
FT   TRANSMEM    336    359
FT   TOPO_DOM    360    377       CYTOPLASMIC.
//
```

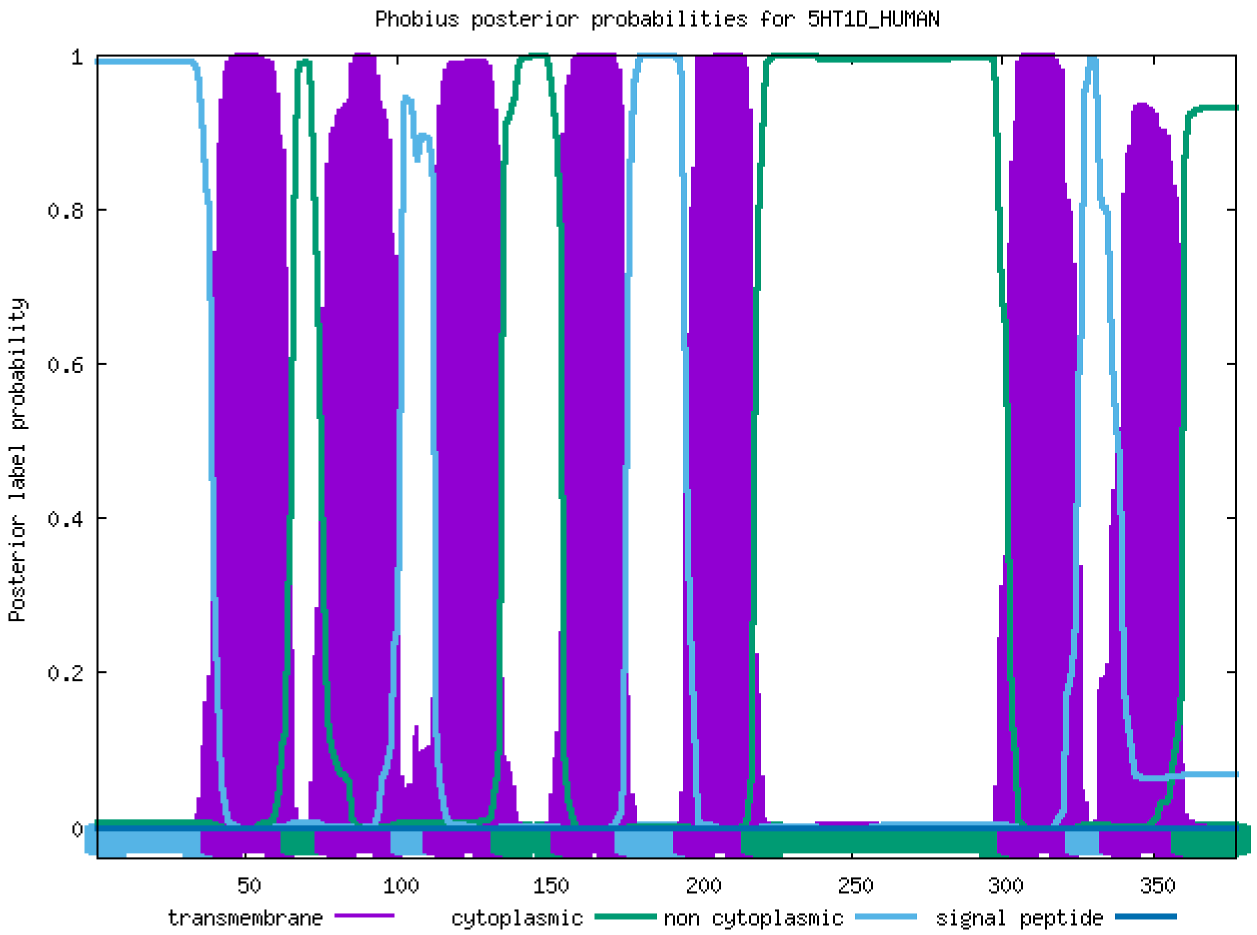

The probability data used in the plot is found here, and the gnuplot script is here.

Prediction of 5HT1E_HUMAN

```
ID   5HT1E_HUMAN
FT   TOPO_DOM      1     21       NON CYTOPLASMIC.
FT   TRANSMEM     22     47
FT   TOPO_DOM     48     58       CYTOPLASMIC.
FT   TRANSMEM     59     85
FT   TOPO_DOM     86     96       NON CYTOPLASMIC.
FT   TRANSMEM     97    118
FT   TOPO_DOM    119    138       CYTOPLASMIC.
FT   TRANSMEM    139    160
FT   TOPO_DOM    161    179       NON CYTOPLASMIC.
FT   TRANSMEM    180    206
FT   TOPO_DOM    207    288       CYTOPLASMIC.
FT   TRANSMEM    289    309
FT   TOPO_DOM    310    328       NON CYTOPLASMIC.
FT   TRANSMEM    329    347
FT   TOPO_DOM    348    365       CYTOPLASMIC.
//
```

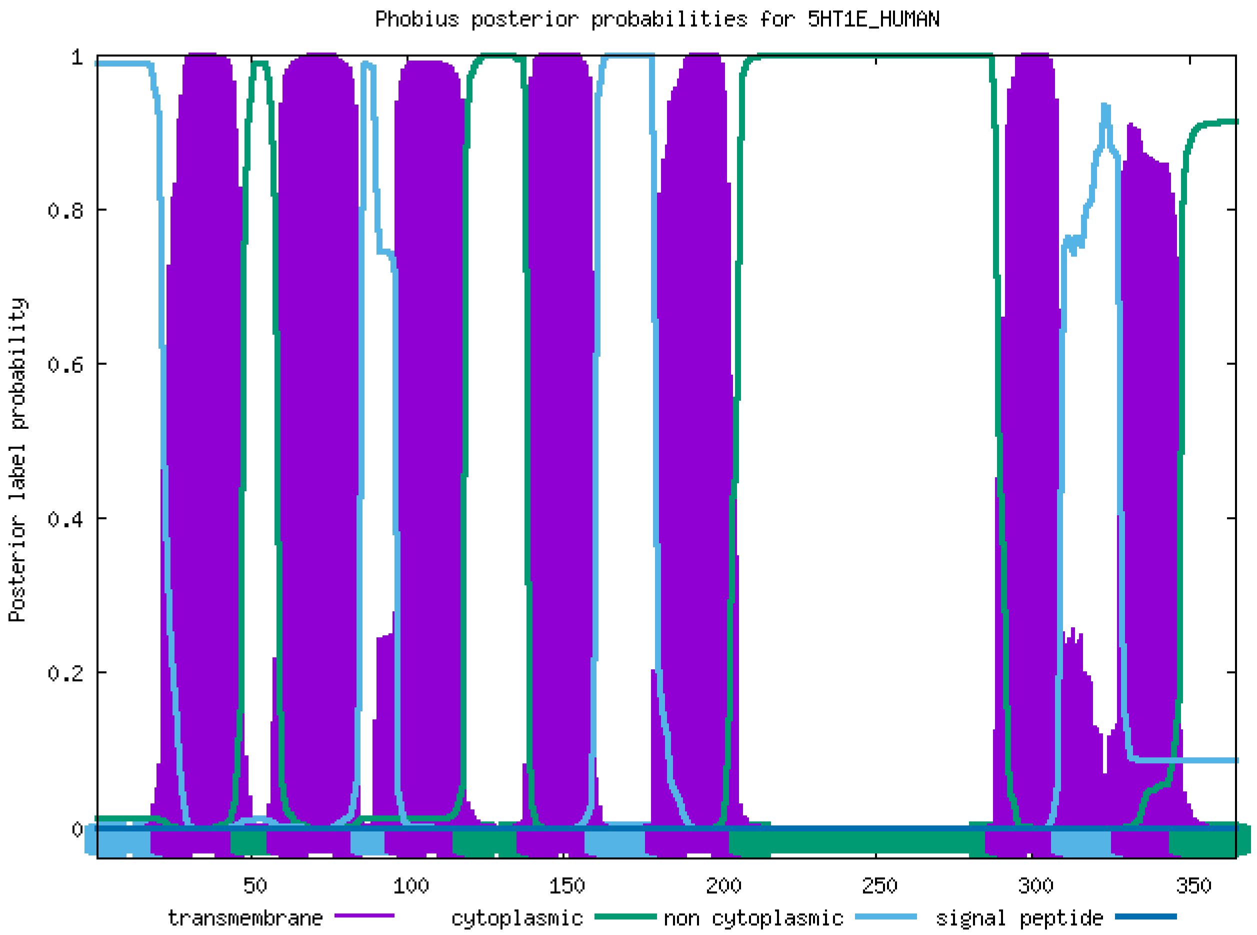

The probability data used in the plot is found here, and the gnuplot script is here.

Prediction of 5HT1F_HUMAN

```
ID    5HT1F_HUMAN
FT    TOPO_DOM      1      23       NON CYTOPLASMIC.
FT    TRANSMEM     24      50
FT    TOPO_DOM     51      61       CYTOPLASMIC.
FT    TRANSMEM     62      86
FT    TOPO_DOM     87      97       NON CYTOPLASMIC.
FT    TRANSMEM     98     119
FT    TOPO_DOM    120     139       CYTOPLASMIC.
FT    TRANSMEM    140     161
FT    TOPO_DOM    162     180       NON CYTOPLASMIC.
FT    TRANSMEM    181     202
FT    TOPO_DOM    203     290       CYTOPLASMIC.
FT    TRANSMEM    291     311
FT    TOPO_DOM    312     330       NON CYTOPLASMIC.
FT    TRANSMEM    331     350
FT    TOPO_DOM    351     366       CYTOPLASMIC.
//
```

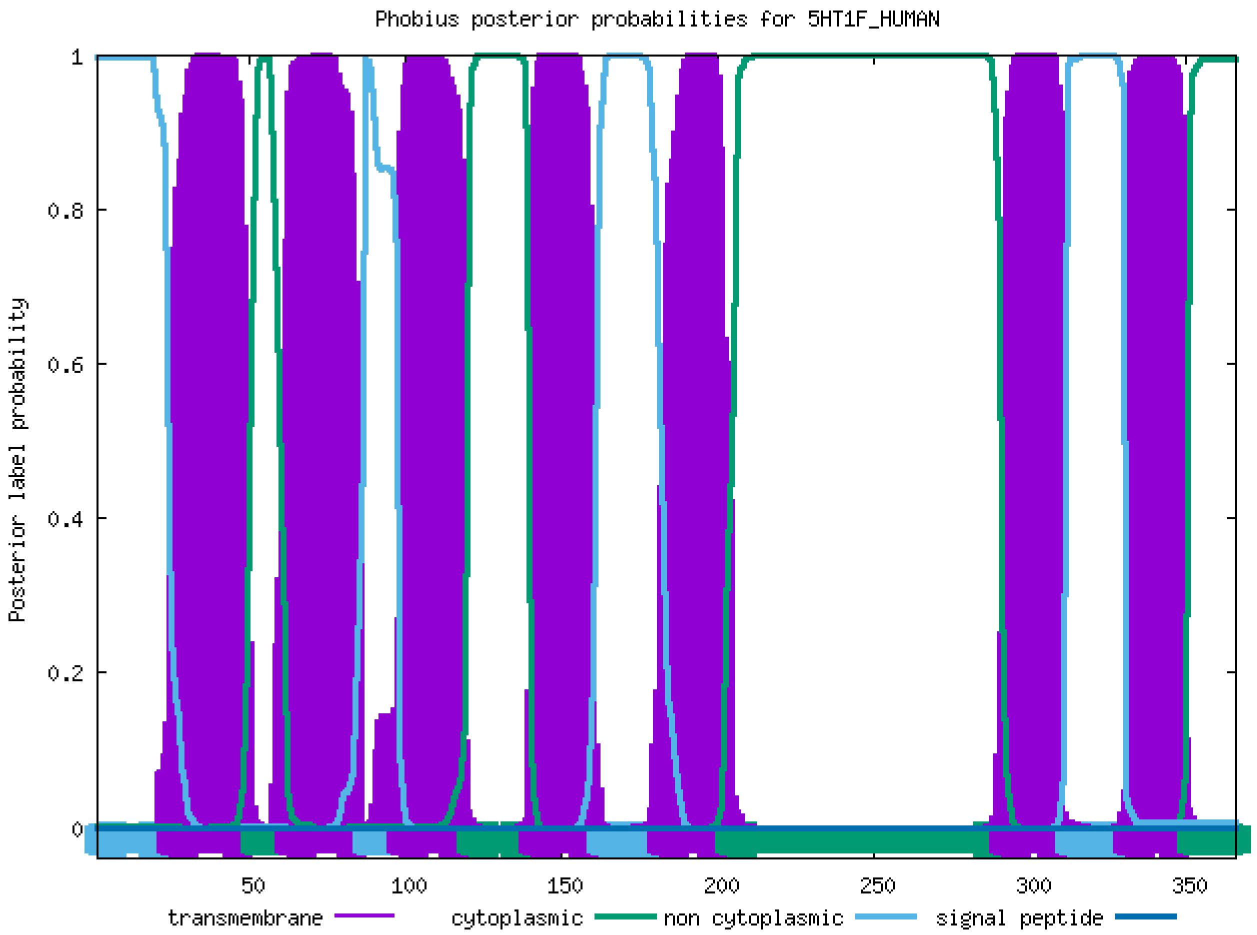

The probability data used in the plot is found here, and the gnuplot script is here.

Prediction of 5HT2A_HUMAN

```
ID    5HT2A_HUMAN
FT    TOPO_DOM      1     75       NON CYTOPLASMIC.
FT    TRANSMEM     76     99
FT    TOPO_DOM    100    110       CYTOPLASMIC.
FT    TRANSMEM    111    139
FT    TOPO_DOM    140    150       NON CYTOPLASMIC.
FT    TRANSMEM    151    171
FT    TOPO_DOM    172    191       CYTOPLASMIC.
FT    TRANSMEM    192    214
FT    TOPO_DOM    215    233       NON CYTOPLASMIC.
FT    TRANSMEM    234    256
FT    TOPO_DOM    257    323       CYTOPLASMIC.
FT    TRANSMEM    324    348
FT    TOPO_DOM    349    359       NON CYTOPLASMIC.
FT    TRANSMEM    360    383
FT    TOPO_DOM    384    471       CYTOPLASMIC.
//
```

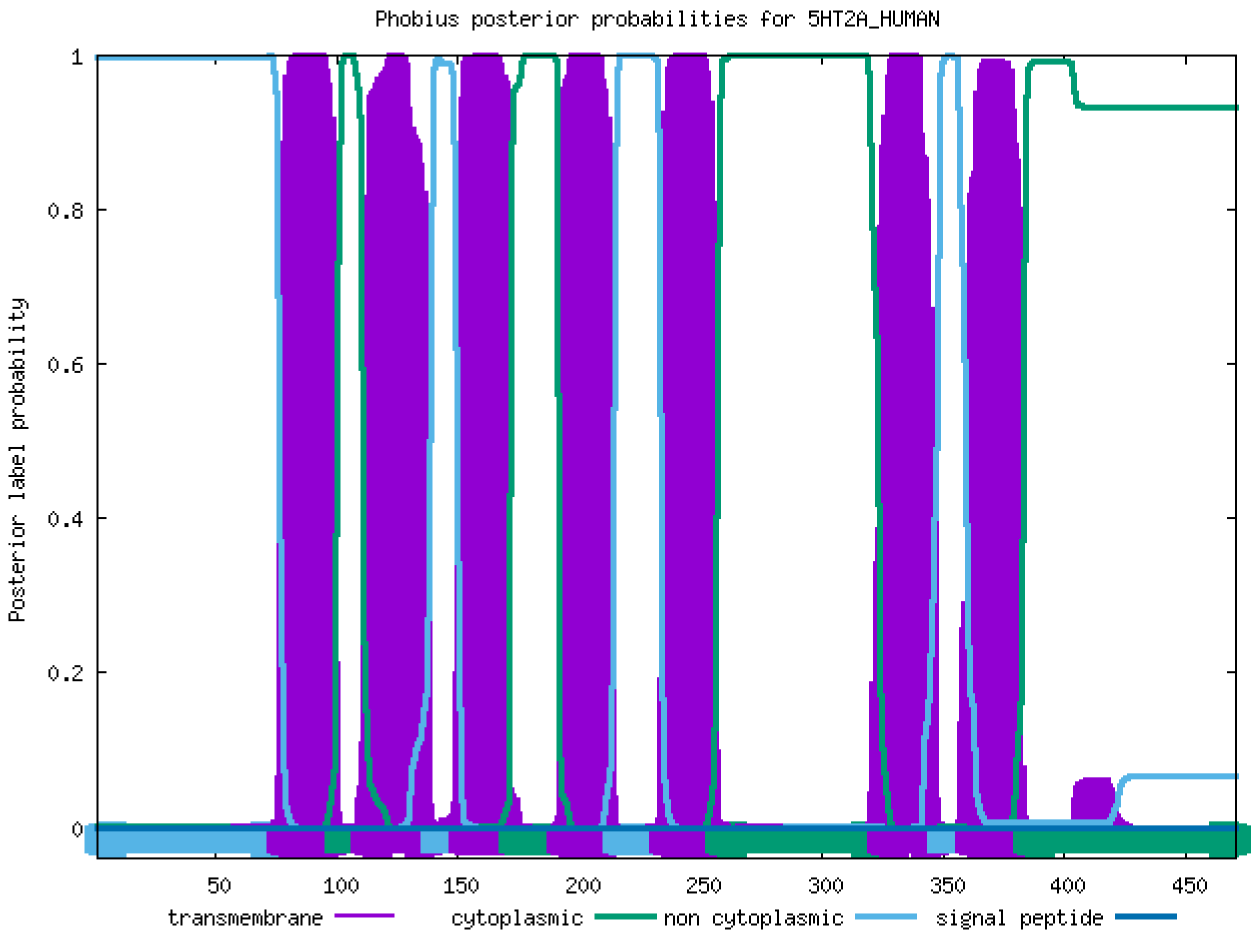

The probability data used in the plot is found here, and the gnuplot script is here.

Prediction of 5HT2B_HUMAN

```
ID   5HT2B_HUMAN
FT   TOPO_DOM      1     55       NON CYTOPLASMIC.
FT   TRANSMEM     56     79
FT   TOPO_DOM     80     90       CYTOPLASMIC.
FT   TRANSMEM     91    117
FT   TOPO_DOM    118    128       NON CYTOPLASMIC.
FT   TRANSMEM    129    151
FT   TOPO_DOM    152    171       CYTOPLASMIC.
FT   TRANSMEM    172    192
FT   TOPO_DOM    193    218       NON CYTOPLASMIC.
FT   TRANSMEM    219    244
FT   TOPO_DOM    245    324       CYTOPLASMIC.
FT   TRANSMEM    325    349
FT   TOPO_DOM    350    363       NON CYTOPLASMIC.
FT   TRANSMEM    364    383
FT   TOPO_DOM    384    481       CYTOPLASMIC.
//
```

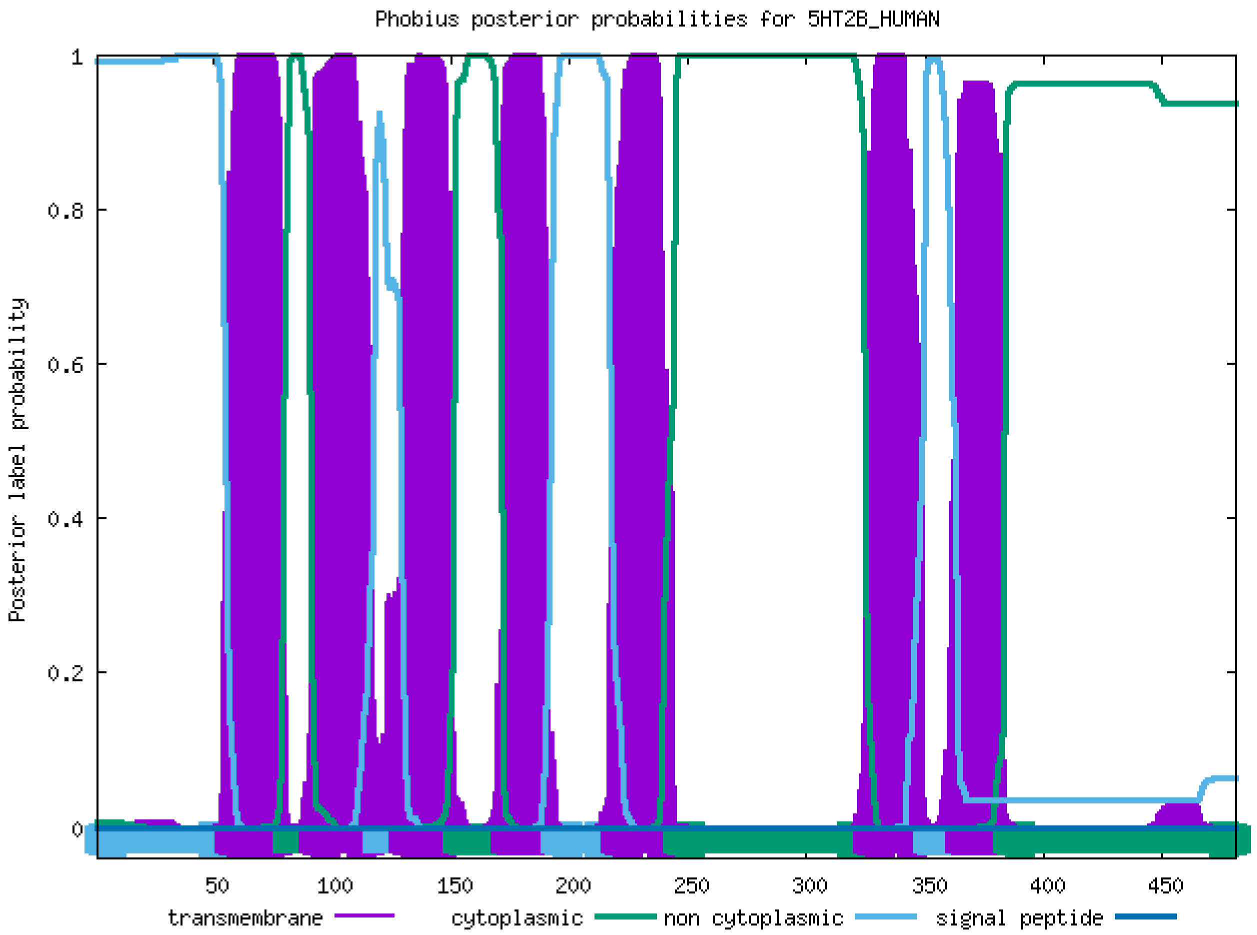

The probability data used in the plot is found here, and the gnuplot script is here.

Prediction of 5HT2C_HUMAN

```
ID   5HT2C_HUMAN
FT   SIGNAL        1     32
FT   REGION        1      9       N-REGION.
FT   REGION       10     21       H-REGION.
FT   REGION       22     32       C-REGION.
FT   TOPO_DOM     33     55       NON CYTOPLASMIC.
FT   TRANSMEM     56     78
FT   TOPO_DOM     79     89       CYTOPLASMIC.
FT   TRANSMEM     90    116
FT   TOPO_DOM    117    127       NON CYTOPLASMIC.
FT   TRANSMEM    128    150
FT   TOPO_DOM    151    170       CYTOPLASMIC.
FT   TRANSMEM    171    194
FT   TOPO_DOM    195    213       NON CYTOPLASMIC.
FT   TRANSMEM    214    241
FT   TOPO_DOM    242    311       CYTOPLASMIC.
FT   TRANSMEM    312    332
FT   TOPO_DOM    333    351       NON CYTOPLASMIC.
FT   TRANSMEM    352    371
FT   TOPO_DOM    372    458       CYTOPLASMIC.
//
```

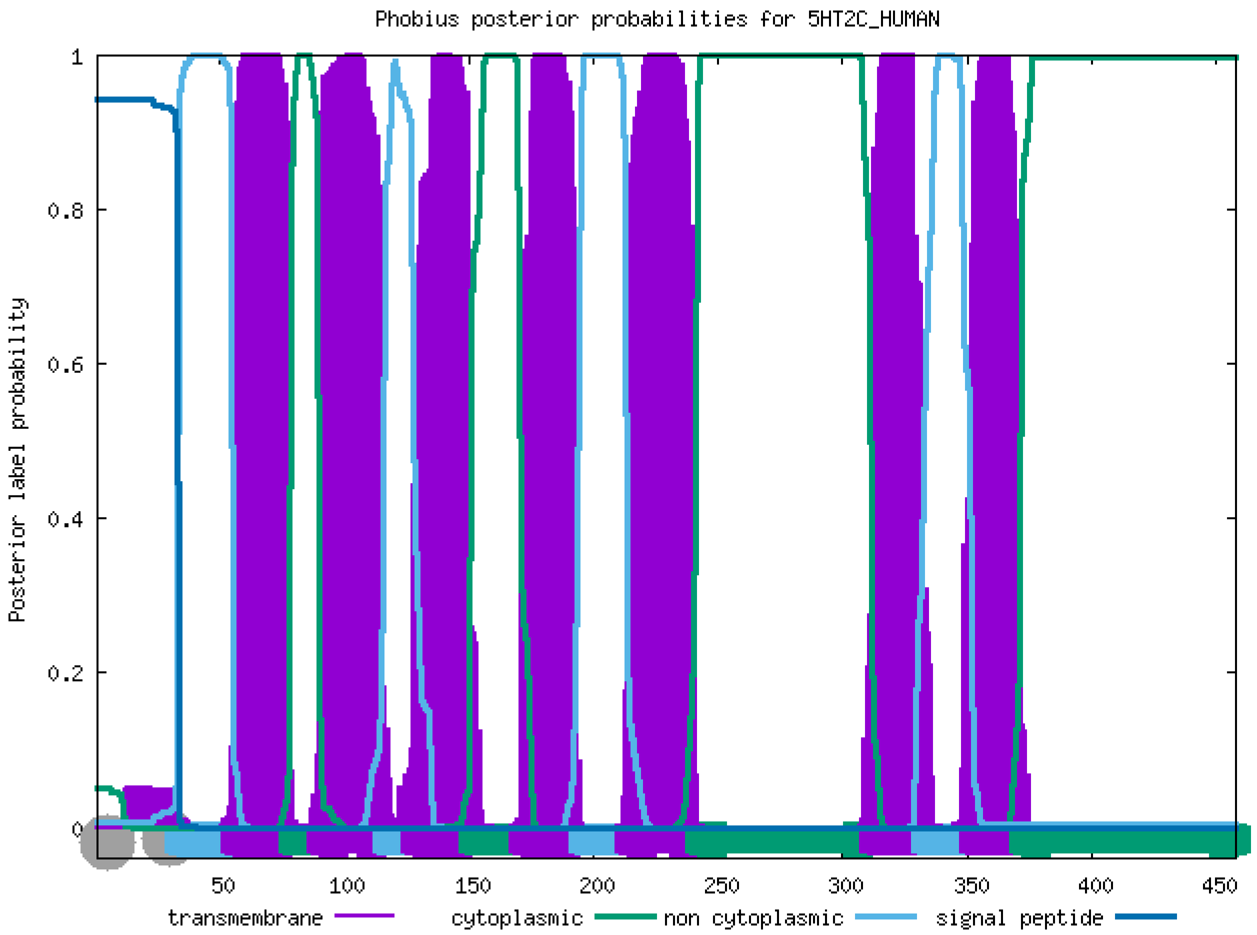

The probability data used in the plot is found here, and the gnuplot script is here.

Prediction of 5HT4R_HUMAN

```
ID    5HT4R_HUMAN
FT    TOPO_DOM      1     19       NON CYTOPLASMIC.
FT    TRANSMEM     20     45
FT    TOPO_DOM     46     56       CYTOPLASMIC.
FT    TRANSMEM     57     79
FT    TOPO_DOM     80     98       NON CYTOPLASMIC.
FT    TRANSMEM     99    117
FT    TOPO_DOM    118    137       CYTOPLASMIC.
FT    TRANSMEM    138    158
FT    TOPO_DOM    159    191       NON CYTOPLASMIC.
FT    TRANSMEM    192    213
FT    TOPO_DOM    214    259       CYTOPLASMIC.
FT    TRANSMEM    260    281
FT    TOPO_DOM    282    292       NON CYTOPLASMIC.
FT    TRANSMEM    293    315
FT    TOPO_DOM    316    388       CYTOPLASMIC.
//
```

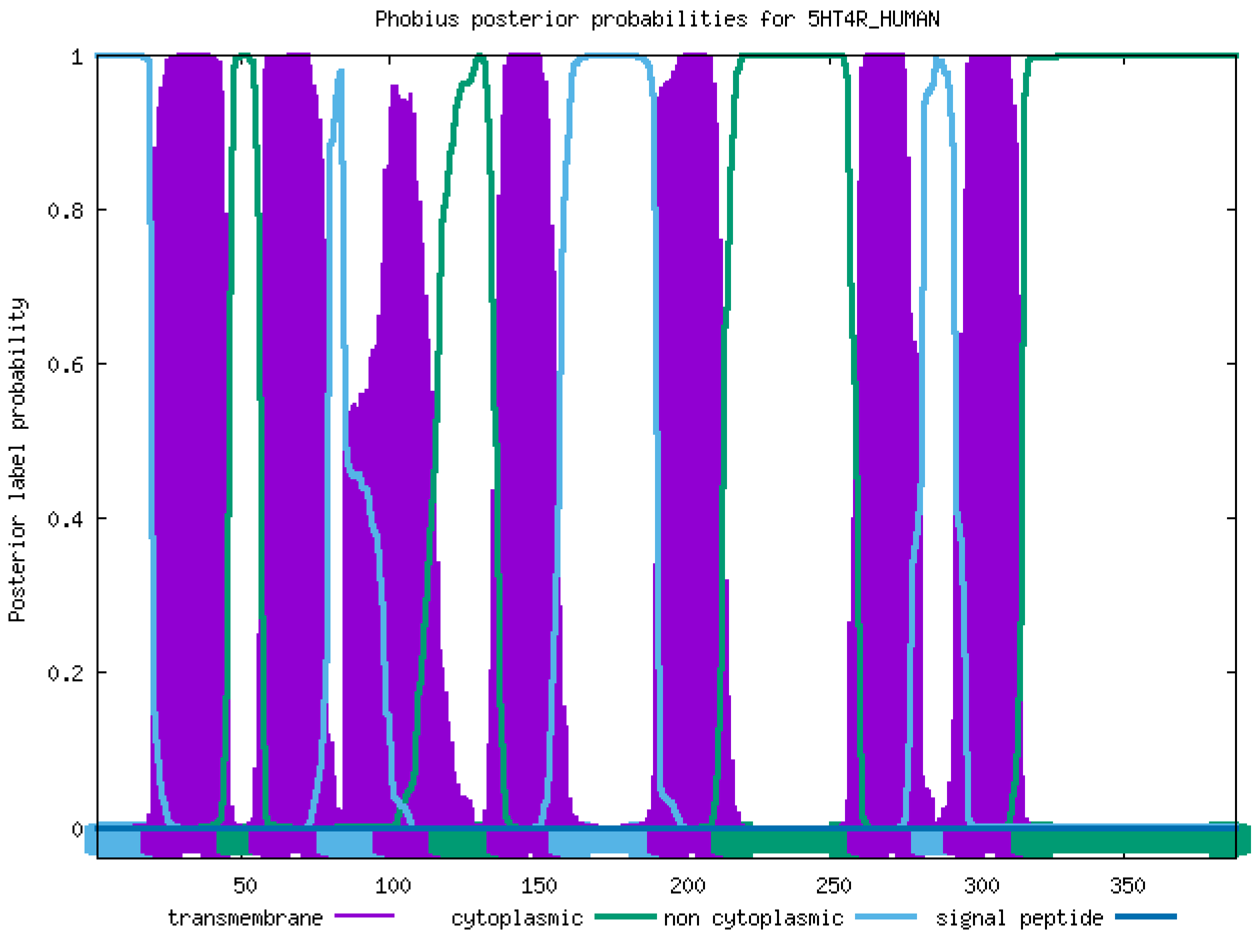

The probability data used in the plot is found here, and the gnuplot script is here.

Prediction of 5HT5A_HUMAN

```
ID    5HT5A_HUMAN
FT    TOPO_DOM      1     37       NON CYTOPLASMIC.
FT    TRANSMEM     38     66
FT    TOPO_DOM     67     77       CYTOPLASMIC.
FT    TRANSMEM     78     96
FT    TOPO_DOM     97    115       NON CYTOPLASMIC.
FT    TRANSMEM    116    137
FT    TOPO_DOM    138    156       CYTOPLASMIC.
FT    TRANSMEM    157    179
FT    TOPO_DOM    180    198       NON CYTOPLASMIC.
FT    TRANSMEM    199    221
FT    TOPO_DOM    222    282       CYTOPLASMIC.
FT    TRANSMEM    283    302
FT    TOPO_DOM    303    321       NON CYTOPLASMIC.
FT    TRANSMEM    322    341
FT    TOPO_DOM    342    357       CYTOPLASMIC.
//
```

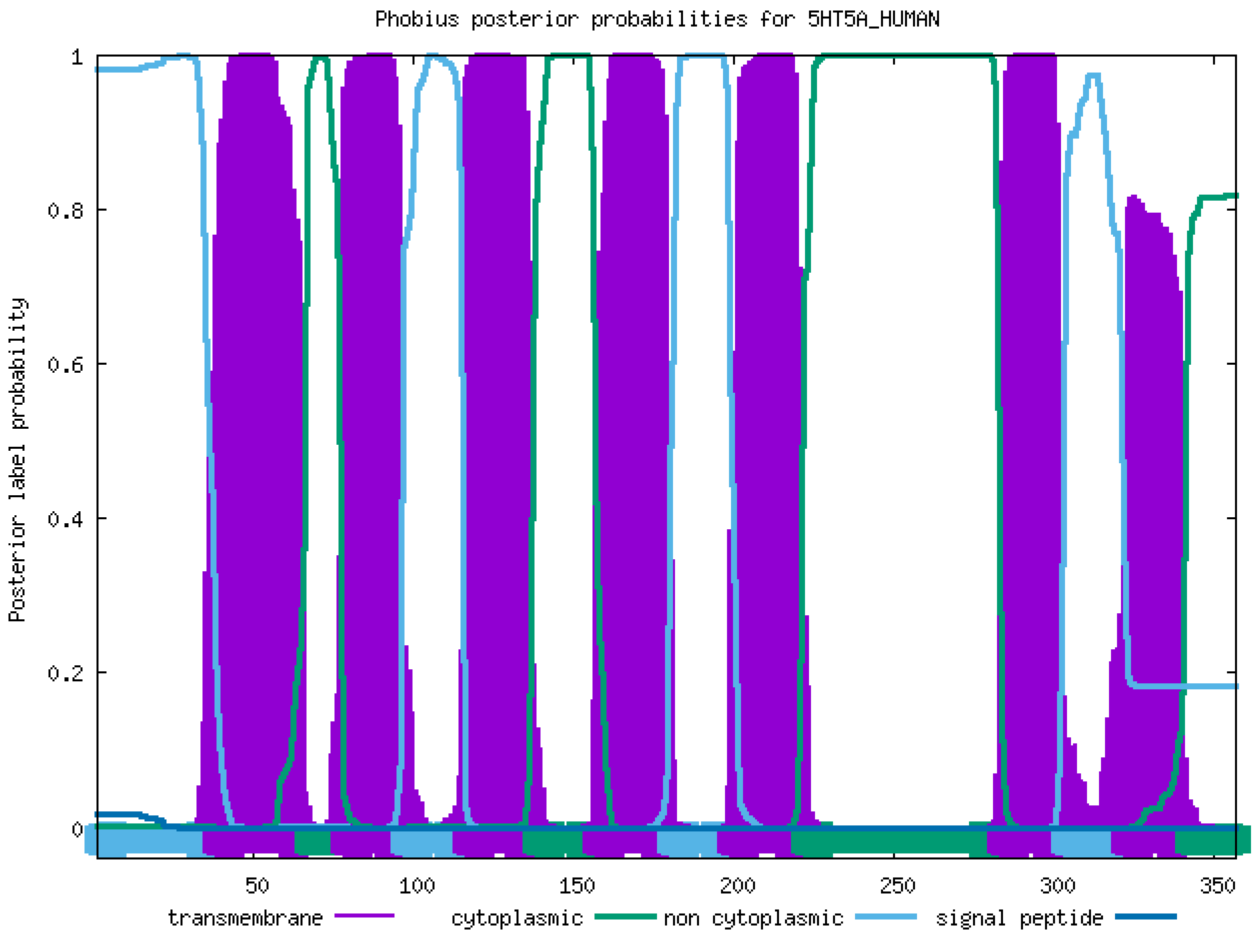

The probability data used in the plot is found here, and the gnuplot script is here.

Prediction of 5HT6R_HUMAN

```
ID   5HT6R_HUMAN
FT   TOPO_DOM      1     27       NON CYTOPLASMIC.
FT   TRANSMEM     28     51
FT   TOPO_DOM     52     62       CYTOPLASMIC.
FT   TRANSMEM     63     80
FT   TOPO_DOM     81     99       NON CYTOPLASMIC.
FT   TRANSMEM    100    122
FT   TOPO_DOM    123    142       CYTOPLASMIC.
FT   TRANSMEM    143    166
FT   TOPO_DOM    167    185       NON CYTOPLASMIC.
FT   TRANSMEM    186    209
FT   TOPO_DOM    210    262       CYTOPLASMIC.
FT   TRANSMEM    263    284
FT   TOPO_DOM    285    303       NON CYTOPLASMIC.
FT   TRANSMEM    304    323
FT   TOPO_DOM    324    440       CYTOPLASMIC.
//
```

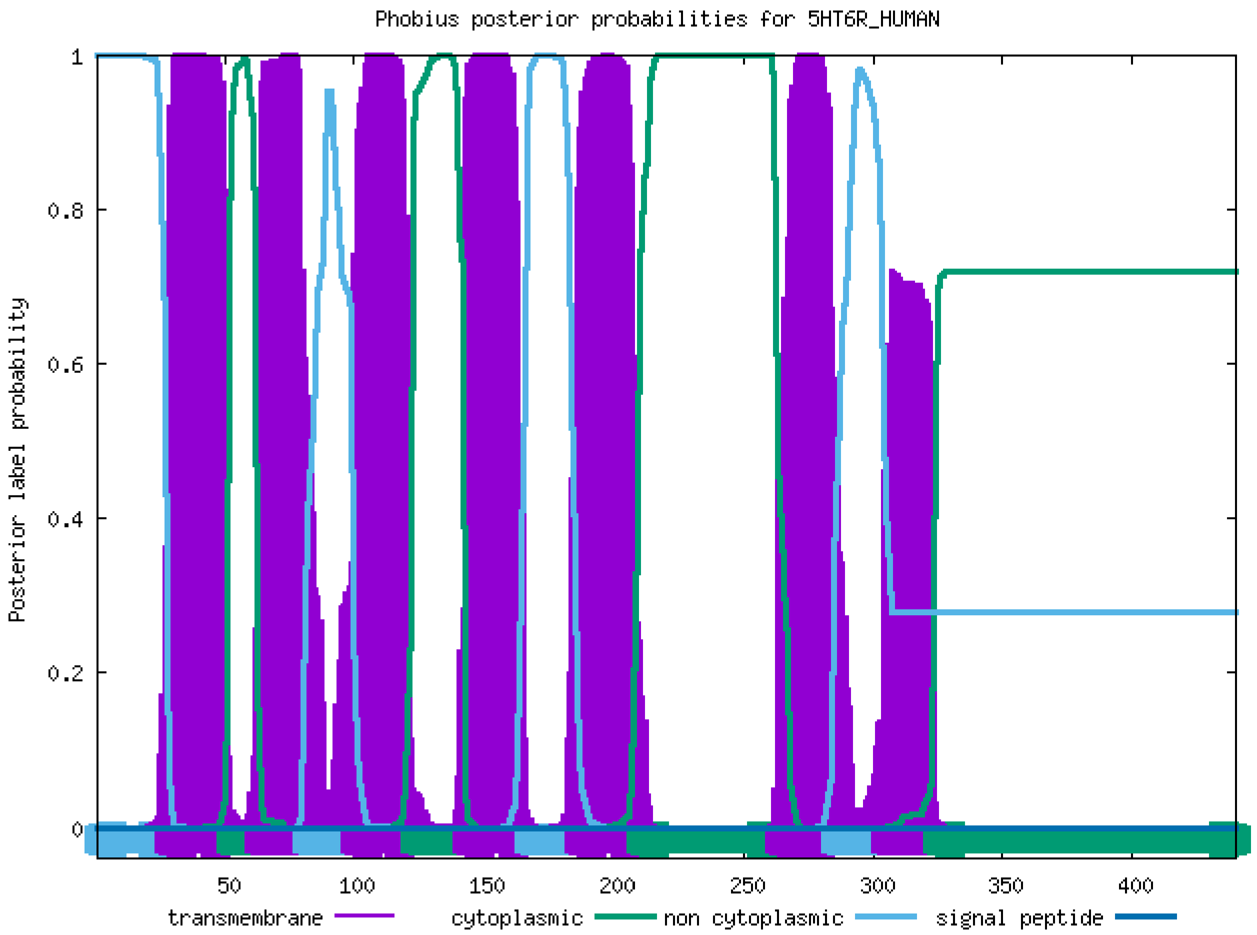

The probability data used in the plot is found here, and the gnuplot script is here.

Prediction of 5HT7R_HUMAN

```
ID   5HT7R_HUMAN
FT   TOPO_DOM      1     81       NON CYTOPLASMIC.
FT   TRANSMEM     82    106
FT   TOPO_DOM    107    117       CYTOPLASMIC.
FT   TRANSMEM    118    138
FT   TOPO_DOM    139    149       NON CYTOPLASMIC.
FT   TRANSMEM    150    178
FT   TOPO_DOM    179    198       CYTOPLASMIC.
FT   TRANSMEM    199    219
FT   TOPO_DOM    220    238       NON CYTOPLASMIC.
FT   TRANSMEM    239    260
FT   TOPO_DOM    261    328       CYTOPLASMIC.
FT   TRANSMEM    329    349
FT   TOPO_DOM    350    368       NON CYTOPLASMIC.
FT   TRANSMEM    369    387
FT   TOPO_DOM    388    479       CYTOPLASMIC.
//
```

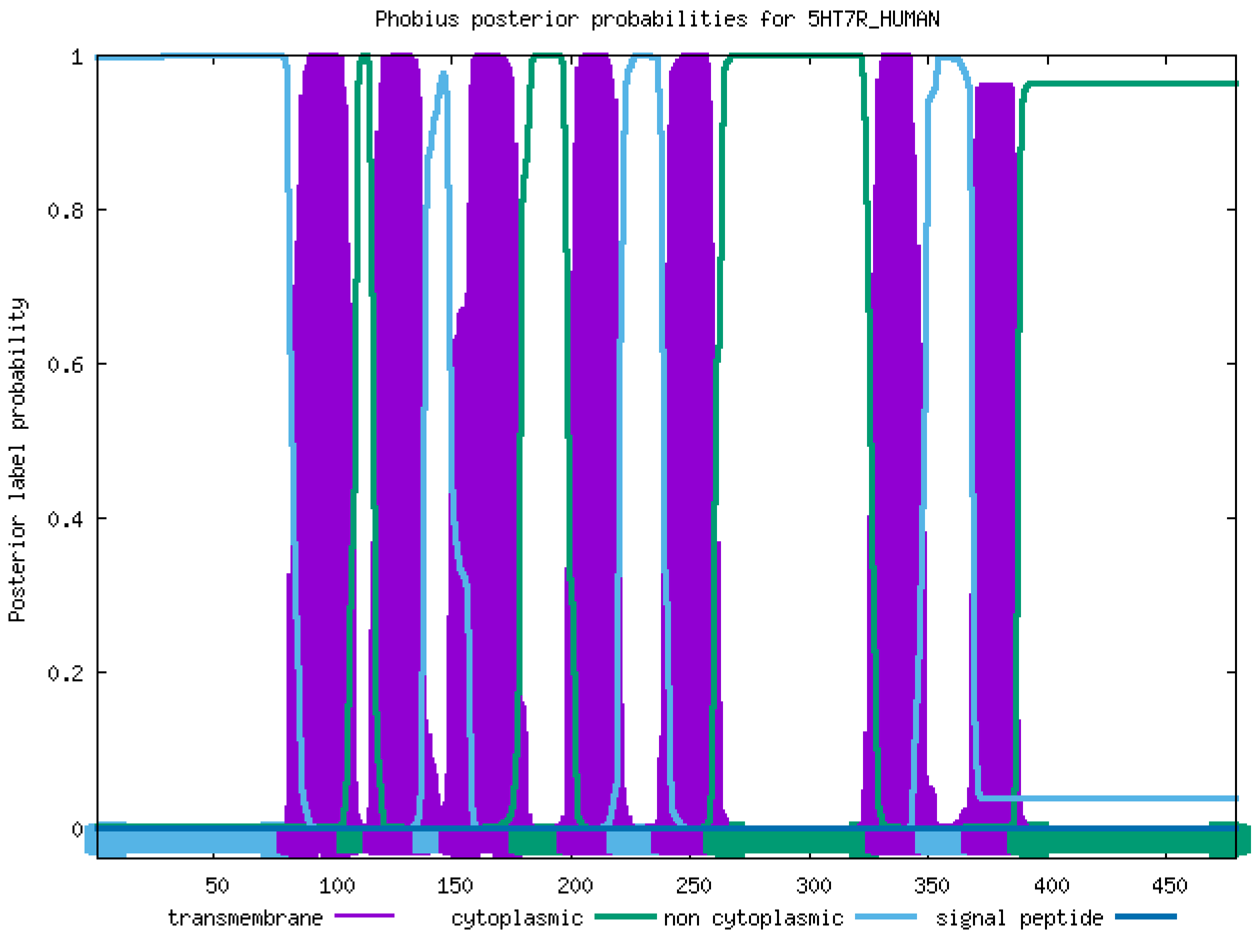

The probability data used in the plot is found here, and the gnuplot script is here.

Prediction of MTR1A_HUMAN

```
ID   MTR1A_HUMAN
FT   TOPO_DOM      1     24       NON CYTOPLASMIC.
FT   TRANSMEM     25     50
FT   TOPO_DOM     51     61       CYTOPLASMIC.
FT   TRANSMEM     62     82
FT   TOPO_DOM     83    101       NON CYTOPLASMIC.
FT   TRANSMEM    102    123
FT   TOPO_DOM    124    143       CYTOPLASMIC.
FT   TRANSMEM    144    165
FT   TOPO_DOM    166    184       NON CYTOPLASMIC.
FT   TRANSMEM    185    215
FT   TOPO_DOM    216    235       CYTOPLASMIC.
FT   TRANSMEM    236    263
FT   TOPO_DOM    264    274       NON CYTOPLASMIC.
FT   TRANSMEM    275    298
FT   TOPO_DOM    299    350       CYTOPLASMIC.
//
```

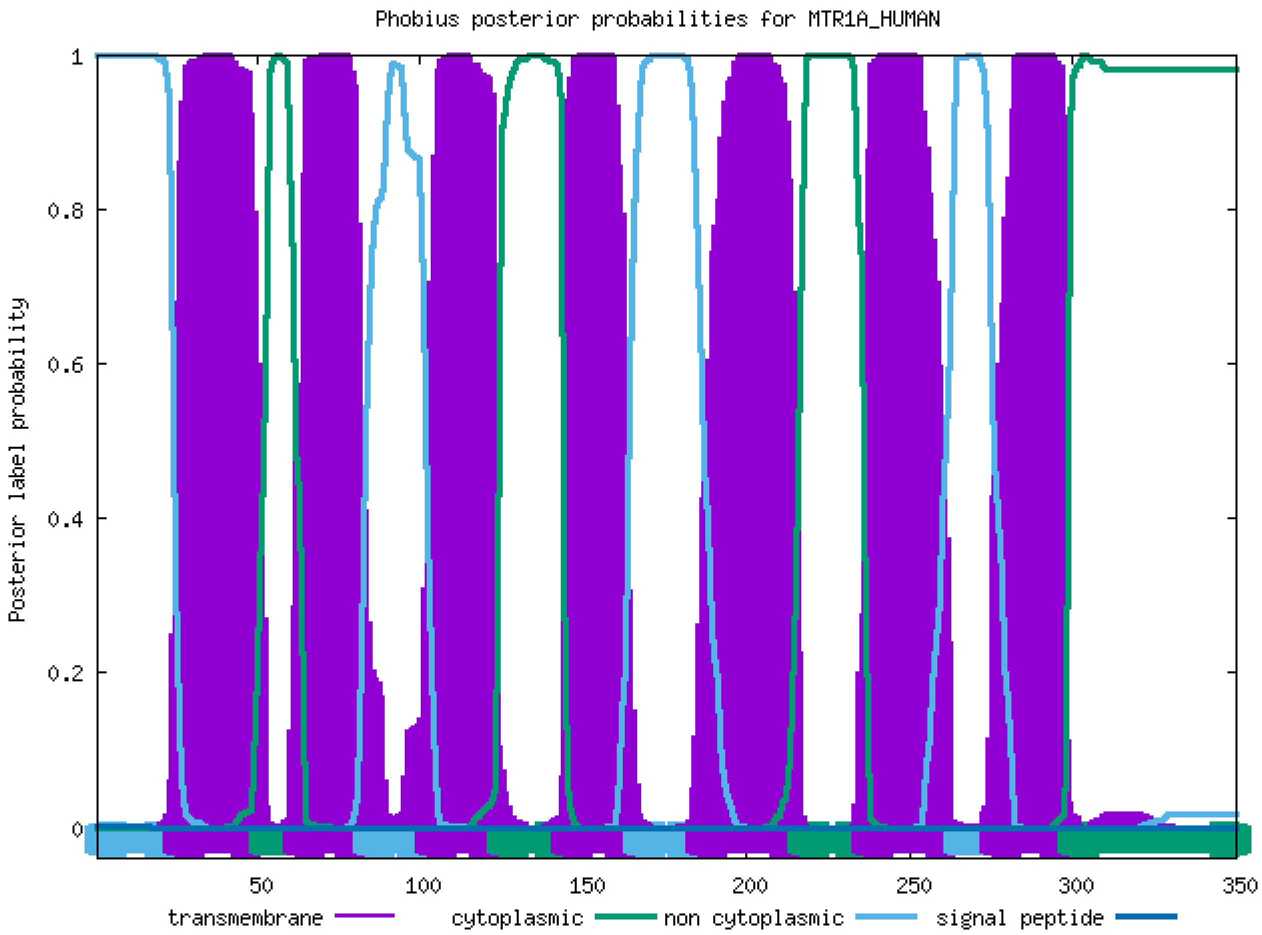

The probability data used in the plot is found here, and the gnuplot script is here.

Prediction of MTR1B_HUMAN

```
ID   MTR1B_HUMAN
FT   TOPO_DOM      1     37       NON CYTOPLASMIC.
FT   TRANSMEM     38     65
FT   TOPO_DOM     66     76       CYTOPLASMIC.
FT   TRANSMEM     77    103
FT   TOPO_DOM    104    114       NON CYTOPLASMIC.
FT   TRANSMEM    115    136
FT   TOPO_DOM    137    156       CYTOPLASMIC.
FT   TRANSMEM    157    180
FT   TOPO_DOM    181    199       NON CYTOPLASMIC.
FT   TRANSMEM    200    228
FT   TOPO_DOM    229    248       CYTOPLASMIC.
FT   TRANSMEM    249    276
FT   TOPO_DOM    277    287       NON CYTOPLASMIC.
FT   TRANSMEM    288    311
FT   TOPO_DOM    312    362       CYTOPLASMIC.
//
```

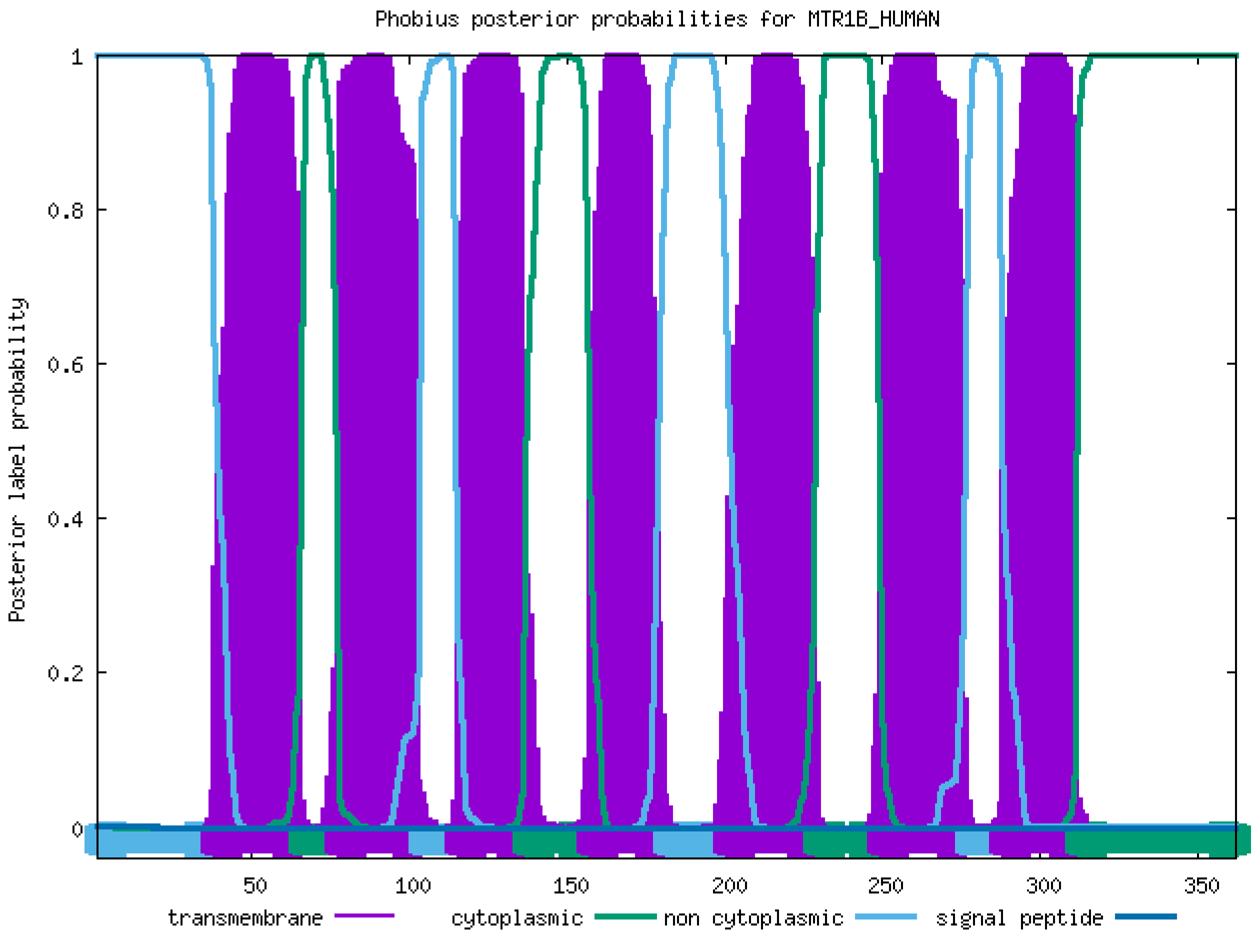

The probability data used in the plot is found here, and the gnuplot script is here.

Prediction of P2Y12_HUMAN

```
ID   P2Y12_HUMAN
FT   TOPO_DOM      1     29       NON CYTOPLASMIC.
FT   TRANSMEM     30     50
FT   TOPO_DOM     51     61       CYTOPLASMIC.
FT   TRANSMEM     62     82
FT   TOPO_DOM     83    101       NON CYTOPLASMIC.
FT   TRANSMEM    102    120
FT   TOPO_DOM    121    140       CYTOPLASMIC.
FT   TRANSMEM    141    161
FT   TOPO_DOM    162    188       NON CYTOPLASMIC.
FT   TRANSMEM    189    213
FT   TOPO_DOM    214    233       CYTOPLASMIC.
FT   TRANSMEM    234    254
FT   TOPO_DOM    255    283       NON CYTOPLASMIC.
FT   TRANSMEM    284    302
FT   TOPO_DOM    303    342       CYTOPLASMIC.
//
```

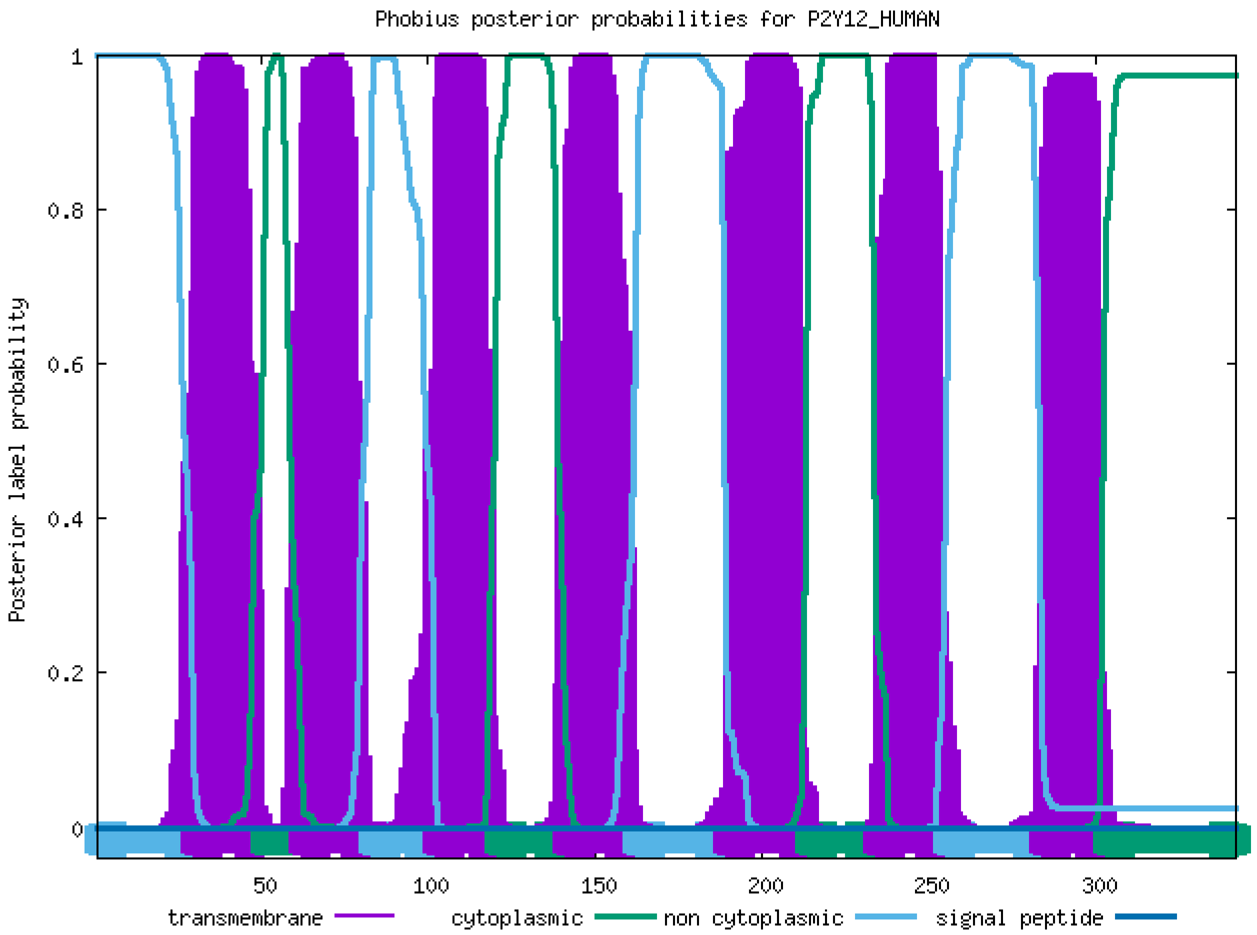

The probability data used in the plot is found here, and the gnuplot script is here.

Prediction of P2RY1_HUMAN

```
ID    P2RY1_HUMAN
FT    TOPO_DOM      1     55       NON CYTOPLASMIC.
FT    TRANSMEM     56     77
FT    TOPO_DOM     78     88       CYTOPLASMIC.
FT    TRANSMEM     89    109
FT    TOPO_DOM    110    128       NON CYTOPLASMIC.
FT    TRANSMEM    129    147
FT    TOPO_DOM    148    167       CYTOPLASMIC.
FT    TRANSMEM    168    189
FT    TOPO_DOM    190    213       NON CYTOPLASMIC.
FT    TRANSMEM    214    246
FT    TOPO_DOM    247    257       CYTOPLASMIC.
FT    TRANSMEM    258    276
FT    TOPO_DOM    277    373       NON CYTOPLASMIC.
//
```

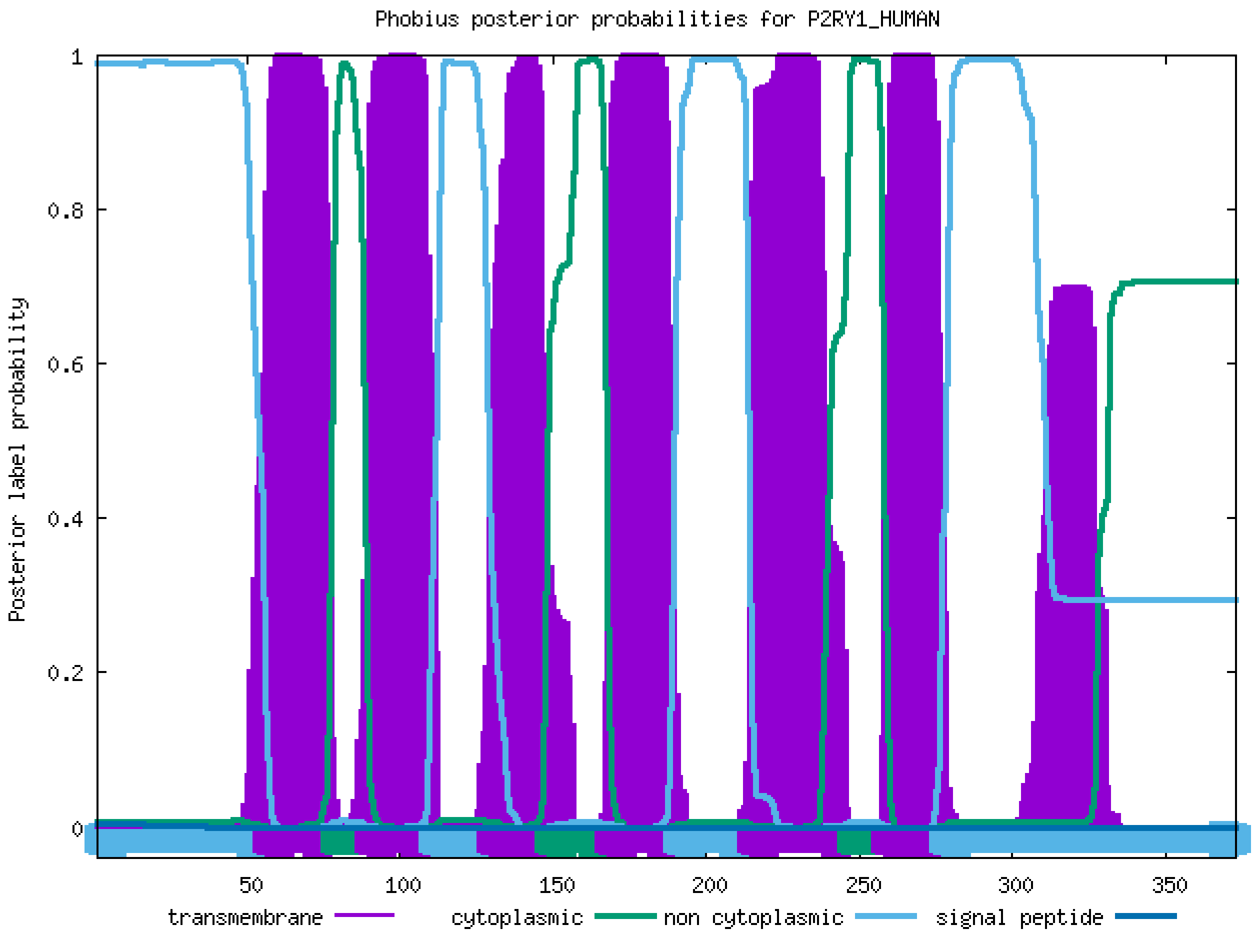

The probability data used in the plot is found here, and the gnuplot script is here.

Prediction of LT4R1_HUMAN

```
ID   LT4R1_HUMAN
FT   TOPO_DOM      1     15       NON CYTOPLASMIC.
FT   TRANSMEM     16     44
FT   TOPO_DOM     45     55       CYTOPLASMIC.
FT   TRANSMEM     56     79
FT   TOPO_DOM     80     98       NON CYTOPLASMIC.
FT   TRANSMEM     99    117
FT   TOPO_DOM    118    136       CYTOPLASMIC.
FT   TRANSMEM    137    159
FT   TOPO_DOM    160    178       NON CYTOPLASMIC.
FT   TRANSMEM    179    201
FT   TOPO_DOM    202    221       CYTOPLASMIC.
FT   TRANSMEM    222    240
FT   TOPO_DOM    241    268       NON CYTOPLASMIC.
FT   TRANSMEM    269    293
FT   TOPO_DOM    294    352       CYTOPLASMIC.
//
```

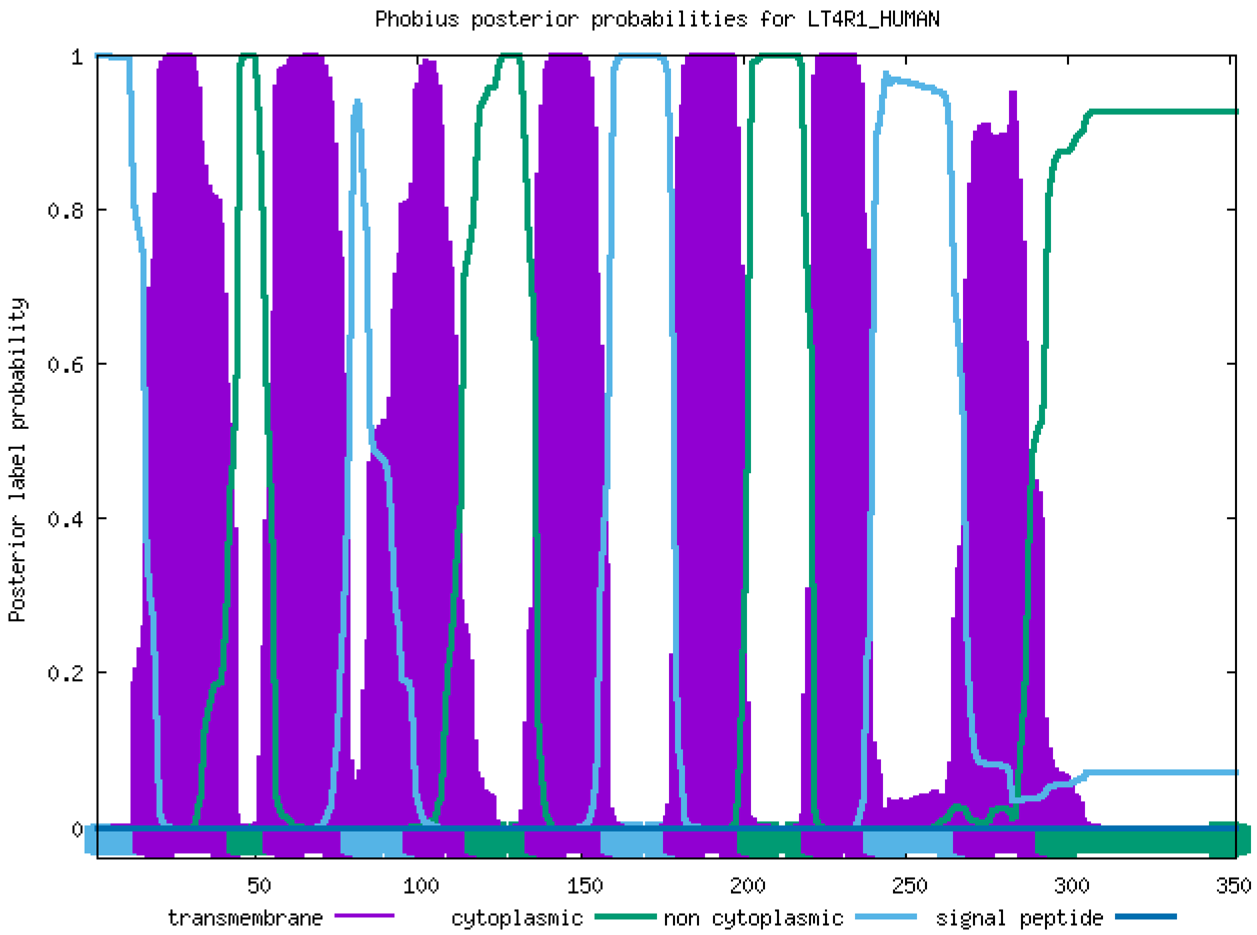

The probability data used in the plot is found here, and the gnuplot script is here.

Prediction of CLTR1_HUMAN

```
ID   CLTR1_HUMAN
FT   TOPO_DOM      1     28       NON CYTOPLASMIC.
FT   TRANSMEM     29     49
FT   TOPO_DOM     50     60       CYTOPLASMIC.
FT   TRANSMEM     61     85
FT   TOPO_DOM     86    104       NON CYTOPLASMIC.
FT   TRANSMEM    105    128
FT   TOPO_DOM    129    139       CYTOPLASMIC.
FT   TRANSMEM    140    160
FT   TOPO_DOM    161    187       NON CYTOPLASMIC.
FT   TRANSMEM    188    212
FT   TOPO_DOM    213    231       CYTOPLASMIC.
FT   TRANSMEM    232    251
FT   TOPO_DOM    252    276       NON CYTOPLASMIC.
FT   TRANSMEM    277    297
FT   TOPO_DOM    298    337       CYTOPLASMIC.
//
```

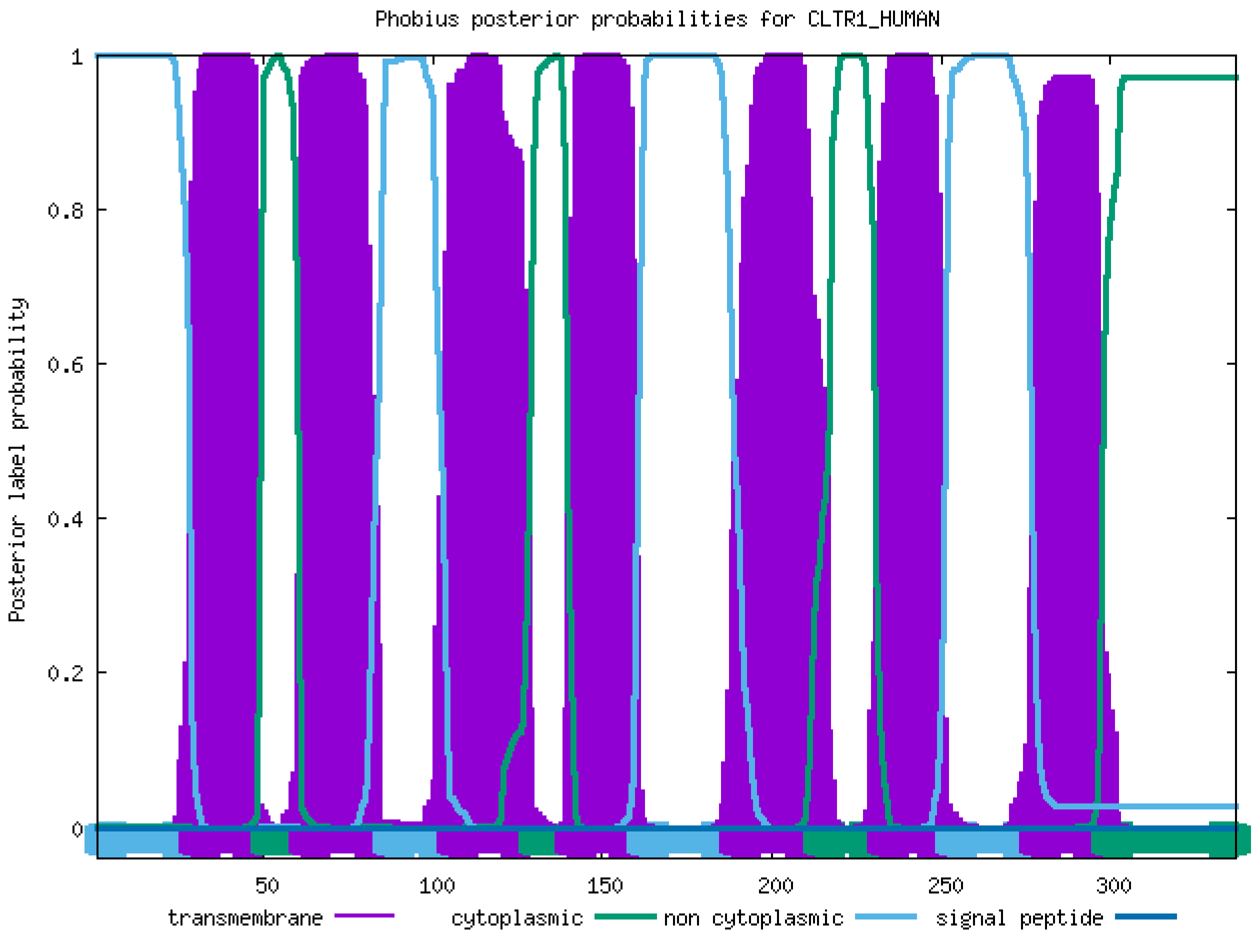

The probability data used in the plot is found here, and the gnuplot script is here.

Prediction of CLTR2_HUMAN

```
ID    CLTR2_HUMAN
FT    TOPO_DOM      1     42       NON CYTOPLASMIC.
FT    TRANSMEM     43     64
FT    TOPO_DOM     65     75       CYTOPLASMIC.
FT    TRANSMEM     76     93
FT    TOPO_DOM     94    112       NON CYTOPLASMIC.
FT    TRANSMEM    113    135
FT    TOPO_DOM    136    154       CYTOPLASMIC.
FT    TRANSMEM    155    174
FT    TOPO_DOM    175    202       NON CYTOPLASMIC.
FT    TRANSMEM    203    224
FT    TOPO_DOM    225    244       CYTOPLASMIC.
FT    TRANSMEM    245    266
FT    TOPO_DOM    267    285       NON CYTOPLASMIC.
FT    TRANSMEM    286    308
FT    TOPO_DOM    309    346       CYTOPLASMIC.
//
```

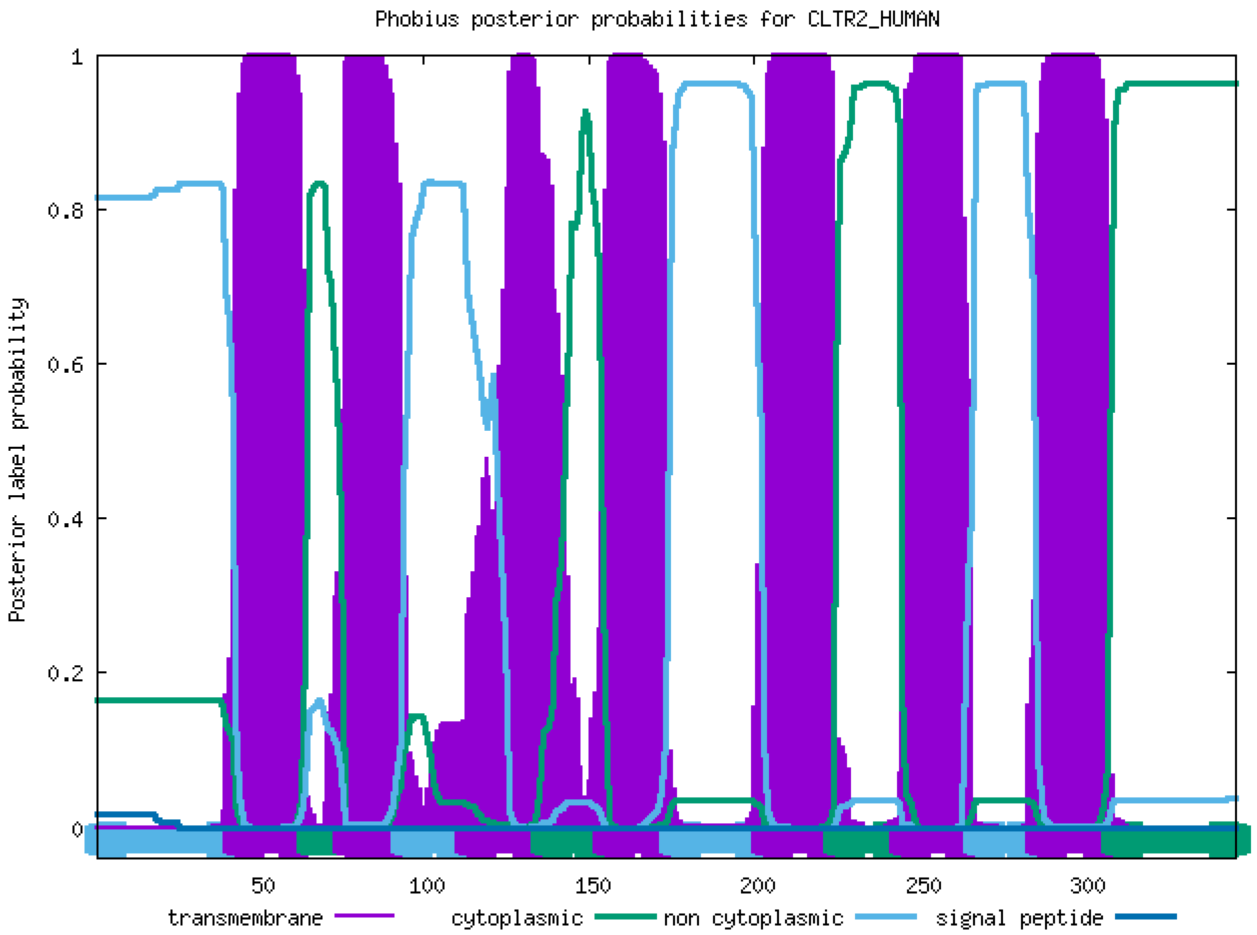

The probability data used in the plot is found here, and the gnuplot script is here.

Prediction of OXYR_HUMAN

```
ID   OXYR_HUMAN
FT   TOPO_DOM      1     42       NON CYTOPLASMIC.
FT   TRANSMEM     43     64
FT   TOPO_DOM     65     75       CYTOPLASMIC.
FT   TRANSMEM     76     94
FT   TOPO_DOM     95    113       NON CYTOPLASMIC.
FT   TRANSMEM    114    135
FT   TOPO_DOM    136    154       CYTOPLASMIC.
FT   TRANSMEM    155    175
FT   TOPO_DOM    176    199       NON CYTOPLASMIC.
FT   TRANSMEM    200    223
FT   TOPO_DOM    224    275       CYTOPLASMIC.
FT   TRANSMEM    276    298
FT   TOPO_DOM    299    309       NON CYTOPLASMIC.
FT   TRANSMEM    310    332
FT   TOPO_DOM    333    389       CYTOPLASMIC.
//
```

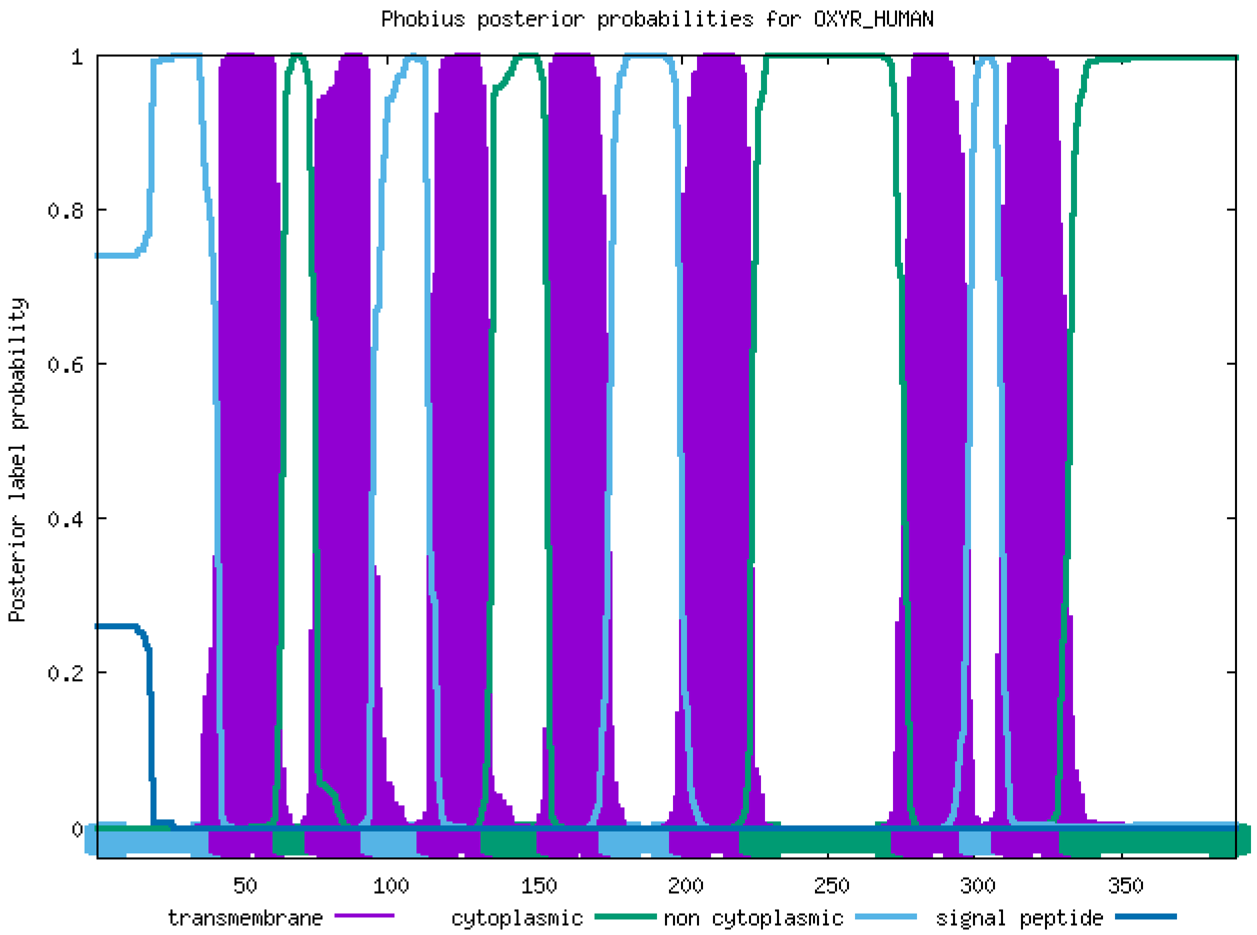

The probability data used in the plot is found here, and the gnuplot script is here.

Prediction of FFAR1_HUMAN

```
ID   FFAR1_HUMAN
FT   TOPO_DOM      1     11       NON CYTOPLASMIC.
FT   TRANSMEM     12     30
FT   TOPO_DOM     31     41       CYTOPLASMIC.
FT   TRANSMEM     42     61
FT   TOPO_DOM     62     80       NON CYTOPLASMIC.
FT   TRANSMEM     81    100
FT   TOPO_DOM    101    120       CYTOPLASMIC.
FT   TRANSMEM    121    144
FT   TOPO_DOM    145    183       NON CYTOPLASMIC.
FT   TRANSMEM    184    210
FT   TOPO_DOM    211    221       CYTOPLASMIC.
FT   TRANSMEM    222    240
FT   TOPO_DOM    241    259       NON CYTOPLASMIC.
FT   TRANSMEM    260    279
FT   TOPO_DOM    280    300       CYTOPLASMIC.
//
```

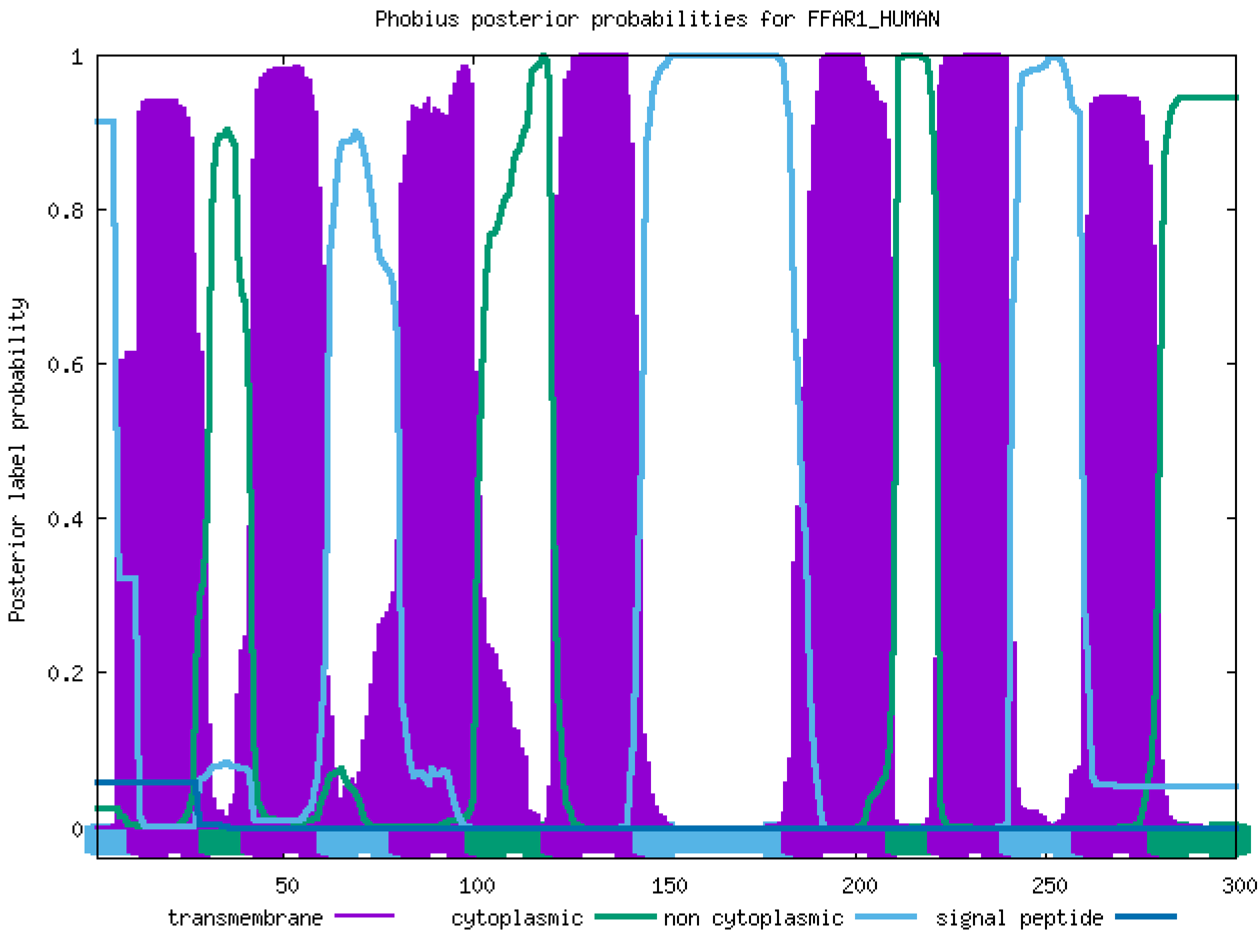

The probability data used in the plot is found here, and the gnuplot script is here.

Prediction of FFAR2_HUMAN

```
ID    FFAR2_HUMAN
FT    TOPO_DOM      1      11       NON CYTOPLASMIC.
FT    TRANSMEM     12      32
FT    TOPO_DOM     33      43       CYTOPLASMIC.
FT    TRANSMEM     44      64
FT    TOPO_DOM     65      75       NON CYTOPLASMIC.
FT    TRANSMEM     76     103
FT    TOPO_DOM    104     123       CYTOPLASMIC.
FT    TRANSMEM    124     150
FT    TOPO_DOM    151     178       NON CYTOPLASMIC.
FT    TRANSMEM    179     200
FT    TOPO_DOM    201     219       CYTOPLASMIC.
FT    TRANSMEM    220     240
FT    TOPO_DOM    241     251       NON CYTOPLASMIC.
FT    TRANSMEM    252     275
FT    TOPO_DOM    276     330       CYTOPLASMIC.
//
```

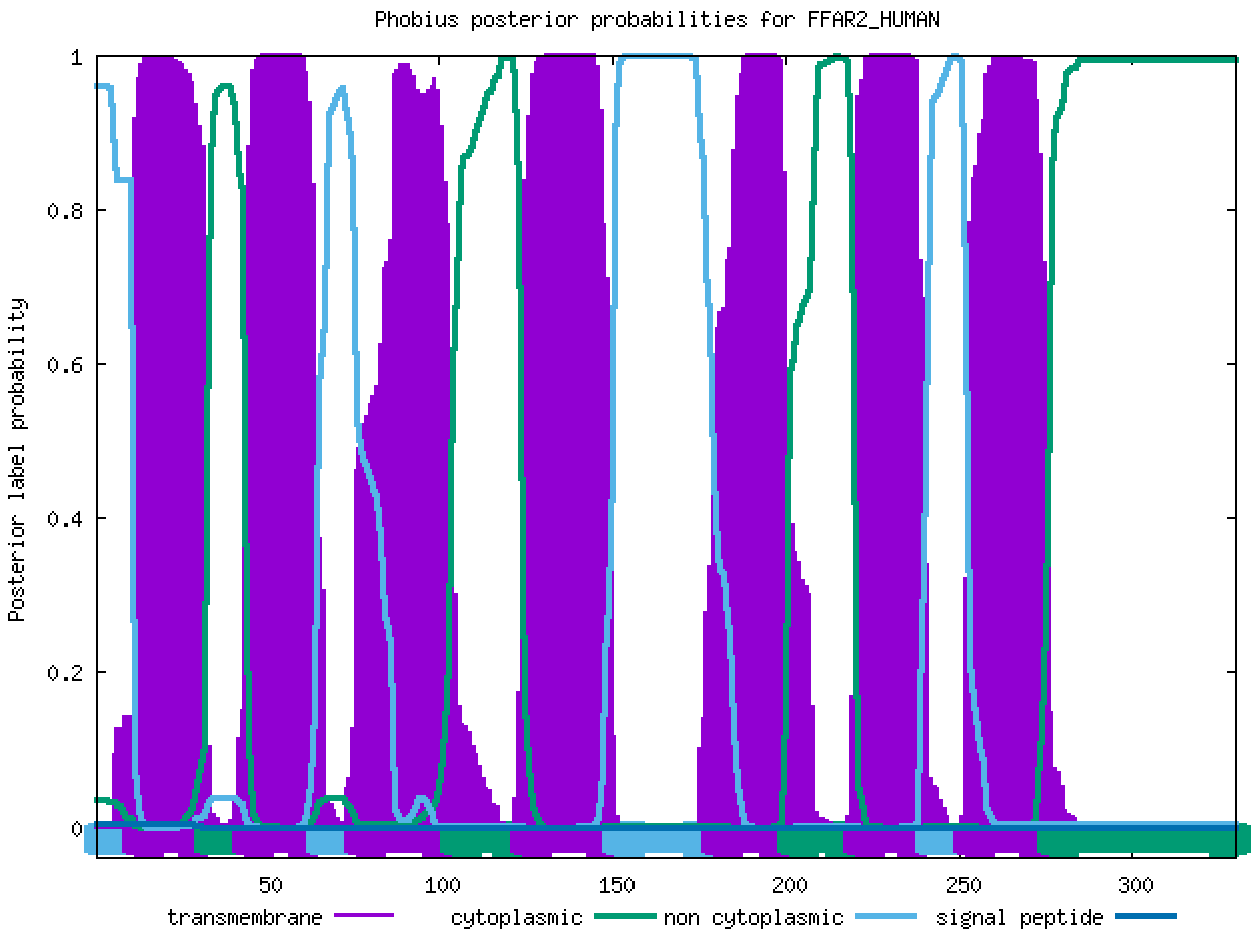

The probability data used in the plot is found here, and the gnuplot script is here.

Prediction of FFAR4_HUMAN

```
ID    FFAR4_HUMAN
FT    TOPO_DOM      1     36       NON CYTOPLASMIC.
FT    TRANSMEM     37     65
FT    TOPO_DOM     66     76       CYTOPLASMIC.
FT    TRANSMEM     77    101
FT    TOPO_DOM    102    112       NON CYTOPLASMIC.
FT    TRANSMEM    113    135
FT    TOPO_DOM    136    155       CYTOPLASMIC.
FT    TRANSMEM    156    180
FT    TOPO_DOM    181    208       NON CYTOPLASMIC.
FT    TRANSMEM    209    230
FT    TOPO_DOM    231    265       CYTOPLASMIC.
FT    TRANSMEM    266    290
FT    TOPO_DOM    291    301       NON CYTOPLASMIC.
FT    TRANSMEM    302    321
FT    TOPO_DOM    322    361       CYTOPLASMIC.
//
```

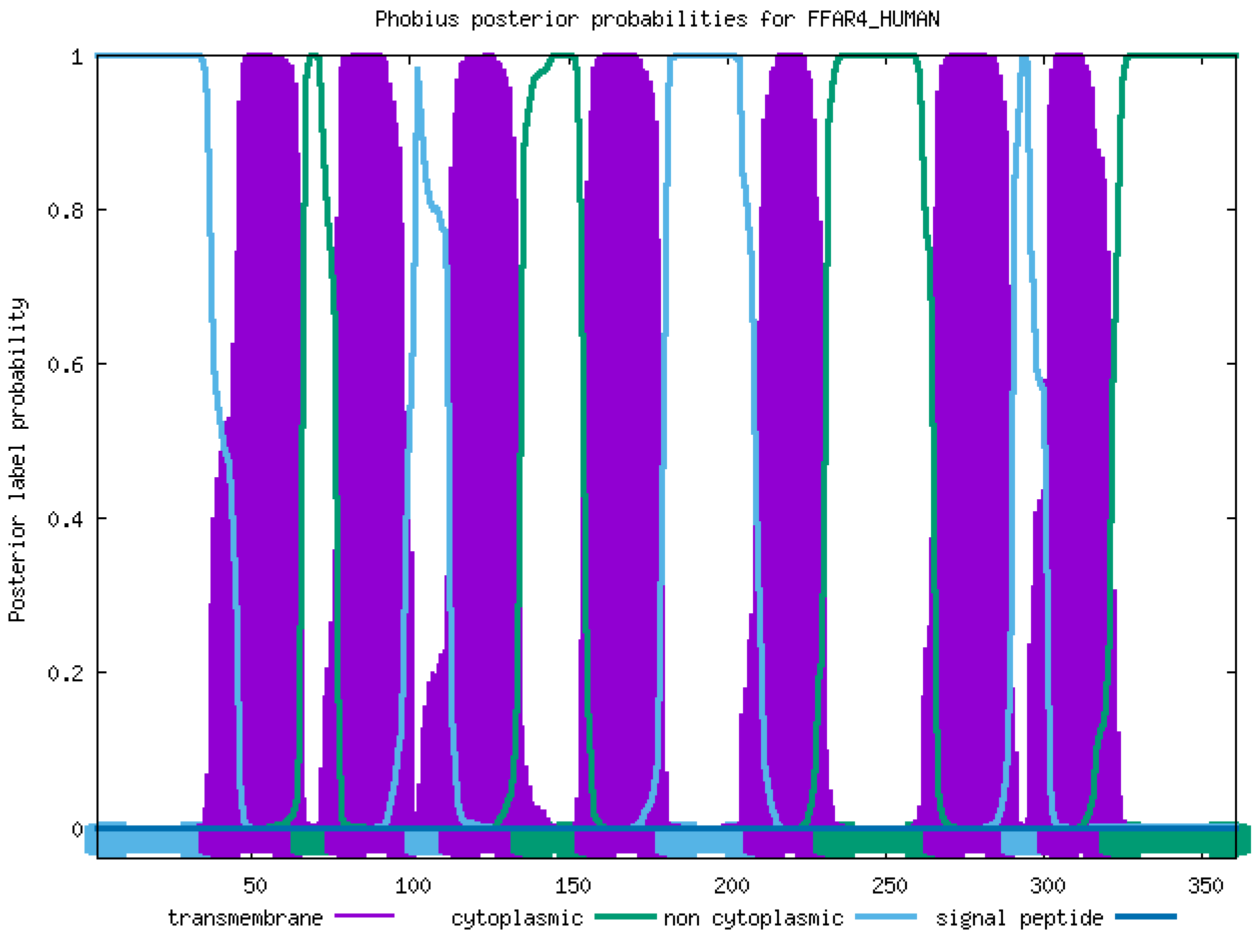

The probability data used in the plot is found here, and the gnuplot script is here.

Prediction of LPAR1_HUMAN

```
ID   LPAR1_HUMAN
FT   TOPO_DOM      1     50       NON CYTOPLASMIC.
FT   TRANSMEM     51     77
FT   TOPO_DOM     78     83       CYTOPLASMIC.
FT   TRANSMEM     84    105
FT   TOPO_DOM    106    124       NON CYTOPLASMIC.
FT   TRANSMEM    125    144
FT   TOPO_DOM    145    163       CYTOPLASMIC.
FT   TRANSMEM    164    186
FT   TOPO_DOM    187    205       NON CYTOPLASMIC.
FT   TRANSMEM    206    232
FT   TOPO_DOM    233    252       CYTOPLASMIC.
FT   TRANSMEM    253    280
FT   TOPO_DOM    281    364       NON CYTOPLASMIC.
//
```

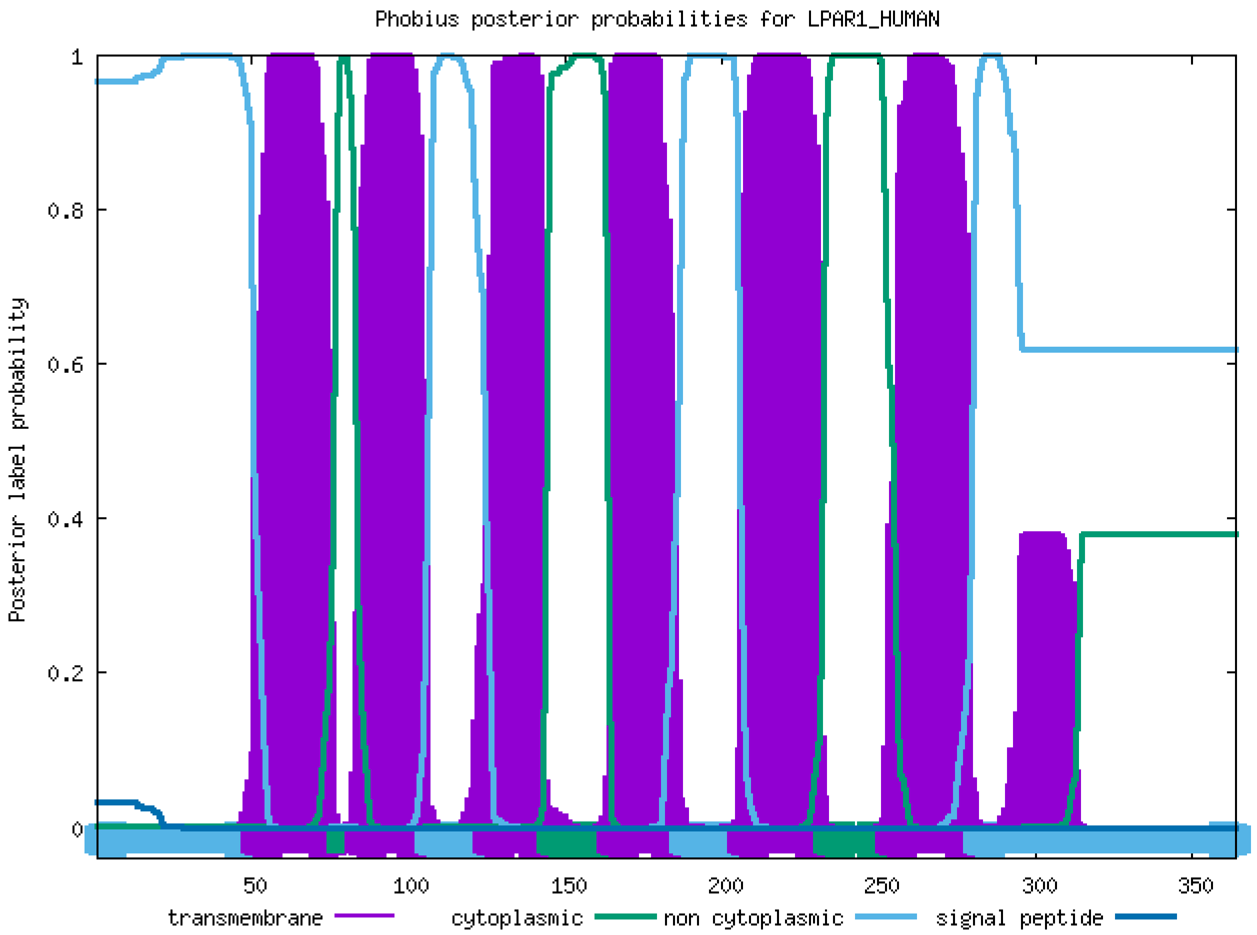

The probability data used in the plot is found here, and the gnuplot script is here.

Prediction of AGTR1_HUMAN

```
ID   AGTR1_HUMAN
FT   TOPO_DOM      1     33       NON CYTOPLASMIC.
FT   TRANSMEM     34     56
FT   TOPO_DOM     57     67       CYTOPLASMIC.
FT   TRANSMEM     68     90
FT   TOPO_DOM     91    109       NON CYTOPLASMIC.
FT   TRANSMEM    110    131
FT   TOPO_DOM    132    142       CYTOPLASMIC.
FT   TRANSMEM    143    165
FT   TOPO_DOM    166    200       NON CYTOPLASMIC.
FT   TRANSMEM    201    218
FT   TOPO_DOM    219    237       CYTOPLASMIC.
FT   TRANSMEM    238    262
FT   TOPO_DOM    263    281       NON CYTOPLASMIC.
FT   TRANSMEM    282    305
FT   TOPO_DOM    306    359       CYTOPLASMIC.
//
```

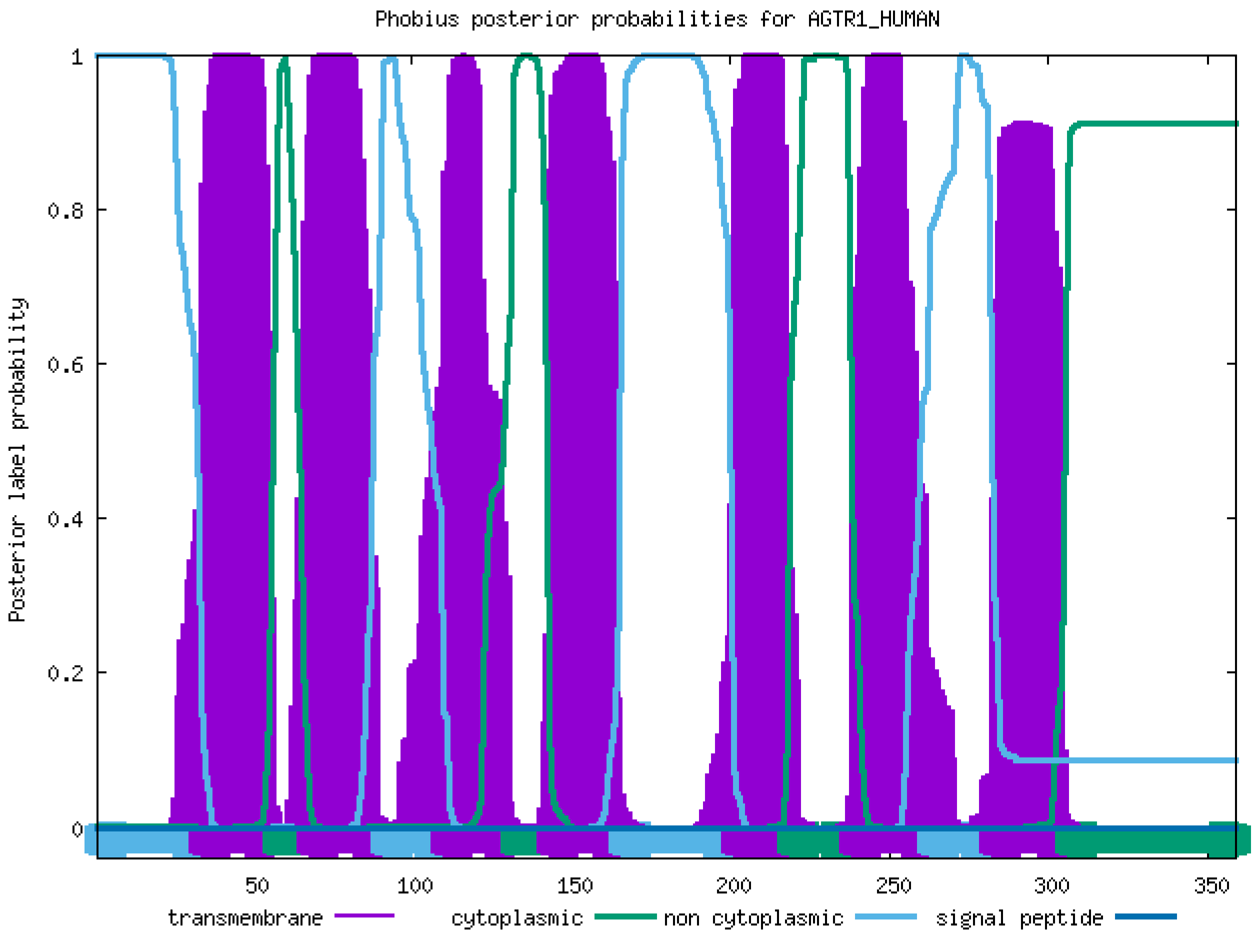

The probability data used in the plot is found here, and the gnuplot script is here.

Prediction of AGTR2_HUMAN

```
ID    AGTR2_HUMAN
FT    TOPO_DOM       1      48       NON CYTOPLASMIC.
FT    TRANSMEM      49      70
FT    TOPO_DOM      71      81       CYTOPLASMIC.
FT    TRANSMEM      82     100
FT    TOPO_DOM     101     119       NON CYTOPLASMIC.
FT    TRANSMEM     120     140
FT    TOPO_DOM     141     159       CYTOPLASMIC.
FT    TRANSMEM     160     181
FT    TOPO_DOM     182     216       NON CYTOPLASMIC.
FT    TRANSMEM     217     234
FT    TOPO_DOM     235     253       CYTOPLASMIC.
FT    TRANSMEM     254     278
FT    TOPO_DOM     279     297       NON CYTOPLASMIC.
FT    TRANSMEM     298     321
FT    TOPO_DOM     322     363       CYTOPLASMIC.
//
```

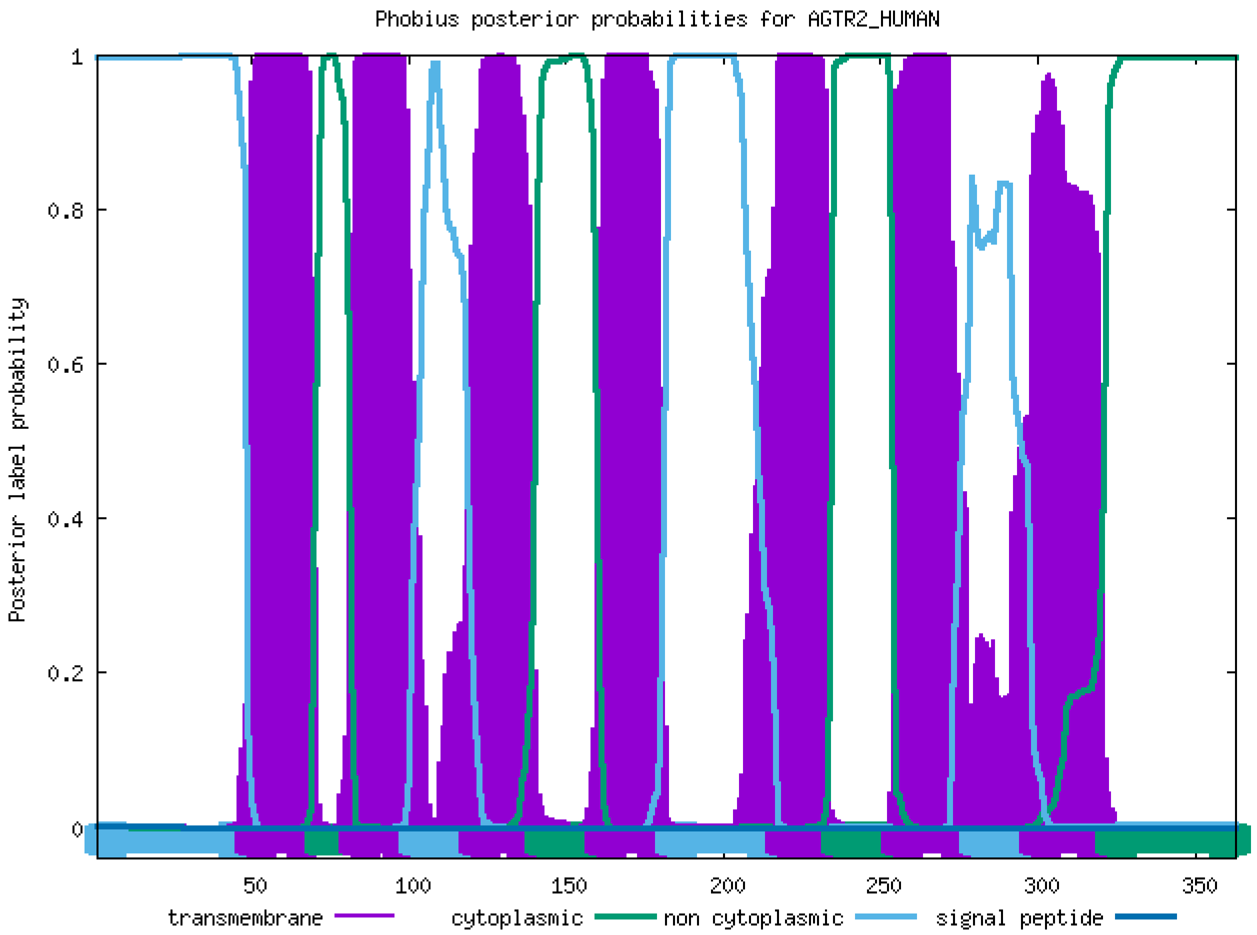

The probability data used in the plot is found here, and the gnuplot script is here.

Prediction of PD2R2_HUMAN

```
ID    PD2R2_HUMAN
FT    TOPO_DOM      1     33       NON CYTOPLASMIC.
FT    TRANSMEM     34     57
FT    TOPO_DOM     58     68       CYTOPLASMIC.
FT    TRANSMEM     69     90
FT    TOPO_DOM     91    109       NON CYTOPLASMIC.
FT    TRANSMEM    110    131
FT    TOPO_DOM    132    150       CYTOPLASMIC.
FT    TRANSMEM    151    169
FT    TOPO_DOM    170    210       NON CYTOPLASMIC.
FT    TRANSMEM    211    232
FT    TOPO_DOM    233    243       CYTOPLASMIC.
FT    TRANSMEM    244    265
FT    TOPO_DOM    266    284       NON CYTOPLASMIC.
FT    TRANSMEM    285    307
FT    TOPO_DOM    308    395       CYTOPLASMIC.
//
```

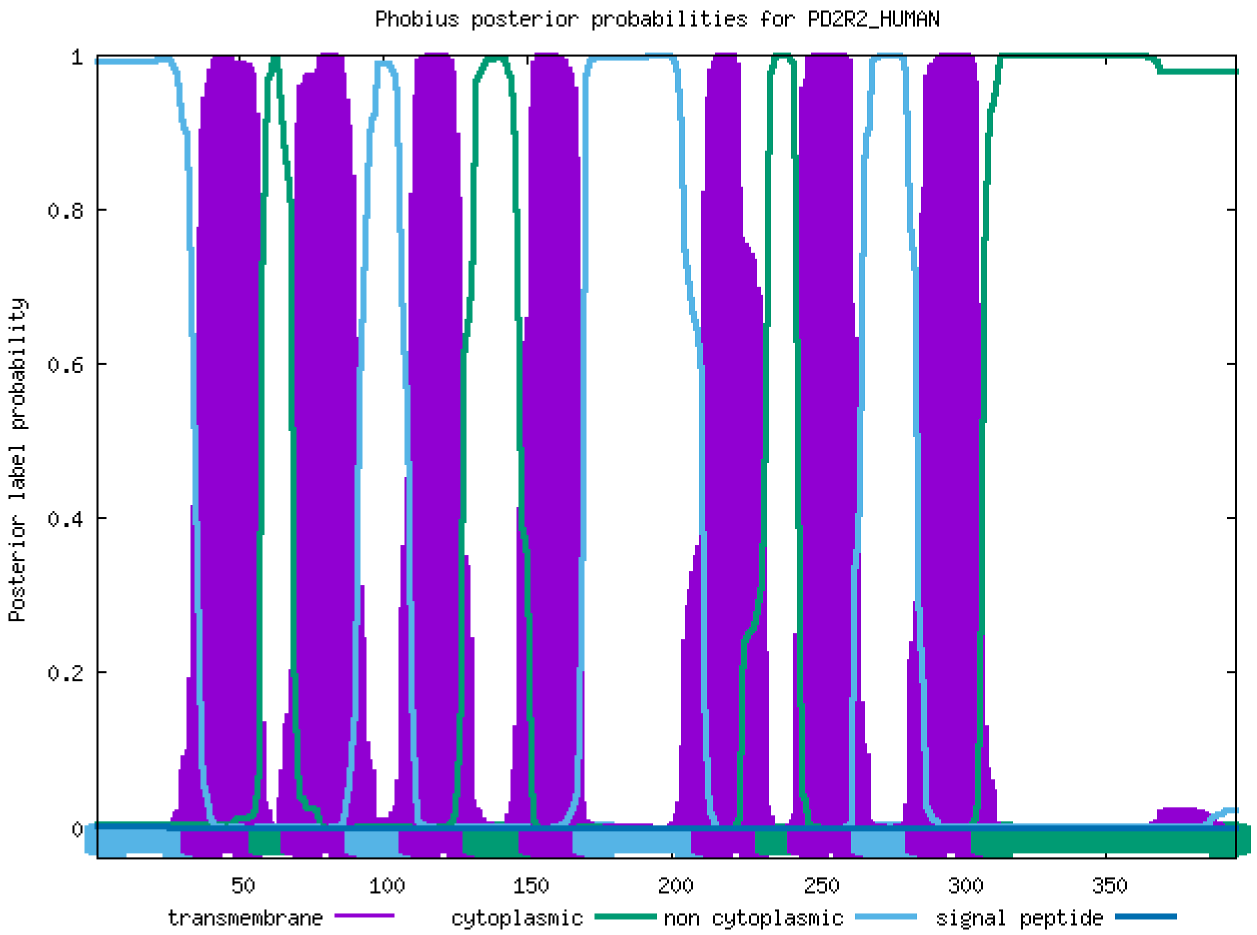

The probability data used in the plot is found here, and the gnuplot script is here.

Prediction of PE2R2_HUMAN

```
ID   PE2R2_HUMAN
FT   TOPO_DOM      1     25       NON CYTOPLASMIC.
FT   TRANSMEM     26     46
FT   TOPO_DOM     47     65       CYTOPLASMIC.
FT   TRANSMEM     66     91
FT   TOPO_DOM     92    110       NON CYTOPLASMIC.
FT   TRANSMEM    111    132
FT   TOPO_DOM    133    152       CYTOPLASMIC.
FT   TRANSMEM    153    173
FT   TOPO_DOM    174    198       NON CYTOPLASMIC.
FT   TRANSMEM    199    223
FT   TOPO_DOM    224    262       CYTOPLASMIC.
FT   TRANSMEM    263    286
FT   TOPO_DOM    287    297       NON CYTOPLASMIC.
FT   TRANSMEM    298    318
FT   TOPO_DOM    319    358       CYTOPLASMIC.
//
```

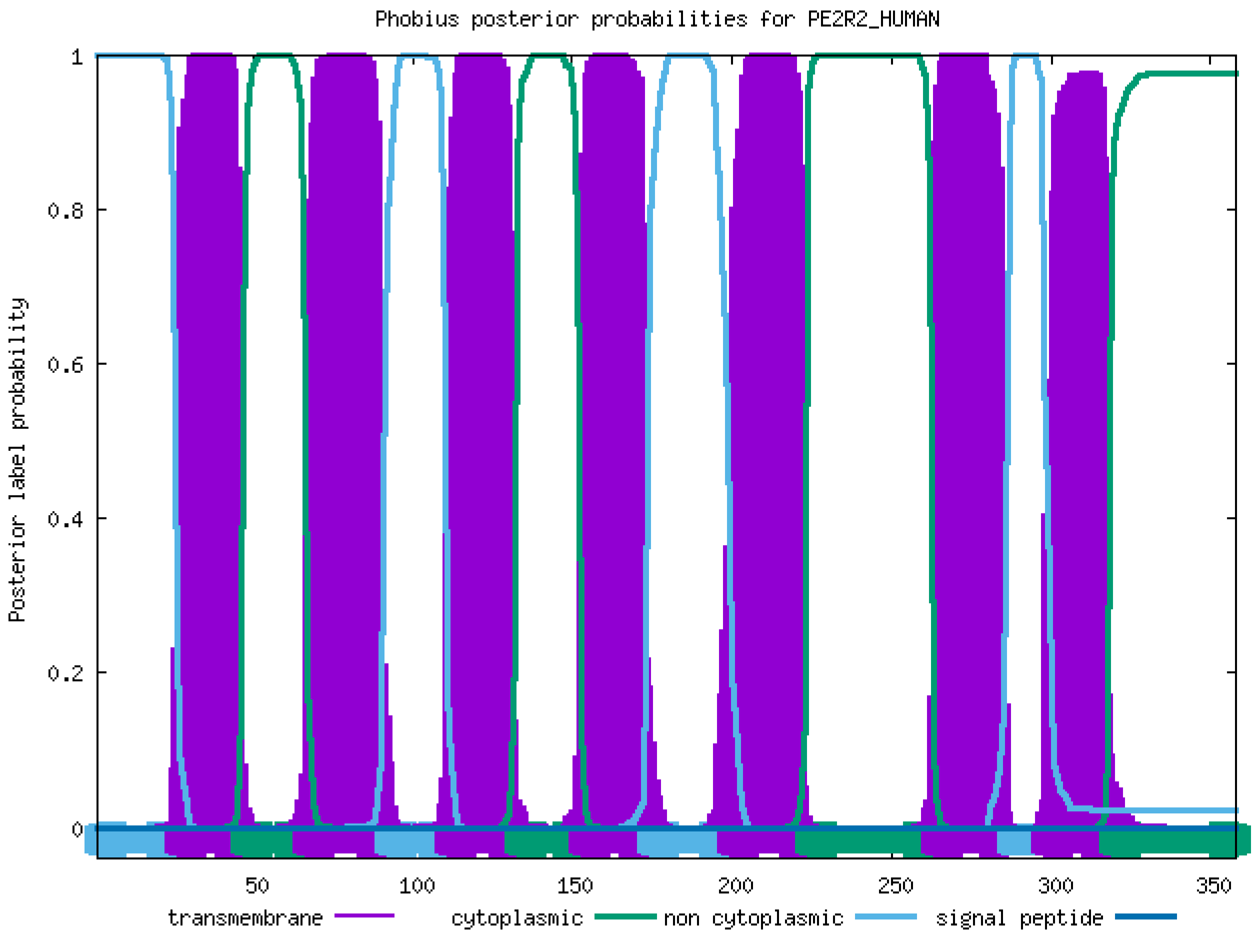

The probability data used in the plot is found here, and the gnuplot script is here.

Prediction of PE2R3_HUMAN

```
ID    PE2R3_HUMAN
FT    TOPO_DOM      1     51       NON CYTOPLASMIC.
FT    TRANSMEM     52     75
FT    TOPO_DOM     76     86       CYTOPLASMIC.
FT    TRANSMEM     87    112
FT    TOPO_DOM    113    131       NON CYTOPLASMIC.
FT    TRANSMEM    132    153
FT    TOPO_DOM    154    172       CYTOPLASMIC.
FT    TRANSMEM    173    194
FT    TOPO_DOM    195    227       NON CYTOPLASMIC.
FT    TRANSMEM    228    253
FT    TOPO_DOM    254    283       CYTOPLASMIC.
FT    TRANSMEM    284    307
FT    TOPO_DOM    308    326       NON CYTOPLASMIC.
FT    TRANSMEM    327    349
FT    TOPO_DOM    350    390       CYTOPLASMIC.
//
```

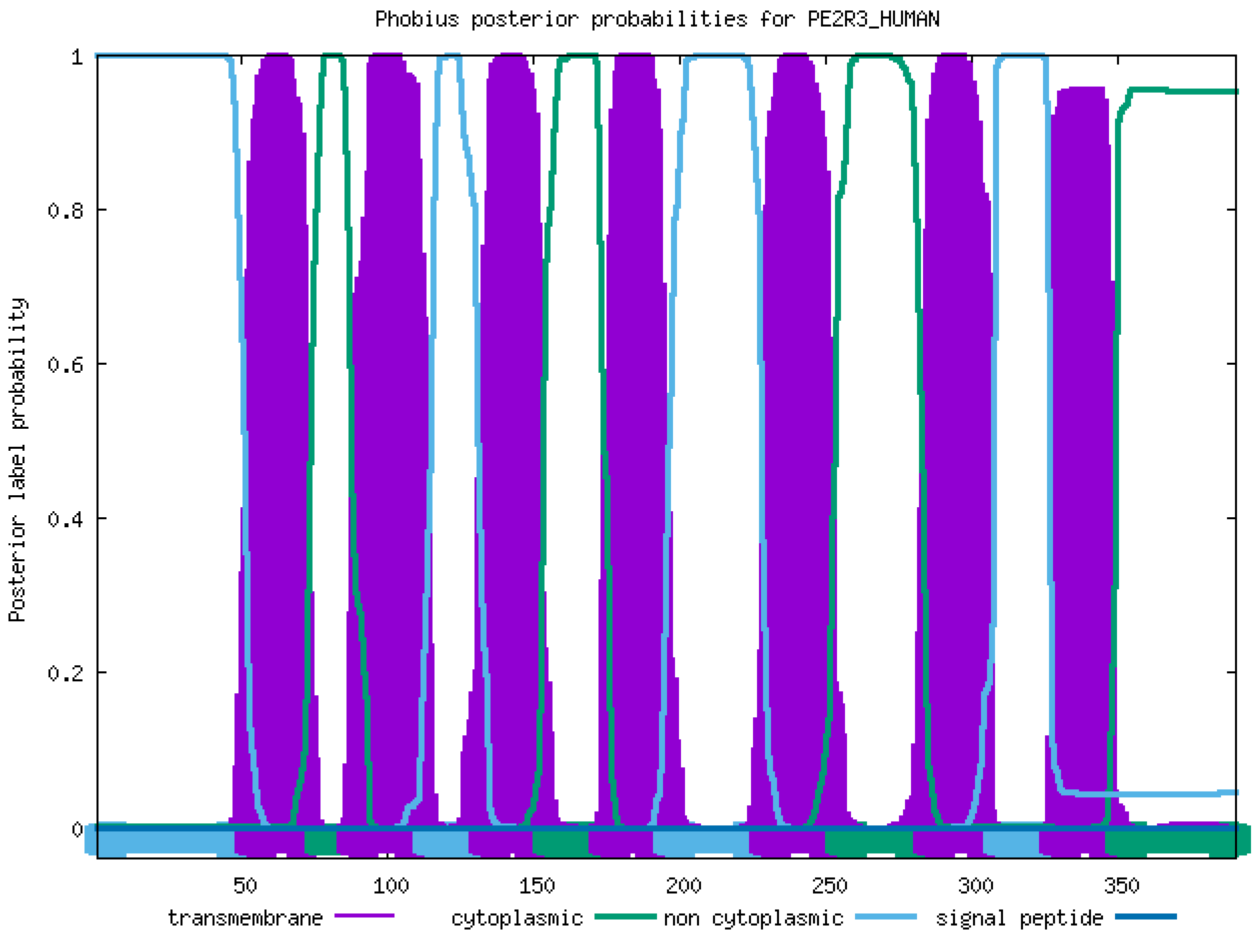

The probability data used in the plot is found here, and the gnuplot script is here.

Prediction of PE2R4_HUMAN

```
ID    PE2R4_HUMAN
FT    TOPO_DOM       1      19       NON CYTOPLASMIC.
FT    TRANSMEM      20      42
FT    TOPO_DOM      43      53       CYTOPLASMIC.
FT    TRANSMEM      54      77
FT    TOPO_DOM      78      96       NON CYTOPLASMIC.
FT    TRANSMEM      97     115
FT    TOPO_DOM     116     134       CYTOPLASMIC.
FT    TRANSMEM     135     156
FT    TOPO_DOM     157     185       NON CYTOPLASMIC.
FT    TRANSMEM     186     211
FT    TOPO_DOM     212     270       CYTOPLASMIC.
FT    TRANSMEM     271     294
FT    TOPO_DOM     295     313       NON CYTOPLASMIC.
FT    TRANSMEM     314     332
FT    TOPO_DOM     333     488       CYTOPLASMIC.
//
```

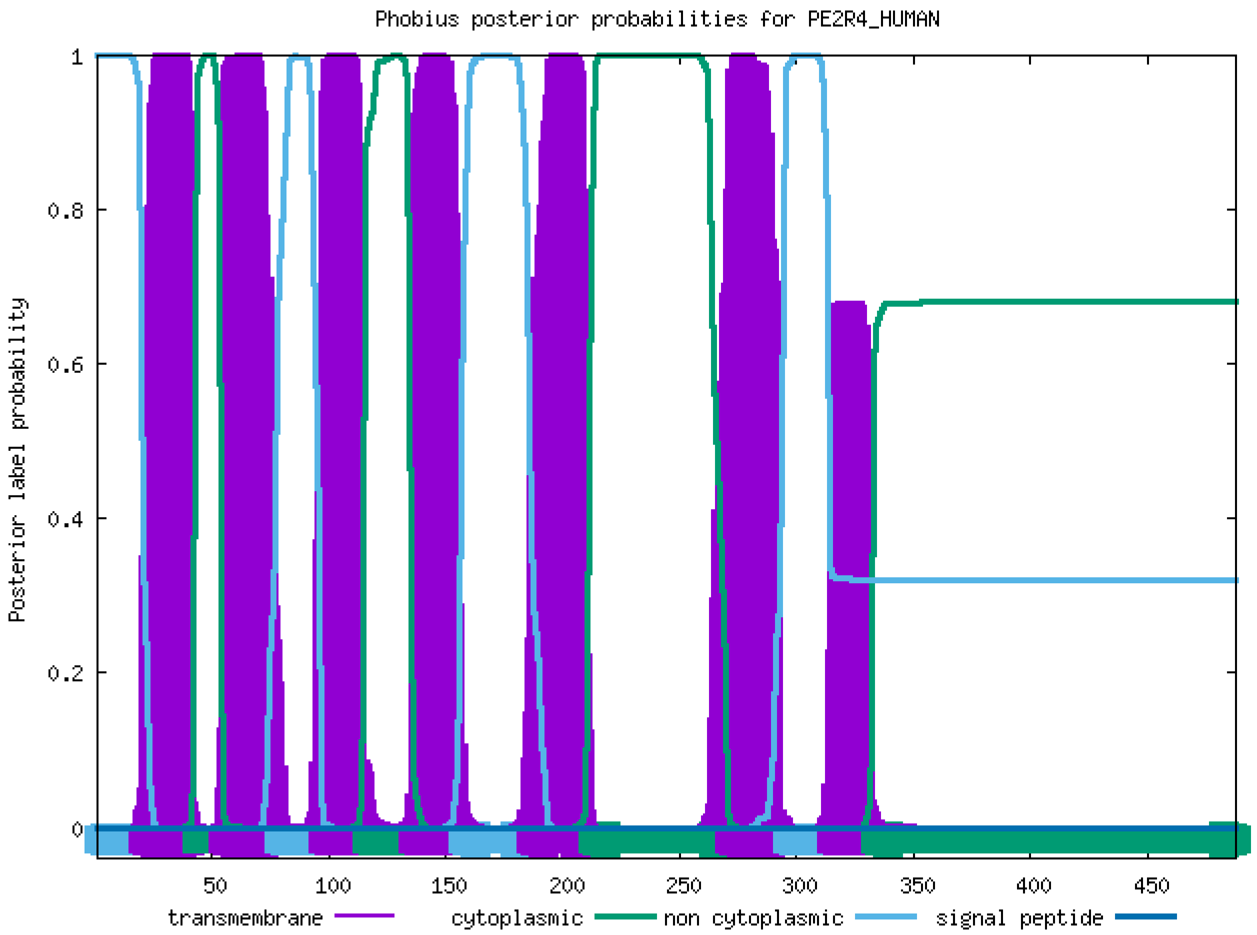

The probability data used in the plot is found here, and the gnuplot script is here.

Prediction of PF2R_HUMAN

```
ID   PF2R_HUMAN
FT   SIGNAL        1     22
FT   REGION        1      8       N-REGION.
FT   REGION        9     17       H-REGION.
FT   REGION       18     22       C-REGION.
FT   TOPO_DOM     23     31       NON CYTOPLASMIC.
FT   TRANSMEM     32     52
FT   TOPO_DOM     53     63       CYTOPLASMIC.
FT   TRANSMEM     64     92
FT   TOPO_DOM     93    103       NON CYTOPLASMIC.
FT   TRANSMEM    104    131
FT   TOPO_DOM    132    151       CYTOPLASMIC.
FT   TRANSMEM    152    172
FT   TOPO_DOM    173    199       NON CYTOPLASMIC.
FT   TRANSMEM    200    228
FT   TOPO_DOM    229    247       CYTOPLASMIC.
FT   TRANSMEM    248    267
FT   TOPO_DOM    268    286       NON CYTOPLASMIC.
FT   TRANSMEM    287    307
FT   TOPO_DOM    308    359       CYTOPLASMIC.
//
```

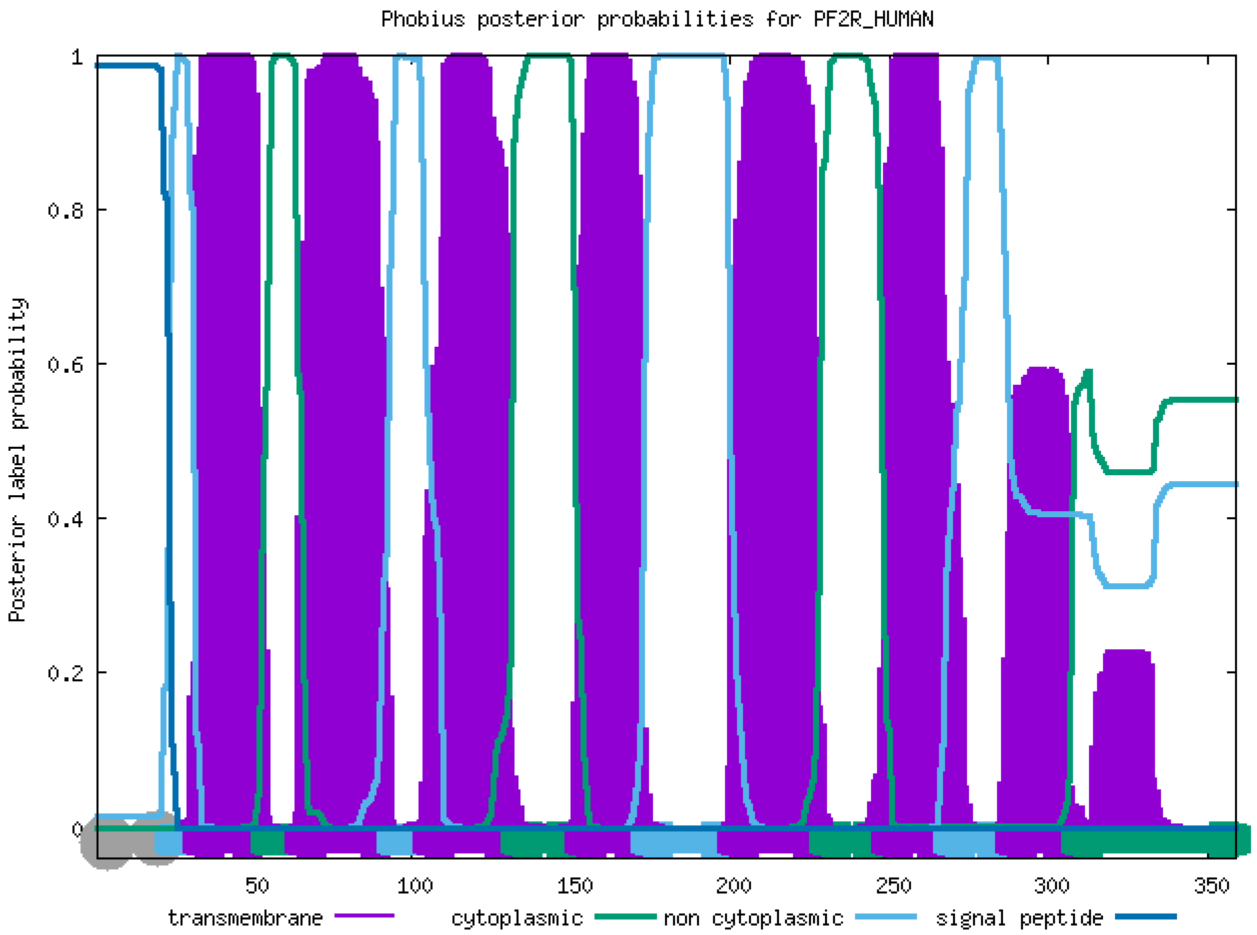

The probability data used in the plot is found here, and the gnuplot script is here.

Prediction of TA2R_HUMAN

```
ID    TA2R_HUMAN
FT    TOPO_DOM       1      28       NON CYTOPLASMIC.
FT    TRANSMEM      29      51
FT    TOPO_DOM      52      62       CYTOPLASMIC.
FT    TRANSMEM      63      87
FT    TOPO_DOM      88     106       NON CYTOPLASMIC.
FT    TRANSMEM     107     128
FT    TOPO_DOM     129     148       CYTOPLASMIC.
FT    TRANSMEM     149     174
FT    TOPO_DOM     175     193       NON CYTOPLASMIC.
FT    TRANSMEM     194     226
FT    TOPO_DOM     227     246       CYTOPLASMIC.
FT    TRANSMEM     247     270
FT    TOPO_DOM     271     289       NON CYTOPLASMIC.
FT    TRANSMEM     290     311
FT    TOPO_DOM     312     343       CYTOPLASMIC.
//
```

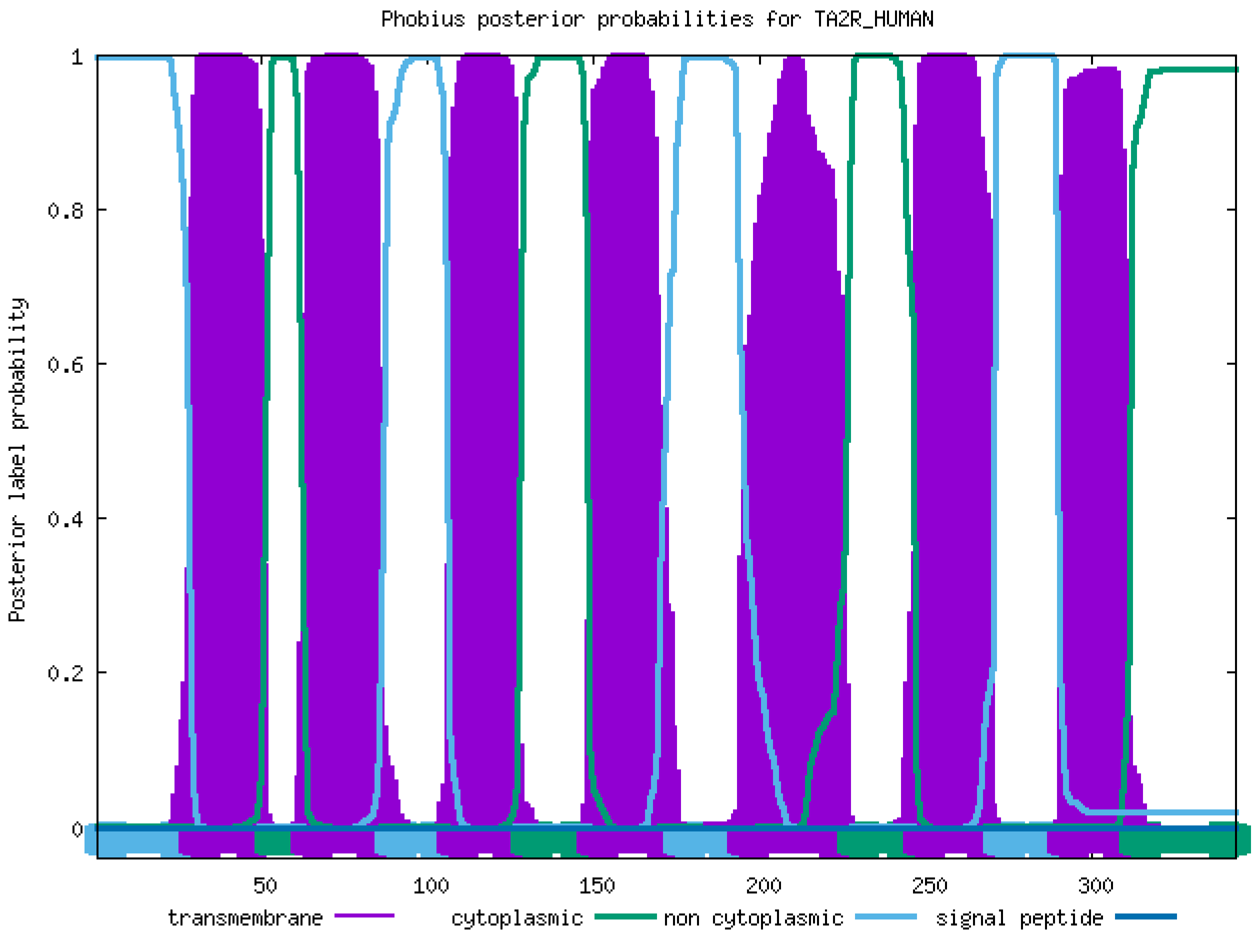

The probability data used in the plot is found here, and the gnuplot script is here.

Prediction of NK1R_HUMAN

```
ID   NK1R_HUMAN
FT   TOPO_DOM      1     31       NON CYTOPLASMIC.
FT   TRANSMEM     32     58
FT   TOPO_DOM     59     69       CYTOPLASMIC.
FT   TRANSMEM     70     90
FT   TOPO_DOM     91    109       NON CYTOPLASMIC.
FT   TRANSMEM    110    128
FT   TOPO_DOM    129    148       CYTOPLASMIC.
FT   TRANSMEM    149    168
FT   TOPO_DOM    169    194       NON CYTOPLASMIC.
FT   TRANSMEM    195    225
FT   TOPO_DOM    226    245       CYTOPLASMIC.
FT   TRANSMEM    246    273
FT   TOPO_DOM    274    284       NON CYTOPLASMIC.
FT   TRANSMEM    285    308
FT   TOPO_DOM    309    407       CYTOPLASMIC.
//
```

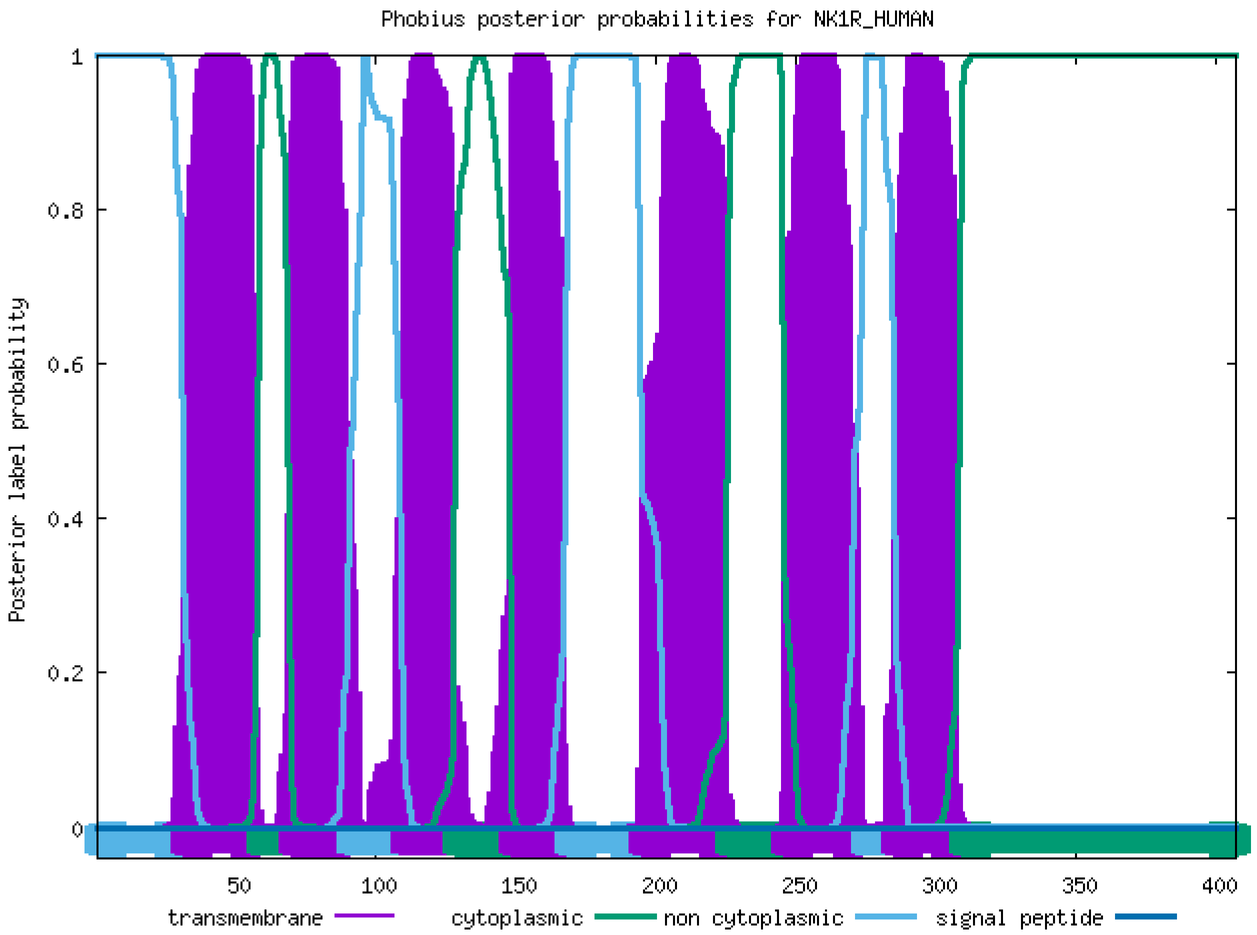

The probability data used in the plot is found here, and the gnuplot script is here.

Prediction of NK3R_HUMAN

```
ID   NK3R_HUMAN
FT   TOPO_DOM      1     84       NON CYTOPLASMIC.
FT   TRANSMEM     85    111
FT   TOPO_DOM    112    122       CYTOPLASMIC.
FT   TRANSMEM    123    148
FT   TOPO_DOM    149    159       NON CYTOPLASMIC.
FT   TRANSMEM    160    181
FT   TOPO_DOM    182    201       CYTOPLASMIC.
FT   TRANSMEM    202    221
FT   TOPO_DOM    222    246       NON CYTOPLASMIC.
FT   TRANSMEM    247    276
FT   TOPO_DOM    277    296       CYTOPLASMIC.
FT   TRANSMEM    297    325
FT   TOPO_DOM    326    336       NON CYTOPLASMIC.
FT   TRANSMEM    337    359
FT   TOPO_DOM    360    465       CYTOPLASMIC.
//
```

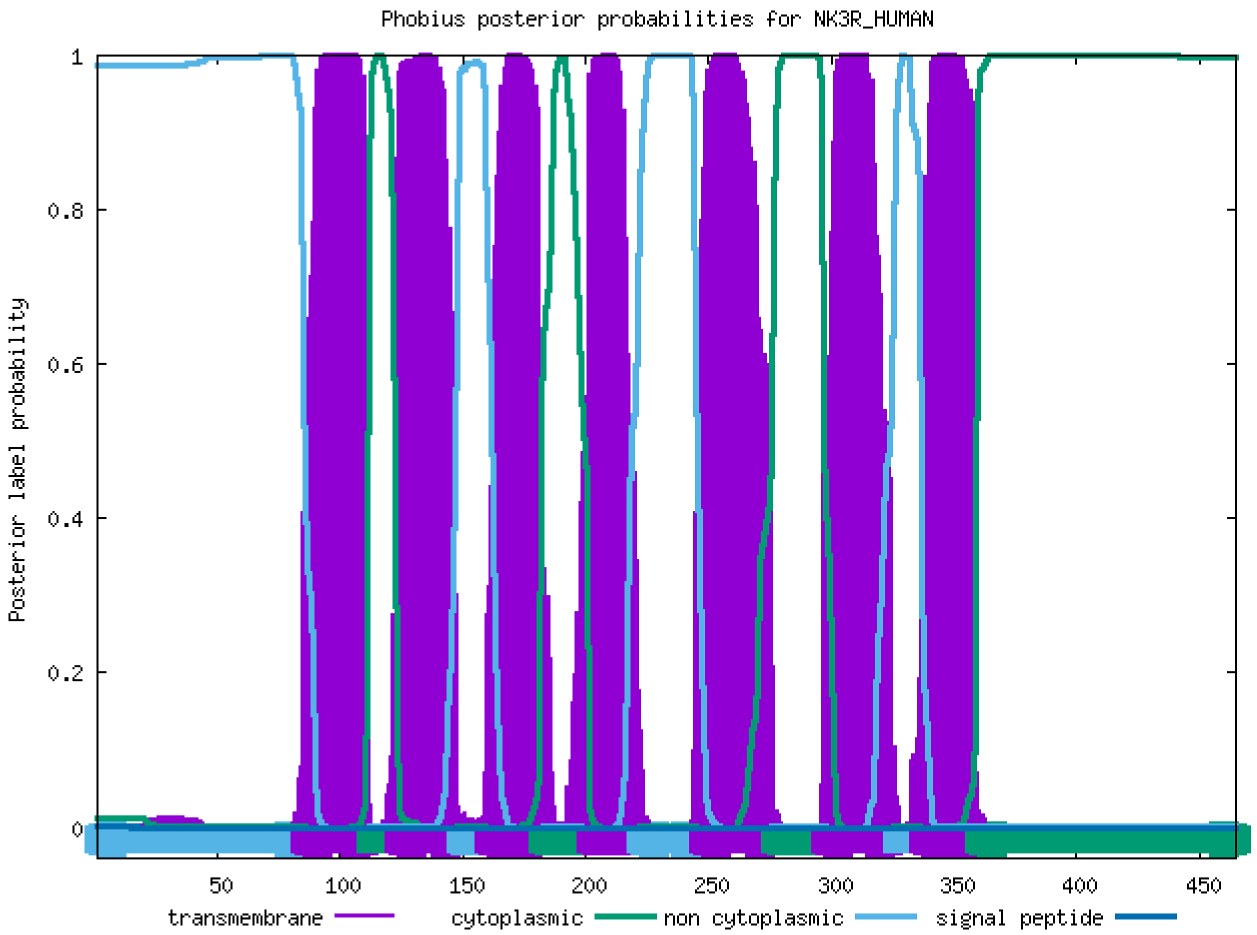

The probability data used in the plot is found here, and the gnuplot script is here.

Prediction of PTAFR_HUMAN

```
ID   PTAFR_HUMAN
FT   TOPO_DOM      1     19       NON CYTOPLASMIC.
FT   TRANSMEM     20     43
FT   TOPO_DOM     44     54       CYTOPLASMIC.
FT   TRANSMEM     55     76
FT   TOPO_DOM     77     87       NON CYTOPLASMIC.
FT   TRANSMEM     88    113
FT   TOPO_DOM    114    133       CYTOPLASMIC.
FT   TRANSMEM    134    154
FT   TOPO_DOM    155    184       NON CYTOPLASMIC.
FT   TRANSMEM    185    209
FT   TOPO_DOM    210    229       CYTOPLASMIC.
FT   TRANSMEM    230    250
FT   TOPO_DOM    251    273       NON CYTOPLASMIC.
FT   TRANSMEM    274    296
FT   TOPO_DOM    297    342       CYTOPLASMIC.
//
```

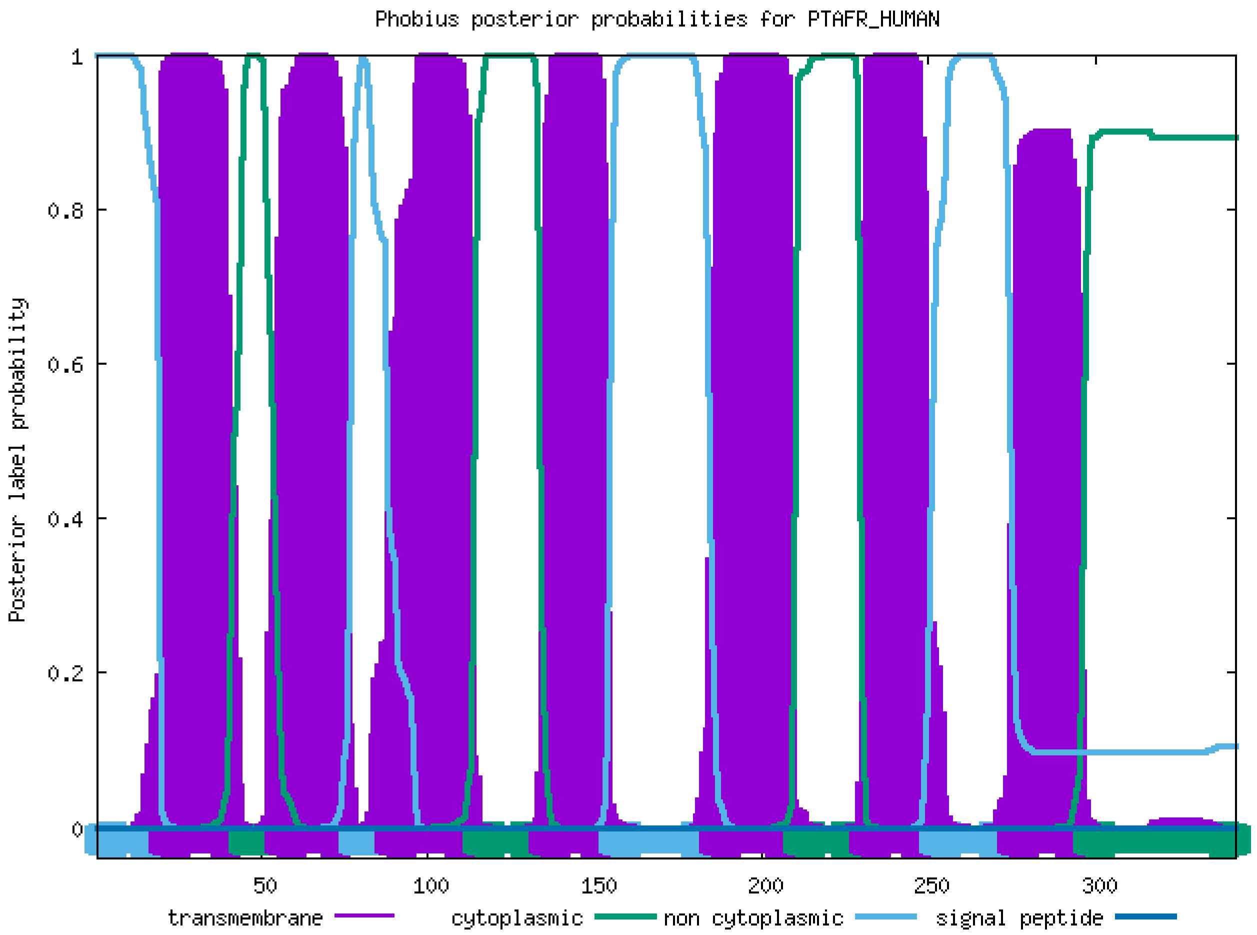

The probability data used in the plot is found here, and the gnuplot script is here.

Prediction of MSHR_HUMAN

```
ID   MSHR_HUMAN
FT   TOPO_DOM      1     43       NON CYTOPLASMIC.
FT   TRANSMEM     44     64
FT   TOPO_DOM     65     75       CYTOPLASMIC.
FT   TRANSMEM     76     99
FT   TOPO_DOM    100    118       NON CYTOPLASMIC.
FT   TRANSMEM    119    140
FT   TOPO_DOM    141    160       CYTOPLASMIC.
FT   TRANSMEM    161    181
FT   TOPO_DOM    182    186       NON CYTOPLASMIC.
FT   TRANSMEM    187    214
FT   TOPO_DOM    215    234       CYTOPLASMIC.
FT   TRANSMEM    235    259
FT   TOPO_DOM    260    278       NON CYTOPLASMIC.
FT   TRANSMEM    279    300
FT   TOPO_DOM    301    317       CYTOPLASMIC.
//
```

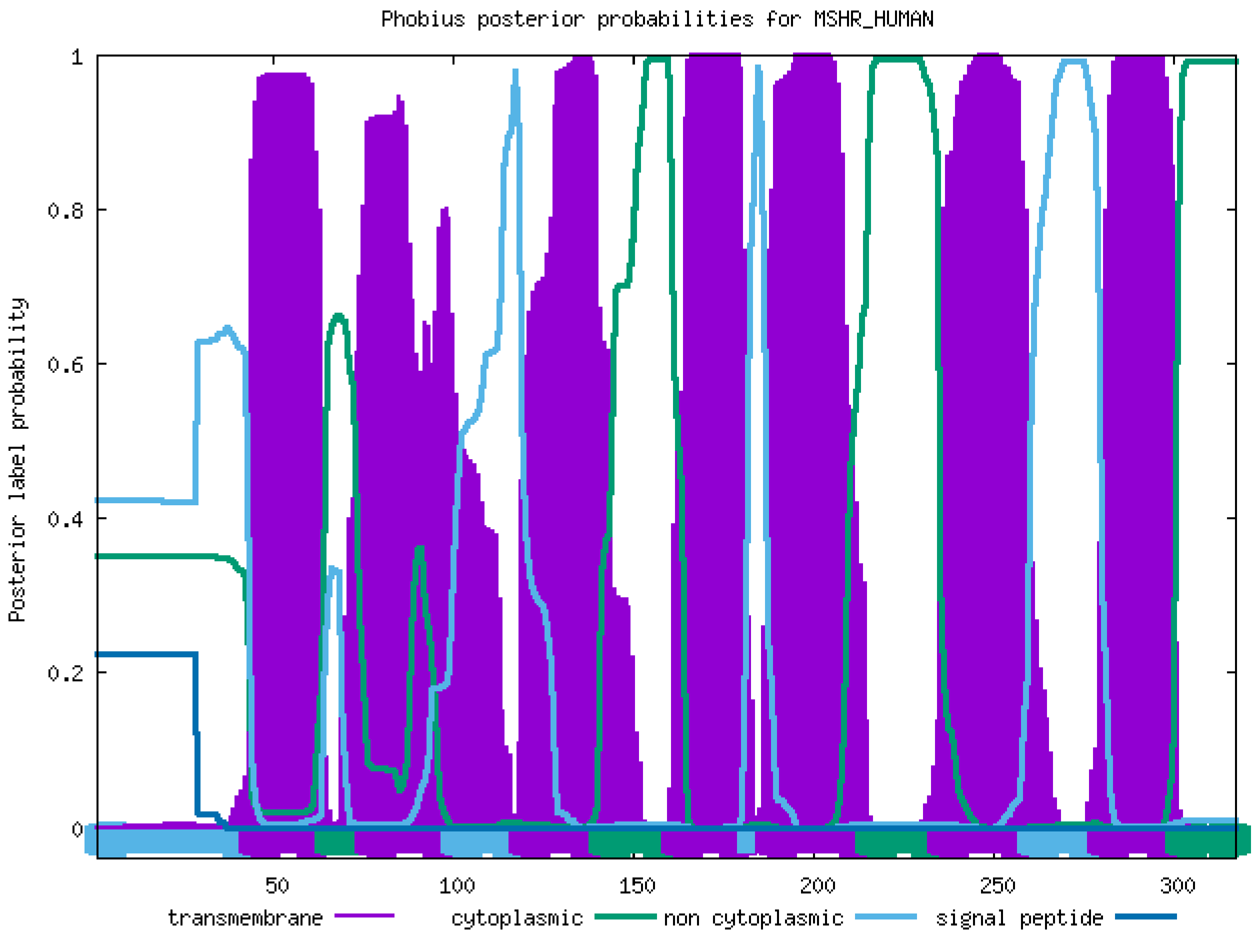

The probability data used in the plot is found here, and the gnuplot script is here.

Prediction of MC3R_HUMAN

```
ID    MC3R_HUMAN
FT    TOPO_DOM      1     43       NON CYTOPLASMIC.
FT    TRANSMEM     44     64
FT    TOPO_DOM     65     75       CYTOPLASMIC.
FT    TRANSMEM     76    100
FT    TOPO_DOM    101    119       NON CYTOPLASMIC.
FT    TRANSMEM    120    140
FT    TOPO_DOM    141    160       CYTOPLASMIC.
FT    TRANSMEM    161    181
FT    TOPO_DOM    182    186       NON CYTOPLASMIC.
FT    TRANSMEM    187    212
FT    TOPO_DOM    213    240       CYTOPLASMIC.
FT    TRANSMEM    241    266
FT    TOPO_DOM    267    280       NON CYTOPLASMIC.
FT    TRANSMEM    281    301
FT    TOPO_DOM    302    323       CYTOPLASMIC.
//
```

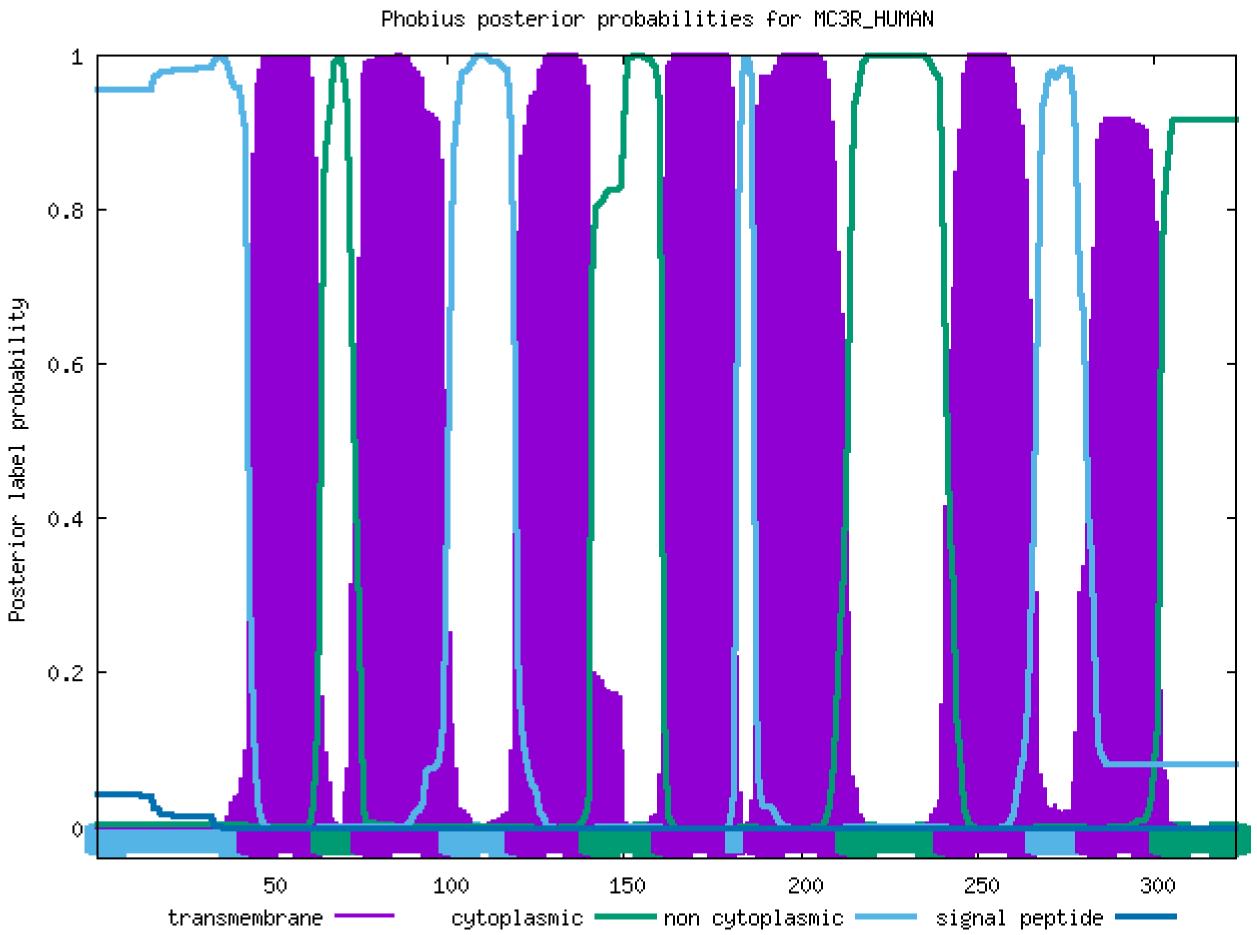

The probability data used in the plot is found here, and the gnuplot script is here.

Prediction of MC4R_HUMAN

```
ID   MC4R_HUMAN
FT   TOPO_DOM      1     49       NON CYTOPLASMIC.
FT   TRANSMEM     50     69
FT   TOPO_DOM     70     80       CYTOPLASMIC.
FT   TRANSMEM     81    104
FT   TOPO_DOM    105    123       NON CYTOPLASMIC.
FT   TRANSMEM    124    145
FT   TOPO_DOM    146    165       CYTOPLASMIC.
FT   TRANSMEM    166    186
FT   TOPO_DOM    187    191       NON CYTOPLASMIC.
FT   TRANSMEM    192    217
FT   TOPO_DOM    218    243       CYTOPLASMIC.
FT   TRANSMEM    244    269
FT   TOPO_DOM    270    283       NON CYTOPLASMIC.
FT   TRANSMEM    284    304
FT   TOPO_DOM    305    332       CYTOPLASMIC.
//
```

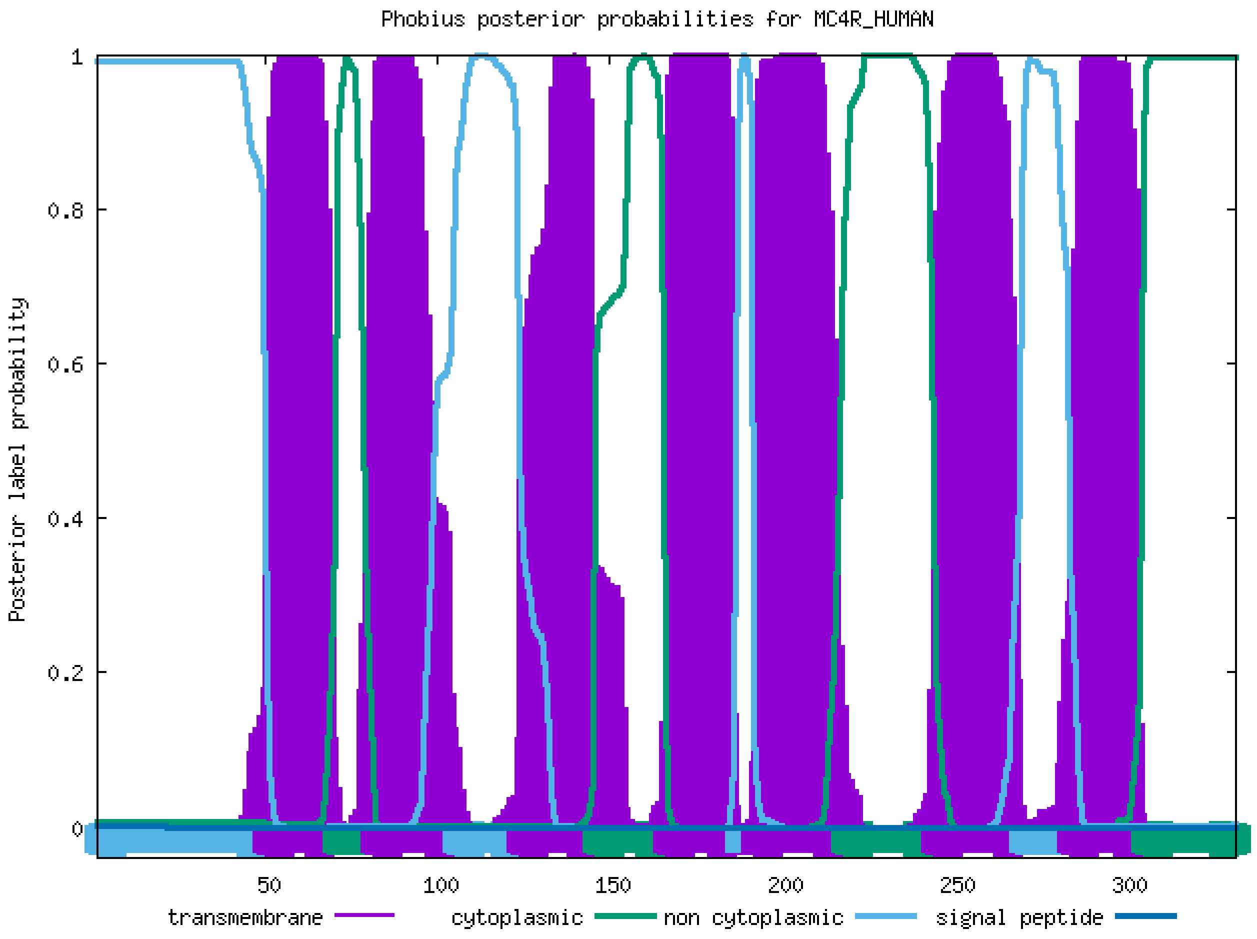

The probability data used in the plot is found here, and the gnuplot script is here.

Prediction of MC5R_HUMAN

```
ID   MC5R_HUMAN
FT   TOPO_DOM      1     37       NON CYTOPLASMIC.
FT   TRANSMEM     38     60
FT   TOPO_DOM     61     71       CYTOPLASMIC.
FT   TRANSMEM     72     98
FT   TOPO_DOM     99    117       NON CYTOPLASMIC.
FT   TRANSMEM    118    138
FT   TOPO_DOM    139    158       CYTOPLASMIC.
FT   TRANSMEM    159    179
FT   TOPO_DOM    180    184       NON CYTOPLASMIC.
FT   TRANSMEM    185    212
FT   TOPO_DOM    213    232       CYTOPLASMIC.
FT   TRANSMEM    233    257
FT   TOPO_DOM    258    276       NON CYTOPLASMIC.
FT   TRANSMEM    277    297
FT   TOPO_DOM    298    325       CYTOPLASMIC.
//
```

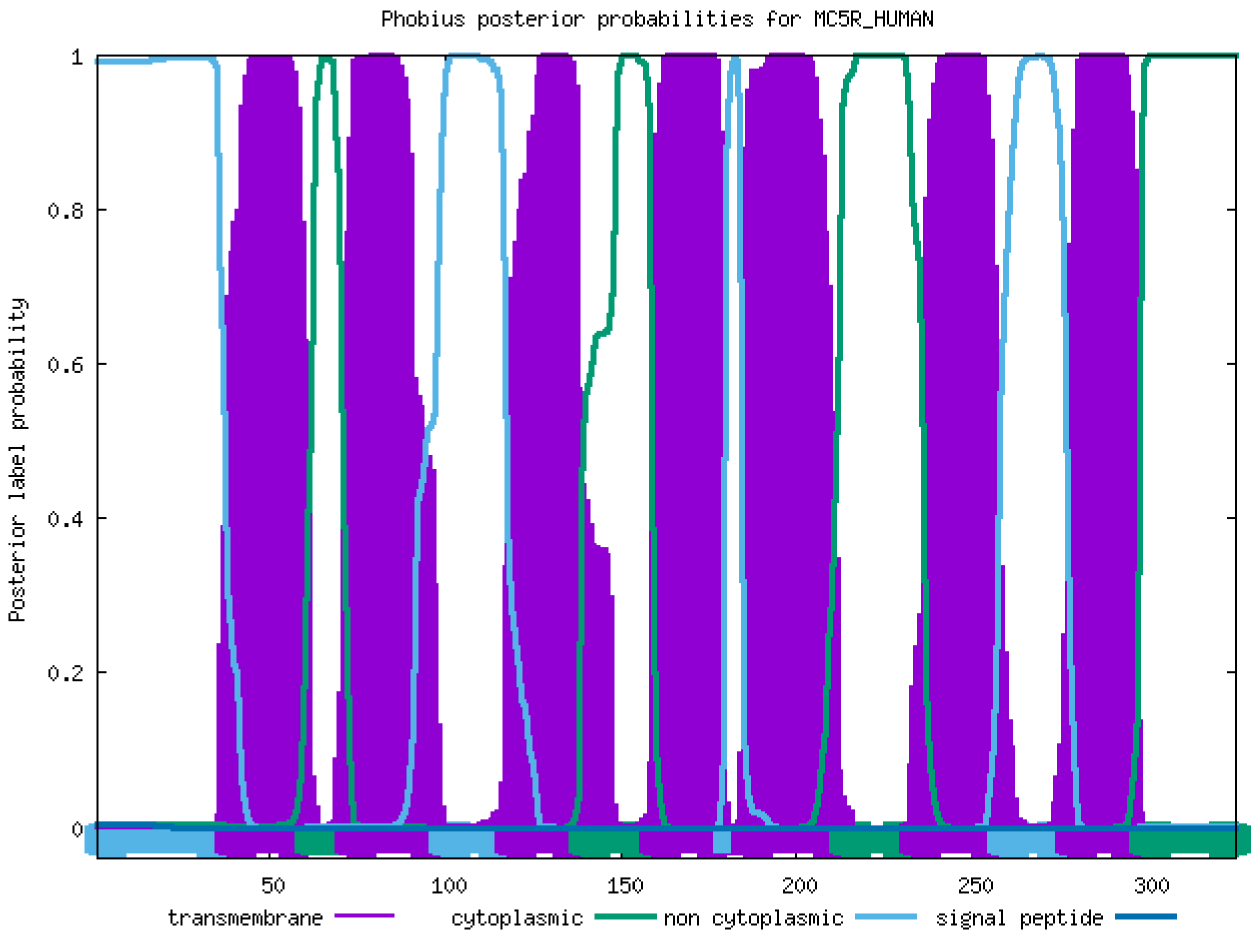

The probability data used in the plot is found here, and the gnuplot script is here.

Prediction of GHSR_HUMAN

```
ID    GHSR_HUMAN
FT    TOPO_DOM      1     41       NON CYTOPLASMIC.
FT    TRANSMEM     42     68
FT    TOPO_DOM     69     79       CYTOPLASMIC.
FT    TRANSMEM     80     98
FT    TOPO_DOM     99    117       NON CYTOPLASMIC.
FT    TRANSMEM    118    139
FT    TOPO_DOM    140    159       CYTOPLASMIC.
FT    TRANSMEM    160    184
FT    TOPO_DOM    185    211       NON CYTOPLASMIC.
FT    TRANSMEM    212    235
FT    TOPO_DOM    236    263       CYTOPLASMIC.
FT    TRANSMEM    264    286
FT    TOPO_DOM    287    305       NON CYTOPLASMIC.
FT    TRANSMEM    306    326
FT    TOPO_DOM    327    366       CYTOPLASMIC.
//
```

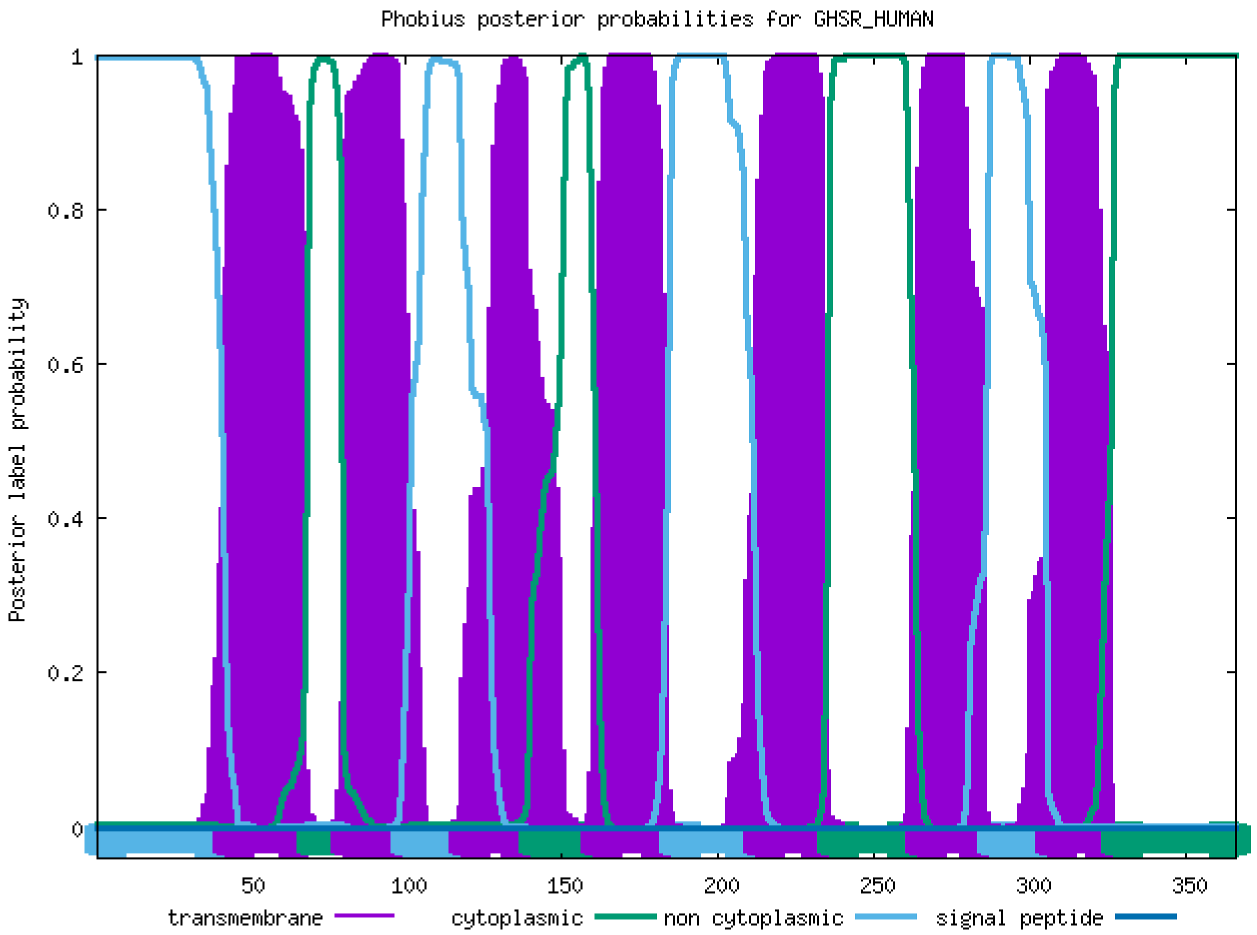

The probability data used in the plot is found here, and the gnuplot script is here.

Prediction of GPBAR_HUMAN

```
ID   GPBAR_HUMAN
FT   TOPO_DOM      1     19       NON CYTOPLASMIC.
FT   TRANSMEM     20     41
FT   TOPO_DOM     42     52       CYTOPLASMIC.
FT   TRANSMEM     53     75
FT   TOPO_DOM     76     80       NON CYTOPLASMIC.
FT   TRANSMEM     81    105
FT   TOPO_DOM    106    125       CYTOPLASMIC.
FT   TRANSMEM    126    145
FT   TOPO_DOM    146    164       NON CYTOPLASMIC.
FT   TRANSMEM    165    186
FT   TOPO_DOM    187    228       CYTOPLASMIC.
FT   TRANSMEM    229    250
FT   TOPO_DOM    251    261       NON CYTOPLASMIC.
FT   TRANSMEM    262    282
FT   TOPO_DOM    283    330       CYTOPLASMIC.
//
```

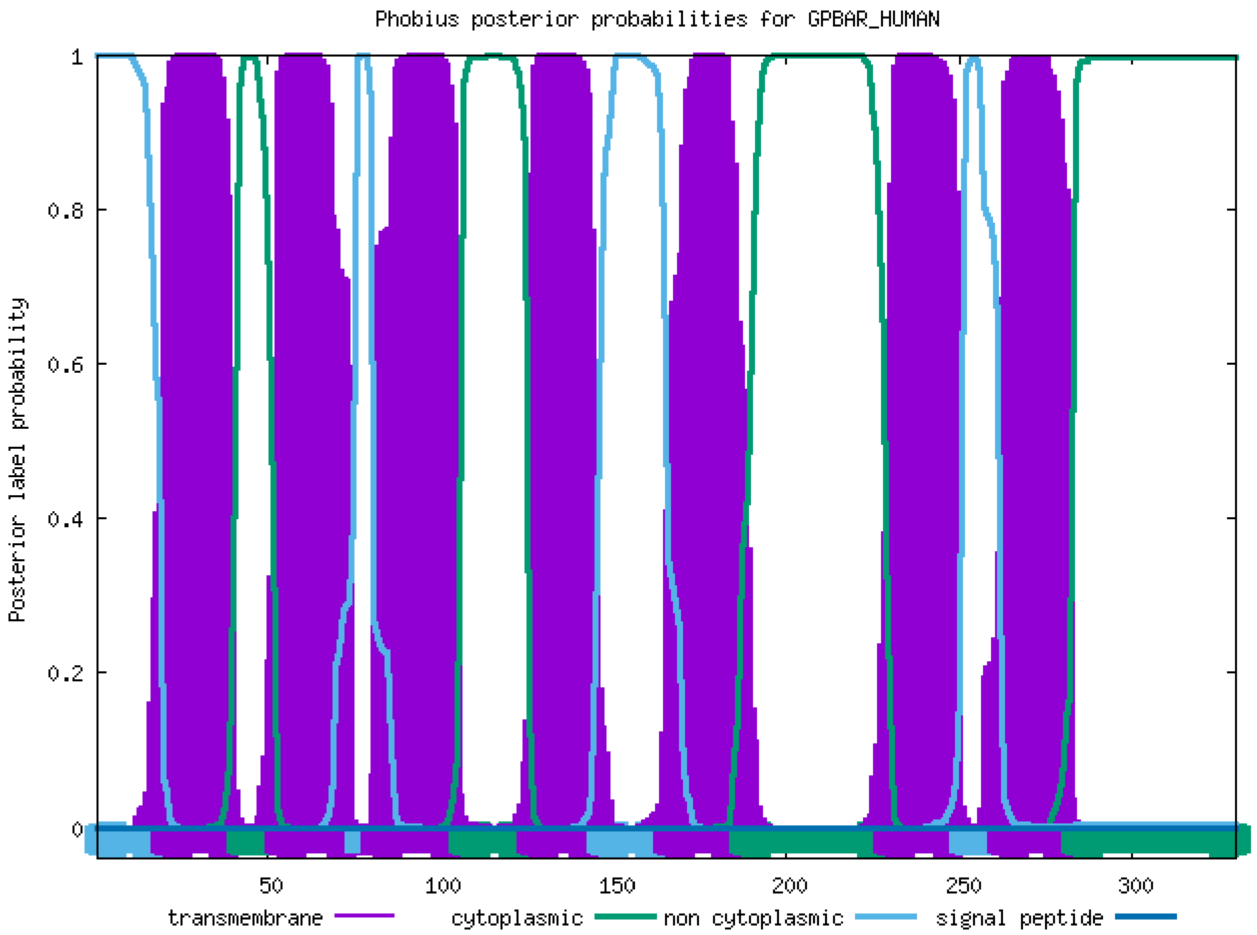

The probability data used in the plot is found here, and the gnuplot script is here.

Prediction of GNRHR_HUMAN

```
ID   GNRHR_HUMAN
FT   TOPO_DOM      1     36       NON CYTOPLASMIC.
FT   TRANSMEM     37     58
FT   TOPO_DOM     59     77       CYTOPLASMIC.
FT   TRANSMEM     78     97
FT   TOPO_DOM     98    116       NON CYTOPLASMIC.
FT   TRANSMEM    117    137
FT   TOPO_DOM    138    157       CYTOPLASMIC.
FT   TRANSMEM    158    178
FT   TOPO_DOM    179    208       NON CYTOPLASMIC.
FT   TRANSMEM    209    230
FT   TOPO_DOM    231    269       CYTOPLASMIC.
FT   TRANSMEM    270    292
FT   TOPO_DOM    293    303       NON CYTOPLASMIC.
FT   TRANSMEM    304    326
FT   TOPO_DOM    327    328       CYTOPLASMIC.
//
```

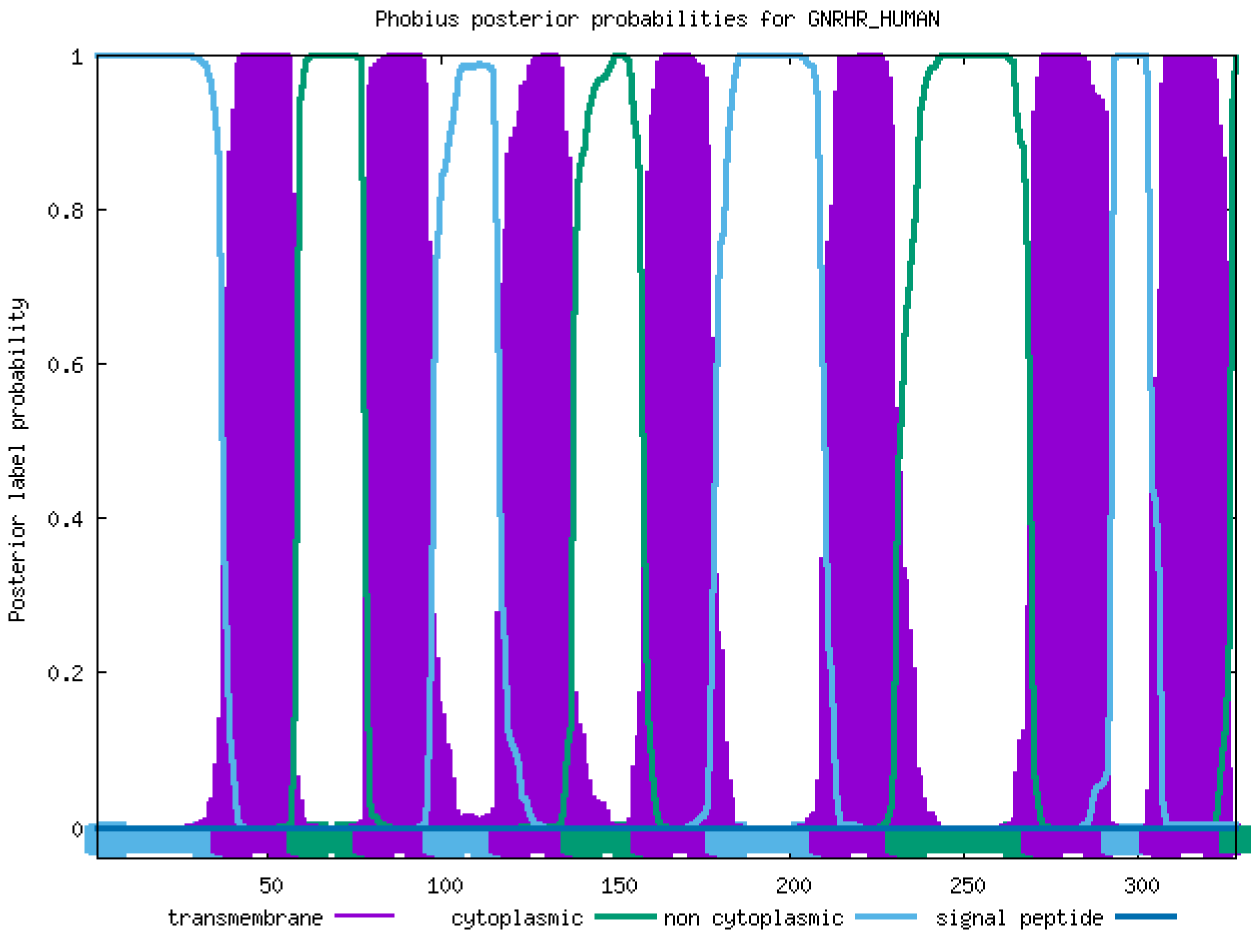

The probability data used in the plot is found here, and the gnuplot script is here.

Prediction of V2R_HUMAN

```
ID    V2R_HUMAN
FT    TOPO_DOM       1      40       NON CYTOPLASMIC.
FT    TRANSMEM      41      62
FT    TOPO_DOM      63      73       CYTOPLASMIC.
FT    TRANSMEM      74      94
FT    TOPO_DOM      95     113       NON CYTOPLASMIC.
FT    TRANSMEM     114     135
FT    TOPO_DOM     136     155       CYTOPLASMIC.
FT    TRANSMEM     156     179
FT    TOPO_DOM     180     204       NON CYTOPLASMIC.
FT    TRANSMEM     205     229
FT    TOPO_DOM     230     273       CYTOPLASMIC.
FT    TRANSMEM     274     296
FT    TOPO_DOM     297     307       NON CYTOPLASMIC.
FT    TRANSMEM     308     328
FT    TOPO_DOM     329     371       CYTOPLASMIC.
//
```

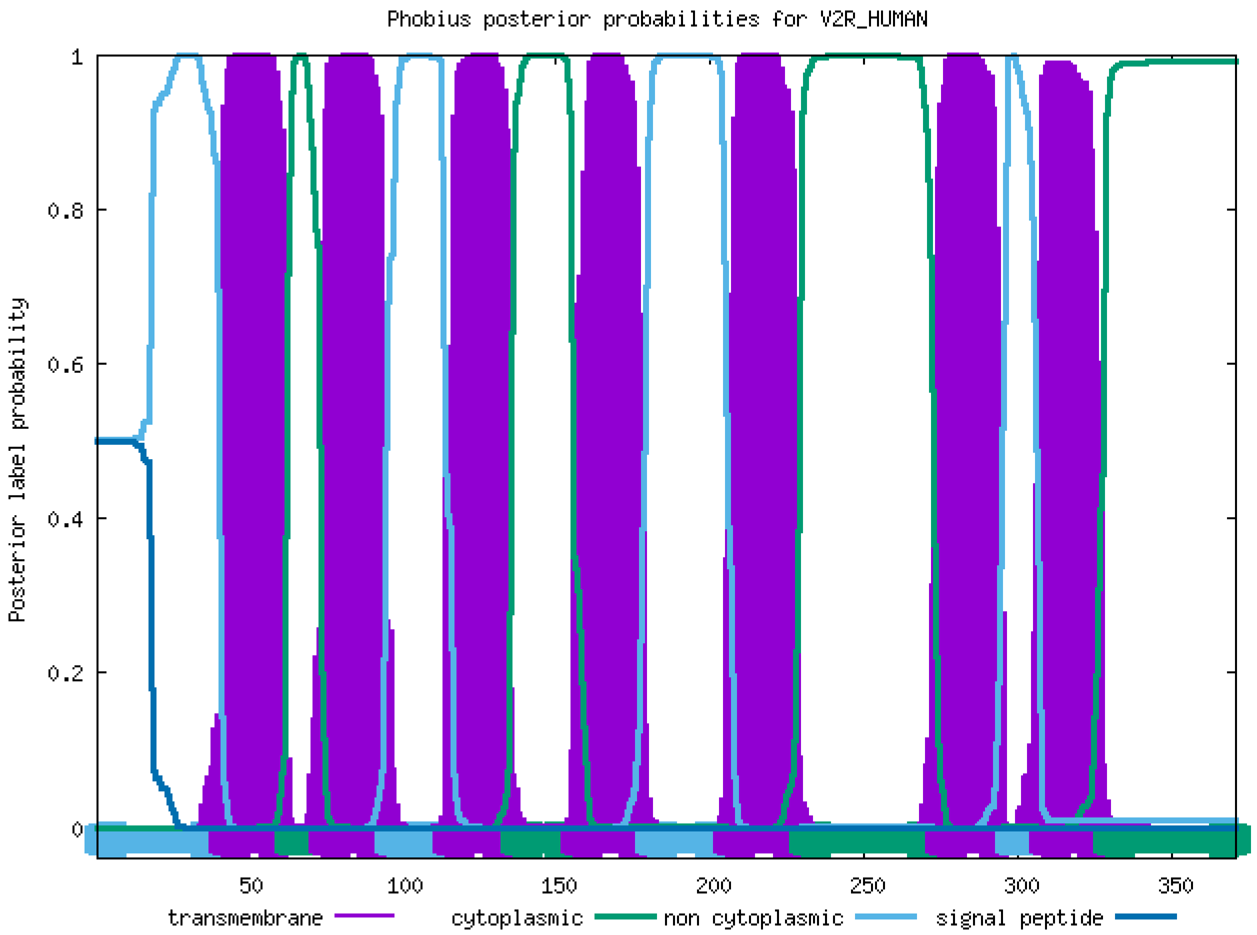

The probability data used in the plot is found here, and the gnuplot script is here.

Prediction of CCKAR_HUMAN

```
ID   CCKAR_HUMAN
FT   TOPO_DOM      1     44       NON CYTOPLASMIC.
FT   TRANSMEM     45     66
FT   TOPO_DOM     67     77       CYTOPLASMIC.
FT   TRANSMEM     78     99
FT   TOPO_DOM    100    118       NON CYTOPLASMIC.
FT   TRANSMEM    119    137
FT   TOPO_DOM    138    157       CYTOPLASMIC.
FT   TRANSMEM    158    179
FT   TOPO_DOM    180    208       NON CYTOPLASMIC.
FT   TRANSMEM    209    232
FT   TOPO_DOM    233    310       CYTOPLASMIC.
FT   TRANSMEM    311    332
FT   TOPO_DOM    333    351       NON CYTOPLASMIC.
FT   TRANSMEM    352    372
FT   TOPO_DOM    373    428       CYTOPLASMIC.
//
```

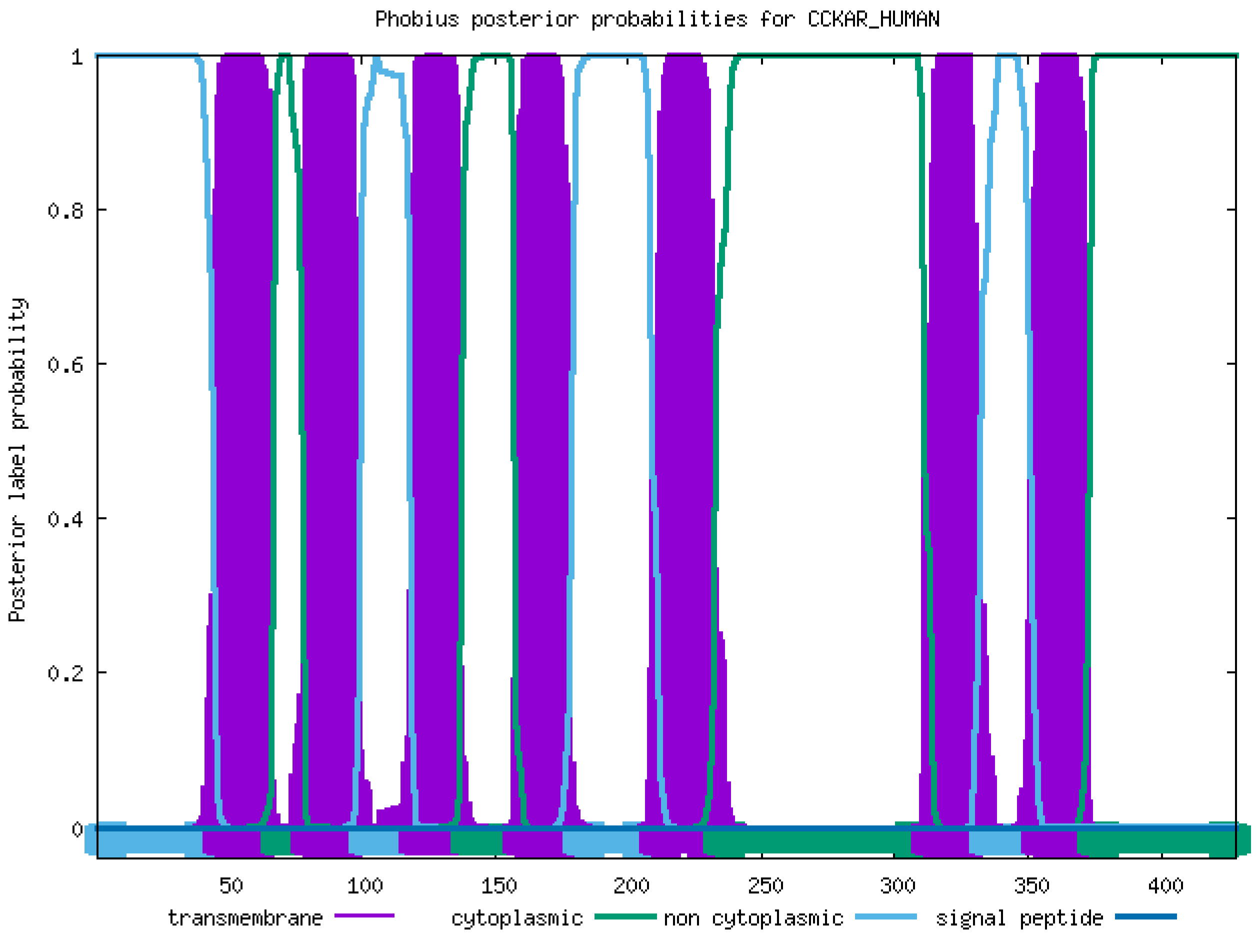

The probability data used in the plot is found here, and the gnuplot script is here.

Prediction of GASR_HUMAN

```
ID   GASR_HUMAN
FT   TOPO_DOM      1     57       NON CYTOPLASMIC.
FT   TRANSMEM     58     79
FT   TOPO_DOM     80     90       CYTOPLASMIC.
FT   TRANSMEM     91    112
FT   TOPO_DOM    113    131       NON CYTOPLASMIC.
FT   TRANSMEM    132    150
FT   TOPO_DOM    151    170       CYTOPLASMIC.
FT   TRANSMEM    171    192
FT   TOPO_DOM    193    217       NON CYTOPLASMIC.
FT   TRANSMEM    218    241
FT   TOPO_DOM    242    330       CYTOPLASMIC.
FT   TRANSMEM    331    350
FT   TOPO_DOM    351    369       NON CYTOPLASMIC.
FT   TRANSMEM    370    392
FT   TOPO_DOM    393    447       CYTOPLASMIC.
//
```

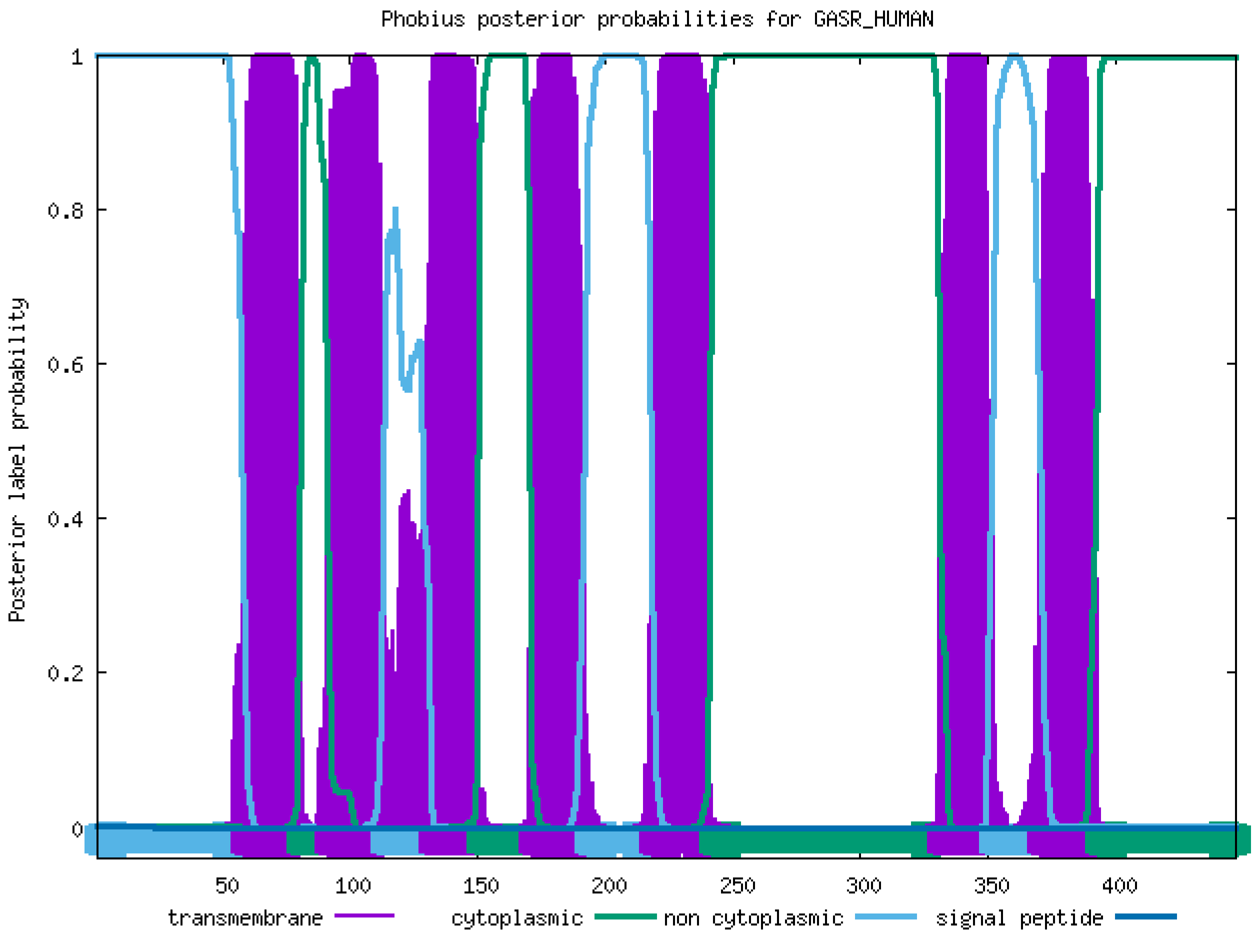

The probability data used in the plot is found here, and the gnuplot script is here.

Prediction of BKRB1_HUMAN

```
ID    BKRB1_HUMAN
FT    TOPO_DOM      1     42       NON CYTOPLASMIC.
FT    TRANSMEM     43     63
FT    TOPO_DOM     64     74       CYTOPLASMIC.
FT    TRANSMEM     75     94
FT    TOPO_DOM     95    105       NON CYTOPLASMIC.
FT    TRANSMEM    106    132
FT    TOPO_DOM    133    152       CYTOPLASMIC.
FT    TRANSMEM    153    175
FT    TOPO_DOM    176    207       NON CYTOPLASMIC.
FT    TRANSMEM    208    229
FT    TOPO_DOM    230    249       CYTOPLASMIC.
FT    TRANSMEM    250    276
FT    TOPO_DOM    277    295       NON CYTOPLASMIC.
FT    TRANSMEM    296    319
FT    TOPO_DOM    320    353       CYTOPLASMIC.
//
```

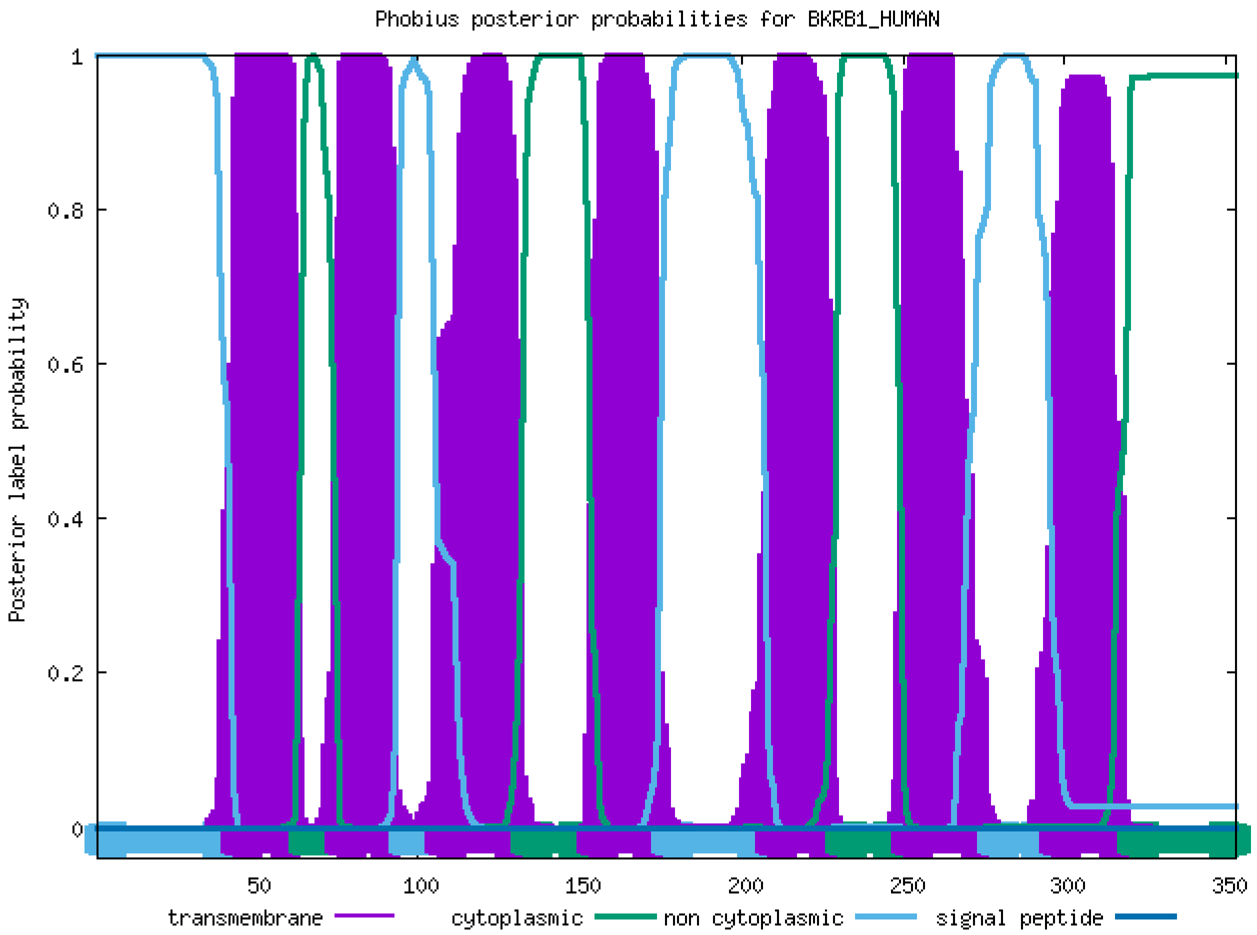

The probability data used in the plot is found here, and the gnuplot script is here.

Prediction of BKRB2_HUMAN

```
ID    BKRB2_HUMAN
FT    TOPO_DOM       1      62       NON CYTOPLASMIC.
FT    TRANSMEM      63      84
FT    TOPO_DOM      85      95       CYTOPLASMIC.
FT    TRANSMEM      96     115
FT    TOPO_DOM     116     134       NON CYTOPLASMIC.
FT    TRANSMEM     135     153
FT    TOPO_DOM     154     173       CYTOPLASMIC.
FT    TRANSMEM     174     195
FT    TOPO_DOM     196     225       NON CYTOPLASMIC.
FT    TRANSMEM     226     247
FT    TOPO_DOM     248     267       CYTOPLASMIC.
FT    TRANSMEM     268     292
FT    TOPO_DOM     293     311       NON CYTOPLASMIC.
FT    TRANSMEM     312     335
FT    TOPO_DOM     336     391       CYTOPLASMIC.
//
```

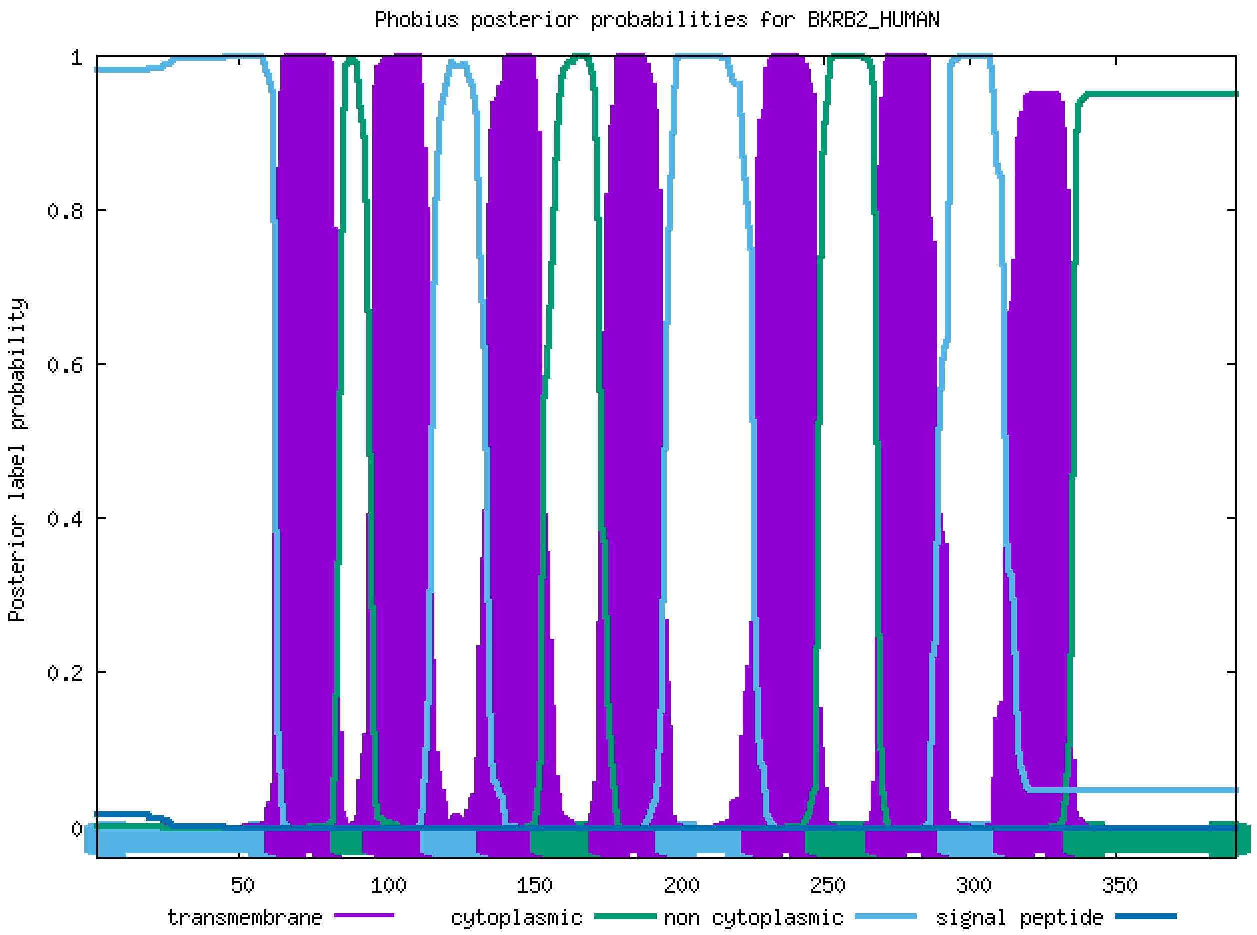

The probability data used in the plot is found here, and the gnuplot script is here.

Prediction of MRGX1_HUMAN

```
ID   MRGX1_HUMAN
FT   TOPO_DOM      1     27       NON CYTOPLASMIC.
FT   TRANSMEM     28     52
FT   TOPO_DOM     53     63       CYTOPLASMIC.
FT   TRANSMEM     64     85
FT   TOPO_DOM     86     96       NON CYTOPLASMIC.
FT   TRANSMEM     97    117
FT   TOPO_DOM    118    137       CYTOPLASMIC.
FT   TRANSMEM    138    166
FT   TOPO_DOM    167    177       NON CYTOPLASMIC.
FT   TRANSMEM    178    202
FT   TOPO_DOM    203    213       CYTOPLASMIC.
FT   TRANSMEM    214    242
FT   TOPO_DOM    243    253       NON CYTOPLASMIC.
FT   TRANSMEM    254    275
FT   TOPO_DOM    276    322       CYTOPLASMIC.
//
```

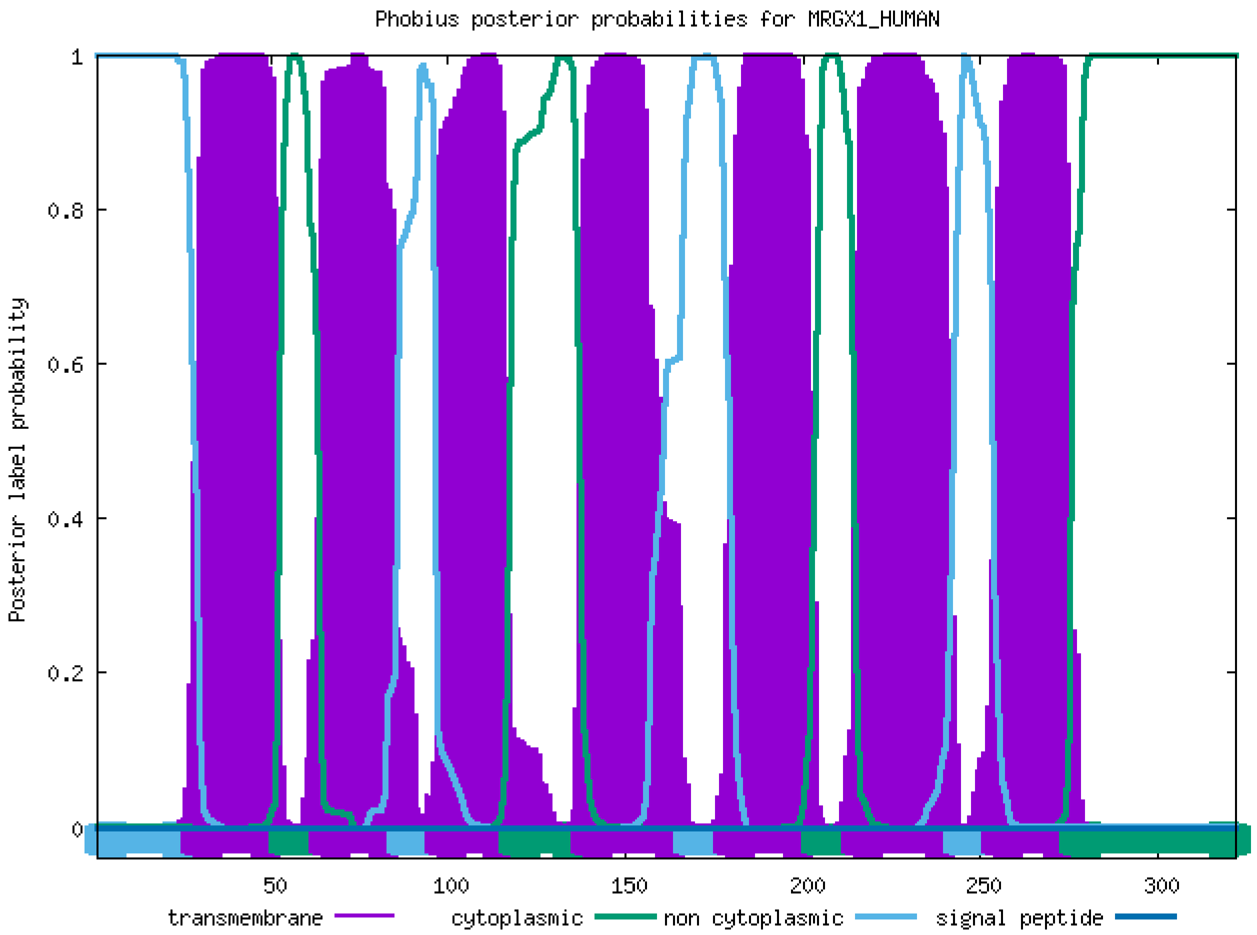

The probability data used in the plot is found here, and the gnuplot script is here.

Prediction of MRGX2_HUMAN

```
ID    MRGX2_HUMAN
FT    TOPO_DOM      1     30       NON CYTOPLASMIC.
FT    TRANSMEM     31     55
FT    TOPO_DOM     56     66       CYTOPLASMIC.
FT    TRANSMEM     67     91
FT    TOPO_DOM     92    102       NON CYTOPLASMIC.
FT    TRANSMEM    103    123
FT    TOPO_DOM    124    143       CYTOPLASMIC.
FT    TRANSMEM    144    163
FT    TOPO_DOM    164    182       NON CYTOPLASMIC.
FT    TRANSMEM    183    209
FT    TOPO_DOM    210    220       CYTOPLASMIC.
FT    TRANSMEM    221    250
FT    TOPO_DOM    251    261       NON CYTOPLASMIC.
FT    TRANSMEM    262    282
FT    TOPO_DOM    283    330       CYTOPLASMIC.
//
```

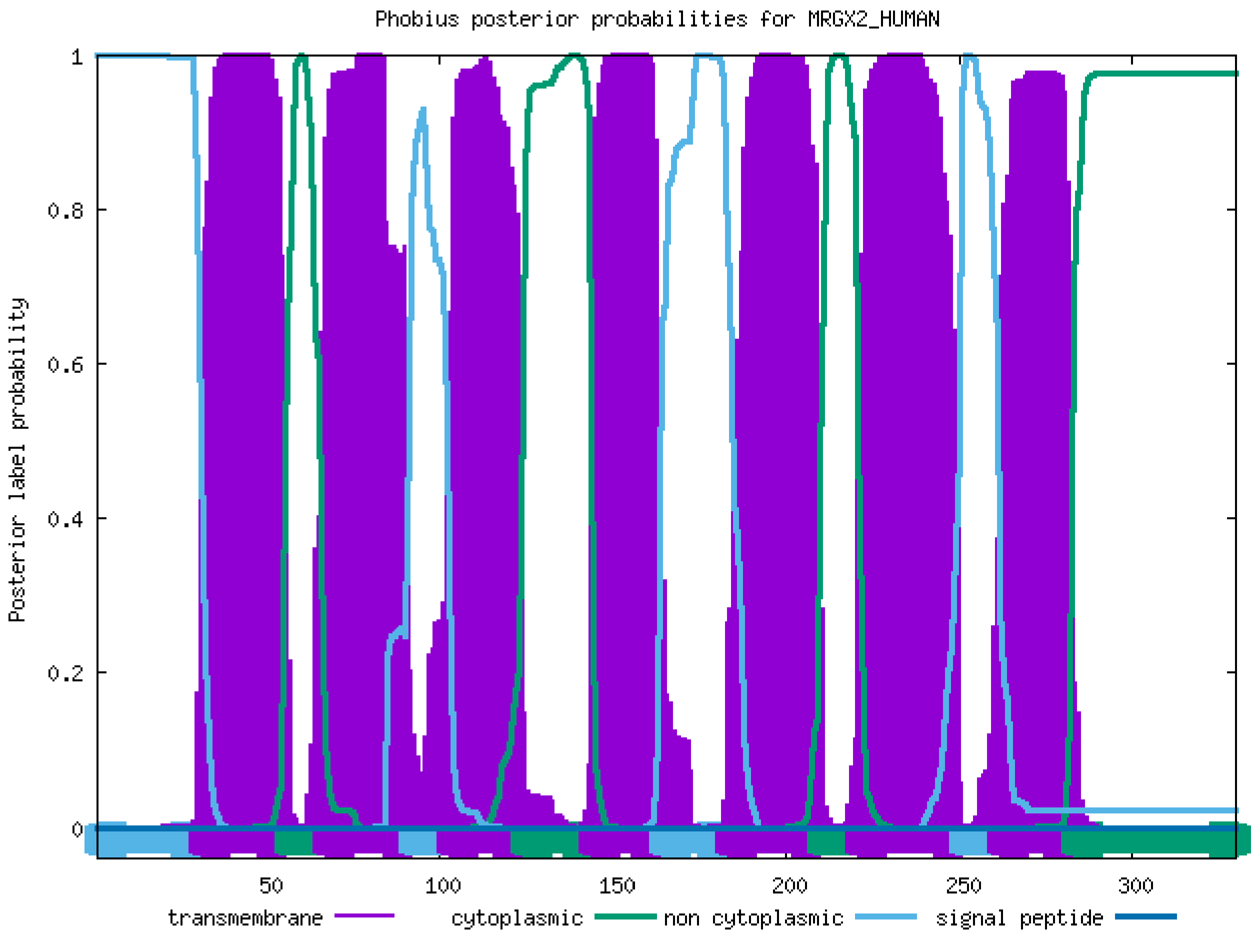

The probability data used in the plot is found here, and the gnuplot script is here.

Prediction of LSHR_HUMAN

```
ID    LSHR_HUMAN
FT    SIGNAL         1      24
FT    REGION         1       5       N-REGION.
FT    REGION         6      17       H-REGION.
FT    REGION        18      24       C-REGION.
FT    TOPO_DOM      25     363       NON CYTOPLASMIC.
FT    TRANSMEM     364     385
FT    TOPO_DOM     386     396       CYTOPLASMIC.
FT    TRANSMEM     397     418
FT    TOPO_DOM     419     441       NON CYTOPLASMIC.
FT    TRANSMEM     442     462
FT    TOPO_DOM     463     482       CYTOPLASMIC.
FT    TRANSMEM     483     506
FT    TOPO_DOM     507     525       NON CYTOPLASMIC.
FT    TRANSMEM     526     551
FT    TOPO_DOM     552     571       CYTOPLASMIC.
FT    TRANSMEM     572     594
FT    TOPO_DOM     595     605       NON CYTOPLASMIC.
FT    TRANSMEM     606     626
FT    TOPO_DOM     627     699       CYTOPLASMIC.
//
```

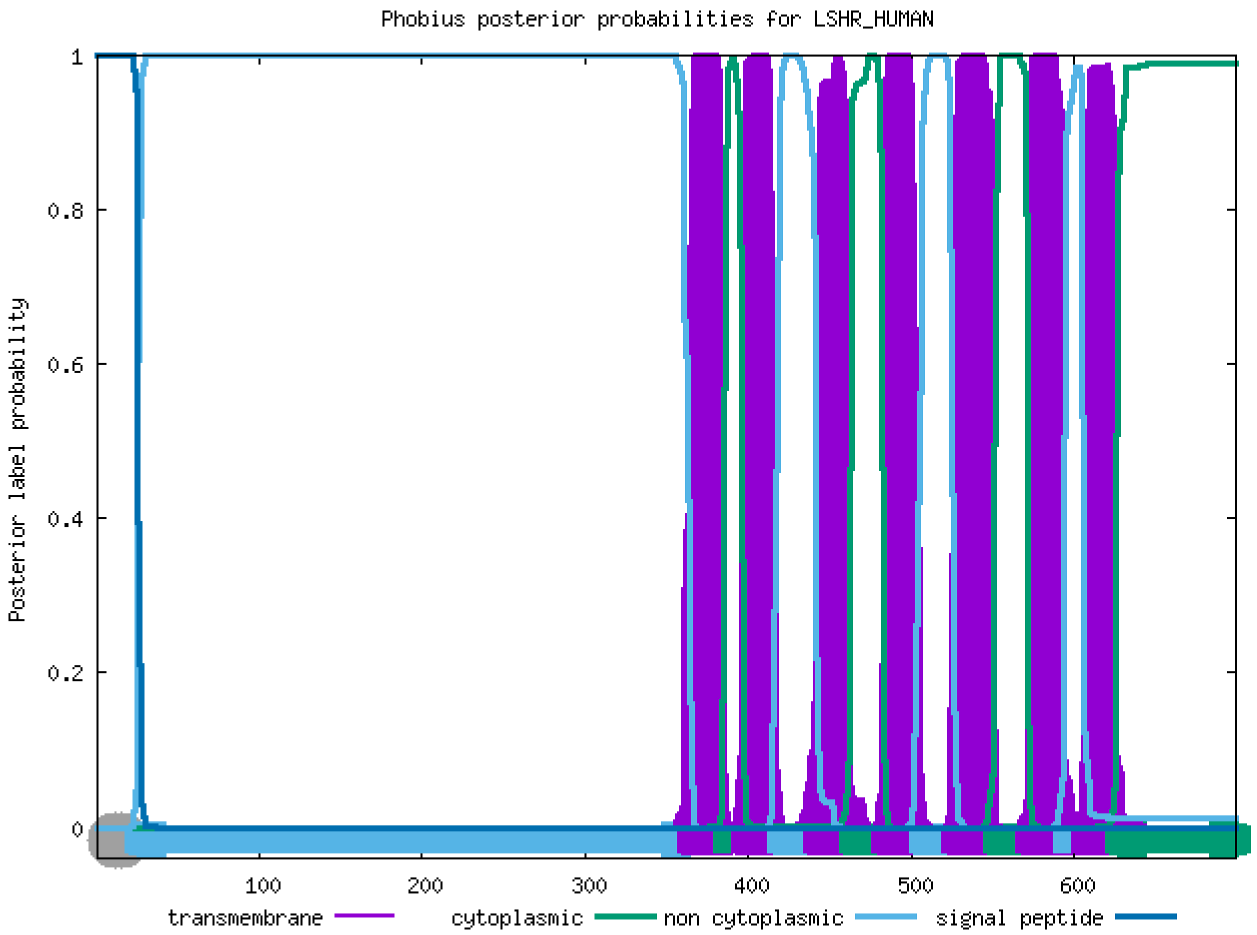

The probability data used in the plot is found here, and the gnuplot script is here.

Prediction of SSR2_HUMAN

```
ID    SSR2_HUMAN
FT    TOPO_DOM       1      47       NON CYTOPLASMIC.
FT    TRANSMEM      48      69
FT    TOPO_DOM      70      80       CYTOPLASMIC.
FT    TRANSMEM      81     104
FT    TOPO_DOM     105     123       NON CYTOPLASMIC.
FT    TRANSMEM     124     143
FT    TOPO_DOM     144     163       CYTOPLASMIC.
FT    TRANSMEM     164     183
FT    TOPO_DOM     184     202       NON CYTOPLASMIC.
FT    TRANSMEM     203     233
FT    TOPO_DOM     234     253       CYTOPLASMIC.
FT    TRANSMEM     254     274
FT    TOPO_DOM     275     293       NON CYTOPLASMIC.
FT    TRANSMEM     294     315
FT    TOPO_DOM     316     369       CYTOPLASMIC.
//
```

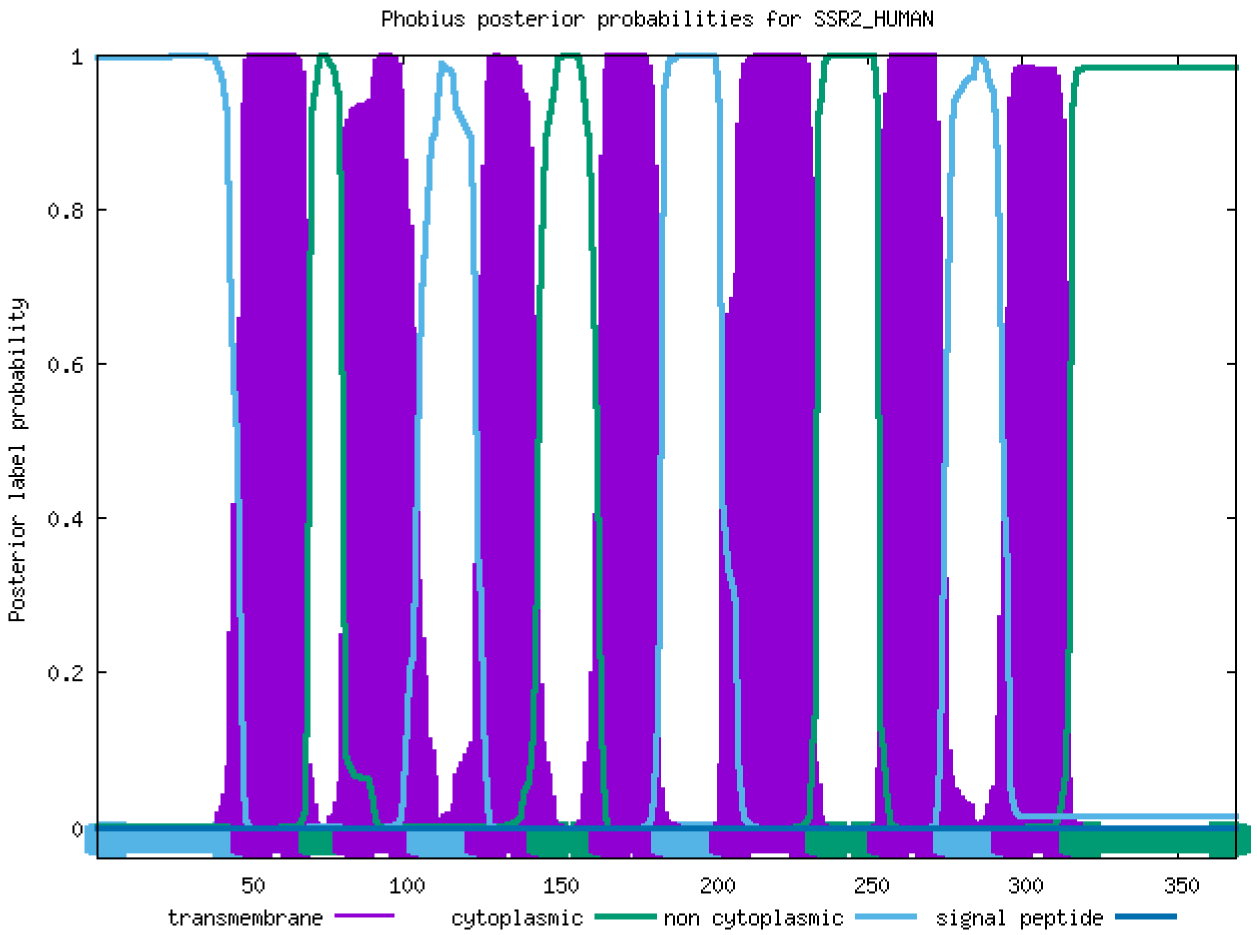

The probability data used in the plot is found here, and the gnuplot script is here.

Prediction of SSR4_HUMAN

```
ID    SSR4_HUMAN
FT    TOPO_DOM      1     47       NON CYTOPLASMIC.
FT    TRANSMEM     48     73
FT    TOPO_DOM     74     84       CYTOPLASMIC.
FT    TRANSMEM     85    108
FT    TOPO_DOM    109    127       NON CYTOPLASMIC.
FT    TRANSMEM    128    147
FT    TOPO_DOM    148    167       CYTOPLASMIC.
FT    TRANSMEM    168    185
FT    TOPO_DOM    186    204       NON CYTOPLASMIC.
FT    TRANSMEM    205    236
FT    TOPO_DOM    237    256       CYTOPLASMIC.
FT    TRANSMEM    257    275
FT    TOPO_DOM    276    294       NON CYTOPLASMIC.
FT    TRANSMEM    295    314
FT    TOPO_DOM    315    388       CYTOPLASMIC.
//
```

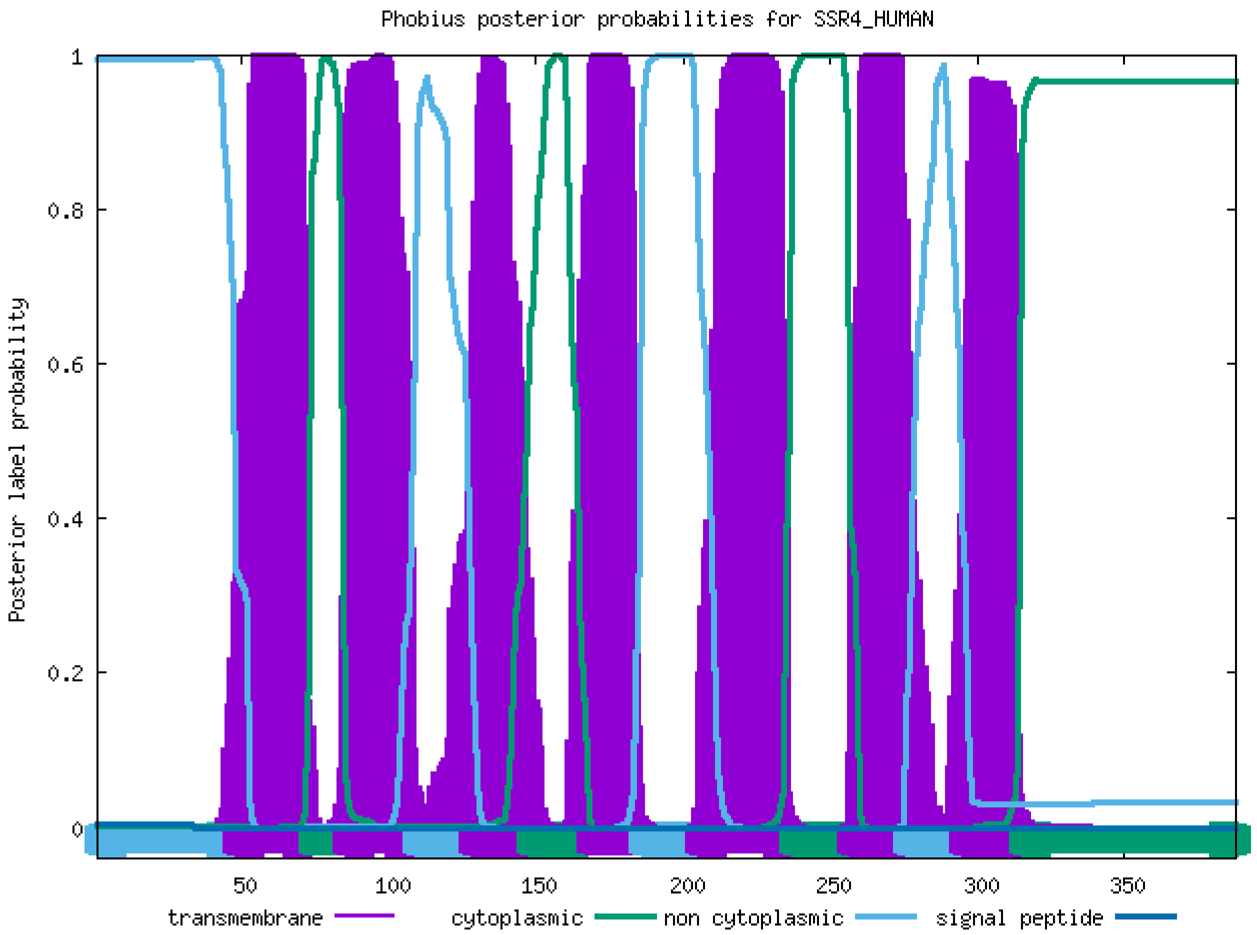

The probability data used in the plot is found here, and the gnuplot script is here.

Prediction of GALR1_HUMAN

```
ID    GALR1_HUMAN
FT    TOPO_DOM      1     33       NON CYTOPLASMIC.
FT    TRANSMEM     34     59
FT    TOPO_DOM     60     70       CYTOPLASMIC.
FT    TRANSMEM     71     98
FT    TOPO_DOM     99    109       NON CYTOPLASMIC.
FT    TRANSMEM    110    131
FT    TOPO_DOM    132    151       CYTOPLASMIC.
FT    TRANSMEM    152    172
FT    TOPO_DOM    173    198       NON CYTOPLASMIC.
FT    TRANSMEM    199    224
FT    TOPO_DOM    225    244       CYTOPLASMIC.
FT    TRANSMEM    245    262
FT    TOPO_DOM    263    281       NON CYTOPLASMIC.
FT    TRANSMEM    282    306
FT    TOPO_DOM    307    349       CYTOPLASMIC.
//
```

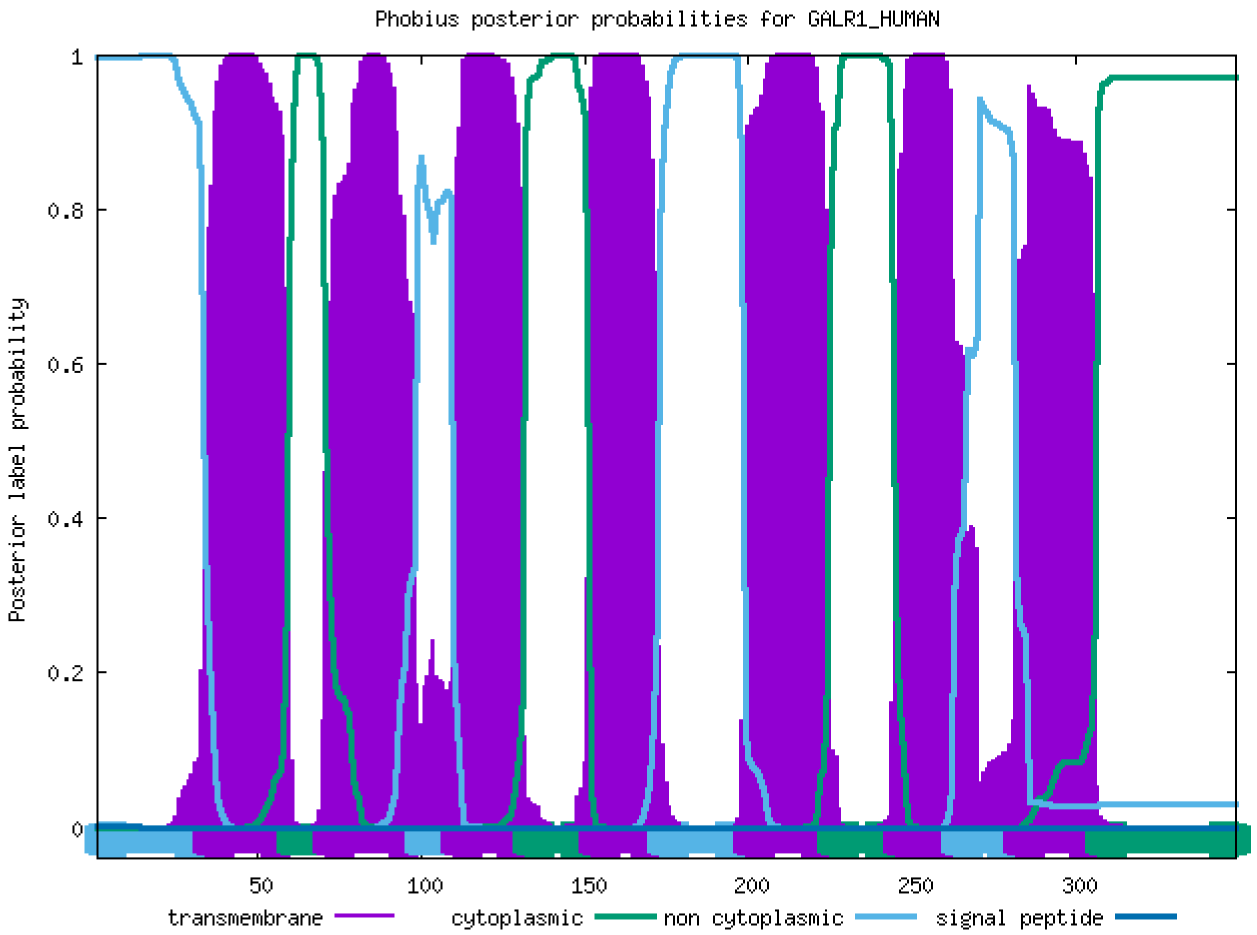

The probability data used in the plot is found here, and the gnuplot script is here.

Prediction of GALR2_HUMAN

```
ID    GALR2_HUMAN
FT    TOPO_DOM      1      25       NON CYTOPLASMIC.
FT    TRANSMEM     26      51
FT    TOPO_DOM     52      62       CYTOPLASMIC.
FT    TRANSMEM     63      88
FT    TOPO_DOM     89      99       NON CYTOPLASMIC.
FT    TRANSMEM    100     121
FT    TOPO_DOM    122     141       CYTOPLASMIC.
FT    TRANSMEM    142     163
FT    TOPO_DOM    164     185       NON CYTOPLASMIC.
FT    TRANSMEM    186     208
FT    TOPO_DOM    209     237       CYTOPLASMIC.
FT    TRANSMEM    238     263
FT    TOPO_DOM    264     274       NON CYTOPLASMIC.
FT    TRANSMEM    275     295
FT    TOPO_DOM    296     387       CYTOPLASMIC.
//
```

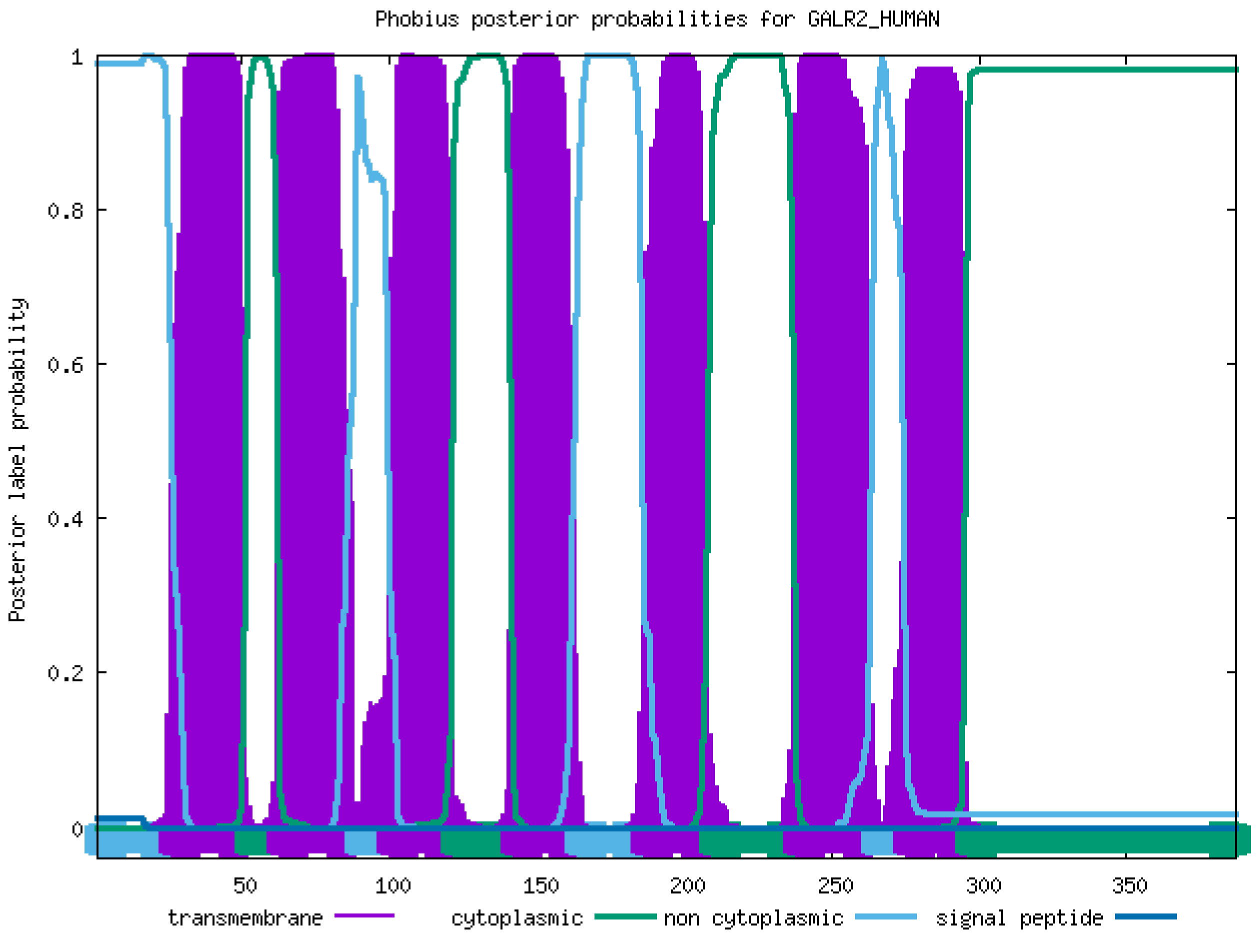

The probability data used in the plot is found here, and the gnuplot script is here.

Prediction of TSHR_HUMAN

```
ID   TSHR_HUMAN
FT   SIGNAL        1     31
FT   REGION        1      5       N-REGION.
FT   REGION        6     13       H-REGION.
FT   REGION       14     31       C-REGION.
FT   TOPO_DOM     32    418       NON CYTOPLASMIC.
FT   TRANSMEM    419    440
FT   TOPO_DOM    441    451       CYTOPLASMIC.
FT   TRANSMEM    452    477
FT   TOPO_DOM    478    496       NON CYTOPLASMIC.
FT   TRANSMEM    497    517
FT   TOPO_DOM    518    537       CYTOPLASMIC.
FT   TRANSMEM    538    560
FT   TOPO_DOM    561    579       NON CYTOPLASMIC.
FT   TRANSMEM    580    606
FT   TOPO_DOM    607    626       CYTOPLASMIC.
FT   TRANSMEM    627    649
FT   TOPO_DOM    650    660       NON CYTOPLASMIC.
FT   TRANSMEM    661    681
FT   TOPO_DOM    682    764       CYTOPLASMIC.
//
```

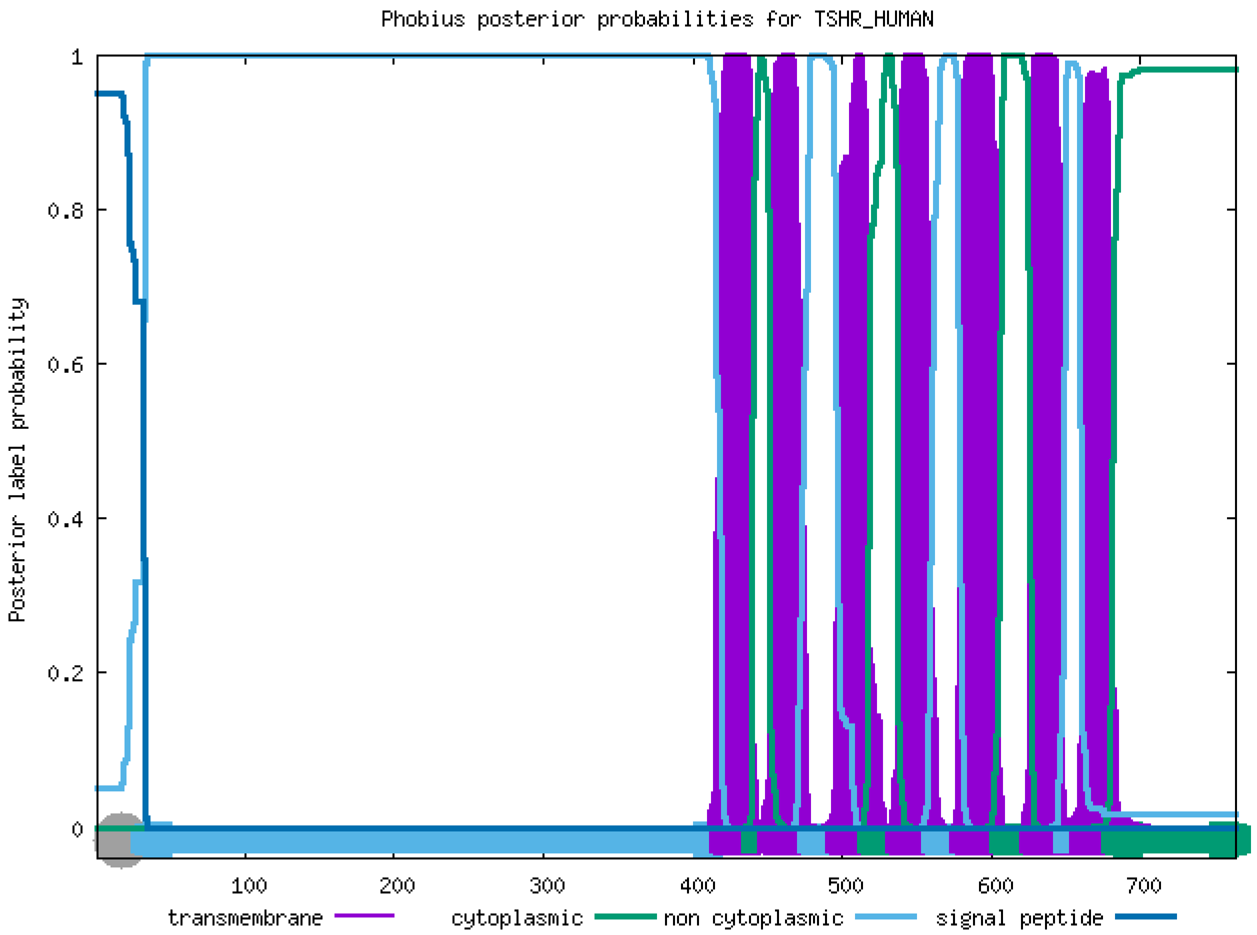

The probability data used in the plot is found here, and the gnuplot script is here.

Prediction of TRFR_HUMAN

```
ID   TRFR_HUMAN
FT   TOPO_DOM      1     25       NON CYTOPLASMIC.
FT   TRANSMEM     26     50
FT   TOPO_DOM     51     61       CYTOPLASMIC.
FT   TRANSMEM     62     80
FT   TOPO_DOM     81     91       NON CYTOPLASMIC.
FT   TRANSMEM     92    109
FT   TOPO_DOM    110    144       CYTOPLASMIC.
FT   TRANSMEM    145    163
FT   TOPO_DOM    164    196       NON CYTOPLASMIC.
FT   TRANSMEM    197    219
FT   TOPO_DOM    220    263       CYTOPLASMIC.
FT   TRANSMEM    264    280
FT   TOPO_DOM    281    299       NON CYTOPLASMIC.
FT   TRANSMEM    300    323
FT   TOPO_DOM    324    398       CYTOPLASMIC.
//
```

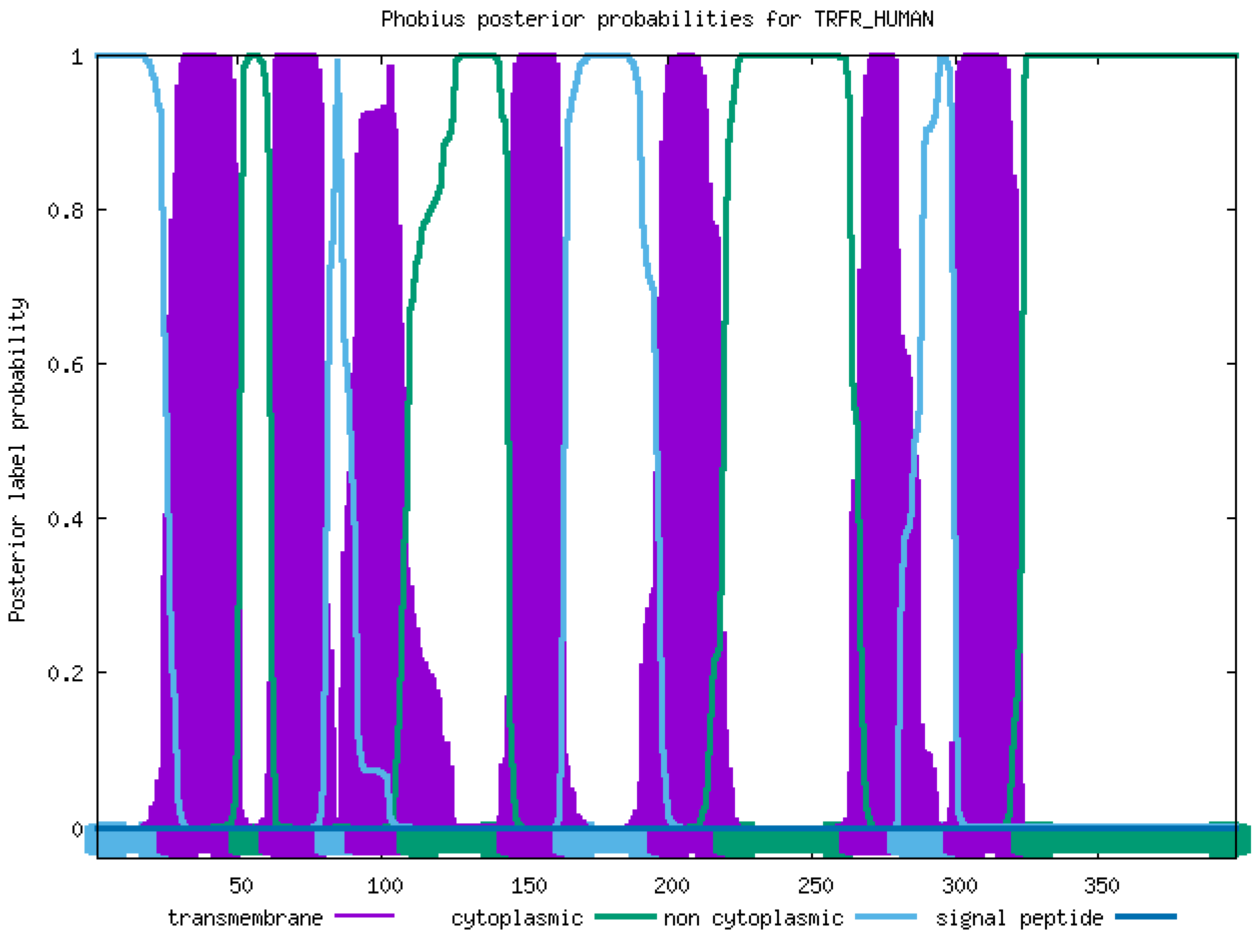

The probability data used in the plot is found here, and the gnuplot script is here.

Prediction of GRPR_HUMAN

```
ID   GRPR_HUMAN
FT   TOPO_DOM      1     38       NON CYTOPLASMIC.
FT   TRANSMEM     39     65
FT   TOPO_DOM     66     76       CYTOPLASMIC.
FT   TRANSMEM     77     98
FT   TOPO_DOM     99    117       NON CYTOPLASMIC.
FT   TRANSMEM    118    136
FT   TOPO_DOM    137    156       CYTOPLASMIC.
FT   TRANSMEM    157    178
FT   TOPO_DOM    179    208       NON CYTOPLASMIC.
FT   TRANSMEM    209    232
FT   TOPO_DOM    233    261       CYTOPLASMIC.
FT   TRANSMEM    262    286
FT   TOPO_DOM    287    297       NON CYTOPLASMIC.
FT   TRANSMEM    298    325
FT   TOPO_DOM    326    384       CYTOPLASMIC.
//
```

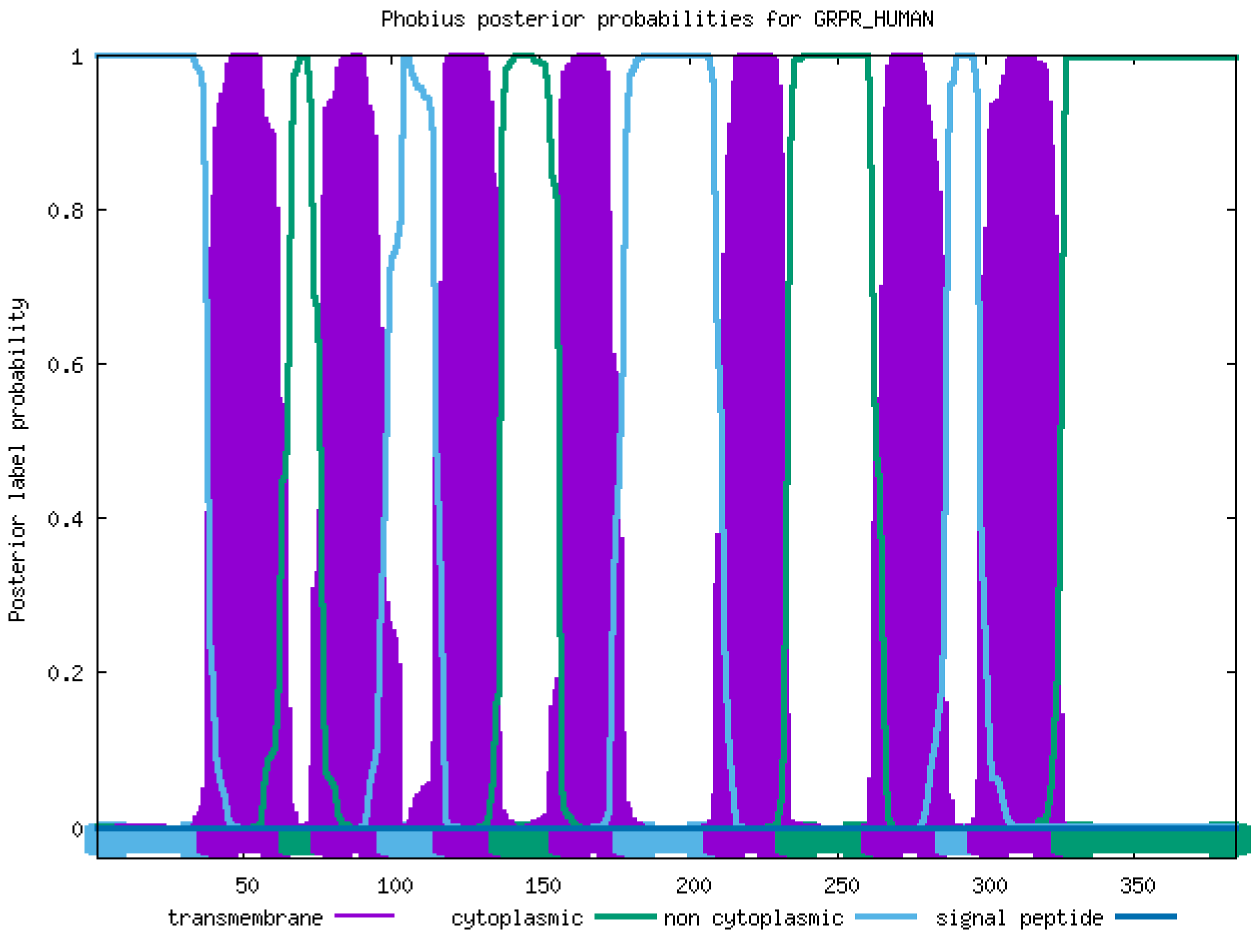

The probability data used in the plot is found here, and the gnuplot script is here.

Prediction of O51E2_HUMAN

```
ID   O51E2_HUMAN
FT   TOPO_DOM      1     23       NON CYTOPLASMIC.
FT   TRANSMEM     24     47
FT   TOPO_DOM     48     58       CYTOPLASMIC.
FT   TRANSMEM     59     86
FT   TOPO_DOM     87     97       NON CYTOPLASMIC.
FT   TRANSMEM     98    119
FT   TOPO_DOM    120    138       CYTOPLASMIC.
FT   TRANSMEM    139    162
FT   TOPO_DOM    163    194       NON CYTOPLASMIC.
FT   TRANSMEM    195    220
FT   TOPO_DOM    221    240       CYTOPLASMIC.
FT   TRANSMEM    241    264
FT   TOPO_DOM    265    269       NON CYTOPLASMIC.
FT   TRANSMEM    270    291
FT   TOPO_DOM    292    320       CYTOPLASMIC.
//
```

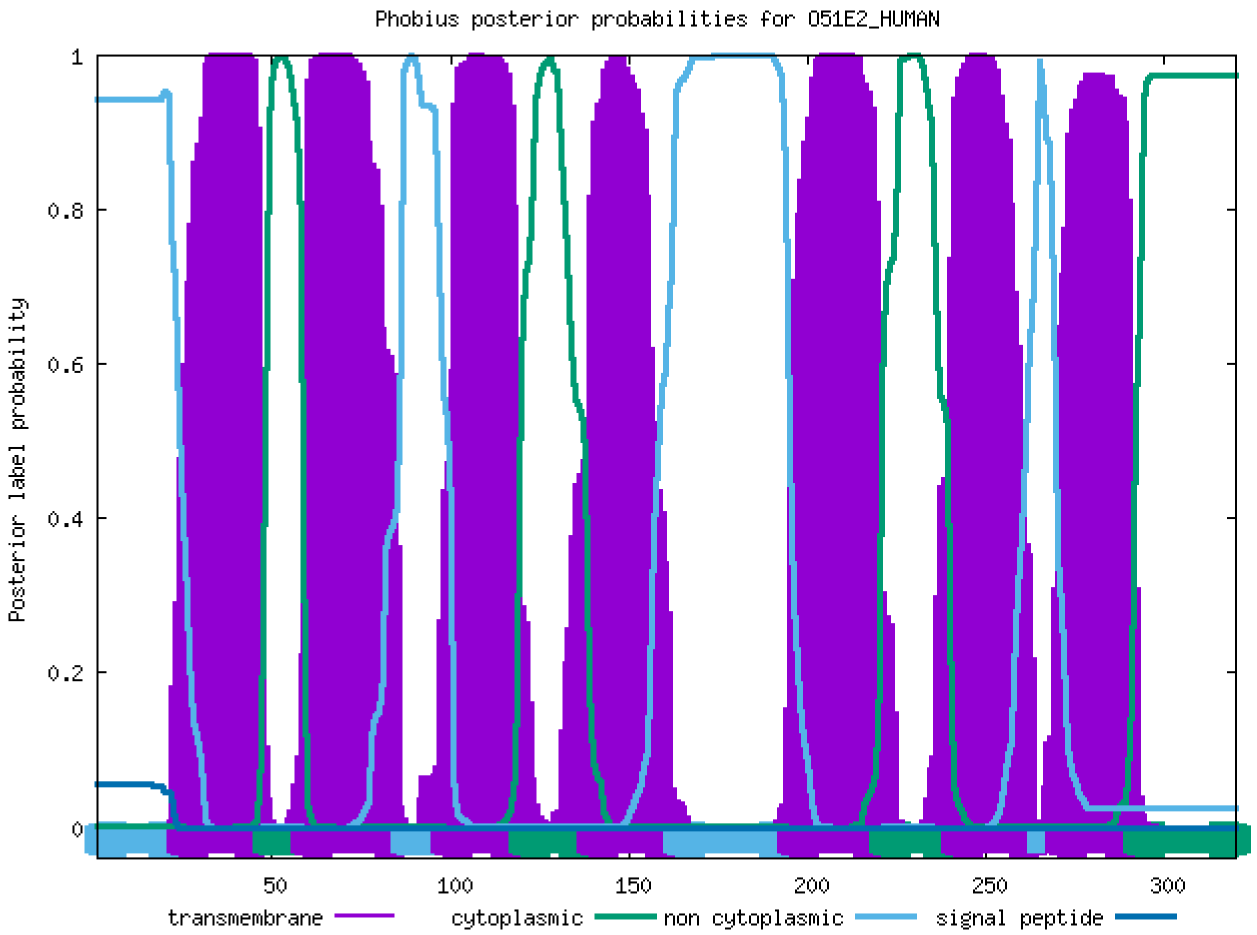

The probability data used in the plot is found here, and the gnuplot script is here.

Prediction of FSHR_HUMAN

```
ID   FSHR_HUMAN
FT   SIGNAL        1     17
FT   REGION        1      1       N-REGION.
FT   REGION        2     12       H-REGION.
FT   REGION       13     17       C-REGION.
FT   TOPO_DOM     18    366       NON CYTOPLASMIC.
FT   TRANSMEM    367    387
FT   TOPO_DOM    388    398       CYTOPLASMIC.
FT   TRANSMEM    399    424
FT   TOPO_DOM    425    443       NON CYTOPLASMIC.
FT   TRANSMEM    444    465
FT   TOPO_DOM    466    485       CYTOPLASMIC.
FT   TRANSMEM    486    509
FT   TOPO_DOM    510    528       NON CYTOPLASMIC.
FT   TRANSMEM    529    554
FT   TOPO_DOM    555    574       CYTOPLASMIC.
FT   TRANSMEM    575    597
FT   TOPO_DOM    598    608       NON CYTOPLASMIC.
FT   TRANSMEM    609    629
FT   TOPO_DOM    630    695       CYTOPLASMIC.
//
```

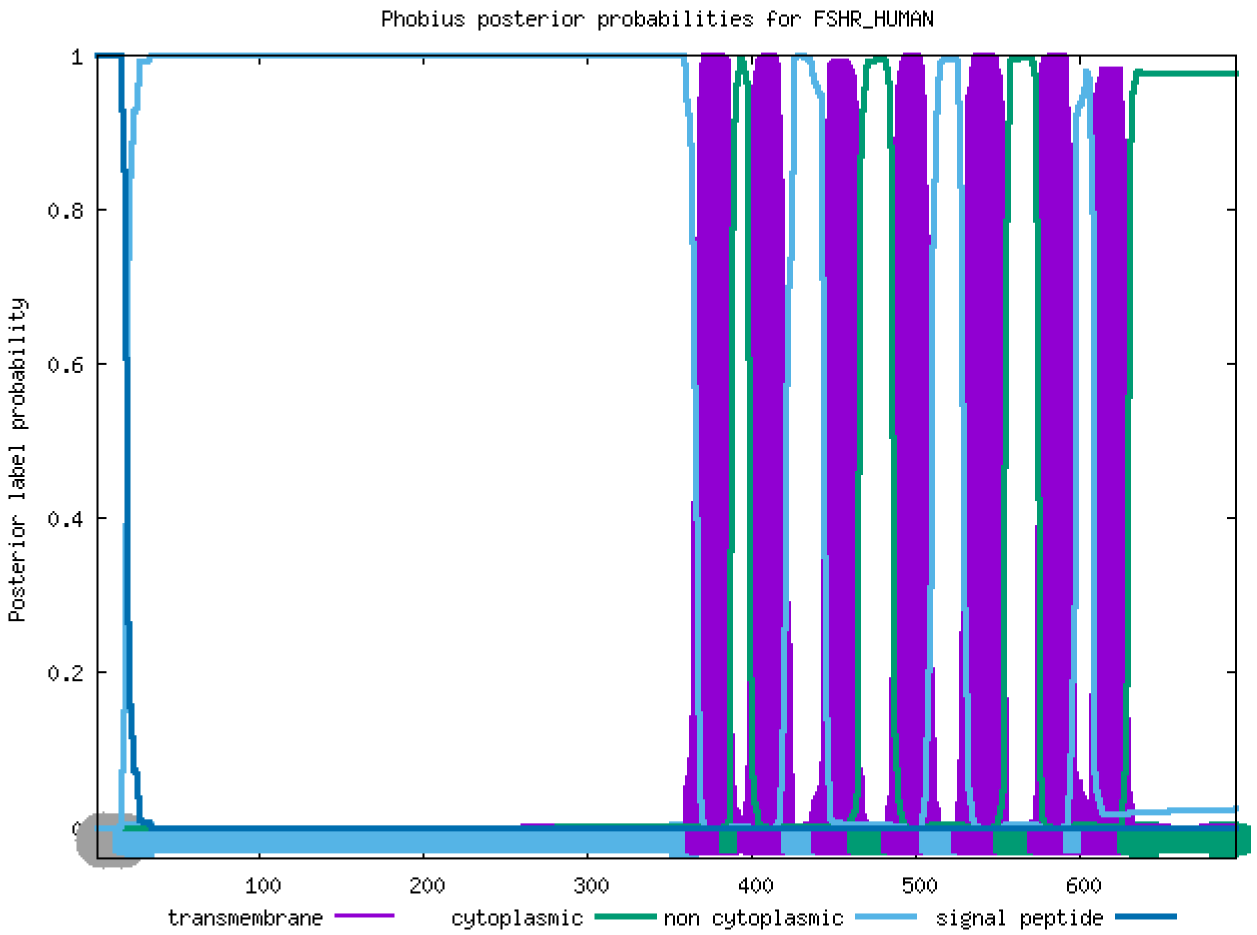

The probability data used in the plot is found here, and the gnuplot script is here.

Prediction of RXFP1_HUMAN

```
ID   RXFP1_HUMAN
FT   TOPO_DOM      1    409       NON CYTOPLASMIC.
FT   TRANSMEM    410    428
FT   TOPO_DOM    429    439       CYTOPLASMIC.
FT   TRANSMEM    440    464
FT   TOPO_DOM    465    486       NON CYTOPLASMIC.
FT   TRANSMEM    487    508
FT   TOPO_DOM    509    528       CYTOPLASMIC.
FT   TRANSMEM    529    547
FT   TOPO_DOM    548    577       NON CYTOPLASMIC.
FT   TRANSMEM    578    599
FT   TOPO_DOM    600    629       CYTOPLASMIC.
FT   TRANSMEM    630    655
FT   TOPO_DOM    656    660       NON CYTOPLASMIC.
FT   TRANSMEM    661    684
FT   TOPO_DOM    685    757       CYTOPLASMIC.
//
```

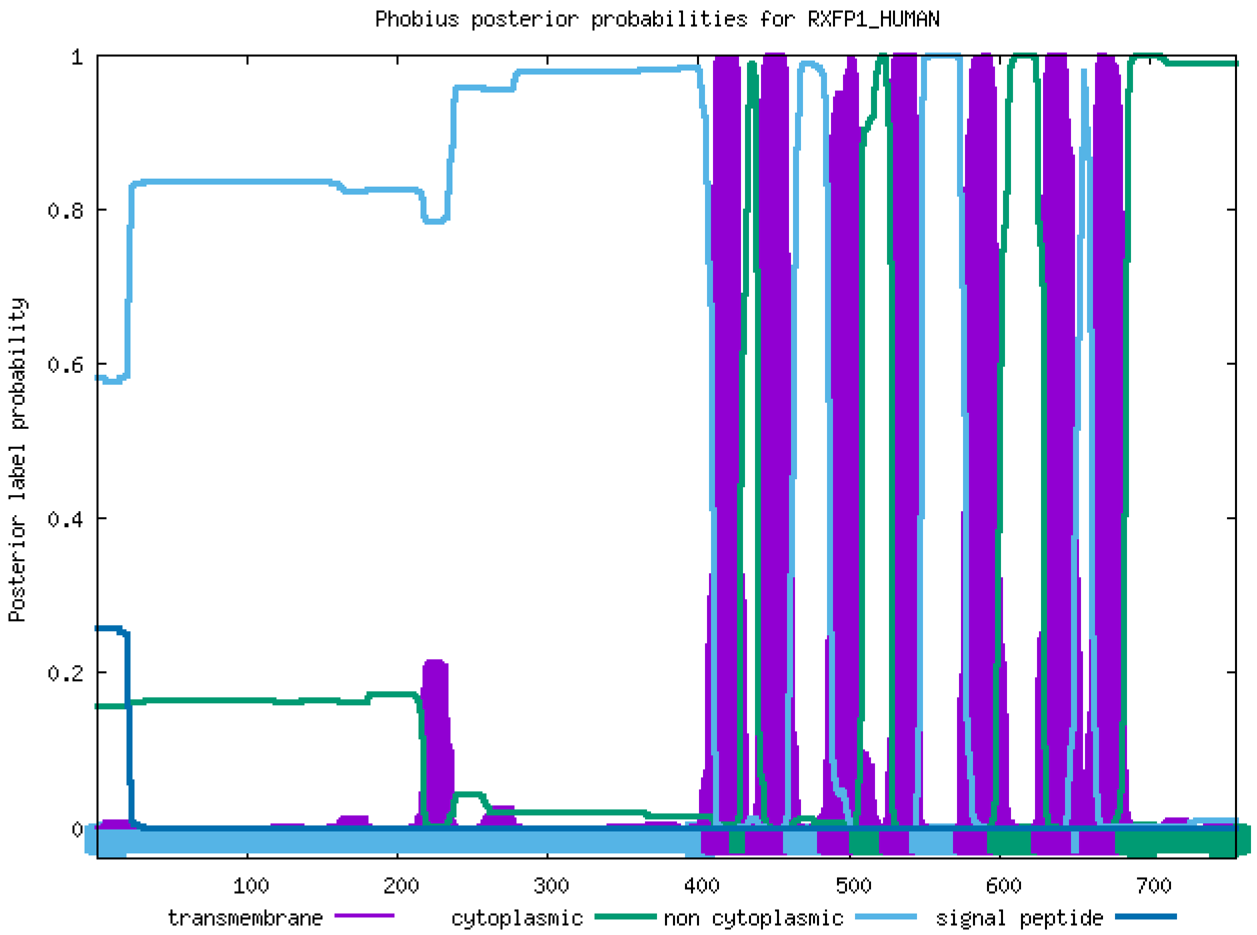

The probability data used in the plot is found here, and the gnuplot script is here.

Prediction of RL3R2_HUMAN

```
ID    RL3R2_HUMAN
FT    TOPO_DOM      1     39       NON CYTOPLASMIC.
FT    TRANSMEM     40     67
FT    TOPO_DOM     68     78       CYTOPLASMIC.
FT    TRANSMEM     79     98
FT    TOPO_DOM     99    117       NON CYTOPLASMIC.
FT    TRANSMEM    118    142
FT    TOPO_DOM    143    153       CYTOPLASMIC.
FT    TRANSMEM    154    174
FT    TOPO_DOM    175    208       NON CYTOPLASMIC.
FT    TRANSMEM    209    230
FT    TOPO_DOM    231    249       CYTOPLASMIC.
FT    TRANSMEM    250    269
FT    TOPO_DOM    270    288       NON CYTOPLASMIC.
FT    TRANSMEM    289    312
FT    TOPO_DOM    313    374       CYTOPLASMIC.
//
```

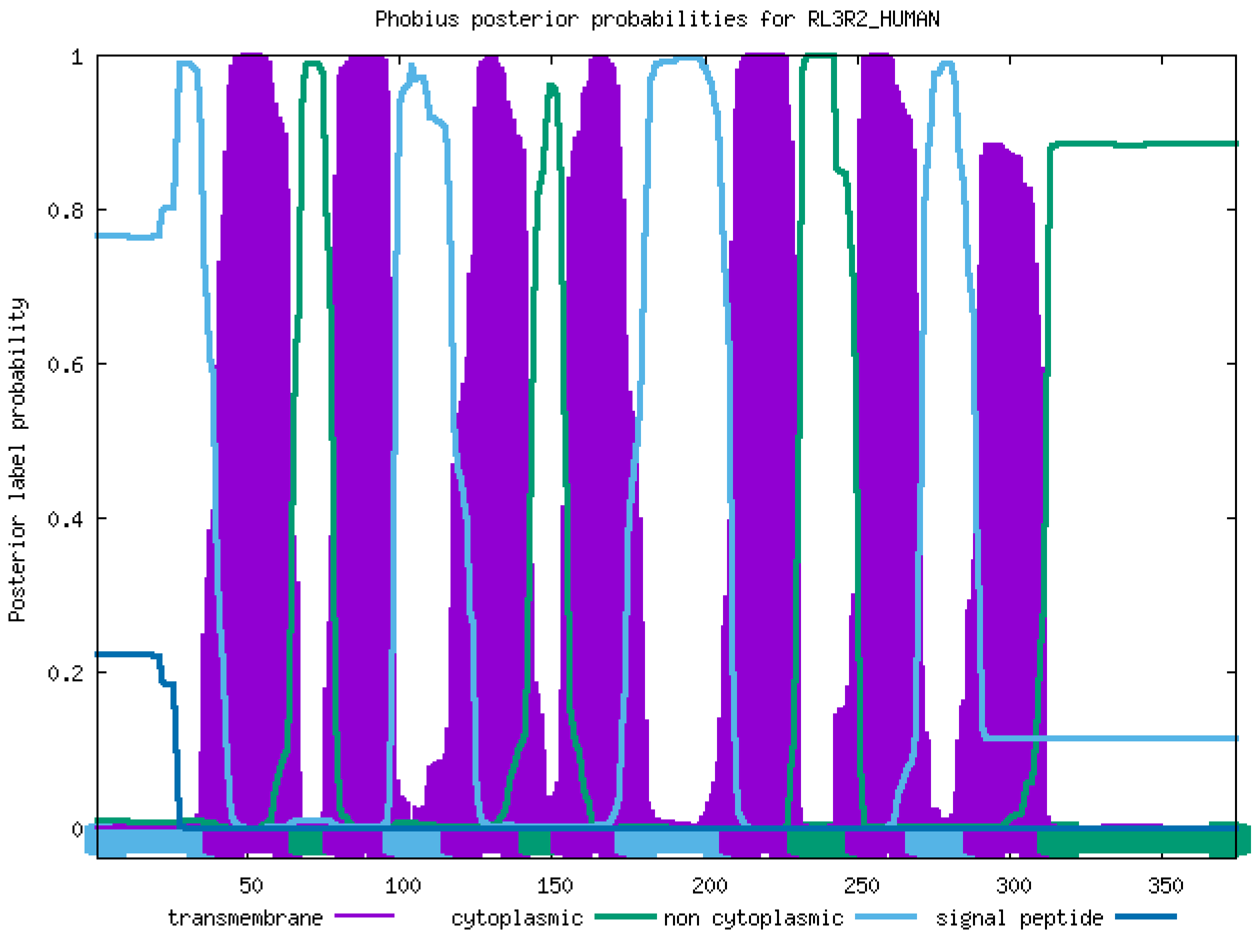

The probability data used in the plot is found here, and the gnuplot script is here.

Prediction of EDNRA_HUMAN

```
ID    EDNRA_HUMAN
FT    SIGNAL        1     20
FT    REGION        1      3       N-REGION.
FT    REGION        4     15       H-REGION.
FT    REGION       16     20       C-REGION.
FT    TOPO_DOM     21     80       NON CYTOPLASMIC.
FT    TRANSMEM     81    106
FT    TOPO_DOM    107    117       CYTOPLASMIC.
FT    TRANSMEM    118    143
FT    TOPO_DOM    144    162       NON CYTOPLASMIC.
FT    TRANSMEM    163    181
FT    TOPO_DOM    182    192       CYTOPLASMIC.
FT    TRANSMEM    193    218
FT    TOPO_DOM    219    256       NON CYTOPLASMIC.
FT    TRANSMEM    257    283
FT    TOPO_DOM    284    303       CYTOPLASMIC.
FT    TRANSMEM    304    324
FT    TOPO_DOM    325    343       NON CYTOPLASMIC.
FT    TRANSMEM    344    372
FT    TOPO_DOM    373    427       CYTOPLASMIC.
//
```

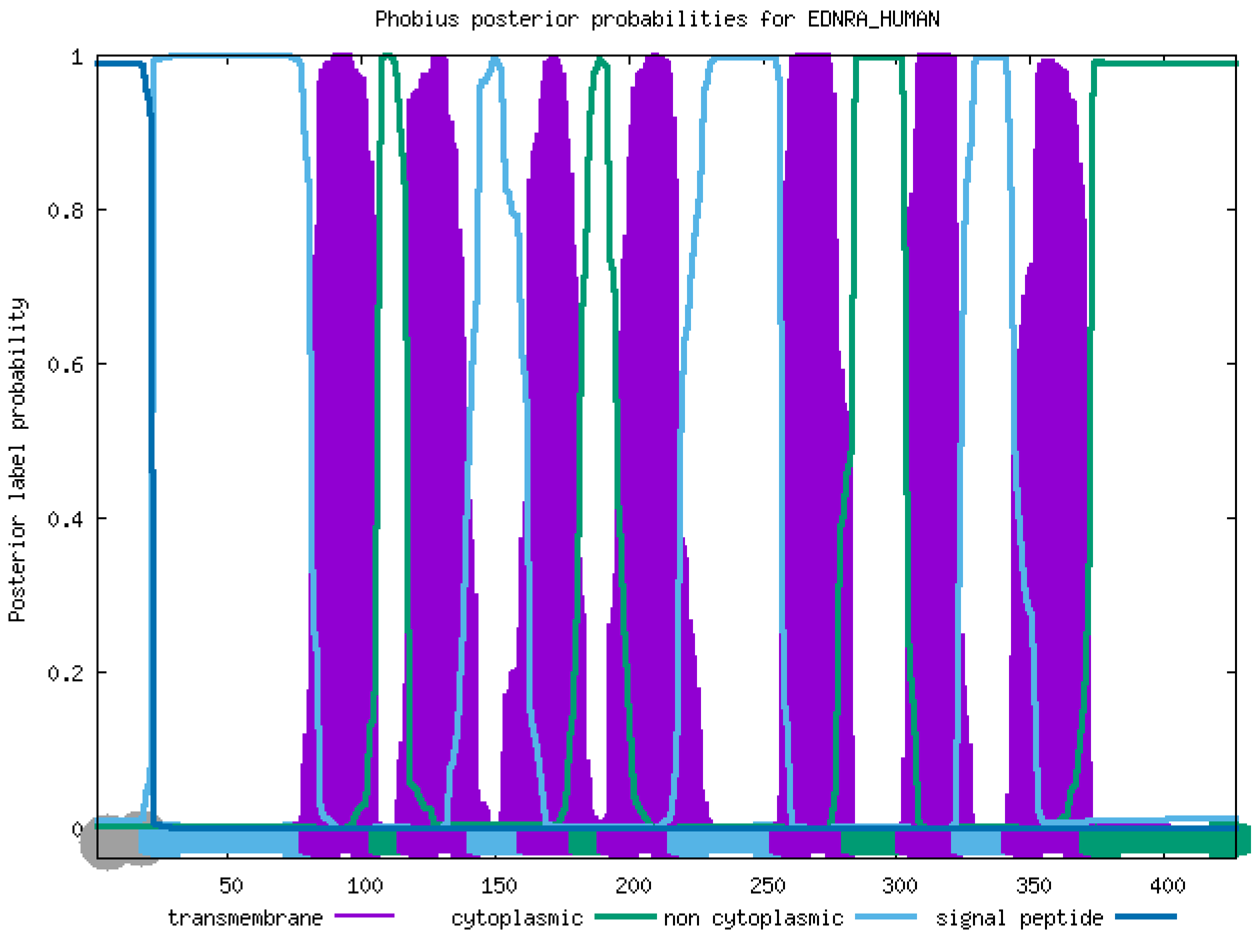

The probability data used in the plot is found here, and the gnuplot script is here.

Prediction of HCAR2_HUMAN

```
ID   HCAR2_HUMAN
FT   TOPO_DOM      1     32       NON CYTOPLASMIC.
FT   TRANSMEM     33     52
FT   TOPO_DOM     53     63       CYTOPLASMIC.
FT   TRANSMEM     64     84
FT   TOPO_DOM     85    103       NON CYTOPLASMIC.
FT   TRANSMEM    104    123
FT   TOPO_DOM    124    143       CYTOPLASMIC.
FT   TRANSMEM    144    163
FT   TOPO_DOM    164    190       NON CYTOPLASMIC.
FT   TRANSMEM    191    212
FT   TOPO_DOM    213    231       CYTOPLASMIC.
FT   TRANSMEM    232    254
FT   TOPO_DOM    255    273       NON CYTOPLASMIC.
FT   TRANSMEM    274    296
FT   TOPO_DOM    297    363       CYTOPLASMIC.
//
```

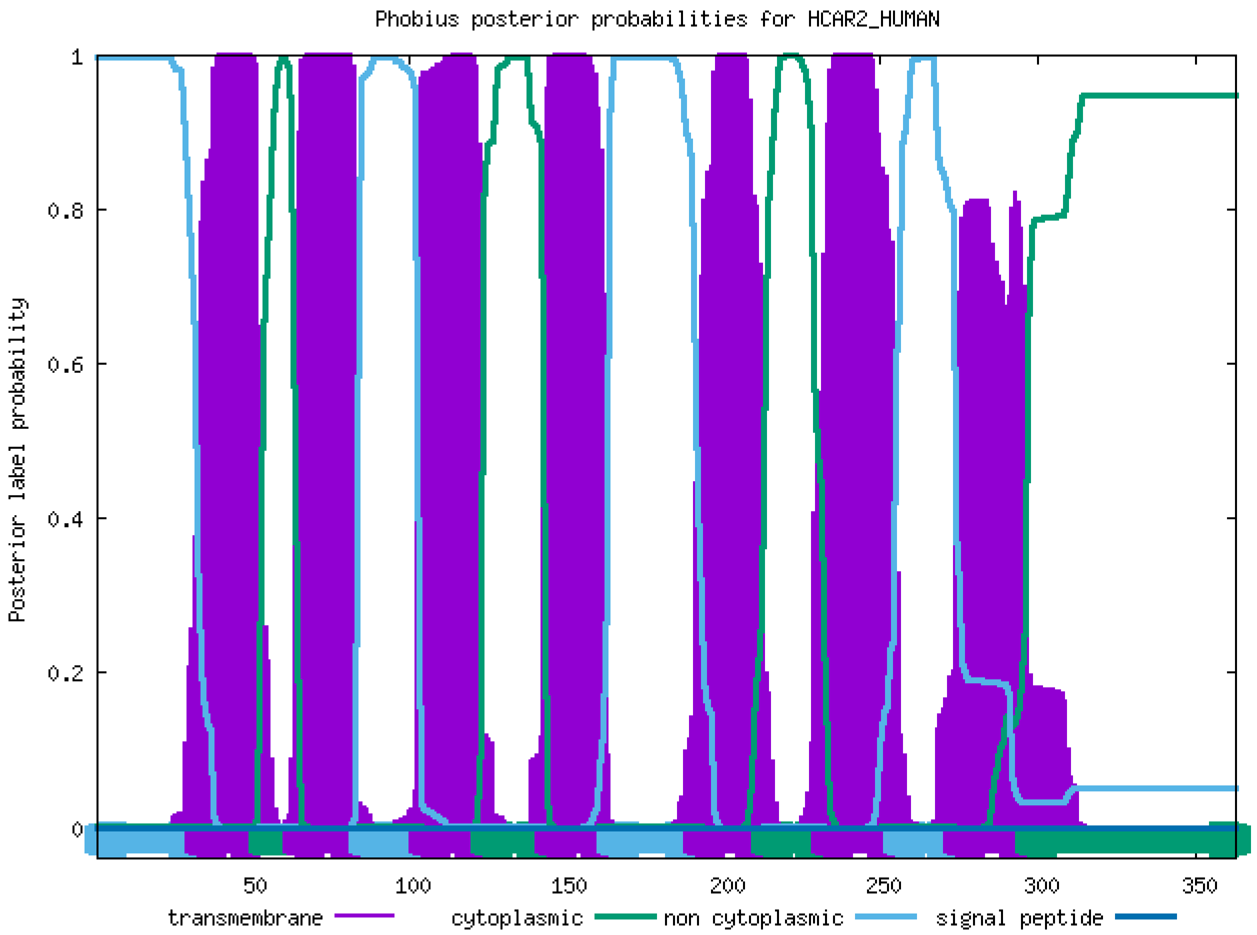

The probability data used in the plot is found here, and the gnuplot script is here.

Prediction of HCAR3_HUMAN

```
ID    HCAR3_HUMAN
FT    TOPO_DOM       1      32       NON CYTOPLASMIC.
FT    TRANSMEM      33      52
FT    TOPO_DOM      53      63       CYTOPLASMIC.
FT    TRANSMEM      64      84
FT    TOPO_DOM      85     103       NON CYTOPLASMIC.
FT    TRANSMEM     104     127
FT    TOPO_DOM     128     138       CYTOPLASMIC.
FT    TRANSMEM     139     163
FT    TOPO_DOM     164     190       NON CYTOPLASMIC.
FT    TRANSMEM     191     212
FT    TOPO_DOM     213     231       CYTOPLASMIC.
FT    TRANSMEM     232     254
FT    TOPO_DOM     255     273       NON CYTOPLASMIC.
FT    TRANSMEM     274     296
FT    TOPO_DOM     297     387       CYTOPLASMIC.
//
```

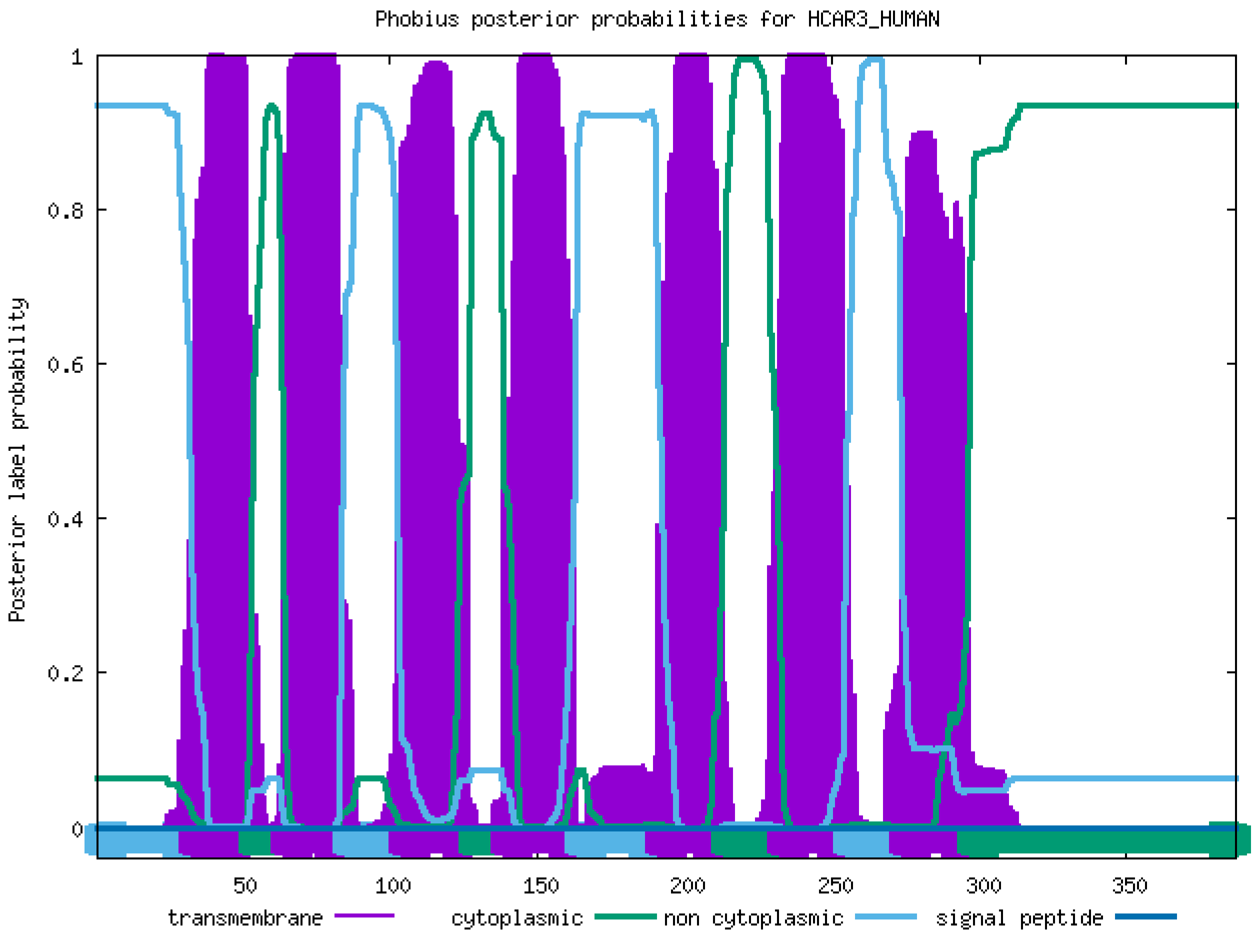

The probability data used in the plot is found here, and the gnuplot script is here.

Prediction of TAAR1_HUMAN

```
ID   TAAR1_HUMAN
FT   TOPO_DOM      1     25       NON CYTOPLASMIC.
FT   TRANSMEM     26     48
FT   TOPO_DOM     49     59       CYTOPLASMIC.
FT   TRANSMEM     60     81
FT   TOPO_DOM     82    103       NON CYTOPLASMIC.
FT   TRANSMEM    104    125
FT   TOPO_DOM    126    136       CYTOPLASMIC.
FT   TRANSMEM    137    161
FT   TOPO_DOM    162    192       NON CYTOPLASMIC.
FT   TRANSMEM    193    213
FT   TOPO_DOM    214    248       CYTOPLASMIC.
FT   TRANSMEM    249    269
FT   TOPO_DOM    270    288       NON CYTOPLASMIC.
FT   TRANSMEM    289    311
FT   TOPO_DOM    312    339       CYTOPLASMIC.
//
```

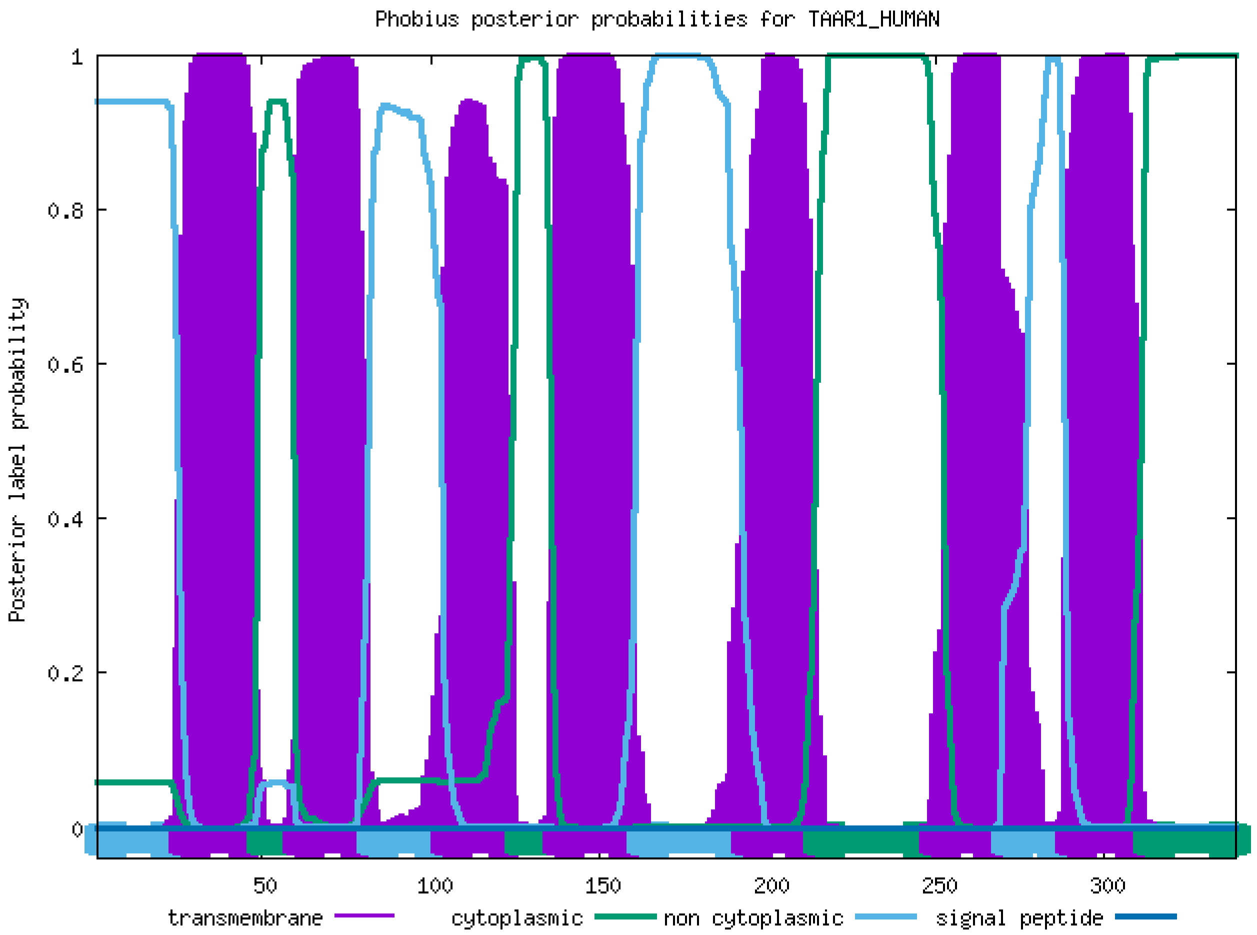

The probability data used in the plot is found here, and the gnuplot script is here.

Prediction of CRFR1_HUMAN

```
ID   CRFR1_HUMAN
FT   SIGNAL        1     23
FT   REGION        1      8       N-REGION.
FT   REGION        9     18       H-REGION.
FT   REGION       19     23       C-REGION.
FT   TOPO_DOM     24    120       NON CYTOPLASMIC.
FT   TRANSMEM    121    142
FT   TOPO_DOM    143    153       CYTOPLASMIC.
FT   TRANSMEM    154    172
FT   TOPO_DOM    173    202       NON CYTOPLASMIC.
FT   TRANSMEM    203    222
FT   TOPO_DOM    223    228       CYTOPLASMIC.
FT   TRANSMEM    229    248
FT   TOPO_DOM    249    269       NON CYTOPLASMIC.
FT   TRANSMEM    270    291
FT   TOPO_DOM    292    311       CYTOPLASMIC.
FT   TRANSMEM    312    332
FT   TOPO_DOM    333    343       NON CYTOPLASMIC.
FT   TRANSMEM    344    366
FT   TOPO_DOM    367    415       CYTOPLASMIC.
//
```

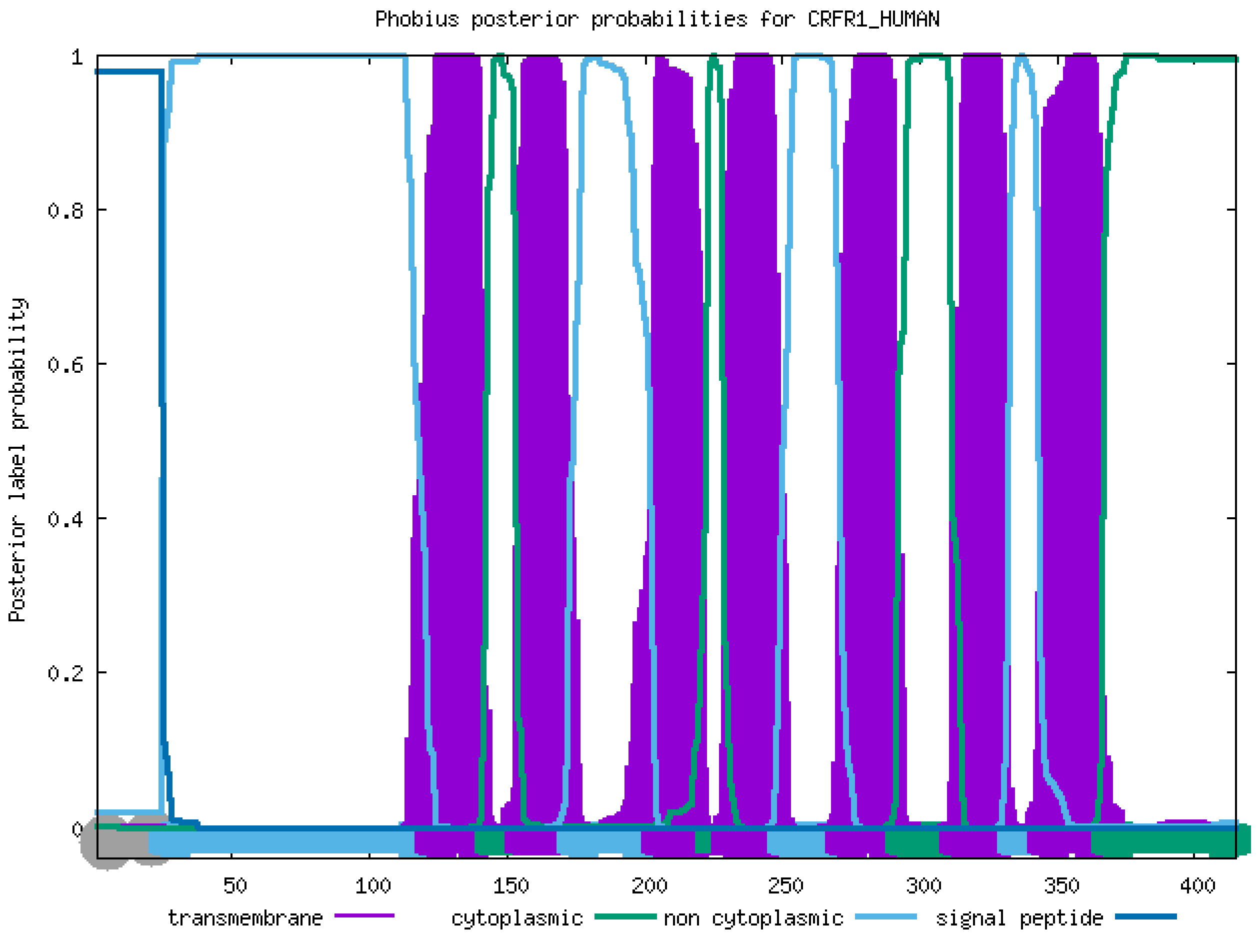

The probability data used in the plot is found here, and the gnuplot script is here.

Prediction of CRFR2_HUMAN

```
ID   CRFR2_HUMAN
FT   SIGNAL         1     19
FT   REGION         1      2       N-REGION.
FT   REGION         3     10       H-REGION.
FT   REGION        11     19       C-REGION.
FT   TOPO_DOM      20    120       NON CYTOPLASMIC.
FT   TRANSMEM     121    141
FT   TOPO_DOM     142    152       CYTOPLASMIC.
FT   TRANSMEM     153    171
FT   TOPO_DOM     172    190       NON CYTOPLASMIC.
FT   TRANSMEM     191    214
FT   TOPO_DOM     215    225       CYTOPLASMIC.
FT   TRANSMEM     226    248
FT   TOPO_DOM     249    267       NON CYTOPLASMIC.
FT   TRANSMEM     268    287
FT   TOPO_DOM     288    307       CYTOPLASMIC.
FT   TRANSMEM     308    328
FT   TOPO_DOM     329    339       NON CYTOPLASMIC.
FT   TRANSMEM     340    362
FT   TOPO_DOM     363    411       CYTOPLASMIC.
//
```

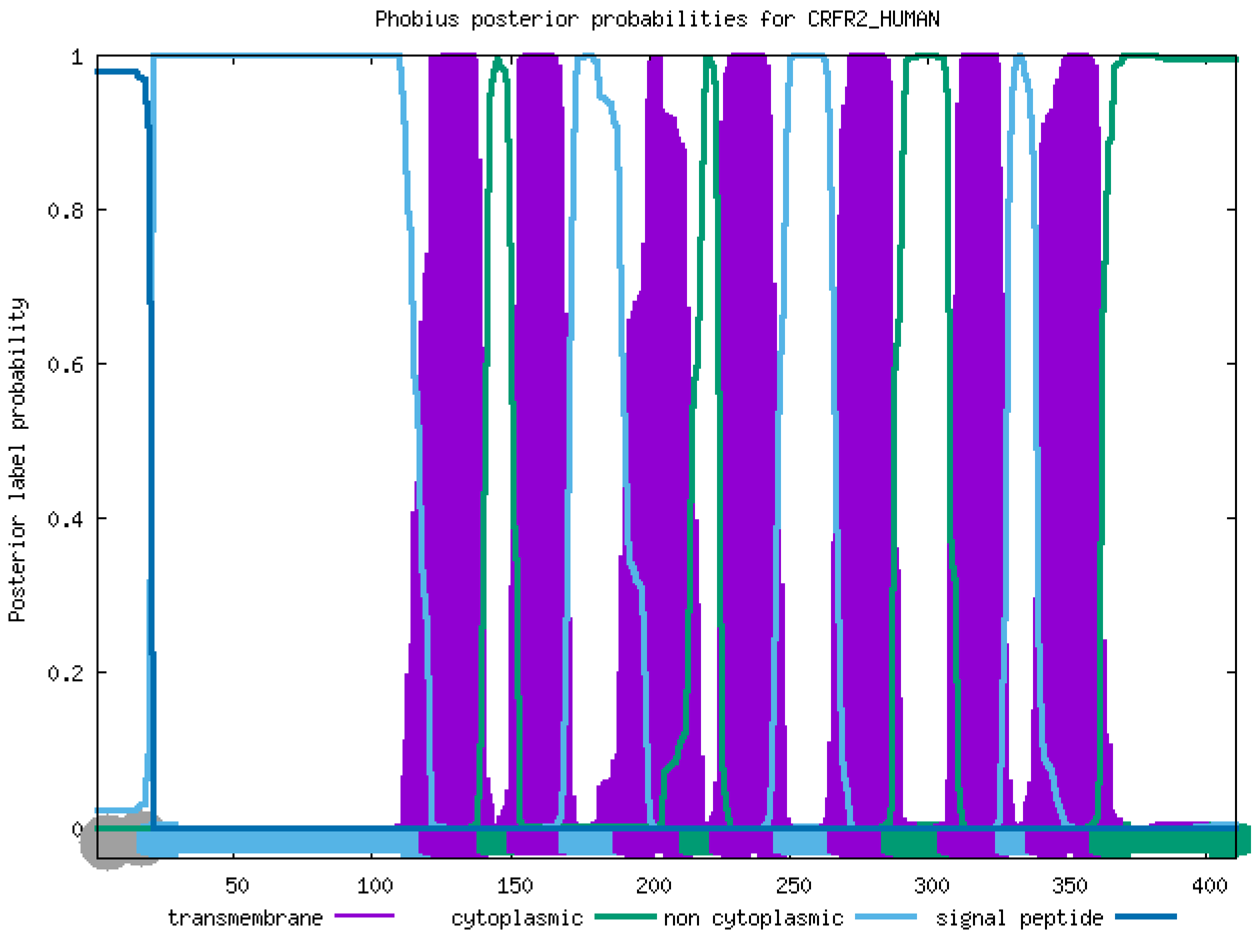

The probability data used in the plot is found here, and the gnuplot script is here.

Prediction of PACR_HUMAN

```
ID   PACR_HUMAN
FT   SIGNAL        1     23
FT   REGION        1      3       N-REGION.
FT   REGION        4     15       H-REGION.
FT   REGION       16     23       C-REGION.
FT   TOPO_DOM     24    154       NON CYTOPLASMIC.
FT   TRANSMEM    155    175
FT   TOPO_DOM    176    186       CYTOPLASMIC.
FT   TRANSMEM    187    205
FT   TOPO_DOM    206    240       NON CYTOPLASMIC.
FT   TRANSMEM    241    260
FT   TOPO_DOM    261    266       CYTOPLASMIC.
FT   TRANSMEM    267    287
FT   TOPO_DOM    288    306       NON CYTOPLASMIC.
FT   TRANSMEM    307    331
FT   TOPO_DOM    332    350       CYTOPLASMIC.
FT   TRANSMEM    351    371
FT   TOPO_DOM    372    382       NON CYTOPLASMIC.
FT   TRANSMEM    383    403
FT   TOPO_DOM    404    468       CYTOPLASMIC.
//
```

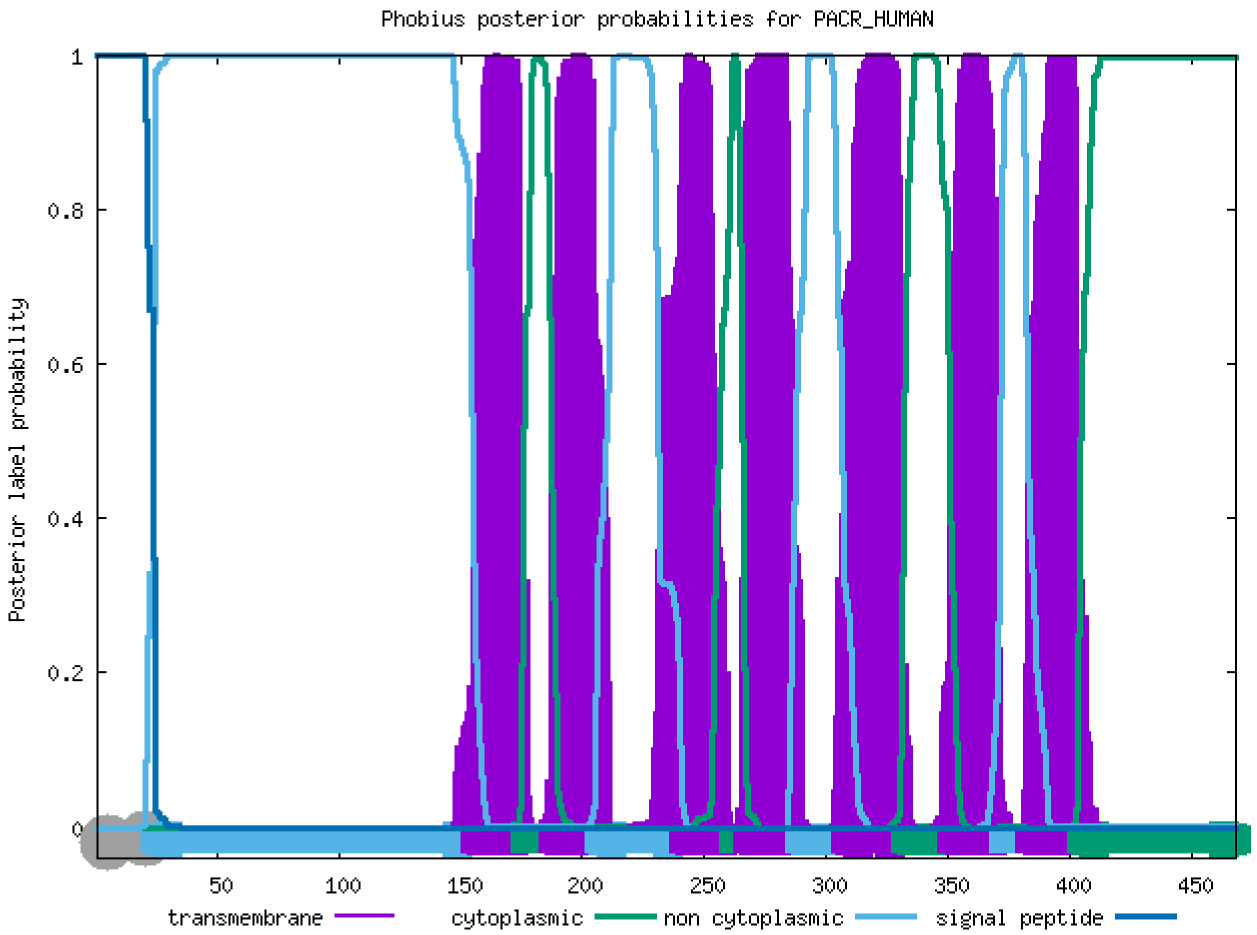

The probability data used in the plot is found here, and the gnuplot script is here.

Prediction of GLR_HUMAN

```
ID   GLR_HUMAN
FT   SIGNAL        1     25
FT   REGION        1      9       N-REGION.
FT   REGION       10     20       H-REGION.
FT   REGION       21     25       C-REGION.
FT   TOPO_DOM     26    146       NON CYTOPLASMIC.
FT   TRANSMEM    147    166
FT   TOPO_DOM    167    177       CYTOPLASMIC.
FT   TRANSMEM    178    199
FT   TOPO_DOM    200    218       NON CYTOPLASMIC.
FT   TRANSMEM    219    244
FT   TOPO_DOM    245    263       CYTOPLASMIC.
FT   TRANSMEM    264    285
FT   TOPO_DOM    286    304       NON CYTOPLASMIC.
FT   TRANSMEM    305    326
FT   TOPO_DOM    327    346       CYTOPLASMIC.
FT   TRANSMEM    347    368
FT   TOPO_DOM    369    379       NON CYTOPLASMIC.
FT   TRANSMEM    380    403
FT   TOPO_DOM    404    477       CYTOPLASMIC.
//
```

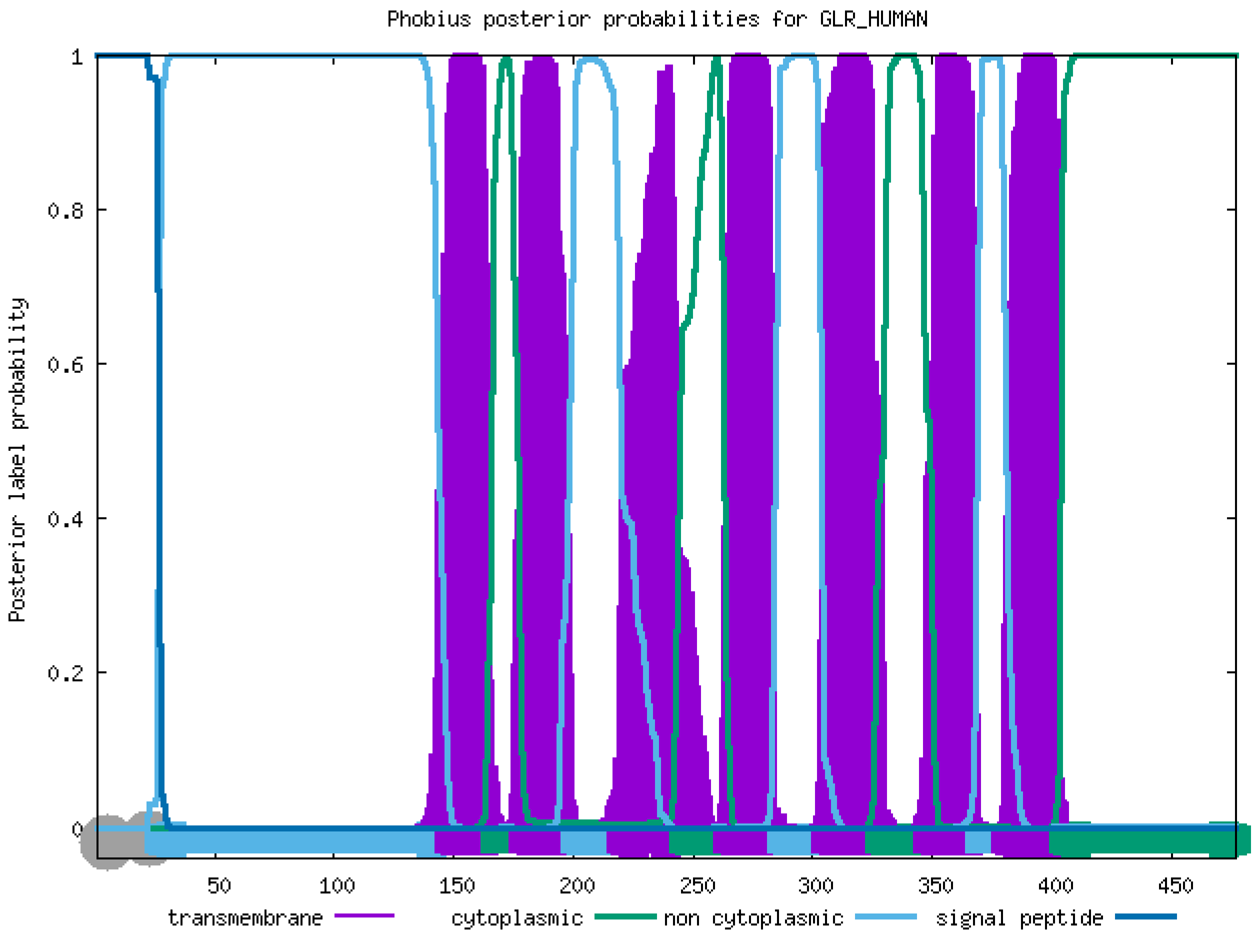

The probability data used in the plot is found here, and the gnuplot script is here.

Prediction of GLP1R_HUMAN

```
ID   GLP1R_HUMAN
FT   SIGNAL        1     21
FT   REGION        1      9       N-REGION.
FT   REGION       10     17       H-REGION.
FT   REGION       18     21       C-REGION.
FT   TOPO_DOM     22    141       NON CYTOPLASMIC.
FT   TRANSMEM    142    167
FT   TOPO_DOM    168    178       CYTOPLASMIC.
FT   TRANSMEM    179    196
FT   TOPO_DOM    197    240       NON CYTOPLASMIC.
FT   TRANSMEM    241    261
FT   TOPO_DOM    262    267       CYTOPLASMIC.
FT   TRANSMEM    268    287
FT   TOPO_DOM    288    306       NON CYTOPLASMIC.
FT   TRANSMEM    307    332
FT   TOPO_DOM    333    352       CYTOPLASMIC.
FT   TRANSMEM    353    370
FT   TOPO_DOM    371    381       NON CYTOPLASMIC.
FT   TRANSMEM    382    405
FT   TOPO_DOM    406    463       CYTOPLASMIC.
//
```

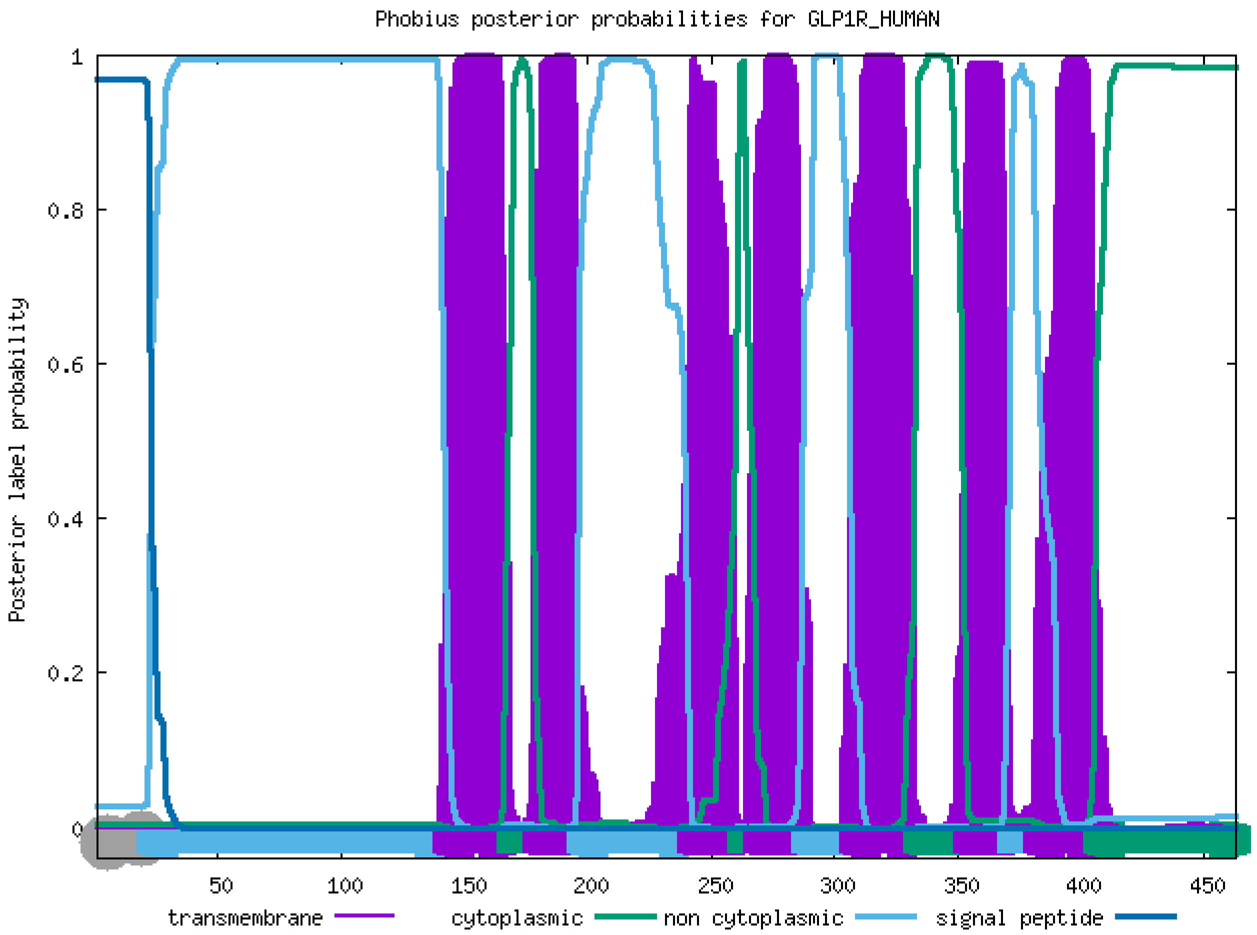

The probability data used in the plot is found here, and the gnuplot script is here.

Prediction of GLP2R_HUMAN

```
ID   GLP2R_HUMAN
FT   TOPO_DOM      1     179       NON CYTOPLASMIC.
FT   TRANSMEM    180     203
FT   TOPO_DOM    204     214       CYTOPLASMIC.
FT   TRANSMEM    215     236
FT   TOPO_DOM    237     265       NON CYTOPLASMIC.
FT   TRANSMEM    266     289
FT   TOPO_DOM    290     300       CYTOPLASMIC.
FT   TRANSMEM    301     321
FT   TOPO_DOM    322     340       NON CYTOPLASMIC.
FT   TRANSMEM    341     362
FT   TOPO_DOM    363     382       CYTOPLASMIC.
FT   TRANSMEM    383     404
FT   TOPO_DOM    405     415       NON CYTOPLASMIC.
FT   TRANSMEM    416     439
FT   TOPO_DOM    440     553       CYTOPLASMIC.
//
```

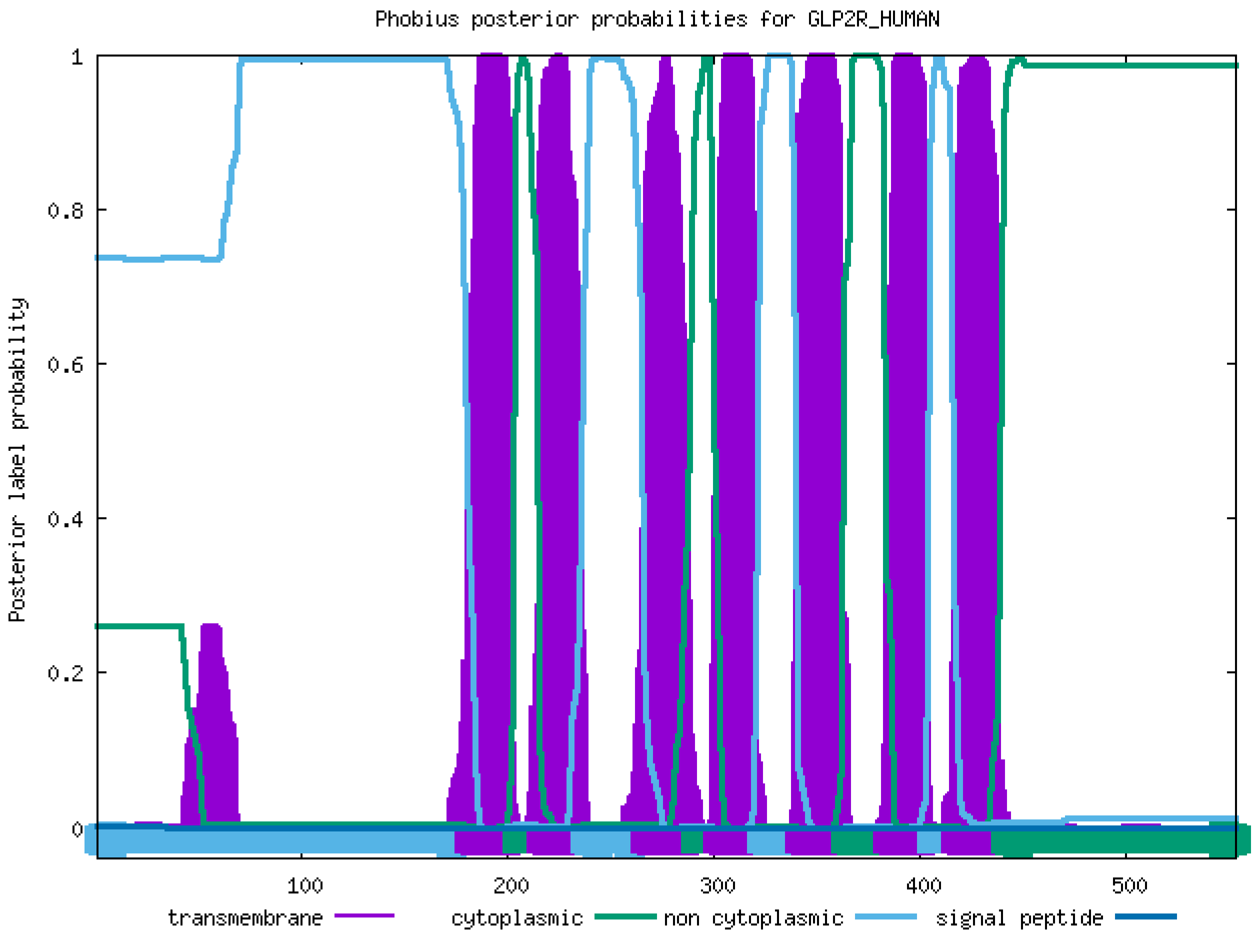

The probability data used in the plot is found here, and the gnuplot script is here.

Prediction of GIPR_HUMAN

```
ID   GIPR_HUMAN
FT   SIGNAL        1     25
FT   REGION        1      8       N-REGION.
FT   REGION        9     20       H-REGION.
FT   REGION       21     25       C-REGION.
FT   TOPO_DOM     26    138       NON CYTOPLASMIC.
FT   TRANSMEM    139    159
FT   TOPO_DOM    160    170       CYTOPLASMIC.
FT   TRANSMEM    171    189
FT   TOPO_DOM    190    230       NON CYTOPLASMIC.
FT   TRANSMEM    231    251
FT   TOPO_DOM    252    257       CYTOPLASMIC.
FT   TRANSMEM    258    277
FT   TOPO_DOM    278    296       NON CYTOPLASMIC.
FT   TRANSMEM    297    318
FT   TOPO_DOM    319    338       CYTOPLASMIC.
FT   TRANSMEM    339    358
FT   TOPO_DOM    359    377       NON CYTOPLASMIC.
FT   TRANSMEM    378    395
FT   TOPO_DOM    396    466       CYTOPLASMIC.
//
```

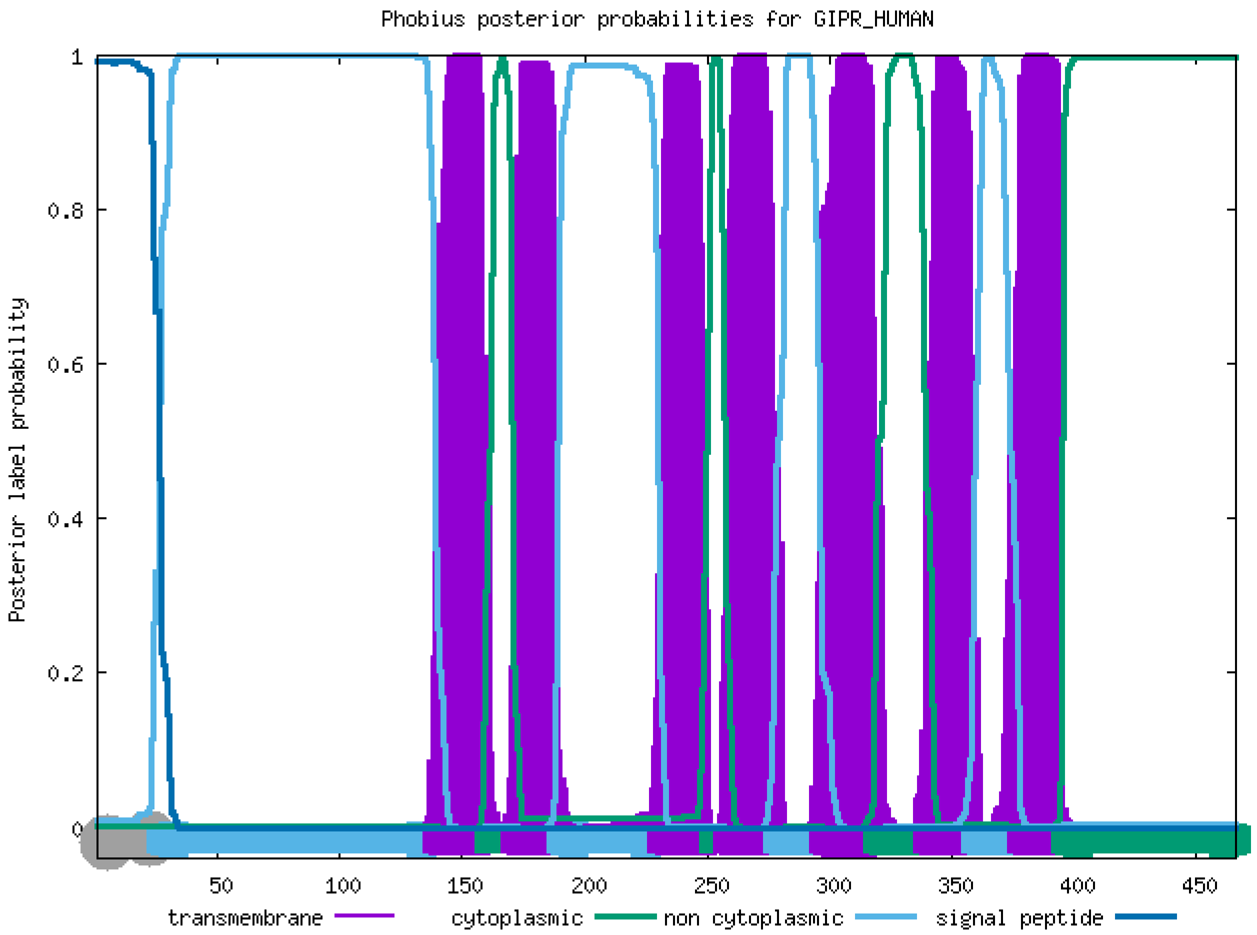

The probability data used in the plot is found here, and the gnuplot script is here.

Prediction of CALCR_HUMAN

```
ID   CALCR_HUMAN
FT   SIGNAL        1      24
FT   REGION        1       8       N-REGION.
FT   REGION        9      16       H-REGION.
FT   REGION       17      24       C-REGION.
FT   TOPO_DOM     25     146       NON CYTOPLASMIC.
FT   TRANSMEM    147     176
FT   TOPO_DOM    177     187       CYTOPLASMIC.
FT   TRANSMEM    188     206
FT   TOPO_DOM    207     233       NON CYTOPLASMIC.
FT   TRANSMEM    234     254
FT   TOPO_DOM    255     260       CYTOPLASMIC.
FT   TRANSMEM    261     284
FT   TOPO_DOM    285     295       NON CYTOPLASMIC.
FT   TRANSMEM    296     320
FT   TOPO_DOM    321     340       CYTOPLASMIC.
FT   TRANSMEM    341     362
FT   TOPO_DOM    363     373       NON CYTOPLASMIC.
FT   TRANSMEM    374     394
FT   TOPO_DOM    395     474       CYTOPLASMIC.
//
```

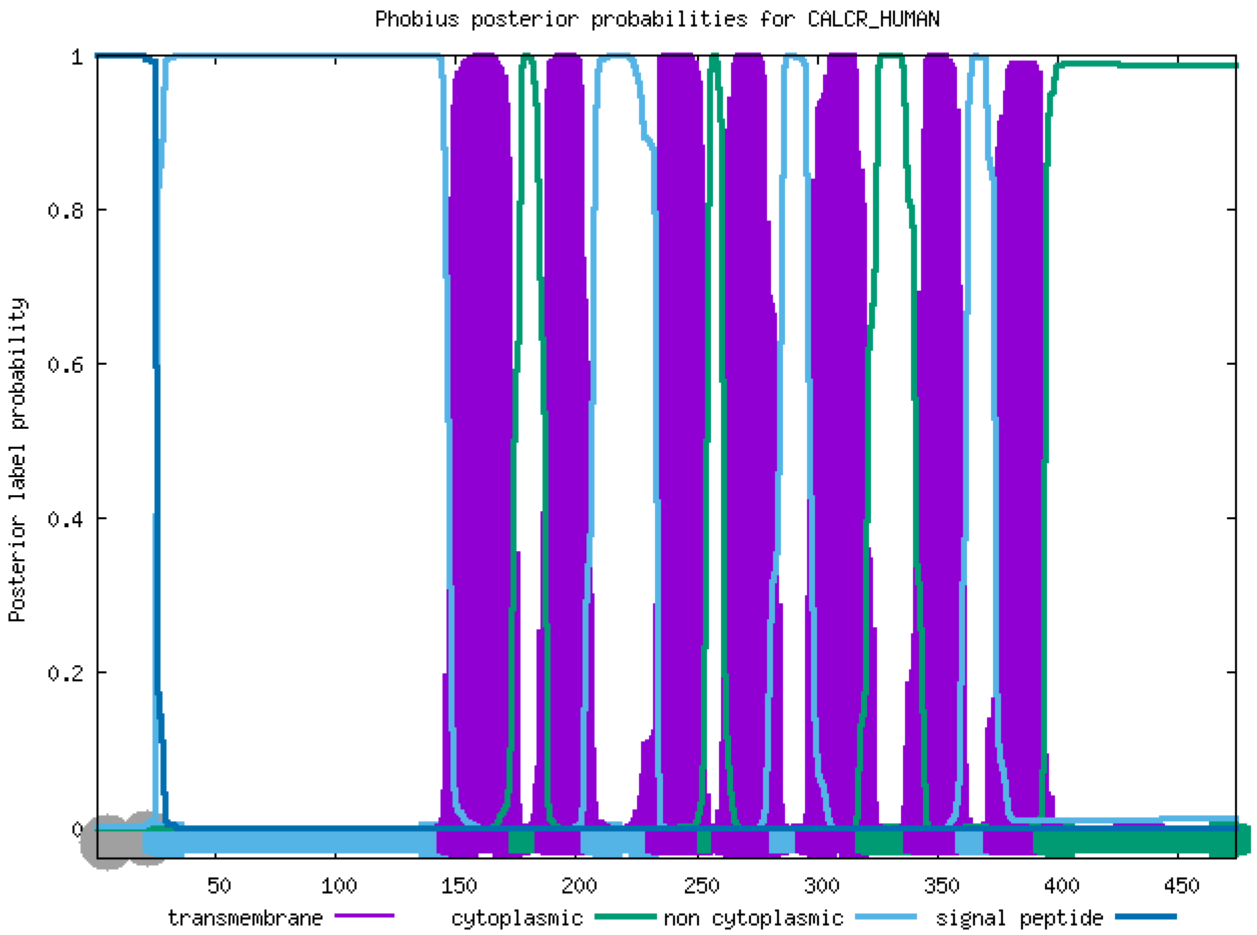

The probability data used in the plot is found here, and the gnuplot script is here.

Prediction of CALRL_HUMAN

```
ID    CALRL_HUMAN
FT    SIGNAL         1      24
FT    REGION         1       4       N-REGION.
FT    REGION         5      16       H-REGION.
FT    REGION        17      24       C-REGION.
FT    TOPO_DOM      25     140       NON CYTOPLASMIC.
FT    TRANSMEM     141     166
FT    TOPO_DOM     167     177       CYTOPLASMIC.
FT    TRANSMEM     178     199
FT    TOPO_DOM     200     226       NON CYTOPLASMIC.
FT    TRANSMEM     227     247
FT    TOPO_DOM     248     253       CYTOPLASMIC.
FT    TRANSMEM     254     277
FT    TOPO_DOM     278     288       NON CYTOPLASMIC.
FT    TRANSMEM     289     313
FT    TOPO_DOM     314     333       CYTOPLASMIC.
FT    TRANSMEM     334     355
FT    TOPO_DOM     356     366       NON CYTOPLASMIC.
FT    TRANSMEM     367     387
FT    TOPO_DOM     388     461       CYTOPLASMIC.
//
```

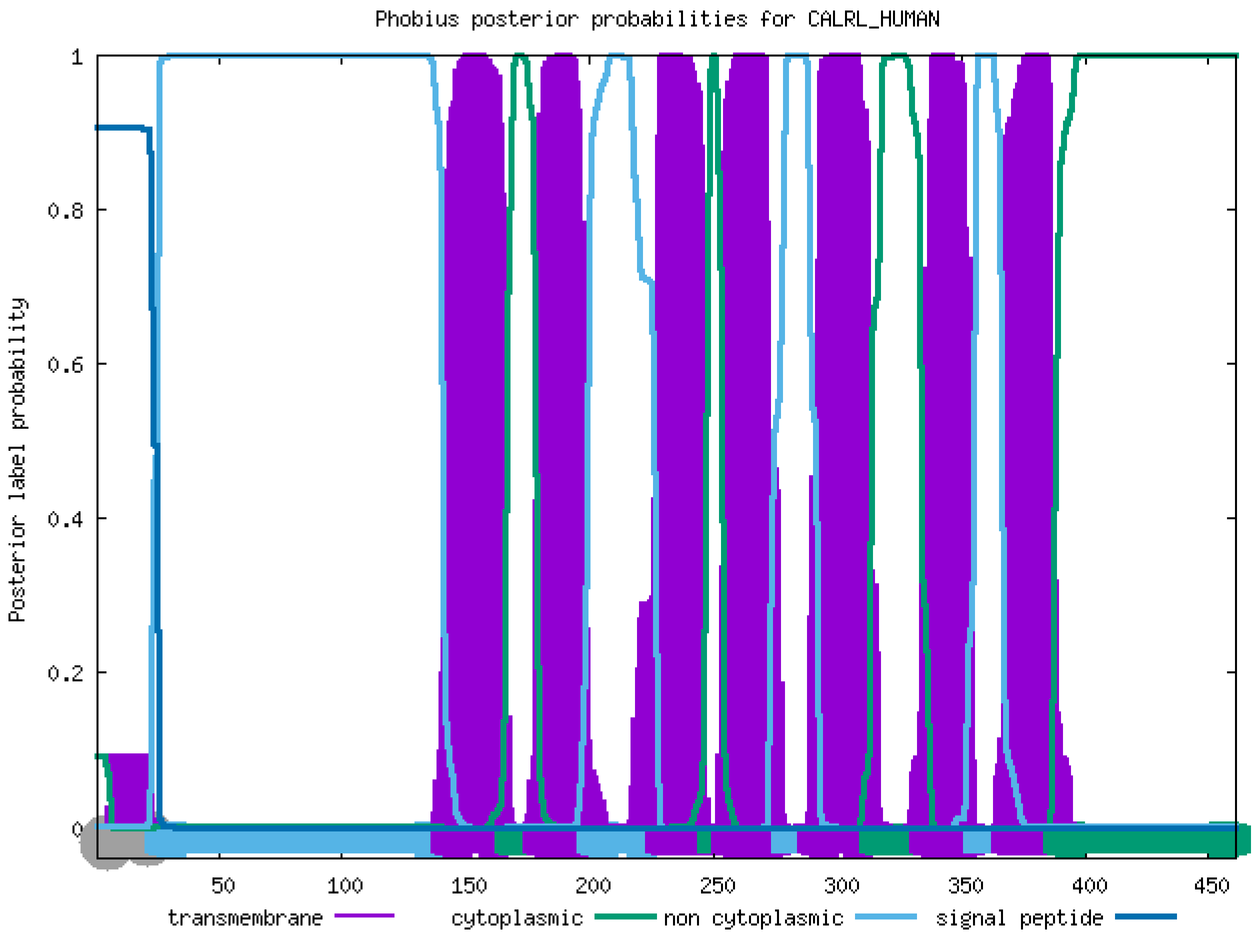

The probability data used in the plot is found here, and the gnuplot script is here.

Prediction of PTH1R_HUMAN

```
ID   PTH1R_HUMAN
FT   SIGNAL        1     24
FT   REGION        1      8       N-REGION.
FT   REGION        9     19       H-REGION.
FT   REGION       20     24       C-REGION.
FT   TOPO_DOM     25    188       NON CYTOPLASMIC.
FT   TRANSMEM    189    212
FT   TOPO_DOM    213    223       CYTOPLASMIC.
FT   TRANSMEM    224    245
FT   TOPO_DOM    246    295       NON CYTOPLASMIC.
FT   TRANSMEM    296    315
FT   TOPO_DOM    316    319       CYTOPLASMIC.
FT   TRANSMEM    320    341
FT   TOPO_DOM    342    360       NON CYTOPLASMIC.
FT   TRANSMEM    361    385
FT   TOPO_DOM    386    405       CYTOPLASMIC.
FT   TRANSMEM    406    426
FT   TOPO_DOM    427    445       NON CYTOPLASMIC.
FT   TRANSMEM    446    466
FT   TOPO_DOM    467    593       CYTOPLASMIC.
//
```

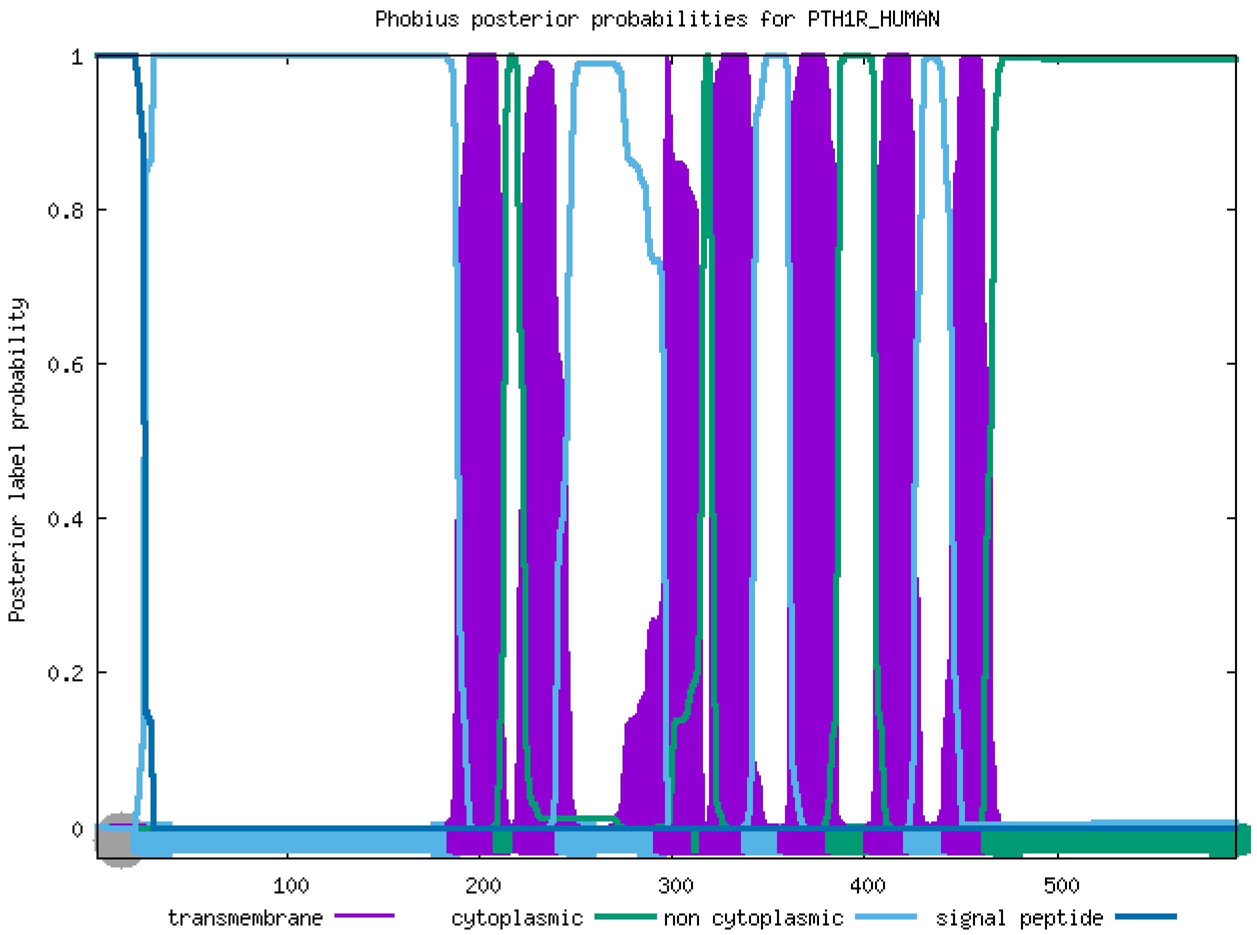

The probability data used in the plot is found here, and the gnuplot script is here.

Prediction of PTH2R_HUMAN

```
ID   PTH2R_HUMAN
FT   SIGNAL        1     24
FT   REGION        1      3       N-REGION.
FT   REGION        4     16       H-REGION.
FT   REGION       17     24       C-REGION.
FT   TOPO_DOM     25    144       NON CYTOPLASMIC.
FT   TRANSMEM    145    166
FT   TOPO_DOM    167    177       CYTOPLASMIC.
FT   TRANSMEM    178    196
FT   TOPO_DOM    197    231       NON CYTOPLASMIC.
FT   TRANSMEM    232    254
FT   TOPO_DOM    255    274       CYTOPLASMIC.
FT   TRANSMEM    275    296
FT   TOPO_DOM    297    315       NON CYTOPLASMIC.
FT   TRANSMEM    316    334
FT   TOPO_DOM    335    364       CYTOPLASMIC.
FT   TRANSMEM    365    387
FT   TOPO_DOM    388    398       NON CYTOPLASMIC.
FT   TRANSMEM    399    416
FT   TOPO_DOM    417    550       CYTOPLASMIC.
//
```

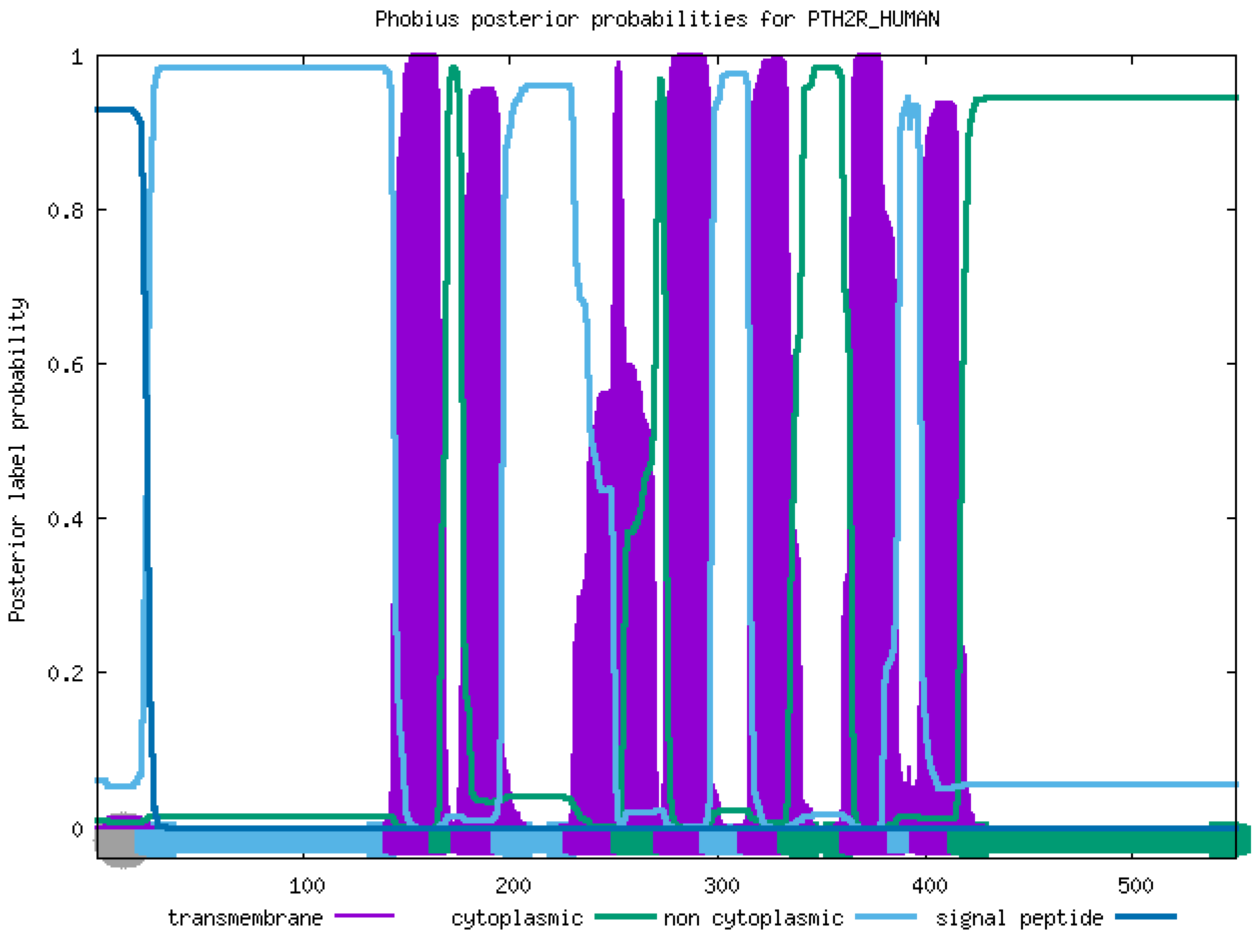

The probability data used in the plot is found here, and the gnuplot script is here.

Prediction of RAMP2_HUMAN

```
ID   RAMP2_HUMAN
FT   SIGNAL        1     42
FT   REGION        1     22       N-REGION.
FT   REGION       23     34       H-REGION.
FT   REGION       35     42       C-REGION.
FT   TOPO_DOM     43    144       NON CYTOPLASMIC.
FT   TRANSMEM    145    166
FT   TOPO_DOM    167    175       CYTOPLASMIC.
//
```

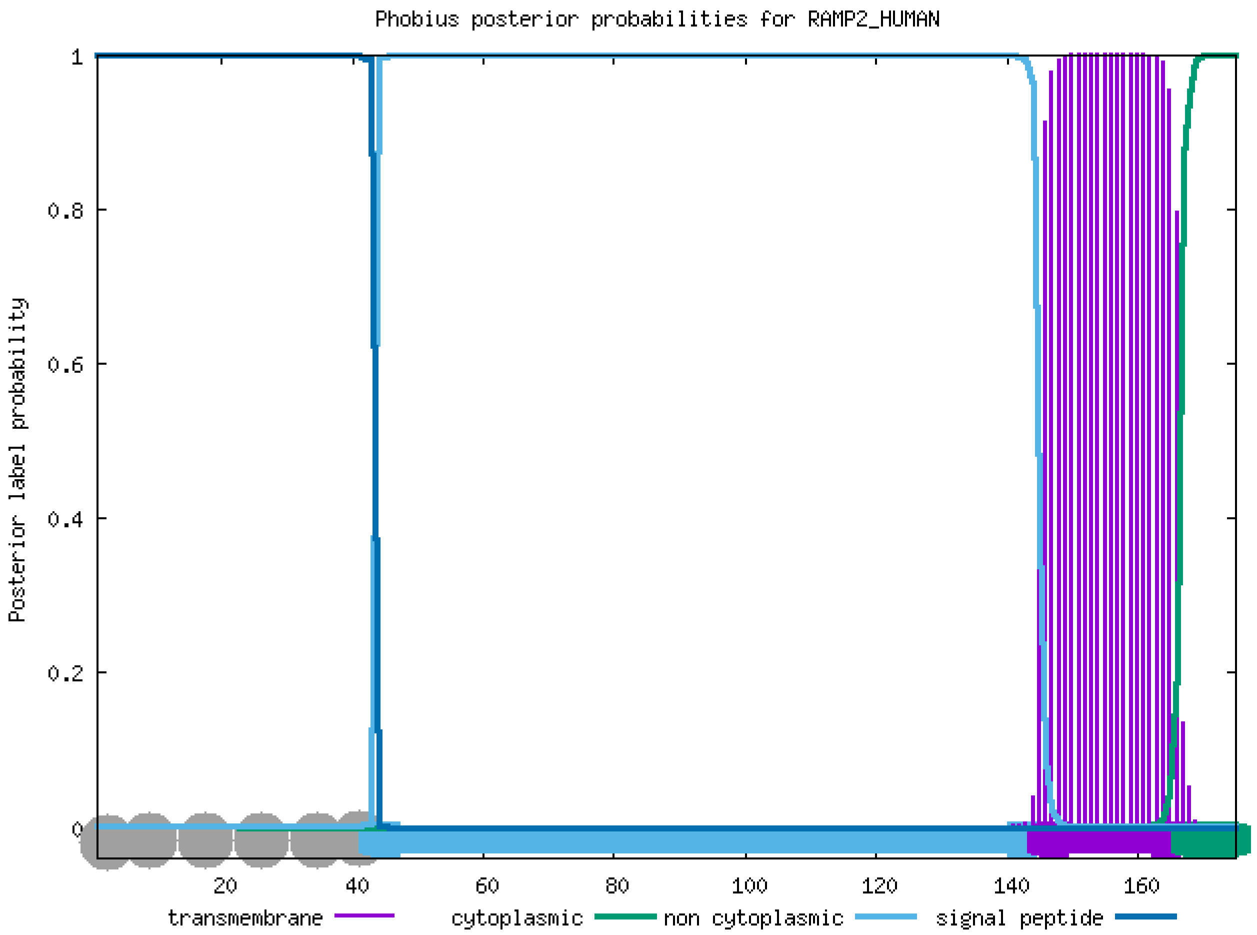

The probability data used in the plot is found here, and the gnuplot script is here.

Prediction of RAMP3_HUMAN

```
ID    RAMP3_HUMAN
FT    SIGNAL          1      27
FT    REGION          1      10       N-REGION.
FT    REGION         11      22       H-REGION.
FT    REGION         23      27       C-REGION.
FT    TOPO_DOM       28     117       NON CYTOPLASMIC.
FT    TRANSMEM      118     139
FT    TOPO_DOM      140     148       CYTOPLASMIC.
//
```

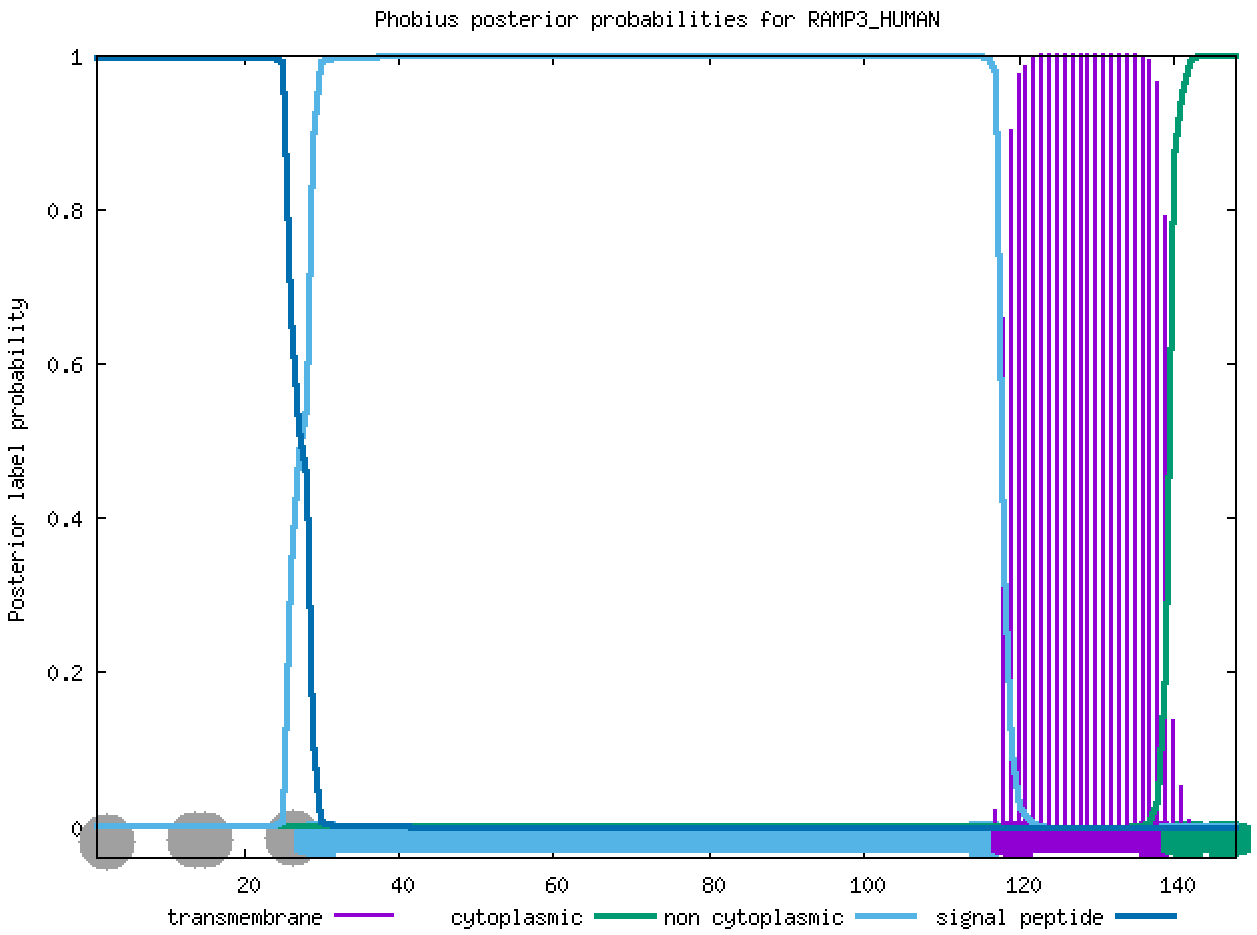

The probability data used in the plot is found here, and the gnuplot script is here.

Prediction of VIPR1_HUMAN

```
ID    VIPR1_HUMAN
FT    SIGNAL        1      30
FT    REGION        1      11       N-REGION.
FT    REGION       12      23       H-REGION.
FT    REGION       24      30       C-REGION.
FT    TOPO_DOM     31     147       NON CYTOPLASMIC.
FT    TRANSMEM    148     167
FT    TOPO_DOM    168     178       CYTOPLASMIC.
FT    TRANSMEM    179     200
FT    TOPO_DOM    201     219       NON CYTOPLASMIC.
FT    TRANSMEM    220     243
FT    TOPO_DOM    244     254       CYTOPLASMIC.
FT    TRANSMEM    255     275
FT    TOPO_DOM    276     294       NON CYTOPLASMIC.
FT    TRANSMEM    295     319
FT    TOPO_DOM    320     338       CYTOPLASMIC.
FT    TRANSMEM    339     359
FT    TOPO_DOM    360     370       NON CYTOPLASMIC.
FT    TRANSMEM    371     391
FT    TOPO_DOM    392     457       CYTOPLASMIC.
//
```

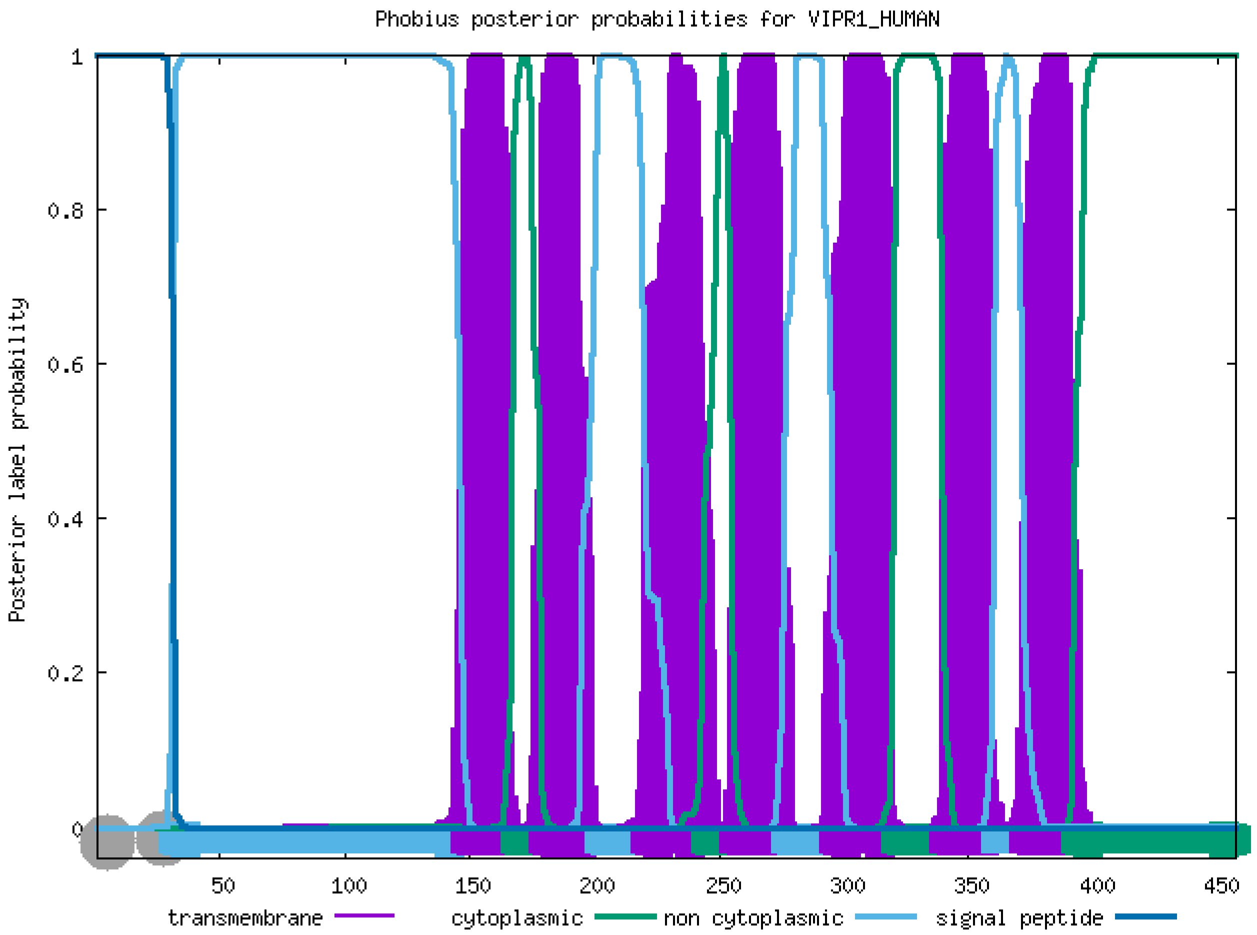

The probability data used in the plot is found here, and the gnuplot script is here.

Prediction of VIPR2_HUMAN

```
ID   VIPR2_HUMAN
FT   SIGNAL         1     20
FT   REGION         1      3       N-REGION.
FT   REGION         4     15       H-REGION.
FT   REGION        16     20       C-REGION.
FT   TOPO_DOM      21    127       NON CYTOPLASMIC.
FT   TRANSMEM     128    151
FT   TOPO_DOM     152    159       CYTOPLASMIC.
FT   TRANSMEM     160    178
FT   TOPO_DOM     179    197       NON CYTOPLASMIC.
FT   TRANSMEM     198    221
FT   TOPO_DOM     222    240       CYTOPLASMIC.
FT   TRANSMEM     241    262
FT   TOPO_DOM     263    281       NON CYTOPLASMIC.
FT   TRANSMEM     282    307
FT   TOPO_DOM     308    330       CYTOPLASMIC.
FT   TRANSMEM     331    351
FT   TOPO_DOM     352    356       NON CYTOPLASMIC.
FT   TRANSMEM     357    378
FT   TOPO_DOM     379    438       CYTOPLASMIC.
//
```

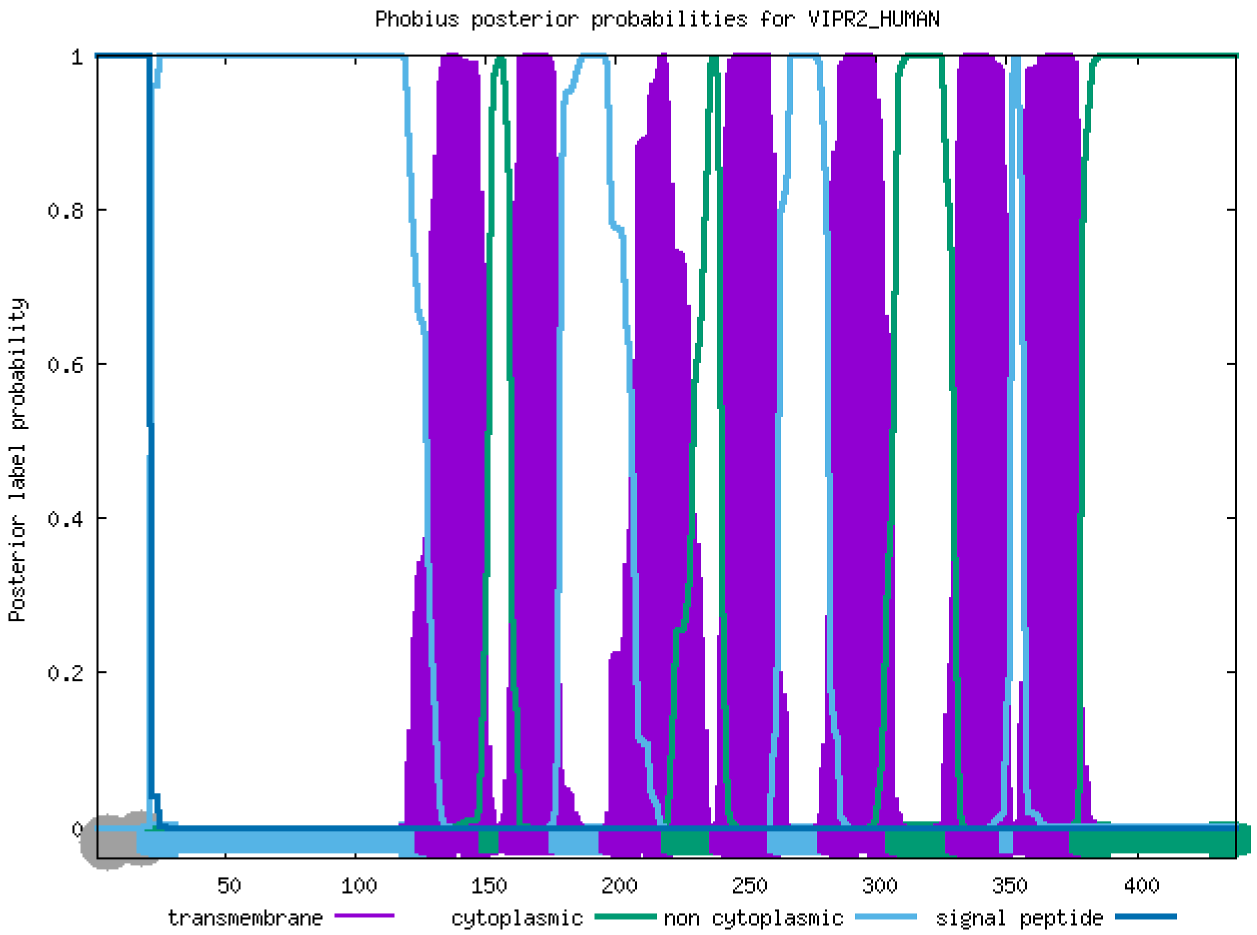

The probability data used in the plot is found here, and the gnuplot script is here.

Prediction of GHRHR_HUMAN

```
ID   GHRHR_HUMAN
FT   SIGNAL        1     22
FT   REGION        1      5       N-REGION.
FT   REGION        6     17       H-REGION.
FT   REGION       18     22       C-REGION.
FT   TOPO_DOM     23    130       NON CYTOPLASMIC.
FT   TRANSMEM    131    154
FT   TOPO_DOM    155    240       CYTOPLASMIC.
FT   TRANSMEM    241    261
FT   TOPO_DOM    262    280       NON CYTOPLASMIC.
FT   TRANSMEM    281    308
FT   TOPO_DOM    309    328       CYTOPLASMIC.
FT   TRANSMEM    329    348
FT   TOPO_DOM    349    359       NON CYTOPLASMIC.
FT   TRANSMEM    360    379
FT   TOPO_DOM    380    423       CYTOPLASMIC.
//
```

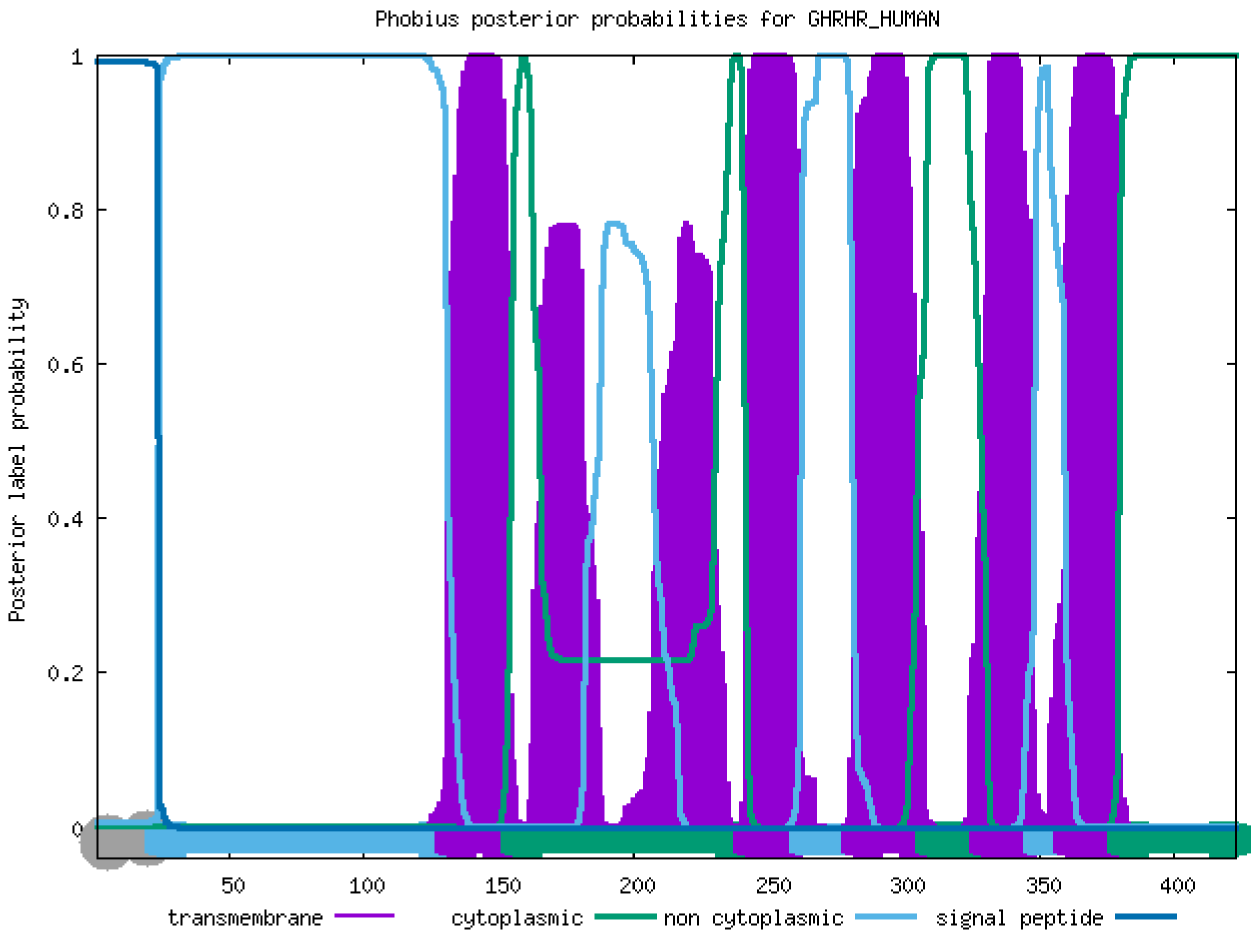

The probability data used in the plot is found here, and the gnuplot script is here.

Prediction of AGRD1_HUMAN

```
ID   AGRD1_HUMAN
FT   SIGNAL        1     25
FT   REGION        1      6       N-REGION.
FT   REGION        7     17       H-REGION.
FT   REGION       18     25       C-REGION.
FT   TOPO_DOM     26    568       NON CYTOPLASMIC.
FT   TRANSMEM    569    592
FT   TOPO_DOM    593    603       CYTOPLASMIC.
FT   TRANSMEM    604    625
FT   TOPO_DOM    626    636       NON CYTOPLASMIC.
FT   TRANSMEM    637    662
FT   TOPO_DOM    663    673       CYTOPLASMIC.
FT   TRANSMEM    674    693
FT   TOPO_DOM    694    712       NON CYTOPLASMIC.
FT   TRANSMEM    713    738
FT   TOPO_DOM    739    758       CYTOPLASMIC.
FT   TRANSMEM    759    780
FT   TOPO_DOM    781    785       NON CYTOPLASMIC.
FT   TRANSMEM    786    809
FT   TOPO_DOM    810    874       CYTOPLASMIC.
//
```

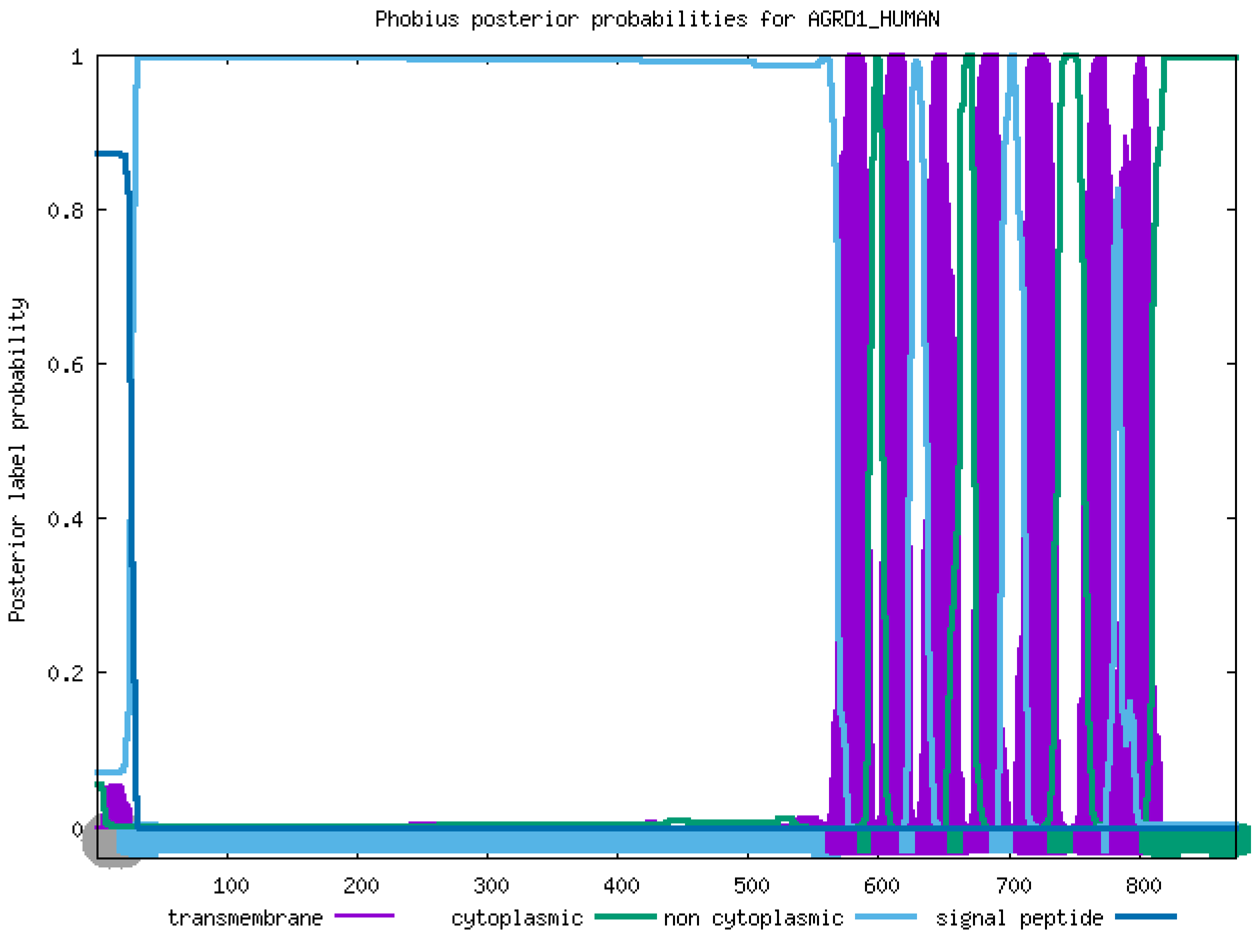

The probability data used in the plot is found here, and the gnuplot script is here.

Prediction of AGRF1_HUMAN

```
ID    AGRF1_HUMAN
FT    SIGNAL          1      19
FT    REGION          1       2       N-REGION.
FT    REGION          3      14       H-REGION.
FT    REGION         15      19       C-REGION.
FT    TOPO_DOM       20     586       NON CYTOPLASMIC.
FT    TRANSMEM      587     606
FT    TOPO_DOM      607     626       CYTOPLASMIC.
FT    TRANSMEM      627     647
FT    TOPO_DOM      648     658       NON CYTOPLASMIC.
FT    TRANSMEM      659     684
FT    TOPO_DOM      685     695       CYTOPLASMIC.
FT    TRANSMEM      696     720
FT    TOPO_DOM      721     743       NON CYTOPLASMIC.
FT    TRANSMEM      744     771
FT    TOPO_DOM      772     791       CYTOPLASMIC.
FT    TRANSMEM      792     812
FT    TOPO_DOM      813     817       NON CYTOPLASMIC.
FT    TRANSMEM      818     841
FT    TOPO_DOM      842     910       CYTOPLASMIC.
//
```

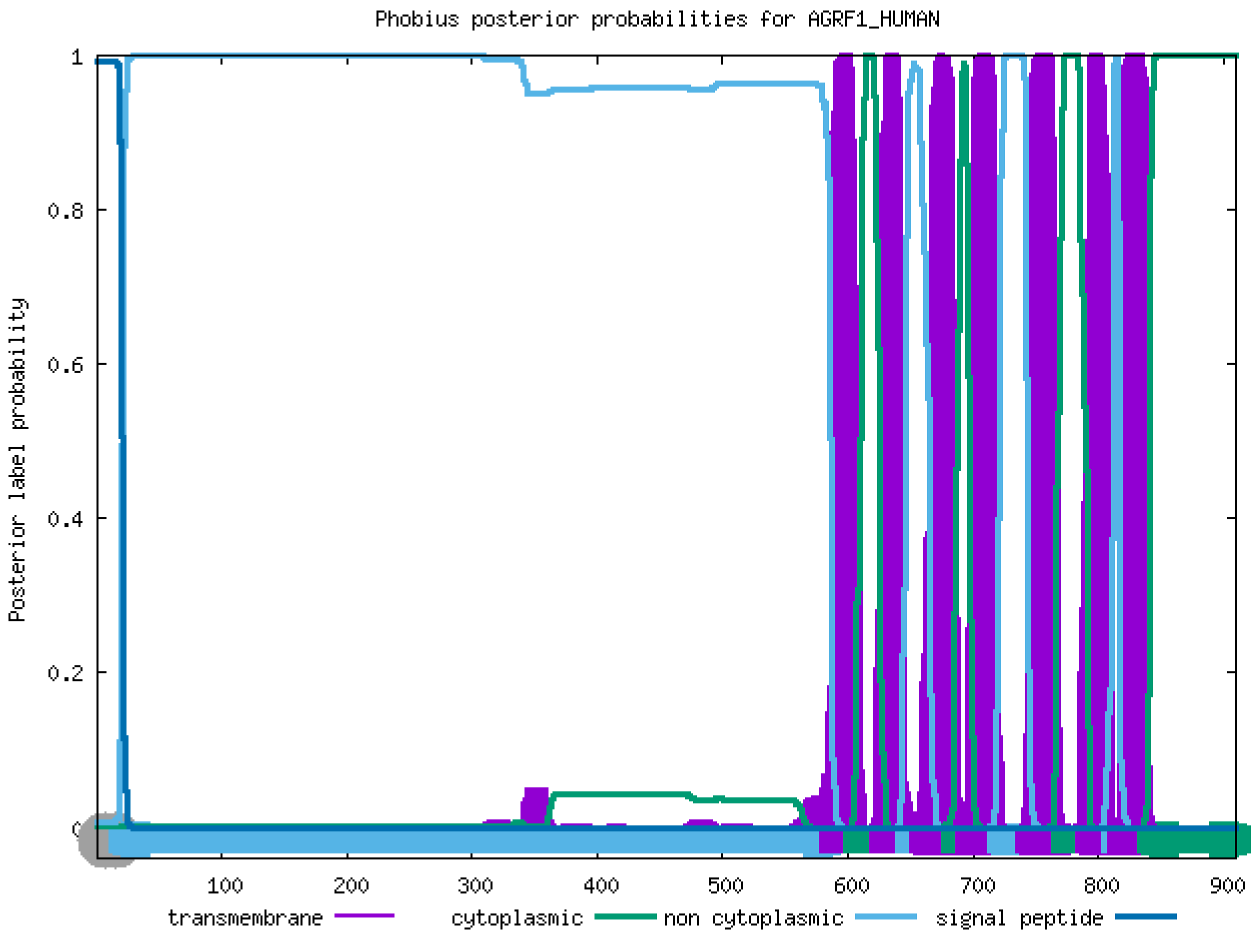

The probability data used in the plot is found here, and the gnuplot script is here.

Prediction of AGRG1_HUMAN

```
ID   AGRG1_HUMAN
FT   SIGNAL        1     22
FT   REGION        1      5       N-REGION.
FT   REGION        6     17       H-REGION.
FT   REGION       18     22       C-REGION.
FT   TOPO_DOM     23    404       NON CYTOPLASMIC.
FT   TRANSMEM    405    428
FT   TOPO_DOM    429    448       CYTOPLASMIC.
FT   TRANSMEM    449    471
FT   TOPO_DOM    472    476       NON CYTOPLASMIC.
FT   TRANSMEM    477    495
FT   TOPO_DOM    496    514       CYTOPLASMIC.
FT   TRANSMEM    515    537
FT   TOPO_DOM    538    574       NON CYTOPLASMIC.
FT   TRANSMEM    575    597
FT   TOPO_DOM    598    608       CYTOPLASMIC.
FT   TRANSMEM    609    631
FT   TOPO_DOM    632    636       NON CYTOPLASMIC.
FT   TRANSMEM    637    659
FT   TOPO_DOM    660    693       CYTOPLASMIC.
//
```

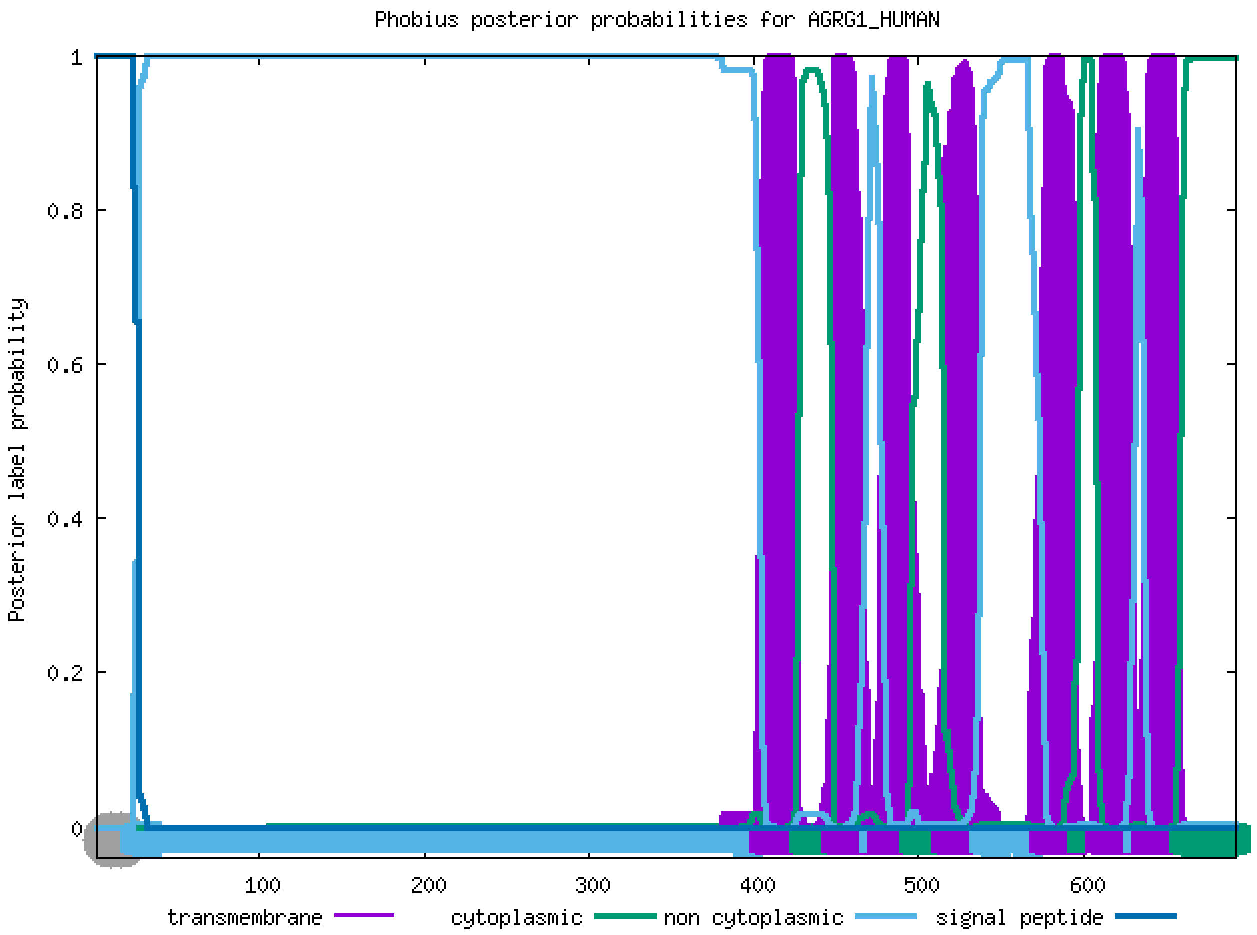

The probability data used in the plot is found here, and the gnuplot script is here.

Prediction of AGRG3_HUMAN

```
ID    AGRG3_HUMAN
FT    SIGNAL         1      20
FT    REGION         1       5       N-REGION.
FT    REGION         6      16       H-REGION.
FT    REGION        17      20       C-REGION.
FT    TOPO_DOM      21     270       NON CYTOPLASMIC.
FT    TRANSMEM     271     293
FT    TOPO_DOM     294     304       CYTOPLASMIC.
FT    TRANSMEM     305     325
FT    TOPO_DOM     326     344       NON CYTOPLASMIC.
FT    TRANSMEM     345     366
FT    TOPO_DOM     367     377       CYTOPLASMIC.
FT    TRANSMEM     378     396
FT    TOPO_DOM     397     428       NON CYTOPLASMIC.
FT    TRANSMEM     429     455
FT    TOPO_DOM     456     475       CYTOPLASMIC.
FT    TRANSMEM     476     498
FT    TOPO_DOM     499     503       NON CYTOPLASMIC.
FT    TRANSMEM     504     526
FT    TOPO_DOM     527     549       CYTOPLASMIC.
//
```

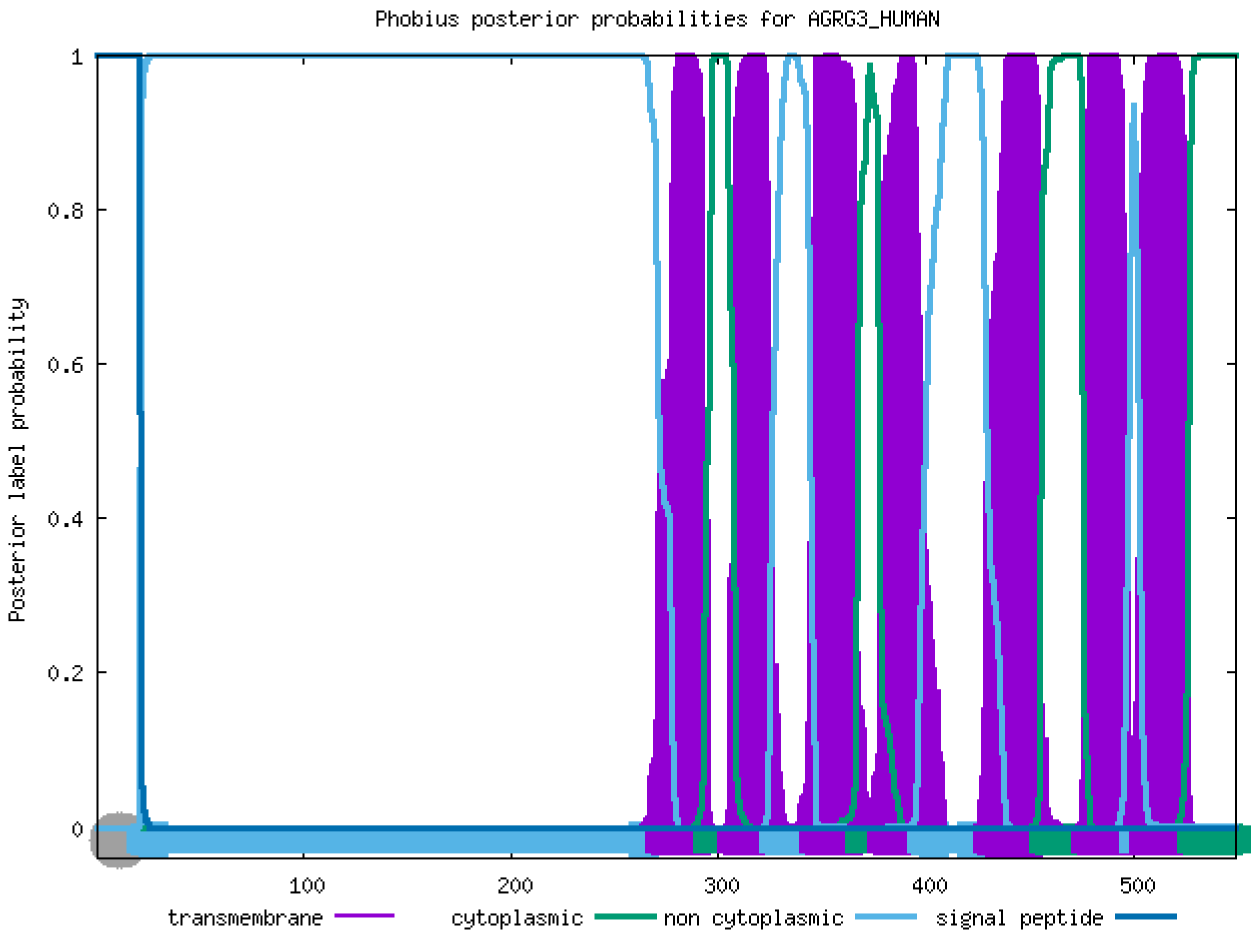

The probability data used in the plot is found here, and the gnuplot script is here.

Prediction of AGRL3_HUMAN

```
ID    AGRL3_HUMAN
FT    SIGNAL         1      22
FT    REGION         1       5       N-REGION.
FT    REGION         6      17       H-REGION.
FT    REGION        18      22       C-REGION.
FT    TOPO_DOM      23     865       NON CYTOPLASMIC.
FT    TRANSMEM     866     889
FT    TOPO_DOM     890     900       CYTOPLASMIC.
FT    TRANSMEM     901     919
FT    TOPO_DOM     920     924       NON CYTOPLASMIC.
FT    TRANSMEM     925     947
FT    TOPO_DOM     948     967       CYTOPLASMIC.
FT    TRANSMEM     968     988
FT    TOPO_DOM     989    1007       NON CYTOPLASMIC.
FT    TRANSMEM    1008    1031
FT    TOPO_DOM    1032    1051       CYTOPLASMIC.
FT    TRANSMEM    1052    1075
FT    TOPO_DOM    1076    1080       NON CYTOPLASMIC.
FT    TRANSMEM    1081    1104
FT    TOPO_DOM    1105    1447       CYTOPLASMIC.
//
```

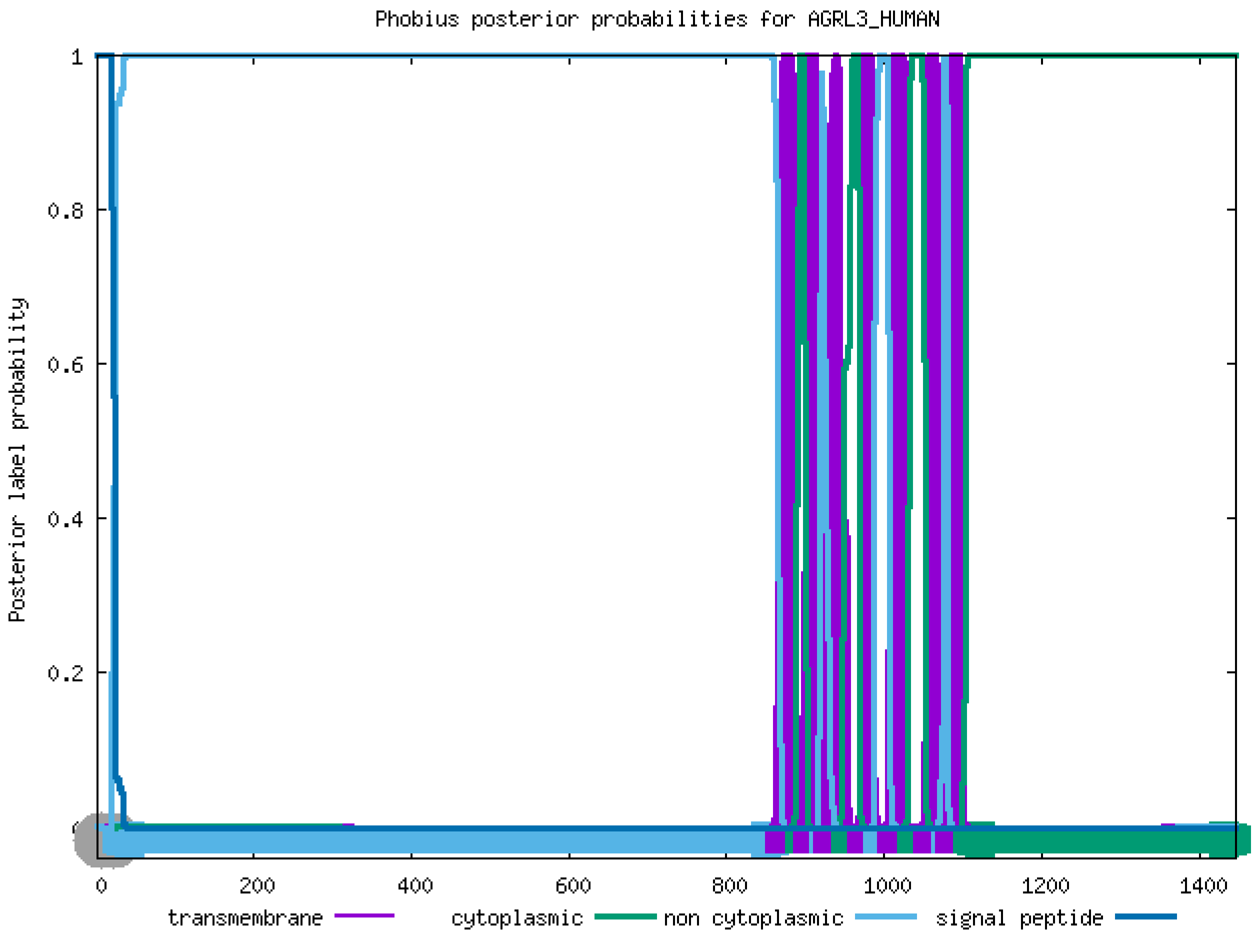

The probability data used in the plot is found here, and the gnuplot script is here.

Prediction of GABR1_HUMAN

```
ID   GABR1_HUMAN
FT   SIGNAL         1     19
FT   REGION         1      1       N-REGION.
FT   REGION         2     12       H-REGION.
FT   REGION        13     19       C-REGION.
FT   TOPO_DOM      20    590       NON CYTOPLASMIC.
FT   TRANSMEM     591    616
FT   TOPO_DOM     617    627       CYTOPLASMIC.
FT   TRANSMEM     628    647
FT   TOPO_DOM     648    666       NON CYTOPLASMIC.
FT   TRANSMEM     667    687
FT   TOPO_DOM     688    707       CYTOPLASMIC.
FT   TRANSMEM     708    731
FT   TOPO_DOM     732    767       NON CYTOPLASMIC.
FT   TRANSMEM     768    788
FT   TOPO_DOM     789    807       CYTOPLASMIC.
FT   TRANSMEM     808    826
FT   TOPO_DOM     827    831       NON CYTOPLASMIC.
FT   TRANSMEM     832    854
FT   TOPO_DOM     855    961       CYTOPLASMIC.
//
```

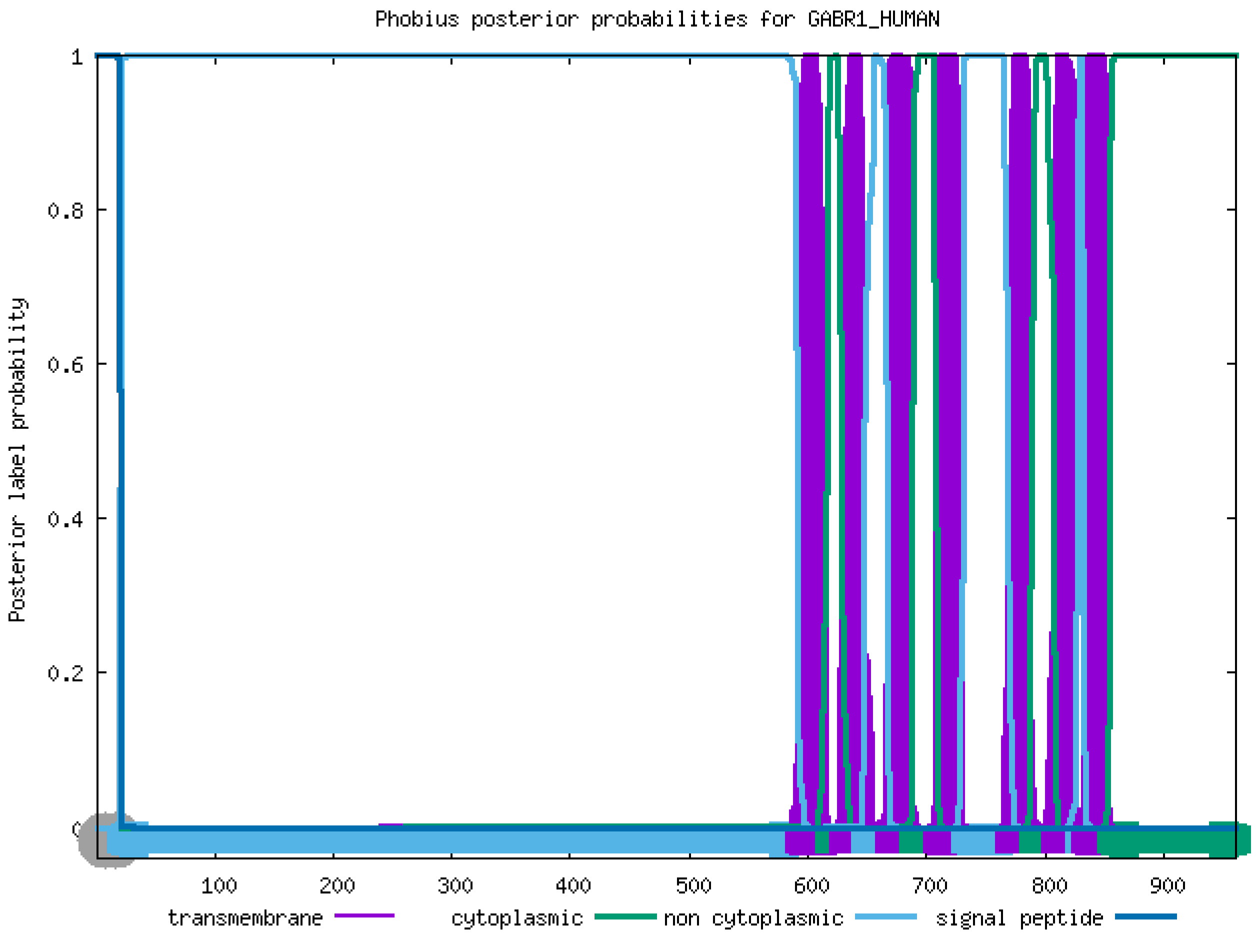

The probability data used in the plot is found here, and the gnuplot script is here.

Prediction of GABR2_HUMAN

```
ID   GABR2_HUMAN
FT   SIGNAL         1     45
FT   REGION         1     22       N-REGION.
FT   REGION        23     33       H-REGION.
FT   REGION        34     45       C-REGION.
FT   TOPO_DOM      46    478       NON CYTOPLASMIC.
FT   TRANSMEM     479    502
FT   TOPO_DOM     503    521       CYTOPLASMIC.
FT   TRANSMEM     522    543
FT   TOPO_DOM     544    554       NON CYTOPLASMIC.
FT   TRANSMEM     555    576
FT   TOPO_DOM     577    596       CYTOPLASMIC.
FT   TRANSMEM     597    618
FT   TOPO_DOM     619    652       NON CYTOPLASMIC.
FT   TRANSMEM     653    676
FT   TOPO_DOM     677    695       CYTOPLASMIC.
FT   TRANSMEM     696    712
FT   TOPO_DOM     713    717       NON CYTOPLASMIC.
FT   TRANSMEM     718    741
FT   TOPO_DOM     742    941       CYTOPLASMIC.
//
```

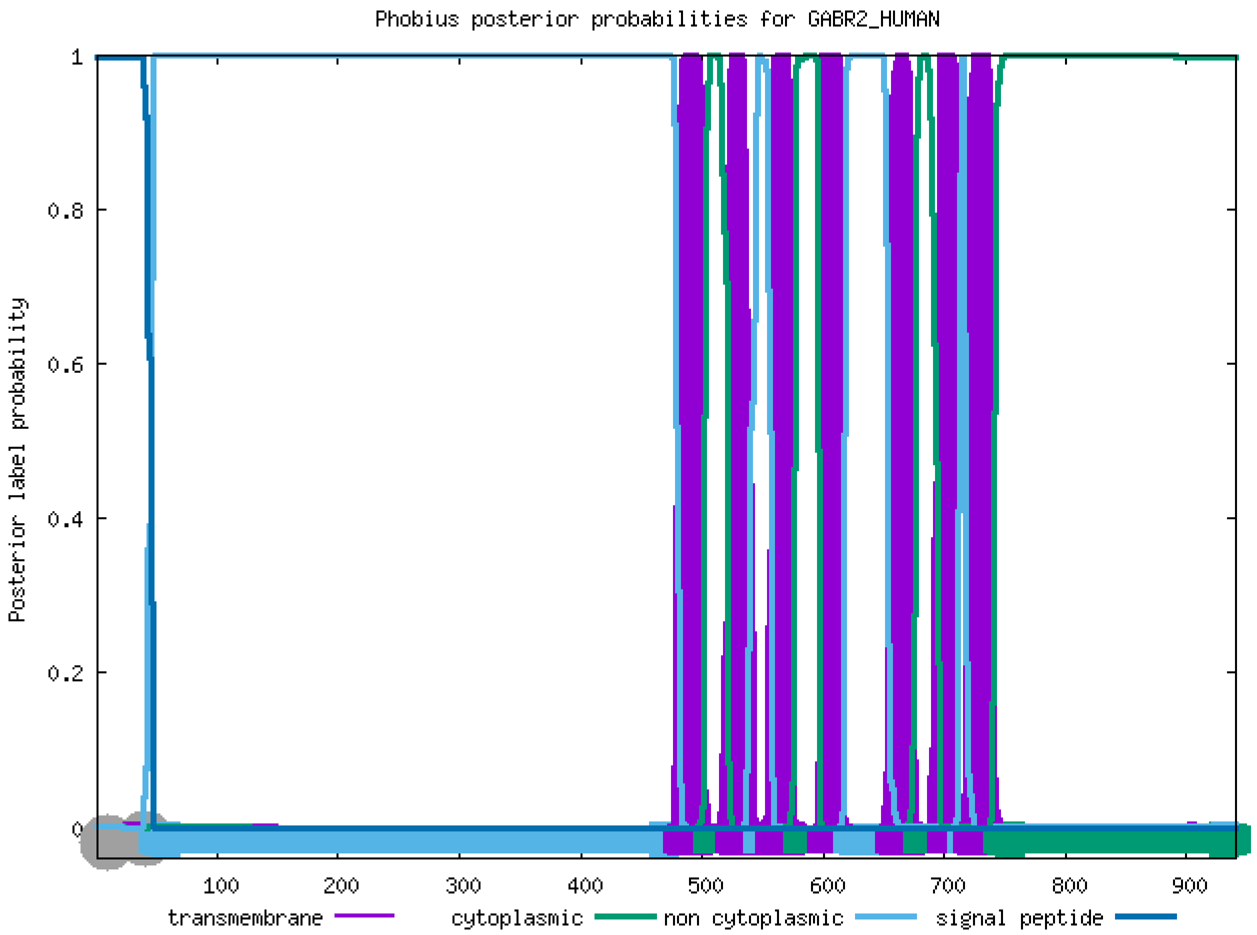

The probability data used in the plot is found here, and the gnuplot script is here.

Prediction of GRM1_HUMAN

```
ID   GRM1_HUMAN
FT   SIGNAL        1     18
FT   REGION        1      2       N-REGION.
FT   REGION        3     14       H-REGION.
FT   REGION       15     18       C-REGION.
FT   TOPO_DOM     19    593       NON CYTOPLASMIC.
FT   TRANSMEM    594    617
FT   TOPO_DOM    618    628       CYTOPLASMIC.
FT   TRANSMEM    629    650
FT   TOPO_DOM    651    661       NON CYTOPLASMIC.
FT   TRANSMEM    662    683
FT   TOPO_DOM    684    703       CYTOPLASMIC.
FT   TRANSMEM    704    727
FT   TOPO_DOM    728    752       NON CYTOPLASMIC.
FT   TRANSMEM    753    772
FT   TOPO_DOM    773    783       CYTOPLASMIC.
FT   TRANSMEM    784    806
FT   TOPO_DOM    807    811       NON CYTOPLASMIC.
FT   TRANSMEM    812    831
FT   TOPO_DOM    832   1194       CYTOPLASMIC.
//
```

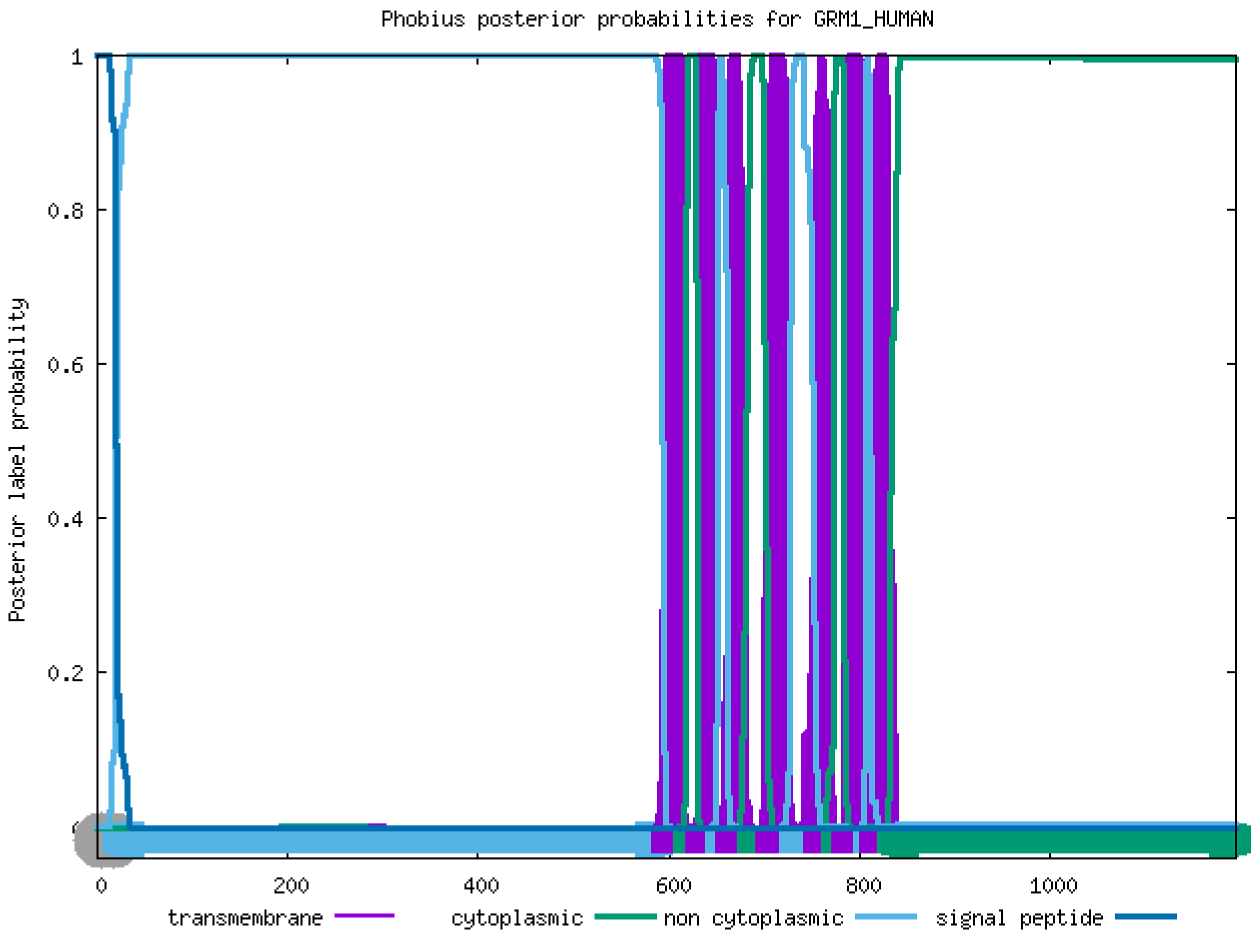

The probability data used in the plot is found here, and the gnuplot script is here.

Prediction of GRM2_HUMAN

```
ID   GRM2_HUMAN
FT   SIGNAL         1     18
FT   REGION         1      2       N-REGION.
FT   REGION         3     13       H-REGION.
FT   REGION        14     18       C-REGION.
FT   TOPO_DOM      19    565       NON CYTOPLASMIC.
FT   TRANSMEM     566    590
FT   TOPO_DOM     591    604       CYTOPLASMIC.
FT   TRANSMEM     605    624
FT   TOPO_DOM     625    635       NON CYTOPLASMIC.
FT   TRANSMEM     636    652
FT   TOPO_DOM     653    672       CYTOPLASMIC.
FT   TRANSMEM     673    700
FT   TOPO_DOM     701    727       NON CYTOPLASMIC.
FT   TRANSMEM     728    747
FT   TOPO_DOM     748    758       CYTOPLASMIC.
FT   TRANSMEM     759    781
FT   TOPO_DOM     782    786       NON CYTOPLASMIC.
FT   TRANSMEM     787    811
FT   TOPO_DOM     812    872       CYTOPLASMIC.
//
```

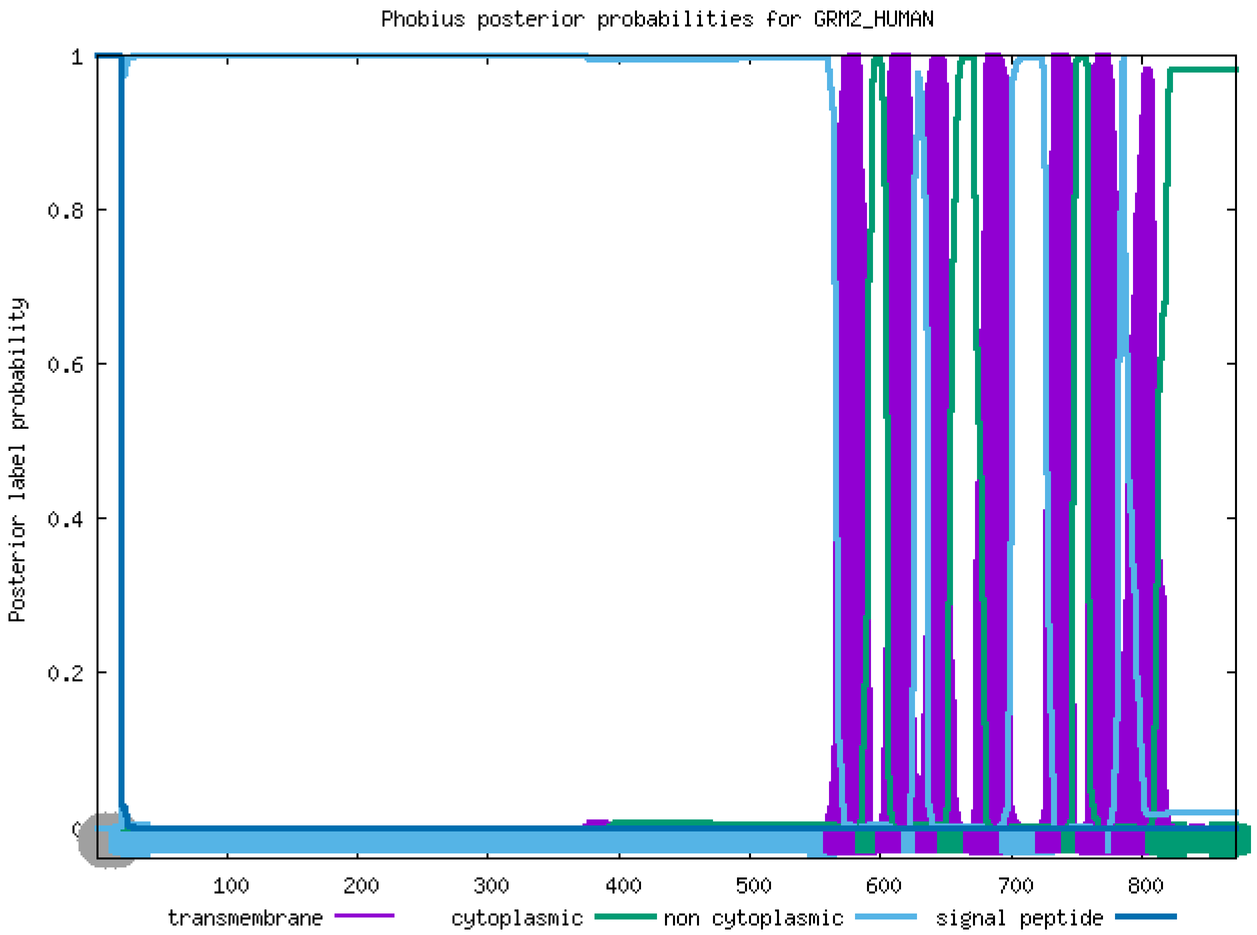

The probability data used in the plot is found here, and the gnuplot script is here.

Prediction of GRM3_HUMAN

```
ID    GRM3_HUMAN
FT    SIGNAL         1     20
FT    REGION         1      6       N-REGION.
FT    REGION         7     15       H-REGION.
FT    REGION        16     20       C-REGION.
FT    TOPO_DOM      21    574       NON CYTOPLASMIC.
FT    TRANSMEM     575    599
FT    TOPO_DOM     600    613       CYTOPLASMIC.
FT    TRANSMEM     614    634
FT    TOPO_DOM     635    645       NON CYTOPLASMIC.
FT    TRANSMEM     646    667
FT    TOPO_DOM     668    687       CYTOPLASMIC.
FT    TRANSMEM     688    709
FT    TOPO_DOM     710    736       NON CYTOPLASMIC.
FT    TRANSMEM     737    756
FT    TOPO_DOM     757    767       CYTOPLASMIC.
FT    TRANSMEM     768    790
FT    TOPO_DOM     791    795       NON CYTOPLASMIC.
FT    TRANSMEM     796    820
FT    TOPO_DOM     821    879       CYTOPLASMIC.
//
```

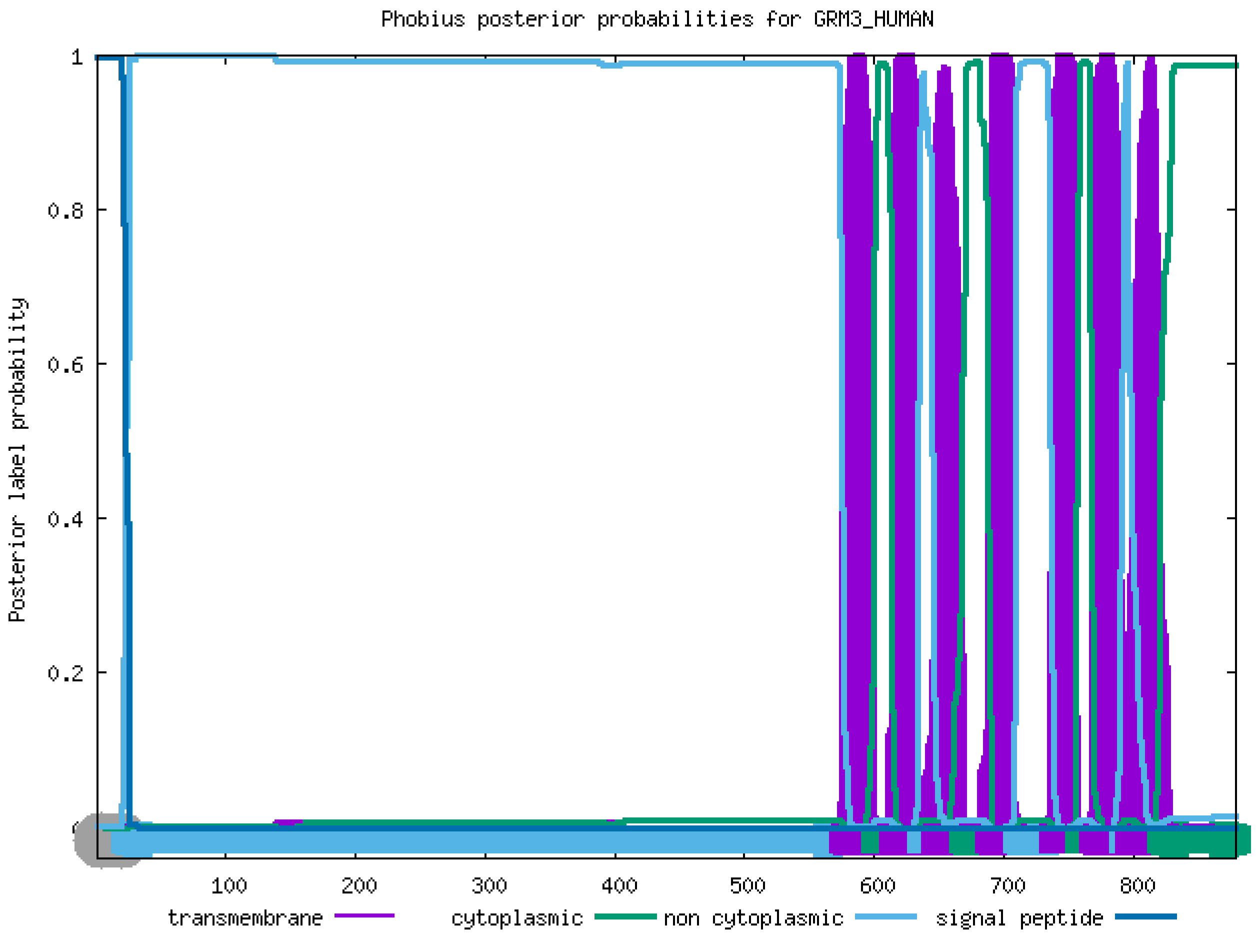

The probability data used in the plot is found here, and the gnuplot script is here.

Prediction of GRM4_HUMAN

```
ID    GRM4_HUMAN
FT    SIGNAL         1      32
FT    REGION         1      13       N-REGION.
FT    REGION        14      24       H-REGION.
FT    REGION        25      32       C-REGION.
FT    TOPO_DOM      33     586       NON CYTOPLASMIC.
FT    TRANSMEM     587     613
FT    TOPO_DOM     614     624       CYTOPLASMIC.
FT    TRANSMEM     625     645
FT    TOPO_DOM     646     656       NON CYTOPLASMIC.
FT    TRANSMEM     657     678
FT    TOPO_DOM     679     698       CYTOPLASMIC.
FT    TRANSMEM     699     720
FT    TOPO_DOM     721     750       NON CYTOPLASMIC.
FT    TRANSMEM     751     772
FT    TOPO_DOM     773     783       CYTOPLASMIC.
FT    TRANSMEM     784     806
FT    TOPO_DOM     807     825       NON CYTOPLASMIC.
FT    TRANSMEM     826     848
FT    TOPO_DOM     849     912       CYTOPLASMIC.
//
```

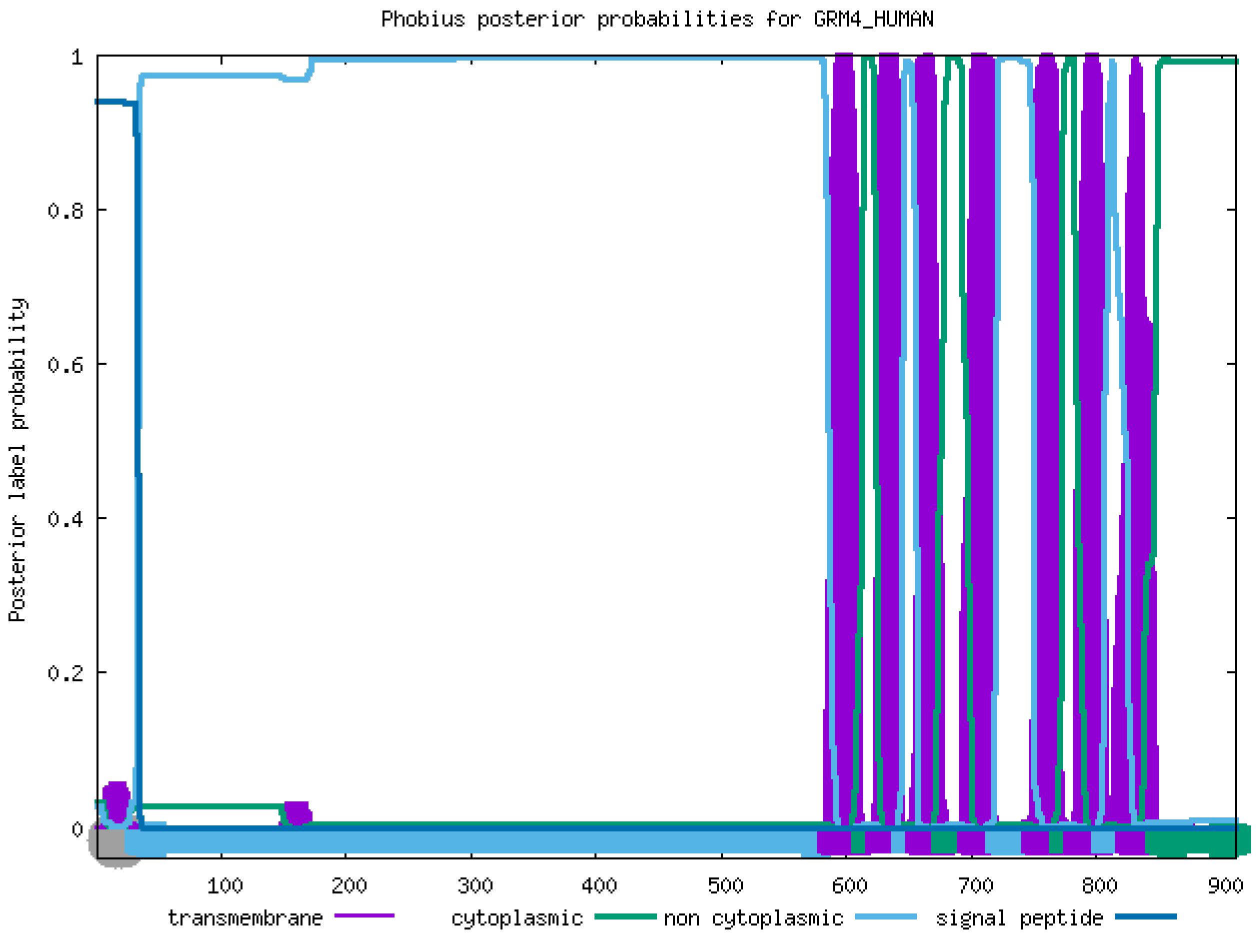

The probability data used in the plot is found here, and the gnuplot script is here.

Prediction of GRM5_HUMAN

```
ID   GRM5_HUMAN
FT   SIGNAL        1     20
FT   REGION        1      1       N-REGION.
FT   REGION        2     12       H-REGION.
FT   REGION       13     20       C-REGION.
FT   TOPO_DOM     21    580       NON CYTOPLASMIC.
FT   TRANSMEM    581    604
FT   TOPO_DOM    605    615       CYTOPLASMIC.
FT   TRANSMEM    616    639
FT   TOPO_DOM    640    644       NON CYTOPLASMIC.
FT   TRANSMEM    645    666
FT   TOPO_DOM    667    686       CYTOPLASMIC.
FT   TRANSMEM    687    714
FT   TOPO_DOM    715    739       NON CYTOPLASMIC.
FT   TRANSMEM    740    759
FT   TOPO_DOM    760    770       CYTOPLASMIC.
FT   TRANSMEM    771    793
FT   TOPO_DOM    794    798       NON CYTOPLASMIC.
FT   TRANSMEM    799    826
FT   TOPO_DOM    827   1212       CYTOPLASMIC.
//
```

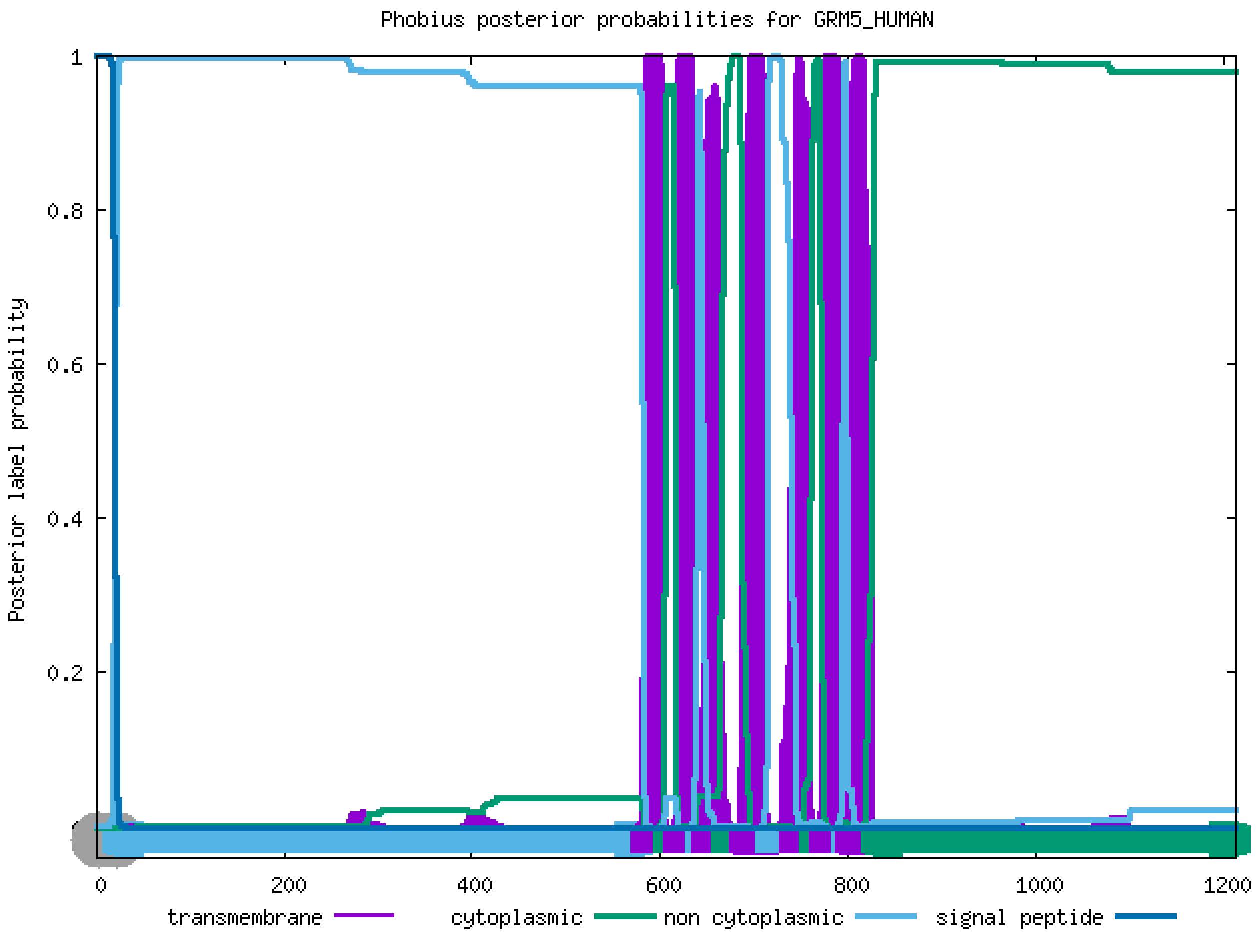

The probability data used in the plot is found here, and the gnuplot script is here.

Prediction of GRM7_HUMAN

```
ID   GRM7_HUMAN
FT   SIGNAL        1     34
FT   REGION        1     17       N-REGION.
FT   REGION       18     29       H-REGION.
FT   REGION       30     34       C-REGION.
FT   TOPO_DOM     35    589       NON CYTOPLASMIC.
FT   TRANSMEM    590    616
FT   TOPO_DOM    617    627       CYTOPLASMIC.
FT   TRANSMEM    628    648
FT   TOPO_DOM    649    659       NON CYTOPLASMIC.
FT   TRANSMEM    660    681
FT   TOPO_DOM    682    701       CYTOPLASMIC.
FT   TRANSMEM    702    723
FT   TOPO_DOM    724    753       NON CYTOPLASMIC.
FT   TRANSMEM    754    775
FT   TOPO_DOM    776    786       CYTOPLASMIC.
FT   TRANSMEM    787    809
FT   TOPO_DOM    810    828       NON CYTOPLASMIC.
FT   TRANSMEM    829    851
FT   TOPO_DOM    852    915       CYTOPLASMIC.
//
```

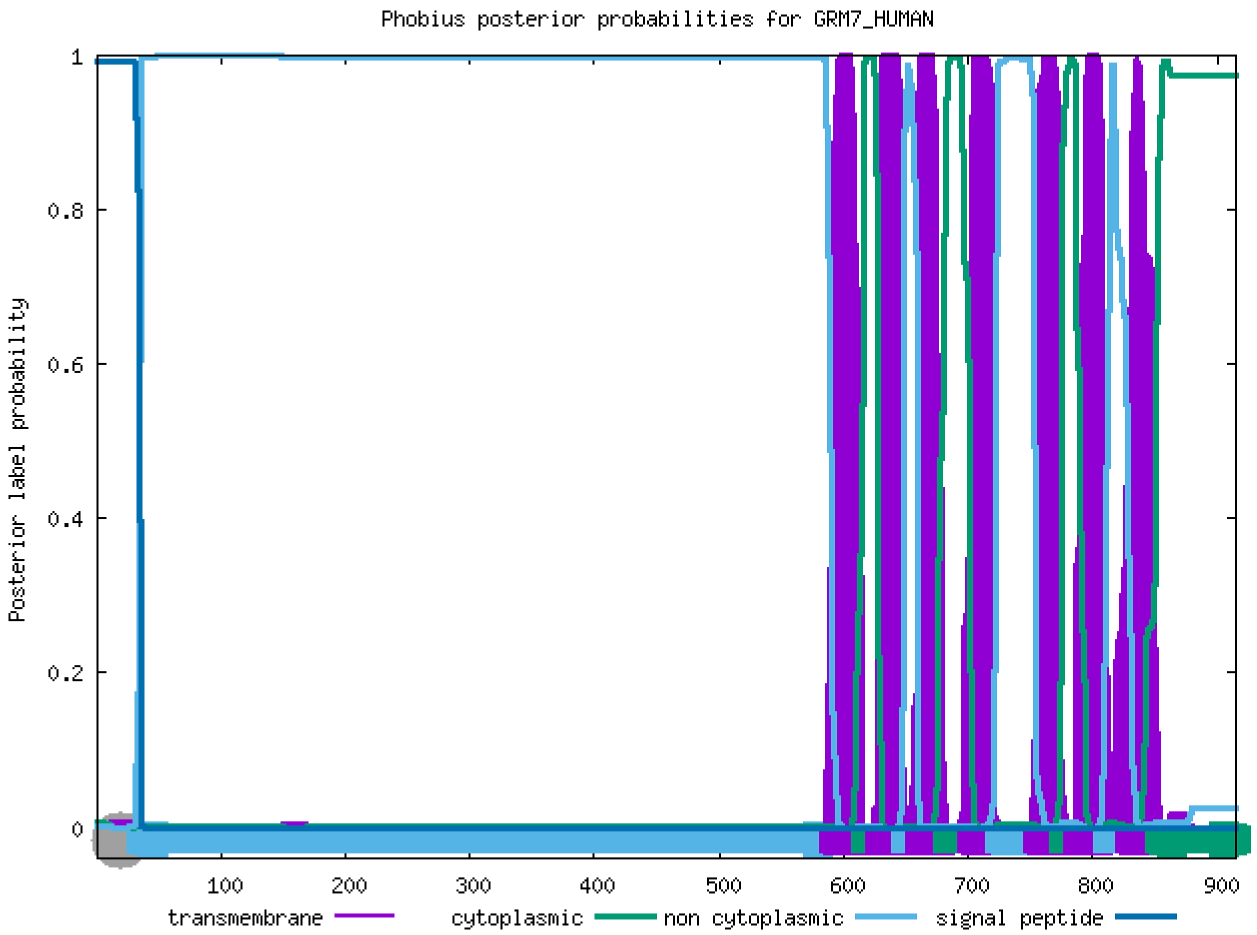

The probability data used in the plot is found here, and the gnuplot script is here.

Prediction of CASR_HUMAN

```
ID   CASR_HUMAN
FT   SIGNAL        1     19
FT   REGION        1      2       N-REGION.
FT   REGION        3     14       H-REGION.
FT   REGION       15     19       C-REGION.
FT   TOPO_DOM     20    611       NON CYTOPLASMIC.
FT   TRANSMEM    612    637
FT   TOPO_DOM    638    648       CYTOPLASMIC.
FT   TRANSMEM    649    670
FT   TOPO_DOM    671    681       NON CYTOPLASMIC.
FT   TRANSMEM    682    704
FT   TOPO_DOM    705    724       CYTOPLASMIC.
FT   TRANSMEM    725    744
FT   TOPO_DOM    745    769       NON CYTOPLASMIC.
FT   TRANSMEM    770    792
FT   TOPO_DOM    793    803       CYTOPLASMIC.
FT   TRANSMEM    804    826
FT   TOPO_DOM    827    831       NON CYTOPLASMIC.
FT   TRANSMEM    832    854
FT   TOPO_DOM    855   1078       CYTOPLASMIC.
//
```

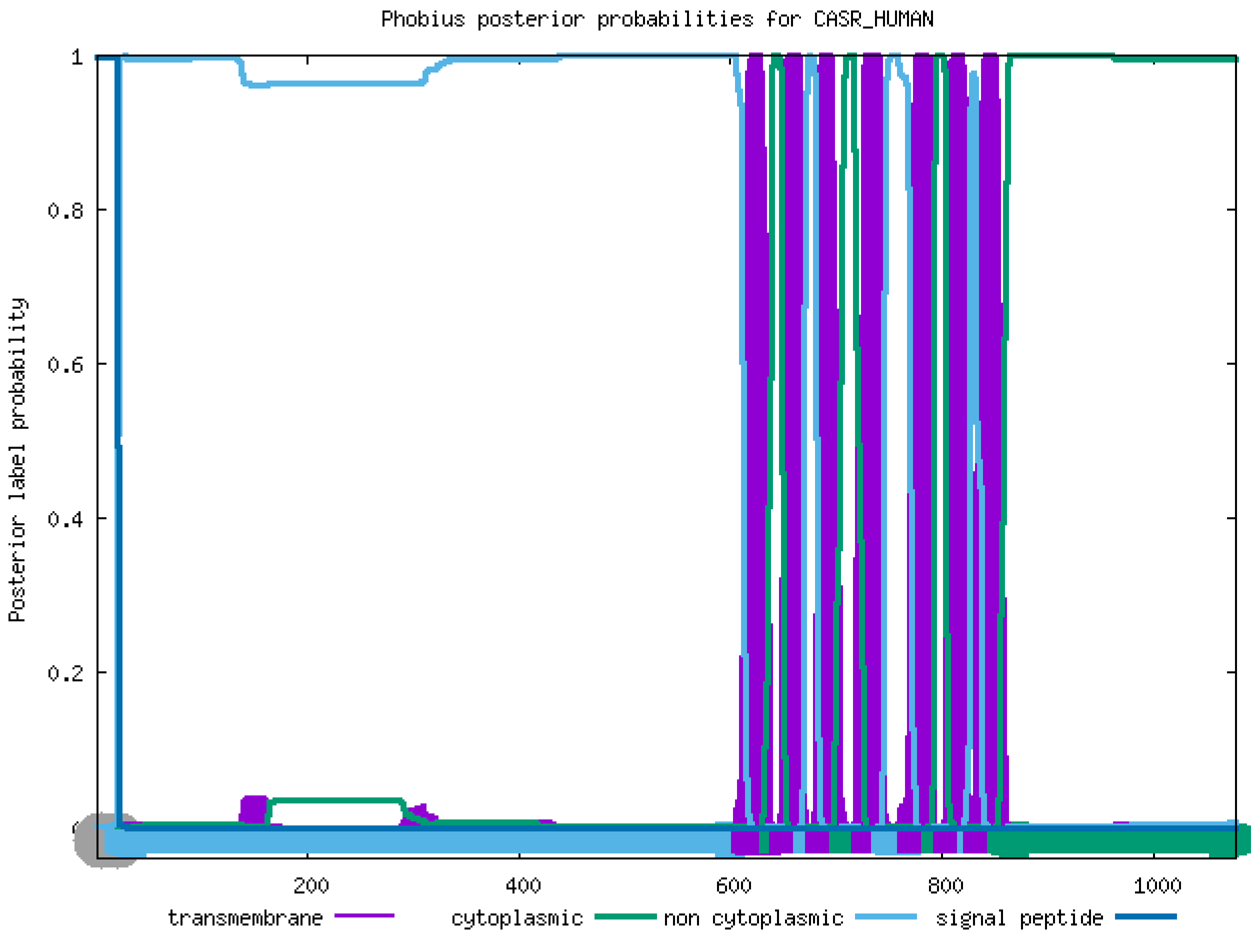

The probability data used in the plot is found here, and the gnuplot script is here.

Prediction of MGLYR_HUMAN

```
ID   MGLYR_HUMAN
FT   SIGNAL        1     24
FT   REGION        1      7       N-REGION.
FT   REGION        8     19       H-REGION.
FT   REGION       20     24       C-REGION.
FT   TOPO_DOM     25    414       NON CYTOPLASMIC.
FT   TRANSMEM    415    439
FT   TOPO_DOM    440    450       CYTOPLASMIC.
FT   TRANSMEM    451    474
FT   TOPO_DOM    475    485       NON CYTOPLASMIC.
FT   TRANSMEM    486    504
FT   TOPO_DOM    505    524       CYTOPLASMIC.
FT   TRANSMEM    525    544
FT   TOPO_DOM    545    579       NON CYTOPLASMIC.
FT   TRANSMEM    580    600
FT   TOPO_DOM    601    611       CYTOPLASMIC.
FT   TRANSMEM    612    630
FT   TOPO_DOM    631    641       NON CYTOPLASMIC.
FT   TRANSMEM    642    664
FT   TOPO_DOM    665   1215       CYTOPLASMIC.
//
```

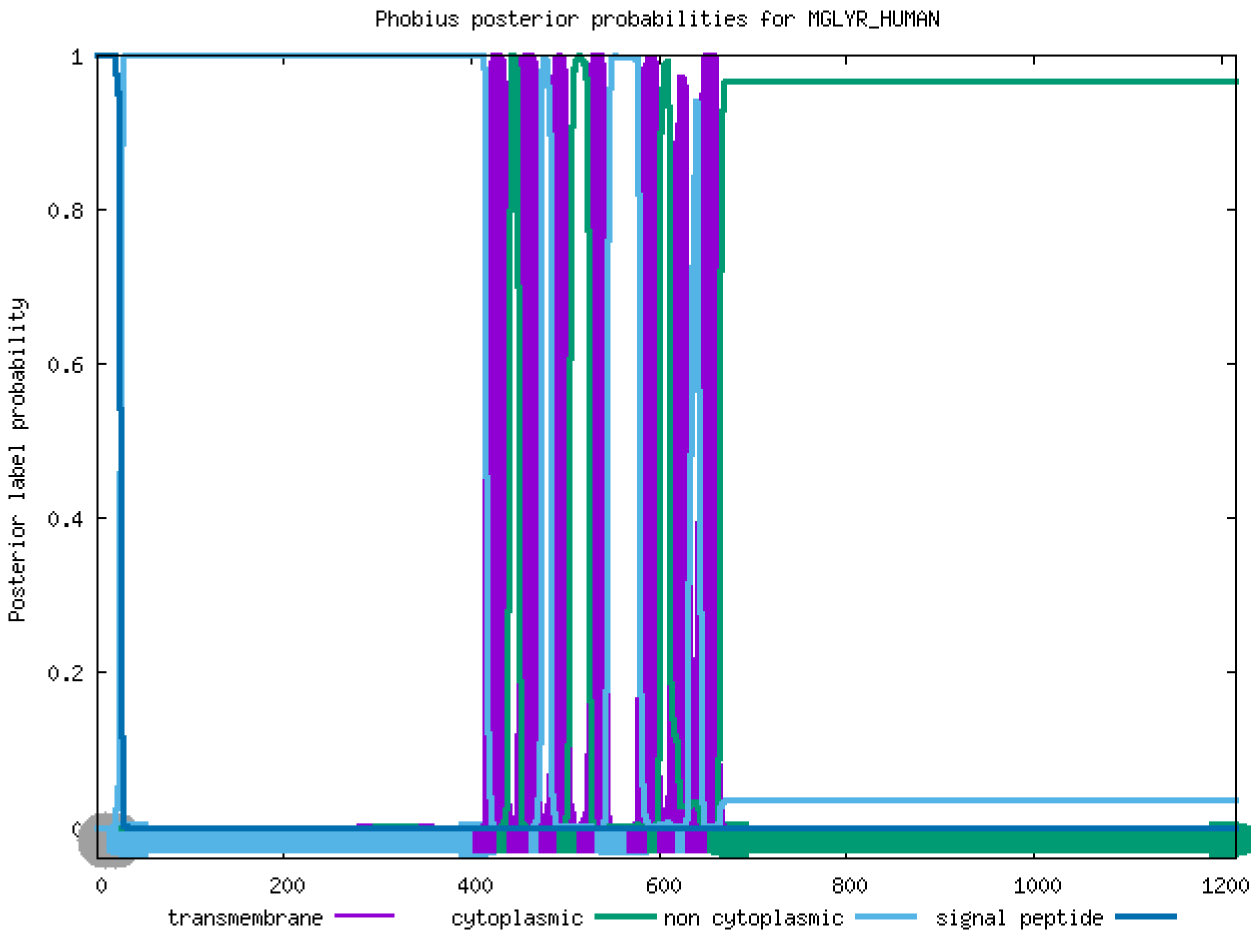

The probability data used in the plot is found here, and the gnuplot script is here.

Prediction of SMO_HUMAN

```
ID   SMO_HUMAN
FT   SIGNAL        1     31
FT   REGION        1     11       N-REGION.
FT   REGION       12     23       H-REGION.
FT   REGION       24     31       C-REGION.
FT   TOPO_DOM     32    232       NON CYTOPLASMIC.
FT   TRANSMEM    233    254
FT   TOPO_DOM    255    265       CYTOPLASMIC.
FT   TRANSMEM    266    283
FT   TOPO_DOM    284    314       NON CYTOPLASMIC.
FT   TRANSMEM    315    339
FT   TOPO_DOM    340    358       CYTOPLASMIC.
FT   TRANSMEM    359    379
FT   TOPO_DOM    380    398       NON CYTOPLASMIC.
FT   TRANSMEM    399    420
FT   TOPO_DOM    421    451       CYTOPLASMIC.
FT   TRANSMEM    452    475
FT   TOPO_DOM    476    519       NON CYTOPLASMIC.
FT   TRANSMEM    520    538
FT   TOPO_DOM    539    787       CYTOPLASMIC.
//
```

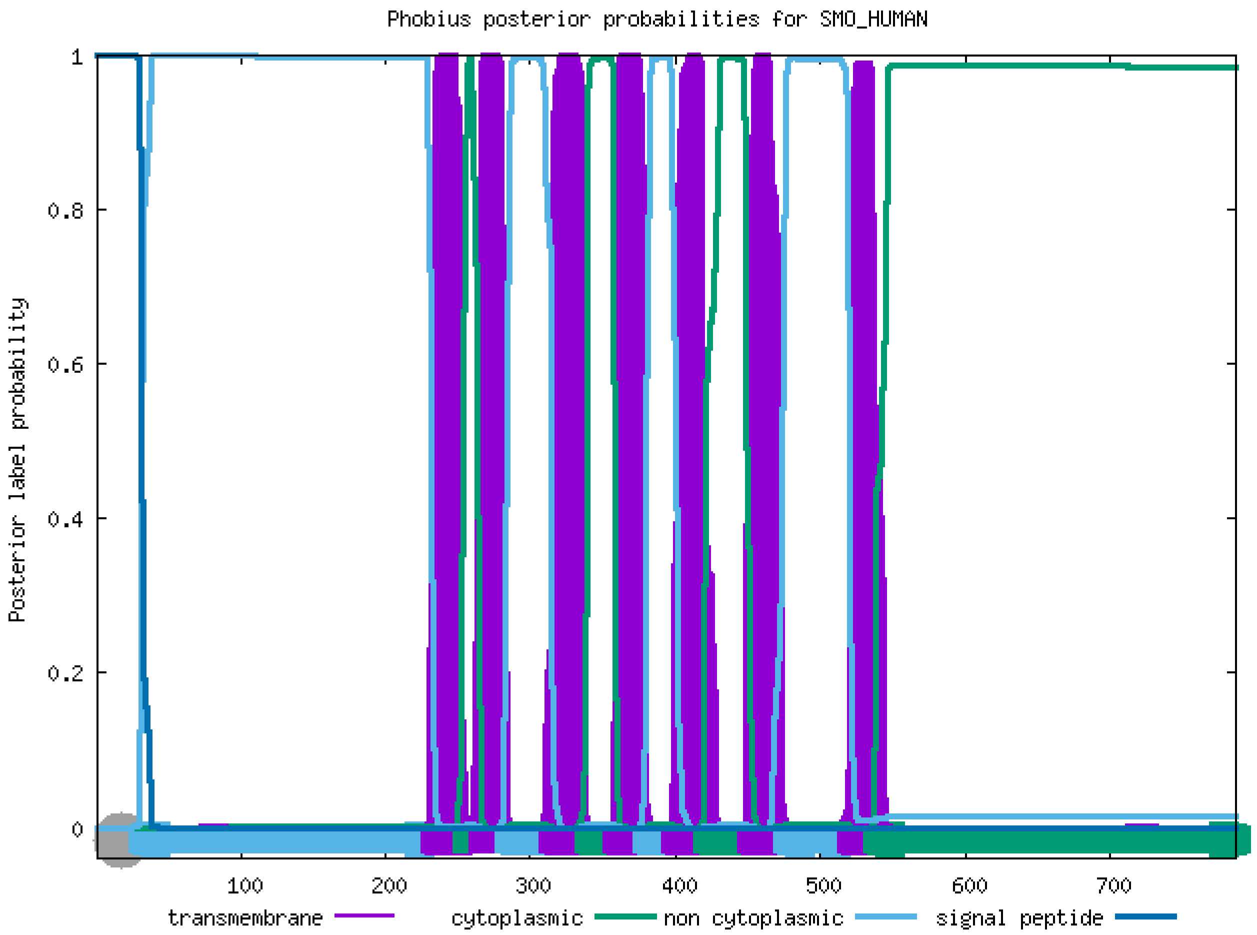

The probability data used in the plot is found here, and the gnuplot script is here.

Prediction of FZD1_HUMAN

```
ID   FZD1_HUMAN
FT   TOPO_DOM      1     50       CYTOPLASMIC.
FT   TRANSMEM     51     70
FT   TOPO_DOM     71    321       NON CYTOPLASMIC.
FT   TRANSMEM    322    343
FT   TOPO_DOM    344    354       CYTOPLASMIC.
FT   TRANSMEM    355    375
FT   TOPO_DOM    376    403       NON CYTOPLASMIC.
FT   TRANSMEM    404    429
FT   TOPO_DOM    430    448       CYTOPLASMIC.
FT   TRANSMEM    449    468
FT   TOPO_DOM    469    487       NON CYTOPLASMIC.
FT   TRANSMEM    488    514
FT   TOPO_DOM    515    534       CYTOPLASMIC.
FT   TRANSMEM    535    558
FT   TOPO_DOM    559    599       NON CYTOPLASMIC.
FT   TRANSMEM    600    622
FT   TOPO_DOM    623    647       CYTOPLASMIC.
//
```

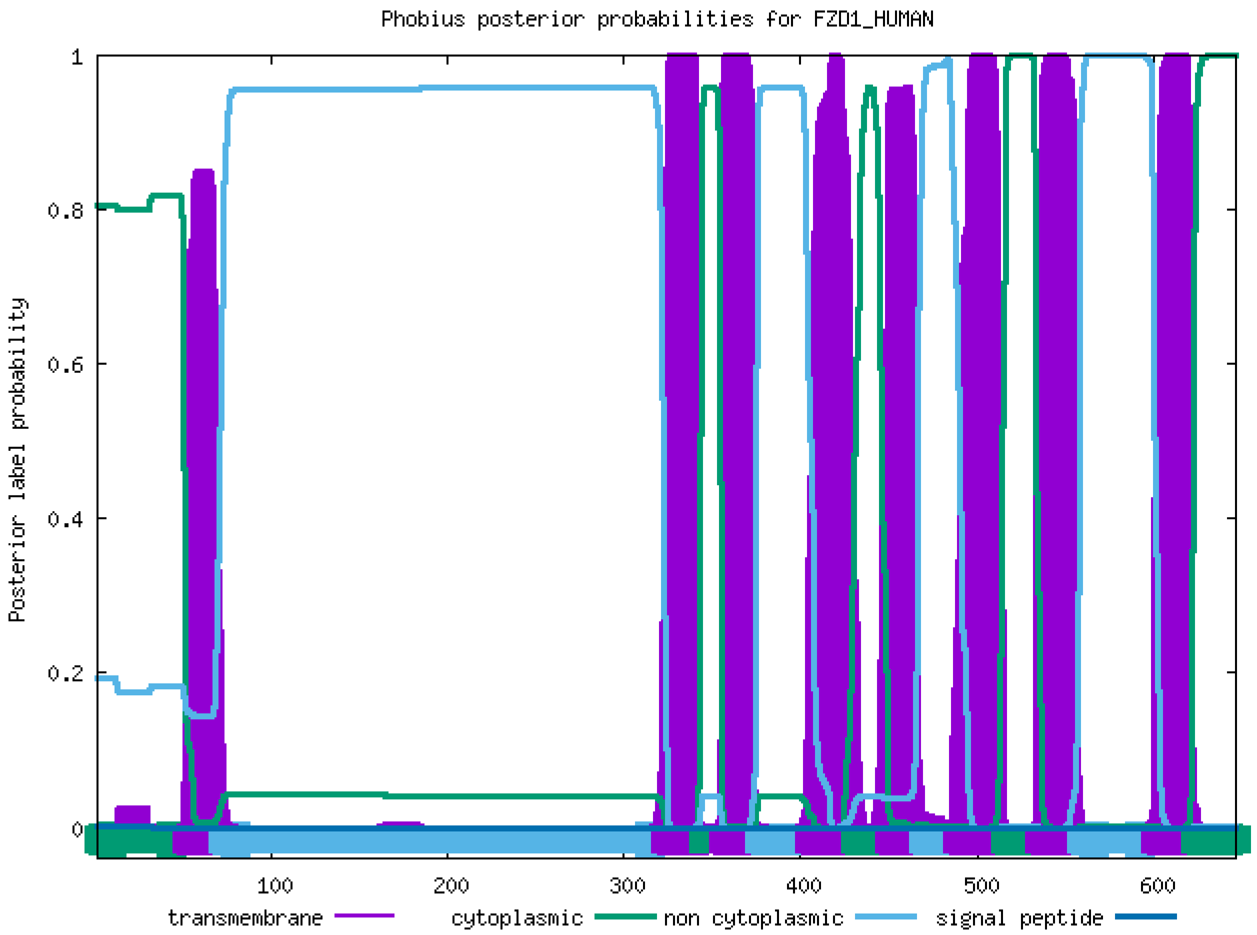

The probability data used in the plot is found here, and the gnuplot script is here.

Prediction of FZD3_HUMAN

```
ID   FZD3_HUMAN
FT   SIGNAL        1     28
FT   REGION        1      4       N-REGION.
FT   REGION        5     16       H-REGION.
FT   REGION       17     28       C-REGION.
FT   TOPO_DOM     29    203       NON CYTOPLASMIC.
FT   TRANSMEM    204    226
FT   TOPO_DOM    227    237       CYTOPLASMIC.
FT   TRANSMEM    238    258
FT   TOPO_DOM    259    290       NON CYTOPLASMIC.
FT   TRANSMEM    291    316
FT   TOPO_DOM    317    327       CYTOPLASMIC.
FT   TRANSMEM    328    350
FT   TOPO_DOM    351    369       NON CYTOPLASMIC.
FT   TRANSMEM    370    397
FT   TOPO_DOM    398    416       CYTOPLASMIC.
FT   TRANSMEM    417    442
FT   TOPO_DOM    443    476       NON CYTOPLASMIC.
FT   TRANSMEM    477    498
FT   TOPO_DOM    499    666       CYTOPLASMIC.
//
```

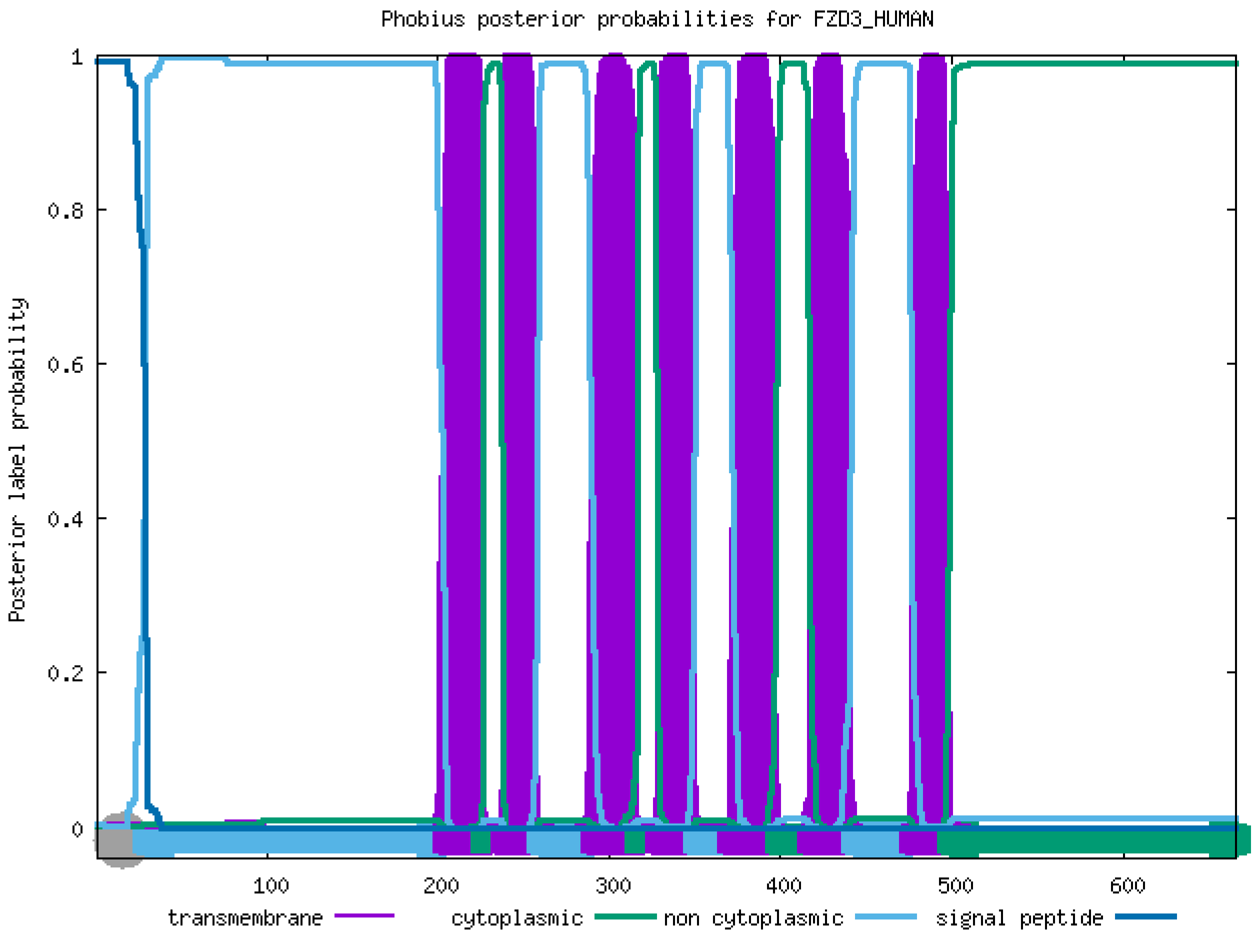

The probability data used in the plot is found here, and the gnuplot script is here.

Prediction of FZD4_HUMAN

```
ID    FZD4_HUMAN
FT    TOPO_DOM      1      11       NON CYTOPLASMIC.
FT    TRANSMEM     12      34
FT    TOPO_DOM     35     220       CYTOPLASMIC.
FT    TRANSMEM    221     243
FT    TOPO_DOM    244     254       NON CYTOPLASMIC.
FT    TRANSMEM    255     275
FT    TOPO_DOM    276     295       CYTOPLASMIC.
FT    TRANSMEM    296     312
FT    TOPO_DOM    313     317       NON CYTOPLASMIC.
FT    TRANSMEM    318     337
FT    TOPO_DOM    338     348       CYTOPLASMIC.
FT    TRANSMEM    349     368
FT    TOPO_DOM    369     387       NON CYTOPLASMIC.
FT    TRANSMEM    388     414
FT    TOPO_DOM    415     434       CYTOPLASMIC.
FT    TRANSMEM    435     458
FT    TOPO_DOM    459     477       NON CYTOPLASMIC.
FT    TRANSMEM    478     496
FT    TOPO_DOM    497     537       CYTOPLASMIC.
//
```

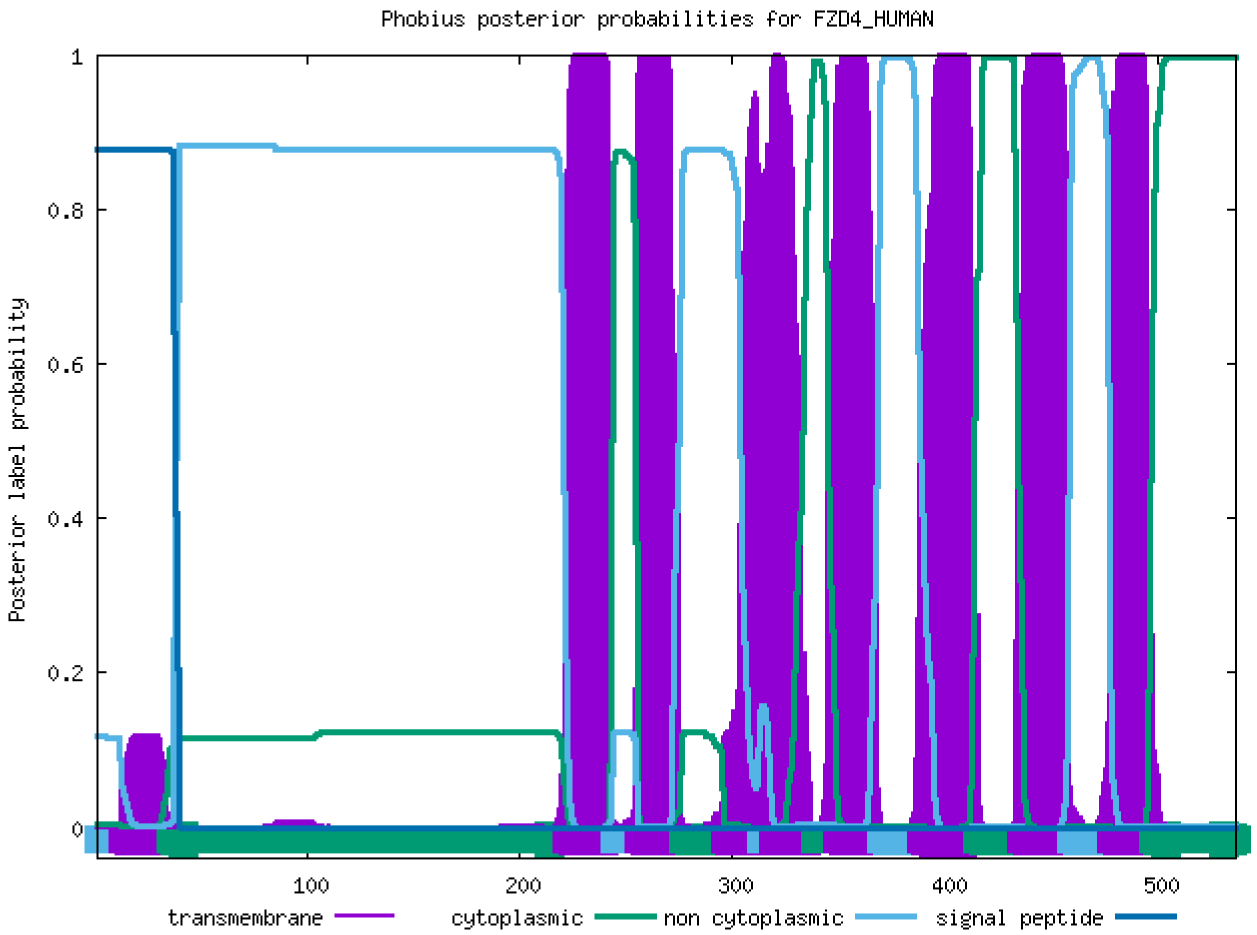

The probability data used in the plot is found here, and the gnuplot script is here.

Prediction of FZD5_HUMAN

```
ID    FZD5_HUMAN
FT    SIGNAL        1      26
FT    REGION        1      10       N-REGION.
FT    REGION       11      21       H-REGION.
FT    REGION       22      26       C-REGION.
FT    TOPO_DOM     27     236       NON CYTOPLASMIC.
FT    TRANSMEM    237     259
FT    TOPO_DOM    260     270       CYTOPLASMIC.
FT    TRANSMEM    271     291
FT    TOPO_DOM    292     310       NON CYTOPLASMIC.
FT    TRANSMEM    311     344
FT    TOPO_DOM    345     355       CYTOPLASMIC.
FT    TRANSMEM    356     378
FT    TOPO_DOM    379     407       NON CYTOPLASMIC.
FT    TRANSMEM    408     429
FT    TOPO_DOM    430     449       CYTOPLASMIC.
FT    TRANSMEM    450     471
FT    TOPO_DOM    472     499       NON CYTOPLASMIC.
FT    TRANSMEM    500     522
FT    TOPO_DOM    523     585       CYTOPLASMIC.
//
```

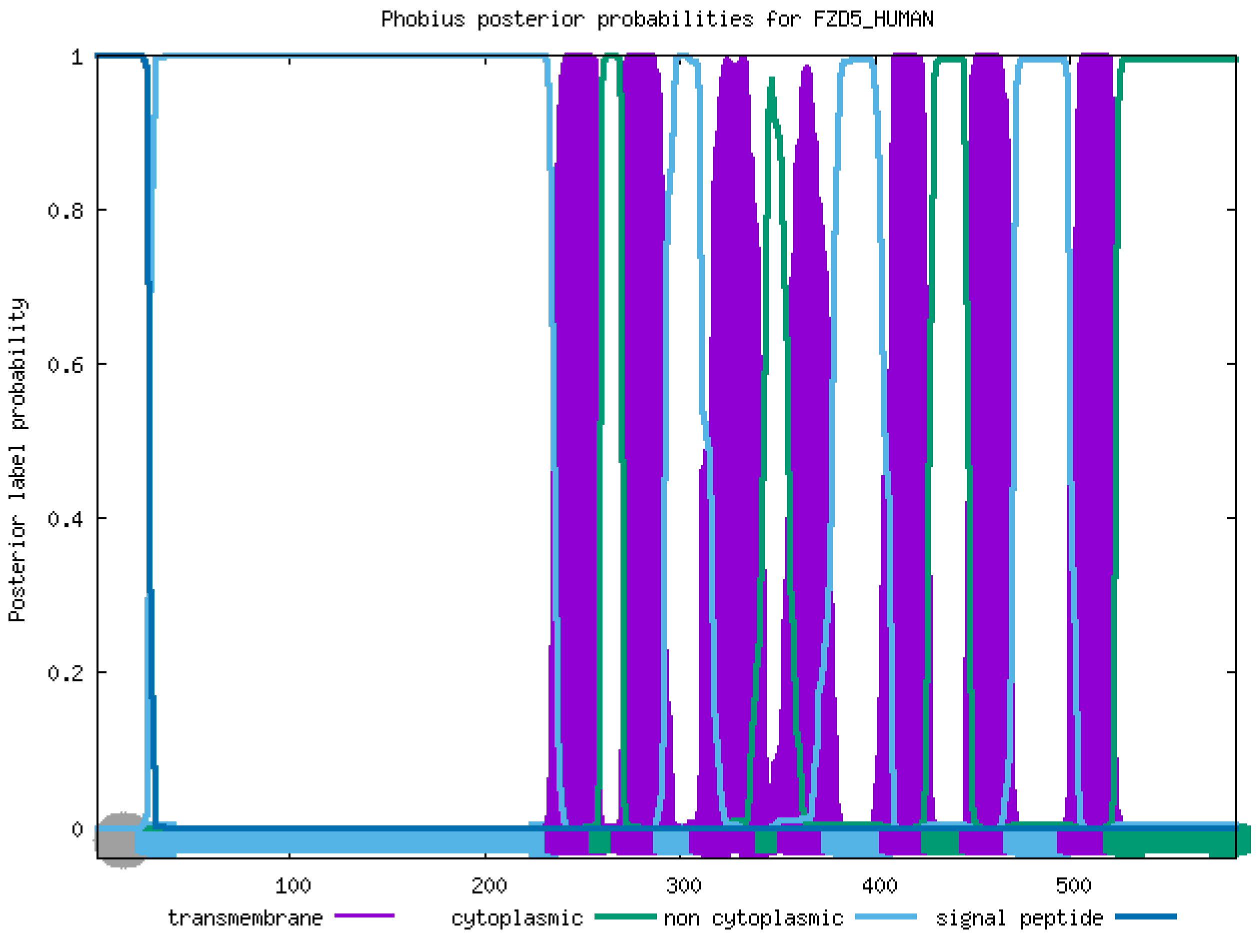

The probability data used in the plot is found here, and the gnuplot script is here.

Prediction of FZD6_HUMAN

```
ID   FZD6_HUMAN
FT   SIGNAL        1     18
FT   REGION        1      3       N-REGION.
FT   REGION        4     13       H-REGION.
FT   REGION       14     18       C-REGION.
FT   TOPO_DOM     19    200       NON CYTOPLASMIC.
FT   TRANSMEM    201    222
FT   TOPO_DOM    223    233       CYTOPLASMIC.
FT   TRANSMEM    234    254
FT   TOPO_DOM    255    283       NON CYTOPLASMIC.
FT   TRANSMEM    284    312
FT   TOPO_DOM    313    323       CYTOPLASMIC.
FT   TRANSMEM    324    345
FT   TOPO_DOM    346    368       NON CYTOPLASMIC.
FT   TRANSMEM    369    393
FT   TOPO_DOM    394    412       CYTOPLASMIC.
FT   TRANSMEM    413    438
FT   TOPO_DOM    439    472       NON CYTOPLASMIC.
FT   TRANSMEM    473    494
FT   TOPO_DOM    495    706       CYTOPLASMIC.
//
```

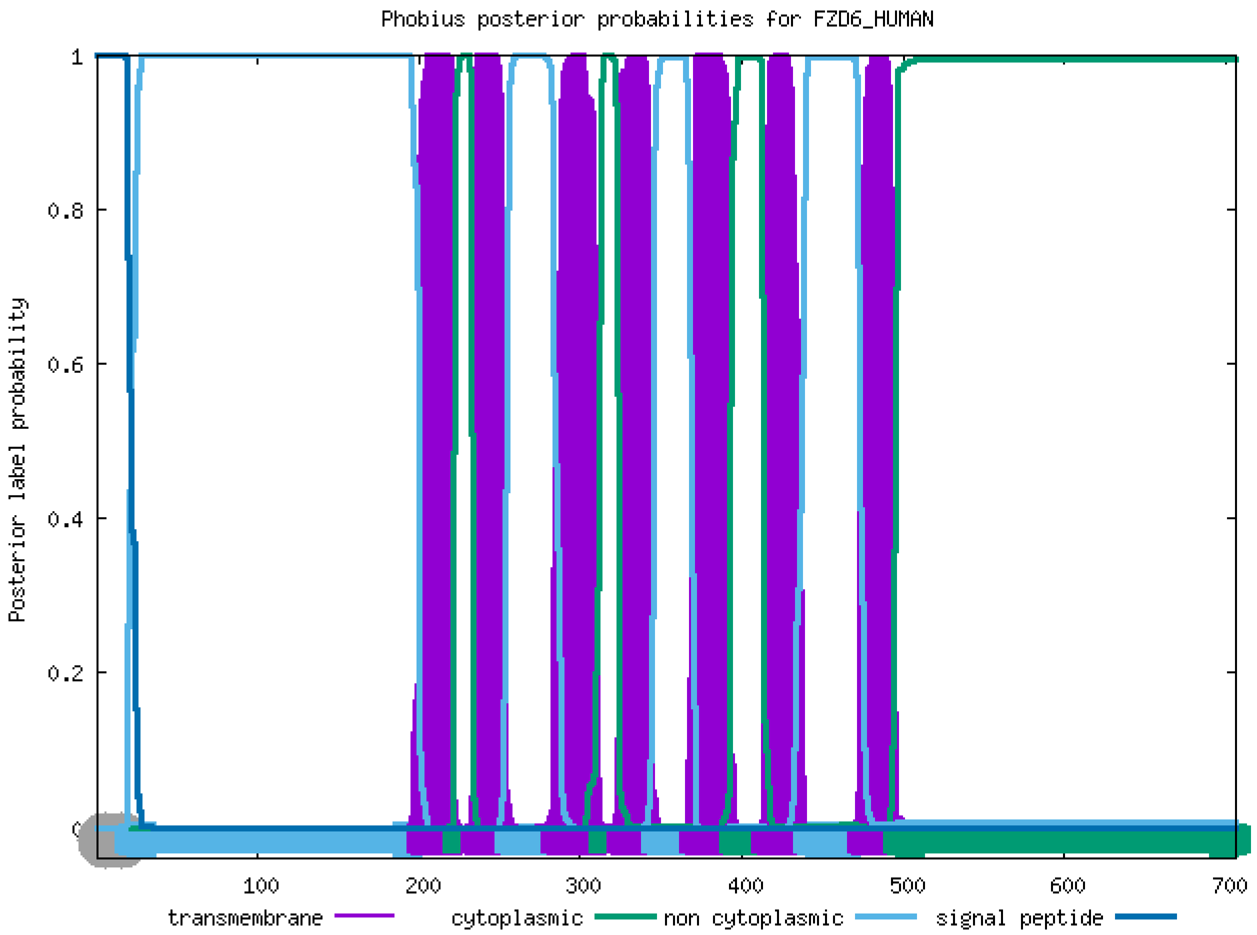

The probability data used in the plot is found here, and the gnuplot script is here.

Prediction of FZD7_HUMAN

```
ID    FZD7_HUMAN
FT    SIGNAL         1     32
FT    REGION         1      5       N-REGION.
FT    REGION         6     24       H-REGION.
FT    REGION        25     32       C-REGION.
FT    TOPO_DOM      33    254       NON CYTOPLASMIC.
FT    TRANSMEM     255    277
FT    TOPO_DOM     278    288       CYTOPLASMIC.
FT    TRANSMEM     289    309
FT    TOPO_DOM     310    337       NON CYTOPLASMIC.
FT    TRANSMEM     338    363
FT    TOPO_DOM     364    382       CYTOPLASMIC.
FT    TRANSMEM     383    402
FT    TOPO_DOM     403    421       NON CYTOPLASMIC.
FT    TRANSMEM     422    448
FT    TOPO_DOM     449    468       CYTOPLASMIC.
FT    TRANSMEM     469    492
FT    TOPO_DOM     493    526       NON CYTOPLASMIC.
FT    TRANSMEM     527    549
FT    TOPO_DOM     550    574       CYTOPLASMIC.
//
```

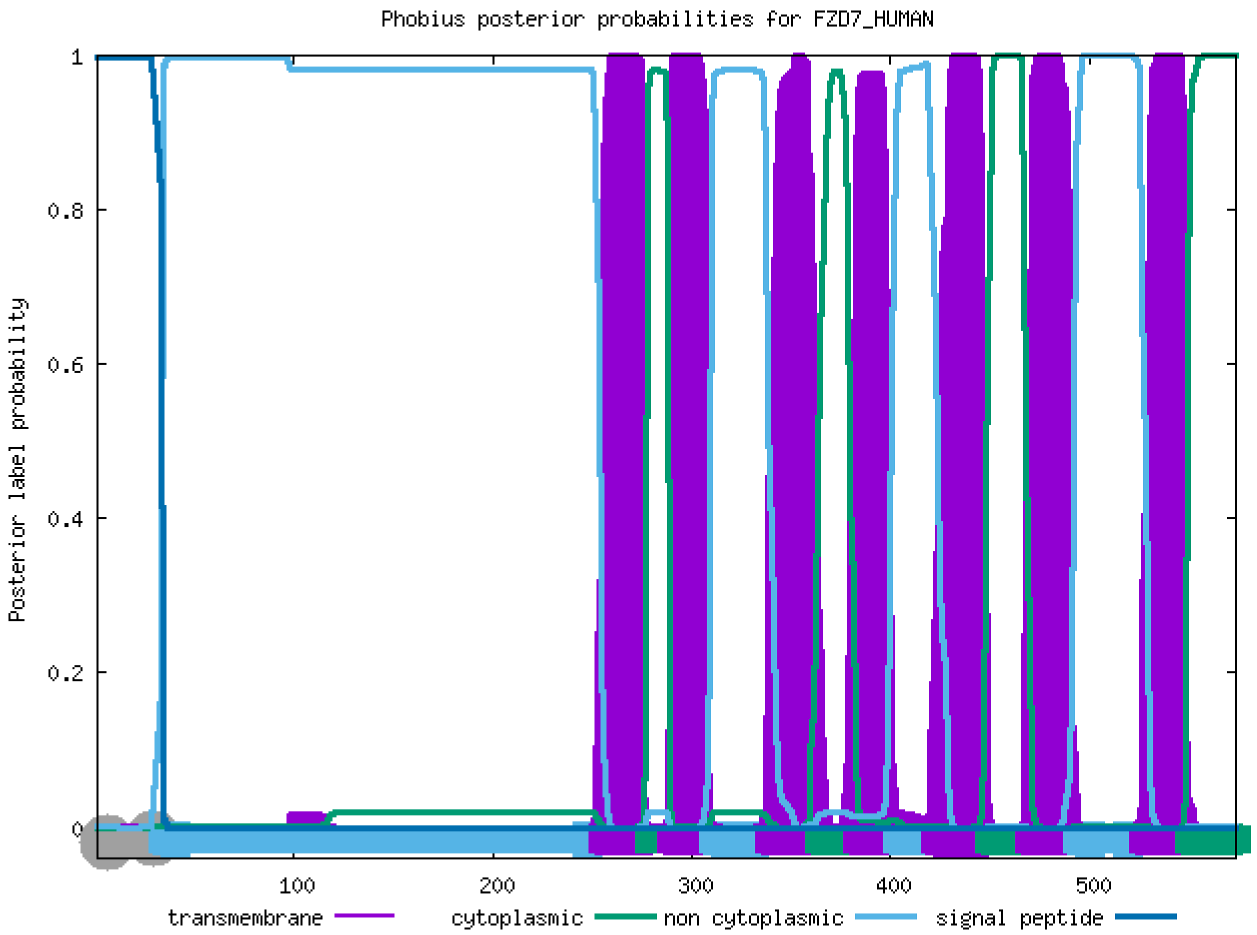

The probability data used in the plot is found here, and the gnuplot script is here.

Prediction of T2R46_HUMAN

```
ID    T2R46_HUMAN
FT    TOPO_DOM      1      5       NON CYTOPLASMIC.
FT    TRANSMEM      6     27
FT    TOPO_DOM     28     46       CYTOPLASMIC.
FT    TRANSMEM     47     67
FT    TOPO_DOM     68     86       NON CYTOPLASMIC.
FT    TRANSMEM     87    108
FT    TOPO_DOM    109    127       CYTOPLASMIC.
FT    TRANSMEM    128    147
FT    TOPO_DOM    148    180       NON CYTOPLASMIC.
FT    TRANSMEM    181    206
FT    TOPO_DOM    207    226       CYTOPLASMIC.
FT    TRANSMEM    227    248
FT    TOPO_DOM    249    259       NON CYTOPLASMIC.
FT    TRANSMEM    260    281
FT    TOPO_DOM    282    309       CYTOPLASMIC.
//
```

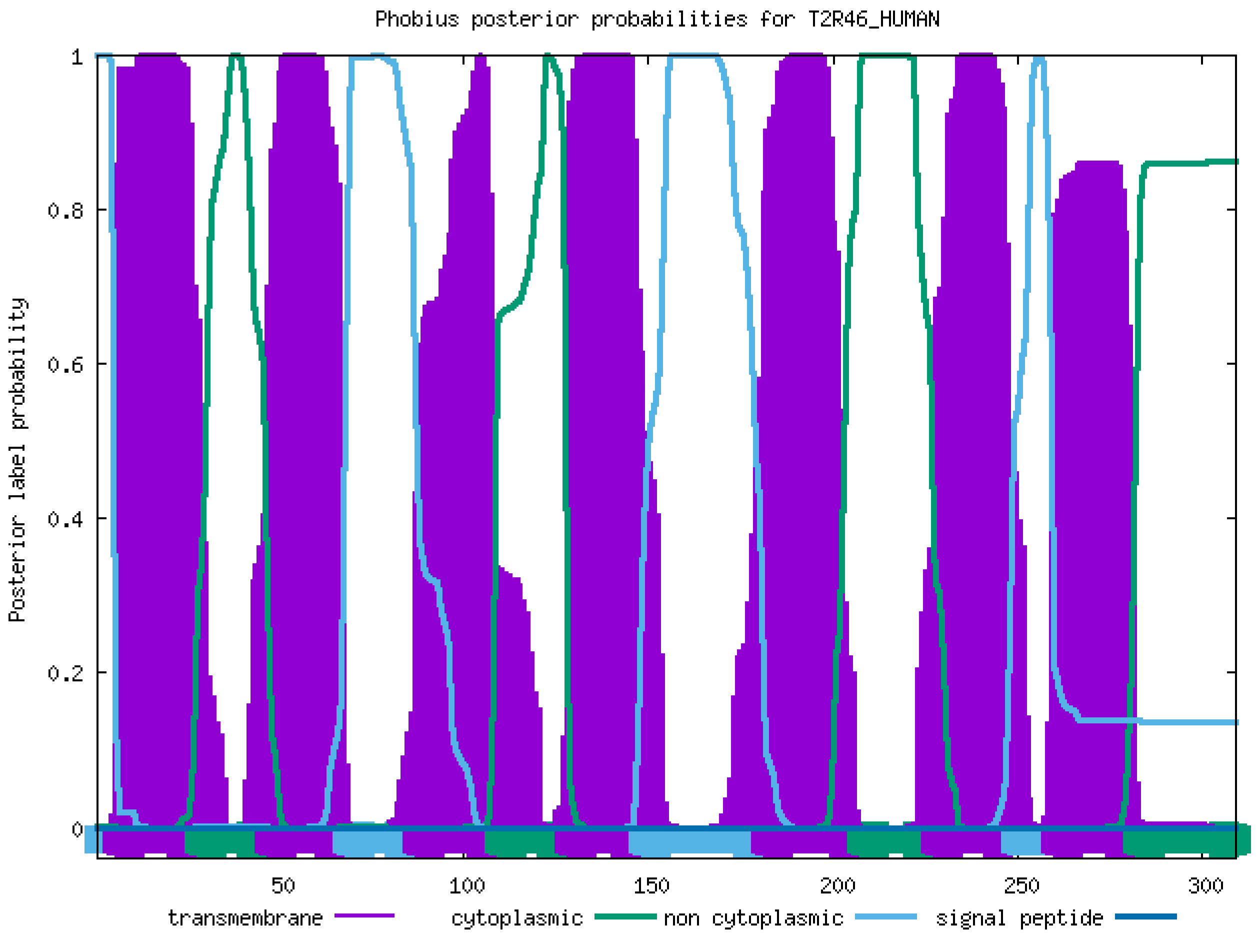

The probability data used in the plot is found here, and the gnuplot script is here.

Prediction of T2R14_HUMAN

```
ID   T2R14_HUMAN
FT   TOPO_DOM      1     11       NON CYTOPLASMIC.
FT   TRANSMEM     12     36
FT   TOPO_DOM     37     56       CYTOPLASMIC.
FT   TRANSMEM     57     76
FT   TOPO_DOM     77     87       NON CYTOPLASMIC.
FT   TRANSMEM     88    109
FT   TOPO_DOM    110    128       CYTOPLASMIC.
FT   TRANSMEM    129    152
FT   TOPO_DOM    153    177       NON CYTOPLASMIC.
FT   TRANSMEM    178    203
FT   TOPO_DOM    204    232       CYTOPLASMIC.
FT   TRANSMEM    233    254
FT   TOPO_DOM    255    259       NON CYTOPLASMIC.
FT   TRANSMEM    260    282
FT   TOPO_DOM    283    317       CYTOPLASMIC.
//
```

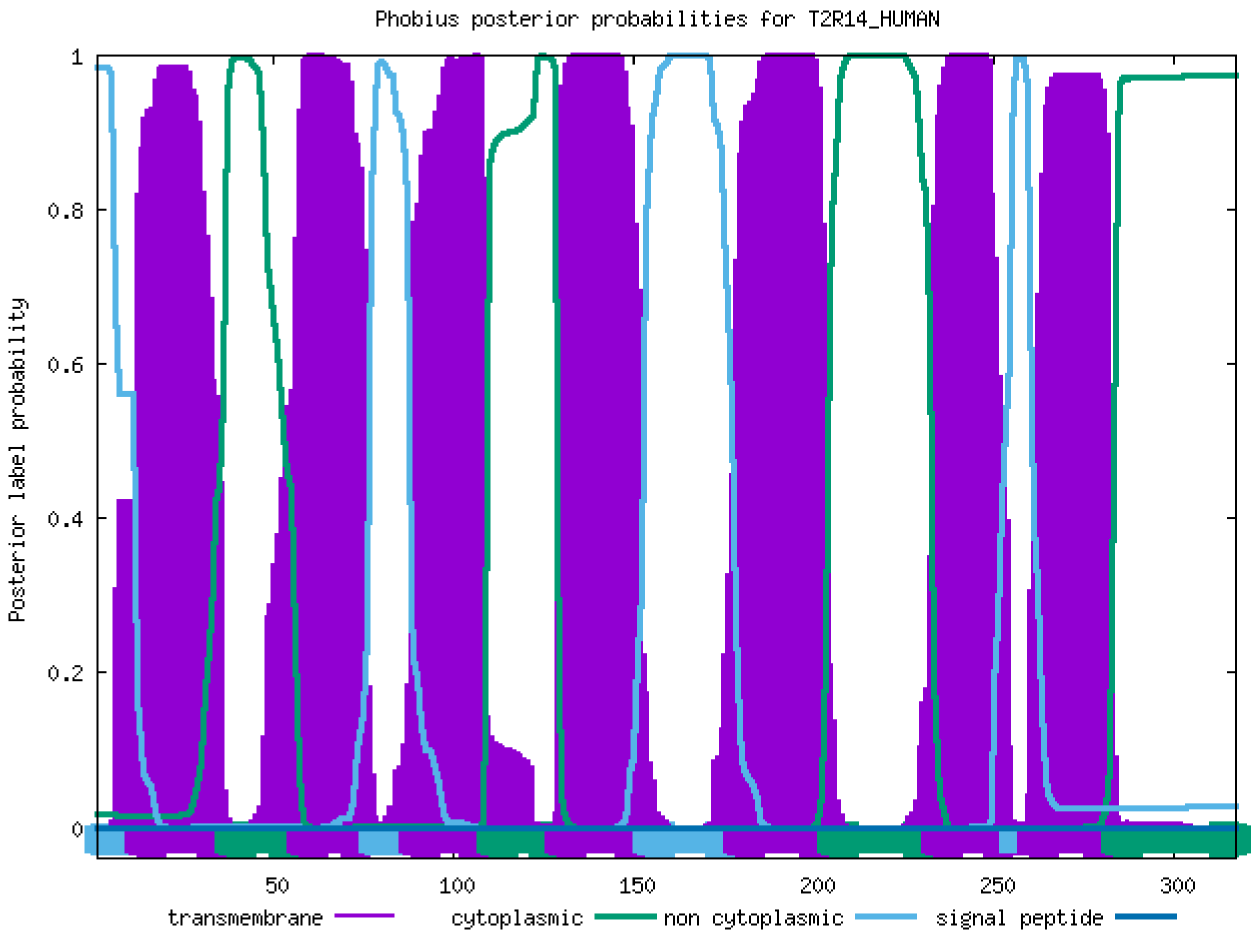

The probability data used in the plot is found here, and the gnuplot script is here.

Prediction of WLS_HUMAN

```
ID    WLS_HUMAN
FT    TOPO_DOM       1      13       CYTOPLASMIC.
FT    TRANSMEM      14      36
FT    TOPO_DOM      37     232       NON CYTOPLASMIC.
FT    TRANSMEM     233     253
FT    TOPO_DOM     254     264       CYTOPLASMIC.
FT    TRANSMEM     265     284
FT    TOPO_DOM     285     295       NON CYTOPLASMIC.
FT    TRANSMEM     296     318
FT    TOPO_DOM     319     338       CYTOPLASMIC.
FT    TRANSMEM     339     359
FT    TOPO_DOM     360     378       NON CYTOPLASMIC.
FT    TRANSMEM     379     407
FT    TOPO_DOM     408     427       CYTOPLASMIC.
FT    TRANSMEM     428     452
FT    TOPO_DOM     453     471       NON CYTOPLASMIC.
FT    TRANSMEM     472     493
FT    TOPO_DOM     494     541       CYTOPLASMIC.
//
```

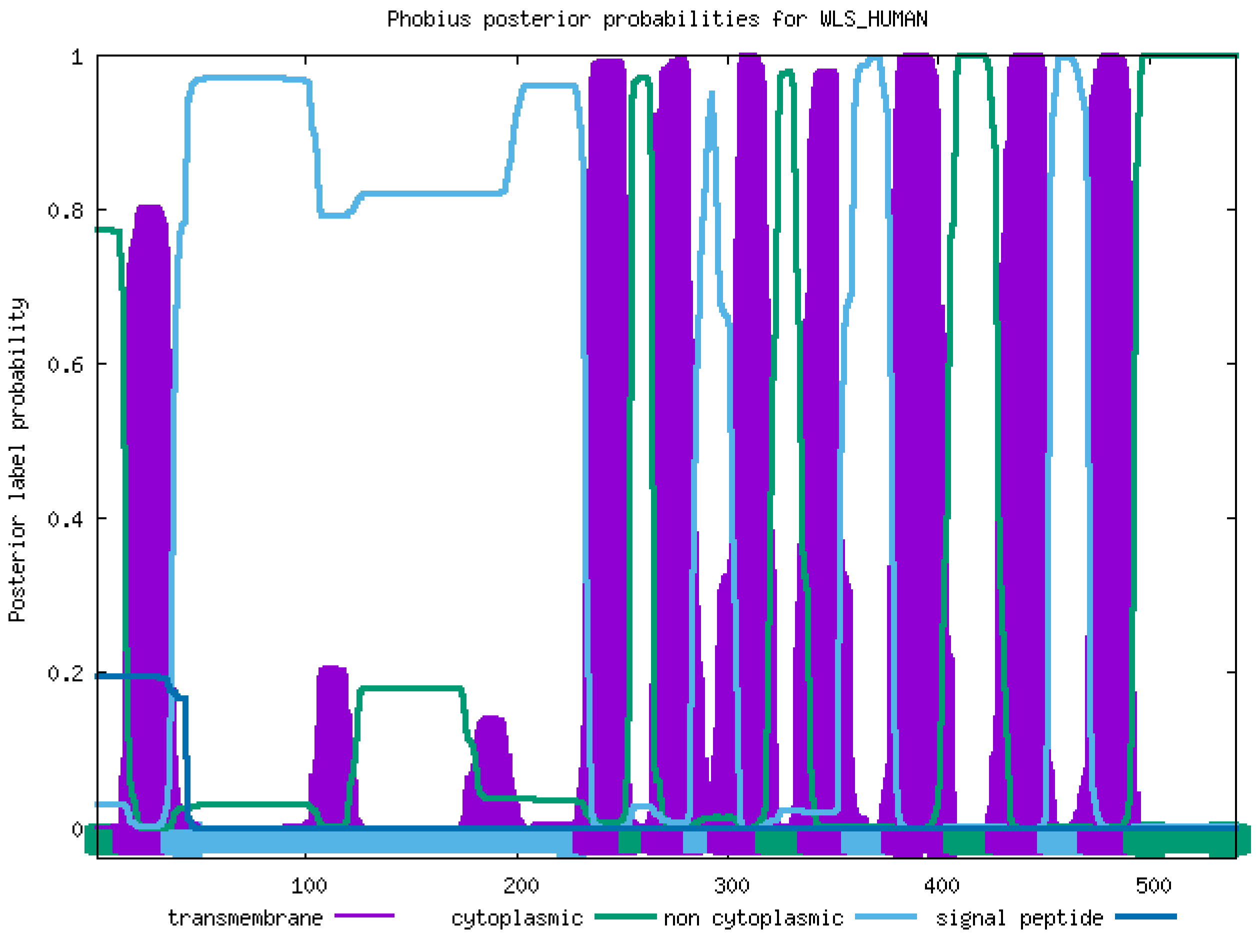

The probability data used in the plot is found here, and the gnuplot script is here.

Prediction of STING_HUMAN

```
ID   STING_HUMAN
FT   TOPO_DOM      1     20       CYTOPLASMIC.
FT   TRANSMEM     21     37
FT   TOPO_DOM     38     42       NON CYTOPLASMIC.
FT   TRANSMEM     43     66
FT   TOPO_DOM     67     86       CYTOPLASMIC.
FT   TRANSMEM     87    107
FT   TOPO_DOM    108    118       NON CYTOPLASMIC.
FT   TRANSMEM    119    136
FT   TOPO_DOM    137    379       CYTOPLASMIC.
//
```

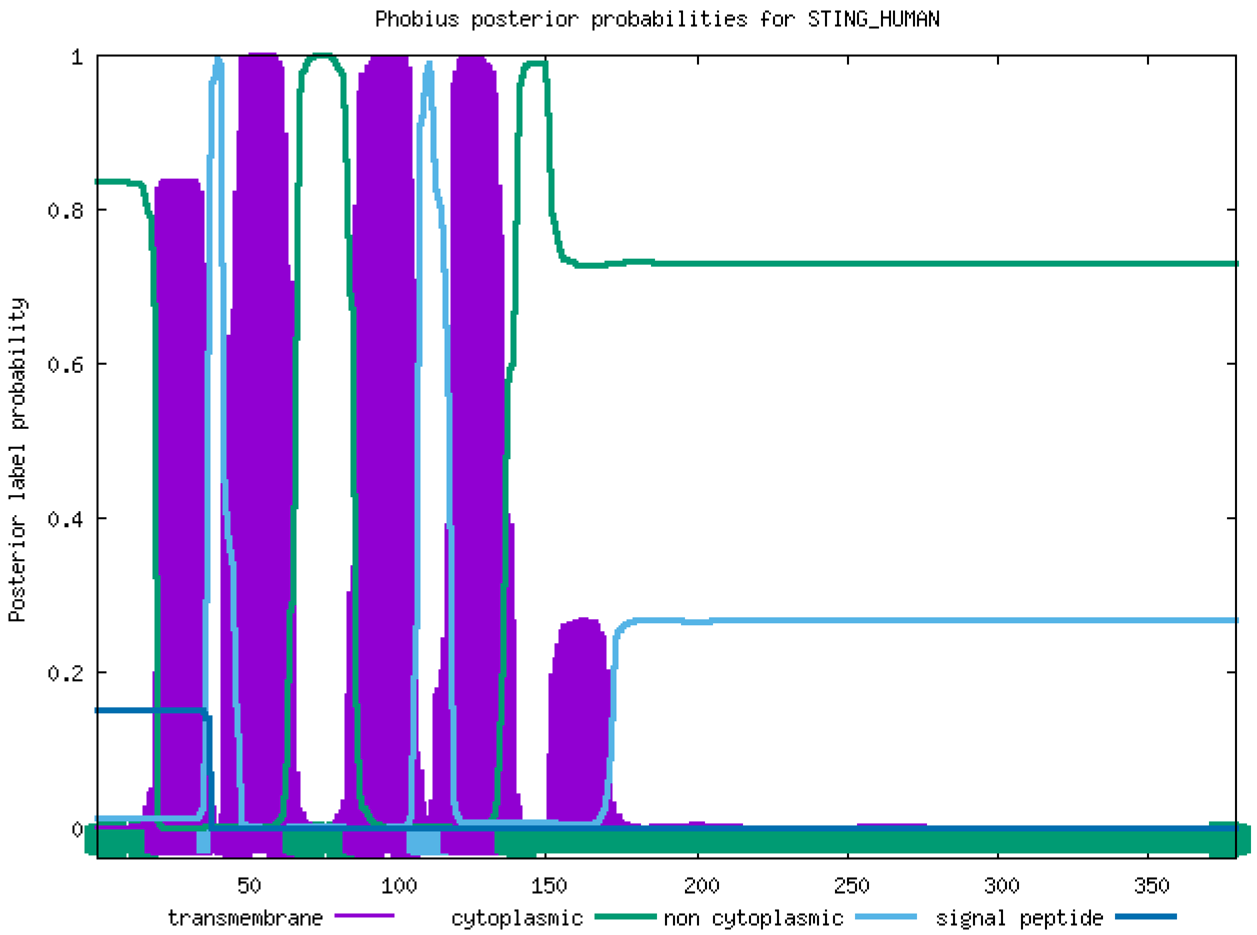

The probability data used in the plot is found here, and the gnuplot script is here.

Prediction of OSTM1_HUMAN

```
ID   OSTM1_HUMAN
FT   SIGNAL         1     31
FT   REGION         1     11       N-REGION.
FT   REGION        12     23       H-REGION.
FT   REGION        24     31       C-REGION.
FT   TOPO_DOM      32    282       NON CYTOPLASMIC.
FT   TRANSMEM     283    305
FT   TOPO_DOM     306    334       CYTOPLASMIC.
//
```

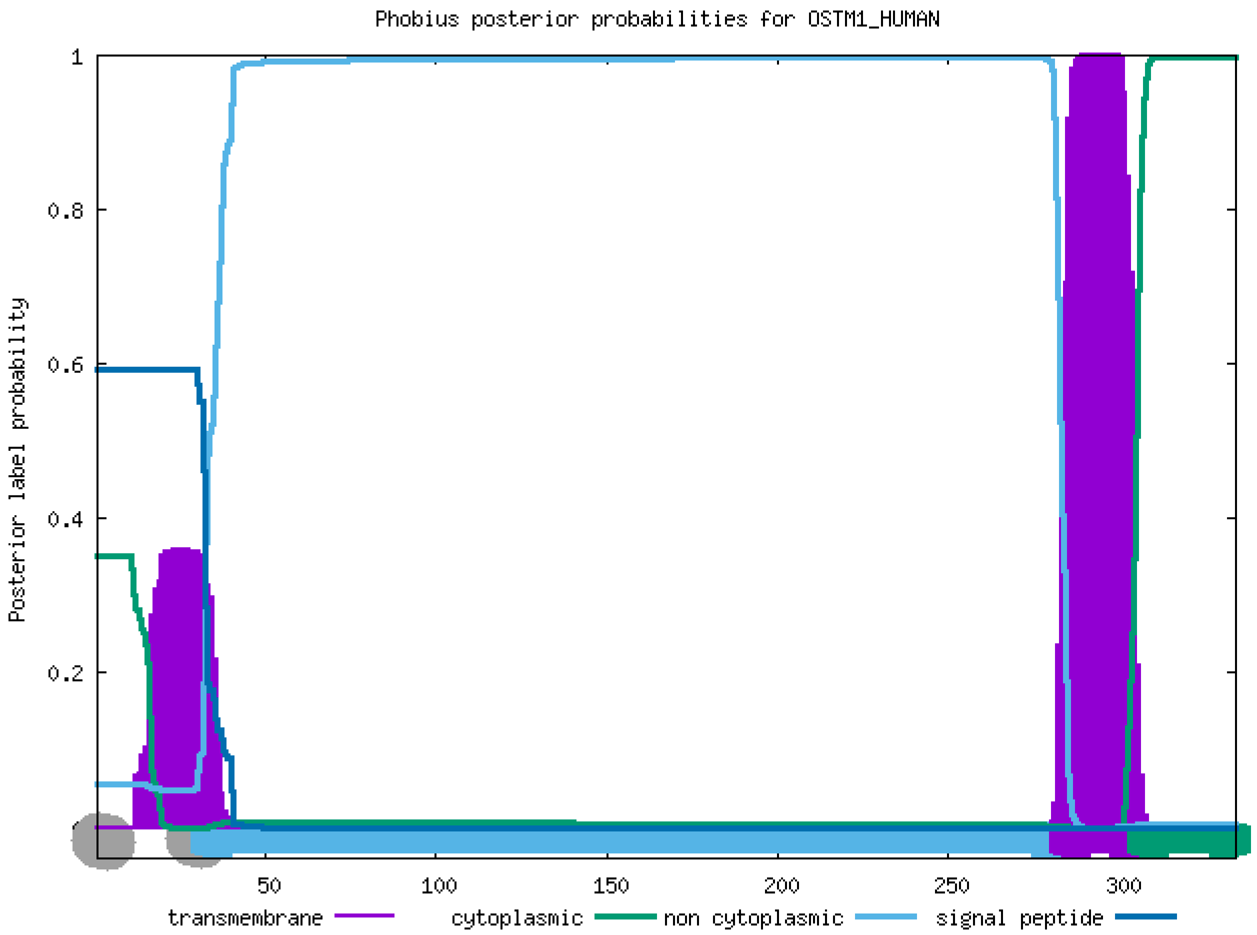

The probability data used in the plot is found here, and the gnuplot script is here.

Prediction of CLCN7_HUMAN

```
ID    CLCN7_HUMAN
FT    TOPO_DOM      1     126       CYTOPLASMIC.
FT    TRANSMEM    127     148
FT    TOPO_DOM    149     175       NON CYTOPLASMIC.
FT    TRANSMEM    176     197
FT    TOPO_DOM    198     226       CYTOPLASMIC.
FT    TRANSMEM    227     245
FT    TOPO_DOM    246     287       NON CYTOPLASMIC.
FT    TRANSMEM    288     311
FT    TOPO_DOM    312     322       CYTOPLASMIC.
FT    TRANSMEM    323     345
FT    TOPO_DOM    346     374       NON CYTOPLASMIC.
FT    TRANSMEM    375     399
FT    TOPO_DOM    400     410       CYTOPLASMIC.
FT    TRANSMEM    411     433
FT    TOPO_DOM    434     487       NON CYTOPLASMIC.
FT    TRANSMEM    488     506
FT    TOPO_DOM    507     517       CYTOPLASMIC.
FT    TRANSMEM    518     541
FT    TOPO_DOM    542     546       NON CYTOPLASMIC.
FT    TRANSMEM    547     569
FT    TOPO_DOM    570     580       CYTOPLASMIC.
FT    TRANSMEM    581     602
FT    TOPO_DOM    603     805       NON CYTOPLASMIC.
//
```

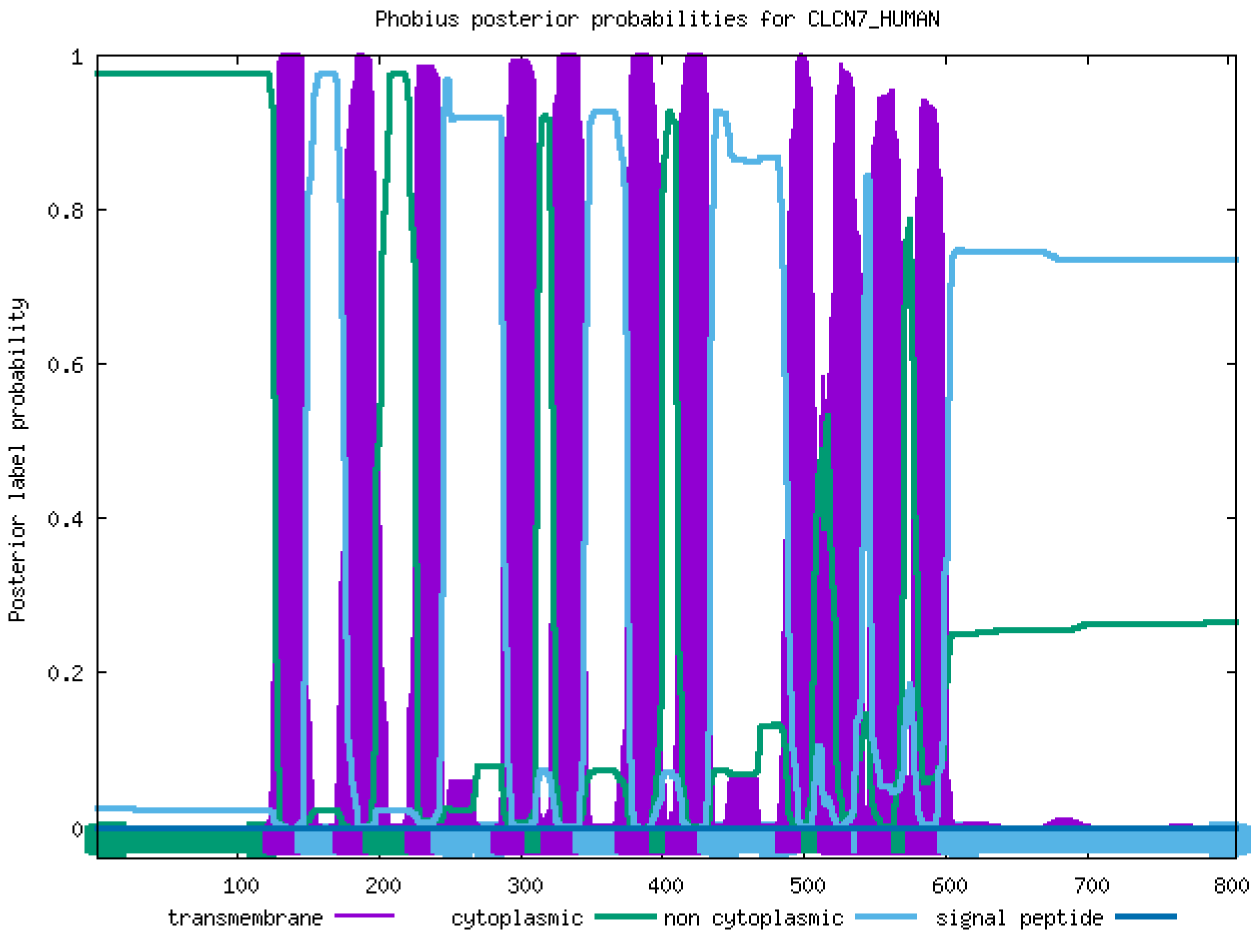

The probability data used in the plot is found here, and the gnuplot script is here.

Prediction of ATAD1_HUMAN

```
ID   ATAD1_HUMAN
FT   TOPO_DOM      1     19       NON CYTOPLASMIC.
FT   TRANSMEM     20     38
FT   TOPO_DOM     39    361       CYTOPLASMIC.
//
```

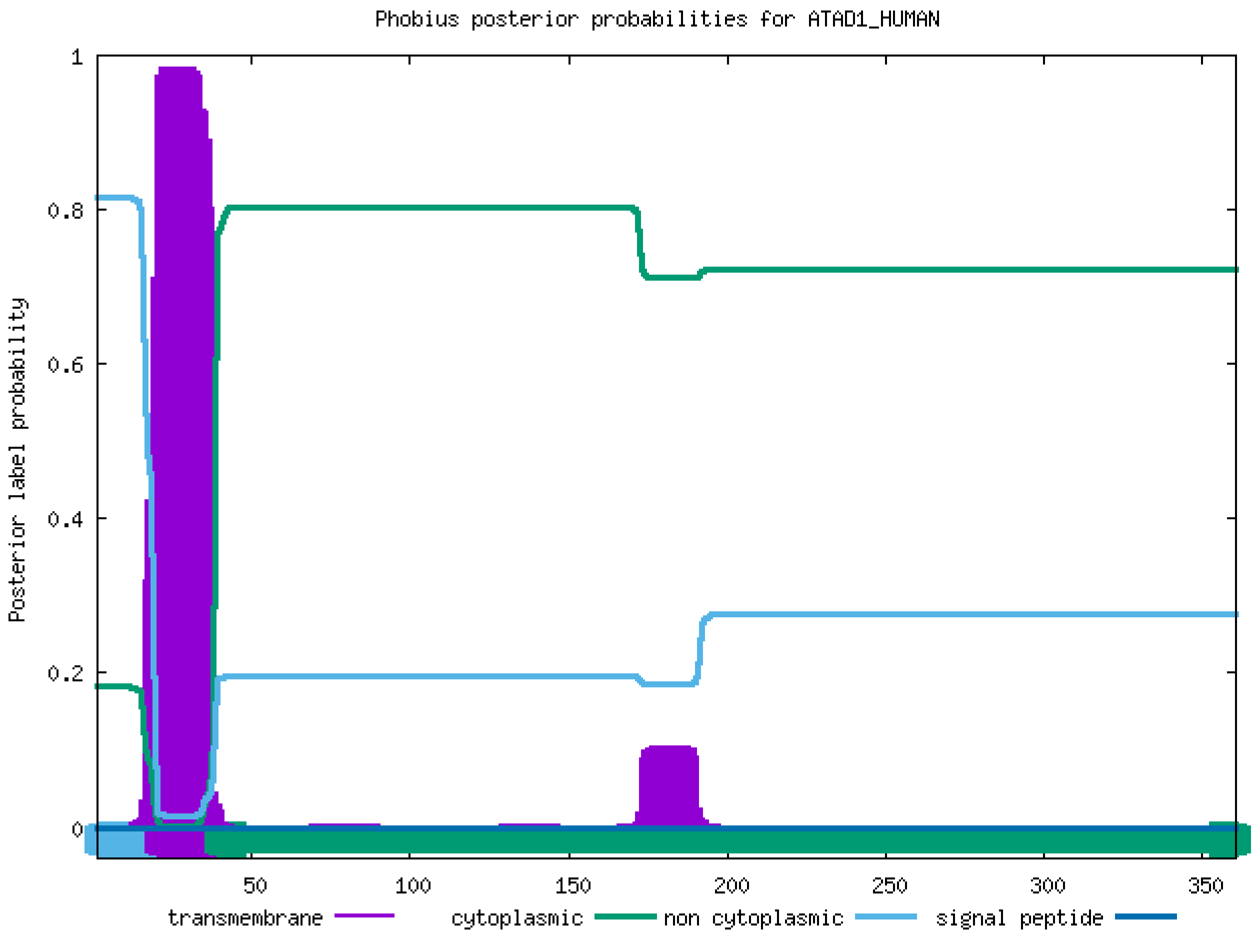

The probability data used in the plot is found here, and the gnuplot script is here.

Prediction of STT3A_HUMAN

```
ID   STT3A_HUMAN
FT   TOPO_DOM      1     19       CYTOPLASMIC.
FT   TRANSMEM     20     41
FT   TOPO_DOM     42     82       NON CYTOPLASMIC.
FT   TRANSMEM     83    103
FT   TOPO_DOM    104    114       CYTOPLASMIC.
FT   TRANSMEM    115    135
FT   TOPO_DOM    136    140       NON CYTOPLASMIC.
FT   TRANSMEM    141    158
FT   TOPO_DOM    159    169       CYTOPLASMIC.
FT   TRANSMEM    170    187
FT   TOPO_DOM    188    198       NON CYTOPLASMIC.
FT   TRANSMEM    199    225
FT   TOPO_DOM    226    236       CYTOPLASMIC.
FT   TRANSMEM    237    256
FT   TOPO_DOM    257    267       NON CYTOPLASMIC.
FT   TRANSMEM    268    286
FT   TOPO_DOM    287    297       CYTOPLASMIC.
FT   TRANSMEM    298    319
FT   TOPO_DOM    320    359       NON CYTOPLASMIC.
FT   TRANSMEM    360    379
FT   TOPO_DOM    380    385       CYTOPLASMIC.
FT   TRANSMEM    386    404
FT   TOPO_DOM    405    409       NON CYTOPLASMIC.
FT   TRANSMEM    410    430
FT   TOPO_DOM    431    450       CYTOPLASMIC.
FT   TRANSMEM    451    473
FT   TOPO_DOM    474    705       NON CYTOPLASMIC.
//
```

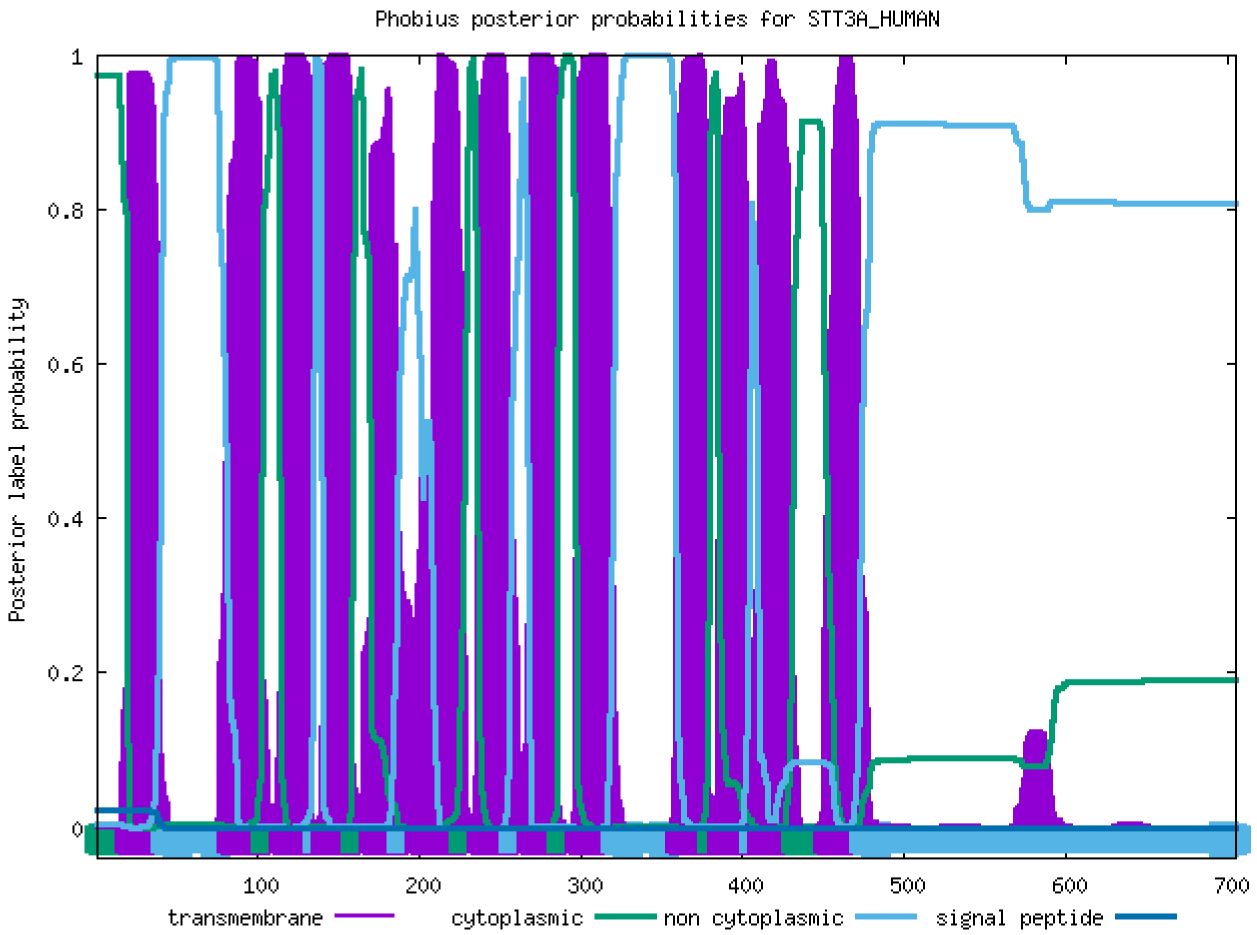

The probability data used in the plot is found here, and the gnuplot script is here.

Prediction of OST4_HUMAN

```
ID   OST4_HUMAN
FT   TOPO_DOM      1      6       CYTOPLASMIC.
FT   TRANSMEM      7     28
FT   TOPO_DOM     29     37       NON CYTOPLASMIC.
//
```

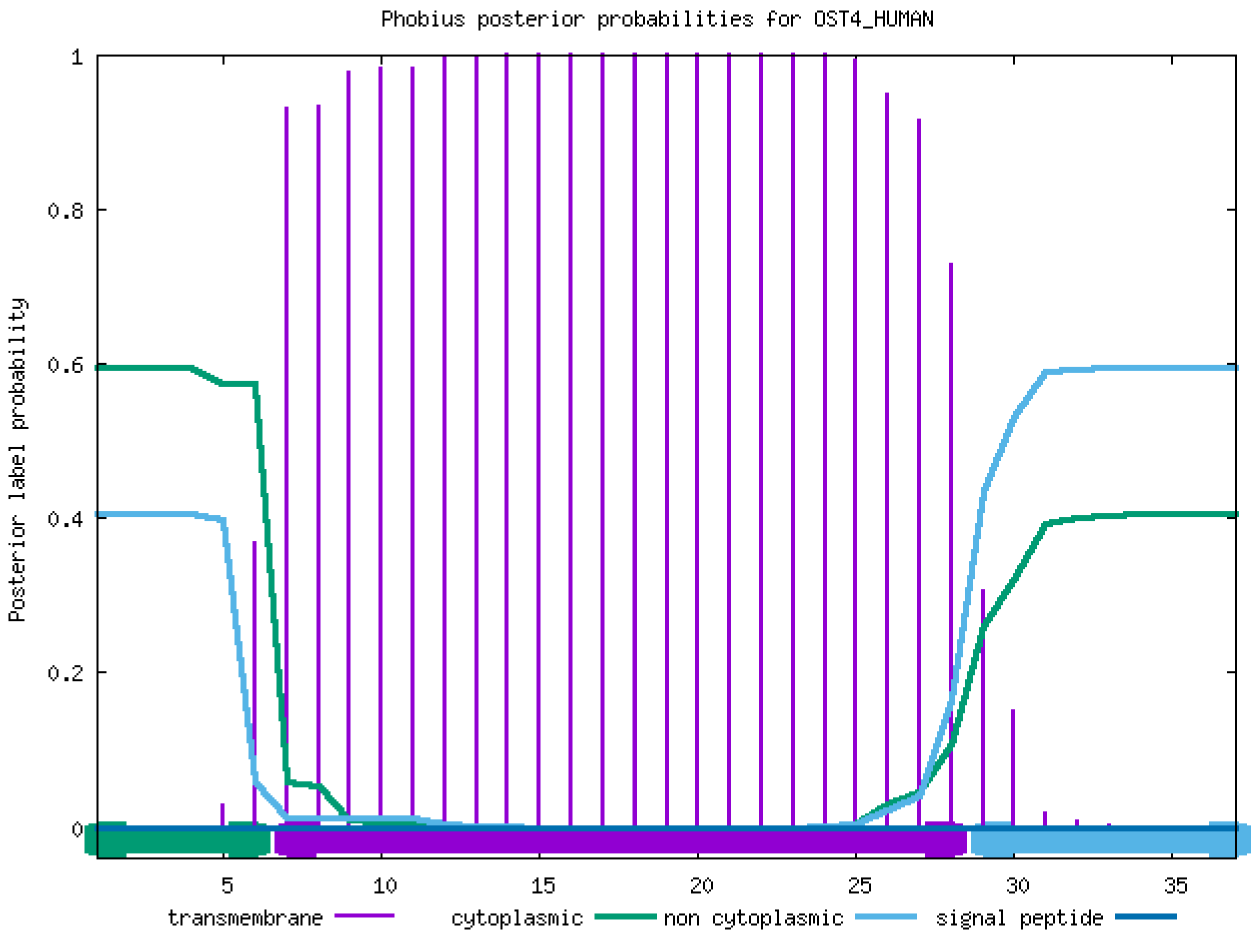

The probability data used in the plot is found here, and the gnuplot script is here.

Prediction of TM258_HUMAN

```
ID   TM258_HUMAN
FT   TOPO_DOM      1     19       NON CYTOPLASMIC.
FT   TRANSMEM     20     42
FT   TOPO_DOM     43     53       CYTOPLASMIC.
FT   TRANSMEM     54     77
FT   TOPO_DOM     78     79       NON CYTOPLASMIC.
//
```

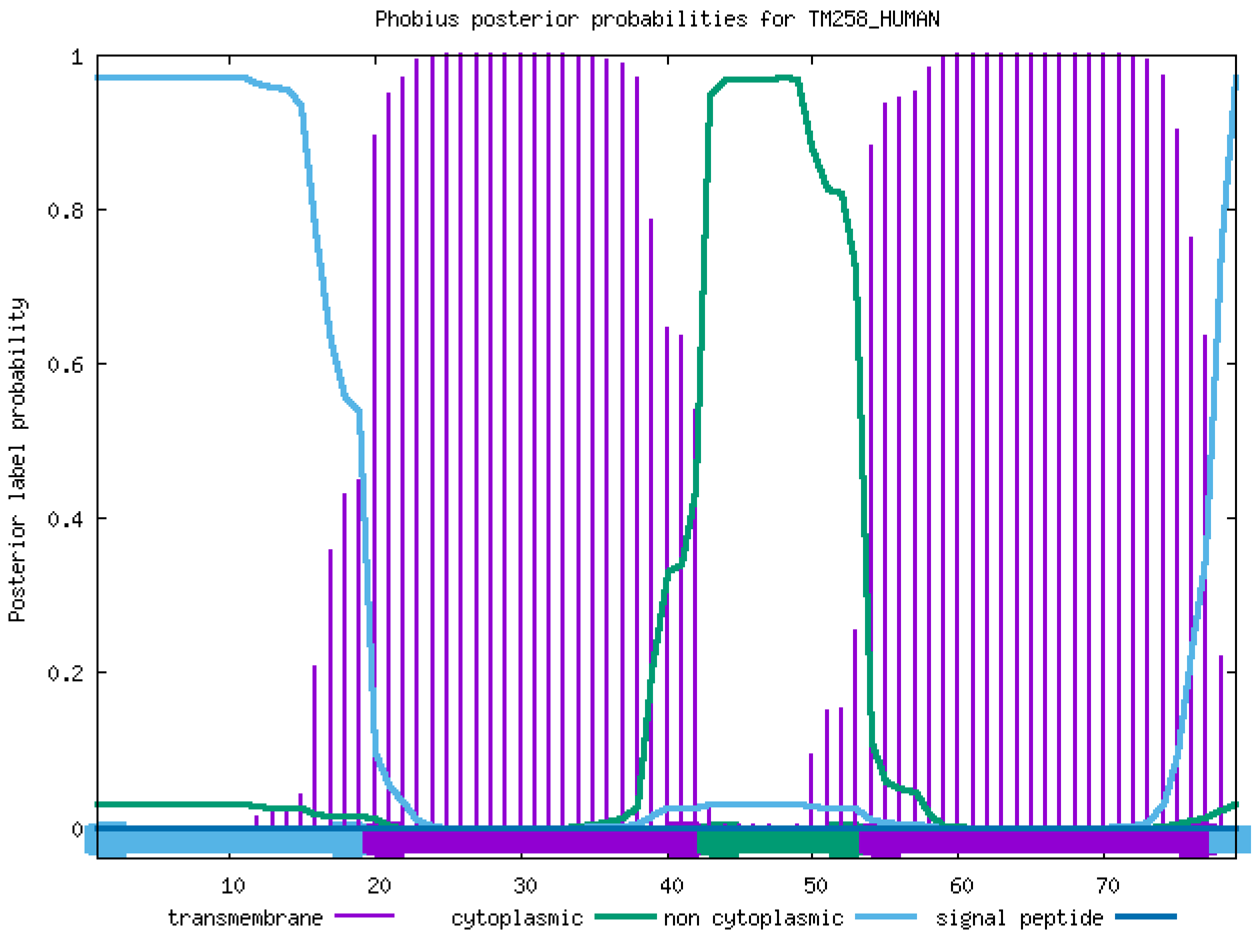

The probability data used in the plot is found here, and the gnuplot script is here.

Prediction of DAD1_HUMAN

```
ID   DAD1_HUMAN
FT   TOPO_DOM      1     29       CYTOPLASMIC.
FT   TRANSMEM     30     49
FT   TOPO_DOM     50     54       NON CYTOPLASMIC.
FT   TRANSMEM     55     73
FT   TOPO_DOM     74     92       CYTOPLASMIC.
FT   TRANSMEM     93    112
FT   TOPO_DOM    113    113       NON CYTOPLASMIC.
//
```

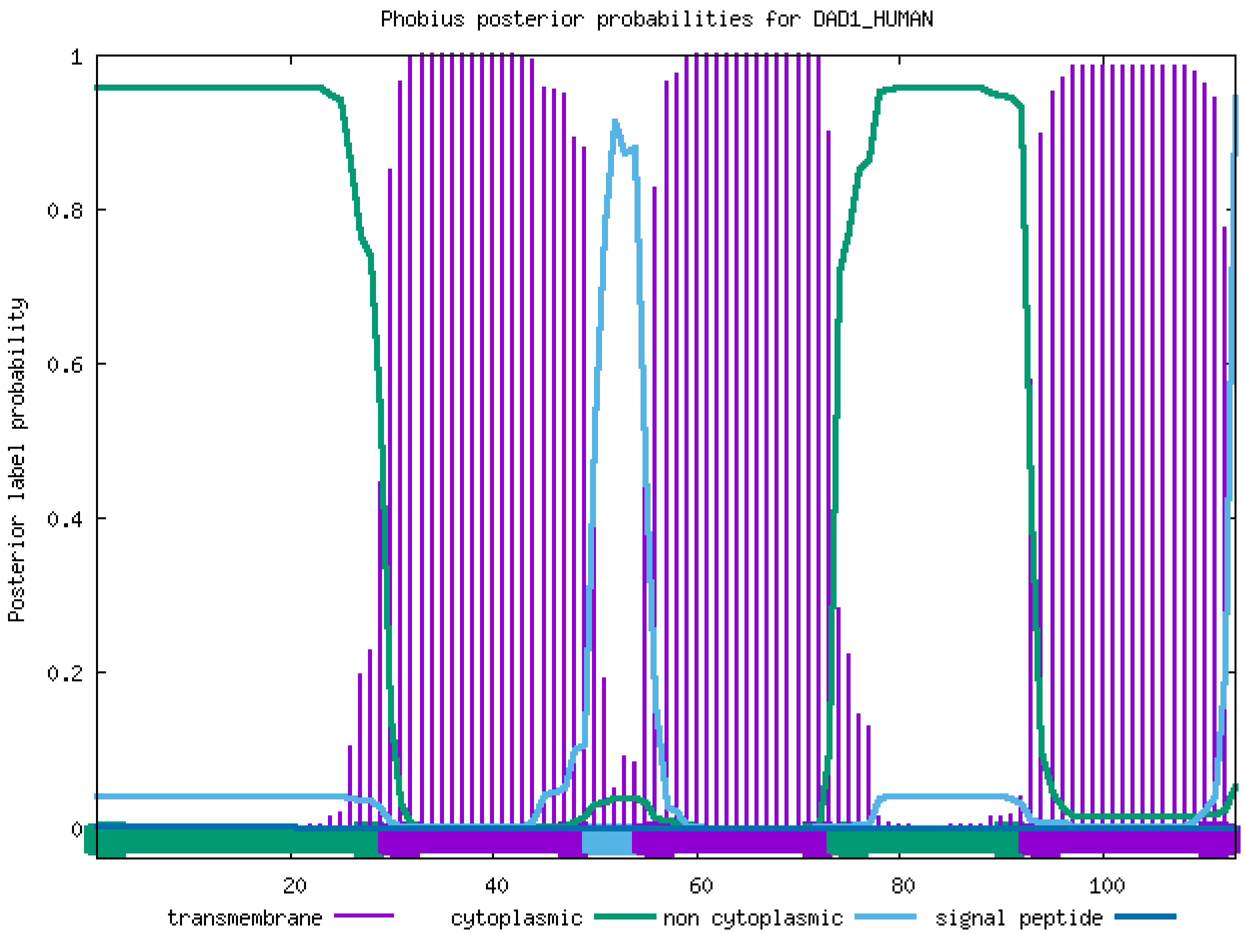

The probability data used in the plot is found here, and the gnuplot script is here.

Prediction of RPN1_HUMAN

```
ID   RPN1_HUMAN
FT   SIGNAL         1     26
FT   REGION         1      2       N-REGION.
FT   REGION         3     14       H-REGION.
FT   REGION        15     26       C-REGION.
FT   TOPO_DOM      27    439       NON CYTOPLASMIC.
FT   TRANSMEM     440    459
FT   TOPO_DOM     460    607       CYTOPLASMIC.
//
```

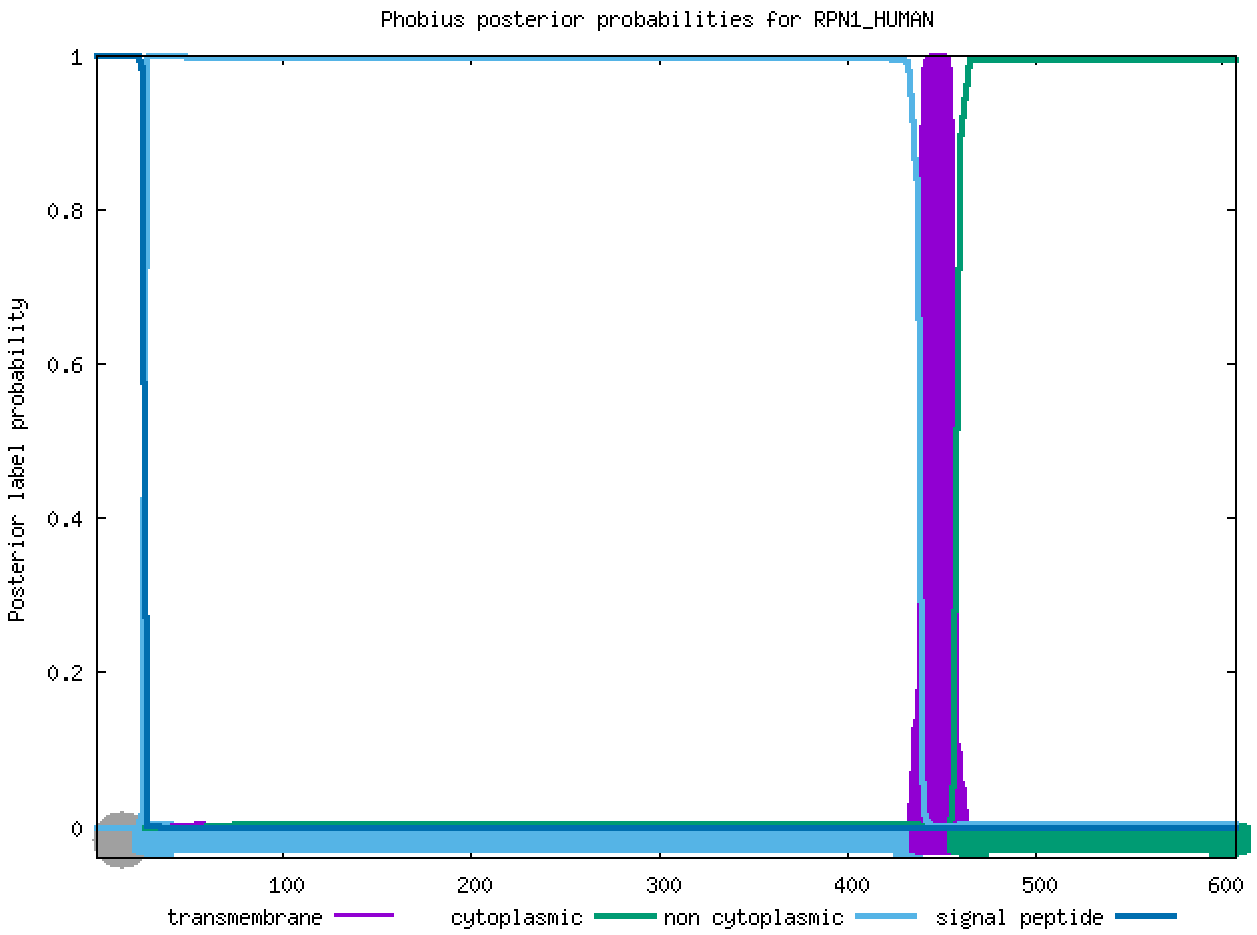

The probability data used in the plot is found here, and the gnuplot script is here.

Prediction of RPN2_HUMAN

```
ID   RPN2_HUMAN
FT   SIGNAL         1     22
FT   REGION         1      5       N-REGION.
FT   REGION         6     17       H-REGION.
FT   REGION        18     22       C-REGION.
FT   TOPO_DOM      23    539       NON CYTOPLASMIC.
FT   TRANSMEM     540    560
FT   TOPO_DOM     561    571       CYTOPLASMIC.
FT   TRANSMEM     572    593
FT   TOPO_DOM     594    604       NON CYTOPLASMIC.
FT   TRANSMEM     605    622
FT   TOPO_DOM     623    631       CYTOPLASMIC.
//
```

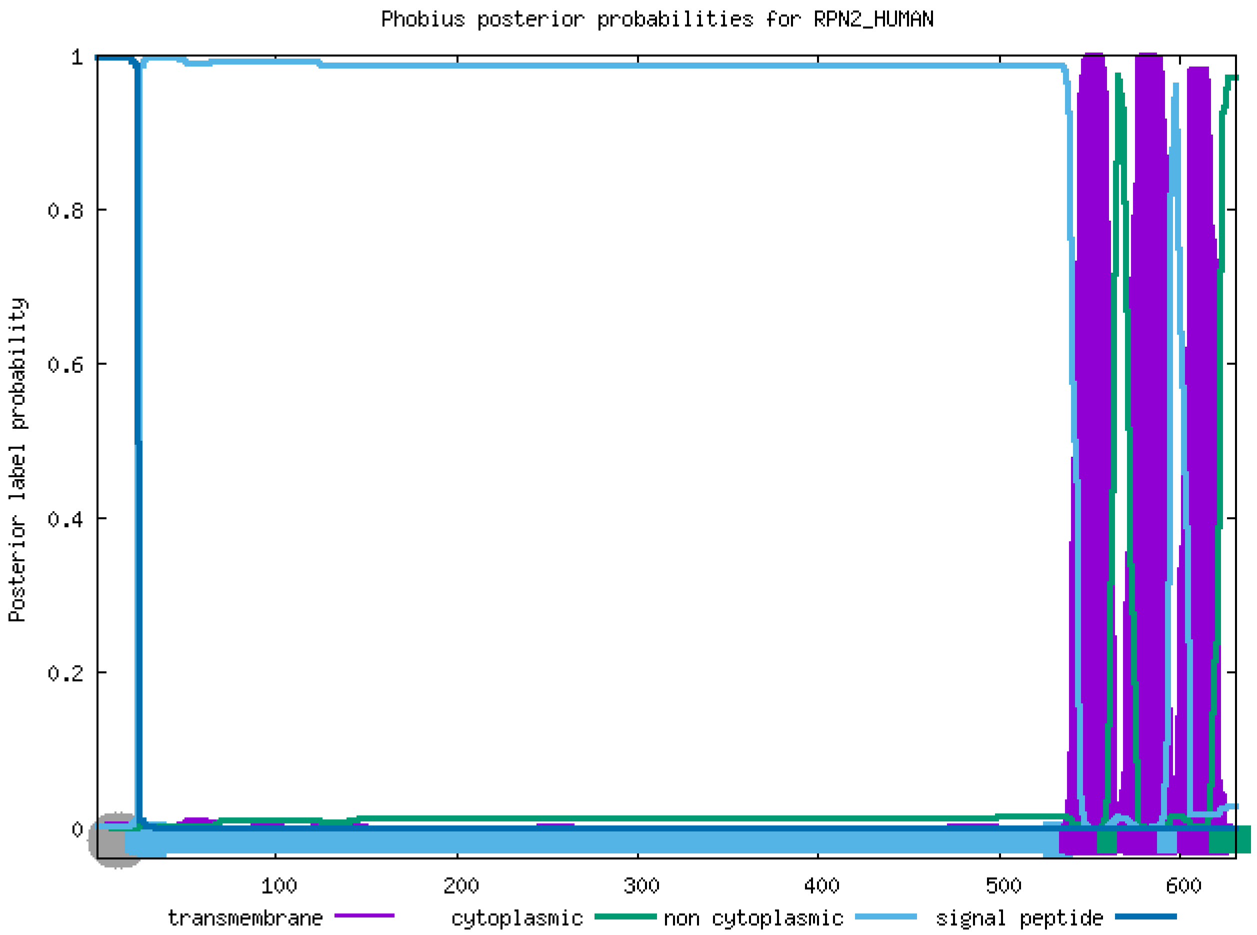

The probability data used in the plot is found here, and the gnuplot script is here.

Prediction of OST48_HUMAN

```
ID   OST48_HUMAN
FT   SIGNAL         1     42
FT   REGION         1     25       N-REGION.
FT   REGION        26     37       H-REGION.
FT   REGION        38     42       C-REGION.
FT   TOPO_DOM      43    425       NON CYTOPLASMIC.
FT   TRANSMEM     426    447
FT   TOPO_DOM     448    456       CYTOPLASMIC.
//
```

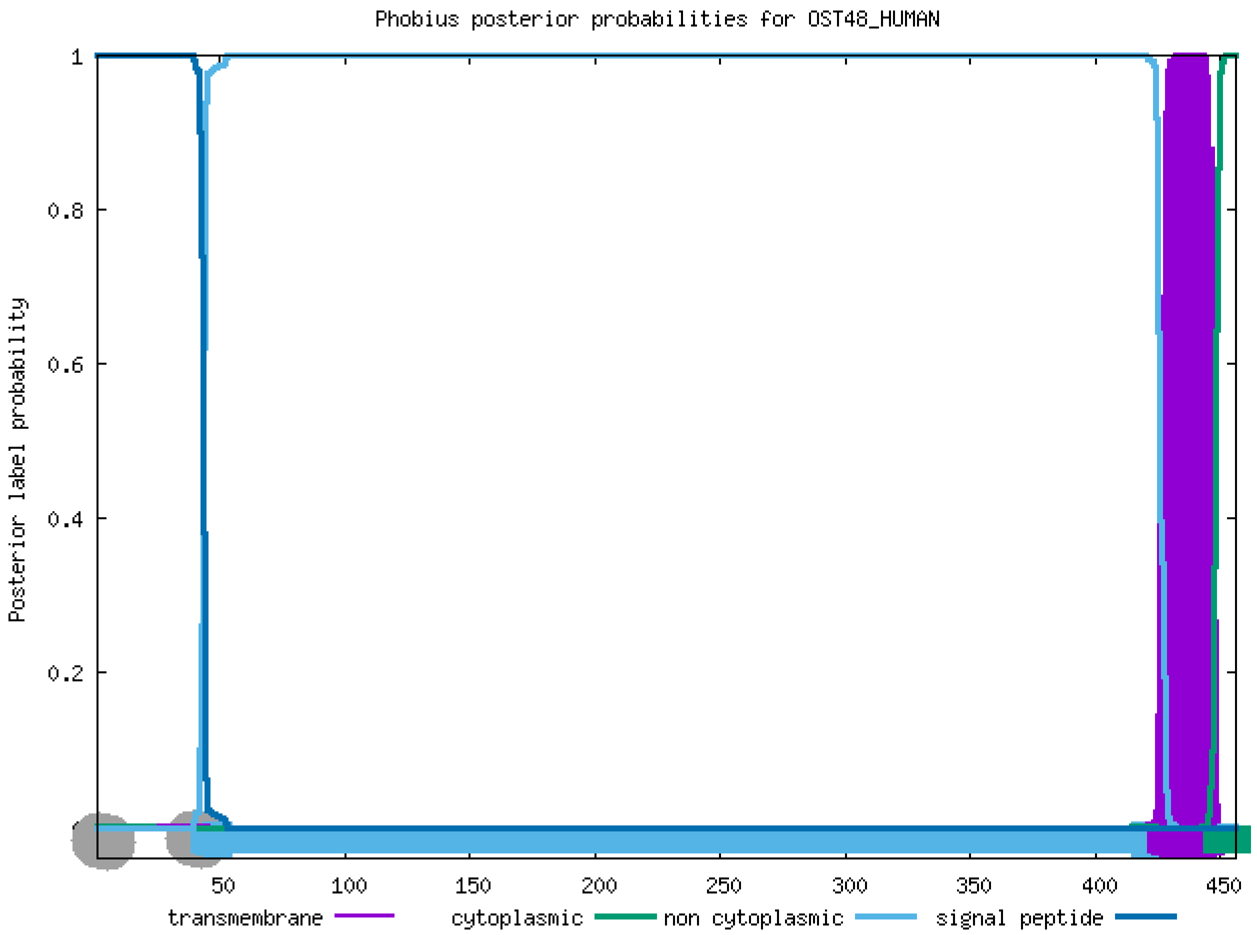

The probability data used in the plot is found here, and the gnuplot script is here.

Prediction of OSTC_HUMAN

```
ID   OSTC_HUMAN
FT   TOPO_DOM      1     30       NON CYTOPLASMIC.
FT   TRANSMEM     31     53
FT   TOPO_DOM     54     82       CYTOPLASMIC.
FT   TRANSMEM     83    103
FT   TOPO_DOM    104    117       NON CYTOPLASMIC.
FT   TRANSMEM    118    139
FT   TOPO_DOM    140    149       CYTOPLASMIC.
//
```

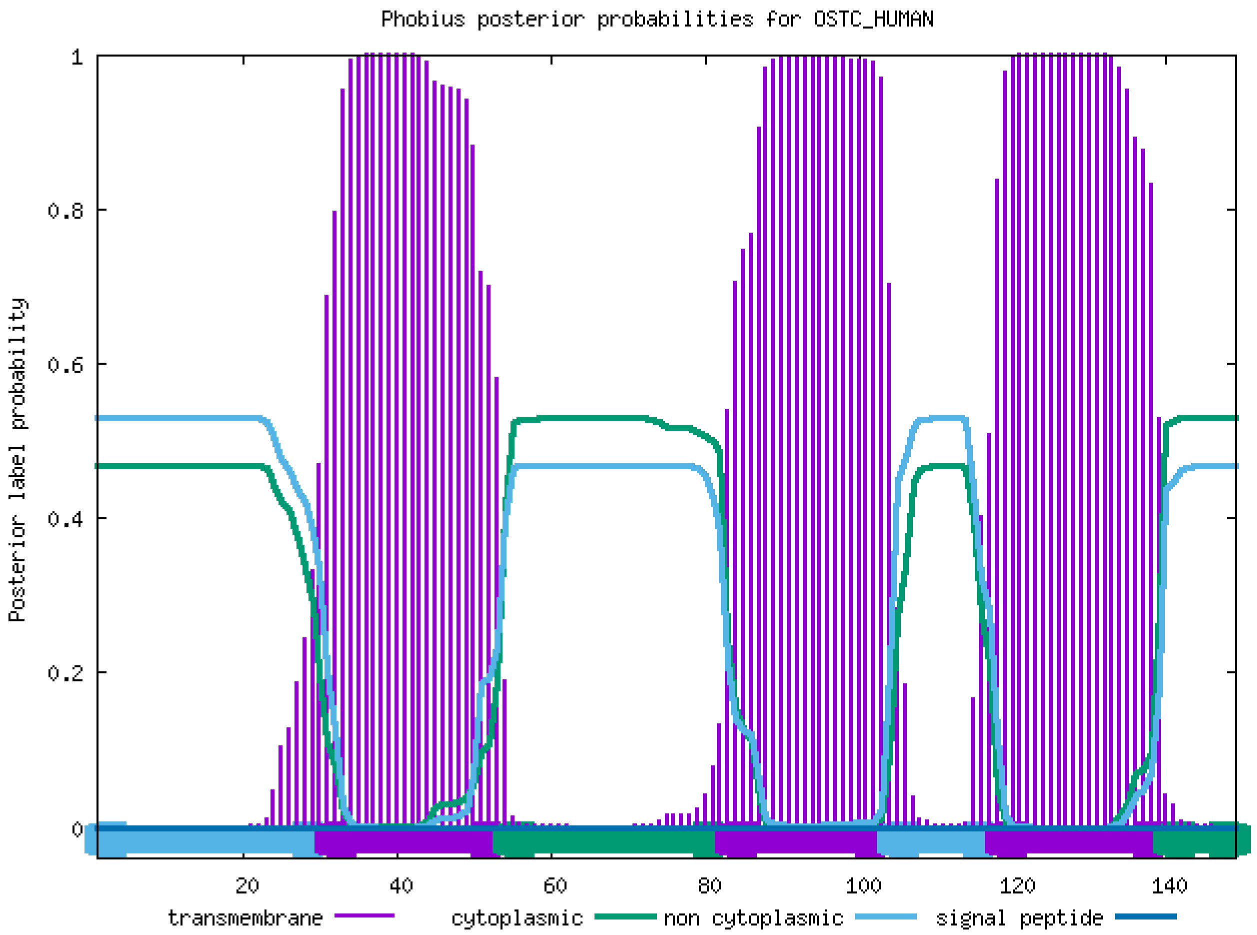

The probability data used in the plot is found here, and the gnuplot script is here.

Prediction of DGAT1_HUMAN

```
ID   DGAT1_HUMAN
FT   TOPO_DOM      1     87       CYTOPLASMIC.
FT   TRANSMEM     88    109
FT   TOPO_DOM    110    128       NON CYTOPLASMIC.
FT   TRANSMEM    129    151
FT   TOPO_DOM    152    162       CYTOPLASMIC.
FT   TRANSMEM    163    186
FT   TOPO_DOM    187    191       NON CYTOPLASMIC.
FT   TRANSMEM    192    210
FT   TOPO_DOM    211    281       CYTOPLASMIC.
FT   TRANSMEM    282    301
FT   TOPO_DOM    302    331       NON CYTOPLASMIC.
FT   TRANSMEM    332    353
FT   TOPO_DOM    354    400       CYTOPLASMIC.
FT   TRANSMEM    401    420
FT   TOPO_DOM    421    425       NON CYTOPLASMIC.
FT   TRANSMEM    426    444
FT   TOPO_DOM    445    455       CYTOPLASMIC.
FT   TRANSMEM    456    474
FT   TOPO_DOM    475    488       NON CYTOPLASMIC.
//
```

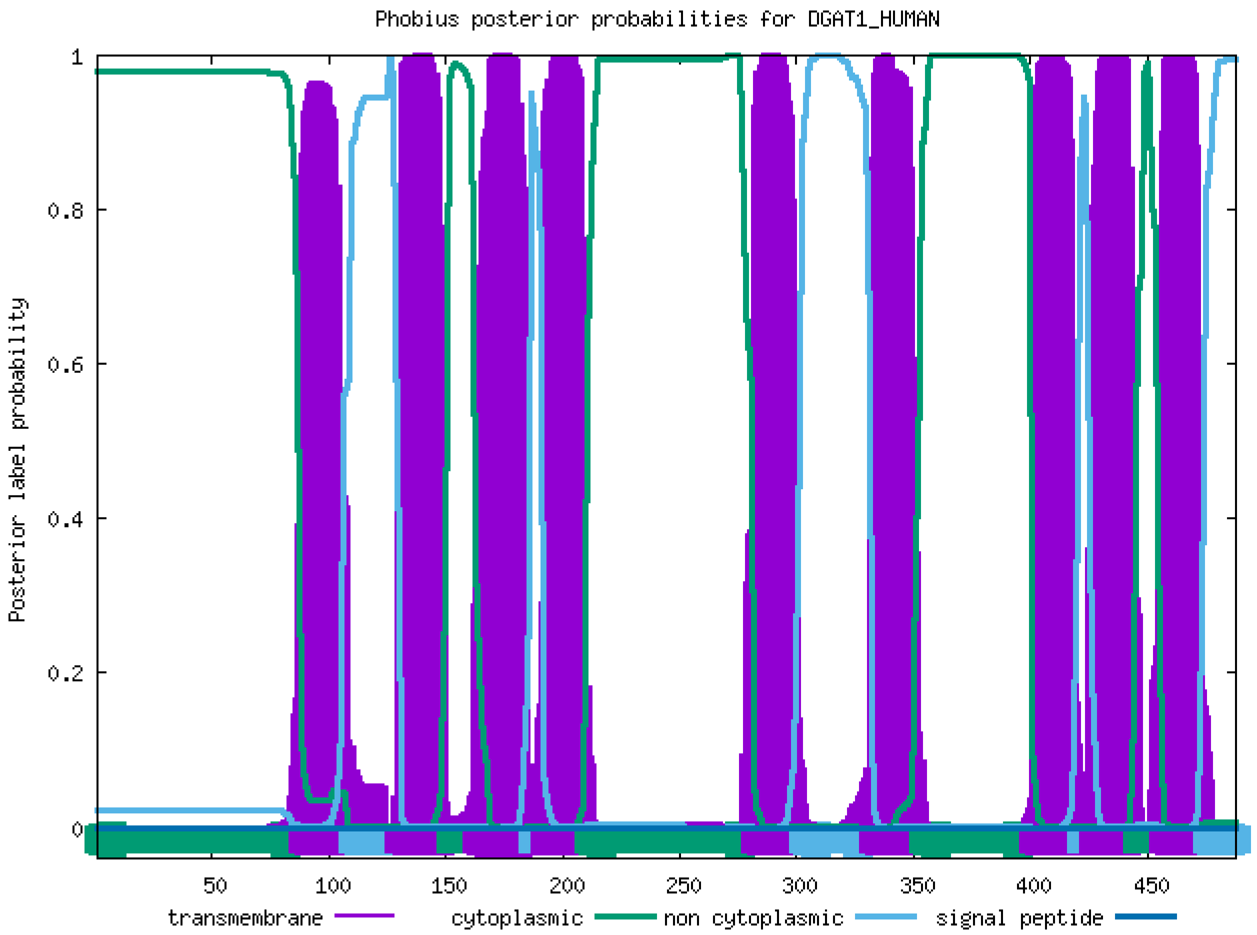

The probability data used in the plot is found here, and the gnuplot script is here.

Prediction of SOAT1_HUMAN

```
ID   SOAT1_HUMAN
FT   TOPO_DOM      1    140       CYTOPLASMIC.
FT   TRANSMEM    141    162
FT   TOPO_DOM    163    181       NON CYTOPLASMIC.
FT   TRANSMEM    182    203
FT   TOPO_DOM    204    223       CYTOPLASMIC.
FT   TRANSMEM    224    244
FT   TOPO_DOM    245    288       NON CYTOPLASMIC.
FT   TRANSMEM    289    308
FT   TOPO_DOM    309    319       CYTOPLASMIC.
FT   TRANSMEM    320    341
FT   TOPO_DOM    342    360       NON CYTOPLASMIC.
FT   TRANSMEM    361    389
FT   TOPO_DOM    390    446       CYTOPLASMIC.
FT   TRANSMEM    447    464
FT   TOPO_DOM    465    469       NON CYTOPLASMIC.
FT   TRANSMEM    470    489
FT   TOPO_DOM    490    500       CYTOPLASMIC.
FT   TRANSMEM    501    520
FT   TOPO_DOM    521    550       NON CYTOPLASMIC.
//
```

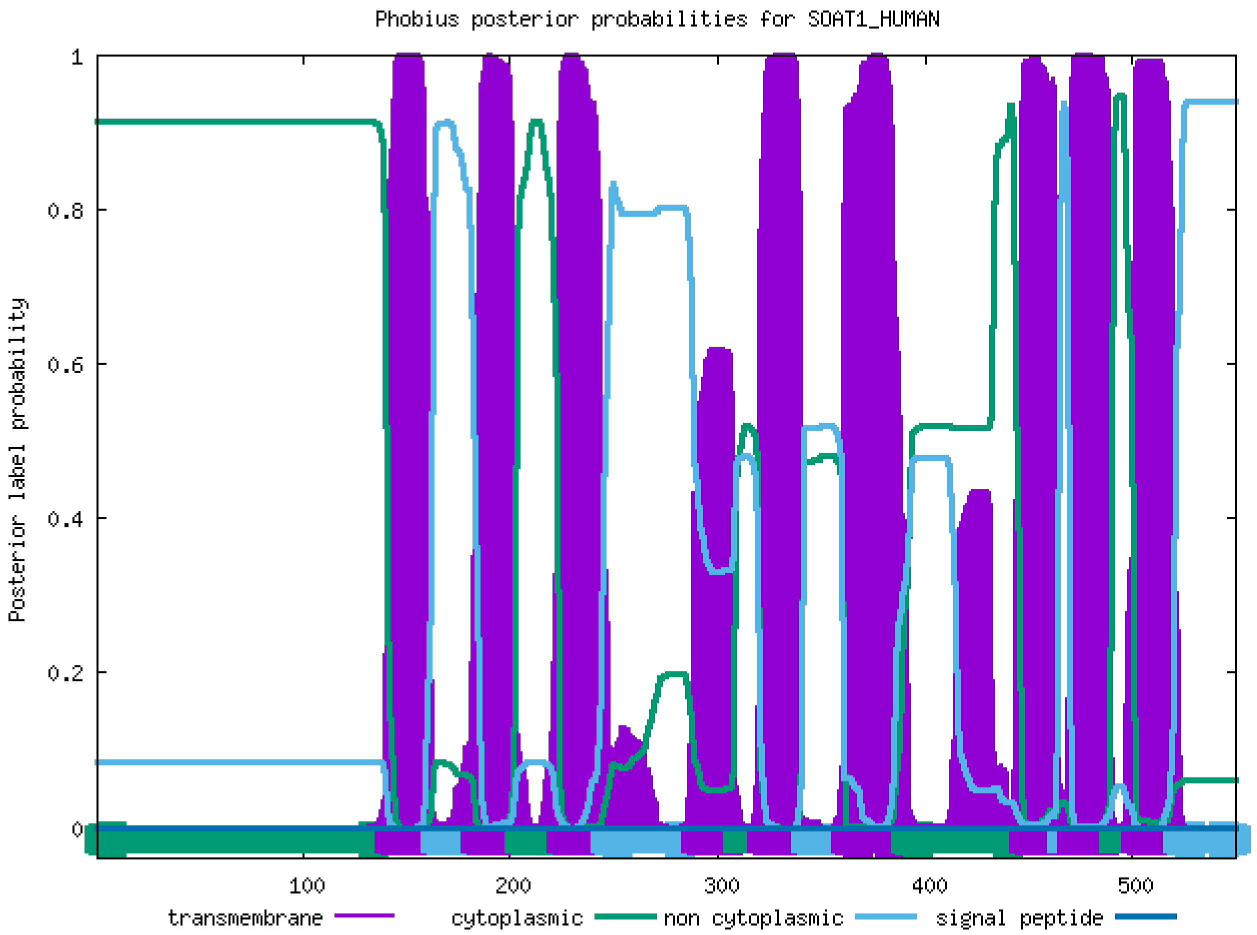

The probability data used in the plot is found here, and the gnuplot script is here.

Prediction of SOAT2_HUMAN

```
ID    SOAT2_HUMAN
FT    TOPO_DOM      1    122       CYTOPLASMIC.
FT    TRANSMEM    123    144
FT    TOPO_DOM    145    155       NON CYTOPLASMIC.
FT    TRANSMEM    156    175
FT    TOPO_DOM    176    195       CYTOPLASMIC.
FT    TRANSMEM    196    218
FT    TOPO_DOM    219    262       NON CYTOPLASMIC.
FT    TRANSMEM    263    282
FT    TOPO_DOM    283    301       CYTOPLASMIC.
FT    TRANSMEM    302    324
FT    TOPO_DOM    325    343       NON CYTOPLASMIC.
FT    TRANSMEM    344    367
FT    TOPO_DOM    368    420       CYTOPLASMIC.
FT    TRANSMEM    421    438
FT    TOPO_DOM    439    443       NON CYTOPLASMIC.
FT    TRANSMEM    444    463
FT    TOPO_DOM    464    474       CYTOPLASMIC.
FT    TRANSMEM    475    492
FT    TOPO_DOM    493    522       NON CYTOPLASMIC.
//
```

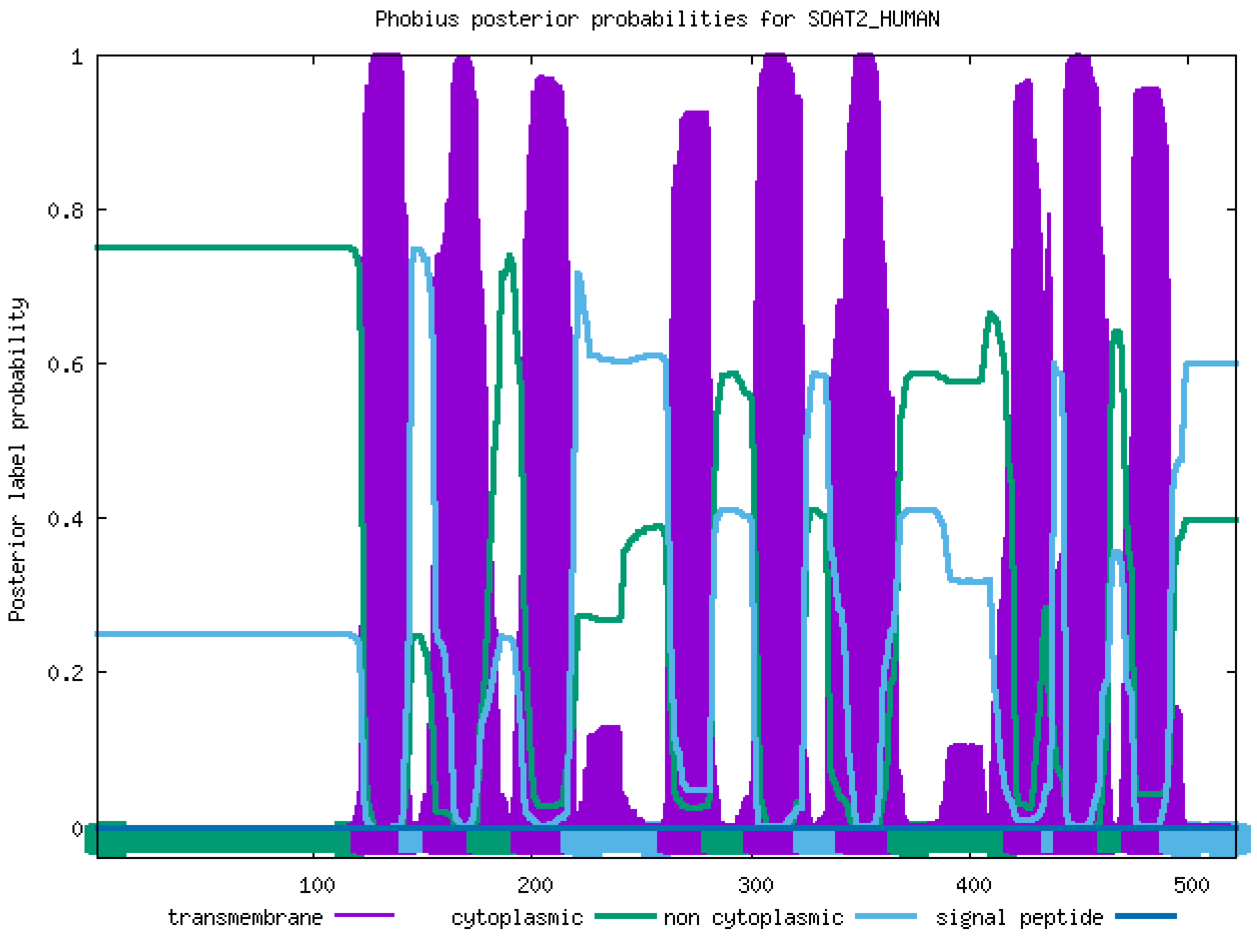

The probability data used in the plot is found here, and the gnuplot script is here.

Prediction of HHAT_HUMAN

```
ID   HHAT_HUMAN
FT   TOPO_DOM      1      6       CYTOPLASMIC.
FT   TRANSMEM      7     23
FT   TOPO_DOM     24     69       NON CYTOPLASMIC.
FT   TRANSMEM     70     89
FT   TOPO_DOM     90     95       CYTOPLASMIC.
FT   TRANSMEM     96    118
FT   TOPO_DOM    119    129       NON CYTOPLASMIC.
FT   TRANSMEM    130    147
FT   TOPO_DOM    148    166       CYTOPLASMIC.
FT   TRANSMEM    167    185
FT   TOPO_DOM    186    204       NON CYTOPLASMIC.
FT   TRANSMEM    205    225
FT   TOPO_DOM    226    236       CYTOPLASMIC.
FT   TRANSMEM    237    257
FT   TOPO_DOM    258    293       NON CYTOPLASMIC.
FT   TRANSMEM    294    313
FT   TOPO_DOM    314    319       CYTOPLASMIC.
FT   TRANSMEM    320    338
FT   TOPO_DOM    339    357       NON CYTOPLASMIC.
FT   TRANSMEM    358    378
FT   TOPO_DOM    379    426       CYTOPLASMIC.
FT   TRANSMEM    427    447
FT   TOPO_DOM    448    466       NON CYTOPLASMIC.
FT   TRANSMEM    467    487
FT   TOPO_DOM    488    493       CYTOPLASMIC.
//
```

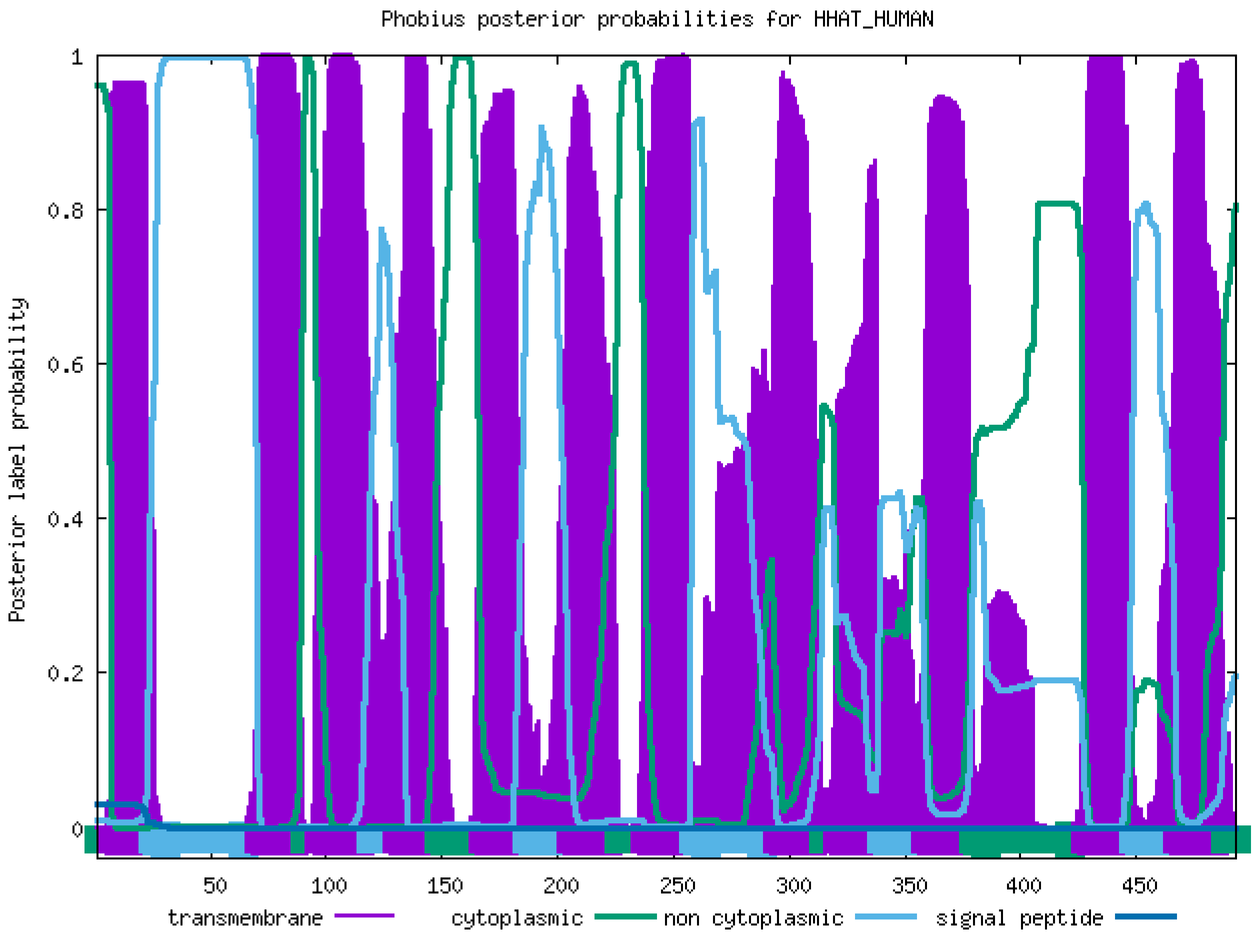

The probability data used in the plot is found here, and the gnuplot script is here.

Prediction of PORCN_HUMAN

```
ID    PORCN_HUMAN
FT    TOPO_DOM      1      28       CYTOPLASMIC.
FT    TRANSMEM     29      47
FT    TOPO_DOM     48      66       NON CYTOPLASMIC.
FT    TRANSMEM     67      89
FT    TOPO_DOM     90      95       CYTOPLASMIC.
FT    TRANSMEM     96     115
FT    TOPO_DOM    116     157       NON CYTOPLASMIC.
FT    TRANSMEM    158     177
FT    TOPO_DOM    178     196       CYTOPLASMIC.
FT    TRANSMEM    197     219
FT    TOPO_DOM    220     337       NON CYTOPLASMIC.
FT    TRANSMEM    338     360
FT    TOPO_DOM    361     396       CYTOPLASMIC.
FT    TRANSMEM    397     417
FT    TOPO_DOM    418     441       NON CYTOPLASMIC.
FT    TRANSMEM    442     460
FT    TOPO_DOM    461     461       CYTOPLASMIC.
//
```

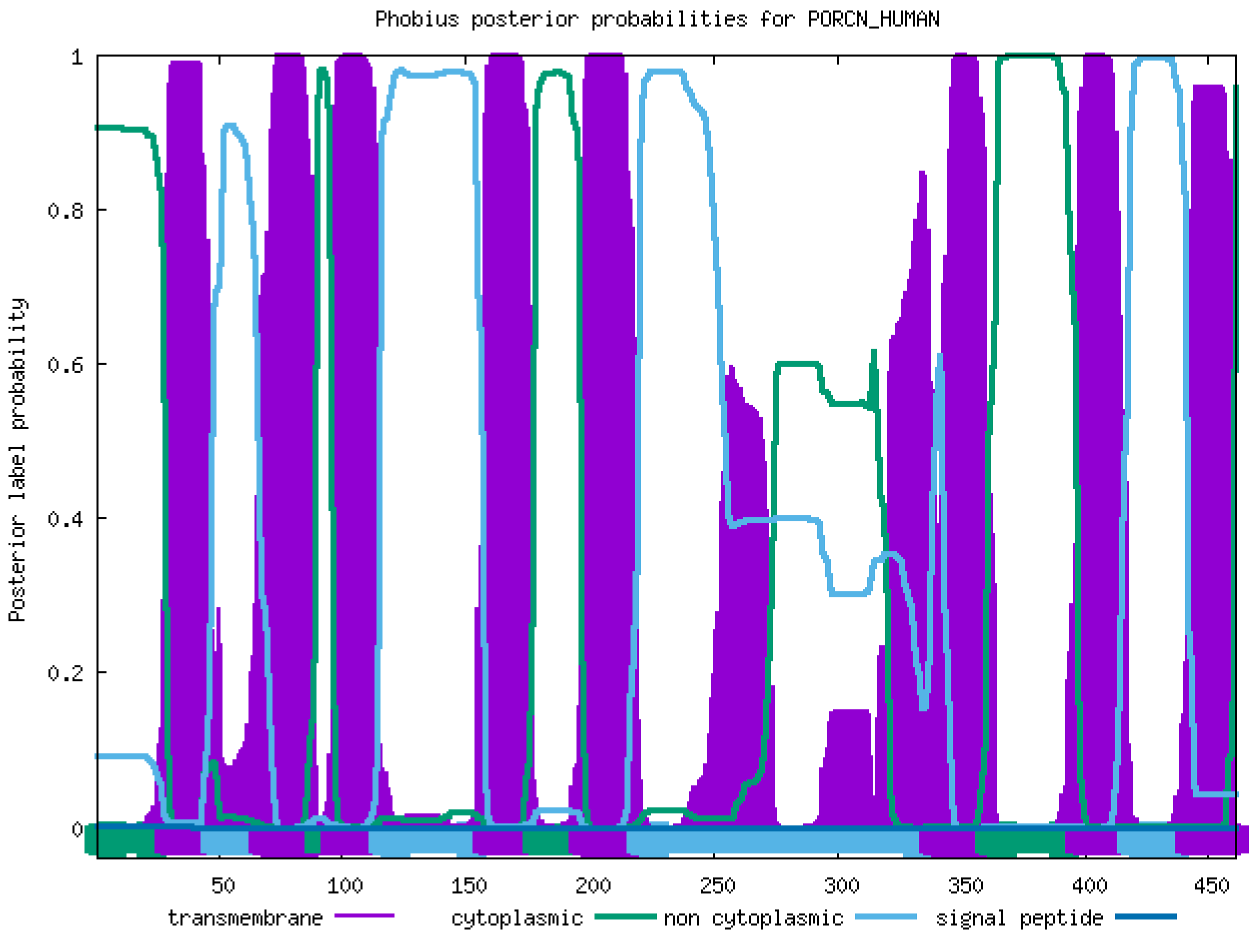

The probability data used in the plot is found here, and the gnuplot script is here.

Prediction of MBOA7_HUMAN

```
ID   MBOA7_HUMAN
FT   TOPO_DOM      1      5       NON CYTOPLASMIC.
FT   TRANSMEM      6     22
FT   TOPO_DOM     23     33       CYTOPLASMIC.
FT   TRANSMEM     34     64
FT   TOPO_DOM     65     75       NON CYTOPLASMIC.
FT   TRANSMEM     76     94
FT   TOPO_DOM     95    194       CYTOPLASMIC.
FT   TRANSMEM    195    212
FT   TOPO_DOM    213    231       NON CYTOPLASMIC.
FT   TRANSMEM    232    252
FT   TOPO_DOM    253    340       CYTOPLASMIC.
FT   TRANSMEM    341    356
FT   TOPO_DOM    357    361       NON CYTOPLASMIC.
FT   TRANSMEM    362    379
FT   TOPO_DOM    380    399       CYTOPLASMIC.
FT   TRANSMEM    400    419
FT   TOPO_DOM    420    424       NON CYTOPLASMIC.
FT   TRANSMEM    425    447
FT   TOPO_DOM    448    472       CYTOPLASMIC.
//
```

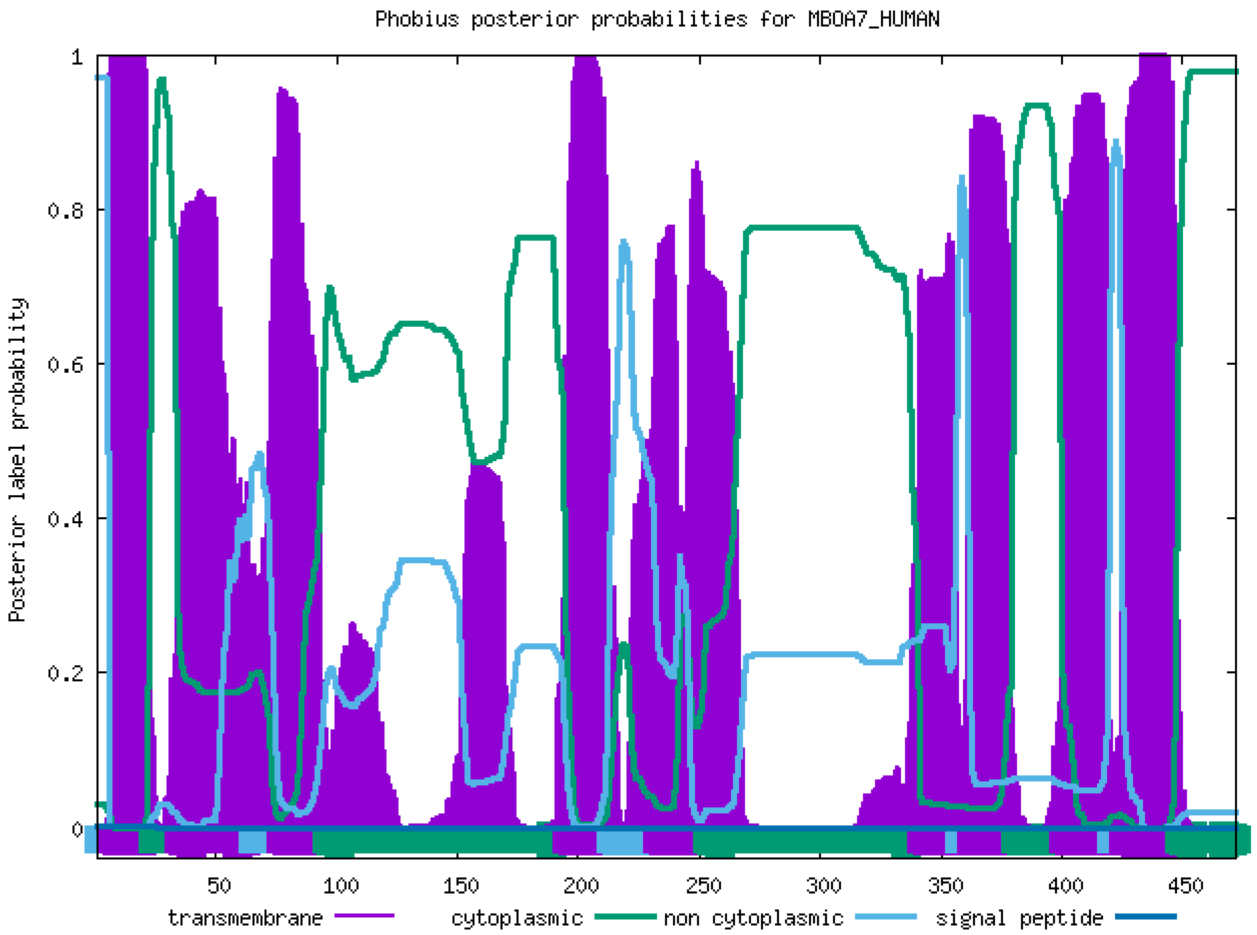

The probability data used in the plot is found here, and the gnuplot script is here.

Prediction of ZDH20_HUMAN

```
ID   ZDH20_HUMAN
FT   TOPO_DOM      1     11       CYTOPLASMIC.
FT   TRANSMEM     12     31
FT   TOPO_DOM     32     50       NON CYTOPLASMIC.
FT   TRANSMEM     51     70
FT   TOPO_DOM     71    169       CYTOPLASMIC.
FT   TRANSMEM    170    190
FT   TOPO_DOM    191    209       NON CYTOPLASMIC.
FT   TRANSMEM    210    236
FT   TOPO_DOM    237    365       CYTOPLASMIC.
//
```

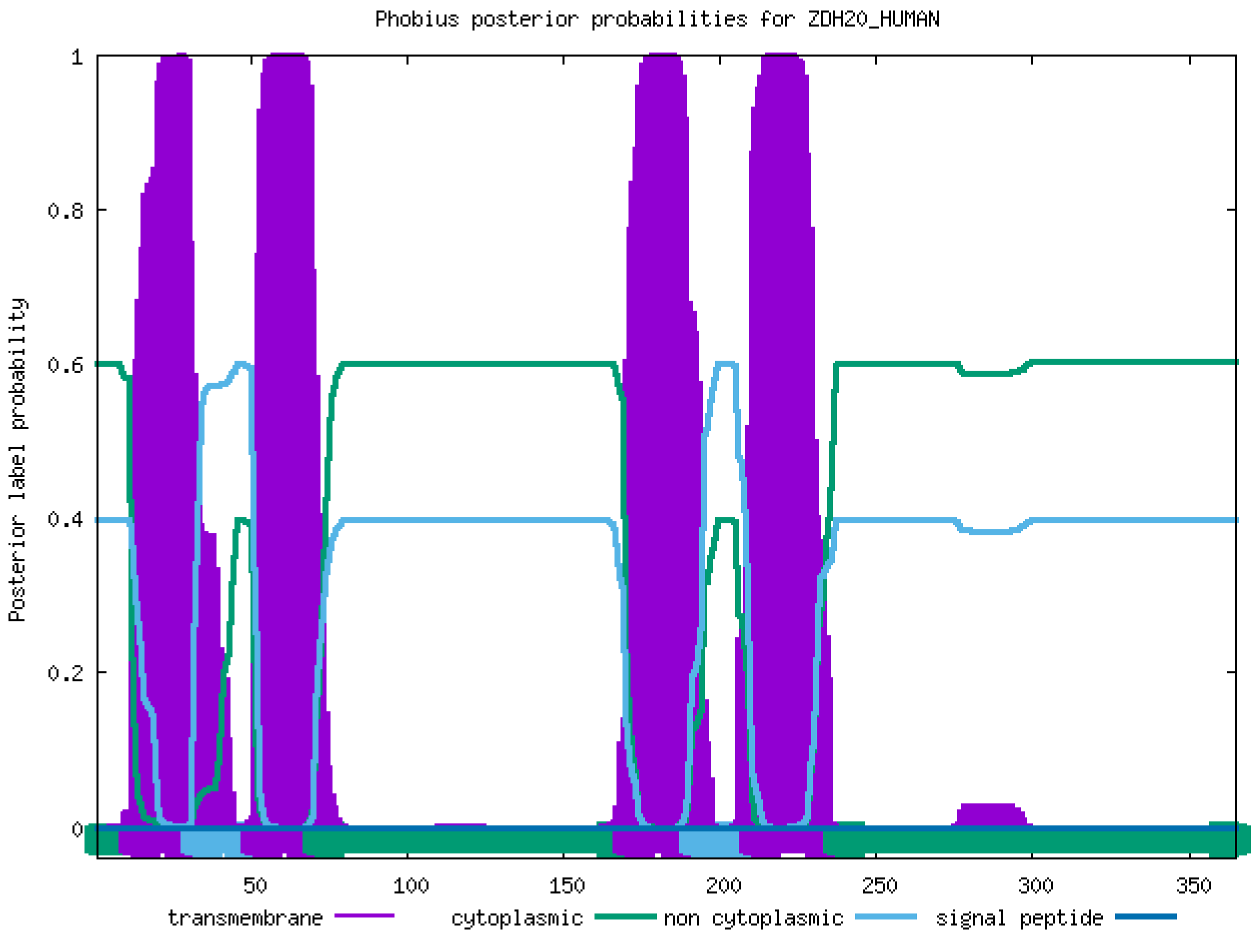

The probability data used in the plot is found here, and the gnuplot script is here.

Prediction of SPTC1_HUMAN

```
ID   SPTC1_HUMAN
FT   TOPO_DOM      1     19       NON CYTOPLASMIC.
FT   TRANSMEM     20     40
FT   TOPO_DOM     41    160       CYTOPLASMIC.
FT   TRANSMEM    161    179
FT   TOPO_DOM    180    473       NON CYTOPLASMIC.
//
```

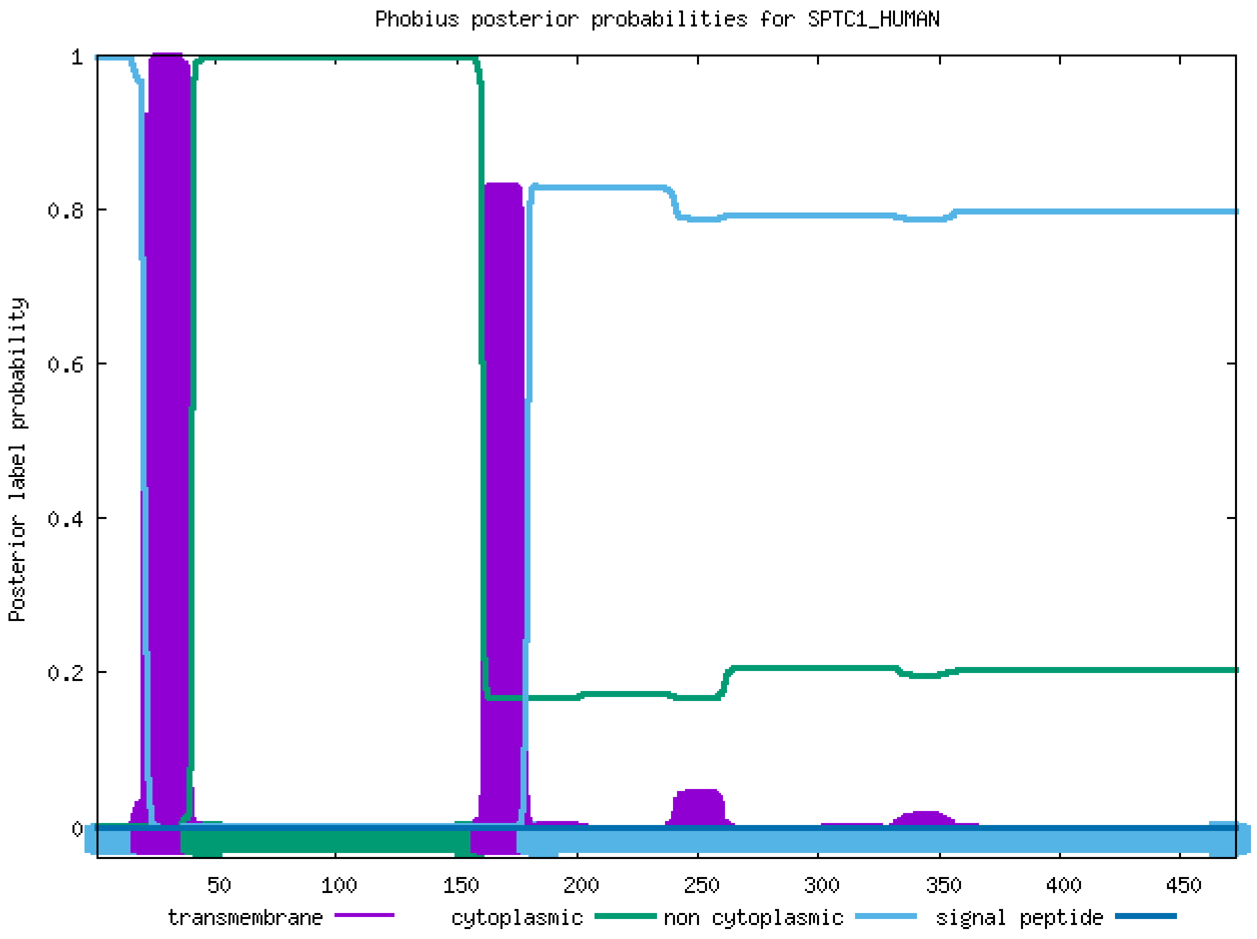

The probability data used in the plot is found here, and the gnuplot script is here.

Prediction of SPTC2_HUMAN

```
ID    SPTC2_HUMAN
FT    TOPO_DOM      1     67       NON CYTOPLASMIC.
FT    TRANSMEM     68     87
FT    TOPO_DOM     88    562       CYTOPLASMIC.
//
```

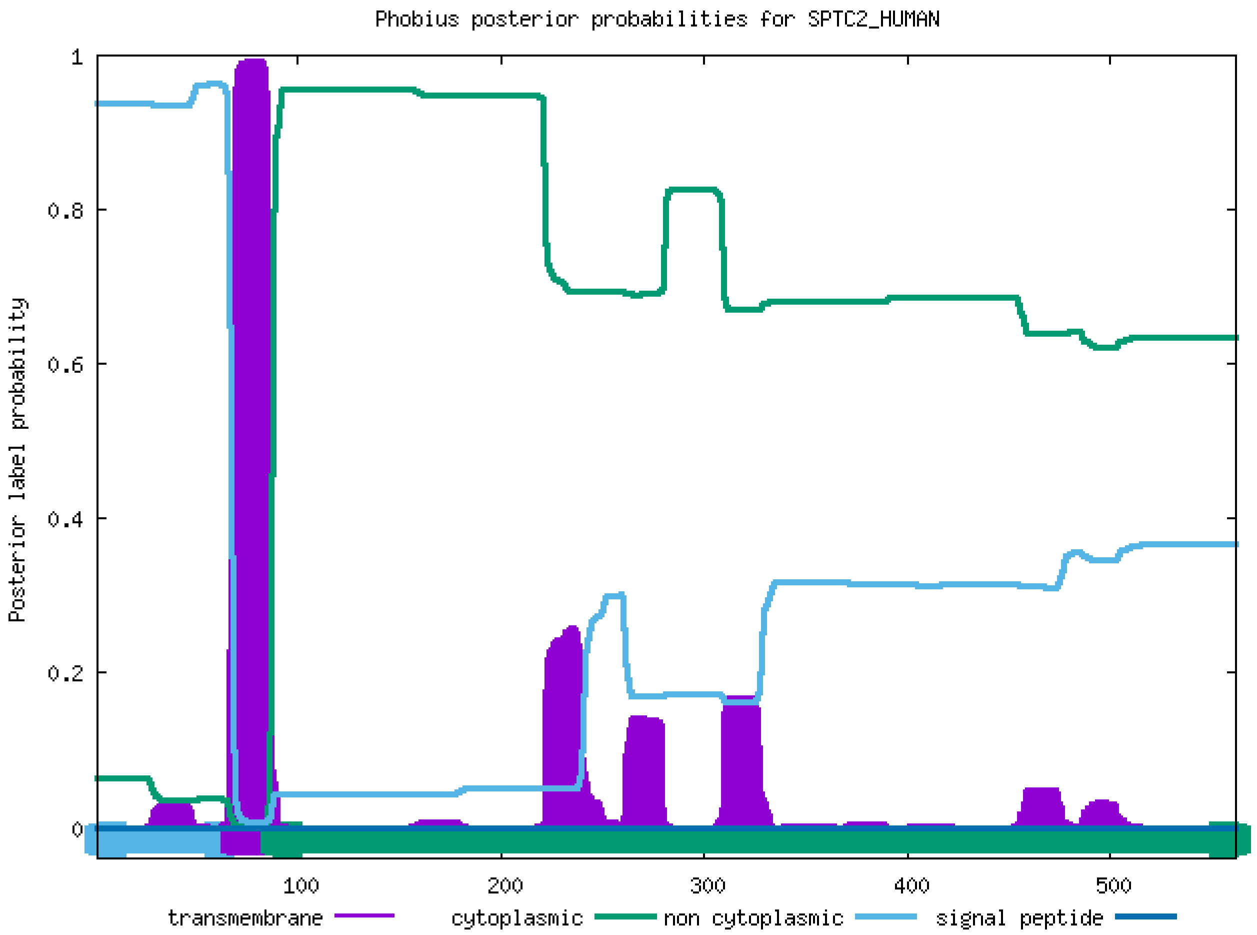

The probability data used in the plot is found here, and the gnuplot script is here.

Prediction of SPTSA_HUMAN

```
ID   SPTSA_HUMAN
FT   TOPO_DOM      1     11       CYTOPLASMIC.
FT   TRANSMEM     12     29
FT   TOPO_DOM     30     34       NON CYTOPLASMIC.
FT   TRANSMEM     35     56
FT   TOPO_DOM     57     71       CYTOPLASMIC.
//
```

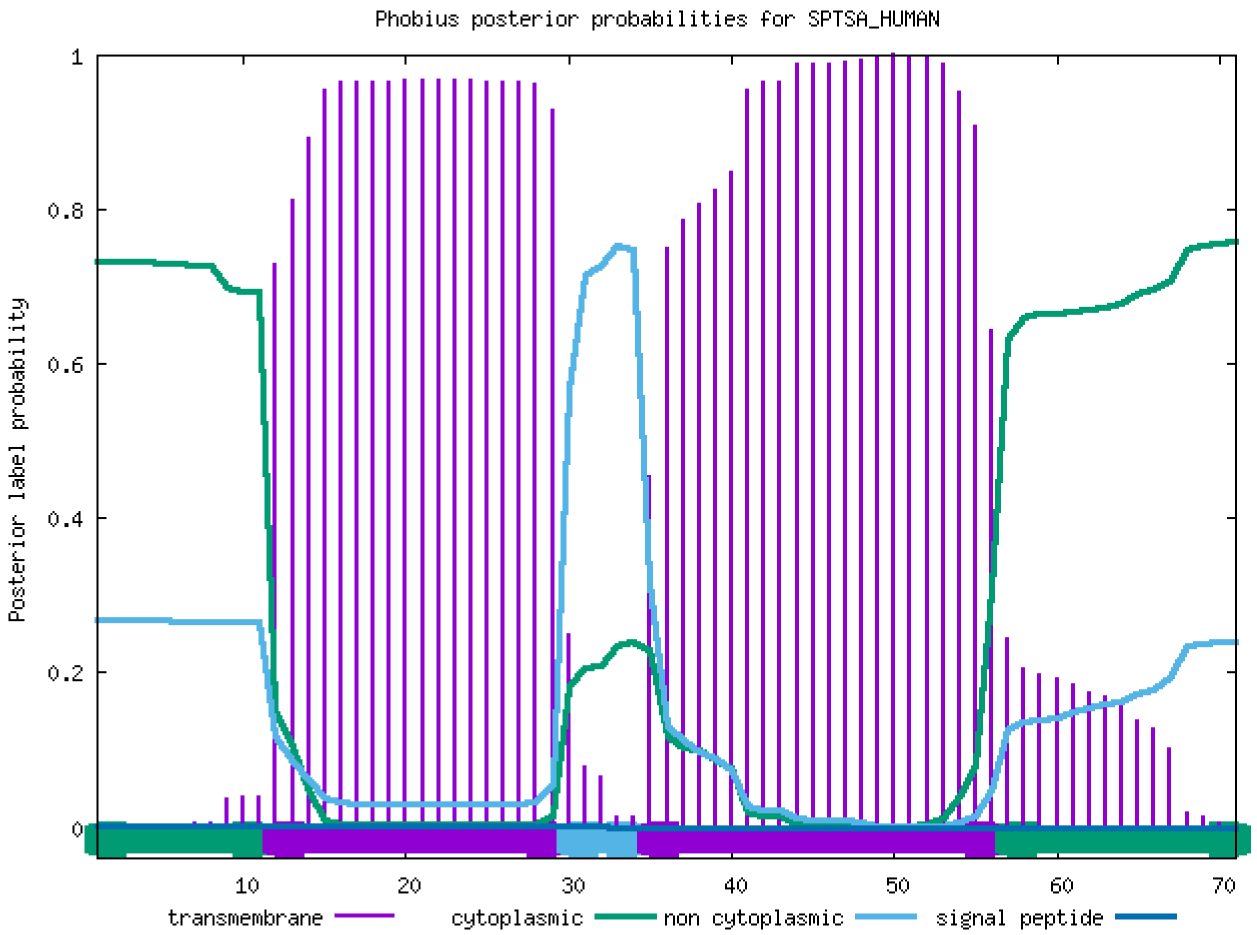

The probability data used in the plot is found here, and the gnuplot script is here.

Prediction of ELOV7_HUMAN

```
ID    ELOV7_HUMAN
FT    TOPO_DOM      1      27       NON CYTOPLASMIC.
FT    TRANSMEM     28      48
FT    TOPO_DOM     49      68       CYTOPLASMIC.
FT    TRANSMEM     69      87
FT    TOPO_DOM     88     117       NON CYTOPLASMIC.
FT    TRANSMEM    118     136
FT    TOPO_DOM    137     142       CYTOPLASMIC.
FT    TRANSMEM    143     162
FT    TOPO_DOM    163     173       NON CYTOPLASMIC.
FT    TRANSMEM    174     194
FT    TOPO_DOM    195     205       CYTOPLASMIC.
FT    TRANSMEM    206     227
FT    TOPO_DOM    228     232       NON CYTOPLASMIC.
FT    TRANSMEM    233     258
FT    TOPO_DOM    259     281       CYTOPLASMIC.
//
```

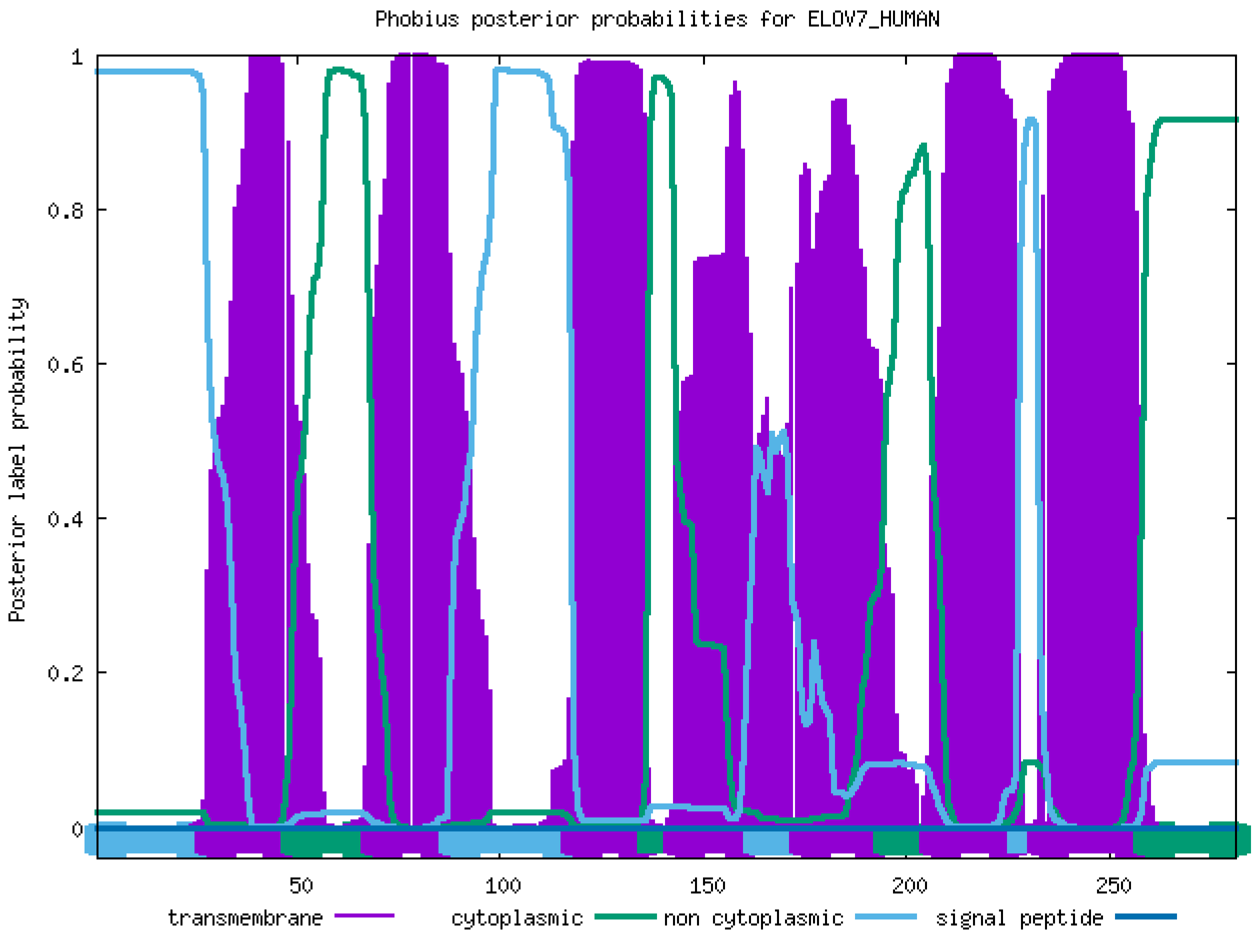

The probability data used in the plot is found here, and the gnuplot script is here.

Prediction of CEPT1_HUMAN

```
ID   CEPT1_HUMAN
FT   TOPO_DOM      1     86       CYTOPLASMIC.
FT   TRANSMEM     87    108
FT   TOPO_DOM    109    113       NON CYTOPLASMIC.
FT   TRANSMEM    114    132
FT   TOPO_DOM    133    180       CYTOPLASMIC.
FT   TRANSMEM    181    201
FT   TOPO_DOM    202    215       NON CYTOPLASMIC.
FT   TRANSMEM    216    235
FT   TOPO_DOM    236    246       CYTOPLASMIC.
FT   TRANSMEM    247    266
FT   TOPO_DOM    267    285       NON CYTOPLASMIC.
FT   TRANSMEM    286    304
FT   TOPO_DOM    305    315       CYTOPLASMIC.
FT   TRANSMEM    316    336
FT   TOPO_DOM    337    370       NON CYTOPLASMIC.
FT   TRANSMEM    371    389
FT   TOPO_DOM    390    416       CYTOPLASMIC.
//
```

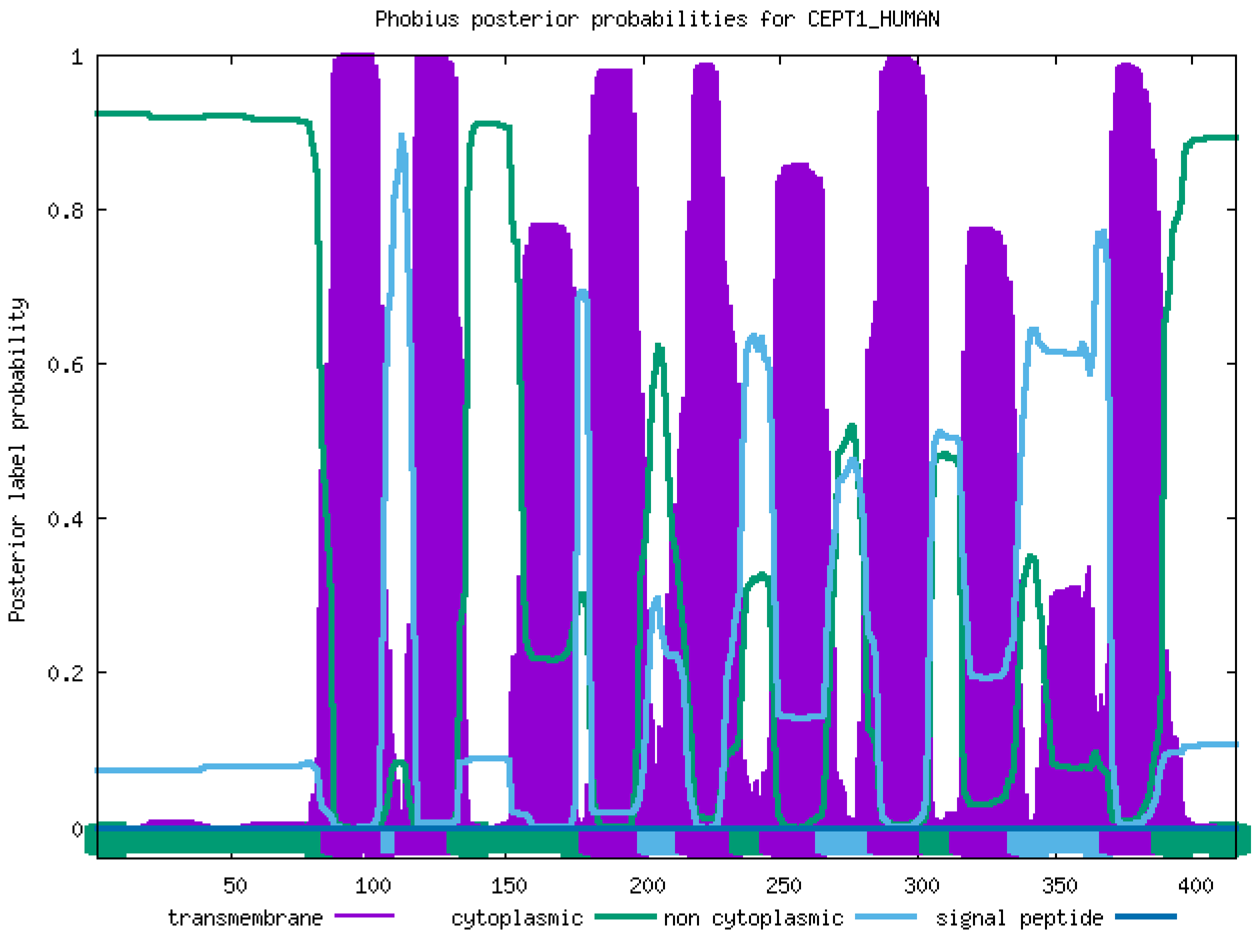

The probability data used in the plot is found here, and the gnuplot script is here.

Prediction of FACE1_HUMAN

```
ID    FACE1_HUMAN
FT    TOPO_DOM      1     19       NON CYTOPLASMIC.
FT    TRANSMEM     20     38
FT    TOPO_DOM     39     76       CYTOPLASMIC.
FT    TRANSMEM     77    102
FT    TOPO_DOM    103    121       NON CYTOPLASMIC.
FT    TRANSMEM    122    144
FT    TOPO_DOM    145    170       CYTOPLASMIC.
FT    TRANSMEM    171    189
FT    TOPO_DOM    190    194       NON CYTOPLASMIC.
FT    TRANSMEM    195    216
FT    TOPO_DOM    217    356       CYTOPLASMIC.
FT    TRANSMEM    357    379
FT    TOPO_DOM    380    384       NON CYTOPLASMIC.
FT    TRANSMEM    385    410
FT    TOPO_DOM    411    475       CYTOPLASMIC.
//
```

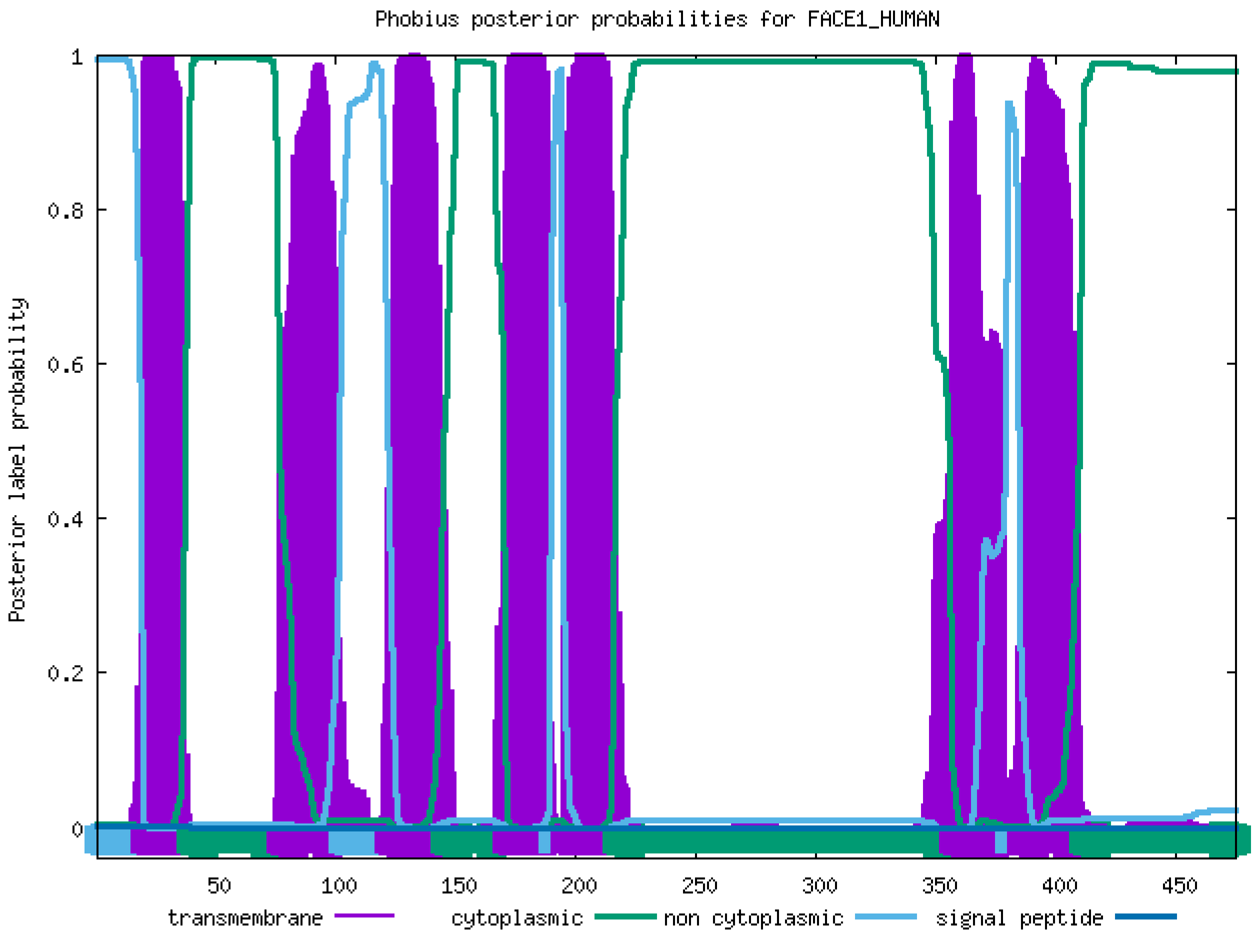

The probability data used in the plot is found here, and the gnuplot script is here.

Prediction of NICA_HUMAN

```
ID   NICA_HUMAN
FT   SIGNAL         1     33
FT   REGION         1     16       N-REGION.
FT   REGION        17     28       H-REGION.
FT   REGION        29     33       C-REGION.
FT   TOPO_DOM      34    669       NON CYTOPLASMIC.
FT   TRANSMEM     670    690
FT   TOPO_DOM     691    709       CYTOPLASMIC.
//
```

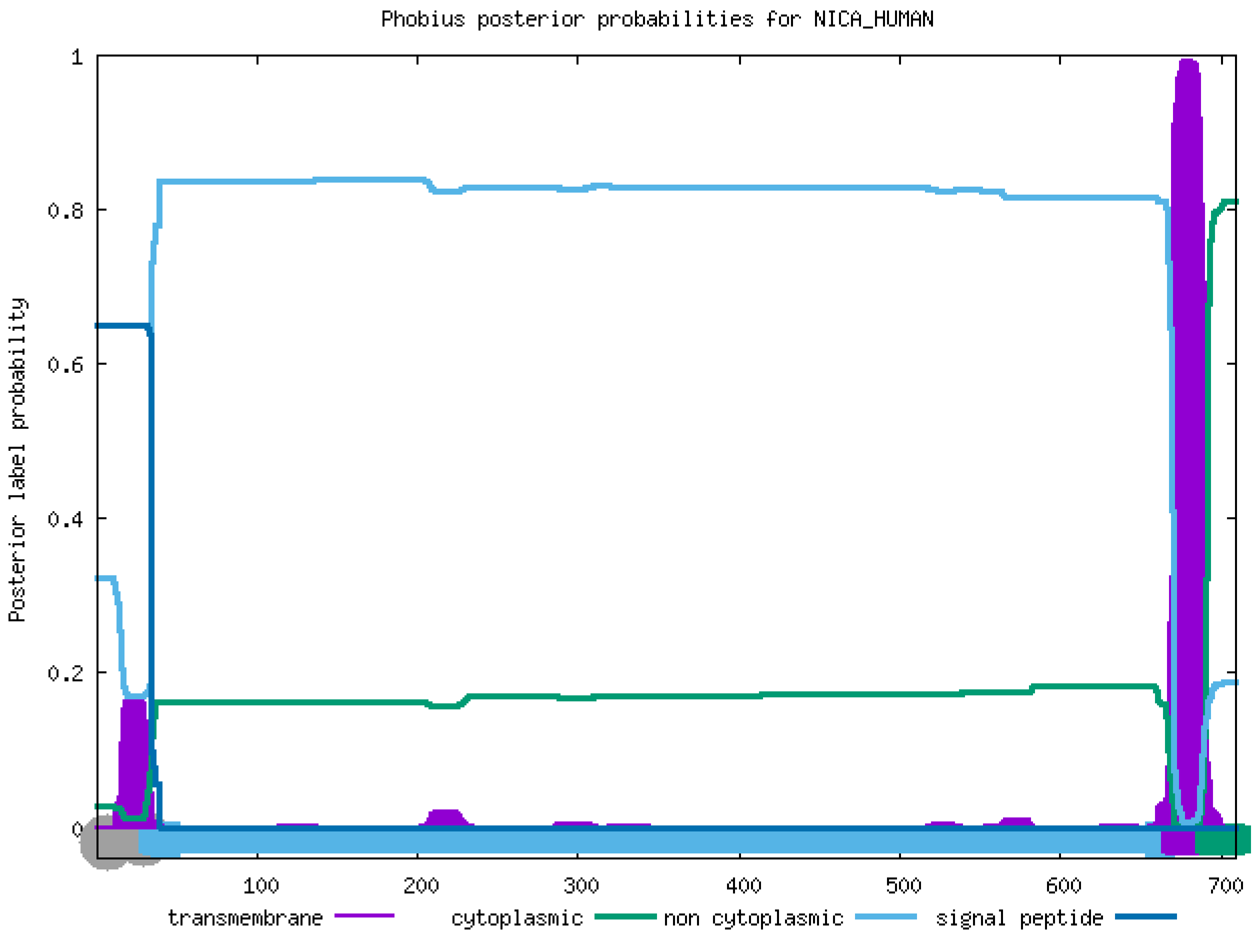

The probability data used in the plot is found here, and the gnuplot script is here.

Prediction of PSN1_HUMAN

```
ID    PSN1_HUMAN
FT    TOPO_DOM      1     81       CYTOPLASMIC.
FT    TRANSMEM     82    100
FT    TOPO_DOM    101    132       NON CYTOPLASMIC.
FT    TRANSMEM    133    154
FT    TOPO_DOM    155    160       CYTOPLASMIC.
FT    TRANSMEM    161    183
FT    TOPO_DOM    184    194       NON CYTOPLASMIC.
FT    TRANSMEM    195    213
FT    TOPO_DOM    214    224       CYTOPLASMIC.
FT    TRANSMEM    225    241
FT    TOPO_DOM    242    246       NON CYTOPLASMIC.
FT    TRANSMEM    247    263
FT    TOPO_DOM    264    407       CYTOPLASMIC.
FT    TRANSMEM    408    428
FT    TOPO_DOM    429    433       NON CYTOPLASMIC.
FT    TRANSMEM    434    453
FT    TOPO_DOM    454    467       CYTOPLASMIC.
//
```

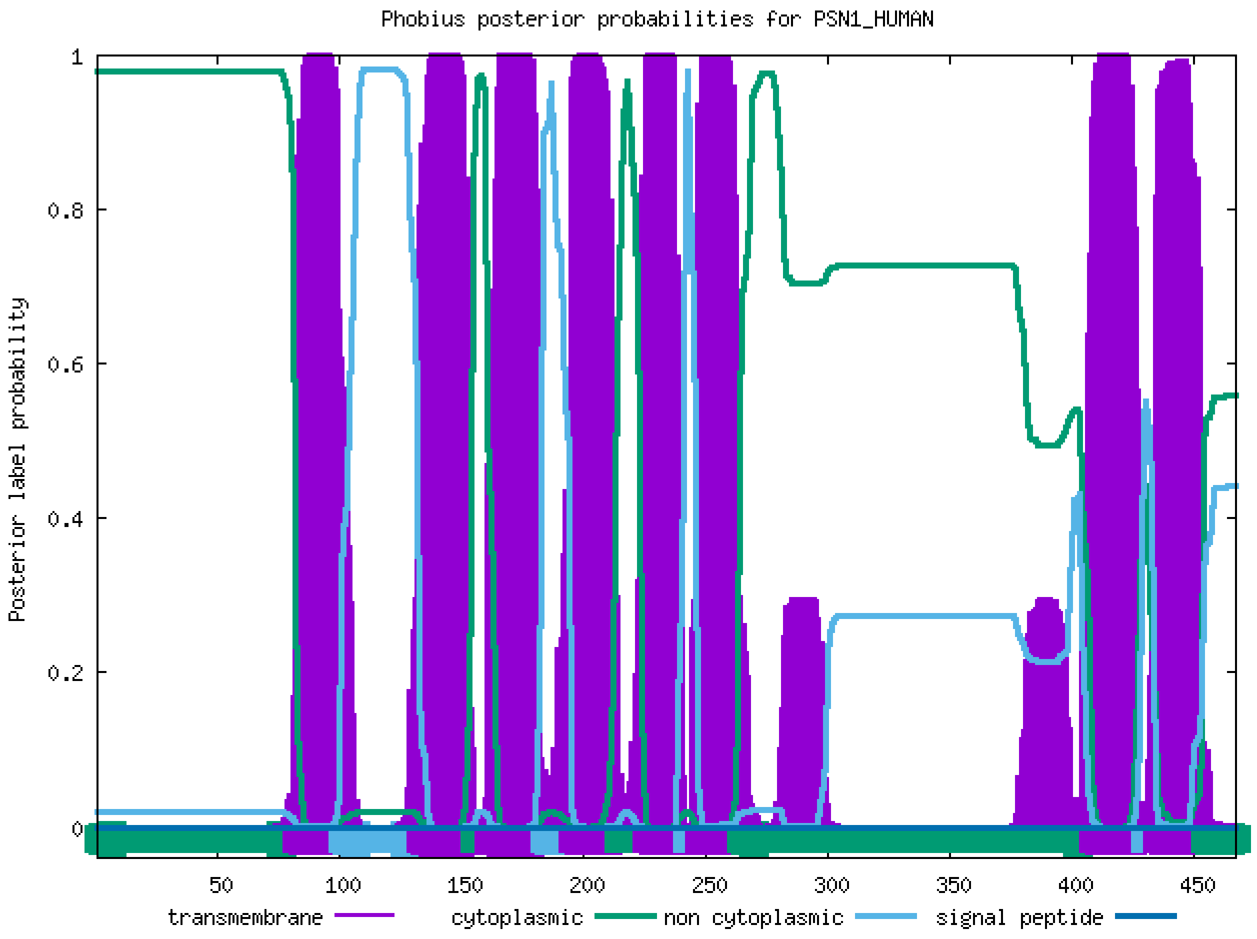

The probability data used in the plot is found here, and the gnuplot script is here.

Prediction of SC11A_HUMAN

```
ID   SC11A_HUMAN
FT   TOPO_DOM      1     17       NON CYTOPLASMIC.
FT   TRANSMEM     18     36
FT   TOPO_DOM     37    137       CYTOPLASMIC.
FT   TRANSMEM    138    156
FT   TOPO_DOM    157    161       NON CYTOPLASMIC.
FT   TRANSMEM    162    178
FT   TOPO_DOM    179    179       CYTOPLASMIC.
//
```

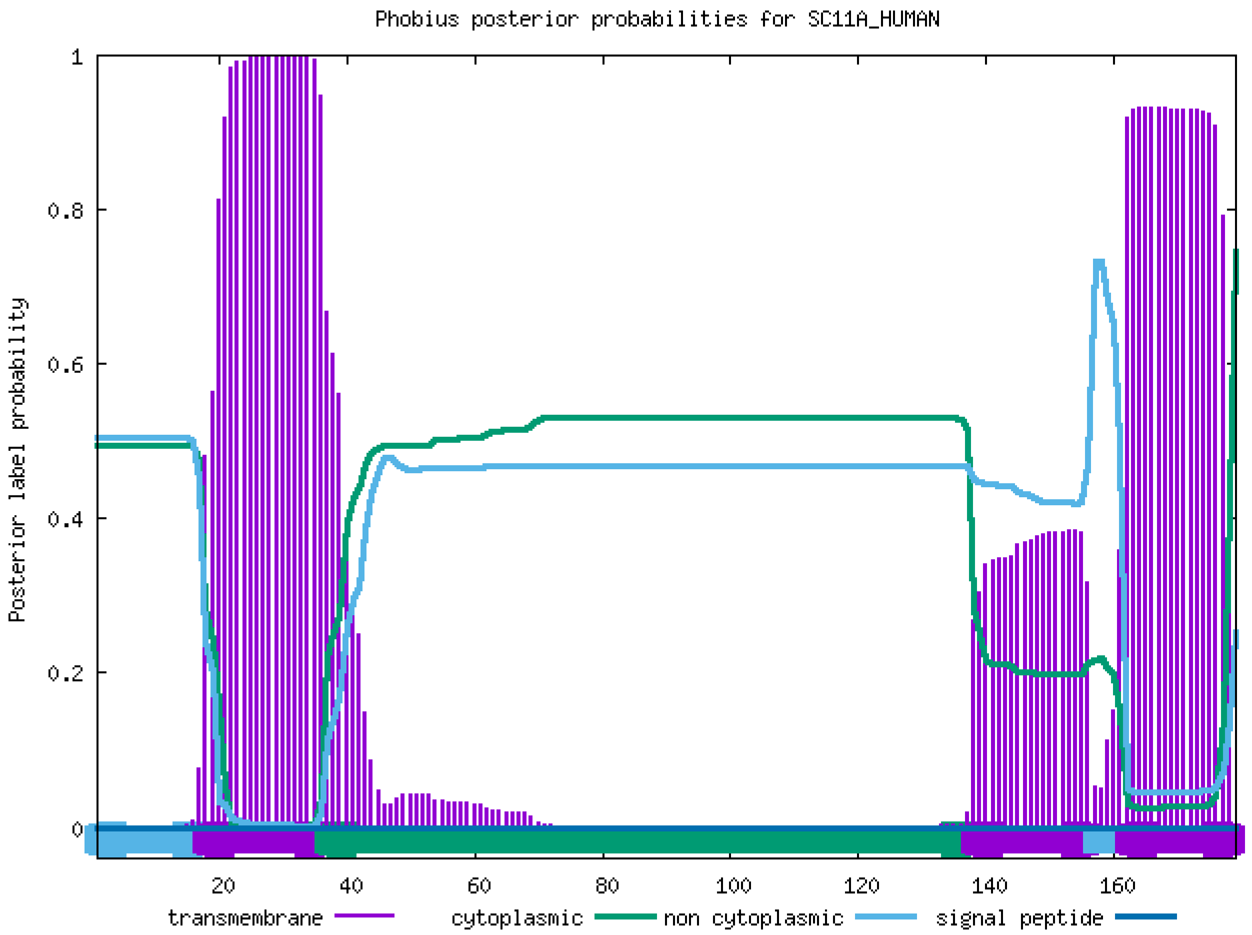

The probability data used in the plot is found here, and the gnuplot script is here.

Prediction of SPCS3_HUMAN

```
ID    SPCS3_HUMAN
FT    TOPO_DOM      1      11       NON CYTOPLASMIC.
FT    TRANSMEM     12      32
FT    TOPO_DOM     33     180       CYTOPLASMIC.
//
```

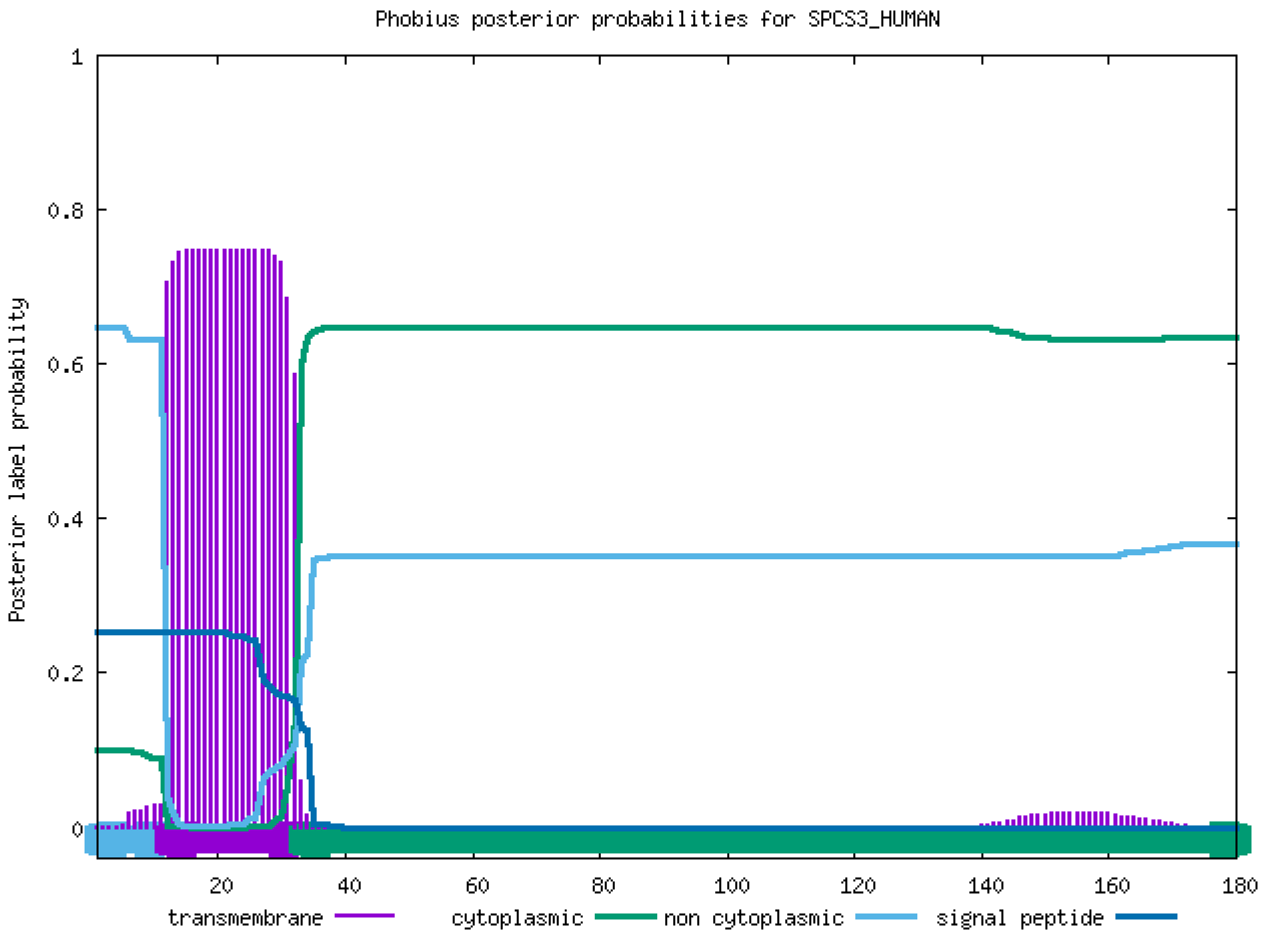

The probability data used in the plot is found here, and the gnuplot script is here.

Prediction of SPCS2_HUMAN

```
ID    SPCS2_HUMAN
FT    TOPO_DOM      1     80       CYTOPLASMIC.
FT    TRANSMEM     81    101
FT    TOPO_DOM    102    112       NON CYTOPLASMIC.
FT    TRANSMEM    113    131
FT    TOPO_DOM    132    226       CYTOPLASMIC.
//
```

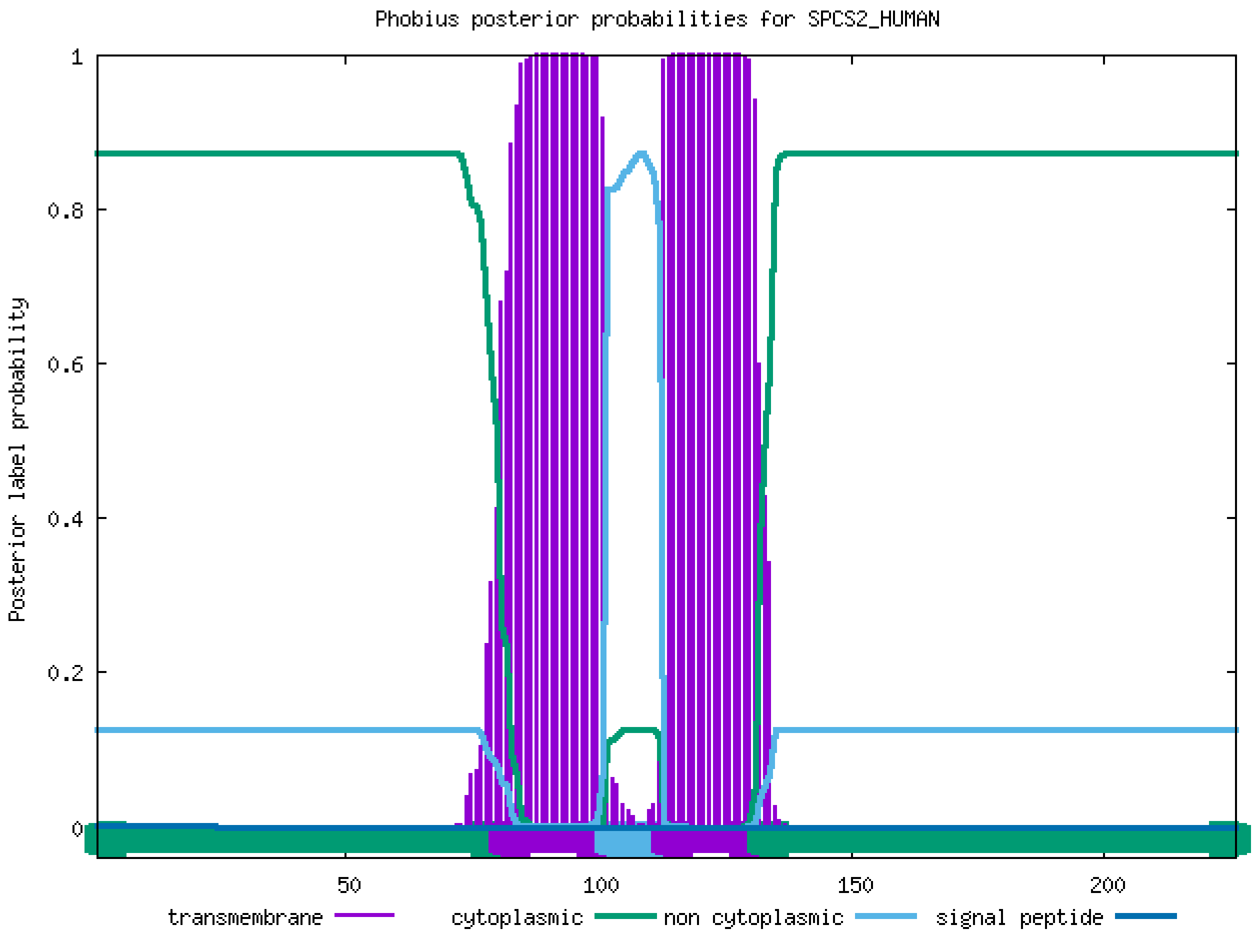

The probability data used in the plot is found here, and the gnuplot script is here.

Prediction of SPCS1_HUMAN

```
ID    SPCS1_HUMAN
FT    TOPO_DOM      1      89       CYTOPLASMIC.
FT    TRANSMEM     90     108
FT    TOPO_DOM    109     113       NON CYTOPLASMIC.
FT    TRANSMEM    114     134
FT    TOPO_DOM    135     169       CYTOPLASMIC.
//
```

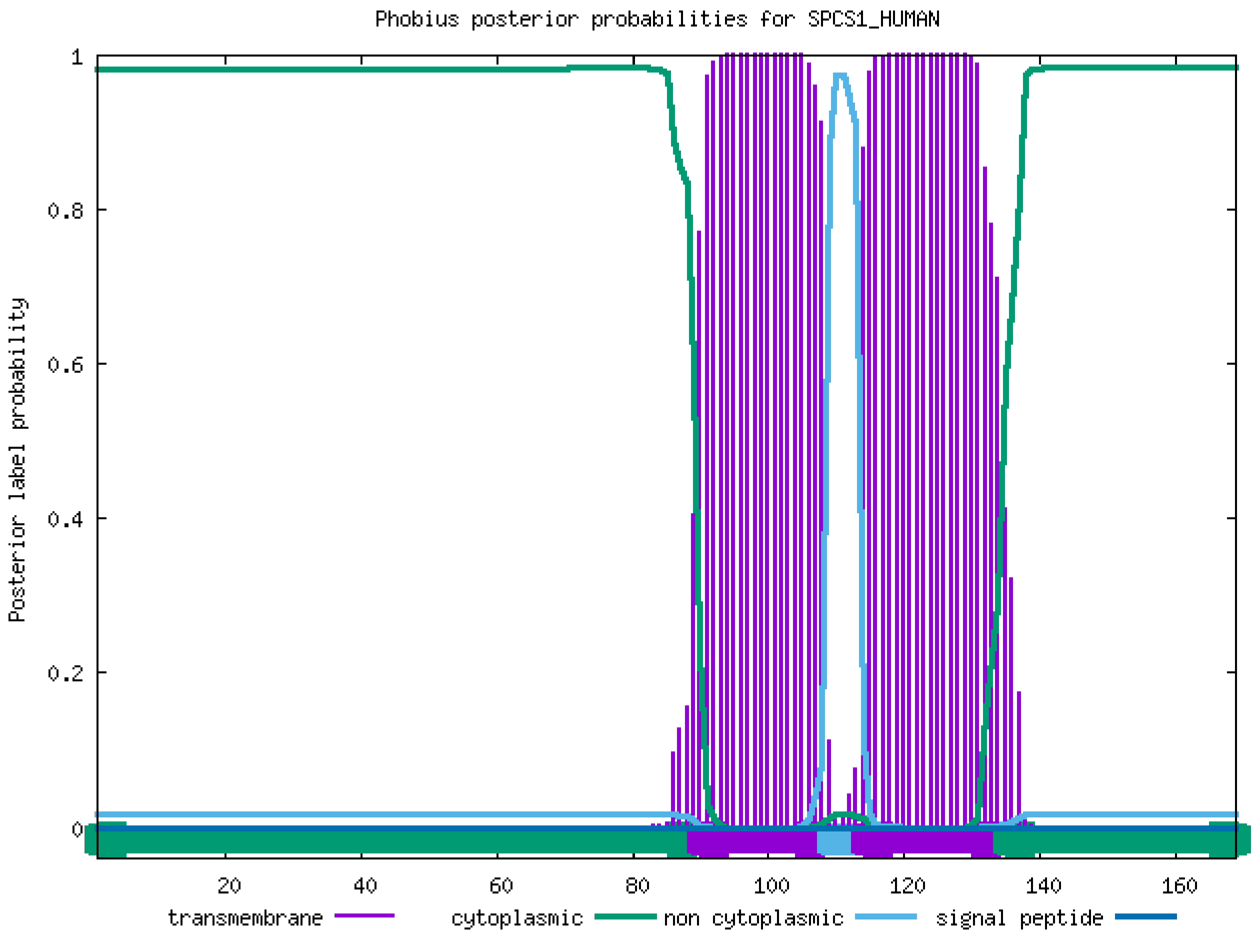

The probability data used in the plot is found here, and the gnuplot script is here.

Prediction of PIGU_HUMAN

```
ID   PIGU_HUMAN
FT   SIGNAL        1     17
FT   REGION        1      1       N-REGION.
FT   REGION        2     13       H-REGION.
FT   REGION       14     17       C-REGION.
FT   TOPO_DOM     18     67       NON CYTOPLASMIC.
FT   TRANSMEM     68     99
FT   TOPO_DOM    100    165       CYTOPLASMIC.
FT   TRANSMEM    166    187
FT   TOPO_DOM    188    192       NON CYTOPLASMIC.
FT   TRANSMEM    193    209
FT   TOPO_DOM    210    229       CYTOPLASMIC.
FT   TRANSMEM    230    251
FT   TOPO_DOM    252    256       NON CYTOPLASMIC.
FT   TRANSMEM    257    278
FT   TOPO_DOM    279    284       CYTOPLASMIC.
FT   TRANSMEM    285    306
FT   TOPO_DOM    307    311       NON CYTOPLASMIC.
FT   TRANSMEM    312    330
FT   TOPO_DOM    331    350       CYTOPLASMIC.
FT   TRANSMEM    351    373
FT   TOPO_DOM    374    384       NON CYTOPLASMIC.
FT   TRANSMEM    385    407
FT   TOPO_DOM    408    435       CYTOPLASMIC.
//
```

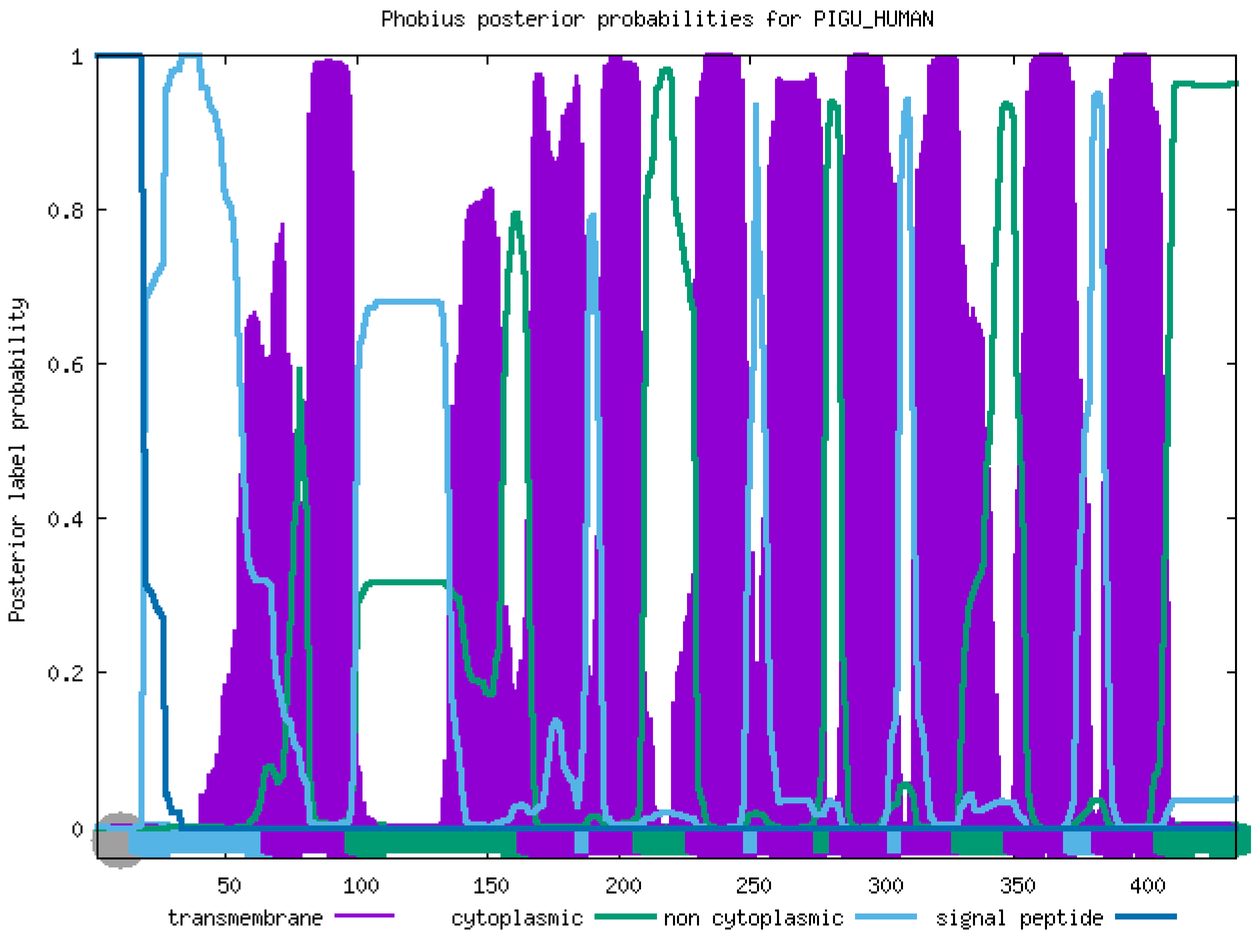

The probability data used in the plot is found here, and the gnuplot script is here.

Prediction of PIGS_HUMAN

```
ID    PIGS_HUMAN
FT    TOPO_DOM      1      18       CYTOPLASMIC.
FT    TRANSMEM     19      36
FT    TOPO_DOM     37     518       NON CYTOPLASMIC.
FT    TRANSMEM    519     542
FT    TOPO_DOM    543     555       CYTOPLASMIC.
//
```

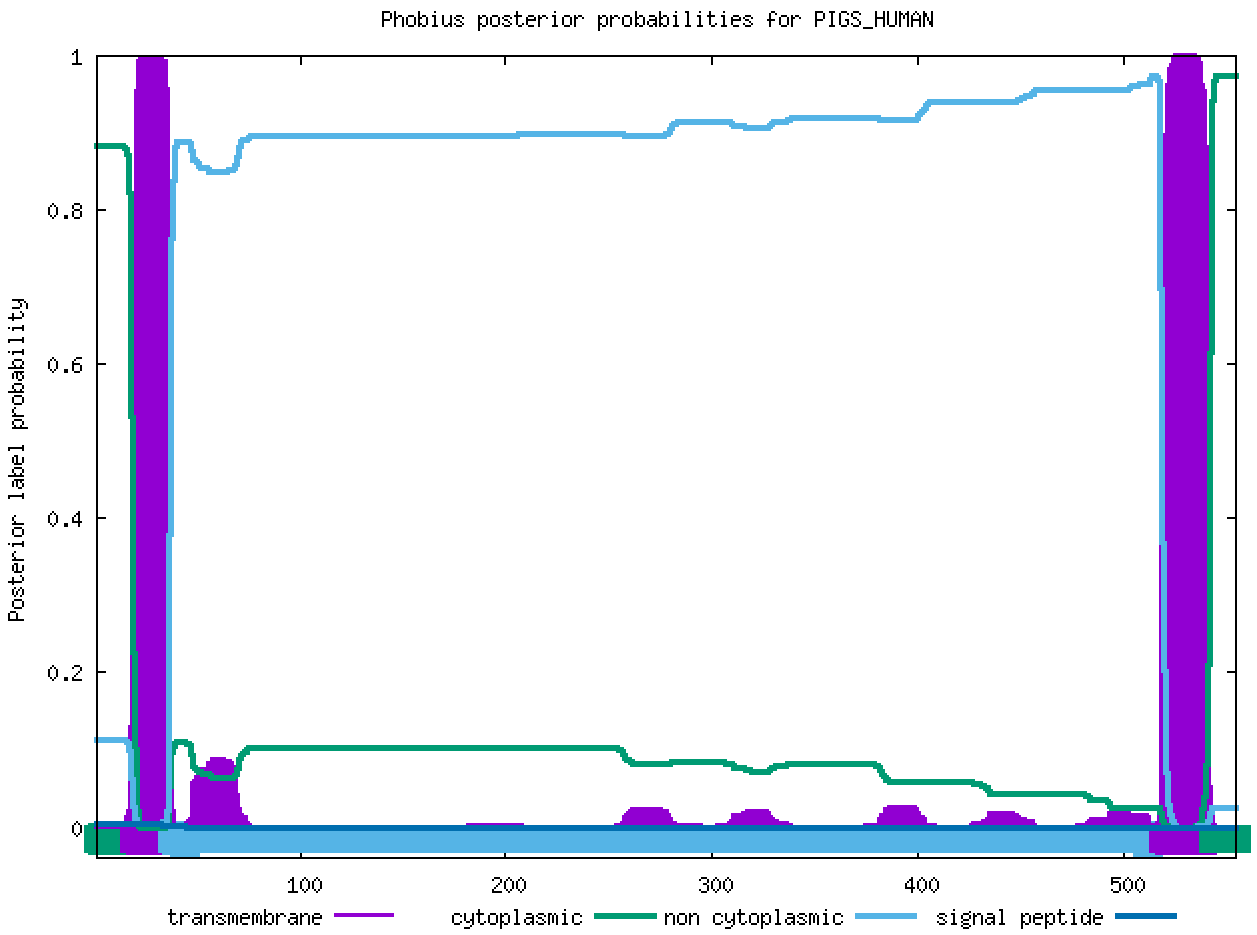

The probability data used in the plot is found here, and the gnuplot script is here.

Prediction of PIGT_HUMAN

```
ID    PIGT_HUMAN
FT    SIGNAL         1      23
FT    REGION         1       6       N-REGION.
FT    REGION         7      15       H-REGION.
FT    REGION        16      23       C-REGION.
FT    TOPO_DOM      24     525       NON CYTOPLASMIC.
FT    TRANSMEM     526     547
FT    TOPO_DOM     548     578       CYTOPLASMIC.
//
```

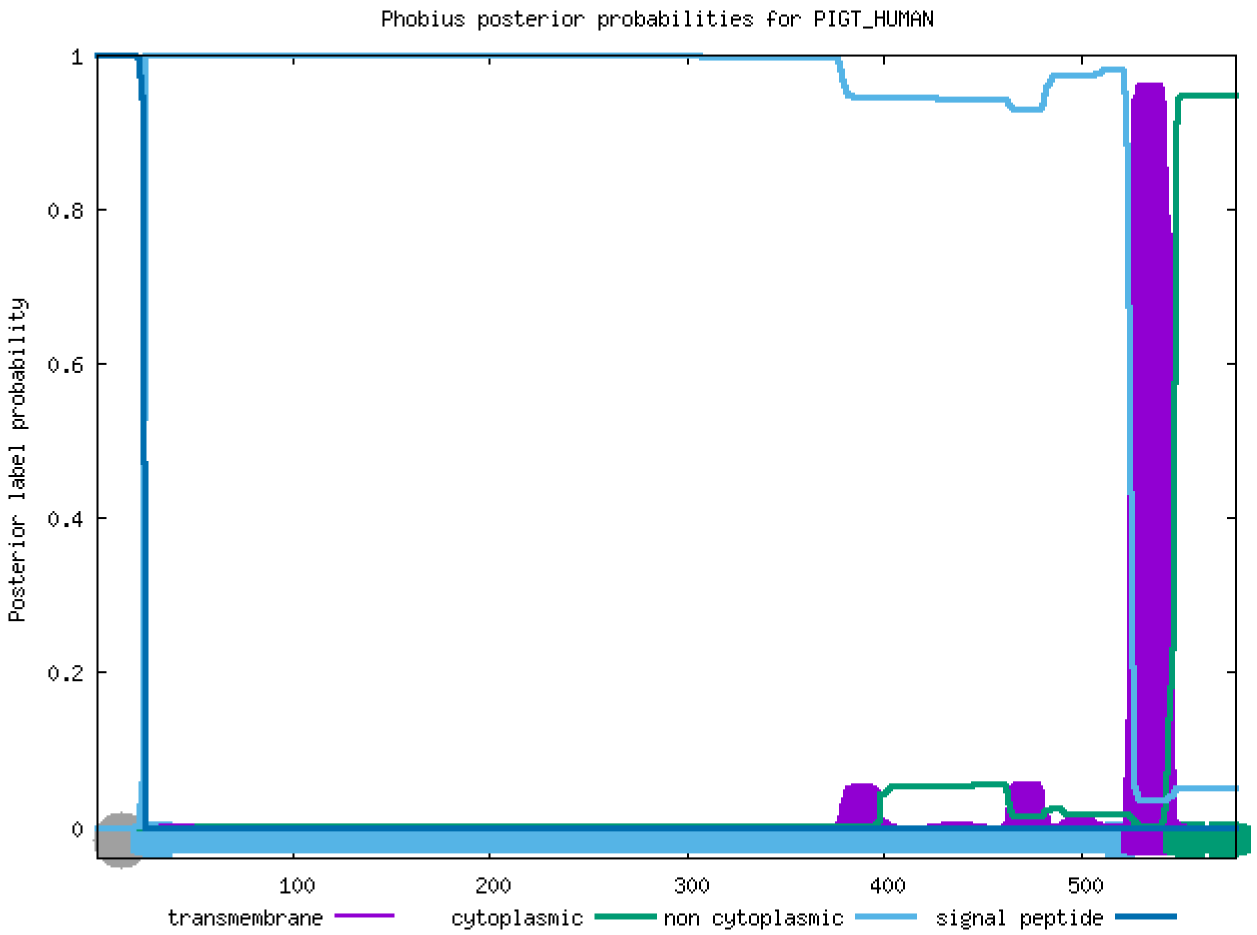

The probability data used in the plot is found here, and the gnuplot script is here.

Prediction of GPI8_HUMAN

```
ID   GPI8_HUMAN
FT   SIGNAL        1     27
FT   REGION        1      9       N-REGION.
FT   REGION       10     22       H-REGION.
FT   REGION       23     27       C-REGION.
FT   TOPO_DOM     28    363       NON CYTOPLASMIC.
FT   TRANSMEM    364    383
FT   TOPO_DOM    384    395       CYTOPLASMIC.
//
```

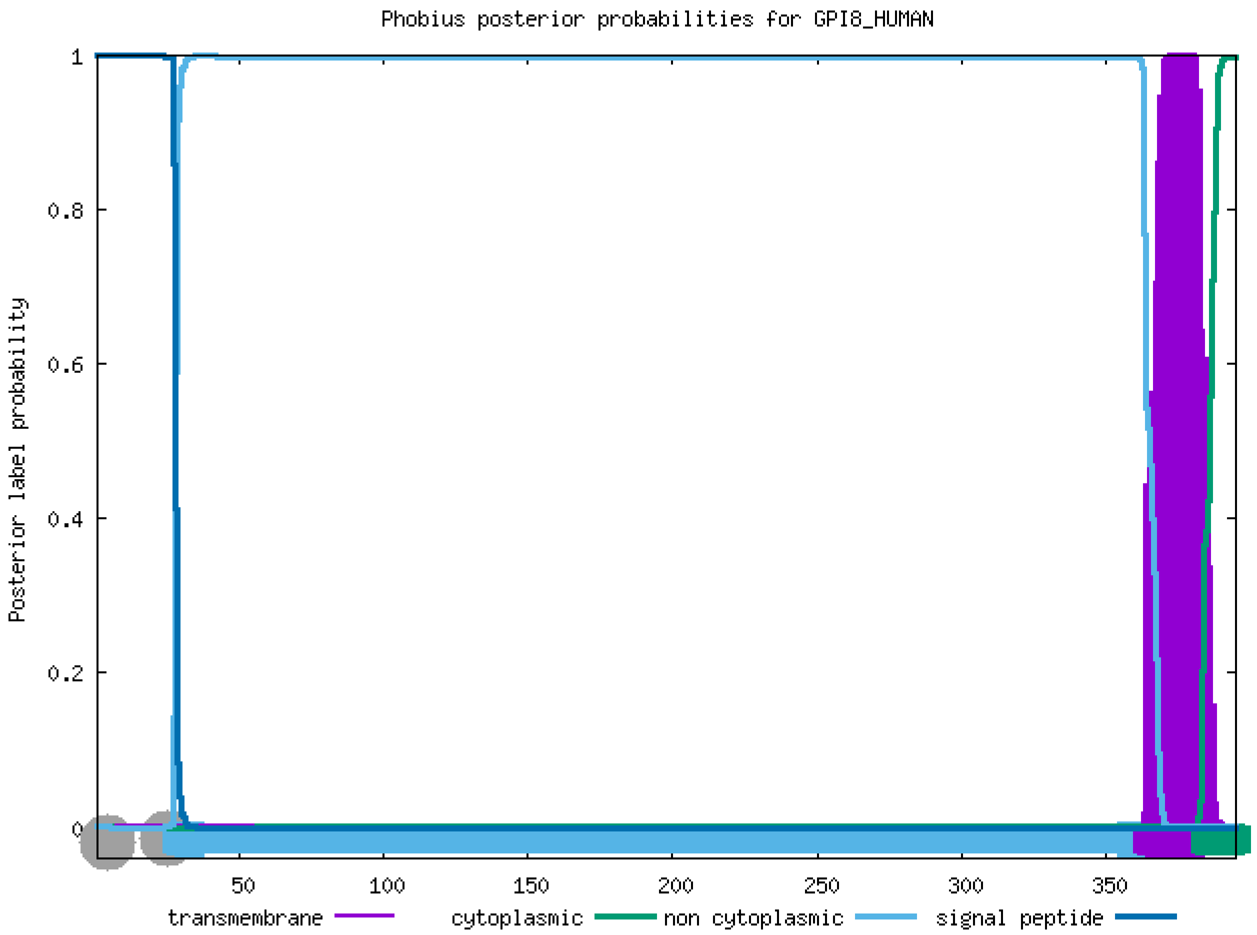

The probability data used in the plot is found here, and the gnuplot script is here.

Prediction of GPAA1_HUMAN

```
ID   GPAA1_HUMAN
FT   TOPO_DOM      1     19       NON CYTOPLASMIC.
FT   TRANSMEM     20     41
FT   TOPO_DOM     42    356       CYTOPLASMIC.
FT   TRANSMEM    357    385
FT   TOPO_DOM    386    421       NON CYTOPLASMIC.
FT   TRANSMEM    422    444
FT   TOPO_DOM    445    455       CYTOPLASMIC.
FT   TRANSMEM    456    478
FT   TOPO_DOM    479    497       NON CYTOPLASMIC.
FT   TRANSMEM    498    523
FT   TOPO_DOM    524    542       CYTOPLASMIC.
FT   TRANSMEM    543    564
FT   TOPO_DOM    565    597       NON CYTOPLASMIC.
FT   TRANSMEM    598    620
FT   TOPO_DOM    621    621       CYTOPLASMIC.
//
```

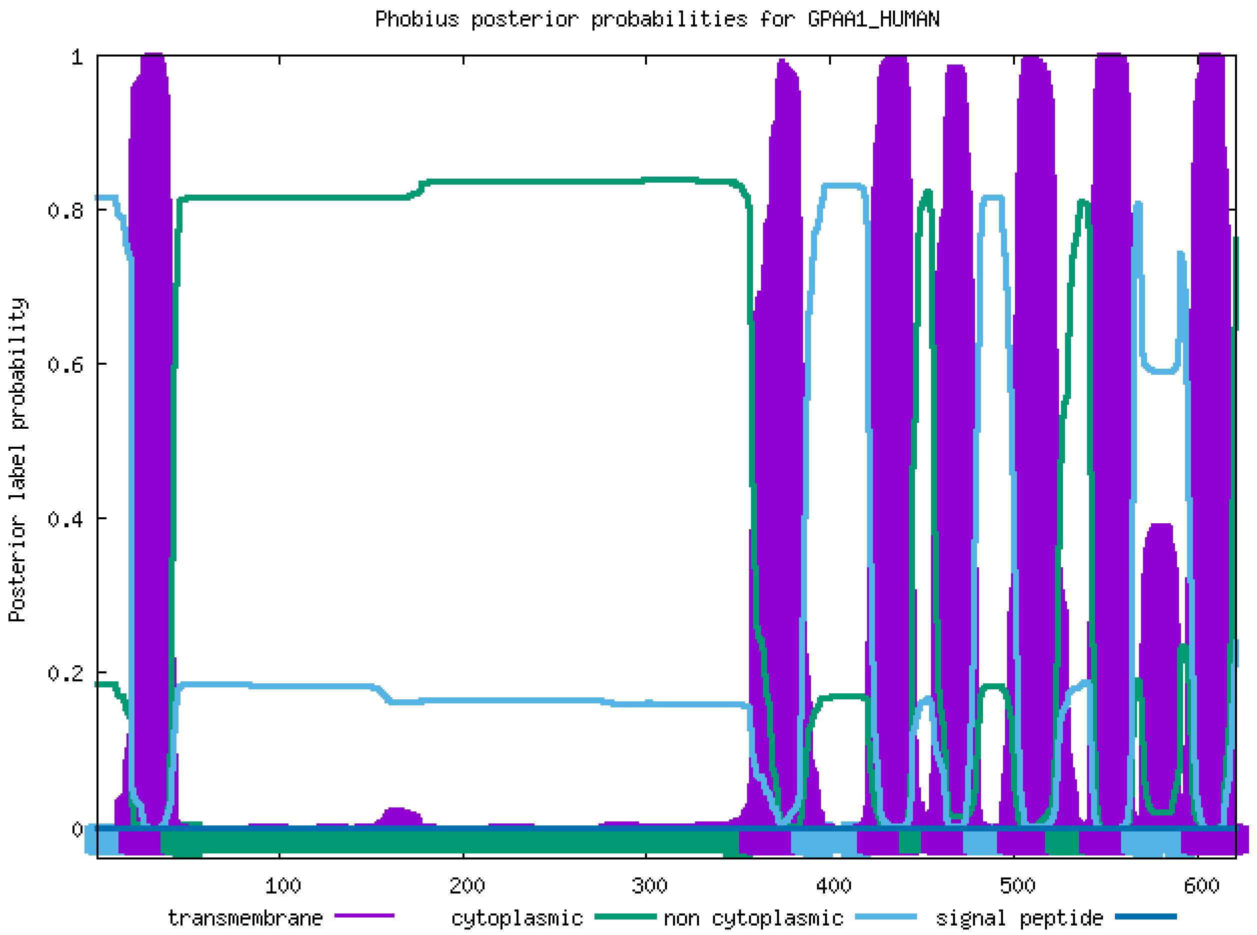

The probability data used in the plot is found here, and the gnuplot script is here.

Prediction of PTC1_HUMAN

```
ID    PTC1_HUMAN
FT    TOPO_DOM      1    438       NON CYTOPLASMIC.
FT    TRANSMEM    439    458
FT    TOPO_DOM    459    469       CYTOPLASMIC.
FT    TRANSMEM    470    495
FT    TOPO_DOM    496    500       NON CYTOPLASMIC.
FT    TRANSMEM    501    523
FT    TOPO_DOM    524    543       CYTOPLASMIC.
FT    TRANSMEM    544    567
FT    TOPO_DOM    568    572       NON CYTOPLASMIC.
FT    TRANSMEM    573    596
FT    TOPO_DOM    597    750       CYTOPLASMIC.
FT    TRANSMEM    751    768
FT    TOPO_DOM    769    787       NON CYTOPLASMIC.
FT    TRANSMEM    788    806
FT    TOPO_DOM    807   1026       CYTOPLASMIC.
FT    TRANSMEM   1027   1048
FT    TOPO_DOM   1049   1053       NON CYTOPLASMIC.
FT    TRANSMEM   1054   1076
FT    TOPO_DOM   1077   1082       CYTOPLASMIC.
FT    TRANSMEM   1083   1109
FT    TOPO_DOM   1110   1120       NON CYTOPLASMIC.
FT    TRANSMEM   1121   1141
FT    TOPO_DOM   1142   1152       CYTOPLASMIC.
FT    TRANSMEM   1153   1178
FT    TOPO_DOM   1179   1447       NON CYTOPLASMIC.
//
```

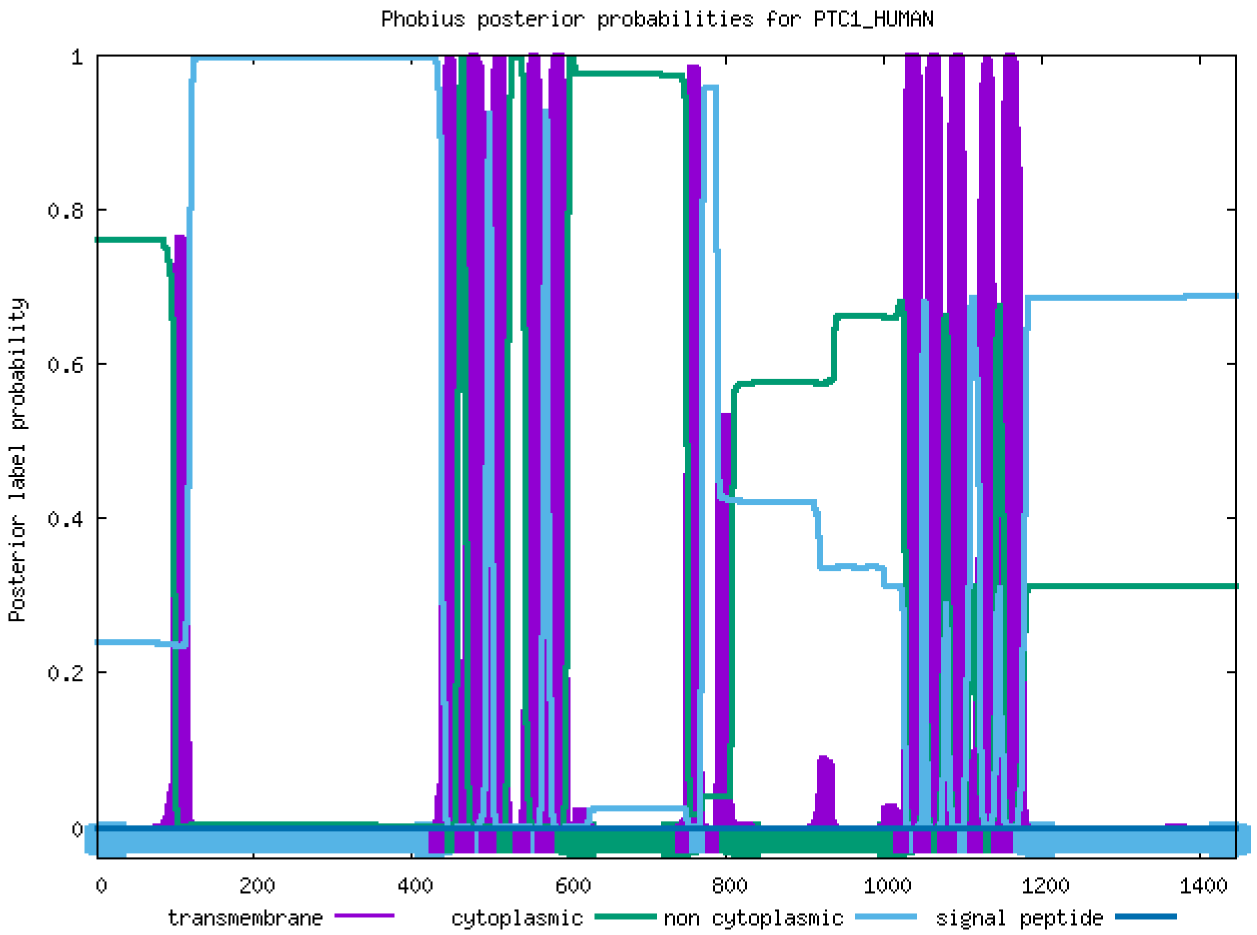

The probability data used in the plot is found here, and the gnuplot script is here.

Prediction of DISP1_HUMAN

```
ID   DISP1_HUMAN
FT   TOPO_DOM      1    187       NON CYTOPLASMIC.
FT   TRANSMEM    188    210
FT   TOPO_DOM    211    498       CYTOPLASMIC.
FT   TRANSMEM    499    518
FT   TOPO_DOM    519    523       NON CYTOPLASMIC.
FT   TRANSMEM    524    545
FT   TOPO_DOM    546    556       CYTOPLASMIC.
FT   TRANSMEM    557    577
FT   TOPO_DOM    578    596       NON CYTOPLASMIC.
FT   TRANSMEM    597    621
FT   TOPO_DOM    622    632       CYTOPLASMIC.
FT   TRANSMEM    633    654
FT   TOPO_DOM    655    718       NON CYTOPLASMIC.
FT   TRANSMEM    719    737
FT   TOPO_DOM    738    984       CYTOPLASMIC.
FT   TRANSMEM    985   1004
FT   TOPO_DOM   1005   1009       NON CYTOPLASMIC.
FT   TRANSMEM   1010   1032
FT   TOPO_DOM   1033   1038       CYTOPLASMIC.
FT   TRANSMEM   1039   1060
FT   TOPO_DOM   1061   1071       NON CYTOPLASMIC.
FT   TRANSMEM   1072   1096
FT   TOPO_DOM   1097   1107       CYTOPLASMIC.
FT   TRANSMEM   1108   1127
FT   TOPO_DOM   1128   1524       NON CYTOPLASMIC.
//
```

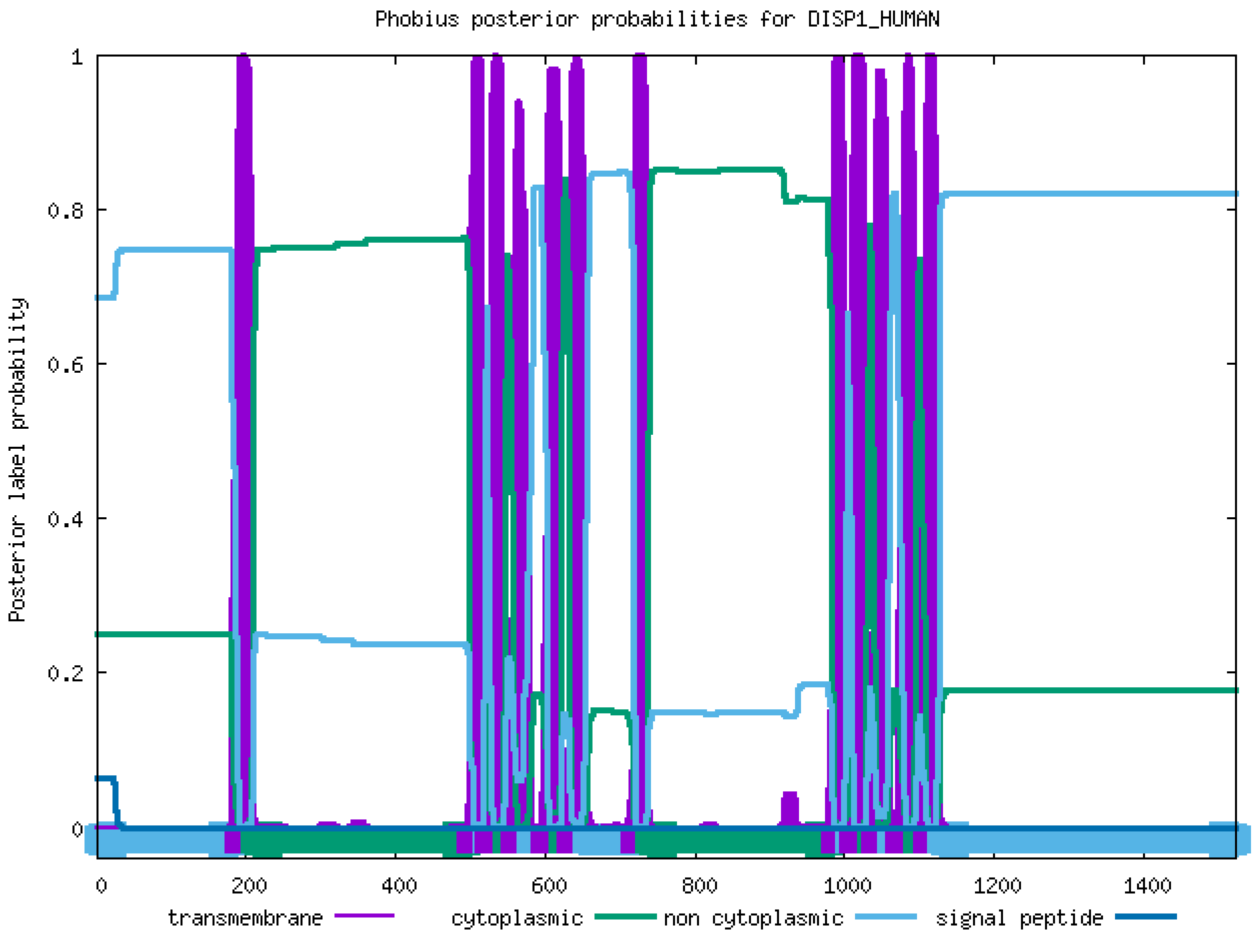

The probability data used in the plot is found here, and the gnuplot script is here.

Prediction of MGST2_HUMAN

```
ID   MGST2_HUMAN
FT   SIGNAL         1     22
FT   REGION         1      5       N-REGION.
FT   REGION         6     17       H-REGION.
FT   REGION        18     22       C-REGION.
FT   TOPO_DOM      23     58       NON CYTOPLASMIC.
FT   TRANSMEM      59     89
FT   TOPO_DOM      90    109       CYTOPLASMIC.
FT   TRANSMEM     110    131
FT   TOPO_DOM     132    147       NON CYTOPLASMIC.
//
```

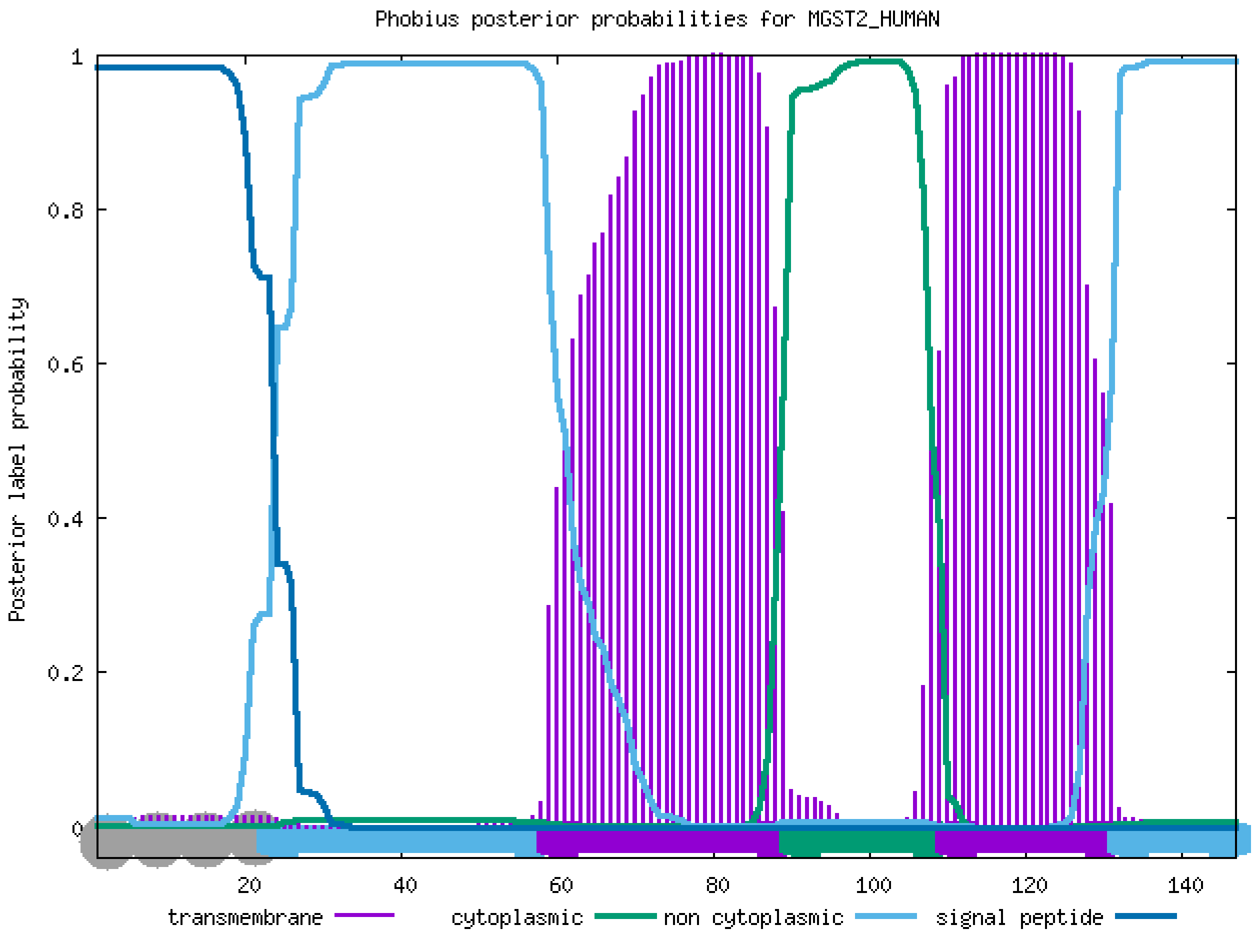

The probability data used in the plot is found here, and the gnuplot script is here.

Prediction of PTGES_HUMAN

```
ID    PTGES_HUMAN
FT    TOPO_DOM      1      11       NON CYTOPLASMIC.
FT    TRANSMEM     12      33
FT    TOPO_DOM     34      75       CYTOPLASMIC.
FT    TRANSMEM     76      92
FT    TOPO_DOM     93      97       NON CYTOPLASMIC.
FT    TRANSMEM     98     118
FT    TOPO_DOM    119     152       CYTOPLASMIC.
//
```

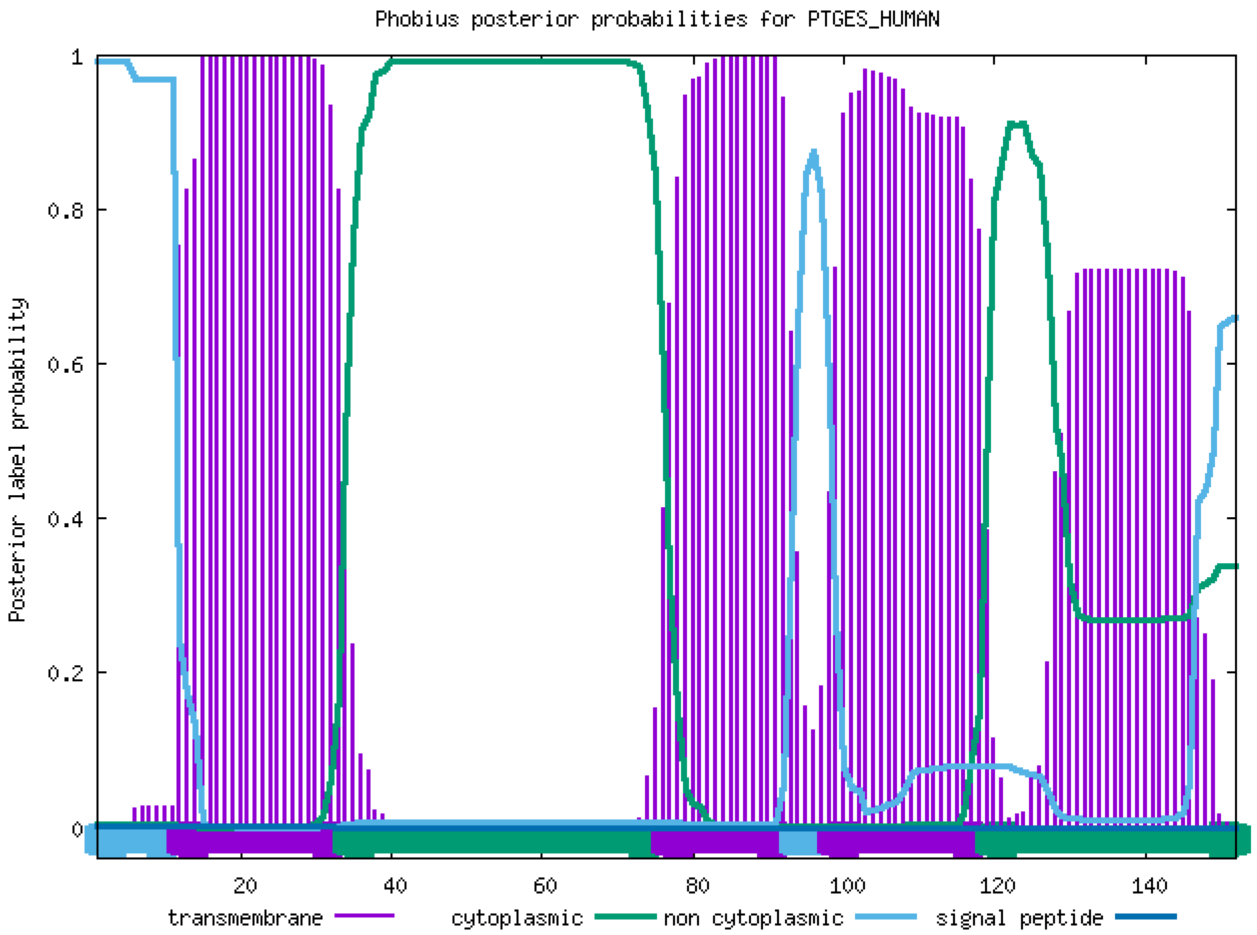

The probability data used in the plot is found here, and the gnuplot script is here.

Prediction of AL5AP_HUMAN

```
ID    AL5AP_HUMAN
FT    TOPO_DOM      1      5       NON CYTOPLASMIC.
FT    TRANSMEM      6     26
FT    TOPO_DOM     27     66       CYTOPLASMIC.
FT    TRANSMEM     67     93
FT    TOPO_DOM     94    117       NON CYTOPLASMIC.
FT    TRANSMEM    118    139
FT    TOPO_DOM    140    161       CYTOPLASMIC.
//
```

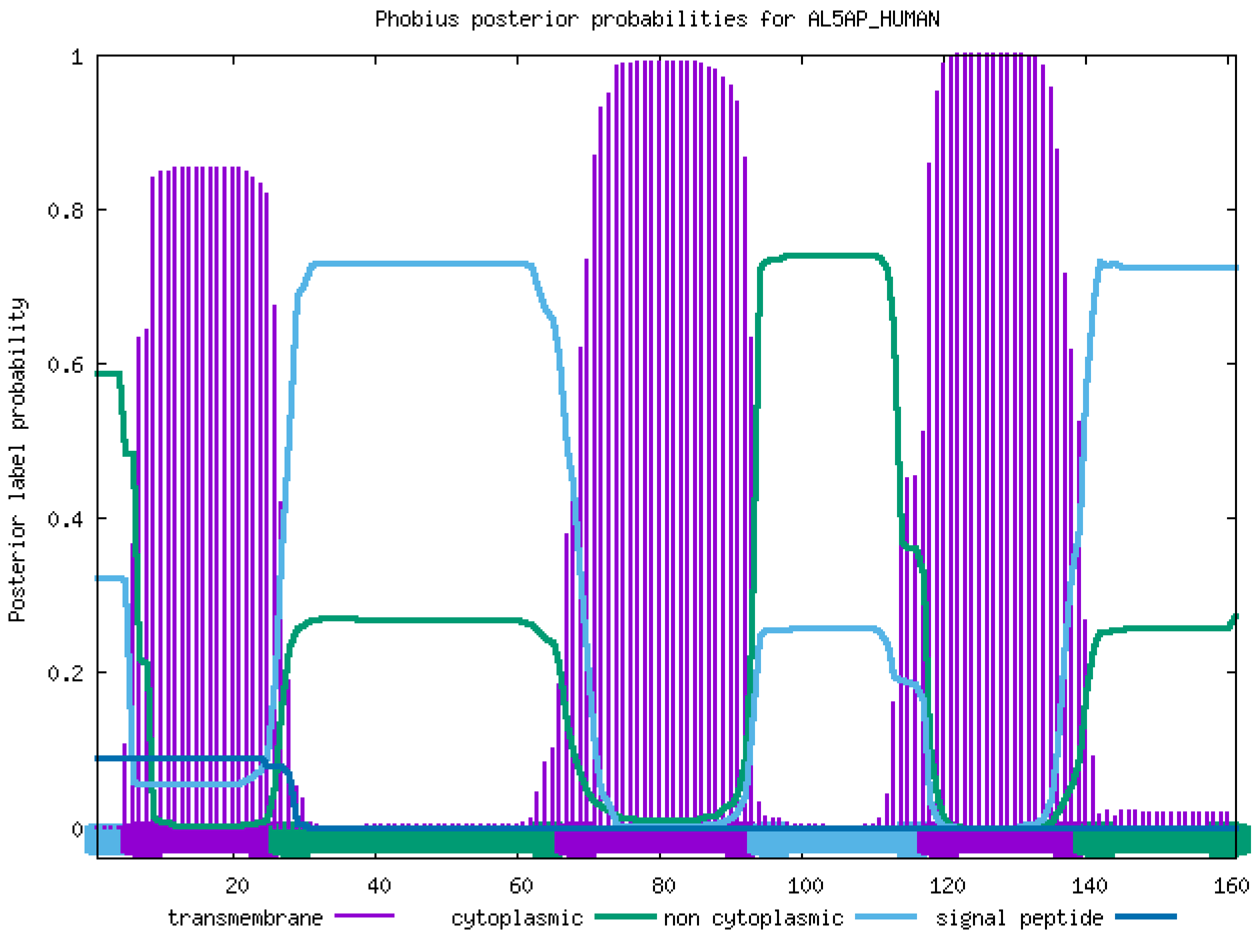

The probability data used in the plot is found here, and the gnuplot script is here.

Prediction of LTC4S_HUMAN

```
ID   LTC4S_HUMAN
FT   SIGNAL         1     34
FT   REGION         1      4       N-REGION.
FT   REGION         5     16       H-REGION.
FT   REGION        17     34       C-REGION.
FT   TOPO_DOM      35     66       NON CYTOPLASMIC.
FT   TRANSMEM      67     89
FT   TOPO_DOM      90    109       CYTOPLASMIC.
FT   TRANSMEM     110    131
FT   TOPO_DOM     132    150       NON CYTOPLASMIC.
//
```

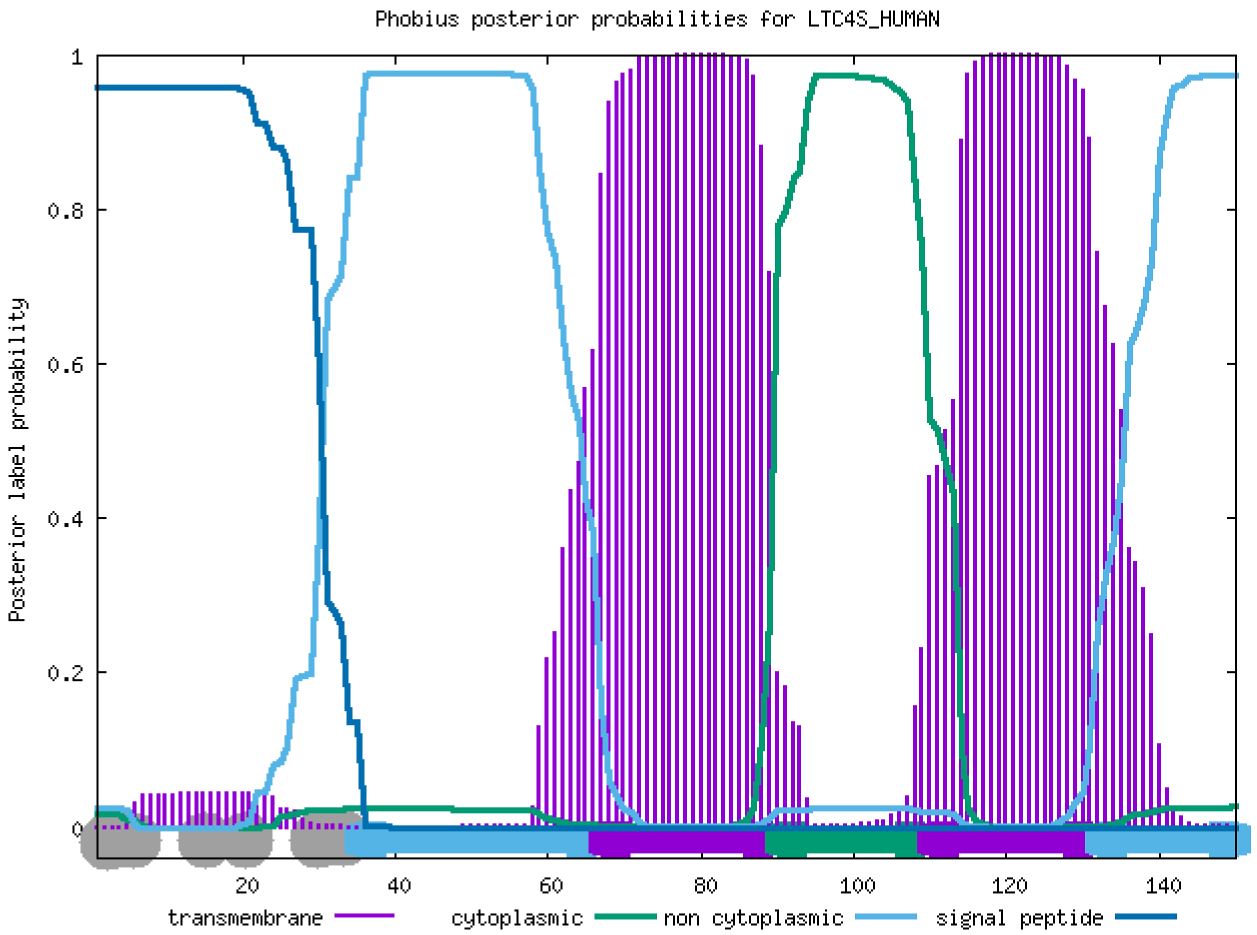

The probability data used in the plot is found here, and the gnuplot script is here.

Prediction of CTNS_HUMAN

```
ID   CTNS_HUMAN
FT   SIGNAL         1     19
FT   REGION         1      5       N-REGION.
FT   REGION         6     14       H-REGION.
FT   REGION        15     19       C-REGION.
FT   TOPO_DOM      20    128       NON CYTOPLASMIC.
FT   TRANSMEM     129    150
FT   TOPO_DOM     151    161       CYTOPLASMIC.
FT   TRANSMEM     162    186
FT   TOPO_DOM     187    205       NON CYTOPLASMIC.
FT   TRANSMEM     206    226
FT   TOPO_DOM     227    232       CYTOPLASMIC.
FT   TRANSMEM     233    256
FT   TOPO_DOM     257    261       NON CYTOPLASMIC.
FT   TRANSMEM     262    282
FT   TOPO_DOM     283    301       CYTOPLASMIC.
FT   TRANSMEM     302    318
FT   TOPO_DOM     319    337       NON CYTOPLASMIC.
FT   TRANSMEM     338    357
FT   TOPO_DOM     358    367       CYTOPLASMIC.
//
```

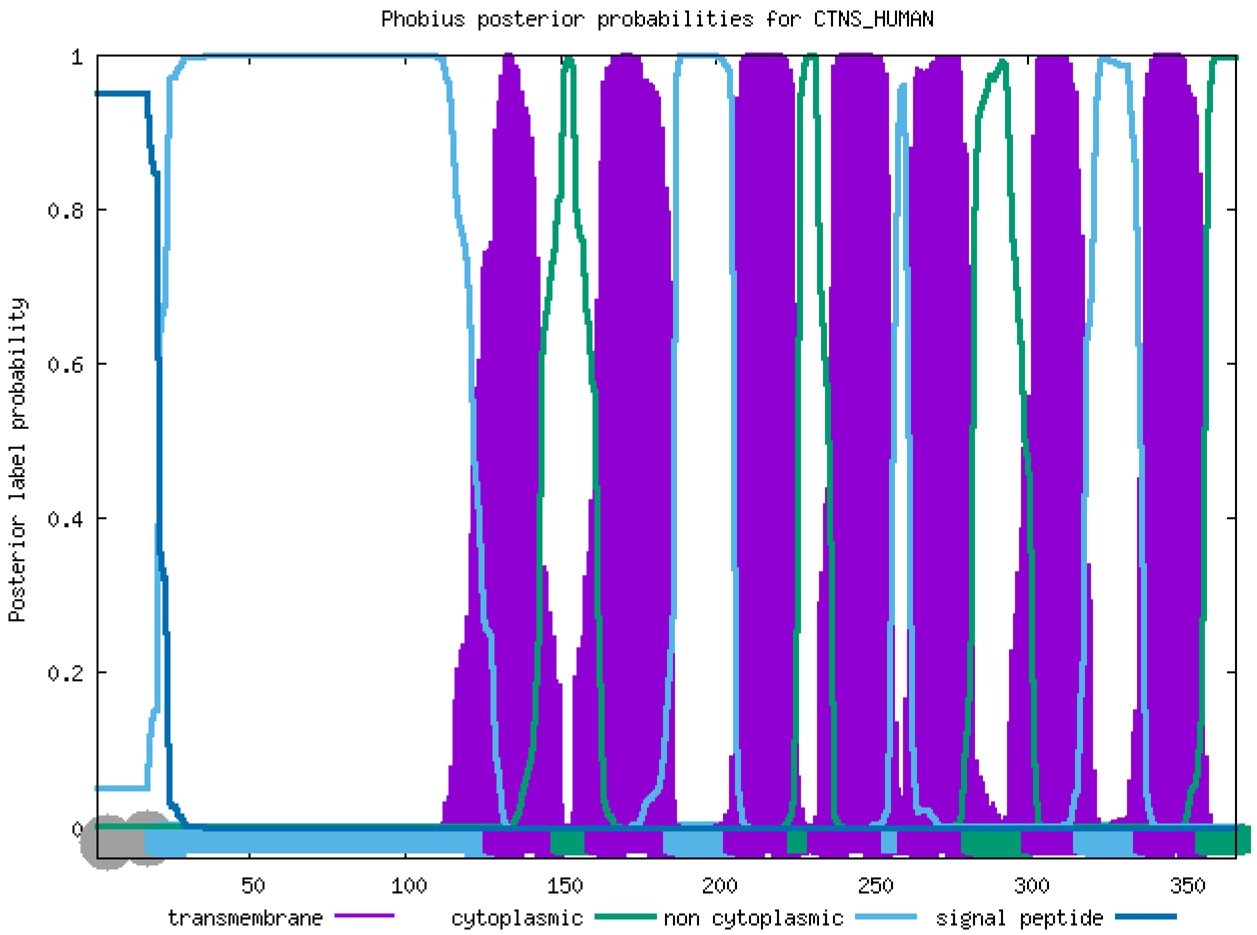

The probability data used in the plot is found here, and the gnuplot script is here.

Prediction of SC6A4_HUMAN

```
ID   SC6A4_HUMAN
FT   TOPO_DOM      1     87       CYTOPLASMIC.
FT   TRANSMEM     88    106
FT   TOPO_DOM    107    117       NON CYTOPLASMIC.
FT   TRANSMEM    118    139
FT   TOPO_DOM    140    159       CYTOPLASMIC.
FT   TRANSMEM    160    188
FT   TOPO_DOM    189    250       NON CYTOPLASMIC.
FT   TRANSMEM    251    268
FT   TOPO_DOM    269    279       CYTOPLASMIC.
FT   TRANSMEM    280    306
FT   TOPO_DOM    307    325       NON CYTOPLASMIC.
FT   TRANSMEM    326    350
FT   TOPO_DOM    351    361       CYTOPLASMIC.
FT   TRANSMEM    362    387
FT   TOPO_DOM    388    406       NON CYTOPLASMIC.
FT   TRANSMEM    407    432
FT   TOPO_DOM    433    464       CYTOPLASMIC.
FT   TRANSMEM    465    489
FT   TOPO_DOM    490    494       NON CYTOPLASMIC.
FT   TRANSMEM    495    516
FT   TOPO_DOM    517    535       CYTOPLASMIC.
FT   TRANSMEM    536    558
FT   TOPO_DOM    559    569       NON CYTOPLASMIC.
FT   TRANSMEM    570    595
FT   TOPO_DOM    596    630       CYTOPLASMIC.
//
```

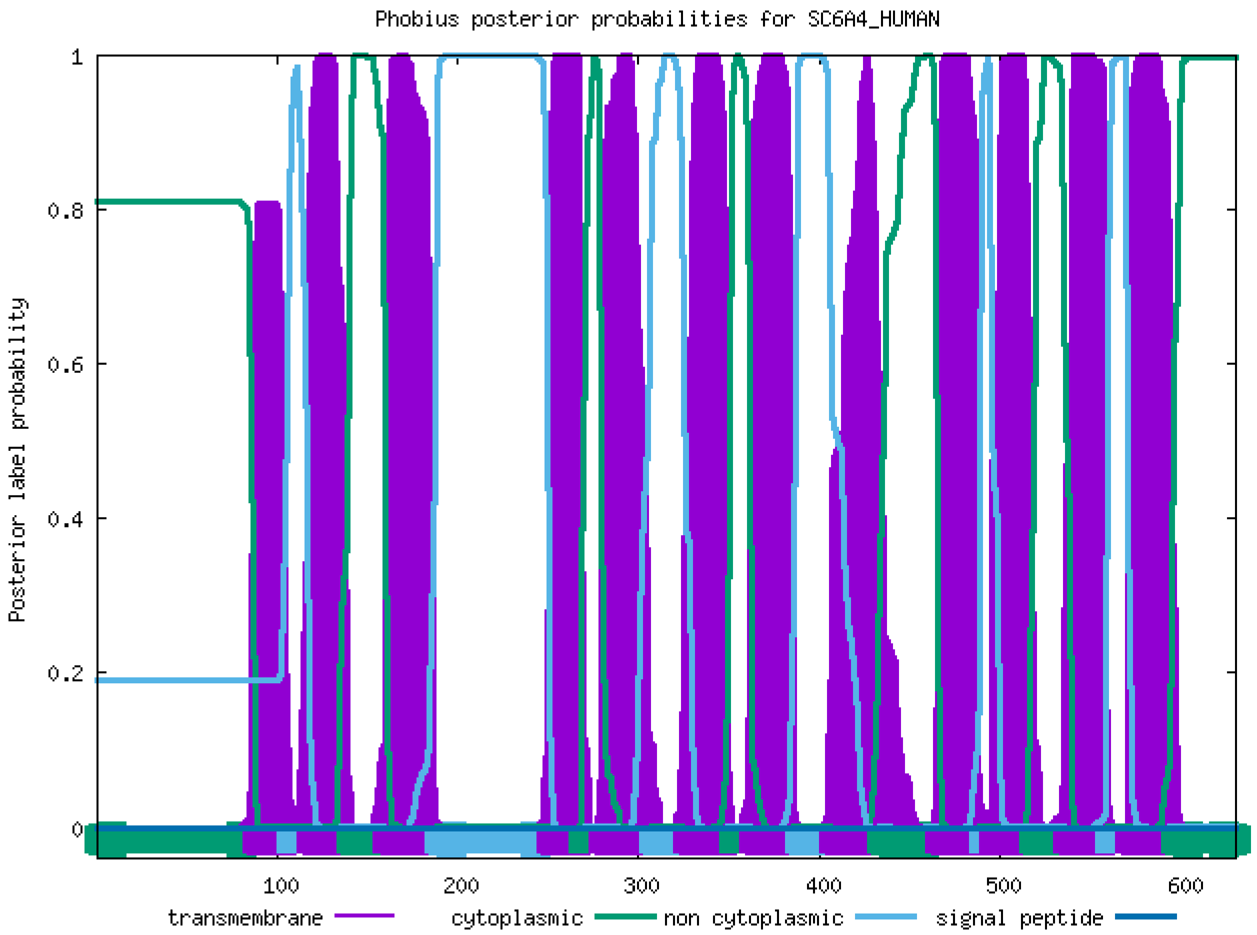

The probability data used in the plot is found here, and the gnuplot script is here.

Prediction of SC6A1_HUMAN

```
ID   SC6A1_HUMAN
FT   TOPO_DOM      1     52       CYTOPLASMIC.
FT   TRANSMEM     53     71
FT   TOPO_DOM     72     82       NON CYTOPLASMIC.
FT   TRANSMEM     83    104
FT   TOPO_DOM    105    124       CYTOPLASMIC.
FT   TRANSMEM    125    152
FT   TOPO_DOM    153    209       NON CYTOPLASMIC.
FT   TRANSMEM    210    229
FT   TOPO_DOM    230    240       CYTOPLASMIC.
FT   TRANSMEM    241    264
FT   TOPO_DOM    265    283       NON CYTOPLASMIC.
FT   TRANSMEM    284    309
FT   TOPO_DOM    310    320       CYTOPLASMIC.
FT   TRANSMEM    321    346
FT   TOPO_DOM    347    379       NON CYTOPLASMIC.
FT   TRANSMEM    380    401
FT   TOPO_DOM    402    421       CYTOPLASMIC.
FT   TRANSMEM    422    447
FT   TOPO_DOM    448    452       NON CYTOPLASMIC.
FT   TRANSMEM    453    477
FT   TOPO_DOM    478    497       CYTOPLASMIC.
FT   TRANSMEM    498    517
FT   TOPO_DOM    518    536       NON CYTOPLASMIC.
FT   TRANSMEM    537    557
FT   TOPO_DOM    558    599       CYTOPLASMIC.
//
```

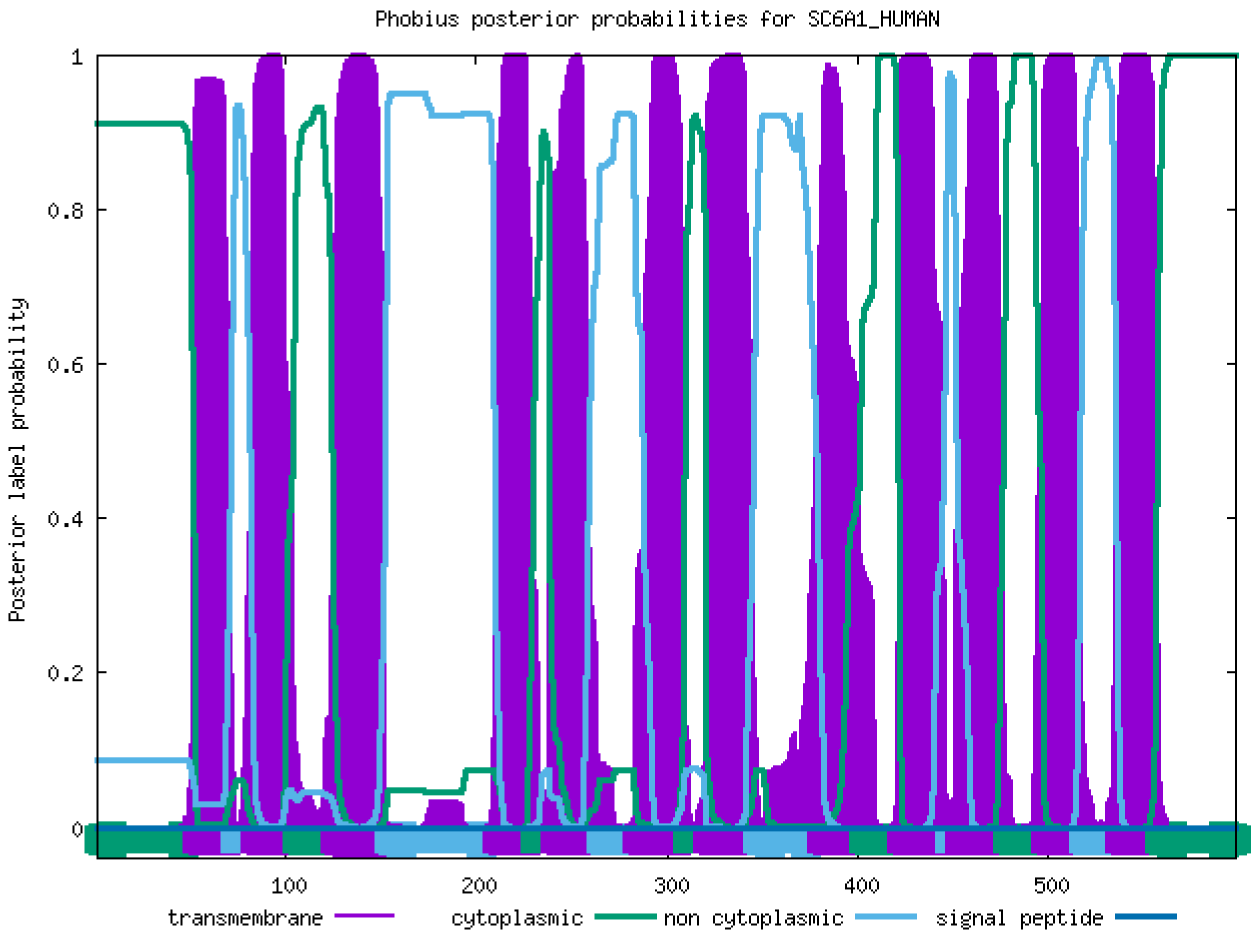

The probability data used in the plot is found here, and the gnuplot script is here.

Prediction of AAAT_HUMAN

```
ID   AAAT_HUMAN
FT   TOPO_DOM      1     52       CYTOPLASMIC.
FT   TRANSMEM     53     79
FT   TOPO_DOM     80     98       NON CYTOPLASMIC.
FT   TRANSMEM     99    119
FT   TOPO_DOM    120    130       CYTOPLASMIC.
FT   TRANSMEM    131    153
FT   TOPO_DOM    154    227       NON CYTOPLASMIC.
FT   TRANSMEM    228    245
FT   TOPO_DOM    246    265       CYTOPLASMIC.
FT   TRANSMEM    266    290
FT   TOPO_DOM    291    301       NON CYTOPLASMIC.
FT   TRANSMEM    302    328
FT   TOPO_DOM    329    334       CYTOPLASMIC.
FT   TRANSMEM    335    357
FT   TOPO_DOM    358    376       NON CYTOPLASMIC.
FT   TRANSMEM    377    402
FT   TOPO_DOM    403    413       CYTOPLASMIC.
FT   TRANSMEM    414    443
FT   TOPO_DOM    444    541       NON CYTOPLASMIC.
//
```

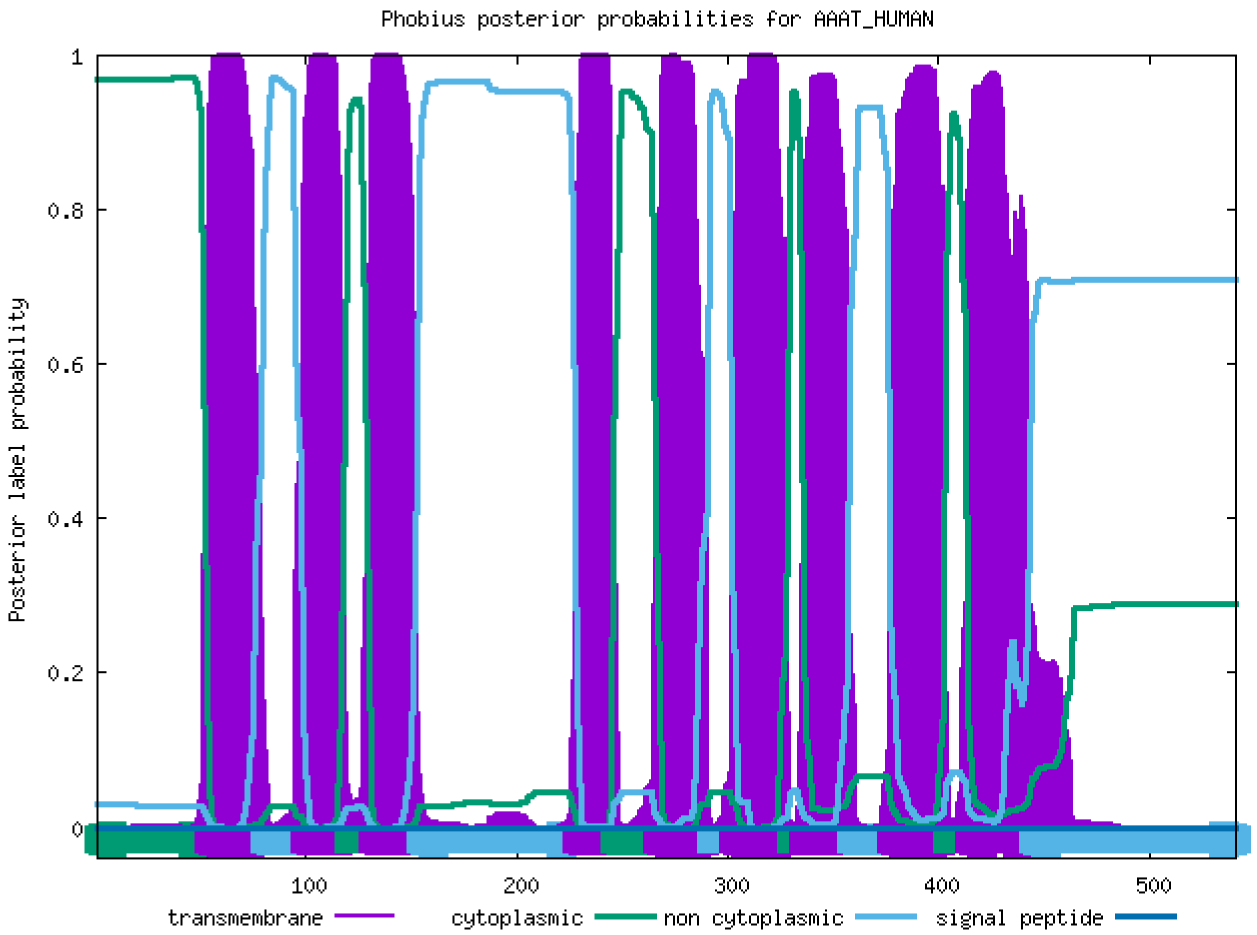

The probability data used in the plot is found here, and the gnuplot script is here.

Prediction of EAA1_HUMAN

```
ID   EAA1_HUMAN
FT   TOPO_DOM      1     48       CYTOPLASMIC.
FT   TRANSMEM     49     71
FT   TOPO_DOM     72     90       NON CYTOPLASMIC.
FT   TRANSMEM     91    111
FT   TOPO_DOM    112    122       CYTOPLASMIC.
FT   TRANSMEM    123    145
FT   TOPO_DOM    146    240       NON CYTOPLASMIC.
FT   TRANSMEM    241    260
FT   TOPO_DOM    261    280       CYTOPLASMIC.
FT   TRANSMEM    281    302
FT   TOPO_DOM    303    313       NON CYTOPLASMIC.
FT   TRANSMEM    314    339
FT   TOPO_DOM    340    345       CYTOPLASMIC.
FT   TRANSMEM    346    365
FT   TOPO_DOM    366    542       NON CYTOPLASMIC.
//
```

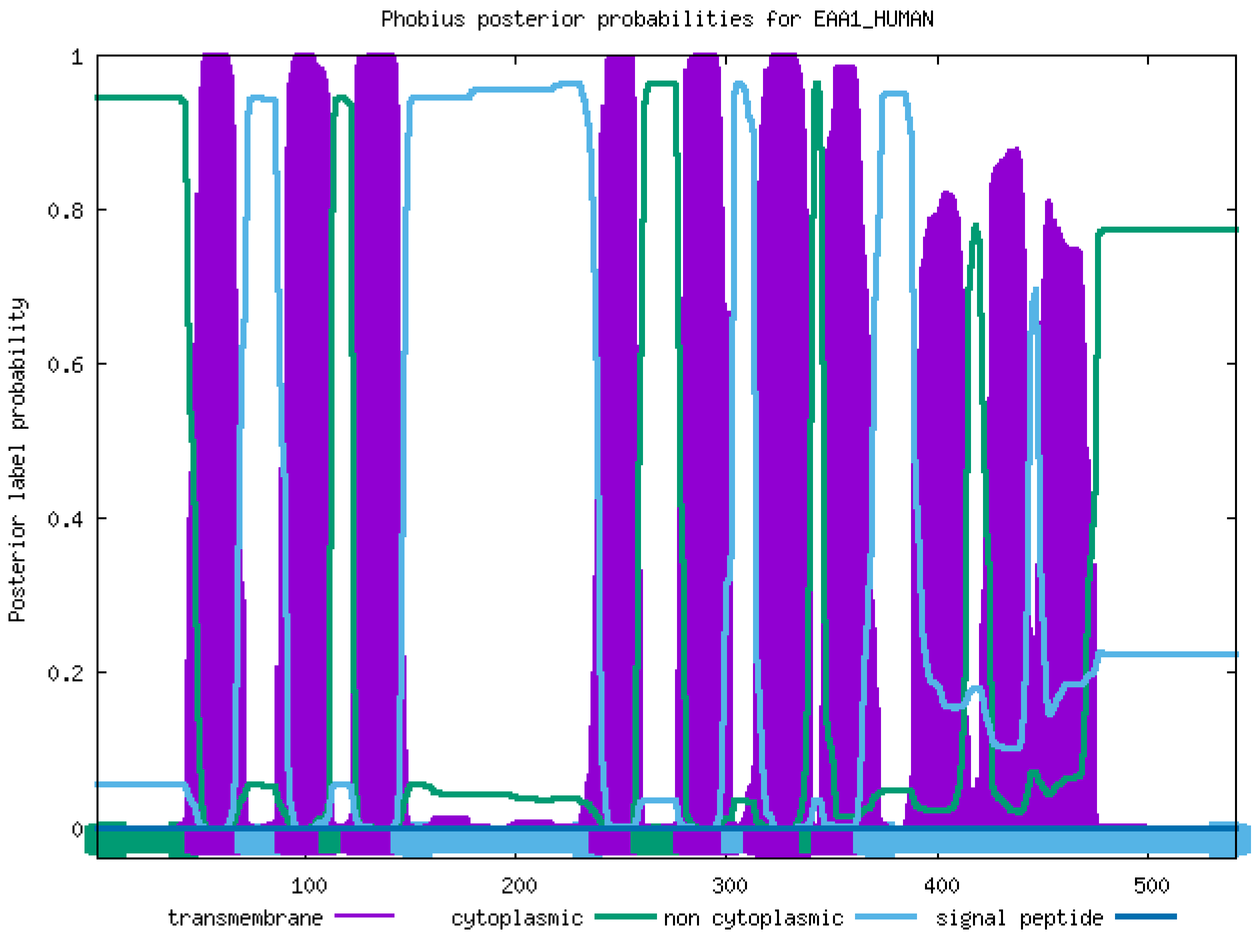

The probability data used in the plot is found here, and the gnuplot script is here.

Prediction of EAA2_HUMAN

```
ID    EAA2_HUMAN
FT    TOPO_DOM      1     44       NON CYTOPLASMIC.
FT    TRANSMEM     45     67
FT    TOPO_DOM     68     87       CYTOPLASMIC.
FT    TRANSMEM     88    108
FT    TOPO_DOM    109    119       NON CYTOPLASMIC.
FT    TRANSMEM    120    142
FT    TOPO_DOM    143    238       CYTOPLASMIC.
FT    TRANSMEM    239    256
FT    TOPO_DOM    257    275       NON CYTOPLASMIC.
FT    TRANSMEM    276    301
FT    TOPO_DOM    302    312       CYTOPLASMIC.
FT    TRANSMEM    313    339
FT    TOPO_DOM    340    344       NON CYTOPLASMIC.
FT    TRANSMEM    345    368
FT    TOPO_DOM    369    387       CYTOPLASMIC.
FT    TRANSMEM    388    413
FT    TOPO_DOM    414    432       NON CYTOPLASMIC.
FT    TRANSMEM    433    457
FT    TOPO_DOM    458    574       CYTOPLASMIC.
//
```

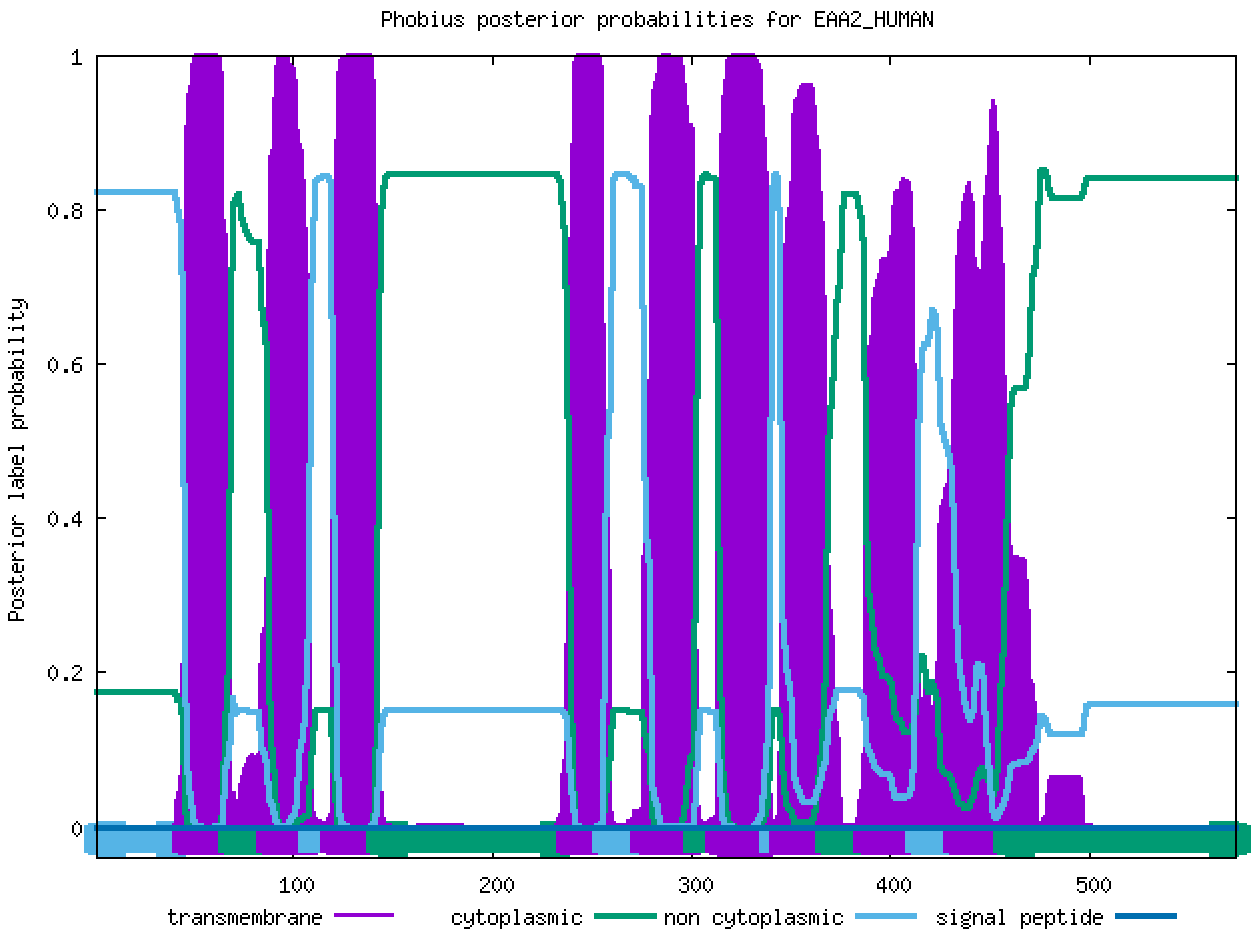

The probability data used in the plot is found here, and the gnuplot script is here.

Prediction of EAA3_HUMAN

```
ID    EAA3_HUMAN
FT    TOPO_DOM      1      19       CYTOPLASMIC.
FT    TRANSMEM     20      38
FT    TOPO_DOM     39      57       NON CYTOPLASMIC.
FT    TRANSMEM     58      82
FT    TOPO_DOM     83      93       CYTOPLASMIC.
FT    TRANSMEM     94     116
FT    TOPO_DOM    117     209       NON CYTOPLASMIC.
FT    TRANSMEM    210     229
FT    TOPO_DOM    230     249       CYTOPLASMIC.
FT    TRANSMEM    250     271
FT    TOPO_DOM    272     282       NON CYTOPLASMIC.
FT    TRANSMEM    283     308
FT    TOPO_DOM    309     319       CYTOPLASMIC.
FT    TRANSMEM    320     341
FT    TOPO_DOM    342     524       NON CYTOPLASMIC.
//
```

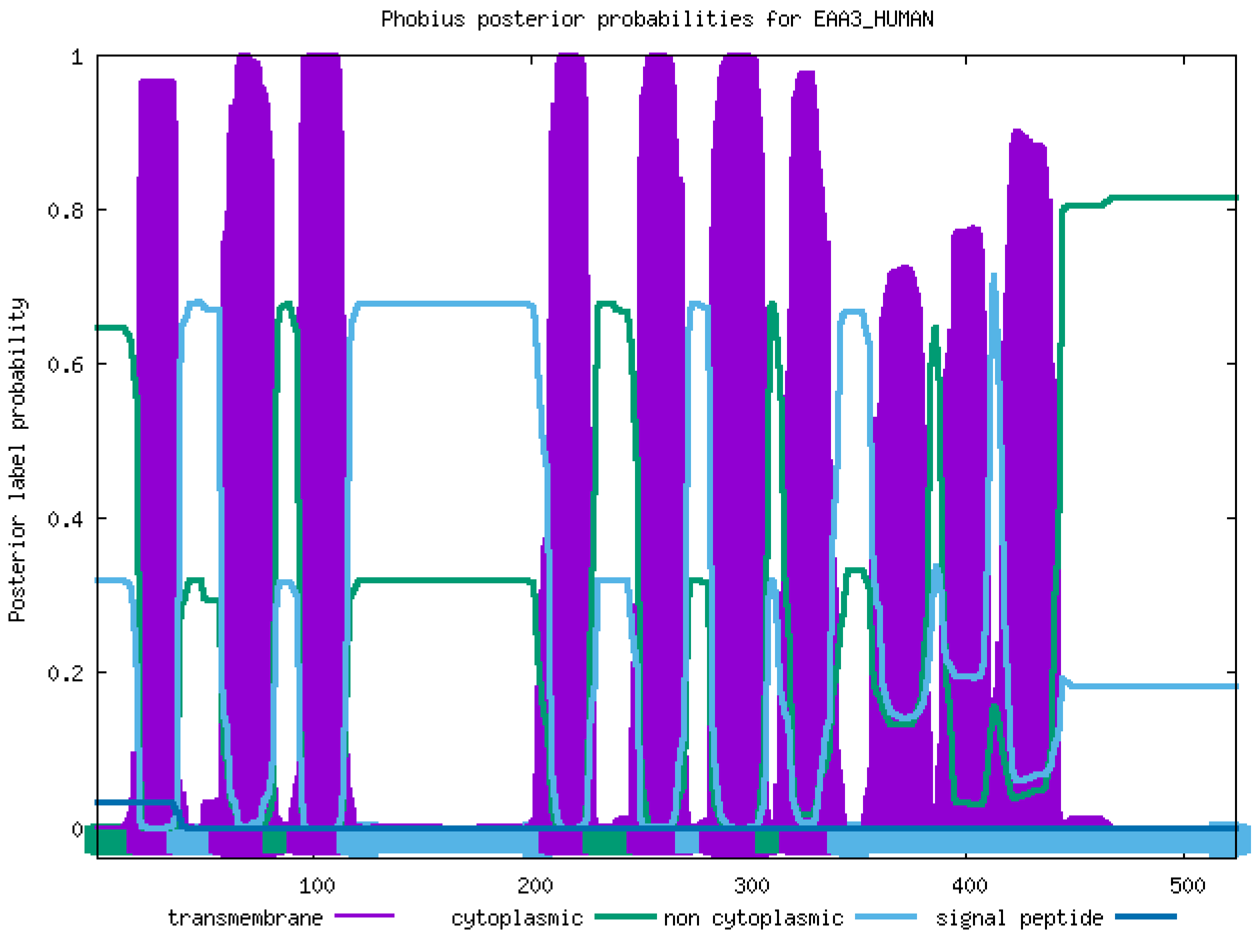

The probability data used in the plot is found here, and the gnuplot script is here.

Prediction of SC6A9_HUMAN

```
ID    SC6A9_HUMAN
FT    TOPO_DOM      1    108       CYTOPLASMIC.
FT    TRANSMEM    109    127
FT    TOPO_DOM    128    138       NON CYTOPLASMIC.
FT    TRANSMEM    139    160
FT    TOPO_DOM    161    180       CYTOPLASMIC.
FT    TRANSMEM    181    208
FT    TOPO_DOM    209    287       NON CYTOPLASMIC.
FT    TRANSMEM    288    309
FT    TOPO_DOM    310    320       CYTOPLASMIC.
FT    TRANSMEM    321    348
FT    TOPO_DOM    349    367       NON CYTOPLASMIC.
FT    TRANSMEM    368    385
FT    TOPO_DOM    386    396       CYTOPLASMIC.
FT    TRANSMEM    397    422
FT    TOPO_DOM    423    455       NON CYTOPLASMIC.
FT    TRANSMEM    456    477
FT    TOPO_DOM    478    497       CYTOPLASMIC.
FT    TRANSMEM    498    524
FT    TOPO_DOM    525    529       NON CYTOPLASMIC.
FT    TRANSMEM    530    550
FT    TOPO_DOM    551    569       CYTOPLASMIC.
FT    TRANSMEM    570    593
FT    TOPO_DOM    594    604       NON CYTOPLASMIC.
FT    TRANSMEM    605    630
FT    TOPO_DOM    631    706       CYTOPLASMIC.
//
```

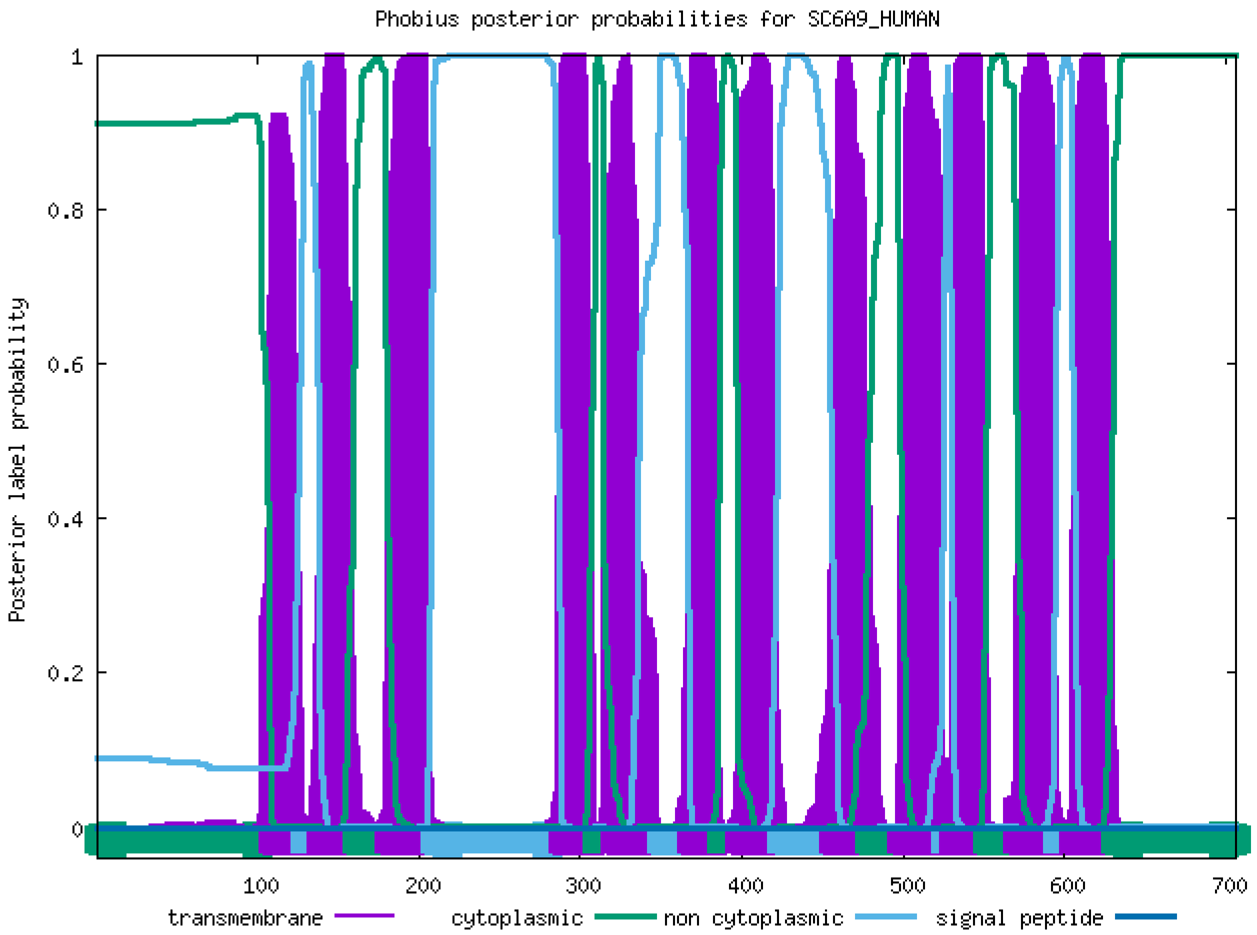

The probability data used in the plot is found here, and the gnuplot script is here.

Prediction of S6A19_HUMAN

```
ID   S6A19_HUMAN
FT   TOPO_DOM      1     40       CYTOPLASMIC.
FT   TRANSMEM     41     58
FT   TOPO_DOM     59     69       NON CYTOPLASMIC.
FT   TRANSMEM     70     92
FT   TOPO_DOM     93    112       CYTOPLASMIC.
FT   TRANSMEM    113    141
FT   TOPO_DOM    142    192       NON CYTOPLASMIC.
FT   TRANSMEM    193    211
FT   TOPO_DOM    212    222       CYTOPLASMIC.
FT   TRANSMEM    223    243
FT   TOPO_DOM    244    270       NON CYTOPLASMIC.
FT   TRANSMEM    271    292
FT   TOPO_DOM    293    303       CYTOPLASMIC.
FT   TRANSMEM    304    325
FT   TOPO_DOM    326    414       NON CYTOPLASMIC.
FT   TRANSMEM    415    436
FT   TOPO_DOM    437    456       CYTOPLASMIC.
FT   TRANSMEM    457    476
FT   TOPO_DOM    477    487       NON CYTOPLASMIC.
FT   TRANSMEM    488    511
FT   TOPO_DOM    512    531       CYTOPLASMIC.
FT   TRANSMEM    532    552
FT   TOPO_DOM    553    580       NON CYTOPLASMIC.
FT   TRANSMEM    581    603
FT   TOPO_DOM    604    634       CYTOPLASMIC.
//
```

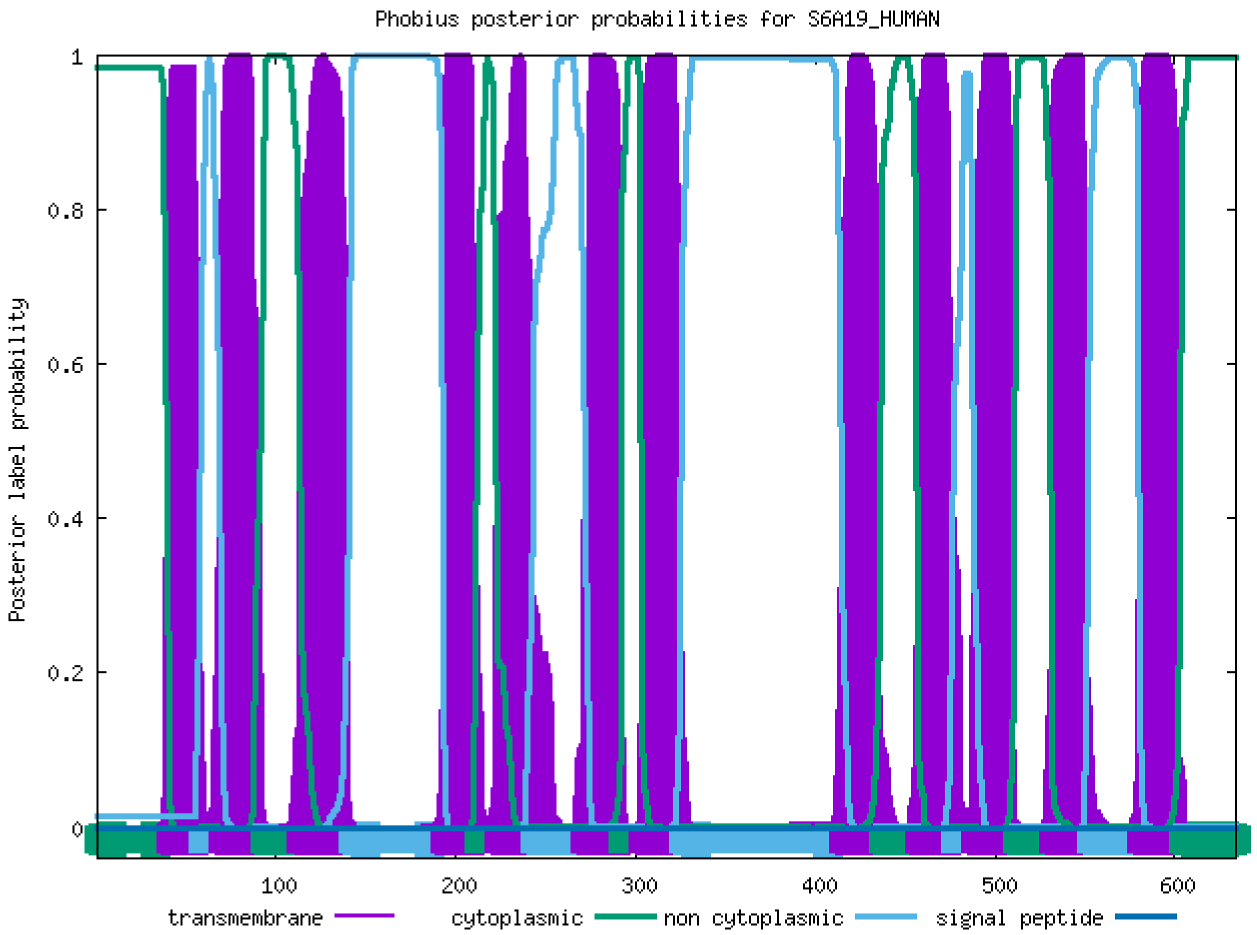

The probability data used in the plot is found here, and the gnuplot script is here.

Prediction of S4A4_HUMAN

```
ID   S4A4_HUMAN
FT   TOPO_DOM      1    468       NON CYTOPLASMIC.
FT   TRANSMEM    469    492
FT   TOPO_DOM    493    503       CYTOPLASMIC.
FT   TRANSMEM    504    533
FT   TOPO_DOM    534    552       NON CYTOPLASMIC.
FT   TRANSMEM    553    571
FT   TOPO_DOM    572    582       CYTOPLASMIC.
FT   TRANSMEM    583    601
FT   TOPO_DOM    602    691       NON CYTOPLASMIC.
FT   TRANSMEM    692    710
FT   TOPO_DOM    711    729       CYTOPLASMIC.
FT   TRANSMEM    730    748
FT   TOPO_DOM    749    776       NON CYTOPLASMIC.
FT   TRANSMEM    777    797
FT   TOPO_DOM    798    817       CYTOPLASMIC.
FT   TRANSMEM    818    842
FT   TOPO_DOM    843    877       NON CYTOPLASMIC.
FT   TRANSMEM    878    897
FT   TOPO_DOM    898    903       CYTOPLASMIC.
FT   TRANSMEM    904    923
FT   TOPO_DOM    924    954       NON CYTOPLASMIC.
FT   TRANSMEM    955    986
FT   TOPO_DOM    987   1079       CYTOPLASMIC.
//
```

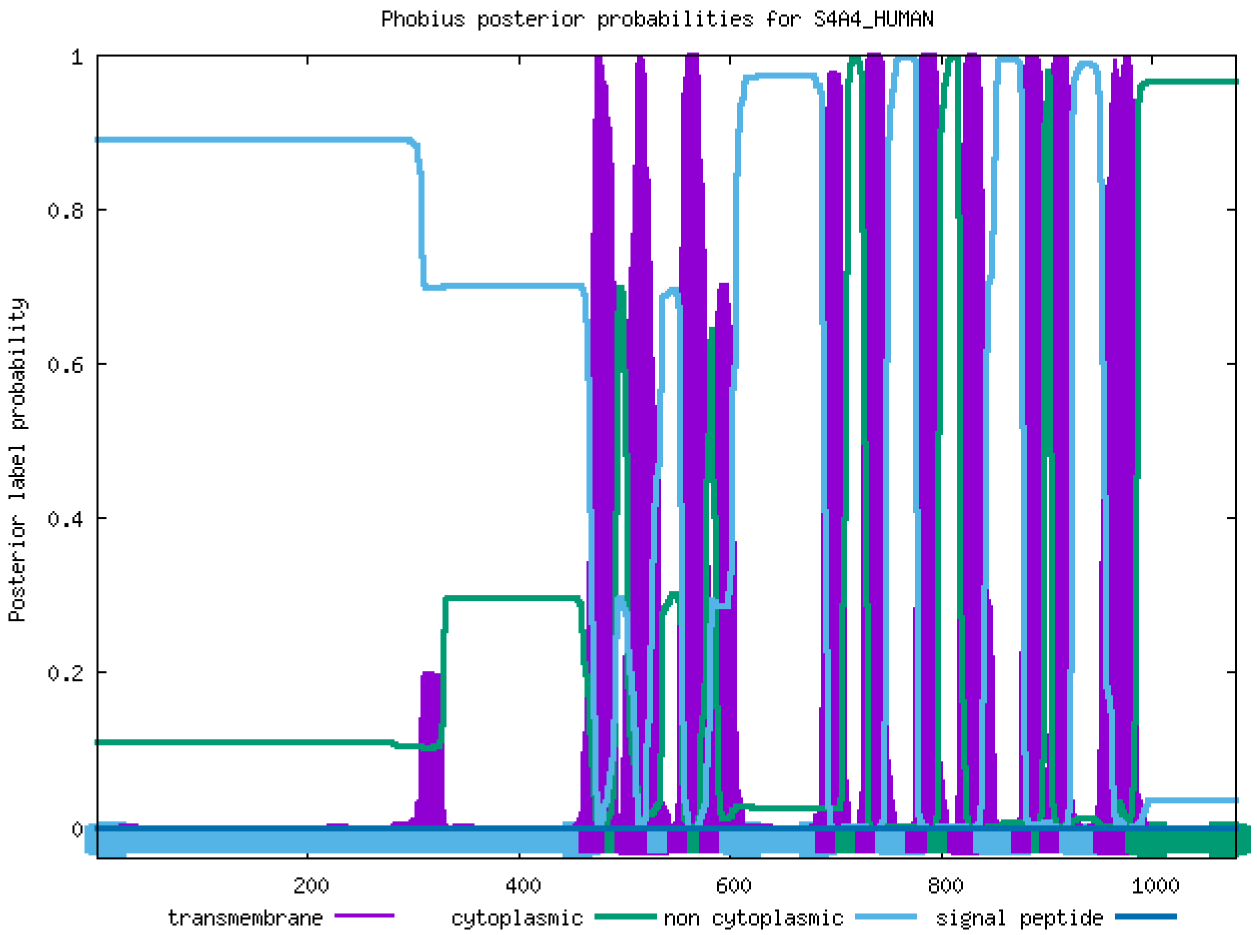

The probability data used in the plot is found here, and the gnuplot script is here.

Prediction of S4A11_HUMAN

```
ID   S4A11_HUMAN
FT   TOPO_DOM      1    358       NON CYTOPLASMIC.
FT   TRANSMEM    359    381
FT   TOPO_DOM    382    401       CYTOPLASMIC.
FT   TRANSMEM    402    428
FT   TOPO_DOM    429    447       NON CYTOPLASMIC.
FT   TRANSMEM    448    470
FT   TOPO_DOM    471    476       CYTOPLASMIC.
FT   TRANSMEM    477    499
FT   TOPO_DOM    500    518       NON CYTOPLASMIC.
FT   TRANSMEM    519    542
FT   TOPO_DOM    543    553       CYTOPLASMIC.
FT   TRANSMEM    554    576
FT   TOPO_DOM    577    595       NON CYTOPLASMIC.
FT   TRANSMEM    596    616
FT   TOPO_DOM    617    636       CYTOPLASMIC.
FT   TRANSMEM    637    658
FT   TOPO_DOM    659    677       NON CYTOPLASMIC.
FT   TRANSMEM    678    702
FT   TOPO_DOM    703    739       CYTOPLASMIC.
FT   TRANSMEM    740    760
FT   TOPO_DOM    761    765       NON CYTOPLASMIC.
FT   TRANSMEM    766    786
FT   TOPO_DOM    787    812       CYTOPLASMIC.
FT   TRANSMEM    813    832
FT   TOPO_DOM    833    837       NON CYTOPLASMIC.
FT   TRANSMEM    838    856
FT   TOPO_DOM    857    875       CYTOPLASMIC.
//
```

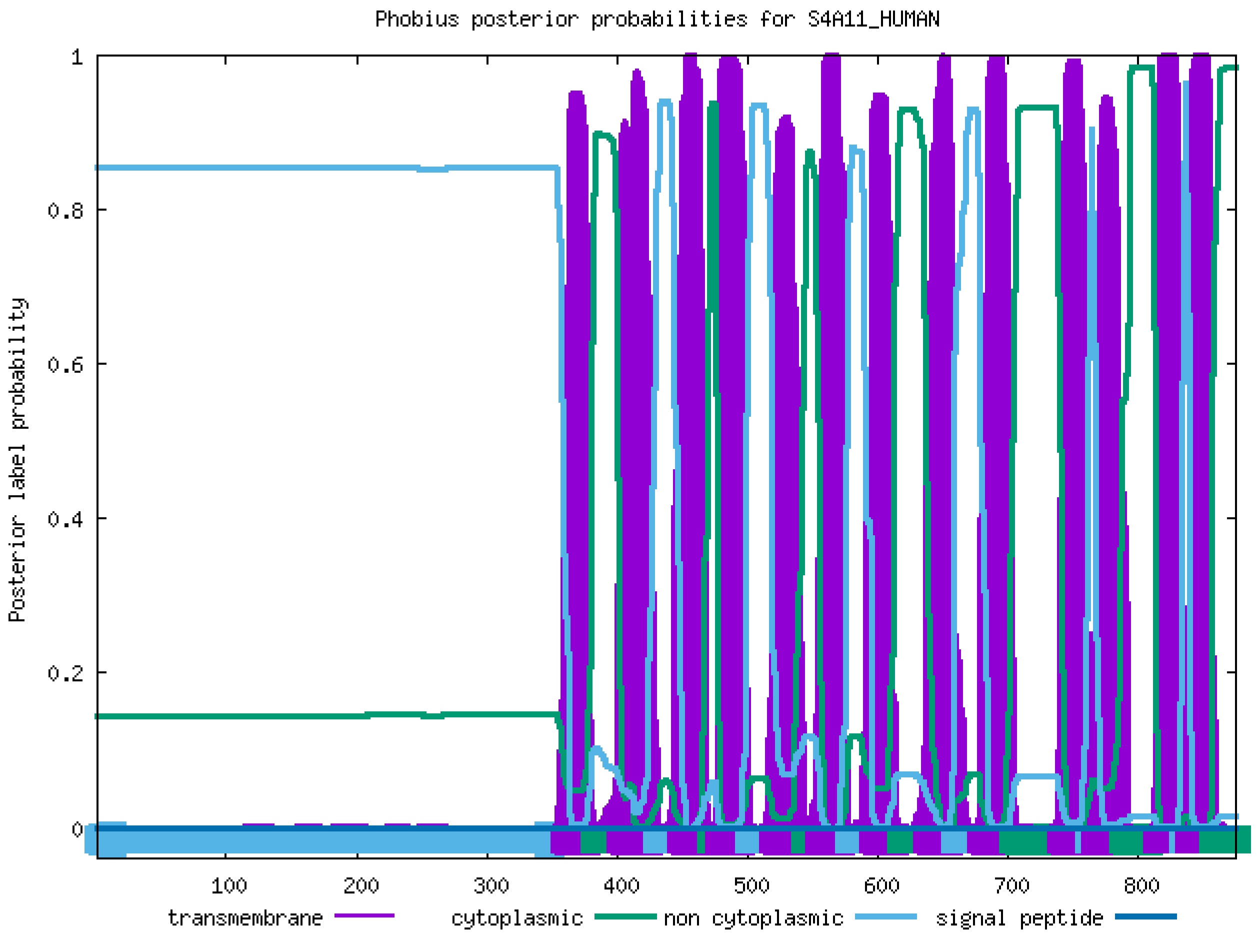

The probability data used in the plot is found here, and the gnuplot script is here.

Prediction of SLC31_HUMAN

```
ID   SLC31_HUMAN
FT   TOPO_DOM      1     87       CYTOPLASMIC.
FT   TRANSMEM     88    110
FT   TOPO_DOM    111    685       NON CYTOPLASMIC.
//
```

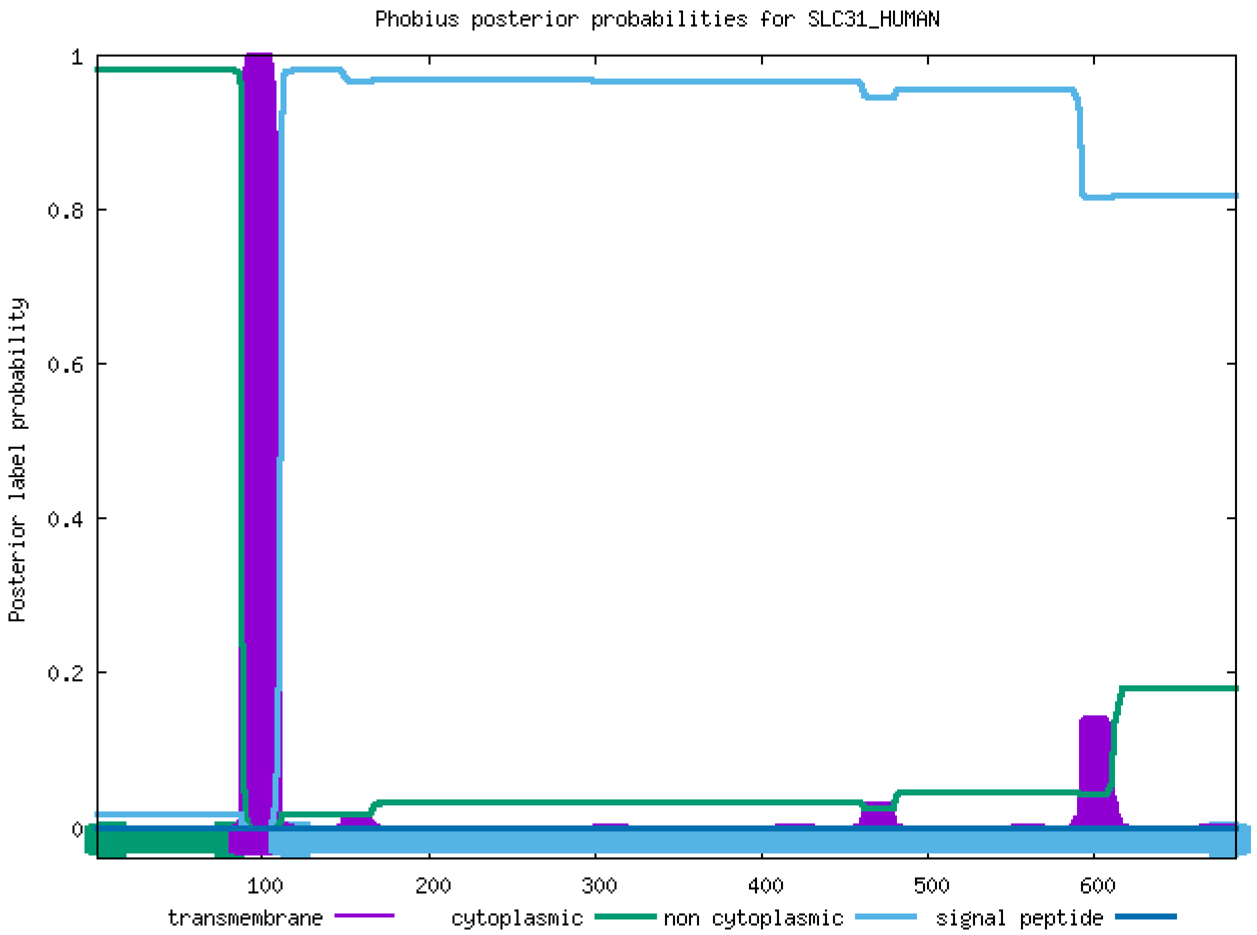

The probability data used in the plot is found here, and the gnuplot script is here.

Prediction of 4F2_HUMAN

```
ID    4F2_HUMAN
FT    TOPO_DOM      1    182       CYTOPLASMIC.
FT    TRANSMEM    183    205
FT    TOPO_DOM    206    630       NON CYTOPLASMIC.
//
```

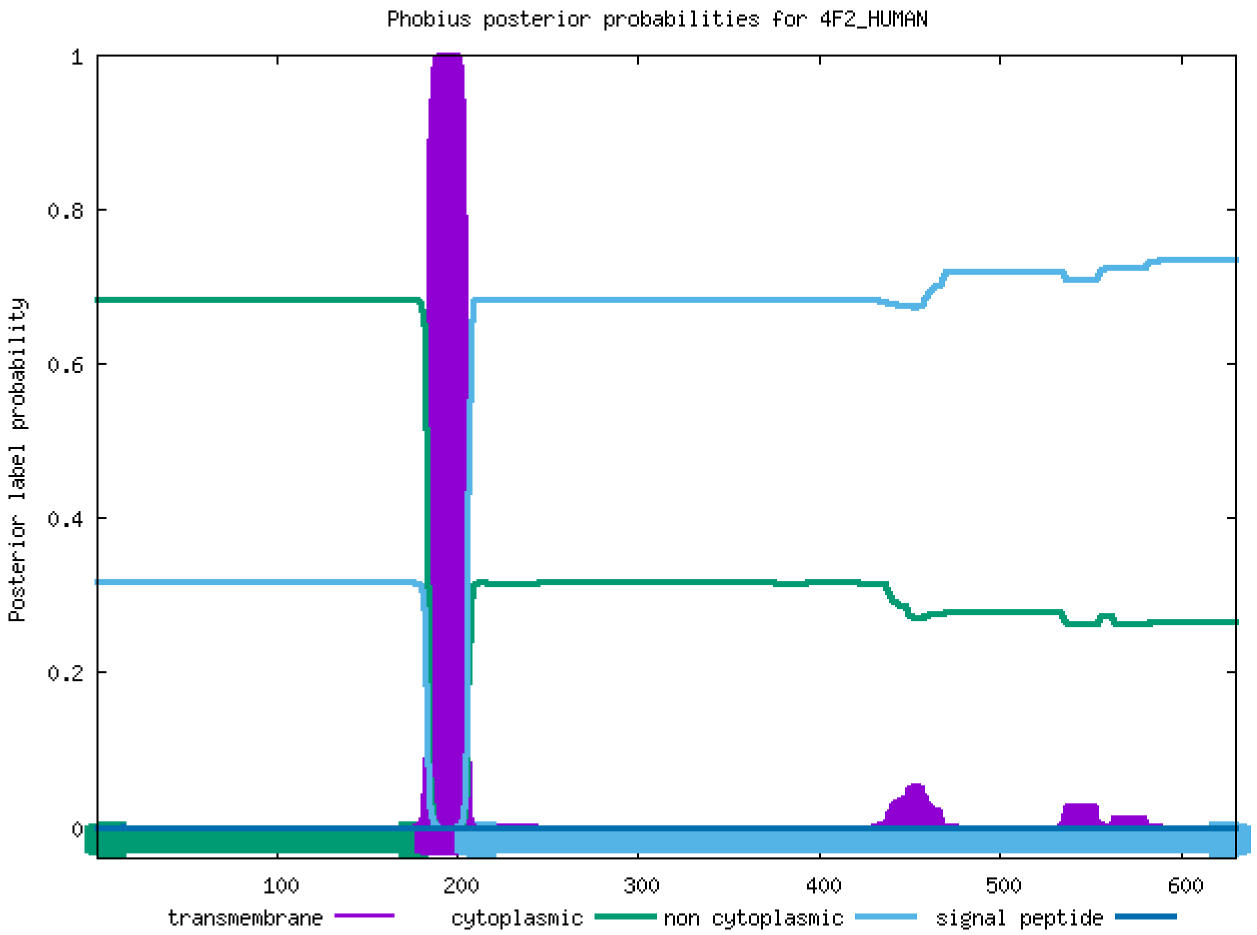

The probability data used in the plot is found here, and the gnuplot script is here.

Prediction of LAT1_HUMAN

```
ID    LAT1_HUMAN
FT    TOPO_DOM      1     51       CYTOPLASMIC.
FT    TRANSMEM     52     71
FT    TOPO_DOM     72     82       NON CYTOPLASMIC.
FT    TRANSMEM     83    104
FT    TOPO_DOM    105    137       CYTOPLASMIC.
FT    TRANSMEM    138    158
FT    TOPO_DOM    159    169       NON CYTOPLASMIC.
FT    TRANSMEM    170    188
FT    TOPO_DOM    189    199       CYTOPLASMIC.
FT    TRANSMEM    200    220
FT    TOPO_DOM    221    239       NON CYTOPLASMIC.
FT    TRANSMEM    240    262
FT    TOPO_DOM    263    273       CYTOPLASMIC.
FT    TRANSMEM    274    298
FT    TOPO_DOM    299    317       NON CYTOPLASMIC.
FT    TRANSMEM    318    339
FT    TOPO_DOM    340    369       CYTOPLASMIC.
FT    TRANSMEM    370    390
FT    TOPO_DOM    391    395       NON CYTOPLASMIC.
FT    TRANSMEM    396    419
FT    TOPO_DOM    420    430       CYTOPLASMIC.
FT    TRANSMEM    431    453
FT    TOPO_DOM    454    458       NON CYTOPLASMIC.
FT    TRANSMEM    459    478
FT    TOPO_DOM    479    507       CYTOPLASMIC.
//
```

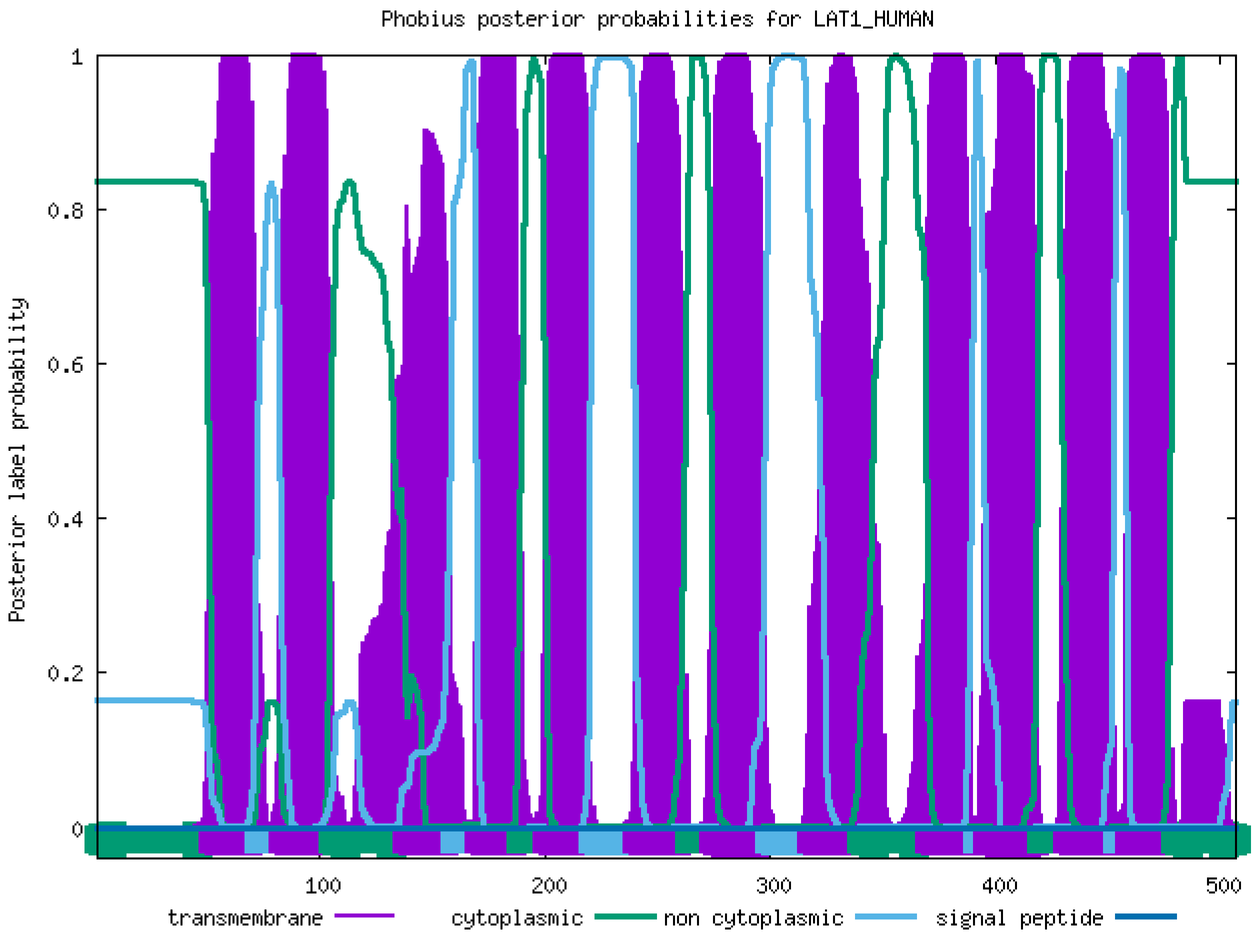

The probability data used in the plot is found here, and the gnuplot script is here.

Prediction of LAT2_HUMAN

```
ID   LAT2_HUMAN
FT   TOPO_DOM      1     39       CYTOPLASMIC.
FT   TRANSMEM     40     60
FT   TOPO_DOM     61     71       NON CYTOPLASMIC.
FT   TRANSMEM     72     93
FT   TOPO_DOM     94    112       CYTOPLASMIC.
FT   TRANSMEM    113    131
FT   TOPO_DOM    132    136       NON CYTOPLASMIC.
FT   TRANSMEM    137    156
FT   TOPO_DOM    157    162       CYTOPLASMIC.
FT   TRANSMEM    163    183
FT   TOPO_DOM    184    188       NON CYTOPLASMIC.
FT   TRANSMEM    189    210
FT   TOPO_DOM    211    230       CYTOPLASMIC.
FT   TRANSMEM    231    248
FT   TOPO_DOM    249    267       NON CYTOPLASMIC.
FT   TRANSMEM    268    290
FT   TOPO_DOM    291    296       CYTOPLASMIC.
FT   TRANSMEM    297    317
FT   TOPO_DOM    318    322       NON CYTOPLASMIC.
FT   TRANSMEM    323    342
FT   TOPO_DOM    343    362       CYTOPLASMIC.
FT   TRANSMEM    363    381
FT   TOPO_DOM    382    386       NON CYTOPLASMIC.
FT   TRANSMEM    387    411
FT   TOPO_DOM    412    422       CYTOPLASMIC.
FT   TRANSMEM    423    444
FT   TOPO_DOM    445    449       NON CYTOPLASMIC.
FT   TRANSMEM    450    469
```

```
FT   TOPO_DOM     470    535       CYTOPLASMIC.
//
```

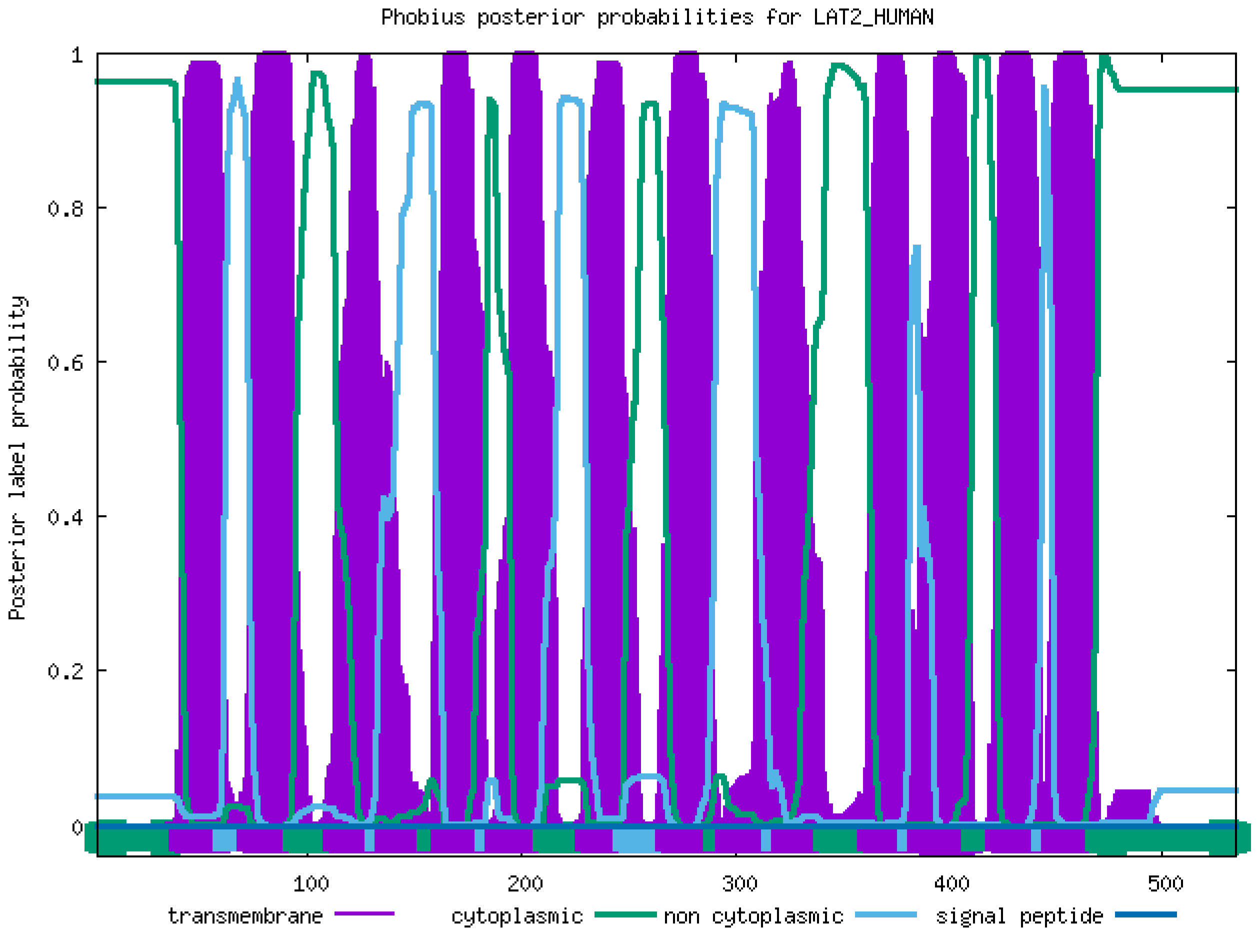

The probability data used in the plot is found here, and the gnuplot script is here.

Prediction of BAT1_HUMAN

```
ID   BAT1_HUMAN
FT   TOPO_DOM       1     29       CYTOPLASMIC.
FT   TRANSMEM      30     49
FT   TOPO_DOM      50     60       NON CYTOPLASMIC.
FT   TRANSMEM      61     86
FT   TOPO_DOM      87    106       CYTOPLASMIC.
FT   TRANSMEM     107    133
FT   TOPO_DOM     134    148       NON CYTOPLASMIC.
FT   TRANSMEM     149    170
FT   TOPO_DOM     171    181       CYTOPLASMIC.
FT   TRANSMEM     182    201
FT   TOPO_DOM     202    212       NON CYTOPLASMIC.
FT   TRANSMEM     213    232
FT   TOPO_DOM     233    251       CYTOPLASMIC.
FT   TRANSMEM     252    270
FT   TOPO_DOM     271    302       NON CYTOPLASMIC.
FT   TRANSMEM     303    329
FT   TOPO_DOM     330    348       CYTOPLASMIC.
FT   TRANSMEM     349    368
FT   TOPO_DOM     369    379       NON CYTOPLASMIC.
FT   TRANSMEM     380    399
FT   TOPO_DOM     400    410       CYTOPLASMIC.
FT   TRANSMEM     411    430
FT   TOPO_DOM     431    435       NON CYTOPLASMIC.
FT   TRANSMEM     436    457
FT   TOPO_DOM     458    487       CYTOPLASMIC.
//
```

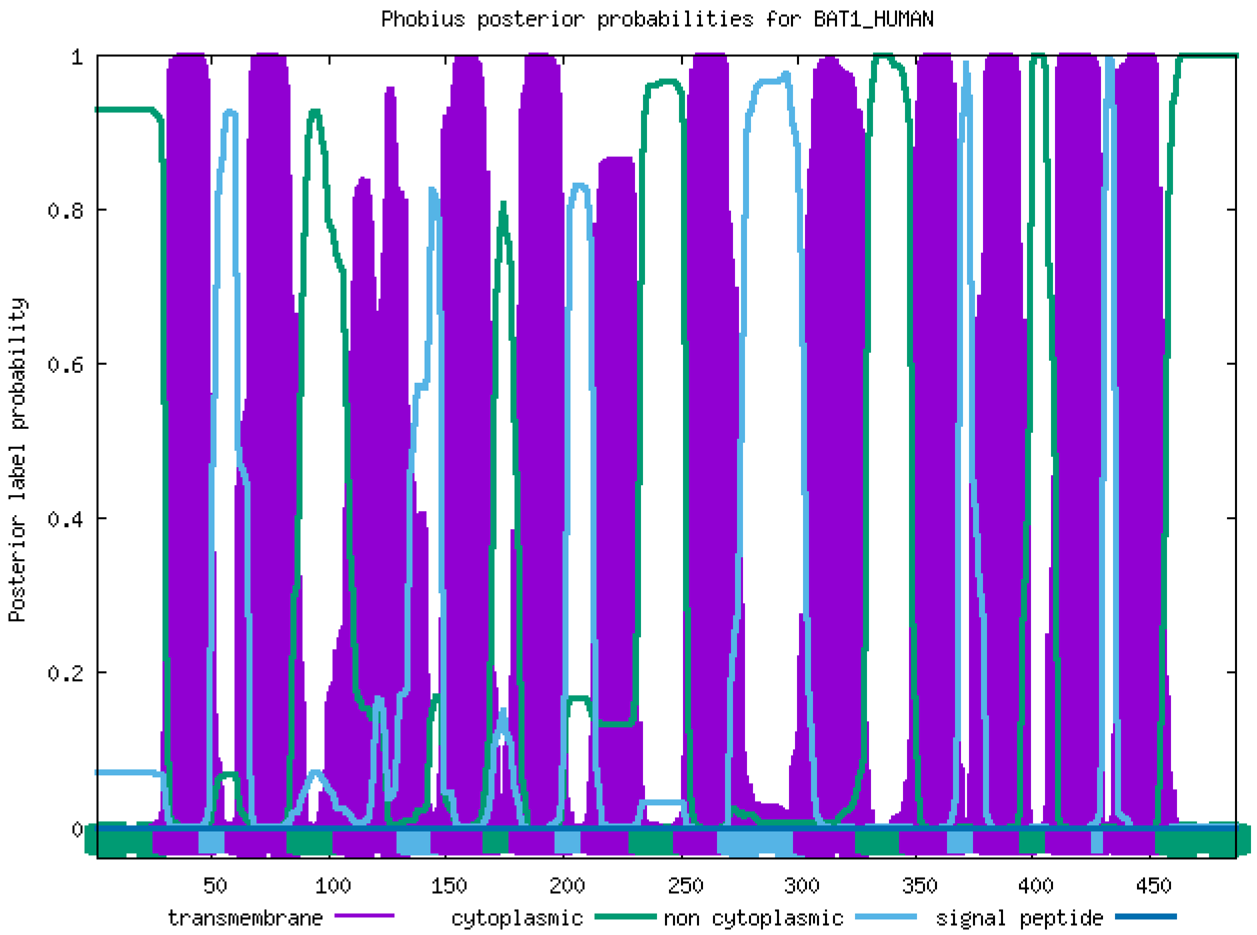

The probability data used in the plot is found here, and the gnuplot script is here.

Prediction of XCT_HUMAN

```
ID   XCT_HUMAN
FT   TOPO_DOM      1     44       CYTOPLASMIC.
FT   TRANSMEM     45     64
FT   TOPO_DOM     65     75       NON CYTOPLASMIC.
FT   TRANSMEM     76     98
FT   TOPO_DOM     99    109       CYTOPLASMIC.
FT   TRANSMEM    110    127
FT   TOPO_DOM    128    132       NON CYTOPLASMIC.
FT   TRANSMEM    133    151
FT   TOPO_DOM    152    162       CYTOPLASMIC.
FT   TRANSMEM    163    186
FT   TOPO_DOM    187    191       NON CYTOPLASMIC.
FT   TRANSMEM    192    214
FT   TOPO_DOM    215    234       CYTOPLASMIC.
FT   TRANSMEM    235    254
FT   TOPO_DOM    255    265       NON CYTOPLASMIC.
FT   TRANSMEM    266    290
FT   TOPO_DOM    291    296       CYTOPLASMIC.
FT   TRANSMEM    297    315
FT   TOPO_DOM    316    320       NON CYTOPLASMIC.
FT   TRANSMEM    321    343
FT   TOPO_DOM    344    363       CYTOPLASMIC.
FT   TRANSMEM    364    382
FT   TOPO_DOM    383    393       NON CYTOPLASMIC.
FT   TRANSMEM    394    412
FT   TOPO_DOM    413    423       CYTOPLASMIC.
FT   TRANSMEM    424    444
FT   TOPO_DOM    445    449       NON CYTOPLASMIC.
FT   TRANSMEM    450    470
```

```
FT   TOPO_DOM    471    501       CYTOPLASMIC.
//
```

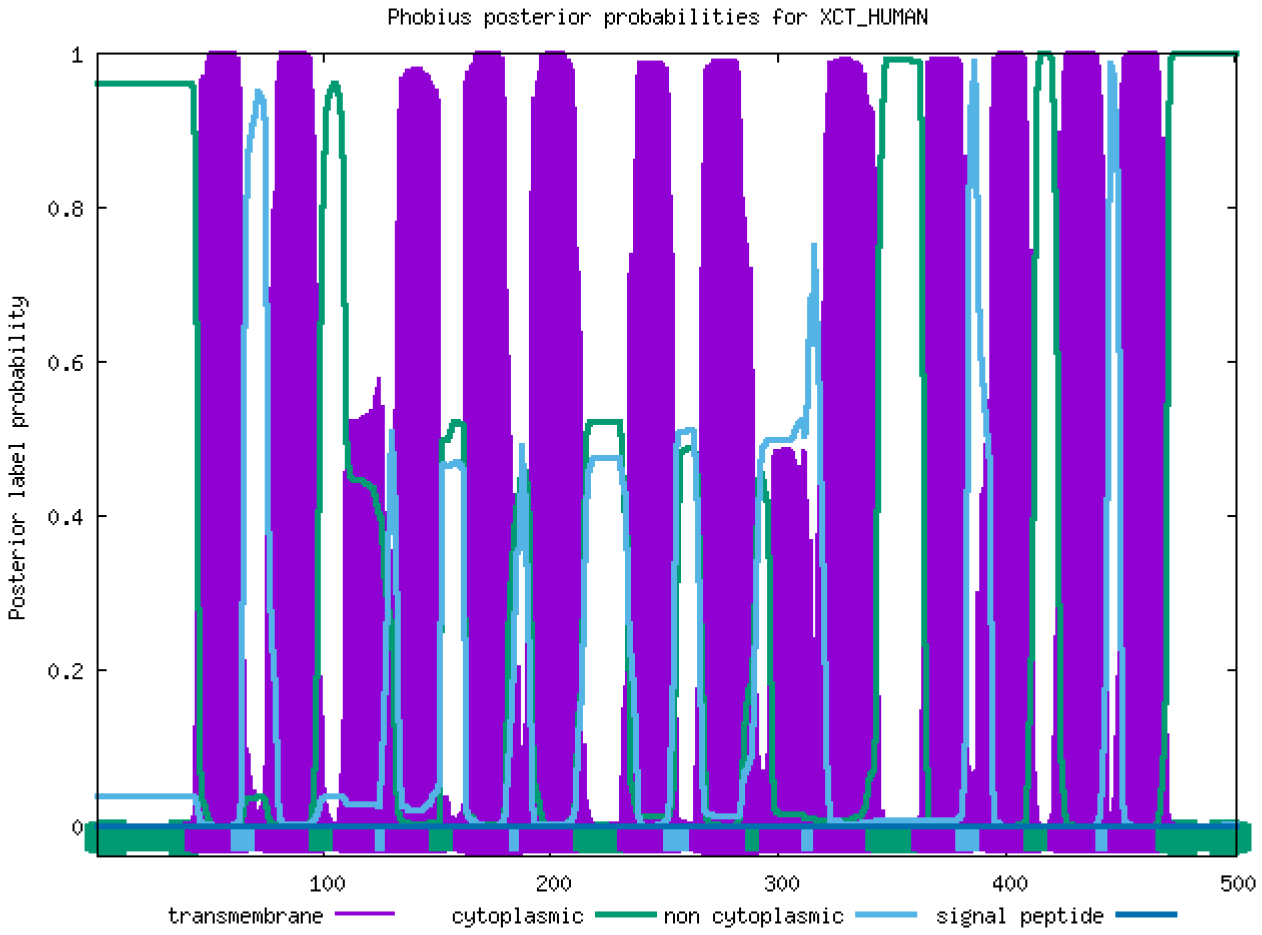

The probability data used in the plot is found here, and the gnuplot script is here.

Prediction of S12A2_HUMAN

```
ID   S12A2_HUMAN
FT   TOPO_DOM      1    290       CYTOPLASMIC.
FT   TRANSMEM    291    311
FT   TOPO_DOM    312    316       NON CYTOPLASMIC.
FT   TRANSMEM    317    343
FT   TOPO_DOM    344    363       CYTOPLASMIC.
FT   TRANSMEM    364    388
FT   TOPO_DOM    389    407       NON CYTOPLASMIC.
FT   TRANSMEM    408    427
FT   TOPO_DOM    428    435       CYTOPLASMIC.
FT   TRANSMEM    436    457
FT   TOPO_DOM    458    486       NON CYTOPLASMIC.
FT   TRANSMEM    487    509
FT   TOPO_DOM    510    520       CYTOPLASMIC.
FT   TRANSMEM    521    541
FT   TOPO_DOM    542    601       NON CYTOPLASMIC.
FT   TRANSMEM    602    622
FT   TOPO_DOM    623    654       CYTOPLASMIC.
FT   TRANSMEM    655    675
FT   TOPO_DOM    676    680       NON CYTOPLASMIC.
FT   TRANSMEM    681    699
FT   TOPO_DOM    700    710       CYTOPLASMIC.
FT   TRANSMEM    711    730
FT   TOPO_DOM    731    735       NON CYTOPLASMIC.
FT   TRANSMEM    736    751
FT   TOPO_DOM    752   1212       CYTOPLASMIC.
//
```

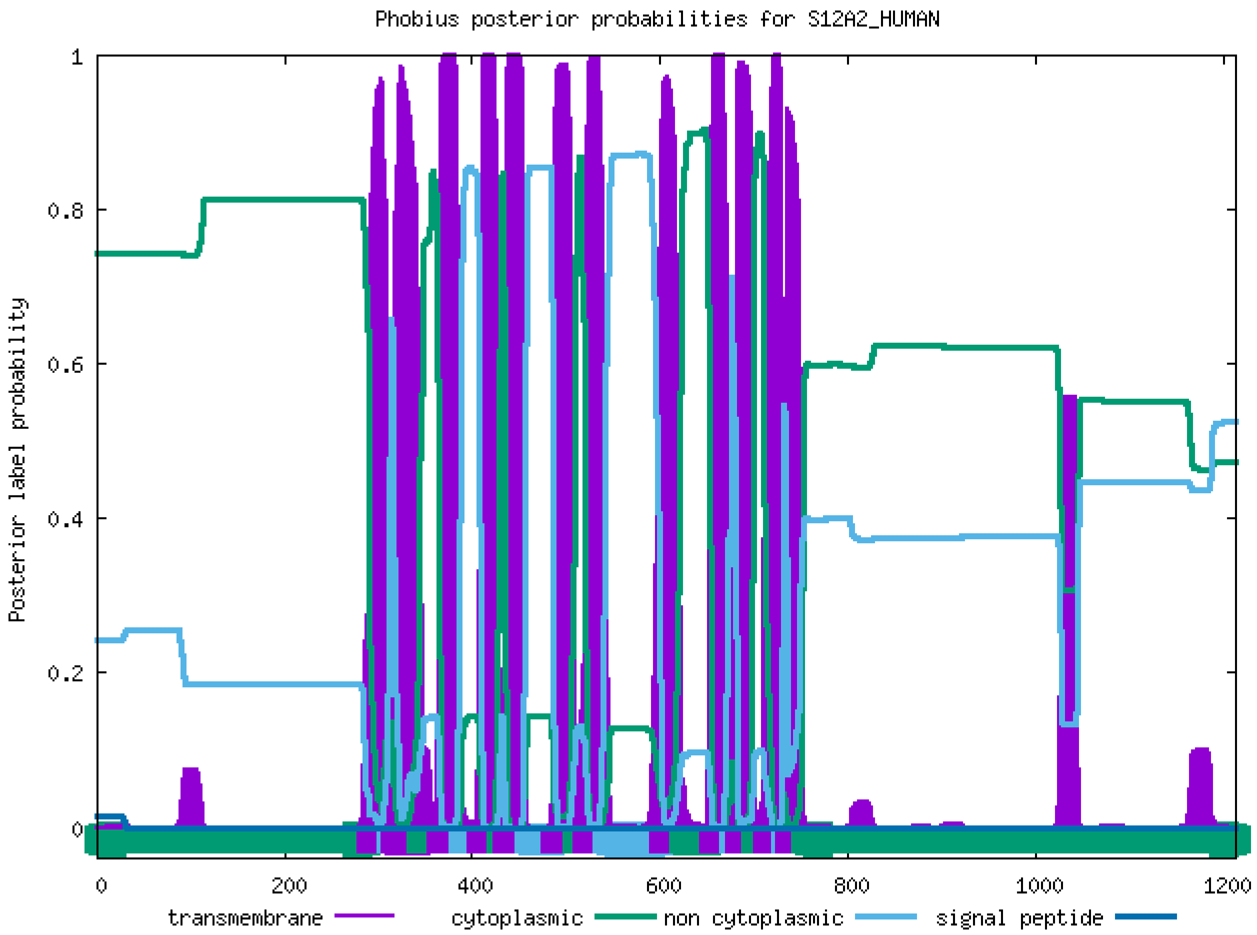

The probability data used in the plot is found here, and the gnuplot script is here.

Prediction of S12A5_HUMAN

```
ID   S12A5_HUMAN
FT   TOPO_DOM      1    125       CYTOPLASMIC.
FT   TRANSMEM    126    152
FT   TOPO_DOM    153    157       NON CYTOPLASMIC.
FT   TRANSMEM    158    182
FT   TOPO_DOM    183    201       CYTOPLASMIC.
FT   TRANSMEM    202    235
FT   TOPO_DOM    236    254       NON CYTOPLASMIC.
FT   TRANSMEM    255    272
FT   TOPO_DOM    273    278       CYTOPLASMIC.
FT   TRANSMEM    279    298
FT   TOPO_DOM    299    418       NON CYTOPLASMIC.
FT   TRANSMEM    419    439
FT   TOPO_DOM    440    458       CYTOPLASMIC.
FT   TRANSMEM    459    485
FT   TOPO_DOM    486    504       NON CYTOPLASMIC.
FT   TRANSMEM    505    523
FT   TOPO_DOM    524    558       CYTOPLASMIC.
FT   TRANSMEM    559    577
FT   TOPO_DOM    578    582       NON CYTOPLASMIC.
FT   TRANSMEM    583    605
FT   TOPO_DOM    606    616       CYTOPLASMIC.
FT   TRANSMEM    617    641
FT   TOPO_DOM    642    850       NON CYTOPLASMIC.
FT   TRANSMEM    851    868
FT   TOPO_DOM    869   1139       CYTOPLASMIC.
//
```

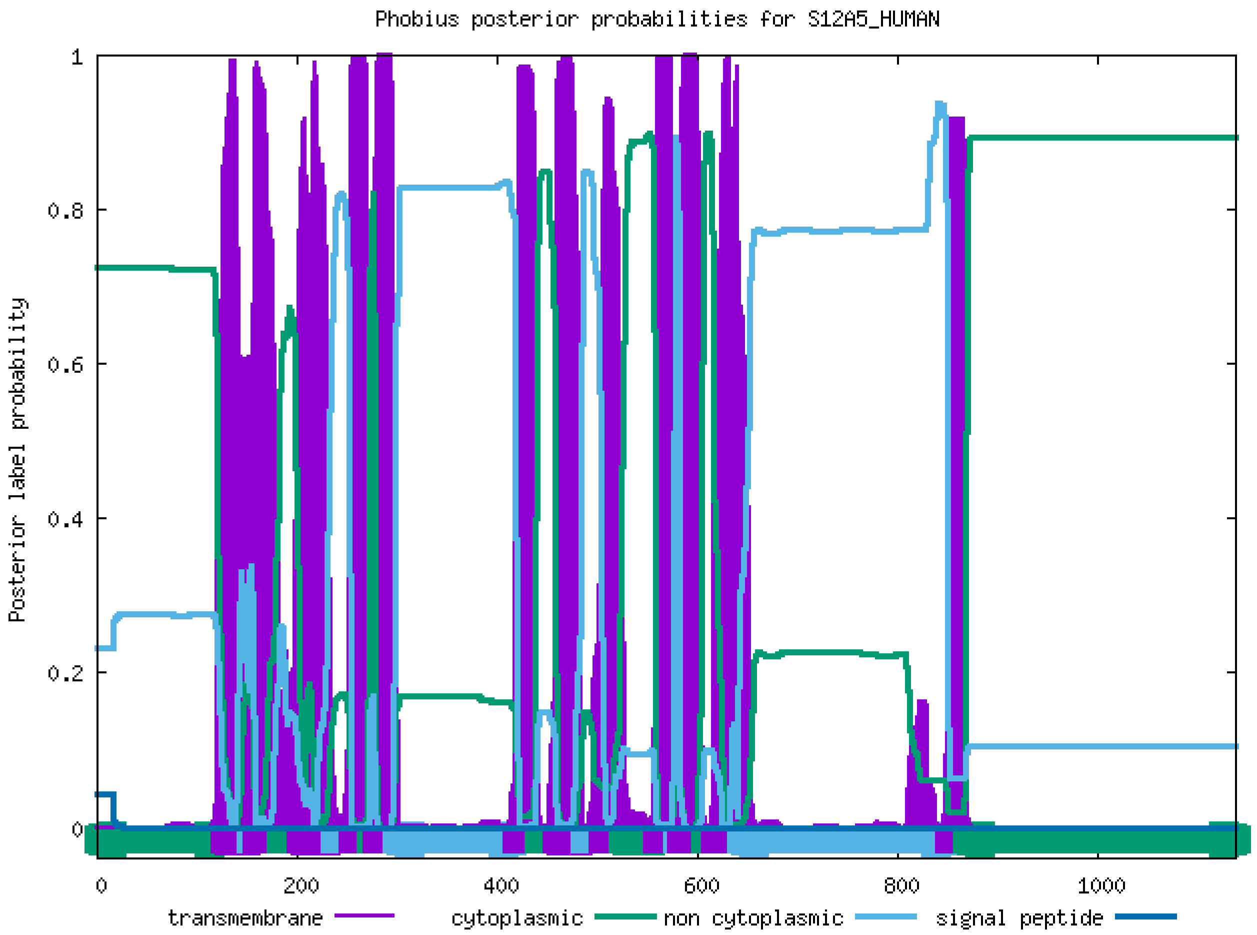

The probability data used in the plot is found here, and the gnuplot script is here.

Prediction of S12A6_HUMAN

```
ID    S12A6_HUMAN
FT    TOPO_DOM       1     185       CYTOPLASMIC.
FT    TRANSMEM     186     206
FT    TOPO_DOM     207     211       NON CYTOPLASMIC.
FT    TRANSMEM     212     234
FT    TOPO_DOM     235     240       CYTOPLASMIC.
FT    TRANSMEM     241     260
FT    TOPO_DOM     261     271       NON CYTOPLASMIC.
FT    TRANSMEM     272     297
FT    TOPO_DOM     298     317       CYTOPLASMIC.
FT    TRANSMEM     318     337
FT    TOPO_DOM     338     342       NON CYTOPLASMIC.
FT    TRANSMEM     343     362
FT    TOPO_DOM     363     475       CYTOPLASMIC.
FT    TRANSMEM     476     496
FT    TOPO_DOM     497     515       NON CYTOPLASMIC.
FT    TRANSMEM     516     542
FT    TOPO_DOM     543     620       CYTOPLASMIC.
FT    TRANSMEM     621     639
FT    TOPO_DOM     640     644       NON CYTOPLASMIC.
FT    TRANSMEM     645     666
FT    TOPO_DOM     667     677       CYTOPLASMIC.
FT    TRANSMEM     678     695
FT    TOPO_DOM     696     700       NON CYTOPLASMIC.
FT    TRANSMEM     701     717
FT    TOPO_DOM     718     874       CYTOPLASMIC.
FT    TRANSMEM     875     893
FT    TOPO_DOM     894     912       NON CYTOPLASMIC.
FT    TRANSMEM     913     930
```

```
FT   TOPO_DOM     931    1150       CYTOPLASMIC.
//
```

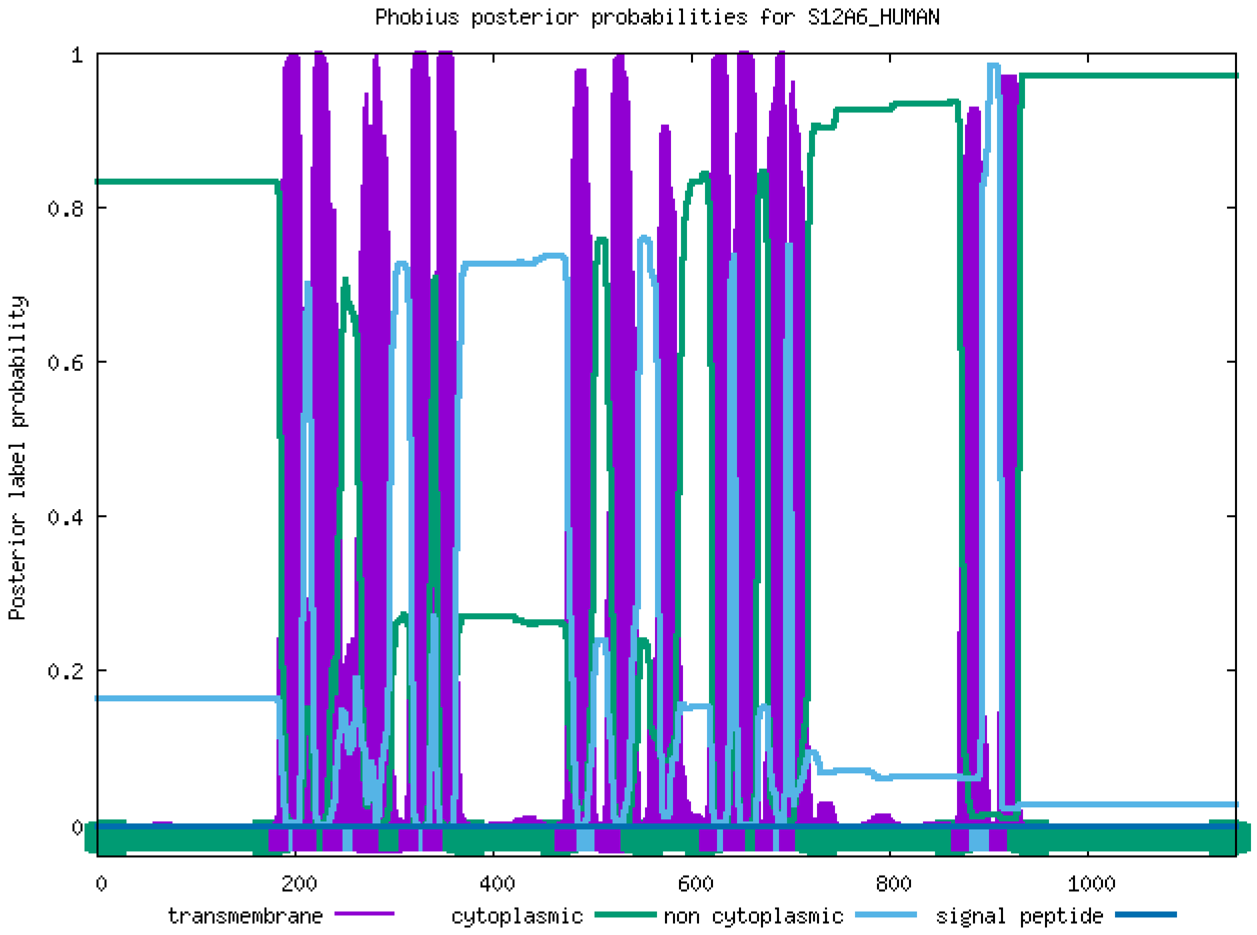

The probability data used in the plot is found here, and the gnuplot script is here.

Prediction of S13A5_HUMAN

```
ID   S13A5_HUMAN
FT   TOPO_DOM       1      11       CYTOPLASMIC.
FT   TRANSMEM      12      33
FT   TOPO_DOM      34      38       NON CYTOPLASMIC.
FT   TRANSMEM      39      67
FT   TOPO_DOM      68      87       CYTOPLASMIC.
FT   TRANSMEM      88     105
FT   TOPO_DOM     106     124       NON CYTOPLASMIC.
FT   TRANSMEM     125     149
FT   TOPO_DOM     150     206       CYTOPLASMIC.
FT   TRANSMEM     207     227
FT   TOPO_DOM     228     246       NON CYTOPLASMIC.
FT   TRANSMEM     247     272
FT   TOPO_DOM     273     308       CYTOPLASMIC.
FT   TRANSMEM     309     331
FT   TOPO_DOM     332     350       NON CYTOPLASMIC.
FT   TRANSMEM     351     370
FT   TOPO_DOM     371     405       CYTOPLASMIC.
FT   TRANSMEM     406     423
FT   TOPO_DOM     424     442       NON CYTOPLASMIC.
FT   TRANSMEM     443     476
FT   TOPO_DOM     477     487       CYTOPLASMIC.
FT   TRANSMEM     488     508
FT   TOPO_DOM     509     527       NON CYTOPLASMIC.
FT   TRANSMEM     528     547
FT   TOPO_DOM     548     568       CYTOPLASMIC.
//
```

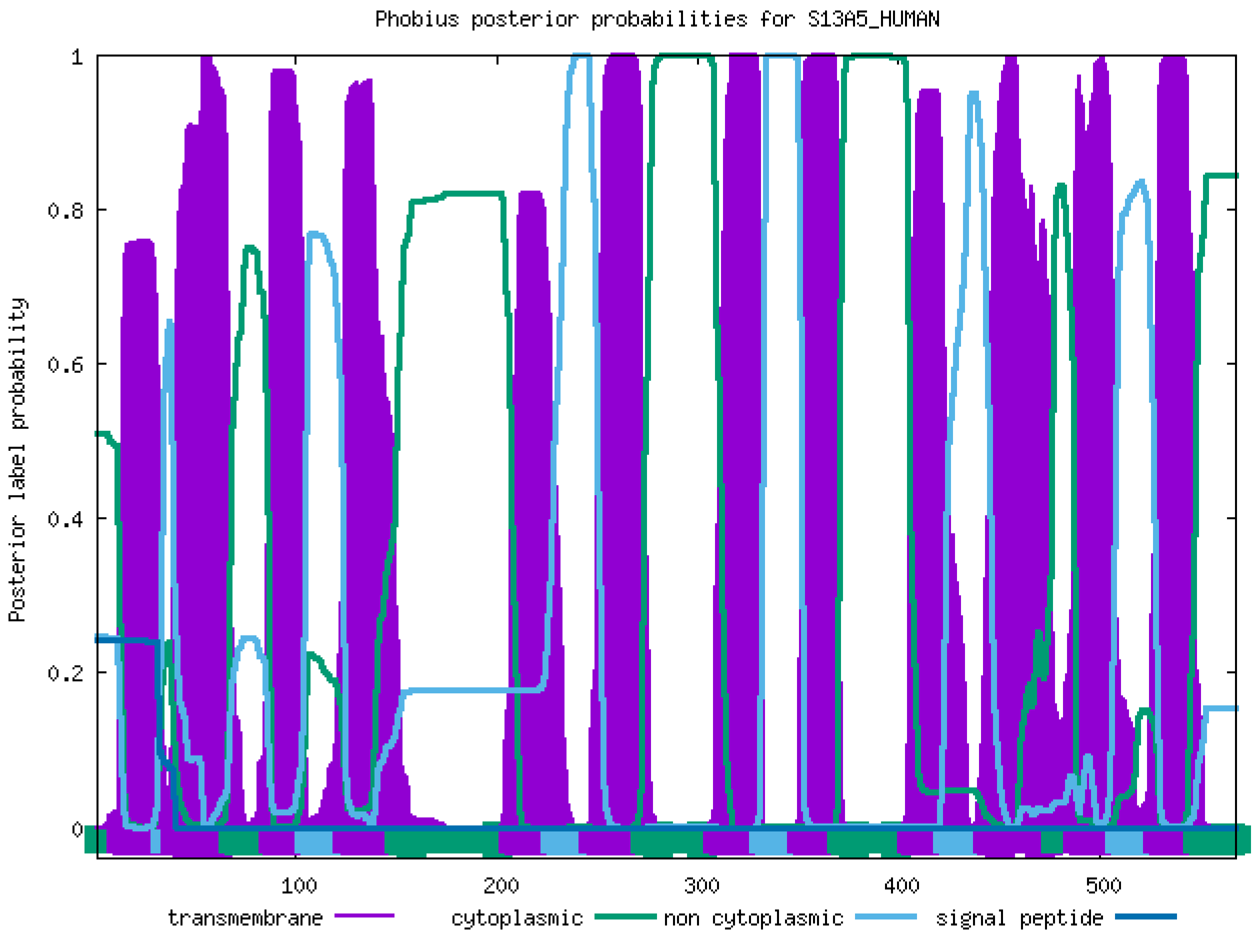

The probability data used in the plot is found here, and the gnuplot script is here.

Prediction of S15A1_HUMAN

```
ID    S15A1_HUMAN
FT    TOPO_DOM      1     5       NON CYTOPLASMIC.
FT    TRANSMEM      6    24
FT    TOPO_DOM     25    30       CYTOPLASMIC.
FT    TRANSMEM     31    47
FT    TOPO_DOM     48    52       NON CYTOPLASMIC.
FT    TRANSMEM     53    74
FT    TOPO_DOM     75    80       CYTOPLASMIC.
FT    TRANSMEM     81    98
FT    TOPO_DOM     99   117       NON CYTOPLASMIC.
FT    TRANSMEM    118   139
FT    TOPO_DOM    140   159       CYTOPLASMIC.
FT    TRANSMEM    160   180
FT    TOPO_DOM    181   199       NON CYTOPLASMIC.
FT    TRANSMEM    200   223
FT    TOPO_DOM    224   278       CYTOPLASMIC.
FT    TRANSMEM    279   297
FT    TOPO_DOM    298   327       NON CYTOPLASMIC.
FT    TRANSMEM    328   349
FT    TOPO_DOM    350   360       CYTOPLASMIC.
FT    TRANSMEM    361   380
FT    TOPO_DOM    381   587       NON CYTOPLASMIC.
FT    TRANSMEM    588   608
FT    TOPO_DOM    609   619       CYTOPLASMIC.
FT    TRANSMEM    620   643
FT    TOPO_DOM    644   648       NON CYTOPLASMIC.
FT    TRANSMEM    649   670
FT    TOPO_DOM    671   708       CYTOPLASMIC.
//
```

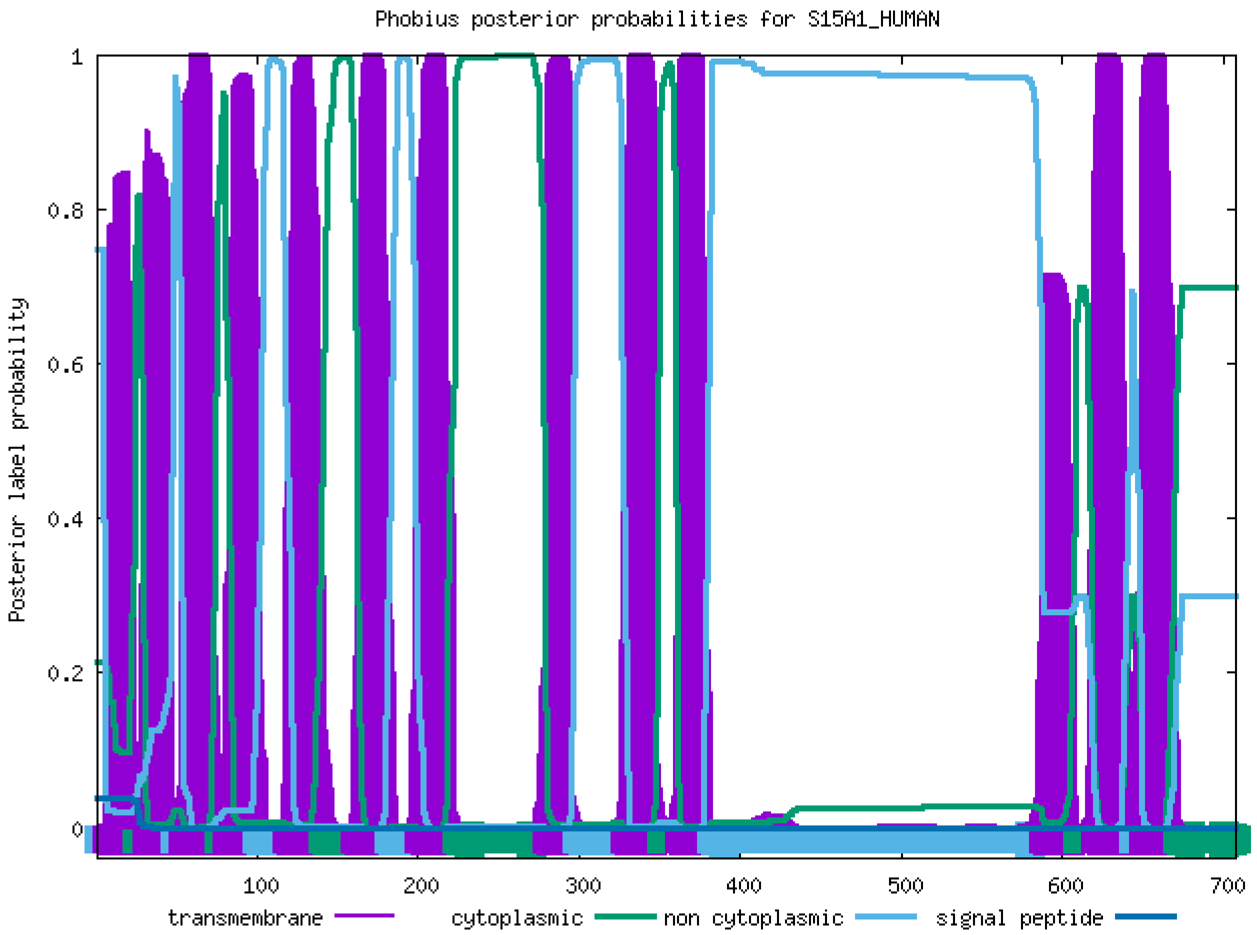

The probability data used in the plot is found here, and the gnuplot script is here.

Prediction of S15A2_HUMAN

```
ID    S15A2_HUMAN
FT    TOPO_DOM      1     57       CYTOPLASMIC.
FT    TRANSMEM     58     78
FT    TOPO_DOM     79     83       NON CYTOPLASMIC.
FT    TRANSMEM     84    104
FT    TOPO_DOM    105    115       CYTOPLASMIC.
FT    TRANSMEM    116    137
FT    TOPO_DOM    138    142       NON CYTOPLASMIC.
FT    TRANSMEM    143    164
FT    TOPO_DOM    165    184       CYTOPLASMIC.
FT    TRANSMEM    185    205
FT    TOPO_DOM    206    216       NON CYTOPLASMIC.
FT    TRANSMEM    217    237
FT    TOPO_DOM    238    297       CYTOPLASMIC.
FT    TRANSMEM    298    316
FT    TOPO_DOM    317    345       NON CYTOPLASMIC.
FT    TRANSMEM    346    367
FT    TOPO_DOM    368    378       CYTOPLASMIC.
FT    TRANSMEM    379    397
FT    TOPO_DOM    398    614       NON CYTOPLASMIC.
FT    TRANSMEM    615    635
FT    TOPO_DOM    636    646       CYTOPLASMIC.
FT    TRANSMEM    647    669
FT    TOPO_DOM    670    674       NON CYTOPLASMIC.
FT    TRANSMEM    675    697
FT    TOPO_DOM    698    729       CYTOPLASMIC.
//
```

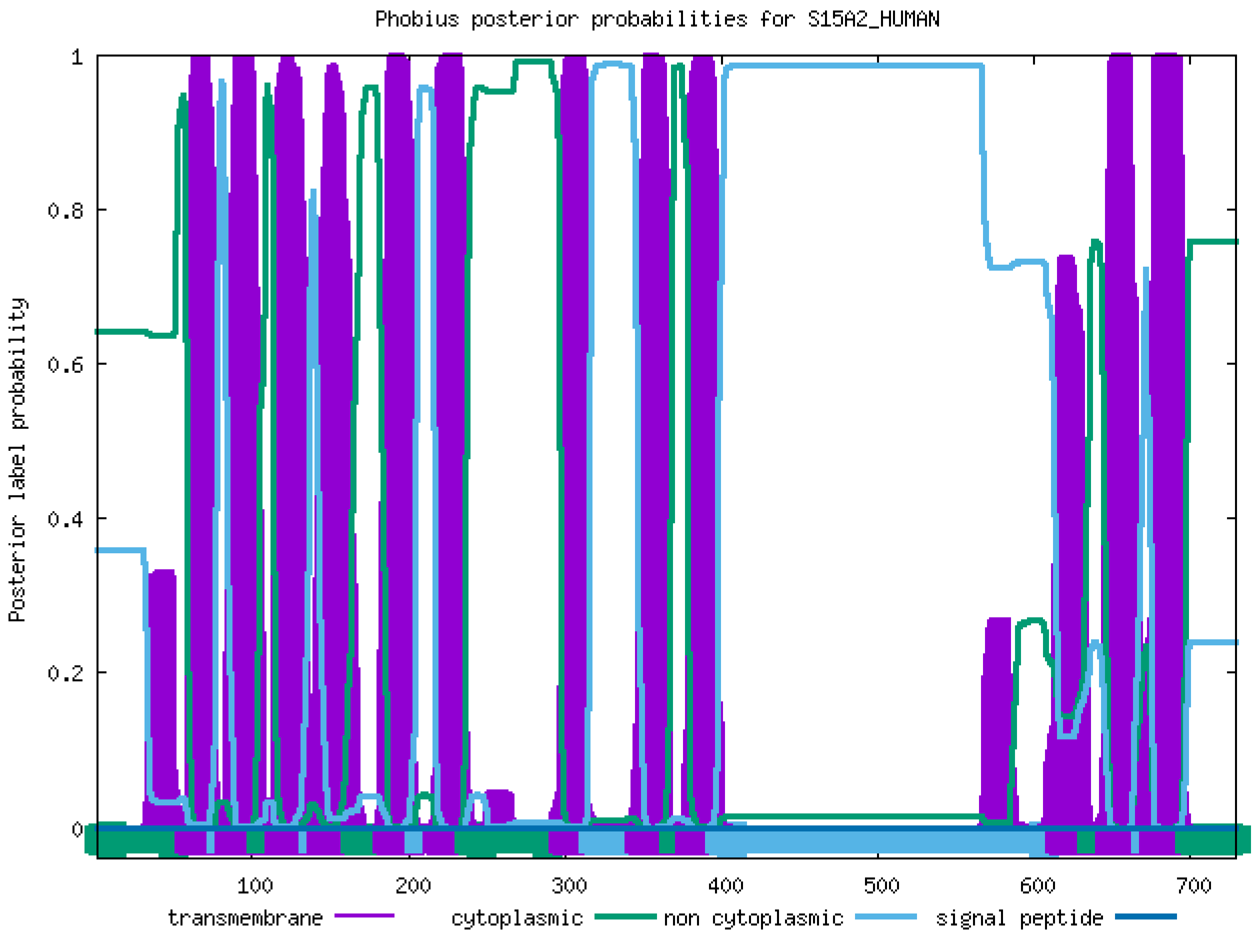

The probability data used in the plot is found here, and the gnuplot script is here.

Prediction of S15A4_HUMAN

```
ID   S15A4_HUMAN
FT   TOPO_DOM      1     19       NON CYTOPLASMIC.
FT   TRANSMEM     20     42
FT   TOPO_DOM     43     48       CYTOPLASMIC.
FT   TRANSMEM     49     65
FT   TOPO_DOM     66     76       NON CYTOPLASMIC.
FT   TRANSMEM     77     96
FT   TOPO_DOM     97    104       CYTOPLASMIC.
FT   TRANSMEM    105    128
FT   TOPO_DOM    129    147       NON CYTOPLASMIC.
FT   TRANSMEM    148    173
FT   TOPO_DOM    174    193       CYTOPLASMIC.
FT   TRANSMEM    194    216
FT   TOPO_DOM    217    221       NON CYTOPLASMIC.
FT   TRANSMEM    222    242
FT   TOPO_DOM    243    316       CYTOPLASMIC.
FT   TRANSMEM    317    338
FT   TOPO_DOM    339    365       NON CYTOPLASMIC.
FT   TRANSMEM    366    383
FT   TOPO_DOM    384    403       CYTOPLASMIC.
FT   TRANSMEM    404    422
FT   TOPO_DOM    423    457       NON CYTOPLASMIC.
FT   TRANSMEM    458    477
FT   TOPO_DOM    478    488       CYTOPLASMIC.
FT   TRANSMEM    489    512
FT   TOPO_DOM    513    531       NON CYTOPLASMIC.
FT   TRANSMEM    532    555
FT   TOPO_DOM    556    577       CYTOPLASMIC.
//
```

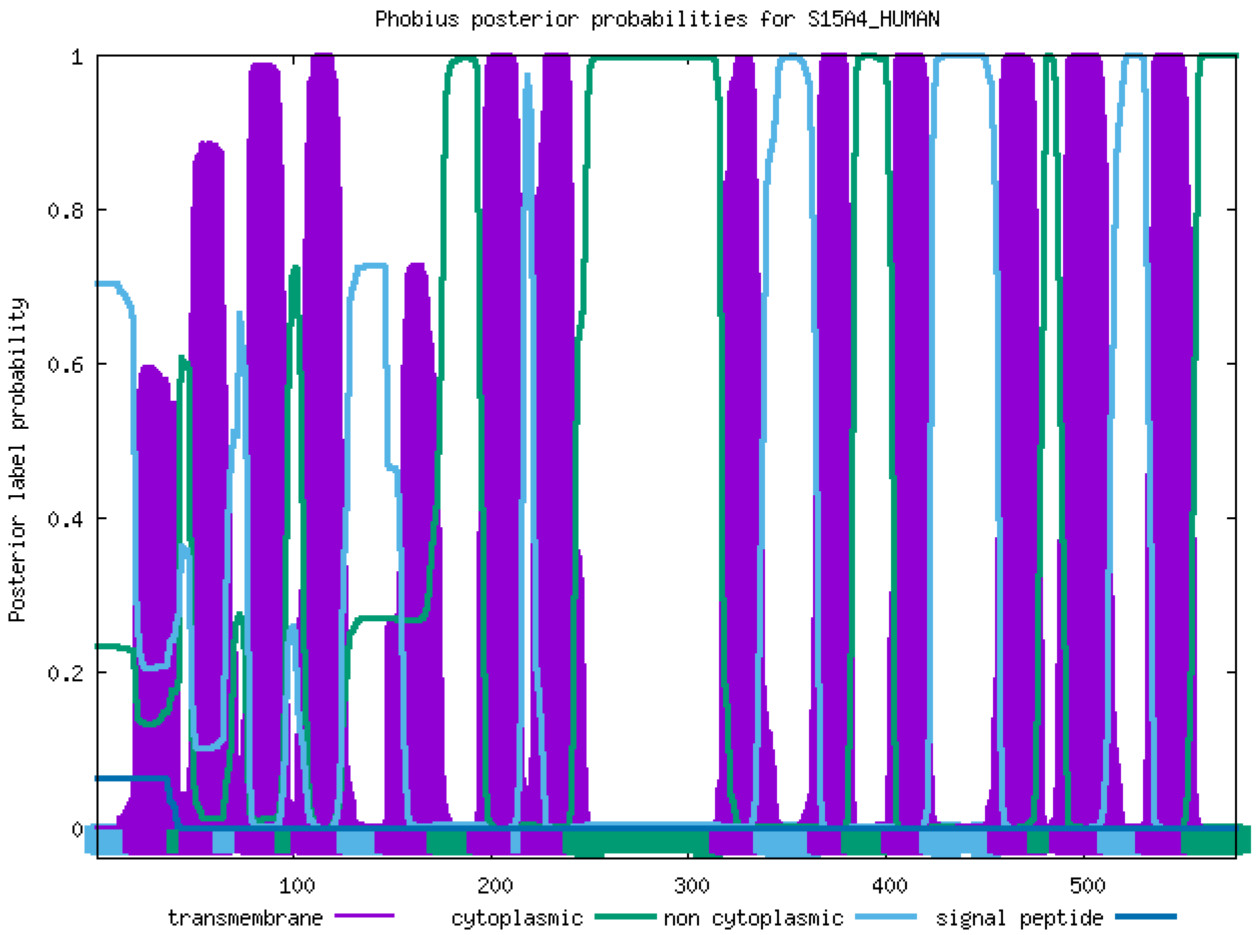

The probability data used in the plot is found here, and the gnuplot script is here.

Prediction of MOT1_HUMAN

```
ID    MOT1_HUMAN
FT    TOPO_DOM      1     16       NON CYTOPLASMIC.
FT    TRANSMEM     17     36
FT    TOPO_DOM     37     86       CYTOPLASMIC.
FT    TRANSMEM     87    105
FT    TOPO_DOM    106    110       NON CYTOPLASMIC.
FT    TRANSMEM    111    132
FT    TOPO_DOM    133    143       CYTOPLASMIC.
FT    TRANSMEM    144    164
FT    TOPO_DOM    165    175       NON CYTOPLASMIC.
FT    TRANSMEM    176    195
FT    TOPO_DOM    196    262       CYTOPLASMIC.
FT    TRANSMEM    263    287
FT    TOPO_DOM    288    298       NON CYTOPLASMIC.
FT    TRANSMEM    299    319
FT    TOPO_DOM    320    330       CYTOPLASMIC.
FT    TRANSMEM    331    347
FT    TOPO_DOM    348    352       NON CYTOPLASMIC.
FT    TRANSMEM    353    375
FT    TOPO_DOM    376    386       CYTOPLASMIC.
FT    TRANSMEM    387    409
FT    TOPO_DOM    410    420       NON CYTOPLASMIC.
FT    TRANSMEM    421    443
FT    TOPO_DOM    444    500       CYTOPLASMIC.
//
```

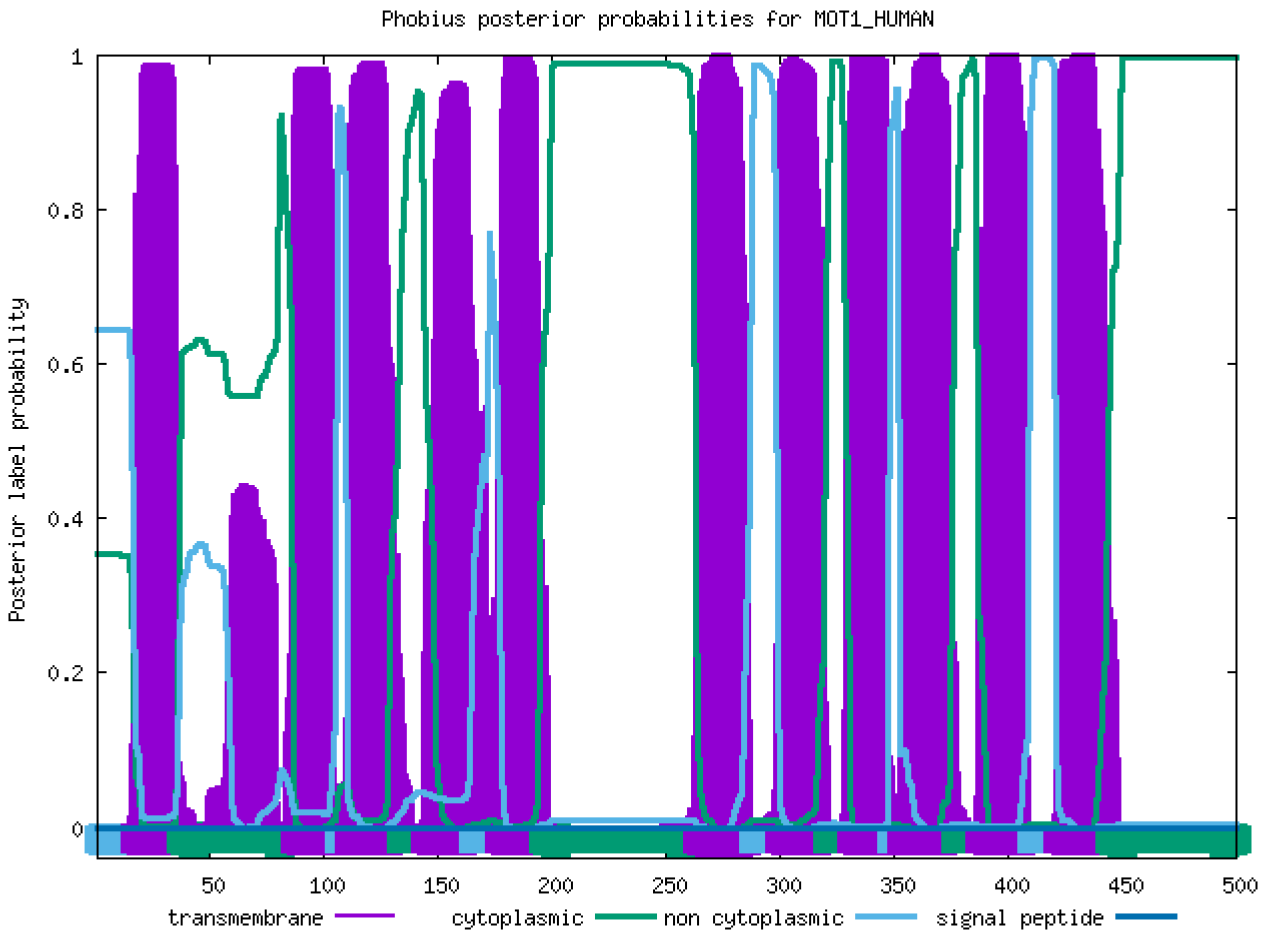

The probability data used in the plot is found here, and the gnuplot script is here.

Prediction of MOT2_HUMAN

```
ID    MOT2_HUMAN
FT    TOPO_DOM      1     17       CYTOPLASMIC.
FT    TRANSMEM     18     36
FT    TOPO_DOM     37     57       NON CYTOPLASMIC.
FT    TRANSMEM     58     80
FT    TOPO_DOM     81     86       CYTOPLASMIC.
FT    TRANSMEM     87    106
FT    TOPO_DOM    107    111       NON CYTOPLASMIC.
FT    TRANSMEM    112    134
FT    TOPO_DOM    135    145       CYTOPLASMIC.
FT    TRANSMEM    146    164
FT    TOPO_DOM    165    175       NON CYTOPLASMIC.
FT    TRANSMEM    176    198
FT    TOPO_DOM    199    246       CYTOPLASMIC.
FT    TRANSMEM    247    268
FT    TOPO_DOM    269    279       NON CYTOPLASMIC.
FT    TRANSMEM    280    303
FT    TOPO_DOM    304    314       CYTOPLASMIC.
FT    TRANSMEM    315    335
FT    TOPO_DOM    336    340       NON CYTOPLASMIC.
FT    TRANSMEM    341    359
FT    TOPO_DOM    360    370       CYTOPLASMIC.
FT    TRANSMEM    371    393
FT    TOPO_DOM    394    404       NON CYTOPLASMIC.
FT    TRANSMEM    405    427
FT    TOPO_DOM    428    478       CYTOPLASMIC.
//
```

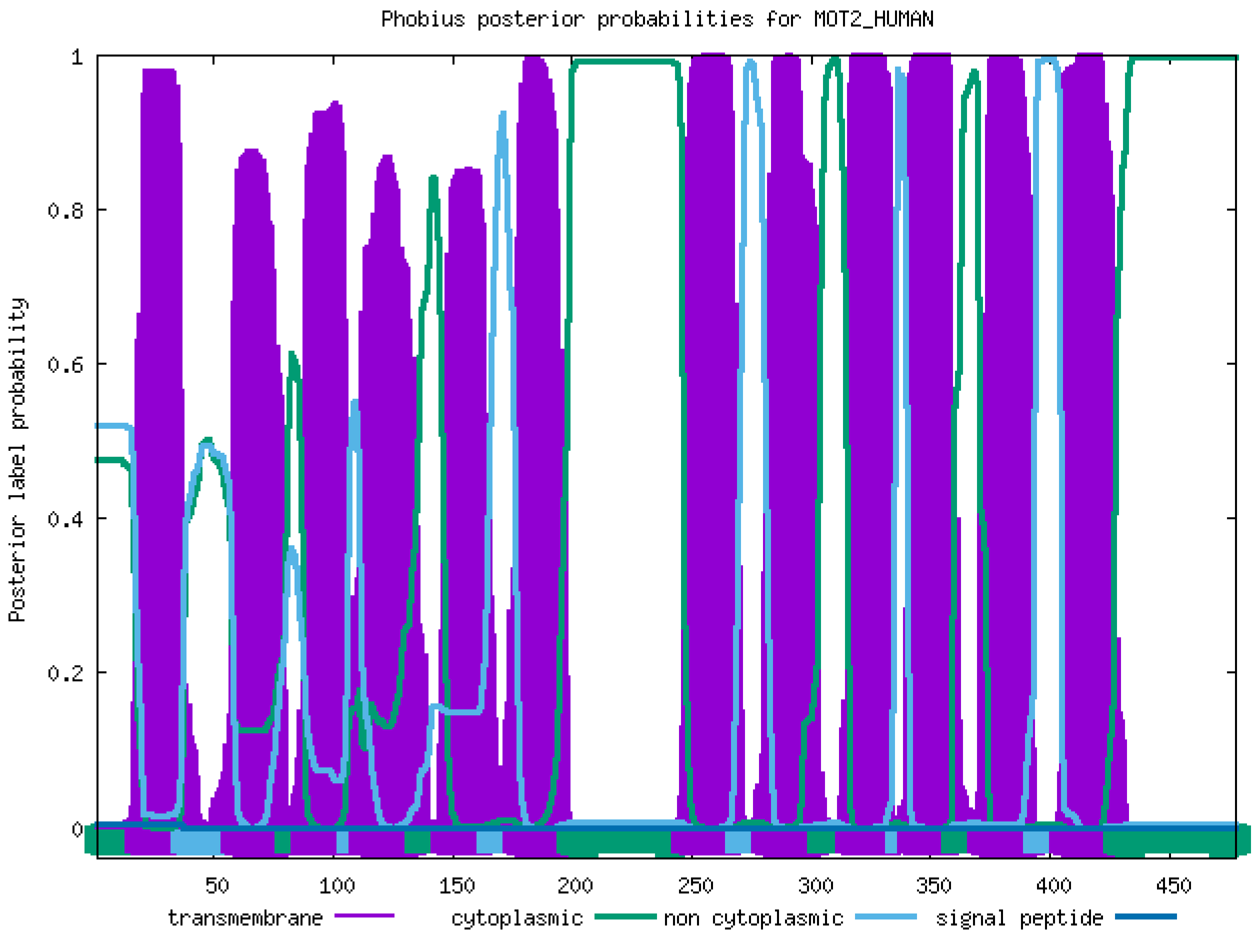

The probability data used in the plot is found here, and the gnuplot script is here.

Prediction of S19A1_HUMAN

```
ID   S19A1_HUMAN
FT   TOPO_DOM      1     67       NON CYTOPLASMIC.
FT   TRANSMEM     68     87
FT   TOPO_DOM     88     93       CYTOPLASMIC.
FT   TRANSMEM     94    112
FT   TOPO_DOM    113    123       NON CYTOPLASMIC.
FT   TRANSMEM    124    144
FT   TOPO_DOM    145    155       CYTOPLASMIC.
FT   TRANSMEM    156    178
FT   TOPO_DOM    179    183       NON CYTOPLASMIC.
FT   TRANSMEM    184    203
FT   TOPO_DOM    204    271       CYTOPLASMIC.
FT   TRANSMEM    272    292
FT   TOPO_DOM    293    303       NON CYTOPLASMIC.
FT   TRANSMEM    304    325
FT   TOPO_DOM    326    336       CYTOPLASMIC.
FT   TRANSMEM    337    355
FT   TOPO_DOM    356    360       NON CYTOPLASMIC.
FT   TRANSMEM    361    383
FT   TOPO_DOM    384    394       CYTOPLASMIC.
FT   TRANSMEM    395    417
FT   TOPO_DOM    418    436       NON CYTOPLASMIC.
FT   TRANSMEM    437    455
FT   TOPO_DOM    456    591       CYTOPLASMIC.
//
```

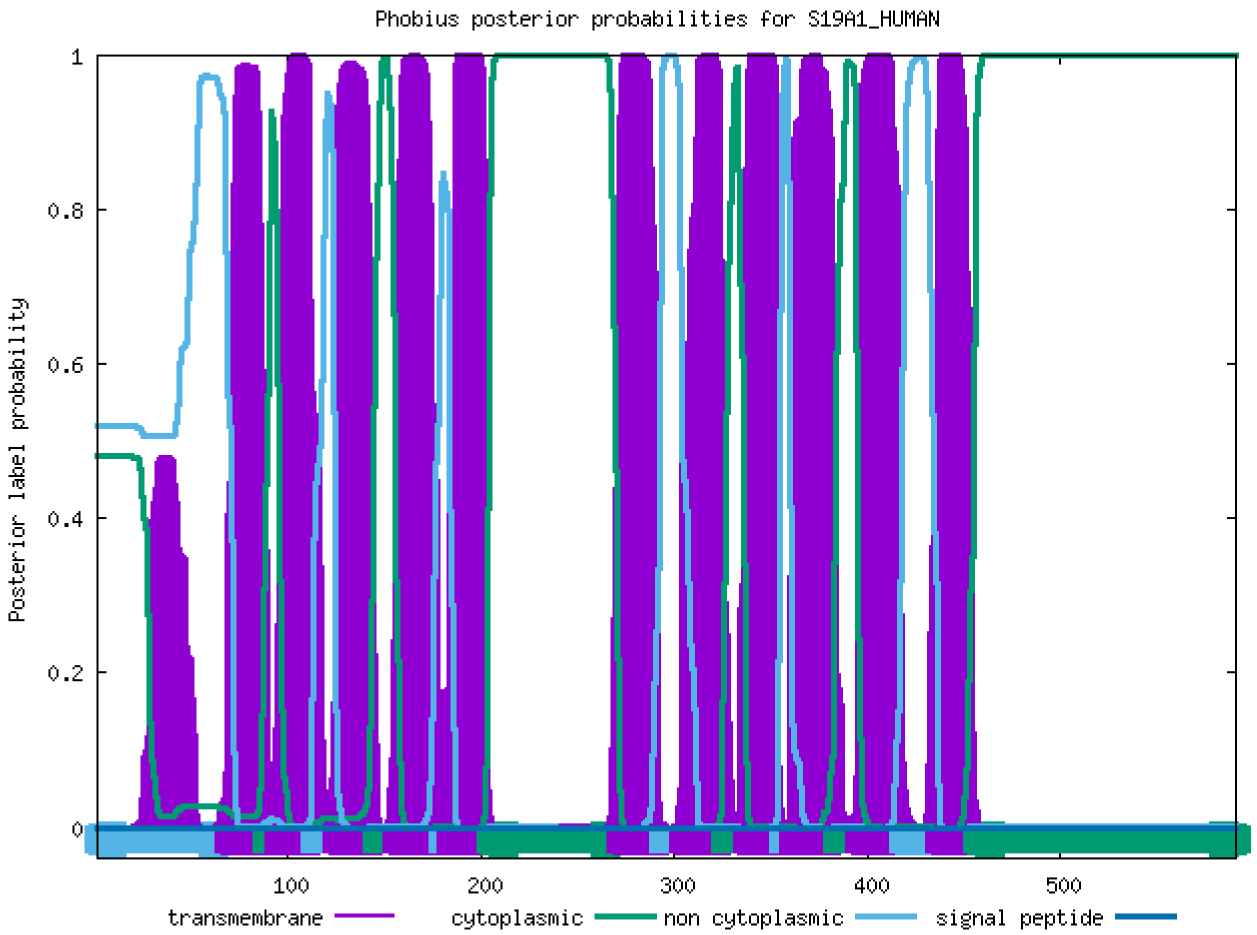

The probability data used in the plot is found here, and the gnuplot script is here.

Prediction of S22A3_HUMAN

```
ID    S22A3_HUMAN
FT    TOPO_DOM      1     20       CYTOPLASMIC.
FT    TRANSMEM     21     42
FT    TOPO_DOM     43    158       NON CYTOPLASMIC.
FT    TRANSMEM    159    176
FT    TOPO_DOM    177    182       CYTOPLASMIC.
FT    TRANSMEM    183    203
FT    TOPO_DOM    204    208       NON CYTOPLASMIC.
FT    TRANSMEM    209    230
FT    TOPO_DOM    231    241       CYTOPLASMIC.
FT    TRANSMEM    242    264
FT    TOPO_DOM    265    269       NON CYTOPLASMIC.
FT    TRANSMEM    270    288
FT    TOPO_DOM    289    351       CYTOPLASMIC.
FT    TRANSMEM    352    374
FT    TOPO_DOM    375    379       NON CYTOPLASMIC.
FT    TRANSMEM    380    401
FT    TOPO_DOM    402    407       CYTOPLASMIC.
FT    TRANSMEM    408    427
FT    TOPO_DOM    428    432       NON CYTOPLASMIC.
FT    TRANSMEM    433    456
FT    TOPO_DOM    457    467       CYTOPLASMIC.
FT    TRANSMEM    468    492
FT    TOPO_DOM    493    497       NON CYTOPLASMIC.
FT    TRANSMEM    498    517
FT    TOPO_DOM    518    556       CYTOPLASMIC.
//
```

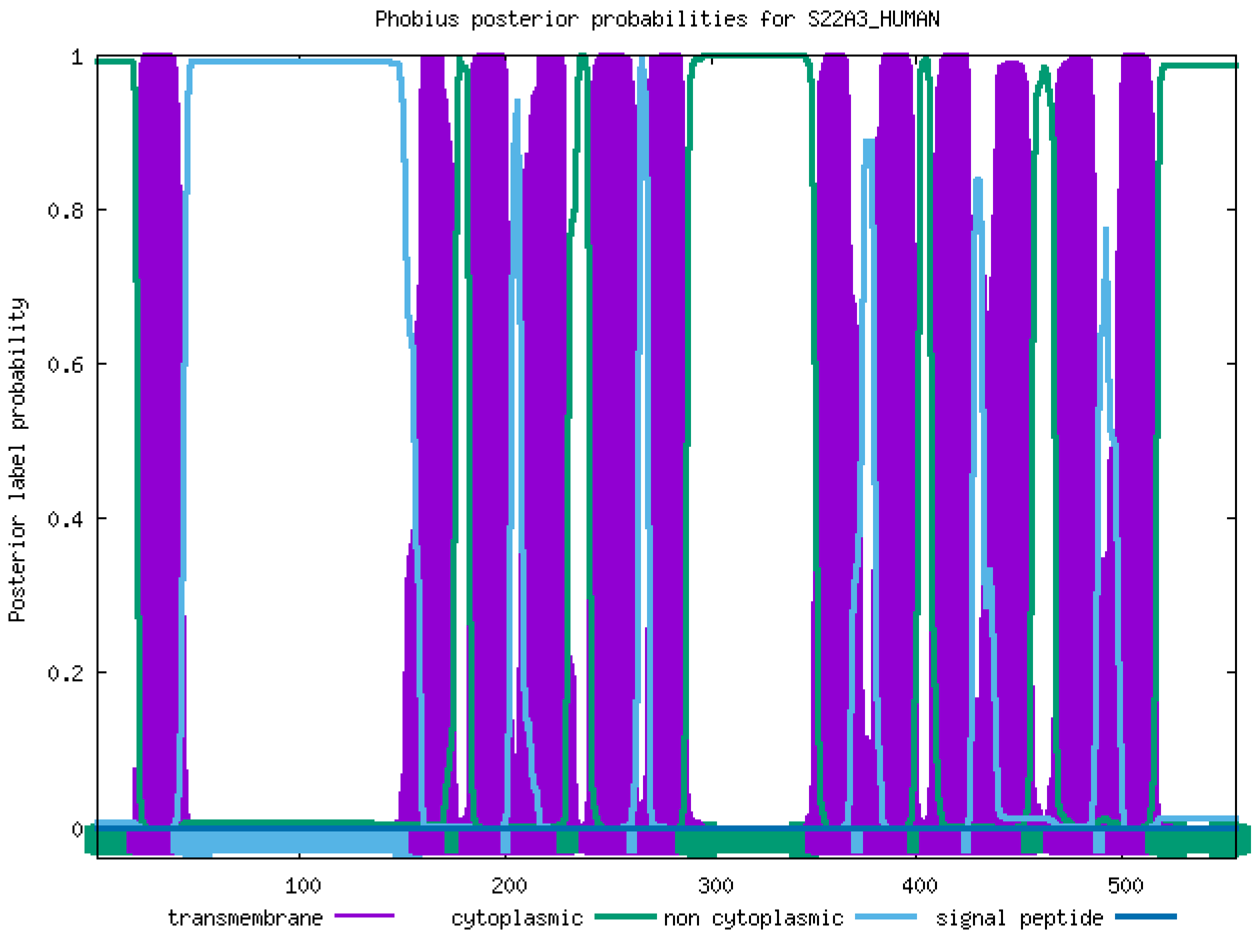

The probability data used in the plot is found here, and the gnuplot script is here.

Prediction of S26A9_HUMAN

```
ID   S26A9_HUMAN
FT   TOPO_DOM      1     45       CYTOPLASMIC.
FT   TRANSMEM     46     63
FT   TOPO_DOM     64     89       NON CYTOPLASMIC.
FT   TRANSMEM     90    117
FT   TOPO_DOM    118    123       CYTOPLASMIC.
FT   TRANSMEM    124    143
FT   TOPO_DOM    144    173       NON CYTOPLASMIC.
FT   TRANSMEM    174    199
FT   TOPO_DOM    200    210       CYTOPLASMIC.
FT   TRANSMEM    211    231
FT   TOPO_DOM    232    250       NON CYTOPLASMIC.
FT   TRANSMEM    251    269
FT   TOPO_DOM    270    336       CYTOPLASMIC.
FT   TRANSMEM    337    356
FT   TOPO_DOM    357    375       NON CYTOPLASMIC.
FT   TRANSMEM    376    397
FT   TOPO_DOM    398    408       CYTOPLASMIC.
FT   TRANSMEM    409    427
FT   TOPO_DOM    428    464       NON CYTOPLASMIC.
FT   TRANSMEM    465    495
FT   TOPO_DOM    496    791       CYTOPLASMIC.
//
```

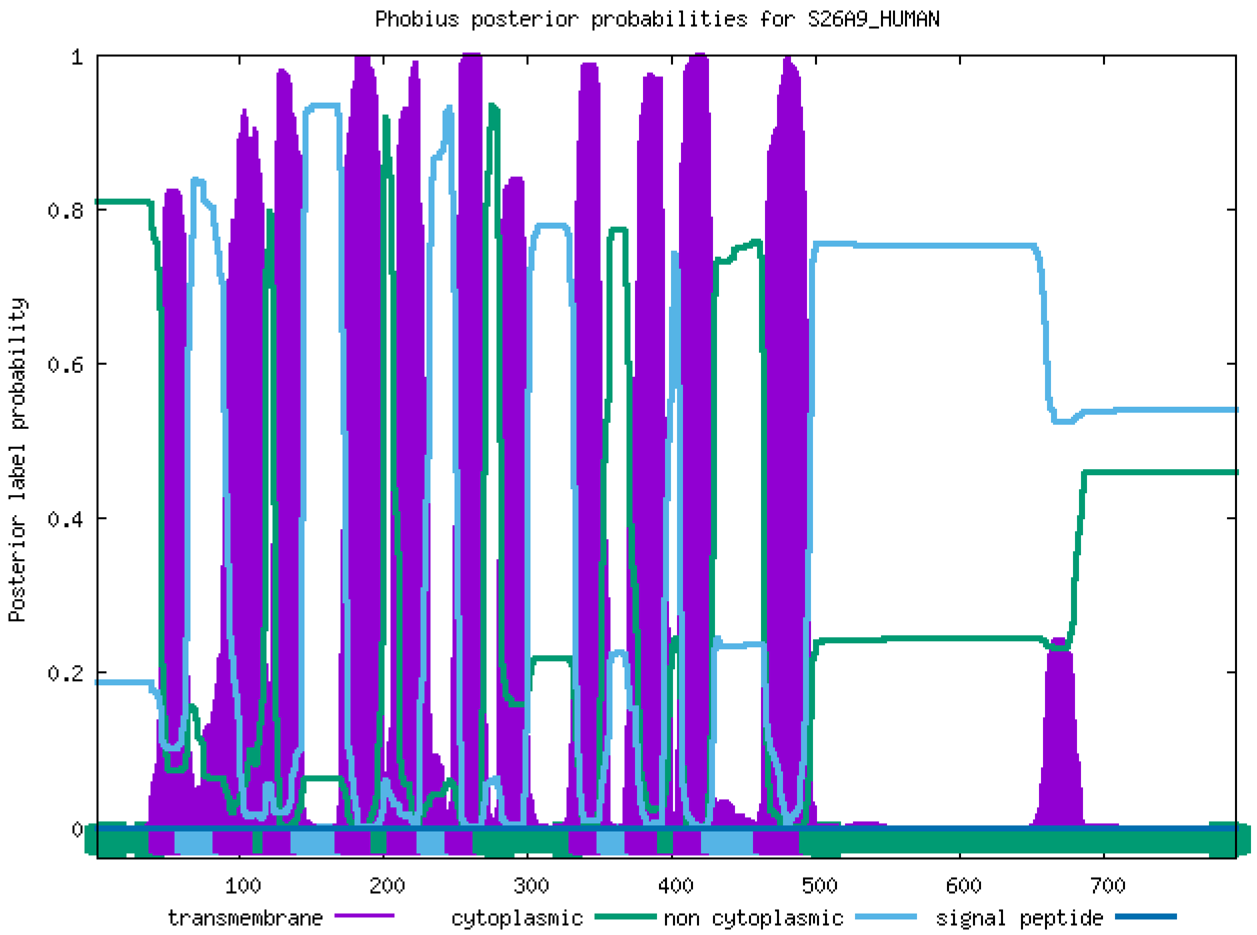

The probability data used in the plot is found here, and the gnuplot script is here.

Prediction of S26A5_HUMAN

```
ID    S26A5_HUMAN
FT    TOPO_DOM       1      98       NON CYTOPLASMIC.
FT    TRANSMEM      99     125
FT    TOPO_DOM     126     131       CYTOPLASMIC.
FT    TRANSMEM     132     152
FT    TOPO_DOM     153     183       NON CYTOPLASMIC.
FT    TRANSMEM     184     205
FT    TOPO_DOM     206     211       CYTOPLASMIC.
FT    TRANSMEM     212     230
FT    TOPO_DOM     231     255       NON CYTOPLASMIC.
FT    TRANSMEM     256     274
FT    TOPO_DOM     275     285       CYTOPLASMIC.
FT    TRANSMEM     286     307
FT    TOPO_DOM     308     334       NON CYTOPLASMIC.
FT    TRANSMEM     335     355
FT    TOPO_DOM     356     374       CYTOPLASMIC.
FT    TRANSMEM     375     399
FT    TOPO_DOM     400     410       NON CYTOPLASMIC.
FT    TRANSMEM     411     432
FT    TOPO_DOM     433     438       CYTOPLASMIC.
FT    TRANSMEM     439     456
FT    TOPO_DOM     457     475       NON CYTOPLASMIC.
FT    TRANSMEM     476     501
FT    TOPO_DOM     502     744       CYTOPLASMIC.
//
```

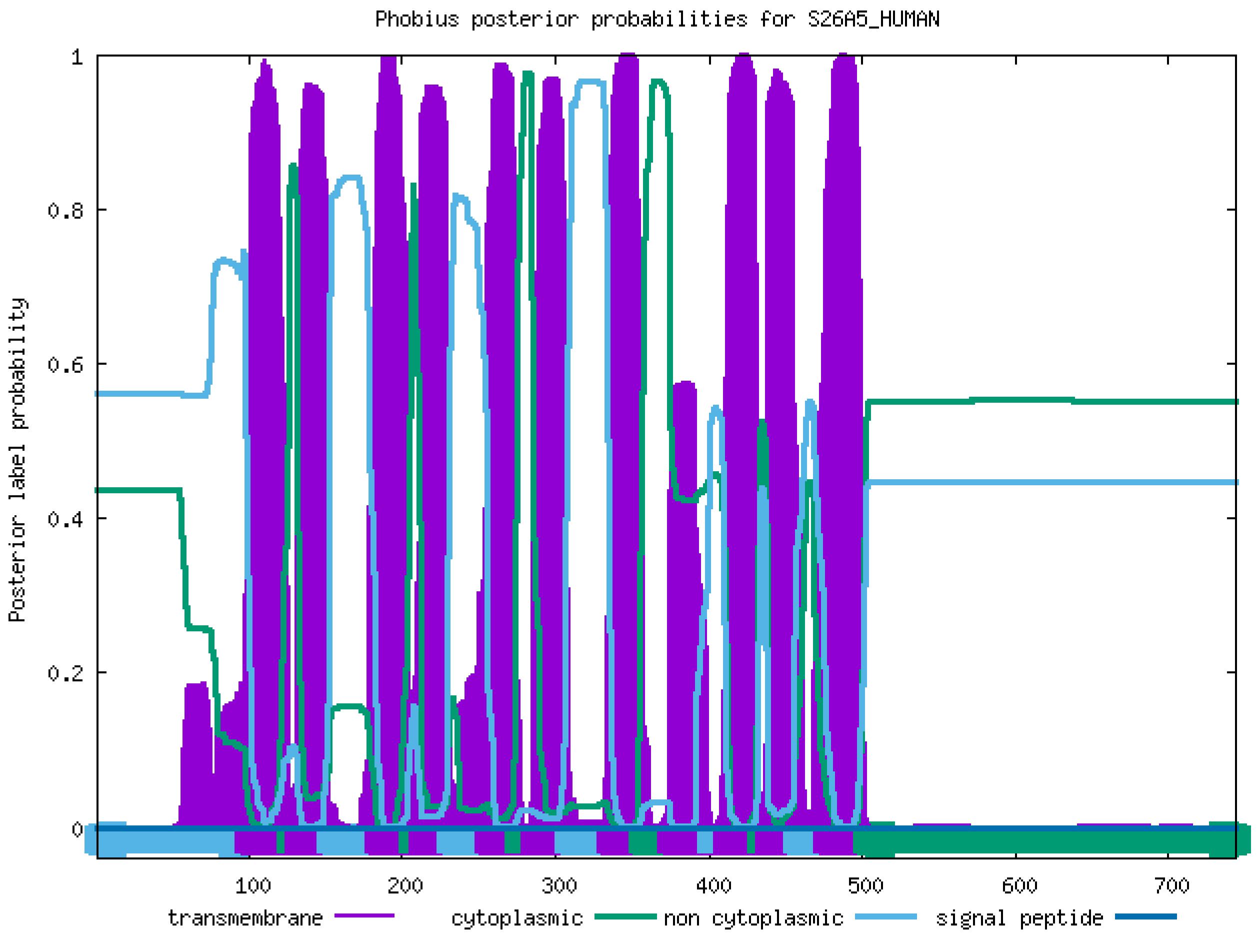

The probability data used in the plot is found here, and the gnuplot script is here.

Prediction of S28A3_HUMAN

```
ID   S28A3_HUMAN
FT   TOPO_DOM      1    103       CYTOPLASMIC.
FT   TRANSMEM    104    125
FT   TOPO_DOM    126    130       NON CYTOPLASMIC.
FT   TRANSMEM    131    149
FT   TOPO_DOM    150    169       CYTOPLASMIC.
FT   TRANSMEM    170    191
FT   TOPO_DOM    192    196       NON CYTOPLASMIC.
FT   TRANSMEM    197    217
FT   TOPO_DOM    218    223       CYTOPLASMIC.
FT   TRANSMEM    224    244
FT   TOPO_DOM    245    282       NON CYTOPLASMIC.
FT   TRANSMEM    283    305
FT   TOPO_DOM    306    316       CYTOPLASMIC.
FT   TRANSMEM    317    340
FT   TOPO_DOM    341    359       NON CYTOPLASMIC.
FT   TRANSMEM    360    382
FT   TOPO_DOM    383    388       CYTOPLASMIC.
FT   TRANSMEM    389    408
FT   TOPO_DOM    409    445       NON CYTOPLASMIC.
FT   TRANSMEM    446    468
FT   TOPO_DOM    469    479       CYTOPLASMIC.
FT   TRANSMEM    480    499
FT   TOPO_DOM    500    554       NON CYTOPLASMIC.
FT   TRANSMEM    555    579
FT   TOPO_DOM    580    590       CYTOPLASMIC.
FT   TRANSMEM    591    612
FT   TOPO_DOM    613    691       NON CYTOPLASMIC.
//
```

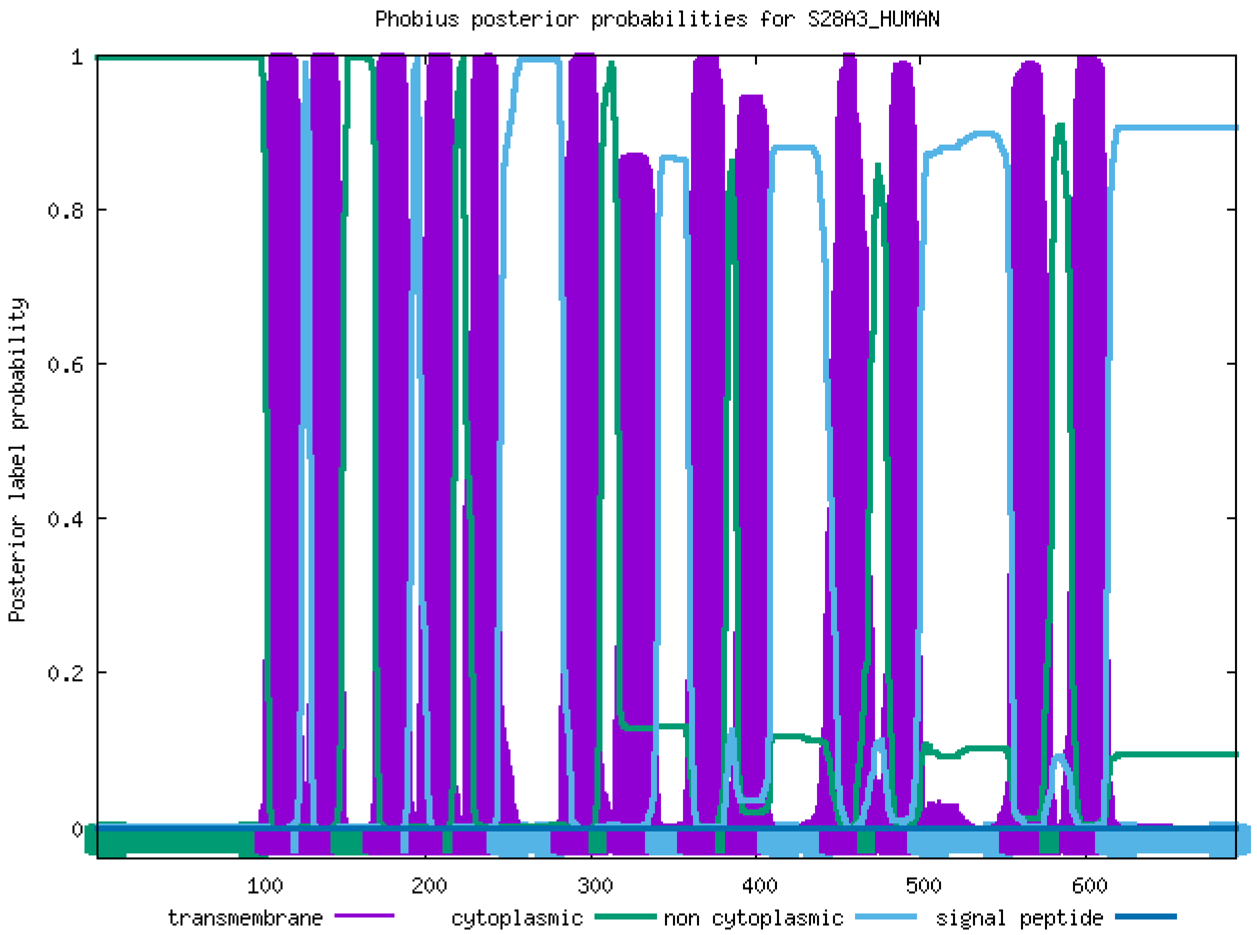

The probability data used in the plot is found here, and the gnuplot script is here.

Prediction of S29A1_HUMAN

```
ID   S29A1_HUMAN
FT   TOPO_DOM      1     11       CYTOPLASMIC.
FT   TRANSMEM     12     32
FT   TOPO_DOM     33     78       NON CYTOPLASMIC.
FT   TRANSMEM     79     98
FT   TOPO_DOM     99    109       CYTOPLASMIC.
FT   TRANSMEM    110    130
FT   TOPO_DOM    131    135       NON CYTOPLASMIC.
FT   TRANSMEM    136    169
FT   TOPO_DOM    170    180       CYTOPLASMIC.
FT   TRANSMEM    181    199
FT   TOPO_DOM    200    204       NON CYTOPLASMIC.
FT   TRANSMEM    205    226
FT   TOPO_DOM    227    288       CYTOPLASMIC.
FT   TRANSMEM    289    307
FT   TOPO_DOM    308    326       NON CYTOPLASMIC.
FT   TRANSMEM    327    344
FT   TOPO_DOM    345    364       CYTOPLASMIC.
FT   TRANSMEM    365    382
FT   TOPO_DOM    383    393       NON CYTOPLASMIC.
FT   TRANSMEM    394    418
FT   TOPO_DOM    419    429       CYTOPLASMIC.
FT   TRANSMEM    430    452
FT   TOPO_DOM    453    456       NON CYTOPLASMIC.
//
```

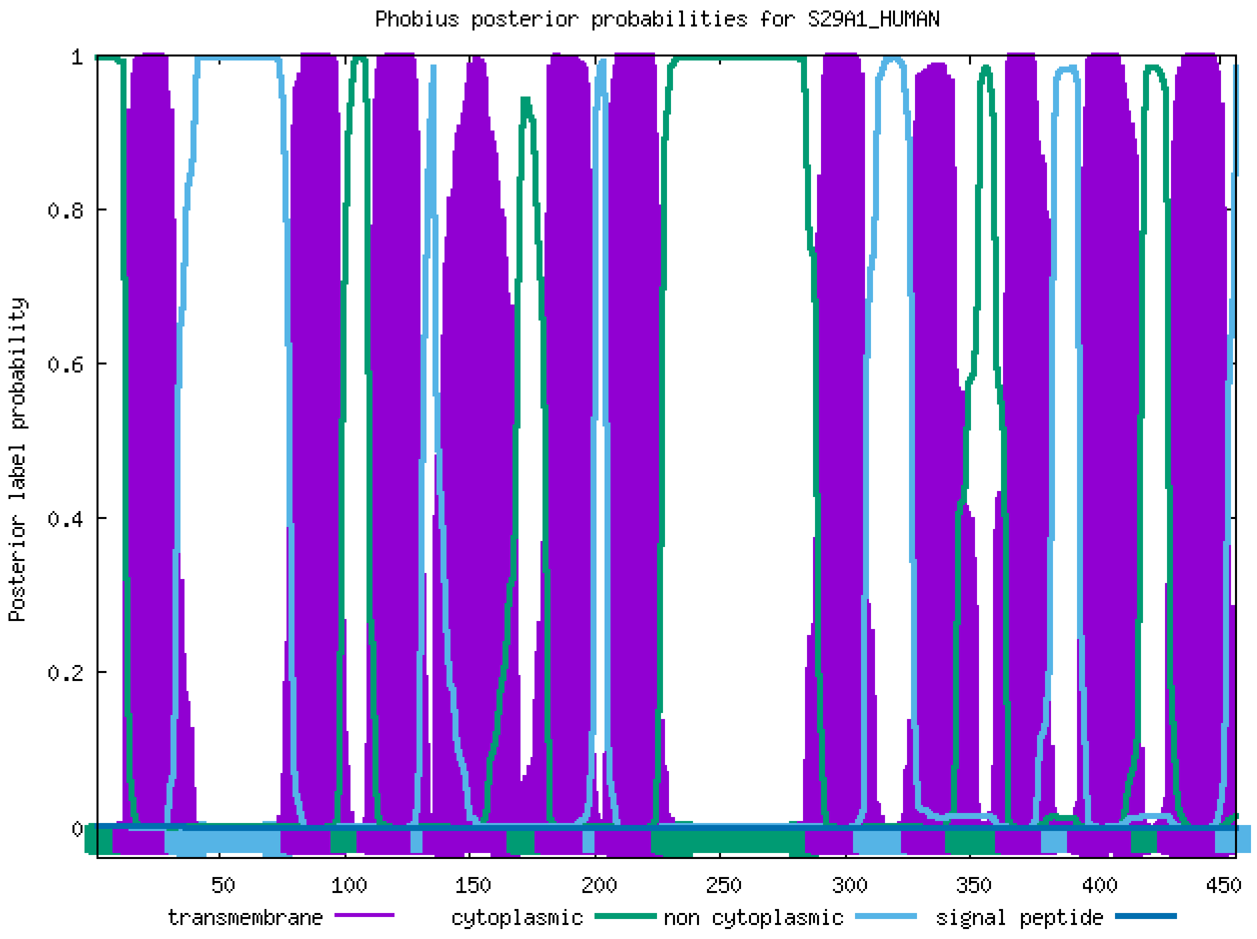

The probability data used in the plot is found here, and the gnuplot script is here.

Prediction of ZNT8_HUMAN

```
ID    ZNT8_HUMAN
FT    TOPO_DOM      1      73       CYTOPLASMIC.
FT    TRANSMEM     74      98
FT    TOPO_DOM     99     103       NON CYTOPLASMIC.
FT    TRANSMEM    104     123
FT    TOPO_DOM    124     143       CYTOPLASMIC.
FT    TRANSMEM    144     161
FT    TOPO_DOM    162     172       NON CYTOPLASMIC.
FT    TRANSMEM    173     196
FT    TOPO_DOM    197     216       CYTOPLASMIC.
FT    TRANSMEM    217     240
FT    TOPO_DOM    241     245       NON CYTOPLASMIC.
FT    TRANSMEM    246     267
FT    TOPO_DOM    268     369       CYTOPLASMIC.
//
```

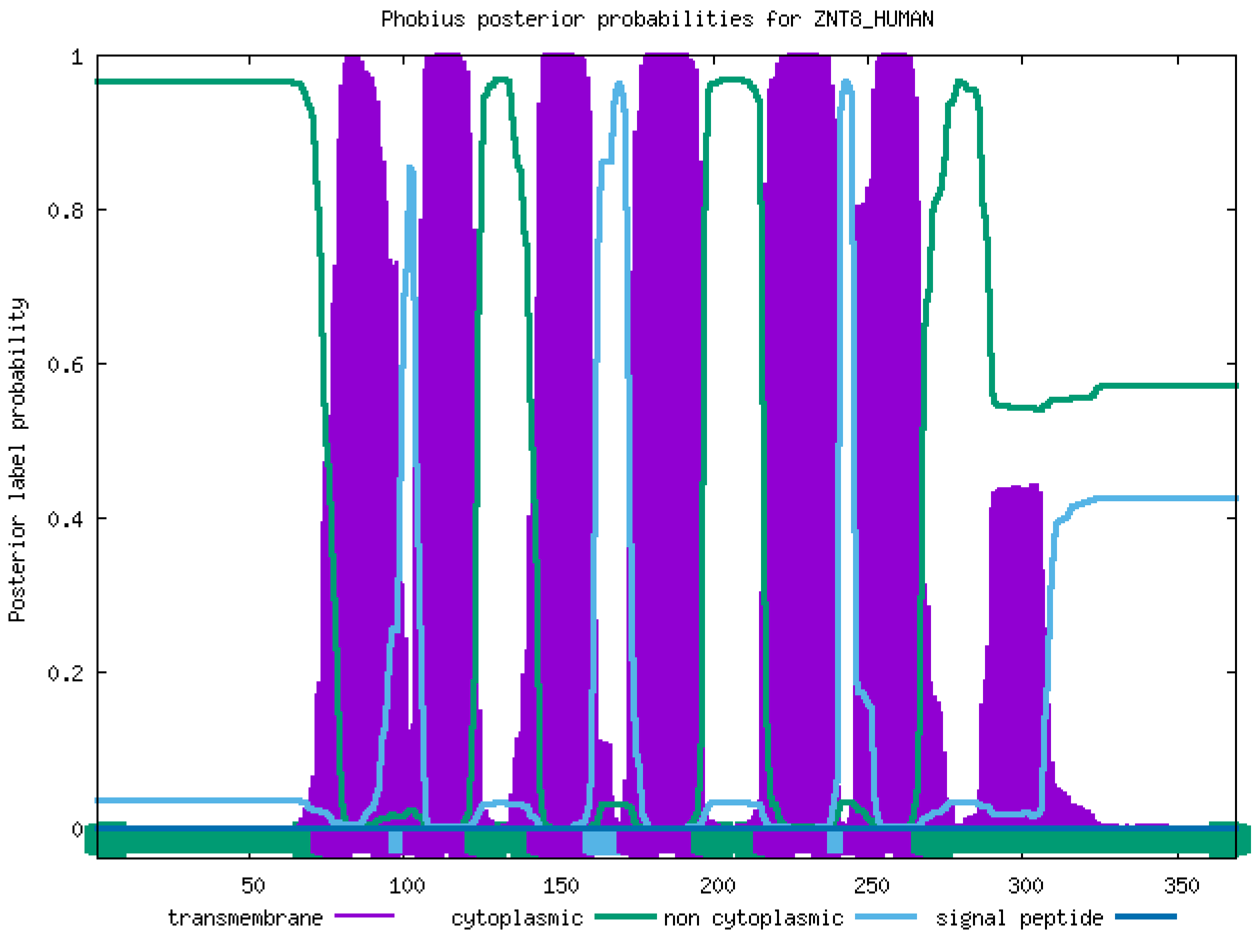

The probability data used in the plot is found here, and the gnuplot script is here.

Prediction of CTL1_HUMAN

```
ID    CTL1_HUMAN
FT    TOPO_DOM      1     28       NON CYTOPLASMIC.
FT    TRANSMEM     29     51
FT    TOPO_DOM     52    213       CYTOPLASMIC.
FT    TRANSMEM    214    233
FT    TOPO_DOM    234    238       NON CYTOPLASMIC.
FT    TRANSMEM    239    263
FT    TOPO_DOM    264    288       CYTOPLASMIC.
FT    TRANSMEM    289    308
FT    TOPO_DOM    309    313       NON CYTOPLASMIC.
FT    TRANSMEM    314    335
FT    TOPO_DOM    336    341       CYTOPLASMIC.
FT    TRANSMEM    342    363
FT    TOPO_DOM    364    382       NON CYTOPLASMIC.
FT    TRANSMEM    383    416
FT    TOPO_DOM    417    537       CYTOPLASMIC.
FT    TRANSMEM    538    559
FT    TOPO_DOM    560    564       NON CYTOPLASMIC.
FT    TRANSMEM    565    587
FT    TOPO_DOM    588    657       CYTOPLASMIC.
//
```

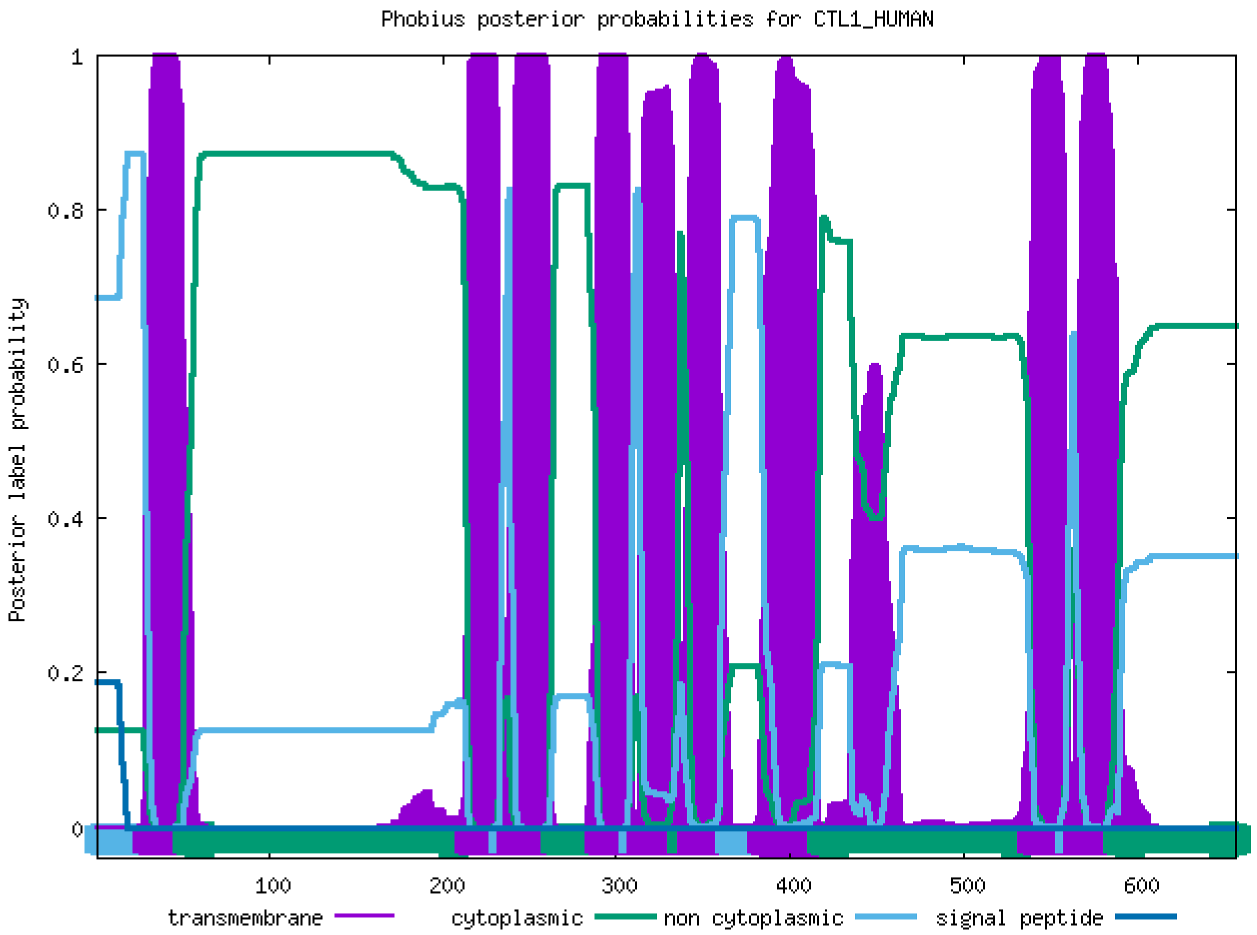

The probability data used in the plot is found here, and the gnuplot script is here.

Prediction of S6A20_HUMAN

```
ID    S6A20_HUMAN
FT    TOPO_DOM      1     11       CYTOPLASMIC.
FT    TRANSMEM     12     31
FT    TOPO_DOM     32     42       NON CYTOPLASMIC.
FT    TRANSMEM     43     61
FT    TOPO_DOM     62     81       CYTOPLASMIC.
FT    TRANSMEM     82    102
FT    TOPO_DOM    103    167       NON CYTOPLASMIC.
FT    TRANSMEM    168    186
FT    TOPO_DOM    187    194       CYTOPLASMIC.
FT    TRANSMEM    195    220
FT    TOPO_DOM    221    239       NON CYTOPLASMIC.
FT    TRANSMEM    240    265
FT    TOPO_DOM    266    276       CYTOPLASMIC.
FT    TRANSMEM    277    298
FT    TOPO_DOM    299    392       NON CYTOPLASMIC.
FT    TRANSMEM    393    415
FT    TOPO_DOM    416    435       CYTOPLASMIC.
FT    TRANSMEM    436    457
FT    TOPO_DOM    458    462       NON CYTOPLASMIC.
FT    TRANSMEM    463    486
FT    TOPO_DOM    487    506       CYTOPLASMIC.
FT    TRANSMEM    507    531
FT    TOPO_DOM    532    550       NON CYTOPLASMIC.
FT    TRANSMEM    551    575
FT    TOPO_DOM    576    592       CYTOPLASMIC.
//
```

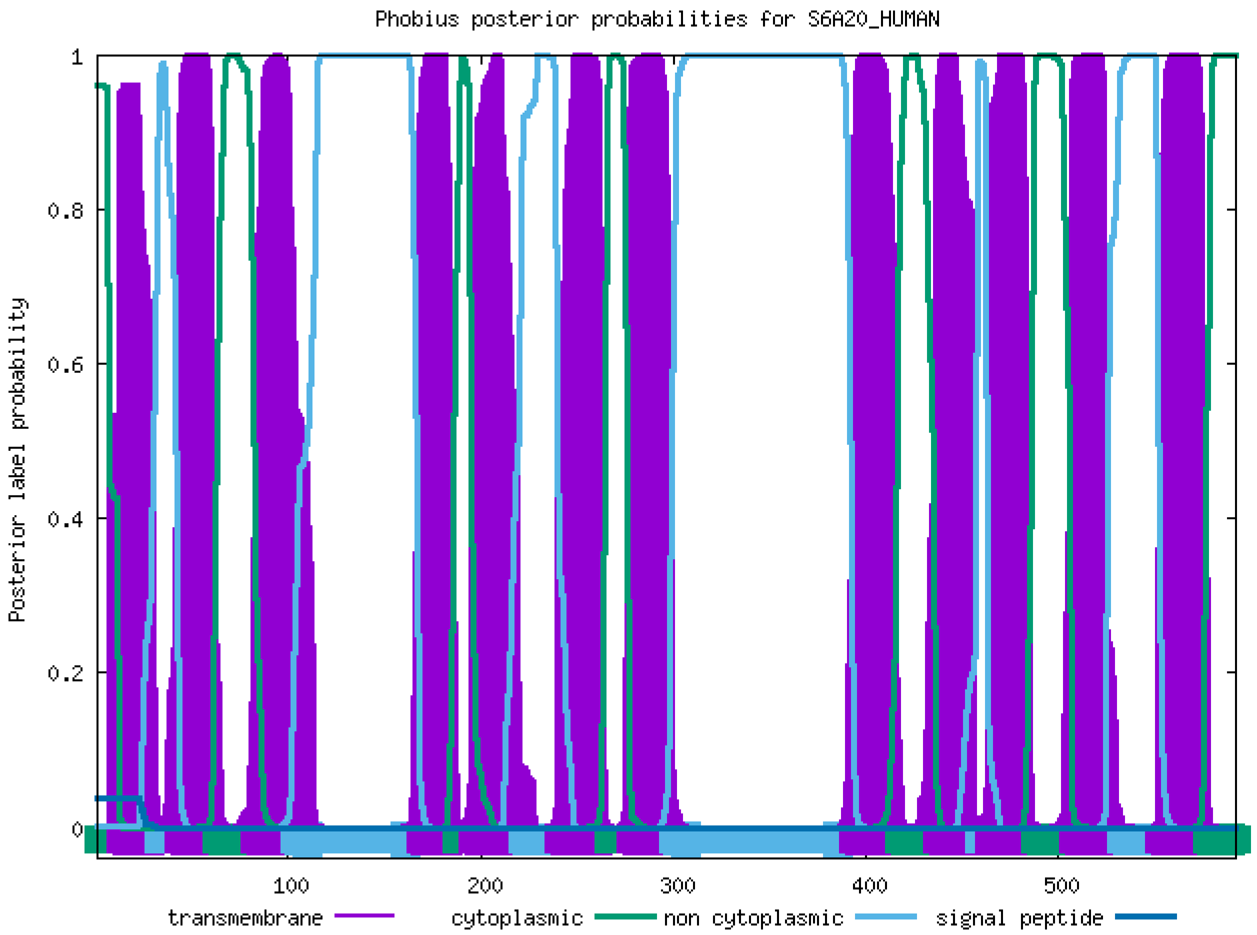

The probability data used in the plot is found here, and the gnuplot script is here.

Prediction of SO1B1_HUMAN

```
ID   SO1B1_HUMAN
FT   TOPO_DOM      1     28       CYTOPLASMIC.
FT   TRANSMEM     29     48
FT   TOPO_DOM     49     67       NON CYTOPLASMIC.
FT   TRANSMEM     68     89
FT   TOPO_DOM     90     95       CYTOPLASMIC.
FT   TRANSMEM     96    117
FT   TOPO_DOM    118    169       NON CYTOPLASMIC.
FT   TRANSMEM    170    195
FT   TOPO_DOM    196    206       CYTOPLASMIC.
FT   TRANSMEM    207    230
FT   TOPO_DOM    231    257       NON CYTOPLASMIC.
FT   TRANSMEM    258    279
FT   TOPO_DOM    280    336       CYTOPLASMIC.
FT   TRANSMEM    337    356
FT   TOPO_DOM    357    375       NON CYTOPLASMIC.
FT   TRANSMEM    376    395
FT   TOPO_DOM    396    406       CYTOPLASMIC.
FT   TRANSMEM    407    428
FT   TOPO_DOM    429    533       NON CYTOPLASMIC.
FT   TRANSMEM    534    560
FT   TOPO_DOM    561    571       CYTOPLASMIC.
FT   TRANSMEM    572    595
FT   TOPO_DOM    596    623       NON CYTOPLASMIC.
FT   TRANSMEM    624    646
FT   TOPO_DOM    647    691       CYTOPLASMIC.
//
```

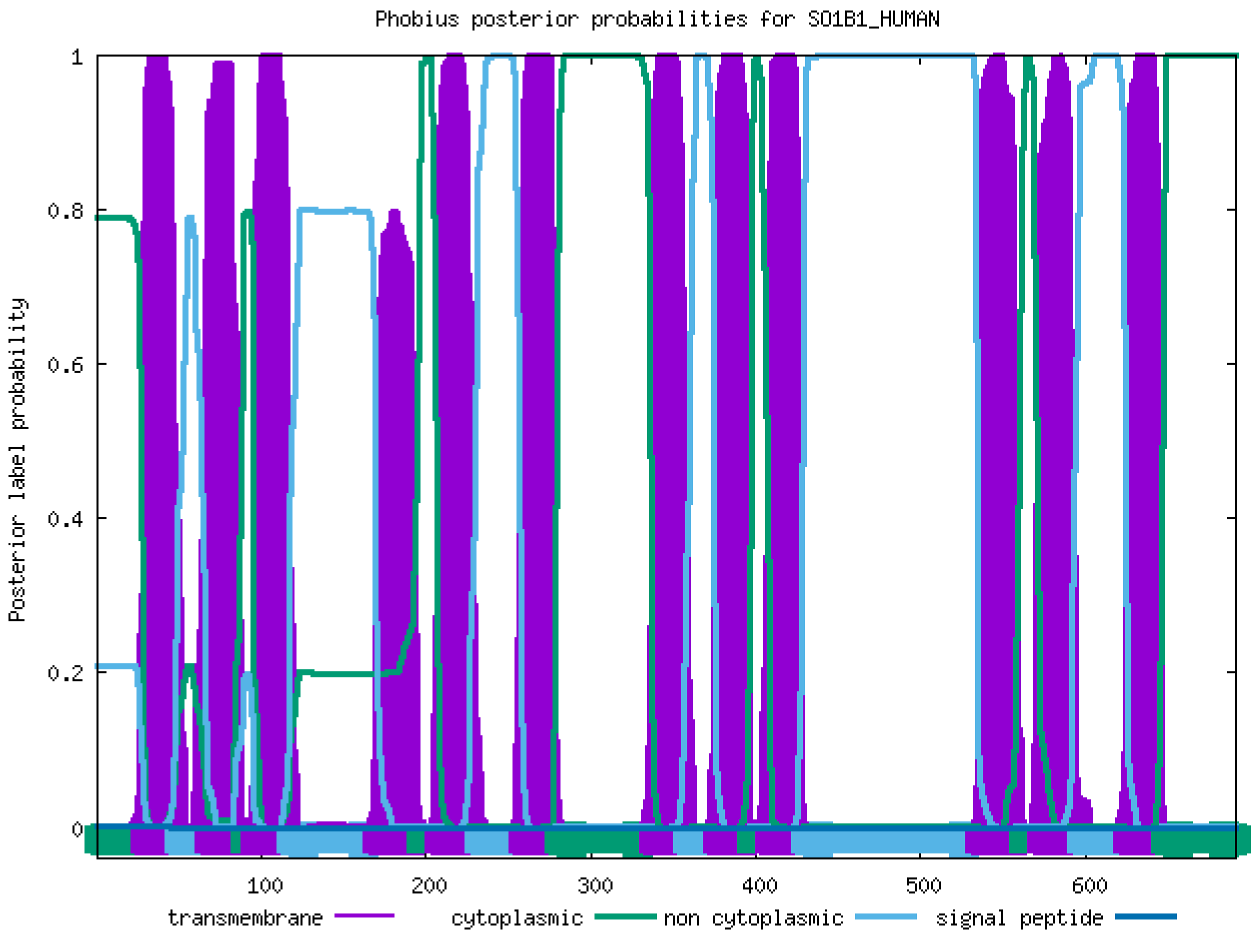

The probability data used in the plot is found here, and the gnuplot script is here.

Prediction of SO1B3_HUMAN

```
ID   SO1B3_HUMAN
FT   TOPO_DOM      1     28       CYTOPLASMIC.
FT   TRANSMEM     29     52
FT   TOPO_DOM     53     63       NON CYTOPLASMIC.
FT   TRANSMEM     64     84
FT   TOPO_DOM     85     95       CYTOPLASMIC.
FT   TRANSMEM     96    117
FT   TOPO_DOM    118    169       NON CYTOPLASMIC.
FT   TRANSMEM    170    195
FT   TOPO_DOM    196    206       CYTOPLASMIC.
FT   TRANSMEM    207    235
FT   TOPO_DOM    236    254       NON CYTOPLASMIC.
FT   TRANSMEM    255    279
FT   TOPO_DOM    280    336       CYTOPLASMIC.
FT   TRANSMEM    337    356
FT   TOPO_DOM    357    375       NON CYTOPLASMIC.
FT   TRANSMEM    376    397
FT   TOPO_DOM    398    403       CYTOPLASMIC.
FT   TRANSMEM    404    425
FT   TOPO_DOM    426    533       NON CYTOPLASMIC.
FT   TRANSMEM    534    558
FT   TOPO_DOM    559    569       CYTOPLASMIC.
FT   TRANSMEM    570    591
FT   TOPO_DOM    592    623       NON CYTOPLASMIC.
FT   TRANSMEM    624    647
FT   TOPO_DOM    648    702       CYTOPLASMIC.
//
```

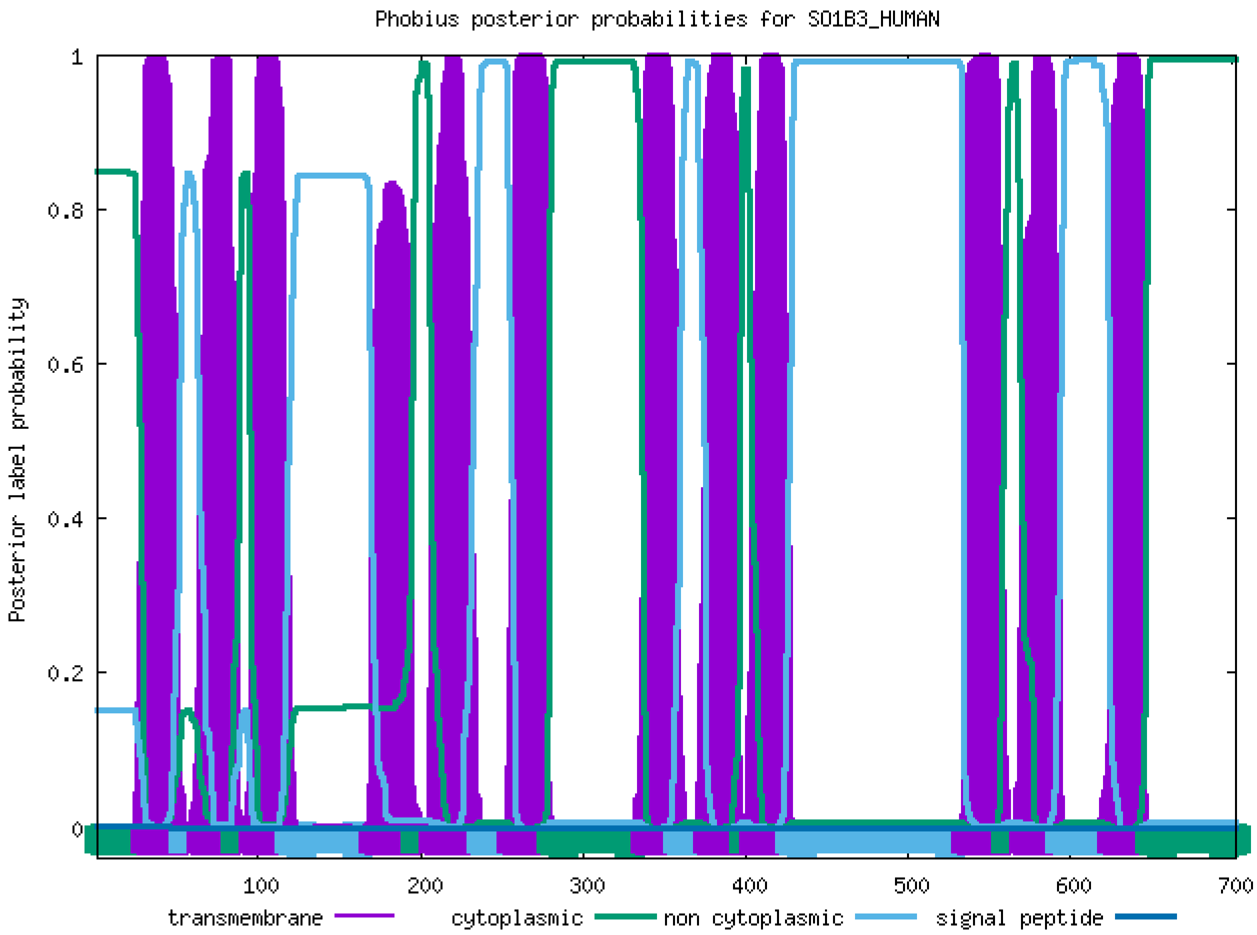

The probability data used in the plot is found here, and the gnuplot script is here.

Prediction of S13A1_HUMAN

```
ID   S13A1_HUMAN
FT   TOPO_DOM      1     11       CYTOPLASMIC.
FT   TRANSMEM     12     32
FT   TOPO_DOM     33     37       NON CYTOPLASMIC.
FT   TRANSMEM     38     61
FT   TOPO_DOM     62     80       CYTOPLASMIC.
FT   TRANSMEM     81     99
FT   TOPO_DOM    100    118       NON CYTOPLASMIC.
FT   TRANSMEM    119    138
FT   TOPO_DOM    139    238       CYTOPLASMIC.
FT   TRANSMEM    239    265
FT   TOPO_DOM    266    284       NON CYTOPLASMIC.
FT   TRANSMEM    285    307
FT   TOPO_DOM    308    342       CYTOPLASMIC.
FT   TRANSMEM    343    362
FT   TOPO_DOM    363    381       NON CYTOPLASMIC.
FT   TRANSMEM    382    399
FT   TOPO_DOM    400    469       CYTOPLASMIC.
FT   TRANSMEM    470    501
FT   TOPO_DOM    502    512       NON CYTOPLASMIC.
FT   TRANSMEM    513    532
FT   TOPO_DOM    533    552       CYTOPLASMIC.
FT   TRANSMEM    553    574
FT   TOPO_DOM    575    595       NON CYTOPLASMIC.
//
```

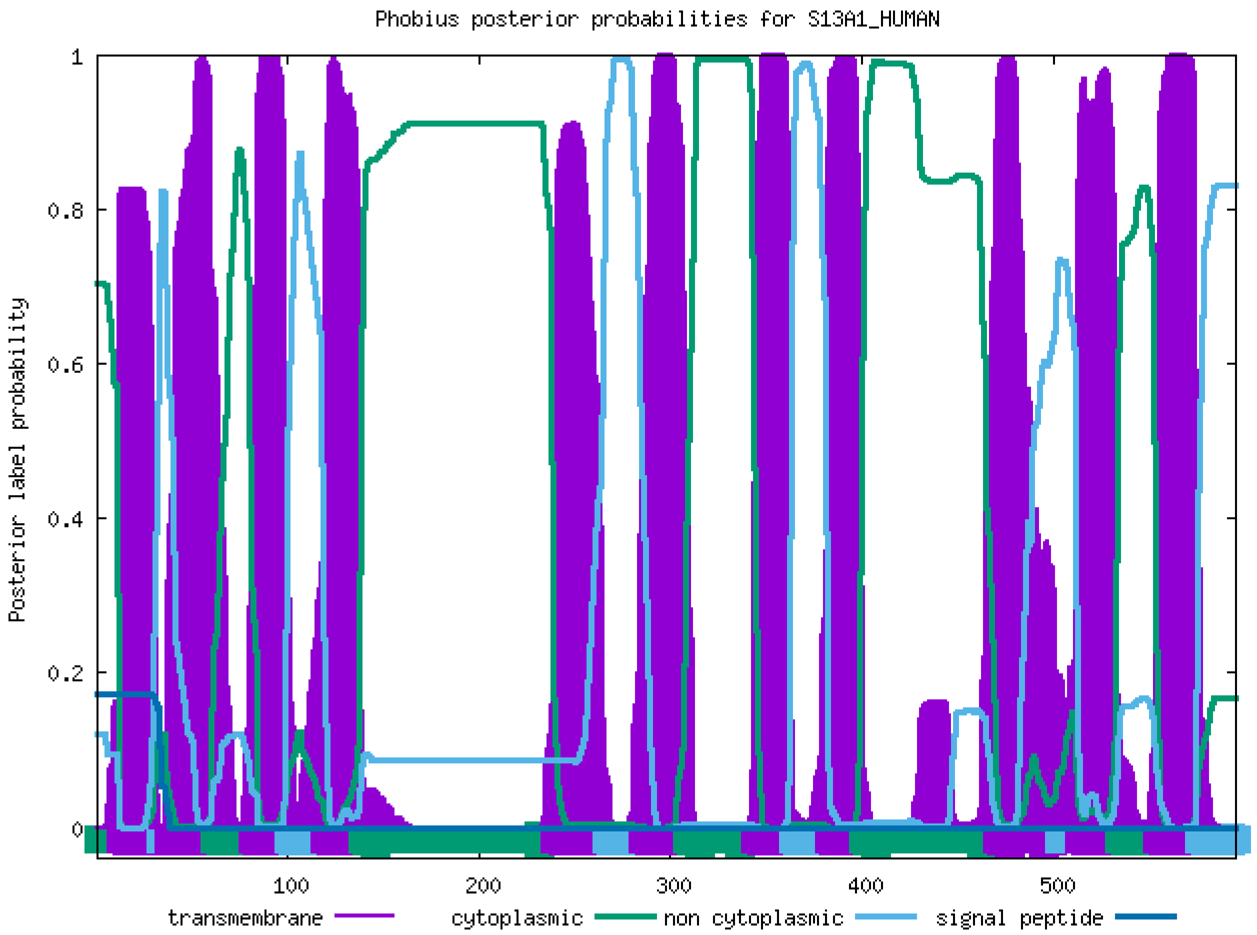

The probability data used in the plot is found here, and the gnuplot script is here.

Prediction of S13A2_HUMAN

```
ID   S13A2_HUMAN
FT   TOPO_DOM      1     11       NON CYTOPLASMIC.
FT   TRANSMEM     12     31
FT   TOPO_DOM     32     37       CYTOPLASMIC.
FT   TRANSMEM     38     67
FT   TOPO_DOM     68     86       NON CYTOPLASMIC.
FT   TRANSMEM     87    105
FT   TOPO_DOM    106    116       CYTOPLASMIC.
FT   TRANSMEM    117    139
FT   TOPO_DOM    140    268       NON CYTOPLASMIC.
FT   TRANSMEM    269    294
FT   TOPO_DOM    295    326       CYTOPLASMIC.
FT   TRANSMEM    327    346
FT   TOPO_DOM    347    365       NON CYTOPLASMIC.
FT   TRANSMEM    366    384
FT   TOPO_DOM    385    416       CYTOPLASMIC.
FT   TRANSMEM    417    432
FT   TOPO_DOM    433    451       NON CYTOPLASMIC.
FT   TRANSMEM    452    470
FT   TOPO_DOM    471    481       CYTOPLASMIC.
FT   TRANSMEM    482    501
FT   TOPO_DOM    502    506       NON CYTOPLASMIC.
FT   TRANSMEM    507    529
FT   TOPO_DOM    530    540       CYTOPLASMIC.
FT   TRANSMEM    541    560
FT   TOPO_DOM    561    592       NON CYTOPLASMIC.
//
```

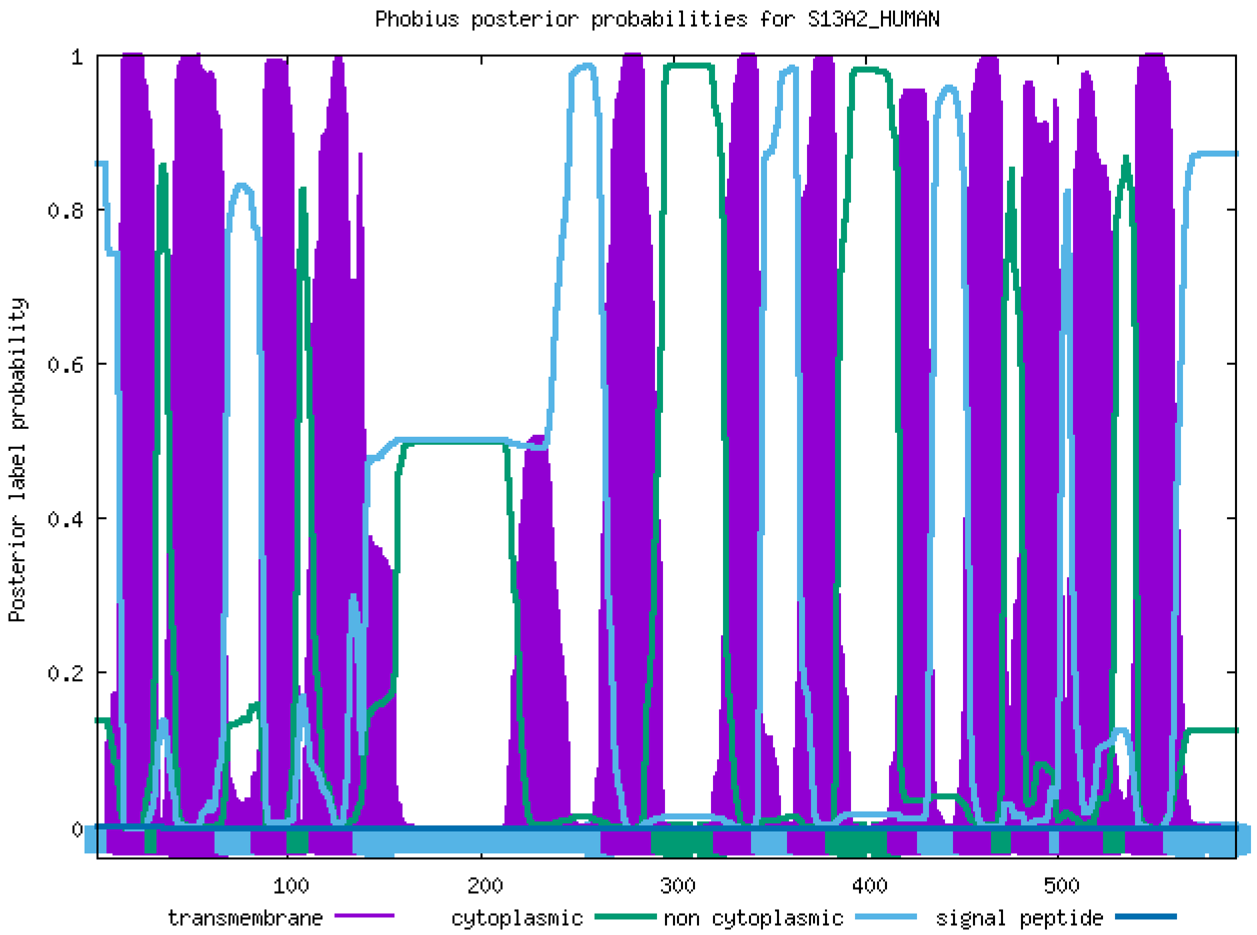

The probability data used in the plot is found here, and the gnuplot script is here.

Prediction of SC5A7_HUMAN

```
ID   SC5A7_HUMAN
FT   TOPO_DOM      1      5       NON CYTOPLASMIC.
FT   TRANSMEM      6     27
FT   TOPO_DOM     28     47       CYTOPLASMIC.
FT   TRANSMEM     48     68
FT   TOPO_DOM     69     79       NON CYTOPLASMIC.
FT   TRANSMEM     80    103
FT   TOPO_DOM    104    128       CYTOPLASMIC.
FT   TRANSMEM    129    157
FT   TOPO_DOM    158    162       NON CYTOPLASMIC.
FT   TRANSMEM    163    182
FT   TOPO_DOM    183    188       CYTOPLASMIC.
FT   TRANSMEM    189    207
FT   TOPO_DOM    208    236       NON CYTOPLASMIC.
FT   TRANSMEM    237    257
FT   TOPO_DOM    258    268       CYTOPLASMIC.
FT   TRANSMEM    269    298
FT   TOPO_DOM    299    317       NON CYTOPLASMIC.
FT   TRANSMEM    318    344
FT   TOPO_DOM    345    376       CYTOPLASMIC.
FT   TRANSMEM    377    398
FT   TOPO_DOM    399    403       NON CYTOPLASMIC.
FT   TRANSMEM    404    427
FT   TOPO_DOM    428    438       CYTOPLASMIC.
FT   TRANSMEM    439    462
FT   TOPO_DOM    463    481       NON CYTOPLASMIC.
FT   TRANSMEM    482    502
FT   TOPO_DOM    503    580       CYTOPLASMIC.
//
```

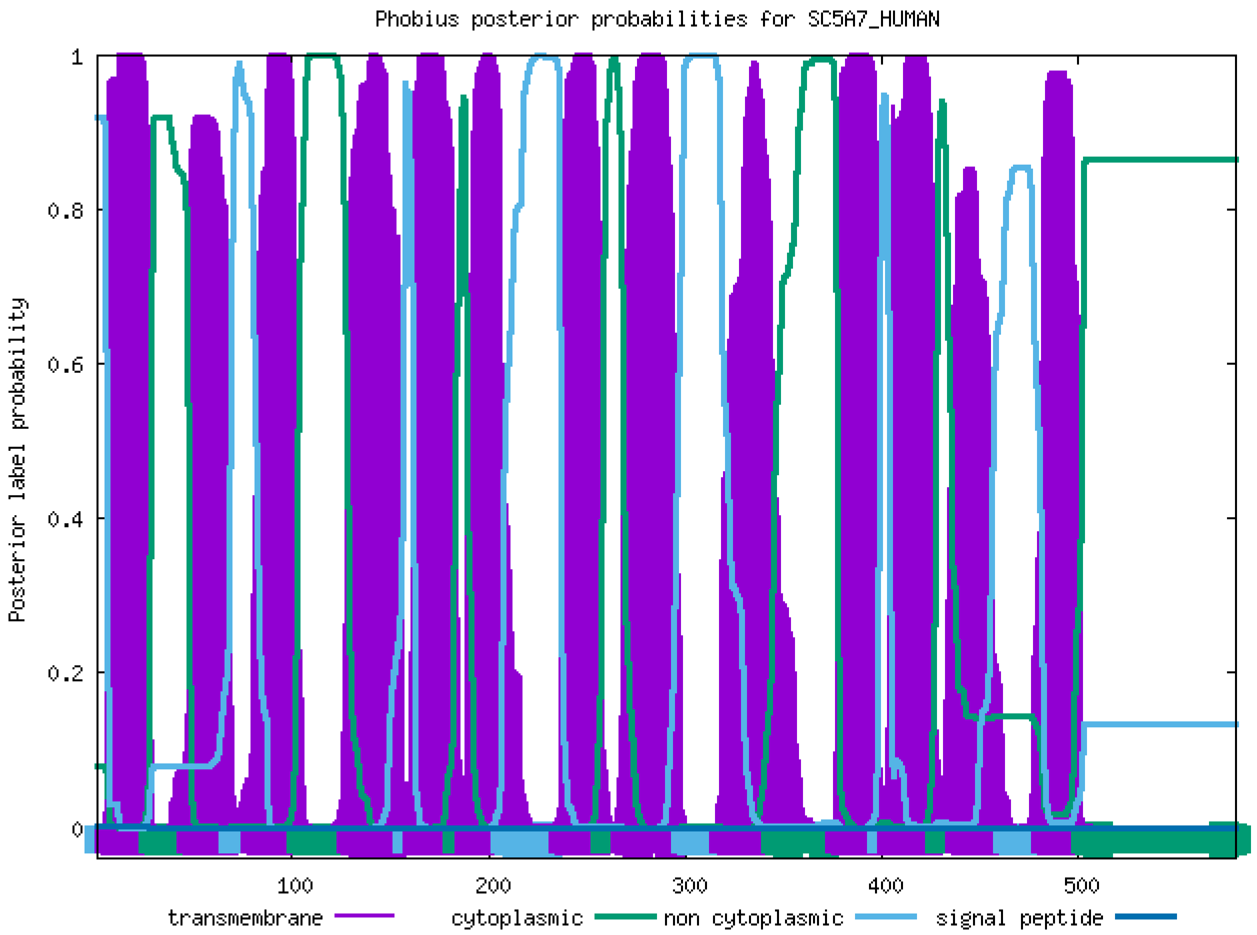

The probability data used in the plot is found here, and the gnuplot script is here.

Prediction of AAA1_HUMAN

```
ID    AAA1_HUMAN
FT    TOPO_DOM      1     39       CYTOPLASMIC.
FT    TRANSMEM     40     60
FT    TOPO_DOM     61     71       NON CYTOPLASMIC.
FT    TRANSMEM     72     96
FT    TOPO_DOM     97    116       CYTOPLASMIC.
FT    TRANSMEM    117    143
FT    TOPO_DOM    144    162       NON CYTOPLASMIC.
FT    TRANSMEM    163    183
FT    TOPO_DOM    184    194       CYTOPLASMIC.
FT    TRANSMEM    195    214
FT    TOPO_DOM    215    233       NON CYTOPLASMIC.
FT    TRANSMEM    234    255
FT    TOPO_DOM    256    266       CYTOPLASMIC.
FT    TRANSMEM    267    290
FT    TOPO_DOM    291    309       NON CYTOPLASMIC.
FT    TRANSMEM    310    336
FT    TOPO_DOM    337    356       CYTOPLASMIC.
FT    TRANSMEM    357    381
FT    TOPO_DOM    382    386       NON CYTOPLASMIC.
FT    TRANSMEM    387    410
FT    TOPO_DOM    411    421       CYTOPLASMIC.
FT    TRANSMEM    422    444
FT    TOPO_DOM    445    449       NON CYTOPLASMIC.
FT    TRANSMEM    450    469
FT    TOPO_DOM    470    523       CYTOPLASMIC.
//
```

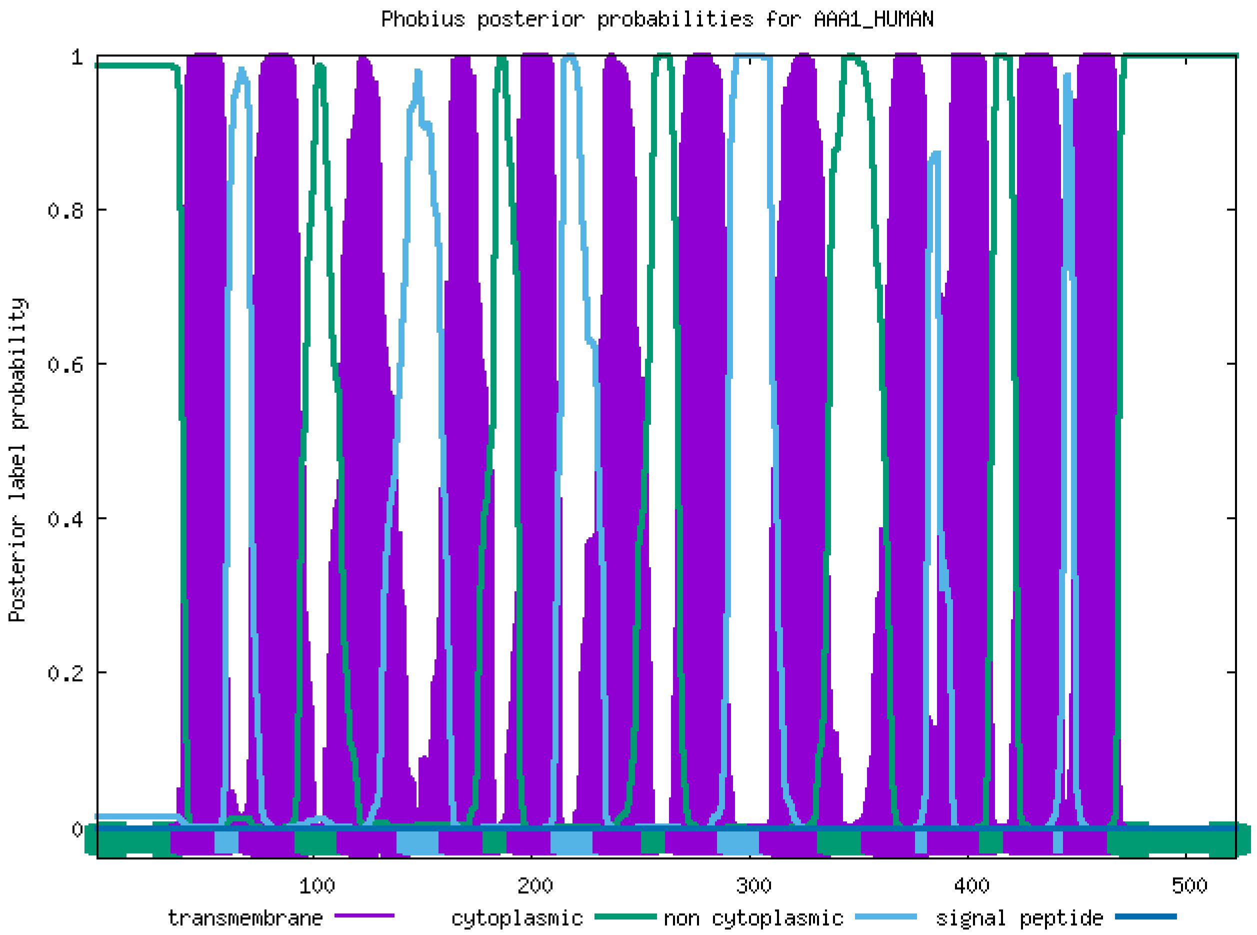

The probability data used in the plot is found here, and the gnuplot script is here.

Prediction of B3AT_HUMAN

```
ID   B3AT_HUMAN
FT   TOPO_DOM      1    404       NON CYTOPLASMIC.
FT   TRANSMEM    405    428
FT   TOPO_DOM    429    448       CYTOPLASMIC.
FT   TRANSMEM    449    471
FT   TOPO_DOM    472    490       NON CYTOPLASMIC.
FT   TRANSMEM    491    516
FT   TOPO_DOM    517    522       CYTOPLASMIC.
FT   TRANSMEM    523    545
FT   TOPO_DOM    546    564       NON CYTOPLASMIC.
FT   TRANSMEM    565    584
FT   TOPO_DOM    585    603       CYTOPLASMIC.
FT   TRANSMEM    604    624
FT   TOPO_DOM    625    660       NON CYTOPLASMIC.
FT   TRANSMEM    661    680
FT   TOPO_DOM    681    700       CYTOPLASMIC.
FT   TRANSMEM    701    724
FT   TOPO_DOM    725    760       NON CYTOPLASMIC.
FT   TRANSMEM    761    780
FT   TOPO_DOM    781    786       CYTOPLASMIC.
FT   TRANSMEM    787    811
FT   TOPO_DOM    812    830       NON CYTOPLASMIC.
FT   TRANSMEM    831    850
FT   TOPO_DOM    851    856       CYTOPLASMIC.
FT   TRANSMEM    857    878
FT   TOPO_DOM    879    911       NON CYTOPLASMIC.
//
```

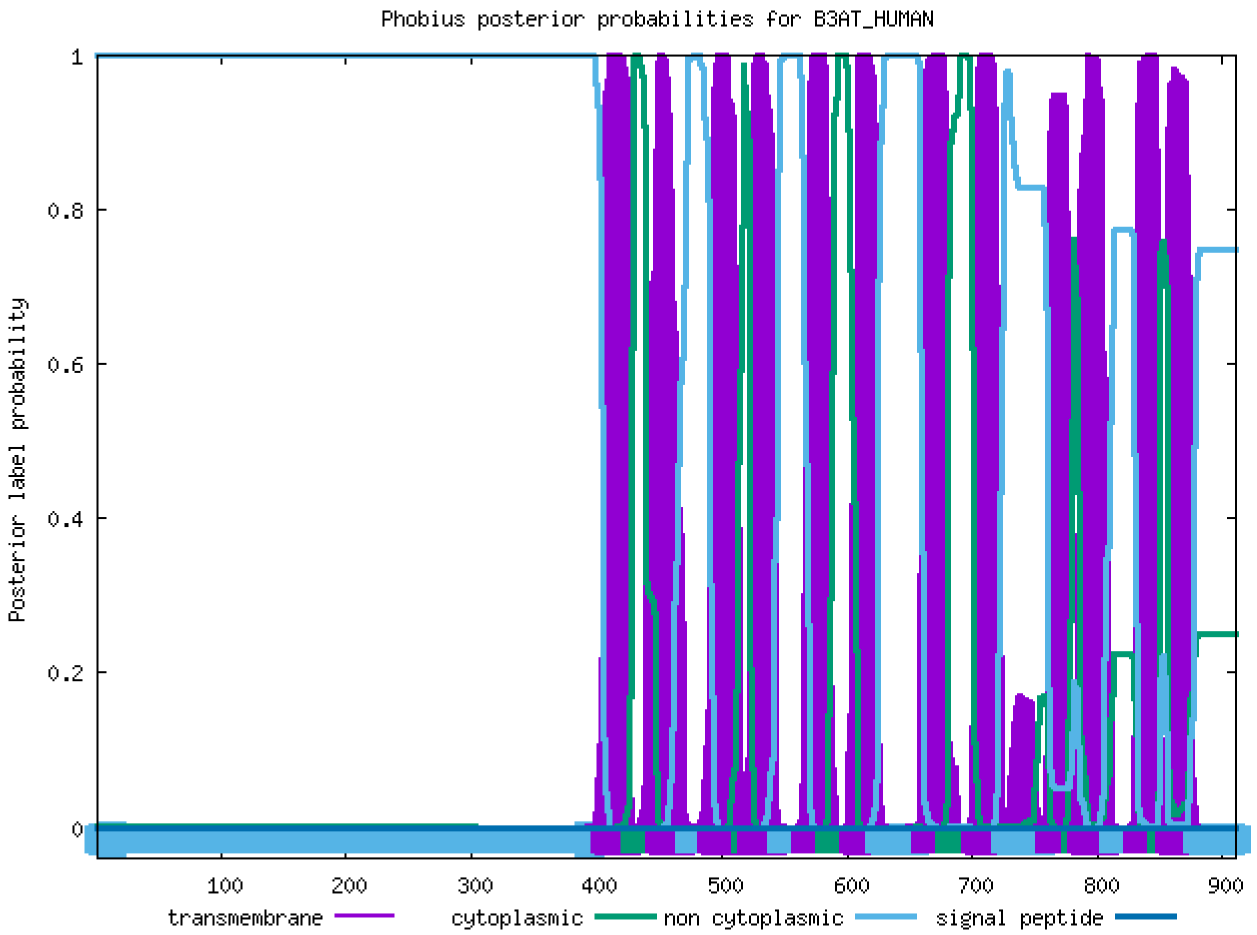

The probability data used in the plot is found here, and the gnuplot script is here.

Prediction of B3A2_HUMAN

```
ID   B3A2_HUMAN
FT   TOPO_DOM      1    708       NON CYTOPLASMIC.
FT   TRANSMEM    709    730
FT   TOPO_DOM    731    750       CYTOPLASMIC.
FT   TRANSMEM    751    774
FT   TOPO_DOM    775    793       NON CYTOPLASMIC.
FT   TRANSMEM    794    819
FT   TOPO_DOM    820    825       CYTOPLASMIC.
FT   TRANSMEM    826    844
FT   TOPO_DOM    845    900       NON CYTOPLASMIC.
FT   TRANSMEM    901    918
FT   TOPO_DOM    919    933       CYTOPLASMIC.
FT   TRANSMEM    934    954
FT   TOPO_DOM    955    990       NON CYTOPLASMIC.
FT   TRANSMEM    991   1010
FT   TOPO_DOM   1011   1030       CYTOPLASMIC.
FT   TRANSMEM   1031   1055
FT   TOPO_DOM   1056   1060       NON CYTOPLASMIC.
FT   TRANSMEM   1061   1079
FT   TOPO_DOM   1080   1090       CYTOPLASMIC.
FT   TRANSMEM   1091   1110
FT   TOPO_DOM   1111   1115       NON CYTOPLASMIC.
FT   TRANSMEM   1116   1136
FT   TOPO_DOM   1137   1162       CYTOPLASMIC.
FT   TRANSMEM   1163   1181
FT   TOPO_DOM   1182   1186       NON CYTOPLASMIC.
FT   TRANSMEM   1187   1208
FT   TOPO_DOM   1209   1241       CYTOPLASMIC.
//
```

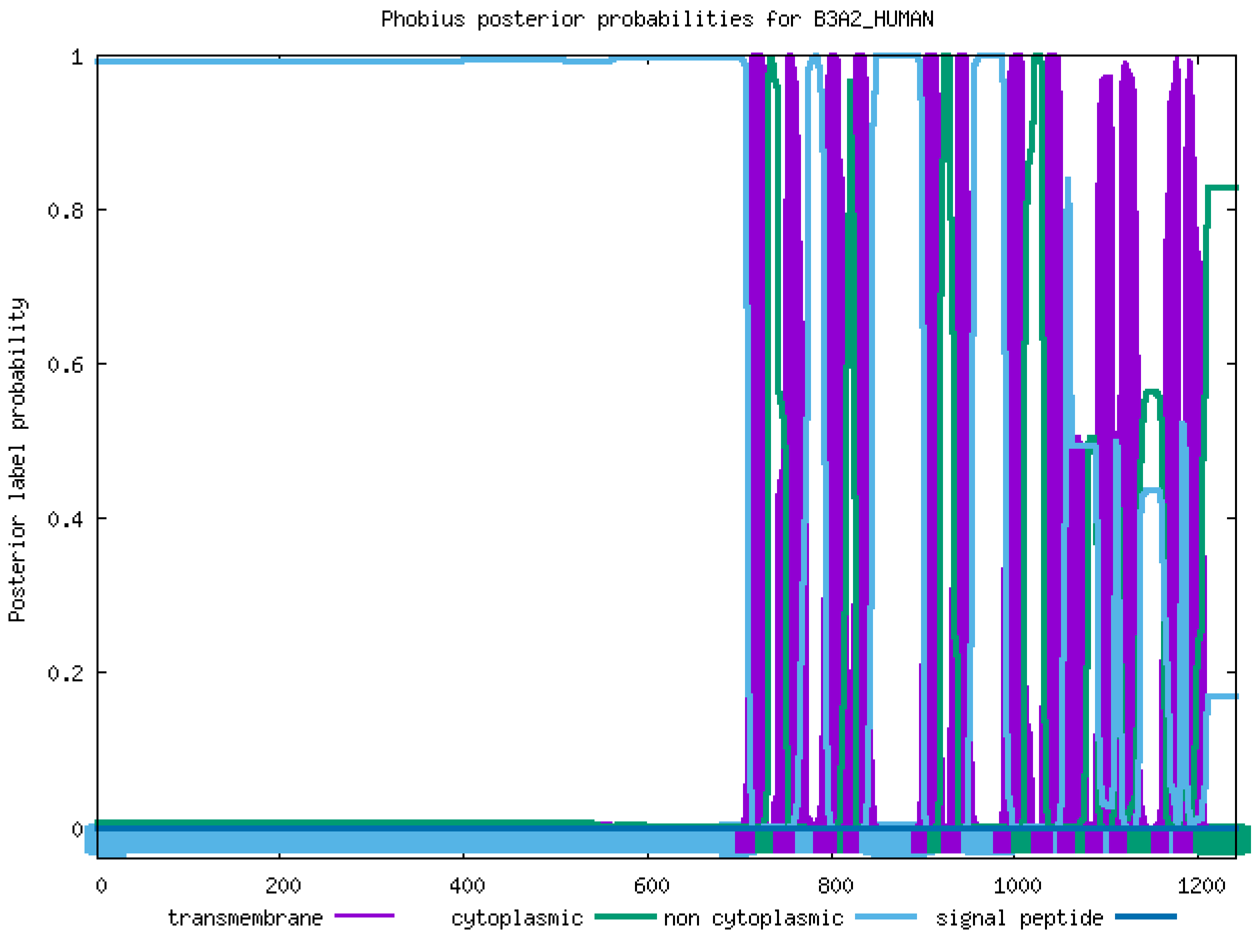

The probability data used in the plot is found here, and the gnuplot script is here.

Prediction of S12A4_HUMAN

```
ID   S12A4_HUMAN
FT   TOPO_DOM      1    118       CYTOPLASMIC.
FT   TRANSMEM    119    141
FT   TOPO_DOM    142    152       NON CYTOPLASMIC.
FT   TRANSMEM    153    176
FT   TOPO_DOM    177    196       CYTOPLASMIC.
FT   TRANSMEM    197    218
FT   TOPO_DOM    219    252       NON CYTOPLASMIC.
FT   TRANSMEM    253    272
FT   TOPO_DOM    273    278       CYTOPLASMIC.
FT   TRANSMEM    279    300
FT   TOPO_DOM    301    409       NON CYTOPLASMIC.
FT   TRANSMEM    410    435
FT   TOPO_DOM    436    455       CYTOPLASMIC.
FT   TRANSMEM    456    482
FT   TOPO_DOM    483    501       NON CYTOPLASMIC.
FT   TRANSMEM    502    520
FT   TOPO_DOM    521    555       CYTOPLASMIC.
FT   TRANSMEM    556    574
FT   TOPO_DOM    575    579       NON CYTOPLASMIC.
FT   TRANSMEM    580    601
FT   TOPO_DOM    602    612       CYTOPLASMIC.
FT   TRANSMEM    613    630
FT   TOPO_DOM    631    635       NON CYTOPLASMIC.
FT   TRANSMEM    636    652
FT   TOPO_DOM    653    809       CYTOPLASMIC.
FT   TRANSMEM    810    828
FT   TOPO_DOM    829    847       NON CYTOPLASMIC.
FT   TRANSMEM    848    865
```

FT   TOPO_DOM   866   1085   CYTOPLASMIC.
//

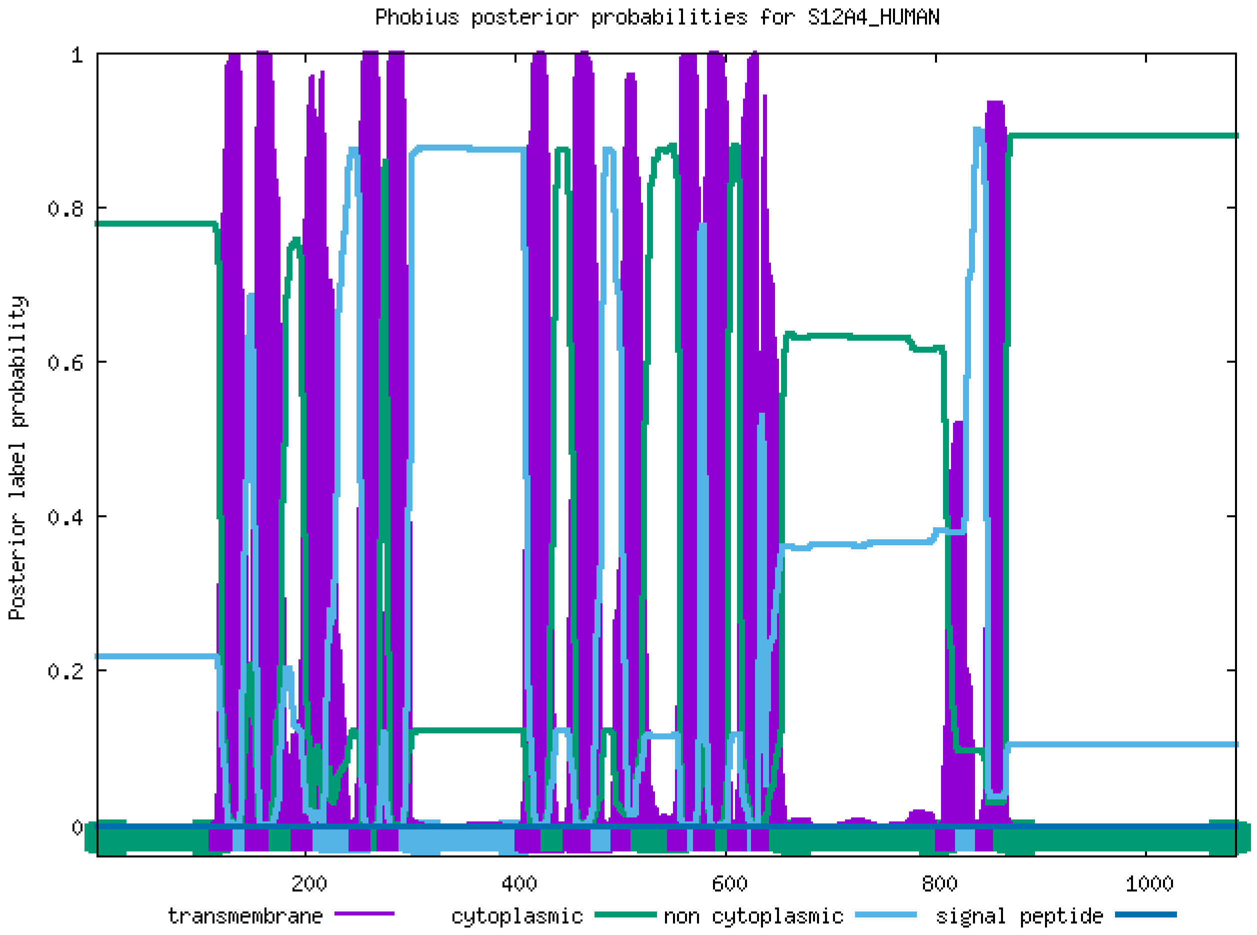

The probability data used in the plot is found here, and the gnuplot script is here.

Prediction of S12A7_HUMAN

```
ID   S12A7_HUMAN
FT   TOPO_DOM       1    117        CYTOPLASMIC.
FT   TRANSMEM     118    141
FT   TOPO_DOM     142    152        NON CYTOPLASMIC.
FT   TRANSMEM     153    176
FT   TOPO_DOM     177    196        CYTOPLASMIC.
FT   TRANSMEM     197    218
FT   TOPO_DOM     219    252        NON CYTOPLASMIC.
FT   TRANSMEM     253    270
FT   TOPO_DOM     271    276        CYTOPLASMIC.
FT   TRANSMEM     277    296
FT   TOPO_DOM     297    415        NON CYTOPLASMIC.
FT   TRANSMEM     416    436
FT   TOPO_DOM     437    455        CYTOPLASMIC.
FT   TRANSMEM     456    482
FT   TOPO_DOM     483    493        NON CYTOPLASMIC.
FT   TRANSMEM     494    520
FT   TOPO_DOM     521    555        CYTOPLASMIC.
FT   TRANSMEM     556    574
FT   TOPO_DOM     575    579        NON CYTOPLASMIC.
FT   TRANSMEM     580    602
FT   TOPO_DOM     603    613        CYTOPLASMIC.
FT   TRANSMEM     614    638
FT   TOPO_DOM     639    847        NON CYTOPLASMIC.
FT   TRANSMEM     848    865
FT   TOPO_DOM     866   1083        CYTOPLASMIC.
//
```

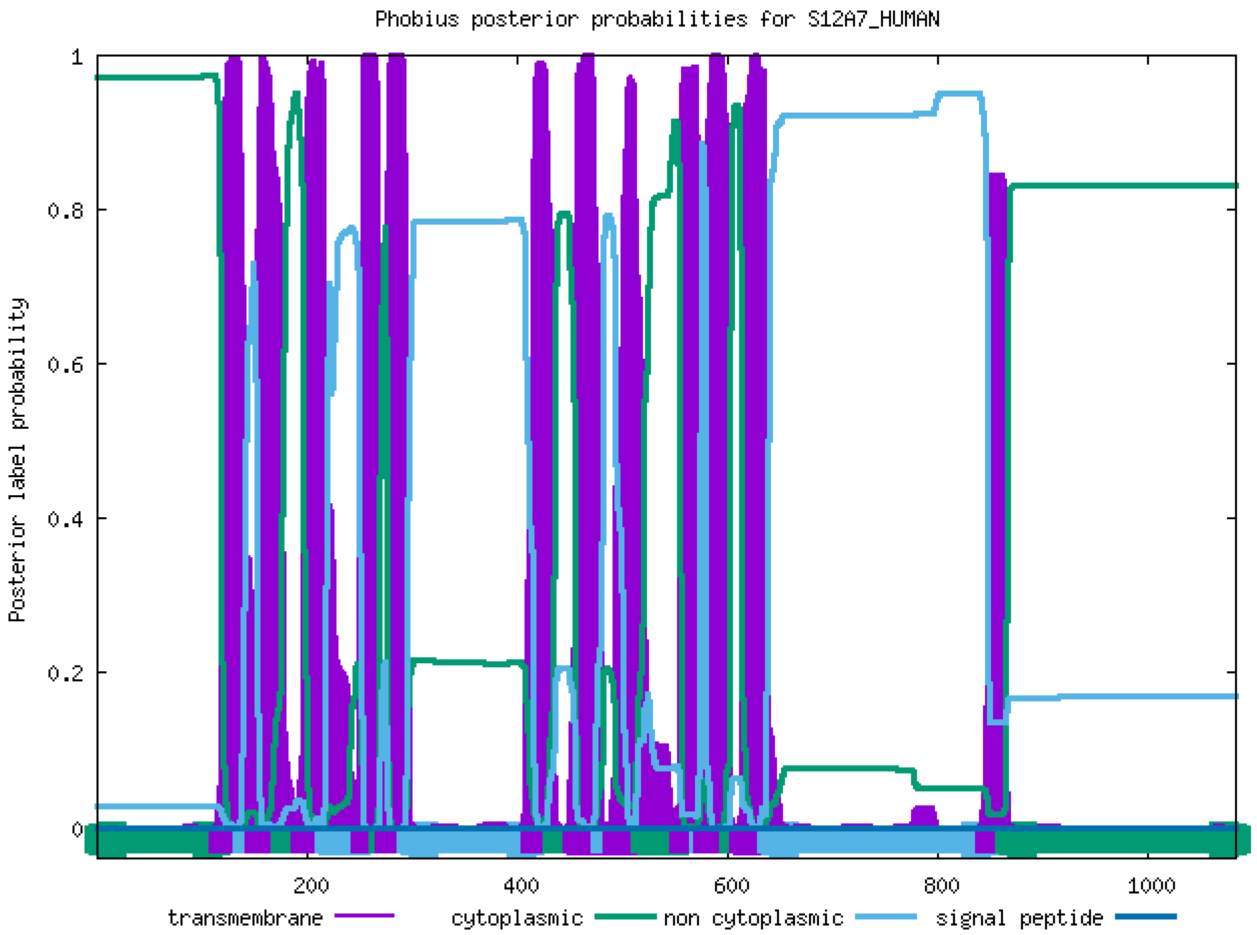

The probability data used in the plot is found here, and the gnuplot script is here.

Prediction of S12A3_HUMAN

```
ID   S12A3_HUMAN
FT   TOPO_DOM      1    137       CYTOPLASMIC.
FT   TRANSMEM    138    157
FT   TOPO_DOM    158    168       NON CYTOPLASMIC.
FT   TRANSMEM    169    194
FT   TOPO_DOM    195    214       CYTOPLASMIC.
FT   TRANSMEM    215    239
FT   TOPO_DOM    240    258       NON CYTOPLASMIC.
FT   TRANSMEM    259    278
FT   TOPO_DOM    279    286       CYTOPLASMIC.
FT   TRANSMEM    287    306
FT   TOPO_DOM    307    339       NON CYTOPLASMIC.
FT   TRANSMEM    340    362
FT   TOPO_DOM    363    373       CYTOPLASMIC.
FT   TRANSMEM    374    393
FT   TOPO_DOM    394    452       NON CYTOPLASMIC.
FT   TRANSMEM    453    474
FT   TOPO_DOM    475    505       CYTOPLASMIC.
FT   TRANSMEM    506    525
FT   TOPO_DOM    526    530       NON CYTOPLASMIC.
FT   TRANSMEM    531    552
FT   TOPO_DOM    553    563       CYTOPLASMIC.
FT   TRANSMEM    564    580
FT   TOPO_DOM    581    585       NON CYTOPLASMIC.
FT   TRANSMEM    586    605
FT   TOPO_DOM    606   1021       CYTOPLASMIC.
//
```

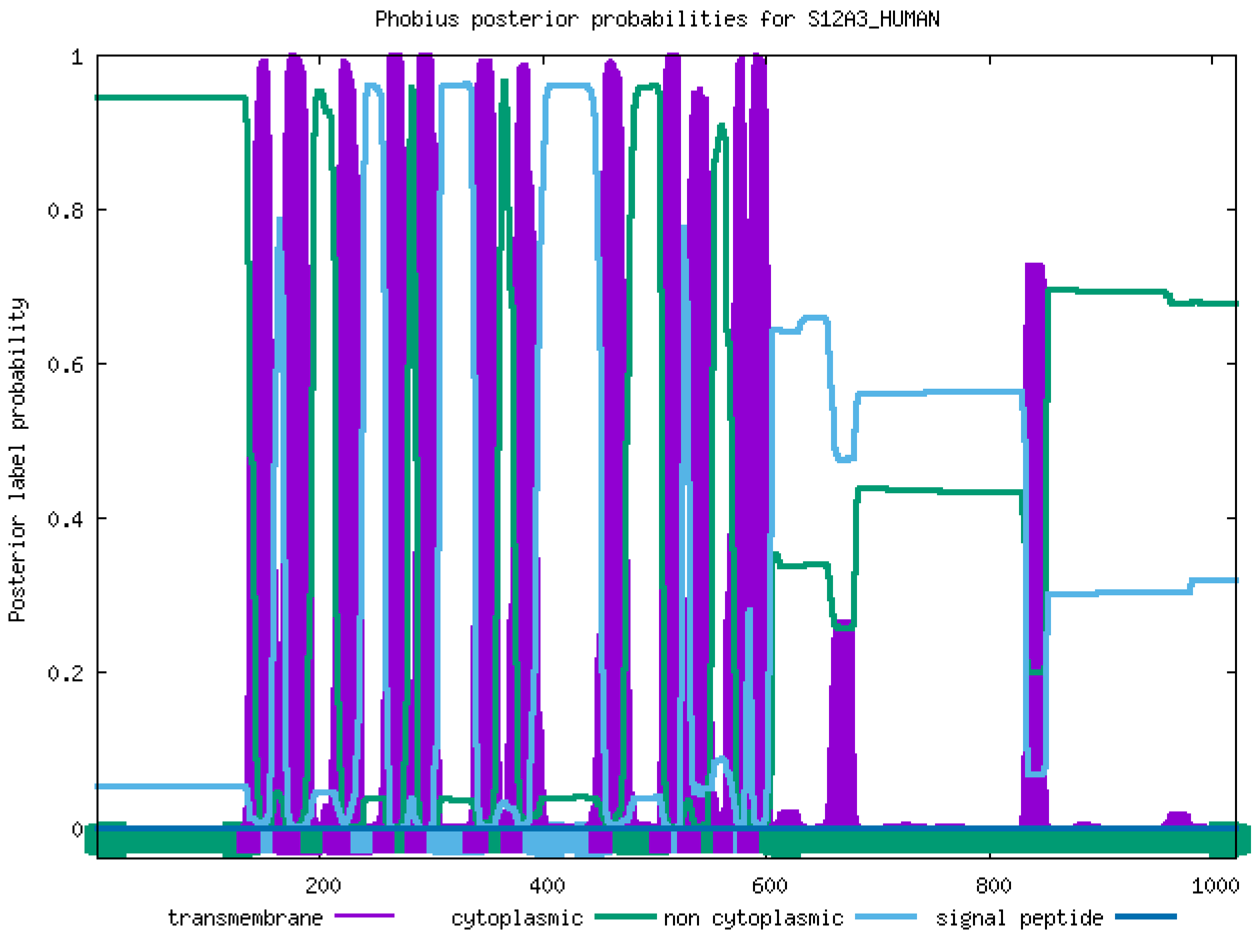

The probability data used in the plot is found here, and the gnuplot script is here.

Prediction of UCP1_HUMAN

```
ID   UCP1_HUMAN
FT   TOPO_DOM      1    307       NON CYTOPLASMIC.
//
```

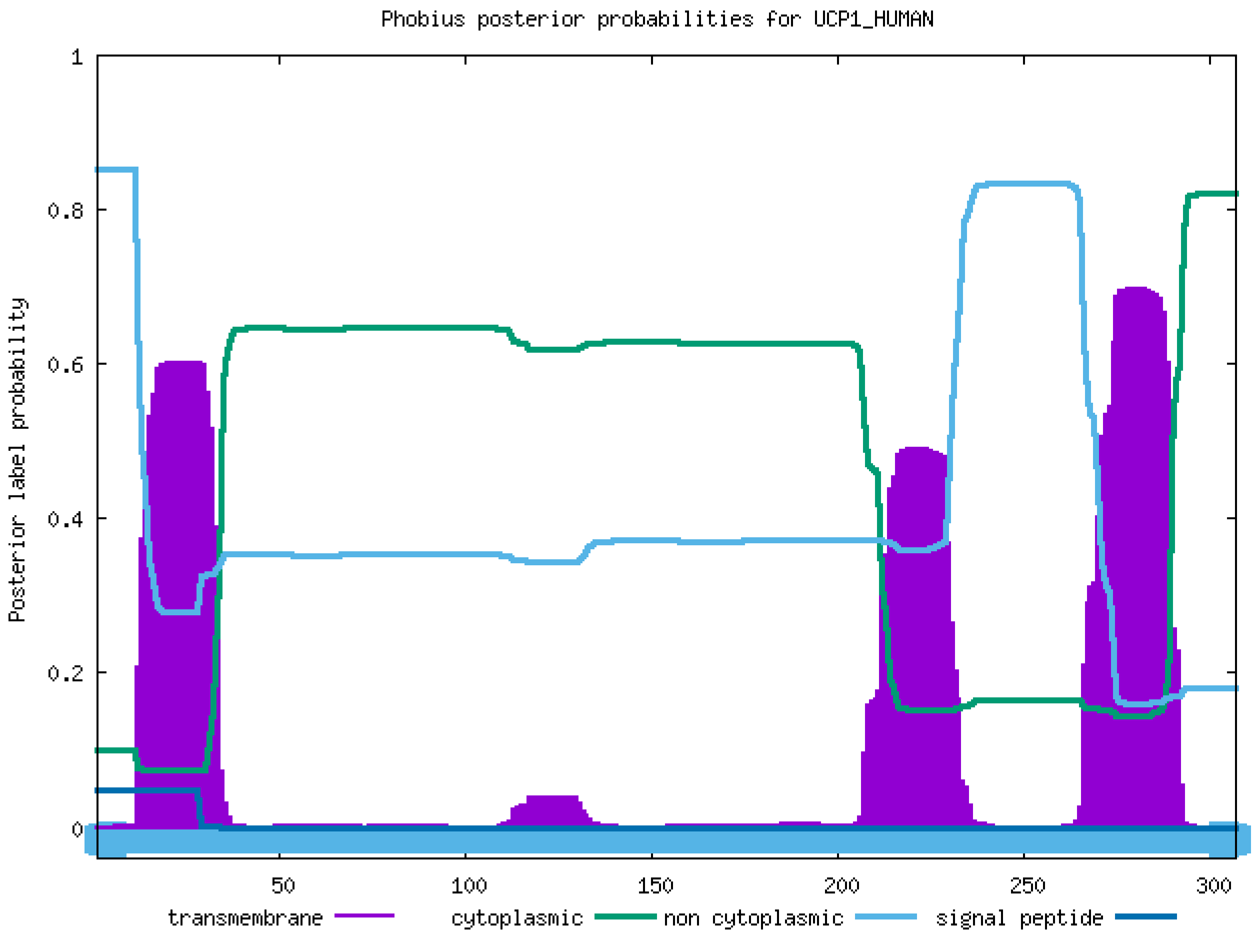

The probability data used in the plot is found here, and the gnuplot script is here.

Prediction of SL9A1_HUMAN

```
ID   SL9A1_HUMAN
FT   TOPO_DOM      1     14       NON CYTOPLASMIC.
FT   TRANSMEM     15     32
FT   TOPO_DOM     33    104       CYTOPLASMIC.
FT   TRANSMEM    105    122
FT   TOPO_DOM    123    127       NON CYTOPLASMIC.
FT   TRANSMEM    128    148
FT   TOPO_DOM    149    159       CYTOPLASMIC.
FT   TRANSMEM    160    177
FT   TOPO_DOM    178    188       NON CYTOPLASMIC.
FT   TRANSMEM    189    216
FT   TOPO_DOM    217    227       CYTOPLASMIC.
FT   TRANSMEM    228    246
FT   TOPO_DOM    247    257       NON CYTOPLASMIC.
FT   TRANSMEM    258    277
FT   TOPO_DOM    278    288       CYTOPLASMIC.
FT   TRANSMEM    289    319
FT   TOPO_DOM    320    338       NON CYTOPLASMIC.
FT   TRANSMEM    339    362
FT   TOPO_DOM    363    382       CYTOPLASMIC.
FT   TRANSMEM    383    405
FT   TOPO_DOM    406    410       NON CYTOPLASMIC.
FT   TRANSMEM    411    437
FT   TOPO_DOM    438    448       CYTOPLASMIC.
FT   TRANSMEM    449    468
FT   TOPO_DOM    469    479       NON CYTOPLASMIC.
FT   TRANSMEM    480    503
FT   TOPO_DOM    504    815       CYTOPLASMIC.
//
```

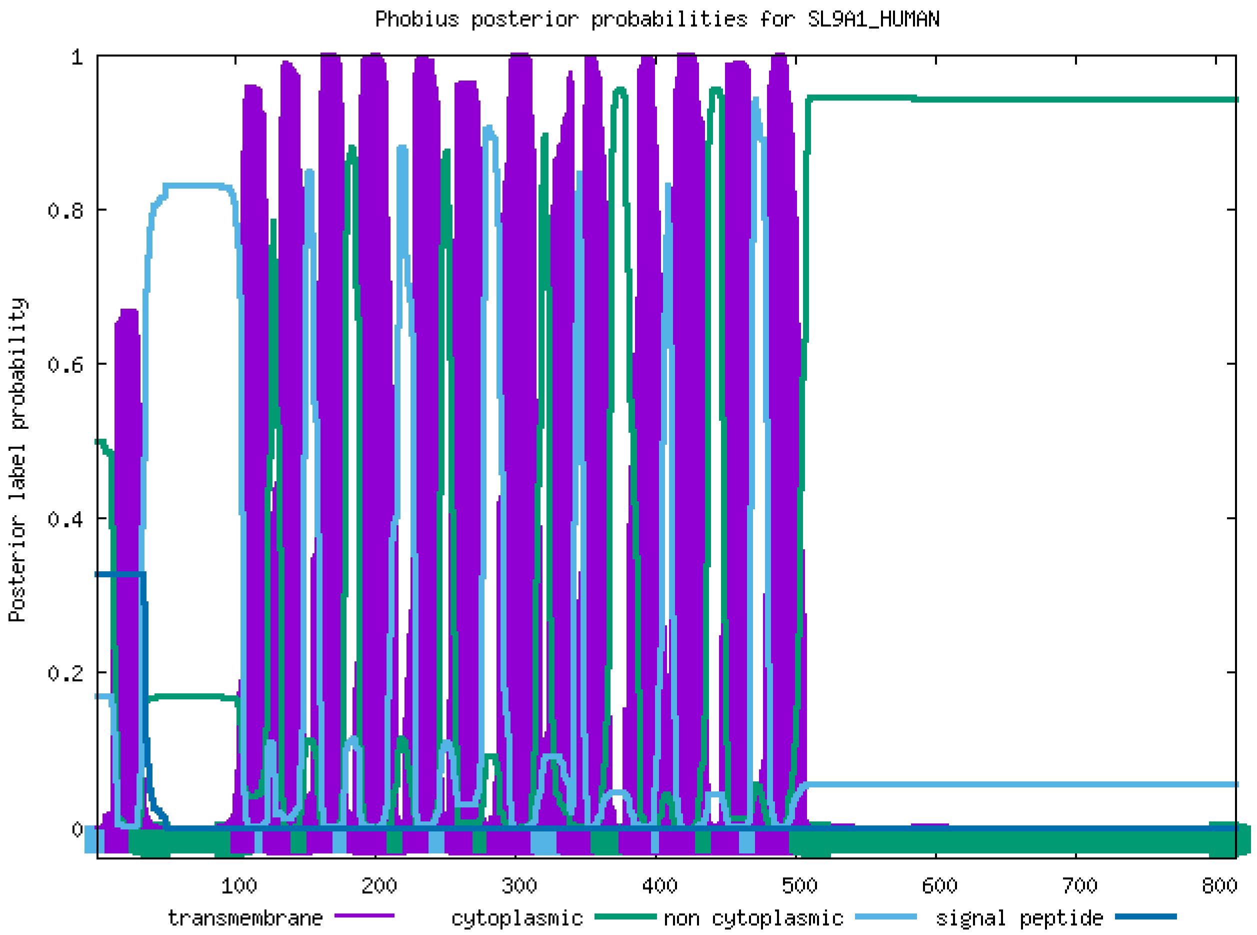

The probability data used in the plot is found here, and the gnuplot script is here.

Prediction of NTCP_HUMAN

```
ID   NTCP_HUMAN
FT   TOPO_DOM      1     24       NON CYTOPLASMIC.
FT   TRANSMEM     25     48
FT   TOPO_DOM     49     59       CYTOPLASMIC.
FT   TRANSMEM     60     79
FT   TOPO_DOM     80     90       NON CYTOPLASMIC.
FT   TRANSMEM     91    111
FT   TOPO_DOM    112    117       CYTOPLASMIC.
FT   TRANSMEM    118    142
FT   TOPO_DOM    143    153       NON CYTOPLASMIC.
FT   TRANSMEM    154    176
FT   TOPO_DOM    177    187       CYTOPLASMIC.
FT   TRANSMEM    188    208
FT   TOPO_DOM    209    227       NON CYTOPLASMIC.
FT   TRANSMEM    228    246
FT   TOPO_DOM    247    257       CYTOPLASMIC.
FT   TRANSMEM    258    276
FT   TOPO_DOM    277    281       NON CYTOPLASMIC.
FT   TRANSMEM    282    307
FT   TOPO_DOM    308    349       CYTOPLASMIC.
//
```

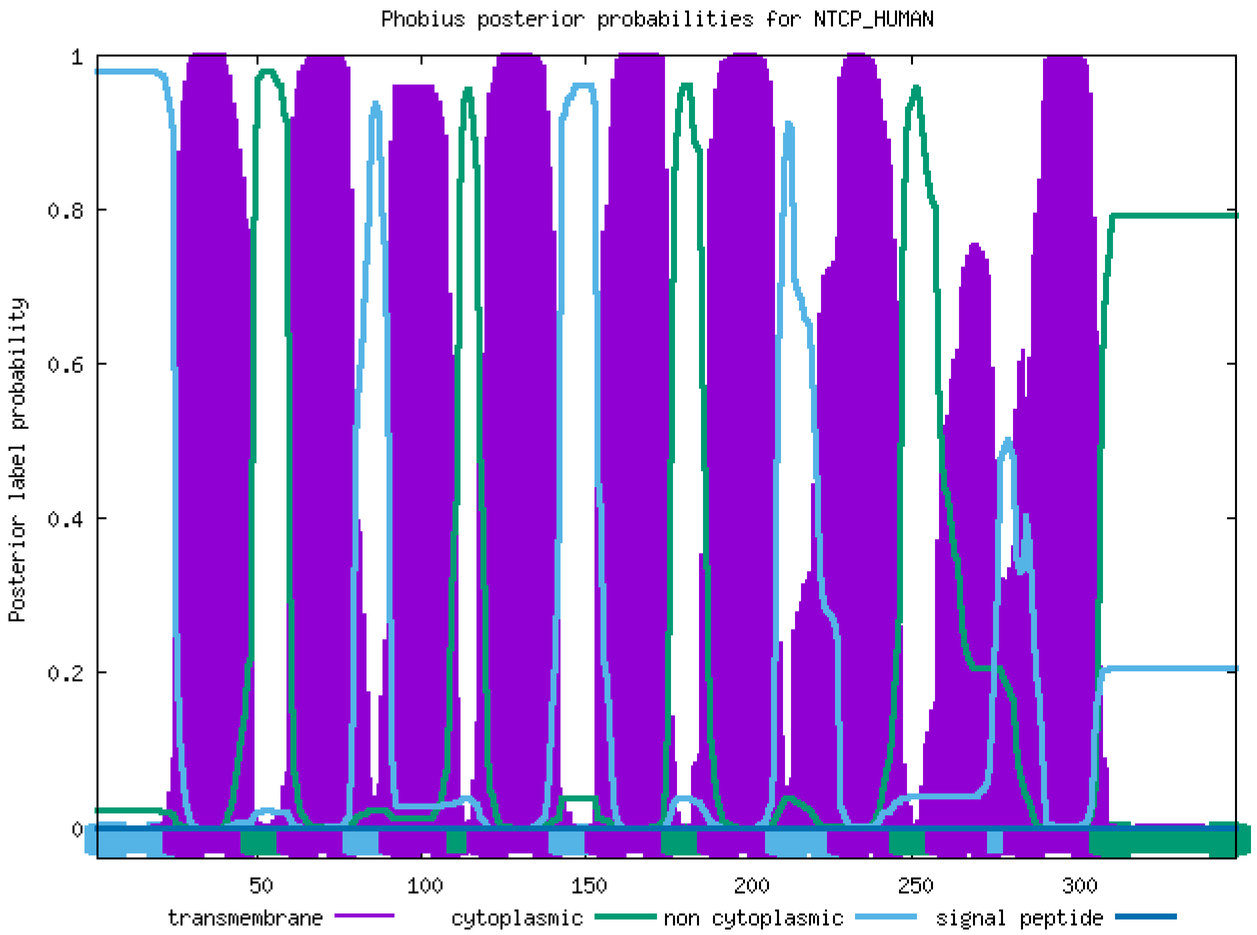

The probability data used in the plot is found here, and the gnuplot script is here.

Prediction of MDR1_HUMAN

```
ID    MDR1_HUMAN
FT    TOPO_DOM      1     48       CYTOPLASMIC.
FT    TRANSMEM     49     72
FT    TOPO_DOM     73    114       NON CYTOPLASMIC.
FT    TRANSMEM    115    139
FT    TOPO_DOM    140    189       CYTOPLASMIC.
FT    TRANSMEM    190    208
FT    TOPO_DOM    209    213       NON CYTOPLASMIC.
FT    TRANSMEM    214    233
FT    TOPO_DOM    234    292       CYTOPLASMIC.
FT    TRANSMEM    293    316
FT    TOPO_DOM    317    327       NON CYTOPLASMIC.
FT    TRANSMEM    328    346
FT    TOPO_DOM    347    707       CYTOPLASMIC.
FT    TRANSMEM    708    733
FT    TOPO_DOM    734    752       NON CYTOPLASMIC.
FT    TRANSMEM    753    777
FT    TOPO_DOM    778    832       CYTOPLASMIC.
FT    TRANSMEM    833    853
FT    TOPO_DOM    854    858       NON CYTOPLASMIC.
FT    TRANSMEM    859    879
FT    TOPO_DOM    880    934       CYTOPLASMIC.
FT    TRANSMEM    935    954
FT    TOPO_DOM    955    973       NON CYTOPLASMIC.
FT    TRANSMEM    974    995
FT    TOPO_DOM    996   1280       CYTOPLASMIC.
//
```

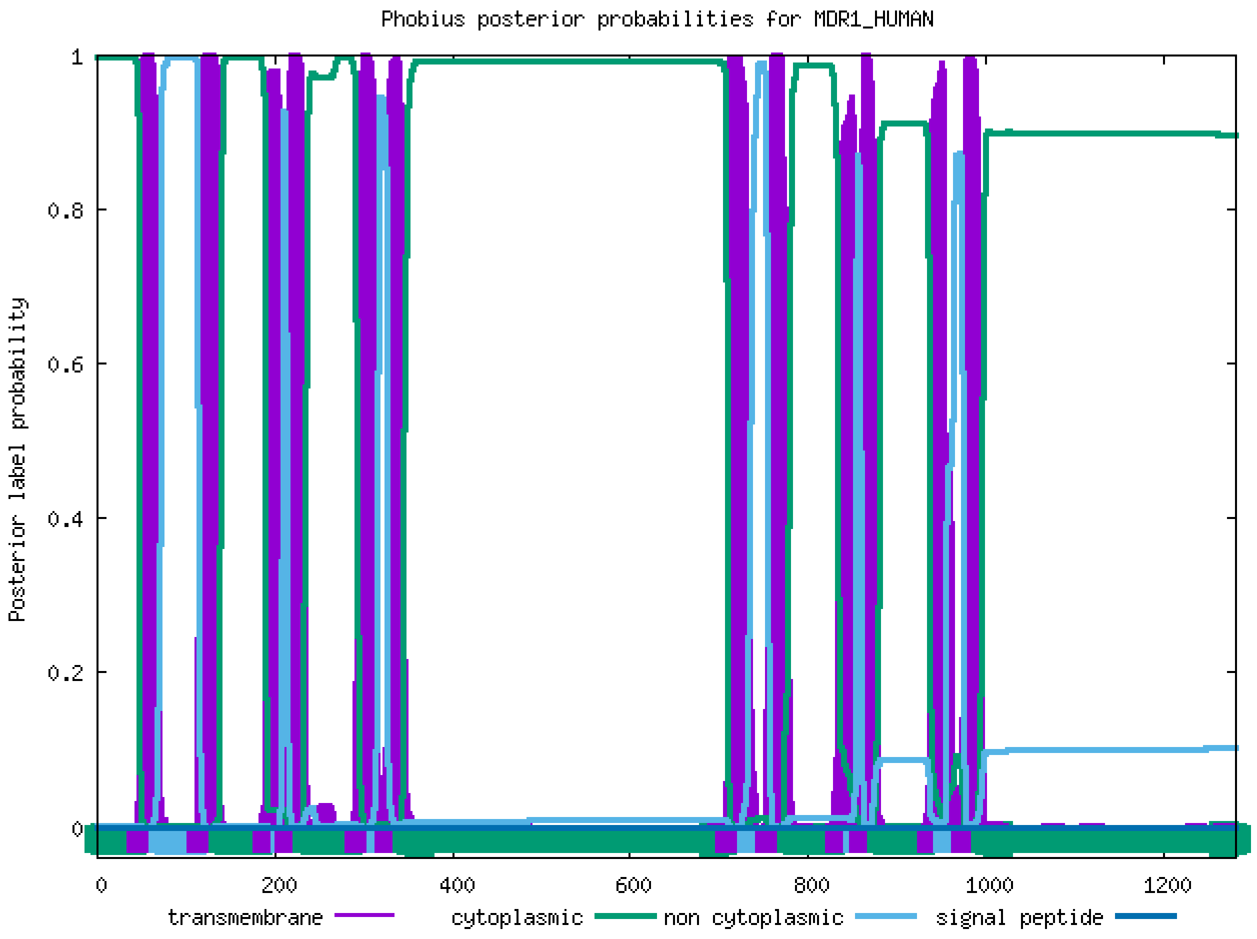

The probability data used in the plot is found here, and the gnuplot script is here.

Prediction of ABCB6_HUMAN

```
ID   ABCB6_HUMAN
FT   TOPO_DOM      1     26       NON CYTOPLASMIC.
FT   TRANSMEM     27     49
FT   TOPO_DOM     50     69       CYTOPLASMIC.
FT   TRANSMEM     70     93
FT   TOPO_DOM     94    104       NON CYTOPLASMIC.
FT   TRANSMEM    105    127
FT   TOPO_DOM    128    147       CYTOPLASMIC.
FT   TRANSMEM    148    166
FT   TOPO_DOM    167    185       NON CYTOPLASMIC.
FT   TRANSMEM    186    206
FT   TOPO_DOM    207    262       CYTOPLASMIC.
FT   TRANSMEM    263    286
FT   TOPO_DOM    287    305       NON CYTOPLASMIC.
FT   TRANSMEM    306    326
FT   TOPO_DOM    327    382       CYTOPLASMIC.
FT   TRANSMEM    383    405
FT   TOPO_DOM    406    410       NON CYTOPLASMIC.
FT   TRANSMEM    411    432
FT   TOPO_DOM    433    502       CYTOPLASMIC.
FT   TRANSMEM    503    521
FT   TOPO_DOM    522    532       NON CYTOPLASMIC.
FT   TRANSMEM    533    551
FT   TOPO_DOM    552    842       CYTOPLASMIC.
//
```

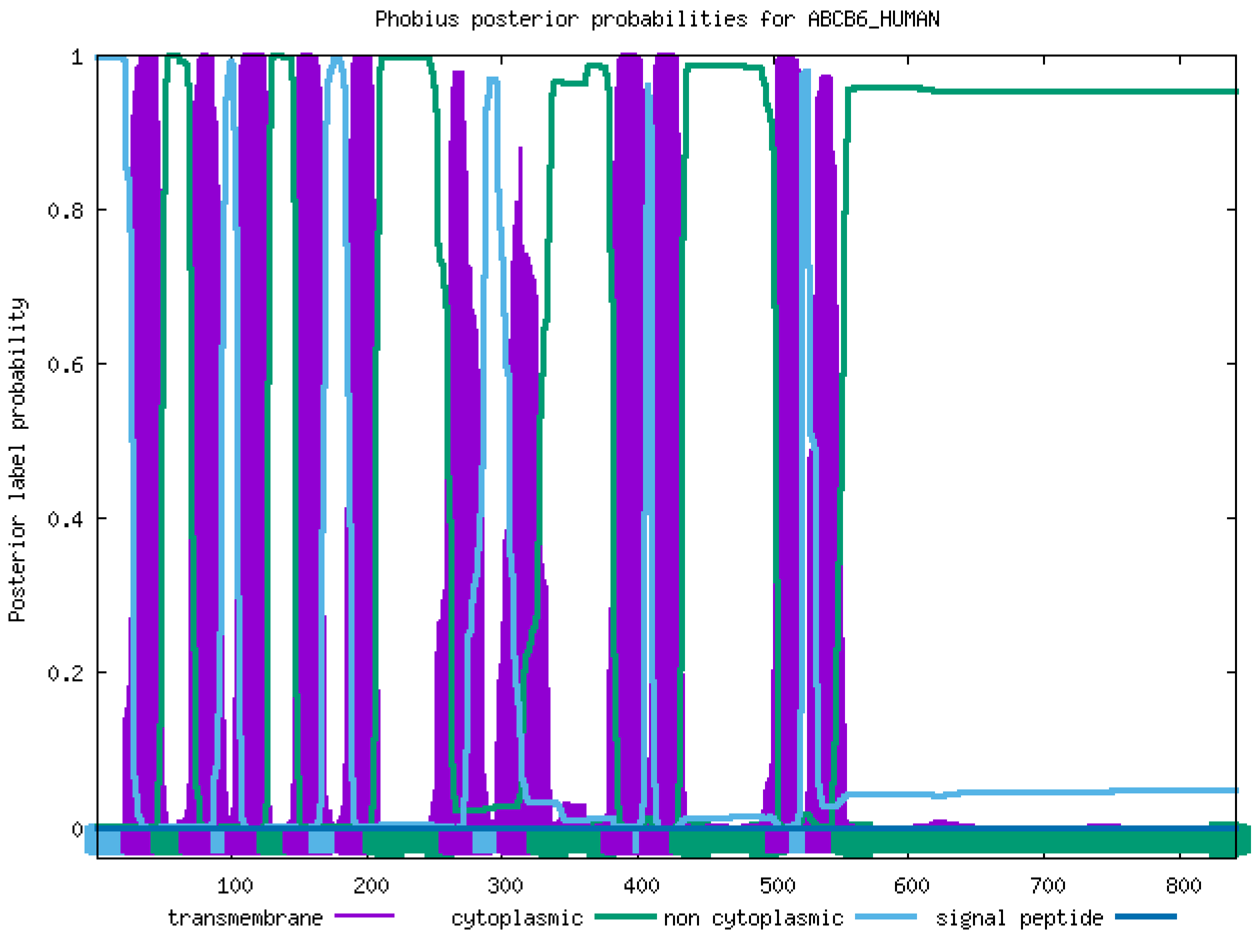

The probability data used in the plot is found here, and the gnuplot script is here.

Prediction of ABCB7_HUMAN

```
ID   ABCB7_HUMAN
FT   TOPO_DOM      1    139       CYTOPLASMIC.
FT   TRANSMEM    140    165
FT   TOPO_DOM    166    184       NON CYTOPLASMIC.
FT   TRANSMEM    185    206
FT   TOPO_DOM    207    259       CYTOPLASMIC.
FT   TRANSMEM    260    287
FT   TOPO_DOM    288    292       NON CYTOPLASMIC.
FT   TRANSMEM    293    314
FT   TOPO_DOM    315    382       CYTOPLASMIC.
FT   TRANSMEM    383    405
FT   TOPO_DOM    406    410       NON CYTOPLASMIC.
FT   TRANSMEM    411    431
FT   TOPO_DOM    432    752       CYTOPLASMIC.
//
```

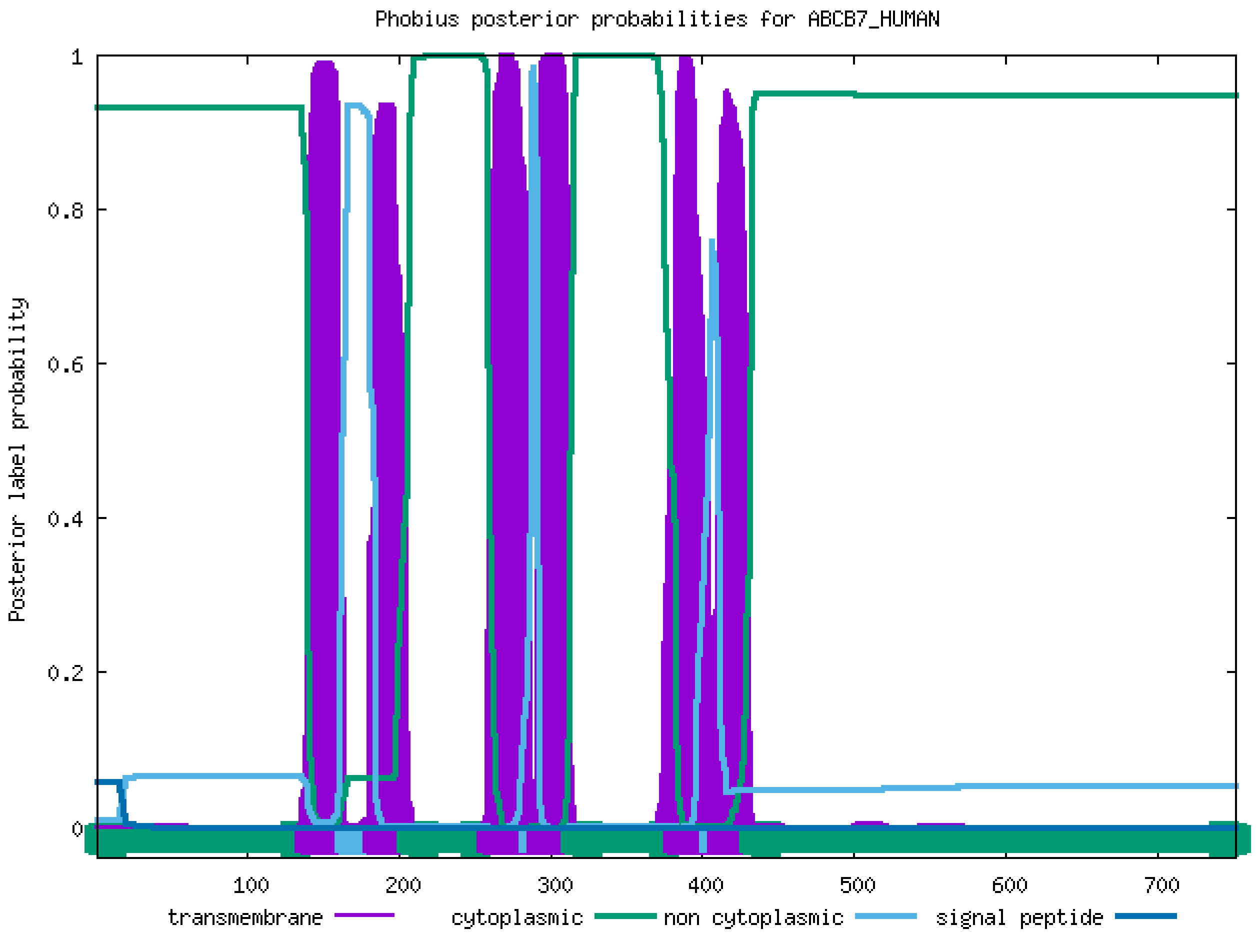

The probability data used in the plot is found here, and the gnuplot script is here.

Prediction of MITOS_HUMAN

```
ID   MITOS_HUMAN
FT   TOPO_DOM      1    144       CYTOPLASMIC.
FT   TRANSMEM    145    165
FT   TOPO_DOM    166    198       NON CYTOPLASMIC.
FT   TRANSMEM    199    219
FT   TOPO_DOM    220    293       CYTOPLASMIC.
FT   TRANSMEM    294    316
FT   TOPO_DOM    317    735       NON CYTOPLASMIC.
//
```

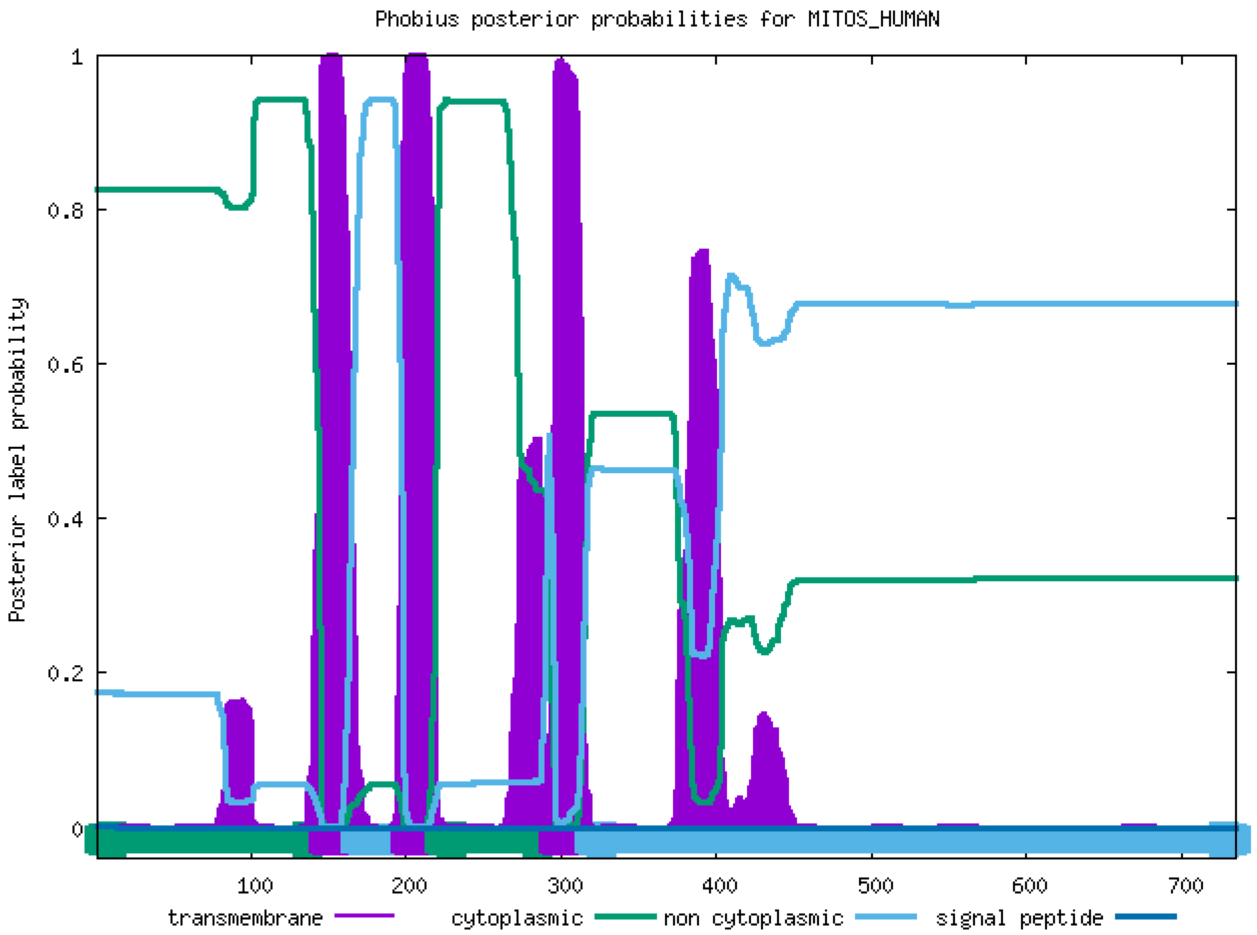

The probability data used in the plot is found here, and the gnuplot script is here.

Prediction of ABCBA_HUMAN

```
ID   ABCBA_HUMAN
FT   TOPO_DOM      1    170       CYTOPLASMIC.
FT   TRANSMEM    171    191
FT   TOPO_DOM    192    210       NON CYTOPLASMIC.
FT   TRANSMEM    211    235
FT   TOPO_DOM    236    292       CYTOPLASMIC.
FT   TRANSMEM    293    311
FT   TOPO_DOM    312    316       NON CYTOPLASMIC.
FT   TRANSMEM    317    336
FT   TOPO_DOM    337    391       CYTOPLASMIC.
FT   TRANSMEM    392    414
FT   TOPO_DOM    415    433       NON CYTOPLASMIC.
FT   TRANSMEM    434    452
FT   TOPO_DOM    453    738       CYTOPLASMIC.
//
```

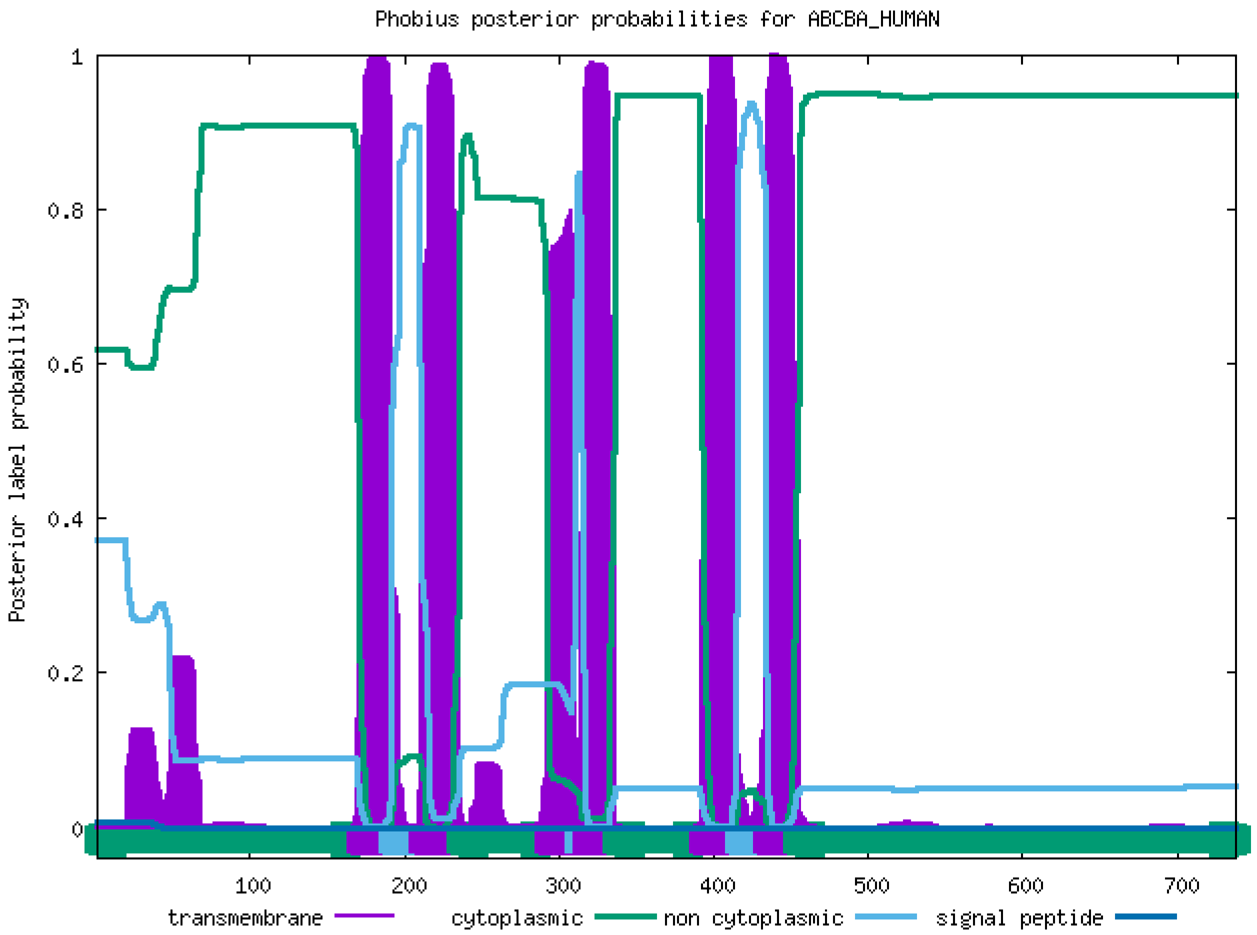

The probability data used in the plot is found here, and the gnuplot script is here.

Prediction of ABCBB_HUMAN

```
ID   ABCBB_HUMAN
FT   TOPO_DOM      1     58       CYTOPLASMIC.
FT   TRANSMEM     59     83
FT   TOPO_DOM     84    143       NON CYTOPLASMIC.
FT   TRANSMEM    144    166
FT   TOPO_DOM    167    216       CYTOPLASMIC.
FT   TRANSMEM    217    236
FT   TOPO_DOM    237    241       NON CYTOPLASMIC.
FT   TRANSMEM    242    263
FT   TOPO_DOM    264    319       CYTOPLASMIC.
FT   TRANSMEM    320    342
FT   TOPO_DOM    343    353       NON CYTOPLASMIC.
FT   TRANSMEM    354    373
FT   TOPO_DOM    374    751       CYTOPLASMIC.
FT   TRANSMEM    752    775
FT   TOPO_DOM    776    794       NON CYTOPLASMIC.
FT   TRANSMEM    795    821
FT   TOPO_DOM    822    875       CYTOPLASMIC.
FT   TRANSMEM    876    896
FT   TOPO_DOM    897    901       NON CYTOPLASMIC.
FT   TRANSMEM    902    922
FT   TOPO_DOM    923    977       CYTOPLASMIC.
FT   TRANSMEM    978    996
FT   TOPO_DOM    997   1015       NON CYTOPLASMIC.
FT   TRANSMEM   1016   1035
FT   TOPO_DOM   1036   1321       CYTOPLASMIC.
//
```

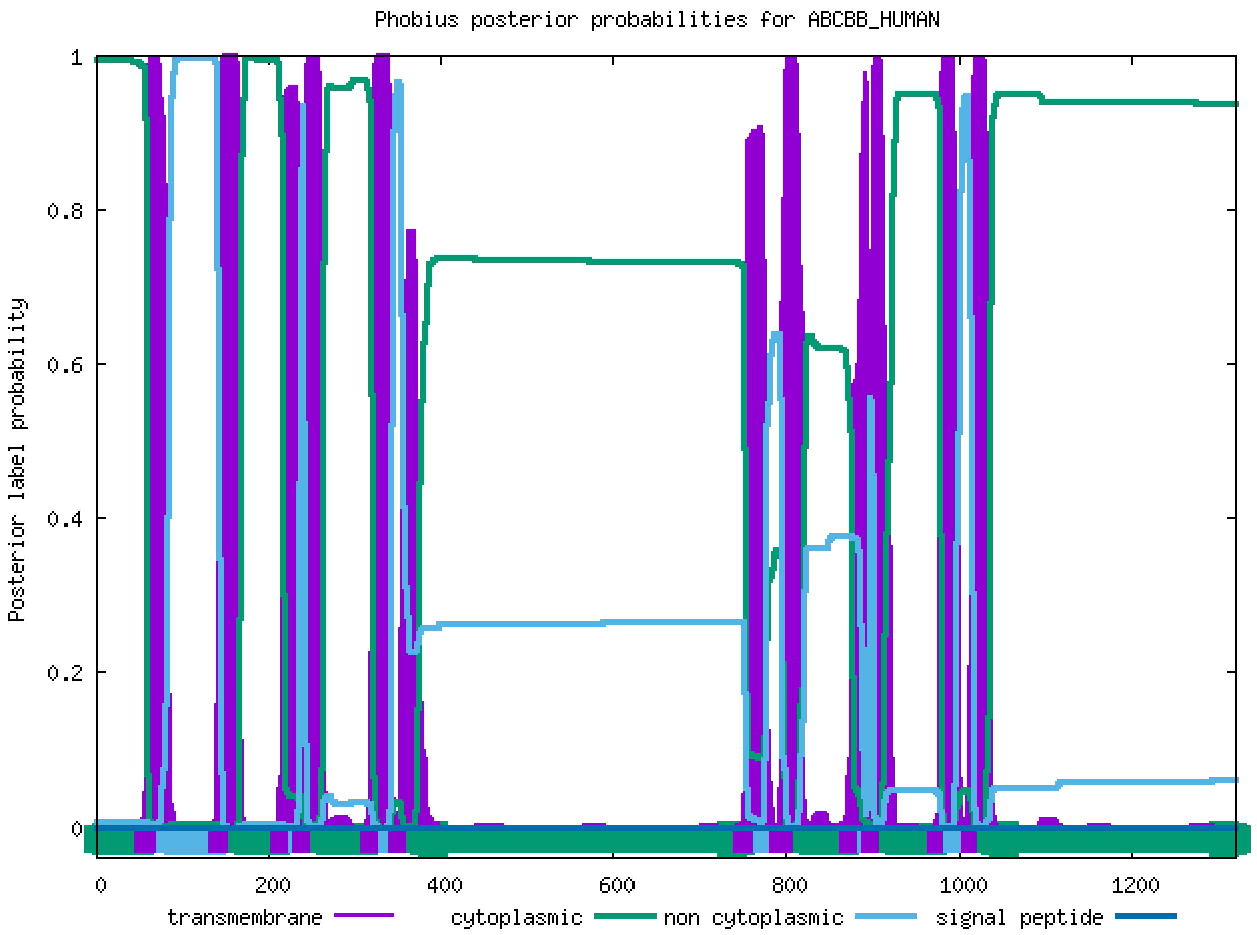

The probability data used in the plot is found here, and the gnuplot script is here.

Prediction of ABCD1_HUMAN

```
ID   ABCD1_HUMAN
FT   SIGNAL       1     34
FT   REGION       1     17       N-REGION.
FT   REGION      18     26       H-REGION.
FT   REGION      27     34       C-REGION.
FT   TOPO_DOM    35     91       NON CYTOPLASMIC.
FT   TRANSMEM    92    113
FT   TOPO_DOM   114    133       CYTOPLASMIC.
FT   TRANSMEM   134    154
FT   TOPO_DOM   155    239       NON CYTOPLASMIC.
FT   TRANSMEM   240    258
FT   TOPO_DOM   259    336       CYTOPLASMIC.
FT   TRANSMEM   337    357
FT   TOPO_DOM   358    745       NON CYTOPLASMIC.
//
```

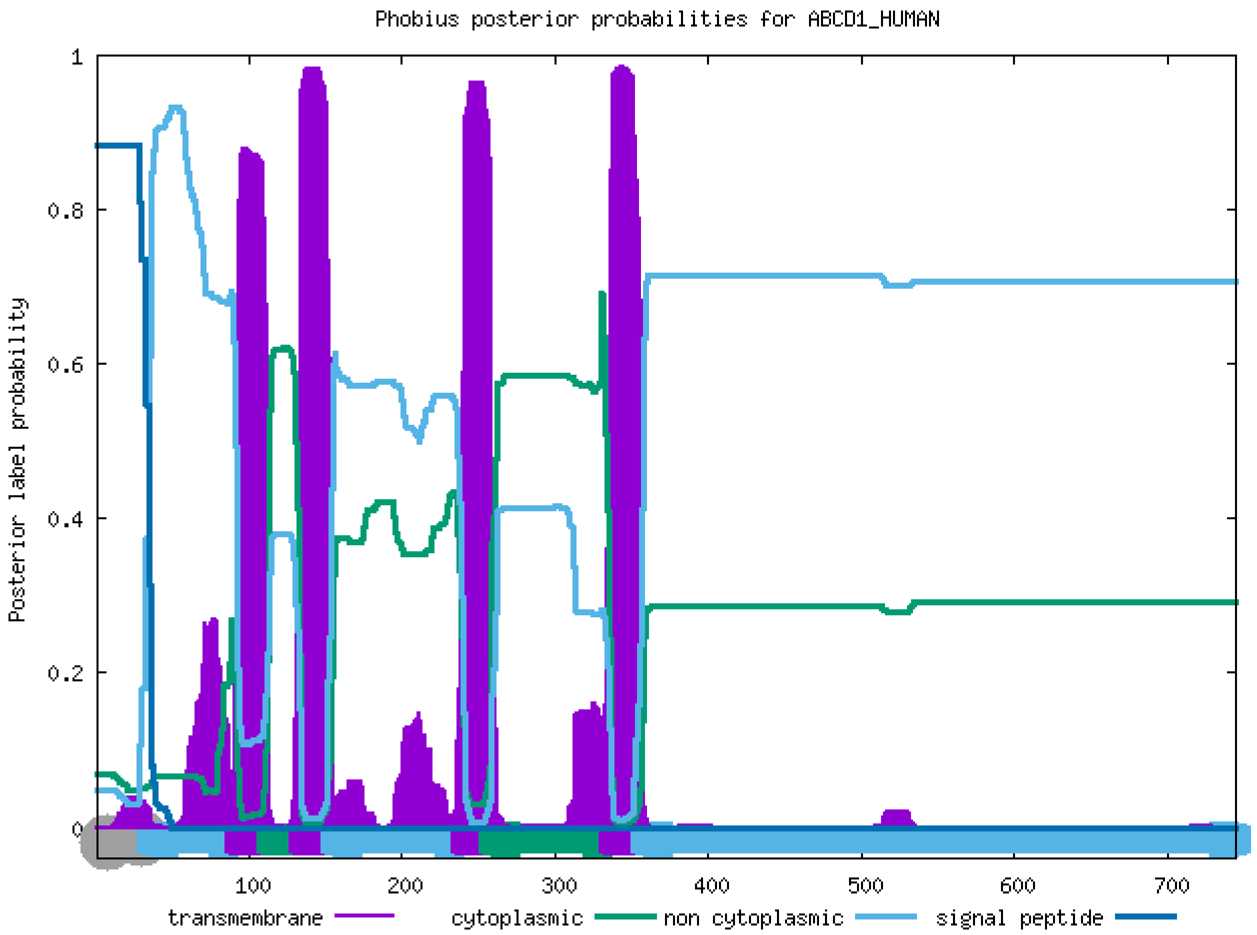

The probability data used in the plot is found here, and the gnuplot script is here.

Prediction of ABCD4_HUMAN

```
ID    ABCD4_HUMAN
FT    TOPO_DOM      1     38       CYTOPLASMIC.
FT    TRANSMEM     39     58
FT    TOPO_DOM     59     77       NON CYTOPLASMIC.
FT    TRANSMEM     78     96
FT    TOPO_DOM     97    156       CYTOPLASMIC.
FT    TRANSMEM    157    176
FT    TOPO_DOM    177    181       NON CYTOPLASMIC.
FT    TRANSMEM    182    201
FT    TOPO_DOM    202    267       CYTOPLASMIC.
FT    TRANSMEM    268    294
FT    TOPO_DOM    295    606       NON CYTOPLASMIC.
//
```

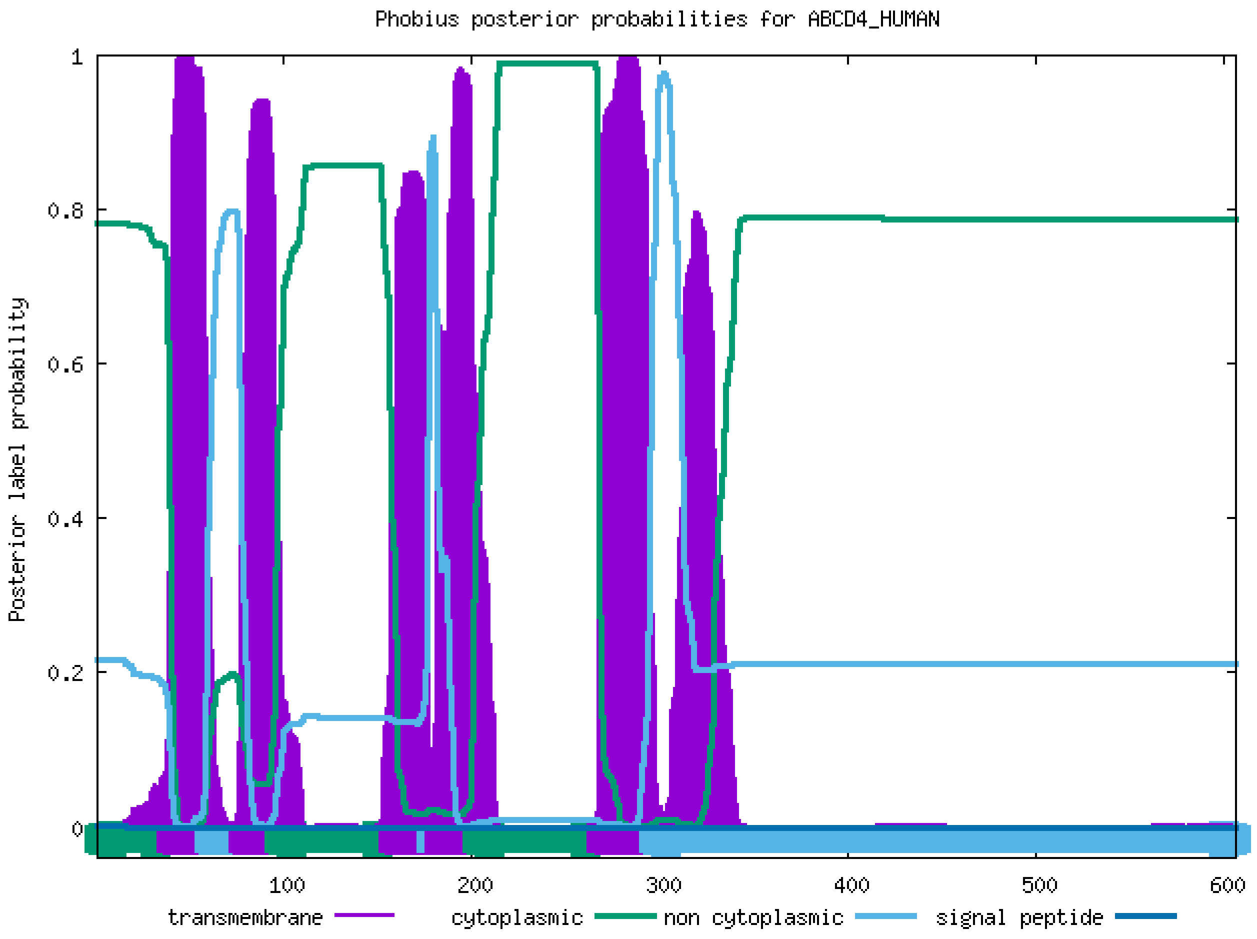

The probability data used in the plot is found here, and the gnuplot script is here.

Prediction of ABCG1_HUMAN

```
ID   ABCG1_HUMAN
FT   SIGNAL         1     20
FT   REGION         1      1       N-REGION.
FT   REGION         2     13       H-REGION.
FT   REGION        14     20       C-REGION.
FT   TOPO_DOM      21    426       NON CYTOPLASMIC.
FT   TRANSMEM     427    444
FT   TOPO_DOM     445    455       CYTOPLASMIC.
FT   TRANSMEM     456    477
FT   TOPO_DOM     478    496       NON CYTOPLASMIC.
FT   TRANSMEM     497    522
FT   TOPO_DOM     523    533       CYTOPLASMIC.
FT   TRANSMEM     534    558
FT   TOPO_DOM     559    563       NON CYTOPLASMIC.
FT   TRANSMEM     564    586
FT   TOPO_DOM     587    597       CYTOPLASMIC.
FT   TRANSMEM     598    616
FT   TOPO_DOM     617    647       NON CYTOPLASMIC.
FT   TRANSMEM     648    670
FT   TOPO_DOM     671    678       CYTOPLASMIC.
//
```

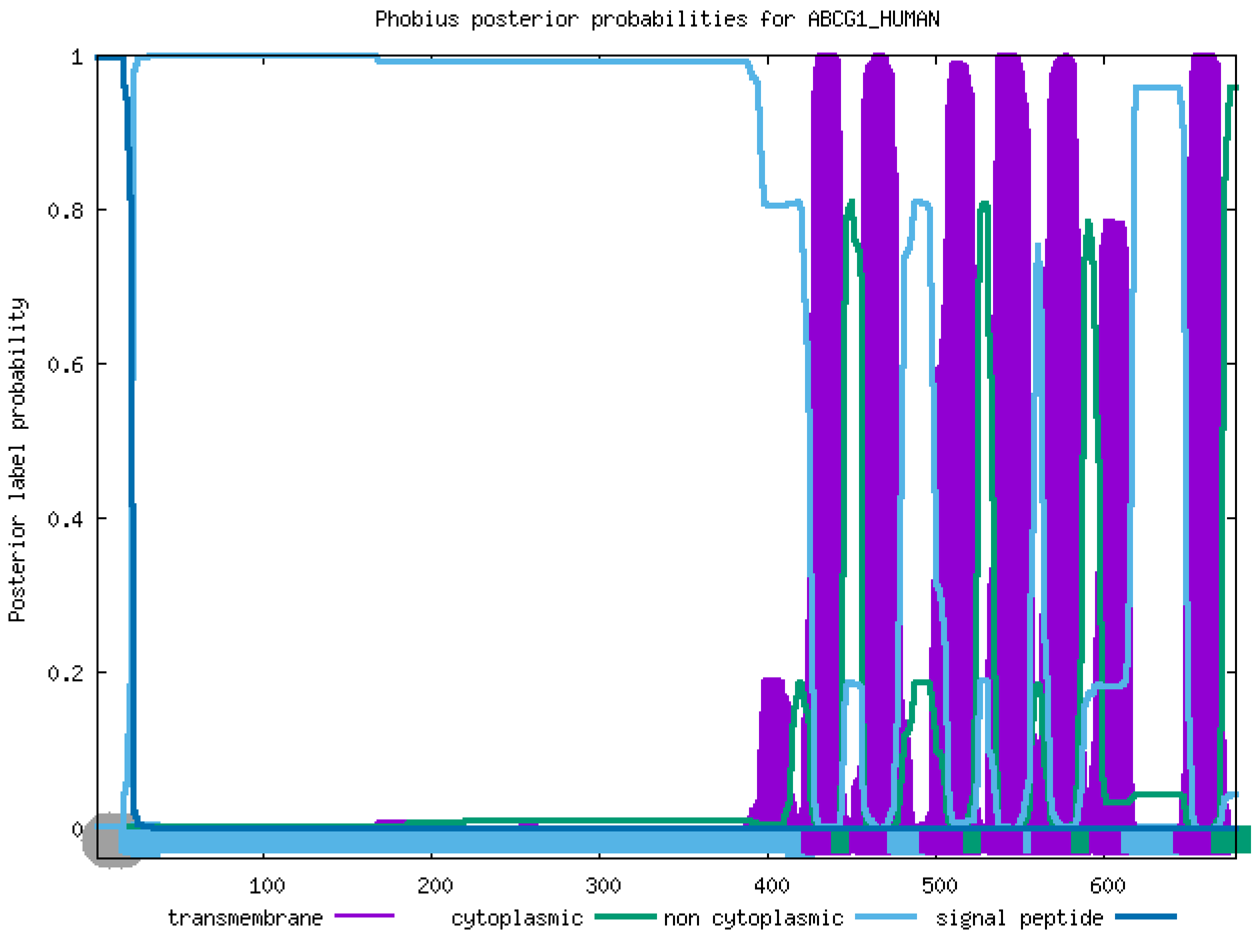

The probability data used in the plot is found here, and the gnuplot script is here.

Prediction of ABCG5_HUMAN

```
ID    ABCG5_HUMAN
FT    TOPO_DOM      1     383       NON CYTOPLASMIC.
FT    TRANSMEM    384     405
FT    TOPO_DOM    406     425       CYTOPLASMIC.
FT    TRANSMEM    426     445
FT    TOPO_DOM    446     464       NON CYTOPLASMIC.
FT    TRANSMEM    465     490
FT    TOPO_DOM    491     501       CYTOPLASMIC.
FT    TRANSMEM    502     523
FT    TOPO_DOM    524     528       NON CYTOPLASMIC.
FT    TRANSMEM    529     549
FT    TOPO_DOM    550     624       CYTOPLASMIC.
FT    TRANSMEM    625     644
FT    TOPO_DOM    645     651       NON CYTOPLASMIC.
//
```

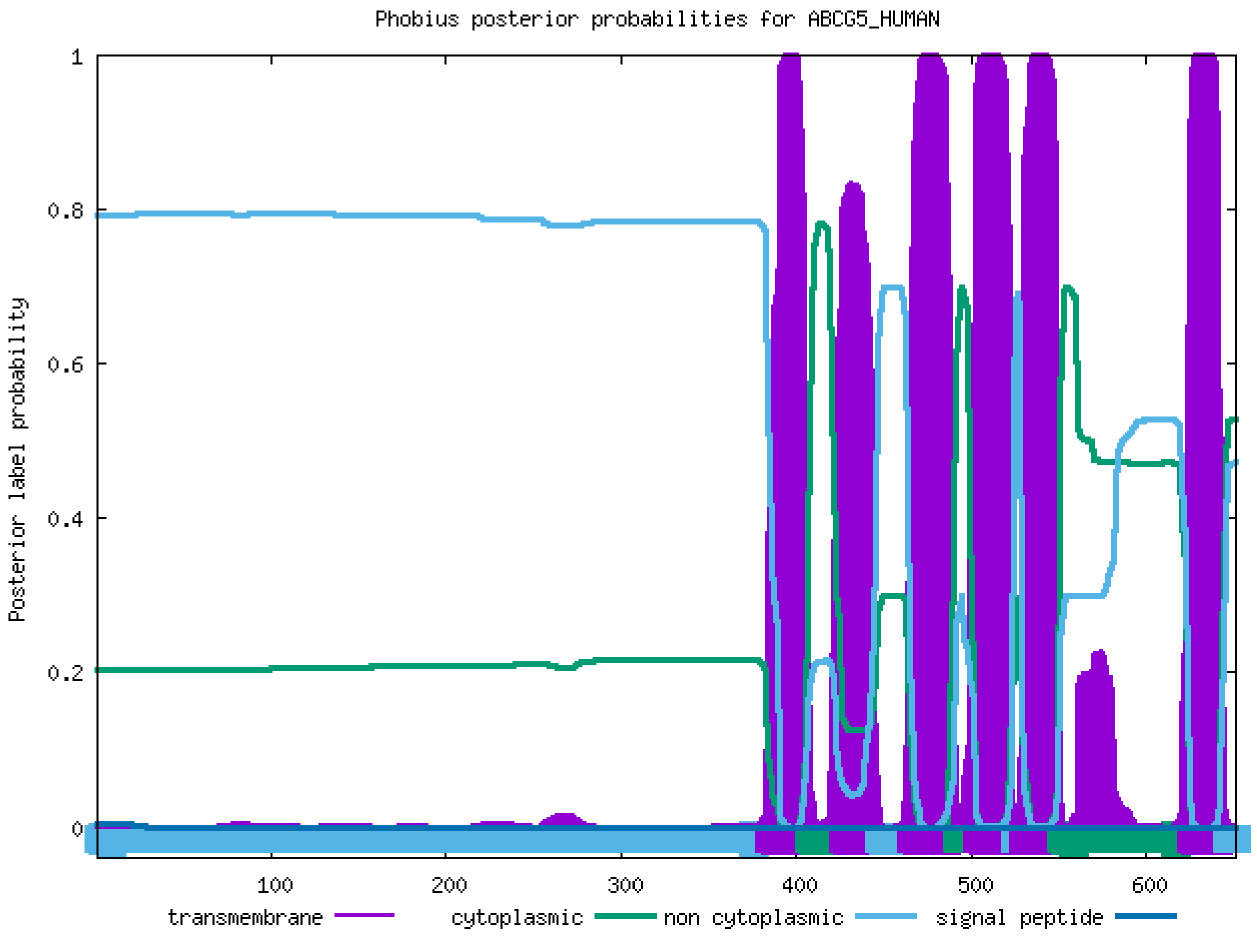

The probability data used in the plot is found here, and the gnuplot script is here.

Prediction of ABCG8_HUMAN

```
ID   ABCG8_HUMAN
FT   TOPO_DOM      1    416       NON CYTOPLASMIC.
FT   TRANSMEM    417    437
FT   TOPO_DOM    438    448       CYTOPLASMIC.
FT   TRANSMEM    449    468
FT   TOPO_DOM    469    527       NON CYTOPLASMIC.
FT   TRANSMEM    528    553
FT   TOPO_DOM    554    564       CYTOPLASMIC.
FT   TRANSMEM    565    590
FT   TOPO_DOM    591    640       NON CYTOPLASMIC.
FT   TRANSMEM    641    660
FT   TOPO_DOM    661    673       CYTOPLASMIC.
//
```

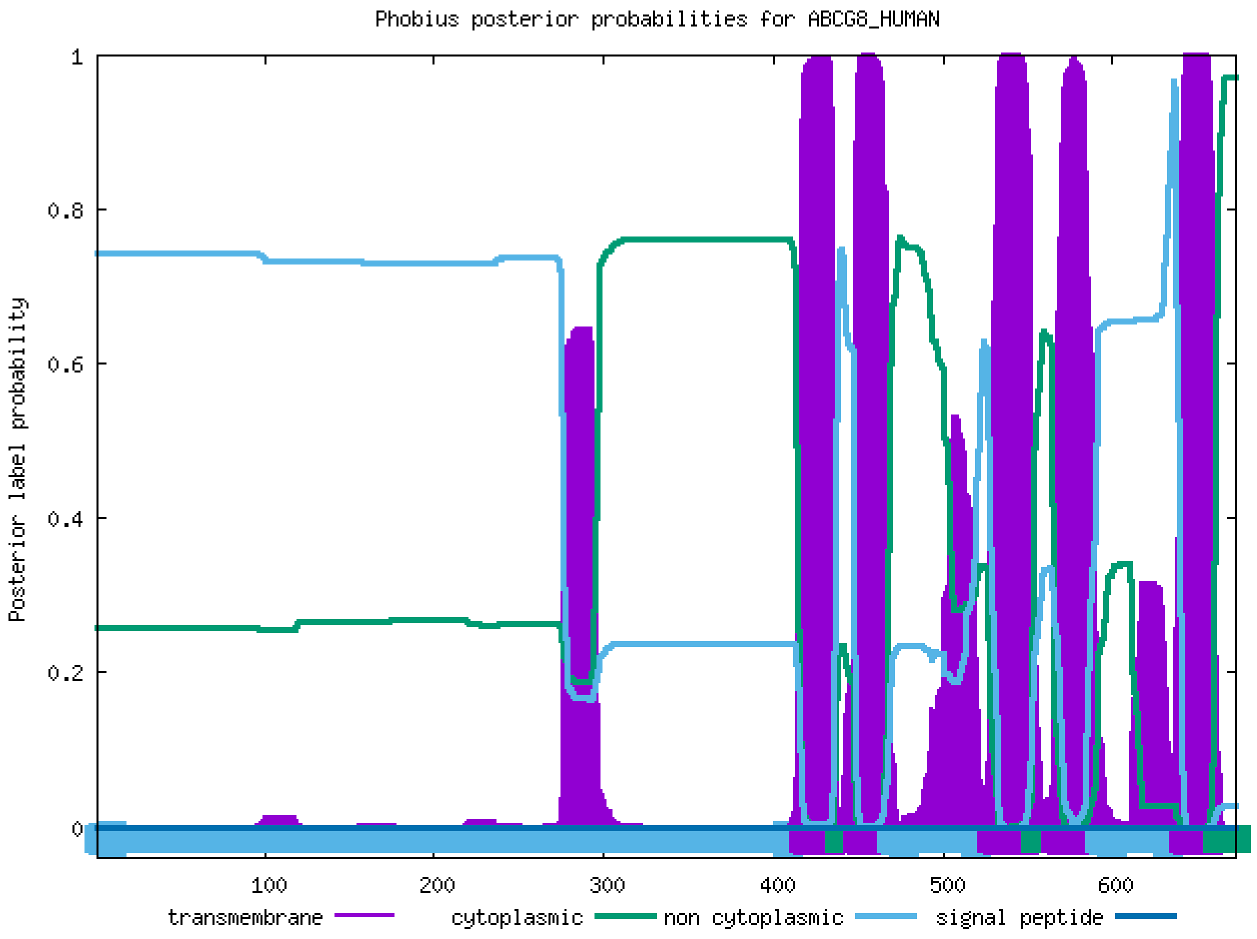

The probability data used in the plot is found here, and the gnuplot script is here.

Prediction of CFTR_HUMAN

```
ID    CFTR_HUMAN
FT    TOPO_DOM       1     71       CYTOPLASMIC.
FT    TRANSMEM      72     91
FT    TOPO_DOM      92    118       NON CYTOPLASMIC.
FT    TRANSMEM     119    143
FT    TOPO_DOM     144    194       CYTOPLASMIC.
FT    TRANSMEM     195    215
FT    TOPO_DOM     216    220       NON CYTOPLASMIC.
FT    TRANSMEM     221    241
FT    TOPO_DOM     242    303       CYTOPLASMIC.
FT    TRANSMEM     304    326
FT    TOPO_DOM     327    331       NON CYTOPLASMIC.
FT    TRANSMEM     332    350
FT    TOPO_DOM     351    858       CYTOPLASMIC.
FT    TRANSMEM     859    883
FT    TOPO_DOM     884    902       NON CYTOPLASMIC.
FT    TRANSMEM     903    927
FT    TOPO_DOM     928    985       CYTOPLASMIC.
FT    TRANSMEM     986   1008
FT    TOPO_DOM    1009   1013       NON CYTOPLASMIC.
FT    TRANSMEM    1014   1033
FT    TOPO_DOM    1034   1104       CYTOPLASMIC.
FT    TRANSMEM    1105   1123
FT    TOPO_DOM    1124   1128       NON CYTOPLASMIC.
FT    TRANSMEM    1129   1147
FT    TOPO_DOM    1148   1480       CYTOPLASMIC.
//
```

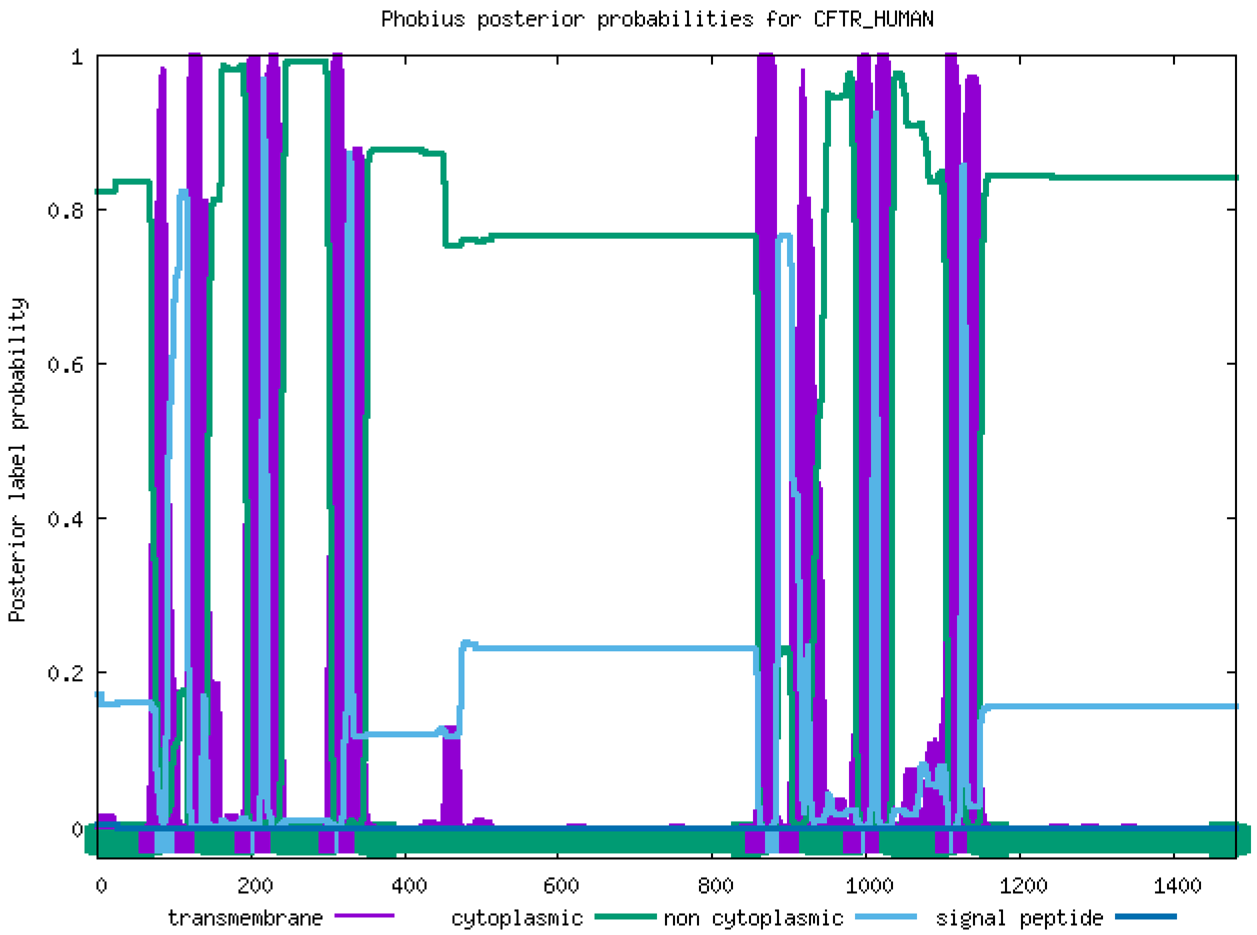

The probability data used in the plot is found here, and the gnuplot script is here.

Prediction of TAP1_HUMAN

```
ID   TAP1_HUMAN
FT   TOPO_DOM      1     11       CYTOPLASMIC.
FT   TRANSMEM     12     35
FT   TOPO_DOM     36     54       NON CYTOPLASMIC.
FT   TRANSMEM     55     80
FT   TOPO_DOM     81     91       CYTOPLASMIC.
FT   TRANSMEM     92    113
FT   TOPO_DOM    114    132       NON CYTOPLASMIC.
FT   TRANSMEM    133    154
FT   TOPO_DOM    155    186       CYTOPLASMIC.
FT   TRANSMEM    187    208
FT   TOPO_DOM    209    227       NON CYTOPLASMIC.
FT   TRANSMEM    228    249
FT   TOPO_DOM    250    313       CYTOPLASMIC.
FT   TRANSMEM    314    341
FT   TOPO_DOM    342    748       NON CYTOPLASMIC.
//
```

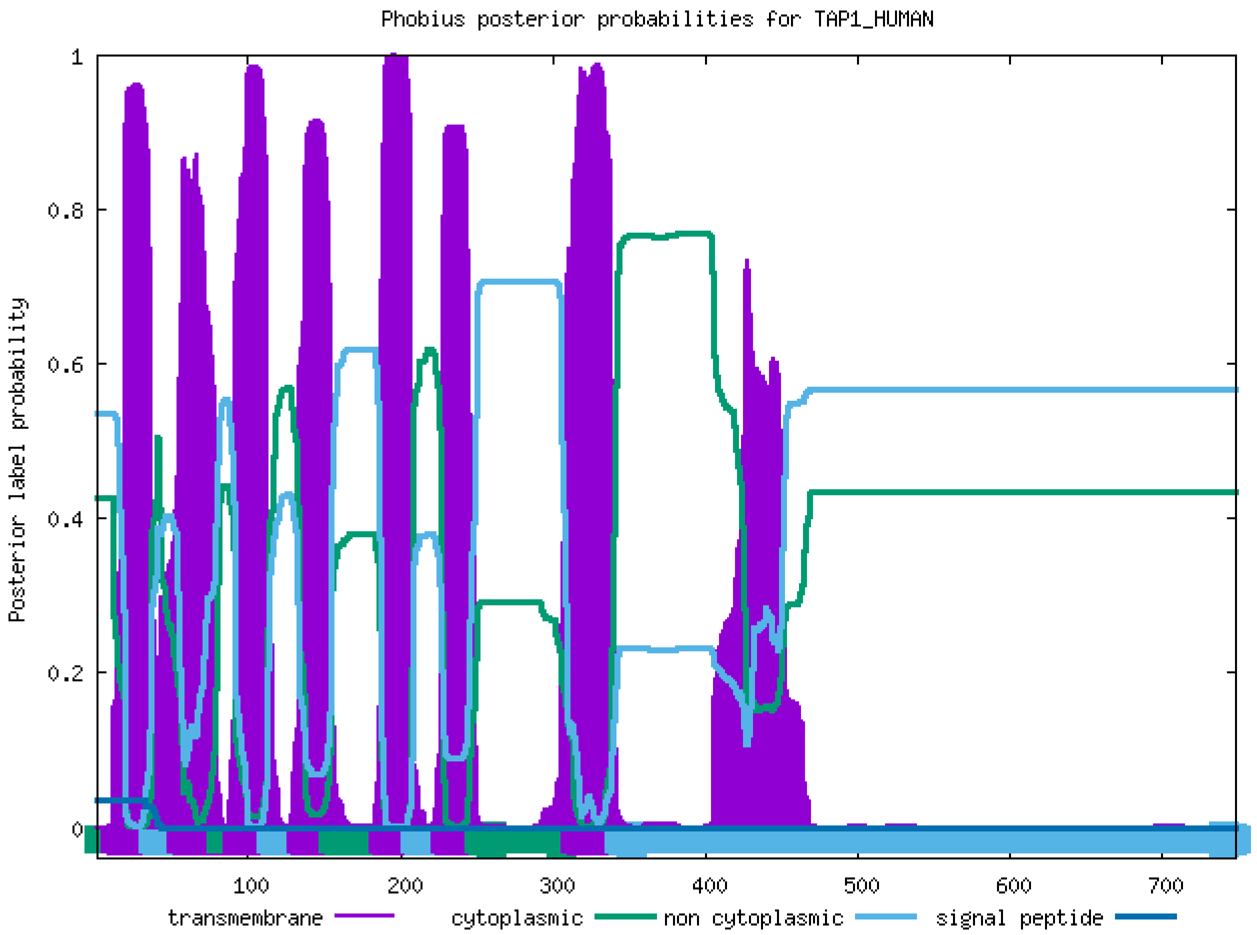

The probability data used in the plot is found here, and the gnuplot script is here.

Prediction of TAP2_HUMAN

```
ID    TAP2_HUMAN
FT    SIGNAL        1     32
FT    REGION        1     11       N-REGION.
FT    REGION       12     23       H-REGION.
FT    REGION       24     32       C-REGION.
FT    TOPO_DOM     33     55       NON CYTOPLASMIC.
FT    TRANSMEM     56     79
FT    TOPO_DOM     80     98       CYTOPLASMIC.
FT    TRANSMEM     99    119
FT    TOPO_DOM    120    148       NON CYTOPLASMIC.
FT    TRANSMEM    149    169
FT    TOPO_DOM    170    188       CYTOPLASMIC.
FT    TRANSMEM    189    208
FT    TOPO_DOM    209    686       NON CYTOPLASMIC.
//
```

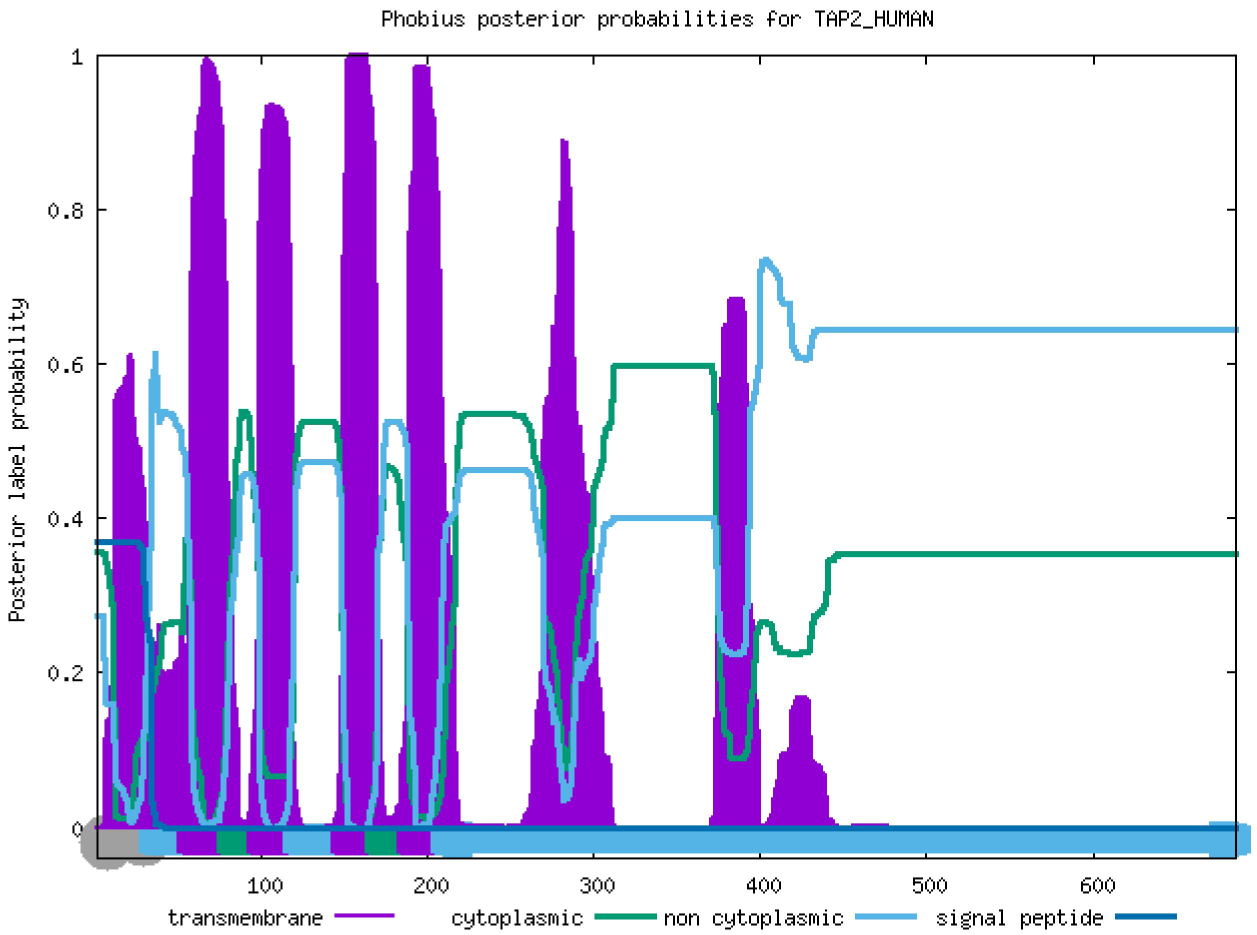

The probability data used in the plot is found here, and the gnuplot script is here.

Prediction of MRP4_HUMAN

```
ID    MRP4_HUMAN
FT    TOPO_DOM      1      91       CYTOPLASMIC.
FT    TRANSMEM     92     114
FT    TOPO_DOM    115     133       NON CYTOPLASMIC.
FT    TRANSMEM    134     157
FT    TOPO_DOM    158     207       CYTOPLASMIC.
FT    TRANSMEM    208     227
FT    TOPO_DOM    228     232       NON CYTOPLASMIC.
FT    TRANSMEM    233     254
FT    TOPO_DOM    255     320       CYTOPLASMIC.
FT    TRANSMEM    321     342
FT    TOPO_DOM    343     347       NON CYTOPLASMIC.
FT    TRANSMEM    348     369
FT    TOPO_DOM    370     710       CYTOPLASMIC.
FT    TRANSMEM    711     730
FT    TOPO_DOM    731     766       NON CYTOPLASMIC.
FT    TRANSMEM    767     791
FT    TOPO_DOM    792     834       CYTOPLASMIC.
FT    TRANSMEM    835     854
FT    TOPO_DOM    855     859       NON CYTOPLASMIC.
FT    TRANSMEM    860     882
FT    TOPO_DOM    883     947       CYTOPLASMIC.
FT    TRANSMEM    948     970
FT    TOPO_DOM    971     975       NON CYTOPLASMIC.
FT    TRANSMEM    976     997
FT    TOPO_DOM    998    1325       CYTOPLASMIC.
//
```

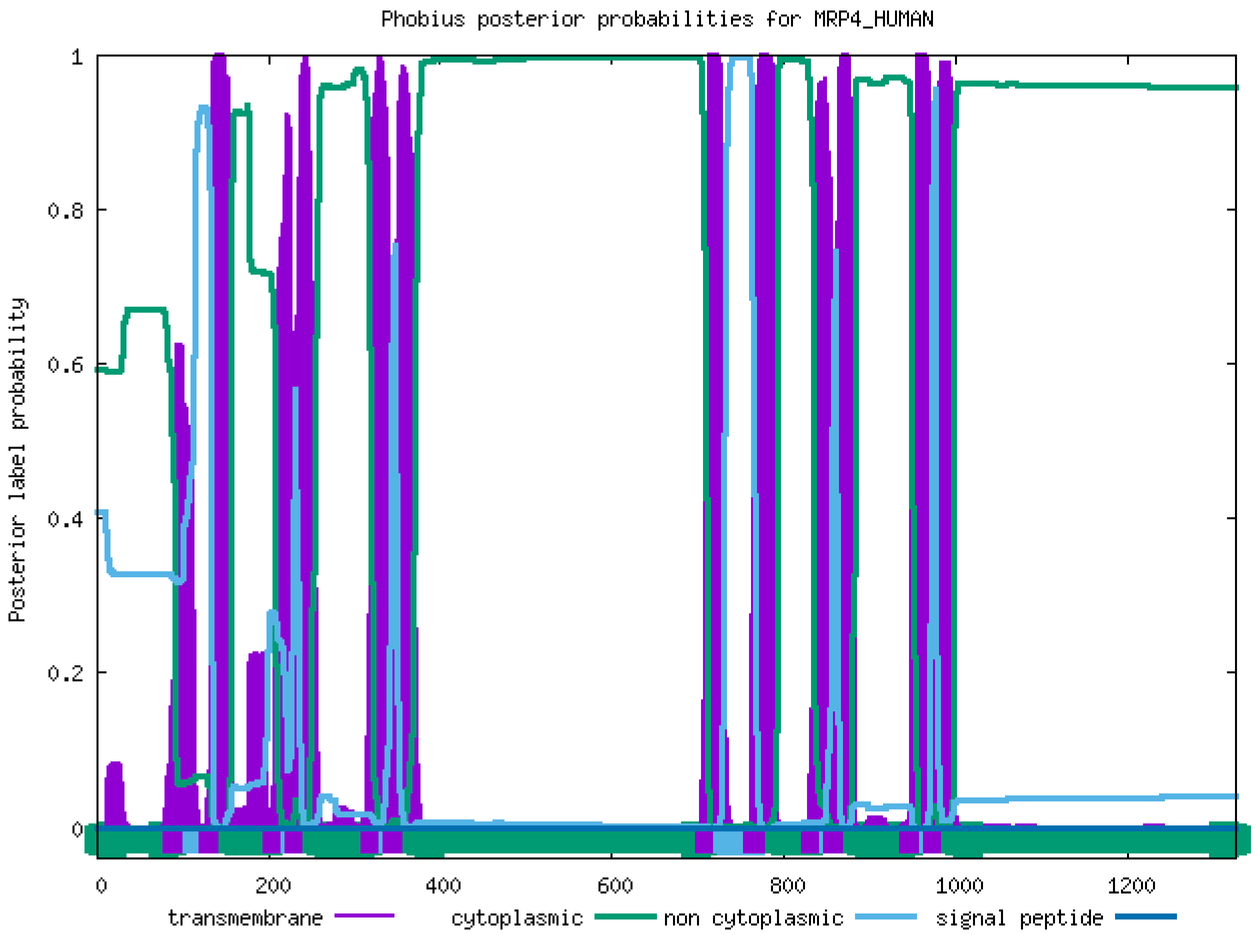

The probability data used in the plot is found here, and the gnuplot script is here.

Prediction of ABCG2_HUMAN

```
ID   ABCG2_HUMAN
FT   TOPO_DOM      1    395       NON CYTOPLASMIC.
FT   TRANSMEM    396    416
FT   TOPO_DOM    417    482       CYTOPLASMIC.
FT   TRANSMEM    483    499
FT   TOPO_DOM    500    504       NON CYTOPLASMIC.
FT   TRANSMEM    505    527
FT   TOPO_DOM    528    538       CYTOPLASMIC.
FT   TRANSMEM    539    565
FT   TOPO_DOM    566    630       NON CYTOPLASMIC.
FT   TRANSMEM    631    650
FT   TOPO_DOM    651    655       CYTOPLASMIC.
//
```

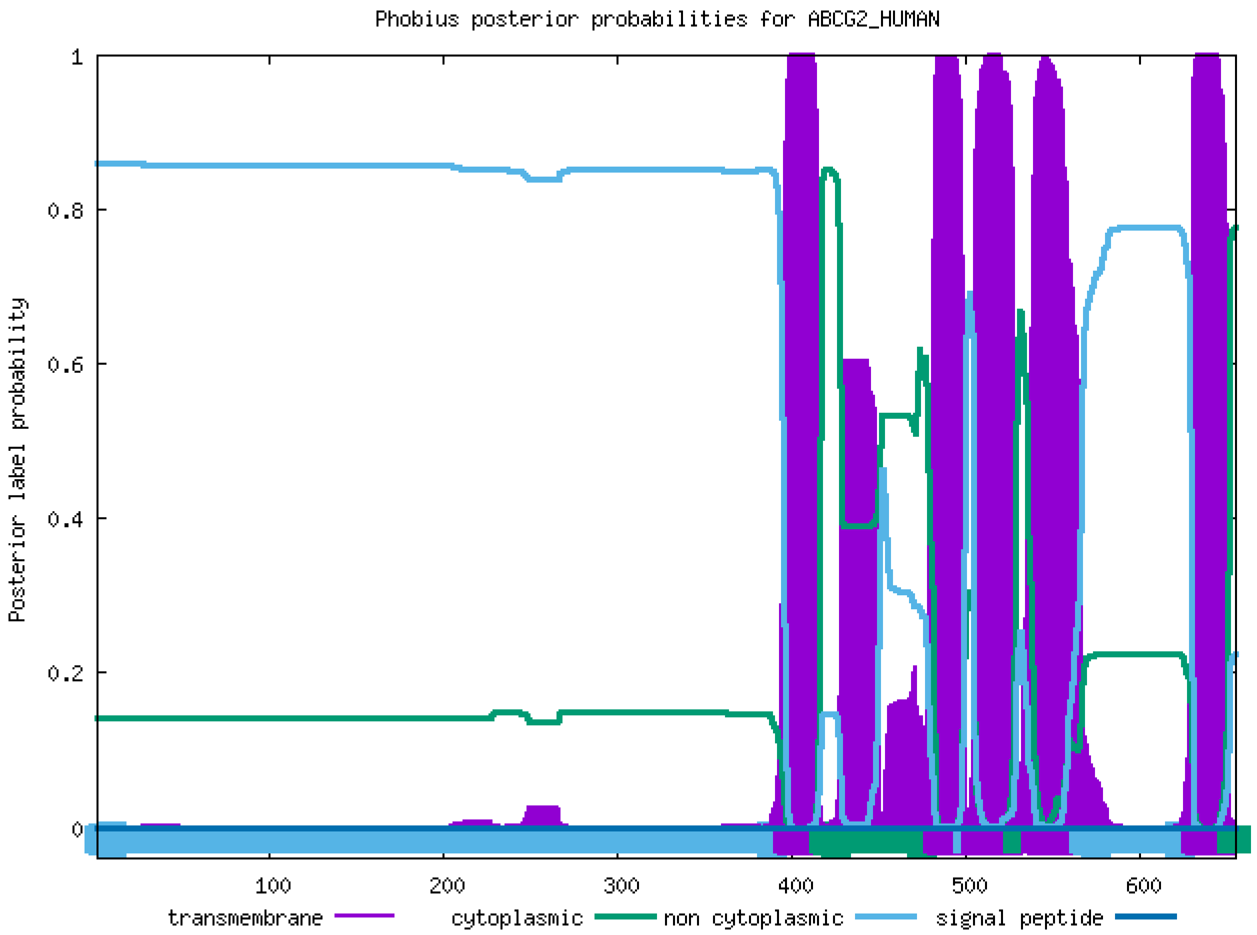

The probability data used in the plot is found here, and the gnuplot script is here.

Prediction of ABCA1_HUMAN

```
ID   ABCA1_HUMAN
FT   TOPO_DOM      1     24       CYTOPLASMIC.
FT   TRANSMEM     25     46
FT   TOPO_DOM     47    639       NON CYTOPLASMIC.
FT   TRANSMEM    640    660
FT   TOPO_DOM    661    679       CYTOPLASMIC.
FT   TRANSMEM    680    704
FT   TOPO_DOM    705    715       NON CYTOPLASMIC.
FT   TRANSMEM    716    736
FT   TOPO_DOM    737    742       CYTOPLASMIC.
FT   TRANSMEM    743    763
FT   TOPO_DOM    764    774       NON CYTOPLASMIC.
FT   TRANSMEM    775    797
FT   TOPO_DOM    798    817       CYTOPLASMIC.
FT   TRANSMEM    818    842
FT   TOPO_DOM    843   1346       NON CYTOPLASMIC.
FT   TRANSMEM   1347   1368
FT   TOPO_DOM   1369   1654       CYTOPLASMIC.
FT   TRANSMEM   1655   1677
FT   TOPO_DOM   1678   1696       NON CYTOPLASMIC.
FT   TRANSMEM   1697   1723
FT   TOPO_DOM   1724   1734       CYTOPLASMIC.
FT   TRANSMEM   1735   1754
FT   TOPO_DOM   1755   1765       NON CYTOPLASMIC.
FT   TRANSMEM   1766   1790
FT   TOPO_DOM   1791   1801       CYTOPLASMIC.
FT   TRANSMEM   1802   1820
FT   TOPO_DOM   1821   1852       NON CYTOPLASMIC.
FT   TRANSMEM   1853   1875
```

```
FT   TOPO_DOM   1876   2261       CYTOPLASMIC.
//
```

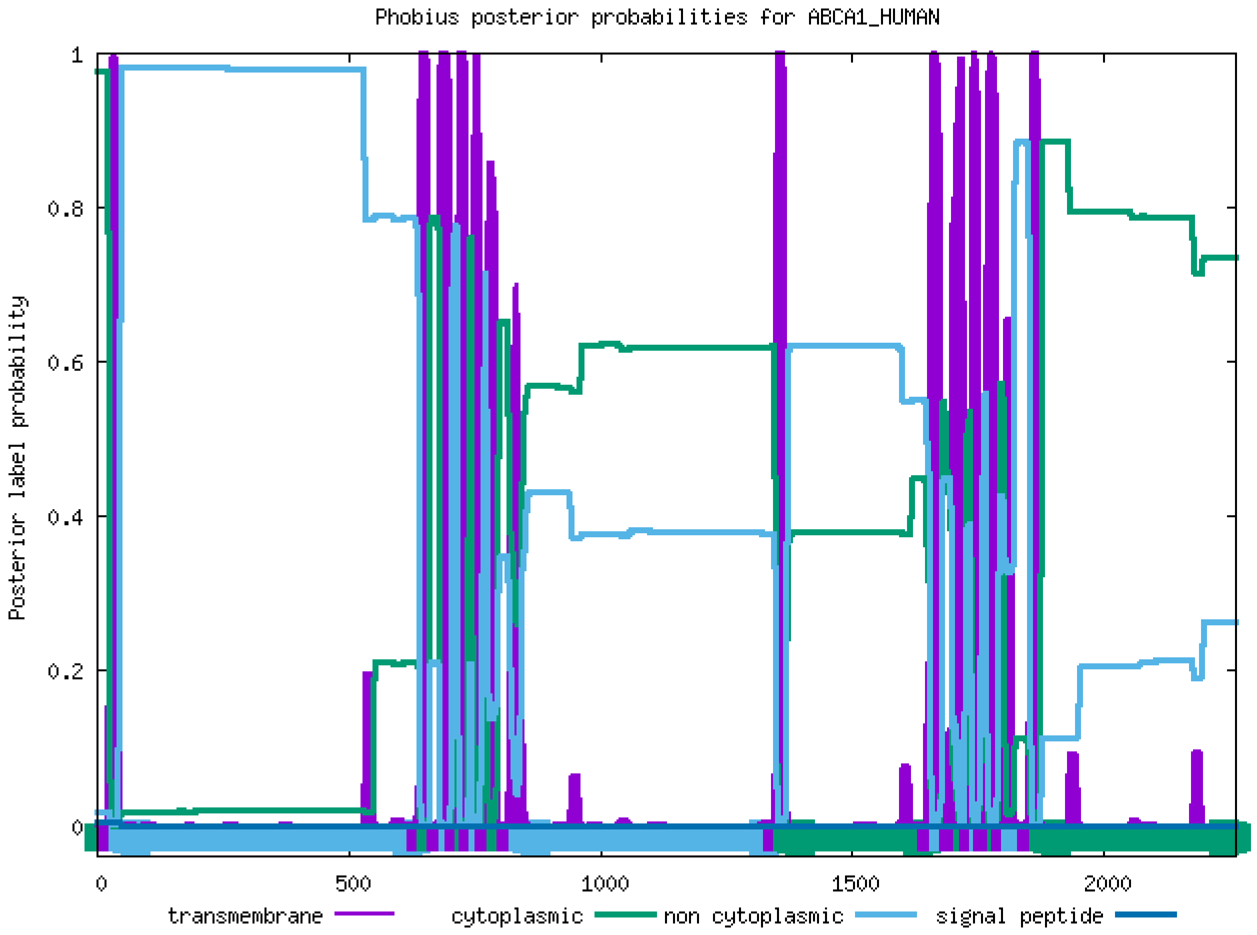

The probability data used in the plot is found here, and the gnuplot script is here.

Prediction of ABCA3_HUMAN

```
ID   ABCA3_HUMAN
FT   TOPO_DOM      1     20       CYTOPLASMIC.
FT   TRANSMEM     21     42
FT   TOPO_DOM     43    262       NON CYTOPLASMIC.
FT   TRANSMEM    263    283
FT   TOPO_DOM    284    302       CYTOPLASMIC.
FT   TRANSMEM    303    327
FT   TOPO_DOM    328    346       NON CYTOPLASMIC.
FT   TRANSMEM    347    368
FT   TOPO_DOM    369    379       CYTOPLASMIC.
FT   TRANSMEM    380    398
FT   TOPO_DOM    399    925       NON CYTOPLASMIC.
FT   TRANSMEM    926    947
FT   TOPO_DOM    948   1099       CYTOPLASMIC.
FT   TRANSMEM   1100   1121
FT   TOPO_DOM   1122   1140       NON CYTOPLASMIC.
FT   TRANSMEM   1141   1164
FT   TOPO_DOM   1165   1184       CYTOPLASMIC.
FT   TRANSMEM   1185   1207
FT   TOPO_DOM   1208   1212       NON CYTOPLASMIC.
FT   TRANSMEM   1213   1233
FT   TOPO_DOM   1234   1244       CYTOPLASMIC.
FT   TRANSMEM   1245   1265
FT   TOPO_DOM   1266   1305       NON CYTOPLASMIC.
FT   TRANSMEM   1306   1324
FT   TOPO_DOM   1325   1704       CYTOPLASMIC.
//
```

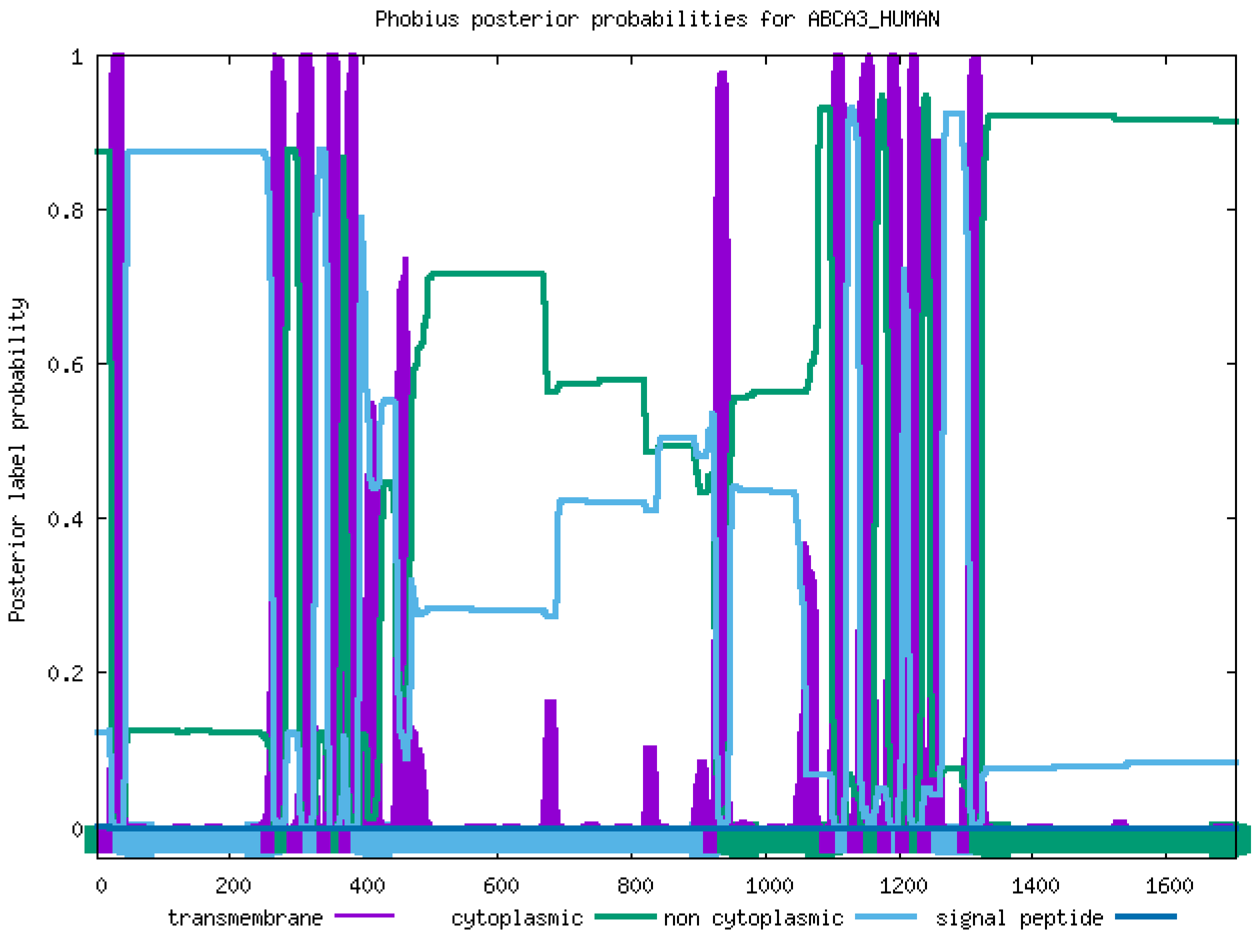

The probability data used in the plot is found here, and the gnuplot script is here.

Prediction of ABCA4_HUMAN

```
ID   ABCA4_HUMAN
FT   TOPO_DOM      1     22       NON CYTOPLASMIC.
FT   TRANSMEM     23     42
FT   TOPO_DOM     43    653       CYTOPLASMIC.
FT   TRANSMEM    654    674
FT   TOPO_DOM    675    693       NON CYTOPLASMIC.
FT   TRANSMEM    694    719
FT   TOPO_DOM    720    730       CYTOPLASMIC.
FT   TRANSMEM    731    754
FT   TOPO_DOM    755    759       NON CYTOPLASMIC.
FT   TRANSMEM    760    782
FT   TOPO_DOM    783    835       CYTOPLASMIC.
FT   TRANSMEM    836    857
FT   TOPO_DOM    858   1372       NON CYTOPLASMIC.
FT   TRANSMEM   1373   1394
FT   TOPO_DOM   1395   1679       CYTOPLASMIC.
FT   TRANSMEM   1680   1702
FT   TOPO_DOM   1703   1727       NON CYTOPLASMIC.
FT   TRANSMEM   1728   1748
FT   TOPO_DOM   1749   1759       CYTOPLASMIC.
FT   TRANSMEM   1760   1779
FT   TOPO_DOM   1780   1790       NON CYTOPLASMIC.
FT   TRANSMEM   1791   1816
FT   TOPO_DOM   1817   1827       CYTOPLASMIC.
FT   TRANSMEM   1828   1846
FT   TOPO_DOM   1847   1878       NON CYTOPLASMIC.
FT   TRANSMEM   1879   1896
FT   TOPO_DOM   1897   2273       CYTOPLASMIC.
//
```

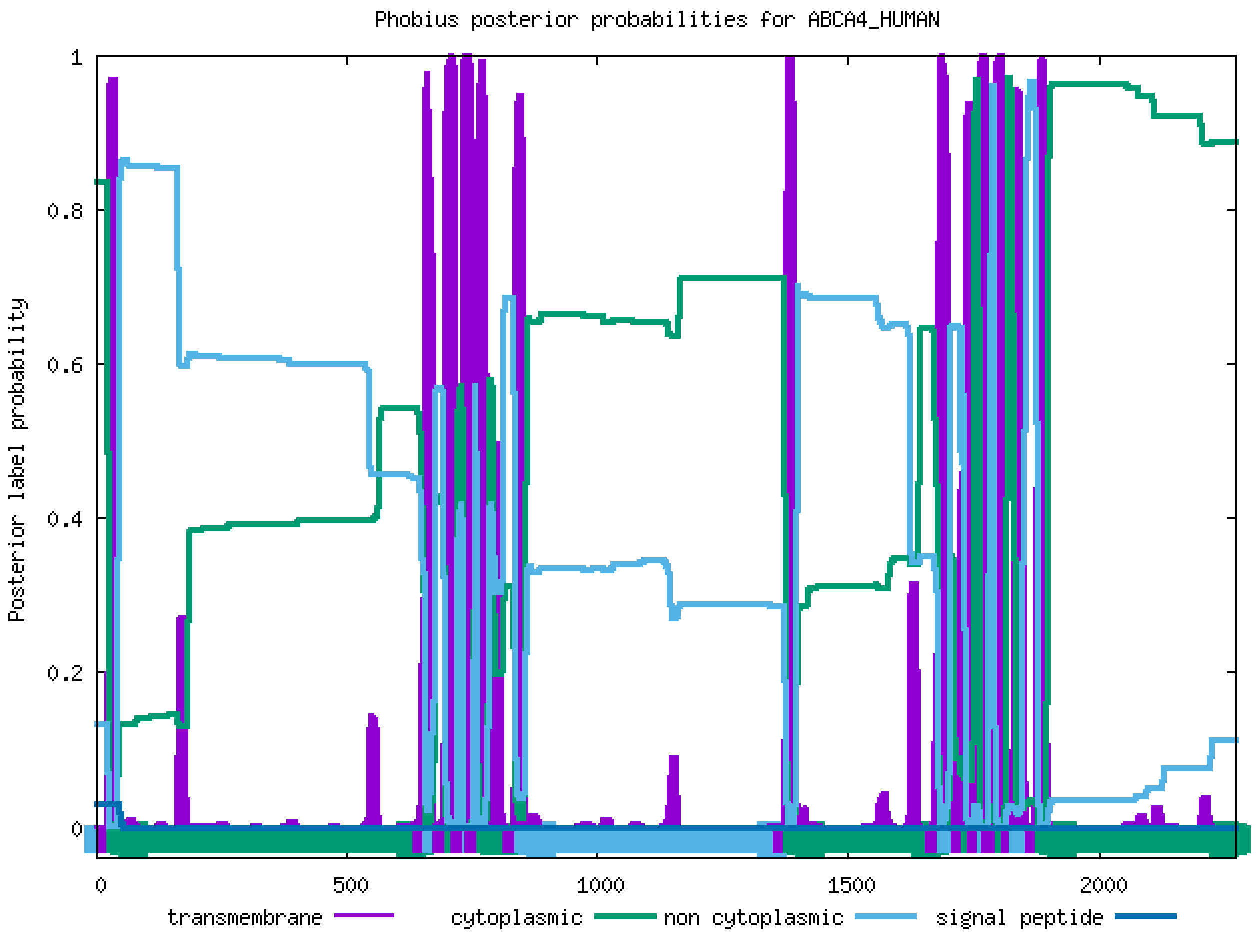

The probability data used in the plot is found here, and the gnuplot script is here.

Prediction of ABCA7_HUMAN

```
ID   ABCA7_HUMAN
FT   SIGNAL        1     45
FT   REGION        1     28       N-REGION.
FT   REGION       29     40       H-REGION.
FT   REGION       41     45       C-REGION.
FT   TOPO_DOM     46    549       NON CYTOPLASMIC.
FT   TRANSMEM    550    570
FT   TOPO_DOM    571    589       CYTOPLASMIC.
FT   TRANSMEM    590    614
FT   TOPO_DOM    615    625       NON CYTOPLASMIC.
FT   TRANSMEM    626    646
FT   TOPO_DOM    647    652       CYTOPLASMIC.
FT   TRANSMEM    653    673
FT   TOPO_DOM    674   1238       NON CYTOPLASMIC.
FT   TRANSMEM   1239   1260
FT   TOPO_DOM   1261   1535       CYTOPLASMIC.
FT   TRANSMEM   1536   1558
FT   TOPO_DOM   1559   1577       NON CYTOPLASMIC.
FT   TRANSMEM   1578   1604
FT   TOPO_DOM   1605   1615       CYTOPLASMIC.
FT   TRANSMEM   1616   1635
FT   TOPO_DOM   1636   1646       NON CYTOPLASMIC.
FT   TRANSMEM   1647   1671
FT   TOPO_DOM   1672   1682       CYTOPLASMIC.
FT   TRANSMEM   1683   1701
FT   TOPO_DOM   1702   1733       NON CYTOPLASMIC.
FT   TRANSMEM   1734   1752
FT   TOPO_DOM   1753   2146       CYTOPLASMIC.
//
```

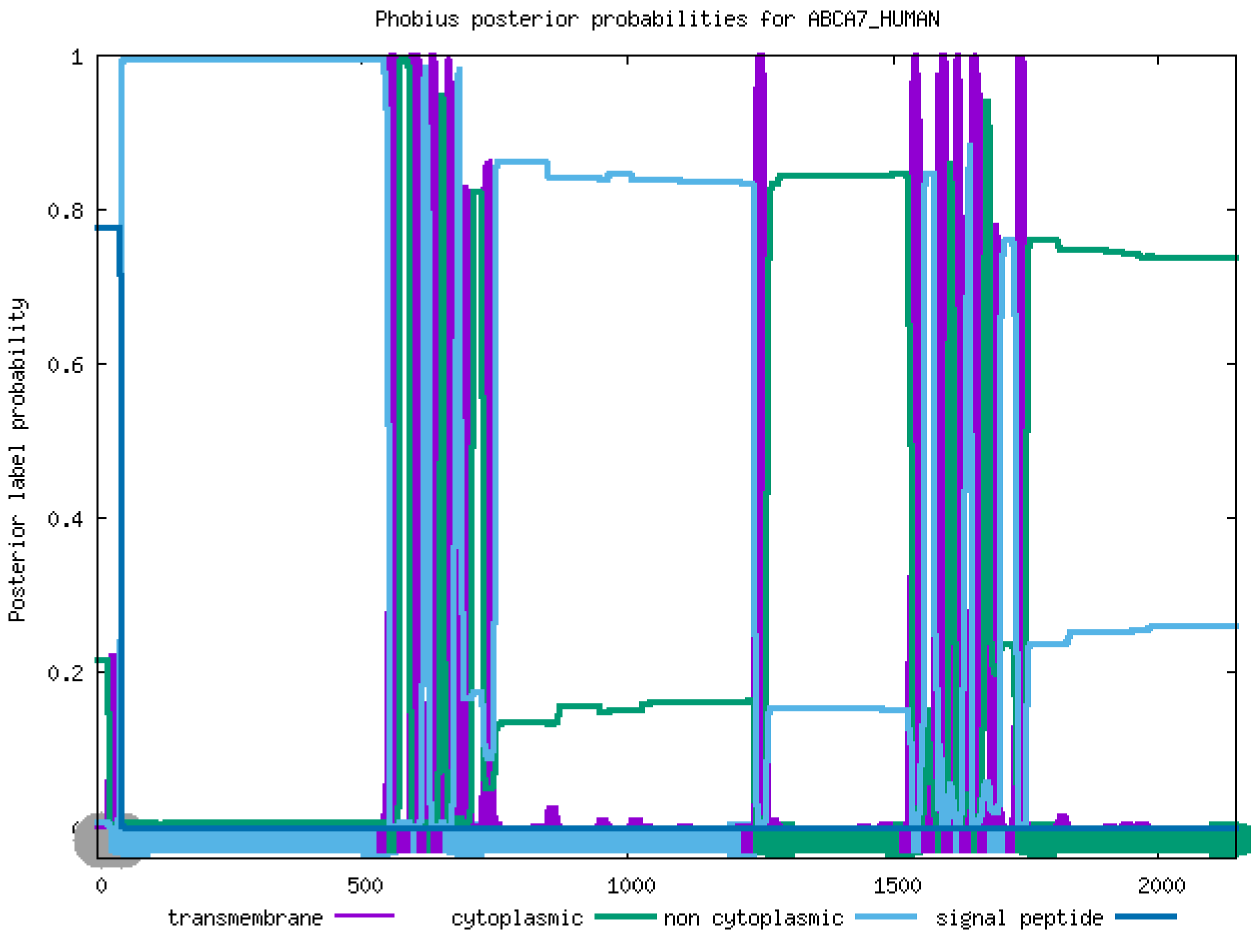

The probability data used in the plot is found here, and the gnuplot script is here.

Prediction of GTR1_HUMAN

```
ID   GTR1_HUMAN
FT   TOPO_DOM      1     11       CYTOPLASMIC.
FT   TRANSMEM     12     28
FT   TOPO_DOM     29     63       NON CYTOPLASMIC.
FT   TRANSMEM     64     87
FT   TOPO_DOM     88     98       CYTOPLASMIC.
FT   TRANSMEM     99    116
FT   TOPO_DOM    117    121       NON CYTOPLASMIC.
FT   TRANSMEM    122    144
FT   TOPO_DOM    145    155       CYTOPLASMIC.
FT   TRANSMEM    156    174
FT   TOPO_DOM    175    185       NON CYTOPLASMIC.
FT   TRANSMEM    186    207
FT   TOPO_DOM    208    271       CYTOPLASMIC.
FT   TRANSMEM    272    295
FT   TOPO_DOM    296    306       NON CYTOPLASMIC.
FT   TRANSMEM    307    328
FT   TOPO_DOM    329    334       CYTOPLASMIC.
FT   TRANSMEM    335    357
FT   TOPO_DOM    358    362       NON CYTOPLASMIC.
FT   TRANSMEM    363    381
FT   TOPO_DOM    382    401       CYTOPLASMIC.
FT   TRANSMEM    402    425
FT   TOPO_DOM    426    430       NON CYTOPLASMIC.
FT   TRANSMEM    431    450
FT   TOPO_DOM    451    492       CYTOPLASMIC.
//
```

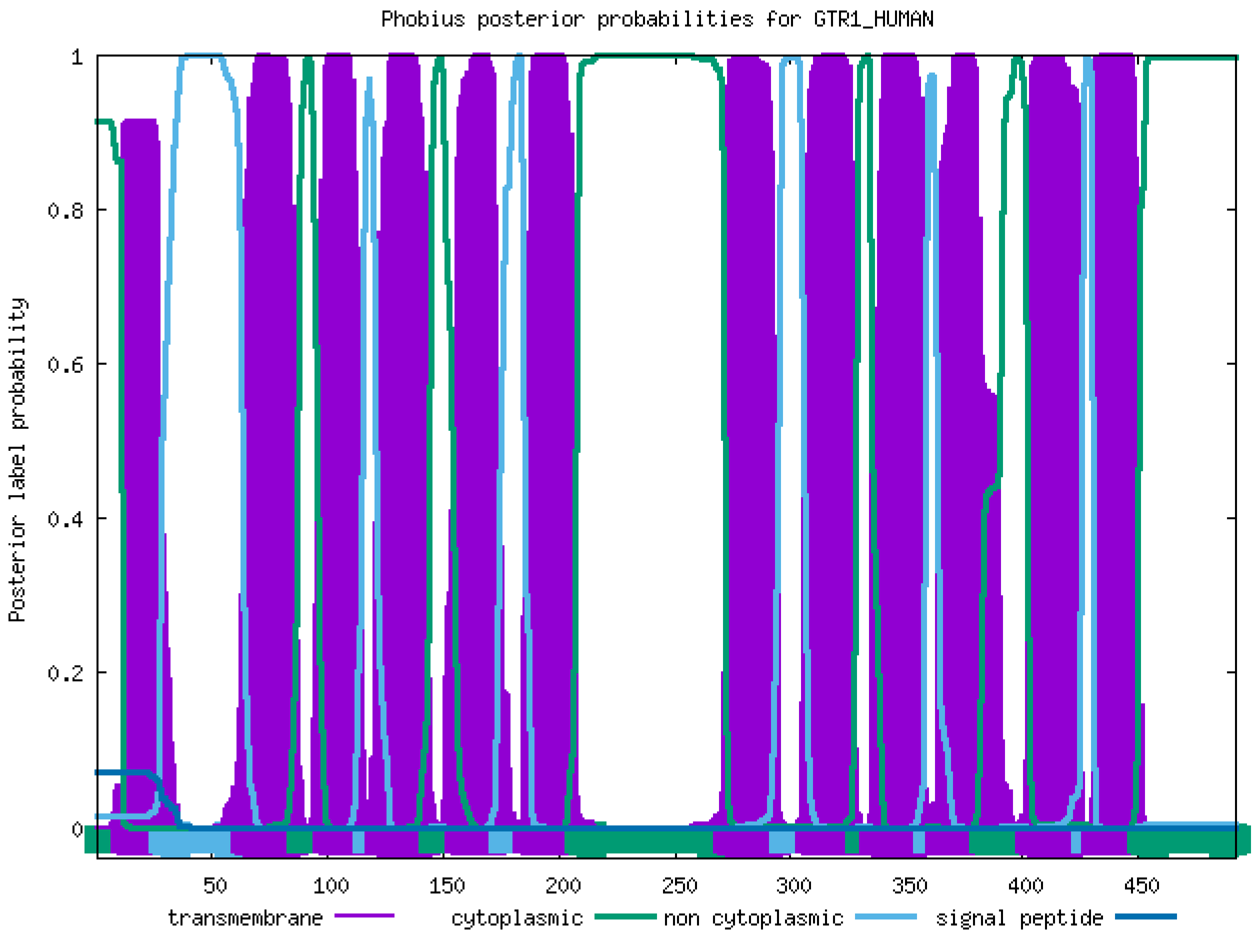

The probability data used in the plot is found here, and the gnuplot script is here.

Prediction of GTR3_HUMAN

```
ID   GTR3_HUMAN
FT   TOPO_DOM      1      6       CYTOPLASMIC.
FT   TRANSMEM      7     26
FT   TOPO_DOM     27     61       NON CYTOPLASMIC.
FT   TRANSMEM     62     84
FT   TOPO_DOM     85     95       CYTOPLASMIC.
FT   TRANSMEM     96    115
FT   TOPO_DOM    116    120       NON CYTOPLASMIC.
FT   TRANSMEM    121    142
FT   TOPO_DOM    143    153       CYTOPLASMIC.
FT   TRANSMEM    154    177
FT   TOPO_DOM    178    182       NON CYTOPLASMIC.
FT   TRANSMEM    183    205
FT   TOPO_DOM    206    269       CYTOPLASMIC.
FT   TRANSMEM    270    293
FT   TOPO_DOM    294    304       NON CYTOPLASMIC.
FT   TRANSMEM    305    326
FT   TOPO_DOM    327    332       CYTOPLASMIC.
FT   TRANSMEM    333    355
FT   TOPO_DOM    356    360       NON CYTOPLASMIC.
FT   TRANSMEM    361    379
FT   TOPO_DOM    380    399       CYTOPLASMIC.
FT   TRANSMEM    400    420
FT   TOPO_DOM    421    425       NON CYTOPLASMIC.
FT   TRANSMEM    426    448
FT   TOPO_DOM    449    496       CYTOPLASMIC.
//
```

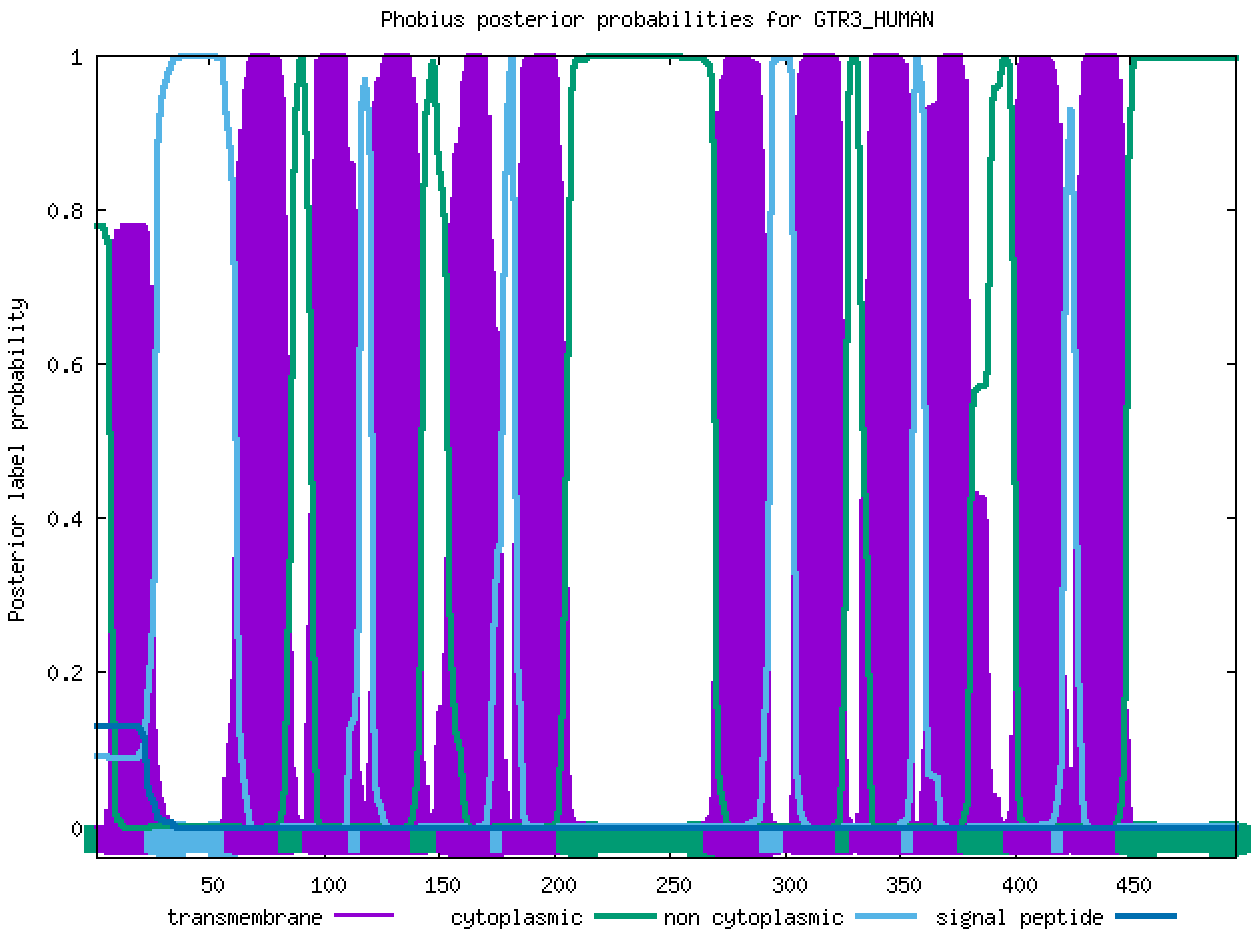

The probability data used in the plot is found here, and the gnuplot script is here.

Prediction of GLUT4_HUMAN

```
ID   GLUT4_HUMAN
FT   TOPO_DOM      1     19       CYTOPLASMIC.
FT   TRANSMEM     20     40
FT   TOPO_DOM     41     79       NON CYTOPLASMIC.
FT   TRANSMEM     80    101
FT   TOPO_DOM    102    112       CYTOPLASMIC.
FT   TRANSMEM    113    133
FT   TOPO_DOM    134    138       NON CYTOPLASMIC.
FT   TRANSMEM    139    160
FT   TOPO_DOM    161    171       CYTOPLASMIC.
FT   TRANSMEM    172    190
FT   TOPO_DOM    191    201       NON CYTOPLASMIC.
FT   TRANSMEM    202    223
FT   TOPO_DOM    224    287       CYTOPLASMIC.
FT   TRANSMEM    288    311
FT   TOPO_DOM    312    322       NON CYTOPLASMIC.
FT   TRANSMEM    323    344
FT   TOPO_DOM    345    350       CYTOPLASMIC.
FT   TRANSMEM    351    373
FT   TOPO_DOM    374    378       NON CYTOPLASMIC.
FT   TRANSMEM    379    397
FT   TOPO_DOM    398    417       CYTOPLASMIC.
FT   TRANSMEM    418    442
FT   TOPO_DOM    443    447       NON CYTOPLASMIC.
FT   TRANSMEM    448    466
FT   TOPO_DOM    467    509       CYTOPLASMIC.
//
```

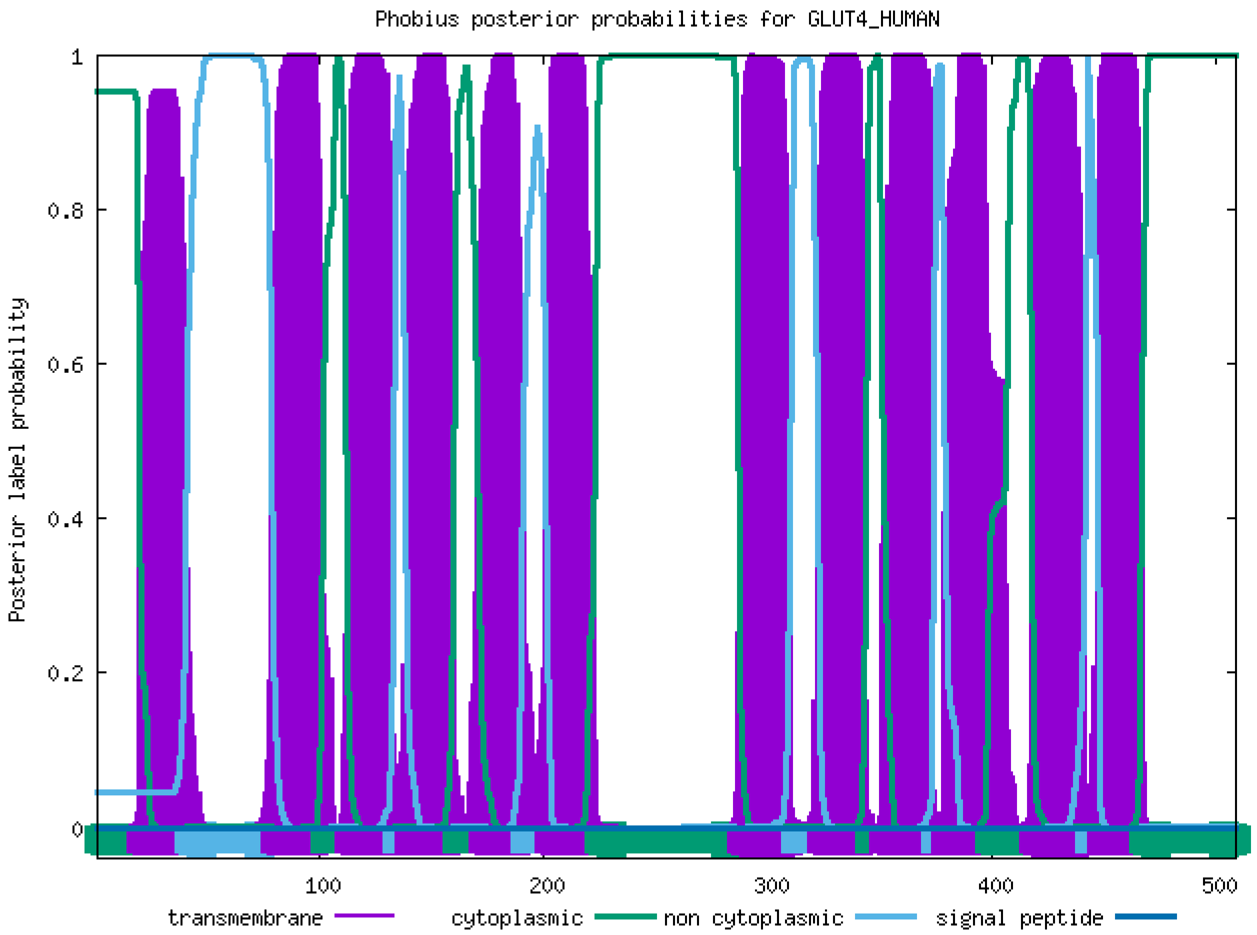

The probability data used in the plot is found here, and the gnuplot script is here.

Prediction of S40A1_HUMAN

```
ID   S40A1_HUMAN
FT   TOPO_DOM      1     57       NON CYTOPLASMIC.
FT   TRANSMEM     58     83
FT   TOPO_DOM     84     94       CYTOPLASMIC.
FT   TRANSMEM     95    115
FT   TOPO_DOM    116    126       NON CYTOPLASMIC.
FT   TRANSMEM    127    146
FT   TOPO_DOM    147    306       CYTOPLASMIC.
FT   TRANSMEM    307    333
FT   TOPO_DOM    334    338       NON CYTOPLASMIC.
FT   TRANSMEM    339    362
FT   TOPO_DOM    363    373       CYTOPLASMIC.
FT   TRANSMEM    374    393
FT   TOPO_DOM    394    449       NON CYTOPLASMIC.
FT   TRANSMEM    450    469
FT   TOPO_DOM    470    489       CYTOPLASMIC.
FT   TRANSMEM    490    513
FT   TOPO_DOM    514    518       NON CYTOPLASMIC.
FT   TRANSMEM    519    539
FT   TOPO_DOM    540    571       CYTOPLASMIC.
//
```

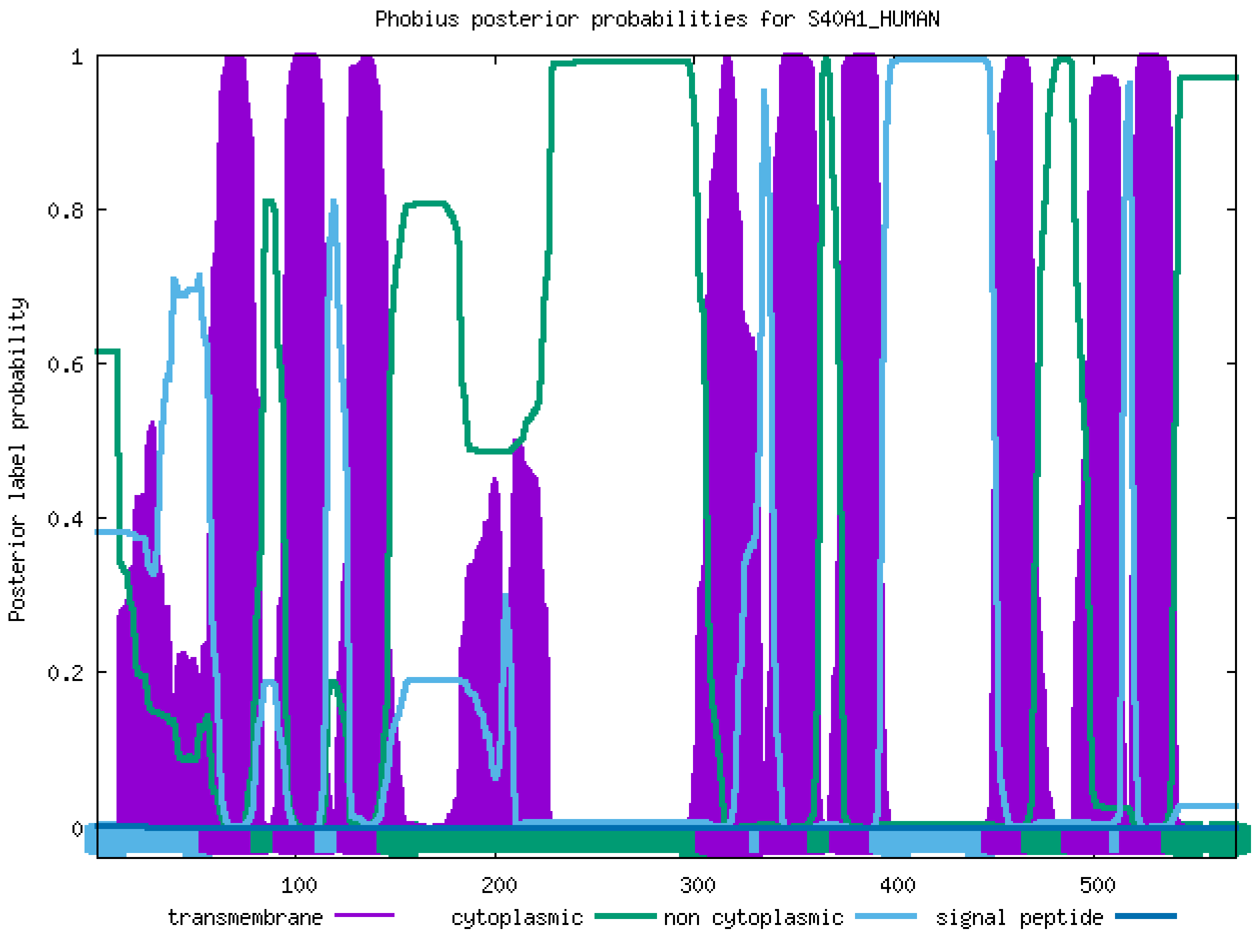

The probability data used in the plot is found here, and the gnuplot script is here.

Prediction of SYCY2_HUMAN

```
ID   SYCY2_HUMAN
FT   SIGNAL        1     15
FT   REGION        1      2       N-REGION.
FT   REGION        3     10       H-REGION.
FT   REGION       11     15       C-REGION.
FT   TOPO_DOM     16    108       NON CYTOPLASMIC.
FT   TRANSMEM    109    129
FT   TOPO_DOM    130    353       CYTOPLASMIC.
FT   TRANSMEM    354    374
FT   TOPO_DOM    375    478       NON CYTOPLASMIC.
FT   TRANSMEM    479    507
FT   TOPO_DOM    508    538       CYTOPLASMIC.
//
```

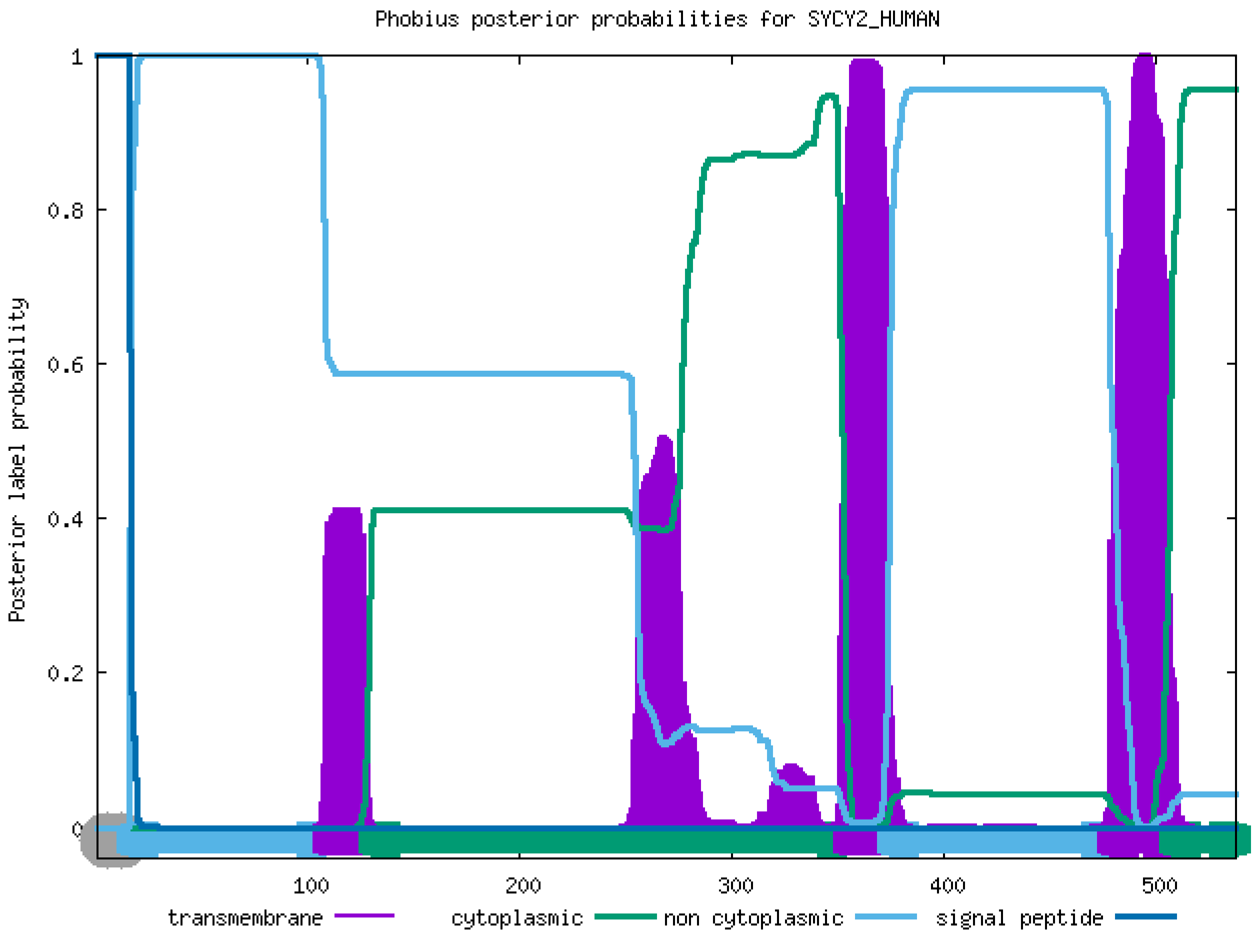

The probability data used in the plot is found here, and the gnuplot script is here.

Prediction of NLS1_HUMAN

```
ID    NLS1_HUMAN
FT    TOPO_DOM      1     46       CYTOPLASMIC.
FT    TRANSMEM     47     71
FT    TOPO_DOM     72    127       NON CYTOPLASMIC.
FT    TRANSMEM    128    149
FT    TOPO_DOM    150    160       CYTOPLASMIC.
FT    TRANSMEM    161    185
FT    TOPO_DOM    186    253       NON CYTOPLASMIC.
FT    TRANSMEM    254    277
FT    TOPO_DOM    278    305       CYTOPLASMIC.
FT    TRANSMEM    306    324
FT    TOPO_DOM    325    343       NON CYTOPLASMIC.
FT    TRANSMEM    344    364
FT    TOPO_DOM    365    370       CYTOPLASMIC.
FT    TRANSMEM    371    389
FT    TOPO_DOM    390    394       NON CYTOPLASMIC.
FT    TRANSMEM    395    419
FT    TOPO_DOM    420    438       CYTOPLASMIC.
FT    TRANSMEM    439    461
FT    TOPO_DOM    462    480       NON CYTOPLASMIC.
FT    TRANSMEM    481    502
FT    TOPO_DOM    503    543       CYTOPLASMIC.
//
```

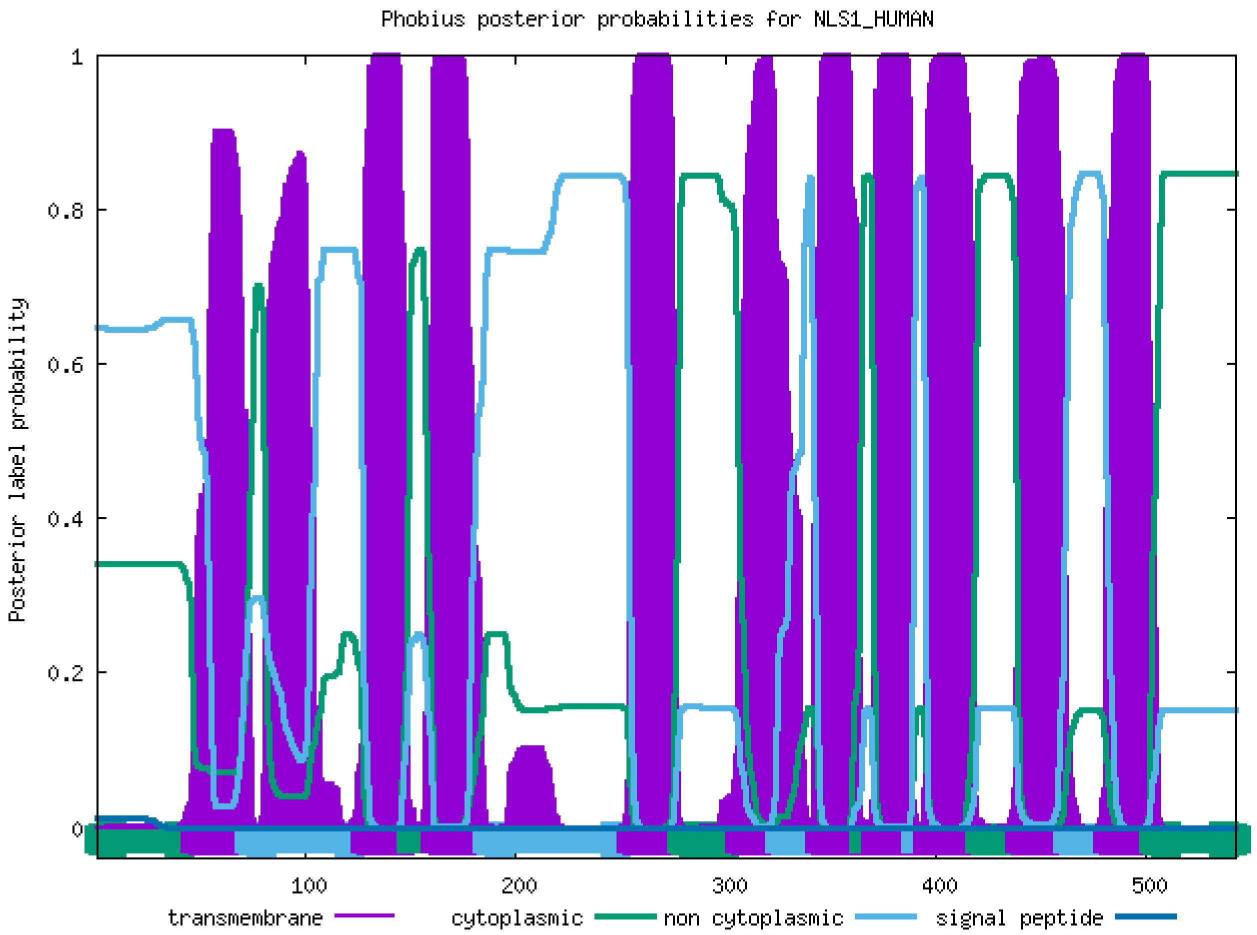

The probability data used in the plot is found here, and the gnuplot script is here.

Prediction of SC5A1_HUMAN

```
ID    SC5A1_HUMAN
FT    TOPO_DOM      1     28       NON CYTOPLASMIC.
FT    TRANSMEM     29     48
FT    TOPO_DOM     49     54       CYTOPLASMIC.
FT    TRANSMEM     55     76
FT    TOPO_DOM     77     95       NON CYTOPLASMIC.
FT    TRANSMEM     96    121
FT    TOPO_DOM    122    141       CYTOPLASMIC.
FT    TRANSMEM    142    171
FT    TOPO_DOM    172    176       NON CYTOPLASMIC.
FT    TRANSMEM    177    202
FT    TOPO_DOM    203    208       CYTOPLASMIC.
FT    TRANSMEM    209    227
FT    TOPO_DOM    228    273       NON CYTOPLASMIC.
FT    TRANSMEM    274    292
FT    TOPO_DOM    293    312       CYTOPLASMIC.
FT    TRANSMEM    313    334
FT    TOPO_DOM    335    380       NON CYTOPLASMIC.
FT    TRANSMEM    381    401
FT    TOPO_DOM    402    421       CYTOPLASMIC.
FT    TRANSMEM    422    443
FT    TOPO_DOM    444    454       NON CYTOPLASMIC.
FT    TRANSMEM    455    477
FT    TOPO_DOM    478    483       CYTOPLASMIC.
FT    TRANSMEM    484    506
FT    TOPO_DOM    507    525       NON CYTOPLASMIC.
FT    TRANSMEM    526    548
FT    TOPO_DOM    549    639       CYTOPLASMIC.
FT    TRANSMEM    640    663
```

```
FT   TOPO_DOM    664    664       NON CYTOPLASMIC.
//
```

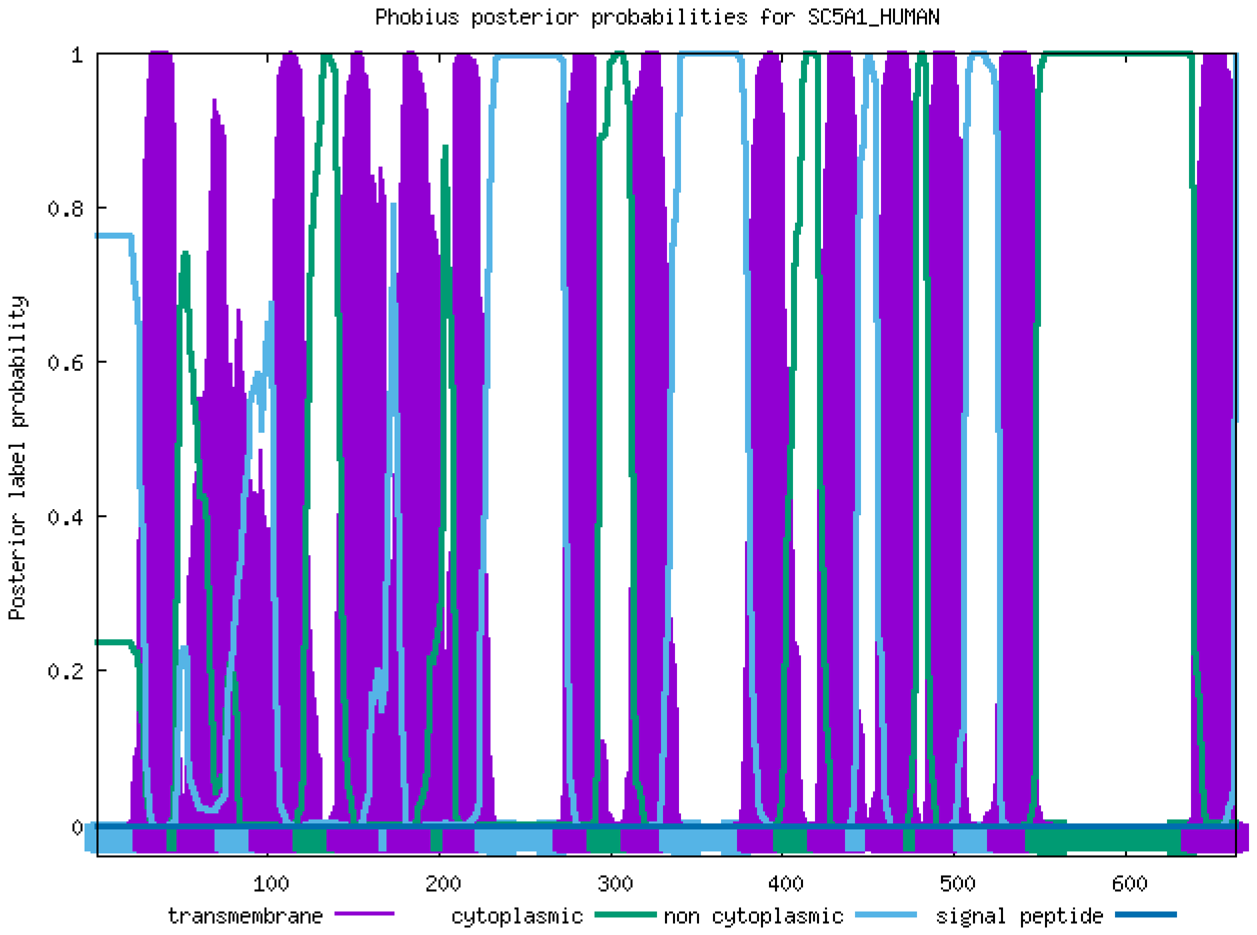

The probability data used in the plot is found here, and the gnuplot script is here.

Prediction of SC5A8_HUMAN

```
ID   SC5A8_HUMAN
FT   TOPO_DOM      1     11       NON CYTOPLASMIC.
FT   TRANSMEM     12     34
FT   TOPO_DOM     35     53       CYTOPLASMIC.
FT   TRANSMEM     54     72
FT   TOPO_DOM     73     83       NON CYTOPLASMIC.
FT   TRANSMEM     84    108
FT   TOPO_DOM    109    128       CYTOPLASMIC.
FT   TRANSMEM    129    150
FT   TOPO_DOM    151    161       NON CYTOPLASMIC.
FT   TRANSMEM    162    182
FT   TOPO_DOM    183    193       CYTOPLASMIC.
FT   TRANSMEM    194    218
FT   TOPO_DOM    219    239       NON CYTOPLASMIC.
FT   TRANSMEM    240    259
FT   TOPO_DOM    260    279       CYTOPLASMIC.
FT   TRANSMEM    280    302
FT   TOPO_DOM    303    339       NON CYTOPLASMIC.
FT   TRANSMEM    340    369
FT   TOPO_DOM    370    389       CYTOPLASMIC.
FT   TRANSMEM    390    410
FT   TOPO_DOM    411    415       NON CYTOPLASMIC.
FT   TRANSMEM    416    436
FT   TOPO_DOM    437    442       CYTOPLASMIC.
FT   TRANSMEM    443    463
FT   TOPO_DOM    464    520       NON CYTOPLASMIC.
FT   TRANSMEM    521    541
```

```
FT   TOPO_DOM    542    610       CYTOPLASMIC.
//
```

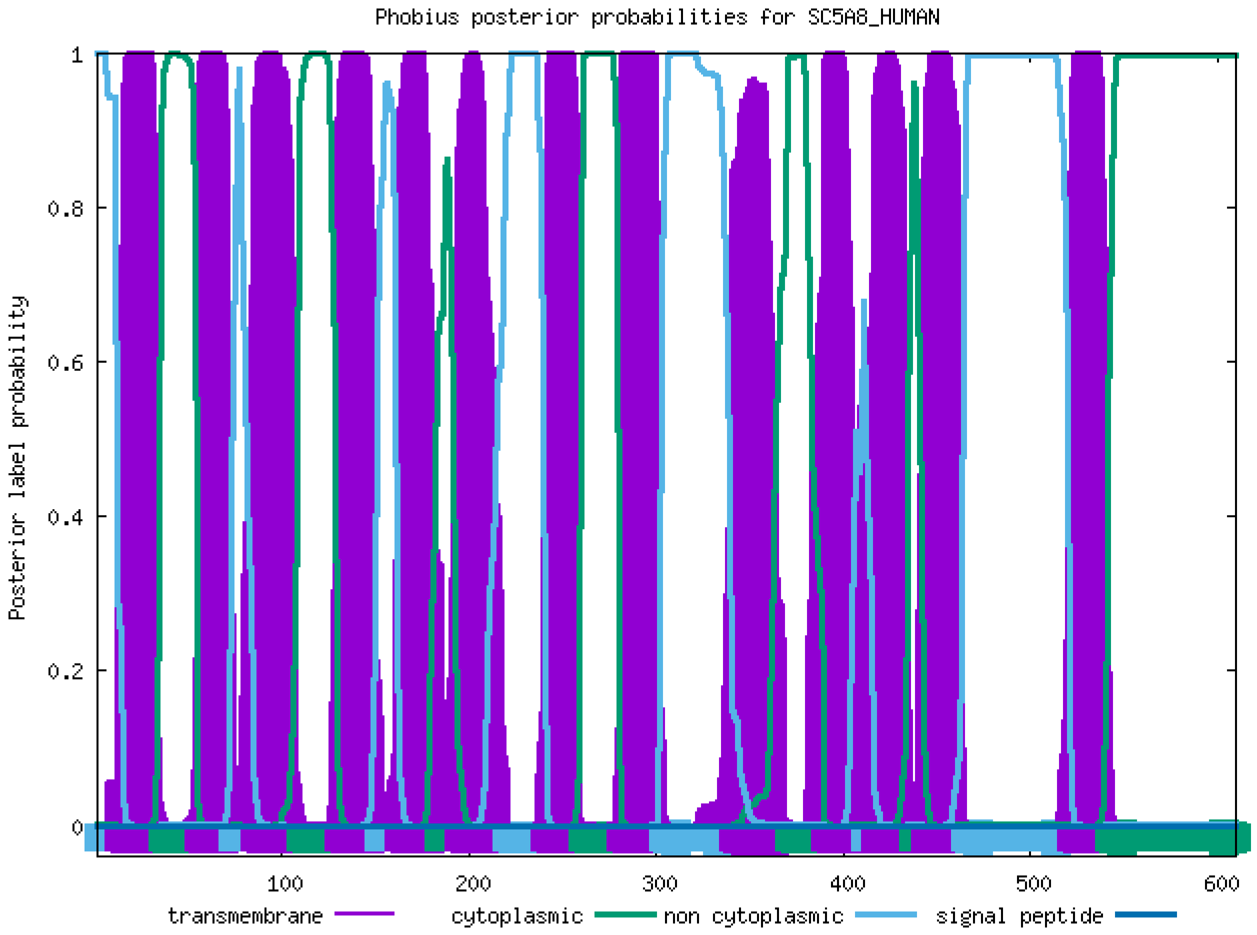

The probability data used in the plot is found here, and the gnuplot script is here.

Prediction of SC5A2_HUMAN

```
ID   SC5A2_HUMAN
FT   TOPO_DOM      1     25       NON CYTOPLASMIC.
FT   TRANSMEM     26     42
FT   TOPO_DOM     43     62       CYTOPLASMIC.
FT   TRANSMEM     63     82
FT   TOPO_DOM     83    101       NON CYTOPLASMIC.
FT   TRANSMEM    102    126
FT   TOPO_DOM    127    137       CYTOPLASMIC.
FT   TRANSMEM    138    157
FT   TOPO_DOM    158    168       NON CYTOPLASMIC.
FT   TRANSMEM    169    190
FT   TOPO_DOM    191    201       CYTOPLASMIC.
FT   TRANSMEM    202    220
FT   TOPO_DOM    221    273       NON CYTOPLASMIC.
FT   TRANSMEM    274    292
FT   TOPO_DOM    293    312       CYTOPLASMIC.
FT   TRANSMEM    313    334
FT   TOPO_DOM    335    380       NON CYTOPLASMIC.
FT   TRANSMEM    381    401
FT   TOPO_DOM    402    421       CYTOPLASMIC.
FT   TRANSMEM    422    443
FT   TOPO_DOM    444    454       NON CYTOPLASMIC.
FT   TRANSMEM    455    477
FT   TOPO_DOM    478    483       CYTOPLASMIC.
FT   TRANSMEM    484    506
FT   TOPO_DOM    507    525       NON CYTOPLASMIC.
FT   TRANSMEM    526    549
```

```
FT   TOPO_DOM    550    647       CYTOPLASMIC.
FT   TRANSMEM    648    671
FT   TOPO_DOM    672    672       NON CYTOPLASMIC.
//
```

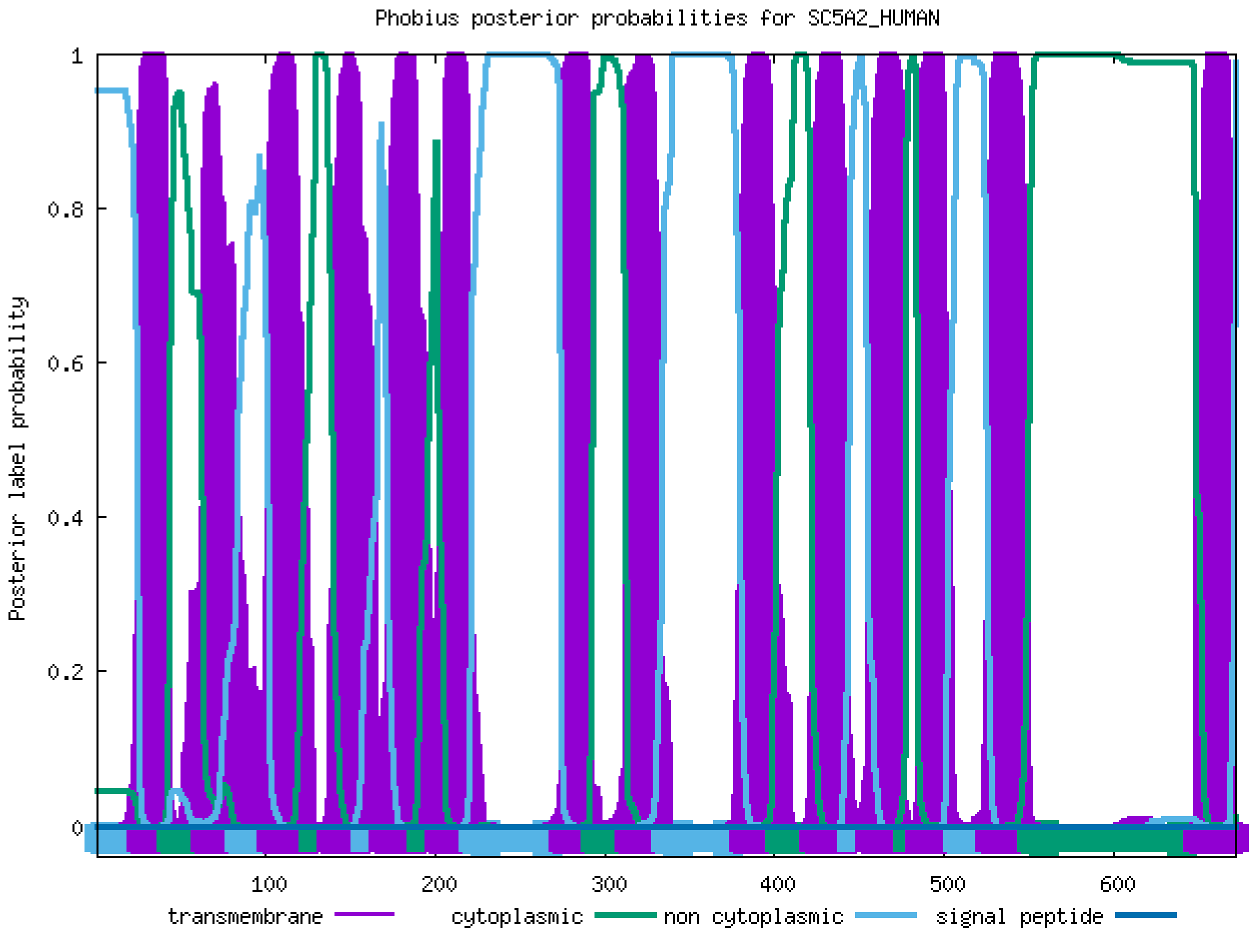

The probability data used in the plot is found here, and the gnuplot script is here.

Prediction of PDZ1I_HUMAN

```
ID   PDZ1I_HUMAN
FT   SIGNAL        1     20
FT   REGION        1      3       N-REGION.
FT   REGION        4     15       H-REGION.
FT   REGION       16     20       C-REGION.
FT   TOPO_DOM     21     29       NON CYTOPLASMIC.
FT   TRANSMEM     30     54
FT   TOPO_DOM     55    114       CYTOPLASMIC.
//
```

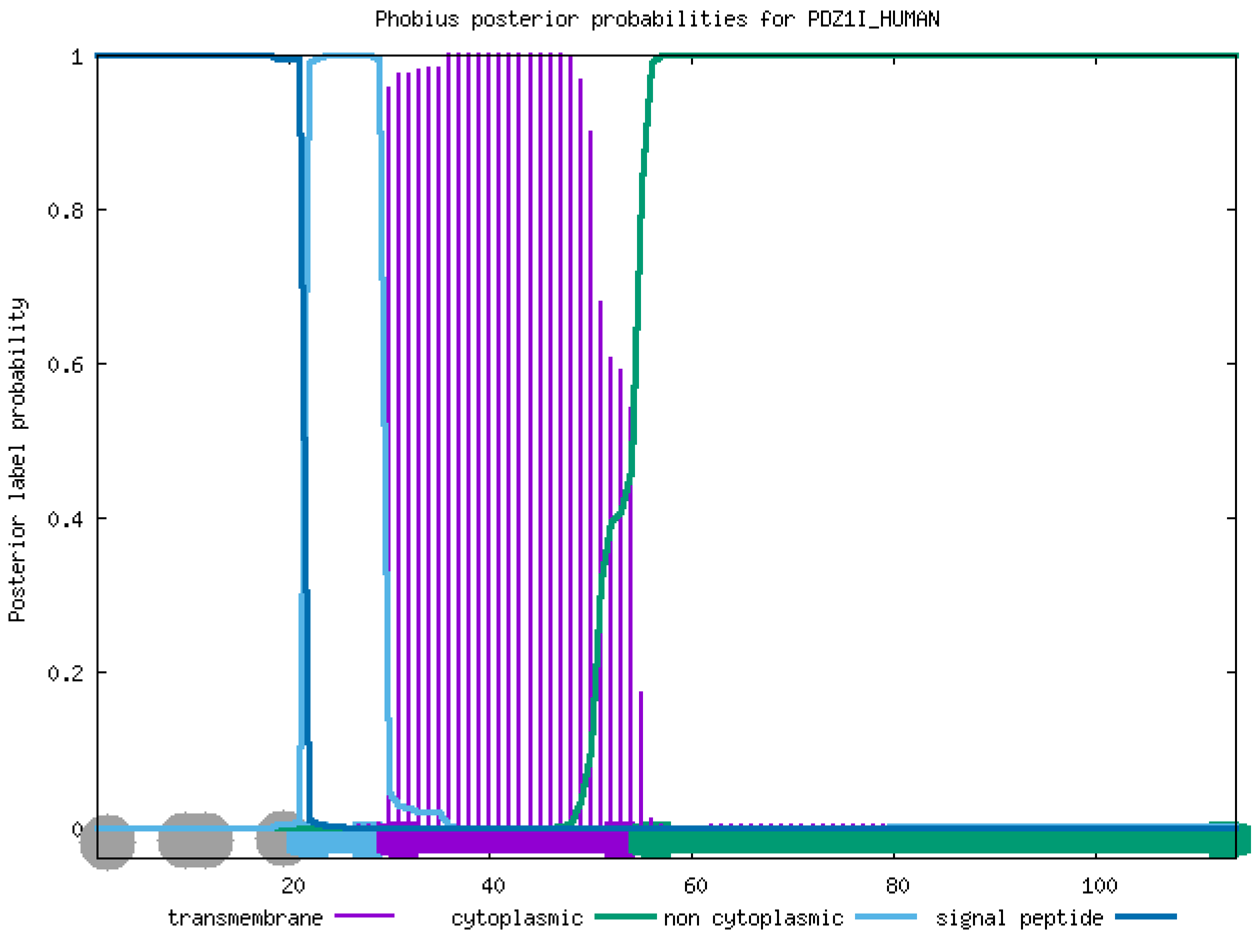

The probability data used in the plot is found here, and the gnuplot script is here.

Prediction of INSI2_HUMAN

```
ID   INSI2_HUMAN
FT   TOPO_DOM      1     27       CYTOPLASMIC.
FT   TRANSMEM     28     49
FT   TOPO_DOM     50     68       NON CYTOPLASMIC.
FT   TRANSMEM     69     88
FT   TOPO_DOM     89    107       CYTOPLASMIC.
FT   TRANSMEM    108    125
FT   TOPO_DOM    126    130       NON CYTOPLASMIC.
FT   TRANSMEM    131    148
FT   TOPO_DOM    149    154       CYTOPLASMIC.
FT   TRANSMEM    155    174
FT   TOPO_DOM    175    185       NON CYTOPLASMIC.
FT   TRANSMEM    186    206
FT   TOPO_DOM    207    225       CYTOPLASMIC.
//
```

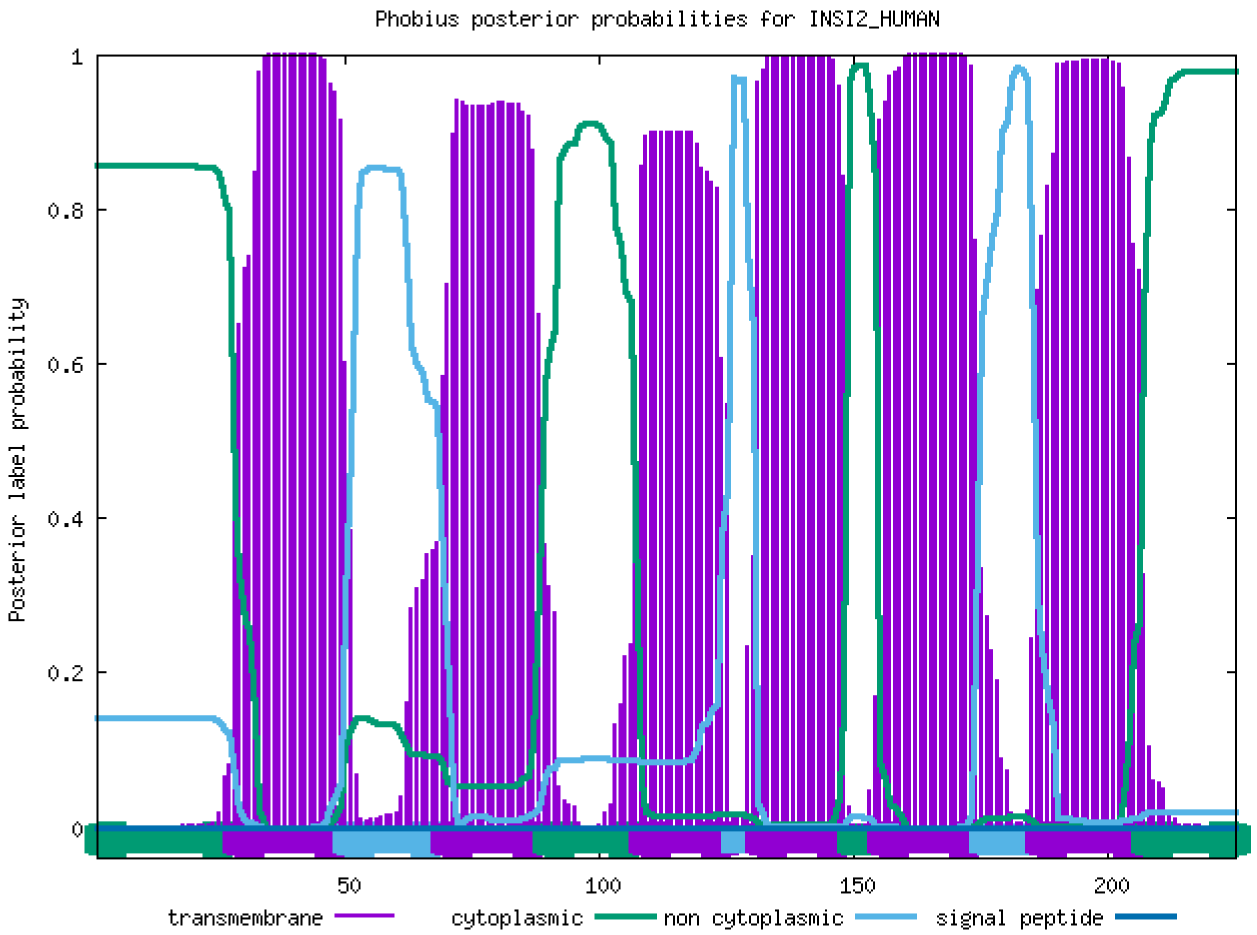

The probability data used in the plot is found here, and the gnuplot script is here.

Prediction of SCAP_HUMAN

```
ID    SCAP_HUMAN
FT    TOPO_DOM      1     19       CYTOPLASMIC.
FT    TRANSMEM     20     45
FT    TOPO_DOM     46    279       NON CYTOPLASMIC.
FT    TRANSMEM    280    301
FT    TOPO_DOM    302    312       CYTOPLASMIC.
FT    TRANSMEM    313    339
FT    TOPO_DOM    340    350       NON CYTOPLASMIC.
FT    TRANSMEM    351    375
FT    TOPO_DOM    376    395       CYTOPLASMIC.
FT    TRANSMEM    396    414
FT    TOPO_DOM    415    419       NON CYTOPLASMIC.
FT    TRANSMEM    420    442
FT    TOPO_DOM    443    710       CYTOPLASMIC.
FT    TRANSMEM    711    733
FT    TOPO_DOM    734   1279       NON CYTOPLASMIC.
//
```

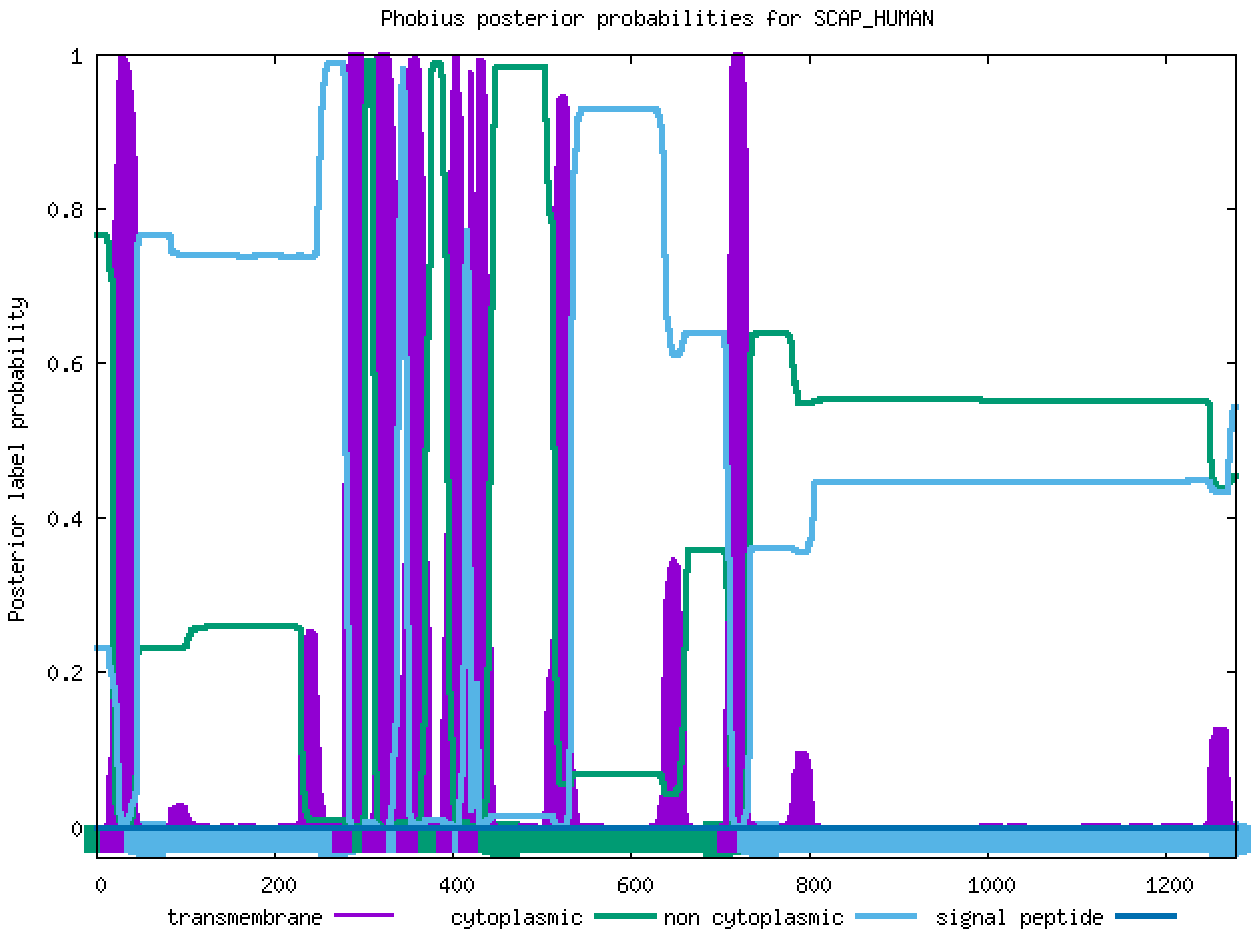

The probability data used in the plot is found here, and the gnuplot script is here.

Prediction of EBP_HUMAN

```
ID    EBP_HUMAN
FT    TOPO_DOM      1     28       NON CYTOPLASMIC.
FT    TRANSMEM     29     54
FT    TOPO_DOM     55     65       CYTOPLASMIC.
FT    TRANSMEM     66     87
FT    TOPO_DOM     88    122       NON CYTOPLASMIC.
FT    TRANSMEM    123    141
FT    TOPO_DOM    142    147       CYTOPLASMIC.
FT    TRANSMEM    148    168
FT    TOPO_DOM    169    182       NON CYTOPLASMIC.
FT    TRANSMEM    183    205
FT    TOPO_DOM    206    230       CYTOPLASMIC.
//
```

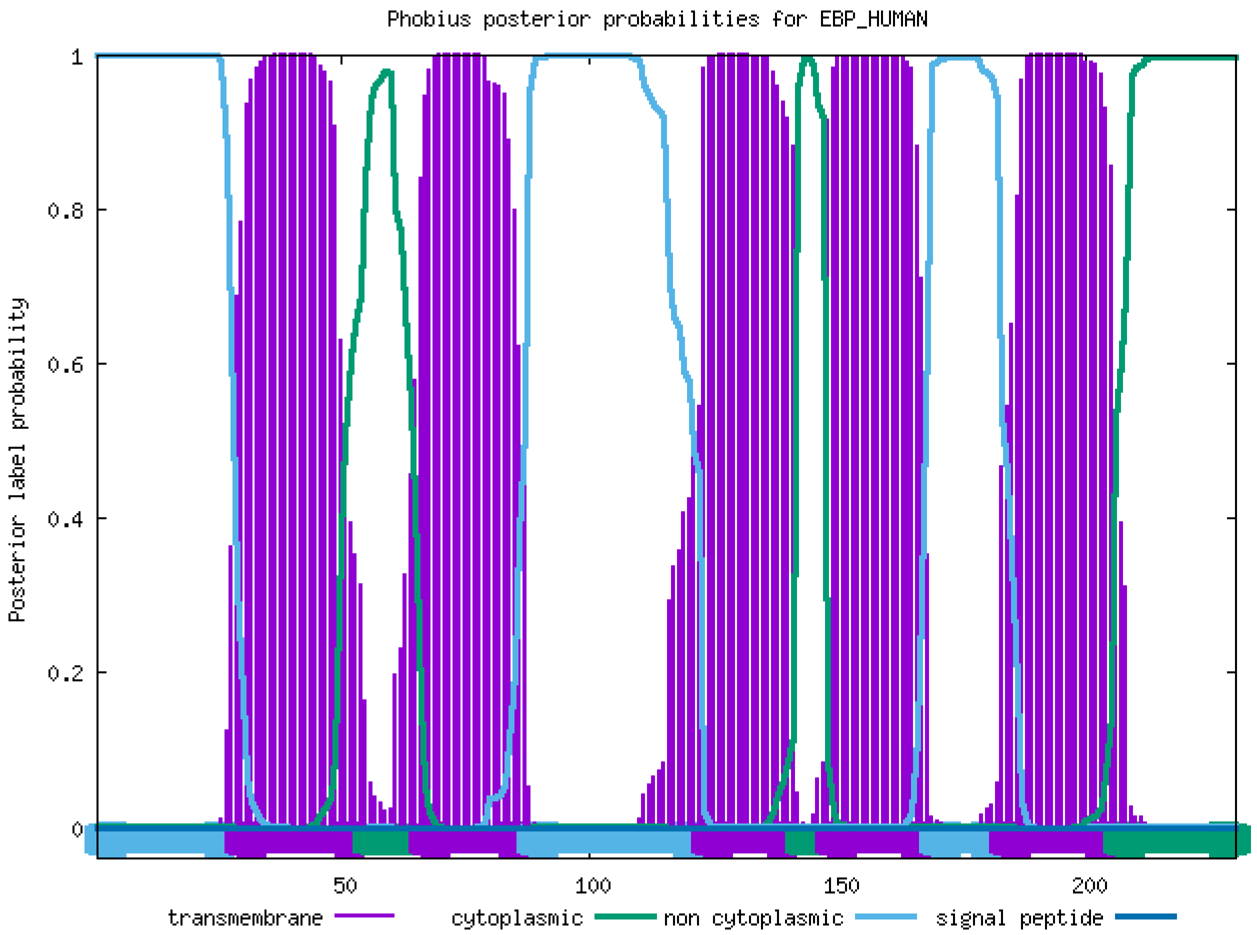

The probability data used in the plot is found here, and the gnuplot script is here.

Prediction of NPC1_HUMAN

```
ID   NPC1_HUMAN
FT   SIGNAL        1     22
FT   REGION        1      4       N-REGION.
FT   REGION        5     16       H-REGION.
FT   REGION       17     22       C-REGION.
FT   TOPO_DOM     23    266       NON CYTOPLASMIC.
FT   TRANSMEM    267    293
FT   TOPO_DOM    294    350       CYTOPLASMIC.
FT   TRANSMEM    351    371
FT   TOPO_DOM    372    620       NON CYTOPLASMIC.
FT   TRANSMEM    621    642
FT   TOPO_DOM    643    653       CYTOPLASMIC.
FT   TRANSMEM    654    678
FT   TOPO_DOM    679    683       NON CYTOPLASMIC.
FT   TRANSMEM    684    706
FT   TOPO_DOM    707    726       CYTOPLASMIC.
FT   TRANSMEM    727    754
FT   TOPO_DOM    755    759       NON CYTOPLASMIC.
FT   TRANSMEM    760    785
FT   TOPO_DOM    786    832       CYTOPLASMIC.
FT   TRANSMEM    833    853
FT   TOPO_DOM    854   1098       NON CYTOPLASMIC.
FT   TRANSMEM   1099   1117
FT   TOPO_DOM   1118   1123       CYTOPLASMIC.
FT   TRANSMEM   1124   1145
FT   TOPO_DOM   1146   1150       NON CYTOPLASMIC.
FT   TRANSMEM   1151   1169
FT   TOPO_DOM   1170   1189       CYTOPLASMIC.
FT   TRANSMEM   1190   1216
FT   TOPO_DOM   1217   1227       NON CYTOPLASMIC.
```

```
FT   TRANSMEM   1228   1251
FT   TOPO_DOM   1252   1278       CYTOPLASMIC.
//
```

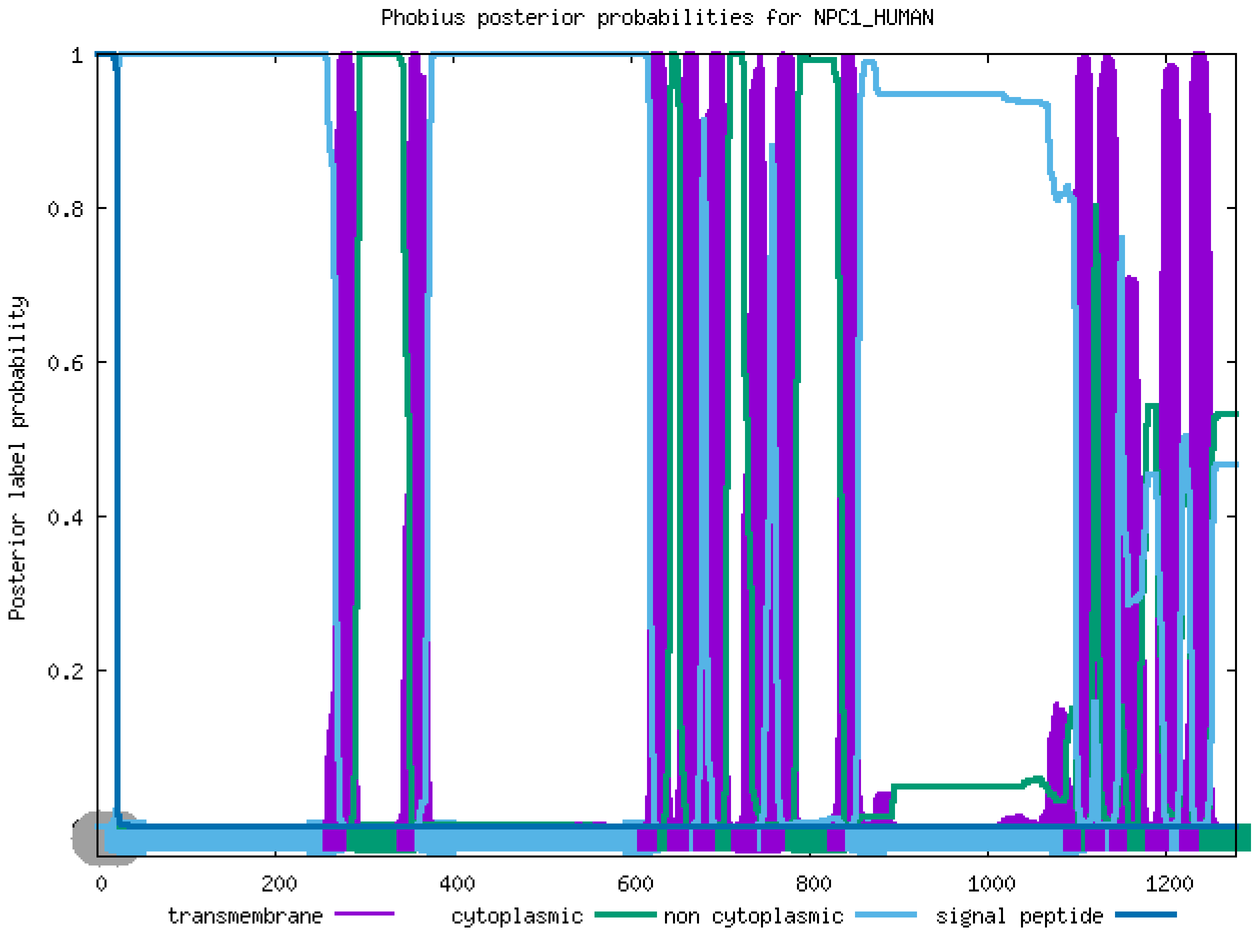

The probability data used in the plot is found here, and the gnuplot script is here.

Prediction of NPCL1_HUMAN

```
ID   NPCL1_HUMAN
FT   SIGNAL         1     21
FT   REGION         1      7       N-REGION.
FT   REGION         8     16       H-REGION.
FT   REGION        17     21       C-REGION.
FT   TOPO_DOM      22    284       NON CYTOPLASMIC.
FT   TRANSMEM     285    305
FT   TOPO_DOM     306    350       CYTOPLASMIC.
FT   TRANSMEM     351    372
FT   TOPO_DOM     373    632       NON CYTOPLASMIC.
FT   TRANSMEM     633    654
FT   TOPO_DOM     655    665       CYTOPLASMIC.
FT   TRANSMEM     666    690
FT   TOPO_DOM     691    695       NON CYTOPLASMIC.
FT   TRANSMEM     696    719
FT   TOPO_DOM     720    738       CYTOPLASMIC.
FT   TRANSMEM     739    761
FT   TOPO_DOM     762    772       NON CYTOPLASMIC.
FT   TRANSMEM     773    797
FT   TOPO_DOM     798    847       CYTOPLASMIC.
FT   TRANSMEM     848    869
FT   TOPO_DOM     870    888       NON CYTOPLASMIC.
FT   TRANSMEM     889    908
FT   TOPO_DOM     909   1139       CYTOPLASMIC.
FT   TRANSMEM    1140   1160
FT   TOPO_DOM    1161   1165       NON CYTOPLASMIC.
```

```
FT   TRANSMEM   1166   1188
FT   TOPO_DOM   1189   1194       CYTOPLASMIC.
FT   TRANSMEM   1195   1213
FT   TOPO_DOM   1214   1232       NON CYTOPLASMIC.
FT   TRANSMEM   1233   1257
FT   TOPO_DOM   1258   1268       CYTOPLASMIC.
FT   TRANSMEM   1269   1292
FT   TOPO_DOM   1293   1359       NON CYTOPLASMIC.
//
```

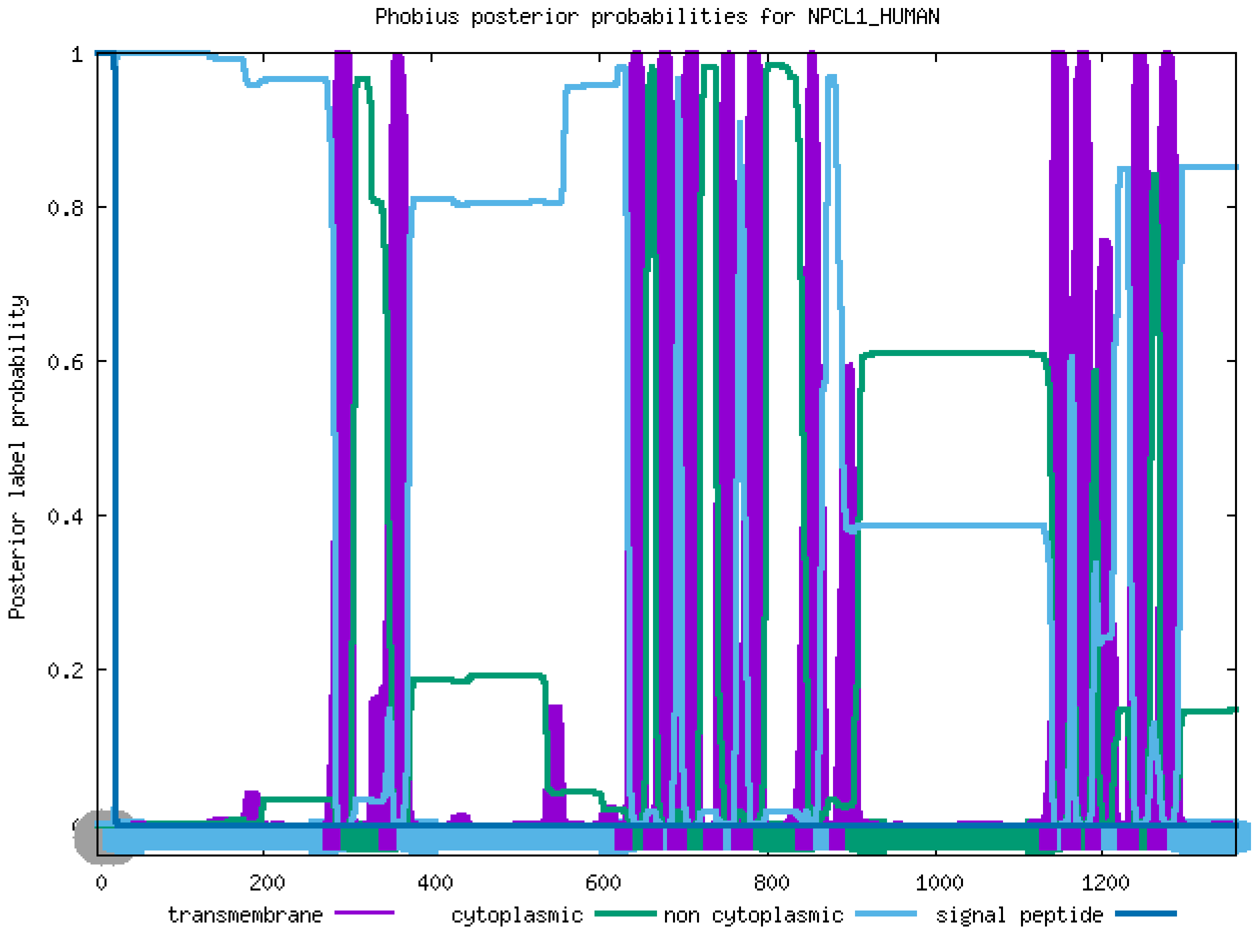

The probability data used in the plot is found here, and the gnuplot script is here.

Prediction of SCD_HUMAN

```
ID   SCD_HUMAN
FT   TOPO_DOM      1     71       CYTOPLASMIC.
FT   TRANSMEM     72     93
FT   TOPO_DOM     94    104       NON CYTOPLASMIC.
FT   TRANSMEM    105    123
FT   TOPO_DOM    124    221       CYTOPLASMIC.
FT   TRANSMEM    222    239
FT   TOPO_DOM    240    250       NON CYTOPLASMIC.
FT   TRANSMEM    251    273
FT   TOPO_DOM    274    284       CYTOPLASMIC.
FT   TRANSMEM    285    301
FT   TOPO_DOM    302    312       NON CYTOPLASMIC.
FT   TRANSMEM    313    334
FT   TOPO_DOM    335    359       CYTOPLASMIC.
//
```

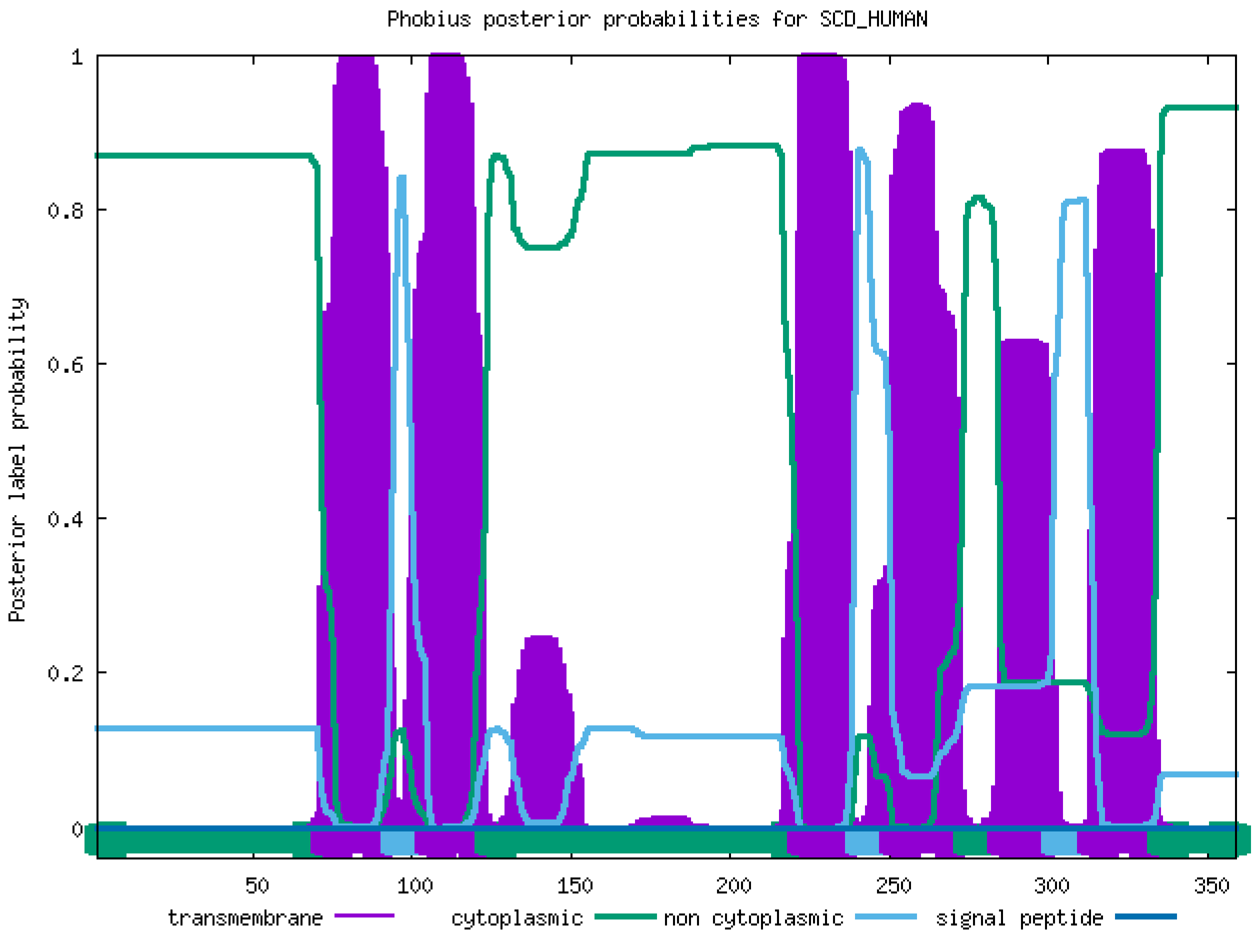

The probability data used in the plot is found here, and the gnuplot script is here.

Prediction of VPP1_HUMAN

```
ID   VPP1_HUMAN
FT   TOPO_DOM      1     400       CYTOPLASMIC.
FT   TRANSMEM    401     425
FT   TOPO_DOM    426     444       NON CYTOPLASMIC.
FT   TRANSMEM    445     464
FT   TOPO_DOM    465     540       CYTOPLASMIC.
FT   TRANSMEM    541     562
FT   TOPO_DOM    563     567       NON CYTOPLASMIC.
FT   TRANSMEM    568     592
FT   TOPO_DOM    593     603       CYTOPLASMIC.
FT   TRANSMEM    604     621
FT   TOPO_DOM    622     632       NON CYTOPLASMIC.
FT   TRANSMEM    633     657
FT   TOPO_DOM    658     740       CYTOPLASMIC.
FT   TRANSMEM    741     764
FT   TOPO_DOM    765     769       NON CYTOPLASMIC.
FT   TRANSMEM    770     793
FT   TOPO_DOM    794     837       CYTOPLASMIC.
//
```

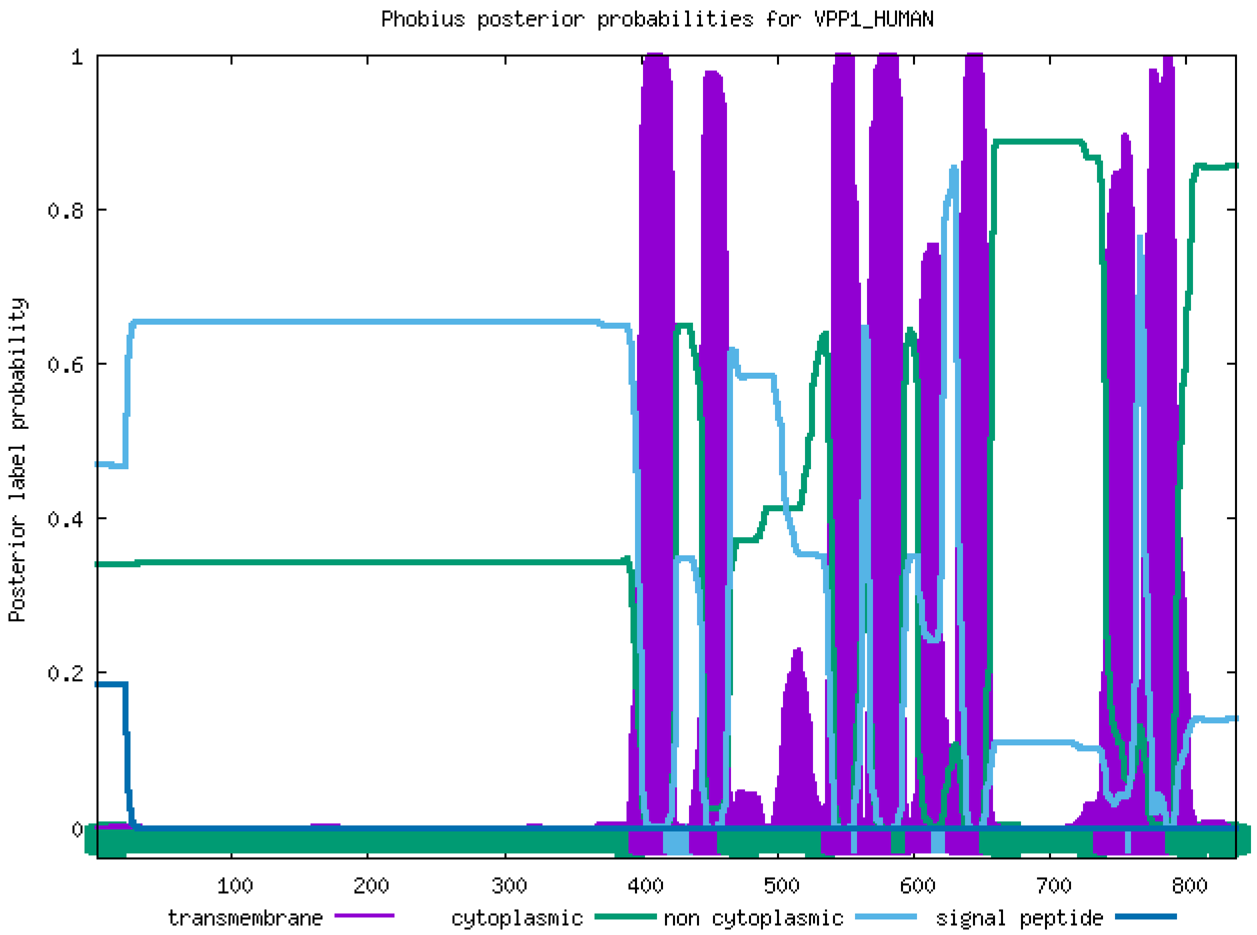

The probability data used in the plot is found here, and the gnuplot script is here.

Prediction of VATO_HUMAN

```
ID   VATO_HUMAN
FT   TOPO_DOM      1      6       CYTOPLASMIC.
FT   TRANSMEM      7     29
FT   TOPO_DOM     30     48       NON CYTOPLASMIC.
FT   TRANSMEM     49     70
FT   TOPO_DOM     71     90       CYTOPLASMIC.
FT   TRANSMEM     91    115
FT   TOPO_DOM    116    134       NON CYTOPLASMIC.
FT   TRANSMEM    135    165
FT   TOPO_DOM    166    171       CYTOPLASMIC.
FT   TRANSMEM    172    196
FT   TOPO_DOM    197    205       NON CYTOPLASMIC.
//
```

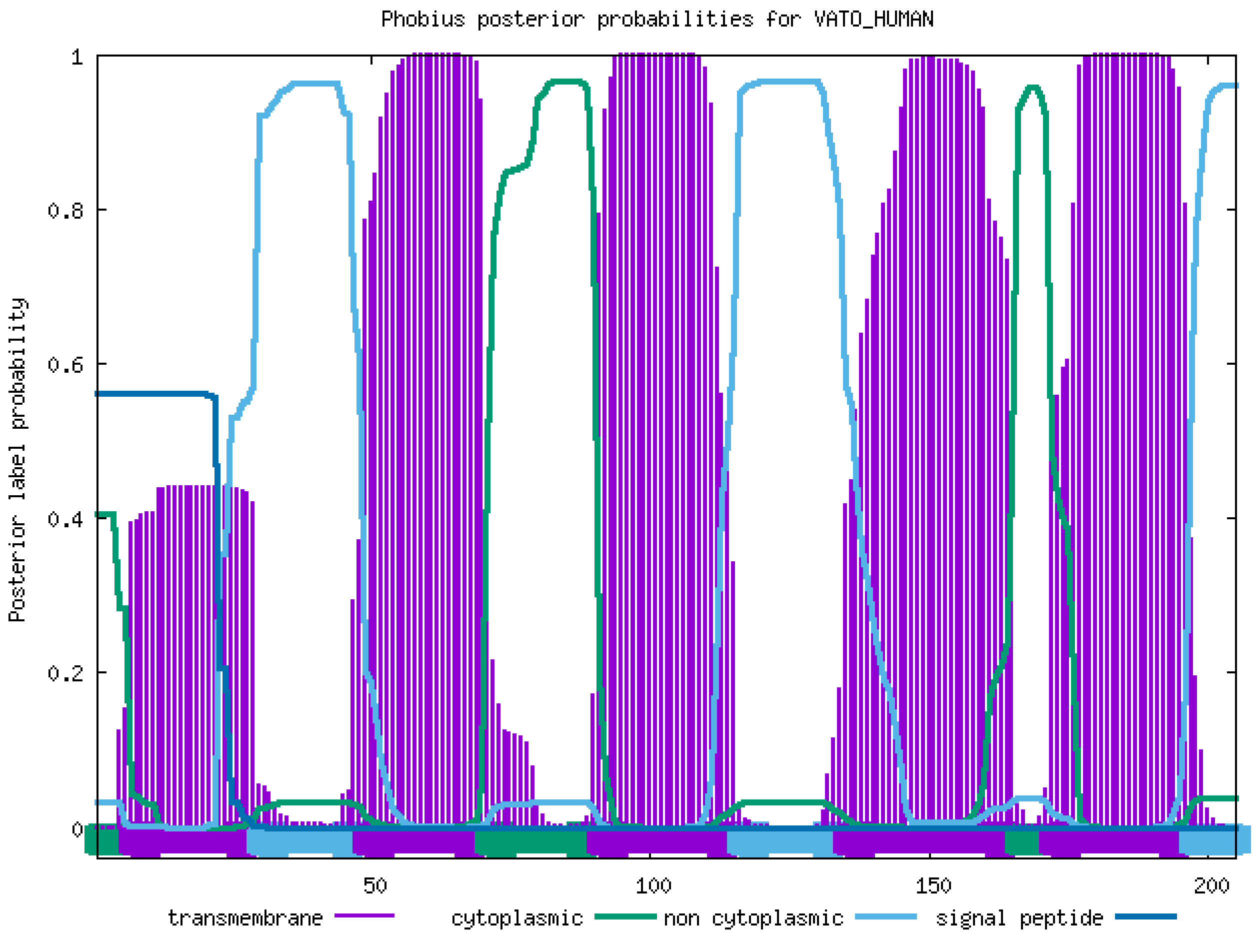

The probability data used in the plot is found here, and the gnuplot script is here.

Prediction of VATL_HUMAN

```
ID    VATL_HUMAN
FT    TOPO_DOM      1      11       NON CYTOPLASMIC.
FT    TRANSMEM     12      35
FT    TOPO_DOM     36      55       CYTOPLASMIC.
FT    TRANSMEM     56      77
FT    TOPO_DOM     78      88       NON CYTOPLASMIC.
FT    TRANSMEM     89     114
FT    TOPO_DOM    115     125       CYTOPLASMIC.
FT    TRANSMEM    126     152
FT    TOPO_DOM    153     155       NON CYTOPLASMIC.
//
```

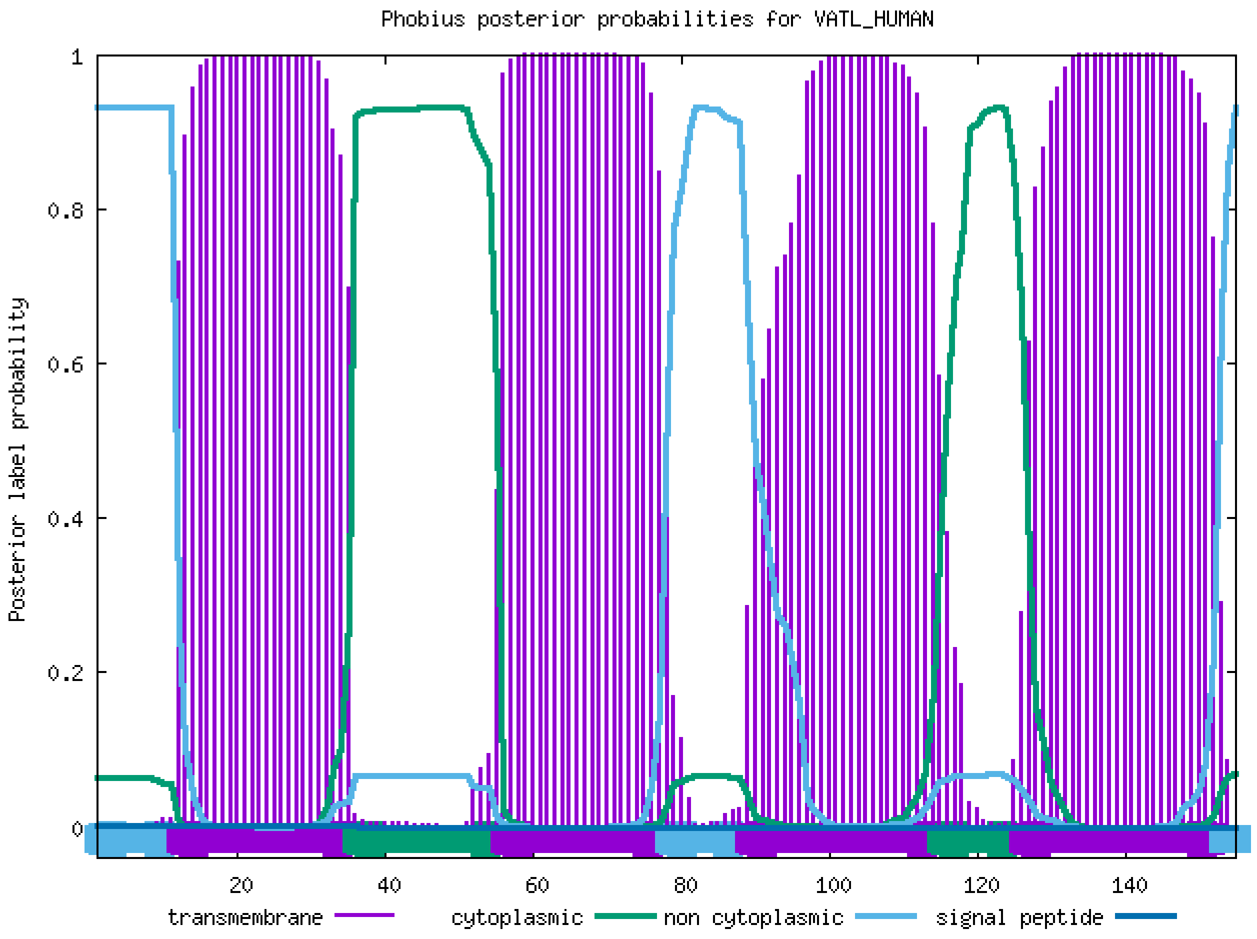

The probability data used in the plot is found here, and the gnuplot script is here.

Prediction of RNK_HUMAN

```
ID    RNK_HUMAN
FT    TOPO_DOM      1     49       CYTOPLASMIC.
FT    TRANSMEM     50     72
FT    TOPO_DOM     73    103       NON CYTOPLASMIC.
FT    TRANSMEM    104    128
FT    TOPO_DOM    129    137       CYTOPLASMIC.
//
```

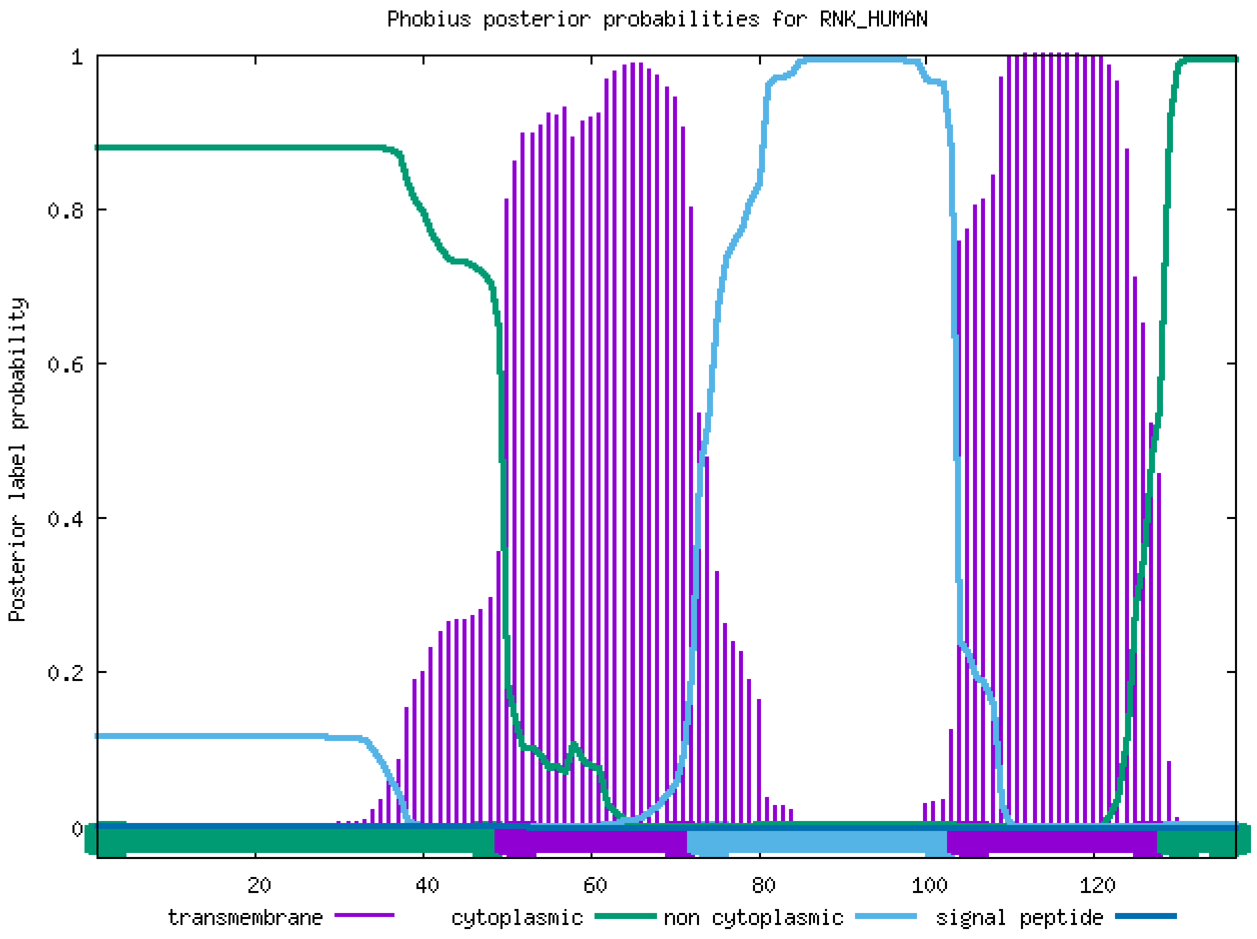

The probability data used in the plot is found here, and the gnuplot script is here.

Prediction of RENR_HUMAN

```
ID   RENR_HUMAN
FT   SIGNAL         1     16
FT   REGION         1      1       N-REGION.
FT   REGION         2     12       H-REGION.
FT   REGION        13     16       C-REGION.
FT   TOPO_DOM      17    308       NON CYTOPLASMIC.
FT   TRANSMEM     309    329
FT   TOPO_DOM     330    350       CYTOPLASMIC.
//
```

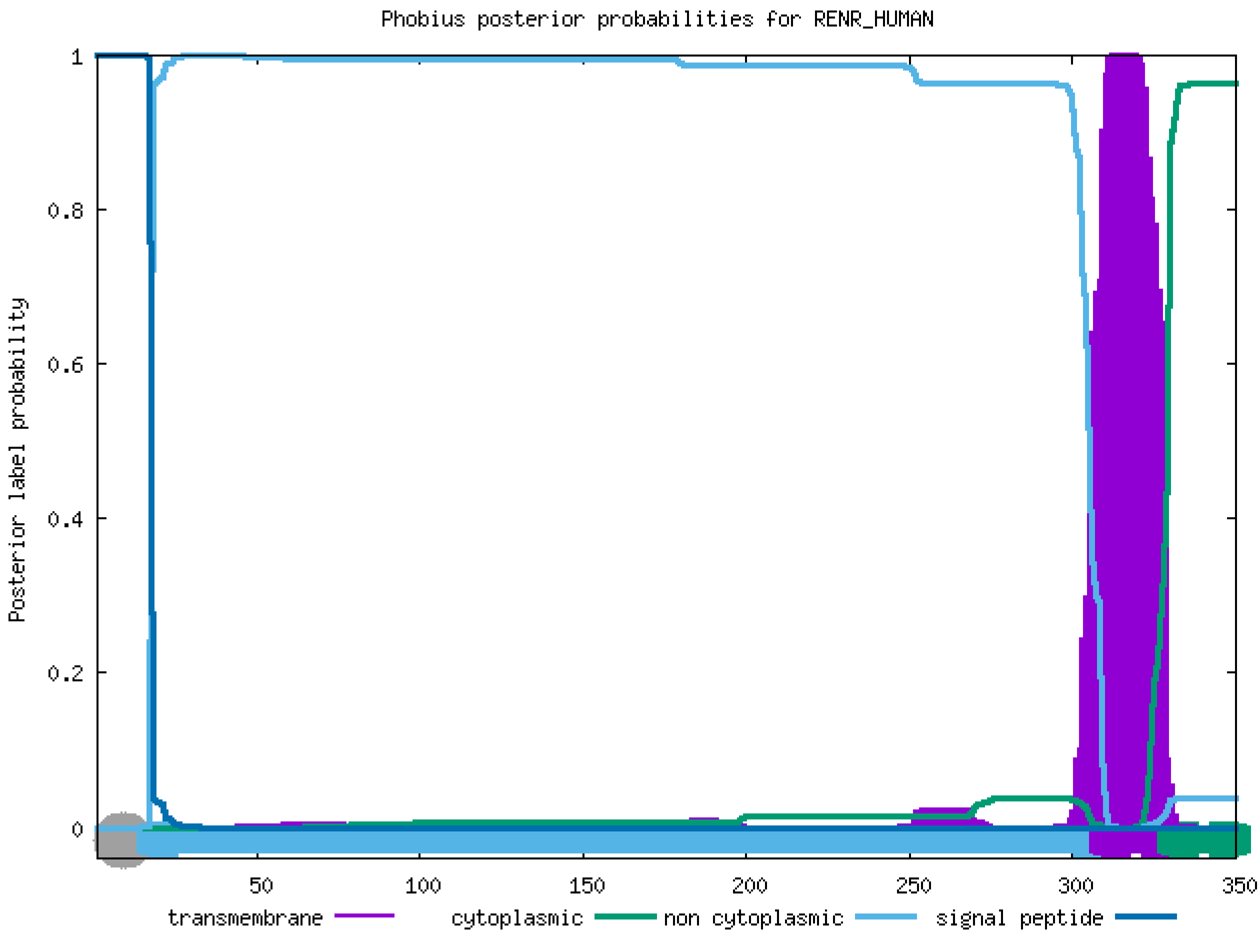

The probability data used in the plot is found here, and the gnuplot script is here.

Prediction of AT5G1_HUMAN

```
ID   AT5G1_HUMAN
FT   TOPO_DOM      1     68       NON CYTOPLASMIC.
FT   TRANSMEM     69     95
FT   TOPO_DOM     96    106       CYTOPLASMIC.
FT   TRANSMEM    107    135
FT   TOPO_DOM    136    136       NON CYTOPLASMIC.
//
```

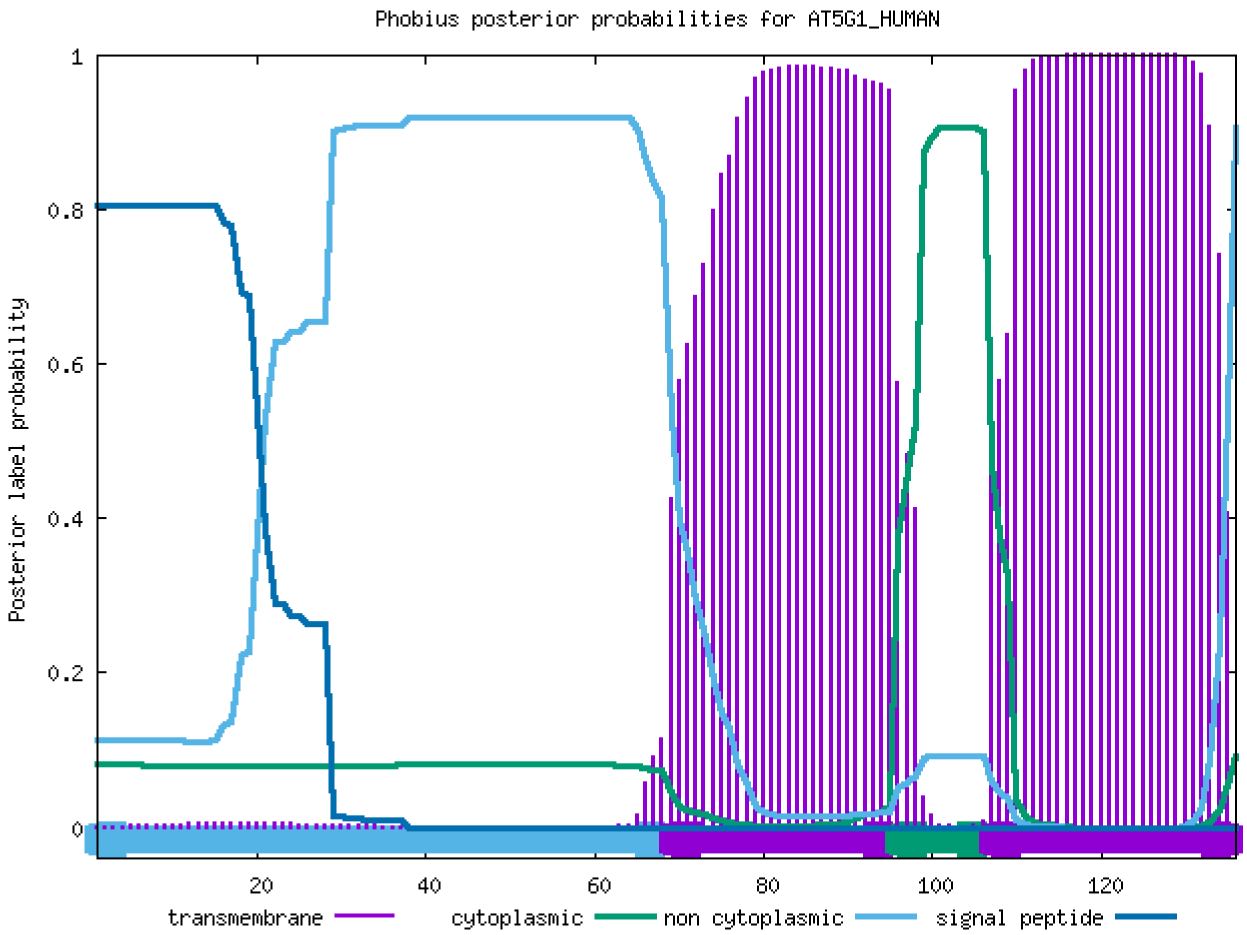

The probability data used in the plot is found here, and the gnuplot script is here.

Prediction of ATP6_HUMAN

```
ID   ATP6_HUMAN
FT   TOPO_DOM      1      5       NON CYTOPLASMIC.
FT   TRANSMEM      6     31
FT   TOPO_DOM     32     66       CYTOPLASMIC.
FT   TRANSMEM     67     86
FT   TOPO_DOM     87     97       NON CYTOPLASMIC.
FT   TRANSMEM     98    117
FT   TOPO_DOM    118    136       CYTOPLASMIC.
FT   TRANSMEM    137    158
FT   TOPO_DOM    159    163       NON CYTOPLASMIC.
FT   TRANSMEM    164    184
FT   TOPO_DOM    185    190       CYTOPLASMIC.
FT   TRANSMEM    191    222
FT   TOPO_DOM    223    226       NON CYTOPLASMIC.
//
```

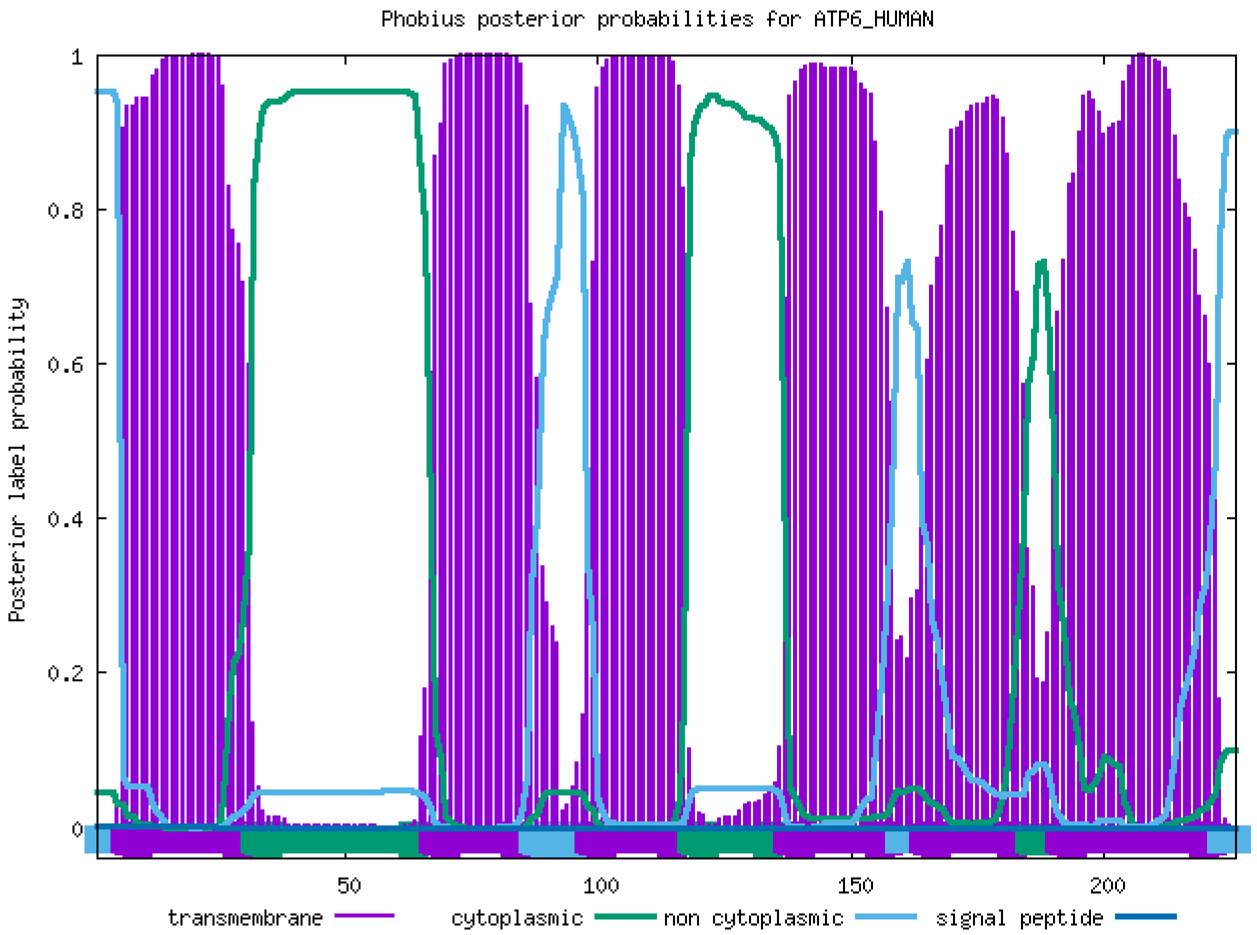

The probability data used in the plot is found here, and the gnuplot script is here.

Prediction of ATP68_HUMAN

```
ID   ATP68_HUMAN
FT   TOPO_DOM      1     19       NON CYTOPLASMIC.
FT   TRANSMEM     20     38
FT   TOPO_DOM     39     58       CYTOPLASMIC.
//
```

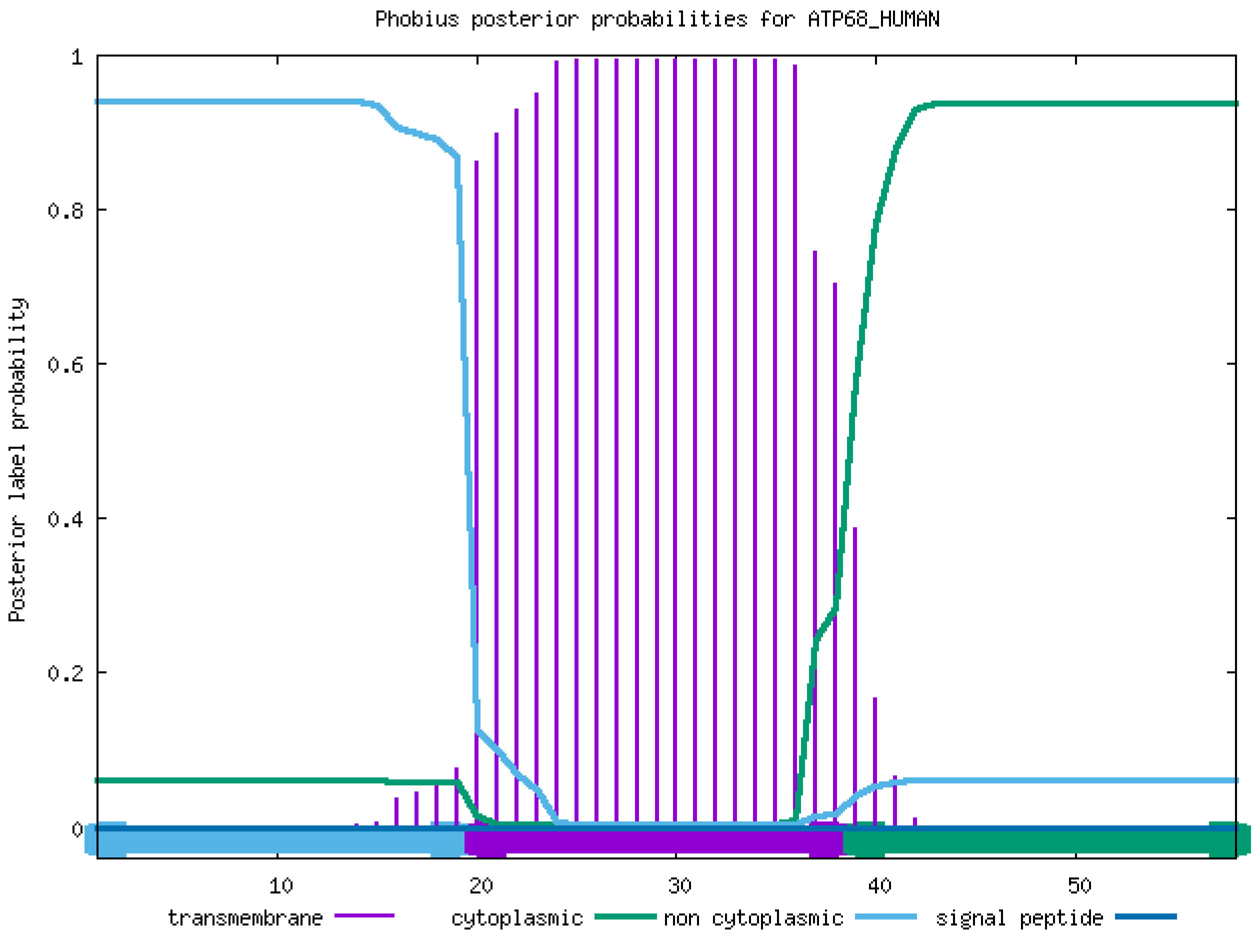

The probability data used in the plot is found here, and the gnuplot script is here.

Prediction of ATP8_HUMAN

```
ID   ATP8_HUMAN
FT   TOPO_DOM      1      11       NON CYTOPLASMIC.
FT   TRANSMEM     12      35
FT   TOPO_DOM     36      68       CYTOPLASMIC.
//
```

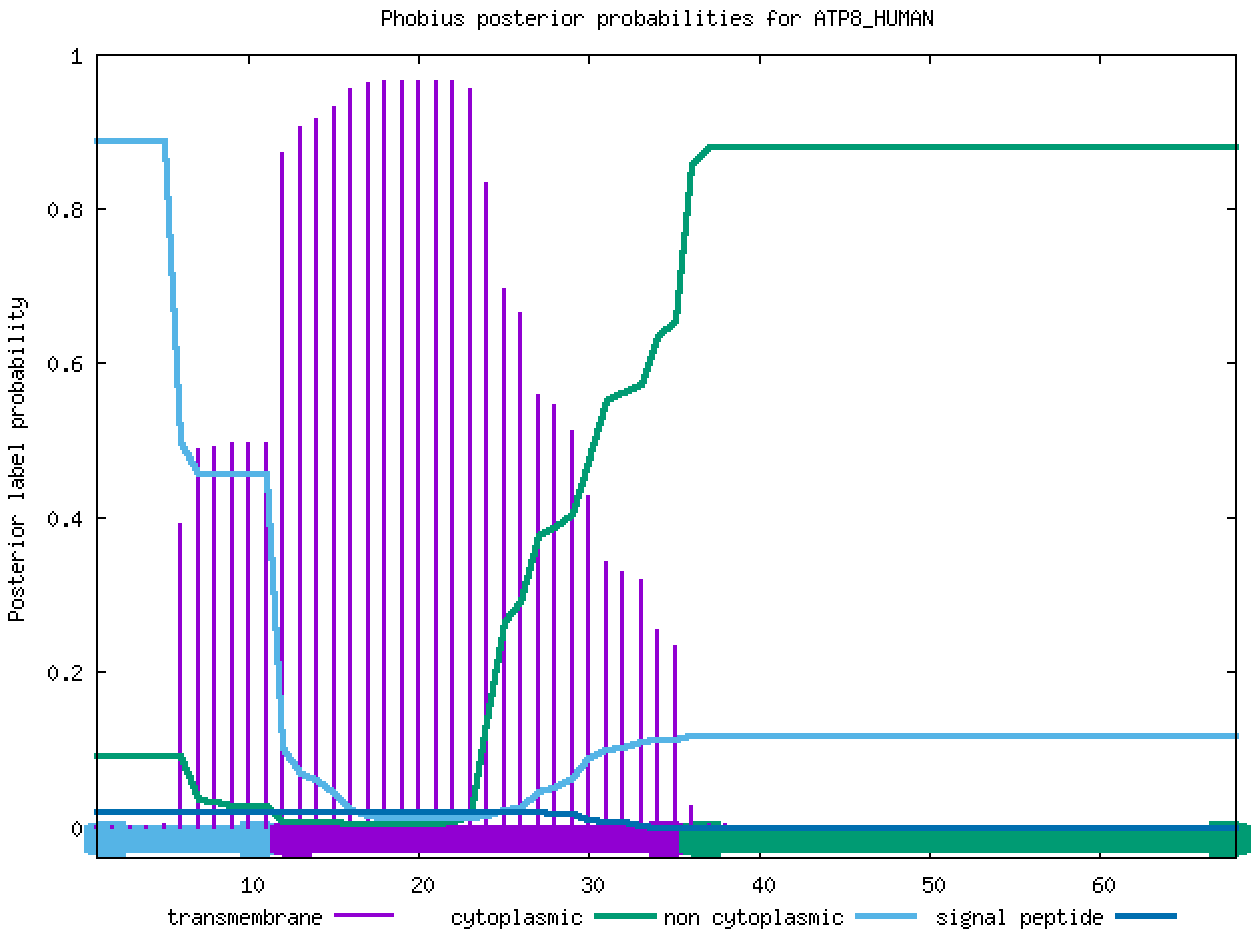

The probability data used in the plot is found here, and the gnuplot script is here.

Prediction of ATPK_HUMAN

```
ID    ATPK_HUMAN
FT    TOPO_DOM      1      61       NON CYTOPLASMIC.
FT    TRANSMEM     62      82
FT    TOPO_DOM     83      94       CYTOPLASMIC.
//
```

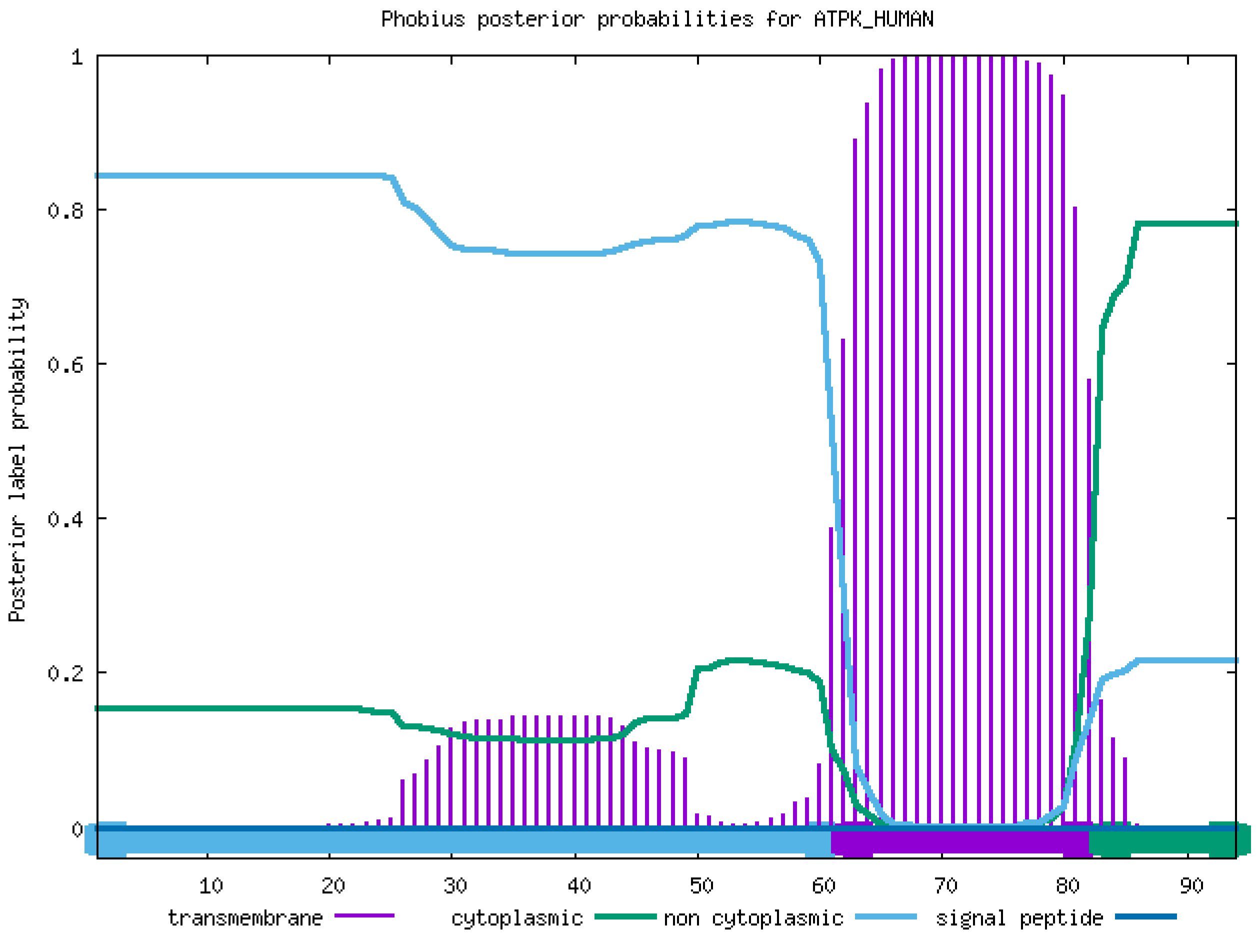

The probability data used in the plot is found here, and the gnuplot script is here.

Prediction of AT2B1_HUMAN

```
ID   AT2B1_HUMAN
FT   TOPO_DOM      1    100       CYTOPLASMIC.
FT   TRANSMEM    101    124
FT   TOPO_DOM    125    152       NON CYTOPLASMIC.
FT   TRANSMEM    153    172
FT   TOPO_DOM    173    377       CYTOPLASMIC.
FT   TRANSMEM    378    399
FT   TOPO_DOM    400    418       NON CYTOPLASMIC.
FT   TRANSMEM    419    445
FT   TOPO_DOM    446    931       CYTOPLASMIC.
FT   TRANSMEM    932    949
FT   TOPO_DOM    950    968       NON CYTOPLASMIC.
FT   TRANSMEM    969    986
FT   TOPO_DOM    987   1006       CYTOPLASMIC.
FT   TRANSMEM   1007   1028
FT   TOPO_DOM   1029   1039       NON CYTOPLASMIC.
FT   TRANSMEM   1040   1061
FT   TOPO_DOM   1062   1220       CYTOPLASMIC.
//
```

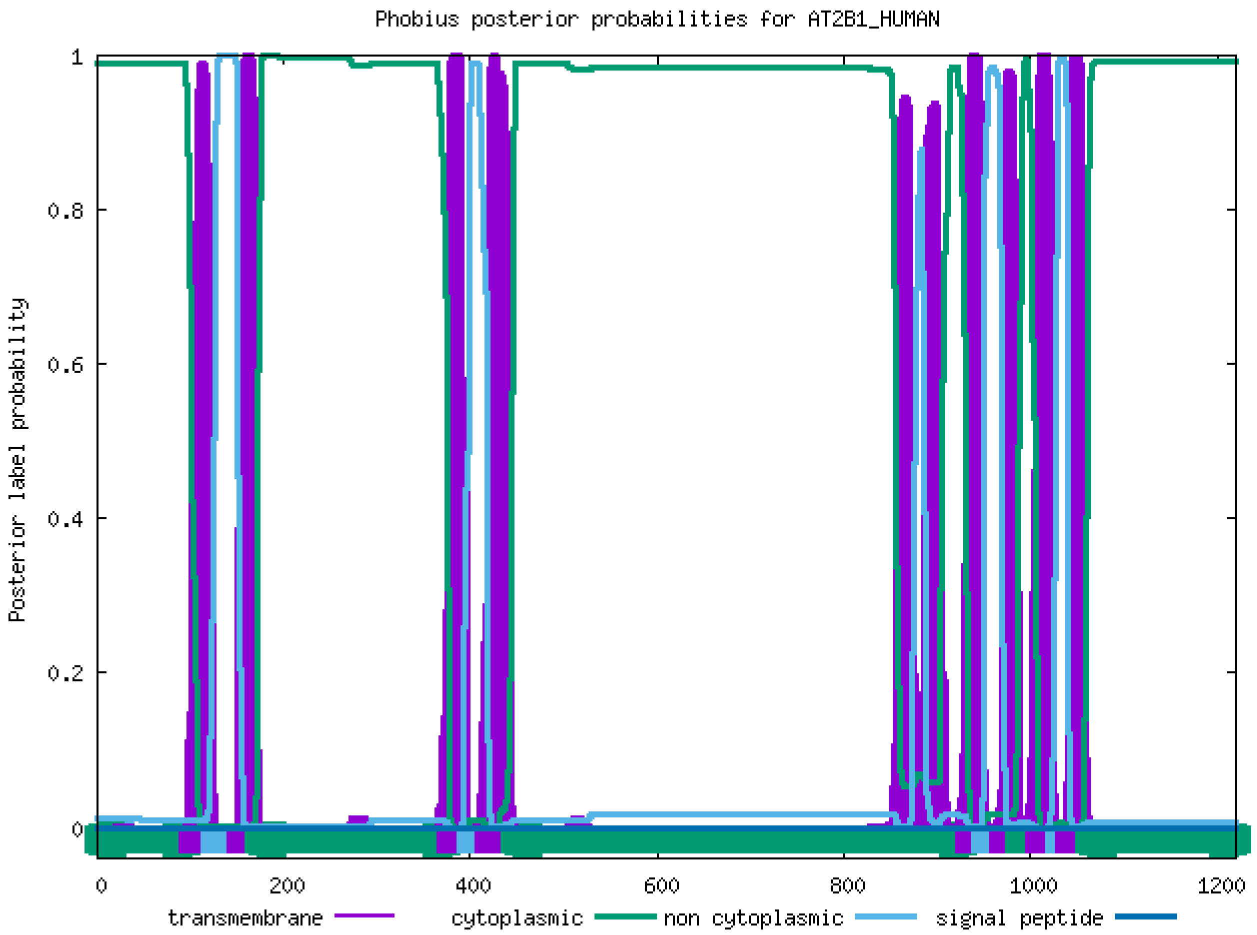

The probability data used in the plot is found here, and the gnuplot script is here.

Prediction of NPTN_HUMAN

```
ID   NPTN_HUMAN
FT   SIGNAL         1     28
FT   REGION         1      5       N-REGION.
FT   REGION         6     17       H-REGION.
FT   REGION        18     28       C-REGION.
FT   TOPO_DOM      29    339       NON CYTOPLASMIC.
FT   TRANSMEM     340    360
FT   TOPO_DOM     361    398       CYTOPLASMIC.
//
```

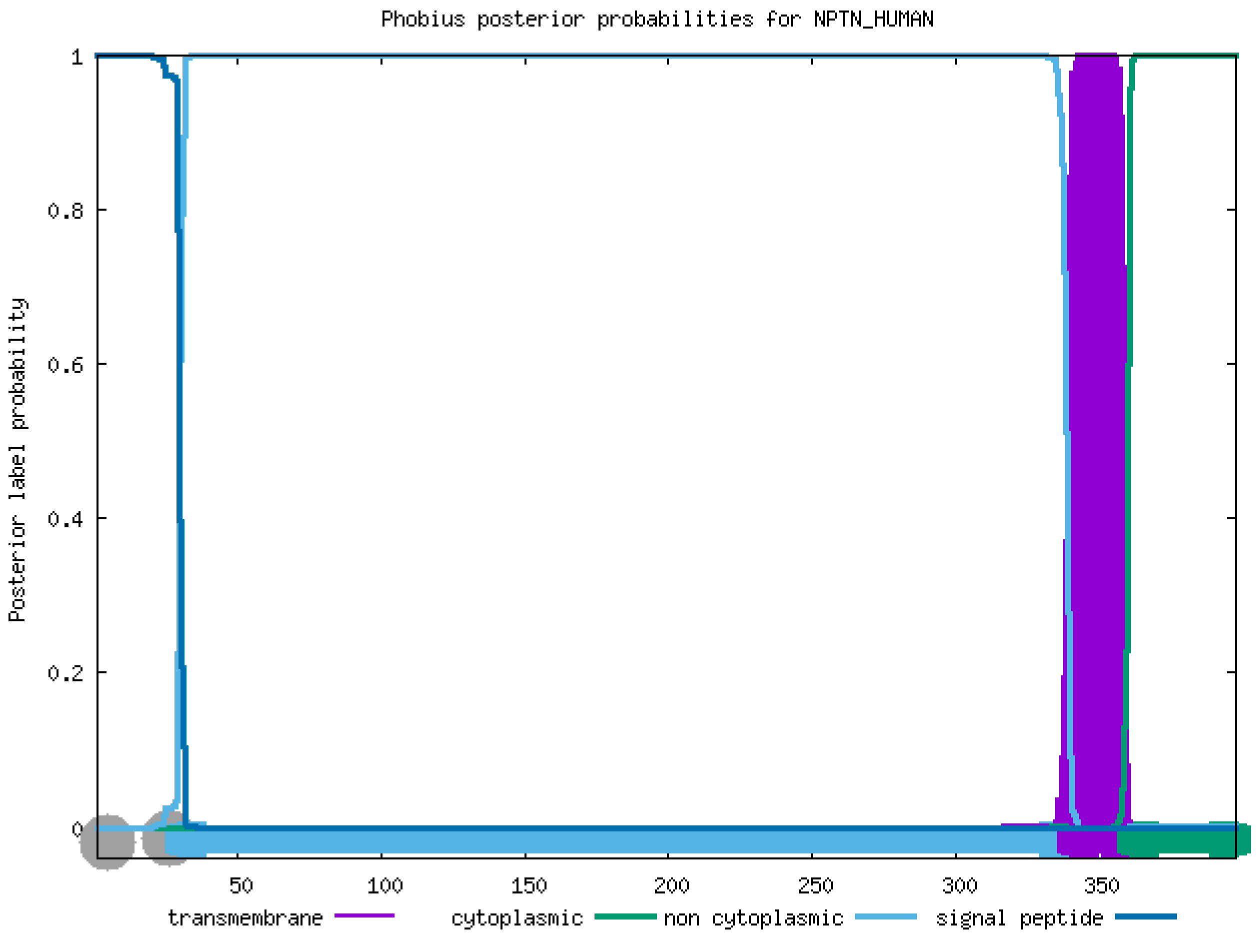

The probability data used in the plot is found here, and the gnuplot script is here.

Prediction of AT2A2_HUMAN

```
ID   AT2A2_HUMAN
FT   TOPO_DOM      1     59       CYTOPLASMIC.
FT   TRANSMEM     60     78
FT   TOPO_DOM     79     83       NON CYTOPLASMIC.
FT   TRANSMEM     84    107
FT   TOPO_DOM    108    259       CYTOPLASMIC.
FT   TRANSMEM    260    280
FT   TOPO_DOM    281    291       NON CYTOPLASMIC.
FT   TRANSMEM    292    321
FT   TOPO_DOM    322    758       CYTOPLASMIC.
FT   TRANSMEM    759    782
FT   TOPO_DOM    783    832       NON CYTOPLASMIC.
FT   TRANSMEM    833    856
FT   TOPO_DOM    857    929       CYTOPLASMIC.
FT   TRANSMEM    930    949
FT   TOPO_DOM    950    954       NON CYTOPLASMIC.
FT   TRANSMEM    955    978
FT   TOPO_DOM    979   1012       CYTOPLASMIC.
FT   TRANSMEM   1013   1031
FT   TOPO_DOM   1032   1042       NON CYTOPLASMIC.
//
```

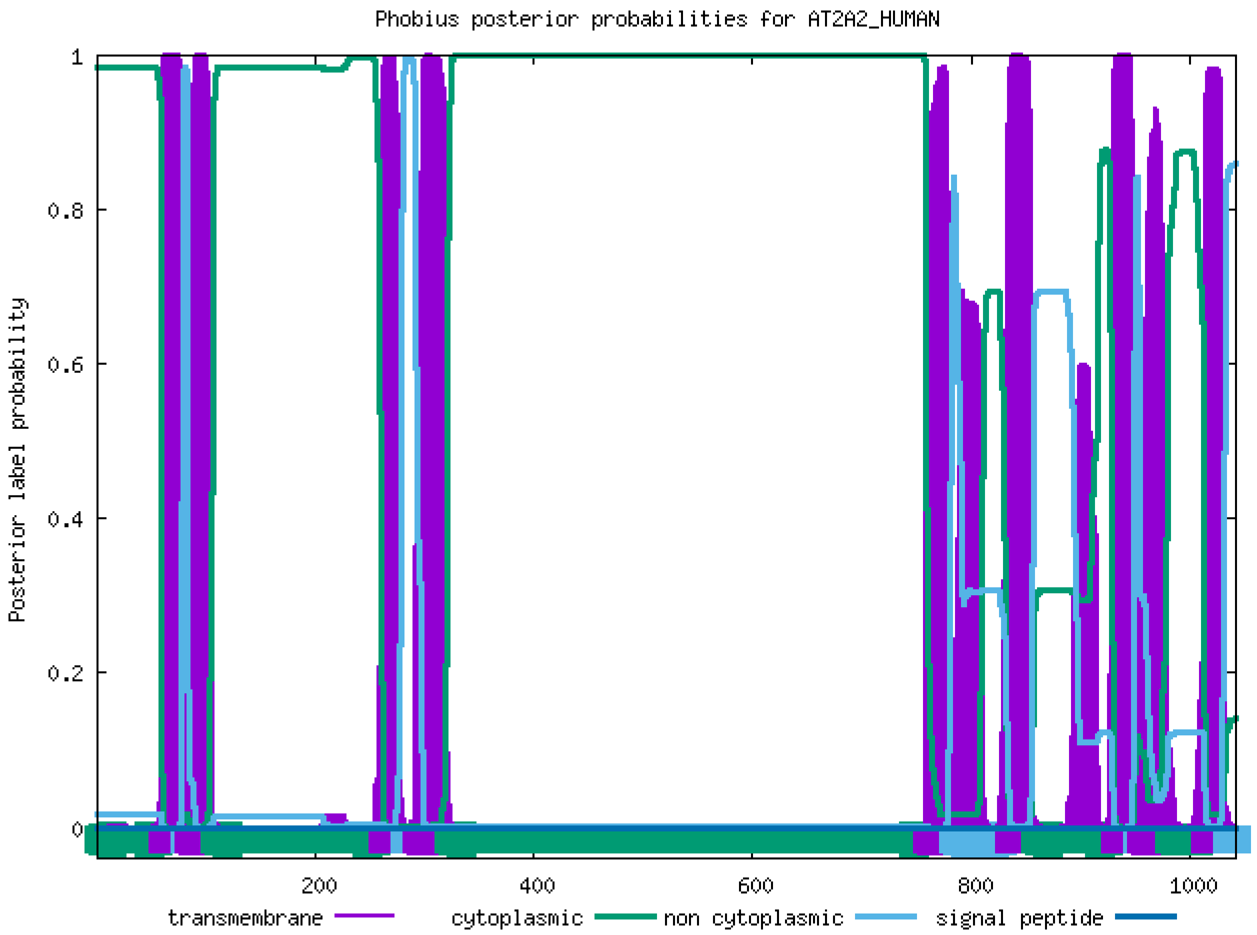

The probability data used in the plot is found here, and the gnuplot script is here.

Prediction of AT1A1_HUMAN

```
ID   AT1A1_HUMAN
FT   TOPO_DOM      1     95       CYTOPLASMIC.
FT   TRANSMEM     96    118
FT   TOPO_DOM    119    129       NON CYTOPLASMIC.
FT   TRANSMEM    130    149
FT   TOPO_DOM    150    290       CYTOPLASMIC.
FT   TRANSMEM    291    315
FT   TOPO_DOM    316    320       NON CYTOPLASMIC.
FT   TRANSMEM    321    344
FT   TOPO_DOM    345    852       CYTOPLASMIC.
FT   TRANSMEM    853    874
FT   TOPO_DOM    875    912       NON CYTOPLASMIC.
FT   TRANSMEM    913    932
FT   TOPO_DOM    933    952       CYTOPLASMIC.
FT   TRANSMEM    953    980
FT   TOPO_DOM    981    985       NON CYTOPLASMIC.
FT   TRANSMEM    986   1004
FT   TOPO_DOM   1005   1023       CYTOPLASMIC.
//
```

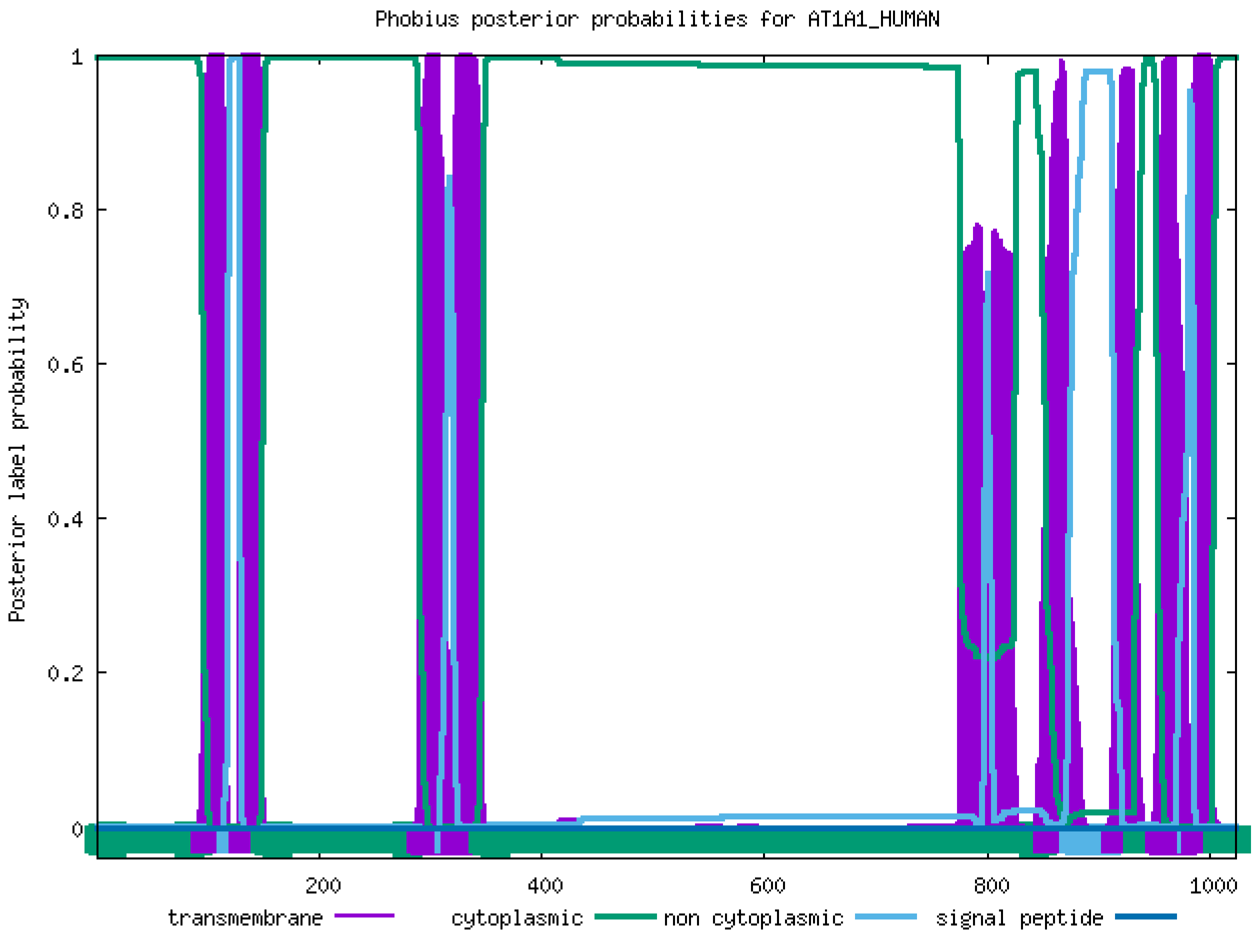

The probability data used in the plot is found here, and the gnuplot script is here.

Prediction of AT1B1_HUMAN

```
ID   AT1B1_HUMAN
FT   TOPO_DOM      1     35       NON CYTOPLASMIC.
FT   TRANSMEM     36     61
FT   TOPO_DOM     62    303       CYTOPLASMIC.
//
```

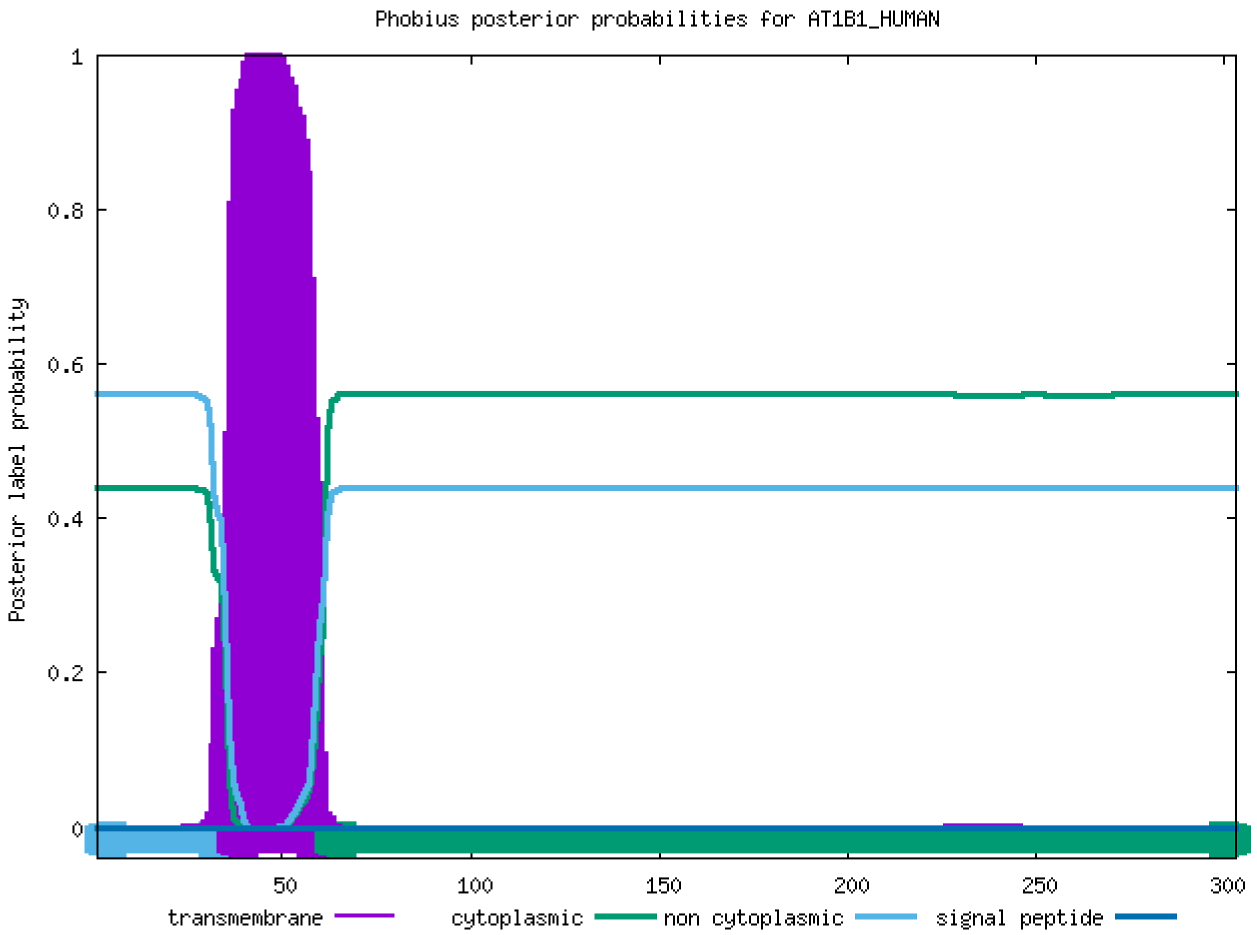

The probability data used in the plot is found here, and the gnuplot script is here.

Prediction of ATNG_HUMAN

```
ID    ATNG_HUMAN
FT    TOPO_DOM      1      29       NON CYTOPLASMIC.
FT    TRANSMEM     30      50
FT    TOPO_DOM     51      66       CYTOPLASMIC.
//
```

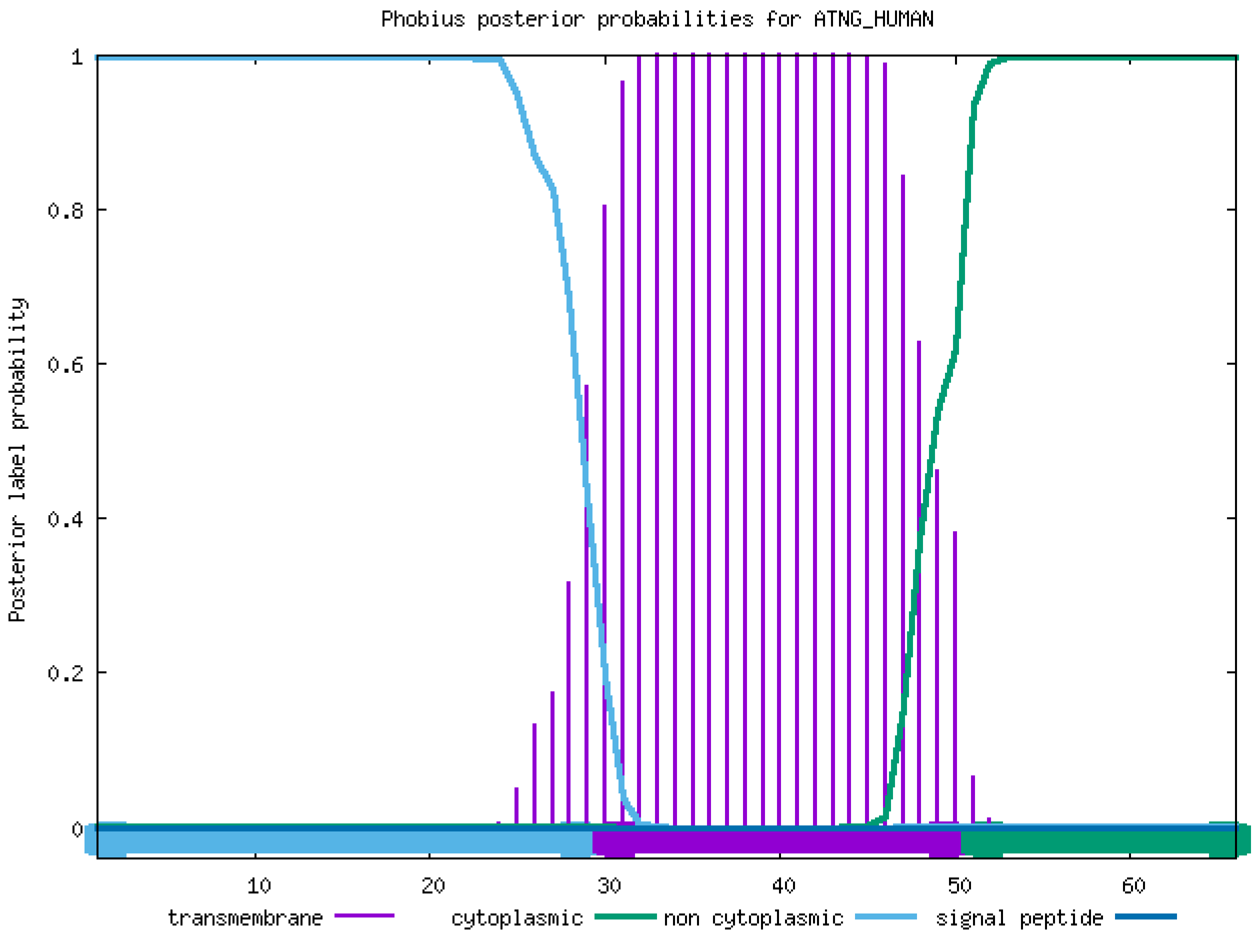

The probability data used in the plot is found here, and the gnuplot script is here.

Prediction of PLM_HUMAN

```
ID    PLM_HUMAN
FT    SIGNAL         1      20
FT    REGION         1       3       N-REGION.
FT    REGION         4      15       H-REGION.
FT    REGION        16      20       C-REGION.
FT    TOPO_DOM      21      35       NON CYTOPLASMIC.
FT    TRANSMEM      36      56
FT    TOPO_DOM      57      92       CYTOPLASMIC.
//
```

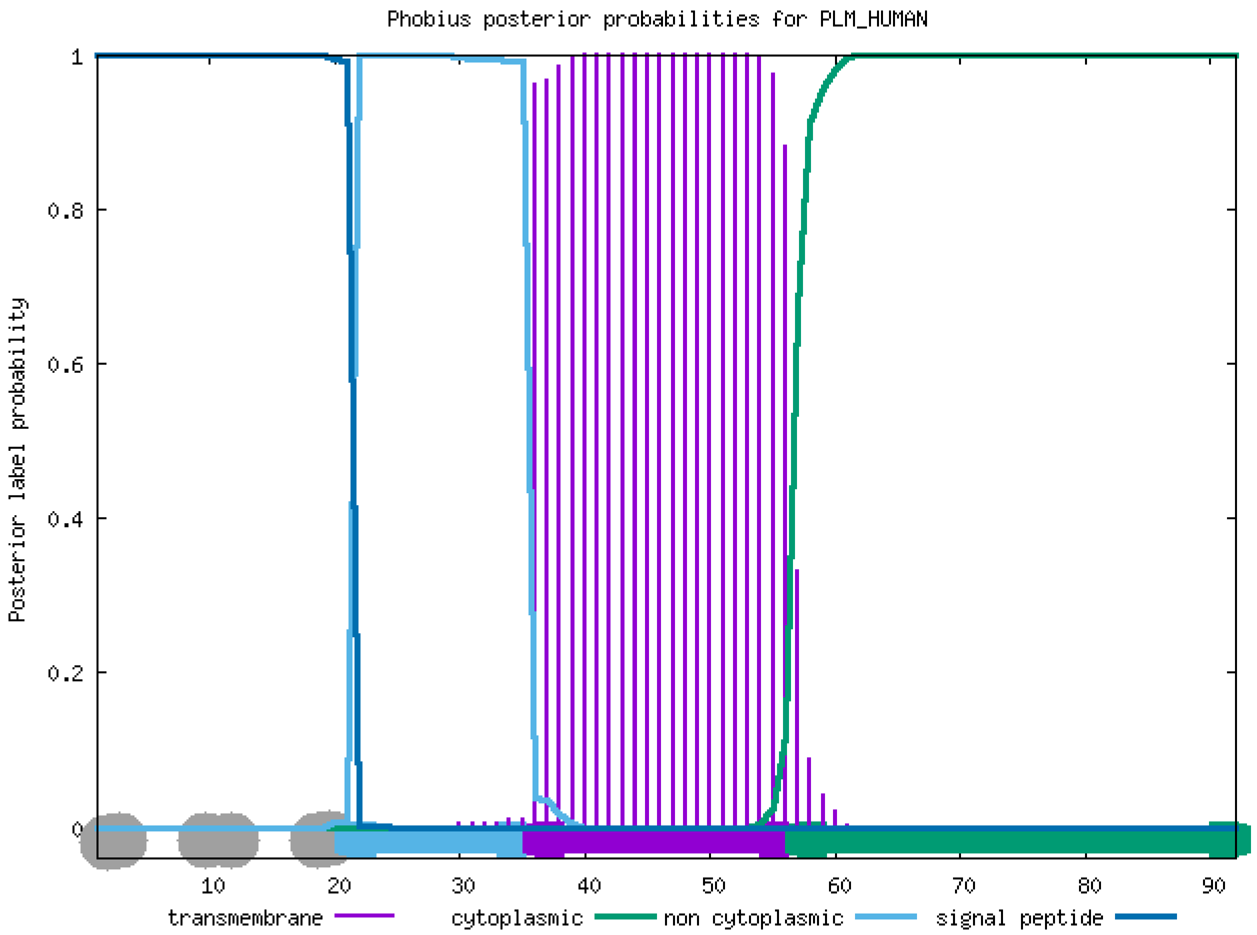

The probability data used in the plot is found here, and the gnuplot script is here.

Prediction of PPLA_HUMAN

```
ID   PPLA_HUMAN
FT   TOPO_DOM      1     30       CYTOPLASMIC.
FT   TRANSMEM     31     51
FT   TOPO_DOM     52     52       NON CYTOPLASMIC.
//
```

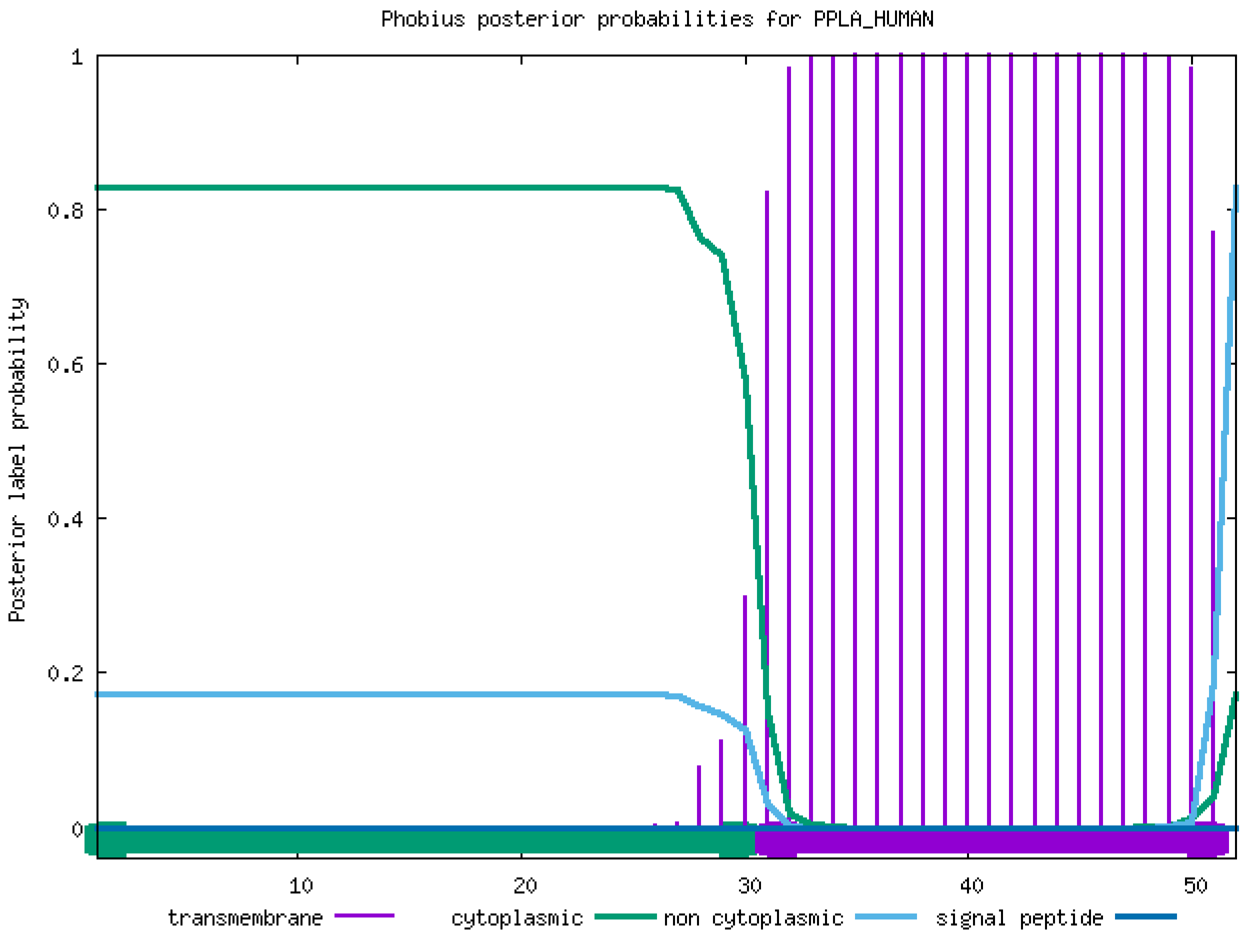

The probability data used in the plot is found here, and the gnuplot script is here.

Prediction of AT8A1_HUMAN

```
ID   AT8A1_HUMAN
FT   TOPO_DOM      1     72       CYTOPLASMIC.
FT   TRANSMEM     73     90
FT   TOPO_DOM     91     95       NON CYTOPLASMIC.
FT   TRANSMEM     96    114
FT   TOPO_DOM    115    297       CYTOPLASMIC.
FT   TRANSMEM    298    320
FT   TOPO_DOM    321    339       NON CYTOPLASMIC.
FT   TRANSMEM    340    363
FT   TOPO_DOM    364    866       CYTOPLASMIC.
FT   TRANSMEM    867    887
FT   TOPO_DOM    888    892       NON CYTOPLASMIC.
FT   TRANSMEM    893    911
FT   TOPO_DOM    912    939       CYTOPLASMIC.
FT   TRANSMEM    940    958
FT   TOPO_DOM    959    977       NON CYTOPLASMIC.
FT   TRANSMEM    978    997
FT   TOPO_DOM    998   1008       CYTOPLASMIC.
FT   TRANSMEM   1009   1029
FT   TOPO_DOM   1030   1048       NON CYTOPLASMIC.
FT   TRANSMEM   1049   1069
FT   TOPO_DOM   1070   1164       CYTOPLASMIC.
//
```

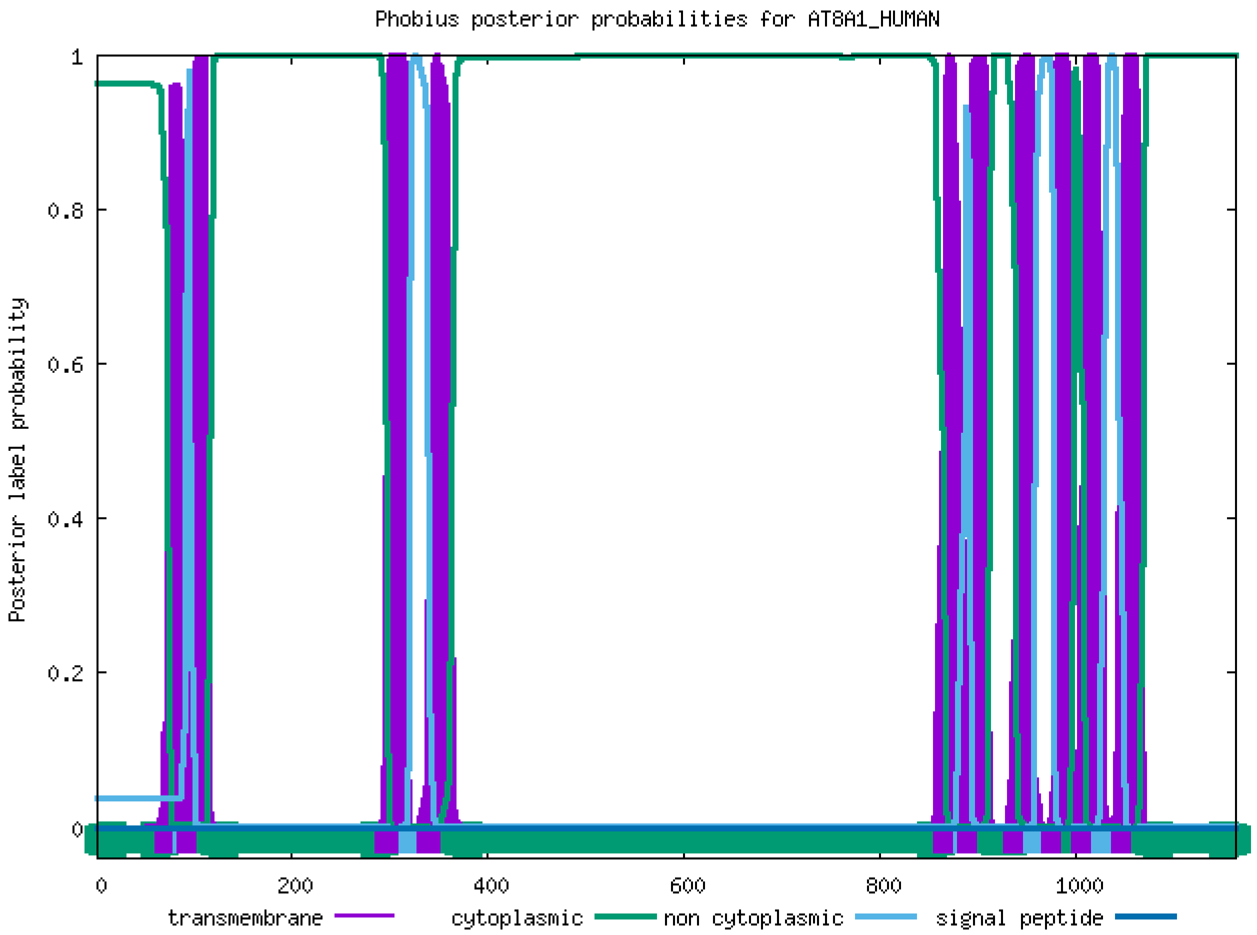

The probability data used in the plot is found here, and the gnuplot script is here.

Prediction of CC50A_HUMAN

```
ID   CC50A_HUMAN
FT   TOPO_DOM      1     43       CYTOPLASMIC.
FT   TRANSMEM     44     70
FT   TOPO_DOM     71    323       NON CYTOPLASMIC.
FT   TRANSMEM    324    346
FT   TOPO_DOM    347    361       CYTOPLASMIC.
//
```

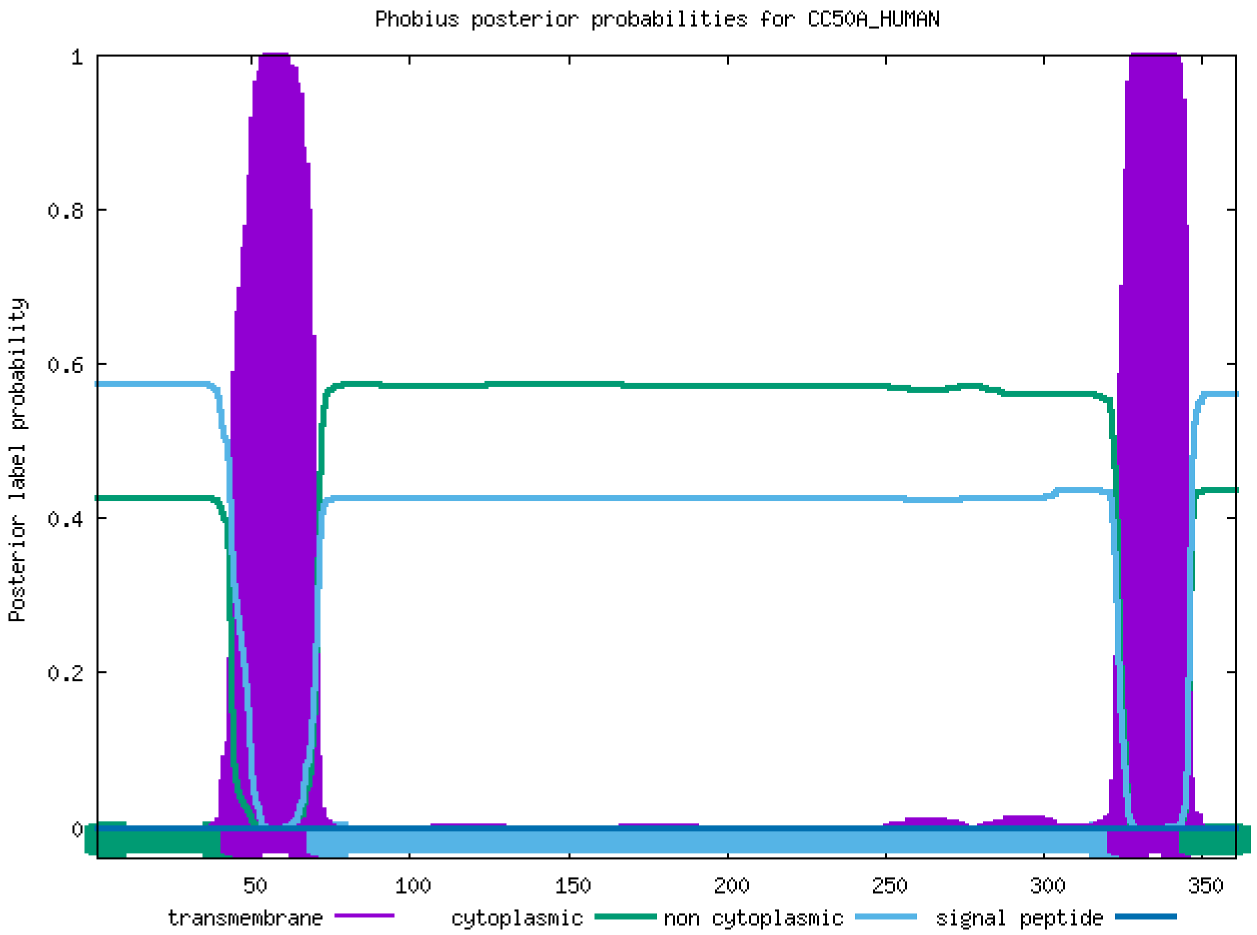

The probability data used in the plot is found here, and the gnuplot script is here.

Prediction of AT132_HUMAN

```
ID    AT132_HUMAN
FT    TOPO_DOM      1     46       NON CYTOPLASMIC.
FT    TRANSMEM     47     67
FT    TOPO_DOM     68    235       CYTOPLASMIC.
FT    TRANSMEM    236    252
FT    TOPO_DOM    253    257       NON CYTOPLASMIC.
FT    TRANSMEM    258    276
FT    TOPO_DOM    277    427       CYTOPLASMIC.
FT    TRANSMEM    428    449
FT    TOPO_DOM    450    460       NON CYTOPLASMIC.
FT    TRANSMEM    461    484
FT    TOPO_DOM    485    935       CYTOPLASMIC.
FT    TRANSMEM    936    955
FT    TOPO_DOM    956    960       NON CYTOPLASMIC.
FT    TRANSMEM    961    978
FT    TOPO_DOM    979    998       CYTOPLASMIC.
FT    TRANSMEM    999   1022
FT    TOPO_DOM   1023   1048       NON CYTOPLASMIC.
FT    TRANSMEM   1049   1066
FT    TOPO_DOM   1067   1077       CYTOPLASMIC.
FT    TRANSMEM   1078   1098
FT    TOPO_DOM   1099   1117       NON CYTOPLASMIC.
FT    TRANSMEM   1118   1138
FT    TOPO_DOM   1139   1180       CYTOPLASMIC.
//
```

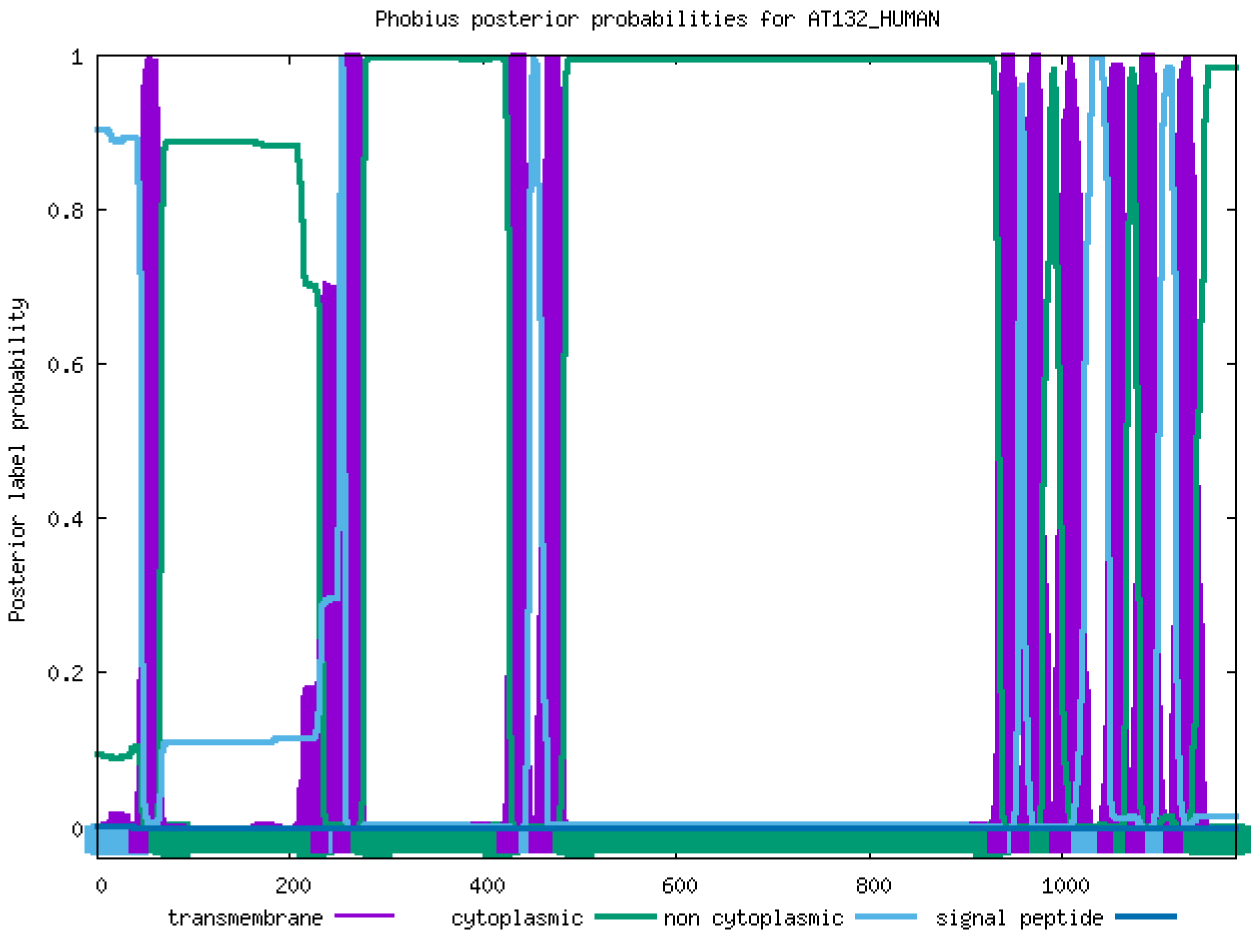

The probability data used in the plot is found here, and the gnuplot script is here.

Prediction of ATP7B_HUMAN

```
ID   ATP7B_HUMAN
FT   TOPO_DOM      1    653       CYTOPLASMIC.
FT   TRANSMEM    654    673
FT   TOPO_DOM    674    692       NON CYTOPLASMIC.
FT   TRANSMEM    693    715
FT   TOPO_DOM    716    726       CYTOPLASMIC.
FT   TRANSMEM    727    753
FT   TOPO_DOM    754    758       NON CYTOPLASMIC.
FT   TRANSMEM    759    780
FT   TOPO_DOM    781    922       CYTOPLASMIC.
FT   TRANSMEM    923    950
FT   TOPO_DOM    951    969       NON CYTOPLASMIC.
FT   TRANSMEM    970    990
FT   TOPO_DOM    991   1324       CYTOPLASMIC.
FT   TRANSMEM   1325   1347
FT   TOPO_DOM   1348   1352       NON CYTOPLASMIC.
FT   TRANSMEM   1353   1373
FT   TOPO_DOM   1374   1465       CYTOPLASMIC.
//
```

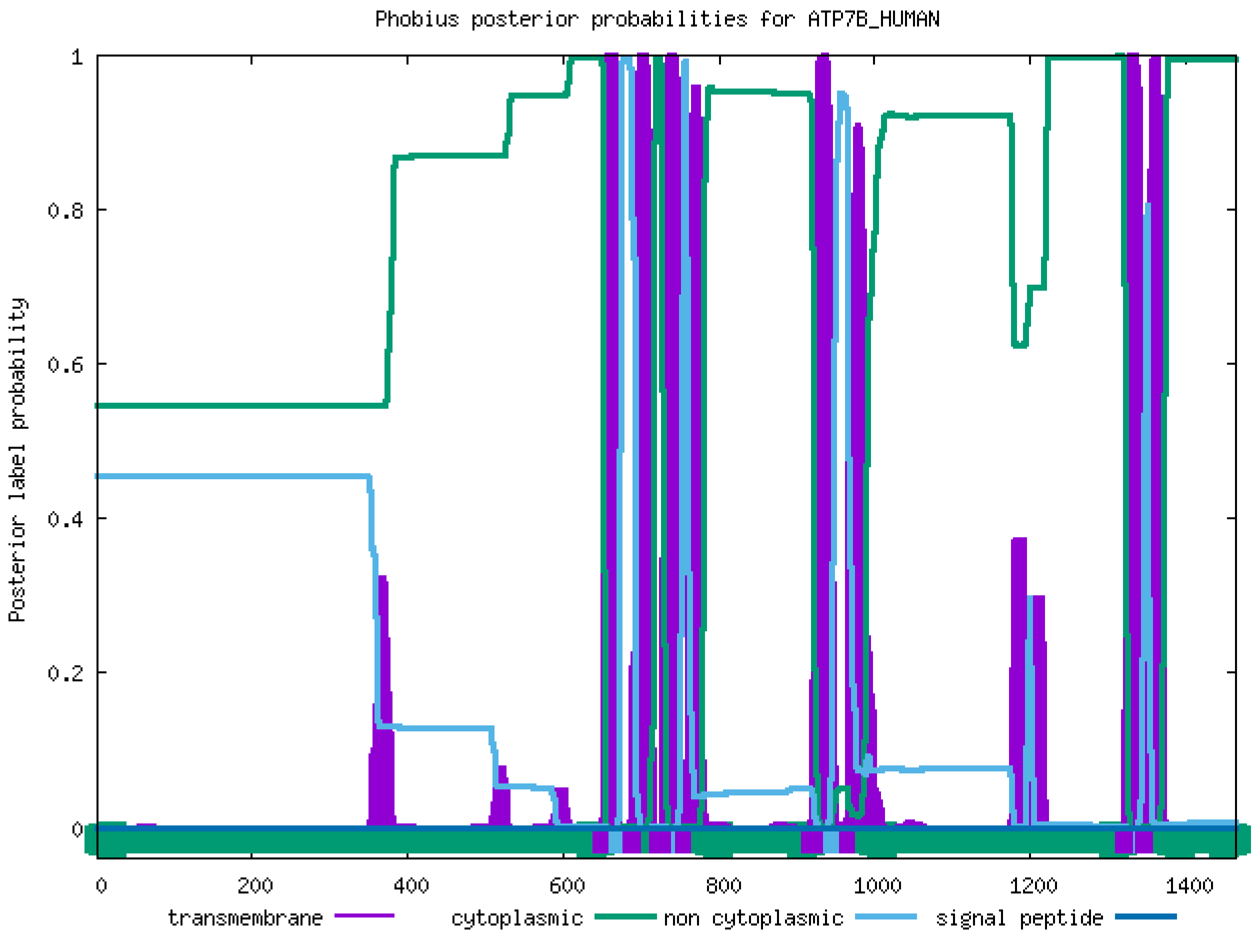

The probability data used in the plot is found here, and the gnuplot script is here.

Prediction of ACER3_HUMAN

```
ID   ACER3_HUMAN
FT   TOPO_DOM      1     33       NON CYTOPLASMIC.
FT   TRANSMEM     34     52
FT   TOPO_DOM     53     63       CYTOPLASMIC.
FT   TRANSMEM     64     84
FT   TOPO_DOM     85     95       NON CYTOPLASMIC.
FT   TRANSMEM     96    112
FT   TOPO_DOM    113    118       CYTOPLASMIC.
FT   TRANSMEM    119    137
FT   TOPO_DOM    138    142       NON CYTOPLASMIC.
FT   TRANSMEM    143    162
FT   TOPO_DOM    163    173       CYTOPLASMIC.
FT   TRANSMEM    174    191
FT   TOPO_DOM    192    217       NON CYTOPLASMIC.
FT   TRANSMEM    218    238
FT   TOPO_DOM    239    267       CYTOPLASMIC.
//
```

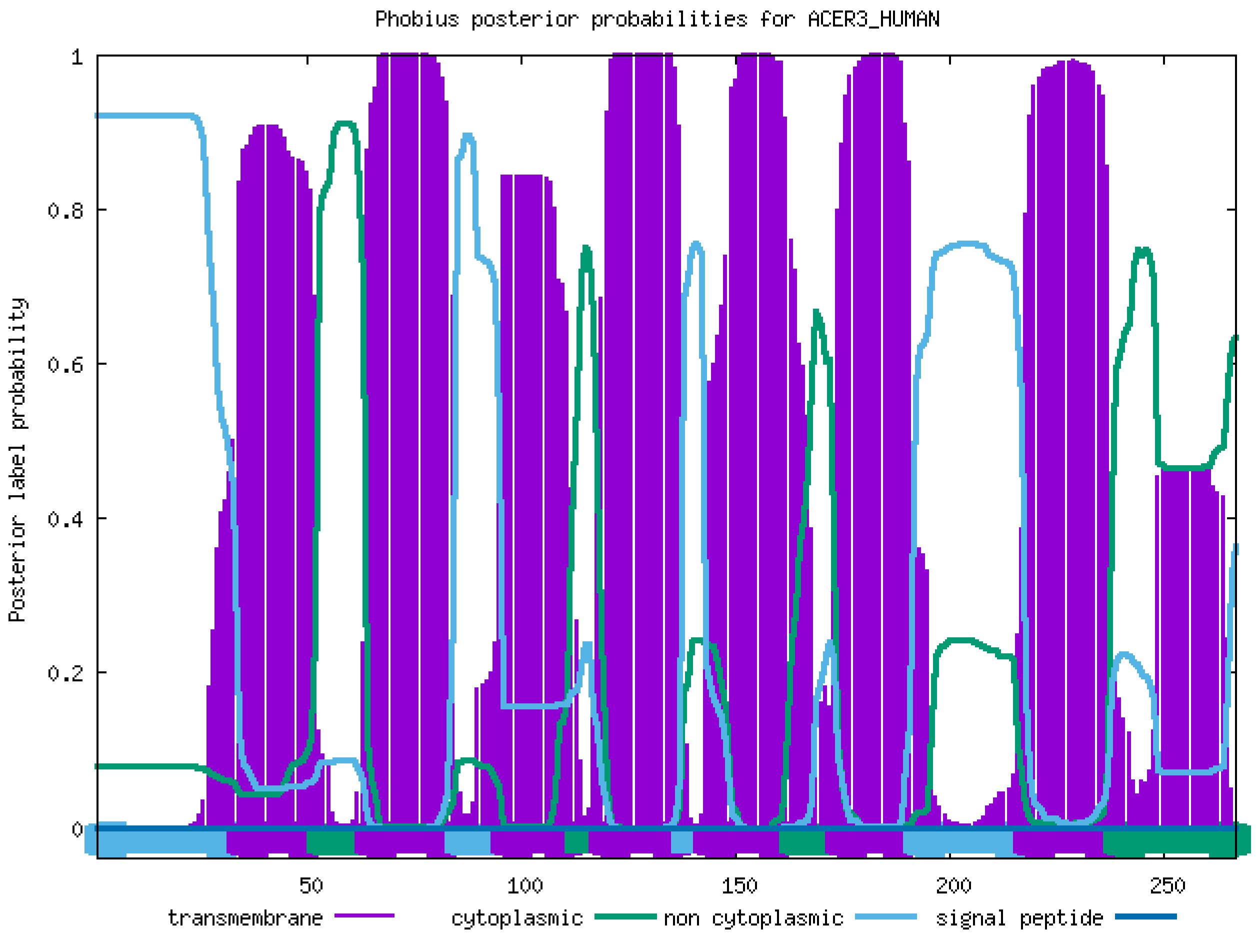

The probability data used in the plot is found here, and the gnuplot script is here.

Prediction of VKOR1_HUMAN

```
ID   VKOR1_HUMAN
FT   TOPO_DOM      1     11       NON CYTOPLASMIC.
FT   TRANSMEM     12     29
FT   TOPO_DOM     30     40       CYTOPLASMIC.
FT   TRANSMEM     41     60
FT   TOPO_DOM     61     79       NON CYTOPLASMIC.
FT   TRANSMEM     80     97
FT   TOPO_DOM     98    103       CYTOPLASMIC.
FT   TRANSMEM    104    122
FT   TOPO_DOM    123    127       NON CYTOPLASMIC.
FT   TRANSMEM    128    150
FT   TOPO_DOM    151    163       CYTOPLASMIC.
//
```

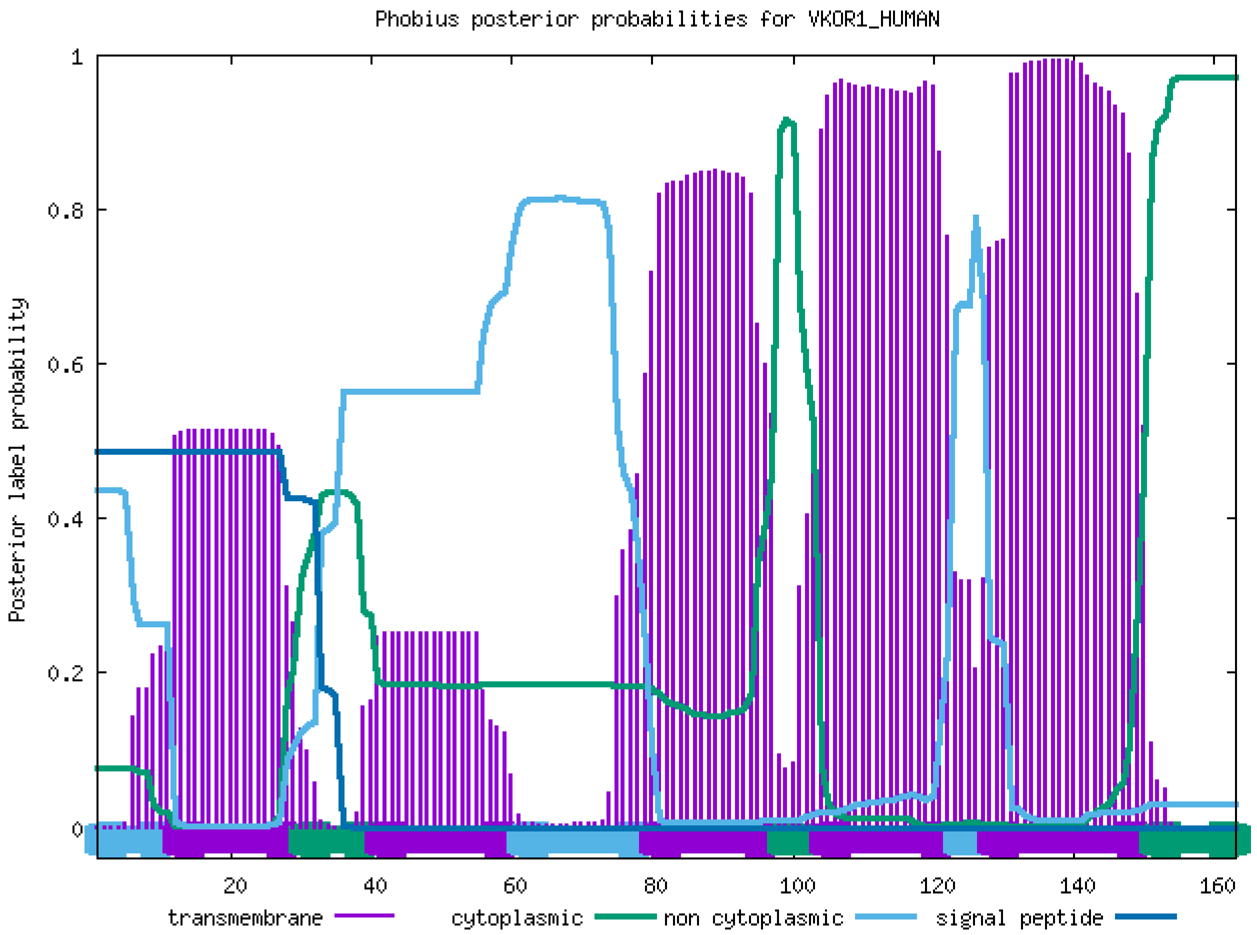

The probability data used in the plot is found here, and the gnuplot script is here.

Prediction of STEA1_HUMAN

```
ID   STEA1_HUMAN
FT   TOPO_DOM      1     72       CYTOPLASMIC.
FT   TRANSMEM     73     91
FT   TOPO_DOM     92    116       NON CYTOPLASMIC.
FT   TRANSMEM    117    143
FT   TOPO_DOM    144    163       CYTOPLASMIC.
FT   TRANSMEM    164    184
FT   TOPO_DOM    185    217       NON CYTOPLASMIC.
FT   TRANSMEM    218    236
FT   TOPO_DOM    237    256       CYTOPLASMIC.
FT   TRANSMEM    257    278
FT   TOPO_DOM    279    289       NON CYTOPLASMIC.
FT   TRANSMEM    290    313
FT   TOPO_DOM    314    339       CYTOPLASMIC.
//
```

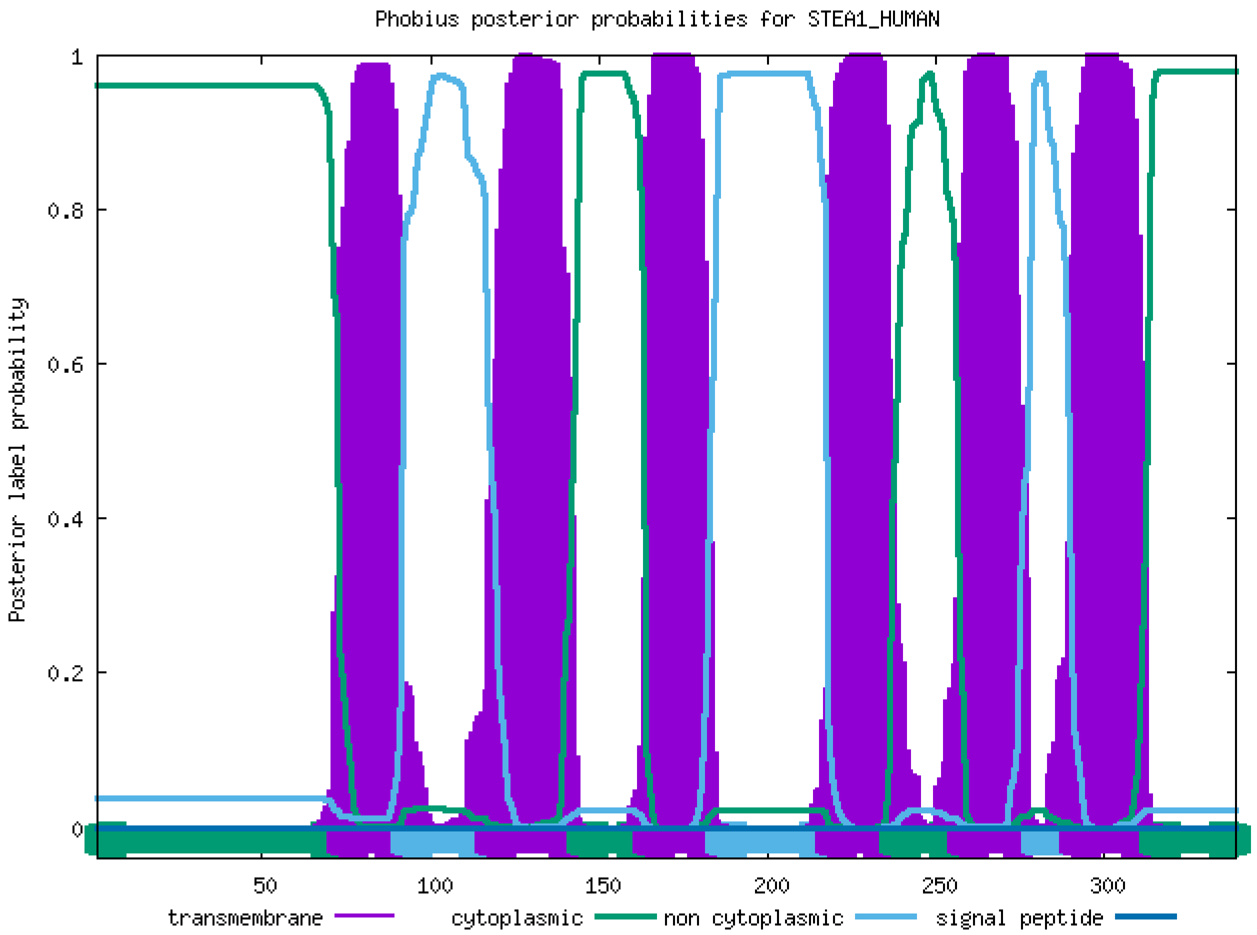

The probability data used in the plot is found here, and the gnuplot script is here.

Prediction of STEA4_HUMAN

```
ID    STEA4_HUMAN
FT    TOPO_DOM      1    203        CYTOPLASMIC.
FT    TRANSMEM    204    229
FT    TOPO_DOM    230    248        NON CYTOPLASMIC.
FT    TRANSMEM    249    271
FT    TOPO_DOM    272    291        CYTOPLASMIC.
FT    TRANSMEM    292    317
FT    TOPO_DOM    318    347        NON CYTOPLASMIC.
FT    TRANSMEM    348    374
FT    TOPO_DOM    375    385        CYTOPLASMIC.
FT    TRANSMEM    386    402
FT    TOPO_DOM    403    413        NON CYTOPLASMIC.
FT    TRANSMEM    414    438
FT    TOPO_DOM    439    459        CYTOPLASMIC.
//
```

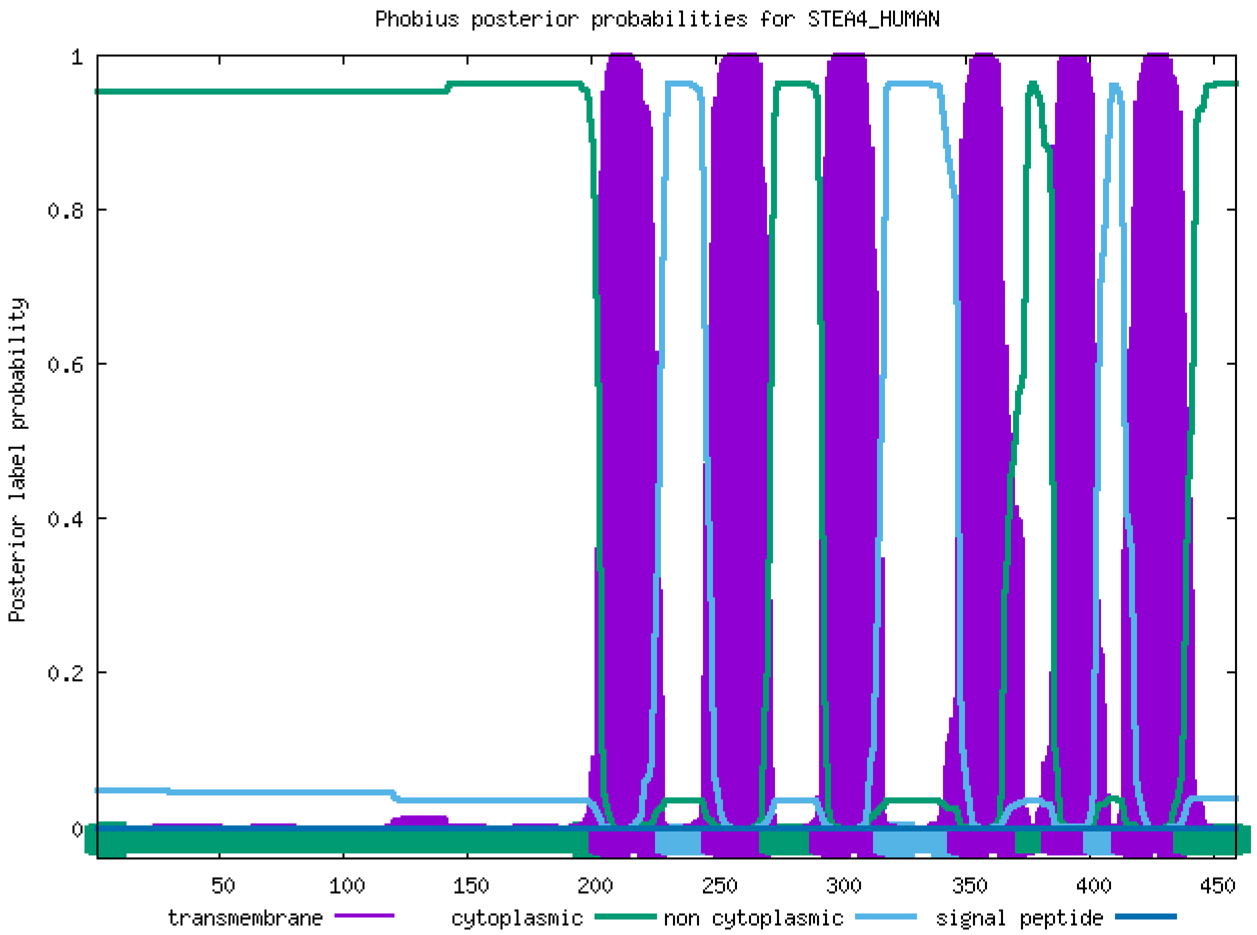

The probability data used in the plot is found here, and the gnuplot script is here.

Prediction of DUOX1_HUMAN

```
ID   DUOX1_HUMAN
FT   SIGNAL        1     21
FT   REGION        1      2       N-REGION.
FT   REGION        3     13       H-REGION.
FT   REGION       14     21       C-REGION.
FT   TOPO_DOM     22    592       NON CYTOPLASMIC.
FT   TRANSMEM    593    617
FT   TOPO_DOM    618   1044       CYTOPLASMIC.
FT   TRANSMEM   1045   1068
FT   TOPO_DOM   1069   1087       NON CYTOPLASMIC.
FT   TRANSMEM   1088   1109
FT   TOPO_DOM   1110   1151       CYTOPLASMIC.
FT   TRANSMEM   1152   1171
FT   TOPO_DOM   1172   1182       NON CYTOPLASMIC.
FT   TRANSMEM   1183   1209
FT   TOPO_DOM   1210   1220       CYTOPLASMIC.
FT   TRANSMEM   1221   1243
FT   TOPO_DOM   1244   1248       NON CYTOPLASMIC.
FT   TRANSMEM   1249   1266
FT   TOPO_DOM   1267   1551       CYTOPLASMIC.
//
```

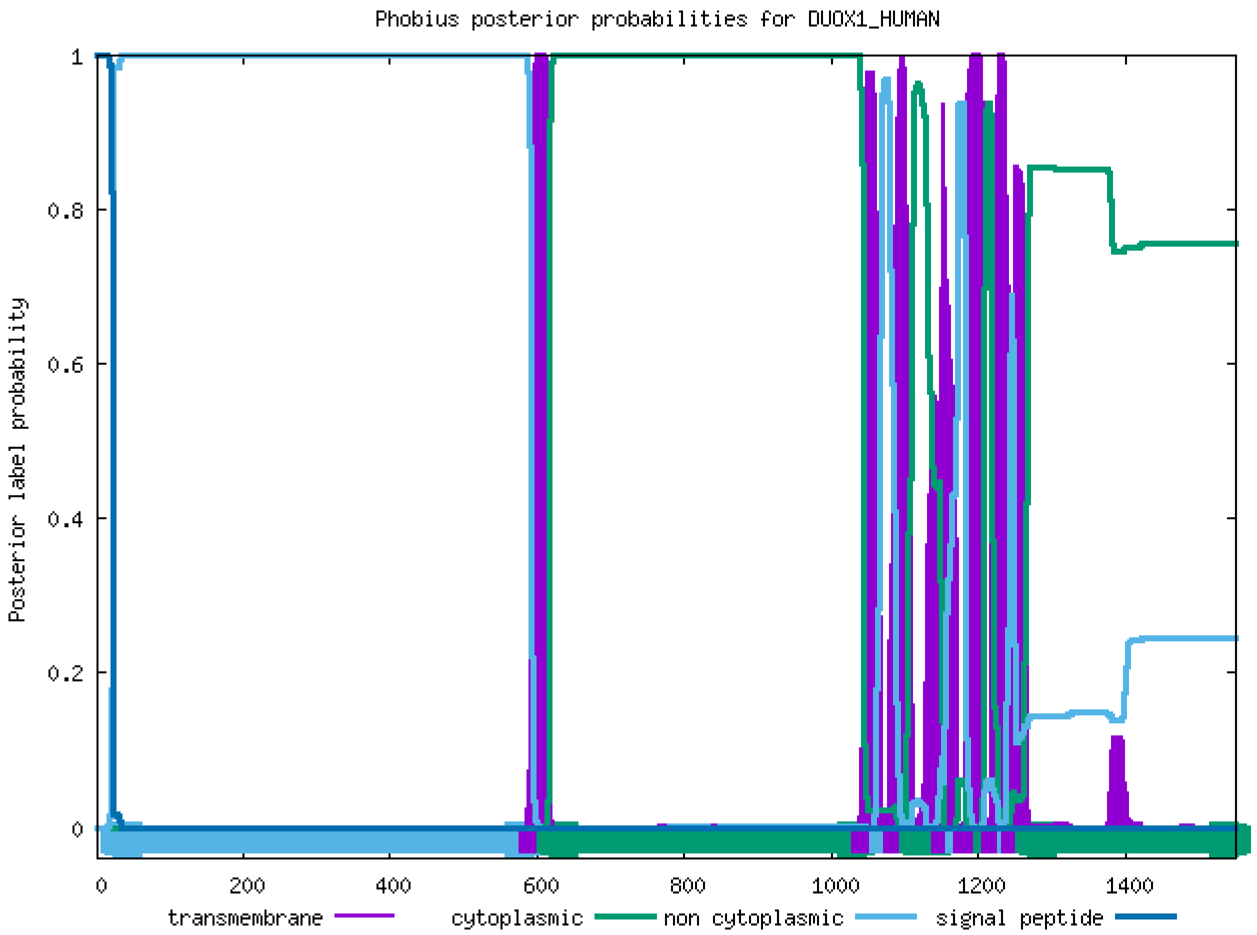

The probability data used in the plot is found here, and the gnuplot script is here.

Prediction of DOXA1_HUMAN

```
ID    DOXA1_HUMAN
FT    TOPO_DOM      1      23       NON CYTOPLASMIC.
FT    TRANSMEM     24      45
FT    TOPO_DOM     46      51       CYTOPLASMIC.
FT    TRANSMEM     52      72
FT    TOPO_DOM     73     178       NON CYTOPLASMIC.
FT    TRANSMEM    179     198
FT    TOPO_DOM    199     209       CYTOPLASMIC.
FT    TRANSMEM    210     230
FT    TOPO_DOM    231     249       NON CYTOPLASMIC.
FT    TRANSMEM    250     271
FT    TOPO_DOM    272     343       CYTOPLASMIC.
//
```

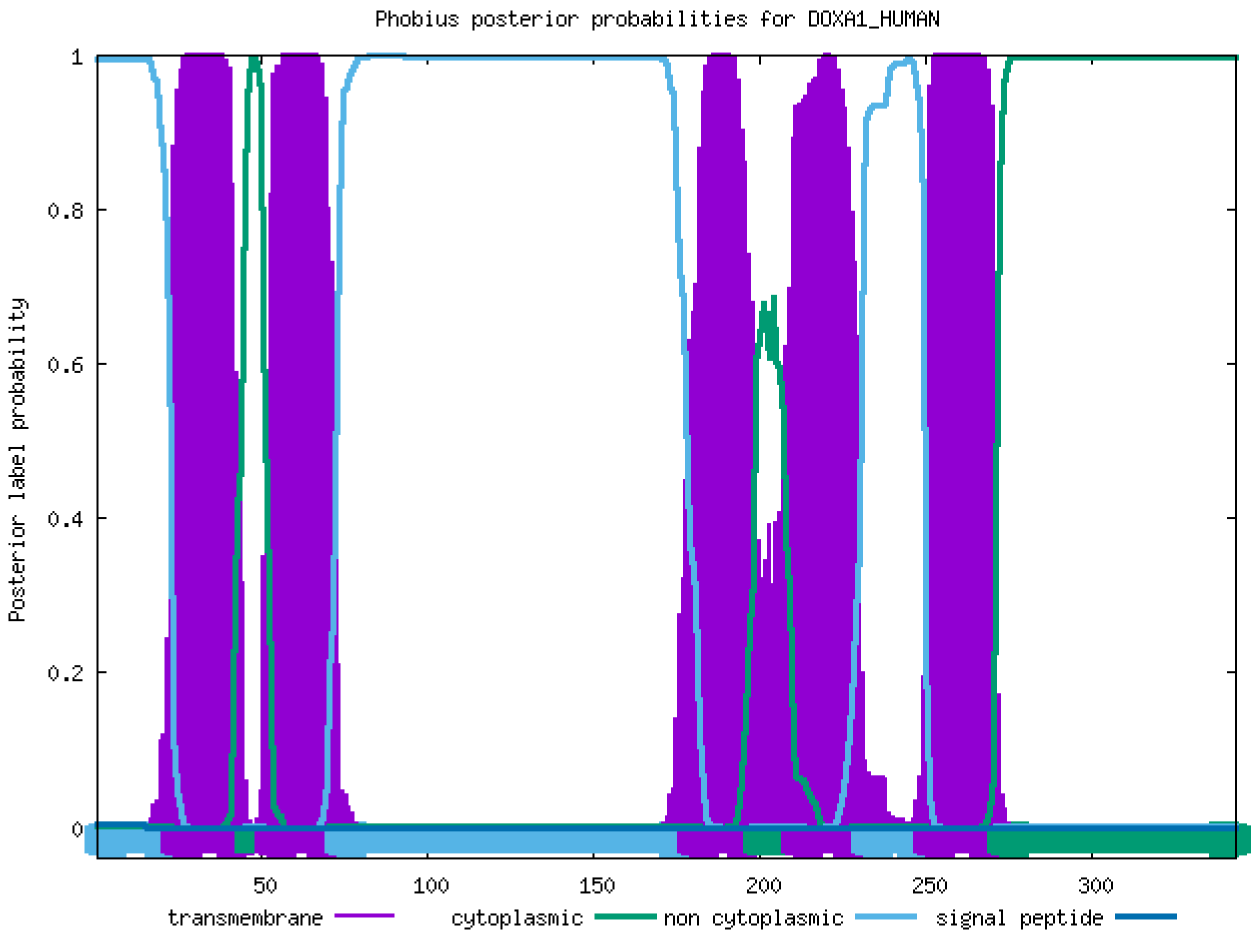

The probability data used in the plot is found here, and the gnuplot script is here.

Prediction of CY24B_HUMAN

```
ID    CY24B_HUMAN
FT    TOPO_DOM      1     11       NON CYTOPLASMIC.
FT    TRANSMEM     12     33
FT    TOPO_DOM     34     44       CYTOPLASMIC.
FT    TRANSMEM     45     69
FT    TOPO_DOM     70    102       NON CYTOPLASMIC.
FT    TRANSMEM    103    121
FT    TOPO_DOM    122    166       CYTOPLASMIC.
FT    TRANSMEM    167    192
FT    TOPO_DOM    193    203       NON CYTOPLASMIC.
FT    TRANSMEM    204    221
FT    TOPO_DOM    222    570       CYTOPLASMIC.
//
```

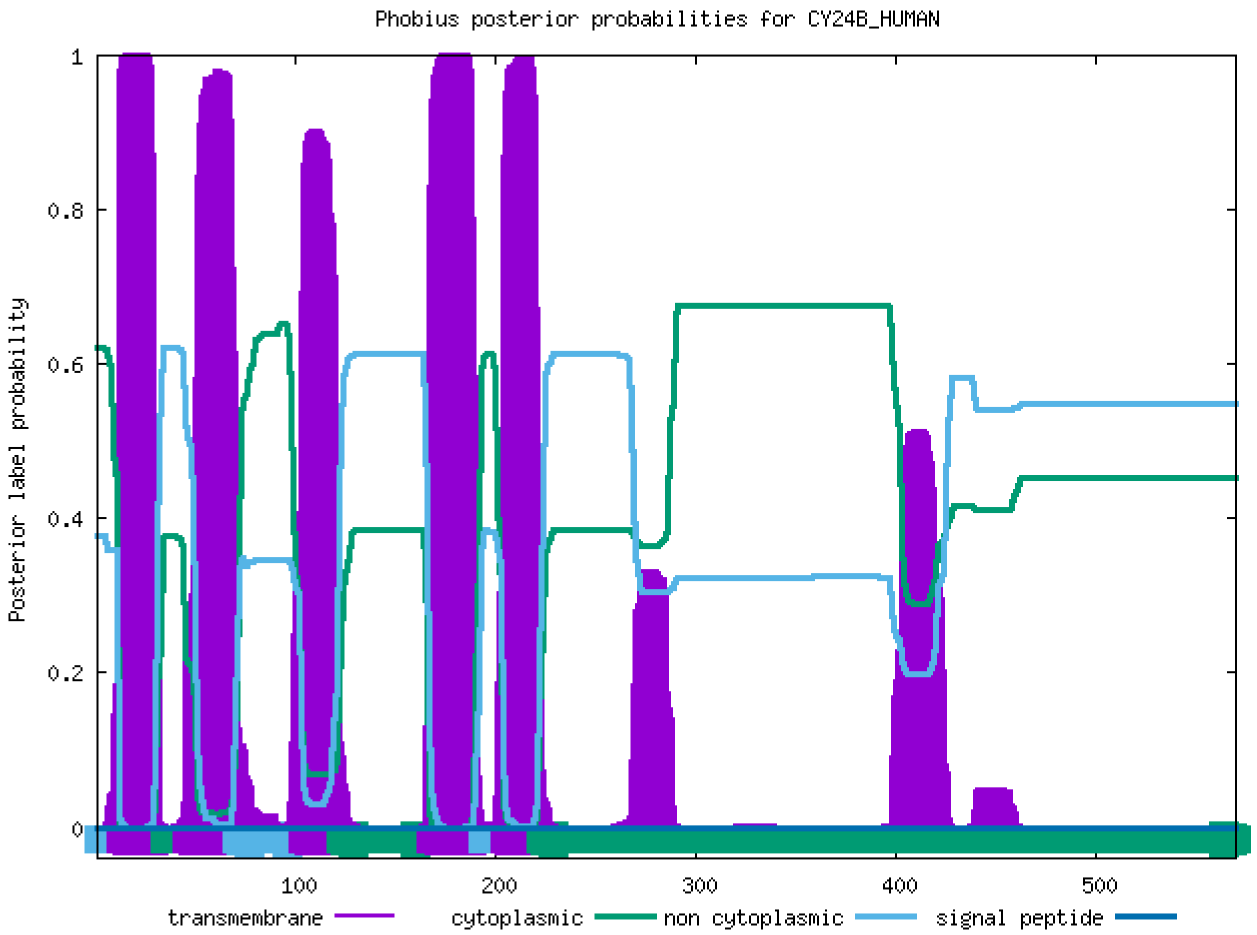

The probability data used in the plot is found here, and the gnuplot script is here.

Prediction of CY24A_HUMAN

```
ID   CY24A_HUMAN
FT   TOPO_DOM      1     11       CYTOPLASMIC.
FT   TRANSMEM     12     30
FT   TOPO_DOM     31     35       NON CYTOPLASMIC.
FT   TRANSMEM     36     54
FT   TOPO_DOM     55     74       CYTOPLASMIC.
FT   TRANSMEM     75     97
FT   TOPO_DOM     98    102       NON CYTOPLASMIC.
FT   TRANSMEM    103    124
FT   TOPO_DOM    125    195       CYTOPLASMIC.
//
```

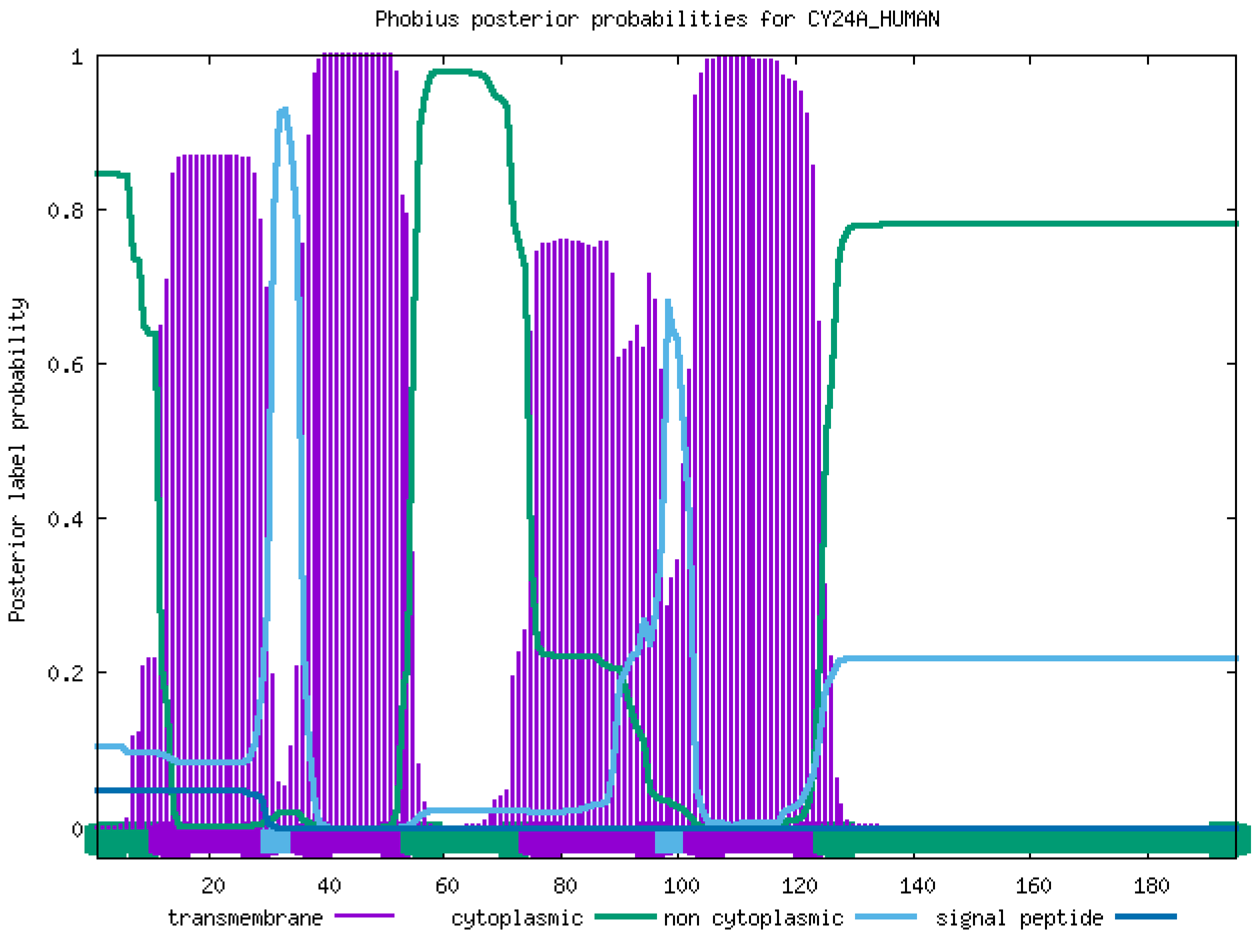

The probability data used in the plot is found here, and the gnuplot script is here.

Prediction of C560_HUMAN

```
ID   C560_HUMAN
FT   TOPO_DOM      1     74       CYTOPLASMIC.
FT   TRANSMEM     75     96
FT   TOPO_DOM     97    107       NON CYTOPLASMIC.
FT   TRANSMEM    108    127
FT   TOPO_DOM    128    147       CYTOPLASMIC.
FT   TRANSMEM    148    168
FT   TOPO_DOM    169    169       NON CYTOPLASMIC.
//
```

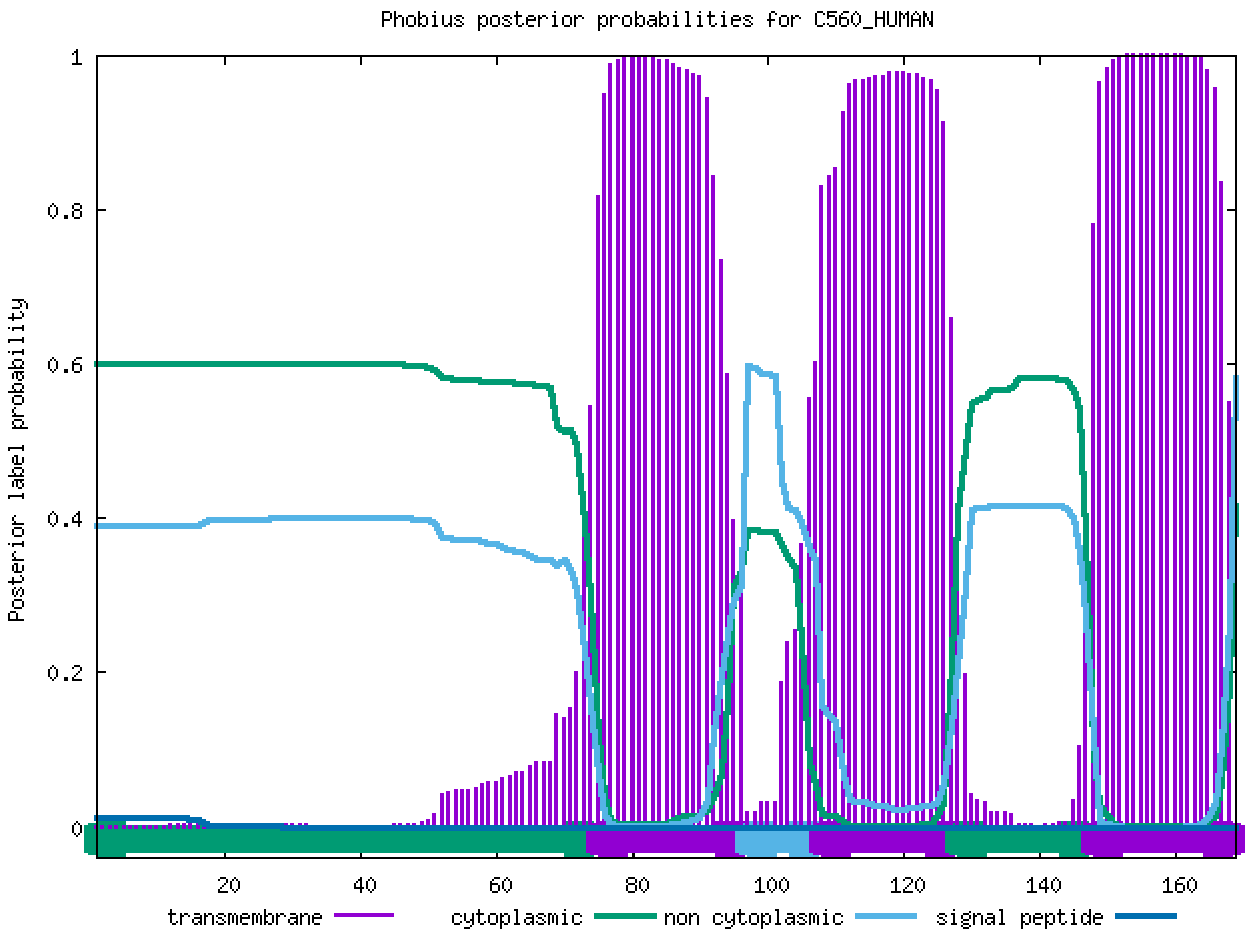

The probability data used in the plot is found here, and the gnuplot script is here.

Prediction of DHSD_HUMAN

```
ID   DHSD_HUMAN
FT   SIGNAL         1     22
FT   REGION         1      2       N-REGION.
FT   REGION         3     14       H-REGION.
FT   REGION        15     22       C-REGION.
FT   TOPO_DOM      23    159       NON CYTOPLASMIC.
//
```

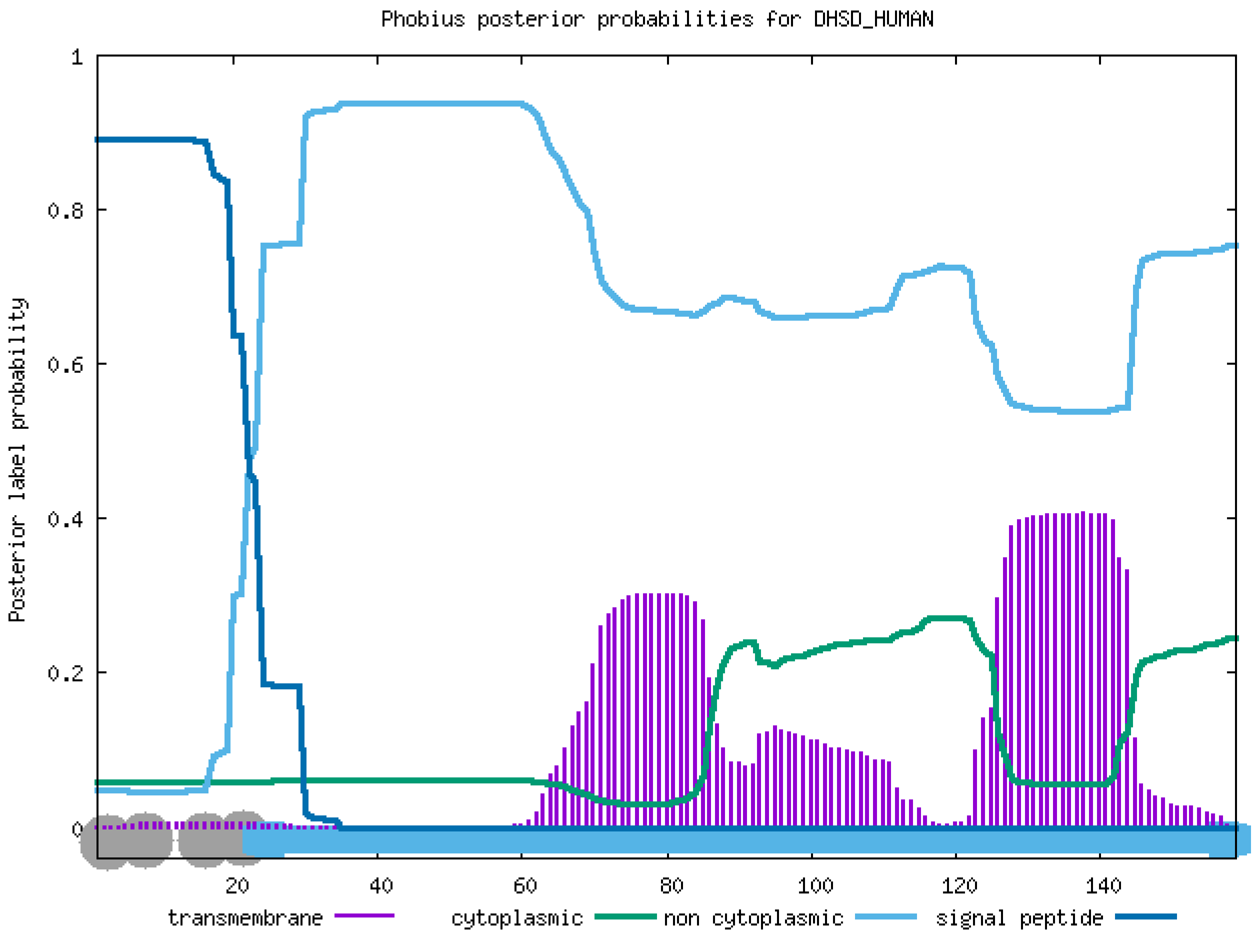

The probability data used in the plot is found here, and the gnuplot script is here.
