# Supplementary material for: Co-evolution of alpha-helical transmembrane protein residues: large-scale variant profiling and complete mutational landscape of 2277 known PDB entries representing 504 unique human protein sequences
Source: J Mol Evol. 2025 Sep 24;93(5):581–99. doi: 10.1007/s00239-025-10262-8 (PMC12579659; doi:10.1007/s00239-025-10262-8)
Supplement: Supplementary file 6 — Supplementary file6 (PDF 61643 kb) [file 239_2025_10262_MOESM6_ESM.pdf]

Supplementary Figure 2. Combined Hydropathy Plots for Studied Proteins

Hydropathy plots generated using the Kyte-Doolittle scale illustrate the hydropathic properties of the protein sequences.

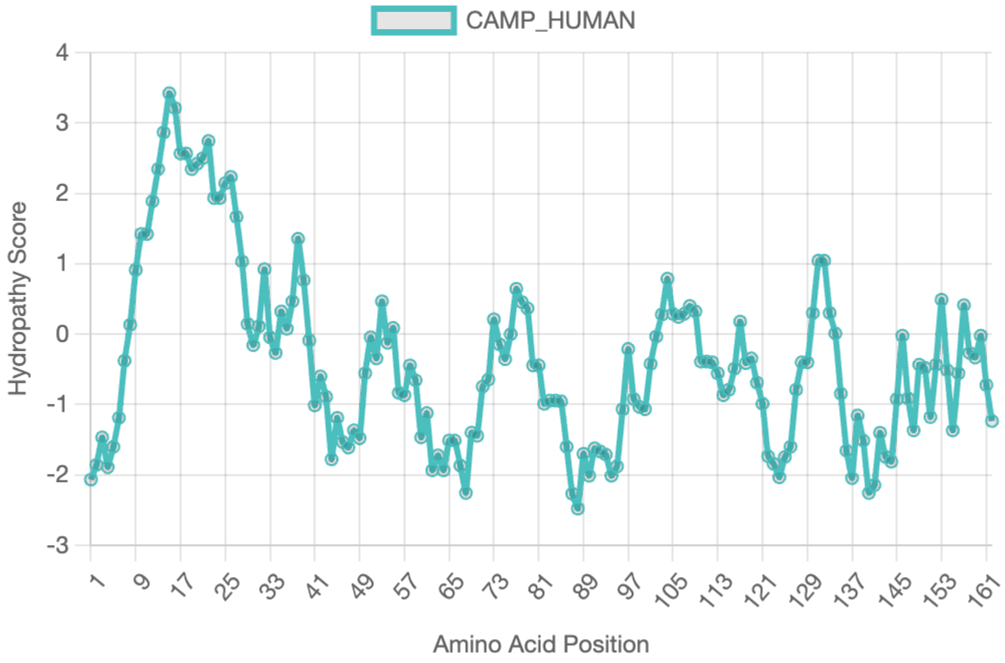

Kyte-Doolittle hydropathy plot for the sequence "CAMP_HUMAN". Positive values indicate hydrophobic regions, while negative values indicate hydrophilic regions.

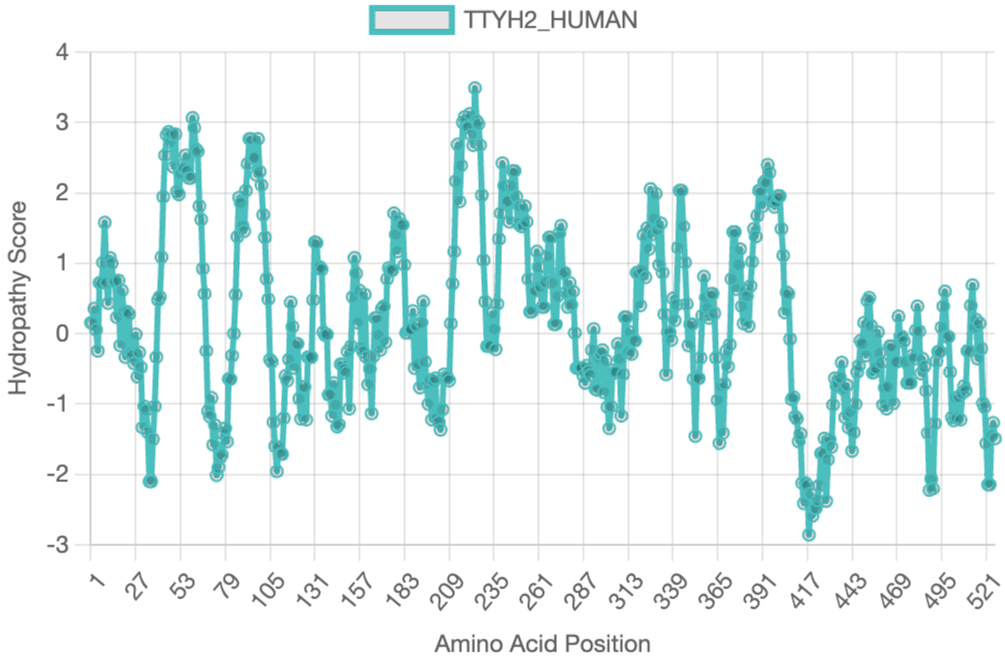

Kyte-Doolittle hydropathy plot for the sequence "TTYH2_HUMAN". Positive values indicate hydrophobic regions, while negative values indicate hydrophilic regions.

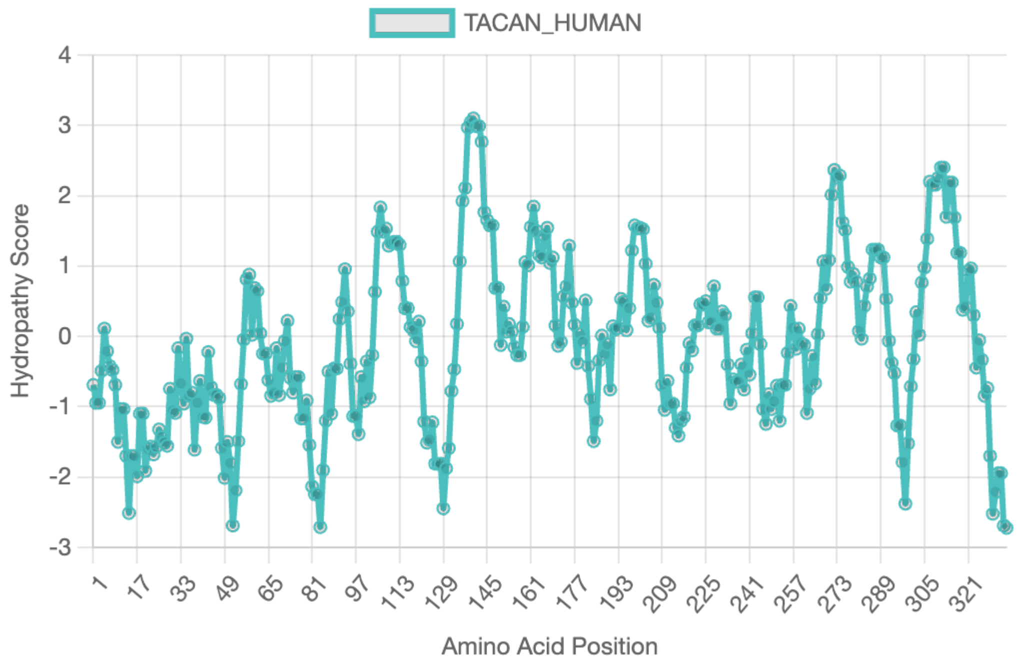

Kyte-Doolittle hydropathy plot for the sequence "TACAN_HUMAN". Positive values indicate hydrophobic regions, while negative values indicate hydrophilic regions.

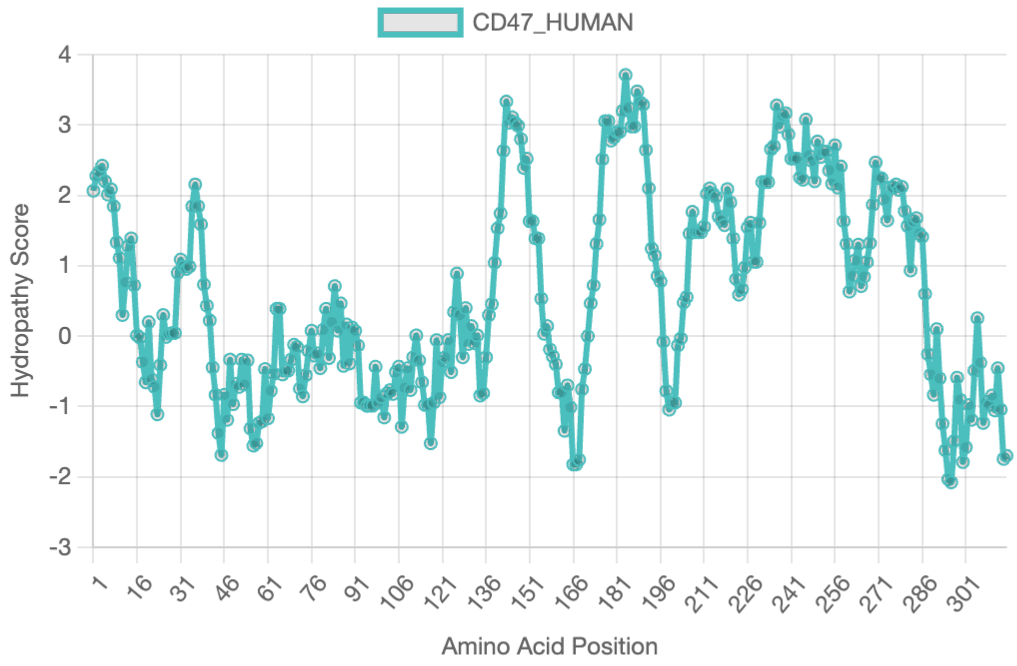

Kyte-Doolittle hydropathy plot for the sequence "CD47_HUMAN". Positive values indicate hydrophobic regions, while negative values indicate hydrophilic regions.

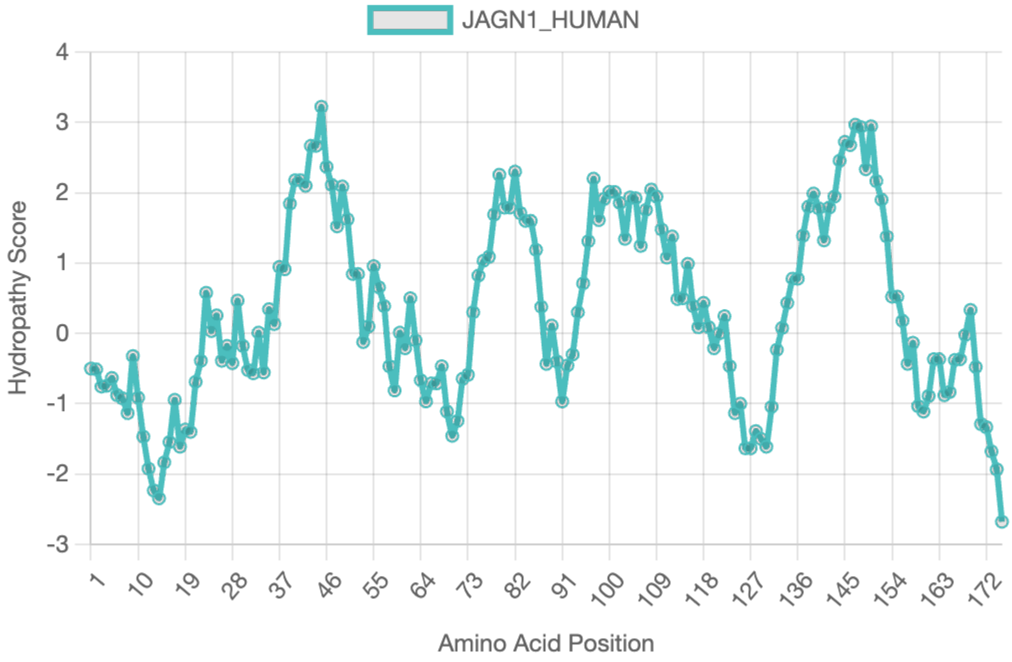

Kyte-Doolittle hydropathy plot for the sequence "JAGN1_HUMAN". Positive values indicate hydrophobic regions, while negative values indicate hydrophilic regions.

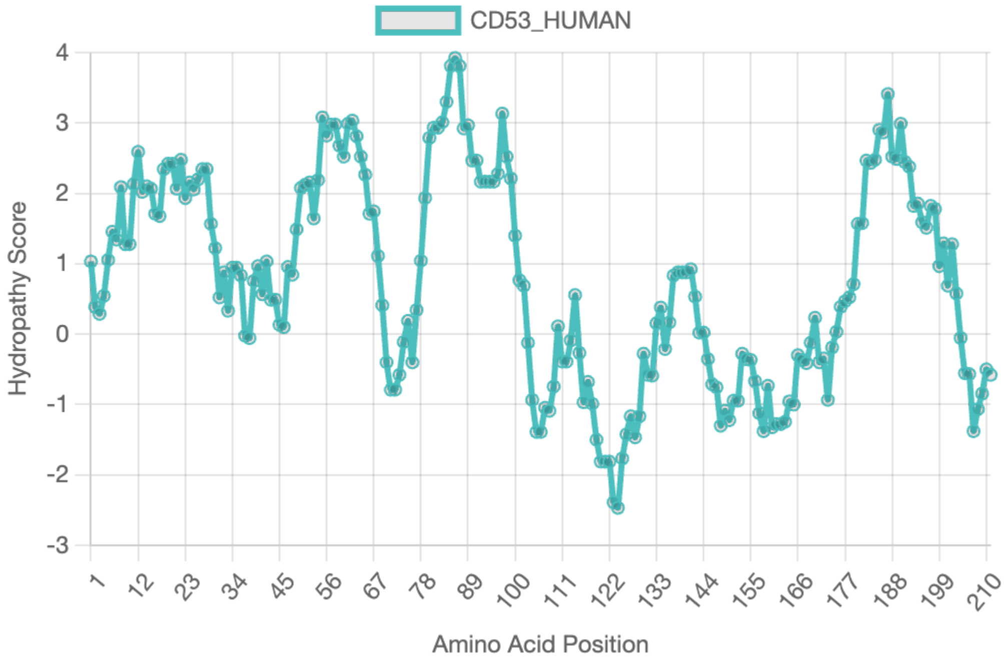

Kyte-Doolittle hydropathy plot for the sequence "CD53_HUMAN". Positive values indicate hydrophobic regions, while negative values indicate hydrophilic regions.

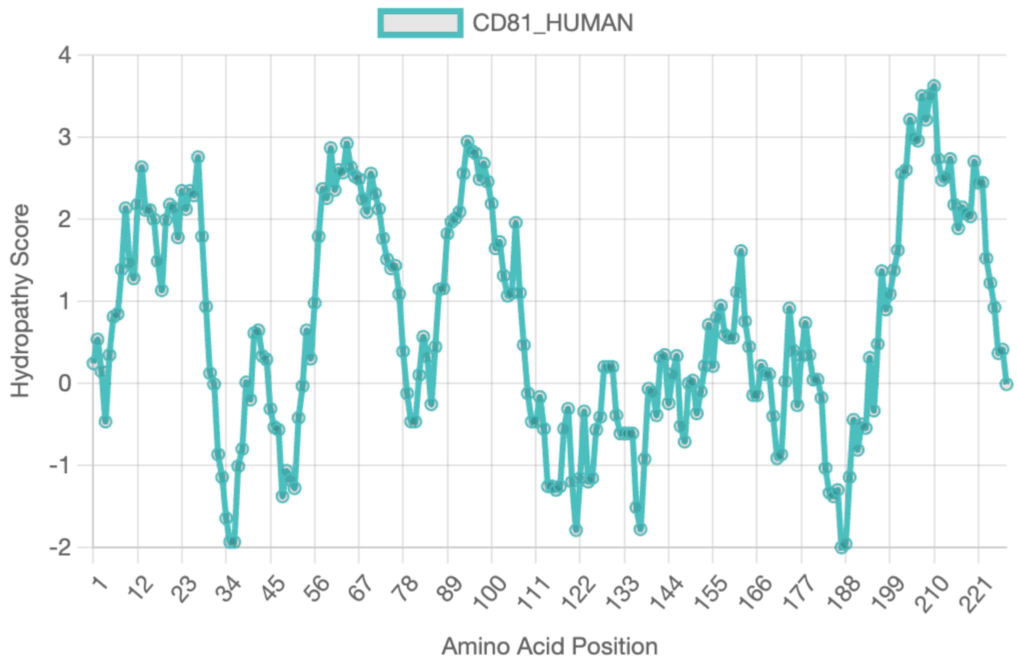

Kyte-Doolittle hydropathy plot for the sequence "CD81_HUMAN". Positive values indicate hydrophobic regions, while negative values indicate hydrophilic regions.

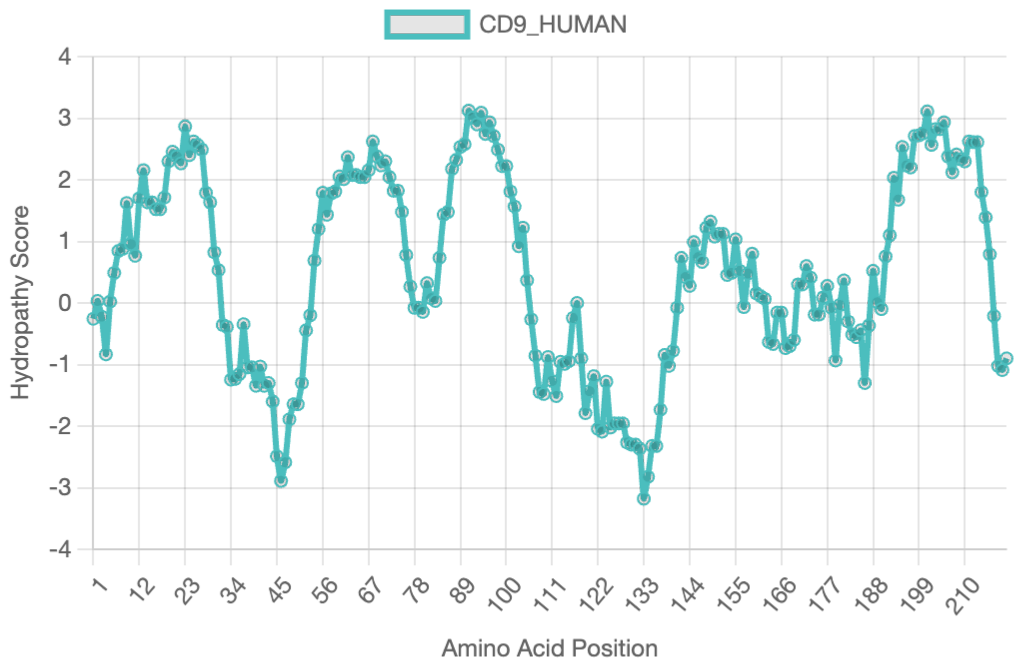

Kyte-Doolittle hydropathy plot for the sequence "CD9_HUMAN". Positive values indicate hydrophobic regions, while negative values indicate hydrophilic regions.

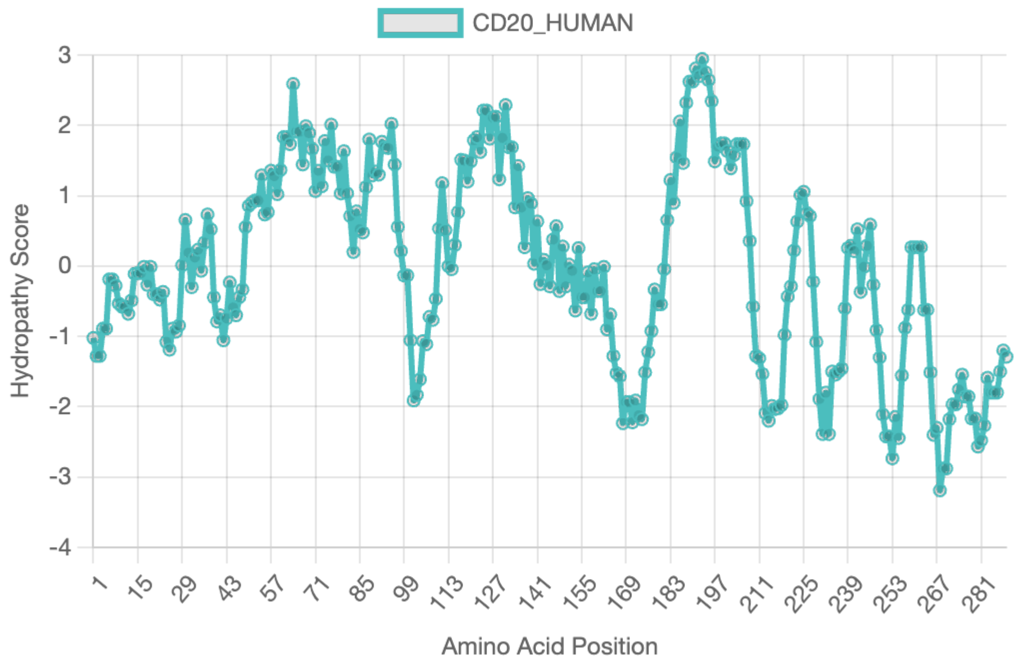

Kyte-Doolittle hydropathy plot for the sequence "CD20_HUMAN". Positive values indicate hydrophobic regions, while negative values indicate hydrophilic regions.

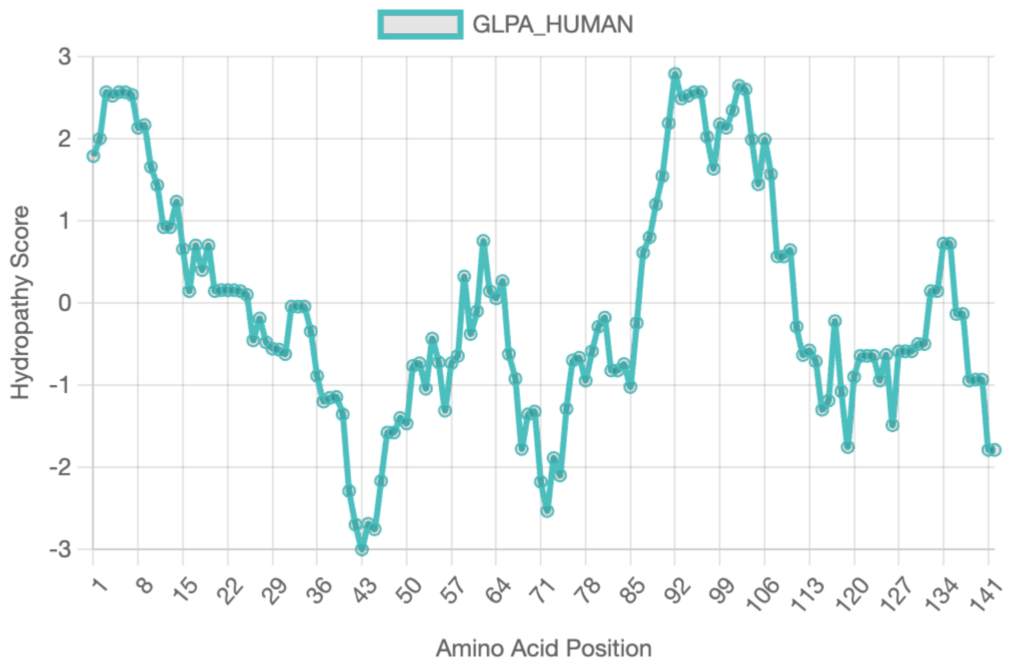

Kyte-Doolittle hydropathy plot for the sequence "GLPA_HUMAN". Positive values indicate hydrophobic regions, while negative values indicate hydrophilic regions.

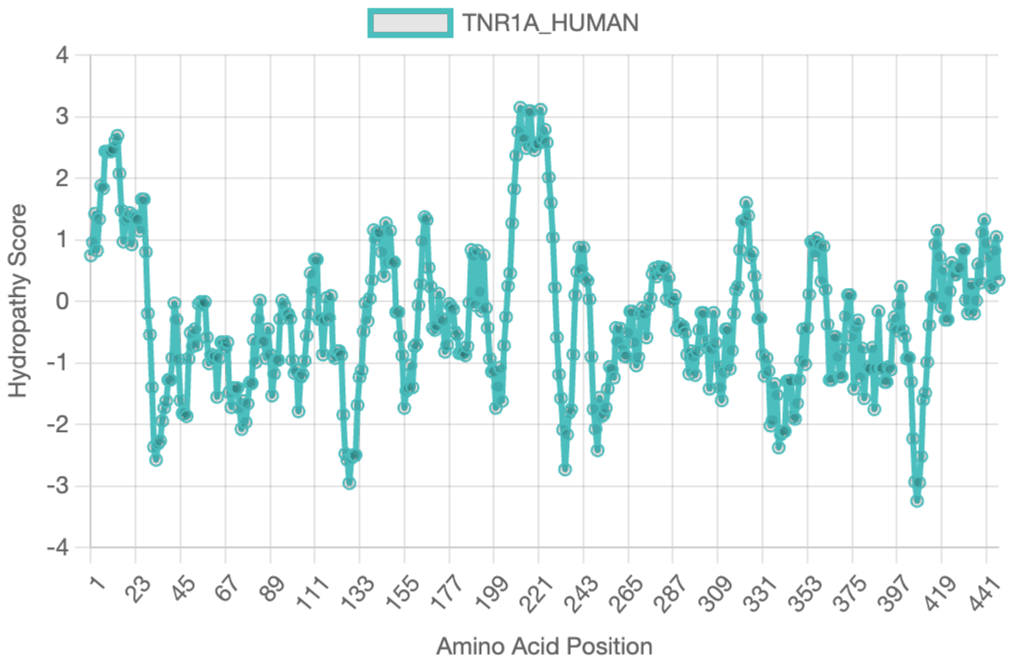

Kyte-Doolittle hydropathy plot for the sequence "TNR1A_HUMAN". Positive values indicate hydrophobic regions, while negative values indicate hydrophilic regions.

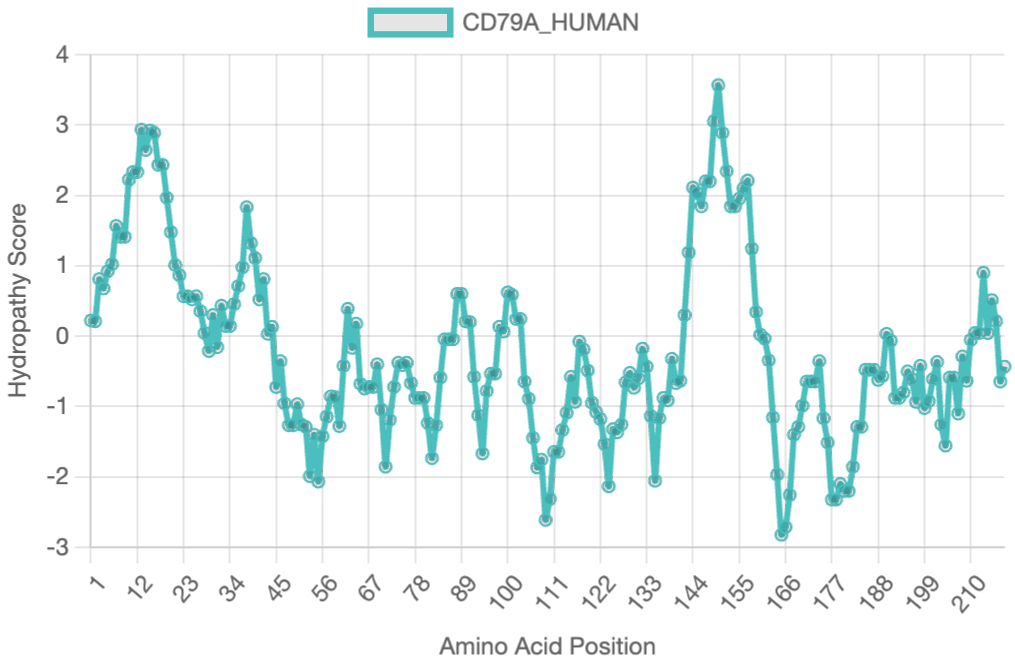

Kyte-Doolittle hydropathy plot for the sequence "CD79A_HUMAN". Positive values indicate hydrophobic regions, while negative values indicate hydrophilic regions.

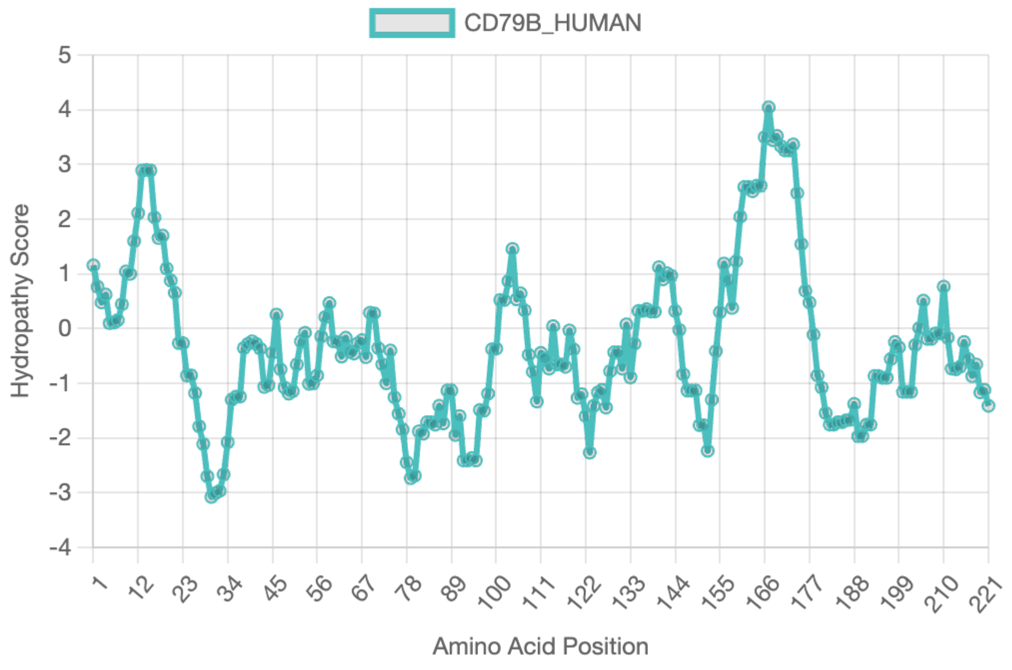

Kyte-Doolittle hydropathy plot for the sequence "CD79B_HUMAN". Positive values indicate hydrophobic regions, while negative values indicate hydrophilic regions.

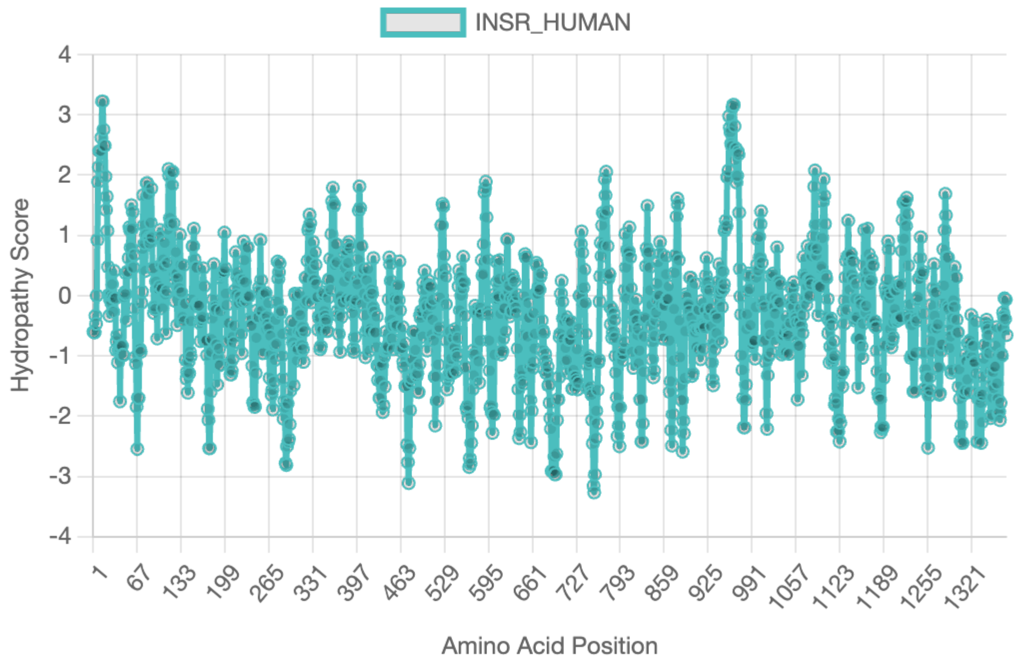

Kyte-Doolittle hydropathy plot for the sequence "INSR_HUMAN". Positive values indicate hydrophobic regions, while negative values indicate hydrophilic regions.

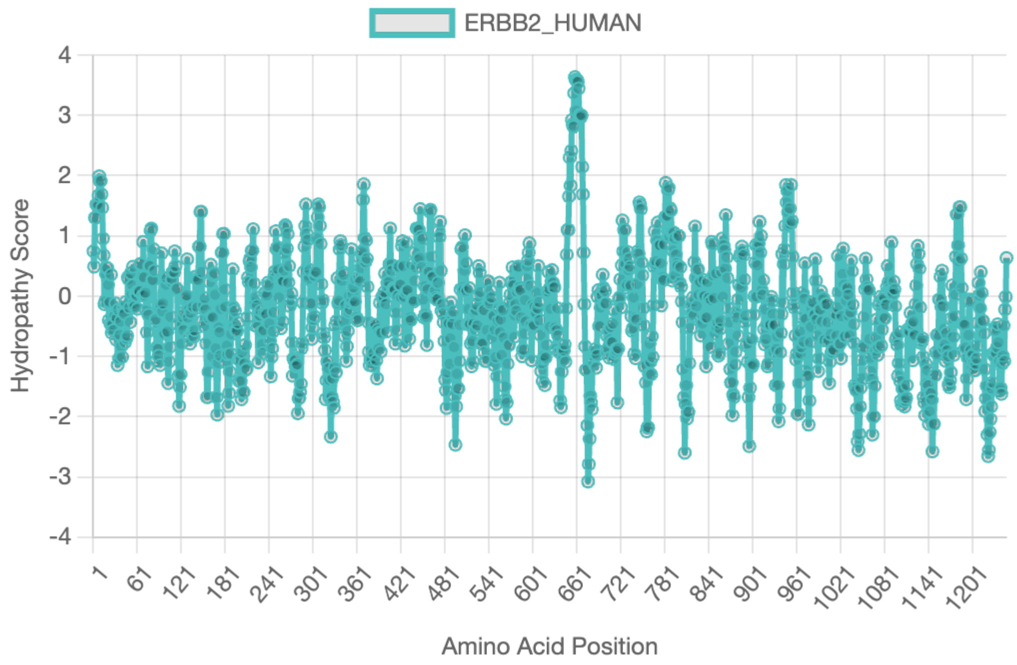

Kyte-Doolittle hydropathy plot for the sequence "ERBB2_HUMAN". Positive values indicate hydrophobic regions, while negative values indicate hydrophilic regions.

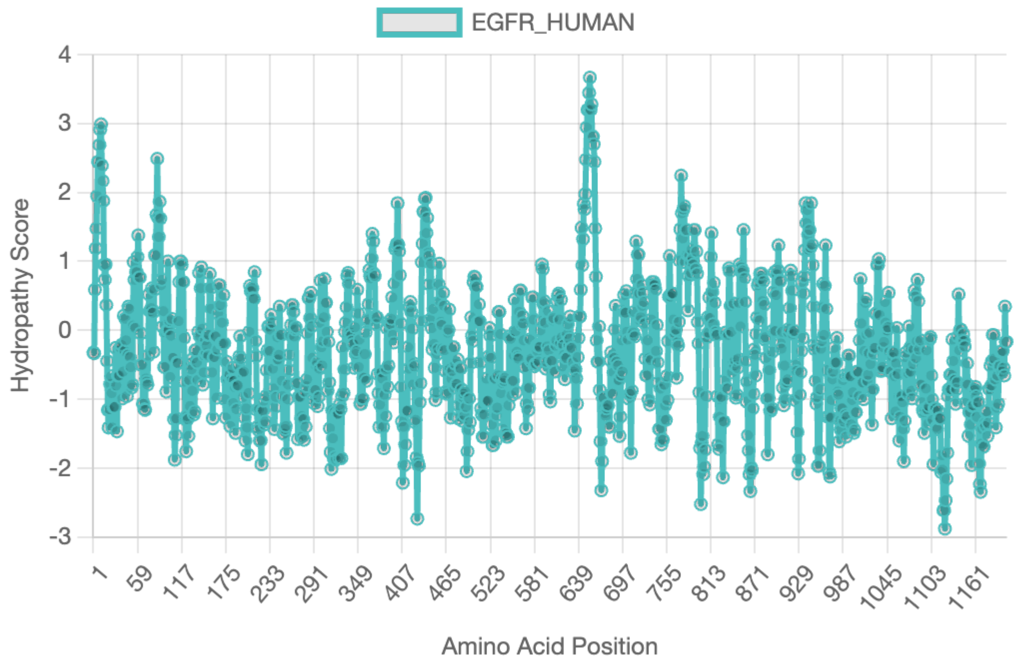

Kyte-Doolittle hydropathy plot for the sequence "EGFR_HUMAN". Positive values indicate hydrophobic regions, while negative values indicate hydrophilic regions.

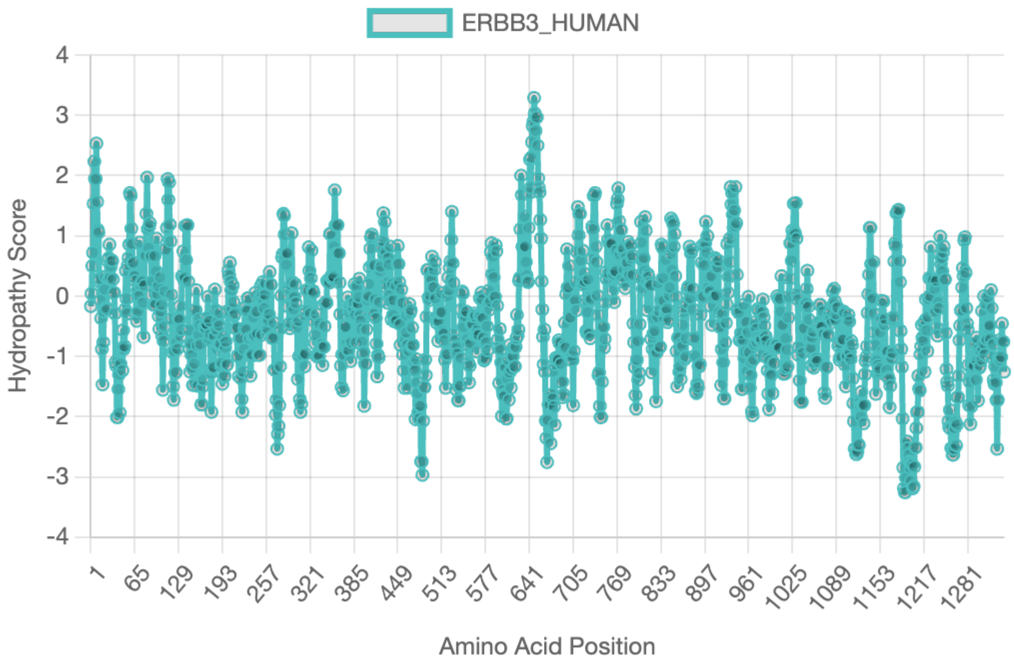

Kyte-Doolittle hydropathy plot for the sequence "ERBB3_HUMAN". Positive values indicate hydrophobic regions, while negative values indicate hydrophilic regions.

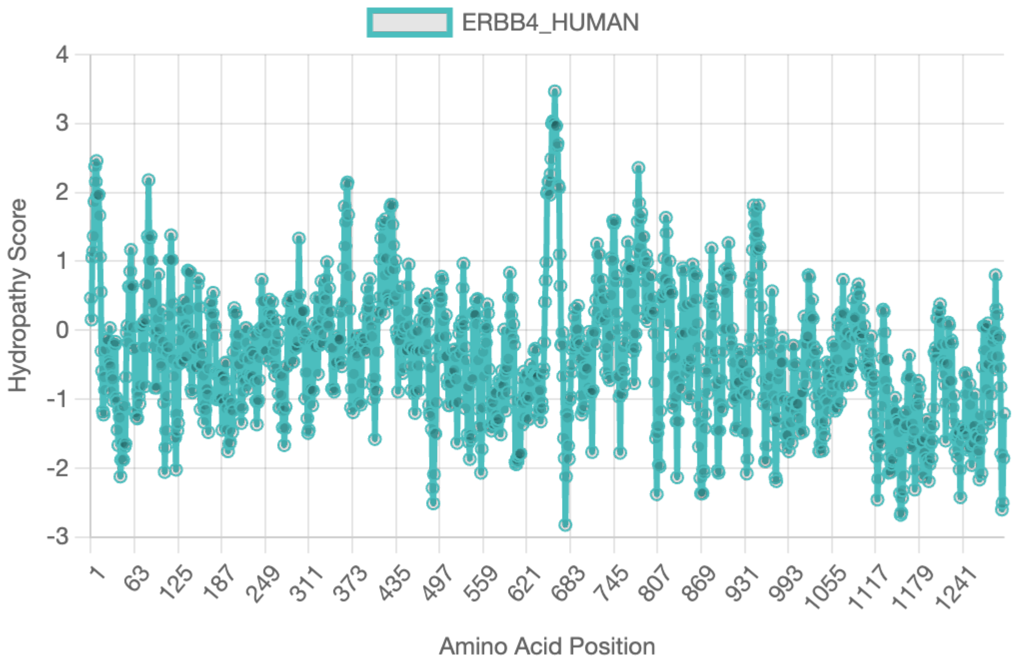

Kyte-Doolittle hydropathy plot for the sequence "ERBB4_HUMAN". Positive values indicate hydrophobic regions, while negative values indicate hydrophilic regions.

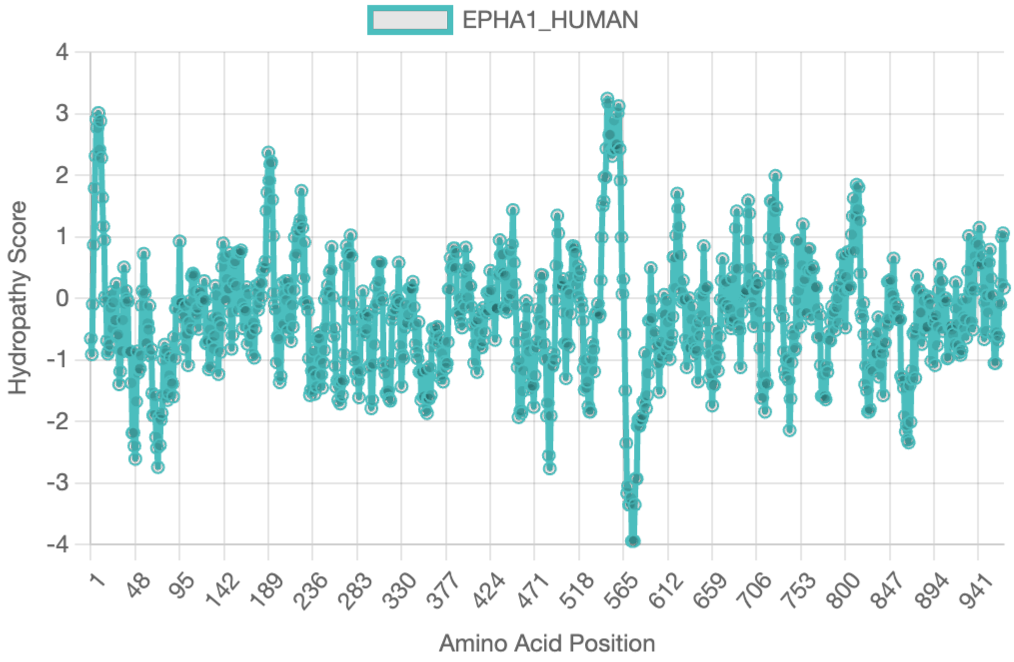

Kyte-Doolittle hydropathy plot for the sequence "EPHA1_HUMAN". Positive values indicate hydrophobic regions, while negative values indicate hydrophilic regions.

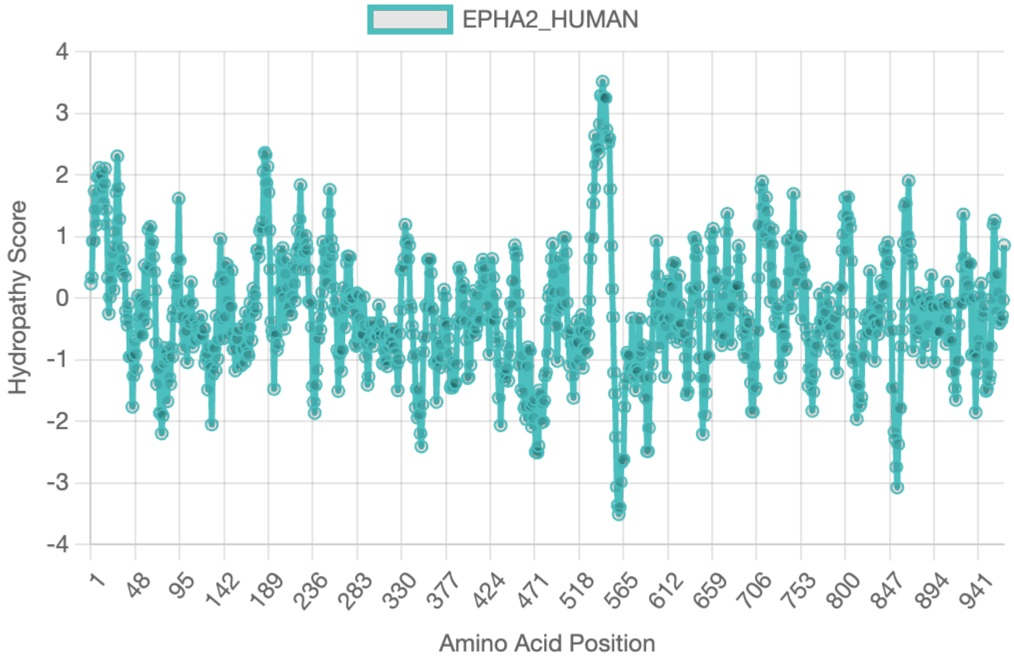

Kyte-Doolittle hydropathy plot for the sequence "EPHA2_HUMAN". Positive values indicate hydrophobic regions, while negative values indicate hydrophilic regions.

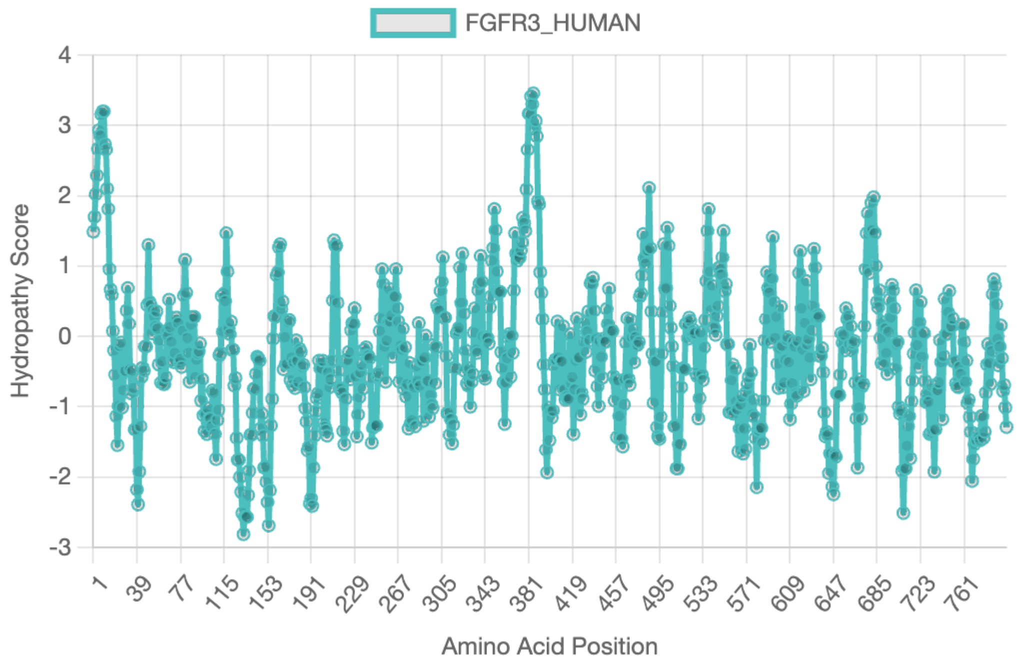

Kyte-Doolittle hydropathy plot for the sequence "FGFR3_HUMAN". Positive values indicate hydrophobic regions, while negative values indicate hydrophilic regions.

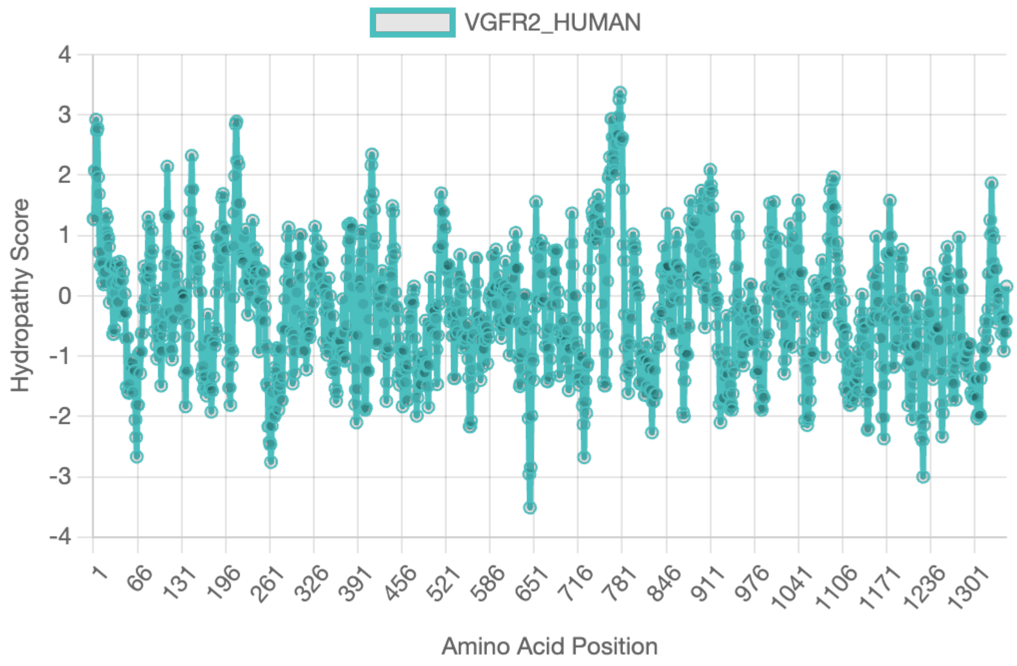

Kyte-Doolittle hydropathy plot for the sequence "VGFR2_HUMAN". Positive values indicate hydrophobic regions, while negative values indicate hydrophilic regions.

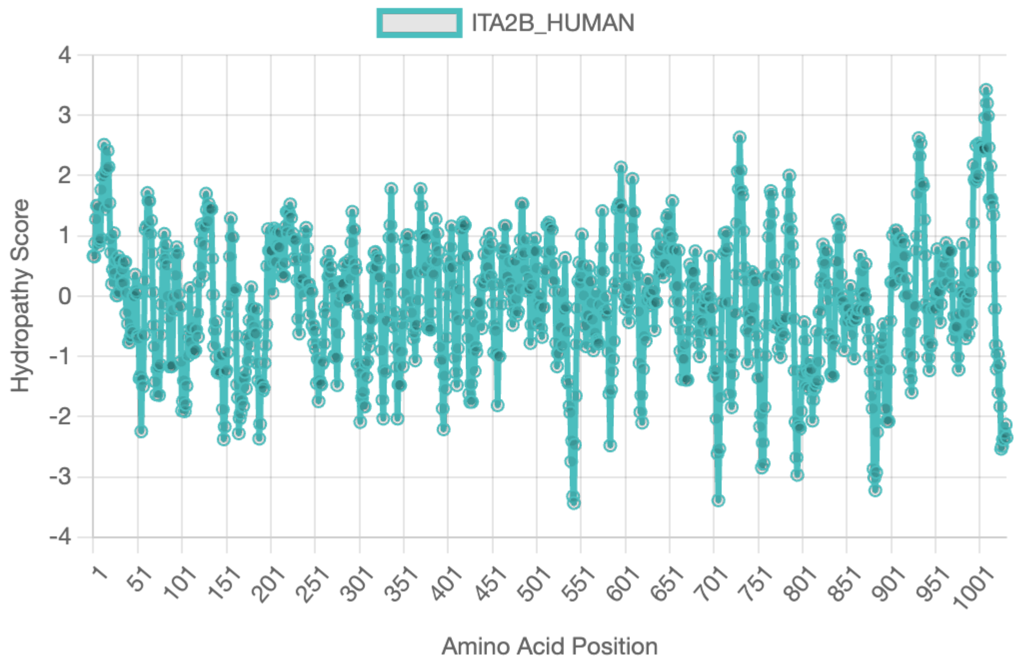

Kyte-Doolittle hydropathy plot for the sequence "ITA2B_HUMAN". Positive values indicate hydrophobic regions, while negative values indicate hydrophilic regions.

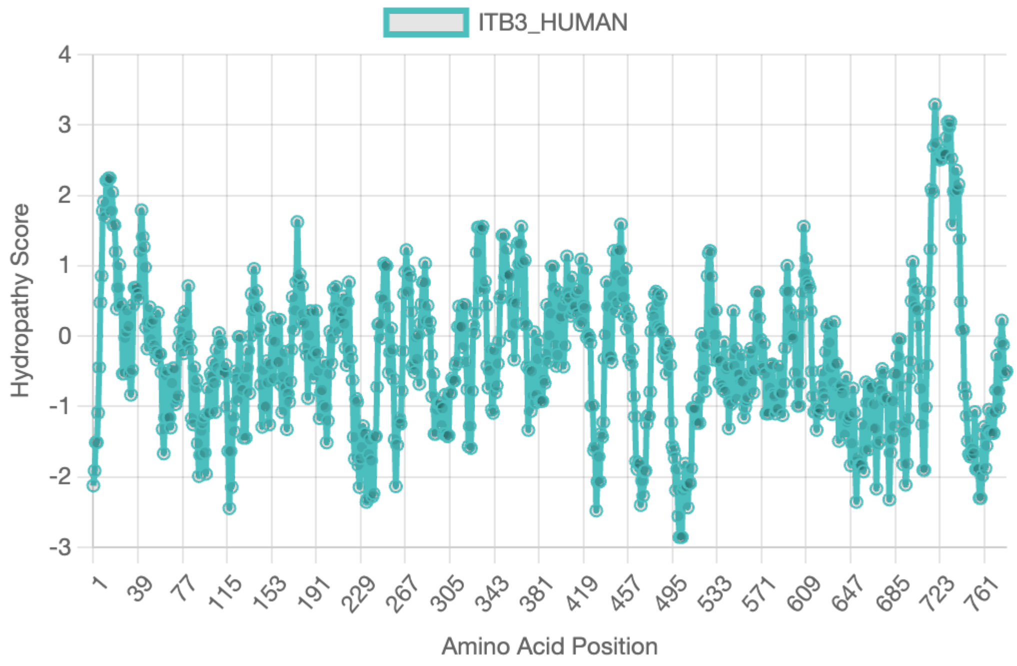

Kyte-Doolittle hydropathy plot for the sequence "ITB3_HUMAN". Positive values indicate hydrophobic regions, while negative values indicate hydrophilic regions.

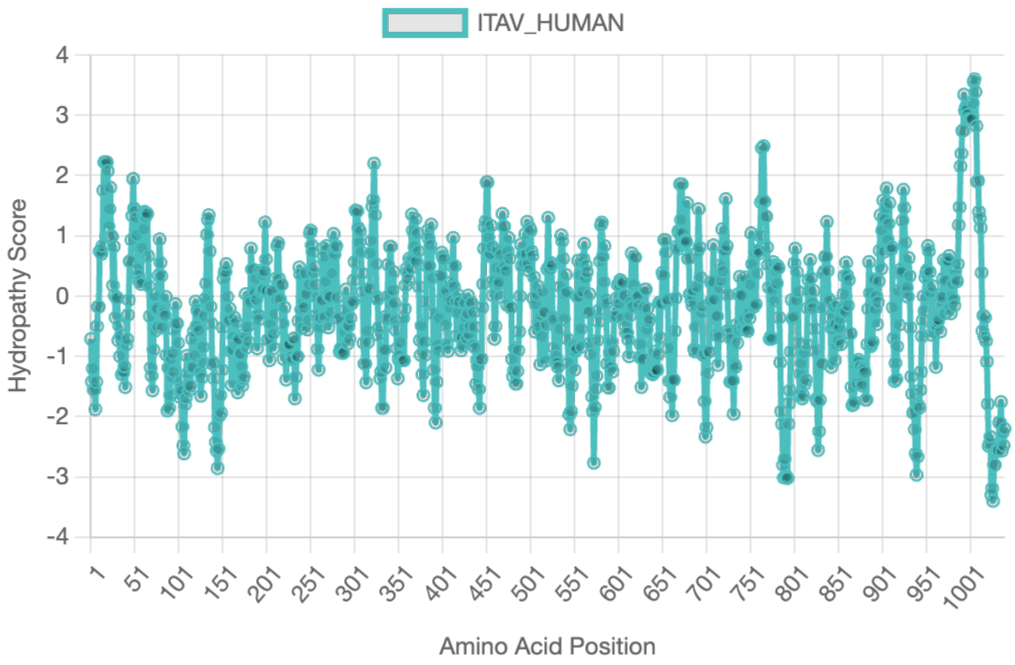

Kyte-Doolittle hydropathy plot for the sequence "ITAV_HUMAN". Positive values indicate hydrophobic regions, while negative values indicate hydrophilic regions.

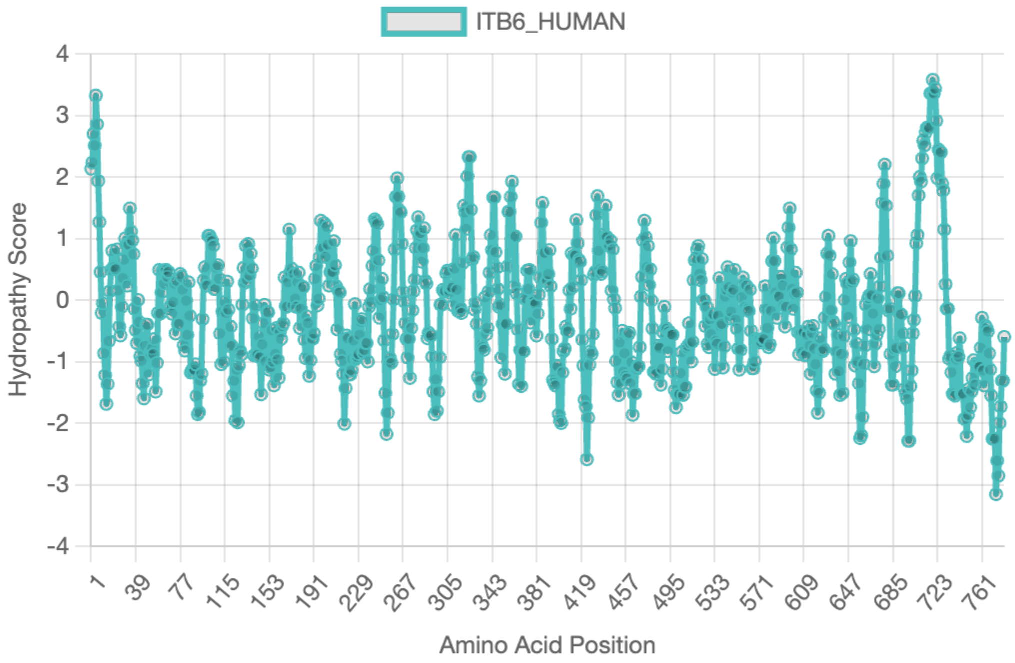

Kyte-Doolittle hydropathy plot for the sequence "ITB6_HUMAN". Positive values indicate hydrophobic regions, while negative values indicate hydrophilic regions.

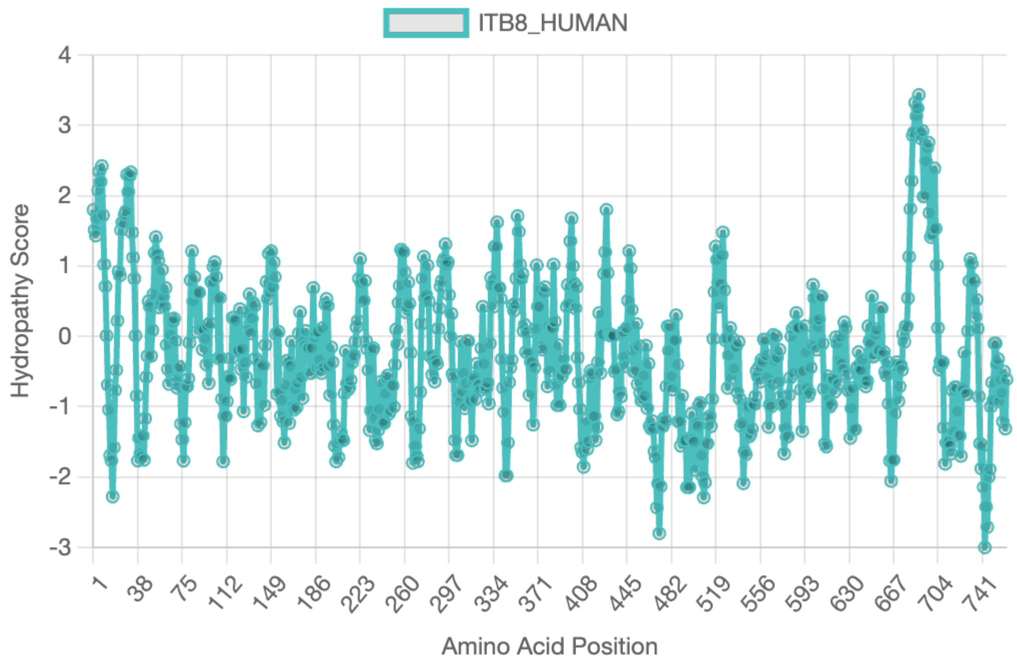

Kyte-Doolittle hydropathy plot for the sequence "ITB8_HUMAN". Positive values indicate hydrophobic regions, while negative values indicate hydrophilic regions.

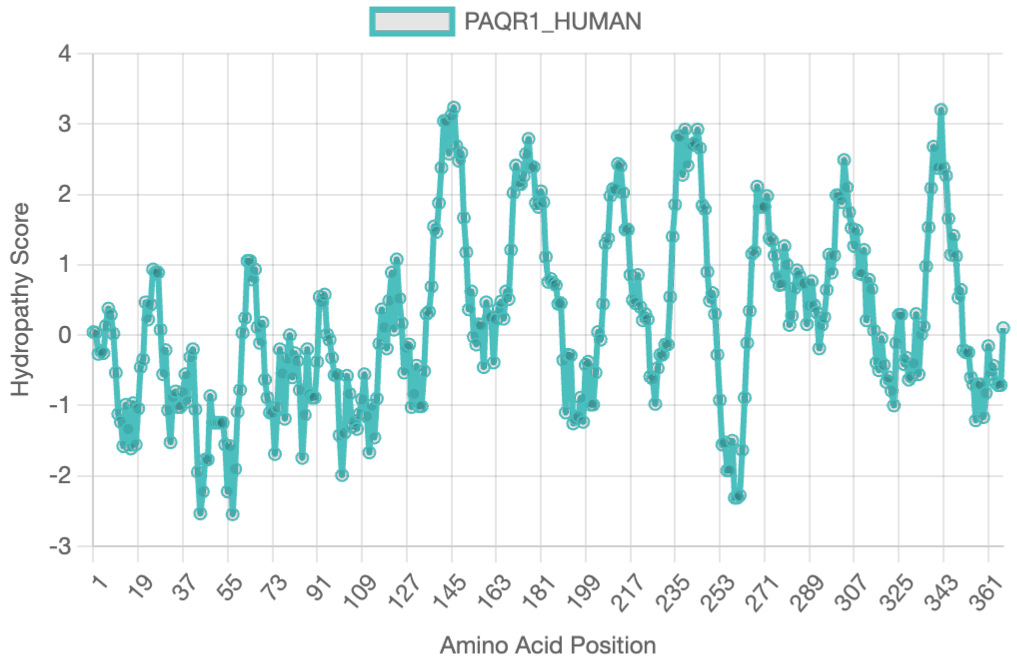

Kyte-Doolittle hydropathy plot for the sequence "PAQR1_HUMAN". Positive values indicate hydrophobic regions, while negative values indicate hydrophilic regions.

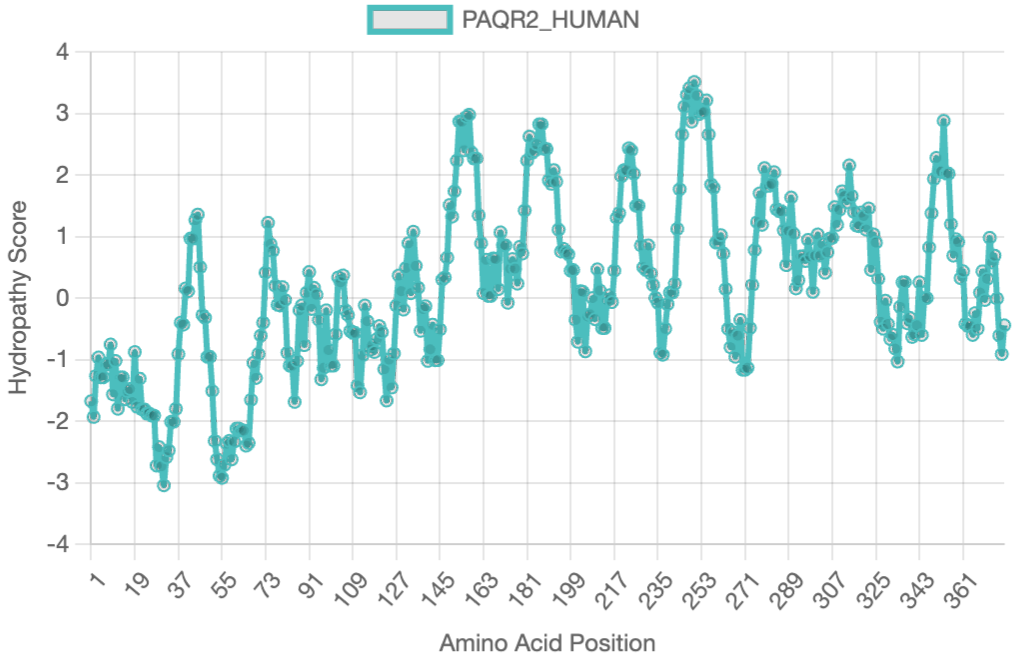

Kyte-Doolittle hydropathy plot for the sequence "PAQR2_HUMAN". Positive values indicate hydrophobic regions, while negative values indicate hydrophilic regions.

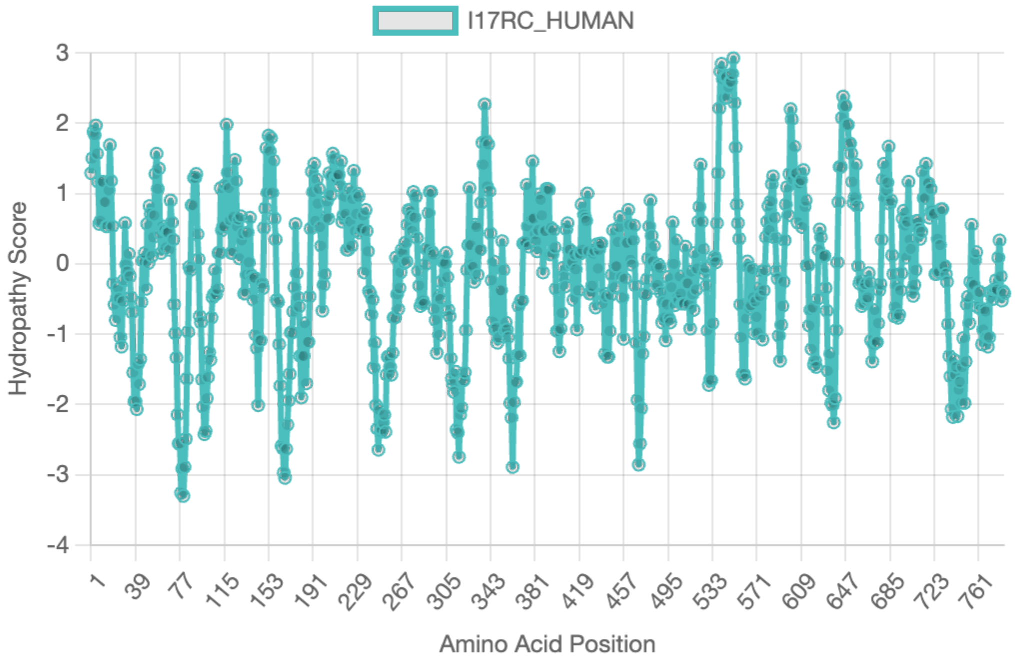

Kyte-Doolittle hydropathy plot for the sequence "I17RC_HUMAN". Positive values indicate hydrophobic regions, while negative values indicate hydrophilic regions.

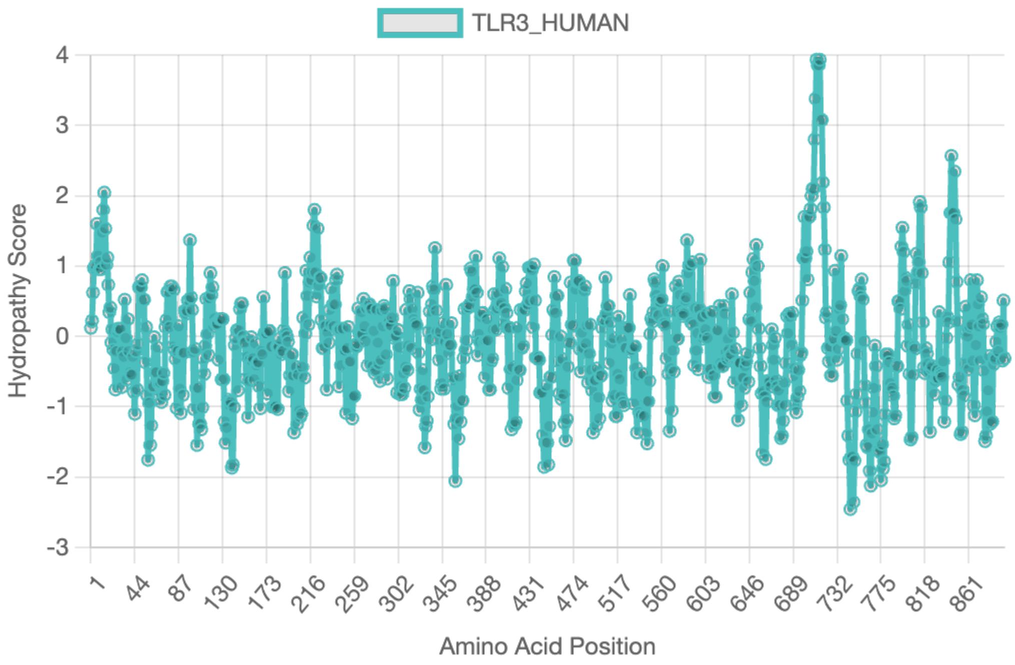

Kyte-Doolittle hydropathy plot for the sequence "TLR3_HUMAN". Positive values indicate hydrophobic regions, while negative values indicate hydrophilic regions.

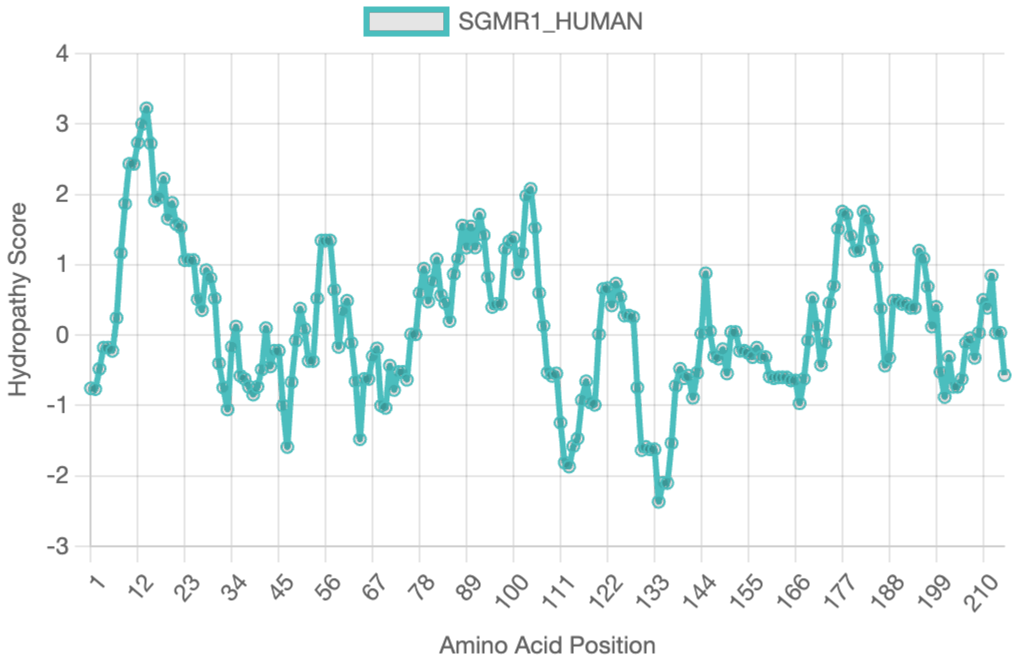

Kyte-Doolittle hydropathy plot for the sequence "SGMR1_HUMAN". Positive values indicate hydrophobic regions, while negative values indicate hydrophilic regions.

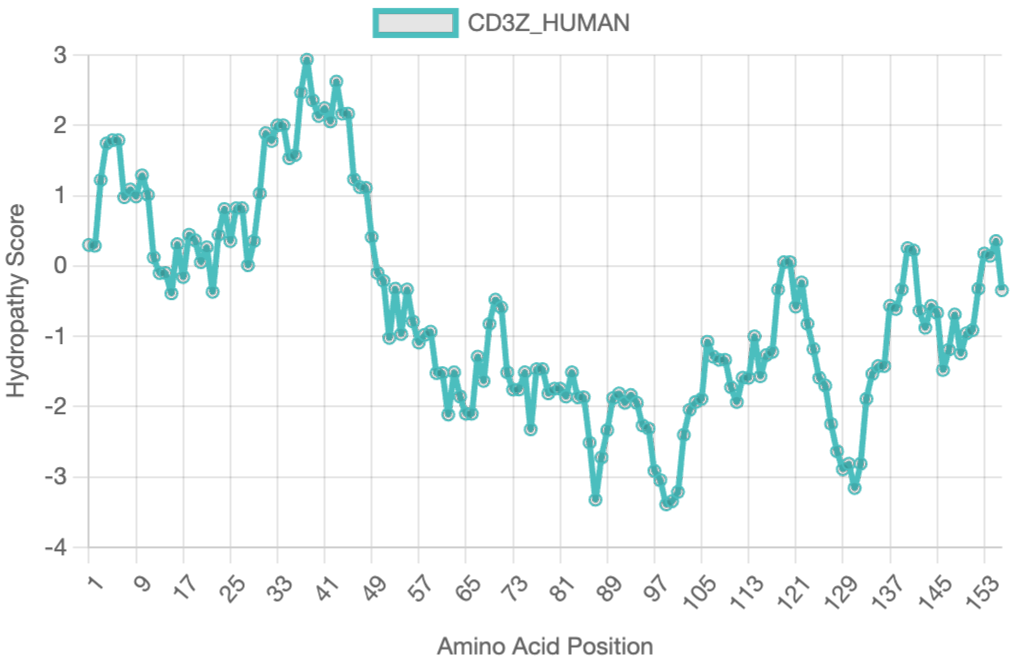

Kyte-Doolittle hydropathy plot for the sequence "CD3Z_HUMAN". Positive values indicate hydrophobic regions, while negative values indicate hydrophilic regions.

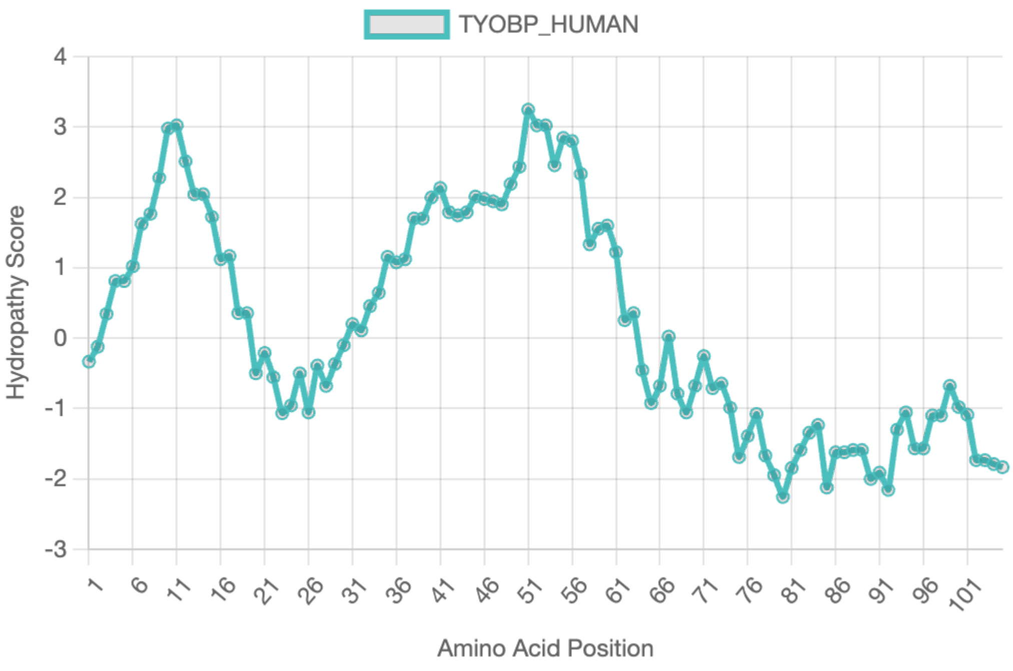

Kyte-Doolittle hydropathy plot for the sequence "TYOBP_HUMAN". Positive values indicate hydrophobic regions, while negative values indicate hydrophilic regions.

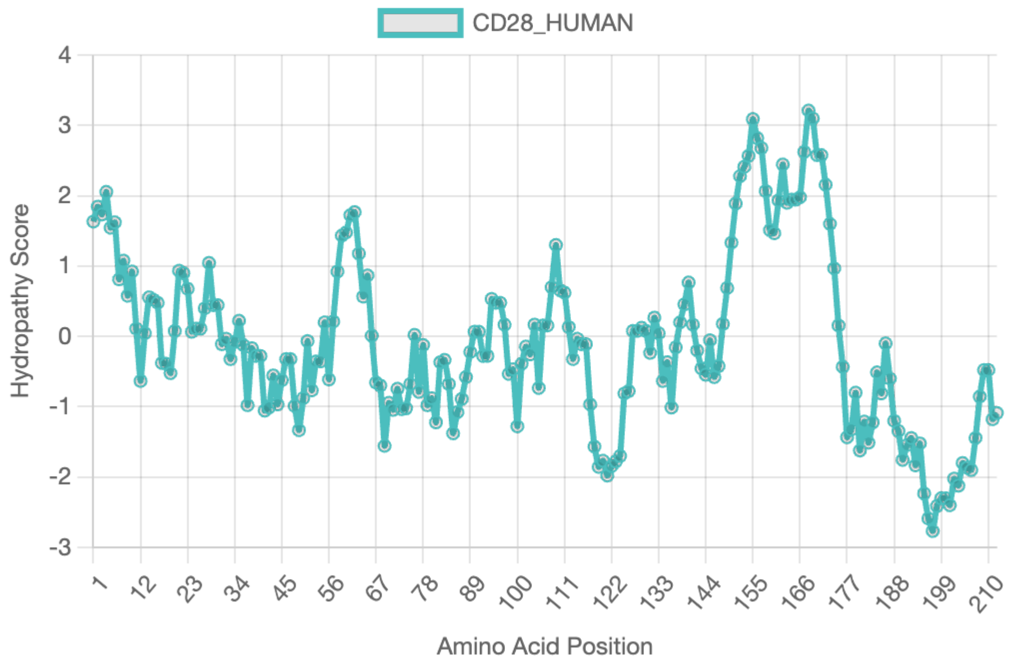

Kyte-Doolittle hydropathy plot for the sequence "CD28_HUMAN". Positive values indicate hydrophobic regions, while negative values indicate hydrophilic regions.

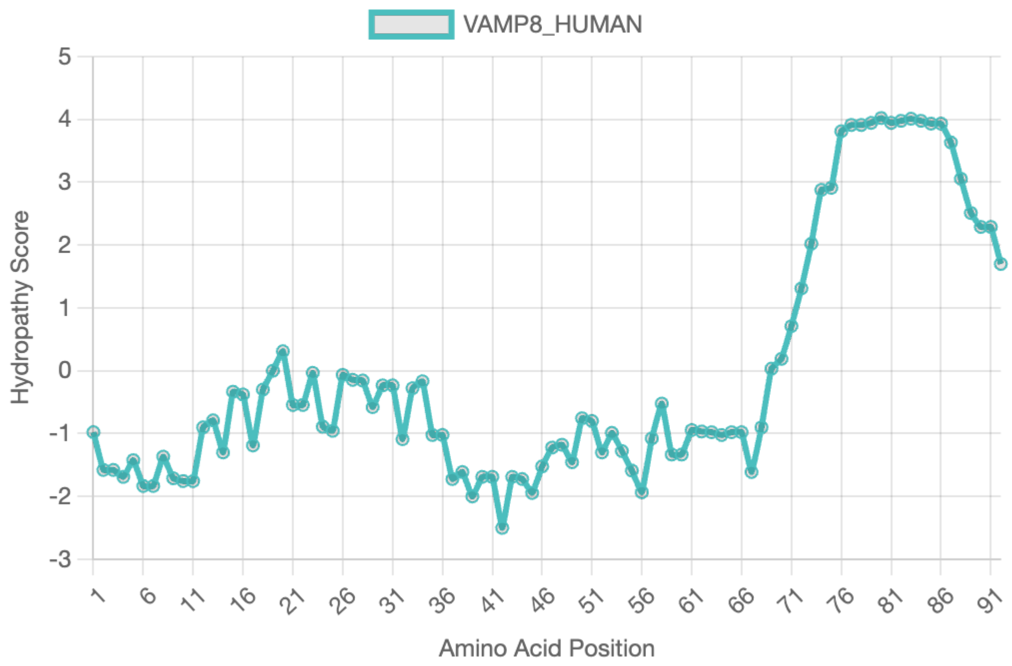

Kyte-Doolittle hydropathy plot for the sequence "VAMP8_HUMAN". Positive values indicate hydrophobic regions, while negative values indicate hydrophilic regions.

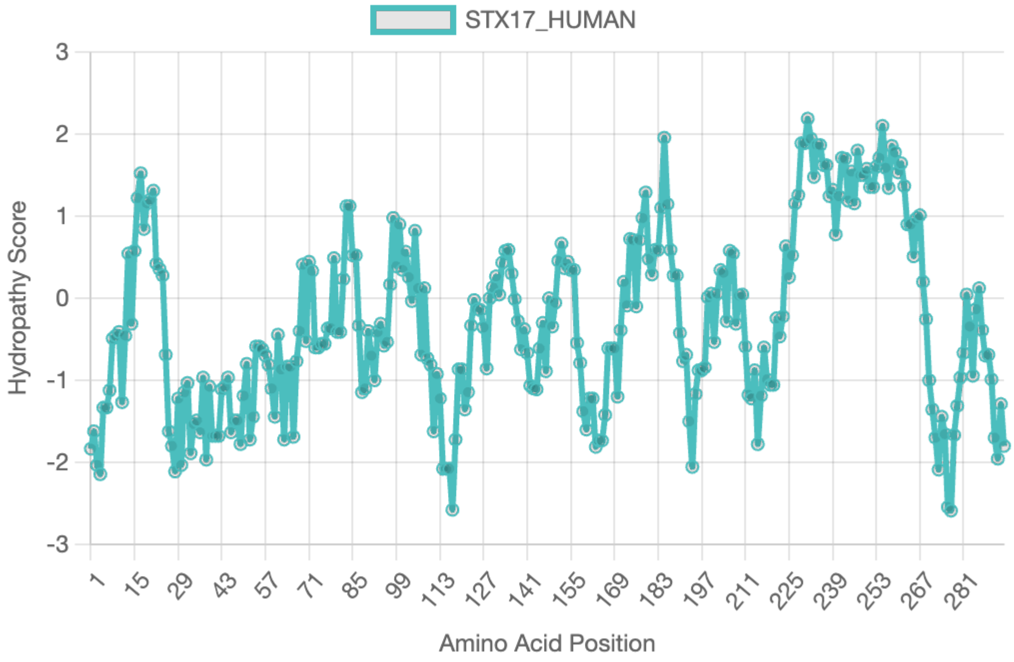

Kyte-Doolittle hydropathy plot for the sequence "STX17_HUMAN". Positive values indicate hydrophobic regions, while negative values indicate hydrophilic regions.

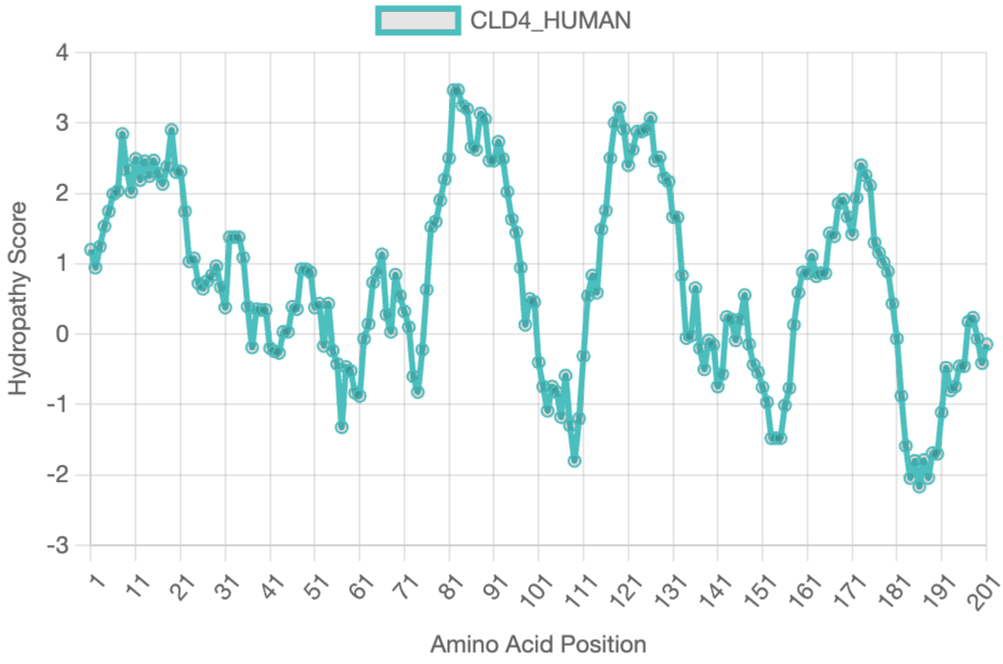

Kyte-Doolittle hydropathy plot for the sequence "CLD4_HUMAN". Positive values indicate hydrophobic regions, while negative values indicate hydrophilic regions.

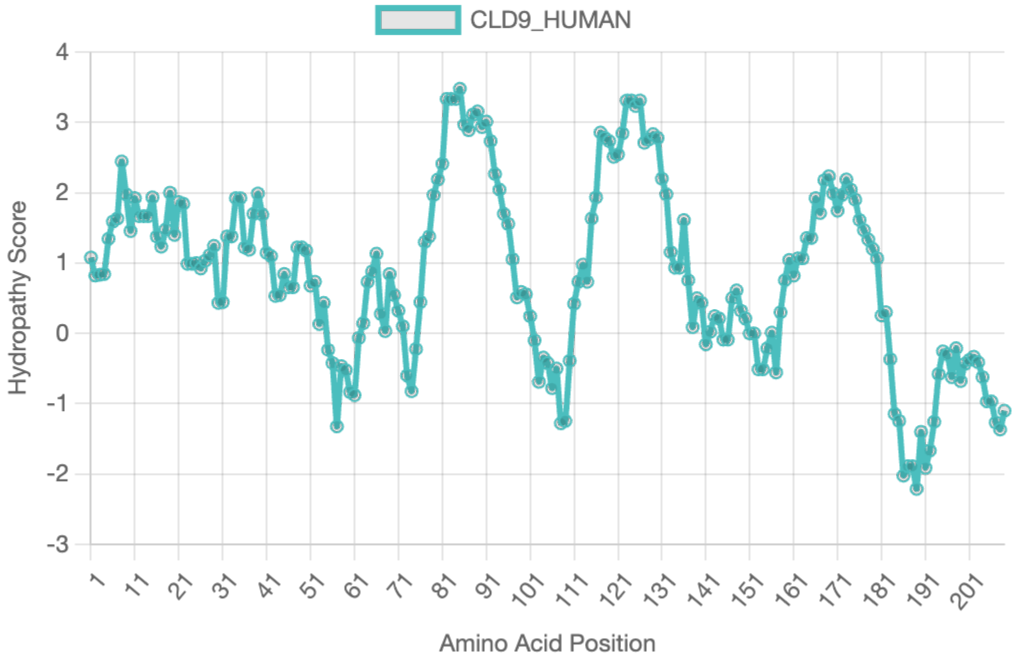

Kyte-Doolittle hydropathy plot for the sequence "CLD9_HUMAN". Positive values indicate hydrophobic regions, while negative values indicate hydrophilic regions.

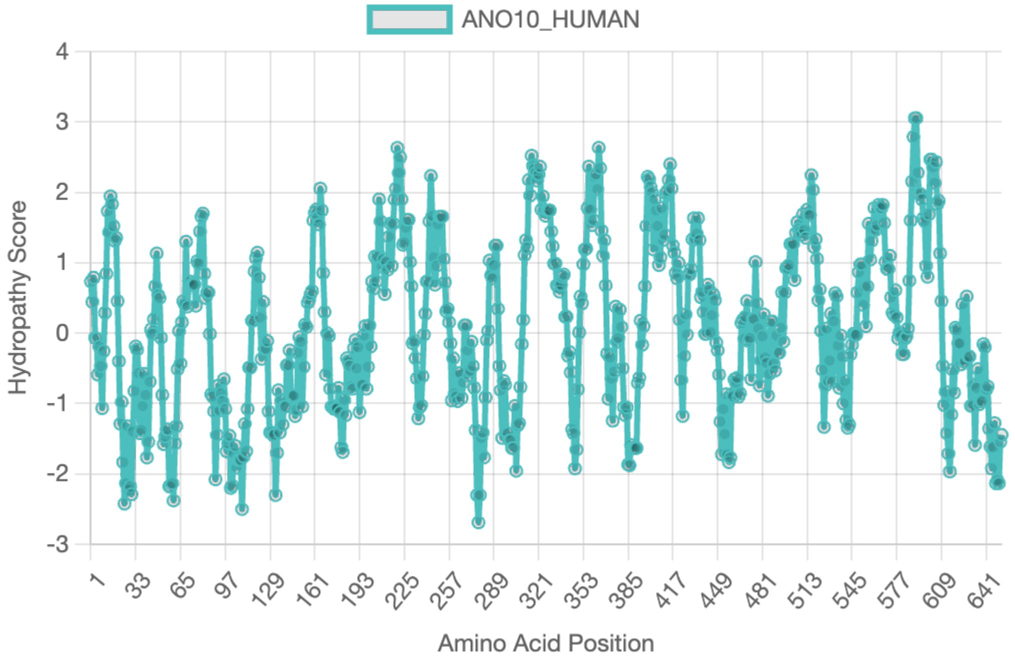

Kyte-Doolittle hydropathy plot for the sequence "ANO10_HUMAN". Positive values indicate hydrophobic regions, while negative values indicate hydrophilic regions.

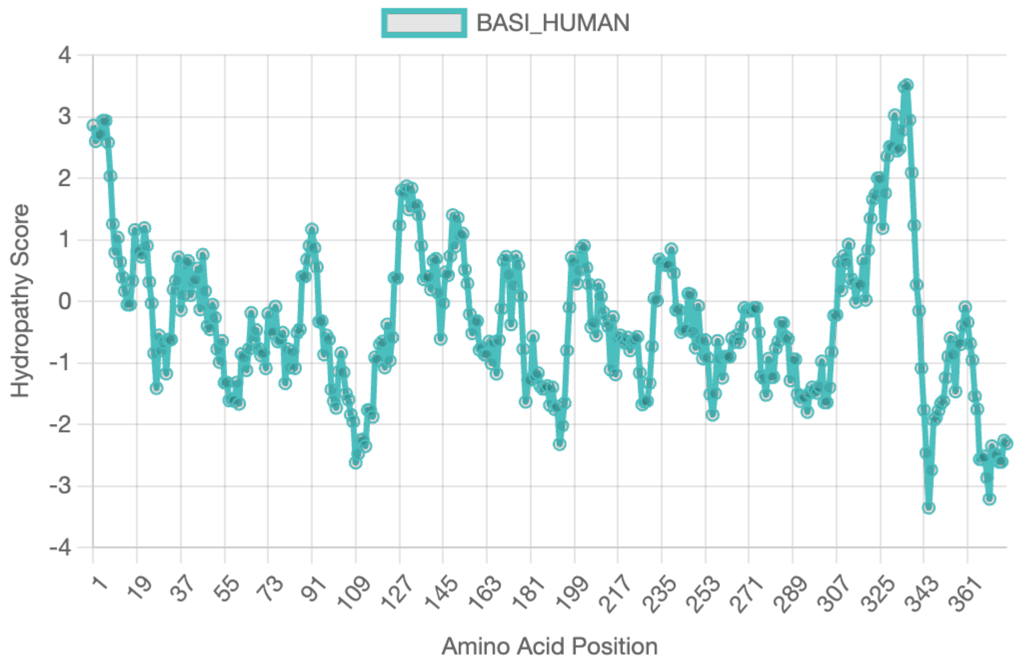

Kyte-Doolittle hydropathy plot for the sequence "BASI_HUMAN". Positive values indicate hydrophobic regions, while negative values indicate hydrophilic regions.

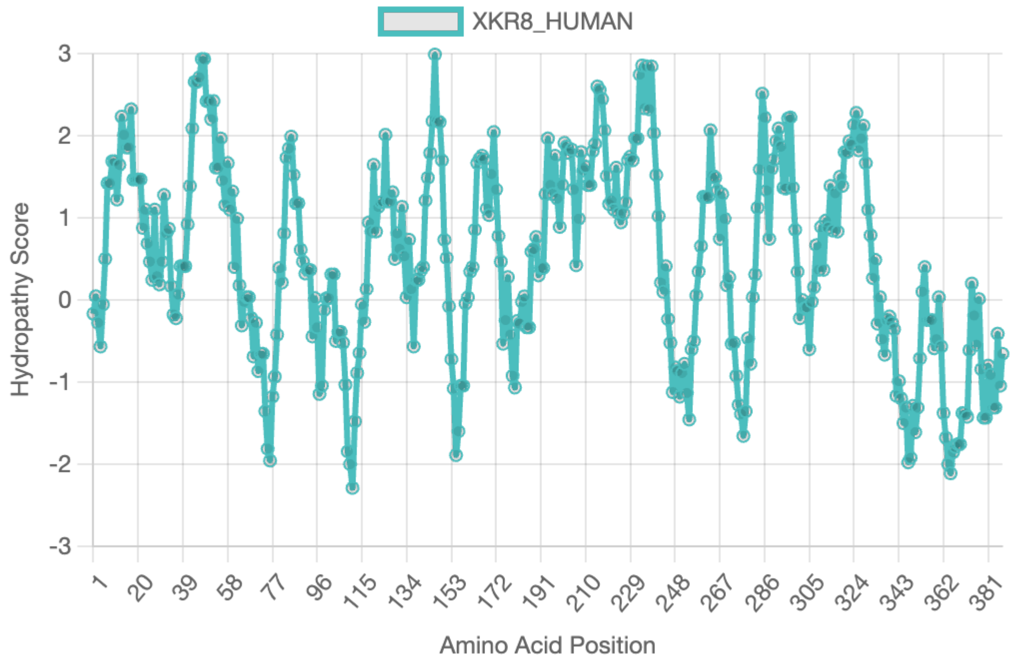

Kyte-Doolittle hydropathy plot for the sequence "XKR8_HUMAN". Positive values indicate hydrophobic regions, while negative values indicate hydrophilic regions.

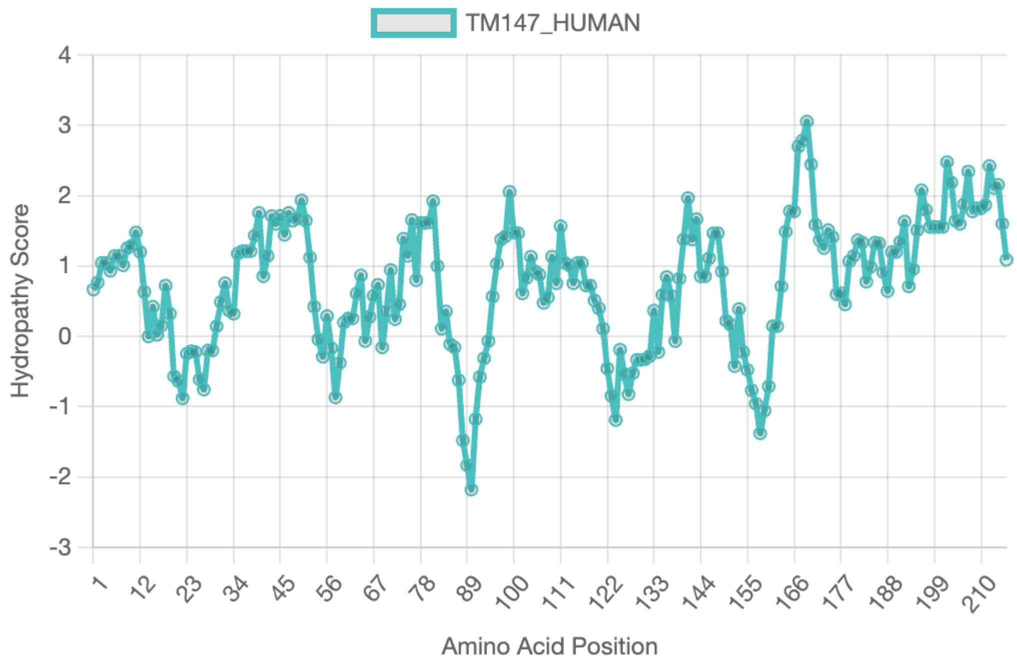

Kyte-Doolittle hydropathy plot for the sequence "TM147_HUMAN". Positive values indicate hydrophobic regions, while negative values indicate hydrophilic regions.

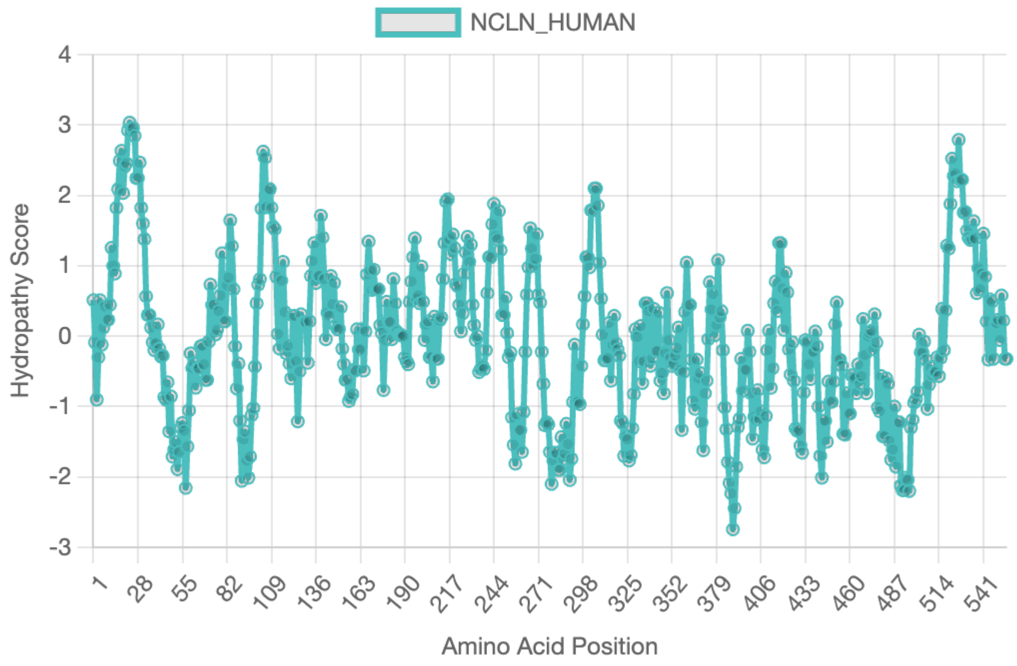

Kyte-Doolittle hydropathy plot for the sequence "NCLN_HUMAN". Positive values indicate hydrophobic regions, while negative values indicate hydrophilic regions.

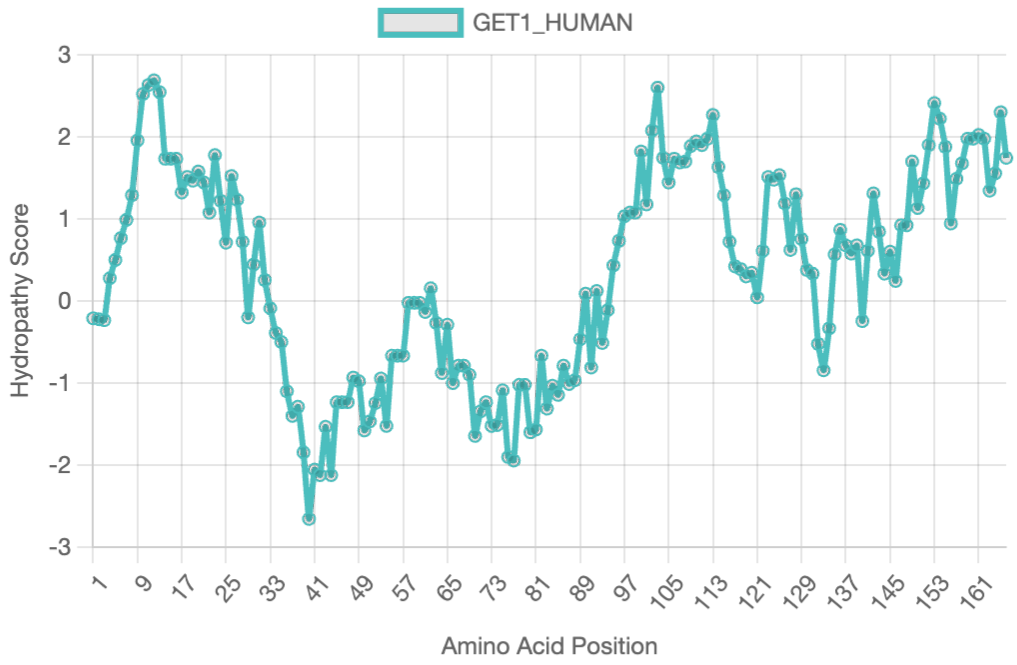

Kyte-Doolittle hydropathy plot for the sequence "GET1_HUMAN". Positive values indicate hydrophobic regions, while negative values indicate hydrophilic regions.

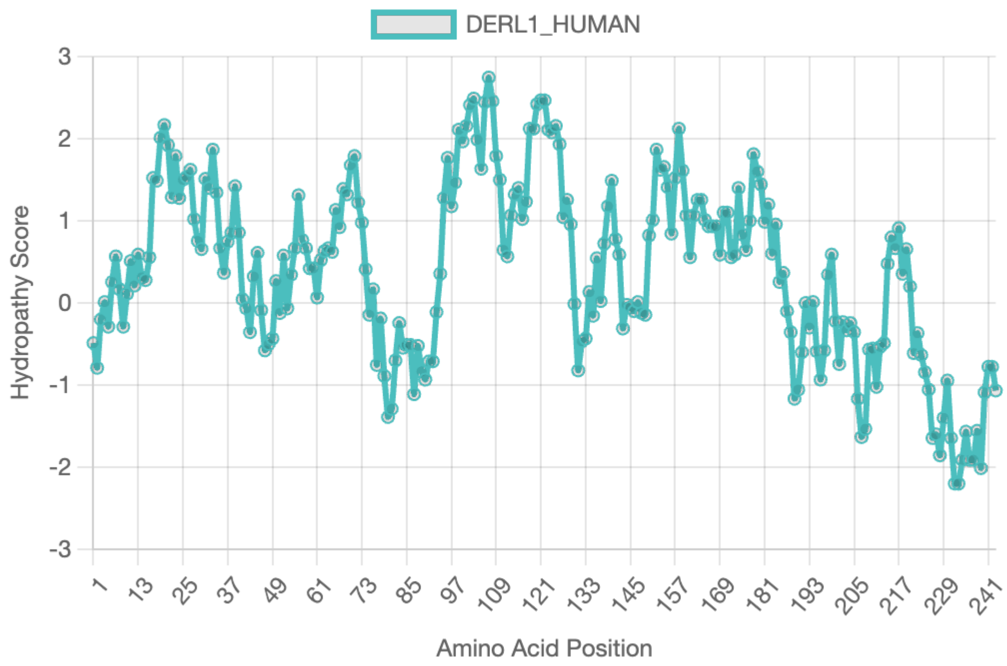

Kyte-Doolittle hydropathy plot for the sequence "DERL1_HUMAN". Positive values indicate hydrophobic regions, while negative values indicate hydrophilic regions.

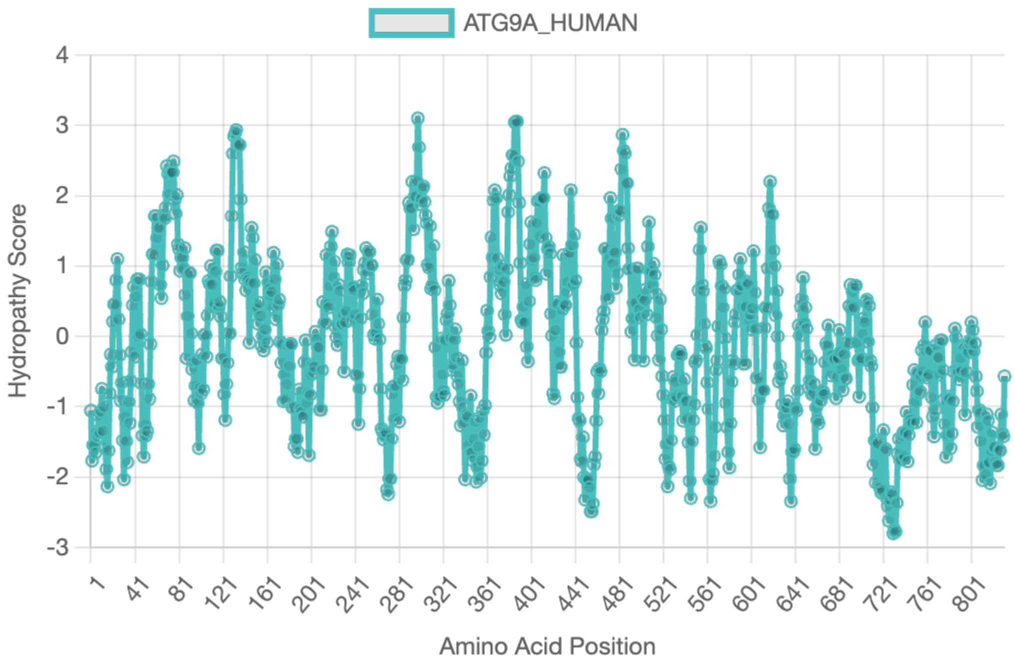

Kyte-Doolittle hydropathy plot for the sequence "ATG9A_HUMAN". Positive values indicate hydrophobic regions, while negative values indicate hydrophilic regions.

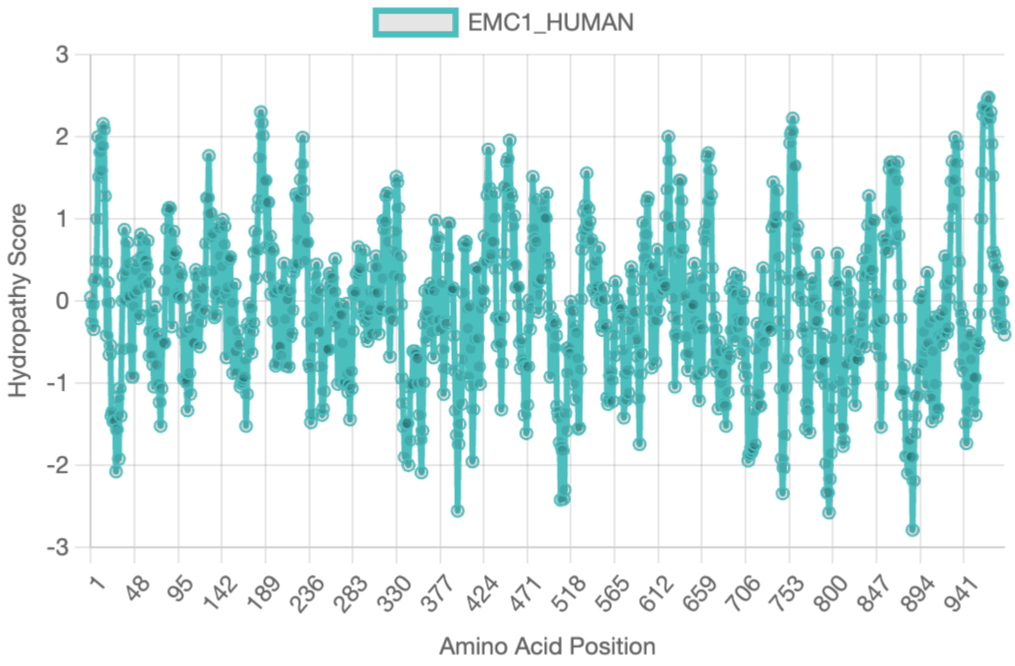

Kyte-Doolittle hydropathy plot for the sequence "EMC1_HUMAN". Positive values indicate hydrophobic regions, while negative values indicate hydrophilic regions.

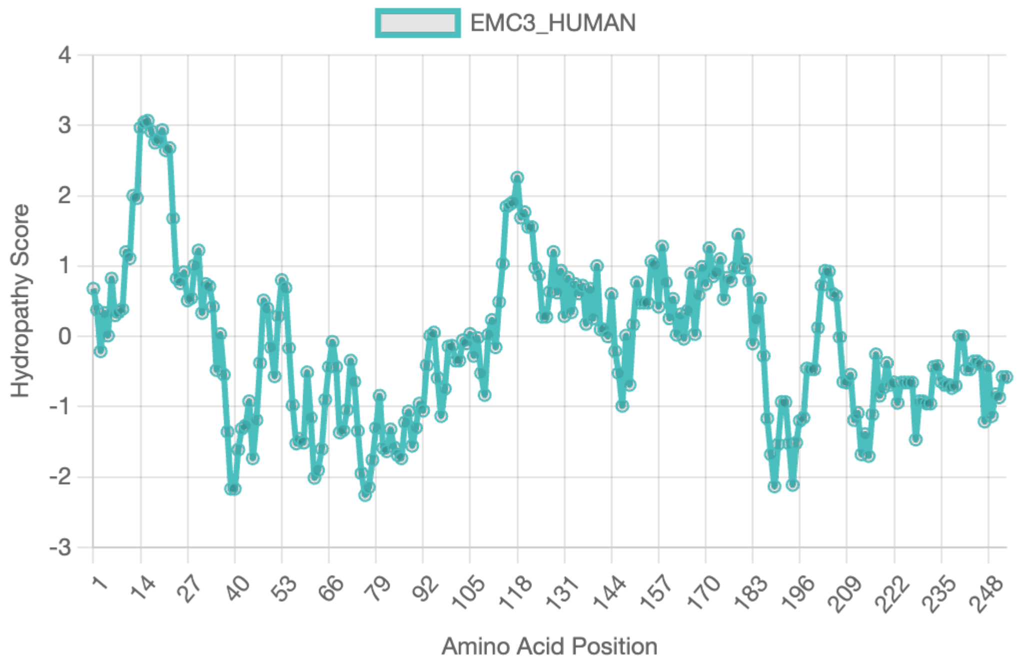

Kyte-Doolittle hydropathy plot for the sequence "EMC3_HUMAN". Positive values indicate hydrophobic regions, while negative values indicate hydrophilic regions.

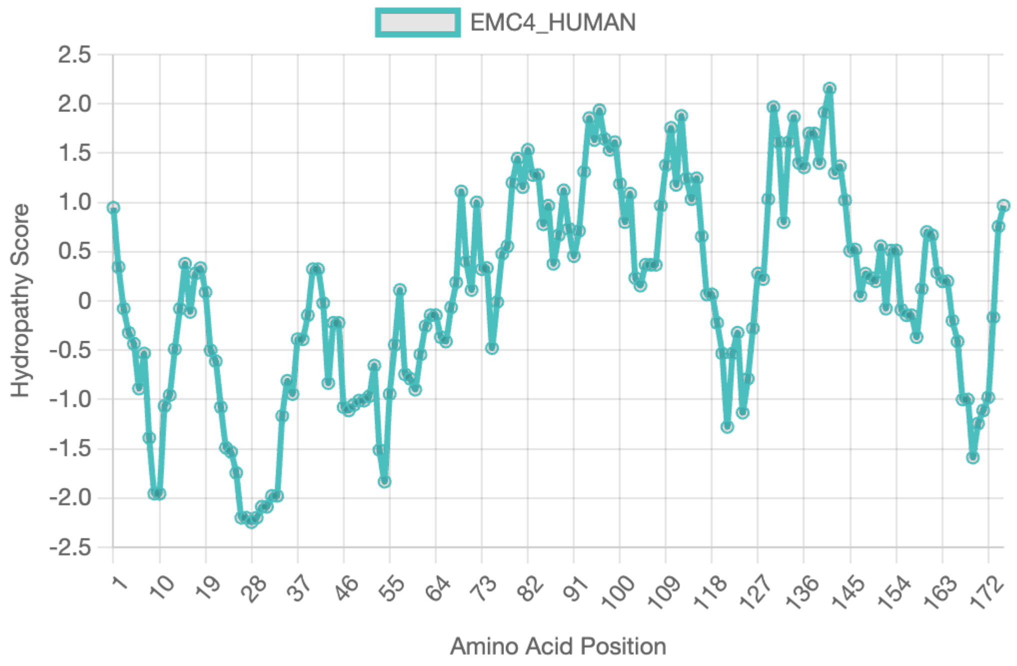

Kyte-Doolittle hydropathy plot for the sequence "EMC4_HUMAN". Positive values indicate hydrophobic regions, while negative values indicate hydrophilic regions.

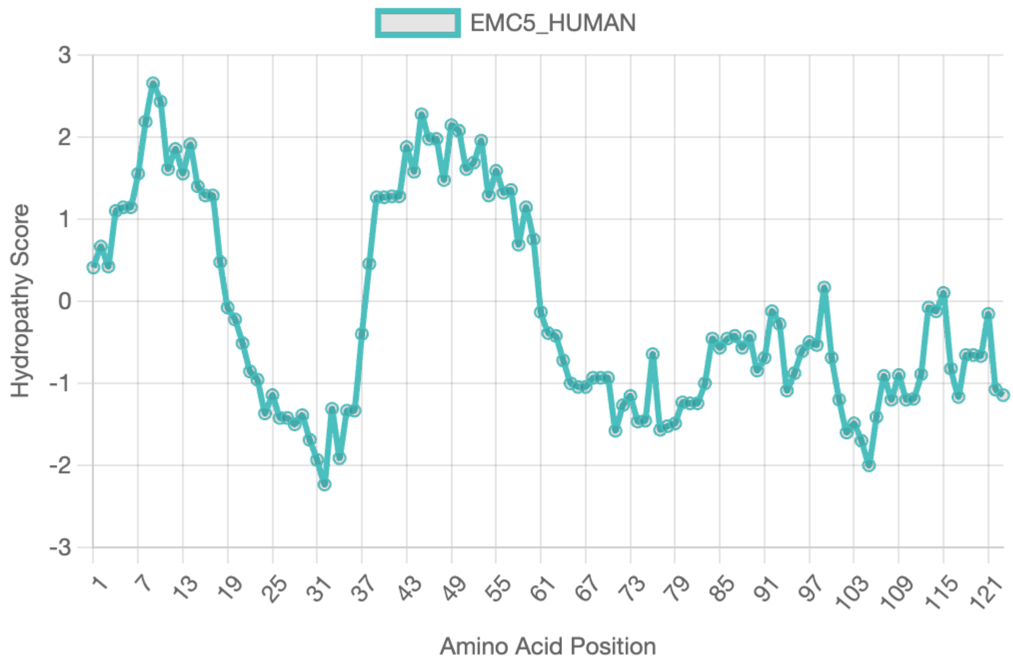

Kyte-Doolittle hydropathy plot for the sequence "EMC5_HUMAN". Positive values indicate hydrophobic regions, while negative values indicate hydrophilic regions.

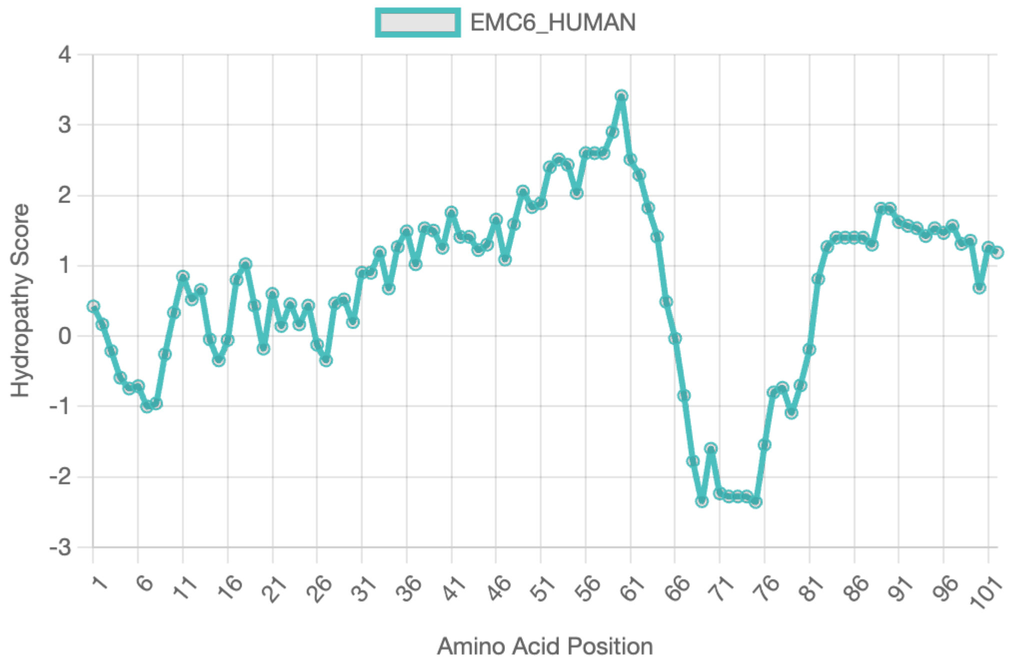

Kyte-Doolittle hydropathy plot for the sequence "EMC6_HUMAN". Positive values indicate hydrophobic regions, while negative values indicate hydrophilic regions.

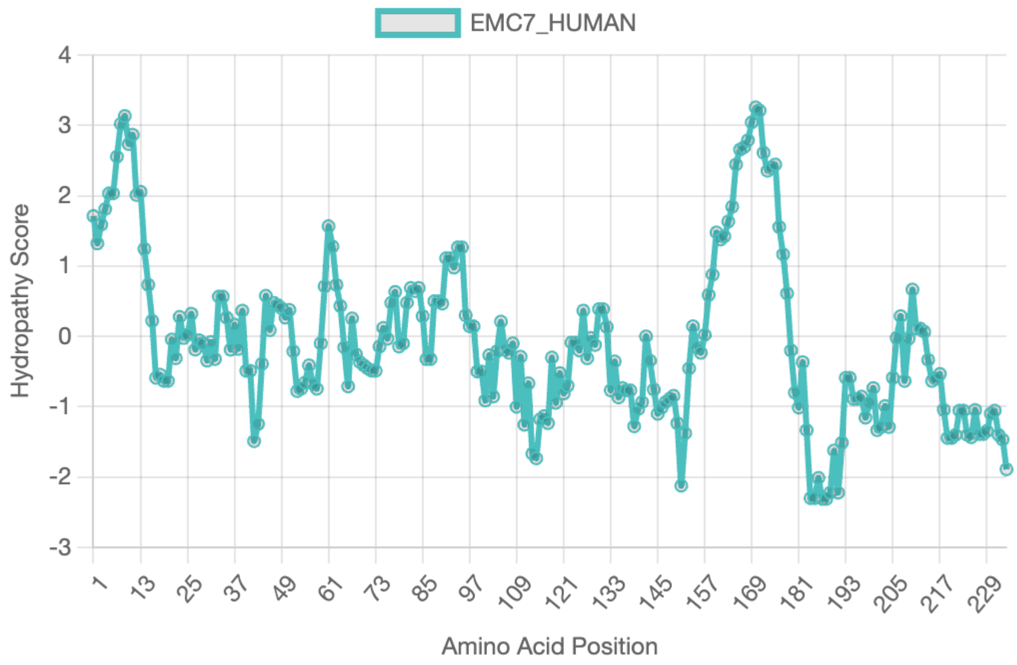

Kyte-Doolittle hydropathy plot for the sequence "EMC7_HUMAN". Positive values indicate hydrophobic regions, while negative values indicate hydrophilic regions.

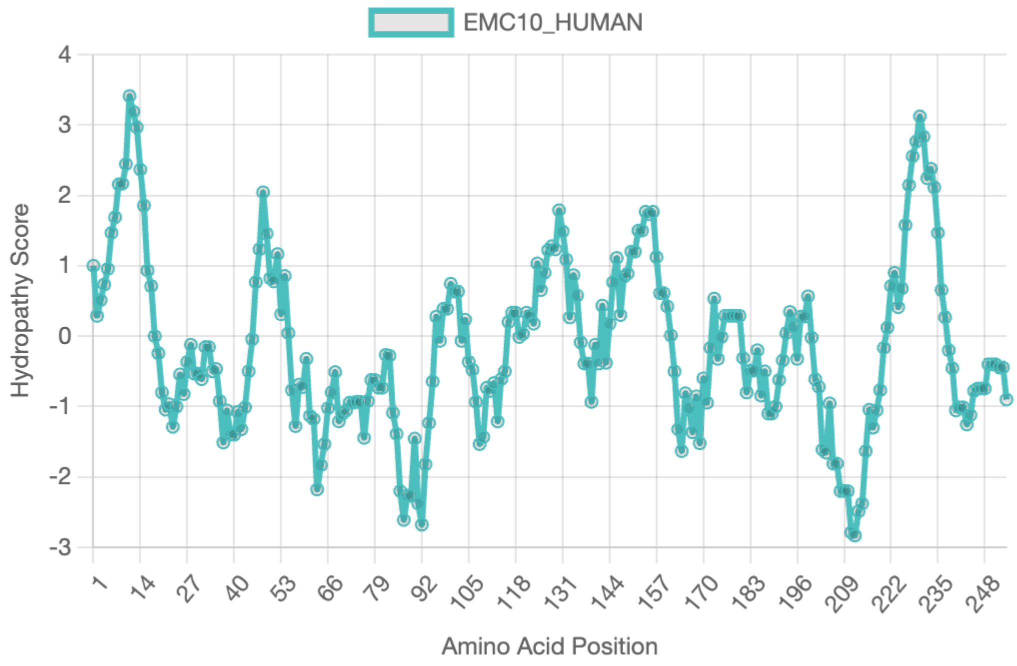

Kyte-Doolittle hydropathy plot for the sequence "EMC10_HUMAN". Positive values indicate hydrophobic regions, while negative values indicate hydrophilic regions.

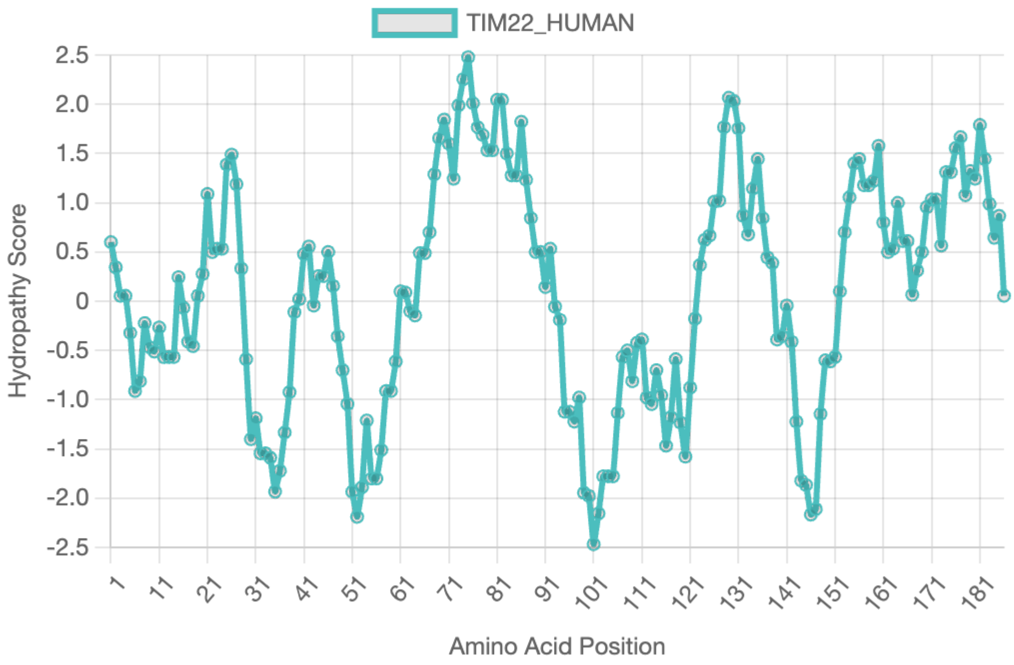

Kyte-Doolittle hydropathy plot for the sequence "TIM22_HUMAN". Positive values indicate hydrophobic regions, while negative values indicate hydrophilic regions.

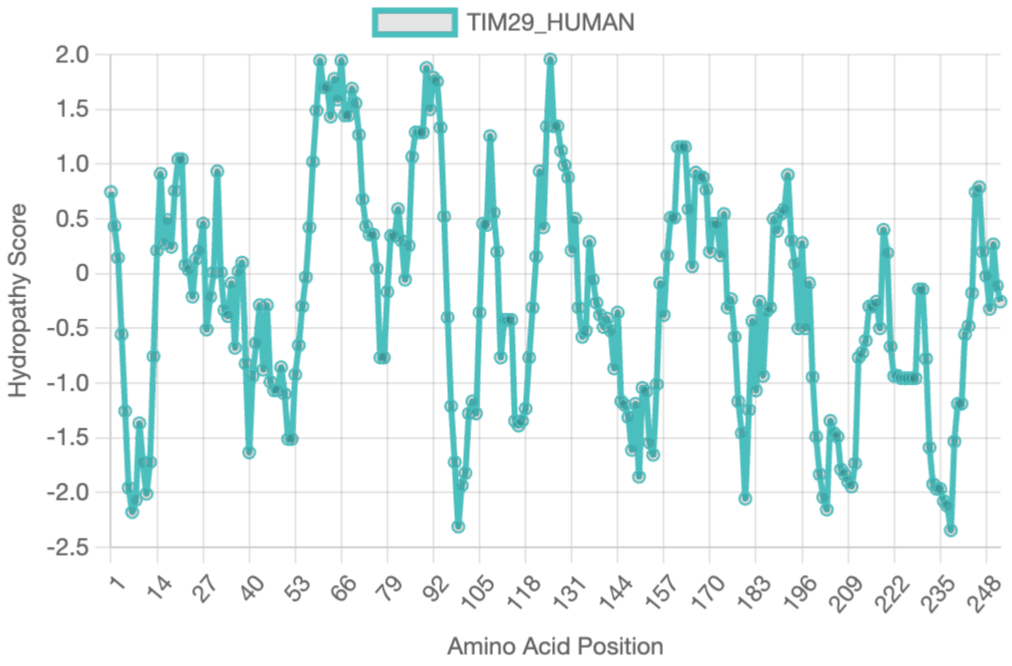

Kyte-Doolittle hydropathy plot for the sequence "TIM29_HUMAN". Positive values indicate hydrophobic regions, while negative values indicate hydrophilic regions.

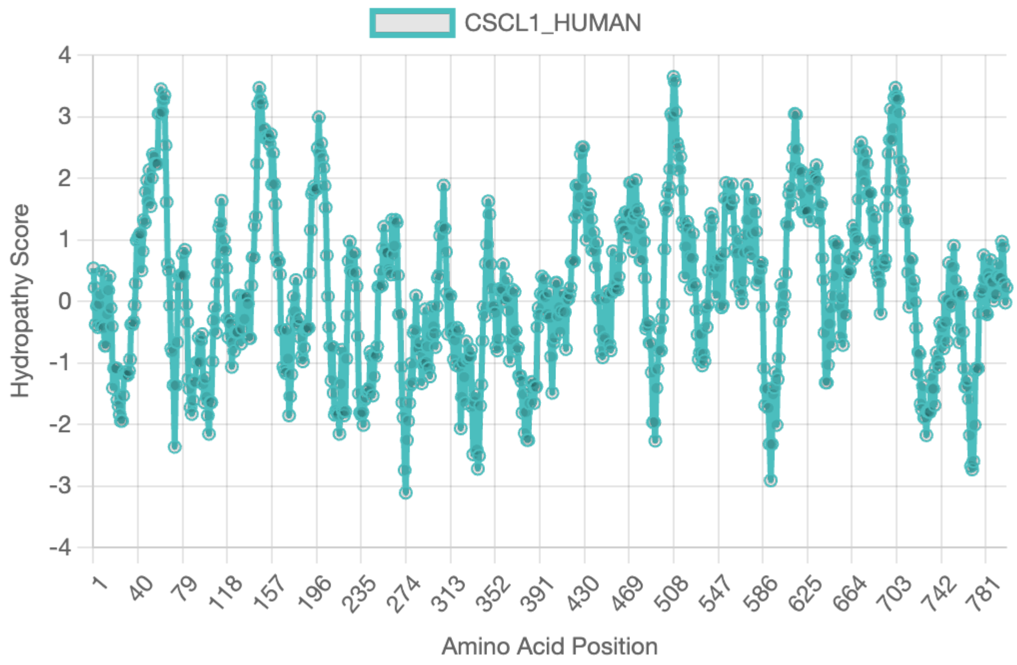

Kyte-Doolittle hydropathy plot for the sequence "CSCL1_HUMAN". Positive values indicate hydrophobic regions, while negative values indicate hydrophilic regions.

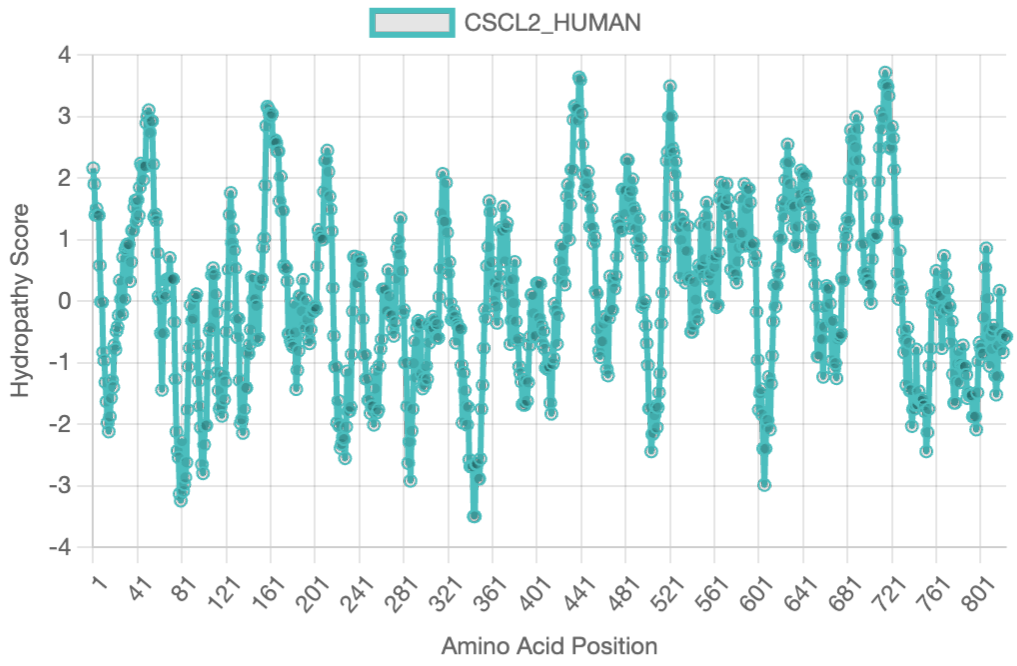

Kyte-Doolittle hydropathy plot for the sequence "CSCL2_HUMAN". Positive values indicate hydrophobic regions, while negative values indicate hydrophilic regions.

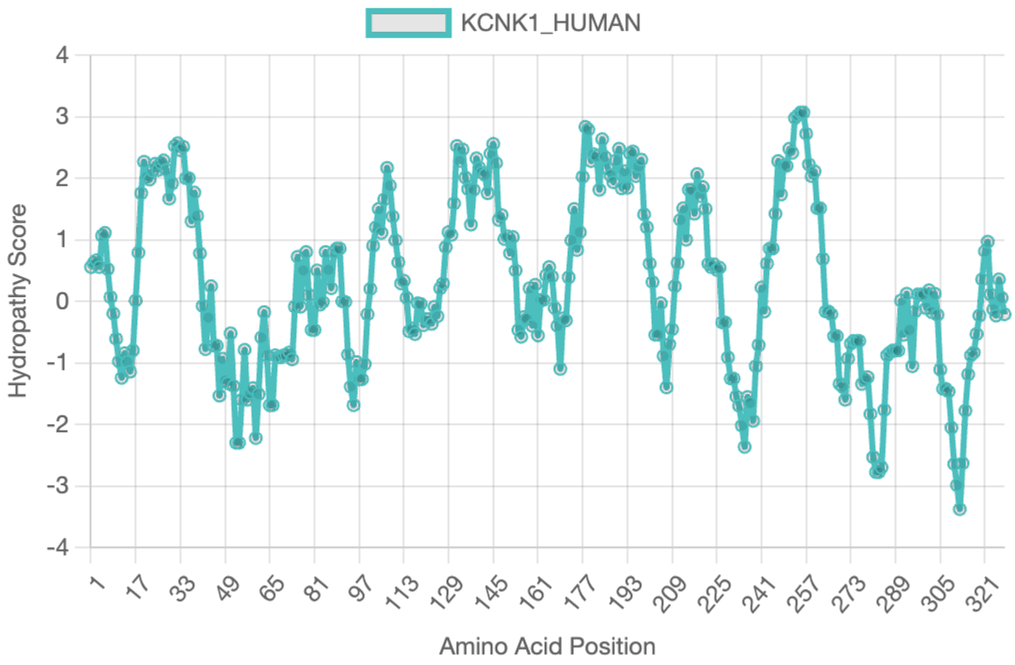

Kyte-Doolittle hydropathy plot for the sequence "KCNK1_HUMAN". Positive values indicate hydrophobic regions, while negative values indicate hydrophilic regions.

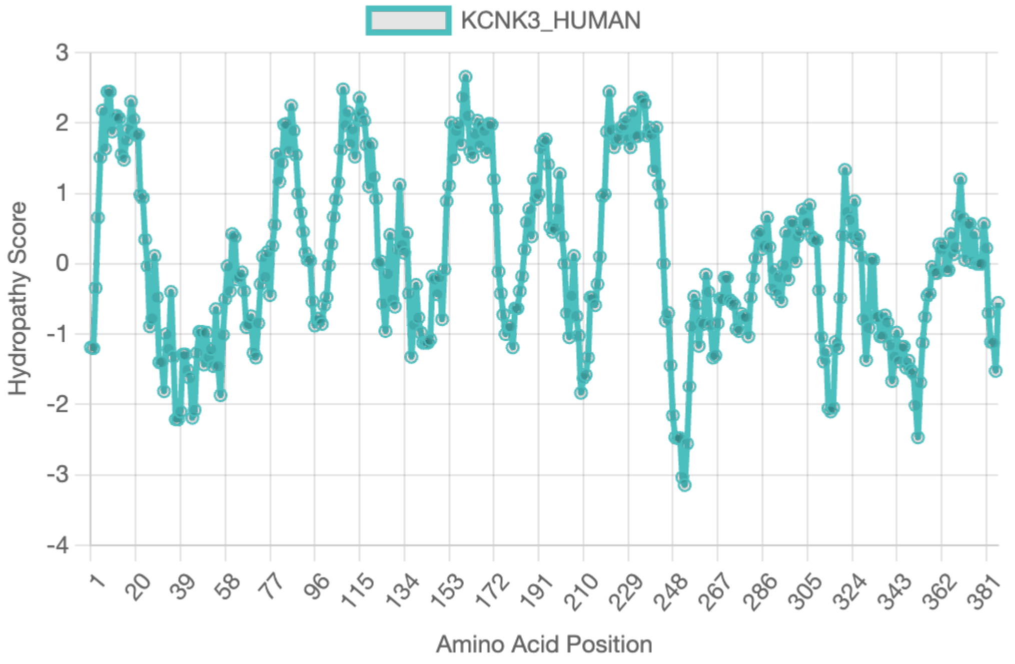

Kyte-Doolittle hydropathy plot for the sequence "KCNK3_HUMAN". Positive values indicate hydrophobic regions, while negative values indicate hydrophilic regions.

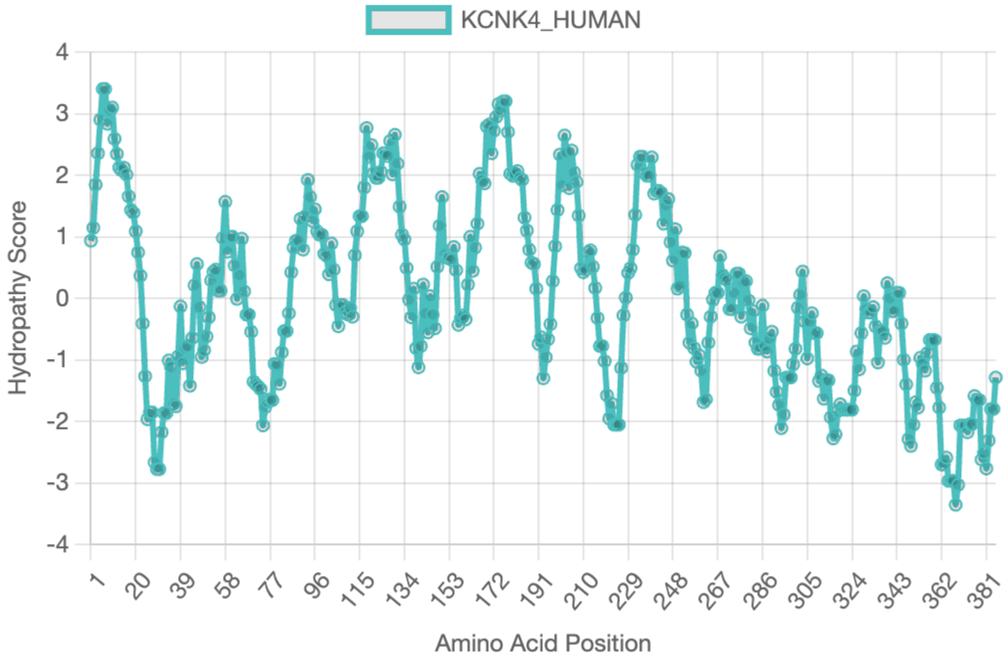

Kyte-Doolittle hydropathy plot for the sequence "KCNK4_HUMAN". Positive values indicate hydrophobic regions, while negative values indicate hydrophilic regions.

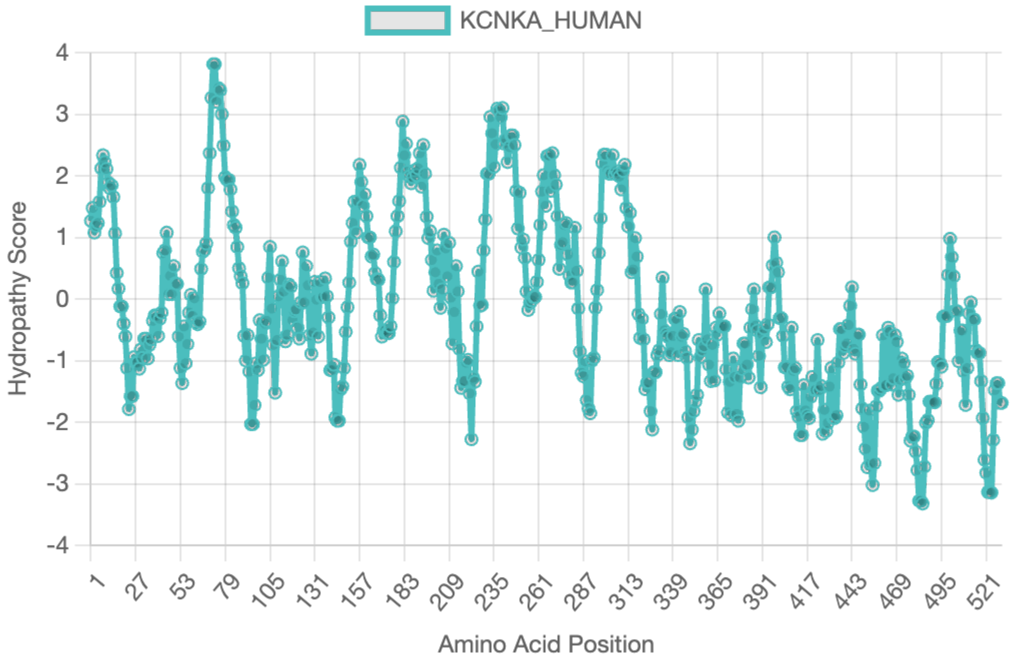

Kyte-Doolittle hydropathy plot for the sequence "KCNKA_HUMAN". Positive values indicate hydrophobic regions, while negative values indicate hydrophilic regions.

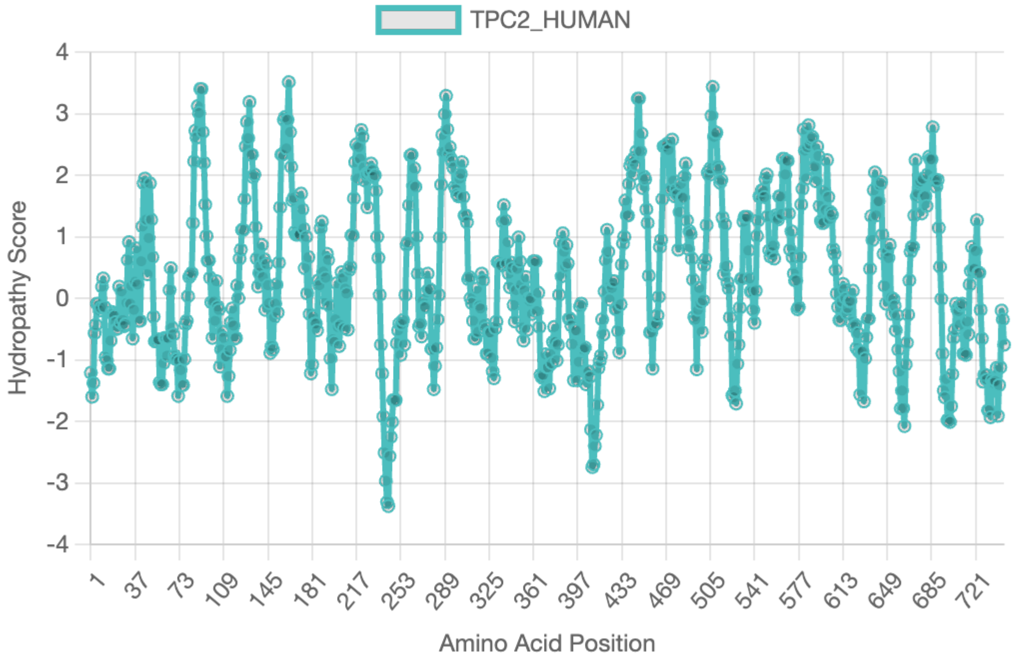

Kyte-Doolittle hydropathy plot for the sequence "TPC2_HUMAN". Positive values indicate hydrophobic regions, while negative values indicate hydrophilic regions.

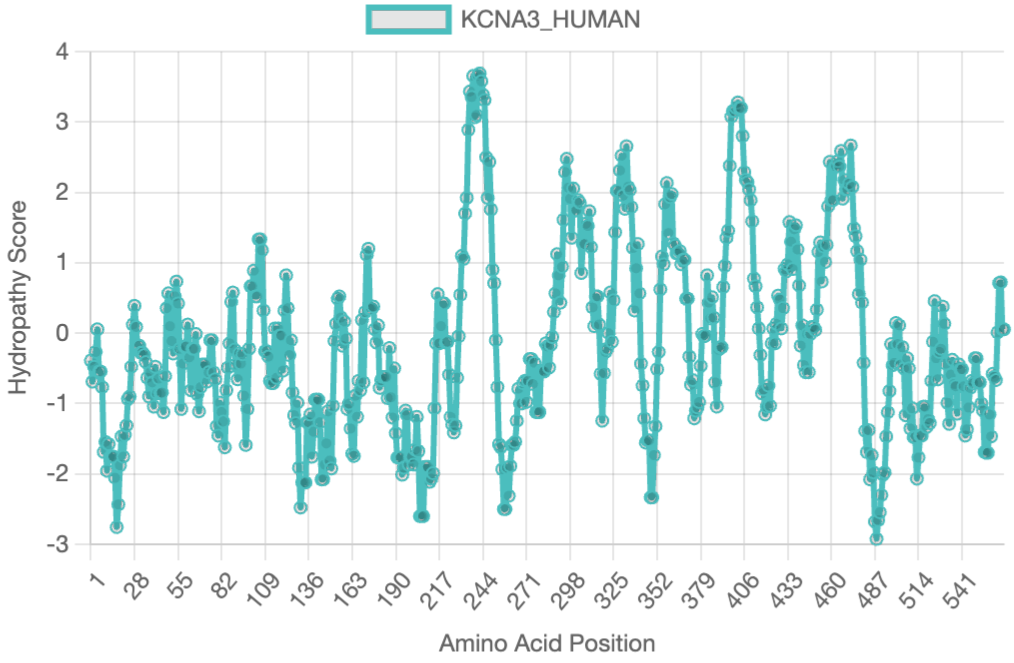

Kyte-Doolittle hydropathy plot for the sequence "KCNA3_HUMAN". Positive values indicate hydrophobic regions, while negative values indicate hydrophilic regions.

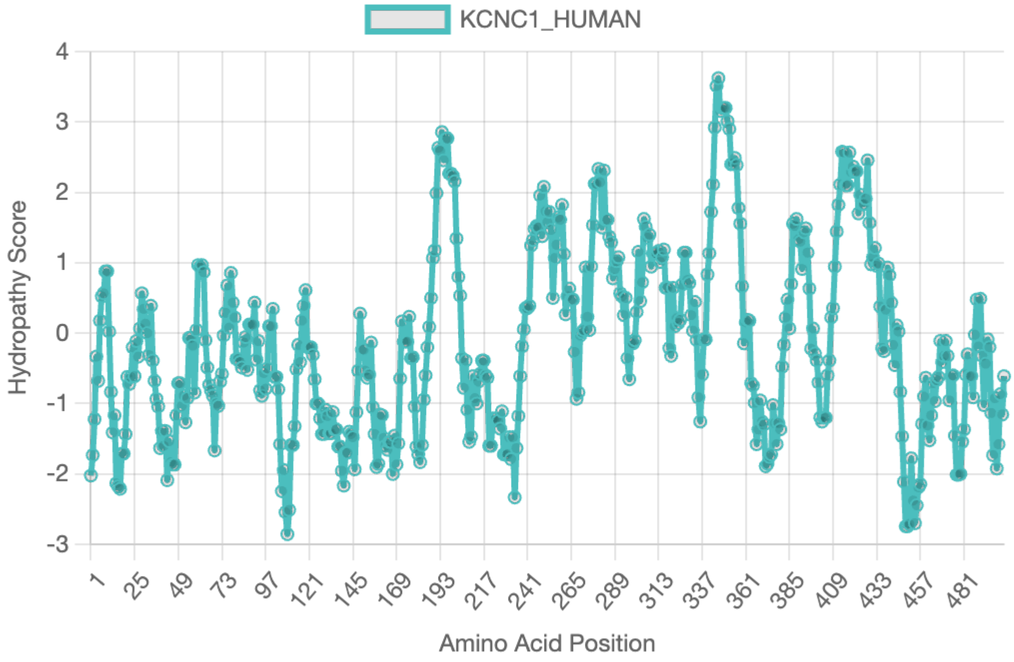

Kyte-Doolittle hydropathy plot for the sequence "KCNC1_HUMAN". Positive values indicate hydrophobic regions, while negative values indicate hydrophilic regions.

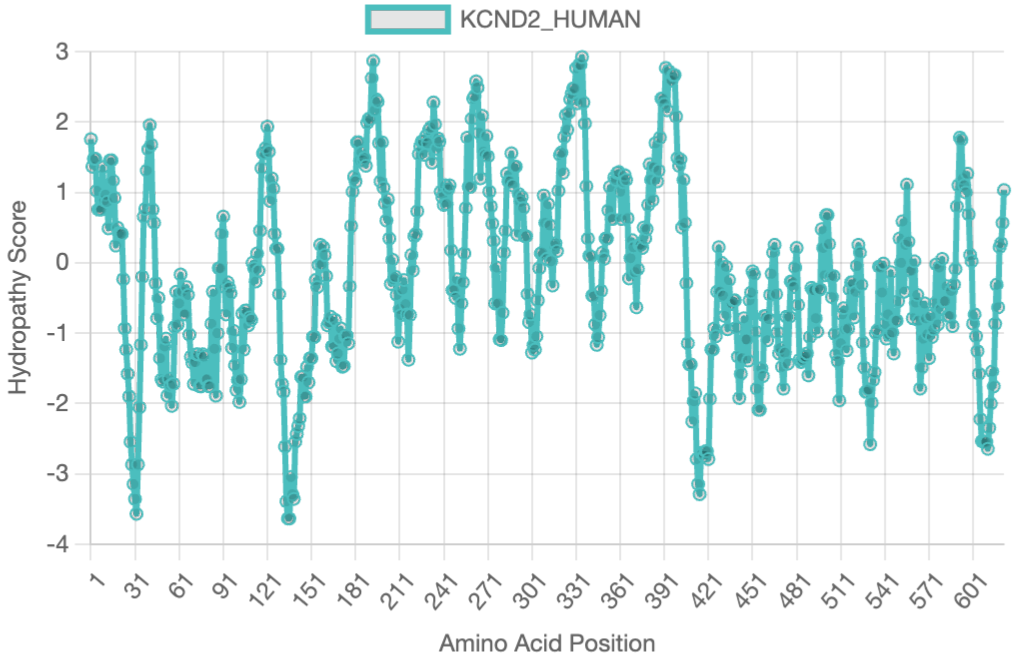

Kyte-Doolittle hydropathy plot for the sequence "KCND2_HUMAN". Positive values indicate hydrophobic regions, while negative values indicate hydrophilic regions.

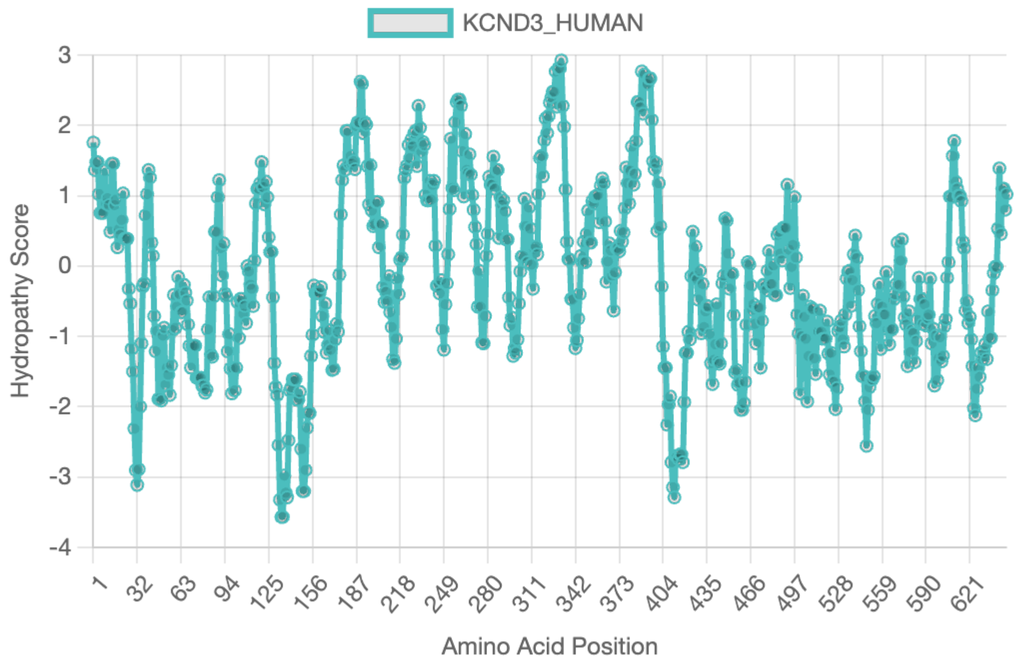

Kyte-Doolittle hydropathy plot for the sequence "KCND3_HUMAN". Positive values indicate hydrophobic regions, while negative values indicate hydrophilic regions.

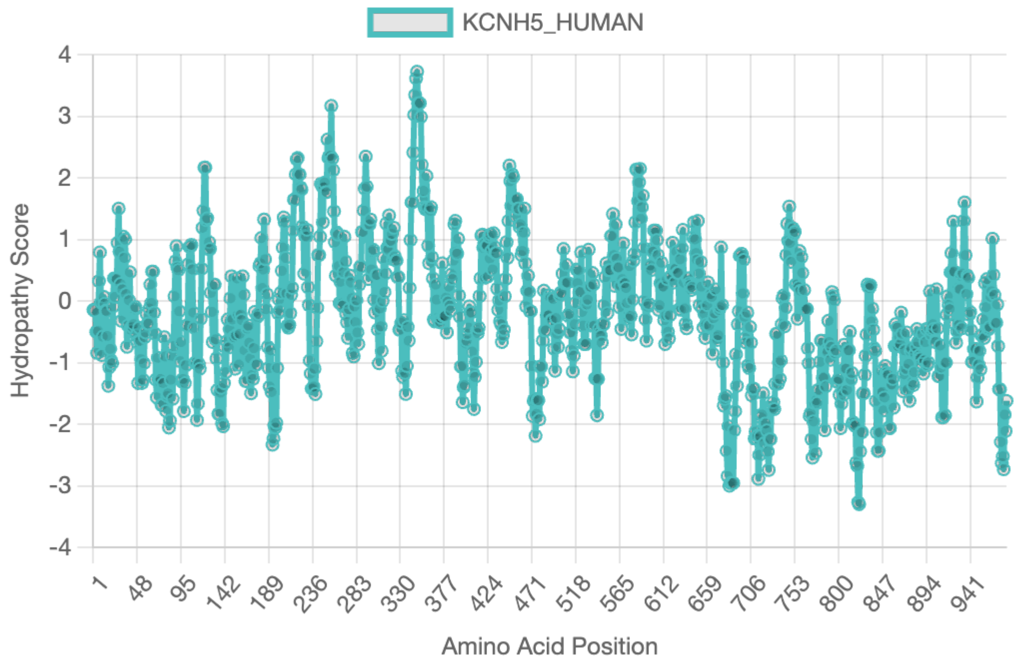

Kyte-Doolittle hydropathy plot for the sequence "KCNH5_HUMAN". Positive values indicate hydrophobic regions, while negative values indicate hydrophilic regions.

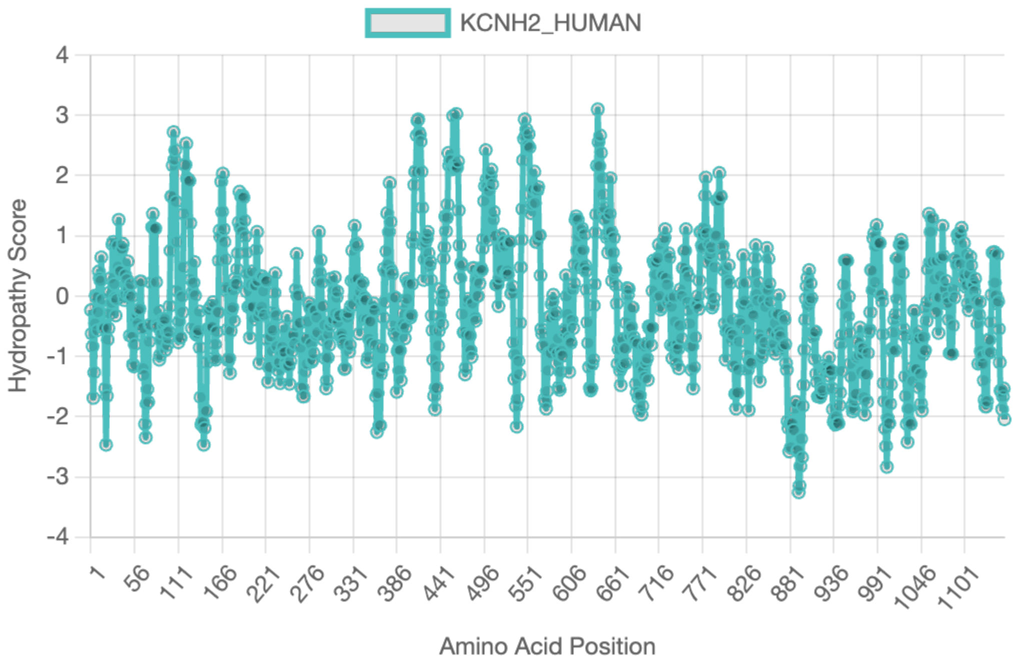

Kyte-Doolittle hydropathy plot for the sequence "KCNH2_HUMAN". Positive values indicate hydrophobic regions, while negative values indicate hydrophilic regions.

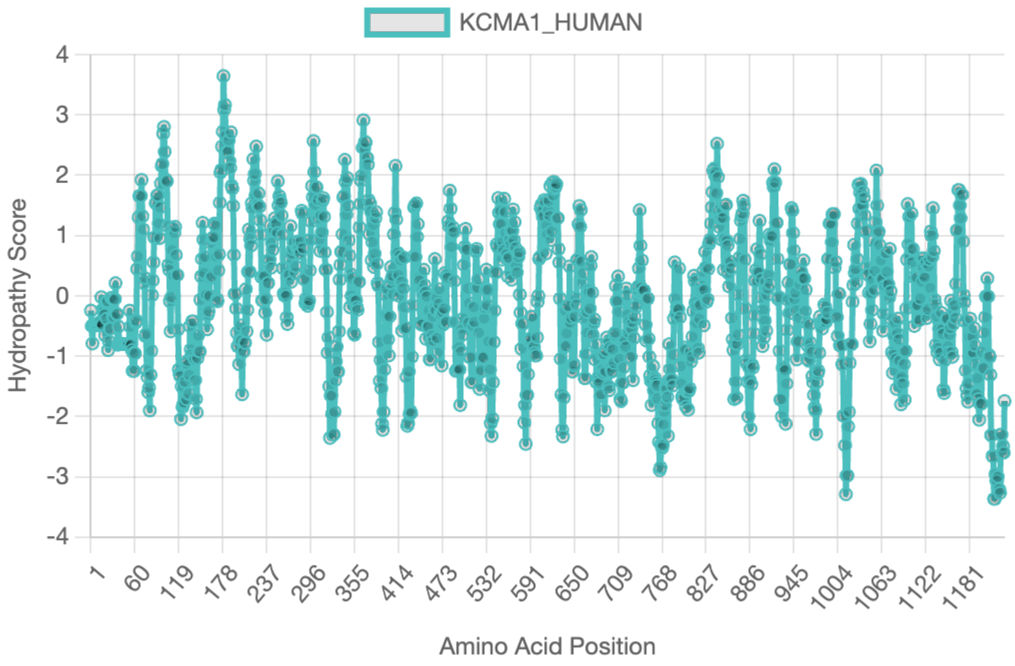

Kyte-Doolittle hydropathy plot for the sequence "KCMA1_HUMAN". Positive values indicate hydrophobic regions, while negative values indicate hydrophilic regions.

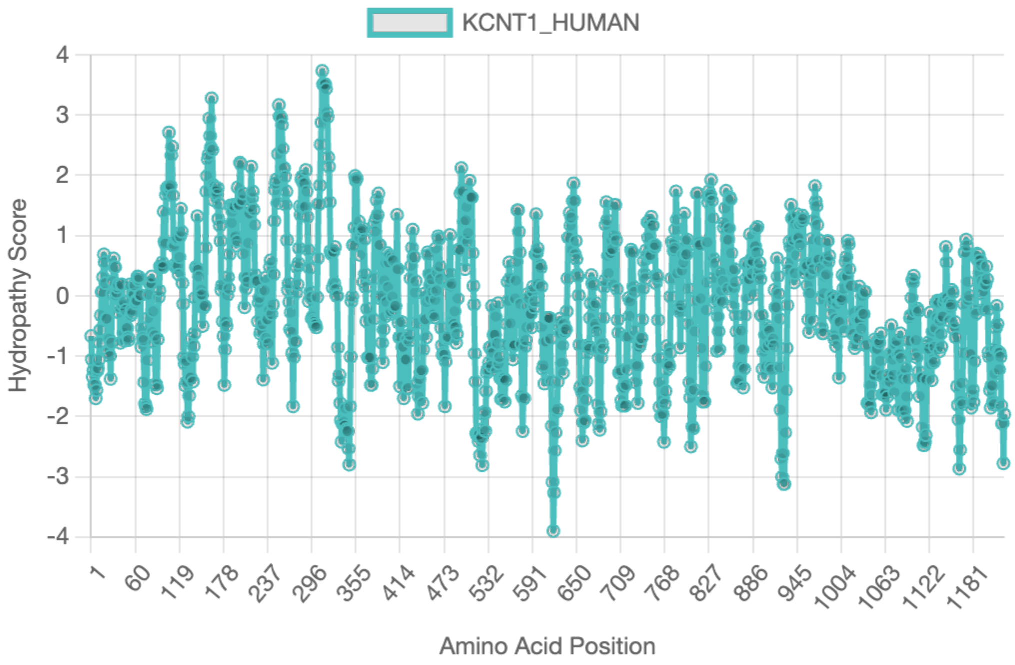

Kyte-Doolittle hydropathy plot for the sequence "KCNT1_HUMAN". Positive values indicate hydrophobic regions, while negative values indicate hydrophilic regions.

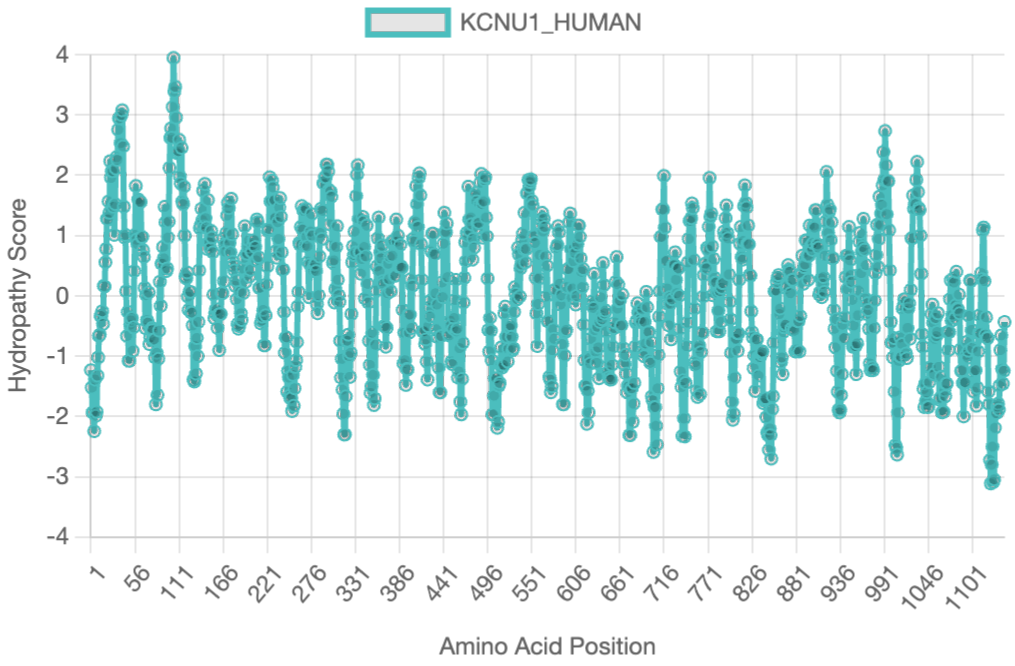

Kyte-Doolittle hydropathy plot for the sequence "KCNU1_HUMAN". Positive values indicate hydrophobic regions, while negative values indicate hydrophilic regions.

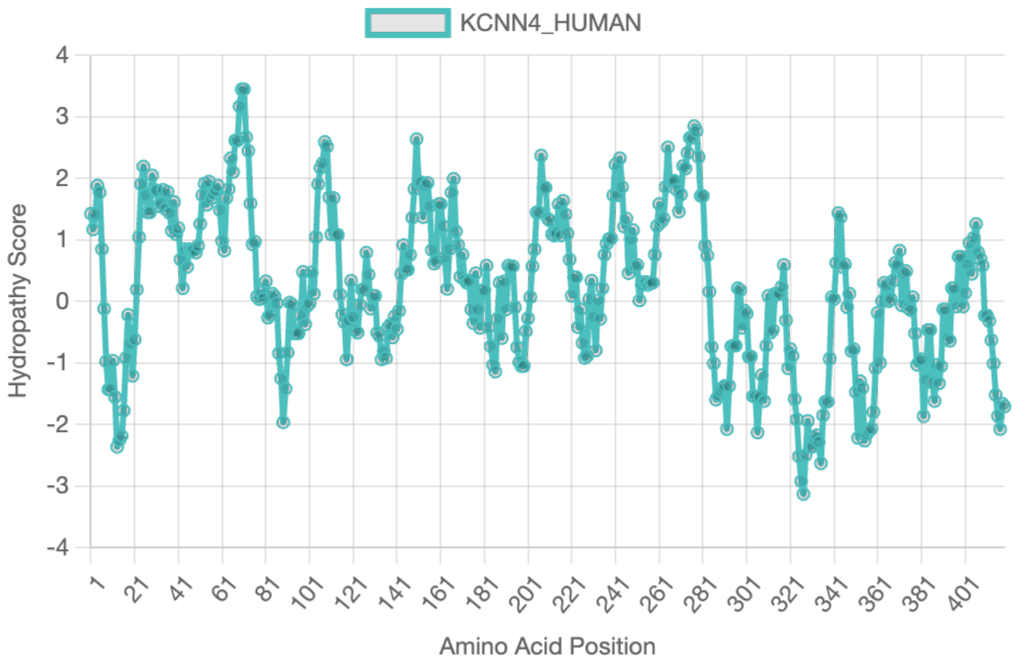

Kyte-Doolittle hydropathy plot for the sequence "KCNN4_HUMAN". Positive values indicate hydrophobic regions, while negative values indicate hydrophilic regions.

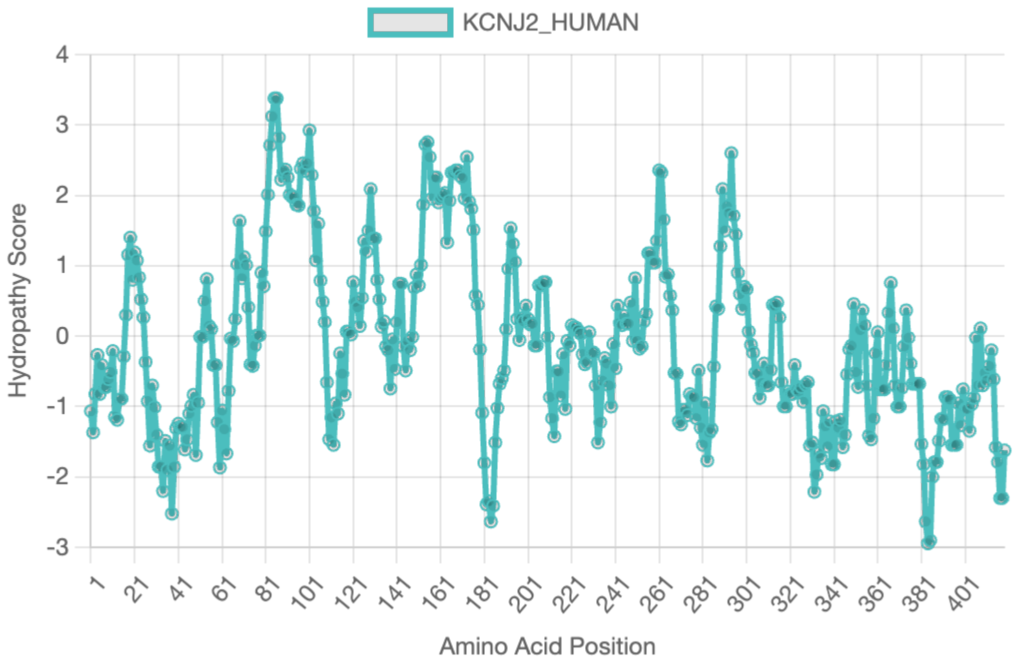

Kyte-Doolittle hydropathy plot for the sequence "KCNJ2_HUMAN". Positive values indicate hydrophobic regions, while negative values indicate hydrophilic regions.

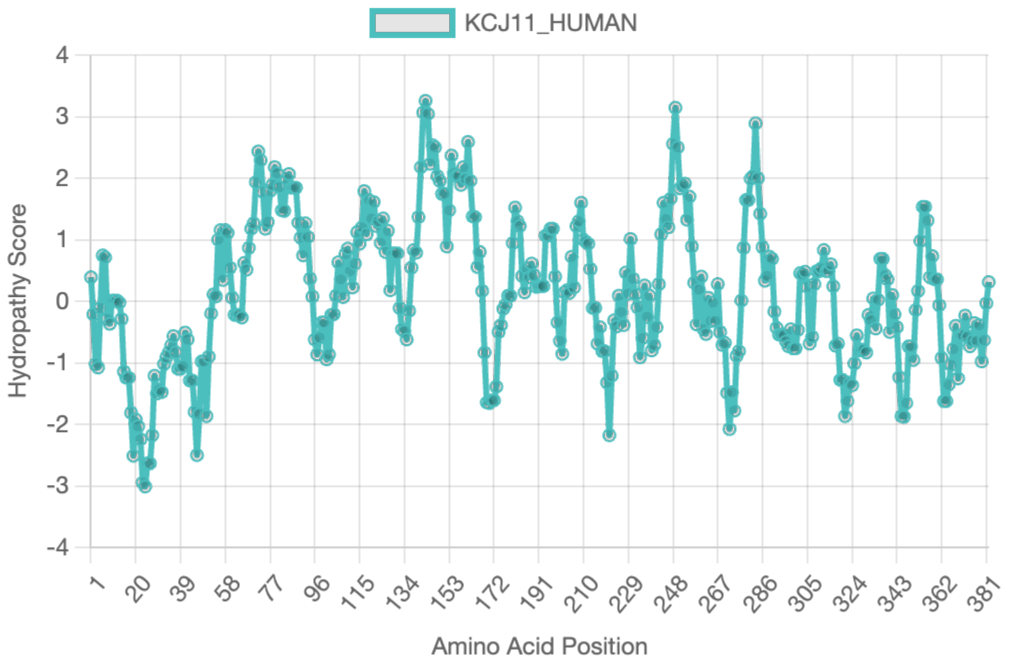

Kyte-Doolittle hydropathy plot for the sequence "KCJ11_HUMAN". Positive values indicate hydrophobic regions, while negative values indicate hydrophilic regions.

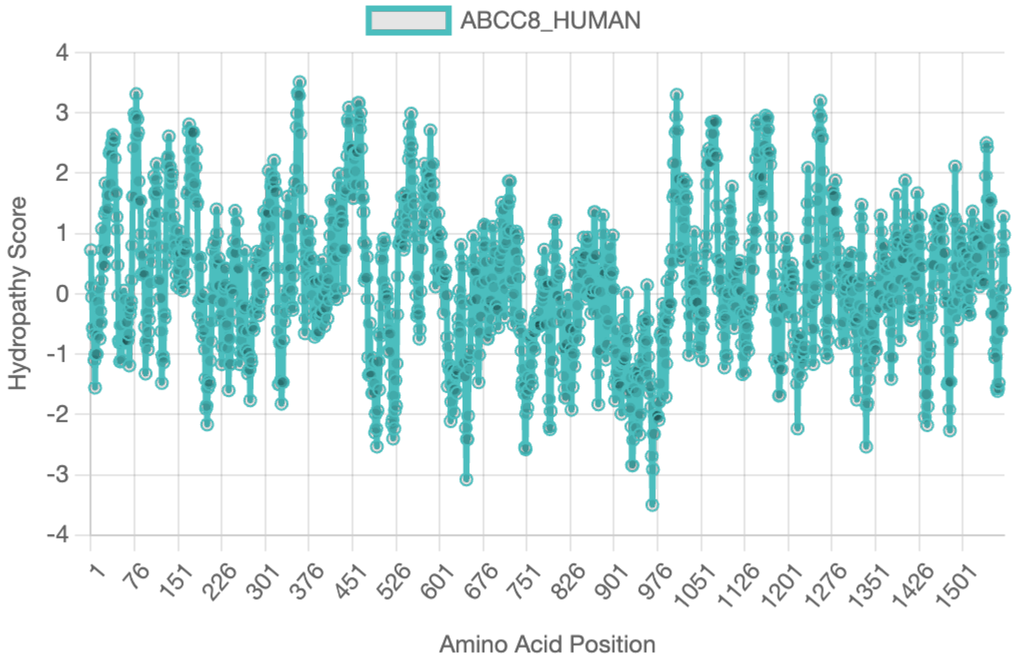

Kyte-Doolittle hydropathy plot for the sequence "ABCC8_HUMAN". Positive values indicate hydrophobic regions, while negative values indicate hydrophilic regions.

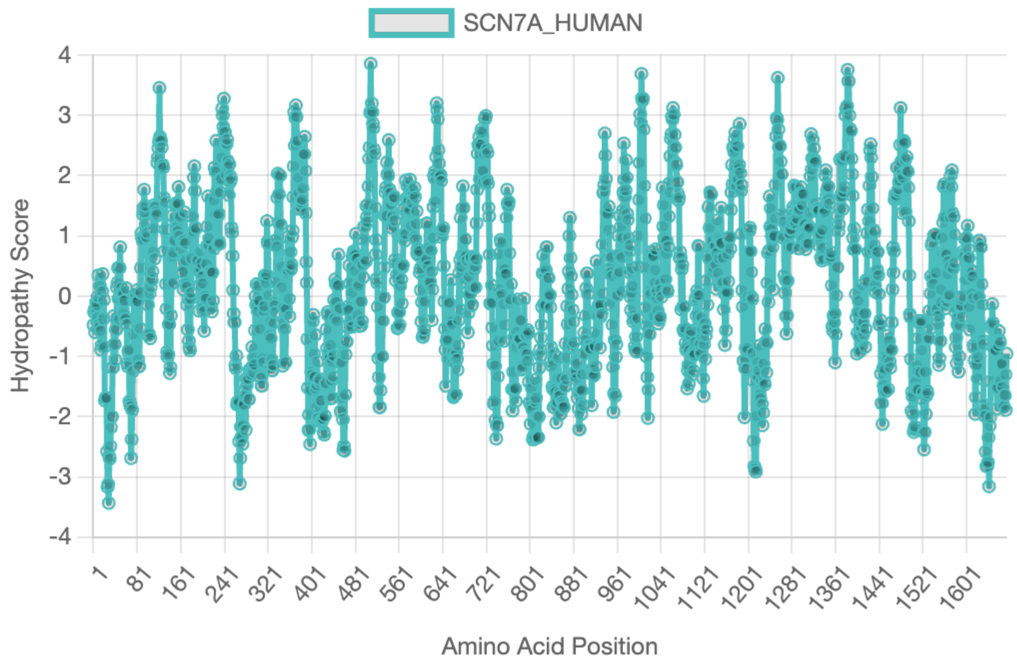

Kyte-Doolittle hydropathy plot for the sequence "SCN7A_HUMAN". Positive values indicate hydrophobic regions, while negative values indicate hydrophilic regions.

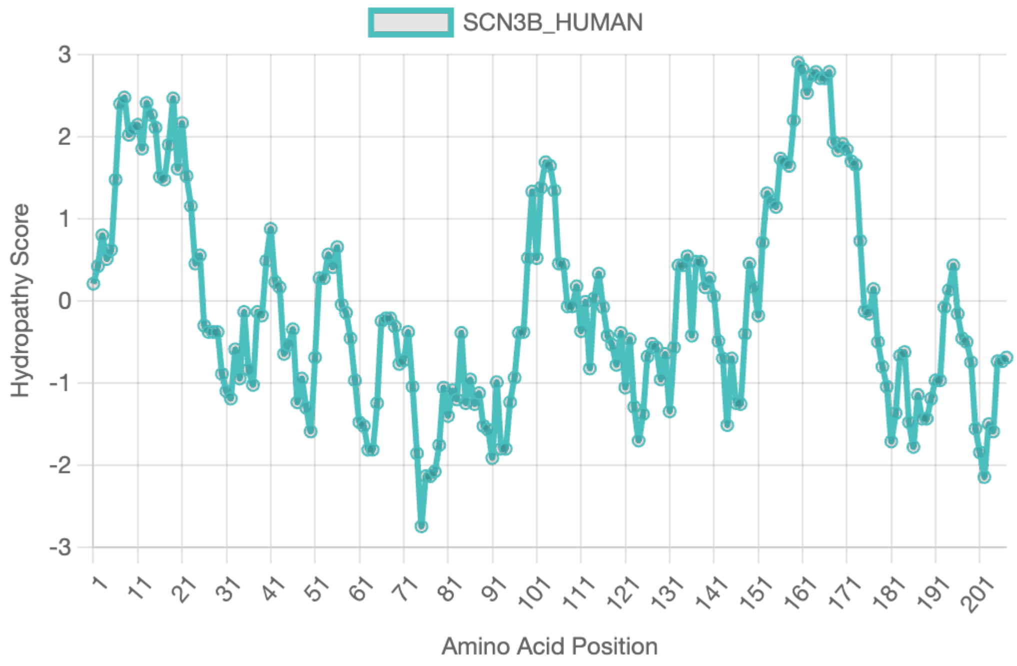

Kyte-Doolittle hydropathy plot for the sequence "SCN3B_HUMAN". Positive values indicate hydrophobic regions, while negative values indicate hydrophilic regions.

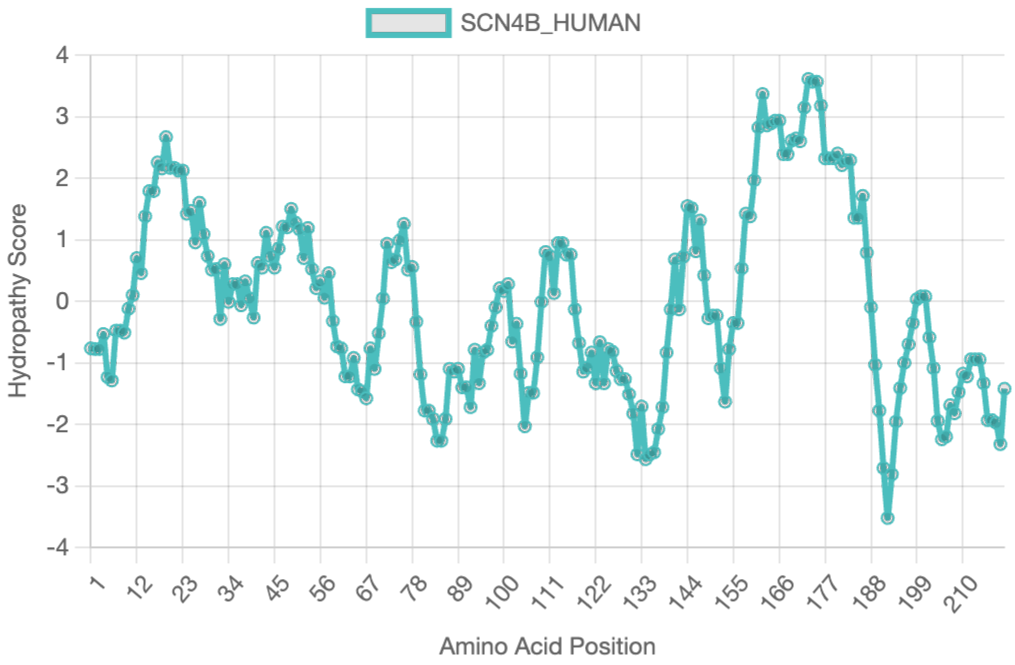

Kyte-Doolittle hydropathy plot for the sequence "SCN4B_HUMAN". Positive values indicate hydrophobic regions, while negative values indicate hydrophilic regions.

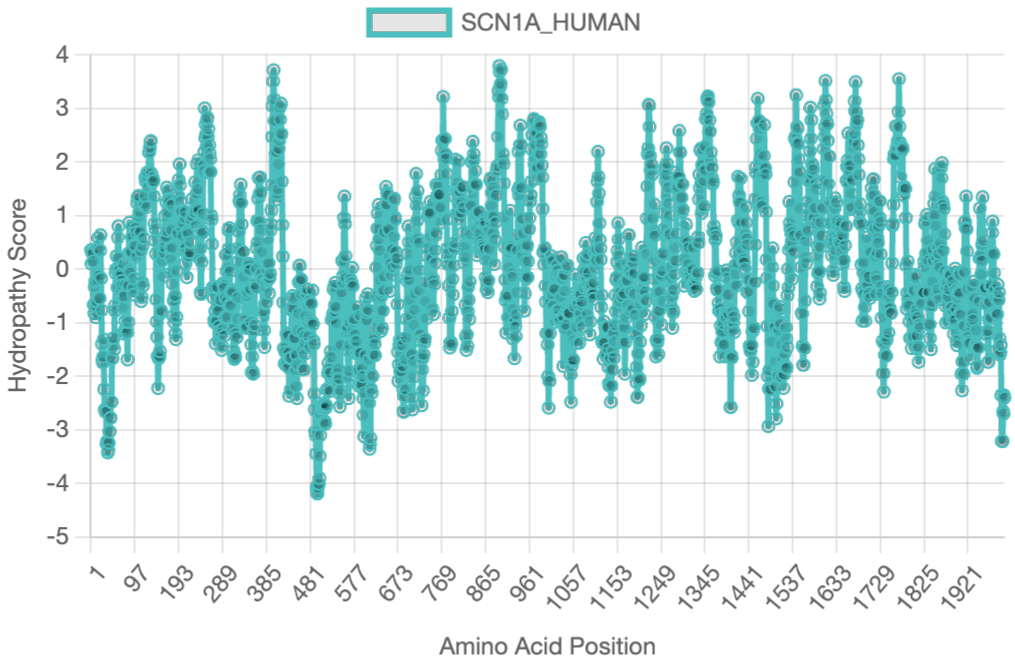

Kyte-Doolittle hydropathy plot for the sequence "SCN1A_HUMAN". Positive values indicate hydrophobic regions, while negative values indicate hydrophilic regions.

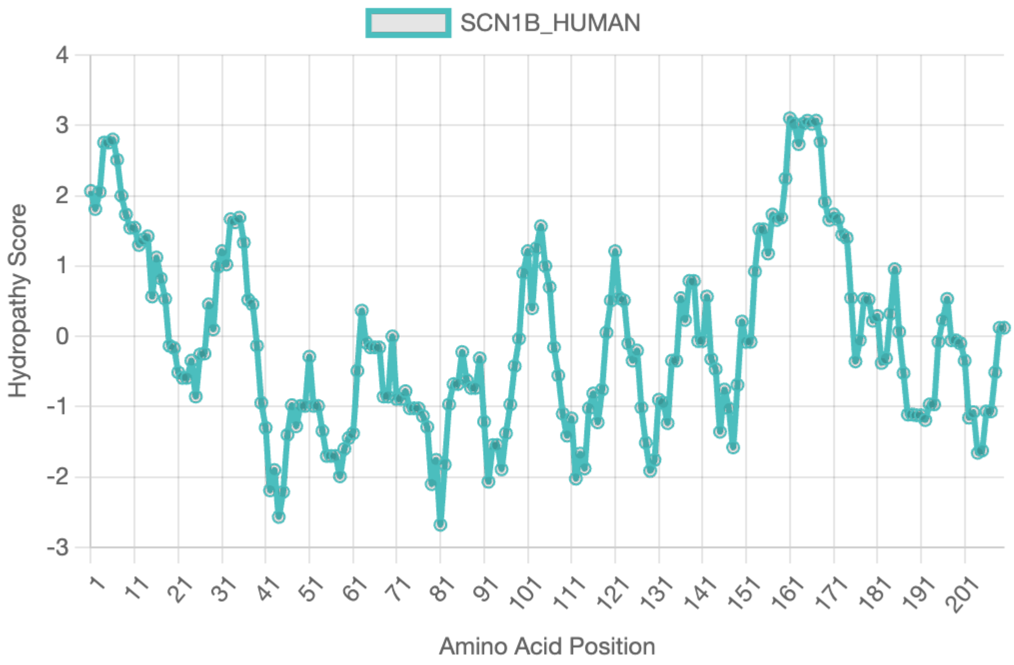

Kyte-Doolittle hydropathy plot for the sequence "SCN1B_HUMAN". Positive values indicate hydrophobic regions, while negative values indicate hydrophilic regions.

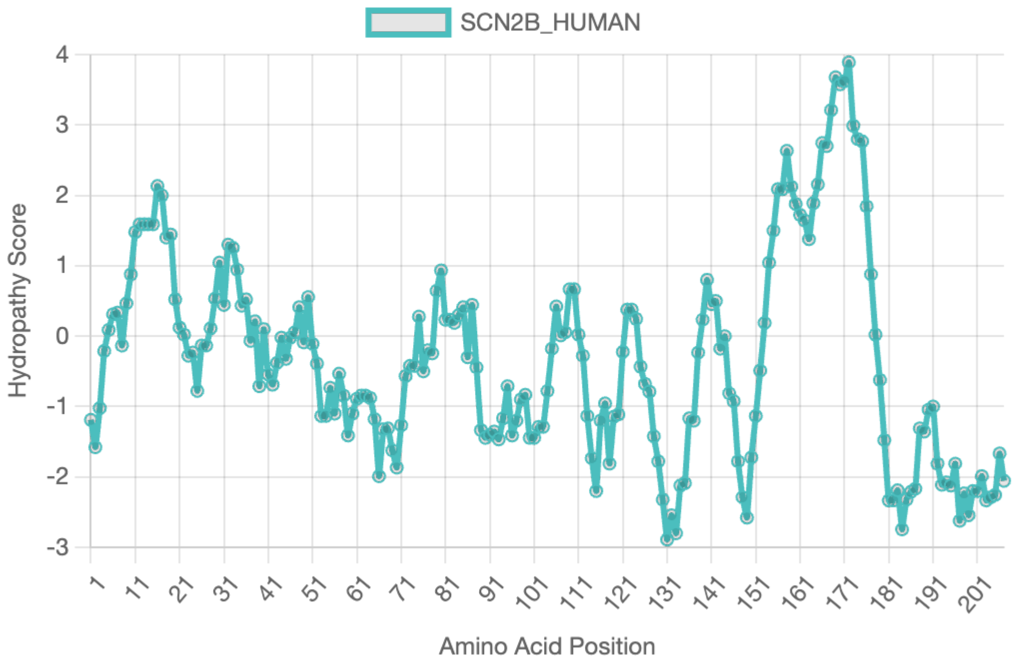

Kyte-Doolittle hydropathy plot for the sequence "SCN2B_HUMAN". Positive values indicate hydrophobic regions, while negative values indicate hydrophilic regions.

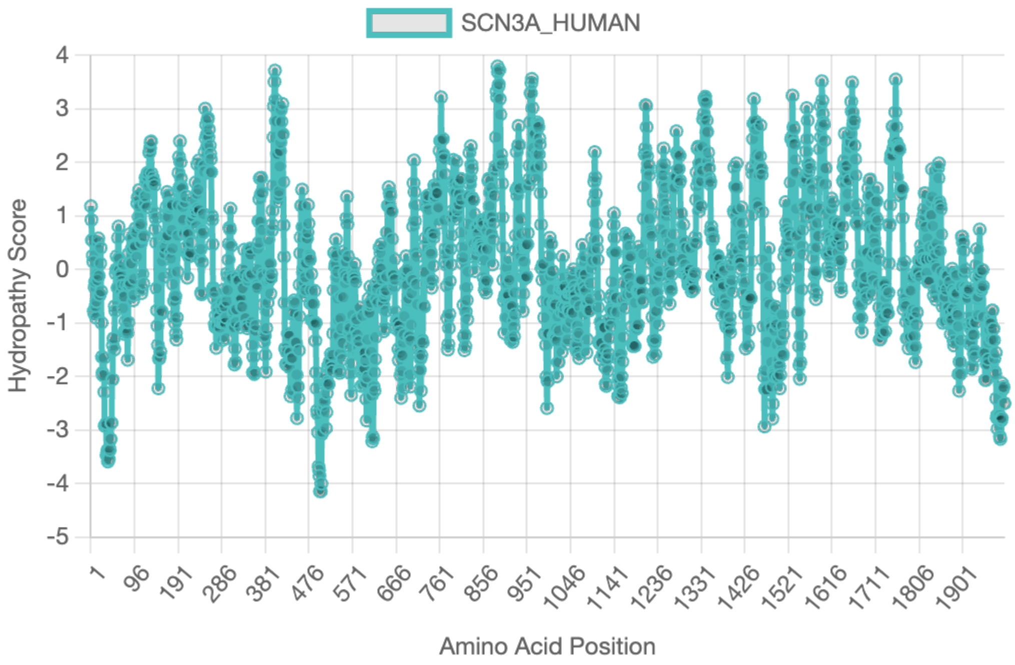

Kyte-Doolittle hydropathy plot for the sequence "SCN3A_HUMAN". Positive values indicate hydrophobic regions, while negative values indicate hydrophilic regions.

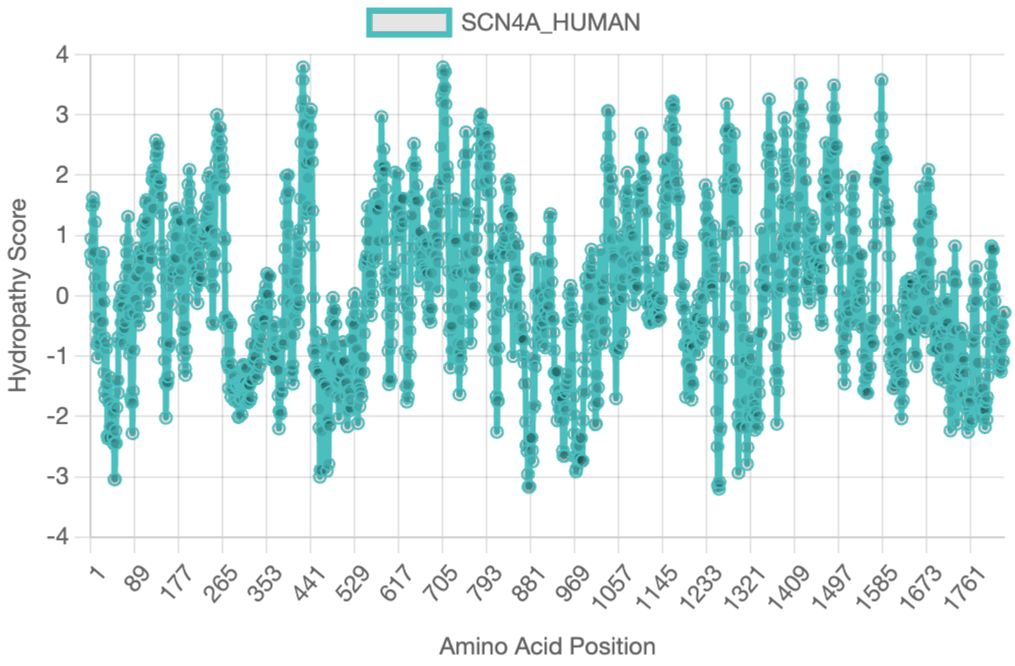

Kyte-Doolittle hydropathy plot for the sequence "SCN4A_HUMAN". Positive values indicate hydrophobic regions, while negative values indicate hydrophilic regions.

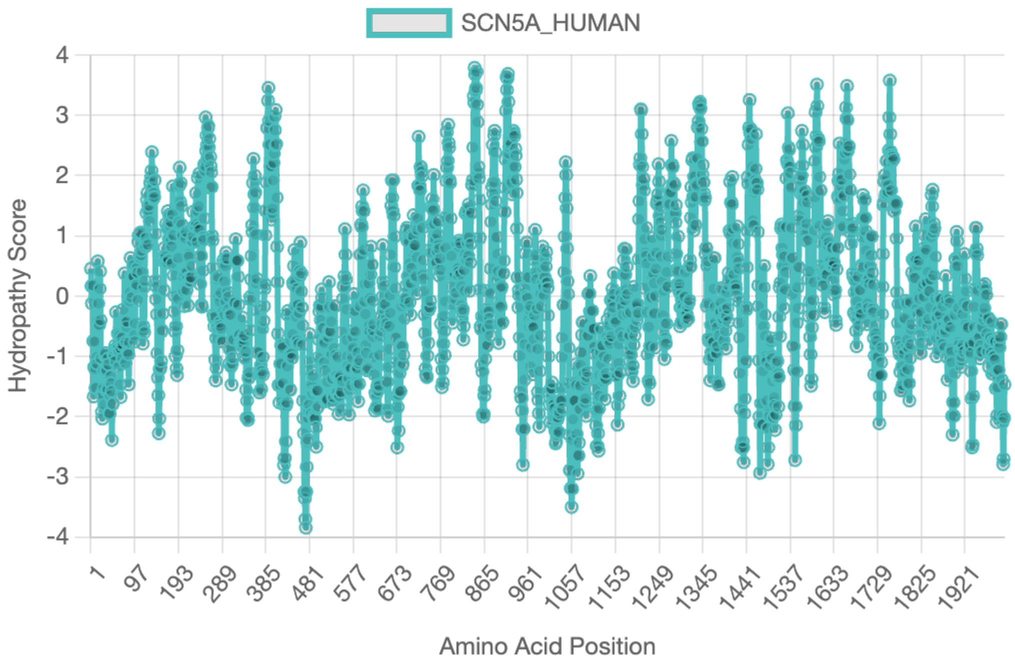

Kyte-Doolittle hydropathy plot for the sequence "SCN5A_HUMAN". Positive values indicate hydrophobic regions, while negative values indicate hydrophilic regions.

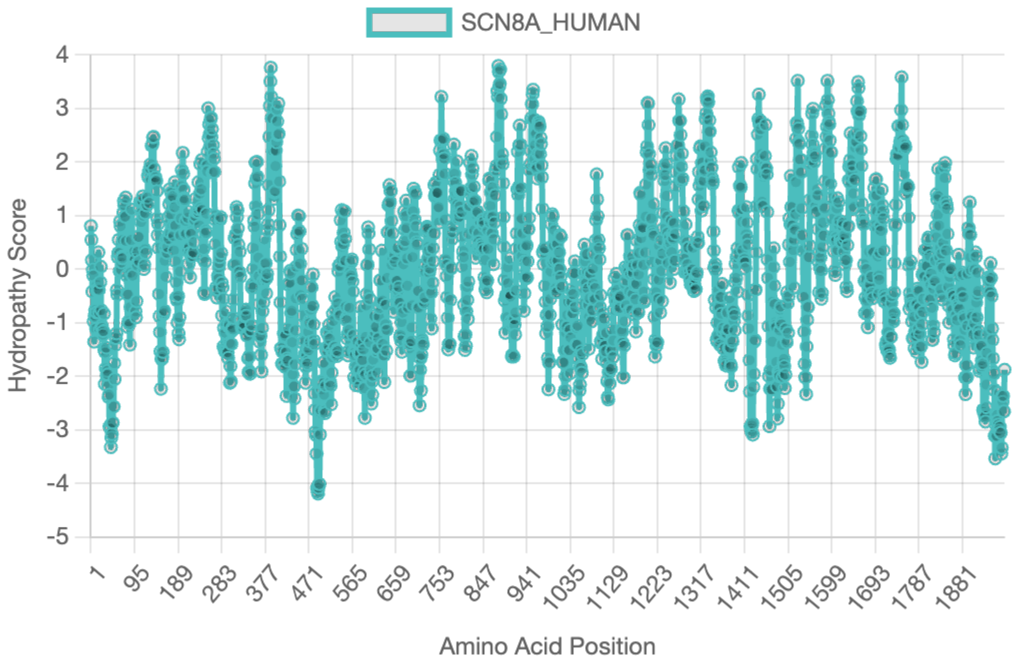

Kyte-Doolittle hydropathy plot for the sequence "SCN8A_HUMAN". Positive values indicate hydrophobic regions, while negative values indicate hydrophilic regions.

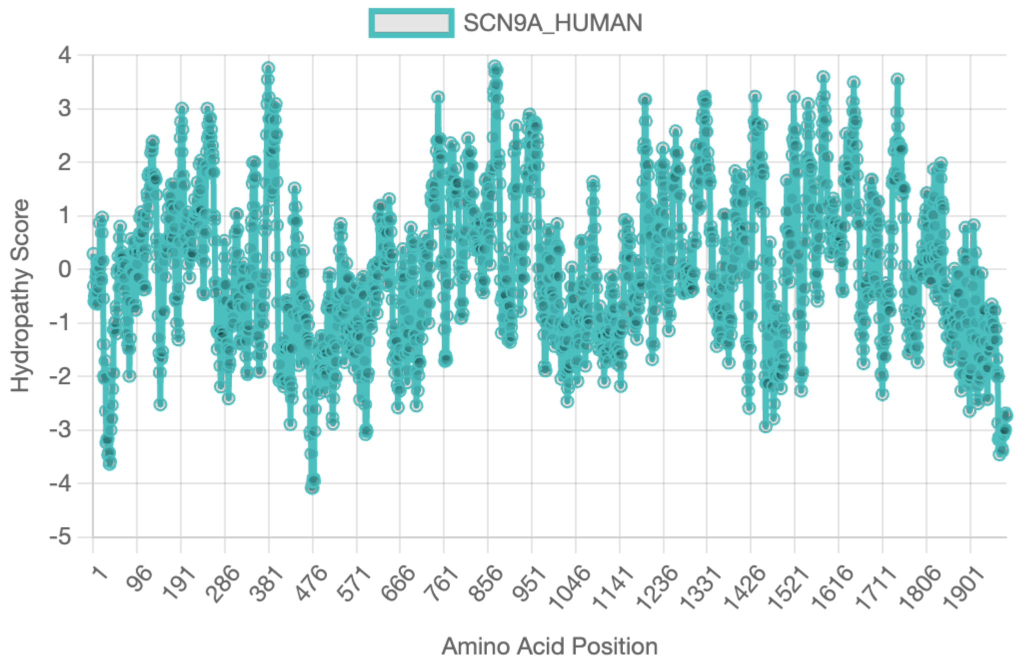

Kyte-Doolittle hydropathy plot for the sequence "SCN9A_HUMAN". Positive values indicate hydrophobic regions, while negative values indicate hydrophilic regions.

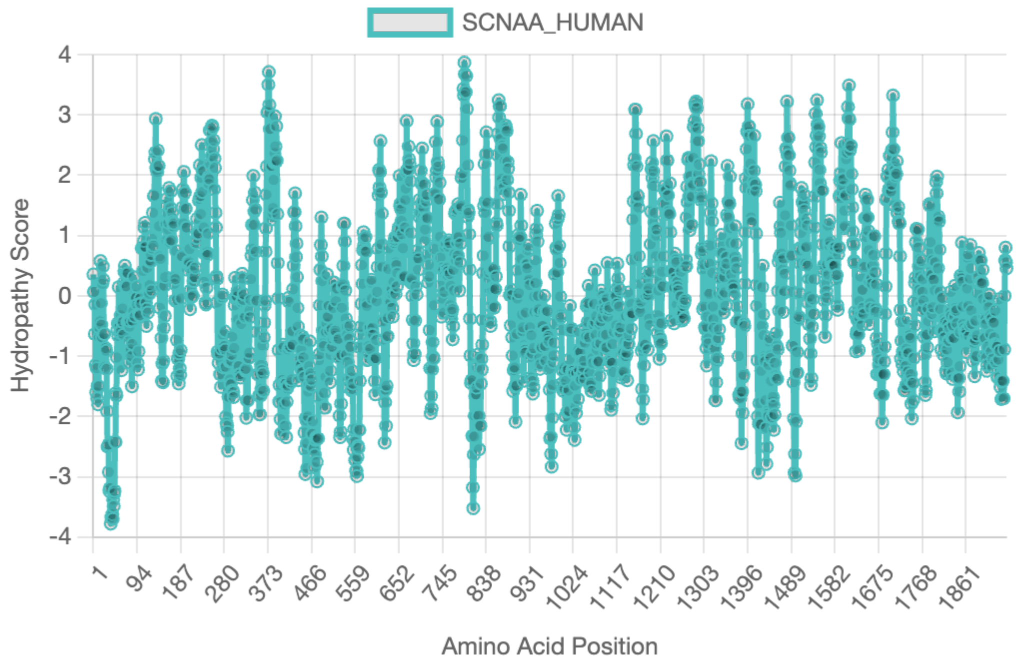

Kyte-Doolittle hydropathy plot for the sequence "SCNAA_HUMAN". Positive values indicate hydrophobic regions, while negative values indicate hydrophilic regions.

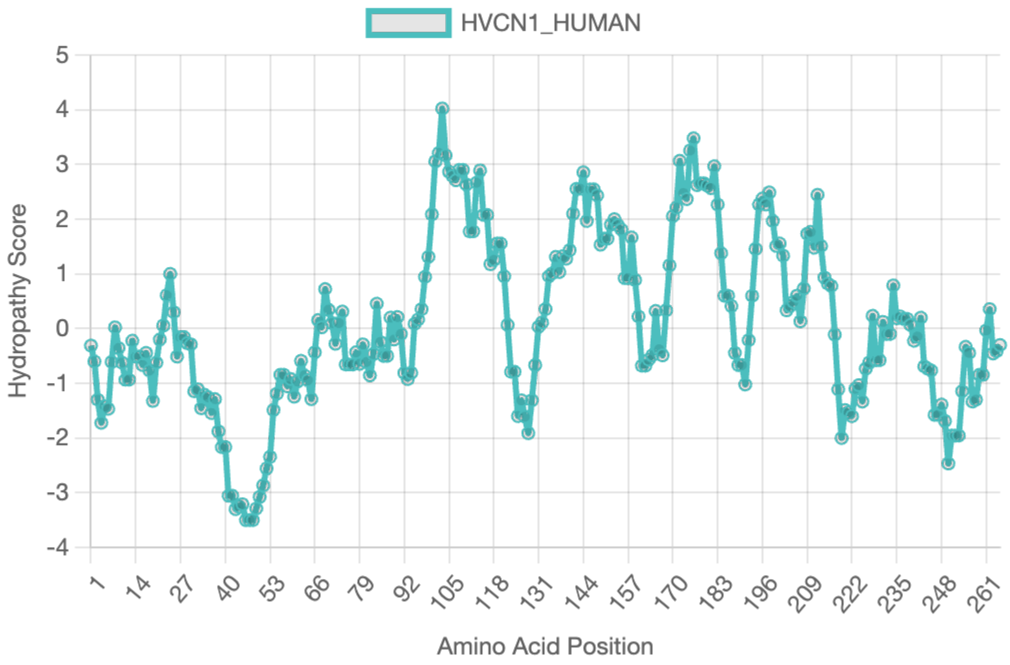

Kyte-Doolittle hydropathy plot for the sequence "HVCN1_HUMAN". Positive values indicate hydrophobic regions, while negative values indicate hydrophilic regions.

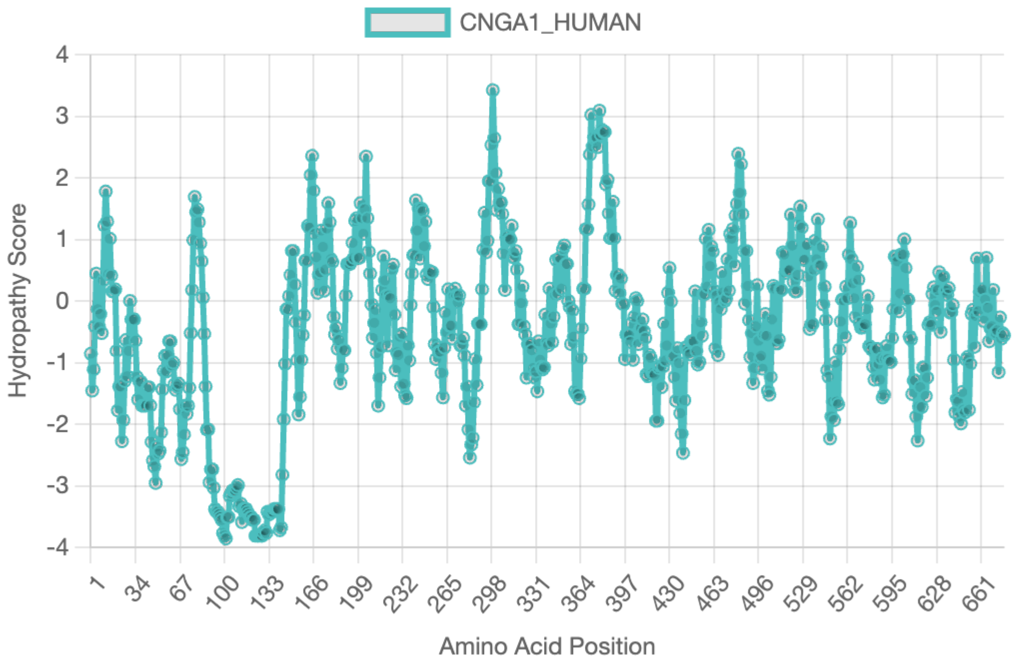

Kyte-Doolittle hydropathy plot for the sequence "CNGA1_HUMAN". Positive values indicate hydrophobic regions, while negative values indicate hydrophilic regions.

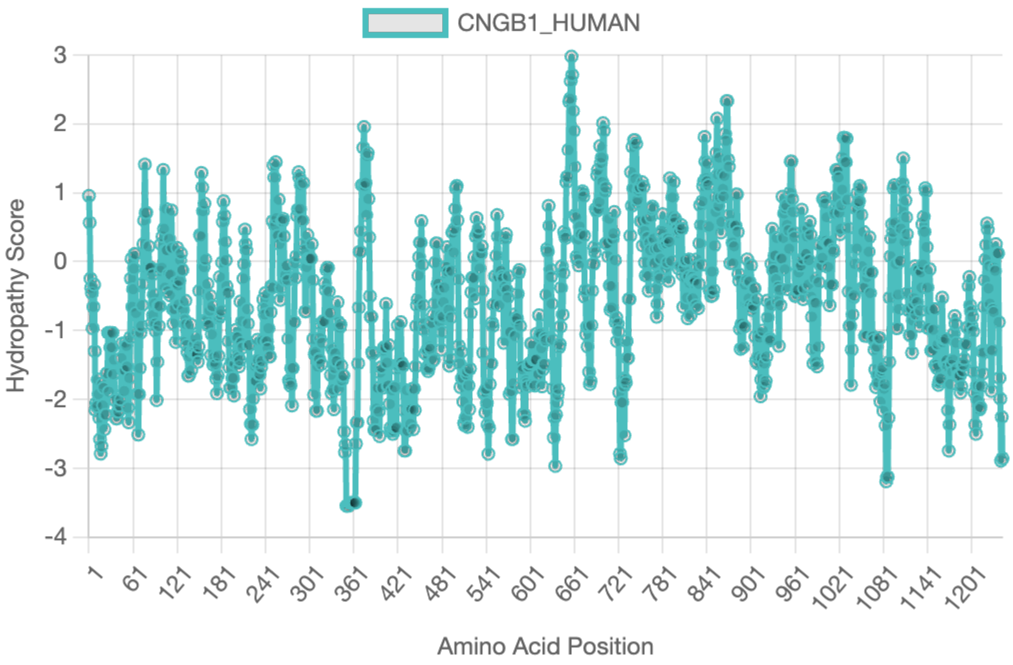

Kyte-Doolittle hydropathy plot for the sequence "CNGB1_HUMAN". Positive values indicate hydrophobic regions, while negative values indicate hydrophilic regions.

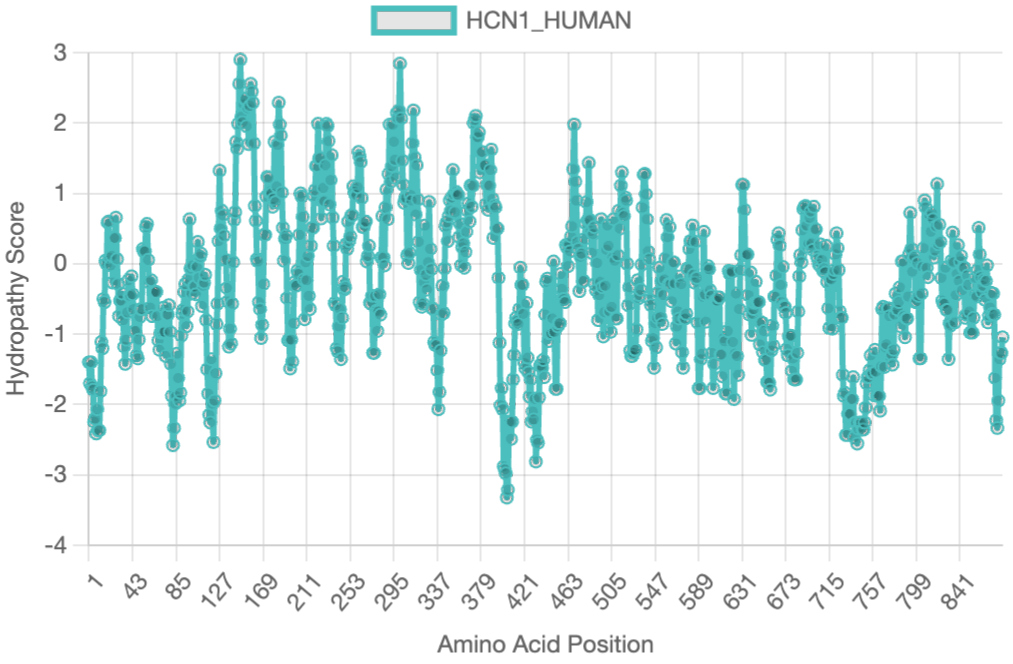

Kyte-Doolittle hydropathy plot for the sequence "HCN1_HUMAN". Positive values indicate hydrophobic regions, while negative values indicate hydrophilic regions.

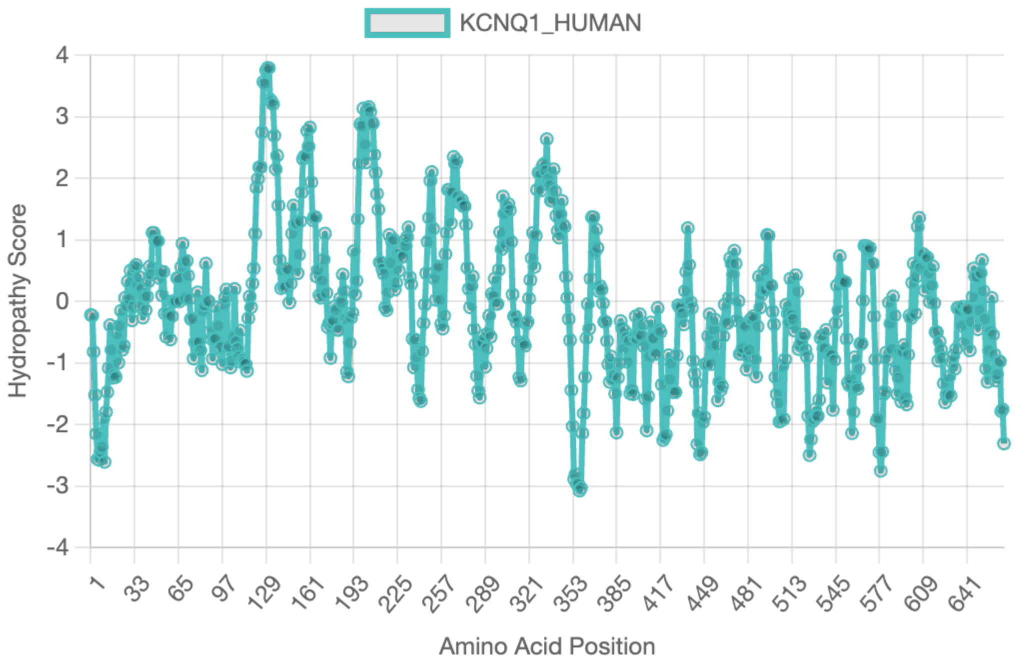

Kyte-Doolittle hydropathy plot for the sequence "KCNQ1_HUMAN". Positive values indicate hydrophobic regions, while negative values indicate hydrophilic regions.

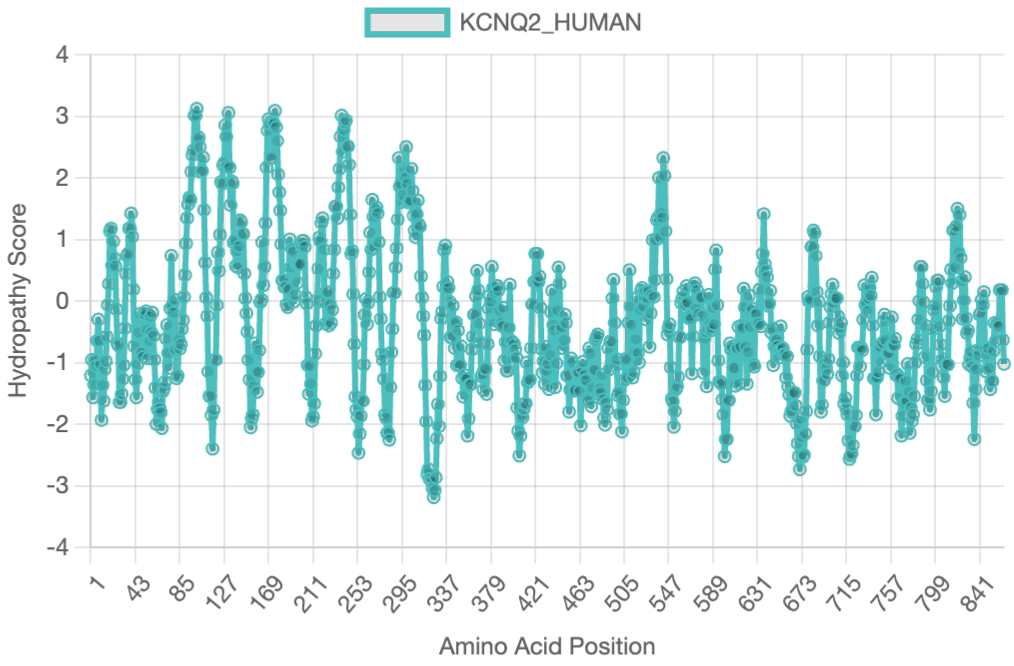

Kyte-Doolittle hydropathy plot for the sequence "KCNQ2_HUMAN". Positive values indicate hydrophobic regions, while negative values indicate hydrophilic regions.

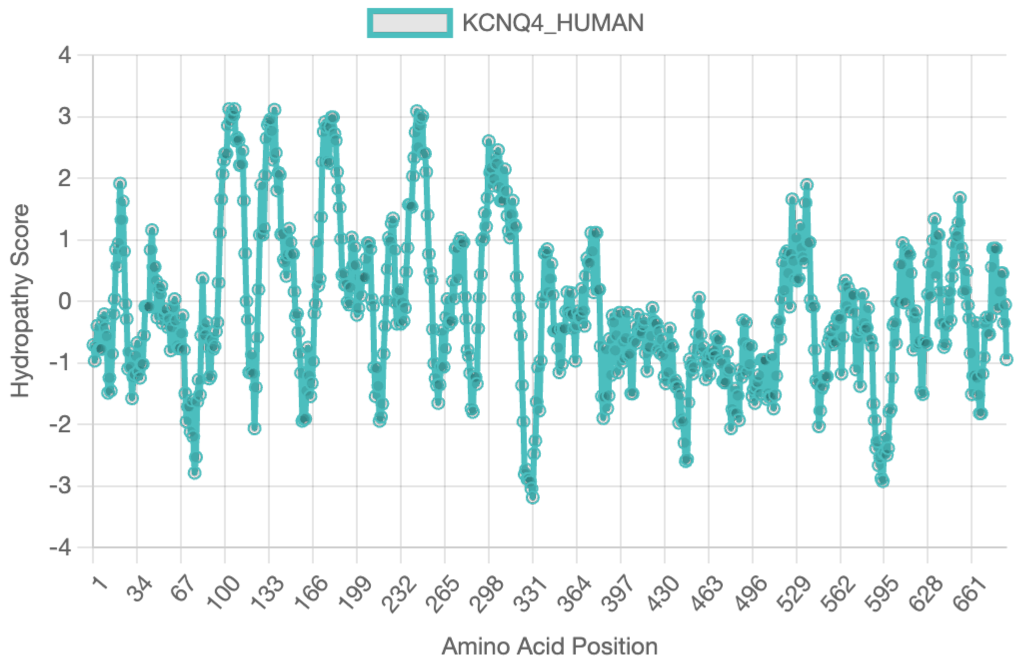

Kyte-Doolittle hydropathy plot for the sequence "KCNQ4_HUMAN". Positive values indicate hydrophobic regions, while negative values indicate hydrophilic regions.

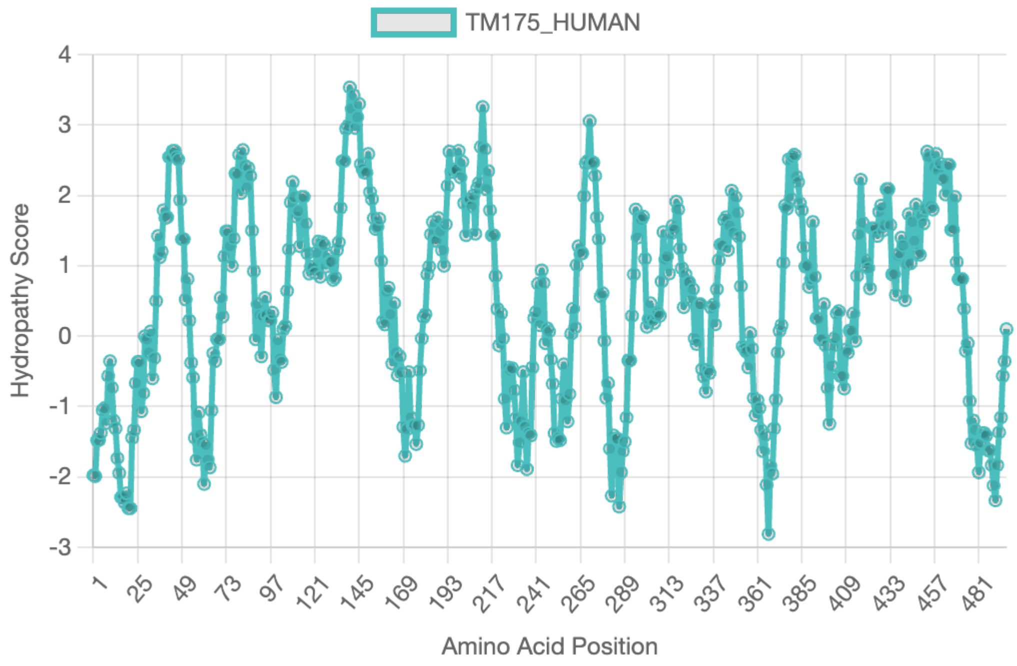

Kyte-Doolittle hydropathy plot for the sequence "TM175_HUMAN". Positive values indicate hydrophobic regions, while negative values indicate hydrophilic regions.

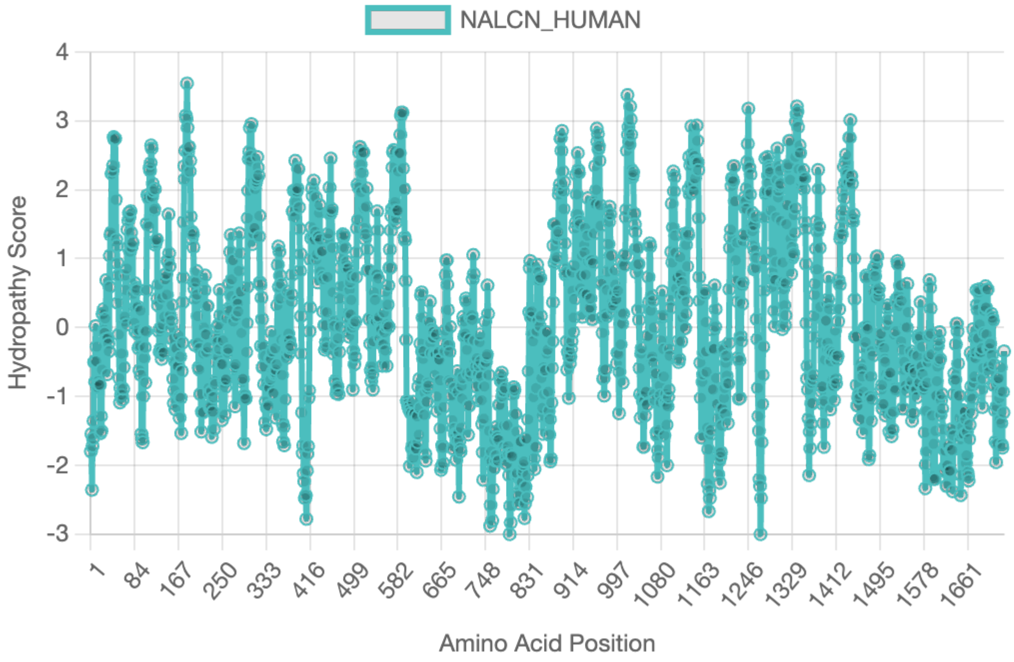

Kyte-Doolittle hydropathy plot for the sequence "NALCN_HUMAN". Positive values indicate hydrophobic regions, while negative values indicate hydrophilic regions.

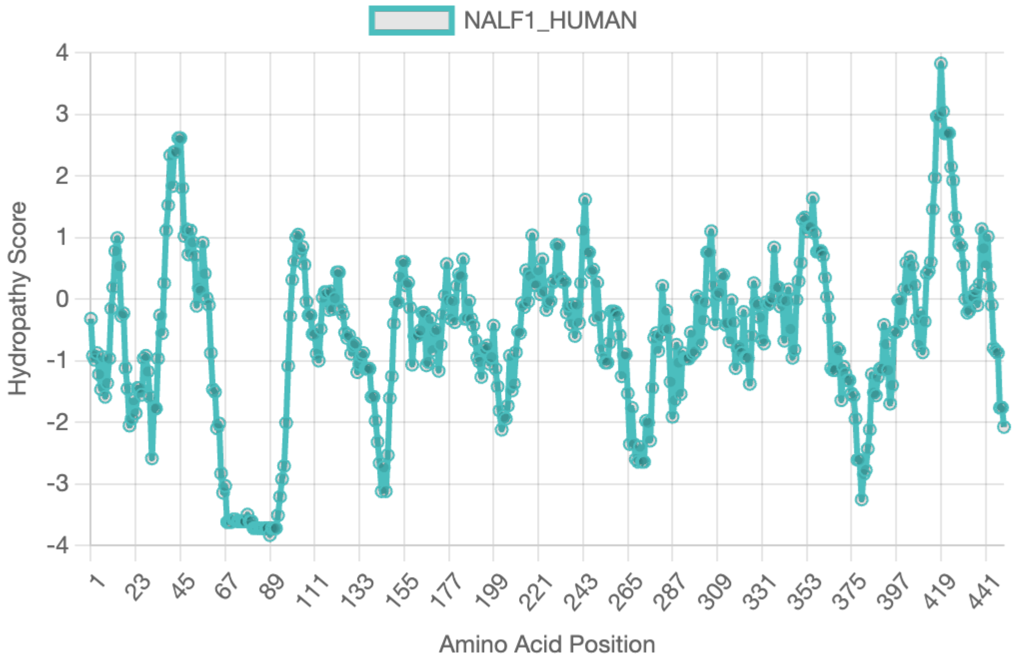

Kyte-Doolittle hydropathy plot for the sequence "NALF1_HUMAN". Positive values indicate hydrophobic regions, while negative values indicate hydrophilic regions.

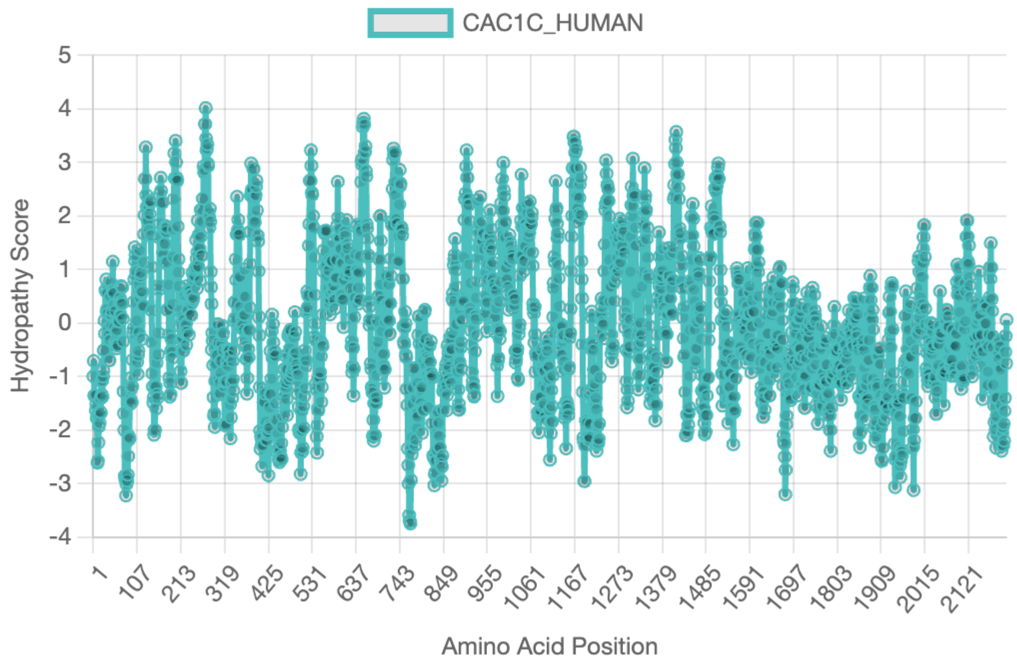

Kyte-Doolittle hydropathy plot for the sequence "CAC1C_HUMAN". Positive values indicate hydrophobic regions, while negative values indicate hydrophilic regions.

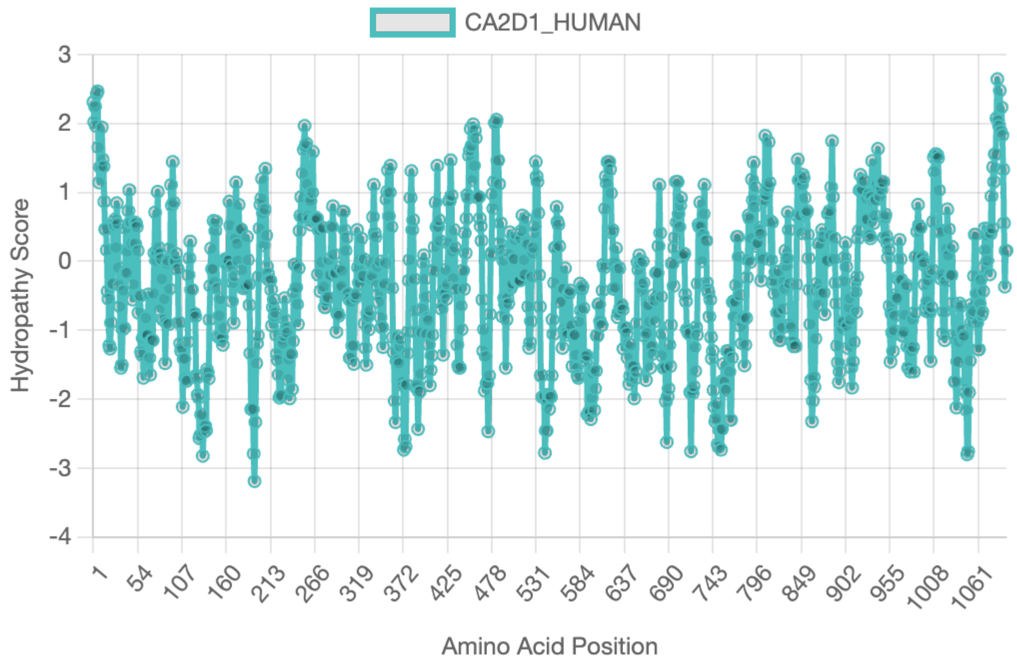

Kyte-Doolittle hydropathy plot for the sequence "CA2D1_HUMAN". Positive values indicate hydrophobic regions, while negative values indicate hydrophilic regions.

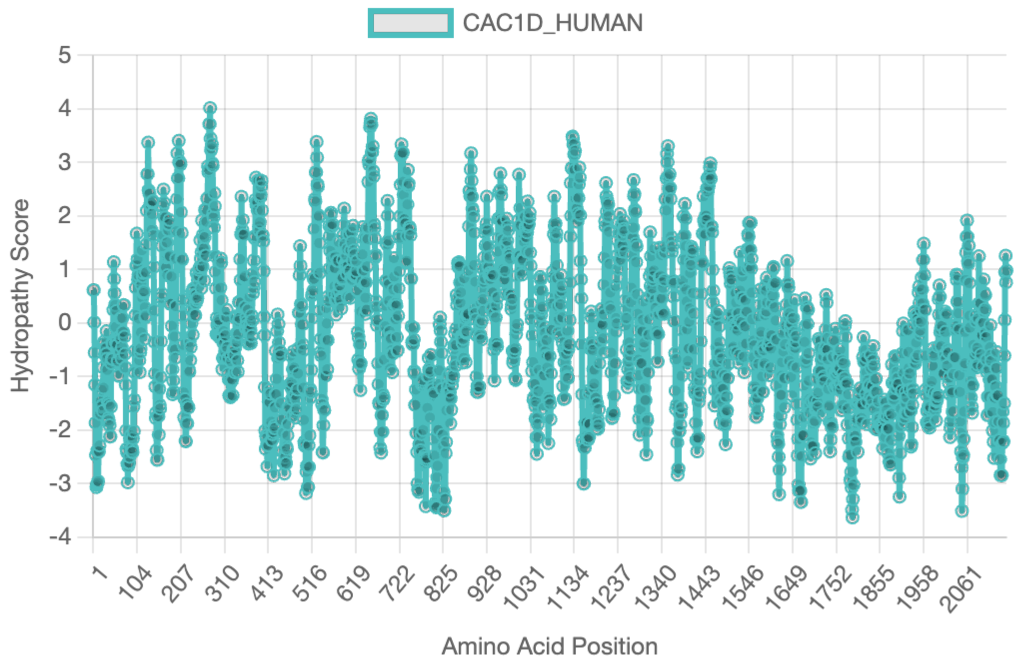

Kyte-Doolittle hydropathy plot for the sequence "CAC1D_HUMAN". Positive values indicate hydrophobic regions, while negative values indicate hydrophilic regions.

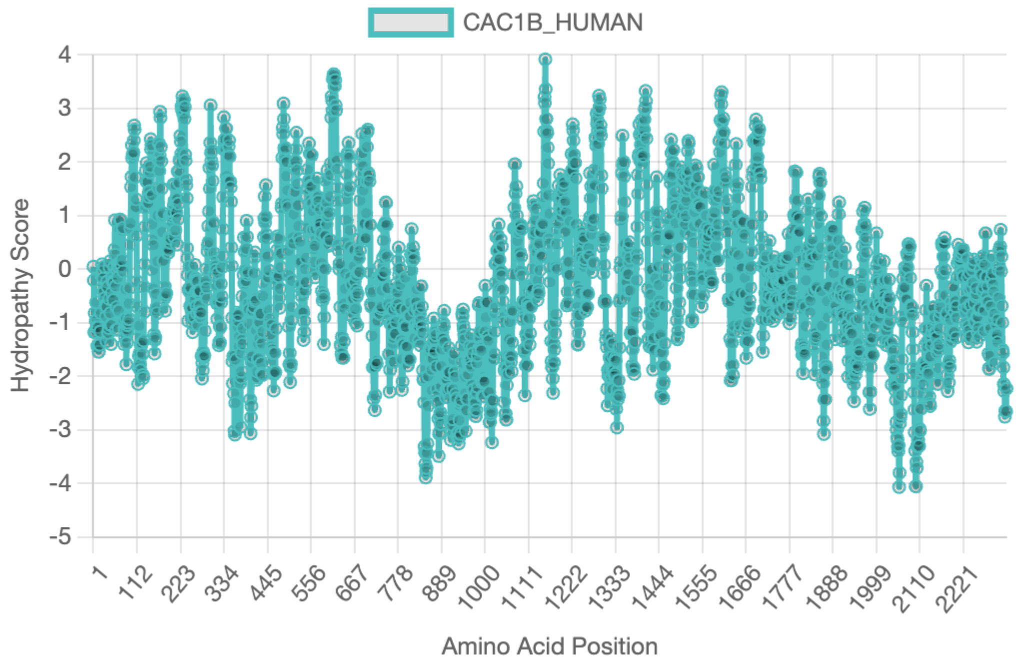

Kyte-Doolittle hydropathy plot for the sequence "CAC1B_HUMAN". Positive values indicate hydrophobic regions, while negative values indicate hydrophilic regions.

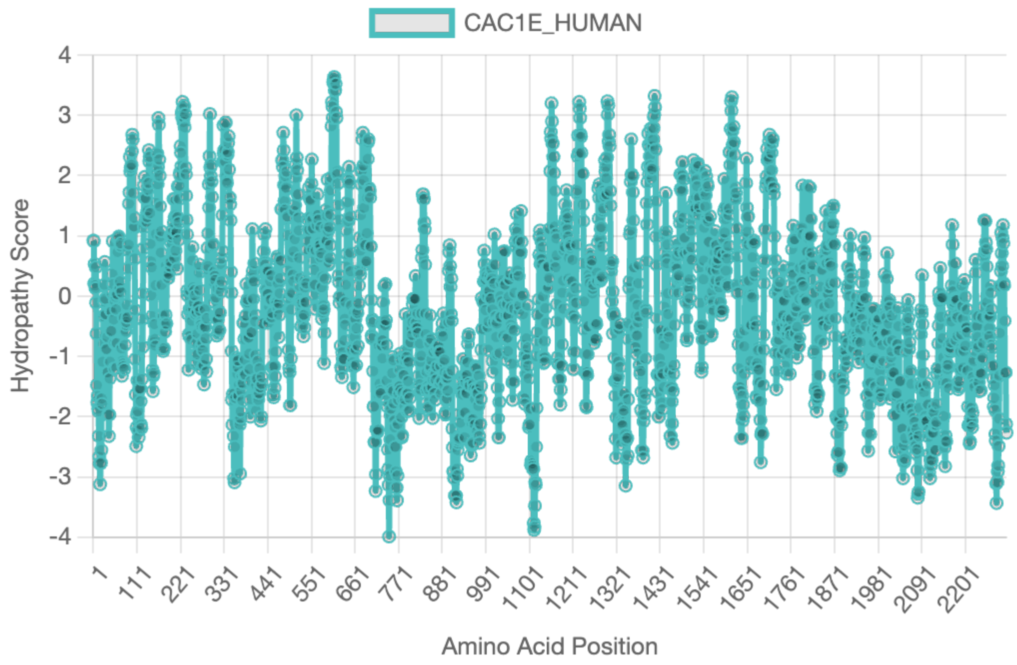

Kyte-Doolittle hydropathy plot for the sequence "CAC1E_HUMAN". Positive values indicate hydrophobic regions, while negative values indicate hydrophilic regions.

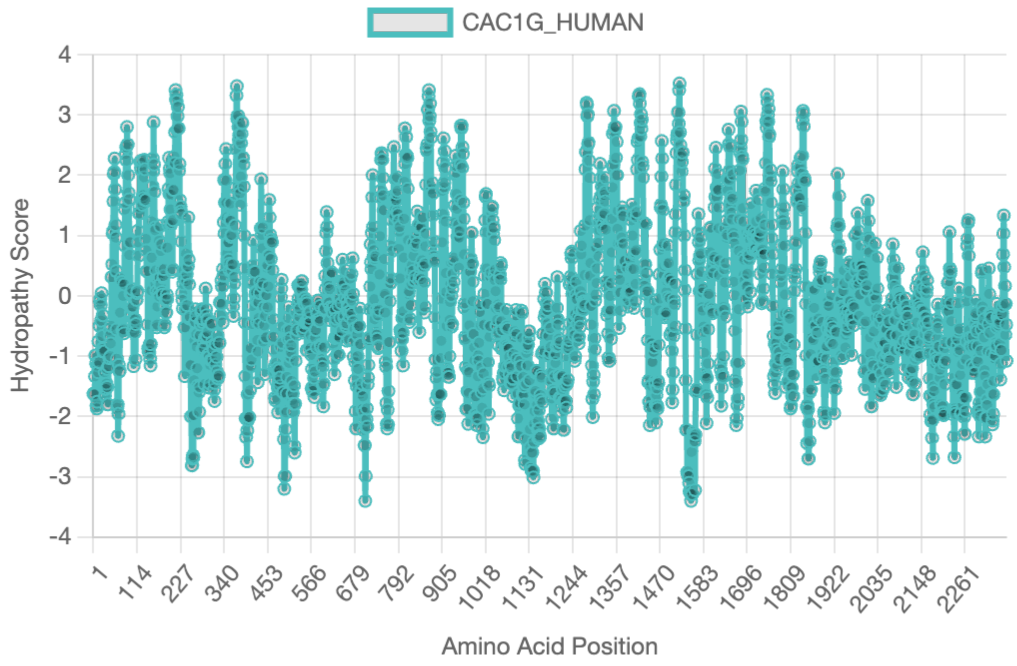

Kyte-Doolittle hydropathy plot for the sequence "CAC1G_HUMAN". Positive values indicate hydrophobic regions, while negative values indicate hydrophilic regions.

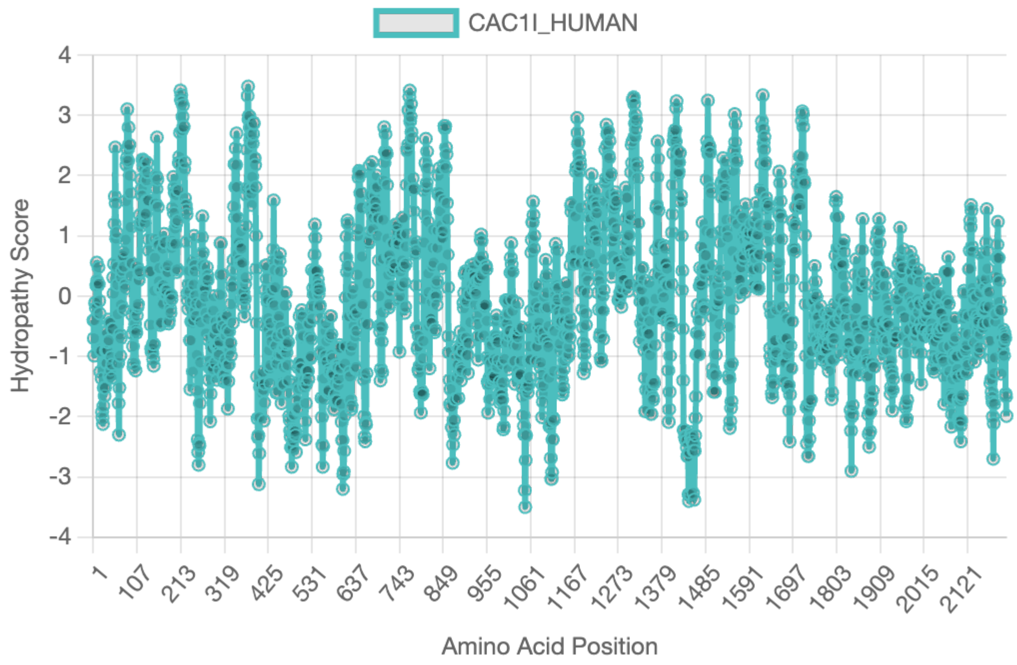

Kyte-Doolittle hydropathy plot for the sequence "CAC1I_HUMAN". Positive values indicate hydrophobic regions, while negative values indicate hydrophilic regions.

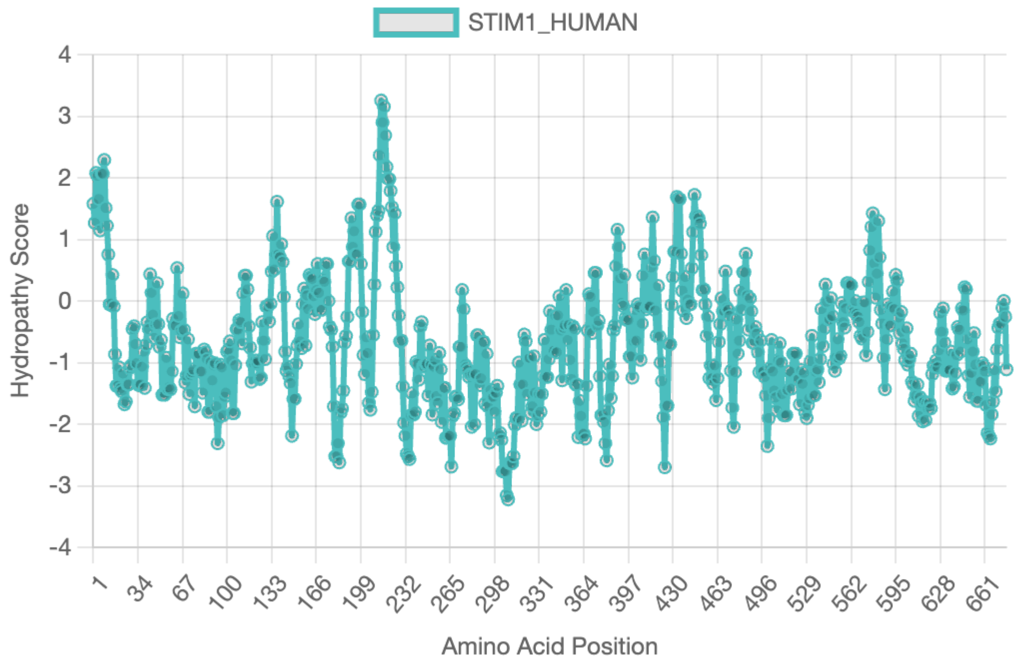

Kyte-Doolittle hydropathy plot for the sequence "STIM1_HUMAN". Positive values indicate hydrophobic regions, while negative values indicate hydrophilic regions.

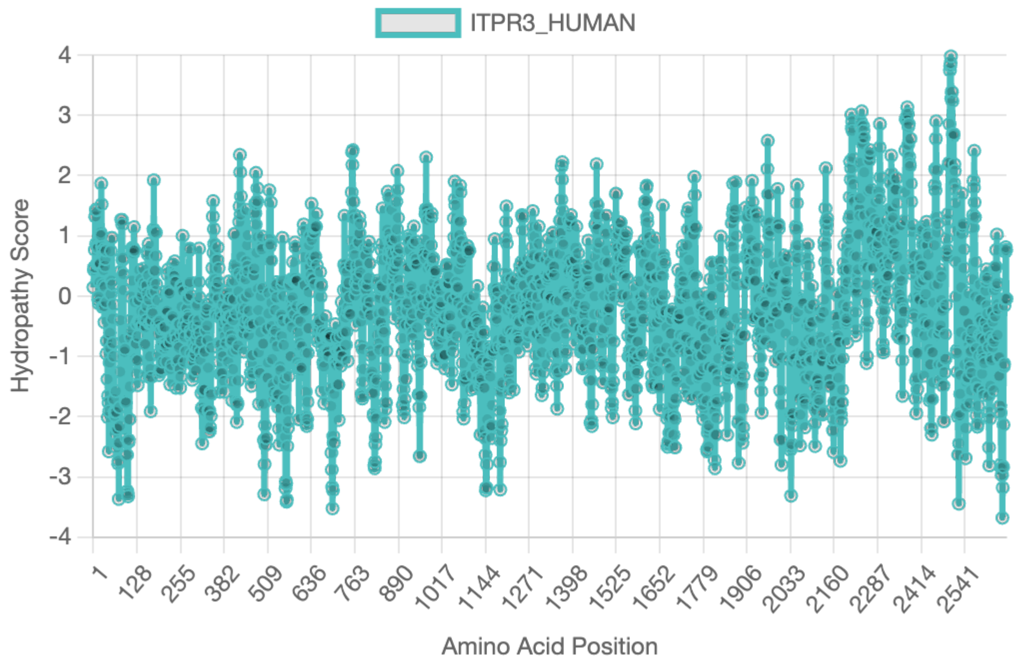

Kyte-Doolittle hydropathy plot for the sequence "ITPR3_HUMAN". Positive values indicate hydrophobic regions, while negative values indicate hydrophilic regions.

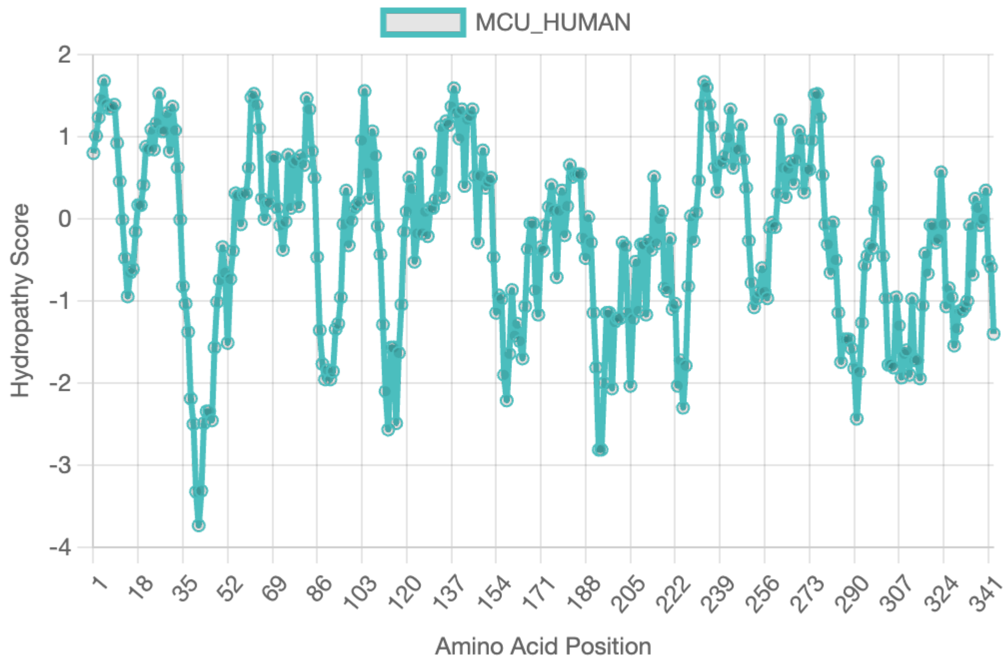

Kyte-Doolittle hydropathy plot for the sequence "MCU_HUMAN". Positive values indicate hydrophobic regions, while negative values indicate hydrophilic regions.

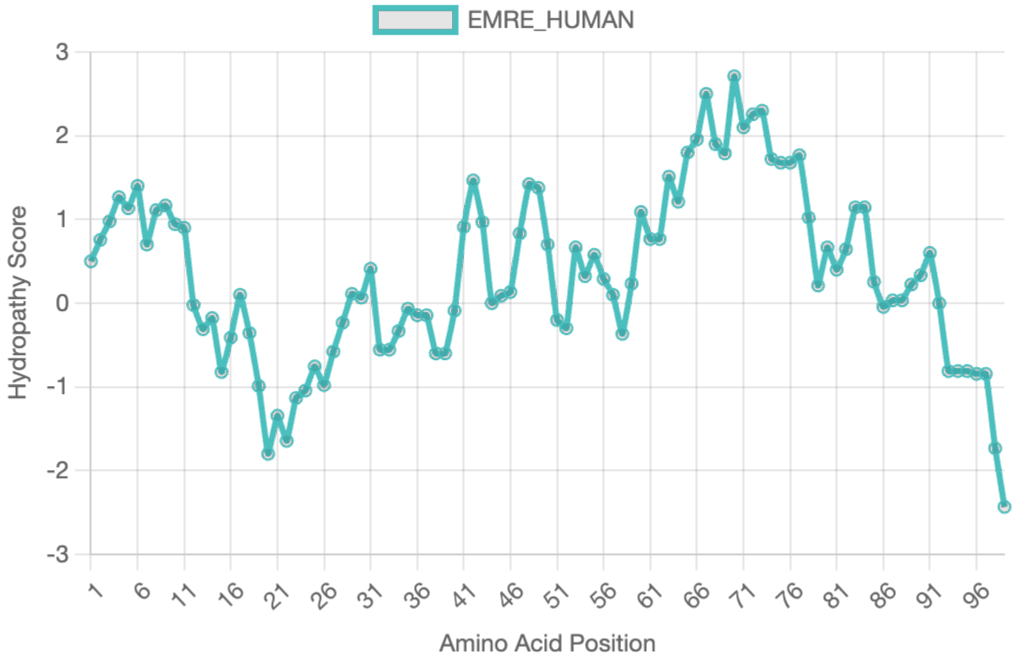

Kyte-Doolittle hydropathy plot for the sequence "EMRE_HUMAN". Positive values indicate hydrophobic regions, while negative values indicate hydrophilic regions.

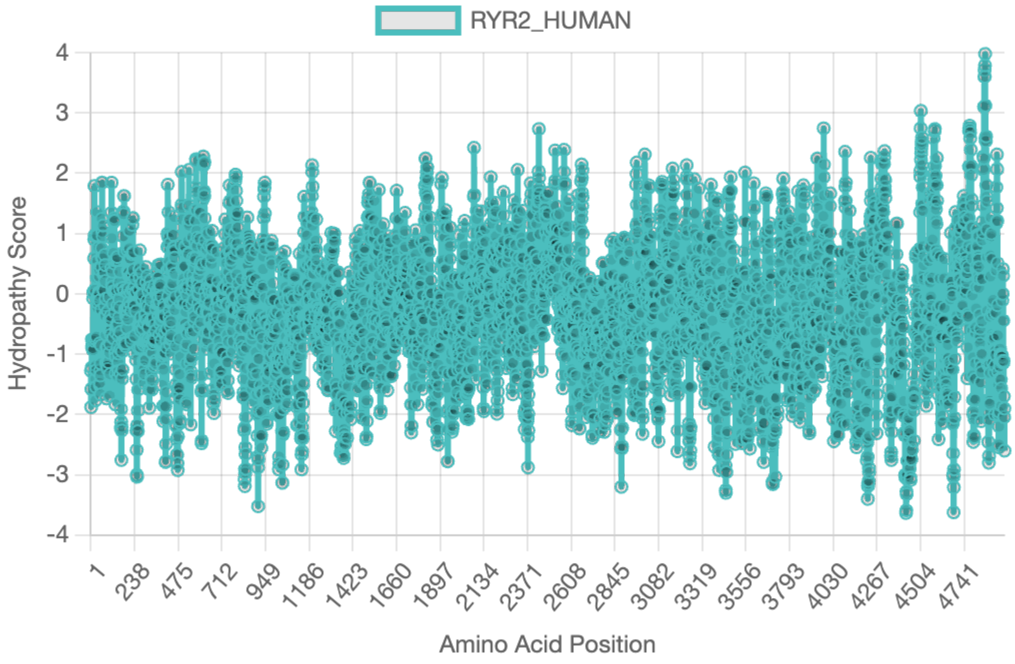

Kyte-Doolittle hydropathy plot for the sequence "RYR2_HUMAN". Positive values indicate hydrophobic regions, while negative values indicate hydrophilic regions.

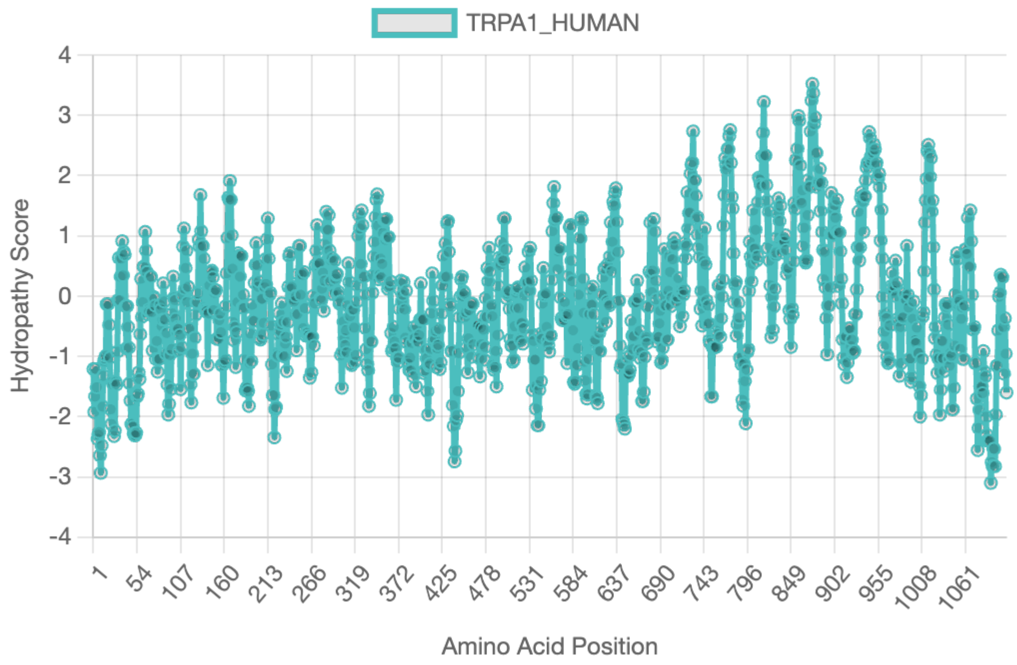

Kyte-Doolittle hydropathy plot for the sequence "TRPA1_HUMAN". Positive values indicate hydrophobic regions, while negative values indicate hydrophilic regions.

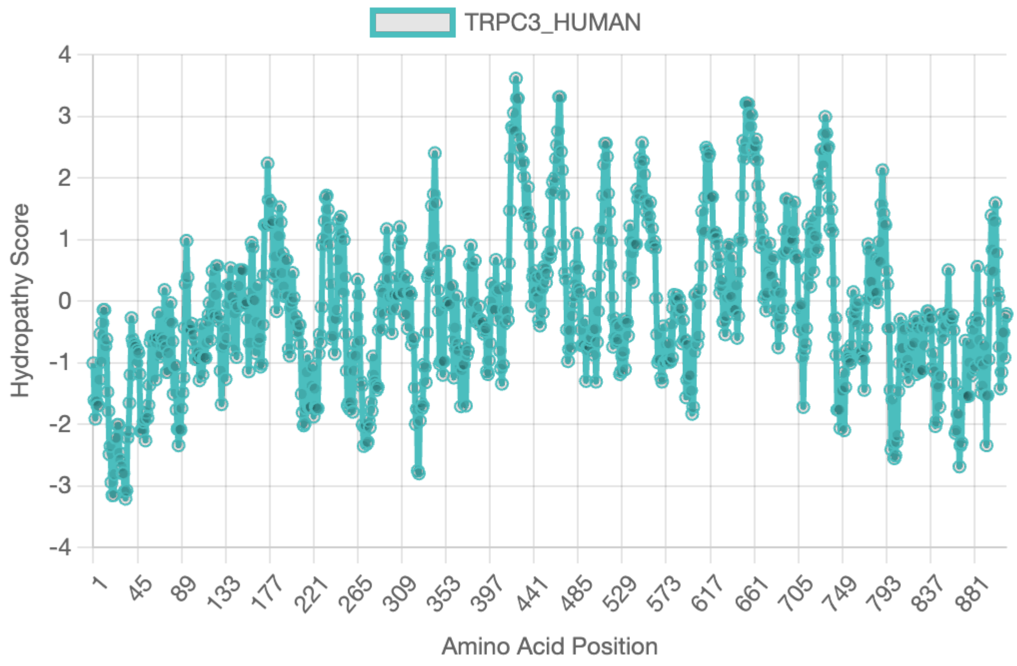

Kyte-Doolittle hydropathy plot for the sequence "TRPC3_HUMAN". Positive values indicate hydrophobic regions, while negative values indicate hydrophilic regions.

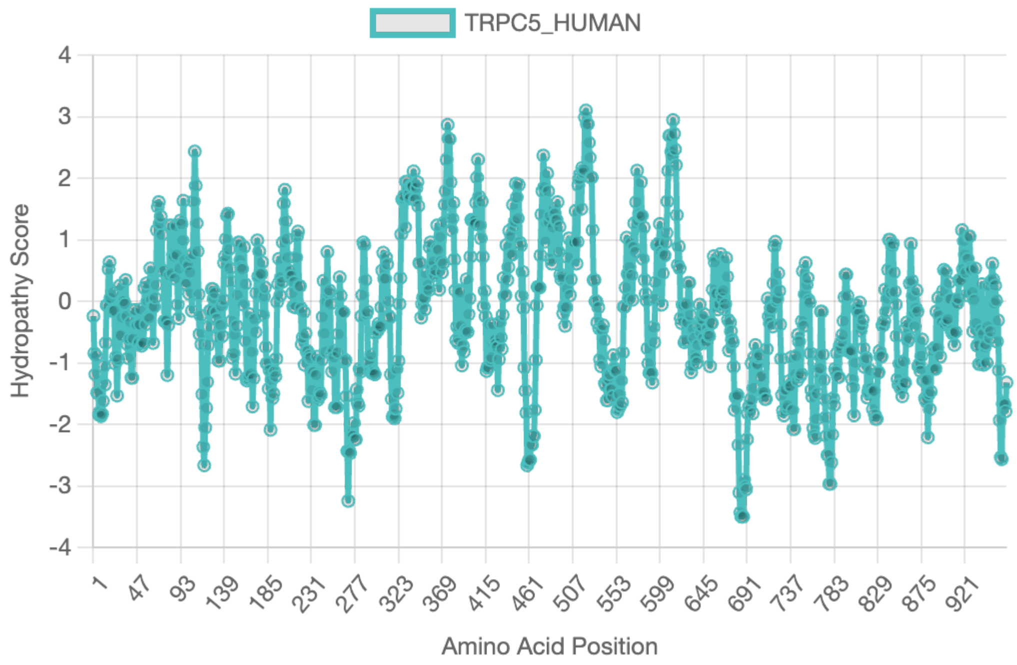

Kyte-Doolittle hydropathy plot for the sequence "TRPC5_HUMAN". Positive values indicate hydrophobic regions, while negative values indicate hydrophilic regions.

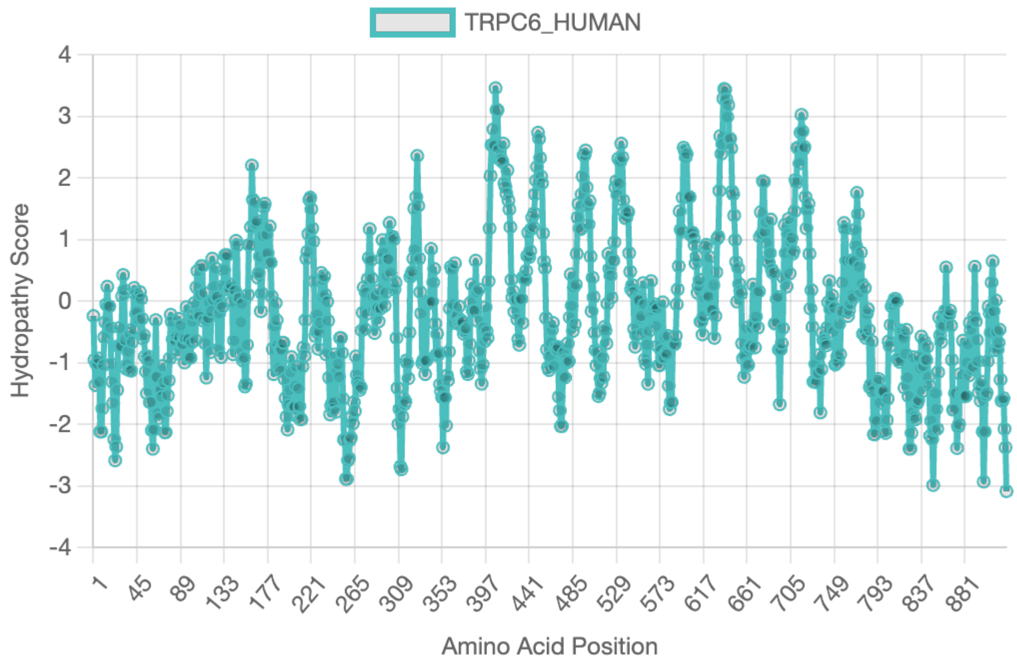

Kyte-Doolittle hydropathy plot for the sequence "TRPC6_HUMAN". Positive values indicate hydrophobic regions, while negative values indicate hydrophilic regions.

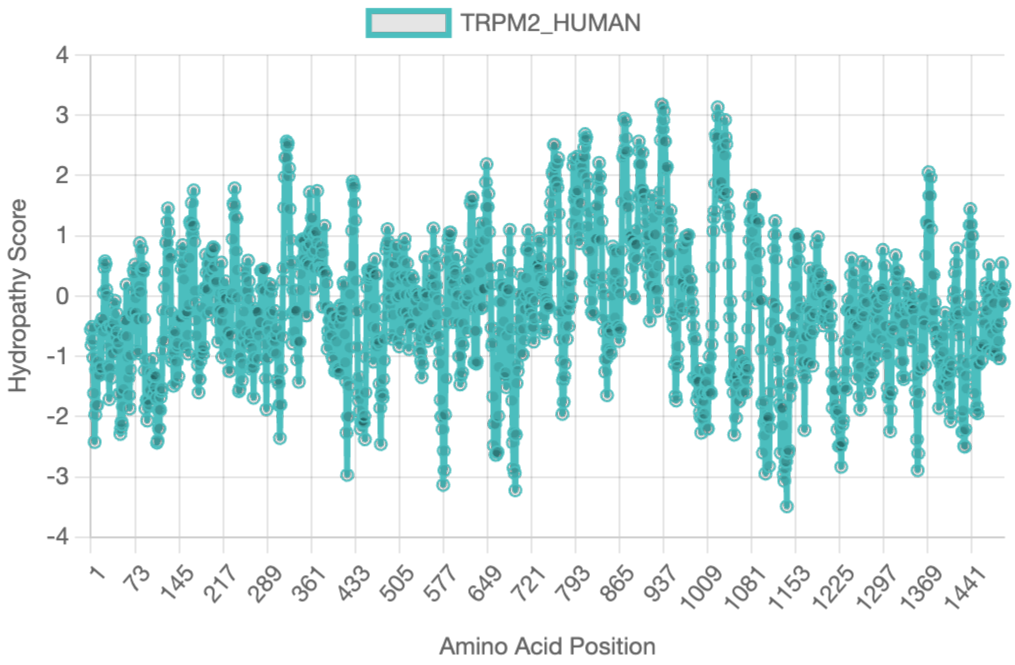

Kyte-Doolittle hydropathy plot for the sequence "TRPM2_HUMAN". Positive values indicate hydrophobic regions, while negative values indicate hydrophilic regions.

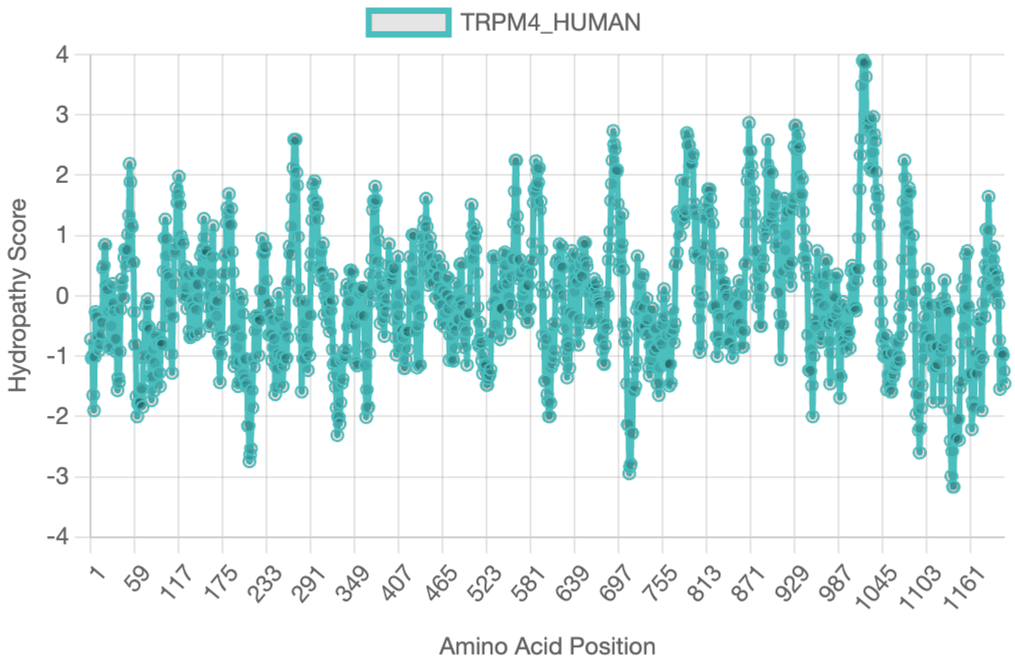

Kyte-Doolittle hydropathy plot for the sequence "TRPM4_HUMAN". Positive values indicate hydrophobic regions, while negative values indicate hydrophilic regions.

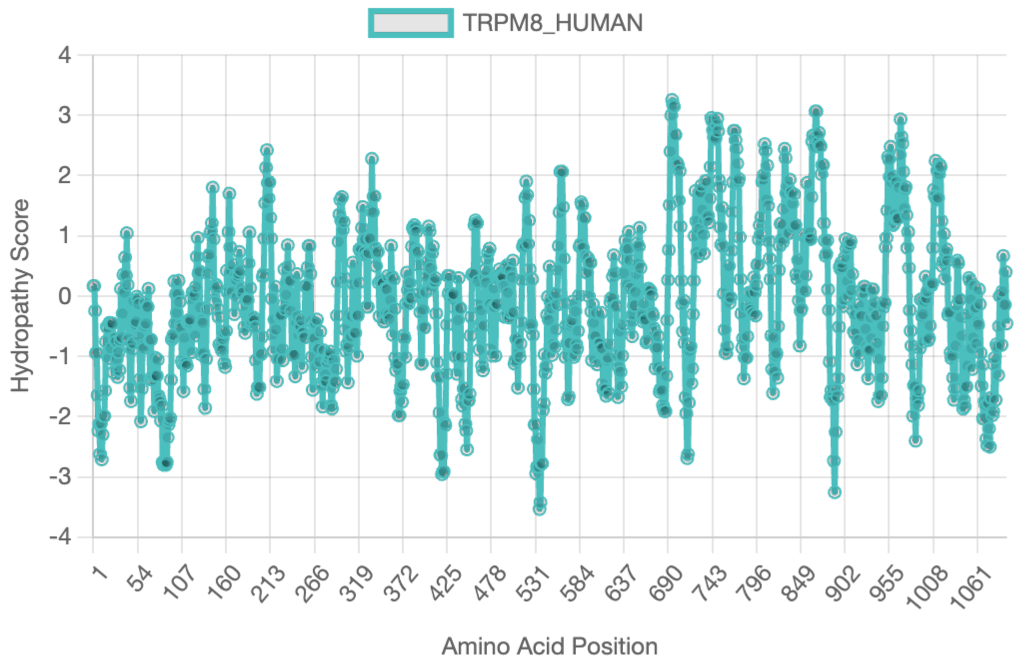

Kyte-Doolittle hydropathy plot for the sequence "TRPM8_HUMAN". Positive values indicate hydrophobic regions, while negative values indicate hydrophilic regions.

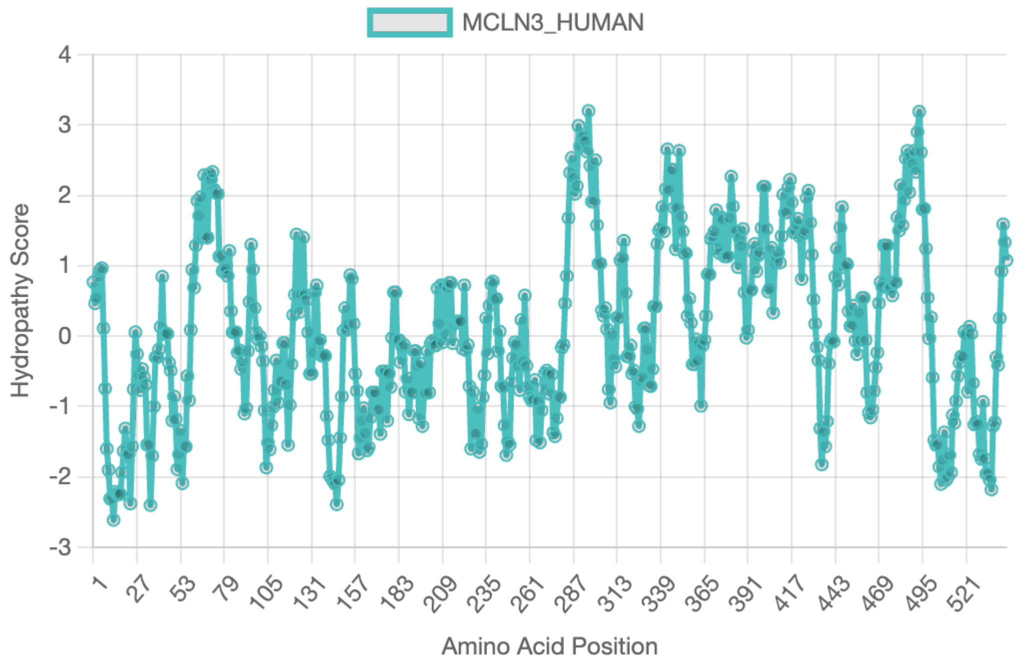

Kyte-Doolittle hydropathy plot for the sequence "MCLN3_HUMAN". Positive values indicate hydrophobic regions, while negative values indicate hydrophilic regions.

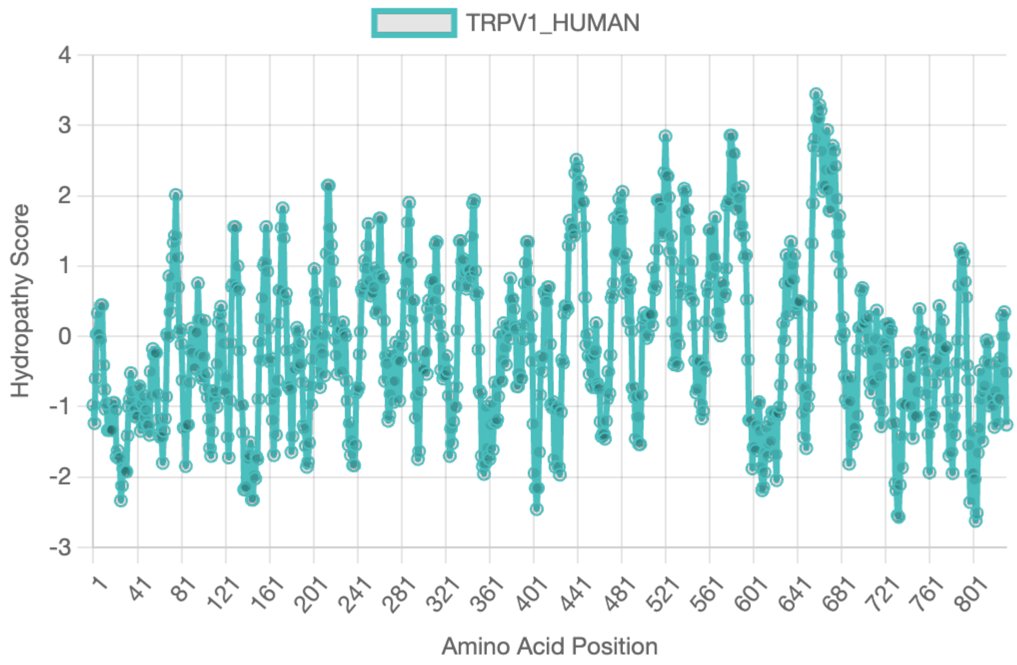

Kyte-Doolittle hydropathy plot for the sequence "TRPV1_HUMAN". Positive values indicate hydrophobic regions, while negative values indicate hydrophilic regions.

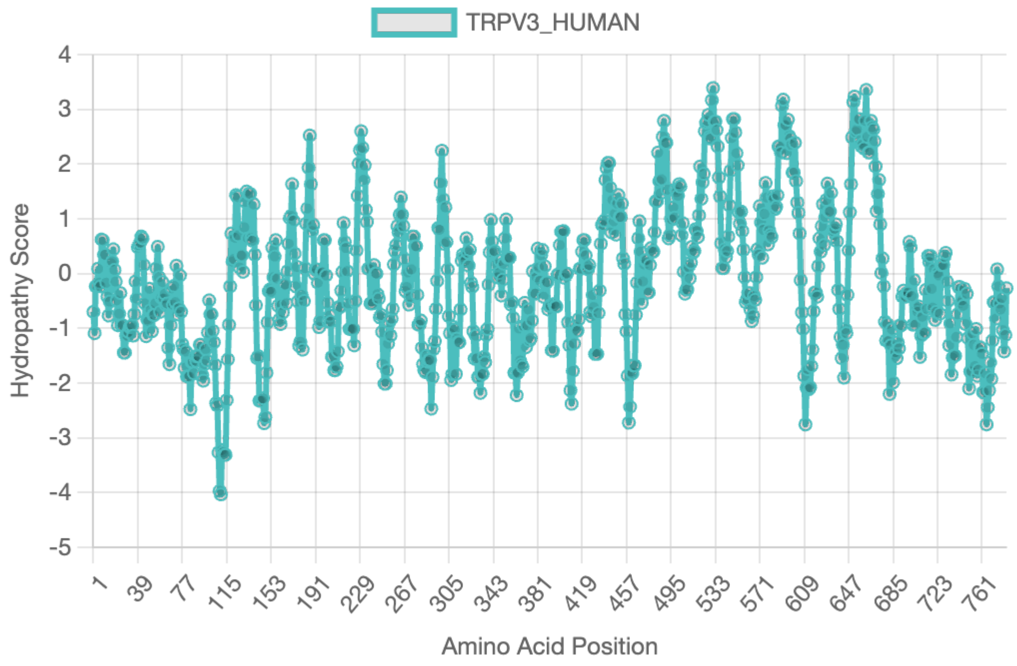

Kyte-Doolittle hydropathy plot for the sequence "TRPV3_HUMAN". Positive values indicate hydrophobic regions, while negative values indicate hydrophilic regions.

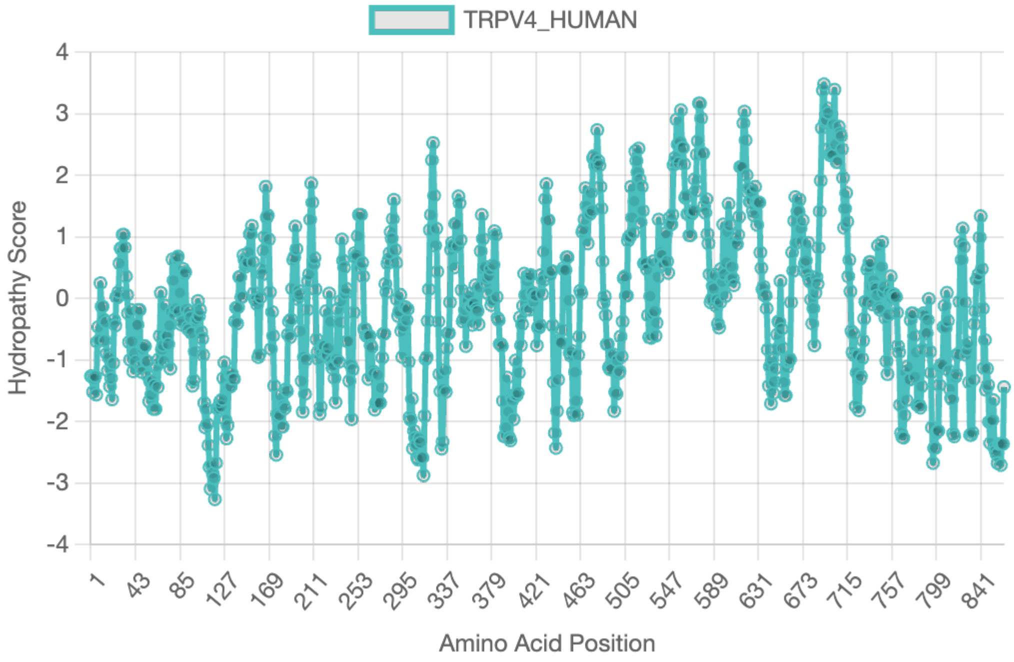

Kyte-Doolittle hydropathy plot for the sequence "TRPV4_HUMAN". Positive values indicate hydrophobic regions, while negative values indicate hydrophilic regions.

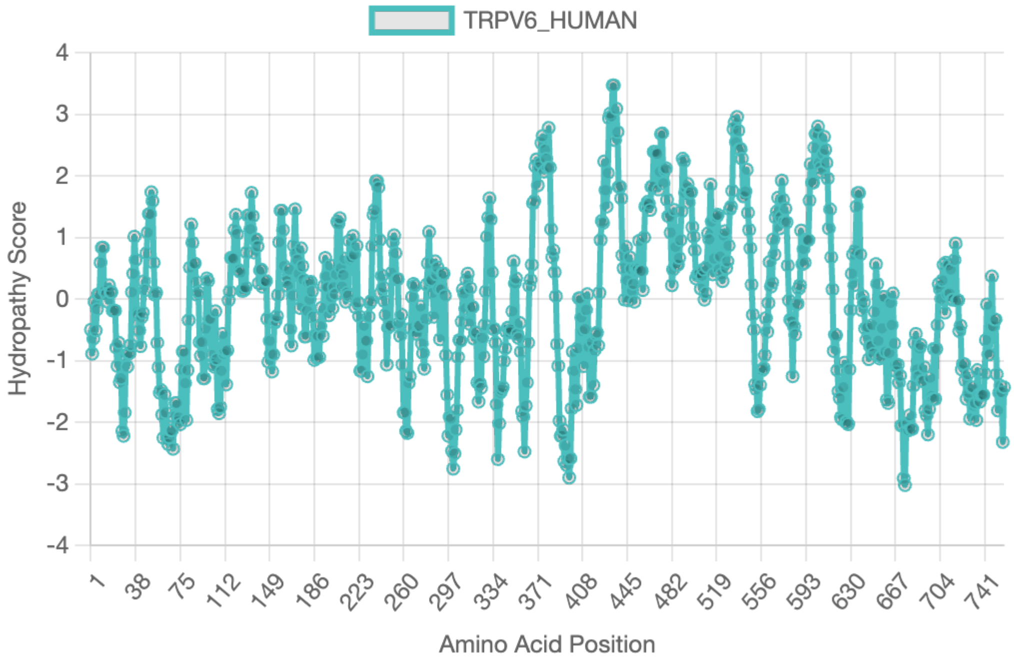

Kyte-Doolittle hydropathy plot for the sequence "TRPV6_HUMAN". Positive values indicate hydrophobic regions, while negative values indicate hydrophilic regions.

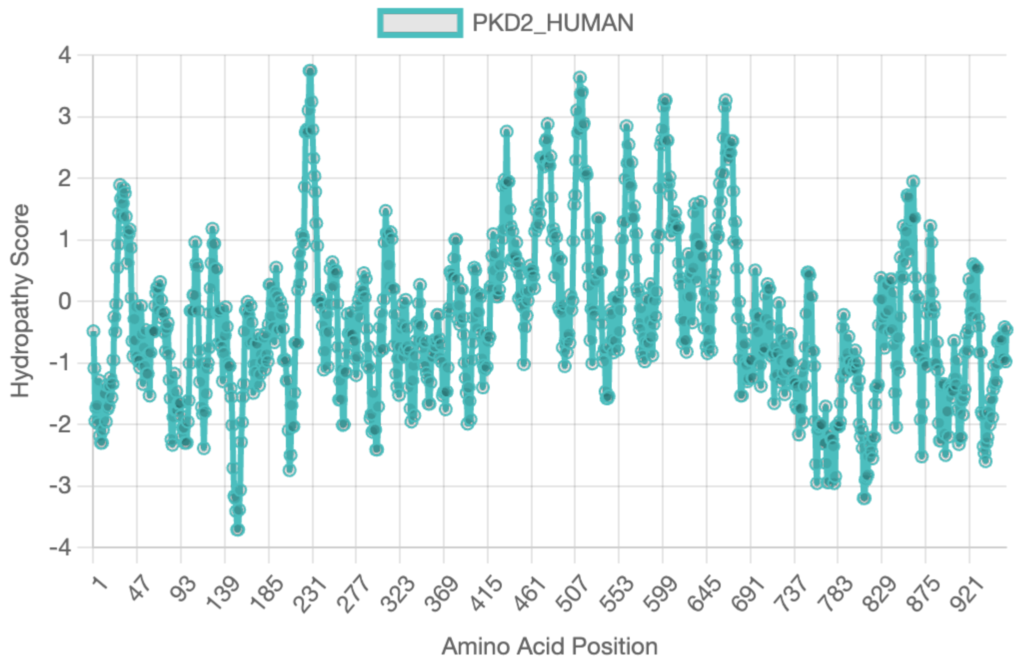

Kyte-Doolittle hydropathy plot for the sequence "PKD2_HUMAN". Positive values indicate hydrophobic regions, while negative values indicate hydrophilic regions.

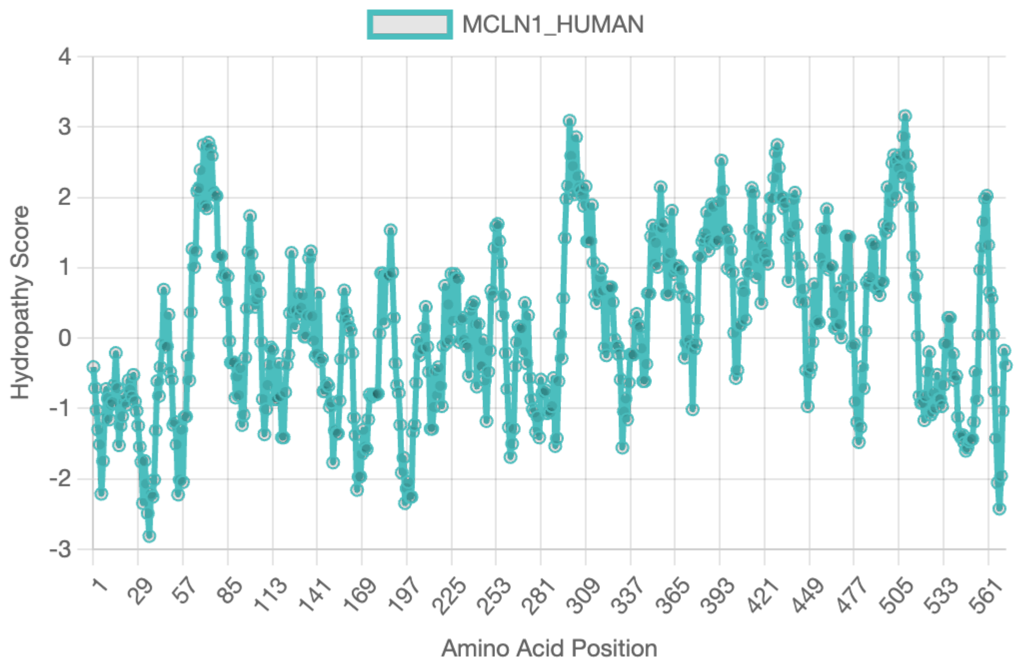

Kyte-Doolittle hydropathy plot for the sequence "MCLN1_HUMAN". Positive values indicate hydrophobic regions, while negative values indicate hydrophilic regions.

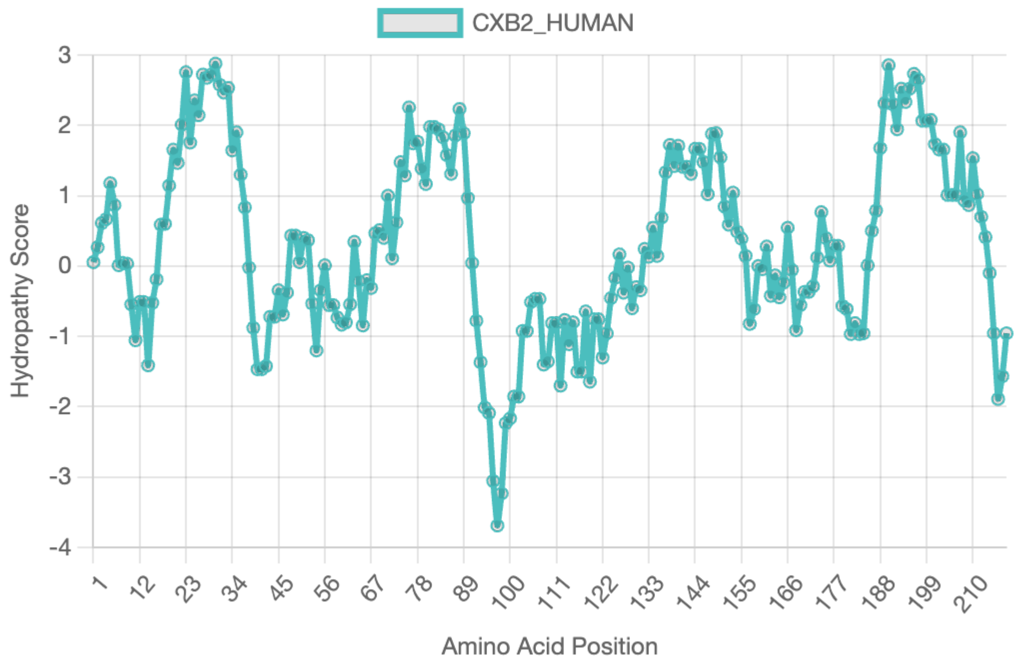

Kyte-Doolittle hydropathy plot for the sequence "CXB2_HUMAN". Positive values indicate hydrophobic regions, while negative values indicate hydrophilic regions.

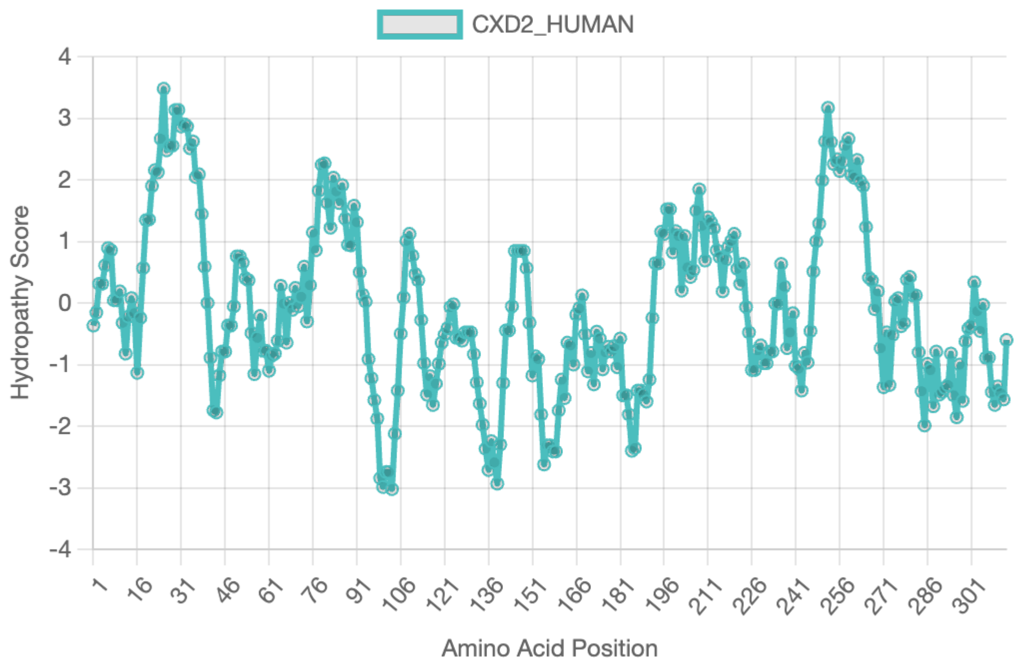

Kyte-Doolittle hydropathy plot for the sequence "CXD2_HUMAN". Positive values indicate hydrophobic regions, while negative values indicate hydrophilic regions.

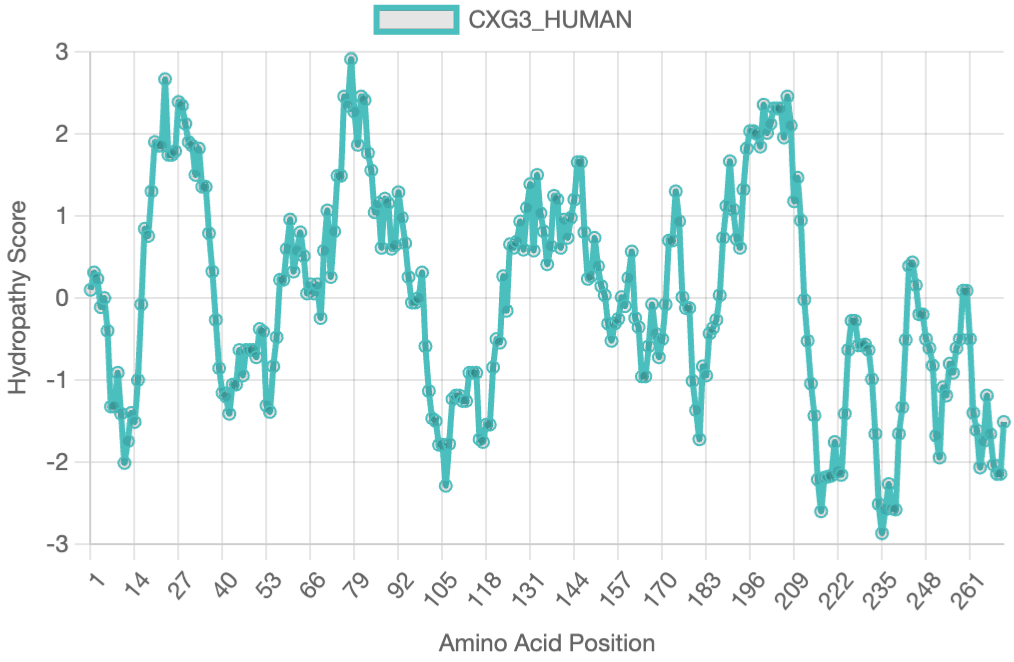

Kyte-Doolittle hydropathy plot for the sequence "CXG3_HUMAN". Positive values indicate hydrophobic regions, while negative values indicate hydrophilic regions.

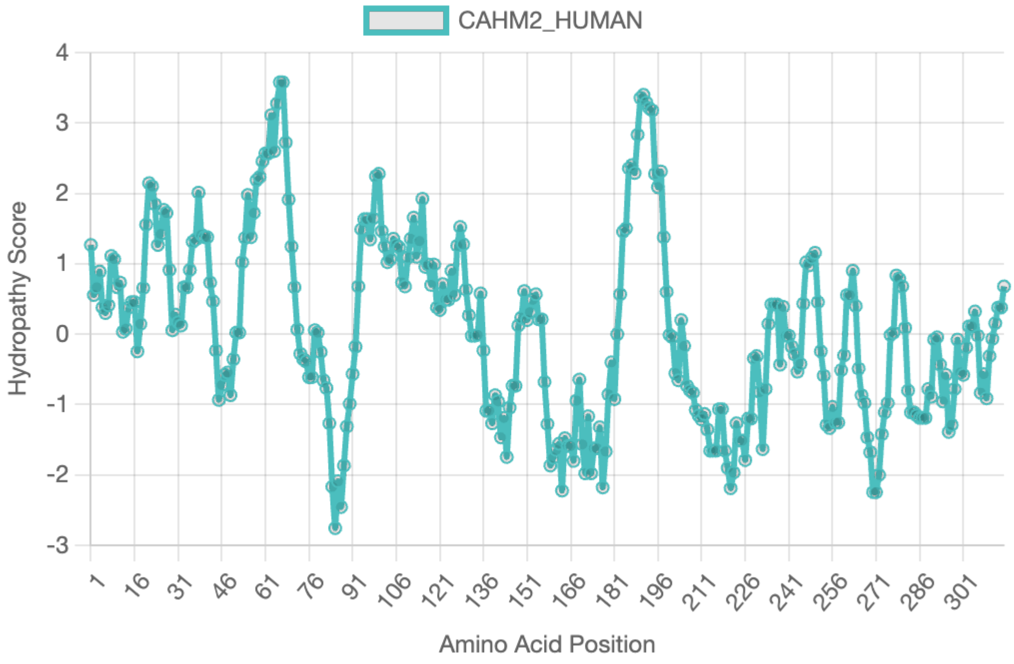

Kyte-Doolittle hydropathy plot for the sequence "CAHM2_HUMAN". Positive values indicate hydrophobic regions, while negative values indicate hydrophilic regions.

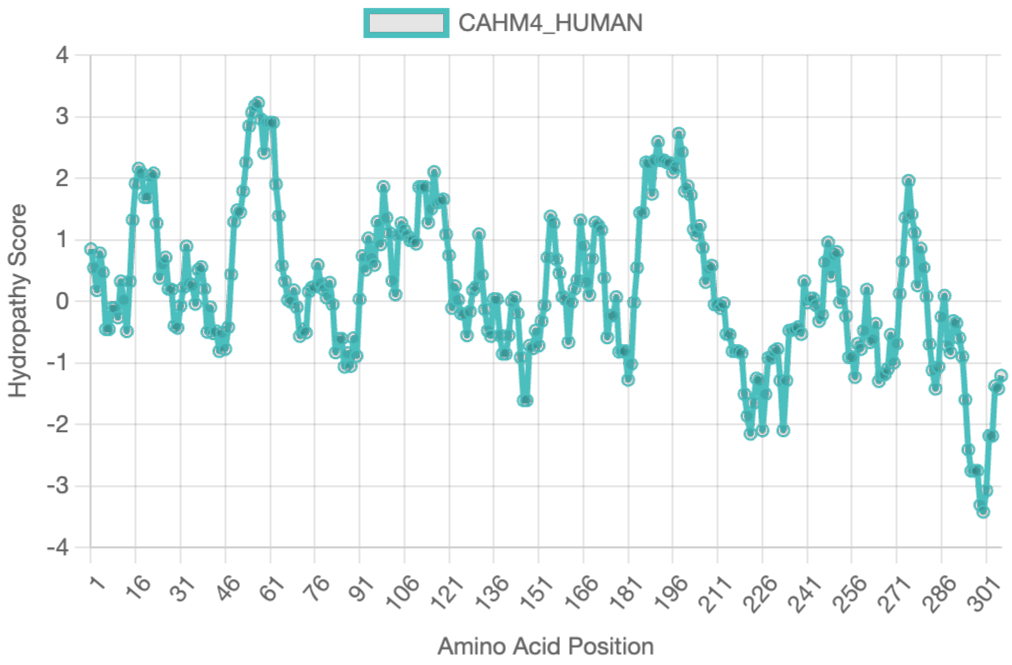

Kyte-Doolittle hydropathy plot for the sequence "CAHM4_HUMAN". Positive values indicate hydrophobic regions, while negative values indicate hydrophilic regions.

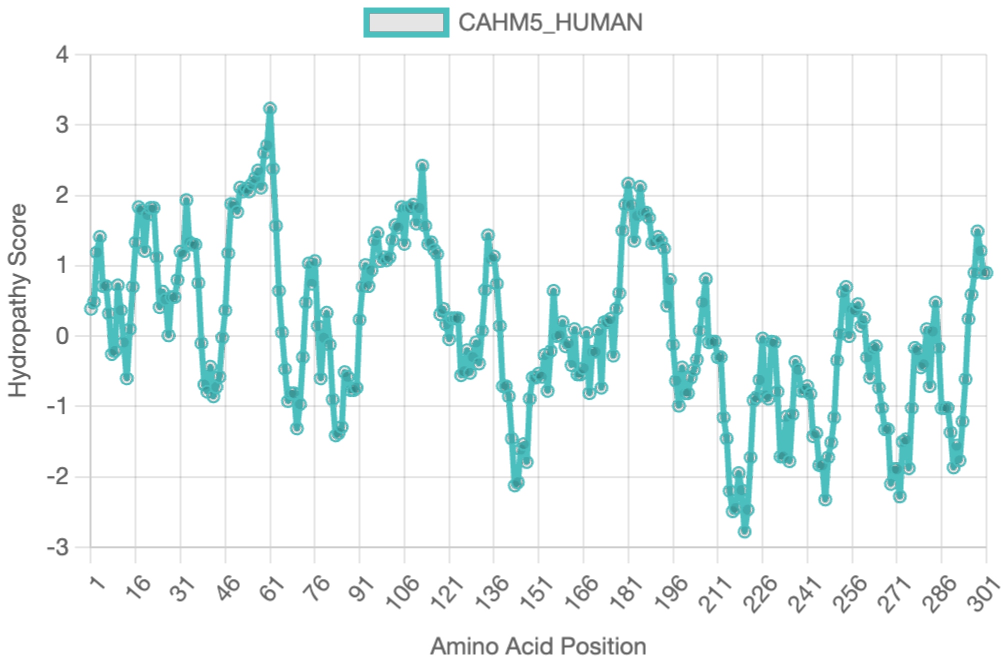

Kyte-Doolittle hydropathy plot for the sequence "CAHM5_HUMAN". Positive values indicate hydrophobic regions, while negative values indicate hydrophilic regions.

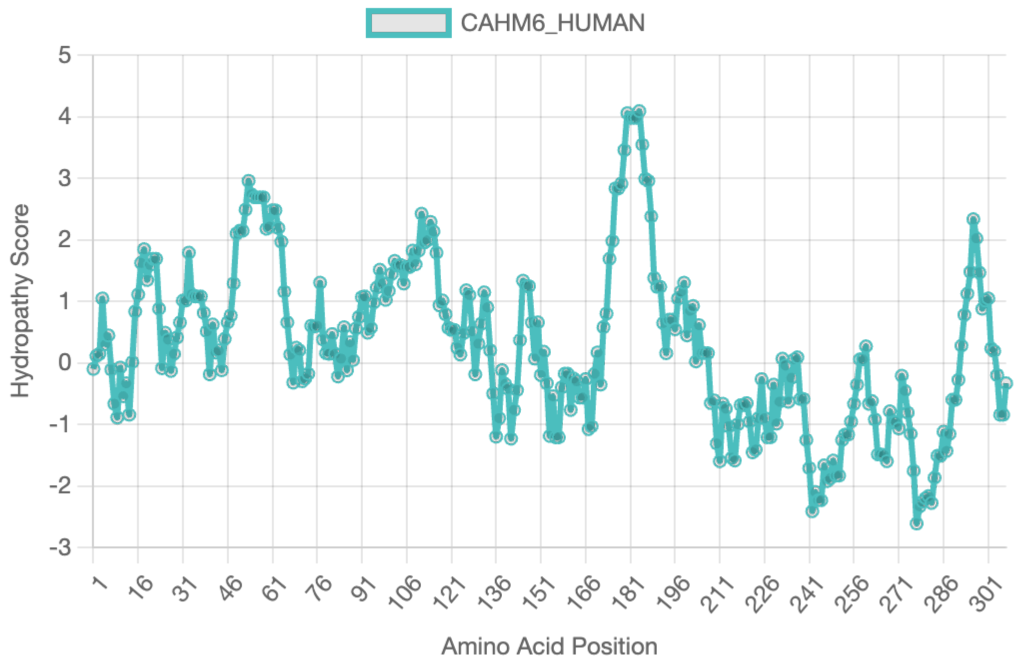

Kyte-Doolittle hydropathy plot for the sequence "CAHM6_HUMAN". Positive values indicate hydrophobic regions, while negative values indicate hydrophilic regions.

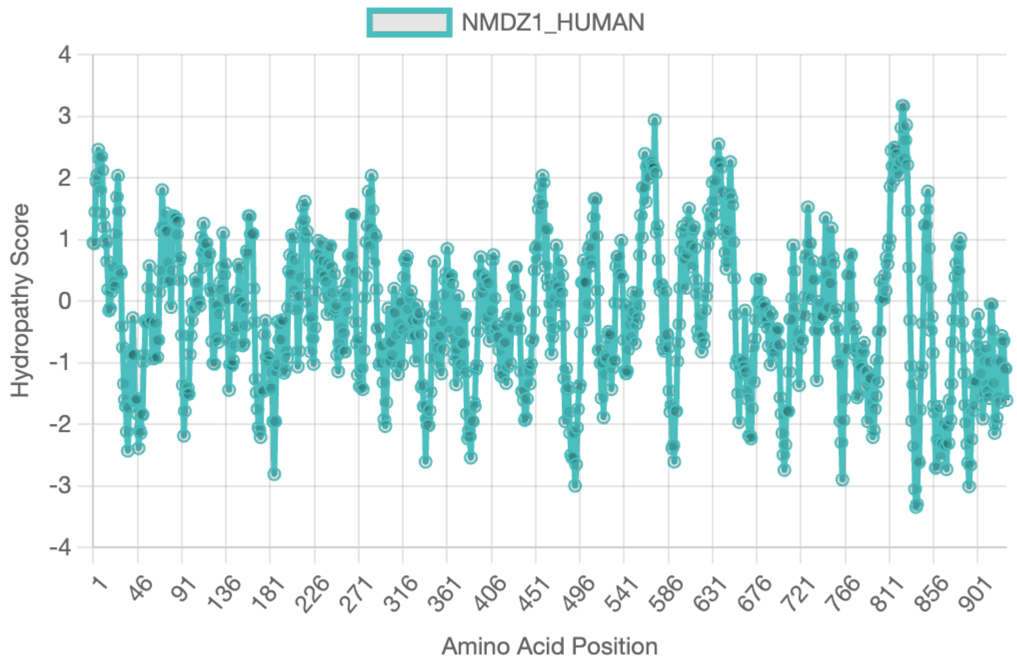

Kyte-Doolittle hydropathy plot for the sequence "NMDZ1_HUMAN". Positive values indicate hydrophobic regions, while negative values indicate hydrophilic regions.

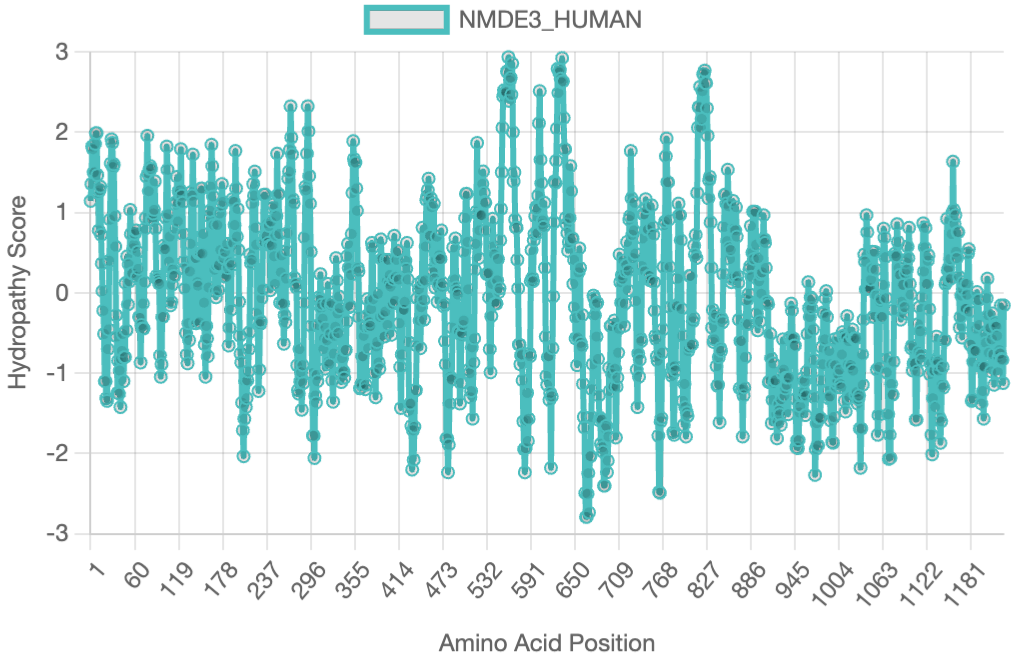

Kyte-Doolittle hydropathy plot for the sequence "NMDE3_HUMAN". Positive values indicate hydrophobic regions, while negative values indicate hydrophilic regions.

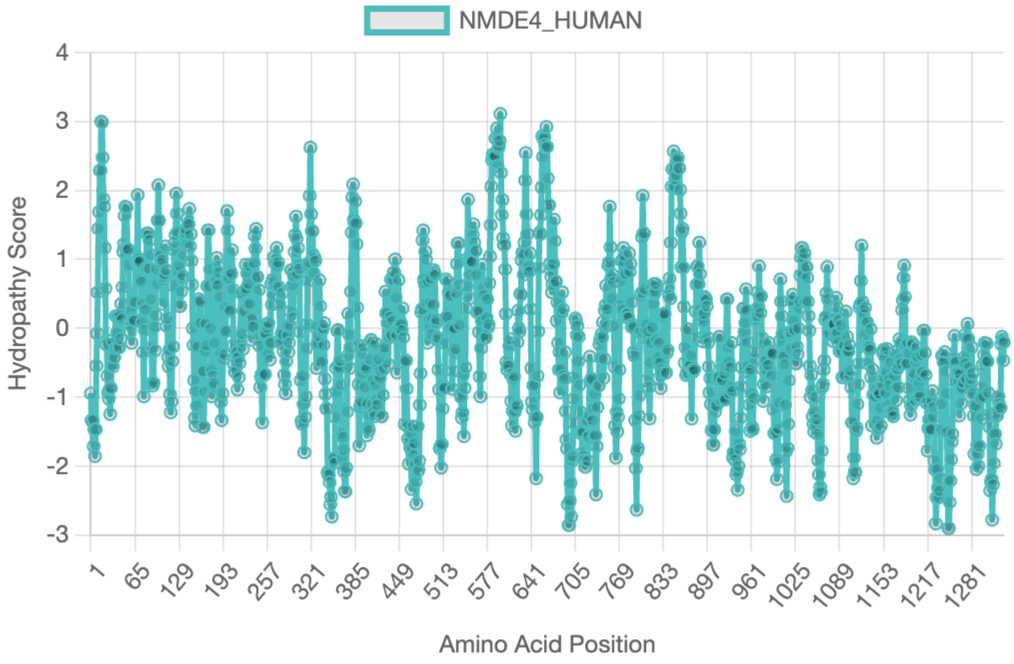

Kyte-Doolittle hydropathy plot for the sequence "NMDE4_HUMAN". Positive values indicate hydrophobic regions, while negative values indicate hydrophilic regions.

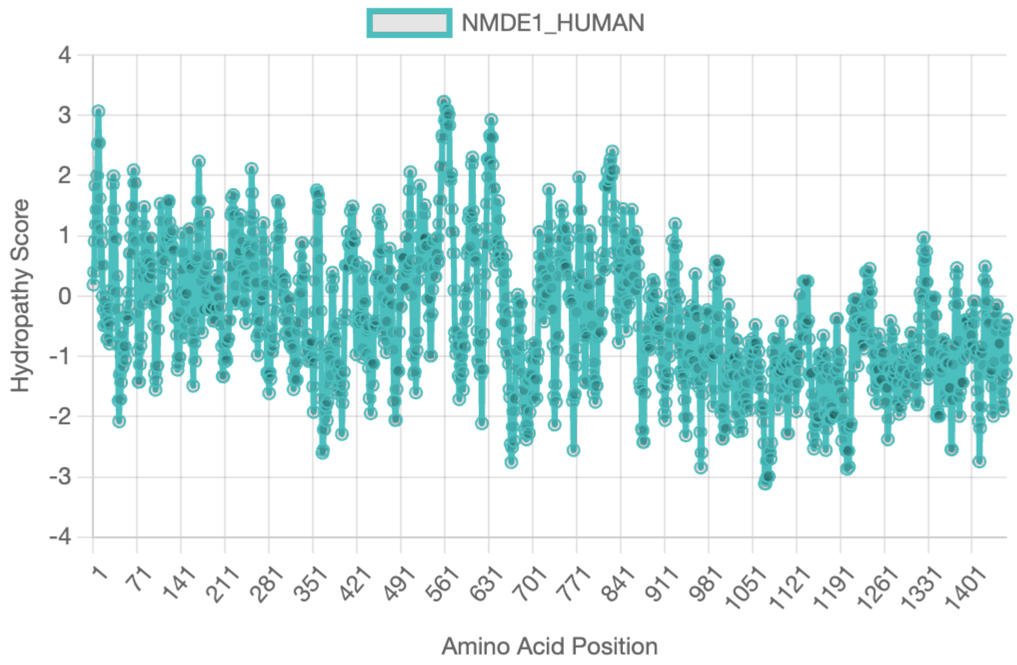

Kyte-Doolittle hydropathy plot for the sequence "NMDE1_HUMAN". Positive values indicate hydrophobic regions, while negative values indicate hydrophilic regions.

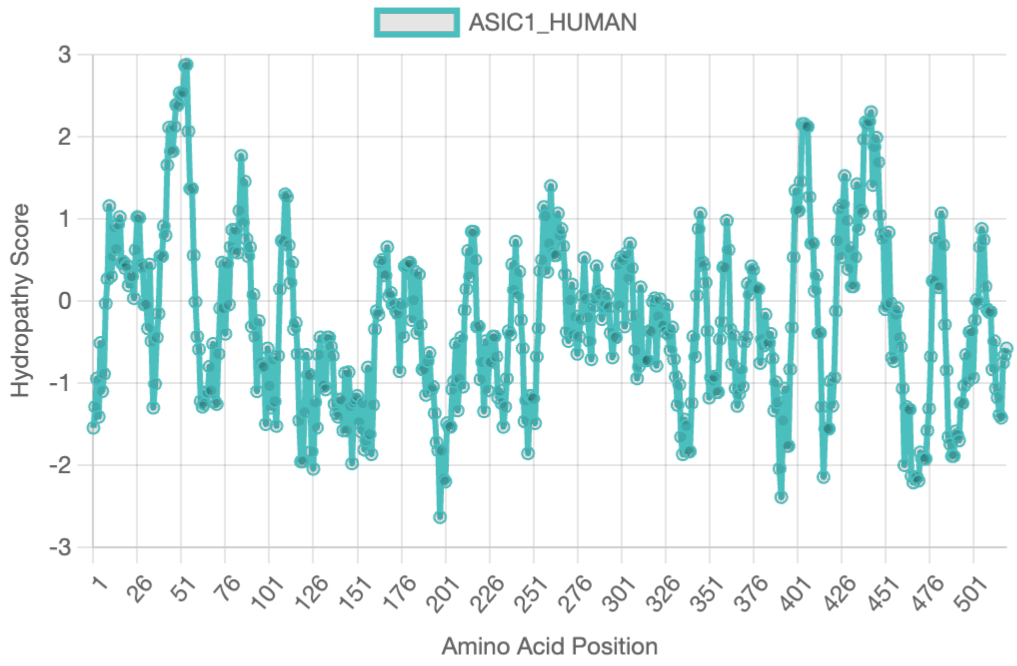

Kyte-Doolittle hydropathy plot for the sequence "ASIC1_HUMAN". Positive values indicate hydrophobic regions, while negative values indicate hydrophilic regions.

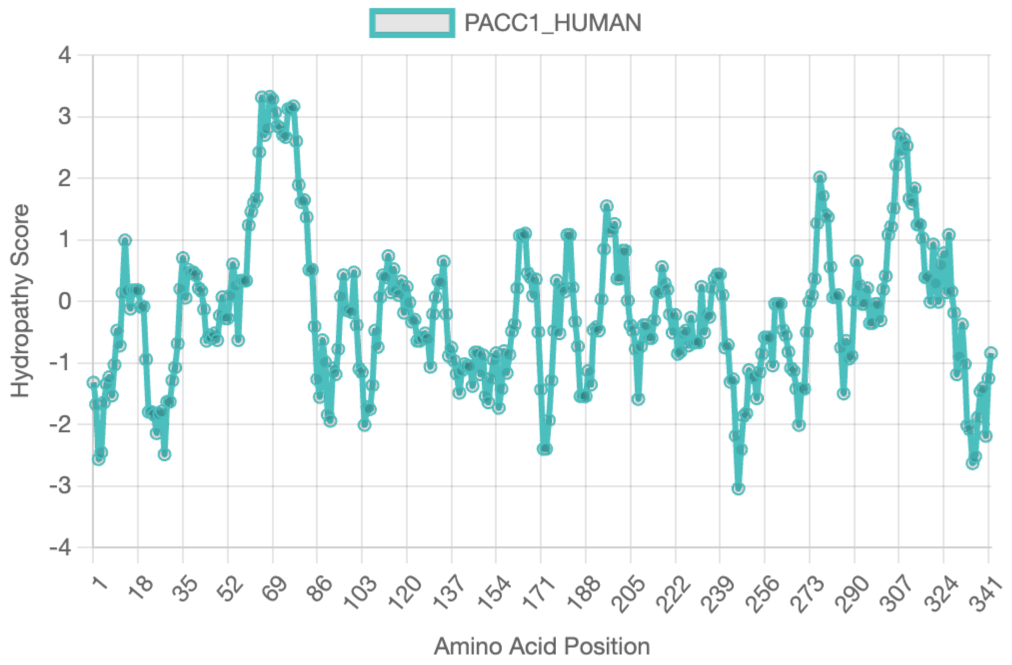

Kyte-Doolittle hydropathy plot for the sequence "PACC1_HUMAN". Positive values indicate hydrophobic regions, while negative values indicate hydrophilic regions.

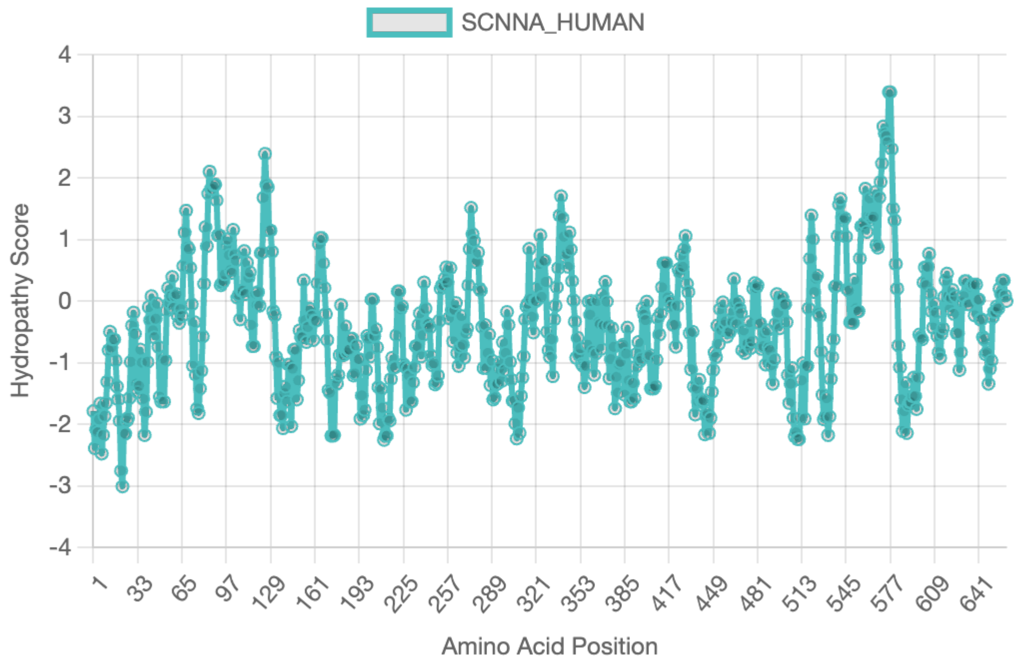

Kyte-Doolittle hydropathy plot for the sequence "SCNNA_HUMAN". Positive values indicate hydrophobic regions, while negative values indicate hydrophilic regions.

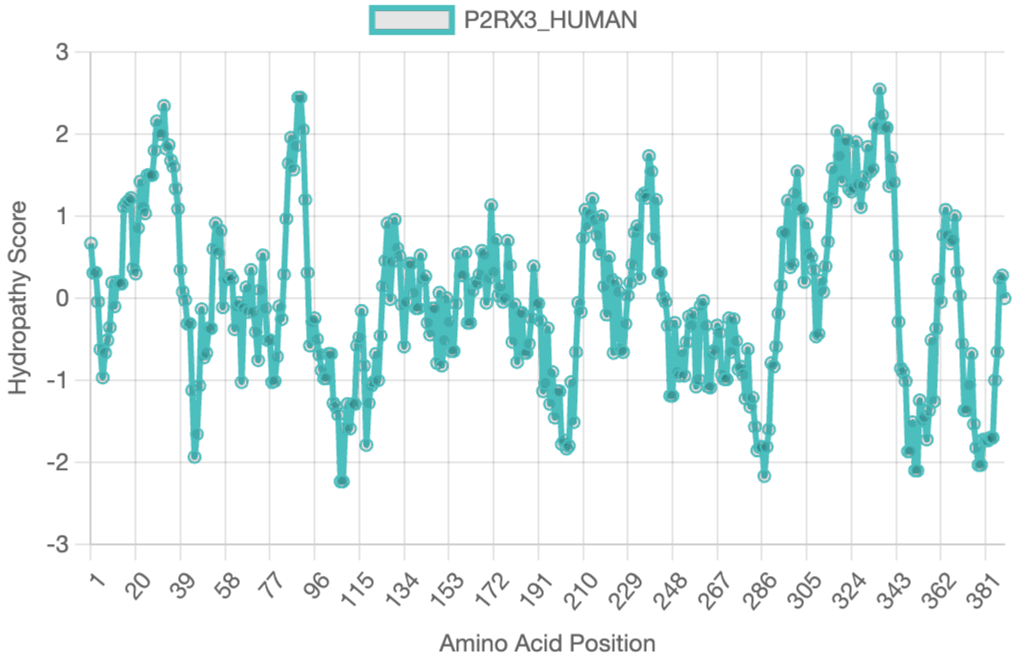

Kyte-Doolittle hydropathy plot for the sequence "P2RX3_HUMAN". Positive values indicate hydrophobic regions, while negative values indicate hydrophilic regions.

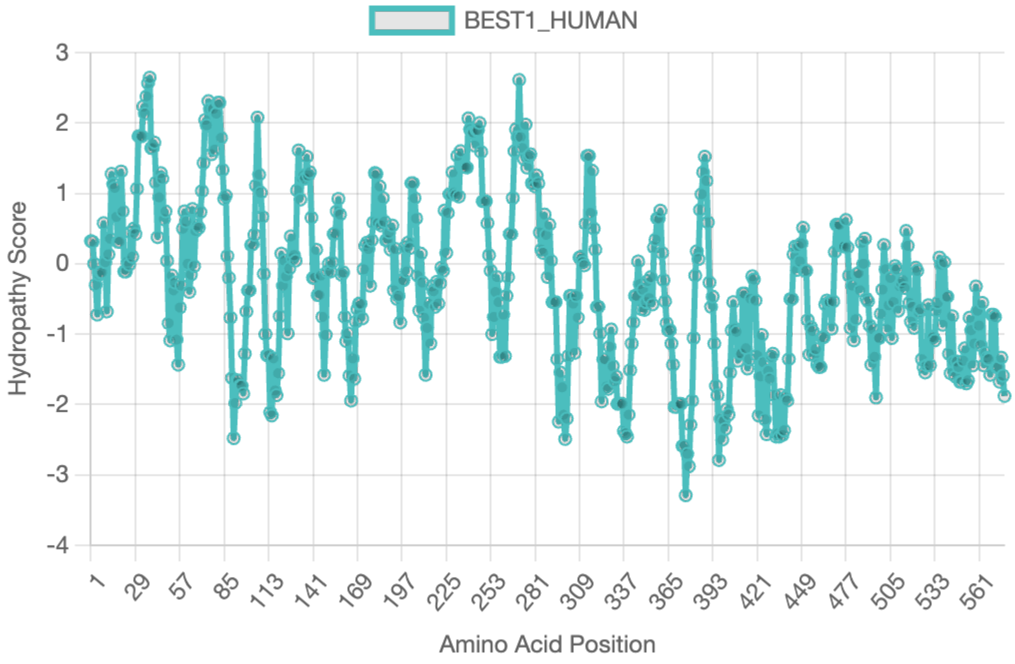

Kyte-Doolittle hydropathy plot for the sequence "BEST1_HUMAN". Positive values indicate hydrophobic regions, while negative values indicate hydrophilic regions.

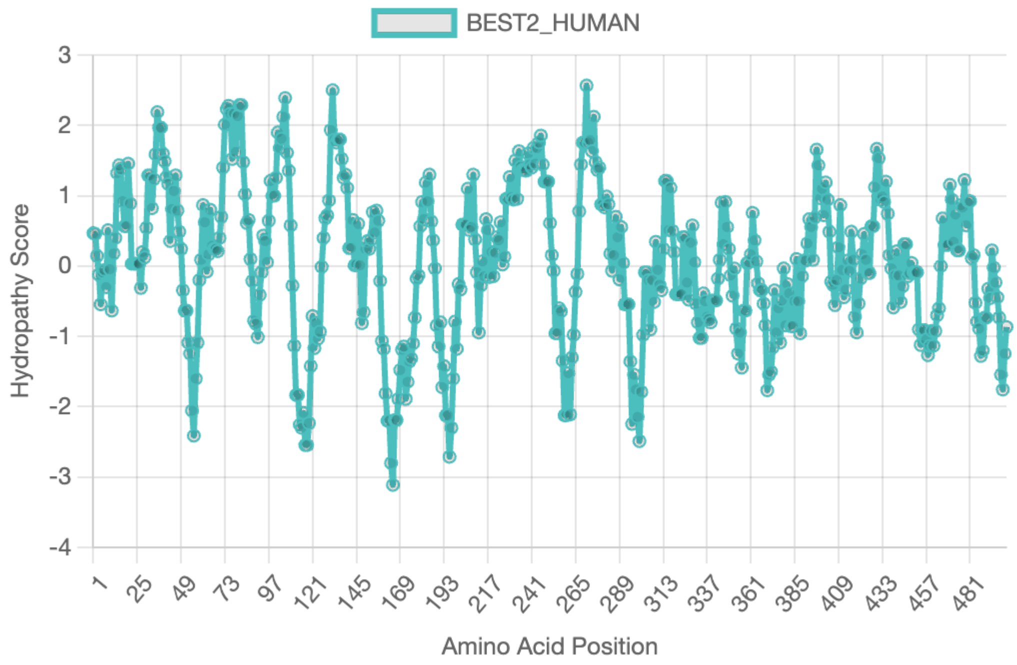

Kyte-Doolittle hydropathy plot for the sequence "BEST2_HUMAN". Positive values indicate hydrophobic regions, while negative values indicate hydrophilic regions.

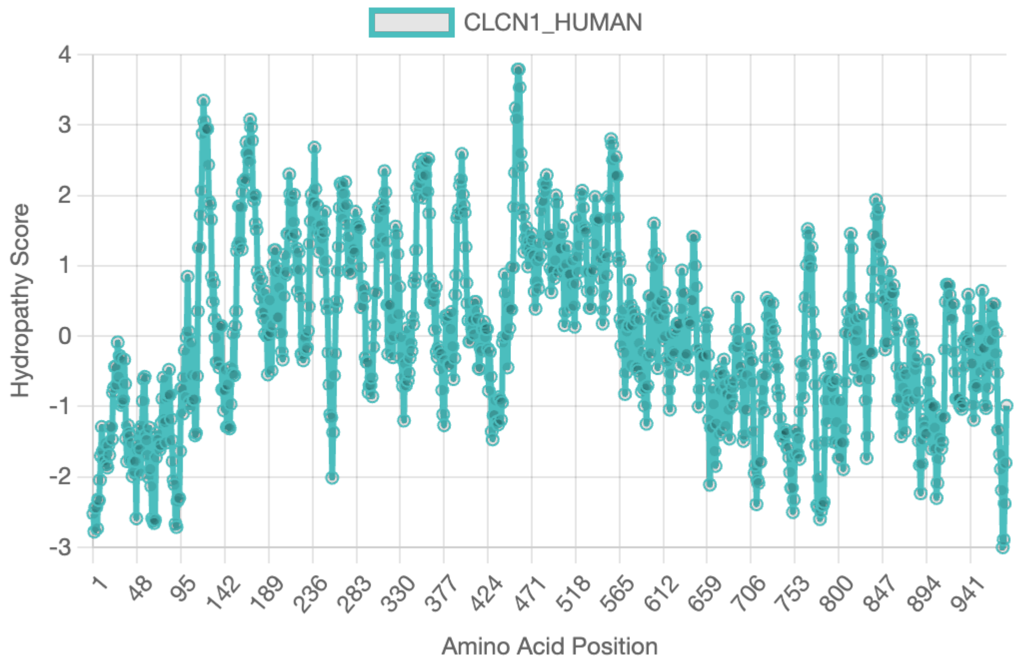

Kyte-Doolittle hydropathy plot for the sequence "CLCN1_HUMAN". Positive values indicate hydrophobic regions, while negative values indicate hydrophilic regions.

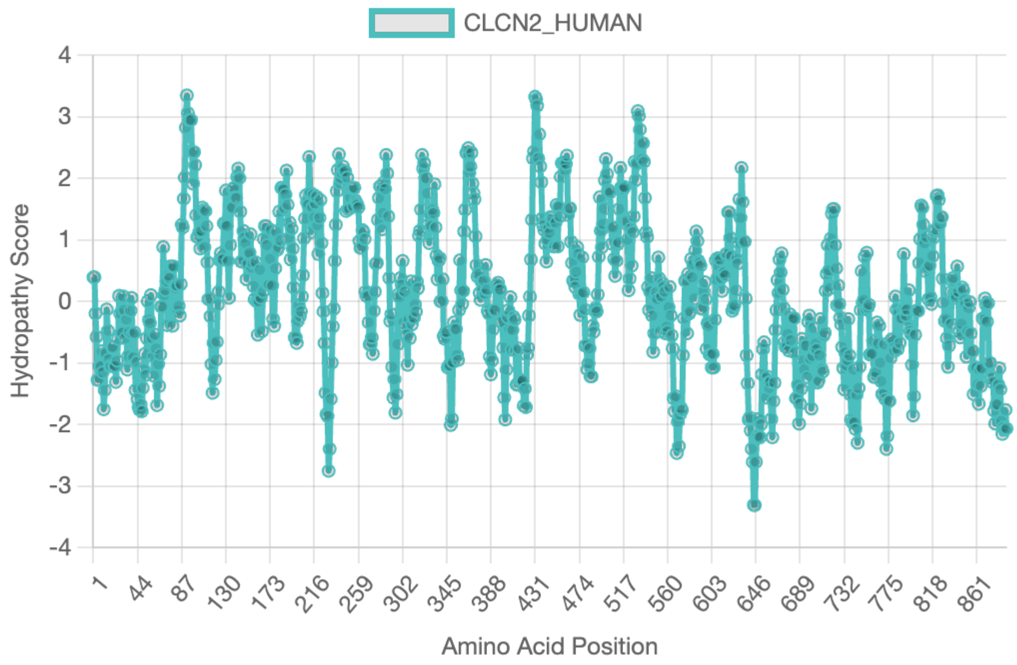

Kyte-Doolittle hydropathy plot for the sequence "CLCN2_HUMAN". Positive values indicate hydrophobic regions, while negative values indicate hydrophilic regions.

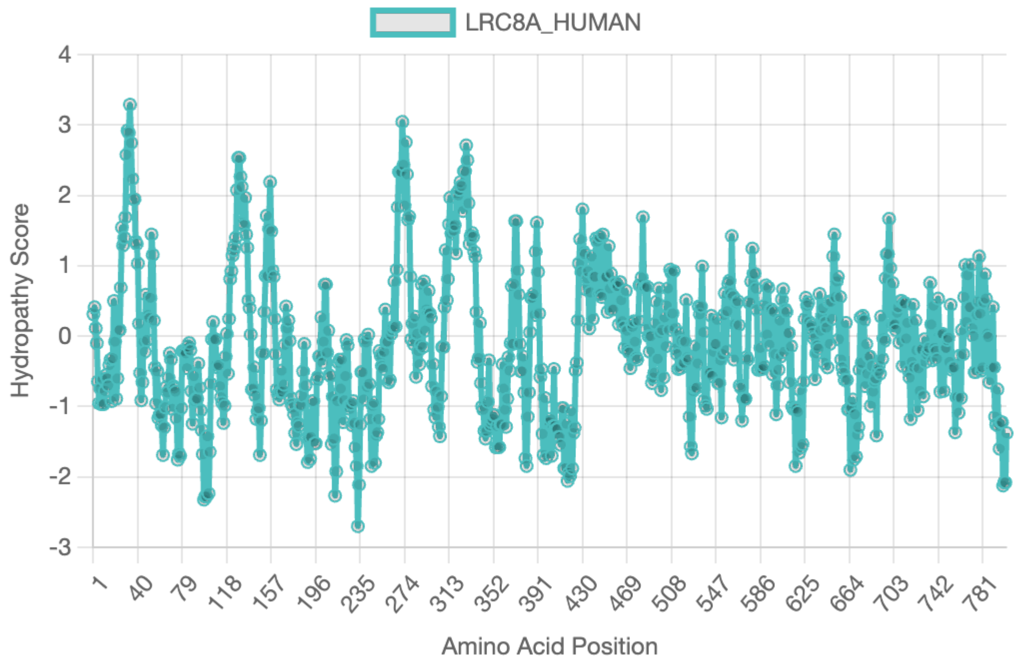

Kyte-Doolittle hydropathy plot for the sequence "LRC8A_HUMAN". Positive values indicate hydrophobic regions, while negative values indicate hydrophilic regions.

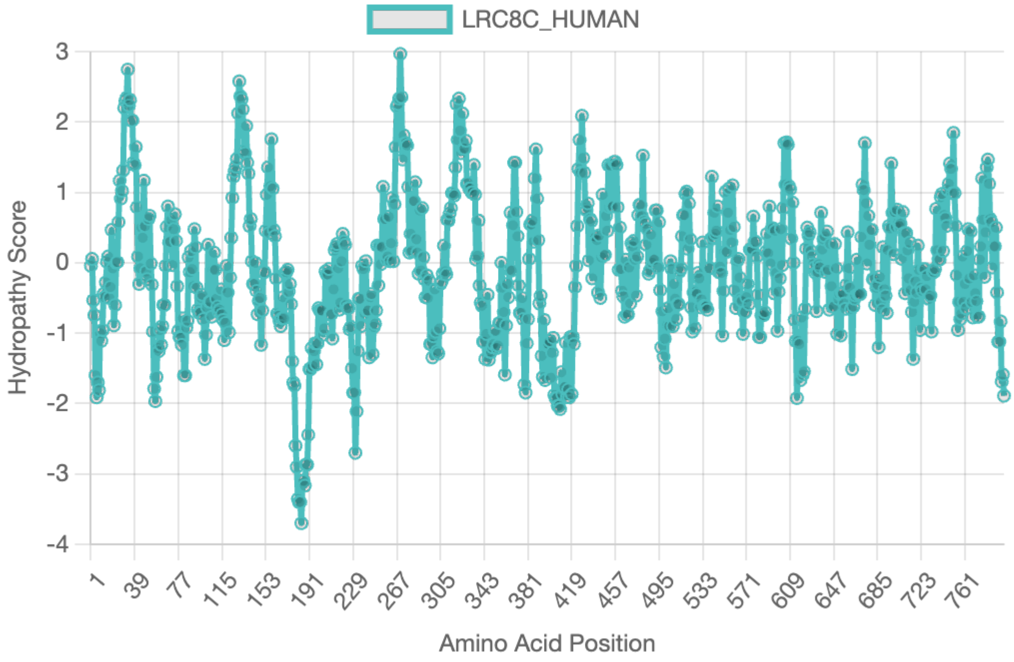

Kyte-Doolittle hydropathy plot for the sequence "LRC8C_HUMAN". Positive values indicate hydrophobic regions, while negative values indicate hydrophilic regions.

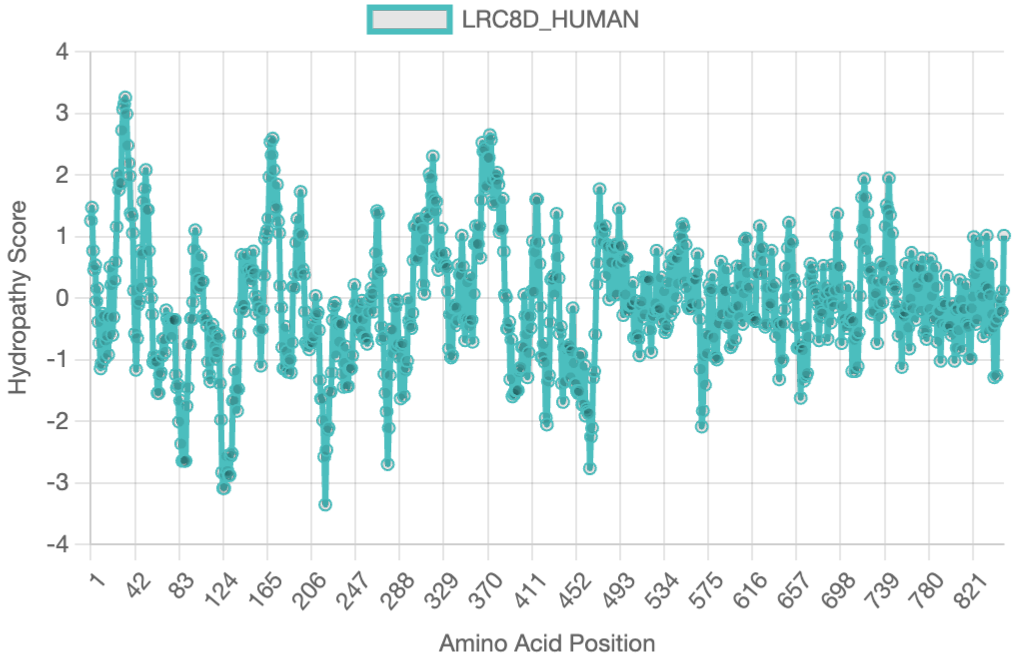

Kyte-Doolittle hydropathy plot for the sequence "LRC8D_HUMAN". Positive values indicate hydrophobic regions, while negative values indicate hydrophilic regions.

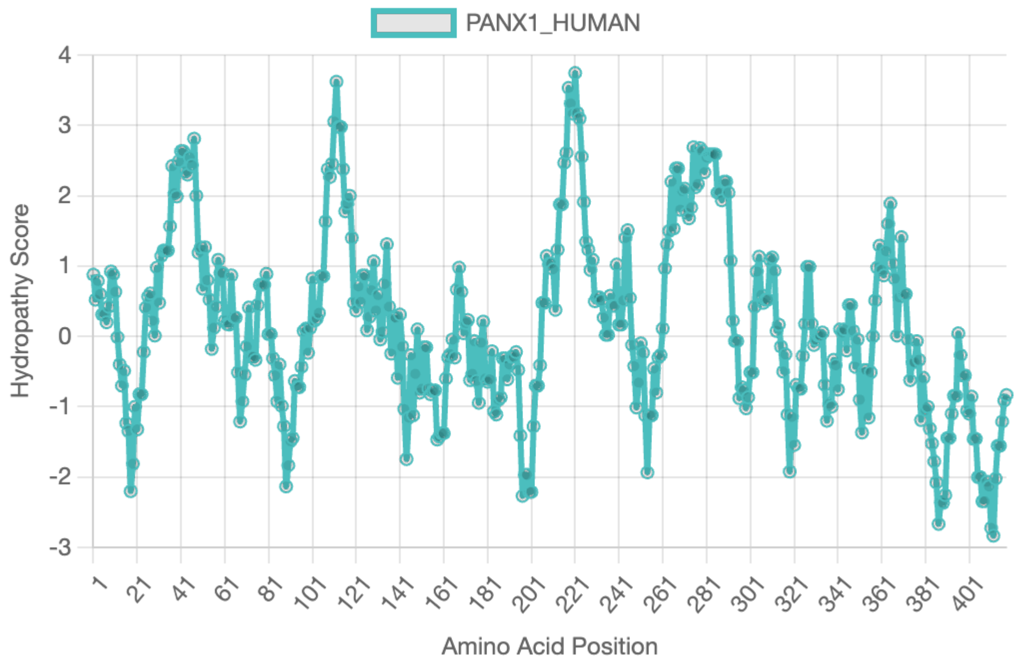

Kyte-Doolittle hydropathy plot for the sequence "PANX1_HUMAN". Positive values indicate hydrophobic regions, while negative values indicate hydrophilic regions.

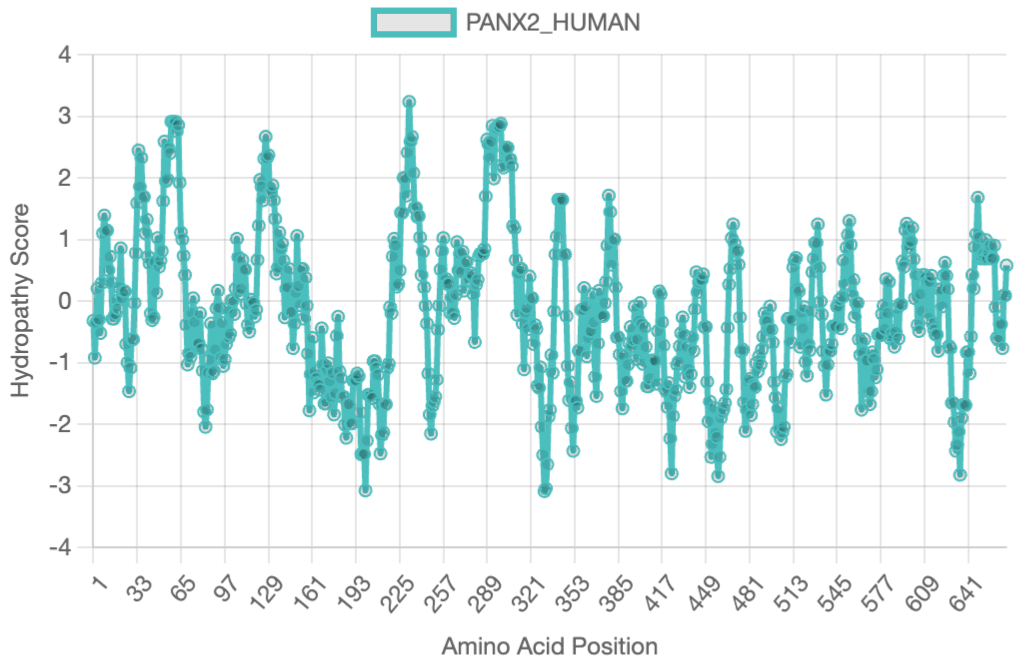

Kyte-Doolittle hydropathy plot for the sequence "PANX2_HUMAN". Positive values indicate hydrophobic regions, while negative values indicate hydrophilic regions.

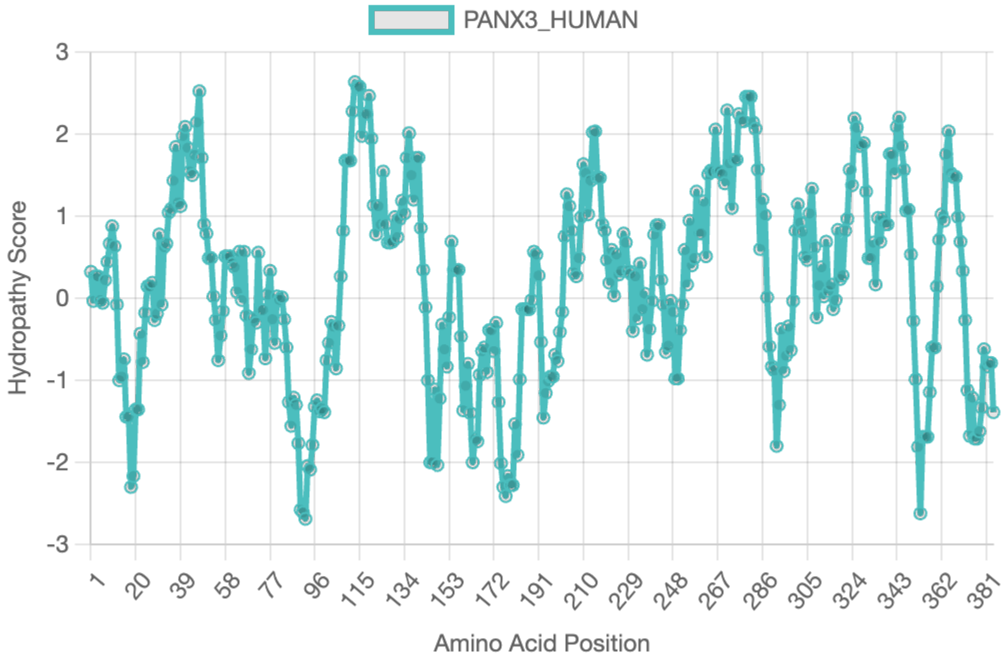

Kyte-Doolittle hydropathy plot for the sequence "PANX3_HUMAN". Positive values indicate hydrophobic regions, while negative values indicate hydrophilic regions.

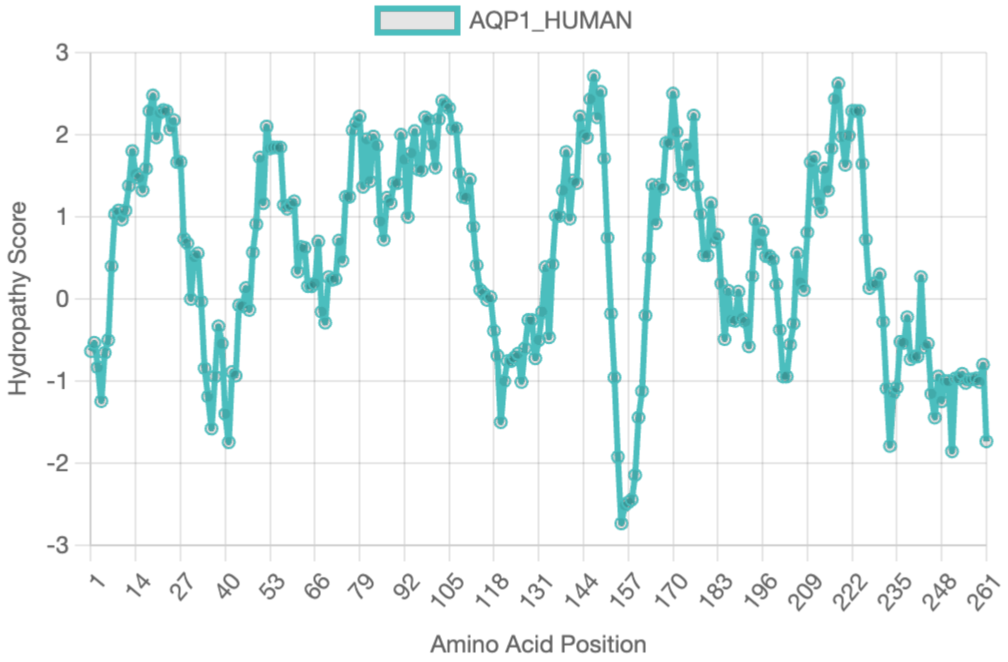

Kyte-Doolittle hydropathy plot for the sequence "AQP1_HUMAN". Positive values indicate hydrophobic regions, while negative values indicate hydrophilic regions.

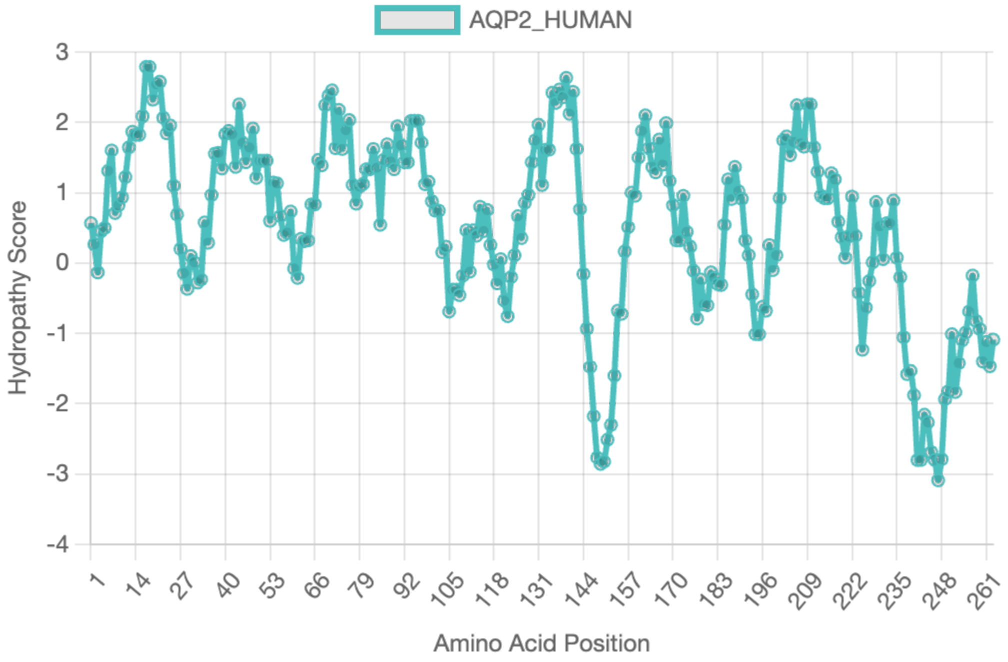

Kyte-Doolittle hydropathy plot for the sequence "AQP2_HUMAN". Positive values indicate hydrophobic regions, while negative values indicate hydrophilic regions.

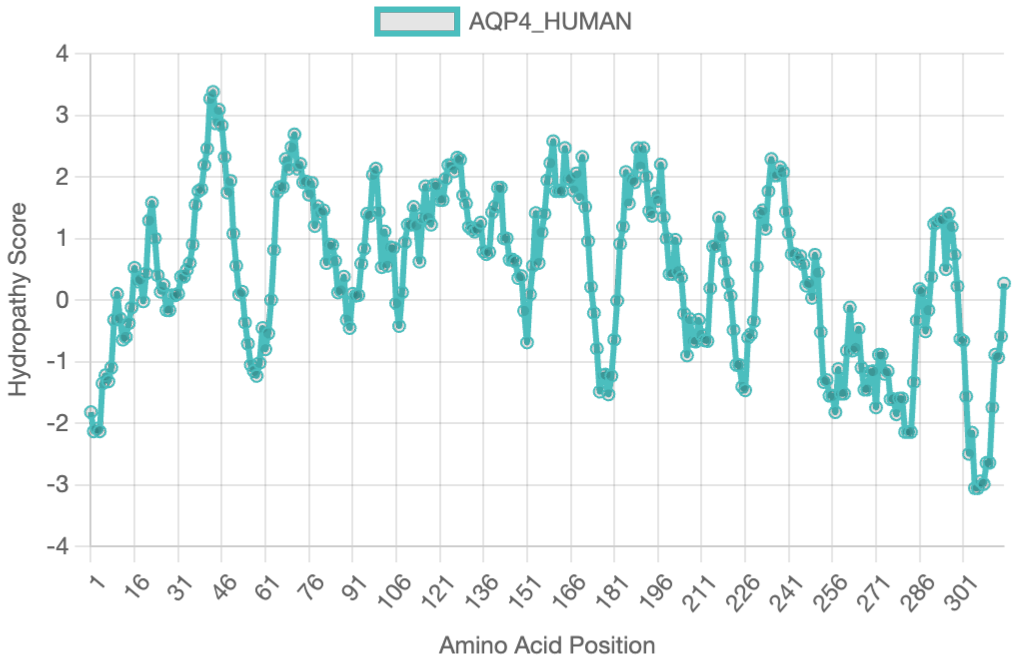

Kyte-Doolittle hydropathy plot for the sequence "AQP4_HUMAN". Positive values indicate hydrophobic regions, while negative values indicate hydrophilic regions.

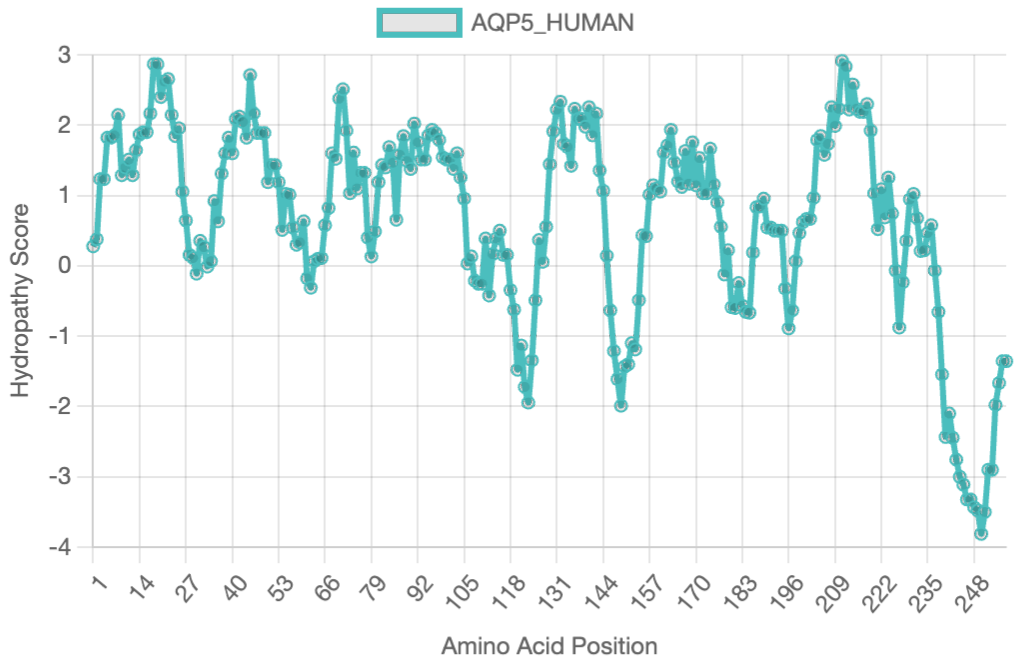

Kyte-Doolittle hydropathy plot for the sequence "AQP5_HUMAN". Positive values indicate hydrophobic regions, while negative values indicate hydrophilic regions.

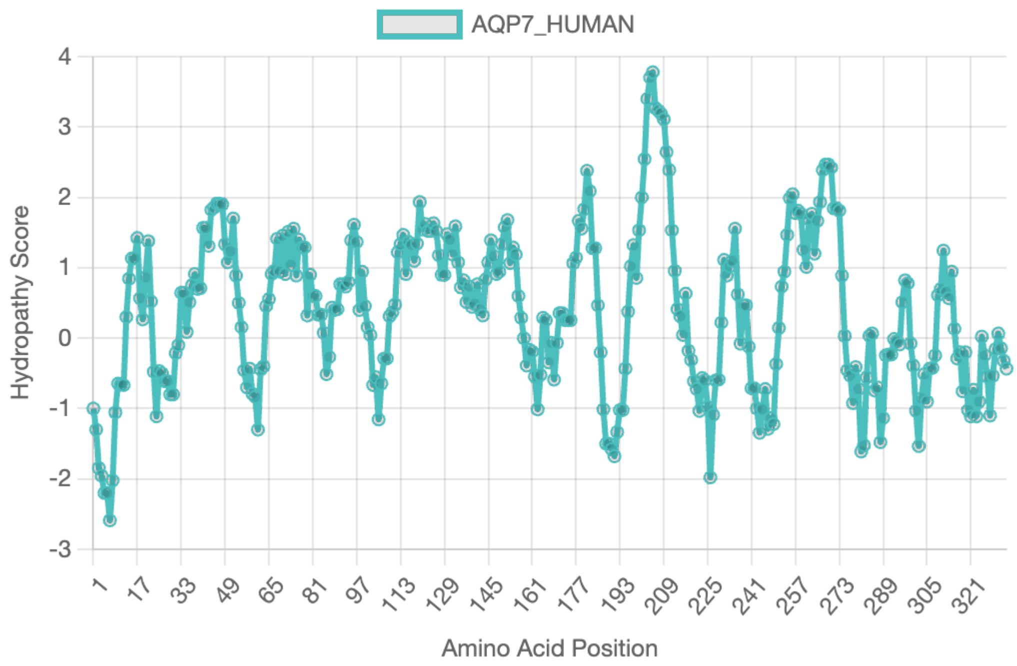

Kyte-Doolittle hydropathy plot for the sequence "AQP7_HUMAN". Positive values indicate hydrophobic regions, while negative values indicate hydrophilic regions.

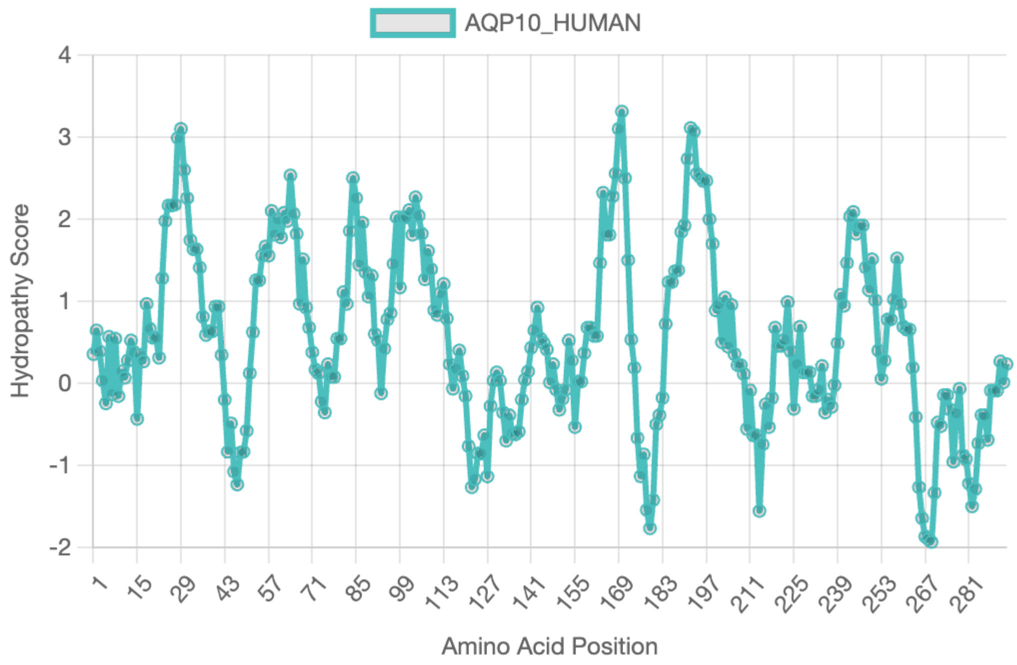

Kyte-Doolittle hydropathy plot for the sequence "AQP10_HUMAN". Positive values indicate hydrophobic regions, while negative values indicate hydrophilic regions.

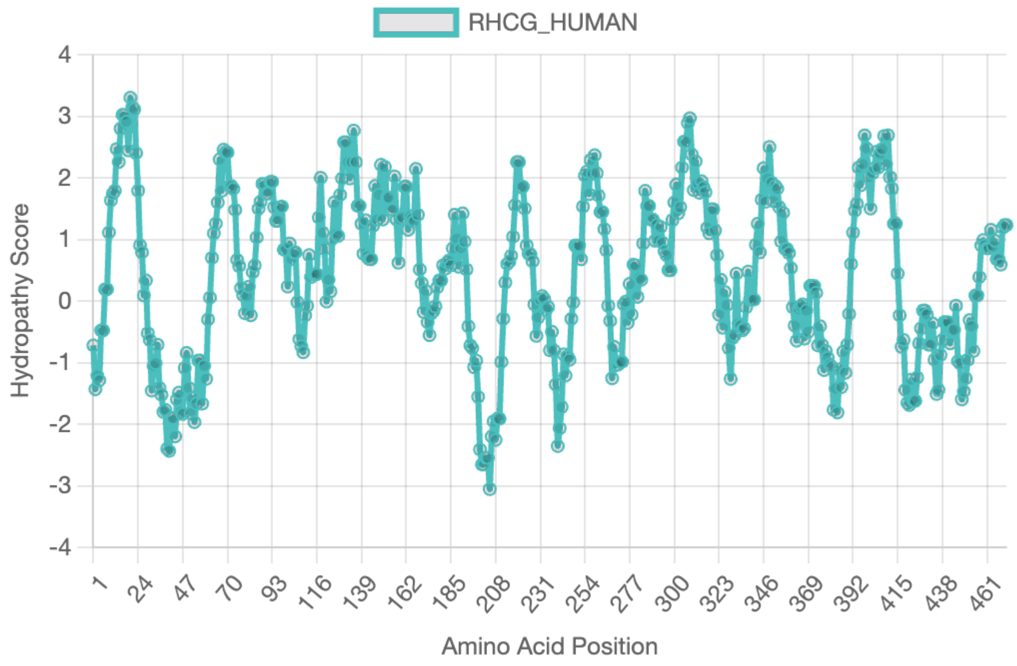

Kyte-Doolittle hydropathy plot for the sequence "RHCG_HUMAN". Positive values indicate hydrophobic regions, while negative values indicate hydrophilic regions.

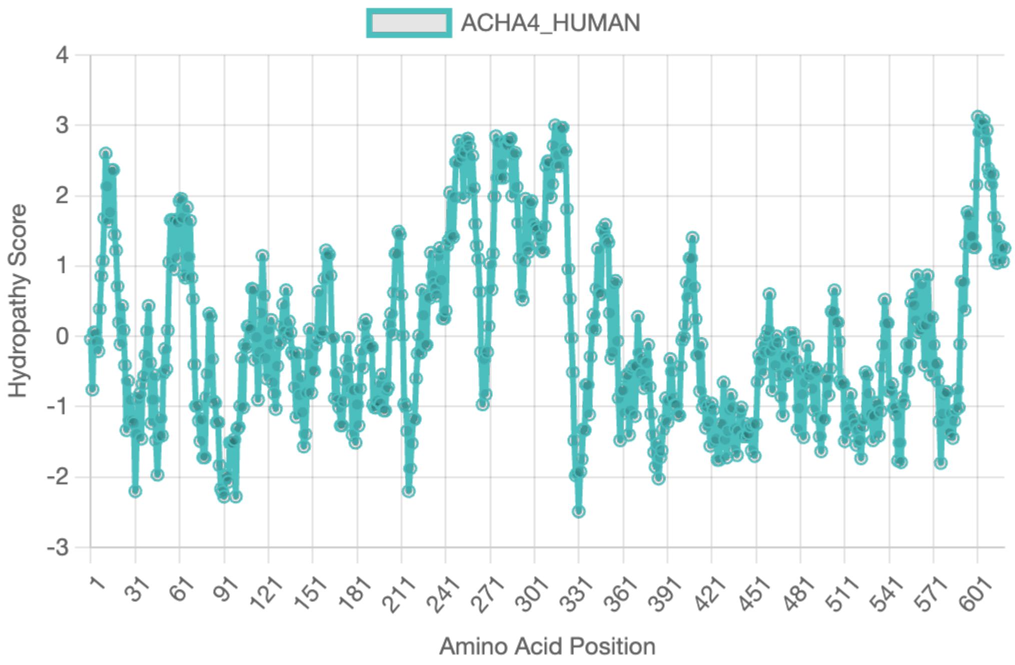

Kyte-Doolittle hydropathy plot for the sequence "ACHA4_HUMAN". Positive values indicate hydrophobic regions, while negative values indicate hydrophilic regions.

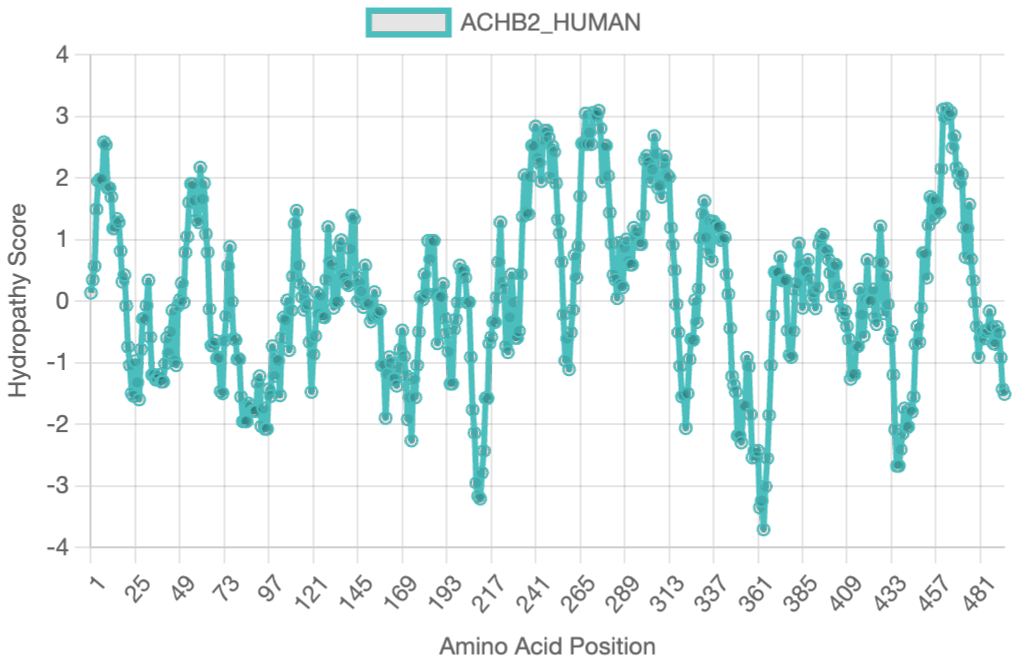

Kyte-Doolittle hydropathy plot for the sequence "ACHB2_HUMAN". Positive values indicate hydrophobic regions, while negative values indicate hydrophilic regions.

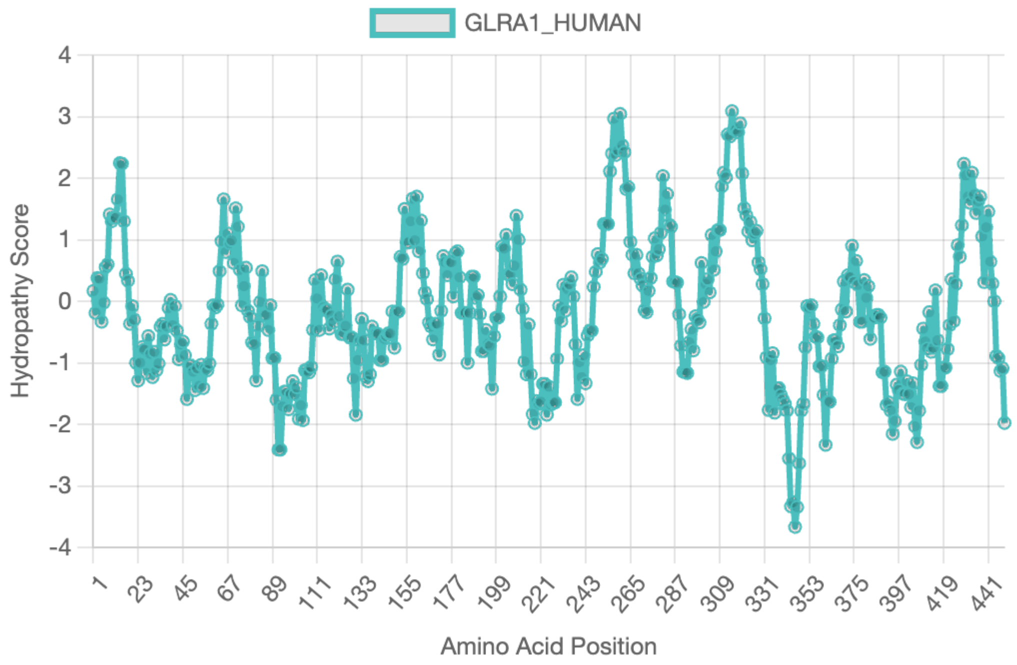

Kyte-Doolittle hydropathy plot for the sequence "GLRA1_HUMAN". Positive values indicate hydrophobic regions, while negative values indicate hydrophilic regions.

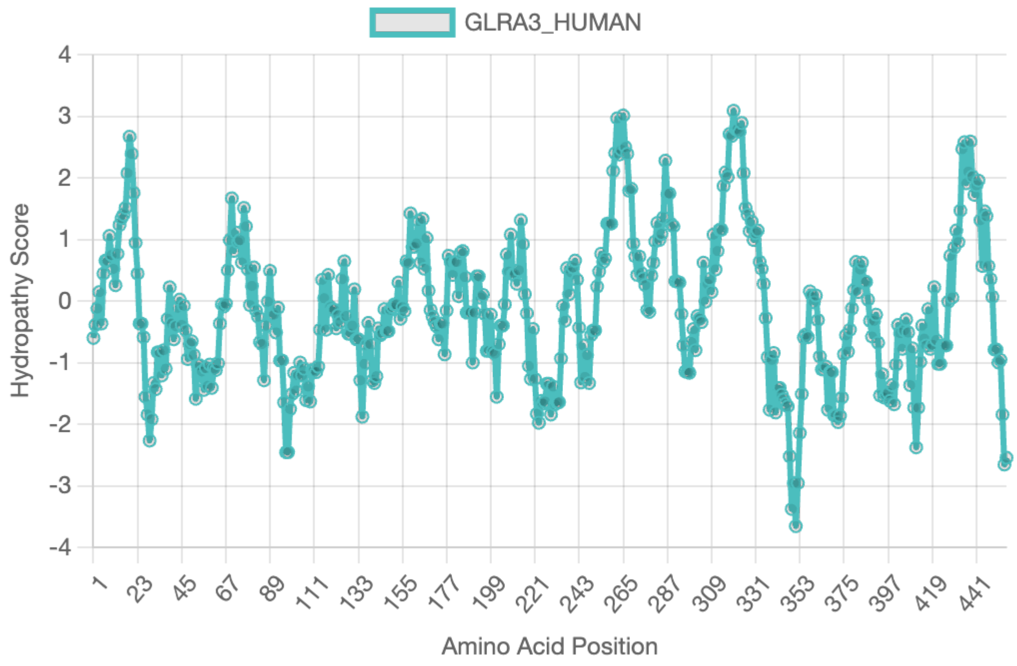

Kyte-Doolittle hydropathy plot for the sequence "GLRA3_HUMAN". Positive values indicate hydrophobic regions, while negative values indicate hydrophilic regions.

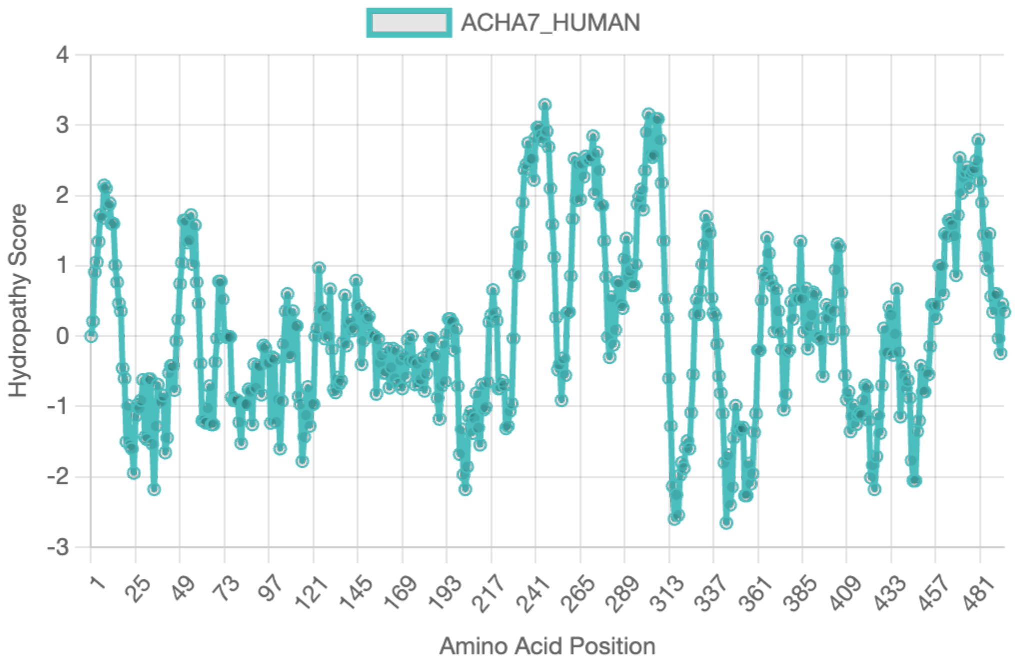

Kyte-Doolittle hydropathy plot for the sequence "ACHA7_HUMAN". Positive values indicate hydrophobic regions, while negative values indicate hydrophilic regions.

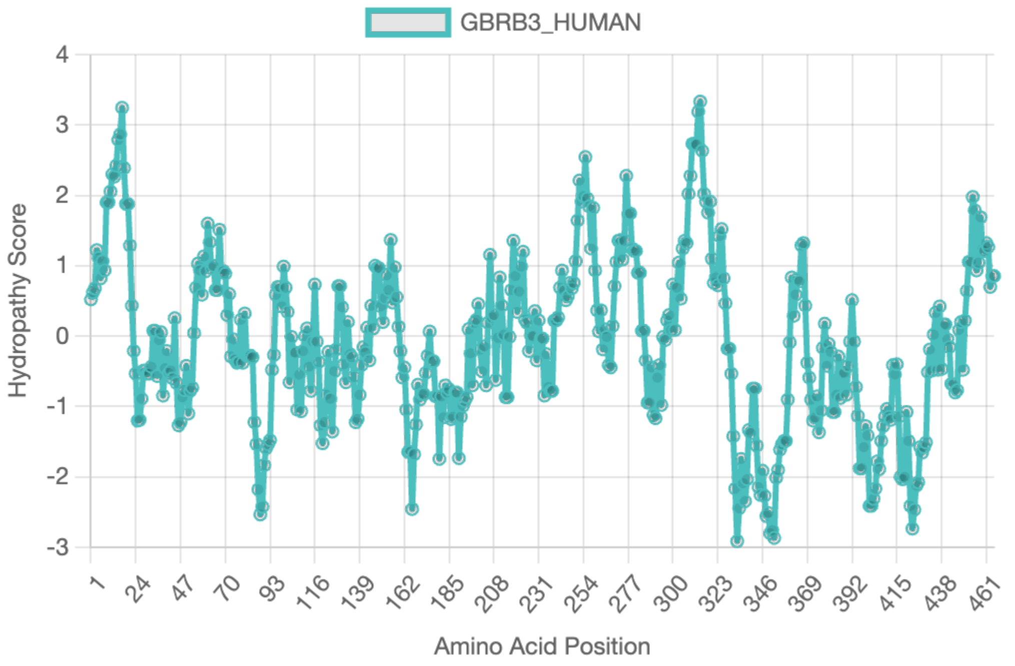

Kyte-Doolittle hydropathy plot for the sequence "GBRB3_HUMAN". Positive values indicate hydrophobic regions, while negative values indicate hydrophilic regions.

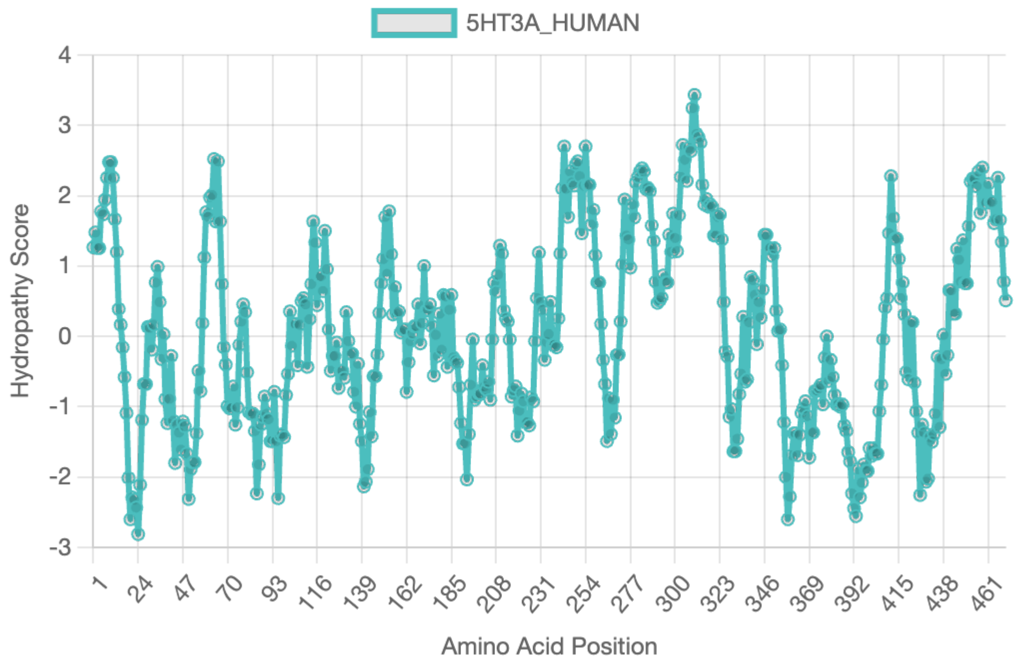

Kyte-Doolittle hydropathy plot for the sequence "5HT3A_HUMAN". Positive values indicate hydrophobic regions, while negative values indicate hydrophilic regions.

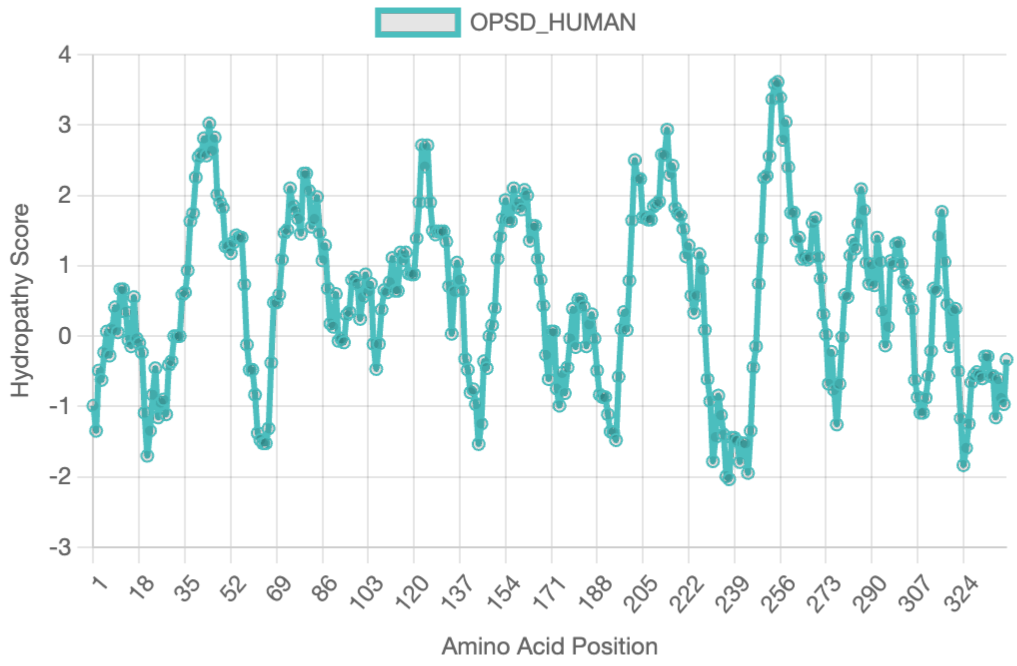

Kyte-Doolittle hydropathy plot for the sequence "OPSD_HUMAN". Positive values indicate hydrophobic regions, while negative values indicate hydrophilic regions.

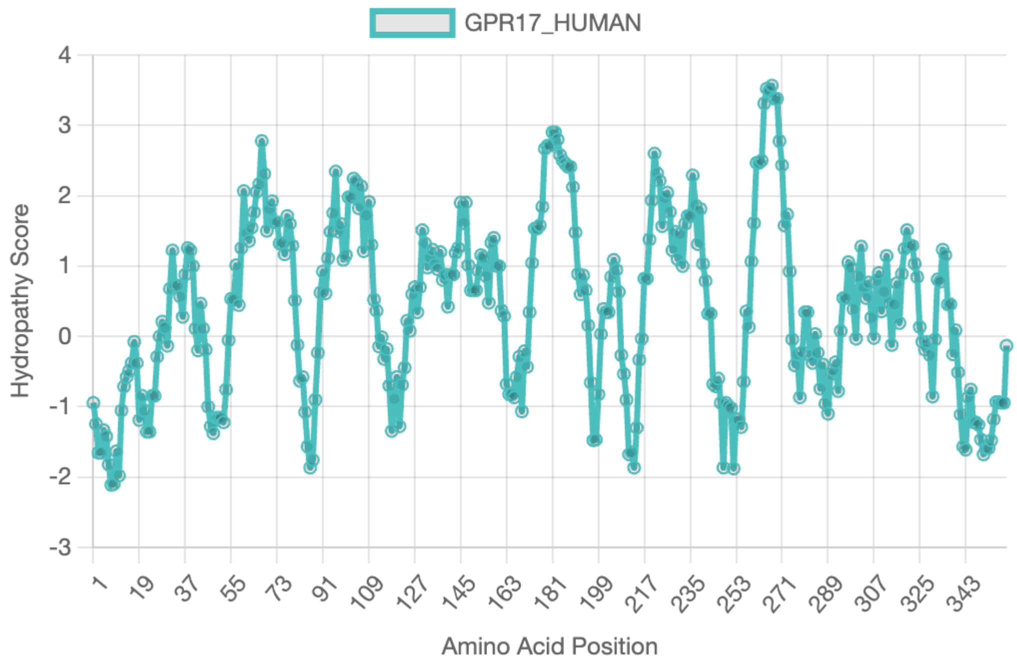

Kyte-Doolittle hydropathy plot for the sequence "GPR17_HUMAN". Positive values indicate hydrophobic regions, while negative values indicate hydrophilic regions.

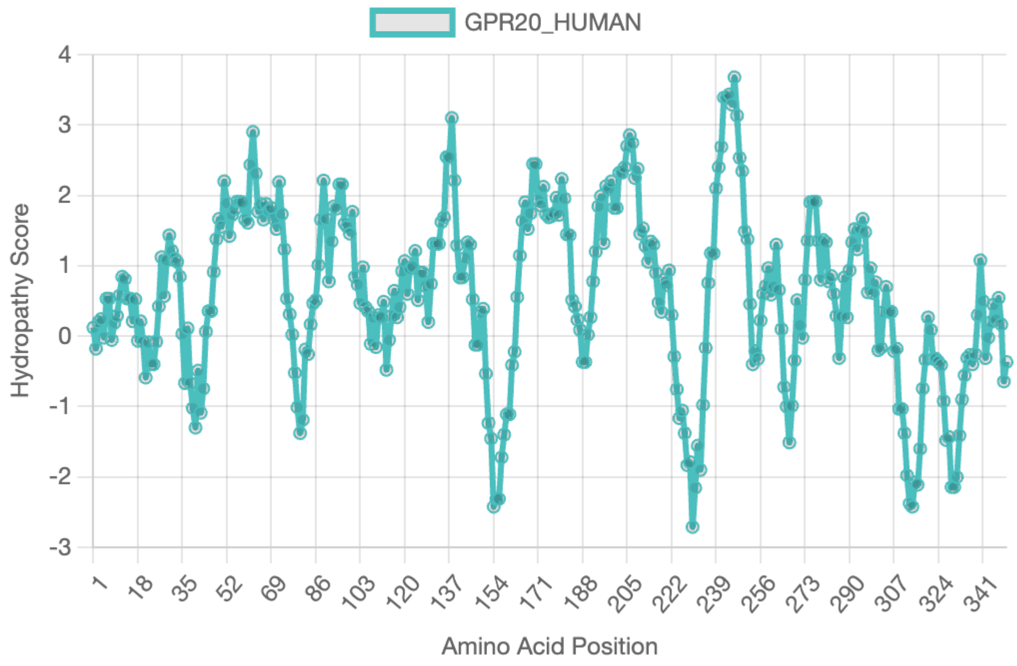

Kyte-Doolittle hydropathy plot for the sequence "GPR20_HUMAN". Positive values indicate hydrophobic regions, while negative values indicate hydrophilic regions.

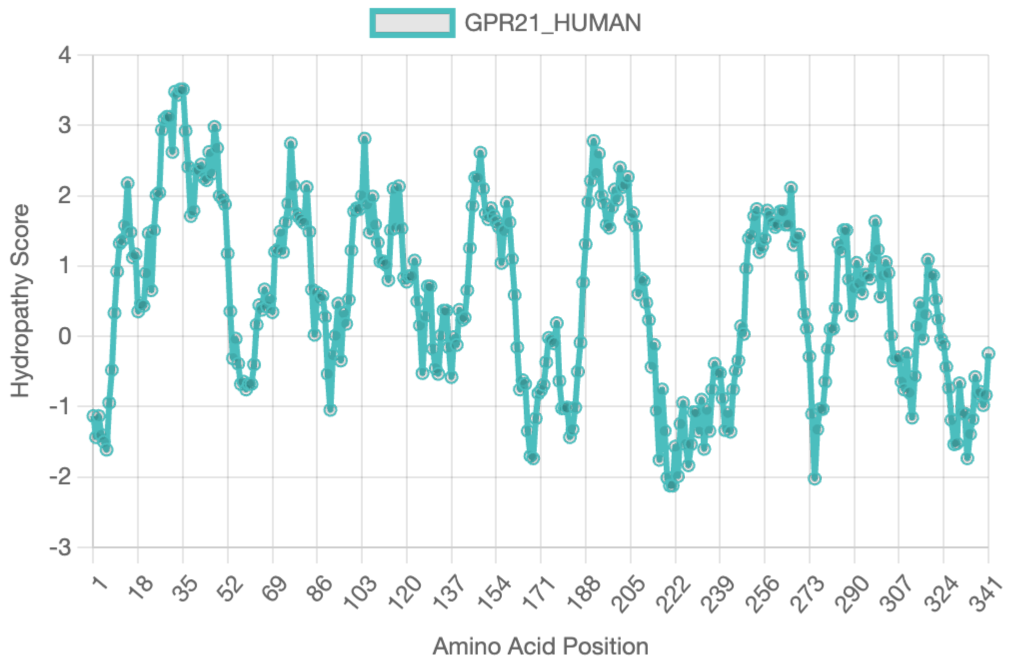

Kyte-Doolittle hydropathy plot for the sequence "GPR21_HUMAN". Positive values indicate hydrophobic regions, while negative values indicate hydrophilic regions.

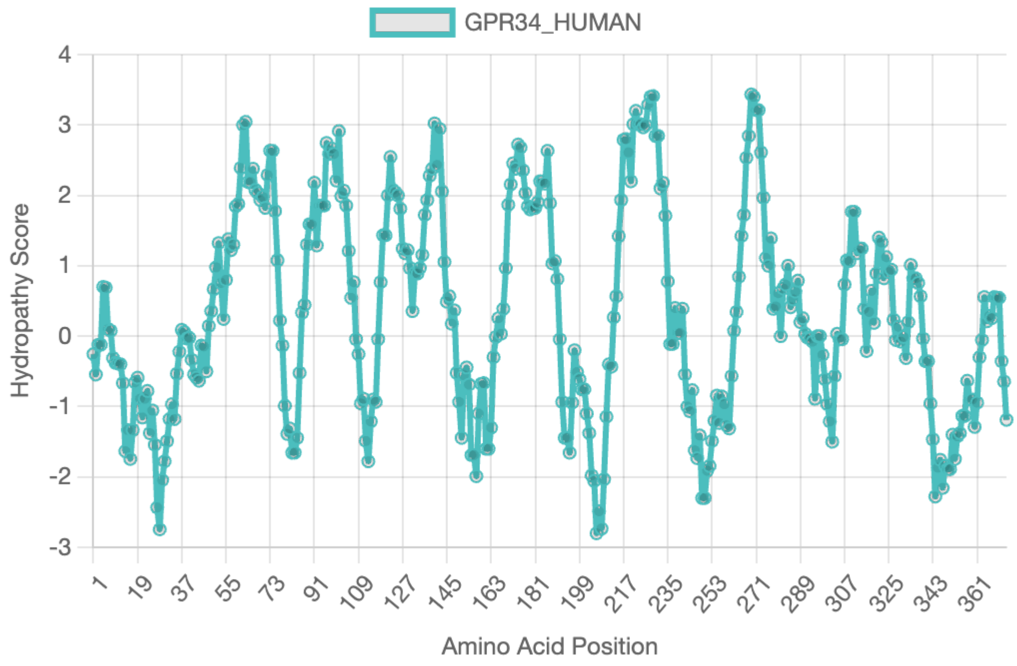

Kyte-Doolittle hydropathy plot for the sequence "GPR34_HUMAN". Positive values indicate hydrophobic regions, while negative values indicate hydrophilic regions.

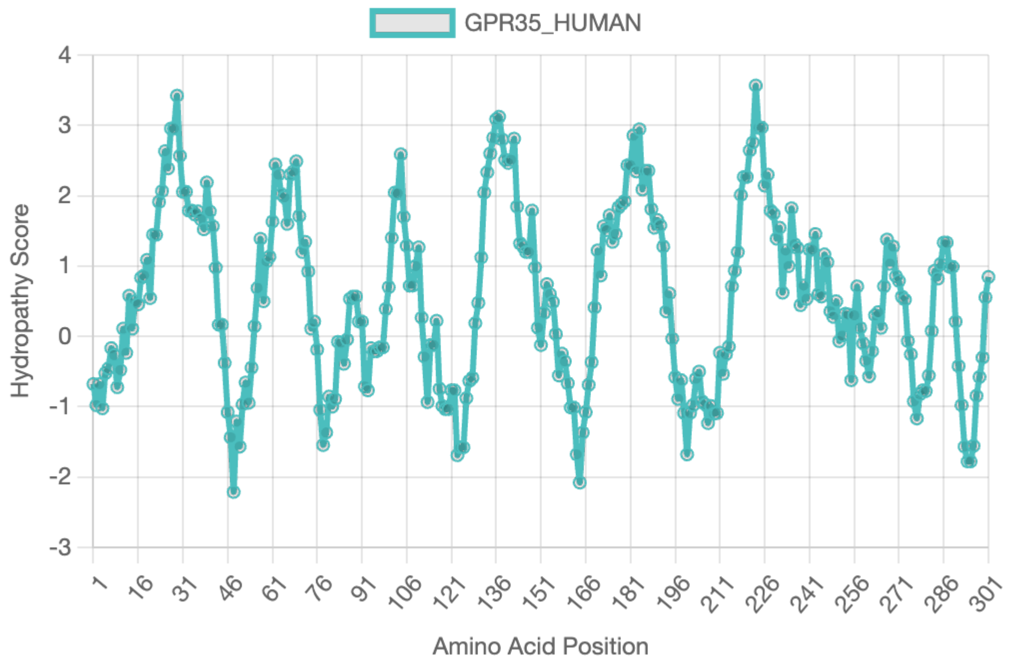

Kyte-Doolittle hydropathy plot for the sequence "GPR35_HUMAN". Positive values indicate hydrophobic regions, while negative values indicate hydrophilic regions.

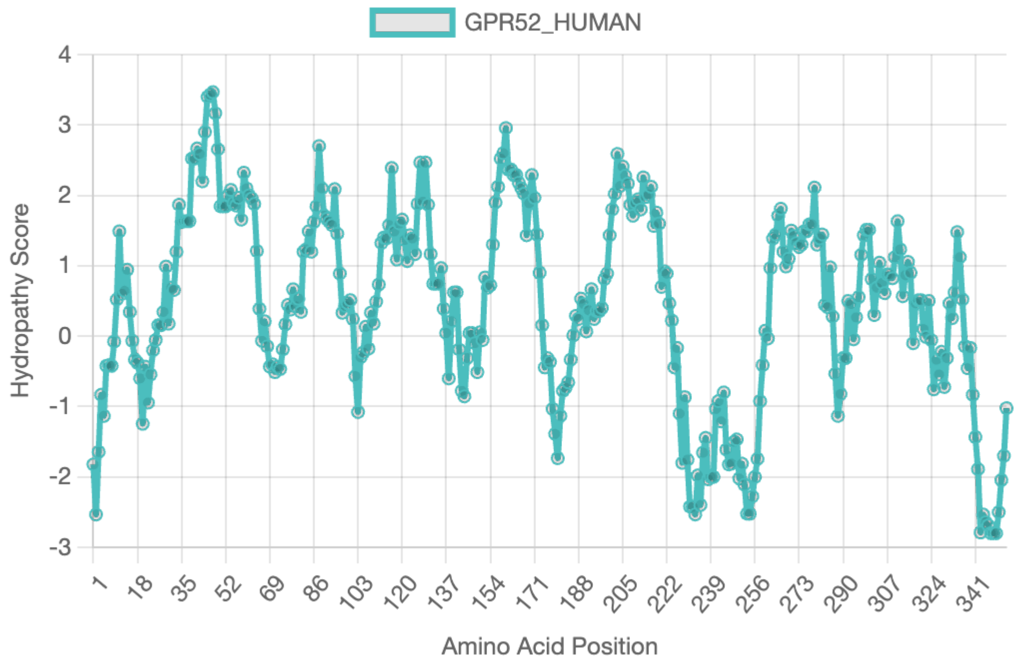

Kyte-Doolittle hydropathy plot for the sequence "GPR52_HUMAN". Positive values indicate hydrophobic regions, while negative values indicate hydrophilic regions.

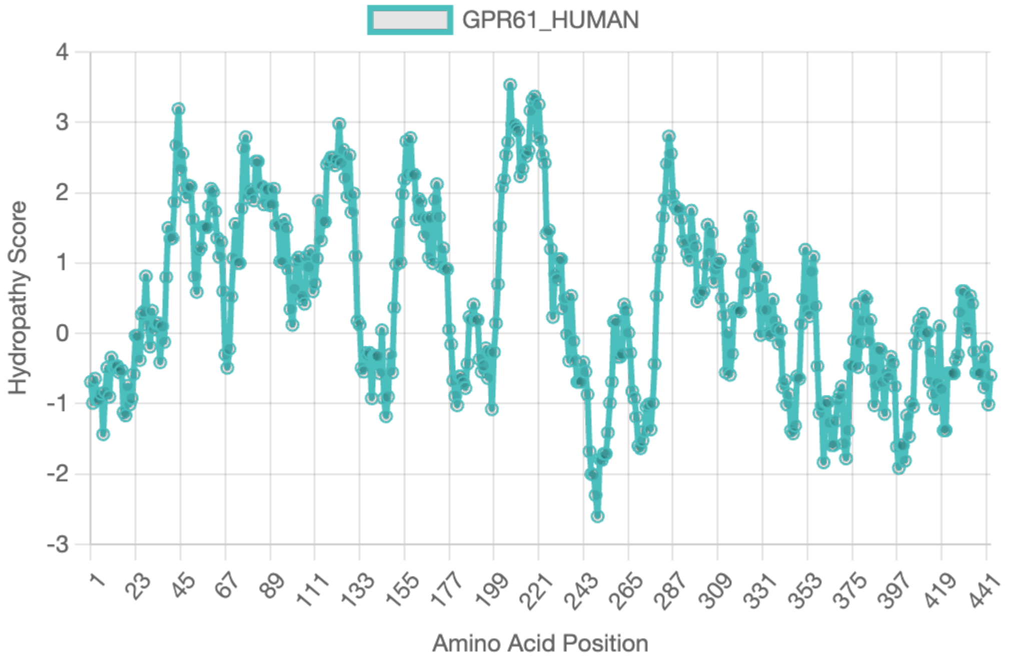

Kyte-Doolittle hydropathy plot for the sequence "GPR61_HUMAN". Positive values indicate hydrophobic regions, while negative values indicate hydrophilic regions.

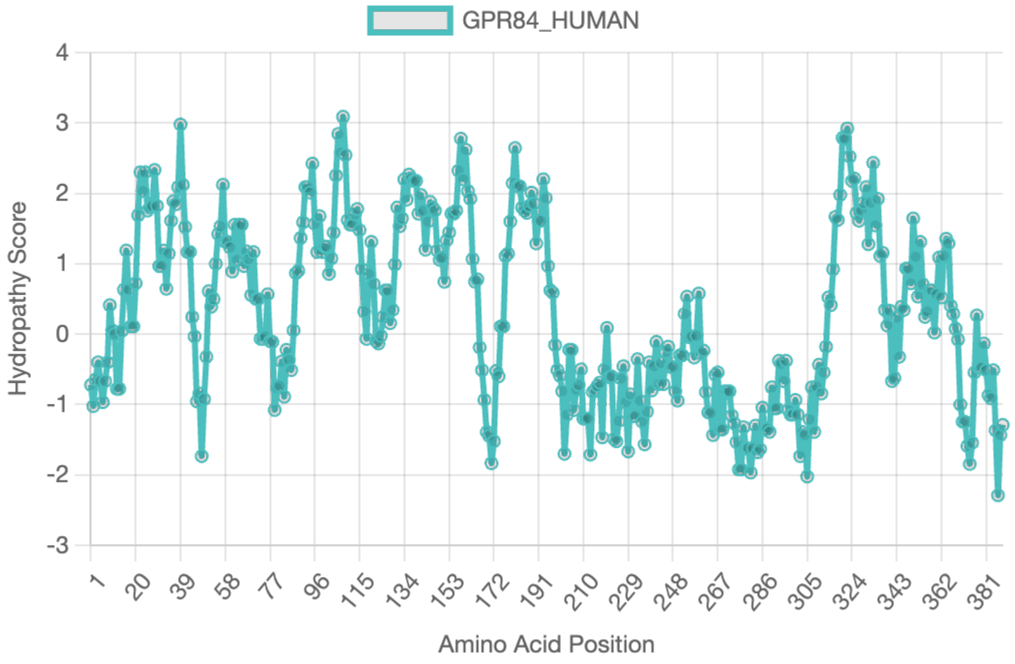

Kyte-Doolittle hydropathy plot for the sequence "GPR84_HUMAN". Positive values indicate hydrophobic regions, while negative values indicate hydrophilic regions.

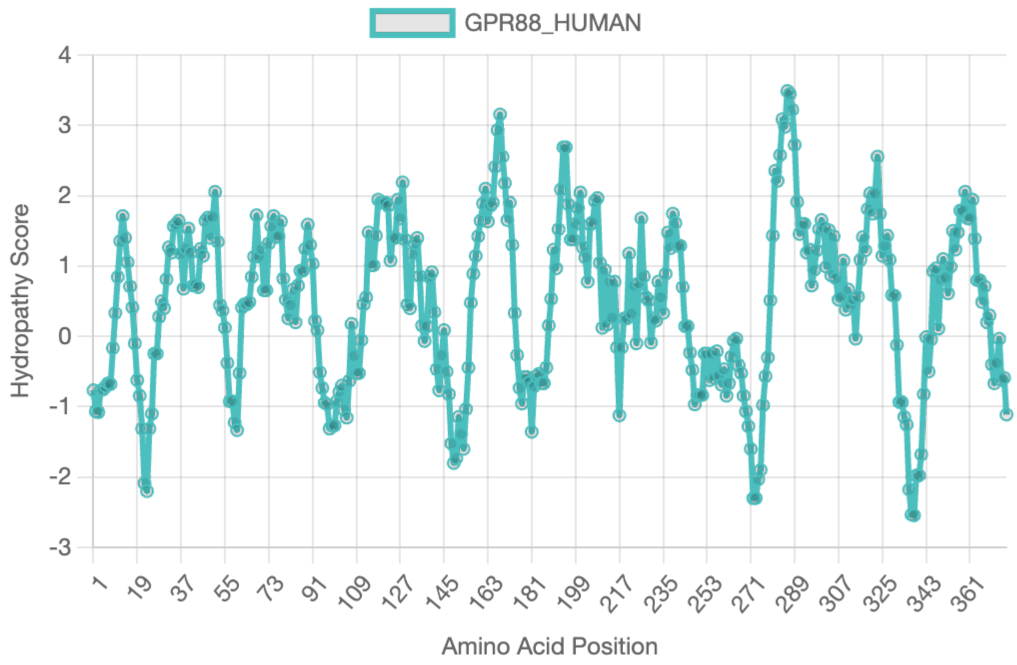

Kyte-Doolittle hydropathy plot for the sequence "GPR88_HUMAN". Positive values indicate hydrophobic regions, while negative values indicate hydrophilic regions.

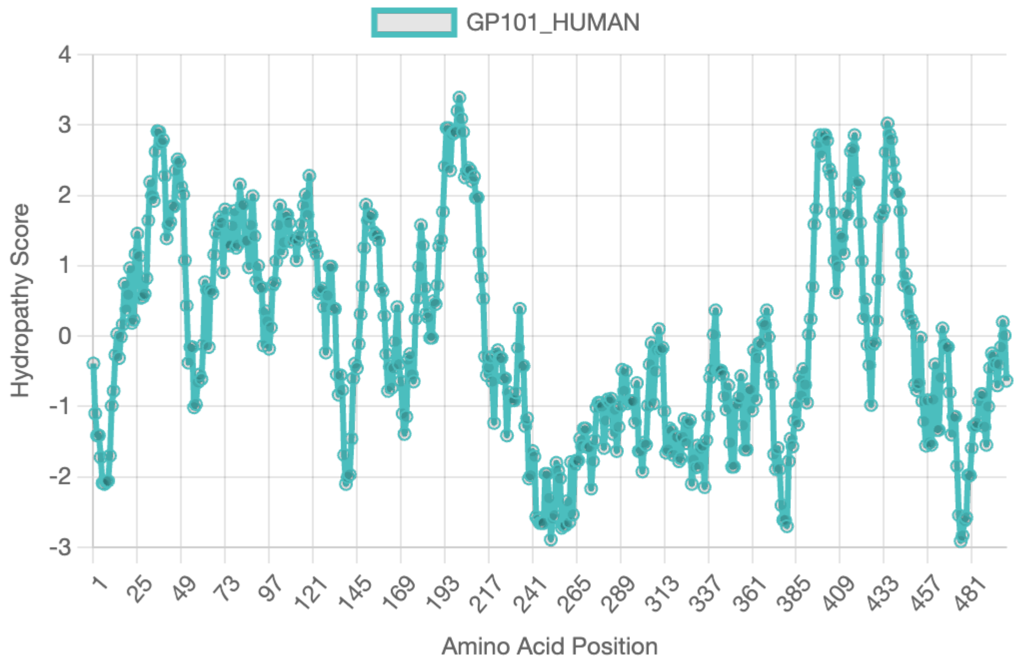

Kyte-Doolittle hydropathy plot for the sequence "GP101_HUMAN". Positive values indicate hydrophobic regions, while negative values indicate hydrophilic regions.

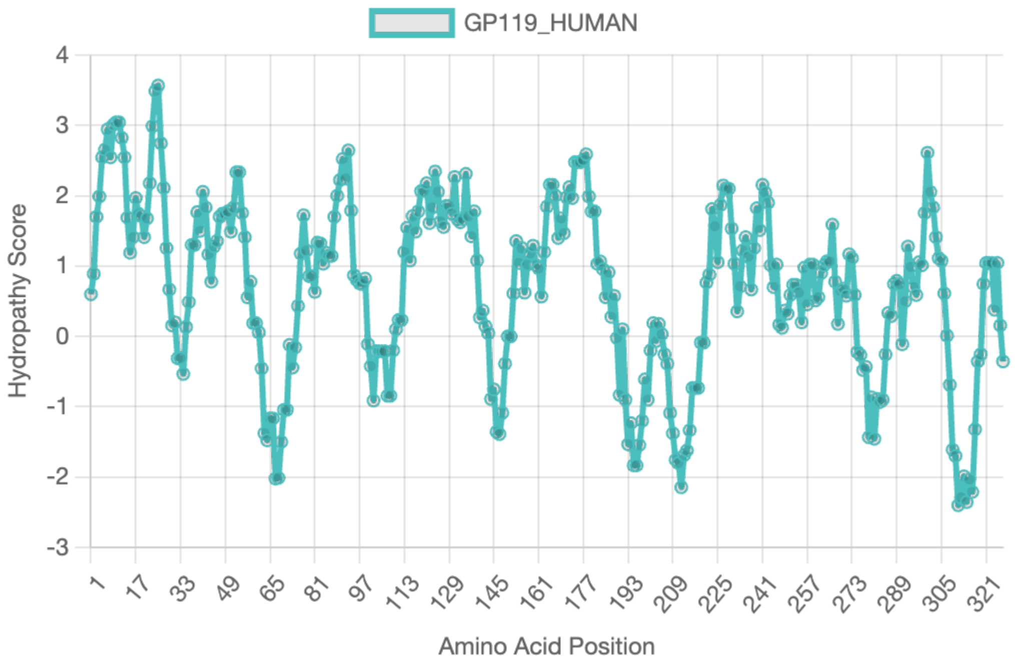

Kyte-Doolittle hydropathy plot for the sequence "GP119_HUMAN". Positive values indicate hydrophobic regions, while negative values indicate hydrophilic regions.

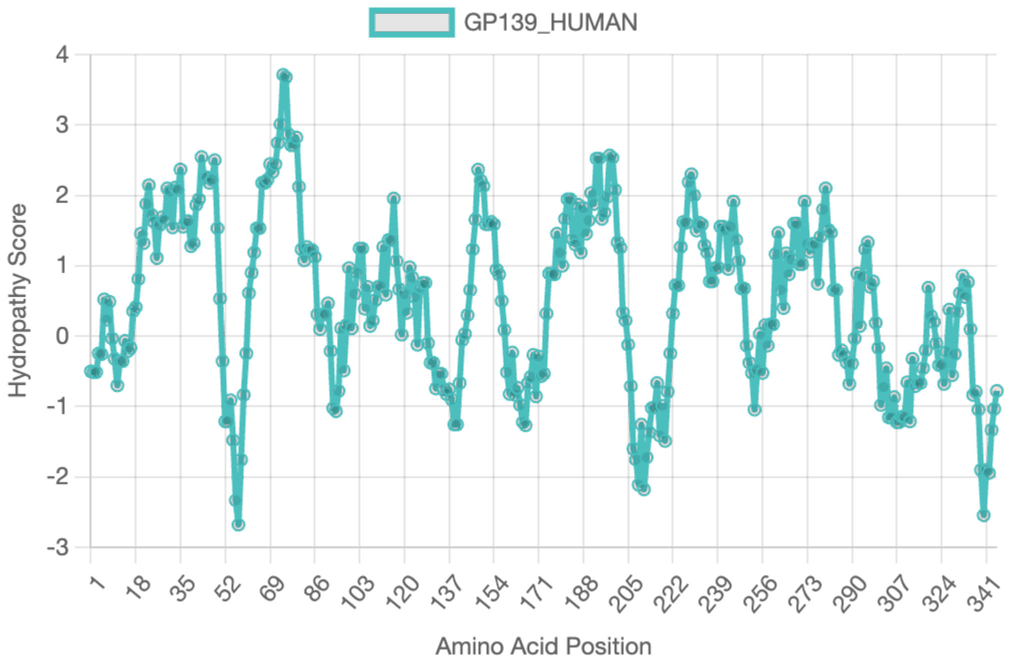

Kyte-Doolittle hydropathy plot for the sequence "GP139_HUMAN". Positive values indicate hydrophobic regions, while negative values indicate hydrophilic regions.

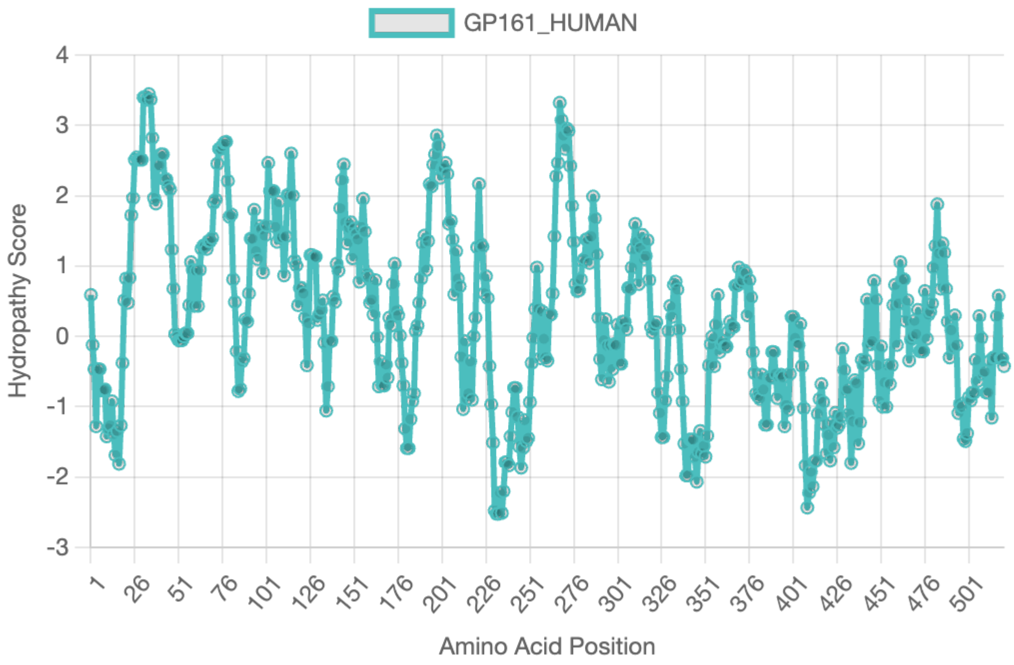

Kyte-Doolittle hydropathy plot for the sequence "GP161_HUMAN". Positive values indicate hydrophobic regions, while negative values indicate hydrophilic regions.

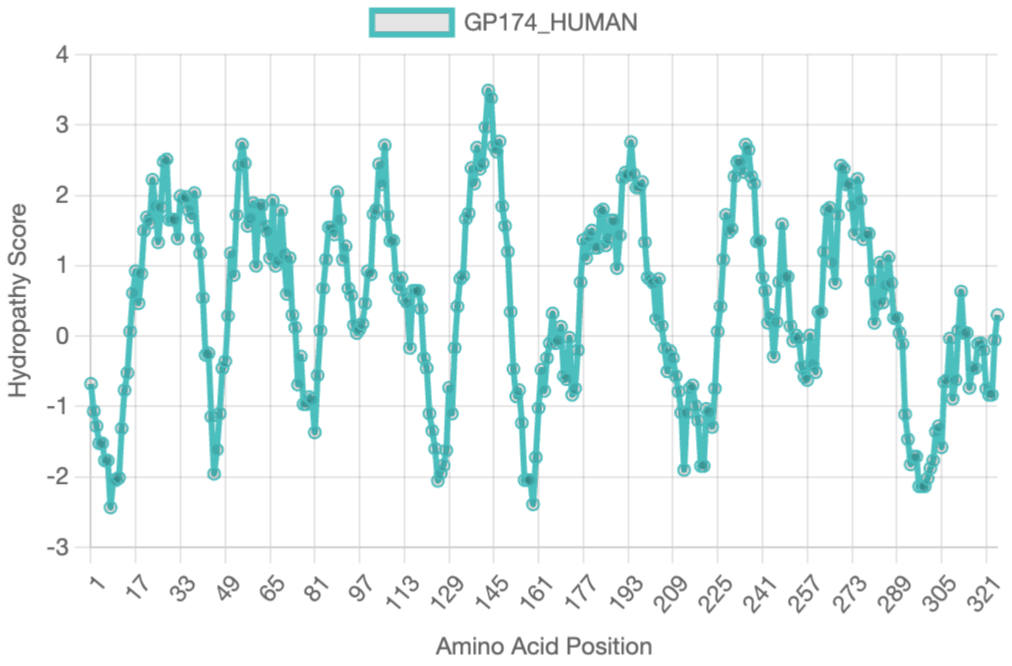

Kyte-Doolittle hydropathy plot for the sequence "GP174_HUMAN". Positive values indicate hydrophobic regions, while negative values indicate hydrophilic regions.

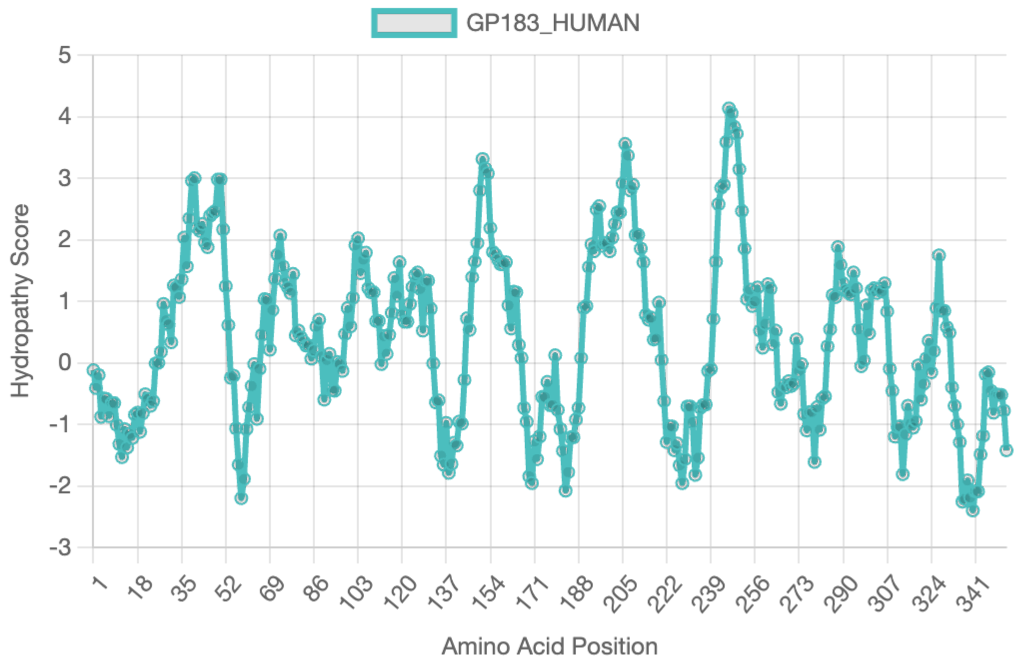

Kyte-Doolittle hydropathy plot for the sequence "GP183_HUMAN". Positive values indicate hydrophobic regions, while negative values indicate hydrophilic regions.

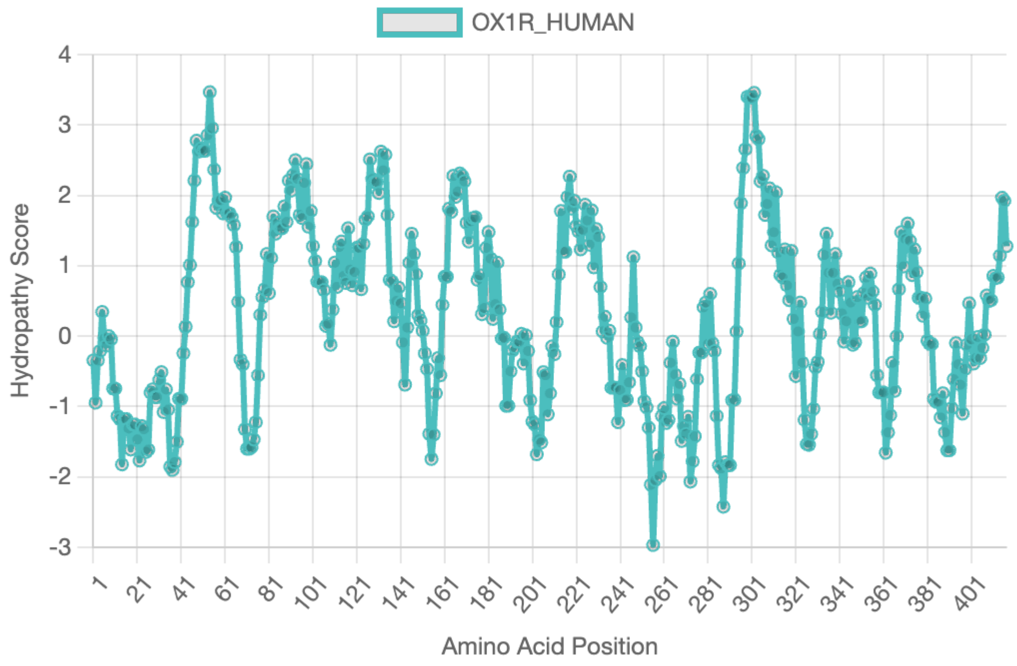

Kyte-Doolittle hydropathy plot for the sequence "OX1R_HUMAN". Positive values indicate hydrophobic regions, while negative values indicate hydrophilic regions.

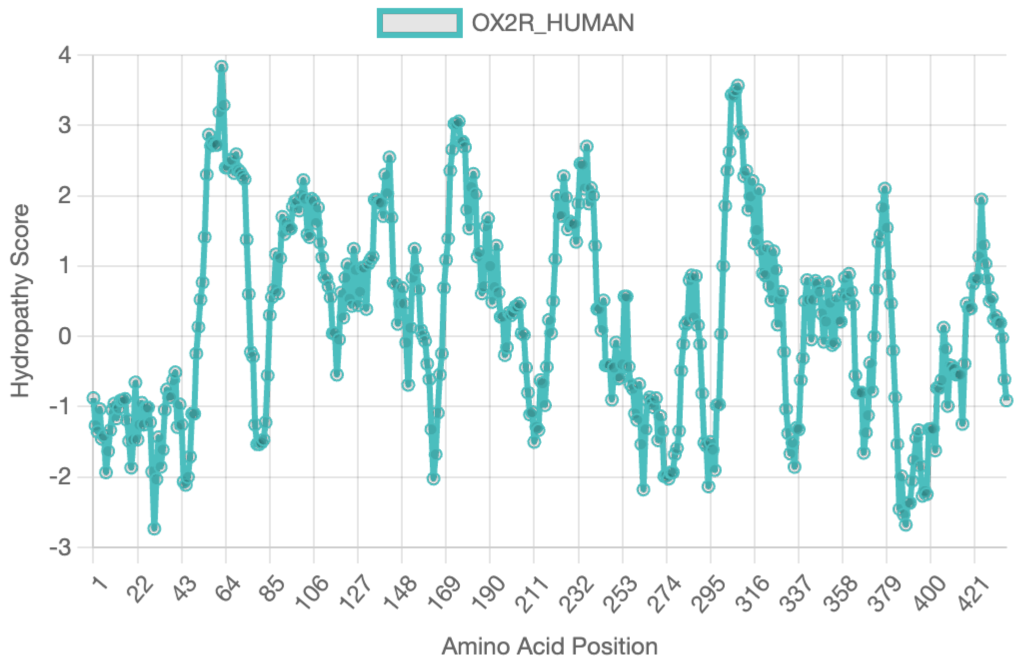

Kyte-Doolittle hydropathy plot for the sequence "OX2R_HUMAN". Positive values indicate hydrophobic regions, while negative values indicate hydrophilic regions.

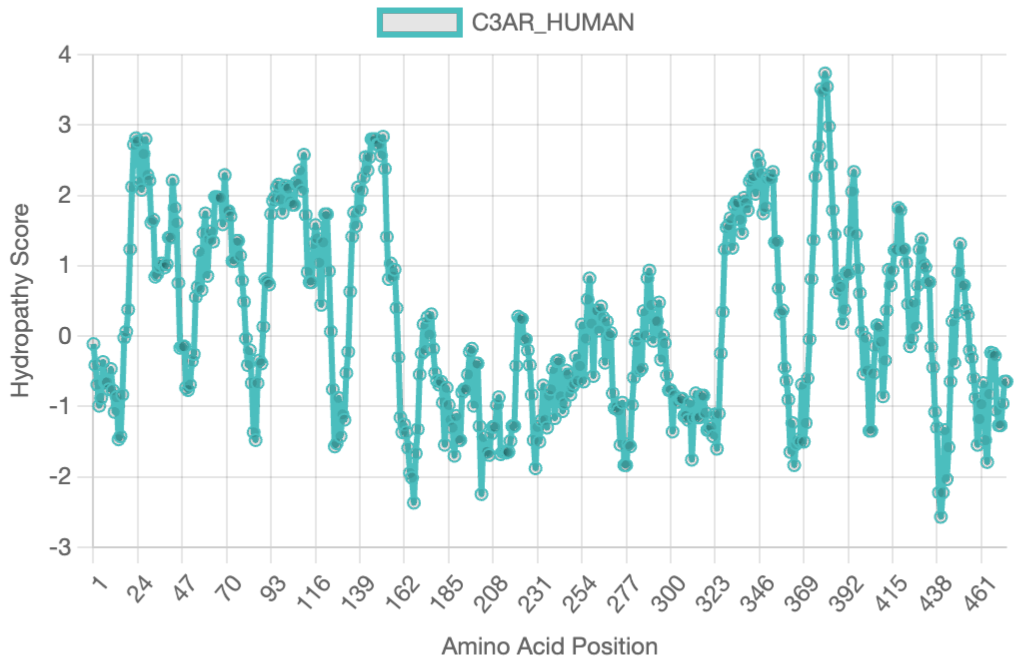

Kyte-Doolittle hydropathy plot for the sequence "C3AR_HUMAN". Positive values indicate hydrophobic regions, while negative values indicate hydrophilic regions.

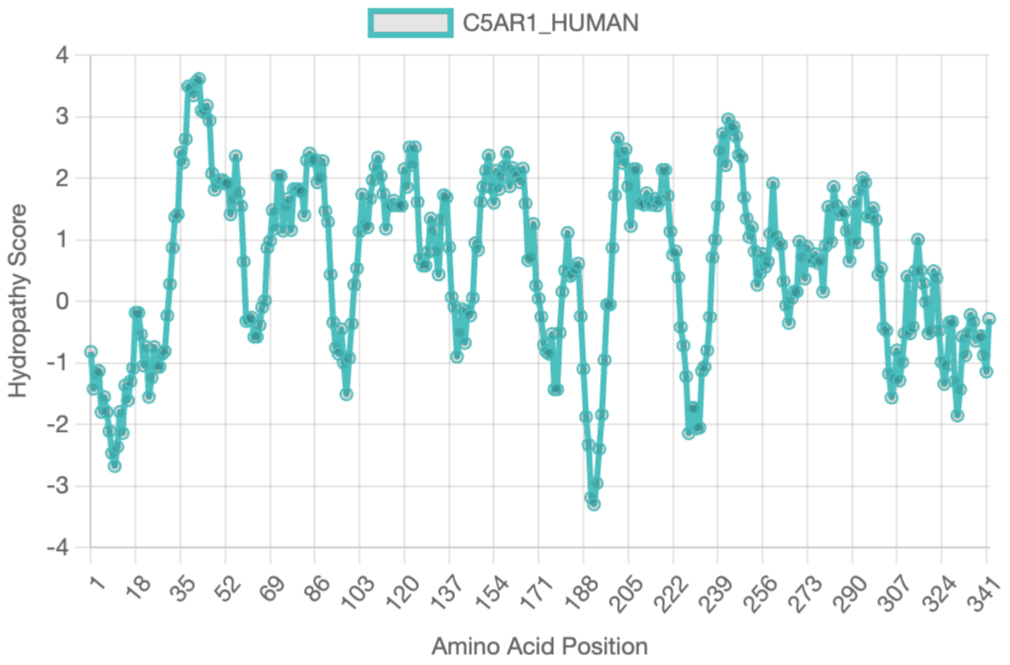

Kyte-Doolittle hydropathy plot for the sequence "C5AR1_HUMAN". Positive values indicate hydrophobic regions, while negative values indicate hydrophilic regions.

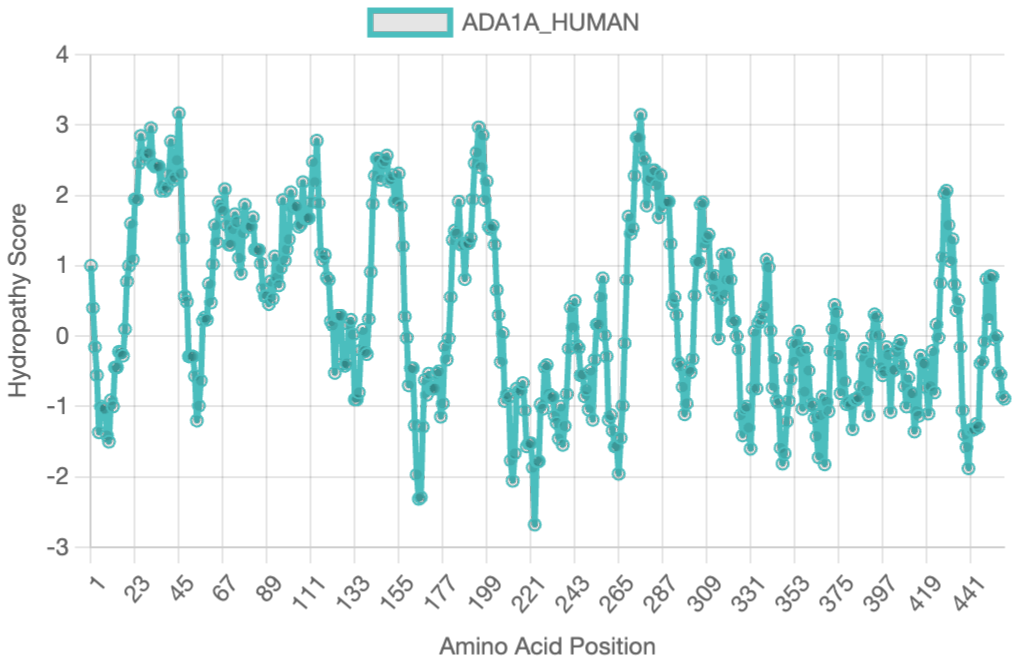

Kyte-Doolittle hydropathy plot for the sequence "ADA1A_HUMAN". Positive values indicate hydrophobic regions, while negative values indicate hydrophilic regions.

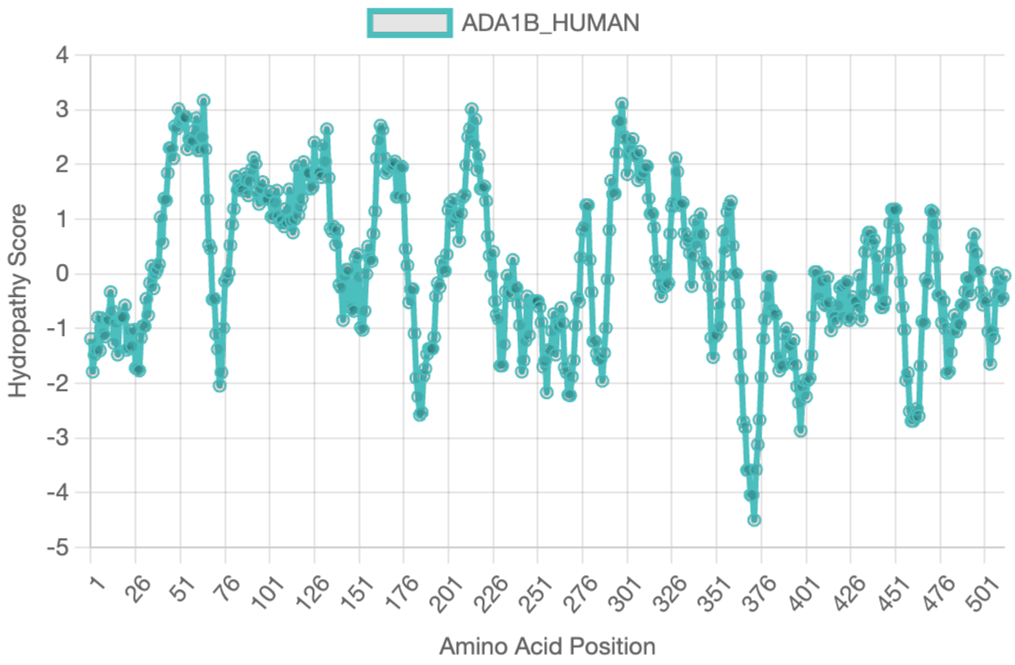

Kyte-Doolittle hydropathy plot for the sequence "ADA1B_HUMAN". Positive values indicate hydrophobic regions, while negative values indicate hydrophilic regions.

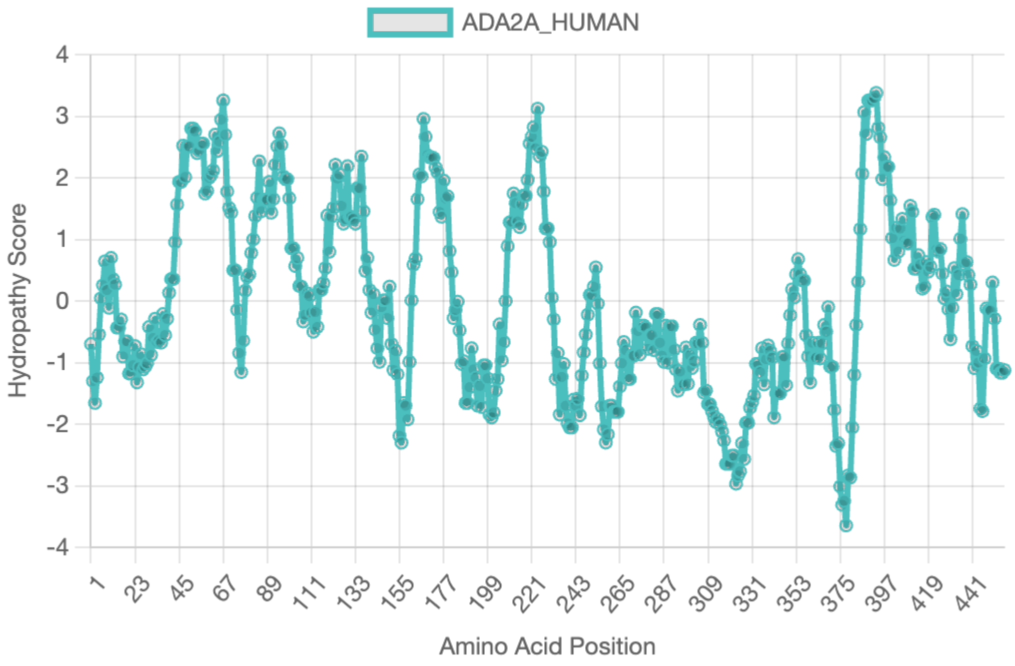

Kyte-Doolittle hydropathy plot for the sequence "ADA2A_HUMAN". Positive values indicate hydrophobic regions, while negative values indicate hydrophilic regions.

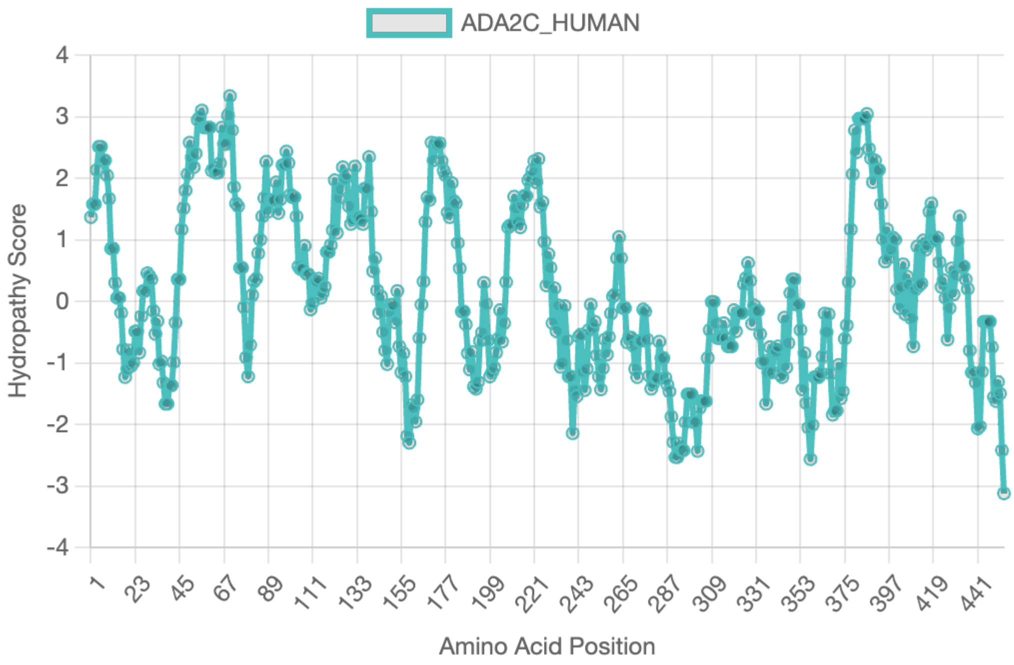

Kyte-Doolittle hydropathy plot for the sequence "ADA2C_HUMAN". Positive values indicate hydrophobic regions, while negative values indicate hydrophilic regions.

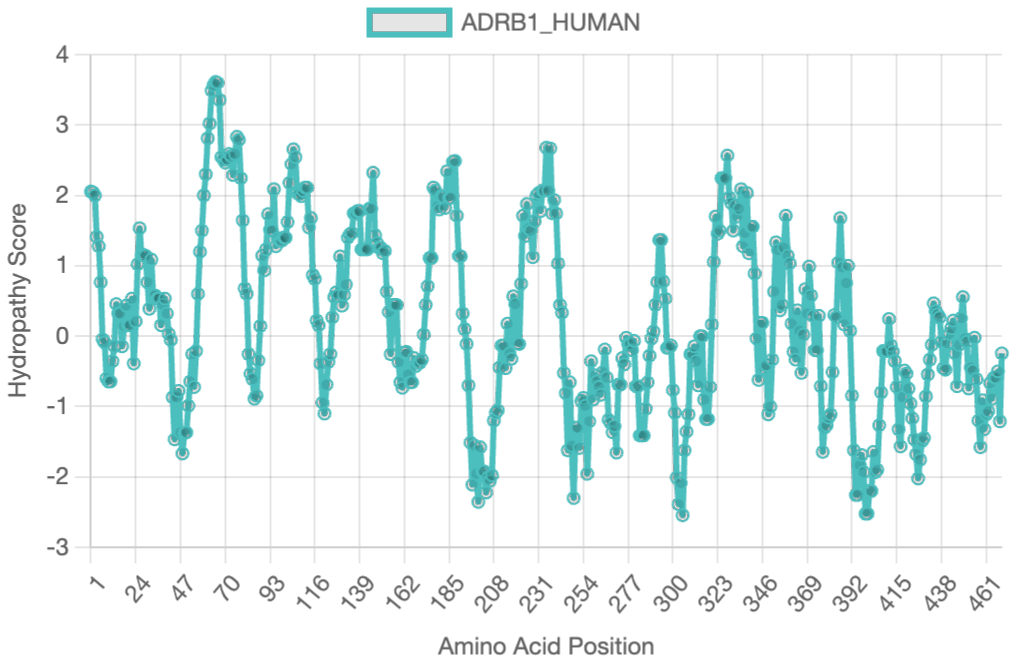

Kyte-Doolittle hydropathy plot for the sequence "ADRB1_HUMAN". Positive values indicate hydrophobic regions, while negative values indicate hydrophilic regions.

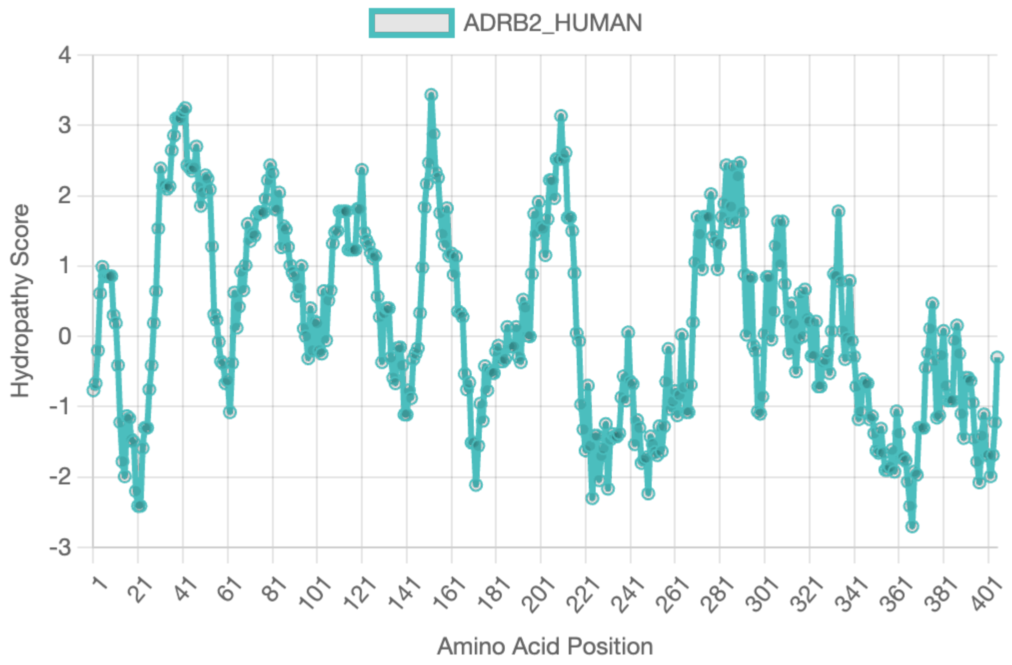

Kyte-Doolittle hydropathy plot for the sequence "ADRB2_HUMAN". Positive values indicate hydrophobic regions, while negative values indicate hydrophilic regions.

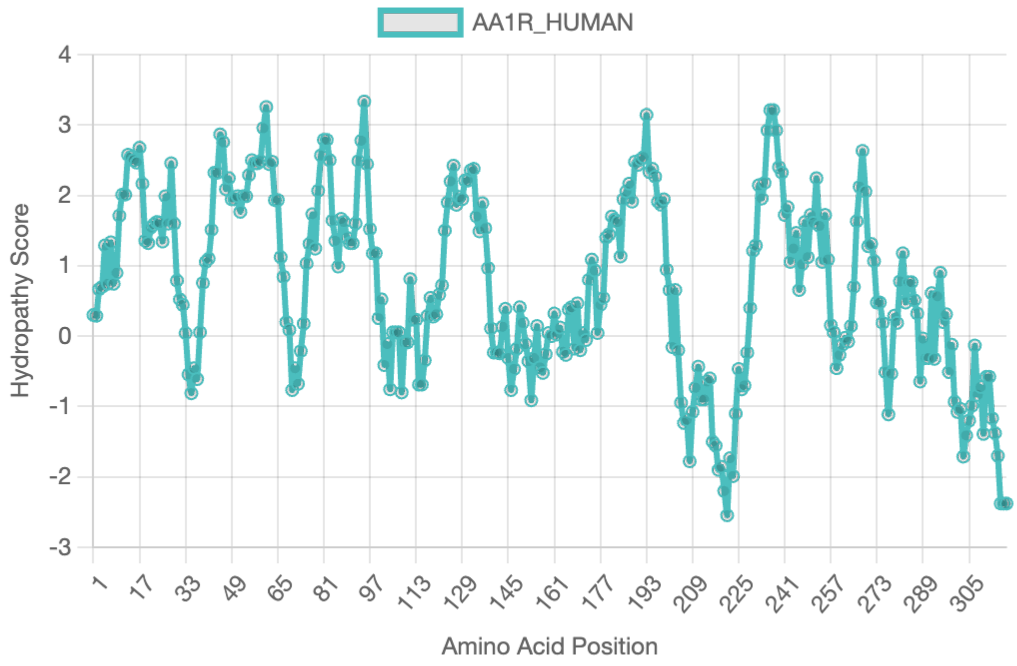

Kyte-Doolittle hydropathy plot for the sequence "AA1R_HUMAN". Positive values indicate hydrophobic regions, while negative values indicate hydrophilic regions.

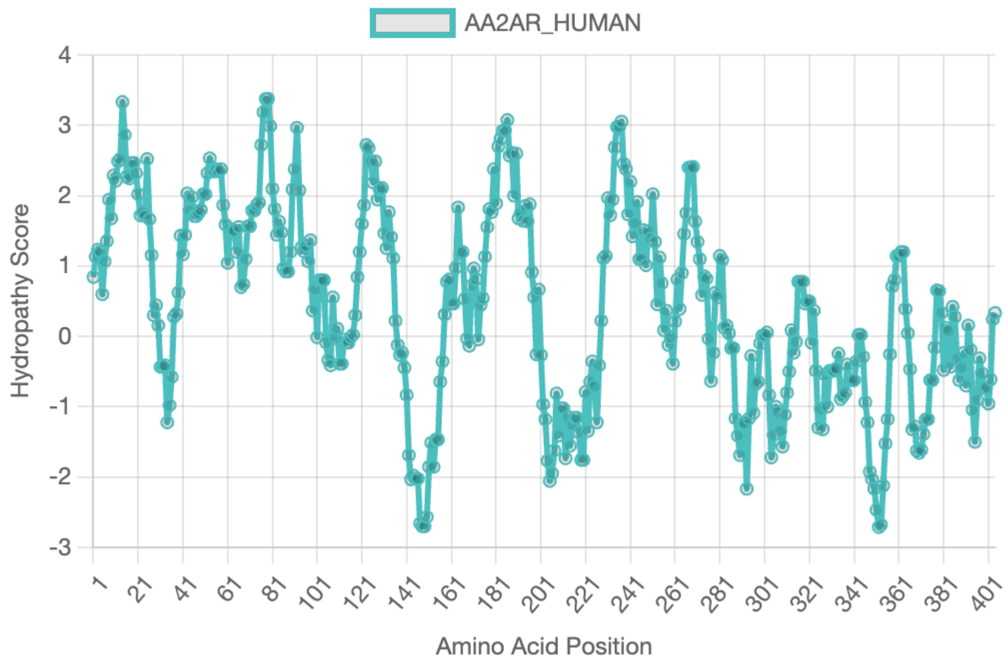

Kyte-Doolittle hydropathy plot for the sequence "AA2AR_HUMAN". Positive values indicate hydrophobic regions, while negative values indicate hydrophilic regions.

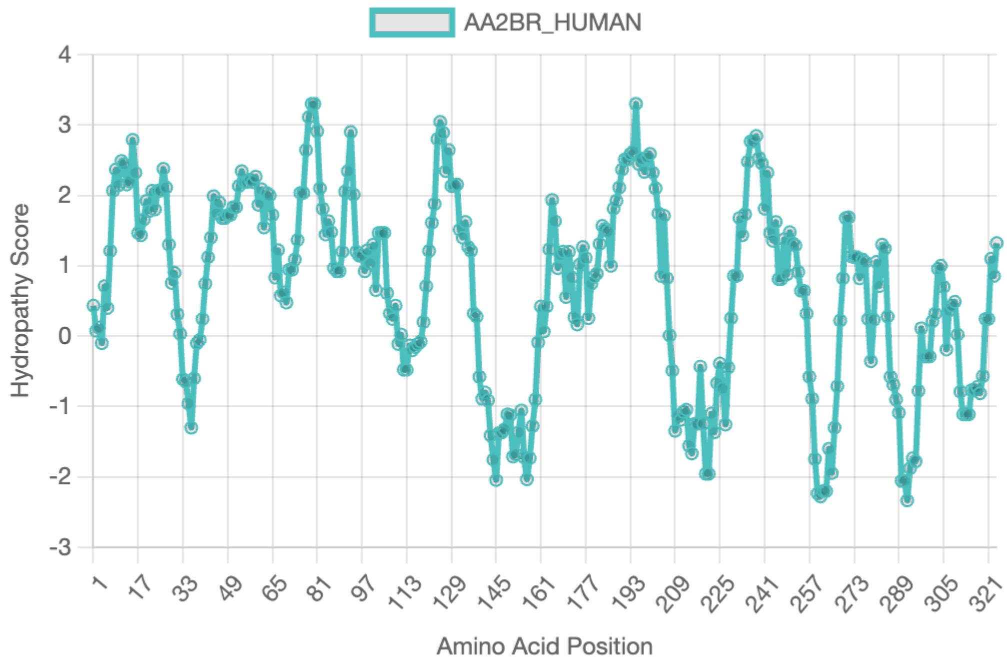

Kyte-Doolittle hydropathy plot for the sequence "AA2BR_HUMAN". Positive values indicate hydrophobic regions, while negative values indicate hydrophilic regions.

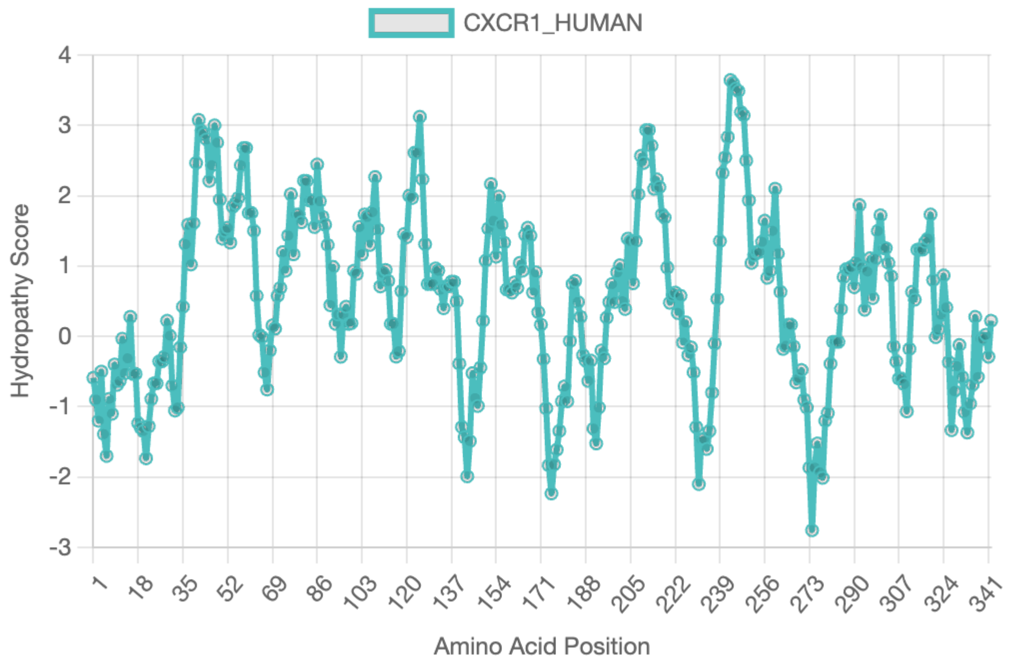

Kyte-Doolittle hydropathy plot for the sequence "CXCR1_HUMAN". Positive values indicate hydrophobic regions, while negative values indicate hydrophilic regions.

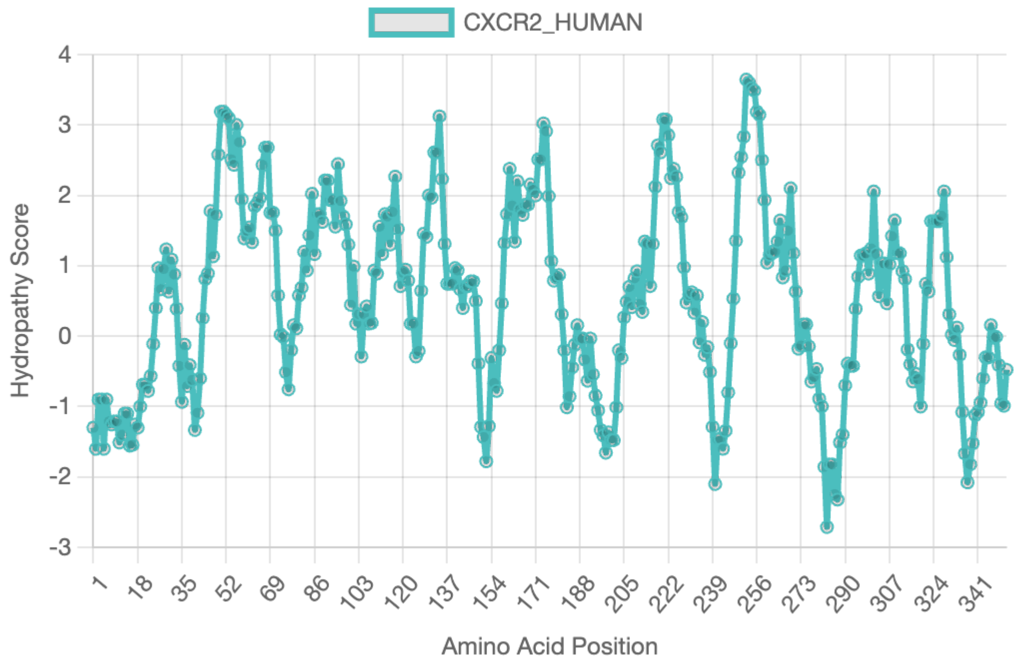

Kyte-Doolittle hydropathy plot for the sequence "CXCR2_HUMAN". Positive values indicate hydrophobic regions, while negative values indicate hydrophilic regions.

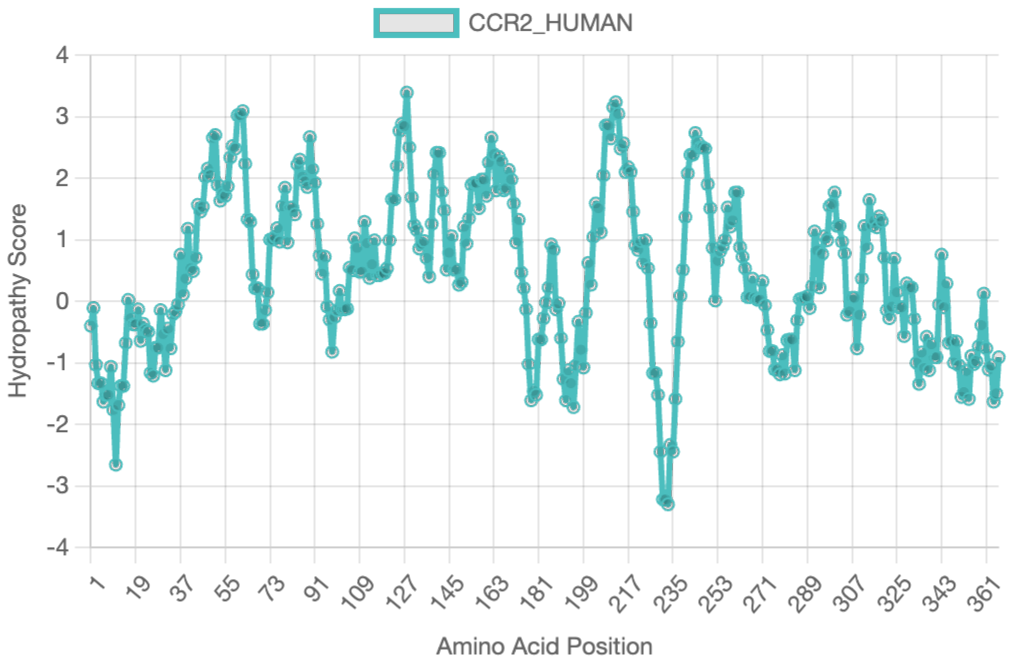

Kyte-Doolittle hydropathy plot for the sequence "CCR2_HUMAN". Positive values indicate hydrophobic regions, while negative values indicate hydrophilic regions.

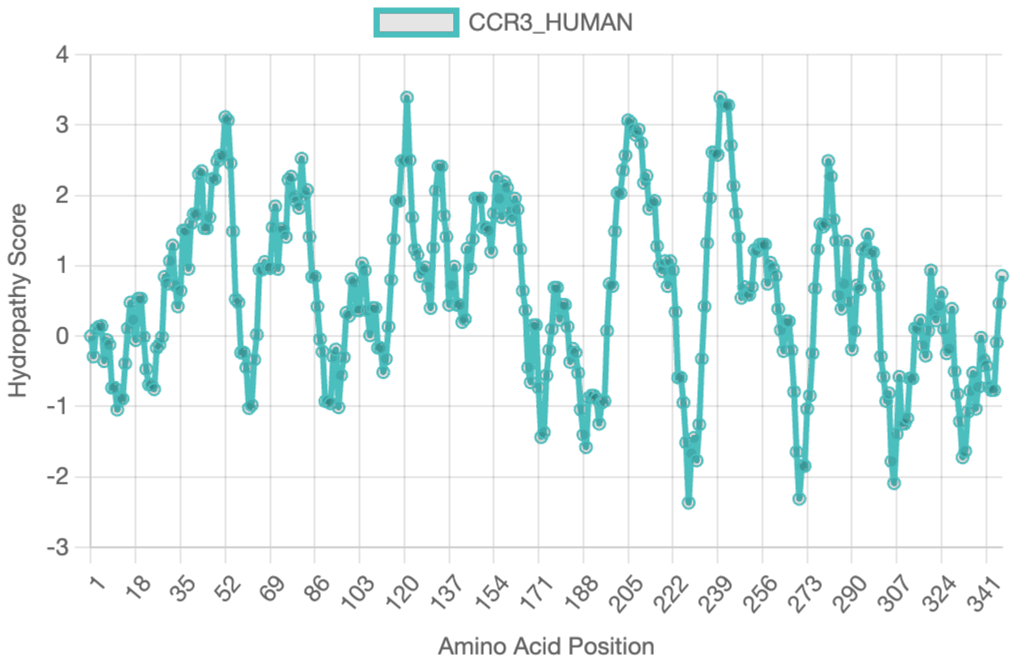

Kyte-Doolittle hydropathy plot for the sequence "CCR3_HUMAN". Positive values indicate hydrophobic regions, while negative values indicate hydrophilic regions.

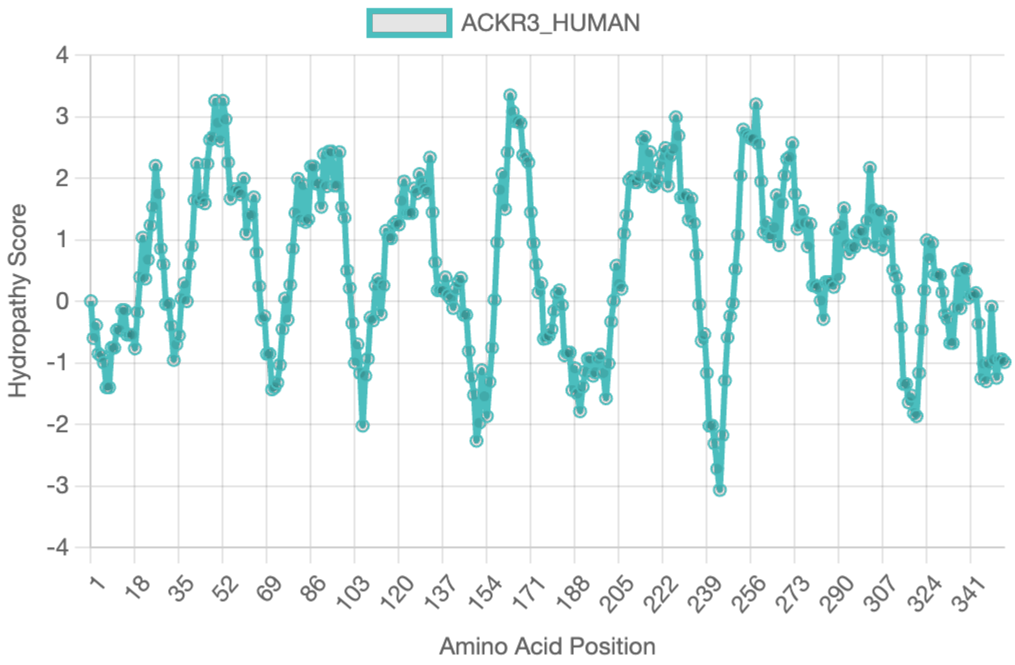

Kyte-Doolittle hydropathy plot for the sequence "ACKR3_HUMAN". Positive values indicate hydrophobic regions, while negative values indicate hydrophilic regions.

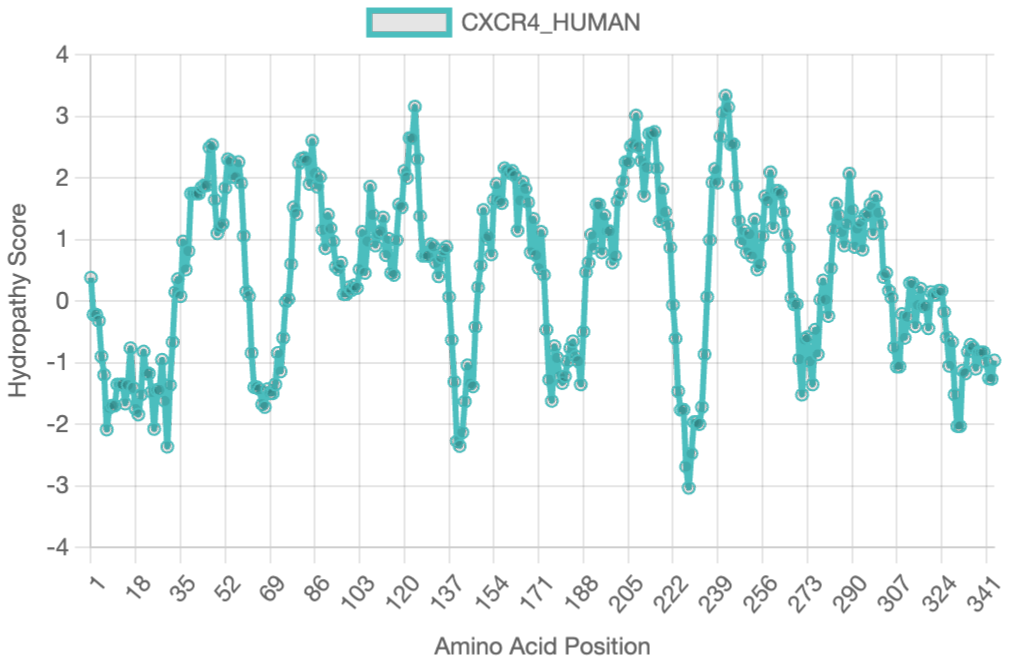

Kyte-Doolittle hydropathy plot for the sequence "CXCR4_HUMAN". Positive values indicate hydrophobic regions, while negative values indicate hydrophilic regions.

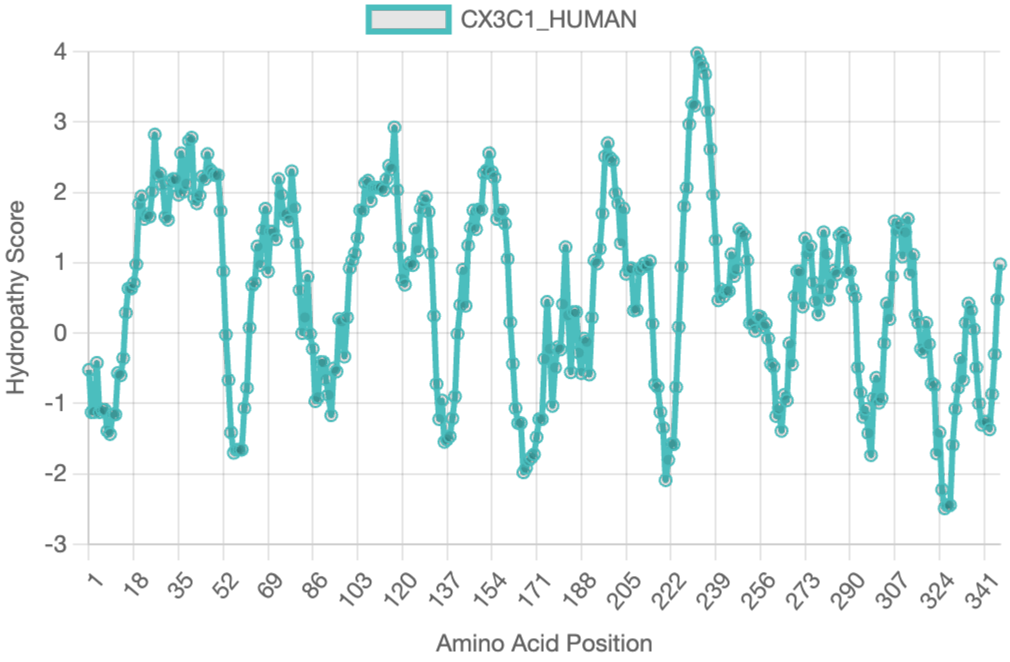

Kyte-Doolittle hydropathy plot for the sequence "CX3C1_HUMAN". Positive values indicate hydrophobic regions, while negative values indicate hydrophilic regions.

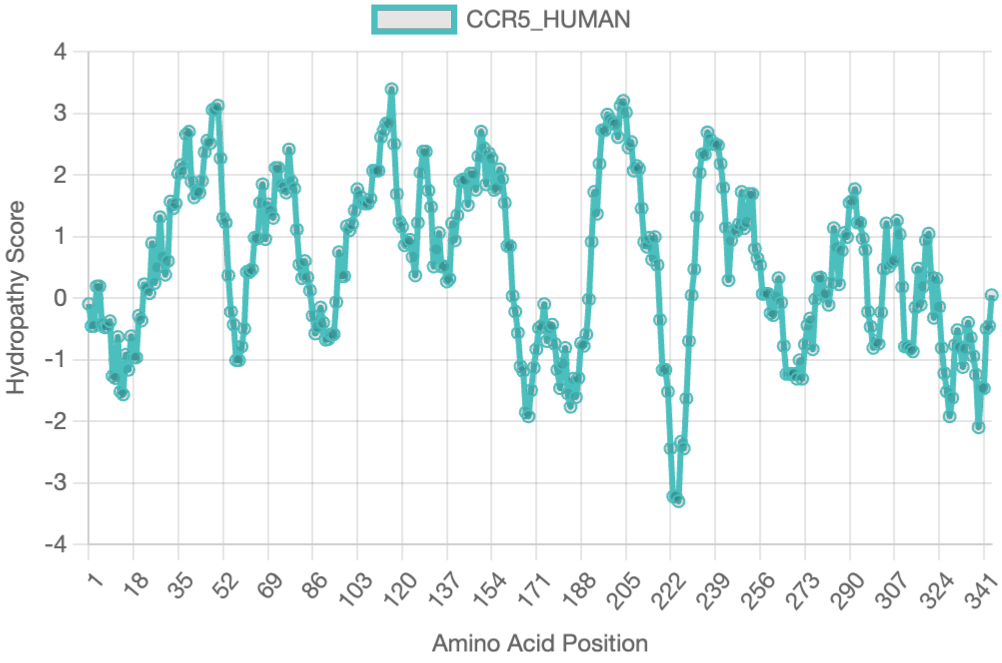

Kyte-Doolittle hydropathy plot for the sequence "CCR5_HUMAN". Positive values indicate hydrophobic regions, while negative values indicate hydrophilic regions.

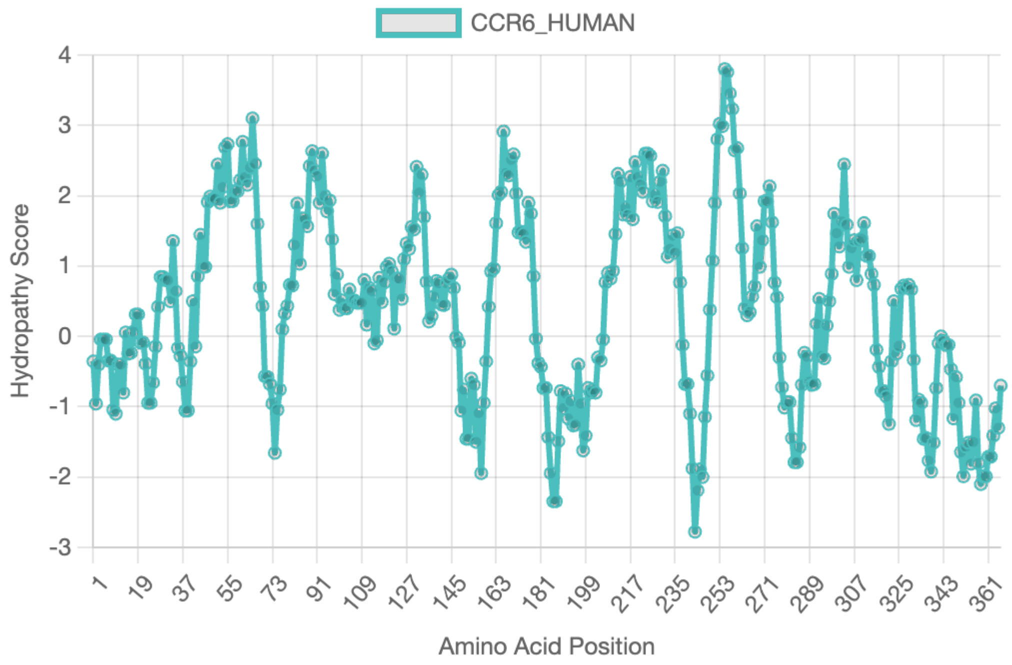

Kyte-Doolittle hydropathy plot for the sequence "CCR6_HUMAN". Positive values indicate hydrophobic regions, while negative values indicate hydrophilic regions.

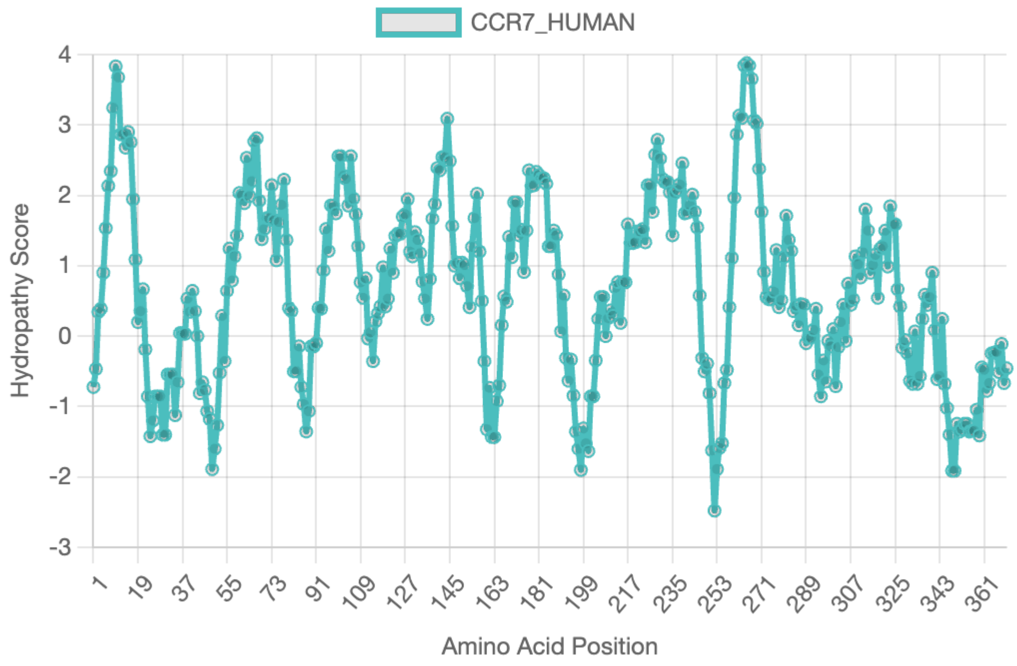

Kyte-Doolittle hydropathy plot for the sequence "CCR7_HUMAN". Positive values indicate hydrophobic regions, while negative values indicate hydrophilic regions.

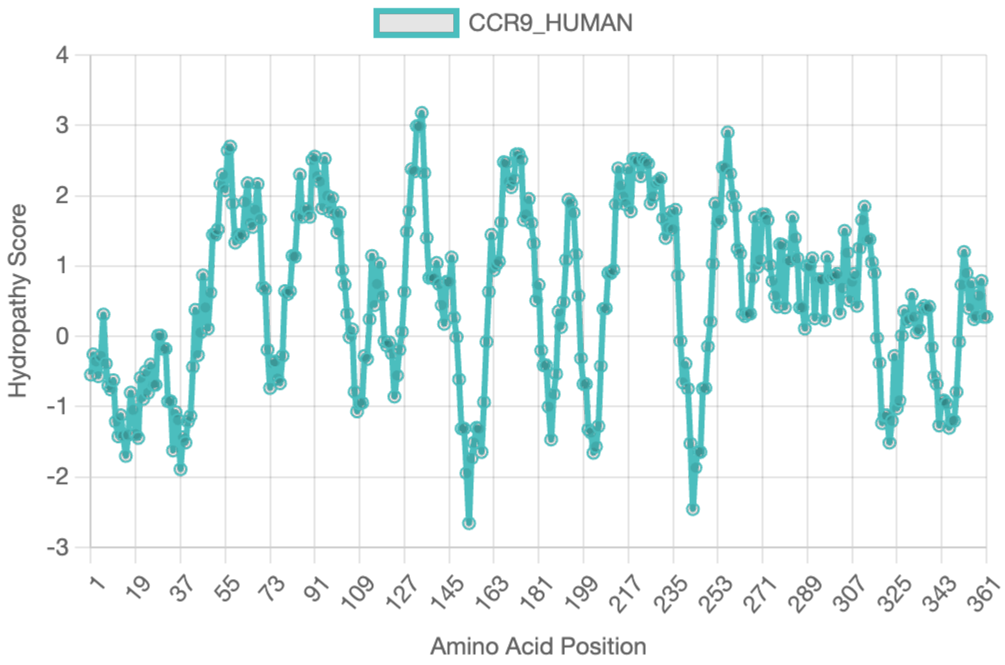

Kyte-Doolittle hydropathy plot for the sequence "CCR9_HUMAN". Positive values indicate hydrophobic regions, while negative values indicate hydrophilic regions.

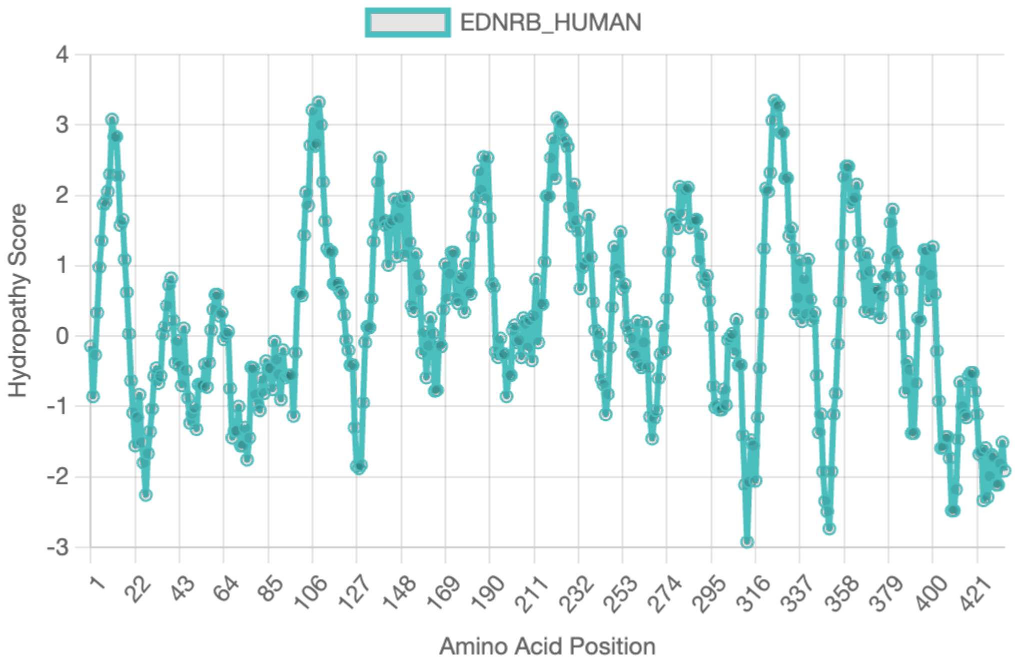

Kyte-Doolittle hydropathy plot for the sequence "EDNRB_HUMAN". Positive values indicate hydrophobic regions, while negative values indicate hydrophilic regions.

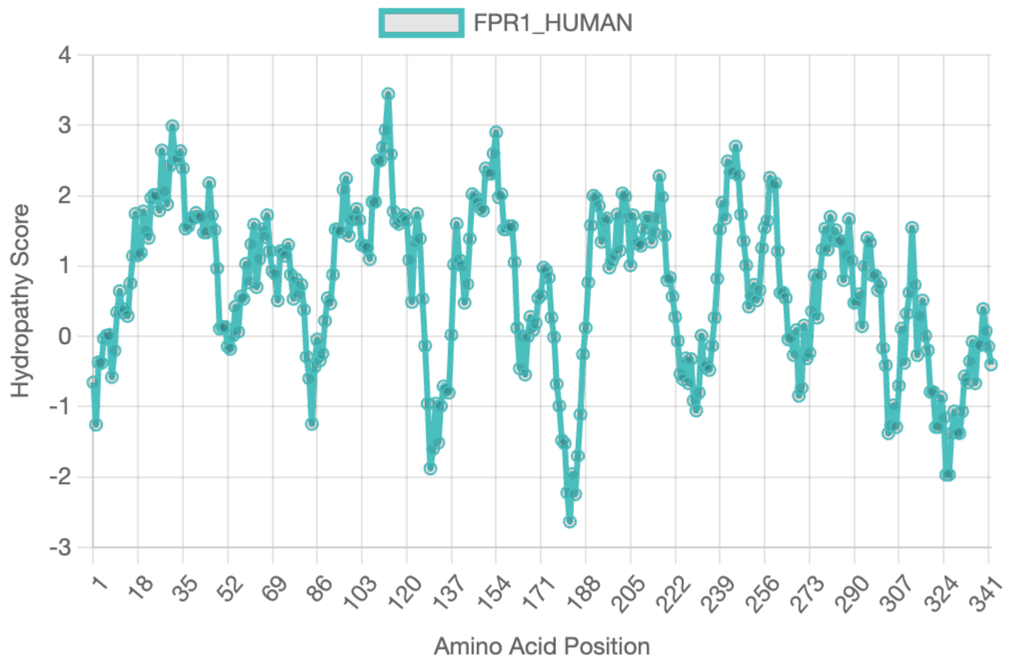

Kyte-Doolittle hydropathy plot for the sequence "FPR1_HUMAN". Positive values indicate hydrophobic regions, while negative values indicate hydrophilic regions.

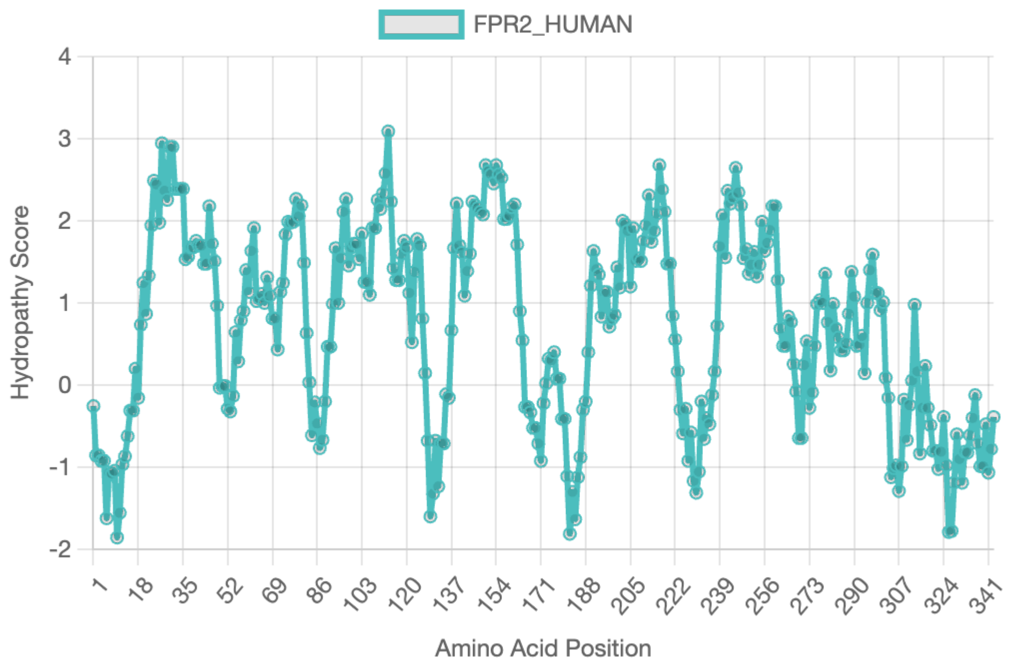

Kyte-Doolittle hydropathy plot for the sequence "FPR2_HUMAN". Positive values indicate hydrophobic regions, while negative values indicate hydrophilic regions.

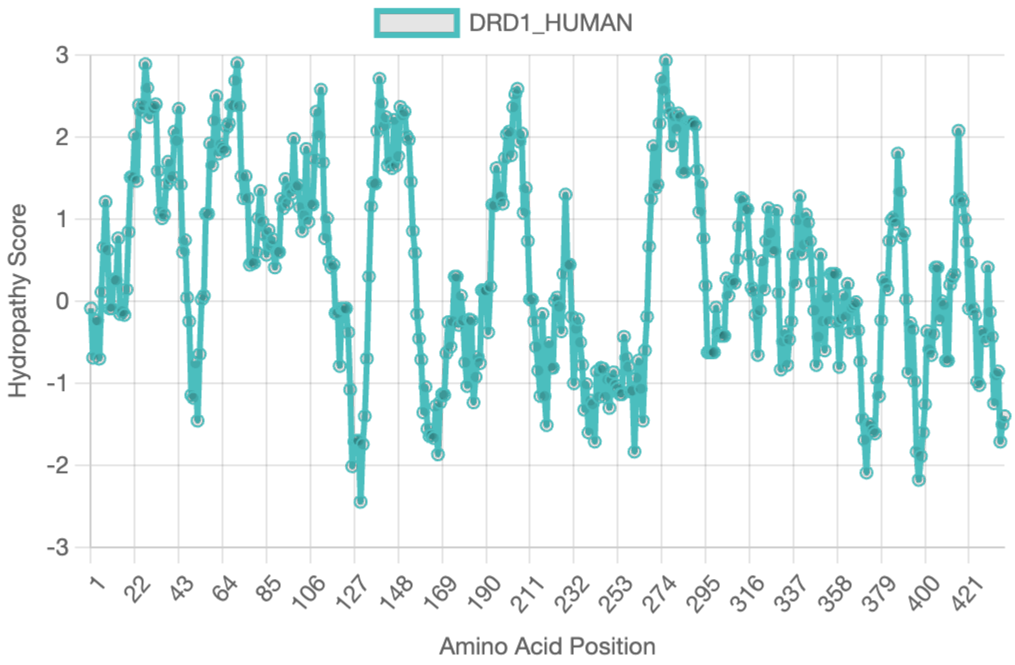

Kyte-Doolittle hydropathy plot for the sequence "DRD1_HUMAN". Positive values indicate hydrophobic regions, while negative values indicate hydrophilic regions.

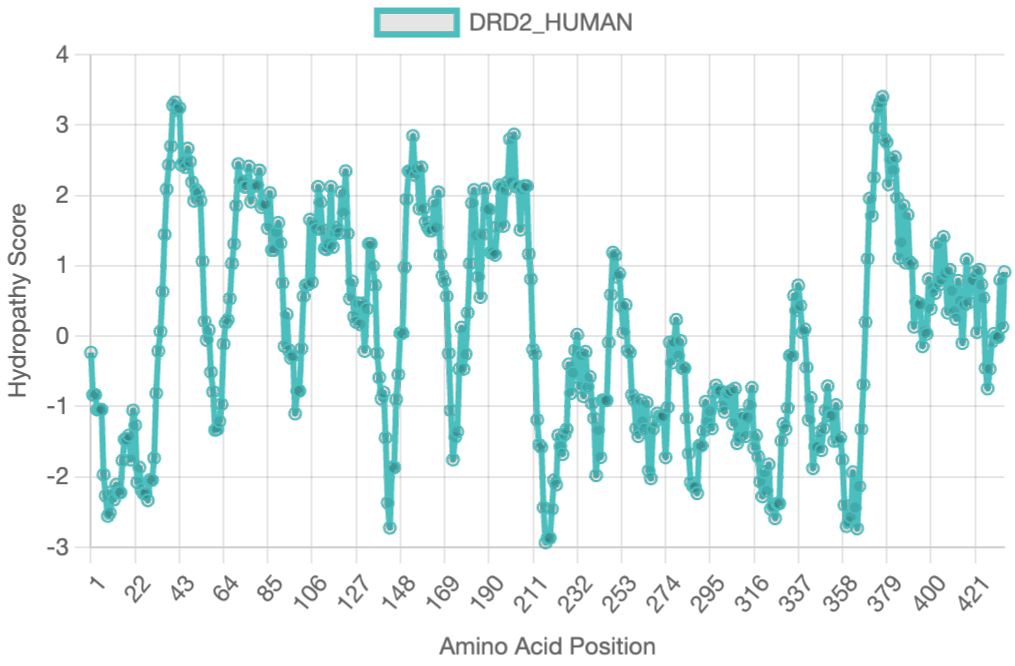

Kyte-Doolittle hydropathy plot for the sequence "DRD2_HUMAN". Positive values indicate hydrophobic regions, while negative values indicate hydrophilic regions.

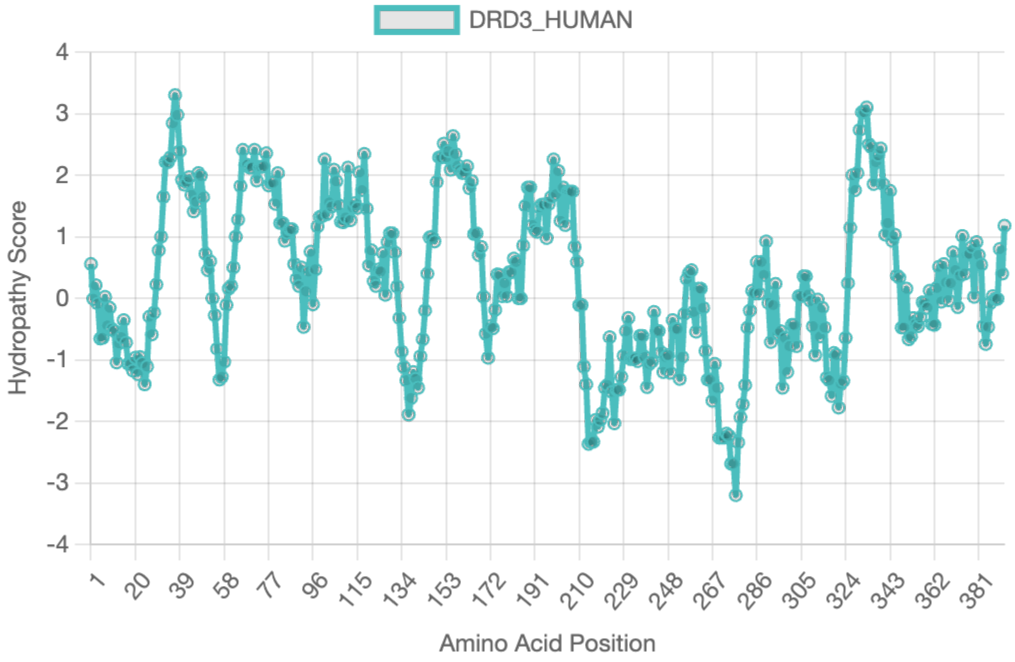

Kyte-Doolittle hydropathy plot for the sequence "DRD3_HUMAN". Positive values indicate hydrophobic regions, while negative values indicate hydrophilic regions.

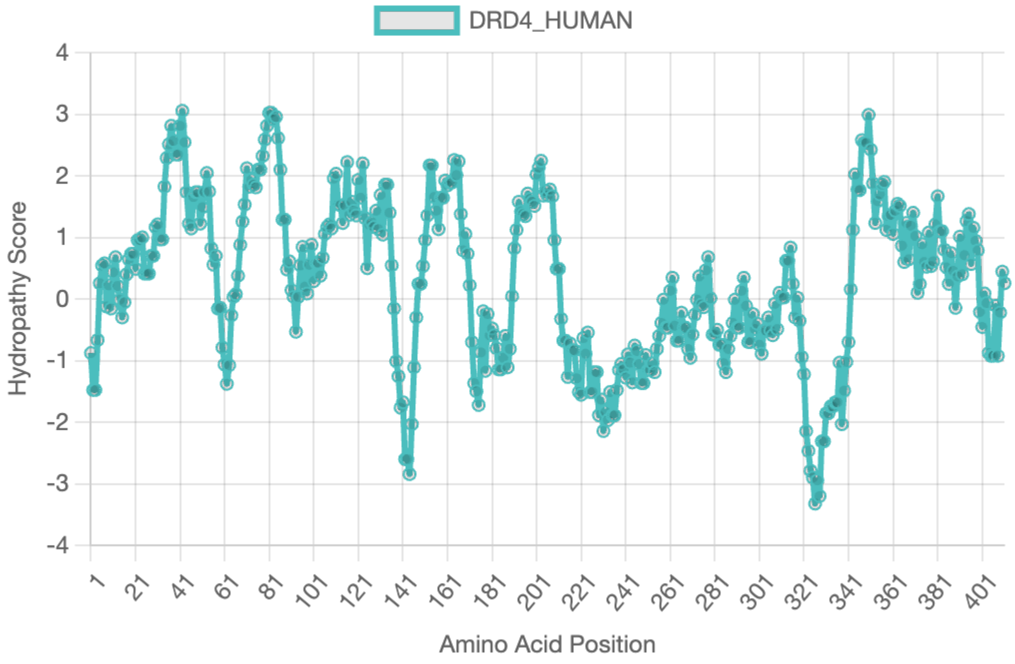

Kyte-Doolittle hydropathy plot for the sequence "DRD4_HUMAN". Positive values indicate hydrophobic regions, while negative values indicate hydrophilic regions.

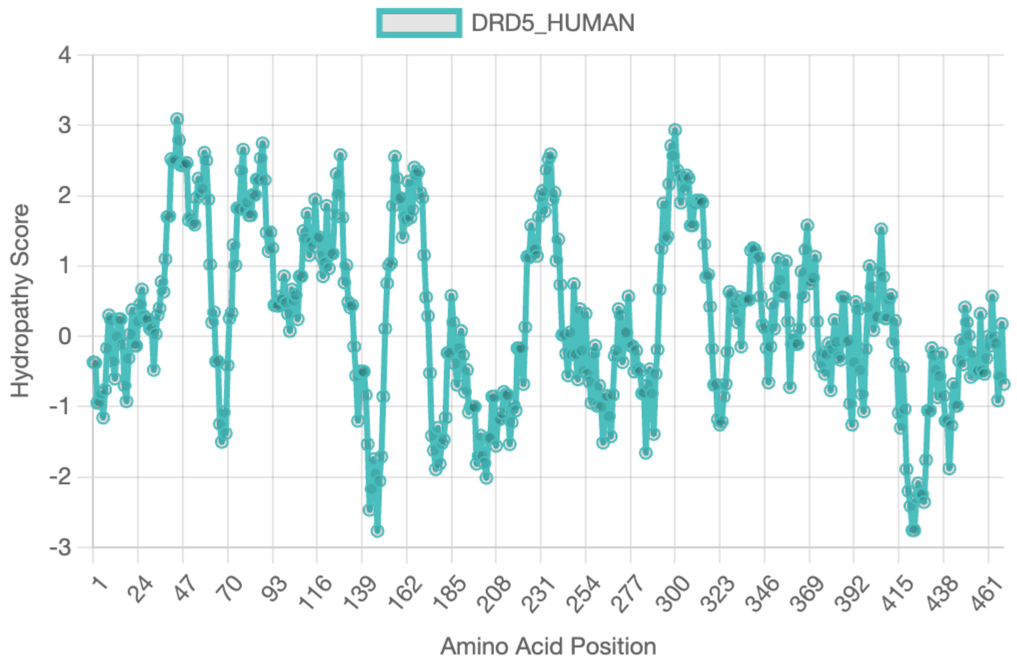

Kyte-Doolittle hydropathy plot for the sequence "DRD5_HUMAN". Positive values indicate hydrophobic regions, while negative values indicate hydrophilic regions.

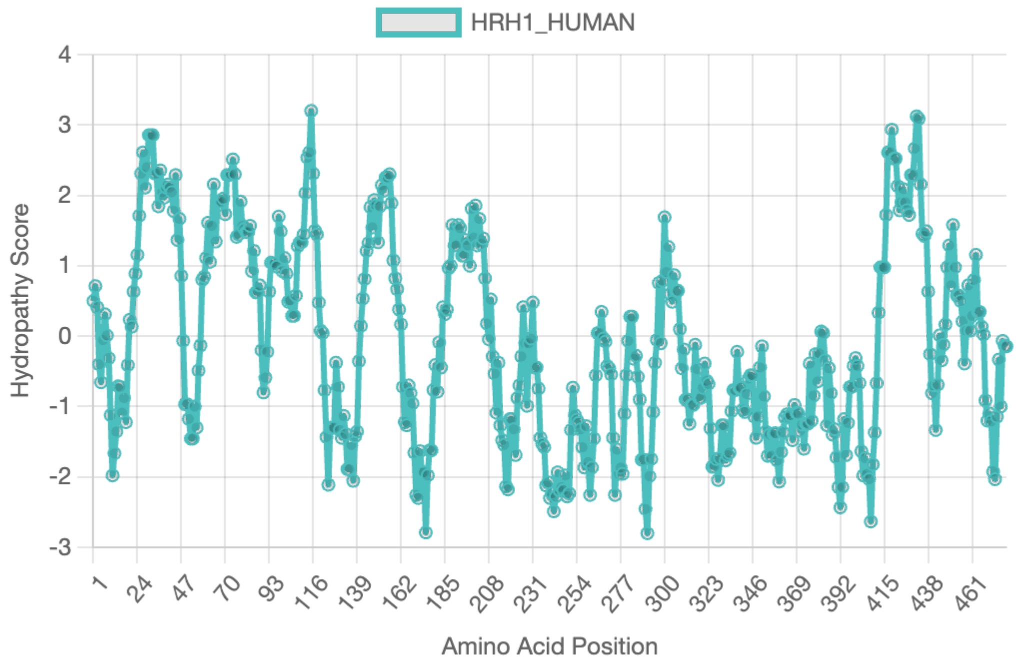

Kyte-Doolittle hydropathy plot for the sequence "HRH1_HUMAN". Positive values indicate hydrophobic regions, while negative values indicate hydrophilic regions.

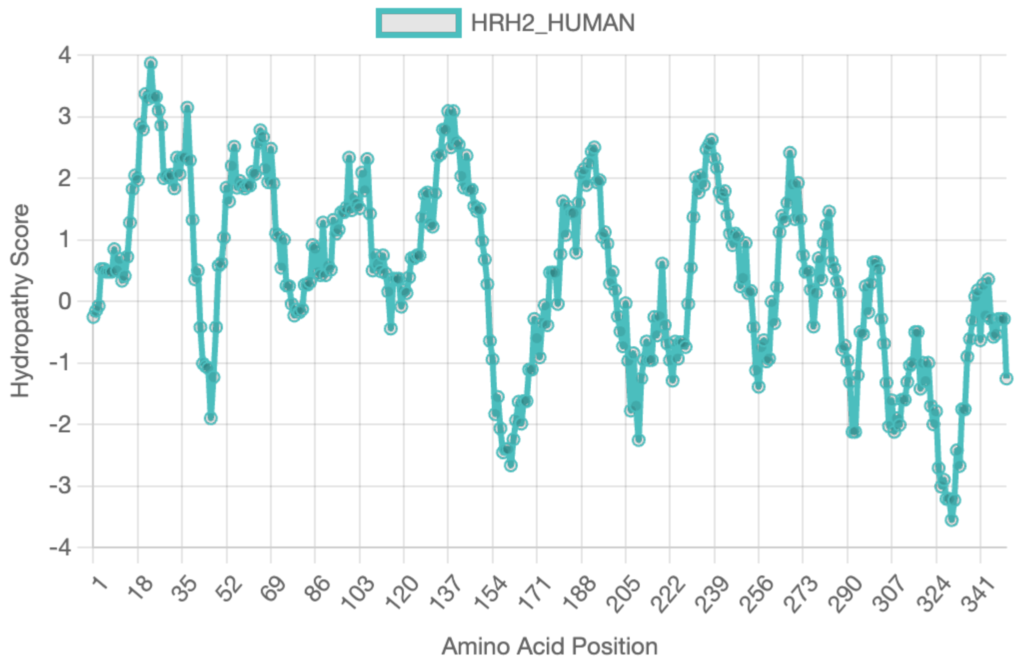

Kyte-Doolittle hydropathy plot for the sequence "HRH2_HUMAN". Positive values indicate hydrophobic regions, while negative values indicate hydrophilic regions.

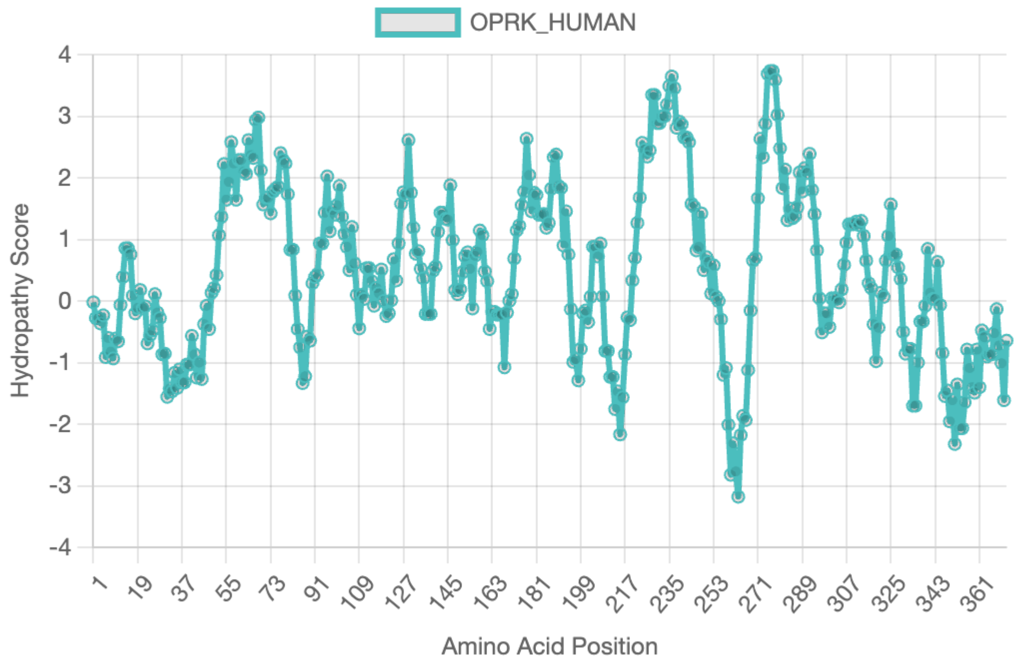

Kyte-Doolittle hydropathy plot for the sequence "OPRK_HUMAN". Positive values indicate hydrophobic regions, while negative values indicate hydrophilic regions.

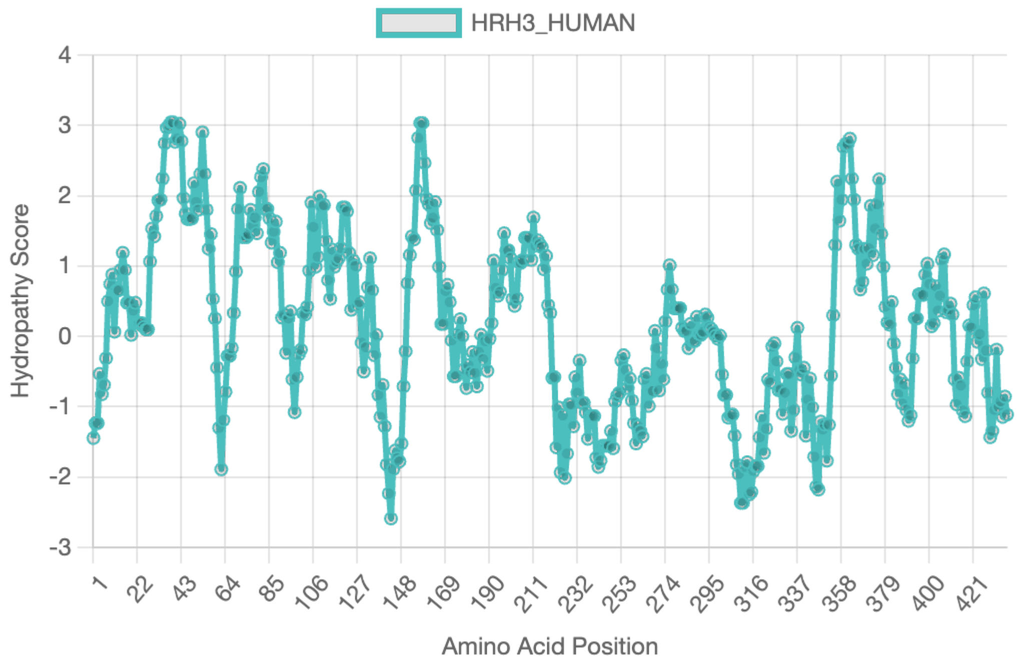

Kyte-Doolittle hydropathy plot for the sequence "HRH3_HUMAN". Positive values indicate hydrophobic regions, while negative values indicate hydrophilic regions.

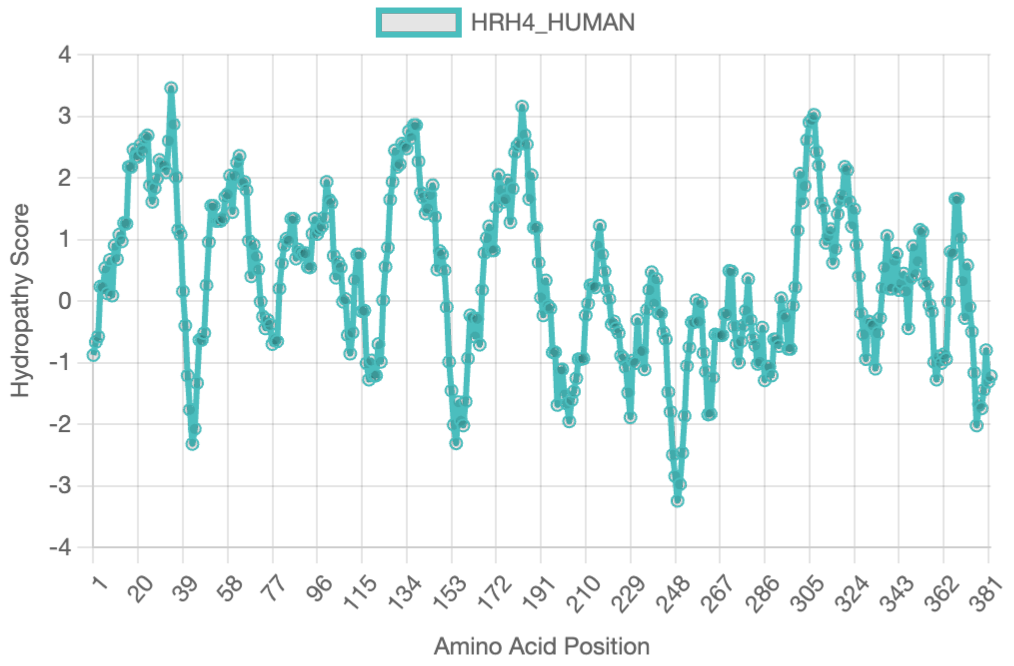

Kyte-Doolittle hydropathy plot for the sequence "HRH4_HUMAN". Positive values indicate hydrophobic regions, while negative values indicate hydrophilic regions.

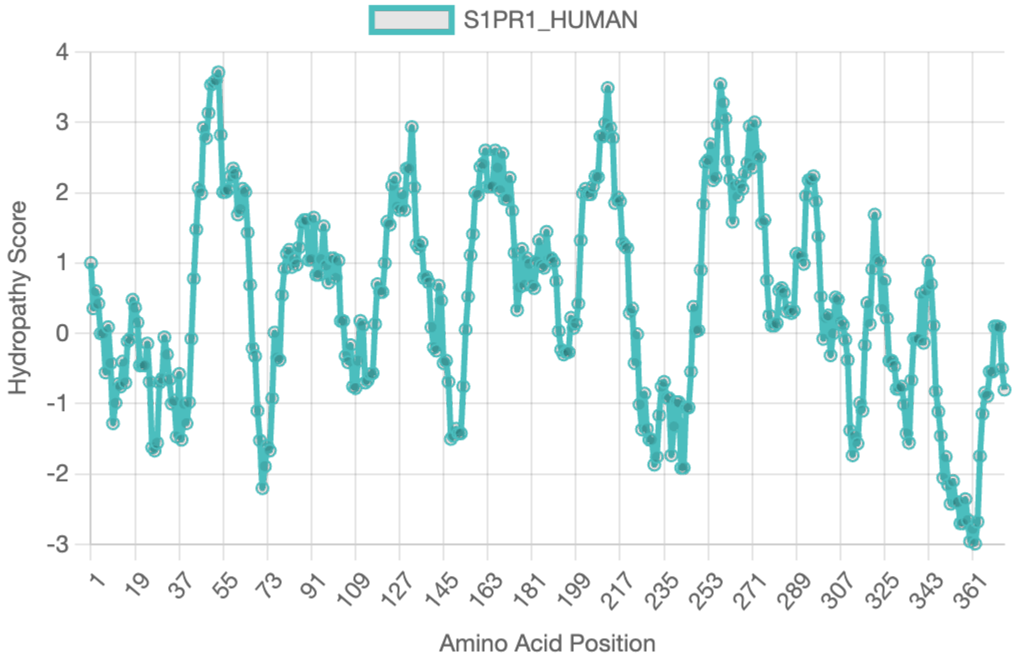

Kyte-Doolittle hydropathy plot for the sequence "S1PR1_HUMAN". Positive values indicate hydrophobic regions, while negative values indicate hydrophilic regions.

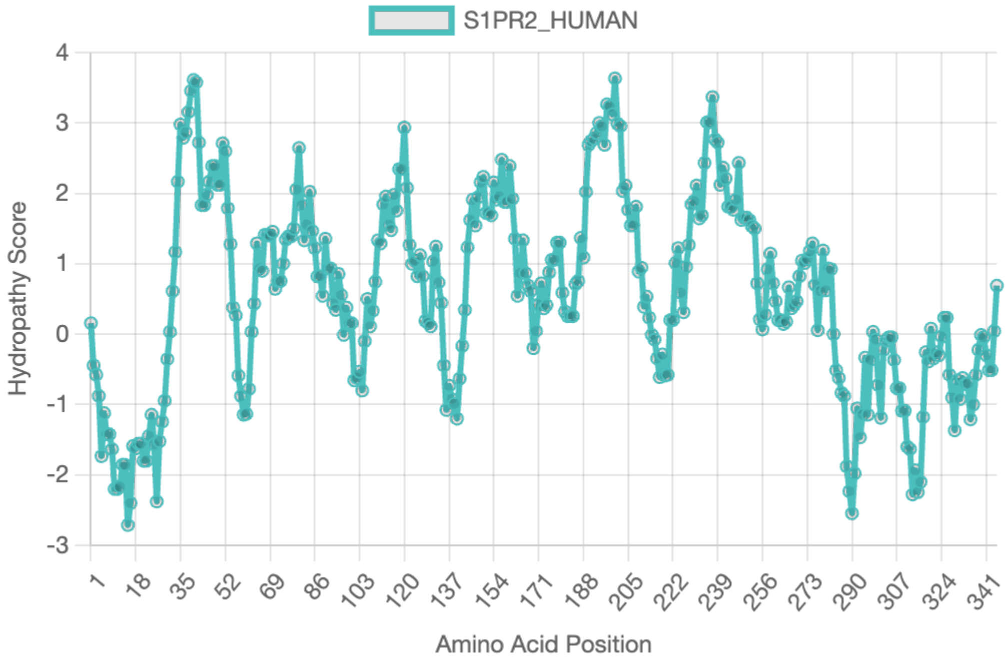

Kyte-Doolittle hydropathy plot for the sequence "S1PR2_HUMAN". Positive values indicate hydrophobic regions, while negative values indicate hydrophilic regions.

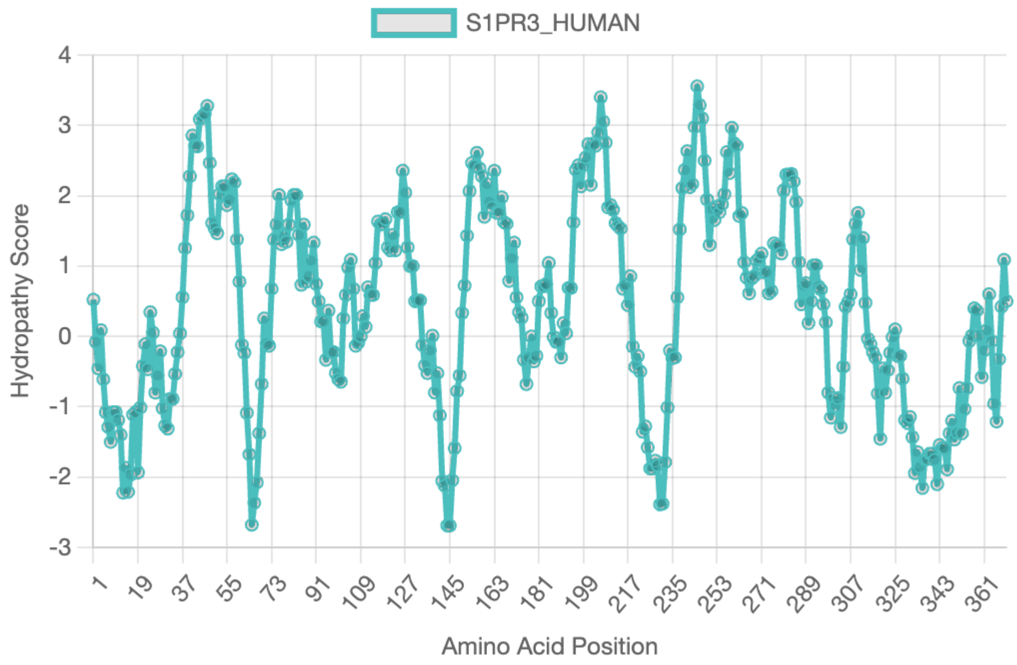

Kyte-Doolittle hydropathy plot for the sequence "S1PR3_HUMAN". Positive values indicate hydrophobic regions, while negative values indicate hydrophilic regions.

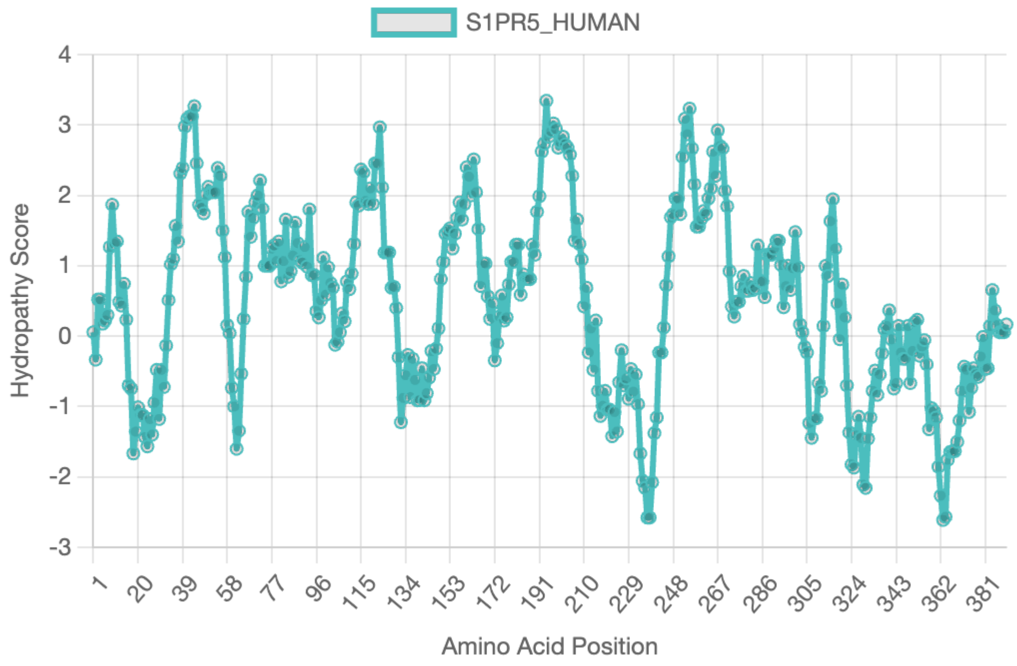

Kyte-Doolittle hydropathy plot for the sequence "S1PR5_HUMAN". Positive values indicate hydrophobic regions, while negative values indicate hydrophilic regions.

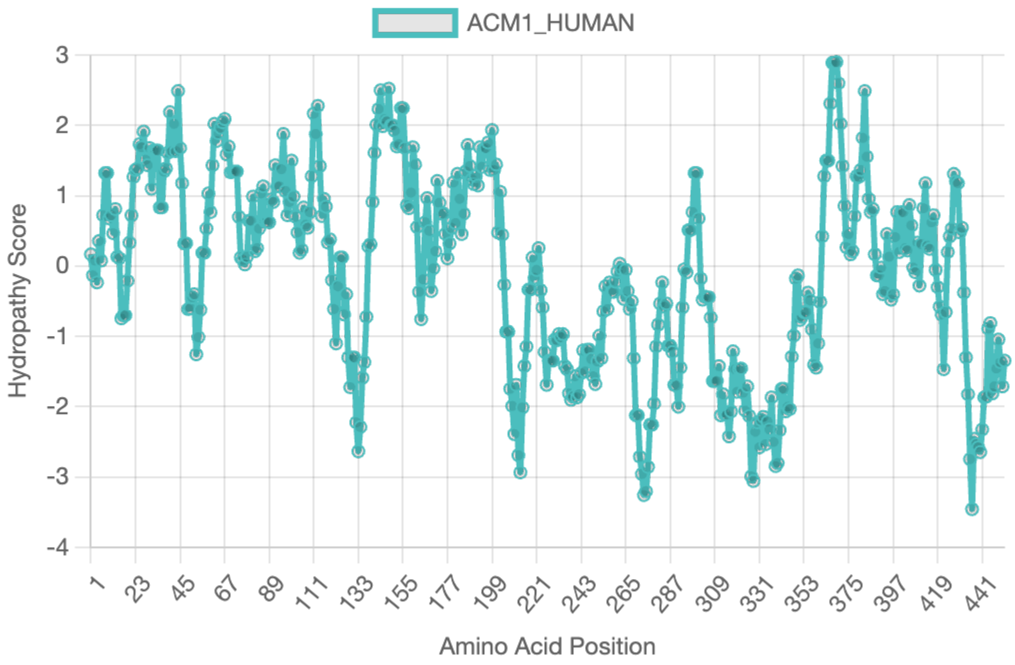

Kyte-Doolittle hydropathy plot for the sequence "ACM1_HUMAN". Positive values indicate hydrophobic regions, while negative values indicate hydrophilic regions.

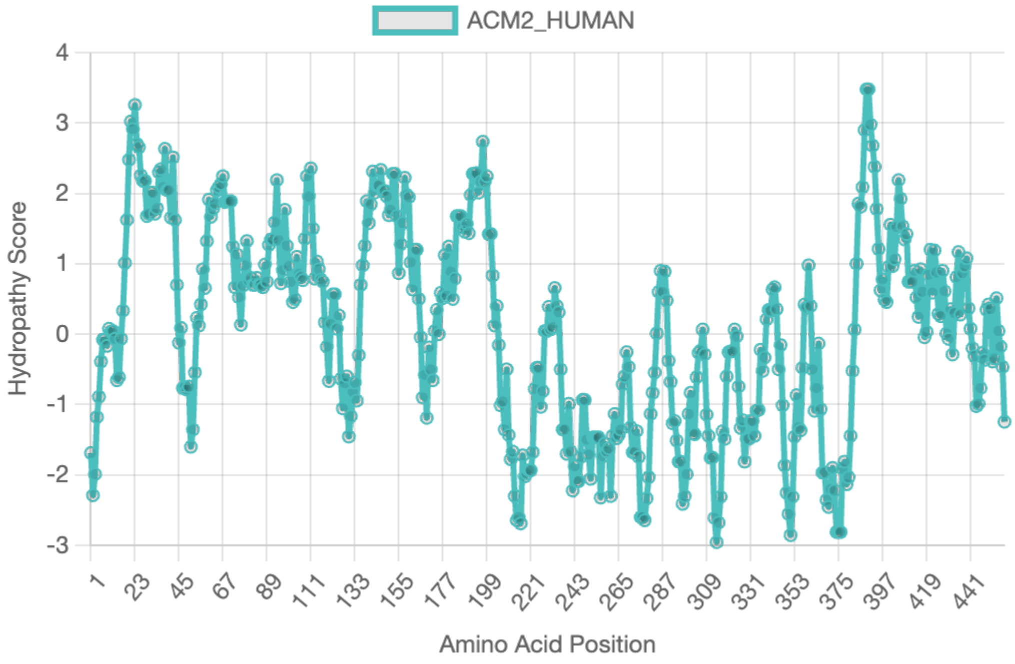

Kyte-Doolittle hydropathy plot for the sequence "ACM2_HUMAN". Positive values indicate hydrophobic regions, while negative values indicate hydrophilic regions.

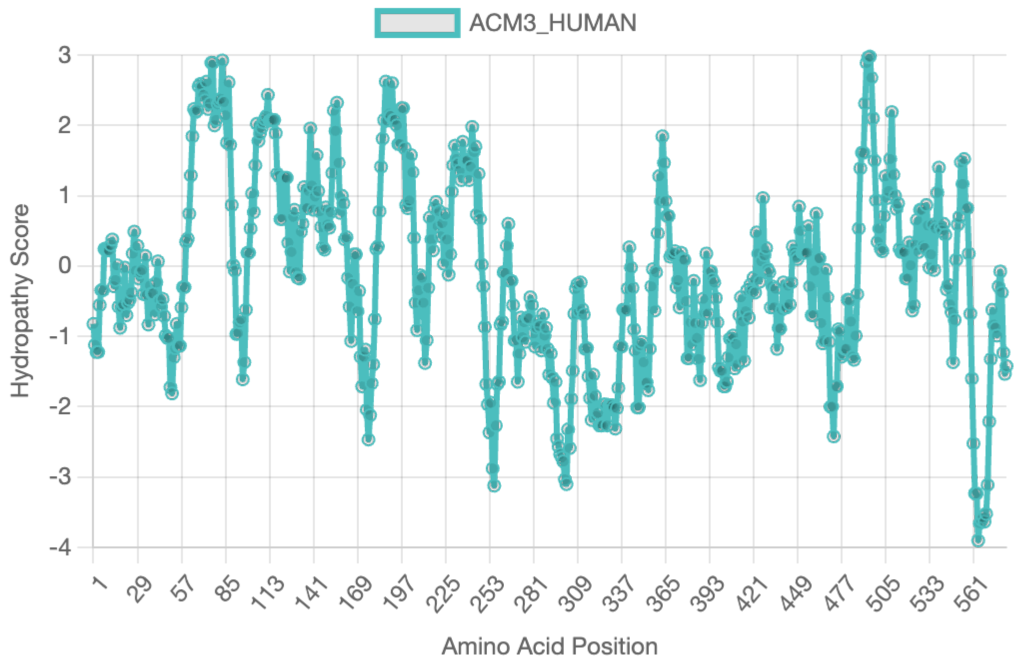

Kyte-Doolittle hydropathy plot for the sequence "ACM3_HUMAN". Positive values indicate hydrophobic regions, while negative values indicate hydrophilic regions.

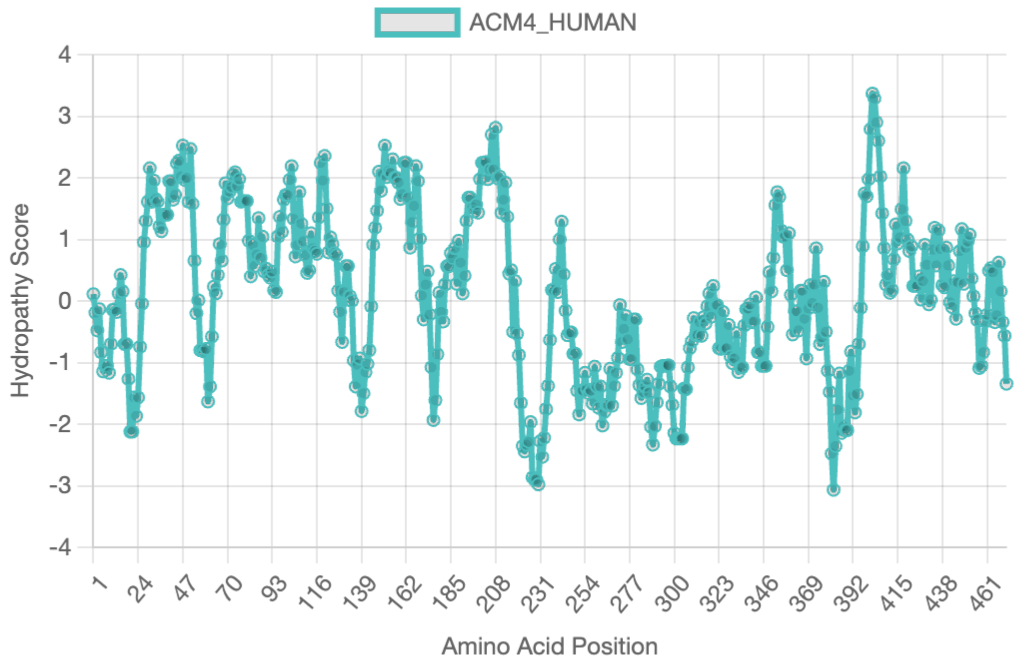

Kyte-Doolittle hydropathy plot for the sequence "ACM4_HUMAN". Positive values indicate hydrophobic regions, while negative values indicate hydrophilic regions.

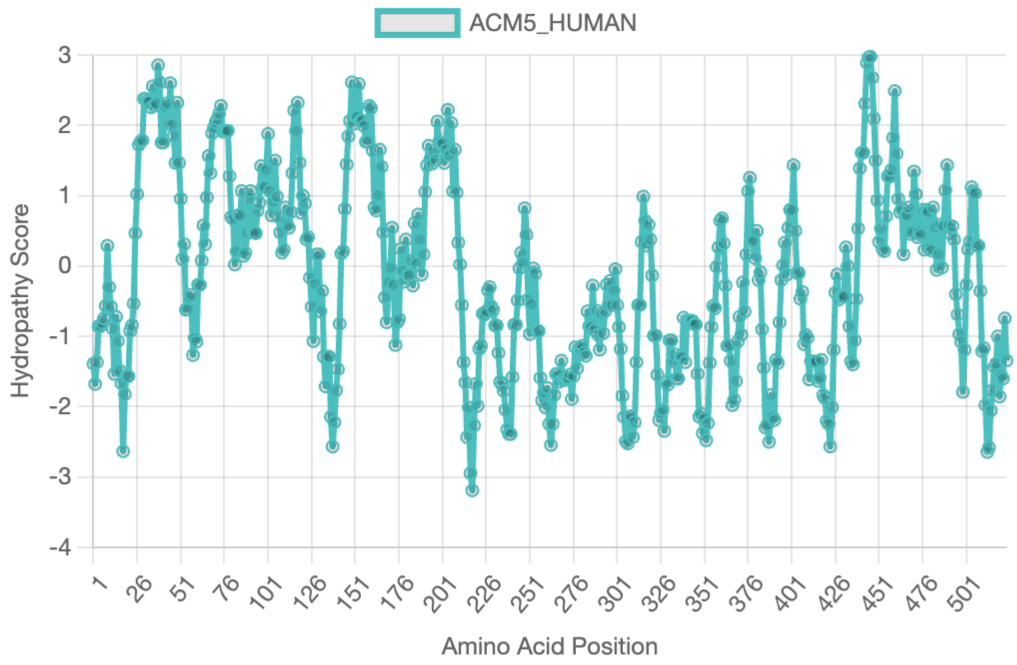

Kyte-Doolittle hydropathy plot for the sequence "ACM5_HUMAN". Positive values indicate hydrophobic regions, while negative values indicate hydrophilic regions.

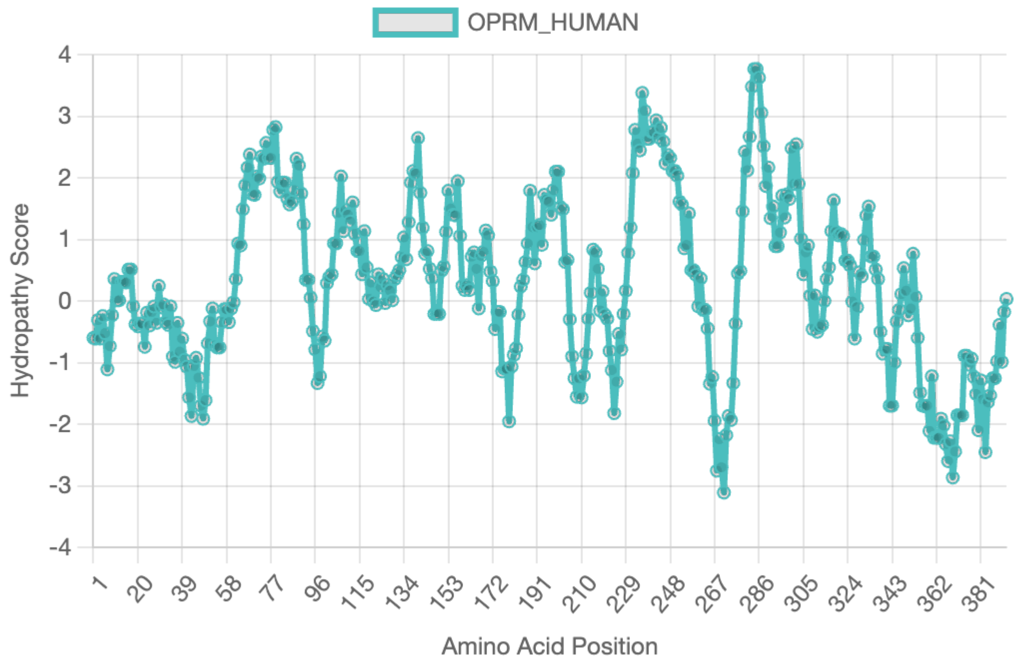

Kyte-Doolittle hydropathy plot for the sequence "OPRM_HUMAN". Positive values indicate hydrophobic regions, while negative values indicate hydrophilic regions.

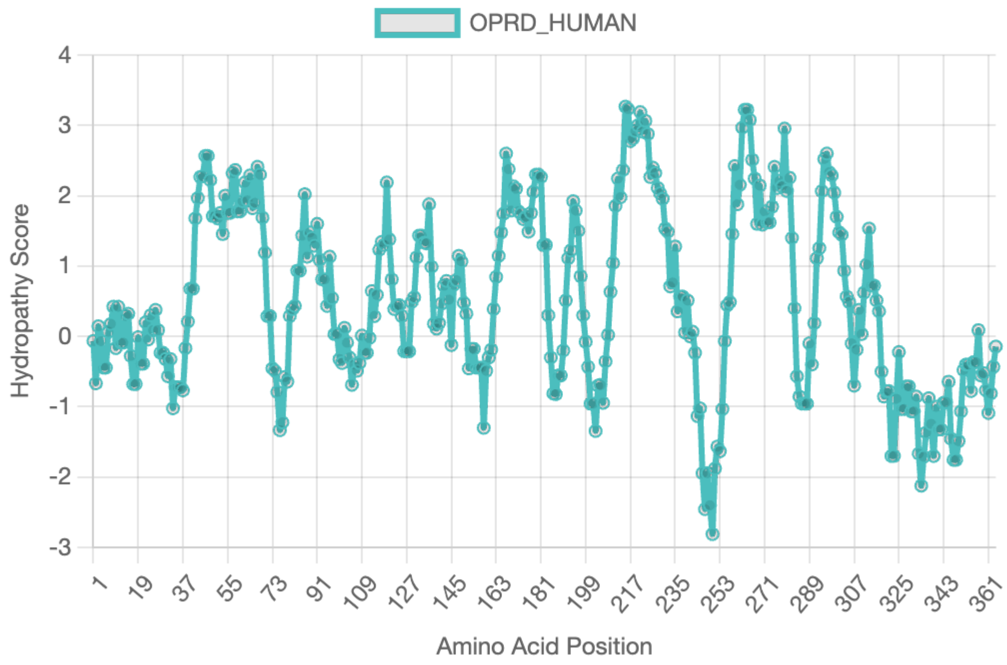

Kyte-Doolittle hydropathy plot for the sequence "OPRD_HUMAN". Positive values indicate hydrophobic regions, while negative values indicate hydrophilic regions.

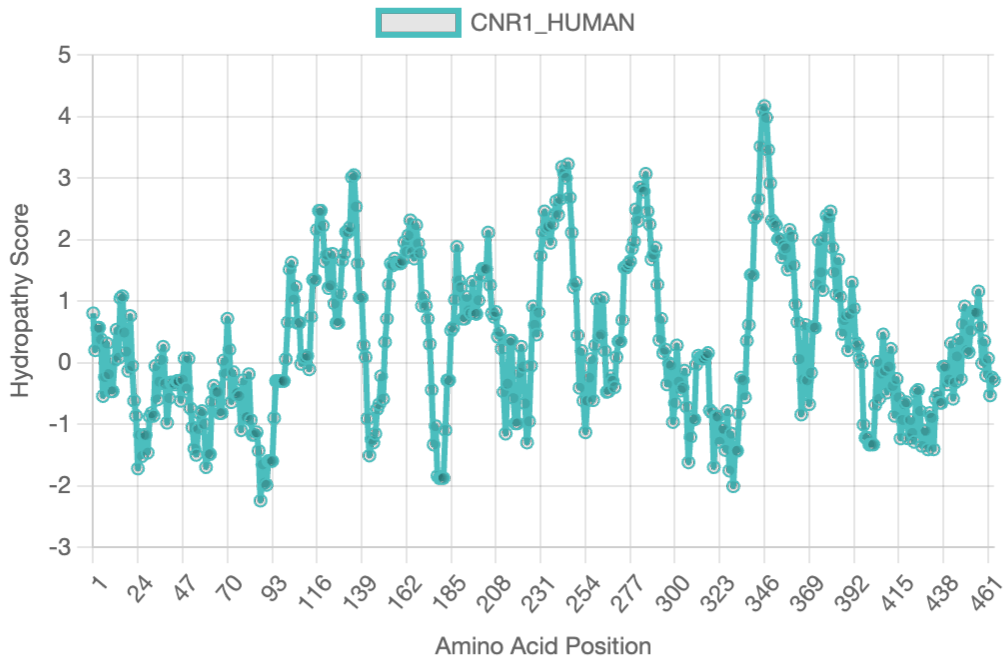

Kyte-Doolittle hydropathy plot for the sequence "CNR1_HUMAN". Positive values indicate hydrophobic regions, while negative values indicate hydrophilic regions.

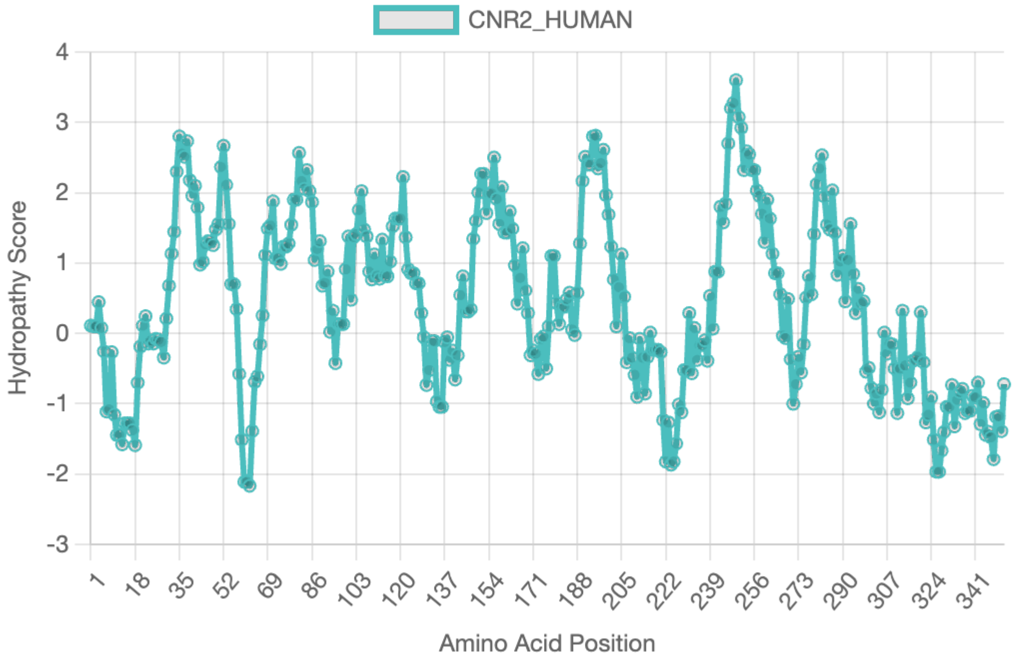

Kyte-Doolittle hydropathy plot for the sequence "CNR2_HUMAN". Positive values indicate hydrophobic regions, while negative values indicate hydrophilic regions.

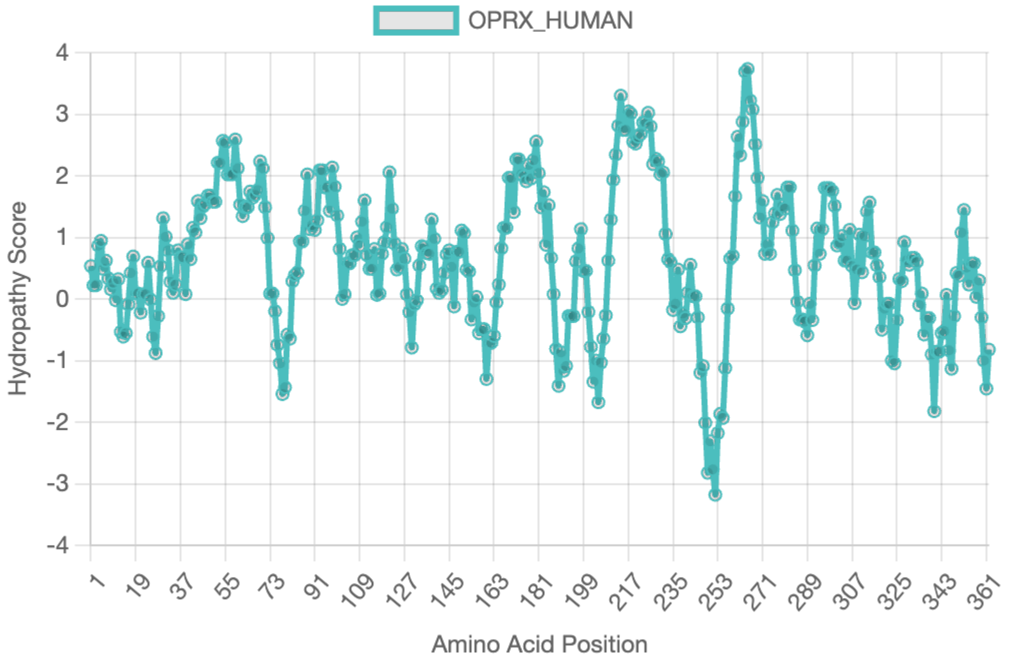

Kyte-Doolittle hydropathy plot for the sequence "OPRX_HUMAN". Positive values indicate hydrophobic regions, while negative values indicate hydrophilic regions.

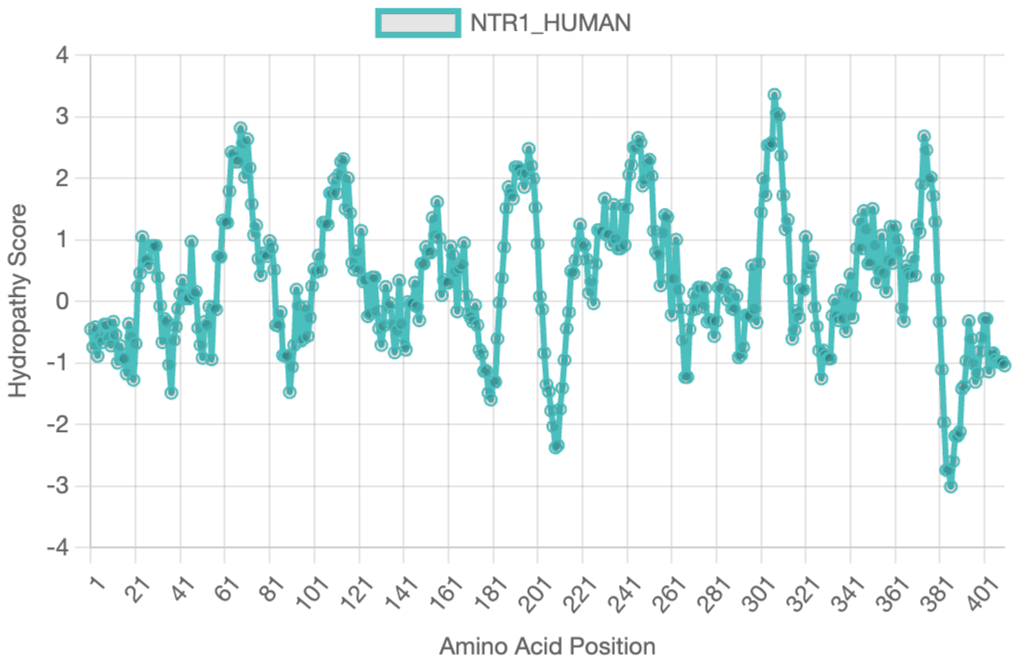

Kyte-Doolittle hydropathy plot for the sequence "NTR1_HUMAN". Positive values indicate hydrophobic regions, while negative values indicate hydrophilic regions.

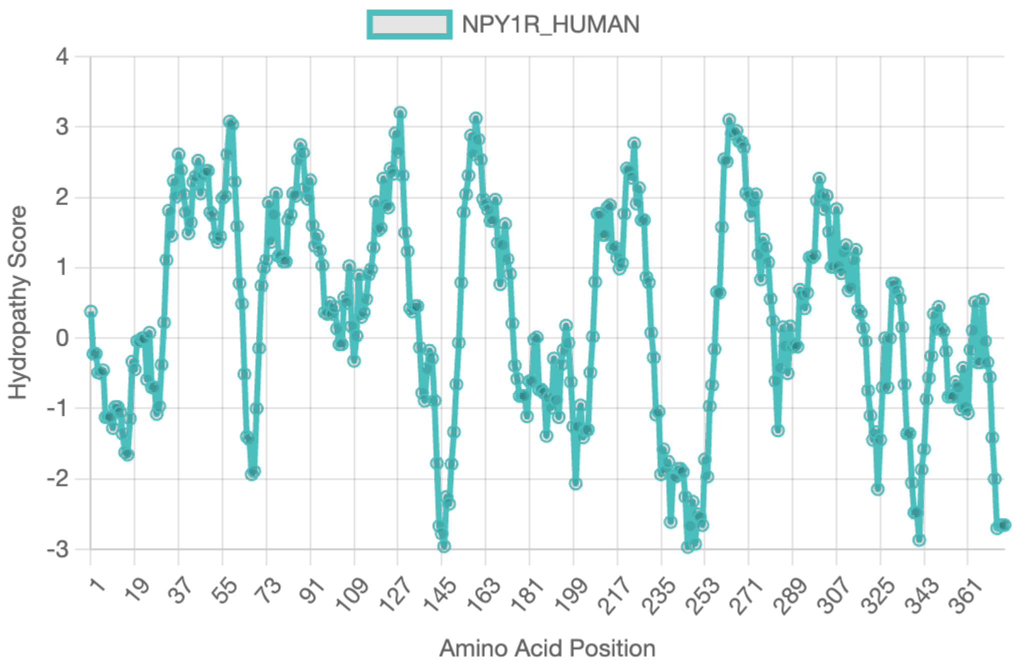

Kyte-Doolittle hydropathy plot for the sequence "NPY1R_HUMAN". Positive values indicate hydrophobic regions, while negative values indicate hydrophilic regions.

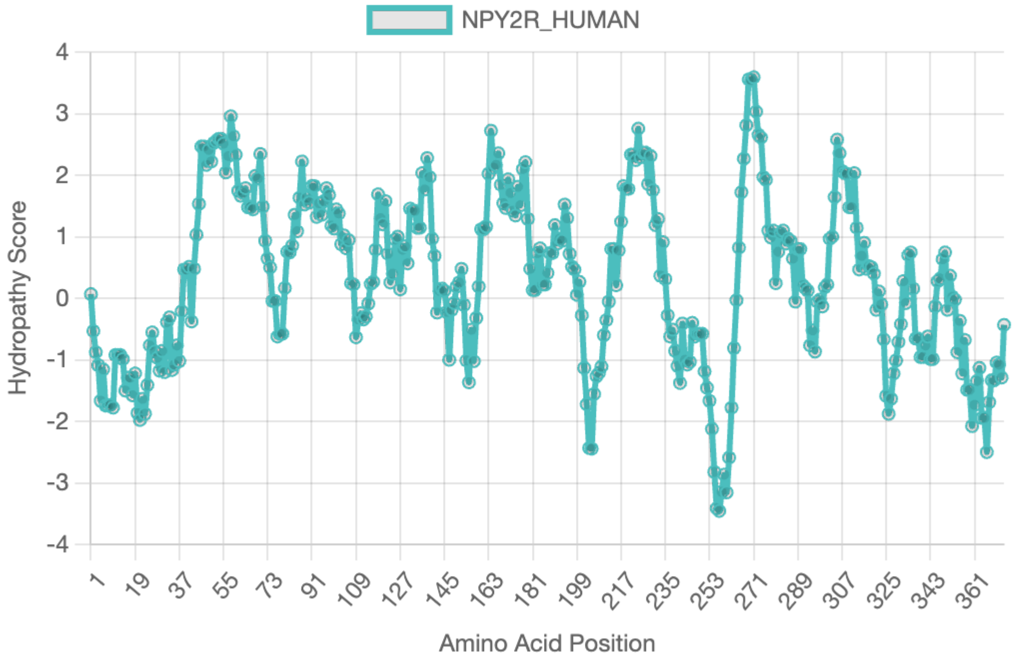

Kyte-Doolittle hydropathy plot for the sequence "NPY2R_HUMAN". Positive values indicate hydrophobic regions, while negative values indicate hydrophilic regions.

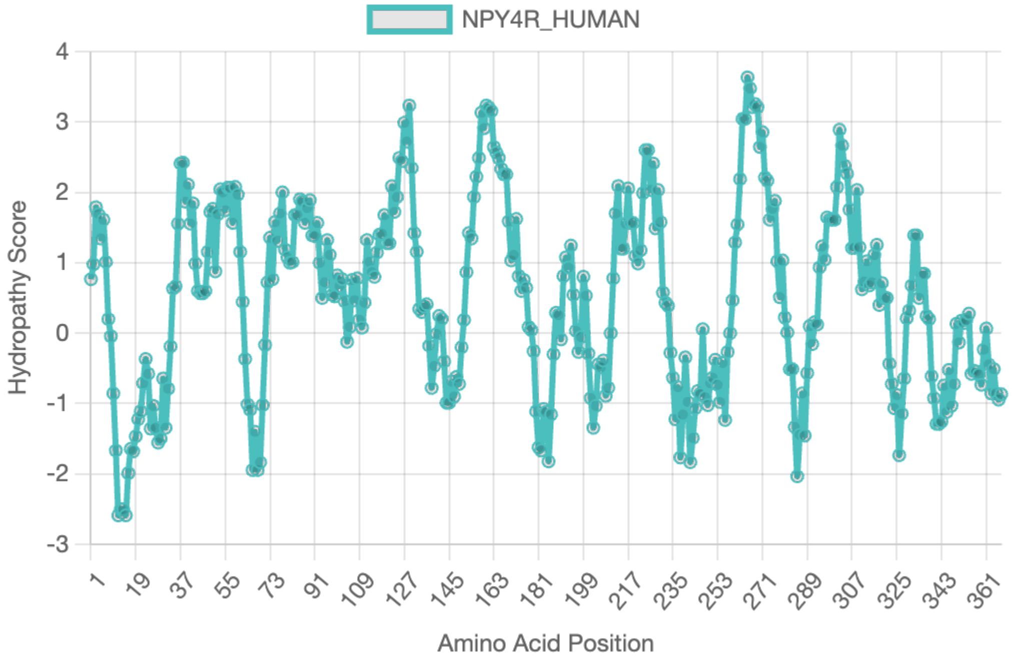

Kyte-Doolittle hydropathy plot for the sequence "NPY4R_HUMAN". Positive values indicate hydrophobic regions, while negative values indicate hydrophilic regions.

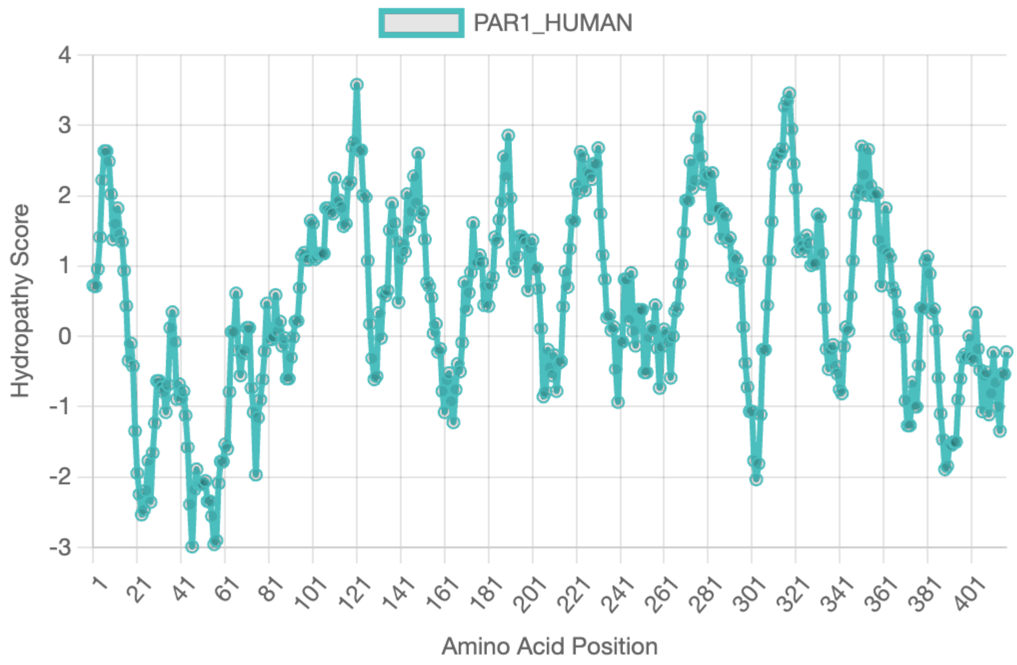

Kyte-Doolittle hydropathy plot for the sequence "PAR1_HUMAN". Positive values indicate hydrophobic regions, while negative values indicate hydrophilic regions.

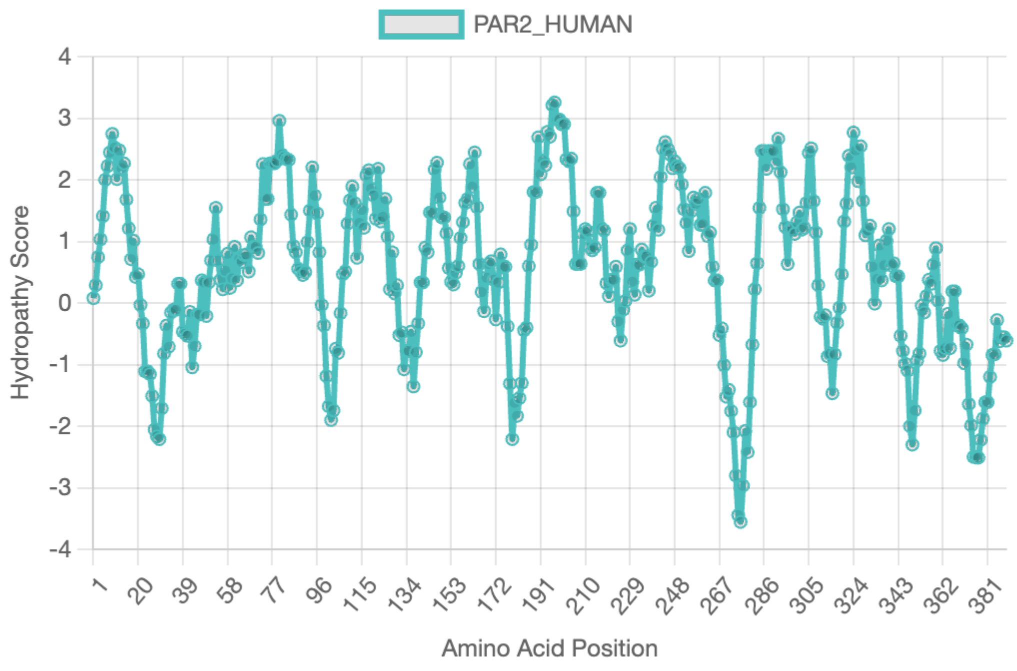

Kyte-Doolittle hydropathy plot for the sequence "PAR2_HUMAN". Positive values indicate hydrophobic regions, while negative values indicate hydrophilic regions.

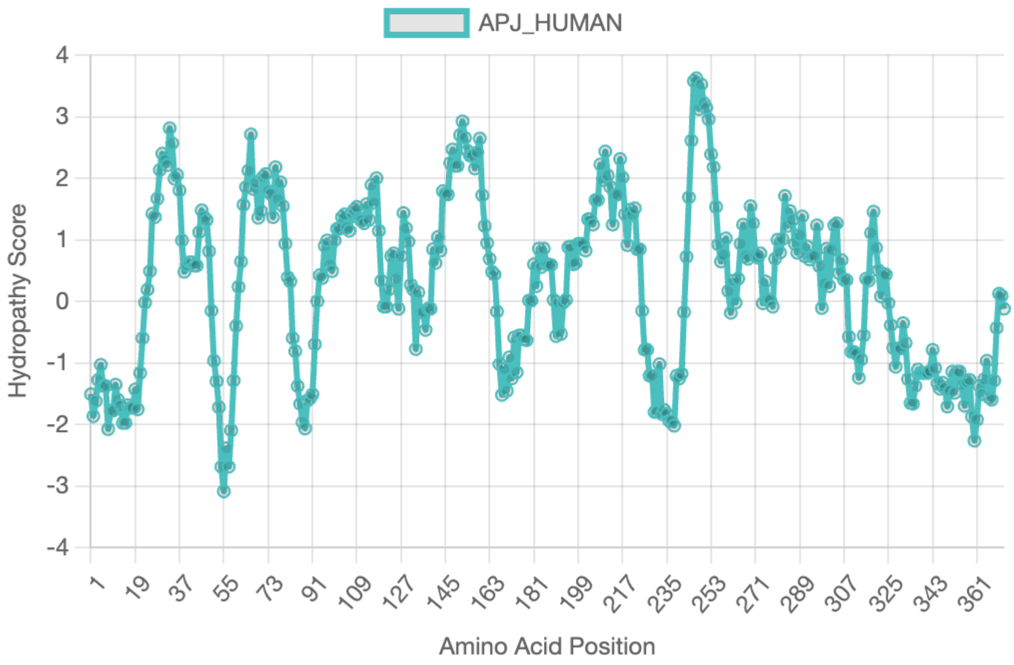

Kyte-Doolittle hydropathy plot for the sequence "APJ_HUMAN". Positive values indicate hydrophobic regions, while negative values indicate hydrophilic regions.

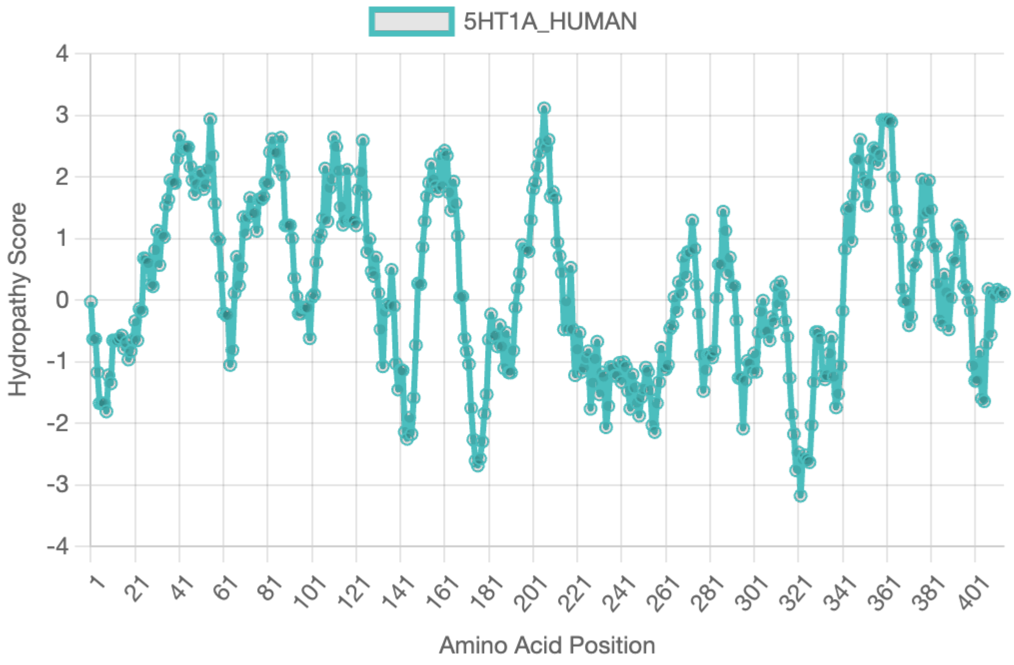

Kyte-Doolittle hydropathy plot for the sequence "5HT1A_HUMAN". Positive values indicate hydrophobic regions, while negative values indicate hydrophilic regions.

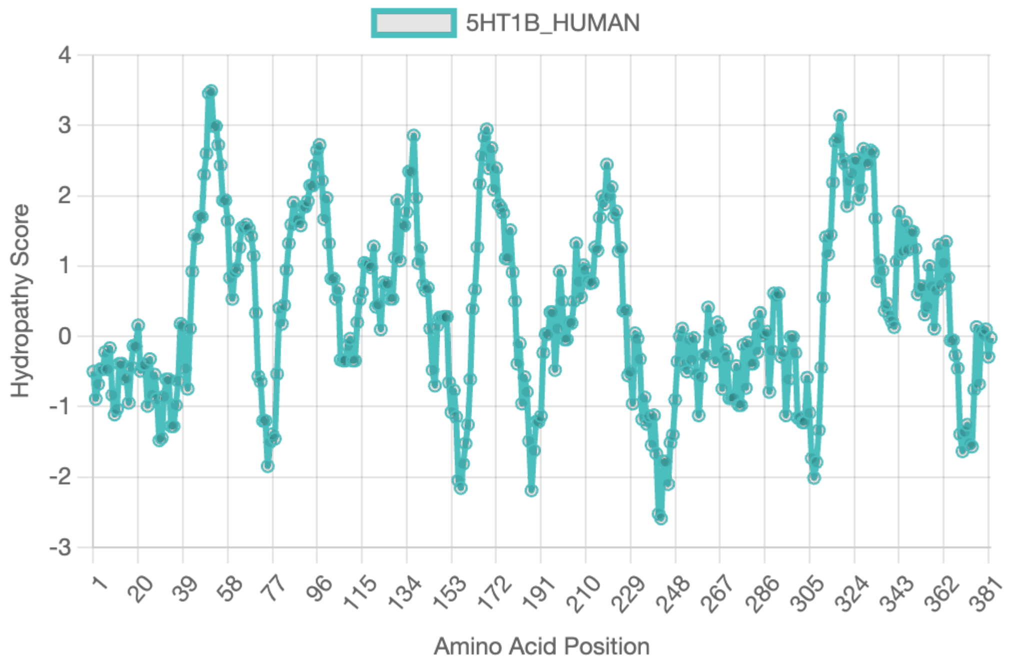

Kyte-Doolittle hydropathy plot for the sequence "5HT1B_HUMAN". Positive values indicate hydrophobic regions, while negative values indicate hydrophilic regions.

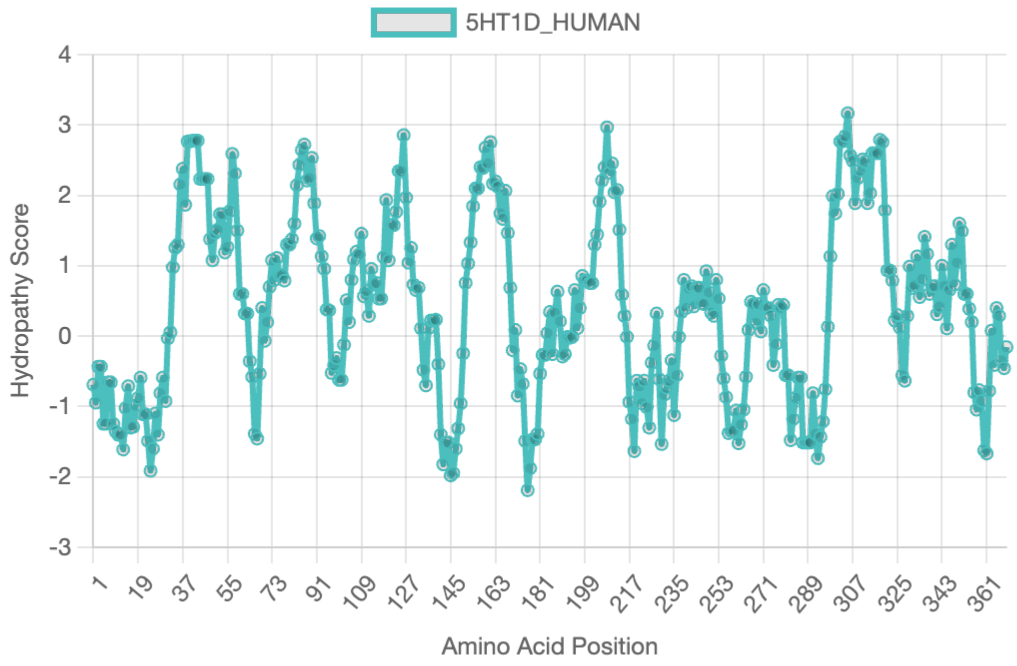

Kyte-Doolittle hydropathy plot for the sequence "5HT1D_HUMAN". Positive values indicate hydrophobic regions, while negative values indicate hydrophilic regions.

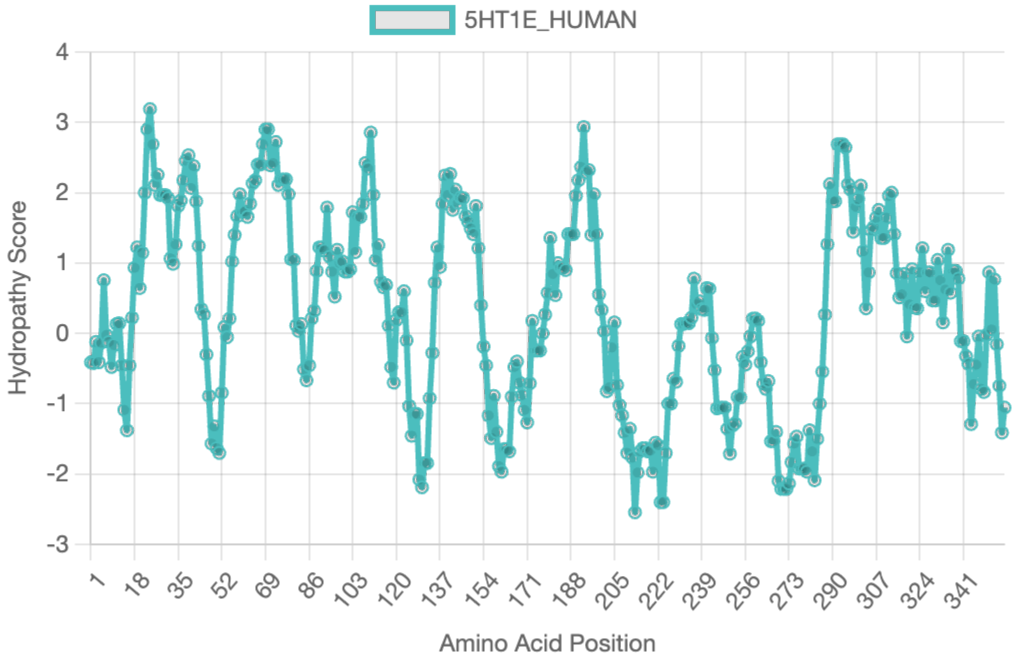

Kyte-Doolittle hydropathy plot for the sequence "5HT1E_HUMAN". Positive values indicate hydrophobic regions, while negative values indicate hydrophilic regions.

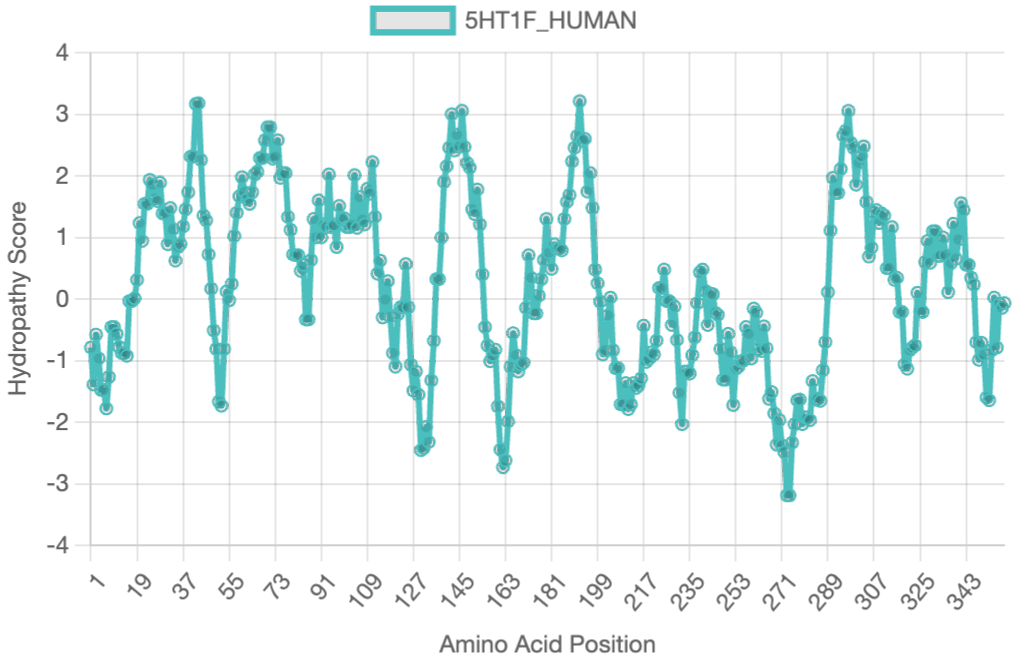

Kyte-Doolittle hydropathy plot for the sequence "5HT1F_HUMAN". Positive values indicate hydrophobic regions, while negative values indicate hydrophilic regions.

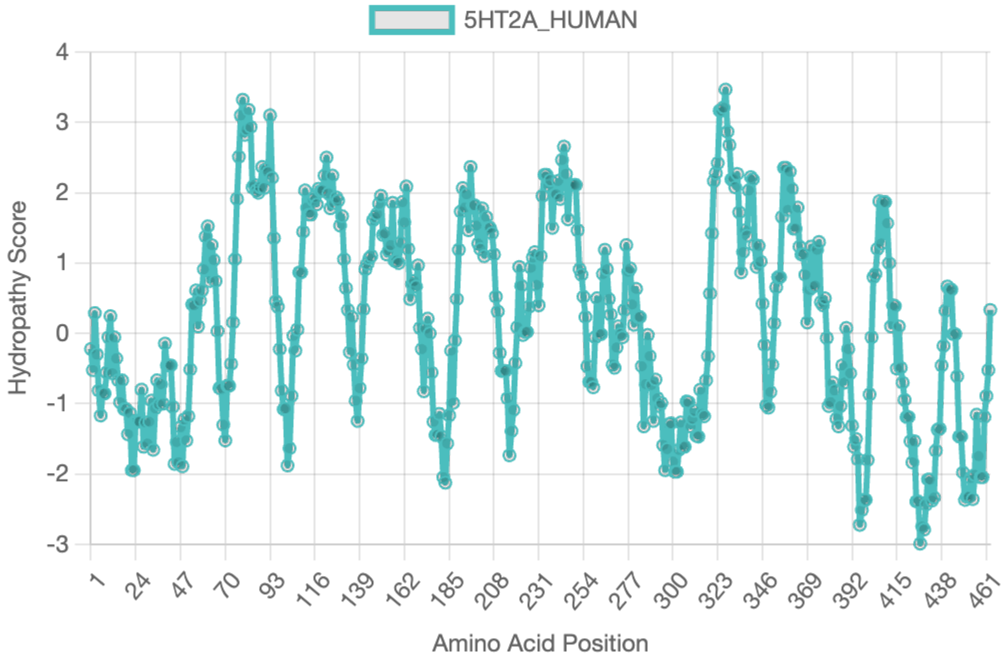

Kyte-Doolittle hydropathy plot for the sequence "5HT2A_HUMAN". Positive values indicate hydrophobic regions, while negative values indicate hydrophilic regions.

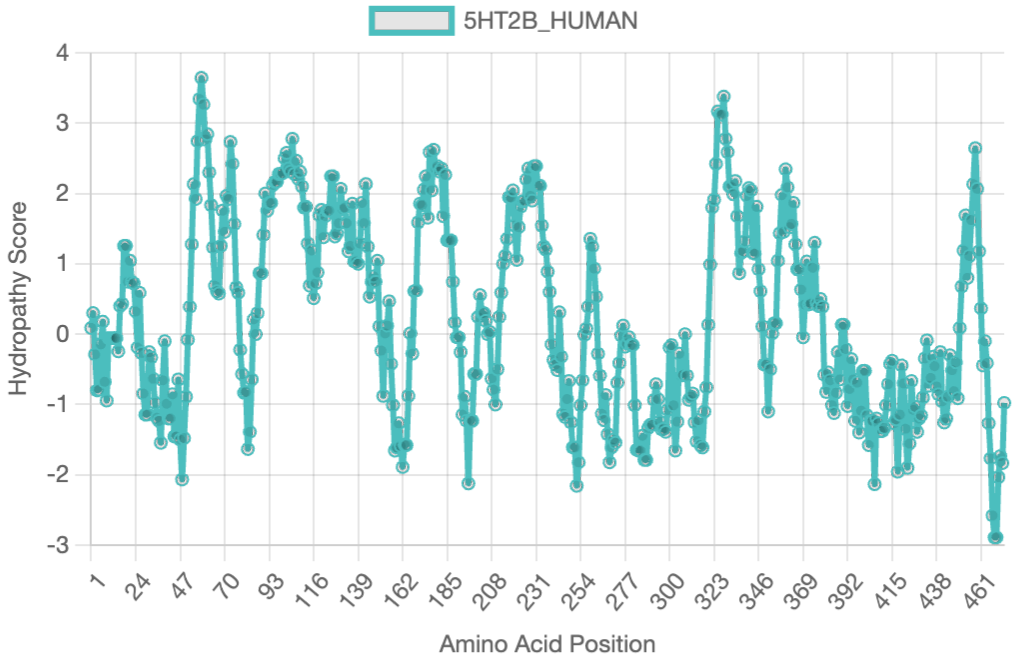

Kyte-Doolittle hydropathy plot for the sequence "5HT2B_HUMAN". Positive values indicate hydrophobic regions, while negative values indicate hydrophilic regions.

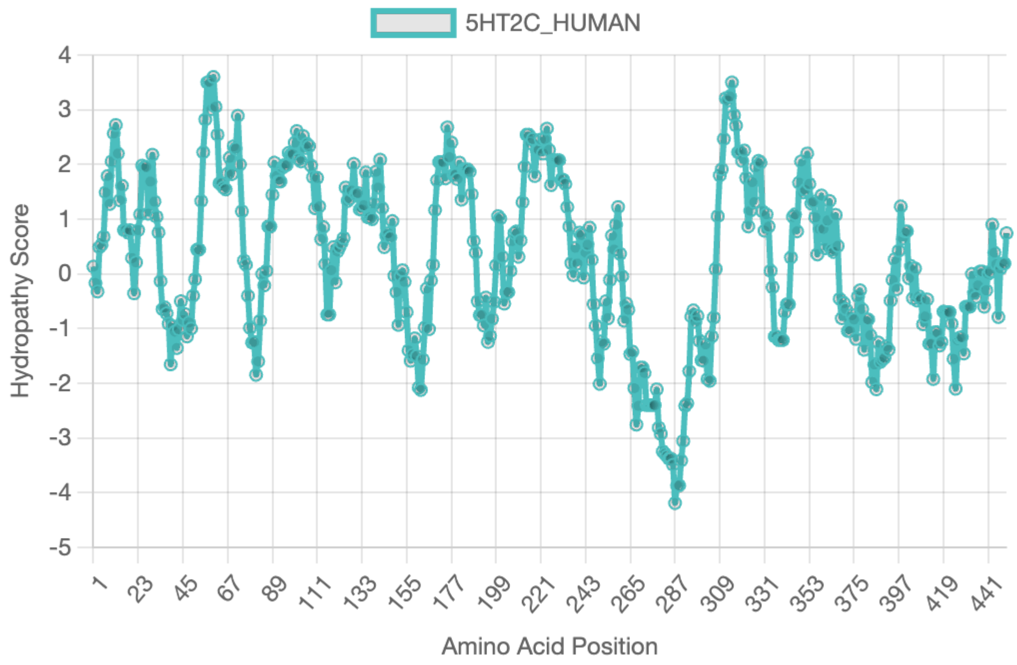

Kyte-Doolittle hydropathy plot for the sequence "5HT2C_HUMAN". Positive values indicate hydrophobic regions, while negative values indicate hydrophilic regions.

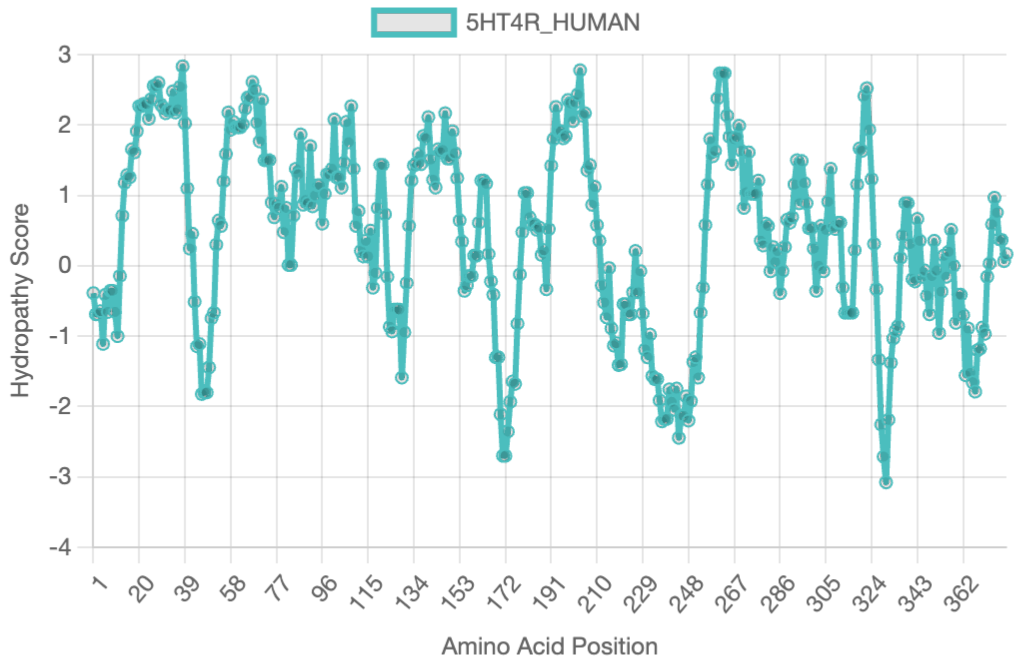

Kyte-Doolittle hydropathy plot for the sequence "5HT4R_HUMAN". Positive values indicate hydrophobic regions, while negative values indicate hydrophilic regions.

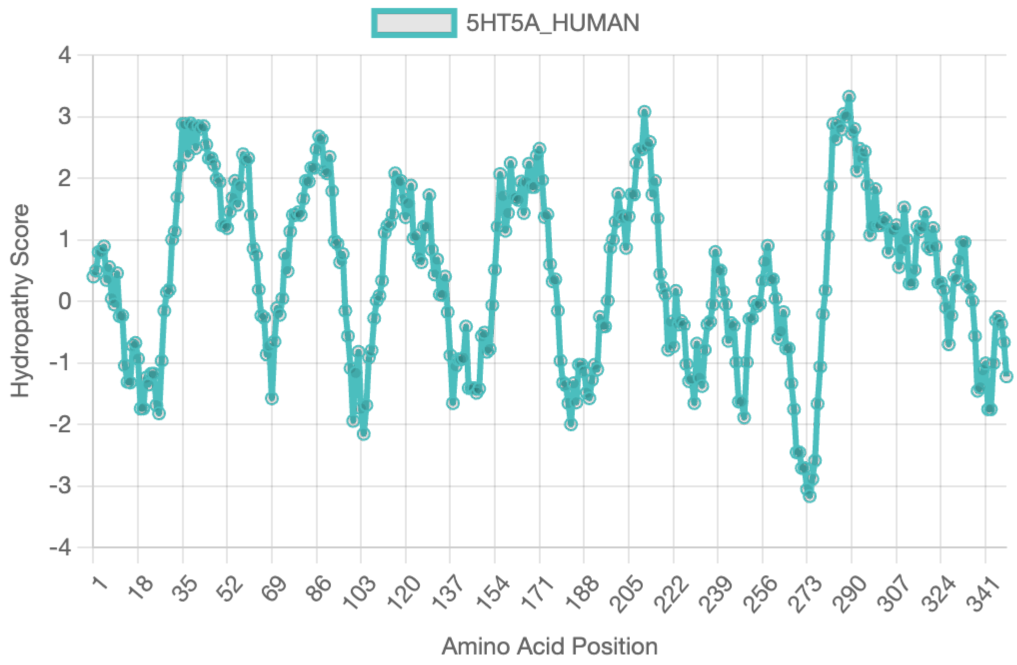

Kyte-Doolittle hydropathy plot for the sequence "5HT5A_HUMAN". Positive values indicate hydrophobic regions, while negative values indicate hydrophilic regions.

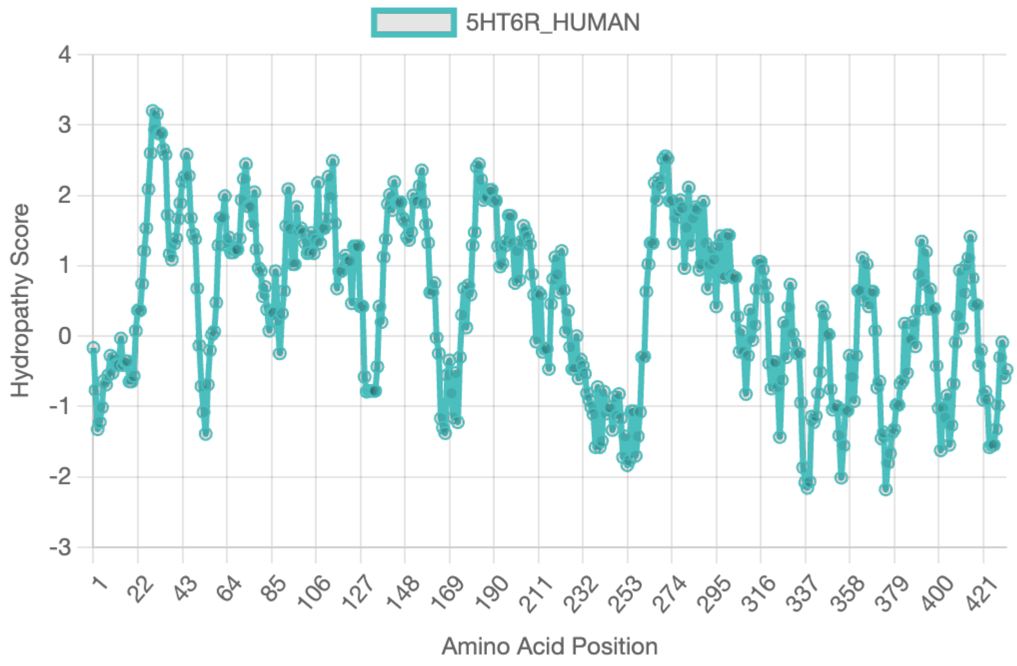

Kyte-Doolittle hydropathy plot for the sequence "5HT6R_HUMAN". Positive values indicate hydrophobic regions, while negative values indicate hydrophilic regions.

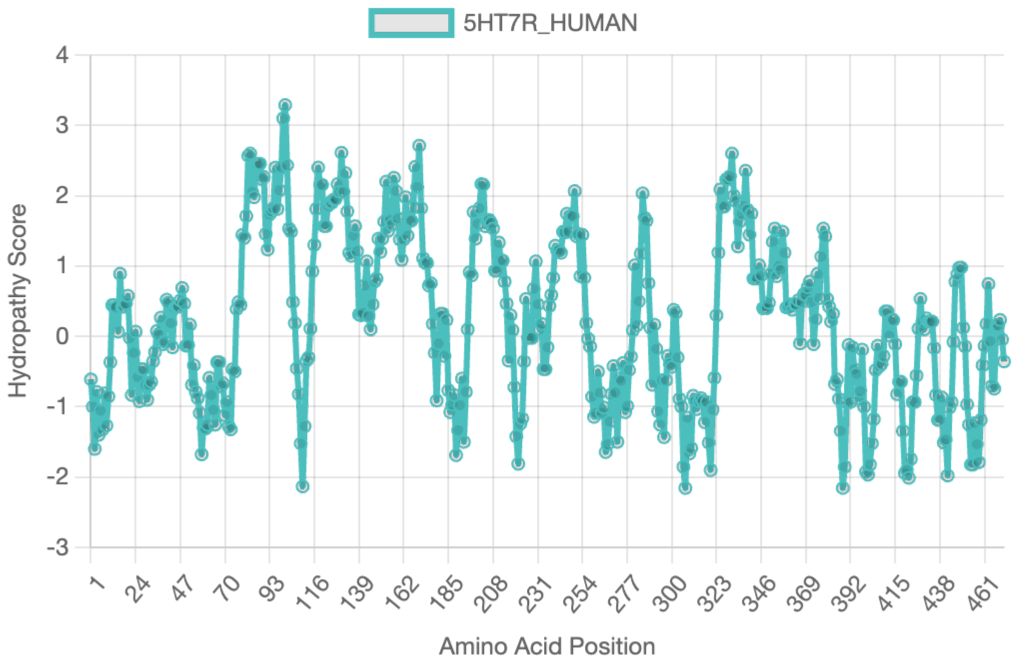

Kyte-Doolittle hydropathy plot for the sequence "5HT7R_HUMAN". Positive values indicate hydrophobic regions, while negative values indicate hydrophilic regions.

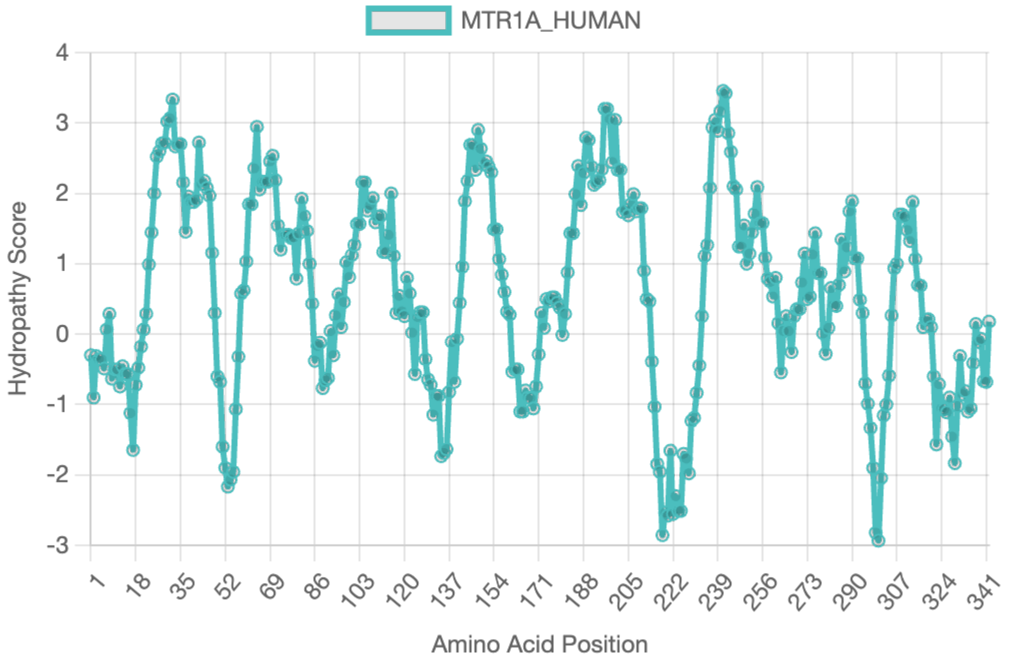

Kyte-Doolittle hydropathy plot for the sequence "MTR1A_HUMAN". Positive values indicate hydrophobic regions, while negative values indicate hydrophilic regions.

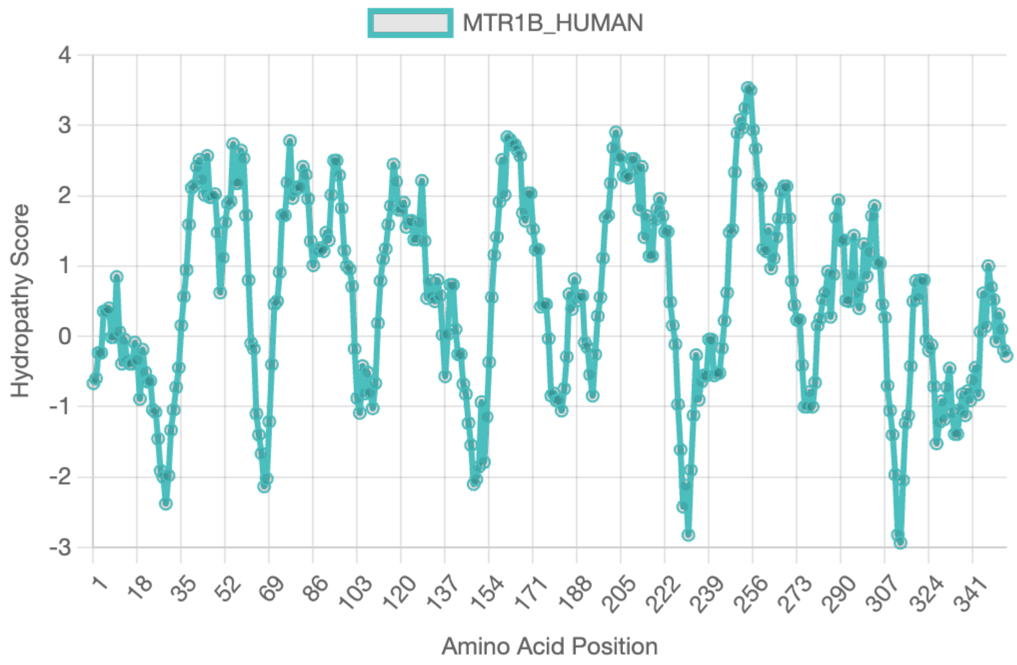

Kyte-Doolittle hydropathy plot for the sequence "MTR1B_HUMAN". Positive values indicate hydrophobic regions, while negative values indicate hydrophilic regions.

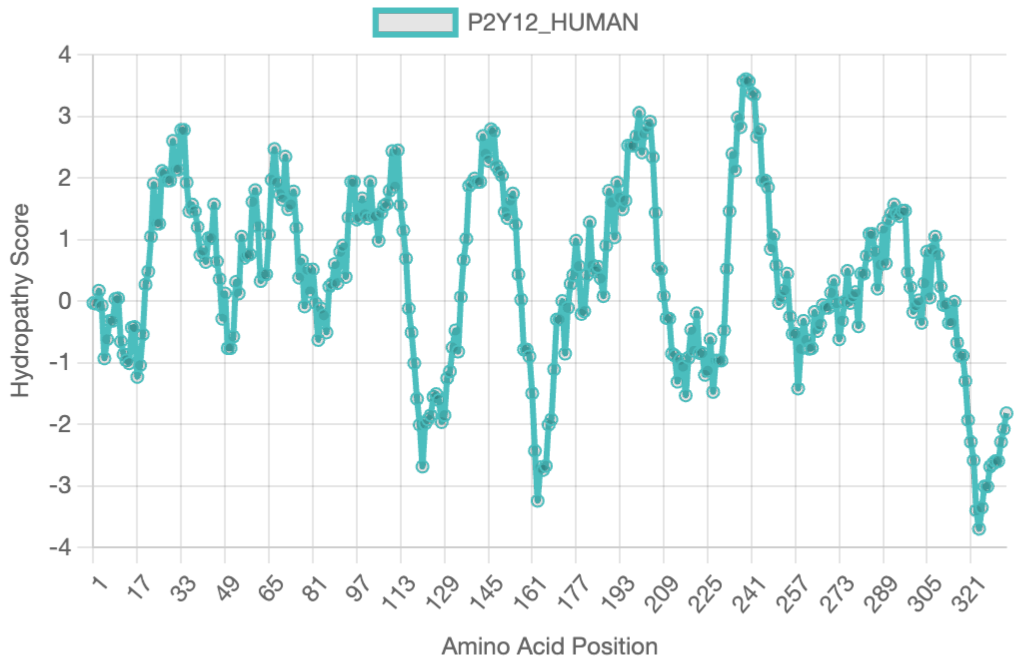

Kyte-Doolittle hydropathy plot for the sequence "P2Y12_HUMAN". Positive values indicate hydrophobic regions, while negative values indicate hydrophilic regions.

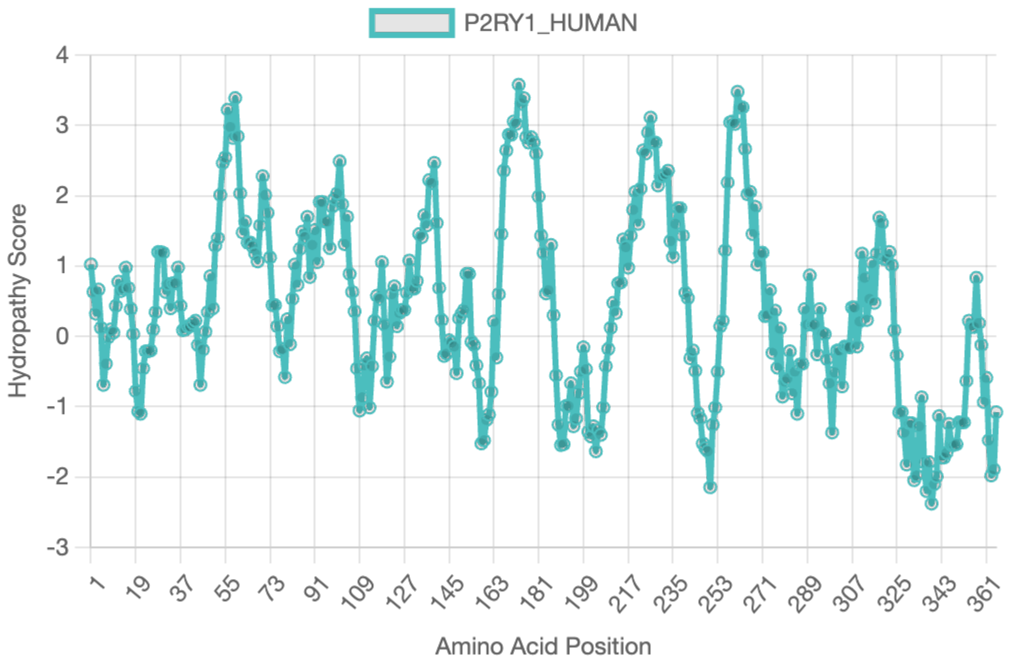

Kyte-Doolittle hydropathy plot for the sequence "P2RY1_HUMAN". Positive values indicate hydrophobic regions, while negative values indicate hydrophilic regions.

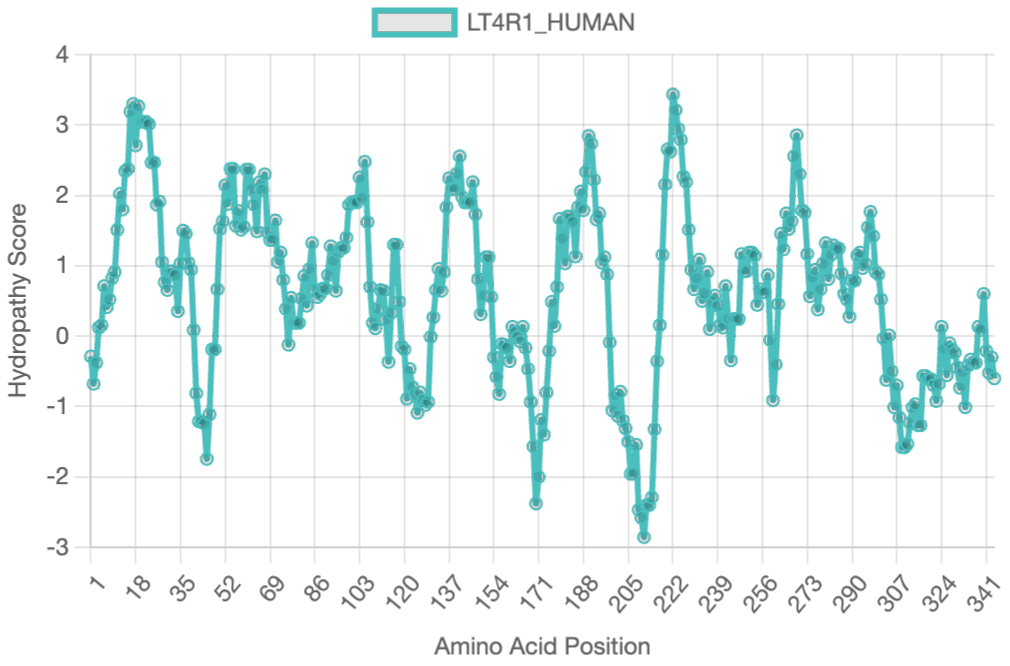

Kyte-Doolittle hydropathy plot for the sequence "LT4R1_HUMAN". Positive values indicate hydrophobic regions, while negative values indicate hydrophilic regions.

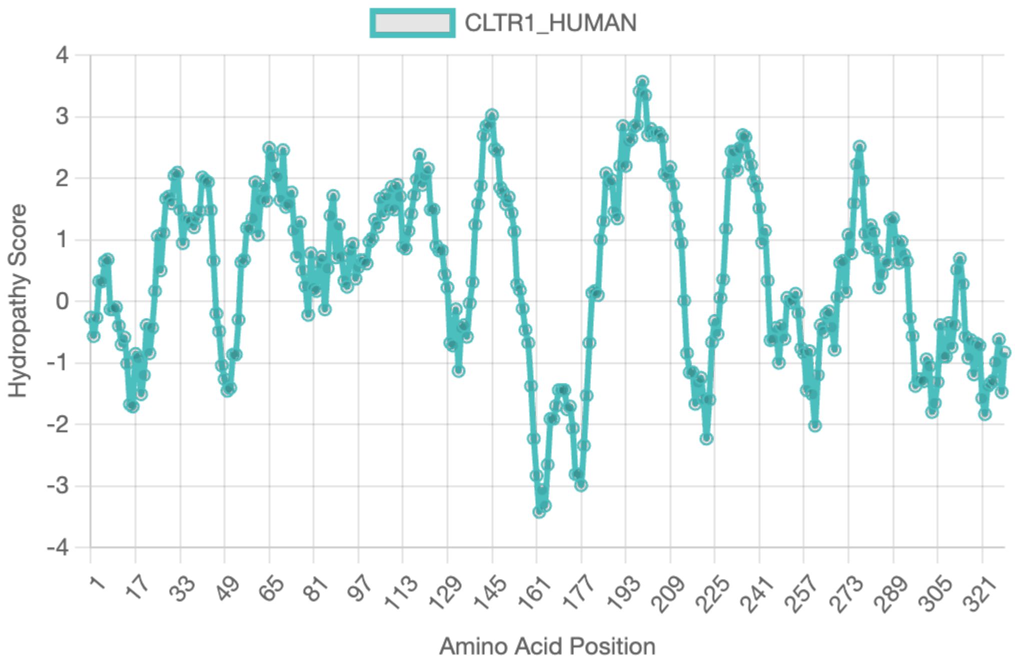

Kyte-Doolittle hydropathy plot for the sequence "CLTR1_HUMAN". Positive values indicate hydrophobic regions, while negative values indicate hydrophilic regions.

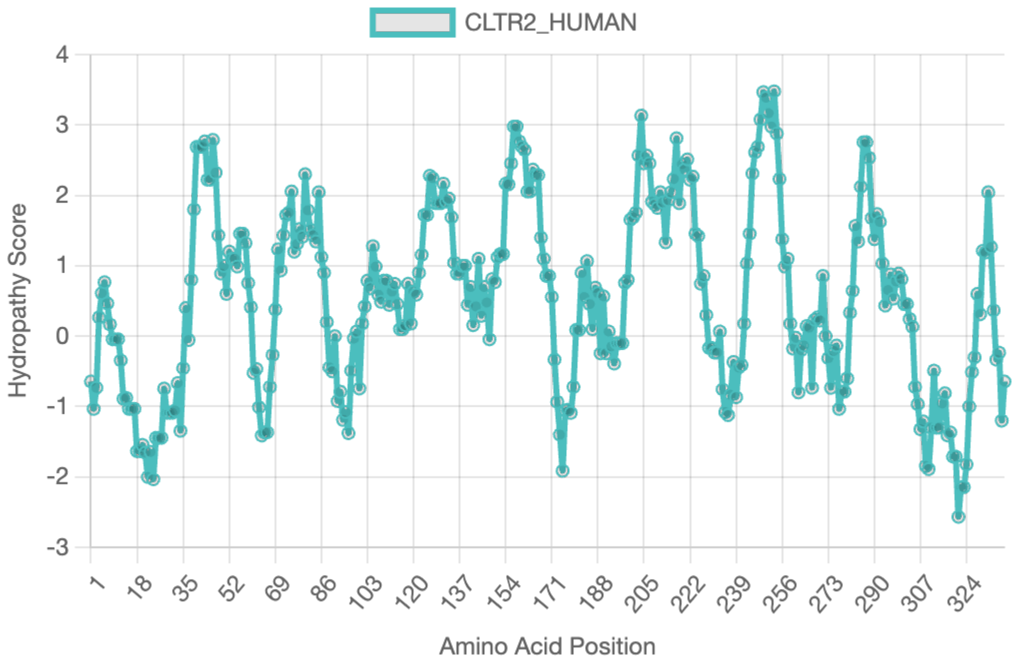

Kyte-Doolittle hydropathy plot for the sequence "CLTR2_HUMAN". Positive values indicate hydrophobic regions, while negative values indicate hydrophilic regions.

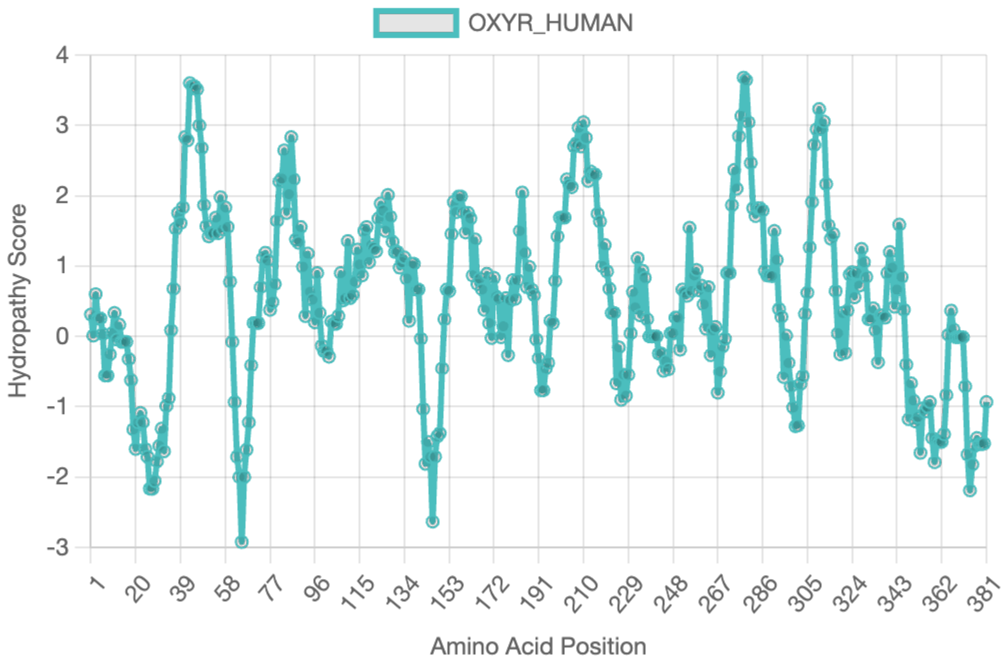

Kyte-Doolittle hydropathy plot for the sequence "OXYR_HUMAN". Positive values indicate hydrophobic regions, while negative values indicate hydrophilic regions.

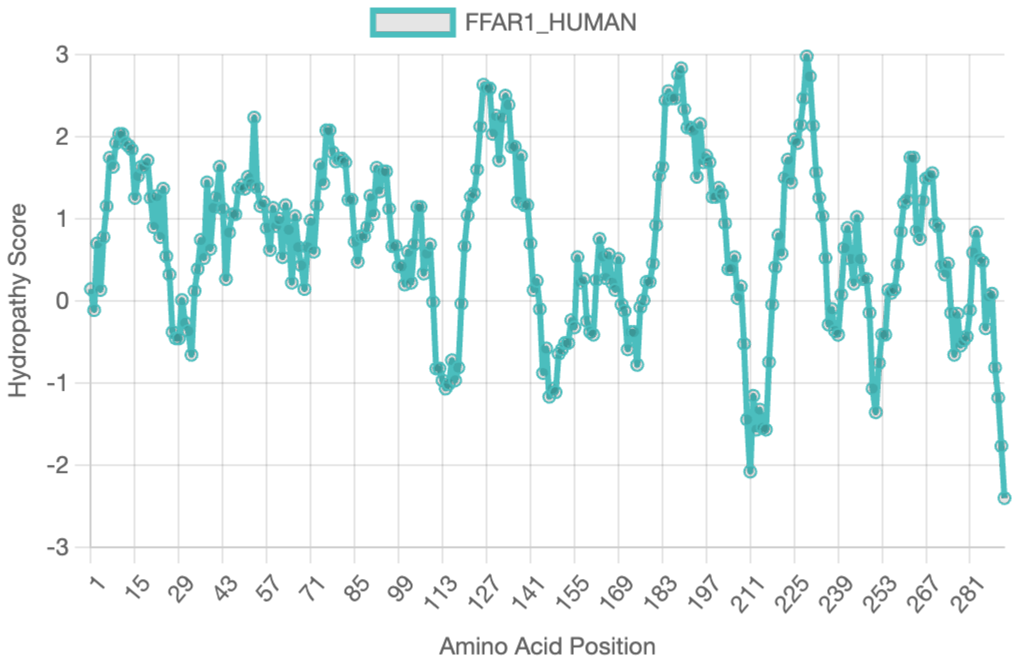

Kyte-Doolittle hydropathy plot for the sequence "FFAR1_HUMAN". Positive values indicate hydrophobic regions, while negative values indicate hydrophilic regions.

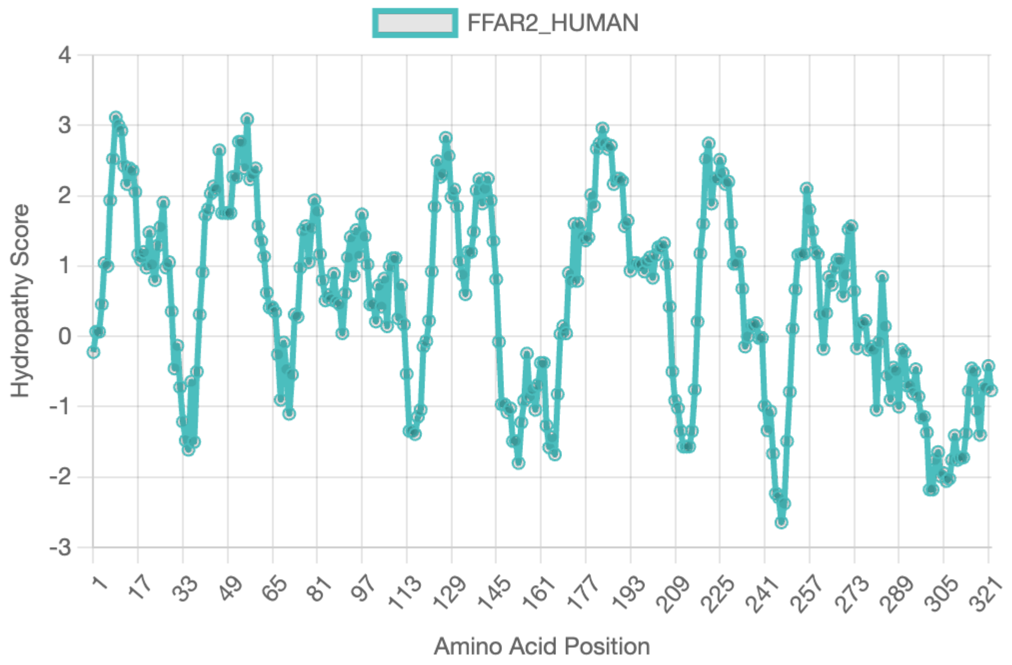

Kyte-Doolittle hydropathy plot for the sequence "FFAR2_HUMAN". Positive values indicate hydrophobic regions, while negative values indicate hydrophilic regions.

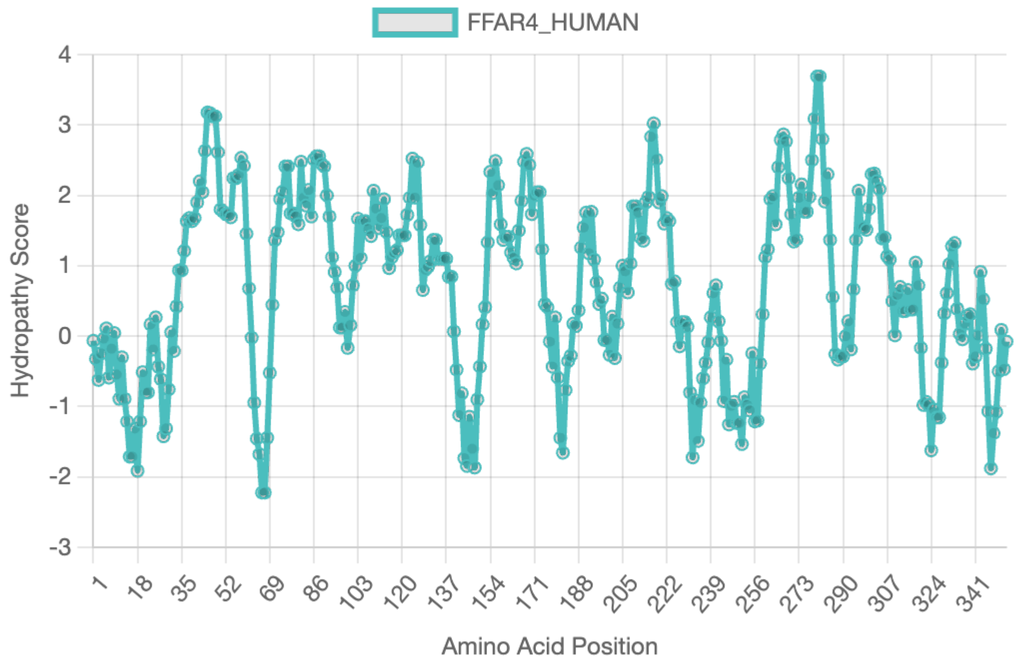

Kyte-Doolittle hydropathy plot for the sequence "FFAR4_HUMAN". Positive values indicate hydrophobic regions, while negative values indicate hydrophilic regions.

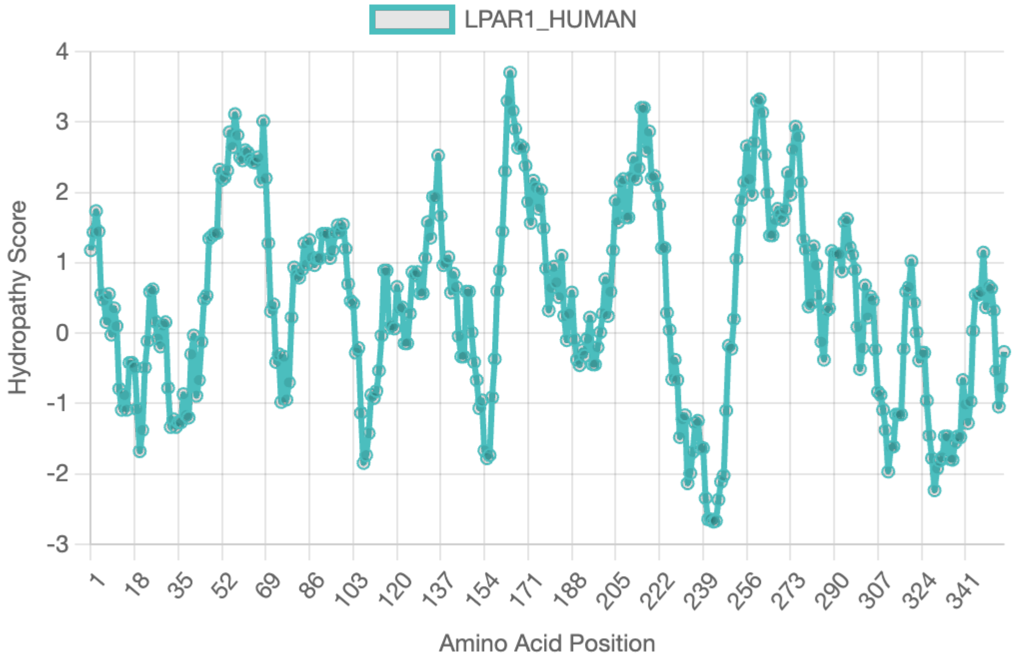

Kyte-Doolittle hydropathy plot for the sequence "LPAR1_HUMAN". Positive values indicate hydrophobic regions, while negative values indicate hydrophilic regions.

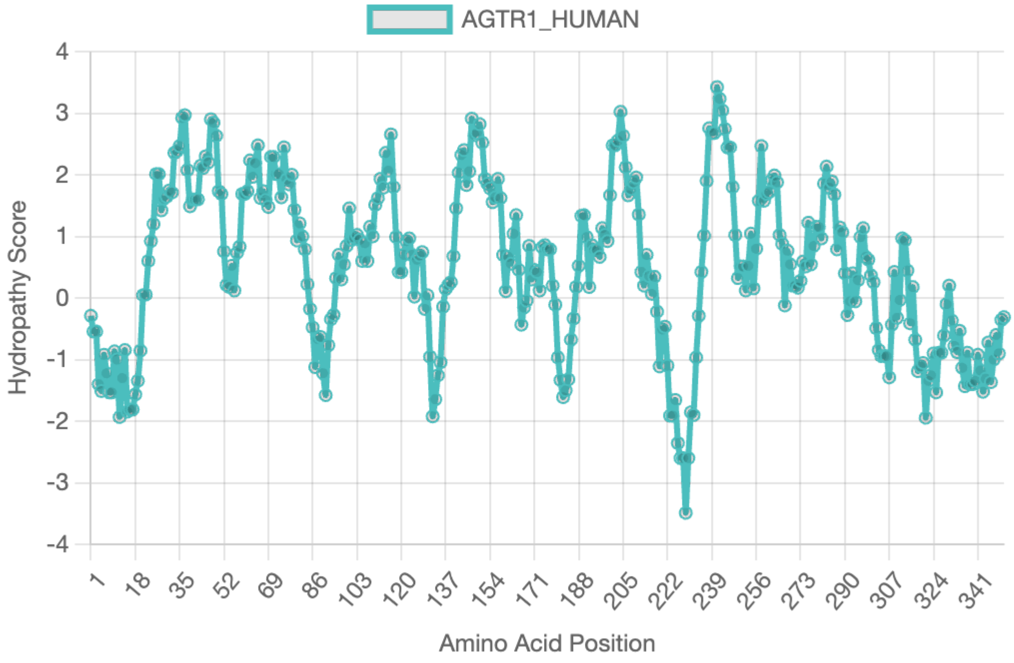

Kyte-Doolittle hydropathy plot for the sequence "AGTR1_HUMAN". Positive values indicate hydrophobic regions, while negative values indicate hydrophilic regions.

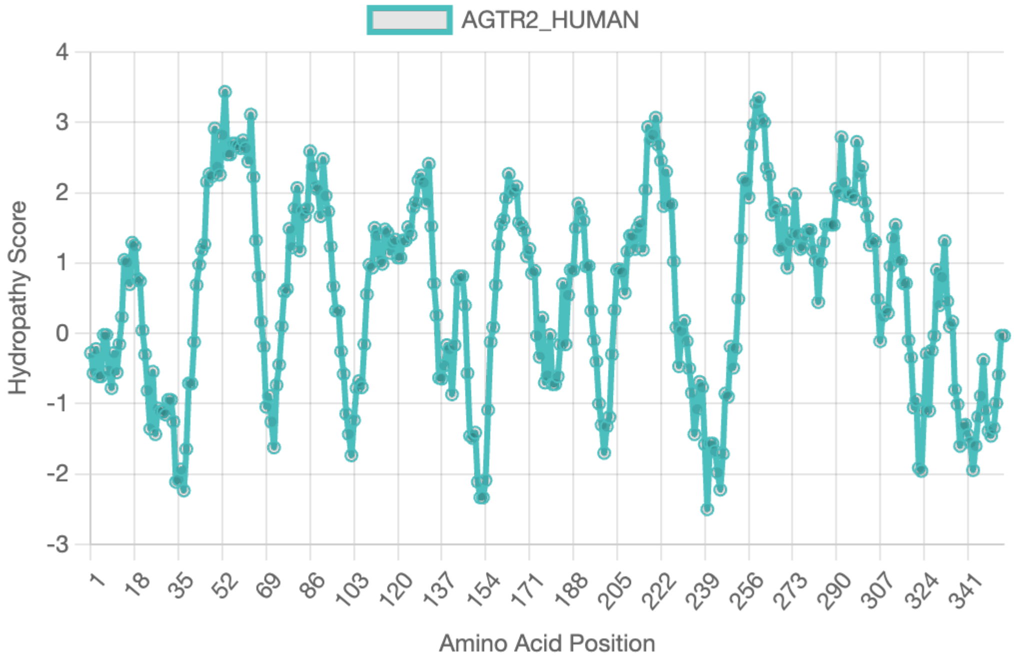

Kyte-Doolittle hydropathy plot for the sequence "AGTR2_HUMAN". Positive values indicate hydrophobic regions, while negative values indicate hydrophilic regions.

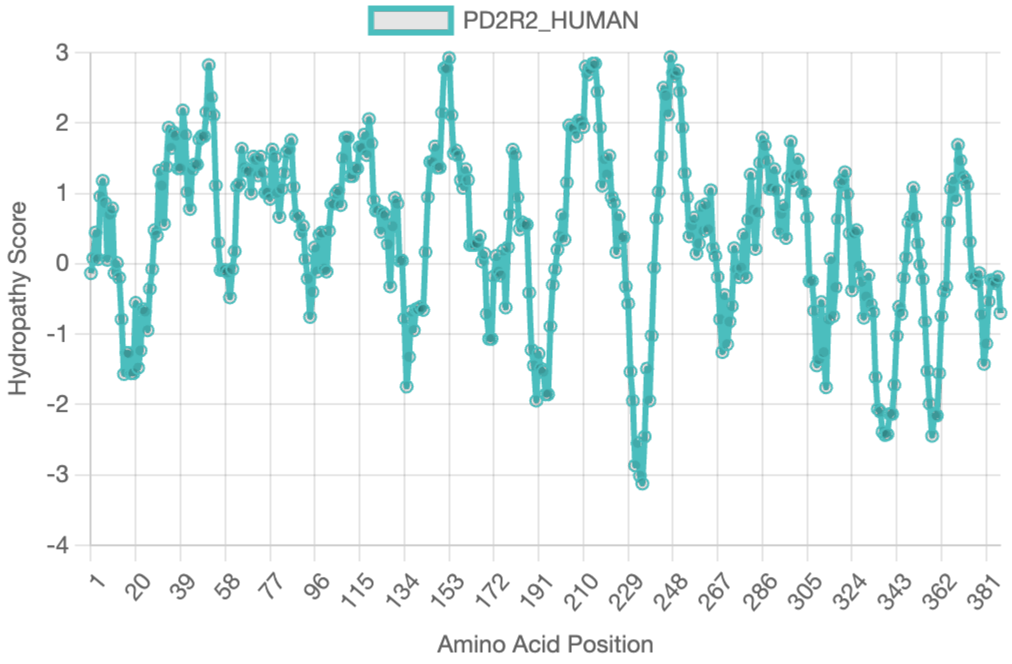

Kyte-Doolittle hydropathy plot for the sequence "PD2R2_HUMAN". Positive values indicate hydrophobic regions, while negative values indicate hydrophilic regions.

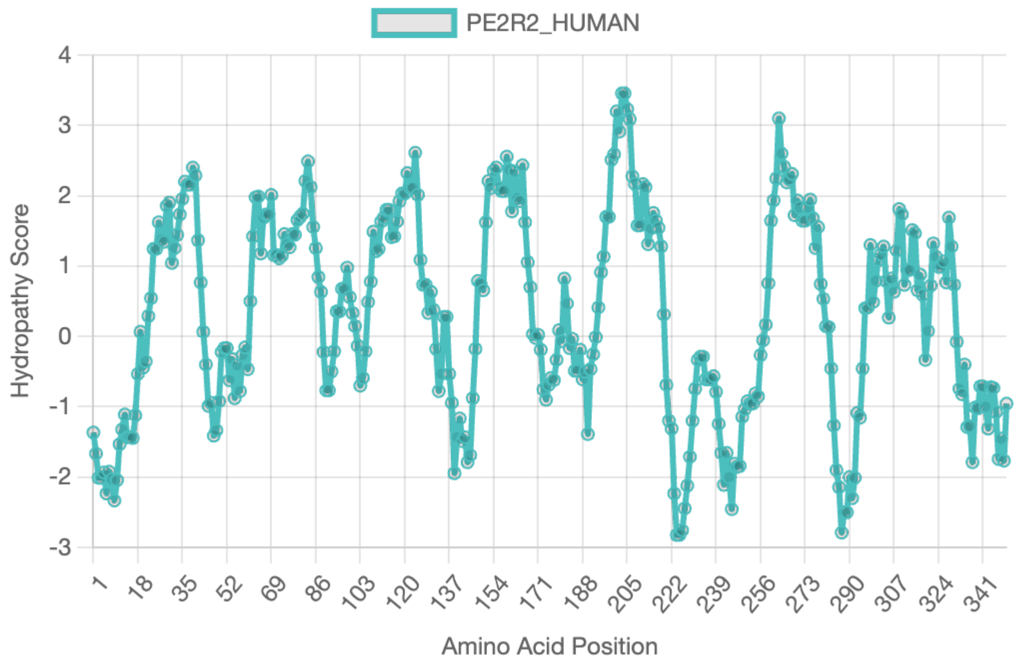

Kyte-Doolittle hydropathy plot for the sequence "PE2R2_HUMAN". Positive values indicate hydrophobic regions, while negative values indicate hydrophilic regions.

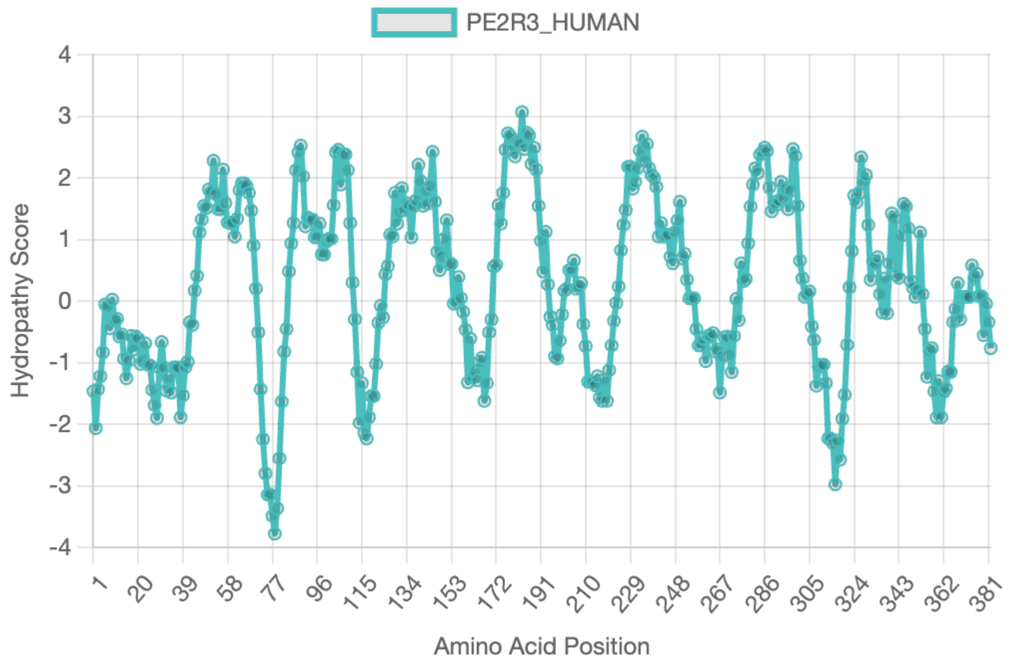

Kyte-Doolittle hydropathy plot for the sequence "PE2R3_HUMAN". Positive values indicate hydrophobic regions, while negative values indicate hydrophilic regions.

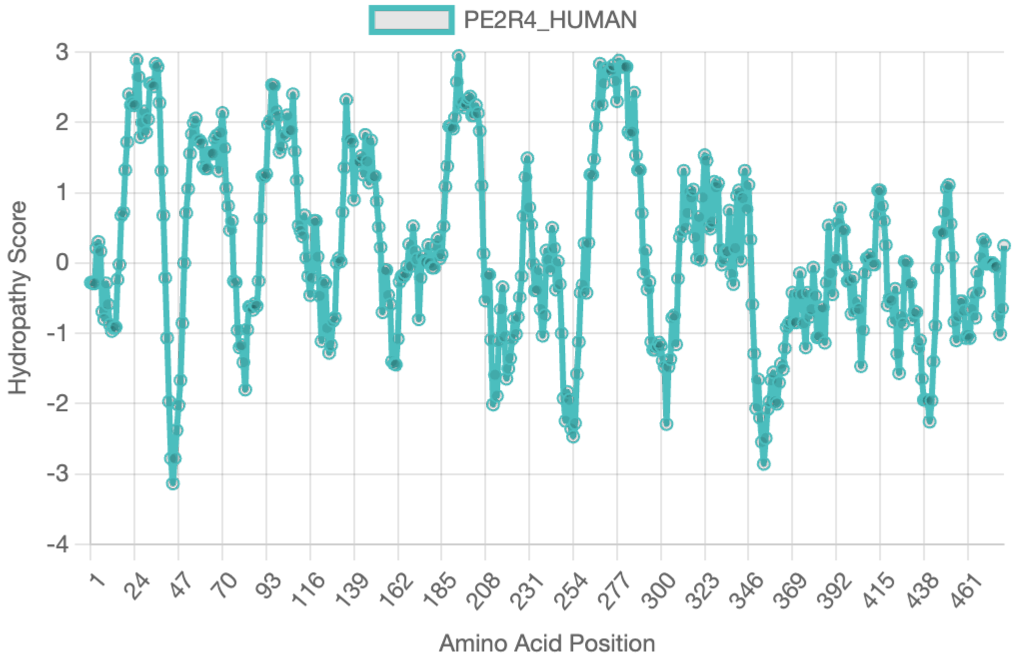

Kyte-Doolittle hydropathy plot for the sequence "PE2R4_HUMAN". Positive values indicate hydrophobic regions, while negative values indicate hydrophilic regions.

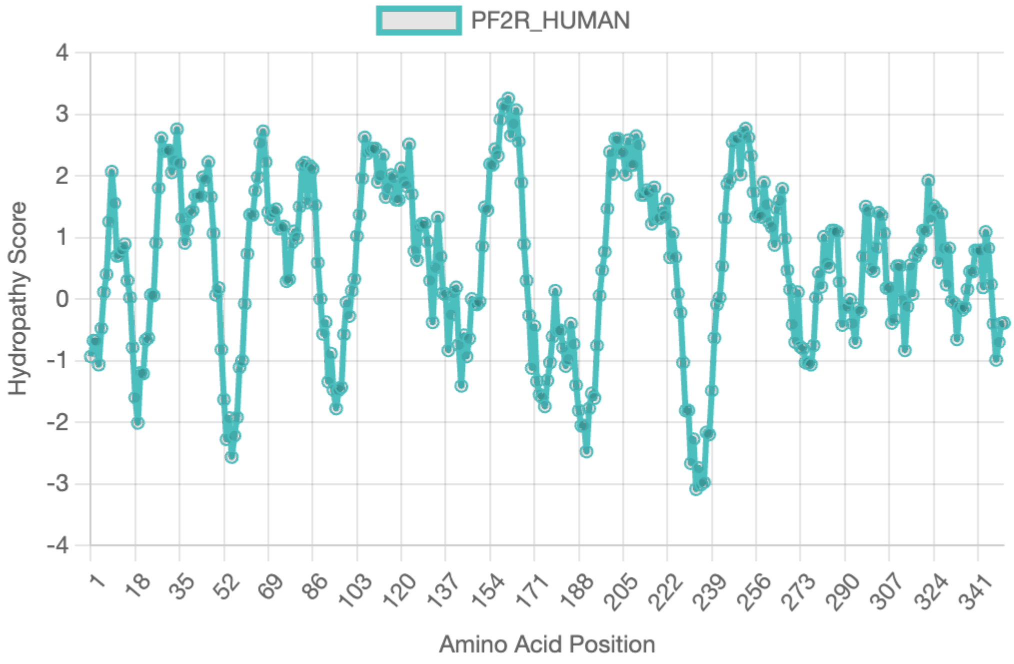

Kyte-Doolittle hydropathy plot for the sequence "PF2R_HUMAN". Positive values indicate hydrophobic regions, while negative values indicate hydrophilic regions.

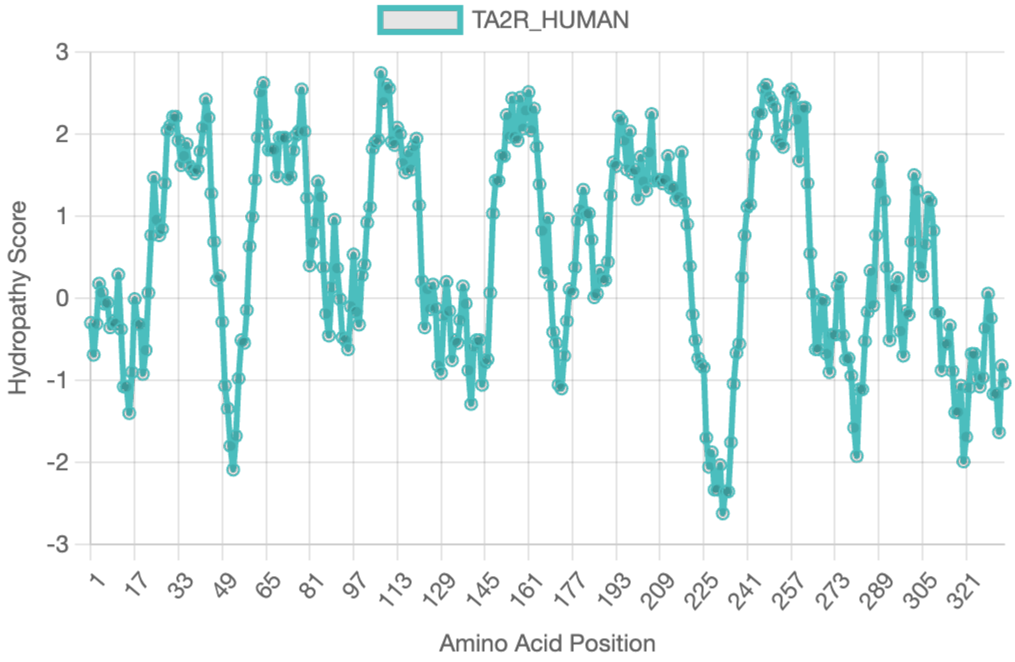

Kyte-Doolittle hydropathy plot for the sequence "TA2R_HUMAN". Positive values indicate hydrophobic regions, while negative values indicate hydrophilic regions.

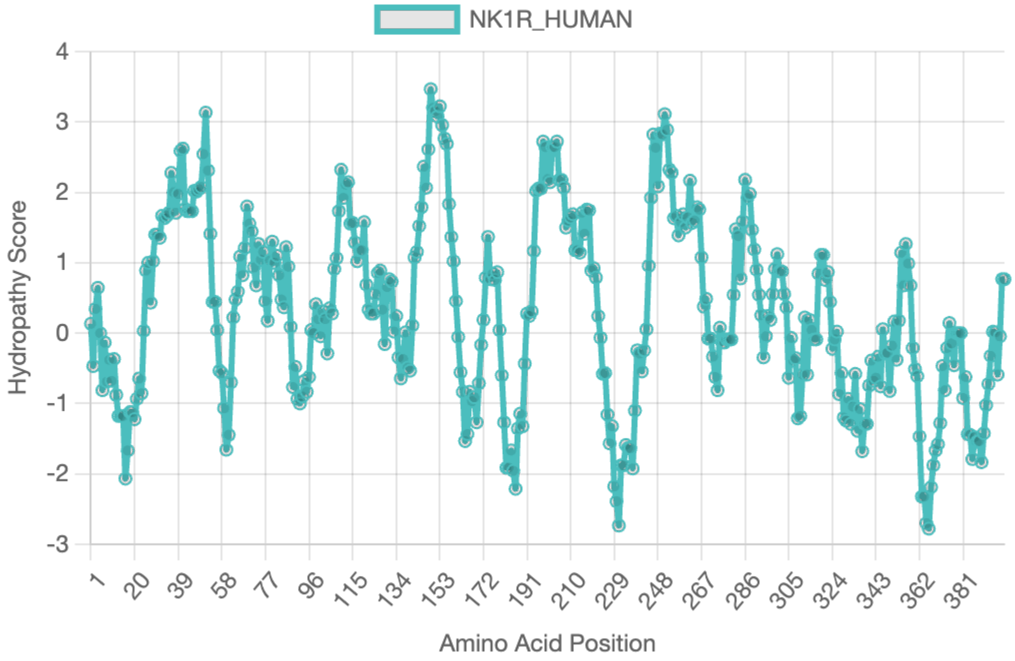

Kyte-Doolittle hydropathy plot for the sequence "NK1R_HUMAN". Positive values indicate hydrophobic regions, while negative values indicate hydrophilic regions.

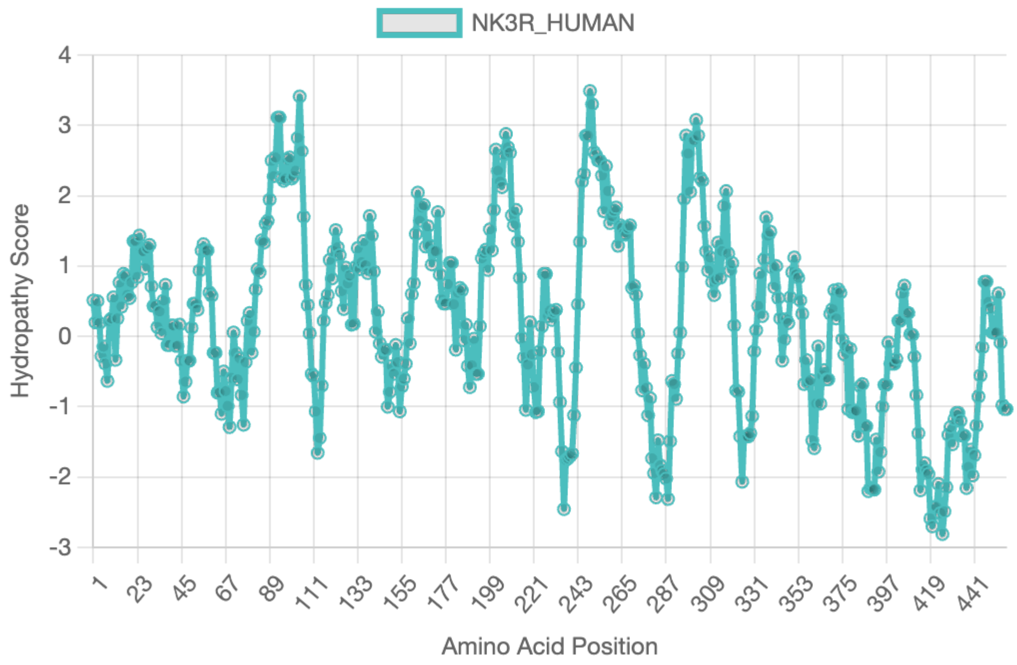

Kyte-Doolittle hydropathy plot for the sequence "NK3R_HUMAN". Positive values indicate hydrophobic regions, while negative values indicate hydrophilic regions.

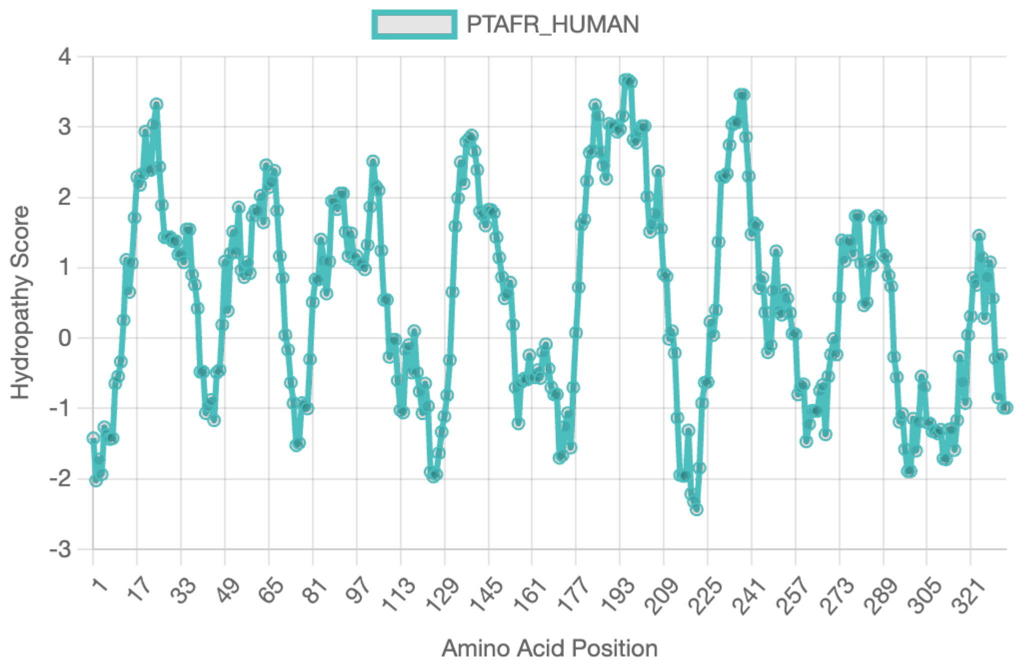

Kyte-Doolittle hydropathy plot for the sequence "PTAFR_HUMAN". Positive values indicate hydrophobic regions, while negative values indicate hydrophilic regions.

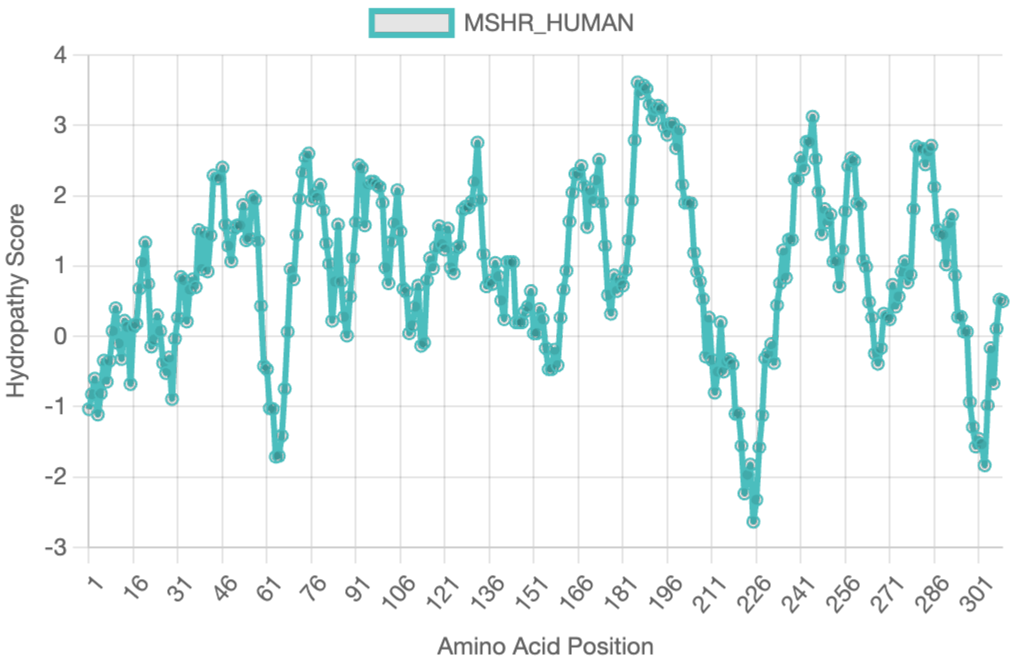

Kyte-Doolittle hydropathy plot for the sequence "MSHR_HUMAN". Positive values indicate hydrophobic regions, while negative values indicate hydrophilic regions.

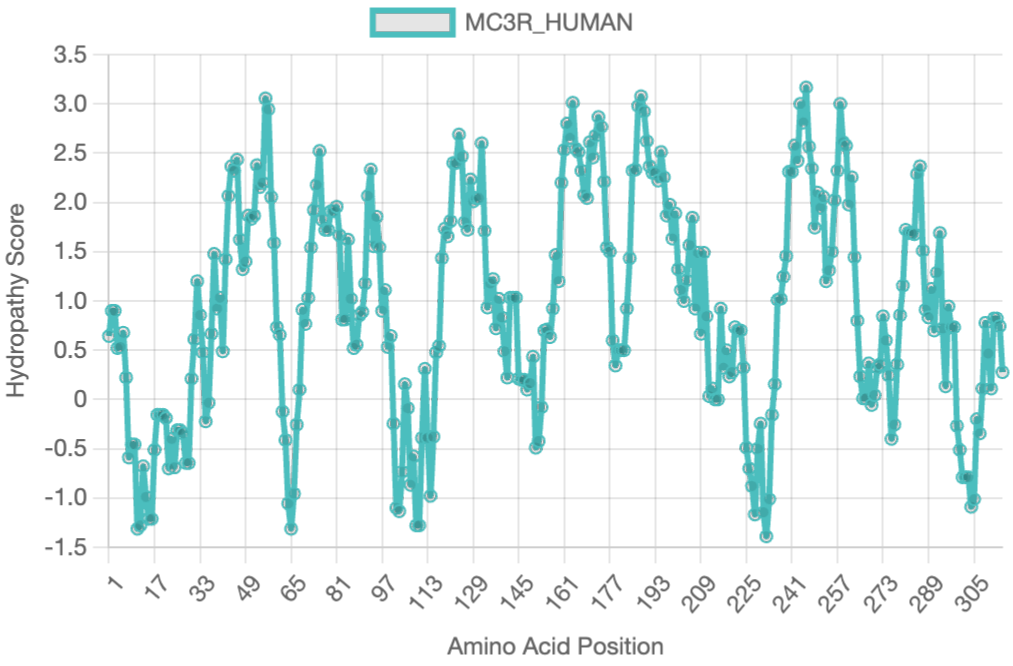

Kyte-Doolittle hydropathy plot for the sequence "MC3R_HUMAN". Positive values indicate hydrophobic regions, while negative values indicate hydrophilic regions.

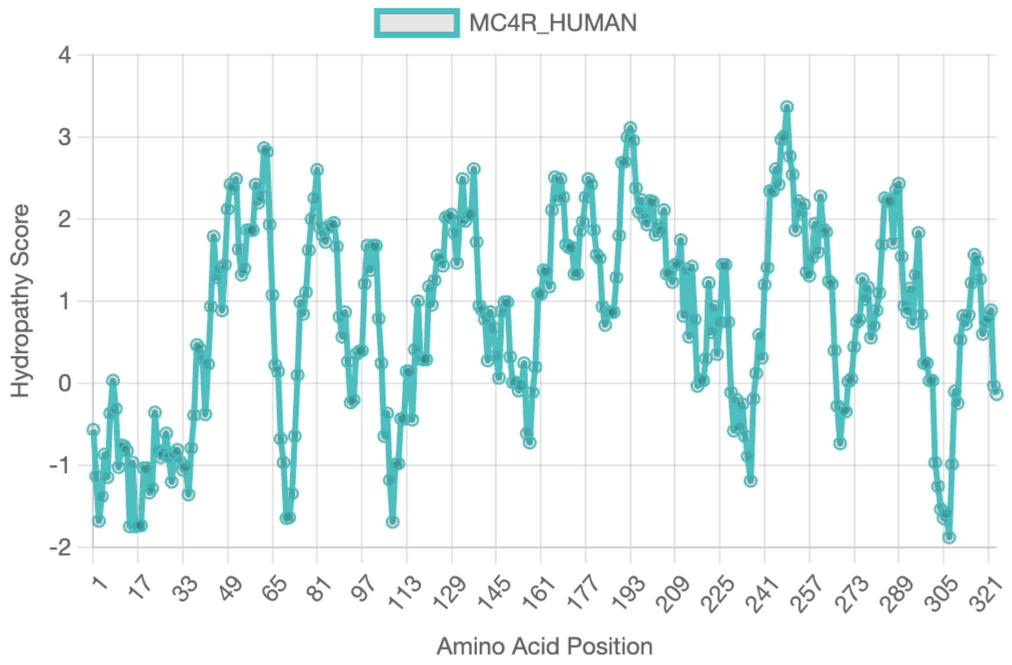

Kyte-Doolittle hydropathy plot for the sequence "MC4R_HUMAN". Positive values indicate hydrophobic regions, while negative values indicate hydrophilic regions.

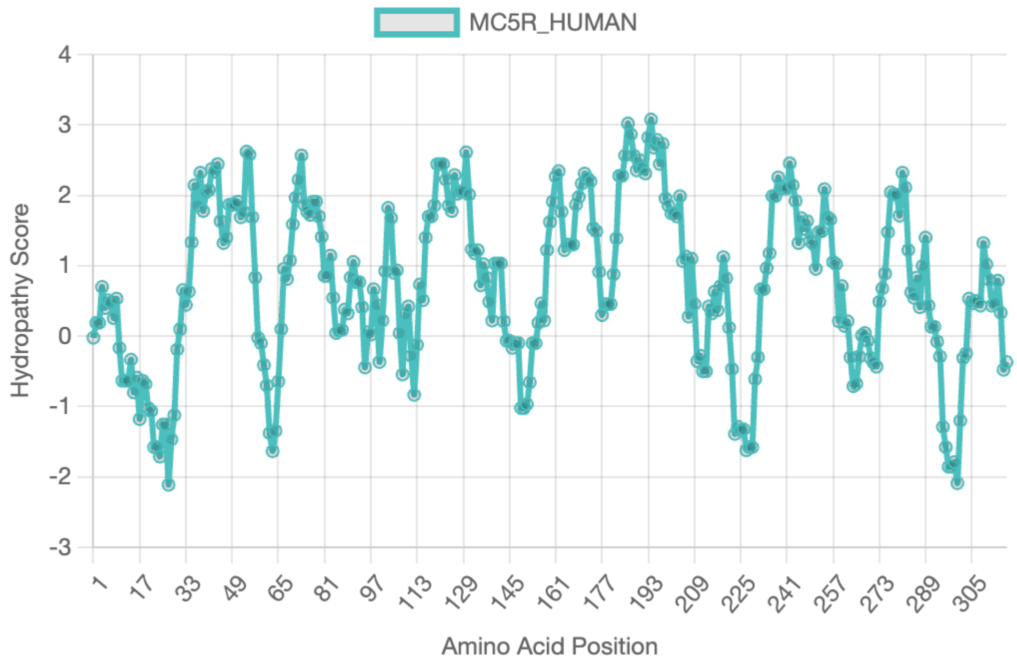

Kyte-Doolittle hydropathy plot for the sequence "MC5R_HUMAN". Positive values indicate hydrophobic regions, while negative values indicate hydrophilic regions.

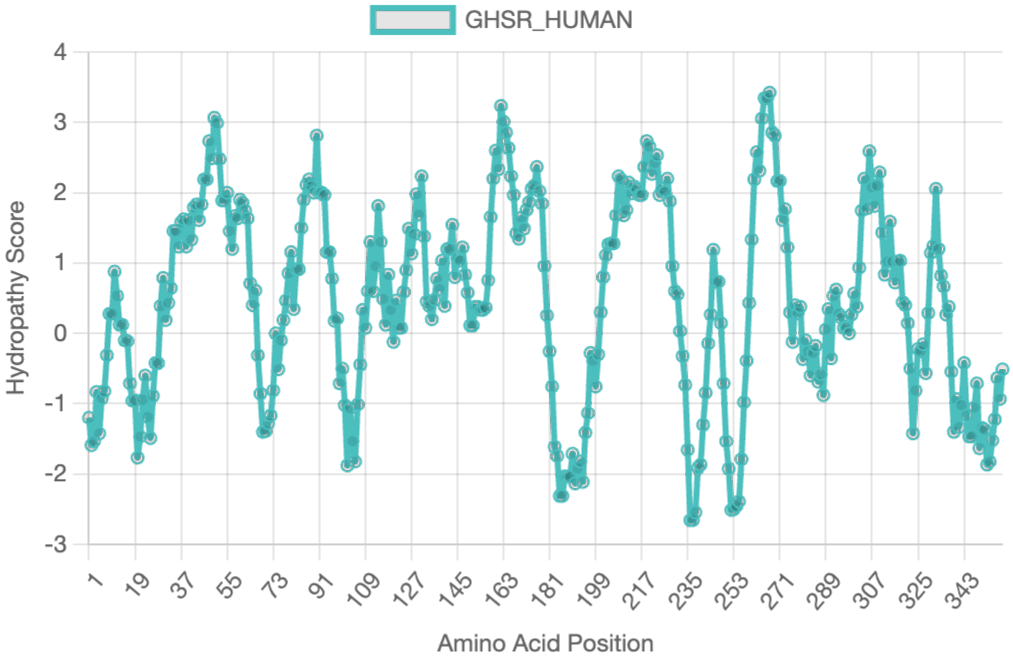

Kyte-Doolittle hydropathy plot for the sequence "GHSR_HUMAN". Positive values indicate hydrophobic regions, while negative values indicate hydrophilic regions.

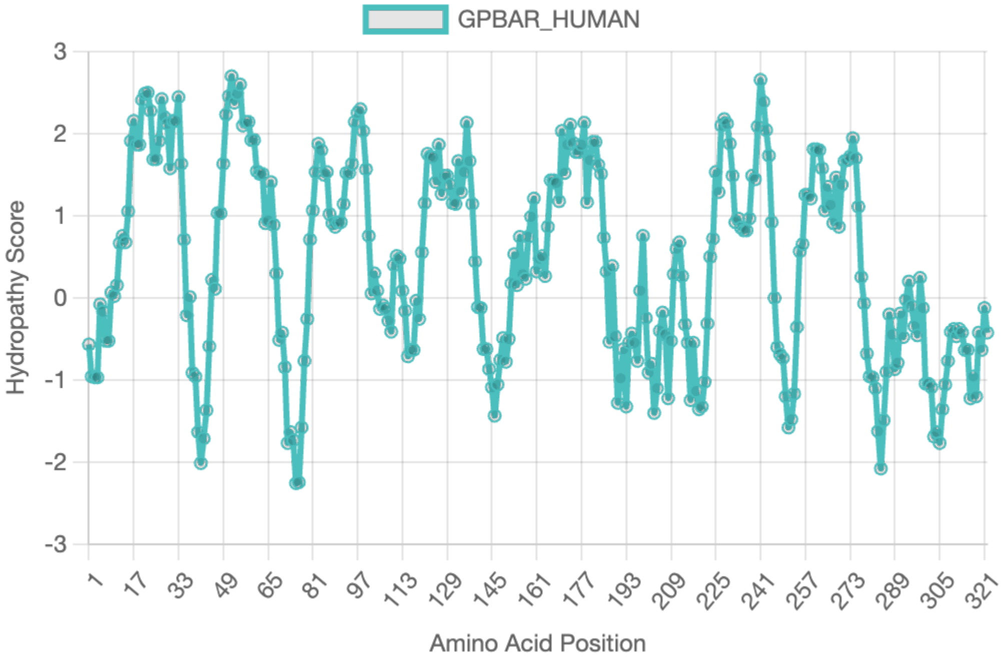

Kyte-Doolittle hydropathy plot for the sequence "GPBAR_HUMAN". Positive values indicate hydrophobic regions, while negative values indicate hydrophilic regions.

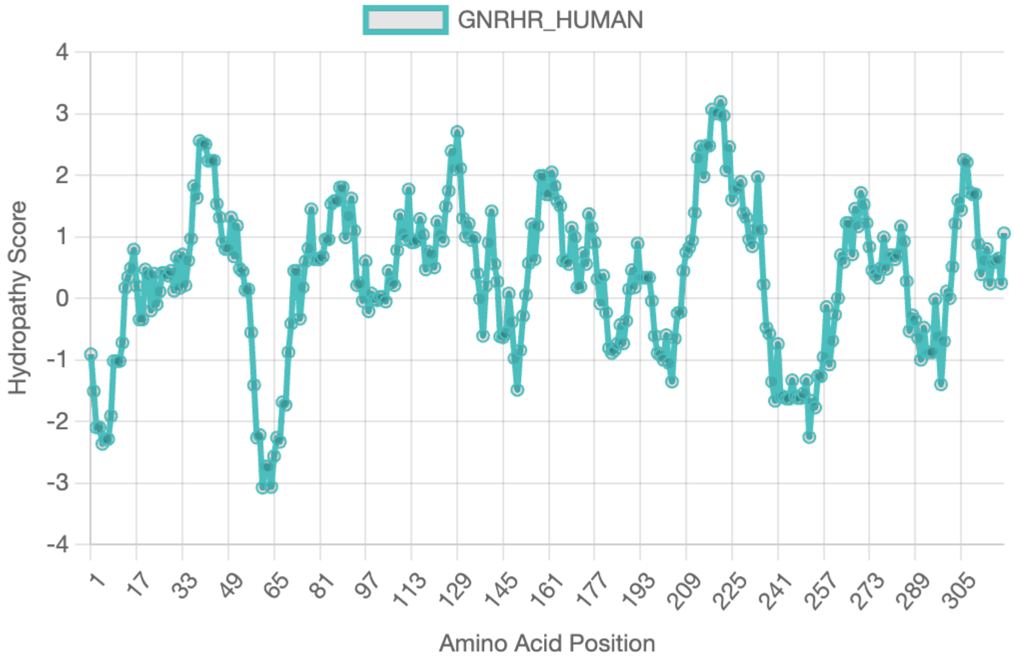

Kyte-Doolittle hydropathy plot for the sequence "GNRHR_HUMAN". Positive values indicate hydrophobic regions, while negative values indicate hydrophilic regions.

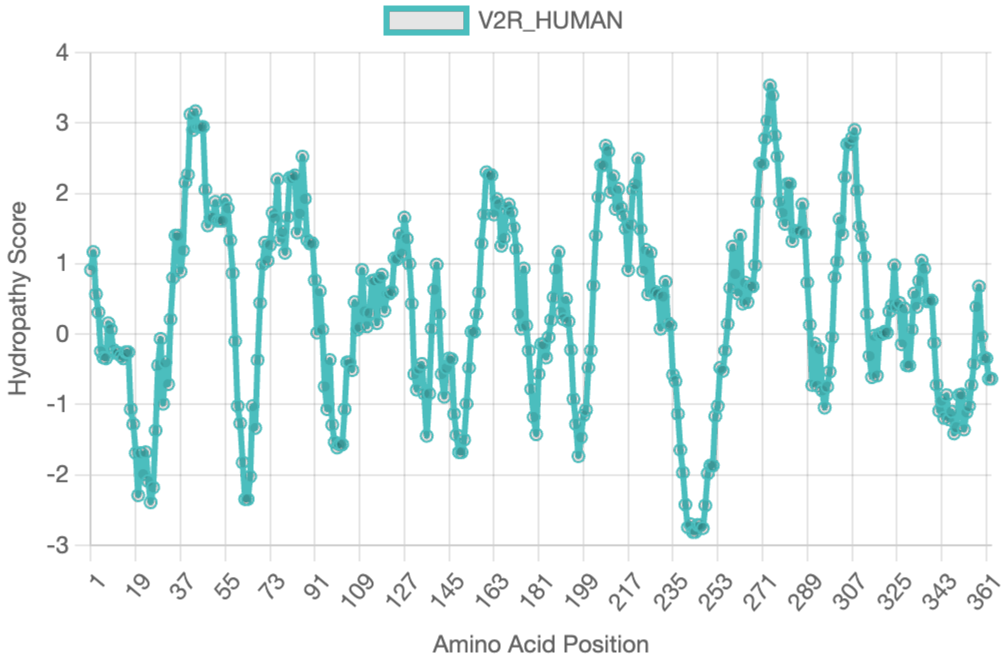

Kyte-Doolittle hydropathy plot for the sequence "V2R_HUMAN". Positive values indicate hydrophobic regions, while negative values indicate hydrophilic regions.

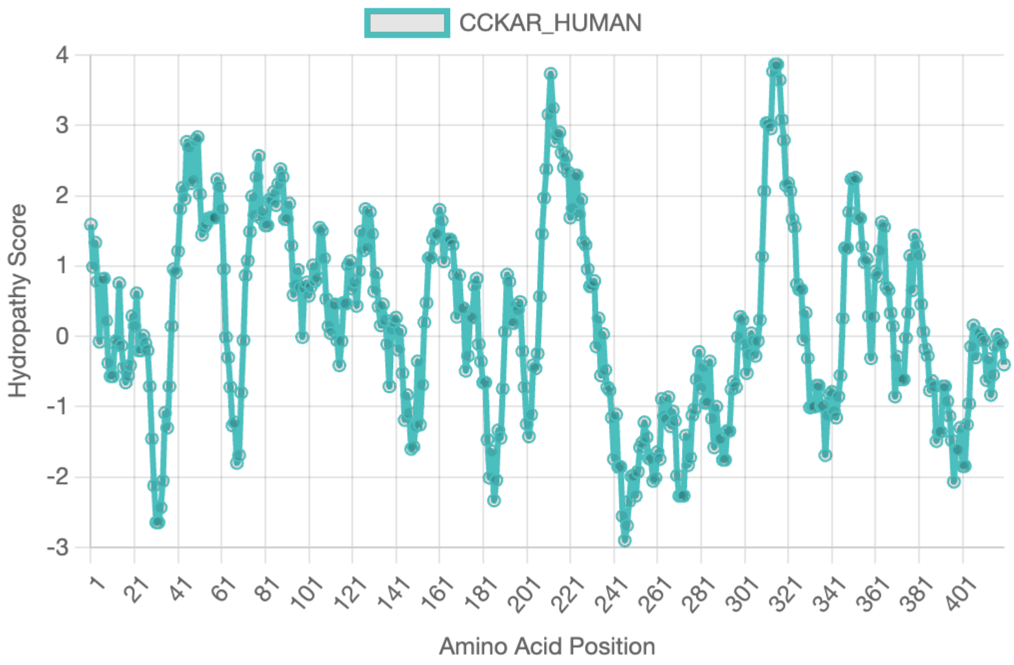

Kyte-Doolittle hydropathy plot for the sequence "CCKAR_HUMAN". Positive values indicate hydrophobic regions, while negative values indicate hydrophilic regions.

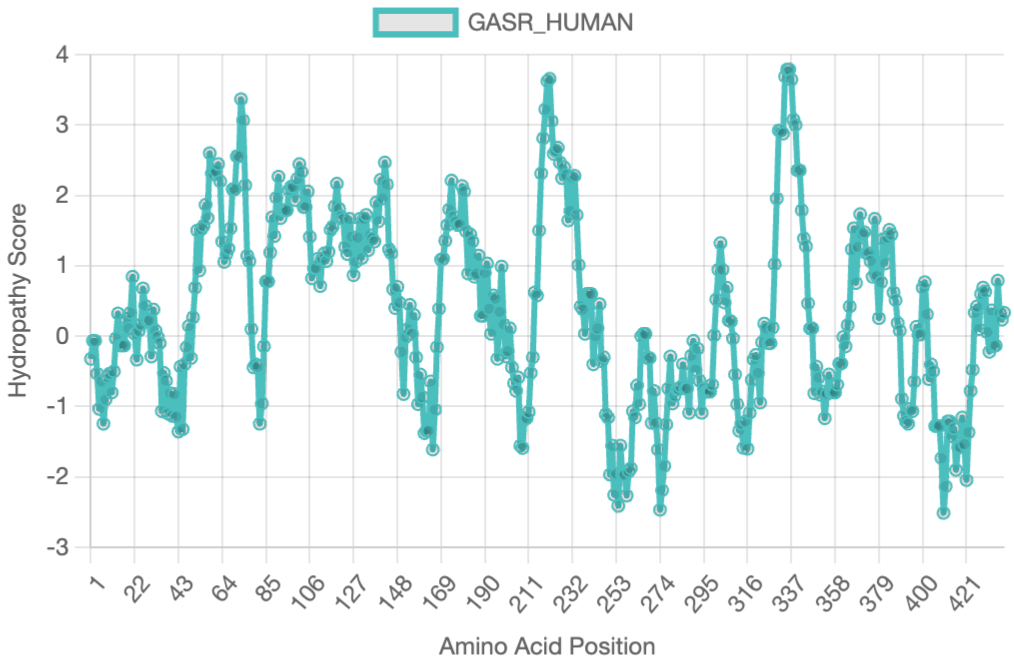

Kyte-Doolittle hydropathy plot for the sequence "GASR_HUMAN". Positive values indicate hydrophobic regions, while negative values indicate hydrophilic regions.

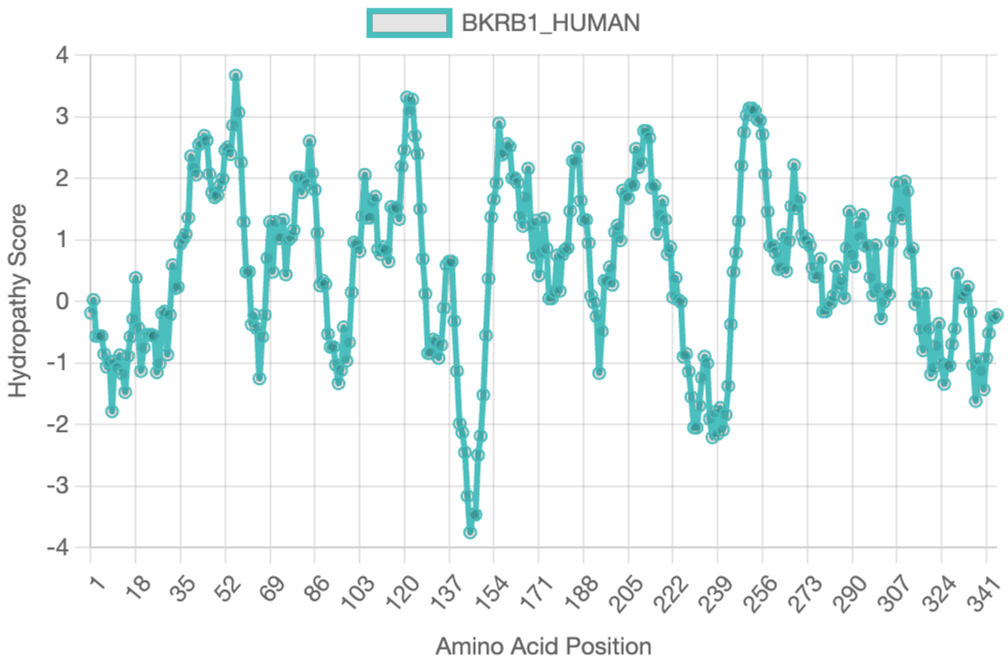

Kyte-Doolittle hydropathy plot for the sequence "BKRB1_HUMAN". Positive values indicate hydrophobic regions, while negative values indicate hydrophilic regions.

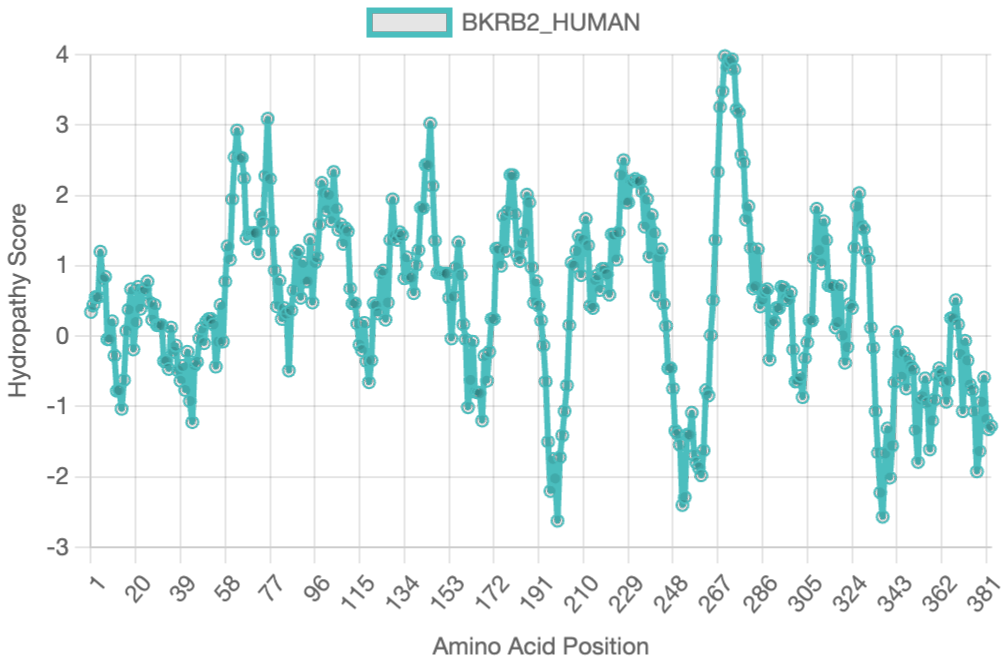

Kyte-Doolittle hydropathy plot for the sequence "BKRB2_HUMAN". Positive values indicate hydrophobic regions, while negative values indicate hydrophilic regions.

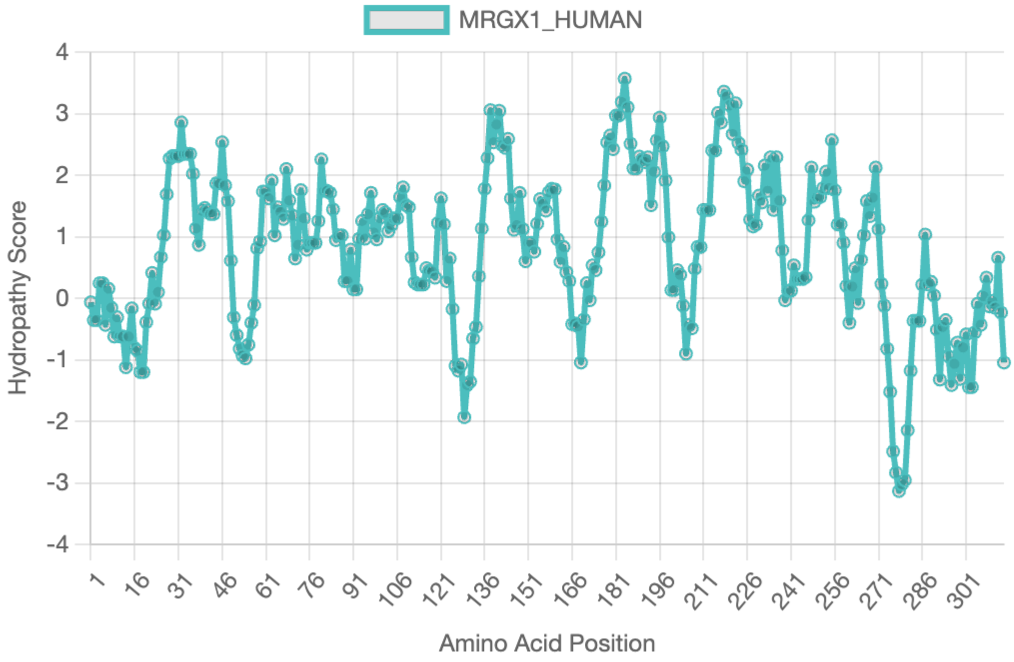

Kyte-Doolittle hydropathy plot for the sequence "MRGX1_HUMAN". Positive values indicate hydrophobic regions, while negative values indicate hydrophilic regions.

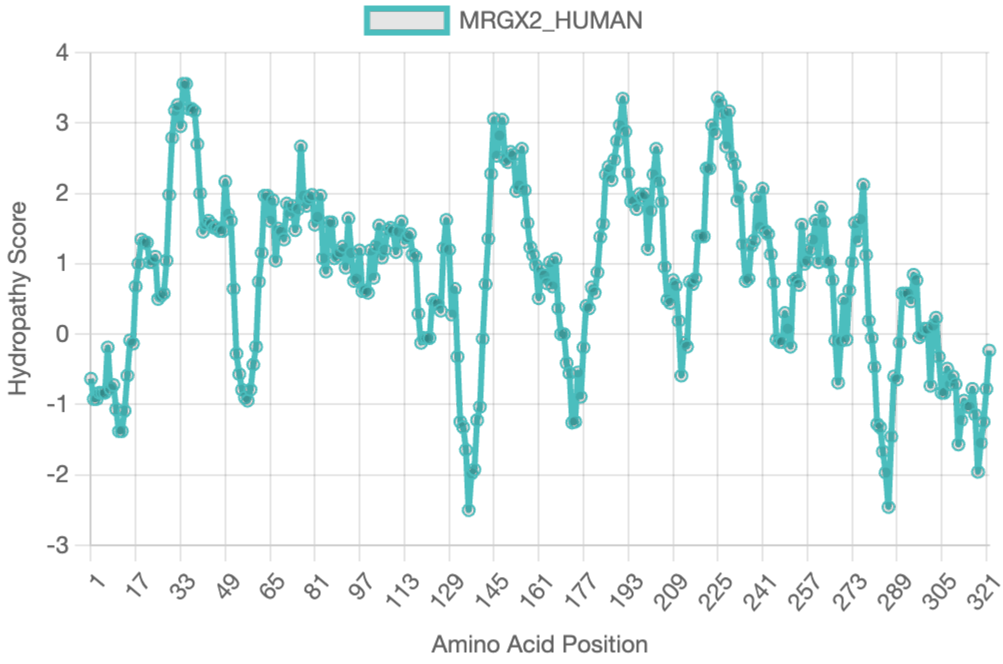

Kyte-Doolittle hydropathy plot for the sequence "MRGX2_HUMAN". Positive values indicate hydrophobic regions, while negative values indicate hydrophilic regions.

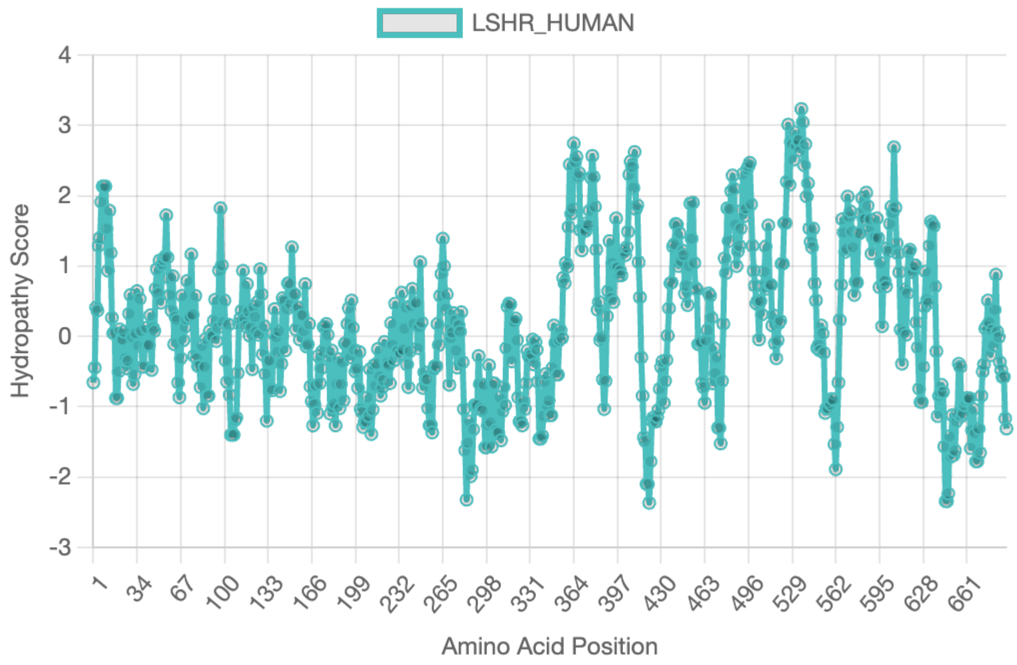

Kyte-Doolittle hydropathy plot for the sequence "LSHR_HUMAN". Positive values indicate hydrophobic regions, while negative values indicate hydrophilic regions.

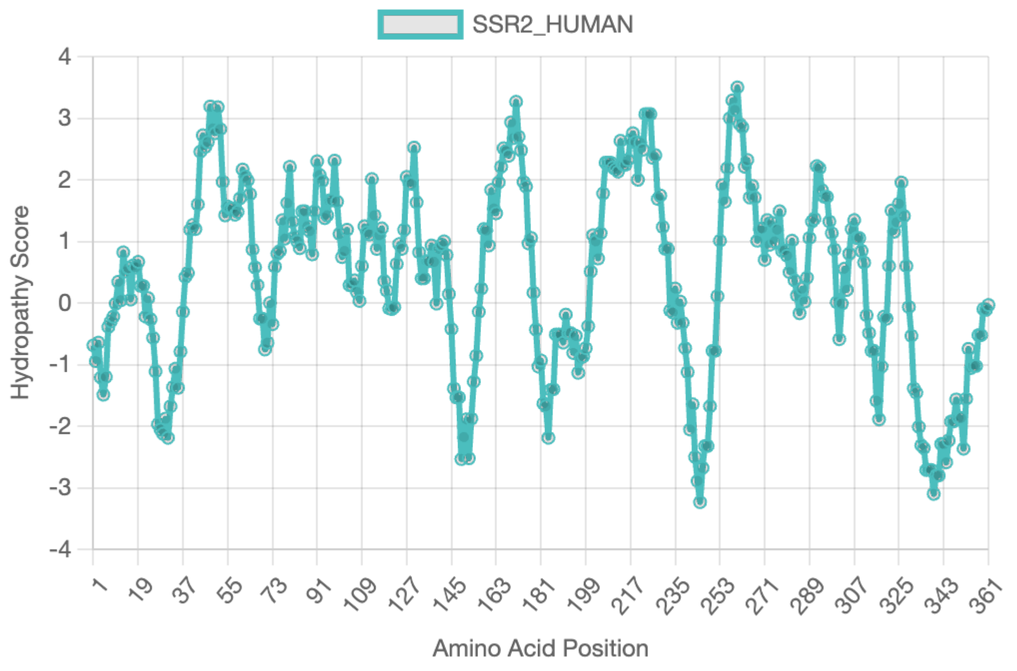

Kyte-Doolittle hydropathy plot for the sequence "SSR2_HUMAN". Positive values indicate hydrophobic regions, while negative values indicate hydrophilic regions.

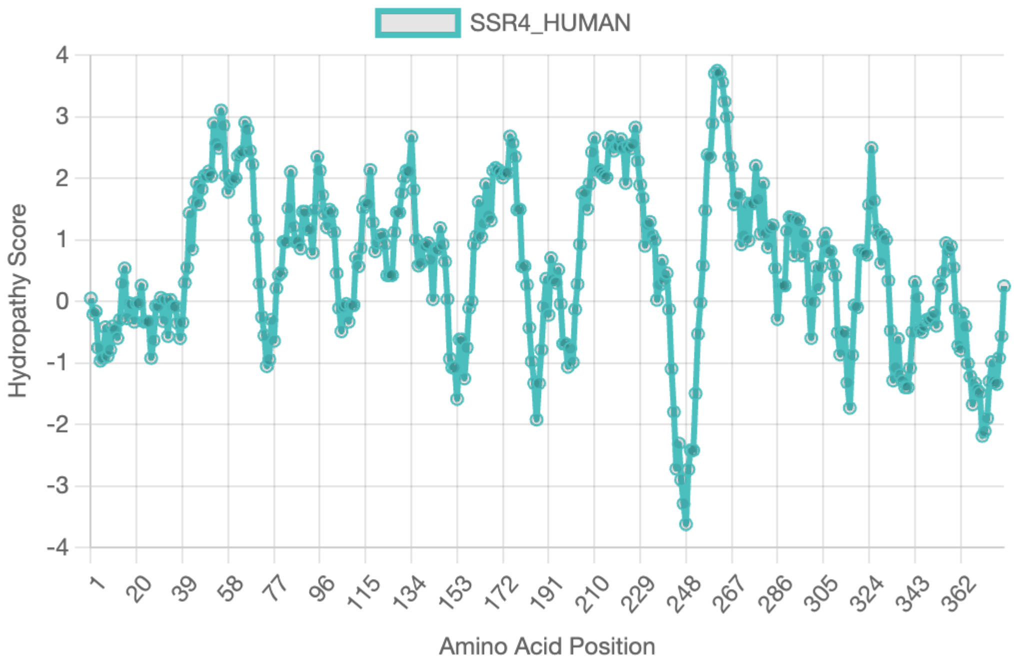

Kyte-Doolittle hydropathy plot for the sequence "SSR4_HUMAN". Positive values indicate hydrophobic regions, while negative values indicate hydrophilic regions.

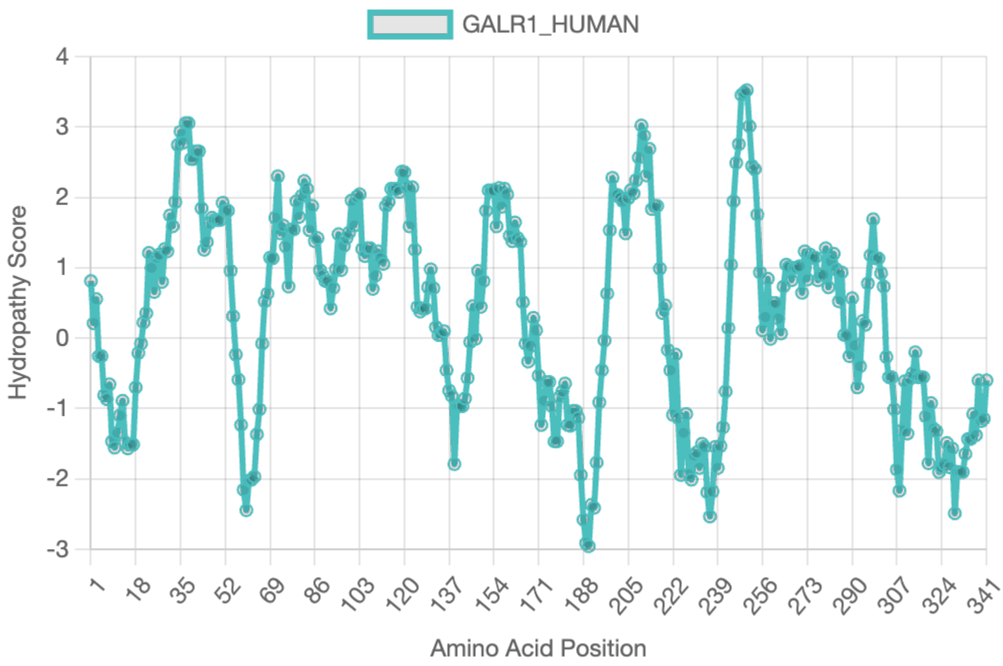

Kyte-Doolittle hydropathy plot for the sequence "GALR1_HUMAN". Positive values indicate hydrophobic regions, while negative values indicate hydrophilic regions.

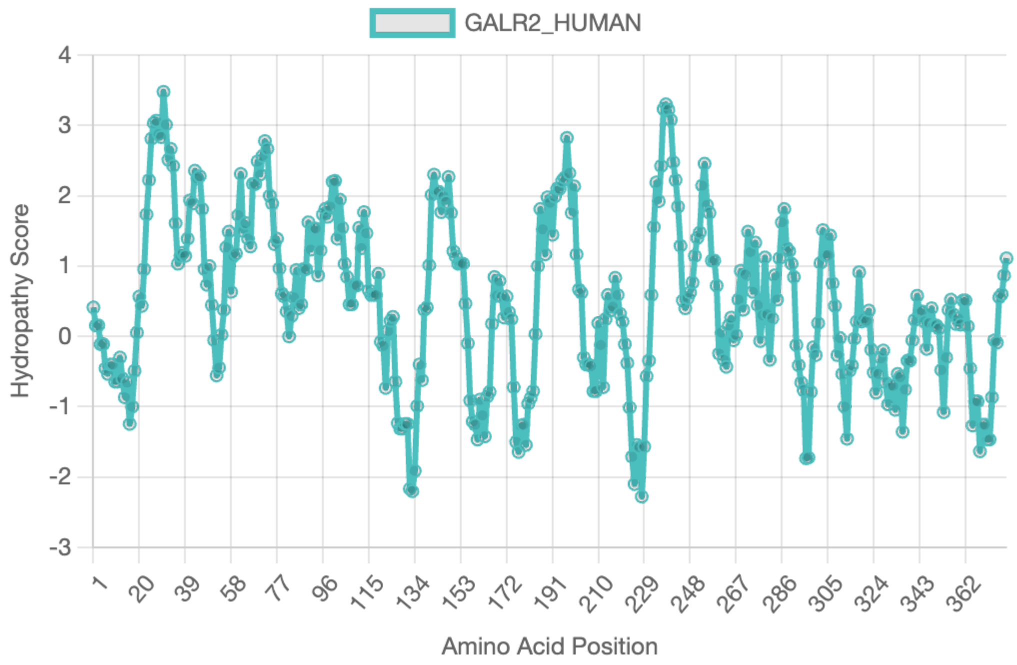

Kyte-Doolittle hydropathy plot for the sequence "GALR2_HUMAN". Positive values indicate hydrophobic regions, while negative values indicate hydrophilic regions.

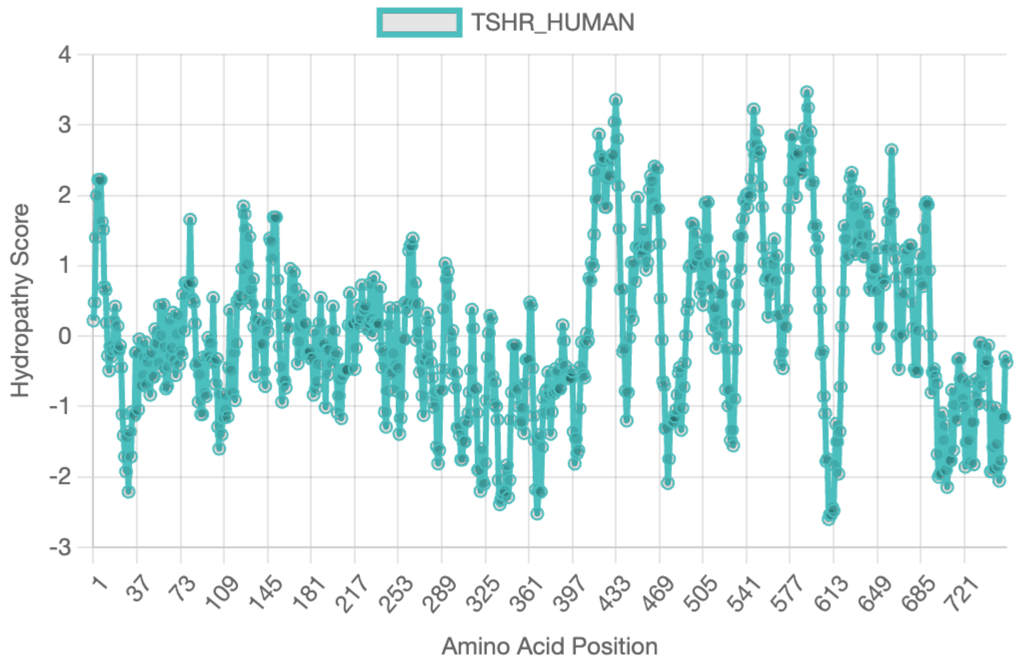

Kyte-Doolittle hydropathy plot for the sequence "TSHR_HUMAN". Positive values indicate hydrophobic regions, while negative values indicate hydrophilic regions.

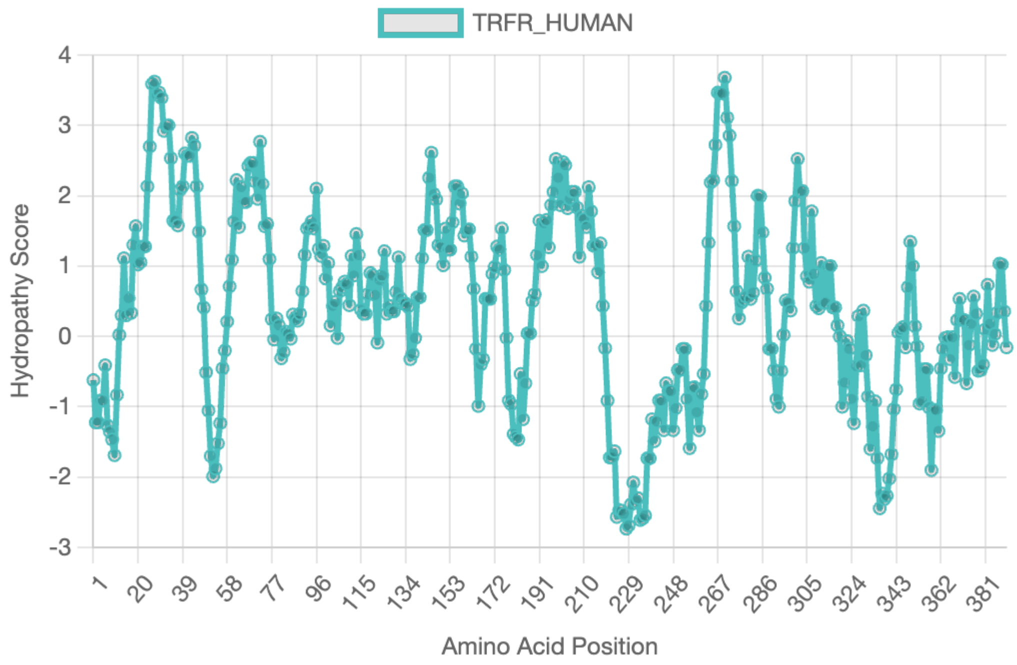

Kyte-Doolittle hydropathy plot for the sequence "TRFR_HUMAN". Positive values indicate hydrophobic regions, while negative values indicate hydrophilic regions.

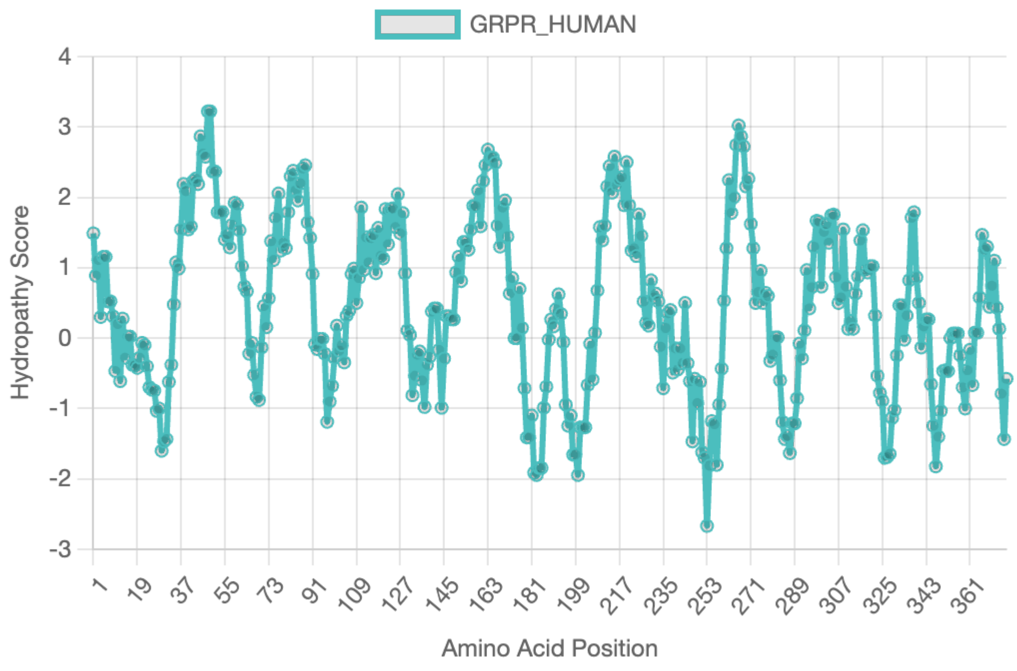

Kyte-Doolittle hydropathy plot for the sequence "GRPR_HUMAN". Positive values indicate hydrophobic regions, while negative values indicate hydrophilic regions.

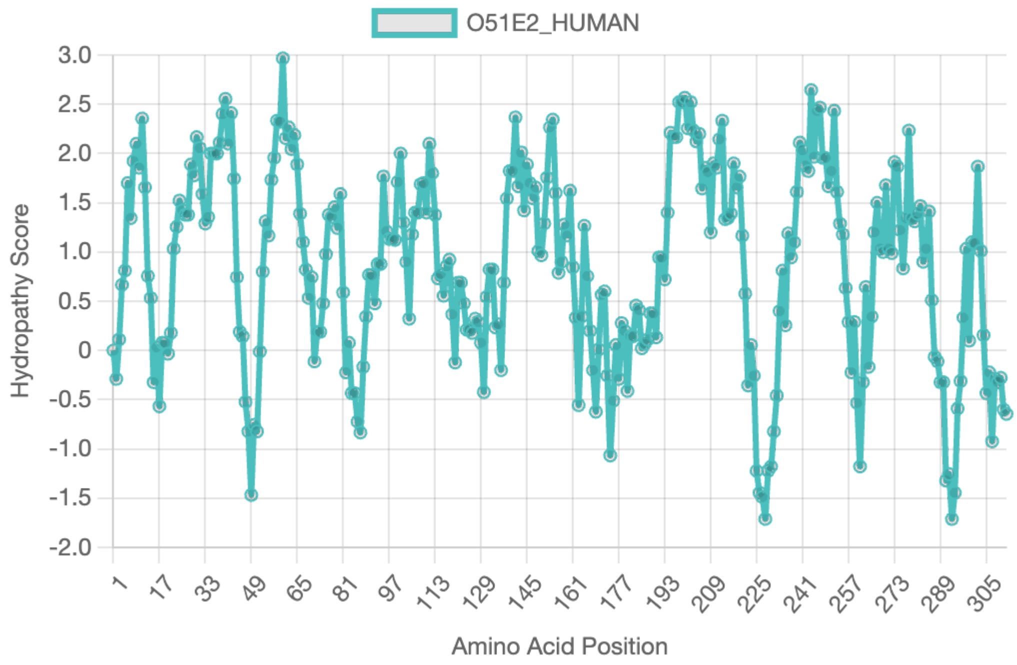

Kyte-Doolittle hydropathy plot for the sequence "O51E2_HUMAN". Positive values indicate hydrophobic regions, while negative values indicate hydrophilic regions.

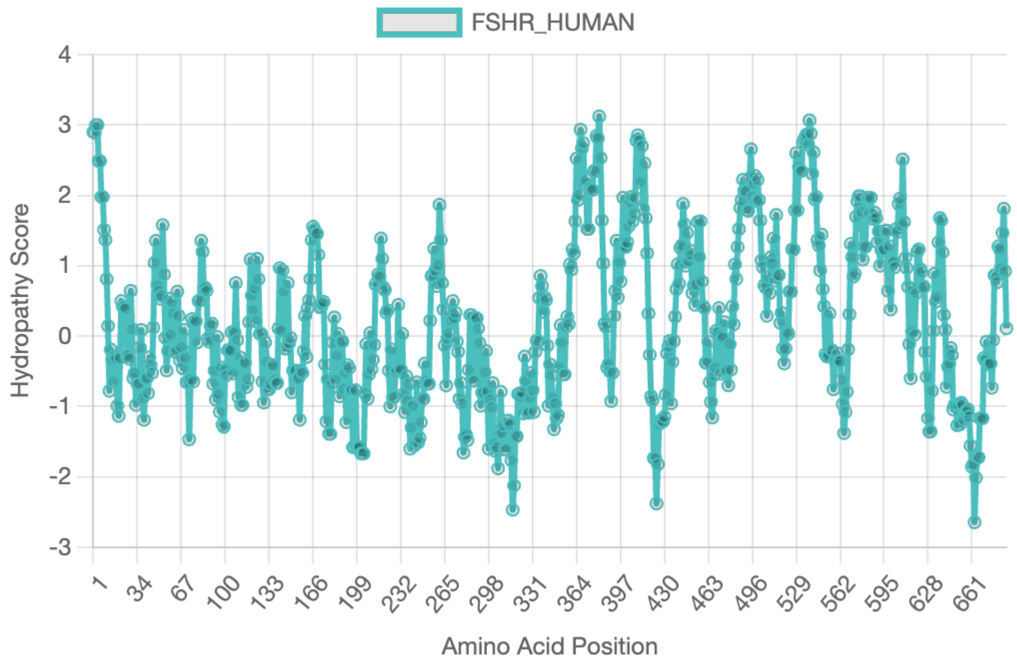

Kyte-Doolittle hydropathy plot for the sequence "FSHR_HUMAN". Positive values indicate hydrophobic regions, while negative values indicate hydrophilic regions.

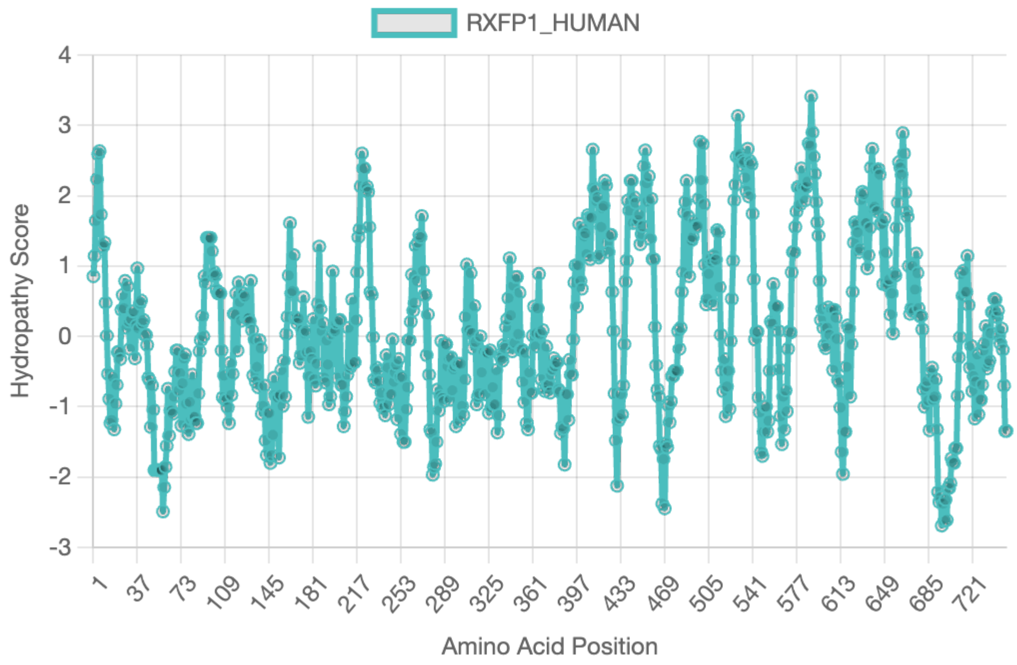

Kyte-Doolittle hydropathy plot for the sequence "RXFP1_HUMAN". Positive values indicate hydrophobic regions, while negative values indicate hydrophilic regions.

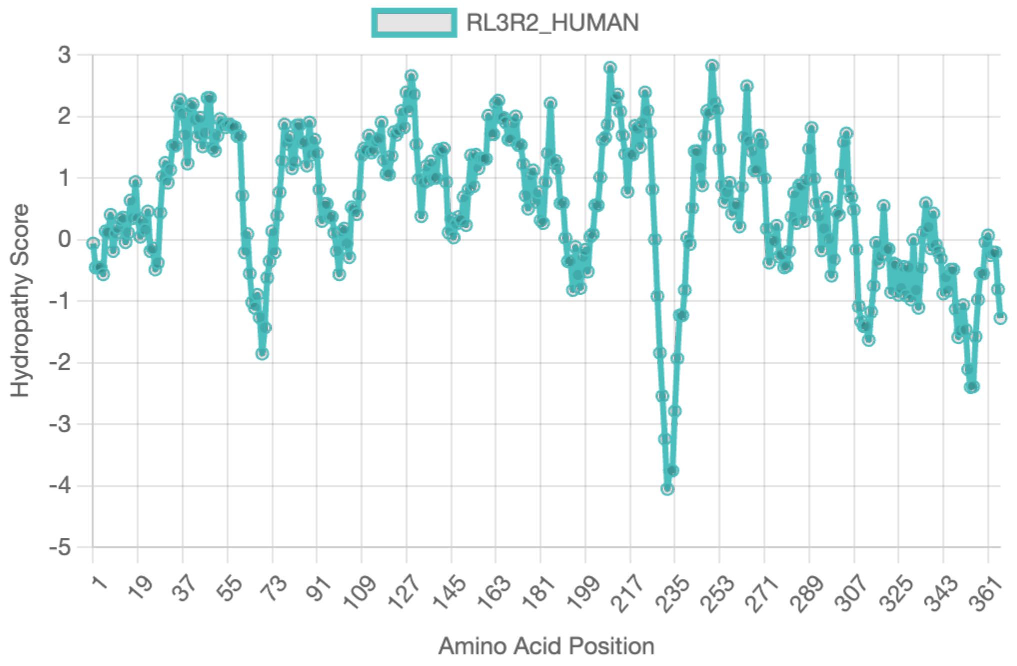

Kyte-Doolittle hydropathy plot for the sequence "RL3R2_HUMAN". Positive values indicate hydrophobic regions, while negative values indicate hydrophilic regions.

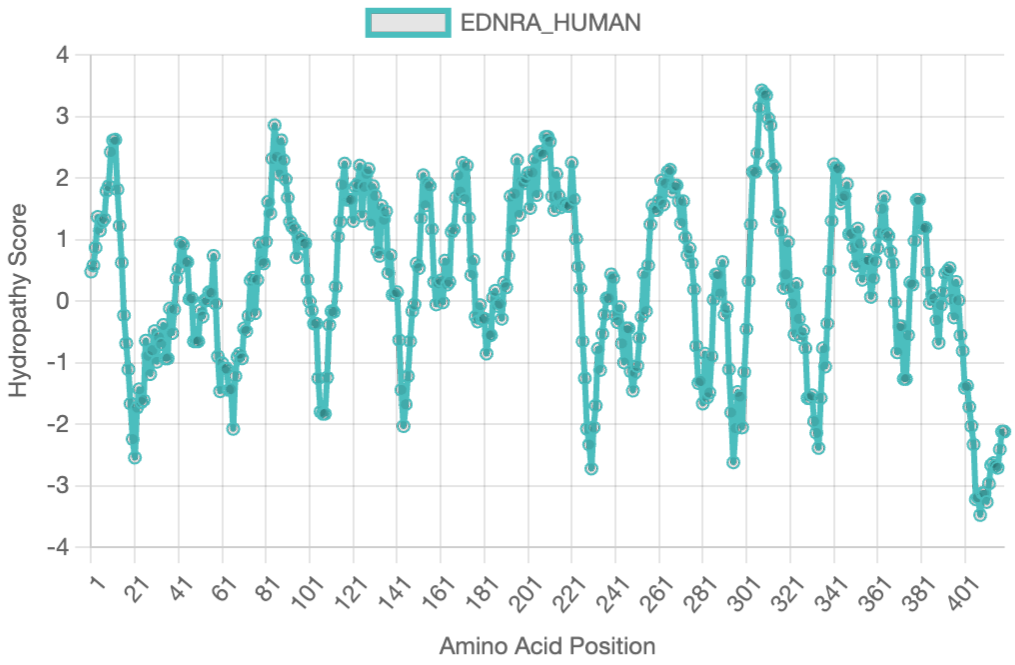

Kyte-Doolittle hydropathy plot for the sequence "EDNRA_HUMAN". Positive values indicate hydrophobic regions, while negative values indicate hydrophilic regions.

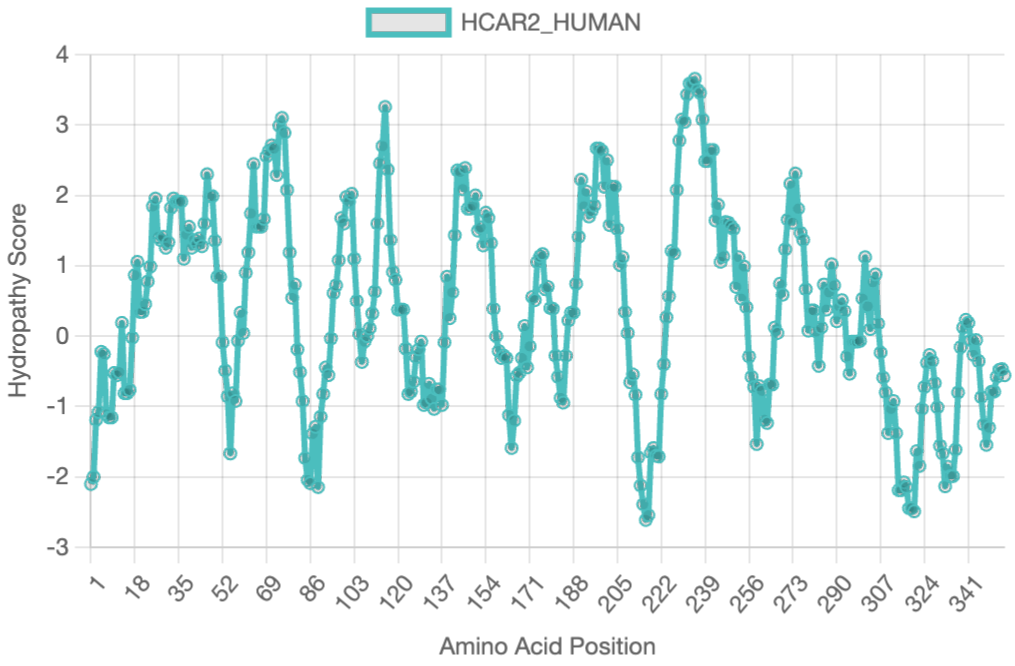

Kyte-Doolittle hydropathy plot for the sequence "HCAR2_HUMAN". Positive values indicate hydrophobic regions, while negative values indicate hydrophilic regions.

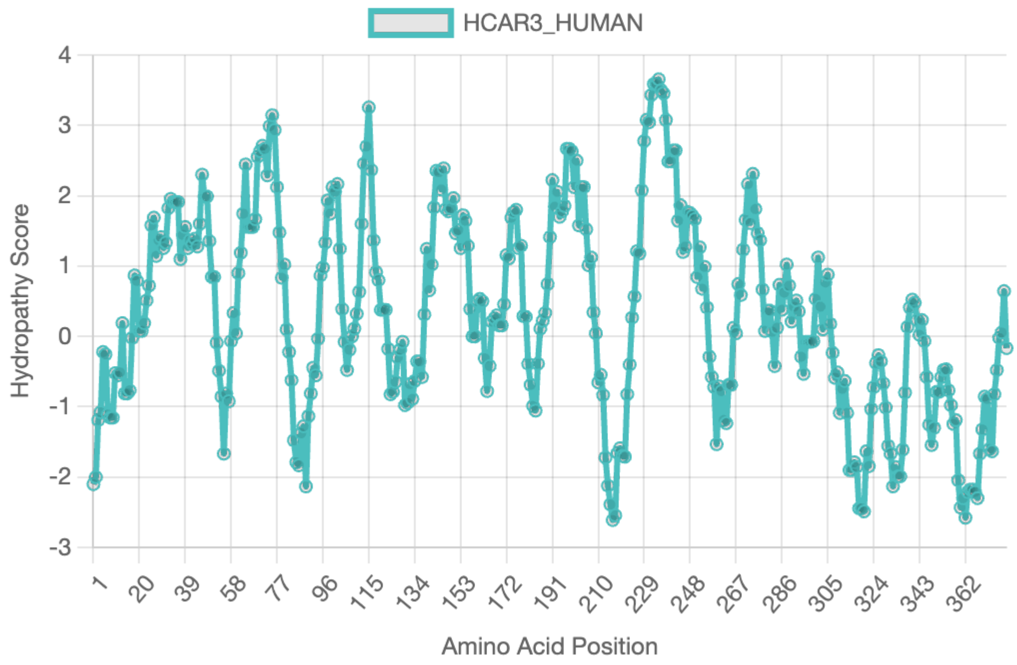

Kyte-Doolittle hydropathy plot for the sequence "HCAR3_HUMAN". Positive values indicate hydrophobic regions, while negative values indicate hydrophilic regions.

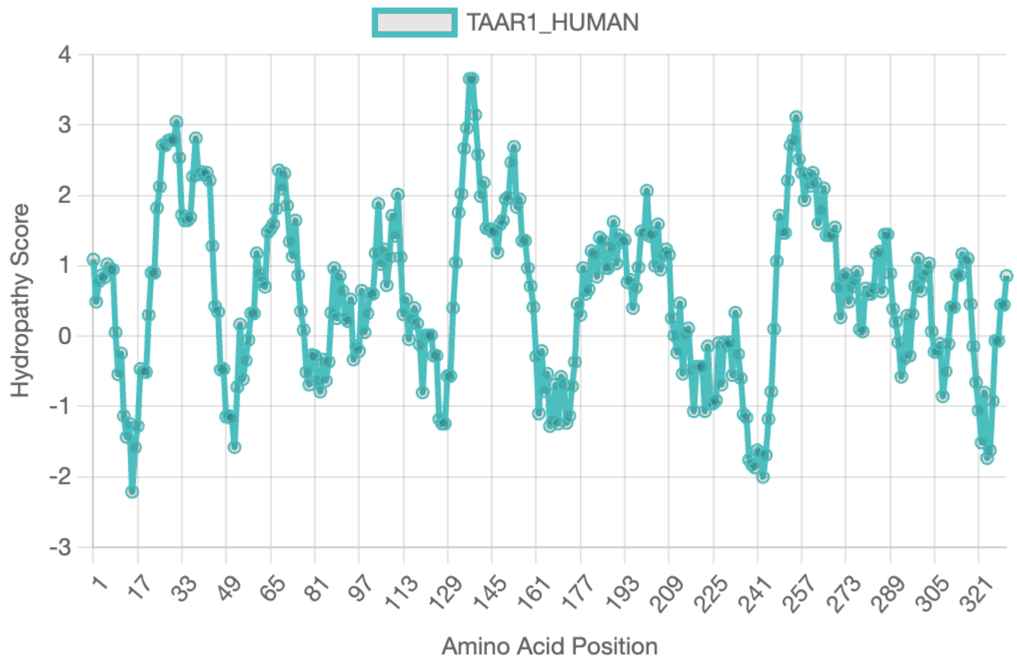

Kyte-Doolittle hydropathy plot for the sequence "TAAR1_HUMAN". Positive values indicate hydrophobic regions, while negative values indicate hydrophilic regions.

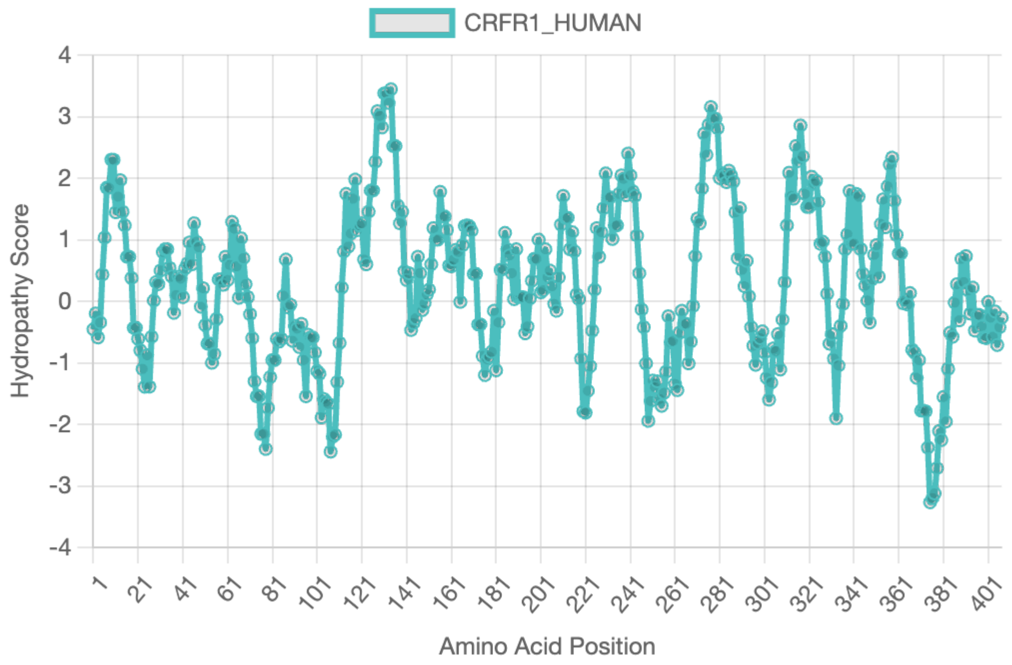

Kyte-Doolittle hydropathy plot for the sequence "CRFR1_HUMAN". Positive values indicate hydrophobic regions, while negative values indicate hydrophilic regions.

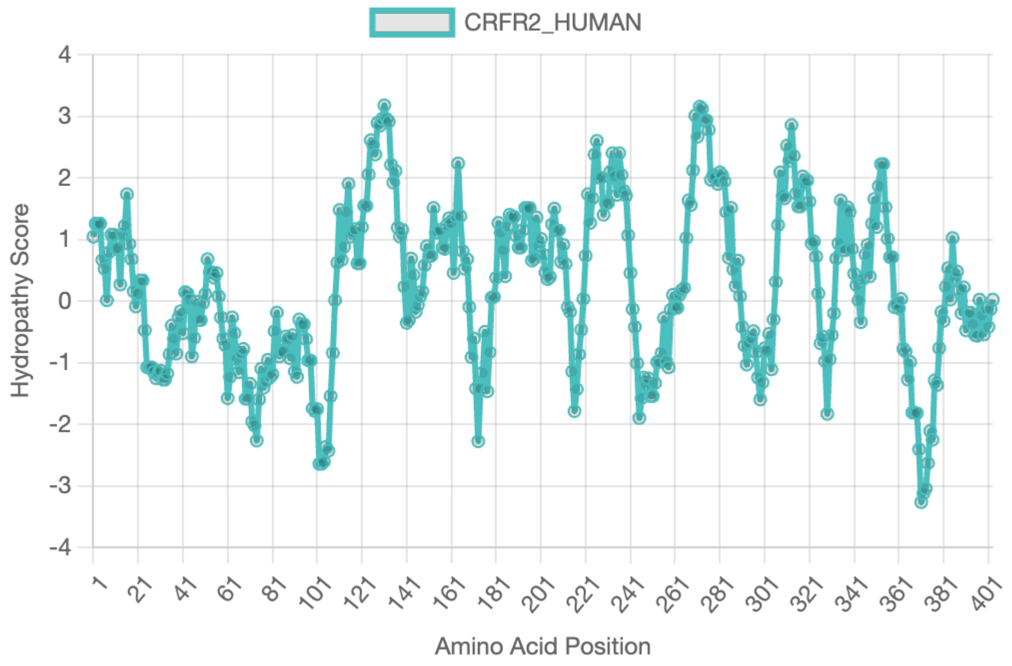

Kyte-Doolittle hydropathy plot for the sequence "CRFR2_HUMAN". Positive values indicate hydrophobic regions, while negative values indicate hydrophilic regions.

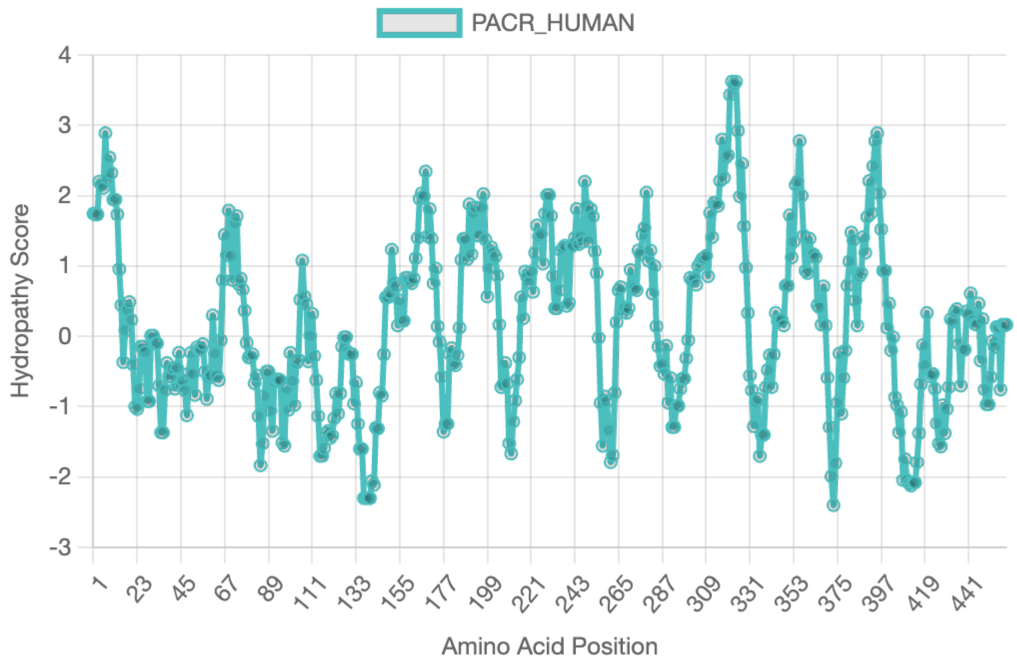

Kyte-Doolittle hydropathy plot for the sequence "PACR_HUMAN". Positive values indicate hydrophobic regions, while negative values indicate hydrophilic regions.

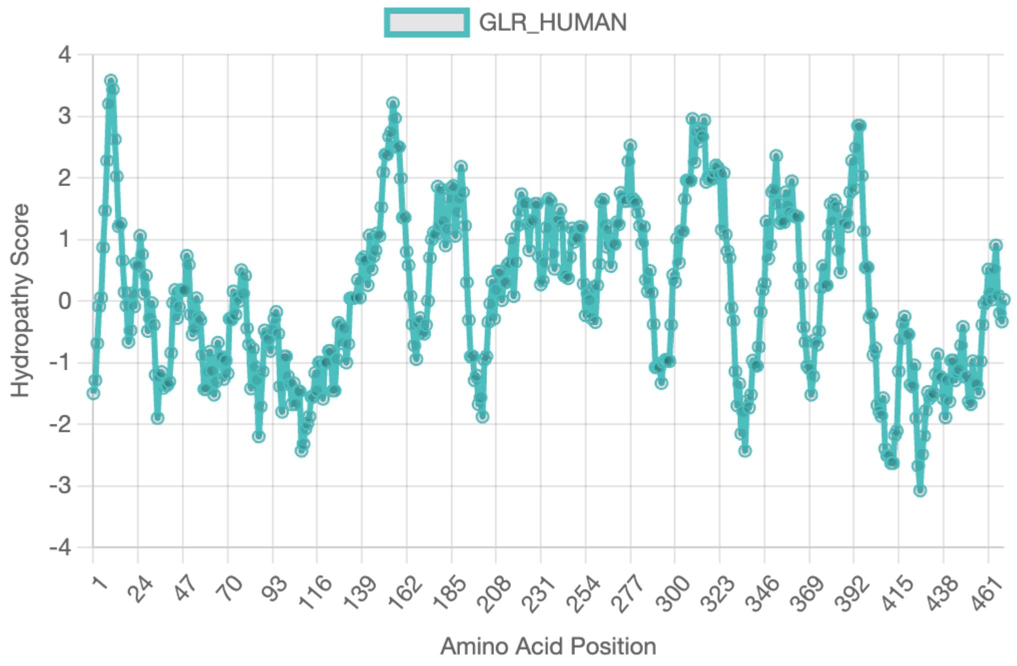

Kyte-Doolittle hydropathy plot for the sequence "GLR_HUMAN". Positive values indicate hydrophobic regions, while negative values indicate hydrophilic regions.

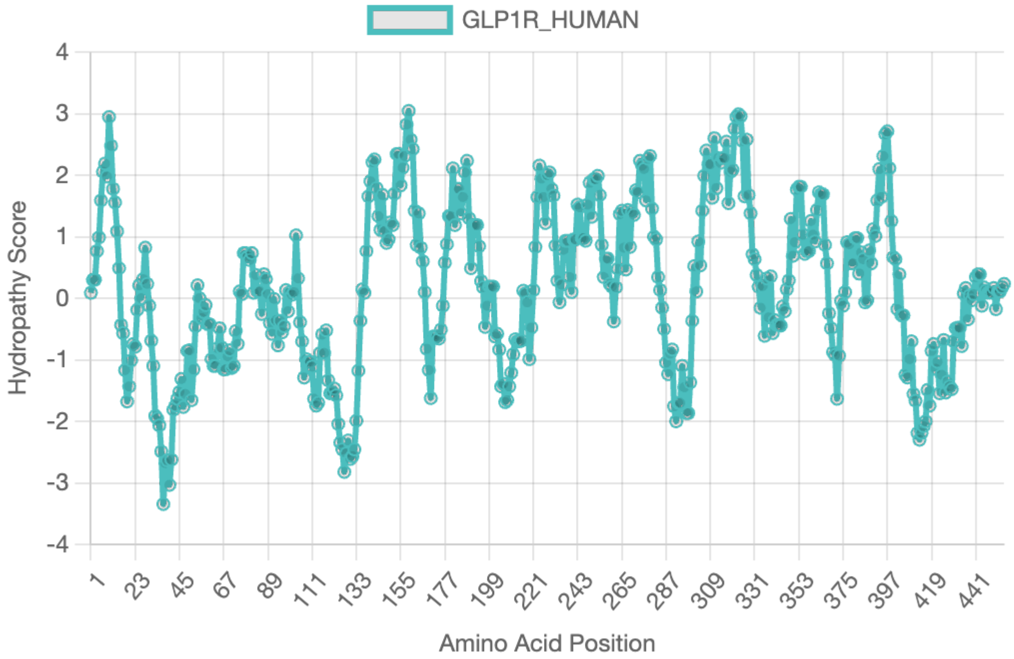

Kyte-Doolittle hydropathy plot for the sequence "GLP1R_HUMAN". Positive values indicate hydrophobic regions, while negative values indicate hydrophilic regions.

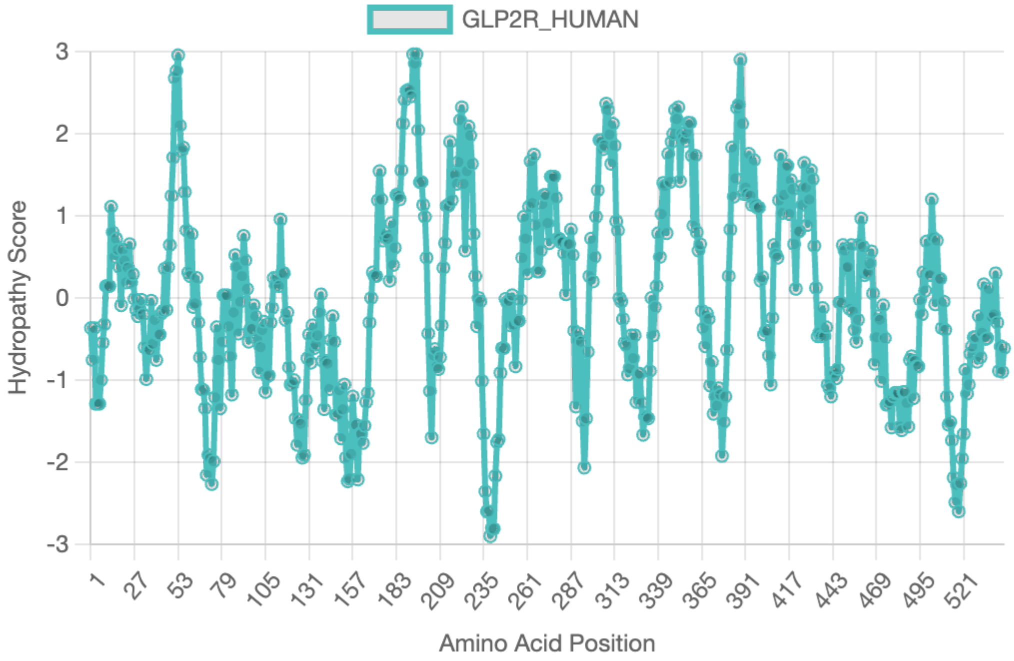

Kyte-Doolittle hydropathy plot for the sequence "GLP2R_HUMAN". Positive values indicate hydrophobic regions, while negative values indicate hydrophilic regions.

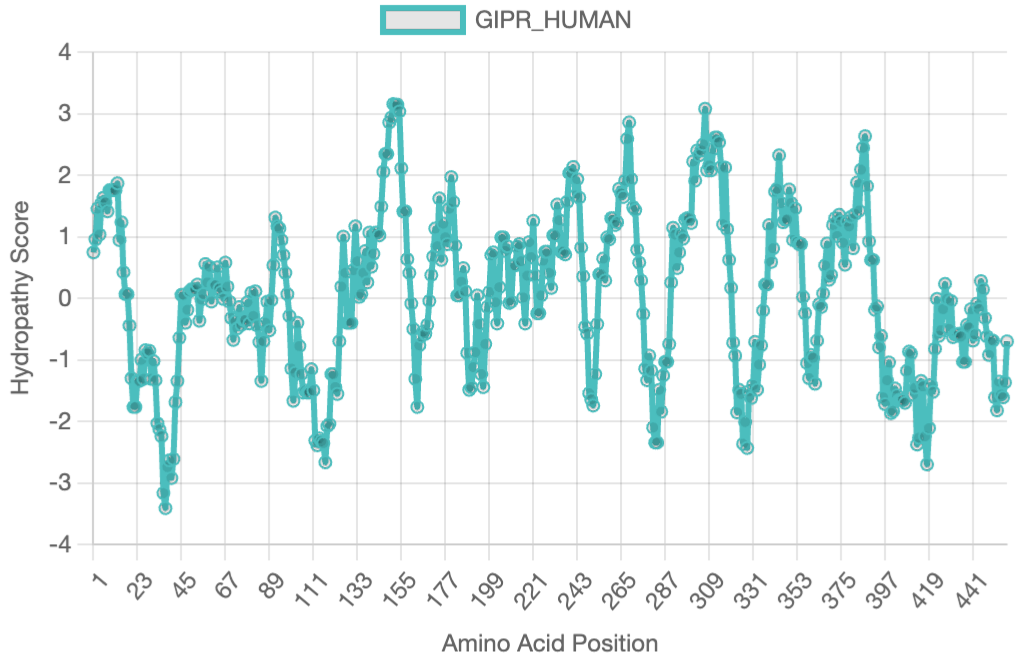

Kyte-Doolittle hydropathy plot for the sequence "GIPR_HUMAN". Positive values indicate hydrophobic regions, while negative values indicate hydrophilic regions.

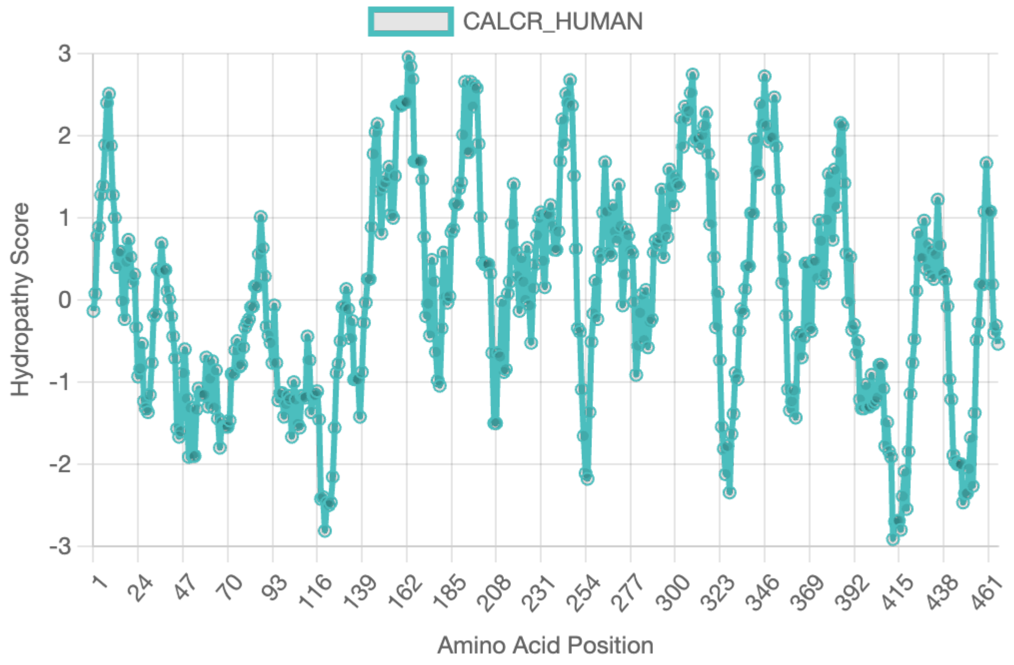

Kyte-Doolittle hydropathy plot for the sequence "CALCR_HUMAN". Positive values indicate hydrophobic regions, while negative values indicate hydrophilic regions.

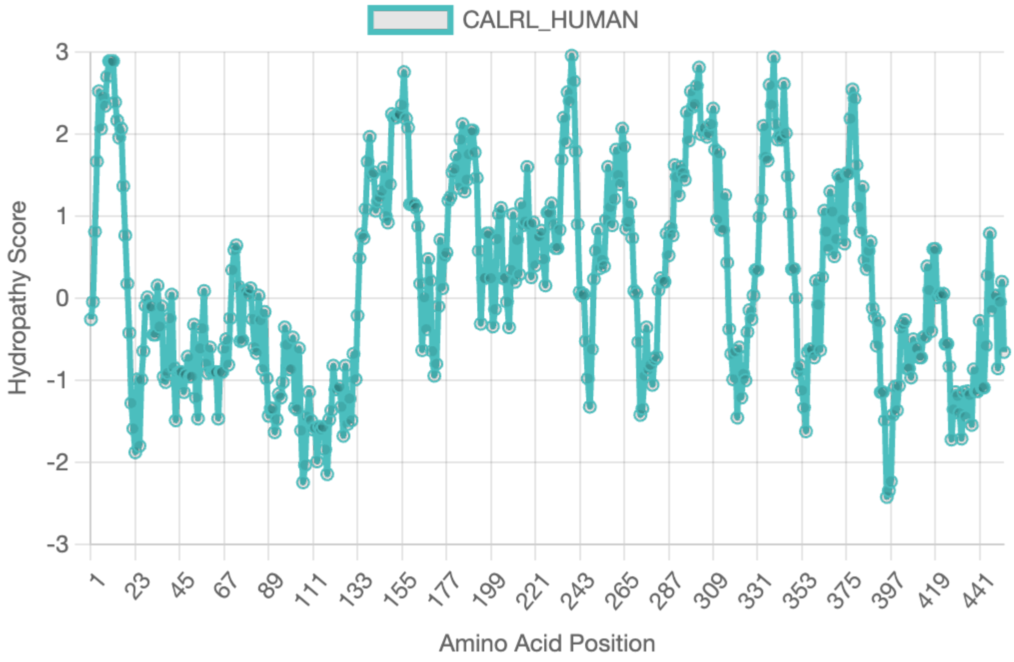

Kyte-Doolittle hydropathy plot for the sequence "CALRL_HUMAN". Positive values indicate hydrophobic regions, while negative values indicate hydrophilic regions.

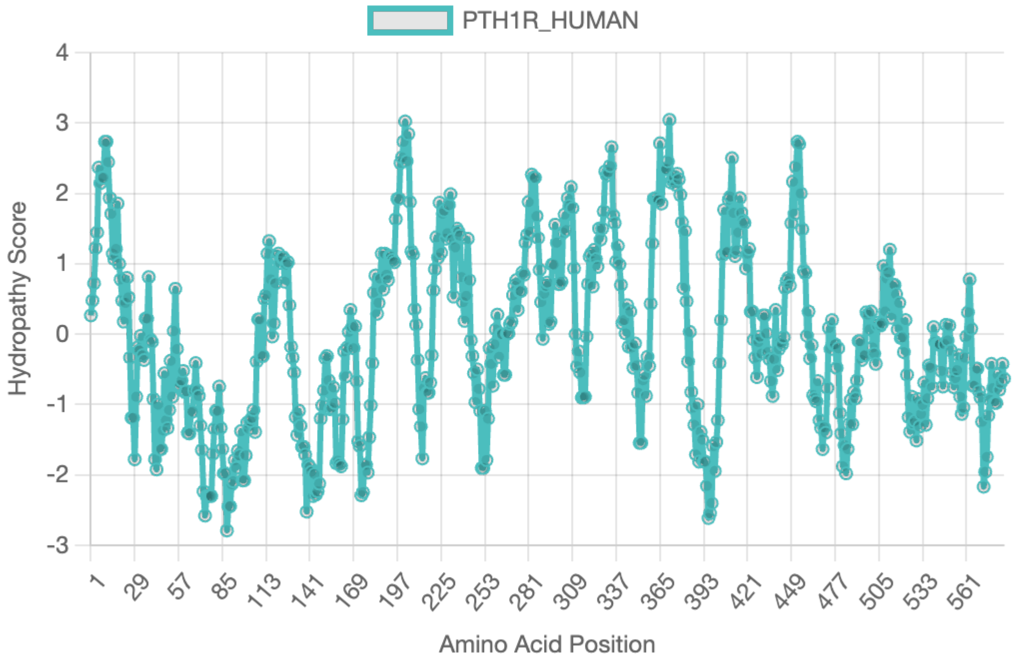

Kyte-Doolittle hydropathy plot for the sequence "PTH1R_HUMAN". Positive values indicate hydrophobic regions, while negative values indicate hydrophilic regions.

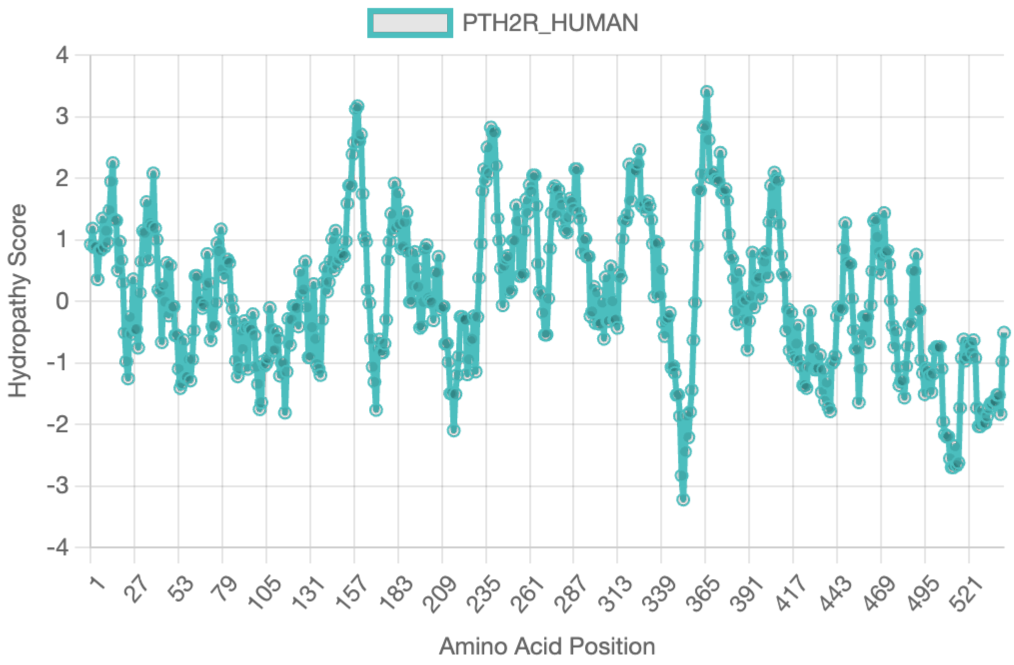

Kyte-Doolittle hydropathy plot for the sequence "PTH2R_HUMAN". Positive values indicate hydrophobic regions, while negative values indicate hydrophilic regions.

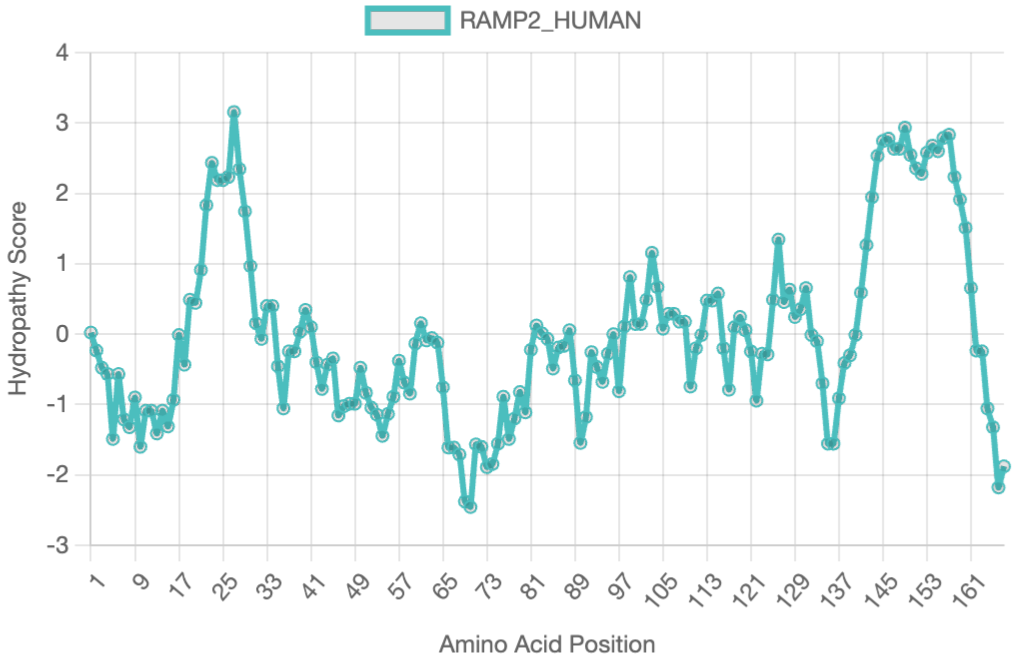

Kyte-Doolittle hydropathy plot for the sequence "RAMP2_HUMAN". Positive values indicate hydrophobic regions, while negative values indicate hydrophilic regions.

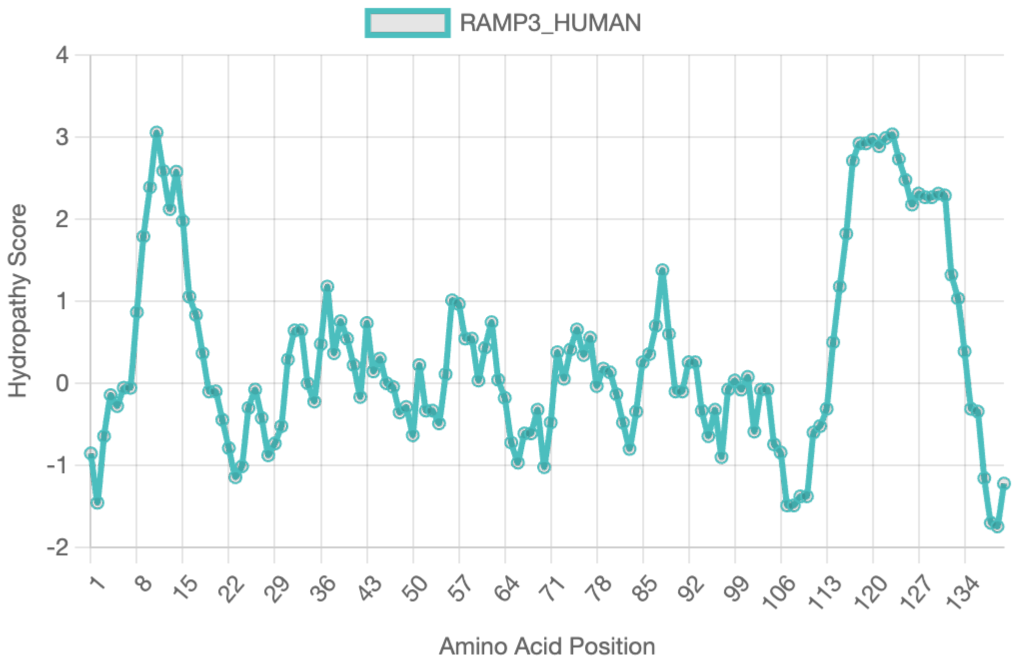

Kyte-Doolittle hydropathy plot for the sequence "RAMP3_HUMAN". Positive values indicate hydrophobic regions, while negative values indicate hydrophilic regions.

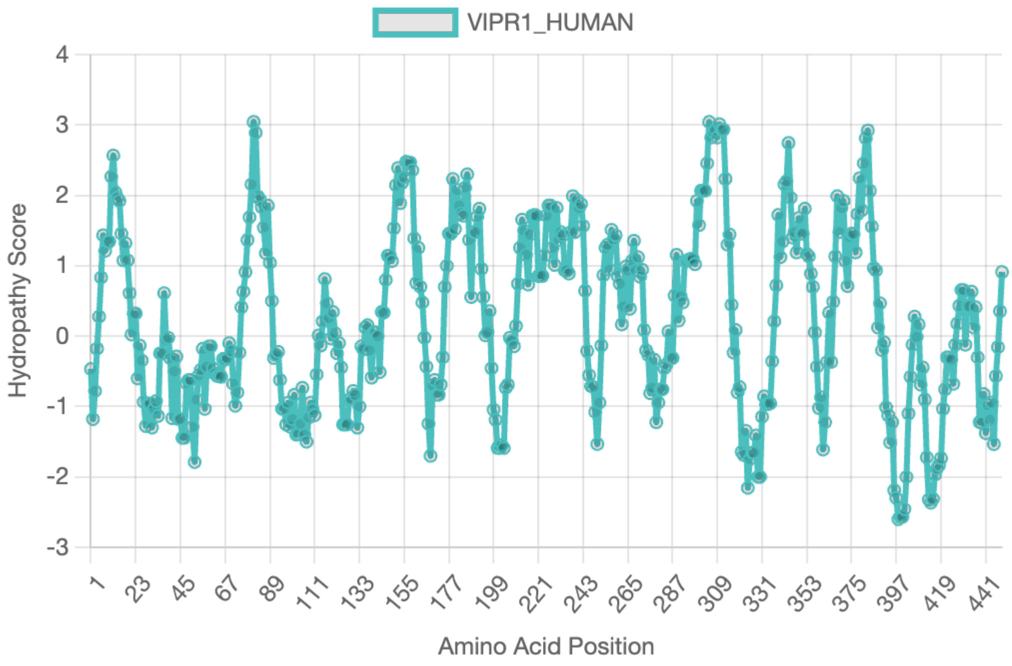

Kyte-Doolittle hydropathy plot for the sequence "VIPR1_HUMAN". Positive values indicate hydrophobic regions, while negative values indicate hydrophilic regions.

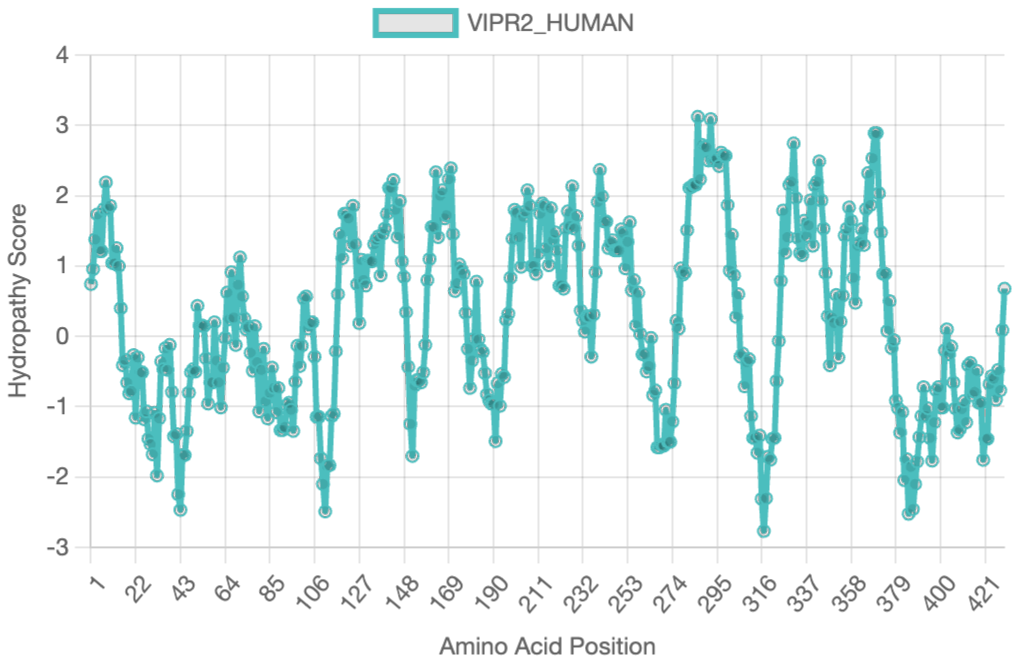

Kyte-Doolittle hydropathy plot for the sequence "VIPR2_HUMAN". Positive values indicate hydrophobic regions, while negative values indicate hydrophilic regions.

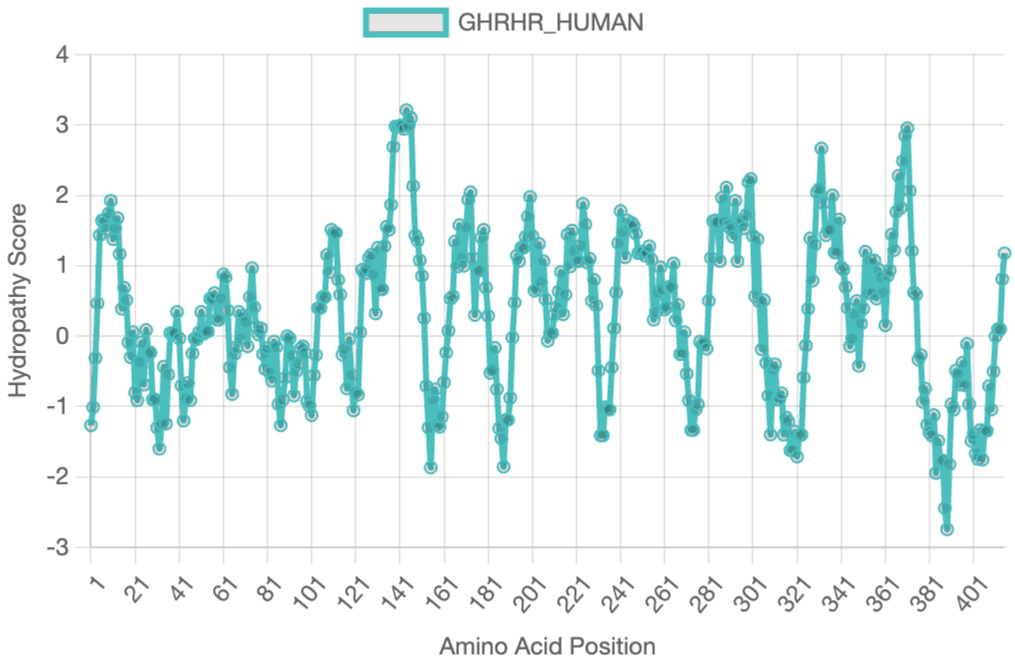

Kyte-Doolittle hydropathy plot for the sequence "GHRHR_HUMAN". Positive values indicate hydrophobic regions, while negative values indicate hydrophilic regions.

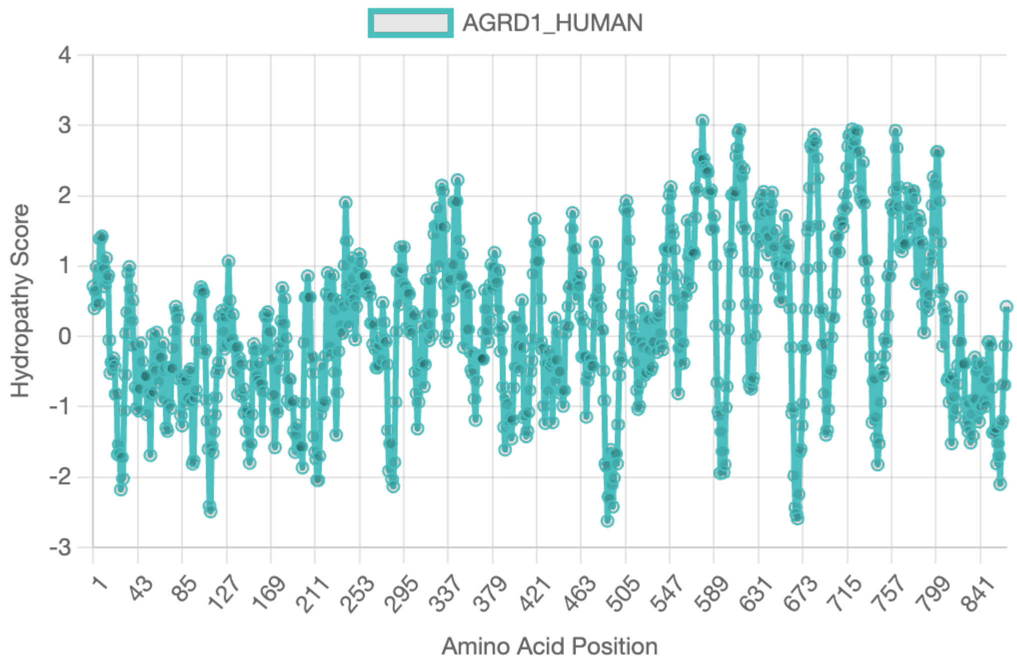

Kyte-Doolittle hydropathy plot for the sequence "AGRD1_HUMAN". Positive values indicate hydrophobic regions, while negative values indicate hydrophilic regions.

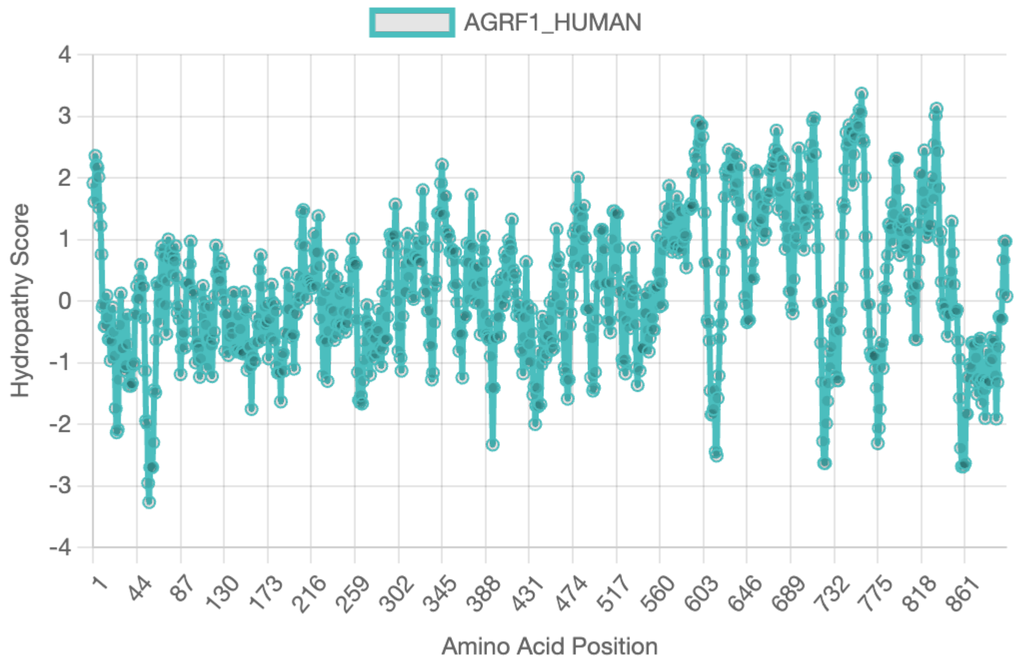

Kyte-Doolittle hydropathy plot for the sequence "AGRF1_HUMAN". Positive values indicate hydrophobic regions, while negative values indicate hydrophilic regions.

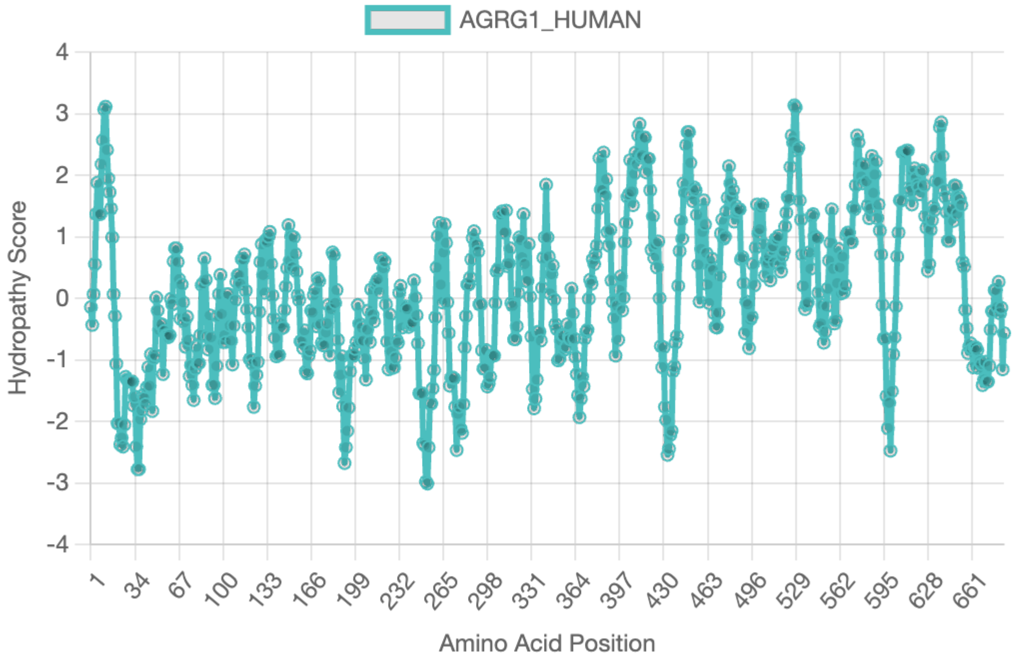

Kyte-Doolittle hydropathy plot for the sequence "AGRG1_HUMAN". Positive values indicate hydrophobic regions, while negative values indicate hydrophilic regions.

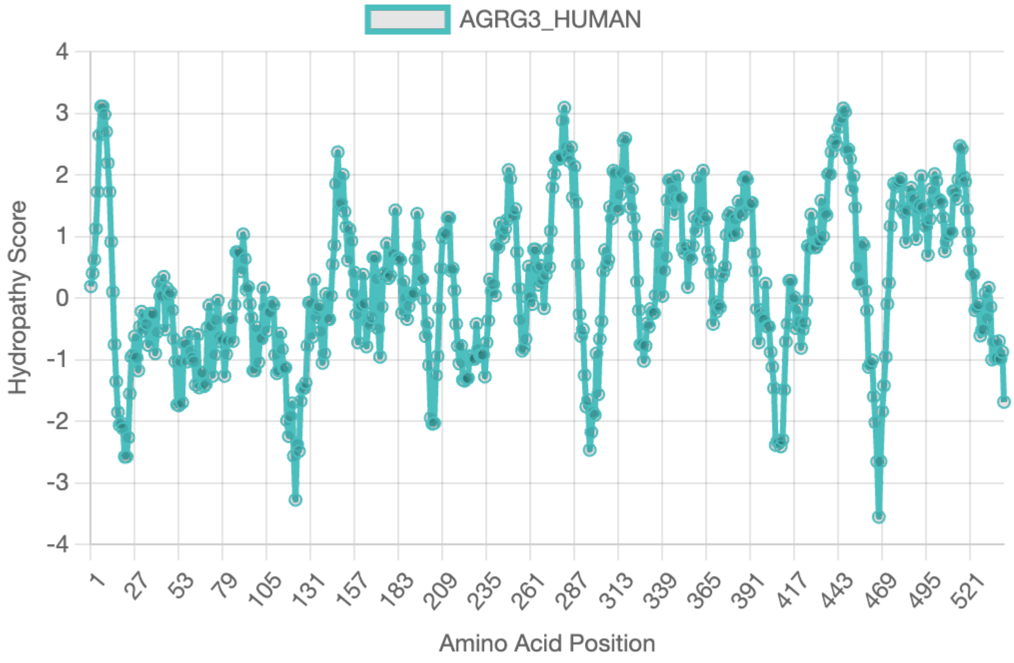

Kyte-Doolittle hydropathy plot for the sequence "AGRG3_HUMAN". Positive values indicate hydrophobic regions, while negative values indicate hydrophilic regions.

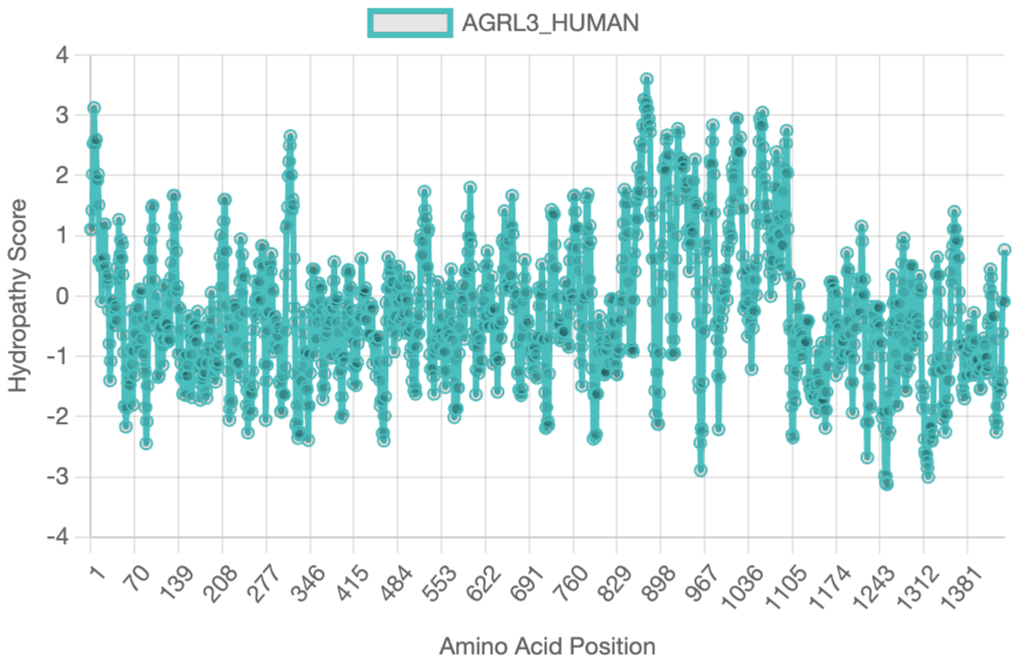

Kyte-Doolittle hydropathy plot for the sequence "AGRL3_HUMAN". Positive values indicate hydrophobic regions, while negative values indicate hydrophilic regions.

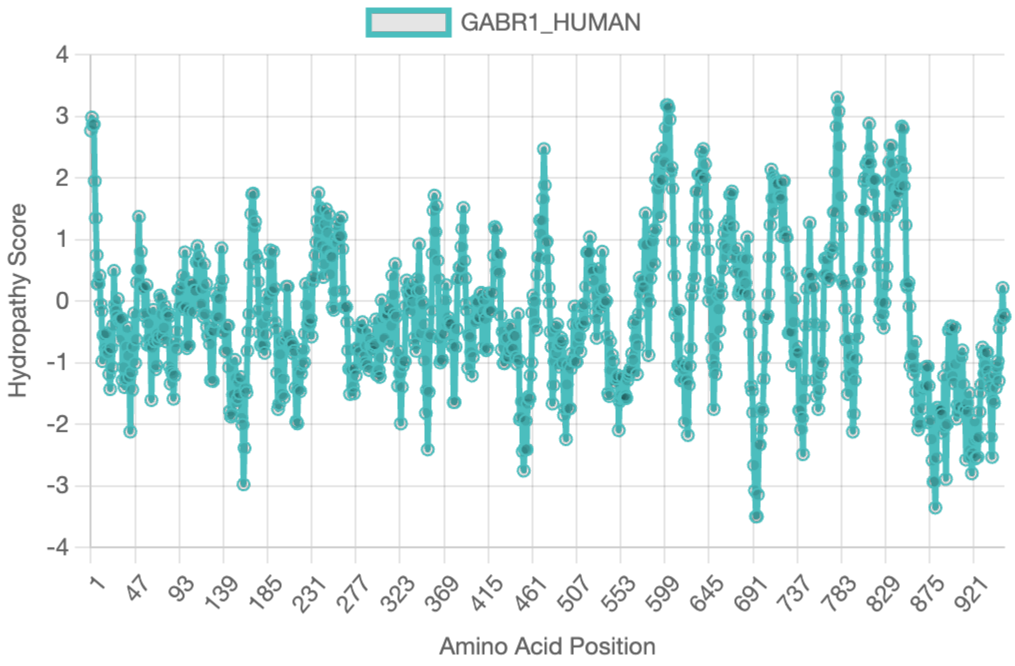

Kyte-Doolittle hydropathy plot for the sequence "GABR1_HUMAN". Positive values indicate hydrophobic regions, while negative values indicate hydrophilic regions.

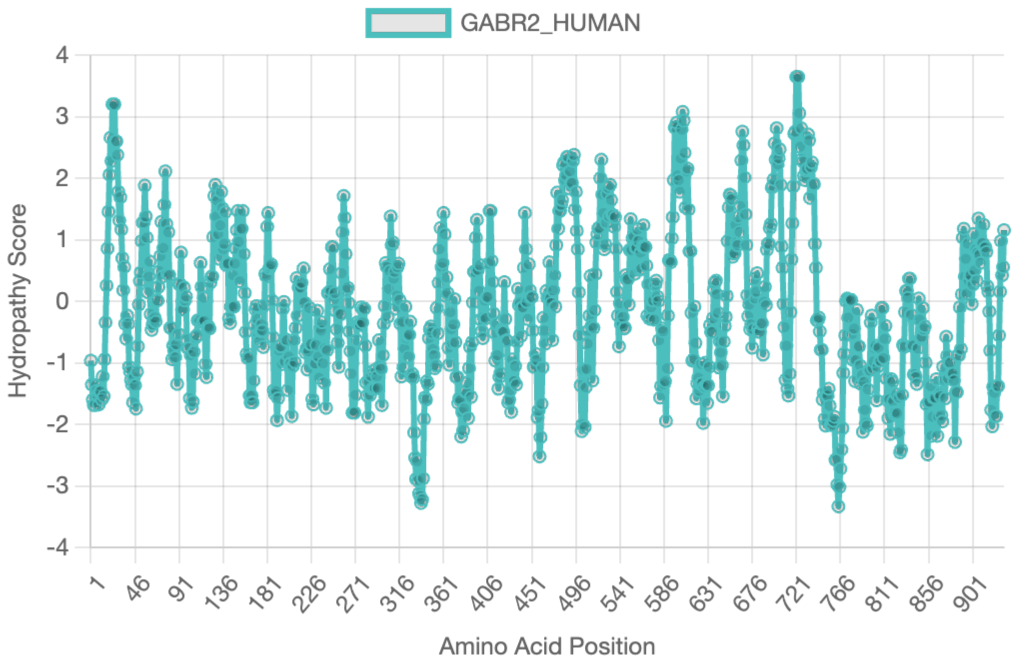

Kyte-Doolittle hydropathy plot for the sequence "GABR2_HUMAN". Positive values indicate hydrophobic regions, while negative values indicate hydrophilic regions.

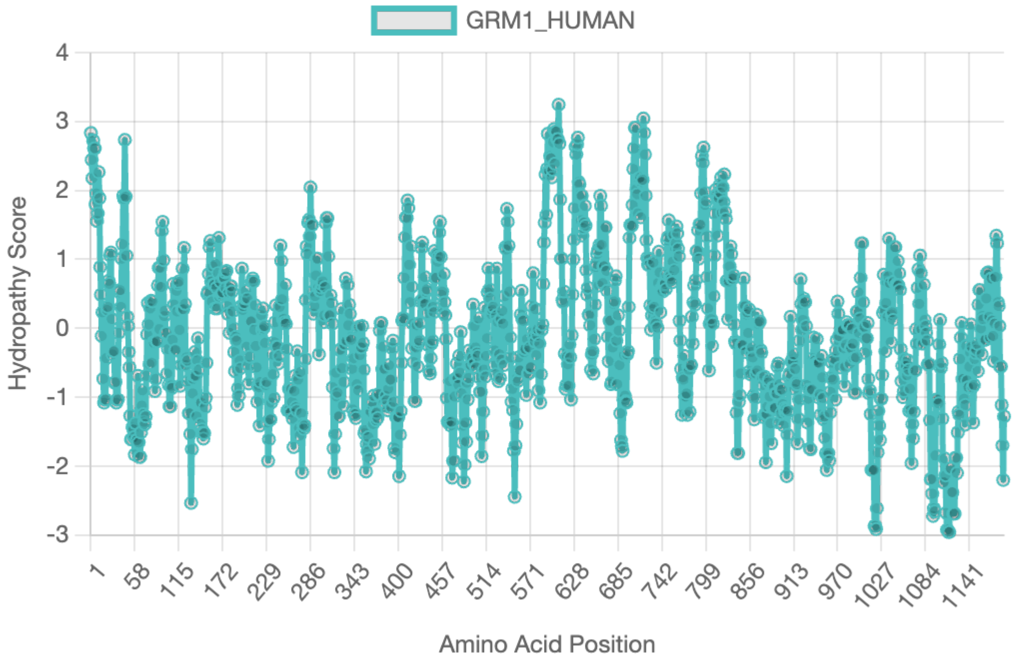

Kyte-Doolittle hydropathy plot for the sequence "GRM1_HUMAN". Positive values indicate hydrophobic regions, while negative values indicate hydrophilic regions.

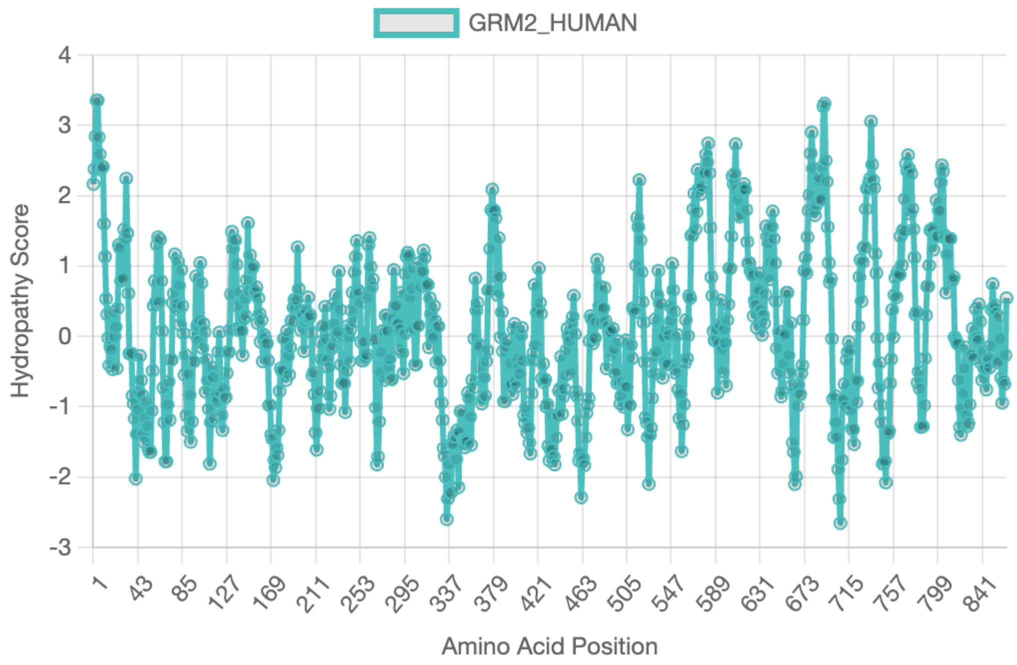

Kyte-Doolittle hydropathy plot for the sequence "GRM2_HUMAN". Positive values indicate hydrophobic regions, while negative values indicate hydrophilic regions.

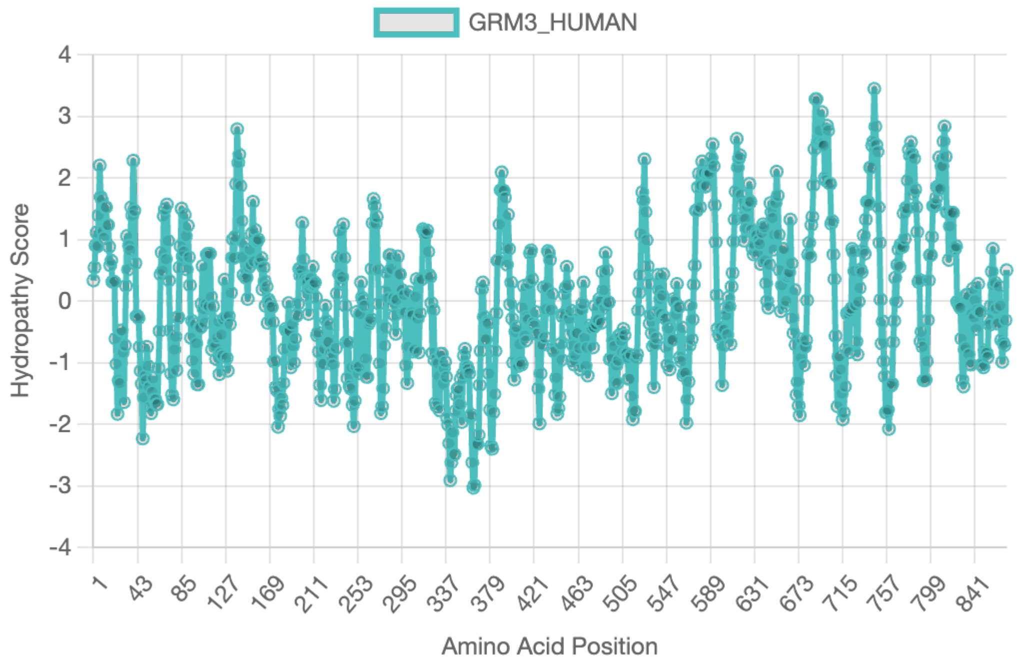

Kyte-Doolittle hydropathy plot for the sequence "GRM3_HUMAN". Positive values indicate hydrophobic regions, while negative values indicate hydrophilic regions.

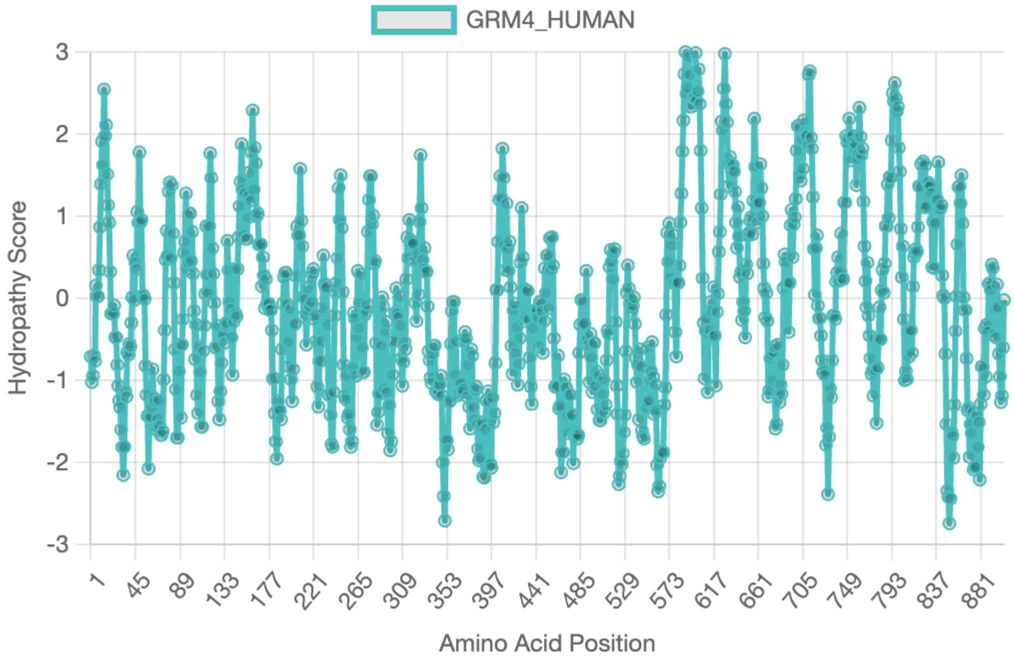

Kyte-Doolittle hydropathy plot for the sequence "GRM4_HUMAN". Positive values indicate hydrophobic regions, while negative values indicate hydrophilic regions.

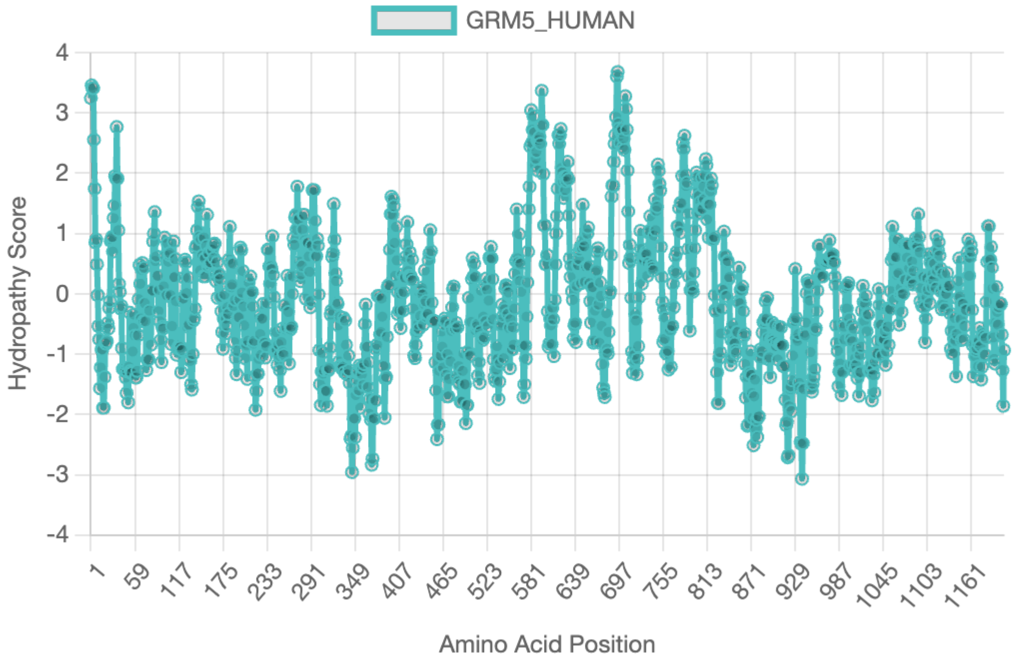

Kyte-Doolittle hydropathy plot for the sequence "GRM5_HUMAN". Positive values indicate hydrophobic regions, while negative values indicate hydrophilic regions.

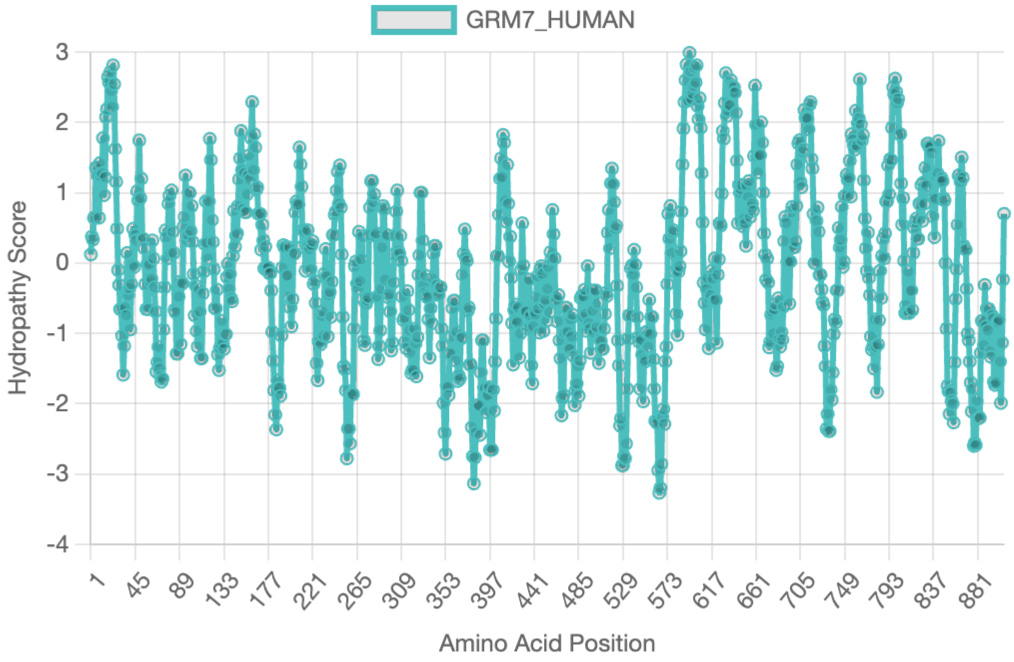

Kyte-Doolittle hydropathy plot for the sequence "GRM7_HUMAN". Positive values indicate hydrophobic regions, while negative values indicate hydrophilic regions.

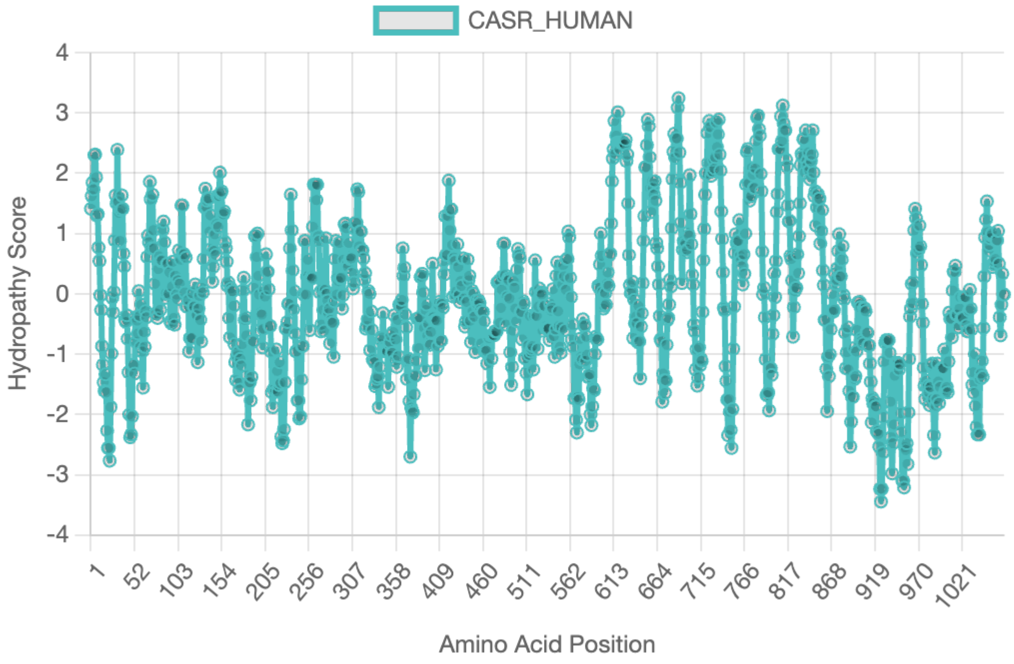

Kyte-Doolittle hydropathy plot for the sequence "CASR_HUMAN". Positive values indicate hydrophobic regions, while negative values indicate hydrophilic regions.

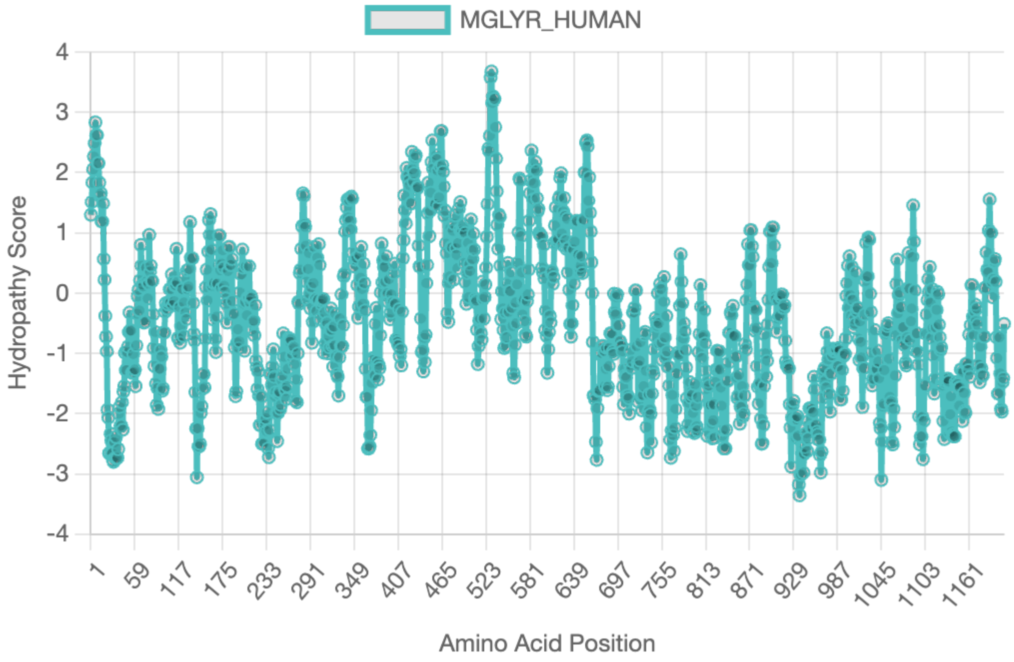

Kyte-Doolittle hydropathy plot for the sequence "MGLYR_HUMAN". Positive values indicate hydrophobic regions, while negative values indicate hydrophilic regions.

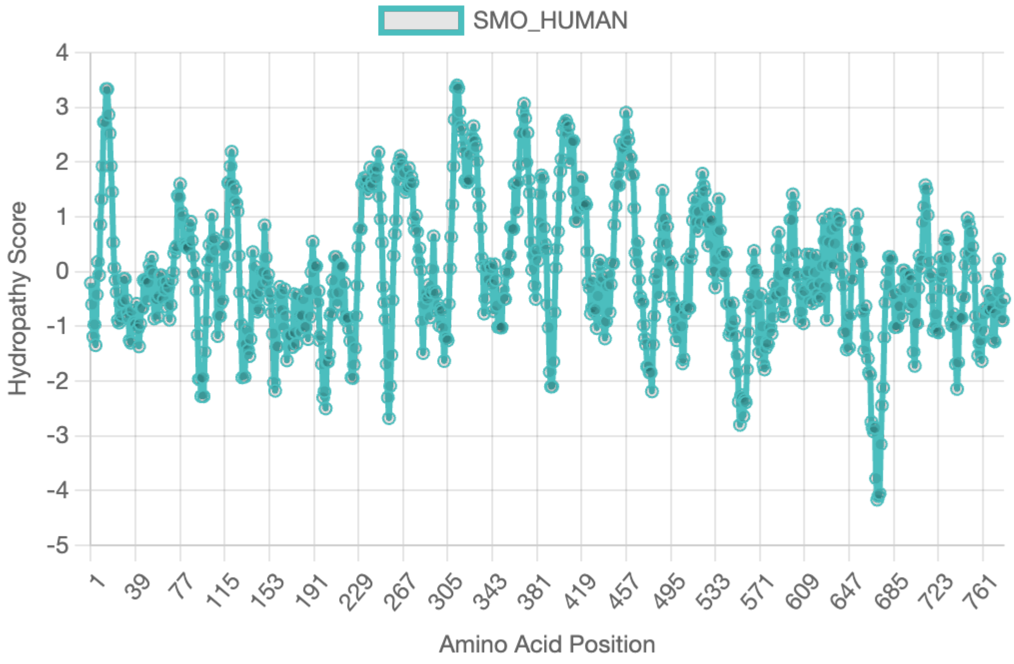

Kyte-Doolittle hydropathy plot for the sequence "SMO_HUMAN". Positive values indicate hydrophobic regions, while negative values indicate hydrophilic regions.

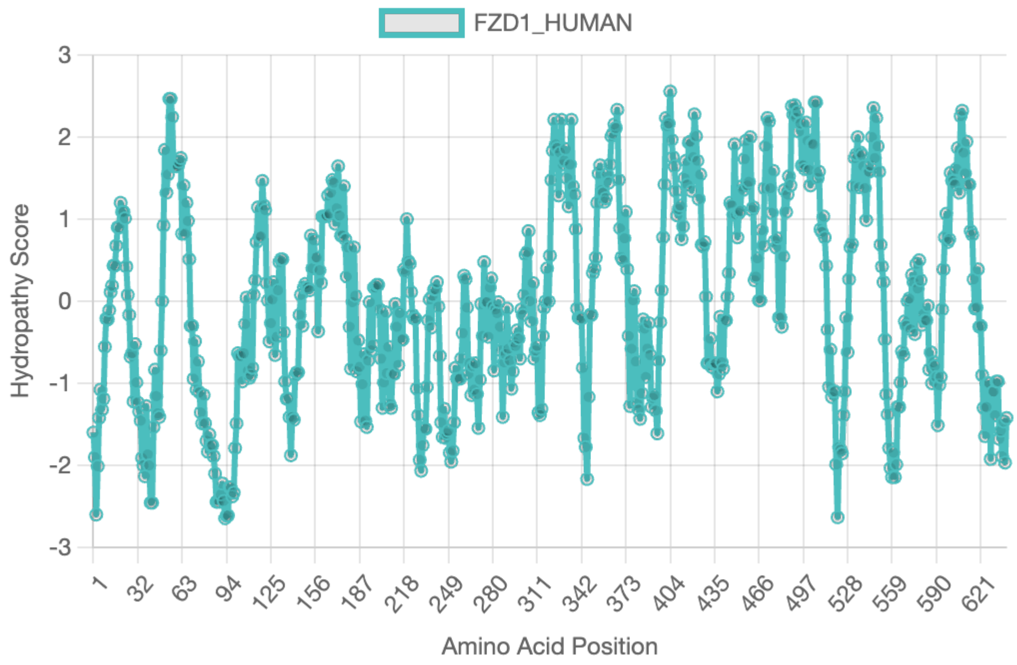

Kyte-Doolittle hydropathy plot for the sequence "FZD1_HUMAN". Positive values indicate hydrophobic regions, while negative values indicate hydrophilic regions.

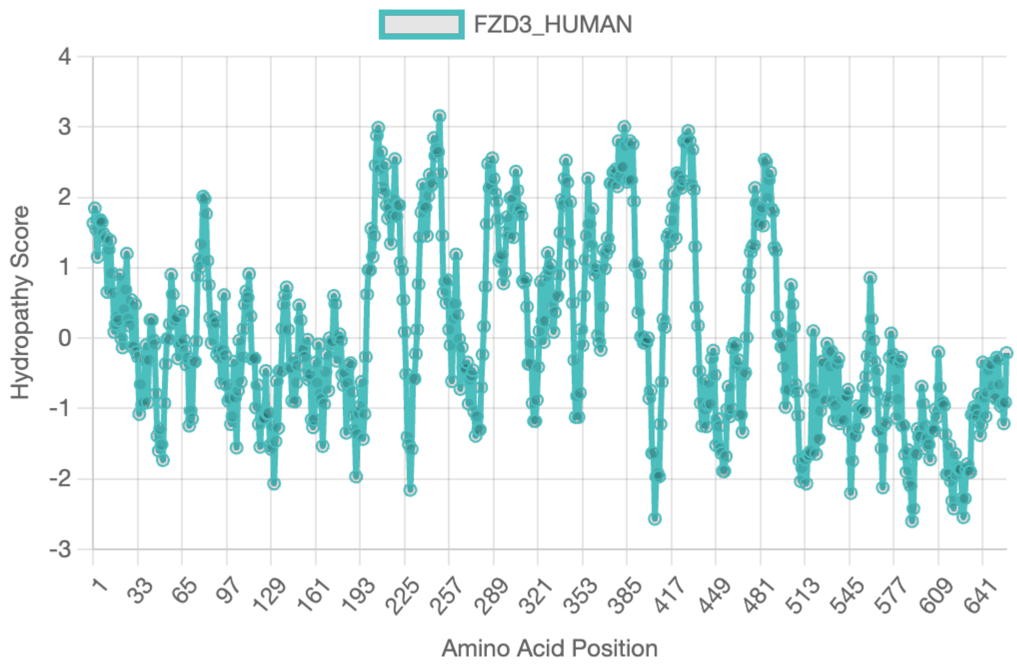

Kyte-Doolittle hydropathy plot for the sequence "FZD3_HUMAN". Positive values indicate hydrophobic regions, while negative values indicate hydrophilic regions.

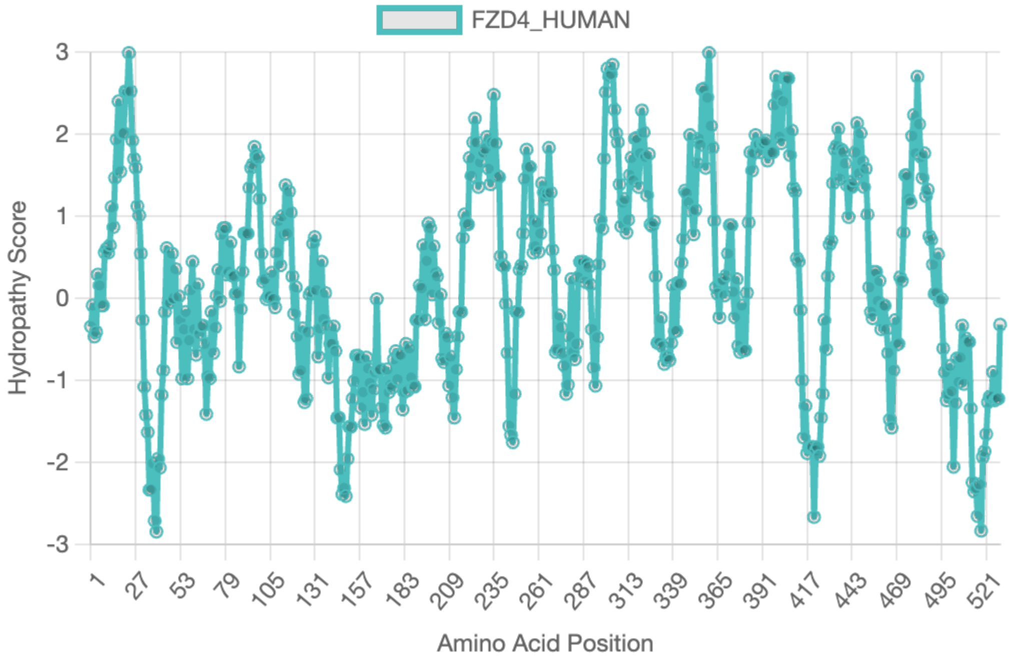

Kyte-Doolittle hydropathy plot for the sequence "FZD4_HUMAN". Positive values indicate hydrophobic regions, while negative values indicate hydrophilic regions.

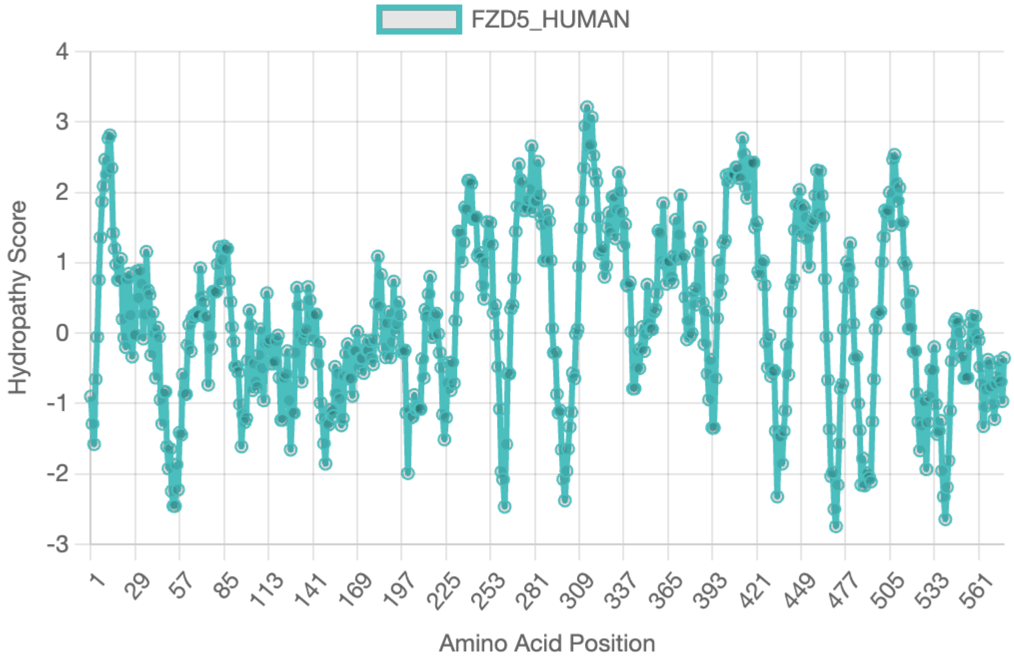

Kyte-Doolittle hydropathy plot for the sequence "FZD5_HUMAN". Positive values indicate hydrophobic regions, while negative values indicate hydrophilic regions.

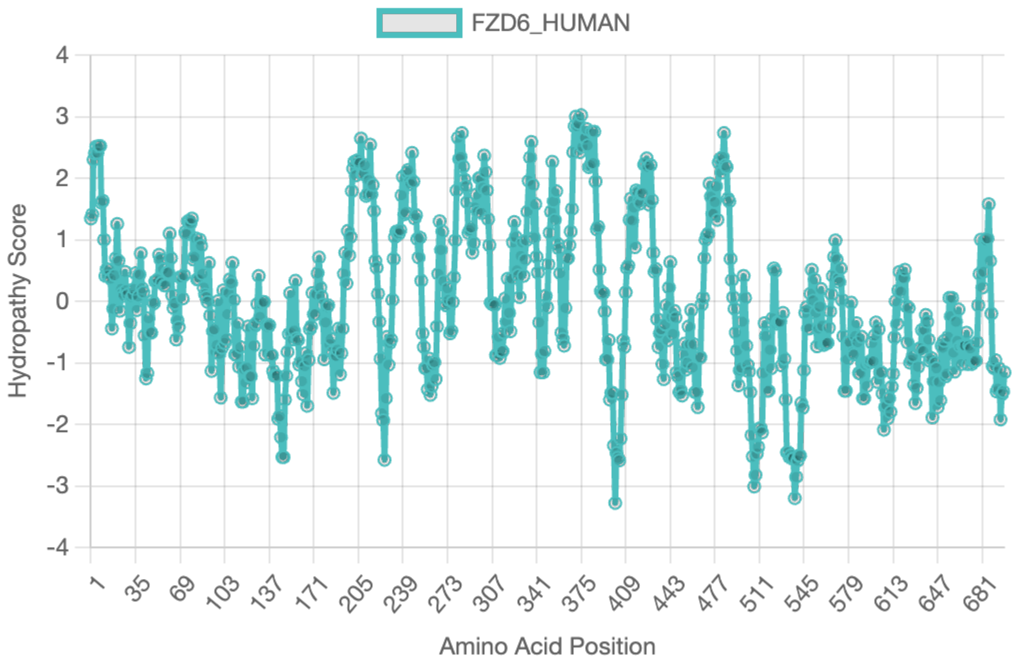

Kyte-Doolittle hydropathy plot for the sequence "FZD6_HUMAN". Positive values indicate hydrophobic regions, while negative values indicate hydrophilic regions.

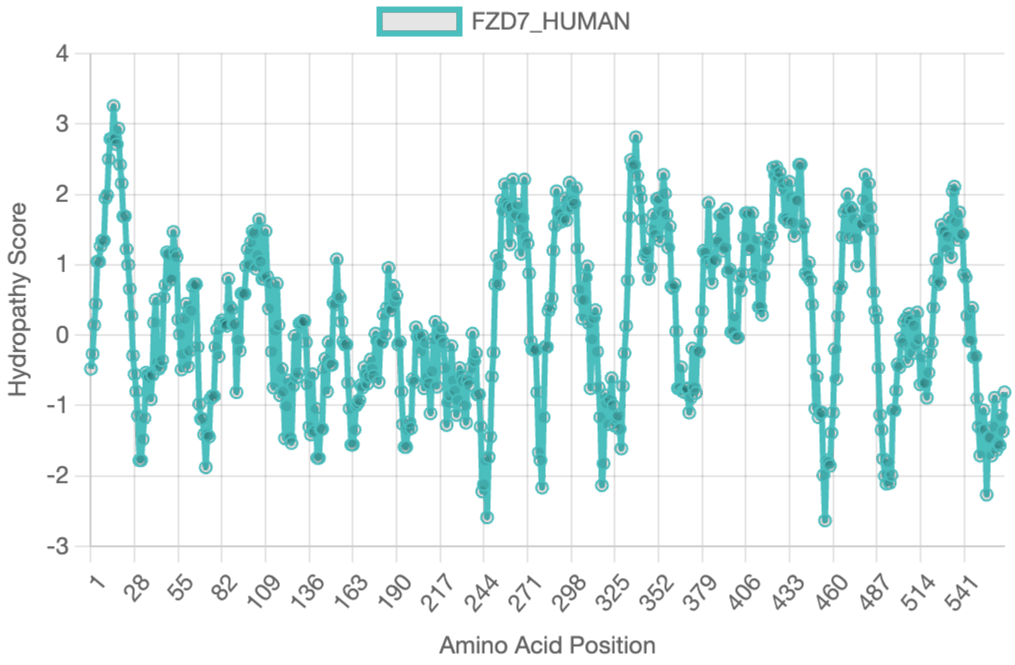

Kyte-Doolittle hydropathy plot for the sequence "FZD7_HUMAN". Positive values indicate hydrophobic regions, while negative values indicate hydrophilic regions.

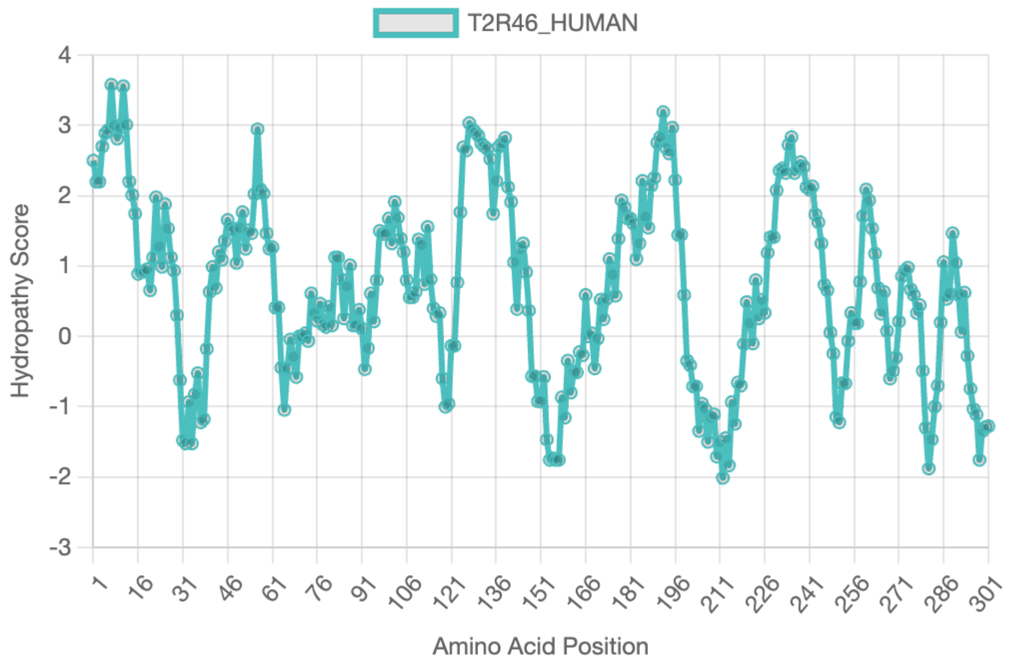

Kyte-Doolittle hydropathy plot for the sequence "T2R46_HUMAN". Positive values indicate hydrophobic regions, while negative values indicate hydrophilic regions.

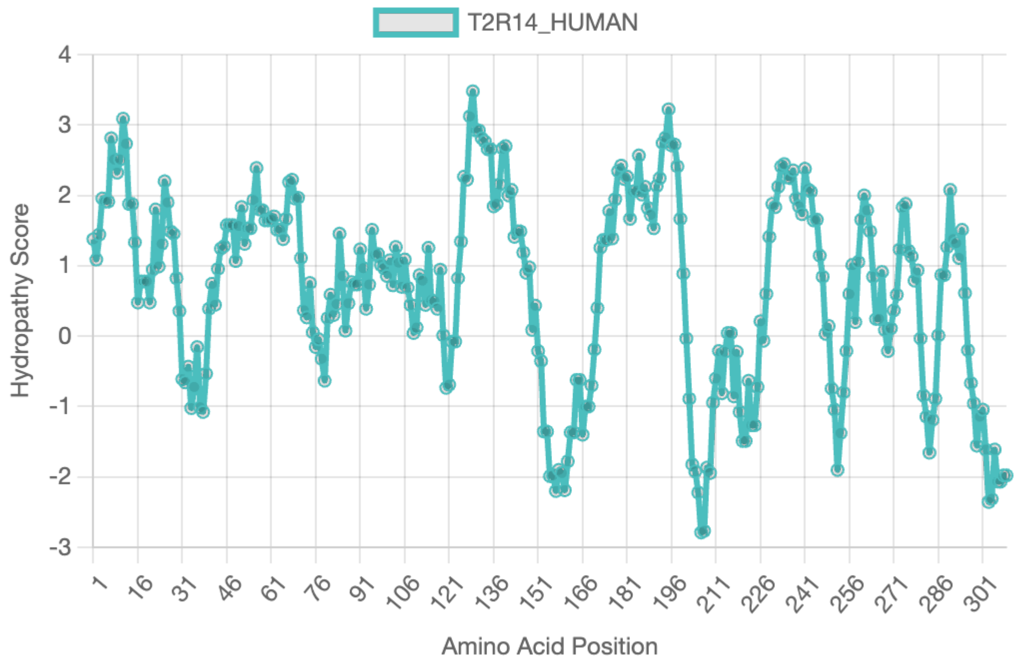

Kyte-Doolittle hydropathy plot for the sequence "T2R14_HUMAN". Positive values indicate hydrophobic regions, while negative values indicate hydrophilic regions.

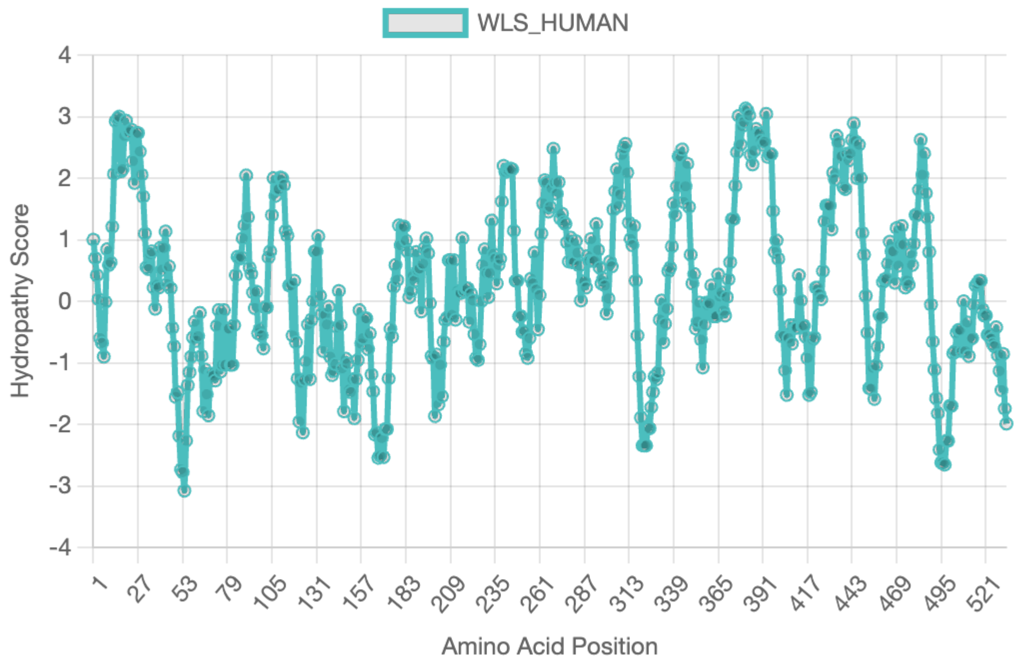

Kyte-Doolittle hydropathy plot for the sequence "WLS_HUMAN". Positive values indicate hydrophobic regions, while negative values indicate hydrophilic regions.

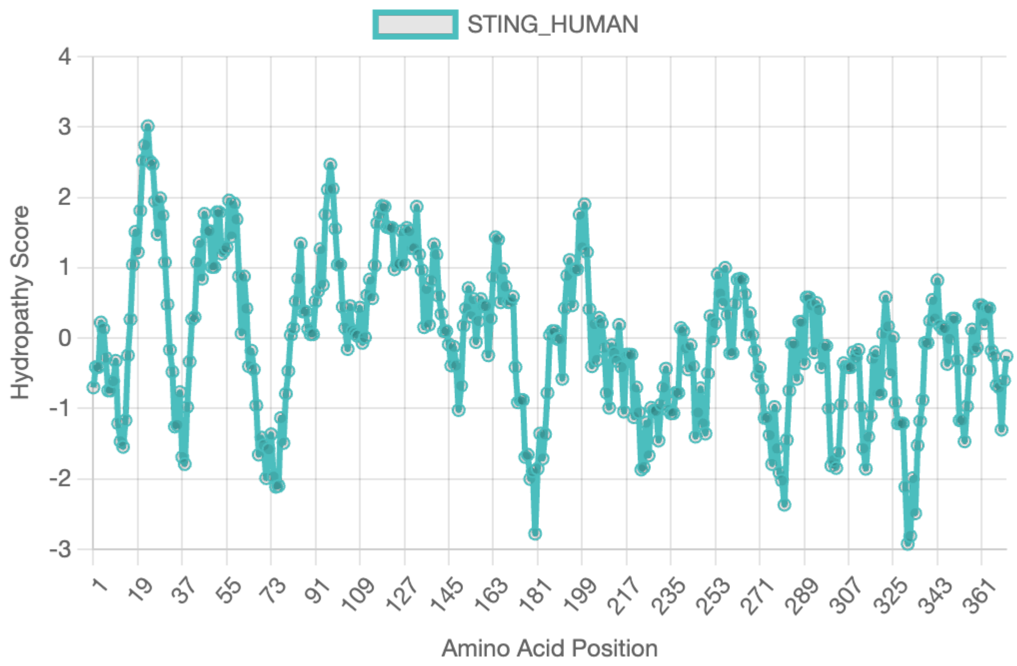

Kyte-Doolittle hydropathy plot for the sequence "STING_HUMAN". Positive values indicate hydrophobic regions, while negative values indicate hydrophilic regions.

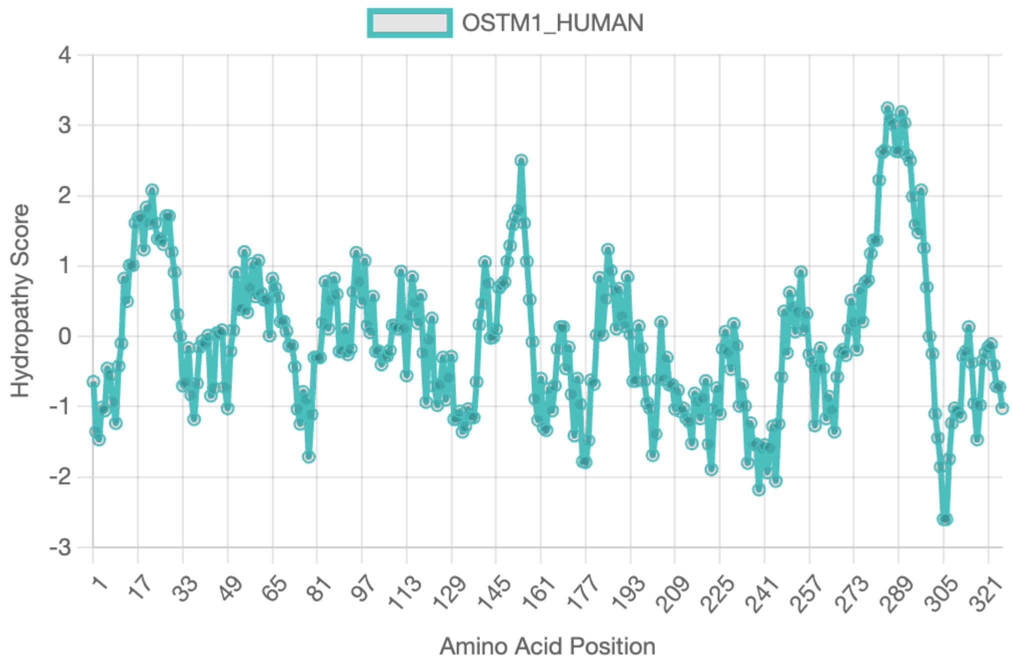

Kyte-Doolittle hydropathy plot for the sequence "OSTM1_HUMAN". Positive values indicate hydrophobic regions, while negative values indicate hydrophilic regions.

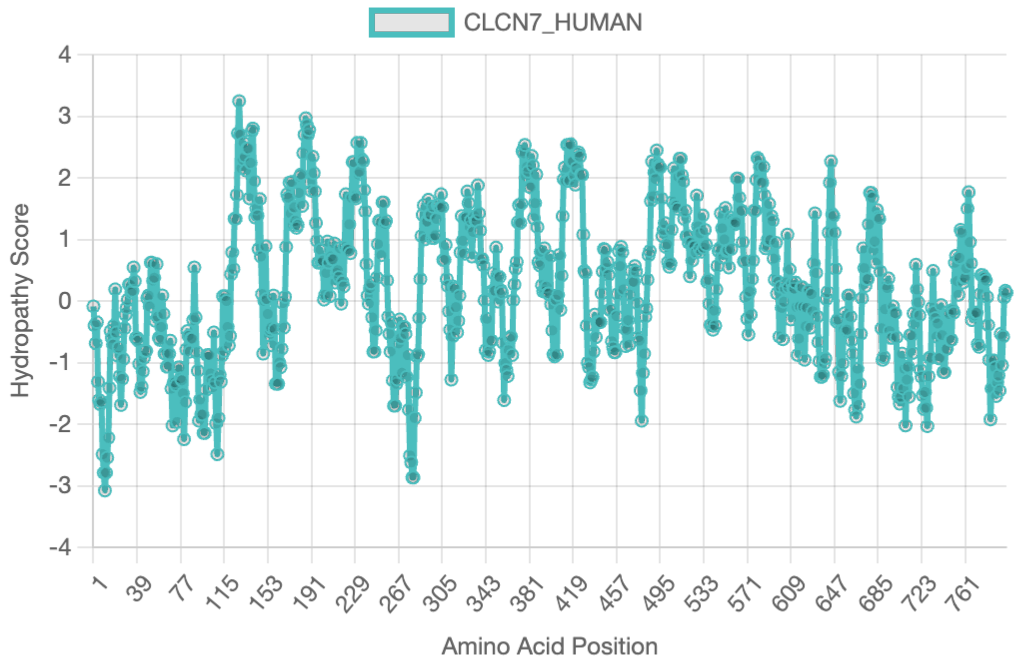

Kyte-Doolittle hydropathy plot for the sequence "CLCN7_HUMAN". Positive values indicate hydrophobic regions, while negative values indicate hydrophilic regions.

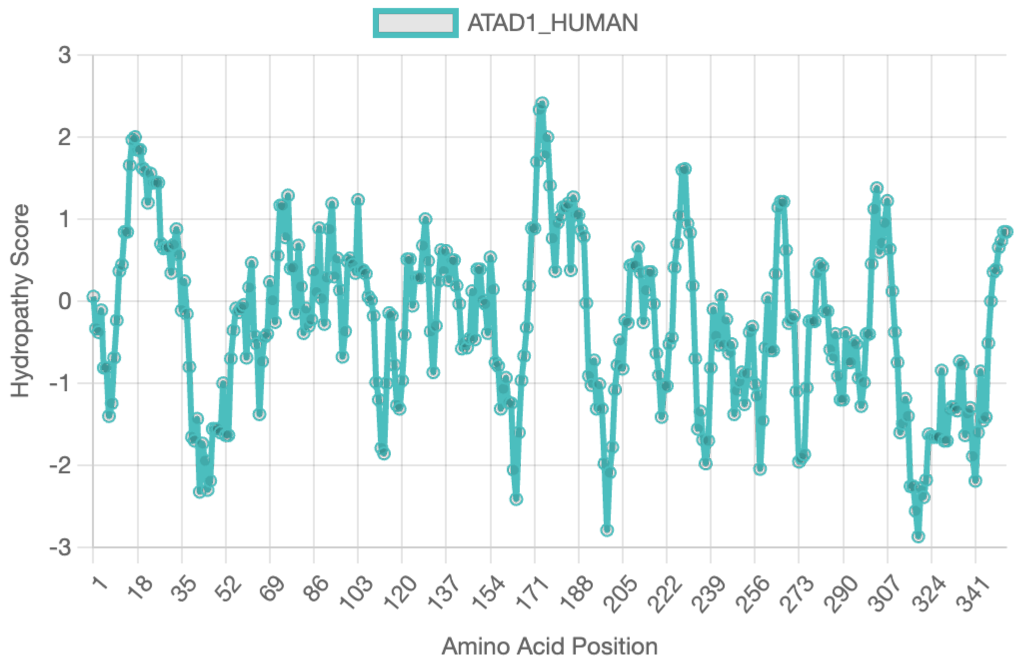

Kyte-Doolittle hydropathy plot for the sequence "ATAD1_HUMAN". Positive values indicate hydrophobic regions, while negative values indicate hydrophilic regions.

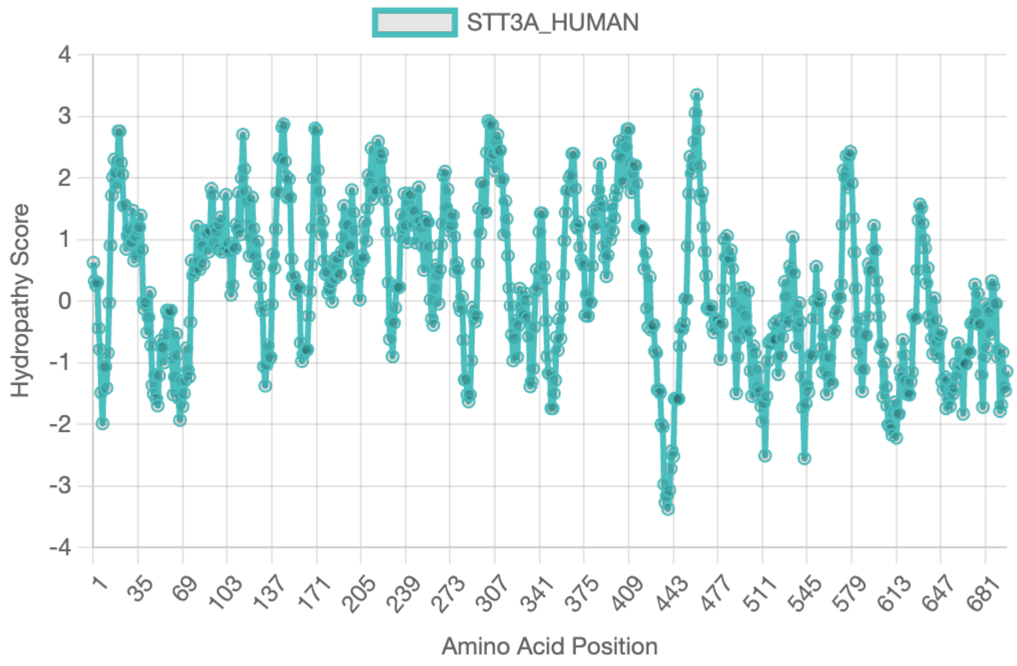

Kyte-Doolittle hydropathy plot for the sequence "STT3A_HUMAN". Positive values indicate hydrophobic regions, while negative values indicate hydrophilic regions.

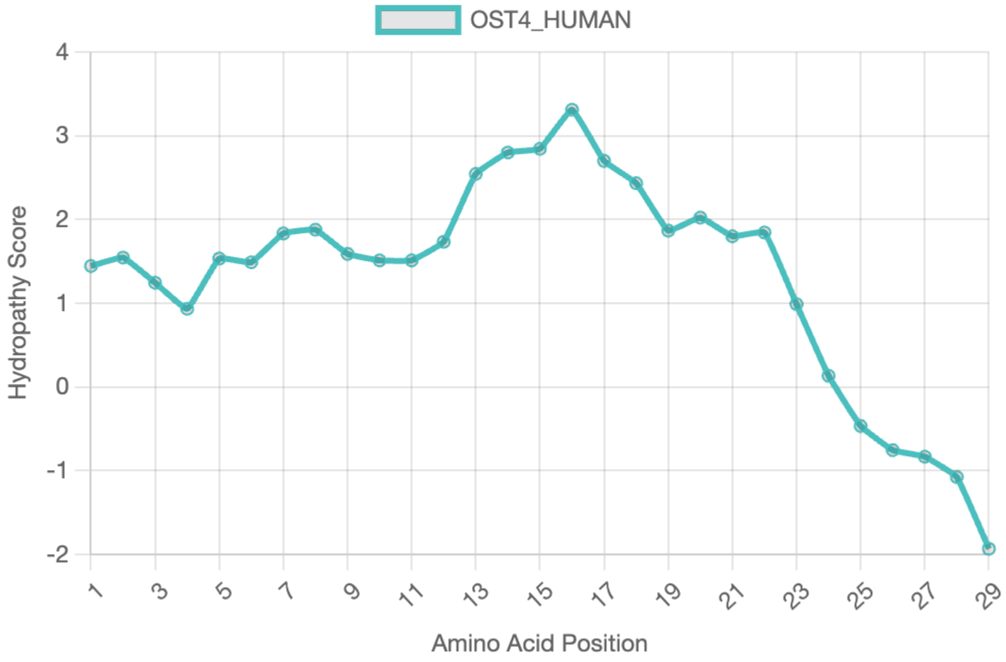

Kyte-Doolittle hydropathy plot for the sequence "OST4_HUMAN". Positive values indicate hydrophobic regions, while negative values indicate hydrophilic regions.

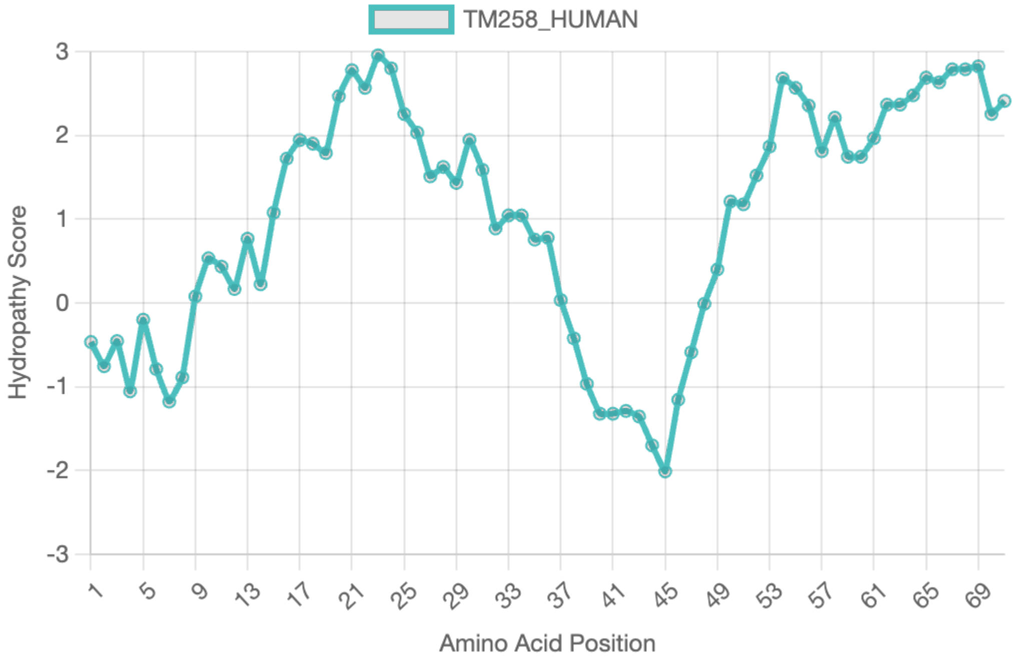

Kyte-Doolittle hydropathy plot for the sequence "TM258_HUMAN". Positive values indicate hydrophobic regions, while negative values indicate hydrophilic regions.

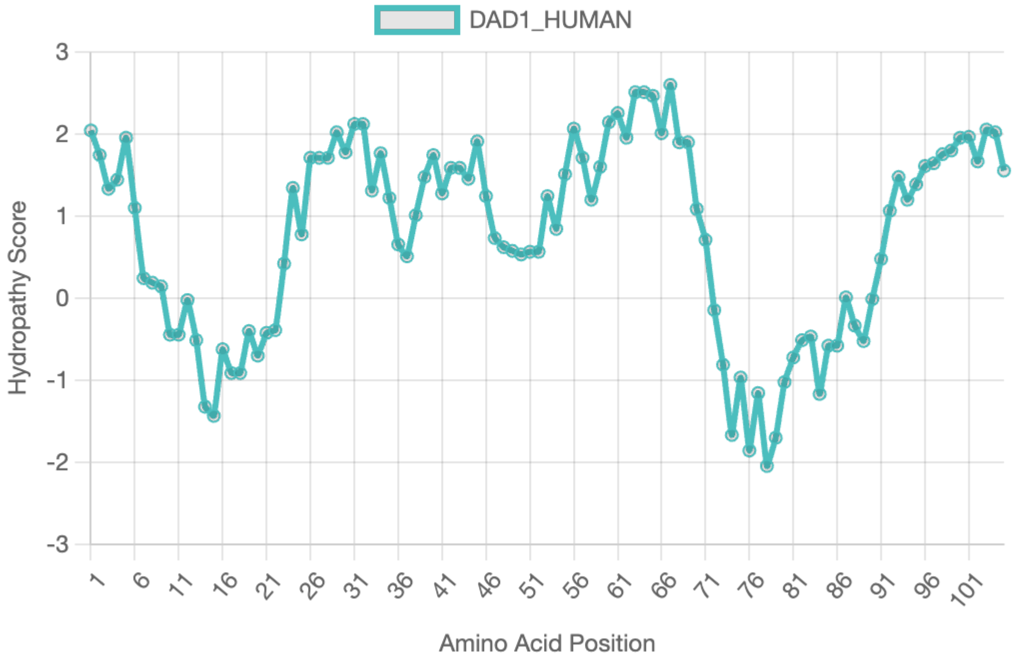

Kyte-Doolittle hydropathy plot for the sequence "DAD1_HUMAN". Positive values indicate hydrophobic regions, while negative values indicate hydrophilic regions.

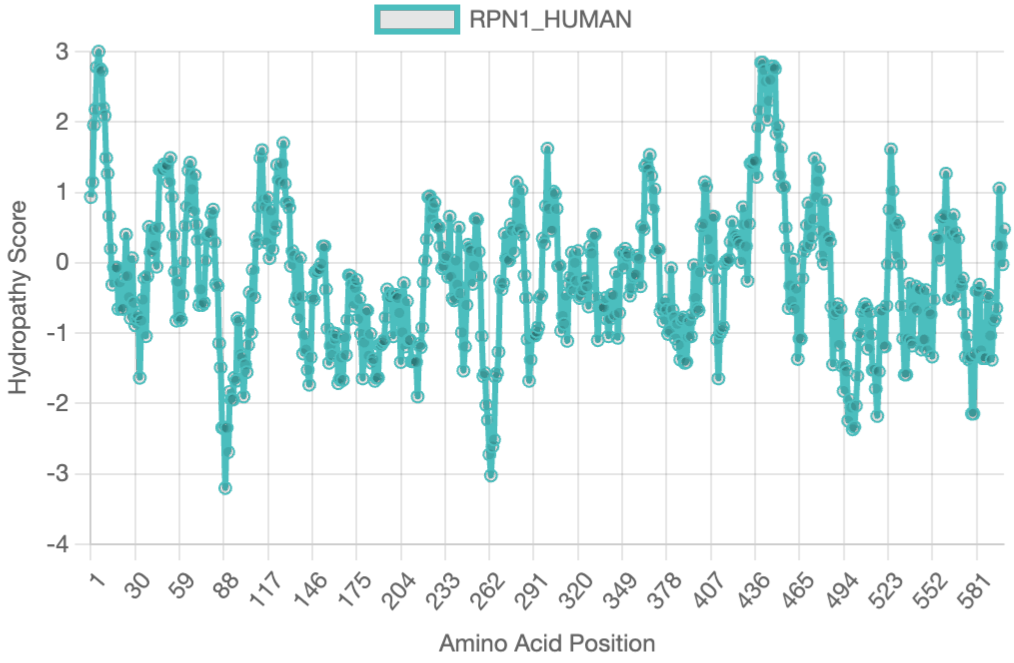

Kyte-Doolittle hydropathy plot for the sequence "RPN1_HUMAN". Positive values indicate hydrophobic regions, while negative values indicate hydrophilic regions.

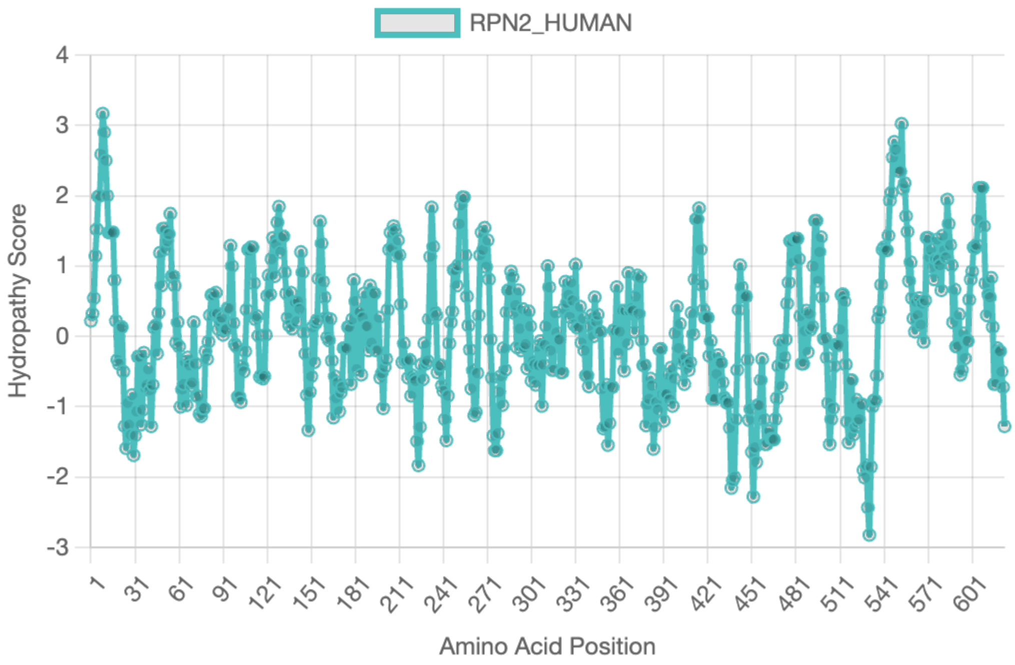

Kyte-Doolittle hydropathy plot for the sequence "RPN2_HUMAN". Positive values indicate hydrophobic regions, while negative values indicate hydrophilic regions.

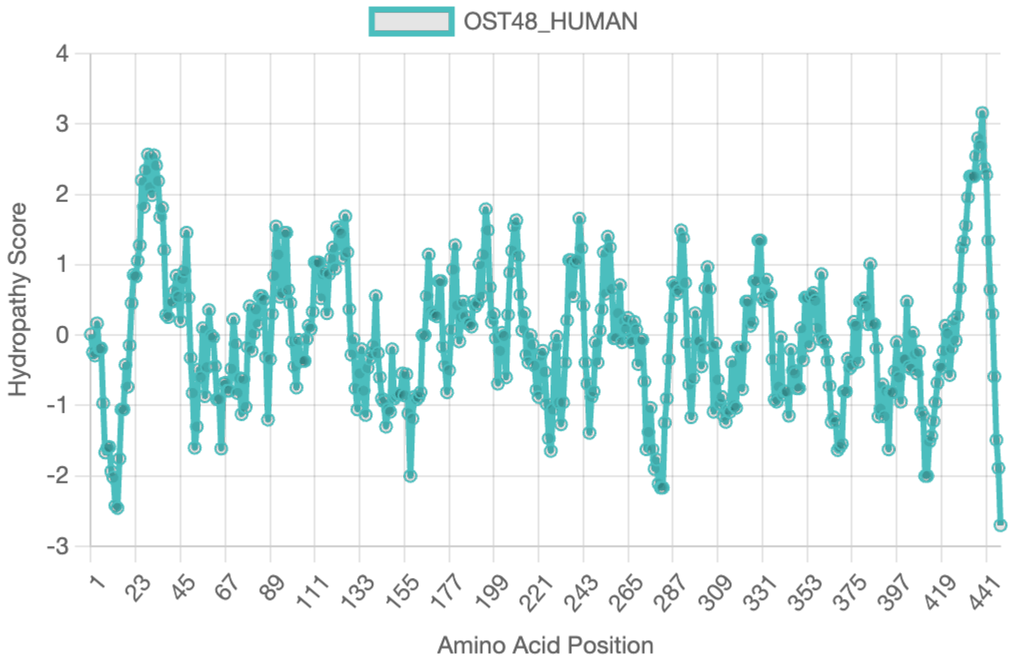

Kyte-Doolittle hydropathy plot for the sequence "OST48_HUMAN". Positive values indicate hydrophobic regions, while negative values indicate hydrophilic regions.

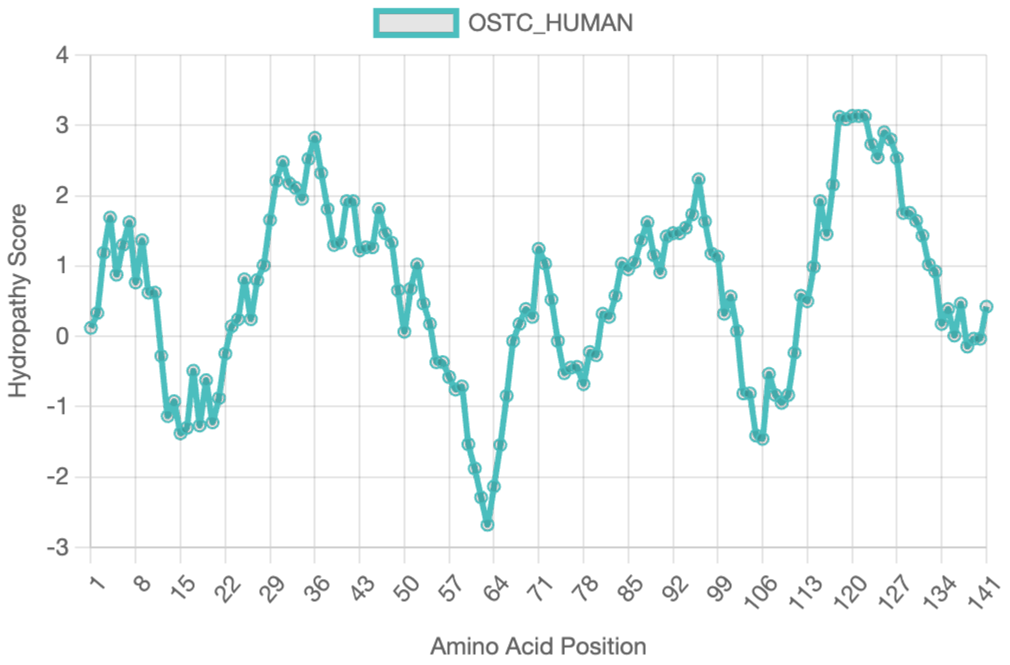

Kyte-Doolittle hydropathy plot for the sequence "OSTC_HUMAN". Positive values indicate hydrophobic regions, while negative values indicate hydrophilic regions.

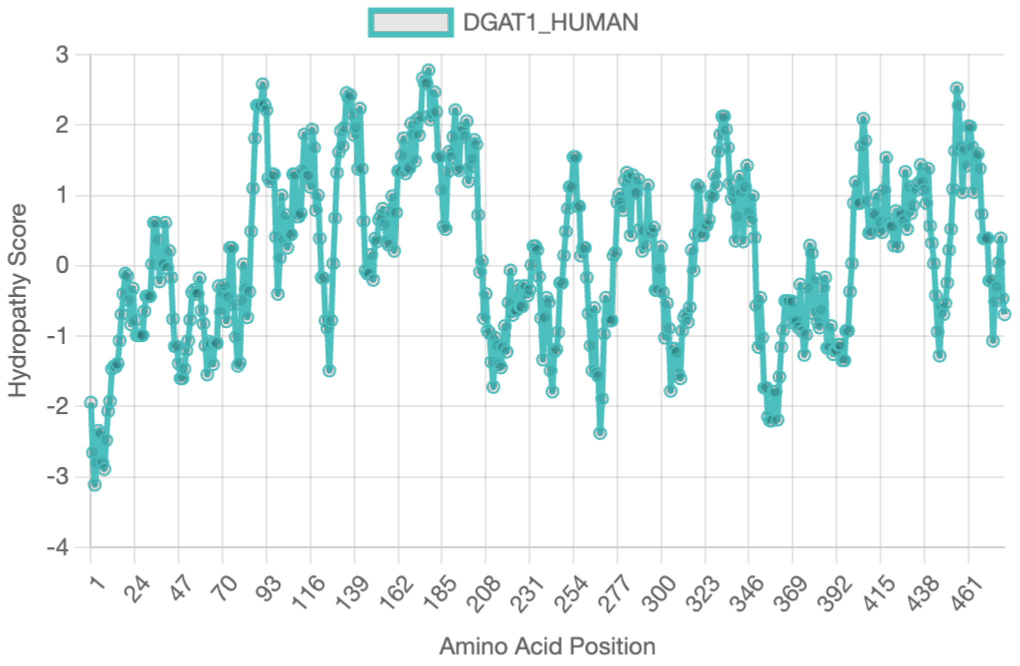

Kyte-Doolittle hydropathy plot for the sequence "DGAT1_HUMAN". Positive values indicate hydrophobic regions, while negative values indicate hydrophilic regions.

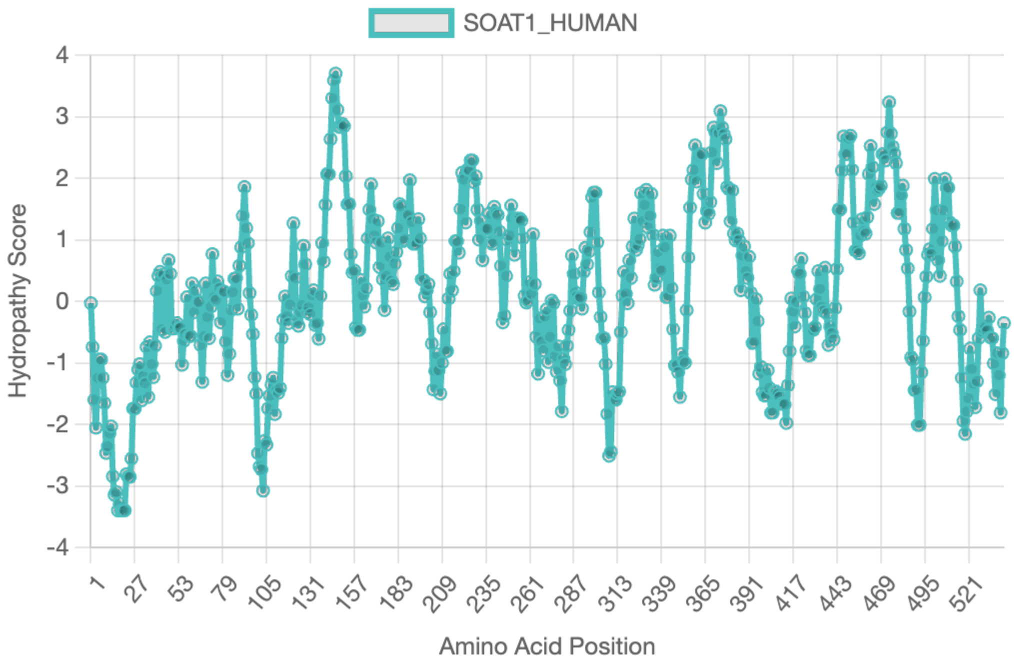

Kyte-Doolittle hydropathy plot for the sequence "SOAT1_HUMAN". Positive values indicate hydrophobic regions, while negative values indicate hydrophilic regions.

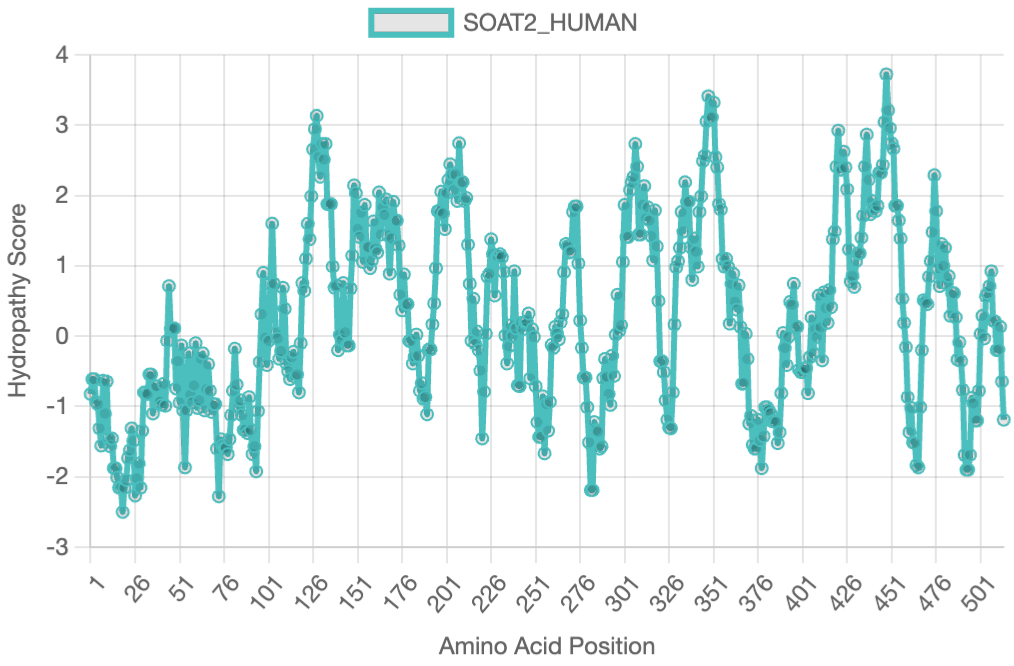

Kyte-Doolittle hydropathy plot for the sequence "SOAT2_HUMAN". Positive values indicate hydrophobic regions, while negative values indicate hydrophilic regions.

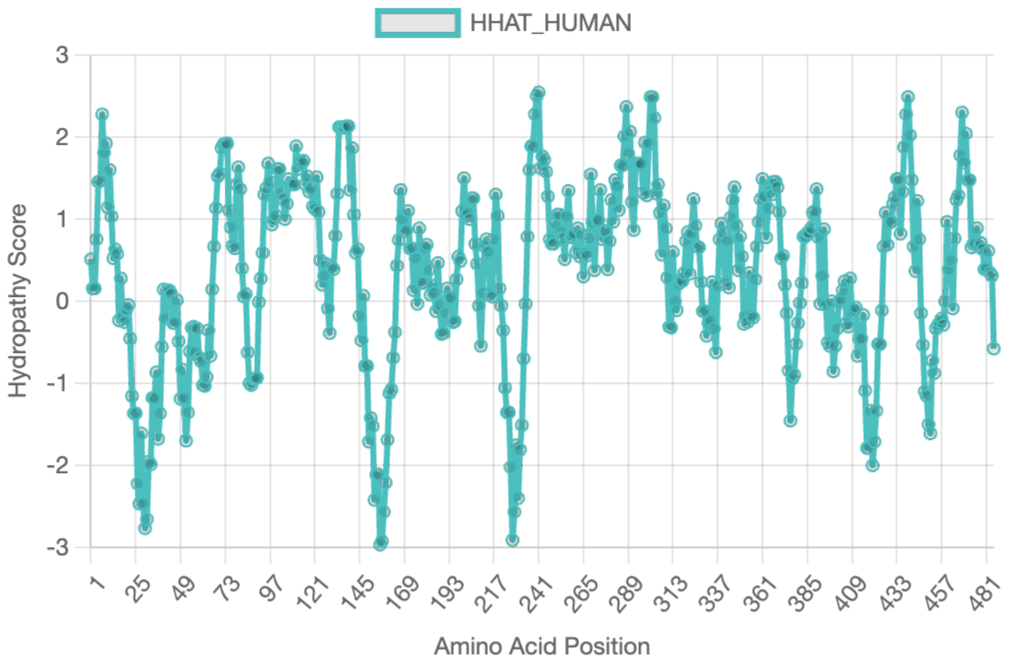

Kyte-Doolittle hydropathy plot for the sequence "HHAT_HUMAN". Positive values indicate hydrophobic regions, while negative values indicate hydrophilic regions.

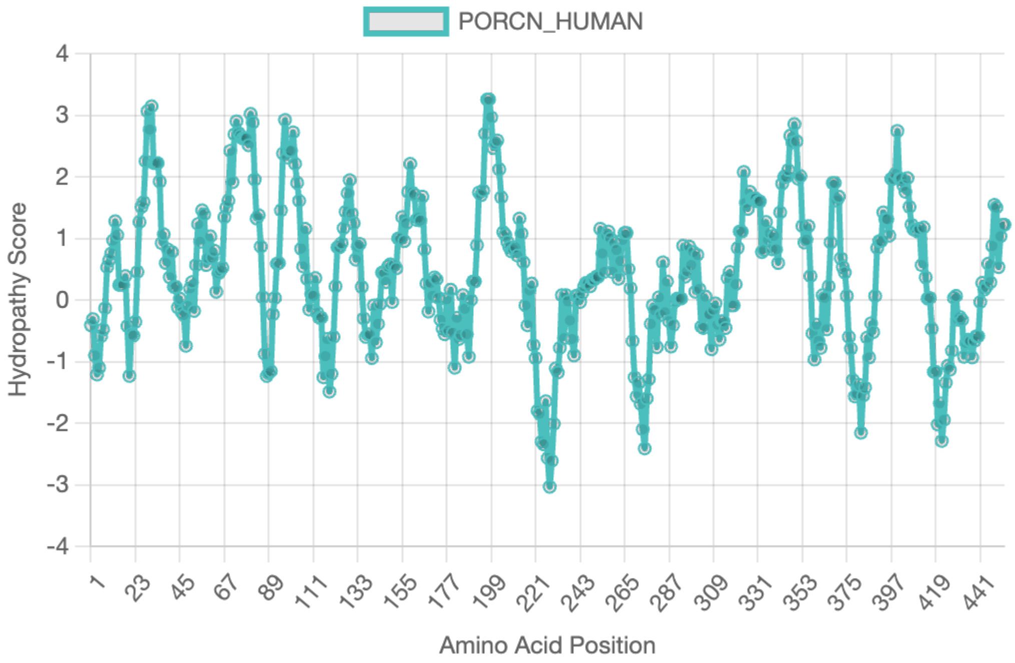

Kyte-Doolittle hydropathy plot for the sequence "PORCN_HUMAN". Positive values indicate hydrophobic regions, while negative values indicate hydrophilic regions.

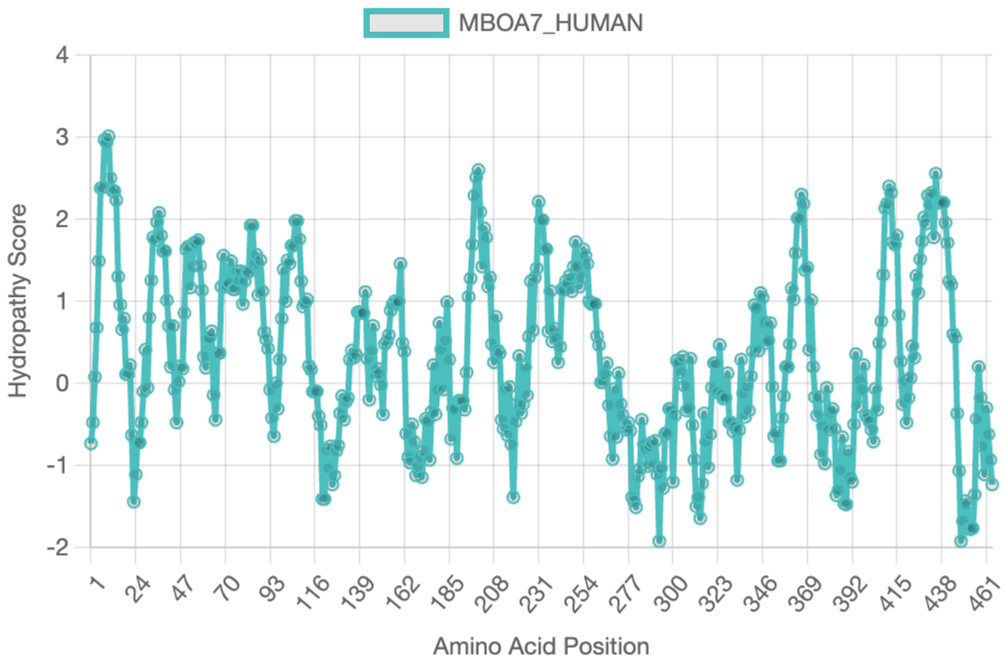

Kyte-Doolittle hydropathy plot for the sequence "MBOA7_HUMAN". Positive values indicate hydrophobic regions, while negative values indicate hydrophilic regions.

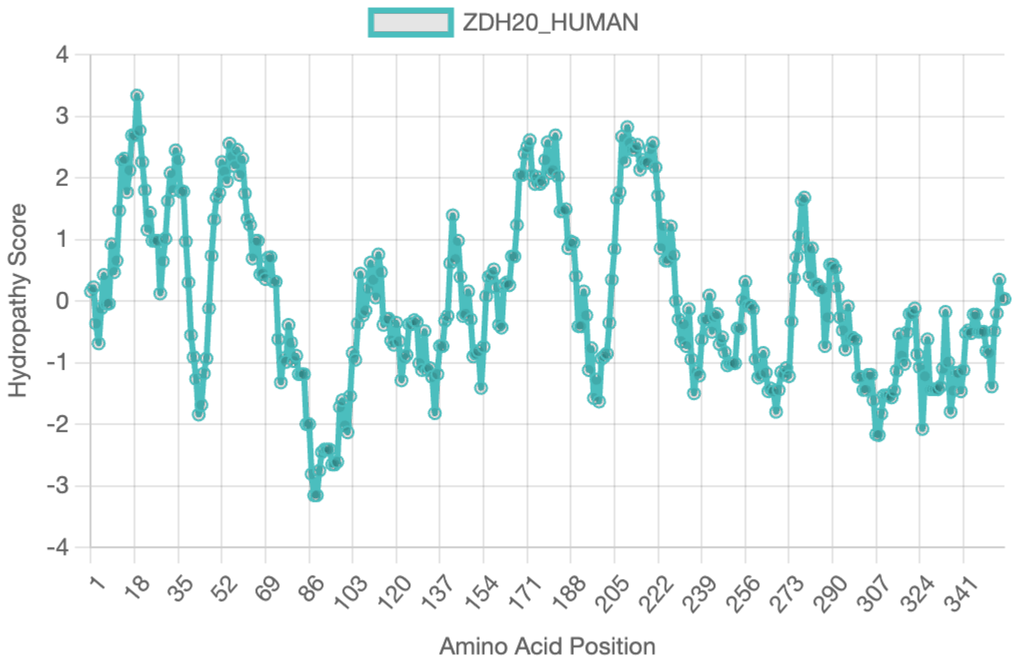

Kyte-Doolittle hydropathy plot for the sequence "ZDH20_HUMAN". Positive values indicate hydrophobic regions, while negative values indicate hydrophilic regions.

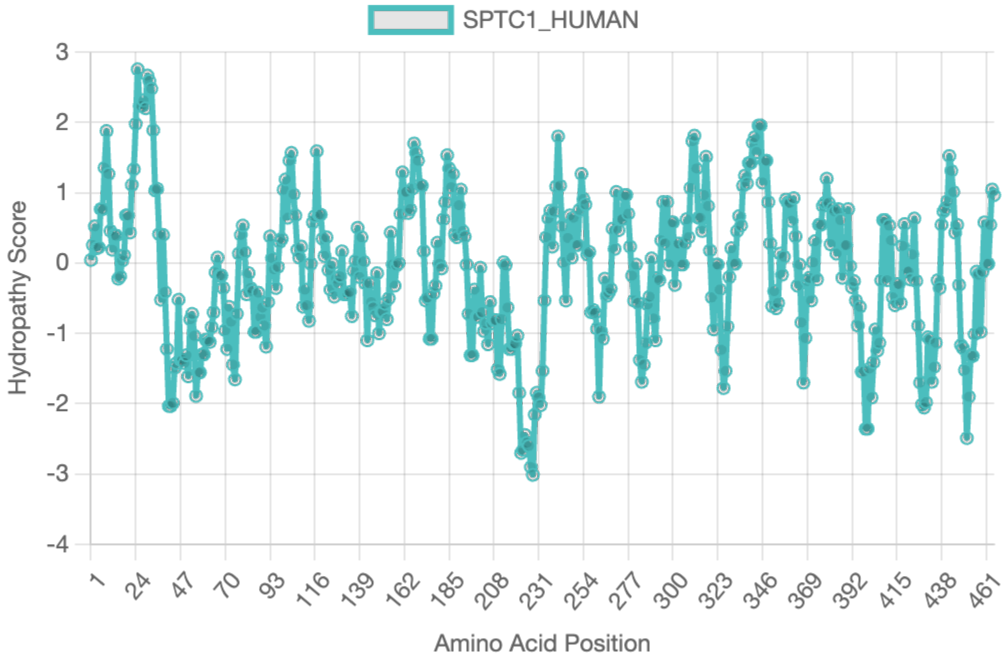

Kyte-Doolittle hydropathy plot for the sequence "SPTC1_HUMAN". Positive values indicate hydrophobic regions, while negative values indicate hydrophilic regions.

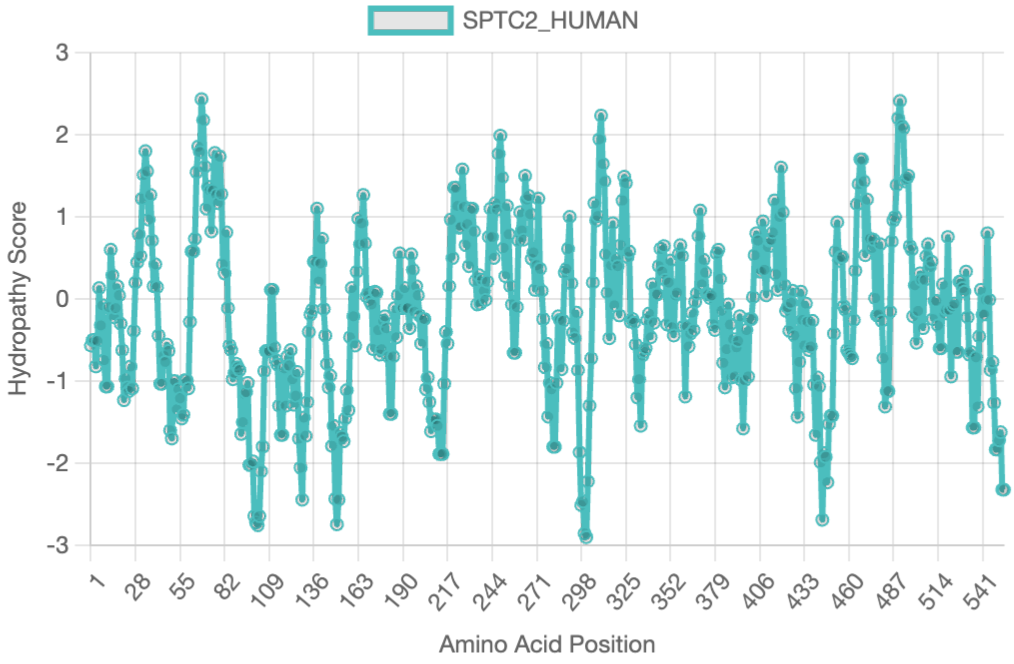

Kyte-Doolittle hydropathy plot for the sequence "SPTC2_HUMAN". Positive values indicate hydrophobic regions, while negative values indicate hydrophilic regions.

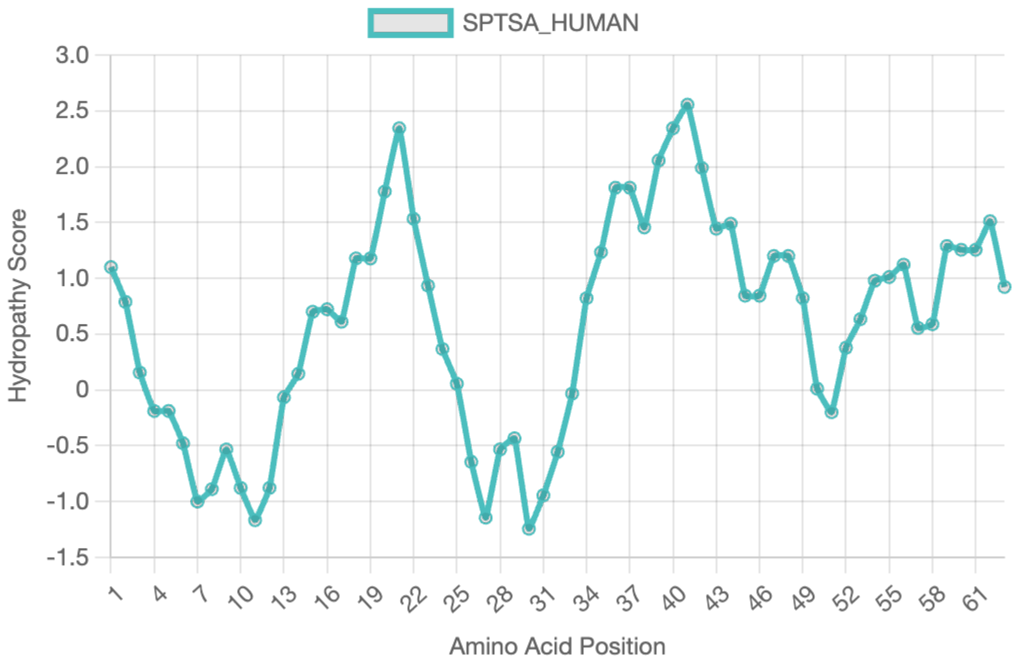

Kyte-Doolittle hydropathy plot for the sequence "SPTSA_HUMAN". Positive values indicate hydrophobic regions, while negative values indicate hydrophilic regions.

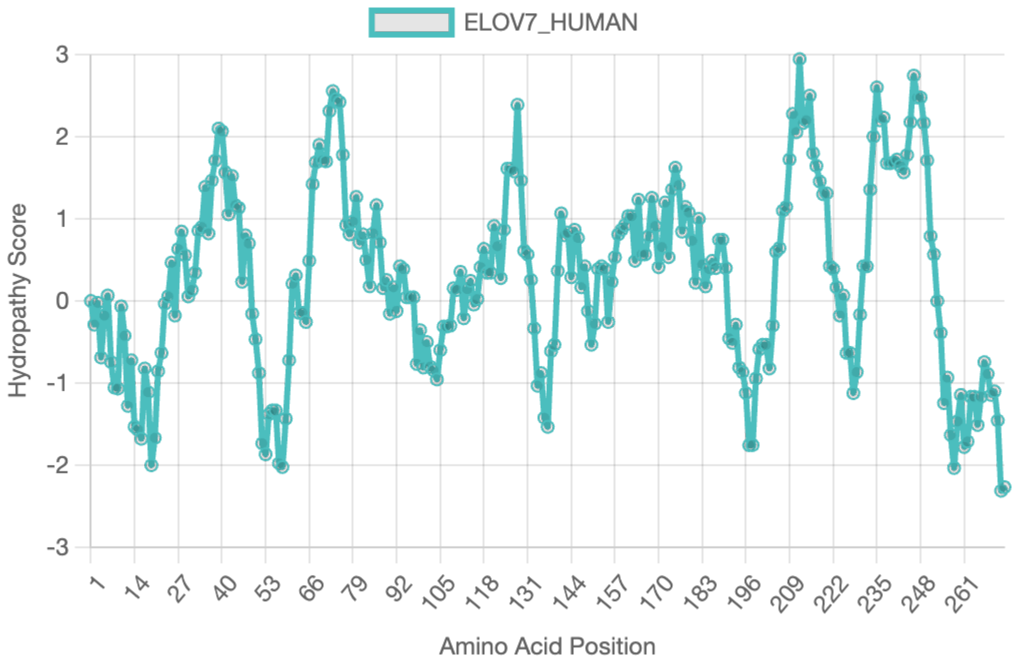

Kyte-Doolittle hydropathy plot for the sequence "ELOV7_HUMAN". Positive values indicate hydrophobic regions, while negative values indicate hydrophilic regions.

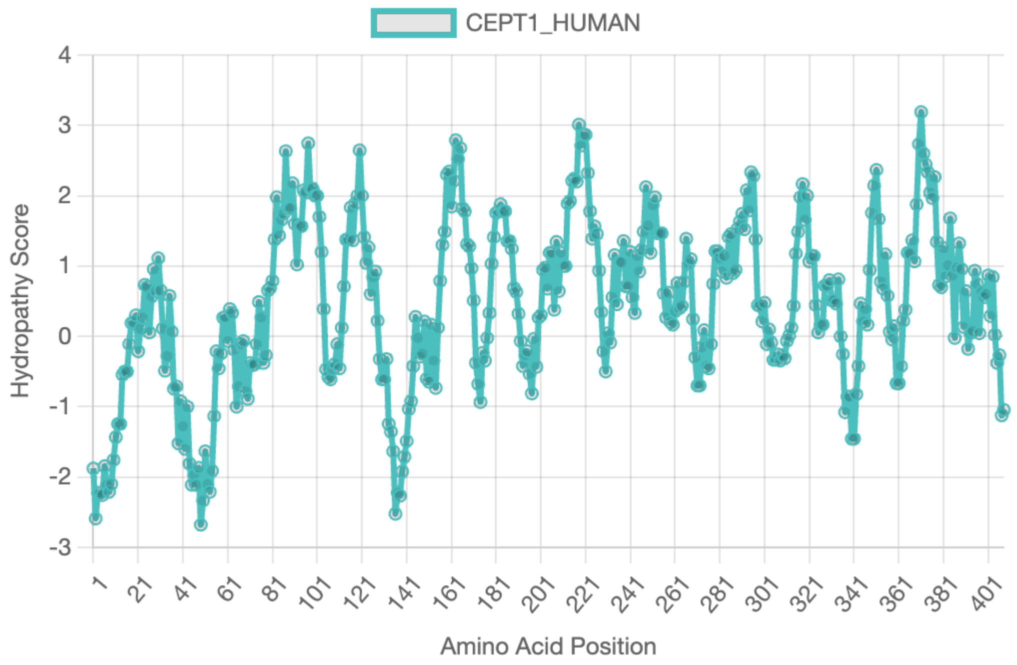

Kyte-Doolittle hydropathy plot for the sequence "CEPT1_HUMAN". Positive values indicate hydrophobic regions, while negative values indicate hydrophilic regions.

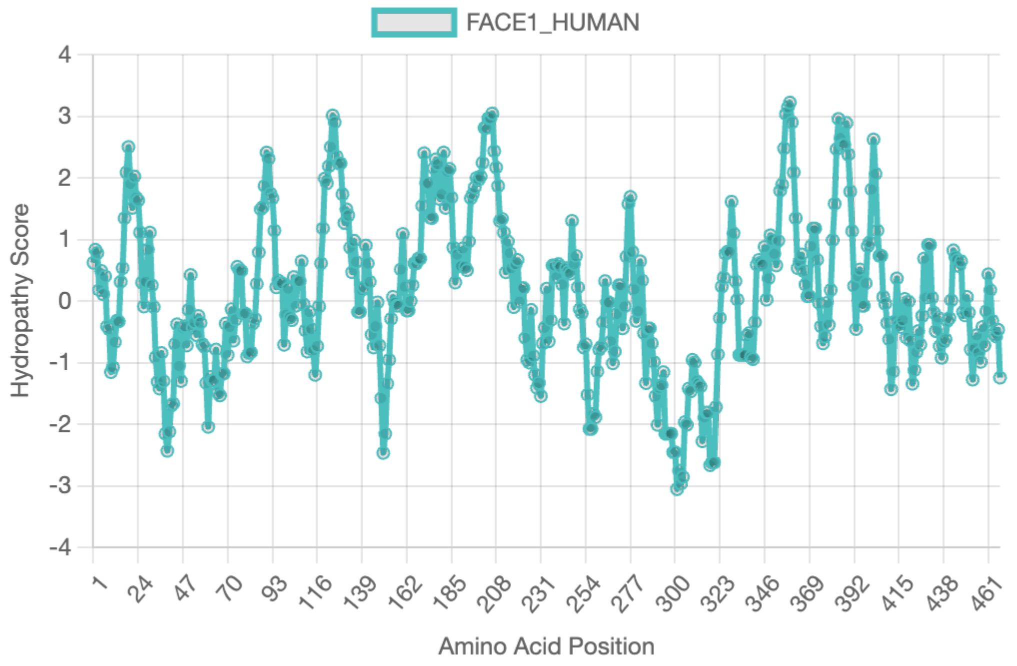

Kyte-Doolittle hydropathy plot for the sequence "FACE1_HUMAN". Positive values indicate hydrophobic regions, while negative values indicate hydrophilic regions.

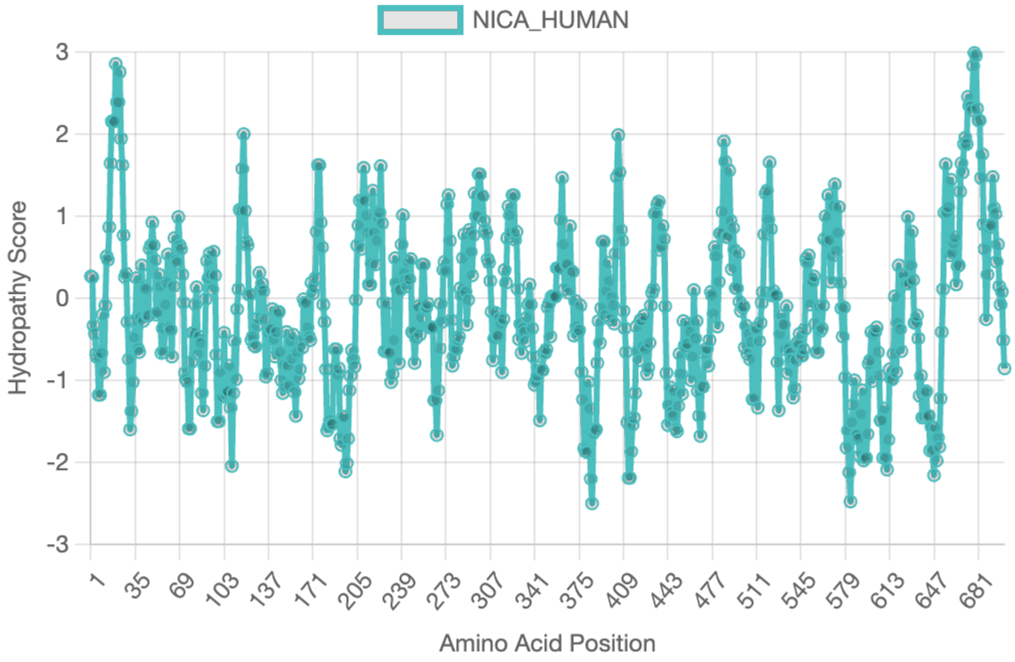

Kyte-Doolittle hydropathy plot for the sequence "NICA_HUMAN". Positive values indicate hydrophobic regions, while negative values indicate hydrophilic regions.

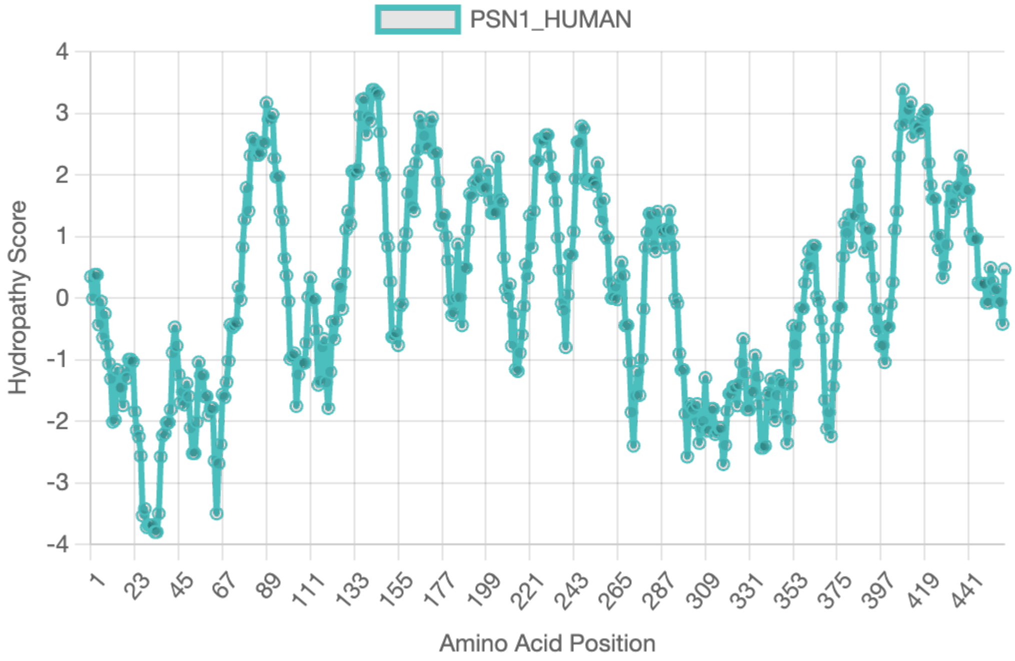

Kyte-Doolittle hydropathy plot for the sequence "PSN1_HUMAN". Positive values indicate hydrophobic regions, while negative values indicate hydrophilic regions.

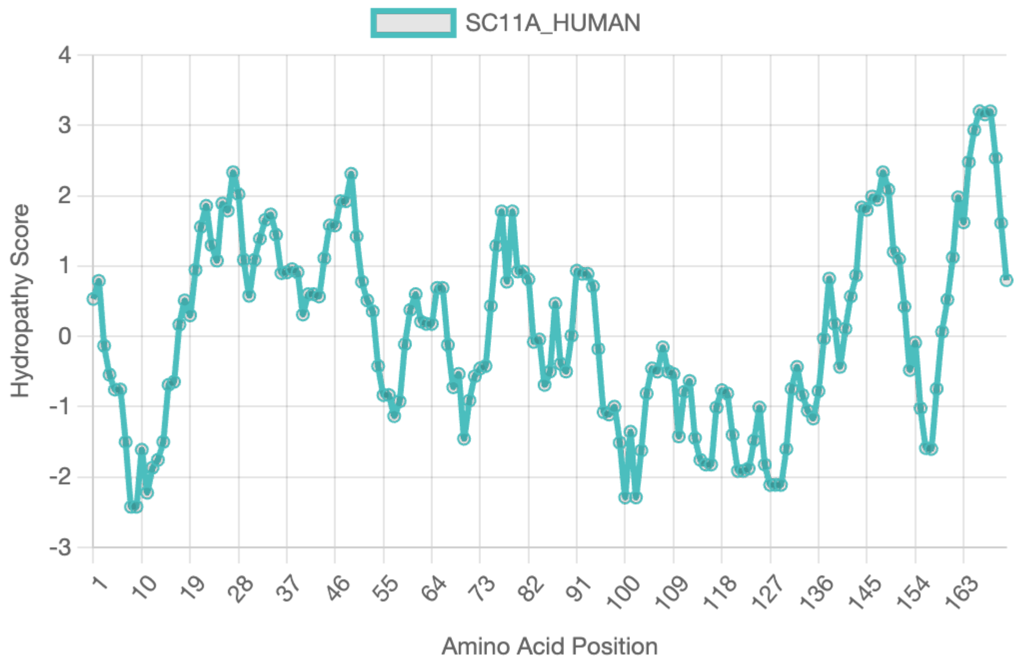

Kyte-Doolittle hydropathy plot for the sequence "SC11A_HUMAN". Positive values indicate hydrophobic regions, while negative values indicate hydrophilic regions.

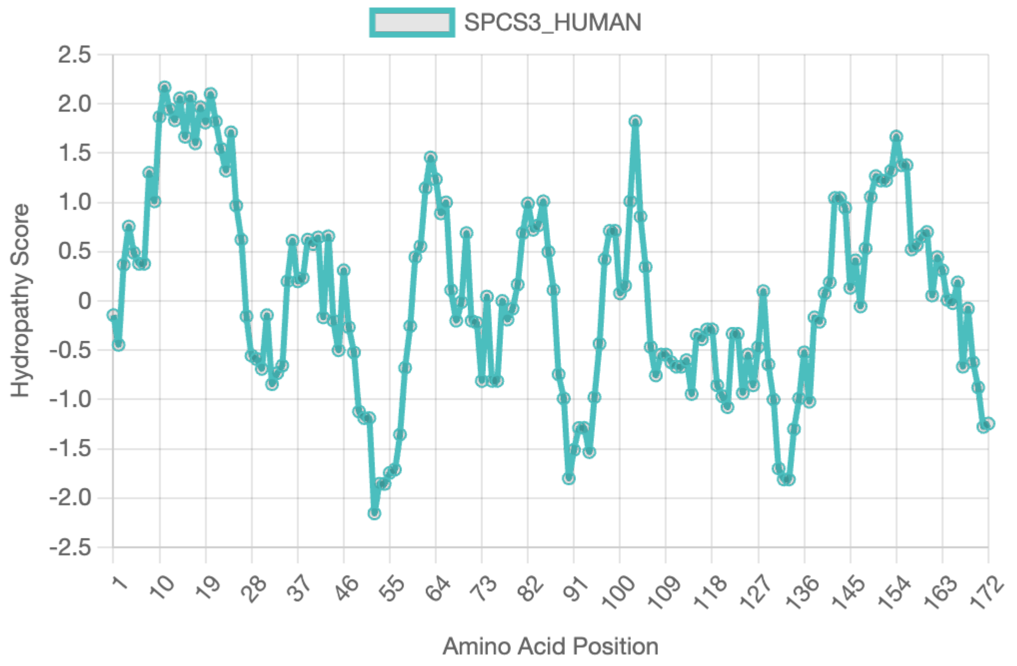

Kyte-Doolittle hydropathy plot for the sequence "SPCS3_HUMAN". Positive values indicate hydrophobic regions, while negative values indicate hydrophilic regions.

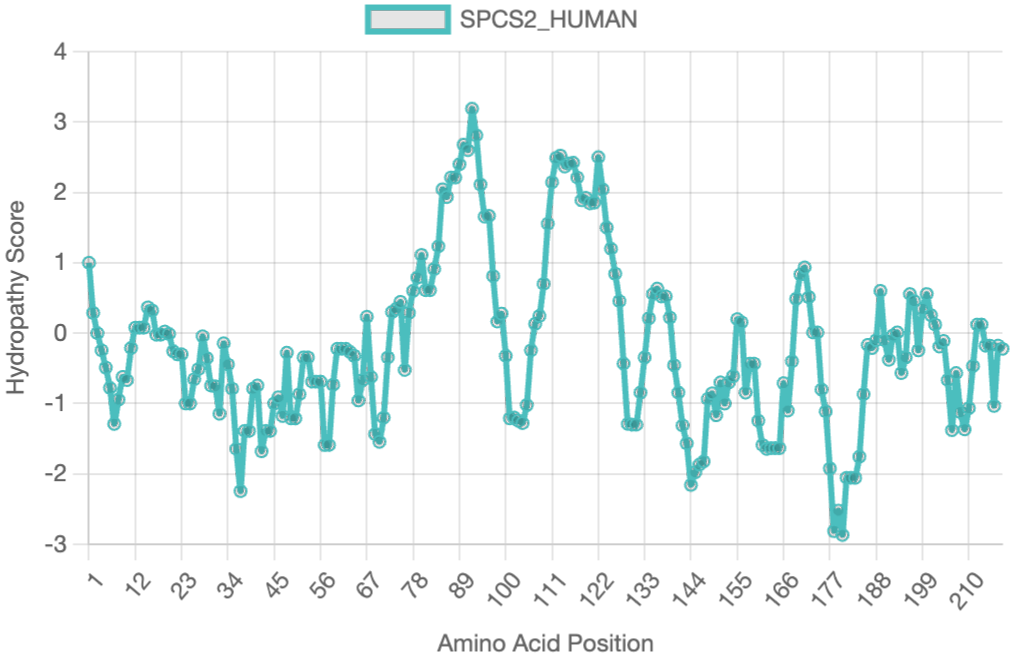

Kyte-Doolittle hydropathy plot for the sequence "SPCS2_HUMAN". Positive values indicate hydrophobic regions, while negative values indicate hydrophilic regions.

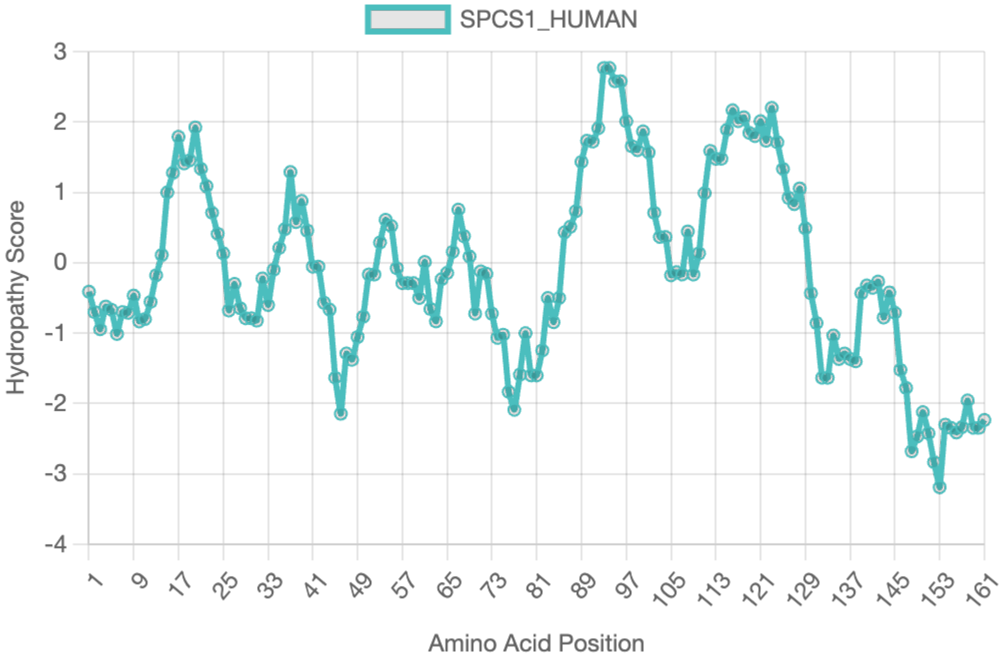

Kyte-Doolittle hydropathy plot for the sequence "SPCS1_HUMAN". Positive values indicate hydrophobic regions, while negative values indicate hydrophilic regions.

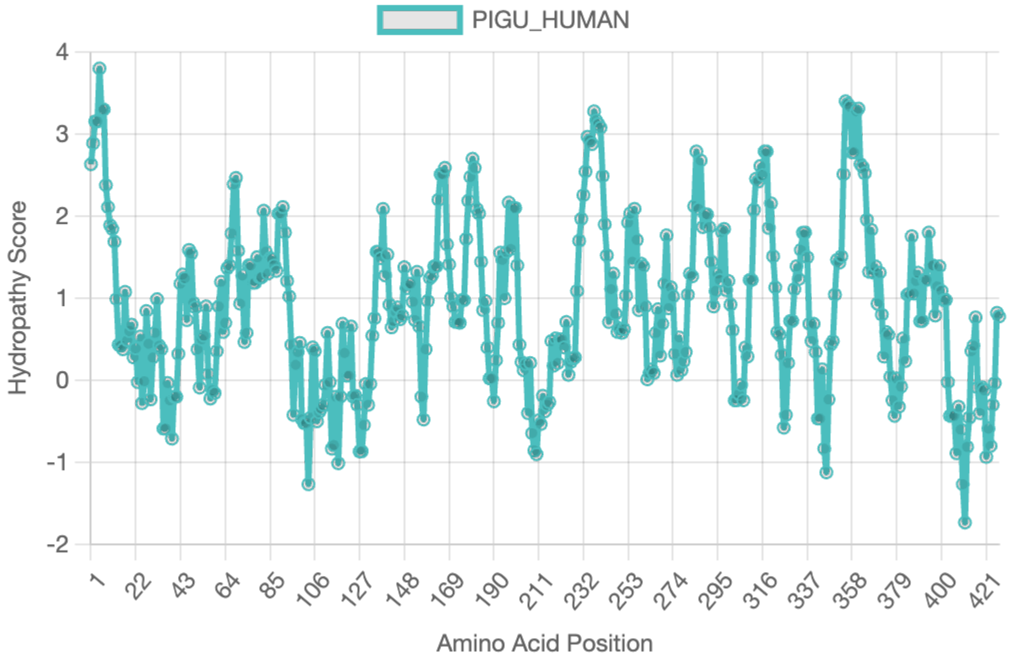

Kyte-Doolittle hydropathy plot for the sequence "PIGU_HUMAN". Positive values indicate hydrophobic regions, while negative values indicate hydrophilic regions.

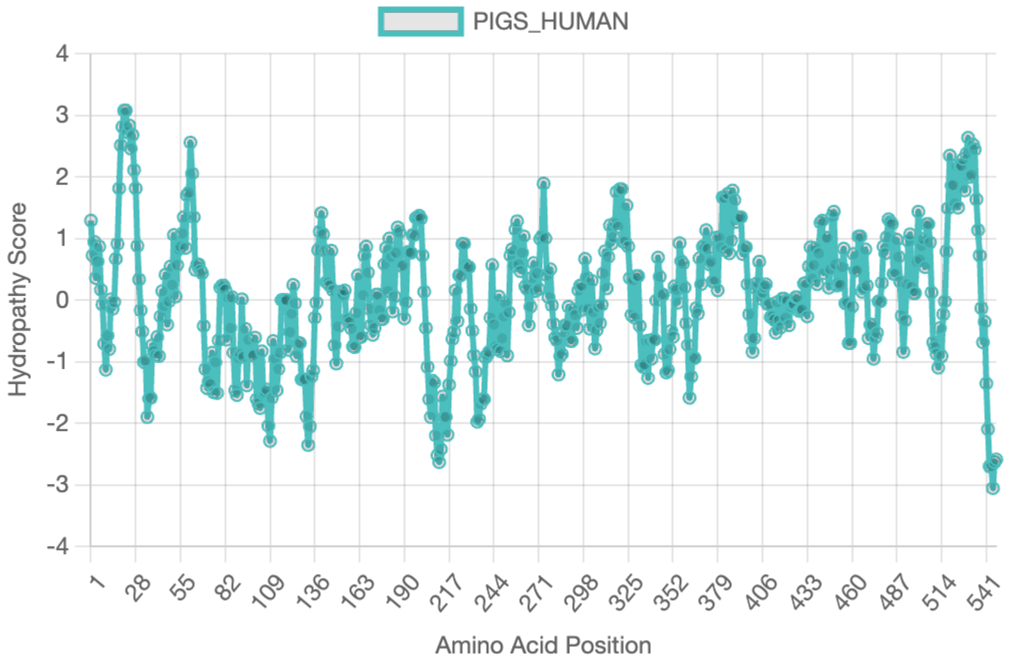

Kyte-Doolittle hydropathy plot for the sequence "PIGS_HUMAN". Positive values indicate hydrophobic regions, while negative values indicate hydrophilic regions.

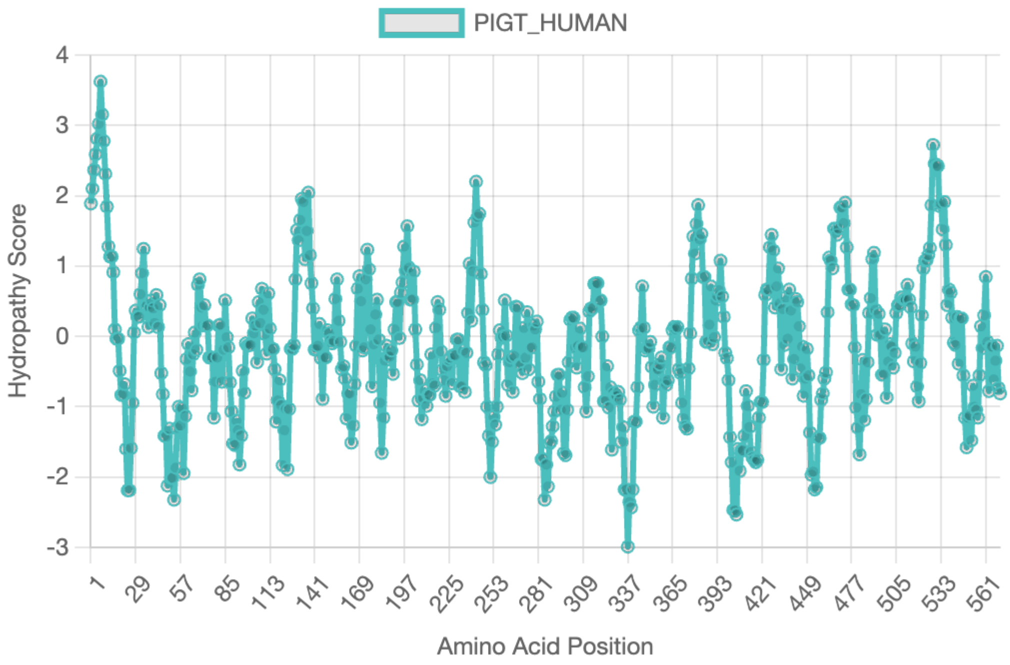

Kyte-Doolittle hydropathy plot for the sequence "PIGT_HUMAN". Positive values indicate hydrophobic regions, while negative values indicate hydrophilic regions.

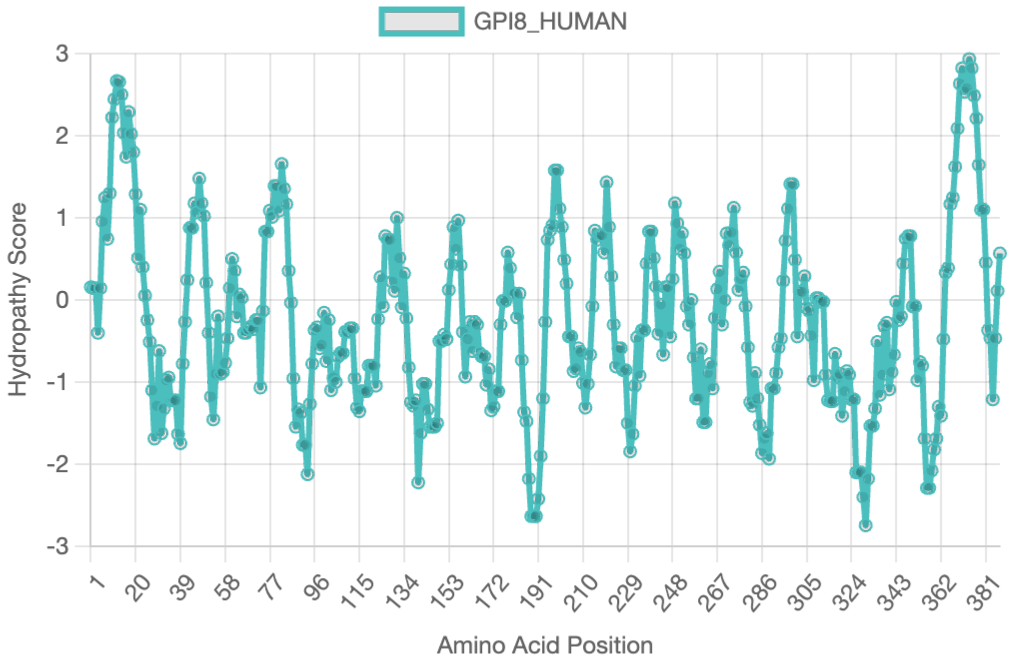

Kyte-Doolittle hydropathy plot for the sequence "GPI8_HUMAN". Positive values indicate hydrophobic regions, while negative values indicate hydrophilic regions.

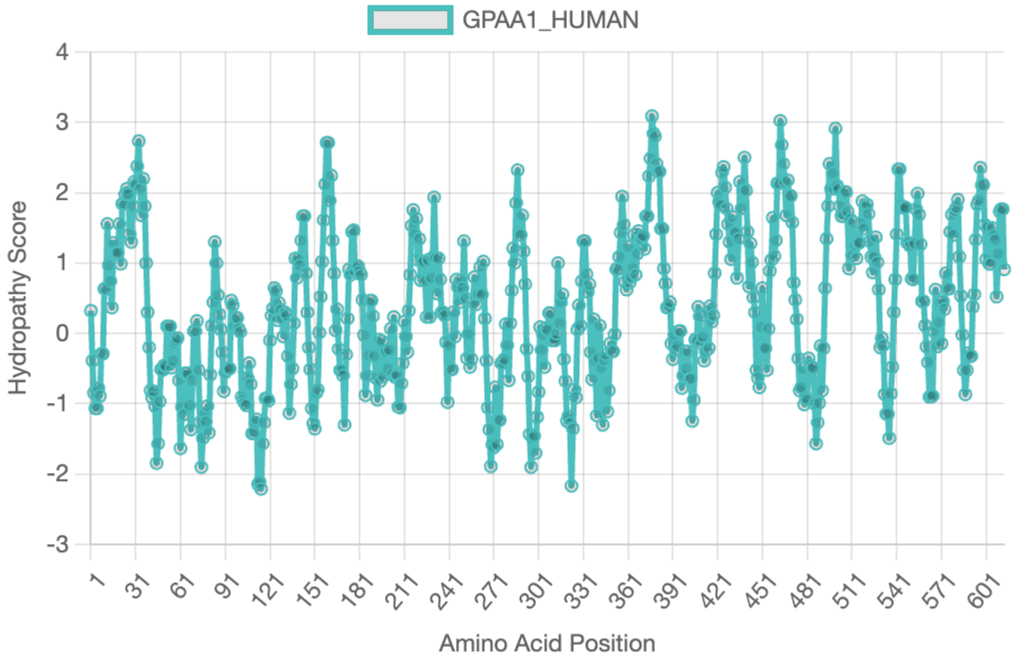

Kyte-Doolittle hydropathy plot for the sequence "GPAA1_HUMAN". Positive values indicate hydrophobic regions, while negative values indicate hydrophilic regions.

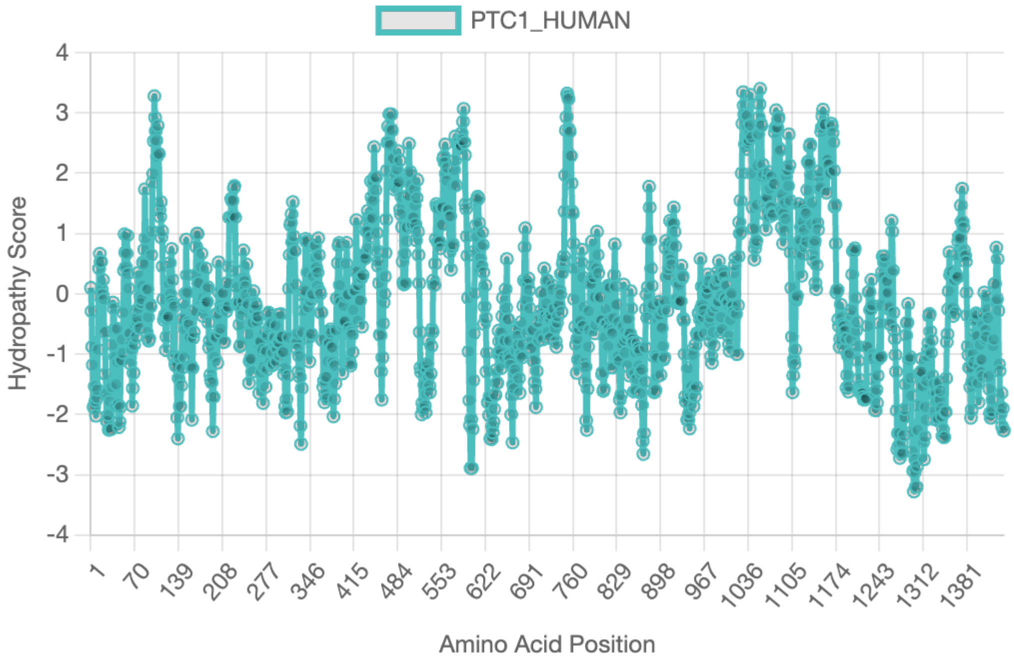

Kyte-Doolittle hydropathy plot for the sequence "PTC1_HUMAN". Positive values indicate hydrophobic regions, while negative values indicate hydrophilic regions.

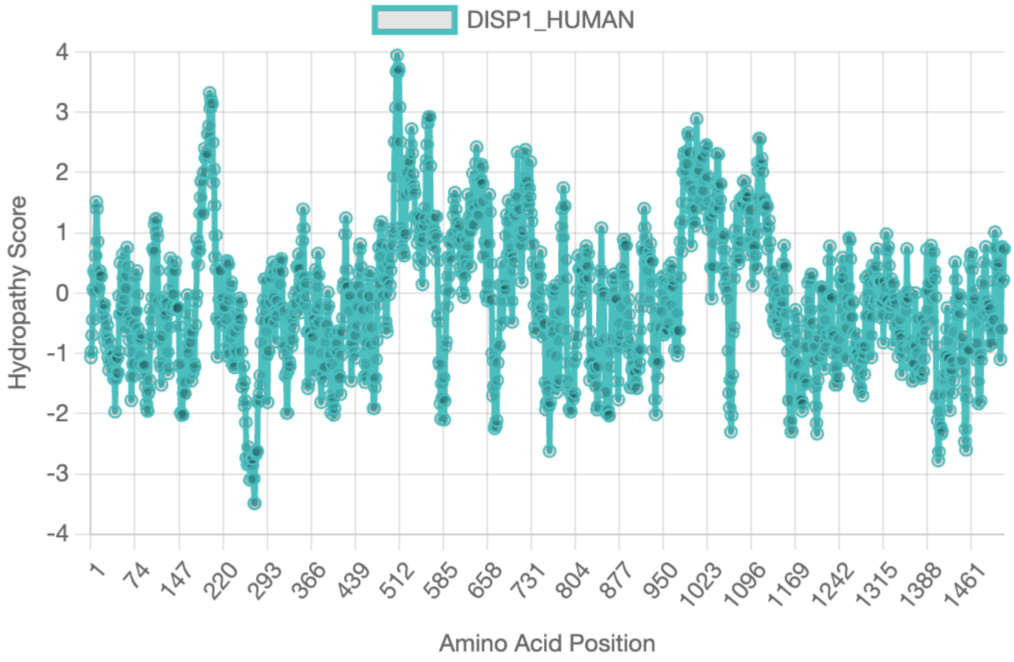

Kyte-Doolittle hydropathy plot for the sequence "DISP1_HUMAN". Positive values indicate hydrophobic regions, while negative values indicate hydrophilic regions.

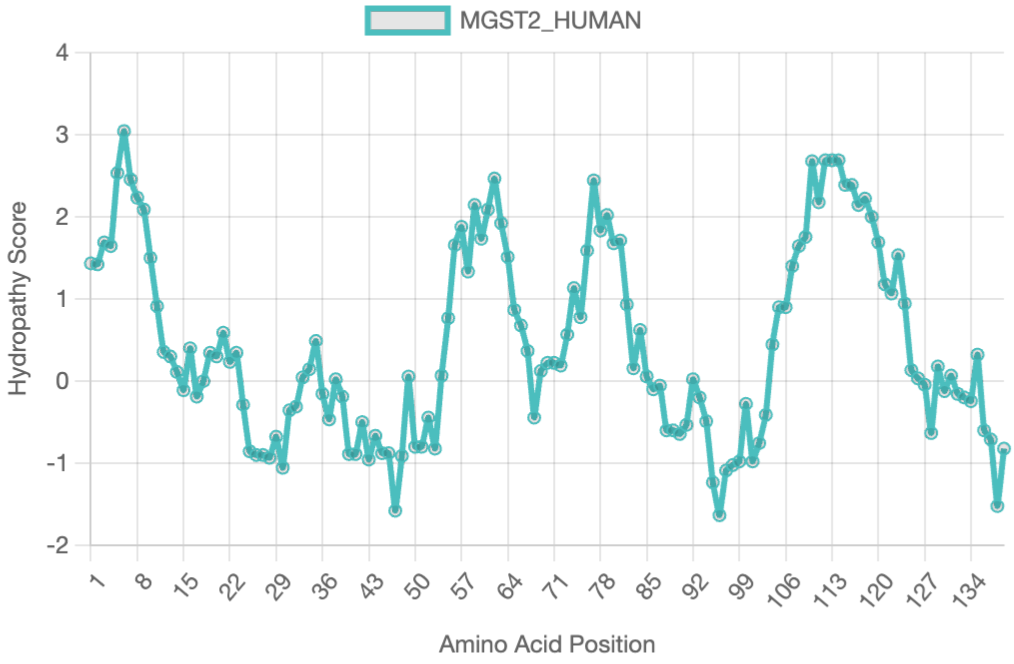

Kyte-Doolittle hydropathy plot for the sequence "MGST2_HUMAN". Positive values indicate hydrophobic regions, while negative values indicate hydrophilic regions.

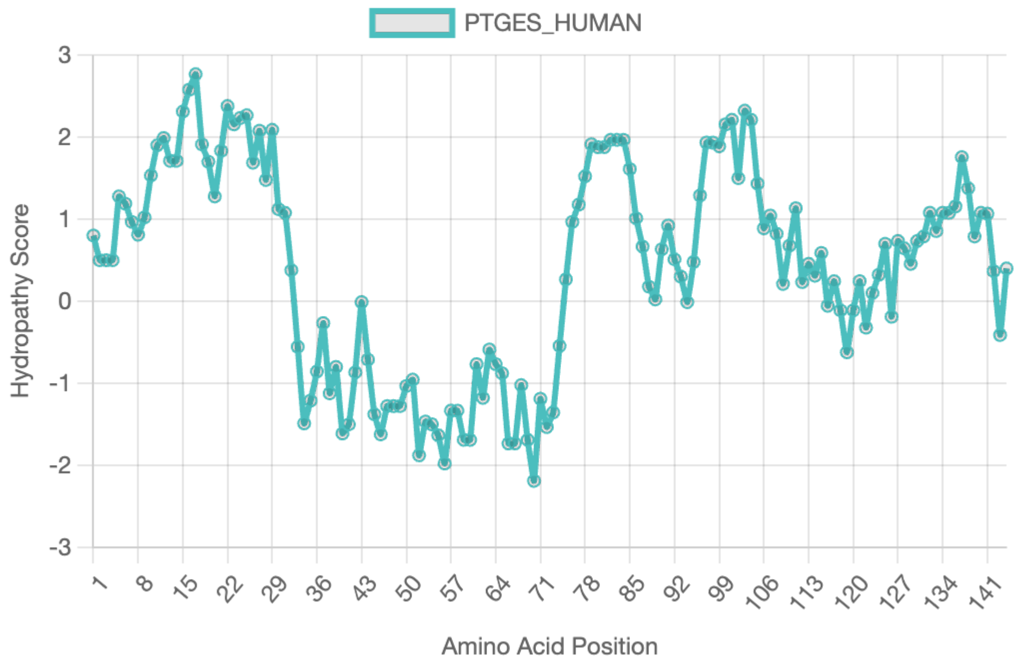

Kyte-Doolittle hydropathy plot for the sequence "PTGES_HUMAN". Positive values indicate hydrophobic regions, while negative values indicate hydrophilic regions.

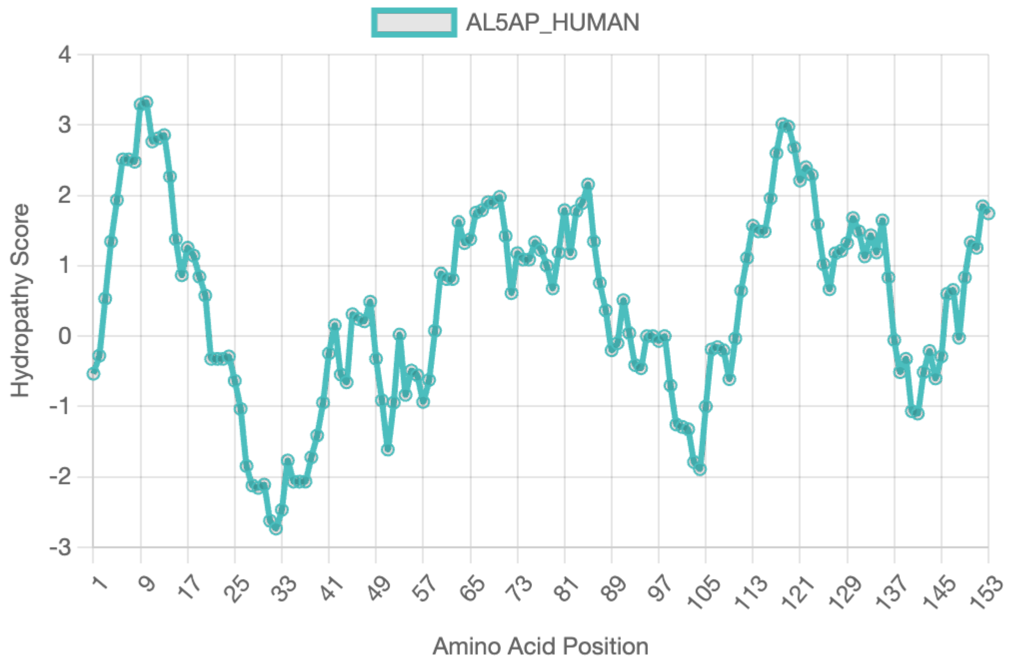

Kyte-Doolittle hydropathy plot for the sequence "AL5AP_HUMAN". Positive values indicate hydrophobic regions, while negative values indicate hydrophilic regions.

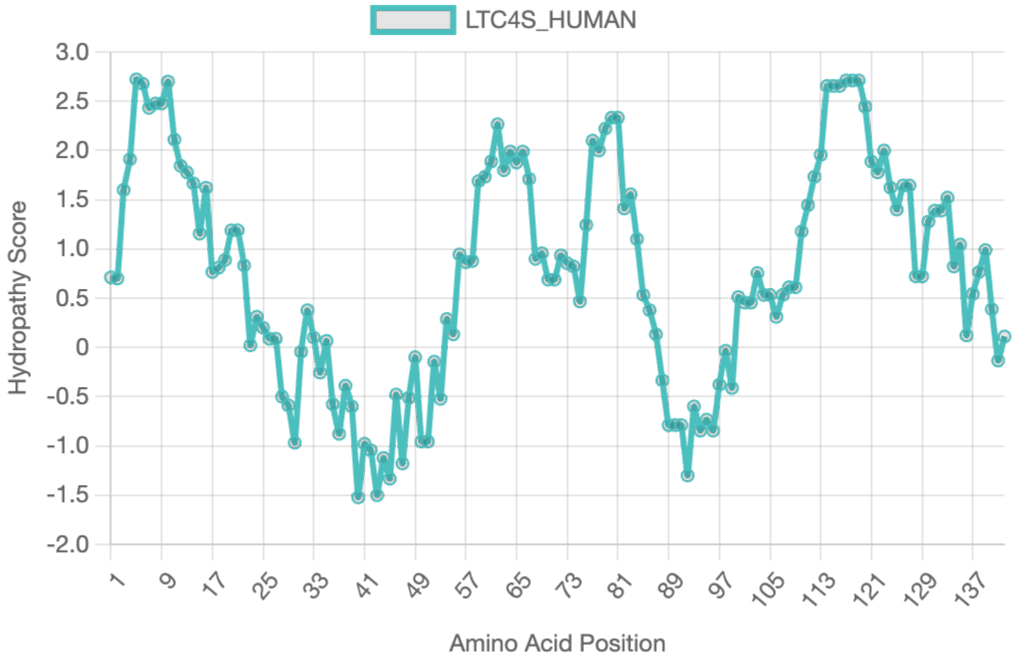

Kyte-Doolittle hydropathy plot for the sequence "LTC4S_HUMAN". Positive values indicate hydrophobic regions, while negative values indicate hydrophilic regions.

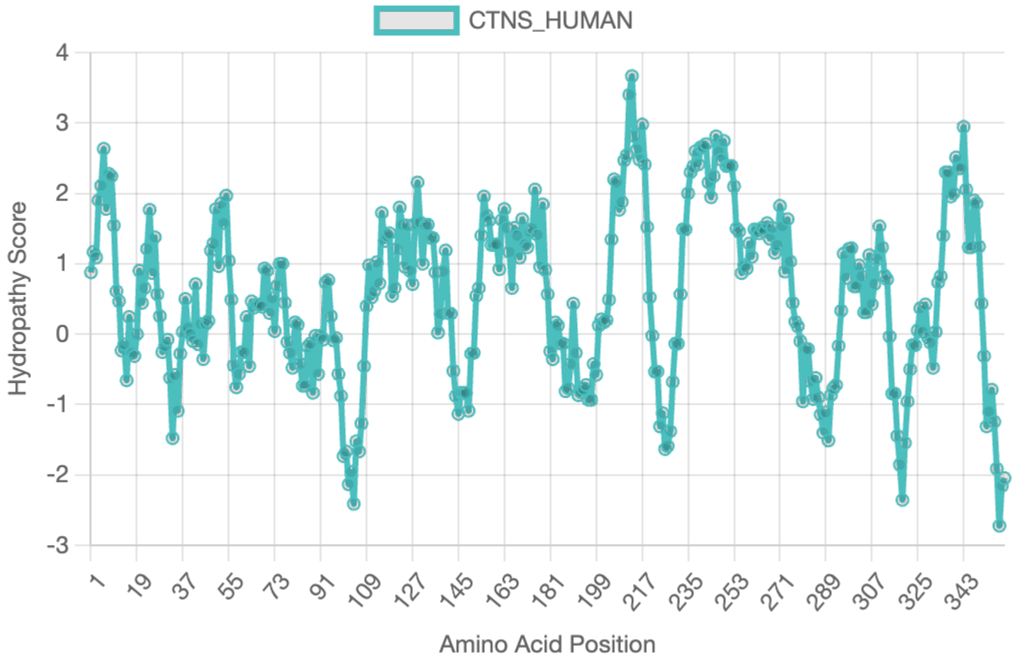

Kyte-Doolittle hydropathy plot for the sequence "CTNS_HUMAN". Positive values indicate hydrophobic regions, while negative values indicate hydrophilic regions.

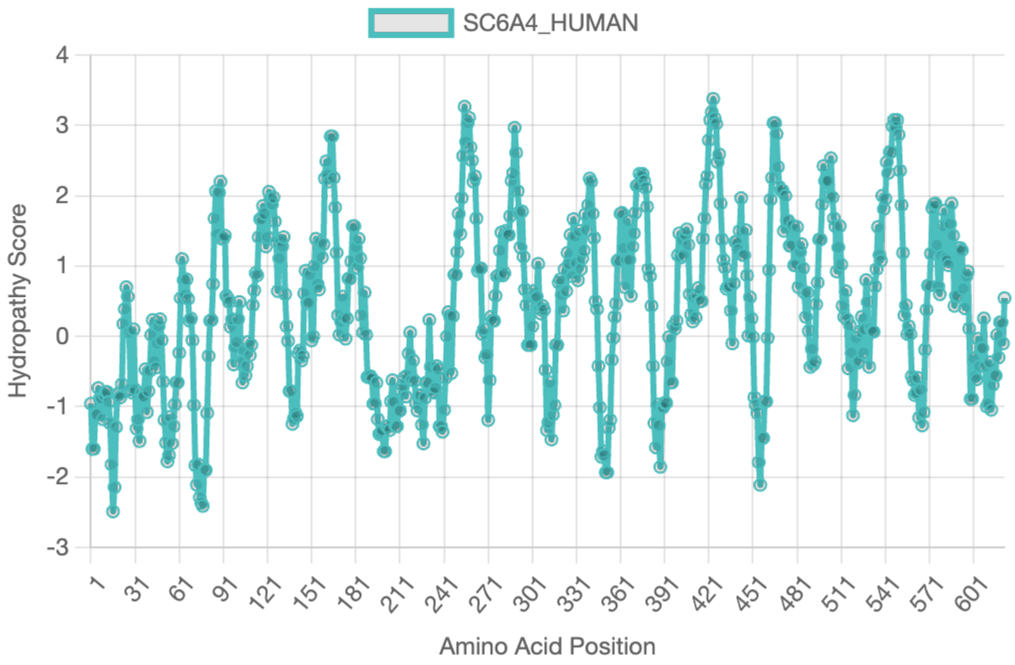

Kyte-Doolittle hydropathy plot for the sequence "SC6A4_HUMAN". Positive values indicate hydrophobic regions, while negative values indicate hydrophilic regions.

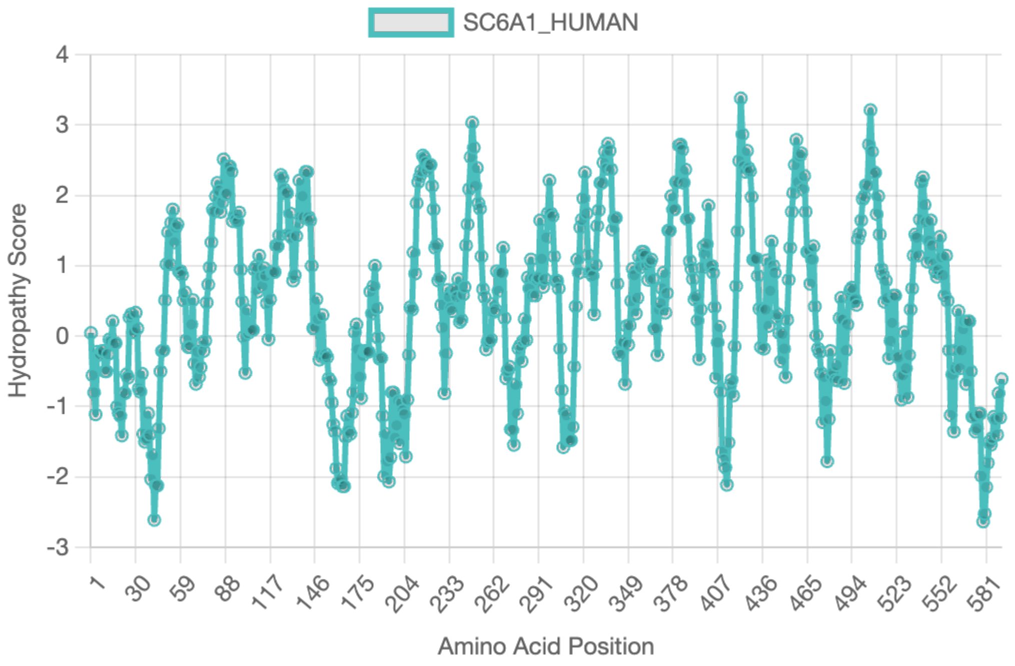

Kyte-Doolittle hydropathy plot for the sequence "SC6A1_HUMAN". Positive values indicate hydrophobic regions, while negative values indicate hydrophilic regions.

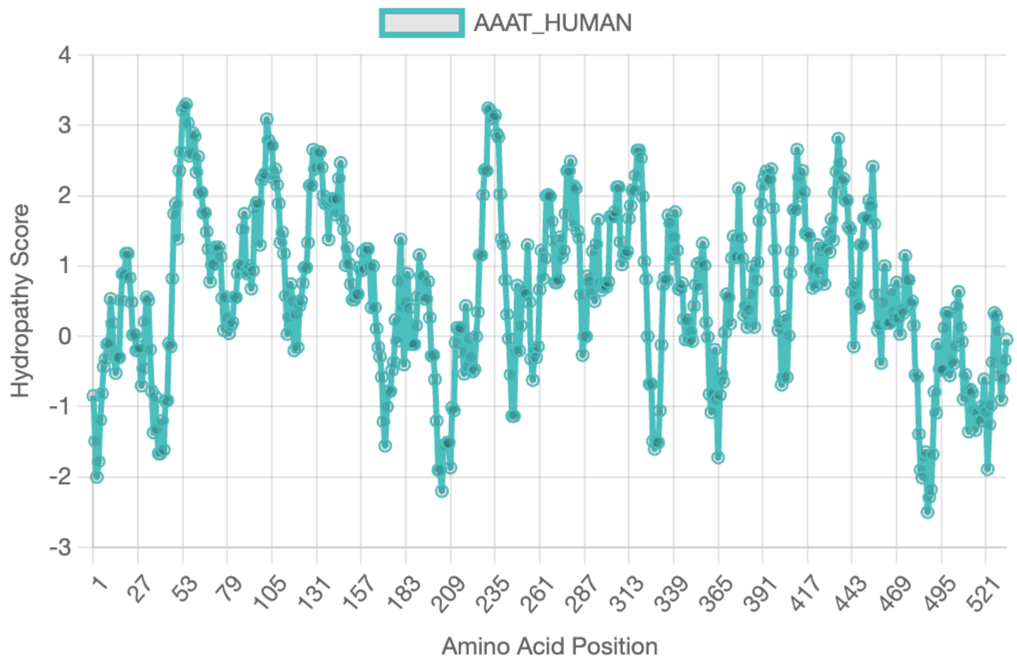

Kyte-Doolittle hydropathy plot for the sequence "AAAT_HUMAN". Positive values indicate hydrophobic regions, while negative values indicate hydrophilic regions.

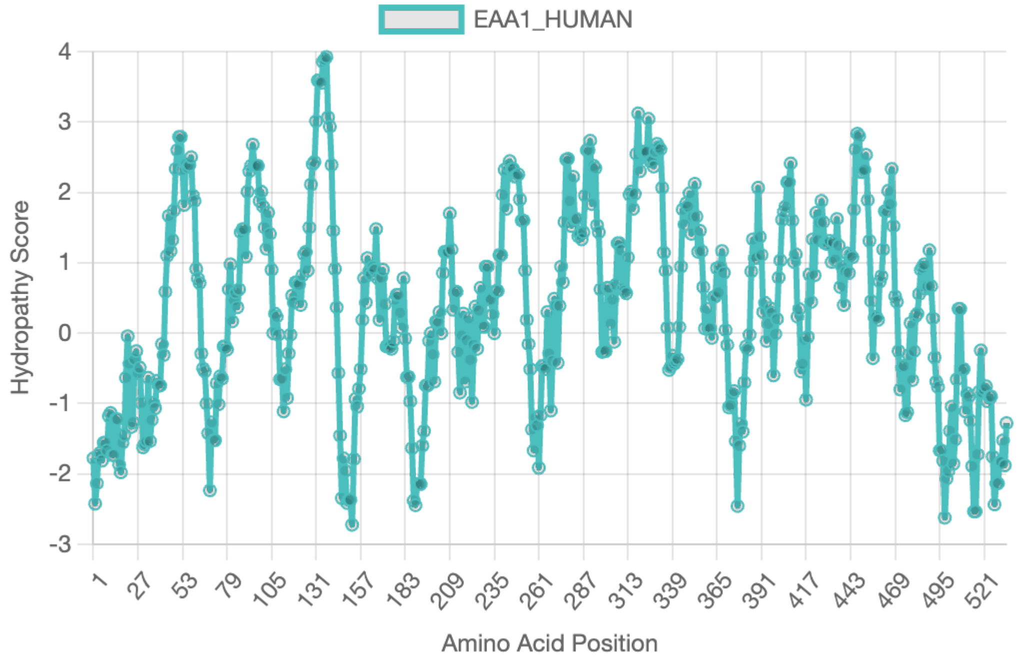

Kyte-Doolittle hydropathy plot for the sequence "EAA1_HUMAN". Positive values indicate hydrophobic regions, while negative values indicate hydrophilic regions.

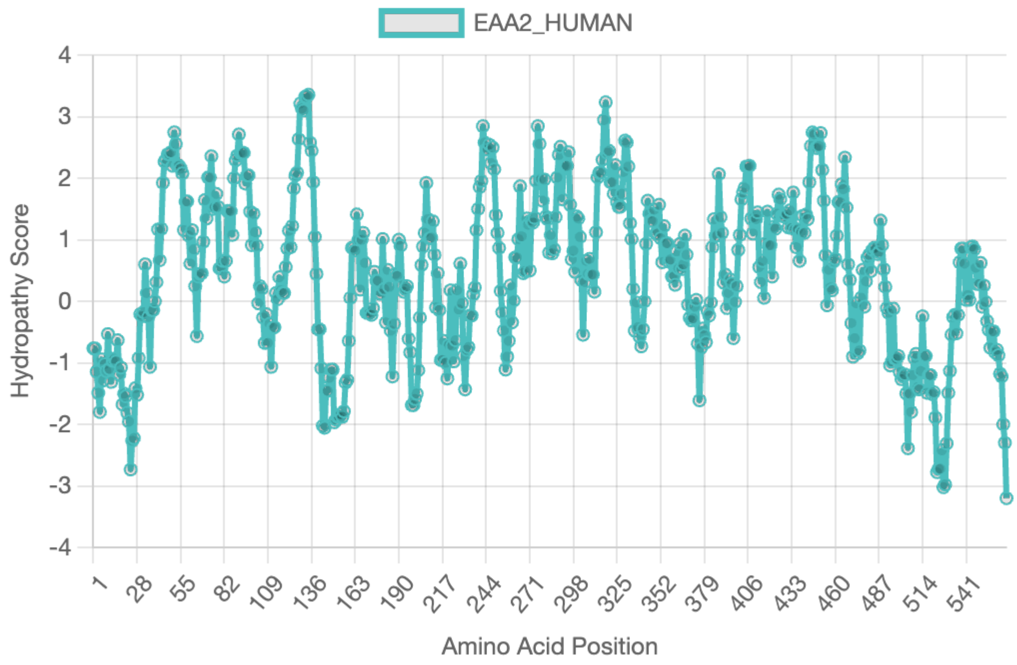

Kyte-Doolittle hydropathy plot for the sequence "EAA2_HUMAN". Positive values indicate hydrophobic regions, while negative values indicate hydrophilic regions.

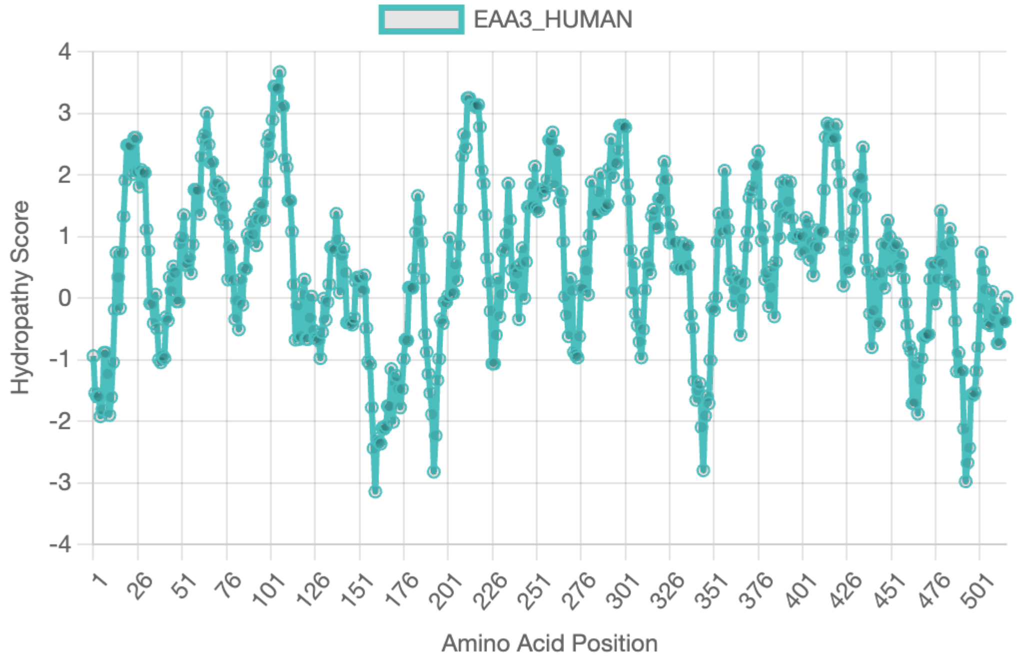

Kyte-Doolittle hydropathy plot for the sequence "EAA3_HUMAN". Positive values indicate hydrophobic regions, while negative values indicate hydrophilic regions.

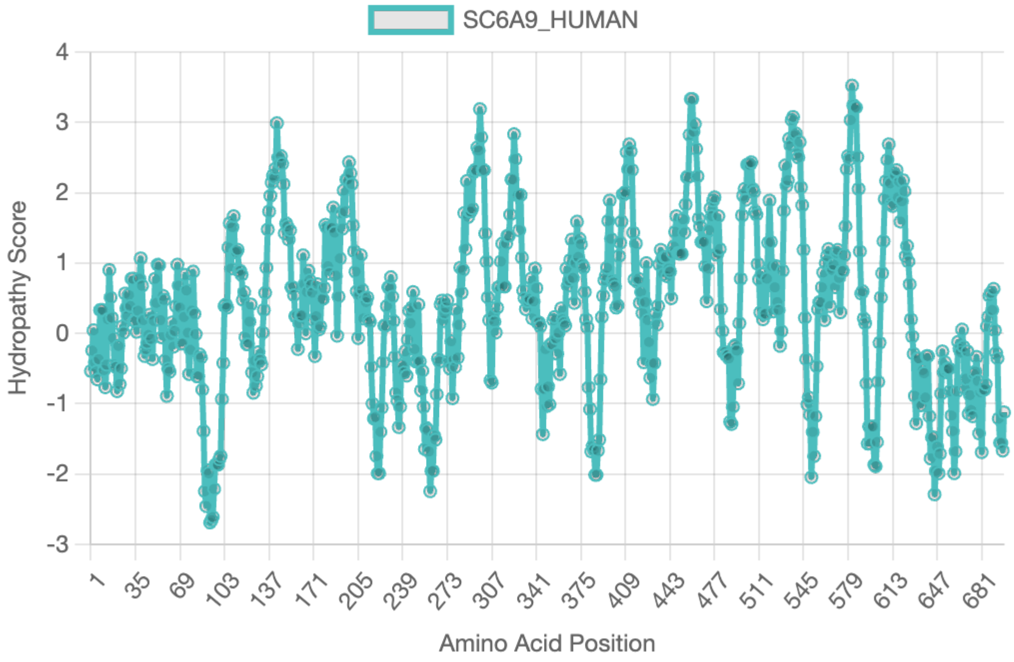

Kyte-Doolittle hydropathy plot for the sequence "SC6A9_HUMAN". Positive values indicate hydrophobic regions, while negative values indicate hydrophilic regions.

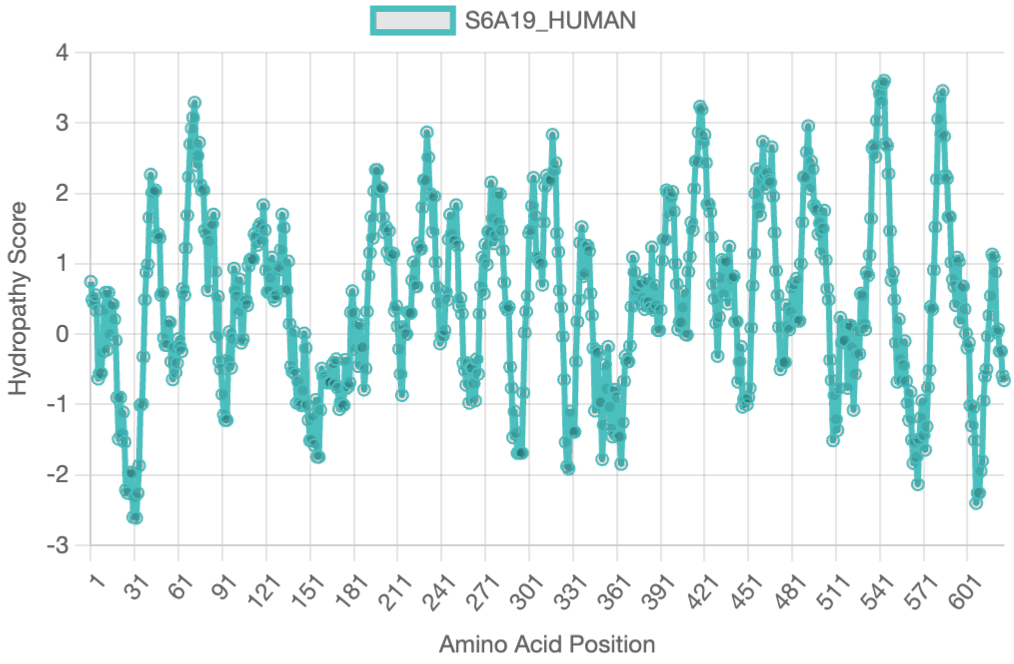

Kyte-Doolittle hydropathy plot for the sequence "S6A19_HUMAN". Positive values indicate hydrophobic regions, while negative values indicate hydrophilic regions.

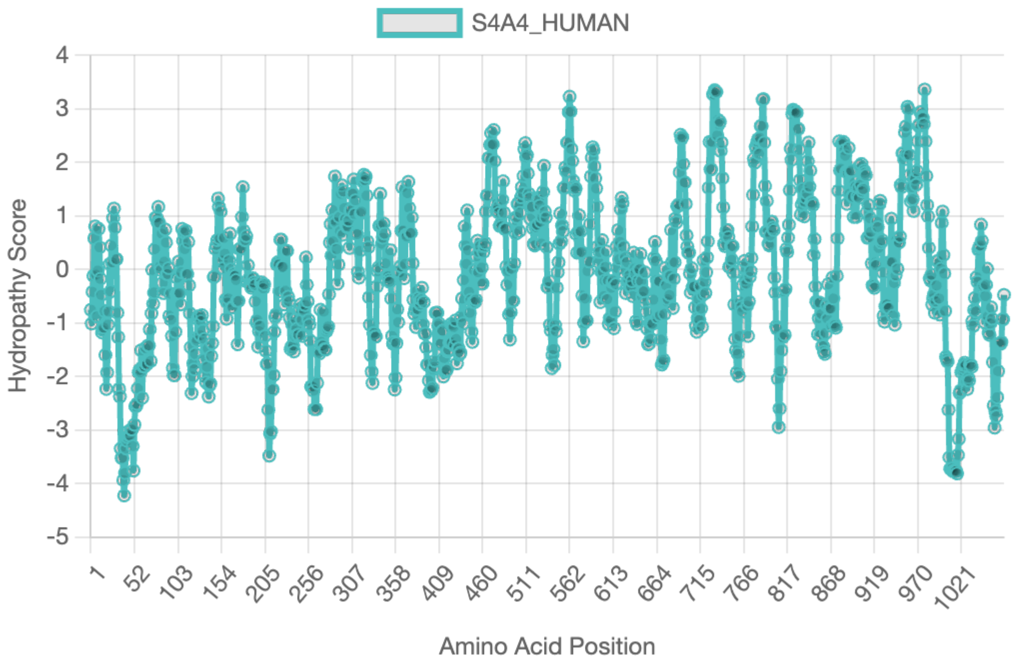

Kyte-Doolittle hydropathy plot for the sequence "S4A4_HUMAN". Positive values indicate hydrophobic regions, while negative values indicate hydrophilic regions.

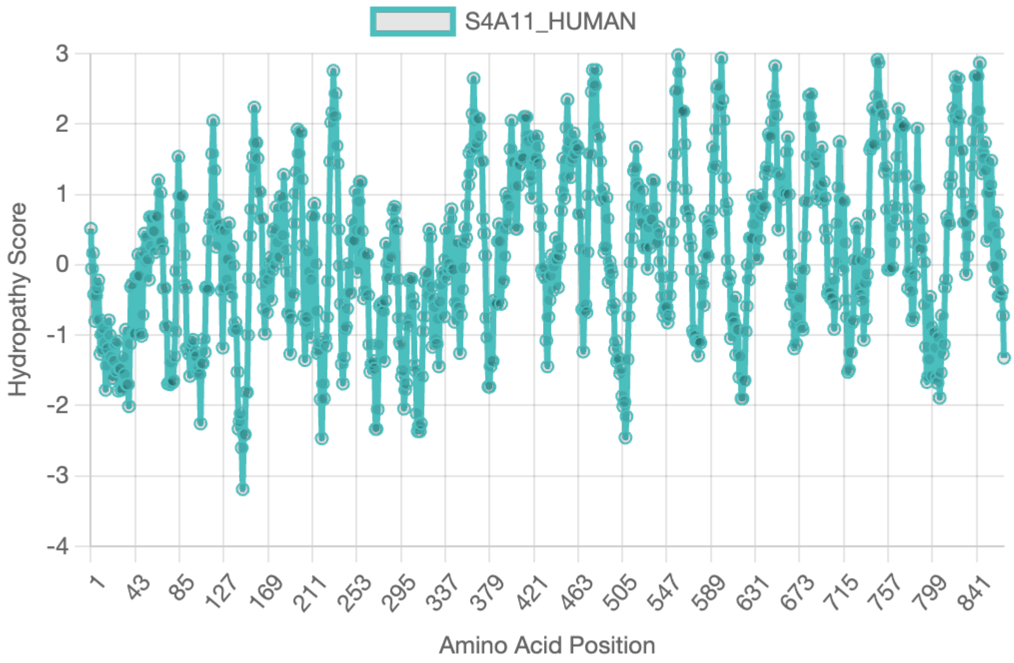

Kyte-Doolittle hydropathy plot for the sequence "S4A11_HUMAN". Positive values indicate hydrophobic regions, while negative values indicate hydrophilic regions.

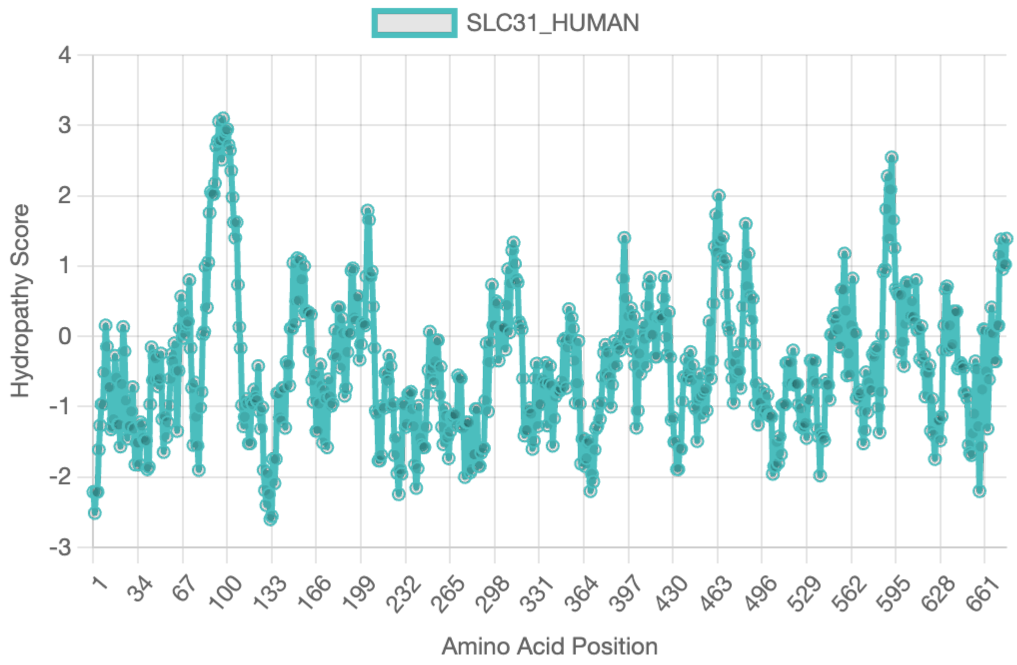

Kyte-Doolittle hydropathy plot for the sequence "SLC31_HUMAN". Positive values indicate hydrophobic regions, while negative values indicate hydrophilic regions.

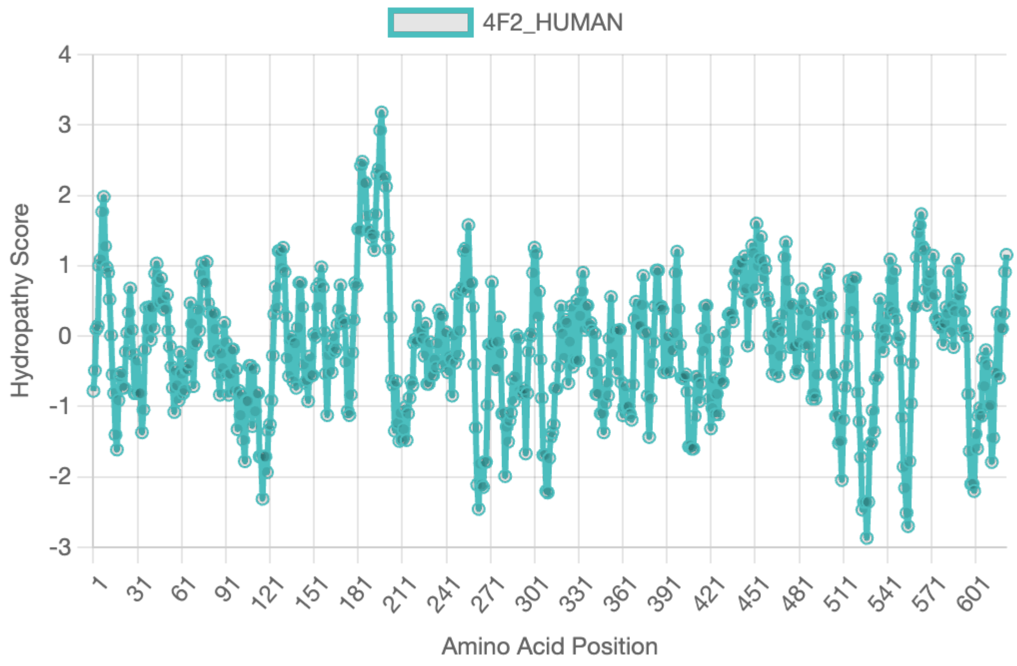

Kyte-Doolittle hydropathy plot for the sequence "4F2_HUMAN". Positive values indicate hydrophobic regions, while negative values indicate hydrophilic regions.

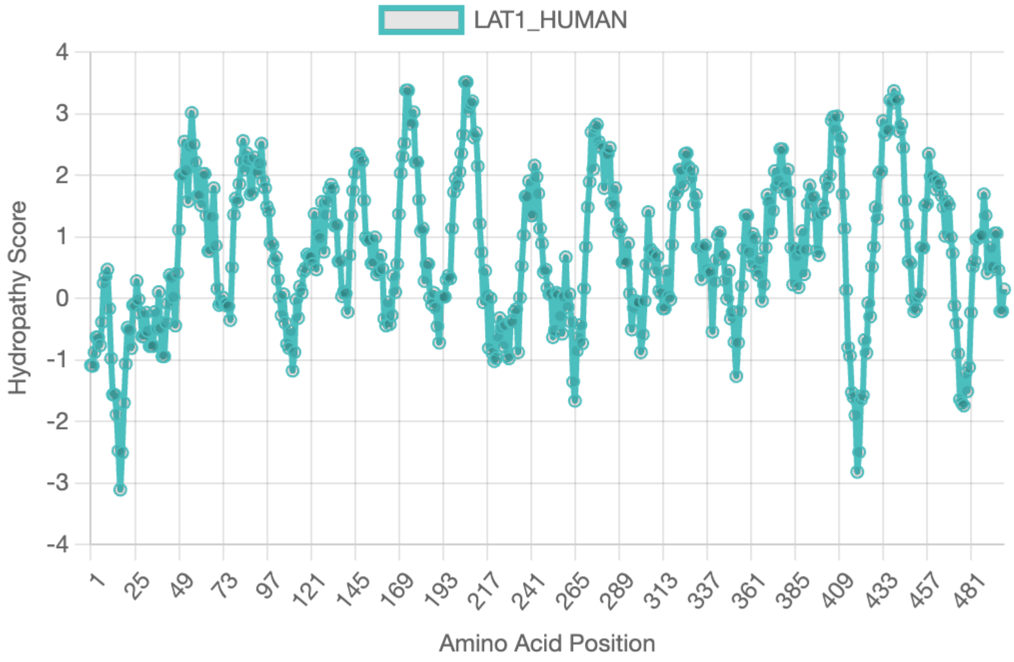

Kyte-Doolittle hydropathy plot for the sequence "LAT1_HUMAN". Positive values indicate hydrophobic regions, while negative values indicate hydrophilic regions.

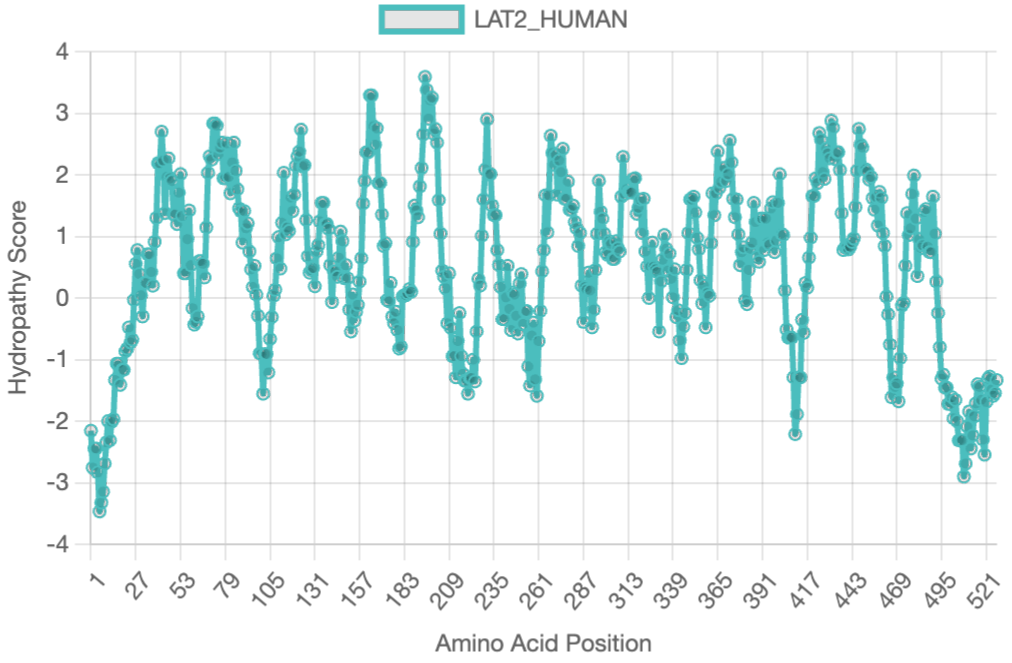

Kyte-Doolittle hydropathy plot for the sequence "LAT2_HUMAN". Positive values indicate hydrophobic regions, while negative values indicate hydrophilic regions.

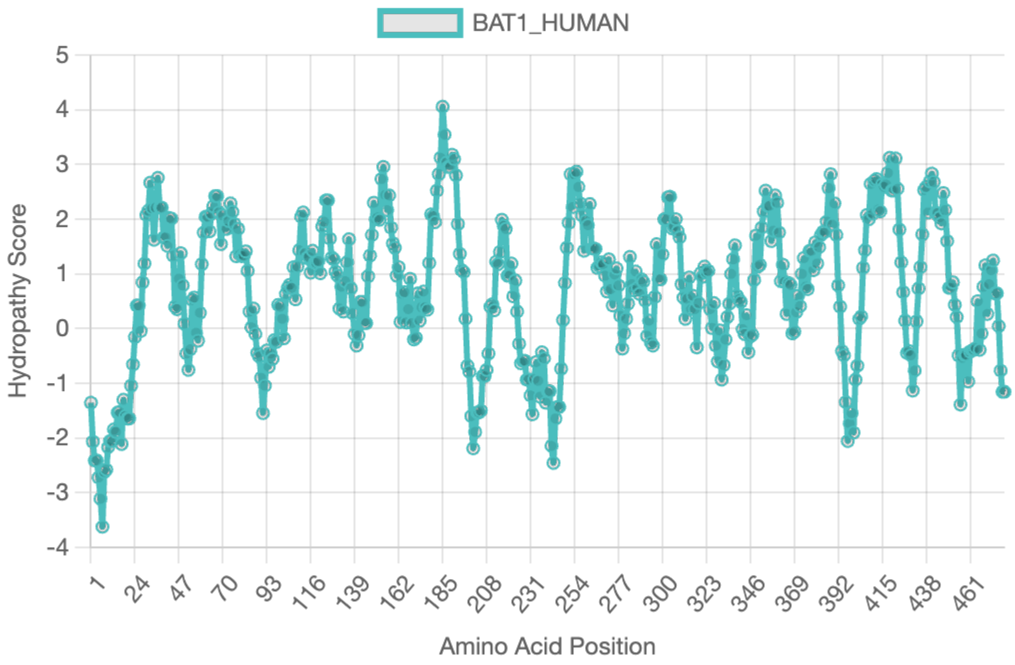

Kyte-Doolittle hydropathy plot for the sequence "BAT1_HUMAN". Positive values indicate hydrophobic regions, while negative values indicate hydrophilic regions.

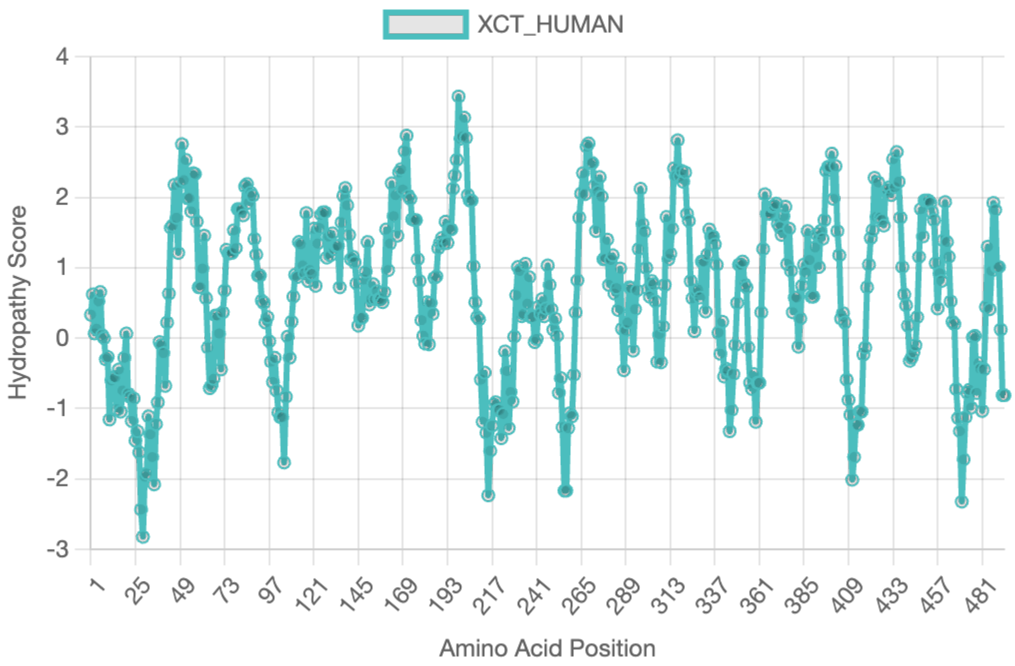

Kyte-Doolittle hydropathy plot for the sequence "XCT_HUMAN". Positive values indicate hydrophobic regions, while negative values indicate hydrophilic regions.

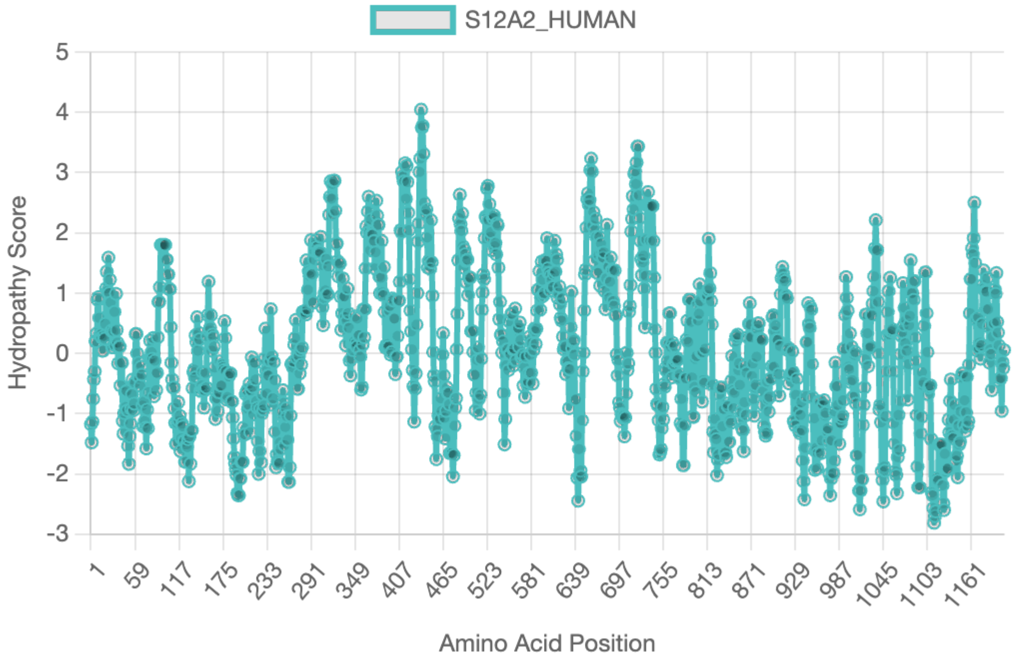

Kyte-Doolittle hydropathy plot for the sequence "S12A2_HUMAN". Positive values indicate hydrophobic regions, while negative values indicate hydrophilic regions.

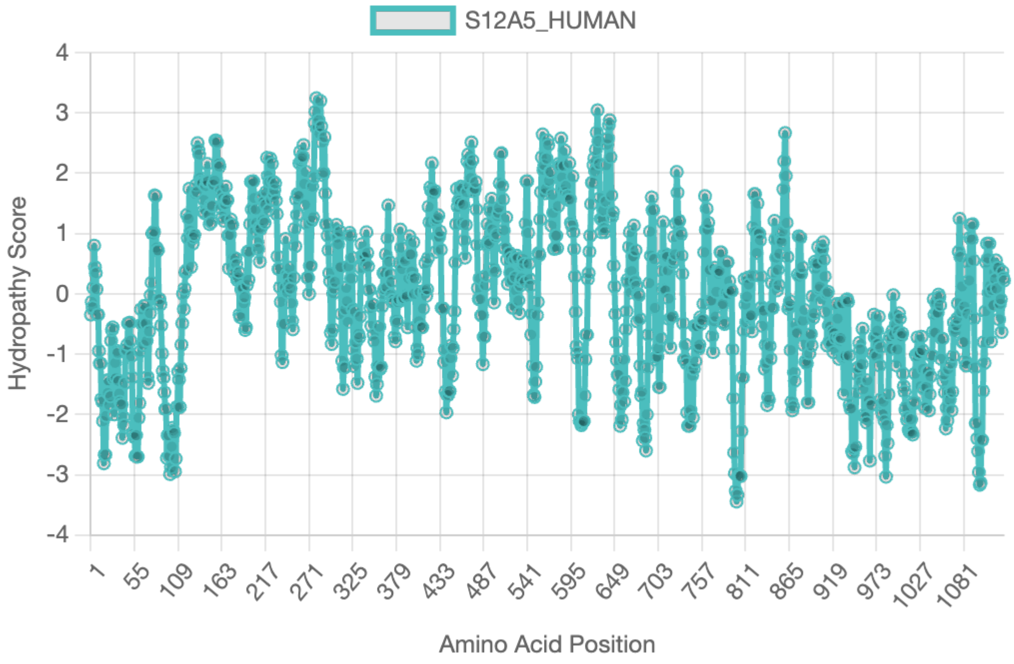

Kyte-Doolittle hydropathy plot for the sequence "S12A5_HUMAN". Positive values indicate hydrophobic regions, while negative values indicate hydrophilic regions.

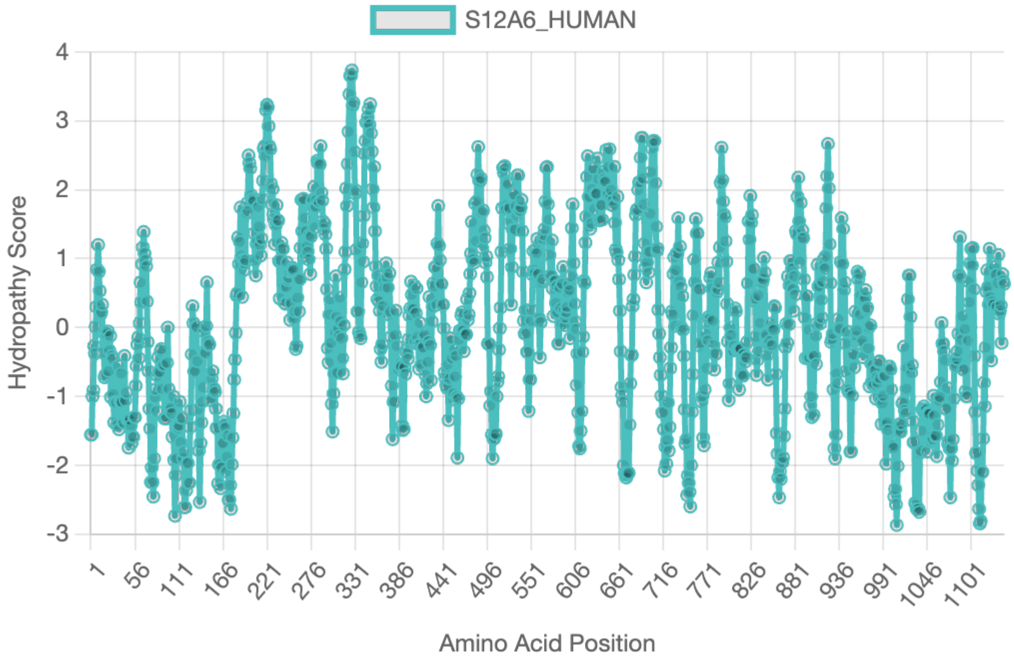

Kyte-Doolittle hydropathy plot for the sequence "S12A6_HUMAN". Positive values indicate hydrophobic regions, while negative values indicate hydrophilic regions.

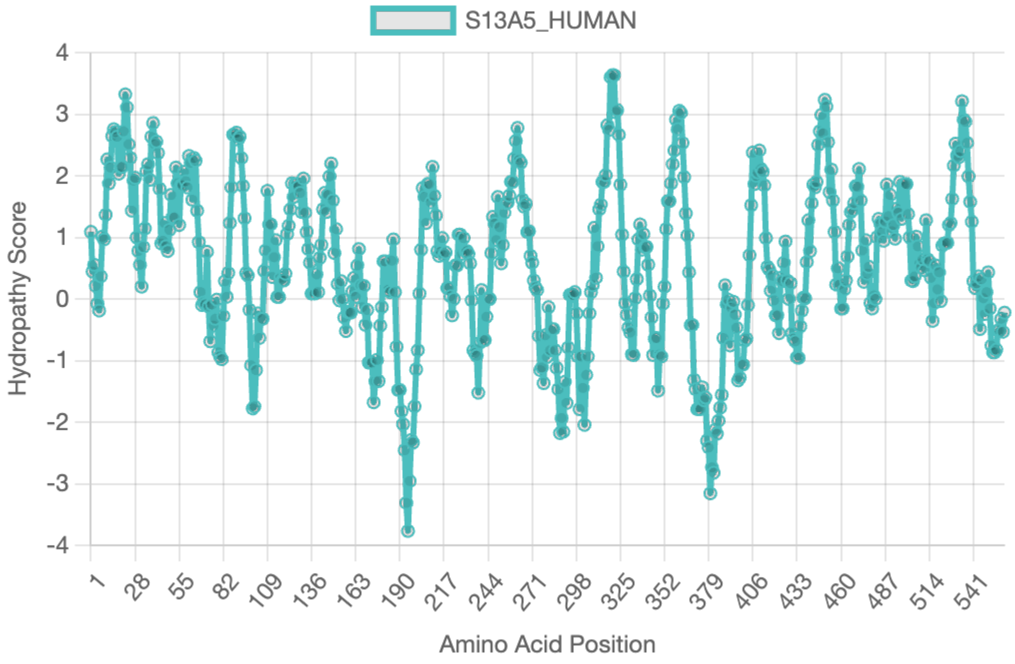

Kyte-Doolittle hydropathy plot for the sequence "S13A5_HUMAN". Positive values indicate hydrophobic regions, while negative values indicate hydrophilic regions.

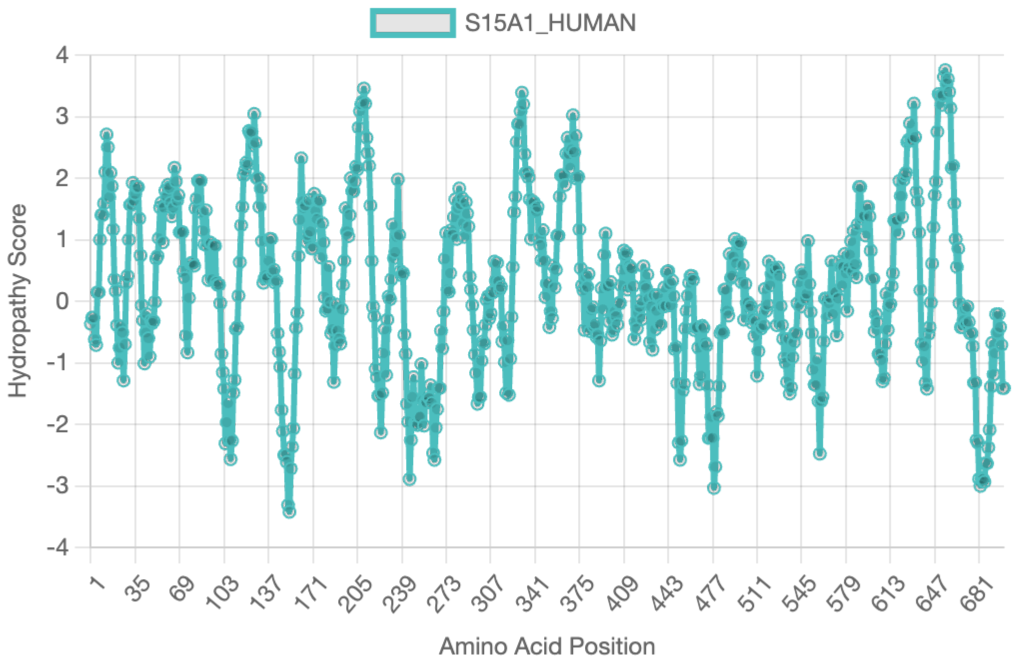

Kyte-Doolittle hydropathy plot for the sequence "S15A1_HUMAN". Positive values indicate hydrophobic regions, while negative values indicate hydrophilic regions.

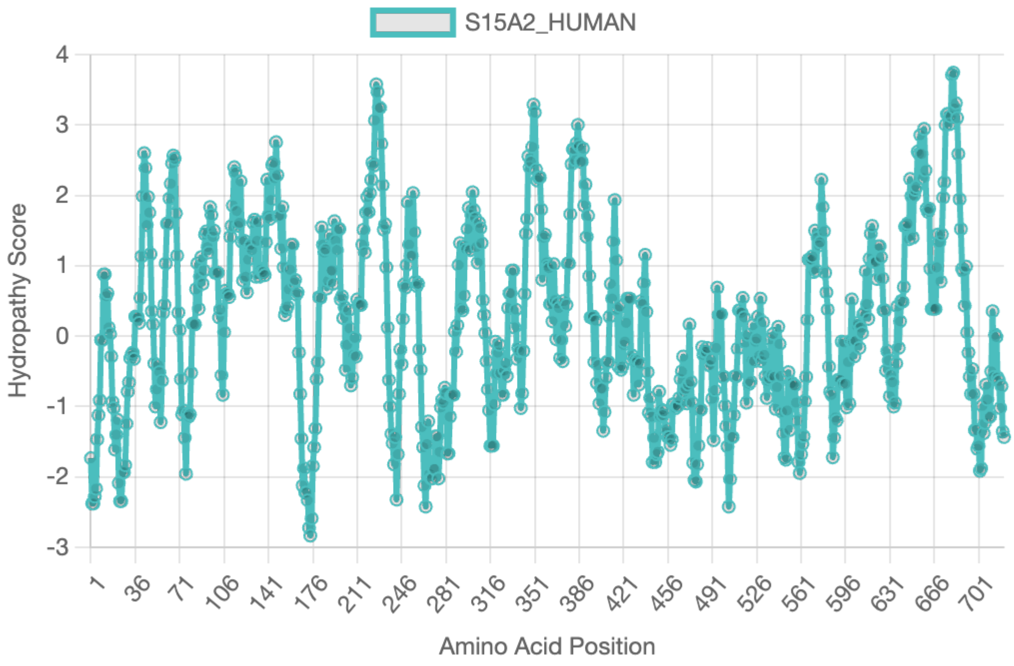

Kyte-Doolittle hydropathy plot for the sequence "S15A2_HUMAN". Positive values indicate hydrophobic regions, while negative values indicate hydrophilic regions.

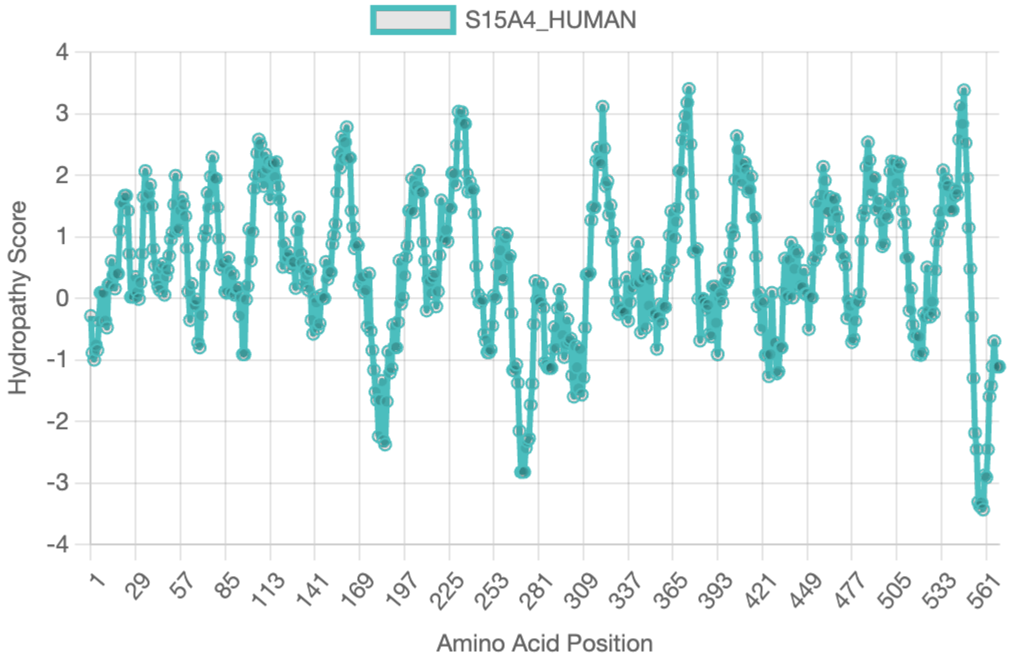

Kyte-Doolittle hydropathy plot for the sequence "S15A4_HUMAN". Positive values indicate hydrophobic regions, while negative values indicate hydrophilic regions.

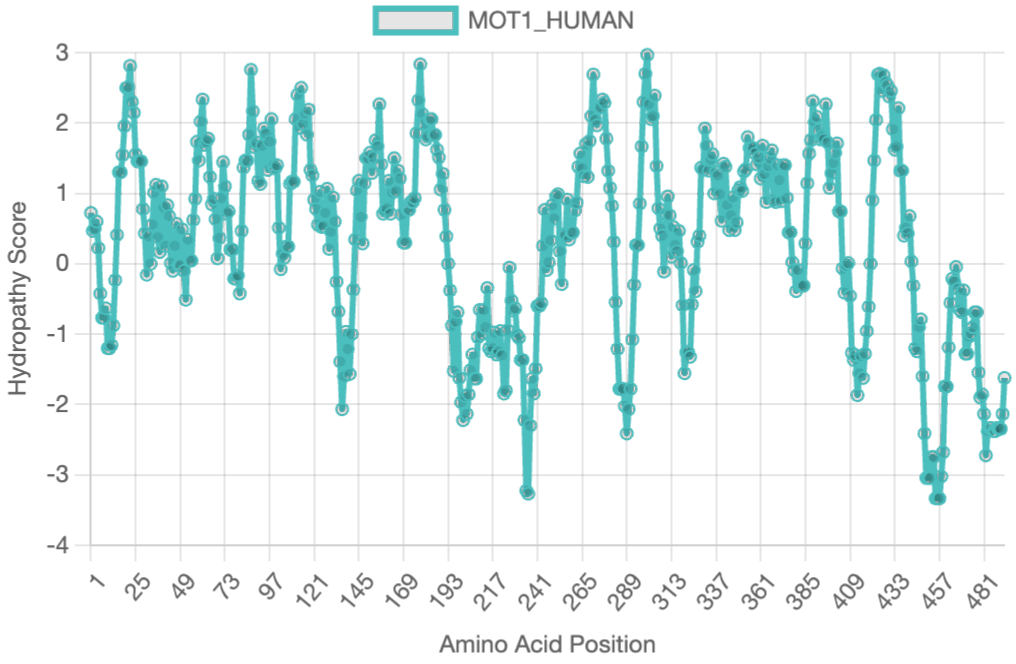

Kyte-Doolittle hydropathy plot for the sequence "MOT1_HUMAN". Positive values indicate hydrophobic regions, while negative values indicate hydrophilic regions.

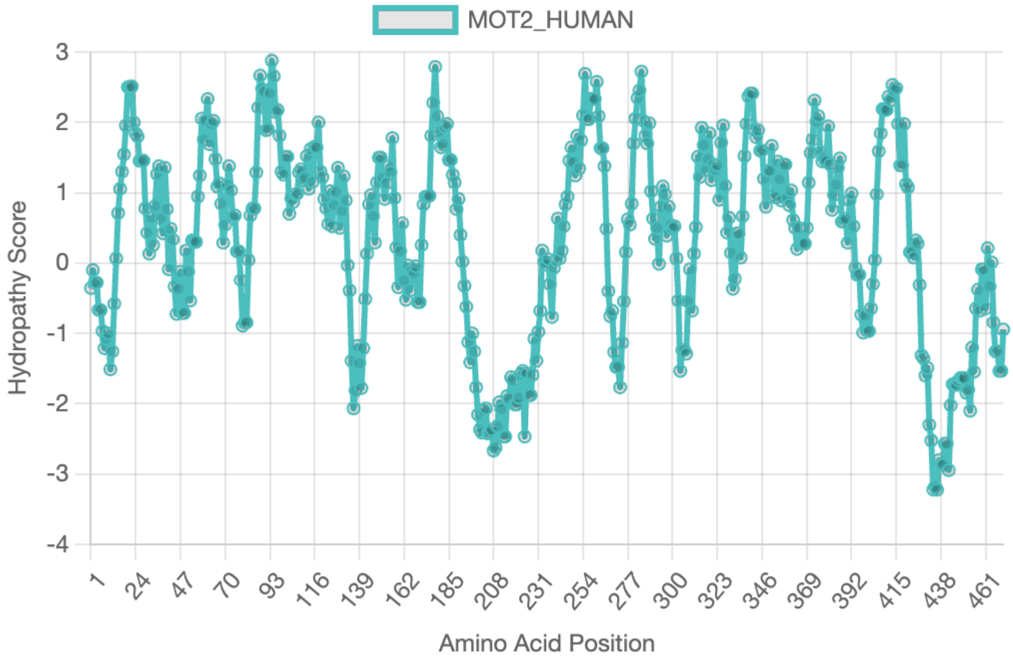

Kyte-Doolittle hydropathy plot for the sequence "MOT2_HUMAN". Positive values indicate hydrophobic regions, while negative values indicate hydrophilic regions.

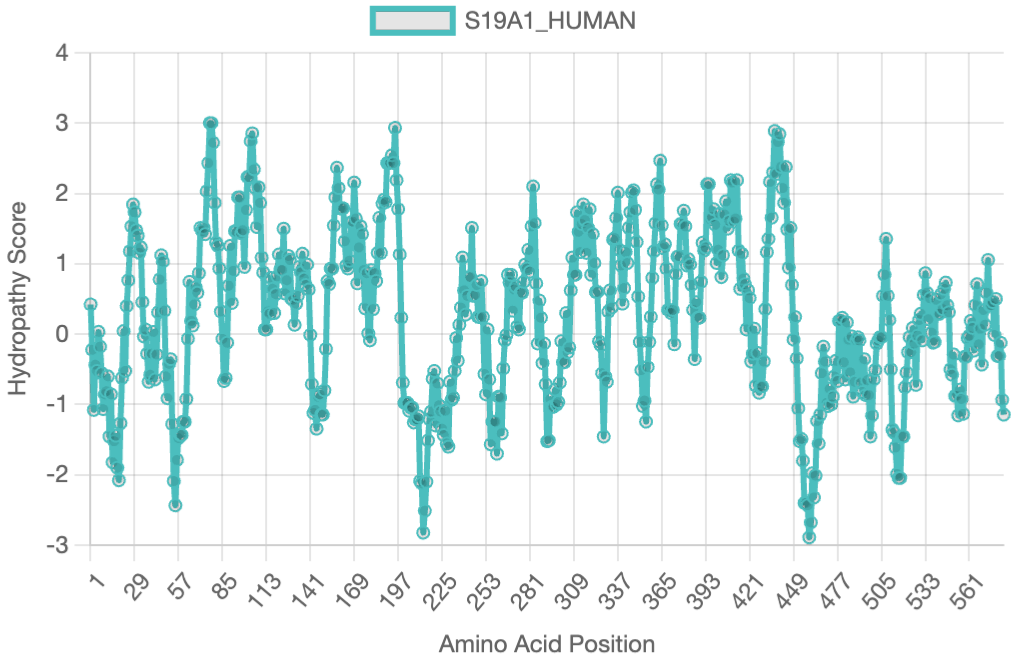

Kyte-Doolittle hydropathy plot for the sequence "S19A1_HUMAN". Positive values indicate hydrophobic regions, while negative values indicate hydrophilic regions.

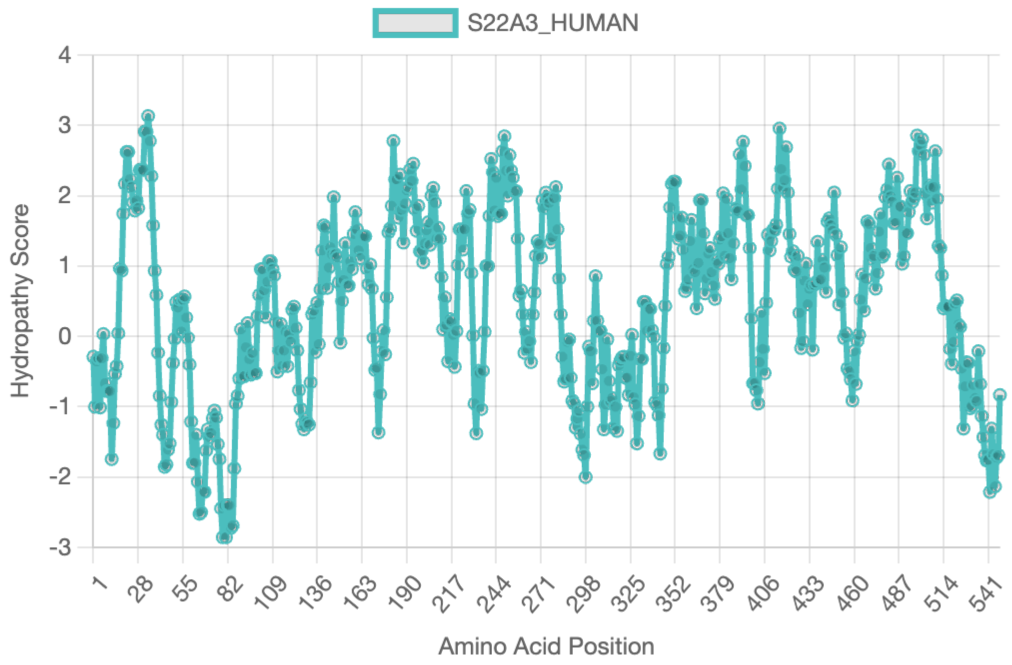

Kyte-Doolittle hydropathy plot for the sequence "S22A3_HUMAN". Positive values indicate hydrophobic regions, while negative values indicate hydrophilic regions.

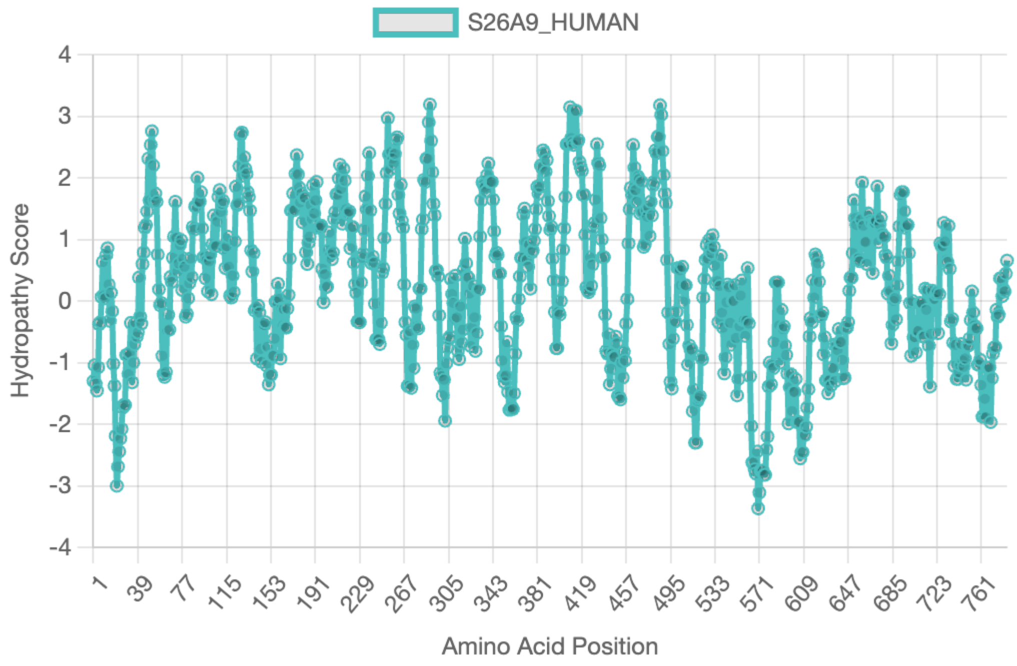

Kyte-Doolittle hydropathy plot for the sequence "S26A9_HUMAN". Positive values indicate hydrophobic regions, while negative values indicate hydrophilic regions.

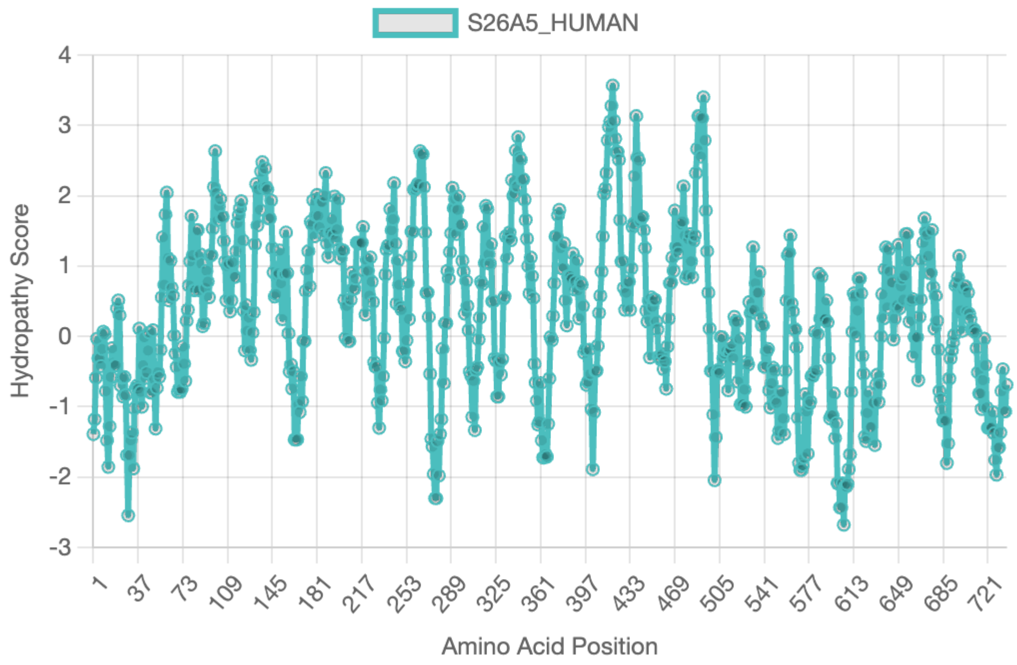

Kyte-Doolittle hydropathy plot for the sequence "S26A5_HUMAN". Positive values indicate hydrophobic regions, while negative values indicate hydrophilic regions.

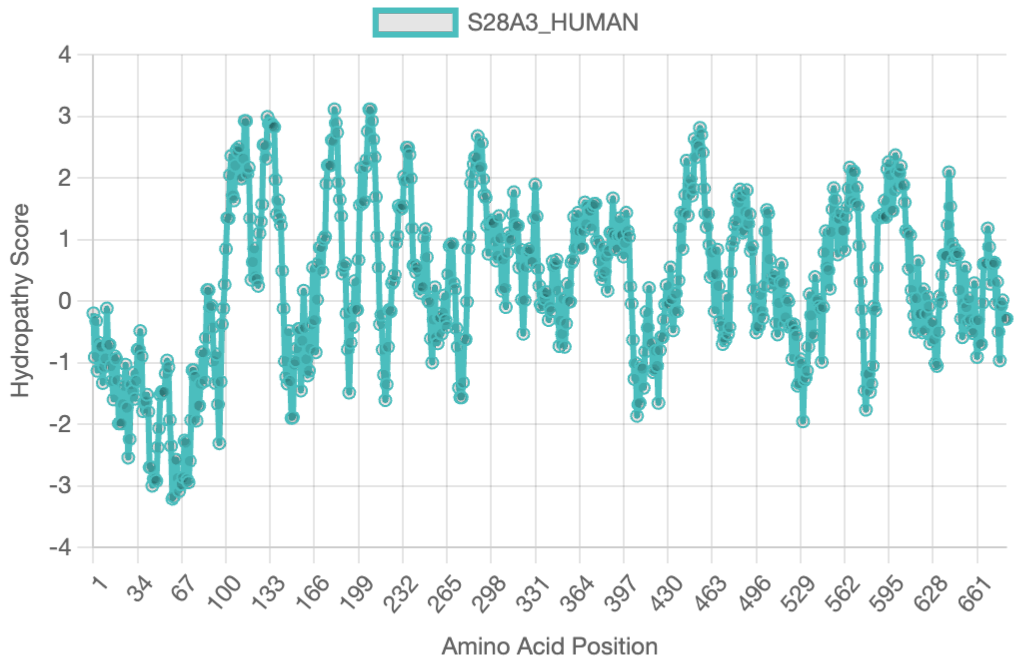

Kyte-Doolittle hydropathy plot for the sequence "S28A3_HUMAN". Positive values indicate hydrophobic regions, while negative values indicate hydrophilic regions.

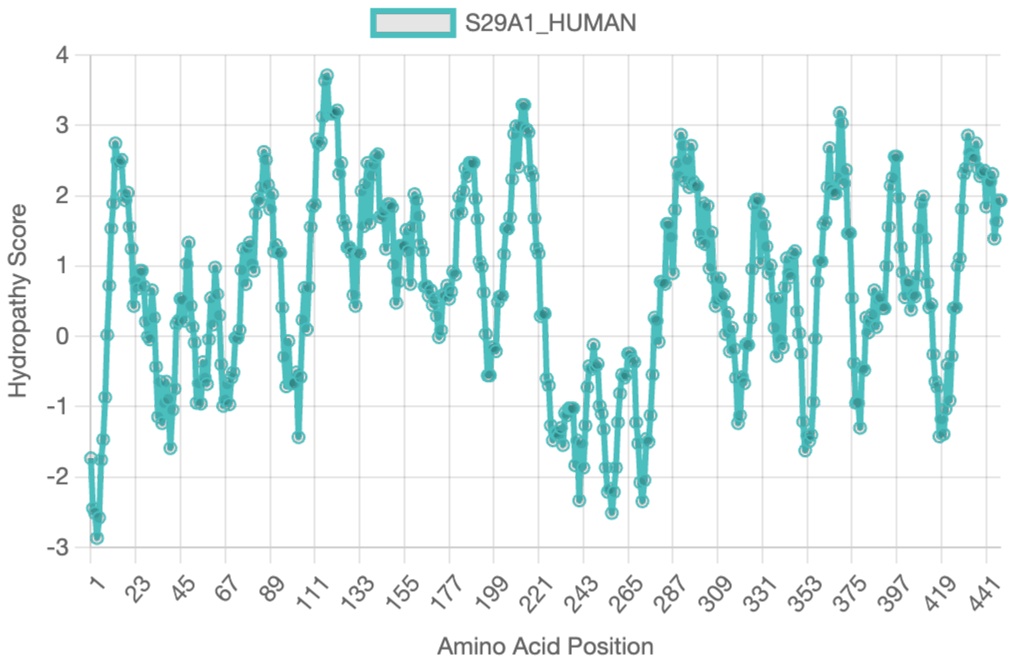

Kyte-Doolittle hydropathy plot for the sequence "S29A1_HUMAN". Positive values indicate hydrophobic regions, while negative values indicate hydrophilic regions.

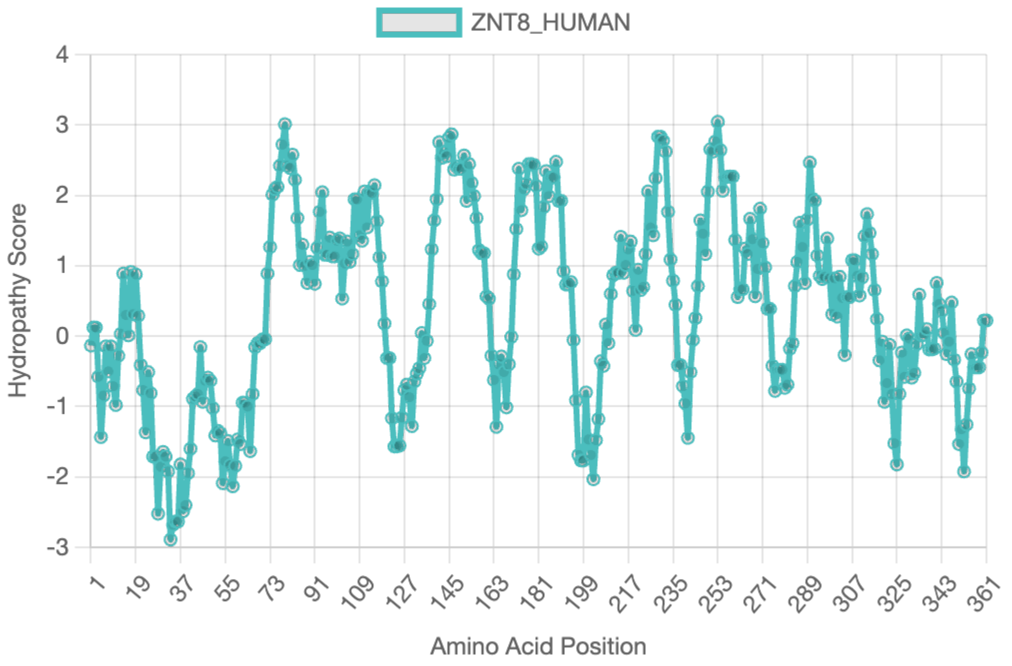

Kyte-Doolittle hydropathy plot for the sequence "ZNT8_HUMAN". Positive values indicate hydrophobic regions, while negative values indicate hydrophilic regions.

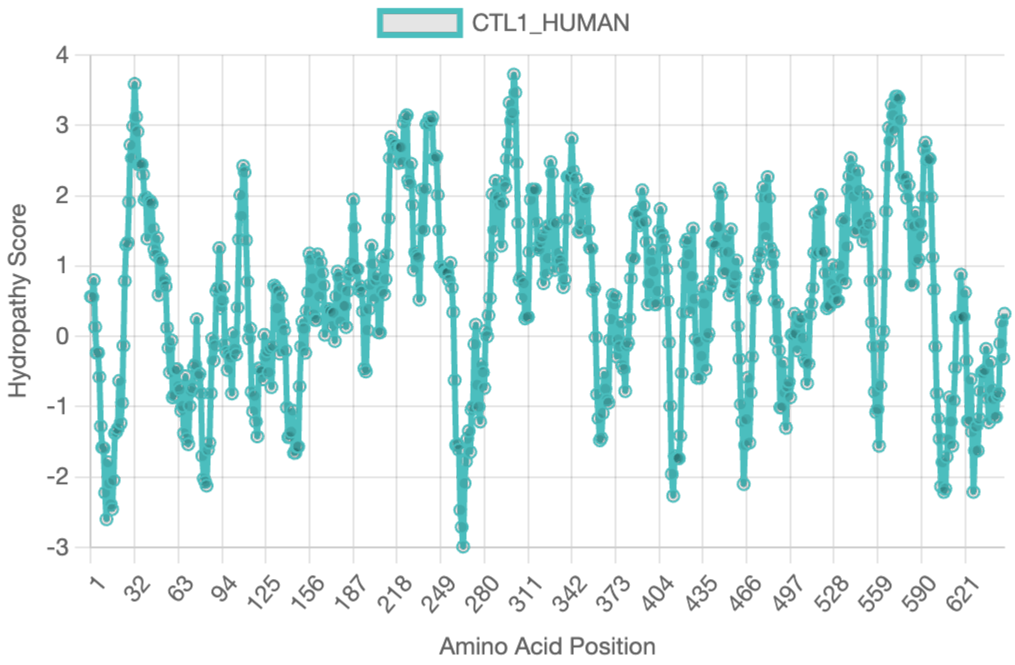

Kyte-Doolittle hydropathy plot for the sequence "CTL1_HUMAN". Positive values indicate hydrophobic regions, while negative values indicate hydrophilic regions.

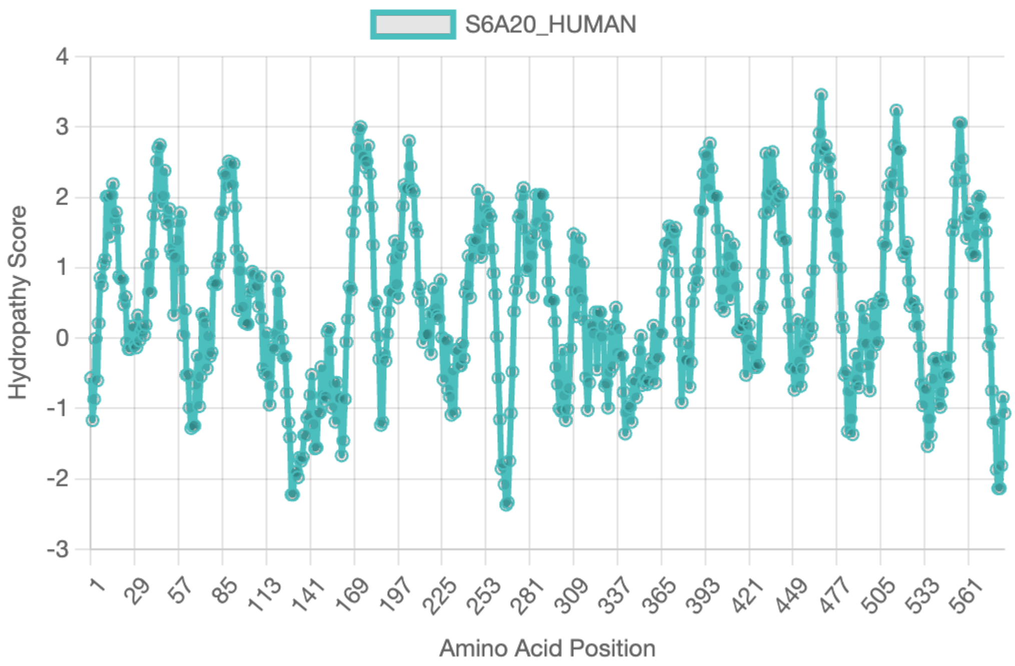

Kyte-Doolittle hydropathy plot for the sequence "S6A20_HUMAN". Positive values indicate hydrophobic regions, while negative values indicate hydrophilic regions.

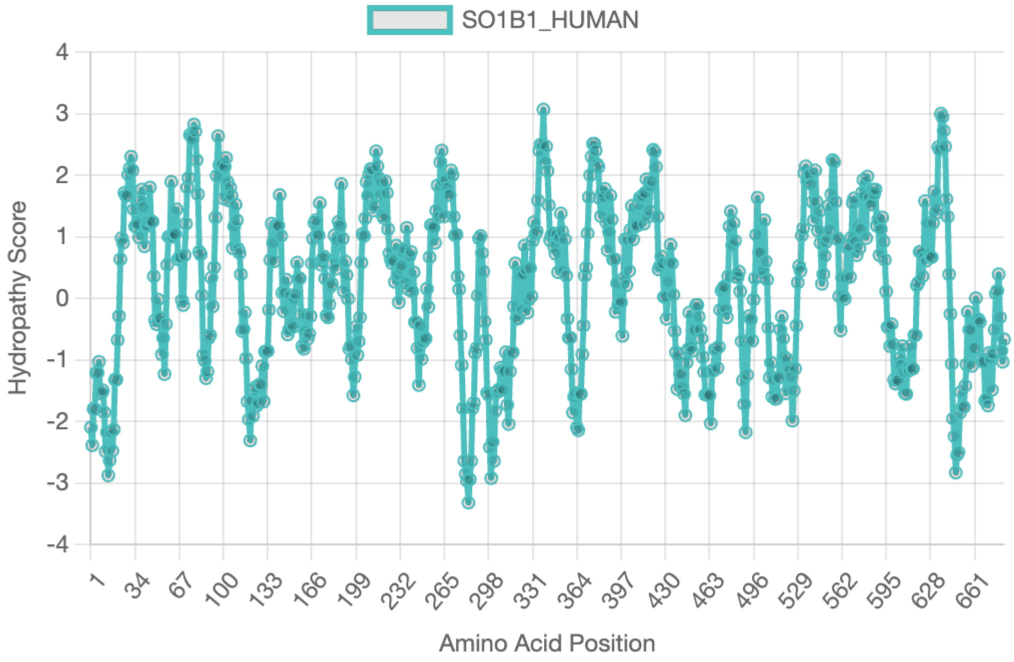

Kyte-Doolittle hydropathy plot for the sequence "SO1B1_HUMAN". Positive values indicate hydrophobic regions, while negative values indicate hydrophilic regions.

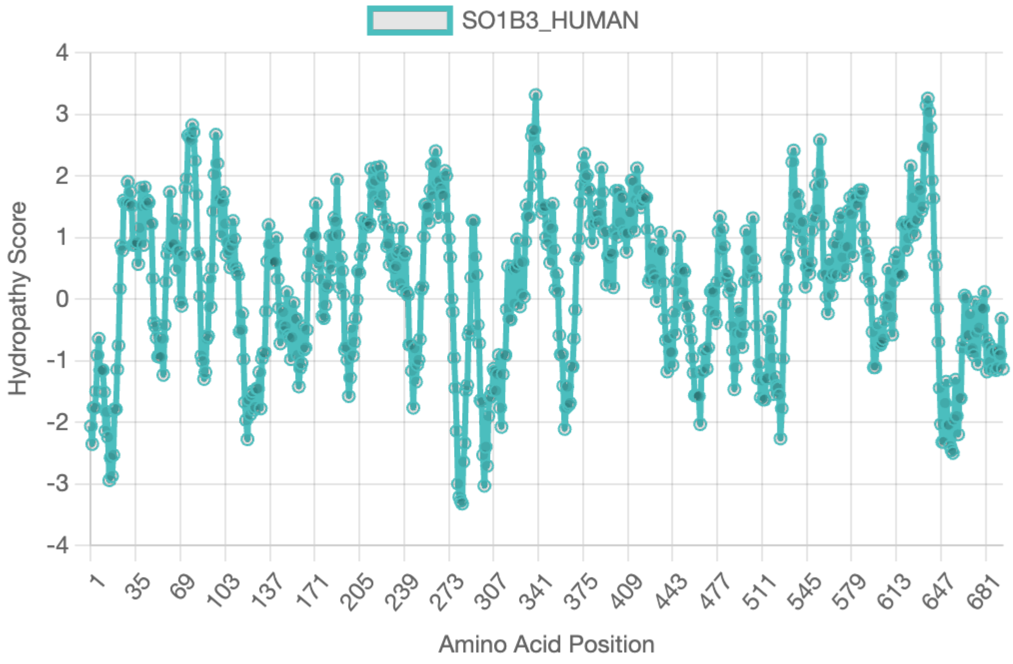

Kyte-Doolittle hydropathy plot for the sequence "SO1B3_HUMAN". Positive values indicate hydrophobic regions, while negative values indicate hydrophilic regions.

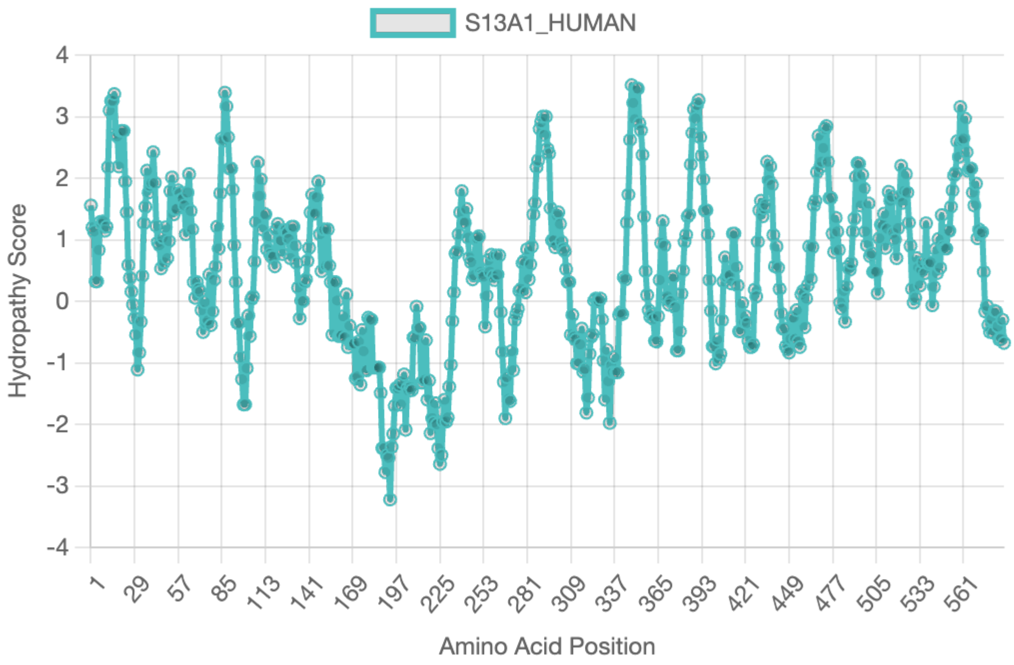

Kyte-Doolittle hydropathy plot for the sequence "S13A1_HUMAN". Positive values indicate hydrophobic regions, while negative values indicate hydrophilic regions.

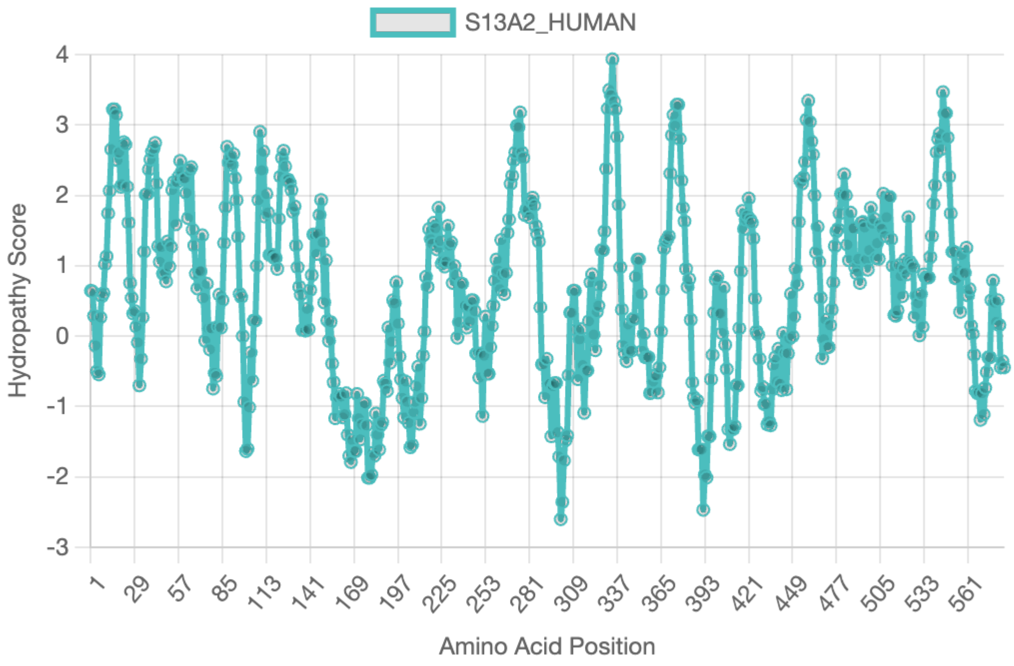

Kyte-Doolittle hydropathy plot for the sequence "S13A2_HUMAN". Positive values indicate hydrophobic regions, while negative values indicate hydrophilic regions.

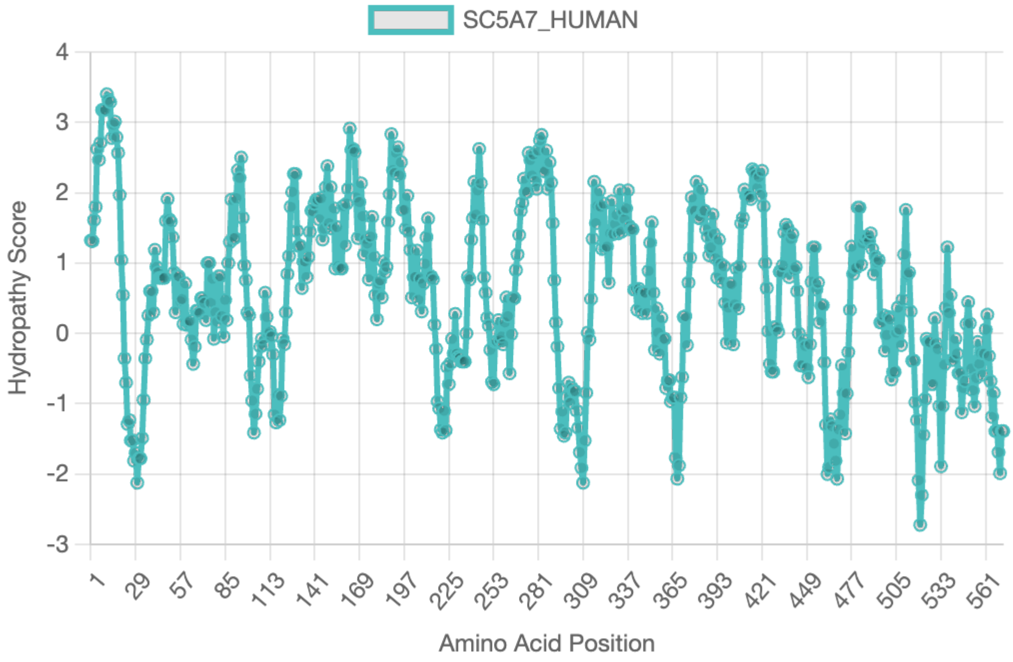

Kyte-Doolittle hydropathy plot for the sequence "SC5A7_HUMAN". Positive values indicate hydrophobic regions, while negative values indicate hydrophilic regions.

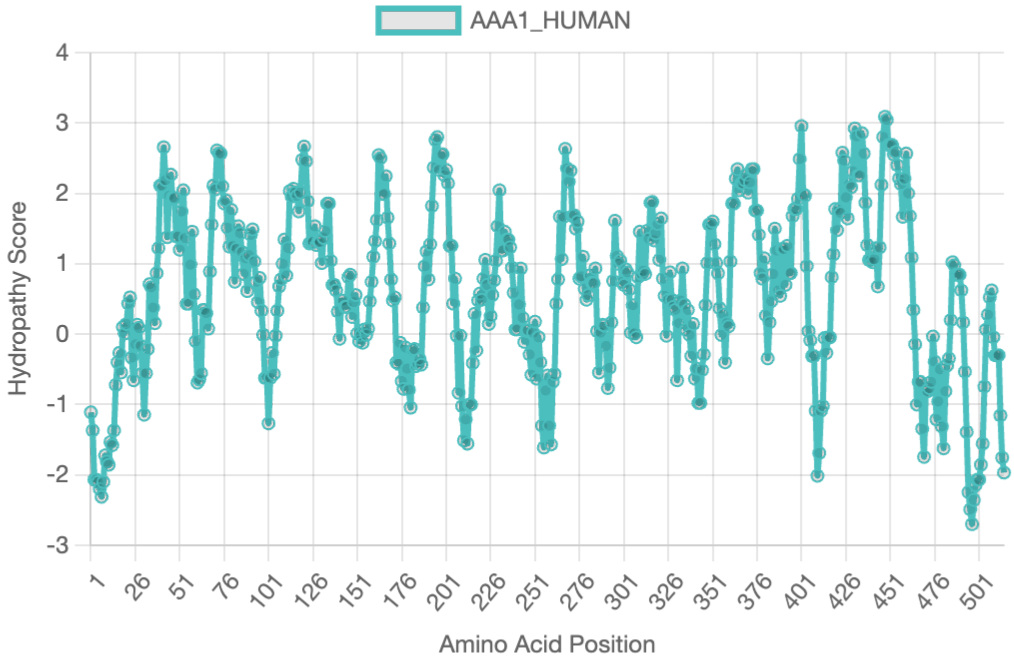

Kyte-Doolittle hydropathy plot for the sequence "AAA1_HUMAN". Positive values indicate hydrophobic regions, while negative values indicate hydrophilic regions.

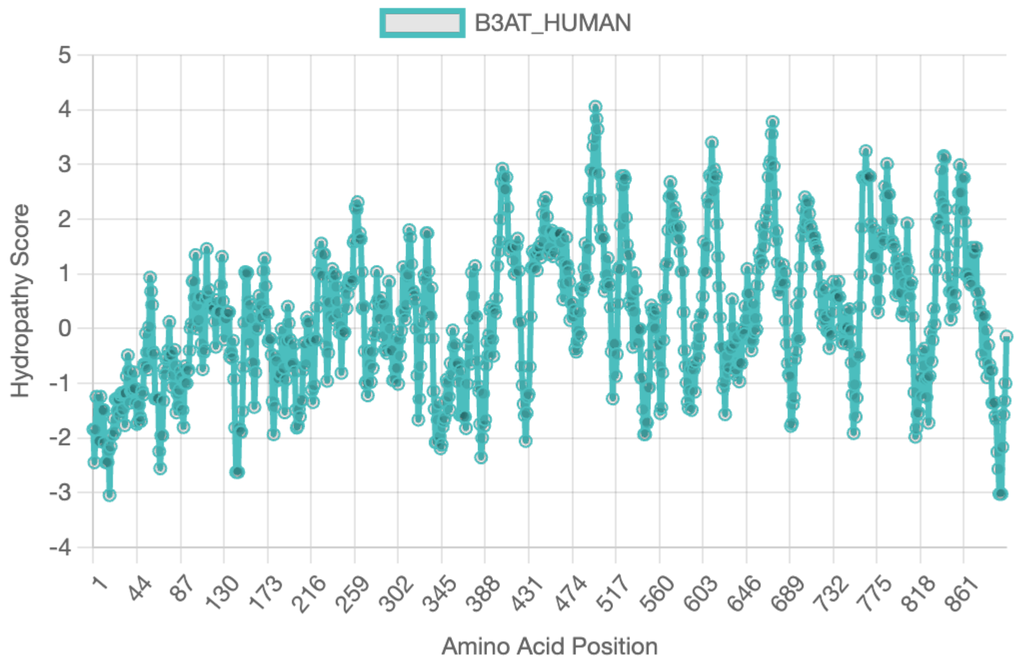

Kyte-Doolittle hydropathy plot for the sequence "B3AT_HUMAN". Positive values indicate hydrophobic regions, while negative values indicate hydrophilic regions.

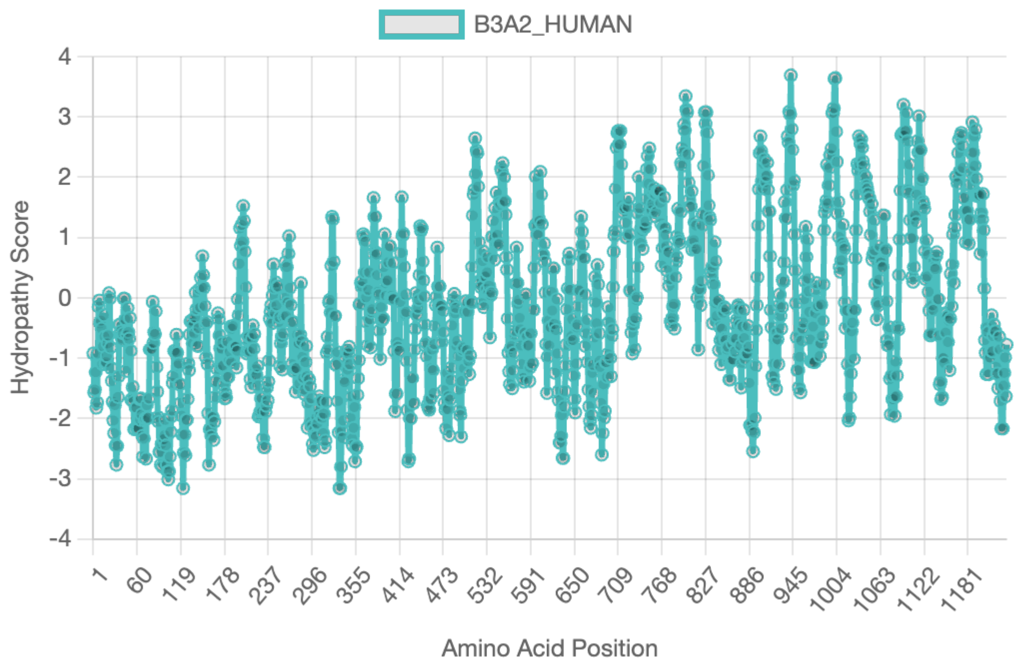

Kyte-Doolittle hydropathy plot for the sequence "B3A2_HUMAN". Positive values indicate hydrophobic regions, while negative values indicate hydrophilic regions.

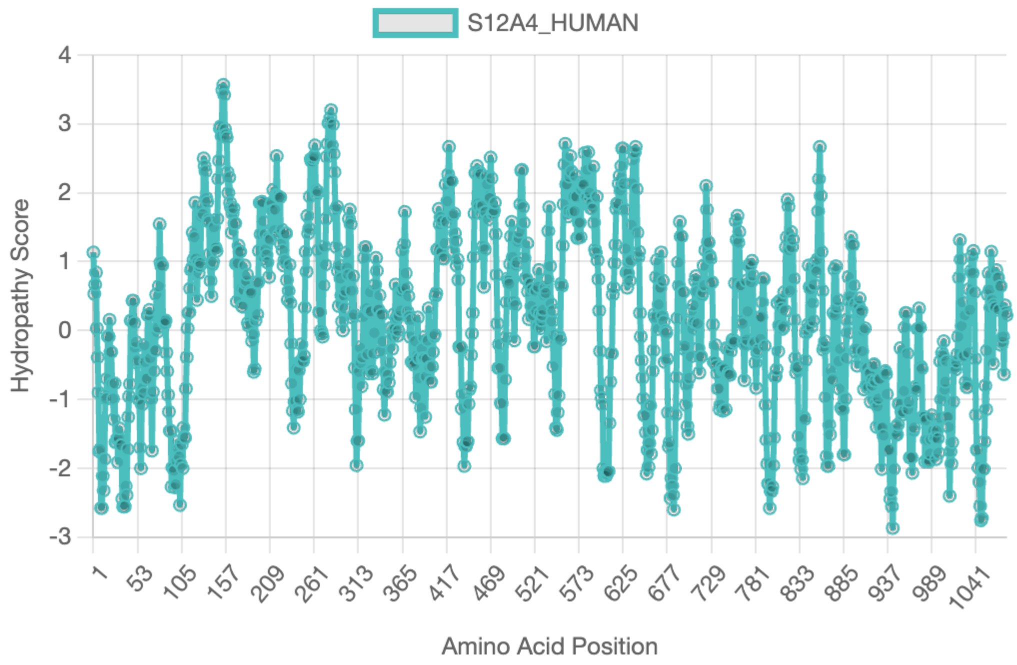

Kyte-Doolittle hydropathy plot for the sequence "S12A4_HUMAN". Positive values indicate hydrophobic regions, while negative values indicate hydrophilic regions.

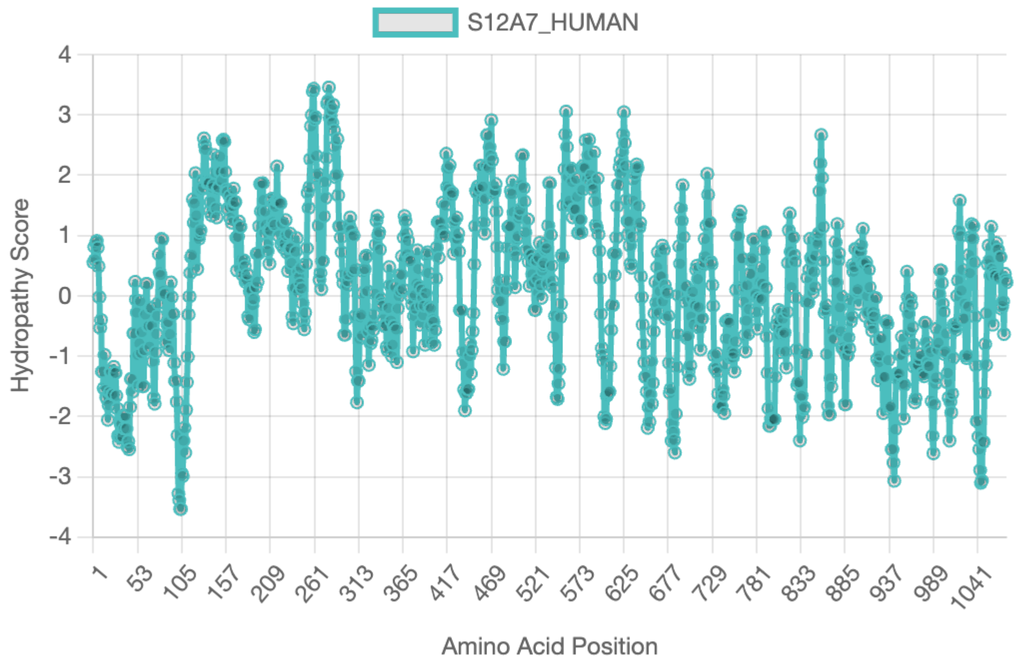

Kyte-Doolittle hydropathy plot for the sequence "S12A7_HUMAN". Positive values indicate hydrophobic regions, while negative values indicate hydrophilic regions.

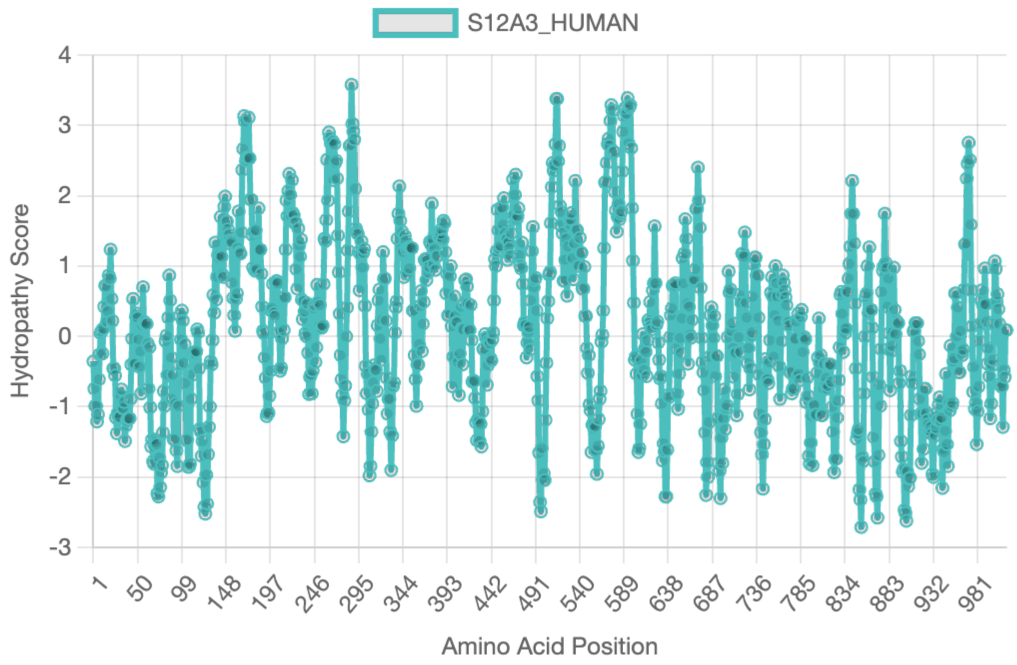

Kyte-Doolittle hydropathy plot for the sequence "S12A3_HUMAN". Positive values indicate hydrophobic regions, while negative values indicate hydrophilic regions.

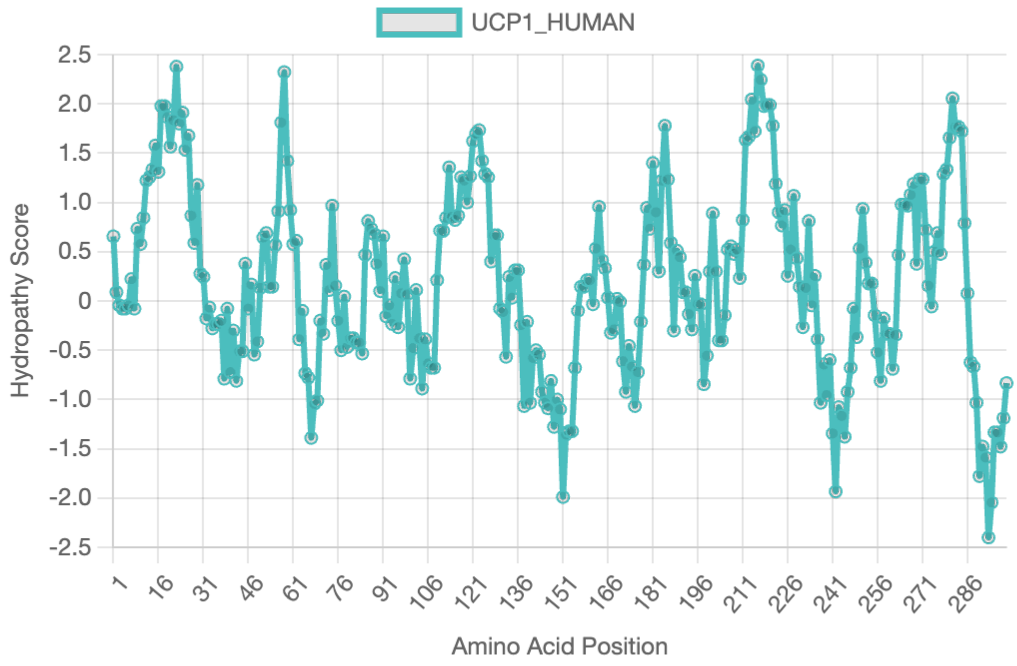

Kyte-Doolittle hydropathy plot for the sequence "UCP1_HUMAN". Positive values indicate hydrophobic regions, while negative values indicate hydrophilic regions.

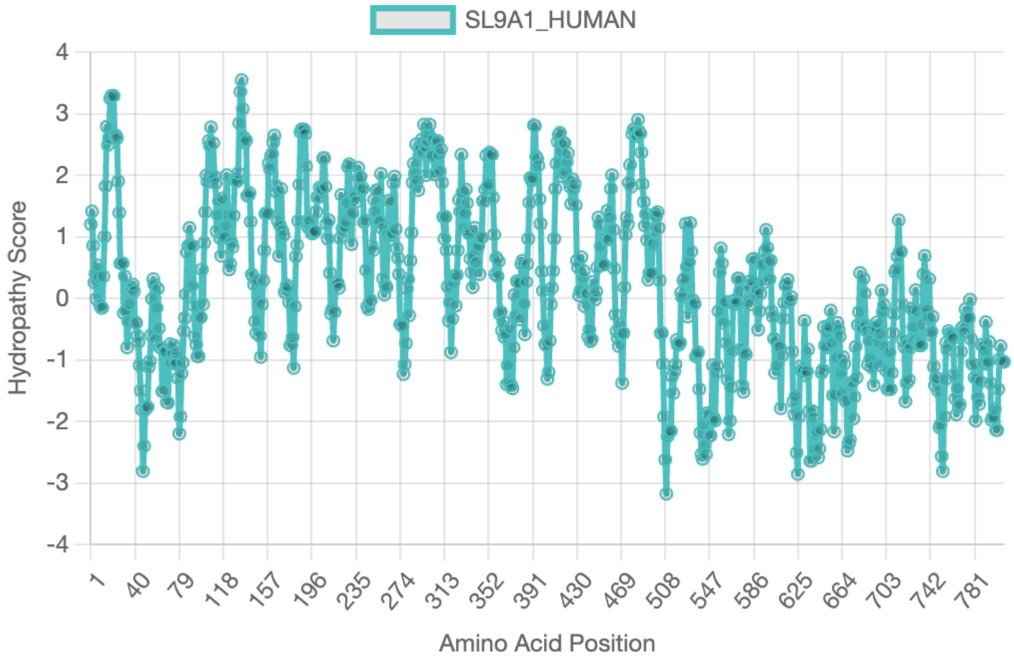

Kyte-Doolittle hydropathy plot for the sequence "SL9A1_HUMAN". Positive values indicate hydrophobic regions, while negative values indicate hydrophilic regions.

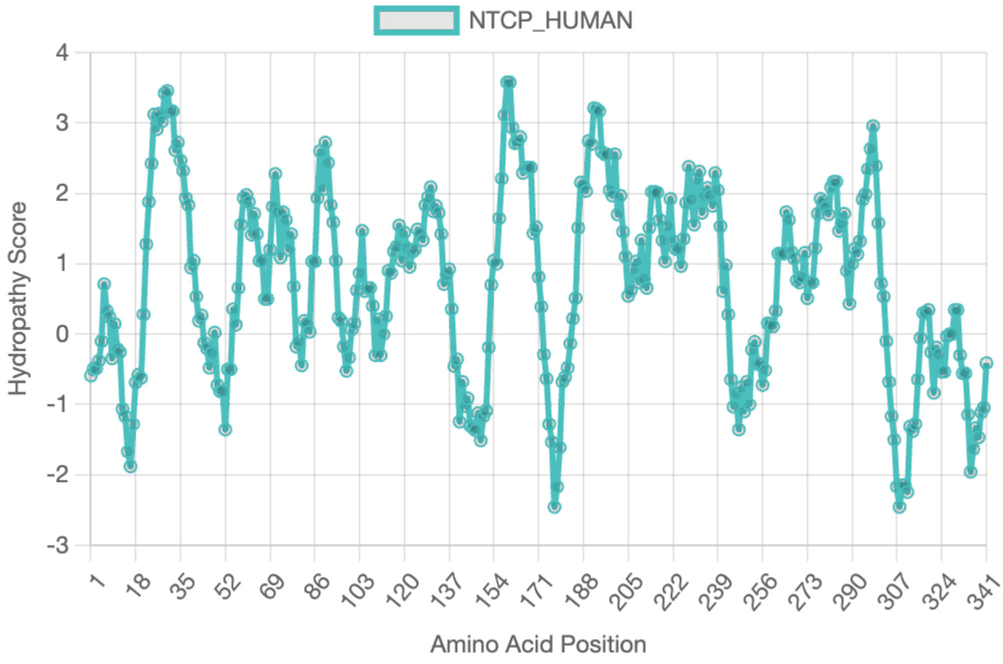

Kyte-Doolittle hydropathy plot for the sequence "NTCP_HUMAN". Positive values indicate hydrophobic regions, while negative values indicate hydrophilic regions.

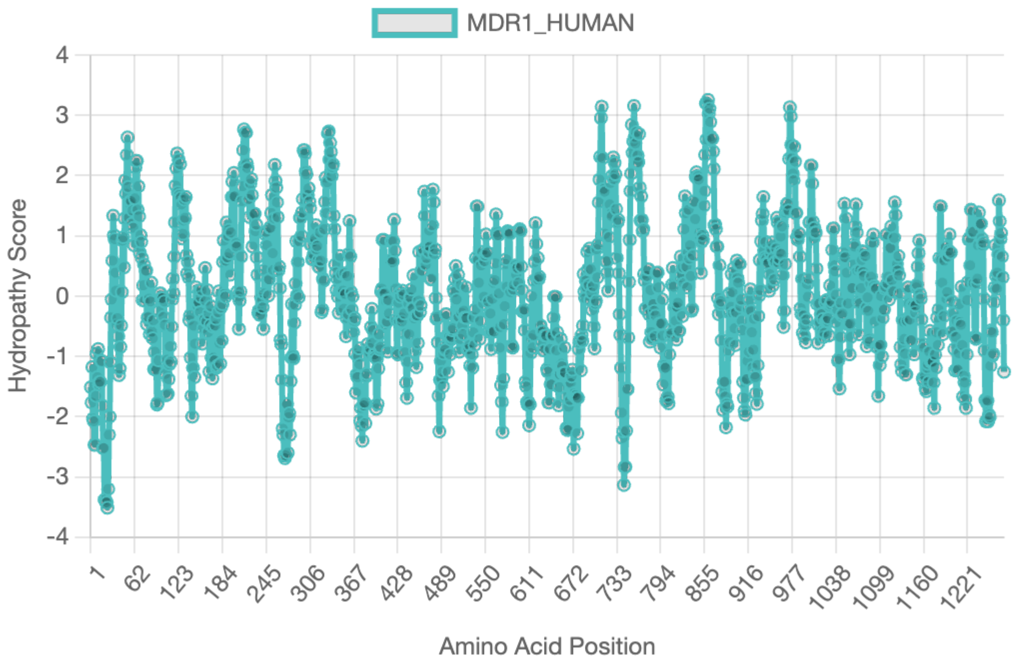

Kyte-Doolittle hydropathy plot for the sequence "MDR1_HUMAN". Positive values indicate hydrophobic regions, while negative values indicate hydrophilic regions.

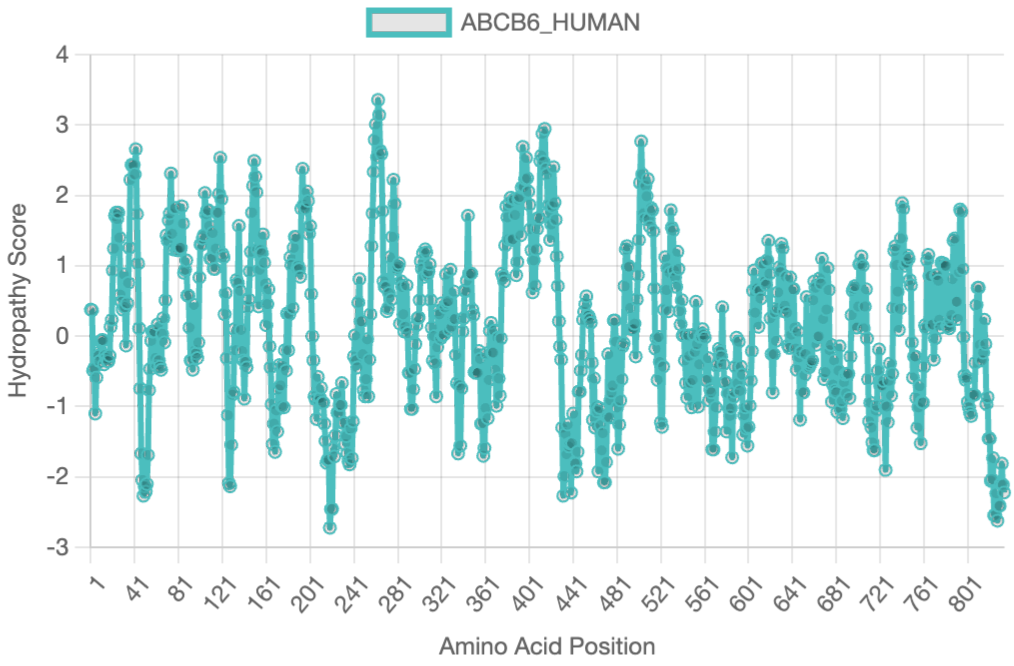

Kyte-Doolittle hydropathy plot for the sequence "ABCB6_HUMAN". Positive values indicate hydrophobic regions, while negative values indicate hydrophilic regions.

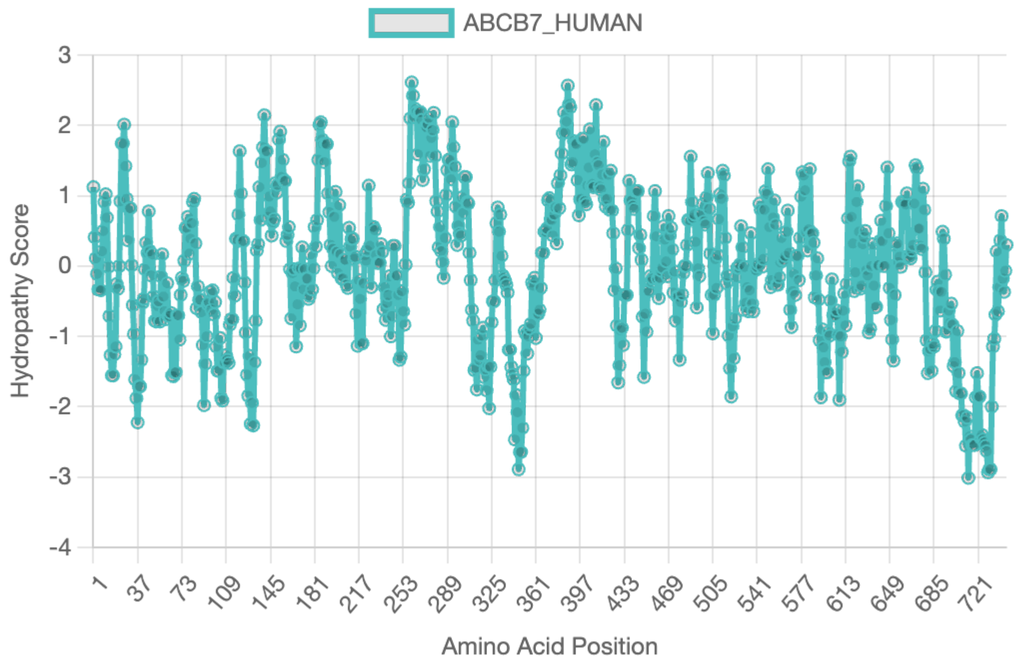

Kyte-Doolittle hydropathy plot for the sequence "ABCB7_HUMAN". Positive values indicate hydrophobic regions, while negative values indicate hydrophilic regions.

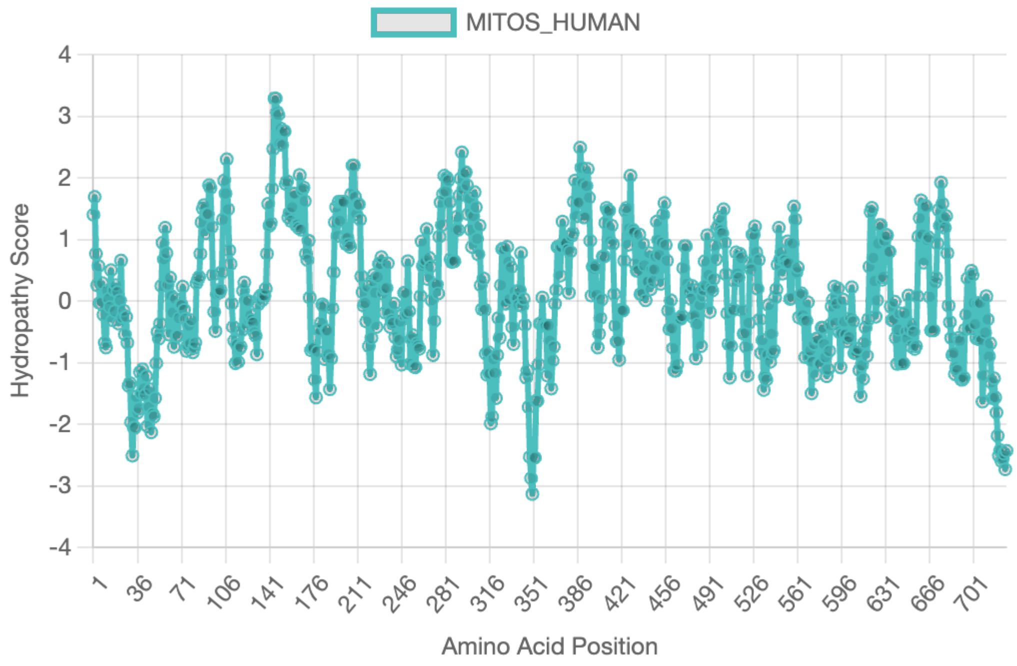

Kyte-Doolittle hydropathy plot for the sequence "MITOS_HUMAN". Positive values indicate hydrophobic regions, while negative values indicate hydrophilic regions.

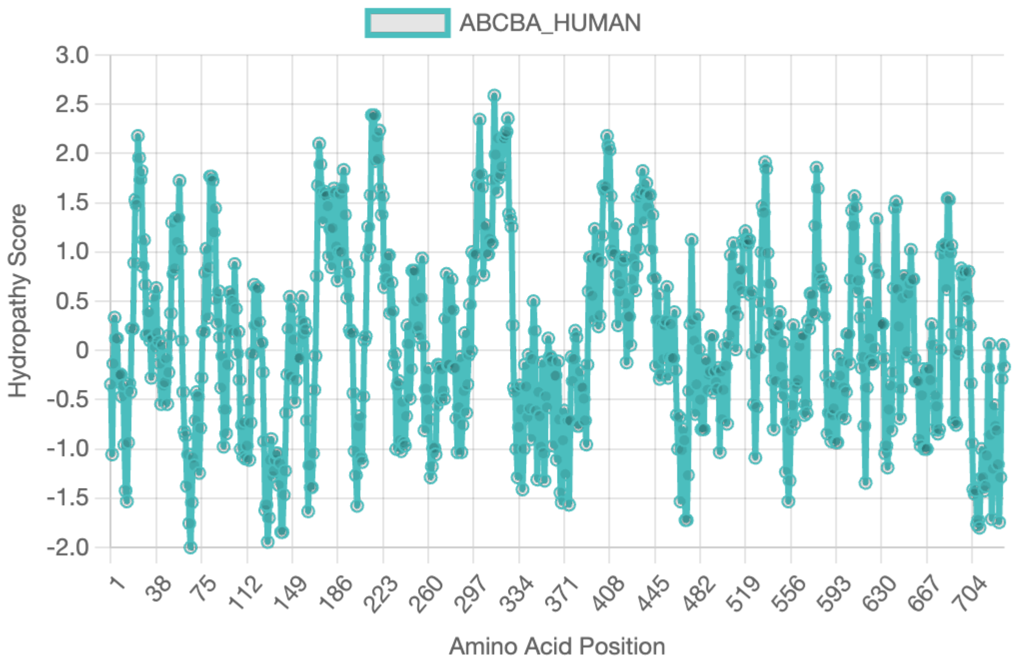

Kyte-Doolittle hydropathy plot for the sequence "ABCBA_HUMAN". Positive values indicate hydrophobic regions, while negative values indicate hydrophilic regions.

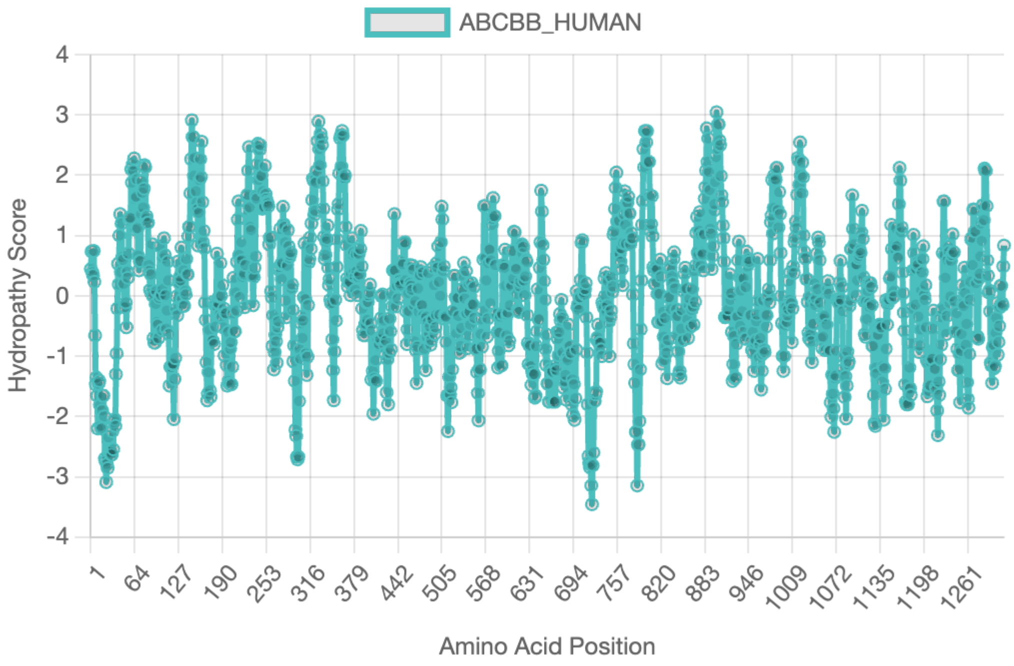

Kyte-Doolittle hydropathy plot for the sequence "ABCBB_HUMAN". Positive values indicate hydrophobic regions, while negative values indicate hydrophilic regions.

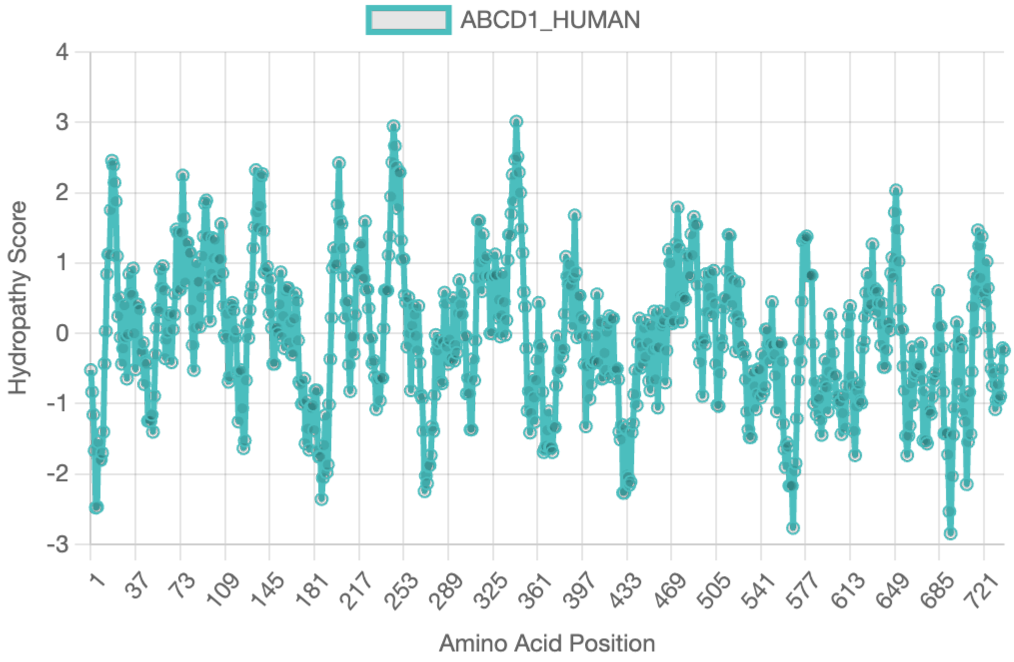

Kyte-Doolittle hydropathy plot for the sequence "ABCD1_HUMAN". Positive values indicate hydrophobic regions, while negative values indicate hydrophilic regions.

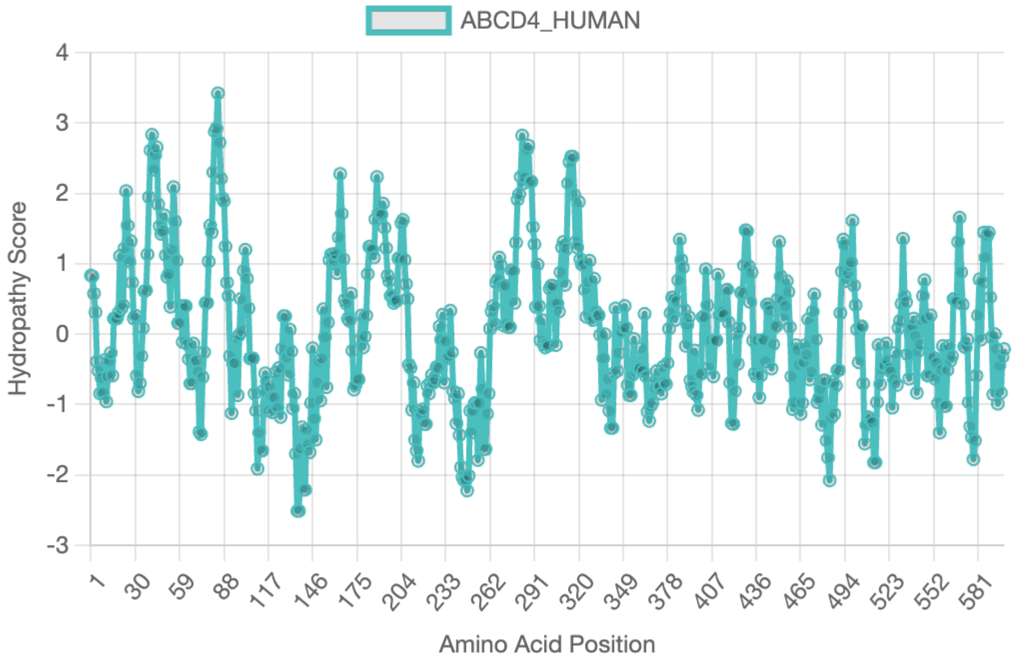

Kyte-Doolittle hydropathy plot for the sequence "ABCD4_HUMAN". Positive values indicate hydrophobic regions, while negative values indicate hydrophilic regions.

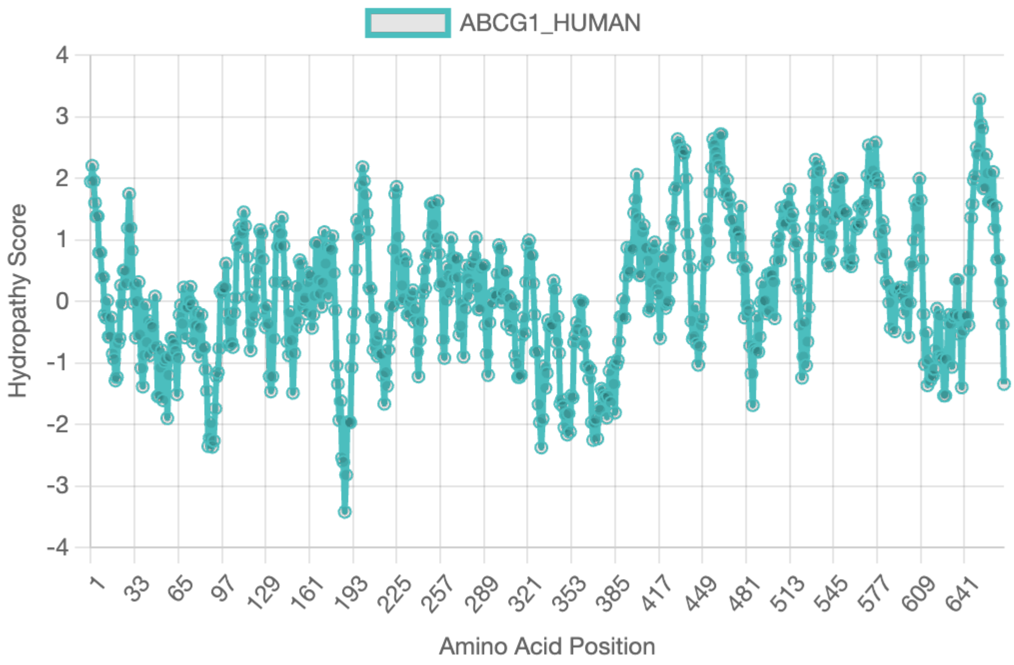

Kyte-Doolittle hydropathy plot for the sequence "ABCG1_HUMAN". Positive values indicate hydrophobic regions, while negative values indicate hydrophilic regions.

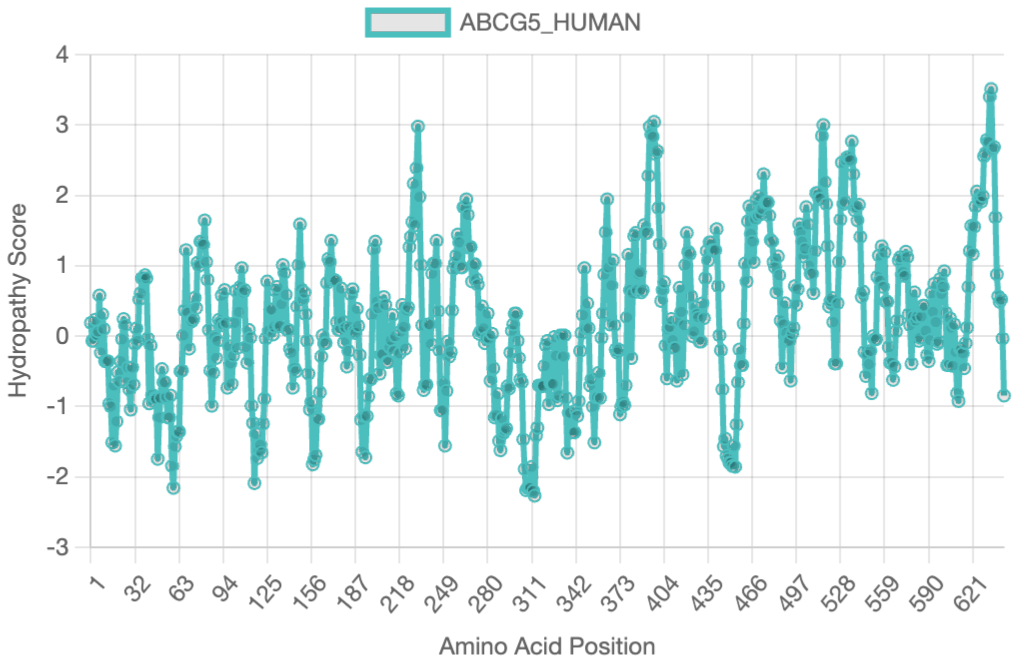

Kyte-Doolittle hydropathy plot for the sequence "ABCG5_HUMAN". Positive values indicate hydrophobic regions, while negative values indicate hydrophilic regions.

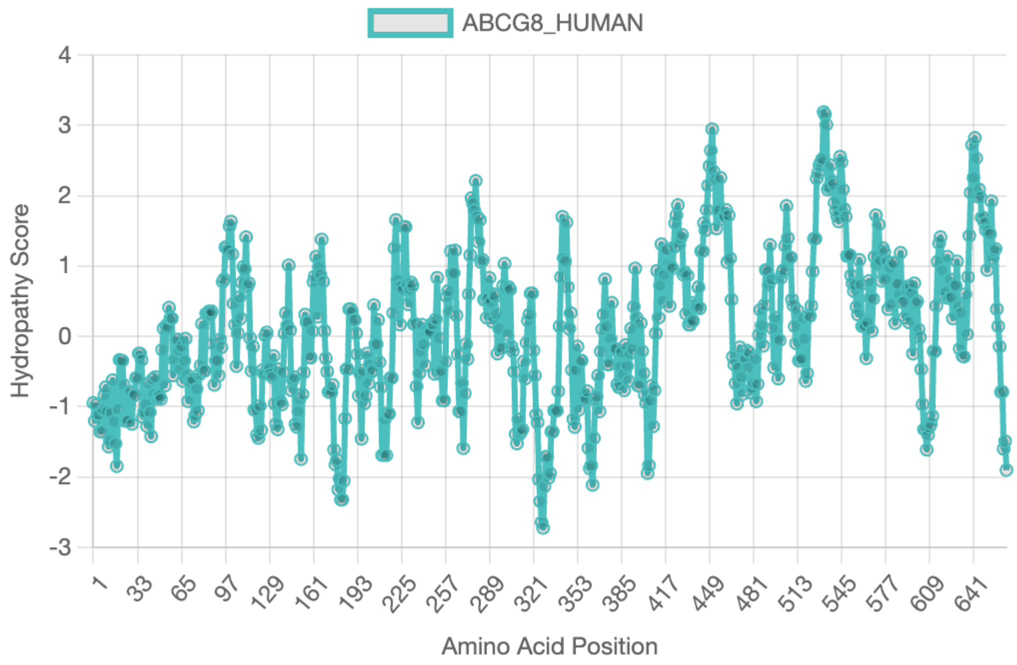

Kyte-Doolittle hydropathy plot for the sequence "ABCG8_HUMAN". Positive values indicate hydrophobic regions, while negative values indicate hydrophilic regions.

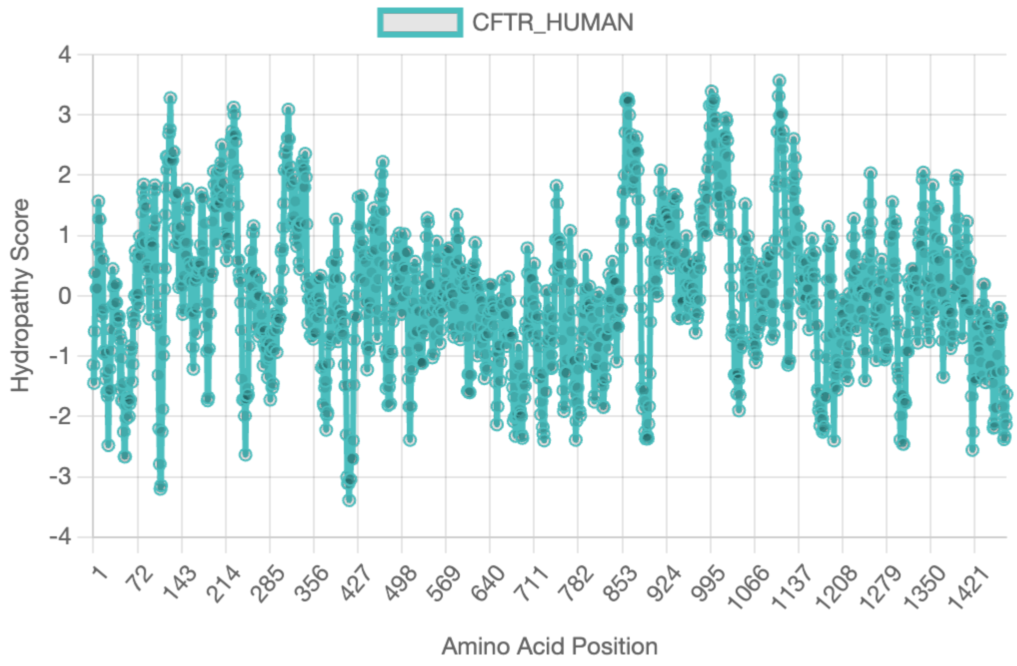

Kyte-Doolittle hydropathy plot for the sequence "CFTR_HUMAN". Positive values indicate hydrophobic regions, while negative values indicate hydrophilic regions.

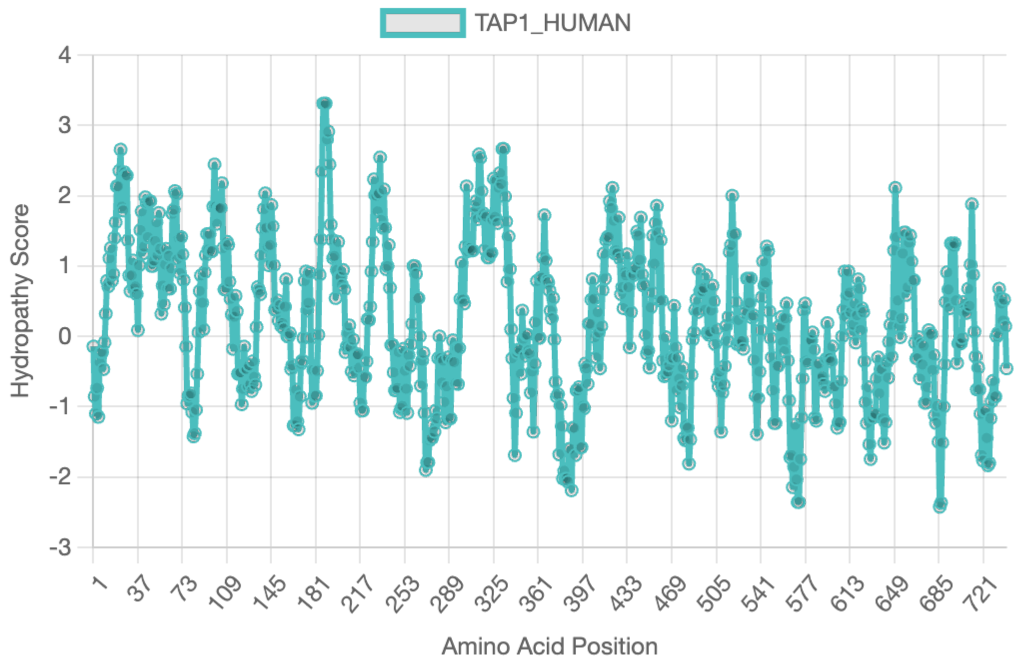

Kyte-Doolittle hydropathy plot for the sequence "TAP1_HUMAN". Positive values indicate hydrophobic regions, while negative values indicate hydrophilic regions.

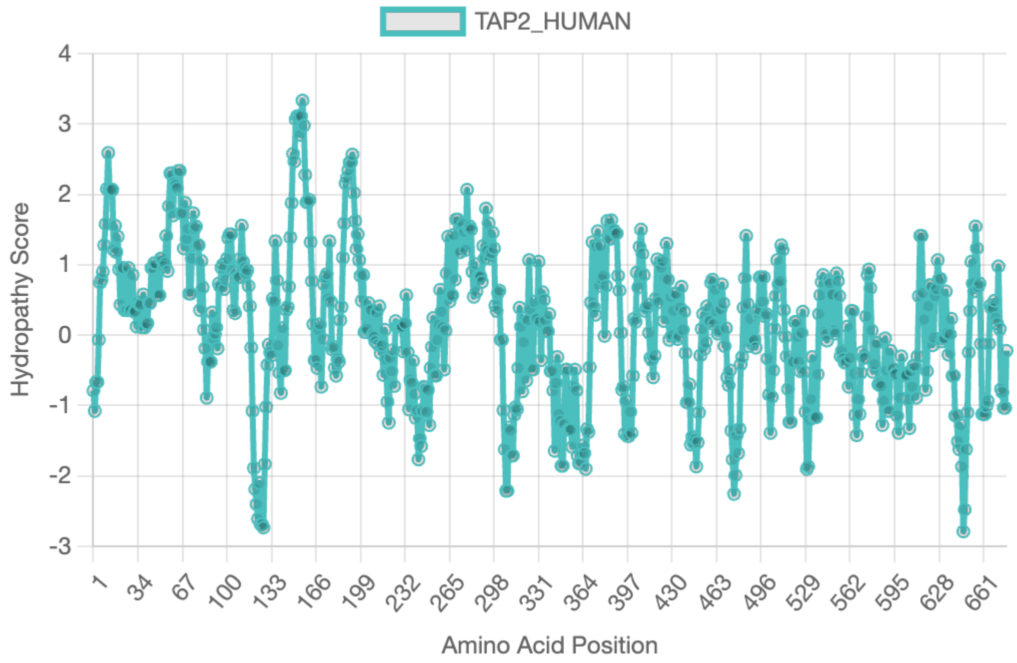

Kyte-Doolittle hydropathy plot for the sequence "TAP2_HUMAN". Positive values indicate hydrophobic regions, while negative values indicate hydrophilic regions.

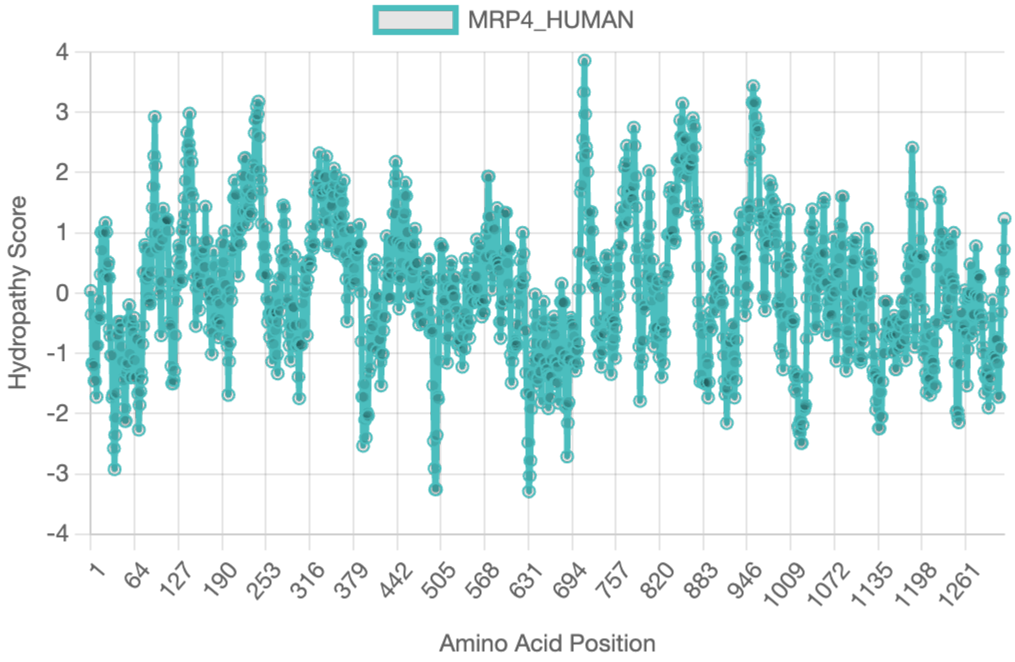

Kyte-Doolittle hydropathy plot for the sequence "MRP4_HUMAN". Positive values indicate hydrophobic regions, while negative values indicate hydrophilic regions.

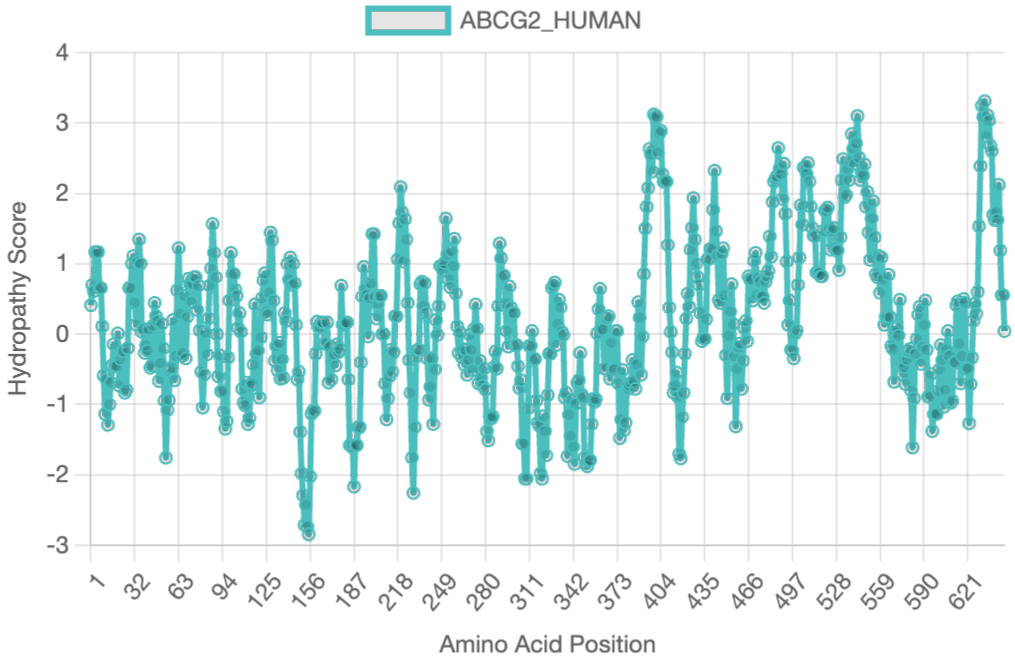

Kyte-Doolittle hydropathy plot for the sequence "ABCG2_HUMAN". Positive values indicate hydrophobic regions, while negative values indicate hydrophilic regions.

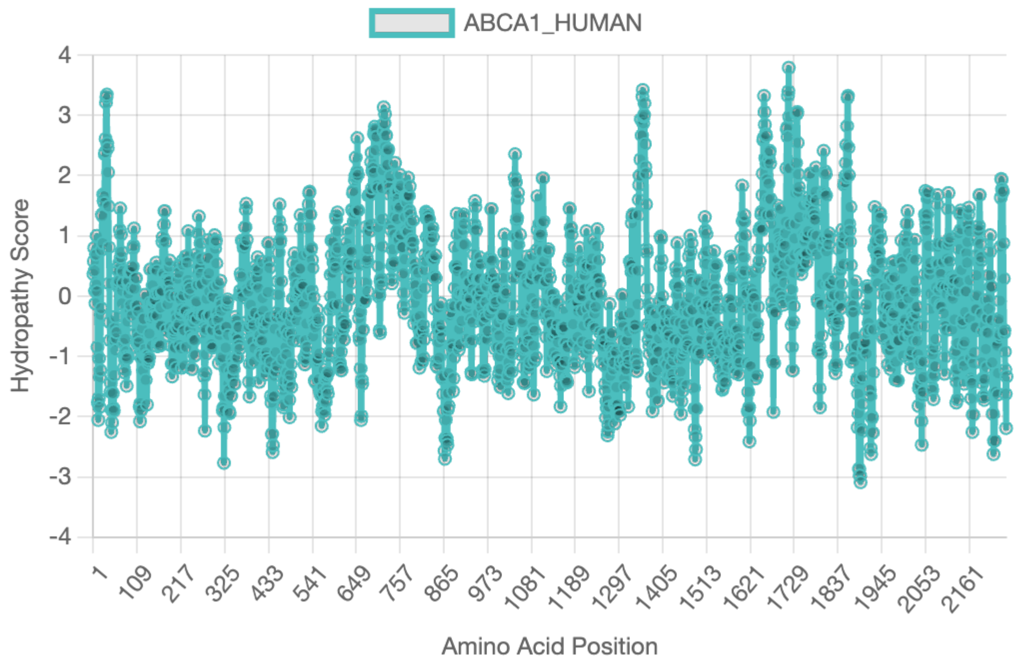

Kyte-Doolittle hydropathy plot for the sequence "ABCA1_HUMAN". Positive values indicate hydrophobic regions, while negative values indicate hydrophilic regions.

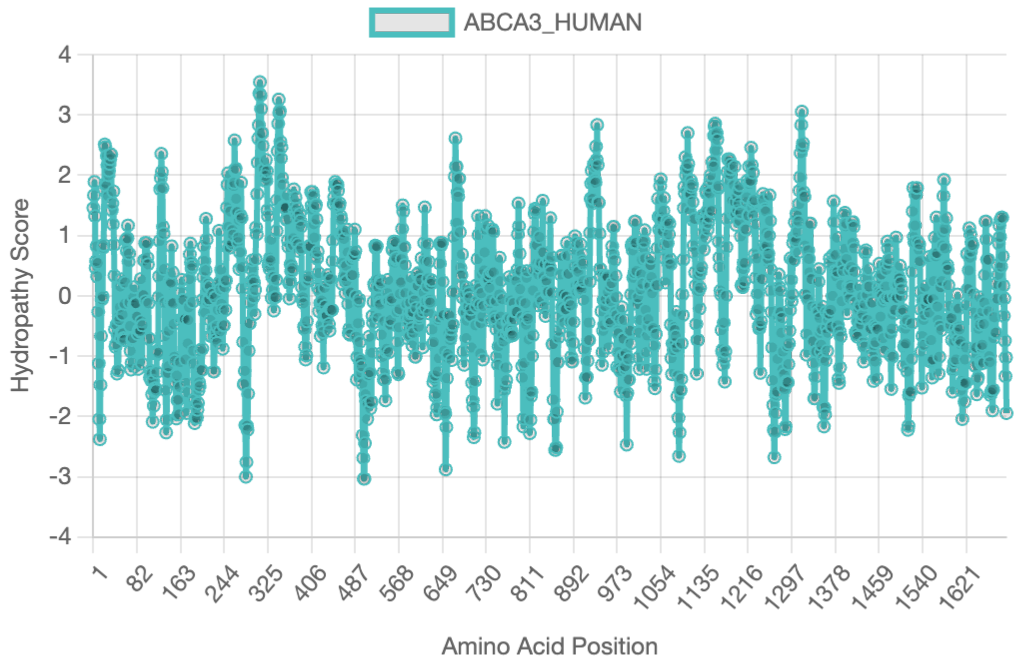

Kyte-Doolittle hydropathy plot for the sequence "ABCA3_HUMAN". Positive values indicate hydrophobic regions, while negative values indicate hydrophilic regions.

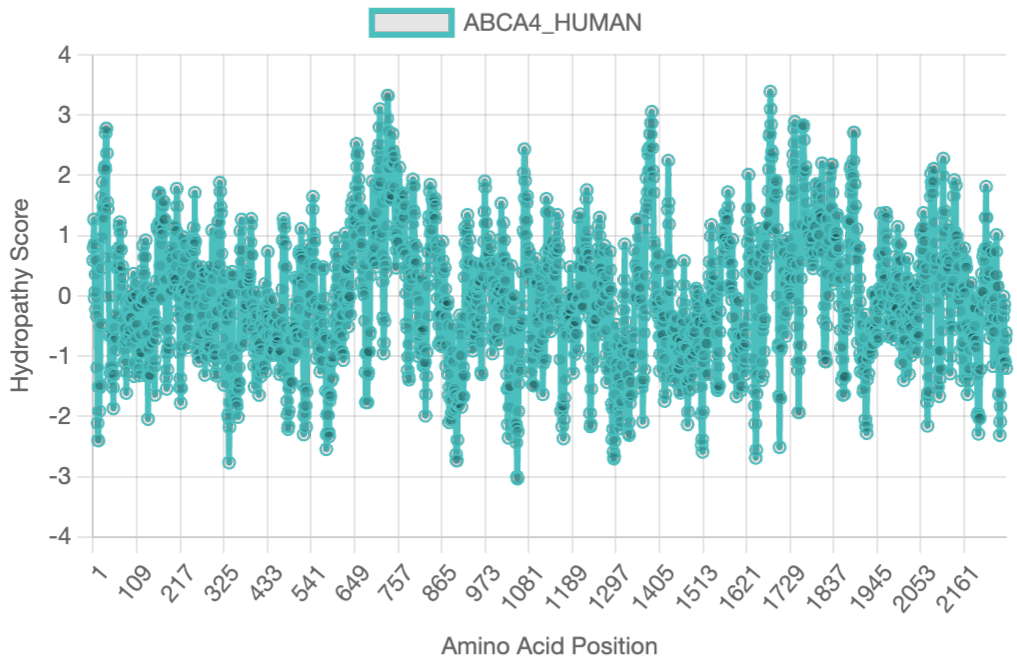

Kyte-Doolittle hydropathy plot for the sequence "ABCA4_HUMAN". Positive values indicate hydrophobic regions, while negative values indicate hydrophilic regions.

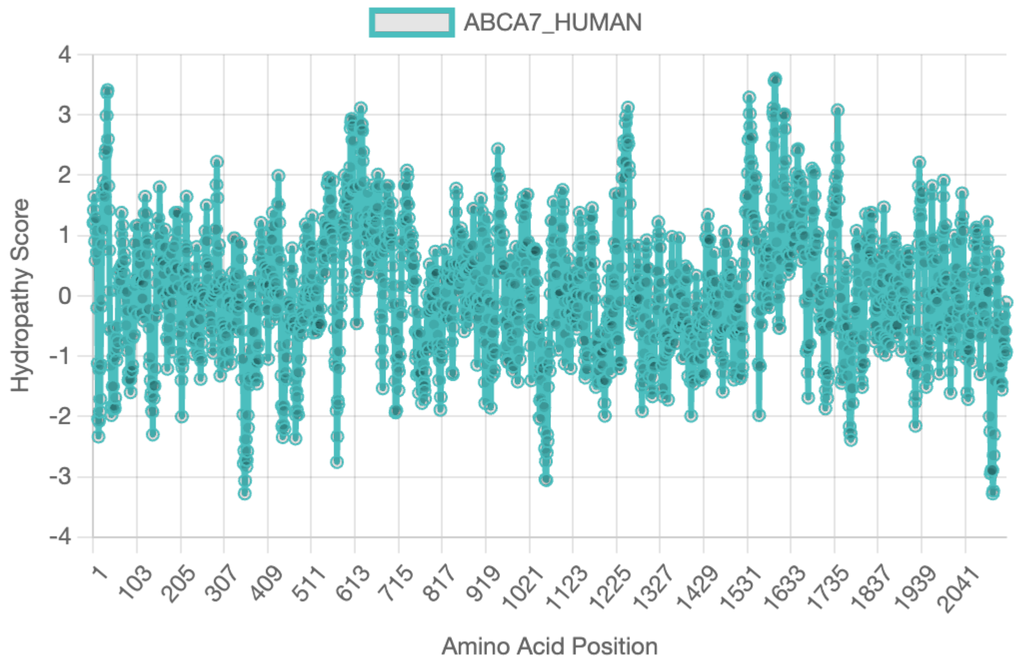

Kyte-Doolittle hydropathy plot for the sequence "ABCA7_HUMAN". Positive values indicate hydrophobic regions, while negative values indicate hydrophilic regions.

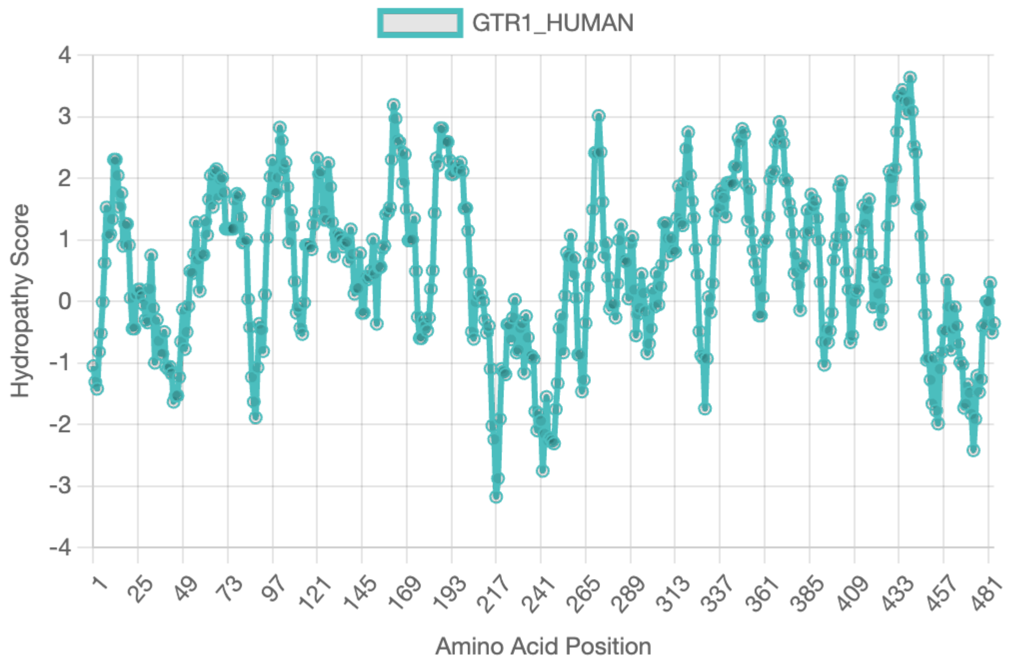

Kyte-Doolittle hydropathy plot for the sequence "GTR1_HUMAN". Positive values indicate hydrophobic regions, while negative values indicate hydrophilic regions.

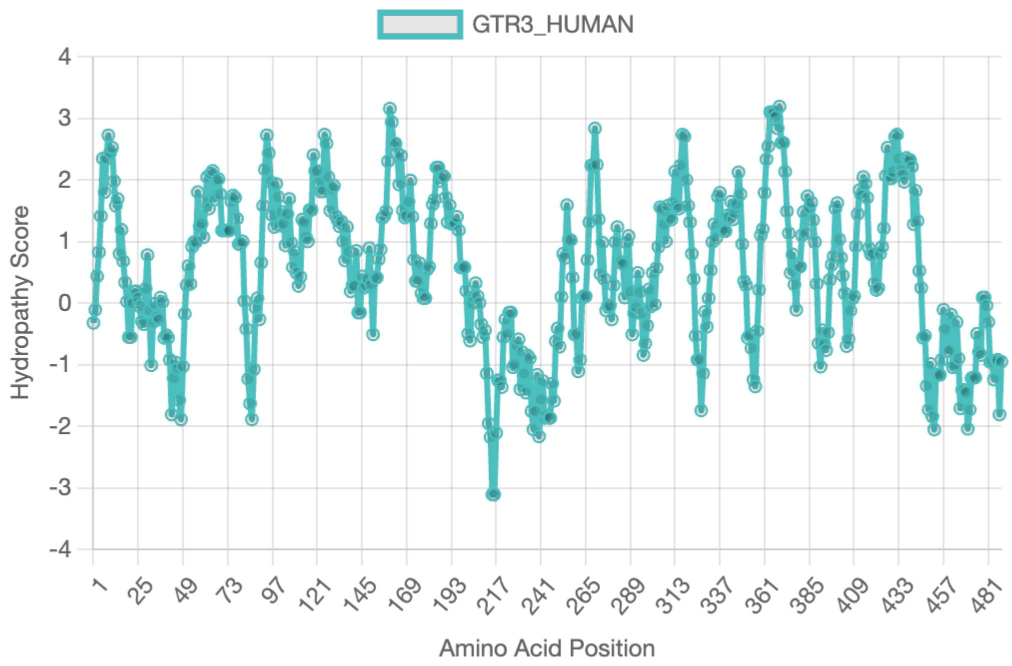

Kyte-Doolittle hydropathy plot for the sequence "GTR3_HUMAN". Positive values indicate hydrophobic regions, while negative values indicate hydrophilic regions.

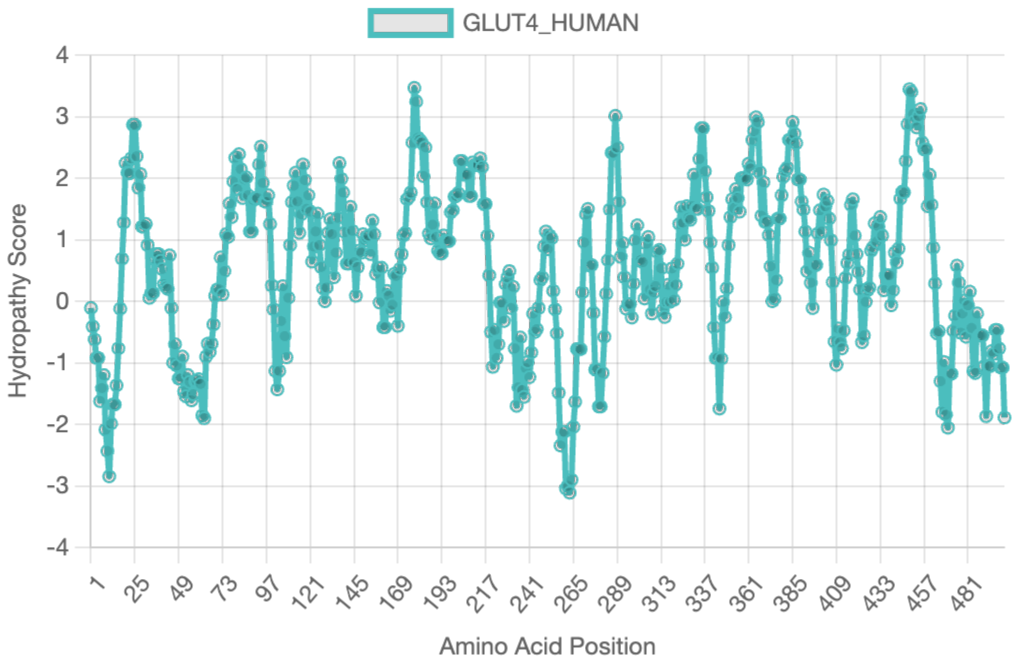

Kyte-Doolittle hydropathy plot for the sequence "GLUT4_HUMAN". Positive values indicate hydrophobic regions, while negative values indicate hydrophilic regions.

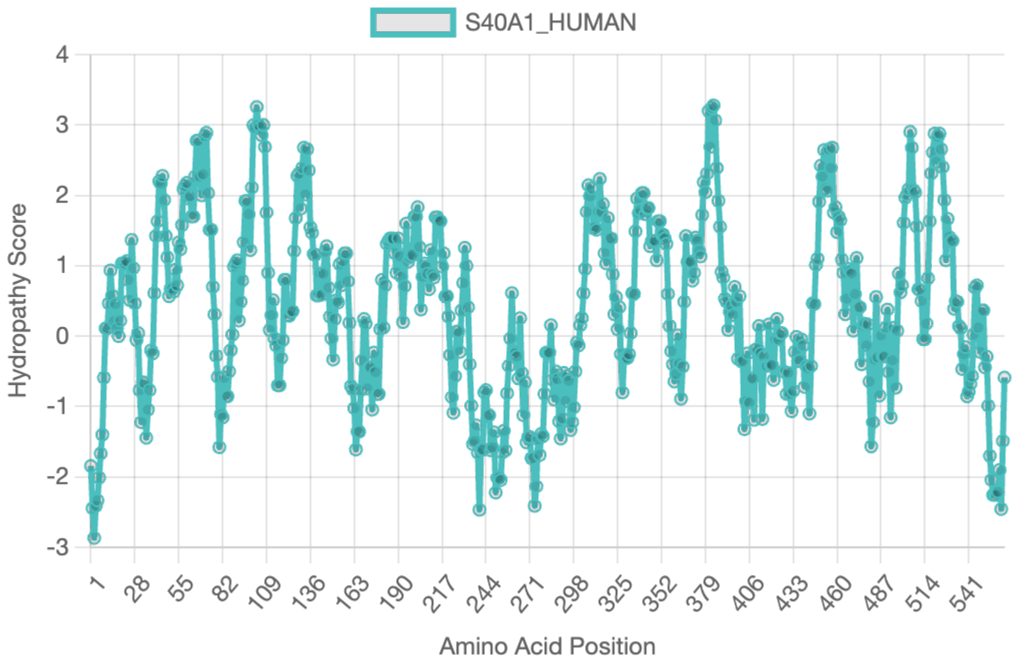

Kyte-Doolittle hydropathy plot for the sequence "S40A1_HUMAN". Positive values indicate hydrophobic regions, while negative values indicate hydrophilic regions.

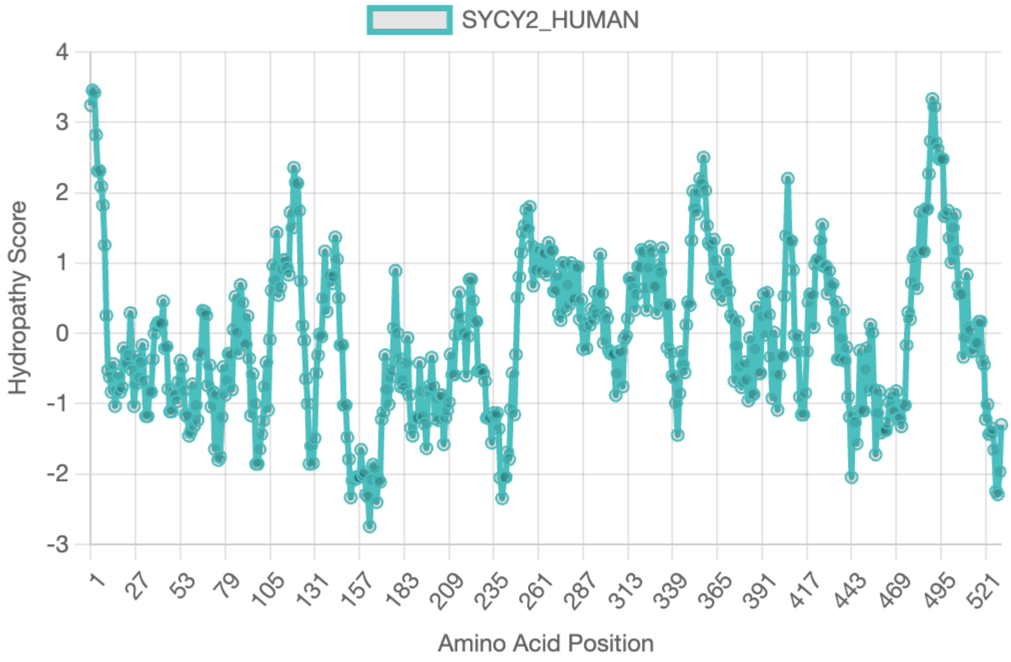

Kyte-Doolittle hydropathy plot for the sequence "SYCY2_HUMAN". Positive values indicate hydrophobic regions, while negative values indicate hydrophilic regions.

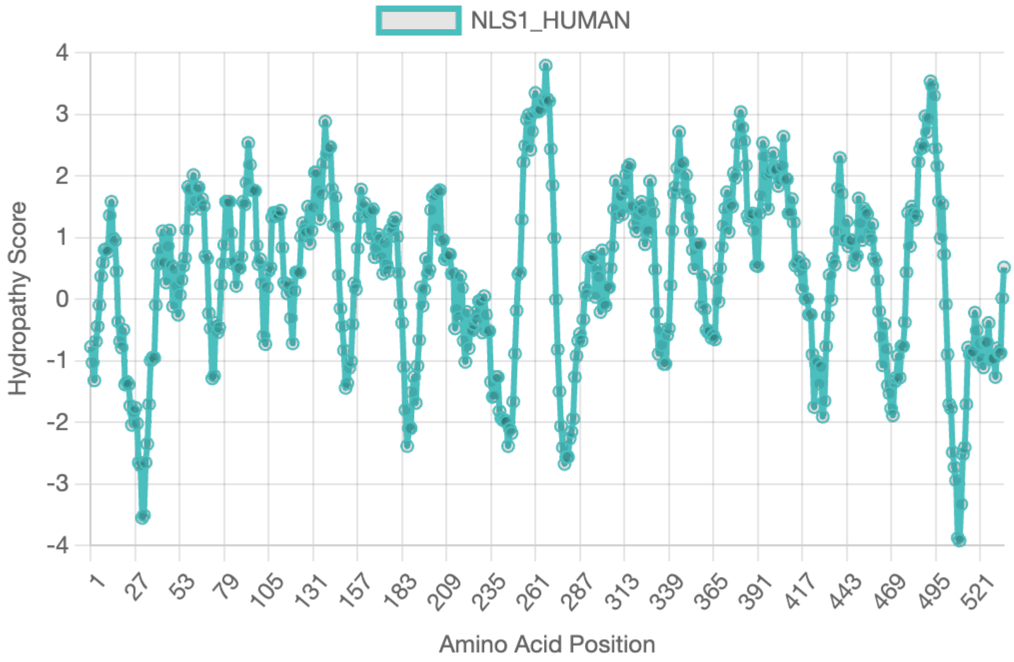

Kyte-Doolittle hydropathy plot for the sequence "NLS1_HUMAN". Positive values indicate hydrophobic regions, while negative values indicate hydrophilic regions.

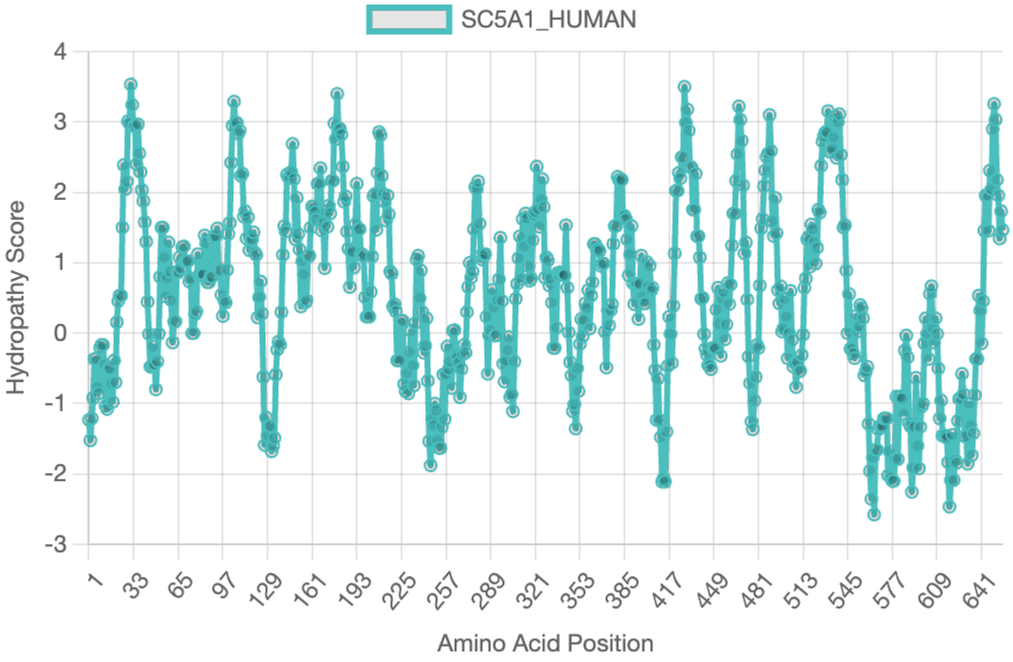

Kyte-Doolittle hydropathy plot for the sequence "SC5A1_HUMAN". Positive values indicate hydrophobic regions, while negative values indicate hydrophilic regions.

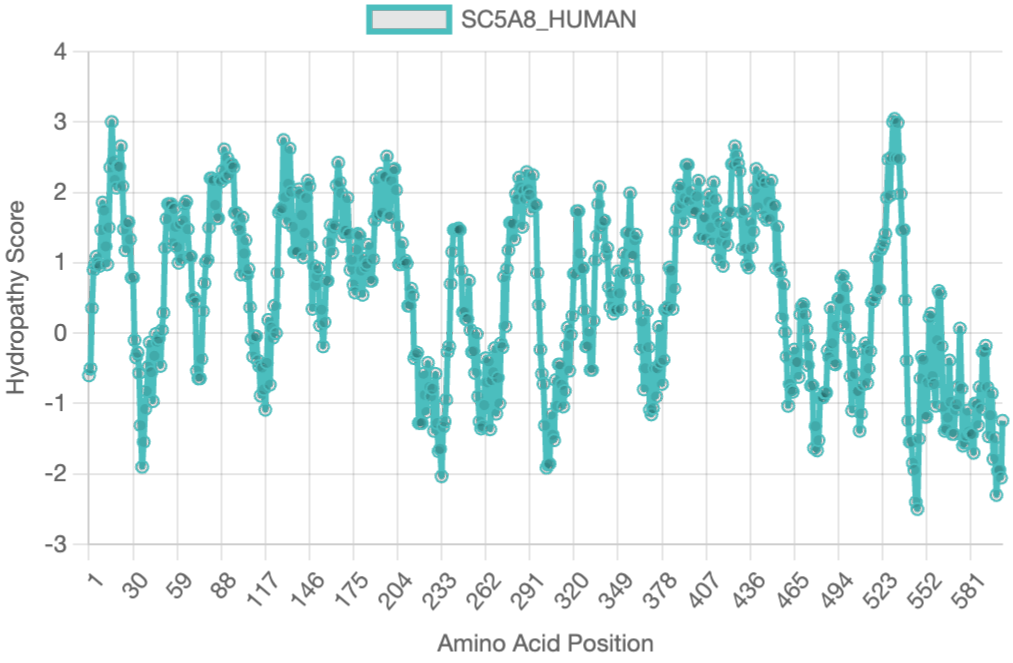

Kyte-Doolittle hydropathy plot for the sequence "SC5A8_HUMAN". Positive values indicate hydrophobic regions, while negative values indicate hydrophilic regions.

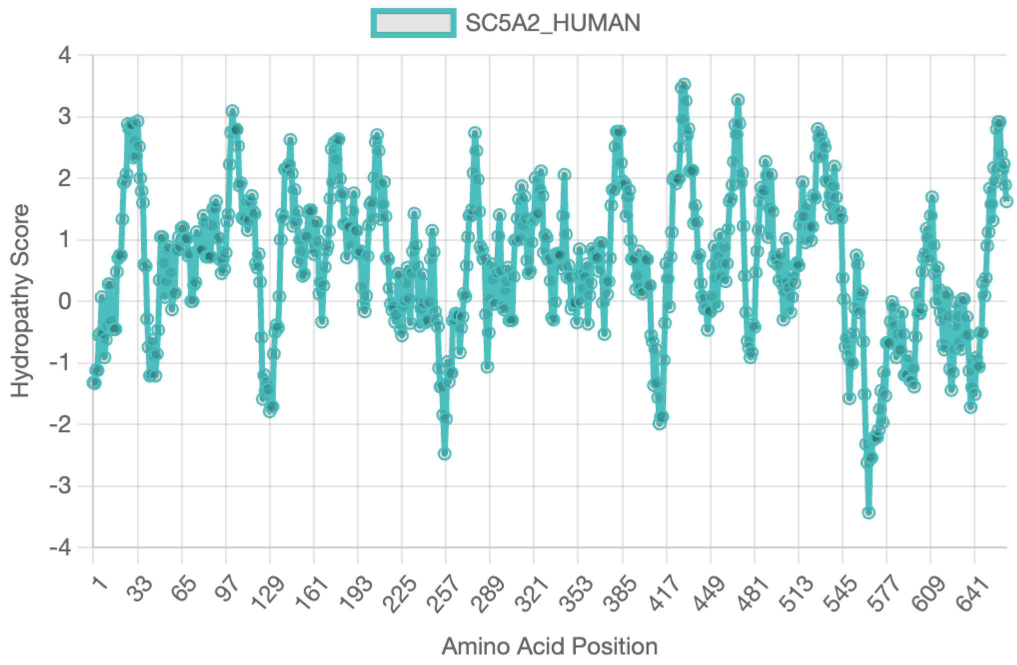

Kyte-Doolittle hydropathy plot for the sequence "SC5A2_HUMAN". Positive values indicate hydrophobic regions, while negative values indicate hydrophilic regions.

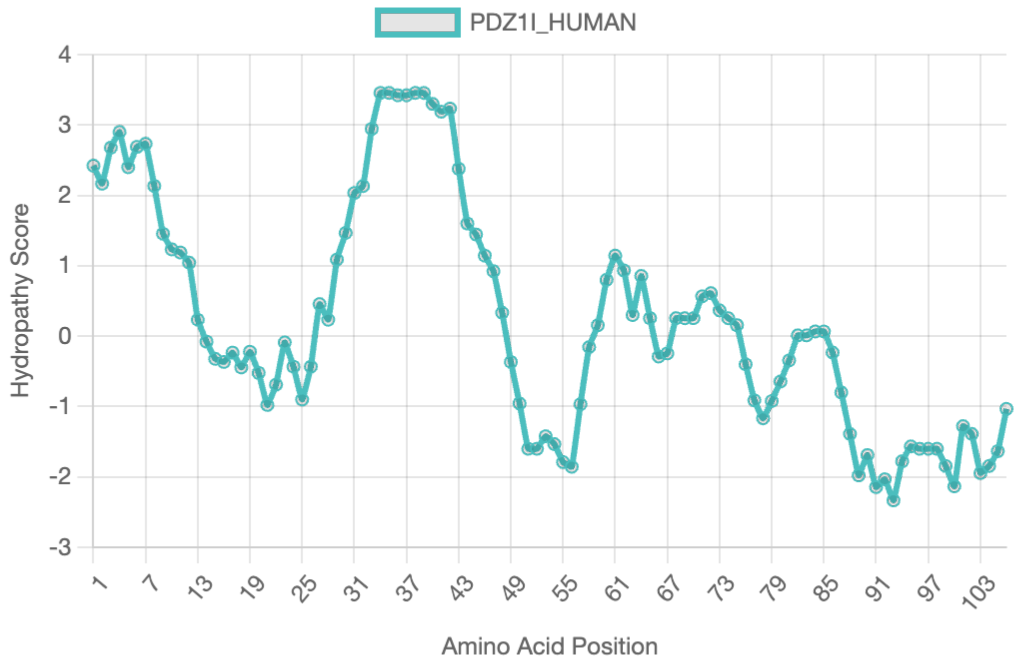

Kyte-Doolittle hydropathy plot for the sequence "PDZ1I_HUMAN". Positive values indicate hydrophobic regions, while negative values indicate hydrophilic regions.

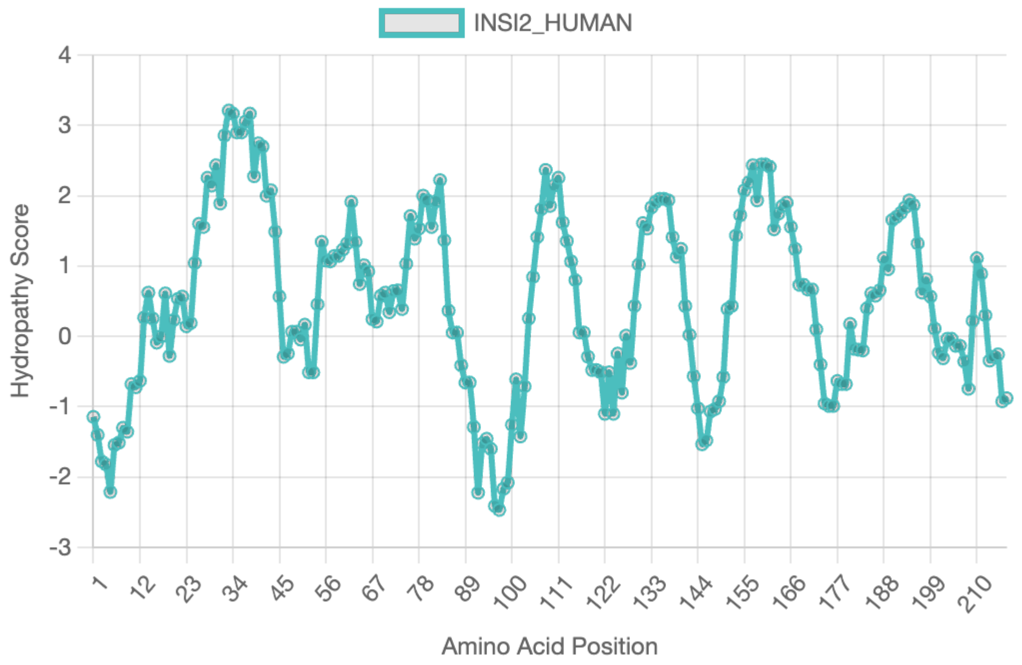

Kyte-Doolittle hydropathy plot for the sequence "INSI2_HUMAN". Positive values indicate hydrophobic regions, while negative values indicate hydrophilic regions.

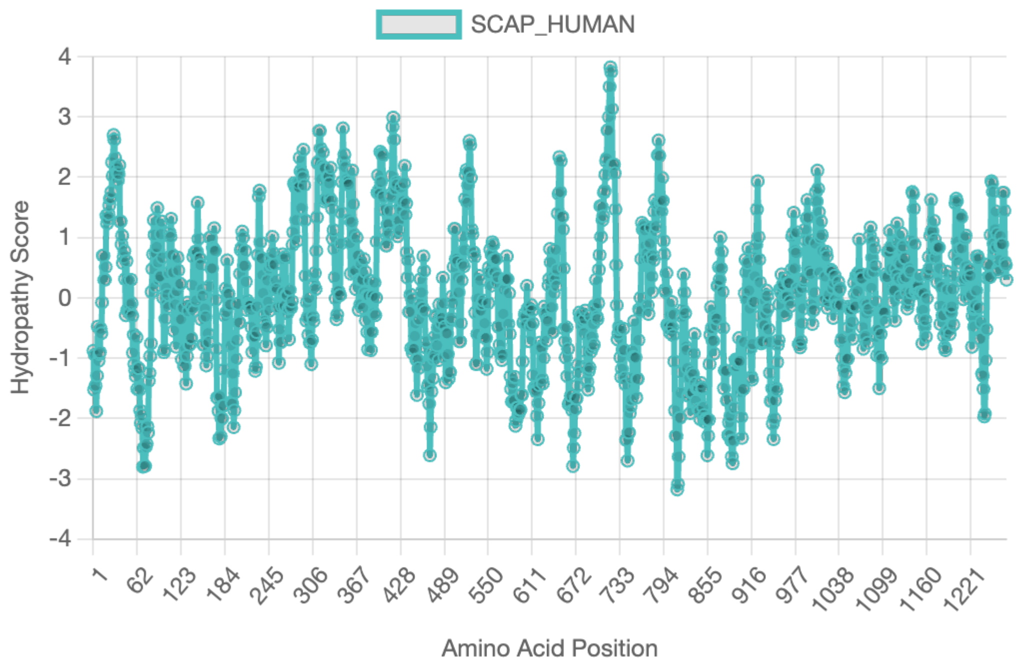

Kyte-Doolittle hydropathy plot for the sequence "SCAP_HUMAN". Positive values indicate hydrophobic regions, while negative values indicate hydrophilic regions.

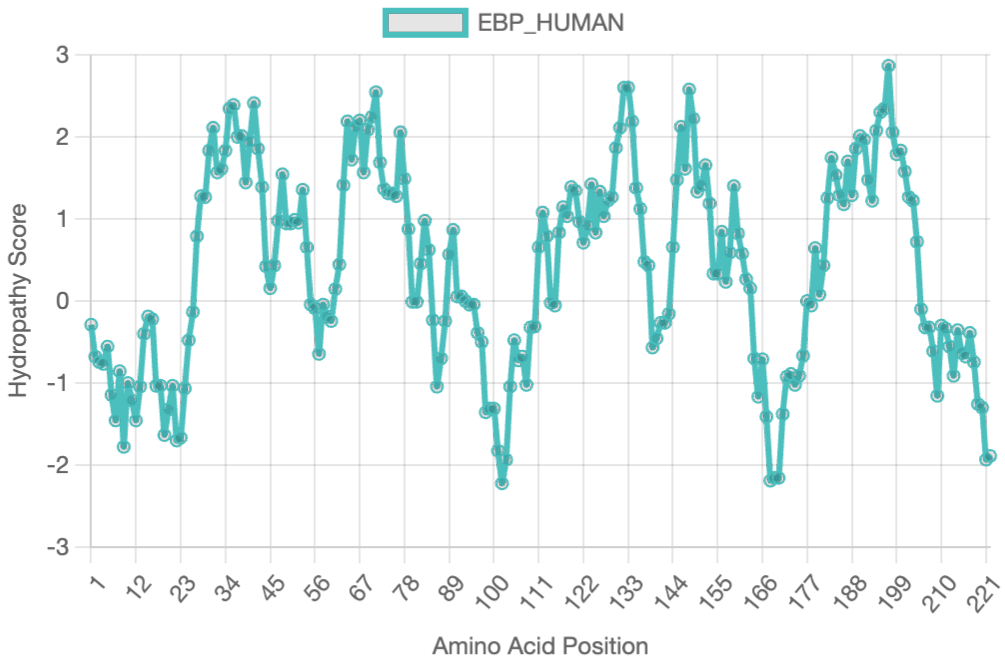

Kyte-Doolittle hydropathy plot for the sequence "EBP_HUMAN". Positive values indicate hydrophobic regions, while negative values indicate hydrophilic regions.

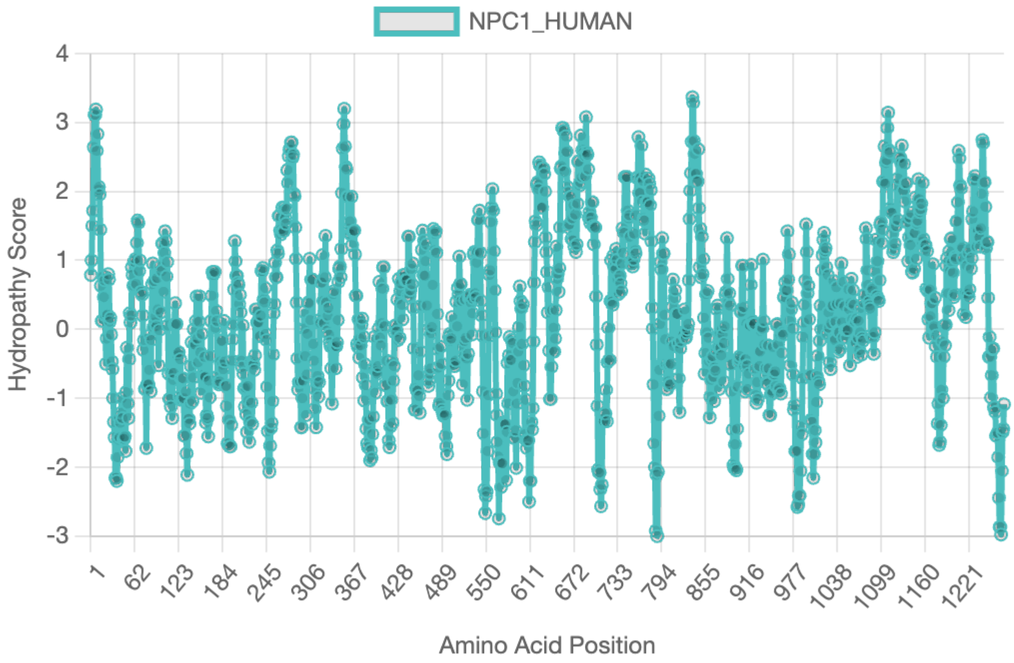

Kyte-Doolittle hydropathy plot for the sequence "NPC1_HUMAN". Positive values indicate hydrophobic regions, while negative values indicate hydrophilic regions.

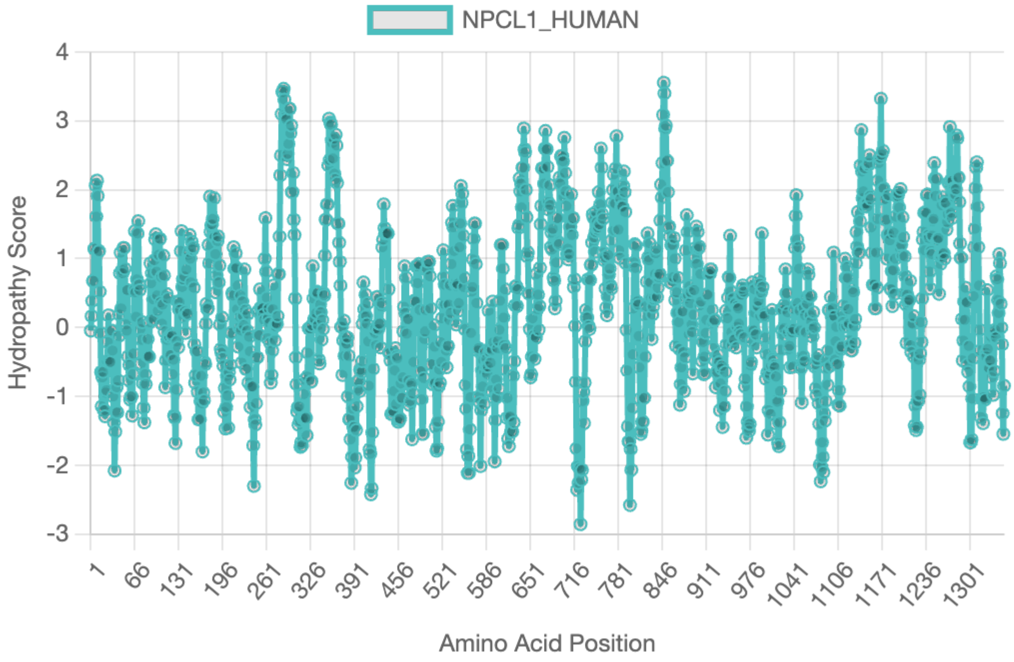

Kyte-Doolittle hydropathy plot for the sequence "NPCL1_HUMAN". Positive values indicate hydrophobic regions, while negative values indicate hydrophilic regions.

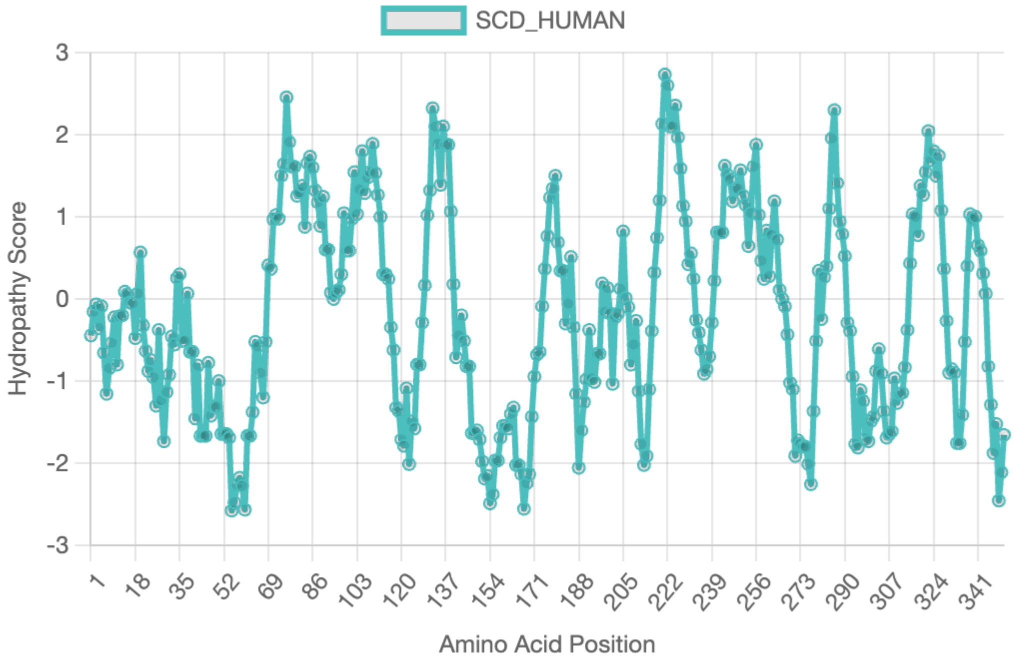

Kyte-Doolittle hydropathy plot for the sequence "SCD_HUMAN". Positive values indicate hydrophobic regions, while negative values indicate hydrophilic regions.

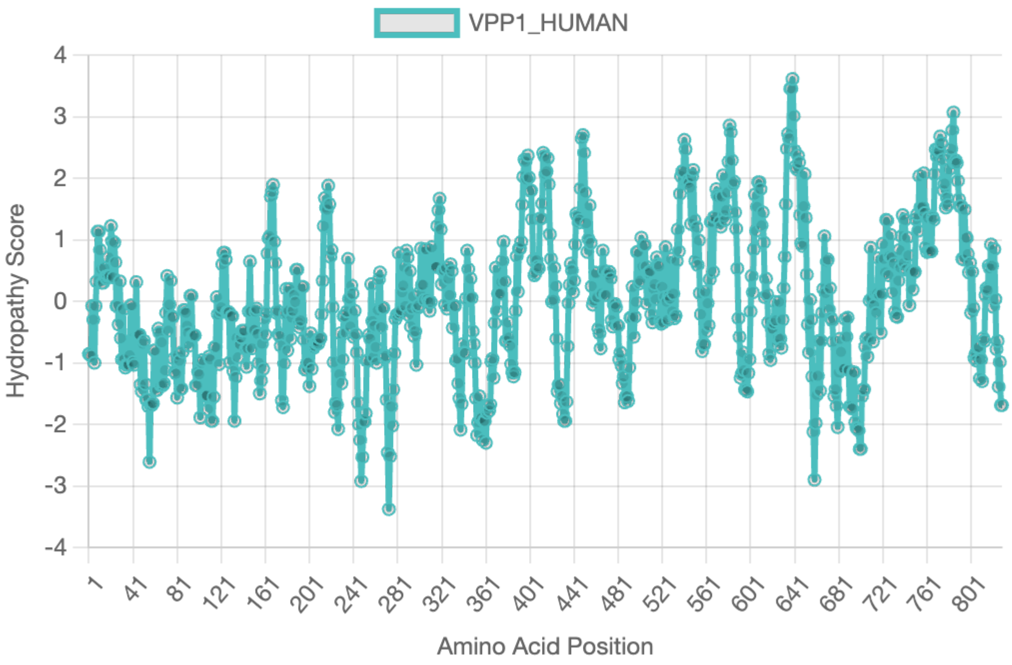

Kyte-Doolittle hydropathy plot for the sequence "VPP1_HUMAN". Positive values indicate hydrophobic regions, while negative values indicate hydrophilic regions.

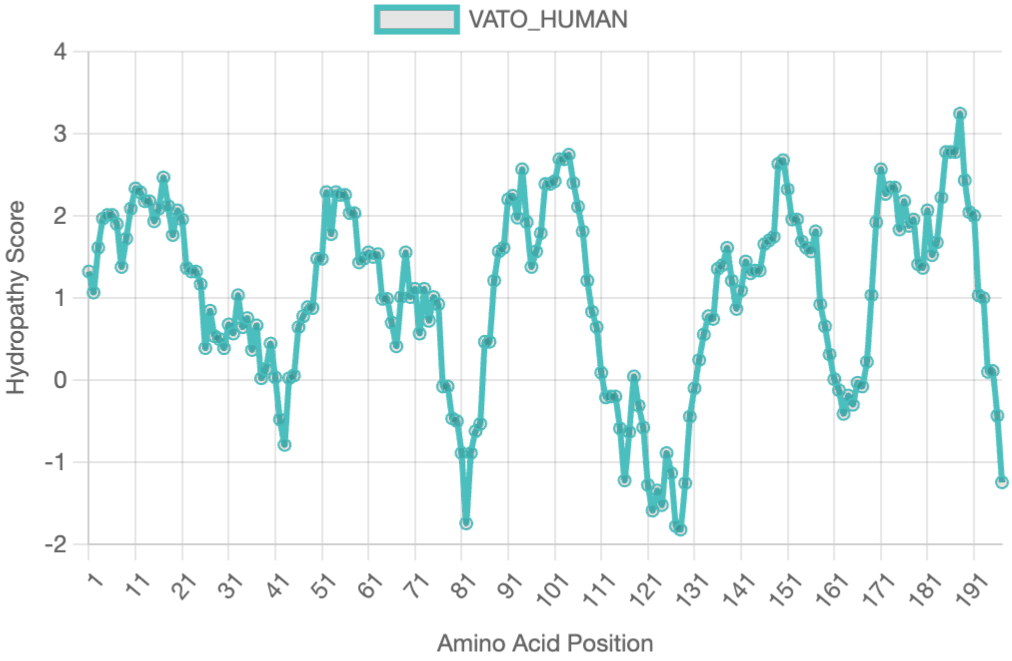

Kyte-Doolittle hydropathy plot for the sequence "VATO_HUMAN". Positive values indicate hydrophobic regions, while negative values indicate hydrophilic regions.

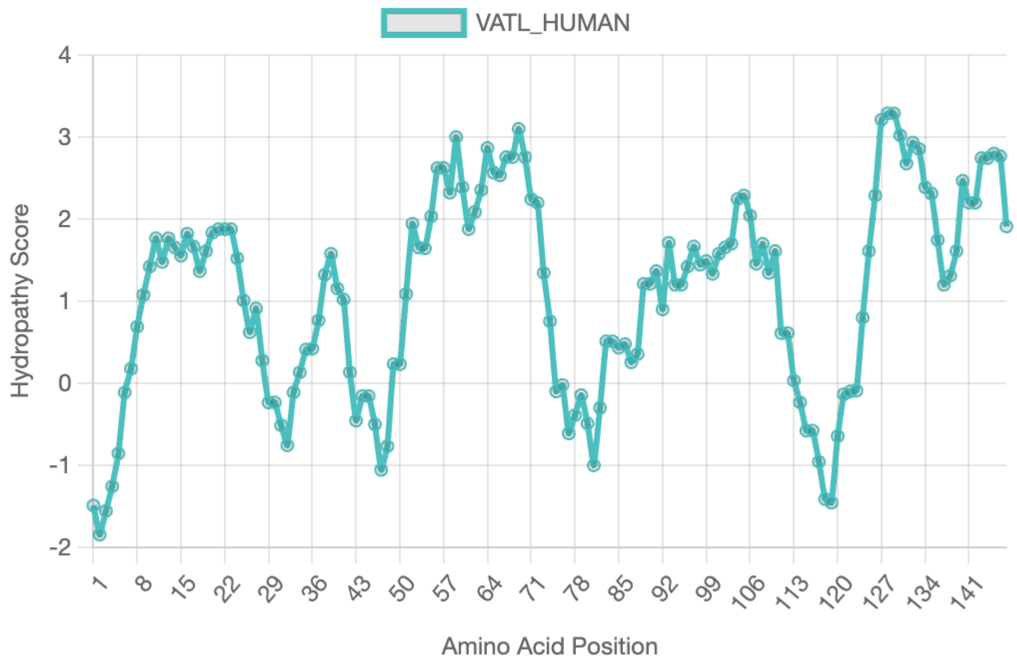

Kyte-Doolittle hydropathy plot for the sequence "VATL_HUMAN". Positive values indicate hydrophobic regions, while negative values indicate hydrophilic regions.

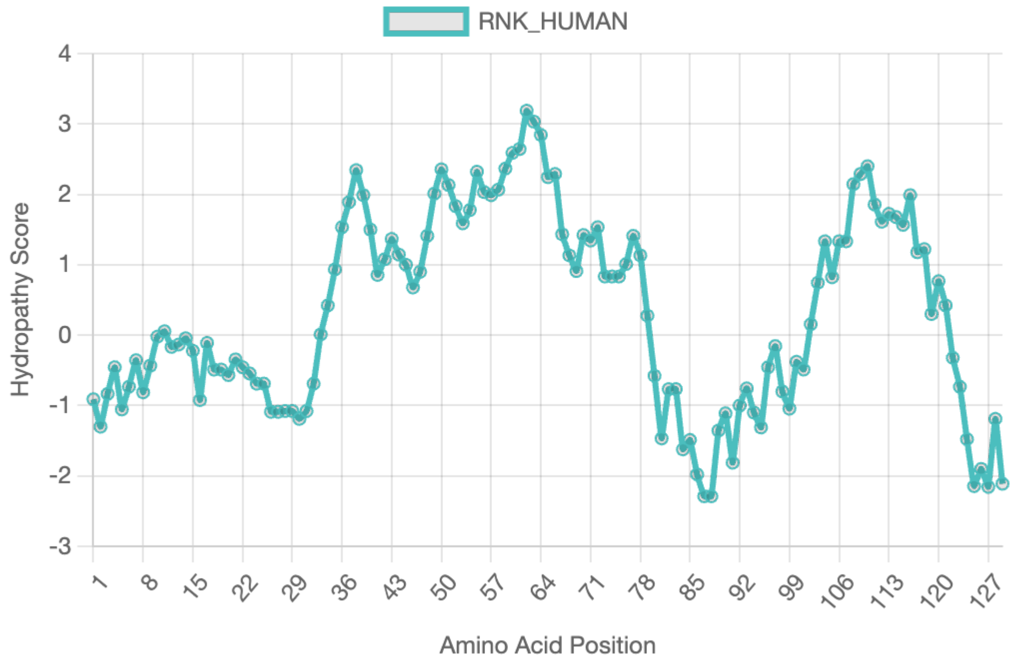

Kyte-Doolittle hydropathy plot for the sequence "RNK_HUMAN". Positive values indicate hydrophobic regions, while negative values indicate hydrophilic regions.

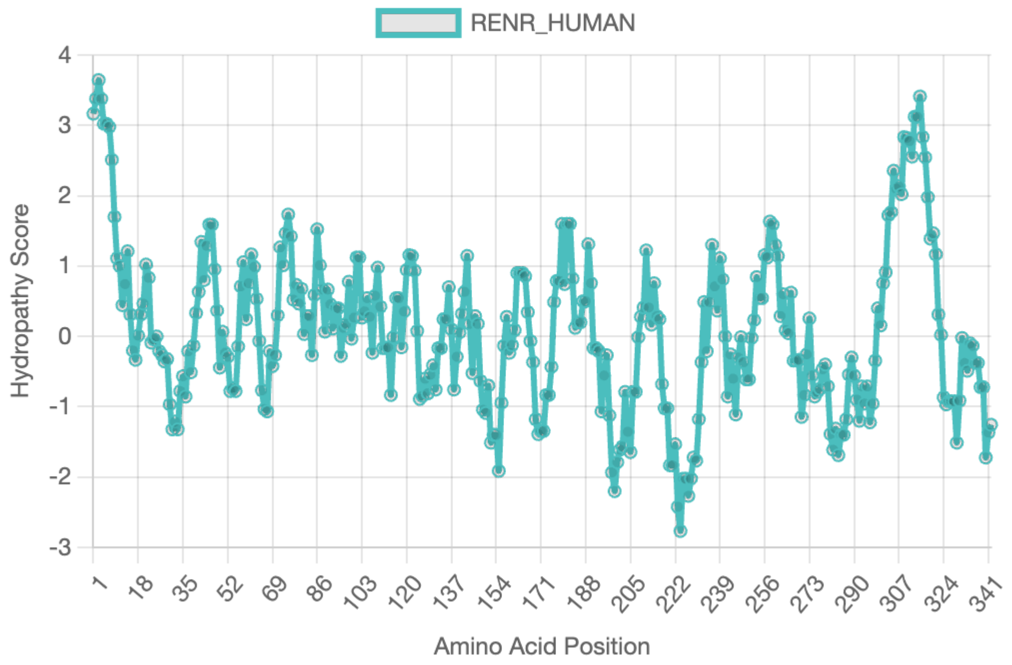

Kyte-Doolittle hydropathy plot for the sequence "RENR_HUMAN". Positive values indicate hydrophobic regions, while negative values indicate hydrophilic regions.

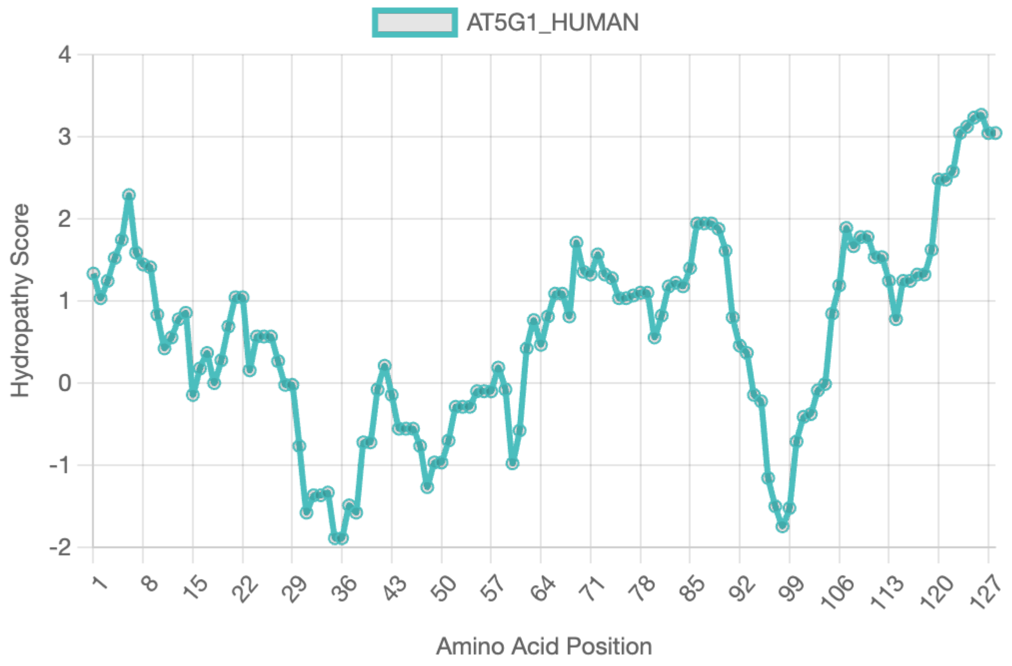

Kyte-Doolittle hydropathy plot for the sequence "AT5G1_HUMAN". Positive values indicate hydrophobic regions, while negative values indicate hydrophilic regions.

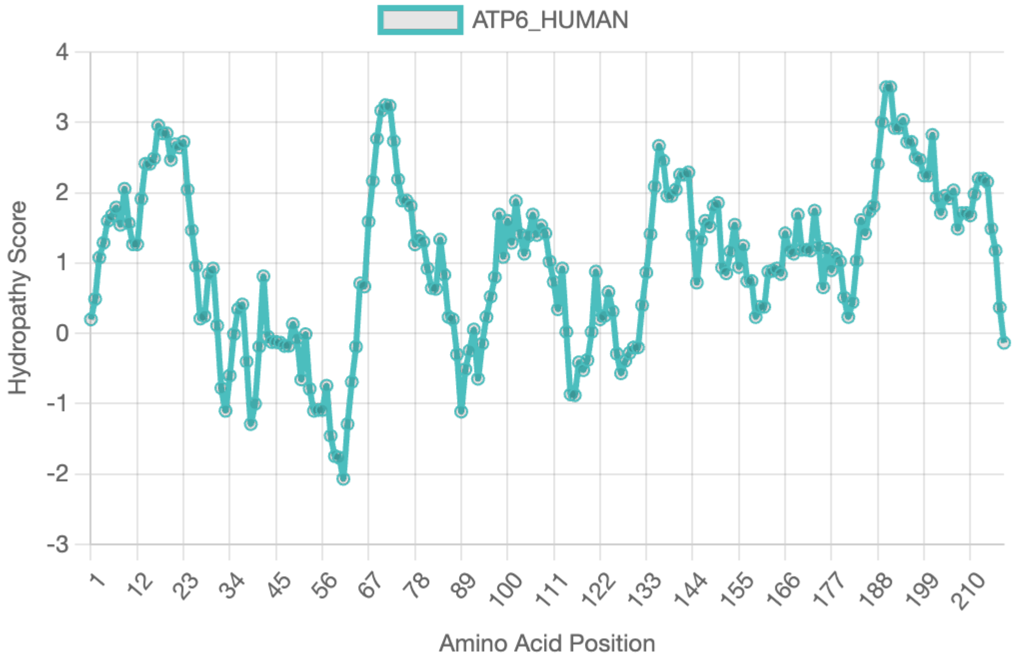

Kyte-Doolittle hydropathy plot for the sequence "ATP6_HUMAN". Positive values indicate hydrophobic regions, while negative values indicate hydrophilic regions.

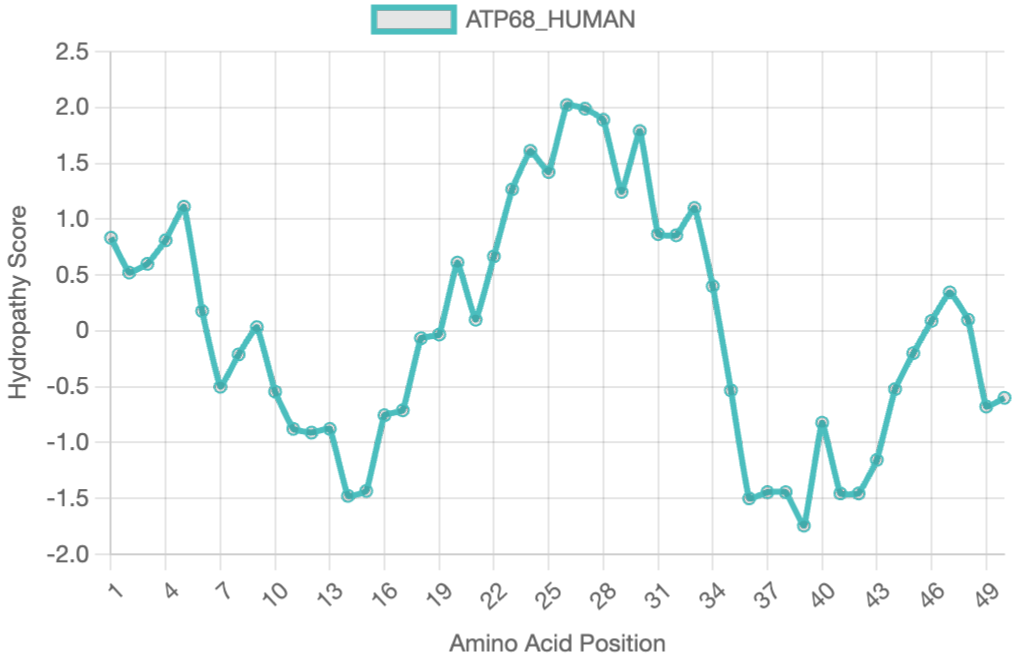

Kyte-Doolittle hydropathy plot for the sequence "ATP68_HUMAN". Positive values indicate hydrophobic regions, while negative values indicate hydrophilic regions.

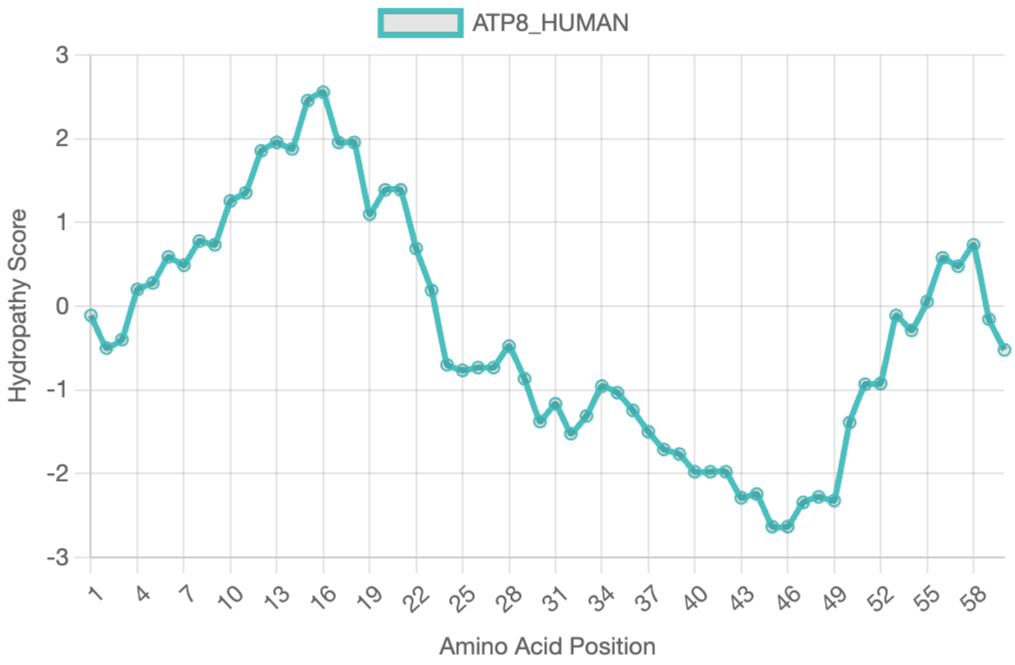

Kyte-Doolittle hydropathy plot for the sequence "ATP8_HUMAN". Positive values indicate hydrophobic regions, while negative values indicate hydrophilic regions.

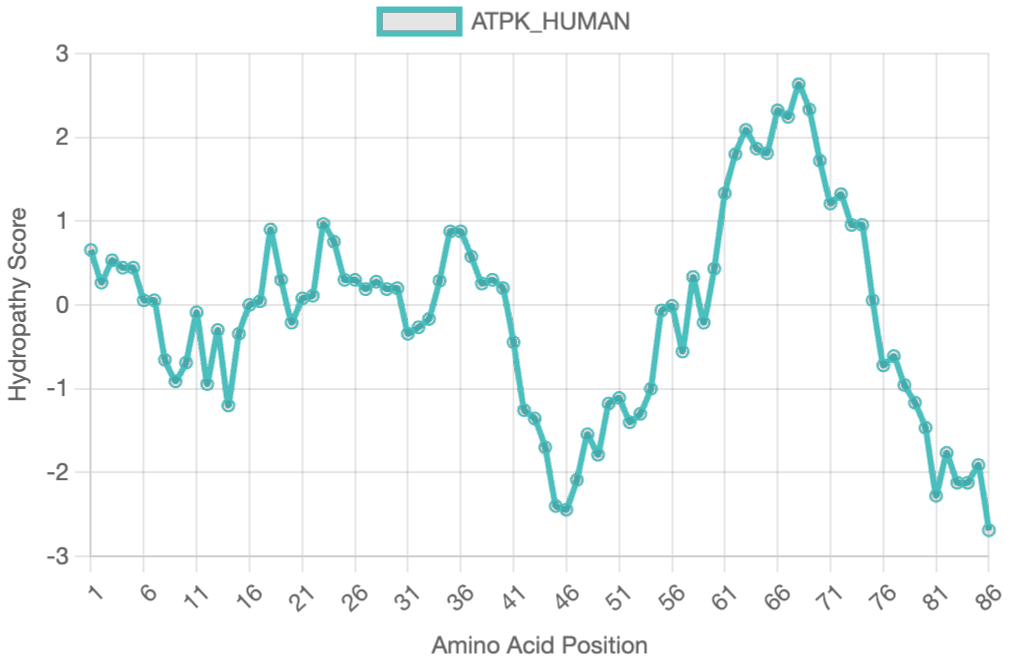

Kyte-Doolittle hydropathy plot for the sequence "ATPK_HUMAN". Positive values indicate hydrophobic regions, while negative values indicate hydrophilic regions.

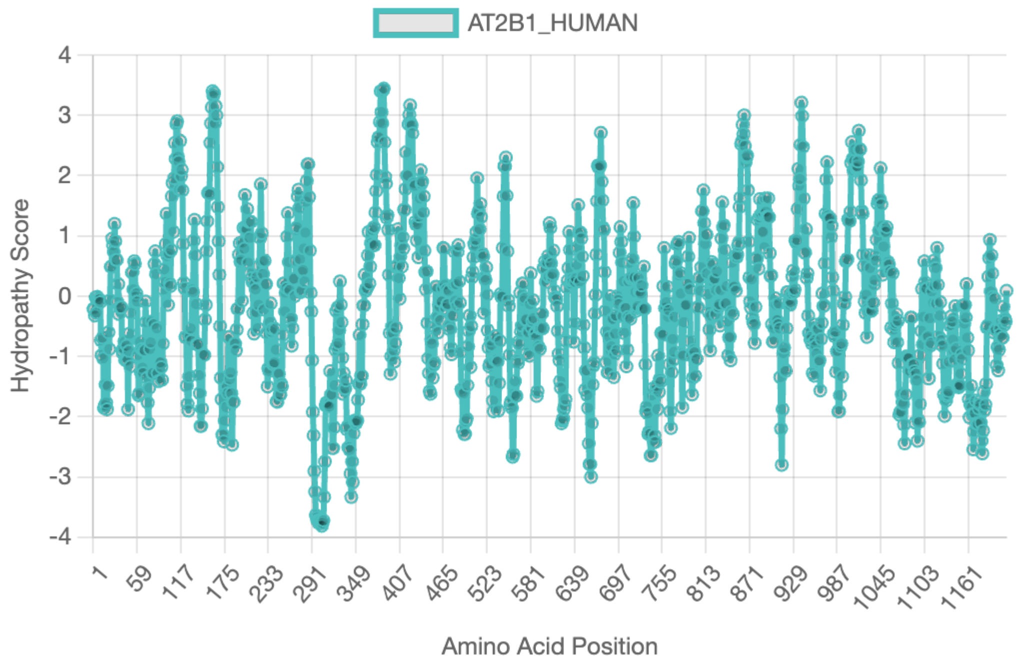

Kyte-Doolittle hydropathy plot for the sequence "AT2B1_HUMAN". Positive values indicate hydrophobic regions, while negative values indicate hydrophilic regions.

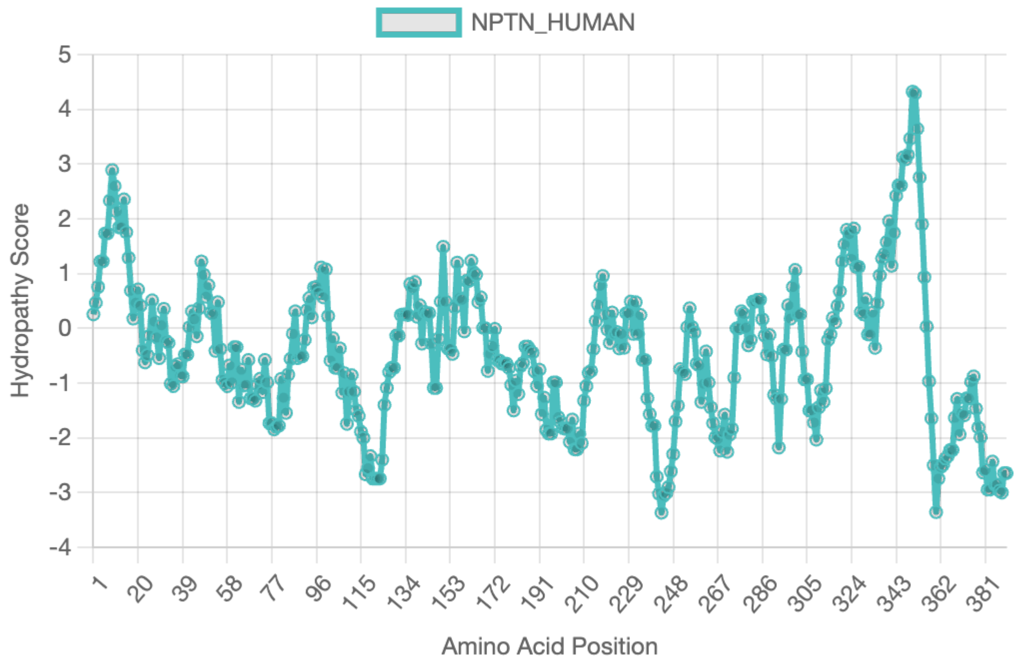

Kyte-Doolittle hydropathy plot for the sequence "NPTN_HUMAN". Positive values indicate hydrophobic regions, while negative values indicate hydrophilic regions.

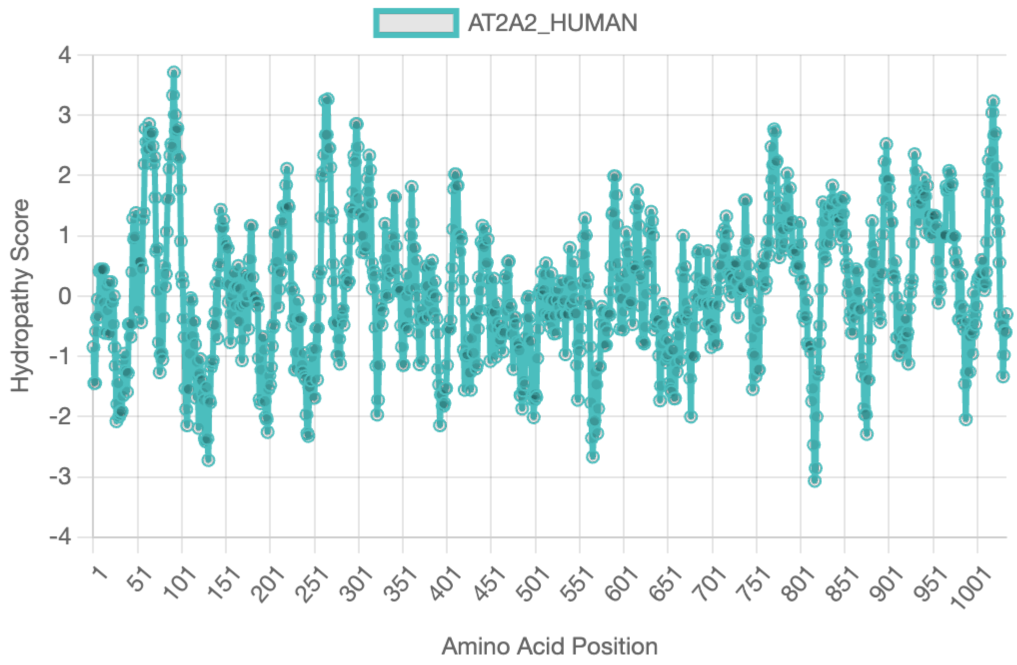

Kyte-Doolittle hydropathy plot for the sequence "AT2A2_HUMAN". Positive values indicate hydrophobic regions, while negative values indicate hydrophilic regions.

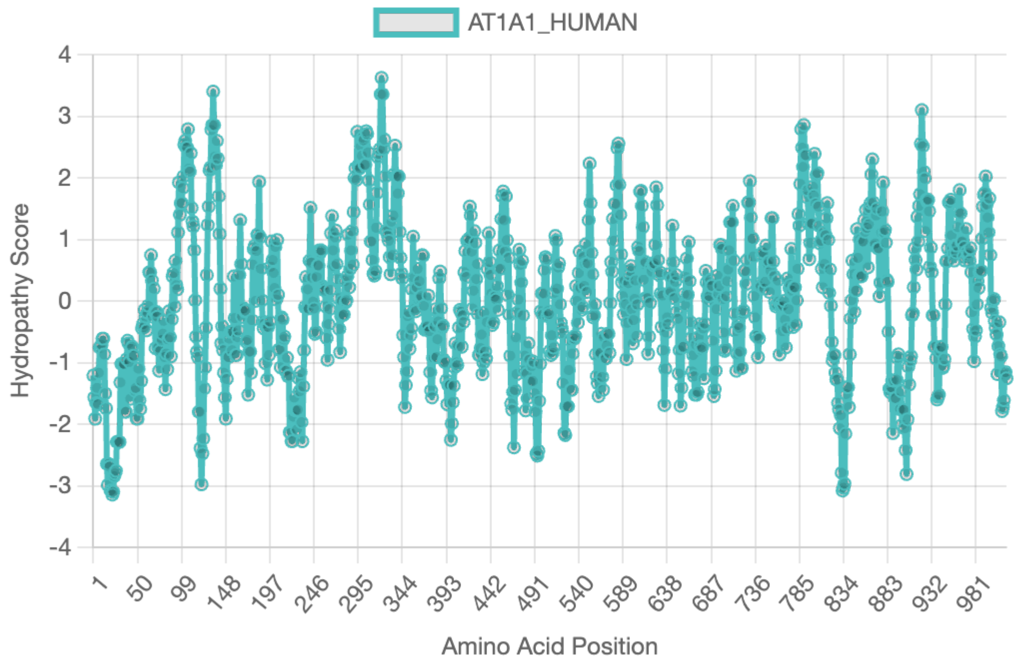

Kyte-Doolittle hydropathy plot for the sequence "AT1A1_HUMAN". Positive values indicate hydrophobic regions, while negative values indicate hydrophilic regions.

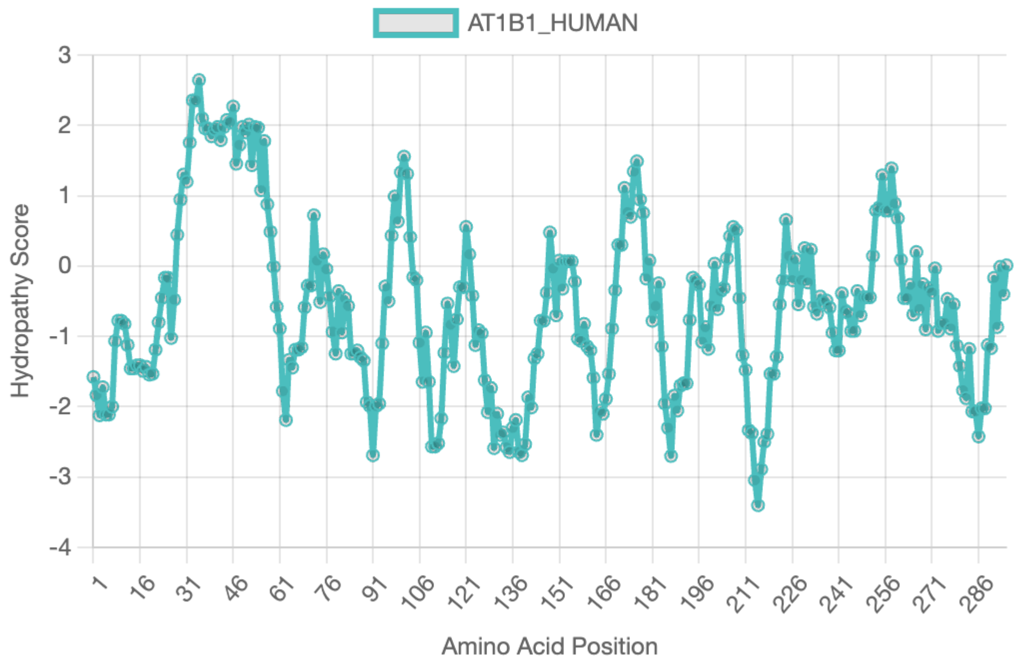

Kyte-Doolittle hydropathy plot for the sequence "AT1B1_HUMAN". Positive values indicate hydrophobic regions, while negative values indicate hydrophilic regions.

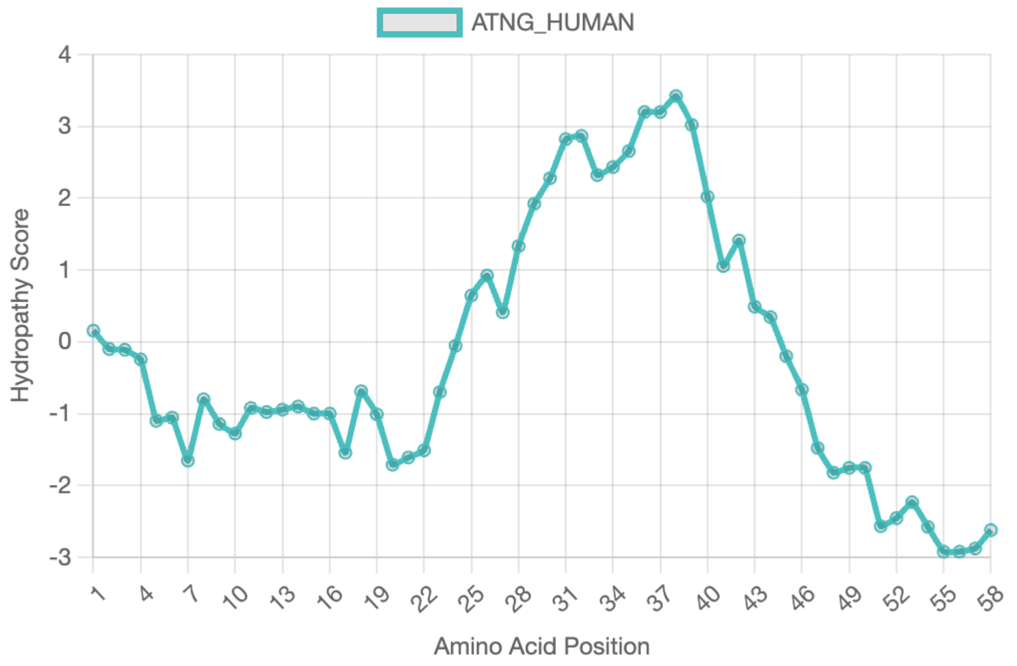

Kyte-Doolittle hydropathy plot for the sequence "ATNG_HUMAN". Positive values indicate hydrophobic regions, while negative values indicate hydrophilic regions.

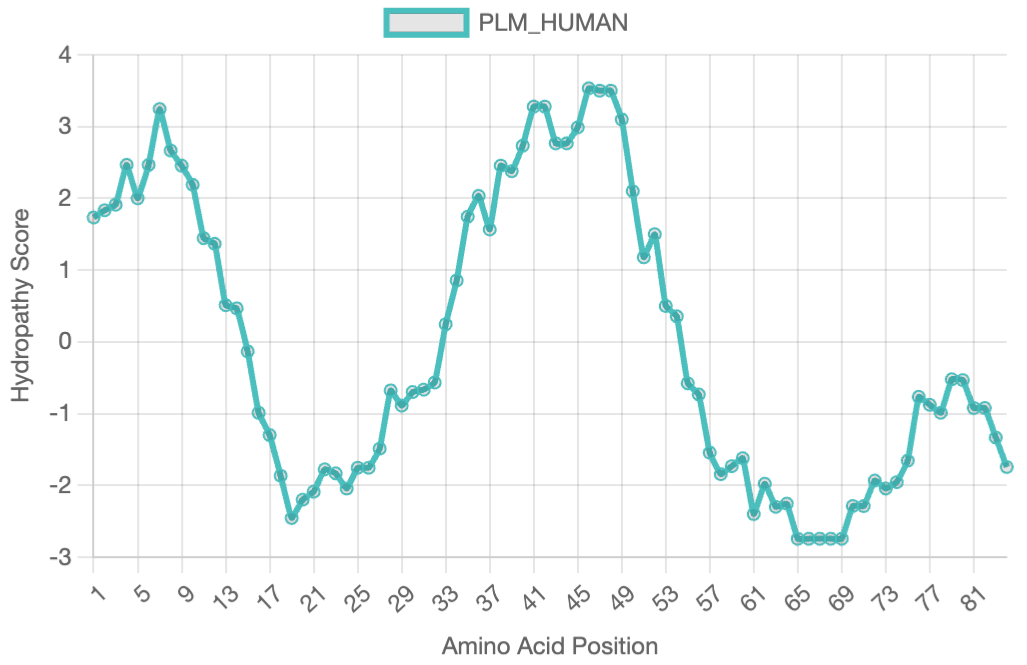

Kyte-Doolittle hydropathy plot for the sequence "PLM_HUMAN". Positive values indicate hydrophobic regions, while negative values indicate hydrophilic regions.

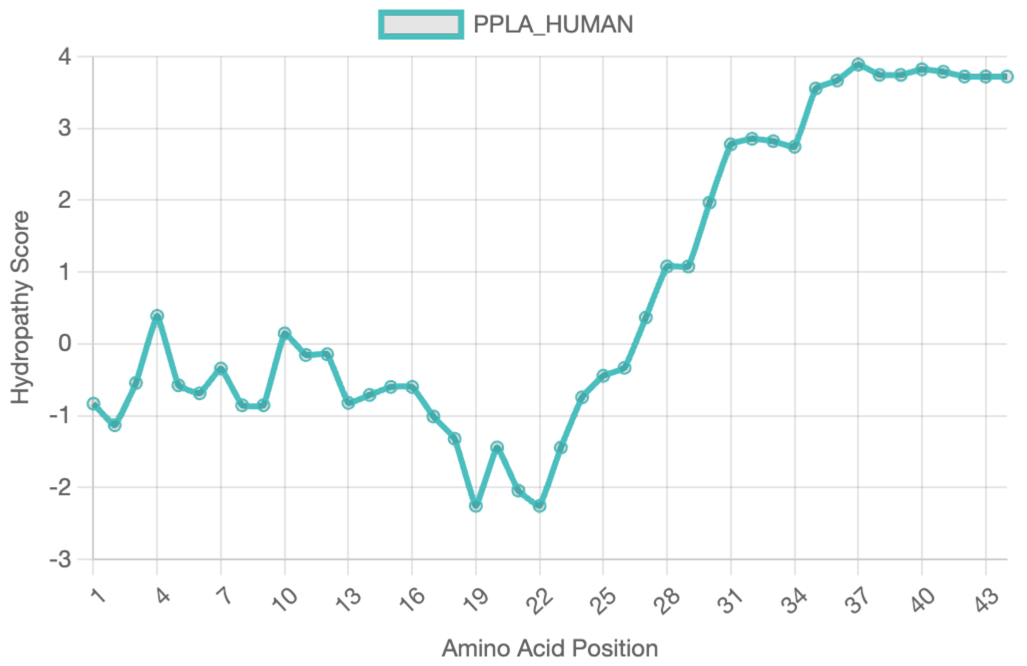

Kyte-Doolittle hydropathy plot for the sequence "PPLA_HUMAN". Positive values indicate hydrophobic regions, while negative values indicate hydrophilic regions.

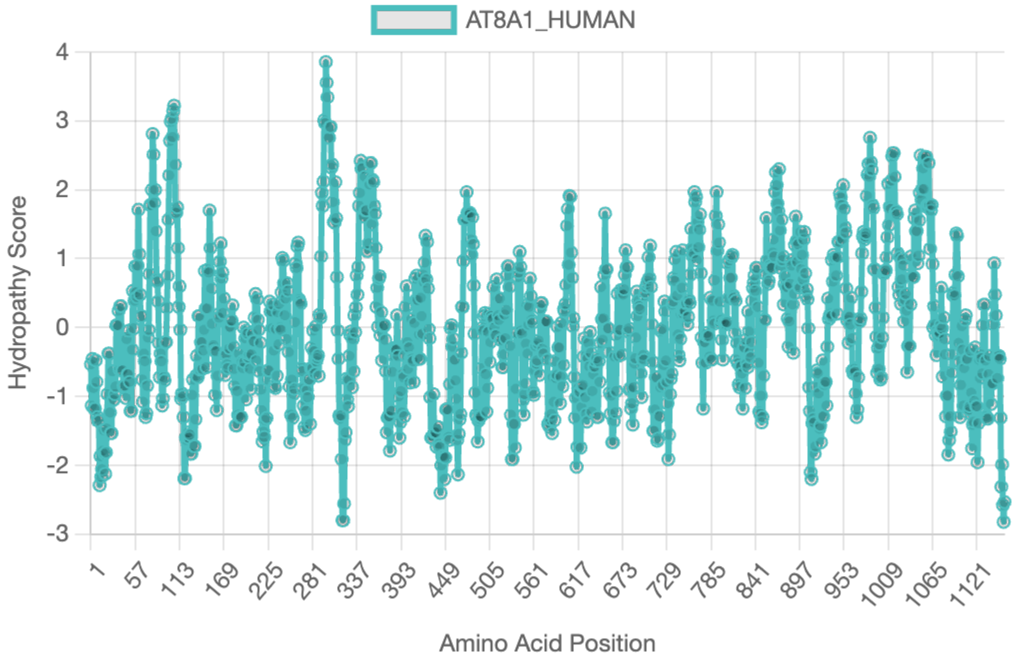

Kyte-Doolittle hydropathy plot for the sequence "AT8A1_HUMAN". Positive values indicate hydrophobic regions, while negative values indicate hydrophilic regions.

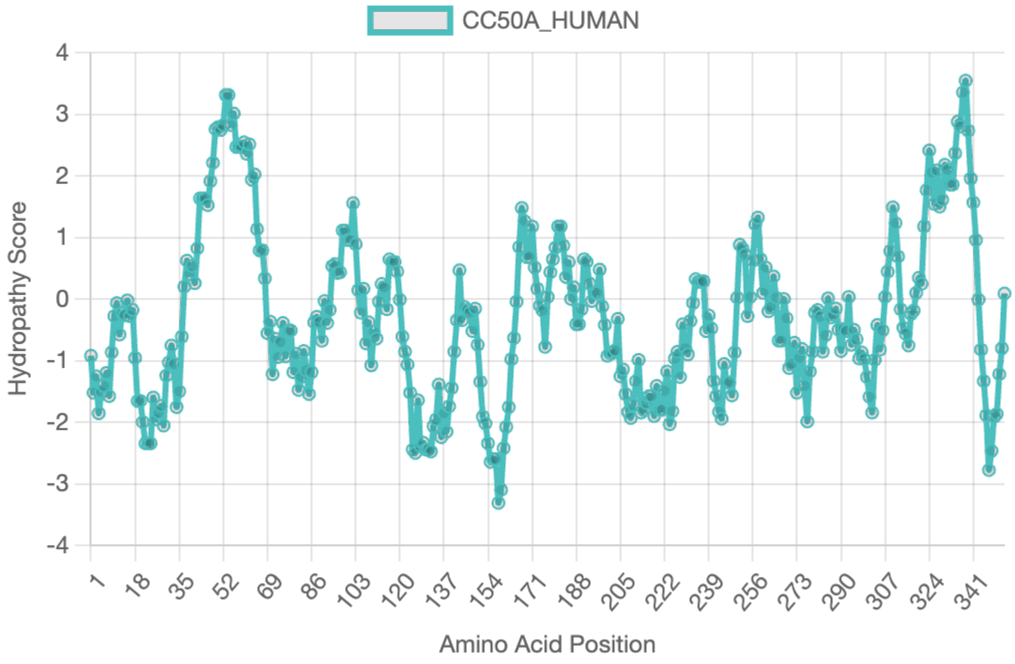

Kyte-Doolittle hydropathy plot for the sequence "CC50A_HUMAN". Positive values indicate hydrophobic regions, while negative values indicate hydrophilic regions.

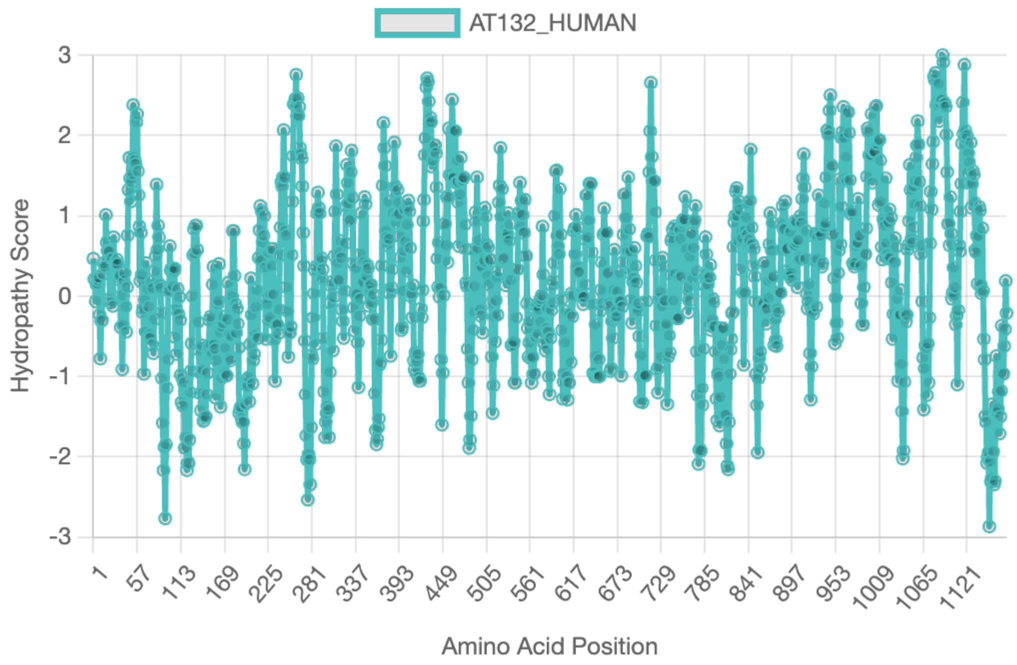

Kyte-Doolittle hydropathy plot for the sequence "AT132_HUMAN". Positive values indicate hydrophobic regions, while negative values indicate hydrophilic regions.

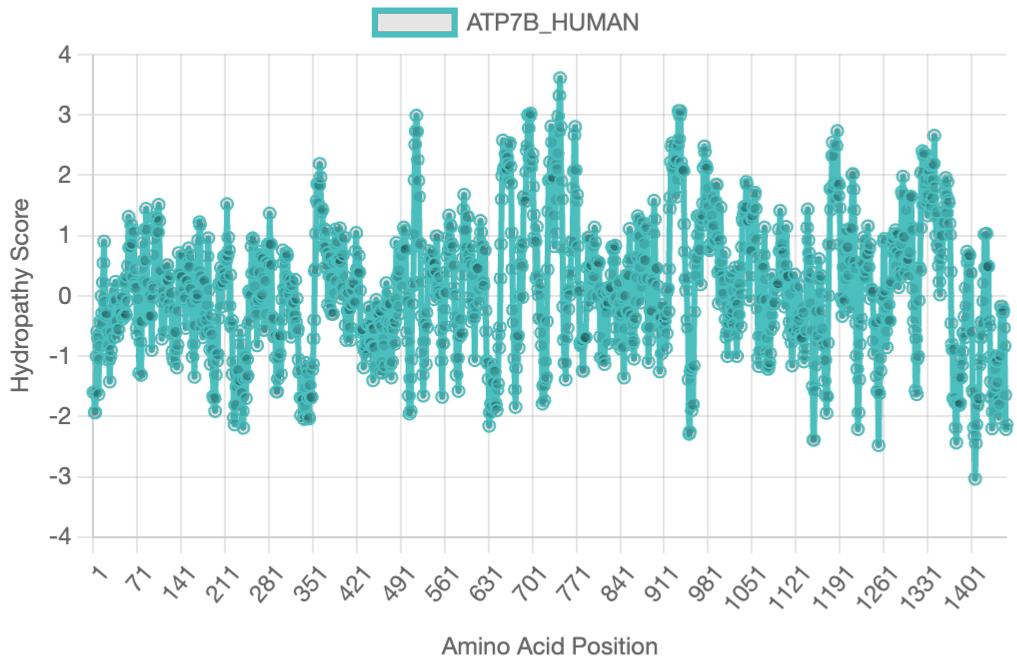

Kyte-Doolittle hydropathy plot for the sequence "ATP7B_HUMAN". Positive values indicate hydrophobic regions, while negative values indicate hydrophilic regions.

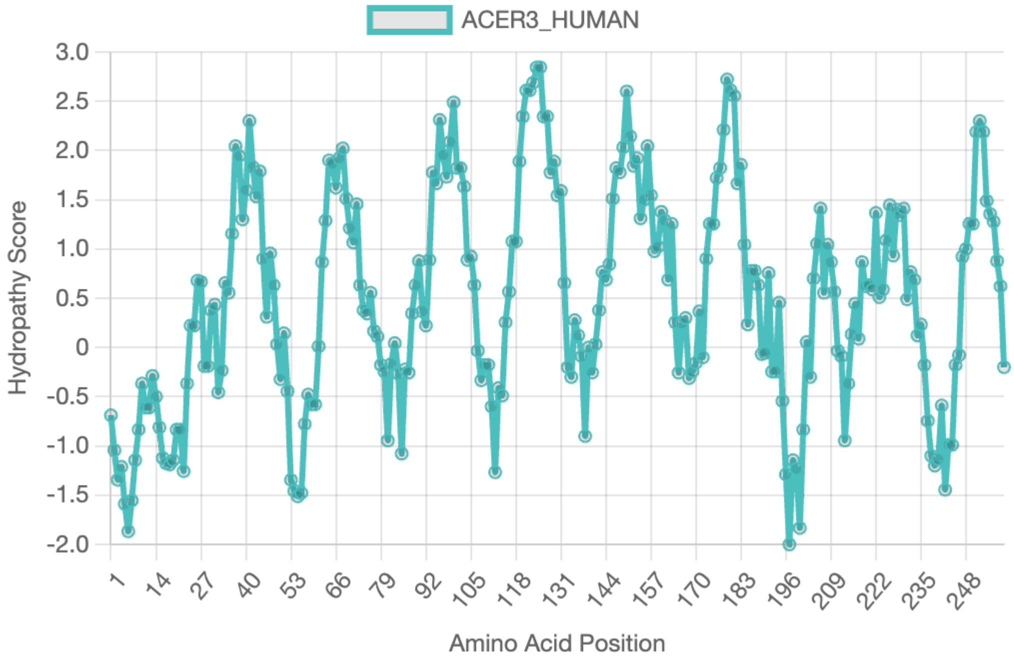

Kyte-Doolittle hydropathy plot for the sequence "ACER3_HUMAN". Positive values indicate hydrophobic regions, while negative values indicate hydrophilic regions.

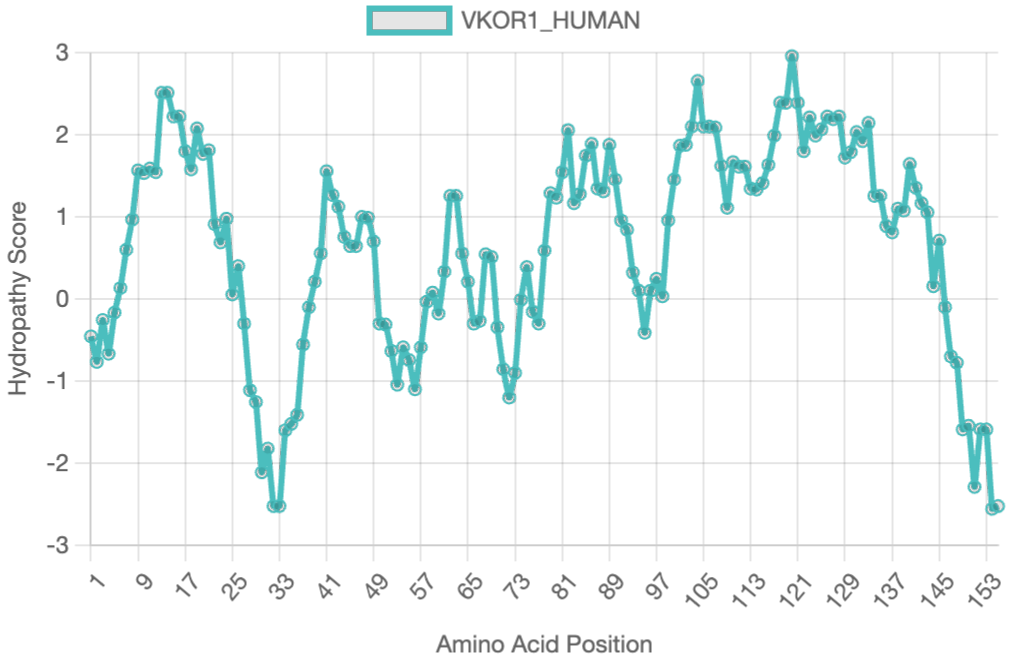

Kyte-Doolittle hydropathy plot for the sequence "VKOR1_HUMAN". Positive values indicate hydrophobic regions, while negative values indicate hydrophilic regions.

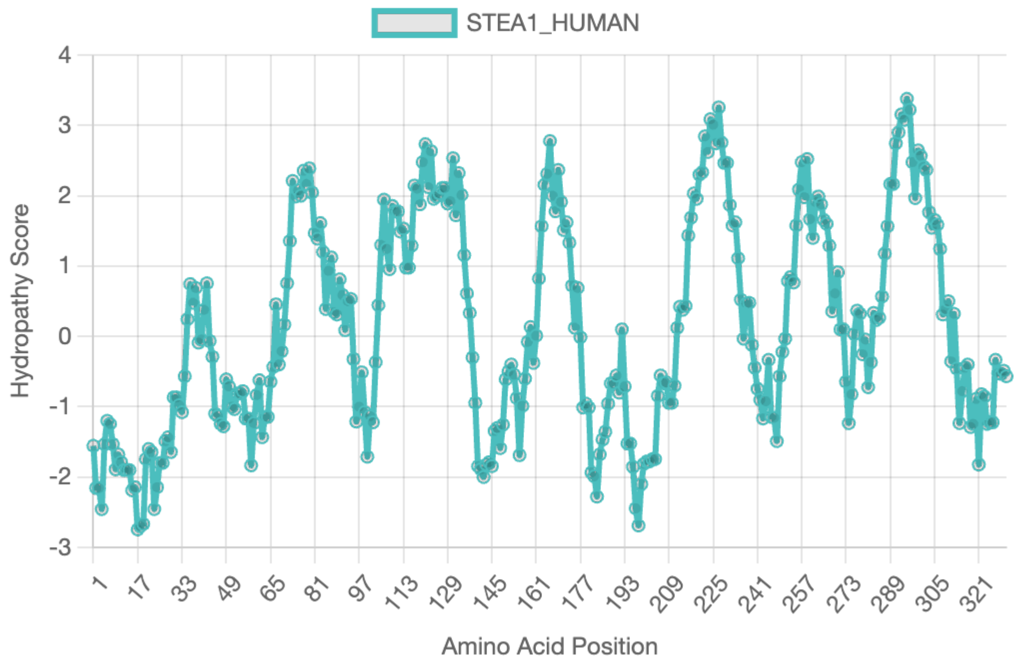

Kyte-Doolittle hydropathy plot for the sequence "STEA1_HUMAN". Positive values indicate hydrophobic regions, while negative values indicate hydrophilic regions.

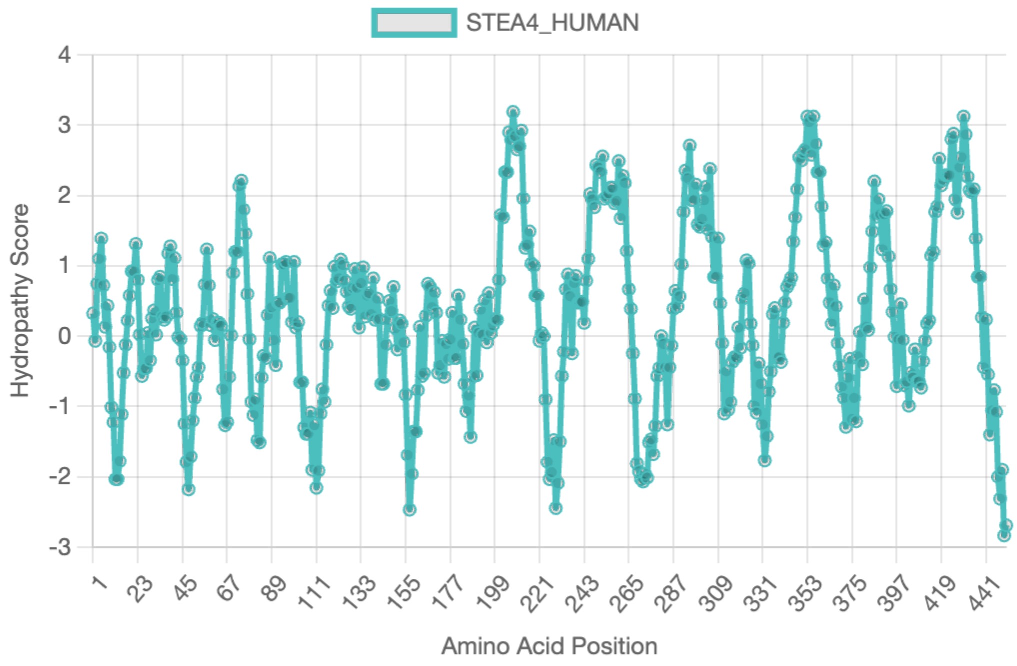

Kyte-Doolittle hydropathy plot for the sequence "STEA4_HUMAN". Positive values indicate hydrophobic regions, while negative values indicate hydrophilic regions.

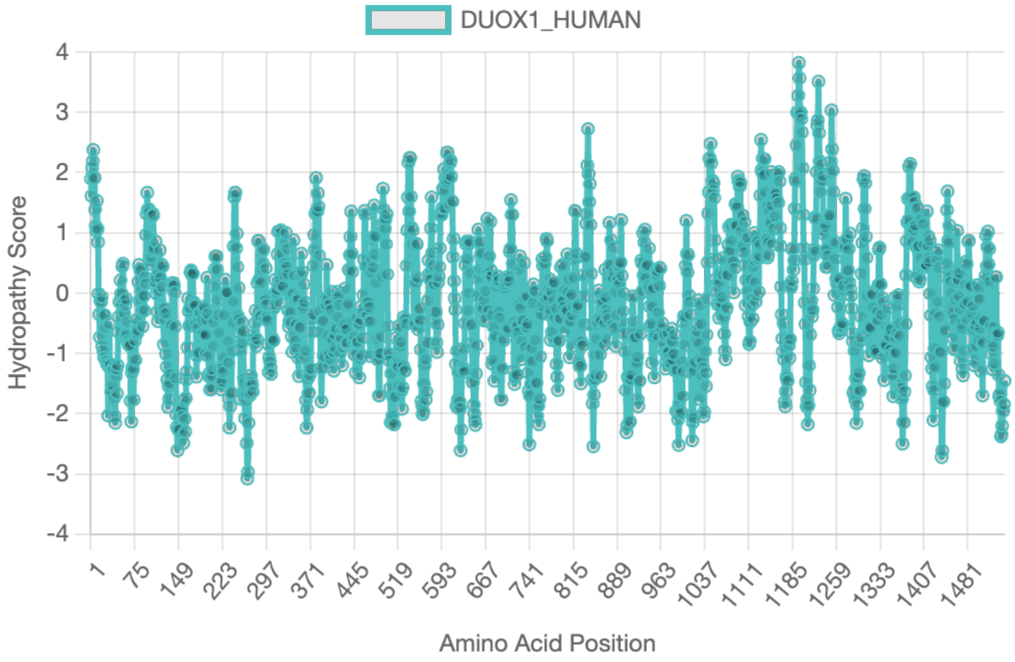

Kyte-Doolittle hydropathy plot for the sequence "DUOX1_HUMAN". Positive values indicate hydrophobic regions, while negative values indicate hydrophilic regions.

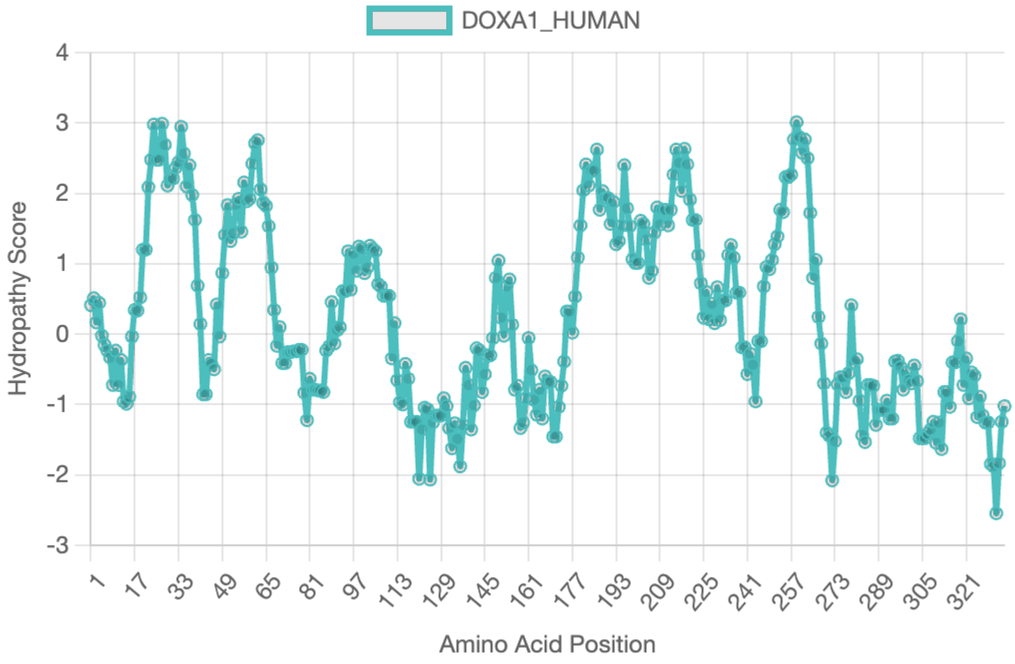

Kyte-Doolittle hydropathy plot for the sequence "DOXA1_HUMAN". Positive values indicate hydrophobic regions, while negative values indicate hydrophilic regions.

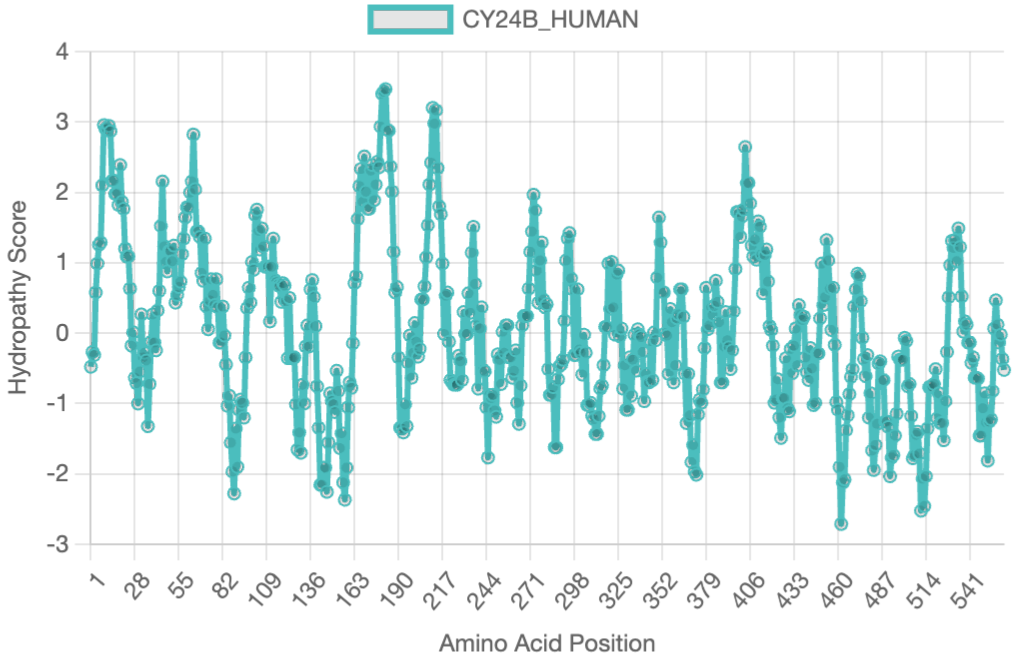

Kyte-Doolittle hydropathy plot for the sequence "CY24B_HUMAN". Positive values indicate hydrophobic regions, while negative values indicate hydrophilic regions.

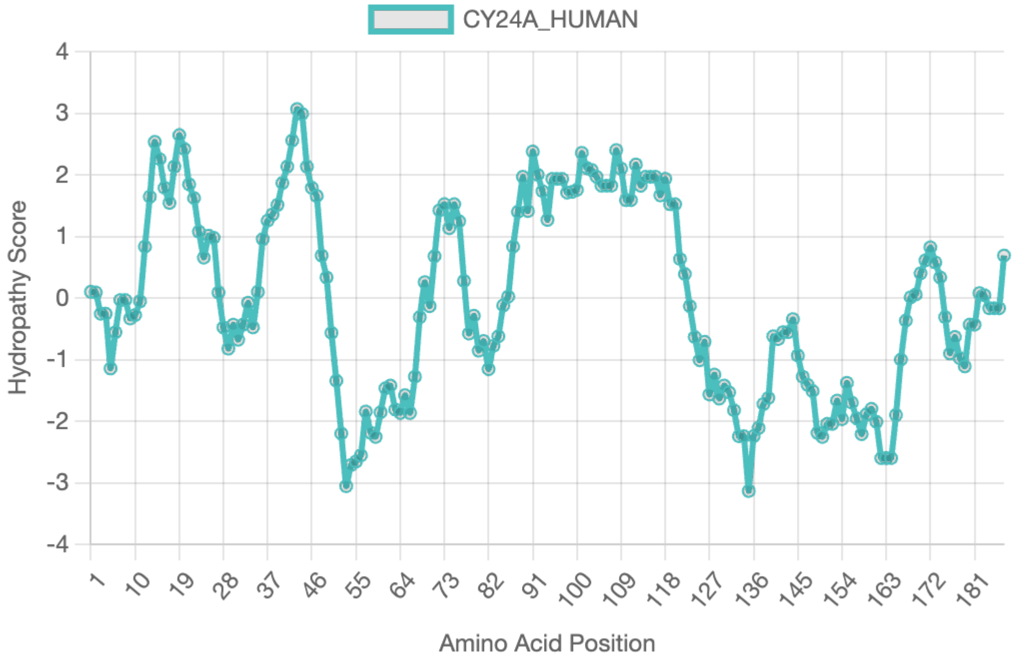

Kyte-Doolittle hydropathy plot for the sequence "CY24A_HUMAN". Positive values indicate hydrophobic regions, while negative values indicate hydrophilic regions.

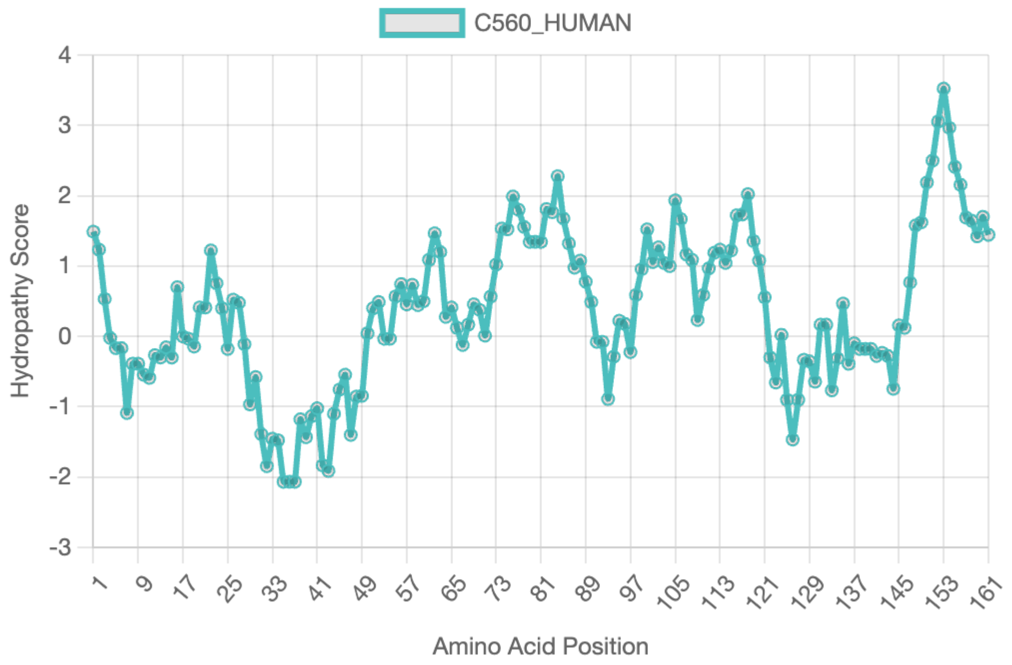

Kyte-Doolittle hydropathy plot for the sequence "C560_HUMAN". Positive values indicate hydrophobic regions, while negative values indicate hydrophilic regions.

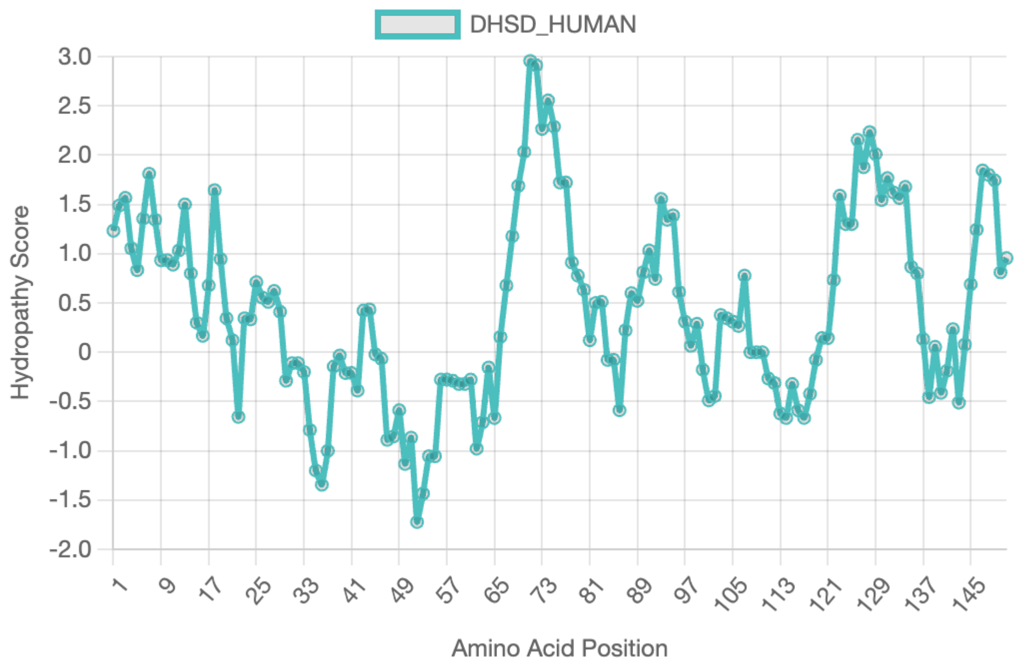

Kyte-Doolittle hydropathy plot for the sequence "DHSD_HUMAN". Positive values indicate hydrophobic regions, while negative values indicate hydrophilic regions.
